# Evolution of the F-Box Gene Family in *Euarchontoglires*: Gene Number Variation and Selection Patterns

**DOI:** 10.1371/journal.pone.0094899

**Published:** 2014-04-11

**Authors:** Ailan Wang, Mingchuan Fu, Xiaoqian Jiang, Yuanhui Mao, Xiangchen Li, Shiheng Tao

**Affiliations:** 1 State Key Laboratory of Crop Stress Biology in Arid Areas and College of Life Sciences, Northwest A & F University, Yangling, Shaanxi, China; 2 Bioinformatics Center, Northwest A&F University, Yangling, Shaanxi, China; University of Lausanne, Switzerland

## Abstract

F-box proteins are substrate adaptors used by the SKP1–CUL1–F-box protein (SCF) complex, a type of E3 ubiquitin ligase complex in the ubiquitin proteasome system (UPS). SCF-mediated ubiquitylation regulates proteolysis of hundreds of cellular proteins involved in key signaling and disease systems. However, our knowledge of the evolution of the F-box gene family in *Euarchontoglires* is limited. In the present study, 559 F-box genes and nine related pseudogenes were identified in eight genomes. Lineage-specific gene gain and loss events occurred during the evolution of *Euarchontoglires*, resulting in varying F-box gene numbers ranging from 66 to 81 among the eight species. Both tandem duplication and retrotransposition were found to have contributed to the increase of F-box gene number, whereas mutation in the F-box domain was the main mechanism responsible for reduction in the number of F-box genes, resulting in a balance of expansion and contraction in the F-box gene family. Thus, the *Euarchontoglire* F-box gene family evolved under a birth-and-death model. Signatures of positive selection were detected in substrate-recognizing domains of multiple F-box proteins, and adaptive changes played a role in evolution of the *Euarchontoglire* F-box gene family. In addition, single nucleotide polymorphism (SNP) distributions were found to be highly non-random among different regions of F-box genes in 1092 human individuals, with domain regions having a significantly lower number of non-synonymous SNPs.

## Introduction

To maintain homeostasis or to undergo specified developmental decisions, an organism must be able to respond rapidly to a variety of environmental changes. Protein turnover plays a critical role in the control of many signaling pathways. More than 80% of all proteins are estimated to be degraded via the ubiqutin-proteasome system (UPS) [1]. Protein ubiquitination is an enzymatic cascade in which ubiquitin is activated by an E1 enzyme, transferred to an E2 ubiquitin-conjugating enzyme and then transferred to a substrate selected by an E3 ubiquitin ligase [2]. An E3 ubiquitin ligase must rapidly and uniquely bind to target proteins in response to stimuli. One of the best characterized E3s are the S phase kinase-associated protein 1 (SKP1)–cullin 1 (CUL1)–F-box protein (SCF) type ubiquitin ligase complexes [3]. CUL1 serves as a scaffold for assembling the ubiquitin-conjugating machinery. The C-terminus of CUL1 interacts with the RING-box protein 1 (RBX1), whereas its N- terminus binds to SKP1, which, in turn, binds to an F-box protein.

F-box proteins contain an N-terminal 48-amino-acid F-box domain (first identified in Cyclin F), which binds to SKP1 to create a link to CUL1. In addition, F-box proteins generally contain C-terminal variable protein-interaction domains, such as Trp–Asp repeats (also called WD40) and leucine-rich repeats (LRR), as well as unknown motifs, which are responsible for binding specific substrates [4]. As a core component of UPS, F-box proteins are involved in a wide range of cellular processes, from cell cycle control to gene transcription and organism development. Given this critical role, misregulation of F-box protein-mediated ubiquitination has been implicated in many human diseases, such as cancers and viral infections [5], [6].

The number of F-box genes varies dramatically even among closely related species [7]. For instance, lineage-specific expansion has been found in annual Arabidopsis but not in the perennial Populus, suggesting an adaptive advantage conferred by F-box genes for particular physiological processes in Arabidopsis [8]. Given the wide involvement of F-box proteins in cellular processes in human cells, pursuing research on the evolution of F-box proteins in humans and other closely related species is very important. However, most previous works in the field have focused on the evolutionary pattern of F-box genes only in plants [9]–[13], where F-box gene expansion was more frequent. Our knowledge of the evolutionary mechanisms responsible for the emergence, maintenance, and loss of F-box gene duplications in animals is rather limited. In the current study, we investigated the variation in the number of F-box genes, as well as underlying mechanisms for such variation, in eight *Euarchontoglire* species with high-quality genome sequences.

Several studies have demonstrated that in nematodes and plants, F-box genes are under strong positive selection pressure at sites in their substrate-binding domains [12]–[14]. In order to find out whether advantageous natural selection has driven F-box genes in *Euarchontoglires* undergoing adaptive evolution, we studied the selection patterns of the F-box gene family. Within a protein, different structural or functional domains are likely to be subject to different functional constraints and evolve at different rates [15]. Therefore, we assessed selective pressures acting on orthologous F-box genes at various levels, such as full-length, partial segments, and single amino acid sites. In addition, mutational burden within different regions of F-box genes was assessed in the human population using 1000 Genomes data. Our results provide insights into the evolutionary regime that has continually reshaped the protein-protein interaction domains responsible for broadening or altering the substrate specificity of F-box genes.

## Materials and Methods

### Genome-wide identification of F-box genes in eight genomes

The hidden Markov model (HMM) profile of the F-box domain (PF00646) was downloaded from Pfam [16]. All sequence data were downloaded from ENSEMBL (version 69, October 2012) [17]. The downloaded HMM profile was used to search the entire set of annotated proteins from eight species, namely, *Callithrix jacchus* (marmoset), *Gorilla gorilla* (gorilla), *Homo sapiens* (human), *Macaca mulatta* (macaque), *Mus musculus* (mouse), *Pan troglodytes* (chimpanzee), *Pongo abelii* (orangutan), and *Rattus norvegicus* (rat) using the hmmsearch program implemented in the HMMER package [18]. We used the default cut-off values to filter the results of these queries. HMMER-predicted proteins were then scanned for F-box and other domains using InterProScan, which is a tool that integrates multiple signature-recognition methods into one resource [19], [20].

For each F-box domain-containing protein identified by the hmmsearch and InterProsScan programs, additional PSI-BLAST [21] searches (with an e-value cut-off of 1e-20) were performed against the entire set of annotated proteins to identify additional F-box proteins that were not found using the HMM profile because of their diverged F-box domains. A second scan for the F-box domain was performed for PSI-BLAST hits.

In order to detect pseudogenes related to F-box genes, all of the F-box protein sequences retrieved from the hmmsearch and PSI-BLAST programs were used as queries to perform a TBLASTN search of the entire set of annotated pseudogenes of each species with an e-value cut-off of 1e-40. Finally, TBLASTN hits were translated and then scanned for presence of F-box domains.

The genomic distribution of identified F-box genes was evaluated by comparing the observed number of genes in each chromosome with its expected number under a Poisson distribution.

### Phylogenetic analysis

Multiple sequence alignments of the F-box protein sequences, which are shown in File S1, were generated using MUSCLE [22] and then manually checked and trimmed with TRIMAL 1.2 (gt = 0.3) [23]. Subsequently, following the Akaike Information Criterion (AIC) computed with ProtTest 3.2 [24], the JTT+F model was chosen to construct a maximum likelihood (ML) tree with PhyML 3.0 [25]. Topological robustness of the phylogenetic tree was assessed by bootstrapping with 100 replicates.

Orthology assignments of F-box genes from the eight species were downloaded from the ENSEMBL database using Biomart. Orthologous groups (also known as orthogroups) were inferred based on phylogenetic relationships and confirmed by reciprocal BLAST. Subsequently, homology relationships of F-box genes in the same orthogroup were checked against the data obtained from ENSEMBL. We nominated human and mouse F-box genes according to the nomenclature proposed by Jin et al. [26]. F-box genes in the other six species considered in this study had the same names as their orthologs in humans.

### Inference of gene-gain and gene-loss events and their underlying mechanisms

Gene-gain and gene-loss events were inferred using the species/gene tree reconciliation approach with NOTUNG software [27]. For these analyses, the reference species tree used was reconstructed according to TIMETREE [28]. The reconciled tree was manually adjusted by applying information on orthologs (from outgroups *Danio rerio* and *Gallus gallus*), because the gene tree may not always be reliable for species/gene tree reconciliation.

We investigated several potential underlying mechanisms responsible for gene-gain events inferred from previous estimations. First, we explored whether retrotransposition may have contributed to such duplications. The nucleotide sequences of duplicated genes were inspected for signatures of retrosequences, such as lack of introns, stretches of poly (A) at the 3′ end, and short direct repeats at both ends. Second, if the duplicated gene was not found to have been generated by retrotransposition, we considered two types of segmental duplications (>90% identity and >1 kb in length) [29]: (*i*) tandem duplication, where the two genes are located within the same chromosomal region (i.e., fewer than 20 genes apart from each other) [30], or (*ii*) interspersed duplication.

Next, we performed a more exhaustive search for genes that are absent in certain lineages. The orthologs present in closely related species were used as queries for BLASTN searches against the genomic sequences in the NCBI database. When a high-identity match (identity >60%) was produced, we examined whether it was annotated as a gene by NCBI; if it wasn't, we manually annotated it using FGENESH+ (www.softberry.com). Next, the proteins annotated were scanned for known functional domains by InterProScan. If an F-box domain was found, the protein was designated as an F-box protein; otherwise, the protein was noted as having lost its F-box domain. If a high-identity match was not obtained, the gene was assumed to have been removed from the genome by deletion.

### Selective pressure analyses

For each orthogroup, orthologous amino-acid sequences with similar length were aligned with MUSCLE [22] and then manually checked and trimmed with TRIMAL 1.2 [23]. TRIMAL removed poorly aligned columns and incomplete sequences, considering the remaining sequences in the MSA using three specified thresholds (- resoverlap 0.75, -seqoverlap 80, -gt 0.7). Subsequently, the sequences that did not pass the sequence overlap threshold were replaced by another transcript of the associated genome. Finally, only columns and sequences that passed the thresholds were retained in the final alignments. These retained protein alignments were used to guide the alignments of the corresponding CDSs using TRIMAL. The multiple coding sequence alignments thus generated are shown in File S3.

Average codon-based evolutionary divergence over all sequence pairs within each orthologous group was measured in terms of *Ka* (the number of non-synonymous substitutions per non-synonymous site) and *Ks* (the number of synonymous substitutions per synonymous site) using MEGA [31]. Next, the codon-based Z-test was used to evaluate the significance of the *Ka*/*Ks* substitution rate.

The distribution of selective pressure across the gene was investigated using the sliding window method in which *Ka*/*Ks* (ω) ratios were calculated by DnaSP with a window length of 30 bp and a step size of 6 bp [32]. The statistic ω was calculated in each window, and its value was assigned to the nucleotide at the midpoint of the window. Values of ω from each window were plotted against the nucleotide position, thus enabling the visualization of distinct selective pressure acting across the gene.

To assess variations in selective pressure among sites, the site-specific models were tested comparatively using M0 (one ratio) and M3 (discrete), both of which are implemented in the PAML software package [33]. To detect positive selection affecting a few sites along particular lineages, we applied branch-site model A for analysis.

The ω value for each codon was calculated using the MEC model that is implemented in the Selecton software package [34]. The MEC model is a combination of empirical and mechanistic model, which accounts for differing empirical amino acid mutation probabilities. Compared with the more conservative M8 model, a smaller proportion of sites (with ω values >1) may be sufficient in the MEC model to indicate positive selection despite a low global ω value for the protein [35]. Program Selecton was run with the MEC model and M8a (a null model that only allows purifying and neutral selection), and their second-order Akaike Information Criterion (AICc) score was compared. To map the detected positive selection sites onto the protein's three-dimensional (3D) structure, we used the homology modeling method to construct a 3D structure of F-box proteins in the SWISS-MODEL workspace [36]. The PyMOL (http://www.pymol.org) graphical interface was used to manipulate and display the F-box protein 3D structure.

### Single-nucleotide polymorphism (SNP) analysis of F-box genes in the human population

Variant calls from the 1000 Genomes project [37] Phase 1 release v3 were obtained from the NCBI ftp server (ftp://ftp-trace.ncbi.nih.gov/1000genomes/ftp/release/20110521/). Variants were annotated using the SNPEff (v3.4) software (http://snpeff.sourceforge.net/). The annotated variants were filtered to retrieve SNP variants in F-box protein coding regions. Statistical analyses were performed using distribution-free non-parametric tests in the R program. The significance of differences in SNP density distributions in different regions of F-box genes was assessed using the Kruskal-Wallis test or the Mann-Whitney U test. The Fisher's exact test was used to compare ratios of non-synonymous to synonymous substitution SNPs between different regions of F-box genes.

## Results

### Identifying F-box proteins and their domain architectures

In order to comprehensively identify F-box genes, we used an integrated hmmsearch–BLAST–InterProScan approach. A total of 559 protein-coding F-box genes were identified in eight genomes (Table S1 A). The F-box gene number in all genomes was approximately 70 (except in the mouse genome), accounting for over 0.3% of the total protein-coding genes (Table S1 B). We also identified nine annotated pseudogenes duplicated from protein-coding F-box genes and 21 corresponding homologous DNA regions (Table S2).

The chromosomal distribution of F-box gene was uniform across all autosomes, except for several chromosomes (Table S3). In five primate genomes, F-box genes were significantly enriched in chromosomes 16 and 19, compared with other chromosomes. Chromosomal translocation may have contributed to the higher F-box gene density in chromosome 16. On the other hand, the high density of F-box genes in human chromosome 19 may be attributed simply to the fact that human chromosome 19 is the most gene-rich chromosome [38]. Interestingly, in all genomes analyzed, F-box genes were absent from chromosome X, and this absence was not random (Table S3). The absence of F-box genes on chromosome X of all the eight species examined is consistent with the fact that mammalian X chromosomes are highly conserved across species [39]. No F-box gene was found on chromosome Y, but the departure from uniformity was statistically insignificant. The mechanisms underlying the absence of F-box genes from sex chromosomes remain unclear.

**Figure 1:**
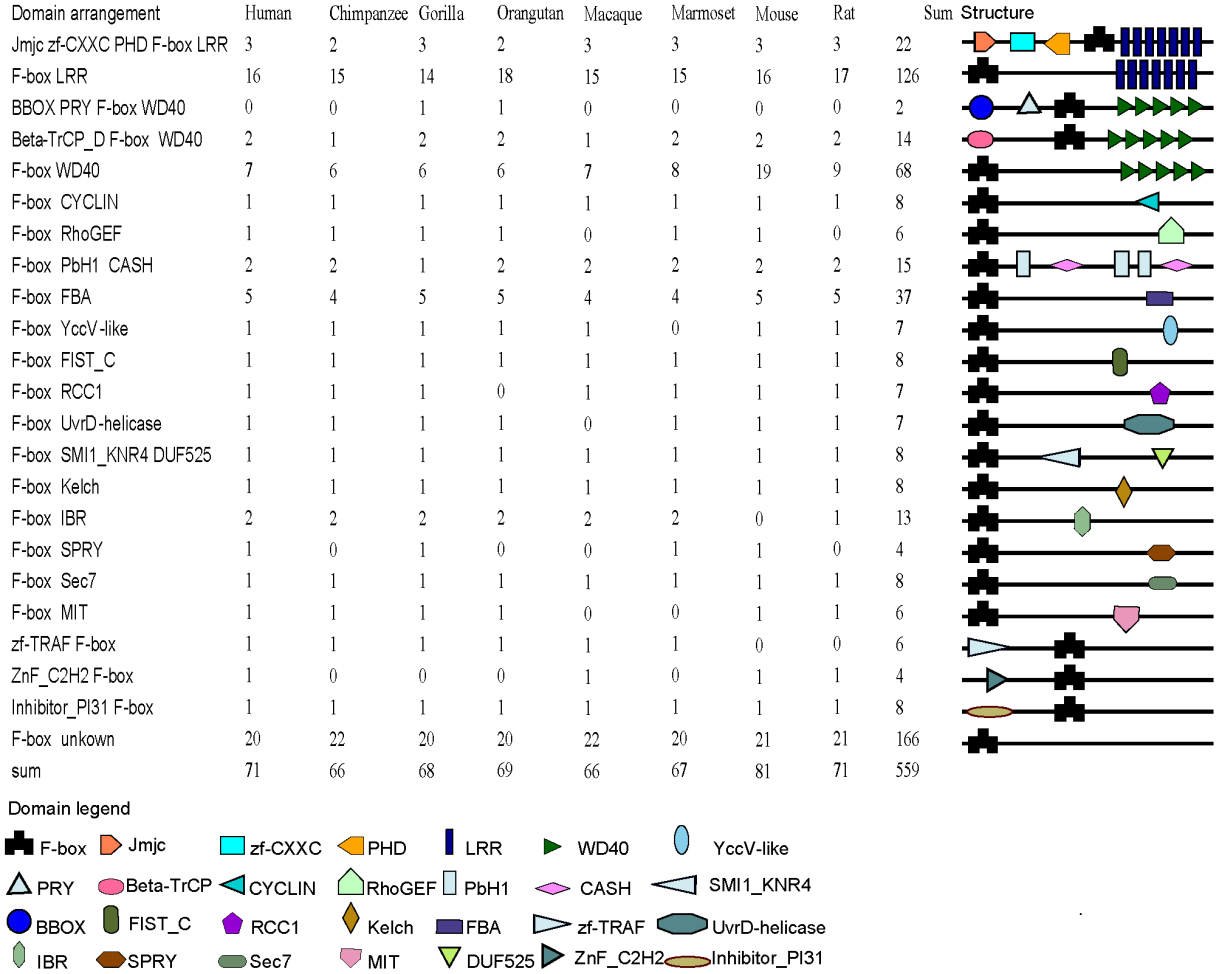
Number and domain structure of F-box proteins from marmoset, gorilla, human, macaque, mouse, chimpanzee, orangutan, and rat.

Twenty-seven types of domains (excluding the F-box domain) were identified within F-box protein sets, and all but nine were located at the C-terminus (Figure 1). Among them, the proportion of the LRR domain was the largest, followed by the WD40 domain. The remaining domains were present in small subsets of F-box proteins. Except the F-box domain, no other recognizable domain was found in 30% of the 559 F-box proteins identified in this study.

### Clustering orthogroups

The 559 F-box genes identified may be clustered into 71 distinct orthogroups (Figure S1). The deep branches were poorly supported by bootstrapping, because F-box genes from different subgroups are of great divergence. However, the vast majority of the single orthogroups reached well-resolved topology with strong bootstrap support. Furthermore, the members in each identified orthogroup were the best blast hits of each other (File S2), and their orthology relationships were confirmed by data obtained from ENSEMBL. These orthogroups include stable and unstable clades based on evolutionary stability. Stable clades are those that contain at least one member of each species (but species-specific duplications could as well result in more copies). Unstable clades in turn do not contain genes of each species. Fifty-seven orthogroups were evolutionarily stable, and the remaining 14 orthogroups were unstable but varied only slightly. The gene orthology relationships among the eight organisms were one-to-one, one-to-many/one-to-one and one-to-zero/one-to-one for 54, 3, and 14 orthogroups, respectively. The one-to-many and one-to-zero orthology relationships are the results of lineage-specific gene duplications and losses, respectively. Although proteins in the majority of the orthogroups contained the same C-terminal domain, species-specific domain accretion or reduction also took place (Figures 1 and S1). For example, the primate FBXL22 lost the LRR domain, and the PRY domain was accreted in the FBXW10 of both gorilla and orangutan. Moreover, orthogroups with the same or similar domain architecture were not necessarily the closest neighbors. For instance, several LRR-domain-containing orthogroups were scattered across the phylogenetic tree.

All F-box genes were directly clustered into their corresponding orthogroups, with the exception of Fbxo6 and Fbxo44. Gene conversion may be a potential source of conflict between a gene tree and a species tree [40]. Hence, gene conversion tests were performed using Geneconv (http://.math.wustl.edu/~sawyer). Statistically significant evidence of a gene conversion event was found between *Fbxo6* and *Fbxo44* of human, chimpanzee, gorilla, orangutan, and macaque at nucleotide 1477 and nucleotide 1707 (site numbering refers to human *Fbxo44* with Ensembl Gene ID ENSG00000132879) (Figure S2). These results suggest that a gene conversion event occurred in the ancestor of these five primates (*Fbxo44* was not found in marmoset, so this species was excluded from our analysis). These gene conversion regions contained sequences of F-box functional domains and partial F-box associated (FBA) region.

### Evolutionary change of F-box gene number and underlying mechanisms

**Figure 2:**
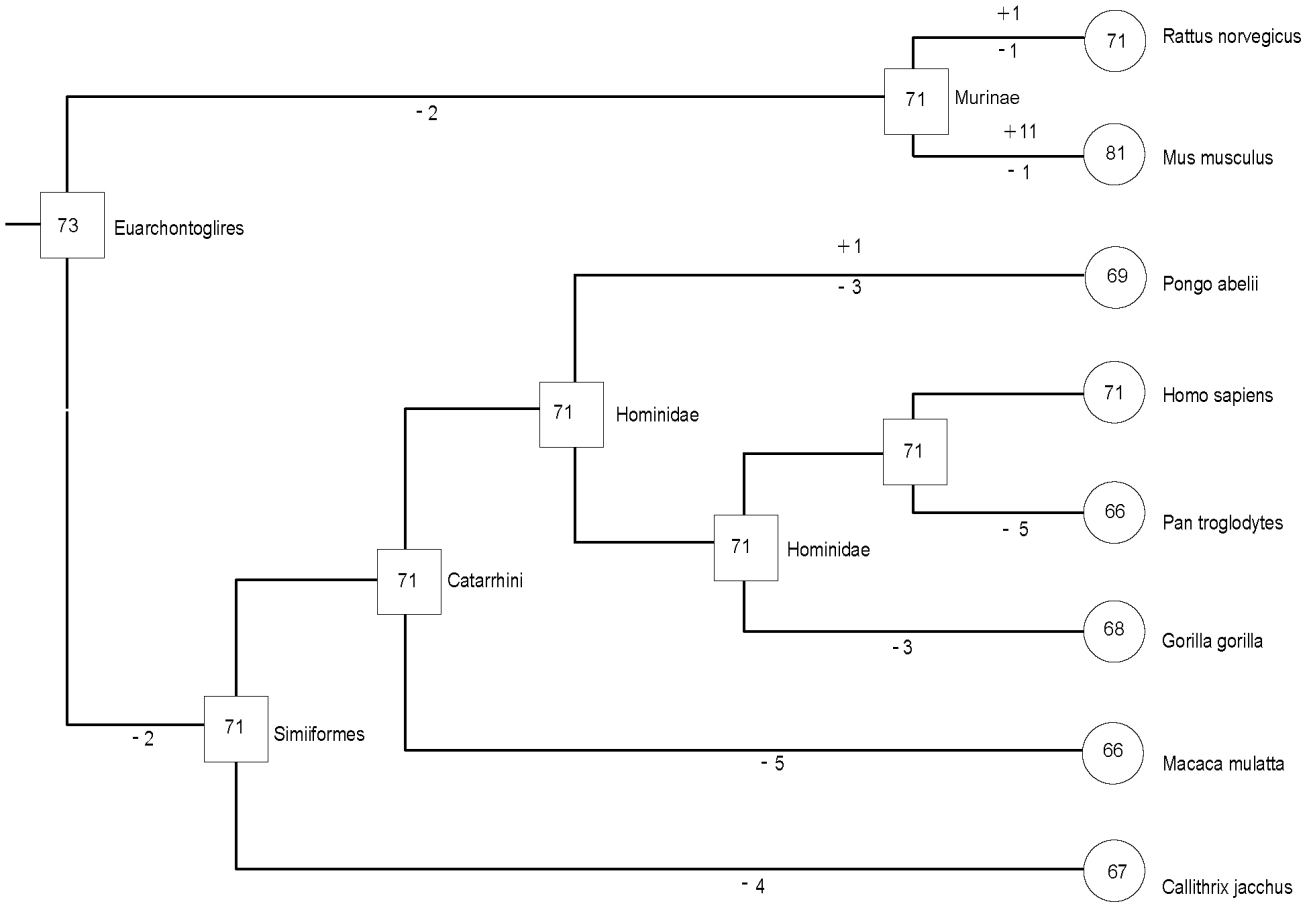
Estimated numbers of ancestral, gained, and lost genes during the evolution of *Euarchontoglire* animals. Names of extant and ancestral species are on the right-hand side of each external and internal node. Numbers within circles and boxes indicate the numbers of genes in each extant and ancestral species, respectively. Branches are not drawn in proportion to their lengths. The numbers above and below each branch are the numbers of genes gained and lost, respectively.

As mentioned above, lineage-specific gene gain and loss events appeared to have given rise to one-to-many or one-to-zero orthologous relationships among the eight organisms. Therefore, we estimated the number of F-box genes in all ancestral organisms and their change at different stages of the evolution of *Euarchontoglire* animals. Evolutionary changes in the number of the F-box genes are shown in Figure 2. Although the number of F-box genes was found to be conserved, as a general trend, during the evolution of *Euarchontoglires*, lineage-specific gene gain and loss events still took place.

**Figure 3:**
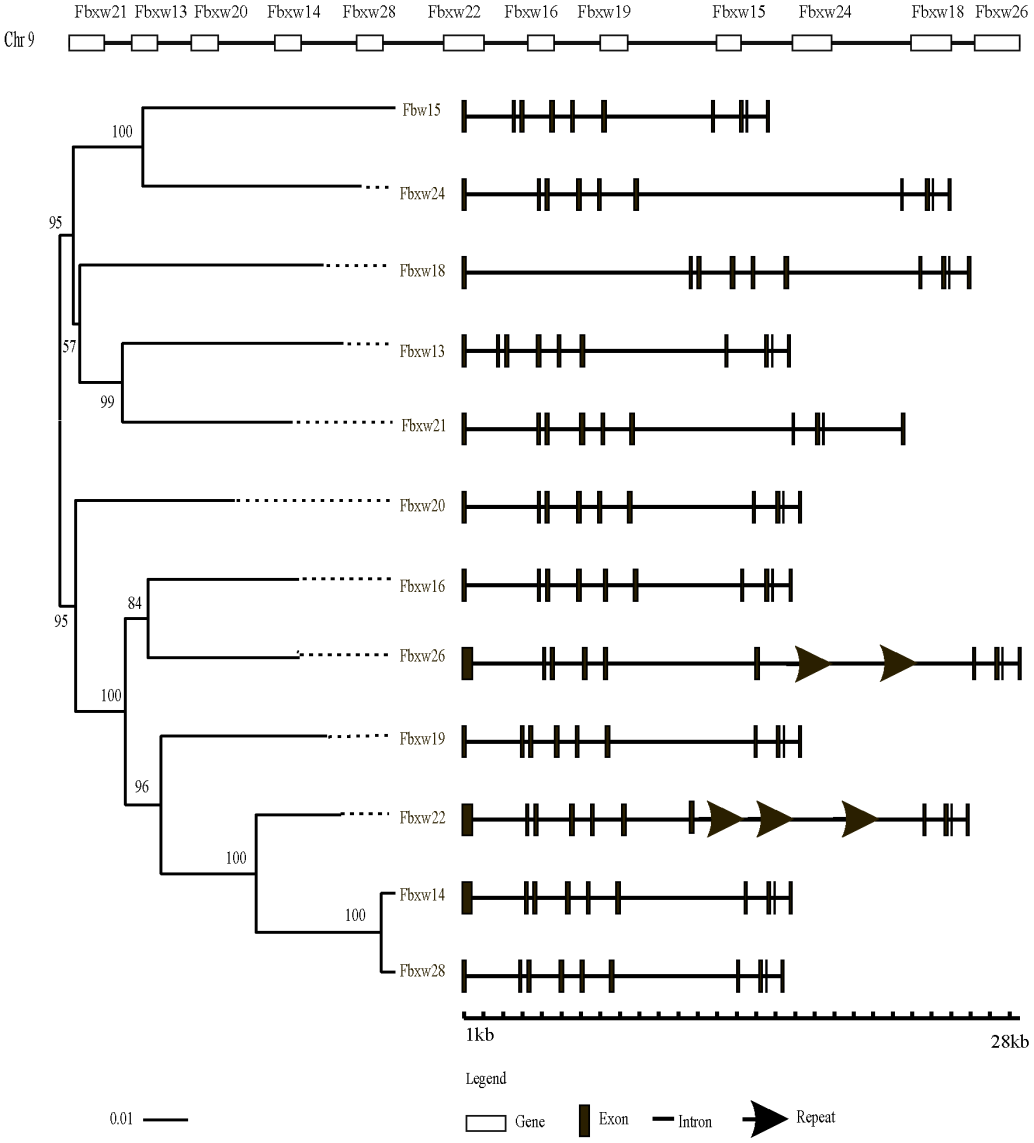
Chromosomal location, phylogenetic relationships and gene structure of the mouse *Fbxw12* gene cluster. These genes diverged at intron length, caused in part by internal sequence repeats.

Multiple mechanisms contributed to F-box gene number variation among these organisms. It should be noticed that a closely linked gene cluster includes 12 neighboring genes in mouse chromosome 9 with similar gene structures and a high level of sequence identity, ranging from 63.7% to 97.9%, in their coding regions (Figure 3). Therefore, this gene cluster likely arose from a series of tandem duplication events. However, these genes appear to have diverged in terms of their intron length, which we identified to be caused by variations in internal intron sequence repeats, such as the 510 bp repeats in intron 6 of *Fbxw22* (Figure 3).

**Figure 4:**
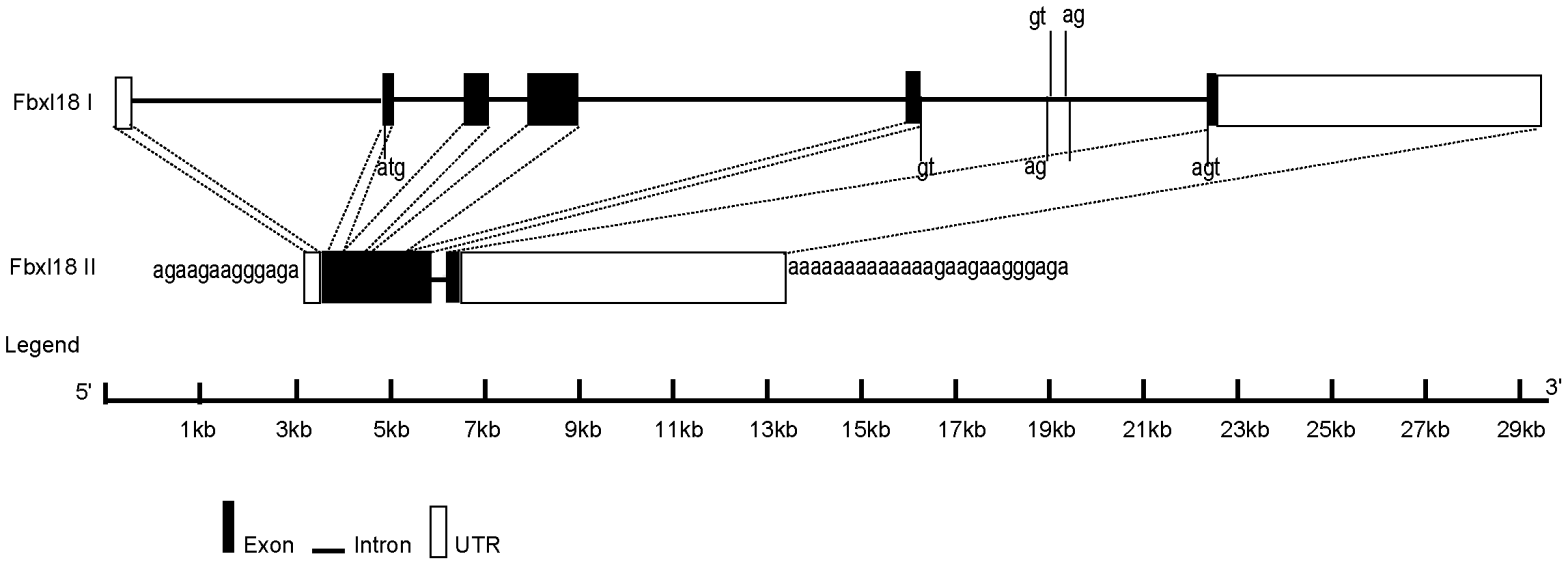
Gene structures of two paralogs *Fbxl18I* (Ensembl Gene ID: ENSRNOG00000001117) and *Fbxl18II* (Ensembl Gene ID: ENSRNOG00000033326). Dotted lines between the two paralogs indicate identical sequence fragments.

Both primates and mice contain a single *Fbxl18* gene, while rats contain two distinct *Fbxl18* genes. A schematic illustration of the gene structures of *Fbxl18I* and its paralogue *Fbxl18II* are shown in Figure 4. Evidences that *Fbxl18II* was formed by retrotransposition of *Fbxl18I* are as follows: (*i*) *Fbxl18I* and *Fbxl18II* are located on chromosomes 5 and 12, respectively; (*ii*) the two sequences are identical over 111 bp in their 5′ UTRs and their 3′ UTR sequences have over 80% similarity; (*iii*) *Fbxl18II* is flanked by a short sequence repeat of ‘agaagaagggaga’; (*iv*) stretches of 12 adenine (A) occur in *Fbxl18II* as relics of poly (A) structure; and (*v*) *Fbxl18II* contains a 114-bp intron in its 5′ UTR. In addition, a 91-bp intron whose counterpart in *Fbxl18I* is flanked by an intron sequence with a GT-AG boundary is also observed (Figure 4). Based on these facts, rat *Fbxl18II* likely represents a semi-processed retrogene. Although functional experimental evidence for rat *Fbxl18II* is lacking, an expressed sequence tag (EST) (GenBank: CB765629.1) with sequence identity with *Fbxl18II*, rather than *Fbxl18I*, was found in the NCBI database. *Fbxl18II* sequence analysis showed that (*i*) the DNA sequence of *Fbxl18II* has a perfect open reading frame (ORF); (*ii*) using RepeatMasker (http://www.repeatmasker.org), a 937 bp sequence upstream of the start codon was predicted as a long interspersed nuclear element (LINE) that provides original evolutionary materials for promoter formation, and *(iii)* a highly likely transcription start site was predicted at about 200 bp upstream of the start codon using FirstEF [41] (*p* = 0.422) and Promoter 2.0 [42] (prediction score  = 1.224) (Figure S3). These findings suggest that the retrogene *Fbxl18II* may indeed be a functional gene.

Absence of a gene in some genomes does not necessarily indicate removal by deletion. In fact, F-box gene sequences without typical F-box domains often remain in the genome, as supported by our findings that they exist as diverged orthologs of their counterparts with intact F-box domains across multiple species. For instance, the rat *Fbxo45* does not contain the typical F-box domain, while its orthologs in mouse and primate do.

### Sequence divergence of orthologs and region- and lineage-specific positive selection

To explore the selection forces driving the evolution of the F-box gene family, we first calculated sequence divergence in the complete coding sequences (CDSs) for each orthogroup. The values of *Ka*, *Ks*, and *ω* showed extensive discrepancy among orthogroups (Figure S4). *Ks* was positively correlated with *Ka*, as assessed using the Kendall test (tau  = 0.419, *p*<0.001). Codon-based Z-test indicated that the *ω* ratios of all orthogroups were significantly lower than 1 (*p*<0.001), except for *Lrrc29* (*p* = 0.107).

**Figure 5:**
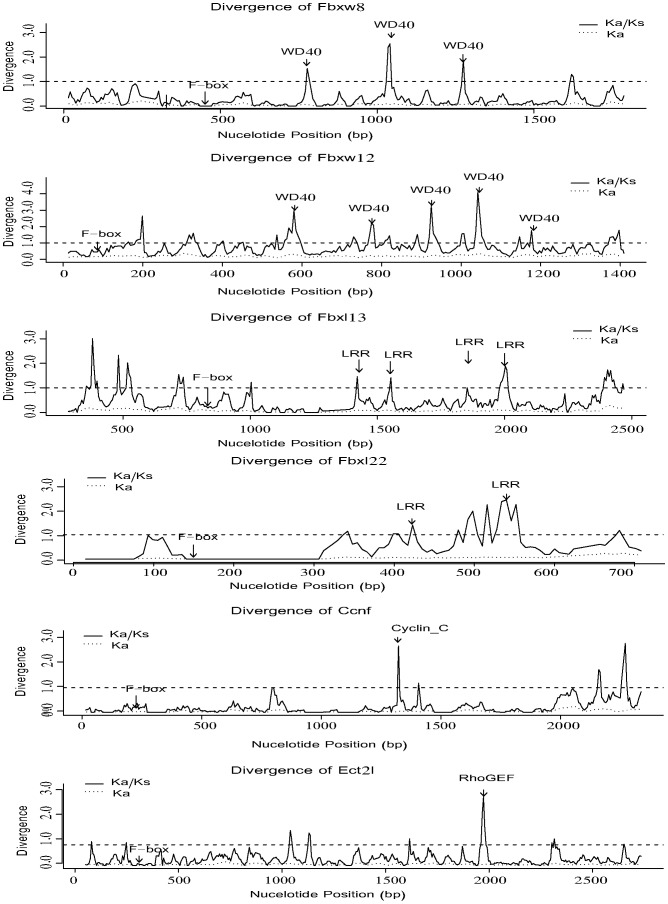
Sliding window analysis of sequence divergence across the protein-coding regions of six orthogroups using a window length of 30 bp and a step size of 6 bp.

To determine the presence of specific regions within F-box genes under positive selection, sliding window analyses were performed. As expected, ω ratios greater than 1 were observed in regions such as WD40, LRR, and Cyclin_C domains (Figure 5), as well as other uncharacterized domains (Figure S5). Contrary to this pattern, the ω ratios of F-box domains were mostly lower than those of other regions within F-box genes. Such functional selective constraints on the F-box domain may be a consequence of co-evolution of the F-box domain and the SKP1 protein since SKP1 residues are highly conserved, particularly in the core portion of the SKP1–F-box protein interface [43]. For many orthogroups, all regions were under selective constraint with ω <1 although the exact ω value varied among regions (Figure S5).

To verify the statistical significance of variation in selective pressure among sites, we applied a pair of models, namely M0 and M3, to perform likelihood ratio tests. Model M0 assigns a homogeneous ω among sites, whereas M3 assumes several ω site classes. The likelihood ratio test indicated that model M3 exhibited significantly better fit with the data than model M0 for all orthogroups, with the exception of *Fbxw7*, *Fbxl7*, *Fbxl14*, *Fbxl15*, *Fbxl18*, *Fbxl20*, *Kdm2A*, *Lrrc29*, *Fbxo2*, *Fbxo4*, *Fbxo11 Fbxo25*, *Fbxo33*, and *Fbxo44*, the *p* values of which were greater than 0.05 (Table S5).

Next, we investigated whether some sites within the F-box gene were under positive selection along specific lineages using branch-site models. The orthogroups that included eight orthologs from the eight species were selected for this analysis. Results showed that some sites were under positive selection along specific lineages. A total of 22 out of 34 orthogroups exhibited site-specific positive selection along a specific branch (Table S6). Furthermore, certain F-box genes, such as *Fbxw12*, underwent adaptive evolution independently in different lineages.

### Molecular adaptation at individual sites and their effects on function

Specific residues may be selected individually, regardless of the pattern of selection governing the global sequence. We calculated ω values at each codon position. Fourteen out of 71 orthogroups underwent adaptive evolution, as indicated by the presence of some positively selected residues in them (Akaike information criterion score < M8a, and posterior mean of ω >1.5) (Table 1). A large number of individual positively selected sites were observed in *Fbxw12* and *Fbxl13.* Although positive selection on these sites were not statistically significant (the lower bound of the confidence interval ω was not >1), the results were in agreement with the interpretation that a large number of sites in the two genes had diverged in *Euarchontoglires* (File S3). Some of these positive selection residues were located in identified substrate-interacting domains.

**Table 1:** Detection of positive selection at individual sites by Bayesian method with MEC model using Selecton.

Gene	AICc of M8a	AICc of MEC	Positive site
Fbxw9	7882	7866	13T **173V**
Fbxw10	16530	16488	352G 359T **772G**
Fbxw12	11143	11075	31H 35I 40Y 44S 45L 57N 85H 95I 97F 98E 99T 100E 101L **121S 127E 146E 147F**
			**148H 150S 151N 166R 167K 187P 188Q 189P 192C 236L 253S 280P 282K 285A 303S**
			**304S 305T 306G 316L 330Y 331E 338A 339A 340H 343C 345I 375R 377E 381A 382A**
			**384N 391C 398E 412H** 422E 426H 427D 430T 431D 447R 450K 451V 452S 453D 464T
Fbxl13	11932	11860	36V 66D 88T 97T 100H 130A 136F 139R 145F 150T 157L 187L 191L 192N 232L **308R**
			**353M** **381L** 420F **427N 470K** **480R 491R 495A 498M 524G 541D** **563E 565Y 566R 570D** **607N**
			**623A** **644L 651E** **670N 673K 674K** 701R 710D 712I 714S 717G 718A 727T 728Y 733Q 735A
Fbxl5	8092	8080	562*
Ccnf	12375	12357	220T* 446A* 691R*
Fbxo5	7538	7506	8C 18S 19A
Fbxo6	5619	5586	212T
Fbxo7	9792	9742	25H 27R 28S 32Q 140L 222L 286C 287K 421T
Fbxo15	9591	9553	65M 267L 269D 270S 275L 276H 288G 291Y 294G 301T 302K 376T 386Q 389N 395A
Fbxo18	14881	14780	190R* 238V* 568G* 1080N*
Fbxo36	2970	2960	100D 107S 132K
Fbxo47	7242	7207	20S 209Q 230R 231S
Fbxo48	2734	2709	2H 4N 9N 11L 15H 18A 28N 36E 40A* 42I 44F 53R 60L

Note: The residues written in bold letters are located in carboxyl-terminal functional domains. Asterisks represents ω confidence interval lower boundary of >1.

**Figure 6:**
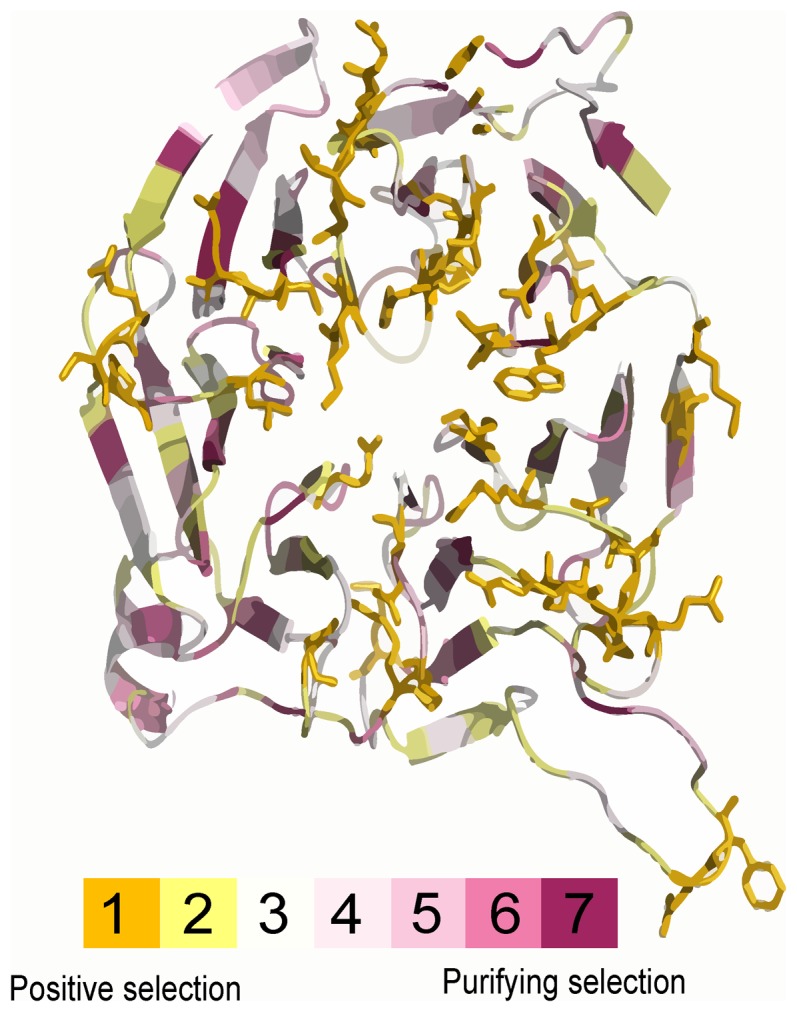
Projection of positive-selection sites onto WD40 domains. The Ka/Ks (ω) value for each residue is color coded on a seven-step scale from violet (<<1) to dark yellow (posterior mean of ω >1.5). Based on the template 3ow8, a 3D structure was constructed for a peptide fragment (residues 89—408) of FBXW12. Most of the residues under strong positive selection (orange-colored stick surface) were mapped to the substrate binding channel.

The relationship between positively selected amino acid sites and their effects on function was preliminarily obtained by mapping the sites onto the 3D structure of the functional domain. A 3D structure was built for FBXW12 in automated mode (Figure 6). Currently known WD40 domain-peptide interaction sites were located on all three major surfaces: top, bottom and circumference [44]. The α-helix region was populated with residues with low ω values, whereby the pivotal corrected 3D structure was safeguarded through purifying selection. By contrast, the majority of residues under positive selection of FBXW12 were located in the substrate-binding channel of the WD40 domain.

### Stronger negative selection on domains than on non-domains of F-box genes in the human population

The 1000 Genomes data provided an opportunity to compare the mutational burden within different regions of the F-box genes. Here, we analyzed SNP density distribution and the difference of the ratios of non-synonymous to synonymous SNP numbers in the domains and non-domains of F-box genes. We mapped 1,254 SNPs genotyped in the 1000 Genomes project to coding regions of human F-box genes, of which 391 mapped to predicted domain regions (File S4). The average SNP density in the F-box genes was 1.080 per 100 bp, indicating that the F-box genes in humans had a high occurrence of single nucleotide substitutions. This result is similar to that found by Clark et al. in *Arabidopsis thaliana* [45]. In the current study, 15 SNPs were found in the coding regions of *Fbxo32* from 1,092 persons. However, only one SNP was found among the coding regions of the same gene from 1,313 cattle [46].

**Figure 7:**
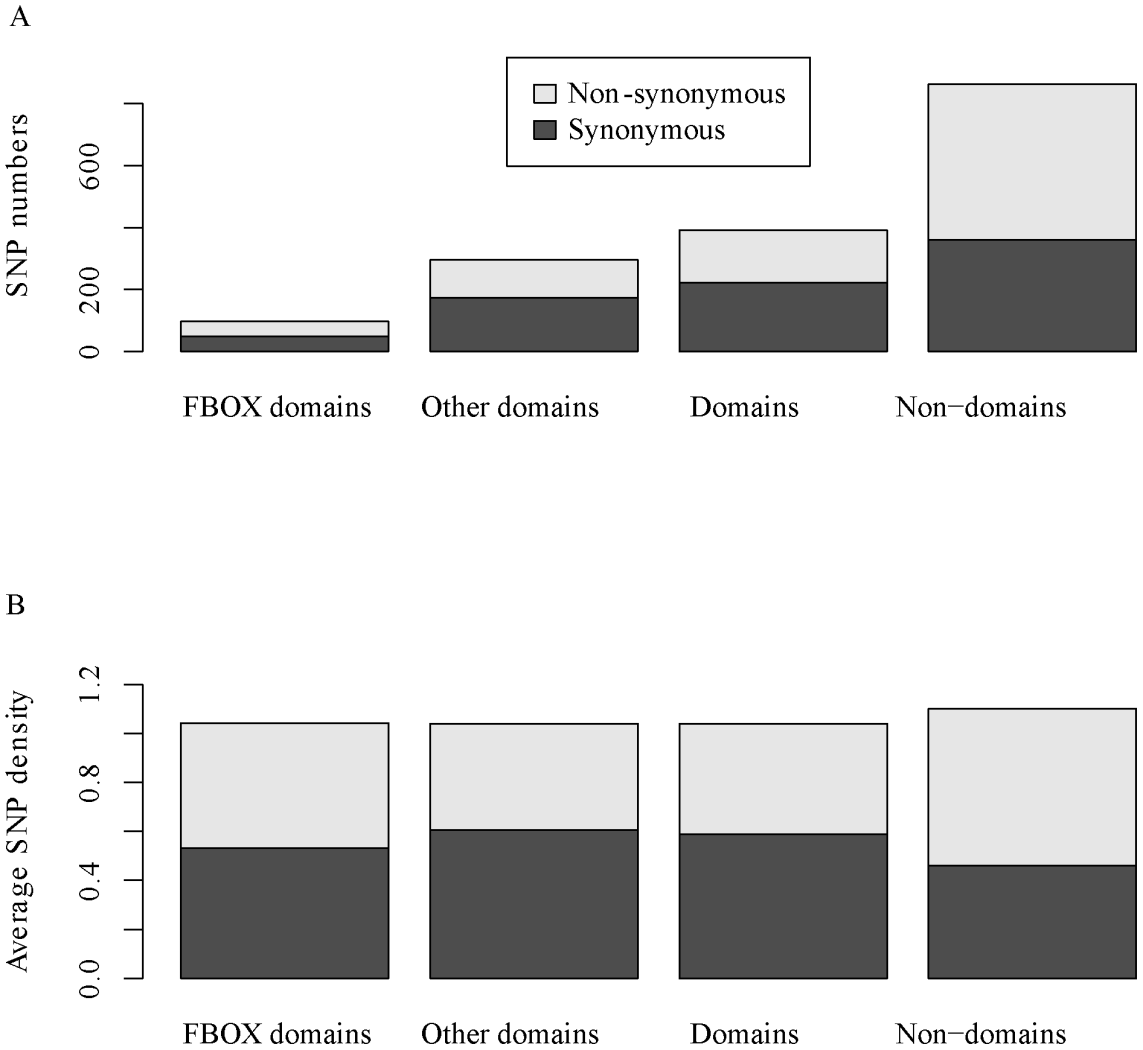
SNP distributions across different regions of F-box genes suggest stronger negative selection on domain regions compared with non-domain regions. A. The total number of synonymous and non-synonymous substitution SNPs in F-box domain, other domain (non F-box domain), domain (including F-box and other domains) and non-domain regions. B. The distributions of average SNP density (SNP numbers per 100 bp) across different regions of F-box genes.

The average SNP density in the F-box domain, other domain and non-domain regions was 1.042, 1.040, and 1.100 SNPs per 100 bp, respectively (Figure 7). The Kruskal-Wallis test for differences in SNP density across regions of F-box genes indicated a significantly uneven distribution (Kruskal-Wallis test *p* = 0.023, Table S7A). Non-synonymous SNP density in domain regions was significantly lower than that in non-domain regions (Mann-Whitney U test *p* = 0.038) (Table S7B). Consistent with this, the ratio of non-synonymous to synonymous SNP numbers in the domain regions (0.769) was significantly lower than that in the non-domain regions (1.391) (Fisher's exact test *p* = 1.403e-06, Table S7C), reflecting stronger purifying selection on domain regions. Contrary to what might have been expected, the F-box domain region was not found to be more conservative than other domain regions based on both SNP density distribution and the ratio of non-synonymous to synonymous SNP number (Figure 7, Table S7C). However, F-box domain regions were the most conservative according to sequence diversity analyses conducted based on the orthogroups (Figures 5 and S5). The differences in results were likely caused by the following factors. First, after the species diverged from *Euarchontoglires*, certain lineages maintained advantageous mutations in the C-terminal functional domains of F-box genes that facilitated adaptation to changing environments by altering their substrate specificities. Second, in humans, C-terminal functional domains of F-box genes have evolved under strong purifying selection to accurately recognize specific substrates.

## Discussion

### Evolutionary conservation and variation in F-box gene number

Conflicting reports exist in the literature regarding the number of F-box genes in humans [26], [47]. We performed a comprehensive search of F-box genes using an integrated method. Approximately 70 F-box genes were identified in each *Euarchontoglire* species, which is much less than the hundreds of F-box genes known to exist in plants. This might be due to the sessile life-style of plants, which prevents them from escaping unfavorable environmental conditions, and necessitates more extensive molecular signaling machineries. Compared with the F-box genes predicted in humans by Jin et al. [26], only *Lmo7* was not detected in our study. A published report indicates that an isoform of LMO7 can bind to SKP1 [48]. The F-box domain of LMO7 contains significant changes compared with the consensus sequence. However, LMO7 can still bind to SKP1 because the mutations occur in non-key residues. In such a case, the F-box domain cannot be predicted by a profile search. The method of identifying functional F-box proteins solely based on the consensus sequence inevitably leads to limitations such as filtering out of diverged F-box proteins like *Fbox45* (Table S4). We also assigned the genes *Ect2l*, *Fbxo47*, and *Fbxo48,* all of which contain F-box domains, to the F-box gene family. In addition, Jin et al. [26] had noted that *Fbxw12*-related sequences are expanded over a cluster of six genes on chromosome 9 in mouse. Surprisingly, we found six other paralogs, namely *Fbxw20*, *Fbxw21*, *Fbxw22*, *Fbxw24*, *Fbxw26*, and *Fbxw28,* in this gene cluster (Figure 3). Unequal crossover at meiosis is one of the possible reasons for tandem gene duplication, which occurs more readily in the presence of tandem-repeating genes [49]. Therefore, we inferred that the *Fbxw12* gene cluster may have been formed by tandem duplication through a series of unequal crossover events. Genes included in a gene cluster often differentiate from each other with respect to expression patterns, such as the mammalian *Hox* gene cluster [50]. Therefore, future experimental investigations should provide insight into the significance of expansion of the *Fbxw12* gene cluster in mouse.

In general, F-box gene expansion or contraction events did not appear to occur massively during the course of *Euarchontoglires* evolution. Hence, F-box gene numbers are conserved among the eight organisms investigated in the current study. Orthologous F-box proteins generally contain conserved domain architectures in these organisms. As such, they are likely to mediate essential biological pathways by interacting with similar substrates. In eukaryotes, although orthologous proteins typically have the same domain architectures and functions, significant exceptions and complications to this generalization may be observed [51]. This assumption can also be applied to F-box genes, since evidence of both lineage-specific domain accretion and reduction was found in this study. Differences in homologous protein domain architectures among species may play a role in the functional diversification of orthologs.

Generally speaking, gene duplication can eventually lead to an imbalance in gene quality, and most of the extra gene copies degenerate via accumulation of mutations and become pseudogenes [49]. For instance, several CYP2D genes and olfactory receptor gene cluster have been reported to be pseudogenes in humans [52], [53]. In *Euarchontoglires*, nine F-box related pseudogenes and their corresponding orthologous DNA regions were found. Certain homologous F-box genes were absent in specific lineages, which may be due to bias caused by incomplete genome sequence, variations in genome assembly quality, or loss of the homologous gene from the genome. Although some homologous genes were still present, their F-box domains could hardly be detected because of long-term divergence. However, it should be noted that loss of the F-box domain in an F-box protein homologue does not necessarily imply loss of function of the ubiquitin ligase. For example, NIPA binds SKP1 and CUL1 despite not having a clear F-box domain, and appears to function as an F-box-like protein [54]. Taken together, our results indicate that although F-box gene gain and loss events do not occur as frequently in *Euarchontoglires* as they do in plants, the evolutionary pattern of the F-box gene family in these species is consistent with the birth-and-death evolution model [30], [55].

### C-terminal domains of F-box proteins have undergone adaptive evolution

C-terminal regions of F-box proteins may have evolved under different selective pressures, such as strong purifying, neutral or positive selection. The protein structure may only be conserved in some parts of an active site responsible for catalysis, while the remaining peripheral regions may have changed considerably, causing change in substrate specificity [56]. Indeed, the analysis of selective pressure at individual sites showed that the vast majority of functional domains are rich in residues with low ω, whereas the residues in the core portion of the protein–protein interface underwent excess amino-acid fixation during the course of *Euarchontoglires* evolution. These results are consistent with previous findings in other organisms. In plants, while the F-box domain appears rather stable, some C-terminal protein-protein interaction domains such as Kelch and FBA show strong signatures of positive selection [12], [13]. Our results are also in agreement with previous findings in nematodes [14]. Co-evolution of the substrate and F-box protein interface may explain the apparent fast evolution of the substrate-binding domain. Alternatively, mutation of residues that are solvent-exposed to a structural fold may be more tolerated than those located at the highly structured core. Therefore, further extensive research is required before the cause of this positive selection can be definitively determined.

Seventy-one F-box gene orthogroups were found to have been under significantly different selective pressure over the course of *Euarchontoglire* evolutionary history. We propose that the orthogroups under strongly purifying selection pressure across the genes still recognize the same or similar targets in the eight organisms, whereas others under positive selection pressure conferring adaptive evolution have evolved to recognize different targets. These findings are in contradiction with previous reports that samples of F-box genes from several specific mammalian families show no evidence of positive selection. This discrepancy is likely caused by sample bias [14]. We suggest that mammalian members of the F-box gene family may be involved in both endogenous and exogenous protein degradation.

### Comparison of F-box genes between animals and plants

F-box genes are small in number and quite conserved in *Euarchontoglires.* Much like in *Euarchontoglires*, no evidence of drastic changes in the total number of F-box genes (42–47) was found in the 12 extant *Drosophila* species considered in a previous study [57]. However, F-box gene family is one of the largest and fastest evolving gene families in plants [12], [13]. For instance, the number of F-box Kelch genes (FBKs) varies dramatically among *Arabidopsis thaliana*, *Oryza sativa*, *Populus trichocarpa* and *Vitis vinifera* [12]. The large number of F-box proteins in plants might be required by their species-specific physiology, such as responses to various hormones [58], [59], the circadian clock and photomorphogenesis [60], [61], flower development [62], and defense responses [63].

For both plants and animals, F-box proteins often carry one of a variety of protein-protein interaction domains in the C-terminal regions in addition to the loosely conserved N-terminal F-box domain [7], [64]. The differences in domain distributions among *Euarchontoglires*, nematodes and plants were striking. Among 27 identified C-terminal domains in *Euarchontoglires*, LRRs, one of the most abundant C-terminal domains in plants [30], was the most common. However, LRRs was rare in nematodes (unpublished data from our laboratory). In C. *elegans*, most F-box proteins contain either the FTH or FBA2 domain [14], both of which are absent in plants and *Euarchontoglires*. In addition to FTH and FBA2, many other distinct domains were also found to be lineage-specific. By contrast, only a single member of the F-box protein family contained the Kelch domain in each species of *Euarchontoglires* as well as in C. *elegans*. However, it has been reported that Kelch-containing F-box proteins expanded dramatically among terrestrial plants [12]. Thus, the very distinctive domain distribution of F-box proteins may reflect their divergent functional roles in plants and animals.

## Conclusions

This study explored the evolutionary forces driving conservation and divergence of the F-box gene family in *Euarchontoglires*. Lineage-specific gene tandem duplication, mRNA-mediated retrotransposition, and gene loss contributed to F-box gene number variation in the eight organisms examined in this study. The evolutionary pattern of the F-box gene family in *Euarchontoglires* was in line with the birth-and-death evolution model, although some genes were found to be subject to concerted evolution. Certain F-box genes undergo adaptive evolution in specific lineages, although the majority of the orthogroups are under strong selective constraint. In addition, population genetic analyses indicated that the evolution of domain regions within F-box genes was shaped by stronger purifying selection compared to that of non-domain regions. Future studies employing proteomics and functional genomics approaches will be essential for the identification of human F-box protein targets and for determination of the cellular biological processes involved. The results of this work significantly improve our understanding of SCF biology. Given the roles of F-box proteins in many diseases, development of new therapies targeting F-box proteins may be expected in the future.

## Supporting Information

Figure S1 **A phylogenetic tree was created using F-box protein sequences of eight species (marmoset, gorilla, human, macaque, mouse, chimpanzee, orangutan, and rat) by the maximum likelihood (ML) method.** Values above branches denote percent support for clades based on 100 bootstrap replicates. The interior colored strip corresponds to the distribution of species in each orthogroup. The outer colored strip represents the C-terminal domain contained in the protein from the corresponding interior species.
(PDF)

Figure S2 **Gene conversion events between** ***Fbxo6*** **and** ***Fbxo44*** **.** HSA, PTR, GGO, PPY, MMU, MUS, and RNO represent the species human, chimpanzee, gorilla, orangutan, macaque, mouse, and rat, respectively. Red box indicates gene conversion tracts.
(PDF)

Figure S3 **Prediction of potential functional gene elements in rat** ***Fbxl18II*** **.**
(PDF)

Figure S4 **Average sequence divergence in protein-coding regions of orthologs from 71 orthogroups.** *Ka*, *Ks*, and ω represent average non-synonymous substitution rate per site, synonymous substitution rate per site, and their ratios between orthologs, respectively.
(PDF)

Figure S5 **Sliding window analysis of sequence divergence across protein-coding regions of 65 orthogroups using a window length of 30 bp and a step size of 6 bp.**
(PDF)

File S1 **Multiple sequence alignment of the F-box protein sequences.**
(PDF)

File S2 **An all-against-all BLAST (-e 1e-50) between every pair of F-box protein sequences from each of the eight organisms.**
(PDF)

File S3 **Multiple sequence alignments of coding region sequences for each orthologous group.**
(PDF)

File S4 **SNPs in F-box genes from the 1000 Genomes Project.**
(XLSX)

Table S1 **F-box gene numbers and their accession numbers in the eight genomes.**
(DOC)

Table S2 **F-box gene-related pseudogenes in the eight genomes.**
(DOC)

Table S3 **Chromosomal distributions of F-box genes in the eight genomes.**
(DOC)

Table S4 **Events causing F-box gene number variation during the evolution of** ***Euarchontoglires.***
(DOC)

Table S5 **Tests of variable ω among sites for 71 orthogroups using models M0-M3 comparison.**
(DOC)

Table S6 **Lineage-specific positive selection was identified using branch-site selection models.**
(DOC)

Table S7 **Statistical tests for differences in SNP distributions across different regions of F-box genes.**
(DOCX)
