# Supplementary figures and images for: Evolution of the F-Box Gene Family in Euarchontoglires: Gene Number Variation and Selection Patterns
Source: PLoS One. 2014 Apr 11;9(4):e94899. doi: 10.1371/journal.pone.0094899 (PMC3984280; doi:10.1371/journal.pone.0094899)

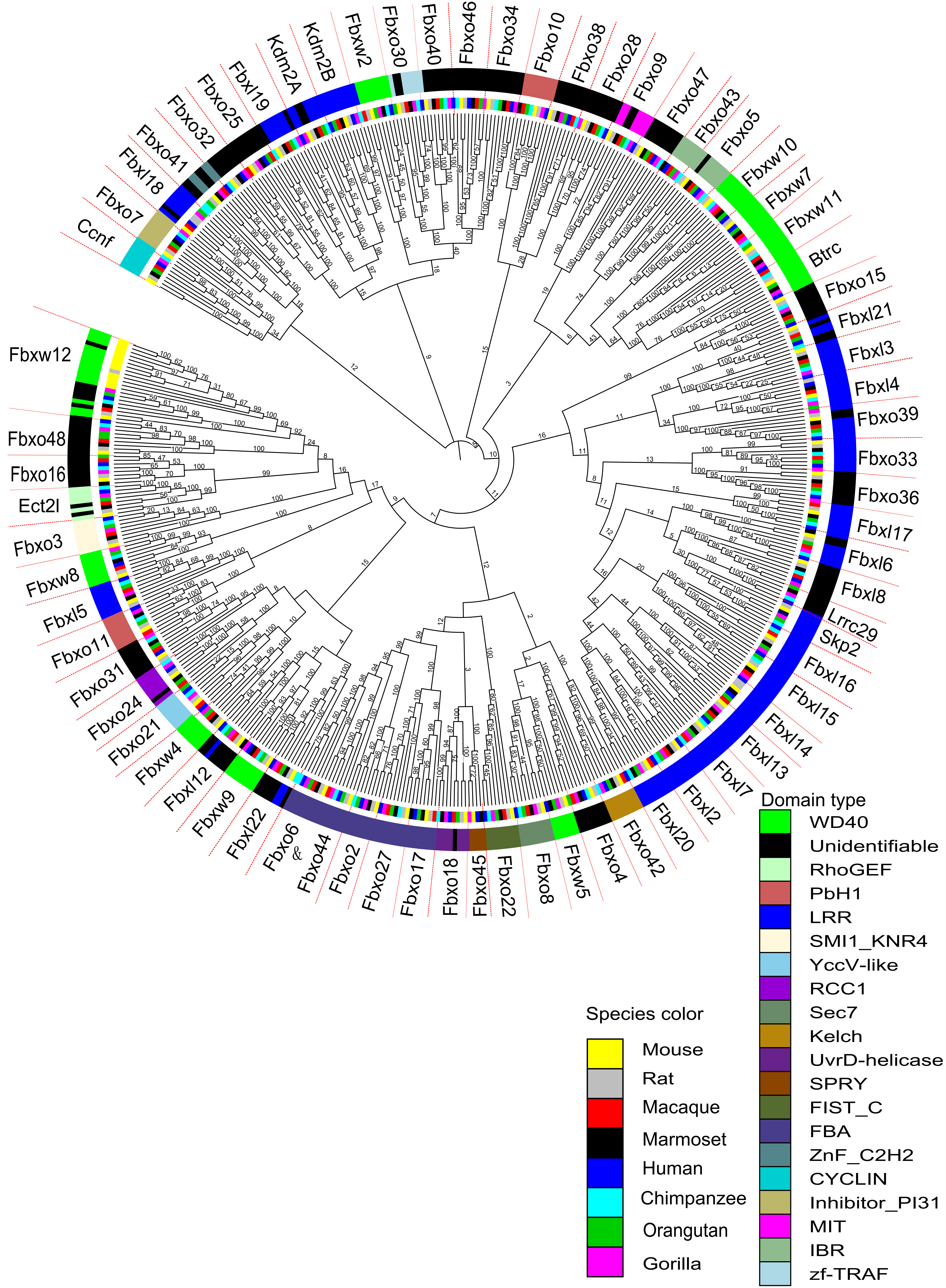

Supplement: Figure S1 — A phylogenetic tree was created using F-box protein sequences of eight species (marmoset, gorilla, human, macaque, mouse, chimpanzee, orangutan, and rat) by the maximum likelihood (ML) method. Values above branches denote percent support for clades based on 100 bootstrap replicates. The interior colored strip corresponds to the distribution of species in each orthogroup. The outer colored strip represents the C-terminal domain contained in the protein from the corresponding interior species. (PDF) [file pone.0094899.s001.pdf]

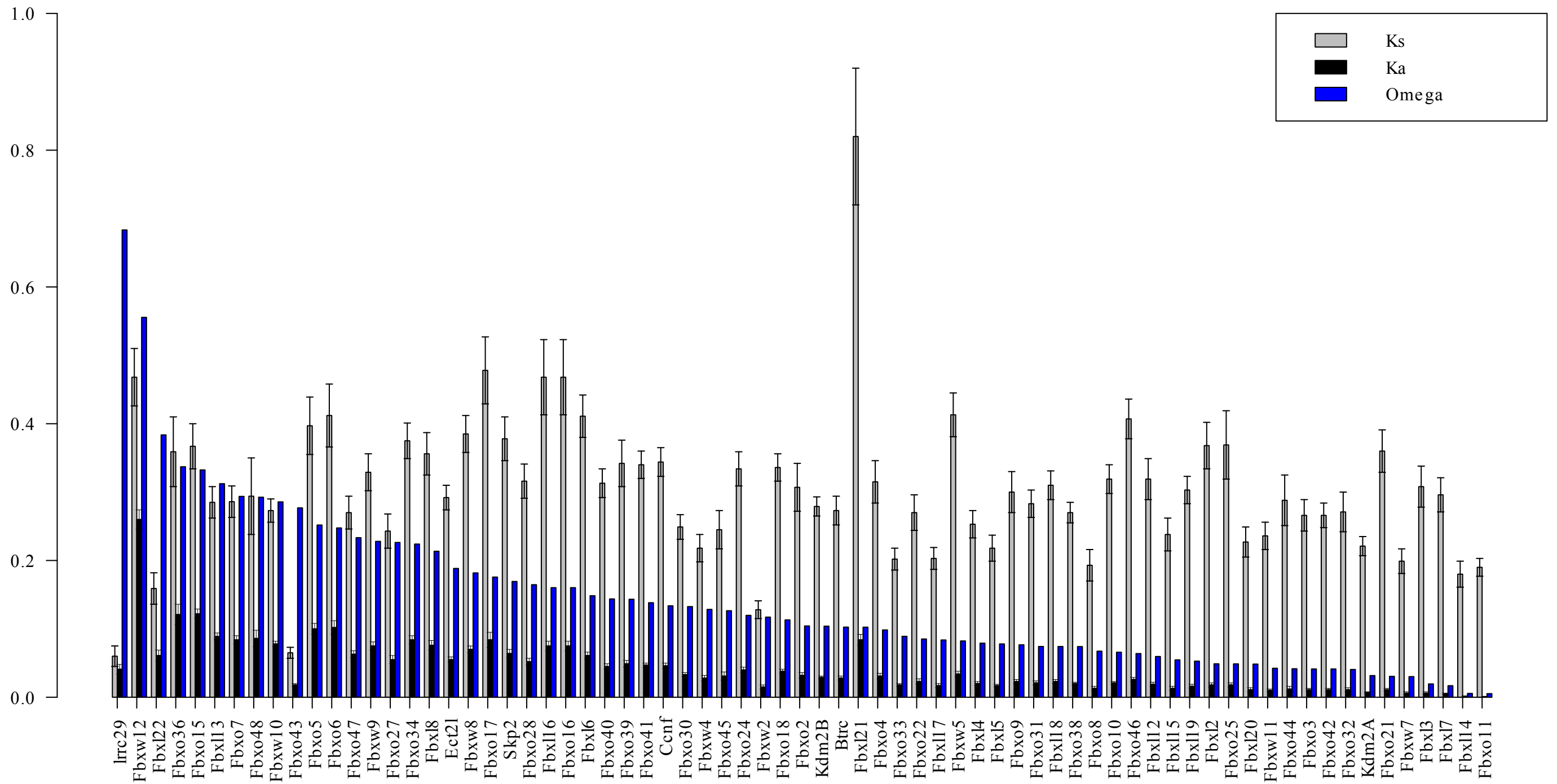

Supplement: Figure S4 — Average sequence divergence in protein-coding regions of orthologs from 71 orthogroups. Ka, Ks, and ω represent average non-synonymous substitution rate per site, synonymous substitution rate per site, and their ratios between orthologs, respectively. (PDF) [file pone.0094899.s004.pdf]
