# Supplementary material for: Evolution of the F-Box Gene Family in Euarchontoglires: Gene Number Variation and Selection Patterns
Source: PLoS One. 2014 Apr 11;9(4):e94899. doi: 10.1371/journal.pone.0094899 (PMC3984280; doi:10.1371/journal.pone.0094899)
Supplement: Figure S2 — Gene conversion events between Fbxo6 and Fbxo44 . HSA, PTR, GGO, PPY, MMU, MUS, and RNO represent the species human, chimpanzee, gorilla, orangutan, macaque, mouse, and rat, respectively. Red box indicates gene conversion tracts. (PDF) [file pone.0094899.s002.pdf]

A. The phylogenetic tree of orthologous groups Fbxo6 and Fbxo44

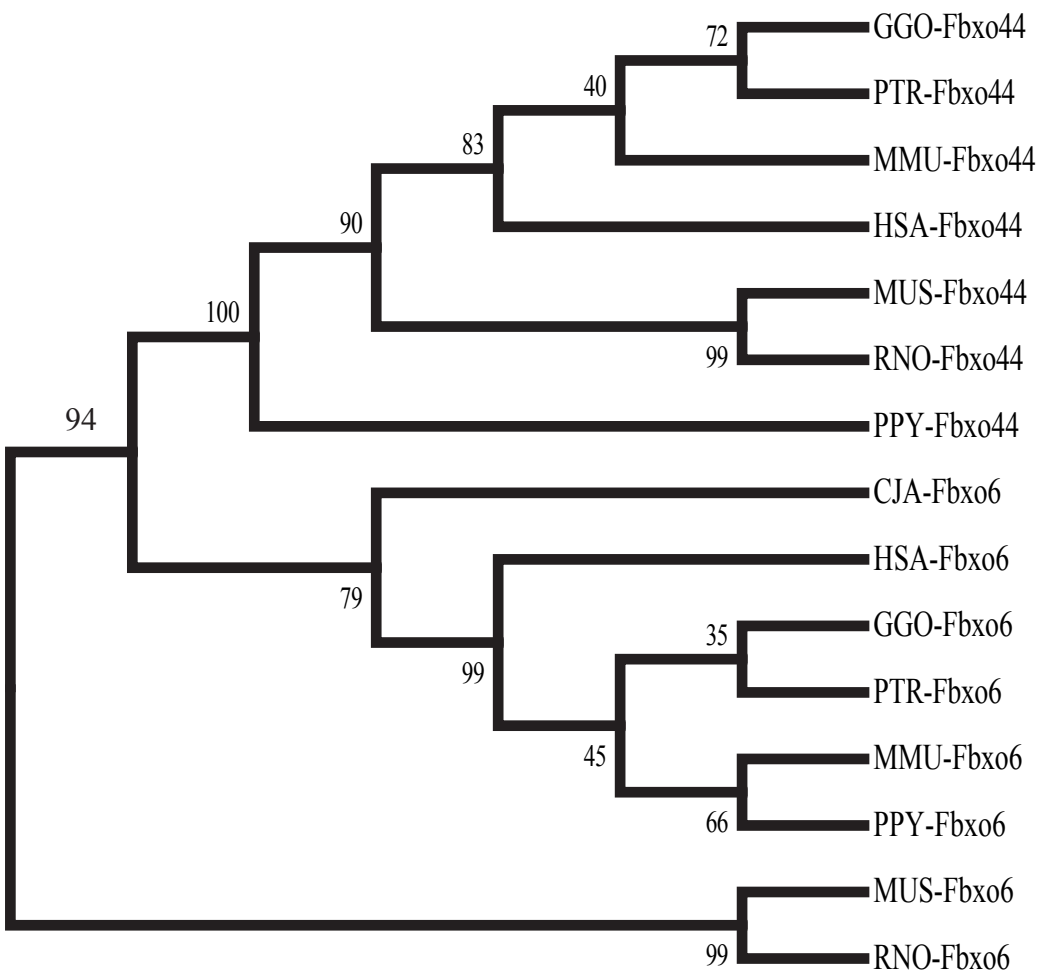

B. Multiple sequence alignment of orthologous groups Fbxo6 and Fbxo44

```
                    10        20        30        40
HSA-Fbxo6   MDAPHSKAALDSI NELPENI LLELFTHVPARQLLLNCRLV
PTR-Fbxo6   ........ ........ ........ ........ ........
GGO-Fbxo6   ........ ........ ........ ........ ........
MMU-Fbxo6   .....p.. ........ ........ ........ ........
PPY-Fbxo6   .....p.. ........ ........ ........ ........
GGO-Fbxo44  -------- m.vgn... ........ ........ ........
PTR-Fbxo44  -------- m.vgn... ........ ........ ........
MMU-Fbxo44  -------- m.vgn... ........ ........ ........
HSA-Fbxo44  -------- m.vgn... ........ ........ ........
PPY-Fbxo44  -------- m.vgn... ........ ........ ........
MUS-Fbxo44  -------- m.vgn... ......i. i..... ...r..p.
RNO-Fbxo44  -------- m.vgn... ......i. ..... ...r..p.
MUS-Fbxo6   --------- mvh.... ......i. i..p.. ...r....
RNO-Fbxo6   --------- mvn.... ........ ..p... ...r....

                    50        60        70        80
HSA-Fbxo6   CSLWRDLI DLMTLWKRKCLREGFI TKDWDQPVADWKI FYF
PTR-Fbxo6   ........ ........ ........ e.....n. .....
GGO-Fbxo6   ........ ........ ........ e.r..... .....
MMU-Fbxo6   ........ ........ ........ e..... .....
PPY-Fbxo6   ........ ........ ........ e..... .....
GGO-Fbxo44  .....v.. ........ ........ e..... .....
PTR-Fbxo44  .....v.. ........ ........ e..... .....
MMU-Fbxo44  .....v.. ........ ........ e..... .....
HSA-Fbxo44  .....v.. ........ ........ e..... .....
PPY-Fbxo44  .....v.. ........ ........ e..... .....
MUS-Fbxo44  .....v.. .....q.. ........ e..... .....
RNO-Fbxo44  .....v.. .....q.. ........ e..... .....
MUS-Fbxo6   .r....vv s....s.. ....f... rce..e.. .v..i
RNO-Fbxo6   .....v.. ....s... ....v... r.e..d.. .....i

                    90        100       110       120
HSA-Fbxo6   LRSLHRNLLRNPCAEEDMFAWQI DFNGGDRWKVESLPGAH
PTR-Fbxo6   ........ ........ ........ ........ ........
GGO-Fbxo6   .q...... ........ ........ ........ ........
MMU-Fbxo6   ........ ........ ........ ........ ........
PPY-Fbxo6   ........ ........ ........ ........ ........
GGO-Fbxo44  .....h.. ...gfef. sl.v...e ...d.srdq
PTR-Fbxo44  .....h.. ...gfef. sl.v...e .g.d.srdq
MMU-Fbxo44  .....h.. ...gfef. sl.v...e ...d.srdq
HSA-Fbxo44  .....h.. ...gfef. sl.v...e ...d.srdq
PPY-Fbxo44  .....h.. ...gfqf. sl.m...e ...d.s.dq
MUS-Fbxo44  ....q... h.....gf ef.sl.v. ...e...d .skdq
RNO-Fbxo44  ....q... h.....gf ef.sl.v. ...e...d .skdq
MUS-Fbxo6   .c..q... ....nl ss.r..s. ....t...sc
RNO-Fbxo6   .c..q... ....nl rs.r..s. ...e....d.
```

C. Gene conversion events

Sequence name	Sim P-value	Aligned begin	Offsets End
HSA-Fbxo6; HSA-Fbxo44	0.0004	13	89
PTR-Fbxo6; PTR-Fbxo44	0.0000	13	89
GGO-Fbxo6; GGO-Fbxo44	0.0004	13	81
PPY-Fbxo6; PPY-Fbxo44	0.0000	13	89
MMU-Fbxo6; MMU-Fbxo44	0.0000	13	89
