# Supplementary material for: Evolution of the F-Box Gene Family in Euarchontoglires: Gene Number Variation and Selection Patterns
Source: PLoS One. 2014 Apr 11;9(4):e94899. doi: 10.1371/journal.pone.0094899 (PMC3984280; doi:10.1371/journal.pone.0094899)
Supplement: Figure S3 — Prediction of potential functional gene elements in rat Fbxl18II . (PDF) [file pone.0094899.s003.pdf]

Prediction of potential functional gene elements in *Fbxl18II*

Positions of predicted functional gene elements:

Exon 1: 1-1748; Exon 2: 1840 bp – 2215 bp

Transcription start site (TSS): -200 bp

Long interspersed nuclear element (LINE): -1520 bp – 586 bp

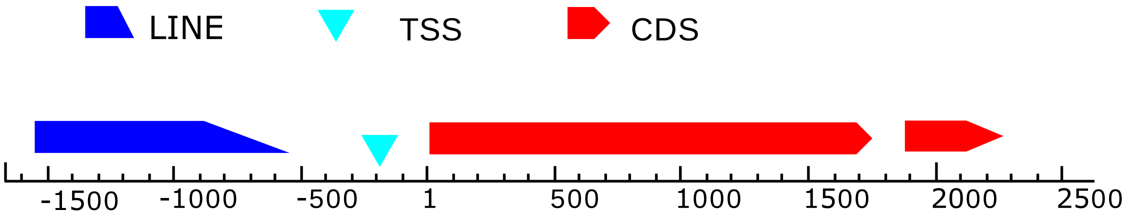

Sequence

>Fbxl18

CCTCAAAGGCAAGTAGGGTTGGGCAGCGGGTAGTTCAGGGCACAAGCAGCATTCATGAGT

GTGTGAATTTATGTCTGGGTCTTCAAATCAATTCCATTGATCAACATGTCTGCTCCTGTA

CCAAAACCATGGCGTTTTTACTACTATAGCTCTGTAGTACAAGTTGAAATCAGGGATGGC

TGCTTTCATATTGAACAGAATTATTATAGTTATACTGGGTTTTTATTTTTCCATATGAAG

TTGAGAATTGTCCTTTTAAGGTCTGTAAAAAAACTGTGTTGGAATTTCAATGAGGATTTC

ATTGACTCTTTAGATTGTTTTTGGTAGGGCGGCCATTTTTTACTATGTTAGTCCAATGGA

TCCATGTACATGGGAGATCTTTCATCTTCTGATATCTTCTTCAATTTCTTCCTTCCAAGA

TTTGAAGATTTTTTATCATCCACGTATTTTACTTGTTTGGTGATATTTTGATTCTTTGAT

TGTAAAGGATGTTTCCCTGATTTTTTTTCAGTCTCCTTGTCATTTGTACATAGGAGGGTT

TTTGAGTTAATCTTGTATCCAGTCACTTTGCAGATAGTGTTCATCAGCTGTAGGTGTTTC

CTACTGAATTTTTATGGACACTTATGTCTACTATCATATCATCTGCAAATACTTTGACTT

CTTCATCTCTATTTTGTATCTCCTTAATCTCCTTTAGTTGTCTTATTGCTCTAGCTAAAA

CTTCAATAGGGCTGGAGAGATGGCTCAGTGGTTAAGAGCACCAACTGCTCTTCCAGAGGT

CCTGAGTTCAAATCCTAGCAACCACATGGTGGCTCAAAGCCAACTGTAAATGAGATCCGA

TGCCCTCTTCAAGTCTACTCATATGTAATAAATAAATAAGTCTTAAGAGAAAAAAAACTT

CAAGTAGTATATTGAATACATCTTGAAACTTTCTCCAAAATTTACCACATACTCAGACAC

AAGGCAAGTCTCAGTGTATACAAGAAAATTGAAATAACTTTCTGCATCCTATCCTATGGA

TAGATATCAACTACAATAGAAACAACAGAAAGCACAGAAAACTTACACACTCATAAAAAC

CAAATCATCCTCTACTAAATGAAATATTAATTAAAGACGAAATGCAGGAGAGAGCTGGAT

GTCTGTCCAAGAGTCTGATCGTTTATGGTGTTGACCAGCCCAGACCGAATTTAAAATGAG

TGATGTTGAAAAAGGCAAAAAGATTTTTGTTCAGAAGTGTGCCCAGTGCCACACTGTGGA

AAAAGGAAGCAAACATAAGACTGGACCAAATCTCCGTGGTCTGTTTGGGCGGAAGACAGA

CCAGGCTGCTGGATTCTCTTACACAGATGCCAACAAGAACAAAGGCATCACCTGAGGAGA

GGATACCCTGATGGAGTATTTGGAAAATCCCAAAAAGTACATCCCTGGAAAAAAATTATC

TTCGCTGGAATTAAGAAGAAGGGAGAGGCCCCTTGCCGGGGTCTCTGTGGAGCTGTGGCC

GCGCCGGCTTGGAGCATCCCTGCGCTGGCAGGCTGGGGGACTGTGAGGCTGCATCTCCGG

CGGCCAGCTCCGGAGAGGAC**ATGTCCAATGATGATGAGGCAGCGGCAGAGGCAGCGGGTG**

ACACCCACCTACTTGGCTTCTCTGATGAGATCCTCCTACATATCCTGAGCCACGTCCCCA

GCACAGACCTGGTTCTCAATGTGCGGCGTACCTGCCGGAAGCTCGCAGCCCTGTGTCTGG

ACAAGAGCCTGGTGCACACTGTGCTACTGCAGAAGGACTACCAGGCCAGTGAGGAGAAGG

TGAAGCAGCTGGTGAAGGAGATTGGCCGGGAGATCCAGCAGCTGAACATGGCAGGCTGCT

ATTGGCTGCCCGGCTCCACCATCGAGCACGTGGCCCGCTGTCACAGCCTGGTGAAGGTGA

ACTCGTCAGGTTGTCACCTCACCTCGCTGCGGCTGTCCAGGGTGCTCTCCTGCCTGCAGC

GCCTGCGCTCACTGGCCATCGACGTGAGCCCTGGCTTCGACGCCAGCCAGCTGAGCAGTG

AGTGCAAGGCCACGCTGAGCCGCGTGCAGGAGCTCAAGCAGACGCTCTTCACACCGTCCT

ACGGCGTGGTGCCCTGCTGTGCCAGCCTGCAGAAGCTGCTGCTGTACTTCGAGATCCTGG

ACCGCACCCGTGAGGGCGCCGTCCTCTCCGGCCAGCTCATGGTGGGCCAGAGCAACGTGC

CCCACTACCAGAACCTGCGTGTCTTCTACGCACGCCTGGCCCCTGGCTACATCAACCAGG

AGGTGGTAAGGCTCTACCTCGCTGTGCTCAGCGACCGCACGCCTGAGAACCTGCATGCCT

TCCTCATCTCCGTCCCCGGCAGCTTCGCAGAGAGCGGGGCCACCAAAAACCTGCTGGACT

CCATGGCCCGCAACGTGGCCCTAGATGCCCTGCAGCTGCCCAAGTCCTGGCTGAATGGTT

CCACCCTCCTCCAGCACATGAAGTTCAACAATCCCTTCTACTTCAGCTTCAGCCGGTGTA

CGCTGTCAGGCGGTCACCTGATCCAGCGACTCATCAATGGGGGGAAGGACCTGCGTAGCC

TGGCCAGCCTCAACCTCAGCGGCTGCGTGCACTGCCTGTCCGCGGACTCATTGCTGCGGA

AGGCCGAGGACGACATCGACAGCAGCATCCTCGAGACTCTGGTGGAGTCCTGCTGCAACC

TGCATCACCTCAATCTCTCAGCCGCCCATCACCACAGCTCTGACGGCCTGGGCCGCCACC

TCTGCCAGCTCCTGGCTCGGCTCTGCCACCTGCGCTCCCTGTCCCTGCCTGTCTGCTCTG

TGGCCGACTCGGCACCCAGGCCTGACCGTGCGCCTGCCCCGCCAGCCATGCATGCAGTAC

CCCGTGGATTTGGCAAGAAGGTGCGCATTGGGGTGCAGACCTGTCCCAACCCCTTTGTGG

GCCAGTCGGCCCCACAGCCTGCCTCTGTGTTTTGGTCTCTGCTGAAGAAGCTCCCATTTC

TGGAGCACCTGGAACTGATCGGGTCCAATTTCTCCTCAGCCATGCCCCGCAATGAGCCAG

CCATCCGCAACTCCCTCCCGCCCTGCAGTCGGGCACAGAACGTGGGGGACTCAGAGGTGG

CCGCCATTGGCCAGCTGACCTTCCTGCGGCACCTGACACTAGCCCAGCTGCCAGGCATGC

TGACGGGCTCTGGACTAGTGAGCATTGGCCTACAGTGCCAGCAGTTGCAGTCCCTGTCGC

TGGCCAACCTGGGCATGATGGGAAAGGTGGTGTACATGCCTGCTCTTGCCGACATGCTGA

AGCACTGCAAGCGTCTGAAGGACCTGAGGTTGCTCGGGCTTACTTTGAACTCACTGTCAT

CCATGAAGGCCTTGAACTCCTGATCCTCCCACCTCAACCCCCAGAGCCTGGGAGCACAGG

CTGGAGCAGCCCTATTTCAACGCCAACGCACAGTTCTTCCAGGCCCTGGGCCAGTGCTCC

TCTCTGCAGCGCCTGTGCCTGGTGTCGCGCAGTGGCACTCTGCAGCCCGATGCCGTGTTG

GCCTTTATGGCCCGTTGCCTGCAGGTCGTCATGTGTCACATGTTCACCGGAGAATCCCTC

ACCACCTGCAAGAGCCTACAGCAGTCGCTCCTCCGGAGTTTCCAGGCCGAGAGACCCGCG

TTGAACGTCGTCATCTTCCCTCTGCTCCACGAGGGCCTGACAGATGTTATCCGAGATGTC

CCCATGGTGCACCTGGACGAGATCACCTTATTCAAAAGCAGAGTGGCCGAGGAACCCCCG

AACCTGTGGTGGTGA
