# Supplementary material for: Evolution of the F-Box Gene Family in Euarchontoglires: Gene Number Variation and Selection Patterns
Source: PLoS One. 2014 Apr 11;9(4):e94899. doi: 10.1371/journal.pone.0094899 (PMC3984280; doi:10.1371/journal.pone.0094899)
Supplement: Figure S5 — Sliding window analysis of sequence divergence across protein-coding regions of 65 orthogroups using a window length of 30 bp and a step size of 6 bp. (PDF) [file pone.0094899.s005.pdf]

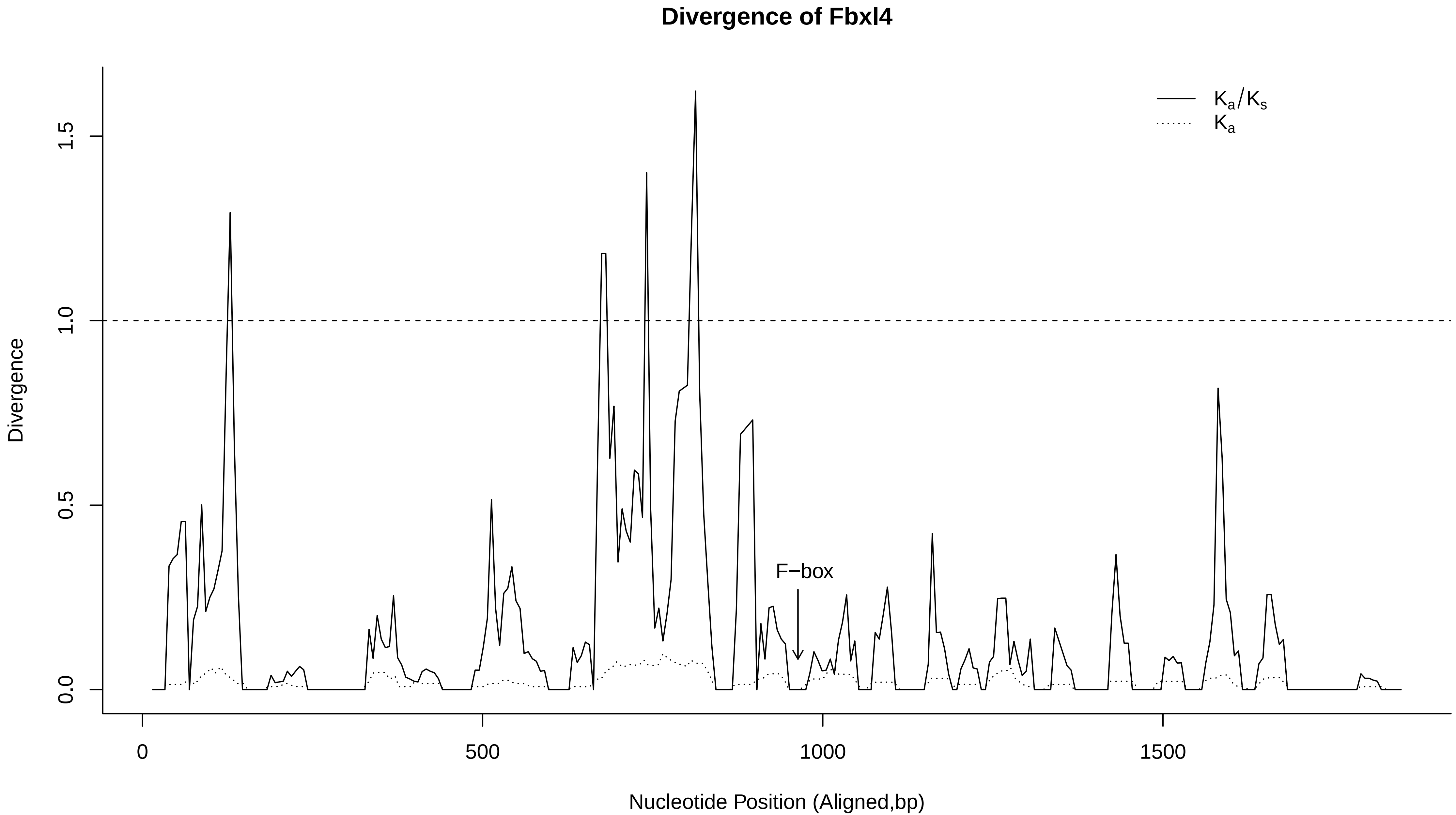

Divergence of Fbxl4
Ka/Ks
Ka
F−box
Divergence
1.5
1.0
0.5
0.0
0
500
1000
1500
Nucleotide Position (Aligned,bp)

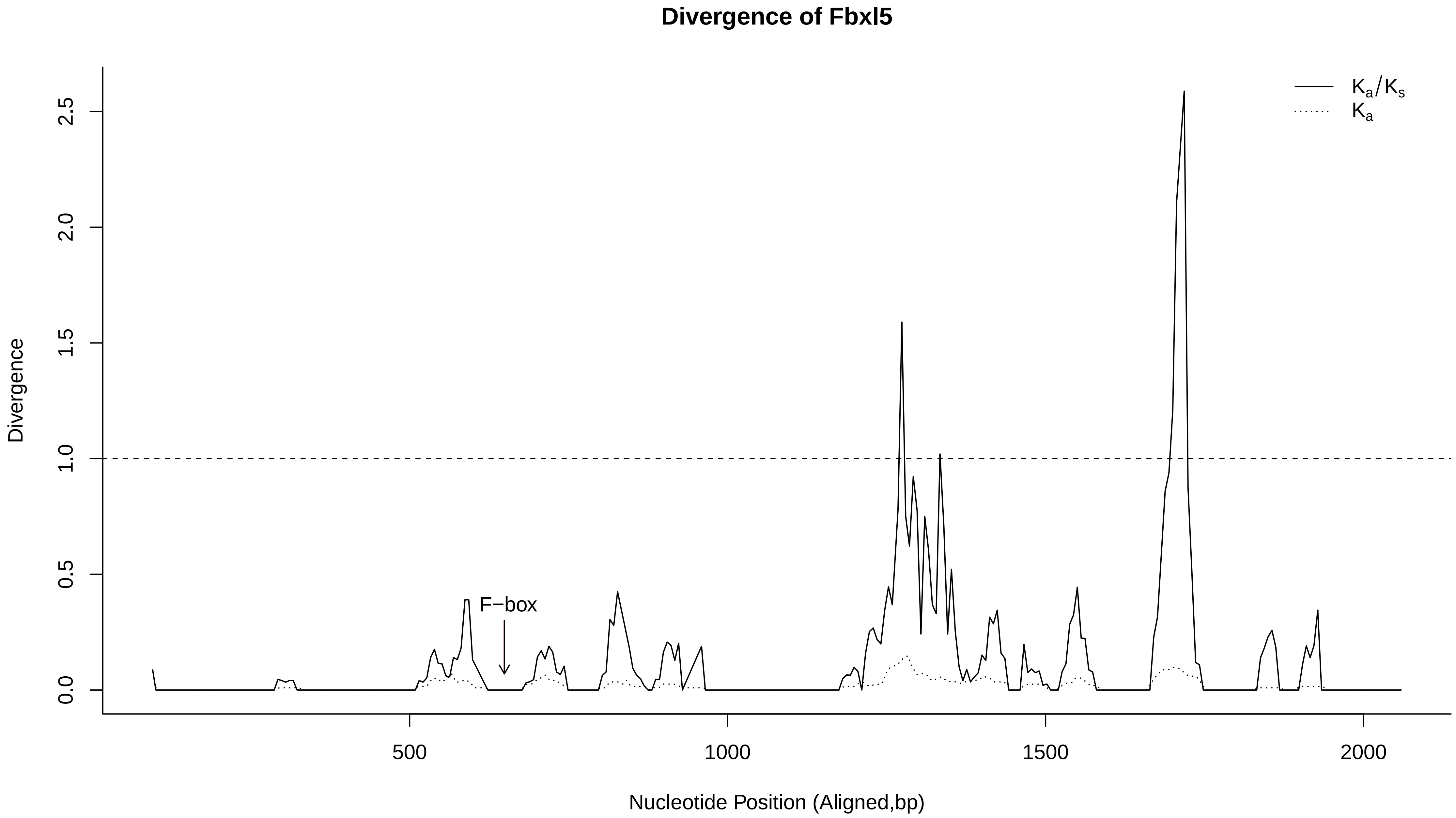

Divergence of Fbxl5
Divergence
Nucleotide Position (Aligned,bp)
0.0
0.5
1.0
1.5
2.0
2.5
500
1000
1500
2000
$K_a/K_s$
$K_a$
F−box

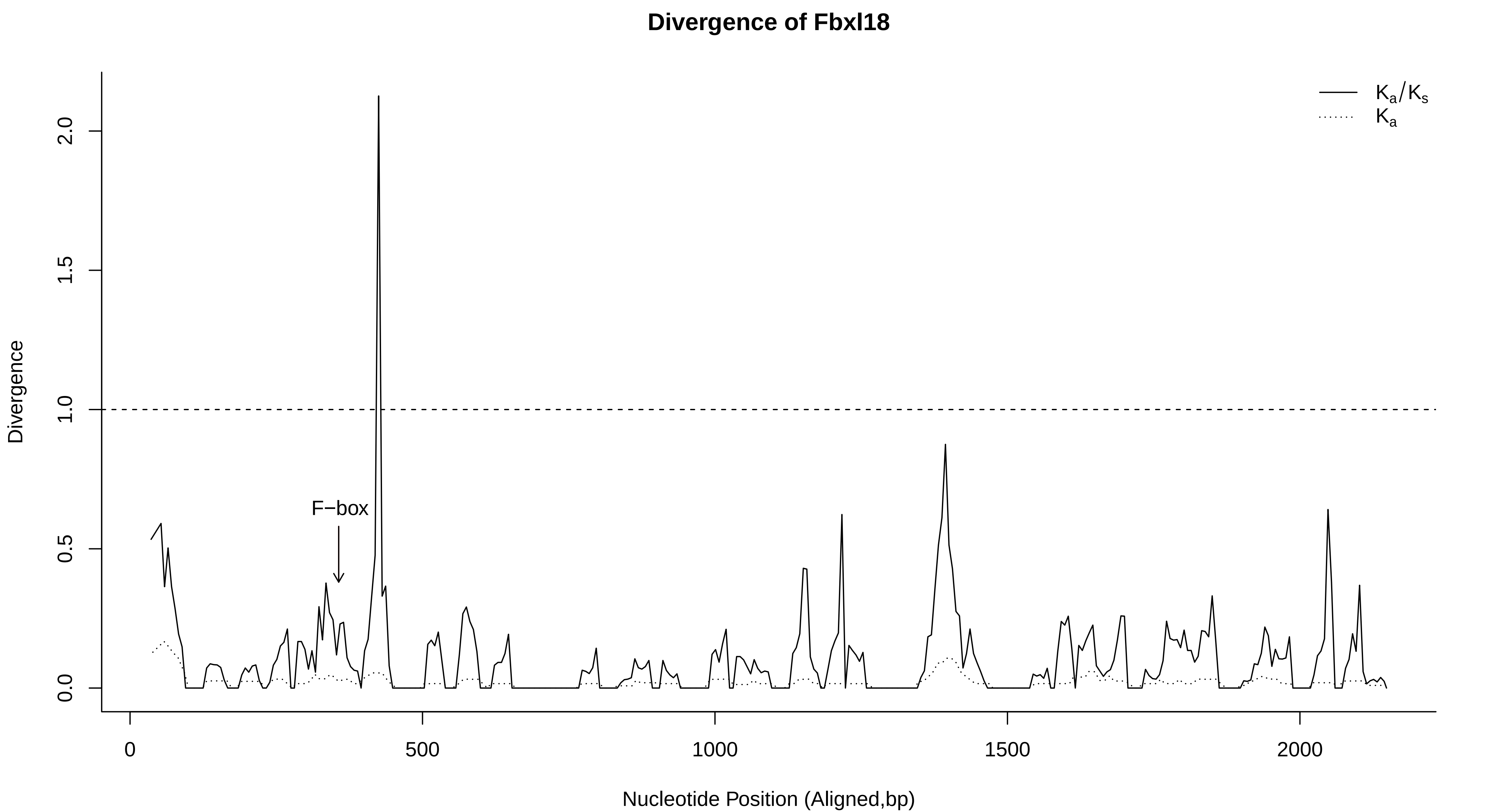

Divergence of Fbxl18
Divergence
Nucleotide Position (Aligned,bp)
$K_a/K_s$
$K_a$
F−box
0.0
0.5
1.0
1.5
2.0
0
500
1000
1500
2000

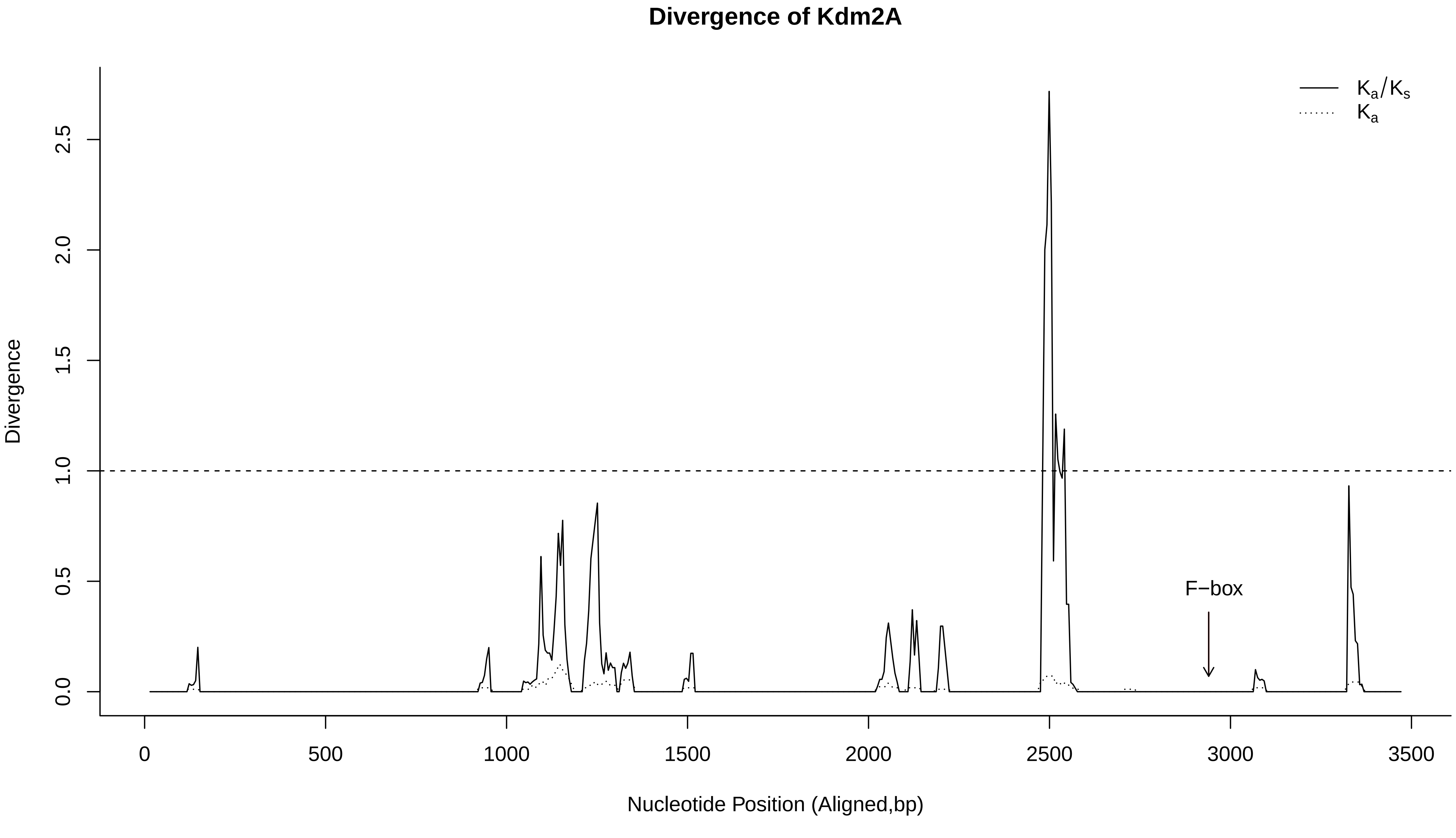
Divergence of Kdm2A
Divergence
0.0
0.5
1.0
1.5
2.0
2.5
0
500
1000
1500
2000
2500
3000
3500
Nucleotide Position (Aligned,bp)
$K_a/K_s$
$K_a$
F−box

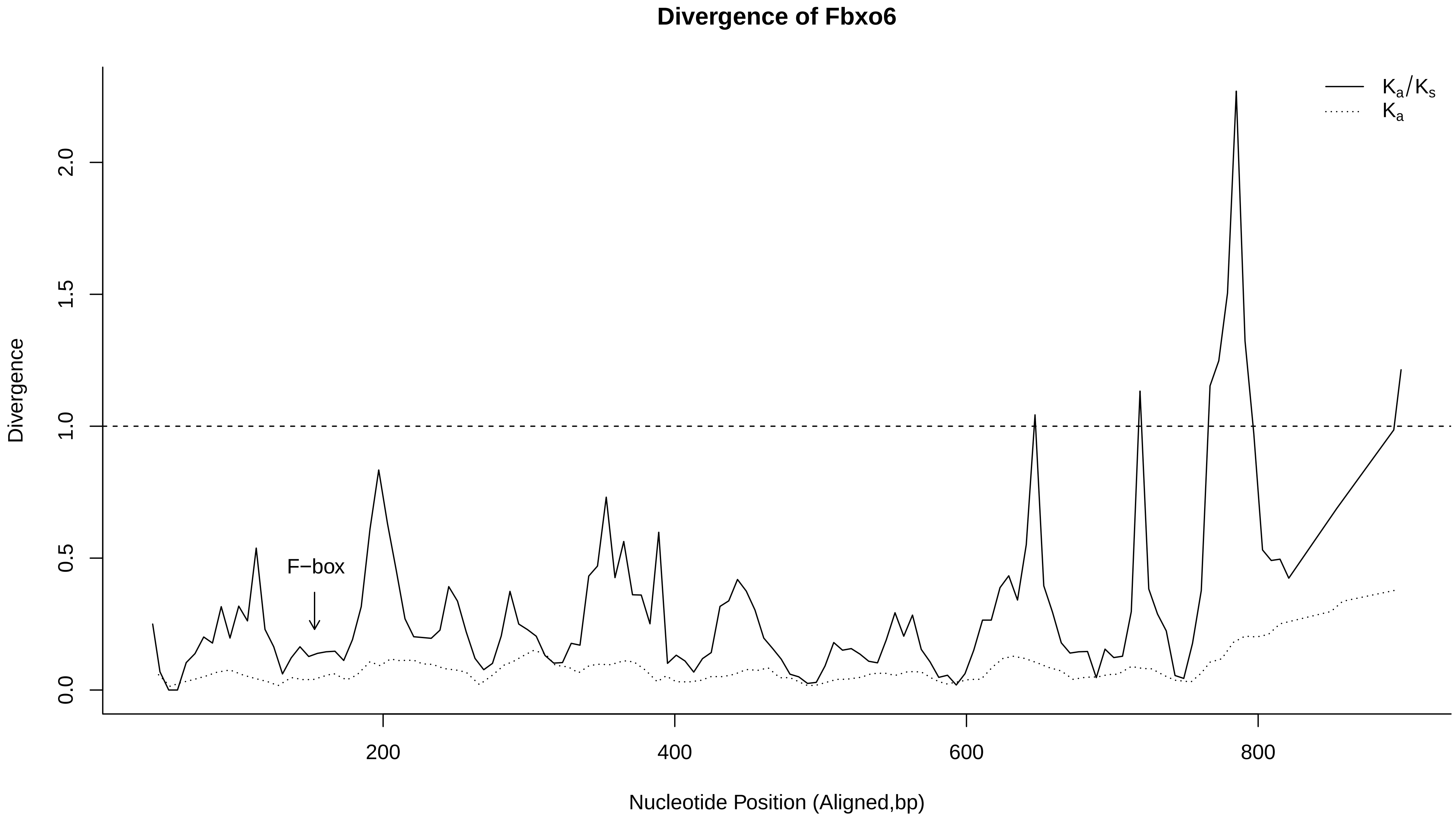

Divergence of Fbxo6
Ka/Ks
Ka
F−box
Divergence
Nucleotide Position (Aligned,bp)
0.0
0.5
1.0
1.5
2.0
200
400
600
800

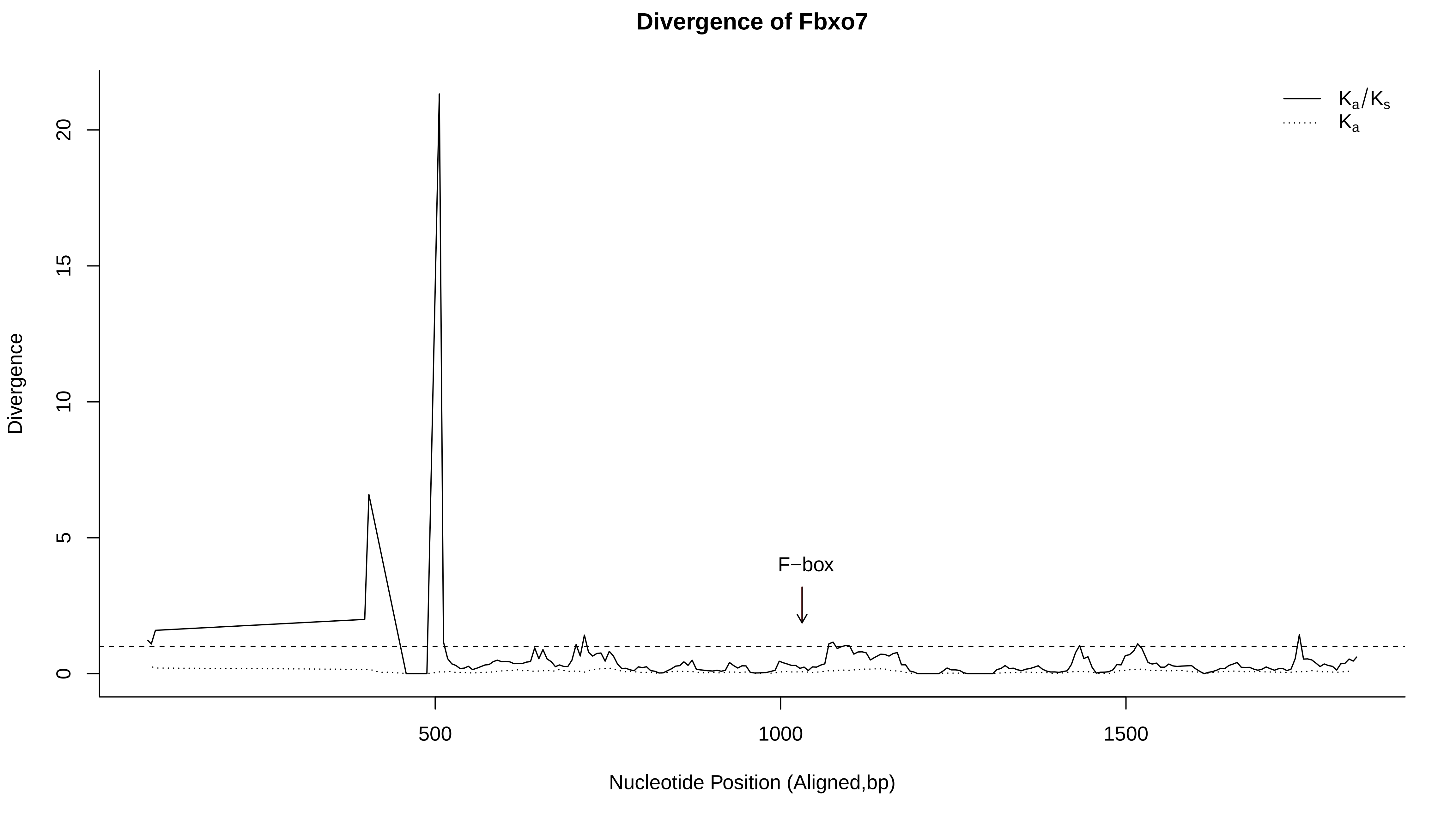
Divergence of Fbxo7
$K_a/K_s$
$K_a$
F−box
Divergence
0
5
10
15
20
500
1000
1500
Nucleotide Position (Aligned,bp)

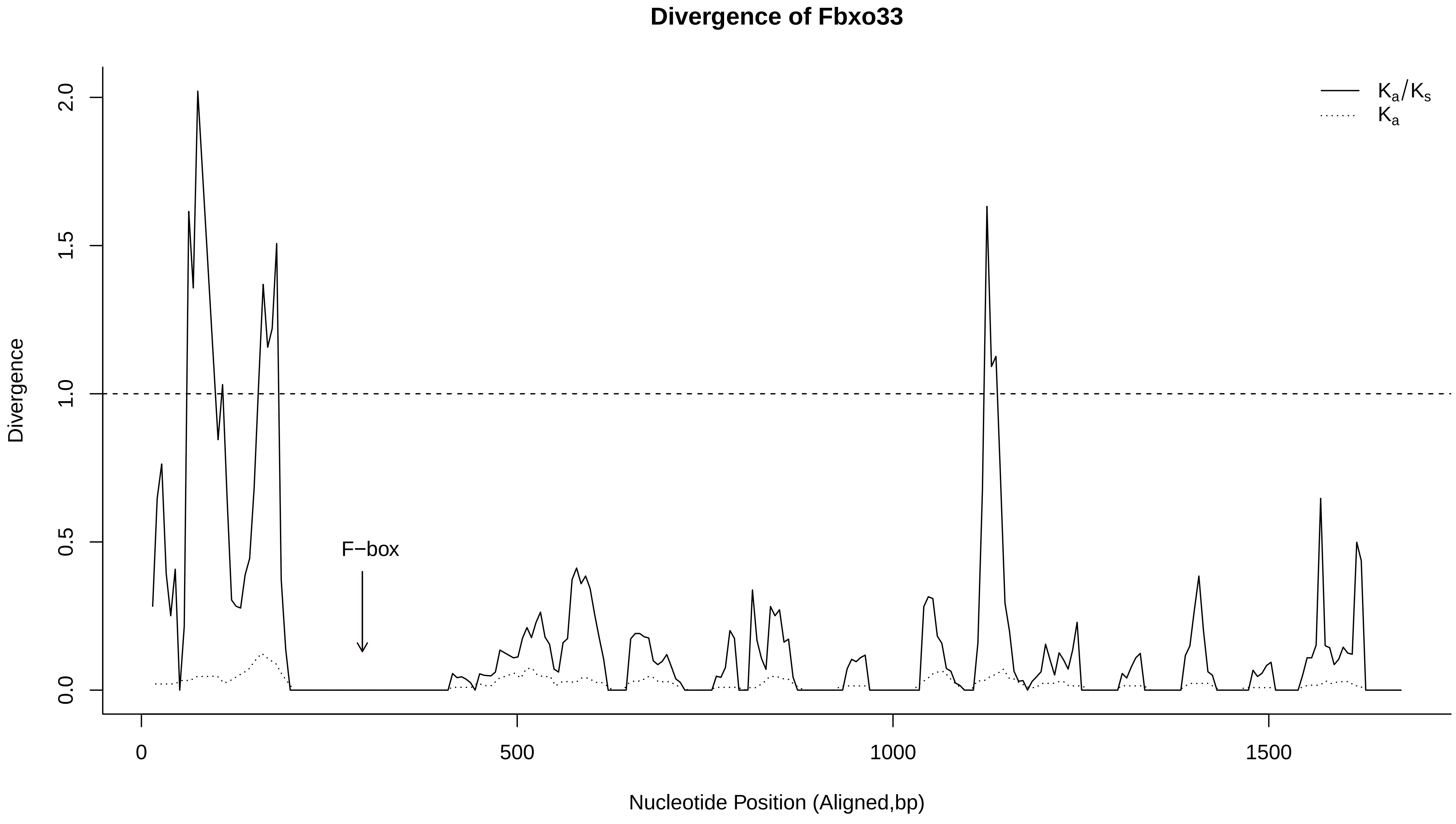
Divergence of Fbxo33
$K_a/K_s$
$K_a$
F−box
Divergence
0.0
0.5
1.0
1.5
2.0
0
500
1000
1500
Nucleotide Position (Aligned,bp)

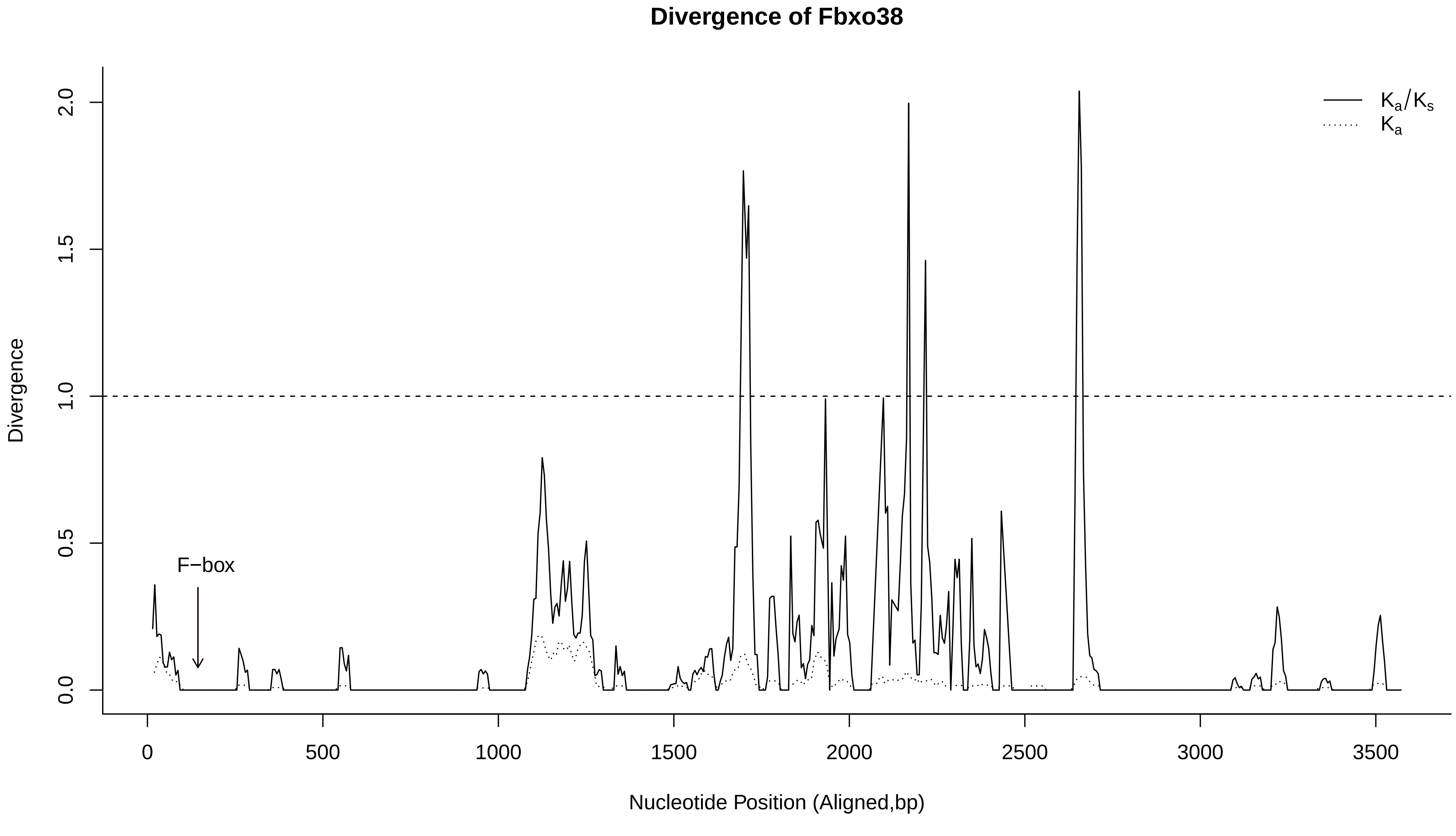

Divergence of Fbxo38
Divergence
Nucleotide Position (Aligned,bp)
0.0
0.5
1.0
1.5
2.0
0
500
1000
1500
2000
2500
3000
3500
F−box
$K_a/K_s$
$K_a$

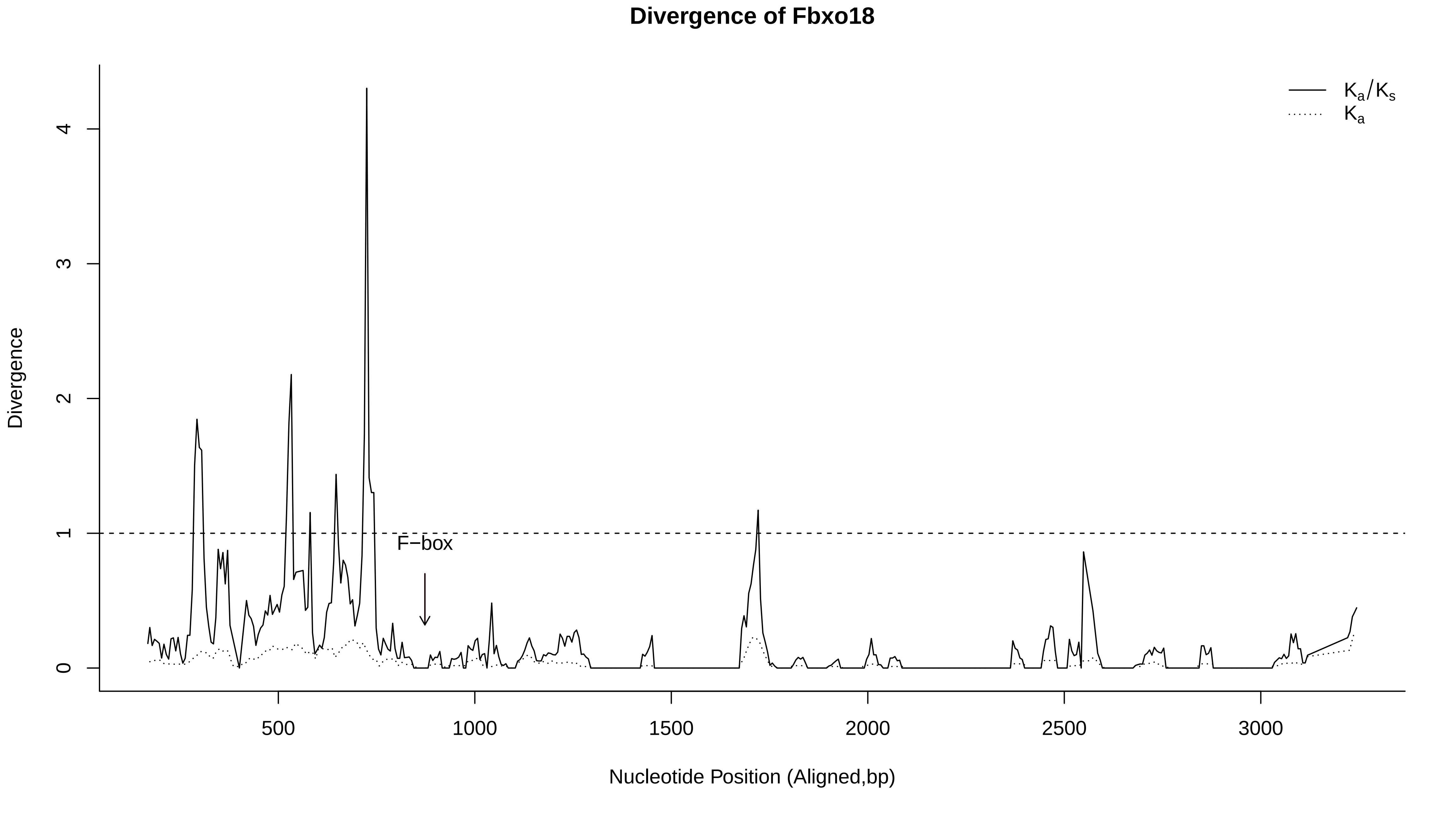

Divergence of Fbxo18
$K_a/K_s$
$K_a$
F−box
Divergence
0
1
2
3
4
500
1000
1500
2000
2500
3000
Nucleotide Position (Aligned,bp)

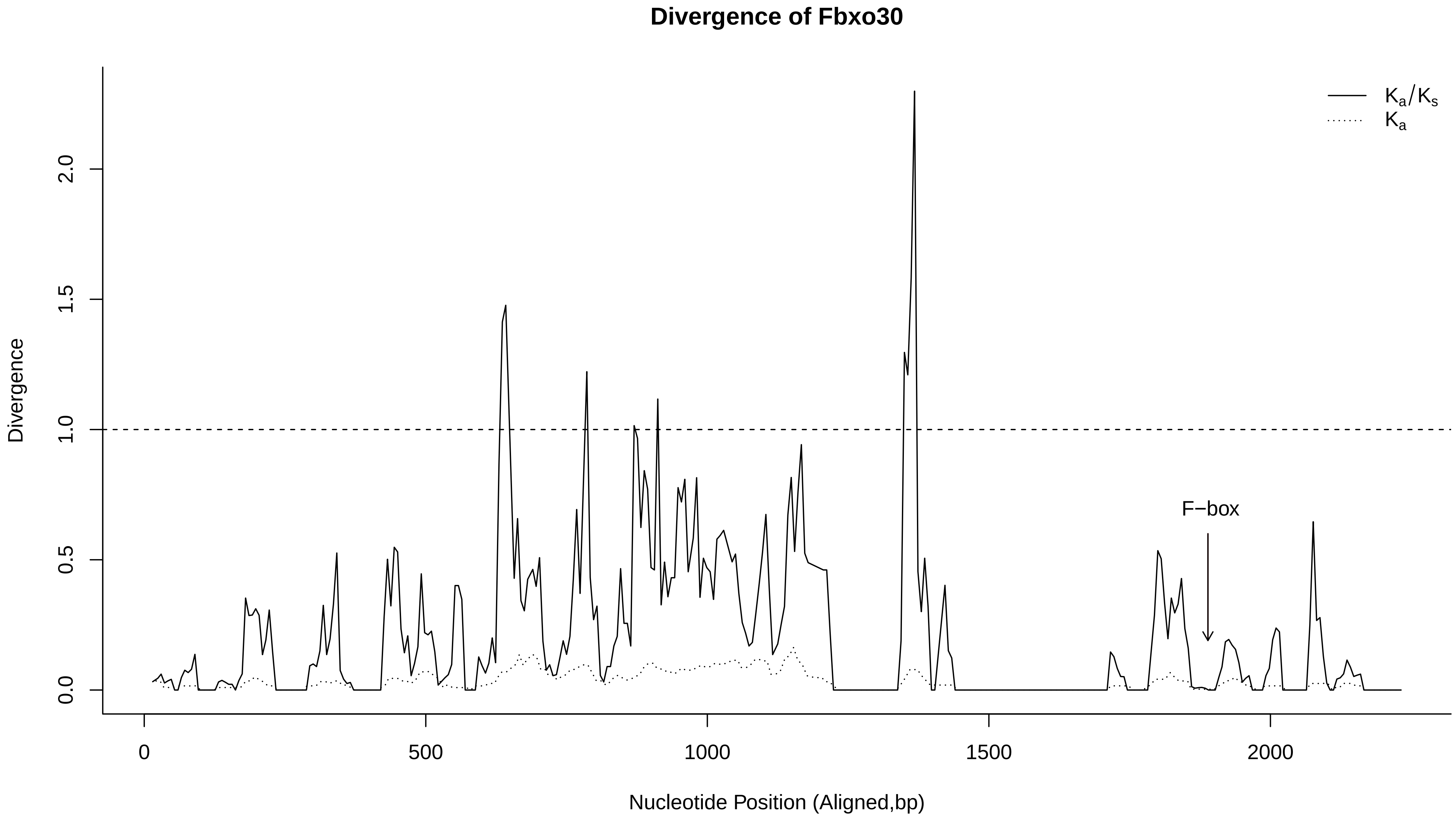
Divergence of Fbxo30
Divergence
Nucleotide Position (Aligned,bp)
$K_a/K_s$
$K_a$
F−box
0.0
0.5
1.0
1.5
2.0
0
500
1000
1500
2000

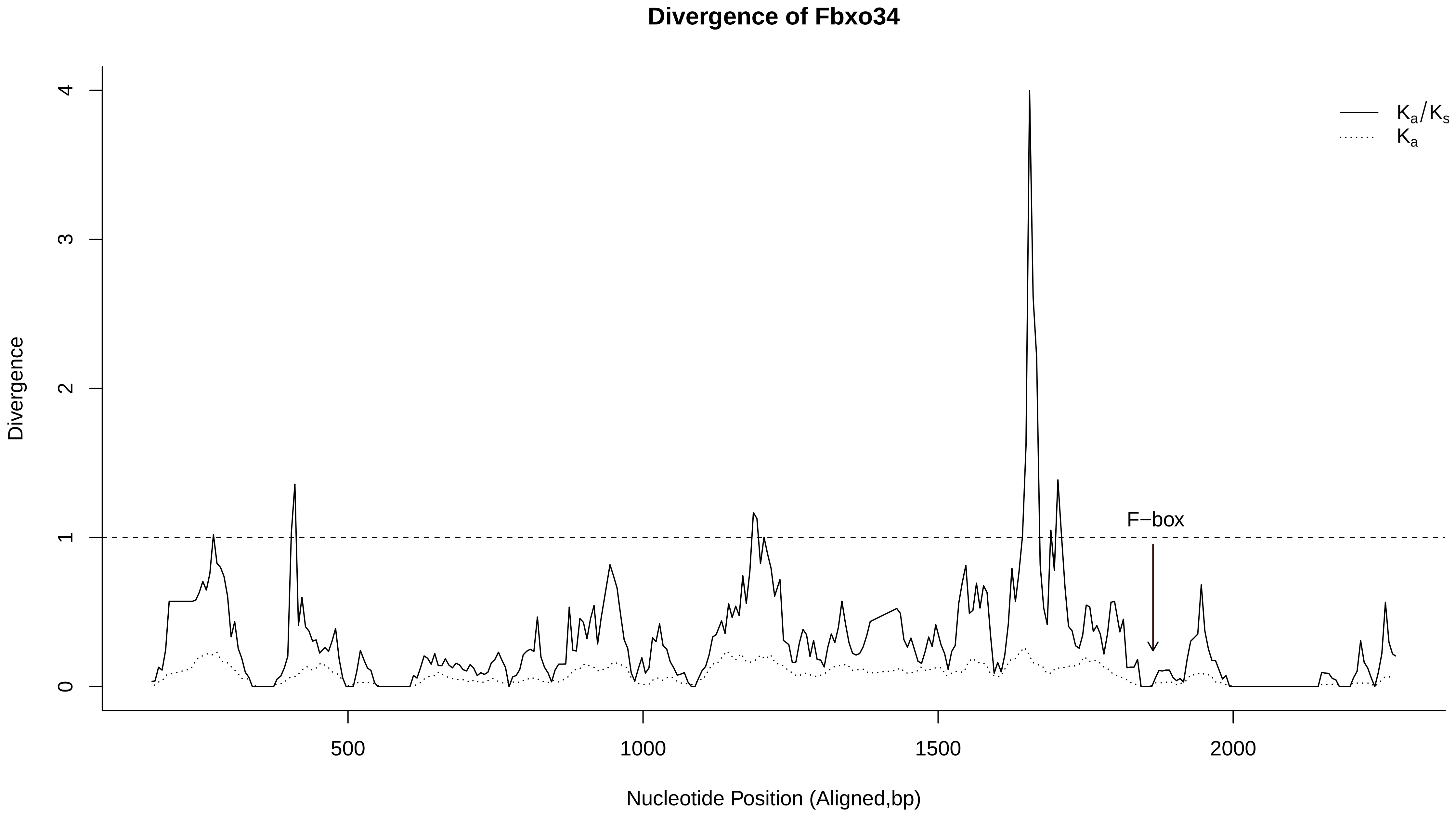
Divergence of Fbxo34
Divergence
Nucleotide Position (Aligned,bp)
0
1
2
3
4
500
1000
1500
2000
$K_a/K_s$
$K_a$
F−box

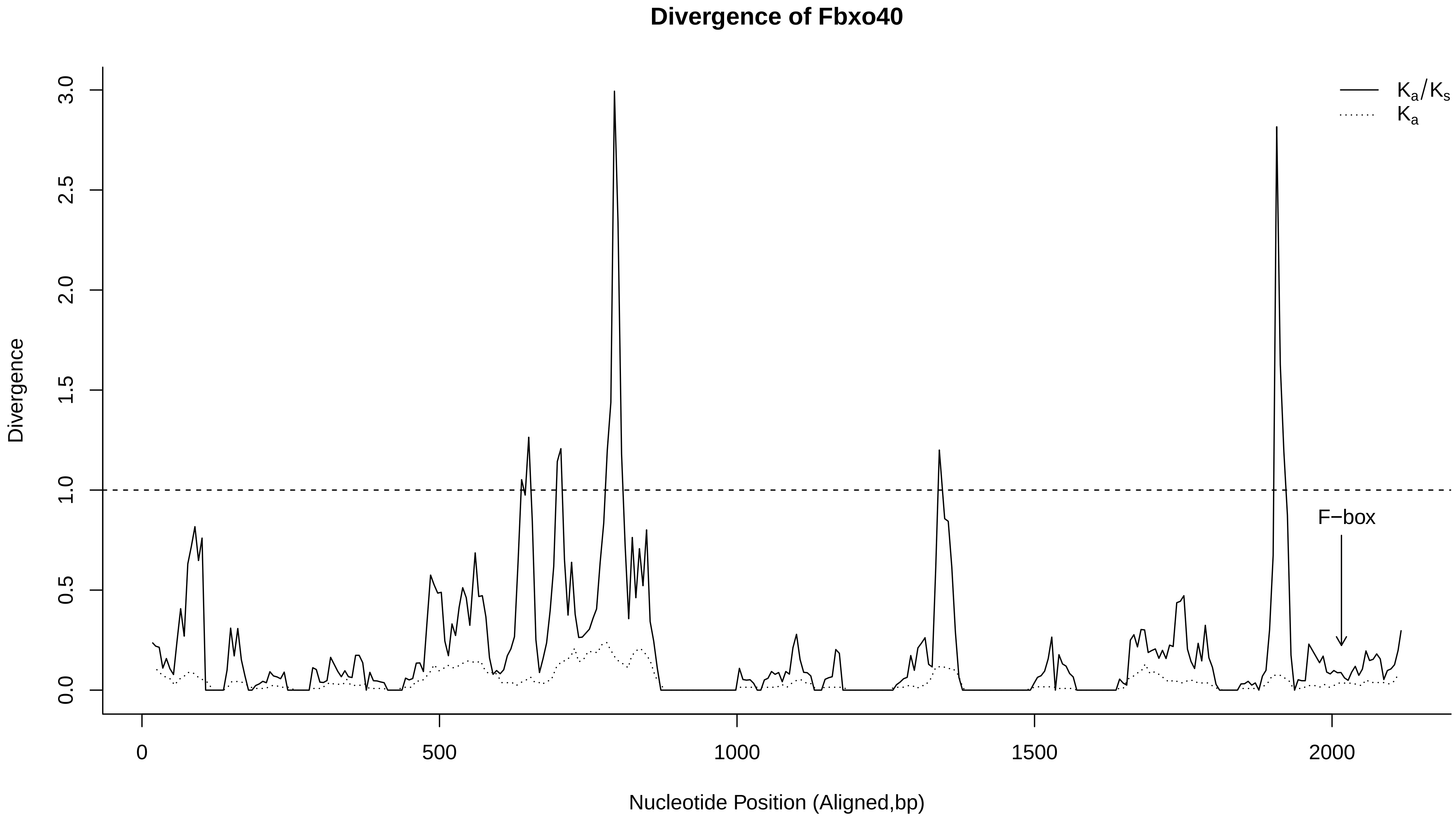
Divergence of Fbxo40
Divergence
Nucleotide Position (Aligned,bp)
$K_a/K_s$
$K_a$
F−box
0.0
0.5
1.0
1.5
2.0
2.5
3.0
0
500
1000
1500
2000

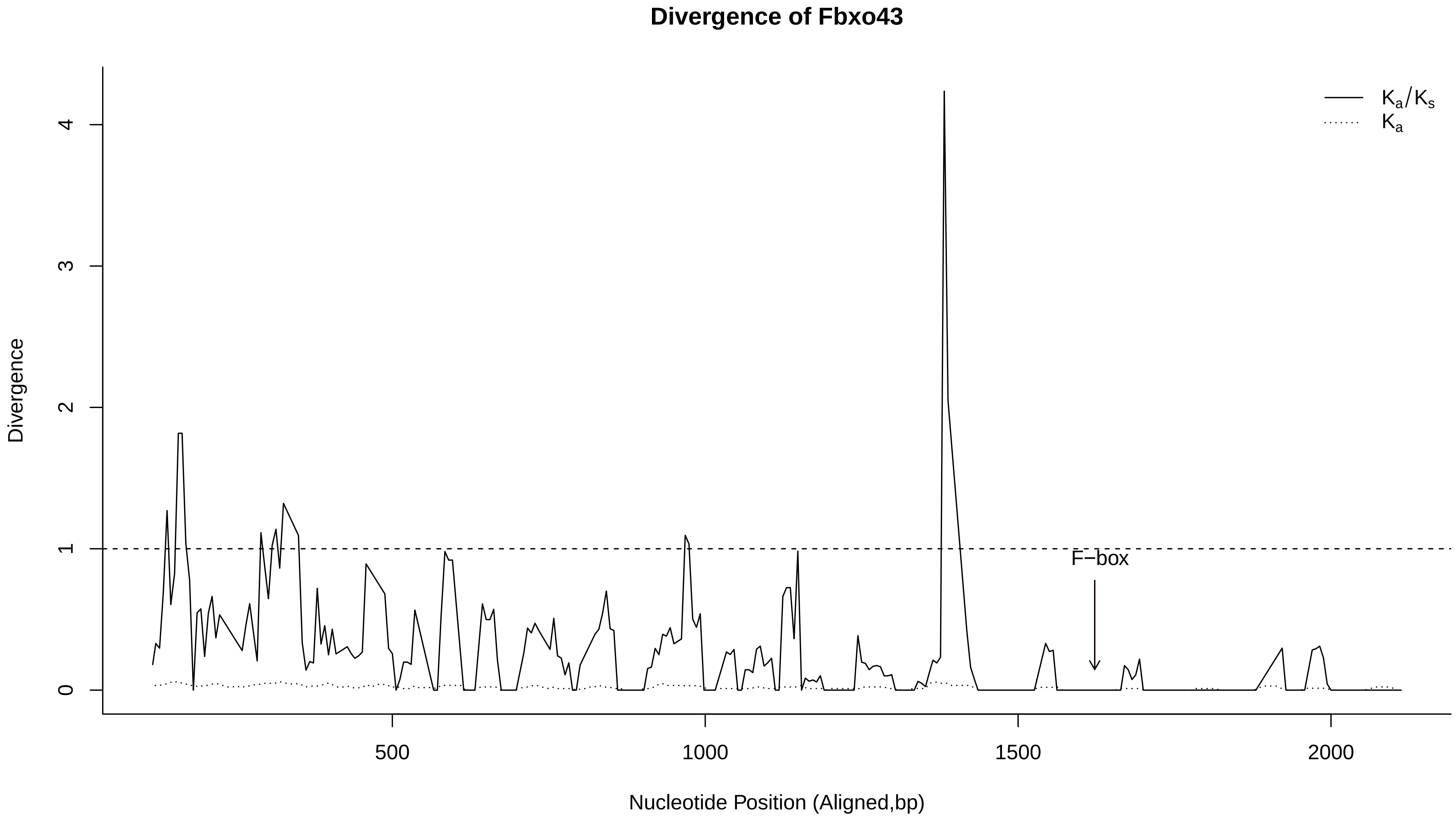
Divergence of Fbxo43
$K_a/K_s$
$K_a$
F−box
Divergence
0
1
2
3
4
500
1000
1500
2000
Nucleotide Position (Aligned,bp)

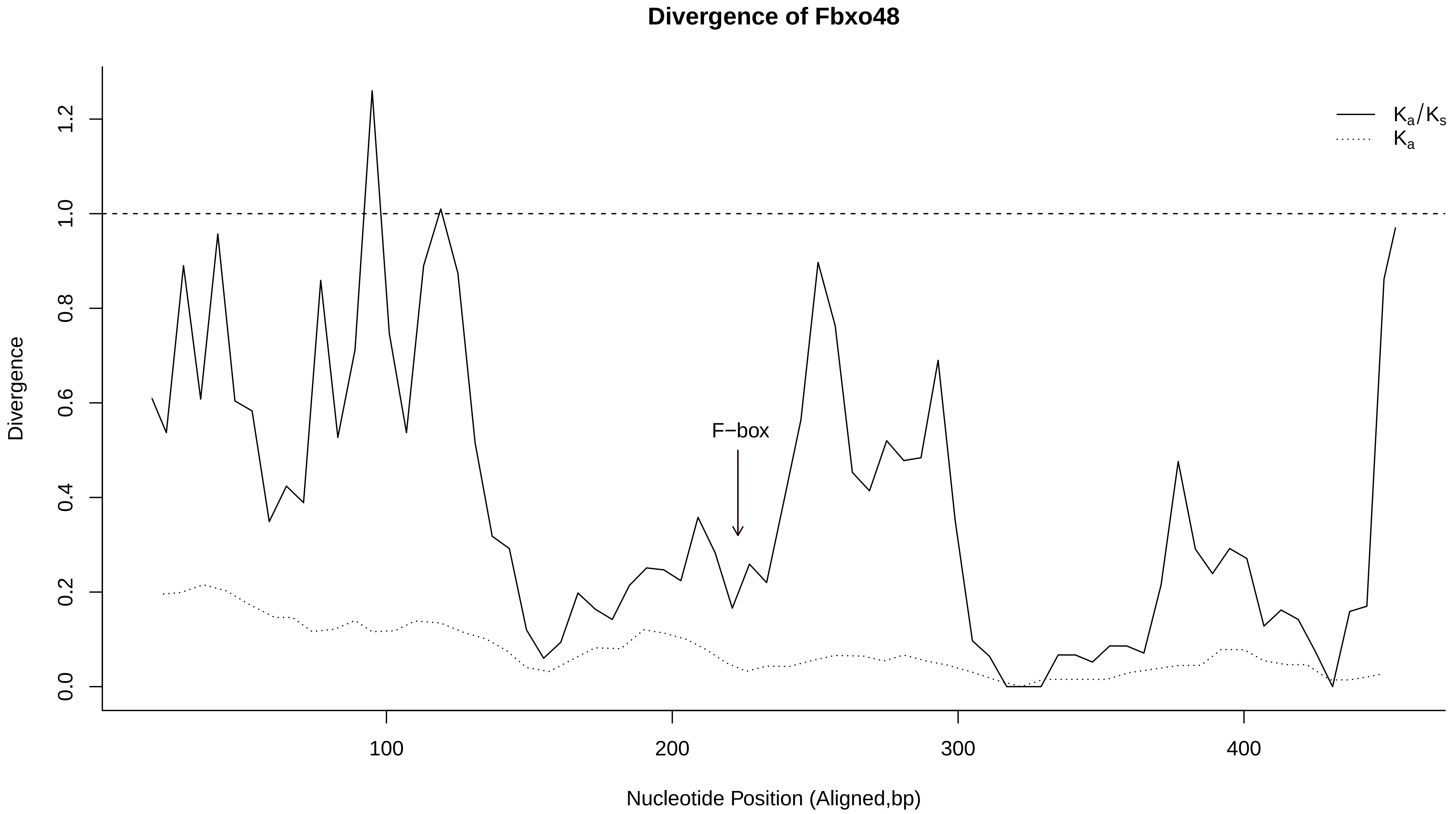
Divergence of Fbxo48
Divergence
Nucleotide Position (Aligned,bp)
Ka/Ks
Ka
F−box
0.0
0.2
0.4
0.6
0.8
1.0
1.2
100
200
300
400

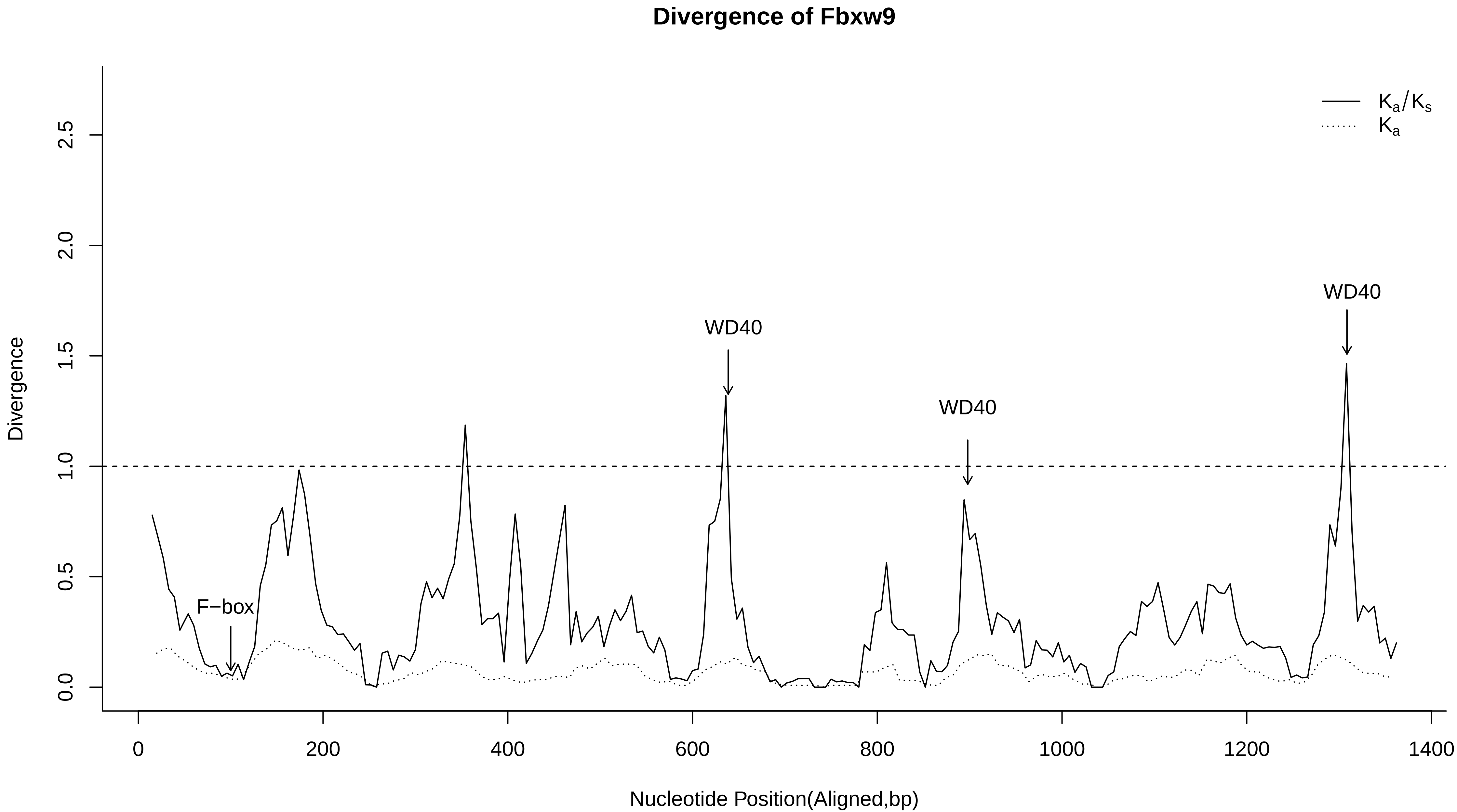

Divergence of Fbxw9
Ka/Ks
Ka
Divergence
0.0
0.5
1.0
1.5
2.0
2.5
F−box
WD40
WD40
WD40
0
200
400
600
800
1000
1200
1400
Nucleotide Position(Aligned,bp)

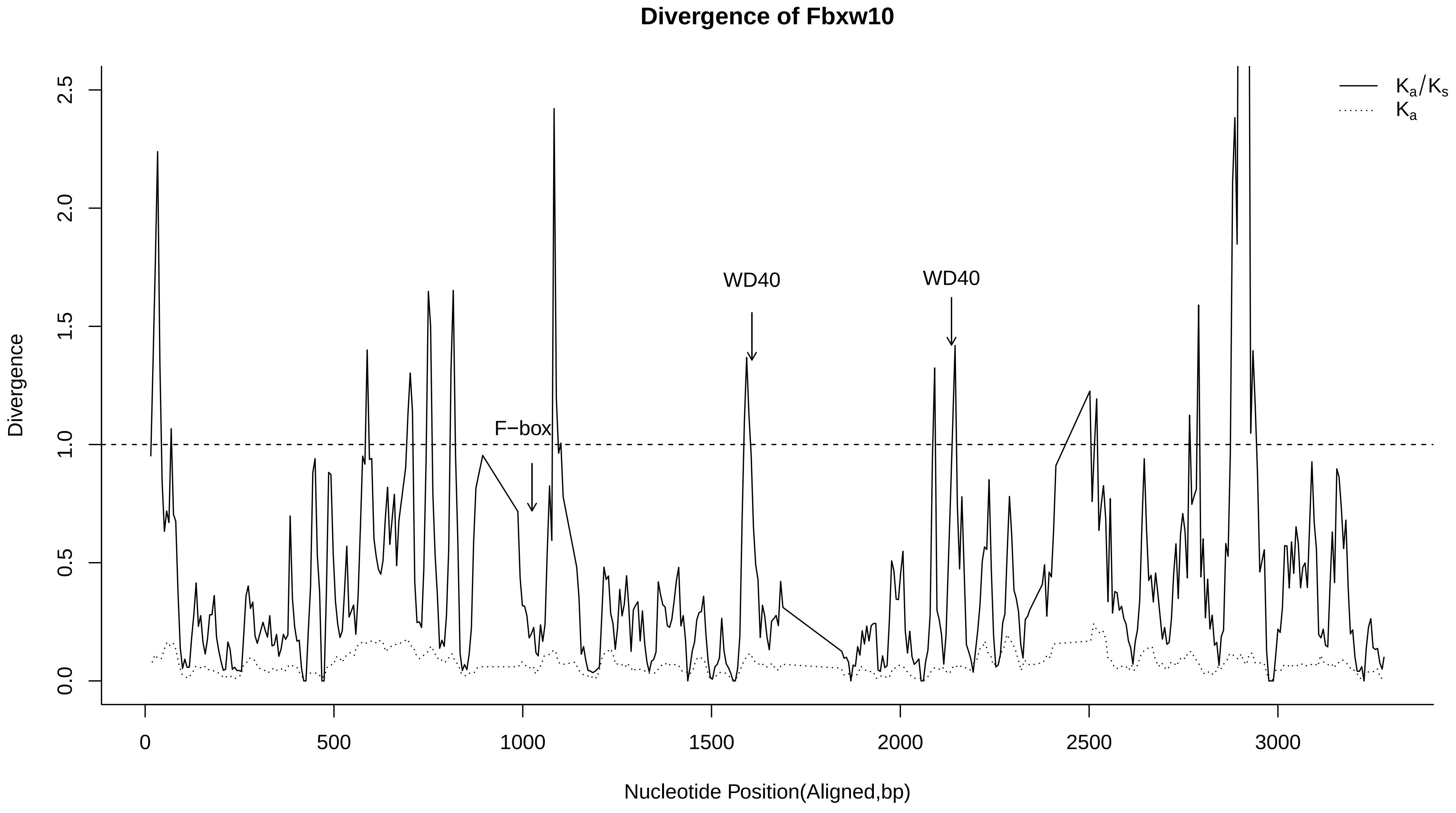

Divergence of Fbxw10
Divergence
Nucleotide Position(Aligned,bp)
0.0
0.5
1.0
1.5
2.0
2.5
0
500
1000
1500
2000
2500
3000
$K_a/K_s$
$K_a$
F−box
WD40
WD40

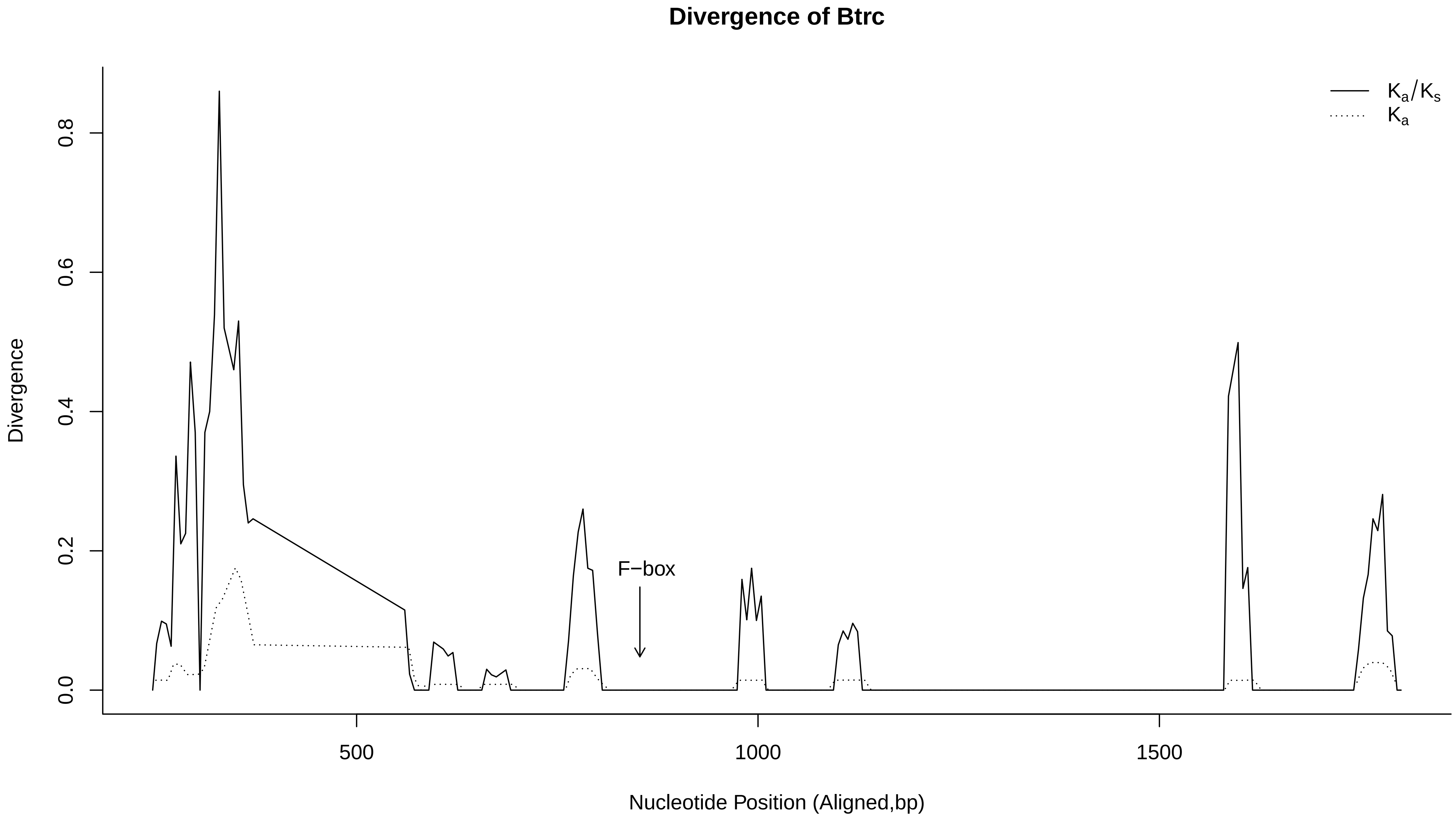
Divergence of Btrc
Ka/Ks
Ka
F−box
0.0
0.2
0.4
0.6
0.8
Divergence
500
1000
1500
Nucleotide Position (Aligned,bp)

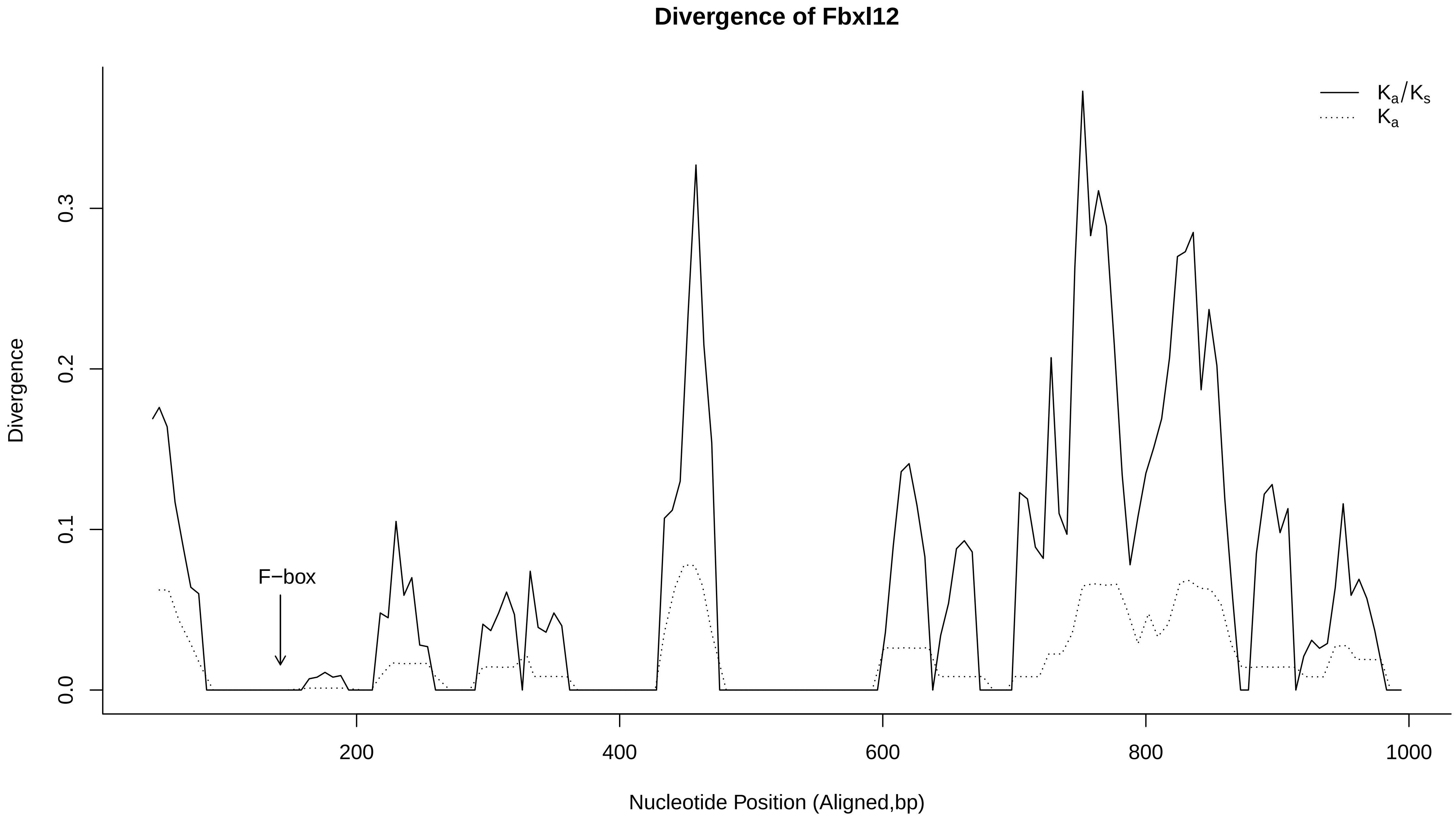

Divergence of Fbxl12
Divergence
0.0
0.1
0.2
0.3
Nucleotide Position (Aligned,bp)
200
400
600
800
1000
F−box
$K_a/K_s$
$K_a$

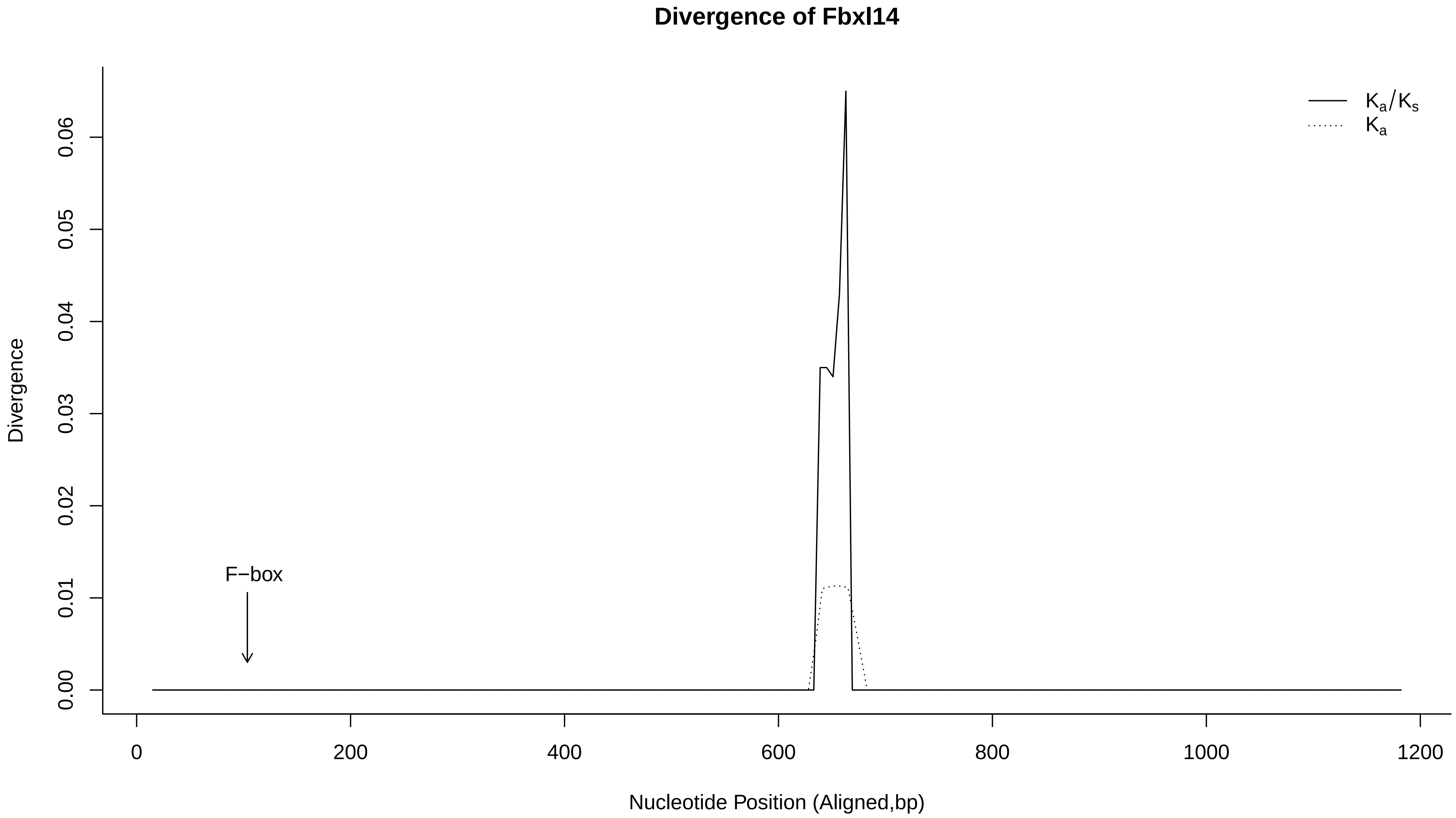

Divergence of Fbxl14
Divergence
0.00
0.01
0.02
0.03
0.04
0.05
0.06
Nucleotide Position (Aligned,bp)
0
200
400
600
800
1000
1200
F−box
Ka/Ks
Ka

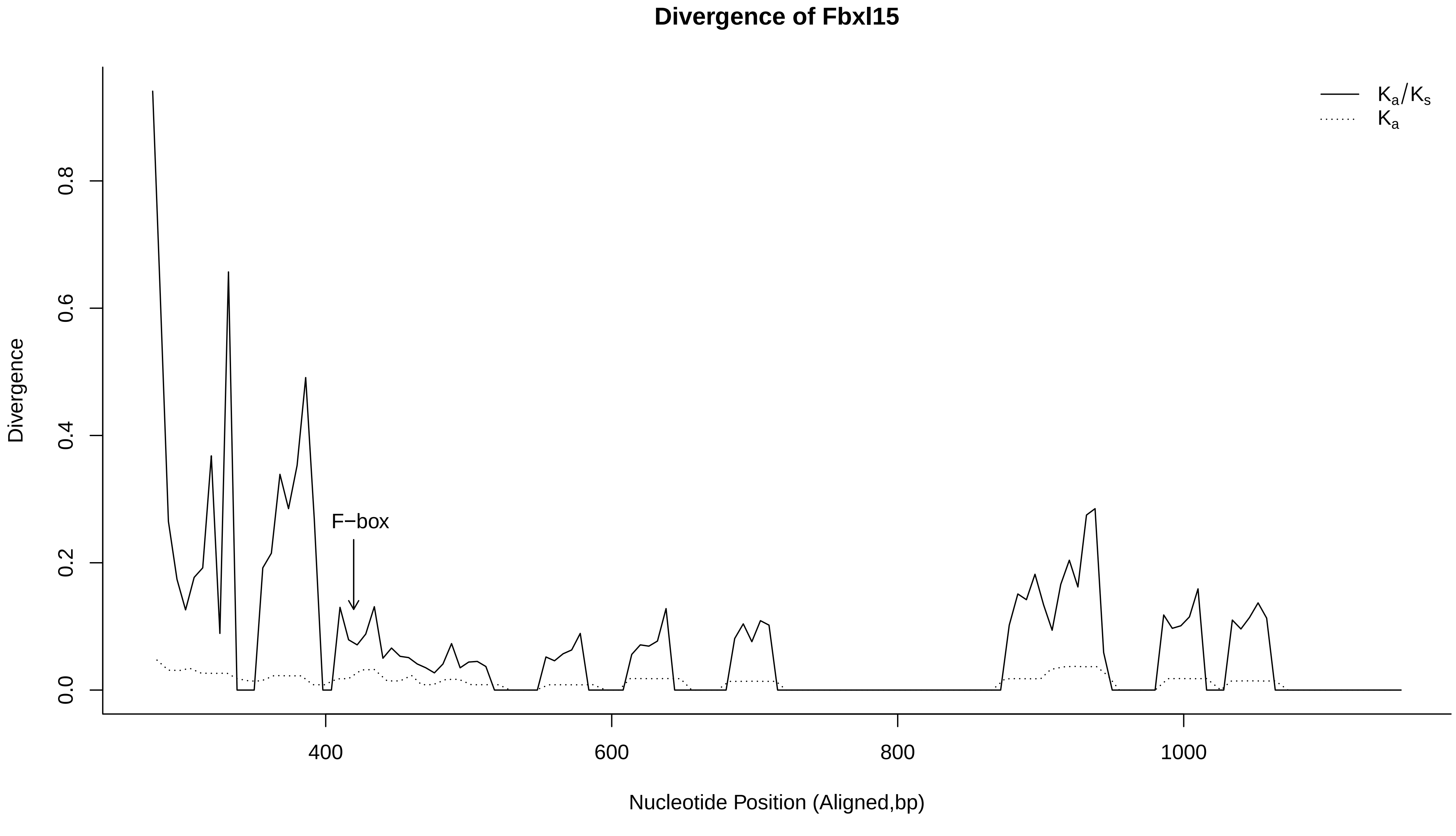
Divergence of Fbxl15
Divergence
0.0
0.2
0.4
0.6
0.8
400
600
800
1000
Nucleotide Position (Aligned,bp)
F−box
$K_a/K_s$
$K_a$

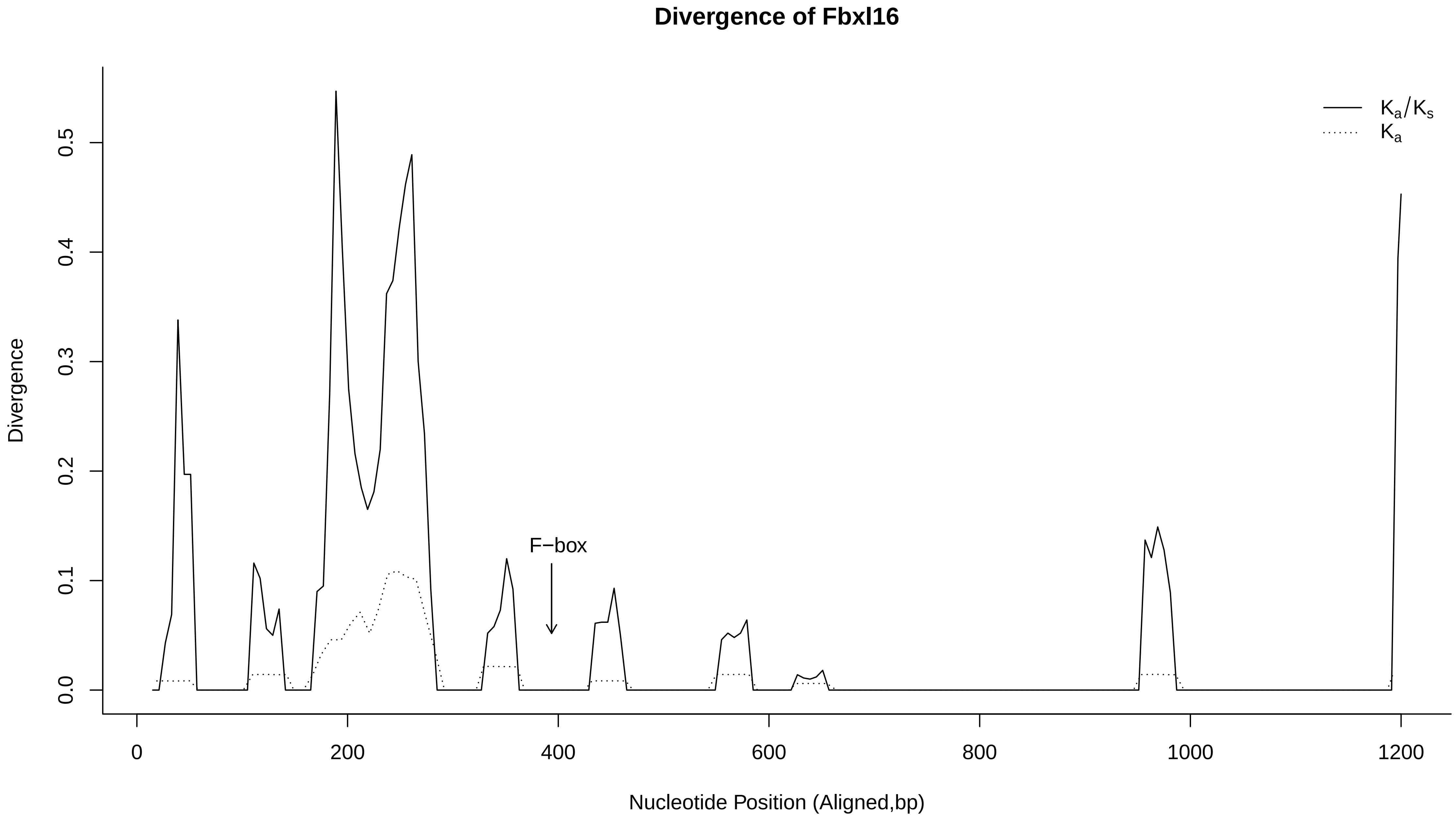

Divergence of Fbxl16
$K_a/K_s$
$K_a$
F−box
Divergence
0.0
0.1
0.2
0.3
0.4
0.5
0
200
400
600
800
1000
1200
Nucleotide Position (Aligned,bp)

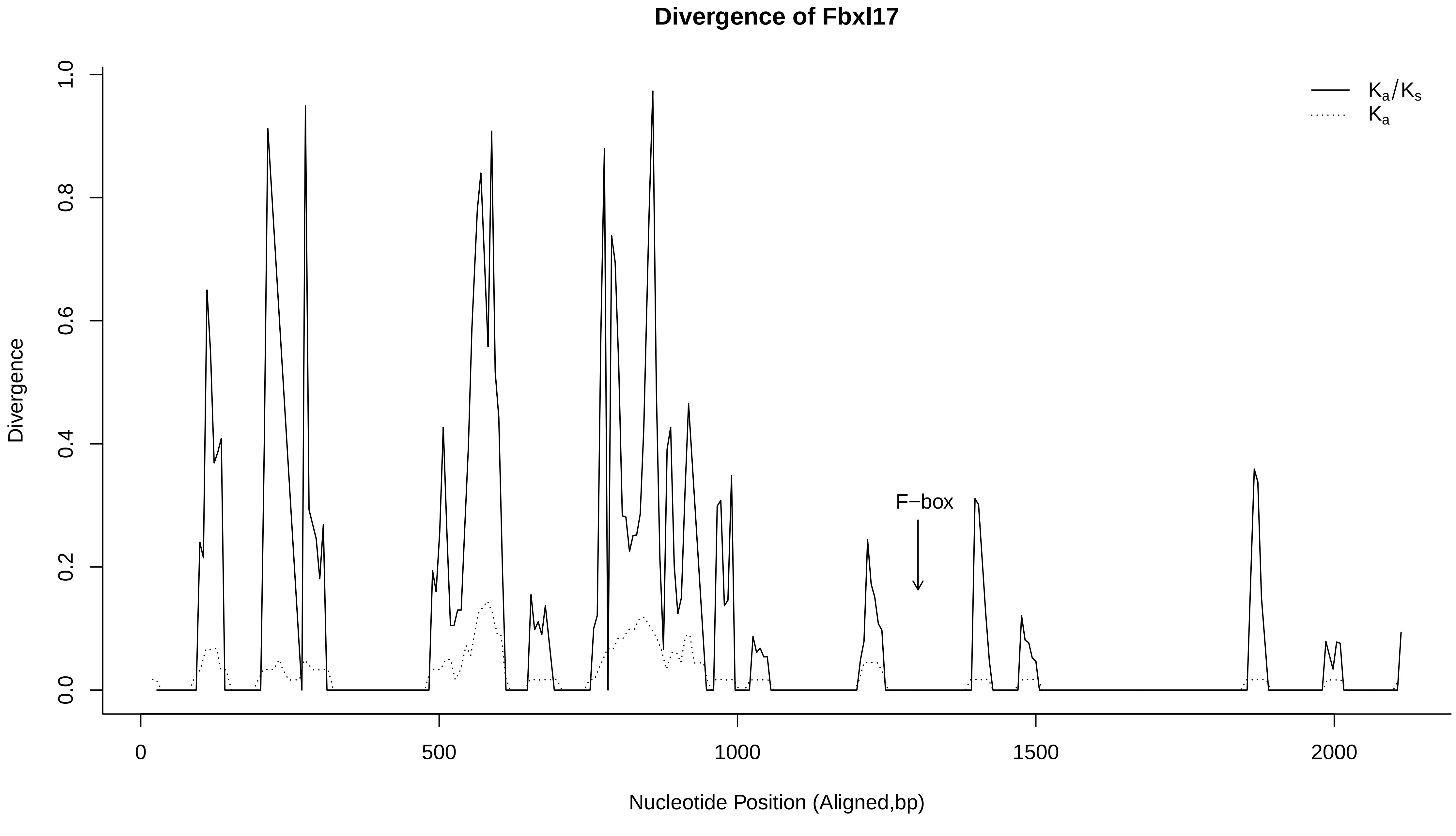
Divergence of Fbxl17
Divergence
Nucleotide Position (Aligned,bp)
Ka/Ks
Ka
F−box
0.0
0.2
0.4
0.6
0.8
1.0
0
500
1000
1500
2000

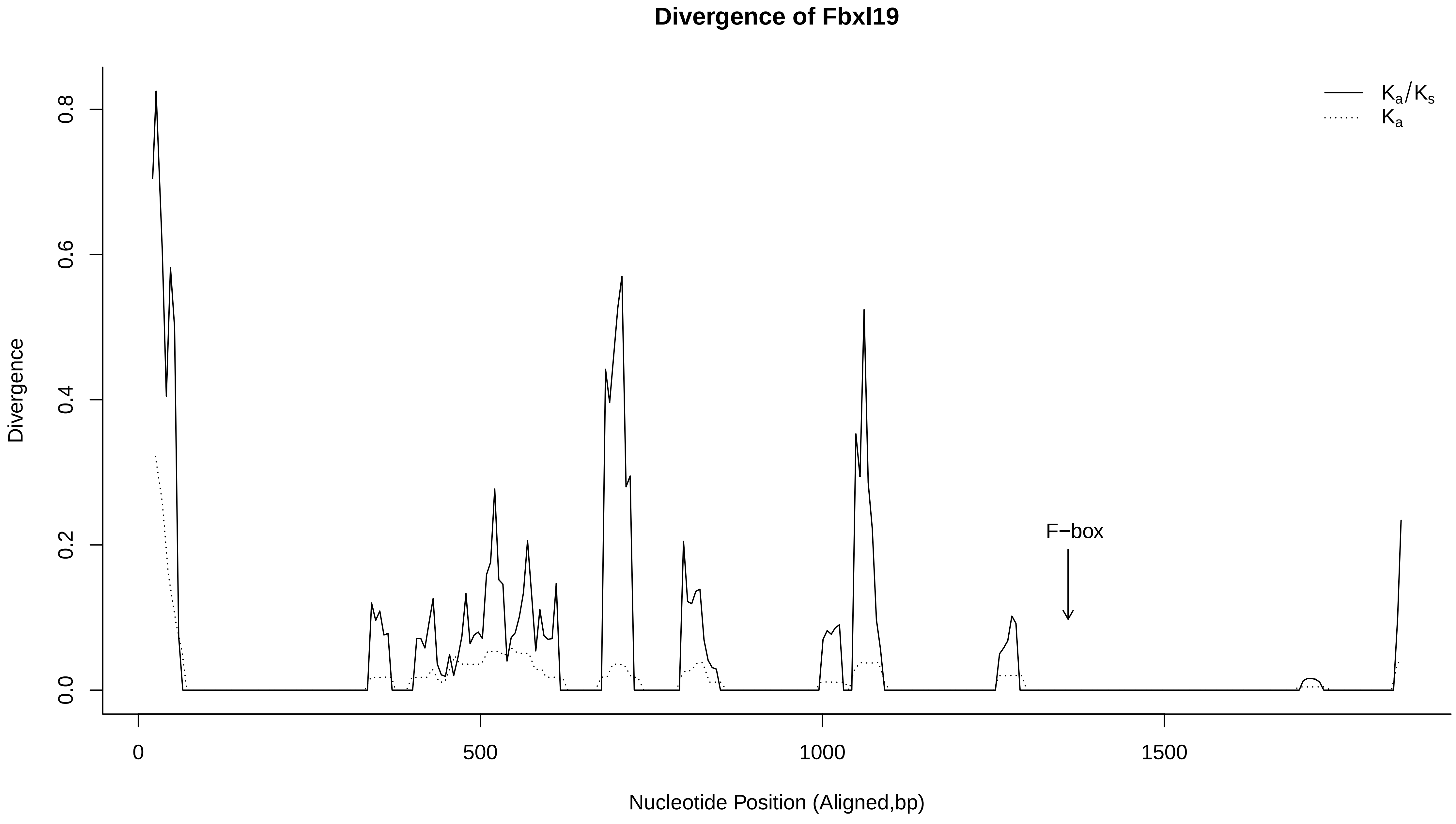

Divergence of Fbxl19
Divergence
Nucleotide Position (Aligned,bp)
0.0
0.2
0.4
0.6
0.8
0
500
1000
1500
$K_a/K_s$
$K_a$
F−box

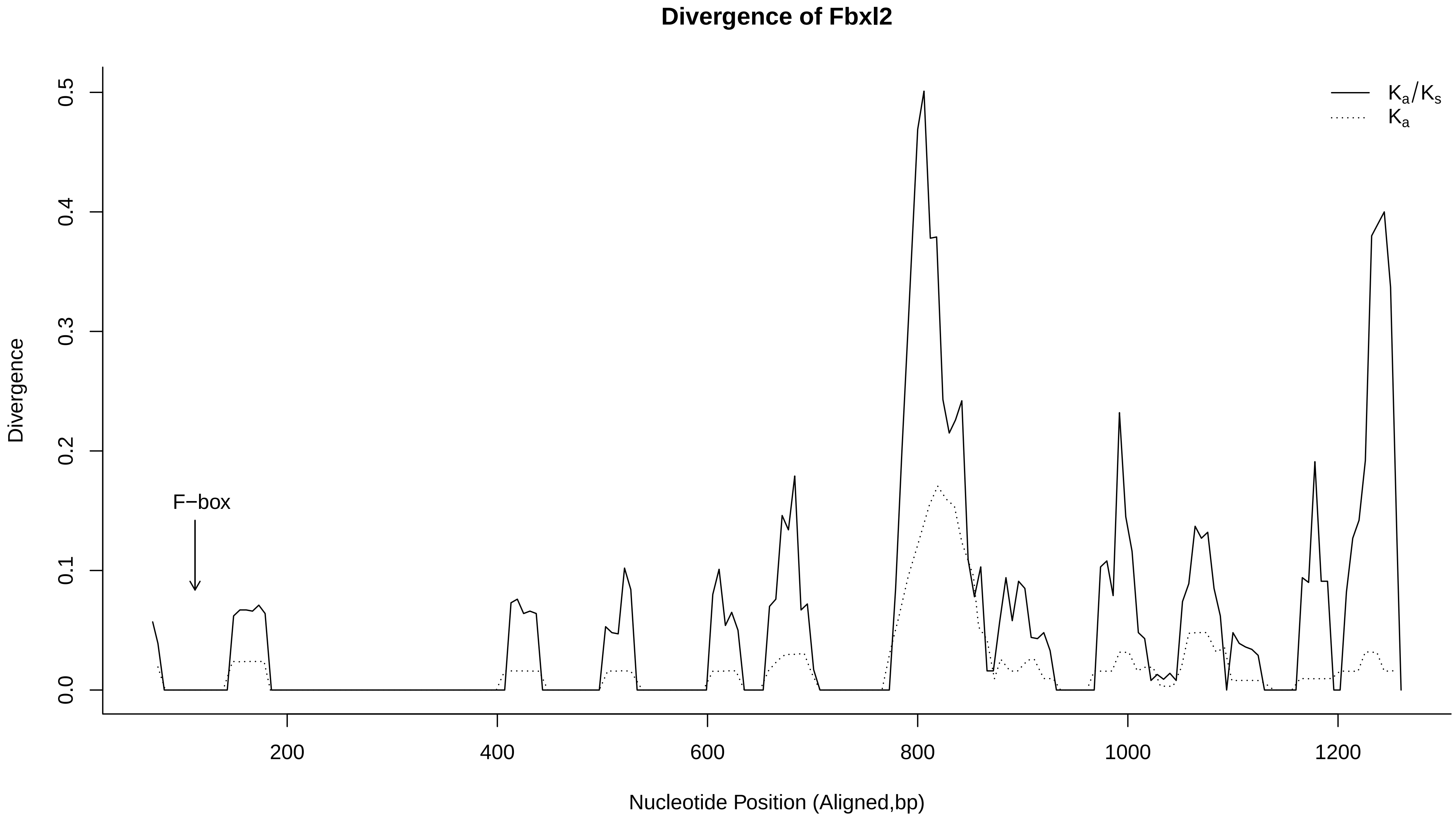

Divergence of Fbxl2
Divergence
Nucleotide Position (Aligned,bp)
0.0
0.1
0.2
0.3
0.4
0.5
200
400
600
800
1000
1200
F−box
$K_a/K_s$
$K_a$

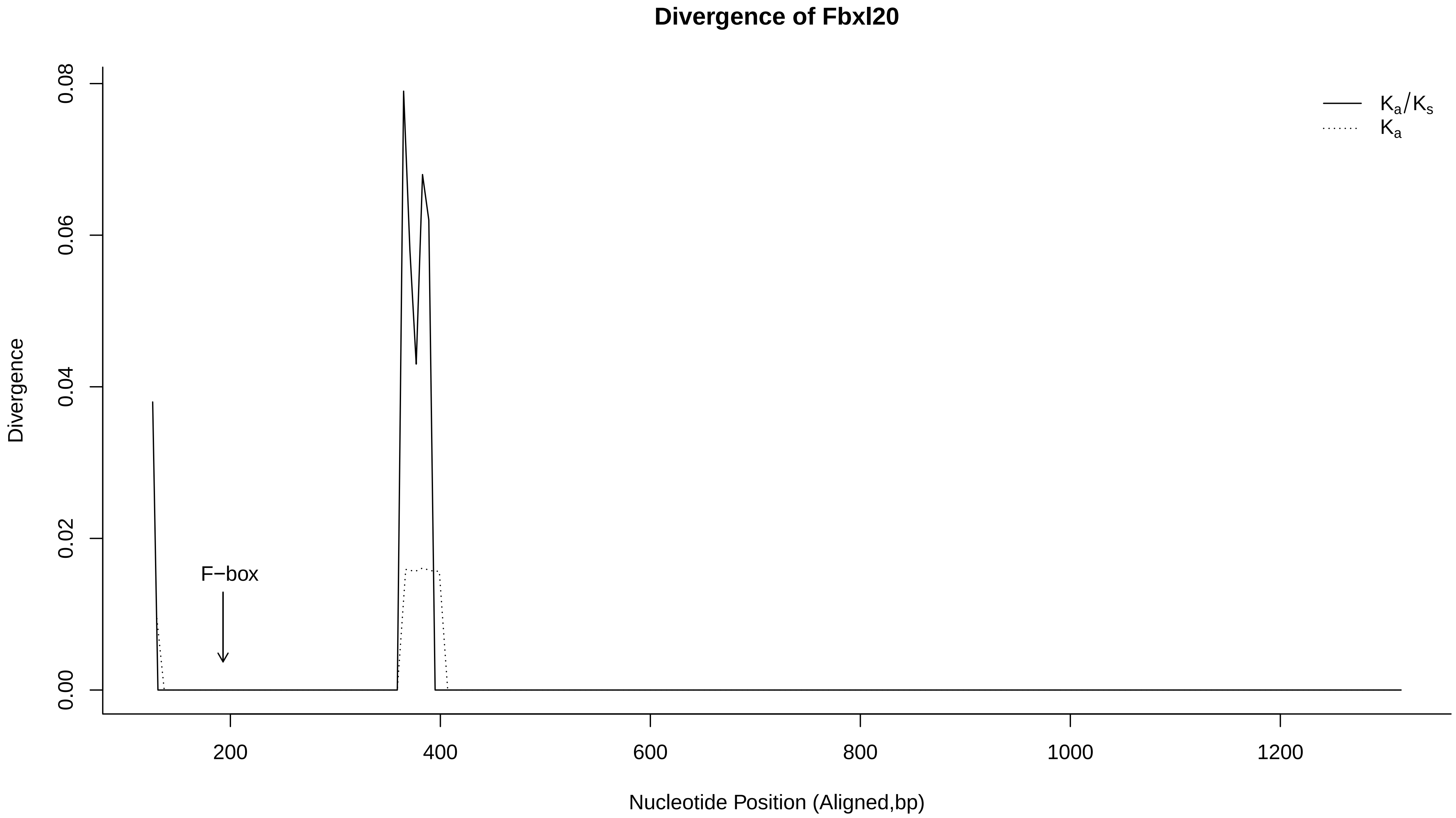

Divergence of Fbxl20
0.00
0.02
0.04
0.06
0.08
Divergence
200
400
600
800
1000
1200
Nucleotide Position (Aligned,bp)
F−box
$K_a/K_s$
$K_a$

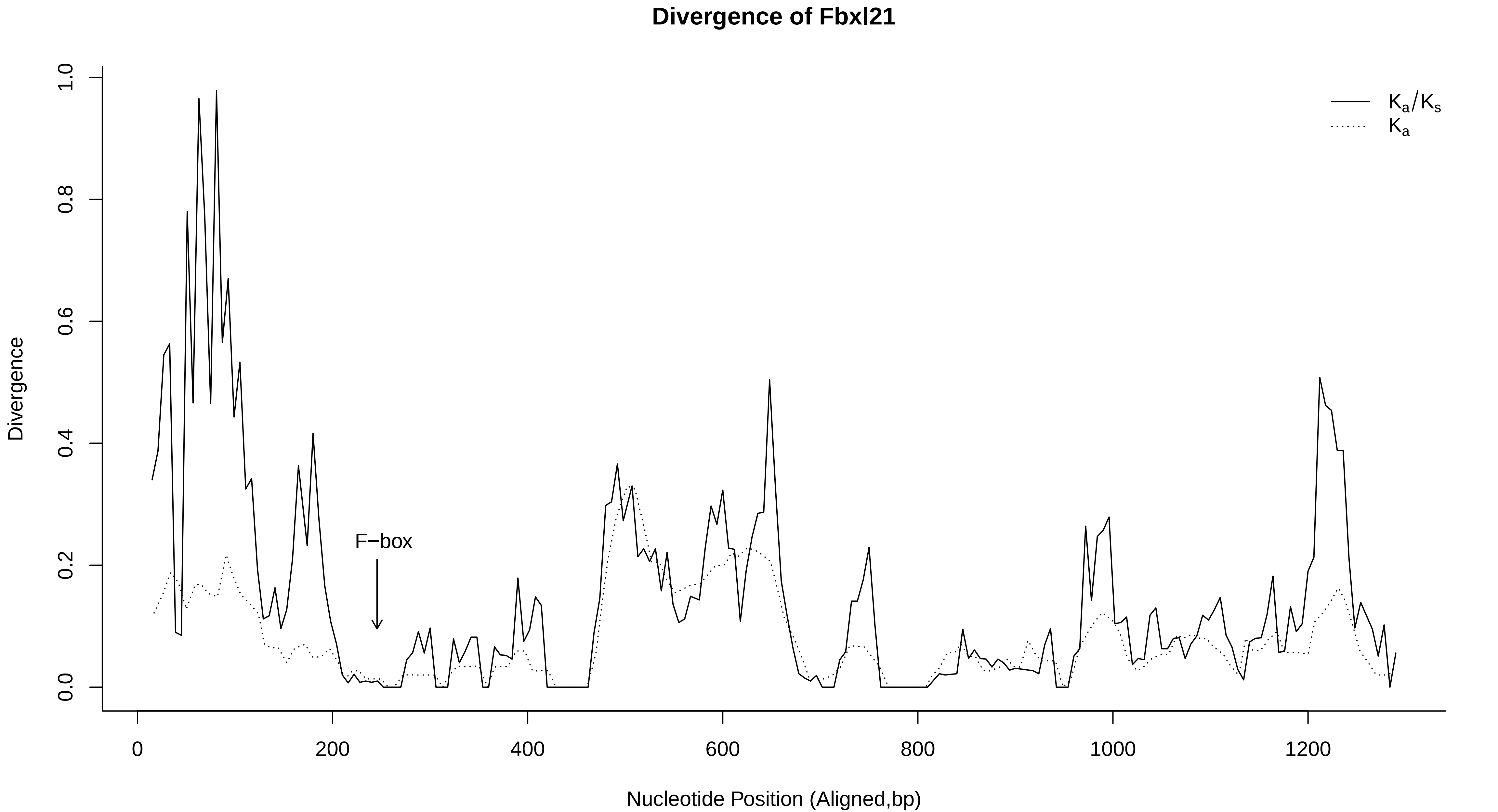
Divergence of Fbxl21
$K_a/K_s$
$K_a$
F−box
Divergence
1.0
0.8
0.6
0.4
0.2
0.0
0
200
400
600
800
1000
1200
Nucleotide Position (Aligned,bp)

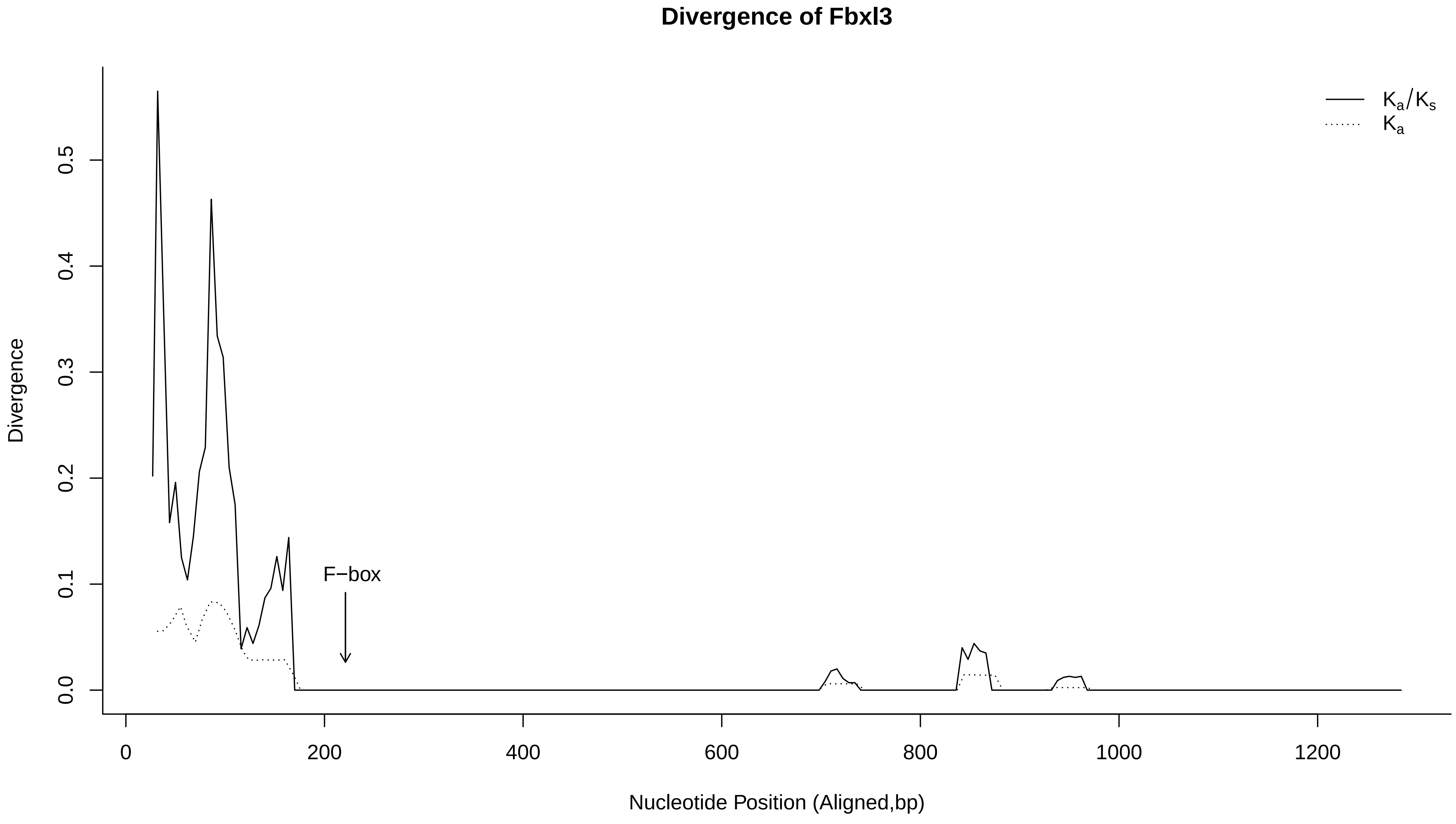
Divergence of Fbxl3
Divergence
0.0
0.1
0.2
0.3
0.4
0.5
0
200
400
600
800
1000
1200
Nucleotide Position (Aligned,bp)
F−box
$K_a/K_s$
$K_a$

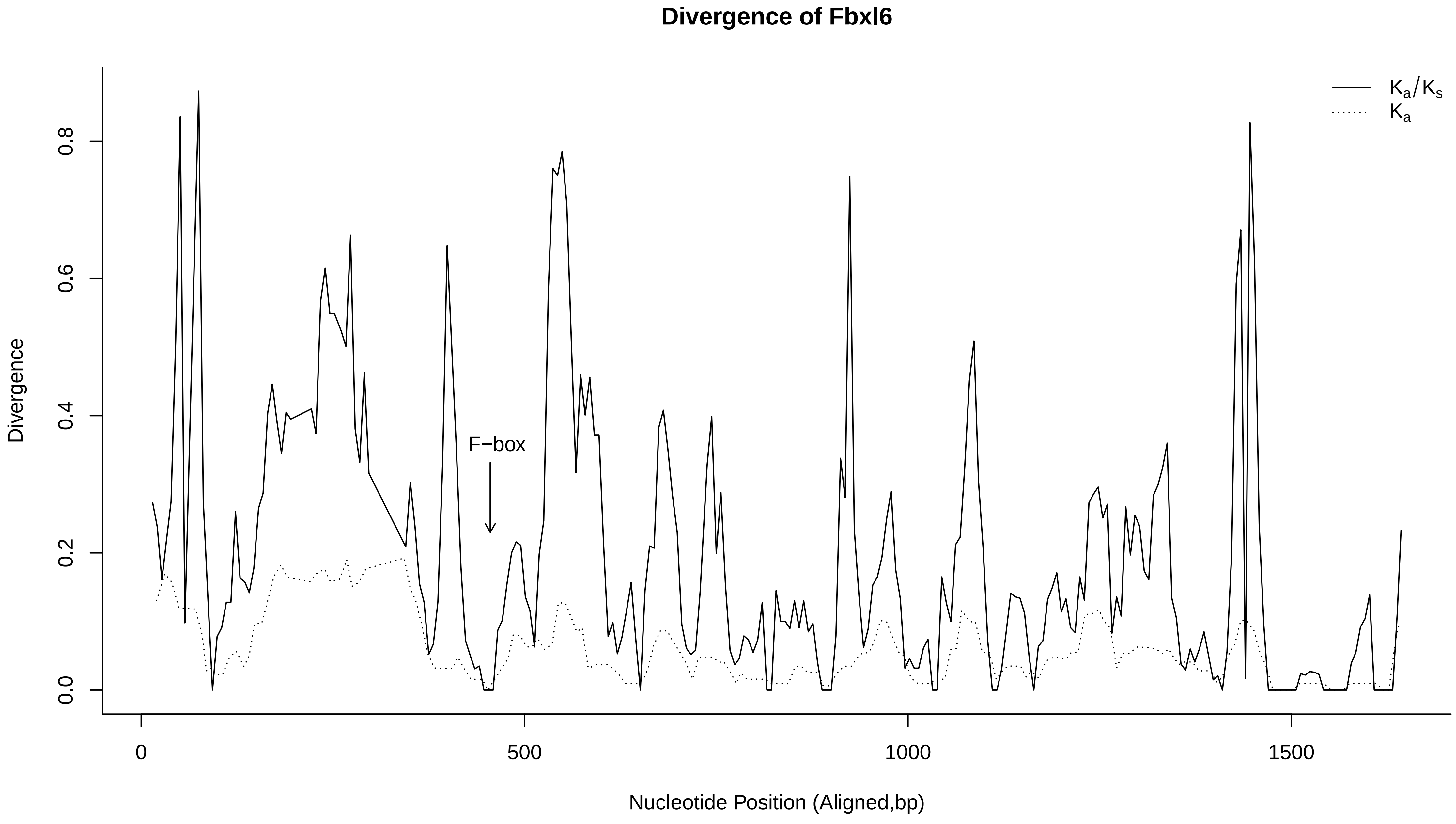

Divergence of Fbxl6
Divergence
0.0
0.2
0.4
0.6
0.8
0
500
1000
1500
Nucleotide Position (Aligned,bp)
F−box
$K_a/K_s$
$K_a$

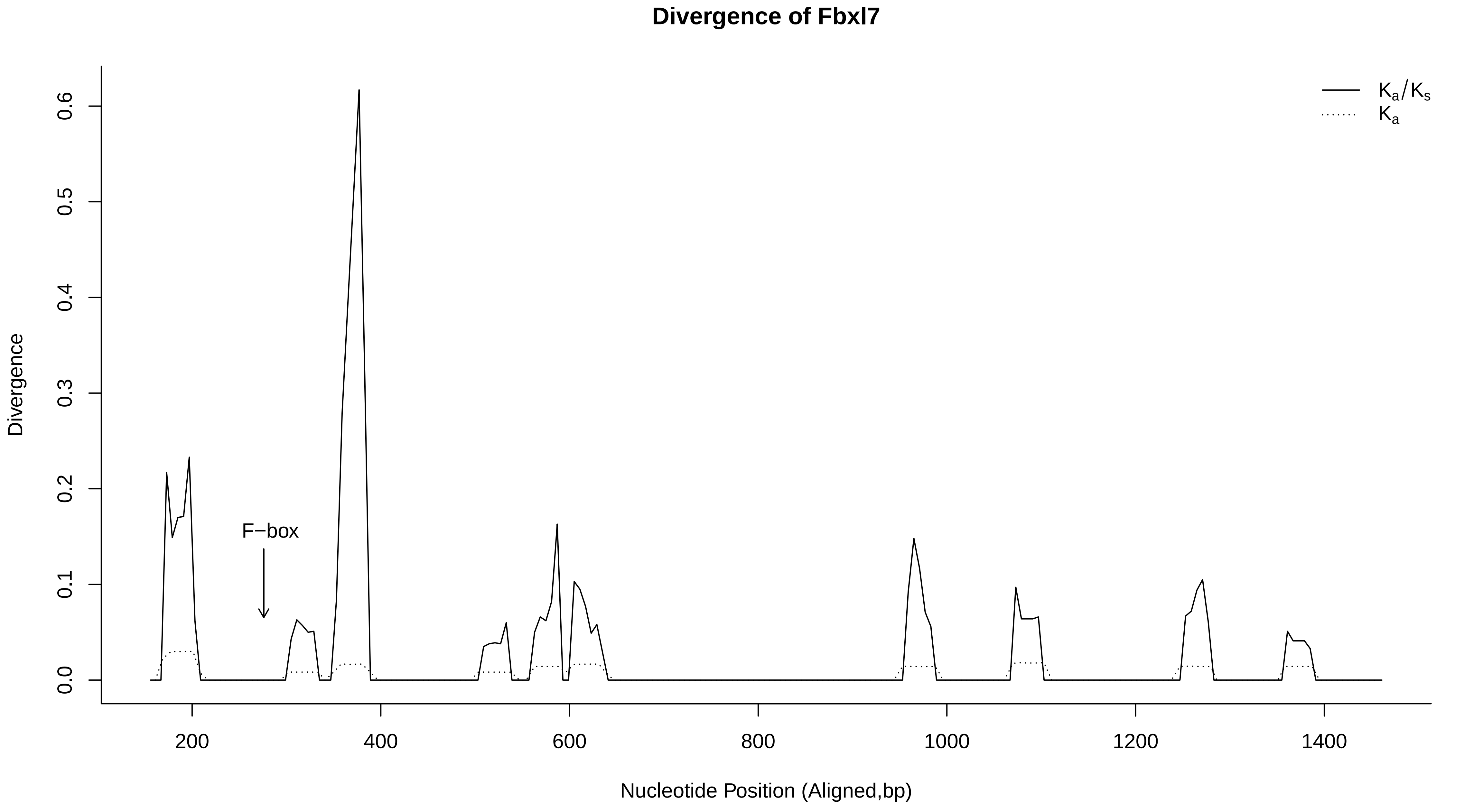
Divergence of Fbxl7
Divergence
Nucleotide Position (Aligned,bp)
0.0
0.1
0.2
0.3
0.4
0.5
0.6
200
400
600
800
1000
1200
1400
F−box
$K_a/K_s$
$K_a$

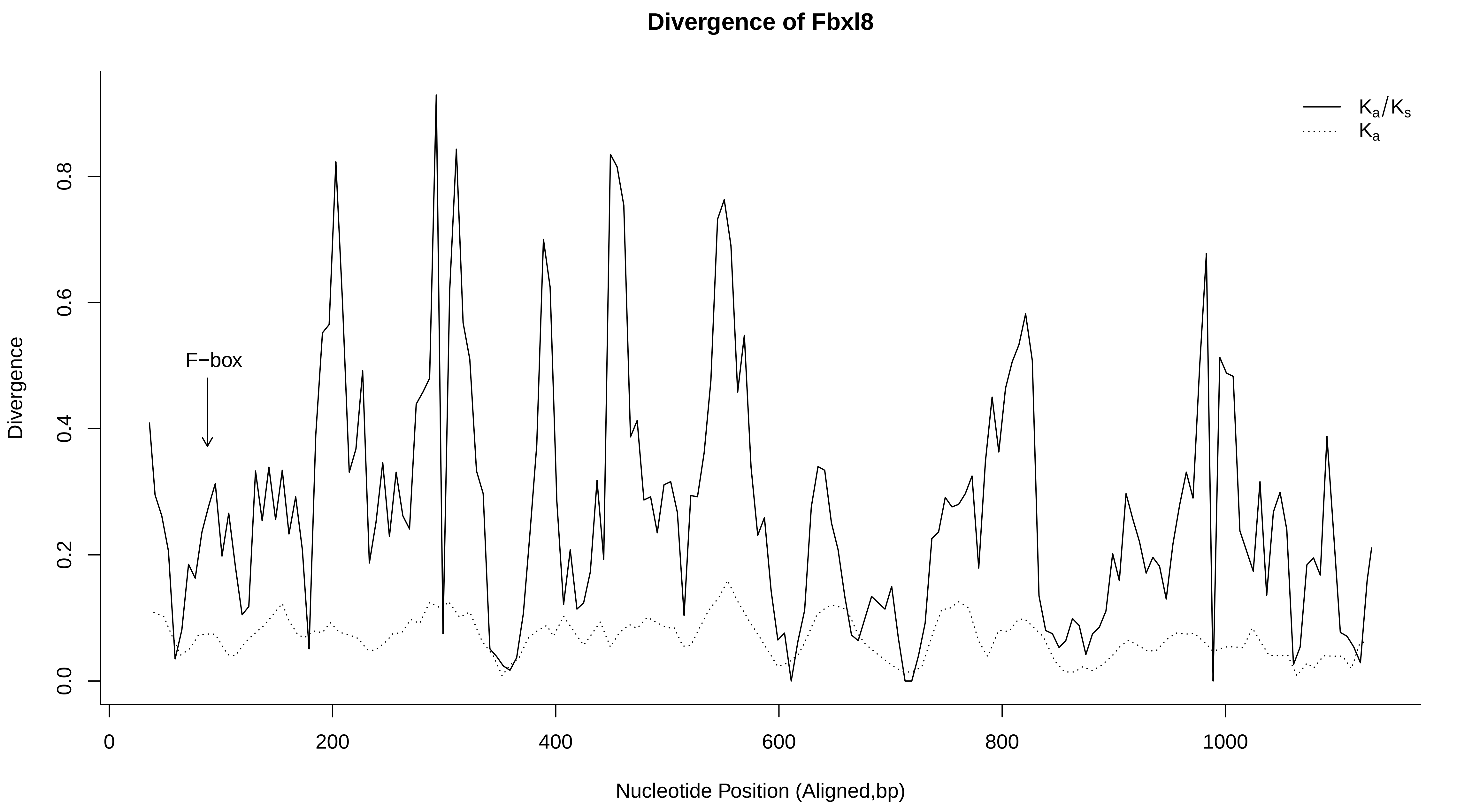
Divergence of Fbxl8
Divergence
Nucleotide Position (Aligned,bp)
F−box
$K_a/K_s$
$K_a$
0.0
0.2
0.4
0.6
0.8
0
200
400
600
800
1000

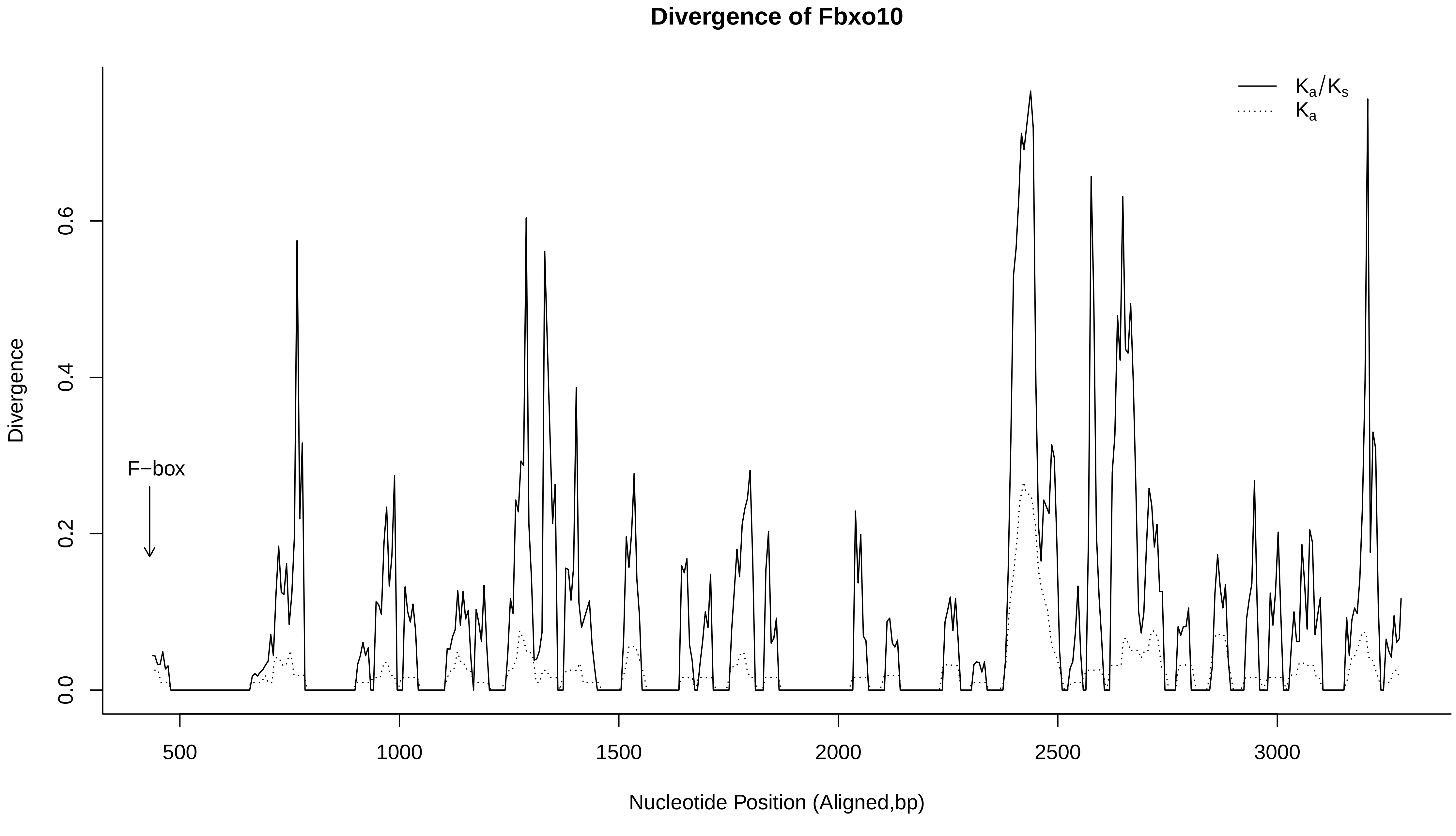
Divergence of Fbxo10
Ka/Ks
Ka
F−box
Divergence
0.0
0.2
0.4
0.6
500
1000
1500
2000
2500
3000
Nucleotide Position (Aligned,bp)

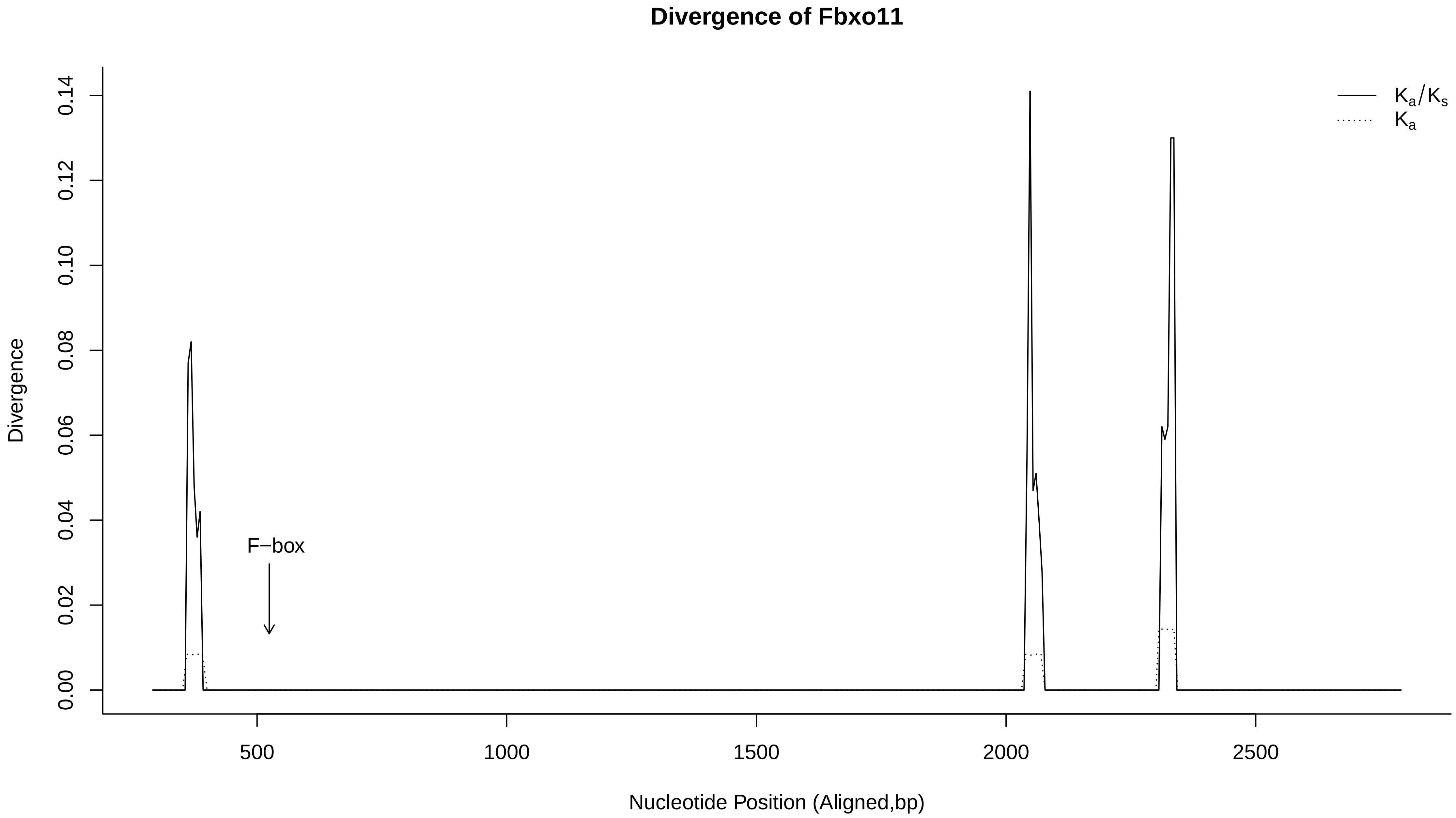

Divergence of Fbxo11
Divergence
0.00
0.02
0.04
0.06
0.08
0.10
0.12
0.14
500
1000
1500
2000
2500
Nucleotide Position (Aligned,bp)
F−box
$K_a/K_s$
$K_a$

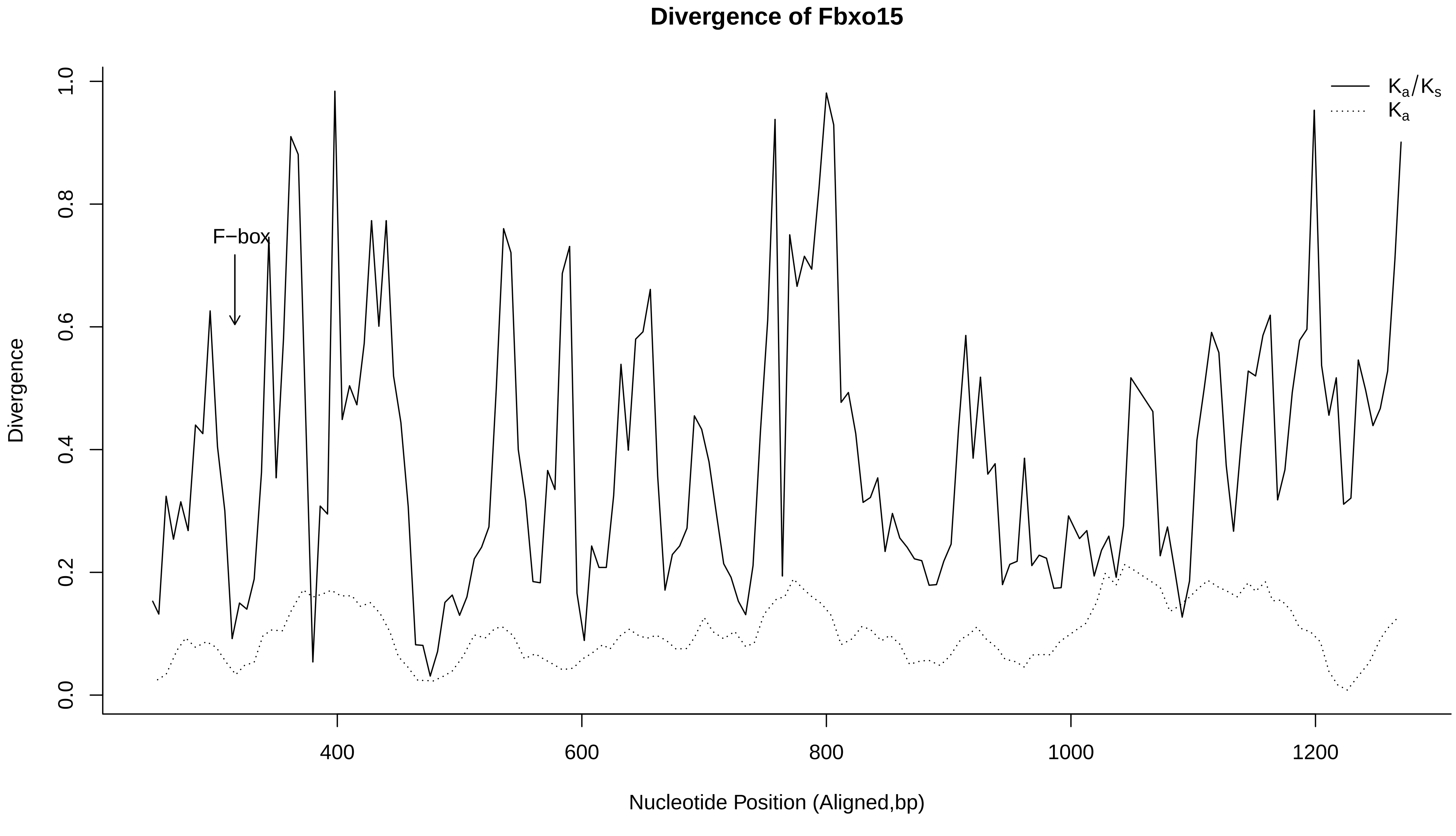
Divergence of Fbxo15
Divergence
Nucleotide Position (Aligned,bp)
$K_a/K_s$
$K_a$
F−box
0.0
0.2
0.4
0.6
0.8
1.0
400
600
800
1000
1200

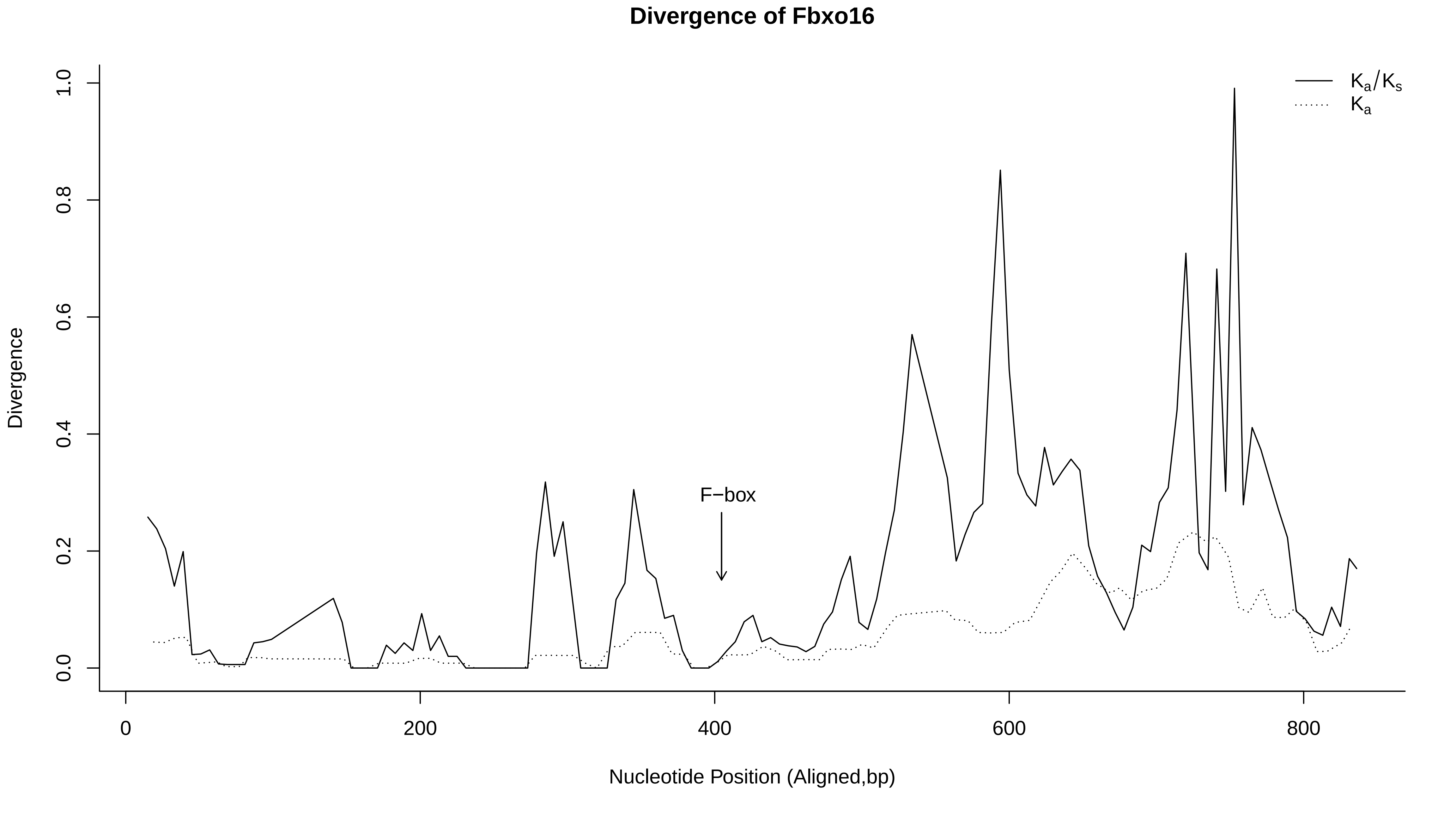

Divergence of Fbxo16
Divergence
Nucleotide Position (Aligned,bp)
0.0
0.2
0.4
0.6
0.8
1.0
0
200
400
600
800
F−box
$K_a/K_s$
$K_a$

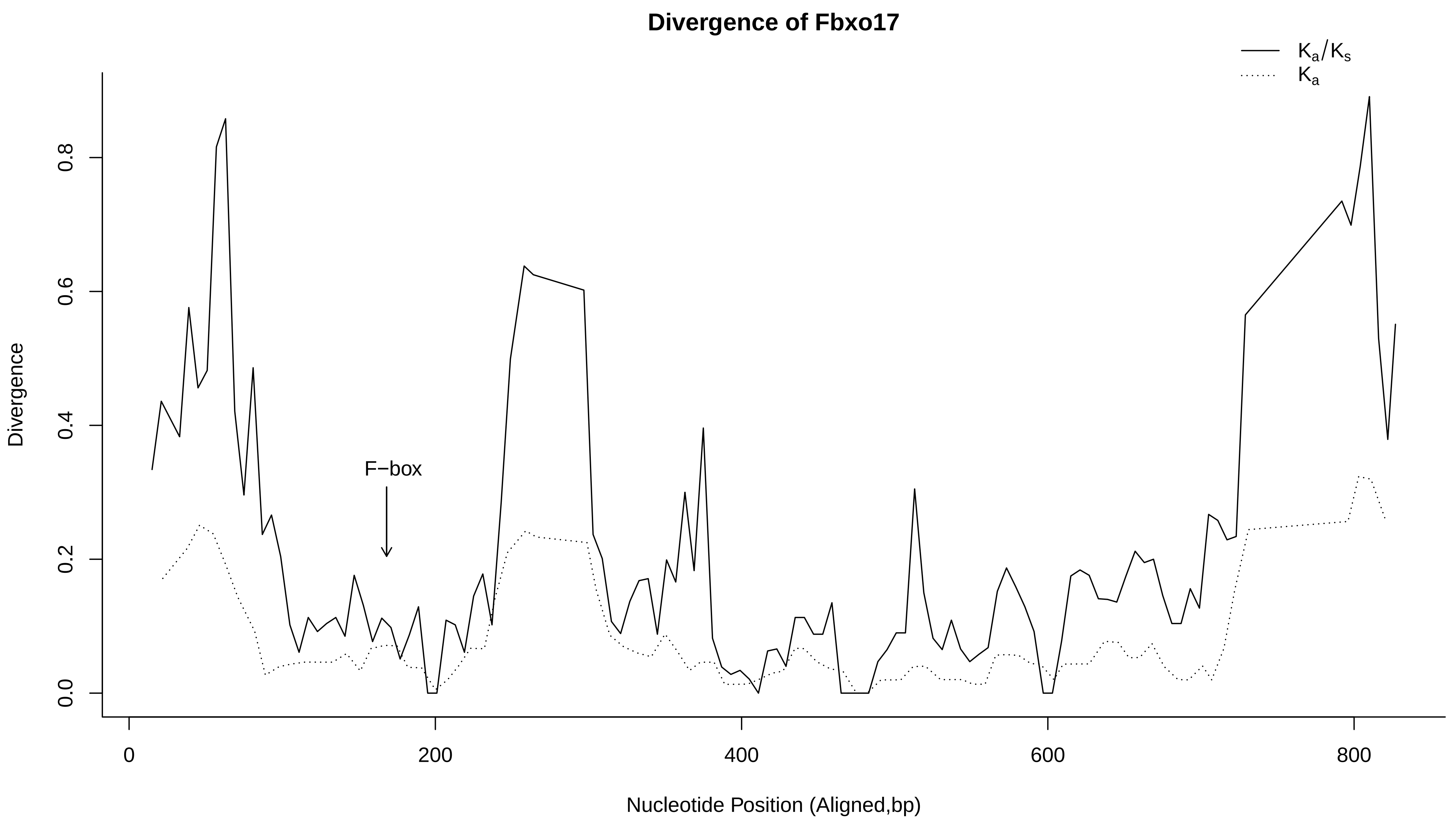
Divergence of Fbxo17
$K_a/K_s$
$K_a$
F−box
Divergence
0.0
0.2
0.4
0.6
0.8
0
200
400
600
800
Nucleotide Position (Aligned,bp)

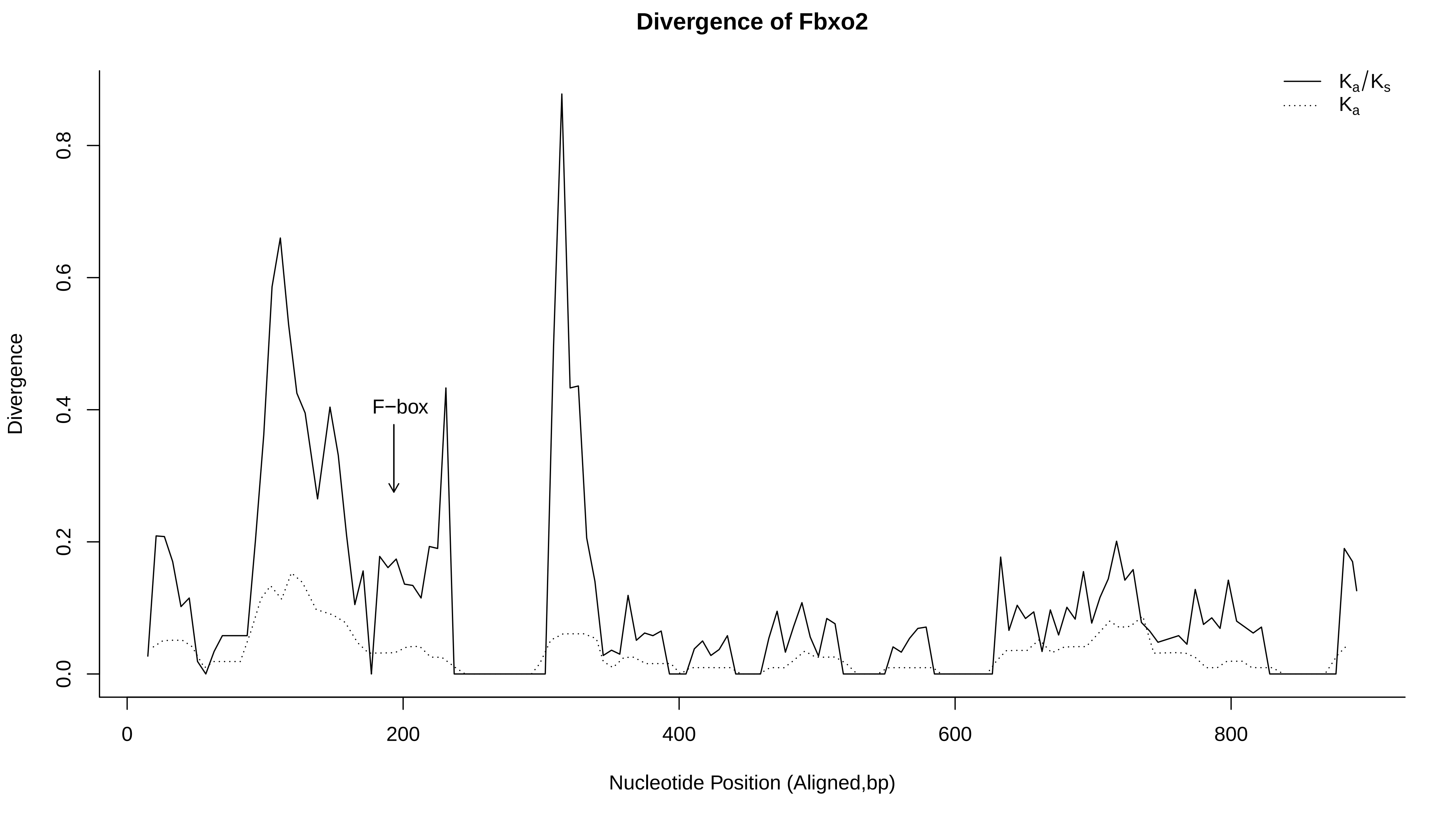
Divergence of Fbxo2
Ka/Ks
Ka
F−box
Divergence
0.0
0.2
0.4
0.6
0.8
0
200
400
600
800
Nucleotide Position (Aligned,bp)

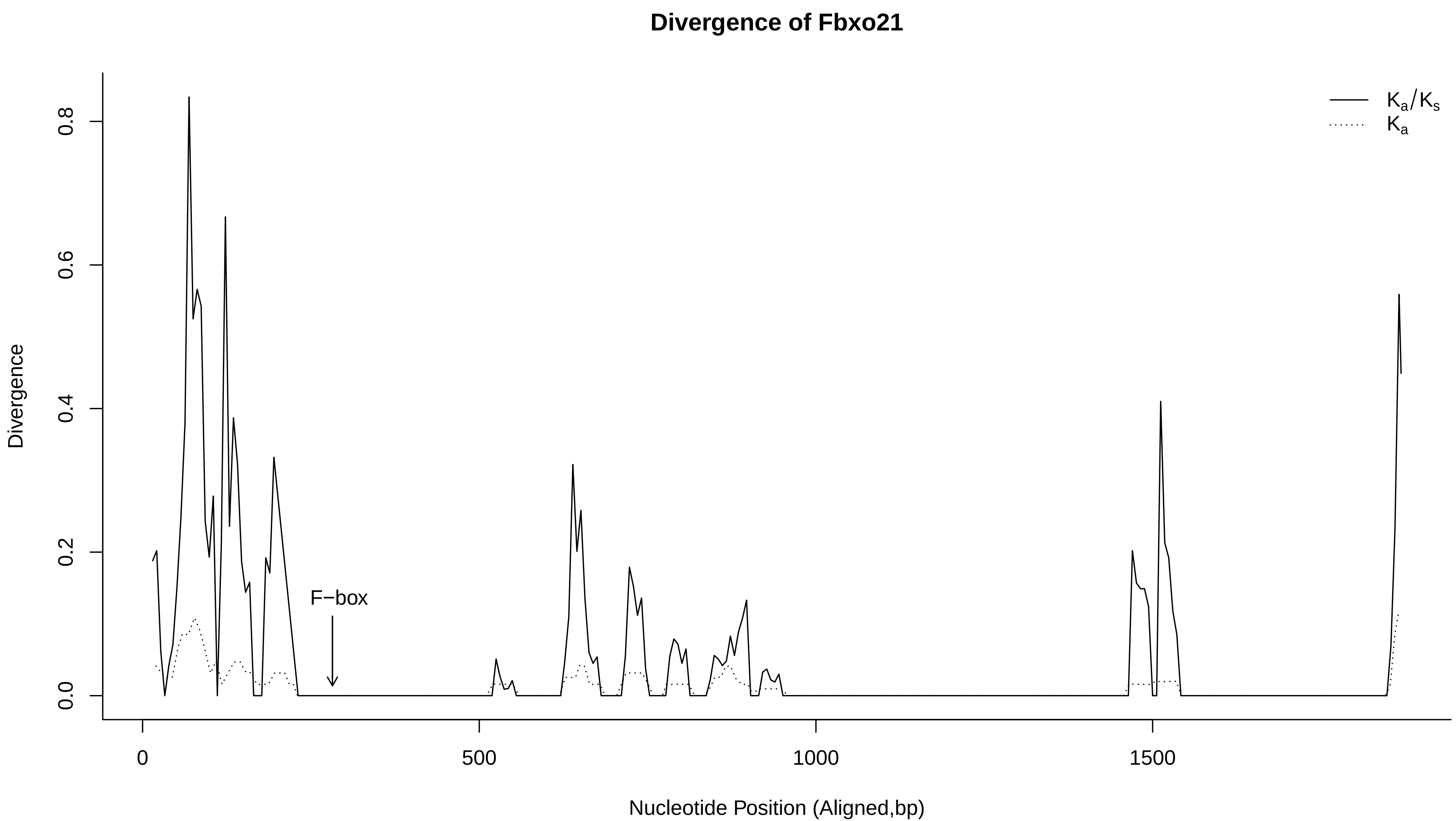

Divergence of Fbxo21
Divergence
Nucleotide Position (Aligned,bp)
0.0
0.2
0.4
0.6
0.8
0
500
1000
1500
$K_a/K_s$
$K_a$
F−box

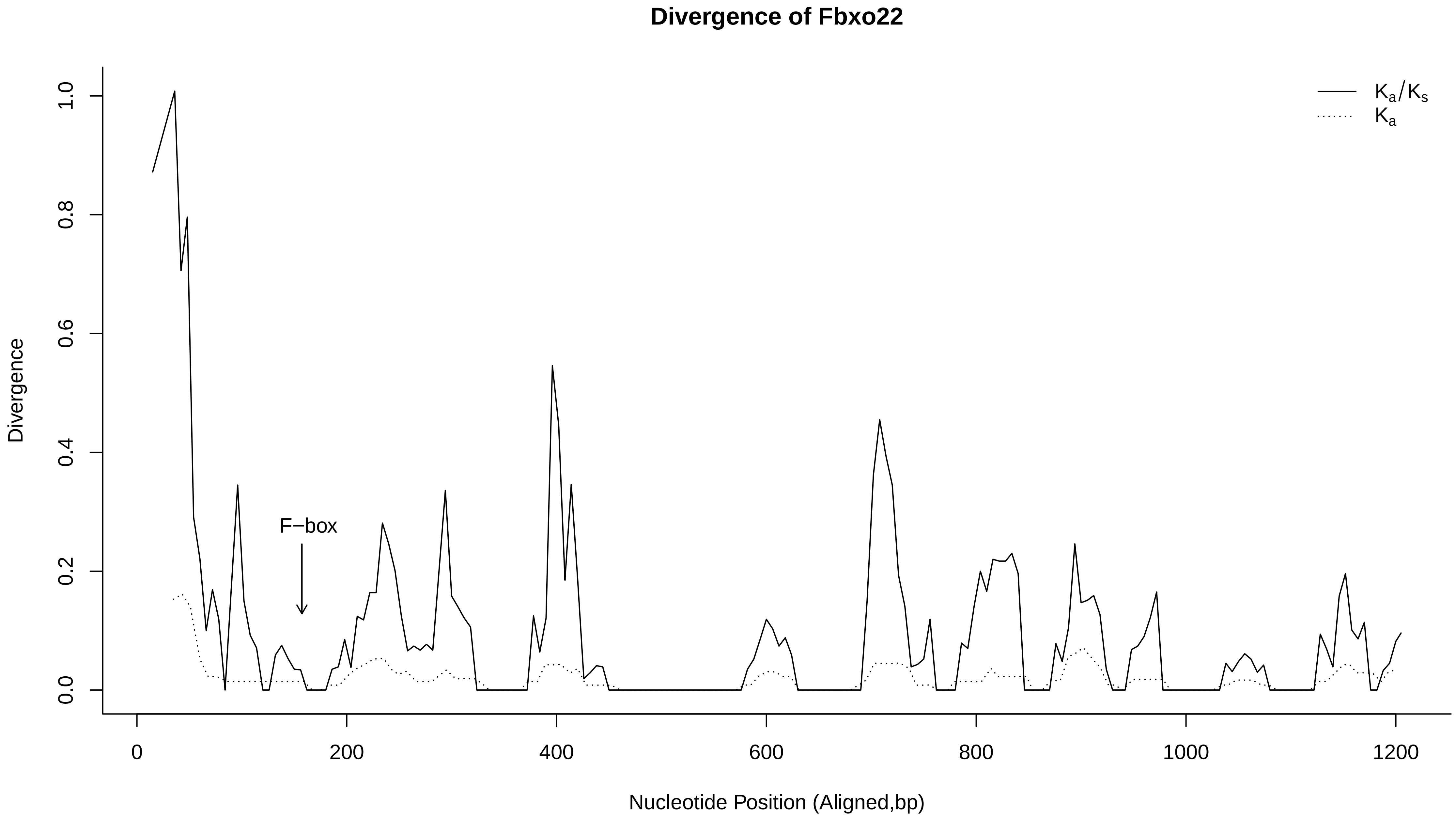

Divergence of Fbxo22
Divergence
Nucleotide Position (Aligned,bp)
F−box
$K_a/K_s$
$K_a$
0.0
0.2
0.4
0.6
0.8
1.0
0
200
400
600
800
1000
1200

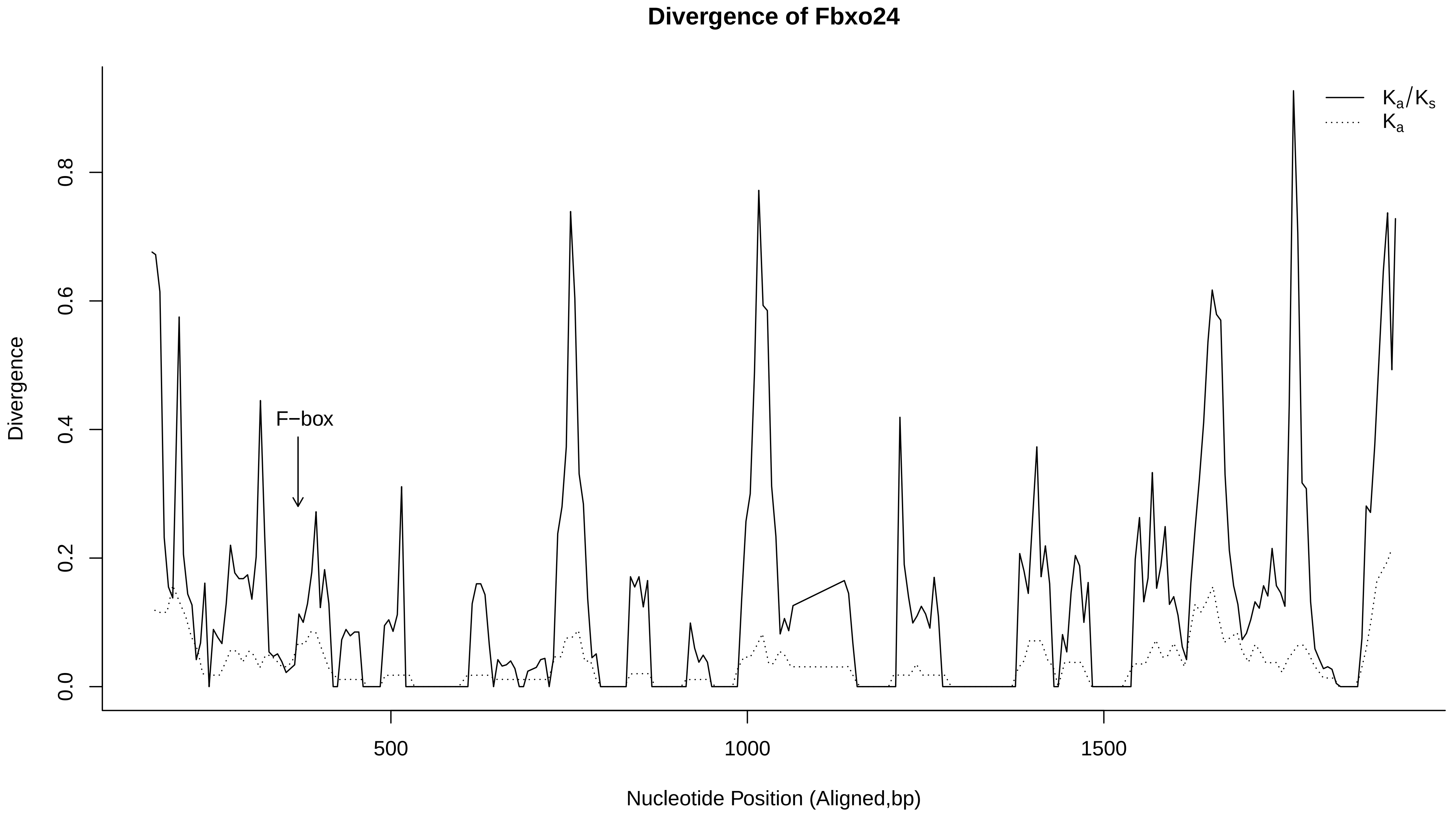

Divergence of Fbxo24
Divergence
Nucleotide Position (Aligned,bp)
0.0
0.2
0.4
0.6
0.8
500
1000
1500
F−box
$K_a/K_s$
$K_a$

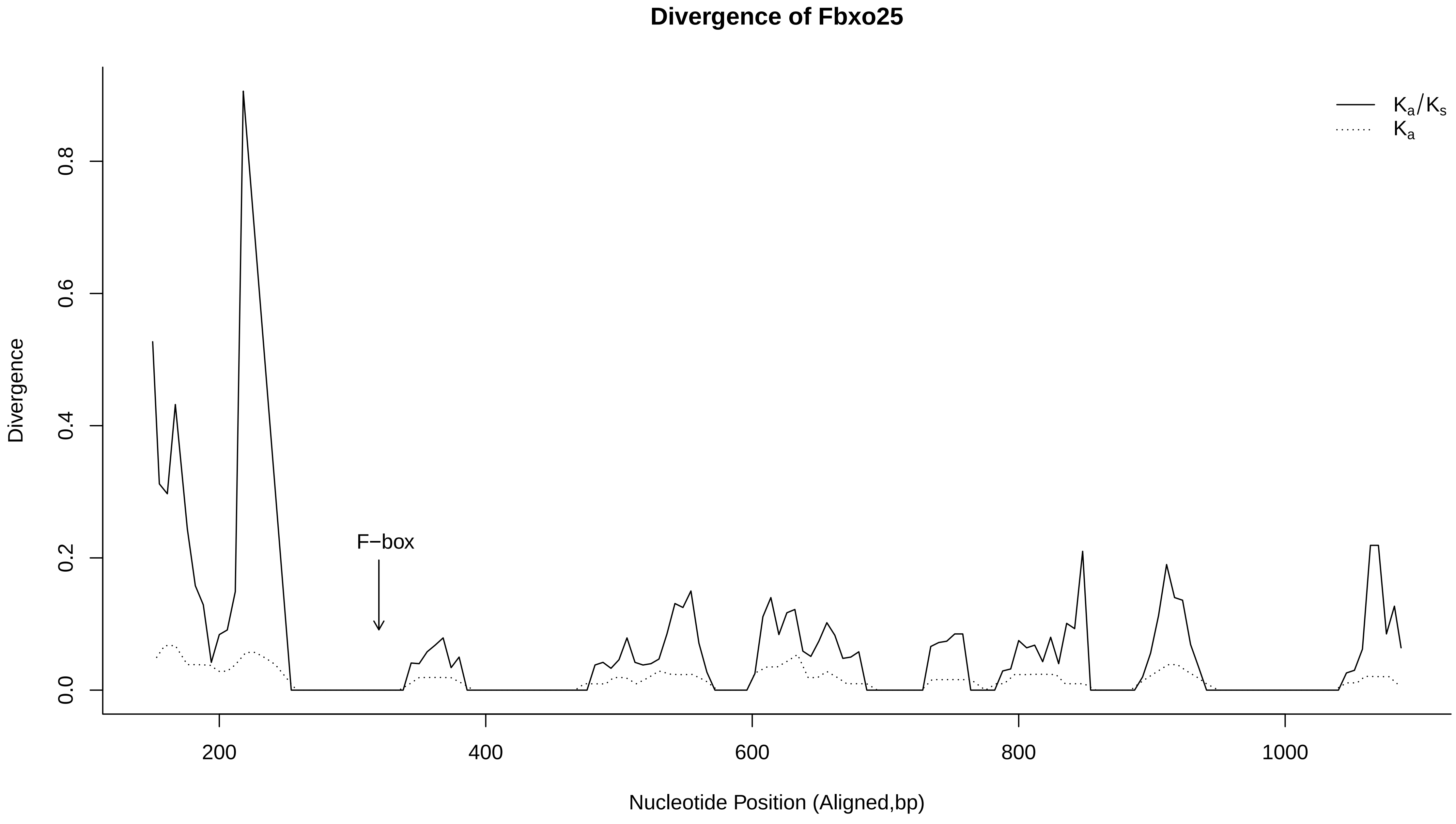
Divergence of Fbxo25
Divergence
0.0
0.2
0.4
0.6
0.8
200
400
600
800
1000
Nucleotide Position (Aligned,bp)
F−box
$K_a/K_s$
$K_a$

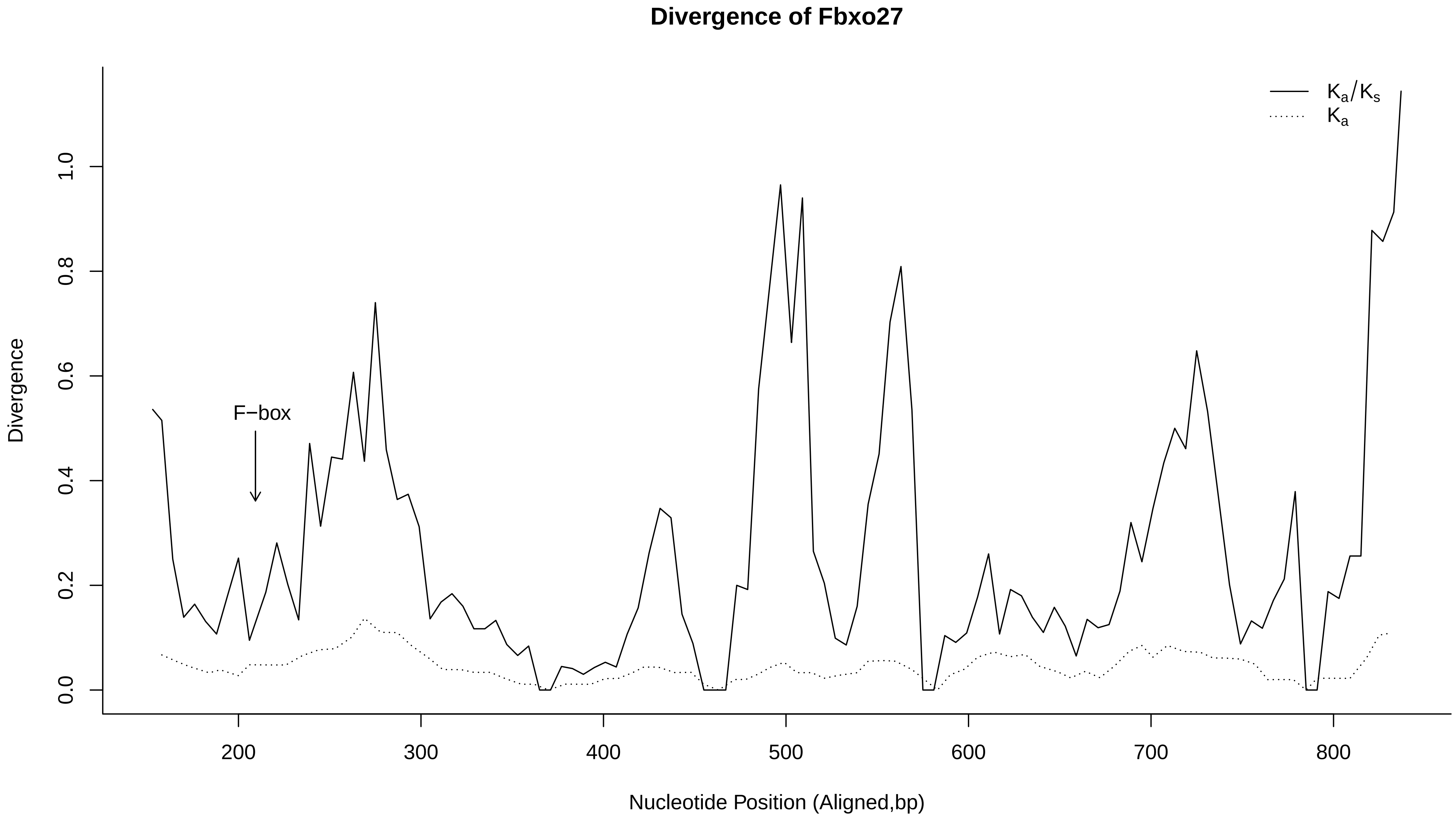
Divergence of Fbxo27
Divergence
Nucleotide Position (Aligned,bp)
$K_a/K_s$
$K_a$
F−box
0.0
0.2
0.4
0.6
0.8
1.0
200
300
400
500
600
700
800

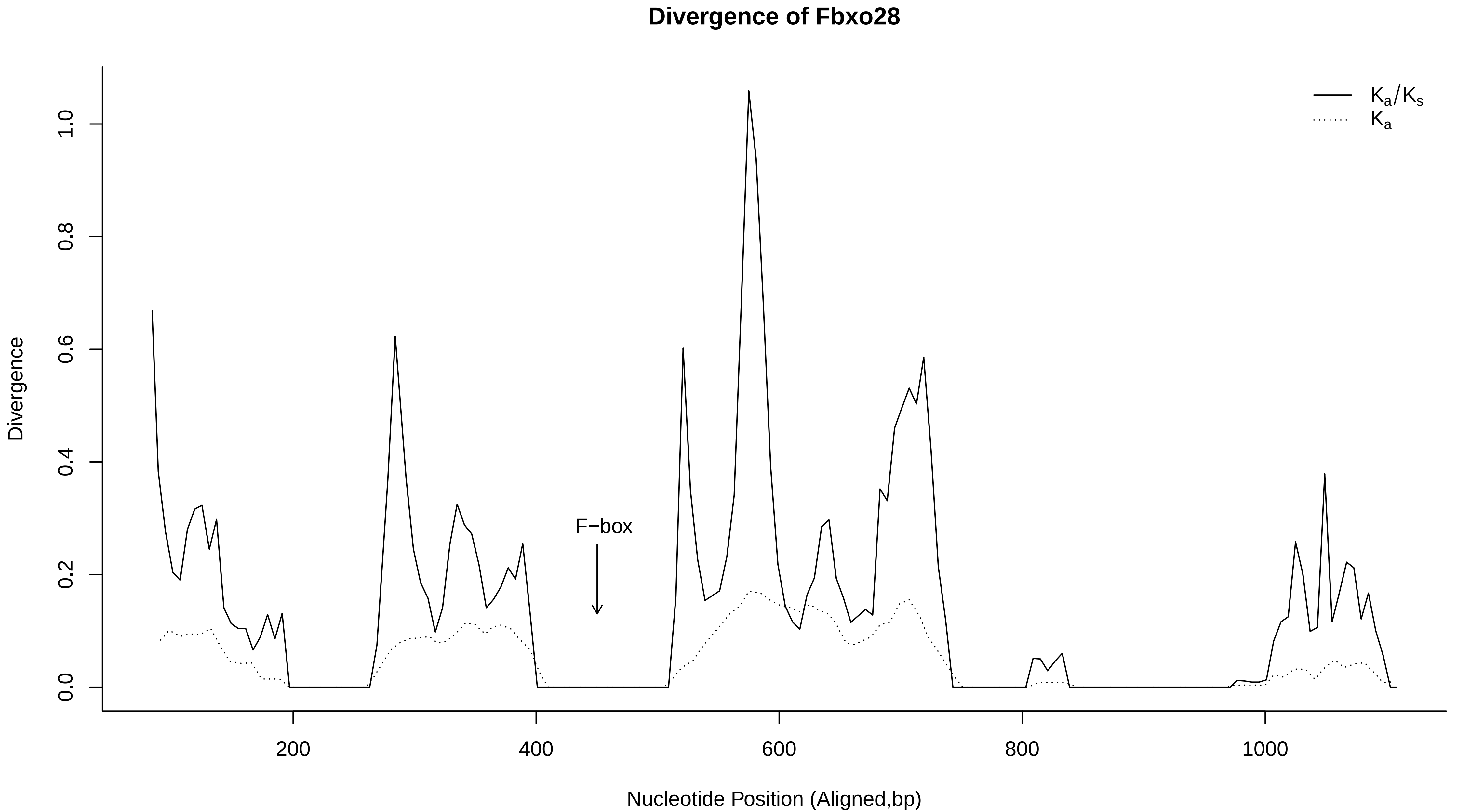

Divergence of Fbxo28
Divergence
Nucleotide Position (Aligned,bp)
$K_a/K_s$
$K_a$
F−box
0.0
0.2
0.4
0.6
0.8
1.0
200
400
600
800
1000

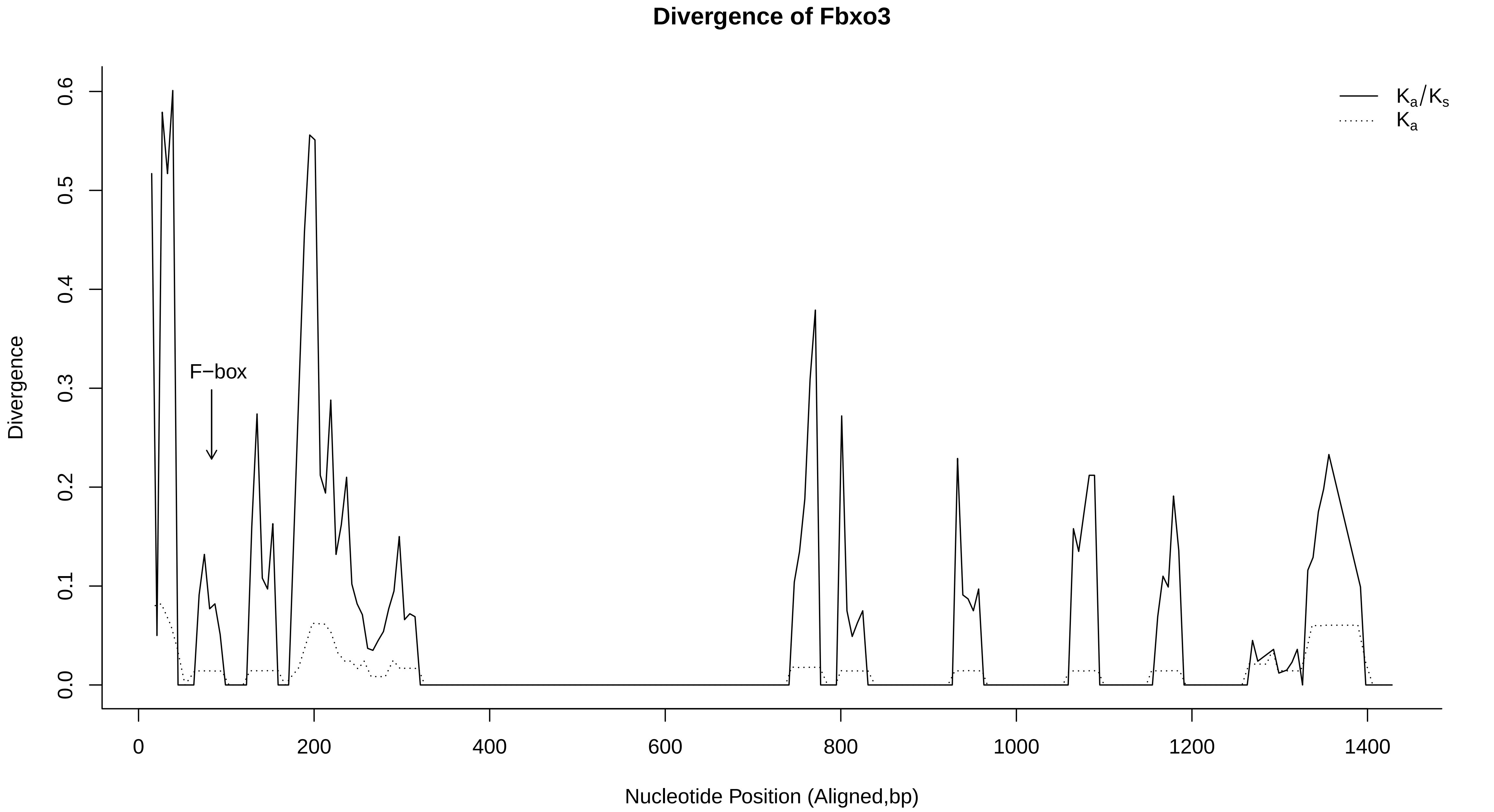

Divergence of Fbxo3
Divergence
Nucleotide Position (Aligned,bp)
F−box
$K_a/K_s$
$K_a$
0.0
0.1
0.2
0.3
0.4
0.5
0.6
0
200
400
600
800
1000
1200
1400

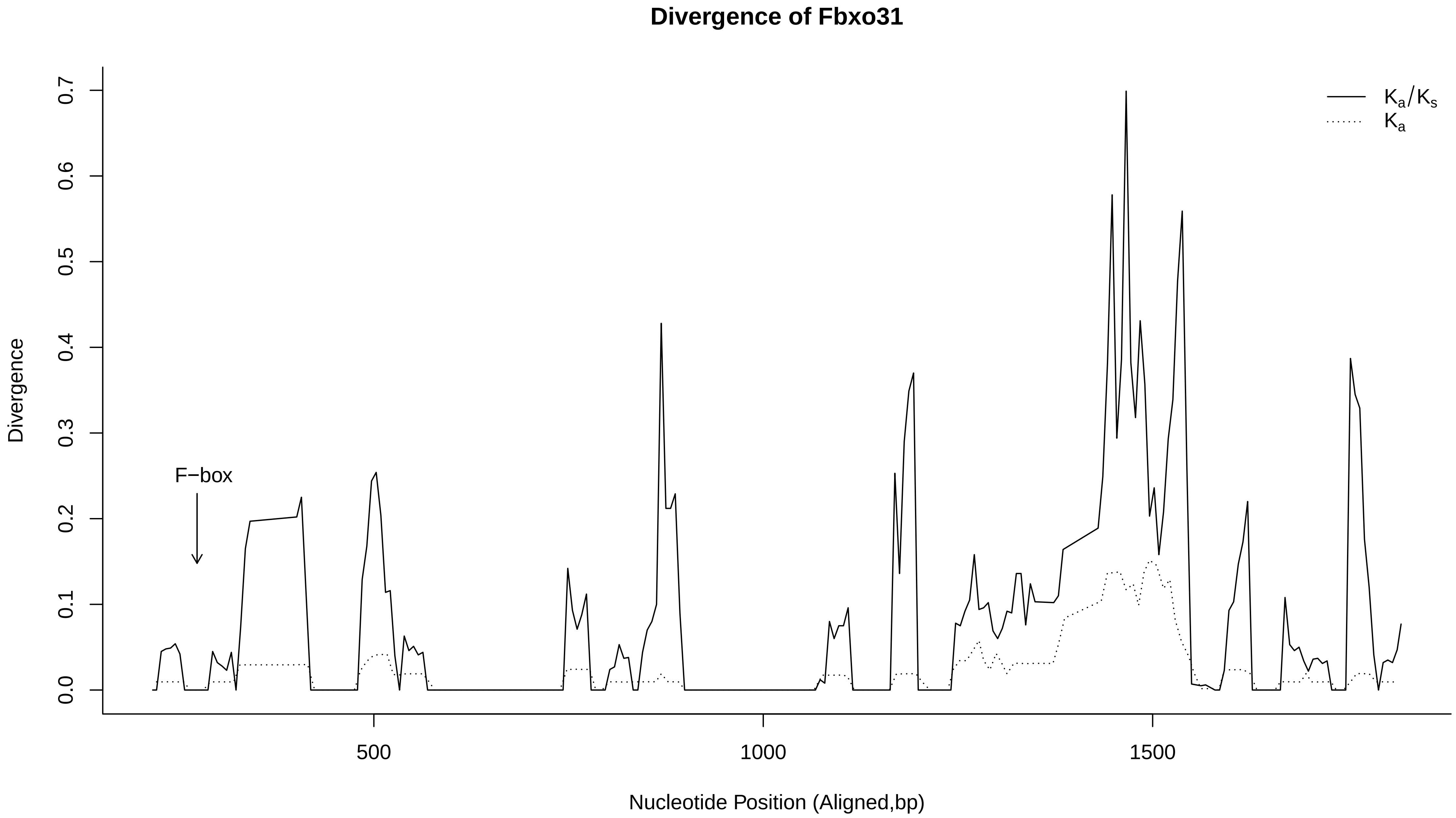

Divergence of Fbxo31
Divergence
Nucleotide Position (Aligned,bp)
0.0
0.1
0.2
0.3
0.4
0.5
0.6
0.7
500
1000
1500
F−box
$K_a/K_s$
$K_a$

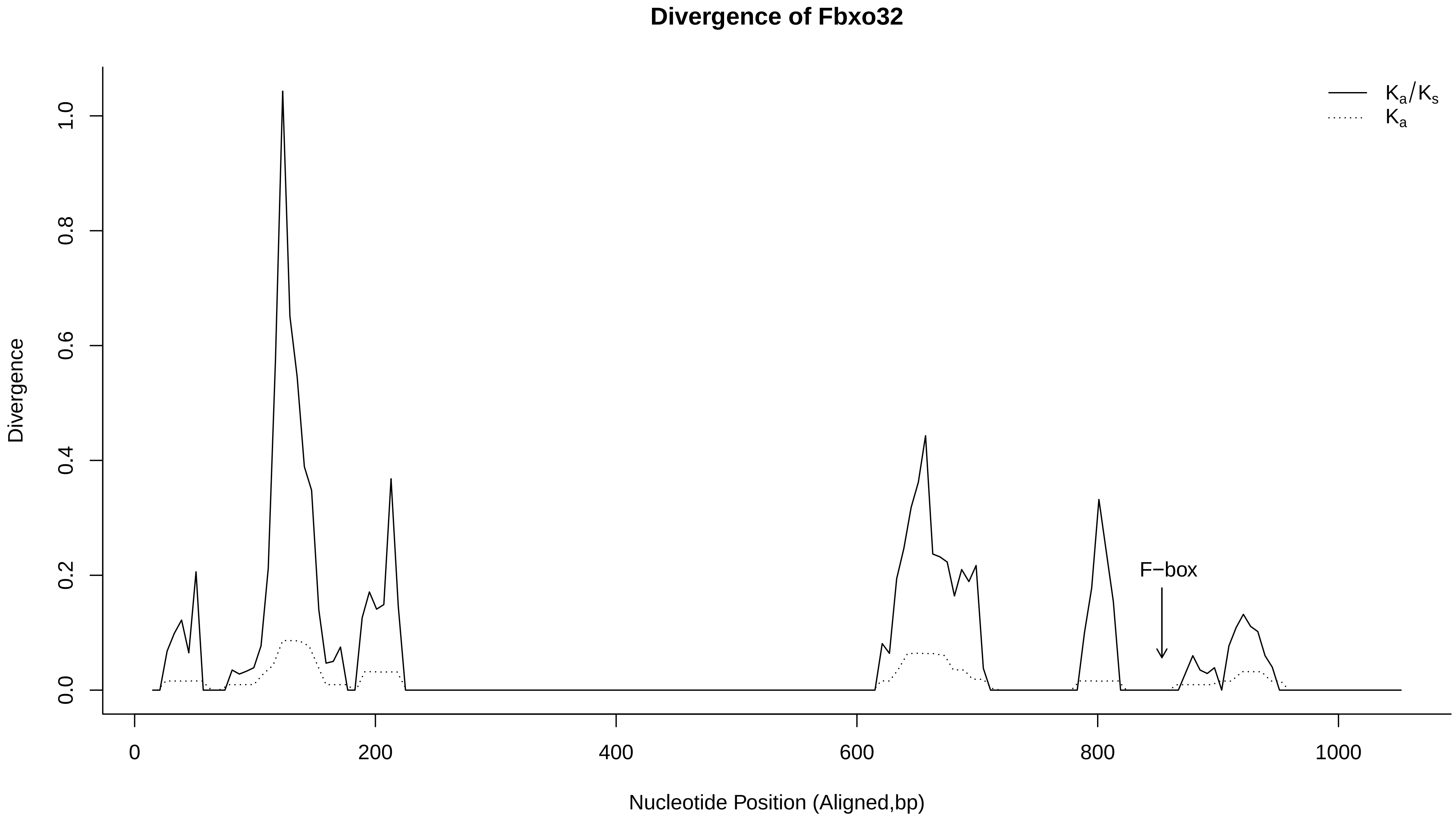

Divergence of Fbxo32
Divergence
Nucleotide Position (Aligned,bp)
$K_a/K_s$
$K_a$
F−box
0.0
0.2
0.4
0.6
0.8
1.0
0
200
400
600
800
1000

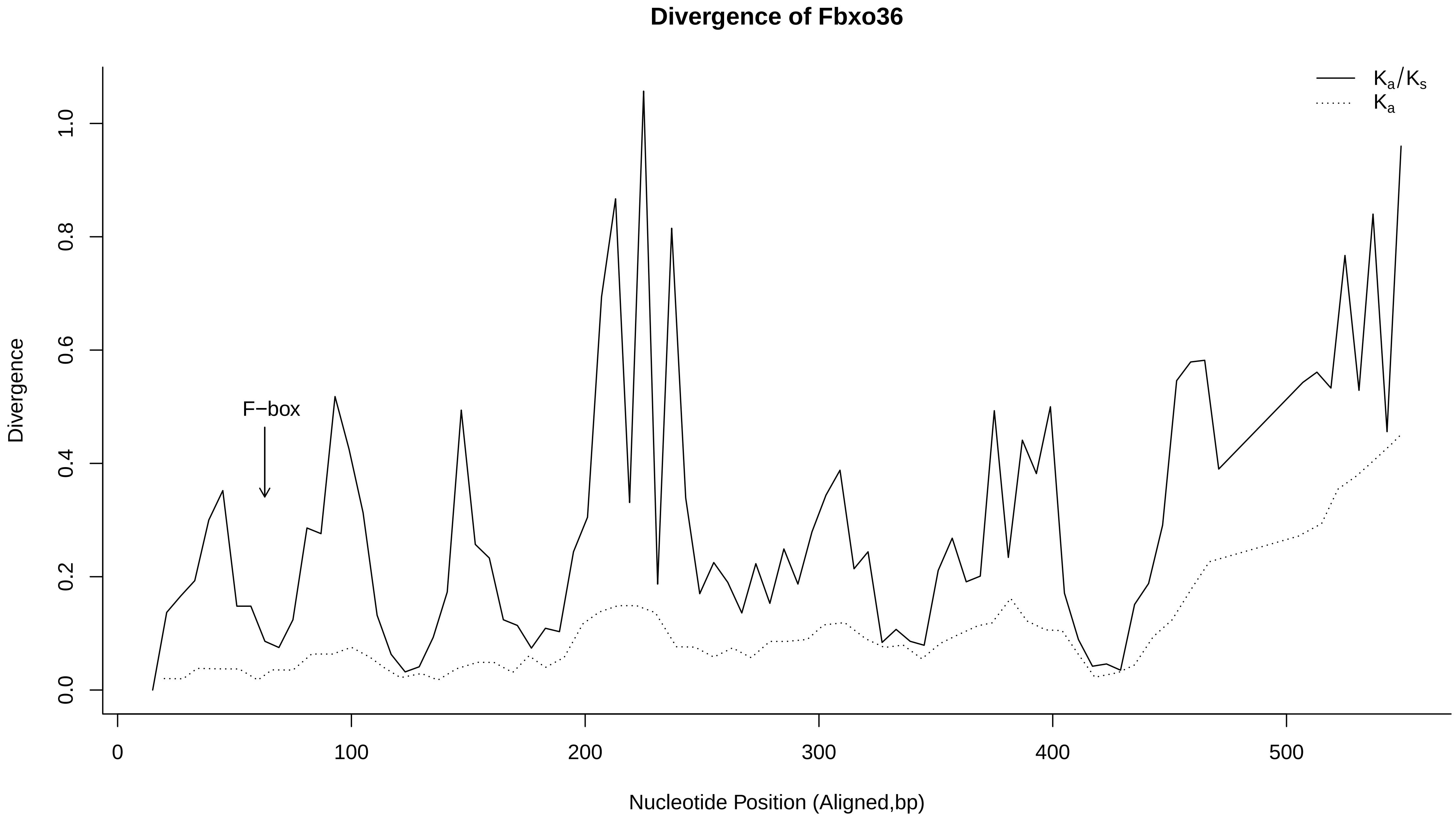

Divergence of Fbxo36
Ka/Ks
Ka
F−box
Divergence
0.0
0.2
0.4
0.6
0.8
1.0
0
100
200
300
400
500
Nucleotide Position (Aligned,bp)

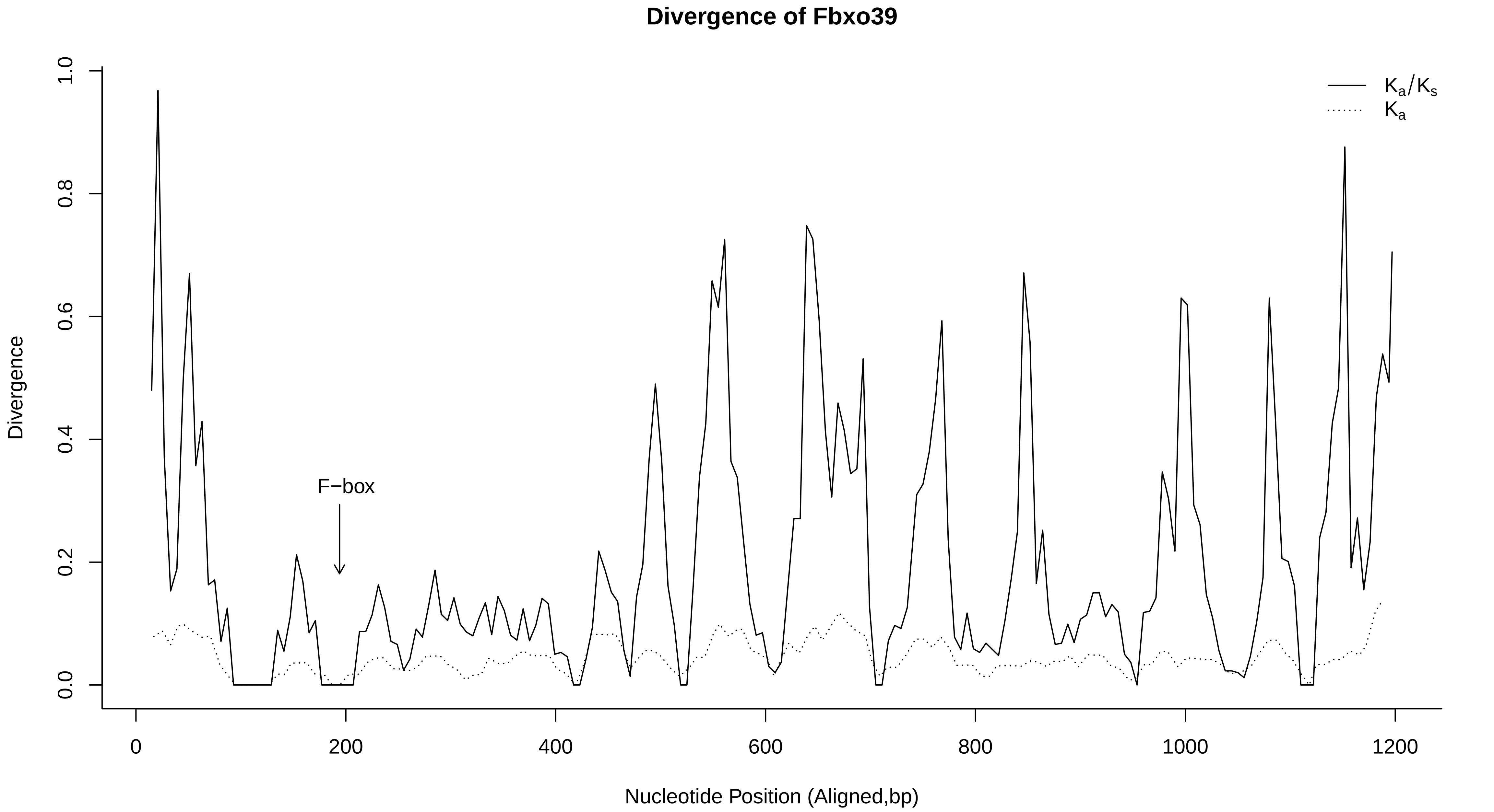

Divergence of Fbxo39
$K_a/K_s$
$K_a$
F−box
Divergence
Nucleotide Position (Aligned,bp)
0.0
0.2
0.4
0.6
0.8
1.0
0
200
400
600
800
1000
1200

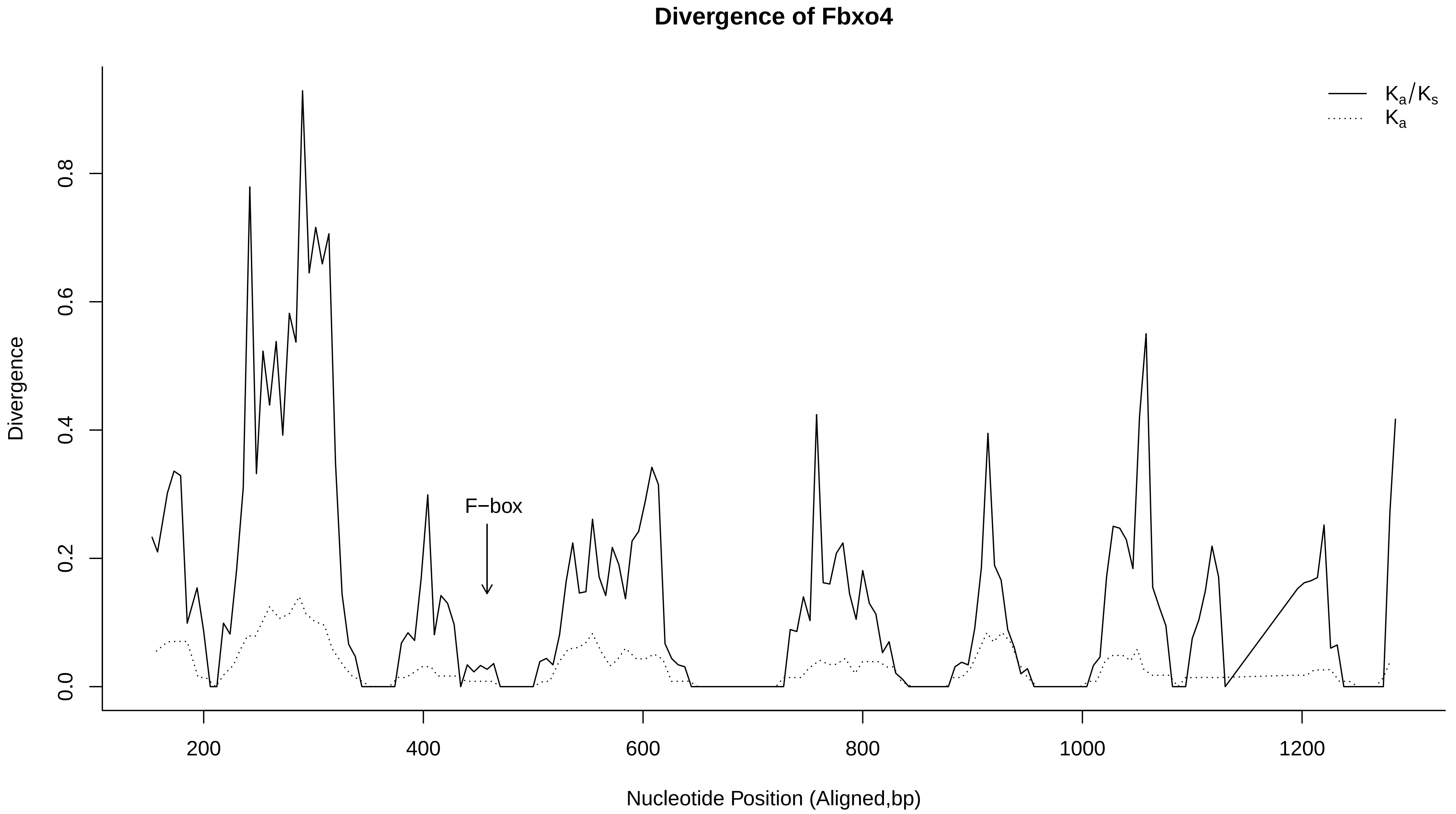

Divergence of Fbxo4
$K_a/K_s$
$K_a$
F−box
Divergence
0.0
0.2
0.4
0.6
0.8
200
400
600
800
1000
1200
Nucleotide Position (Aligned,bp)

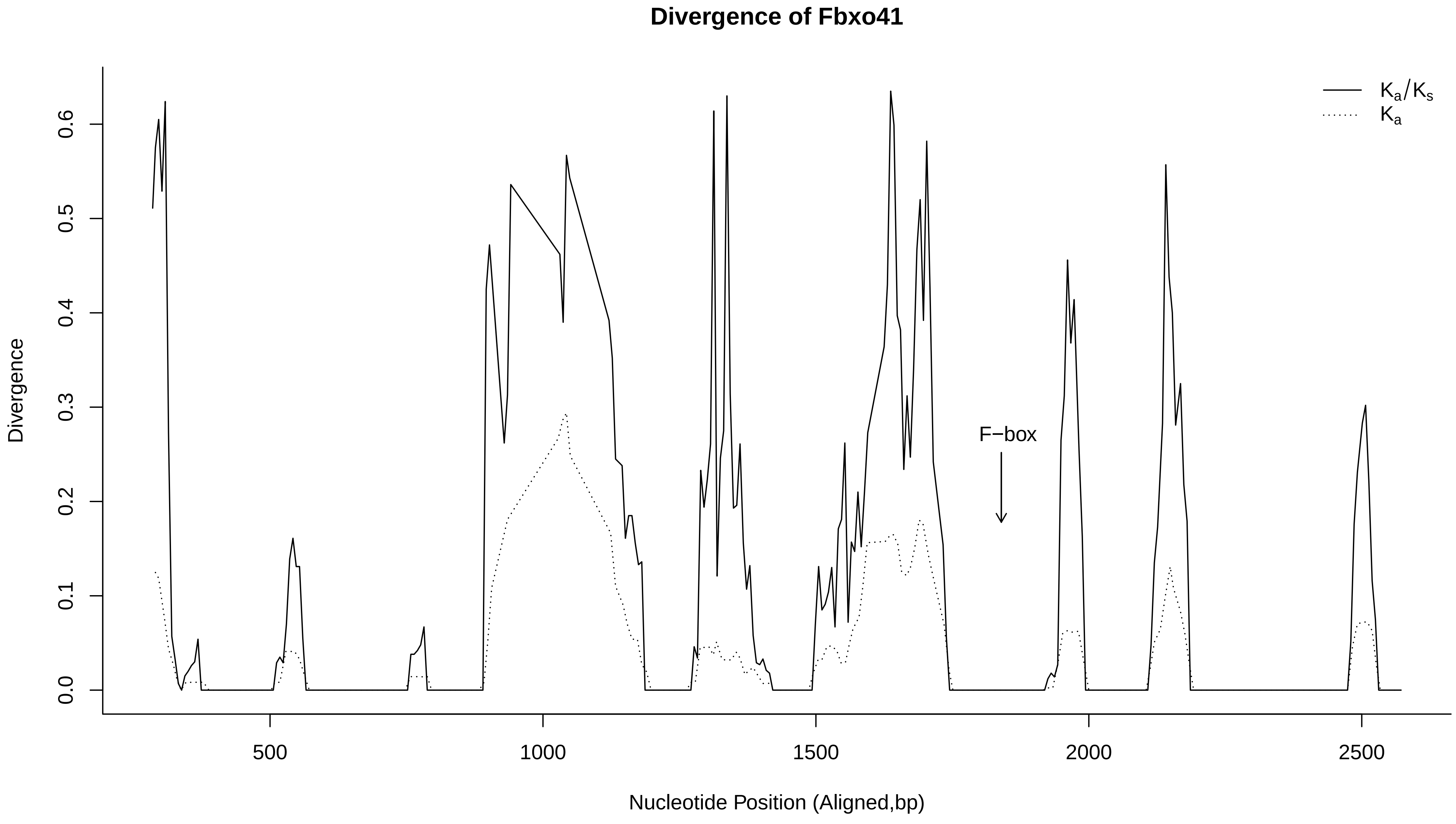

Divergence of Fbxo41
$K_a/K_s$
$K_a$
F−box
Divergence
0.0
0.1
0.2
0.3
0.4
0.5
0.6
500
1000
1500
2000
2500
Nucleotide Position (Aligned,bp)

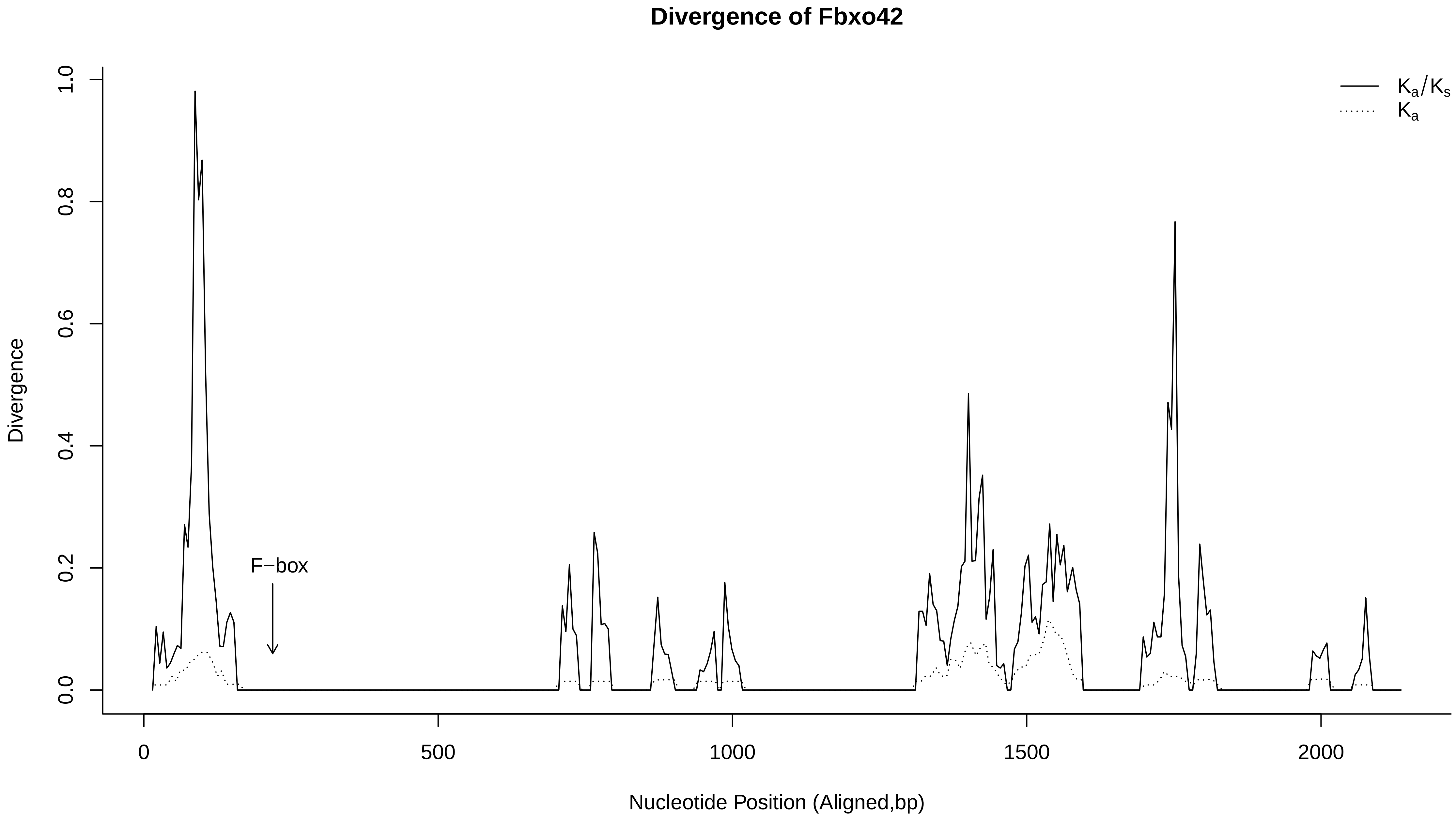

Divergence of Fbxo42
Ka/Ks
Ka
F−box
Divergence
1.0
0.8
0.6
0.4
0.2
0.0
0
500
1000
1500
2000
Nucleotide Position (Aligned,bp)

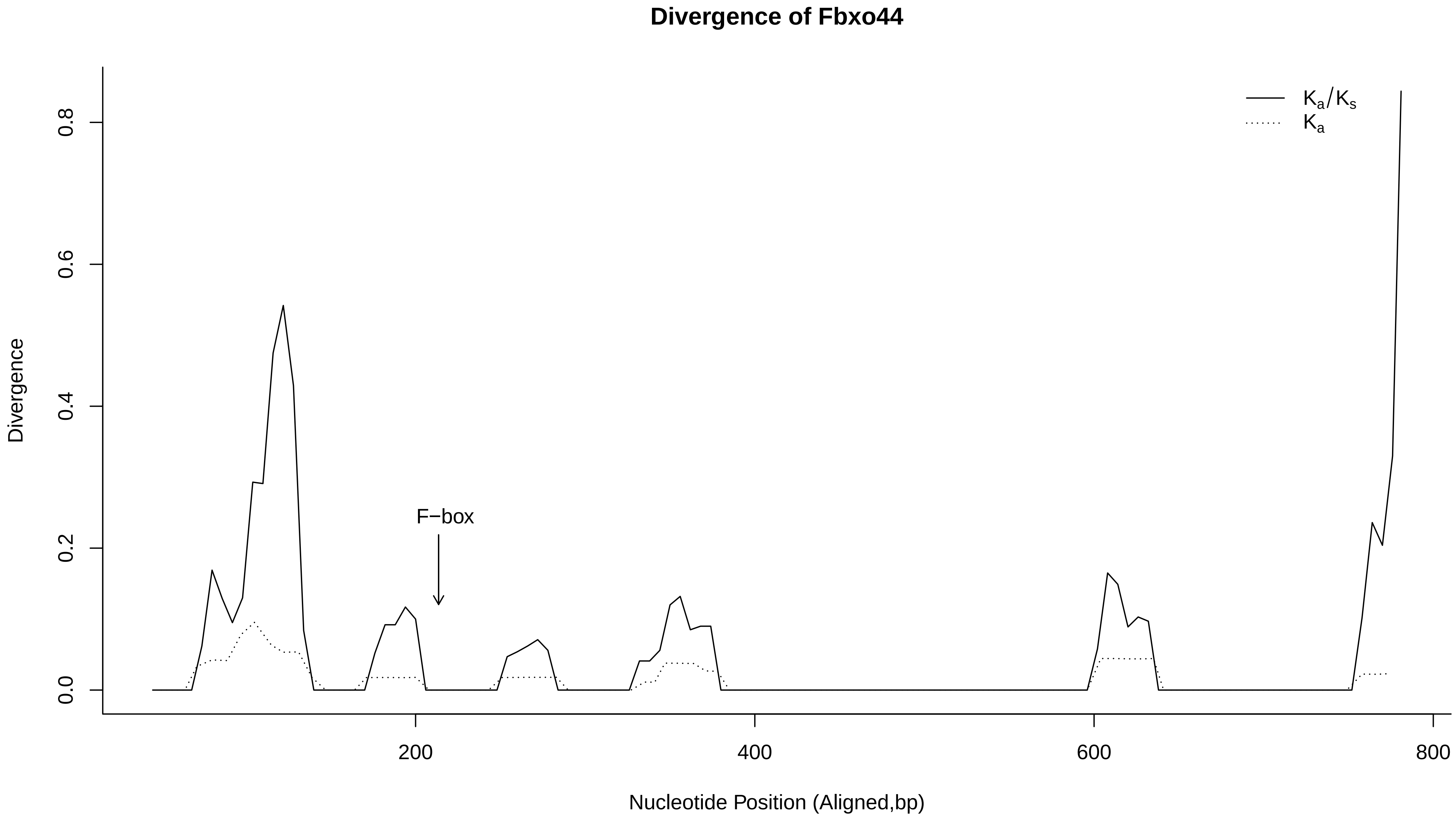

Divergence of Fbxo44
Divergence
0.0
0.2
0.4
0.6
0.8
F−box
200
400
600
800
Nucleotide Position (Aligned,bp)
$K_a/K_s$
$K_a$

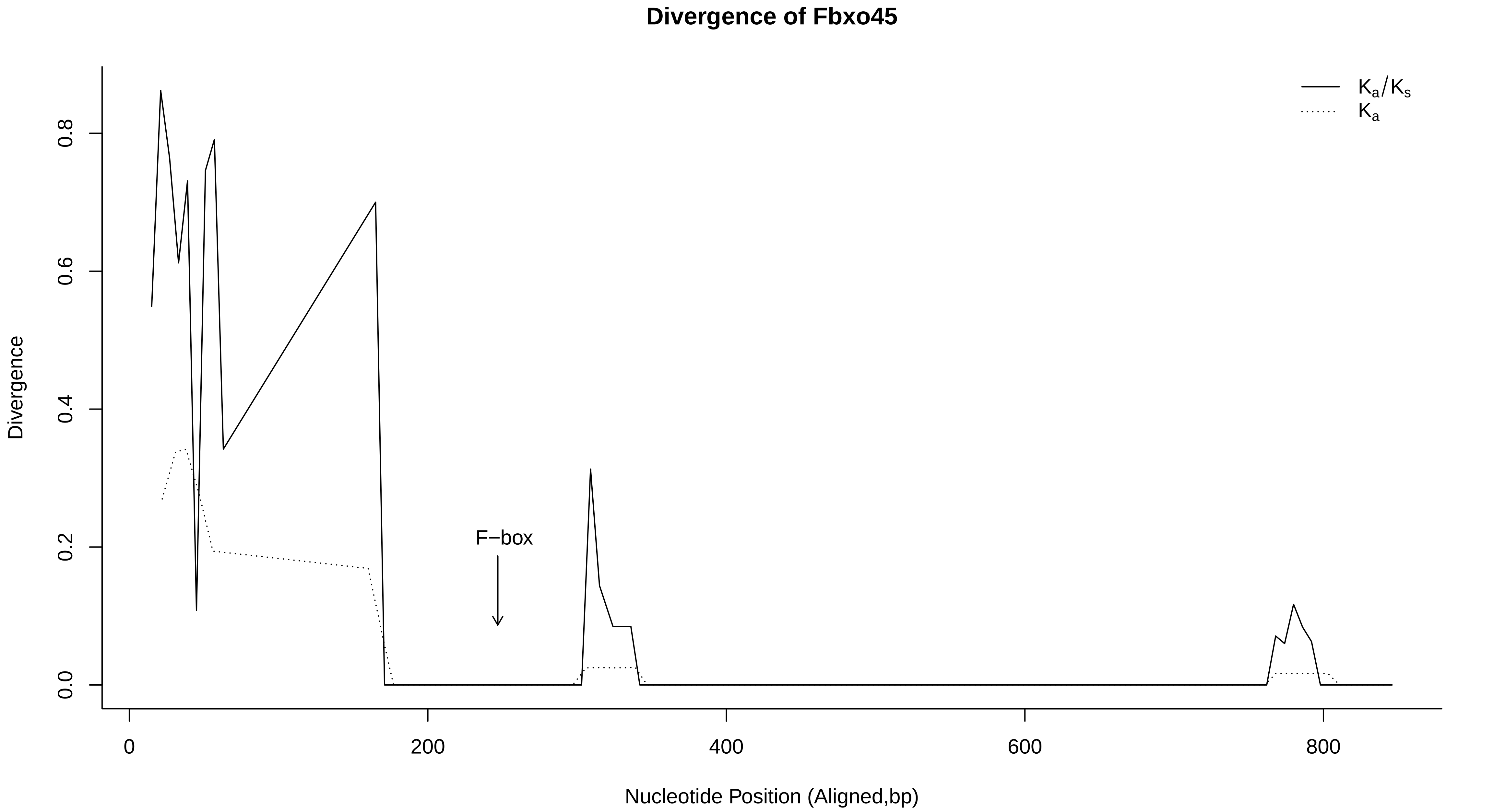
Divergence of Fbxo45
$K_a/K_s$
$K_a$
F−box
Divergence
0.0
0.2
0.4
0.6
0.8
0
200
400
600
800
Nucleotide Position (Aligned,bp)

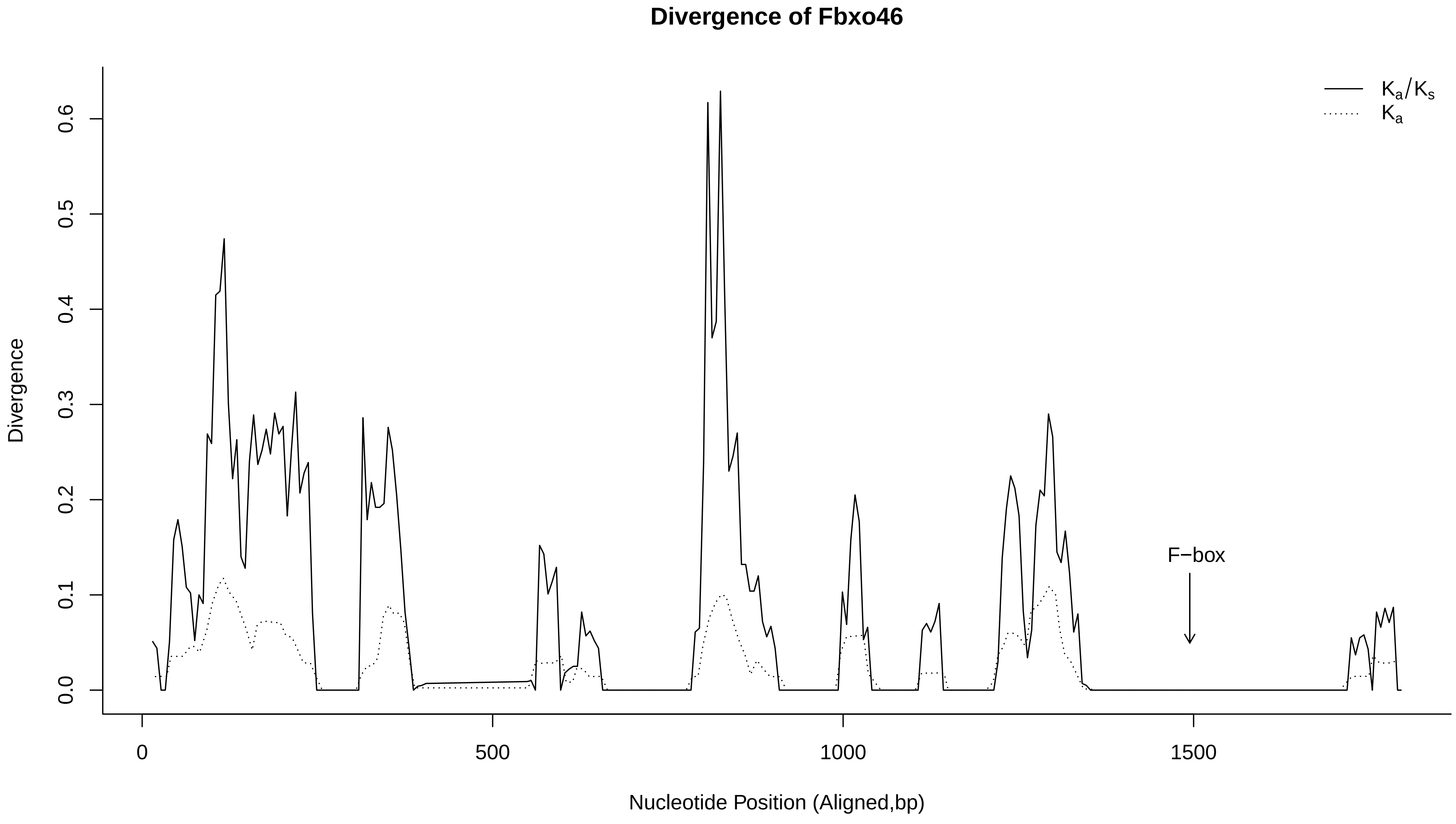

Divergence of Fbxo46
Divergence
Nucleotide Position (Aligned,bp)
0.0
0.1
0.2
0.3
0.4
0.5
0.6
0
500
1000
1500
$K_a/K_s$
$K_a$
F−box

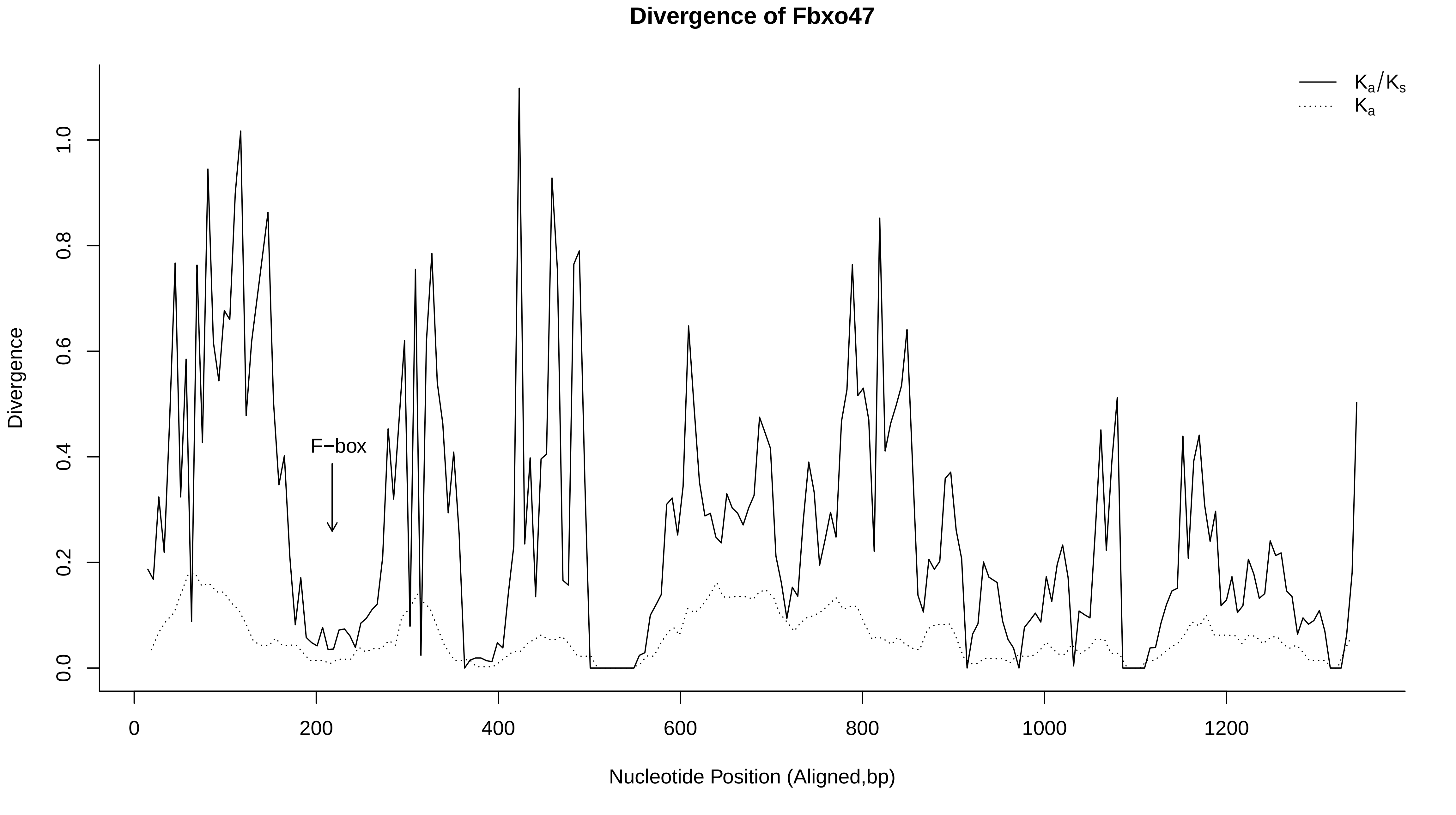

Divergence of Fbxo47
Divergence
Nucleotide Position (Aligned,bp)
$K_a/K_s$
$K_a$
F−box
0.0
0.2
0.4
0.6
0.8
1.0
0
200
400
600
800
1000
1200

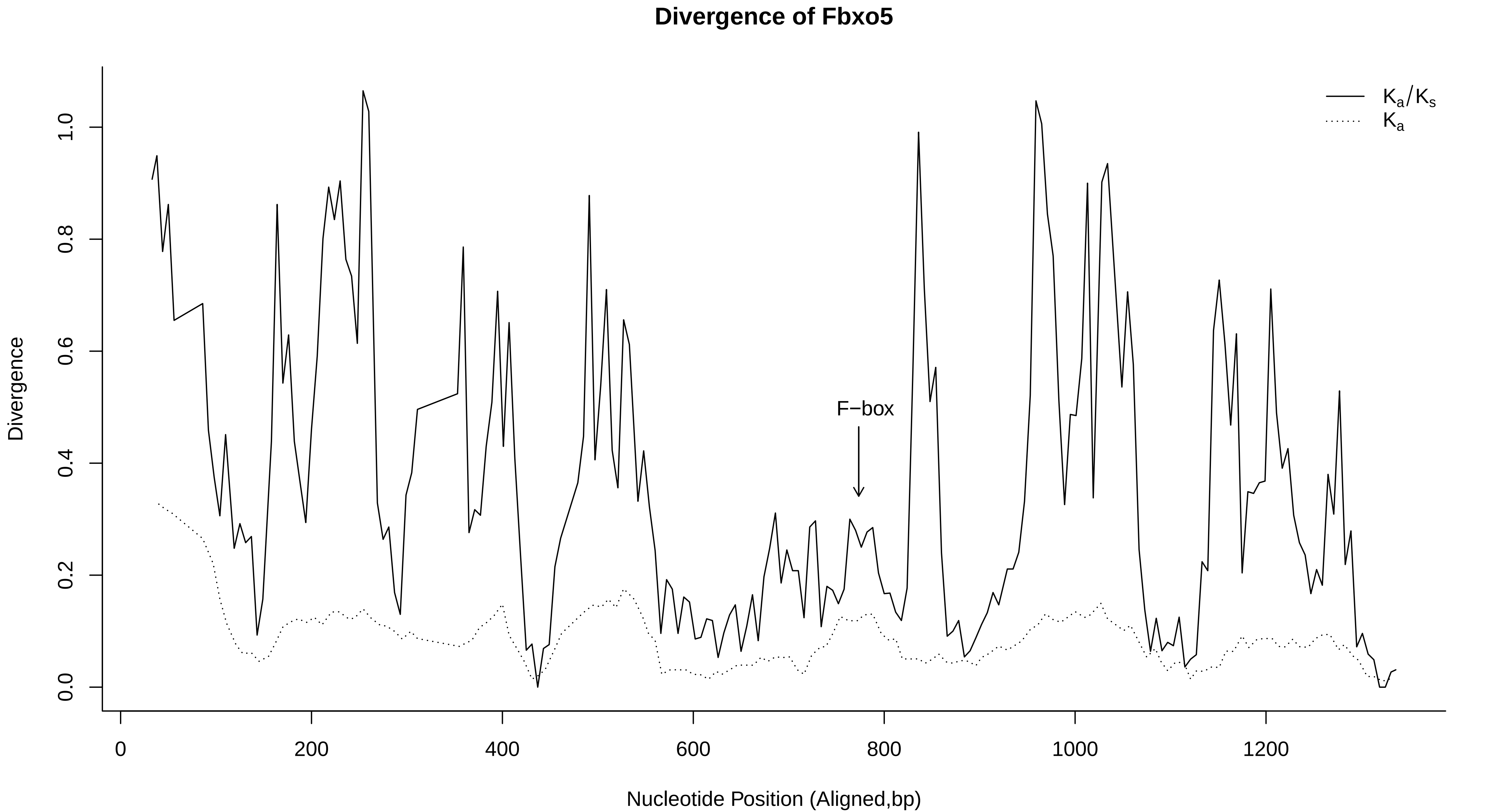

Divergence of Fbxo5
Ka/Ks
Ka
F−box
Divergence
0.0
0.2
0.4
0.6
0.8
1.0
0
200
400
600
800
1000
1200
Nucleotide Position (Aligned,bp)

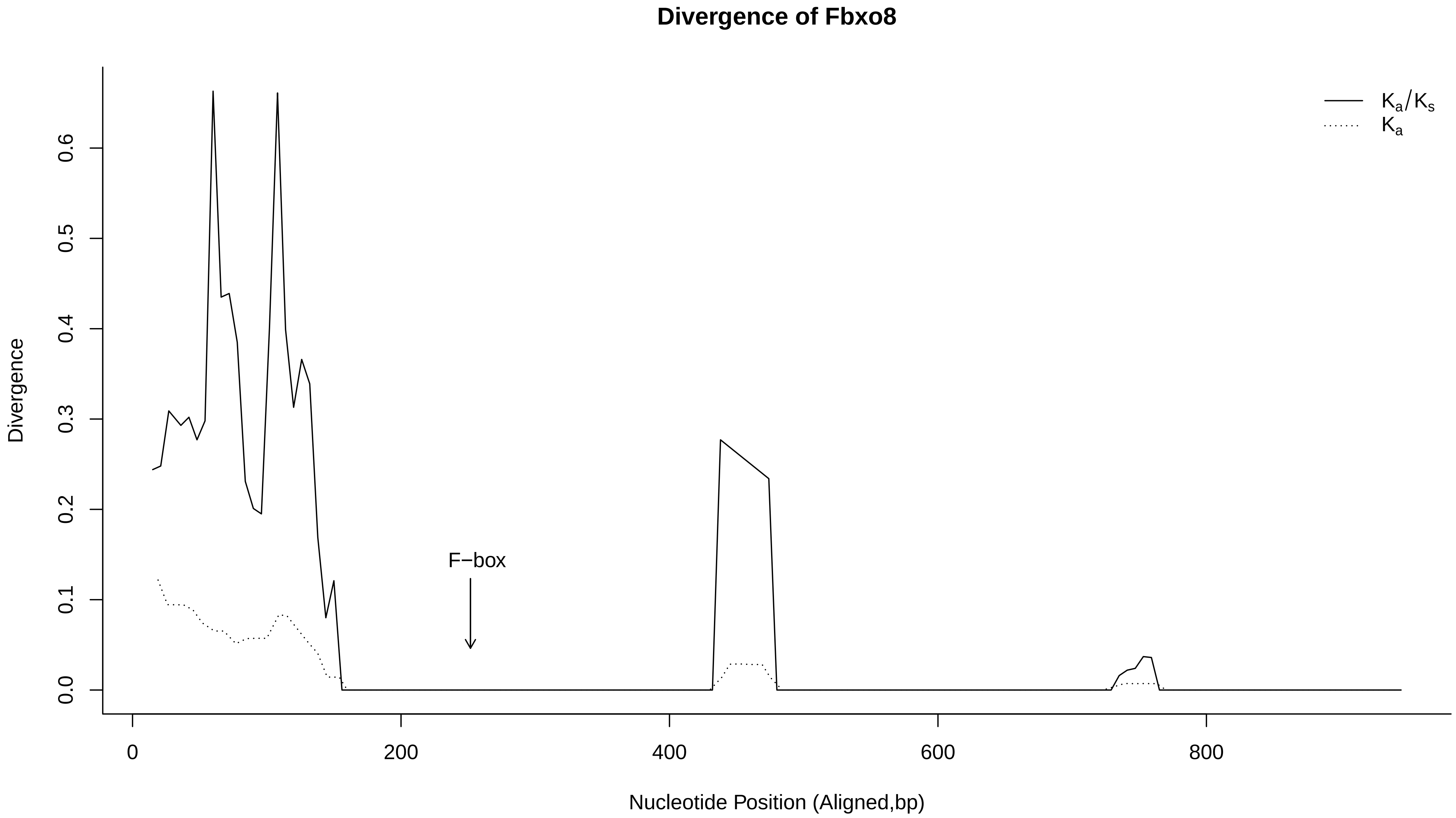
Divergence of Fbxo8
Divergence
Nucleotide Position (Aligned,bp)
0.0
0.1
0.2
0.3
0.4
0.5
0.6
0
200
400
600
800
$K_a/K_s$
$K_a$
F−box

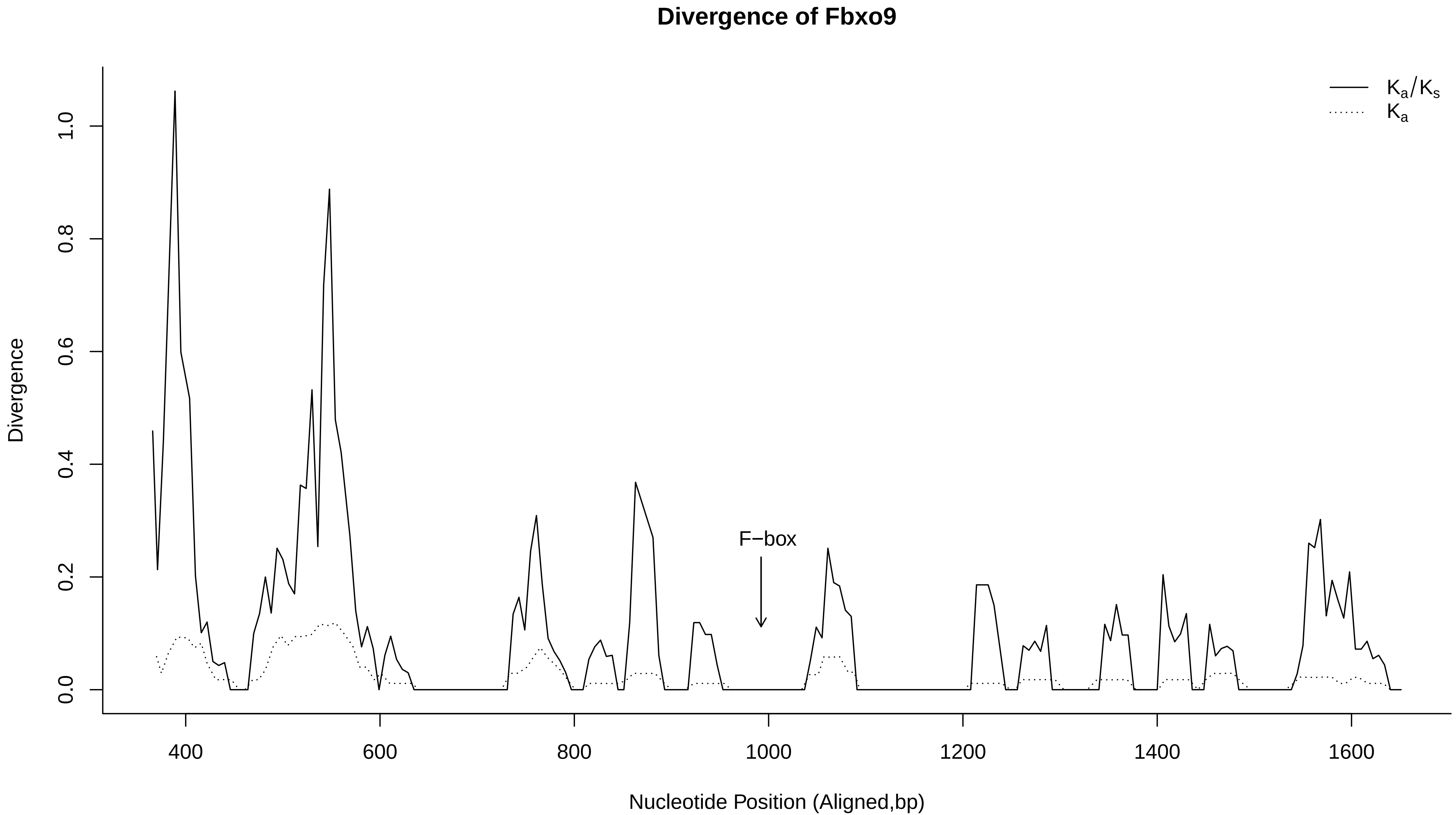

Divergence of Fbxo9
$K_a/K_s$
$K_a$
F−box
Divergence
0.0
0.2
0.4
0.6
0.8
1.0
400
600
800
1000
1200
1400
1600
Nucleotide Position (Aligned,bp)

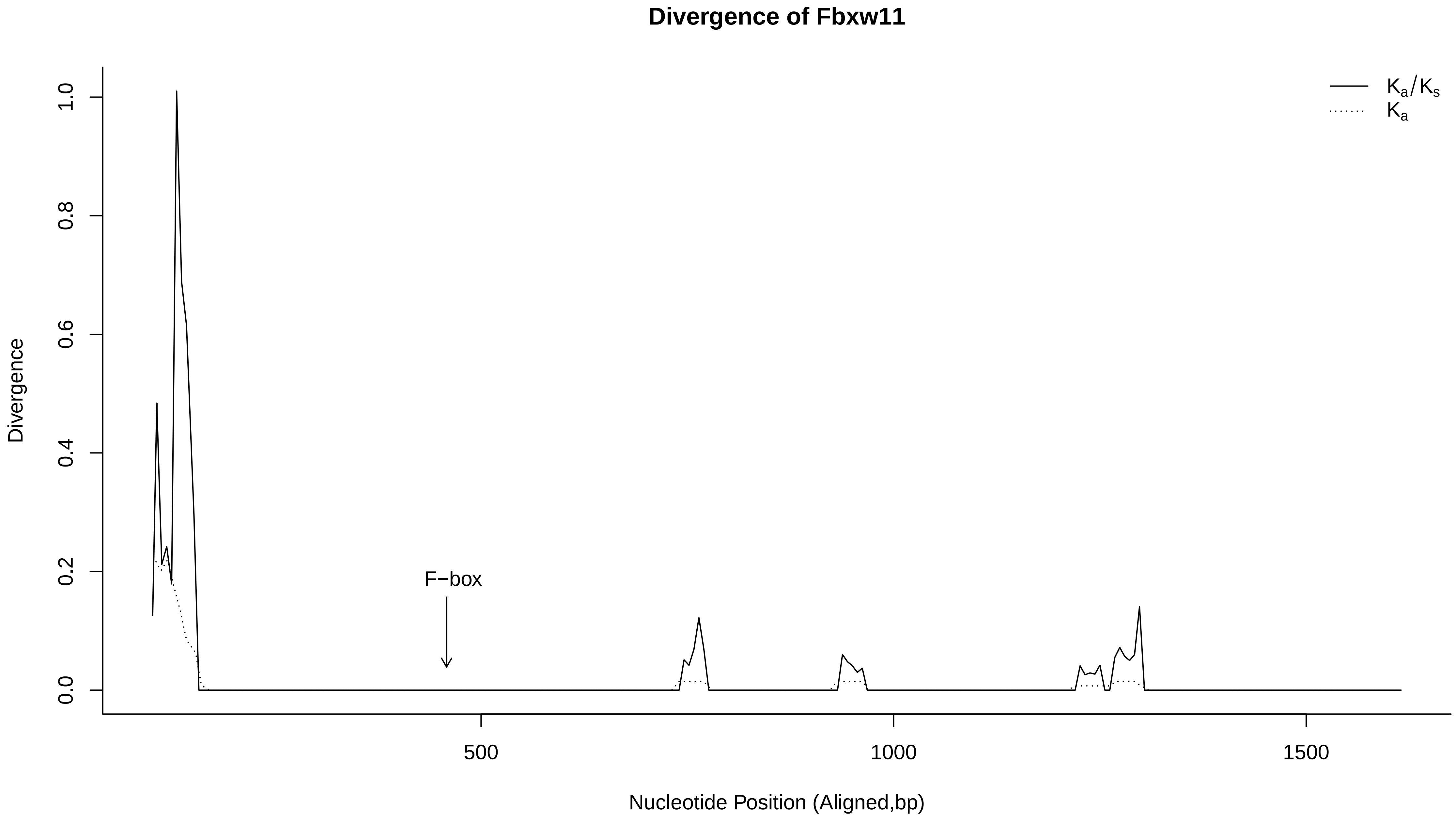

Divergence of Fbxw11
Divergence
Nucleotide Position (Aligned,bp)
0.0
0.2
0.4
0.6
0.8
1.0
500
1000
1500
F−box
$K_a/K_s$
$K_a$

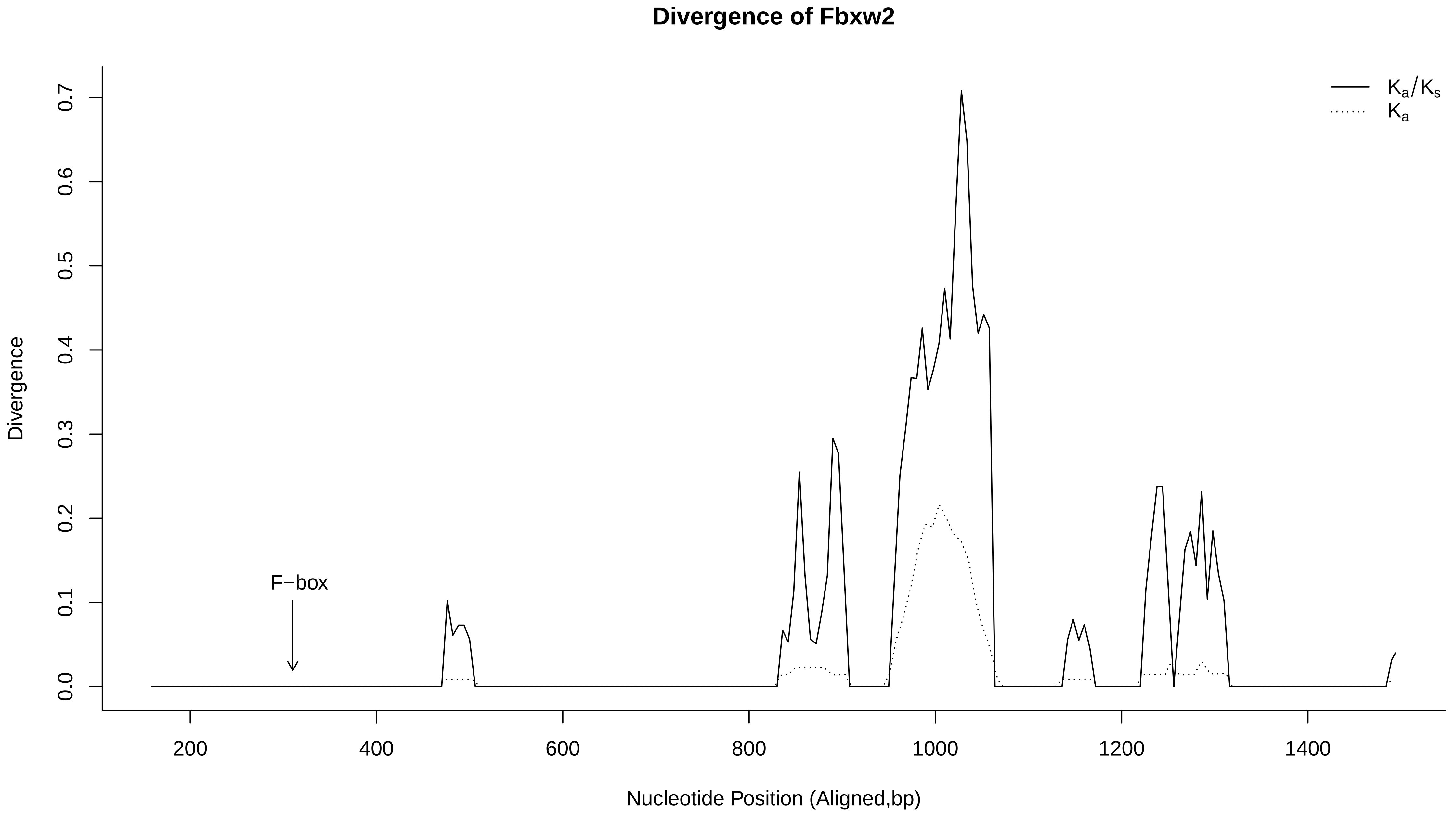

Divergence of Fbxw2
Divergence
0.0
0.1
0.2
0.3
0.4
0.5
0.6
0.7
200
400
600
800
1000
1200
1400
Nucleotide Position (Aligned,bp)
F−box
$K_a/K_s$
$K_a$

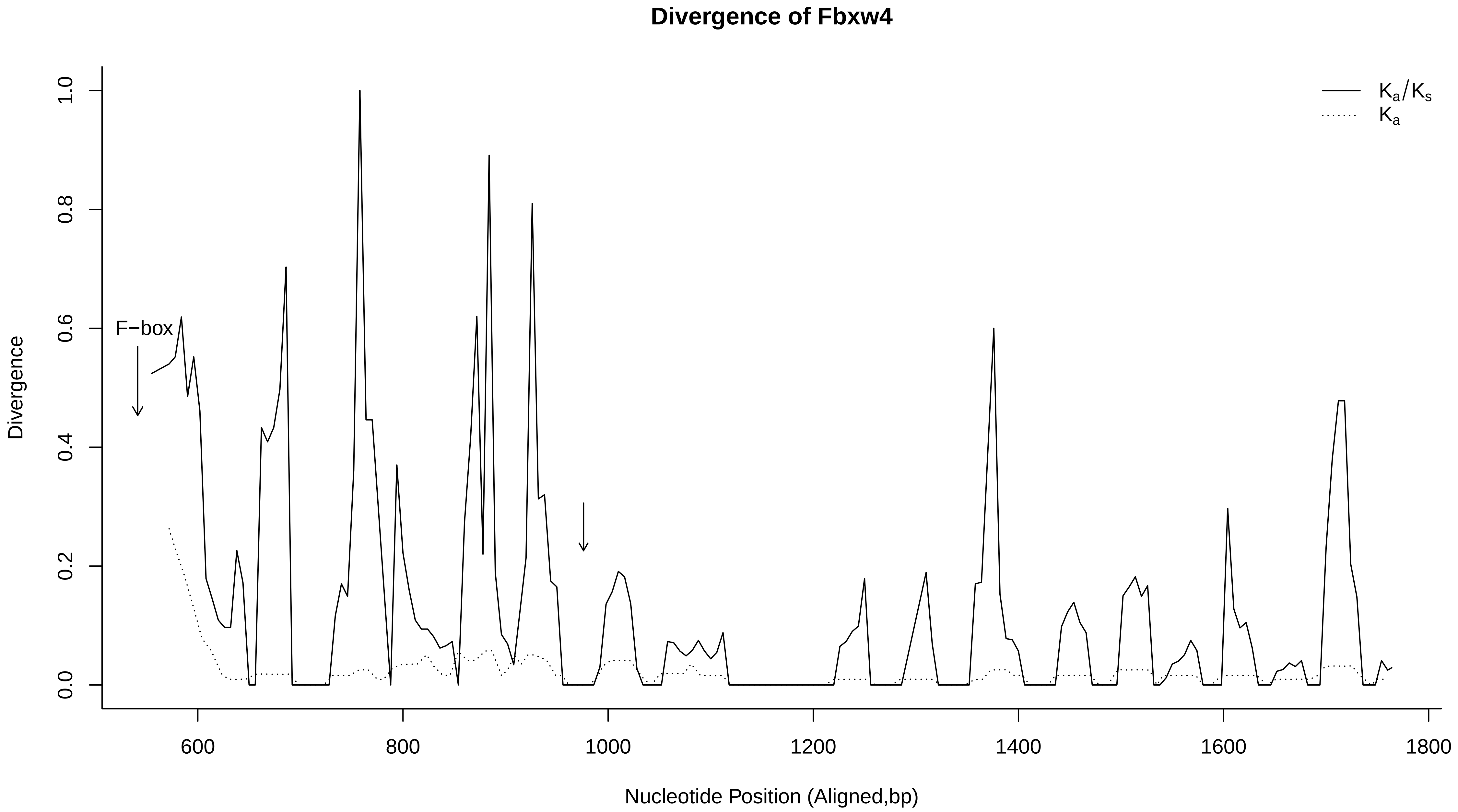
Divergence of Fbxw4
Divergence
Nucleotide Position (Aligned,bp)
F−box
$K_a/K_s$
$K_a$
0.0
0.2
0.4
0.6
0.8
1.0
600
800
1000
1200
1400
1600
1800

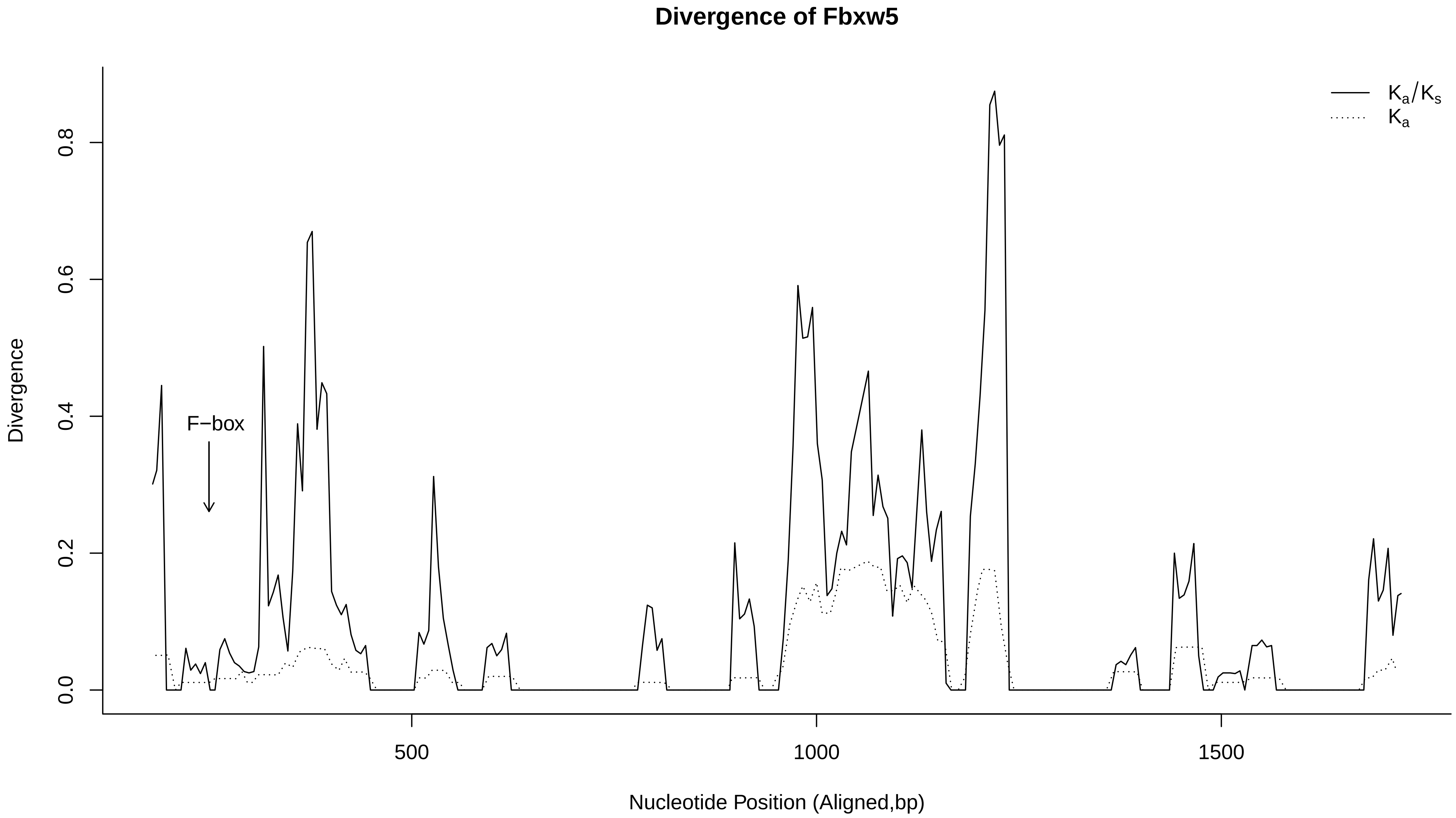

Divergence of Fbxw5
Ka/Ks
Ka
F−box
Divergence
0.0
0.2
0.4
0.6
0.8
500
1000
1500
Nucleotide Position (Aligned,bp)

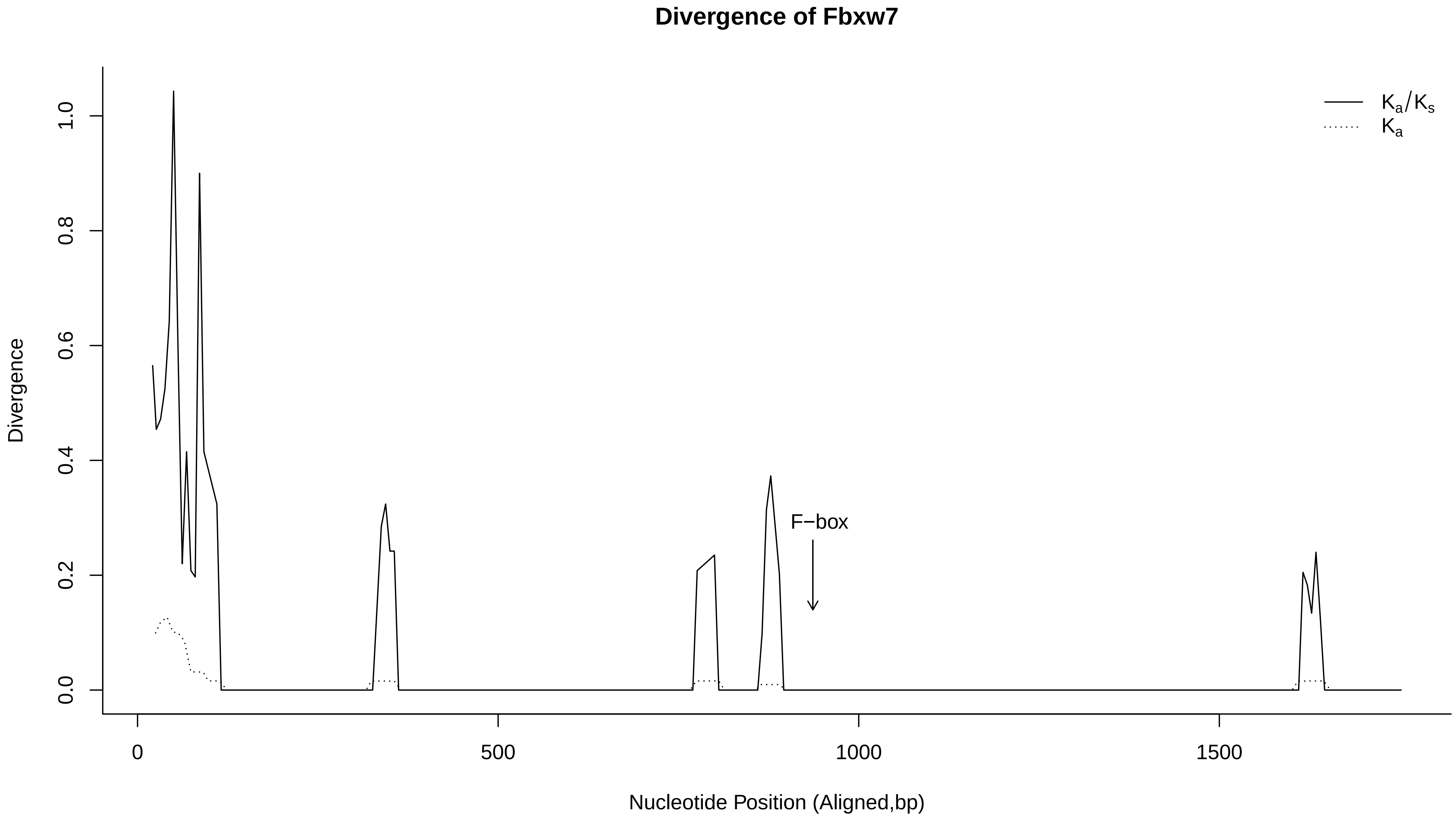

Divergence of Fbxw7
Divergence
Nucleotide Position (Aligned,bp)
0.0
0.2
0.4
0.6
0.8
1.0
0
500
1000
1500
$K_a/K_s$
$K_a$
F−box

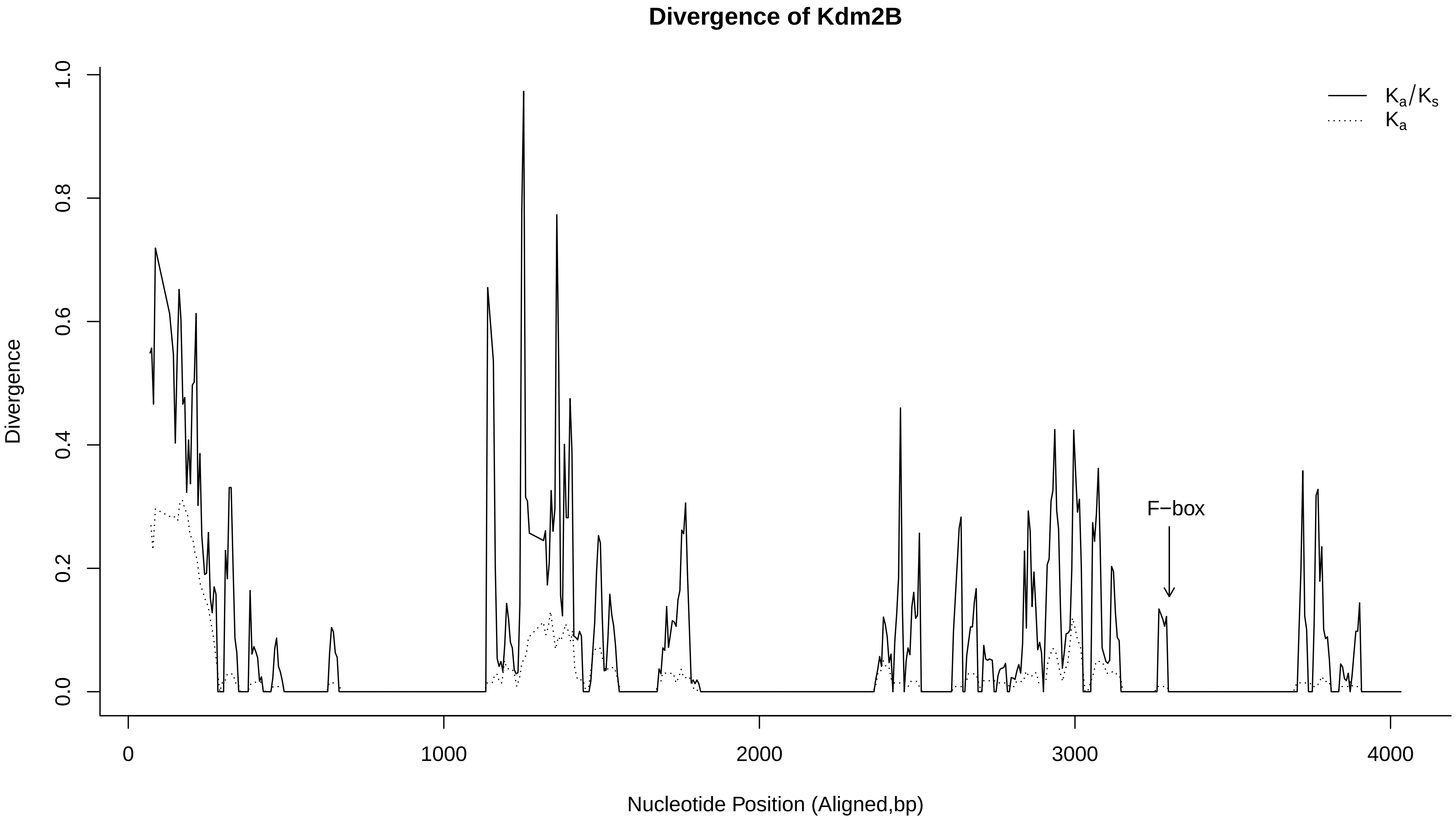

Divergence of Kdm2B
Divergence
1.0
0.8
0.6
0.4
0.2
0.0
Ka/Ks
Ka
F−box
0
1000
2000
3000
4000
Nucleotide Position (Aligned,bp)

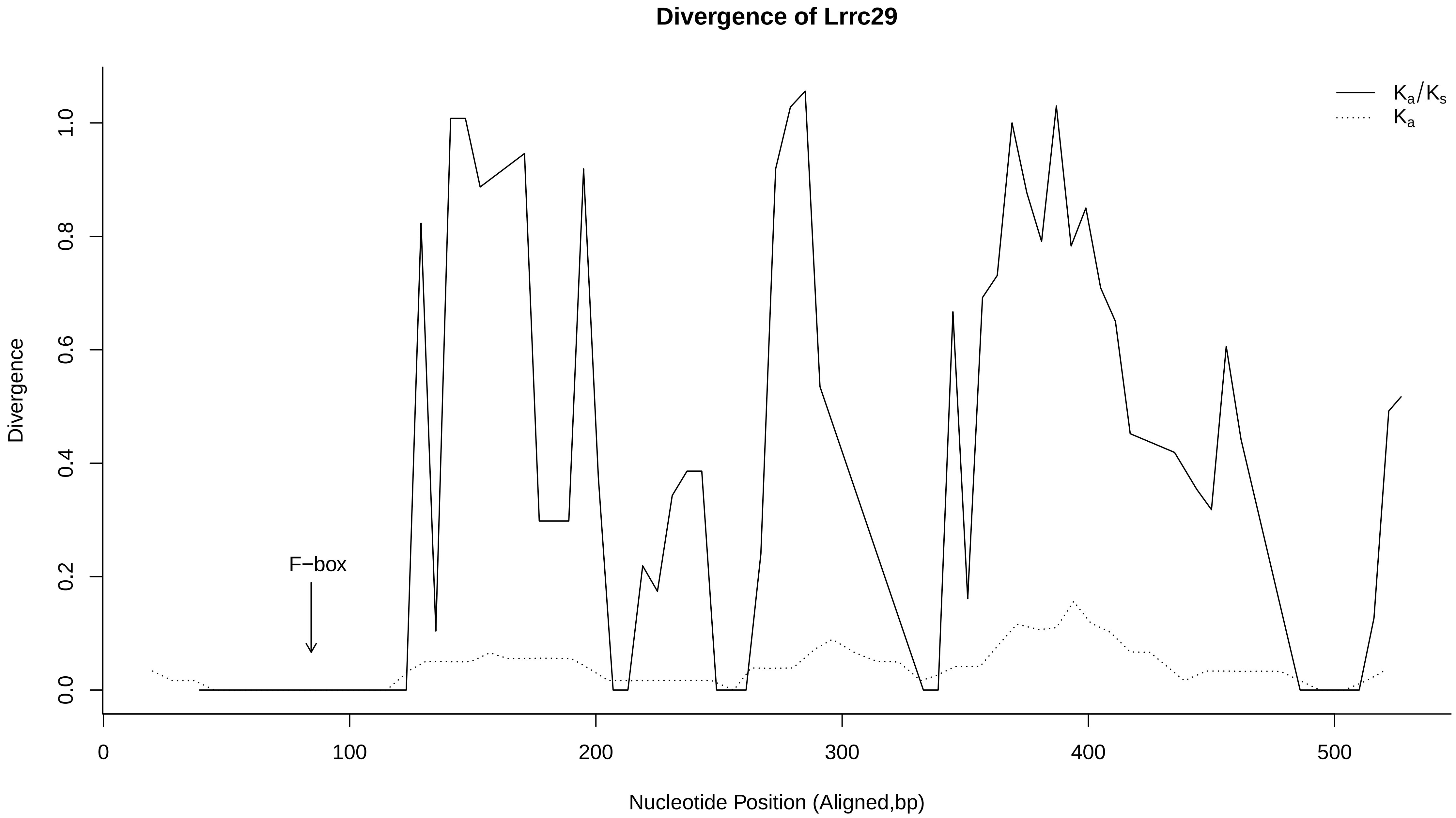

Divergence of Lrrc29
Divergence
1.0
0.8
0.6
0.4
0.2
0.0
0
100
200
300
400
500
Nucleotide Position (Aligned,bp)
$K_a/K_s$
$K_a$
F−box

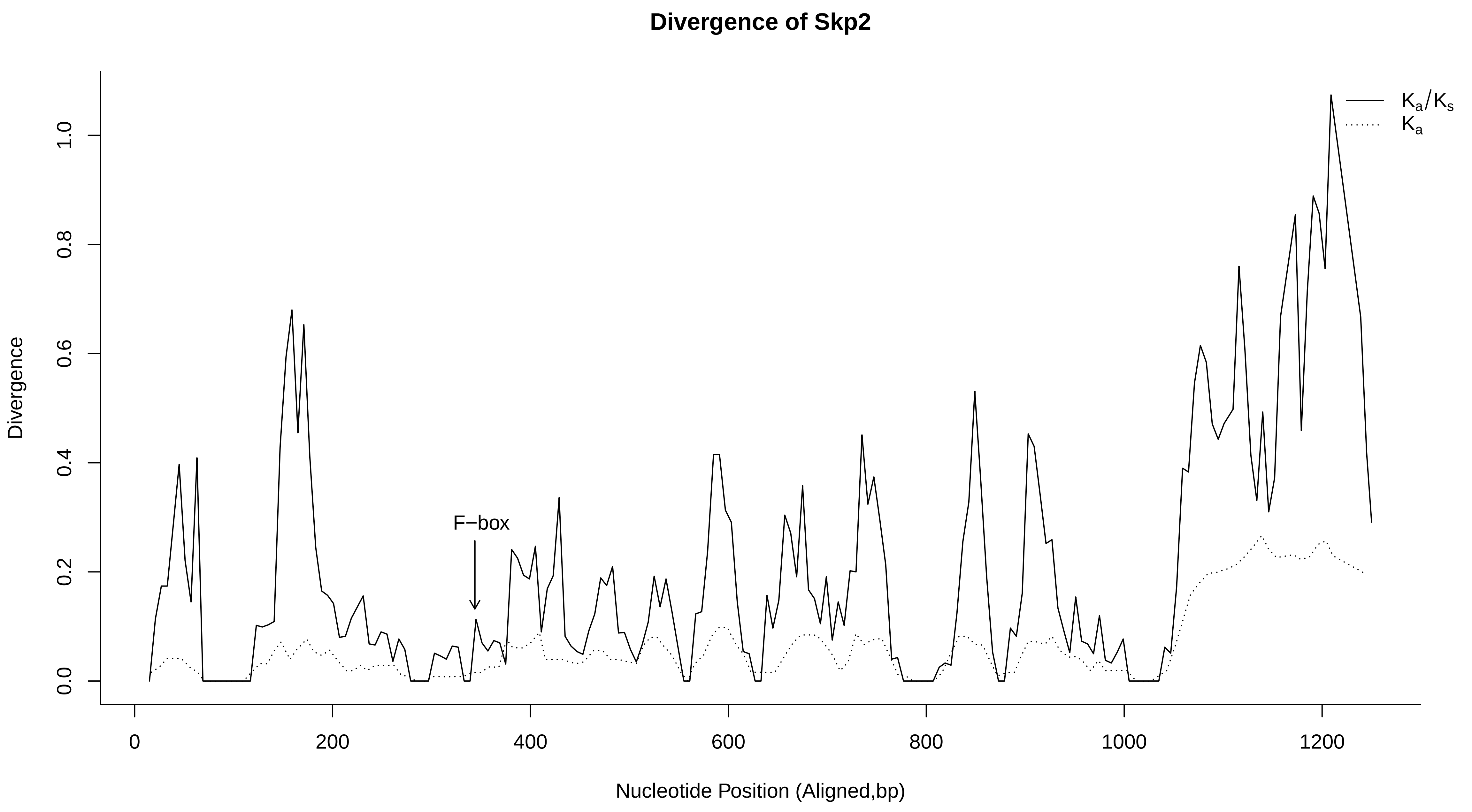

Divergence of Skp2
Divergence
Nucleotide Position (Aligned,bp)
F−box
$K_a/K_s$
$K_a$
0.0
0.2
0.4
0.6
0.8
1.0
0
200
400
600
800
1000
1200
