# Supplementary material for: Evolution of the F-Box Gene Family in Euarchontoglires: Gene Number Variation and Selection Patterns
Source: PLoS One. 2014 Apr 11;9(4):e94899. doi: 10.1371/journal.pone.0094899 (PMC3984280; doi:10.1371/journal.pone.0094899)
Supplement: File S1 — Multiple sequence alignment of the F-box protein sequences. (PDF) [file pone.0094899.s006.pdf]

559 576

```
CJA-Fbxo5     ------------------------------------------PLQLRPTATPCSCSAVTA
MMU-Fbxo5     ----------------------------------MSRRPCSCAPRPPRCCCSASPSAVTA
PPY-Fbxo5     -----------------------------------MSRRPCSCALRPPRCSCSASPSAVT
HSA-Fbxo5     -----------------------------------MSRRPCSCALRPPRCSCSASPSAVT
GGO-Fbxo5     -----------------------------------MSRRPCSCALRPPRCSCSASPSAVT
PTR-Fbxo5     -----------------------------------MSRRPCSCALRPPRCSCSASPSAVT
MUS-Fbxo5     -------------------------------------------MSRRTCSDLRRPSSCPC
RNO-Fbxo5     -------------------------------------------MSRRACGDLPRPSSCLC
MUS-Fbxw5     -----------------------MLSAVEFSGGQLVGLARTAMSGTPDYQSLPGVGDEEA
RNO-Fbxw5     -----------------------MLSAMEFSAGQLVGLARTAMSGTPDHQSLPGVGGEEA
CJA-Fbxw5     ------------------------------------------------------------
PPY-Fbxw5     ------------------------------------------------------------
GGO-Fbxw5     ------------------------------------------------------------
HSA-Fbxw5     ------------------------------------------------------------
CJA-Fbxw12    ------------------------------------------------------------
MMU-Fbxw12    ------------------------------------------------------------
PPY-Fbxw12    ------------------------------------------------------------
GGO-Fbxw12    ------------------------------------------------------------
PTR-Fbxw12    ------------------------------------------------------------
HSA-Fbxw12    ------------------------------------------------------------
CJA-Fbxo3     ------------------------------------------------------------
MMU-Fbxo3     ------------------------------------------------------------
GGO-Fbxo3     ------------------------------------------------------------
PPY-Fbxo3     ------------------------------------------------------------
PTR-Fbxo3     ------------------------------------------------------------
HSA-Fbxo3     ------------------------------------------------------------
MUS-Fbxo3     ------------------------------------------------------------
RNO-Fbxo3     ------------------------------------------------------------
MUS-Fbxw17    ------------------------------------------------------------
RNO-Fbxw17    ------------------------------------------------------------
MUS-Ccnf      ----------------MGSGGGECWREVGDRSYGTEAGGAWTRAPVTRECASHPPKHGVV
RNO-Ccnf      ----------------MGSGGGECWREAGDGSHGTETGGERTRAPMTGEGASHPPKHGVV
CJA-Ccnf      ------------------------------------------------------MGSGGV
MMU-Ccnf      ------------------------------------------------------MGSGGV
PPY-Ccnf      ------------------------------------------------------MGSGGV
GGO-Ccnf      ------------------------------------------------------MGSGGV
PTR-Ccnf      ------------------------------------------------------MGSGGV
HSA-Ccnf      ------------------------------------------------------MGSGGV
MUS-Fbxl22    ------------------------------------------------------------
RNO-Fbxl22    ------------------------------------------------------------
CJA-Fbxl22    ------------------------------------------------------------
MMU-Fbxl22    ------------------------------------------------------------
PPY-Fbxl22    ------------------------------------------------------------
GGO-Fbxl22    ------------------------------------------------------------
```

```
HSA-Fbxl22   ------------------------------------------------------------
PTR-Fbxl22   ------------------------------------------------------------
CJA-Fbxo43   SEAGNGVDCPPVVNSKYSTFRDCCSTSSLQDNGYSELKSCSFDNTDKDYLGKKEKGPALL
MMU-Fbxo43   TEAGNGVDSPPIVNSKYSTFRDFCSTSSFQDSGYNELKSCSFDNTDKEYLGKKVKGPTLL
GGO-Fbxo43   TEAGNGADSPLIVNSKYSTFRDFRSTSSFQDSGYNELKSCSFDNIDKEYLGKKEKGPTLL
PPY-Fbxo43   TEAGNGADSPPIVNSKYSTFRDFCSTSSFQDSGYNELKSCSFDNIDKEYLGKKEKGPPLL
PTR-Fbxo43   TEAGNGADSPPIVNSKYSTFRDFCSTSSFQDSGYNELKSCSFDNIDKEYLGKKEKGPTLL
HSA-Fbxo43   TEAGNGADSPPIVNSKYSTFRDFCSTSSFQDSGYNELKSCSFDNIDKEYLGKKEKGPTLL
CJA-Fbxo42   --------------------------------------MASSSDSEDDSFMAVDQEETVL
MMU-Fbxo42   --------------------------------------MASSSDSEDDSFMAVDQEETVL
PPY-Fbxo42   --------------------------------------MASSSDSEDDSFMAVDQEETVL
GGO-Fbxo42   --------------------------------------MASSSDSEDDSFMAVDQEETVL
PTR-Fbxo42   --------------------------------------MASSSDSEDDSFMAVDQEETVL
HSA-Fbxo42   --------------------------------------MASSSDSEDDSFMAVDQEETVL
MUS-Fbxo42   --------------------------------------MASSSDSEDDSVMAVDQEETAL
RNO-Fbxo42   --------------------------------------MASSSDSEDDSFMAVDQEETAL
MUS-Fbxl18   -----MPHPTWRPHRVHPGASGSTSRRAPQPRPLPGSLWSCGRAGFEHPYAGRLGDCEAA
RNO-Fbxl18   ILLHILSHVPSTDLVLNVRRTCRKLAALCLDKSLVHTVLLQKDYQASEEKVKQLVKEIGR
RNO-Fbxl18   ILLHILSHVPSTDLVLNVRRTCRKLAALCLDKSLVHTVLLQKDYQASEEKVKQLVKEIGR
PPY-Fbxl18   ILLHILSHVPSTDLILNVRRTCRKLAALCLDKSLIHTVLLQKDYQASEDKVRQLVKEIGR
PTR-Fbxl18   ILLHILSHVPSTDLILNVRRTCRKLAALCLDKSLIHTVLLQKDYQASEDKVRQLVKEIGR
MMU-Fbxl18   ILLHILSHVPSTDLILNVRRTCRKLAALCLDKSLIHTVLLQKDYQASEDKVRQLVKEIGR
GGO-Fbxl18   ILLHILSHVPSTDLILNVRRTCRKLAALCLDKSLIHTVLLQKDYQASEDKVRQLVKEIGR
HSA-Fbxl18   ILLHILSHVPSTDLILNVRRTCRKLAALCLDKSLIHTVLLQKDYQASEDKVRQLVKEIGR
MUS-Fbxo38   IFRYLPLQDIMCMECLSRKLKEAVTLYLRVVRVVDLCAGRWWEYMPSGFTDSSFLTLLKK
RNO-Fbxo38   IFRYLPLQDIMCMECLSRKLKEAVTLYLRVVRVVDLCAGRWWEYMPSGFTDCSFLTLLKK
GGO-Fbxo38   IFRYLPLQDIMCMECLSRKLKEAVTLYLRVVRVVDLCAGRWWEYMPSGFTDSSFLTLLKK
PPY-Fbxo38   IFRYLPLQDIMCMECLSRKLKEAVTLYLRVVRVVDLCAGRWWEYMPSGFTDSSFLTLLKK
CJA-Fbxo38   IFRYLPLQDIMCMECLSRKLKEAVTLYLRVVRVVDLCAGRWWEYMPSGFTDSSFLTLLKK
MMU-Fbxo38   IFRYLPLQDIMCMECLSRKLKEAVTLYLRVVRVVDLCAGRWWEYMPSGFTDSSFLTLLKK
PTR-Fbxo38   IFRYLPLQDIMCMECLSRKLKEAVTLYLRVVRVVDLCAGRWWEYMPSGFTDSSFLTLLKK
HSA-Fbxo38   IFRYLPLQDIMCMECLSRKLKEAVTLYLRVVRVVDLCAGRWWEYMPSGFTDASFLTLLKK
GGO-Fbxo28   ------------------------------------------SSLASGSTQRQPPPPAPQ
MUS-Fbxo28   ---------------------MAAASEERMAEEGGGGHGDGGSCSAAGSAQRQPPAPPSQ
RNO-Fbxo28   ---------------------MAAASEERMAEEGGGGHGDGGSCSAASSAQRQPPTPPSQ
CJA-Fbxo28   -------------------MAAAAAAEERMAEEGGGGHGDGSSSLAAGSTQRQPPPPPPQ
MMU-Fbxo28   --------------------MAAAAAEDRMAEEGGGSHGDGGSSLASGSTQRQPPPPPPQ
PPY-Fbxo28   ---------------------MAAAAEERMAEEGGGGQGDGGSSLASGSTQRQPPPPPPQ
PTR-Fbxo28   ---------------------MAAAAEERMAEEGGGGQGDGGSSLASGSTQRQPPPPAPQ
HSA-Fbxo28   ---------------------MAAAAEERMAEEGGGGQGDGGSSLASGSTQRQPPPPAPQ
PPY-Fbxo41   SGFPLAPEPAALLAVPGARREVFESTSFQGKEQAAGPSPAAPHLLHHHHHHAPLAHFPGD
RNO-Fbxo41   SGFPLAPEPAALLAVPGARREVFESTSFQGKEQAAGPAPAGPHLLHHHHHHAPLAHFPGD
MUS-Fbxo41   SGFPLAPEPAALLAVPGARREVFESTSFQGKEQATGPSPAGPHLLHHHHHHAPLAHFPAD
CJA-Fbxo41   SDFPLAPEPAALLAVPGARREVFESTSSRARSRRSVLSPAAPHLHHHHHHHAPLAHFPGD
MMU-Fbxo41   SGFPLAPEPAALLAVPGARREVFESTSFQGKEQAAGPSPAAPHLLHHHHHHAPLAHFPGD
```

```
GGO-Fbxo41   RLTGGAAEPAALLAVPGARREVFESTSFQGKEQAAGPSPAAPHLLHHHHHHAPLAHFPGD
PTR-Fbxo41   GDLVPASLPCEELAEPGLVPAAAARYALREIEIPLGELFARKSVASSACSTPPPGPVPGP
HSA-Fbxo41   SGFPLAPEPAALLAVPGARREVFESTSFQGKEQAAGPSPAAPHLLHHHHHHAPLAHFPGD
CJA-Lrrc29   ------------------------------------------------------------
GGO-Lrrc29   ------------------------------------------------------------
PTR-Lrrc29   ------------------------------------------------------------
HSA-Lrrc29   ------------------------------------------------------------
CJA-Fbxo30   -------------------------------MEEELQHSHCVNCVSRRCMTRPEPGISCD
MMU-Fbxo30   -------------------------------MEEQLQHSHCVNCVSRRCMTRPEPGISCD
PPY-Fbxo30   -------------------------------MEEELQHSHCVNCVSRRCMTRPEPGISCD
PTR-Fbxo30   -------------------------------MEEELQHSHCVNCVSRRCMTRPEPGISCD
GGO-Fbxo30   -------------------------------MEEELQHSHCVNCVSRRCMTRPEPGISCD
HSA-Fbxo30   -------------------------------MEEELQHSHCVNCVSRRCMTRPEPGISCD
MUS-Fbxo30   ------------------------------MEEEVQQHSHCMNCVSRRCMTRPEPGVSCD
RNO-Fbxo30   ------------------------------MEEEVQQHSHCVNCVSRRCMTRPEPGVSCD
CJA-Fbxo40   --------------------------MGKARRTSPGQHRHCEGCFNRHCHIPVEPNISCL
MMU-Fbxo40   --------------------------MGKARRPPPGHHRHCEGCFNRHCHIPAEPNVSCL
PPY-Fbxo40   --------------------------MGKARRPPPGHHRHCEGCFNRHCHIPVEPNTSCL
GGO-Fbxo40   --------------------------MGKARRSPPGHHRHCEGCFNRHCHIPVEPNTSCL
PTR-Fbxo40   --------------------------MGKARRSPPGHHRHCEGCFNRHCHIPVEPNTSCL
HSA-Fbxo40   --------------------------MGKARRSPPGHHRHCEGCFNRHCHIPVEPNTSCL
MUS-Fbxo40   --------------------------MGRARKPPPALHRHCEGCFNRHCHVPVEPSVSCL
RNO-Fbxo40   ---------------------------GRARRPPPALHRHCEGCIDRHCRVPAEPSVSCL
CJA-Fbxo9    -------------------------------------------------MAEAEEDCHSD
PPY-Fbxo9    ------------------------------------------------MAEAEEDCHSDT
HSA-Fbxo9    ---------------------------------------------MPDIIWVFPPQAEAE
GGO-Fbxo9    HPSAPRSGPLPREDGCRTPGPQLLPLPGALLRPRTLLSSAAETGRSRHPDTQHPSSGGRC
PTR-Fbxo9    ---------------------------------------------------------MAE
MMU-Fbxo9    -------------MQLVPDIEFKITYTRSPDGDGVGNSYIEDNDDDSKMADLLSYFQQQL
MUS-Fbxo9    ------------------------------------------------MSAEAEEDCHSD
RNO-Fbxo9    -------------------------------------------------MAEAEEDCHSD
CJA-Fbxo7    QVEVPEAEPTLGQLRTHLSLALLPTWGYSSDTRFAITLNNKDALTGDEETLASYGIVSGD
PTR-Fbxo7    PLEVPETEPTLGHLRSRLRQSLLCTWGYSSNTRFTITLNYKDPLTGDEETLASYGIVSGD
HSA-Fbxo7    PLEVPETEPTLGHLRSHLRQSLLCTWGYSSNTRFTITLNYKDPLTGDEETLASYGIVSGD
GGO-Fbxo7    PLEVPETEPTLGHLRSHLRQSLLCTWGYSSNTRFTITLNYKDPLTGDEETLASYGIVSGD
MMU-Fbxo7    FTGGLGSSGRRGAGGCRRLDARVVGWGPAPGEHGAASRGLRSPSGSDTRFTITLNYKDAL
PPY-Fbxo7    --------------------------------------------------MAWPPGGSGP
MUS-Fbxo7    PLEVPESEPTLGQLRAHLSQVLLPTLGFSSDTRFAITLNNKDALTGDEETLASYGIVSGD
RNO-Fbxo7    PLEVPESEPTLGQLRAHLIQDLLPTLGFSSDTRFAITLNNKDALTGDEETLASYGIVSGD
CJA-Fbxo47   ------------------------------------------MASRINTSFTLIPNQKHR
PPY-Fbxo47   ------------------------------------------MASRINTNFTLIPNQKLR
MMU-Fbxo47   ------------------------------------------MASRINTNFTLIPNQKLR
HSA-Fbxo47   ------------------------------------------MASRINTNFTLIPNQKLR
GGO-Fbxo47   ------------------------------------------MASRINTNFTLIPNQKLR
PTR-Fbxo47   ------------------------------------------MASRINTNFTLIPNQKLR
```

```
MUS-Fbxo47   ------------------------------------------MASRVNTSFTLIPKQKCR
RNO-Fbxo47   ------------------------------------------MASRVNTTFTLIPSSKCR
GGO-Fbxl4    -----------MSPVFPMLTVLTMFYYICLRRRARTATRGEMMNSHRTIESNSRTSPLNA
PTR-Fbxl4    -----------MSPVFPMLTVLTMFYYICLRRRARTATRGEMMNTHRTIESNSRTSPLNA
HSA-Fbxl4    -----------MSPVFPMLTVLTMFYYICLRRRARTATRGEMMNTHRAIESNSQTSPLNA
PPY-Fbxl4    -----------MSPVFPMLTVLTMFYYICLRRRARTATRGEMMNSHRTIESNSRPSPLNA
CJA-Fbxl4    -----------MSPVFPMLTVLTMFYYICLRRRARTATRGEMMNSHRTIESNSRTSPLNA
MMU-Fbxl4    -----------MSPVFPMLTVLTMFYYICLRRRARTATRGEMMNSHRTIESNSRTSPLNA
MUS-Fbxl4    -----------MSPVFPMLTVLTMFYYMCLRRRARTATRGDMMNSHRTIVSNSRTSPLNA
RNO-Fbxl4    -----------MSPVFPMLTVLTMFYYMCLRRRARTATRGDMMSSHRTIVSNSRTSPLNA
CJA-Fbxo4    ---------------------------MAGSEPRSGTSSPPPPFSDWGRLEAAILSGWRT
MMU-Fbxo4    ---------------------------MAGSEPRSGTSSPPPPFSDWGRLEAAILSGWKT
PPY-Fbxo4    ---------------------------MAGSEPRSGTSSPPPPFSDWGRLEAAILSGWKT
GGO-Fbxo4    ---------------------------MAGSEPRRGSNSPPPPFSDWGRLEAAILSGWKT
PTR-Fbxo4    ---------------------------MAGSEPRRGTNSPPPPFSDWGRLEAAILSGWKT
HSA-Fbxo4    ---------------------------MAGSEPRSGTNSPPPPFSDWGRLEAAILSGWKT
MUS-Fbxo4    -----------------------------MAGSEPRGAGSPPPASDWGRLEAAILSGWRT
RNO-Fbxo4    APSRPAWLVPAGRAETEAERLGNRCARPCLAEPAAMAGSEPRGAGSPPPASDWGRLEAAI
CJA-Fbxo48   -------------------------------------------------MQKNSKRSNNS
MMU-Fbxo48   -------------------------------------------------MHKNSKRNSNS
GGO-Fbxo48   -------------------------------------------------MHKNSKRNSNS
PTR-Fbxo48   -------------------------------------------------MHKNSKRNNNS
HSA-Fbxo48   -------------------------------------------------MHKNSKRNNNL
PPY-Fbxo48   -------------------------------------------------MHKNSKRNNNS
PPY-Fbxo48   -------------------------------------------------MHKNSKRNNNS
MUS-Fbxo48   -------------------------------------------------MKKTSKKNNNF
RNO-Fbxo48   --------------------------------------------------MKKTSKNSNA
GGO-Fbxl3    ----------------------------------------------FRNRMKRGGRDSDR
MMU-Fbxl3    --------------------------------------------------MKRGGRDSDR
CJA-Fbxl3    --------------------------------------------------MKRGGRDSDH
PPY-Fbxl3    --------------------------------------------------MKRGGRDSDR
PTR-Fbxl3    --------------------------------------------------MKRGGRDSDR
HSA-Fbxl3    --------------------------------------------------MKRGGRDSDR
MUS-Fbxl3    --------------------------------------------------MKRGGRDSDQ
RNO-Fbxl3    --------------------------------------------------MKRGGRDSDQ
RNO-Fbxl21   --------------------------------------------MKRNNFSTVNKVVHSS
MUS-Fbxl21   ------------------MGVGDYPMAVSFHWLSSNCNLEATFRMKRNNFSAVNKVVQSS
HSA-Fbxl21   --------------------------------------------MKRNSLSVENKIVQLS
PTR-Fbxl21   --------------------------------------------MKRNSLSVGNKIVQLS
MMU-Fbxl21   --------------------------------------------MKRNSLSVENKIVQLS
PPY-Fbxl21   ------------------------------------------------------------
PPY-Fbxo11   -------------MVAEESGPGAQNSPYQLRRKTLLPKRTACPTKNSMEGASTSTTENFG
PTR-Fbxo11   -------------MVAEESGPGAQNSPYQLRRKTLLPKRTACPTKNSMEGASTSTTENFG
MMU-Fbxo11   PPPPPPPPPPLPQERNNVGERDDDVPADMVAEESGPGAQNSPYQLRRKTLLPKRTACPTK
CJA-Fbxo11   PPLPQERNNVGERDDDVPADMVAEESGPGAQNSPYQLRRKTLLPKRTACPTKNSMEGAST
```

```
GGO-Fbxo11   PPLPQERNNVGERDDDVPADMVAEESGPGAQNSPYQLRRKTLLPKRTACPTKNSMEGAST
HSA-Fbxo11   PPLPQERNNVGERDDDVPADMVAEESGPGAQNSPYQLRRKTLLPKRTACPTKNSMEGAST
MUS-Fbxo11   QPPPPPPPPPPPPQDRNNAGERDDVPADMVAEESGPGAQNSPYQLRRKTLLPKRTACPTK
RNO-Fbxo11   -------DDVPADMVAEESGPGAQNSPYQLRRKTLLPKRTACPTKNSMEGASTSTTENFG
PPY-Fbxw11   SVMEDQNEDESPKKNTLWQISNGTSSVIVSRKRPSEGNYQKEKDLCIKYFDQWSESDQVE
MMU-Fbxw11   ESMCALSCLQSMPSVRCLQISNGTSSVIVSRKRPSEGNYQKEKDLCIKYFDQWSESDQVE
MUS-Fbxw11   SVMEDQNEDESPKKSALWQISNGTSSVIVSRKRPSEGNYQKEKDLCIKYFDQWSESDQVE
RNO-Fbxw11   ESMCALSCLQSMPSVRCLQISNGTSSVIVSRKRPSEGNYQKEKDLCIKYFDQWSESDQVE
CJA-Fbxw11   ESMCALSCLQSMPSVRCLQISNGTSSVIVSRKRPSEGNYQKEKDLCIKYFDQWSESDQVE
GGO-Fbxw11   ESMCALSCLQSMPSVRCLQISNGTSSVIVSRKRPSEGNYQKEKDLCIKYFDQWSESDQVE
HSA-Fbxw11   ESMCALSCLQSMPSVRCLQISNGTSSVIVSRKRPSEGNYQKEKDLCIKYFDQWSESDQVE
PTR-Fbxw11   ESMCALSCLQSMPSVRCLQISNGTSSVIVSRKRPSEGNYQKEKDLCIKYFDQWSESDQVE
MMU-Btrc     REDCNNGEPPRKIIPEKNSLRQTYNSCARLCLNQETVCLASTAMKTENCVAKVIFLLRAL
RNO-Btrc     TVCLTSTAMKTENCVAKTKLANGTSSMIVPKQRKLSASYEKEKELCVKYFEQWSESDQVE
MUS-Btrc     TVCLTSTAMKTENCVAKAKLANGTSSMIVPKQRKLSASYEKEKELCVKYFEQWSESDQVE
PPY-Btrc     TVCLASTAMKTENCVAKTKLANGTSSMIVPKQRKLSASYEKEKELCVKYFEQWSESDQVE
PTR-Btrc     CSSLADSMPSLRCLYNPGTGALTAFQNSSEREDCNNGEPPRKIIPEKNSLRQTYNSCARL
CJA-Btrc     TVCLASTAMKTENCVAKTKLANGTSSMIVPKQRKLSASYEKEKELCVKYFEQWSESDQVE
GGO-Btrc     TVCLASTAMKTENCVAKTKLANGTSSMIVPKQRKLSASYEKEKELCVKYFEQWSESDQVE
HSA-Btrc     TVCLASTAMKTENCVAKTKLANGTSSMIVPKQRKLSASYEKEKELCVKYFEQWSESDQVE
RNO-Fbxw12   ------------------------------------------------------------
MUS-Fbxw15   ------------------------------------------------------------
MUS-Fbxw24   ------------------------------------------------------------
MUS-Fbxw12   ------------------------------------------------------------
MUS-Fbxw18   ------------------------------------------------------------
MUS-Fbxw21   ------------------------------------------------------------
MUS-Fbxw20   ------------------------------------------------------------
MUS-Fbxw22   ------------------------------------------------------------
MUS-Fbxw14   ------------------------------------------------------------
MUS-Fbxw28   ------------------------------------------------------------
MUS-Fbxw19   ------------------------------------------------------------
MUS-Fbxw16   ------------------------------------------------------------
MUS-Fbxw26   ------------------------------------------------------------
MUS-Fbxw8    GERRSPRRPEAGARGEPASGYLGLAQGLLEGAGRPPAPRPGRGGDRKDTSSRSRSPPDRD
RNO-Fbxw8    EAGERRPRRPEAGARGEPASGYLGLAQGLLEGAGRPPAPRPGRTDRKDVSSRSRSPPDRD
CJA-Fbxw8    QEELAQAQAPRKWRRSDAAERRARRPEVAPGRSEQALGDPALAQGLLEGAGRPPAARATR
MMU-Fbxw8    QAPRKRRRPEAAERWARRPEVVPGRGEQASGDPALAQGLLEGAGRPPAARATRTEGQDVA
PPY-Fbxw8    EVAERRARRPEVGSGHGEQASGDPTLAQGLLEGAGRPPAARATRAEGQVTSRSRSPLARE
GGO-Fbxw8    ------------------------------------------------------------
PTR-Fbxw8    AAERRARRPEVGSGRGEQASGDPALAQGLLEGAGRPPAARATRAEGQDVASRSRSLAREG
HSA-Fbxw8    RRRPEAAERRARRPEVGSGRGEQASGDPALAQRLLEGAGRPPAARATRAEGQDVASRSRS
GGO-Fbxw10   PRSRASQMAELDLMAPGPLPRATAQPPAPLSPDSGSPSPDSGSASPVEEEDVGSSEKLGR
MUS-Fbxw10   PVCQKCEACVLAWKIFATKEWFRRVNDISQRRFLVSILGQLNSLYLLQYFQNILETTQGK
RNO-Fbxw10   KGSNWVPVCHKCEACVLAWKIFATKEWFRRVNDTSQRRFLVSILVQLNSLYLLQYFQNIL
CJA-Fbxw10   CRINDVSQRRFLVSILKQLNSLYLLHYFQNILQTTQGKDFIYNRSLVDLSKKEGKVAKSS
```

```
MMU-Fbxw10   CRISDISQRRFLVSILKQLNSLYLLHYFQNILQTTQGKDFIYNRSRIDLSKKERKVVKSF
PPY-Fbxw10   CRINDISQRRFLVGILKQLNSLYLLHYFQNILQTTQGKDFIYSRSRIDLSKKEGKVVKSS
HSA-Fbxw10   CRINDISQRRFLVGILKQLNSLYLLHYFQNILQTTQGKDFIYNRSRINLSKKEGKVVKSS
PTR-Fbxw10   CRINDISQRRFLVGILKQLNSLYLLHYFQNILQTTQGKDFIYNRSRINLSKKEGKVVKSS
MMU-Fbxw2    ------------------------------MERKDFETWLDNISVTFLSLTDLQKNETLD
GGO-Fbxw2    ------------------------------MERKDFETWLDNISVTFLSLTDLQKNETLD
PPY-Fbxw2    ------------------------------MERKDFETWLDNISVTFLSLTDLQKNETLD
CJA-Fbxw2    ------------------------------MERKDFETWLDNISVTFLSLTDLQKNETLD
PTR-Fbxw2    ------------------------------MERKDFETWLDNISVTFLSLTDLQKNETLD
HSA-Fbxw2    ------------------------------MERKDFETWLDNISVTFLSLTDLQKNETLD
RNO-Fbxw2    ------------------------------MERKDFETWLDNISVTFLSLTDLQKNETLD
MUS-Fbxw2    ------------------------------MERKDFETWLDNISVTFLSLTDLQKNETLD
GGO-Fbxo8    ---------------MGQGLWRVVRNQQLQQEGYSEQGYLTREQSRRMAASNISNTNHRK
PPY-Fbxo8    ---------------MGQGLWRVVRNQQLQQEGYSEQGYLTREQSRRMAASNISNTNHRK
PTR-Fbxo8    ---------------MGQGLWRVVRNQQLQQEGYSEQGYLTREQSRRMAASNISNTNHRK
HSA-Fbxo8    ---------------MGQGLWRVVRNQQLQQEGYSEQGYLTREQSRRMAASNISNTNHRK
CJA-Fbxo8    ---------------MGQGLWRVVRNQQLQQEGYSEQGYLTREQSRRMAANNISNTSHRK
MMU-Fbxo8    ---------------MGQGLWRVVRNQQLQQEGYSEQGYLTREQSRRMAANNISNSNHRK
MUS-Fbxo8    ---------------MGQGLWRVARNHHLQQEAYSETGYLSREQSRRVASSNISHTSHRK
RNO-Fbxo8    ---------------VSQGLWTNLQXQHLQQEAYGETGYLSREQSRRVASSNISHTSHRK
CJA-Fbxl5    -----------MKEEEEVFQPMLMEYFTYEELKDIKKKVIAQHCSQKDTAELLRGLSLWN
GGO-Fbxl5    LEAFTRDFLPHMKEEEEVFQPMLMEYFTYEELKDIKKKVIAQHCSQKDTAELLRGLSLWN
MMU-Fbxl5    LEAFTRDFLPHMKEEEEVFQPMLMEYFTYEELKDIKKKVIAQHCSQKDTAELLRGLSLWN
PPY-Fbxl5    LEAFTRDFLPHMKEEEEVFQPMLMEYFTYEELKDIKKKVIAQHCSQKDTAELLRGLSLWN
PTR-Fbxl5    LEAFTRDFLPHMKEEEEVFQPMLMEYFTYEELKDIKKKVIAQHCSQKDTAELLRGLSLWN
HSA-Fbxl5    LEAFTRDFLPHMKEEEEVFQPMLMEYFTYEELKDIKKKVIAQHCSQKDTAELLRGLSLWN
MUS-Fbxl5    LEAFTRDFLPHMKEEEEVFQPMLMEYFTYEELKDIKKKVIAQHCSQKDTAELLRGLSLWN
RNO-Fbxl5    LEAFTRDFLPHMKEEEEVFQPMLMEYFTYEELKDIKKKVIAQHCSQKDTAELLRGLSLWN
PPY-Fbxw7    SEYTSTTGLVPCSATPTTFGDLRAANGQGQQRRRITSVQPPTGLQEWLKMFQSWSGPEKL
RNO-Fbxw7    SDYTSTTGLVPCSATPTTFGDLRAANGQGQQRRRITSVQPPTGLQEWLKMFQSWSGPEKL
MUS-Fbxw7    SDYTSTTGLVPCSATPTTFGDLRAANGQGQQRRRITSVQPPTGLQEWLKMFQSWSGPEKL
CJA-Fbxw7    SEYTSTTGLVPCSATPTTFGDLRAANGQGQQRRRITSVQPPTGLQEWLKMFQSWSGPEKL
MMU-Fbxw7    SEYTSTTGLVPCSATPTTFGDLRAANGQGQQRRRITSVQPPTGLQEWLKMFQSWSGPEKL
GGO-Fbxw7    SEYTSTTGLVPCSATPTTFGDLRAANGQGQQRRRITSVQPPTGLQEWLKMFQSWSGPEKL
PTR-Fbxw7    SEYTSTTGLVPCSATPTTFGDLRAANGQGQQRRRITSVQPPTGLQEWLKMFQSWSGPEKL
HSA-Fbxw7    SEYTSTTGLVPCSATPTTFGDLRAANGQGQQRRRITSVQPPTGLQEWLKMFQSWSGPEKL
CJA-Ect2l    -----------MESFHMRFSAWTPFNNKSLNRQLFQERVALISHWFDLWTNKQRQEFLFA
HSA-Ect2l    -----------MESFHTRFSAWTPFSNKSLNRQLFQERVALISHWFDLWTNKQRQEFLFA
PTR-Ect2l    -----------MESFHTRFSAWTPFSNKSLNRQLFQERVALISHWFDLWTNKQRQEFLFA
PPY-Ect2l    -----------MESFHTRFSAWTPFSNKSLNRQLFQERVALISHWFDLWTNKQRQEFLFT
GGO-Ect2l    -----------MESFHTRFSAWTPFSNKPLNRQLFQERVALISHWFDLWTNKQRQEFLFA
MMU-Ect2l    ------------------------------------------------------------
RNO-Ect2l    ------------------------------------------------------------
MUS-Ect2l    WPWKFLTEQDCLWMPKCTKFGWFLPYTPTQNEYGAWKHHYIACVSSLDWLTPREAAAVYG
CJA-Fbxo16   MMAFAPPKNTDGPKMQTKMSTWTPLNHQLLNDRVFEERRALLGKWFDKWTDSQRRRILTG
```

```
MMU-Fbxo16   ------------MMAFAPPKNLDGPKMQTKMSTWTPLNHQLLNDRFDKWTDSQRRRILTG
HSA-Fbxo16   MMAFAPPKNTDGPKMQTKMSTWTPLNHQLLNDRVFEERRALLGKWFDKWTDSQRRRILTG
PPY-Fbxo16   ------------MMAFAPPKNTDGPKMHTKMSTWTPLNHQLLNDRFDKWTDSQRRRILTG
GGO-Fbxo16   MMAFAPPKSTDGPKMQTKMSTWTPLNHQLLNDRVFEERRALLGKWFDKWTDSQRRRILTG
PTR-Fbxo16   MMAFAPPKNTDGPKMQTKMSTWTPLNHQLLNDRVFEERRALLGKWFDKWTDSQRRRILTG
MUS-Fbxo16   MMAFAPPKSIDGPKMQTKMSTWTPLNHQLLNDQVFEERRALLGKWFDKWTDSQRRRILTG
RNO-Fbxo16   MMAFAPPKSIDGPKMQTKMSTWTPLNHQLLNDQVFEERRALLGKWFDKWTDSQRRRILTG
MMU-Fbxo18   VPECDNGPHCGPLGHLHRRCQRTSAHLLIFMEHPEMRRFKRKHLTAIDCQHLARSHLAVT
GGO-Fbxo18   PQEGSGEAGLGSSGSAPPSRKRSWSSEEESNQATGTSRWDGVSKKAPRHHLSVPCTRPRE
CJA-Fbxo18   PQEGSGEARLGASGSVPPSRKRARSFEEESNQATGTSRWDGVSKKAPRHHLSVPCTRPRE
PPY-Fbxo18   PQEGSGEAWLGSSGSAPPSRKRSRSSEEESNQATGTSQWDGVSKKALRHHLSVPCTRPRE
PTR-Fbxo18   PQEGSGEAGLGSSGSAPPSRKRSWSSEEESNQATGTSWWDGVSKKAPRHHLSVPCTRPRE
HSA-Fbxo18   PQEGSAGPGSPGSAPPSRKRSWSSEEESNQATGTSRWDGVSKKAPRHHLSVPCTRPREAR
MUS-Fbxo18   QVDNGEARLGSSGSAQPARKRAHCFEEATESGQWDGVTKKTPRHRLFPSCSRLREARQGA
RNO-Fbxo18   QVENGEVRLGSPGSAQPARKRPRSLEEATESGQWDGVTKKTPRHRLFPSCARLREVRQGA
CJA-Fbxo33   ----------------MLLFLSVPQRHPGARTRAGAARVARWRRRRRLRLQQLRRLRGLL
MMU-Fbxo33   -------------------MLLFLSVPQRPPGARTRAGAARVARWRWLRLQQLRRLRGLL
PPY-Fbxo33   -------------------MLLFLSVLQRPPGARTRAGAARVARWRRLRLQQLRRLRGLL
HSA-Fbxo33   -------------------MLLFLSVPQRPPGARTRAGAARVARWRRLRLQQLRRLRGLL
GGO-Fbxo33   -------------------MLLFLSVPQRPPGARTRAGAARVARWRRLRLQQLRRLRGLL
PTR-Fbxo33   -----------------MLLFLSVPQRPPGARTRAGAARVARWRRLRLRLQQLRRLRGLL
MUS-Fbxo33   ----------------MLLFLSVPQRPPGARTRAGAARLVRWRRRQRLRLLQLRRLRGLL
RNO-Fbxo33   ----------------MLLFLSVPQRPPGARTRAGAARVVRWRRRQRLRLRQLRRLRGLL
MMU-Fbxo21   -------------------------------------------------------MAAAA
GGO-Fbxo21   -------------------------------------------------------MAAAA
PTR-Fbxo21   -------------------------------------------------------MAAAA
HSA-Fbxo21   -------------------------------------------------------MAAAA
PPY-Fbxo21   -------------------------------------------------------MAAAA
MUS-Fbxo21   -------------------------------------------------------MASVA
RNO-Fbxo21   -------------------------------------------------------MAAVA
MUS-Fbxo24   ------------------------------------------------------------
RNO-Fbxo24   ------------------------------------------------------------
CJA-Fbxo24   -------------MVWESQQERGPGPRRQEGRSRERAARMRRSQRRKLQKFTGLCRQEEG
PPY-Fbxo24   -----------------------------------------------MGEKAVPLLRRRR
HSA-Fbxo24   ---------MVWESQQERGGQGPRRQEGRSREGAIRMVRGSQRRKLQKFTRLCGREEGLR
GGO-Fbxo24   ----------MVWESQQEGGQGPRRQEGRSREGAIRMVRGSQRGKLQKFTRLCGREEGLR
MMU-Fbxo24   ---------------------------------------------MGEKAVPLLRRRRVK
PTR-Fbxo24   -----------------------------------------------MGEKAVPLLRRRR
PTR-Fbxo32   ------------------------------------------------------------
HSA-Fbxo32   ------------------------------------------------------------
CJA-Fbxo32   ------------------------------------------------------------
GGO-Fbxo32   ------------------------------------------------------------
MMU-Fbxo32   ------------------------------------------------------------
MUS-Fbxo32   ------------------------------------------------------------
RNO-Fbxo32   ------------------------------------------------------------
```

```
CJA-Fbxo25   ---------------------------------------------MPFLGQDWRSPGWSW
GGO-Fbxo25   ------------------------------------------------------------
HSA-Fbxo25   ---------------------------------------------MPFLGQDWRSPGWSW
MMU-Fbxo25   ---------------------------------------------MPFLGQDWRSPGWSW
PTR-Fbxo25   ---------------------------------------------MPFLGQDWRSPGWSW
PPY-Fbxo25   ---------------------------------------------MPFLGQDWRSPGWSW
MUS-Fbxo25   ---------------------------------------------MPFLGQDWRSPGWSW
RNO-Fbxo25   ------------------------------------------------------------
MUS-Fbxl19   ---------------------------MSSSSRGPGAGARRRRTRCRRCRACVRTECGDC
RNO-Fbxl19   ---------------------------MSSSSRGPGAGARRRRTRCRRCRACVRTECGDC
CJA-Fbxl19   -------MGLEFPGKGESGPSALLTPPMSSSSRGPGAGARRRRTRCRRCRACVRTECGDC
GGO-Fbxl19   -------------------------MAAVDEKVGGAGGRKRRRTRCRRCRACVRTECGDC
PPY-Fbxl19   ---------------------------MSSSSRGPGAGARRRRTRCRRCRACVRTECGDC
HSA-Fbxl19   -------MGMKVPGKGESGPSALLTPPMSSSSRGPGAGARRRRTRCRRCRACVRTECGDC
PTR-Fbxl19   ------------------------------------------------------------
MMU-Fbxl19   ----------MKQSCLLRQCTAPVLPHTAVCLLCGEAGKEDTVEGEEEKFGLSLMECTIC
GGO-Kdm2A    GVPIVQWPKRDKKPQASMKPAPRLTPVRPAAASPIVSGARRRRVRCRKCKACVQGECGVC
MMU-Kdm2A    TRPKVRVPTIPITKPHTMKPAPRLTPVRPAAASPIVSGARRRRVRCRKCKACVQGECGVC
CJA-Kdm2A    TRPKVRVPTIPITKPHTMKPAPRLTPVRPAAASPIVSGARRRRVRCRKCKACVQGECGVC
PPY-Kdm2A    TRPKVRVPTIPITKPHTMKPAPRLTPVRPAAASPIVSGARRRRVRCRKCKACVQGECGVC
PTR-Kdm2A    TRPKVRVPTIPITKPHTMKPAPRLTPVRPAAASPIVSGARRRRVRCRKCKACVQGECGVC
HSA-Kdm2A    TRPKVRVPTIPITKPHTMKPAPRLTPVRPAAASPIVSGARRRRVRCRKCKACVQGECGVC
MUS-Kdm2A    TRPKVRVPTIPITKPHTMKPAPRLTPVRPAAASPIVSGARRRRVRCRKCKACVQGECGVC
RNO-Kdm2A    TRPKVRVPTIPITKPHTMKPAPRLTPVRPAAASPIVSGARRRRVRCRKCKACVQGECGVC
MMU-Kdm2B    PVVTWPKKTPKNRAVGRPKGKLGPASAVKLAANRTTAGARRRRTRCRKCEACLRTECGEC
CJA-Kdm2B    PVVTWPKKTPKNRAVGRPKGKLGPASAVKLAANRTTAGARRRRTRCRKCEACLRTECGEC
PPY-Kdm2B    PVVTWPKKTPKNRAVGRPKGKLGPASAVKLAANRTTAGARRRRTRCRKCEACLRTECGEC
GGO-Kdm2B    PVVTWPKKTPKNRAVGRPKGKLGPASAVKLAANRTTAGARRRRTRCRKCEACLRTECGEC
PTR-Kdm2B    PVVTWPKKTPKNRAVGRPKGKLGPASAVKLAANRTTAGARRRRTRCRKCEACLRTECGEC
HSA-Kdm2B    PVVTWPKKTPKNRAVGRPKGKLGPASAVKLAANRTTAGARRRRTRCRKCEACLRTECGEC
MUS-Kdm2B    PVVSWPKKTAKNRVVGRPKGKLGPASAVKLAANRTTAGARRRRTRCRKCEACLRTECGEC
RNO-Kdm2B    PVVSWPKKTPKNRVVGRPKGKLGPASAVKLAANRTTAGARRRRTRCRKCEACLRTECGEC
CJA-Fbxo46   SLLPFQLWCPRPFGTYSQNQPRPASAALKPSACAEPGGGVEPDHGPAHSENTPPALATEA
PTR-Fbxo46   SLLPFQLWCPRPFGTYSQNQPRPPSAALKPSACPEPGGGAEPDHGPAHSENTPPALATEV
MMU-Fbxo46   SLLPFQLWCPRPFGTYSQNQPRPPSAALKPSACPEPGGGAEPDHGPAHSENTPPALATEV
PPY-Fbxo46   SLLPFQLWCPRPFGTYSQNQPRPPSAALKPSACPEPGGGAEPDHGPAHSENTPPALATEV
GGO-Fbxo46   SLLPFQLWCPRPFGTYSQNQPRPPSAALKPSACPEPGGGAEPDHGPAHSENTPPALAAEV
HSA-Fbxo46   SLLPFQLWCPRPFGTYSQNQPRPPSAALKPSACPEPGGGAEPDHGPAHSENTPPALATEV
MUS-Fbxo46   SLLPFQLWCPRPFSKYSQNQPRPPSTALKPPVCPDTSSGTEPDHRPAHLESTPPAVAAEA
RNO-Fbxo46   SLLPFQLWCPRPFSKYSQNQPRPPSATLKPPVCPDTSSGTEPDHRPAHLESTPPALAAEA
CJA-Fbxo34   QRTPMNHQKAVNDETCNPSHITPSVFPSASLGKTLSRKPFGILSPNVLCSMSGKSPVESS
MMU-Fbxo34   QRTPMNHQKAVNDETCKASHVTPSVFPSASLGKASSRKPFGILSPNVLCSMSGKSPVESS
PPY-Fbxo34   ---------------------MKIKSSWDIDGRATKRRKKSGDLKKAKVQVERMREVNSR
HSA-Fbxo34   QRTPMNHQKAVNDETCKASHITSSVFPSASLGKASSRKPFGILSPNVLCSMSGKSPVESS
GGO-Fbxo34   QRTPMNHQKAVNDETCKASHITPSVFPSASLSKASSRKPFGILSPNVLCSMSGKSPVESS
```

```
PTR-Fbxo34   QRTPMNHQKAVNDETCKASHITPSVFPSASLGKASSRKPFGILSPNVLCSMSGKSPVESS
MUS-Fbxo34   GLRRSALSSWSASVSGSESGLPPPHRAAAAPGQALARQKHRASVMHLKLYWKLQKKERPL
RNO-Fbxo34   SVSGSESGLPPPHRAAAAPGQAPAGQTHRASVMHLKPYWKLQKKERPLSHGNTDGDAKGR
MUS-Fbxo36   ------------------------------------------------------------
RNO-Fbxo36   ------------------------------------------------------------
CJA-Fbxo36   ------------------------------------------------------------
MMU-Fbxo36   ------------------------------------------------------GQTALI
GGO-Fbxo36   -------------------------------------------------------QTALI
PPY-Fbxo36   ------------------------------------------------------------
HSA-Fbxo36   ------------------------------------------------------------
PTR-Fbxo36   ------------------------------------------------------------
CJA-Fbxl8    ------------------------------------------------------------
MMU-Fbxl8    ------------------------------------------------------------
GGO-Fbxl8    ------------------------------------------------------------
PPY-Fbxl8    ------------------------------------------------------------
PTR-Fbxl8    ------------------------------------------------------------
HSA-Fbxl8    ------------------------------------------------------------
MUS-Fbxl8    ------------------------------------------------------------
RNO-Fbxl8    ------------------------------------------------------------
MUS-Fbxo15   ------------------------------------------MEESELEIFRSKFVRGSS
RNO-Fbxo15   ------------------------------------------------------------
CJA-Fbxo15   ------------------------------------------------------------
MMU-Fbxo15   ------------------------------------------------------------
PPY-Fbxo15   ------------------------------------------------------------
HSA-Fbxo15   ---------------MATGRGRILQQHWLGLQTLRGPSRGGGAARGRARAFGCRKGPGVK
PTR-Fbxo15   ---------------MATGRGRILRQHWLGLQTLRGPSRGGGAARGRARAFGCRKGPGVK
GGO-Fbxo15   -----------------------------------------------------------K
MUS-Fbxl6    RSSKRPDARGRSAEDWWWDRLAPRGSGYHLLQADSMLLVLPDLEPPRARAHRRARRRAPR
RNO-Fbxl6    RGSKRPDARGRSAEDWWWDRLAPRGSGYHLLQADSMLLVLPDLEPTRARAHRRAPRRAPR
CJA-Fbxl6    SAEDWWWDRLAPSGSGYHLLQSDSMLLVLSEPGSARSRAQRRATRRAPRPPPGPRAAAKP
MMU-Fbxl6    AEDWWWDRLAPSGSGYHLLPSDSMLLVLSEPGPARTRAQRRAARRTPRAARRTPRQPPPG
PPY-Fbxl6    --------MAAPASGQVRRRARAAPRPRKAEDWWWDRLAPRGSGYHLLQSDNMLLAQAQP
PTR-Fbxl6    WWDRLAPRGSGYHLLQSDSMLLVLSEPGPARPRAQRRASRRTPRQAPPGPSAAAKPKAGL
GGO-Fbxl6    DRLAPRGSGYHLLQSDSMLLVLSEPGPARPRAQRRASRRTPRQPPPGPSAAAKPKARLRP
HSA-Fbxl6    WWDRLAPRGSGYHLLQSDSMLLVLSEPGPARPRAQRRASRRTPRQPPRGPSAAAKPKAGL
MUS-Skp2     QSGNVTTSFTWGWDSSKTSELLSGMGVSALEKEEVDSENIPHGLLSNLGHPQSPPRKRVK
RNO-Skp2     QSSNVTTSFTWGWDSSKTSELLSGMGVSALEKEEVDSENIPHGLLSNLGHPQSPPRKRLK
PTR-Skp2     LSSNVATSFTWGWDSSKTSELLSGMGVSALEKEEPDSENIPQELLSNLGHPESPPRKRLK
HSA-Skp2     LSSNVATSFTWGWDSSKTSELLSGMGVSALEKEEPDSENIPQELLSNLGHPESPPRKRLK
CJA-Skp2     LSSNVATSFTWGWDSSKTSELLSGMGVSALEKEEPDSENIPQELLSNLGIPQSPPRKRLK
GGO-Skp2     LSSNVATSFTWGWDSSKTSELLSGMGVSALEKEEPDSENIPRELLSNLGHPESPPRKRLK
PPY-Skp2     LSSNVATSFTWGWDSSKTSELLSGMGVSALEKEEPDSENIPQELLSNLGHPESPPRKRLK
MUS-Fbxl16   GIDGDPKPPCLPRNGLVKLPGQPNGLGAASITKGTPAAKNRPCQPPPPPTLPPPSLATPL
RNO-Fbxl16   GIDGDPKPSCLPRNGLVKLPGQPNGLGAASITKGTPAAKNRPCQPPPPPTLPPPSLATPL
PTR-Fbxl16   GIDGDPKPPCLPRNGLVKLPGQPNGLGAASITKGTPATKNRPCQPPPPPTLPPPSLAAPL
```

```
CJA-Fbxl16   GIDGDPKPPCLPRNGLVKLPGQPNGLGAASITKGTPATKNRPCQPPPPPTLPPPSLAAPL
MMU-Fbxl16   GIDGDPKPPCLPRNGLVKLPGQPNGLGAASITKGTPATKNRPCQPPPPPTLPPPSLAAPL
PPY-Fbxl16   GIDGDPKPPCLPRNGLVKLPGQPNGLGAASITKGTPATKNRPCQPPPPPTLPPPSLAAPL
GGO-Fbxl16   GIDGDPKPPCLPRNGLVKLPGQPNGLGAASITKGTPATKNRPCQPPPPPTLPPPSLAAPL
HSA-Fbxl16   GIDGDPKPPCLPRNGLVKLPGQPNGLGAASITKGTPATKNRPCQPPPPPTLPPPSLAAPL
MUS-Fbxo39   ------------------------------------------------------------
RNO-Fbxo39   ------------------------------------------------------------
CJA-Fbxo39   ------------------------------------------------------------
MMU-Fbxo39   ------------------------------------------------------------
PPY-Fbxo39   ------------------------------------------------------------
GGO-Fbxo39   ------------------------------------------------------------
PTR-Fbxo39   ------------------------------------------------------------
HSA-Fbxo39   ------------------------------------------------------------
PPY-Fbxl17   RPRCCSWCRRRRPLLRLPRRTPAKVPPQPAAPRSRDCFFRGPCMLCFIVHSPGAPAPAGP
MMU-Fbxl17   ------------------------------------------------------------
RNO-Fbxl17   --PLAASSCPRPPPPPPPRRPRPASPASECAPIEAAAGDAVRAGGTAPSSAQQQPESGDS
MUS-Fbxl17   PSQKRPRCCSWCRRRRPLLRLPRRALAKASPQPAAPRSRDCFFRGPCMLCFIVHSPGAPA
CJA-Fbxl17   PSQKRPRCCSWCRRRRPLLRLPRRTPAKVPPQPAAPRSRDCFFRGPCMLCFIVHSPGAPA
PTR-Fbxl17   PSQKRPRCCSWCRRRRPLLRLPRRTPAKVPPQPAAPRSRDCFFRGPCMLCFIVHSPGAPA
HSA-Fbxl17   PSQKRPRCCSWCRRRRPLLRLPRRTPAKVPPQPAAPRSRDCFFRGPCMLCFIVHSPGAPA
MUS-Fbxw9    ------MDLSSGRSGDPRSCEEESDPEPDPDPDTQAEAYVARVLTPPKLGLTPRRSSLQS
RNO-Fbxw9    ------MELPSGQCGNPRSCEDESDPEPEPDPDAQAEAYVARVLTPPKPGMTPRRSSLQS
CJA-Fbxw9    ------MELPLGPCDDSRGWDDDSDPESETDPDAQAEAYVARVLSPPKSELATPRPSQLS
MMU-Fbxw9    ------MELPLGQCHDSRSWDDDSDPESETDPDAQAEAYVARVLSPPKSGLAFPRPSQLS
PPY-Fbxw9    ------MELPLGPCDDSRAWDDDSDPESETDPDAQAEAYVARVLSPPKSGLAFSRPSQLS
HSA-Fbxw9    ------MELPLGRCDDSRTWDDDSDPESETDPDAQAKAYVARVLSPPKSGLAFSRPSQLS
GGO-Fbxw9    ------MELPLGRCDDSRTWDDDSDPESETDPDAQAKAYVARVLSPPKSGLAFSRPSQLS
PTR-Fbxw9    -----MELPLGRCDDSRTWDNDESDPESETDPDAQAKAYVARVLSPPKSGLAFSRPSQLS
MUS-Fbxo22   ------------------------------------------------------------
RNO-Fbxo22   ------------------------------------------------------------
CJA-Fbxo22   ------------------------------------------------------------
MMU-Fbxo22   -----------------------------------------------------------M
PPY-Fbxo22   ------------------------------------------------------------
GGO-Fbxo22   ------------------------------------------------------------
PTR-Fbxo22   ------------------------------------------------------------
HSA-Fbxo22   ------------------------------------------------------------
CJA-Fbxl7    --------------DSDLSMRTLSTPSPALICPPNLPGFQNGRGSSTSSSSITGETVAMV
MUS-Fbxl7    HTPTKAQRNVATSEDSDLSMRTLSTPSPALICPPTLPGFQNGRGSSTSSSSITGETVAMV
RNO-Fbxl7    --------------DSDLSMRTLSTPSPALICPPALPGFQNGRGSSTSSSSITGETVAMV
PPY-Fbxl7    --------------DSDLSMRTLSTPSPALICPPNLPGFQNGRGSSTSSSSITGETVAMV
GGO-Fbxl7    --------------DSDLSMRTLSTPSPALICPPNLPGFQNGRGSSTSSSSITGETVAMV
MMU-Fbxl7    HTPTKAQKNVATSEDSDLSMRTLSTPSPALICPPNLPGFQNGRGSSTSSSSITGETVAMV
PTR-Fbxl7    HTPTKAQKNVATSEDSDLSMRTLSTPSPALICPPNLPGFQNGRGSSTSSSSITGETVAMV
HSA-Fbxl7    HTPTKAQKNVATSEDSDLSMRTLSTPSPALICPPNLPGFQNGRGSSTSSSSITGETVAMV
MMU-Fbxl2    ------------------------------------------------------------
```

```
CJA-Fbxl2    ------------------------------------------------------------
GGO-Fbxl2    ------------------------------------------------------------
PPY-Fbxl2    ------------------------------------------------------------
PTR-Fbxl2    ------------------------------------------------------------
HSA-Fbxl2    ------------------------------------------------------------
MUS-Fbxl2    ------------------------------------------------------------
RNO-Fbxl2    ------------------------------------------------------------
MMU-Fbxl20   ------------------------------------------------------------
RNO-Fbxl20   -----------------------------------------------------------G
MUS-Fbxl20   ------------------------------------------------------------
GGO-Fbxl20   ------------------------------------------------------------
PPY-Fbxl20   ------------------------------------------------------------
CJA-Fbxl20   ------------------------------------------------------------
PTR-Fbxl20   ------------------------------------------------------------
HSA-Fbxl20   ------------------------------------------------------------
MUS-Fbxl15   ------------------------------------------------------------
RNO-Fbxl15   ------------------------------------------------------------
PPY-Fbxl15   --------------MRSGFRCNEPAEAVAPKERQTVSGAVALASFTSPHTHPVDDGGRHY
CJA-Fbxl15   ------------------------------------------------------------
MMU-Fbxl15   ------------------------------------------------------------
GGO-Fbxl15   ------------------------------------------------------------
HSA-Fbxl15   ------------------------------------------------------------
PTR-Fbxl15   ------------------------------------------------------------
RNO-Fbxl14   ----------------------------------------------------------LG
HSA-Fbxl14   ------------------------------------------------------------
PPY-Fbxl14   ------------------------------------------------------------
MMU-Fbxl14   ------------------------------------------------------------
RNO-Fbxl14   ------------------------------------------------------------
MUS-Fbxl14   ------------------------------------------------------------
CJA-Fbxl14   ------------------------------------------------------------
CJA-Fbxl13   KEKLKNMVLRIQRIIYCHKLTIILTKWRNKARCKNKKKEDELVLKHELQLKKWRNRLLLK
GGO-Fbxl13   KEKLKNMLLRIQQIIYCHKLTIILTKWRNTARHKSKKKEDELILKHELQLKKWKNRLILK
MMU-Fbxl13   KEKLKNTLLRIQQIIYCHKLSIILTKWRNRARLKSKKKEDELILKHELQLKKWKNRLILK
PPY-Fbxl13   KEKLKNMLLRIQQIIYCHKLTIILTKWRNTARHKSKKKEDELILKHELQLKKWKNRLILK
HSA-Fbxl13   KEKLKNILLRIQQIIYCHKLTIILTKWRNTARHKSKKKEDELILKHELQLKKWKNRLILK
PTR-Fbxl13   KEKIKNMLLRIQQIIYCHKLTIILTKWRNTARHKSKKKEDELILKHELQLKKWKNRLILK
MUS-Fbxl13   FAYTVMSRERLITTLLRLRHLFYMQRQRIILAKWKERARHKSKTREDDLISKHELQLKKW
RNO-Fbxl13   -------MASLRNAPPRLRNYFKDNYIPQICEALLCGLLVTCPEDPLKYLESMILAIIEN
CJA-Fbxo45   --------------------------------------------------RAGRAVSAPA
MUS-Fbxo45   ---------------------------------------------------MAAPGPGAG
GGO-Fbxo45   ------------------------------------------------------------
HSA-Fbxo45   -------------------------------------------------MAAPAPGAGAA
MUS-Fbxl12   ------------------------------------------------------------
RNO-Fbxl12   ------------------------------------------------------------
CJA-Fbxl12   ------------------------------------------------------------
```

```
GGO-Fbxl12   ------------------------------------------------------------
MMU-Fbxl12   ------------------------------------------------------------
PPY-Fbxl12   ------------------------------------------------------------
PTR-Fbxl12   ------------------------------------------------------------
HSA-Fbxl12   ------------------------------------------------------------
PPY-Fbxw4    ----------------------------------------------------------MA
GGO-Fbxw4    ---------------------------------------------------------MAA
MMU-Fbxw4    -----------------------------------------------------------M
HSA-Fbxw4    ---------------------------------------------------------MAA
CJA-Fbxw4    -------------------------------------------------------MAAAA
MUS-Fbxw4    -----------------------------------------------------------M
RNO-Fbxw4    ------------------------------------------------------------
MUS-Fbxo10   --------METGGLPLELWRMILAYLHLPDLGRCSLVCRAWYELILSLDSTRWRQLCLGC
RNO-Fbxo10   --------METGGLPLELWRVILAYLHLPDLGRCSLVCRAWYELILSLDSTRWRQLCLGC
CJA-Fbxo10   --------METGGLPLELWRMILAYLHLPDLGRCSLVCRAWYELILSLDSTRWRQLCLGC
MMU-Fbxo10   --------MEAGGLPLELWRMILAYLHLPDLGRCSLVCRAWYELILSLDSTRWRQLCLGC
PPY-Fbxo10   --------MEAGGLPLELWRMILAYLHLPDLGRCSLVCRAWYELILSLDSTRWRQLCLGC
PTR-Fbxo10   --------MEAGGLPLELWRMILAYLHLPDLGRCSLVCRAWYELILSLDSTRWRQLCLGC
GGO-Fbxo10   --------MEAGGLPLELWRMILAYLHLPDLGRCSLVCRAWYELILSLDSTRWRQLCLGC
HSA-Fbxo10   --------MEAGGLPLELWRMILAYLHLPDLGRCSLVCRAWYELILSLDSTRWRQLCLGC
MUS-Fbxo31   ---------------------------------MAVCARLCGVGPARGCRRRQQRRGPAE
RNO-Fbxo31   ---------------------------------MAVCARLCGVGPARGCRRRQQRRGPAE
CJA-Fbxo31   --------------MAVCARLCGVGPSRGCRRRQQRRGPAETAAADSEPDTDPEEERIEA
MMU-Fbxo31   --------------MAVCARLCGVGPSRGCRRRQQRRGPAETAAADSEPDTDPEEERIEA
PTR-Fbxo31   --------------MAVCARLCGVGPSRGCRRRQQRRGPAETAAADSEPDTDPEEERIEA
PPY-Fbxo31   --------------MAVCARLCGVGPSRGCRRRQQRRGPAETAAADSEPDTDPEEERIEA
GGO-Fbxo31   --------------MAVCARLCGVGPSRGCRRRQQRRGPAETAAADSEPDTDPEEERIEA
HSA-Fbxo31   ------------------MAVCARLCGVGPSRGCRRRQQRRGPAETAAADSEPDTDPEEE
RNO-Fbxo17   ------------------------------------------------------------
MUS-Fbxo17   ------------------------------------------------------------
CJA-Fbxo17   ------------------------------------------------------------
MMU-Fbxo17   ------------------------------------------------------------
PPY-Fbxo17   ------------------------------------------------------------
HSA-Fbxo17   ----------------------------------------------------------FK
GGO-Fbxo17   ------------------------------------------------------------
CJA-Fbxo27   ------------------------------------------------------------
MMU-Fbxo27   ------------------------------------------------------------
PPY-Fbxo27   ------------------------------------------------------------
GGO-Fbxo27   ------------------------------------------------------------
HSA-Fbxo27   ------------------------------------------------------------
PTR-Fbxo27   ------------------------------------------------------------
MUS-Fbxo27   ------------------------------------------------------------
RNO-Fbxo27   ------------------------------------------------------------
CJA-Fbxo2    ----------------------------------------MDGDGDPESVGQPEEASPEE
GGO-Fbxo2    ----------------------------------------MDGDGDPDSVGQPEEASPEE
```

```
PPY-Fbxo2     ----------------------------------------MDGDGDPESVGQPKEASPEE
PTR-Fbxo2     ----------------------------------------MDGDGDPESVGQPEEASPEE
HSA-Fbxo2     ----------------------------------------MDGDGDPESVGQPEEASPEE
MUS-Fbxo2     -----------------------------------MDGDGDPESVSHPEEASPEEQPEEA
RNO-Fbxo2     -----------------------------------MDGDGDPESVSHPEEASPEEQPEEA
HSA-Fbxo44    ------------------------------------------------------------
PPY-Fbxo44    ------------------------------------------------------------
MUS-Fbxo44    ------------------------------------------------------------
RNO-Fbxo44    ------------------------------------------------------------
GGO-Fbxo44    ------------------------------------------------------------
PTR-Fbxo44    ------------------------------------------------------------
MMU-Fbxo44    ------------------------------------------------------------
CJA-Fbxo6     ------------------------------------------------------------
GGO-Fbxo6     ------------------------------------------------------------
HSA-Fbxo6     ------------------------------------------------------------
PTR-Fbxo6     ------------------------------------------------------------
MMU-Fbxo6     ------------------------------------------------------------
PPY-Fbxo6     ------------------------------------------------------------
MUS-Fbxo6     ------------------------------------------------------------
RNO-Fbxo6     ------------------------------------------------------------

AGRPRPSDSCKEEESSTLSVKMKCDFN---CNHVHSGLKLVKPDD----IG---RR---V
AGRPRPSDSCKEEESSTLSVKMKCDFN---CNHVHSGLKLVKPDD----IGRL------V
AAERPRPSDSCKEESSTLSVKMKCDFN---CNNVHSGLKLVKPDD----IGRL------V
AAGRPRPSDSCKEESSTLSVKMKCDFN---CHVHS-GLKLVKPDD----IGRL------V
AAGRPRPSDSCKEESSTLSVKMKCDFN---CHVHS-GLKLVKPDD----IGRL------V
AAGRPRPSDSCKEESSTLSVKMKCDFN---CHVHS-GLKLVKPDD----IGRL------V
RLGARTTVDGCKEESPVLSVTMKCFNC-NP-DLSE--LEVVKPEDGIEAYSPVC--LEPS
PLDARTTVDGCKEESPVLSVTMKCFNC-NPDLELE-VVKTEDSGR-EGSYSPVC--LEPS
WVQSRHWWVSGPSGQNVTMDEGGLPL--LPDSLVYQIFLSLGPAD-VLAAGLVCRQWQAV
WVESSHWWVSGPSGQNVTMDEGGMPL--LPDSLVYQIFLSLGPAD-VLAAGLVCRQWQAV
------------------MDEGGTPL--LPDSLVYQIFLSLGPAD-VLAAGLVCRQWLAV
------------------MDEGNTPL--LPDSLVYQIFLSLGPTD-VLAAGLVCRQWQAV
------------------MDEGGTPL--LPDSLVYQIFLSLGPAD-VLAAGLVCRQWQAV
------------------MDEGGTPL--LPDSLVYQIFLSLGPAD-VLAAGLVCRQWQAV
-----------------------MEIR-LPDLALKRIFSFLDVFS-LLQVSQVNKDWNRV
-----------------------MEIQ-LPL-ALKQIFSFLDLFG-LLQASRTNNHWNRI
-----------------------MEIR-LPDLALKRIFSFLDLFG-LLQVSQVNKHWNRI
-----------------------MEIR-LPDLALKRIFSFLDLFG-LLQVSQVNKHWNRI
-----------------------MEIR-LPDLALKRIFSFLDLFG--LLVSQVNKHWNRI
-----------------------MEIR-LPDLALKRIFSFLDLFG-LLQVSQVNKHWNRI
-------------MAAMETETAPLTLESLPTPLLL-ILSFLDYRD-LINCCYVSRRLSQL
-------------MAAMETDTAQLTLESLPTPLLL-ILSFLDYRD-LINCCYVSRRLSQL
-------------MAAMETETAPLTLESLPTPLLL-ILSFLDYRD-LINCCYVSRRLSQL
-------------MAAMETETAPLTLESLPTPLLL-ILSFLDYRD-LINCCYVSRRLSQL
```

-------------MAAMETETAPLTLESLPTPLLL-ILSFLDYRD-LINCCYVSRRLSQL
-------------MAAMETETAPLTLESLPTPLLL-ILSFLDYRD-LINCCYVSRRLSQL
-------------MAAVEAETGLLTLESLPTPLLL-ILSFVDYRD-LINCCYVSRRLSQL
-------------MAAVEAETGLLTLESLPTPLLL-ILSFVDYRD-LINCCYVSRRLSQL
----------------MAPQLGPHELQ--------HIFSFLEARD-LLRAAQVNKVWNEV
----------------MALQLGPHELQ--------HVFSFLEARD-LLRAAQVNKVWNEV
IHCRCAKCFCYPTKRRIKRRPRNLTILSLPEDVLFHILKWLSVGDSHLKYLVDNSVWASA
IHCRCAKCFCYPTKRRIKRRPRNLTIL-LPEDVLFHILKWLSVGDSHLKYLVDNHAWASA
VHCRCAKCFCYPTKRRVRRRPRNLTIL-LPEDVLFHILKWLSVEDVHSQLKDLVHAWACA
VHCRCAKCFCYPTKRRIRRRPRNLTIL-LPEDVLFHILKWLSVEDSQLKDLVDNSVWACA
VHCRCAKCFCYPTKRRIRRRPRNLTIL-LPEDVLFHILKWLSVEDSQLKDLVDNTVWACA
VHCRCAKCFCYPTKRRIRRRPRNLTIL-LPEDVLFHILKWLSVEDSQLKDLVDNSVWACA
VHCRCAKCFCYPTKRRIRRRPRNLTIL-LPEDVLFHILKWLSVEDSQLKDLVDNSVWACA
VHCRCAKCFCYPTKRRIRRRPRNLTIL-LPEDVLFHILKWLSVEDSQLKDLVDNSVWACA
--------------MHITQLNRECLLC---------LFSFLDKDS-RRSLSRTCSQLRDV
--------------MHITQLNRECLLR---------LFSFLDKDS-RKSLSRTCSQLRDV
--------------MHITQLNRECLLH---------LFSFLDKDS-RKSLARTCPQLREV
--------------MHITQLNRECLLH---------LFSFLDKDS-RKSLARTCSQLHDV
--------------MHITQLNRECLLH---------LFSFLDKDS-RKSLARTCSQLHDV
--------------MHITQLNRECLLH---------LFSFLDKDS-RKSLARTCSQLHDV
--------------MHITQLNRECLLH---------LFSFLDKDS-RKSLARTCSQLHDV
--------MWPLLTMHITQLNRECLLH---------LFSFLDKDS-RKSLARTCSQLHDV
HEHPETSSLDLTHPLESPTQKKKFILLKTP-----ELCETPKISDRRLNLSFSLGDFESV
HEHPETSGLGLTHSLESPTQKKKFI---LPE-LCEKISGKKCLPRLHVSFSLLKGDFESV
YEHPETSGLGLTHPLESPTQKKKCILPKTPE-LCEKISGKKCLPRLNVSFALLKGDFESV
YEHPETSGLGLKHPLESPTQKKKCILPKTPE-LCEKISGKKCLPRLNVSFALLKGDFESV
YEHPETSGLGLTHPLESPTQKKKCILPKTPE-LCEKISGKKCLPRLNVSFALLKGDFESV
YEHPETSGLGLTHPLESPTQKKKCILPKTP-----ELCETPKISGLNVSFALLKGDFESV
EGTMEQDEEPHLALEAEETRHNRSMLE-LPEEVLEYILSFLSPYQEHKTAALVCKQWYRL
EGTMEQDEEPHPVLEADETRHNRSMSE-LPEEVLEYILSFLSPYQEHKTAALVCKQWYRL
EGTMEQDEEPHPVLEAEETRHNRSMSE-LPEEVLEYILSFLSPYQEHKTAALVCKQWYRL
EGTMDQDEEPHPLLEAEETRHNRSMSE-LPEEVLEYILSFLSPYQEHKTAALVCKQWYRL
EGTMDQDEEPHPVLEAEETRHNRSMSE-LPEEVLEYILSFLSPYQEHKTAALVCKQWYRL
EGTMDQDEEPHPVLEAEETRHNRSMSE-LPEEVLEYILSFLSPYQEHKTAALVCKQWYRL
EGTMEQDEDPHPVLEVEETRHNRSMSE-LPEEVLEYILSFLSPYQEHKTAALVCKQWYRL
EGTMEQDEDPHPVLEVEETRHNRSMSE-LPEEVLEYILSFLSPYQEHKTAALVCKQWYRL
APAASSGEDMSSEEEAAAEAAGDTHLLGFSDEILLHILSHVPSTDRTCRLAALCKSLQKL
EIQQLNMAGCYWLPGSTIEHVARCHSL-VKSRLSR-VLSCLDVSPSECK-ATLSQELKQC
EIQQLNMAGCYWLPGSTIEHVARCHSL-VKSLRLSRVLSCLDVSPSECKATLSRQELKQC
EIQQLSMAGCYWLPGSTVEHVARCRSLNLSSKMLS-ALQHLDVSPSECK-ATLSRELKQC
EIQQLSMAGCYWLPGSTVEHVARCRSLNLSSKMLS-ALQHLDVSPSECKATLSRRELKQC
EIQQLSMAGCYWLPGSTVEHVARCRSLNLSSKMLS-ALQHLDVSPSECK-ATLSRELKQC
EIQQLSMAGCYWLPGSTVEHVARCRSLNLSSKMLS-ALQHLDVSPSECK-ATLSRELKQC
EIQQLSMAGCYWLPGSTVEHVARCRSLNLSSKMLS-ALQHLDVSPSECK-ATLSRELKQC
MPDVEQLYGLHPRYLERRRVRGQEAFS-IPC-PNLEIWTYMPENKAKIQTLHLVNVWVRL

MPDVEQLYGLHPRYLERRRVRGHEAFSACPLELVESIWTYMIPPEAKIQTLHLVNVWVRL
MPDVEQLYGLHPRYLERRRVRGHEAFSACPLELVESIWTYMIPPEAKIQTLHLVNVWVRL
MPDVEQLYGLHPRYLERRRVRGHEAFSACPLELVESIWTYMIPPEAKIQTLHLVNVWVRL
MPDVEQLYGLHPRYLERRRVRGHEAFSACPLELVESIWTYMIPPEAKIQTLHLVNVWVRL
MPDVEQLYGLHPRYLERRRVRGHEAFSACPLELVESIWTYMPENKAKIQTLHLVNVWVRL
MPDVEQLYGLHPRYLERRRVRGHEAFSACPLELVESIWTYMPENKAKIQTLHLVNVWVRL
MPDVEQLYGLHPRYLERRRVRGHEAFSACPLELVESIWTYMPENKAKIQTLHLVNVWVRL
HPQPGSQALPAPALAPDQLPQNNTLVA-LPIAIEN-ILSFMSYDECRRDKSNFCRR---I
APPPGSQAPAAPALAPDHLPQNNTLVA-LPIVAIENILSFMSYDELVCKMDLVCQR---M
ALQPGSQAPAAPALAPDHLPQNNTLVA-LPIVAIENILSFMSYDE-ISQLRLVCKRMDLV
HPQPGSQALPAPALAPDQLPQNNTLVA-LPIVAIENILSFMSYDELVCKMDLVCQR---M
HPQPGSQALPAPALAPDQLPQNNTLVA-LPIVAIENILSFMSYDELVCKMDLVCQR---M
HPQPGSQALPAPALAPDQLPQNNTLVA-LPIVAIENILSFMSYDE-ISQLRLVCKRMDLV
HPQPGSQALPAPALAPDQLPQNNTLVA-LPIVAIENILSFMSYDE-ISQLRLVCKRMDLV
HPQPGSQALPAPALAPDQLPQNNTLVA-LPIVAIENILSFMSYDE-ISQLRLVCKRMDLV
LVPASLPCEELAEPGLVPAAAARYALREIPS-VASSACSTPPPGPGPASASPASPSPADV
LVPASLPCEELAEPGLVPAARYALREI-IPS-VASSACSTPPPGPGPTSASPASPSPADV
LVPASLPCEELAEPGLVPAARYALREI-IPS-VASSACSTPPPGPGPSSASPASPSPADV
LVPASLPCEELAEPGLVPAAAARYALREIPS-VASSACSTPPPGPGPASASPASPSPADV
LVPASLPCEELAEPGLVPAAAARYALREIPKSVASSACSTPPPGPGPASASPASPSPADV
LVPASLPCEELAEPGLVPAAAARYALREIPKSVASSACSTPPPGPGPASASPASPSPADV
CPGPASASPASPSPADVAYEEGLARLKKLEEEVEQKIAGQVGRLQELETARQESARLGRL
LVPASLPCEELAEPGLVPAAAARYALREIPKSVASSACSTPPPGPGPASASPASPSPADV
-----------------------MAES-LPQEMLTYILSFLPLSD-QKEASLVSWAWYRA
-----------------------MAKS-LPLEMLTYILSFLPLSD-QKEASLVSWAWYRA
-----------------------MAES-LPLEMLTYILSFLPLSD-QKEASLVSWAWYRA
-----------------------MAES-LPLEMLTYILSFLPLSD-QKEASLVSWAWYRA
LIGCPLVCGAVFHSCKADEHRLLCPFERVPCLNSDFGCPFTMARNEMCPASVVCMEWNRW
LIGCPLVCGAVFHSCKAEEHRLLCPFERVPCLNSDFGCPFTMARNEMCPASVVCMEWNRW
LIGCPLVCGAVFHSCKADEHRLLCPFERVPCLNSDFGCPFTMARNEMCPASVVCMEWNRW
LIGCPLVCGAVFHSCKADEHRLLCPFERVPCLNSDFGCPFTMARNEMCPASVVCMEWNRW
LIGCPLVCGAVFHSCKADEHRLLCPFERVPCLNSDFGCPFTMARNEMCPASVVCMEWNRW
LIGCPLVCGAVFHSCKADEHRLLCPFERVPCLNSDFGCPFTMARNEMCPASVVCMEWNRW
LIGCPLVCGAVFHSCKADEHRLLCPFERVACNRNF-GCPFTLARNEMCPASVVCMEWNRW
LIGCPLVCGAVFHSCKADEHRLLCPFERVPCLNSNFGCPFTLARNEMCPASVVCMEWNRW
VISCHLLCGATFHMCKEAEHQLLCPLEQVPCNSKY-GCPLSMSRHQVCPASVVCMEWNRW
VISCHLLCGATFHMCKEAEHQLLCPLEQVPCNSEY-GCPLSMSRHQVCPASVVCMEWNRW
VISCHLLCGATFHMCKEAEHQLLCPLEQVPCNSEY-GCPLSMSRHQVCPASVVCMEWNRW
VISCHLLCGATFHMCKEAEHQLLCPLEQVPCNSEY-GCPLSMSRHQVCPASVVCMEWNRW
VISCHLLCGATFHMCKEAEHQLLCPLEQVPCNSEY-GCPLSMSRHQVCPASVVCMEWNRW
VISCHLLCGATFHMCKEAEHQLLCPLEQVPCNSEY-GCPLSMSRHQVCPASVVCMEWNRW
VISCHLLCGATFHMCKESEHTLLCPLEQVPCNSEY-GCPLSMARHQVCPASVVCMEWIRW
VISCHLLCGATFHMCKESEHTLLCPLEQVPCNSEY-GCPLSMARHQVCPASVVCMEWNRW
PLRADEDDEENESLAETDLQAQLQMFR---AQWMFEGVSSSNLENRACRATRGS--LQKT
VRADDDEENESPAETDLQAQLQMFRAQ--------WMFELAPGVS-SSNENRPCRAARGS

EDCHSDTVRADDDEENESPAETDLQAQ-LQ---MFRMFELAPGVS-SSNENRPCRAARGS
RGGTESPSSASGRPASMAEAEEDCHSD---------TVRADDDEELQAQLQMFRAQWMFE
AEEDCHSDTVRADDDEENESPAETDLQ---ALQMFRMFELAPGVS-SSNENRPCRAARGS
TFQESVLKLCQPELESSQTHISVLPME----VLMY-IFRWVVSSDSLEQLSLVCRGFYIC
ADRVGDEGNESPAERDLQAQLQMFRAQ--------WMFELTPGVG-SSHETRPCGRSSML
AVRVGDEGHESPAERDLQAQLQMFRAQ--------WMFELTPGVG-SSNESRPCGRSSIL
LICLVLEDAIPAPNLPSSTDSEHSSLQDQP------SLATSSNQT-SIQGEQLSDSFQGQ
LICLILQDDIPAPNIPSSTDSEHSSLQEQP------SLATISNQT-SMQDEQPSDSFQGQ
LICLILQDDIPAPNIPSSTDSEHSSLQEQPSLATSSSMQDEQPSD-SFQQAAQSGVWND-
LICLILQDDIPAPNIPSSTDSEHSPLQEQP------SLATSSNQT-SIQDEQPSDSFQGQ
TGDEETLASYGIVSGDLICLILQDDIPNLPSSTDS-EHSSLQNNDNETSIQDEQDSFQGQ
LLDSEHSSLQNNEQPSLATSSNQTSIQ---DEQPSDSFQGQAAQS---------DVWND-
LICLVLEDDMPAPNLPSSTDTEHSSLQDQP----S-LAATPSQTNTDSSQGQATDAWTD-
LICLVLEDEMPAPNLPSSTDSEHSSLQ-PP-LAA--TSSQANIPDSDSSHGQVTDAWTD-
RSNHQTSCYSKTLGSGFQPISTFGNFKALPLEIFQIILKYLSVKDMLSMSKTVSHHIVNY
RSNRQTSCYSKTLGSGFQPTSTFGNFKALPLEIFQIILKYLSVKDMLSMSKTVSQHIINY
RSNRQTSCCSKTLGSGFQPISTFGNFKALPLEIFQIILKYLSVKDMLSMSKTVSQHIINY
RSNRQTSCYSKTLGSGFQPISTFGNFKALPLEIFQIILKYLSVKDMLSMSKTVSQHIINY
RSNRQTSCYSKTLGSGFQPISTFGNFKALPLEIFQIILKYLSVKDMLSMSKTVSQHIINY
RSNRQSSCYSKTLGSGFQPISTFGNFKALPLEIFQIILKYLSVKDMLSMSKTVSQHIINY
RSNHHSSYFCNTLDSDPQLLSTLGNFKVLPLILHI-ILRYLSVKDMLSMSKTVSQHIINY
RLNHQSSDFCNTLDSDPQLLSTLGNFK-LPLEILHIILRYLSVKDMLSMVSKTVHIINYI
EVVQYAKEVVDFSSHYGNENSMSYTMWGVP-----NVFPSSGDFT-QTAVFRTYGTWWDT
EVVQYAKEVVDFSSHYGSENSMSYTMWGVP-----NVFPSSGDFT-QTAVFRTYGTWWDT
EVVQYAKEVVDFSSHYGSENSMSYTMWGVP-----NVFPSSGDFT-QTAVFRTYGTWWDT
EVVQYAKEVVDFSSHYGSENSMSYTMWGVP-----NVFPSSGDFT-QTAVFRTYGTWWDT
EVVQYAKEVVDFSSHYGSENSMSYTMWGVP-----NVFPSSGDFT-QTAVFRTYGTWWDT
EVVQYAKEVVDFSSHYGSENSMSYTMRGVP-----NVFPSSGDFT-QTAVFRTYGTWWDT
EVVQYAKEVVDFSSHYGSENSMSYTMWGVP-----NVFPSSGDFT-QTAVFRTYGTWWDT
EVVQYAKEVVDFSSHYGSENSMSYTMWGVP-----NVFPSSGDFT-QTAVFRTYGTWWDT
FWQSVSKERVARTASREEVDEAASTLTRLPIDVQLYILSFLSPHD-LCQLGSTNHYWNET
FWQSMSKERVARTASREEVDEAASTLTRLPIDVQLYILSFLSPHD-LCQLGSTNHYWNET
FWQSVSKERVARTTSREEVDEAASTLTRLPIDVQLYILSFLSPHD-LCQLGSTNHYWNET
FWQSVSKERVARTTSREEVDEAASTLMRLPIDVQLYILSFLSPHD-LCQLGSTNHYWNET
FWQSVSKERVARTTSREEVDEAASTLTRLPIDVQLYILSFLSPHD-LCQLGSTNHYWNET
FWQSVSKERVARTTSREEVDEAASTLTRLPIDVQLYILSFLSPHD-LCQLGSTNHYWNET
FWYSVAKERATPTASRKEAAEETSALTRLPVDVQLYILSFLSPHD-LCQLGSTDHYWNKT
LSGWRTFWNSVGKERATRTASRKEEAERLPIDVQLYILSFLSPHD-LCQLGSTDHYWNET
RVSLTDTNSVDAEKENHESQNNFVDL--LPEEISLKIFSQLDMRS-LCRASLTCRSWNDT
RVSHTEVNSVDAEKEKNESENNFVEL--LPQELTFKIFSQLDIRS-LCRASLTCRSWNYT
RVSHTEANSVDAEKEKNESQNNFFEL--LPAEITFKIFSQLDIRS-LCRASLTCRSWNDT
GVSHTEANSVDAEKEKNESQNNFFEL--LPAEITFKIFSQLDIRS-LCRASLTCRSWNDT
RVSHTEANSVDAEKEKNESQNNFFEL--LPAEITFKIFSQLDIRS-LCRASLTCRSWNDT
RVSHTEVNSVDAEKEKNESQNNFVEL--LPTEITFKIFSQLDIRS-LCRASLTCRSWNDR
RVSHTEVNSVDAEKEKNESQNNFVEL--LPTEITFKIFSQLDIRS-LCRASLTCRSWNDR

KIPGTELNSADAERGKEESQRNFVEL--LPE-VTYKIFSQLDIQS-LCRASRTCTGWNCA
KVPGMELNSEDAEGGRKENQSNFVDL--LPLEVTFNIFSQLDIPS-LCSASRTCTSWNHA
NSSEEGTAEKSKKLRTTNEHSQTCDWGNLLQDIILQVFKYLPLLD-RAHASQVCRNWNQV
NSSEEGTTEKSKKLRTTNEHSQTCDWGNLLQDIILQVFKYLPLLD-RAHASQVCRNWNQV
NSSEEGTAEKSKKLRTTNEHSQTCDWGNLLQDIILQVFKYLPLLD-RAHASQVCRNWNQV
NSSEEGTAEKSKKLRTTNEHSQTCDWGNLLQDIILQVFKYLPLLD-RAHASQVCRNWNQV
NSSEEGTAEKSKKLRTTNEHSQTCDWGNLLQDIILQVFKYLPLLD-RAHASQVCRNWNQV
NSSEEGTAEKSKKLRTTNEHSQTCDWGNLLQDIILQVFKYLPLLD-RAHASQVCRNWNQV
DSAEEGTAEKPKRPRTTQERSQPCDWGNLLQDIVLHVFKYLPLLD-RAHASQVCRNWNQV
DSPEEGTAEKPKKPRTTHECSQPCDWGNLLQDIVLHVFKYLPLLD-RAHASQVCRNWNQV
PAVKQPKRGLCTSLRQTQMLSALLDWG-LPHHVILRIFQYLPLVD-RARASSVCRSWNEV
PVVKQPKRGLCSSLRQTHALSVLLDWG-LPHHVILQIFQYLPLID-RARASSVCRRWNEV
GAAKQPKVGFYSSLNQTHTHTVLLDWGSLPHHVVLQIFQYLPLLD-RACASSVCRRWNEV
GAAKQPKVGFYSSLNQTHTHTVLLDWG-LPHHVVLQIFQYLPLLD-RACASSVCRRWNEV
GAVKQPKLGFYSSLNQTHTHTVRLDWG-LPHHVVLRIFQYLPLLD-RARASSVCRRWNEV
---------------------------LP--LLD-----------RARASSVCRRWNEV
HRAKRARVSGKSQDLSAAPAEQYLQEK-LPDEVVLKIFSYLLEQD-LCRAACVCKRFSEL
HRAKRARVSGKSQDLSAAPAEQYLQEK-LPDEVVLKIFSYLLEQD-LCRAACVCKRFSEL
NSMEGASTSTTENFGHRAKRARVSGKSKLPDEVVLKIFSYLLEQD-LCRAACVCKRFSEL
STTENFGHRAKRARVSGKSQDLSAAPAKLPDEVVLKIFSYLLEQD-LCRAACVCKRFSEL
STTENFGHRAKRARVSGKSQDLSAAPAKLPDEVVLKIFSYLLEQD-LCRAACVCKRFSEL
STTENFGHRAKRARVSGKSQDLSAAPAKLPDEVVLKIFSYLLEQD-LCRAACVCKRFSEL
SSMEGASTSTTENFGHRAKRARVSGKSKLPDEVVLKIFSYLLEQD-LCRAACVCKRFSEL
HRAKRARVSGKSQDLSAAPAEQYLQEK-LPDEVVLKIFSYLLEQD-LCRAACVCKRFSEL
FVEHLISRMCHYQHGHINSYLKPMLQRALPEGLDHIILSYLDARS-LCAAELVCKEWQRV
FVEHLISRMCHYQHGHINSYLKPMLQRALPEGLDHIILSYLDARS-LCAAELVCKEWQRV
FVEHLISRMCHYQHGHINSYLKPMLQRALPEGLDHIILSYLDARS-LCAAELVCKEWQRV
FVEHLISRMCHYQHGHINSYLKPMLQRALPEGLDHIILSYLDARS-LCAAELVCKEWQRV
FVEHLISRMCHYQHGHINSYLKPMLQRALPEGLDHIILSYLDARS-LCAAELVCKEWQRV
FVEHLISRMCHYQHGHINSYLKPMLQRALPEGLDHIILSYLDARS-LCAAELVCKEWQRV
FVEHLISRMCHYQHGHINSYLKPMLQRALPEGLDHIILSYLDARS-LCAAELVCKEWQRV
FVEHLISRMCHYQHGHINSYLKPMLQRALPEGLDHIILSYLDARS-LCAAELVCKEWQRV
IVEHTRKRNKKLKAVNKMCLKLCPEWAARPPSVFFNILSYLDAKS-LCAAELVCKEWYRV
FVEHLISQMCHYQHGHINSYLKPMLQRALPAGLDHIILSYLDARS-LCAAELVCKEWYRV
FVEHLISQMCHYQHGHINSYLKPMLQRALPDHIAENILSYLDAKS-LCAAELVCKEWYRV
FVEHLISQMCHYQHGHINSYLKPMLQRALPDHIAENILSYLDAKS-LCAAELVCKEWYRV
CLNQETVCLGSTAMKTENCVAKTKLANIVPDHIAENILSYLDAKS-LCAAELVCKEWYRV
FVEHLISQMCHYQHGHINSYLKPMLQRALPDHIAENILSYLDAKS-LCAAELVCKEWYRV
FVEHLISQMCHYQHGHINSYLKPMLQRALPDHIAENILSYLDAKS-LCAAELVCKEWYRV
FVEHLISQMCHYQHGHINSYLKPMLQRALPDHIAENILSYLDAKS-LCAAELVCKEWYRV
-----------------------MEFH-LPSPMRK-ILSYLDAFS-LLQIAQVNKRWNTF
-----------------------MAIH-LPCPMMK-ILSYLDAYS-LLQAAQVNKDWNEL
--------------------MEIHLSS-FP--MME-IFSYLDAYS-LLQVAQVNKNWNAL
-----------------------MEIH-LPSPLMK-IFSYLDAYS-LLQAAQVNKDWNEL
---------------------METHLPSLPSPMMK-ILSYLDAYS-LLQVAQVNKNWNAL

```
-----------------------MEIH-LPRPLME-IFSYLDAYS-LLQVAQVNKNWNEL
-----------------------MEVH-LPRPLMK-IFSYLDAYS-LLQAAQVNKNWNEL
-----------------------MEVY-LPSPMMK-ILSYLDAYS-LLQVAQVNKNWNEL
-----------------------MEIH-LPSPMME-ILSYLDAYS-LLQAAQVNKNWNEL
-----------------------MEIH-LPSPMME-ILSYLDAYS-LLQAAQVNKNWNEL
-----------------------MEIH-LPSPMMK-ILSYLDAYS-LLQAAKVNKNWNEL
-----------------------MEIH-LPSLPMMEILSYLDAYS-LLQVAQVNKNWNAL
-----------------------MQTH-LPSPMME-IFSYLDAYS-LLQVAQVNKNWNAL
ATEPEPLVDQLIRDLNELDDVPFFDVR-LPE-LAINIFQYLNRREGLCA--QVSKTWKVI
AAEPEPLVDQLIRDLNEMDDVPFFDVH-LPYELAINIFQYLNRREGLCA--QVSKTWKVI
AEAQDVASRSGFQQLVDQLIRDLNEMNQLPYELAINIFQYLDRKE-LGRCAQVSKTWKVI
SRSRSPLAREGAGGGEQLVDQLIRDLN-VPE-LAINIFQYLDRKEGRCA--QVSKTWKVI
GAGGEQLVDQLIRDLNEMNDVPFFDIQ-LPYELAINIFQYLDRKE-LGRCAQVSKTWKVI
---MDDYSLDEFRRRWQNEMNDVPFFDQLPYELAINIFQYLDRKE-LGRCAQVSKTWKVI
AGGGEQLVDQLIRDLNEMNDVPFFDIQ-LPYELAINIFQYLDRKE-LGRCAQVSKTWKVI
PLAREGAGGGEQLVDQLIRDLNEMNDVQLPYELAINIFQYLDRKE-LGRCAQVSKTWKVI
ETEEQDSDSAEQGDPAGEGKEVLCDFC-LDSCLTC-MVNYCEEHLLQSHLTEPVHNWRYC
DFIYNRSRIKLSRKGGKEEEVVKSSLNQMLDTVERKILYWFGNSTLLLL--QMCNLWNSM
ETTQGKDFIYNRSRIKLNRKGGKEEEV-LNS-KMKEILCWFGNSTYTLLLLQMCNLWNNI
LNQMSDKTVEQKMKELLYWFVNSTHWTLLLPKLLLTVIRVLFLREGLNQITDVCFSWRTA
LNQMLDKTVEQKMKEILYWFGNSTHWTKANNKLLLTVIRVLFLREGLNQITDVCFSWTAA
LNQMLDETVEQKMKEILYWFANSTQWTLLLPKLLLTVIRVLFLREGLNQIRDVCFSWTAA
LNQMLDKTVEQKMKEILYWFANSTQWTLLLPKLLLTVIRVLFLREGLNQITDVCFSWTAA
LNQMLDKTVERKMKEILYWFANSTQWTLLLPKLLLTVIRVLFLRELNQDITDVCFSWTAA
HLISLSGAVQLRHLSNNLETLLKRDFLLLPLELSFYLLKWLDPQT-LLTCCLVSKQWNKV
HLISLSGAVQLRHLSNNLETLLKRDFLLLPLELSFYLLKWLDPQT-LLTCCLVSKQWNKV
HLISLSGAVQLRHLSNNLETLLKRDFLLLPLELSFYLLKWLDPQT-LLTCCLVSKQWNKV
HLISLSGAVQLRHLSNNLETLLKRDFLLLPLELSFYLLKWLDPQT-LLTCCLVSKQWNKV
HLISLSGAVQLRHLSNNLETLLKRDFLLLPLELSFYLLKWLDPQT-LLTCCLVSKQWNKV
HLISLSGAVQLRHLSNNLETLLKRDFLLLPLELSFYLLKWLDPQT-LLTCCLVSKQWNKV
HLISLSGAVQLRHLSNNLETLLKRDFLLLPLELSFYLLKWLDPQT-LLTCCLVSKQWNKV
HLISLSGAVQLRHLSNNLETLLKRDFLLLPLELSFYLLKWLDPQT-LLTCCLVSKQWNKV
QVQGGIDIYHLLKARKSKEQEGFINLEMLPPELSFTILSYLNATD-LCLASCV---WQDL
QVQGGIDIYHLLKARKSKEQEGFINLEMLPPELSFTILSYLNATD-LCLASCV---WQDL
QVQGGIDIYHLLKARKSKEQEGFINLEMLPPELSFTILSYLNATD-LCLASCV---WQDL
QVQGGIDIYHLLKARKSKEQEGFINLEMLPPELSFTILSYLNATD-LCLASCV---WQDL
QVQGGIDIYHLLKARKSKEQEGFINLEMLPPELSFTILSYLNATD-LCLASCV---WQDL
QVQGGIDIYHLLKARKSKEQEGFINLEMLPPELSFTILSYLNATD-LCLASCV---WQDL
QAQGGIDIYHLLKARKSKEQEGFINLEMLPPELSFTILSYLNATD-LCLASCV---WQDL
QAQGGIDIYHLLKARKSKEQEGFINLEMLPPELSFTILSYLNATD-LCLASCV---WQDL
HAEERQKFFKYSVDEKSDKEAEVSEHSHLPPEVMLSIFSYLNPQE-LCRCSQVSTKWSQL
HAEERQKFFKYSVDEKSDKEAEVSEHSHLPPEVMLSIFSYLNPQE-LCRCSQVSMKWSQL
HAEERQKYFKYSVDEKSDKEAEVSEQSHLPPEVMLSIFSYLNPQE-LCRCSQVSMKWSQL
HAEERQKFFKYSVDEKSDKEAEVSEHSHLPPEVMLSIFSYLNPQE-LCRCSQVSMKWSQL
HAEERQKFFKYSVDEKSDKEAEVSEHSHLPPVMLS-IFSYLNPQE-LCRCSQVSMKWSQL
```

```
HAEERQKFFKYSVDEKSDKEAEVSEHSHLPPVMLS-IFSYLNPQE-LCRCSQVSMKWSQL
QAEERQKVLKYSVDEKADTEAEVSEHSHLPPEVMLSIFSYLNPQE-LCRCSQVSTKWSQL
QAEERQKVFKYSVDEKSDAEAEVSEHSLPPE-VMLSIFSYLNPQE-LCRCSQVSTKWSQL
LALDELIDSCEPTQVKHMMQVIEPQFQLLPKELALYVLSFLEPKD-LLQAAQTCRYWRIL
LALDELIDSCEPTQVKHMMQVIEPQFQLLPKELALYVLSFLEPKD-LLQAAQTCRYWRIL
LALDELIDSCEPTQVKHMMQVIEPQFQLLPKELALYVLSFLEPKD-LLQAAQTCRYWRIL
LALDELIDSCEPTQVKHMMQVIEPQFQLLPKELALYVLSFLEPKD-LLQAAQTCRYWRIL
LALDELIDSCEPTQVKHMMQVIEPQFQLLPKELALYVLSFLEPKD-LLQAAQTCRYWRIL
LALDELIDSCEPTQVKHMMQVIEPQFQLLPKELALYVLSFLEPKD-LLQAAQTCRYWRIL
LALDELIDSCEPTQVKHMMQVIEPQFQLLPKELALYVLSFLEPKD-LLQAAQTCRYWRIL
LALDELIDSCEPTQVKHMMQVIEPQFQLLPKELALYVLSFLEPKD-LLQAAQTCRYWRIL
IFLRCTKSQLRFVQDWFSERKQVAKLDVLPRISLY-IFSFLSPKD-LCAAAQVSWAWKFL
IFLRCTKSQLRFVQDWFSERMQVAKVDVLPRISLY-IFSFLSPKD-LCAAAQVSWPWKFL
IFLRCTKSQLRFVQDWFSERMQVAKVDVLPRISLY-IFSFLSPKD-LCAAAQVSWPWKFL
IFLRCSKSQLRFVQDWFSERMQVARVDVLPRFISLYIFSFLSPKD-LCAAAQVSWPWKFL
IFLRCTKSQLRFVQDWFSERMQVAKVDVLPRISLY-IFSFLSPKD-LCAAAQVSWPWKFL
------------------------DFSVLPRISLY-IFSFLSPKD-LCAAAQVSWPWKFL
----------------------DFSTV-LPC-ISLYIFSFLNPKD-LCAAAQVSWPWKFL
TLNEPKTEDEEFQERRREKCLRKIIWE----NIAFRLFKARPPWL-SGTCSRLLTS---L
LLELCSLSQQKFCCRKLQEKIPAEALDKLPRVLSLYIFSFLDPRS-LCRCAQVCWHWKNL
LLERCSLSQQKFCCRKLQEKIPAEALDKLPRVLSLYIFSFLDPRS-LCRCAQVCWHWKNL
LLERCSLSQQKFCCRKLQEKIPAEALDKLPRVLSLYIFSFLDPRS-LCRCAQVCWHWKNL
LLERCSLSQQKFCCRKLQEKIPAEALDKLPRVLSLYIFSFLDPRS-LCRCAQVCWHWKNL
LLERCSLSQQKFCCRKLQEKIPAEALDKLPRVLSLYIFSFLDPRS-LCRCAQVCWHWKNL
LLERCSLSQQMFCCRKLQEKIPAEALD-LPRVLSLYIFSFLDPRS-LCRCAQVCWHWKNL
LLERCSLSQQKFCCRKLQEKIPAEALDKLPRVLSVYIFSFLDPRS-LCRCAQVSWYWKSL
LLERCSLSQQKFCCRKLQEKIPAEALDKLPRVLSVYIFSFLDPRS-LCRCAQVTXYWKSL
QPFGQRWTNRDPNHGLYPKPRTKRGSRCIPEFFLAGCTNDMAKSN--SVGQDSCQDSEGL
ARQEAEDSTSRLSAESGETDQDAGDMGSLPSEVLRHVFAFLPVED-LYWLSLVCHLWREI
ARREAEDSTSRLSAESGETDQDAGDLGSLPSEVLRHVFAFLPVED-LYWLSLVCHLWREI
ARQEAEDSMSRLSAESGETDQDAGDMGSLPSEVLRHVFAFLPVED-LYWLSLVCHLWREI
ARQEAEDSTSRLSAESGETDQDAGDMGSLPSEVLRHVFAFLPVED-LYWLSLVCHLWREI
QEAEDSTSRLSAESGETDQDAGDVGPDSLPSEVLRHVFAFLPVED-LYWLSLVCHLWREI
EDSLSQCSPVPGEAGRDIEDIGPDPLPRLPSEVLRHIFAFLPVED-LYWLSLVCHLWREI
EDGLSQSSPVHGEAAQDIEDIGPDPLPRLPSEVLRHIFAFLPVED-LYWLSLVCHLWREI
RGLRGRPGAGSQRRGRMALCGQAAGAA-LPE-LIVHIFSFLPAPD-RLRASASCSHWREC
RVLRGRPEAGTRRRGRMALCGQAAGAA-LPE-LIVHIFSFLPAPD-RLRASASCSHWREC
RVLRGRPGTGSRRRGRMALCGQAAGAA-LPE-LIVHIFSFLPAPD-RLRASASCSHWREC
RVLRGRPGAGSRRRGRMALCGQAAGAASLPSELIVHIFSFLPAPD-RLRASASCSHWREC
RVLRGRPGAGSRRRGRMALCGQAAGAA-LPSELIVHIFSFLPAPD-RLRASASCSHWREC
RVLRGRPGAGSRRRGRMALCGQAAGAA-LPSELIVHIFSFLPAPD-RLRASASCSHWREC
RGLRRRPGTGGRRPSRMALCGQAAGAA-LPE-LIVHIFSFLPAPD-RLRASASCSHWREC
RGLRGRSGTSGRRRSRMALCGQAAGAA-LPSELIVHIFSFLPAPD-RLRASASCSHWREC
VDSAMEVVPALPEEAAPEIAGLSCLVN-LPVEYIL-CCGSLTAAD-IGRVSSTCRRLREL
VDSAMEVVPALAEEAAPEVAGLSCLVN-LPGEVLEYCCGSLTAAD-IGRVSSTCRRLREL
```

VDSAMEVVPALAEEAAPEVAGLSCLVN-LPGEVLEYCCGSLTAAD-IGRVSSTCRRLREL
VDSAMEVVPALAEEAAPEVAGLSCLVN-LPGEVLEYCCGSLTAAD-IGRVSSTCRRLREL
VDSAMEVVPALAEEAAPEVAGLSCLVN-LPGEVLEYCCGSLTAAD-IGRVSSTCRRLREL
GDSAMEVVPALAEEAAAEATGPSCLVQ-LPGEVLEYILCSGSLTADIGRVSSTCRRLREV
GDSAMEVVPALAEEAAAEASGPSCLVQ-LPGEVLEYILCSGSLTADIGRVSSTCRRLREV
MVKRSCPSCGLEAGSEKKERGNPISVQLFPPELVEHIVSFLPVKD-LVALGQTCHYFHEV
MVKRSCPSCGLEAGGEKKERGNPISVQLFPPELVEHIVSFLPVKD-VVALGQTCHYFHEV
LWVKRSCPSCGPELGGEEKKGRGNPISLFPPELVEHIISFLPVRD-LVALGQTCRYFHKV
VKRSCPSCGSELGVEEKRGKGNPISIQLFPPELVEHIISFLPVRD-LVALGQTCRYFHEV
VKRSCPSCGSELGVEEKRGKGNPISIQLFPPELVEHIISFLPVRD-LVALGQTCRYFHEV
VKRSCPSCGSELGVEEKRGKGNPISIQLFPPELVEHIISFLPVRD-LVALGQTCRYFHEV
RSCPSCGPELGVEEKKGKGNPISIQLF-PPE-LVEHIISFLPVRD-LVALGQTCRYFHEV
VKRSCPSCGSELGVEEKRGKGNPISIQLFPPELVEHIISFLPVRD-LVALGQTCRYFHEV
-----------MPFLGQDWRSPGQNWV---KTADG-WKRFLDEKSFVSDLSSYCEVYNK-
----MPFLGQDWRSPGQNWVKTADGWK-----------RFLDEKSFVSDLSSYCEVYNK-
----MPFLGQDWRSPGQSWVKTADGWK-----------RFLDEKSFVSDLSSYCEVYNK-
----MPFLGQDWRSPGQSWVKTADGWK-----------RFLDEKSFVSDLSSYCEVYNK-
----MPFLGQDWRSPGQSWVKTADGWK-----------RFLDEKSFVSDLSSYCEVYNK-
-----------MPFLGQDWRSPGQSWV---K-TADGWKRFLDEKSFVSDLSSYC--NKEV
----MPFLGQDWRSPGQSWVKTADGWK-----------RFLDEKSFVSDLSSYC--FKRY
IKTEDGWKRCESCSQKLERENNHCNIS---HSIIL------NSED---------EE---I
-----------------------------------IL---NSED---------GE---I
IKTEDGWKRCESCSQKLERENNRCNIS---HSIIL------NSED---------GE---I
IKTEDGWKRCESCSQKLERENNHCNIS---HSIIL------NSED---------GE---I
IKTEDGWKRCESCSQKLERENNHCNIS---HSIIL------NSED---------GE---I
IKTEDGWKRCESCSQKLERENNHCNIS---HSIIL------NSED---------GE---I
IKTEDGWKRCDPCSHELRSEDSQYTIN---HSIIL------NSGE---------EE---I
-----------------QVNTGLTLSD-LPLMLNNILYRFSDGWD-IVTLGQVTPTLYML
HFCRDMKKFGGPGRMKQSCLLRQCTAP-LPC-LLCEAGKEDTVEGLSLMECTICNEIVHV
HFCRDMKKFGGPGRMKQSCLLRQCTAPVLPHTAVCLLCGEAGKEDFSLSLMECTNEIVHV
HFCRDMKKFGGPGRMKQSCLLRQCTAP-LPC-LLCEAGKEDTVEGLSLMECTICNEIVHV
HFCRDMKKFGGPGRMKQSCLLRQCTAPVLPHTAVCLLCGEAEDTVLSLMECTICNEIVHV
HFCRDMKKFGGPGRMKQSCLLRQCTAP-LPN-AEIPNCWECPRCTGRTSKDSGEGRWK-L
HFCRDMKKFGGPGRMKQSCLLRQCTAP-LPC-LLCEAGKEDTVEGLSLMECTICNEIVHV
------CHPGLPQGQVAKPKPPLASAE-GP-----AVPSPSPQRE---------KL----
NEIVHPGCLKMGKAEGVINAEIPNCWE-CPRCTQE-GRTSKDSGENGEEGASLGSGWK-L
HYCRDMKKFGGPGRMKQSCVLRQCLAP-LPS-VTCSLCGEVDQNEKKLMECCICNEIVHL
HYCRDMKKFGGPGRMKQSCVLRQCLAPRLPHSVTCSLCGEVDQNEKKLMECCICNEIVHL
HYCRDMKKFGGPGRMKQSCVLRQCLAPRLPHSVTCSLCGEVDQNEKKLMECCICNEIVHL
HYCRDMKKFGGPGRMKQSCVLRQCLAPRLPHSVTCSLCGEVDQNEKKLMECCICNEIVHL
HYCRDMKKFGGPGRMKQSCVLRQCLAP-LPHSVTCSLCGEVDQNEKKLMECCICNEIVHL
HYCRDMKKFGGPGRMKQSCVLRQCLAP-LPHSVTCSLCGEVDQNEKKLMECCICNEIVHL
HYCRDMKKFGGPGRMKQSCVLRQCLAPRLPHSVTCSLCGEVDQNEKKLMECCICNEIVHL
HYCRDMKKFGGPGRMKQSCVLRQCLAP-LPHSVTCSLCGEVDQNEKKLMECCICNEIVHL
HFCKDMKKFGGPGRMKQSCIMRQCIAP-LPT-AVCLVCGEATVEELMLMECSICNEIIHV

HFCKDMKKFGGPGRMKQSCIMRQCIAP-LPT-AVCLVCGEATVEELMLMECSICNEIIHV
HFCKDMKKFGGPGRMKQSCIMRQCIAP-LPC-LVCEAGKEDTVEELMLMECSICNEIIHV
HFCKDMKKFGGPGRMKQSCIMRQCIAPVLPHTAVCLVCGEAEDTVLMLMECSICNEIIHV
HFCKDMKKFGGPGRMKQSCIMRQCIAPVLPHTAVCLVCGEAEDTVLMLMECSICNEIIHV
HFCKDMKKFGGPGRMKQSCIMRQCIAP-LPHTAVCLVCGEATVEELMLMECSICNEIIHV
HFCKDMKKFGGPGRMKQSCIMRQCIAPVLPHTAVCLVCGEATVEELMLMECSICNEIIHV
HFCKDMKKFGGPGRMKQSCIMRQCIAPVLPHTAVCLVCGEAEDTVLMLMECSICNEIIHV
PASQPAPLLSAAAAGDEGRVLLDTWYVIKPGNTKE-KVAFFVAHQGGSRASSMKGHWGSD
PASQPAPLLSAAAAGDEGRVLLDTWYVIKPGNTKE-KVAFFVAHQGGSRASSIRVRWGS-
PASQPAPLLSAAAAGDEGRVLLDTWYVIKPGNTKE-KVAFFVAHQGGSRASSMKGHWGSD
PASQPAPLLSAAAAGDEGRVLLDTWYVIKPGNTKE-KVAFFVAHQGGSRASSMKGHWGSD
PASQPAPLLSAAAAGDEGRVLLDTWYVIKPGNTKE-KVAFFVAHQGGSRASSMKGHWGSD
PASQPAPLLSAAAAGDEGRVLLDTWYV-IKPNTKE-KVAFFVAHQGGSRASSMKGHWGSD
PTSQPAPLLSTAASGDEGRVLLDTWYVIKPGNTKE-KVAFFVAHQGSSRASSMKGHWGSD
PTSQHAPLLSTAASGDEGRVLLDTWYV-IKPGNTKEKVAFFVAHQSRASSMKVKGHWGSD
LNVKTTKNAPSATIHQGEEEGPLDIWAVKPGNTKE-KIAFFAAHQNRIGSMKIKSSWDID
LNVKTKKNAPSATIHQGEEEGPLDIWAVKPGNTKE-KIAFFAAHQNRIGSMKIKSSWDID
CYQPEPFACGIEHCSVHYVSDSGDGVYGRPLSVIQ-MVAFLEQRALLASCSKNCTN----
LNVKTKKNAPSATIHQGEEEGPLDIWAVKPGNTKE-KIAFFASHQ-CSNIGSMKSSWDID
LNVKTKKNAPSATIHQGEEEGPLDIWAVKPGNTKE-KIAFFASHQ-CSNIGSMKSSWDID
LNVKTKKNAPSATIHQGEEEGPLDIWAVVKPNTKE-KIAFFASHQ-CSNIGSMKSSWDID
EVSRDTLRTPMSHGKANGDVKARASYMVLPSSLVKSPFGILSPNV-LCSSGKSPNSLNVH
ANCMKPTPSPSASPVKASSRKPFGILS--P-NVLCSMSGKSPVENNAPSAVQQTGLWAIF
----------------------MASW--LP-ETLFEIVGQGPAPS---------KDYYQL
----------------------MASW--LP-ETLFEIVGQGPAPS---------KDYYQL
----------------------MASW--LP-ETLFEIVGQGPPPS---------KDYYQL
FGTRILNYVINLCKGKFDFLERLSD-----N-LLLNIISYLDLED-IARLSQTSHRFAKL
FGARILDYVINLCKGKFDFLERLSD-----N-LLLNIISYLDLED-IARLCQTSHRFAKL
------MASWLPETLFETVGQGPPPSK---D-----YYQLLVTRS----QAKVIYRWWKI
----------------------MASW--LP-ETLFETVGQGPPPS---------KDYYQL
----------------------MASW--LP-ETLFETVGQGPPPS---------KDYYQL
----------------MAEPGER-----LPEEVLALIFRHLSLKD-RAAAARVCRAWADA
--------------------MAETGEG-LPEEVLALIFRHLPLRD-RAAAARVCRAWAAA
-------------HPSRIWAMAEPGEG-LPEEVLALIFRHLSLRD-RAAAARVCRAWAAA
--------------------MAEPGEG-LPEEVLALIFRHLSLRD-RAAAARVCRAWAAA
--------------------MAEPGEG-LPEEVLALIFRHLSLRD-RAAAARVCRAWAAA
--------------------MAEPGEG-LPEEVLALIFRHLSLRD-RAAAARVCRAWAAA
--------------------MGELVDN-LPEEVLALIFRDLPLRD-LAVATRVCRAWAAA
---------------------MVELIEKLPEVLGL-IFRDLPLRD-RAVAARVCRAWAAA
VTKQHAWRNQHSEKRCSSSISSISLDR-MPSEILVKILSYLDAVT-LVCIGCVSRRFYHL
------MTQCARPGQHSEKPSSVCFISRMPSEILLNIFSYLDVVS-LLCVGCVNRRFYHL
----------------------------MPE-ILLKIFSYLDAVS-LLCTGCVSRRFYHL
----------------------------MPE-ILLKIFSYLDAVSLLCA---GCRRFYHL
----------------------------MPE-ILLKIFSYLDAVSLLCT---GCRRFYHL
LSAGSAALRCHAGGGQHWESSFSCCSG-MPE-ILLKIFSYLDAVSLLCT---GCRRFYHL

LSAGSAALRCHARGGQHWESSFSCCSGGMPSEILLKIFSYLDAVS-LLCTGCVSRRFYHL
LSAGSAALRCHARGGQHWESSFSCCSGGMPSEILLKIFSYLDAVS-LLCTGCVSRRFYHL
SLARGPTAVAKPRTKPRPEPSLDQGLDRIPLEVLVHIFGLLVAAHFLGRAARVCRHWHEA
SLARGPTAVAKPRAKPRPEPSLDQGLDRIPLEVLVHIFGLLVAAHFLGRAARVCRHWHEA
KPKPKARLRPEPAAAPEPADGLDAGWGRIPLEILVQIFRLLVAADFLGRAARVCRRWQEA
RAAAKPKATLRPDPAPSPEEGPDAGWGRIPLEILVQIFGLLVAADFLGRAARVCRRWQEA
PPPPVAARAPAPAPTPTPEEGQDAGWGRIPLEILVQIFGLLVAADFLGRAARVCRRWQEA
RPEAAAAPAPAPAPTPTPEEGPDAGWGRIPLEILVQIFGLLVAADFLGRAARVCRRWQEA
ESAAAPAPAPAPAPTPTPEERPDAGWGRIPLEILVQIFGLLVAADFLGRAARVCRRWQEA
RSEAAAAPAPAPAPTPTPEEGPDAGWGRIPLEILVQIFGLLVAADFLGRAARVCRRWQEA
GKGSDKDFVIIRRPKLSRENFPGVSWDSLPDELLLGIFSCLCLPE-LLRVSGVCKRWYRL
SKGSDKDFVIIRRPKLNRENFPGVSWDSLPDELLLGIFSCLCLPE-LLRVSGVCKRWYRL
SKGSDKDFVIVRRPKLNRENFPGVSWDSLPDELLLGIFSCLCLPE-LLKVSGVCKRWYRL
SKGSDKDFVIVRRPKLNRENFPGVSWDSLPDELLLGIFSCLCLPE-LLKVSGVCKRWYRL
SKGSDKDFVIIRRPKLNRENFPGVSWDSLPDELLLGIFSCLCLPE-LLKVSGVCRRWYRL
SKGSDKDFVIVRRPKLNRENFPGVSWDSLPDELLLGIFSCLCLPE-LLKVSGVCKRWYRL
SKGSDKDFVIVRRPKLNRENFPGVSWDSLPDELLLGIFSCLCLPE-LLKVSGVCKRWYRL
SRVALAGGPCPPASGPASGPVSGPPVE-RPDKILNGLFWYFSACE-KCILAQVCKAWRRV
SRVALAGGPCPPASGPASGPVPGPPVE-RPDKILNGLFWYFSACE-KCILAQVCKAWRRV
SRAALAGGPCPPAGGPASALAPGHPVE-RPEKILNGLFWYFSACE-KCVLAQVCKAWRRV
PRAALAGGPCSPAGGPASALTPGPPAE-RPDKILNGLFWYFSACE-KCVLAQVCKAWRRV
PRAALAGGPCPLAGGPASALAPGPPAE-RPDKILNGLFWYFSACE-KCVLAQVCKAWRRV
PRAALAGGPCPPAGGPASALAPGPPAE-RPDKILNGLFWYFSACE-KCVLAQVCKAWRRV
SRAALAGGPCPPAGGPASALAPGHPAE-RPDKILNGLFWYFSACE-KCVLAQVCKAWRRV
SRAALAGGPCTPAGGPASALAPGHPAE-RPDKILNGLFWYFSACE-KCVLAQVCKAWRRV
---------MDEDCEVTQLQEQSCWAT-LPDVCLRRVFWWLGDRD-RSRAALVCRKWNQI
---------MDEDSEVTQPQDQSCWAT-LPDCLRR-VFWWLGDRD-RSRAALVCRKWNQI
---------MDEESELIQPEDESCWAS-LPDLCLCRVFWWLGDRD-RSRAALVCRKWNQI
---------MDEESELIQPQDQSCWAA-LPDVCLCRVFWWLGDRD-RSRAALVCRKWNQM
---------MDEESELIQPQDQSCWAT-LPDVCLCRVFWWLGDRD-RSRAALVCRKWNQM
---------MDEESELIQPQDQSCWAF-LPDLCLCRVFWWLGDRD-RSRAALVCRKWNQM
---------MDEESELIQPQDQSCWAF-LPDLCLCRVFWWLGDRD-RSRAALVCRKWNQM
---------MDEESELIQPQDQSCWAF-LPDLCLCRVFWWLGDRD-RSRAALVCRKWNQM
EEEPPLSPPPPPRDGAYAAASSSQHLALAAR-FLLAAAAAAAAASLLQRVGAGCPPWQAW
------------------------------------IFSNLSLDE-RCLASLVCKYWRDL
DCQEPPENPCDCHREPPPEIPDINQLP--P-SILLKIFSNLSLDE-RCLASLVCKYWRDL
SAGLEEEPPLSPPPPPPRDGAYAAVSS---QHLAR-RYAALAAEDLLSSAAAAASSWEQQ
PAGPEEEPPFSPPPPPRDGAYAAAASSHLADCAAA-ARRFLLSSASASSPASCCKEWEQQ
PAGPEEEPPLSPPPRDGAYAAASSSQH-LADCAAA-ARRFLLSSASASSPASCCKEWEQQ
PAGPEEEPPLSPPPRDGAYAAASSSQH-LADCAAA-ARRFLLSSASASSPASCCKEWEQQ
MFSASLGVPERKAASKVPAVRLPGLLS-LPPELLLEICAYLDARV-VLQLPCVCQALHDL
MFSASLGMPERKDASKVPAVSLPGLLS-LPPELLLEICAYLDARVVLHVLPCVCQALHNL
TPASSPSASEPRAASKVSTVSEPGLLS-LPPELLLEICSYLDARL-VLHLSRVCHALRNL
TPAASPSASEPRAASKVSAVSEPGLLS-LPPELLLEICSYLDARL-VLHLSRVCHALRDL
TPAASPSASEPRAASKVSAVSEPGLLS-LPPELLLEICSYLDARL-VLHLSRVCHALRDL

TPAASPSASEPRAASRVSAVSEPGLLS-LPPELLLEICSYLDARL-VLHLSRVCHALRDL
TPAASPSASEPRAASKVSAVSEPGLLS-LPPELLLEICSYLDARL-VLHLSRVCHALRDL
TPAASPSASEPRAASKVSAVSEPGLLS-LPPELLLEICSYLDARLVLHVLSRVCHALRDL
---MEPAGGGGGVSSSTDPRSTYVLSN-LA-EVVERVFTFLPAKA-LLRVAGVCRLWREC
------MEPAGGGSSSTDPRGTYVLSN-LA-EVVERVFTFLPAKA-LLRVAGVCRLWREC
-MEPVGGCGDSCGSSSVDPRSTFVLSN-LA-EVVERVLTFLPAKA-LLRVACVCRLWREC
ELVGSSGERCGSSSVDPRSTFVLSNLA----EVVERVLTFLPAKA-LLRVACVCRLWREC
--MESVGCCGDCRGSSVDPRSTFVLSN-LA-EVVERVLTFLPAKA-LLRVACVCRLWREC
--METVGCCGECRGSSVDPRSTFVLSN-LA-EVVERVLTFLPAKA-LLRVACVCRLWREC
--MEPVGCCGECRGSSVDPRSTFVLSN-LA-EVVERVLTFLPAKA-LLRVACVCRLWREC
--MEPVGCCGECRGSSVDPRSTFVLSN-LA-EVVERVLTFLPAKA-LLRVACVCRLWREC
HSPPPTRLTHPLIRLASRPQKEQASIDRLPDHSVVHIFSFLPTNQ-LCRCARVCRRWYNL
HSPPPTRLTHPLIRLASRPQKEQASIDRLPDHSMVQIFSFLPTNQ-LCRCARVCRRWYNL
HSPPPTRLTHPLIRLASRPQKEQASIDRLPDHSMVQIFSFLPTNQ-LCRCARVCRRWYNL
HSPPPTRLTHPLIRLASKPQKEQASIDRLPDHSMVQIFSFLPTNQ-LCRCARVCRRWYNL
HSPPPTRLTHPLIRLASRPQKEQASIDRLPDHSMVQIFSFLPTNQ-LCRCARVCRRWYNL
HSPPPTRLTHPLIRLASRPQKEQASIDRLPDHSMVQIFSFLPTNQ-LCRCARVCRRWYNL
HSPPPTRLTHPLIRLASRPQKEQASIDRLPDHSMVQIFSFLPTNQ-LCRCARVCRRWYNL
HSPPPTRLTHPLIRLASRPQKEQASIDRLPDHSMVQIFSFLPTNQ-LCRCARVCRRWYNL
--------------ISLTSSKVICIV--CPQ-LLNIIFSFLDIVT-LCRCAQISKAWNIL
------------------------LYR---------IFSFLDIVT-LCRCAQISKAWNIL
-------------MVFSNNDEGLINKK-LPKELLLRIFSFLDIVT-LCRCAQISKAWNIL
-------------MVFSNNDEGLINKK-LPKELLLRIFSFLDIVT-LCRCAQISKAWNIL
-------------MVFSNNDEGLINKK-LPKELLLRIFSFLDIVT-LCRCAQISKAWNIL
-------------MVFSNNDEGLINKK-LPKELLLRIFSFLDIVT-LCRCAQISKAWNIL
-------------MVFSNSDDGLINKK-LPKELLLRIFSFLDIVT-LCRCAQISKAWNIL
--------------VFSNNDDGLINKK-LPKELLLRIFSFLDIVT-LCRCAQISKAWNIL
-------------QMFSNSDEAVINKK-LPKELLLRIFIFFSRYL-QCDCSEFYRAWNVL
ESRDCIFTFGTILQMFSNSDEAVINKK-LPKELLLRIFSFLDVVT-LCRCAQVSRAWNVL
MRRDVNGVTKSRFEMFSNSDEAVINKK-LPKELLLRIFSFLDVVT-LCRCAQVSRAWNVL
--------------MFSNSDEAVINKK-LPKELLLRIFSFLDVVT-LCRCAQVSRAWNVL
-------------MRRDVNGVTKSRFE---------IFSFLDVVT-LCRCAQVSRAWNVL
-MRRDVNGVTKSRFEMFSNSDEAVINKKLPKELLLRIFSFLDVVT-LCRCAQVSRAWNVL
-MRRDVNGVTKSRFEMFSNSDEAVINKKLPKELLLRIFSFLDVVT-LCRCAQVSRAWNVL
-MRRDVNGVTKSRFEMFSNSDEAVINKKLPKELLLRIFSFLDVVT-LCRCAQVSRAWNVL
--MEPPMEQSGGEQEPGAVRLLDLPWELLP-----HVLNWVPLRQ-LLRLQRVSRAFRAL
--MEPPMEQSGGEQEPGAVRLLDLPWELLP-----HVLNWVPLRQ-LLRLQRVSRAFRAL
SWGSPKRILGLGPGDAAKEAGLAPPLQ---EKILLRRQPTEPPMEGAIRLLDLP--WEDV
--KEPPMEPSGGEQEPGAVRLLDLPWELLP-----HVLNRVPLRQ-LLRLQRVSRAFRAL
--MEPPMEPSGGEQEPGAVRLLDLPWELLP-----HVLNRVPLCQ-LLRLQRVSRAFRAL
--MEPPTEPSGGEQEPGAVRFLDLPWELLP-----HVLNRVPLRQ-LLRLQRVSRAFRSL
--MEPPMEPSGGEQEPGAVRFLDLPWELLP-----HVLNRVPLRQ-LLRLQRVSRAFRSL
--MEPPMEPSGGEQEPGAVRFLDLPWELLP-----HVLNRVPLRQ-LLRLQRVSRAFRSL
RLRPRLLHPARRSGGRKMETHISCLFP---ELLAM-IFGYLDVRD-KGRAVQVCRAWRDA
-----------------METHISCLFP----ELLAMIFGYLDVRD-KGRAAQVCTAWRDA

```
-----------------METHISCLFP----ELLAMIFGYLDVRD-KGRAAQVCTAWRDA
-----------------METHISCLFP----ELLAMIFGYLDVRD-KGRAAQVCTAWRDA
-----------------METHISCLFP----ELLAMIFGYLDVRD-KGRAAQVCTAWRDA
-----------------METHISCLFP----ELLAMIFGYLDVRD-KGRAAQVCTAWRDA
-----------------METHISCLFP----ELLAMIFGYLDVRD-KGRAAQVCTAWRDA
GASAEKSNFPEQSSSEVCLVDETLECDQLPEAILQ-IFFYLSLKD-VLICGQVNHAWMLM
RAAAEESNFPERSSSEVSLVDETLKCDLLPEAILQ-IFFYLSLKD-VIICGQVNHAWMLM
RATAEESNFPEQSSSEGSLVDETLKCDLLPEAILQ-IFFYLSLKD-VIICGQVNRAWMLM
RAAAEESNFPEQSSSEVSLVDETLKCDLLPEAILQ-IFFYLSLKD-VIICGQVSHAWMLM
RAAAEESNFPERSSSEVFLVDETLKCDLLPEAILQ-IFFYLSLKD-VIICGQVNHAWMLM
RAAAEESNFPERSSSEVSLVDETLKCDLLPEAILQ-IFFYLSLKD-VIICGQVNHAWMLM
KFKLGKPISLEGSLSDIAVENRRIAFDVLPEQAILQIFLYLTFKD-MMACSRVNRSWMAM
GLENLLWDMCIHPSLKPKVRRLSETYL---DELFG-LDDQLMTPELMIKACTFYHL---V
LSRGGGAEDAGAAGASALSAGSGAGGR-LPSVLEL-VFSYLELSE-LRSCALVCKHWYRC
AASGGASGGGAGAGGGASAGSGSSGVGRLPSVLEL-VFSYLELSE-LRSCALVCKHWYRC
--------MAAPAPGAGAASGGAGCSG--------------GGSE-LRSCALVCKHWYRC
SGGAGCSGGGAGAGAGSGSGAAGAGGR-LPSVLEL-VFSYLELSE-LRSCALVCKHWYRC
---------------------MATLFD-LPDLVLLEIFSYLPVRD-RIRISRVCHRWKRL
---------------------MATLFD-LPDLVLLEIFSYLPVRD-RIRISRVCHRWKRL
---------------------MATLVE-LPDSVLLEIFSYLPVRD-RIRISRVCHRWKRL
-----------PEAEVRVGGIMATLIE-LPDVLLE-IFSYLPVRD-RIRISRVCHRWKRL
---------------------MATLVE-LPDVLLE-IFSYLPVRD-RIRISRVCHRWKRL
---------------------MATLVE-LPDVLLE-IFSYLPVRD-RIRISRVCHRWKRL
---------------------MATLVE-LPDVLLE-IFSYLPVRD-RIRISRVCHRWKRL
---------------------MATLVE-LPDVLLE-IFSYLPVRD-RIRISRVCHRWKRL
AAAGEEEKEEAARESAARLAAGPALWR-LPEELLLLICSYLDMRA-LGRLAQVCR-WLRR
AAGEEEEEEEAAREAAARPAAGPALWR-LPEELLLLICSYLDIRA-LGRLAQVCR-WLRR
AAEAGEEEEEAARESAARPAAGPALWR-LPEELLLLICSYLDMRA-LGRLAQVCR-WLRR
AAGEEEEEEEAARESAARPAAGPALWR-LPEELLLLICSYLDMRA-LGRLAQVCR-WLRR
GEEEEEEEEEAARESAARPAAGPALWR-LPEELLLLICSYLDMRA-LGRLAQVCR-WLRR
AEDAAEDAAAAAVEPATRPAAGPALWR-LPEELLLLICSYLDTRA-LGRLAQVCR-WLRR
-MAAAAEDGAAAVEPATRPAAGPALWR-LPEELLLLICSYLDMRA-LGRLAQVCR-WLRR
TECRHPNWPNQPDVEPESWREAFKQHY-LADLESS-ICFSLFRRK-KERTLSVGHEFDSL
TECRHPNWPNQPDVEPESWREAFKQHY-LADLESSICFSLFRRKK-ERRTLSVGHEFDSL
TKCRHPNWPNQPDVEPESWREAFKQHY-LADLESSVCFSLFRRRK-ERRTLSVGHEFDSL
TECRHPNWPNQPDVEPESWREAFKQHY-LAL-DLEVCFSLFRRRRVGPG-----HEFDSL
TECRHPNWPNQPDVEPESWREAFKQHY-LAL-DLEICFSLFRRRRVGPG-----HEFDSL
TECRHPNWPNQPDVEPESWREAFKQHY-LAL-DLEICFSLFRRRRVGPG-----REFDSL
TECRHPNWPNQPDVEPESWREAFKQHY-LADLESSICFSLFRRRR-ERRTLSVGREFDSL
TECRHPNWPNQPDVEPESWREAFKQHY-LADLESSICFSLFRRRR-ERRTLSVGREFDSL
TAAADSEADTDPEEERIEAGPARCSLLELPPLLVE-IFASLPGTD-LPSLAQVCSRFRRI
TAAADSEADTDPEEERIEAGPARCSLLELPPELLVEIFASLPGTD-LPSLAQVCSRFRRI
GAAVLAGVGGGMCPGPSPSPPRCSLLE-LPPELLVEIFASLPGTD-LPSLAQVCTKFRRI
SAAVLAGVGGGLCAGPSSPPPRCSLLE-LPPELLVEIFASLPGTD-LPSLAQVCTKFRRI
SAAVLAGVGGGLCAGPSPPPPRCSLLE-LPPELLVEIFASLPGTD-LPSLAQVCTKFRRI
```

```
SAAVLAGVGGGLCAGPSPPPPRCSLLE-LPPELLVEIFASLPGTD-LPSLAQVCTKFRHI
SAAVLAGVGGGLCAGPSPPPPRCSLLE-LPPELLVEIFASLPGTD-LPSLAQVCTKFRRI
RIEASAGVGGGLCAGPSPPPPRCSLLE-LPPELLVEIFASLPGTD-LPSLAQVCTKFRRI
-------MGAQPSRRRMTEAQHMALAE-LPPELLLQVLSHVPPRA-LVTCRPVCRAWRDL
-------MGARPSRRQMTEARRLALTK-LPPELLVQVLSHVPPRA-LVTCRPVCRAWRDL
------MGARPSRRQPRLPADPPLALDALPPELLVQVLSHVPPRA-LVMCRPVCRAWRDV
--LRLLEMGARLSRRRLPADPSLALDA-LPPELLVQVLSHVPPRA-LVTCRPVCRAWRDI
-GLWLLEMGARLSRRRLPADPSLTLDA-LP-ELLVQLLSHVPPRALVTRCRPVCRAWRDI
QGLWLLEMGARLSRRRLPADPSLALDA-LP-ELLVQVLSHVPPRSLVTRCRPVCRAWRDI
-------MGARLSRRRLPADPSLALDA-LP-ELLVQVLSHVPPRSLVTRCRPVCRAWRDI
MSASASRCRAARVPAPEPEPEGALDLSQMPPELLLVVLSHVPPRT-LLGCRQVCRGWRDL
----------------------------------------------LGRCRQVCRGWRAL
-------------ALELEDEEALDLSQ-SPELLLV-VLSHVPPRT-LLGCRQVCRGWRAL
MGASVSRGRAARVPAPEPEPEEALDLSQLPPELLLVVLSHVPPRTLLGRCRQVCRGWRAL
MGASVSRGRAARVPAPEPEPEEALDLSQLPPELLLVVLSHVPPRTLLGRCRQVCRGWRAL
MGASVSRGRAARVPAPEPEPEEALDLS-LPPELLLVVVSHVPPRTGRCR--QVCRGWRAL
---MGAWISRTRVPTPEPDPQEVLDLSRLPPELLLLVLSHVPPRT-LLMCRRVCRAWRAL
---MGAWTSRTRIPMPEPDPQEALDLSRLPPELLLLVLSHVPPRTLLVHCRRVCRAWRAL
QPEEVSTEEERPEDQEEEEEEEAAAAYELPEPLLLRVLAALPAAELVQACRLVCLRWKEL
QPEEASAEEERPEDQEEEEAAAAAYLDELPEPLLLRVLAALPAAELVQACRLVCLRWKEL
QPEEASAEEERPEDQEEEEAAAAAYLDELPEPLLLRVLAALPAAELVRACRLVCLRWKEL
QPEEASAEEERLEDQEEEEAAAAAYLDELPEPLLLRVLAALPAAELVQACRLVCLRWKEL
QPEEASAEEERPEDQQEEEAAAAAAYL-LPEPLLLRVLAALPAAEQACR--LVCLRWKEL
GAEASAEEEQLREAEEEEEAEAVEYLA-LPP-LLLRVLAELPATEQACR--LVCLRWKEL
GAEASAEEEQPREEEEEETEAVEYLAE-LPEPLLLRVLAELPATELVQACRLVCLRWKEL
-------------------MAVGNINE-LPENILLELFTHVPARQLLLNCRLVCSLWRDL
-------------------MAVGNINE-LPENILLELFTHVPARQLLLNCRLVCSLWRDL
-------------------MAVGNINE-LPENILLELFIHIPARQLLLRCRPVCSLWRDL
---ASAEHKGPLQQERTDTMAVGNINE-LPENILLELFTHIPARQLLLRCRPVCSLWRDL
---------LQEGVQKPQAMAVGNINE-LPENILLELFTHVPARQLLLNCRLVCSLWRDL
-------------------MAVGNINE-LPENILLELFTHVPARQLLLNCRLVCSLWRDL
-------------------MAVGNINE-LPENILLELFTHVPARQLLLNCRLVCSLWRDL
------------MDAPHPTTALASINE-LPENILLELFLHVPARQLLLNCRPVCSLWRDL
------------MDAPHSKAALDSINE-LPENILLELFTHVPARQLLLNCRLVCSLWRDL
------------MDAPHSKAALDSINE-LPENILLELFTHVPARQLLLNCRLVCSLWRDL
------------MDAPHSKAALDSINE-LPENILLELFTHVPARQLLLNCRLVCSLWRDL
------------MDAPHPKAALDSINE-LPENILLELFTHVPARQLLLNCRLVCSLWRDL
------------MDAPHPKAALDSINE-LPENILLELFTHVPARQLLLNCRLVCSLWRDL
---------------------MVHINE-LPENILLELFIHIPAPQLLRNCRLVCRLWRDL
----------------------MVNINELPENILLELFTHVPAPQLLRNCRLVCSLWRDL

SYTPGYLEGSCK---DCINKDYERLSCIGSPVVSPRIVELETESKPLHNKENQ-------
SYTPAYLEGSCK----DCIKDYERLSCIGSPIVSPRIVELETESKPLHNKENQ-------
SYTPAYLEGSCK----DCIKDYERLSCIGSPIVSPRTVELETESKPLHNKENQ-------
SYTPAYLEGSCK----DCIKDYERLSCIGSPIVSPRIVQLETESKRLHNKENQ-------
```

SYTPAYLEGSCK----DCIKDYERLSCIGSPIVSPRIVELETESKPLHNKENQ-------
SYTPAYLEGSCK----DCIKDYERLSCIGSPIVSPRIVELETESKPLHNKENQ-------
CND-------CV----RNHERLSFIDSPIVGHDNKENQRVQNTL----------------
CDNVGNHERLSFVEVGHDNKENQRVQNILDSSKEVEELEASRLYEDSGYSSFIQSDSDDG
SRDEFLWKEQFY----RYYQVARDVPRHPAATSWYEEFRRLYDMVPCVEVQTLKEHTDQV
SRDEFLWREQFY----RYYQVARDVPRHPAATSWYEEFRRLYDMVPCVEVQTLKEHTDQV
SRDEFLWREQFY----RYYQVARDVPRHPAATSWLEEFQRLYDMVPCVEVQTLREHTDQV
SRDEFLWREQFY----RYYRVARDLPRHPAATSWYEEFQRLYDTVPCVEVQTLREHTDQV
SRDEFLWREQFY----RYYQVARDVPRHPAAMSWYEEFQRLYDTVPCVEVQTLREHTDQV
SRDEFLWREQFY----RYYQVARDVPRHPAAMSWYEEFQRLYDTVPCVEVQTLREHTDQV
AESNYLWRAHSL----QRWDCNNVTDQHLGAHTWKQFFLHQRRKEL--------------
AESLQRWDCSNF----TNQHLGTHTWKKFFLHQRRKELRLALAQPHNFIY----------
ADSDYLWRSLSL----QRWDCSNFTYQHLGTHTWKQFFLHQRRK----------------
ADSDYLWRSLSL----QRWDCSNFTNQHLGTHTWKQFFLHQRRK----------------
ADSDYLWRSLSL----QRWDCSNFTNQHLGTHTWKQFFLHQRRKELRLALAQ--------
ADSDYLWRSLSL----QRWDCSNFTNQHLGTHTWKQFFLHQRRK----------------
SSHDPLWRRHCKKYSEDEKTQKNQCWKSLFIDTYSDVGRYIDHYAAIKKAWDDLKK----
SSHDPLWRRHCKKYSEEEKTQKNQCWKSLFIDTYSDVGRYIDHYAAIKKAWDDLKK----
SSHDPLWRRHCKKYSEEEKTQKNQCWKSLFIDTYSDVGRYIDHYAAIKKAWDDLKK----
SSHDPLWRRHCKKYSEEEKTQKNQCWKSLFIDTYSDVGRYIDHYAAIKKAWDDLKK----
SSHDPLWRRHCKKYSEEEKTQKNQCWKSLFIDTYSDVGRYIDHYAAIKKAWDDLKK----
SSHDPLWRRHCKKYSEEEKTQKNQCWKSLFIDTYSDVGRYIDHYAAIKKAWDDLKK----
STHDPLWRRHCKKYSEEEKAGKSQCWRSLFIETYSDVGRYIDHYAAIKKAWRDLKK----
STHDPLWRRHCKKYTEEEKARKNQCWKSLFIATYSDVGRYINHYAAIKKAWDDLKK----
SMTKELWRQLCL----RRWASCKAFPVVVGTQTWKKYYFCRSEL----------------
SMTNELWRQLCL----RRWAPCKALPVVLGTQTWRQYYFCRSELEFR-------------
SFQ-ELWPSPQNYN--EGLSVSDEACAEVNGLKASRFFSMAERLNTGSEPFIWLFIRPPW
SFQ-ELWPSPQNYN--EGLSVSDEACAEVNGLKASRFFSMAERLNTGSDPFIWLFIRPPW
SFQ-ELWPSPGNYN--EGLSVSDEARAEVNGLKASHFFSLTERLNAGAVPFIWLFIRPPW
SFQ-ELWPSPGNYN--EGLSVSDEARAEVNGLKASRFFSLAERLNVGAAPFIWLFIRPPW
SFQ-ELWPSPGNYN--EGLSVSDEARAEVNGLKASRFFSLAERLNVGAAPFIWLFIRPPW
SFQ-ELWPSPGNYN--EGLSVSDEARAEVNGLKASRFFSLAERLNVGAAPFIWLFIRPPW
SFQ-ELWPSPGNYN--EGLSVSDEARAEVNGLKASRFFSLAERLNVGAAPFIWLFIRPPW
SFQ-ELWPSPGNYN--EGLSVSDEARAEVNGLKASRFFSLAERLNVGAAPFIWLFIRPPW
FEDPTLWPLLHF--------HSLAELKKDNFRLSPALRSLSICWHS--------------
FEDPTLWSLLHF--------HSLTELKKDNFRLSPALRSLSICWHS--------------
FEDPTLWSLLHF--------RSLTELQKDNFLLGPALRSLSICWHS--------------
FEDPALWSLLHF--------RSLTELQKDNFLLGPALRSLSICWHS--------------
FEDPALWSLLHF--------RSLTELQKDNFLLGPALRSLSICWHS--------------
FEDPALWSLLHF--------RSLTELQKDNFLLGPALRSLSICWHS--------------
FEDPALWSLLHF--------RSLTELQKDNFLLGPALRSLSICWHS--------------
FEDPALWSLLHF--
LNLPSSASGLSR----PNNFSPLVTSTLKTEEVTLSSQKLRLNFSQQKTSTIDDSKDDCN
LNLPSSASGFSR----ANNFSPLVTSTLKTEEVTSCSQKLRLNFSQQKTSTIDDSKDDCS
INLPSSASGFSR----ANNFSPLVTSTLKTEEVTSCSQKLRLNFSQQKTSTIDDSKHDCS

```
INLPSSASGFSR----ANNFSPLVTSTLKTEEVTSCSQKLRLNFSQQKTSTIDDSKDDCS
INLPSSASGFPR----ANNFSPLVTSTLKTEEVTSCSQKLRLNFSQQKTSTIDDSKDDCS
INLPSSASGFSR----ANNFSPLVTSTLKTEEVTSCSQKLRLNFSQQKTSTIDDSKDDCS
IKGNIQWESRTYQS--SCNAAFNDLWRLDLNSKEWIRPLASGSYPSPKAGATLVVYKDLL
IKGNIQWESRTYQS--SCNAAFNDLWRLDLNSKEWIRPLASGSYPSPKAGATLVVYKDLL
IKGNIQWESRTYQS--SCNAAFNDLWRLDLNSKEWIRPLASGSYPSPKAGATLVVYKDLL
IKGNIQWESRTYQS--SCNAAFNDLWRLDLNSKEWIRPLASGSYPSPKAGATLVVYKDLL
IKGNIQWESRTYQS--SCNAAFNDLWRLDLNSKEWIRPLASGSYPSPKAGATLVVYKDLL
IKGNIQWESRTYQS--SCNAAFNDLWRLDLNSKEWIRPLASGSYPSPKAGATLVVYKDLL
IKGNIQWESRTYQS--SCNAAFNDLWRLDLNSKEWIRPLASGSYPSPKAGATLVVYKDLL
IKGNIQWESRTYQS--SCNAAFNDLWRLDLNSKEWIRPLASGSYPSPKAGATLVVYKDLL
VKEGCYWLSGST---IEHVARCHSLVKVNLSGCHLTSLRLSKVLSALQHLRSLAIDVSPG
CASLLYFEILDR----TREGAVLSGQLMVGQSNVPHYQNLRVFYARLAPGYINQEVEVVR
CASLLYFEILDR----TREGAVLSGQLMVGQSNVPHYQNLRVFYARLAPGYINQEVVRLY
CTSLLYFEILDR----TREGAILSGQLMVGQSNVPHYQNLRVFYARLAPGYINQEVVRLY
CTSLLYFEILDR----TREGAILSGQLMVGQSNVPHYQNLRVFYARLAPGYINQEVVRLY
CTSLLYFEILDR----TREGAILSGQLMVGQSNVPHYQNLRVFYARLAPGYINQEVVRLY
CTSLLYFEILDR----TREGAILSGQLMVGQSNVPHYQNLRVFYARLAPGYINQEVVRLY
CTSLLYFEILDR----TREGAILSGQLMVGQSNVPHYQNLRVFYARLAPGYINQEVVRLY
TKPQPFKDFLCILA--SARNLEHLEMVRVPFLGGLIQHVVEDSWRSGGFRNLHTIVLGAC
TKPQPFKDFLCIPLLASARNLEHLEMVRVPFLGGLIQHVVEDSWRSGGFRNLHTIVLGAC
TKPQPFKDFLCIPLLASARNLEHLEMVRVPFLGGLIQHVVEDSWRSGGFRNLHTIVLGAC
TKPQPFKDFLCIPLLASARNLEHLEMVRVPFLGGLIQHVVEDSWRSGGFRNLHTIVLGAC
TKPQPFKDFLCIPLLASARNLEHLEMVRVPFLGGLIQHVVEDSWRSGGFRNLHTIVLGAC
TKPQPFKDFLCIPLLASARNLEHLEMVRVPFLGGLIQHVVEDSWRSGGFRNLHTIVLGAC
TKPQPFKDFLCIPLLASARNLEHLEMVRVPFLGGLIQHVVEDSWRSGGFRNLHTIVLGAC
TKPQPFKDFLCIPLLASARNLEHLEMVRVPFLGGLIQHVVEDSWRSGGFRNLHTIVLGAC
LNQIQRYRSYKL----CKRLLKSLKERRESERRNHSLARHADILAAVETRLSLLNM----
LNQVERFHNLCQ------KQVKAQLPRRESERRNHSLARHADILAAVETRLSLLNM----
CQRVERYHNLCQ------KQVKAQLPRRESERRNHSLARHADILAAVETRLSLLNM----
LNQVERYHNLCQ------KQVKAQLPRRESERRNHSLARHADILAAVETRLSLLNM----
LNQVERYHNLCQ------KQVKAQLPRRESERRNHSLARHADILAAVETRLSLLNM----
CQRVERYHNLCQ------KQVKAQLPRRESERRNHSLARHADILAAVETRLSLLNM----
CQRVERYHNLCQ------KQVKAQLPRRESERRNHSLARHADILAAVETRLSLLNM----
CQRVERYHNLCQ------KQVKAQLPRRESERRNHSLARHADILAAVETRLSLLNM----
AYERLKIRALEKEL--ERKAAELETARQESARLGREKEELEERASELSRQVDVSVELLAS
AYERLKIRALEKEL--ERKAAELETARQESARLGREKEELEERASELSRQVDVSVELLAS
AYERLKIRALEKEL--ERKAAELETARQESARLGREKEELEERASELSRQVDVSVELLAS
AYERLKIRALEKEL--ERKAAELETARQESARLGREKEELEERASELSRQVDVSVELLAS
AYEEGLARLKIRGRELERKAAELETARQESARLGREKEELEERASELSRQVDVSVELLAS
AYEEGLARLKIRGRELERKAAELETARQESARLGREKEELEERASELSRQVDVSVELLAS
SRQVELLASLKQQEKQQEVVQIDQFLKETAAREASAKLRLQQFIEELLERADRAERQLQ-
AYEEGLARLKIRGRELERKAAELETARQESARLGREKEELEERASELSRQVDVSVELLAS
AQN-ALRESLGL----KSVCCISLTNLDDSPASHQVLQSVAYHLGPHLQ-----------
AQN-ALRESLGL----RGICCISLTNLDGS------------------------------
```

```
AQN-ALRESLGL----RGICCISLTNLDGSLASNQVLQSV--------------------
AQN-ALRESLGL----RGICCISLTNLDGSLASHQVLQSV--------------------
PVSRKSYENLSR--DVDEVAQLDMALALQDQRMLLESLKVATMMSKGTDKVSKPREQISV
PVSRKSYENLSR--DVDEVAQLDMALALQDQRMLLESLKVATMMSKATDKVSKPREQISV
PVSRKSYENLSR--DVDEVAQLDMALALQDQRMLLESLKVATMMSKATDKVSKPREQISV
PVSRKSYENLSR--DVDEVAQLDMALALQDQRMLLESLKVATMMSKATDKVSKPREQISV
PVSRKSYENLSR--DVDEVAQLDMALALQDQRMLLESLKVATMMSKATDKVSKPREQISV
PVSRKSYENLSR--DVDEVAQLDMALALQDQRMLLESLKVATMMSKATDKVSKPREQISV
PVSRKSYESLSR--DVDEVAQLDMALALQDQRMLLESLKVATMMSKATDKISEPREQISV
PVSRKSYESLSR--DADEVSQLDMALALQDQRMLLESLKVATMMSKATGKISKPREQISV
PNVTTLHENIMK--ENPNEECLDTALALQDQKVLFRSLKMVELFPETREATEEEPTMNGE
PNVTTLHENIMK--ETPSEECLDTALALQDQKVLFRSLKMVELFPETREATEEEPTMNGE
PNVTTLHENIMK--ETPSEECLDTALALQDQKVLFRSLKMVELFPETREATEEEPTVNGE
PNVTTLHENIMK--ETPSEECLDTALALQDQKVLFRSLKMVELFPETREATEEEPTMNGE
PNVTTLHENIMK--ETPSEECLDTALALRDQKVLFRSLKMVELFPETREATEEEPTMNGE
PNVTTLHENIMK--ETPSEECLDTALALQDQKVLFRSLKMVELFPETREATEEEPTMNGE
PNVTFLHENIMK--ETPSEECLDTALALQDQKVLFRSLKMVELFPETRDATEEEPDMNGD
PNVTVLHENIMK--ESPSEECLDTALALQDQKVLFRSLKMVELFPETRDATEEEPAMNGD
AADQEQAKEEKL----SIIDARELFLKAVEEEQNGALYEAIKFYRRAMQLVPDIE-----
LQKDTKGKQEQA----KEEKARELFLKAVEEEQNGALYEAIKFYRRAMQLVPDIE-----
LQKDTKGKQEQA----KEEKARELFLKAVEEEQNGALYEAIKFYRRAMQLVPDIE-----
LAPSNLENRPCRKGQAKEEKARELFLKAVEEEQNGALYEAIKFYRRAMQLVPDIE-----
LQKDTKGKQEQA----KEEKARELFLKAVEEEQNGALYEAIKFYRRAMQLVPDIE-----
ARDPEIWRLACL-------KVWGRSCIKLVPYTSWREMFL--------------------
KAADTKGRQELA----KEEKARELFLQAVEEEQNGALYEAIKFYRRAMQLVPDIE-----
KAAADKGRQELA----KEEKARELFLKAVEEEQNGALYEAIKFYRRAMQLVPDIE-----
AAQSDVWNDDNMSGNFEAESIQDIAVMEESTGFYPSEPMLCSESVEGQVPHSLETL----
AAQSGVWNDDSMLGNFEAESIQDNAHMAEGTGFYPSEPMLCSESVEGQVPHSLETL----
--DSMLGPSQNF----EAESIQDNAHMAEGTGFYPSEPMLCSESVEGQVPHSLETL----
AAQSGVWNDDSMLGNFEAESIQDNAHMAEGTGFYPSEPMLCSESVEGQVPHSLETL----
AAQSDVWNDDSTLGNFEAESIQDIEDMAEGTGFYPSEPMLCSESVEGQVPHSLETL----
---STLGPSQNF----EAESIQDNADMAEGTGFCLSEPMLCSESVEGQVPHSLETL----
--DSMEGPSQNV----EAESIQDAMSMEEVSGFHPLEPMLCNETEDGQVPHSLETL----
--DSMEGPSHSA----EAVSIQDAMSVEEASGFHPLEPMLCSETEDGQVPHSLEAL----
ISTSSGRKRLLL----QDFHNLELPGRRQDSAILEHYRSLGLLFKRCTLLLPTKERLK--
ISTSSGSKRLLL----QDFHNLELPDGRQDSAILEHYRSLGLLFKRCTLLLPTKERLK--
ISTSSGSKRLLL----QDFHNLELPDRRQDSAILEHYRSLGLLFKRCTLLLPTKERLK--
ISTSSGSKRLLL----QDFHNLELPDRRQDSAILEHYRSLGLLFKRCTLLLPTKERLK--
ISTSSGSKRLLL----QDFHNLELPDRRQDSAILEHYRSLGLLFKRCTLLLPTKERLK--
ISTSSGSKRLLL----QDFHNLELPDRRQDSAILEHYRSLGLLFKRCTLLLPTKERLK--
ISTSSGSRRLLL----QNFHDLDLPGTKEETALLEHYRALGLLFKRCTLLLPTKERLK--
STS-SGSRRLLL----QNFHDLDLPGTKEETALLEHYRALGLLFKRCTLLLPTKERLK--
PPNSQDYVELTF----EQQVYPTAVHVLETYHPGAVIRILACSANPYSPNPPAEVRWEIL
PPNSQDYVELTF----EQQVYPTALHVLETYHPGAVIRILACSANPYSPNPPAEVRWEIL
PPNSQDYVELTF----EQQVYPTAVHVLETYHPGAVIRILACSANPYSPNPPAEVRWEIL
```

```
PPNSQDYVELAF----EQQVYPTAVHVLETYHPGAVIRILACSANPYSPNPPAEVRWEIL
PPNSQDYVELTF----EQQVYPTAVYVLETYHPGAVIRILACSANPYSPNPPAEVRWEIL
PPNSQDYVELTF----EQQVYPTAVHVLETYHPGAVIRILACSANPYSPNPPTEVRWEIL
PSSSQDYVELTF----EQQVYPTAVHVLETYHPGAVIRILACSANPYSPNPPAEVRWEIL
PSSSQDYVELTF----EQQVYPTAVHVLETYHPGAVIRILACSANPYSPNPPAEVRWETL
VRDPILWRYFLL--------RDLPSWSSVDWKSLPDLEILKKPISEVTDGAFFDYMA---
VRDPILWRYFLL--------RDLPSWSSVDWKSLPDLEILKKPVSEVTDGAFFDYMA---
VRDPILWRYFLL--------RDLPSWSSVDWKSLPDLEILKKPISEVTDGAFFDYMA---
VRDPILWRYFLL--------RDLPSWSSVDWKSLPDLEILKKPISEVTDGAFFDYMA---
VRDPILWRYFLL--------RDLPSWSSVDWKSLPDLEILKKPISEVTDGAFFDYMA---
VRDPILWRYFLL--------RDLPSWSSVDWKSLPDLEILKKPISEVTDGAFFDYMA---
IRDPILWRYFLL--------RDLPSWSSVDWKSLPDLEILKKPISEVTDSTCLDYME---
VRDPILWRYFLF--------RDLPSWSSVDWKSLPDLEILKKPISEVTDSTCFDYM----
VRNDSLWKPHCM------------------------------------------------
IRNDSLWKPHCM------------------------------------------------
IRNDSLWKPHCM------------------------------------------------
IRNDSLWKPHCM------------------------------------------------
IRNDSLWKPHCM------------------------------------------------
IRNDSLWKPHCM------------------------------------------------
IRNDSLWKPHCM------------------------------------------------
IRNDSLWKPHCL------------------------------------------------
IRNDSLWKPHCL------------------------------------------------
FHMPDLWRCFEF----ELNQPATSYLKATHPELIKQIIKRHSNHLQYVSFKVDSS-----
FHMPDLWRCFEF----ELNQPATSYLKATHPELIKQIIKRHSNHLQYVSFKVDSS-----
FHMPDLWRCFEF----ELNQPATSYLKATHPELIKQIIKRHSNHLQYVSFKVDSS-----
FHMPDLWRCFEF----ELNQPATSYLKATHPELIKQIIKRHSNHLQYVSFKVDSS-----
FHMPDLWRCFEF----ELNQPATSYLKATHPELIKQIIKRHSNHLQYVSFKVDSS-----
FHMPDLWRCFEF----ELNQPATSYLKATHPELIKQIIKRHSNHLQYVSFKVDSS-----
FHMPDLWRCFEF----ELNQPATSYLKATHPELIKQIIKRHSNHLQYVSFKVDSS-----
FHMPDLWRCFEF----ELNQPATSYLKATHPELIKQIIKRHSNHLQYVSFKVDSS-----
FHIPDLWRKFEF----ELNQSATSYFKSTHPDLIQQIIKKHAAHLQYVSFKVDSS-----
FHIPDLWRKFEF----ELNQSATSYFKSTHPDLIQQIIKKHAAHLQYVSFKVDSS-----
FHISDLWRKFEF----ELNQSATSSFKSTHPDLIQQIIKKHFAHLQYVSFKVDSS-----
FHIPDLWRKFEF----ELNQSATSSFKSTHPDLIQQIIKKHSAHLQYVSFKVDSS-----
FHIPDLWRKFEF----ELNQSATSYFKSTHPDLIQQIIKKHSAHLQYVSFKVDSS-----
FHIPDLWRKFEF----ELNQSATSYFKSTHPDLIQQIIKKHSAHLQYVSFKVDSS-----
ANDPILWKRLYMGKINPEEYEHPNPWKESFQQLYKGAHVKPGFAEHFYSNPARYKGRENM
ANDPILWKRLYMGKINPEEYEHPNPWKESFQQLYKGAHVKPGFAEHFYSNPARYKGRENM
ANDPILWKRLYMGKINPEEYEHPNPWKESFQQLYKGAHVKPGFAEHFYSNPARYKGRENM
ANDPILWKRLYMGKINPEEYEHPNPWKESFQQLYKGAHVKPGFAEHFYSNPARYKGRENM
ANDPILWKRLYMGKINPEEYEHPNPWKESFQQLYKGAHVKPGFAEHFYSNPARYKGRENM
ANDPILWKRLYMGKINPEEYEHPNPWKESFQQLYKGAHVKPGFAEHFYSNPARYKGRENM
ANDPILWKRLYMGKINPEEYEHPNPWKESFQQLYKGAHVKPGFAEHFYSNPARYKGRENM
ANDPILWKRLYMGKINPEEYEHPNPWKESFQQLYKGAHVKPGFAEHFYSNPARYKGRENM
ISEGMLWKKLIE-----RMVRTDPLWKGLSERRGWDQYLFKNRPTDGPPNSFYRS-----
```

```
ISEGMLWKKLIE-----RMVRTDPLWKGLSERRGWDQYLFKNRPTDGPPNSFYRS-----
ISEGMLWKKLIE-----RMVRTDPLWKGLSERRGWDQYLFKNRPTDGPPNSFYRS-----
ISEGMLWKKLIE-----RMVRTDPLWKGLSERRGWDQYLFKNRPTDGPPNSFYRS-----
ISEGMLWKKLIE-----RMVRTDPLWKGLSERRGWDQYLFKNRPTDGPPNSFYRS-----
ISEGMLWKKLIE-----RMVRTDPLWKGLSERRGWDQYLFKNRPTDGPPNSFYRS-----
ISEGMLWKKLIE-----RMVRTDPLWKGLSERRGWDQYLFKNRPTDGPPNSFYRS-----
ISEGMLWKKLIE-----RMVRTDPLWKGLSERRGWDQYLFKNRPTDGPPNSFYRS-----
TSDGMLWKKLIE-----RMVRTDSLWRGLAERRGWGQYLFKNKPPDGNAPPNSFYRA---
TSDGMLWKKLIE-----RMVRTDSLWRGLAERRGWGQYLFKNKPPDENAPPNSFYRA---
TSDGMLWKKLIE-----RMVRTDSLWRGLAERRGWGQYLFKNKPPDENAPPNSFYRA---
TSDGMLWRKLIE-----RMVRTDSLWRGLAERRGWGQYLFKNKPPDGNAAPNSFYRA---
TSDGMLWKKLIE-----RMVRTDSLWRGLAERRGWGQYLFKNKPPDGNAPPNSFYRA---
TSDGMLWKKLIE-----RMVRTDSLWRGLAERRGWGQYLFKNKPPDGNTPPNSFYRA---
TSDGMLWKKLIE-----RMVRTDSLWRGLAERRGWGQYLFKNKPPDGNAPPNSFYRA---
TSDGMLWKKLIE-----RMVRTDSLWRGLAERRGWGQYLFKNKPPDGNAPPNSFYRA---
ASTDVLWRKFCL----KRWFFYEVVTLELLGTESWKQFFVCRTRQ---------------
ASSDVLWRKLCQ----KRWLYCDMDTLQLQGKETWKQFFIDRIWQERA------------
ASNDFLWRKLCQ----ERWLFCDMVTLQLLGKETWKQFFVYRTWQ---------------
ASSDVLWRRLCQ----KRWLFCDMVTLQLLGTETWKEFFVSRTWQ---------------
ASSDVLWRKLCQ----KRWLYCDTVILQLHDKETWKQFFVNRTYQ---------------
ASSDVLWRKLCQ----KRWFYCNMVTQQLLGKETWKEFFIYRTWQ---------------
ASSDVLWRKLCQ----KRWLFCDMVTLQLLGTETWKQFFVFRTWQ---------------
ASSDVLWRRLCQ----KRWFYCDMVTLPLHGKETWKQFFIYRTWQ---------------
ASSDVLWRKLCQ----KRWLYCYMFTLPLHGLETWKQFFFNKTWQ---------------
ASSDVLWRKLCQ----KRWLYCYMFTLPLHGLETWKQFFFDKTWQ---------------
ASSDVLWRKLCQ----KRWLYCDRVTLQLHGLETWKQFFISRTWQ---------------
ASSDVLWRKLCQ----KRWLYCDMVTLQLHGKKTWKQFFFYRTWQ---------------
ASSDVLWRKLCQ----KRWYYCDMDTLQLHGKETWKQFFVYRTWQ---------------
AEDEVLWYRLCRLP--HSRFSDYTCWKLILQECLAKEHTLRANWKNRKGAVSELEHVPDA
AEDEVLWYRLCRLP--HSRFSDYTCWKLILQECLAPVHLIRPSWMNRKGAVSELEHVPDA
AEDEVLWYRLCQ-QLPGSSISDCSCWKLIFQECQAKEHMLRTNWKNRKGAVSELEHVPDA
AEDEVLWYRLCQLP--DSSISDYSCWKLIFQECRAKEHMLRTNWKNRKGAVSELEHIPDA
AEDEVLWYRLCQ-QLPDSSISDYSCWKLIFQECRAKEHMLRTNWKNRKGAVSELEHVPDA
AEDEVLWYRLCQ-QLPDSSISDYSCWKLIFQECRAKEHMLRSNWKNRKGAVSELEHVPDA
AEDEVLWYRLCQ-QLPDSSISDYSCWKLIFQECRAKEHMLRTNWKNRKGAVSELEHVPDA
AEDEVLWYRLCQLP--DSSISDYSCWKLIFQECRAKEHMLRTNWKNRKGAVSELEHVPDT
PAHHSPLSAFCC---PDQQCICQDCCQEHSGHTIVSLDAARRDKEVSVSEVKAVDEMQFG
FFPYVSWAARPKMNFGKGGMSRLGDDPCNLLLSLDHVQLLSSGYSKYRDFIRDLPLHLSK
SFPYISWSARPKFG--RGGTSRLGDEPCNLLLSLDHVQLLSSGFSKYRDFIRDLPLHLSK
LENEGPWRRSLQMNSEKGDLTKPGFDPCNLLVDLDDIRDLSSGFSKYRDFIRYLPIHLSK
PENEEPWRNSLQMNSEKGGITKPGYDPCNLLVDLDDLRDLSSGFSKYRDFIRYLPIHLSK
PENEEPWRNSLRMNSGKGDITKPGYDPCNLLVDLDDIRDLSSGFSKYRDFIRYLPIHLSK
PENEEPWRNSLRMNSGKGDITKPGYDPCNLLVDLDDIRDLSSGFSKYRDFIRYLPIHLSK
PENEEPWRNSLRMNSGKGDITKPGYDPCNLLVDLDDIRDLSSGFSKYRDFIRYLPIHLAK
ISATEVWQTACK-NQIDDSVQDALHWKKVYLKAILRMKQLEDHEAFETSSLIGHSAR---
```

ISATEVWQTACK-NQIDDSVQDALHWKKVYLKAILRMKQLEDHEAFETSSLIGHSAR---
ISATEVWQTACK-NQIDDSVQDALHWKKVYLKAILRMKQLEDHEAFETSSLIGHSAR---
ISATEVWQTACK-NQIDDSVQDALHWKKVYLKAILRMKQLEDHEAFETSSLIGHSAR---
ISATEVWQTACK-NQIDDSVQDALHWKKVYLKAILRMKQLEDHEAFETSSLIGHSAR---
ISATEVWQTACK-NQIDDSVQDALHWKKVYLKAILRMKQLEDHEAFETSSLIGHSAR---
ISATEVWQTACK-NQIDDSVQDALHWKKVYLKAILRMKQLEDHEAFETSSLIGHSAR---
ISATEVWQTACK-NQIDDSVQDSLHWKKVYLKAILRMKQLEDHEAFETSSLIGHSAR---
ANDELLWQGLCK----STWGHCSIYNKNPPLGFSFRKLYMQLDEG---------------
ANDELLWQGLCK----STWGHCSIYNKNPPLGFSFRKLYMQLDEG---------------
ANDELLWQGLCK----STWGHCSIYNKNPPLGFSFRKLYMQLDEG---------------
ANDELLWQGLCK----STWGHCSIYNKNPPLGFSFRKLYMQLDEG---------------
ANDELLWQGLCK----STWGHCSIYNKNPPLGFSFRKLYMQLDEG---------------
ANDELLWQGLCK----STWGHCSIYNKNPPLGFSFRKLYMQLDEG---------------
ANDELLWQGLCK----STWGHCSIYNKNPPLGFSFRKLYMQLDEG---------------
ANDELLWQGLCK----STWGHCSIYNKNPPLGFSFRKLYMQLDEG---------------
AKTGSLWKHLYPARYSGPATELDTEPDEEWVKNRKDESRAFHDWDEDADIDESEESAEE-
TKTIPLWLHFDSCIYSGPATELDTEPDEEWVKNRKDESRAFHEWDEDADIDESEESAEE-
TKAGSLWKHLYPARYSGPATELDTEPDEEWVKNRKDESRAFHEWDEDADIDESEESPEE-
TKTGSLWKHLYPARYSGPATELDTEPDEEWVKNRKDESRAFHEWDEDADIDESEESAEE-
TKTGSLWKHLYPARYSGPATELDTEPDEEWVKNRKDESRAFHEWDEDADIDESEESAEE-
TKTGSLWKHLYPARYSGPATELDTEPDDEWVKNRKDESRAFHEWDEDADIDESEESAEE-
AKTGSLWKHLYPARYSGPATELDTEPDEEWVRNRKDESRAFQEWDEDADIDESEESAEE-
AKTGSLWKHLYPYS--GPATELDTEPDEEWVRSRKDESRAFQEWDEDADIDESEESAEE-
AEDNLLWREKCKDEIKRRKVIKPGFIHSPWKSAYIRQHRIDTNWRRGELKSPKVLKG---
AEDNLLWREKCKDEIKRRKIIKPGFIHSPWKSAYIRQHRIDTNWRRGELRSPKVLKG---
AEDNLLWREKCKDEIKRRKIIKPGFIHSPWKSAYIRQHRIDTNWRRGELKSPKVLKGHD-
AEDNLLWREKCKDEIKRRKVIKPGFIHSPWKSAYIRQHRIDTNWRRGELKSPKVLKG---
AEDNLLWREKCKDEIKRRKVIKPGFIHSPWKSAYIRQHRIDTNWRRGELKSPKVLKG---
AEDNLLWREKCKDEIKRRKVIKPGFIHSPWKSAYIRQHRIDTNWRRGELKSPKVLKG---
AEDNLLWREKCKDEIKRRKVIKPGFIHSPWKSAYIRQHRIDTNWRRGELKSPKVLKG---
AEDNLLWREKCKDEIKRRKVIKPGFIHSPWKSAYIRQHRIDTNWRRGELKSPKVLKG---
TEQDCLWMPKCVPRATYGTLNEPKTEDEELLERHREKCLRKRIWEKIALRRKELFKVRPP
TEQDCLWMPKCVPRATYGTLNEPKTEDEELLERQREKCLRKRIWEKIALRKKELFKVRPP
TEQDCLWMPKCVPRATYGTLNEPKTEDEELLERQREKCLRKRIWEKIALRKKELFKVRPP
TEQDCLWMPKCIPRATYGTLNEPKTEDEELLERQREKCLRKRIWEKIVLRKKELFKVRPP
TEQDCLWMPKCVPRATYGTLNEPKTEDEELLERQREKCLRKRIWEKIALRKKELFKVRPP
TEQDCLWMPKCV--
TEQDCLWMPKCT--
PGGPQLWRDGTGYERQFLRASLDALPKRSNISGSHSYPLLLKKHHHGVGRTDALPLHAIL
AELDQLWMLKCL-----RFNWCINFSPTPFEQGIWKKHYIQMVKELH-------------
AELDQLWMLKCL-----RFNWYINFSPTPFEQGIWKKHYIQMVKELHVTKPKTP------
AELDQLWMLKCL-----RFNWYINFSPTPFEQGIWKKHYIQMVKELHITKPKTP------
AELDQLWMLKCL-----RFNWYINFSPTPFEQGIWKKHYIQMVKELHVTKPKTP------
AELDQLWMLKCL-----RFNWYINFSPTPFEQGIWKKHYIQMVKELHITKPKTP------
AELDQLWMLKCL--------------RFNWYINFSPTPFEQGIWKKHYIQMVKELH----

```
AELDQLWMLKCL-----RFNWYISFSPTPFEQGVWKKHYIQMVRELHVTK----------
AELDQLWMLKCL-----RFNWYISFSPTPFEQGVWKKHYIQMVRELHVTK----------
PQEGEAWLG-------SSGSAPPSRKRSRSSEEESNQATGTSRWDGVSKKAPRHHLIVPC
ISDFIPWKKLYHNCKESDLCVLNLIRYTATTKCSPSVDPERVLWSLRDHPLLPEAEACVR
ISDFIPWKKLYHNCKESDLCVLNLIRYAATTKCSPSVDPERVLWSLRDHPLLPEAKACMR
ISDFIPWKKLYHNCKESDLCVLNLIRYTATTKCSPSVDPERVLWSLRDHPLLPEAQACVR
ISDFIPWKKLYHNCKESDLCVLNLIRYTATTKCSSSVDPERVLWSLRDHPLLPEAEACVR
ISDFIPWKKLYHNCKESDLCVLNLIRYTATTKCSPSVDPERVLWSLRDHPLLPEAEACVR
INDFIPWKKLYHSHKDSDLCVLNLIRYTATTKCSPSVDPERVLWSLRDHPLLLEAEACMR
VNDFIPWKKLYHSHKDSDLCVLNLIRYTATTKCSPSVDPERVLWSLRDHPLLLEAEACMR
LFYPALWPQLRIPA--EQPRLEFLMRKCGWFVRELRVEFAAENYLSGCGPGDGGGADT--
LFYPALWPQLRIPA--EQPRLEFLMRKCGWFVRELHVEFAAENYLSGGGPGDGGGADT--
LFYPALWPQLRIPA--EQPRLEFLMRKCGWFVRELRVEFAAENYLSGGGPGDGGGVDT--
LFYPALWPQLRICLPAEQPRLEFLMRKCGWFVRELRVEFAAENYLSGGGPGDGGGADT--
LFYPALWPQLRIPA--EQPRLEFLMRKCGWFVRELRVEFAAENYLSGGGPGDGGGADS--
LFYPALWPQLRIPA--EQPRLEFLMRKCGWFVRELRVEFAAENYLSGSGPGDGGGADT--
LFYPALWPQLRIPA--EQPRLEFLMRKCGWFVRELRVEFAAENYLSGGGGPGDGGSGGGT
LFYPALWPQLRIPA--EQPRLEFLMRKCGWFVRELRVEFAAENYLSGGGGPGDGGGSGGG
CQSGKVWKEQFR----VRWPSLMKHYSPTDYVNWLEEYKVRQKAGLEARKIVASFSKRFF
CQSGKVWKEQFR----VRWPSLMKHYSPTDYVNWLEEYKVRQKAGLEARKIVASFSKRFF
CQSGKVWKEQFR----VRWPSLMKHYSPTDYVNWLEEYKVRQKAGLEARKIVASFSKRFF
CQSGKVWKEQFR----VRWPSLMKHYSPTDYVNWLEEYKVRQKAGLEARKIVASFSKRFF
CQSGKVWKEQFR----VRWPSLMKHYSPTDYVNWLEEYKVRQKAGLEARKIVASFSKRFF
CQSGQVWKEQFR----VRWPSLMKHYSPTDYVNWLEEYKVRQKAGLEARKIVASFSKRFF
CQSGQVWKEQFR----VRWPSLMKHYSPTDYVNWLEEYKVRQKAGLEARKIVASFSKRFF
CDAEGVWRRICR-RRIRDQSSGARPWKRAAILNYTKGLYFQAFGGRRRCLSKSVAPMLAH
CDAEGVWRRICR-RRIRDQGSGARPWKRAAILNYTKGLYFQAFGGRRRCLSKSVAPMLAH
CDAEGVWRLICR-RRLRDQGSGVRPWKRAAILNYTKGLYFQAFGGRRRCLSKSVAPLLAH
CDGEGVWRRICR--RLSPRLRDQDTKGLYFQAFGGRRRCLSKSVAPLLAH----------
CDGEGVWRRICR-RRLQDQGSGVRPWKRAAILNYTKGLYFQAFGGRRRCLSKSVAPLLAH
CDGEGVWRRICR-RRLQDQGSGVRPWKRAAILNYTKGLYFQAFGGRRRCLSKSVAPLLAH
CDAEGVWRRICRRL--SPRLRDQGSGVRPWKRAAILNYTKGLYFQAFGGRRR--------
CDGEGVWRRICR-RRLQDQGSGVRPWKRAAILNYTKCLYFQAFGGRRRCLSKSVAPLLAH
---ENLFNSLNY------DVAAKKRKKDMLNSKTKTQYFHQEKWIYVHKGSTK-------
---ENLFNSLNY------DVAAKKRKKDMLNSKTKTQYFHQEKWIYVHKGSTK-------
---ENLFNSLNY------DVAAKKRKKDMLNSKTKTQYFHQEKWIYVHKGSTK-------
---ENLFNSLNY------DVAAKKRKKDMLNSKTKTQYFHQEKWIYVHKGSTK-------
---ENLFNSLNY------DVAAKKRKKDMLNSKTKTQYFHQEKWIYVHKGSTK-------
YSKENLFSSLNY------DVAAKKRKKDIQNSKTKTQYFHQEKWIYVHKGSTK-------
CNKENLFNSLNY------DVAAKKRKKDIQNSKTKTQYFHQEKWIYVHKGSTK-------
FSN------------EEHEYASKKRKKDHFRNDTNTQSFYREKWIYVHKESTK-------
FNN------------EEHEYASKKRKKDHFRNDTNTQSFYREKWIYVHKESTK-------
FNN------------EEHEYASKKRKKDHFRNDTNTQSFYREKWIYVHKESTK-------
FNN------------EEHEYASKKRKKDHFRNDTNTQSFYREKWIYVHKESTK-------
FNN------------EEHEYASKKRKKDHFRNDTNTQSFYREKWIYVHKESTK-------
```

```
FNN------------EEHEYASKKRKKDHFRNDTNTQSFYREKWIYVHKESTK-------
FNN-------------ECEYAAKKRKKEHFGNDTAAHSFYREKWIYVHKESTK-------
SEDRRLWKRLCQ--HFAEKQFCRHLILSEKGHIEWKLMYFTL------------------
INSPNCWECPRCLG--GGWKLTEEPPPPPPLPRRKGPLPAGPTPDDVPGPPKRKEREGGN
INSPNCWECPRCEELGGGWKLTEEPPPPPPLPRRKGPLPAGPPPDDVPGPPKRKEREGGN
INAPNCWECPRCLG--SGWKLTEEPPLPPPPPRRKGPLPAGPPAEDLPGPPKRKEREAGN
INAPNCWECPRC----TQEGRTSKDSGEGPGRRRADNGEEGASLGSGWKLTEEPPLPPPP
TEEPPPPRRKGPER--EAGNEPPTPRKKVKGGRERHLKKVGGDACLLRGSDPGGPGLLPP
INAPNCWECPRCLG--SGWKLTEEPPLPPPPPRRKGPLPAGPPPEDVPGPPKRKEREAGN
----ERFKRMCQ----LLERVPDTSSSSSDSDSDSDSSGTSLSEDEAPGEARNG------
TEEPPLPPPPPRPKREAGNEPPTPRKKVKGGRERHLKKKPKPPLASAEGPAVPSPSPQRE
LNEPNCWECPKCMC--HVEAAPPPERTSAMTLAHRRLTRQDQLWTPTGGDGFPSTSLPRT
NEEPNCWECPKC--YQEDNSEKAQKRKMEESDEEAVQAKVLRPLRSCDEPLTPPPHSPTS
NEEPNCWECPKC--YQEDSSEKAQKRKMEESDEEAVQAKVLRPLRSCDEPLTPPPHSPTS
NEEPNCWECPKC--YQEDSSEKAQKRKMEESDEEAVQAKVLRPLRSCDEPLTPPPHSPTS
NEEPNCWECPKCYQ--EDSSEKAQKRKMEESDEEAVQAKVLRPLRSCDEPLTPPPHSPTS
NEEPNCWECPKCYQ--EDSSEKAQKRKMEESDEEAVQAKVLRPLRSCDEPLTPPPHSPTS
NEEPNCWECPKC--YQEDSSDKAQKRKIEESDEEAVQAKVLRPLRSCEEPLTPPPHSPTS
NEEPNCWECPKCYQ--EDSSEKAQKRKIEESDEEAVQAKVLRPLRSCEEPLTPPPHSPTS
VNDPNCWECPKCRR--RSDEHPKKVPPDGLLRRKSDDVHLRKKRKYEKPQELSGRKRASS
VNDPNCWECPKCEA--RSDEHPKKAPPDGILRRKSDDVHLRKKRKYEKPQELSGRKRASS
VNDPNCWECPKCRR--RSDEHPKKVPPDGLLRRKSDDVHLRKKRKYEKPQELSGRKRASS
VNDPNCWECPKCRR--RSDEHPKKVPPDGLLRRKSDDVHLRKKRKYEKPQELSGRKRASS
VNDPNCWECPKCRR--RSDEHPKKVPPDGLLRRKSDDVHLRKKRKYEKPQELSGRKRASS
VNDPNCWECPKCRR--RSDEHSKKVPPDGLLRRKSDDVHLRKKRKYEKPQELSGRKRASS
VNDPNCWECPKCRR--RSDEHPKKVPADGILRRKSDDVHLRRKRKYEKPQELSGRKRASS
VNDPNCWECPKCRR--RSDEHPKKVPTDGILRRKSDDVHLRRKRKYEKPQELSARKRAST
SSK-AKRRRRCLPPEGPSSAGEDVDLLSVAEMVALVEQRAALALQSYPRPATPAPVVFVS
---DSSKAKRRR----RAALALQSYPRPTTPAPVVFVSAEQGGPAKGVGSERR-------
SSK-AKRRRRCLLAEGPASAGEDVDLLSVAEMVALVEQRAALALQSYPRPATPAPVVFVS
SSK-AKRRRRCLLPEGPTSAGEDVDLLSVAEMVALVEQRAALALQSYPRPTTPAPVVFVS
SSK-AKRRRRCLPPEGPASAGDDVDLLSVAEMVALVEQRAALALQSYPRPTTPAPVVFVS
SSK-AKRRRRCLPPEGPASAGEDVDLLSVAEMVALVEQRAALALQSYPRPTTPAPVVFVS
SSK-AKRRRRCLTPVTPTSSGDDVDLVSVAEMVALVEQRAALALQSYPRPSTPAPVVFVS
SSK-AKRRRRCLVA--PTSSGDDVDLLSVAEMVALVEQRAALALQSYPRPSTPAPVVFVS
GRAGDLKKAKVQSVSDSCDGVYAGRPLSVIQMVAFLEQRASALLASCTRNCTNSPAIVRF
GRAGDLRKAKVQSVSDSGDGVYAGRPLSVIQMVAFLEQRASALLASCSKNCTNSPAIVRF
--S-VRFSGQSR----GVPAASESYSAPGACEEAAERGKLEVGEPQSEPVRVLDMVA---
GRAGDLKKAKVQVNYQPEPFACGIEHCSVHYVSDSGDGVYAGRPLSVIQMVAFLEQRASA
GRAGDLKKAKVQVNYQPEPFACGIEHCSVHYVSDSGDGVYAGRPLSVIQMVAFLEQRASA
GRAGDLKKAKVQ--VERMREVNSRCYQPEPFACGIEHCSVHYVSDSGDGVYAGRPLSVIQ
QSEPGTWAIVKPIKDIDGRATKRRKKSGDLKKAKLQLEMMREINSQCYQSEPFVCGVEHC
FAAKSSWDIDGRSVSDSGDGVYAARPLSVIQMVAFLEQRATALLASCTKNCTNSPAIVKI
LITIFRWWKISL--------RSEYRSAKPGETKESHEDFLDNSHLQVQ------------
LITIFRWWKISL--------RSEYRSAKPGETKETHEDFLDNSHLQVQ------------
```

```
LVTIFRWWTISL--------RSEYRSTKPGETKETHEDFLENAHLQSQ------------
CMSDKLWEQIVQ------------------------------------------------
CMSDKLWEQIVQ------------------------------------------------
SLR------------------SEYRSTKPGEAKETHEDFLENSHLQGQ------------
LVTIFRWWKISL--------RSEYRSTKPGEAKETHEDFLENSHLQGQ------------
LVTIFRWWKISL--------RSEYRSTKPGEAKETHEDFLENSHLQGQ------------
ANSSAVWHDTKI----SCECELEGMLPPYLSACLDHVHNLRLEFEPSREPSRRAAIKLLM
ATCSTVWHDTKI----SCECELEGMLPLYLSTCLDHVHNLRLEFEPSREVSRRAAIELLM
ATCSAVWHDTKI----SCECELEGMLPPYLSACLDHVHNLRLEFEPSRKPSRRAAIELLM
ATCSAVWHDTKI----SCECELEGMLPPYLSACLDHVHKLRLEFEPSRKPSRRAAIELLM
ATCSAVWHDTKI----SCECELEGMLPPYLSACLDHVHNLRLEFEPSRKPSRRAAIELL-
ATCSAVWHDTKI----SCECELEGMLPPYLSACLDHIHNLRLEFEPSRKPSRRAAIELLM
AANSTVWSDKSI----SCDCELEDLLPPYLSSCLDHIHNLRLEYEPSKKPSRGTATELLT
ATNSAVWYDTSI----SCDCELENLLPPGLSACLDHIHNLSLEYEPSKKPSRRTATELLT
ADDNLIWVRKYA----AAFRSKRSRWKATSVEETATSLSLLSVWDKEDGYWKKEYIT---
TSDNLIWVKIYS----TAFSSKRRYWKLTSVEETATCVSLLSVEDKEVGYWKKE------
ANDNFIWIRIYSTR--SNRKVSSVQKIATSMSFLSVQNKEAGYWKKEYITKQIASVKAAL
ANDNFIWIRIYSTR--SNWKVNSVEKIAVSVSFLSVQDKEAGYWKKEYITKQIASVKAAL
ANDNFIWIGIYSTR--SNWKVNSVEKIAMSMSFLSVQDKEAGYWKKEYITKQIASVKAAL
ANDNFIWIGIYSAR--SNWKFNSVEKIAMSMSFLSVQDKEAGYWKKEYITKQIASVKAAL
ANDNFIWIGIYS----TAFSPARSNWKFNSVEKIAMSMSFLSVQDKEAGYWKKEYIT---
ANDNFIWIRIYS----TAFSPTRSNWKFNSVEKIAMSMSFLSVQDKEAGYWKKEYIT---
TSHPSLWHTVTLKG--EKKLLACLEWLVPNRFSQLQSLTLIHWKSQVHSVLE--------
TSHPSLWHTVTLKG--EKKLLACLEWLIPNRFSQLQRLTLIHWKSQVHSVLE--------
ASQPALWHTVTLANKAEKKLLASLEWLMPSRFSQLQLTLI--HWKSQVHPVLK-------
ASQPALWHTVTLAKKAEKKLLASLEWLMPNRFSQLQRLTLIHWKSQVHPVLK--------
ASQPALWHTVTL-------SSPLVGRPAKGGVKAEKKLLASLEW----------------
ASQPALWHTVTLAKKAEKKLLASLEWLMPNRFSQLQRLTLIHWKSQVHPVLK--------
ASQPALWHTVTLAKKAEKKLLASLEWLMPNRFSQLQRLTLIHWKSQVHPVLK--------
ASQPALWHTVTLAKKAEKKLLASLEWLMPNRFSQLQRLTLIHWKSQVHPVLK--------
SLDESLWQSLDL----AGKNLHPDVTVRLLSRGVVAFRCPRSFMEQPLGESFSSFRVQHM
SLDESLWQSLDL----AGKNLHPDVTVRLLSRGVVAFRCPRSFMEQPLGESFSSFRVQHM
ASDESLWQTLDL----TGKNLHPDVTGRLLSQGVIAFRCPRSFMDQPLAEHFSPFRVQHM
ASDESLWQTLDL----TGKNLHPDVTGRLLSQGVIAFRCPRSFMDQPLAEHFSPFRVQHM
AFDESLWQTLDL----TGKNLHPDVTGRLLSQGVIAFRCPRSFMDQPLAEHFSPFRVQHM
ASDESLWQTLDL----TGKNLHPDVTGRLLSQGVIAFRCPRSFMDQPLAEHFSPFRVQHM
ASDESLWQTLDL----TGKNLHPDVTGRLLSQGVIAFRCPRSFMDQPLAEHFSPFRVQHM
LYQPKFWAGLTP-------VLHAKELYNVLPGGEKEFVNLQGFAARGFEG----------
LYQPKFWAGLTP-------VLHAKELYNVLPGGEKEFVNLQGFAARGFEG----------
LYQPKFWAGLTP-------VLHAKELYNVLPGGEKEFVNLQGFAARGFEG----------
LYQPKFWAGLTP-------VLHAKELYNALPGGEKEFVNLQGFAARGFEG----------
LYQPKFWAGLTP-------VLHAKELYNVLPGGEKEFVNLQGFAARGFEG----------
LYQPKFWAGLTP-------VLHAKELYNVLPGGEKEFVNLQGFAARGFEG----------
LYQPKFWAGLTP-------VLHAKELYNVLPGGEKEFVNLQGFAARGFEG----------
LYQPKFWAGLTP-------VLHAKELYNVLPGGEKEFVNLQGFAARGFEG----------
```

MYSADLWRYRTI----TFSGRPSRVHASEFESALWYIKKFGRYLEHLEIKFLNPYN----
MYSADLWRYRTI----TFSGRPSRVHASEFESALWYVKKFGRYLEHLEIKFLNPYN----
MYSADLWRYRTI----TFSGRPSRVHASEFESALWYVKKFGRYLEHLEVKFLN-------
MYSADLWRYRTI----TFSGRPSRVHASEVESALWYVKKFGRYLERLEVKFLNPYN----
MYSADLWRYRTI----TFSGRPSRVHASEVESALWYVKKFGRYLERLEVKFLNPYN----
MYSAELWRYRTI----TFSGRPSRVHASEVESAVWYVKKFGRYLEHLEVKFMNPYN----
MYSAELWRYRTI----TFSGRPSRVHASEVESAVWYVKKFGRYLEHLEVKFMNPYN----
MYSAELWRYRTI----TFSGRPSRVHASEVESAVWYVKKFGRYLEHLEVKFMNPYN----
VXXXXXXXXXXXSP--PKWCANGRGPGSPPAPPASSPAAAAGLWRRRRRRWWGGPAGGGA
CLDFQFWKQLDL------SSRQQVTDELLEKIASRSQNIIEINISDCRSMSDTGVC----
CLDFQFWKQLDL--
GRSVQLFRAPPAPQATALEMVCKRKGAGVPACTPCKQPRCGCGGCGGGGGGGGGPAGGGA
GRSVQLFRGPPP----PAEPLTPLEMVCKRKGAGVPACTPCKQPRCGGGGCGGGGGGGGG
GRSVQLFRGPTPAG--VPACTPCKQPRCGGGGCGGGGGGGGGGGPAGGGASPPRP-----
GRSVQLFRGPTPAG--VPACTPCKQPRCGGGGCGGGGGGGGGGGPAGGGASPPRP-----
VRDRVTWRLRAQ--RVRAPYPVVEEENFDWPAACIELEQHLARWAEDGQ-----------
VRDRVTWRLRAQ--RVRSPYPVVEEENFDWPAACIELEQHLARWAEDGQ-----------
VLDHVTWRLRAQ--RVRAPYPVVEGKNFDWPAACTELEQHLSRWAEDGRWAEHFC-----
VYDRVTWRLRAL--RRKNFDWPAACIALEEHLSRWAEDGRRAEYFCLADGHLAA------
VSDHVTWRLRTL--RVRAPYPVVEEKNFDWPAACIALEQHLSRWAEDGR-----------
VSDHVTWRLRAL--RVRAPYPVVEEKNFDWPAACIALEQHLSRWAEDGRWVEYFC-----
VSDHVTWRLRAL--RVRAPYPVVEEKNFDWPAACIALEQHLSRWAEDGRWVEYFC-----
VSDHVTWRLRAL--RVRAPYPVVEEKNFDWPAACIALEQHLSRWAEDGR-----------
VRR--VLRTHRS----VTWISAGVAEAGHLEGHCLVRVVAEALENVRILPQ---------
VRR--VLRTHRS----VTWISAGVAEAGHLEGHCLVRVVAEALENVRILPR---------
VRR--VLRTHRS----LTWISAGLADAGHLERHCLVRVVAEELENVRILPH---------
VRR--VLRTHRS----VTWISAGLAEAGHLERHCLVRVVAEELEAKNVRILPH-------
VRR--VLRTHRS----VTWISAGLAEASHLERHCLVRVVAEELEAINVRILPH-------
VRR--VLRTHRS----VTWISAGLAEAGHLERHCLVRVVAEELENVRILPH---------
VRR--VLRTHRS----VTWISAGVAEAGHLERHCLVRVVAEELENVRILPH---------
VRR--VLRTHRS----VTWISAGLAEAGHLEGHCLVRVVAEELENVRILPH---------
AWDPRLWRTIRL---TGETINVDRALKVLSRRLCQDTPNVCLMLETVTVSGCRRLTDRGL
AWDPRLWRTIRL---TGETINVDRALKVLTRRLCQDTPNVCLMLETVIVSGCRRLTDRGL
AWDPRLWRTIRL---TGETINVDRALKVLTRRLCQDTPNVCLMLETVIVSGCRRLTDRGL
AWDPRLWRTIRL---TGETINVDRALKVLTRRLCQDTPNVCLMLETVTVSGCRRLTDRGL
AWDPRLWRTIRL---TGETINVDRALKVLTRRLCQDTPNVCLMLETVTVSGCRRLTDRGL
AWDPRLWRTIRL---TGETINVDRALKVLTRRLCQDTPNVCLMLETVTVSGCRRLTDRGL
AWDPRLWRTIRL---TGETINVDRALKVLTRRLCQDTPNVCLMLETVTVSGCRRLTDRGL
AWDPRLWRTIRL---TGETINVDRALKVLTRRLCQDTPNVCLMLETVTVSGCRRLTDRGL
ALDGSNWQRIDL-----FNFQTDVEVKVLYNSKILIYLSVNICGDSSPPSLELPCKHIPE
ALDGSNWQRIDL-----FNFQTDVEGRVVENISKRCGGFLRKLSLRGCIGVGDSSLK---
ALDGSNWQRIDL-----FNFQTDVEGRVVENISKRCGGFLRKLSLRGCIGVGDSSLK---
ALDGSNWQRIDL-----FNFQTDVEGRVVENISKRCGGFLRKLSLRGCIGVGDSSLK---
ALDGSNWQRIDL-----FNFQTDVEGRVVENISKRCGGFLRKLSLRGCIGVGDSSLK---
ALDGSNWQRIDL-----FNFQTDVEGRVVENISKRCGGFLRKLSLRGCIGVGDSSLK---

```
ALDGSNWQRVDL-----FNFQTDVEGRVVENISKRCGGFLRKLSLRGCIGVGDSSLK---
ALDGSNWQRVDL-----FNFQTDVEGRVVENISKRCGGFLRKLSLRGCIGVGDSSLK---
ALDGSNWQRIDL-----FDFQRDIEGRVVENISKRCGGFLRKLSLRGCLGVGDNALR---
ALDGSNWQRIDL-----FDFQRDIEGRVVENISKRCGGFLRKLSLRGCLGVGDNALR---
ALDGSNWQRIDL-----FDFQRDIEGRVVENISKRCGGFLRKLSLRGCLGVGDNALR---
ALDGSNWQRIDL-----FDFQRDIEGRVVENISKRCGGFLRKLSLRGCLGVGDNALRQR-
ALDGSNWQRIDL-----FDFQRDIEGRVVENISKRCGGFLRKLSLRGCLGVGDNALR---
ALDGSNWQRIDL-----FDFQRDIEGRVVENISKRCGGFLRKLSLRGCLGVGDNALR---
ALDGSNWQRIDL-----FDFQRDIEGRVVENISKRCGGFLRKLSLRGCLGVGDNALR---
ALDGSNWQRIDL-----FDFQRDIEGRVVENISKRCGGFLRKLSLRGCLGVGDNALR---
VQL-----------------HLARLRRFDAAQVGPQIPRAALA-----------------
VQL-----------------HLARLRRFDAAQVGPQIPRAALV-----------------
LLP-HVLNRVPL----RQLLRLQRVSRAFRALVQLHLAGLRRFDAAQVGPQIPRAALA--
VQL-----------------HLAGLRRFDAAQVGPQIPRAALA-----------------
VQL-----------------HLAGLRRFDAAQVGLQIPRAALA-----------------
VQL-----------------HLAGLRRFDAAQVGPQIPRAALA-----------------
VQL-----------------HLAGLRRFDAAQVGPQIPRAALA-----------------
VQL-----------------HLAGLRRFDAAQVGPQIPRAALA-----------------
ASHKSVWRGVEI------LSLRRSLSYVIQGMANIESLNLSGCYNLTDKAH---------
AYHKSVWRGVEA--KLHLRRANPSLFPSLQARGIRRVQILSLRRSLS-------------
AYHKSVWRGVEA--KLHLRRANPSLFPSLQARGIRRVQILSLRRSLS-------------
AYHKSVWRGVEA--KLHLRRANPSLFPSLQARGIRRVQILSLRRSLS-------------
AYHKSVWRGVEA--KLHLRRANPSLFPSLQARGIRRVQILSLRRSLS-------------
AYHKSVWRGVEA--KLHLRRANPSLFPSLQARGIRRVQILSLRRSLS-------------
AYHKSVWRGVEA--KLHLRRANPSLFPSLQARGIRRVQILSLRRSLS-------------
TQLSSLWNAIDF----STVKHAIPDKYIVSTLQRWHLNVLRLNFRGCLLRPKTF------
TQLNSLWNAVSR-------YLNYVFTLGCIAEVADCLNMLRLILEDCRVKRAQ-------
TQLNSLWNAIDF----STVKNVIPDKYILSTLQRWRLNVLRLNFHGCLLRPKTF------
TQLNSLWNAIDF----STVKNVIPDKYIVSTLQRWRLNVLRLNFRGCLLRPKTF------
TQLNSLWNAIDF----SSVKNVIPDKYIVSTLQRWRLNVLRLNFRGCLLRPKTF------
TQLNSLWNAIDF----SSVKNVIPDKYIVSTLQRWRLNVLRLNFRGCLLRPKTF------
IQRGSLWNSIDF-----STVKNIADKCVVTTLQKWRLNVLRLNFRGCDFRTKTL------
KSHFGVWKEIAI--ANQEAIMAEKMDKAIAHDNFRCQKHIFNRWFSYTVKRRERLIATLL
LHGSEVWRSLCA----RSLAEEALRTDILCNLPSYKAKVIRAFQHA--------------
LHGSEVWRSLCA------RSLAEEALRTDILCNLPSYKAKVRAFQHA-------------
LHGSEVWRSLCA------RSLAEEALRTDILCNLPSYKAKIRAFQHA-------------
LHGSEVWRSLCA------RSLAEEALRTDILCNLPSYKAKIRAFQHA-------------
VDDRWLWRHVDL---TLYTMRPKVMWHLLRRYMASRLYSL--------------------
VDDRWLWRHVDL---TLYTMRPKVMWHLLRRYMASRLHSL--------------------
VDDRWLWRHVDL---TLYTMRPKVMWHLLRRYMGSRLHSL--------------------
VDDRWLWRHVDL---TLYTMRPKVMWHLLRRYMASRLHSL--------------------
VDDRWLWRHVDL---TLYTMRPKVMWHLLRRYMASRLHSL--------------------
VDDRWLWRHVDL---TLYTMRPKVMWHLLRRYMASRLHSL--------------------
VDDRWLWRHVDL---TLYTMRPKVMWHLLRRYMASRLHSL--------------------
VDDRWLWRHVDL---TLYTMRPKVMWHLLRRYMASRLHSL--------------------
```

```
FTSDLLWRRIAR--ASLNSGFTRLGTDLMTSVPVKERVKVSQNWRLGRCREGI-------
FTSDLLWRRIAR--ASLNSGFTRLGTDLMTSVPVKERVKVSQNWRLGRCREGI-------
FTSDLLWRRIAR--ASLNSGFTRLGTDLMTSVPVKERVKVSQNWRLGRCREAI-------
FTSDLLWRRIAR--ASLNSGFTRLGTDLMTSVPVKERVKVSQNWRLGRCREGI-------
FTSDLLWRRIAR--ASLNSGFTRLGTDLMTSVPVKERVKVSQNWRLGRCREGI-------
FTSDLLWRPIAR--ASLNTGFTRLGTDLMAGIPVKERVKLSQNWRLGRCRDRI-------
FTSDLLWRPIARAS--LNSGFTRLGTDLMTSIPVKERVKLSQNWRLGRCRDRI-------
GSAASLYDRIVLIVKLGEVALLASIDQHCSTTRVCNLVFMPAWFSPIMYKTTSGHIQFDN
GSAASLYDRIVLIVKLGEVVLLASIDQHCSTTRVCNLVFMPAWFSPIMYKTTSGHIQFDN
GSAASLYDRIVLKL--GEVALLASIDQHCSTTRLCNLVFMPAWFSPFMYKTTAGHVQFDN
GSAASLYDRIVLKL--GEVALLASIDQHCSTTRLCNLVFMPAWFSPIMYKTTSGHVQFDN
GSAASLYDRIVLKL--GEVALLASIDQHCSTTRLCNLVFMPAWFSPIMYKTTSGHVQFDN
GSAASLYDRIVLKL--GEVALLASIDQHCSTTRLCNLVFTPAWFSPIMYKTTSGHVQFDN
GSAASLYDRIVLIVKLGEVALLASIDQHCSTTRLCNLVFTPAWFSPIMYKTTSGHVQFDN
GSAASLYDRIVLIVKLGEVALLASIDQHCSTTRLCNLVFTPAWFSPIMYKTTSGHVQFDN
LHTDTIWRRRCRYGNLRKLEITGVSCRDVYAKLLHRYRHILGLWQPDIGPYGGLLNVVVD
LHTDTIWRRRCR------------------------------------------------
LHTDTIWRRRCRYGNLRKLEITGVSCRDVYAKLLHRYRHILGLWQPDIGPYGGLLNVVVD
LHTDTIWRRRCRYGNLRKLEITGVSCRDVYAKLLHRYRHILGLWQPDIGPYGGLLNVVVD
LHTDTIWRRRCRYGNLRKLEITGVSCRDVYAKLLHRYRHILGLWQPDIGPYGGLLNVVVD
LHTDTIWRRRCRYGNLRKLEITGVSCRDVYAKLLHRYRHILGLWQPDIGPYGGLLNVVVD
LHTDTIWRRRCRYGNLRKLEITGVSCRDVYAKLLHRYRHILGLWQPDIGPYGGLLNVVVD
LHTDTIWRRRCRYGNLRKLEITGVSCRDVYAKLLHRYRHILGLWQPDIGPYGGLLNVVVD
VDGPSVWLLQLA----RDRSAEGRALYALAQSCPADRNGH--------------------
VDGPSIWLLQLA----RDRSAEGRALYALAQRCPADADADAD------------------
VDGPTVWLLQLA----RDRSAEGRALYAVVQRCL--------------------------
VDGPTVWLLQLA------------------------------------------------
VDGPTVWLLQLA----RDRSAEGRALYAVAQRCLPSKEDK--------------------
VDGPTVWLLQLA----RDRSAEGRALYAVAQRCLPSNEDK--------------------
VDGPTVWLLQLA----RDRSAEGRALYAVAQRCLPSNEDK--------------------
VDSQALWLLILA----RDHSATGRALLQLARSCQSAS-----------------------
VDGQALWLLILA----RDHSATGRALLHLARSCQSPA-----------------------
VDGQALWLLIAR----------------DHGATGRALLHLARSCQSPA------------
VDGQALWLLILA----RDHGATGRALLHLARSCQSPA-----------------------
VDGQALWLLILA----RDHGATGRALLHLARSCQSPA-----------------------
VDGQALWLLILA------------------------------------------------
VDGQALWLLLLA----RDHSAAGRALLTLARRCLPPA-----------------------
VDGQALWLLLLA----RDHSAAGRALLTLARRCLPPA-----------------------
VDGAPLWLLKCQ----QEGLVPEG--GAEEERDHWQRFYFLSKRRRN-------------
VDGAPLWLLKCQ----QEGLVPEG--GVEEERDHWQQFYFLSKRRRN-------------
VDGAPLWLLKCQ----QEGLVPEG--GAEEERDHWQQFYFLSKRRRN-------------
VDGAPLWLLKCQ----QEGLVPEG--GVEEERDHWQQFYFLSKRRRN-------------
VDGAPLWLLKCQ----QEGLVPEG--GVEEERDHWQQFYFLSKRRRN-------------
VDGAPLWLLKCQ----QEGLVPEGSADEERDHWQQFYFLSKRRRN---------------
VDGAPLWLLKCQ----QEGLVPEG--SADDERDHWQQFYFLSKRRRN-------------
```

```
IDLVTLWKRKCLIT--EDWDQPVADWKIFYFLRSLHRN----------------------
IDLVTLWKRKCL-RITEDWDQPVADWKIFYFLRSLHRN----------------------
IDLVTLWKRKCL-QITEDWDQPVADWKIFYFLRSLQRN----------------------
IDLVTLWKRKCL-QITEDWDQPVADWKIFYFLRSLQRN----------------------
IDLVTLWKRKCL-RITEDWDQPVADWKIFYFLRSLHRN----------------------
IDLVTLWKRKCL-RITEDWDQPVADWKIFYFLRSLHRN----------------------
IDLVTLWKRKCL-RITEDWDQPVADWKIFYFLRSLHRN----------------------
IDLMILWKRKCLIT--KDWDQPVEDWKICYFLRSLQRN----------------------
IDLMTLWKRKCLIT--EDRDQPVADWKIFYFLQSLHRN----------------------
IDLMTLWKRKCL-RITKDWDQPVADWKIFYFLRSLHRN----------------------
IDLMTLWKRKCL-RITEDWDQPVANWKIFYFLRSLHRN----------------------
IDLMTLWKRKCLIT--EDWDQPVADWKIFYFLRSLHRN----------------------
IDLMTLWKRKCL-RITEDWDQPVADWKIFYFLRSLHRN----------------------
IDVVSLWKRKSLFT--KDRCEPVEDWKVFYILCSLQRN----------------------
IDVMTLWKRKSL-------REGFVTKDRDEPVDDWKIFYILCSLQRN-------------

-HVQQTNNNTNEIEVLETSRLYED-----------------SGYSSFSQLSGLSEHEEGS
-HVQQTLNSTNEIEALETSRLYED-----------------SGYSSFSQQSGLSEHEEGS
-HVQQTLNSTNEIEALETSRLYED-----------------SGYSSFSQQSGLSEHEEGS
-HVQQTLNSTNEIEALETSRLYED-----------------SGYSSFSLQSGLSEHEEGS
-HVQQTLNSTNELEALETSRLYED-----------------SGYSSFSQQSGLSEHEEGS
-HVQQTLNSTNEIEALETSRLYED-----------------SGYSSFSQQSGLSEHEEGS
-DSSNETEELEASRLYEDSG----------------------YSSFTQSDRDDGILILE
IILENFRNSSQAHLLLSSQSPDQH--------PNKNLLPALHFERVVCSTLKKNGKRNSK
LHLSFSHSGYQFASCSKDCTVKIWADMRPYNWSYTQFSQFNQDDSLLLASGVFLGPHNSS
LHLSFSHSGYQFASCSKDCTVKIWADMRPYNWSYTQFSQFNQDDSLLLASGVFLGPHNSS
LHLSFSHSGYQFASCSKDCTVKIWADMRPYNWSYTQFSQFNQDDSLLLASGVFLGPHNSS
LHLSFSHSGYQFASCSKDCTVKIWADMRPYNWSYTQFSQFNKDDSLLLASGVFLGPHNSS
LHLSFSHSGYQFASCSKDCTVKIWADMRPYNWSYTQFSQFNKDDSLLLASGVFLGPHNSS
LHLSFSHSGYQFASCSKDCTVKIWADMRPYNWSYTQFSQFNKDDSLLLASGVFLGPHNSS
-RLSLAQPDNFICKITENTAFEAG--------------------LAYLSGNSFTMDEQEK
-KVTKNIAYDMELAYLSGNSLTMD-----------------EQEKSVICGVSPKQ-----
-ELRLALAQPRNFIYKVTKNIAFE------------------TQLAYLSGNSLTMDEQEK
-ELQLALAQPHNFVYKVTKNIAFE------------------TELAYLSGNRLTVDEQEK
-PHNFICKVTKNIAFETELA--------------------------YLSGNRLTVDEQEK
-ELRLALAQPHNFIYKVTKNIAFE------------------TELAYLSGNRLTVDEQEK
-YLEPRCPRMVLSLKEGAREEDLD---------AVEAQIGCKLPDDYRCSYRIHNGQKLV
-YLEPRCPRMVLSLKEGAREEDLD---------AVEAQIGCKLPDDYRCSYRIHNGQKLV
-YLEPRCPRMVLSLKEGAREEDLD---------AVEAQIGCKLPDDYRCSYRIHNGQKLV
-YLEPRCPRMVLSLKEGAREEDLD---------AVEAQIGCKLPDDYRCSYRIHNGQKLV
-YLEPRCPRMVLSLKEGAREEDLD---------AVEAQIGCKLPDDYRCSYRIHNGQKLV
-YLEPRCPRMVLSLKEGAREEDLD---------AVEAQIGCKLPDDYRCSYRIHNGQKLV
-YLEPRCPRMVLSLKEGAREEDLD---------AVEAQIGCKLPDDYRCSYRIHNGQKLV
-YLEPRCPRMVLSLKEGAREEDLD---------AVEAQIGCKLPDDYRCSYRIHNGQKLV
-EFRMESGRPQDFICKAISG-----------------HTGMIDQLAYVSPHEYRFDEGAR
```

-VKSGRPQDFICKAISGHTGTIDQ--------------------LAYVSPHEYRFDEKAR
SVSGSCCKAVVHDSLRAECQLQRSGRCLHSFRKLRDYAAKGCWEAQLALAKACAGGSQLG
SVSGSCCKAVVHDSLRAECQLQRSGRCLHSFRKLRDYAAKGCWEAQLALAKACAGGSQLG
SVSGSCCKAVVHESLRAESQLQRTGRSLHSFRKLRDYAAKGCWEAQLSLAKACANGNQLG
SVSGSCCKAVVHESLRAECQLQRTGRCLHSFRKVRDYAAKGCWEAQLSLAKACANGNQLG
SVSGSCCKAVVHESLRAECQLQRTGRCLHSFRKLRDYAAKGCWEAQLSLAKACANANQLG
SVSGSCCKAVVHESLRAECQLQRTGRCLHSFRKLRDYAAKGCWEAQLSLAKACANANQLG
SVSGSCCKAVVHESLRAECQLQRTGRCLHSFRKLRDYAAKGCWEAQLSLAKACANANQLG
SVSGSCCKAVVHESLRAECQLQRTGRCLHSFRKLRDYAAKGCWEAQLSLAKACANANQLG
-SRVQVCSIEDWLKSALQRS--------------------------------ICSQHESL
-SRVQVCSIEDWLKSAFQRS-------------------------ICSQHENLVNDFLLQ
-SRVQVCSIEDWLKSRSICS--
-SRVQVCSIEDWLKSAFQRS--
-SRVQVCSIEDWLKSAFQRS--
-SRVQVCSIEDWLKSAFQRS--
-SRVQVCSIEDWLKSAFQRS--
-RSLTELQKDNFLLGPALRS--
LIQGNNFKDSITHDFSDSSLCINDSPSPEVKGNISTPEDSGFNSLSLEKSEYSLSDQEGS
LIQGNDFKDSITHDFSDSSLSINDSPSPEVRGNISTPEDSGFNSLSLEKSEDSLSDQEGS
LIQGNNFKDSITHDFSDSSLCINDSPSPEVRGSISTPEDSGFNSLSLEKSEDSLSDQEGS
LIQGNNFKDSITHDFSDSSLCINDSPSPEVRGNISTPEDSGFNSLSLEKSEDSLSDQEGS
LIQGNNFKDSITHDFSDSSLCINDSPSPEVRGSISTPEDSGFNSLSLEKSEDSLSDQEGS
LIQGNNFKDSITHDFSDSSLCINDSPSPEVRGSISTPEDSGFNSLSLEKSEDSLSDQEGS
VPYPLHQPERFFDEIHTYSPSKNWLDLEQWAWSKPNISGPSPHPRGGQSQIVIDDATILI
VPYPLHQPERFFDEIHTYSPSKNWLDLEQWAWSKPNISGPSPHPRGGQSQIVIDDATILI
VPYPLHQPERFFDEIHTYSPSKNWLDLEQWAWSKPNISGPSPHPRGGQSQIVIDDATILI
VPYPLHQPERFFDEIHTYSPSKNWLDLEQWAWSKPNISGPSPHPRGGQSQIVIDDATILI
VPYPLHQPERFFDEIHTYSPSKNWLDLEQWAWSKPNISGPSPHPRGGQSQIVIDDATILI
VPYPLHQPERFFDEIHTYSPSKNWLDLEQWAWSKPNISGPSPHPRGGQSQIVIDDATILI
VPYPLHQPERFFDEIHTYSPSKNWLDLEQWAWSKPNISGPSPHPRGGQSQIVIDDTTLLI
VPYPLHQPERFFDEIHTYSPSKNWLDLEQWAWSKPNISGPSPHPRGGQSQIVIDDATILI
FATLSRVQELKQTLFTPSYGVVPCEVVRLYLAVLSDRTPENLHAFLISVPGSFAESGATK
LVLSDRTPENLHAFLISVPGSFAEQRLINGGKDLRSLASLNLSGCVHCLSADSLLRKAED
LVLSDRTPENLHAFLISVPGSFAEQRLINGGKDLRSLASLNLSGCVHCLSADSLLRKAED
LVLSDRTPQNLHAFLISVPGSFAEQQVINGGKDLRSLASLNLSGCVHCLSPDSLLRKAED
LVLSDRTPQNLHAFLISVPGSFAEQQVINGGKDLRSLASLNLSGCVHCLSPDSLLRKAED
LVLSDRTPQNLHAFLISVPGSFAEQQVINGGKDLRSLASLNLSGCVHCLSPDSLLRKAED
LVLSDRTPQNLHAFLISVPGSFAEQQVINGGKDLRSLASLNLSGCVHCLSPDSLLRKAED
LVLSDRTPQNLHAFLISVPGSFAEQQVINGGKDLRSLASLNLSGCVHCLSPDSLLRKAED
KIQPSLTKDGVFSALKMAELEFPQYNCPHLHNPYNWISDHSRWMRLVDINLVRCHALKLD
KIQPSLTKDGVFSALKMAELEFPQYNCPHLHNPYNWISDHSRWTRLVDINLVRCHALKLD
KIQPSLTKDGVFSALKMAELEFPQYTCPHWAPRTSFYSDHSRWTRLVDINLVRCHALKLD
KIQPSLTKDGVFSALKMAELEFPQYNCPHLHNPYNWISDHSRWTRLVDINLVRCHALKLD
KIQPSLTKDGVFSALKMAELEFPQYNCPHLHNSYNWLSDHSRWTRLVDINLVRCHALKLD
KIQPSLTKDGVFSALKMAELEFPQYNCPHLHNPYNWISDHSRWTRLVDINLVRCHALKLD

```
KIQPSLTKDGVFSALKMAELEFPQYNCPHLHNPYNWISDHSRWTRLVDINLVRCHALKLD
KIQPSLTKDGVFSALKMAELEFPQYNCPHLHNPYNWISDHSRWTRLVDINLVRCHALKLD
-TFMKYVDSNLCCFIPGKLL---------------------SELRCYRMLAYSFQTYMPY
-TFMKYVDSNLCCFIPGKVIDEIY-----------------RVLRYVNSTRAPQRAHEVL
-TFMKYVDSNLCCFIPGKVIDEIY-----------------RVLRYVNSTRAPQRAHEVL
-TFMKYVDSNLCCFIPGKVIDEIY-----------------RVLRYVNSTRAPQRAHEVL
-TFMKYVDSNLCCFIPGKVIDEIY-----------------RVLRYVNSTRAPQRAHEVL
-TFMKYVDSNLCCFIPGKVIDEIY-----------------RVLRYVNSTRAPQRAHEVL
-TFMKYVDSNLCCFIPGKVIDEIY-----------------RVLRYVNSTRAPQRAHEVL
-TFMKYVDSNLCCFIPGKVIDEIY-----------------RVLRYVNSTRAPQRAHEVL
LQFLQVCLTMLVVLCLHGSSRVSHHHAGPAVPNTYAVSRHGSSPSTGASSRVPAASQSSG
LKQQRIYTPPSIIKRSQSRNSRQEHHAGPAVPSTYAVSRHGSSPSTGASSRVPAASQSSG
LRKQQEVVQIDQFLKETAAREASAHHAGSAVPSTYAVSRHGSSPSTGASSRVPAASQSSG
LRKQQEVVQIDQFLKETAAREASAHHAGPAMPSTYAVSRHGSSPSTGASSRVPAASQSSG
LRKQQEVVQIDQFLKETAAREASAHHAGPAVPSTYAVSRHGSSPSTGASSRVPAASQSSG
LRKQQEVVQIDQFLKETAAREASAHHVGPAVPNTYAVSRHGSSPSTGASSRVPAASQSSG
-VISSSCGSTPSASLGRGGGPNARHHVGPAVPNTYAVSRHGSSPSTGASSRVPAASQSSG
LRKQQEVVQIDQFLKETAAREASAHHVGPAVPNTYAVSRHGSSPSTGASSRVPAASQSSG
-SLSLGGGSPTEASFVDLIL----------------------------------------
-PASHQVLQSVAYHLGPYLQSLSL------------------------------------
-AYHLGPHLQSLSLGGGSPT----------------------------------------
-AYHLGPHLQSLSLGGGSPT----------------------------------------
KPHANGLVSVDEESYGALYQATVEDQNLKDQDHLYEEEIGAVGGIDHNDTSQNARSERNG
KPHTNGLVSVDEESYGALYQATVEDQNLKDQDHLYEEEIGAVGGIDYNDTDQNAQSEQNG
KPHTNGLVSVDEESYGALYQATVEDQNLKDQDHLYEEEIGAVGGIDYNDTNQNAQSEQNG
KPHANGLVSVDEESYGALYQATVEDQNLKDQDHLYEEEIGAVGGIDYNDTNQNAQSEQNG
KPHTNGLVSVDEESYGALYQATVEDQNLKDQDHLYEEEIGAVGGIDYNDTNQNAQSEQNG
KPHANGLVSVDEESYGALYQATVEDQNLKDQDHLYEEEIGAVGGIDYNDTNQNAQSEQNG
KPRTNGLVSVDEESYGALYQATVEDKNLKDQDHLDEGEIGAVGGVDYSGTSQNAQAEQNG
NPRANGLVSADEESYGALYEATVEDRNAKDQDHLDEGEIGAVGGIDYTGTSQNAQAEQNG
TPHDLSATNEEMAELSQEEREMLALDKFGQWENIFSKEHAASALTNSSVSCESKNKIGPE
TPHGLSATNGEMAELSQEEREVLALAKFGQWENIFSKEHAASALTNSSASCESKNKNGPE
NPHGLSATNGEMAELSQEEREVLALVKFGQWENIFSKEHAASALTNSSASCESKNKNDPE
TPHGLSATNGEMAELSQEEREVLALVKFGQWENIFSKEHAASALTNSSASCESKNKNDSK
TPHGLSATNGEMAELSQEEREVLALVKFGQWENIFSKEHAASALTNSSVSCESKNKNDSE
TPHGLSATNGEMAELSQEEREVLALVKFGQWENIFSKEHAASALTNSSASCESKNKNDSE
TLAPNSCLPATSRQMMELSQEERDASVLTGSLGKSEDKNGDVAGKEQCSSNVRIGDAEGS
TLVPNSCLPATNGQTMELSQEERDASVLTGSSGKSEDKNENVSGKEQRSGDGSTGDAECS
-FKITYTRSPDGDGVGNSYRILKL-----------FNIHLKAQESVLKLCQPELESSQTH
-FKITYTRSPDGDGVGNSYIEDNDDDSKMADLLSYFQQQLTFQESVLKLCQPELESSQTH
-FKITYTRSPDGDGVGNSYIEDNDDDSKMADLLSYFQQQLTFQESVLKLCQPELESSQIH
-FKITYTRSPDGDGVGNSYIEDNDDDSKMADLLSYFQQQLTFQESVLKLCQPELESSQTH
-FKITYTRSPDGDGVGNSYIEDNDDDSKMADLLSYFQQQLTFQESVLKLCQPELESSQTH
-ERPRVRFDGVYISKTTYIRQGEQ------------------------------------
-FKITYTRSPDGDGVGSGYIEENEDASKMADLLSYFQQQLTLQESVLKLCQPELETSQTH
```

```
-FKITYTRSPDGDGVGSSYIEDNEDASKMADLLSYFQQQLTFQESVLKLCQPELETSQTH
-YQSANCSDASDALIVLIHLLMLE--------SGYTPQGTEAKAMSMPEKWKSSGVYKLQ
-YQSADCSDANDALIVLIHLLMLE--------SGYIPQGTEAKALSMPEKWKSSGVYKLQ
-YQSADCSDANDALIVLIHLLMLE--------SGYIPQGTEAKALSMPEKWKLSGVYKLQ
-YQSADCSDANDALIVLIHLLMLE--------SGYIPQGTEAKALSMPEKWKSSGVYKLQ
-YQSADCSDANDALIVLIHLLMLE--------SGYMPQGTEAKALSMPEKWKLSGVYKLQ
-YQSADCSDANDALIVLIHLLMLE--------SGYIPQGTEAKALSMPEKWKLSGVYKLQ
-YQSAGCSNISDALIVLVHLLMLE--------SGYIPQGTETKAVTMPEKWKSSGVYKLQ
-YQSAGCSTVSDALIVLVHLLMLE--------SGYIPQGTEAKAASMPEKWKSSGVYKLQ
-YIHKILTEVSCFKFSGCAAPMQC------------LGFTCYGMFLQTLTAGWDELECHR
-YIHKILTEVSCFKFNGCAAPMQC------------LGLTCYGMFLQTLTAGWDELECHR
-YIHKILTEVSCFKFNGCAAPMQC------------LGLTCYGMFLQTLTAGWDELECHR
-YIHKILTEVSCFKFNGCAAPMQC------------LGLTCYGMFLQTLTAGWDELECHR
-YIHKILTEVSCFKFNGCAAPMQC------------LGLTCYGMFLQTLTAGWDELECHR
-YIHKILTEVSCFKFNGCAAPMQC------------LGLTCYGMFLQTLTAGWDELECHR
-YIHKILSEVSCFKFSGCSVPLQC------------LGLSCYGMFLQTLTAGWDELECHR
-YIQKILSEVPCFKFSGCSAPLQC-----------------LGLSCYGMFLQT-------
WERPTKVNASQARQFKPCIKQINFSLLDYYTELDAVVLHGVKDKPVLSHKTSLIDMNDIE
WERPTKVNASQARQFKPCIKQINFSLLDYYTELDAVVLHGVKDKPVISLKTSLIDINDRE
WERPTKVNASQARQFKPCIKQINFSLLEYYTELDAVVLHGVKDKPVLSLKTSLIDMNDIE
WERPTKVNASQARQFKPCIKQINFSLLDYYTELDAVVLHGVKDKPVLSLKTSLIDMNDIE
WERPTKVNASQARQFKPCIKQINFSLLDYYTELDAVVLHGVKDKPVLSLKTSLIDMNDIE
WERPTKLNASQARQFKPCIKQINFSLLDYYTELDAVVLHGVKDKPVLSLKTSLIDMNDID
WERPMKVNASQARQFKPCIKQINFSLLDYYTELDAVVLHGTKDKPLLSLKTALVDMNDLE
WERPTKVNASQARQFKPRIKQINFSLLDYYTELDAVVLHGTKDKPLLSLKTALVDMNDLE
-VYKMCCPYTRKASKTSRPM--------------------------YGAVTSFLHSLIIQ
-VYRMCCPYTRRASKSSRPM--------------------------YGAVTSFLHSLIIQ
-VYRMCCPYTRRASKSSRPM--------------------------YGAVTSFLHSLIIQ
-VYRMCCPYTRRASKSSRPM--------------------------YGAVTSFLHSLIIQ
-VYRMCCPYTRRASKSSRPM--------------------------YGAVTSFLHSLIIQ
-VYRMCCPYTRRASKSSRPM--------------------------YGAVTSFLHSLIIQ
-VYKMCCPYTRRALKASRPM--------------------------YGVVTSFLHSLIIQ
-EVYKMCCPYTRRALKASRP-------------------------MYGAVTSFLHSLIIQ
-TLRAVCRREIDDDLESGYSWRVI------------------------------------
-TVRAVCRREIDDDLESGYSWRVI------------------------------------
-TVRAVCRREIDDDLESGYSWRVI------------------------------------
-TVRAVCRREIDDDLESGYSWRVI------------------------------------
-TVRAVCRREIDDDLESGYSWRVI------------------------------------
-TVRAVCRREIDDDLESGYSWRVI------------------------------------
-TVRAVCRREIDDDLESGYSWRVI------------------------------------
-TIRAVCQREIDDDIKSGYTWRVI------------------------------------
-SIRAVCQREVDDDIENGYTWRVI------------------------------------
-KESAEAACDILSQLVNCSL----------------------KTLGLISTARPSFMDLPK
-KESAEAACDILSQLVNCSL----------------------KTLGLISTARPSFMDLPK
-KESAEAACDILSQLVNCSL----------------------KTLGLISTARPSFMDLPK
```

-KESAEAACDILSQLVNCSL----------------------KTLGLISTARPSFMDLPK
-KESAEAACDILSQLVNCSL----------------------KTLGLISTARPSFMDLPK
-KESAEAACDILSQLVNCSL----------------------KTLGLISTARPSFMDLPK
-KESAEAACDILSQLVNCSL----------------------KTLGLISTARPSFMDLPK
-KESAEAACDILSQLVNCSL----------------------KTLGLISTARPSFMDLPK
-TESAEAACHILSQLVNCSIQTLGAKPSFMNMPKVRILLLALTSYFLVYKAQPSEQPEET
-TESAEAACDILSQLVNCSI----------------------QTLGLISTAKPSFMNVPK
-AESAEAACDILSQLVNCSI----------------------QTLGLISTAKPSFMNVSE
-AESAEAACDILSQLVNCSIQTLG----------------------LISTAKPSFMNVSE
-VESAEAACGILSQLVNCSI----------------------QTLGLISTAKPSFMNVSE
-AESAEAACDILSQLVNCSVQTLG----------------------LISTAKPSFMNVSE
LVQEAHFDGLIFVHSGIYTDEWIYMTIRFNPDDKSAQHHNAHHCLEITVNCSPIIDHCII
LVQEAHFDGLIFVHSGIYTDEWIYMTIRFNPDDKSAQHHNAHHCLEITVNCSPIIDHCII
LVQEAHFDGLIFVHSGIYTDEWIYMTIRFNPDDKSAQHHNAHHCLEITVNCSPIIDHCII
LVQEAHFDGLIFVHSGIYTDEWIYMTIRFNPDDKSAQHHNAHHCLEITVNCSPIIDHCII
LVQEAHFDGLIFVHSGIYTDEWIYMTIRFNPDDKSAQHHNAHHCLEITVNCSPIIDHCII
LVQEAHFDGLIFVHSGIYTDEWIYMTIRFNPDDKSAQHHNAHHCLEITVNCSPIIDHCII
LVQEAHFDGLIFVHSGIYTDEWIYMTIRFNPDDKSAQHHNAHHCLEITVNCSPIIDHCII
LVQEAHFDGLIFVHSGIYTDEWIYMTIRFNPDDKSAQHHNAHHCLEITVNCSPIIDHCII
-LYPKIIQDIETIESNWRCGRHNL-------------------QRIQCRSENSKGVYCLQ
-LYPKIIQDIETIESNWRCGRHNL-------------------QRIQCRSENSKGVYCLQ
-LYPKIIQDIETIESNWRCGRHNL-------------------QRIQCRSENSKGVYCLQ
-LYPKIIQDIETIESNWRCGRHNL-------------------QRIQCRSENSKGVYCLQ
-LYPKIIQDIETIESNWRCGRHNL-------------------QRIQCRSENSKGVYCLQ
-LYPKIIQDIETIESNWRCGRHNL-------------------QRIQCRSENSKGVYCLQ
-LYPKIIQDIETIESNWRCGRHNL-------------------QRIQCRSENSKGVYCLQ
-LYPKIIQDIETIESNWRCGRHNL-------------------QRIQCRSENSKGVYCLQ
-LYPKIIQDIETIESNWRCGRHSL-------------------QRIHCRSETSKGVYCLQ
-LYPKIIQDIETIESNWRCGRHSL-------------------QRIHCRSETSKGVYCLQ
-LYPKIIQDIETIESNWRCGRHSL-------------------QRIHCRSETSKGVYCLQ
-LYPKIIQDIETIESNWRCGRHSL-------------------QRIHCRSETSKGVYCLQ
-LYPKIIQDIETIESNWRCGRHSL-------------------QRIHCRSETSKGVYCLQ
-LYPKIIQDIETIESNWRCGRHSL-------------------QRIHCRSETSKGVYCLQ
-LYPKIIQDIETIESNWRCGRHSL-------------------QRIHCRSETSKGVYCLQ
-LYPKIIQDIETIESNWRCGRHSL-------------------QRIHCRSETSKGVYCLQ
-ERAKSRAKPEDFTYKEIPVEIGA-----------------PRNAYYISGCGLTRDGQGK
-KFRAKAKDFTYKEIPLMCGLFGY--------------------ACYISGCGLTRKGQDK
-EHVKSRAIPEDFTYKEIPLECGV-----------------RGYAGYISGCALTRNGQGK
-EHAKSRAIREDFIYKEIPA-----------------EYGVRAHPCYISGRGLTRNGQGR
-EHTKTRAKPEDFTYKEICA-----------------ETGIWAYACYISGRGLTRNGQGT
-EHAKSRAKPEDFIYKEIPA-----------------EYGIQAYACYISEHGLTRNGQGR
-EHAKTRAKPEDFTYKEIPAEYGF-----------------RANACYISGCGLTRNVQDR
-EHAKSRAKPEDFTYKEIPVKFEF-----------------WAHPCYISRHGLTRNGQGK
-EHAKTRAKPEDFTYKEFPMEFEF-----------------RAHPWYISRHGLTRNGQGK
-EHAKTRAKPEDFTYKEFPVEFEF-----------------RAHPWYISRHGLTRNGQEK

```
-EHAKTRAKPEDFNYKEIPVAFEF-----------------RAHPCYISRHGLIRNGQGK
-EHAKSRAKPEDFTYKEIPVEFEF-----------------RAYAYYISRHGLTRNGQGK
-EHAKTRAKPEDFTYKEIPVEFEF-----------------RAYAYYISKHGLKRNGQGK
VLCDVRSHDGVVIAGYTSGDVRVW----------------DTRTWDYVAPFLESESEEED
VLCDVRSHDGVVIAGYTSGEVRVW----------------DTRTWDYVAPFLESESEEED
VLCDVHSHDGVVIAGYTSGDVRVW----------------DTRTWDYVAPFLESEDEEDE
VLCDVHSHDGVVIAGYTSGDVRVW----------------DTRTWDYVAPFLESEDEEDE
VLRDVHSHDGVVIAGYTSGDVRVW----------------DTRTWDYVAPFLESEDEEDE
VLCDVHSHDGVVIAGYTSGDVRVW----------------DTRTWDYVAPFLESEDEEDE
VLCDVHSHDGVVIAGYTSGDVRVW----------------DTRTWDYVAPFLESEDEEDE
VLCDVHSHDGVVIAGYTSGDVRVW----------------DTRTWDYVAPFLESEDEEDE
ENVMLFLEEKEQAALSQANGIKAHVQFLEEYCKFKNTEDITFPSVYIGLKDKLSGHKVIT
YLDKHSLNRCIFVSQHWATLVDQSLRTKTDYNLWNAYQNQETQLVQMEERNVFCGTYNIR
YLDKNSLNKCVFVSQHWATLVQQVLRTKNDYNLWTAYQNQETQLIQMEERNVFCGTYNIR
YHTLNKCASVSQHWAALAQQVKVELRTKTDYNLWTAYQNQETQQVLMEERNVFCGTYNVR
YHTLNKCASVSQHWAAMAQQVKMDLRTKNEYNLWTAYQNQETQQVLMEERNIFCGTYNVR
YHTLNKCASVSQHWAAMAQQVKMDLRTKNEYTLWTAYQNQETQQVLMEERNVFCGTYNVR
YHTLNKCASVSQHWAAMAQQVKMDLRTKNEYNLWTAYQNEETQQVLIEERNVFCGTYNVR
YHTLNKCASVSQHWAAMAQQVKMDLRTKNEYNLWTAYQNEETQQVLMEERNVFCGTYNVR
-VYALYYKDGLLCTGSDDLSAKLW--------------DVSTGQCVYGIQTHTCAAVKFD
-VYALYYKDGLLCTGSDDLSAKLW--------------DVSTGQCVYGIQTHTCAAVKFD
-VYALYYKDGLLCTGSDDLSAKLW--------------DVSTGQCVYGIQTHTCAAVKFD
-VYALYYKDGLLCTGSDDLSAKLW--------------DVSTGQCVYGIQTHTCAAVKFD
-VYALYYKDGLLCTGSDDLSAKLW--------------DVSTGQCVYGIQTHTCAAVKFD
-VYALYYKDGLLCTGSDDLSAKLW--------------DVSTGQCVYGIQTHTCAAVKFD
-VYALYYKDGLLCTGSDDLSAKLW--------------DVSTGQCVYGIQTHTCAAVKFD
-VYALYYKDGLLCTGSDDLSAKLW--------------DVSTGQCVYGIQTHTCAAVKFD
-SLTFNANPDEGVNYFMSKGILDD------------------------------------
-SLTFNANPDEGVNYFMSKGILDD------------------------------------
-SLTFNANPDEGVNYFMSKGILDD------------------------------------
-SLTFNANPDEGVNYFMSKGILDD------------------------------------
-SLTFNANPDEGVNYFMSKGILDD------------------------------------
-SLTFNANPDEGVNYFMSKGILDD------------------------------------
-SLTFNANPEEGVSYFMSKGILDD------------------------------------
-SLTFNANPEEGVSYFMSKGILDD------------------------------------
-SIAISIAQMEKRLLHGLIHNILP-YVGTSVKTLVLAYSSAVSSKMVRQILELCPNLEHL
-SIAISIAQMEKRLLHGLIHNVLP-YVGTSVKTLVLAYSSAVSSKMVRQILELCPNLEHL
-SIAISIAQMEKRLLHGLIHNVLP-YVGTSVKTLVLAYSSAVSSKMVRQILELCPNLEHL
-SIAISIAQMEKRLLHGLIHNVLP-YVGTSVKTLVLAYSSAVSSKMVRQILELCPNLEHL
-SIAISIAQMEKRLLHGLIHNVLP-YVGTSVKTLVLAYSSAVSSKMVRQILELCPNLEHL
-SIAISIAQMEKRLLHGLIHNVLP-YVGTSVKTLVLAYSSAVSSKMVRQILELCPNLEHL
-SVAISIAQMEKRVLHGLIHNVLP-YVGTSVKTLVLAYSSAVSSKMVRQILELCPNLEHL
-SVAISIAQMEKRLLHGLIHNVLP-YVGTSVKTLVLAYSSAVSSKMVRQILELCPNLEHL
-HDDHVITCLQFCGNRIVSGSDDN--------------TLKVWSAVTGKCLRTLVGHTGG
-HDDHVITCLQFCGNRIVSGSDDN--------------TLKVWSAVTGKCLRTLVGHTGG
```

```
-DHVITCLQFCGNRIVSGSDDNTL----------------KVWSAVTGKCLRTLVGHTGG
-HDDHVITCLQFCGNRIVSGSDDN--------------TLKVWSAVTGKCLRTLVGHTGG
-HDDHVITCLQFCGNRIVSGSDDN--------------TLKVWSAVTGKCLRTLVGHTGG
-HDDHVITCLQFCGNRIVSGSDDN--------------TLKVWSAVTGKCLRTLVGHTGG
-HDDHVITCLQFCGNRIVSGSDDN--------------TLKVWSAVTGKCLRTLVGHTGG
-HDDHVITCLQFCGNRIVSGSDDN--------------TLKVWSAVTGKCLRTLVGHTGG
WVLKPRCQPRLSQTVRERAGSHEAPAYEMVVESVKSGVISVVYEHSVTLESLLYLIEKAL
WVLKPRCQPRLSQTVRERVGLHEAPAYEMVMESVKAGVVSVVYEHSVTLESLLYLIEKAL
WVLKPRCQPRLSQTVRERVGLHEAPAYEMVVESVKAGVVSVVYEHSVTLESLLYLIEKAL
WVLKPRCQPRLSQTVREQVGLHEAPAYEMVVESVKAGVISVVYEHNVTLESLLYLIEKAL
WVLKPRCQPRLSQTVRERVGLHEAPAYEMVVESVKAGVVSVVYEHSVTLESLLYLIEKAL
-KFGWFLPYTPTDN----------------------------------------------
-RFGWFLPYAPTRN----------------------------------------------
ISSRIPAYEMVMESIKPDVVAVLYDLLQGYKICIKNVLWPEVRDFWEKLGSRVATEEEGG
-VTKPKTPPKDGFVITSNSP----------------------------------------
-PKDGFVIADVQLVTSNSPEEKQS------------------------------------
-PKDGFVIADVQLVTSNSPEEKQS------------------------------------
-PKDGFVIADVQLVTSNSPEEKQS------------------------------------
-PKNGFVIADVQLVTSNSPEEKQS------------------------------------
-ITKPKTPPKDGFVIADVQLVTSN------------------------------------
-PKTPPKDEFTTADVQPIPGNSPD------------------------------------
-PKTPPKDGFITADVQPIPGSSPE------------------------------------
TREARQEAEDSTSRLSEESGETDQ----DAGDMGLDPIPDSYYGLLGTLPCQEAPSHICS
QQRLLFCLRRPSSTVTMPDVTETLKEEPSVWPGKKTIQLTHEQQLILNHKMEPLQVVKIM
QSSVNDIQRLLFCLRRPSSTVTMPKEEPSVWPGKKTIQLTHEQQLILNHKMEPLQVVKIM
QSSVNDIQRLLFCLRRPSSTVTMPKEEPSVWPGKKTIQLTHEQQLILNHKMEPLQVVKIM
QSSVNDIQRLLFCLRRPSSTVTMPKEEPSVWPGKKTIQLTHEQQLILNHKMEPLQVVKIM
QQRLLFCLRRPSSTVTMPDVTETLKEEPSVWPGKKTIQLTHEQQLILNHKMEPLQVVKIM
QVLLSSCVNDIQHLLFCLRRTMPDEEPSVWPGKKTSIQLTHEQQLILNHKMEPLQVVKIM
QVLLSSCVNDIQHLLFCLRRTMPDEEPSVWPGKKTSIQLTHEQQLILNHKMEPLQVVKIM
-GTGGEEVEALQLSARWLEVLRTY-----------------LELVLCVLVSIRNNRNLQK
-GTGGEEVEALQLSARWLEVLRTY-----------------LELVLCVLVSIRNNRNLQK
-GTGGEEVEALQLSARWLEVLRTY-----------------LELVLCVLVSIRNNRNLQK
-GTGGEEVEALQLSARWLEVLRTY-----------------LELVLCVLVSIRNNRNLQK
-GTGGEEVEALQLSARWLEVLRTY-----------------LELVLCVLVSIRNNRNLQK
-GTGGEEVEALQLSARWLEVLRTY-----------------LELVLCVLVSIRNNRNLQK
DGTGGEDGEALQLSSRWLEVLRIY-----------------LELVLCVLLSIRNNRNLQK
TGNGGEESEALQLSSRWLEVLRIY-----------------LELVLCVLLSIRNNRNLQK
-SEHVPCNGFSDIENLEGPEIFFENNLKAFLQQPDDYESYLEGAVYIDQYCNPLSDISLK
-SEHVPCNGFSDIENLEGPEIFFENNLKAFLQQPDDYESYLEGAVYIDQYCNPLSDISLK
-SEHVPCNGFSDIENLEGPEIFFENNLKAFLQQPDDYESYLEGAVYIDQYCNPLSDISLK
-SEHVPCNGFSDIENLEGPEIFFENNLKAFLQQPDDYESYLEGAVYIDQYCNPLSDISLK
-SEHVPCNGFSDIENLEGPEIFFENNLKAFLQQPDDYESYLEGAVYIDQYCNPLSDISLK
-SEHVPCNGFSDIENLEGPEIFFENNLKAFLQQPDDYESYLEGAVYIDQYCNPLSDISFR
-SEHVPCNGFSDIENLEGPEIFFENNLKAFLQQPDDYESYLEGAVYIDQYCNPLSDISLR
```

-GYRRFLPTKDHVFILDYVGTLFFNALVSSTLGQIQWKRACRYVVLCRGAKDFASDPRCD
-GYRRFLPTKDHVFILDYVGTLFFNALVSSTLGQIQWKRACRYVVLCRGAKDFASDPRCD
-GYRRFLPTKDHVFILDYVGTLFFNALVSSTLGQMQWKRACRYVVLCCGAKDFASDPRCD
-GYRRFLPTKDHVFILDYVGTLFF------------------------------------
-GYRRFLPTKDHVFILDYVGTLFFKNALVSTLGQMQWKRACRYVVLCRGAKDFASDPRCD
-GYRRFLPTKDHVFILDYVGTLFFKNALVSTLGQMQWKRACRYVVLCRGAKDFASDPRCD
-CLSKSVAPLLAHGYRRFLPTKDH-------------------VFILDYVGTLFF-----
-GYRRFLPTKDHVFILDYVGTLFFKNALVSTLGQMQWKRACRYVVLCRGAKDFASDPRCD
-ERHGYCTLGEAFNRLDFSTAILD----------SRRFNYVVRLLELIAKSQLTSLSGIA
-ERHGYCTLGEAFNRLDFSTAILD----------SRRFNYVVRLLELIAKSQLTSLSGIA
-ERHGYCTLGEAFNRLDFSTAILD----------SRRFNYVVRLLELIAKSQLTSLSGIA
-ERHGYCTLGEAFNRLDFSTAILD----------SRRFNYVVRLLELIAKSQLTSLSGIA
-ERHGYCTLGEAFNRLDFSTAILD----------SRRFNYVVRLLELIAKSQLTSLSGIA
-ERHGYCTLGEAFNRLDFSTAILD----------SRRFNYVVRLLELIAKSQLTSLSGIA
-ERHGYCTLGEAFNRLDFSTAILD----------SRRFNYVVRLLELIAKSQLTSLSGIA
-ERHGYCTLGEAFNRLDFSSAIQD----------IRRFNYVVKLLQLIAKSQLTSLSGVA
-ERHGYCTLGEAFNRLDFSSAIQD----------TRRFNYVVKLLQLIAKSQLTSLSGVA
-ERHGYCTLGEAFNRLDFSSAIQD----------IRRFNYVVKLLQLIAKSQLTSLSGVA
-ERHGYCTLGEAFNRLDFSSAIQD----------IRRFNYVVKLLQLIAKSQLTSLSGVA
-ERHGYCTLGEAFNRLDFSSAIQD----------IRRFNYVVKLLQLIAKSQLTSLSGVA
-ERHGYCTLGEAFNRLDFSSAIQD----------IRRFNYVVKLLQLIAKSQLTSLSGVA
-ERHGYCTLGEAFNRLDFSSAIQD----------IRRFTYVVKLLQLIAKSQLTSLSGVA
-QKYYPTKEQYGDTLHFCRH--
EVKGGRERHLKKVGGDACLLRGADAVPSPSPQREKLERFKRMCQLLERVPDTSSSSSDSD
EERHLKKVGGDACLLRGADPLPPRRVPDTSSSSSDSDSDSDSSGTSLSEDEAPGEARNGR
EVKGGRERHLKKVGGDACLLRGSDVVPSPSPQREKLERFKRMCQLLERVPDTSSSSSDSD
PRRKGPLPAGPPPEDVPGPPKRKEAGNEPPTPRKKVKGGRERHLKKVGGDACLLRGSDPG
-RVLNPSQAFSSCHPGLPPENWEKSDSDSDSDSSGTSLSEDEAPGEARNGRRPTRGSSGE
EVKGGRERHLKKVGGDACLLRGSDAVPSPSPQREKLERFKRMCQLLERVPDTSSSSSDSD
-RRPARGSSGEKENRGGRRAVRPG-----------------SGGPLLSWPLGPAP-----
KRFKRMCQLLERVPDTSSSSSDSD----SDSDSSGTSLSEDEAPGEARNGRRPARGSSGE
LIFLLFISSHLDTKIDGCVLVLPSSRDERFKRRQLLRLQATERTMVREKENNPSGKKELS
MIHDPVSPRGMVTRSSPGAGPSDHSRDERFKRRQLLRLQATERTMVREKENNPSGKKELS
MIHDPVSPRGVVTRSSPGAGPSDHSRDERFKRRQLLRLQATERTMVREKENNPSGKKELS
MIHDPVSPRGMVTRSSPGAGPSDHSRDERFKRRQLLRLQATERTMVREKENNPSGKKELS
MIHDPVSPRGMVTRSSPGAGPSDHSRDERFKRRQLLRLQATERTMVREKENNPSGKKELS
MIHDPVSPRGMVTRSSPGAGPSDHSRDERFKRRQLLRLQATERTMVREKENNPSGKKELS
MIHDPVSPRGMVTRSSPGAGPSDHSRDERFKRRQLLRLQATERTMVREKENNPSGKKELS
MIHDPVSPRGMVTRSSPGAGPSDHSRDERFKRRQLLRLQATERTMVREKENNPSGKKELS
LSHLSPRPPLGSSLSPWWRSSLTYDLPEAPPKTRESDHSRSSSPTAGPSTEGTEGPEEKK
LSHLSPRPPLGSSLSPWWRSSLTYLPEAAPPKTRESDHSRSSSPTAGPSTEGAEGPEEKK
LSHLSPRPPLGSSLSPWWRSSLTYDLPEAPPKTRESDHSRSSSPTAGPSTEGAEGPEEKK
LSHLSPRPPLGSSLSPWWRSSLTYELPEAPPKTRESDHSRSSSPTAGPSTEGAEGPEEKK
LSHLSPRPPLGSSLSPWWRSSLTYELPEAPPKTRESDHSRSSSPTAGPSTEGAEGPEEKK
LSHLSPRPPLGSSLSPWWRSSLTYELPEAPPKTRESDHSRSSSPTAGPSTEGAEGPEEKK

```
LSHLSPRPPLGSSLSPWWRSSLTYDLPEAPPKTRESDQSRSSSPTAGPSTEGAEGPEEKK
LSHLSPRPPLGSSLSPWWRSSLTYDLPEAPPKTRESDQSRSSSPTAGPSTEGAEGPEEKR
AGSERRSGGGDCSRVAEAVAHFEAQRDSPPTKGLRKEERPGPGPGEVRIAFRISNGREPR
-SGGGDCSRVAEAVAHFEAQRDSP-----PTKGLRKEERPGPGPGEVRIAFRISNGREPR
AGSERRSGGGDCSRVAEAVAHFEAQRDSPPTKGLRKEERPGPGPGEVRIAFRISNGREPR
AGSERRSGGGDCSRVAEAVAHFEAQRDSPPTKGLRKEERPGPGPGEVRIAFRISNGREPR
AGSERRSGGGDCSRVAEAVAHFEAQRDSPPTKGLRKEERPGPGPGEVRIAFRISNGREPR
AGSERRSGGGDCSRVAEAVAHFEAQRDSPPTKGLRKEERPGPGPGEVRIAFRISNGREPR
AGSERRSGGGDCSRVAEAVAHFEAQRDSPPTKGLRKEERPGPGPGEVRIAFRISNVREPH
AGSERRSGGGDCSRVAEAVAHFEAQRDSPPTKGLRKEERPGPGPGEVRIAFRISNVREPQ
SSAPGACEEPTERGNPQAVEPQSEMVAKLESECLKRQSHREPGSLSRNNSFRRNVGRVLL
SQSRGVPAATESCSAPGACEERGN-QREPGSLSRNNSFRRNVGRVLLANSTQADEGKTKK
-KLESECLKRQGQREPGSLSRNNS-------------FRRNVGRVLLANSTQADEGKTKK
LSPAIVRFSGQSRGVPAVSESYSA-QREPGSLSRNNSFRRNVGRVLLANSTQADEGKTKK
LSPAIVRFSGQSRGVPAVSESYSA-QREPGSLSRNNSFRRNVGRVLLANSTQADEGKTKK
MFLEQRASALLASCSKNCTNRFSGLEVGEPQSEPVRVLDMVAKLESECLKRQGQREPGSL
SFLEQKATALLASCTKNCTNKISGGQREPGSLSRNNSFRRNVGRVLLTNGSQASDKSEEG
SQSRGMPPVPEPFSAPDTCEESKEGQREPGSLSRNNSFRRNVGRVLLTNGSQASDKNGEG
-VAVVFGTKILDYVFNLCEG----------------------------------------
-VAVVFGAKILDYVFNLCEGKFDY-------------------------LERLSDKLLLK
-TAIIFGVRILDYVINLCKGQVDF-------------------------LERLSDDLLLN
----------------STCDTITP------------------------------------
----------------STCDTITP------------------------------------
-TALIFGARILDYVINLCKGKFDF-------------------------LERLSDDLLLN
-TALIFGARILDYVINLCKGKFDF-------------------------LERLSDDLLLT
-TALIFGARILDYVINLCKGKFDF-------------------------LERLSDDLLLN
TADRALGLRGLCLECRGEKPLFDS------------GHDVLEAVHAVCGAARELRHLDLR
VAGRALGLRGLRLECRGEKPLFDA------------GRDVLEAVHAVCGAARELRHLDLR
VAGRAPGLRGLRLECRGEKPLFDA------------GRDVLEAVHAVCGAASQLRHLDLR
VAGRALGLRGLRLECRGEKPLFDA------------GRDILEAVHAVCGAASQLRHLDLR
-MVLAGRAPGLRGLRLECRGEKPL---------FYAGRDVLEAVHAVCGAASQLRHLDLR
VAGRAPGLRGLRLECRGEKPLFDA------------GRDVLEAVHAVCGAASQLRHLDLR
AASRAPRLRGLRLECRGEKPLFDA------------GQDILGAVHAVCGAAHQLRHLDLR
AASRAPRLRGLRLECRGEKPLFDA------------GRDILGALHTVCGAAHQLRHLDLR
-KQISSVKAALTNSLSPVKRRTSL-PSKTKESLRISGLGWTIILREASGKEHIMQHSNLS
-YITRQISSLKSALAHIIMPVNPY-------TGLPVKTKEALRVFGLGWAIILREKNGKE
ADILKPVNPYTGLPVKTKEALRIF----------------GLGWAIILKEKSGKEHIME
ADILKPVNPYTGLPVKTKEALRMF----------------GLGWAIILKEKSGKEYIME
ADILKPVNPYTGLPVKTKEALRIF----------------GLGWAIILKEKNGKEYIME
ADILKPVNPYTGLPVKTKEALRIF----------------GLGWAIILKEKGGKEYIME
-KQIASVKAALADILKPVNPYTGL----------PVKTKEALRIFGLGWAIILKEKSGKE
-KQIASVKAALADILKPVNPYTGL----------PVKTKEALRIFGLGWAIILKEKSGKE
-LVSKFCPRLTFLKLSDCHTVTAE---------------TLVMLARACCQLHSLDLHHSM
-LVSKFCPRLTFLKLSDCHGVTAE---------------TLVMLAKACCQLHSLDLHHSM
-LVGESCPRLTFLKLSGCHGVTAD---------------ALVMVAKACCQLHSLDLQHSM
```

```
-LVGESCPRLTFLKLSGCHGVTAD---------------ALVMLAKACCQLHSLDLQHSM
-LMPNRFSQLQRLTLIHWKSQVHP-------------------VLKVESTAVVSFLEEAG
-LVGECCPRLTFLKLSGCHGVTAD---------------ALVMLAKACCQLHSLDLQHSM
-LVGECCPRLTFLKLSGCHGVTAD---------------ALVMLAKACCQLHSLDLQHSM
-LVGECCPRLTFLKLSGCHGVTAD---------------ALVMLAKACCQLHSLDLQHSM
DKILSECSKLQNLSLEGLQL-----------------SDPIVKTLAQNENLVRLNLCGCS
DGILSECSKLQNLSLEGLQL-----------------SDPIVTTLAQNENLVRLNLCGCS
DGILSQCSKLQNLSLEGLRL-----------------SDPIVNTLAKNSNLVRLNLSGCS
DGILSQCSKLQNLSLEGLRL-----------------SDPIVNTLAKNSNLVRLNLSGCS
DGILSQCSKLQNLSLEGLRL-----------------SDPIVNNLAQNSNLVRLNLCGCS
DGILSQCSKLQNLSLEGLRL-----------------SDPIVNTLAKNSNLVRLNLSGCS
DGILSQCSKLQNLSLEGLRL-----------------SDPIVNNLAQNSNLVRLNLSGCS
-FCLVGVSDLDICEFIDNYSLSKK------------------GVKAMSLKRSTITDAGLE
-FCLVGVSDLDICEFIDNYSLSKK------------------GVKAMSLKRSTITDAGLE
-FCLVGVSDLDICEFIDNYALSKK------------------GVKAMSLKRSTITDAGLE
-FCLVGVSDLDICEFIDNYALSKK------------------GVKAMSLKRSTITDAGLE
-FCLVGVSDLDICEFIDNYALSKK------------------GVKAMSLKRSTITDAGLE
-FCLVGVSDLDICEFIDNYALSKK------------------GVKAMSLKRSTITDAGLE
-FCLVGVSDLDICEFIDNYALSKK------------------GVKAMSLKRSTITDAGLE
-FCLVGVSDLDICEFIDNYALSKK------------------GVKAMSLKRSTITDAGLE
-AVLTKKFQVTMRGLLSCLGKSNN--------RLRSLSIQHLELDRLVWRNSIRGSLIKS
-AVLTKKFQVTMRGLLSCLGKSNN--------RLRSLSIQHLELDRLVWRNSIRGSLIKS
-PYNAVLTKMFQVTMQGLLSRLGK-----SNNRLKSLSIQHLELDRLVWRNSIRRSFVRS
-AVLTKKFQVTMRGLLSCLSKSNN--------RLKSLSIQHLELDRLVWRNSIRSSFIRS
-AVLTKKFQVTMRGLLSCLSKSNN--------RLKSLSIQHLELDRLVWRNSIRSSFIRS
-AVLTKKFQVTMRGLLSCLSKSNN--------RLKALSIQYLELDRLVWRNSIRSSLISS
-AVLTKKFQVTMRGLLSCLSKSNN--------RLKSLSIQYLELDRLVWRNSIRSSFISS
-AVLTKKFQVTMRGLLSCLSKSNN--------RLKSLSIQYLELDRLVWRNSIRSSFISS
SPDAGCCQAPEQPPQPLCPPPSSPEGAPTEAGGDAVRTGGTAPSSTQQQQHECGDADCR
-VLAFKCPGLLRYTAYRCKQLSDT------------------SIIAVASHCPLLQKVHVG
-SSRQQVTDELLEKIASRSQ----------------------------------------
SPDAGCCQAPEQPPPPLCPAPASPAPIVAAAGDTVRAGGTAPSSAQQQPESGDADCQEPP
GPDAGCCQGPEQPQQPLCPPPSSPGAPTEAGGDVVRVGGTAPSSAQQQRECGDADCLEPP
-PDAGCCQAPEQPPQPLCPPPSSPGAPTEAGGDAVRAGGTAPSSAQQQHECGDADCREPP
-PDAGCCQAPEQPPQPLCPPPSSPGAPTEAGGDAVRAGGTAPLSAQQQHECGDADCRESP
-RTEYFCLADGHFASIDAVLLLQG-----------------GALCLSGSRDRNVNLWDLR
-RTEYFCLADGHFASIDAVLLLQG------------------GTLCLSGSRDR-------
-LANGHISSIDSVLLLQGGALCLSRDRNVNLWDLRQLGMEPSQVLVKTLGTDRFSTHEGW
-VDSVLLLQGGSLCLSGSRDRTVN------LWDLRQLGTEPSQVLVKTLGTKRNSTHEGW
-WVEYFCLADGHVASVDSVLLLQG-----------------GSLCLSGSRDRNVNLWDLR
-LAEGHVASVDSVLLLQGGSLCLSRDRNVNLWDLRQLGTESNQVLIKTLGTKRNSTHEGW
-LADGHVASVDSVLLLQGGSLCLSRDRNVNLWDLRQLGTESSQVLVKTLGTKRNSTHEGW
-WVEYFCLAEGHVASVDSVLLLQG------------------GSLCLSGSRDR-------
-TVLYMADSETFISLEECRGHKRA-------RKRTTMETACALEKLFPKQCQVLGIVTPG
-TVLYMADSETFISLEECRGHKRA-------RKRTTMETACALEKLFPKQCQVLGIVTPG
```

```
-TVLYMADSETFISLEECRGHKRA-------RKRTSMETALALEKLFPKRCQVLGIVTPG
-TVLYMADSETFISLEECRGHKRA-------RKRTSMETALALEKLFPKQCQVLGIVTPG
-TVLYMADSETFISLEECRGHKRA-------RKRTSMETALALEKLFPKQCQVLGIVTPG
-TVLYMADSETFISLEECRGHKRA-------RKRTSMETALALEKLFPKQCQVLGIVTPG
-TVLYMADSETFISLEECRGHKRA-------RKRTSMETALALEKLFPKQCQVLGIVTPG
-TVLYMADSETFISLEECRGHKRA-------RKRTSMETALALEKLFPKQCQVLGIVTPG
YIISQCCPELRRLEVSGCYNISNE---------------AVFDVVSLCPNLEHLDVSGCS
YTIAQCCPELRRLEVSGCYNISNE---------------AVFDVVSLCPNLEHLDVSGCS
YTIAQCCPELRRLEVSGCYNISNE---------------AVFDVVSLCPNLEHLDVSGCS
YTIAQCCPELRRLEVSGCYNISNE---------------AVFDVVSLCPNLEHLDVSGCS
YTIAQCCPELRRLEVSGCYNISNE---------------AVFDVVSLCPNLEHLDVSGCS
YTIAQCCPELRRLEVSGCYNISNE---------------AVFDVVSLCPNLEHLDVSGCS
YTIAQCCPELRRLEVSGCYNISNE---------------AVFDVVSLCPNLEHLDVSGCS
YTIAQCCPELRRLEVSGCYNISNE---------------AVFDVVSLCPNLEHLDVSGCS
-ILKSPCTKGEANLWFTRKHFMDS-----------------LKNMHFGKEIIVWNFASKN
-TFAQNCRNIEHLNLNGCTKITDS---------------TCYSLSRFCSKLKHLDLTSCV
-TFAQNCRNIEHLNLNGCTKITDS---------------TCYSLSRFCSKLKHLDLTSCV
-TFAQNCRNIEHLNLNGCTKITDS---------------TCYSLSRFCSKLKHLDLTSCV
-TFAQNCRNIEHLNLNGCTKITDS---------------TCYSLSRFCSKLKHLDLTSCV
-TFAQNCRNIEHLNLNGCTKITDS---------------TCYSLSRFCSKLKHLDLTSCV
-TFAQNCRNIEHLNLNGCTKITDS---------------TCYSLSRFCSKLKHLDLTSCV
-TFAQNCRNIEHLNLNGCTKITDS---------------TCYSLSRFCSKLKHLDLTSCV
-TFAQNCRNIEVLNLNGCTKTTDA------PPTIFSKFYIDISFIVIPKNIKYIRMNVIK
-TFAQNCRNIEVLSLNGCTKTTDA---------------TCTSLSKFCSKLRHLDLASCT
-TFAQNCRNIEVLSLNGCTKTTDA---------------TCTSLSKFCSKLRHLDLASCT
-TFAQNCRNIEVLNLNGCTKTTDA---------------TCTSLSKFCSKLRHLDLASCT
-TFAQNCRNIEVLNLNGCTKTTDA---------------TCTSLSKFCSKLRHLDLASCT
-TFAQNCRNIEVLNLNGCTKTTDA---------------TCTSLSKFCSKLRHLDLASCT
-TFAQNCRNIEVLNLNGCTKTTDA---------------TCTSLSKFCSKLRHLDLASCT
-TFAQNCRNIEVLNLNGCTKTTDA---------------TCTSLSKFCSKLRHLDLASCT
-RLLRDAEGLQELALAPCHEWLSD--------------EDLVPVLARNPQLRSVALAGCG
-RLLRDAEGLQELALAPCHEWLLD--------------EDLVPVLARNPQLRSVALAGCG
-RLLRDAEGLQELALAPCHEWLSD--------------EDLVPVLARNPQLRSVALGGCG
-RLLRDAEGLQELALAPCHEWLSD--------------EDLVPVLARNPQLRSVALAGCG
-RLLRDAEGLQELALAPCHEWLSD--------------EDLVPVLARNPQLRSVALGGCG
-RLLRDAEGLQELALAPCHEWLSD--------------EDLVPVLARNPQLRSVALGGCG
-RLLRDAEGLQELALAPCHEWLSD--------------EDLVPVLARNPQLRSVALGGCG
-RLLRDAEGLQELALAPCHEWLSD--------------EDLVPVLARNPQLRSVALGGCG
-AFVQEIGSLRALNLSPCKQITDS------------------------------------
-YVIQGMANIESLNLSGCYNLTDN-----GLGHAFVQEIGSLRALNLSLCKQITDSSLGR
-YVIQGMANIESLNLSGCYNLTDN-----GLGHAFVQEIGSLRALNLSLCKQITDSSLGR
-YVIQGMANIESLNLSGCYNLTDN-----GLGHAFVQEIGSLRALNLSLCKQITDSSLGR
-YVIQGMANIESLNLSGCYNLTDN-----GLGHAFVQEIGSLRALNLSLCKQITDSSLGR
-YVIQGMANIESLNLSGCYNLTDN-----GLGHAFVQEIGSLRALNLSLCKQITDSSLGR
-YVIQGMANIESLNLSGCYNLTDN-----GLGHAFVQEIGSLRALNLSLCKQITDSSLGR
```

-RSASHCRNLQELNVSDCPTFTDE--------SMRHISEGCPGVLYLNLSNTTITNRTMR
-RDLGHCRNLQELNVSGCPTFTDE--------SMRHISEGCPGVLCLNLSNTTITNRMMR
-RSVSHCRNLQELNVSDCPTFTDE--------SMRHISEGCPGVLYLNLSNTTITNRTMR
-RSVSHCRNLQELNVSDCPTFTDE--------SMRHISEGCPGVLYLNLSNTTITNRTMR
-RSVSHCRNLQELNVSDCPTFTDE--------SMRHISEGCPGVLCLNLSNTTITNRTMR
-RSVSHCRNLQELNVSDCPTFTDE--------SMRHISEGCPGVLCLNLSNTTITNRTMR
-KAVSHCKNLQELNVSDCQSFTDE--------SMRHISEGCPGVLYLNLSNTTITNRTMR
RRHLFYTQKQRIILAKWKEKARHK--SKKREDDLITKHELQLKKWKFKLSLEKPINIEGS
-FSTNDCSRNVYIKKNGFTLHRNP----------------------------------IA
-FSTNDCSRNVYIKKNGFTLHRNP----------------------------------IA
-FSTNDCSRNVYIKKNGFTLHRNP----------------------------------IA
-FSTNDCSRNVYIKKNGFTLHRNP----------------------------------IA
-RMGGYLFSGSQAPQLSPALMRAL------------------------------------
-RMGGYLFSGSQAPQLSPALMRAL------------------------------------
-RMGGYLFSGSQAPQLSPALLRAL------------------------------------
-RMGGYLFSGSQAPQLSPALLRAL------------------------------------
-RMGGYLFSGSQAPQLSPALLRAL------------------------------------
-RMGGYLFSGSQAPQLSPALLRAL------------------------------------
-RMGGYLFSGSQAPQLSPALLRAL------------------------------------
-RMGGYLFSGSQAPQLSPALLRAL------------------------------------
-LLKWRCSQMPWMQLEDDSLYISQ---------ANFILAYQFRPDGASLNRRPLGVFAGH
-LLKWRCSQMPWMQLEDDSLYISQ---------ANFILAYQFRPDGASLNRRPLGVFAGH
-LLKWRCSQMPWMQLEDDSLYISQ---------ANFILAYQFRPDGASLNRRPLGVFAGH
-LLKWRCRQMPWMQLEDDSLYISQ---------ANFILAYQFRPDGASLNRRPLGVFAGH
-LLKWRCSQMPWMQLEDDSLYISQ---------ANFILAYQFRPDGASLNRRPLGVFAGH
-LLKWRYSQMPWMQLQDASLYLSQ---------ANFILAYQFRPDGASLNRRPFRVFSGH
-LLKWRYSQMPWMQLEDSSLYLSQ---------ANFILAYQFRPDGASLNRRPFRVFAGH
CVHGPGTCQVKFCTFKNTHVFLHNSPIFLPAEDHDFLMSLDLESRDQAWSPRTCDIVIEG
CVHGPGTCQVKFCTFKNTHVFLHNSPIFLPAEDNDFLMSLDLESQDQAWSPRTCDIVIEG
CVRGPGTCQVKFCTFRNTHVFLHN------------------VPLCVLENCEFVGSENNS
CQVHGPGTCQVKFCTFKNTHIFLHSPTFLPTEDSDFLMSLDLESRDQAWSPKTCDIVIEG
CQVHGPGTCQVKFCTFKNTHIFLHSPTFLPTEDSDFLMSLDLESRDQAWSPKTCDIVIEG
CQVHGPGTCQVKFCTFKNTHIFLHSPTFLPTEDSDFLMSLDLESRDQAWSPKTCDIVIEG
CVHGPGTCQVKFCTFKNTHIFLHNSPTFLPTEDSDFLMSLDLESRDQAWSPKTCDIVIEG
CVHGPGTCQVKFCTFKNTHIFLHNSPTFLPTEDSDFLMSLDLESRDQAWSPKTCDIVIEG
GIIGWMYLPPHDPHVGDPMRFKPLVECMYGHKGPHNGHIQIVKRDEFSTKCNQTDHHRMS
-EEYGVCENLRKLEITGVSC--
GIIGWMYLPPHDPHVNDPMRFKPLVECMYGHKGPHHGHIQIVKKDEFSTKCNQTDHHRMS
GIIGWMYLPPHDPHVDDPMRFKPLVECMYGHKGPHHGHIQIVKKDEFSTKCNQTDHHRMS
GIIGWMYLPPHDPHVDDPMRFKPLVECMYGHKGPHHGHIQIVKKDEFSTKCNQTDHHRMS
GIIGWMYLPPHDPHVDDPMRFKPLVECMYGHKGPHHGHIQIVKKDEFSTKCNQTDHHRMS
GIIGWMYLPPHDPHVDDPMRFKPLVECMYGHKGPHHGHIQIVKKDEFSTKCNQTDHHRMS
GIIGWMYLPPHDPHVDDPMRFKPLVECMYGHKGPHHGHIQIVKKDEFSTKCNQTDHHRMS
-DDFPLCALARFCLLAPLGR---------------------------NLIYNSCGEQGFR
-ADGNRHDEFPFCALARFCL---------------------RAPFGRNLIHNSCGEQGFR

```
-PSNEDEEEFPLCALARYCL---------------------RAPFGRNLIFNSCGEQGFK
-RDRSAEGRALYAVAQRCLP----------------------------------------
-EEFPLCALARYCL----RA-----------------------PFGRNLIFNSCGEQGFR
-EEFPLCALARYCL----RA-----------------------PFGRNLIFNSCGEQGFR
-EEFPLCALARYCL----RA-----------------------PFGRNLIFNSCGEQGFR
-RNARPCPLGRFCARRPIGR---------------------------NLIRNPCGQEGLR
-RNARPCPLGRFCARRPIGR---------------------------NLIRNPCGQEGLR
-RNARPCPLGRFCARRPIGR---------------------------NLIRNPCGQEGLR
-RNARPCPLGRFCARRPIGR---------------------------NLIRNPCGQEGLR
-RNARPCPLGRFCARRPIGR---------------------------NLIRNPCGQEGLR
-RDHGATGRALLHLARSCQSPARN------------------------------------
-HEDTPCPLGQFCALRPLGR---------------------------NLISNPCGQEGLR
-REGTPCPLGQFCALRPLGR----------------------------------------
-LLRNPCGEEDLEGWCDVEH----------------------------------------
-LLRNPCGEEDLEGWCDVEH----------------------------------------
-LLRNPCGEEDLEGWCDVEH----------------------------------------
-LLRNPCGEEDLEGWCDVEH----------------------------------------
-LLRNPCGEEDLEGWCDVEH----------------------------------------
-LLRNPCGEEDLEGWSDVEH----------------------------------------
-LLRNPCGEEDLEGWCDVEH----------------------------------------
-LLHNPCAEEGFEFWSLDVN----------------------------------------
-LLHNPCAEEGFQFWSLDMN----------------------------------------
-LLHNPCAEEGFEFWSLDVN----------------------------------------
-LLHNPCAEEGFEFWSLDVN----------------------------------------
-LLHNPCAEEGFEFWSLDVN----------------------------------------
-LLHNPCAEEGFEFWSLDVN----------------------------------------
-LLHNPCAEEGFEFWSLDVN----------------------------------------
-LLRNPCAEEGMLAWQIDTE----------------------------------------
-LLRNPCAEEDMFAWQIDFN----------------------------------------
-LLRNPCAEEDMFAWQIDFN----------------------------------------
-LLRNPCAEEDMFAWQIDFN----------------------------------------
-LLRNPCAEEDMFAWQIDFN----------------------------------------
-LLRNPCAEEDMFAWQIDFN----------------------------------------
-LLRNPCAEENLSSWRIDSN----------------------------------------
-LLRNPCAEENLRSWRIDSN----------------------------------------

LLEENFSDS-PQSCLQYPNKNLLP------VLHFEKVVCSTLKRNPKLDREMLKEIIAKN
LLEENFSDS-PQSCLQYPNKNLLP------VLHFEKVVCSTLKRNPKVDREMLKEIIARN
LLEENFSDG-LQSCLQYPNKNLLP------VLHFEKVVCSTLKRNPKVDREMLKEIIARN
LLEENFGDSLQSCLLQIQSPDQYP----LPVLHFEKVVCSTLKRNPKVDREMLKEIIARN
LLEENFGDSLQSCLLQIQSPDQYP----LPVLHFEKVVCSTLKRNPKVDREMLKEIIARN
LLEENFGDSLQSCLLQIQSPDQYP----LPVLHFEKVVCSTLKRNPKVDREMLKEIIARN
NFRNSPQARLLPSQSPDQHPNKTL----LPVLHFERVVCSTLKRNPKVDREMLKEVIASN
VDQEMLKEVIASGNITLQNIIGKK----DILAELSRRGCMHLLANIFMKLSGMDLINLSV
SGEIAVISLDVFGCWLTETSLISG----NLHRIGDITSCSVLWNVNVVKRLFKIQNLNAT
```

```
SGEIAVISLDVFGCWLTETSLISG----NLHRIGDVTSCSVLWNNAFQDVESENVNVVKL
SGEIAVISLDVFGCWLTETSLISG----NLHRIGDITSCSVLWNNAFQDVESENVNVVKL
SGEIAVISLDVFGCWLTETSLISG----NLHRIGDITSCSVLWNNAFQDVESENVNVVKL
SGEIAVISLDVFGCWLTETSLISG----NLHRIGDITSCSVLWNNAFQDVESENVNVVKL
SGEIAVISLDVFGCWLTETSLISG----NLHRIGDITSCSVLWNNAFQDVESENVNVVKL
SIICSVSPKKELCAWDVQEGTMIW----SSPVQKFQISNLVTL---PQMHLVITEDKSKT
----------ELCAWDVQEGTMIWSSPVHEFYFLN--LVTLPQ-----MHLAITMDQKKT
SIICSVSPKQELCAWDVQEGTIIWSSPVQEFHLSNLVTLPQMH-------LAVTMDRKKT
SIICSVSPKQELCAWDVQEGTMIWSSPVQEFHFSNLVTLPQMH-------LAITMDRKKT
SIICSVSPKQELCAWDVQEDTMIWSSPVQEFHFSNLVTLPQMH-------LAITMDRKKT
SIICSVSPKQELCAWDVQEGTMIWSSPVFHFSN----LVTLPQ-----MHLAITMDRKKT
VPGLLGSMALSEDLLDVDTAAGGF----GLKYCLPLTFCIHTG-LSQYIAVEAAEGRNKN
VPGLLGSMALSEDLLDVDTAAGGF----GLKYCLPLTFCIHTG-LSQYIAVEAAEGRNKN
VPGLLGSMALSEDLLDVDTAAGGF----GLKYCLPLTFCIHTG-LSQYIAVEAAEGRNKN
VPGLLGSMALSEDLLDVDTAAGGF----GLKYCLPLTFCIHTG-LSQYIAVEAAEGRNKN
VPGLLGSMALSEDLLDVDTAAGGF----GLKYCLPLTFCIHTG-LSQYIAVEAAEGRNKN
VPGLLGSMALSEDLLDVDTAAGGF----GLKYCLPLTFCIHTG-LSQYIAVEAAEGRNKN
VPGLLGSMALSEDLLDVDTAAGGF----GLKYCLPLTFCIHTG-LSQYIAVEAAEGRNKN
VPGLLGSMALSEDLLDVDTAAGGF----GLKYCLPLTFCIHTG-LSQYIAVEAAEGRNKN
SVVCTVSSDCTVRAWDLQEGSEIW----QPAPLVNVVAYPQLQ---LVVTVDIQGLIMGK
SVVCTVSSDCTVRAWDLQEGTEMWSSPVQPAPLVNLVAYPQLQ-------LVVTVDTQGL
LEGKACSESVKGLSDTMRYILIDWTMKDRYKLQLLGIACMVIC-EDLVRVMGEIISALEK
LEGKACSESVKGLSDTMRYILIDWTMKDRYKLQLLGIACMVIC-EDLVRVMGEIISALEK
LEVRASSEIVKGLNDTMRYILIDWTMKDRYTLQLLGIACMVIC----LVRMMGEIVSALE
LEVRASNEIVKGLNDTMRYILIDWTMKDRYRLQLLGIACMVIC---LVRMMGEIVSALEK
LEVRASNEIVKGLNDTMRYILIDWTMKDRYRLQLLGIACMVIC---LVRMMGEIVSALEK
LEVRASSEIVKGLNDTMRYILIDWTMKDRYRLQLLGIACMVIC---LVRMMGEIVSALEK
LEVRASSEIVKGLNDTMRYILIDWTMKDRYRLQLLGIACMVIC---LVRMMGEIVSALEK
LEVRASSEIVKGLNDTMRYILIDWTMKDRYRLQLLGIACMVIC-EDLVRMMGEIVSALEK
VNDFLLQV--CNRCPNLTSVTLSG--------------CGHVT-DDCLARLLLSCPRLRT
V---------CNRCPNLASVTLSG--------------CGHVT-DDCLARLLLGCPRLRA
---------------RHESLVNDF----------LLRVCDSLC-AVRPQRRREAPPSSGL
------------ICSRHESLVNDF----------LLQVCDRLSAVRSPRRREAPAPFSGP
------------ICSRHESLVNDF----------LLRVCDRLSPARSPRRREAPAPSSGP
------------ICSRHESLVNDF----------LLRVCDRLSAVRSPRRREAPAPSSGP
------------ICSRHESLVNDF----------LLRVCDRLSAVRSPRRREAPAPSSGP
----------LSICWHSSRVQ----------------VCSIED-----------------
FQELFQKHK-LKRSRRLSTLREQG----SQSETEEEKQIVHPGEKRAAAASAISEGQLNS
FQELLQKHK-LGRSRRLSTLREQSSQSETEEEK----QIVHPDEKRAAAASAISGGQLSS
FQELLQKHK-LGRSRRLSTLREQSSQSETEEEK----QIVHPDEKRAAAASAISEGQLSS
FQELLQKHK-LGRSRRLSTLREQSSQSETEEEK----QIVHPDEKRAAAASAISEGQLSS
FQELLQKHK-LGRSRRLSTLREQSSQSETEEEK----QIVHPDEKRAAAASAISEGQLSS
FQELLQKHK-LGRSRRLSTLREQS----SQSETEEEKQIVHPDEKRAAAASAISEGQLSS
LGGCGGPNALFKDAWLLHMHSGPWWQPLNEEHGAPELWCHPAC-NSRPSPISATPPALVE
LGGCGGPNALFKDAWLLHMHSGPWWQPLNEEHGAPELWCHPAC-NSRPSPISATPPALVE
```

```
LGGCGGPNALFKDAWLLHMHSGPWWQPLNEEHGAPELWCHPAC-NSRPSPISATPPALVE
LGGCGGPNALFKDAWLLHMHSGPWWQPLNEEHGAPELWCHPAC-NSRPSPISATPPALVE
LGGCGGPNALFKDAWLLHMHSGPWWQPLNEEHGAPELWCHPAC-NSRPSPISATPPALVE
LGGCGGPNALFKDAWLLHMHSGPWWQPLNEEHGAPELWCHPAC-NSRPSPISATPPALVE
LGGCGGPNALFKDAWLLHMHPGPWWQPLNEDHGAPELWCHPAC-NSRPSPISATPPALVE
LGGCGGPNALFKDAWLLHMHPGPWWQPLNEDHGAPELWCHPAC-NSRPSPISATPPALVE
NLLDSMARNVLPKSWQHMKFNNPFCTLSRSLASLNLSGCVHCLADSLLRKAEDDIDSSIE
DIDSSILETLVESCCNLHHLNLSA----HSSDGLGRHLCQLLALSLPVCSVADSAPRPDA
DIDSSILETLVESCCNLHHLNLSAAHHHRHLCQLLARLCHLRSLSLPVCSVADSAPRPDA
DIDSSILETLVASCCNLRHLNLSA----HSSEGLGRHLCQLLARLRHLRSLSLPVCSVAD
DIDSSILETLVASCCNLRHLNLSA----HSSEGLGRHLCQLLARLRHLRSLSLPVCSVAD
DIDSSILETLVASCCNLRHLNLSAEGLGRHLRSLSLPVCSVADRAPAQPAMHAVPRGFGK
DIDSSILETLVASCCNLRHLNLSA----HSSEGLGRHLCQLLARLRHLRSLSLPVCSVAD
DIDSSILETLVASCCNLRHLNLSA----HSSEGLGRHLCQLLALSLPVCSVADSAPRADA
SFGQFVELLPNHHHPDDSDDDNDFRPDLGNGMPAHNREVLPVDEEDSLELQEVWAPKNGT
SFGQFVELLPNHHHPDDSDDDNDFGEAQSGLQRVVKPTPIAVHEEDSLELQEVWIPKNGA
SFGQFIELLPHHHPDDSDEENDFREQQFSGLQRVVKPTPITVHEEDSLELQEVWIPKNGT
SFGQFIELLPHHHPDDSDEENDFREQQFSGLQRVVKPTPITVHEEDSLELQEVWIPKNGT
SFGQFIELLPNHHHPDLQPGEQQF----DALNEMEDIVQEDGEEEDSLELQEVWIPKNGT
SFGQFIELLPHHHPDDSDEENDFREQQFSGLQRVVKPTPITVHEEDSLELQEVWIPKNGT
SFGQFIELLPHHHPDDSDEENDFREQQFSGLQRVVKPTPITVHEEDSLELQEVWIPKNGT
SFGQFIELLPHHHPDDSDEENDFREQQFSGLQRVVKPTSITVHEEDSLELQEVWIPKNGT
KEYKILDALKFLNSYSLGYLDMKV-------------ILKNK----YHGKRVSGTPLNY
QELR-----------DISSMAMEY---------FDEKIVPILK--RKLPGSDVSGRLMGS
QELR-----------DISSMAMEY---------FDEKIVPILK--RKLPGSDVSGRLMGS
QELR-----------DISSMAMEY---------FDEKIVPVLK--RKLPGSDVSGRLMGS
QELR-----------DISSMAMEY---------FDEKIVPILK--RKLPGSDVSGRLMGS
QELR-----------DISSMAMEY---------FDEKIVPILK--RKLPGSDVSGRLMGS
QELR-----------DISSMAMEY---------FDEKIVPILK--RKLPGSDVSGRLMGS
QELR-----------DISSMAMEY---------FDEKIVPILK--RKLPGSDVSGRLMGS
CYDSDSLELPLGTRAQAANGGSERSQPPGLRRQAIQNWQRRPR-LDAPRPGPAMAGPLSS
CYDSDSLELPLGSRAQATNGGSERSQPPGLRRQAIQNWQRRPR-SDVPRPGPAVAGPLNS
CYDSDSLELPLGSRAQATNGGSERSQAPGLRRQAIQNWQRRPR-SDVPRPGPAVAGPLNS
CYDSDSLELPLGTRAQAANGGSERSQPPGLRRQAIQNWQRRPR-LDAPRPGPAMAGPLSS
CYDSDSLELPLGTRAQATNGGSERSQPPGLRRQAIQNWQRRPR-LDAPRPGPAMAGPLSS
CYDSDSLELPLGTRAQAANGGSEWSQPPGLRRQAIQNWQRRPR-LDAPRPGPAMAGPLSS
CYDSDSLELPLGTRAQAANGGSERSQPPGLRRQAIQNWQRRPR-LDAPRPGPAMAGPLSS
CYDSDSLELPLGTRAQAANGGSERSQPPGLRRQAIQNWQRRPR-LDAPRPGPAMAGPLSS
------------GCPTLRVLDLSG-------------CNSLF----------------T
----------GGGSPEASFVALIL-------------GCPALC----------------V
---------------EASSVALIL-------------GCPALC----------------V
---------------EASSVALIL-------------GCPALC----------------V
SSDLLCDLNTKNICPQVIDQNHNL----SKQSNLTNGACVASDVAAQLREVIPSNALLNT
SSDLLCDLNTENICTQVIDQNQNLHGDSTNGDRVASDGTSKPSVAAQLREIIPSSALPNT
SSDLLCDLNAENICTQVIDQNQNL----SKQSNLTNGDCVASSVAAQLREIIPSSALPNT
```

SSDLLCDLNTENICTQVIDQNQNL----SKQSNLTNGDCVASSVAAQLREIIPSSALPNT
SSDLLCDLNTENICTQVIDQNQNL----SKQSNLTNGDCVASSVAAQLREIIPSSALPNT
SSDLLCDLNTENICTQVIDQNQNLHGD-SKQSNLTNGDCVASSVAAQLREIIPSSALPNT
SSDLLCDLNPEKMCIQVKGQDQNF----STESNITNGDCVEADVPEQLREISPFSALPDT
SSDLLCNLNTEKMCTQVKDQDQNFHGD-STESNITNGDCVEADPDEDDDEDLCWKKDLGS
KEQISSGNNTGLAPWQDGVLERLK----TAVDAKDYNMYLVHNMLIHFGQMPACTPKERD
KEQISSGNNMGLAPWQDGVLERLK----TAVDAKDYNMYLVHNMLIHFGQMPACTPKERD
KEQISSGNNMGLAPWQDGVLERLK----TAVDAKDYNMYLVHNMLIHFGQMPACTPKERD
KEQISSGHNMGLAPWQDGVLERLK----TAVDAKDYNMYLVHNMLIHFGQMPACTPKERD
KEQISSGHNMGLAPWQDGVLERLK----TAVDAKDYNMYLVHNMLIHFGQMPACTPKERD
KEQISSGHNMGLAPWQDGVLERLK----TAVDAKDYNMYLVHNMLIHFGQMPACTPKERD
AERRGPQESQGLAPWQDGVLERLK----TAVDAKDYNMYLVHNMLIHFGQMPACTPKERD
TERRGPQESPGLAPWQDGVLERLK----TAVDAKDYNMYLVHNMLIHFGQMPACTPKERD
ISVLPMEVLMYIFRWDLDLRSLEQ----LSLVCRGFYICARDPEIWRLACLKVWGRSCIK
ISVLPMEVLMYIFRWDLDLRSLEQ----LSLVCRGFYICARDPEIWRLACLKVWGRSCVK
ISVLPMEVLMYIFRWDLDLRSLEQ----LSLVCRGFYICARDPEIWRLACLKVWGRSCIK
ISVLPMEVLMYIFRWDLDLRSLEQ----LSLVCRGFYICARDPEIWRLACLKVWGRSCIK
ISVLPMEVLMYIFRWDLDLRSLEQ----LSLVCRGFYICARDPEIWRLACLKVWGRSCIK
---------------SLDGFYRAW-----HQVEYYRYIRFFP-DGHVMMLTTPEEPQSV
ISVLPMEVLMYIFRWDLDLRSLEQ----LSLVCRGFYICARDPEIWRLACLKVWGRSCMK
ISVLPMEVLMYIFRWDLDLRSLEQ----LSLVCRGFYICARDPEIWRLACLKVWGRSCMK
YMHPLCEGGSALTCVPLGNLIVVN----ATLKINSEIRSVKRL--QLLPESFICKEKLGE
YMHPLCEGSSTLTCVPLGNLIVVN----ATLKINNEIRSVKRL--QLLPESFICKEKLGE
YMHPLCEGSSTLTCVPLGNLIVVN----ATLKINNEIRSVKRL--QLLPESFICKEKLGE
YMHPLCEGSSTLTCVPLGNLIVVN----ATLKINNEIRSVKRL--QLLPESFICKEKLGE
YMHPLCEGSSTLTCVPLGNLIVVN----ATLKINNEIRSVKRL--QLLPESFICKEKLGE
YMHPLCEGSSTLTCVPLGNLIVVN----ATLKINNEIRSVKRL--QLLPESFICKEKLGE
YTHPLCEGGFVLTCVPLGNLIIIN----ATIKVNGGIKNVKSV--QLQPGSYVAAGVEPE
YTHPLCEGGSVLTCVPLGKLIMIN----ATIKVNGGIKNVKSV---QLKPGAYVRRAEPE
VYNFLCEL--TNLCRKMQMVVCSK----RKLELRIRLFCRNVL-LDHWTHQNDSAFWLTI
VYNFLCEL--TNLCRKMQMAVCSK----RKLELRIRLFCRNVL-LDHWTHQSDSAFWLTI
VYNFLCEL--TNLCRKMQMAVCSK----RKLELRIRLFCRNVL-LDHWTHRSDSAFWLTI
VYNFLCEL--TNLCRKIQMAVCSK----QKLELRIRLFCRNVL-LDHWTHRSDSAFWLTI
VYNFLCEL--TNLCRKMQMAVCSK----RKLELRIRLFCRNVL-LDHWTHRSDSAFWLTI
VYNFLCEL--TNLCRKMQMAVCSK----RKLELRIRLFCRNVL-LDHWTHRSDSAFWLTI
VYNFLCEL--TNLSRKMQTVVCNK----RKLELRVRLFCRNVL-LDHWTHRSDSAFWLTI
----------LTAGWDELECHRVYNFLCNLSRKMQTVVCNKPG--KDFQVESRVSFLLGK
DDAYEEKDGCGPNNGELIQLILNH--------LTLPDLCRLAQ-TCKLLSQHCCDPLQYH
DDAYAEKDGCGPNNGELIQLILNH--------LTLPDLCRLAQ-TCKLLSQHCCDPLQYH
DDAYAEKDGCFSSAVKLPYELIQL----ILNHLTLPDLCRLAQ-TCKLLSQHCCDPLQYH
DDAYEEKDGCGPNNGELIQLILNH--------LTLPDLCRLAQ-TCKLLSQHCCDPLQYH
DDAYEEKNGCFSSAVKLPYELIQL----ILNHLTLPDLCRLAQ-TCKLLSQHCCDPLQYH
DDAYEEKDGCFSSAVKLPYELIQL----ILNHLTLPDLCRLAQ-TCKLLSQHCCDPLQYH
DDDYEEKDGCGPHNGELIQLILNH--------LSLPDLCRLAQ-TCRLLHQHCCDPLQYH
DDDYEEKDGCLGDGPKLPYELIQL----ILNHLSLPDLCRLAQ-TCRLLHQHCCDPLQYH

```
NEPRFAMFGP-----GLEELNTSL----VLSLMSSEELCPTAG----LPQRQIDGIGSGN
NEPRFAMFGP-----GLEELNTSL----VLSLMSSEELCPTAG----LPQRQIDGIGSGN
NEPRFAMFGP-----GLEELNTSL----VLSLMSSEELCPTAG----LPQRQIDGIGSGN
NEPRFAMFGP-----GLEELNTSL----VLSLMSSEELCPTAG----LPQRQIDGIGSGN
NEPRFAMFGP-----GLEELNTSL----VLSLMSSEELCPTAG----LPQRQIDGIGSGN
NEPRFAMFGP-----GLEELNTSL----VLSLMSSEELCPTAG----LPQRQIDGIGSGN
NEPRFAMFGP-----GLEELNTSL----VLSLMSSEDLCPTAG----LPHRQIDGIGSGN
NEPRFAMFGP-----GLEELNTSL----VLSLMSSEDLCPTAG----LPHTQIEGIGSGS
----------LLRNYQKSKVKHEW------LSGRYSNICSSIS---LPEKIMYPMDADT-
----------LLRNYQKSKVKHEW------LSGRYSNICSPIS---LPEKTMYPMDADT-
----------LLRNYQKSKVKHEW------LSGRYSNICSPIS---LPEKIMYPMDADT-
----------LLRNYQKSKVKHEW------LSGRYSNICSPIS---LPEKIMYPMDADT-
----------LLRNYQKSKVKHEW------LSGRYSNICSPIS---LPEKIMYPMDADT-
----------LLRNYQKSKVKHEW------LSGRYSNICSPIS---LPEKIMYPMDADT-
----------LLRNYQKSKVKHEW------LSGRYSNICSPIS---LPEKIMYPMDADT-
----------LLRNYQKSKVKYEW-------------LSGRYS-----------------
----------LLRNYQKSKVKNEW-----------------------------LSGRYSN
SHFISALTVVFVNSKSLSSLKIDD----TPVDD----PSLKVL-------VANNSDTLKL
SHFISALTVVFVNSKSLSSLKIDD----TPVDD----PSLKVL-------VANNSDTLKL
SHFISALTVVFVNSKSLSSLKIDD----TPVDD----PSLKVL-------VANNSDTLKL
SHFISALTVVFVNSKSLSSLKIDD----TPVDD----PSLKVL-------VANNSDTLKL
SHFISALTVVFVNSKSLSSLKIDD----TPVDD----PSLKVL-------VANNSDTLKL
SHFISALTVVFVNSKSLSSLKIDD----TPVDD----PSLKVL-------VANNSDTLKL
SHFISALTVVFVNSKSLSSLKIDD----TPVDD----PSLKVL-------VANNSDTLKL
SHFISALTVVFVNSKSLSSLKIDD----TPVDD----PSLKVL-------VANNSDTLKL
LWDPFLVSLSGKKGPTLQFLKGTY----RKHVAACRILCVADH-CQGLRELALNYYILSE
SHFVSALTVVFVNSKSLSSIKIED----TPVDD----PSLKIL-------VANNSDTLRL
SHFVSALTVVFINSKSLSSIKIED----TPVDD----PSLKIL-------VANNSDTLRL
VRILCHLTVVFINSKSLSSIKIED----TPVDD----PSLKIL-------VANNSDTLRL
SHFVSALTVVFINSKSLSSIKIED----TPVDD----PSLKIL-------VANNSDTLRL
SRFVSALTVVFINSKSLSSIKIED----TPVDD----PSLNIL-------VANNSDTLRL
RSTCTVGSAVLAGIWIIRRNHIHHGRDVKAYANPTVVRCEIHHKIYANNFAGVWITSNSD
RSTCTVGSAVLAGIWIIRRNHIHHGRDVKAYANPTVVRCEIHHKIYANNFAGVWITSNSD
RSTCTVGSAVLAGIWIIRRNHIHHGRDVKAYANPTVVRCEIHHKIYANNFAGVWITSNSD
RSTCTVGSAVLAGIWIIRRNHIHHGRDVKAYANPTVVRCEIHHKIYANNFAGVWITSNSD
RSTCTVGSAVLAGIWIIRRNHIHHGRDVKAYANPTVVRCEIHHKIYANNFAGVWITSNSD
RSTCTVGSAVLAGIWIIRRNHIHHGRDVKAYANPTVVRCEIHHKIYANNFAGVWITSNSD
RSTCTVGSAVLAGIWIIRRNHIHHGRDVKAYANPTVVRCEIHHKIYANNFAGVWITSNSD
RSTCTVGSAVLAGIWIIRRNHIHHGRDVKAYANPTVVRCEIHHKIYANNFAGVWITSNSD
YDDEKIISGLSIKVWDVNTGEVLN----TLIHHNEAVLHLRFS-----NGLMVTCSKDRS
YDDEKIISGLSIKIWDKTSLECLK----VLTGHTGSVLCLQYD-----ERVIVTGSSDST
YDDDKIISGLSIKIWDKSSLECLK----VLTGHTGSVLCLQYD-----ERVIVTGSSDST
YDDDKIISGLSIKIWDKSSLECLK----VLTGHTGSVLCLQYD-----ERVIVTGSSDST
YDDEKIISGLSIKIWDKTSLECLK----VLTGHTGSVLCLQYD-----ERVIVTGSSDST
YDDEKIISGLSIKIWDKTSLECLK----VLTGHTGSVLCLQYD-----ERVIVTGSSDST
```

```
YDDEKIISGLSIKIWDKTSLECLK----VLTGHTGSVLCLQYD-----ERVIVTGSSDST
YDDEKIISGLSIKIWDKSSLECLK----VLTGHTGSVLCLQYD-----ERVIVTGSSDST
YDDQKIVSGLTIKIWDKNTLECKR----ILTGHTGSVLCLQYD-----ERVIITGSSDST
YDDQKIVSGLTIKIWDKSTLECKR----ILTGHTGSVLCLQYD-----ERVIITGSSDST
YDDQKIVSGLTIKIWDKSTLECKR----ILTGHTGSVLCLQYD-----ERVIITGSSDST
YDDQKIVSGLTIKIWDKNTLECKR----ILTGHTGSVLCLQYD-----ERVIITGSSDST
YDDQKIVSGLTIKIWDKNTLECKR----ILTGHTGSVLCLQYD-----ERVIITGSSDST
YDDQKIVSGLTIKIWDKNTLECKR----ILTGHTGSVLCLQYD-----ERVIITGSSDST
YDDQKIVSGLTIKIWDKNTLECKR----ILTGHTGSVLCLQYD-----ERVIITGSSDST
YDDQKIVSGLTIKIWDKNTLECKR----ILTGHTGSVLCLQYD-----ERVIITGSSDST
SVICVVTSMNTISTWDLHKGAVTW----VSPVQPSYIKILATL---PEMHIAVTVDIEST
SVVCMVNSKNTISTWDVHKSVITW----KSPEQPASIKLLTTL---PEMHIAVTVDIQST
SVVCMVSSKNKISTWDISESVITWVSPVASIKL----LTTLPD-----MHIAVTVDIQST
PVVCMVTSVNRISTWDIQEGVLTW----SPVQQVGIKLLTTLP----EMHIAVTVDIQST
SVVCMLTSMTKISTWDIHEGVMTW------VSPVQPTTIKLLN-TLPEMHIAVTVDIHST
SVICMATSMNRISTWDIHEGVLTW----VSPEQPASIKLLTTL---PEMYIAVTVDMEST
SVICMVSSMTKLSTWDIREGIMTW----VSTEQPAYIKLLTTL---PEMHIAVTVDTHST
SAICMVTSVNRISTWDIHEGAMTW------VSPVQPSYIMQMT-TLPEMHIAVTVDMQST
SAVCMTSMN-RISTWDIHEGAMTW----SPKQPSYIVWMTTLP----EMQIAVTIDMQST
SAVCMTSMN-RISTWDIHEGAMTW----SPKQPSYIVWMTTLP----EMQIAVTIDMQST
SAVCMVTSTNRISTWDVHEGAMTW------VSPVQPSYITRMT-TLPEMHIAVTIDMQSN
SAVCMVTSTNRISTWDIHEGAMTW----SPVQESYIELMTTLP----EMHIAVTVDISST
SAVCMVTSMNRISTWDIHEGTMTW----SPVQEFYIELMTTLP----EMHIVVTVDIRST
PGMQPYVSFVILNIWDLRTGRFPIFRFEHDARIQALALSQEKPIVATASAFDVVMLYPNE
PGMQPYVSFVILNVWDLRTGRFPI----FRFEHDARIQALALSQEKPVVATASAFDVVMY
PGLQPNVSFVFLNIWDLRTGRYPV----HRFEHDARIQALALSQDDAAVATASAFDIVMS
PGMQPNVSFVFLNIWDLRTGKYPVHRFEHDARI----QALALSTVATASAFDVVMLSPSE
PGMQPNVSFVFLNIWDLRTGKYPV----HRFEHDARIQALALSQDDATVATASAFDVVMS
PGMQPNVSFVFLNIWDLRTGKYPV----HRFEHDARIQALALSQDDATVATASAFDVVMS
PGMQPNVSFVFLNIWDLRTGKYPV----HRFEHDARIQALALSQDDATVATASAFDVVMS
PGMQPNVSFVFLNIWDLRTGKYPV----HRFEHDARIQALALSQDDATVATASAFDVVMS
ESTVHLIQLLNTTPWDLPSRFLHWQSLYFRRPSLGTGFCLVSLSKSATSQVYWTAKTQHS
VLSDTFDQNRSIRYWDVKTGACVR----IFYGHQGTITCLDVY-----KNRLVSGAKDGQ
ILSDTFDQNRSIRYWDIKTGACIR----IFYGHQGTITCLDLY-----KNRLVSGAKDGQ
ILSDRWDQNRSIRYWDLRSGACTR----IFNGHQGTVTCMDLC-----KNRLVSGARDCQ
ILSDTWDQKRSIRYWDLKSGACTR----IFGGHQGTITCIDLC-----KNRLVSGGKDCQ
ILSDTWDQNRSIRYWDLKSGACTR----IFGGHQGTITCMDLC-----KNRLVSGGRDCQ
ILSDTWDQNRSIRYWDLKSGVCTR----IFGGHQGTITCMDLC-----KNRLVSGGRDCQ
ILSDTWDQNRSIRYWDLKSGVCAR----IFGGHQGTITCMDLCGAVKCLFFDQWHLLSGT
EQKLVTGSFDTVACWEWSSGARTQ----HFRGHTGAVFSVDYN---DELDVLVSGSADFT
EQKLVTGSFDTVACWEWSSGARTQ----HFRGHTGAVFSVDYN---DELDILVSGSADFT
EQKLVTGSFDTVACWEWSSGARTQ----HFRGHTGAVFSVDYN---DELDILVSGSADFT
EQKLVTGSFDTVACWEWSSGARTQ----HFRGHTGAVFSVDYN---DELDILVSGSADFT
EQKLVTGSFDTVACWEWSSGARTQ----HFRGHTGAVFSVDYN---DELDILVSGSADFT
EQKLVTGSFDTVACWEWSSGARTQ----HFRGHTGAVFSVDYN---DELDILVSGSADFT
```

```
EQKLVTGSFDTVACWEWSSGARTQ----HFRGHTGAVFSVDYS---DELDILVSGSADFA
EQKLVTGSFDTVACWEWSSGARTQ----HFRGHTGAVFSVDYS---DELDILVSGSADFA
---------------SPKEIAKFI-------------FCTRTL---NWKKLRIYLDESR-
---------------SPKEIAKFI-------------FCTRTL---NWKKLRIYLDERR-
---------------SPKEIAKFI-------------FCTRTL---NWKKLRIYLDERR-
---------------SPKEIAKFI-------------FCTRTL---NWKKLRIYLDERR-
---------------SPKEIAKFI-------------FCTRTL---NWKKLRIYLDERR-
---------------SPKEIAKFI-------------FCTRTL---NWKKLRIYLDERR-
---------------SPKEIAKFI-------------FCTRTL---NWKKLRIYLDERR-
---------------SPKEIAKFI-------------FCTRTL---NWKKLRIYLDERR-
DLTQTDISDSAFDSWSLRHLDLSGCEKIRALGILTSHQSGFLKTSMSKITSTTWKNKDIT
DLTQTDISDSAFDSWSLRHLDLSGCEKIRALGILTSHQSGFLKTSTSKITSTTWKNKDIT
DLTQTDISDSAFDSWSLRHLDLSGCEKIRALGILTSHQSGFLKTSTSKITSTTWKNKDIT
DLTQTDISDSAFDSWSLRHLDLSGCEKIRALGILTSHQSGFLKTSTSKITSTTWKNKDVT
DLTQTDISDSAFDSWSLRHLDLSGVALERALGILTSHQSGFLKTSTSKITSTTWKNKDIT
DLTQTDISDSAFDSWSLRHLDLSGVALERALGILTSHQSGFLKTSTSKITSTAWKNKDIT
DLTQTDISDSAFDSWSLRHLDLSGCEKIRALGVLTSHQSGFLK-SAGKAASTPWTSKDIT
DLTQTDISDSAFDSWSLRHLDLSGCEKITDMALEKISRALGVL---TSHQSGVLKSAGKT
VWSSQMRDNITLKVWNAETGECIH----TLYGHTSTVRCMHLH-----EKRVVSGSRDAT
VWSSQMRDNITLKVWNAETGECIH----TLYGHTSTVRCMHLH-----EKRVVSGSRDAT
VWSSQMRDNITLKVWNAETGECIH----TLYGHTSTVRCMHLH-----EKRVVSGSRDAT
VWSSQMRDNITLKVWNAETGECIH----TLYGHTSTVRCMHLH-----EKRVVSGSRDAT
VWSSQMRDNITLKVWNAETGECIH----TLYGHTSTVRCMHLH-----EKRVVSGSRDAT
VWSSQMRDNITLKVWNAETGECIH----TLYGHTSTVRCMHLH-----EKRVVSGSRDAT
VWSSQMRDNITLKVWNAETGECIH----TLYGHTSTVRCMHLH-----EKRVVSGSRDAT
VWSSQMRDNITLKVWNAETGECIH----TLYGHTSTVRCMHLH-----EKRVVSGSRDAT
DGQKAQSMGITEVLSQLSQLTGTFFTAPDTLKEVRKQLCPLFKQALADGLMELSKEDSEN
DGQKAQSIGIIEVLSQLSQLTGTFFTAPETLKTVRKQLYPFFKQALADGLMELSKEDSEN
DGQKAQSIGIIEVLSQLSQLTGTFFTAPETLKTVRKQLYPFFKQALADGLMELSKEDSEN
AGQKAQSMGIIEVLSQLSQLTGTFFTAPETLKTVRKQLYPLFKQALADGLMELSKEDSEN
DGQKAQSIGIIEVLSQLSQLTGTFFTAPETLKTVRKQLYPFFKQALADGLMELSKEDSES
----------EYGAWKRHYVACVS----------------HLD-----------------
----------EYGAWKQHYI-----------------ACVSS------------------
HVDLFVPLGALTGTWSYQHILSDWRTPLYFSESKLQAWSSFTELEDTLKSVRKTLNPLFN
---------------EEKQSPLSA---------------------------SQSSSSLKR
---------------PLSAFRSSS-------------------------SLRKKNNSGQA
---------------PLSAFRSSS-------------------------SLRKKNNSGEA
---------------PLSAFRSSS-------------------------SLRKKNNSGEA
---------------PLSAFRSSS-------------------------SLRKKNNSGEA
---------------SPEEKQSPL-------------SAFRSS-----SSLRKKNNSGEA
---------------EKQSPSLAF----RSSSSLRKKNNPGEK----------------E
---------------EKQSPSLAF----RSSSSLRKKNNPGEK----------------E
LPSEVLRHVFEDLYWNLSLVCHLWREIIDPLFIPWKKLYHRYL-MNEEQAVSKVDGILSN
AFAGTGKTSTNVICKTFHSMAYGHSKKKKGGFIRAKLVCKTLEEELTIDHVPIWCEKLNV
AFAGTGKTSTNVICKTFHSMAYGHSKKKKGGFIRAKLVCKTLEEELTIDHVPIWCEKLNV
```

AFAGTGKTSTNVICKTFHSMAYGHSKKKKGGFIRAKLVCKTLEEELTIDHVPIWCEKLNV
AFAGTGKTSTNVICKTFHSMAYGHSKKKKGGFIRAKLVCKTLEEELTIDHVPIWCEKLNV
AFAGTGKTSTNVICKTFHSMAYGHSKKKKGGFIRAKLVCKTLEEELTIDHVPIWCEKLNV
AFAGTGKTSTNVICKTFHSMAYSHLKKKKGGFIRAKLVCKTLEEELTIDHVPIWCEKLNV
AFAGTGKTSTFKLTPMVNSVLAEG----KGGFIRAKLVCKTLEEELTIDHVPIWCEKLNV
FSLFGDISILDPDGKKIKQIQQLFEEILSNSRQMKWLSCGFMLLSNAVANTMEHLSLLDN
FSLFGDISVLDPDGKKIKQIQQLFEEILSNSRQLKWLSCGFMLLSNAVASTMEHLSLLDN
FSLFGDISVLDPDGKKIKQIQQLFEEILSNSRQLKWLSCGFMLLSNAVANTMEHLSLLDN
FSLFGDISVLDPDGKKIKQIQQLFEEILSNSRQLKWLSCGFMLLSNAVANTMEHLSLLDN
FSLFGDISVLDPDGKKIKQIQQLFEEILSNSRQLKWLSCGFMLLSNAVANTMEHLSLLDN
FSLFGDISVLDPDGKKIKQIQQLFEEILSNSRQLKWLSCGFMLLSNAVANTMEHLSLLDN
FSLFGDISVVDPDGKKIKQIQQLFEEILSNSRQLKWLSCGFMLLSNPIANTMEHLSLLDN
FSLFGDISVVDPDGKKIKQIQQLFEEILSNSRQLKWLSCGFMLLSNSIANTMEHLSLLDN
DIQAQIDSIVELVCKTLRGINSRH----PSLAFKAGESSMIMEQVLDAMNYVLYDQLKFG
DIQAQIDSIVELVCKTLRGINSRH----PSLAFKAGESSMIMEQVLDAMNYVLYDQLKFG
DIQAQIDSIVELVCKTLRGINSRH----PSLAFKAGESSMIMEQVLDAMNYVLYDQLKFG
DIQAQIDSIVELVCKTLRGINSRH----PSLAFKAGESSMIMEQVLDAMNYVLYDQLKFG
DIQAQIDSIVELVCKTLRGINSRH----PSLAFKAGESSMIMEQVLDAMNYVLYDQLKFG
DIQAQIHSIVELVCKTLRGINSRH----PSLTFRAGESSMIMEQVLDAINYVLYDQLKFG
DIQAQIHSIVELVCKTLRGINSRH----PSLTFRAGESSMIMEQVLDAINYVLYDQLKFG
TVYRKYLYVLACDCVGQRVFKMTF----HHSMSFKQIVLVGQEEEGKIYSLVVNETQLDQ
TVYRKYLYVLACDCVGQRVFKMTF----HHSMSFKQIVLVGQEEEGKIYSLVVNETQLDQ
TAYRKYLYVLTCDCVGQRVFKMTF----HHSMSFKQIVLVGQEEEGKIYSLIVNETQLDQ
----------LKNALVSTLGQMQW-----KRACRYVVLCRGAK---DFASDPRCDTVYRY
TVYRKYLYVLSRACDRVFKMTFHH----SMTFKQIVLVGQETQEEGKIYSLVVNETQLDQ
TVYRKYLYVLSRACDRVFKMTFHH----SMTFKQIVLVGQETQEEGKIYSLVVNETQLDQ
----------LKNALVSTLGQMQWKRACRYVV-----LCRGAKFASDPRCDTVYRKYLYV
TVYRKYLYVLSRACDRVFKMTFHH----SMTFKQIVLVGQETQEEGKIYSLVVNETQLDQ
QKNFMNILEKLKVLEDQQNIRLIR----ELLQTLYTSLCTLVQKSVLVGNINMWVYRMET
QKNFMNILEKLKVLEDQQNIRLIR----ELLQTLYTSLCTLVQKSVLVGNINMWVYRMET
QKNFMNILEKLKVLEDQQNIRLIR----ELLQTLYTSLCTLVQKSVLVGNINMWVYRMET
QKNFMNILEKLKVLEDQQNIRLIR----ELLQTLYTSLCTLVQKSVLVGNINMWVYRMET
QKNFMNILEKLKVLEDQQNIRLIR----ELLQTLYTSLCTLVQKSVLVGNINMWVYRMET
QKNFMNILEKLKVLEDQQNIRLIR----ELLQTLYTSLCTLVQKSVLVGNINMWVYRMET
QKNFMNILEKLKVLEDQQNIRLIR----ELLQTLYTSLCTLVQKSVLVGNINMWVYRMET
QKNYFNILDKQKVLDDHHNPRLIK----DLLQDLSSTLCILIRKSVLVGNINIWICRLET
QKNYFNILDKQKVLDDHHNPRLIK----DLLQDLSSTLCILIRKSVLVGNINIWICRLET
QKNYFNILDKQKVLDDHHNPRLIK----DLLQDLSSTLCILIRKSVLVGNINIWICRLET
QKNYFNILDKQKVLDDHHNPRLIK----DLLQDLSSTLCILIRKSVLVGNINIWICRLET
QKNYFNILDKQKVLDDHHNPRLIK----DLLQDLSSTLCILIRKSVLVGNINIWICRLET
QKNYFNILDKQKVLDDHHNPRLIK----DLLQDLSSTLCILIRKSVLVGNINIWICRLET
QKNYFNILDKQKVLDDHQNPRLIK----GLLQDLSSTLGILVRKSVLVGNINIWICRLET
-------------------------------------CSILF-----------------
SDSDSSGTSLPARGSSGEKENRGGRRAITGGPLLSWPLGPAPPGSDHPLPRAAWLRVFQH
RPARGSSGEKPLLSWPLGPAPPPRPPQLRPPPRSPEPDTLPLAGSDHPLPRAAWLRVFQH

```
SDSDSSGTSLPARGSSGEKENRGGRRAVSGGPLLSWPLGPAPPGSDHPLPRAAWLRVFQH
GPGLLPPRVLPPENWEKPKPPLASAEGPREKLERFKRMCQLLETSSSSSDSDSDSDSSGS
KENRGGRRAVPLLSWPLGPAPPPRPPQLERHVVRPPPRSPEPDGSDHPLPRAAWLRVFQH
SDSDSSGTSLPARGSSGEKENRGGRRAVSGGPLLSWPLGPAPPGSDHPLPRAAWLRVFQH
----------PPRPPQLERHVVRPPPRSPEPDT----LPLAAG-SDHPLPRAAWLRVFQH
KENRGGRRAVPLLSWPLGPAPPPRPPQLRPPPRSPEPDTLPLAGSDHPLPRAAWLRVFQH
EVEKAKIRGSTSIVPKLQAITASSANLRRVLVQ----HCPARTGDESWMQREVWMSVFRY
EVEKAKIRGSTSIVPKLQAITASSANL-RHSPRVLVQHCPARTGDESWMQREVWMSVFRY
EVEKAKIRGSTSIVPKLQAITASSANL-RHSPRVLVQHCPARTGDESWMQREVWMSVFRY
EVEKAKIRGSTSIVPKLQAITASSANL-RHSPRVLVQHCPARTGDESWMQREVWMSVFRY
EVEKAKIRGSTSIVPKLQAITASSANL-RHSPRVLVQHCPARTGDESWMQREVWMSVFRY
EVEKAKIRGSTSIVPKLQAITASSANL-RHSPRVLVQHCPARTGDESWMQREVWMSVFRY
EVEKAKIRGSTSIVPKLQAITASSANL-RPNPRVLMQHCPARNGDESWMQREVWMSVFRY
EVEKAKIRGSTSIVPKLQAITASSANLRRVLMQ----HCPARNGDESWMQREVWMSVFRY
KVKMRRKRRLLNGTPRELRHQLGPSPPRRPPPSVSPPKCIQMEGAAHVMHREVWMAVFSY
KVKIRRKRRLLNGTPRELRHQLGPSPPRRPPPSVSPPKCIQMEGAAHVMHREVWMAVFSY
KVKMRRKRRLHEIQRTENSLANENQQPIKRPPG----ICERPH----RFSKGLNGTPREL
KVKMRRKRRLLNGTPRELRHQLGPSLRSRPPPSVSPPKCIQME-AAHVMHREVWMAVFSY
KVKMRRKRRLLNGTPRELRHQLGPSLRSRPPPSVSPPKCIQME-AAHVMHREVWMAVFSY
KVKMRRKRRLLNGTPRELRHQLGPSLRSRPPPSVSPPKCIQMEGAAHVMHREVWMAVFSY
KVKMRRKRRLLNGTPRELRHSLGPGLRSRPPPSASPPKCIQMEGAAHVMHREVWMAVFSY
KVKMRRKRRLLNGTPRELRHSLGPGLRSRPPPSTSPPKCIQME-AAHVMHREVWMAVFSY
APDSGLPSG-GGGRPGCAYPGSPG----PGARAKDKITCDLYQRDALPSNVEFLLARADE
APDSGLPSG-GGGRPGCAYPGSPG----PGARAKDKITCDLYQRDALPSNVEFLLARADE
APDSGLPTG-GGGRPGCAYPGSPG----PGARAKDKITCDLYQRDALPSNVEFLLARADE
APDSGLPSG-GGGRPGCAYPGSPG----PGARAKDKITCDLYQRDALPSNVEFLLARADE
APDSGLPSG-GGGRPGCAYPGSPG----PGARAKDKITCDLYQRDALPSNVEFLLARADE
APDSGLPSG-GGGRPGCAYPGSPG----PGARAKDKITCDLYQRDALPSNVEFLLARADE
SPDGNLPNG-GGGRPGCAYPGSPG----PGTRAKDKITCDLYQRDALPSNVEFLLARADE
SPDGSLANG-GGGRPACPYPGSPG----PGTRAKDKITCDLYQRDALPSNVEFLLARADE
ANSTQADGGKPMGSVRADHYSPKE----DQAWDGASQDCPSLP-AGVSFHIDSAELELGQ
GALEAPDTQVADHCS--PKEDQAW----DSASQ----DCPPLP-AGVSFHIDSAELEPGQ
GVLEAPDTQVADRCS--PKEDQAW----DGASQ----DCPPLP-AGVSFHIDSAELEPGQ
GVLEAPDTQVSVDCGPSRADRCSP----DQAWDGASQDCPPLP-AGVSFHIDSAELEPGQ
GVLEAPDTQVSVDCGPSRADRCSP----DQAWDGASQDCPPLP-AGVSFHIDSAELEPGQ
SRNNSFRRNVADEGKQVNPVGSVS----VDCGPSRADRCSPKEQDCPPLPAGVSFHIDSE
SADTADPQENGEQAWDGTSQSCPSFHMDEPGQQTAMKSCSRDD-VEMVEEFDELPTDAVR
SADTPGTQVHGEQAWDGTSQGCPSFHTDEPALHTAVKHCSRNDEFDELPTDAVSCRELVT
---------------KFDYLERLS------DRLLLKIICYLDL--EDIASLSQTSSKFEL
IMNYLDLE-------DIARLSQTS--------SRFEKLCKSDS---------LWEQIVQT
IISYLDLE-------DIARLSQTS--------HRFAKLCMS--------------DKLSQ
---------------DVRALAEDT----------------------------GWRQLFFT
---------------DVRALAEDT----------------------------GWRQLFFT
IISYLDLE-------DIARLCQTS--------HRFAKLCMSDK---------LWEQIVQ-
IISYLDLE-------DIARLCQTS--------HRFAKLCMSDK---------LWEQIVQ-
```

IISYLDLE-------DIARLCQTS--------HRFAKLCMSDK---------LWEQIVQ-
RLPFTLDDALARSCPELHSLFLDNSTLVPGSVLELLGACPRLR------ALGLHLASLSH
RLPFTLDDALARGCPELHSLFLDNSTLVPGSVLELLEACPRLR------ALGLHLASLSH
RLPFTLDNALARSCPELHSLFLDNSTLVPGSVLELLEACPRLR------ALGLHLASLSH
CLPFTLDDALARSCPELHSLFLDNSTLVPGSVLKLLEACPRLR------ALGLHLASLSH
RLPFTLDDALARSCPELHSLFLDNSTLVPGSVLELLEACPRLR------ALGLHLASLSH
RLSFTLDDALARSCPELHSLFLDNSTLVPGSVLELLEACPRLR------ALGLHLASLSH
HLPYTLDDTLAAGCPELRSLFLDNHALVPTSVLKLLEACPHLR------ALGLHLASMSR
HLPYTVDDTLAGGCPELRSLFLDN----ALVNSVQPASVLRLLACPHLRALGLHLASMSR
VNDNSVTVFWHDKNWHVDTLSTLD------LYGATPIFMEQYK--GPNTSCPRWLSLIEK
YIMRHADHSV-----NDTSVTVVW----KNWPHLATLSTLNLYYLTPDPNSPRWLSLIGK
HVDLSINDTSVTVIWLASLSTLDLCGVTPVFMD----WYKTPT-----KHRLRWHSLIAK
HVDLSVNDTSVTVIWLASLSTLDLCGVTPVFMD----WYKTPT-----KHRLRWHSLIAK
HADLSINDTSVTVIWLASLSTLDLCGMTPVFTD----WYKTPT-----KHRLRWHSLIAK
HVDLSINDTSVTVIWLASLSTLDLCGMTPVFTD----WYKTPT-----KHRLRWHSLIAK
YIMEHVDLSI-----NDTSVTVIWYGKKCLASLSTLDLCGMTP------VFTDWYKTPTH
YIMEHVDLSI-----NDTSVTVIW----KKWPCLASLSTLDLC--GMTPVFTDWYKTPTH
VESTAVVSFLEEAGSRMRKLWLTYSSQTAILGALLDNCCPQLQ--VLQVSTGMNCNNTPQ
VESTAVVSFLEEAGSRMRRLWLTYSSQTAILGALLGNCCSQLQVLEVSAGMSCNNTPLQL
VESTAVVSFLEEAGPRMRKLWLTY----AILSALLGSCCPQLQ------VLEVSTDINRS
VESTAVVSFLEEAGSRMRKLWLTYSSQTAILGALLGSCCPQLQ--VLEVSSGINRNSIPQ
SR--------MRRLW-LTYSSQTT----AILGALLGSCCPQLQ--VLEVSTGINRNSIPQ
VESTAVVSFLKEAGSRMRKFWLTYSSQTAILGALLGSCCPQLQ--VLEVSTGINRNSIPQ
VESTAVVSFLEEAGSRMRKLWLTYSSQTAILGALLGSCCPQLQ--VLEVSTGINRNSIPQ
VESTAVVSFLEEAGSRMRKLWLTYSSQTAILGALLGSCCPQLQ--VLEVSTGINRNSIPQ
GFSESAVATLLSSCSRLDELNLSW-------------CFDFT-EKHVQAAVAHLPNTIQ
GFSESAVATLLSSCSRLDELNLSW-------------CFDFT-EKHVQAAVAHLPDTLQ
GFSEFALQTLLSSCSRLDELNLSW-------------CFDFT-EKHVQVAVAHVSETIQ
GFSEFALQTLLSSCSRLDELNLSW-------------CFDFT-EKHVQVAVAHVSETIQ
GFSEFALQTLLSGCSRLDELNLSW-------------CFDFT-EKHVQVAVAHVSETIQ
GFSEFALQTLLSSCSRLDELNLSW-------------CFDFT-EKHVQVAVAHVSETIQ
GFSEFALQTLLSSCSRLDELNLSW-------------CFDFT-EKHVQVAVAHVSETIQ
VMLEQMQGVVLSGCNDFTEAGLWS----ARITSLSVSDCINVA-DDAIAAISQLLPNLAE
VMLEQMQGVVLSGCNDFTEAGLWS----ARITSLSVSDCINVA-DDAIAAISQLLPNLAE
VMLEQMQGVVLSGCNDFTEAGLWS----ARITSLSVSDCINVA-DDAIAAISQLLPNLAE
VMLEQMQGVVLSGCNDFTEAGLWS----ARITSLSVSDCINVA-DDAIAAISQLLPNLAE
VMLEQMQGVVLSGCNDFTEAGLWS----ARITSLSVSDCINVA-DDAIAAISQLLPNLAE
VMLEQMQGVVLSGCNDFTEAGLWS----ARITSLSVSDCINVA-DDAIAAISQLLPNLAE
VMLEQMQGVVLSGCNDFTEAGLWS----ARITSLSVSDCINVA-DDAIAAISQLLPNLAE
VMLEQMQGVVLSGCNDFTEAGLWS----ARITSLSVSDCINVA-DDAIAAISQLLPNLAE
LSFFLKKMGK-----HLDHLSLKG------ARLTVEQGCHILN-----SLSYMQNENMAE
LSFFLKKMGK-----HLDHLSLKG------ARLTVEQGCHILNRNENVASELNIEDFFSH
LSFFLKKIGK-----HLDYLNLKG------ARLTVEQGCHVLN----SLSYMRNKNVISE
LSFFLKKMGK-----HLDYLNLKG------ARLTVEQGCHILD----SLSHLRNENVISE
LSFFLKKMGK-----RLDYLNLKG------ARLTVEQGCQILD----SLSCMRNENVISE

```
LSFFLKKMGK-----RLDYLNLKG------ARLTVEQGCQILD----SLSYMRNENVISE
LSFFLKKMGK-----RLDYLNLKG------ARLTVEQGCQILD----SLSYVRNENVISE
LSFFLKKMGK-----RLDYLNLKG------ARLTVEQGCQILD----SLSYMRNENVISE
EPPENPCDCHPPETPDINQLPPSILLKILSLDE----RCLSAS-----LVCKYWRDLCLD
NQDKLTDEGLGSKCRELKDIHFGQCYKIDEGMIVIAKGCLKLQ------RIYMQENKLVD
---------------NIVEINISD-------------CRSMS--------DSGVCVLA-
ENPCDCHREPPPEIPDINQLPPSINLSLSLVCKYWRDLCLDFQTDELLEKIASRSQNIIE
ENPCDCHREPPPETPDINQLPPSILLKISLVCKYWRDLCLDFQTDELLEKIASRSQNIIE
ENPCDCHREPPPETPDINQLPPSILLKILSLDE----RCLSAS-----LVCKYWRDLCLD
ENPCDCHREPPPETPDINQLPPSILLKILSLDE----RCLSAS-----LVCKYWRDLCLD
HLGKDPSRV------LVKALGTQG-----NSTHKGWVWSLAAQ-----DHRVCSGSWDST
----------NVNLWDLRHLGKEPSRVLGNSTHKGWVWSLAAQ-----DHRVCSGSWDST
VWSLAAQDHCTVKLWDMAADGQQF----GEIKANSAVLCLSYL-----PDILVTGTYDKK
VWSLAAQDHRTVKLWDLAADGQQF----GEIKTSSAVLCLSYL-----SDVLVTGTYDKK
QLGTESNQ-------VLVKTLGTK----RNSTHEGWVWSLAAQ------DHRVCSGSWDT
VWSLAAQDHRCSGSWDMAADGQQF----GEIKASSAVLCLSYL-----PDILVTGTYDKK
VWSLAAQDHRCSGSWDMAADGQQF----GEIKASSAVLCLSYL-----PDILVTGTYDKK
----------NVNLWDLRQLGTESNQVLRNSTHEGWVWSLAAQ-----DHRVCSGSWDST
IVVTPMGSG-SNRPQEIEIGESGF----LLFPQIEGIKIQPFHFIKDSKNLTLERHQLTE
IVVTPMGSG-SNRPQEIEIGESGF----LLFPQIEGIKIQPFHFIKDPKNLTLERHQLTE
IVVTPMGSG-SNRPQEIEIGESGF----LLFPQIEGIKIQPFHFIKDPKNLALERHQLTE
IVVTPMGSG-SNRPQEIEIGESGF----LLFPQIEGIKIQPFHFIKDPKNLTLERHQLTE
IVVTPMGSG-SNRPQEIEIGESGF----LLFPQIEGIKIQPFHFIKDPKNLTLERHQLTE
IVVTPMGSG-SNRPQEIEIGESGF----LLFPQIEGIKIQPFHFIKDPKNLTLERHQLTE
IVVTPMGSG-SNRPQEIEIGESGF----LLFPQIEGIKIQPFHFIKDPKNLTLERHQLTE
IVVTPMGSG-SNRPQEIEIGESGF----LLFPQIEGIKIQPFHFIKDPKNLTLERHQLTE
KVTCISLT--REASIKLSPLHGKQ----ISIRYLDMTDCFVLE-DEGLHTIAAHCTQLTH
KVTCISLT--REASIKLSPLHGKQ----ISIRYLDMTDCFVLE-DEGLHTIAAHCTQLTH
KVTCISLT--REASIKLSPLHGKQ----ISIRYLDMTDCFVLE-DEGLHTIAAHCTQLTH
KVTCISLT--REASIKLSPLHGKQ----ISIRYLDMTDCFVLE-DEGLHTIAAHCTQLTH
KVTCISLT--REASIKLSPLHGKQ----ISIRYLDMTDCFVLE-DEGLHTIAAHCTQLTH
KVTCISLT--REASIKLSPLHGKQ----ISIRYLDMTDCFVLE-DEGLHTIAAHCTQLTH
KVTCISLT--REASIKLSPLHGKQ----ISIRYLDMTDCFVLE-DEGLHTIAAHCTQLTH
KVTCISLT--REASIKLSPLHGKQ----ISIRYLDMTDCFVLE-DEGLHTIAAHCTQLTH
INNSTRNRRKIPSCHVPYTGITTM----AHFCPSREILCAMKS---DCLSLSLSSLSFTH
SITNSSLKGISEGCRNLEYLNLSWDGIERGLKALLLRGCTQLE-DEALKHIQNYCHELVS
SITNSSLKGISEGCRNLEYLNLSWDGIERGLKALLLRGCTQLE-DEALKHIQNYCHELVS
SITNSSLKGISEGCRNLEYLNLSWDGIERGLKALLLRGCTQLE-DEALKHIQNYCHELVS
SITNSSLKGISEGCRNLEYLNLSWDGIERGLKALLLRGCTQLE-DEALKHIQNYCHELVS
SITNSSLKGISEGCRNLEYLNLSWDGIERGLKALLLRGCTQLE-DEALKHIQNYCHELVS
SVTNSSLKGISEGCRNLEYLNLSWEGIERGLKALLLRGCTQLE-DEALKHIQNHCHELVS
SVTNSSLKGISEGCRNLEYLNLSWEGIERGLKALLLRGCTQLE-DEALKHIQNHCHELVS
IIEGCPLLEQLNISWGIQALVRGC----GGLKALFLKGCTQLE-DEALKYIGAHCPELVT
SITNMSLKALSEGCPLLEQLNISWDGIQGGLKALFLKGCTQLE-DEALKYIGAHCPELVT
SITNMSLKALSEGCPLLEQLNISWDGIQGGLKALFLKGCTQLE-DEALKYIGAHCPELVT
```

SITNMSLKALSEGCPLLEQLNISWDGIQGGLKALFLKGCTQLE-DEALKYIGAHCPELVT
SITNMSLKALSEGCPLLEQLNISWDGIQGGLKALFLKGCTQLE-DEALKYIGAHCPELVT
SITNMSLKALSEGCPLLEQLNISWDGIQGGLKALFLKGCTQLE-DEALKYIGAHCPELVT
SITNMSLKALSEGCPLLEQLNISWDGIQGGLKALFLKGCTQLE-DEALKYIGAHCPELVT
SITNMSLKALSEGCPLLEQLNISWDGIQGGLKALFLKGCTQLE-DEALKYIGAHCPELVT
QLSRRALGALAEGCPRLQRLSLAH--------------CDWVD-GLALRGLADRCPALEE
QLSRRALGALAEGCPRLQRISLAH--------------CDWVD-GLALRGLADRCPALEE
QLSRRALGALAEGCPRLQRLSLAH--------------CDWVD-GLALRGLADRCPALEE
QLSRRALGALAEGCPRLQRLSLAH--------------CDWVD-GLALRGLADRCPALEE
QLSRRALGALAEGCPRLQRLSLAH--------------CDWVD-GLALRGLADRCPALEE
QLSRRALGALAEGCPRLQRLSLAH--------------CDWVD-GLALRGLADRCPALEE
QLSRRALGALAEGCPRLQRLSLAH--------------CDWVD-GLALRGLADRCPALEE
QLSRRALGALAEGCPRLQRLSLAH--------------CDWVD-GLALRGLADRCPALEE
---------------SLDRIAQYL----KGLEVLELGGCSNIT-NTGLLLIAWGLQRLKS
IAQYLKGLEVLGGCSNITNTGLLL----QRLKSLNLRSCRHLSHLAGMTRSAAEGCLGLQ
IAQYLKGLEVLGGCSNITNTGLLL----QRLKSLNLRSCRHLSHLAGMTRSAAEGCLGLQ
IAQYLKGLEVLGGCSNITNTGLLL----QRLKSLNLRSCRHLSHLAGMTRSAAEGCLGLQ
IAQYLKGLEVLGGCSNITNTGLLL----QRLKSLNLRSCRHLSHLAGMTRSAAEGCLGLQ
IAQYLKGLEVLGGCSNITNTGLLL----QRLKSLNLRSCRHLSHLAGMTRSAAEGCLGLQ
IAQYLKGLEVLGGCSNITNTGLLL----QRLKSLNLRSCRHLSHLAGMTRSAAEGCLGLQ
LLPRHFHNLQLAYCRRFTDKGLQY----HKLIYLDLSGCTQIS-VQGFRYIANSCTGILH
LLPRHFHNLQLAYCRRFTDKGLQY----HKLIYLDLSGCTQIS-VQGFRYIANSCTGIMH
LLPRHFHNLQLAYCRGFTDKGLQY----HKLIYLDLSGCTQIS-VQGFRYIANSCTGITH
LLPRHFHNLQLAYCRRFTDKGLQY----HKLIYLDLSGCTQIS-VQGFRYIANSCTGVMH
LLPRHFHNLQLAYCRRFTDKGLQY----HKLIYLDLSGCTQIS-VQGFRYIANSCTGIMH
LLPRHFHNLQLAYCRRFTDKGLQY----HKLIYLDLSGCTQIS-VQGFRYISNSCTGIMH
LLPRYFHNLQGNGCHKLIYLDLSGCTQV---------KCPRISSVVLIGSPHISDSAFKA
FSDFGVTDKRDISVLPDQAVVQIF----VYLTFKDLVSCSQVN--------RSWMSMIQR
QSTDGARTKI-----GFSEGRHAW--------------------------EVWWEGPLGT
QSTDGARTKI-----GFSEGRHAW--------------------------EVWWEGPLGT
QSTDGARTKI-----GFSEGRHAW--------------------------EVWWEGPLGT
QSTDGARTKI-----GFSEGRHAW--------------------------EVWWEGPLGT
----------GQKCPNLKRL------------------CLHVA-DLSMVPITSLPSTLRT
----------GQKCPNLKRL------------------CLHVA-DLSMVPITSLPSTLRT
----------GQKCPNLQR------------------LCLHVA-DLSMVPITSLPSTLRT
----------GQKCPNLKRL------------------CLHVA-DLSMVPITSLPSTLRT
----------GQKCPNLKRL------------------CLHVA-DLSMVPITSLPSTLRT
----------GQKCPNLKRL------------------CLHVA-DLSMVPITSLPSTLRT
----------GQKCPNLKRL------------------CLHVA-DLSMVPITSLPSTLRT
----------GQKCPNLKRL------------------CLHVA-DLSMVPITSLPSTLRT
DEDVCHFVLAGDGKIGVHKIHSTF----KYSAHEQEVNCVDCK-----GGIIVSGSRDRT
DEDVCHFVLAGDGKIGVHKIHSTF----KYSAHEQEVNCVDCK-----GGIIVSGSRDRT
DEDVCHFVLAGDGKIGVHKIHSTF----KYSAHEQEVNCVDCK-----GGIIVSGSRDRT
DEDVCHFVLAGDGKIGIHKIHSTF----KYSAHEQEVNCVDCK-----GGIIVSGSRDRT
DEDVCHFVLAGDGKIGIHKIHSTF----KYSAHEQEVNCVDCK-----GGIIVSGSRDRT

```
DEDVCHFVLAGDGKIGVHKIHSTF----KYSAHEQEVNCVDCK-----GGIIVSGSRDRT
DEDVCHFVLAGDGKIGVHKIHSTF----KYSAHEQEVNCVDCK-----GGIIVSGSRDRT
SQSPTSPVCSSDGGLDDEQLTYRLPRPVLPGASIQLPSCLVLNVFRNLTYAVRCIHNSKV
SQSPTSPVSSSDGGLDDEQLTYRLPRPVLPGASIQLPSCLVLNVFRNLTYAVRCIHNSKV
VTVEGHPS--ADKNWAYKYLLGLIKSTPTFLPTEDSDFLMSLD---LESRDQAWSPKTCD
SQSPTSPASSSDGGLDEDQLMYRLSYQVANSVQ----GCLIRKLTYAVRCIHNSKIIMLR
SQSPTSPASSSDGGLDEDQLMYRLSYQVANSVQ----GCLIRKLTYAVRCIHNSKIIMLR
SQSPTSPASSSDGGLDEDQLMYRLSYQVANSVQ----GCLIRKLTYAVRCIHNSKIIMLR
SQSPTSPASSSDGGLDEDQLMYRLSYQVLPGASIQLPSCLVLNIFRNLTYAVRCIHNSKI
SQSPTSPASSSDGGLDEDQLMYRLSYQVLPGASIQLPSCLVLNIFRNLTYAVRCIHNSKI
GGRQEEFRTWLREEWTLEDIFHEH----KFIYTSQYDNCLTYR------RIYLPPSHPDL
------------------------------------------------------------
GGRQEEFRTWLREEWTLEDIFHEH----KFIYTSQYDNCLTYR------RIYLPPSRPDL
GGRQEEFRTWLREEWTLEDIFHEH----KFIYTSQYDNCLTYR------RIYLPPSRPDL
GGRQEEFRTWLREEWTLEDIFHEH----KFIYTSQYDNCLTYR------RIYLPPSHPDL
GGRQEEFRTWLREEWTLEDIFHEH----KFIYTSQYDNCLTYR------RIYLPPSRPDL
GGRQEEFRTWLREEWTLEDIFHEH----KFIYTSQYDNCLTYR------RIYLPPSRPDL
GGRQEEFRTWLREEWTLEDIFHEH----KFIYTSQYDNCLTYR------RIYLPPSRPDL
GWEVEH----GGNGWAVEKNLTMV----QTCFVTSFEWCFKRQ-LVDLVKEGVWQELLDS
GWEVEH----GGNGWAVEKNLTLV----QTCFVTSFEWCSKRQ-LVDLVKEGVWQELLDS
GWEVEH----GGNGWAIEKNLTLV----QTCFVTSFEWCFKRQ-LVDLVMEGVWQELLDS
----------------------------SNEDKEEFPLCALAR-----------------
GWEVEH----GGNGWAIEKNLTPV----QTCFVTSFEWCSKRQ-LVDLVMEGVWQELLDS
GWEVEH----GGNGWAIEKNLTPV----QTCFVTSFEWCSKRQ-LVDLVMEGVWQELLDS
GWEVEH----GGNGWAIEKNLTPV----QTCFVTSFEWCSKRQ-LVDLVMEGVWQELLDS
KWMVQH----GGDGWTVRGAPSQT------CFVTSFSWCRKKQ-VLDLEEEGLWPELLDS
KWMVQH----GGDGWVVEENRTTV----QTCFVTSFSWCRKKQ-VLDLEEEGLWPELLDS
KWMVQH----GGDGWVVEENRTTVPGAPQTCFVTSFSWCCKKQ-VLDLEEEGLWPELLDS
KWMVQH----GGDGWVVEENRTTVPGAPQTCFVTSFSWCCKKQ-VLDLEEEGLWPELLDS
KWMVQH----GGDGWVVEENRTTVPGAPQTCFVTSFSWCCKKQ-VLDLEEEGLWPELLDS
----------ARPCP------LGR-------------FCARRP---------IGRNLIR-
KWMVRH----GGDGWVVEKNRKPVPGAPQTCFVTSFSWCRKKQ-VVDLVEKGLWPELLD-
----------NLLCN----------------------PCGQAR--------------HD-
----------GGDGWRVEELPGDSGVEFKKYFASSFEWCRKAQ-VIDLHAEGYWEELLDT
----------GGDGWRVEELPGDSGVEFKKYFASSFEWCRKAQ-VIDLQAEGYWEELLDT
----------GGDGWRVEELPGDSGVEFKKYFASSFEWCRKAQ-VIDLQAEGYWEELLDT
----------GGDGWRVEELPGDSGVEFKKYFASSFEWCRKAQ-VIDLQAEGYWEELLDT
----------GGDGWRVEELPGDSGVEFKKYFASSFEWCRKAQ-VIDLQAEGYWEELLDT
----------GGDGWRVEELPGDNGVEFKKYFASSFEWCRKAQ-VIDLQAEGYWEELLDT
----------GGDGWRVEELPGDSDDSVKKYFASSFEWCRKAQ-VIDLQAEGYWEELLDT
----------GGDEWKVEDLSRDQ----RKEFPNDQHLPQVPG--GGPQGRRVLGGADG-
----------GGDEWKVEDLSGDQ------------------RKEFPNDQVRSQARLR-
----------GGDEWKVEDLSKDQRKEFKKYFVTSYYTCLKSQ-VVDLKAEGYWEELMDT
----------GGDEWKVEDLSKDQRKEFKKYFVTSYYTCLKSQ-VVDLKAEGYWEELMDT
----------GGDEWKVEDLSRDQRKEFKKYFVTSYYTCLKSQ-VVDLKAEGYWEELMDT
```

```
----------GGDEWKGEDLSRDQRKEFKKYFVTSYYTCLKSQ-VVDLKAEGYWEELMDT
----------GGDEWKVEDLSRDQRKEFKKYFVTSYYTCLKSQ-VVDLKAEGYWEELMDT
----------GGDKWKVESLPGAHRTAFKNYFVTSYGMCLKSQ-LVDLVAKGYWEELLDT
----------GGDRWKVESLPGAHGTDFKKYFVTSYEMCLKSQ-LVDLVAEGYWEELLDT
----------GGDRWKVESLPGAHGTDFKKYFVTSYEMCLKSQ-LVDLVAEGYWEELLDT
----------GGDRWKVESLPGAHGTDFKKYFVTSYEMCLKSQ-LVDLVAEGYWEELLDT
----------GGDRWKVESLPGAHGTDFKKYFVTSYEMCLKSQ-LVDLVAEGYWEELLDT
----------GGDRWKVESLPGAHGTDFKKYFVTSYEMCLKSQ-LVDLVAEGYWEELLDT
----------GGDRWKVETLPGSCGTSFKKYFVTSFEMCLKSQ-MVDLKAEGYCEELMDT
----------GGDEWKVESLPGDHGTSFKKYFVTSYGMCLKSQ-MVDLKAEGYSEKLLDT

FRLQNIIG-RKMGLECVDILSELFLGLRHLLATILAQLSDMDLINVSK----------VS
FRLQNIIG-RKMGLECVDILSELFRGLRHLLATILAQLSDMDLINVSK----------VS
FRLQNIIG-RKMGLECVDILSELFRGLRHLLATILAQLSDMDLINVSK----------VS
FRLQNIIG-RKMGLECVDILSELFRGLRHVLATILAQLSDMDLINVSK----------VS
FRLQNIIG-RKMGLECVDILSELFRGLRHVLATILAQLSDMDLINVSK----------VS
FRLQNIIG-RKMGLECVDILSELFRGLRHVLATILAQLSDMDLINVSK----------VS
FRLQNIIG-KKMGLEHLDILAELSRGFVHLLANILTKLSGMDLVNLSK-----VSRIWKK
SRIWKKIL----ESDKGAFQQYSKAMQRVIESNKLSLHASTRGYVVGR------------
IRTADCSRSPDLLLDASDQAGLPCRVFDLGGDTEEEATDPGLHTSGSD--KGLRRVFDSV
FKIQNLNA--STIRTVMVADCSRFDSPDLLLDAGDQAGLPCRVFDLGG--DTEEEATDPG
FKIQNLNA--STIRTVMVADCSRFDSPDLLLDAGDPGTSPCRVFDLGS--PAPAHAKEGL
FKIQNLNA--STIRTVMVADCSRFDSPDLLLEAGDPATSPCRIFDLGS-DPAPAHASEGF
FKIQNLNA--STVRTVMVADCSRFDSPDLLLEAGDPATSPCRIFDLGS--PAPAHAKEGL
FKIQNLNA--STVRTVMVADCSRFDSPDLLLEAGDPATSPCRIFDLGS--PAPAHAKEGL
IKVWNCQD--RDSLATLPMPETCYC-------MEAHLTKDGPFLMVGD--------AAGD
IKVWNCQD--RDALAILRMPESCYG-------MEAYLTKDGPFLTVGDAN--------LP
IKVWNCQD--RDALAVLPMPEPCYC-------MEAYLTKDGPFLMVGDAD--------LP
IKVWNCQD--RDALAVLPMPQPCYC-------MEAYLTKDGPFLMVGDAD--------LP
IKVWNCQD--RDALAVLPMPQPCYC-------MEAYLTKDGPFLMVGDAD--------LP
IKVWNCQD--RDALAVLPMPQPCYC-------MEAYLTKDGPFLMVGDAD--------LP
EVFYQCPDQMARNPAAIDMFIIGATFTDWFTSYVNNVVSGGFPIIRDQ------------
EVFYQCPDQMARNPAAIDMFIIGATFTDWFTSYVKNVVSGGFPIIRDQ------------
EVFYQCPDQMARNPAAIDMFIIGATFTDWFTSYVKNVVSGGFPIIRDQ------------
EVFYQCPDQMARNPAAIDMFIIGATFTDWFTSYVKNVVSGGFPIIRDQ------------
EVFYQCPDQMARNPAAIDMFIIGATFTDWFTSYVKNVVSGGFPIIRDQ------------
EVFYQCPDQMARNPAAIDMFIIGATFTDWFTSYVKNVVSGGFPIIRDQ------------
EVFYQCPDQMARNPAAIDMFIIGATFTDWFTSYVNNVVSGGFPIIRDQ------------
EVFYQCPDQMARNPAAIDMFIIGATFTDWFTSYVNNVVSGGFPIIRDQ------------
AKTWAFFS-----LPASCSSMEACDHPEGPFLLVACDEGTLYTMTVPQ------LQVVSR
IRGWNTDTWAFFSLPTSCSSMEACDHPEGSFLLVACAEGTLYTMTVPQQVPVSLMCSPDK
IRIPTVVDLLTLVPVAPRTQHLCSFLCEL---TLLHTSLSIYAPARLAALTQPWTTHLWD
IRIPTVVDLLTLVPVAPRTQHLCSFLCEL---TLLHTSLSVYAPARLAALTQPWTTQLWD
GKIRVPTVLLTLVPVEPRTQHLCSFLCEL---SLLHTSLSAYAPARLAALTQPWTTQLWD
IRVPTVVDLLALVPVELRTQHLCSFLCEL---SLLHTSLSTYAPARLAALTQPWTTQLWD
```

IRVPTVVDLLTLVPVELRTQHLCSFLCEL---SLLHTSLSTYAPARLAALTQPWTTQLWD
IRVPTVVDLLTLVPVELRTQHLCSFLCEL---SLLHTSLSAYAPARLAALTQPWTTQLWD
IRVPTVVDLLTLVPVELRTQHLCSFLCEL---SLLHTSLSAYAPARLAALTQPWTTQLWD
IRVPTVVDLLTLVPVELRTQHLCSFLCEL---SLLHTSLSAYAPARLAALTQPWTTQLWD
LRLENCAR---VTNRTLAAVAAHGRALQTLHVDFCRNVSAAGLLRLRA------------
LRLENCAR----------------------------VTNRTLAAVAA------------
LAVWKSPR----------WGGPHHS---------ELALRAGVREARAA------------
IAV---------ESKSPRWGGPDHS-------EFAGLRAGVTGARAAA------------
IAVGSKSP---------RWGGHDHS-------EFAALRAGVTGARAAA------------
IAVGSKSP---------RWGGPDHS-------EFADLRAGVTGARAAP------------
IAV---------GPKSPRWGGPDHS-------EFADLRSGVTGARAAA------------
---WLKSA----------FQRSICS-----------------------------------
DESGDSTFKNLSKTPALQLVHELFMKSKR---KRSQQNSGHEFLEEGDGILIGKKMGIEK
DESGDLTFKNLSKTPALQLVHELFMKSKR---KRLQENSGHEFLEQGNEILIGKKMGIEK
DESGDLTFKNLSKTPALQLVHELLMKSKR---KRLQENSGHEFLEQGDEILIGKKMGIEK
DESGDLTFKNLSKTPALQLVHELFMKSKR---KRFQENSGHEFLEQGDEILIGKKMGIEK
DESGDLTFKNLSKTPALQLVHELFMKSKR---KRLQENSGHEFLEQGDEILIGKKMGIEK
DESGDLTFKNLSKTPALQLVHELFMKSKR---KRLQENSGHEFLEQGDEILIGKKMGIEK
TREYRSQSMDEAPCVNGRWGTLRPRRGDGSPILNGGSLSPGTAAVGGSLSPSAAEGCDLK
TREYRSQSMDEAPCVNGRWGTLRPRRGDGSPILNGGSLSPGTAAVGGSLSPSAAEGYDLK
TREYRSQSMDEAPCVNGRWGTLRPRRGDGSPILNGGSLSPGTAAVGGSLSPSAAEGYDLK
TREYRSQSMDEAPCVNGRWGTLRPRRGDGSPILNGGSLSPGTAAVGGSLSPSAAEGYDLK
TREYRSQSMDEAPCVNGRWGTLRPRRGDGSPILNGGSLSPGTAAVGGSLSPSAAEGYDLK
TREYRSQSMDEAPCVNGRWGTLRPRRGDGSPILNGGSLSPGTAAVGGSLSPSAPEGYDLK
TREYRSQSMDEAPCVNGRWGTLRPRRGDGSPILNGGNLSPGTVAVGGALSPSASDGYDLK
TREYRSQSMDEAPCVNGRWGTLRPRRGDGSPILNGGNLSPGTAAVGGALSPSASDGYDLK
TLVESCCN---LHHLNLSAAHHHSSLCQLLARLCHLRSLSLPVCSVADRPRGFGKKVRIG
PAPHAVPR---GFGKKVRIGVQTCP--NPFVGQSAPQPASVFWSLLKK----LPFLEHLE
PAPHAVPR---GFGKKVRIGVQTCP--NPFVGQSAPQPASVFWSLLKK------LPFLEH
SAPRADRA---PAQPAMHAVPRGFGKVRVGVQSCPSPFSGQAGPQPSS--LLKNLPFLEH
SAPRADRA---PAQPAMHAVPRGFGKVRVGVQSCPSPFSGQAGPQPSS--LLKNLPFLEH
VRVQSCPSQAGPQPSSVFWSLLKNLHLELIGSNFSSAMPRNEPAIRNSASGQLAFLRHLT
SAPRADRA---PAQPAMHAVPRGFGKVRVGVQSCPSPFSGQAGPQPSS--LLKNLPFLEH
PAQHAVPRRVGVQSCPSPFSGQACPHLELIGSNFSSAMPRNEPAIRNSASGQLAFLRHLT
RRYSEREEVSGKGKTPLRKRCNNSHGCQVTSEQIKADMKAARDVSEKKSDSSTAASTAGN
RRYEEKTGVSGKGKTPLRKRYNNSHGCQVTSEQIKADMKAARDISEKKPCNSSSPSTASQ
RRYEEKTGGKGKTPLRKRYNSHQMGGCQVTSEQIKADMKAARDIPEKKPCNSSSHNTASQ
RRYEEKTGSSRGKGKTPLRKRYNSHGCQVTSEQIKADMKAARDIPEKKPCNSSSHSTASQ
RRYEEKTGAVSGKGKTPLRKRYNSHGCQVTSEQIKADMKAARDIPEKKPCNSSSHSTASQ
RRYEEKTGSVSGKGKTPLRKRYNSHGCQVTSEQIKADMKAARDIPEKKPCNSSSHSTASQ
RRYEEKTGSVSGKGKTPLRKRYNSHGCQVTSEQIKADMKAARDIPEKKPCNSSSHNTASQ
RRYEEKTGSVSGKGKTPLRKRYNSHGCQVTSEQIKADMKAARDIPEKKPCNSSSHNTASQ
FPVPGPSA---ALTTMQLFSKQNPSRQEVTKLQQQVKTNGAGVTVLRR------------
PPVPGPSA---ALTTMQLFSKQNPSRQEVTKLQQQVRTNGAGVTVLRR------------
PPVPGPSA---ALTTMQLFSKQNPSRQEVTKLQQQVKTNGAGVTVLRR------------

PPVPGPSA---ALTTMQLFSKQNPSRQEVTKLQQQVKTNGAGVTVLRR------------
PPVPGPSA---ALTTMQLFSKQNPSRQEVTKLQQQVKTNGAGVTVLRR------------
PPVPGPSA---ALTTMQLFSKQNPSRQEVTKLQQQVKTNGAGVTVLRR------------
PPVPGPSA---ALTTMQLFSKQNPSRQEVTKLQQQVKTNGAGVTVLRR------------
PPVPGPSA---ALTTMQLFSKQNPSRQEVTKLQQQVKTNGAGVTVLRR------------
CRLSAPFK----PGAGTLVTGQGCSGTSV---LRLSVAQGCHIRGAGGILVARHPAVWTR
CRLSARPEGRGRRMERGSPSRSNEVISPEILKMRAALFCIFTYLDTRTLAVARHPAVWTR
CRLSARPEGRGRRVERGSPSRSNEVISPEILKMRAALFCIFTYLDTRTLAVARHPAVWTR
CRLSARPEGRGRRAERGSPSRSNEVISPEILKMRAALFCIFTYLDTRTLAVARHPAVWTR
CRLSARPEGRGRRAERGSPSRSNEVISPEILKMRAALFCIFTYLDTRTLAVARHPAVWTR
CRLSARPEGRGRRAERVSPSRSNEVISPEILKMRAALFCIFTYLDTRTLAVARHPAVWTR
CRLSARPEGRGRRAERVSPSRSNEVISPEILKMRAALFCIFTYLDTRTLAVARHPAVWTR
CRLSARPEGRGRRAERVSPSRSNEVISPEILKMRAALFCIFTYLDTRTLAVARHPAVWTR
SGTWLAQP-------------ETTH-------SIRQTLSGLHELNLTG------------
FDLSGCNS---LFTSGTLLAQPEMA------QSVQQALSSLRELNLAG----------LD
LDLSGCNS---LFTSGILLAQPEMA------QSVQQALSGLCELNLAG------------
LDLSGCNS---LFTSGTLLAQPEMA------QSVQQALSGLRELNLAG------------
VQHPDDEDLSFSSAPSFNFLSNSCWAVDTSDLEVAEDPMGLQGIDLITALRGISDSRMVD
VQHPDDEDLSFSHAPSFNFLSNSCWAVDTSDLEVAEDPMGLQGIDLITALRGISDSRMAD
VQHPDDEGLSFSHAPSFNFLSNSSWAVDTSDLEVAEDPMGLQGIDLITALRGISDSRMAD
VQHPDDEGLSFSHAPSFNFLSNSCWAVDTSDLEVAEDPMGLQGIDLITALRGISDSRMAD
VQHPDDEGLSFSHAPSFNFLSNSCWAVDTSDLEVAEDPMGLQGIDLITALRGISDSRMAD
VQHPDDEGLSFSHAPSFNFLSNSCWAVDTSDLEVAEDPMGLQGIDLITALRGISDSRMAD
FQQPDEDDSPFSHAPSFKFLSNSWYAVDTSDLEVAEDPMGLQGIDLITALRGISDSRMTD
KDVNGSPLTSFKFLSNSWYIPKEDKAVDTSDLEVAEDPMGLQGIDLITALRGISDSRMVD
FVYGKLEAQEVKTVYTFKVPVSYCGRARLGDAMLSCKPSEHKAVDTSD--LGITVEDLPK
FVYGKLEAQEVKTVYTFKVPVSYCGRARLGDAMLSCKPSEHKAVDTSD--LGITVEDLPK
FVYGKLEAQEVKTVYTFKVPVSYCGRARLGDAMLSCKPSEHKAVDTSD--LGITVEDLPK
FVYGKLEAQEVKTVYTFKVPVSYCGRARLGDAMLSCKPSEHKAVDTSD--LGITVEDLPK
FVYGKLEAQEVKTVYTFKVPVSYCGRARLGDAMLSCKPSEHKAVDTSD--LGITVEDLPK
FVYGKLEAQEVKTVYTFKVPVSYCGRARLGDAMLSCKPSEHKAVDTSD--LGITVEDLPK
FVYGNLEAQEVKTVYTFKIPVSYCGRARLGDAMLKCRPSEHKAVDTSD--LGISVEDLPK
FVYGNLEAQEVKTVYTFKIPVSYCGRARLGDAMLRCRPSEHKAVDTSD--LGISVEDLPK
LVPWREMF---LERPRVRFDGVYIS--------KTTYIRQGEQSLDGF------YRAWHQ
LVPWREMF---LERPRVRFDGVYIS--------KTTYIRQGEQSLDGF------YRAWHQ
LVPWREMF---LERPRVRFDGVYIS--------KTTYIRQGEQSLDGF------YRAWHQ
LVPWREMF---LERPRVRFDVGVYI-------SKTTYIRQGEQSLDGF------YRAWHQ
LVPWREMF---LERPRVRFDGVYIS--------KTTYIRQGEQSLDGF------YRAWHQ
PRLRTRNT----RTDAILLGHYRLS-------QDTDNQTKVFAVITKK------------
LVPWREMF---LERPRVRFDGVYIS--------KTTYIRQGEQSLDGF------YRAWHQ
LVPWREMF---LERPRVRFDGVYIS--------KTTYIRQGEQSLDGF------YRAWHQ
NVAKIYKDLSRLFKDQLVYPLLAFT-------RQALNLPDVFGLVVLPEK--------RL
NVAKDLQKLSRLFKDQLVYPLLAFT-------RQALNLPDVFGLVVLP--LELKLRIFRL
NVAKDLQKLSRLFKDQLVYPLLAFT-------RQALNLPDVFGLVVLP--LELKLRIFRL
NVAKDLQKLSRLFKDQLVYPLLAFT-------RQALNLPDVFGLVVLP--LELKLRIFRL

```
HVAKDLQKLSRLFKDQLVYPLLAFT-------RQALSLPDVFGLVVLP--LELKLRIFRL
NVAKDLQKLSRLFKDQLVYPLLAFT-------RQALNLPDVFGLVVLP--LELKLRIFRL
SAAKDLKKLSRLFKDQLVYPLLAFT-------RQVLNLPDVFGLVVLP--LELKLRIFRL
SAAKDLKKLSRLFKDQLVYPLLAFT-------RQVLNLPDVFGLVVLP--LELKLRIFRL
LKPWPMVN-----QARLLYIIFGPISPQDGQVIWQKMIEEPADEFNLK------------
LKPWPMVN-----QARLLYIIFGPISPQDGQVVWQKMIEEPTDEFSLK------------
LKPWPMVN-----QARLLYIIFGPISPQDGQVVWQKMIEEPTDEFSLK------------
LKPWPMVN-----QARLLYIIFGPISPQDGQVVWQEMIEEPTDEFSLK------------
LKPWPMVN-----QARLLYIIFGPISPQDGQVVWQEMIEEPTDEFSLK------------
LKPWPMVN-----QARLLYIIFGPISPQDGQVVWQEMIEEPTDEFSLK------------
LKPWPMVN-----QARLLYIIFGPTSPHDGQVIWQEMIEGPTDESSLK------------
GLLKGLAH----TSDPRIWKKRIIKPWRNVKQLFILYISFHPSFPIPGVW--------IE
LNLWAKLD-----DTSLEFLQSRCTLVQW---LNLSWTGNRGFISVAGFRVRLELSCSHF
LNLWAKLG-----DTSLEFLQSRCTLVQW---LNLSWTGNRGFISVAGFRVRLELSCSHF
LNLWAKLD-----DTSLEFLQSRCTLVQWLNLSWTGNRGFISVAGFSR--VRLELSCSHF
LNLWAKLD-----DTSLEFLQSRCTLVQW---LNLSWTGNRGFISVAGFRVRLELSCSHF
LNLWAKLN-----DTSLEFLQARCTLVQWLNLSWTGNRGFISVTGFSR--VRLELSCSHF
LNLWAKLD-----DTSLEFLQSRCTLVQWLNLSWTGNRGFISVAGFSR--VRLELSCSHF
LNLWARLD-----DTSLEFLQARCVLVQW---LNLSWTGNRGFISVSGFRVRLELSCSHF
LNLWAKLD-----DTSLEFLQARCANLSWTGNRGFISVSGFSRFLKVC--VRLELSCSHF
FQLSSQHK----FNILILYSTTRKERDRAREEHTSAVNKIFSRQNEGD------------
FQLNNQHK----FNILILYSTTRKERDRAREEHTSAVNKIFSRQNEGD------------
FQLNNQHK----FNILILYSTTRKERDRAREEHTSAVNKMFSRQNEGD------------
FQLNNQHK----FNILILYSTTRKERDRAREEHTSAVNKMFSRHNEGD------------
FQLNNQHK----FNILILYSTTRKERDRAREEHTSAVNKMFSRHKEGD------------
FQLNNQHK----FNILILYSTTRKERDRAREEHTSAVNKMFSRHNEGD--------DQQG
FQLNNQQK----FNILILYSTTRKERDRAREEHTSTVNKMFSLQSEGD------------
FQLSNQHK----FNILILYSTTRKERDRAREEHTSTVNKMFSLQSEGD------------
---WGEIL----------------------------------------------------
---WGEIL----------------------------------------------------
---WGEIL----------------------------------------------------
---WGEIL----------------------------------------------------
---WGEIL----------------------------------------------------
---WGEIL----------------------------------------------------
---WGEIL----------------------------------------------------
-------------------------NIRS-------------------------------
IRS---------------------------------------------------------
LKMSSCPH---VSPAGILCVADQCHGLRELALNYHLLSDELLLALSSE--------KHVR
LKMSSCPH---VSPAGILCVADQCHGLRELALNYHLLSDELLLALSSE--------KHVR
LKMSSCPH---VSPAGILCVADQCHGLRELALNYHLLSDELLLALSSE--------KHVR
LKMSSCPH---VSPAGILCVADQCHGLRELALNYHLLSDELLLALSSE--------KHVR
LKMSSCPH---VSPAGILCVADQCHGLRELALNYHLLSDELLLALSSE--------KHVR
LKMSSCPH---VSPAGILCVADQCHGLRELALNYHLLSDELLLALSSE--------KHVR
LKMSSCPH---VSPAGILCVADQCHGLRELALNYHLLSDELLLALSSE--------KHVR
LKMSSCPH---VSPAGILCVADQCHGLRELALNYHLLSDELLLALSSE--------KHVR
```

```
LLLALSSE------THVNLEHLRID-------VVSENPGQIKFHSIKK------------
LKMSSCPH---VSSDGILCVADHCQGLRELALNYYILSDEILLALSSE--------THVN
PKMSSCPH---VSSDGILCVADRCQGLRELALNYYILTDELFLALSSE--------THVN
LKMSSCPH---VSSDGILCVADHCQGLRELALNYYILTDELFLALSSEH---------VN
LKISSCPH---VSSDGILCVADHCQGLRELALNYYILTDELLFALSSE--------THVN
LKMSSCPH---VSSDGILCVADHCQGLRELALNYYILTDELLLALSSEH---------VN
PTIRGNSIGNDIYGNALAGIQIRTNSCPIVRHNKIHDGQHGGIYVHEKQVTLAGVWVTTG
PTIRGNSIGNDIYGNALAGIQIRTNSCPIVRHNKIHDGQHGGIYVHEKQVTLAGVWVTTG
PTIRGNSIGNDIYGNALAGIQIRTNSCPIVRHNKIHDGQHGGIYVHEKQVTLAGVWVTTG
PTIRGNSIGNDIYGNALAGIQIRTNSCPIVRHNKIHDGQHGGIYVHEKQVTLAGVWVTTG
PTIRGNSIGNDIYGNALAGIQIRTNSCPIVRHNKIHDGQHGGIYVHEKQVTLAGVWVTTG
PTIRGNSIGNDIYGNALAGIQIRTNSCPIVRHNKIHDGQHGGIYVHEKQVTLAGVWVTTG
PTIRGNSIGNDIYGNALAGIQIRTNSCPIVRHNKIHDGQHGGIYVHEKQVTLAGVWVTTG
PTIRGNSIGNDIYGNALAGIQIRTNSCPIVRHNKIHDGQHGGIYVHEKQVTLAGVWVTTG
IAVWDMAS---ATDITLRRVLVGHR-----AAVNVVDFDDKYIVSASG------------
VRVWDVNT-----GEVLNTLIHHNE------AVLHLRFSNGLMVTCSK-------DRSIA
VRVWDVNT-----GEVLNTLIHHNE------AVLHLRFSNGLMVTCSK-------DRSIA
VRVWDVNT-----GEVLNTLIHHNE------AVLHLRFSNGLMVTCSK-------DRSIA
VRVWDVNT-----GEVLNTLIHHNE------AVLHLRFSNGLMVTCSK-------DRSIA
VRVWDVNT-----GEVLNTLIHHNE------AVLHLRFSNGLMVTCSK-------DRSIA
VRVWDVNT-----GEVLNTLIHHNE------AVLHLRFSNGLMVTCSK-------DRSIA
VRVWDVNT-----GEVLNTLIHHNE------AVLHLRFSNGLMVTCSK-------DRSIA
VRVWDVNT-----GEMLNTLIHHCE------AVLHLRFNNGMMVTCSK-------DRSIA
VRVWDVNA-----GEMLNTLIHHCE------AVLHLRFNNGMMVTCSK-------DRSIA
VRVWDVNA-----GEMLNTLIHHCE------AVLHLRFNNGMMVTCSK-------DRSIA
VRVWDVNT-----GEMLNTLIHHCE------AVLHLRFNNGMMVTCSK-------DRSIA
VRVWDVNT-----GEMLNTLIHHCE------AVLHLRFNNGMMVTCSK-------DRSIA
VRVWDVNT-----GEMLNTLIHHCE------AVLHLRFNNGMMVTCSK-------DRSIA
VRVWDVNT-----GEMLNTLIHHCE------AVLHLRFNNGMMVTCSK-------DRSIA
VRVWDVNT-----GEMLNTLIHHCE------AVLHLRFNNGMMVTCSK-------DRSIA
IKLWDCHN---REALATNNMLSSCQ------SLKAVFTKDGPVVLAGD--RIPDLHLISR
IKLWDCHN---REALATNNLKSPCK------SLKAVFTKDGPIVLIGD--RIPDLYLIST
IKLWDCHN-----REALATNNLESP-CKS---LKAVISKDGPIVLAGDLN----------
IKLWDCQN---SEALATSNLESPCK------LLKAVFTKDGPMVLIGD--RIPDLHLISK
IKLWDCNSSDALATNNLFFPCQTLKTKDAAIVLVSDTLGNLYIFRIPD------LHLIST
IKLWDCHN---SEALATNSLISPCQ------SLKAVITKDGPIVLIGD--RIPDLYHITR
IKLWDCHN---REALATNCLFSSCK------LLKAVFSKDDPIVLVGD--RIPDLHLISK
IKLWDCHN---REVLATKALSFSCQ------LLQAEFTKDGPIVLVGD--RIPDLYLISK
IKLWDCHN--KEVLASKTGLFFSCK------LLQSEFTKDGPIVLVGD--RIPDLHLISK
IKLWDCHN--KEVLASKTGLFFGCK------LLQSEFTKDGPIVLVGD--RIPDLHLISK
IKLWDCHN---RKVLATTGLLSSCQ------LLQAVFTNDSPIVLVGD--RIPDLHLISK
IKLWDCHN---RDALATKSIFSPCQ------ILKAVFTKDGPIVLVGD--RIPDLHLISR
IKLWDCHN---SEALATKRMVSSCQ------LLKAVITKDGPIVLIGD--RIPDLHLISE
EGHWHVAS-EFEVQKLVDYLEIVPNTGRY---PVAIATAGDLVYLLKADA--------LH
PNEWHVAS-EFEVQKLVDYLEIVPNTGRY---PVAIATAGDLVYLLKADA--------LH
```

PNEWQIAA----EFEVPKLVQRLEIVPETRRYPVAIAAAGDLMYLLKA----------ED
EGYWQIAA-EFEVPKLVQYLEIVPETGRY---PVAVAAAGDLMYLLKADA--------LL
PNEWQIAA-EFEVPKLVQYLEIVPE---TRRYPVAVAAAGDLMYLLKA----------ED
PNEWQIAA-EFEVPKLVQYLEIVPE---TRRYPVVVAAAGDLMYLLKA----------ED
PNEWQIAA-EFEVPKLVQYLEIVPETRRY---PVAVAAAGDLMYLLKA----------ED
PNEWQIAAPKLVQYLEIVPETRRYP-------VAVAAAGDLMYLLKAESR--------LL
LPLSKAPEGAASNPEEPWRNSLRCIKPGYDPCNLLVDLDDIRDLSSGF------SKYRDF
VKEWDIETPILAAKISETYIVSSCELTGHEGAVKCLFFNEWHLVSGGAASMAFKHPKEVL
VKEWDIET-----GKCLKTFKHKDP-------ILAARISETYIVSSCE----------RG
VKVWDVDTPILATRINDTYIVSACELSGHEGAVKCLFFDQWHLLSGSAWMAFKHPREVLH
VKVWDVDTPILATRINDTYIVSSCE--RGVVKVWHIVMAQLVKTLNGHLFTDGLVMAWSM
VKVWDVDTPILATRINDTYIVSSCELNGHEGAVKCLFFDQWHLLSGSTWMAFKHPKEVLD
VKVWDVDTPILATRINDTYIVSSCELSGHEGAVKCLFFDQWHLLSGSTWMAFKHPKEVLD
DGLWSMVGVLDVSLLFLRVISACADKANGRGDPVLSFFIQGNRMVVNTSVWQYAVEKTKQ
VKVWALSACLNTLTGHTEWVTKVGRLRRNLLVRLFEGGSGGCMMVYNS---------DQQ
VKVWALSALNTLTGHTEWVTKVVLQKCKV---KSLLHSPGDHILLSAD-------KYEIK
VKVWALSALNTLTGHTEWVTKVVLQKCKV---KSLLHSPGDYILLSAD-------KYEIK
VKVWALSALNTLTGHTEWVTKVVLQKCKV---KSLLHSPGDYILLSAD-------KYEIK
VKVWALSALNTLTGHTEWVTKVVLQKCKV---KSLLHSPGDYILLSAD-------KYEIK
VKVWALSALNTLTGHTEWVTKVVLQKCKV---KSLLHSPGDYILLSAD-------KYEIK
VKVWALSALNTLTGHTEWVTKVVLQQCKV---KSLLHSPGDYILLSAD-------KYEIK
VKVWALSALNTLTGHTEWVTKVVLQKCKV---KSLLHSPGDYILLSAD-------KYEIK
---RDVLD------DLVTLHNFRNQ-------FLPNALREFFRHIHAP------------
---DVLDD-------LVTLHNFRNQ-------FLPNALREFFRHIHAP------------
---DVLDD-------LVTLHNFRNQ-------FLPNALREFFRHIHAP------------
---DVLDD-------LVTLHNFRNQ-------FLPNALREFFRHIHAP------------
---DVLDD-------LVTLHNFRNQ-------FLPNALREFFRHIHAP------------
---DVLDD-------LVTLHNFRNQ-------FLPNALREFFRHIHAP------------
---DVLDD-------LVTLHNFRNQ-------FLPNALREFFRHIHAP------------
---DVLDD-------LVTLHNFRNQ-------FLPNALREFFRHIHAP------------
MQSFTCLHIGEEIDNEHPWSKPVSSDIEDAVEWRHRNVESLCVMETAS--TSCCYNKDIV
MQSYACLHIGEEIDNEHPWTKPVSSDIEDTVEWRHRNVESLCVMETAS--TSGCFSKDIV
MQSYACLHIGEEIDNEHPWTKPVSSDIEDTVEWKHRNVESLCVMETAS--TSGCYSKDIV
MQSYACLHIGEEIDNEHPWTKPVSSDIEDTVEWRHRNVESLCVMETAS--TSGCFSKDIV
MQSYACLHIGEEIDNEHPWTKPVSSDIEDTVEWRHRNVESLCVMETAS--TSGCFSKDIV
MQSYACLHIGEEIDNEHPWTKPVSSDIEDTVEWRHRNVESLCVMETAS--TSGCFSKDIV
MPSYACLHIGEEIDNEHSWTEPVSSDIEDAVEWRHRNVESLCVMETAS--SSGCYSKDIV
STPWTNKDPSTTQYACLHNLTNKGVHTWTEPVSSESFTPPYVWMLDAELDRNVESLCVME
LRVWDIET-----GQCLHVLMGHVA-------AVRCVQYDGRRVVSGA---------YDF
LRVWDIET-----GQCLHVLMGHVA-------AVRCVQYDGRRVVSGA---------YDF
LRVWDIET-----GQCLHVLMGHVA-------AVRCVQYDGRRVVSGA---------YDF
LRVWDIET-----GQCLHVLMGHVA-------AVRCVQYDGRRVVSGA---------YDF
LRVWDIET-----GQCLHVLMGHVA-------AVRCVQYDGRRVVSGA---------YDF
LRVWDIET-----GQCLHVLMGHVA-------AVRCVQYDGRRVVSGA---------YDF
LRVWDIET-----GQCLHVLMGHVA-------AVRCVQYDGRRVVSGA---------YDF

LRVWDIET-----GQCLHVLMGHVA-------AVRCVQYDGRRVVSGA---------YDF
GIEWDTKSKNDLNFEALINLERMLQQSERKYVQMLEIVRDVYVTPLKALSIQILFSDILR
VVEWDTKSKNDLNFEALINLERILQQSERKYVQILEIVRDVYVAPLKALSIQIIFCDILQ
VVEWDTKSKNDLNFEALINLERILQQSERKYVQILEIVRDVYVAPLKALSIQIIFCDILQ
VVEWDTKSKNDLNFEALINLERILQQSERKYVQMLEIVRNVYVAPLKALSIQIIFSDILQ
VVEWDTKSKNDLNFEALINLERILQQSERKYVQILEIVRDVYVAPLKAASIQIIFSDILQ
---WLTPR--

LQKGRMIGFDTLGMDSILNNQGPAQALADGLMELSKEGSEGSAIEDNS----QDSKSSPG
IKS--------KAQADPMFTLSHTH-------NIFILFYDFEKNLDGS------------
LPPWRSSD--KHPTDIIRFNYLDNC-------------DPLETVWQGR------------
LPPWRSSD--KHPTDIIRFNYLDNR-------------DPMETVQQGR------------
LPPWRSSD---------------------------KHPTDIIRFNYLD------------
LPPWRSSD-----KHPTDIIRFNYL-----------------------------------
LPPWRSSD-----KHPTDIIRFNYL-----------------------------------
LPPWRSSD--KHPTDIIRFNYLDNC--------------DPELFRLGR------------
LPPWRSSD--KHPTDIIRFNYLDNC--------------DPELFRLGR------------
CGISDLCV-----LNLIRYTATTKCSPSVDPERVLWSLRDHPLLPEAECRAGGVNVWALV
LEAWDNMRLGECTEEAYQMTHDGYLFVDEAQDCTPAIMNIVLSQPCGKHQALFTVPHTHV
LEAWDNMRLGECTEEAYQMTHDGYLFVDEAQDCTPAIMNIVLSQPCGKHQALFTVPHTHV
LEAWDNMRLGECTEEAYQMTHDGYLFVDEAQDCTPAIMNIVLSQPCGKHQALFTVPHTHV
LEAWDNMRLGECTEEAYQMTHDGYLFVDEAQDCTPAIMNIVLSQPCGKHQALFTVPHTHV
LEAWDNMRLGECTEEAHQMTHDGYLFVDEAQDCTPAIMNIVLSQPCGKHQALFTVPHTHV
LEAWDNMRLGECKEEAYQMTHDGYLFVDEAQDCTPAIMNIVLSQPCGKHQALFTVPHTHV
LEAWDNMRLGECKEEAYQMTHDGYLFVDEAQDCTPAIMNIVLSQPCGKHQALFTVPHTHV
NIPGNSTLVELERFVNLHSLALDFCDFTA---EMARVLTDSNHVPLQRSL----------
NIPGNSTLVELERFVNLHSLALDFCDFTA---EMARVLTDSNHVPLQRSL----------
NIPGNSTLVELERFVNLHSLALDFCDFTA---EMARVLTDSNHVPLQRSL----------
NIPGNSTLVELERFVNLHSLALDFCDFTA---EMARVLTDSNHVPLQR------------
NIPGNSTLVELERFVNLHSLALDFCDFTA---EMARVLTDSNHVPLQRSL----------
NIPGNSTLVELERFVNLHSLALDFCDFTA---EMARVLTDSNHVPLQRSL----------
NIPGNSTLVELERFVNLRSLALDFCDFTA---EMARVLTDSNHVPLQRSL----------
NIPGNSTLVELERFVNLRSLALDFCDFTA---EMARVLTDSNHVPLQRSL----------
NRMNALNLQVLIRRTGIPISMSLLYLTIARQLGVPLEPVNFPSHFLLRGTDAFGKGKQLT
NRMNALNLQVLIRRTGIPISMSLLYLTIARQLGVPLEPVNFPSHFLLRGTDAFGKGKQLT
NRMNALNLQVLIRRTGIPISMSLLYLTIARQLGVPLEPVNFPSHFLLRGTDAFGKGKQLT
NRMNALNLQVLIRRTGIPISMSLLYLTIARQLGVPLEPVNFPSHFLLRGTDAFGKGKQLT
NRMNALNLQVLIRRTGIPISMSLLYLTIARQLGVPLEPVNFPSHFLLRGTDAFGKGKQLT
NRMNALNLQVLTRRTGIPISMSLLYLTVARQLGVPLEPVNFPSHFLLRGTDAFGKGKQLT
NRMNALNLQVLTRRTGIPISMSLLYLTIARQLGVPLEPVNFPSHFLLRGTDAFGKGKQLT
PRSYTVQLALRKVSRCLPHLRVTCMGGTLIPRKQVPKAAELRAPDPPDGYRDLFGTLQAF
PRSYTVQLALRKVSRCLPHLRVTCMGKGFTPRKWVPKAAELRAPDPPDGYRDLFGTLQAF
PRSYTVQLALRKVSHCLPHLRVACMTSNQSSTLYITEQGGVYFEVHTP--RDLFGTLQAF
LYVLATRE---QQEVVGTTSSRACD------------CVEVYLQSSGQ-----------R
PRSYTVQLALRKVSHYLPHLRVACMVTDQGGVYFEVHTPGVYRDLFGT-------LQAFD

```
PRSYTVQLALRKVSHYLPHLRVACMVTDQGGVYFEVHTPGVYRDLFGTFPSLPAKILFCA
LATREQQE------VVGTTSSRACDCVEVYLQSSGQRVFKMTFHHSMTKI--------VG
PRSYTVQLALRKVSHYLPHLRVACMVTDQGGVYFEVHTPGVYRDLFGTFPSLPAKILFCA
ILHWQQQL------NNIQISRPFLKGLTFTDLPLCLQLNIMQRLSDGR------------
ILHWQQQL------NNIQITRPAFKGLTFTDLPLCLQLNIMQRLSDGR------------
ILHWQQQL------NNIQITRPAFKGLTFTDLPLCLQLNIMQRLSDGR------------
ILHWQQQL------NNIQITRPAFKGLTFTDLPLCLQLNIMQRLSDGR------------
ILHWQQQL------NNIQITRPAFKGLTFTDLPLCLQLNIMQRLSDGR------------
ILHWQQQL------NSIQISRPAFKGLTITDLPVCLQLNIMQRLSDGRL-----------
ILHWQQQL------NSIQISRPAFKGLTITDLPVCLQLNIMQRLSDGR------------
ILTWQQQL------QDLQMTKQVNNGLTLSDLPLHMLNNILYRFSDGW------------
ILAWQQQL------QDLQMTKQVNNGLTLSDLPLHMLNNILYRFSDGW------------
ILAWQQQL------QDLQMTKQVNNGLTLSDLPLHMLNNILYRFSDGW------------
ILAWQQQL------QDLQMTKQVNNGLTLSDLPLHMLNNILYRFSDGW------------
ILAWQQQL------QDLQMTKQVNNGLTLSDLPLHMLNNILYRFSDGW------------
ILAWQQQL------QDLQMTKQVNNGLTLSDLPLHMLNNILYRFSDGW------------
VLSWQQQL------QNLQVTKVNTG-LTLSDLPLHMLNNILYRFSDGW------------
---WKDYH--------LAWLLKDSG-----------------------------------
LGPRELCV---CMRVCRTWSRWCYDRRKS---LTPPMLSGVVRRQPRADS--------TG
LGPRELCV---CMRVCRTWSRWCYDDLSRRKSLTPPMLSGVVRRQPRA-----LDLSWTG
LGPRELCV---CMRVCRTWSRWCYDRRKS---LTPPMLSGVVRRQPRADS--------TG
LSEDEAPGRNGRRPARGSSGEKENRPLGPAPPPRPPQLERHVVRPPPRLLRAAWLRVFQH
LGPRELCI---CMRVCRTWSRWCYDRRKS---LTPPMLSGVVRRQPRADS--------TG
LGPRELCI---CMRVCRTWSRWCYDRRKS---LTPPMLSGVVRRQPRADS--------TG
LGPRELCI---CMRVCRTWSRWCYDRRKS---LTPPMLSGVVRRQPRADS--------TG
LGPRELCI---CMRVCRTWSRWCYDDLSRRKSLTPPMLSGVVRRQPRA-----LDLSWTG
LSRRELCE---CMRVCKTWYKWCCDRCKA---IVPQALSGIIKRQPVSDS--------TN
LSRRELCE---CMRVCKTWYKWCCDRCKA---IVPQALSGIIKRQPVSDS--------TN
LSRRELCE---CMRVCKTWYKWCCDRCKA---IVPQALSGIIKRQPVSDS--------TN
LSRRELCE---CMRVCKTWYKWCCDRCKA---IVPQALSGIIKRQPVSDS--------TN
LSRRELCE---CMRVCKTWYKWCCDRCKA---IVPQALSGIIKRQPVSDS--------TN
LSRRELCE---CMRVCKTWYKWCCDRCKA---IVPQALSGIIKRQPVSDS--------TN
LSRKELCE---CMRVCKTWYKWCCDRCKA---IVPQALSGIIKRQPVSDS--------TN
LSRRELCE---CMRVCKTWYKWCCDRCKA---IVPQALSGIIKRQPVSDS--------TN
LSHQDLCV---CMRVCRTWNRWCCDHCKS---ITPVMLSGIIRRQPVS----------LD
LSHQDLCV---CMRVCRTWNRWCCDHCKS---ITPLMLSGIIRRQPVS----------LD
RHQLGPSLPRVISRPPPSVSPPKCIQMERHVIRPPPISPPPDSLPLDDAH--------AV
LSHQDLCV---CMRVCRTWNRWCCDHCKS---ITPLMLSGIIRRQPVSDS--------TN
LSHQDLCV---CMRVCRTWNRWCCDHCKS---ITPLMLSGIIRRQPVSDS--------TN
LSHQDLCV---CMRVCRTWNRWCCDHCKS---ITPLMLSGIIRRQPVSDS--------TN
LSHRDLCV---CMRVCRTWNRWCCDRCKS---ITPLMLSGIIRRQPVSDS--------TN
LSHQDLCV---CMRVCRTWNRWCCDHCKS---ITPLMLSGIIRRQPVS-----LDLSWTN
ASEGESPAPEDTPPAPPPPPARDCGASGFHVDVVVTGVVDECIFFGKD---GTKNVKEET
ASEGDSPAPEDTPPAPPPPPARDCGASGFHVDVVVTGVVDECIFFGKD---GTKNVKEET
ASEGESPAPEDTPPAPPPPPARDCGASGFHVDVVVTGVVDECIFFGKD---GTKNVKEET
```

```
ASEGDSPAPEDTPPAPPPPPARDCGASGFHVDVVVTGVVDECIFFGKD---GTKNVKEET
ASEGDSPAPEDTPPAPPPPPARDCGASGFHVDVVVTGVVDECIFFGKD---GTKNVKEET
ASEGDSPAPEDTPPAPPPPPARDCGASGFHVDVVVTGVVDECIFFGKD---GTKNVKEET
ASEGETPAPEDTPPAPPPPPARDCGASGFHVDVVVTGVVDACIFFGKD---GTKNVKEET
ASEGETPAPEDTPPAPPPPPARDCGASGFHVDVVVTGVVDACIFFGKDTN--------ET
TAMKNSNREMTDELVGLAFSSHTYSASELPTDAVDCMSRELVSLTSQNELEEDQPSSLNS
TAIKNSNREMTDELVGLPFSSHTYSASELPTDAVDCMSRELVSLTSHN-DSITVSKVEKD
TAVKNSNREMTDELVGLPFSSHTYSASELPTDAVDCMSRELVSLTSQN-DSITVSKVEKD
TAVKNSNREMTDELVGLPFSSHTYSASELPTDAVDCMSRELVSLTSRN-PSITVSKVDKD
TAVKNSNREMTDELVGLPFSSHTYSASELPTGAVDCMSRELVSLTSQN-PSITVSKIDKD
LEPTAVKNEMTDELVGLPFSSHTYSQASELPTNAVDCMSRELVSLTSQELDKDQPSNLNS
IRRTVTKHSPEQRQDPLCISITVCTDRPAALDSLEEPLPGMLFFLSSG--REHPAPEASE
LTSHSPEQ----RQEPLCVSITVCTAQPSALDPLEEPLPGMLFFLSPG--SEHPAPEATE
CKSWEQIV------------QSTCD-----------------------------------
YDT---------------------------------------------------------
IVQSTCDT----------------------------------------------------
NKLQLQRQ----------------------------------------------------
NKLQLQRQ----------------------------------------------------
---STCDT----------------------------------------------------
---STCDT----------------------------------------------------
---STCDT----------------------------------------------------
ATLEVLAA---PDRSPFALLTLRCACPED---ARASPLPNETWAALRRHG--------VE
AILEALAA---PDRAPFALLALRCACPED---ARASPLPNEAWAALRRHG--------VE
AILEALAA---PDRAPFALLALRCACPED---ARASPLPNEAWVSLRRHG--------VE
AILEALAA---PDRAPFALLALRCACPED---ARASPLPNEAWVALRRHG--------VE
AILEALAA---PDRAPFALLALRCACPED---ARASPLPNEAWVALRRHG--------VE
AILEALAA---PDRAPFALLALRCACPED---ARASPLPNEAWVALRR----RHPGLAVE
AALELLAA---PHRSPFALLALRCACPED---ARASPLPDEAWATLSCHG--------VE
AALELLAA---PHRAPFTLLALKCA---CPEDARASPLPDEAWATLTC------------
YDLSNLRK--SAMIGCDRHVRVFCV--NPGLLVGLWQENGGLAFVMAN----IHSHGLFE
YDLSNLSE--STMMGCDGLIRIFCLNPGLLVGLWQKEDNLAFVMANLH---------FHH
YNLSHLTE--STMIGCDRLIRIFCLHPGL---LVGLWKKEEELAFLMALF--------VE
YNLSHLTV--STMIGCDRLIRIFCLHPGL---LVGVWKKEEELAFVMALF--------VE
YDLSHLTV--STMIGCDRLIRIFCLHPGL---LVGVWKKEEELAFVMALF--------VE
YNLSHLTI--STMIGCDRLIRIFCLHPGL---LVGVWKKEEELAFVMALF--------VE
RLRWHSLI----AKYNLSHLTVSTM-------IGCDRLIRIFCLHPGL----------LV
RLRWHSLIAKYNLSHLTVSTMIGCD-------RLIRIFCLHPGLLVGV------------
LPVEALQKLQVLRLLNLIWLPKPCG----RGVPQGPGFPSLEELCLAG-------STCNF
PVERGCPQLQVLRLLNLIWLPKPCGRGA----PQGPGFPSLEELCLAGT---------SF
IPL---------QLPVEALQKGCPQLQVPGSPPLPPVTPSPSYSLGS--AAAEPDVAAQ
LPVEALQKLQVLRLLNLMWLPKPLG-RGVAPGPGFPSLEELCLASSAC----------NF
LPVEALQKLQVLRLLNLMWLPKPPG-RGAAPGPGFPSLEELCLASSTC----------NF
LPVEALQKLQVLRLLNLMWLPKPLG-RGVAPGPGFPSLEELCLASSTC----------NF
LPVEALQKLQVLRLLNLMWLPKPPG-RGVAPGPGFPSLEELCLASSTC----------NF
LPVEALQKLQVLRLLNLMWLPKPPG-RGVAPGPGFPSLEELCLASSTC----------NF
```

```
LNLSGYRK--NLQKTDLCTIIKRCP------NLIRLDLSDSIMLKNDC------------
LNLSGYRK--NLQKTDLCTLIKRCP--------------NLVRLDLSD------------
LNLSGYRK--NLQKSDLSTLVRRCP------NLVHLDLSDSVMLKNDC------------
LNLSGYRK--NLQKSDLSTLVRRCP------NLVHLDLSDSVMLKNDC------------
LNLSGYRK--NLQKSDLSTLVRRCP------NLVHLDLSDSIMLKNDC------------
LNLSGYRK--NLQKSDLSTLVRRCP------NLVHLDLSDSVMLKNDC------------
LNLSGYRK--NLQKSDLSTLVRRCP------NLVHLDLSDSVMLKNDC------------
LSLQAYHV---TDTALAYFTARQGHSTHTLRLLSCWEITNHGVVNVVH------------
LSLQAYHV---TDTALAYFTARQGHSTHTLRLLSCWEITNHGVVNVVH------------
LSLQAYHV---TDTALAYFTARQGH---STHTLRLLSCWEITNHGVVN------------
LSLQAYHV---TDTALAYFTARQGHSTHTLRLLSCWEITNHGVVNVVH------------
LSLQAYHV---TDTALAYFTARQGHSTHTLRLLSCWEITNHGVVNVVH------------
LSLQAYHV---TDTALAYFTARQGHSTHTLRLLSCWEITNHGVVNVVH------------
LSLQAYHV---TDTALAYFTARQGHSTHTLRLLSCWEITNHGVVNVVH------------
LSLQAYHV---TDTALAYFTARQGHSTHTLRLLSCWEITNHGVVNVVH------------
LNIEDFFS-----HHLAVYGSSQFN--KAMATFRNLTFLTLNYNCISD-----------E
LAVYGSSQMATFHNLTFLTLNYNCISDELLETLSENNAGTLRTMNIKC---HVHDPHGQV
LNIEDYFS-----HHLAVYSSAQFK-------KTMSTFHNLVSLTLNY-----------N
LNIEDYFS-----HHLAVYSSPQFK-------KTMSTFHNLVSLNLNY------------
LNIEDYFS-----HHLAVYSSPQFK-------KTMSTFHNLVSLNLNY-------NCISD
LNIEDYFS-----HHLAVYNSPQFK-------KTMSTFHNLVSLNLNY----------NC
LNIEDYFS-----HHLAVYNSPQFK-------KTMSTFHNLVSLNLNY-------NCISD
LNIEDYFS-----HHLAVYNSPQFK-------KTMSTFHNLVSLNLNY-------NCISD
FQFWKQLDIASRSQNIIEINISDCRMSDNGVCVLAFKCPGLLRYTAYRKLSHCPLLQKVH
QSVKAFAE----HCPELQYVGFMGCSVTSKGVIHLTKLRNLSSLDLRH---------ITE
---FKCPG-------LLRYTAYRCK-----------QLSDTSIIAVAS------------
INISDCRS---LSDSGVCVLAFKCPGLLRYTAYRCKQLSDTSIIAVAS--QKVHVGNQDK
INISDCRS---MSDTGVCVLAFKCPGLLRYTAYRCKQLSDTSIIAVASCL---------DK
FQFWKQLDIASRSQNIIEINISDCRMSDNGVCVLAFKCPGLLRYTAYRKLSHCPLLQKVH
FQFWKQLDIASRSQNIIEINISDCRMSDNGVCVLAFKCPGLLRYTAYRKLSHCPLLQKVH
VKLWDMAA------DGQQFGEIKGK-----AAVLCLSYQPDILVTGTY-------DKKVT
VKLWDMAA------DGQQFGEIKGK----AAVLCLSYQPDILVTGTYD----------KK
VTIYDPRDRMETQDAPLGWGPLRGS---DFPPPWPIAHEAGPALLKSQ------------
VAIYDPREGMETRDAPMGWERGPSRGSDIPPPRPITHEASPALLKCQQ------------
VKLWDMAA-DGQQFGEIKASSAVLC-LSYLPDILVTGTYDKKVTIYDP----------RA
VTIYDPRDRMETRDALMGWGGPSRG--SDIPPHRPITHEAGPALLKHQ------------
VTIYDPRDRMETRDAPVGWGGPSRG--SDIPPRRPITHEAGPALLKHQ------------
VKLWDMAA-DGQQFGEIKASSAVLC-LSYLPDILVTGTYDKKVTIYDP----------RA
VGLLDNPE----LRVVLVFGYNCCKGASNYLHRVVSTFSDMNIILAGG-----------Q
VGLLDNPE----LRVVLVFGYNCCKGASNYLHRVVSTFSNMNIILAGG-----QVDNLSS
VGLLDNPE----LRVVLVFGYNCCKGANNFLQQVVSTFSDMNIILAGG-----QVDNLSS
VGLLDNPE----LRVVLVFGYNCCKGASNYLQQVVSTFSDMNIILAGG-----QVDNLSS
VGLLDNPE----LRVVLVFGYNCCKGASNYLQQVVSTFSDMNIILAGG-----QVDNLSS
VGLLDNPE----LRVVLVFGYNCCKGASNYLQQVVSTFSDMNIILAGG-----QVDNLSS
VGLLDNPE----LRVVLVFGYNCCKGASNYLQQVVSTFSDMNIILAGG-----QVDNLSS
```

```
VGLLDNPE----LRVVLVFGYNCCKGASNYLQQVVSTFSDMNIILAGG-----QVDNLSS
LYLRRCVR---LTDEGLRYLVIYCASIKELSVSDCRFVSDFGLREIAK------------
LYLRRCVR---LTDEGLRYLVIYCTSIKELSVSDCRFVSDFGLREIAK------------
LYLRRCVR---LTDEGLRYLVIYCTSIKELSVSDCRFVSDFGLREIAK------------
LYLRRCVR---LTDEGLRYLVIYCASIKELSVSDCRFVSDFGLREIAK------------
LYLRRCVR---LTDEGLRYLVIYCASIKELSVSDCRFVSDFGLREIAK------------
LYLRRCVR---LTDEGLRYLVIYCASIKELSVSDCRFVSDFGLREIAK------------
LYLRRCVR---LTDEGLRYLVIYCASIKELSVSDCRFVSDFGLREIAK------------
LYLRRCVR---LTDEGLRYLVIYCASIKELSVSDCRFVSDFGLREIAK------------
THPHHIHGQTALTIDYVLLFPRGCT-------LLLSYIASSSSFYIDCSS--------LN
LNLQSCSR---ITDEGVVQVCRGCHRLQALCLSGCSNLTDASLTALGL------------
LNLQSCSR---ITDEGVVQICRGCHRLQALCLSGCSNLTDASLTALGL------------
LNLQSCSR---ITDEGVVQICRGCHRLQALCLSGCSNLTDASLTALGL------------
LNLQSCSR---ITDEGVVQICRGCHRLQALCLSGCSNLTDASLTALGL------------
LNLQSCSR---ITDEGVVQICRGCHRLQALCLSGCSNLTDASLTALGL------------
LNLQSCSR---ITDDGVVQICRGCHRLQALCLSGCSNLTDASLTALGL------------
LNLQSCSR---ITDDGVVQICRGCHRLQALCLSGCSNLTDASLTALGL------------
LNLQTCLQ---ITDEGLITICRGCHKLQSLCASGCSNITDAILNALGQ------------
LNLQTCLQ---ITDEGLITICRGCHKLQSLCASGCSNITDAILNALGQ------------
LNLQTCLQ---ITDEGLITICRGCHKLQS---LCASGCSNITDAILNA------------
LNLQTCLQ---ITDEGLITICRGCHKLQSLCASGCSNITDAILNALGQ------------
LNLQTCLQ---ITDEGLITICRGCHKLQSLCASGCSNITDAILNALGQ------------
LNLQTCLQ---ITDEGLITICRGCHKLQSLCASGCSNITDAILNALGQ------------
LNLQTCLQ---ITDEGLITICRGCHKLQSLCASGCSNITDAILNALGQ------------
LNLQTCLQ---ITDEGLITICRGCHKLQSLCASGCSNITDAILNALGQ------------
LDLTACRQ---LKDEAIVYLAQRRG-------AGLRSLSLAVNANVGD------------
LDLTACRQ---LKDEAIVYLAQRRG-------AGLRSLSLAVNANVGD------------
LDLTACRQ---LKDEAIVYLAQRRG-------AGLRSLSLAVNANVGD------------
LDLTACRQ---LKDEAIVYLAQRRG-------AGLRSLSLAVNANVGD------------
LDLTACRQ---LKDEAIVYLAQRRG-------AGLRSLSLAVNANVGD------------
LDLTACRQ---LKDEAIVYLAQRRG-------AGLRSLSLAVNANVGD------------
LDLTACRQ---LKDEAIVYLAQRRG-------AGLRSLSLAVNANVGD------------
LDLTACRQ---LKDEAIVYLAQRRGGLRSLSLAVNANVGDAAVQELAR------------
LNPRSCRH---LSDVGIGHLAGMTR-------SAAEGCLGLEQLTLQD---------CQK
LTLQDCQK---LTDLSLKHISRGLTGLRLLNLSFCGGISDAGLLHLSH------------
LTLQDCQK---LTDLSLKHISRGLTGLRLLNLSFCGGISDAGLLHLSH------------
LTLQDCQK---LTDLSLKHISRGLTGLRLLNLSFCGGISDAGLLHLSH------------
LTLQDCQK---LTDLSLKHISRGLTGLRLLNLSFCGGISDAGLLHLSH------------
LTLQDCQK---LTDLSLKHISRGLTGLRLLNLSFCGGISDAGLLHLSH------------
LTLQDCQK---LTDLSLKHISRGLTGLRLLNLSFCGGISDAGLLHLSH------------
LIINDMPT---LTDNCVKALVEKCSGAPHISDCTFKALSTCKLRKIRFTAPDLSHIYMAD
LTINDMPT---LTDNCVKALVEKCSGAPHISDCTFRALSACKLRKIRFTAPNLSHIYMAD
LTINDMPT---LTDNCVKALVEKCSGAPHISDRTFKALSTCKLRKIRFTAPNLSHIYMAD
LTINDMPT---LTDNCVKALVEKCSGAPHITDCTFKALSTCKLRKIRFTAPNLSHIYMAD
LTINDMPT---LTDNCVKALVEKCSGAPHISDCTFRALSACKLRKIRFTAPNLSHIYMAD
```

```
LTINDMPT---LTDNCVKALVEKCSGAPHISDCTFRALSACKLRKIRFTAPNLSHIYMAD
LSSCDLKK--IRFEGNKRISDACFK-------SIDRNYPGINHIYMVD----------CK
GSLWNSID-----FSTVKNIADRCVVTTLQKWRLNVLRLNFRGCVLRA------------
VAVIGIAT----KRAPMQCQGYVAL-LGSDDQSWGWNLVDNNLLHNGE------------
VAVIGIAT----KRAPMQCQGYVAL-LGSDDQSWGWNLVDNNLLHNGE------------
VAVIGIAT----KRAPMQCQGYVAL-LGSDDQSWGWNLVDNNLLHNGE------------
VAVIGIAT----KRAPMQCQGYVAL-LGSDDQSWGWNLVDNNLLHNGE------------
LELHSCEIWLQKEQDPTVLPLLECIVLDRVPAFRDEHLQGLTRFRALR---------SLV
LELHSCEIWLQKEQDPTVLPLLECIVLDRVPAFRDEHLQGLTRFRALR---------SLV
LELHSCEIWLLKQQDPTVLPLLECIVLDRVPAFRDEHLQGLTRFRALR---------SLV
LELHSCEI-------SMAWLHKQQD-PTVLPLLECIVLDRVPAFRDEH------------
LELHSCEI-------SMAWLHKQQD-PTVLPLLECIVLDRVPAFRDEH------------
LELHSCEI-------SMAWLHKQQD-PTVLPLLECIVLDRVPAFRDEH------------
LELHSCEI-------SMAWLHKQQD-PTVLPLLECIVLDRVPAFRDEH------------
LELHSCEI-------SMAWLHKQQD-PTVLPLLECIVLDRVPAFRDEH------------
AKVWPLAS--GRLGQCLHTIQTEDR-------VWSIAISPLLSSFVTG------------
AKVWPLAS--GRLGQCLHTIQTEDR-------VWSIAISPLLSSFVTG------------
AKVWPLAS--GRLGQCLHTIQTEDR-------VWSIAISPLLSSFVTG------------
AKVWPLAS--GRLGQCLHTIQTEDR-------VWSIAISPLLSSFVTG------------
AKVWPLAS--GRLGQCLHTIQTEDR-------VWSIAISPLLSSFVTG------------
AKVWPLAS--GRLGQCLHTIQTEDR-------VWSIAISPLLSSFVTG------------
AKVWPLAS--GQLGQCLHTIQTEDR-------VWSIAISPVLSSFVTG------------
MLRYRCRAFLRLEGGGLIAGNNIYHGVDIRKKSNPLILCNQIHHGLRSNKSNKEAGIYIL
MLRYRCRAFLRLEGGGLIAGNNIYHGVDIRKKSNPLILCNQIHHGLRSNKSNKEAGIYIL
IVIEGSQSASSSPKPASKAGSQEAEGSDGERGAQTPDSSDGGLSPSGELYHPVLGGSFLG
NDIYRCRAFLRLEGGGLIAGNNIYHLCNQ---IHHGLRSGIVVLGNGKIRAGIYILYHGN
NDIYRCRAFLRLEGGGLIAGNNIYHLCNQ---IHHGLRSGIVVLGNGKIRAGIYILYHGN
NDIYRCRAFLRLEGGGLIAGNNIYHLCNQ---IHHGLRSGIVVLGNGKIRAGIYILYHGN
MLRYRCRAFLRLEGGGLIAGNNIYHGVDIRKKSNPLILCNQIHHGLRSNKSNKEAGIYIL
MLRYRCRAFLRLEGGGLIAGNNIYHGVDIRKKSNPLILCNQIHHGLRSNKSNKEAGIYIL
IKPKGTYGSHGLEIVMLSFHGSRARGTKITGDPNIPAGQQTVEIDLQR---------RIQ
------------------------------------------------------------
IKPKGTYGSHGLEIVMLSFHGRRARGTKITGDPNIPAGQQTVEIDLRH---------QIQ
IKPKGTYGSHGLEIVMLSFHGRRARGTKITGDPNIPAGQQTVEIDLRH---------RIQ
IKPKGTYGSHGLEIVMLSFHGRRARGTKITGDPNIPAGQQTVEIDLRH---------RIQ
IKPKGTYGSHGLEIVMLSFHGRRARGTKITGDPNIPAGQQTVEIDLRH---------RIQ
IKPKGTYGSHGLEIVMLSFHGRRARGTKITGDPNIPAGQQTVEIDLRH---------RIQ
IKPKGTYGSHGLEIVMLSFHGRRARGTKITGDPNIPAGQQTVEIDLRH---------RIQ
AQI--------EIYIADWWGARENCGCIYRLRVRLLDEHENEVVKFSA------------
GQIEICIA--------DWWGARENCGCIYRLRVRLLDEYENEVVKFSA------------
AQIEICVA--------DWWGARENCGCVYRLRVRLLDVYENEVVKFSA------------
---YCLRA----------------------------------------------------
AQIEICVA--------DWWGARENCGCVY---QLRVRLLDVYEKEVVK------------
AQIEICVA--------DWWGARENCGCVY---QLRVRLLDVYEKEVVK------------
AQIEICVA--------DWWGARENCGCVY---QLRVRLLDVYEKEVVK------------
```

```
GRIEICVS--------DWWGARHDSGCRY---RLLVQLLDANQTVLDK------------
GRIEICVS--------DWWGARHDSGCMY---RLLVQLLDANQTVLDK------------
GRIEICVS--------DWWGARHDSGCMY---RLLVQLLDANQTVLDK------------
GRIEICVS--------DWWGARHDSGCMY---RLLVQLLDANQTVLDK------------
GRIEICVS--------DWWGARHDSGCMY---RLLVQLLDANQTVLDK------------
-------------NPCGQWGARHDSGCMY---RLLVQLLDANQTVLDK------------
---SGGVE----IAVSDWWGARHDSGCKY---RLFVTLLDAHQNVIDK------------
---SGCKY------------------------RLFVTLLDAHQNVIEK------------
TQPAIVVK--------DWYSGRSDAGCLY---ELTVKLLSEHEDVLAE------------
TQPAIVVK--------DWYSGRSDAGCLY---ELTVKLLSEHEDVLAE------------
TQPAIVVK--------DWYSGRSDAGCLY---ELTVKLLSEHEDVLAE------------
TQPAIVVK--------DWYSGRSDAGCLY---ELTVKLLSEHENVLAE------------
TQPAIVVK--------DWYSGRSDAGCLY---ELTVKLLSEHENVLAE------------
TQPAIVVK--------DWYSGRTDAGSLY---ELTVRLLSENEDVLAE------------
TQPAIVVK--------DWYSGRTDAGSLYELTVRLLSEHEDVLAEFNT------------
---YHTAG---HRGQGLVRSQARLR-------VQVPAVRSAPVVRARA------------
--------------------------------VQVPSVRSAPVVHVRA------------
TRP--------DIEVKDWFAARPDCGSKY---QLCVQLLSSAHAPLGT------------
TRP--------DIEVKDWFAARPDCGSKY---QLCVQLLSSAHAPLGT------------
TRP--------DIEVKDWFAARPDCGSKY---QLCVQLLSSAHAPLGT------------
TRP--------DIEVKDWFAARPDCGSKY---QLCVQLLSSAHAPLGT------------
TRP--------DIEVKDWFAARPDCGSKY---QLCVQLLSSAHAPLGT------------
FRPDIVVK--------DWFAARHDCGCTY---QLKVYLASADYFVLDS------------
FRPDIVVK--------DWFAARADCGCTY---QLKVQLASADYFVLAS------------
FRPDIVVK--------DWFAARADCGCTY---QLKVQLASADYFVLAS------------
FRPDIVVK--------DWFAARADCGCTY---QLKVQLASADYFVLAS------------
FRPDIVVK--------DWFAARADCGCTY---QLKVQLTSADYFVLAS------------
FRPDIVVK--------DWFAARADCGCTY---QLKVQLASADYFVLAS------------
FRPDIVVK--------DWVAPRADCGCTY---QLRVQLASADYIVLAS------------
VRPDIVVK--------DWFAPRADCGCTY---HLRVQLASADYIVLAS------------

TTWKKILEDDKGAFQLYSKAIQ---RVTENN--KFSPHASTREYVMFRTPLASVQKSAAQ
TTWKKILEDDKGAFQLYSKAIQRVTENNNKF----SPHASTREFVMFRTPLASVQKSAAQ
TTWKKILEDDKGAFQLYSKAIQRVTENNNKF----SPHASTREYVMFRTPLASVQKSAAQ
TTWKKILEDDKGAFQLYSKAIQRVTENNNKF----SPHASTREYVMFRTPLASVQKSAAQ
TTWKKILEDDKGAFQLYSKAIQRVTENNNKF----SPHASTREYVMFRTPLSSVQKSAAQ
TTWKKILEDDKGAFQLYSKAIQRVTENNNKF----SPHASTREYVMFRTPLASVQKSAAQ
ILENNKGAFQLYSKTMQRVIESSKLSLHATT-RGYVVGRAALTCVQKSSTWAPPKKDVQI
-------TALTCVQKSSTWAPPKNVQPSQRG--------------QRGSVYSRHNEFLEV
LDGHGQLSDCALETKVAELLAQGHTKPPECN------DADTRNKYLIFTTGCLTYSPHQI
LHTSGSGHVKEGLRRVFDSVLDGHGQLSDCA----LETKVAELLAQGHTKPPECPDADSR
RHFLDSVLEGRAQPQLSDCVLETKVELLAQGKPPEHSDTGTRSKYLIFTTGCLTYSPHQI
RHFLDRVLEGRAQPQLSERVLETKVELLAQGKPPERSATGARSKYLIFTTGCLTYSPHQI
RHFLDRVLEGRAQPQLSERVLETKVELLAQGKPPERSATGARSKYLIFTTGCLTYSPHQI
RHFLDRVLEGRAQPQLSERMLETKVELLAQGKPPERSATGAKSKYLIFTTGCLTYSPHQI
```

IYTFTLPELRNISKVTAFQCAIVLLRCSPDKKWVFACGTYSRTFPQVFLAEDLLRPSEGS
GLRDVSKVTASQYGIVFLHCSPDKKIFACGT--------YRRTLPQVFLTESLLRPSEGS
GLRDVSKVTAFQYGIVLLHCSPDKKVFACGT--------YSRTLPQVFLTESLLRPSEGS
GLRDISKVTAFQYGIVLLHCSPDKKVFACGT--------YSRTLPQVFLTESLLRPSEGS
GLRDVSKVTAFQYGIVLLHCSPDKKVFACGT--------YSRTLPQVFLTESLLRPSEGS
GLRDVSKVTAFQYGIVLLHCSPDKKVFACGT--------YSRTLPQVFLTESLLRPSEGS
--IFRYVHDPECVATTGDITVSVSTSFLPEL-----SSVHPPHYFFTYRIRIEMSKDALP
--IFRYVHDPECVATTGDITVSVSTSFLPEL-----SSVHPPHYFFTYRIRIEMSKDALP
--IFRYVHDPECVATTGDITVSVSTSFLPEL-----SSVHPPHYFFTYRIRIEMSKDALP
--IFRYVHDPECVATTGDITVSVSTSFLPEL-----SSVHPPHYFFTYRIRIEMSKDALP
--IFRYVHDPECVATTGDITVSVSTSFLPEL-----SSVHPPHYFFTYRIRIEMSKDALP
--IFRYVHDPECVATTGDITVSVSTSFLPEL-----SSVHPPHYFFTYRIRIEMSKDALP
--IFRYIHDPECVATTGDITVSVSTSFLPEL-----SSVHPPHYFFTYRIRIEMSRDALP
--IFRYIHDPECVATTGDITVSVSTSFLPEL-----SSVHPPHYFFTYRIRIEMSRDALP
VPTFPHSSVSLTCSPDTQWVFVQGSDLGPKV--FYTQPLLHPSEDRLPVSTTLPIRLSSR
QWVLVSAQDSDLGPKVFYTQPLPSEDRLPASLPIRLSSRACWAPDEAARLMVTHRDDSGV
LTGFSYSDLVPCVLSLHKKCFHDDAPKDYRQKQRFEDKCYEEISREEVLSYADLCSTIGV
LTGFSYSDLTPCVLSLHKKCFHDDAPKDYRQKQRFEDKCYEEISQEEVLSYAELCSALGV
LTGFSSEDLIPCVLSLHKKCFHDDAPKDYRQKQRFEDKRYGEISQEEVLSYSQLCAALGV
LTGFSYEDLIPCVLSLHKKCFHDDAPKDYRQKQRFEDKRYGEISQEKVLSYSQLCAALGV
LTGFSYEDLIPCVLSLHKKCFHDDAPKDYRQKQRFEDKRYGEISQEEVLSYSQLCAALGV
LTGFSYEDLIPCVLSLHKKCFHDDAPKDYRQKQRFEDKRYGEISQEEVLSYSQLCAALGV
LTGFSYEDLIPCVLSLHKKCFHDDAPKDYRQKQRFEDKRYGEISQEEVLSYSQLCAALGV
LTGFSYEDLIPCVLSLHKKCFHDDAPKDYRQKQRFEDKRYGEISQEEVLSYSQLCAALGV
-----------ACPNLRLSAER--S-----------------------------------
-----------HGRALQTFHVDFCR----------------------------NVSAAGL
-----------VRRGLESLRAERPLETQPAP----------------GVSWGP-------
---------RRGLESLRAERPRETRSAPGVP-----------------------------
---------RRGLGSFRAERPSETRPAPGVS-----------------------------
---------RRGLGSLRAERPSETPPAPGVS-----------------------------
---------RRGLGSLRAERPSETPPAPGVS-----------------------------
----------------------RHESLVNDF-----------------------------
LDILTELKYRNLKHILAMVLDSLTAESLCSV--------WKVSRNWREIVVQDNANRRRK
LDILTELKYRNLKHILAMVLDSLTAESLCSV--------WKVSRNWREIVVQDNANRRRK
LDILTELKYRNLKHILAMVLESLTAESLCSV--------WKVSRNWREIVVQDNANRRRK
LDILTELKYRNLKHILAMVLDSLTAESLCSV--------WKVSRNWREIVVQDNANRRRK
LDILTELKYRNLKHILAMVLESLTAESLCSV--------WKVSRNWREIVVQDNANRRRK
LDILTELKYRNLKHILAMVLESLTAESLCSVREIVVQDKNANRRRKFYITQLKTDSEGAV
MGLSLAPRRGSLPDQKDLRLGSIDLDLKPAS-----GSNPMDSMDNRTVGGSMRHPPEQT
MGLSLAPRRGSLPDQKDLRLGSIDLDLKSASPMDGMDNRTVGSMRHPPEQTNGVHTPPHV
IGLSLAPRRGSLPDQKDLRLGSVDLDLKPAS-----SSNPMDGMDNRTVGGSMRHPPEQT
IGLSLAPRRGSLPDQKDLRLGSIDLDLKPAS-----SSNPMDGMDNRTVGGSMRHPPEQT
IGLSLAPRRGSLPDQKDLRLGSIDLDLKPAS-----SSNPMDGMDNRTVGGSMRHPPEQT
IGLSLAPRRGSLPDQKDLRLGSIDLDLKPAS-----SSNPMDGMDNRTVGGSMRHPPEQT
VGLSLAPRRGSLPDQKDLRLSSIDLDLKSAS-----SSSHVDSIDNRTVAGSVRHPPEQT

```
VGLSLVPRRGSLPDQKDLRLSSIDVDLKSAS-----TSSRVDSTDSRTVAGSVRHPPEQT
VQTCPNPFVGQSAPQPASVFWSLLKSLPPCSSEVAAIGQLTFLRHLTLAQLPGMLTGSGL
LIGSNFSSAMPRNEPAIRNSLPPCSRAQNVGSEVAAIGQLTFLRHLTLAQLPGMLTGSGL
LELIGSNFSSAMPRNEPAIRNSPCSRAQNVGSEVAAIGQLTFLRHLTLAQLPGMLTGSGL
LELIGSNFSSAMPRNEPAIRNSPCSRAQSVGSEVAAIGQLAFLRHLTLAQLPSVLTGSGL
LELIGSNFSSAMPRNEPAIRNSPCSRAQSVGSEVAAIGQLAFLRHLTLAQLPSVLTGSGL
LAQLPSVLTGSGLVSIGLQCQQLRSSLANLGYMPALSDMLKHCKRLRDLRLEQFSANAQF
LELIGSNFSSAMPRNEPAIRNSPCSRAQSVGSEVAAIGQLAFLRHLTLAQLPSVLTGSGL
LAQLPSVLTGSGLVNIGLQCQQLRSSLANLGYMPALSDMLKHCKRLRDLRLEQFSANAQF
ASSPSTASQSPDFARTVTSSGSSEPPEVDVSSVCPRCCCLRPQESQRRTGRCSERPSTSR
SPDFVRTVTSGGSSEPSPPEVDRQCVCSPGGAMEEGDAESSVCPRCCCLRPQESQRRTSR
SPDFVRTVNSGGSSEPSPTEVDRQCACSPGGAESSVCPRCCCHRPQESQRRTSRCSDEER
SPDFVRTVNSGGSSEPSPTEVDRQCACSPGGAESSVCPRCCCHRPQESQRRTSRCSDEER
SPDFVRTVNSGGSSEPSPTEVDRQCACSPGGAESSVCPRCCCHRPQESQRRTSRCSDEER
SPDFVRTVNSGGSSEPSPTEVDRQCACSPGGAESSVCPRCCCHRPQESQRRTSRCSDEER
SPDFVRTVNSGGSSEPSPTEVDRQCACSPGGAESSVCPRCCCHRPQESQRRTSRCSDEER
SPDFVRTVNSGGSSEPSPTEVDRQCACSPGGAESSVCPRCCCHRPQESQRRTSRCSDEER
-----------EISELRTKVQEQQKQLQDQD-------------------------QKLL
-----------EISELRTKVQEQQKQLQDQD-------------------------QKLL
-----------EISELRTKVQEQQKQLQDQD-------------------------QKLL
-----------EISELRTKVQEQQKQLQDQD-------------------------QKLL
-----------EISELRTKVQEQQKQLQDQD-------------------------QKLL
-----------EISELRTKVQEQQKQLQDQD-------------------------QKLL
-----------EISELRTKVQEQQKQLQDQD-------------------------QKLL
-----------EISELRTKVQEQQKQLQDQD-------------------------QKLL
VLLENARVCSKFLAMLAQWCTQAHSNLKPRQEEYARSTRGCLEAGLESPAEGQLGGNLLI
VLLENARVCSKFLAMLAQWCTQAHSNLKPRQEEYARSTRGCLEAGLESLLKAA-GGNLLI
VLLENARVCSKFLAMLAQWCTQAHSTLQNLKEEYARSTRGCLEAGLESLLKAA-GGNLLI
VLLENARVCSKFLAMLAQWCTQAHSTLQNLKEEYARSTRGCLEAGLESLLKAA-GGNLLI
VLLENARVCSKFLAMLAQWCTQAHSRQRGKKEEYARSTRGCLEAGLESLLKAA-GGNLLI
VLLENARVCSKFLAMLAQWCTQAHSRQRGKKEEYARSTRGCLEAGLESLLKAA-GGNLLI
VLLENARVCSKFLAMLAQWCTQAHSRQRGKKEEYARSTRGCLEAGLESLLKAA-GGNLLI
VLLENARVCSKFLAMLAQWCTQAHSRQRGKKEEYARSTRGCLEAGLESLLKAA-GGNLLI
------------LRDLADLSFN---RLSSCA-----------------------------
LADLSFNRRSSCAPSLERLSLAYCH-----------------------------------
------------LRDLADLSFN---RLSSCA-----------------------------
---------LRDLADLSFNQLSSCAPSLERL-----------------------------
VYHIDFGTQTFSLPSAILATNTMVGEIASAS-----ACDHANPQLSNPSPFQTLGLDLVL
IYHIDVGTQTFSLPSAILATNTMVGEIASAS-----ACDHANPQLSNPSPFQTLGLDLVL
IYHIDVGTQTFSLPSAILATSTMVGEIASAS-----ACDHANPQLSNPSPFQTLGLDLVL
IYHIDVGTQTFSLPSAILATSTMVGEIASAS-----ACDHANPQLSNPSPFQTLGLDLVL
IYHIDVGTQTFSLPSAILATSTMVGEIASAS-----ACDHANPQLSNPSPFQTLGLDLVL
IYHIDVGTQTFSLPSAILATSTMVGEIASAS-----ACDHANPQLSNPSPFQTLGLDLVL
VYHVDFGTQTFSLPSAILATNTMVGEIASAS-----ACDHANPQLSNPSPFQTLGLDLVL
VYHVDFGTQTFSLPSAILATNTMVGEIASAS-----ACDHANPQLSNPSPFQTLGLDLVL
```

```
SDLIKTTLQCALERELKGHVISESRSIDGLF--------MDFATQTYNFEPEQFSSGTVL
SDLIKTTLQCALERELKGHVISESRSIDGLF--------MDFATQTYNFEPEQFSSGTVL
SDLIKTTLQCALERELKGHVISESRSIDGLF--------MDFATQTYNFEPEQFSSGTVL
SDLIKTTLQCALERELKGHVISESRSIDGLFFEPEQFSSGTVLADLTAATPGGLHVELHS
SDLIKTTLQCALERELKGHVISESRSIDGLFFEPEQFSSGTVLADLTAATPGGLHVELHS
SDLIKTTLQCALERELKGHVISESRSIDGLFFEPEQFSSGTVLADLTAATPGGLHVELHS
SDLIKTTLQCALERELKGHVISESRSIDGLFDLATQTYNFEPEQFSSETVLADLLGTAQP
SDLIKTTLQCALERELKGHVISESRSIDGLFDLATQTYNFESEQFSSETVLADLLGTTQS
VEYYRYIRFFPDGHVMMLTTPEEPQSIVPRL------------RTRNTRTDAILLGHYRL
VEYYRYIRFFPDGHVMMLTTPEEPQSIVPRL------------RTRNTRTDAILLGHYRL
VEYYRYIRFFPDGHVMMLTTPEEPQSIVPRL------------RTRNTRTDAILLGHYRL
VEYYRYIRFFPDGHVMMLTTPEEPQSIVPRL------------RTRNTRTDAILLGHYRL
VEYYRYIRFFPDGHVMMLTTPEEPQSIVPRL-----RTRNTRTDAILLGHYRLSQDTDNQ
KEEKPLDYKYRYFRRVPVQEADQSFHVGLQL---------------------CSSGHQR
VEYYRYMRFFPDGHVMMLTTPEEPPSIVPRL------------RTRNTRTDAILLGHYRL
VEYYRYIRFFPDGHVMMLTTPEEPPSIVPRL-----RTRNTRTDAILLGHYRLSQDADNQ
LDVRSILSLSAVCRDLFTASNDPLLRFLYLR------DFRDSTVRVQDTDWKELYRKRHI
LDVRSVLSLSAVCRDLFTASNDPLLRFLYLR------DFRDNTVRVQDTDWKELYRKRHI
LDVRSVLSLSAVCRDLFTASNDPLLRFLYLR------DFRDNTVRVQDTDWKELYRKRHI
LDVRSVLSLSAVCRDLFTASND---PLLWRF--LYLRDFRDNTVRIQDTDWKELYRKRHI
LDVRSVLSLSAVCRDLFTASND---PLLWRF--LYLRDFRDNTVRVQDTDWKELYRKRHI
LDVRSVLSLSAVCRDLFTASNDPLLRFLYLR------DFRDNTVRVQDTDWKELYRKRHI
LDVHSVLALSAVCHDLLIASNDRCLYLRDFR---------DGTVRGPDTDWKELYRKKHI
LDVHSVLALSAVCHDLLIASNDRCLYLRDFR---------DSTIRGPDTDWKELYRKKHI
--GLADAIKLLYDSGTKEWTADDVISLVDELPHEWLLENNARLLMLTGNNICLFMASKAV
--GLADAIKLLYDASTKEWTADDVISLVDDLPREWLLENNARLLMLSGNNICFFMASKAV
--GLADAIKLLYDASTEEWTADDVISLVDELPREWLLENNARLLMLSGNNICFFMASKAV
--GLADAIKLLYDASTKEWTADDVISLVDELPREWLLENNARLLMLSGNNICFFMASKAV
--GLADAIKLLYDASTKEWTADDVISLVDELPREWLLENNARLLMLSGNNICFFMASKAV
--GLADAIKLLYDASTKEWTADDVISLVDELPREWLLENNARLLMLSGNNICFFMASKAV
--GLANAIKLLYDTGAKGWTADDVISLVDELPREWLLENNARLLILSGNNICFFMASKAV
GPTDESSLKGLANAIKLLYDTGAKGTADDVI--------------------------SLV
LNETCLEVISEMCPNLQALNLSSCDKLPPQA----FNHIAKLCSLKRLVLYRTKVEQTAL
LNETCLEVISEMCPNLQALNLSSCDKLPPQA----FNHIAKLCSLKRLVLYRTKVEQTAL
LNETCLEVISEMCPNLQALNLSSCDKLPPQA----FNHIAKLCSLKRLVLYRTKVEQTAL
LNETSLEVISEMCPNLQALNLSSCDKLPPQA----FNHISKLCSLKRLVLYRTKVEQTAL
LNETCLEVISEMCPNLQALNLSSCDKLPPQA----FNHISKLCSLKRLVLYRTKVEQTAL
LNETCLEVISEMCPNLQALNLSSCDKLPPQA----FNHIAKLCSLKRLVLYRTKVEQTAL
LNDTCLEVISEMCPNLQDLNLSSCDKLPPQA----FGHIAKLCSLKRLVLYRTKVEQTAL
LNDACLEVISEMCPNLQDLNLSSCDKLPPQA----FGHIAKLRSLKRLILYRTKVEQTAL
---DQQGSRYSVIPQIQKVCEVGFIYVANGEAHKRHKWQDEFSHIMAMTDPAFGSSGRPL
---DQQGSRYSVIPQIQKVCEVGFIYVANAEAHKRHEWQDEFSHIMAMTDPAFGSSGRPL
---DQQGSRYSVIPQIQKVCEVGFIYVANAEAHKRHEWQDEFSHIMAMTDPAFGSSGRPL
---DQQGSRYSVIPQIQKVCEVGFIYVANAEAHKRHEWQDEFSHIMAMTDPAFGSSGRPL
---DQQGSRYSVIPQIQKVCEVVDG-FIYVA--------------NAEAHKRHEWQDEFS
```

```
SRYSVIPQIQKVCEVVDGFIYVANAEAHKRH-----EWQDEFSHIMAMTDPAFGSSGRPL
---EQQGSRYSVIPQIQKVCEVGFIYVANAEAHRRHEWQDEFSRIMAMTDPAFGSSGRPM
---EQQGSRYSVIPQIQKVCEVGFIYVANAEAHRRHEWQDEFSRIMAMTDPAFGSSGRPM
------------------------------------------------------------
------------------------------------------------------------
------------------------------------------------------------
------------------------------------------------------------
------------------------------------------------------------
------------------------------------------------------------
------------------------------------------------------------
---------PVNLPEKAMCPMDADTEILDAE-----------------------------
---------PVSLPEKAMCPMDADT-----------------------------------
LEHLRIDVVSENPGQTHFHTIQKSSPKVNLVYEEEFDPFFRYEIPATHLYFGRSVSKDVL
LEHLRIDVVSENPGQTHFHTIQKSSPKVNLVYEEEFDPFFRYEIPATHLYFGRSVSKDVL
LEHLRIDVVSENPGQTHFHTIQKSSPKVNLVYEEEFDPFFRYEIPATHLYFGRSVSKDVL
LEHLRIDVVSENPGQTHFHTIQKSSPKVNLVYEEEFDPFFRYEIPATHLYFGRSVSKDVL
LEHLRIDVVSENPGQTHFHTIQKSSPKVNLVYEEEFDPFFRYEIPATHLYFGRSVSKDVL
LEHLRIDVVSENPGQTHFHTIQKSSPKVNLVYEEEFDPFFRYEIPATHLYFGRSVSKDVL
LEHLRIDVVSENPGQTHFHTIQKSSPKVNLVYEEEFDPFFRYEIPATHLYFGRSVSKDVL
LEHLRIDVVSENPGQTHFHTIQKSSPKVNLVYEEEFDPFFRYEIPATHLYFGRSVSKDVL
PSWDALVKHSPGVNVVMYFFLYEEE----------FDTFFKEETPVTHLYFGRSVSRTIL
LEHLRIDVVSENPGQIKFHSIKKRSPRVNVVYEEEFEAFFKEETPVTHLYFGRSVSRAIL
LEHLRIDVVSENPGQIKFHAVKKHSPRVNVVYEEEFETFFKEETPVTHLYFGRSVSKVVL
LEHLRIDVVSENPGQIKFHAIKKHSPRVNVVYEEEFETFFKEETPVTHLYFGRSVSRVVL
LEHLRIDVVSENPGQIKFHAIKKHSPRVNVVCEEEFEMFFKEETPVTHLYFGCSVSKVVL
LEHLQIDVVSENPGQIKFHAIKKHSPRVNVVYEEEFETFFKEETPVTYLYFGRSVSKSVL
STPVLRRNRIHSGKQVGVYFYDNGHGVLEDNYSGVQIRTGSNPKIRRNKIWGGLVYNSGL
STPVLRRNRIHSGKQVGVYFYDNGHGVLEDNYSGVQIRTGSNPKIRRNKIWGGLVYNSGL
STPVLRRNRIHSGKQVGVYFYDNGHGVLEDNYSGVQIRTGSNPKIRRNKIWGGLVYNSGL
STPVLRRNRIHSGKQVGVYFYDNGHGVLEDNYSGVQIRTGSNPKIRRNKIWGGLVYNSGL
STPVLRRNRIHSGKQVGVYFYDNGHGVLEDNYSGVQIRTGSNPKIRRNKIWGGLVYNSGL
STPVLRRNRIHSGKQVGVYFYDNGHGVLEDNYSGVQIRTGSNPKIRRNKIWGGLVYNSGL
STPVLRRNRIHSGKQVGVYFYDNGHGVLEDNYSGVQIRTGSNPKIRRNKIWGGLVYNSGL
STPVLRRNRIHSGKQVGVYFYDNGHGVLEDNYSGVQIRTGSNPKIRRNKIWGGLVYNSGL
------------DRTIKVWSTSTCE----------------------------FVRTLNG
VWDMASATDITLRRVLVGHRAAVNVDFDDKY---IVSASGDRTIKVWSTSTCEFVRTLNG
VWDMASATDITLRRVLVGHRAAVNVDFDDKY---IVSASGDRTIKVWSTSTCEFVRTLNG
VWDMASATDITLRRVLVGHRAAVNVDFDDKY---IVSASGDRTIKVWSTSTCEFVRTLNG
VWDMASATDITLRRVLVGHRAAVNVDFDDKY---IVSASGDRTIKVWSTSTCEFVRTLNG
VWDMASATDITLRRVLVGHRAAVNVDFDDKY---IVSASGDRTIKVWSTSTCEFVRTLNG
VWDMASATDITLRRVLVGHRAAVNVDFDDKY---IVSASGDRTIKVWSTSTCEFVRTLNG
VWDMASATDITLRRVLVGHRAAVNVDFDDKY---IVSASGDRTIKVWSTSTCEFVRTLNG
VWDMASPTDITLRRVLVGHRAAVNVDFDDKY---IVSASGDRTIKVWNTSTCEFVRTLNG
VWDMASPTDITLRRVLVGHRAAVNVDFDDKY---IVSASGDRTIKVWNTSTCEFVRTLNG
VWDMASPTDITLRRVLVGHRAAVNVDFDDKY---IVSASGDRTIKVWNTSTCEFVRTLNG
```

```
VWDMASPTDITLRRVLVGHRAAVNVDFDDKY---IVSASGDRTIKVWNTSTCEFVRTLNG
VWDMASPTDITLRRVLVGHRAAVNVDFDDKY---IVSASGDRTIKVWNTSTCEFVRTLNG
VWDMASPTDITLRRVLVGHRAAVNVDFDDKY---IVSASGDRTIKVWNTSTCEFVRTLNG
VWDMASPTDITLRRVLVGHRAAVNVDFDDKY---IVSASGDRTIKVWNTSTCEFVRTLNG
VWDMASPTDITLRRVLVGHRAAVNVDFDDKY---IVSASGDRTIKVWNTSTCEFVRTLNG
VHVFQYGINELYCSPQKKWVFLKHSQILPKVSLLRTSEFSAPASTILSFPLCQPRREDRI
VNVLPYGFDGIYCSPQKKWVLLKHPHILPKVEFSAPVSTVLKLSLYERVFWTPRREDRIT
------IPDLHLISTVNVFPCGFDKDCSPQK------KWVLLSQNHPCIYPKVFYMSSLL
LKVFQCSINQLNCSPQKKWIFLKHPHILPKVSLLRTSEFSAPVSTILKFSLCQRMEDRIT
INVFPYGINELYCSPQKKWVFLKHPHILPKVSLLRRSEFSAPVSTVLNFSLCDPRKEDRI
LKVFPYGISELYCSPQKKWIFLKHPHILPKVSLLRTSEFSAPVSTDLKFSLCQPRREDRI
VNVFPYGIDELHCSPQKKWVFLKHPRVLTKVSLLRTSEFSAPVSTILKFSLCDRREDRIT
VNVFPYSIDELYCSPQKKWVFLMGKRLHDLIEFSAPVSTVLEVPLIRKVFWTPRREDRIT
VNVFPYSIDELYFSPQKKWVFLIGHRLNDLTEFSAPVSTVLEVPLIRKVFWTPRREDRIT
VNVFPYSIDELYFSPQKKWVFLIGHRLNDLTEFSAPVSTVLKVPVIRKVFWTPRREDRIT
VNVFPYGIDELHCSPQKKWVLLRHVSLLRTSEFSAPVSTDLKFSLCQRAFWTPRREDRIT
HNVFPYAIDELHCSPQNKWVLLRNPQVLTKVEFSDPVSTSLKFPLCQGVFWTPRREDRIT
VNVFPFAIDELHCSPQKKWVFLKHPHVLTKVSLLRPTEFSDPVSTVLEFSLCKRREDRIT
YVYGQPATCLDVSASQVAFGVKSLGYEGNKI------LVYSLEAERCLSKLGNALGDFTC
YVYGQPATCLDVSASQVAFGVKSLGVYEGNK-----ILVYSLEAERCLSKLGNALGDFTC
SARTLLYAHGPPVTCLDVSANQVAFGVQGLGYEGSKILVYSLEAGCHLLKLGNVLRDFTC
YAHGPPVTCLDVSANQVAFGVQGLGVYEGSK-----ILVYSLEAGRRLLKLGNVLRDFTC
SARTLLYAHGPPVTCLDVSANQVAFGVQGLGYEGSKILVYSLEAGCRLLKLGNVLRDFTC
SARTLLYAHGPPVTCLDVSANQVAFGVQGLGYEGSKILVYSLEAGRRLLKLGNVLRDFTC
SARTLLYAHGPPVTCLDVSANQVAFGVQGLGYEGSKILVYSLEAGRRLLKLGNVLRDFTC
YAHGPPVTCLDVSANQVAFGVQGLGVYEGSK-----ILVYSLEAGRRLLKLGNVLRDFTC
IRYLPIHLSKYILRMLDRHTLNKCASVSQHWMDLSAQGFIQNQITFLQGSYTRGIDPNYA
QVSLLYLRVISACGDGKIRIYNNCLKVIKVDRMVAHTDSNILVFQFENVKWQYSSDKNKV
IVKVWHVVTAQLQKTLTGHEGAVKCLFFNQW------HLVSGGADGLVMAWSM-VGKYER
VFLLFLRVISACADGKIRIYNFGNCRGDPVLQFEHIKWQYAMEKIKQEKNKEKVHSPRES
VGKYERCLMAFKHPKMVVNTESNVLKQKNKK-EKEEEKEENSLMEILSKSNMQVHSPRES
VSLLFLRVISACADGKIRIYNFGNCRGDPVLQFEHIKWQYAVEKTKQKKNKEKVHSPRES
VSLLFLRVISACADGKIRIYNFGNCRGDPVLQFEHIKWQYAVEKTKQKKNKEKVHSPRES
KKNKEKEEEKEENSLMEILSKCNIQVHSPRESKQTVIQELLPGKPPKSRVLLKKSPRRDA
IWPIGREINCKCLKTLSVSEDRSIC-LQPRL---HFDGKYIVCSSALGLYQWDFASYDIL
IWPIGREINCKCLKTLSVSEDRSIC-LQPRL---HFDGKYIVCSSALGLYQWDFASYDIL
IWPIGREINCKCLKTLSVSEDRSIC-LQPRL---HFDGQYIVCSSALGLYQWDFASYDIL
IWPIGREINCKCLKTLSVSEDRSIC-LQPRL---HFDGKYIVCSSALGLYQWDFASYDIL
IWPIGREINCKCLKTLSVSEDRSIC-LQPRL---HFDGKYIVCSSALGLYQWDFASYDIL
IWPIGREINCKCLKTLSVSEDRSIC-LQPRL---HFDGKYIVCSSALGLYQWDFASYDIL
IWPIGREINCKCLKTLSVSEDRSIC-LQPRL---HFDGKYIVCSSALGLYQWDFASYDIL
IWPIGREINCKCLKTLSVSEDRSIC-LQPRL---HFDGKYIVCSSALGLYQWDFASYDIL
------EERGEYLETLITKFSHRFC----------------------------ACNPDLM
------EERGEYLETLITKFSHRFC----------------------------ACNPDLM
------EERGEYLETLITKFSHRFC----------------------------ACNPDLM
```

```
------EERGEYLETLITKFSHRFC----------------------------ACNPDLM
------EERGEYLETLITKFSHRFC----------------------------ACNPDLM
------EERGEYLETLITKFSHRFC----------------------------ACNPDLM
------EERGEYLETLITKFSHRFC----------------------------ACNPDLM
------EERGEYLETLITKFSHRFC----------------------------ACNPDLM
GLRTSVCWQQHCASPAFAYCGH---SFCCTGALRTMSALPESSAMCRKASRTRLPREKDL
GLRTSVCWQQHCASPAFAYCGH---SFCCTGALRTMSSLPESYAMCRKVARTRLPRGKDL
GLRTSVCWQQHCASPAFAYCGH---SFCCTGALRTMSTLPESSAMCRKASRTRLPRGKDL
GLRTSVCWQQHCASPAFAYCGH---SFCCTGALRTMSALPESSAMCRKASRTRLPRGKDL
GLRTSVCWQQHCASPAFAYCGH---SFCCTGALRTMSSLPESSAMCRKAARTRLPRGKDL
GLRTSVCWQQHCASPAFAYCGH---SFCCTGALRTMSSLPESSAMCRKAARTRLPRGKDL
GLRTSVCWQQHCASPAFAYCGH---SFCCTGALRTMTTLPATSAMCRKALRTTLPRGKDL
TASNFGCSSSGCYSKDIVGLRTSVCAFAYCGALRTMSALPAASAVCRKALRTTLPRGKDL
MVKVWDPETETCLHTLQGHTNRVYSQFDGIH---VVSGSLDTSIRVWDVETGNLTGHQSL
MVKVWDPETETCLHTLQGHTNRVYSQFDGIH---VVSGSLDTSIRVWDVETGNLTGHQSL
MVKVWDPETETCLHTLQGHTNRVYSQFDGIH---VVSGSLDTSIRVWDVETGNLTGHQSL
MVKVWDPETETCLHTLQGHTNRVYSQFDGIH---VVSGSLDTSIRVWDVETGNLTGHQSL
MVKVWDPETETCLHTLQGHTNRVYSQFDGIH---VVSGSLDTSIRVWDVETGNLTGHQSL
MVKVWDPETETCLHTLQGHTNRVYSQFDGIH---VVSGSLDTSIRVWDVETGNLTGHQSL
MVKVWDPETETCLHTLQGHTNRVYSQFDGIH---VVSGSLDTSIRVWDVETGNLTGHQSL
MVKVWDPETETCLHTLQGHTNRVYSQFDGIH---VVSGSLDTSIRVWDVETGNLTGHQSL
ILSLNRQFLDNLRDRLQEWGPAHCIEIVTKFPVVLKTIDKCREMIPAFRTFLKRHDKTIA
ILSLNRQFLDNLRDRLQEWGPAHCVEIVTKFPVILKTIEKCREMIPAFRTFLKRHDKTIV
ILSLNRQFLDNLRDRLQEWGPAHCVEIVTKFPVILKTIEKCREMIPAFRTFLKRHDKTIV
ILSLNRQFLDNLRDRLQEWGPTHCVSQLNTYPVILKTIEKCREMIPAFRTFLKRHDKTIV
ILNLNRQFLDNLRDRLQEWGPALCVSQLNTYPVILKTIEKCREMIPAFRTFLKRHDKTIV
---------------------E--------------------------------------
------------------------------------------------------------
QSNLNVEVLVKLERKLQMNSVETRAQVVREL--VQSERRYVQMLRIVQDVYAKPLRAALS
--------------KIKYTKRRKRNEMTPDF-----------------------------
------KKRNEMTPDFSRQSHDKKNKLQDRT-----------------------------
------RKRNQMTPDFSRQSHDKKNKLQDRT----------------------RLRKAQS
----------NCDPMETVWQGGRKR-----------------------------------
---------DNCDPMETVWQGRRKRNQMTPD-----------------------------
---------DNCDPMETVWQGRRKRNQMTPD-----------------------------
------RKRSEVTPDFKRQLRDKKNKLQDRA----------------------RLRKAQS
------RKRKEVTPDFKQQFRDKKNKLQDRA-----------------------------
AAMVLLSSSVNDIQQLLFCLRRPSSTVTMPD---VTETLYCIAVLLYAMREKGINISNRI
FYLTQSFRFGVEIAYVGATILDVCKRVRKKTHQSGIRGDAKGQVALLSRTNANVFDEAVR
FYLTQSFRFGVEIAYVGATILDVCKRVRKKTHQSGIRGDVKGQVALLSRTNANVFDEAVR
FYLTQSFRFGVEIAYVGATILDVCKRVRKKTHQSGIRGDAKGQVALLSRTNANVFDEAVR
FYLTQSFRFGVEIAYVGATILDVCKRVRKKTHQSGIRGDAKGQVALLSRTNANVFDEAVR
FYLTQSFRFGVEIAYVGATILDVCKRVRKKTHQSGIRGDAKGQVALLSRTNANVFDEAVR
FYLTQSFRFGVEIAYVGATILDVCKRVRKKTHQSGIRGDIKGQVALLSRTNANVFDEAVR
FYLTQSFRFGVEIAYVGATILDVCKRVRKKTHQSGIRGDVKGQVALLSRTNANVFDEAVR
```

```
------VHNVSVMHKSLENMPNDEHKALSRKRVYIMAFDTKSEDMLKILKPSMPLERIHF
------VHSVSIMHKSLDNMPNDEHKALSRKRVYIMAFDIKSEDMLKILKPSIPLERIHF
------VHNVSIMHKSLDNMPNDEHKALSRKRVYIMAFDIKSEDMLKILKPSIPLERIHF
--LSLLVHNVSVMHKSLDNMPNDEHKALSRKRVYIMAFDIKSEDMLKILKPSIPLERIHF
------VHNVSVMHKSLDNMPNDEHKALSRKRVYIMAFDIKSEDMLKILKPSIPLERIHF
------VHNVSVMHKSLDNMPNDEHKALSRKRVYIMAFDIKSEDMLKILKPSIPLERIHF
------VHNASVMLKSLDNMPNDEHALSRKSRVYLMVFDIKSEDMLKILKPSIPLERVHF
------VHNASVMLKSLDNMPNDEHKALSRKRVYLMAFDVKSEDMLKILKPSIPLERVHF
VKECEYLIGQHVTAALYGVVNVQRMNLLSLGKREGIDQSYQLLRDSLDLYLA-MYPDQVQ
VKECEYLIGQHVTAALYGVVNVVLQRMVGNLKREGIDQSYQLLRDSLDLYLAMYPDQVQL
VKECEYLIGQHVTAALYGVVNVVLQRMVGNLKREGIDQSYQLLRDSLDLYLAMYPDQVQL
VKECEYLIGQHVTAALYGVVNVVLQRMVGNLKREGIDQSYQLLRDSLDLYLAMYPDQVQL
VKECEYLIGQHVTAALYGVVNVVLQRMVGNLKREGIDQSYQLLRDSLDLYLAMYPDQVQL
VKECEYLIGQHVTAALYGVVNVVLQRMVGNLKREGIDQSYQLLRDSLDLYLAMYPDQVQL
VKECEYLIGQHVTAALYGVVNVVLQRMVGNLKREGIDQSYQLLRDSLDLYLAMYPDQVQL
DPLDHQMPLALSLPAKVLFCALNHLGLVDEFNRYGQLGTGDKMDRGEPTQVHYLQRPIAL
DPLDHQMPLALSLPAKVLFCALNHLGLVDEFNRYGQLGTGDKMDRGEPTQVHYLHRPIAL
DPLDQQMPLALSLPAKILFCALNHLGLVDEFNRYGQLGTGDKMDRGEPTQVRYLQRPIAL
VFKMTFHHSMAFKQIVLVGQETALLLLTEEG---------------------KIYSLVV
PLDQQMPLALSLPAKILFCALGGLVDEFGRINRYGQLGTGDKMDRGEPTQVCYLQRPITL
LGYNHLGLVDEFGRIFMQGNNRGTGDKMDRG-----EPTQVRYLQRPITLWC-GLNHSLV
QETQRALLLLTEEGKIYSLVVNETQLDQPRS---------------------YTVQLAL
LGYNHLGLVDEFGRIFMQGNNRGTGDKMDRG-----EPTQVRYLQRPVTLWC-GLNHSLV
----DLVSLGQAAPDLHVLSEDRLLKKLCQY---------------------HFSERQI
----DLVSLGQAAPDLHVLSEDRLLKKLCQY---------------------HFSERQI
----DLVSLGQAAPDLHVLSEDRLLKKLCQY---------------------HFSERQI
----DLVSLGQAAPDLHVLSEDRLLKKLCQY---------------------HFSERQI
----DLVSLGQAAPDLHVLSEDRLLKKLCQY---------------------HFSERQI
------VSLGQAAPDLHVLSEDRLLKRLCQY---------------------HFSERQI
----DLVSLGQAAPDLHVLSEDRLLKRLCQY---------------------HFSERQI
----DIITLGQVTPTLYMLSEDRQLKKLCQY---------------------HFAEKQF
----DIVTLGQVTPTLYMLSEDRQLKKLCQY---------------------HFAEKQF
----DIITLGQVTPTLYMLSEDRQLKKLCQY---------------------HFAEKQF
----DIITLGQVTPTLYMLSEDRQLKKLCQY---------------------HFAEKQF
----DIITLGQVTPTLYMLSEDRQLKKLCQY---------------------HFAEKQF
----DIITLGQVTPTLYMLSEDRQLKKLCQY---------------------HFAEKQF
----DIVTLGQVTPTLYMLSEDRRLKRLCQY---------------------HFAEQQF
-------------HPCTAADPDSCF-----------------------------------
VSKKQLMWLLNRLQGLQELVLSGCSSVSALG------SAPLPALRLLDLRWIEDVKDSQL
VSKKQLMWLLNRLQGLQELVLSGCSSVSALG------SAPLPALRLLDLRWIEDVKDSQL
VSKKQLMWLLNRLQGLQELVLSGCSSVSALG------SAPLPALRLLDLRWIEDVKDSQL
LGPRELCICMRVCRTWSRWCYDKRLPRMDLSTPPMLSGVVRRQPRALDLSWTG-VSKKQL
VSKKQLMWLLNRLQGLQELVLSGCSSVSALG------SAPLPALRLLDLRWIEDVKDSQL
VSKKQLMWLLNRLQGLQELVLSGCSSVSALG------SAPLPALRLLDLRWIEDVKDSQL
VSKKQLMWLLNRLQGLQELVLSGCSSVSALG------SAPLPALRLLDLRWIEDVKDSQL
```

VSKKQLMWLLNRLQGLQELVLSGCSSVSALG------SAPLPALRLLDLRWIEDVKDSQL
ISKKQLTWLVNRLPGLKDLLLAGCSAVSALS------TSSCPLLRTLDLRWAVGIKDPQI
ISKKQLTWLVNRLPGLKDLLLAGCSSAVSAL-----STSSCPLLRTLDLRWAVGIKDPQI
ISKKQLTWLVNRLPGLKDLLLAGCSSAVSAL-----STSSCPLLRTLDLRWAVGIKDPQI
ISKKQLTWLVNRLPGLKDLLLAGCSSAVSAL-----STSSCPLLRTLDLRWAVGIKDPQI
ISKKQLTWLVNRLPGLKDLLLAGCSSAVSAL-----STSSCPLLRTLDLRWAVGIKDPQI
ISKKQLTWLVNRLPGLKDLLLAGCSSAVSAL-----STSSCPLLRTLDLRWAVGIKDPQI
ISKKQLTWLVNRLPGLKDLLLAGCSSAVSAL-----STSSCPLLRTLDLRWAVGIKDPQI
ISKKQLTWLVNRLPGLKDLLLAGCSSAVSAL-----STSSCPLLRTLDLRWAVGIKDPQI
VSW-------TICKKQLSWLINRLLRDLVLSAVSALCSSSCPLLRTLDVQWVEGLKDAQM
LSW------TNISKKQLSWLINRLPRDLVLSAVSALCSSSCPLLRTLDVQWVEGLKDAQM
FSYLSHQDLCVCMRVCRTWNRLRDLVLSGCSAVSALCSSSCPLLRTLDVQWVEGLKDAQM
ISKKQLSWLINRLPGLRDLVLSGCSIAVSAL-----CSSSCPLLRTLDVQWVEGLKDAQM
ISKKQLSWLINRLPGLRDLVLSGCSIAVSAL-----CSSSCPLLRTLDVQWVEGLKDAQM
ISKKQLSWLINRLPGLRDLVLSGCSIAVSAL-----CSSSCPLLRTLDVQWVEGLKDAQM
ISKKQLSWLINRLPGLRDLVLSGCSIAVSAL-----CSSSCPLLRTLDVQWVEGLKDAQM
ISKKQLSWLINRLPGLRDLVLSGCSIAVSAL-----CSSSCPLLRTLDVQWVEGLKDAQM
VCLTVSPEEPPPPGQLFFLQNRDGPPEPPPADSPATAPGPDDAEGTADTSLCRLYRHVSH
VCLTVSPEEPPPPGQLFFLQNRDGPPEPPPADSPATAPGPDDAEGTADTSLCRLYRHVSH
VCLTVSPEEPPPPGQLFFLQNRDGPPEPPPADSPATAPGPDDAEGTADTSLCRLYRHVSH
VCLTVSPEEPPPPGQLFFLQNRDGPPEPPPADSPATAPGPDDAEGTADTSLCRLYRHVSH
VCLTVSPEEPPPPGQLFFLQNRDGPPEPPPADSPATAPGPDDAEGTADTSLCRLYRHVSH
VCLTVSPEEPPPPGQLFFLQNRDGPPEPPPADSPATAPGPDDAEGTADTSLCRLYRHVSH
VCLTVSPEEPPPPGQLFFLQSREGPPEPPPADIPSTVPGPDDSEGTTDTSLCRLYRHVSH
VCLTVSPEEPPPPGQLFFLQSRGPEPPEPPPDTASKVPGPEDSEGTTDTSLCRLYRHVSH
CEDPLPGMLFFLPPGQQSSDCSQLNGST--------TKEASEASQLEDAAEGDSASEEKS
QPSSLNSCEDPLPGMLFFLPPGDCSQLNESTQLEDAAGGDSASEEKSGSAEPFVPPVSSV
QPSTLNSCEDPVPGMLFFLPPGDCSQLNESTQLEDAAGGDSASEEKSGSAEPFVSPASSV
QPSILNSCEDPVPGMLFFLPPGDYSQLNESTQLEDAAGGDSASEEKSGSAEPFVLPASSV
QPSNLNSCEDPVPGMLFFLPPGDYSQLNESTQLEDAAGGDSASEEKSGSAEPFVLPASSV
CEDPVPGMLFFLPPGQHLSDYSSTTKESSEASAEPFVPPASSVESTLPVLEASSWKKQVS
ASQPQDAAEGSSAGEEKDASVE---PLLPAASPGGSTSQVLEAATCKKQVSQDFLETRFK
ASQPPDAAEGSSAREEKDASLE---PLVPAASGEGSTSQGLEAATCKKQVSQDFLETRFK
----------TITPDMRALAKNMGW-----------------------------------
-----------ITPDMRALANDVGW-----------------------------------
-----------ITPDMKALAAD--------------------------------------

-----------ITPDVRALAED--------------------------------------
-----------ITPDVRALAED--------------------------------------
-----------ITPDVRALAED--------------------------------------
LELEPALPAESVTRILQPAVPVAALRLNLSG------------DTIGPVRFAAHHYAATL
LELEPALPFESVTRVLQPAVPVAALRLNLSG------------DTVGPVRFAAHHYAATL
LELEPALPAESVTRVLQPAVPVAALRLNLSG------------DTVGPLRFAAHHYAATL
LELEPALPAESVTRVLQPAVPVAALRLNLSG------------DTVGPVRFAAHHYAATL

```
LELEPALPAESVTRVLQPAVPVAALRLNLSG------------DTVGPVRFAAHHYAATL
LELEPALPAESVTRVLQPAVPVLRLNLSGDT--------------VGPVRFAAHHYAATL
LELEPVLPDEAVTRILQPAVPVLRLNLSGDT--------------VGPVRFATRHYAETL
-YHPGLKVELELEPVLPDEAVSRILPAVPVA----VLRLNLSGDTVGPVRFAARHYAETL
RSIMGSDTIPYTLPPDTTFVDNYPDSMTFYGFQLHIDIHGSKTYFLCSTFHNLFCRRAGI
LVERSTLGSATLLPSMLPSHSPLCYWFPRYGVYQLYIDIHKVSIFSLCSTFRVLFHRREC
RSTLGSATIPYELPPHSPFLDDSPEGLHGYQ----------------------LHVDLHS
RSTLGSATIPYELPPHSPFLDDSPEGLHGYQ----------------------LHVDLHS
RSTLGSATIPYELPPHSPFLDDGPEGLHGYQ----------------------LHVDLHS
RSTLGSATIPYELPPHSPFLDDSPEGLHGYQ----------------------LHVDLHS
GVWKKEEELAFVMANLHFHHLVERSTLGSAT----------------------IPYELPP
--WKKEEELAFVMANLHFHHLVERSTLGSATYELPPHSPFLDDSPEYGLHGYQLHVDLHS
VSNEVLGRLLHRSPKLRMLDLRGCARVTPSG----LCHLPCQELEQLYLGLYGISDGLTL
VSNEVLGRLLHCSPKLRLLDLRGCARITPTG----LCHLPCQELEQLYLGLYGMSDGLAL
ASWNEGQLPGQASPQLMLTPLTAELEQLHLGGTSDRLTLAKEGSPLLTQKWCRTLRELDL
VSNEVLGRLLHGSPNLRLLDLRGCARITPAG----LQDLPCRELEQLHLG---LYGTSDR
VSNEVLGRLLHGSPNLRLLDLRGCARITPAG----LQDLPCRELEQLHLG---LYGTSDR
VSNEVLGRLLHGSPNLRLLDLRGCARITPAG----LQDLPCRELEQLHLG---LYGTSDR
VSNEVLGRLLHGSPNLRLLDLRGCARITPAG----LQDLPCRELEQLHLG---LYGTSDR
VSNEVLGRLLHGSPNLRLLDLRGCARITPAG----LQDLPCRELEQLHLG---LYGTSDR
------FPEFFQLNYLQHLSLSRCYDIIPDT----------------LLELGEIPTLKTL
----SIMLKNDCFPEFFQLNYLQHLSLSRCY----------------------DIIPETL
------FQEFFQLNYLQHLSLSRCYDIIPET----------------LLELGEIPTLKTL
------FQEFFQLNYLQHLSLSRCYDIIPET----------------LLELGEIPTLKTL
------FPEFFQLNYLQHLSLSRCYDIIPET----LLLIWISQNQNSHNISQISHEGFKV
------FQEFFQLNYLQHLSLSRCYDIIPETLLLAMIYQNQNSHSNYNVSQV-SHEGFKV
------FQEFFQLNYLQHLSLSRCYDIIPET-----------------LLLVTRAGVRIR
-----------SLPNLTSLSLSGCSKVTDDG--VELVAENLRKLRSLDLSWCPRITDMAL
-----------SLPNLTSLSLSGCSKVTDDG--VELVAENLRKLRSLDLSWCPRITDMAL
--------VVHSLPNLTALSLSGCSKVTDDG--VELVAENLRKLRSLDLSWCPRITDMAL
-----------SLPNLTALSLSGCSKVTDDG--VELVAENLRKLRSLDLSWCPRITDMAL
-----------SLPNLTALSLSGCSKVTDDG--VELVAENLRKLRSLDLSWCPRITDMAL
-----------SLPNLTALSLSGCSKVTDDG--VELVAENLRKLRSLDLSWCPRITDMAL
-----------SLPNLTALSLSGCSKVTDDG--VELVAENLRKLRSLDLSWCPRITDMAL
-----------SLPNLTALSLSGCSKVTDDG--VELVAENLRKLRSLDLSWCPRITDMAL
LLETLSENNAGTLRTMNIKCHVHDPHGQVVWSWAKLARQASNLKVNFFFERVMKYERLAR
VWGMSWAKLARQASNLKVNFFFKYERLARILQEIPVRSISLRSCYFSDPDWSMTLTDLLP
CISDELLENLCENNASTLWTINIKCHIHDPHSWAKLGREATNLKVNFFFERIMKYECLAR
NCISDELLENLCENASTLWTINIKCHIHDPHSWAKLARQATNLKVNFYFERIMKYERLAR
ELLENLCENASTLQTINIKCHIHDPHGQVIWSWAKLARQATNLKVNFFFERIMKYERLAR
ISDELLENLCENASTLQTINIK-CHVHDPHG------------QVIWGMSWAKLARQATN
ELLENLCENASTLRTINIKCHVHDPHGQVIWSWAKLARQATNLKVNFFFERIMKYERLAR
ELLENLCENASTLRTINIKCHVHDPHGQVIWSWAKLARQATNLKVNFFFERIMKYERLAR
VGN-QDKLTDEGLKQLGSKCRELKDHFGQCYEGMIVIAKGCLKLQRIYMQENKLVTDQSM
LDNETVMEIVKRCKNLSSLNLCLNWIINDRC--VEVIAKEGQNLKELYLVSCK-ITDYAL
```

--------HCPLLQKVHVGNQD-------------------------------KLTDEGL
LTDEGLKQLGSRCRELKDIHFGQCYKISDEG--MIVIAKSCLKLQRIYMQENKLVTDQSV
LTDEGLKQLGSKCRELKDIHFGQCYKISDEG--MIVIAKGCLKLQRIYMQENKLVTDQSV
VGN-QDKLTDEGLKQLGSKCRELKDHFGQCYEGMIVIAKGCLKLQRIYMQENKLVTDQSV
VGN-QDKLTDEGLKQLGSKCRELKDHFGQCYEGMIVIAKGCLKLQRIYMQENKLVTDQSV
IYDPRAGLALVKSRRLHSSAVLDDRHVISGS----EDHSLVVFDRRANSVLQRLQLDSYL
VTIYDPRAGLTLVKSRRLHSSAVLAVLADDRISGSEDHSLVVFDRRANSVLQRLQLDSYL
--------RLHSSPVLALLADDQHI----------ISGSEDRTLVVVDRRANRVLQRLQL
---------LHSRPVLALMADDQHI-ISGSE-----DRTLVVVDRRANRVLQRLQLDSYL
GPALLKRQQLHSRPVLTLLADDRHI-ISGSE-----DHTLVVVDRRANSVLQRLQLDSYL
--------QLHSRPVLTLLADDRHI-ISGSE-----DHTLVVVDRRANSVLQRLQLDSYL
--------QLHSRPVLTLLADDRHI-ISGSE-----DHTLVVVDRRANSVLQRLQLDSYL
GPALLKHQQLHSRPVLTLLADDRHI-ISGSE-----DHTLVVVDRRANSVLQRLQLDSYL
VDNLSSLTCEKNPLDIDATGVVSGHRIQSATLTEDVNDAKTVEAAMQRLKAANIPEQNTI
LTCEKNPLDIDATGVVGLSFSGSATVLLNED----VNDTKTAEAAMQRLKAANIPEQNTI
LTSEKNPLDIDATGVVGLSFSGSATVLLSED----VNDEKTAEAAMQRLKAANIPEQNTI
LTSEKNPLDIDAAGVVGLSFSGSATVLLNED----VSDEKTAEAAMQRLKAANIPEQNTI
LTSEKNPLDIDASGVVGLSFSGSATVLLNED----VSDEKTAEAAMQRLKAANIPEQNTI
LTSEKNPLDIDASGVVGLSFSGSATVLLNED----VSDEKTAEAAMQRLKAANIPEHNTI
LTSEKNPLDIDASGVVGLSFSGSATVLLNED----VSDEKTAEAAMQRLKAANIPEHNTI
LTSEKNPLDIDASGVVGLSFSGSATVLLNED----VSDEKTAEAAMQRLKAANIPEHNTI
-----------LESRLRYLSIAHCGRVTDVG--IRYVAKYCSKLRYLNARGCEGITDHGV
-----------LESRLRYLSIAHCGRITDVG--IRYVAKYCSKLRYLNARGCEGITDHGV
-----------LESRLRYLSIAHCGRITDVG--IRYVAKYCSKLRYLNARGCEGITDHGV
-----------LESRLRYLSIAHCGRVTDVG--IRYVAKYCSKLRYLNARGCEGITDHGV
-----------LESRLRYLSIAHCGRITDVG--IRYVAKYCSKLRYLNARGCEGITDHGV
-----------LESRLRYLSIAHCGRVTDVG--IRYVAKYCSKLRYLNARGCEGITDHGV
-----------LESRLRYLSIAHCGRVTDVG--IRYVAKYCSKLRYLNARGCEGITDHGV
-----------LESRLRYLSIAHCGRVTDVG--IRYVAKYCSKLRYLNARGCEGITDHGV
CIHFRTKKFGRVKHRNNALGETVRSVFDMEN--------------LRELHNCHLDLSLGI
----------NCPRLQMYWESVTCTKMSVMH--PSLLSTNCHELEKMDLEECILITDSTL
-----------NCPRLQILEAARCSHLTDAG--FTLLARNCHELEKMDLEECILITDSTL
-----------NCPRLQILEAARCSHLTDAG--FTLLARNCHELEKMDLEECILITDSTL
-----------NCPRLQILEAARCSHLTDAG--FTLLARNCHELEKMDLEECILITDSTL
-----------NCPRLQILEAARCSHLTDAG--FTLLARNCHELEKMDLEECILITDSTL
-----------NCPRLQVLEAARCSHLTDAG--FTLLARNCHELEKMDLEECVLITDSTL
-----------NCPRLQVLEAARCSHLTDAG--FTLLARNCHDLEKMDLEECVLITDSTL
-----------NCPRLRILEVARCSQLTDVG--FTTLARNCHELEKMDLEECVQITDSTL
-----------NCPRLRILEVARCSQLTDVG--FTTLARNCHELEKMDLEECVQITDSTL
--------LGQNCPRLRILEVARCSQLTDVG--FTTLARNCHELEKMDLEECVQITDSTL
----------NCPRLSRILEVARCSQLTDVG--FTTLARNCHELEKMDLEECVQITDSTL
-----------NCPRLRILEVARCSQLTDVG--FTTLARNCHELEKMDLEECVQITDSTL
-----------NCPRLRILEVARCSQLTDVG--FTTLARNCHELEKMDLEECVQITDSTL
-----------NCPRLRILEVARCSQLTDVG--FTTLARNCHELEKMDLEECVQITDSTL
-----------NCPRLRILEVARCSQLTDVG--FTTLARNCHELEKMDLEECVQITDSTL

```
----------TAVQELARNCPQLEHDLTGCL----------------------RVGSDGV
---TAVQELARNCPQLEHLDLTGCL----------------------------RVGSDGV
----------AAVQELARNCPELQHDLTGCL----------------------RVGSDGV
----------TAVQELARNCPELQHDLTGCL----------------------RVGSDGV
----------AAVQELARNCPELQHDLTGCL----------------------RVGSDGV
----------AAVQELARNCPELQHDLTGCL----------------------RVGSDGV
----------AAVQELARNCPELHHDLTGCL----------------------RVGSDGV
-----------NCPELQHLDLTGCL----------------------------RVGSDGV
LTDLSLKHISLHMGSLRSLNLRPCD-ISDTG--IMHLAMSSLRLSGLDVSFCDKVGDQSL
------------MGSLRSLNLRSCDNISDTG--IMHLAMGSLRLSGLDVSFCDKVGDQSL
------------MGSLRSLNLRSCDNISDTG--IMHLAMGSLRLSGLDVSFCDKVGDQSL
------------MGSLRSLNLRSCDNISDTG--IMHLAMGSLRLSGLDVSFCDKVGDQSL
------------MGSLRSLNLRSCDNISDTG--IMHLAMGSLRLSGLDVSFCDKVGDQSL
------------MGSLRSLNLRSCDNISDTG--IMHLAMGSLRLSGLDVSFCDKVGDQSL
------------MGSLRSLNLRSCDNISDTG--IMHLAMGSLRLSGLDVSFCDKVGDQSL
CKGITDSSLRSLSPLKQLTVLNNCVRIGDVGLRQFLDGPASIRIRELNLSNCVQLSDVSV
CKGITDSSLRSLSPLKQLTVLNNCVRIGDMGLKQFLDGPASIRIRELNLSNCVRLSDASV
CKGITDSSLRSLSPLRQLTVLNNCVRIGDMGLRQFLDGPASIRIRELNLSNCVRLSDVSV
CKGITDSSLRSLSPLKQLTVLNNCVRIGDMGLKQFLDGPASIKIRELNLSNCVQLSDASV
CKGITDSSLRSLSPLKQLTVLNNCVRIGDMGLKQFLDGPASMRIRELNLSNCVRLSDASV
CKGITDSSLRSLSPLKQLTVLNNCVRIGDMGLKQFLDGPASIKIRELNLSNCVRLSDASV
GLTDSSLKSLSLLKQLTVLNLTNCIRIGDIGLKHFFDGPASIRLRELNLTNCSLLGDSSV
----KTLKSVSHCKNLQELNVSDCPSFTDES--MRHISEGCPGVLYLNLSNTT-ITNRTM
------------VNGSFPQCNNAPK----------------------------YQIGERI
------------VNGSFPQCNNAPK----------------------------YQIGERI
------------VNGSFPQCNNAPK----------------------------YQIGERI
------------VNGSFPQCNNAPK----------------------------YQIGERI
LGGTYRVTETGLDASLQELSYLQRLEVLGCT----------------------LSADSTL
LGGTYRVTETGLDSSLQELSYLQRLEVLGCT----------------------LSADSTL
LGGTYRVTETGLDASLQELSYLQRLEVVGCT----------------------LSADSTL
------LQGLTRFRALRSLVLGGTYRVTETG--LDAGLQELSYLQRLEVLGCTLSADSTL
------LQGLTRFRALRSLVLGGTYRVTETG--LDAGLQELSYLQRLEVLGCTLSADSTL
------LQGLTRFRALRSLVLGGTYRVTETG--LDAGLQELSYLQRLEVLGCTLSADSTL
------LQGLTRFRALRSLVLGGTYRVTETG--LDAGLQELSYLQRLEVLGCTLSADSTL
------LQGLTRFRALRSLVLGGTYRVTETG--LDAGLQELSYLQRLEVLGCTLSADSTL
------TACCGHFSPLRIWDLNQLMTHLGSD----------------------FPPGAGV
------TACCGHFSPLRIWDLNQLMTHLGSD----------------------FPPGAGV
------TACCGHFSPLRIWDLNQLMTHLGSD----------------------FPPGAGV
------TACCGHFSPLRIWDLNQLMTHLGSD----------------------FPPGAGV
------TACCGHFSPLRIWDLNSGQTHLGSD----------------------FPPGAGV
------TACCGHFSPLRIWDLNQLITHLGSD----------------------FPPGAGV
------TACCGHFSPLRIWDLTSGQTHLGSD----------------------FPPGAGV
YHGNPIVSGNHIFKGRAAGIAVNGKGLITENQWGGVDIRRGGVPILRSNLICFGYSDGVV
YHGNPIVSGNHIFKGRAAGIAVNGKGLITENQWGGVDIRRGGVPILRSNLICFGYSDGVV
PPLPGASIQLPSCLVLNSLQQELQKDKEAMA--LANSVQGCLIRKCLFRDGKGGVFVCSY
```

PIVSGNHIFKGRAAGIAVNENGKGLENVIREQWGGVDIRRGGIPVLRSNLICFGYSDGVV
PVVSGNHIFKGRAAGIAVNENGKGLENVIREQWGGVDIRRGGIPVLRSNLICFGYSDGVV
PVVSGNHIFKGRAAGIAVNENGKGLENVIREQWGGVDIRRGGIPVLRSNLICFGYSDGVV
YHGNPVVSGNHIFKGRAAGIAVNENGGIPVLCFGYSDGVVVGDEGKGLIEGNTIYANKGC
YHGNPVVSGNHIFKGRAAGIAVNENGGIPVLCFGYSDGVVVGDEGKGLIEGNTIYANKGC
LPDVENLRNFNELSRIVLEVREQVRQEQEAG-------EGAAPPREPSAKAADGPPAKDG

LPDLENQRNFNELSRIVLEVRERVRQEQQQE---YEAGEGRGRQGLRESQPSPAQPRAEA
LPDLENQRNFNELSRLVLEVRERVRQEQQEGGPRESQPSPAQPRVEAPSRGPDGTPGEDS
LPDLENQRNFNELSRIVLEVRE--RVRQEQQ--GHEAGEGRGRQGPRESQPSPAQPRAEA
LPDLENQRNFNELSRIVLEVRERVRQEQQEGGPRESQPSPAQPRAEAPSKGPDGTPGEDV
LPDLENQRNFNELSRIVLEVRERVRQEQQEG--GHEAGEGRGRQGPRESQPSPAQPRAEA
LPDLENQRNFNELSRIVLEVRERVRQEQQEG--GHEAGEGRGRQGPRESQPSPAQPRAEA
-----------SPNPVLQWTERSCR-----------------------------------
-----------SPNPVLQWTERSCRQVSHVF------------------------TNFGK
-----------SPNPVLQWTERGCRQVSHVF------------------------TNFGK
----------------------PFR-----------------------------------
--------FSASPDPVLQWTERGCRQVSHVF------------------------TNFGK
--------FSASPDPVLQWTERGCRQVSHVF------------------------TNFGK
--------FSASPDPVLQWTERGCRQVSHVF------------------------TNFGK
--------FSAVPDPIPQWNNNVCLHVTHVF------------------------SNIKM
--------FSAVPDPIPQWNNNACLHVTHVF------------------------SNIKM
--------FSAVPDPIPQWNNNACLHVTHVF------------------------SNIKM
--------FSAVPDPIPQWNNNACLHVTHVF------------------------SNIKM
--------FSAVPDPIPQWNNNACLHVTHVF------------------------SNIKM
---------SAVPDPIPQWNNNACLHVTHVF------------------------SNIKM
--------FSAVPDPIEQWNNDIYLQVTHVF----------------------SGIRRGI
--------FSSVPDPIEQWNNDVYLQVTHVF------------------------SNIRR
---------FNSGQVAVPQDSDGGGMEISHT-----------------------------
---------FSSGQVAVPQDSDGGGMEISHT-----------------------------
---------FSSGQVAVPQDNDGGGMEISHT-----------------------------
---------FSSGQVAVPQDSDGGGMEISHT-----------------------------
---------FSSGQVAVPQDSDGGGMEISHT-----------------------------
------------FATGQVAVPEDGSMEISHT-----------------------------
--------GQVAVPEDGSWMEISHT-----------------------------------
--------SGDLPARPGDHPAEERC----------------------------QVEGGLP
--------SGDLPARPSDHPAEERCQVEGGL--------------------------PHI
-----FQPDPVMIQQKSDAKWR---EVCGQG----------------------PHGNLGL
---------FQPDPVMIQQKSDAKWREVSHT-----------------------------
----------FQPDPATIQQKSDAKREVSHT-----------------------------
----------FQPDPATIQQKSDAKREVSHT-----------------------------
----------FQPDPATIQQKSDAKREVSHT-----------------------------
----------FEPPPVTINQWSDATTEVSHT-----------------------------
----------FEPPPVTIQQWNNATTEVSYT-----------------------------
----------FEPPPVTIQQWNNATTEVSYT-----------------------------

```
----------FEPPPVTIQQWNNATTEVSYT------------------------------
----------FEPPPVTIQQWNNAATEVSYT------------------------------
----------FEPPPVTIQQWNNAATEVSYT------------------------------
----------FEPPPVTFQQWNDAKQEISHT------------------------------
----------FEPPPVTIEQWNDASQEISHT------------------------------

TSLKKDAQ----AKVSSQGD----QKGSTY-------SRHNEFSEVAKTLKKNESLKACI
TSLKKDAQ----TKVSNQGD----QKGSTY-------SRHNEFSEVAKTLKKNESLKACI
TSLKKDAQ----TKLSSQGD----QKGSTY-------SRHNEFSEVAKTLKKNESLKACI
TSLKKDAQ----TKLSNQGD----QKGSTY-------SRHNEFSEVAKTLKKNESLKACI
TSLKKDAQ----TKLSNQGD----QKGSTY-------SRHNEFSEVAKTLKKNESLKACI
TSLKKDAQ----TKLSNQGD----QKGSTY-------SRHNEFSEVAKTLKKNESLKACI
KSSSQRGQ----RVSTYSRH----NEFVEV-------AKTLK----NNESLKACVRCNFP
AKTLKNNE----SLKACVRC----NFPAKY-------DHYLERAVCKRESCKFEYCTKCL
GIKQILPH----QMTTAGPV----LGEGRGVIDVHGHIIGMGLS--PDNRYLYVNSRAWP
NKYLIFTT----GCLTYSPH----QIGIKQMTTAGPVGHIIGMGLSPDNRYLYVNSRAWP
GIKQILPH----QMTTAGPV----LGEGRGVIDVHGHIIGMGLS--PDNRYLYVNSRAWP
GIKQILPH----QMTTAGPV----LGEGRGVIDVHGHIIGMGLS--PDNRYLYVNSRAWP
GIKQILPH----QMTTAGPV----LGEGRGVIDIHGHIIGMGLS--PDNRYLYVNSRAWP
GIKQILPH----QMTTAGPV----LGEGRGVIDIHGHIIGMGLS--PDNRYLYVNSRAWP
APLSTFLP----HKLCTTACWNPKKKNRITLMSHNRRYEISSFQLPPRLEHPTWIGASDG
APLSTSLP----HKLCVSACWTPKVKNRLTLMSQSNPHKIASFQLAAHLESPVWMGANDG
APLSTFLP----HKLCASACWAPKVKNRITLMSQSSTHEVASFQVAAHLKCPIWMGASDG
APLSTFLP----HKLCASACWTPKVKNRITLMSQSSTYEIASFQVAAHLKCPIWMGASDG
VPLSTFLP----HKLCASACWTPKVKNRITLMSQSSTYEIASFQVAAHLKCPIWMGASDG
VPLSTFLP----HKLCASACWTPKVKNRITLMSQSSTYEIASFQVAAHLKCPIWMGASDG
EKACQLDS----RYWRITNA----KGDVEE-------VQGPGVVGEPIISPGRVYEYTSC
EKACQLDS----RYWRITNA----KGDVEE-------VQGPGVVGEPIISPGRVYEYTSC
EKACQLDS----RYWRITNA----KGDVEE-------VQGPGVVGEPIISPGRVYEYTSC
EKACQLDS----RYWRITNA----KGDVEE-------VQGPGVVGEPIISPGRVYEYTSC
EKACQLDS----RYWRITNA----KGDVEE-------VQGPGVVGEPIISPGRVYEYTSC
EKACQLDS----RYWRITNA----KGDVEE-------VQGPGVVGEPIISPGRVYEYTSC
EKACQLDS----RYWRITNA----KGDVEE-------VQGPGVVGEPIISPGRIYEYTSC
EKACQLDS----RYWRITNA----KGDVEE-------VQGPGVVGEPIISPGRIYEYTSC
ACAPDEAA----RLMVIHRD----DGGVHLKSRDRLSQQLASFHLPDTMTPHLMQGHGSQ
HLVITTFE----LKANKSRD----RVSILA-------QQLASFPLPDTMTPHLMQGHGSQ
KQESPEPPSGEIHTFLSSPSEETLLGSLLD-WSLECCLDVTVVYLNPEDKIHPAPGTQAP
KQESPEPPSGEIHTFLSSPSEETLLGSLLD-WSLDCCLDVTVVYLNPEDKSHPTPGTQAP
TQDSPDPP----IHAFLSSPAELSSQEETLDWSLDCCQESSDEEACPEDEGPQDPQVSVL
TQDSPDPPTGEIHAFLSSPSEETLLGSFLD-WSLDCCQESSDEEACPEDERPQDPQVPAL
TQDSPDPPTGEIHAFLSSPSEETLLGSFLD-WSLDCCQESSDEEACPEDRGPQDPQALAL
TQDSPDPPTGEIHAFLSSPSEETLLGSFLD-WSLDCCQESSDEEACPEDKGPQDPQALAL
TQDSPDPPTGEIHAFLSSPSEETLLGSFLD-WSLDCCQESSDEEACPEDKGPQDPQALAL
TQDSPDPPTGEIHAFLSSPSAELSSQEETLDWSLDCCQESSDEEACPEDKGPQDPQALAL
--AAMIPD----QPPRARVC-------------------------AASAFSAAPGRQSM
```

```
LRLRAACP----NLILSAEH----SAAMIP-------DQPPRARASPASVFSPAPGRQSA
--SPPGPP----VVISVKQE-----------------EGKWGAPPC-GLPPRALACSPLL
--WGRPSP----------------GPPVVI----------------SVKQEEGKQGQKEP
--WGPPPP--------------------------------------GAPAVISVKQERRG
--WGPPPP----------------GAPVVI----------------SVKQEEGKQGRTGR
--WGPPPP----------------GAPVVI----------------SVKQEEGKQGRTGR
--LLRVCD----RVRGLNDT----------------------------------------
FYITQLKTDSEGAALNVEDAQLLNRSALRSVQAQARISQREQGSTFSPWGEVLTPLASSS
FYITQLKTDSEGAVLNVEDAQLLNRSALRSVQAQARISQREQGSTLSPWGEVLTPLASSS
FYITQLKTDSEGAVLNVEDAQLLNRSALRSVQAQARISQREQGSTLSPWGEVLTPLASSS
FYITQLKTDSEGAVLNVEDAQLLNRSALRSVQAQARISQREQGSTLSPWGEVLTPLASSS
FYITQLKTDSEGAVLNVEDAQLLNRSALRSVQAQARISQREQGSTLSPWGEVLTPLASSS
LNVEDAAT----RLQLLNRS------ALRSVQAQARISQREQGSTLSPWGEVLTPLASSS
NGVHTPPHAGAVSPGALRRSSQSLSSGETVPIPRPGPGDGHSLPPIARRLGHHPPQSLNV
ASALAGAV----SPGALRRSSQSLSSGETVPIPRPGPGDGHSLPPIARRLGHHPPQSLNV
NGVHTPPHAGAVSPGALRRSSQSLSSGETVPIPRPGPGDGHSLPPIARRLGHHPPQSLNV
NGVHTPPHAGAVSPGALRRSSQSLSSGETVPIPRPGPGDGHSLPPIARRLGHHPPQSLNV
NGVHTPPHAGAVSPGALRRSSQSLSSGETVPIPRPGPGDGHSLPPIARRLGHHPPQSLNV
NGVHTPPHAGAVSPGALRRSSQSLSSGETVPIPRPGPGDGHSLPPIARRLGHHPPQSLNV
NGVHTPPHAGAVSPGALRRSSQSLSSGETVPNSRPGPGDGHSLPPIARRLGHHPPQSLNV
NGVHTPPHAGAVSPGALRRSSQSLSSGETVPIPRPGPGDGHSLPPIARRLGHHPPQSLNV
VSIGLQCQ----HLQSLSLA----NLGMMGMLKHCKRQFFQALGQCQRLCLVSRSGTLQP
VSIGLQCQ----QLQSLSLA----NLGMMGMLKHCKRQFFQALGQCQRLCLVSRSGTLQP
VSIGLQCQ----QLQSLSLA----NLGMMGPALADMLQFFQALGQCQRLCLVSRSGTLQP
VSIGLQCQ----QLRSLSLA----NLGMMGMLKHCKRQFFQALSQCQRLCLVSRSGTLQP
VNIGLQCQ----QLRSLSLA----NLGMMGPALSDMLQFFQALSQCQRLCLVSRSGTLQP
FQALSQCP--SLQRLCLVSR----SGTLQPLHVVMCHFTGESLATCQSLLRRWGEVTGRR
VNIGLQCQ----QLRSLSLA----NLGMMGMLKHCKRQALSQCPSLQRLCLVSRSGTLQP
FQALSQCP--SLQRLCLVSR----SGTLQPLQVVMCHFTGESLATCQSLLRRWGEVTGRR
ACVVNGADAFSFRTLPQGGSDVQSNEDYPRRPLTRARITDLVLKDCPKMMFIHATRCRVL
RSDEERPSGADGRTLPQGGSDVQSRARSRLIPESEIAITDLVLKDCPKMMFIHATRCRVL
PSTSRACVSAFSRTLPQGGSDVQSRARSRLVAKTKPRITDLVLKDCPKMMFIHATRCRVL
PSTSRACVSAFSRTLPQGGSDVQSRARSRLVAKTKPRITDLVLKDCPKMMFIHATRCRVL
PSTSRACVSAFSRTLPQGGSDVQSRARSRLVAKTKPRITDLVLKDCPKMMFIHATRCRVL
PSTSRACVSAFSRTLPQGGSDVQSRARSRLVAKTKPRITDLVLKDCPKMMFIHATRCRVL
PSTSRACVSAFSRTLPQGGSDVQSRARSRLVAKTKPRITDLVLKDCPKMMFIHATRCRVL
PSTSRACVSAFSRTLPQGGSDVQSRARSRLVAKTKPRITDLVLKDCPKMMFIHATRCRVL
EQTQIIGE----QNARLAEL--------------------------ERKLREVMESAVGN
EQTQIIGE----QNARLAEL--------------------------ERKLREVMESAVGT
EQTQIIGE----QNARLAEL--------------------------ERKLREVMESAVGT
EQTQIIGE----QNARLAEL--------------------------ERKLREVMESAVGN
EQTQIIGE----QNARLAEL--------------------------ERKLREVMESAVGN
EQTQIIGE----QNARLAEL--------------------------ERKLREVMESAVGN
EQTQIIGE----QNARLAEL--------------------------ERKLREVMESAVGN
EQTQIIGE----QNARLAEL--------------------------ERKLREVMESAVGN
```

```
LRISHCPNILTDRSLWLASCVAQDCREDSSPFKWAPLVCREGLKGLEMLVLTATPVTPKA
LRISHCPNILTDRSLWLASCTDPVGHEVIWALGAGCRRCLQMIGRCPHLRALGVGGAGCG
LRISHCPNILTDRSLWLASCDPVGHEVIWA-LGAGCRRCLQMIGRCPHLRALGVGGAGCG
LRISHCPNILTDRSLWLASCDPVGHEVIWA-LGAGCRRCLQMIGRCPHLRALGVGGAGCG
LRISHCPNILTDRSLWLASCTDPVGHEVIWALGAGCRRCLQMIGRCPHLRALGVGGAGCG
LRISHCPNILTDRSLWLASCTDPVGHEVIWALGAGCRRCLQMIGRCPHLRALGVGGAGCG
LRISHCPNILTDRSLWLASCTDPVGHEVIWALGAGCRRCLQMIGRCPHLRALGVGGAGCG
LRISHCPNILTDRSLWLASCTDPVGHEVIWALGAGCRRCLQMIGRCPHLRALGVGGAGCG
----------------------------------------------PSLERLSLAYCHLT
------------LTFELGPA----------------------------------------
----------------------------------------------PSLERLSLAYCHLT
--SLAYCH----LTFELGPA----------------------------------------
ECVARYQPKQRSMFTFVCGQWMEQRCPLAY-YGCTYSLRSFGVQPCTVLVEPARNCVLGL
ECVARYQPKQRSMFTFVCGQWMEQRCPLAY-YGCTYSLRSFGVQPCVLVEPARNCVLGLH
ECVARYQPKQRSMFTFVCGQWMEQRCPLAY-YGCTYSLRSFGVQPCTVLVEPARNCVLGL
ECVARYQPKQRSMFTFVCGQWMEQRCPLAY-YGCTYSLRSFGVQPCTVLVEPARNCVLGL
ECVARYQPKQRSMFTFVCGQWMEQRCPLAY-YGCTYSLRSFGVQPCTVLVEPARNCVLGL
ECVARYQPKQRSMFTFVCGQWMEQRCPLAY-YGCTYSLRSFGVQPCTVLVEPARNCVLGL
ECVARYQP----RSMFTFVCWMEQRCPLAY-YGCTYSLRSFGVQPCTVLEEPSRNCVLGL
ECVARYQPKQRSMFTFVCGQWMEQRCPLAY-YGCTYSLRSFGVQPCTALEEPSRNCVLGL
ADLAPATPSECVSSAFTFTCWFQHRCPLAY-MGCTFVEVAPELSEGRKNNHLLGHGGKSE
ADLTAATPSECVSSAFTFTCWFQHRCPLAY-LGCTFVEVAPELSERRKNNHLLGHGGKSQ
ADLTTATPSECVSSAFTFTCWFQHRCPLAY-LGCTFVEVASELSEGRKNNHLLGHGGKSQ
ECVTRRHNKSSSAFTFTCNKWFQHRCPLAY-LGCTFVEVAPELSEGRKNNHLLGHGGKSQ
ECVTRRHNKSSSAFTFTCNKWFQHRCPLAY-LGCTFVEVAPELSEGRKNNHLLGHGGKSQ
ECVTRRHNKSSSAFTFTCNKWFQHRCPLAY-LGCTFVEVAPELSEGRKNNHLLGHGGKSQ
GGLHVELHNKSSSAFTFTCNWFQHRCPLAY-LGCTFVTFAIKPEVAPELSEKWKSDHLSG
GGLHVELHNKSSSAFTFTCNWFQHRCPLAY-LGCTFVTFAIKPEVAPELSEKWKSDHLSG
SQDTDNQT----KVFAVITKEKPLDYKYRY-------FRRVPVQEADQSFHVGLQLCSSG
SQDTDNQT----KVFAVITKEKPLDYKYRY-------FRRVPVQEADQSFHVGLQLCSSG
SQDTDNQT----KVFAVITKEKPLDYKYRY-------FRRVPVQEADQSFHVGLQLCSSG
SQDTDNQT----KVFAVITKEKPLDYKYRY-------FRRVPVQEADQSFHVGLQLCSSG
TKVFAVIT----KKKEEKPL----DYKYRY-------FRRVPVQEADQSFHVGLQLCSSG
FN----------KLIWIHHS--------------------------CHITYKSTGETAVS
SQDADNQT----KVFAVITKEKPLDHKYRY-------FRRVPVQEADHSFHVGLQLCSSG
TKVFAVIT----KKKEEKPL----DHKYRY-------FRRVPVQEADHNFHVGLQLCSSG
QRKESPRG----RFVLLLPSNPLHPRPFSSSLLPPGIGEYDQRPILPYVGDPINSLIPGP
QRKESPKG----RFVMLRPS----STHTIP-------FYPNPLHPRPFPSSRLPPGIIGG
QRKESPKG----RFVMLLPS----STHTIP-------FYPNPLHPRPFPSSRLPPGIIGG
QRKESPKG----RFVMLLPS----STHTIP-------FYPNPLHPRPFPSSRLPPGIIGG
QRKESPKG----RFVMLLPS----STHGIP-------FYPNPLHPRPFPSSRLPPGIIGG
QRKESPKG----RFVMLLPSNPLHPRPFPSGIIGGEYDQRPTLPYVGDPINSLIPGPGET
QRKEAQRM----RHAMFLPS-----------------AHPIPFCPIPVYPRAYLPTSLLP
QRKEAQRM----RHVMYLPS-----------------VHPIPFCPIPVYPRPYLPTTLLP
NGRAIELA----RFIVFLAL-----------------VCEKELYCMDWTVKMMQRVCKIF
```

```
NGRTIELA----RLIVFLAL-----------------VCEKELYCMDWTVKMMQKVCKVF
NGRTIELA----RLIVFLAL-----------------VCEKELYCMDWTVKMMQKVCKVF
NGRTIELA----RLVVFLAL-----------------VCEKELYCMDWTVKMMQKVCKVF
NGRTIELA----RLVVFLAL-----------------VCEKELYCMDWTVKMMQKVCKVF
NGRTIELA----RLVVFLAL-----------------VCEKELYCMDWTVKMMQKVCKVF
NGRAVELA----RLVVFLAL-----------------VCEKELYCMDWTVRMMQKVCKVF
DELSEVVP----REWLLENNLILSGNNICFTFMASKAGRAIELARLIVFLALVCEKELYC
LSILNFCS--ELQHLSLGSCVMIEDYDVIASMIGAKCELASGCPLLEELDLGWCPTLQSS
LSILNFCS--ELQHLSLGSCVMIEDYDVIASMIGAKCELASGCPLLEELDLGWCPTLQSS
LSILNFCS--ELQHLSLGSCVMIEDYDVIAMIGAKCKELASGCPLLEELDLGWCPTLQSS
LSILNFCS--ELQHLSLGSCVMIEDYDVIASMIGAKCELASGCPLLEELDLGWCPTLQSS
LSILNFCS--ELQHLSLGSCVMIEDYDVIAMIGAKCKELASGCPLLEELDLGWCPTLQSS
LSILNFCS--ELQHLSLGSCVMIEDYDVIAMIGAKCKELASGCPLLEELDLGWCPTLQSS
LSILNFCA--ELQHLSLGSCVMIEDYDVIASMIGAKCELASGCVLLEELDLGWCPTLQSS
LSILNFCA--ELQHLSLGSCVMIEDYDVIAMIGAKCKELASGCALLEELDLGWCPTLQSS
LVLSCISQ----GDVKRMPC-----------------FYLAHELRLSLLNHPWMVQDIEA
LVLSCISQ----GDVKRMPC-----------------FYLAHELHLNLLNHPWLVQDTEA
LVLSCISQ----GDVKRMPC-----------------FYLAHELHLNLLNHPWLVQDTEA
LVLSCISQ----GDVKRMPC-----------------FYLAHELHLNLLNHPWLVQDTEA
HIMAMTDP----AFGSS--------------------GRPLLVLSCISLNHPWLVQDTEA
LVLSCISQ----GDVKRMPC-----------------FYLAHELHLNLLNHPWLVQDTEA
LVLSCISQ----ADVKRMPC-----------------FYLAHELHLSLLNHPWMVQDTEA
LVLSCISQ----ADVKRMPC-----------------FYLAHELRLSLLNHPWMVQDTEA
------------------------EAELER------------------------------
------------------------EAELER------------------------------
------------------------EAELER------------------------------
------------------------EAELER------------------------------
------------------------EAELER------------------------------
------------------------EAELER------------------------------
------------------------EAELER------------------------------
--LEREVE----KLQ---------------------------------------------
----------------------------------------------------GEILDAELG
GRVGMTCP----RLVELVVC----ANGLRPIRIAERCLSAIGLGECEVSCSAFVEFVKMC
GRVGMTCP----RLVELVVC----ANGLRPIRIAERCLSAIGLGECEVSCSAFVEFVKMC
GRVGMTCP----RLVELVVC----ANGLRPIRIAERCLSAIGLGECEVSCSAFVEFVKMC
GRVGMTCP----RLVELVVC----ANGLRPIRIAERCLSAIGLGECEVSCSAFVEFVKMC
GRVGMTCP----RLVELVVC----ANGLRPIRIAERCLSAIGLGECEVSCSAFVEFVKMC
GRVGMTCP----RLVELVVC----ANGLRPIRIAERCLSAIGLGECEVSCSAFVEFVKMC
GRVGMTCP----RLVELVVC----ANGLRPIRIAERCLSAIGLGECEVSCSAFVEFVKMC
GRVGMTCP----RLVELVVC----ANGLRPIRIAERCLSAIGLGECEVSCSAFVEFVKMC
GRIGLNCP----RLIELVVC----ANGLQPIRIAEHCLTALGLSECEVSCSAFVEFVRLC
GRIGLNCP----RLIELVVC----ANGLLPIRIAKHCLTSLGLSECEVSCSAFVEFVRLC
GRVGLNCP----RLIELVVC----ANDLQPICIAEHCLTALGLSKCEVSCSAFIRFVRLC
GRVGLNCP----RLIELVVCLQPLDNELIC-IAEHCTLTALGLSKCEVSCSAFIRFVRLC
GRIALNCP----RLTELVVC----ANGLQPICVAEHCLTALGLSECEVSCSAFVEFVRLC
```

GRVGLNCP----RLIELVVCLQPLDNELIC-IVEHCTLTALGLSECEVSCSAFIKFVRLC
GCIEDNEIMAGVPTLRRNKIVLISTNSHPIIFDGFAAGIEITNHATATLEGNQIFNNRFG
GCIEDNEIMAGVPTLRRNKIVLISTNSHPIIFDGFAAGIEITNHATATLEGNQIFNNRFG
GCIEDNEIMAGVPTLRRNKIVLISTNSHPIIFDGFAAGIEITNHATATLEGNQIFNNRFG
GCIEDNEIMAGVPTLRRNKIVLISTNSHPIIFDGFAAGIEITNHATATLEGNQIFNNRFG
GCIEDNEIMAGVPTLRRNKIVLISTNSHPIIFDGFAAGIEITNHATATLEGNQIFNNRFG
GCIEDNEIMAGVPTLRRNKIVLISTNSHPIIFDGFAAGIEITNHATATLEGNQIFNNRFG
GCIEDNEIMAGVPTLRRNKIVLISTNSHPVIFDGFAAGIEITNHATATLEGNQIFNNRFG
GCIEDNEIMAGVPTLRRNKIVLISTNSHPVIFDGFAAGIEITNHATATLEGNQIFNNRFG
HKRGIACLQYRDRLVVSGSS----DNTIRL-WDIECGACLRVLEGHEELVRCIRFDNKRI
HKRGIACLQYRDRLVVSGSS----DNTIRL-WDIECGACLRVLEGHEELVRCIRFDNKRI
HKRGIACLQYRDRLVVSGSS----DNTIRL-WDIECGACLRVLEGHEELVRCIRFDNKRI
HKRGIACLQYRDRLVVSGSS----DNTIRL-WDIECGACLRVLEGHEELVRCIRFDNKRI
HKRGIACLQYRDRLVVSGSS----DNTIRL-WDIECGACLRVLEGHEELVRCIRFDNKRI
HKRGIACLQYRDRLVVSGSS----DNTIRL-WDIECGACLRVLEGHEELVRCIRFDNKRI
HKRGIACLQYRDRLVVSGSS----DNTIRL-WDIECGACLRVLEGHEELVRCIRFDNKRI
HKRGIACLQYRDRLVVSGSS----DNTIRL-WDIECGACLRVLEGHEELVRCIRFDNKRI
HKRGIACLQYRDRLVVSGSS----DNTIRL-WDIECGACLRVLEGHEELVRCIRFDNKRI
HKRGIACLQYRDRLVVSGSS----DNTIRL-WDIECGACLRVLEGHEELVRCIRFDNKRI
HKRGIACLQYRDRLVVSGSS----DNTIRL-WDIECGACLRVLEGHEELVRCIRFDNKRI
HKRGIACLQYRDRLVVSGSS----DNTIRL-WDIECGACLRVLEGHEELVRCIRFDNKRI
HKRGIACLQYRDRLVVSGSS----DNTIRL-WDIECGACLRVLEGHEELVRCIRFDNKRI
HKRGIACLQYRDRLVVSGSS----DNTIRL-WDIECGACLRVLEGHEELVRCIRFDNKRI
HKRGIACLQYRDRLVVSGSS----DNTIRL-WDIECGACLRVLEGHEELVRCIRFDNKRI
HKRGIACLQYRDRLVVSGSS----DNTIRL-WDIECGACLRVLEGHEELVRCIRFDNKRI
TLMSIKGS----KKITTFVTIRVKEYAIAN-------FSLKKFEDTLEWMGVSDKNVIVC
LMSRSGFP----QVKMFETY----DIKLEELIASFELQCHKVNPQRFGVSDKNVIVCSTE
RTSEFSDPVSTVLEFSLCKRWTPRREDRITLMSRRGRDLVASFSLQDYNENPKWMGVSDK
LMSIRRTQ----KITKFITF----DMKLKEIQNKMTVQFVASFSVPDDMGRPKWFGVSGK
TLMSISAP----KVTKFVTF----DMKLEELIASFSLTDYEGRLECFGVSDKDVIVCSTG
TLMSIHGP----KIKKFITF----DMELEKFFAS---FSLQNYEERPEWYGVSDKDVIVC
QMSRGVPP----RPTKFATF----DMKLEELVAS---FSLQDYEASPEWMGVSNKDVIVC
LISTVSPQ----NNTKFDTF----DMKLEGLTAS---FSLAEREGKPDYFGVSDKDVIVC
LMSLILPE----NITKFDTF----DMKLEGLTAS---FSLAEDEGTPDHFGVSDKDVIVC
LMSLILPK----NITKFDTF----DMKLEGLTAS---FSLAEDEGRPDYFGVSDKDVIVC
LMSSTIPP----DPTKFATF----DMKLEELVAS---FSLQDCKERAEWMGVSDKDVIVC
LMARNAPP----RPAKFGTF----DMKLEE-IGNKVTHLVASFSLQDYKERAEWMGVSEK
LMSRNVPP----SHTKFGIF----DMKLEELTASFSLAEHEGRPECFGVSDKDVFVCSTG
VNIRDSPPSGNMRRVRIHDLDKIALSLSAHQLGVSAVDDWKVVSGGEEGLVSVWDYRMNQ
VNIRDSPPDRRVRLHDLRTD----KIALSLQLGVSAVDDWKIVSGGEEGLVSVWDYRMNQ
VNLDDSPPDGRVRIHDLRTS----NVTLSLQLGVSAVNDWKIVSGKEDLVSVWDYRMNQK
VNLSDSPPSGNMRIHDLRSG-NIALSLSAHQLGVSAVDDWKIVSGGEEGLVSVWDYRTNQ
VNLSDSPPDGRVRIHDLRSG----NIALSLQMD----DWKIVSGGEEGLVSVWDYRTNQK
VNLSDSPPDGRVRIHDLRSG----NIALSLQMD----DWKIVSGGEEGLVSVWDYRTNQK
VNLSDSPPDGRVRIHDLRSG----NIALSLRVSAVQMDDWKIVSGGEEGLVSVWDYRMNQ

VNLSDSPPDGRVRIHDLRSG----NIALSLRVSAVQMDDWKIVSGGEEGLVSVWDYRMNQ
NNVSIPVP----KMVDDGKSWDLKHQGTITSGGRDCQVWDVDTGKCLNTFRHKDPILATR
KKSKDKEEDEHSSLKDSVSSHPLQRFLLTIEFAYPHREIQKLQPNLPSLIRPKVSDSLRG
CLMAFKHPQVSLLYLRVISA--CGDGKIRI-YNFLNGNCLKVIKVDARGDPVLSFFYQGN
VSLPKKPPKSRVLQVKKNEIHPKRRFLLTVEFTYPCREIRKLQPNLPMIICSRFSGSLKG
VSLPSKPPLLKPLIEELQSQHPKKQFLLTVKFAYPCREIQKLQPNLPMIIRSRFSGSLKG
VSLPGKPPLLKPLTEELQSQHPKKQFLLTVEFAYPCREIQKLQPNLPMIIRSRFSGSLKG
VSLPGKPPLLKPLIEELQSQHPKKQFLLTVEFAYPCREIQKLQPNLPMIIRSRFSGSLKG
DDLETPGKSWKIDQFLLTVCYPRKRAVDRLRLSNPPILHSPRVQSTPMIIRSRFSGSLKG
RVIKTPEI----ANLALLGF----GDIFAL-LFDNRYLYIMDLRTESLISRWPLPEYRKS
RVIKTPEI----ANLALLGF----GDIFAL-LFDNRYLYIMDLRTESLISRWPLPEYRKS
RVIKTPEI----ANLALLGF----GDIFAL-LFDNRYLYIMDLRTESLISRWPLPEYRKS
RVIKTPEI----ANLALLGF----GDIFAL-LFDNRYLYIMDLRTESLISRWPLPEYRKS
RVIKTPEI----ANLALLGF----GDIFAL-LFDNRYLYIMDLRTESLISRWPLPEYRKS
RVIKTPEI----ANLALLGF----GDIFAL-LFDNRYLYIMDLRTESLISRWPLPEYRKS
RVIKTPEV----ANLALLGF----GDVFAL-LFDNHYLYIMDLRTESLISRWPLPEYRKS
RVIKTPEV----ANLALLGF----GDVFAL-LFDNHYLYIMDLRTESLISRWPLPEYRKS
RELGLSPD----AVYVLCYS-----------------LILLS----IDLTSPHVKNKMSK
RELGLSPD----AVYVLCYS-----------------LILLS----IDLTSPHVKNKMSK
RELGLSPD----AVYVLCYS-----------------LILLS----IDLTSPHVKNKMSK
RELGLSPD----AVYVLCYS-----------------LILLS----IDLTSPHVKNKMSK
RELGLSPD----AVYVLCYS-----------------LILLS----IDLTSPHVKNKMSK
RELGLSPD----AVYVLCYS-----------------LILLS----IDLTSPHVKNKMSK
RELGLSPD----AVYVLCYS-----------------LILLS----IDLTSPHVKNKMSK
RELGLSPD----AVYVLCYS-----------------LILLS----IDLTSPHVKNKMSK
IYFGSEKSGRVLLFLSLSGCYQITDHGLRVLTLGGGLLEHLNLSGCLTITGAGLQDLVSA
IYFGSEKSGRVLLFLSLSGCYQITDHGLRVLTLGGGLLEHLNLSGCLTITGAGLQDLVSA
IYFGSEKSGRVLLFLSLSGCYQITDHGLRVLGGGLPYLEHLNLSGCLTITGAGLQDLVSA
IYFGSEKSGRVLLFLSLSGCYQITDHGLRVLGGGLPYLEHLNLSGCLTITGAGLQDLVSA
IYFGSEKSGRVLLFLSLSGCYQITDHGLRVLGGGLPYLEHLNLSGCLTITGAGLQDLVSA
IYFGSEKSGRVLLFLSLSGCYQITDHGLRVLGGGLPYLEHLNLSGCLTITGAGLQDLVSA
IYFGSEKSGRVLLFLSLSGCYQITDHGLRALTLGGGLLEHLNLSGCLTVTGAGLQDLVSA
IYFGSEKSGRVLLFLSLSGCYQITDHGLRVLTLGGGL---------PYLEHLNLSGCLTV
TSGMELKD----NILVSGNA----DSTVKI-WDIKTGQCLQTLQG-PNKHQSAVTCLQFN
TSGMELKD----NILVSGNA----DSTVKI-WDIKTGQCLQTLQG-PSKHQSAVTCLQFN
TSGMELKD----NILVSGNA----DSTVKI-WDIKTGQCLQTLQG-PSKHQSAVTCLQFN
TSGMELKD----NILVSGNA----DSTVKI-WDIKTGQCLQTLQG-PNKHQSAVTCLQFN
TSGMELKD----NILVSGNA----DSTVKI-WDIKTGQCLQTLQG-PNKHQSAVTCLQFN
TSGMELKD----NILVSGNA----DSTVKI-WDIKTGQCLQTLQG-PNKHQSAVTCLQFN
TSGMELKD----NILVSGNA----DSTVKI-WDIKTGQCLQTLQG-PNKHQSAVTCLQFN
TSGMELKD----NILVSGNA----DSTVKI-WDIKTGQCLQTLQG-PNKHQSAVTCLQFN
TKMLSLPEFE--EYLNLLYATPAEIDQIKKNINMKDRDIQRIIWGCPTLSEVNRYLIRVQ
TKMLSLPEFE--EYLNLLYATPAEIDQIKKNITMKDHDIQRIIWGCPTLSEVNRYLIRVQ
TKMLSLPEFE--EYLNLLYATPAEIDQIKKNINMKDHDIQRIIWGCPTLSEVNRYLIRVQ
TKMLSLPEFE--EYLNLLYAEHVDRGDLTT-------DIQRIIWGCPTISEVNRYLIRVQ

```
TKMLSLPEFE--EYLNLLYATPAEIDQIKKNINMKDHDIQRIIWGCPTLSEVNRYLIRVQ
------------------------------------------------------------
------------LDWLTPRE----------------------------------------
SNHIIFSDREFLQEWSPAHCNFFNNYPVVLKMREQLSDVQTLICGCPTLSEANRYLIRTQ
--SRQSPD----KKNKLQDR--------------------------TRLRKAQSMVSTAY
--RLRKAQ----SMMSLSSS-----SPLKV----------------PAHLAWPPPESVGL
MVMGRGFS----VPFASAGE-----------------RQQHGECPVPTPWKAVPPGSPGV
----------------------------------------------NQMTPDFSRQSHDK
--FSRQSH----DKKNKLQD--------------------------RTRLRKAQSMMSRR
--FSRQSH----DKKNKLQD--------------------------RTRLRKAQSMMSRR
LISLSSPP----------------KVPVRL-------AWPLHLPVAPSDREAATEALLEH
--RLRKAQ----SLMSLSS-----PPKVPV----------------RLAWPLHLPGAPSD
HYNIFYC-----LYLQENSCHKMEPLQVVKIMAFAGTGKTSTLVKYEKWSQSRFLYVTFN
VTEGEFPSKSFGRIIDIWILFKRYAKIAVVVQRIEKCAAMEKFTIVPPFPCFWFTCLQIF
VTEGEVPSKSFGRIIDIWILFKRYAKIAVVVQRIEKCHFRVESFSEDEWNLLYVAVTRAK
VTEGEVPSKSFGRIIDIWILFKRYAKIAVVVQRIEKCFAEYILGTVHKAKGLEFDTVHVL
VTEGEFPSKSFGRIIDIWILFKRYAKIAVVVQRIEKCHFRVESFSEDEWNLLYVAVTRAK
VTEGEFPSKSFGRIIDIWILFKRYAKIAVVVQRIEKCHFRVESFSEDEWNLLYVAVTRAK
VTEGESPAKSFGRIIDIWTLFKRYAKIAVVVERIERCHFRVESFSEDEWNLLYVAVTRAK
VTEGESPAKSFGRIIDIWTLFKRYAKIAVVVERIERCHFRVESFSEDEWNLLYVAVTRAK
DSVDLISR----QYDKFLTHFILMNDVIDT-SGFPDLLSLLAIHGYTVWAHNLIAIARLR
DSVDLISR----QYDKFLTHFILMNDVIDT-SGFPDLLSLLAIHGYTVWAHNLIAIARLR
DSVDLISR----QYDKFLTHFILMNDVIDT-SGFPDLLSLLAVHGYTVWAHNLIAIARLR
DSVDLISR----QYDKFLTHFILMNDVIDT-SGFPDLLSLLAIHGYTVWAHNLIAIARLR
DSVDLISR----QYDKFLTHFILMNDVIDT-SGFPDLLSLLAIHGYTVWAHNLIAIARLR
DSVDLISR----QYDKFLTHFILMNDVIDT-SGFPDLLSLLAIHGYTVWAHNLIAIARLR
DSVDLISR----QYDKFLTHFILMNDMIDT-SGFPDLLTLLAIHGYTVWAHNLIAIARLR
DSVDLISR----QYDKFLTHFILMNDMIDT-SGFPDLLTLLAIHGYTVWAHNLIAIARLR
LLLGIWPE----KSFCLVLKLVQHEVGVEVVCYSIGLIMKHKRYGYNCVIYGWDPTCMMG
--LLLQAR----LYFHLGIWYLVQHTLEHIEVGVEVKIMKHKRYGYNCVIYGWDPTCMMG
--LLLQAR----LYFHLGIWYLVQHTLEHIEVGVEVKIMKHKRYGYNCVIYGWDPTCMMG
--LLLQAR----LYFHLGIWYLVQHTLEHIEVGVEVKIMKHKRYGYNCVIYGWDPTCMMG
--LLLQAR----LYFHLGIWYLVQHTLEHIEVGVEVKIMKHKRYGYNCVIYGWDPTCMMG
--LLLQAR----LYFHLGIWYLV-QHTLEHEVKLRSEVMKHKRYGYNCVIYGWDPTCMMG
--LLLQAR----LYFHLGIWYLVQHTLEHIEVGVEVKVMKHKRYGYNCVIYGWDPTCMMG
WCQTSDFS----KELLGCGC----GAGGRL-PGWPKGASFVKLHIKVPLCACSLCSTREC
WCQTSDFS----KELLGCGC----GAGGRLGWPKGS-ASFVKLHIKVPLCACSLCSTREC
WCQSSDFS----KELLGCGC----GAGGRLGWPKGSAQVKVPLCACALCATRDCLYMLSS
NETQLDQP----RSYTVQLA--------------------------LRKVSHYLPHLRVA
WCQSSEFS----KELLGCGC----GAGGRLGWPKGSAQVKVPLCACALCATRECLYILSS
LSQSSEFS----KELLGCGC----GAGGRLGWPKGSAQVKVPLCACALCATRECLYILSS
RKVSHYLPHLRVACMTSNQSYVTDQGGVYFEVHTPGVDLFGTLQAFDPLDQQMPLALSLP
LSQSSEFS----KELLGCGC----GAGGRLGWPKGSAQVKVPLCACALCATRECLYILSS
RKRLILSDDWKKMYFKLVRC-----------------YPRKEQYGDTLQLCKHCHILSWK
RKRLILSDDWKKMYFKLVRC-----------------YPRKEQYGDTLQLCKHCHILSWK
```

```
RKRLILSDDWKRMYFKLVRC-----------------YPRKEQYGDTLQLCKHCHILSWK
RKRLILSDDWKKMYFKLVRC-----------------YPRKEQYGDTLQLCKHCHILSWK
RKRLILSDDWKKMYFKLVRC-----------------YPRKEQYGDTLQLCKHCHILSWK
RKRLILSDDWKKMYFKLVRC-----------------YPRREQYGVTLQLCKHCHILSWK
RKRLILSDDWKKMYFKLVRC-----------------YPRREQYGVTLQLCKHCHILSWK
CRHLILSEEWKLMYFALKKH-----YPAKE-------QYGDTLHFCRHCSILFWKDYHLA
CRHLILSEEWKLMYFALQKH-----YPAKE-------QYGDTLHFCRHCSILFWKDYHLA
CRHLILSEEWKLMYFALQKH-----YPAKE-------QYGDTLHFCRHCSILFWKDYHLA
CRHLILSEEWKLMYFALQKH-----YPAKE-------QYGDTLHFCRHCSILFWKDYHLA
CRHLILSEEWKLMYFALQKH-----YPAKE-------QYGDTLHFCRHCSILFWKDYHLA
CRHLILSKEWKLMYFALQKH-----YPAKE-------QYGDTLHFCRHCSILFWKDYHLA
CRHLILSEEWKLMYFTLQKY-----YPTKE-------QYGDTLHFCRHCSILFWKDYHLA
--TPVSPE----HFIDLFKF----------------------------------------
RELLLPPPGQTESRGRLQGVLELTDASLRLLLRHAPQLTAPTSPLRETLVHLNLAGCHRL
RELLLPPPSRGRRLAGLELT----DASLRLLLRHAPQLTAPTSPLRETLVHLNLAGCHRL
RELLLPPPGQTESRGRLQGVLELTDASLRLLLRHAPQLTAPTSPLRETLVHLNLAGCHRL
MWLLNRLQ--GLQELVLSGC----------SWLSVSALGSAPLPALRLLDLRWIEDVKDS
RELLLPPPGQTESRGRLQGVLELTDASLRLLLRHAPQLTAPTSPLRETLVHLNLAGCHRL
RELLLPPPGQTESRGRLQGVLELTDASLRL-LLRHAPLTAPTSPLRETLVHLNLAGCHRL
RELLLPPPGQTESRGRLQGVLELTDASLRL-LLRHAPLTAPTSPLRETLVHLNLAGCHRL
RELLLPPPSRGRRLAGLELT----DASLRLLLRHAPQLTAPTSPLRETLVHLNLAGCHRL
RDLLTPPAQDNRSKLRNMTDLDITDATLRLIIRHMPLLTAVGSSTRYSLTELNMAGCNKL
RDLLTPPASKLRNMTDFRLALDITDATLRLIIRHMPLLTAVGSSTRYSLTELNMAGCNKL
RDLLTPPASKLRNMTDFRLALDITDATLRLIIRHMPLLTAVGSSTRYSLTELNMAGCNKL
RDLLTPPASKLRNMTDFRLALDITDATLRLIIRHMPLLTAVGSSTRYSLTELNMAGCNKL
RDLLTPPASKLRNMTDFRLALDITDATLRLIIRHMPLLTAVGSSTRYSLTELNMAGCNKL
RDLLTPPASKLRNMTDFRLALDITDATLRLIIRHMPLLTAVGSSTRYSLTELNMAGCNKL
RDLLTPPTSKLRNMTDFRLALDITDATLRLIIRHMPLLTAVGSSTRYSLTELNMAGCNKL
RDLLTPPTSKLRNMTDFRLALDITDATLRLIIRHMPLLTAVGSSTRYSLTELNMAGCNKL
RDLLSPPTGQMDNRSKLRNILDITDASLRLIIRHMPLLTAVGTTTRDSLTEINLSDCNKV
RDLLSPPTGQMDNRSKLRNILDITDASLRLIIRHMPLLTAVGTTTRDSLTEINLSDCNKV
RDLLSPPTGQMDNRSKLRNI--ITDASLRLIIRHMPLLTAVGTTTRDSLTEINLSDCNKV
RDLLSPPTNRSKRLAGLDIT----DASLRLIIRHMPLLTAVGTTTRDSLTEINLSDCNKV
RDLLSPPTNRSKRLAGLDIT----DASLRLIIRHMPLLTAVGTTTRDSLTEINLSDCNKV
RDLLSPPTNRSKRLAGLDIT----DASLRLIIRHMPLLTAVGTTTRDSLTEINLSDCNKV
RDLLSPPTNRSKRLAGLDIT----DVSLRLIIRHMPLLTAVGTTTRDSLTEVNLSDCNKV
RDLLSPPTNRSKRLAGLDIT----DVSLRLIIRHMPLLTAVGTTTRDSLTEINLSDCNKV
DFLEIRFKLEPRQYMLLLPESFLPTRALAA-LKCTCHDPLYRDDPCKQCRKRYEKGDVSL
DFLEIRFKLEPRQYMLLLPESFLPTRALAA-LKCTCHDPLYRDDPCKQCRKRYEKGDVSL
DFLEIRFKLEPRQYMLLLPESFLPTRALAA-LKCTCHDPLYRDDPCKQCRKRYEKGDVSL
DFLEIRFKLEPRQYMLLLPESFLPTRALAA-LKCTCHDPLYRDDPCKQCRKRYEKGDVSL
DFLEIRFKLEPRQYMLLLPESFLPTRALAA-LKCTCHDPLYRDDPCKQCRKRYEKGDVSL
DFLEIRFKLEPRQYMLLLPE----HVLVKIALKCTCHKGIIEAFGVRATDSRWSRDPLYR
DFLEIRFKLEPRQYMLLLPE----HVLVKIALKCTCHDPLYRDDPCKQCRKRYEKGDVSL
DFLEIRFKLEPRQYMLLLPESFLPTRALAA-LKCTCHSRWSRDPLYKQCRKRYEKGDVSL
```

```
GSAEPFASVESTSKKQVSHDRLLPTKSLVA-LKCTCCKFIIEYYNIRPADSRWVRDPRYR
ESTLPVLEKKQVSHDFLETRRLLPTKSLVA-LKCTCCKFIIEYYNIRPADSRWVRDPRYR
ESTLPVLEKKQVSHDFLETRRLLPTKSLVA-LKCTCCKFIIEYYNIRPADSRWVRDPRYR
ESTLPVLEKKQVSHDFLETRRLLPTKSLVA-LKCTCCKFIIEYYNIRPADSRWVRDPRYR
ESTLPVLEKKQVSHDFLETRRLLPTKSLVA-LKCTCCKFIIEYYNIRPADSRWVRDPRYR
HDQLLEPQ----QYMAFLPH----HIMVKIALKCTCCKFIIEYYNIRPADSRWVRDPRYR
IQQLLEPQ----QYMACLPH----HIIVKIILKCTCRKSIIEYYNIRPADSRWVRDPRYR
IQQLLEPQ----QYMACLPH----HIMVKIILKCTCRKSIIEYYNIRPADSRWVRDPRYR
------------RQMFLTNN--------------------------IQLQRQTRKKKQRQ
------------RQMFFTNK--------------------------IQLQRQIRKKKQRQ
--VGRCAS--------------------------------------PTSSSPSSAGGNKS
--LRKRKQ----KYGNLREK----------------------------------------
--LRKRKQ----KYGNLREK----------------------------------------
----------------------------------------------TGWRQLFFTNKLQL
----------------------------------------------TGWRQLFFTNKLQL
----------------------------------------------TGWRQLFFTNKLQL
RALEVRAA----ASAGLNAA----LEELAA--------------RCAGLREVHCFCVVSP
RALEVRAA----ASAKLNAA----LEELAA--------------RCAALREVHCFCVVSH
CALEVRAA----ASAELNAA----LEELAA--------------RCAALREVHCFCVVSH
CALEVRAA----ASAELNAA----LEELAA--------------RCAALREVHCFCVVSH
CALEVRAA----ASAELNAA----LEELAA--------------RCAALREVHCFCVVSH
CALEVRAA----ASAELNAA----LEELAA--------------RCAALREVHCFCVVSH
RALEVRAS----ASPELHTA----LEELAA--------------RCAGLREIHCFCVVRP
RALEVRAS----ASTELHTA----LEELAA--------------RCAGLREIHCFCVVRP
NN-GYVKF----LMINLKNN----REHLPL-------VGKVGLEWRTDCLNGRIESCIVV
IENGYVKF----VVISLKNN----REHLPL-------IGKVGLAWRTNVFDGFIESCFVV
GGVFYLCG--TFRNLFTKKG--NIENGHVKLIVINLKREHLPLIGKLSWKTDIFDGCIKS
GGVFYLCG--TFRNLFTKKG--NIENGHVKLIVIHLKREHLPLIGKLSWKTDILDGCIKS
GGVFFLCG--TFRNLFTKKG--NIENGHVKLIVIHLRREHLPLIGKLSWKTDIFDGCIKS
GGVFYLCG--TFRNLFTKRG--NIENGHVKLIVIHLKREHLPLIGKLSWKTDIFDGCIKS
HSPFLDDS----PEYGLHGY----QLHVDL-------HSGGVFYLCGTFRNLFTKRGNIE
GGVFYLCGRGNIIVIHLKNN----REHLPL-------IGKVGLSWKTDIFDGCIKSCSMM
AKDGSPLLYHTLRELDFSGQ----GFSEKDLEQALAVFSGTPGGLHPALCSLNLRGTRVT
AKDGSPLLYHTLRELDFSGQ-GFSEKDLEQ-ALAVFSGTTEGLP--PALCSLNLRGTRVT
SG----------QGFSEKDL----EQALAA-------FLSTPGGLHPALCSLNLRGTRVT
LTLAKEGS----PLLTQKWCLDLSGQGFSEKDLEQALAAFLSTPGGPALCSLNLRGTRVT
LTLAKEGS----PLLTQKWCQWFSEKDLEQ-------AAFLSTPGSPALCSLNLRGTRVT
LTLAKEGS----PFLTQKWCLDLSGQGFSEKDLEQALAAFLSTPGGPALCSLNLRGTRVT
LTLAKEGS----PFLTQKWCLDLSGQGFSEKDLEQALAAFLSTPGGPALCSLNLRGTRVT
LTLAKEGS----PFLTQKWCLDLSGQGFSEAL--AAFLSTPGGSH-PALCSLNLRGTRVT
QVFGIVPE----GTLQLLRE------ALPR----------------LQINCAYFTTIARP
LELGEIPT----------------LKTLQV--------FGIVPDGTLQLLREALPRLQIN
QVFGIVPD----GTLQLLKE------ALPH----------------LQINCSHFTTIARP
QVFGIVPD----GTLQLLKE------ALPH----------------LQINCSHFTTIARP
GA----------GLLSLLVT----RAGVRI----------------QLDSDIGCPQTYRT
```

```
GA----------GLLSSLVT----RAGVRI----------------RLDSDIGCPQTYRT
LDSDIGCP----QTYRTSKL--------------------------KSSHKLFCQHVRVI
EYVACDLH--RLEELVLDRC----DTGLSY-LSTMSSLRSLYLRWCCQVQDFGLKHLLAM
EYVACDLH--RLEELVLDRC----DTGLSY-LSTMSSLRSLYLRWCCQVQDFGLKHLLAM
EYVACDLH--RLEELVLDRCVRITDTGLSY-------LSTMSSLR-SLYITTGLYGLRQH
EYVACDLH--RLEELVLDRC----DTGLSY-LSTMSSLRSLYLRWCCQVQDFGLKHLLAL
EYVACDLH--RLEELVLDRC-----------------ITDTGLSYLSTMSSLRSLYLRWC
EYVACDLH----EELVLDRC----DTGLSY-LSTMSSLRSLYLRWCCQVQDFGLKHLLAL
EYVACDLH--RLEELVLDRCVRITDTGLSY-LSTMSSLRSLYLRWCCQVQDFGLKHLLAL
EYVACDLH--RLEELVLDRCVRITDTGLSY-LSTMSSLRSLYLRWCCQVQDFGLKHLLAL
ILLQEIPV----RSISLRSCWSMRPTLTDLLPTFRNTLQKLTFEF-NNNHESLDEQLHLL
TFRNTLQK----LTFEFNNN----HESLDE-------QLHLLILACRKLFYFKIWAFLDV
ILLQEIPV----RSLSLRSCFSDPDCSMRPLPTFRHTLQKLTFEF-NNNHESLDEELHVL
ILLQEIPV----RSISLRSCWSMRPTLIDLLPTFRHTLQKLTFEF-NNNHESLDEELHLL
ILLQEIPI----RSISLRSCWSMRPTLIDLLPSFRHTLQKLTCEF-NNNHESLDEELHLL
LKVNFFFE----RIMKYERL----ARILLQ----EIPIRSISLRSC-YFSDPDYSMRPTL
ILLQEIPI----RSISLRSCFSDPDYSMRPLPTFRHTLQKLTCEF-NNNHESLDDELHLL
ILLQEIPI----RSISLRSCFSDPDCSMRPLPTFRHTLQKLTCEF-NNNHESLDEELHLL
KAFAEHCP--ELQYVGFMGCTELDNETVMEIVKRCKNVEVIAKEG-QNLKELYLVSCKIT
IAIGRYSM--TIETVDVGWCKEITDQGATLIAQSSKSLRYLGLMRCDKVRVDYQVVCFLH
KQLGSKCR--ELKDIHFGQC-------------------------YKISDEGMVVIAKS
KAFAEHCP--ELQYVGFMGCRNLSSLDLRHEIVKRCKVEVIAKEG-QNLKELYLVSCKIT
KAFAEHCP--ELQYVGFMGCTELDNETVME-IVKRCKVEVIAKEG-QNLKELYLVSCKIT
KAFAEHCP--ELQYVGFMGCTELDNETVME-IVKRCKVEVIAKEG-QNLKELYLVSCKIT
KAFAEHCP--ELQYVGFMGCTELDNETVME-IVKRCKVEVIAKEG-QNLKELYLVSCKIT
LCMSYQEP----QLWAGDNQ------GLLH--------VFANRDGCFQLVRSFDVGHQSQ
LCMSYQEP----QLWAGDNQ------GLL--------HVFANQDGCFQLVRTFDVGHQSQ
DSLLCMSH----QDMQLWTG----DNEGLL-------HVFANRNGCFQHVRSFNVGHRSH
LCMSYQEP----QLWAGDNH------GLLH--------VFVNRNGCLQLIRSFDVGHSFP
LCMSYQEP----QLWAGDNQ------GLLH--------VFANRNGCFQLIRSFDVGHSFP
LCMSYQEP----QLWAGDNQ------GLLH--------VFANRNGCFQLIRSFDVGHSFP
LCMSYQEP----QLWAGDNQ------GLLH--------VFANRNGCFQLIRSFDVGHSFP
LCMSYQEP----QLWAGDNQ------GLL--------HVFANRNGCFQLIRSFDVGHSFP
GFMFACVG----RGFQYYRA----KGNVEA-------DAFRKFFPSVPLFGFFGNGEIGC
GFMFACVG----RGFQYYRA----KGNVEA-------DAFRKFFPNVPLFGFFGNGEIGC
GFMFACVG----RGFQYYRA----KGNVEA-------DAFRKYFPSVPLFGFFGNGEIGC
GFMFACVG----RGFQYYRA----KGNVEA-------DAFRKFFPSVPLFGFFGNGEIGC
GFMFACVG----RGFQYYRA----KGNVEA-------DAFRKFFPSVPLFGFFGNGEIGC
GFMFACVG----RGFQYYRA----KGNVEA-------DAFRKFFPSVPLFGFFGNGEIGC
GFMFACVG----RGFQYYRA----KGNVEA-------DAFRKFFPSVPLFGFFGNGEIGC
GFMFACVG----RGFQYYRA----KGNVEA-------DAFRKFFPSVPLFGFFGNGEIGC
EYLAKNCT--KLKSLDIGKCPLVSDTGLEC-LALNCFLKRLSLKSCESITGQGLQIVAAN
EYLAKNCT--KLKSLDIGKCPLVSDTGLES-LALNCFLKRLSLKSCESITGQGLQIVAAN
EYLAKNCT--KLKSLDIGKCPLVSDTGLES-LALNCFLKRLSLKSCESITGQGLQIVAAN
EYLAKNCT--KLKSLDIGKCPLVSDTGLEC-LALNCFLKRLSLKSCESITGQGLQIVAAN
```

```
EYLAKNCT--KLKSLDIGKCPLVSDTGLEC-LALNCFLKRLSLKSCESITGQGLQIVAAN
EYLAKNCT--KLKSLDIGKCPLVSDTGLEC-LALNCFLKRLSLKSCESITGQGLQIVAAN
EYLAKNCT--KLKSLDIGKCPLVSDTGLEC-LALNCFLKRLSLKSCESITGQGLQIVAAN
EYLAKNCT--KLKSLDIGKCPLVSDTGLEC-LALNCFLKRLSLKSCESITGQGLQIVAAN
RVLQTFTN----LFQSLSHCELITDDGILHLSNSTCGLERLELYDCQQVTRAGIKRMRAQ
IQLSIHCP--KLQALSLSHCELITDDGILHLSNSTCGLRVLELDNCLLITDVALEHLENC
IQLSIHCP--KLQALSLSHCELITDDGILHLSNSTCGLRVLELDNCLLITDVALEHLENC
IQLSIHCP--KLQALSLSHCELITDDGILHLSNSTCGLRVLELDNCLLITDVALEHLENC
IQLSIHCP--KLQALSLSHCELITDDGILHLSNSTCGLRVLELDNCLLITDVALEHLENC
IQLSIHCP--KLQALSLSHCELITDDGILHLSNSTCGLRVLELDNCLLITDVALEHLENC
VQLSIHCP--KLQALSLSHCELITDEGILHLSSSTCGLRVLELDNCLLVTDASLEHLENC
IQLSIHCP--KLQALSLSHCELITDEGILHLSSSTCGLRVLELDNCLLVTDASLEHLENC
IQLSIHCP--RLQVLSLSHCELITDDGIRH-LGNGACLEVIELDNCPLITDASLEHLKSC
IQLSIHCP--RLQVLSLSHCELITDDGIRH-LGNGACLEVIELDNCPLITDASLEHLKSC
IQLSIHCP--RLQVLSLSHCELITDDGIRH-LGNGACLEVIELDNCPLITDASLEHLKSC
IQLSIHCP--RLQVLSLSHCELITDDGIRH-LGNGACLEVIELDNCPLITDASLEHLKSC
IQLSIHCP--RLQVLSLSHCELITDDGIRH-LGNGACLEVIELDNCPLITDASLEHLKSC
IQLSIHCP--RLQVLSLSHCELITDDGIRH-LGNGACLEVIELDNCPLITDASLEHLKSC
IQLSIHCP--RLQVLSLSHCELITDDGIRH-LGNGACLEVIELDNCPLITDASLEHLKSC
IQLSIHCP--RLQVLSLSHCELITDDGIRH-LGNGACLEVIELDNCPLITDASLEHLKSC
RTLAEYCP--ALRSLRVRHC--------------------------HHVAEPSLSRLRKR
RTLAEYCP--ALRSLRVRHC--------------------------HHVAEPSLSRLRKR
RTLAEYCP--ALRSLRVRHC--------------------------HHVAESSLSRLRKR
RTLAEYCP--ALRSLRVRHC--------------------------HHVAESSLSRLRKR
RTLAEYCP--ALRSLRVRHC--------------------------HHVAESSLSRLRKR
RTLAEYCP--VLRSLRVRHC--------------------------HHVAESSLSRLRKR
RTLAEYCP--VLRSLRVRHC--------------------------HHVAESSLSRLRKR
RTLAEYCP----RSLRVRHC--------------------------HHVAESSLSRLRKR
AYIAQGLD--SLKSLSLCSC----DDGINRMVRQMHGLRTLNIGQCVRITDKGLELIAEH
AYIAQGLD--GLKSLSLCSC----DDGINRMVRQMHGLRTLNIGQCVRITDKGLELIAEH
AYIAQGLD--GLKSLSLCSC----DDGINRMVRQMHGLRTLNIGQCVRITDKGLELIAEH
AYIAQGLD--GLKSLSLCSC----DDGINRMVRQMHGLRTLNIGQCVRITDKGLELIAEH
AYIAQGLD--GLKSLSLCSC----DDGINRMVRQMHGLRTLNIGQCVRITDKGLELIAEH
AYIAQGLD--GLKSLSLCSC----DDGINRMVRQMHGLRTLNIGQCVRITDKGLELIAEH
AYIAQGLD--GLKSLSLCSC----DDGINRMVRQMHGLRTLNIGQCVRITDKGLELIAEH
LKLSERCP----NYLSLRNCFSLVNEDLNV-LSRHKKLECLDVSYCSQLSDMIIKALAIY
MKLSERCP----NYLSLRNCFSLVNEGLNV-LSRHKKLEHLDVSYCSQLSDMIIKALAIY
MKLSERCP----NYLSLRNCFSLVNEGLNV-LSKHKKLEHLDVSYCSQLSDMIIKALAIY
MKLSERCP----NYLSLRNCFSLVNEGLNV-LSRHKKLEHLDVSYCSQLSDMIIKALAIY
MKLSERCP----NYLSLRNCFSLVNEGLNV-LSRHKKLEHLDVSYCSQLSDMIIKALAIY
MKLSERCP--NLNYLSLRNCFSLVNEGLNV-LSRHKKLEHLDVSYCSQLSDMIIKALAIY
IRLSERCP--NLHYLNLRNCTLISNEGMTI-LSRHRKLEHLDVSYCSQLTDDIIKTIAIF
RLLPRYFH--NLQNLSLAYCRKFTDKGLQYNLGNGCHLIYLDLSGCTQISVQGFRNIANS
RVILDMED----KTLAFERG--------------------------YEFLGVAFRGLPKV
RVILDMED----KTLAFERG--------------------------YEFLGVAFRGLPKA
```

```
RVILDMED----KTLAFERG--------------------------YEFLGVAFRGLPKV
RVILDMED----KTLAFERG--------------------------YEFLGVAFRGLPKV
LAISRHLR----DVRKIRLT----VGGLSA-QGLVFLEGMPVLESLCFQGPLITPDMPTP
LAISRHLR----DVRKIRLT----VGGLSA-QGLVFLEGMPVLESLCFQGPLITPEMPTP
LAISRHLR----DVRKIRLT----VRGLSA-PGLAVLEGMPALESLCLQGPLVTPEMPTP
LAISRHLR----DVRKIRLT----VRGLSA-------PGLAVLEGMPALESLCLQGPLIT
LAISRHLR----DVRKIRLT----VRGLSA-------PGLAVLEGMPALESLCLQGPLIT
LAISRHLR----HVRKIRLT----VRGLSA-------PGLAVLEGMPALESLCLQGPLVT
LAISRHLR----DVRKIRLT----VRGLSA-------PGLAVLEGMPALESLCLQGPLVT
LAISRHLR----DVRKIRLT----VRGLSA-------PGLAVLEGMPALESLCLQGPLVT
LDVMYESPDTYVRYWDLRTSEEPHDSTLYCTDGNHLLATGSSYYGVVRLWDRRQRACLHA
LDVMYESPDTYVRYWDLRTSEEPHDSTLYCTDGNHLLATGSSYYGVVRLWDRRQRACLHA
LDVMYESPDTYVRYWDLRTSEEPHDSTLYCTDGNHLLATGSSYYGVVRLWDRRQRACLHA
LDVMYESPDTYVRYWDLRTSEEPHDSTLYCTDGNHLLATGSSYYGVVRLWDRRQRACLHA
LDVMYESPDTYVRYWDLRTSEEPHDSTLYCLQTDGNHATGSSYYGVVRLWDRRQRACLHA
LDVMYESPDTYVRYWDLRTSEEPHDSTFYCLQTDGNHATGSSYYGLVRLWDRRQRACLHA
LDVMYESPDTYVRYWDLRTSEEPHDSTFYCLQTDGNHATGSSYYGLVRLWDRRQRACLHA
VGNTIYANSSSLPHVTSNHVDGDADDPLRRSINHNGAQCKVELRGNGIYDNRGHGIITKG
VGNTIYANSSSLPHVSSNHVDGDADDPLRRLVESNSIQCKVELRGNGIYDNRGHGIITKG
GRAKMEGN----IFRNLTYAVMLRNDIYRCFLRLEGGKKSNPLILCNQIHHGLRSGIVVL
VGDEGKGLIEGNTIYANKGCAVFSQKDGSSELPRGHRLRGNGIYDNHGIITKGDSTIVIE
VGDEGKGLIEGNTIYANKGCAVFSQKDGSSELPRGHRLRGNGIYDNHGIITKGDSTIVIE
VGDEGKGLIEGNTIYANKGCAVFSQKDGSSELPRGHRLRGNGIYDNHGIITKGDSTIVIE
GVVTSNHV----SYNGLYGVWETEDDPLRRLVESNSIQCKVELRGNSTIVIENDIIGNRG
GVVTSNHV----SYNGLYGVWETEDDPLRRLVESNSIQCKVELRGNSTIVIENDIIGNRG
KEPGGGAE----AAEQSASS----GQGQPF-------VLPVGVSSRNEDYPRTCRLCFYG
--RDVYAK----------------------------------------------------
PSRGPDGTGDAAVAEQPAQS----GQGQPF-------VLPVGVSSRNEDYPRTCRMCFYG
GKPGDASA----AAEPPAQC----GQGQPF-------VLPVGVSSRNEDYPRTCRMCFYG
PSKGPDGTGDAVAAEQPAQC----GQGQPF-------VLPVGVSSRNEDYPRTCRMCFYG
GEPGDAVA----AAEQPAQC----GQGQPF-------VLPVGVSSRNEDYPRTCRMCFYG
PSKGPDGTGDAVAAEQPAQC----GQGQPF-------VLPVGVSSRNEDYPRTCRMCFYG
PSKGPDGTGDAVAAEQPAQC----GQGQPF-------VLPVGVSSRNEDYPRTCRMCFYG
---QVSPD--------------------------------------CSLNPGLLQGLSRL
GI----------RYVSFEQY--------------------------GRDTRSWVGHYGAL
GI----------RYVSFEQY--------------------------GRDTRSWVGHYGAL
------------RNLIFNSC--------------------------GE------------
GI----------RYVSFEQY--------------------------GRDVSSWVGHYGAL
GI----------RYVSFEQY--------------------------GRDVSSWVGHYGAL
GI----------RYVSFEQY--------------------------GRDVSSWVGHYGAL
GI----------RFVSFEHW--------------------------GQDTQFWAGHYGAR
GV----------RFVSFEHW--------------------------GQDTQFWAGHYGAR
GV----------RFVSFEHR--------------------------GQDTQFWAGHYGAR
GV----------RFVSFEHR--------------------------GQDTQFWAGHYGAR
GV----------RFVSFEHR--------------------------GQDTQFWAGHYGAR
```

```
GV----------RFVSFEHR--------------------------GQDTQFWAGHYGAR
------------RFVSFEHW--------------------------GQDTQFWAGHYGAR
GV----------RFVSFEHW--------------------------GQDTQFWAGHYGAR
--FTDYGP--GVRFVRFEHG--------------------------DSVYWKGWFGARVT
--FTDYGP--GVRFVRFEHG--------------------------DSVYWKGWFGARVT
--FTDYGP--GVRFVRFEHG------GQDS-------VYWKGWFGARVTNSSVWVEP---
--FTDYGP--GVRFVRFEHG------GQDS-------VYWKGWFGARVTNSSVWVEP---
--FTDYGP--GVRFVRFEHG------GQDS-------VYWKGWFGARVTNSSVWVEP---
--FIDYGPGVRFVRFEHGGQ-----------------VYWKGWFGA----------RVTN
--FTDYGP--GVRYVRFEHG------GQDS-------VYWKGWFGARVTNSSVWVEP---
HILQLPAR----RPLHLVSA--------------------------RRRGHSLLGRLVRP
LQLPARRP--------------------------------------PHLVSARRRGHSLL
TCFSRRIG----VSFQLRQQ--------------------------SRETERAPTQKQFP
--FSNYPP--GVRYIWFQHG------GVDT-------HYWAGWYG-PRVTNSSVIIGPPL
--FSNYPP--GVRYIWFQHG------GVDT-------HYWAGWYG-PRVTNSSITI----
--FSNYPP--GVRYIWFQHG------GVDT-------HYWAGWYG-PRVTNSSITI----
--FSNYPP--GVRYIWFQHG------GVDT-------HYWAGWYG-PRVTNSSITIGPPL
--FSDYPP--GVRYILFRHG------GQDT-------QFWAGWYG-PGVTNSSIVVSHKM
--FSDYPR--GVRYILFQHG------GRDT-------QYWAGWYG-PRVTNSSIVVSPKM
--FSDYPR--GVRYILFQHG------GRDT-------QYWAGWYG-PRVTNSSIVVSPKM
--FSDYPR--GVRYILFQHG------GRDT-------QYWAGWYG-PRVTNSSIVVSPKM
--FSDYPR--GVRYILFQHG------GSDT-------QYWAGWYG-PRVTNSSIVVSHKR
--FSDYPR--GVRYILFQHG------GRDT-------QYWAGWYG-PRVTNSSIVVSPKM
--FSDYPP--GVRHILFQHG------GQDT-------QFWKGWYG-PRVTNSSIIISHRT
--FSNYPP--GVRHILFQHG------GKDT-------QFWKGWYG-PRVTNSSIIVSHRT

RCNSPAKYDCYL------------QRATCKREGCGFDYCTRCLCNYHTTKDCSDGKFLKA
RCNSPAKYDCYL------------QRATCKREGCGFDYCTRCLCNYHTTKDCSDGKLLKA
RCNSPAKYDCYL------------QRATCKREGCGFDYCTKCLCNYHTTKDCSDGKLLKA
RCNSPAKYDCYL------------QRATCKREGCGFDYCTKCLCNYHTTKDCSDGKLLKA
RCNSPAKYDCYL------------QRATCKREGCGFDYCTKCLCNYHTTKDCSDGKLLKA
RCNSPAKYDCYL------------QRATCKREGCGFDYCTKCLCNYHTTKDCSDGKLLKA
AKYDHYL-----------------ERAVCKRESCQFEYCTKCLCAYHNNKDCLNGKILKA
CAYHNNK------------------------------------------DCLNSKNLKA
PGSVVADPMQPPPIAEEIDLLVFDLRAHRAYTPNDECFFIFLDVSRDFVASGAEDRHGYI
PGSVVADPMQPPPIAEEIDLLVFDLRAHRAYTPNDECFFIFLDVSRDFVASGAEDRHGYI
SGAVVADPMQPPPIAEEIDLLVFDLRAHRAYTPNDECFFIFLDVSRDFVASGAEDRHGYI
SGAVVADMQPPPIAEEGLLVFDLKLRAHRAYTPNDECFFIFLDVSRDFVASGAEDRHGYI
NGAVVADPMQPPPIAEEIDLLVFDLRAHRAYTPNDECFFIFLDVSRDFVASGAEDRHGYI
NGAVVADPMQPPPIAEEIDLLVFDLRAHRAYTPNDECFFIFLDVSRDFVASGAEDRHGYI
YLIVFPSPDLLLFSITGFLLQQFQPLYVLTTSADSVHLYMWEEGGCPPYLRSCCHLDSTW
YTIVFTNPYLLL-----FSITGFLDPRYVLTTSKNSVHVYMWEEGGRHPYLRSCYRLENI
YMIVFTSPYLLLFSITGFLLQRFEPCYVLTTSENSVHVYVWEEGGRHPYLRSCCHLENTW
YMIVFTSPYLLLFSITGFLLQRFEPCYVLTTSENSVHVYMWEEGGRHPYLRSCCHLENTW
YMIVFTSPYLLLFSITGFLLQRFEPCYVLTTSENSVHVYMWEEGGRHPYLRSCCHLENTW
```

```
YMIVFTSPYLLL-----FSITGFLPCYVLTTSENSVHVYMWEEGGRHPYLRSCCHLENTW
TTFSTTSGYMEGYYTFHFLYFKDKNVAIPRFHMACPTFRVSIARLEMGPDEYEEMEEEEE
TTFSTTSGYMEGYYTFHFLYFKDKNVAIPRFHMACPTFRVSIARLEMGPDEYEEMEEEEE
TTFSTTSGYMEGYYTFHFLYFKDKNVAIPRFHMACPTFRVSIARLEMGPDEYEEMEEEEE
TTFSTTSGYMEGYYTFHFLYFKDKNVAIPRFHMACPTFRVSIARLEMGPDEYEEMEEEEE
TTFSTTSGYMEGYYTFHFLYFKDKNVAIPRFHMACPTFRVSIARLEMGPDEYEEMEEEEE
TTFSTTSGYMEGYYTFHFLYFKDKNVAIPRFHMACPTFRVSIARLEMGPDEYEEMEEEEE
TTFSTTSGYMEGYYTFHFLYFKDKACPTFRVSIARLEMGPDEYEEMEEEAEEEEEEENDD
TTFSTTSGYMEGYYTFHFLYFKDKACPTFRVSIARLEMGPDEYEEMEEEAEEEEEEENDD
TILLTSRSELVLFTIHGLQLMAFQPDRVITAALDLSLRVYVWNKKNTSPVLKSCYHLLGG
VILLTSGSELVLFNIHGLQLMAFQPDRVITAALDLSLCVYVWNKENTSPILKSCYHLLGG
PASAPRPLLCNRGDRAKDITTSGYSLDGGMGGSPQSTSVLSVGSHSSTKPCHHQAKKSCL
PASAPWPLPCNRGDPGKDVTTSGYSLDGGMVGSPRSTSVLSVGSHSSTKPCYHQAKKSCL
DTQIPAVGPESPLCTSQQPGKDITSVDGSLGVLPRSTSVLSLDGDLRTQPYHHQARKPCL
DTQIPATGPKSLLRTSPEPGKDVTSVDGGLGVLPRPTSVLSLDSDSHTQPCHHQARKSCL
DTQIPATGPKPLLCTSREPGKDVTPTSSVDGGLGALPRPTSVLSLDSDSHTKPCHHQARK
DTQIPATGPKPLVRTSREPGKDVTSVDGGLGALPRPTSVLSLDSDSHTQPCHHQARKSCL
DTQIPATGPKPLVRTSREPGKDVTSVDGGLGALPRPTSVLSLDSDSHTQPCHHQARKSCL
DTQIPATGPKPLVRTSREPGKDVTSVDGGLGALPQPTSVLSLDSDSHTQPCHHQARKSCL
PHPTGSY-----------------------------------------------------
PHPTGFY-----------------------------------------------------
GADEFPE-----------------------------------------------------
PSRFART-----------------------------------------RGCLPTFLGAD
RKSHRAA---------------------------------------------PPAVCRTR
RSHRAAP------------------------------------------PCGFARTRVC
RSHRAAP------------------------------------------PCGFARTRVC
--VAPGT-----------------------------------------------------
VTHLSSKEEYVKVAKTLFIDEAIKPCPRCQSPAKYQPYKKRGLCSRTACGFDFCVLCLCA
VTHLSSKEEYVKVAKTLFIDEALKPCPRCQSPAKYQPYKKRGLCSRTACGFDFCVLCLCA
VTHLSSKEEYVKVAKTLFTDEALKPCPRCQSPAKYQPYKKRGLCSRTACGFDFCVLCLCA
VTHLSSKEEYVKVAKTLFTDEALKPCPRCQSPAKYQPYKKRGLCSQTACGFDFCVLCLCA
VTHLSSKEEYVKVAKTLFTDEALKPCPRCQSPAKYQPYKKRGLCSRTACGFDFCVLCLCA
VTHLSSKEEYVKVAKTLFTDEALKPCPRCQSPAKYQPYKKRGLCSRTACGFDFCVLCLCA
GKPLYQSMNCKPMQMYVLDIKDTKEKGRVKWKVFNSSSVV----------GPPETSLHTV
GKPLYQSMNCKPMQMYVLDIKDTKEKGRVKWKVFNSSSVV----------GPPETSLHTV
GKPLYQSMNCKPMQMYVLDIKDTKEKGRVKWKVFNSSSVV----------GPPETSLHTV
GKPLYQSMNCKPMQMYVLDIKDTKEKGRVKWKVFNSSSVV----------GPPETSLHTV
GKPLYQSMNCKPMQMYVLDIKDTKEKGRVKWKVFNSSSVV----------GPPETSLHTV
GKPLYQSMNCKPMQMYVLDIKDTKEKGRVKWKVFNSSSVV----------GPPETSLHTV
GKPLYQSMNCKPMQMYVLDIKDTKEKGRVKWKVFTSSSVV----------GPPETSLHTV
GKPLYQSMNCKPMQMYVLDIKDTKEKGRVKWKVFTSSSVV----------GPPETSLHTV
DAVLAFMARCLQVVMCHMFTGESLTCKSLQQSLLRSFQAERPALNVVIFPLLHEGLTDVI
DAVLAFMARCLQVVMCHMFTGESLTCKSLQQSLLRSFQAERPALNVVIFPLLHEGLTDVI
DAVLAFMARCLQVVMCHMFTGESLTCKSLQQSLLRSFQAERPALNVVIFPLLHEGLTDVI
DAVLAFMTRCLHVVMCHLFTGESLTCKSLQQSLLRSFQAERPALNVIIFPLLHEGLTDVI
```

DAVLAFMARCLQVVMCHLFTGESLTCKSLQQSLLRSFQAERPALNVVVFPLLHEGLTDVI
PQLFTELREEPPARRSRAIGSRQPSGITLVGVSPSLVVKTTCVYVLFKNLDYASTFFFFF
DAVLAFMARCLQVVMCHLFTGESLVTGRRPQLFTELREEPSARMSRATGRRQPCLPDSGV
PQLFTELREEPSARTSRATGRRQPVCCPCGRPLAVSGIILVGVSPSLVVKTTCVYRVLFK
KHLKVENFSNPPNVRNKVRIRNWMKYPKYPWGREIYTLEGVVDGAPYSMISDFPWLRSLR
KHLKVENFSNPPNVRNKVRIRNWMKYPKYPWGREIYTLEGVVDGAPYSMISDFPWLRSLR
KHLKVENFSNPPNVRNKVRIRSWMKYPKYPWGREIYTLEGVVDGAPYSMISDFPWLRSLR
KHLKVENFSNPPNVRNKVRIRSWMKYPKYPWGREIYTLEGVVDGAPYSMISDFPWLRSLR
KHLKVENFSNPPNVRNKVRIRSWMKYPKYPWGREIYTLEGVVDGAPYSMISDFPWLRSLR
KHLKVENFSNPPNVRNKVRIRSWMKYPKYPWGREIYTLEGVVDGAPYSMISDFPWLRSLR
KHLKVENFSNPPNVRNKVRIRSWMKYPKYPWGREIYTLEGVVDGAPYSMISDFPWLRSLR
KHLKVENFSNPPNVRNKVRIRSWMKYPKYPWGREIYTLEGVVDGAPYSMISDFPWLRSLR
SSGSGQNEESPR--------------------------------------KRKKATEAID
SSGSGQSEESPR--------------------------------------KRRKATEAID
SSGSGQSEESPR--------------------------------------KRRKATEAID
SSGSGQNEESPR--------------------------------------KRKKAGEAID
SSGSGQNEESPR--------------------------------------KRKKATEAID
SSGSGQNEESPR--------------------------------------KRKKATEAID
SSGSGQNEESPR--------------------------------------KRKKATEAID
SSGSGQNEESPR--------------------------------------KRKKATEAID
LLHFNSIRNLKSIGGSRLGLADYFEGLAFSKILHIKVEWRLLTRGRGRQGPCQPSPPGHS
VQGLASLRNCMR-----LQVLELDHVSEITQEVAAEVCREGLKGLEMLVLTATPVTPKAL
VQGLASLRNCMRLQVLELDHVSEIEMLVLTATPVTPKALLHFNSICRNLKSIVVQIGIAD
VQGLASLRNCMRLQVLELDHVSEIEMLVLTATPVTPKALLHFNSICRNLKSIVVQIGIAD
VQGLASLRNCMRLQVLELDHVSEIEMLVLTATPVTPKALLHFNSICRNLKSIVVQIGIAD
VQGLASLRNCMRLQVLELDHVSEIEMLVLTATPVTPKALLHFNSICRNLKSIVVQIGIAD
VQGLASLRNCMRLQVLELDHVSEIEMLVLTATPVTPKALLHFNSICRNLKSIVVQIGIAD
VQGLASLRNCMRLQVLELDHVSEIEMLVLTATPVTPKALLHFNSICRNLKSIVVQIGIAD
FEPDPAR---

FELGPAR---

HSDHLSSFEVLQHIAGFLDGFSLCMRDVCGSLLQSRGMVILQWGKRKYPEGNSSWQIKEK
NDHLSSLFEVLQHIAGFLDGFSLCMRDVCGSLLQSRGMVILQWGKRKYPEGNSSWQIKEK
HNDHLSSFEVLQHIAGFLDGFSLCLSCVSKVWRFSTAFCSVNEWKFADILSMADHLKKCS
HNDHLSSFEVLQHIAGFLDGFSLCMRDVCGSLLQSRGMVILQWGKRKYPEGNSSWQIKEK
HNDHLSSFEVLQHIAGFLDGFSLCMRDVCGSLLQSRGMVILQWGKRKYPEGNSSWQIKEK
HNDHLSSFEVLQHIAGFLDGFSLCMRDVCGSLLQSRGMVILQWGKRKYPEGNSSWQIKEK
RSDHLSSFEVLQHIAGFLDGFSLCMRDVCGSLLQSRGMVILQWGKKKYPEGNSSWQIKEK
RSDHLSSFEVLQHIAGFLDGFSLCMRDICGSLLQSRGMVILQWGKKKYPEGNSSWQIKEK
NSLTSLPLEILQYIAGFLDSVSLAMRNICATLLQERGMVLLQWKKKRYSHGGFSWRVHRE
NSLTSLPLEILQYIAGFLDSVSLAMRNICATLLQERGMVLLQWKKKRYSHGGTSWRVHRE
NSLTSLPLEILKYIAGFLDSVSLAMRNICATLLQERGMVLLQWKKKRYSHGGTSWRVHRE
NSLTSLPLEILKYIAGFLDSVSLAMRNICATLLQERGMVLLQWKKKRYSHGGTSWRVHRE
NSLTSLPLEILKYIAGFLDSVSLAMRNICATLLQERGMVLLQWKKKRYSHGGTSWRVHRE

```
NSLTSLPLEILKYIAGFLDSVSLAMRNICATLLQERGMVLLQWKKKRYSHGGTSWRVHRE
RDGKSLNLEVLQYIAGFLDSISLSMRNICATLLQERGMVLSQWKKKRYSHGGTSWKVHNQ
RDGKSLNLEVLQYIAGFLDSISLSMRNICATLLQERGMVLLQWKKKRYSHGGTSWKVHNQ
HQRFNKL-------------------------------------------IWIHHSCHIT
HQRFNKL-------------------------------------------IWIHHSCHIT
HQRFNKL-------------------------------------------IWIHHSCHIT
HQRFNKL-------------------------------------------IWIHHSCHIT
HQRFNKL-------------------------------------------IWIHHSCHIT
AFEIDKM---------------------------------------------YTPLFFAR
HQRFNKL-------------------------------------------IWIHHSCHIT
HQRFNKL-------------------------------------------IWIHHSCHIT
GETPSQFPLRPR-----FDPIGPL--------------------------PGPNPILPGR
EYDQRPTLPYVGDPIN--------SLIPGPGETPSQFAPLRPRFDPVGPLPGPNPILPGR
EYDQRPTLPYVGDPIS--------SLIPGPGETPSQFPPLRPRFDPVGPLPGPNPILPGR
EYDQRPTLPYVGDPIN--------SLIPGPGETPSQFPPLRPRFDPIGPLPGPNPILPGR
EYDQRPTLPYVGDPIN--------SLIPGPGETPSQFPPLRPRFDPIGPLPGPNPILPGR
PSQFPPL--RPR-----FDPIGPL--------------------------PGPNPILPGR
PGIIGGEYDERPILPSVGDPVT--SLIPRPGELPGQFRPLRPRFDPVDPLPGPHSLLPGR
PGIIGGEYDERPILPSVGDPVT--SLIPRPGEPPSQFRPVRPRFDPVGPLPGSNSLLPGR
STPVERN--------------------NFLQNVANAFACITMEMLQSIMSGDRDQDDRSF
STPVERN--------------------NFLQSVANAFACVIMEMLQSIMSGDRDEDDRSF
STPVERN--------------------NFLQNVANAFACVIMEMLQSIMSGDRDEDDRSF
STPVERK--------------------NFLQNVANAFACVIMEMLQSIMSGDRDEDDRSF
STPVERK--------------------NFLQNVANAFACVIMEMLQSIMSGDRDEDDRSF
STPVERK--------------------NFLQNVANAFACVIMEMLQSIMSGDRDEDDRSF
STAAERK--------------------SFLQSIANAFACVTMEMLQPVMSGDRDDDDRGF
MDWTVRMQKVCKVFNTATERKSFL------QSVANAFACVTMEMLQPVMSGERDDDDRGF
TGCFTRLRQLPNLQKLFLTANRSVCDTDIDELACNCTRLQQLDILGTRMVSPASLRKLLE
TGCFTRLRQLPNLQKLFLTANRSVCDTDIDELACNCTRLQQLDILGTRMVSPASLRKLLE
TGCFTRLHQLPNLQKLFLTANRSVCDTDIDELACNCTRLQQLDILGTRMVSPASLRKLLE
TGCFTRLRQLPNLQKLFLTANRSVCDTDIEELACNCTRLQQLDILGTRMVSPASLRKLLE
TGCFTRLRQLPNLQKLFLTANRSVCDTDIEELACNCTRLQQLDILGTRMVSPASLRKLLE
TGCFTRLRQLPHLQKLFLTANRSVCDTDIEELACNCTRLQQLDILGTRMVSPASLRKLLE
TGCFVRLRQLPNLQKLFLTANRSVCDTDIEELASNCTRLQQLDILGTRMVSPASLRKLLE
TGCFARLRQLPNLQKLFLTANRSVCDTDIEELASNCTRLQQLDILGTRMVSPASLRKLLE
ETLTGFL-------------------------------------------NGIEWILEEV
ETLTGFL-------------------------------------------NGIEWILEEV
ETLTGFL-------------------------------------------NGIEWILEEV
ETLTGFL-------------------------------------------NGIEWILEEV
ETLTGFL-------------------------------------------NGIEWILEEV
ETLTGFL-------------------------------------------NGIEWILEEV
ETLTGFL-------------------------------------------NGIEWILEEV
ETLTGFL-------------------------------------------NGIEWILEEV
------------------------------------------------------------
------------------------------------------------------------
```


REVEKLQ---
GGRLSQLEEVLI--------------------------------------PDQKYSLEQI
GGRLSQLEEVLI--------------------------------------PDQKYSLEQI
GGRLSQLEEVLI--------------------------------------PDQKYSLEQI
GGRLSQLEEVLI--------------------------------------PDQKYSLEQI
GGRLSQLEEVLI--------------------------------------PDQKYSLEQI
GGRLSQLEEVLI--------------------------------------PDQKYSLEQI
GGRLSQLEEVLI--------------------------------------PDQKYSLEQI
GGRLSQLEEVLI--------------------------------------PDQKYSLEQI
GRRLTQLEEVLV--------------------------------------PDDRYTPDEV
GRRLTQLEEVLV--------------------------------------PDDRYTPDEV
ERRLTQLEEVLI--------------------------------------PDEDYSLDEI
GRRLTQLEEVLI--------------------------------------PDEDYSLDEI
GRRLTQLEEVLI--------------------------------------PDEDYSLDEI
GRRLTQLEEVLI--------------------------------------PDEDYSLDEI
GLFLASGVNVTMKDNKIMNNQDAISYPMHDFYRCHTCNTTDRNAICVNCIKKCHQGHDVE
GLFLASGVNVTMKDNKIMNNQDAISYPMHDFYRCHTCNTTDRNAICVNCIKKCHQGHDVE
GLFLASGVNVTMKDNKIMNNQDAISYPMHDFYRCHTCNTTDRNAICVNCIKKCHQGHDVE
GLFLASGVNVTMKDNKIMNNQDAISYPMHDFYRCHTCNTTDRNAICVNCIKKCHQGHDVE
GLFLASGVNVTMKDNKIMNNQDAISYPMHDFYRCHTCNTTDRNAICVNCIKKCHQGHDVE
GLFLASGVNVTMKDNKIMNNQDAISYPMHDFYRCHTCNTTDRNAICVNCIKKCHQGHDVE
GLFLASGVNVTMKDNKIMNNQDAISYPMHDFYRCHTCNTTDRNAICVNCIKKCHQGHDVE
GLFLASGVNVTMKDNKIMNNQDAISYPMHDFYRCHTCNTTDRNAICVNCIKKCHQGHDVE
VSGAYDG------KIKVWDLQAALDPRAPASTLCLR--------------TLVEHSGRVF
VSGAYDG------KIKVWDLQAALDPRAPASTLCLR--------------TLVEHSGRVF
VSGAYDG------KIKVWDLQAALDPRAPASTLCLR--------------TLVEHSGRVF
VSGAYDG------KIKVWDLQAALDPRAPASTLCLR--------------TLVEHSGRVF
VSGAYDG------KIKVWDLQAALDPRAPASTLCLR--------------TLVEHSGRVF
VSGAYDG------KIKVWDLQAALDPRAPASTLCLR--------------TLVEHSGRVF
VSGAYDG------KIKVWDLQAALDPRAPASTLCLR--------------TLVEHSGRVF
VSGAYDG------KIKVWDLQAALDPRAPASTLCLR--------------TLVEHSGRVF
VSGAYDG------KIKVWDLVAALDPRAPAGTLCLR--------------TLVEHSGRVF
VSGAYDG------KIKVWDLMAALDPRAPAGTLCLR--------------TLVEHSGRVF
VSGAYDG------KIKVWDLMAALDPRAPAGTLCLR--------------TLVEHSGRVF
VSGAYDG------KIKVWDLVAALDPRAPAGTLCLR--------------TLVEHSGRVF
VSGAYDG------KIKVWDLVAALDPRAPAGTLCLR--------------TLVEHSGRVF
VSGAYDG------KIKVWDLVAALDPRAPAGTLCLR--------------TLVEHSGRVF
VSGAYDG------KIKVWDLVAALDPRAPAGTLCLR--------------TLVEHSGRVF
VSGAYDG------KIKVWDLVAALDPRAPAGTLCLR--------------TLVEHSGRVF

STGSSLLYSITGLHLQTFPYGPGEVDPLHVIVTFIDGSLELFAWEERSPLLRRCYRLYNK
SSLLLFDINGLRLKTFQYCPEMIVVDPLHVIVICNTGSMDVYAWEERSLLLRKCYRLHIE
SVIVCSTSSLLL-----FSMKGLHVDPVHVIITCNDGSIDVYMWEERSLLLKMCYRLQNG
DVIVCSTSSLLLFNIKGVYLQTIMMDPIYVIVTFNNGSLVVYAWEERCQQLNGCYQLQSR
SSLLLFSIYGVCLQTFDYCSEEILVDPFHVIVTFIDGSLDVYAWEERCQQLSKCYRLQNR
STRFSLLFDINGHCLQAFQYCPEQVDPLHVIVSCNDGFLDVYAWEERSQQLNKCYRLQYS
STGSSLLFNIHGLRLQTFHYCPEEVDPLHVIVTCNDGSMDVYAWEERSLLLRKCYRLQNR
STGSSLLFDINGLRLQTFQYCPEEVDPVHVIVTCNDGSLDVYAWEERSLLLRKCYRLLNR
STRFSLLFDINGLRLQTFHYCPEEVDPVHVIVTCDAGSLDIYAWEKRSLLLRKCYRLRNR
STRFSLLFDINGLRLQTFHYCPEEVDPVHVIVTCDAGSLDIYAWEKRSLLLRKCYRLRNR
STGSSLLFDINGLRLQTFQYCPEEVDPVHVIVTCNNGSLDVYVWEERSPLLRRCYRLRKR
DTIVCST----GSSLLLFDMNGLRVDPVHVIVTCNDGSLDIYAWEERSPLLRKCYRLQNR
SSLLLFDGLCLQSFQYCPEMITRLVDPVHVIITCNNGSLDVYAWEERSPLLRKCYRLQNR
KLWEVHSHPVRY-----LSFNSHSTANVPYEKVLRNSDLDNFACHRRHGLIHAYEFAVD
KLWEVHSRHPVR--YISFNSHSLITANVPYEKVLRNSDLDNFACHRRHGLIHAYEFAVD
LWEVYSGHPVKHISFSSRSLI---TANVPYQPVVRNADRDNFTPHRRHQGLIRAYEFAVD
KLWEVYSHPVQH-----ISFSSHSTANVPYQTVMRNVDLDSFTTHRRHRGLIRAYEFAVD
LWEVYSGHPVQHISFSSHSLI---TANVPYQTVMRNADLDSFTTHRRHRGLIRAYEFAVD
LWEVYSGHPVQHISFSSHSLI---TANVPYQTVMRNADLDSFTTHRRHRGLIRAYEFAVD
KLWEV--YSGHPVQHISFSSHSLITANVPYQTVMRNADLDSFTTHRRHRGLIRAYEFAVD
KLWEVYSHPVQH---ISFSSHSLITANVPYQTVMRNADLDSFTTHRRHRGLIRAYEFAVD
INDTYIVSSCERGLVKVWHVAMAQTLNGHEGAVKCLFFDQWHLLSGSTDGLVMAWSMVGK
DEHLTSSRAGPLTSMQVIKPNRMLRPRFYTTLDPLRMNTGFMLMTVKEEKEFAEAKMKEY
RMVVHTDSSILV-----FQFENVKWQYSSDKNKTKKNKDKEEEREETSLGDTHSKSSLQV
GDQVTSSSTGPLTSMQVIKPNRMLRPCMYTALDPLRVNTEFMLLTVKEEKEYQEAKMKEY
GDQVTSSVCSPLTSMQVIKPNRMLRPRIYTALDPLRVNTEFVLLTVKEEKEHQEAKMKEY
GDQMTSSSTGPLTSMQVIKPNRMLRPRIYTALDPLRVNTEFVLLTVKREKEHQEAKMKEY
GDQVTSSSTGPLTSMQVIKPNRMLRPRIYTALDPFRVNTEFVLLTVKEEKEHQEAKMKEY
GDQVTSSSTGPLTSMQVIKPNRMLRPRIYTALDPFRVNTEFVLLTVKEEKEHQEAKMKEY
KRGSSFL--AGEASWLNGLD
KRGSSFL--AGEASWLNGLD
KRGSSFL--AGEASWLNGLD
KRGSSFL--AGEASWLNGLD
KRGSSFL--AGEASWLNGLD
KRGSSFL--AGEASWLNGLD
KRGSSFL--AGEASWLNGLD
KRGSSFL--AGEASWLNGLD
REFIRNTRRAAQ--------------------------------------NISEDFVGHL
REFIRNTRRAAQ--------------------------------------NISEDFVGHL
REFIRNTRRAAQ--------------------------------------NISEDFVGHL
REFIRNTRRAAQ--------------------------------------NISEDFVGHL
REFIRNTRRAAQ--------------------------------------NISEDFVGHL
REFIRNTRRAAQ--------------------------------------NISEDFVGHL
REFIRNTRRAAQ--------------------------------------NISEDFVGHL
REFIRNTRRAAQ--------------------------------------NISEDFVGHL

```
CPSLNDEYYCDN---------------------------------INGPHADTASGCQNL
CPSLNDEYYCDN---------------------------------INGPHADTASGCQNL
CPSLNDEYYCDN---------------------------------INGPHADTASGCQNL
CPSLNDEYYCDN---------------------------------INGPHADTASGCQNL
CPSLNDE-------------------------------YFYYCDNINGPHADTASGCQNL
CPSLNDE-------------------------------YFYYCDNINGPHADTASGCQNL
CPSLNDEYYCDN---------------------------------INGPHADTASGCQNL
TGAGLQDSACPS-----LNDEYFYYCDNINGPH-----------------ADTASGCQNL
KNFVITSSDDGT---------------VKLWDLKTGEFIRNLVTLESGGSGGVVWRIRAS
KNFVITS---------------------------------------SDDGTVKLWDLKTG
KNFVITSSDDGT---------------VKLWDLKTGEFIRNLVTLESGGSGGVVWRIRAS
KNFVITSSDDGT---------------VKLWDLKTGEFIRNLVTLESGGSGGVVWRIRAS
KNFVITSSDDGT---------------VKLWDLKTGEFIRNLVTLESGGSGGVVWRIRAS
KNFVITSSDDGT---------------VKLWDLKTGEFIRNLVTLESGGSGGVVWRIRAS
KNFVITSSDDGT---------------VKLWDLKTGEFIRNLVTLESGGSGGVVWRIRAS
KNFVITS-----------------DGTVKLWDLKTGEFIRNLVTLESGGSGGVVWRIRAS
DVAQLHCDEEISFSLRLYEHICDLALLVSSRGTSHTPFERTSKTTYQFIASVALHRLLIE
DVAQLHCDEEISFSLRLYEHIHDLALLVSSRGTSHTPFERTSKTTYQFIASVALHRLLIE
DVAQLHCDEEISFSLRLYEHIHDLALLVSSRGTSHTPFERTSKTTYQFIASVALHRLLIE
DVAQLHCDEEISFSLRLYEHIHDLALLVSSRGTSHTPFERTSKTTYQFIASVALHRLLIE
DVAQLHCDEEISFSLRLYEHIHDLALLISSRGTSHTPFERTSKTTYQFIASVALHQLLIE
------------------------------------------------------------
------------------------------------------------------------
DVVQLHCDEKMDFSLRLYEQIRDLVLLVSSRSTSHTPFEKTSKTTYQFTASVALPRLLIE
EIFVTI------------------------------------------------------
PATATTA--------------------------------------------------EIL
GTQCLGG-------------------------------------------ALGCPTLGAT
KNKLQDR-----------------------------------------TRLRKAQSMMSR
NPFPLCP-----------------------------------------------------
NPFPLCP-----------------------------------------------------
LQKHPGL-----------------------------------------------------
HEATTEV-------------------------------------------LTEHHQNHPG
KSIAKQAERVFPSNESHVLFIGDGYCRYQSKKKLNLFKLTPFMVNSVLAEGKGGFIRAKL
FSPLLCL---ESFSEDEWNLLYVARCCVGQCNNAIPVDTVLTMKKLPITYSNRKENKGGY
KRLIMTKENILTLAGEYFLQAELTRCCVGQCNNAIPVDTVLTMKKLPITYSNRKENKGGY
DDFVKVPPQLPHFRVESFSEDEWNAVTRAKKRLIMTKSLENILTLAGEYFLQAELTSNVL
KRLIMTKENILTLAGEYFLQAELTRCCVGQCNNAIPVDTVLTMKKLPITYSNRKENKGGY
KRLIMTKENILTLAGEYFLQAELTRCCVGQCNNAIPVDTVLTMKKLPITYSNRKENKGGY
KRLIMTKENILTLAGEYFLQAELTHCCVGQCNNTIPVDTILTMKKLPITYSNRKENKGGY
KRLIMTKENILTLAGEYFLQAELTHCCVGQCNNTIPVDTILTMRKLPITYSNRKENKGGY
GSDLKVLVTEES-----IDFDQGELADQDVDPVH----------------NLIEQVSLGL
GSDLKVLVTEES-----IDFDQGELADQDVDPVH----------------NLIEQVSLGL
GSDLKVLVTEES-----IDFDQGELADQDVDPVH----------------NLIEQVSLGL
GSDLKVL--------------------EVTEESIDFDQGELADQDVDPVHNLIEQVSLGL
GSDLKVL--------------------EVTEESIDFDQGELADQDVDPVHNLIEQVSLGL
```

```
GSDLKVL--------------------EVTEESIDFDQGELADQDVDPVHNLIEQVSLGL
GSDLKVLVTEES-----IDFDQGELADQDVDPVQ----------------NLIEQVSLGL
GSDLKVLVTEES-----IDFDQGELADQDVDPVH----------------NLLEQVSLGL
HEWIRNMHSLPHGHHQPF------YNVLVEDGSCRYAAQENLEYNVEPQEISHPDVGRYF
HEWIRNMHSLPHGHHQPF------YNVLVEDGSCRYAAQENLEYNVEPQEISHPDVGRYF
HEWIRNMHSLPHGHHQPF------YNVLVEDGSCRYAAQENLEYNVEPQEISHPDVGRYF
HEWIRNMHSLPHGHHQPF------YNVLVEDGSCRYAAQENLEYNVEPQEISHPDVGRYF
HEWIRNMHSLPHGHHQPF------YNVLVEDGSCRYAAQENLEYNVEPQEISHPDVGRYF
HEWIRNMHSLPHGHHQPF------YNVLVEDGSCRYAAQENLEYNVEPQEISHPDVGRYF
HEWIRNMHSLPHGHHQPF------YNVLVEDGSCRYAAQENLEYNVEPQEISHPDVGRYF
LYMLSSHEQCPVYRDLPASRVGGSAPQDPGGTAQACEEYLSQIHSCPTLQDRMEKMKEIV
LYMLSSHEQCPAYRDLPASRVGGSAPQDPGGTAQACEEYLSQIHSCPTLQDRMEKMKEIV
HDIEEHTRDLPASRVMGTPEPSLGAPQDPGGTARACEEYLSQIHSCHTWQDRMEKMKEII
CMTSNQS-------------------------------------------STLYVTDPT
HDIEQHARHLPASRVVGTPEPSLGAPQDPGGMAQACEEYLSQIHSCQTLQDRTEKMKEIV
HDIEQHARDLPANRVVGTPEPSLGAPQDPGGMAQACEEYLSQIYSCQTLQDRMEKMKEIV
AKILFCAGYNHLGLVDEFGRIFMQ--------------------------GNNRYGQLGT
HDIEQHARDLPASRVVGTPEPSLGAPQDPGGMAQACEEYLSQIHSCQTLQDRMEKMKEIV
GTDHPCT-------------------------------------------ANNPESCSVS
GTDHPCT-------------------------------------------ANNPESCSVS
GTDHPCT-------------------------------------------ANNPESCSVS
GTDHPCT-------------------------------------------ANNPESCSVS
GTDHPCT-------------------------------------------ANNPESCSVS
GTDHPCT-------------------------------------------ANNPESCSVS
GTDHPCT-------------------------------------------ANNPESCSVS
LLFKDSGHPCTA-------------------------------------ADPDSCFTP
LLFKDSGHPCTA-------------------------------------ADPDSCFMP
LLFKDSGHPCTA-------------------------------------ADPDSCFTP
LLFKDSGHPCTA-------------------------------------ADPDSCFTP
LLFKDSGHPCTA-------------------------------------ADPDSCFTP
LLFKDSGHPCTA-------------------------------------ADPDSCFTP
WLLKDSGHPCTA-------------------------------------ADPDSCFTP
------------------------------------------------------------
TDHCLPLRRCPR-----LRRLDLRSCRQLSPEACAR--------------LAAAGPPGPF
TDHCLPLRRCPR-----LRRLDLRSCRQLSPEACAR--------------LAAAGPPGPF
TDHCLPLRRCPR-----LRRLDLRSCRQLSPEACAR--------------LAAAGPPGPF
QLRELLLPPPDTKPGQTESRGRLQGVAELRLAGLELTDASLRLLLRHAPQLSALDLSHCA
TDHCLPLRRCPR-----LRRLDLRSCRQLSPEACAR--------------LAAAGPPGPF
TDHCLPLRRCPR-----LRRLDLRSCRQLSPEACAR--------------LAAAGPPGPF
TDHCLPLRRCPR-----LRRLDLRSCRQLSPEACAR--------------LAAAGPPGPF
TDHCLPLRRCPR-----LRRLDLRSCRQLSPEACAR--------------LAAAGPPGPF
TDQTLIYRRIAN-----VTLIDLRGCKQITRKACEH--------------FISDLSINSL
TDQTLIYRRIAN-----VTLIDLRGCKQITRKACEH--------------FISDLSINSL
TDQTLIYRRIAN-----VTLIDLRGCKQITRKACEH--------------FISDLSINSL
TDQTLIYRRIAN-----VTLIDLRGCKQITRKACEH--------------FISDLSINSL
```

```
TDQTLIYRRIAN-----VTLIDLRGCKQITRKACEH--------------FISDLSINSL
TDQTLIYRRIAN-----VTLIDLRGCKQITRKACEH--------------FISDLSINSL
TDQTLFFRRIAN-----VTLIDLRGCKQITRKACEH--------------FISDLSINSL
TDQTLFFRRIAN-----VTLIDLRGCKQITRKACEH--------------FISDLSINSL
TDQCLSFKRCGN-----ICHIDLRYCKQVTKEGCEQF-------------IAEMSVSVQF
TDQCLSFKRCGN-----ICHIDLRYCKQVTKEGCEQF-------------IAEMSVSVQF
TDQCLSFKRCGN-----ICHIDLRYCKQVTKEGCEQF-------------IAEMSVSVQF
TDQCLSFKRCGN-----ICHIDLRYCKQVTKEGCEQF-------------IAEMSVSVQF
TDQCLSFKRCGN-----ICHIDLRYCKQVTKEGCEQF-------------IAEMSVSVQF
TDQCLSFKRCGN-----ICHIDLRYCKQVTKEGCEQF-------------IAEMSVSVQF
TDLCLSFKRCGN-----ICHIDLRYCKQVTKEGCEQF-------------IAEMSVSVQF
TDQCLSFKRCGN-----ICHIDLRYCKQVTKEGCEQF-------------IAEMSVSVQF
CRWHPKPHHDLPYGRSYWMCCR--RADRETPGCRLGLHDNNWVLPCNGPGGGRAGREEGR
CRWHPKPYHHDL----PYGRSYWMCCRRADRETP----------------GCRLGLHDNN
CRWHPKPYHHDL----PYGRSYWMCCRRADRETP----------------GCRLGLHDNN
CRWHPKPYHHDL----PYGRSYWMCCRRADRETP----------------GCRLGLHDNN
CRWHPKPYHHDL----PYGRSYWMCCRRADRETP----------------GCRLGLHDNN
DDPCKQC--RKRYEKGDVSLCRWHPKPYHHDLPYGRSYWMCCRRADRETPGCRLGLHDNN
CRWHPKPHHDLPYGRSYWM-----CCRRADRETP----------------GCRLGLHDNN
CRWHPKPHHDLP-----YGRSYWMCCRRADRETP----------------GCRLGLHDNN
EDPCKQC--KKKYVKGDVSLCRWHPKPYCQALPYGPGYWMCCHRSQKGFPGCKLGLHDNH
EDPCKQC--KKKYVKGDVSLCRWHPKPYCQALPYGPGYWMCCHRSQKGFPGCKLGLHDNH
EDPCKQC--KKKYVKGDVSLCRWHPKPYCQALPYGPGYWMCCHRSQKGFPGCKLGLHDNH
EDPCKQC--KKKYVKGDVSLCRWHPKPYCQALPYGPGYWMCCHRSQKGFPGCKLGLHDNH
EDPCKQC--KKKYVKGDVSLCRWHPKPYCQALPYGPGYWMCCHRSQKGFPGCKLGLHDNH
EDPCKQC--KKKYVKGDVSLCRWHPKPYCQALPYGPGYWMCCHRSQKGFPGCKLGLHDNH
EDPCKQC--KKKYVKGDVSLCRWHPKPYCQALPYGPGYWMCCHQSQKGFPGCKLGLHDNH
EDPCKQC--KKKYVKGDVSLCRWHPKPYCQALPYGPGYWMCCHQSQKGFPGCKLGLHDNH
ENQAEKL-----------------------------------------------------
GSETTKP-----------------------------------------------------
TEA---------------------------------------------------------
-----QP-----------------------------------------------------
-----QP-----------------------------------------------------
QRQLRKR-----------------------------------------------------
QRQLRKR-----------------------------------------------------
QRQLRKR-----------------------------------------------------
SVLDAFRAHCPR---------------------------------------LRNYTLKLT
SVLEAFRAHCPR---------------------------------------LRTYTLKLT
SVLDAFRAHCPR---------------------------------------LRTYTLKLK
SVLDAFRAHCPR---------------------------------------LRTYTLKLT
SVLDAFRAHCPR---------------------------------------LRTYTLKLT
SVLDAFRAHCPR---------------------------------------LRTYTLKLT
SVLDAFRAHCPR---------------------------------------LRSYTLKLK
SVLDAFRAHCPR---------------------------------------LRSYTLKLK
DMTLLDEDKKPIWYVS--------SPVCLRSACLPDFPQPAYSFEYMDSVGGVCADLGWF
```

DVTLLDEHRKPFWCVSSP------VCMRSPAYPSDGSSFLGNTYYVDYMDKEGGVHAELV
CSMMDVTHGRPF-----WCFSSP-VCMRLPATPSDGPSLLGQTYSVDYVDAEGRVHVELV
CSMMDVTHGKPF-----WCFSSP-VCMRSPATPADGPSFLGQTYSVDYVDAEGRVHVELV
CSMMDITHGKPF-----WCFSSP-VCMRSPATPSDSPSFLGQTYNVDYVDAEGRVHVELV
CSMMDVTLDEHG-----KPFWCFSVCLRSPATPSDSSSFLGQTYNVDYVDAEGRVHVELV
NGHVKLIVIHLK----------------------------------NNREHLPLIGKVGL
DVTLLDEHGKPF-----WCFSSP-VCLRSPATPSDSSSFLGQTYNVDYVDAEGRVHVELV
PSTVSSVSGCPG-----LLYLNLESCRCLPRGLK----------------RVYRGLEEVQ
PSTVSSVSSCPG-----LLYLNLESCRCLPRGLK----------------RAYRGLEEVQ
PSTVSSVSSCPGLLYLNLESCRCL---------------------PRGLKRAYRGLEEVQ
PSTVSSVSSCPGLLYLNLE-----SCRCLPRGLK----------------RAYRGLEEVQ
PSTVSSVSSCPGLLYLNLE-----SCRCLPRGLK----------------RAYRGLEEVQ
PSTVSSVSSCPGLLYLNLE-----SCRCLPRGLK----------------RAYRGLEEVQ
PSTVSSVSSCPGLLYLNLE-----SCRCLPRGLK----------------RAYRGLEEVQ
PSTVSSVSSCPGLLYLNLE-----SCRCLPRGLK----------------RAYRGLEEVQ
TMDSKKN---LEIWGIKCR
CAYFTSI---ARPTMDNKKNPEI
TIGNKKN---QEIWGIKCR
TIGNKKN---QEIWGIKCR
SKLKSSH---KLFCQHVRVICIF
SKLKSSH---KLFCQHVRVICIF
CIFVCDF---
RNLRLLSAGCPLLTTTGLSGLVQL--------------QELEELELTNCPGATPELFKYF
RSLRLLSAGCPLLTTTGLSGLVQL--------------QELEELELTNCPGATPELFKYF
QEVEEQE---LTTCPGSTELK
GSLRLLSAGCPLLTTTGLSGLVQL--------------QELEELELTNCPGATPELFKYF
CQVQDFG---LKHLL
GSLRLLSAGCPLLTTTGLSGLVQL--------------QELEELELTNCPGATPELFKYF
GSLRLLSAGCPLLTTTGLSGLVQL--------------QELEELELTNCPGATPELFKYF
GSLRLLSAGCPLLTTTGLSGLVQL--------------QELEELELTNCPGATPELFKYF
ILACRKLFYFKI-----WAFLDVKILKSQEEGQCSLHTLKVRIYTNRYETNEEDRTLREI
KFVERIL-------------------KSQEEGQCSLRTLKVRIYTNRYETNEEDRTLREI
ILSCRKLFYFKI-----WAFLDVRILKSQKEGKCALRTLKVRIYTNRYETNEEDRTLREI
IVSCRKLFYFKI-----WAFLDVRILKSQKERQCALRTLKVRIYTNRYETNEEDRTLREI
IISCRKLFYFKI-----WAFLDVSILKSQKERQCALRIFKVRIYTNRYETNEEENTLREI
IDLLPTFRHTLQKLTCEFNNNHESLDEELHLLIISCRKLFYFKIWAFLDVSFVERILKSQ
IISCRKLFYFKI-----WAFLDVSILKSQKERQCALRVFKARIYTNRYETNEEDKTLQEI
IISCRKLFYFKIWAFLDVSFVER-ILKSQKERQCALRVFKARIYTNRYETNEEDKTLQEI
DYALIAIRYSMT-----IETVDVGWCKEITDQGATLIAQSSKSLRYLGLMRCDKVNEVTV
ISIVNSLMSYPS------------------------FSIPTPIYSILYIHYIYIYTIIAM
CLKLQRI---
DYALIAIGRYSVTIETVDVG----WCKEITDQGATLIAQSSKSLRYLGLMRCDKVNELTV
DYALIAIRYSMT-----IETVDVGWCKEITDQGATLIAQSSKSLRYLGLMRCDKVNEVTV
DYALIAIRYSMT-----IETVDVGWCKEITDQGATLIAQSSKSLRYLGLMRCDKVNEVTV
DYALIAIRYSMT-----IETVDVGWCKEITDQGATLIAQSSKSLRYLGLMRCDKVNEVTV

```
ITGIKHS-----------------LGTLYTTSTDKTIRVHVPTDPPRTICTRSHHNVLNG
ITGIKHS-----------------LGTLYTTSTDKTIRVHVPTDPPKTICTRRHHNVLNG
ITGIQYS-----------------LGALYTASTDKTLRVHVPTDPPRTICTRRHDNGFTK
ITGIQYS-----------------VGALYTTSTDRSIRVHVPTDPPRTICTRKYDSTLNR
ITGIQYS-----------------VGALYTTSTDKTIRVHVPTDPPRTICTRKHDNGLNR
ITGIQYS-----------------VGALYTTSTDKTIRVHVPTDPPRTICTRRHDNGLNR
ITGIQYS-----------------VGALYTTSTDKTIRVHVPTDPPRTICTRRHDNGLNR
ITGIQYS-----------------VGALYTTSTDKTIRVHVPTDPPRTICTRRHDNGLNR
DRIVTGN---------------------------------------FILRRCNEVKEEDL
DRIVTGN---------------------------------------FILRRCNEVKEEDL
DRIVTGN---------------------------------------FILRKCNEVKDDDL
DRIVTGN---------------------------------------FILRKCNEVKDDDL
DRIVTGN---------------------------------------FILRKCNEVKDDDL
DRIVTGN---------------------------------------FILRKCNEVKDDDL
DRIVTGN---------------------------------------FILRKCNEVKDDDL
DRIVTGN---------------------------------------FILRKCNEVKDDDL
CFDLQTLQDCEV--------------------------------------SVEALRFVKR
CFDLQMLQDCEV--------------------------------------SVEALRFVKR
CFDLQMLQDCEV--------------------------------------SVEALRFVKR
CFDLQTLQDCEV--------------------------------------SVEALRFVKR
CFDLQTLQDCEV--------------------------------------SVEALRFVKR
CFDLQTLQDCEV--------------------------------------SVEALRFVKR
CFDLQTLQDCEV--------------------------------------SVEALRFVKR
CFDLQTLQDCEV--------------------------------------SVEALRFVKR
LPHVKVHYFAPV-----TPPTAVA--------------------------GSGQRLCRCC
RGLERLEYDCQQVTRAGIKRMRAQ--------------LPHVKVHAYFAPVTPPTAVAGS
RGLERLEYDCQQVTRAGIKRMRAQ--------------LPHVKVHAYFAPVTPPTAVAGS
RGLERLEYDCQQVTRAGIKRMRAQ--------------LPHVKVHAYFAPVTPPTAVAGS
RGLERLEYDCQQVTRAGIKRMRAQ--------------LPHVKVHAYFAPVTPPTAVAGS
RGLERLEYDCQQVTRAGIKRMRAQ--------------LPHVKVHAYFAPVTPPTAVAGS
RGLERLEYDCQQVTRAGIKRMRAQ--------------LPHVKVHAYFAPVTPPPAVAGS
RGLERLEYDCQQVTRAGIKRMRAQ--------------LPRVKVHAYFAPVTPPPAVAGS
HSLERIEYDCQQITRAGIKRLRTH--------------LPNIKVHAYFAPVTPPPSVGGS
HSLERIEYDCQQITRAGIKRLRTH--------------LPNIKVHAYFAPVTPPPSVGGS
HSLERIEYDCQQITRAGIKRLRTH--------------LPNIKVHAYFAPVTPPPSVGGS
HSLERIEYDCQQITRAGIKRLRTH--------------LPNIKVHAYFAPVTPPPSVGGS
HSLERIEYDCQQITRAGIKRLRTH--------------LPNIKVHAYFAPVTPPPSVGGS
HSLERIEYDCQQITRAGIKRLRTH--------------LPNIKVHAYFAPVTPPPSVGGS
HSLERIEYDCQQITRAGIKRLRTH--------------LPNIKVHAYFAPVTPPPSVGGS
HSLERIEYDCQQITRAGIKRLRTH--------------LPNIKVHAYFAPVTPPPSVGGS
GVDIDVE---PP--------------------------------------LHQALVLLQD
GVDIDVE-----------------------------------------PPLHQALVLLQD
GVDIDVE---PP--------------------------------------LHQALVLLQD
GVDIDVE---PP--------------------------------------LHQALVLLQD
GVDIDVE---PP--------------------------------------LHQALVLLQD
```

```
GVDIDVE---PP--------------------------------------LHQALVLLQD
GVDIDVE---PP--------------------------------------LHQALVLLQD
GVDIDVE-----------------------------------------PPLHQALVLLQD
LSQLTGI----------------------------------------DLYGCTRITKRGL
LSQLTGIYGCTRITKRGLERITQL--------------------------PCLKVLNLGL
LSQLTGIYGCTRITKRGLERITQL--------------------------PCLKVLNLGL
LSQLTGIYGCTRITKRGLERITQL--------------------------PCLKVLNLGL
LSQLTGIYGCTRITKRGLERITQL--------------------------PCLKVLNLGL
LSQLTGIYGCTRITKRGLERITQL--------------------------PCLKVLNLGL
LSQLTGIYGCTRITKRGLERITQL--------------------------PCLKVLNLGL
CINLTSLAGCPKITDSVMEMLSAKGCVLLTDQILDDLQIGCKQLRILRMQYCTNISKNAA
CINLTSLAGCPKITDSAMEMLSAKGCVLLTDQILEDLQIGCKQLRILKMQYCTNISKKAA
CINLTSLAGCPKITDSAMEMLSAKGCVLLTDQILEDLQIGCKQLRILKMQYCTNISKKAA
CINLTSLAGCPKITDSAMEMLSAKGCVLLTDQILEDLQIGCKQLRILKMQYCTNISKKAA
CINLTSLAGCPKITDSAMEMLSAKGCVLLTDQILEDLQIGCKQLRILKMQYCTNISKKAA
CINLTSLAGCPKITDSAMEMLSAKGCVLLTDQILEDLQIGCKQLRILKMQYCTNISKKAA
CTRITSLAGCPKITDAGMEILSARGCIQLTDQIIQDLQIGCKQLRILKMQFCKSISPAAA
CTGIMHL----------------------------------------------TINDMPT
CLYPAVS----------------------------------------AVYGNTEVTLVYL
CLYPAVS----------------------------------------AVYGNTEVTLVYL
CLYPAVS----------------------------------------AVYGNTEVTLVYL
CLYPAVS----------------------------------------AVYGNTEVTLVYL
TQIVSSCLTMPKLRVLEVQGLGWE--------------------------GQEAEKILCK
AQIVSSCLTMPKLRVLEVQGLGWE--------------------------GQEAEKVLCK
TEILSSCLTMPKLRVLELQGLGWE--------------------------GQEAEKILCK
PEMPSPT--------------------EILSSCLTMPKLRVLELQGLGWEGQEAEKILCK
PEMPSPT--------------------EILSSCLTMPKLRVLELQGLGWEGQEAEKILCK
PEMPSPT--------------------EILSSCLTMPKLRVLELQGLGWEGQEAEKILCK
PEMPSPT--------------------EILSSCLTMPKLRVLELQGLGWEGQEAEKILCK
PEMPSPT--------------------EILSSCLTMPKLRVLELQGLGWEGQEAEKILCK
FPLTSTPLSSPV------------YCLR----------------------LTTKHLYAAL
FPLTSTPLSSPV------------YCLR----------------------LTTKHLYAAL
FPLTSTPLSSPV------------YCLR----------------------LTTKHLYAAL
FPLTSTPLSSPV------------YCLR----------------------LTTKHLYAAL
FPLTSTPLSSPV------------YCLR----------------------LTTKHLYAAL
FSLTSTPLSSPV------------YCLR----------------------FTTRHLYAAL
FPLTSTPLSSPV------------YCLR----------------------FTTRHLYAAL
DGTAVVENDIIGNRGSGLQLLPRSGIAVRGRVKALVQENIIFQGKTNKTIFQQITNNREC
DGTAVVENDIIGNRGSGLQLLPRSGIAVRGRVKALVQENIIFQGKTNKTIFQQITNNREC
GNGKGIIRNNQIFSNKEAGIYILYGNPVVSGNHIFKGRAAGIAVNENGKGLITENVIREN
NDIIGNRSGLQL-----LPRSDTKGIAVRGRAKALVQENIIFQGKTNKTIFQQISNNREC
NDIIGNRSGLQL-----LPRSDTKGIAVRGRAKALVQENIIFQGKTSKTIFQQISNNREC
NDIIGNRSGLQL-----LPRSDTKGIAVRGRAKALVQENIIFQGKTSKTIFQQISNNREC
SGLQLLPRSDTKVIKNRIHSFRAYGIAVRGRAKALVQENIIFQGKTSKTIFQQISNNREC
SGLQLLPRSDTKVIKNRIHSFRAYGIAVRGRAKALVQENIIFQGKTSKTIFQQISNNREC
```

```
TGLIAGHFTSPERTPGVFVLF---DEDRFGFLWLELKSFSLYSRVQATFQNAAAPSPQAF
------------------------------------------------------------
TGLIAGHFTSPERTPGVFILF---DEDRFGFVWLELKSFSLYSRVQATFRNADAPSPQAF
TGLIAGHFTSPERTPGVFILF---DEDRFGFVWLELKSFSLYSRVQATFRNADAPSPQAF
TGLIAGHFTSPERTPGVFILF---DEDRFGFVWLELKSFSLYSRVQATFRNADAPSPQAF
TGLIAGHFTSPERTPGVFILF---DEDRFGFVWLELKSFSLYSRVQATFRNADAPSPQAF
TGLIAGHFTSPERTPGVFILF---DEDRFGFVWLELKSFSLYSRVQATFRNADAPSPQAF
TGLIAGHFTSPERTPGVFILF---DEDRFGFVWLELKSFSLYSRVQATFRNADAPSPQAF
H-----------------------------------------------------------
VTHSSVR-----------------------------------------------------
VTHSSVR-----------------------------------------------------
------------------------------------------------------------
VTHSSVR-----------------------------------------------------
VTHSSVR-----------------------------------------------------
VTHSSVR-----------------------------------------------------
LTNSSVM-----------------------------------------------------
VTNSSVI-----------------------------------------------------
VTNSSVI-----------------------------------------------------
VTNSSVI-----------------------------------------------------
VTNSSVI-----------------------------------------------------
VTNSSVIRVRLS------------------------------------------------
VTNSSVI-----------------------------------------------------
MTNSSVIRVCPS------------------------------------------------
NSSVQLVPARP-------------------------------------------------
NSSVQLGPALP-------------------------------------------------
------------------------------------------------------------
------------------------------------------------------------
------------------------------------------------------------
SSVWVEP-----------------------------------------------------
------------------------------------------------------------
EGHQQQHHHRAP-----------------------------------------------A
GRLVRPE-----------------------------------------GHQQQHHHRAPA
ERMPPWC----------------------------------------------TDCLASI
P-----------------------------------------------------------
-------GPPLP------------------------------------------------
-------GPPLP------------------------------------------------
P-----------------------------------------------------------
SRNLASSEAQPG------------------------------------------------
TRNQASSEAQPG----------------------------------------QKHGQEEA
TRNQASSEAQPG----------------------------------------QKHGQEEA
TRNQASSEAQPG----------------------------------------QKHGQEEA
TRNQASSEAQPG----------------------------------------KKHGQEEA
TRNQASSETQPG----------------------------------------QKHGQEEA
AKNPPPARTLPE------------ETVVIGRRRR----------------ASDSNTHEGF
AKNPAPARTLPE----------------------------------------EDTSNRRK
```

SCKIGPLPGTKKSKKNLRRL----------------
SCKIGPLPGTKKSKKNLRRL----------------
SCKIGPLPGTKKSKKNLRRL----------------
SCKIGPLPGTKKSKKNLRRL----------------
SCKIGPLPGTKKSKKNLRRL----------------
SCKIGPLPGTKKSKKNLRRL----------------
SCKVGPLPGTKKSKKNLQRL----------------
SCKMGPLPGTKKSKKNLQRL----------------
WDRHYNICLAKLHEDVVNSVAFSPQEQELLDSQRRC
WDRHYNICLAKLRHEDVVNSVAF-------------
WDRHYNICLAKLSDDATIKAWRSPRTVRILQAPRPR
WDRHYNICLAKLSDDATIKAWRSPRTVRVLQAPRPR
WDRHYNICLARLRHEDVVNSVVFSPQEQELLLTASD
WDRHYNICLARLSDDATIKAWRSPRTMRVLQAPRPR
RDHISHRFISCVMCDNASIVLKVRNITESSIVVMYS
WHDPTTDSCISSVMCDNASIVLTVTASEYSMLVMYS
HDHTTDSCISSVMCDNASIVLRLRKVSDSSILVMYS
HDHTTDSCISSVMCDNASIVLTVTKVSDSSILVMYS
HDHTTDSCISSVMCDNASIVLRVRKVSDSSILVMYS
HDHTTDSCISSVMCDNASIVLRVRKVSDSSILVMYS
EEEEEDEDDDSADMDESDEEDEEERRRRVFDVPIRR
EEEEEDEDDDSADMDESDEDDEEERRRRVFDVPIRR
EEEEEDEDDDSADMDESDEDDEEERRRRVFDVPIRR
EEEEEDEDDDSADMDESDEDDEEERRRRVFDVPIRR
EEEEEDEDDDSADMDESDEDDEEERRRRVFDVPIRR
EEEEEDEDDDSADMDESDEDDEEERRRRVFDVPIRR
SADMDESDESDADENESDEGEGEARRRRVFDVPIRR
SADMDESDESDEDENESDEGEGEERRRRVFDVPIRR
SHRWASGFTHVK-SDSRSIAGVEARSTGTSILRSYC
SHRWASGFTHVK-SDSRSIAGVEARSTGTSILRSYC
QCRPPNSPESGVHQQPVKRQNLSVHSDKDMHLAS--
QCRPPNPPESGAHQQPVKRQNLSVHSDEDTNLGFLK
QCRPPSPPEGSVPQQQVKRKNLCVHSEEEGMNWSLR
QCRPPSPPESSVPRQQVKRINLCIHSEEEDTNLGLV
SCLQCRPPESSVPRQQVKRINLCVHSEEEDMNLGLV
QCRPPSPPESSGPRQQVKRINLCVHSEEEDMNLGLV
QCRPPSPPESSVPQQQVKRINLCIHSEEEDMNLGLV
QCRPPSPPESSVPQQQVKRINLCIHSEEEDMNLGLV

ALPE--------------------------------
VCRPPSWGDAFP------------------------
PPTFPGADAFPE------------------------

```
PPTFPGADAFPQ------------------------
------------------------------------
YHGSEECSRGAAKPRNRKDAVPGSAQSKRNLKRL--
YHGSEECSRGAAKPRNRKDALPGSAQSKRNLKRL--
YHGSEECSRGAAKPRNRKDTLPGSAQSKRNLKRL--
YHGSEECSRGAAKPRNRKDALPGSAQSKRNLKRL--
YHGSEECSRGAAKPRNRKDALPGSAQSKRNLKRL--
YHGSEECSRGAAKPRNRKDALPGSAQSKRNLKRL--
VQGRGELIIFGGLMDKKQNVKYYPKTNALYFVRAKR
VQGRGELIIFGGLMDKKQNVKYYPKTNALYFVRAKR
VQGRGELIIFGGLMDKKQNVKYYPKTNALYFVRAKR
VQGRGELIIFGGLMDKKQNVKYYPKTNALYFVRAKR
VQGRGELIIFGGLMDKKQNVKYYPKTNALYFVRAKR
VQGRGELIIFGGLMDKKQNVKYYPKTNALYFVRAKR
VQGRGELIVFGGLMDKKQNVKYYPKTNALYFVRAKR
VQGRGELIIFGGLMDKKQNVKYYPKTNALYFVRAKR
RDVPMLHLDEITAEEPPNLWW---------------
RDVPMVHLDEITAEEPPNLWW---------------
RDVPMVHLDEITAEEPPNLWW---------------
RDVPLVHLDEITAEEPPNLWW---------------
RDVPLVHLDEITAEEPPNLWW---------------
WRESHSVVQAGVQWRDLSSLQPLLSDESCQFSLENQ
VCCPCGLPLAVSGIILVGVSPSLVVKTTCVYRVVFK
NLDYASIFFLVCQPLLSGLQPQPPEQLENELEIGFS
TAEPNSFARYDFMETIGEEISEMRQMKRGIFQRVVA
AAEPNSFARYDFMETIGEEISEMRQMKRGVFQRVVA
AAEPNSFARYDFMETIGEEISEMRQMKKGVFQRVVA
AAEPNSFARYDFMETIGEEISEMRQMKKGVFQRVVA
AAEPNSFARYDFMETIGEEISEMRQMKKGVFQRVVA
AAEPNSFARYDFMETIGEEISEMRQMKKGVFQRVVA
AAEPNSFARYDFMETIGEEISEMRQMKKGVFQRVVA
AAEPNSFARYDFMETIGEEISEMRQMKKGVFQRVVA
SLRKSKRLRNRK------------------------
SLRKSKRLRNRK------------------------
SLRKSKRLRNRK------------------------
CLRKSKRLRNRK------------------------
SLRKSKRLRNRK------------------------
SLRKSKRLRNRK------------------------
SLRKSKRLRNRK------------------------
SLRKSKRLRNRK------------------------
LWTSEGPWFXTRPLEAECYPWLLEGDRQVSCPPPGX
LHFNSICRNLKSIVVQIGIADYFKEPSSPEAQKLFE
YFKEPSSPEAQKLFEDMVTKLQALRRRPGFSKILHI
YFKEPSSPEAQKLFEDMVTKLQALRRRPGFSKILHI
YFKEPSSPEAQKLFEDMVTKLQALRRRPGFSKILHI
```

```
YFKEPSSPEAQKLFEDMVTKLQALRRRPGFSKILHI
YFKEPSSPEAQKLFEDMVTKLQALRRRPGFSKILHI
YFKEPSSPEAQKLFEDMVTKLQALRRRPGFSKILHI
------------------------------------
------------------------------------
------------------------------------
------------------------------------
VWRFSTAFCSVNEWRFADILSMADHLKKCSYNVVEK
VWRFSTAFCSVNEWKFADILSMADHLKKCSYNVVEK
YNVVEKREEAIPLPCMCVTRELTKEGRSLRSVLKPV
VWRFSTAFCSVNEWKFADILSMADHLKKCSYNVVEK
VWRFSTAFCSVNEWKFADILSMADHLKKCSYNVVEK
VWRFSTAFCSVNEWKFADILSMADHLKKCSYNVVEK
VWRFSTAFCSVNDWKFADILSMADHLKNCSYNVIEK
VWRFSTAFCSVNDWKFADILSMADHLKKCSYNVIEK
IWQFSSLFSKIKSWEFNEVTSMSEHLKSCPFNIVEH
IWQFSSLFSKIKSWEFNEVTSMSEHLKSCPFNIVEH
IWQFSSLFSKIKSWEFNEVTSMSEHLKSCPFNIVEH
IWQFSSLFSKIKSWEFNEVTSMSEHLKSCPFNIVEH
IWQFSSLFSKIKSWEFNEVTSMSEHLKSCPFNIVEH
IWQFSSLFSKIKSWEFNEVTSMSEHLKSCPFNIVEH
IWQFSSLFSKINSWEFNDVTSMSEHLKTCPFNIVER
IWQFSSLFSKIKSWEFNDVTSMSEHLKTCPFNVVEH
YKSTGETAVSAFEIDKMYTPLFFARIYEQISVT---
YKSTGETAVSAFEIDKMYTPLFFARVRSYTAFSERP
YKSTGETAVSAFEIDKMYTPLFFARVRSYTAFSERP
YKSTGETAVSAFEIDKMYTPLFFARVRSYTAFSERP
YKSTGETAVSAFEIDKMYTPLFFARVRSYTAFSERP
VRSYTAFSERPL------------------------
YKATGETAVSAFEIDKMYTPLLFARVRSYTAFSERP
YRSTGETAVSAFDIDKMYTPLFFARVRSYTAFSERP
GSPSDRFPFRPSRGRPTDSRLSFM------------
GGPNDRFPFRPSRGRPTDGRLSFM------------
GGPNDRFPFRPSRGRPTDGRLSFM------------
GGPNDRFPFRPSRGRPTDGRLSFM------------
GGPNDRFPFRPSRGRPTDGRLSFM------------
GGPNDRFPFRPSRGRPTDGRLSFM------------
AIPNNRFPFRPGRGRSADSRLPFL------------
ASPNNRFPFRPGRGRSADNRLPYL------------
LNLFHLVHAQANFHKEVLYLTMNTPLST--------
LNLFHLVHAQANFHKEVLYLTMNTPLST--------
LNLFHLVHAQANFHKEVLYLTMNTSLSA--------
LNLFHLVHAQANFHKEVLYLTMNTPLST--------
LNLFHLVHAQANFHKEVLYLTMNTPLST--------
LNLFHLVHAQANFHKEVLYLTMNTPLST--------
```

```
LNLFHLLHAQANFHKEVLYLTMNAISS---------
LNLFHLLHAQANFHKEVLYLTMNAVSS---------
SCKDLSLLDVSFQIDNRAVLELNASFPKVFIKKSFT
SCKDLSLLDVSFQIDNRAVLELNASFPKVFIKKSFT
SCKDLSLLDVSFQIDNRAVLELNASFPKVFIKKSFT
SCKDLSLLDVSFQIDNRAVLELNASFPKVFIKKSFT
SCKDLSLLDVSFQIDNRAVLELNASFPKVFIKKSFT
SCKDLSLLDVSFQIDNRAVLELNASFPKVFIKKSFT
SCKDLSLLDVSFQIDNKAVLELNASFPKVFIKKSFT
SCKDLSLLDVSFQIDNRAVLELNASFPKVFIKKSFT
ESKRAR------------------------------
ESKRAR------------------------------
ESKHSR------------------------------
ESKRAR------------------------------
ESKRAR------------------------------
ESKRAR------------------------------
ESKRAK------------------------------
ESKHAK------------------------------
------------------------------------
------------------------------------
------------------------------------
------------------------------------
------------------------------------
------------------------------------
------------------------------------
------------------------------------
------------------------------------
HWEVSKHLGRVWFPDMMPTW----------------
HWEVSKHLGRVWFPDMMPTW----------------
HWEVSKHLGRVWFPDMMPTW----------------
HWEVSKHLGRVWFPDMMPTW----------------
HWEVSKHLGRVWFPDMMPTW----------------
HWEVSKHLGRVWFPDMMPTW----------------
HWEVSKHLGRVWFPDMMPTW----------------
HWEVSKHLGRVWFPDMMPTW----------------
HTEVSKHLGRVWFPDVMPVW----------------
HTEVSKHLGRVWFPDVMPIW----------------
HTEVSKYLGRVWFPDVMPLW----------------
HTEVSKYLGRVWFPDVMPLW----------------
HTEVSKYLGRVWFPDVMPLW----------------
HTEVSKYLGRVWFPDVMPL-----------------
FIRHDRFFCDCGLSNPCTLAGEPTHDTDTLYDSAPP
FIRHDRFFCDCGLSNPCTLAGEPTHDTDTLYDSAPP
FIRHDRFFCDCGLSNPCTLAGEPTHDTDTLYDSAPP
FIRHDRFFCDCGLSNPCTLAGEPTHDTDTLYDSAPP
```

FIRHDRFFCDCGLSNPCTLAGEPTHDTDTLYDSAPP
FIRHDRFFCDCGLSNPCTLAGEPTHDTDTLYDSAPP
FIRHDRFFCDCGLSNPCTLAGEPTHDTDTLYDSAPP
FIRHDRFFCDCGLSNPCTLAGEPTHDTDTLYDSAPP
RLQFDEFQIISSSHDDTILIWDFLNVPPSAQNETRS
RLQFDEFQIISSSHDDTILIWDFLNVPPSAQNETRS
RLQFDEFQIISSSHDDTILIWDFLNVPPSAQNETRS
RLQFDEFQIISSSHDDTILIWDFLNVPPSAQNETRS
RLQFDEFQIISSSHDDTILIWDFLNVPPSAQNETRS
RLQFDEFQIISSSHDDTILIWDFLNVPPSAQNETRS
RLQFDEFQIISSSHDDTILIWDFLNVPPSAQNETRS
RLQFDEFQIISSSHDDTILIWDFLNVPPSAQNETRS
RLQFDEFQIVSSSHDDTILIWDFLNDPAAQAEPPRS
RLQFDEFQIVSSSHDDTILIWDFLNDPATHAEPPRS
RLQFDEFQIVSSSHDDTILIWDFLNDPAAHAEPPRS
RLQFDEFQIVSSSHDDTILIWDFLNDPAAQAEPPHS
RLQFDEFQIVSSSHDDTILIWDFLNDPAAQAEPPRS
RLQFDEFQIVSSSHDDTILIWDFLNDPAAQAEPPRS
RLQFDEFQIVSSSHDDTILIWDFLNDPAAQPEPPRS
RLQFDEFQIVSSSHDDTILIWDFLNDPAAQAEPPRS
RHMLHRRFVNKTLCDDVSIVRVMTNVPSSSFLMVYT
RPLPLYGFIYKAACDDVSIIQLITDELSLSSLTSYA
RHLPPFGFIKNLLCDDVSIVQLMIDRQGPCFLMAYT
RRLPQQSFINKTLCDDMSIIRVMTTHSIPSFLMAYI
RRLPRQSCFEKTLCDEVSIIRMVRNGRNPCYLMTYT
KHLPSSGLINKTLSDDVSIIQVITIRTTPCFLMAFI
RNLPPESFIFKTLCDDVSIIGVMTNSPAPCFLMAYT
RYQPPSAFIIETLCDDVSIILVMTNSPNPCCLIAYT
NYQQMSGYIIKTLCDDMSIISVANNSPKPCCLMAYT
NYQQMSGYIIKTLCDDMSIISVANNSPKPCCLMAYT
GYLPLSGFIIKTLCDESSIILVMTSSPIPCFLMAYT
RYLLPSSFINNALCDDVSIIGVMISSPSFCYLMAYT
RYLPPSSFINKALCDDVSIIGVMTNSPAPCYLMAYT
QLAFQSPLPVCRLPRDIMAGYSYDLALSFPHDSI--
QLAFQSPLPICRLPRDTVAGYSYDLALSFPYDSI--
QLAFQSPLPACRSSCDTMATHYYDLALAFPYNHV--
QLAFQSPLPVCRSSCDAMATHYYDLALAFPYNHV--
QLAFQSPLPVCRSSCDAMATHYYDLALAFPYNHV--
QLAFQSPLPVCHSSCDAMATHYYDLALAFPYNHV--
QLAFQSPLPVCRSSCDAMATHYYDLALAFPYNHV--
QLAFQSPLPVCRSSCDAMATHYYDLALAFPYNHV--
YERCLMAFKHPKLNNQSKCLAHSQGSTALPCTSARS
EASVSTKEVDPGKASKAAWIRKIKGLPIDNFMKEGK
HSLRDSGSIKQEFSKSRVLLKQTRNLSS--------
QARESTGVVDPEKARKAAWIRKIKGLPIDNFMKRGK

```
QARESTGVVDPGKASKAAWIRKIKGLPIDNFMKQGK
QTRESTGVVDPGKASKAAWIRKIKGLPIDNFMKQGK
QARESTGVVDPGKVSKAAWIRKIKGLPIDNFTKQGK
QARESTGVVDPGKASKAAWIRKIKGLPIDNFTKQGK
GHNDTGLVFATSMPDHSIHLVLWKEHG---------
GHNDTGLVFATSMPDHSIHLVLWKEHG---------
GHNDTGLVFATSMPDHSIHLVLWKEHG---------
GHNDTGLVFATSMPDHSIHLVLWKEHG---------
GHNDTGLVFATSMPDHSIHLVLWKEHG---------
GHNDTGLVFATSMPDHSIHLVLWKEHG---------
GHNDTGLVFATSMPDHSIHLVLWKEHG---------
GHNDTGLVFATSMPDHSIHLVLWKEHC---------
YDNIYLIGHVAA------------------------
YDNIYLIGHVAA------------------------
YDNIYLIGHVAA------------------------
YDNIYLIGHVAA------------------------
YDNIYLIGHVAA------------------------
YDNIYLIGHVAA------------------------
YDNIYLIGHVAA------------------------
YDNIYLIGHVAA------------------------
QCGFRACCRSGE------------------------
QCGFRACCRSGE------------------------
QCGFRACCRSGE------------------------
QCGFRACCRSGE------------------------
QCGFRACCRSGE------------------------
QCGFRACCRSGE------------------------
QCGFRACCRSGE------------------------
QCGFRACCRSGE------------------------
NTKLVCAVGSRNGTEETKLLVLDFDVDMK-------
EFIRNLVTLESG------------------------
NTKLVCAVGSRNGTEETKLLVLDFDVDMK-------
NTKLVCAVGSRNGTEETKLLVLDFDVDMK-------
NTKLVCAVGSRNGTEETKLLVLDFDVDMK-------
NTKLVCAVGSRNGTEETKLLVLDFDVDMK-------
NTKLVCAVGSRNGTEETKLLVLDFDVDMK-------
NTKLVCAVGSRNGTEETKLLVLDFDVDMK-------
NIPDSKYVKNAFILQGPKYKWICATEAEDDKFLWLS
NIPDSKYVKNAFILQGPKYKWICATEIEDDKFLWLS
NIPDSKYVKNAFILQGPKYKWICATEIEDDKFLWLS
NIPDSKYVKNAFILQGPKYKWICATEIEDDKFLWLS
NIPDSKYVKNAFILQGPKYKWICATEIEEDKFLWLS
------------------------------------
------------------------------------
DIPDSKYIKNAFILRGSKREWICATEVSDDKFLWLS
------------------------------------
```

```
MQHLQRHPGPQA------------------------
ARRGRSPAFHHL------------------------
RNPFPLCP----------------------------
------------------------------------
------------------------------------
-QSPSPRLQSQS------------------------
LPSPSLRLQSQS------------------------
VCKTLENFFASATIDHVPIWCKNSQGQRVMVEQSEK
LCHSCAEQRIGPLAFLTASPEQVRAMERTVENIVLP
LCHSCAEQRIGPLAFLTASPEQVRAMERTVENIVLP
KTGVVRCCVGQCNAIPVDTVLTMKKLPITY------
LCHSCAEQRIGPLAFLTASPEQVRAMERTVENIVL-
LCHSCAEQRIGPLAFLTASPEQVRAMERTVENIVLP
LCHSCAEQRIGPLAFLTASPEQVRAMERTVEDIVLP
LCHSCAEQRIGPLAFLTASPEQVRAMERTVEDLVLP
GQPWHAVMDIESLSVFTEPNRHFYREMQSFSEDI--
GQPWHAVMDIESLSVFTEPNRHFYREMQSFSEDI--
GQPWHAVMDIESLSVFTEPNRHFYREMQSFSEDI--
GQPWHAVMDIESLSVFTEPNRHFYREMQSFSEDI--
GQPWHAVMDIESLSVFTEPNRHFYREMQSFSEDI--
GQPWHAVMDIESLSVFTEPNRHFYREMQSFSEDI--
GQSWHAVLDIESLSVFTEPNRHFYREMQSFSEDI--
GQSWHAVLDIESLSVFTEPNRHFYREMQSFSEDI--
SEFTGTHYIPNAIRYPEDLEFVYETVQNIYSAKKEN
SEFTGTHYIPNAIRYPEDLEFVYETVQNIYSAKKEN
SEFTGTHYIPNAIRYPEDLEFVYETVQNIYSAKKEN
SEFTGTHYIPNAIRYPEDLEFVYETVQNIYSAKKEN
SEFTGTHYIPNAIRYPEDLEFVYETVQNIYSAKKEN
SEFTGTHYIPNAIRYPEDLEFVYETVQNIYSAKEDT
SEFTGTHYIPNAIRYPEDLEFVYETVQNIYSAKEDT
GWMPLMAAQKDFFWEALDMLQRAAGGAGPDTSTPES
GWMPLMAAQKDFFWEALDMLQRAASGGGPGTSTPES
GWMPLMAAQKDFFWEALDMLQQDVGGGVAPPVPES-
LCSWLQPWG---------------------------
GWMPLMAAQKDFFWEALDMLQRAEGGGGGVGPPAPE
GWMPLMAAQKDFFWEALDMLQRAEGGGGGVGPPAPE
GDKMDRGEPTQV------------------------
GWMPLMAAQKDFFWEALDMLQRAEGGGGGVGPPAPE
LSPQDFINLFKF------------------------
LSPQDFINLFKF------------------------
LSPQDFINLFKF------------------------
LSPQDFINLFKF------------------------
LSPQDFINLFKF------------------------
LSPQDFINLFKF------------------------
LSPQDFINLFKF------------------------
```

```
VSPQHFIDLFKF------------------------
VSPQHFIDLFKF------------------------
VSPQHFIDLFKF------------------------
VSPQHFIDLFKF------------------------
VSPQHFIDLFKF------------------------
VSPQHFIDLFKF------------------------
VSPEHFIDLFKF------------------------
------------------------------------
RCPEEKLLLKDS------------------------
RCPEEKLLLKDS------------------------
RCPEEKLLLKDS------------------------
HVGDPSVHLLTAPTSPLRETLVHLNLAGKH------
RCPEEKLLLKDS------------------------
RCPEEKLLLKDS------------------------
RCPEEKLLLKDS------------------------
RCPEEKLLLKDS------------------------
YCLSDEKLIQKI------------------------
YCLSDEKLIQKI------------------------
YCLSDEKLIQKI------------------------
YCLSDEKLIQKI------------------------
YCLSDEKLIQKI------------------------
YCLSDEKLIQKI------------------------
YCLSDEKLIQKI------------------------
YCLSDEKLIQKI------------------------
GQVEEKLLQKLS------------------------
GQVEEKLLQKLS------------------------
GQVEEKLLQKLS------------------------
GQVEEKLLQKLS------------------------
GQVEEKLLQKLS------------------------
GQVEEKLLQKLS------------------------
GQVEEKLLQKLS------------------------
GQVEEKLLQKLS------------------------
VQQSSDPIKHLQXPPGSRGSGLGRPIPPLQAALIPP
WVLPCNGPGGGRAGREEGR-----------------
WVLPCNGPGGGRAGREEGR-----------------
WVLPCNGPGGGRAGREEGR-----------------
WVLPCNGPGGGRAGREEGR-----------------
WVLPCNGPGGGRAGREEGR-----------------
WVLPCNGVGGGRAGREEGR-----------------
WVLPCNGVGGGRAGREEGR-----------------
WVPACHSFNRAIHKKAKGTEAEEEY-----------
WVPACHSFNRAIHKKAKGTEAEEEY-----------
WVPACHSFNRAIHKKAKGTEAEEEY-----------
WVPACHSFNRAIHKKAKGTEAEEEY-----------
WVPACHSFNRAIHKKAKGTEAEEEY-----------
```

```
WVPACHSFNRAIHKKAKGTEAEEEY-----------
WLPACHSFNRAIHKKTRGSETEEEY-----------
WLPACHSFNRAIHKKSRGSETEEEY-----------
-----------A------------------------
-----------T------------------------
------------------------------------
------------------------------------
------------------------------------
KQKYGNLREKQP------------------------
KQKYGNLREKQP------------------------
KQKYGNLREKQP------------------------
REPHPWRPTPVA------------------------
REPHPWRPTLVARLGDFSPPSPQT------------
REPHPWRPTLLA------------------------
REPHPWRPTLVA------------------------
REPHPWRPTLVA------------------------
REPHPWRPTLVA------------------------
REPHPWRPTLVR------------------------
REPHPWRPTLMR------------------------
ENTDEYFIVRLDIYLSVAKLQQWFGRQ---------
WIEETEEYFIVSLALYLSVAKINHWFGTKY------
WIRETEEYFIVSLVLYLSVAKINHWFGTEY------
WIRETEEYLIVNLVLYLSVAKINRWFGTEY------
WIRETEEYLIVNLVLYLSIAKINHWFGTEY------
WIRETEEYLIVNLVLYLSIAKINHWFGTEY------
SWKTDIFDGCIK------------------------
WIRETEEYLIVNLVLYLSIAKINHWFGTEY------
WCLEQLLTSPPSAKEPT-------------------
WCLEQLLTSPPSSREST-------------------
WCLEQLLTSPSP------------------------
WCLEQLLTSPLP------------------------
WCLEQLLTSPSP------------------------
WCLEQLLTSPSP------------------------
WCLEQLLTSPSP------------------------
WCLEQLLTSPSP------------------------
LTLQKPSCL---------------------------
WGIKCRLTLQKP------------------------
LTLQKPSCL---------------------------
LTLQKPSCL---------------------------
VCDFYFYRLVLK------------------------
VCDFYFYRLVLK------------------------
---YFYRLVLKQ------------------------
SQHLPRCLVIE-------------------------
SQHLPRCLVIE-------------------------
YSQHPRCVE---------------------------
```

```
SQHLPRCLVIE-------------------------
ALGSWRLLSLAG------------------------
SQHLPRCLVIE-------------------------
SQHLPRCLVIE-------------------------
SQHLPRCLVIE-------------------------
YRKYRKLIDSELNYFVVAYPMM--------------
YRKYRKLIDSELNYFVIAYPMM--------------
YRKYRKLIDSELSYFVIAYPMM--------------
YRKYRKLIDSELSYFVIAYPMM--------------
YRKYRKLIQSELNYFVMAYSKM--------------
KERQCALRVFKV------------------------
YRKYRKLIESELSYFVIVYSVM--------------
YRKYRKLIESELSYFVIVYSVM--------------
EQLVQQYPHITFSTVLQDCKRTLERAYQMGWTPNMS
HCSPAFVN----------------------------
YMQENKLVS---------------------------
EQLVQQYPHITFSTVLQDCKRTLERAYQMGWTPNMS
EQLVQQYPHITFSTVLQDCKRTLERAYQMGWTPNMS
EQLVQQYPHITFSTVLQDCKRTLERAYQMGWTPNMS
EQLVQQYPHITFSTVLQDCKRTLERAYQMGWTPNMS
ICAEGNVVVAASGGLSLEVWRLLA------------
ICAEGNIVVAASGGLSLEVWRLLA------------
VCAEGNLVVAASGGLSLEVWRLQA------------
VCAEGNLVVAGSGDVSLEVWRLPA------------
VCAEGNLVVAGSGDLSLEVWRLQA------------
VCAEGNLVVAGSGDLSLEVWRLQA------------
VCAEGNLVVAGSGDLSLEVWRLQA------------
VCAEGNLVVAGSGDLSLEVWRLQA------------
FHSYTTIMALVHLGTSK-------------------
FHSYTTIMALVHLGASK-------------------
FHSYTTIMALIHLGSSK-------------------
FHSYTTIMALIHLGSSK-------------------
FHSYTTIMALIHLGSSK-------------------
FHSYTTIMALIHLGSSK-------------------
FHSYTTIMALIHLGSSK-------------------
FHSYTTIMALIHLGSSK-------------------
HCKRCVIEHTNPF-----------------------
HCKRCVIEHTNPF-----------------------
HCKRCVIEHTNPF-----------------------
HCKRCVIEHTNPF-----------------------
HCKRCVIEHTNPF-----------------------
HCKRCVIEHTNPF-----------------------
HCKRCVIEHTNPF-----------------------
HCKRCVIEHTNPF-----------------------
VIL---------------------------------
```

```
GQRLCRCCVIL-------------------------
GQRLCRCCVIL-------------------------
GQRLCRCCVIL-------------------------
GQRLCRCCVIL-------------------------
GQRLCRCCVIL-------------------------
GHRLCRCCVIL-------------------------
GHRLCRCCVIL-------------------------
RQRFCRCCIIL-------------------------
RQRFCRCCIIL-------------------------
RQRFCRCCIIL-------------------------
RQRFCRCCIIL-------------------------
RQRFCRCCIIL-------------------------
RQRFCRCCIIL-------------------------
RQRFCRCCIIL-------------------------
RQRFCRCCIIL-------------------------
MAGFAPFVNLQV------------------------
MAGFAPFVNLQV------------------------
MAGFAPFVNLQV------------------------
MAGFAPFVNLQV------------------------
MAGFAPFVNLQV------------------------
MAGFAPFVNLQV------------------------
MAGFAPFVNLQV------------------------
MAGFAPFVNLQV------------------------
ERI---------------------------------
WQMTDSEKEARGDFSPLFTVRTRGSSRR--------
WQMTDSEKEARGDFSPLFTVRTRGSSRR--------
WQMTDSEKEARGDFSPLFTVRTRGSSRR--------
WQMTDSEKVR--------------------------
WQMTDSEKVR--------------------------
WQMTDSEK----------------------------
ERMSSKVQQQEYNSNDPPRWFRYDREGNPLTQPENI
QRMSSKVQQQEYNTNDPPRWFGYDREGNPVTDLDNI
QRMSSKVQQQEYNSNDPPRWFGYDREGDPLTELDNV
QRMSSKVQQQEYNSNDPPRWFGYDREGNRVTELDNI
QRMSSKVQQQEYNTNDPPRWFGYDREGNPVTELDNI
QRMSSKVQQQEYNTNDPPRWFGYDREGNPVTELDNI
QKMSSVVQHQEY-NSDNPPHWFGYDSEGNPLDKIHS
LTDNCVKVGREK------------------------
GKPLDG------------------------------
GKPLDG------------------------------
GKPLDG------------------------------
GKPLDG------------------------------
GLPHCIVIVRAC-PKESMDWWM--------------
GLPHCLVIVRAC-PKESMDWWM--------------
GLPHCMVIVRAC-PKESMDWWM--------------
```

```
GLPHCMVIVRAC-PKESMDWWM--------------
GLPHCMVIVRCP--KESMDWWM--------------
GLPHCMVIVRAC-PKESMDWWM--------------
GLPHCMVIVRAC-PKESMDWWM--------------
GLPHCMVIVRAC-PKESMDWWM--------------
SYNLHVLDFQNP------------------------
SYNLHVLDFQNP------------------------
SYNLHVLDFQNP------------------------
SYNLHVLDFQNP------------------------
SYNLHVLDFQNP------------------------
SYNLHVLDFQNP------------------------
SYNLHVLDFENP------------------------
IMQNNKFLVFKK-KSDTWRLVNPPARPHLENSLRGS
IMQNNKFLVFKK-KSDTWRLVNPPARPHLENSLRGS
QWGGVDIRRGGI-PVLRSNLICFGYSDGVVVGDEGK
VMQNNKFLVFKK-KSDTWRLVNPPARPHLETSLRRP
IMQNNKFLVFKK-KSDTWRLVNPPARPHLENSLRRP
IMQNNKFLVFKK-KSDTWRLVNPPARPHLENSLRRP
VMQNNKFLVFKK-KSDTWRLVNPPARPHLENSLRRP
IMQNNKFLVFKK-KSDTWRLVNPPARPHLENSLRRP
DEMLRNIQSLTS------------------------
------------------------------------
DEMLKNIQSLTS------------------------
DEMLKNIQSLTS------------------------
DEMLKNIQSLTS------------------------
DEMLKNIQSLTS------------------------
DEMLKNIQSLTS------------------------
DEMLKNIQSLTS------------------------
------------------------------------
------VRIRLS------------------------
------VRIRLS------------------------
------------------------------------
------VRIRLS------------------------
------VRIRLS------------------------
------VRIRLS------------------------
------VQVRPP------------------------
------VRVHLS------------------------
------VRVRLS------------------------
------VRVRLS------------------------
------VRVRLS------------------------
------------------------------------
------IRVCQS------------------------
------------------------------------
------------------------------------
------------------------------------
```

```
------------------------------------
------------------------------------
------------------------------------
------------------------------------
------------------------------------
ALTPPEPPSAEP------------------------
ALTPPEPPSAEP------------------------
HRALCSIPSAKK------------------------
------------------------------------
------------------------------------
------------------------------------
------------------------------------
QTQLPYKVVLPI------------------------
AQSPYRAVVQIF------------------------
AQSPYRAVVQIF------------------------
AQSPYRAVVQIF------------------------
AQSPYQAVLQIF------------------------
AQSPYQAVLQIF------------------------
FWQGLWQRLRR-------------------------
ILSFGSWEDLSP------------------------
```
