# Supplementary material for: Evolution of the F-Box Gene Family in Euarchontoglires: Gene Number Variation and Selection Patterns
Source: PLoS One. 2014 Apr 11;9(4):e94899. doi: 10.1371/journal.pone.0094899 (PMC3984280; doi:10.1371/journal.pone.0094899)
Supplement: File S2 — An all-against-all BLAST (-e 1e-50) between every pair of F-box protein sequences from each of the eight organisms. (PDF) [file pone.0094899.s007.pdf]

```
# BLASTP 2.2.20 [Feb-08-2009]
# Query: CJA-Btrc
# Database: 559_protein.db
# Query id, Subject id, % identity, alignment length, mismatches, gap openings, q. start, q. end, s. start, s. end, e-value, bit score
CJA-Btrc	CJA-Btrc	100.00	579	0	0	1	579	1	579	0.0	1208
CJA-Btrc	HSA-Btrc	95.37	605	2	1	1	579	1	605	0.0	1190
CJA-Btrc	PPY-Btrc	94.88	605	5	1	1	579	1	605	0.0	1183
CJA-Btrc	MUS-Btrc	94.05	605	10	1	1	579	35	639	0.0	1176
CJA-Btrc	RNO-Btrc	93.89	589	10	1	17	579	17	605	0.0	1144
CJA-Btrc	GGO-Btrc	99.62	528	2	0	52	579	31	558	0.0	1098
CJA-Btrc	MMU-Btrc	84.49	593	61	4	13	579	1	588	0.0	1008
CJA-Btrc	PTR-Btrc	84.31	580	54	9	1	579	1	544	0.0	964
CJA-Btrc	MUS-Fbxw11	80.00	575	100	3	5	579	4	563	0.0	955
CJA-Btrc	PTR-Fbxw11	79.30	575	83	3	5	579	4	542	0.0	949
CJA-Btrc	HSA-Fbxw11	79.30	575	83	3	5	579	4	542	0.0	949
CJA-Btrc	GGO-Fbxw11	79.30	575	83	3	5	579	4	542	0.0	949
CJA-Btrc	CJA-Fbxw11	79.30	575	83	3	5	579	4	542	0.0	949
CJA-Btrc	MMU-Fbxw11	80.18	565	76	3	15	579	1	529	0.0	939
CJA-Btrc	RNO-Fbxw11	80.46	563	74	3	17	579	1	527	0.0	937
CJA-Btrc	PPY-Fbxw11	51.38	508	181	5	84	579	22	475	3e-148	517
# BLASTP 2.2.20 [Feb-08-2009]
# Query: GGO-Btrc
# Database: 559_protein.db
# Query id, Subject id, % identity, alignment length, mismatches, gap openings, q. start, q. end, s. start, s. end, e-value, bit score
GGO-Btrc	GGO-Btrc	100.00	558	0	0	1	558	1	558	0.0	1164
GGO-Btrc	PTR-Btrc	99.76	419	1	0	140	558	126	544	0.0	874
GGO-Btrc	HSA-Btrc	99.64	554	2	0	5	558	52	605	0.0	1152
GGO-Btrc	PPY-Btrc	99.10	554	5	0	5	558	52	605	0.0	1145
GGO-Btrc	MUS-Btrc	98.19	554	10	0	5	558	86	639	0.0	1137
GGO-Btrc	RNO-Btrc	98.01	554	11	0	5	558	52	605	0.0	1134
GGO-Btrc	CJA-Btrc	99.62	528	2	0	31	558	52	579	0.0	1098
GGO-Btrc	MMU-Btrc	88.45	554	59	3	5	558	40	588	0.0	997
GGO-Btrc	HSA-Fbxw11	83.74	529	80	3	30	558	20	542	0.0	920
GGO-Btrc	GGO-Fbxw11	83.74	529	80	3	30	558	20	542	0.0	920
GGO-Btrc	CJA-Fbxw11	83.74	529	80	3	30	558	20	542	0.0	920
GGO-Btrc	PTR-Fbxw11	83.74	529	80	3	30	558	20	542	0.0	920
GGO-Btrc	MMU-Fbxw11	83.74	529	80	3	30	558	7	529	0.0	920
GGO-Btrc	MUS-Fbxw11	84.59	519	78	1	40	558	47	563	0.0	919
GGO-Btrc	RNO-Fbxw11	83.74	529	80	3	30	558	5	527	0.0	918
GGO-Btrc	PPY-Fbxw11	51.38	508	181	5	63	558	22	475	4e-148	516
# BLASTP 2.2.20 [Feb-08-2009]
# Query: MMU-Btrc
# Database: 559_protein.db
# Query id, Subject id, % identity, alignment length, mismatches, gap openings, q. start, q. end, s. start, s. end, e-value, bit score
MMU-Btrc	MMU-Btrc	100.00	588	0	0	1	588	1	588	0.0	1189
```

MMU-Btrc	HSA-Btrc	89.04	593	60	3	1	588	13	605	0.0	1071
MMU-Btrc	PPY-Btrc	88.53	593	63	3	1	588	13	605	0.0	1065
MMU-Btrc	MUS-Btrc	87.86	593	67	3	1	588	47	639	0.0	1059
MMU-Btrc	RNO-Btrc	87.80	590	67	3	4	588	16	605	0.0	1054
MMU-Btrc	PTR-Btrc	87.41	588	18	2	1	588	13	544	0.0	1039
MMU-Btrc	CJA-Btrc	84.49	593	61	4	1	588	13	579	0.0	1001
MMU-Btrc	GGO-Btrc	88.45	554	59	3	40	588	5	558	0.0	990
MMU-Btrc	MUS-Fbxw11	70.90	591	130	6	1	588	12	563	0.0	822
MMU-Btrc	MMU-Fbxw11	69.78	589	115	6	3	588	1	529	0.0	799
MMU-Btrc	PTR-Fbxw11	75.43	529	119	6	65	588	20	542	0.0	791
MMU-Btrc	HSA-Fbxw11	75.43	529	119	6	65	588	20	542	0.0	791
MMU-Btrc	GGO-Fbxw11	75.43	529	119	6	65	588	20	542	0.0	791
MMU-Btrc	CJA-Fbxw11	75.43	529	119	6	65	588	20	542	0.0	791
MMU-Btrc	RNO-Fbxw11	75.43	529	119	6	65	588	5	527	0.0	789
MMU-Btrc	PPY-Fbxw11	71.37	489	95	4	103	588	29	475	0.0	687

BLASTP 2.2.20 [Feb-08-2009]
Query: MUS-Btrc
Database: 559_protein.db
Query id, Subject id, % identity, alignment length, mismatches, gap openings, q. start, q. end, s. start, s. end, e-value, bit score

MUS-Btrc	MUS-Btrc	100.00	639	0	0	1	639	1	639	0.0	1302
MUS-Btrc	HSA-Btrc	98.68	605	8	0	35	639	1	605	0.0	1249
MUS-Btrc	PPY-Btrc	98.18	605	11	0	35	639	1	605	0.0	1242
MUS-Btrc	RNO-Btrc	99.49	589	3	0	51	639	17	605	0.0	1225
MUS-Btrc	CJA-Btrc	94.05	605	10	1	35	639	1	579	0.0	1176
MUS-Btrc	GGO-Btrc	98.19	554	10	0	86	639	5	558	0.0	1137
MUS-Btrc	MMU-Btrc	87.86	593	67	3	47	639	1	588	0.0	1065
MUS-Btrc	PTR-Btrc	78.88	606	65	9	35	639	1	544	0.0	969
MUS-Btrc	MUS-Fbxw11	76.86	605	98	4	35	639	1	563	0.0	955
MUS-Btrc	PTR-Fbxw11	83.43	531	78	4	111	639	20	542	0.0	919
MUS-Btrc	HSA-Fbxw11	83.24	531	79	4	111	639	20	542	0.0	918
MUS-Btrc	GGO-Fbxw11	83.24	531	79	4	111	639	20	542	0.0	918
MUS-Btrc	CJA-Fbxw11	83.24	531	79	4	111	639	20	542	0.0	918
MUS-Btrc	MMU-Fbxw11	83.24	531	79	4	111	639	7	529	0.0	918
MUS-Btrc	RNO-Fbxw11	83.43	531	78	4	111	639	5	527	0.0	917
MUS-Btrc	PPY-Fbxw11	50.79	508	184	5	144	639	22	475	1e-146	511

BLASTP 2.2.20 [Feb-08-2009]
Query: PPY-Btrc
Database: 559_protein.db
Query id, Subject id, % identity, alignment length, mismatches, gap openings, q. start, q. end, s. start, s. end, e-value, bit score

PPY-Btrc	PPY-Btrc	100.00	605	0	0	1	605	1	605	0.0	1263
PPY-Btrc	HSA-Btrc	99.50	605	3	0	1	605	1	605	0.0	1257
PPY-Btrc	MUS-Btrc	98.18	605	11	0	1	605	35	639	0.0	1241
PPY-Btrc	RNO-Btrc	97.96	589	12	0	17	605	17	605	0.0	1208
PPY-Btrc	CJA-Btrc	94.88	605	5	1	1	605	1	579	0.0	1183
PPY-Btrc	GGO-Btrc	99.10	554	5	0	52	605	5	558	0.0	1145

```
PPY-Btrc    MMU-Btrc    88.53    593    63    3    13    605    1    588    0.0    1071
PPY-Btrc    PTR-Btrc    79.21    606    63    9    1    605    1    544    0.0    963
PPY-Btrc    MUS-Fbxw11    76.53    605    100    4    1    605    1    563    0.0    952
PPY-Btrc    PTR-Fbxw11    83.18    529    83    3    77    605    20    542    0.0    916
PPY-Btrc    HSA-Fbxw11    83.18    529    83    3    77    605    20    542    0.0    916
PPY-Btrc    GGO-Fbxw11    83.18    529    83    3    77    605    20    542    0.0    916
PPY-Btrc    CJA-Fbxw11    83.18    529    83    3    77    605    20    542    0.0    916
PPY-Btrc    MMU-Fbxw11    83.18    529    83    3    77    605    7    529    0.0    914
PPY-Btrc    RNO-Fbxw11    83.18    529    83    3    77    605    5    527    0.0    913
PPY-Btrc    PPY-Fbxw11    50.79    508    184    5    110    605    22    475    4e-146    510
# BLASTP 2.2.20 [Feb-08-2009]
# Query: PTR-Btrc
# Database: 559_protein.db
# Query id, Subject id, % identity, alignment length, mismatches, gap openings, q. start, q. end, s. start, s. end, e-value, bit score
PTR-Btrc    PTR-Btrc    100.00    544    0    0    1    544    1    544    0.0    1130
PTR-Btrc    MMU-Btrc    87.41    588    18    2    13    544    1    588    0.0    1038
PTR-Btrc    CJA-Btrc    84.31    580    54    9    1    544    1    579    0.0    964
PTR-Btrc    HSA-Btrc    79.70    606    60    9    1    544    1    605    0.0    939
PTR-Btrc    PPY-Btrc    79.21    606    63    9    1    544    1    605    0.0    933
PTR-Btrc    MUS-Btrc    78.88    606    65    9    1    544    35    639    0.0    928
PTR-Btrc    RNO-Btrc    77.97    590    67    9    17    544    17    605    0.0    893
PTR-Btrc    GGO-Btrc    99.76    419    1    0    126    544    140    558    0.0    874
PTR-Btrc    MUS-Fbxw11    73.59    568    121    9    1    544    1    563    0.0    827
PTR-Btrc    HSA-Fbxw11    73.62    561    112    9    1    544    1    542    0.0    816
PTR-Btrc    GGO-Fbxw11    73.62    561    112    9    1    544    1    542    0.0    816
PTR-Btrc    CJA-Fbxw11    73.62    561    112    9    1    544    1    542    0.0    816
PTR-Btrc    PTR-Fbxw11    73.62    561    112    9    1    544    1    542    0.0    816
PTR-Btrc    MMU-Fbxw11    74.59    547    104    8    15    544    1    529    0.0    805
PTR-Btrc    RNO-Fbxw11    74.86    545    102    8    17    544    1    527    0.0    803
PTR-Btrc    PPY-Fbxw11    79.29    425    43    3    120    544    96    475    0.0    682
# BLASTP 2.2.20 [Feb-08-2009]
# Query: RNO-Btrc
# Database: 559_protein.db
# Query id, Subject id, % identity, alignment length, mismatches, gap openings, q. start, q. end, s. start, s. end, e-value, bit score
RNO-Btrc    RNO-Btrc    100.00    605    0    0    1    605    1    605    0.0    1263
RNO-Btrc    MUS-Btrc    99.49    589    3    0    17    605    51    639    0.0    1224
RNO-Btrc    HSA-Btrc    98.47    589    9    0    17    605    17    605    0.0    1214
RNO-Btrc    PPY-Btrc    97.96    589    12    0    17    605    17    605    0.0    1208
RNO-Btrc    CJA-Btrc    93.89    589    10    1    17    605    17    579    0.0    1144
RNO-Btrc    GGO-Btrc    98.01    554    11    0    52    605    5    558    0.0    1134
RNO-Btrc    MMU-Btrc    87.80    590    67    3    16    605    4    588    0.0    1059
RNO-Btrc    PTR-Btrc    87.97    590    67    9    17    605    17    544    0.0    993
RNO-Btrc    MUS-Fbxw11    78.27    589    87    3    17    605    16    563    0.0    944
RNO-Btrc    PTR-Fbxw11    83.62    531    77    4    77    605    20    542    0.0    919
RNO-Btrc    HSA-Fbxw11    83.43    531    78    4    77    605    20    542    0.0    918
```

```
RNO-Btrc    GGO-Fbxw11  83.43   531   78    4   77    605   20    542   0.0      918
RNO-Btrc    CJA-Fbxw11  83.43   531   78    4   77    605   20    542   0.0      918
RNO-Btrc    RNO-Fbxw11  83.62   531   77    4   77    605   5     527   0.0      917
RNO-Btrc    MMU-Fbxw11  83.43   531   78    4   77    605   7     529   0.0      917
RNO-Btrc    PPY-Fbxw11  50.98   508   183   5   110   605   22    475   1e-146   511
# BLASTP 2.2.20 [Feb-08-2009]
# Query: HSA-Btrc
# Database: 559_protein.db
# Query id, Subject id, % identity, alignment length, mismatches, gap openings, q. start, q. end, s. start, s. end, e-value, bit score
HSA-Btrc    HSA-Btrc    100.00  605   0     0   1     605   1     605   0.0      1263
HSA-Btrc    PPY-Btrc    99.50   605   3     0   1     605   1     605   0.0      1257
HSA-Btrc    MUS-Btrc    98.68   605   8     0   1     605   35    639   0.0      1248
HSA-Btrc    RNO-Btrc    98.47   589   9     0   17    605   17    605   0.0      1214
HSA-Btrc    CJA-Btrc    95.37   605   2     1   1     605   1     579   0.0      1190
HSA-Btrc    GGO-Btrc    99.64   554   2     0   52    605   5     558   0.0      1152
HSA-Btrc    MMU-Btrc    89.04   593   60    3   13    605   1     588   0.0      1077
HSA-Btrc    PTR-Btrc    86.70   606   60    9   1     605   1     544   0.0      969
HSA-Btrc    MUS-Fbxw11  77.02   605   97    4   1     605   1     563   0.0      958
HSA-Btrc    PTR-Fbxw11  83.74   529   80    3   77    605   20    542   0.0      922
HSA-Btrc    HSA-Fbxw11  83.74   529   80    3   77    605   20    542   0.0      922
HSA-Btrc    GGO-Fbxw11  83.74   529   80    3   77    605   20    542   0.0      922
HSA-Btrc    CJA-Fbxw11  83.74   529   80    3   77    605   20    542   0.0      922
HSA-Btrc    MMU-Fbxw11  83.74   529   80    3   77    605   7     529   0.0      921
HSA-Btrc    RNO-Fbxw11  83.74   529   80    3   77    605   5     527   0.0      920
HSA-Btrc    PPY-Fbxw11  51.38   508   181   5   110   605   22    475   3e-148   516
# BLASTP 2.2.20 [Feb-08-2009]
# Query: CJA-Ccnf
# Database: 559_protein.db
# Query id, Subject id, % identity, alignment length, mismatches, gap openings, q. start, q. end, s. start, s. end, e-value, bit score
CJA-Ccnf    CJA-Ccnf    100.00  785   0     0   1     785   1     785   0.0      1522
CJA-Ccnf    PPY-Ccnf    93.23   783   50    1   1     783   1     780   0.0      1407
CJA-Ccnf    PTR-Ccnf    93.87   783   48    0   1     783   1     783   0.0      1395
CJA-Ccnf    HSA-Ccnf    93.74   783   49    0   1     783   1     783   0.0      1394
CJA-Ccnf    GGO-Ccnf    93.61   783   50    0   1     783   1     783   0.0      1392
CJA-Ccnf    MMU-Ccnf    93.74   783   49    0   1     783   1     783   0.0      1373
CJA-Ccnf    RNO-Ccnf    83.20   774   125   1   4     777   42    810   0.0      1190
CJA-Ccnf    MUS-Ccnf    82.69   774   129   1   4     777   42    810   0.0      1182
# BLASTP 2.2.20 [Feb-08-2009]
# Query: GGO-Ccnf
# Database: 559_protein.db
# Query id, Subject id, % identity, alignment length, mismatches, gap openings, q. start, q. end, s. start, s. end, e-value, bit score
GGO-Ccnf    GGO-Ccnf    100.00  786   0     0   1     786   1     786   0.0      1449
GGO-Ccnf    PTR-Ccnf    99.36   786   5     0   1     786   1     786   0.0      1442
GGO-Ccnf    HSA-Ccnf    99.24   786   6     0   1     786   1     786   0.0      1441
GGO-Ccnf    PPY-Ccnf    98.22   786   11    1   1     786   1     783   0.0      1427
```

```
GGO-Ccnf    MMU-Ccnf    97.20     786     22     0    1     786    1     786    0.0    1406
GGO-Ccnf    CJA-Ccnf    93.61     783     50     0    1     783    1     783    0.0    1345
GGO-Ccnf    RNO-Ccnf    84.67     783     114    2    4     786    42    818    0.0    1188
GGO-Ccnf    MUS-Ccnf    83.44     779     123    2    4     782    42    814    0.0    1169
# BLASTP 2.2.20 [Feb-08-2009]
# Query: MMU-Ccnf
# Database: 559_protein.db
# Query id, Subject id, % identity, alignment length, mismatches, gap openings, q. start, q. end, s. start, s. end, e-value, bit score
MMU-Ccnf    MMU-Ccnf    100.00    786     0      0    1     786    1     786    0.0    1462
MMU-Ccnf    PPY-Ccnf    97.07     786     20     1    1     786    1     783    0.0    1420
MMU-Ccnf    PTR-Ccnf    97.58     786     19     0    1     786    1     786    0.0    1419
MMU-Ccnf    HSA-Ccnf    97.46     786     20     0    1     786    1     786    0.0    1417
MMU-Ccnf    GGO-Ccnf    97.20     786     22     0    1     786    1     786    0.0    1415
MMU-Ccnf    CJA-Ccnf    93.74     783     49     0    1     783    1     783    0.0    1356
MMU-Ccnf    RNO-Ccnf    83.91     783     120    2    4     786    42    818    0.0    1191
MMU-Ccnf    MUS-Ccnf    83.20     774     125    1    4     777    42    810    0.0    1173
# BLASTP 2.2.20 [Feb-08-2009]
# Query: MUS-Ccnf
# Database: 559_protein.db
# Query id, Subject id, % identity, alignment length, mismatches, gap openings, q. start, q. end, s. start, s. end, e-value, bit score
MUS-Ccnf    MUS-Ccnf    100.00    815     0      0    1     815    1     815    0.0    1536
MUS-Ccnf    RNO-Ccnf    94.72     814     43     0    1     814    1     814    0.0    1445
MUS-Ccnf    PPY-Ccnf    83.44     779     120    3    42    814    4     779    0.0    1198
MUS-Ccnf    HSA-Ccnf    83.70     779     121    2    42    814    4     782    0.0    1182
MUS-Ccnf    GGO-Ccnf    83.44     779     123    2    42    814    4     782    0.0    1180
MUS-Ccnf    PTR-Ccnf    83.57     779     122    2    42    814    4     782    0.0    1179
MUS-Ccnf    MMU-Ccnf    83.20     774     125    1    42    810    4     777    0.0    1172
MUS-Ccnf    CJA-Ccnf    82.69     774     129    1    42    810    4     777    0.0    1172
# BLASTP 2.2.20 [Feb-08-2009]
# Query: PPY-Ccnf
# Database: 559_protein.db
# Query id, Subject id, % identity, alignment length, mismatches, gap openings, q. start, q. end, s. start, s. end, e-value, bit score
PPY-Ccnf    PPY-Ccnf    100.00    783     0      0    1     783    1     783    0.0    1483
PPY-Ccnf    PTR-Ccnf    98.35     786     10     1    1     783    1     786    0.0    1435
PPY-Ccnf    HSA-Ccnf    98.22     786     11     1    1     783    1     786    0.0    1433
PPY-Ccnf    GGO-Ccnf    98.22     786     11     1    1     783    1     786    0.0    1432
PPY-Ccnf    MMU-Ccnf    97.07     786     20     1    1     783    1     786    0.0    1418
PPY-Ccnf    CJA-Ccnf    93.23     783     50     1    1     780    1     783    0.0    1365
PPY-Ccnf    RNO-Ccnf    84.55     783     112    3    4     783    42    818    0.0    1216
PPY-Ccnf    MUS-Ccnf    83.44     779     120    3    4     779    42    814    0.0    1194
# BLASTP 2.2.20 [Feb-08-2009]
# Query: PTR-Ccnf
# Database: 559_protein.db
# Query id, Subject id, % identity, alignment length, mismatches, gap openings, q. start, q. end, s. start, s. end, e-value, bit score
PTR-Ccnf    PTR-Ccnf    100.00    786     0      0    1     786    1     786    0.0    1441
```

```
PTR-Ccnf    HSA-Ccnf    99.87     786     1      0     1     786    1     786    0.0    1439
PTR-Ccnf    GGO-Ccnf    99.36     786     5      0     1     786    1     786    0.0    1434
PTR-Ccnf    PPY-Ccnf    98.35     786     10     1     1     786    1     783    0.0    1423
PTR-Ccnf    MMU-Ccnf    97.58     786     19     0     1     786    1     786    0.0    1402
PTR-Ccnf    CJA-Ccnf    93.87     783     48     0     1     783    1     783    0.0    1340
PTR-Ccnf    RNO-Ccnf    84.80     783     113    2     4     786    42    818    0.0    1186
PTR-Ccnf    MUS-Ccnf    83.57     779     122    2     4     782    42    814    0.0    1163
# BLASTP 2.2.20 [Feb-08-2009]
# Query: RNO-Ccnf
# Database: 559_protein.db
# Query id, Subject id, % identity, alignment length, mismatches, gap openings, q. start, q. end, s. start, s. end, e-value, bit score
RNO-Ccnf    RNO-Ccnf    100.00    818     0      0     1     818    1     818    0.0    1545
RNO-Ccnf    MUS-Ccnf    94.72     814     43     0     1     814    1     814    0.0    1445
RNO-Ccnf    PPY-Ccnf    84.55     783     112    3     42    818    4     783    0.0    1220
RNO-Ccnf    PTR-Ccnf    84.80     783     113    2     42    818    4     786    0.0    1203
RNO-Ccnf    HSA-Ccnf    84.67     783     114    2     42    818    4     786    0.0    1201
RNO-Ccnf    GGO-Ccnf    84.67     783     114    2     42    818    4     786    0.0    1198
RNO-Ccnf    MMU-Ccnf    83.91     783     120    2     42    818    4     786    0.0    1191
RNO-Ccnf    CJA-Ccnf    83.20     774     125    1     42    810    4     777    0.0    1180
# BLASTP 2.2.20 [Feb-08-2009]
# Query: HSA-Ccnf
# Database: 559_protein.db
# Query id, Subject id, % identity, alignment length, mismatches, gap openings, q. start, q. end, s. start, s. end, e-value, bit score
HSA-Ccnf    HSA-Ccnf    100.00    786     0      0     1     786    1     786    0.0    1441
HSA-Ccnf    PTR-Ccnf    99.87     786     1      0     1     786    1     786    0.0    1439
HSA-Ccnf    GGO-Ccnf    99.24     786     6      0     1     786    1     786    0.0    1433
HSA-Ccnf    PPY-Ccnf    98.22     786     11     1     1     786    1     783    0.0    1422
HSA-Ccnf    MMU-Ccnf    97.46     786     20     0     1     786    1     786    0.0    1401
HSA-Ccnf    CJA-Ccnf    93.74     783     49     0     1     783    1     783    0.0    1338
HSA-Ccnf    RNO-Ccnf    84.67     783     114    2     4     786    42    818    0.0    1184
HSA-Ccnf    MUS-Ccnf    83.70     779     121    2     4     782    42    814    0.0    1165
# BLASTP 2.2.20 [Feb-08-2009]
# Query: CJA-Ect2l
# Database: 559_protein.db
# Query id, Subject id, % identity, alignment length, mismatches, gap openings, q. start, q. end, s. start, s. end, e-value, bit score
CJA-Ect2l   CJA-Ect2l   100.00    904     0      0     1     904    1     904    0.0    1878
CJA-Ect2l   PTR-Ect2l   93.58     904     58     0     1     904    1     904    0.0    1768
CJA-Ect2l   HSA-Ect2l   93.36     904     60     0     1     904    1     904    0.0    1763
CJA-Ect2l   PPY-Ect2l   92.37     904     69     0     1     904    1     904    0.0    1749
CJA-Ect2l   GGO-Ect2l   92.37     904     68     1     1     904    1     903    0.0    1741
CJA-Ect2l   MUS-Ect2l   62.29     838     227    10    70    903    1     753    0.0     982
# BLASTP 2.2.20 [Feb-08-2009]
# Query: GGO-Ect2l
# Database: 559_protein.db
# Query id, Subject id, % identity, alignment length, mismatches, gap openings, q. start, q. end, s. start, s. end, e-value, bit score
```

```
GGO-Ect2l   GGO-Ect2l   100.00      903         0           0           1           903         1           903         0.0         1816
GGO-Ect2l   PTR-Ect2l   97.68       904         20          1           1           903         1           904         0.0         1783
GGO-Ect2l   HSA-Ect2l   97.46       904         22          1           1           903         1           904         0.0         1781
GGO-Ect2l   PPY-Ect2l   96.02       904         35          1           1           903         1           904         0.0         1761
GGO-Ect2l   CJA-Ect2l   91.70       904         74          1           1           903         1           904         0.0         1685
GGO-Ect2l   MUS-Ect2l   75.08       610         145         3           71          678         2           606         0.0          892
GGO-Ect2l   MUS-Ect2l   72.55       153         42          0           750         902         601         753         3e-065       241
# BLASTP 2.2.20 [Feb-08-2009]
# Query: MUS-Ect2l
# Database: 559_protein.db
# Query id, Subject id, % identity, alignment length, mismatches, gap openings, q. start, q. end, s. start, s. end, e-value, bit score
MUS-Ect2l   MUS-Ect2l   100.00      753         0           0           1           753         1           753         0.0         1526
MUS-Ect2l   CJA-Ect2l   62.29       838         227         10          1           753         70          903         0.0          952
MUS-Ect2l   HSA-Ect2l   76.39       610         138         2           2           606         71          679         0.0          949
MUS-Ect2l   HSA-Ect2l   74.51       153         39          0           601         753         751         903         1e-057       216
MUS-Ect2l   PTR-Ect2l   76.23       610         139         2           2           606         71          679         0.0          948
MUS-Ect2l   PTR-Ect2l   74.51       153         39          0           601         753         751         903         8e-058       217
MUS-Ect2l   PPY-Ect2l   75.74       610         142         2           2           606         71          679         0.0          946
MUS-Ect2l   PPY-Ect2l   73.20       153         41          0           601         753         751         903         1e-057       216
MUS-Ect2l   GGO-Ect2l   75.90       610         140         3           2           606         71          678         0.0          937
MUS-Ect2l   GGO-Ect2l   72.55       153         42          0           601         753         750         902         1e-056       213
# BLASTP 2.2.20 [Feb-08-2009]
# Query: PPY-Ect2l
# Database: 559_protein.db
# Query id, Subject id, % identity, alignment length, mismatches, gap openings, q. start, q. end, s. start, s. end, e-value, bit score
PPY-Ect2l   PPY-Ect2l   100.00      904         0           0           1           904         1           904         0.0         1844
PPY-Ect2l   PTR-Ect2l   97.46       904         23          0           1           904         1           904         0.0         1807
PPY-Ect2l   HSA-Ect2l   97.35       904         24          0           1           904         1           904         0.0         1805
PPY-Ect2l   GGO-Ect2l   96.68       904         29          1           1           904         1           903         0.0         1787
PPY-Ect2l   CJA-Ect2l   92.37       904         69          0           1           904         1           904         0.0         1716
PPY-Ect2l   MUS-Ect2l   75.74       610         142         2           71          679         2           606         0.0          944
PPY-Ect2l   MUS-Ect2l   73.20       153         41          0           751         903         601         753         2e-057       215
# BLASTP 2.2.20 [Feb-08-2009]
# Query: PTR-Ect2l
# Database: 559_protein.db
# Query id, Subject id, % identity, alignment length, mismatches, gap openings, q. start, q. end, s. start, s. end, e-value, bit score
PTR-Ect2l   PTR-Ect2l   100.00      904         0           0           1           904         1           904         0.0         1850
PTR-Ect2l   HSA-Ect2l   99.56       904         4           0           1           904         1           904         0.0         1843
PTR-Ect2l   GGO-Ect2l   98.34       904         14          1           1           904         1           903         0.0         1815
PTR-Ect2l   PPY-Ect2l   97.46       904         23          0           1           904         1           904         0.0         1811
PTR-Ect2l   CJA-Ect2l   93.58       904         58          0           1           904         1           904         0.0         1741
PTR-Ect2l   MUS-Ect2l   76.23       610         139         2           71          679         2           606         0.0          929
PTR-Ect2l   MUS-Ect2l   74.51       153         39          0           751         903         601         753         2e-066       246
# BLASTP 2.2.20 [Feb-08-2009]
# Query: HSA-Ect2l
```

```
# Database: 559_protein.db
# Query id, Subject id, % identity, alignment length, mismatches, gap openings, q. start, q. end, s. start, s. end, e-value, bit score
HSA-Ect2l   HSA-Ect2l   100.00      904         0           0           1           904         1           904         0.0           1877
HSA-Ect2l   PTR-Ect2l   99.56       904         4           0           1           904         1           904         0.0           1869
HSA-Ect2l   GGO-Ect2l   98.12       904         16          1           1           904         1           903         0.0           1838
HSA-Ect2l   PPY-Ect2l   97.35       904         24          0           1           904         1           904         0.0           1837
HSA-Ect2l   CJA-Ect2l   93.36       904         60          0           1           904         1           904         0.0           1763
HSA-Ect2l   MUS-Ect2l   76.39       610         138         2           71          679         2           606         0.0            951
HSA-Ect2l   MUS-Ect2l   74.51       153         39          0           751         903         601         753         2e-066         246
# BLASTP 2.2.20 [Feb-08-2009]
# Query: CJA-Fbxl12
# Database: 559_protein.db
# Query id, Subject id, % identity, alignment length, mismatches, gap openings, q. start, q. end, s. start, s. end, e-value, bit score
CJA-Fbxl12  CJA-Fbxl12  100.00      326         0           0           1           326         1           326         0.0            649
CJA-Fbxl12  HSA-Fbxl12  98.16       326         6           0           1           326         1           326         0.0            639
CJA-Fbxl12  PTR-Fbxl12  98.16       326         6           0           1           326         1           326         0.0            639
CJA-Fbxl12  GGO-Fbxl12  97.55       326         8           0           1           326         11          336         0.0            639
CJA-Fbxl12  PPY-Fbxl12  97.85       326         7           0           1           326         1           326         0.0            636
CJA-Fbxl12  MMU-Fbxl12  97.55       326         7           1           1           326         1           325         0.0            632
CJA-Fbxl12  MUS-Fbxl12  92.94       326         23          0           1           326         1           326         1e-177         613
CJA-Fbxl12  RNO-Fbxl12  92.64       326         24          0           1           326         1           326         1e-177         613
# BLASTP 2.2.20 [Feb-08-2009]
# Query: GGO-Fbxl12
# Database: 559_protein.db
# Query id, Subject id, % identity, alignment length, mismatches, gap openings, q. start, q. end, s. start, s. end, e-value, bit score
GGO-Fbxl12  GGO-Fbxl12  100.00      336         0           0           1           336         1           336         0.0            670
GGO-Fbxl12  HSA-Fbxl12  99.39       326         2           0           11          336         1           326         0.0            649
GGO-Fbxl12  PTR-Fbxl12  99.39       326         2           0           11          336         1           326         0.0            649
GGO-Fbxl12  PPY-Fbxl12  99.08       326         3           0           11          336         1           326         0.0            647
GGO-Fbxl12  MMU-Fbxl12  99.39       326         1           1           11          336         1           325         0.0            643
GGO-Fbxl12  CJA-Fbxl12  97.55       326         8           0           11          336         1           326         0.0            639
GGO-Fbxl12  RNO-Fbxl12  93.25       326         22          0           11          336         1           326         2e-178         616
GGO-Fbxl12  MUS-Fbxl12  93.56       326         21          0           11          336         1           326         2e-178         616
# BLASTP 2.2.20 [Feb-08-2009]
# Query: MMU-Fbxl12
# Database: 559_protein.db
# Query id, Subject id, % identity, alignment length, mismatches, gap openings, q. start, q. end, s. start, s. end, e-value, bit score
MMU-Fbxl12  MMU-Fbxl12  100.00      325         0           0           1           325         1           325         0.0            648
MMU-Fbxl12  GGO-Fbxl12  99.39       326         1           1           1           325         11          336         0.0            643
MMU-Fbxl12  HSA-Fbxl12  99.39       326         1           1           1           325         1           326         0.0            643
MMU-Fbxl12  PTR-Fbxl12  99.39       326         1           1           1           325         1           326         0.0            643
MMU-Fbxl12  PPY-Fbxl12  99.08       326         2           1           1           325         1           326         0.0            640
MMU-Fbxl12  CJA-Fbxl12  97.55       326         7           1           1           325         1           326         0.0            632
MMU-Fbxl12  MUS-Fbxl12  93.25       326         21          1           1           325         1           326         1e-176         610
MMU-Fbxl12  RNO-Fbxl12  92.94       326         22          1           1           325         1           326         2e-176         609
```

```
# BLASTP 2.2.20 [Feb-08-2009]
# Query: MUS-Fbxl12
# Database: 559_protein.db
# Query id, Subject id, % identity, alignment length, mismatches, gap openings, q. start, q. end, s. start, s. end, e-value, bit score
MUS-Fbxl12  MUS-Fbxl12  100.00      326         0           0           1           326         1           326         0.0         655
MUS-Fbxl12  RNO-Fbxl12  98.16       326         6           0           1           326         1           326         0.0         647
MUS-Fbxl12  GGO-Fbxl12  93.56       326         21          0           1           326         11          336         2e-178      616
MUS-Fbxl12  HSA-Fbxl12  93.25       326         22          0           1           326         1           326         2e-178      615
MUS-Fbxl12  PTR-Fbxl12  93.25       326         22          0           1           326         1           326         2e-178      615
MUS-Fbxl12  CJA-Fbxl12  92.94       326         23          0           1           326         1           326         1e-177      613
MUS-Fbxl12  PPY-Fbxl12  92.94       326         23          0           1           326         1           326         2e-177      613
MUS-Fbxl12  MMU-Fbxl12  93.25       326         21          1           1           326         1           325         2e-176      610
# BLASTP 2.2.20 [Feb-08-2009]
# Query: PPY-Fbxl12
# Database: 559_protein.db
# Query id, Subject id, % identity, alignment length, mismatches, gap openings, q. start, q. end, s. start, s. end, e-value, bit score
PPY-Fbxl12  PPY-Fbxl12  100.00      326         0           0           1           326         1           326         0.0         650
PPY-Fbxl12  HSA-Fbxl12  99.69       326         1           0           1           326         1           326         0.0         647
PPY-Fbxl12  PTR-Fbxl12  99.69       326         1           0           1           326         1           326         0.0         647
PPY-Fbxl12  GGO-Fbxl12  99.08       326         3           0           1           326         11          336         0.0         647
PPY-Fbxl12  MMU-Fbxl12  99.08       326         2           1           1           326         1           325         0.0         640
PPY-Fbxl12  CJA-Fbxl12  97.85       326         7           0           1           326         1           326         0.0         636
PPY-Fbxl12  MUS-Fbxl12  92.94       326         23          0           1           326         1           326         2e-177      613
PPY-Fbxl12  RNO-Fbxl12  92.64       326         24          0           1           326         1           326         2e-177      613
# BLASTP 2.2.20 [Feb-08-2009]
# Query: PTR-Fbxl12
# Database: 559_protein.db
# Query id, Subject id, % identity, alignment length, mismatches, gap openings, q. start, q. end, s. start, s. end, e-value, bit score
PTR-Fbxl12  HSA-Fbxl12  100.00      326         0           0           1           326         1           326         0.0         650
PTR-Fbxl12  PTR-Fbxl12  100.00      326         0           0           1           326         1           326         0.0         650
PTR-Fbxl12  GGO-Fbxl12  99.39       326         2           0           1           326         11          336         0.0         649
PTR-Fbxl12  PPY-Fbxl12  99.69       326         1           0           1           326         1           326         0.0         647
PTR-Fbxl12  MMU-Fbxl12  99.39       326         1           1           1           326         1           325         0.0         643
PTR-Fbxl12  CJA-Fbxl12  98.16       326         6           0           1           326         1           326         0.0         639
PTR-Fbxl12  MUS-Fbxl12  93.25       326         22          0           1           326         1           326         2e-178      615
PTR-Fbxl12  RNO-Fbxl12  92.94       326         23          0           1           326         1           326         3e-178      615
# BLASTP 2.2.20 [Feb-08-2009]
# Query: RNO-Fbxl12
# Database: 559_protein.db
# Query id, Subject id, % identity, alignment length, mismatches, gap openings, q. start, q. end, s. start, s. end, e-value, bit score
RNO-Fbxl12  RNO-Fbxl12  100.00      326         0           0           1           326         1           326         0.0         654
RNO-Fbxl12  MUS-Fbxl12  98.16       326         6           0           1           326         1           326         0.0         647
RNO-Fbxl12  GGO-Fbxl12  93.25       326         22          0           1           326         11          336         2e-178      616
RNO-Fbxl12  HSA-Fbxl12  92.94       326         23          0           1           326         1           326         3e-178      615
RNO-Fbxl12  PTR-Fbxl12  92.94       326         23          0           1           326         1           326         3e-178      615
```

```
RNO-Fbxl12  CJA-Fbxl12  92.64       326         24          0           1           326         1           326         1e-177        613
RNO-Fbxl12  PPY-Fbxl12  92.64       326         24          0           1           326         1           326         2e-177        613
RNO-Fbxl12  MMU-Fbxl12  92.94       326         22          1           1           326         1           325         2e-176        609
# BLASTP 2.2.20 [Feb-08-2009]
# Query: HSA-Fbxl12
# Database: 559_protein.db
# Query id, Subject id, % identity, alignment length, mismatches, gap openings, q. start, q. end, s. start, s. end, e-value, bit score
HSA-Fbxl12  HSA-Fbxl12  100.00      326         0           0           1           326         1           326         0.0           650
HSA-Fbxl12  PTR-Fbxl12  100.00      326         0           0           1           326         1           326         0.0           650
HSA-Fbxl12  GGO-Fbxl12  99.39       326         2           0           1           326         11          336         0.0           649
HSA-Fbxl12  PPY-Fbxl12  99.69       326         1           0           1           326         1           326         0.0           647
HSA-Fbxl12  MMU-Fbxl12  99.39       326         1           1           1           326         1           325         0.0           643
HSA-Fbxl12  CJA-Fbxl12  98.16       326         6           0           1           326         1           326         0.0           639
HSA-Fbxl12  MUS-Fbxl12  93.25       326         22          0           1           326         1           326         2e-178        615
HSA-Fbxl12  RNO-Fbxl12  92.94       326         23          0           1           326         1           326         3e-178        615
# BLASTP 2.2.20 [Feb-08-2009]
# Query: CJA-Fbxl13
# Database: 559_protein.db
# Query id, Subject id, % identity, alignment length, mismatches, gap openings, q. start, q. end, s. start, s. end, e-value, bit score
CJA-Fbxl13  CJA-Fbxl13  100.00      735         0           0           1           735         1           735         0.0          1493
CJA-Fbxl13  PTR-Fbxl13  90.88       735         67          0           1           735         1           735         0.0          1375
CJA-Fbxl13  PPY-Fbxl13  91.02       735         66          0           1           735         1           735         0.0          1372
CJA-Fbxl13  HSA-Fbxl13  90.88       735         67          0           1           735         1           735         0.0          1371
CJA-Fbxl13  MMU-Fbxl13  90.20       735         72          0           1           735         1           735         0.0          1356
CJA-Fbxl13  GGO-Fbxl13  86.53       735         95          3           1           735         1           731         0.0          1280
CJA-Fbxl13  MUS-Fbxl13  61.63       735         238         5           1           733         91          783         0.0           886
CJA-Fbxl13  RNO-Fbxl13  66.76       376         117         3           1           374         91          460         7e-149        519
# BLASTP 2.2.20 [Feb-08-2009]
# Query: GGO-Fbxl13
# Database: 559_protein.db
# Query id, Subject id, % identity, alignment length, mismatches, gap openings, q. start, q. end, s. start, s. end, e-value, bit score
GGO-Fbxl13  GGO-Fbxl13  100.00      731         0           0           1           731         1           731         0.0          1512
GGO-Fbxl13  PTR-Fbxl13  94.01       735         40          3           1           731         1           735         0.0          1411
GGO-Fbxl13  HSA-Fbxl13  94.01       735         40          3           1           731         1           735         0.0          1410
GGO-Fbxl13  PPY-Fbxl13  92.11       735         54          3           1           731         1           735         0.0          1385
GGO-Fbxl13  MMU-Fbxl13  90.09       737         65          4           1           731         1           735         0.0          1350
GGO-Fbxl13  CJA-Fbxl13  86.53       735         95          3           1           731         1           735         0.0          1304
GGO-Fbxl13  MUS-Fbxl13  59.62       733         251         5           1           728         91          783         0.0           870
GGO-Fbxl13  RNO-Fbxl13  60.84       383         124         6           1           370         91          460         3e-133        467
# BLASTP 2.2.20 [Feb-08-2009]
# Query: MMU-Fbxl13
# Database: 559_protein.db
# Query id, Subject id, % identity, alignment length, mismatches, gap openings, q. start, q. end, s. start, s. end, e-value, bit score
MMU-Fbxl13  MMU-Fbxl13  100.00      735         0           0           1           735         1           735         0.0          1494
MMU-Fbxl13  PPY-Fbxl13  95.10       735         36          0           1           735         1           735         0.0          1426
```

```
MMU-Fbxl13  HSA-Fbxl13  94.97       735         37          0           1           735         1           735         0.0          1421
MMU-Fbxl13  PTR-Fbxl13  94.69       735         39          0           1           735         1           735         0.0          1420
MMU-Fbxl13  CJA-Fbxl13  90.20       735         72          0           1           735         1           735         0.0          1357
MMU-Fbxl13  GGO-Fbxl13  90.09       737         65          4           1           735         1           731         0.0          1325
MMU-Fbxl13  MUS-Fbxl13  63.24       740         218         6           1           733         91          783         0.0           939
MMU-Fbxl13  RNO-Fbxl13  68.62       376         110         3           1           374         91          460         1e-153        535
# BLASTP 2.2.20 [Feb-08-2009]
# Query: MUS-Fbxl13
# Database: 559_protein.db
# Query id, Subject id, % identity, alignment length, mismatches, gap openings, q. start, q. end, s. start, s. end, e-value, bit score
MUS-Fbxl13  MUS-Fbxl13  100.00      790         0           0           1           790         1           790         0.0          1517
MUS-Fbxl13  PPY-Fbxl13  62.99       735         228         5           91          783         1           733         0.0           878
MUS-Fbxl13  MMU-Fbxl13  63.24       740         218         6           91          783         1           733         0.0           876
MUS-Fbxl13  HSA-Fbxl13  63.55       727         222         4           91          776         1           725         0.0           874
MUS-Fbxl13  PTR-Fbxl13  63.27       727         224         4           91          776         1           725         0.0           873
MUS-Fbxl13  CJA-Fbxl13  61.63       735         238         5           91          783         1           733         0.0           844
MUS-Fbxl13  GGO-Fbxl13  59.62       733         251         5           91          783         1           728         0.0           805
MUS-Fbxl13  RNO-Fbxl13  81.58       456         75          2           1           447         1           456         0.0           751
# BLASTP 2.2.20 [Feb-08-2009]
# Query: PPY-Fbxl13
# Database: 559_protein.db
# Query id, Subject id, % identity, alignment length, mismatches, gap openings, q. start, q. end, s. start, s. end, e-value, bit score
PPY-Fbxl13  PPY-Fbxl13  100.00      735         0           0           1           735         1           735         0.0          1522
PPY-Fbxl13  PTR-Fbxl13  97.28       735         20          0           1           735         1           735         0.0          1486
PPY-Fbxl13  HSA-Fbxl13  97.14       735         21          0           1           735         1           735         0.0          1485
PPY-Fbxl13  MMU-Fbxl13  95.10       735         36          0           1           735         1           735         0.0          1452
PPY-Fbxl13  CJA-Fbxl13  91.02       735         66          0           1           735         1           735         0.0          1400
PPY-Fbxl13  GGO-Fbxl13  92.11       735         54          3           1           735         1           731         0.0          1385
PPY-Fbxl13  MUS-Fbxl13  62.99       735         228         5           1           733         91          783         0.0           944
PPY-Fbxl13  RNO-Fbxl13  68.35       376         111         3           1           374         91          460         5e-155        539
# BLASTP 2.2.20 [Feb-08-2009]
# Query: RNO-Fbxl13
# Database: 559_protein.db
# Query id, Subject id, % identity, alignment length, mismatches, gap openings, q. start, q. end, s. start, s. end, e-value, bit score
RNO-Fbxl13  RNO-Fbxl13  100.00      460         0           0           1           460         1           460         0.0           957
RNO-Fbxl13  MUS-Fbxl13  81.58       456         75          2           1           456         1           447         0.0           781
RNO-Fbxl13  PPY-Fbxl13  68.35       376         111         3           91          460         1           374         3e-155        539
RNO-Fbxl13  PTR-Fbxl13  68.35       376         111         3           91          460         1           374         4e-155        539
RNO-Fbxl13  HSA-Fbxl13  68.62       376         110         3           91          460         1           374         3e-154        536
RNO-Fbxl13  MMU-Fbxl13  68.62       376         110         3           91          460         1           374         7e-154        535
RNO-Fbxl13  CJA-Fbxl13  66.76       376         117         3           91          460         1           374         4e-149        519
RNO-Fbxl13  GGO-Fbxl13  60.84       383         124         6           91          460         1           370         2e-133        467
# BLASTP 2.2.20 [Feb-08-2009]
# Query: HSA-Fbxl13
# Database: 559_protein.db
```

```
# Query id, Subject id, % identity, alignment length, mismatches, gap openings, q. start, q. end, s. start, s. end, e-value, bit score
HSA-Fbxl13  HSA-Fbxl13  100.00      735         0           0           1           735         1           735         0.0           1519
HSA-Fbxl13  PTR-Fbxl13  99.05       735         7           0           1           735         1           735         0.0           1508
HSA-Fbxl13  PPY-Fbxl13  97.14       735         21          0           1           735         1           735         0.0           1485
HSA-Fbxl13  MMU-Fbxl13  94.97       735         37          0           1           735         1           735         0.0           1447
HSA-Fbxl13  GGO-Fbxl13  94.01       735         40          3           1           735         1           731         0.0           1410
HSA-Fbxl13  CJA-Fbxl13  90.88       735         67          0           1           735         1           735         0.0           1397
HSA-Fbxl13  MUS-Fbxl13  63.08       734         228         4           1           732         91          783         0.0            940
HSA-Fbxl13  RNO-Fbxl13  68.62       376         110         3           1           374         91          460         5e-154         536
# BLASTP 2.2.20 [Feb-08-2009]
# Query: PTR-Fbxl13
# Database: 559_protein.db
# Query id, Subject id, % identity, alignment length, mismatches, gap openings, q. start, q. end, s. start, s. end, e-value, bit score
PTR-Fbxl13  PTR-Fbxl13  100.00      735         0           0           1           735         1           735         0.0           1521
PTR-Fbxl13  HSA-Fbxl13  99.05       735         7           0           1           735         1           735         0.0           1508
PTR-Fbxl13  PPY-Fbxl13  97.28       735         20          0           1           735         1           735         0.0           1486
PTR-Fbxl13  MMU-Fbxl13  94.69       735         39          0           1           735         1           735         0.0           1444
PTR-Fbxl13  GGO-Fbxl13  94.01       735         40          3           1           735         1           731         0.0           1411
PTR-Fbxl13  CJA-Fbxl13  90.88       735         67          0           1           735         1           735         0.0           1400
PTR-Fbxl13  MUS-Fbxl13  62.81       734         230         4           1           732         91          783         0.0            938
PTR-Fbxl13  RNO-Fbxl13  68.35       376         111         3           1           374         91          460         7e-155         539
# BLASTP 2.2.20 [Feb-08-2009]
# Query: CJA-Fbxl14
# Database: 559_protein.db
# Query id, Subject id, % identity, alignment length, mismatches, gap openings, q. start, q. end, s. start, s. end, e-value, bit score
CJA-Fbxl14  HSA-Fbxl14  100.00      398         0           0           1           398         1           398         0.0            793
CJA-Fbxl14  PPY-Fbxl14  100.00      398         0           0           1           398         1           398         0.0            793
CJA-Fbxl14  MMU-Fbxl14  100.00      398         0           0           1           398         1           398         0.0            793
CJA-Fbxl14  CJA-Fbxl14  100.00      398         0           0           1           398         1           398         0.0            793
CJA-Fbxl14  RNO-Fbxl14a 100.00      398         0           0           1           398         1           398         0.0            793
CJA-Fbxl14  MUS-Fbxl14  100.00      398         0           0           1           398         1           398         0.0            793
CJA-Fbxl14  RNO-Fbxl14b 79.84       377         23          4           1           377         20          343         2e-160         556
# BLASTP 2.2.20 [Feb-08-2009]
# Query: MMU-Fbxl14
# Database: 559_protein.db
# Query id, Subject id, % identity, alignment length, mismatches, gap openings, q. start, q. end, s. start, s. end, e-value, bit score
MMU-Fbxl14  HSA-Fbxl14  100.00      418         0           0           1           418         1           418         0.0            836
MMU-Fbxl14  PPY-Fbxl14  100.00      418         0           0           1           418         1           418         0.0            836
MMU-Fbxl14  MMU-Fbxl14  100.00      418         0           0           1           418         1           418         0.0            836
MMU-Fbxl14  RNO-Fbxl14a 100.00      398         0           0           1           398         1           398         0.0            793
MMU-Fbxl14  MUS-Fbxl14  100.00      398         0           0           1           398         1           398         0.0            793
MMU-Fbxl14  CJA-Fbxl14  100.00      398         0           0           1           398         1           398         0.0            793
MMU-Fbxl14  RNO-Fbxl14b 79.84       377         23          4           1           377         20          343         2e-160         557
# BLASTP 2.2.20 [Feb-08-2009]
# Query: MUS-Fbxl14
```

```
# Database: 559_protein.db
# Query id, Subject id, % identity, alignment length, mismatches, gap openings, q. start, q. end, s. start, s. end, e-value, bit score
MUS-Fbxl14  RNO-Fbxl14a 100.00      400         0           0           1           400         1           400         0.0           796
MUS-Fbxl14  MUS-Fbxl14  100.00      400         0           0           1           400         1           400         0.0           796
MUS-Fbxl14  HSA-Fbxl14  100.00      398         0           0           1           398         1           398         0.0           793
MUS-Fbxl14  PPY-Fbxl14  100.00      398         0           0           1           398         1           398         0.0           793
MUS-Fbxl14  MMU-Fbxl14  100.00      398         0           0           1           398         1           398         0.0           793
MUS-Fbxl14  CJA-Fbxl14  100.00      398         0           0           1           398         1           398         0.0           793
MUS-Fbxl14  RNO-Fbxl14b 79.84       377         23          4           1           377         20          343         2e-160        556
# BLASTP 2.2.20 [Feb-08-2009]
# Query: PPY-Fbxl14
# Database: 559_protein.db
# Query id, Subject id, % identity, alignment length, mismatches, gap openings, q. start, q. end, s. start, s. end, e-value, bit score
PPY-Fbxl14  HSA-Fbxl14  100.00      418         0           0           1           418         1           418         0.0           836
PPY-Fbxl14  PPY-Fbxl14  100.00      418         0           0           1           418         1           418         0.0           836
PPY-Fbxl14  MMU-Fbxl14  100.00      418         0           0           1           418         1           418         0.0           836
PPY-Fbxl14  RNO-Fbxl14a 100.00      398         0           0           1           398         1           398         0.0           793
PPY-Fbxl14  MUS-Fbxl14  100.00      398         0           0           1           398         1           398         0.0           793
PPY-Fbxl14  CJA-Fbxl14  100.00      398         0           0           1           398         1           398         0.0           793
PPY-Fbxl14  RNO-Fbxl14b 79.84       377         23          4           1           377         20          343         2e-160        557
# BLASTP 2.2.20 [Feb-08-2009]
# Query: RNO-Fbxl14a
# Database: 559_protein.db
# Query id, Subject id, % identity, alignment length, mismatches, gap openings, q. start, q. end, s. start, s. end, e-value, bit score
RNO-Fbxl14a RNO-Fbxl14a 100.00      400         0           0           1           400         1           400         0.0           796
RNO-Fbxl14a MUS-Fbxl14  100.00      400         0           0           1           400         1           400         0.0           796
RNO-Fbxl14a HSA-Fbxl14  100.00      398         0           0           1           398         1           398         0.0           793
RNO-Fbxl14a PPY-Fbxl14  100.00      398         0           0           1           398         1           398         0.0           793
RNO-Fbxl14a MMU-Fbxl14  100.00      398         0           0           1           398         1           398         0.0           793
RNO-Fbxl14a CJA-Fbxl14  100.00      398         0           0           1           398         1           398         0.0           793
RNO-Fbxl14a RNO-Fbxl14b 79.84       377         23          4           1           377         20          343         2e-160        556
# BLASTP 2.2.20 [Feb-08-2009]
# Query: RNO-Fbxl14b
# Database: 559_protein.db
# Query id, Subject id, % identity, alignment length, mismatches, gap openings, q. start, q. end, s. start, s. end, e-value, bit score
RNO-Fbxl14b RNO-Fbxl14b 100.00      329         0           0           15          343         15          343         0.0           632
RNO-Fbxl14b CJA-Fbxl14  82.49       377         13          4           20          343         1           377         1e-159        553
RNO-Fbxl14b HSA-Fbxl14  82.49       377         13          4           20          343         1           377         1e-159        553
RNO-Fbxl14b PPY-Fbxl14  82.49       377         13          4           20          343         1           377         1e-159        553
RNO-Fbxl14b MMU-Fbxl14  82.49       377         13          4           20          343         1           377         1e-159        553
RNO-Fbxl14b RNO-Fbxl14a 82.49       377         13          4           20          343         1           377         1e-159        553
RNO-Fbxl14b MUS-Fbxl14  82.49       377         13          4           20          343         1           377         1e-159        553
# BLASTP 2.2.20 [Feb-08-2009]
# Query: HSA-Fbxl14
# Database: 559_protein.db
```

```
# Query id, Subject id, % identity, alignment length, mismatches, gap openings, q. start, q. end, s. start, s. end, e-value, bit score
HSA-Fbxl14  HSA-Fbxl14  100.00      418         0           0           1           418         1           418         0.0         836
HSA-Fbxl14  PPY-Fbxl14  100.00      418         0           0           1           418         1           418         0.0         836
HSA-Fbxl14  MMU-Fbxl14  100.00      418         0           0           1           418         1           418         0.0         836
HSA-Fbxl14  RNO-Fbxl14a 100.00      398         0           0           1           398         1           398         0.0         793
HSA-Fbxl14  MUS-Fbxl14  100.00      398         0           0           1           398         1           398         0.0         793
HSA-Fbxl14  CJA-Fbxl14  100.00      398         0           0           1           398         1           398         0.0         793
HSA-Fbxl14  RNO-Fbxl14b 79.84       377         23          4           1           377         20          343         2e-160      557
# BLASTP 2.2.20 [Feb-08-2009]
# Query: CJA-Fbxl15
# Database: 559_protein.db
# Query id, Subject id, % identity, alignment length, mismatches, gap openings, q. start, q. end, s. start, s. end, e-value, bit score
CJA-Fbxl15  CJA-Fbxl15  100.00      300         0           0           1           300         1           300         7e-152      528
CJA-Fbxl15  PPY-Fbxl15  98.33       300         5           0           1           300         89          388         2e-149      519
CJA-Fbxl15  PTR-Fbxl15  98.33       299         5           0           2           300         2           300         7e-149      518
CJA-Fbxl15  MMU-Fbxl15  98.66       299         4           0           2           300         2           300         1e-148      516
CJA-Fbxl15  GGO-Fbxl15  97.99       299         6           0           2           300         2           300         3e-148      516
CJA-Fbxl15  HSA-Fbxl15  97.99       299         6           0           2           300         2           300         3e-148      516
CJA-Fbxl15  MUS-Fbxl15  97.99       299         6           0           2           300         2           300         1e-147      513
CJA-Fbxl15  RNO-Fbxl15  96.99       299         9           0           2           300         2           300         1e-146      510
# BLASTP 2.2.20 [Feb-08-2009]
# Query: GGO-Fbxl15
# Database: 559_protein.db
# Query id, Subject id, % identity, alignment length, mismatches, gap openings, q. start, q. end, s. start, s. end, e-value, bit score
GGO-Fbxl15  GGO-Fbxl15  100.00      300         0           0           1           300         1           300         3e-152      529
GGO-Fbxl15  PTR-Fbxl15  99.67       300         1           0           1           300         1           300         1e-151      526
GGO-Fbxl15  HSA-Fbxl15  99.33       300         2           0           1           300         1           300         6e-151      525
GGO-Fbxl15  MMU-Fbxl15  98.00       300         6           0           1           300         1           300         1e-148      517
GGO-Fbxl15  PPY-Fbxl15  97.67       300         7           0           1           300         89          388         1e-148      516
GGO-Fbxl15  CJA-Fbxl15  97.99       299         6           0           2           300         2           300         2e-148      516
GGO-Fbxl15  MUS-Fbxl15  96.00       300         12          0           1           300         1           300         2e-145      506
GGO-Fbxl15  RNO-Fbxl15  95.00       300         15          0           1           300         1           300         2e-144      503
# BLASTP 2.2.20 [Feb-08-2009]
# Query: MMU-Fbxl15
# Database: 559_protein.db
# Query id, Subject id, % identity, alignment length, mismatches, gap openings, q. start, q. end, s. start, s. end, e-value, bit score
MMU-Fbxl15  MMU-Fbxl15  100.00      300         0           0           1           300         1           300         3e-163      565
MMU-Fbxl15  PTR-Fbxl15  98.33       300         5           0           1           300         1           300         5e-160      555
MMU-Fbxl15  PPY-Fbxl15  98.33       300         5           0           1           300         89          388         6e-160      554
MMU-Fbxl15  CJA-Fbxl15  98.66       299         4           0           2           300         2           300         2e-159      553
MMU-Fbxl15  HSA-Fbxl15  98.00       300         6           0           1           300         1           300         2e-159      553
MMU-Fbxl15  GGO-Fbxl15  98.00       300         6           0           1           300         1           300         2e-159      552
MMU-Fbxl15  MUS-Fbxl15  96.67       300         10          0           1           300         1           300         9e-156      540
MMU-Fbxl15  RNO-Fbxl15  95.67       300         13          0           1           300         1           300         1e-154      537
# BLASTP 2.2.20 [Feb-08-2009]
```

```
# Query: MUS-Fbxl15
# Database: 559_protein.db
# Query id, Subject id, % identity, alignment length, mismatches, gap openings, q. start, q. end, s. start, s. end, e-value, bit score
MUS-Fbxl15  MUS-Fbxl15  100.00      300         0           0           1           300         1           300         4e-158      548
MUS-Fbxl15  RNO-Fbxl15  99.00       300         3           0           1           300         1           300         5e-157      545
MUS-Fbxl15  CJA-Fbxl15  97.99       299         6           0           2           300         2           300         6e-153      531
MUS-Fbxl15  PTR-Fbxl15  96.33       300         11          0           1           300         1           300         2e-151      526
MUS-Fbxl15  MMU-Fbxl15  96.67       300         10          0           1           300         1           300         4e-151      525
MUS-Fbxl15  HSA-Fbxl15  96.33       300         11          0           1           300         1           300         4e-151      525
MUS-Fbxl15  PPY-Fbxl15  96.33       300         11          0           1           300         89          388         5e-151      525
MUS-Fbxl15  GGO-Fbxl15  96.00       300         12          0           1           300         1           300         9e-151      524
# BLASTP 2.2.20 [Feb-08-2009]
# Query: PPY-Fbxl15
# Database: 559_protein.db
# Query id, Subject id, % identity, alignment length, mismatches, gap openings, q. start, q. end, s. start, s. end, e-value, bit score
PPY-Fbxl15  PPY-Fbxl15  100.00      388         0           0           1           388         1           388         0.0         708
PPY-Fbxl15  CJA-Fbxl15  98.33       300         5           0           89          388         1           300         3e-149      520
PPY-Fbxl15  PTR-Fbxl15  98.00       300         6           0           89          388         1           300         3e-149      519
PPY-Fbxl15  MMU-Fbxl15  98.33       300         5           0           89          388         1           300         4e-149      519
PPY-Fbxl15  GGO-Fbxl15  97.67       300         7           0           89          388         1           300         1e-148      517
PPY-Fbxl15  HSA-Fbxl15  97.67       300         7           0           89          388         1           300         1e-148      517
PPY-Fbxl15  MUS-Fbxl15  96.33       300         11          0           89          388         1           300         6e-146      508
PPY-Fbxl15  RNO-Fbxl15  95.33       300         14          0           89          388         1           300         8e-145      504
# BLASTP 2.2.20 [Feb-08-2009]
# Query: PTR-Fbxl15
# Database: 559_protein.db
# Query id, Subject id, % identity, alignment length, mismatches, gap openings, q. start, q. end, s. start, s. end, e-value, bit score
PTR-Fbxl15  PTR-Fbxl15  100.00      300         0           0           1           300         1           300         5e-152      528
PTR-Fbxl15  GGO-Fbxl15  99.67       300         1           0           1           300         1           300         1e-151      526
PTR-Fbxl15  HSA-Fbxl15  99.67       300         1           0           1           300         1           300         2e-151      526
PTR-Fbxl15  MMU-Fbxl15  98.33       300         5           0           1           300         1           300         2e-149      519
PTR-Fbxl15  PPY-Fbxl15  98.00       300         6           0           1           300         89          388         2e-149      519
PTR-Fbxl15  CJA-Fbxl15  98.33       299         5           0           2           300         2           300         6e-149      518
PTR-Fbxl15  MUS-Fbxl15  96.33       300         11          0           1           300         1           300         5e-146      508
PTR-Fbxl15  RNO-Fbxl15  95.33       300         14          0           1           300         1           300         4e-145      505
# BLASTP 2.2.20 [Feb-08-2009]
# Query: RNO-Fbxl15
# Database: 559_protein.db
# Query id, Subject id, % identity, alignment length, mismatches, gap openings, q. start, q. end, s. start, s. end, e-value, bit score
RNO-Fbxl15  RNO-Fbxl15  100.00      300         0           0           1           300         1           300         3e-163      565
RNO-Fbxl15  MUS-Fbxl15  99.00       300         3           0           1           300         1           300         6e-162      561
RNO-Fbxl15  CJA-Fbxl15  96.99       299         9           0           2           300         2           300         1e-156      543
RNO-Fbxl15  PTR-Fbxl15  95.33       300         14          0           1           300         1           300         3e-155      538
RNO-Fbxl15  MMU-Fbxl15  95.67       300         13          0           1           300         1           300         5e-155      538
RNO-Fbxl15  HSA-Fbxl15  95.33       300         14          0           1           300         1           300         6e-155      538
```

```
RNO-Fbxl15  PPY-Fbxl15  95.33   300   14   0   1   300   89   388   7e-155   537
RNO-Fbxl15  GGO-Fbxl15  95.00   300   15   0   1   300   1    300   2e-154   536
# BLASTP 2.2.20 [Feb-08-2009]
# Query: HSA-Fbxl15
# Database: 559_protein.db
# Query id, Subject id, % identity, alignment length, mismatches, gap openings, q. start, q. end, s. start, s. end, e-value, bit score
HSA-Fbxl15  HSA-Fbxl15  100.00  300   0    0   1   300   1    300   3e-152   528
HSA-Fbxl15  PTR-Fbxl15  99.67   300   1    0   1   300   1    300   2e-151   526
HSA-Fbxl15  GGO-Fbxl15  99.33   300   2    0   1   300   1    300   5e-151   525
HSA-Fbxl15  MMU-Fbxl15  98.00   300   6    0   1   300   1    300   9e-149   517
HSA-Fbxl15  PPY-Fbxl15  97.67   300   7    0   1   300   89   388   2e-148   516
HSA-Fbxl15  CJA-Fbxl15  97.99   299   6    0   2   300   2    300   2e-148   516
HSA-Fbxl15  MUS-Fbxl15  96.33   300   11   0   1   300   1    300   8e-146   507
HSA-Fbxl15  RNO-Fbxl15  95.33   300   14   0   1   300   1    300   6e-145   504
# BLASTP 2.2.20 [Feb-08-2009]
# Query: GGO-Fbxl16
# Database: 559_protein.db
# Query id, Subject id, % identity, alignment length, mismatches, gap openings, q. start, q. end, s. start, s. end, e-value, bit score
GGO-Fbxl16  GGO-Fbxl16  100.00  479   0    0   1   479   1    479   0.0   817
GGO-Fbxl16  HSA-Fbxl16  99.79   479   1    0   1   479   1    479   0.0   815
GGO-Fbxl16  PPY-Fbxl16  99.58   479   2    0   1   479   1    479   0.0   814
GGO-Fbxl16  CJA-Fbxl16  98.96   479   5    0   1   479   1    479   0.0   810
GGO-Fbxl16  MUS-Fbxl16  96.66   479   16   0   1   479   1    479   0.0   810
GGO-Fbxl16  RNO-Fbxl16  96.87   479   15   0   1   479   1    479   0.0   807
GGO-Fbxl16  MMU-Fbxl16  99.28   417   3    0   1   417   1    417   0.0   754
GGO-Fbxl16  PTR-Fbxl16  89.92   476   12   4   1   476   1    440   0.0   729
# BLASTP 2.2.20 [Feb-08-2009]
# Query: MUS-Fbxl16
# Database: 559_protein.db
# Query id, Subject id, % identity, alignment length, mismatches, gap openings, q. start, q. end, s. start, s. end, e-value, bit score
MUS-Fbxl16  MUS-Fbxl16  100.00  479   0    0   1   479   1    479   0.0   822
MUS-Fbxl16  RNO-Fbxl16  99.37   479   3    0   1   479   1    479   0.0   818
MUS-Fbxl16  GGO-Fbxl16  96.66   479   16   0   1   479   1    479   0.0   814
MUS-Fbxl16  HSA-Fbxl16  96.45   479   17   0   1   479   1    479   0.0   813
MUS-Fbxl16  PPY-Fbxl16  96.66   479   16   0   1   479   1    479   0.0   806
MUS-Fbxl16  CJA-Fbxl16  96.24   479   18   0   1   479   1    479   0.0   802
MUS-Fbxl16  MMU-Fbxl16  96.62   414   14   0   1   414   1    414   0.0   733
MUS-Fbxl16  PTR-Fbxl16  87.61   476   23   4   1   476   1    440   0.0   728
# BLASTP 2.2.20 [Feb-08-2009]
# Query: PPY-Fbxl16
# Database: 559_protein.db
# Query id, Subject id, % identity, alignment length, mismatches, gap openings, q. start, q. end, s. start, s. end, e-value, bit score
PPY-Fbxl16  GGO-Fbxl16  99.58   479   2    0   1   479   1    479   0.0   817
PPY-Fbxl16  HSA-Fbxl16  99.37   479   3    0   1   479   1    479   0.0   815
PPY-Fbxl16  PPY-Fbxl16  100.00  479   0    0   1   479   1    479   0.0   814
```

```
PPY-Fbxl16  CJA-Fbxl16  99.37   479  3   0  1  479  1  479  0.0  810
PPY-Fbxl16  MUS-Fbxl16  96.66   479  16  0  1  479  1  479  0.0  810
PPY-Fbxl16  RNO-Fbxl16  96.87   479  15  0  1  479  1  479  0.0  807
PPY-Fbxl16  MMU-Fbxl16  99.76   417  1   0  1  417  1  417  0.0  754
PPY-Fbxl16  PTR-Fbxl16  89.50   476  14  4  1  476  1  440  0.0  729
# BLASTP 2.2.20 [Feb-08-2009]
# Query: PTR-Fbxl16
# Database: 559_protein.db
# Query id, Subject id, % identity, alignment length, mismatches, gap openings, q. start, q. end, s. start, s. end, e-value, bit score
PTR-Fbxl16  PTR-Fbxl16  100.00  441  0   0  1  441  1  441  0.0  813
PTR-Fbxl16  GGO-Fbxl16  89.92   476  12  4  1  440  1  476  0.0  731
PTR-Fbxl16  HSA-Fbxl16  89.71   476  13  4  1  440  1  476  0.0  729
PTR-Fbxl16  MMU-Fbxl16  98.76   404  5   0  1  404  1  404  0.0  727
PTR-Fbxl16  PPY-Fbxl16  89.50   476  14  4  1  440  1  476  0.0  725
PTR-Fbxl16  MUS-Fbxl16  87.61   476  23  4  1  440  1  476  0.0  723
PTR-Fbxl16  CJA-Fbxl16  88.87   476  17  4  1  440  1  476  0.0  722
PTR-Fbxl16  RNO-Fbxl16  87.61   476  23  4  1  440  1  476  0.0  719
# BLASTP 2.2.20 [Feb-08-2009]
# Query: HSA-Fbxl16
# Database: 559_protein.db
# Query id, Subject id, % identity, alignment length, mismatches, gap openings, q. start, q. end, s. start, s. end, e-value, bit score
HSA-Fbxl16  GGO-Fbxl16  99.79   479  1   0  1  479  1  479  0.0  817
HSA-Fbxl16  HSA-Fbxl16  100.00  479  0   0  1  479  1  479  0.0  815
HSA-Fbxl16  PPY-Fbxl16  99.37   479  3   0  1  479  1  479  0.0  814
HSA-Fbxl16  CJA-Fbxl16  98.96   479  5   0  1  479  1  479  0.0  810
HSA-Fbxl16  MUS-Fbxl16  96.45   479  17  0  1  479  1  479  0.0  810
HSA-Fbxl16  RNO-Fbxl16  96.66   479  16  0  1  479  1  479  0.0  807
HSA-Fbxl16  MMU-Fbxl16  99.04   417  4   0  1  417  1  417  0.0  754
HSA-Fbxl16  PTR-Fbxl16  89.71   476  13  4  1  476  1  440  0.0  729
# BLASTP 2.2.20 [Feb-08-2009]
# Query: RNO-Fbxl16
# Database: 559_protein.db
# Query id, Subject id, % identity, alignment length, mismatches, gap openings, q. start, q. end, s. start, s. end, e-value, bit score
RNO-Fbxl16  RNO-Fbxl16  100.00  479  0   0  1  479  1  479  0.0  817
RNO-Fbxl16  MUS-Fbxl16  99.37   479  3   0  1  479  1  479  0.0  815
RNO-Fbxl16  GGO-Fbxl16  96.87   479  15  0  1  479  1  479  0.0  809
RNO-Fbxl16  HSA-Fbxl16  96.66   479  16  0  1  479  1  479  0.0  808
RNO-Fbxl16  PPY-Fbxl16  96.87   479  15  0  1  479  1  479  0.0  800
RNO-Fbxl16  CJA-Fbxl16  96.45   479  17  0  1  479  1  479  0.0  797
RNO-Fbxl16  MMU-Fbxl16  96.64   417  14  0  1  417  1  417  0.0  726
RNO-Fbxl16  PTR-Fbxl16  87.61   476  23  4  1  476  1  440  0.0  712
# BLASTP 2.2.20 [Feb-08-2009]
# Query: CJA-Fbxl16
# Database: 559_protein.db
# Query id, Subject id, % identity, alignment length, mismatches, gap openings, q. start, q. end, s. start, s. end, e-value, bit score
```

```
CJA-Fbxl16  CJA-Fbxl16  100.00      479         0           0           1           479         1           479         0.0             827
CJA-Fbxl16  PPY-Fbxl16  99.37       479         3           0           1           479         1           479         0.0             827
CJA-Fbxl16  GGO-Fbxl16  98.96       479         5           0           1           479         1           479         0.0             825
CJA-Fbxl16  HSA-Fbxl16  98.96       479         5           0           1           479         1           479         0.0             823
CJA-Fbxl16  RNO-Fbxl16  96.45       479         17          0           1           479         1           479         0.0             818
CJA-Fbxl16  MUS-Fbxl16  96.24       479         18          0           1           479         1           479         0.0             818
CJA-Fbxl16  MMU-Fbxl16  99.04       417         4           0           1           417         1           417         0.0             755
CJA-Fbxl16  PTR-Fbxl16  88.87       476         17          4           1           476         1           440         0.0             732
# BLASTP 2.2.20 [Feb-08-2009]
# Query: MMU-Fbxl16
# Database: 559_protein.db
# Query id, Subject id, % identity, alignment length, mismatches, gap openings, q. start, q. end, s. start, s. end, e-value, bit score
MMU-Fbxl16  HSA-Fbxl16  99.04       417         4           0           1           417         1           417         0.0             757
MMU-Fbxl16  GGO-Fbxl16  99.28       417         3           0           1           417         1           417         0.0             756
MMU-Fbxl16  PPY-Fbxl16  99.76       417         1           0           1           417         1           417         0.0             756
MMU-Fbxl16  MMU-Fbxl16  100.00      417         0           0           1           417         1           417         0.0             756
MMU-Fbxl16  CJA-Fbxl16  99.04       417         4           0           1           417         1           417         0.0             756
MMU-Fbxl16  MUS-Fbxl16  96.64       417         14          0           1           417         1           417         0.0             752
MMU-Fbxl16  RNO-Fbxl16  96.64       417         14          0           1           417         1           417         0.0             748
MMU-Fbxl16  PTR-Fbxl16  98.76       404         5           0           1           404         1           404         0.0             726
# BLASTP 2.2.20 [Feb-08-2009]
# Query: CJA-Fbxl17
# Database: 559_protein.db
# Query id, Subject id, % identity, alignment length, mismatches, gap openings, q. start, q. end, s. start, s. end, e-value, bit score
CJA-Fbxl17  CJA-Fbxl17  100.00      704         0           0           1           704         1           704         0.0            1038
CJA-Fbxl17  PTR-Fbxl17  95.46       705         27          4           1           704         1           701         0.0            1027
CJA-Fbxl17  HSA-Fbxl17  95.60       705         26          4           1           704         1           701         0.0            1023
CJA-Fbxl17  MUS-Fbxl17  86.60       709         82          6           1           704         1           701         0.0             969
CJA-Fbxl17  PPY-Fbxl17  97.94       436         7           1           271         704         316         751         0.0             841
CJA-Fbxl17  MMU-Fbxl17  95.38       346         13          1           335         680         1           343         0.0             633
CJA-Fbxl17  RNO-Fbxl17  93.28       238         15          1           271         507         27          264         1e-130          458
# BLASTP 2.2.20 [Feb-08-2009]
# Query: MMU-Fbxl17
# Database: 559_protein.db
# Query id, Subject id, % identity, alignment length, mismatches, gap openings, q. start, q. end, s. start, s. end, e-value, bit score
MMU-Fbxl17  MMU-Fbxl17  100.00      380         0           0           1           380         1           380         0.0             708
MMU-Fbxl17  HSA-Fbxl17  95.09       346         14          1           1           343         332         677         0.0             632
MMU-Fbxl17  CJA-Fbxl17  95.38       346         13          1           1           343         335         680         0.0             632
MMU-Fbxl17  PTR-Fbxl17  95.09       346         14          1           1           343         332         677         0.0             631
MMU-Fbxl17  PPY-Fbxl17  94.80       346         15          1           1           343         382         727         0.0             629
MMU-Fbxl17  MUS-Fbxl17  93.64       346         19          1           1           343         332         677         1e-180          624
MMU-Fbxl17  RNO-Fbxl17  97.11       173         5           0           1           173         92          264         7e-099          352
# BLASTP 2.2.20 [Feb-08-2009]
# Query: MUS-Fbxl17
# Database: 559_protein.db
```

```
# Query id, Subject id, % identity, alignment length, mismatches, gap openings, q. start, q. end, s. start, s. end, e-value, bit score
MUS-Fbxl17  MUS-Fbxl17  100.00      701         0           0           1           701         1           701         0.0          1053
MUS-Fbxl17  PTR-Fbxl17  96.73       428         14          0           274         701         274         701         0.0           822
MUS-Fbxl17  CJA-Fbxl17  96.50       428         15          0           274         701         277         704         0.0           818
MUS-Fbxl17  HSA-Fbxl17  96.26       428         16          0           274         701         274         701         0.0           816
MUS-Fbxl17  PPY-Fbxl17  95.58       430         17          1           274         701         322         751         0.0           812
MUS-Fbxl17  MMU-Fbxl17  93.64       346         19          1           332         677         1           343         2e-180        624
MUS-Fbxl17  RNO-Fbxl17  96.15       234         9           0           271         504         31          264         2e-134        471
# BLASTP 2.2.20 [Feb-08-2009]
# Query: PTR-Fbxl17
# Database: 559_protein.db
# Query id, Subject id, % identity, alignment length, mismatches, gap openings, q. start, q. end, s. start, s. end, e-value, bit score
PTR-Fbxl17  PTR-Fbxl17  100.00      701         0           0           1           701         1           701         0.0          1058
PTR-Fbxl17  HSA-Fbxl17  99.43       701         4           0           1           701         1           701         0.0          1051
PTR-Fbxl17  CJA-Fbxl17  94.47       705         34          4           1           701         1           704         0.0          1010
PTR-Fbxl17  MUS-Fbxl17  85.71       707         89          6           1           701         1           701         0.0           972
PTR-Fbxl17  PPY-Fbxl17  98.85       436         3           1           268         701         316         751         0.0           850
PTR-Fbxl17  MMU-Fbxl17  95.09       346         14          1           332         677         1           343         0.0           631
PTR-Fbxl17  RNO-Fbxl17  94.12       238         13          1           268         504         27          264         7e-132        462
# BLASTP 2.2.20 [Feb-08-2009]
# Query: RNO-Fbxl17
# Database: 559_protein.db
# Query id, Subject id, % identity, alignment length, mismatches, gap openings, q. start, q. end, s. start, s. end, e-value, bit score
RNO-Fbxl17  RNO-Fbxl17  100.00      238         0           0           27          264         27          264         2e-139        486
RNO-Fbxl17  MUS-Fbxl17  96.22       238         9           0           27          264         267         504         5e-137        478
RNO-Fbxl17  PTR-Fbxl17  94.12       238         13          1           27          264         268         504         7e-132        461
RNO-Fbxl17  CJA-Fbxl17  93.28       238         15          1           27          264         271         507         3e-130        455
RNO-Fbxl17  HSA-Fbxl17  93.28       238         15          1           27          264         268         504         4e-130        455
RNO-Fbxl17  PPY-Fbxl17  92.50       240         15          2           27          264         316         554         2e-129        452
RNO-Fbxl17  MMU-Fbxl17  97.11       173         5           0           92          264         1           173         7e-099        351
# BLASTP 2.2.20 [Feb-08-2009]
# Query: HSA-Fbxl17
# Database: 559_protein.db
# Query id, Subject id, % identity, alignment length, mismatches, gap openings, q. start, q. end, s. start, s. end, e-value, bit score
HSA-Fbxl17  HSA-Fbxl17  100.00      701         0           0           1           701         1           701         0.0          1058
HSA-Fbxl17  PTR-Fbxl17  99.43       701         4           0           1           701         1           701         0.0          1052
HSA-Fbxl17  CJA-Fbxl17  94.47       705         34          4           1           701         1           704         0.0          1006
HSA-Fbxl17  MUS-Fbxl17  85.57       707         90          6           1           701         1           701         0.0           967
HSA-Fbxl17  PPY-Fbxl17  98.39       436         5           1           268         701         316         751         0.0           845
HSA-Fbxl17  MMU-Fbxl17  95.09       346         14          1           332         677         1           343         0.0           630
HSA-Fbxl17  RNO-Fbxl17  93.28       238         15          1           268         504         27          264         3e-130        457
# BLASTP 2.2.20 [Feb-08-2009]
# Query: PPY-Fbxl17
# Database: 559_protein.db
# Query id, Subject id, % identity, alignment length, mismatches, gap openings, q. start, q. end, s. start, s. end, e-value, bit score
```

```
PPY-Fbxl17  PPY-Fbxl17  100.00      436         0           0           316         751         316         751         0.0          864
PPY-Fbxl17  PTR-Fbxl17  98.85       436         3           1           316         751         268         701         0.0          850
PPY-Fbxl17  HSA-Fbxl17  98.39       436         5           1           316         751         268         701         0.0          843
PPY-Fbxl17  CJA-Fbxl17  97.94       436         7           1           316         751         271         704         0.0          838
PPY-Fbxl17  MUS-Fbxl17  94.74       437         20          2           316         751         267         701         0.0          811
PPY-Fbxl17  MMU-Fbxl17  94.80       346         15          1           382         727         1           343         0.0          629
PPY-Fbxl17  RNO-Fbxl17  92.50       240         15          2           316         554         27          264         2e-129       454
# BLASTP 2.2.20 [Feb-08-2009]
# Query: GGO-Fbxl18
# Database: 559_protein.db
# Query id, Subject id, % identity, alignment length, mismatches, gap openings, q. start, q. end, s. start, s. end, e-value, bit score
GGO-Fbxl18  GGO-Fbxl18  100.00      751         0           0           1           751         1           751         0.0          1441
GGO-Fbxl18  HSA-Fbxl18  99.47       751         4           0           1           751         1           751         0.0          1429
GGO-Fbxl18  MMU-Fbxl18  97.86       746         15          1           6           751         1           745         0.0          1391
GGO-Fbxl18  PPY-Fbxl18  98.08       678         10          1           1           678         1           675         0.0          1256
GGO-Fbxl18  PTR-Fbxl18  98.51       673         7           1           6           678         1           670         0.0          1253
GGO-Fbxl18  MUS-Fbxl18  89.51       677         64          2           2           678         59          728         0.0          1140
GGO-Fbxl18  RNO-Fbxl18b 90.96       653         56          1           26          678         15          664         0.0          1137
GGO-Fbxl18  RNO-Fbxl18a 90.84       655         55          2           26          678         15          666         0.0          1134
# BLASTP 2.2.20 [Feb-08-2009]
# Query: MMU-Fbxl18
# Database: 559_protein.db
# Query id, Subject id, % identity, alignment length, mismatches, gap openings, q. start, q. end, s. start, s. end, e-value, bit score
MMU-Fbxl18  MMU-Fbxl18  100.00      795         0           0           1           795         1           795         0.0          1535
MMU-Fbxl18  HSA-Fbxl18  95.88       800         28          3           1           795         6           805         0.0          1445
MMU-Fbxl18  GGO-Fbxl18  97.86       746         15          1           1           745         6           751         0.0          1392
MMU-Fbxl18  PPY-Fbxl18  98.37       673         8           1           1           673         6           675         0.0          1248
MMU-Fbxl18  PTR-Fbxl18  98.22       673         9           1           1           673         1           670         0.0          1244
MMU-Fbxl18  RNO-Fbxl18b 91.12       653         55          1           21          673         15          664         0.0          1134
MMU-Fbxl18  RNO-Fbxl18a 90.99       655         54          2           21          673         15          666         0.0          1132
MMU-Fbxl18  MUS-Fbxl18  91.27       653         54          1           21          673         79          728         0.0          1132
# BLASTP 2.2.20 [Feb-08-2009]
# Query: MUS-Fbxl18
# Database: 559_protein.db
# Query id, Subject id, % identity, alignment length, mismatches, gap openings, q. start, q. end, s. start, s. end, e-value, bit score
MUS-Fbxl18  MUS-Fbxl18  100.00      771         0           0           1           771         1           771         0.0          1450
MUS-Fbxl18  RNO-Fbxl18b 98.85       693         8           0           79          771         15          707         0.0          1314
MUS-Fbxl18  RNO-Fbxl18a 98.71       695         7           1           79          771         15          709         0.0          1313
MUS-Fbxl18  PPY-Fbxl18  92.50       693         52          0           79          771         26          718         0.0          1251
MUS-Fbxl18  PTR-Fbxl18  92.50       693         52          0           79          771         21          713         0.0          1250
MUS-Fbxl18  GGO-Fbxl18  91.12       653         55          1           79          728         26          678         0.0          1142
MUS-Fbxl18  HSA-Fbxl18  91.12       653         55          1           79          728         26          678         0.0          1141
MUS-Fbxl18  MMU-Fbxl18  91.27       653         54          1           79          728         21          673         0.0          1139
# BLASTP 2.2.20 [Feb-08-2009]
# Query: PPY-Fbxl18
```

```
# Database: 559_protein.db
# Query id, Subject id, % identity, alignment length, mismatches, gap openings, q. start, q. end, s. start, s. end, e-value, bit score
PPY-Fbxl18  PPY-Fbxl18  100.00      718         0           0           1           718         1           718         0.0         1375
PPY-Fbxl18  PTR-Fbxl18  99.02       713         7           0           6           718         1           713         0.0         1355
PPY-Fbxl18  GGO-Fbxl18  98.08       678         10          1           1           675         1           678         0.0         1255
PPY-Fbxl18  HSA-Fbxl18  97.94       678         11          1           1           675         1           678         0.0         1251
PPY-Fbxl18  MMU-Fbxl18  98.37       673         8           1           6           675         1           673         0.0         1248
PPY-Fbxl18  MUS-Fbxl18  90.93       717         61          1           2           718         59          771         0.0         1247
PPY-Fbxl18  RNO-Fbxl18b 92.50       693         52          0           26          718         15          707         0.0         1246
PPY-Fbxl18  RNO-Fbxl18a 92.37       695         51          1           26          718         15          709         0.0         1244
# BLASTP 2.2.20 [Feb-08-2009]
# Query: PTR-Fbxl18
# Database: 559_protein.db
# Query id, Subject id, % identity, alignment length, mismatches, gap openings, q. start, q. end, s. start, s. end, e-value, bit score
PTR-Fbxl18  PTR-Fbxl18  100.00      713         0           0           1           713         1           713         0.0         1365
PTR-Fbxl18  PPY-Fbxl18  99.02       713         7           0           1           713         6           718         0.0         1355
PTR-Fbxl18  GGO-Fbxl18  98.51       673         7           1           1           670         6           678         0.0         1253
PTR-Fbxl18  HSA-Fbxl18  98.37       673         8           1           1           670         6           678         0.0         1248
PTR-Fbxl18  RNO-Fbxl18b 92.50       693         52          0           21          713         15          707         0.0         1245
PTR-Fbxl18  MMU-Fbxl18  98.22       673         9           1           1           670         1           673         0.0         1244
PTR-Fbxl18  MUS-Fbxl18  92.50       693         52          0           21          713         79          771         0.0         1243
PTR-Fbxl18  RNO-Fbxl18a 92.37       695         51          1           21          713         15          709         0.0         1243
# BLASTP 2.2.20 [Feb-08-2009]
# Query: RNO-Fbxl18a
# Database: 559_protein.db
# Query id, Subject id, % identity, alignment length, mismatches, gap openings, q. start, q. end, s. start, s. end, e-value, bit score
RNO-Fbxl18a RNO-Fbxl18a 100.00      709         0           0           1           709         1           709         0.0         1325
RNO-Fbxl18a RNO-Fbxl18b 99.58       709         1           1           1           709         1           707         0.0         1315
RNO-Fbxl18a MUS-Fbxl18  98.31       709         10          1           1           709         65          771         0.0         1296
RNO-Fbxl18a PPY-Fbxl18  92.37       695         51          1           15          709         26          718         0.0         1233
RNO-Fbxl18a PTR-Fbxl18  92.37       695         51          1           15          709         21          713         0.0         1231
RNO-Fbxl18a GGO-Fbxl18  90.84       655         55          2           15          666         26          678         0.0         1122
RNO-Fbxl18a MMU-Fbxl18  90.99       655         54          2           15          666         21          673         0.0         1119
RNO-Fbxl18a HSA-Fbxl18  90.84       655         55          2           15          666         26          678         0.0         1119
# BLASTP 2.2.20 [Feb-08-2009]
# Query: RNO-Fbxl18b
# Database: 559_protein.db
# Query id, Subject id, % identity, alignment length, mismatches, gap openings, q. start, q. end, s. start, s. end, e-value, bit score
RNO-Fbxl18b RNO-Fbxl18b 100.00      707         0           0           1           707         1           707         0.0         1322
RNO-Fbxl18b RNO-Fbxl18a 99.58       709         1           1           1           707         1           709         0.0         1315
RNO-Fbxl18b MUS-Fbxl18  98.44       707         11          0           1           707         65          771         0.0         1299
RNO-Fbxl18b PTR-Fbxl18  92.50       693         52          0           15          707         21          713         0.0         1234
RNO-Fbxl18b PPY-Fbxl18  92.50       693         52          0           15          707         26          718         0.0         1234
RNO-Fbxl18b GGO-Fbxl18  90.96       653         56          1           15          664         26          678         0.0         1123
RNO-Fbxl18b HSA-Fbxl18  90.96       653         56          1           15          664         26          678         0.0         1123
```

```
RNO-Fbxl18b MMU-Fbxl18  91.12   653     55      1       15      664     21      673     0.0     1121
# BLASTP 2.2.20 [Feb-08-2009]
# Query: HSA-Fbxl18
# Database: 559_protein.db
# Query id, Subject id, % identity, alignment length, mismatches, gap openings, q. start, q. end, s. start, s. end, e-value, bit score
HSA-Fbxl18  HSA-Fbxl18  100.00  805     0       0       1       805     1       805     0.0     1494
HSA-Fbxl18  GGO-Fbxl18  99.47   751     4       0       1       751     1       751     0.0     1430
HSA-Fbxl18  MMU-Fbxl18  94.63   800     38      3       6       805     1       795     0.0     1415
HSA-Fbxl18  PPY-Fbxl18  97.94   678     11      1       1       678     1       675     0.0     1252
HSA-Fbxl18  PTR-Fbxl18  98.37   673     8       1       6       678     1       670     0.0     1249
HSA-Fbxl18  MUS-Fbxl18  89.51   677     64      2       2       678     59      728     0.0     1138
HSA-Fbxl18  RNO-Fbxl18b 90.96   653     56      1       26      678     15      664     0.0     1135
HSA-Fbxl18  RNO-Fbxl18a 90.84   655     55      2       26      678     15      666     0.0     1132
# BLASTP 2.2.20 [Feb-08-2009]
# Query: CJA-Fbxl19
# Database: 559_protein.db
# Query id, Subject id, % identity, alignment length, mismatches, gap openings, q. start, q. end, s. start, s. end, e-value, bit score
CJA-Fbxl19  CJA-Fbxl19  100.00  694     0       0       1       694     1       694     0.0     1019
CJA-Fbxl19  HSA-Fbxl19  98.85   694     8       0       1       694     1       694     0.0      995
CJA-Fbxl19  MUS-Fbxl19  96.90   646     20      0       49      694     29      674     0.0      985
CJA-Fbxl19  RNO-Fbxl19  97.37   646     17      0       49      694     29      674     0.0      970
CJA-Fbxl19  GGO-Fbxl19  98.98   590     6       0       49      638     31      620     0.0      922
CJA-Fbxl19  PPY-Fbxl19  92.26   646     6       1       49      694     29      630     0.0      867
CJA-Fbxl19  MMU-Fbxl19  92.34   627     5       1       68      694     1       584     0.0      866
CJA-Fbxl19  PTR-Fbxl19  98.34   422     7       0       273     694     1       422     1e-178   618
CJA-Fbxl19  MUS-Kdm2B   59.59   245     99      0       398     642     1012    1256    3e-079   287
CJA-Fbxl19  MUS-Kdm2B   71.90   121     33      1       49      169     596     715     4e-053   201
CJA-Fbxl19  HSA-Kdm2B   59.35   246     100     0       398     643     1039    1284    2e-078   285
CJA-Fbxl19  HSA-Kdm2B   71.90   121     33      1       49      169     623     742     5e-053   201
CJA-Fbxl19  PTR-Kdm2B   59.35   246     100     0       398     643     1039    1284    2e-078   285
CJA-Fbxl19  PTR-Kdm2B   71.90   121     33      1       49      169     623     742     5e-053   201
CJA-Fbxl19  GGO-Kdm2B   59.35   246     100     0       398     643     955     1200    2e-078   285
CJA-Fbxl19  GGO-Kdm2B   71.90   121     33      1       49      169     539     658     4e-053   201
CJA-Fbxl19  CJA-Kdm2B   59.35   246     100     0       398     643     1037    1282    3e-078   285
CJA-Fbxl19  CJA-Kdm2B   70.73   123     34      1       49      169     617     739     6e-053   200
CJA-Fbxl19  RNO-Kdm2B   58.94   246     101     0       398     643     1008    1253    4e-078   284
CJA-Fbxl19  RNO-Kdm2B   71.90   121     33      1       49      169     592     711     4e-053   201
CJA-Fbxl19  MMU-Kdm2B   58.13   246     100     2       398     643     1038    1280    3e-074   271
CJA-Fbxl19  MMU-Kdm2B   71.90   121     33      1       49      169     622     741     6e-053   200
CJA-Fbxl19  HSA-Kdm2A   51.62   277     131     3       419     694     888     1162    5e-069   254
CJA-Fbxl19  PTR-Kdm2A   51.62   277     131     3       419     694     888     1162    5e-069   254
CJA-Fbxl19  RNO-Kdm2A   51.62   277     131     3       419     694     887     1161    6e-069   253
CJA-Fbxl19  GGO-Kdm2A   51.62   277     131     3       419     694     1007    1281    7e-069   253
CJA-Fbxl19  CJA-Kdm2A   51.62   277     131     3       419     694     886     1160    7e-069   253
CJA-Fbxl19  MMU-Kdm2A   51.62   277     131     3       419     694     888     1162    8e-069   253
```

CJA-Fbxl19	MUS-Kdm2A	51.26	277	132	3	419	694	887	1161	9e-069	253
CJA-Fbxl19	PPY-Kdm2A	51.62	277	131	3	419	694	395	669	2e-068	252
CJA-Fbxl19	PPY-Kdm2B	71.90	121	33	1	49	169	623	742	4e-053	201

BLASTP 2.2.20 [Feb-08-2009]
Query: GGO-Fbxl19
Database: 559_protein.db
Query id, Subject id, % identity, alignment length, mismatches, gap openings, q. start, q. end, s. start, s. end, e-value, bit score

GGO-Fbxl19	GGO-Fbxl19	100.00	588	0	0	33	620	33	620	0.0	913
GGO-Fbxl19	HSA-Fbxl19	99.83	588	1	0	33	620	51	638	0.0	909
GGO-Fbxl19	CJA-Fbxl19	98.98	588	6	0	33	620	51	638	0.0	905
GGO-Fbxl19	RNO-Fbxl19	97.45	588	15	0	33	620	31	618	0.0	898
GGO-Fbxl19	MUS-Fbxl19	96.94	588	18	0	33	620	31	618	0.0	895
GGO-Fbxl19	MMU-Fbxl19	92.29	571	1	1	50	620	1	528	0.0	798
GGO-Fbxl19	PPY-Fbxl19	92.18	588	2	1	33	620	31	574	0.0	795
GGO-Fbxl19	PTR-Fbxl19	98.36	366	6	0	255	620	1	366	1e-161	561
GGO-Fbxl19	MUS-Kdm2B	59.66	238	96	0	380	617	1012	1249	9e-076	276
GGO-Fbxl19	MUS-Kdm2B	72.27	119	32	1	33	151	598	715	4e-052	197
GGO-Fbxl19	GGO-Kdm2B	59.66	238	96	0	380	617	955	1192	3e-075	274
GGO-Fbxl19	GGO-Kdm2B	72.27	119	32	1	33	151	541	658	3e-052	198
GGO-Fbxl19	HSA-Kdm2B	59.66	238	96	0	380	617	1039	1276	4e-075	274
GGO-Fbxl19	HSA-Kdm2B	72.27	119	32	1	33	151	625	742	3e-052	197
GGO-Fbxl19	PTR-Kdm2B	59.66	238	96	0	380	617	1039	1276	4e-075	274
GGO-Fbxl19	PTR-Kdm2B	72.27	119	32	1	33	151	625	742	4e-052	197
GGO-Fbxl19	CJA-Kdm2B	59.66	238	96	0	380	617	1037	1274	5e-075	273
GGO-Fbxl19	CJA-Kdm2B	71.07	121	33	1	33	151	619	739	5e-052	197
GGO-Fbxl19	RNO-Kdm2B	59.24	238	97	0	380	617	1008	1245	7e-075	273
GGO-Fbxl19	RNO-Kdm2B	72.27	119	32	1	33	151	594	711	3e-052	198
GGO-Fbxl19	MMU-Kdm2B	58.40	238	96	2	380	617	1038	1272	6e-071	260
GGO-Fbxl19	MMU-Kdm2B	72.27	119	32	1	33	151	624	741	5e-052	197
GGO-Fbxl19	RNO-Kdm2A	56.42	218	93	2	401	618	887	1102	2e-065	241
GGO-Fbxl19	MMU-Kdm2A	56.42	218	93	2	401	618	888	1103	2e-065	241
GGO-Fbxl19	CJA-Kdm2A	56.42	218	93	2	401	618	886	1101	2e-065	241
GGO-Fbxl19	HSA-Kdm2A	56.42	218	93	2	401	618	888	1103	2e-065	241
GGO-Fbxl19	PTR-Kdm2A	56.42	218	93	2	401	618	888	1103	2e-065	241
GGO-Fbxl19	GGO-Kdm2A	56.42	218	93	2	401	618	1007	1222	3e-065	241
GGO-Fbxl19	MUS-Kdm2A	55.96	218	94	2	401	618	887	1102	5e-065	240
GGO-Fbxl19	PPY-Kdm2A	56.42	218	93	2	401	618	395	610	6e-065	240
GGO-Fbxl19	PPY-Kdm2B	72.27	119	32	1	33	151	625	742	3e-052	198

BLASTP 2.2.20 [Feb-08-2009]
Query: MMU-Fbxl19
Database: 559_protein.db
Query id, Subject id, % identity, alignment length, mismatches, gap openings, q. start, q. end, s. start, s. end, e-value, bit score

MMU-Fbxl19	MMU-Fbxl19	100.00	584	0	0	1	584	1	584	0.0	863
MMU-Fbxl19	HSA-Fbxl19	92.34	627	5	1	1	584	68	694	0.0	841
MMU-Fbxl19	CJA-Fbxl19	91.55	627	10	1	1	584	68	694	0.0	837

MMU-Fbxl19	RNO-Fbxl19	90.43	627	17	1	1	584	48	674	0.0	830
MMU-Fbxl19	MUS-Fbxl19	89.95	627	20	1	1	584	48	674	0.0	827
MMU-Fbxl19	GGO-Fbxl19	91.42	571	6	1	1	528	50	620	0.0	782
MMU-Fbxl19	PPY-Fbxl19	85.17	627	6	2	1	584	48	630	0.0	727
MMU-Fbxl19	PTR-Fbxl19	100.00	406	0	0	179	584	17	422	2e-173	600
MMU-Fbxl19	MUS-Kdm2B	59.18	245	100	0	288	532	1012	1256	1e-078	285
MMU-Fbxl19	PTR-Kdm2B	58.94	246	101	0	288	533	1039	1284	5e-078	283
MMU-Fbxl19	HSA-Kdm2B	58.94	246	101	0	288	533	1039	1284	6e-078	283
MMU-Fbxl19	GGO-Kdm2B	58.94	246	101	0	288	533	955	1200	7e-078	283
MMU-Fbxl19	CJA-Kdm2B	58.94	246	101	0	288	533	1037	1282	8e-078	283
MMU-Fbxl19	RNO-Kdm2B	58.54	246	102	0	288	533	1008	1253	2e-077	281
MMU-Fbxl19	MMU-Kdm2B	57.72	246	101	2	288	533	1038	1280	1e-073	269
MMU-Fbxl19	RNO-Kdm2A	51.62	277	131	3	309	584	887	1161	1e-068	252
MMU-Fbxl19	MMU-Kdm2A	51.62	277	131	3	309	584	888	1162	1e-068	252
MMU-Fbxl19	HSA-Kdm2A	51.62	277	131	3	309	584	888	1162	1e-068	252
MMU-Fbxl19	PTR-Kdm2A	51.62	277	131	3	309	584	888	1162	1e-068	252
MMU-Fbxl19	GGO-Kdm2A	51.62	277	131	3	309	584	1007	1281	1e-068	252
MMU-Fbxl19	CJA-Kdm2A	51.62	277	131	3	309	584	886	1160	2e-068	252
MMU-Fbxl19	MUS-Kdm2A	51.26	277	132	3	309	584	887	1161	2e-068	251
MMU-Fbxl19	PPY-Kdm2A	51.62	277	131	3	309	584	395	669	4e-068	251

BLASTP 2.2.20 [Feb-08-2009]
Query: MUS-Fbxl19
Database: 559_protein.db
Query id, Subject id, % identity, alignment length, mismatches, gap openings, q. start, q. end, s. start, s. end, e-value, bit score

MUS-Fbxl19	MUS-Fbxl19	100.00	646	0	0	29	674	29	674	0.0	1041
MUS-Fbxl19	RNO-Fbxl19	99.54	646	3	0	29	674	29	674	0.0	1017
MUS-Fbxl19	CJA-Fbxl19	96.90	646	20	0	29	674	49	694	0.0	1017
MUS-Fbxl19	HSA-Fbxl19	97.37	646	17	0	29	674	49	694	0.0	1004
MUS-Fbxl19	GGO-Fbxl19	96.95	590	18	0	29	618	31	620	0.0	947
MUS-Fbxl19	PPY-Fbxl19	90.71	646	16	1	29	674	29	630	0.0	894
MUS-Fbxl19	MMU-Fbxl19	90.75	627	15	1	48	674	1	584	0.0	858
MUS-Fbxl19	PTR-Fbxl19	97.87	422	9	0	253	674	1	422	2e-178	617
MUS-Fbxl19	MUS-Kdm2B	59.59	245	99	0	378	622	1012	1256	6e-079	286
MUS-Fbxl19	MUS-Kdm2B	72.13	122	33	1	28	149	595	715	7e-054	203
MUS-Fbxl19	GGO-Kdm2B	59.35	246	100	0	378	623	955	1200	2e-078	285
MUS-Fbxl19	GGO-Kdm2B	72.13	122	33	1	28	149	538	658	7e-054	203
MUS-Fbxl19	HSA-Kdm2B	59.35	246	100	0	378	623	1039	1284	2e-078	285
MUS-Fbxl19	HSA-Kdm2B	72.13	122	33	1	28	149	622	742	7e-054	203
MUS-Fbxl19	PTR-Kdm2B	59.35	246	100	0	378	623	1039	1284	3e-078	285
MUS-Fbxl19	PTR-Kdm2B	72.13	122	33	1	28	149	622	742	7e-054	203
MUS-Fbxl19	CJA-Kdm2B	59.35	246	100	0	378	623	1037	1282	3e-078	284
MUS-Fbxl19	CJA-Kdm2B	70.97	124	34	1	28	149	616	739	8e-054	203
MUS-Fbxl19	RNO-Kdm2B	58.94	246	101	0	378	623	1008	1253	6e-078	283
MUS-Fbxl19	RNO-Kdm2B	72.13	122	33	1	28	149	591	711	7e-054	203
MUS-Fbxl19	MMU-Kdm2B	58.13	246	100	2	378	623	1038	1280	5e-074	270

MUS-Fbxl19	MMU-Kdm2B	72.13	122	33	1	28	149	621	741	1e-053	202
MUS-Fbxl19	MMU-Kdm2A	51.62	277	131	3	399	674	888	1162	5e-069	253
MUS-Fbxl19	CJA-Kdm2A	51.62	277	131	3	399	674	886	1160	6e-069	253
MUS-Fbxl19	HSA-Kdm2A	51.62	277	131	3	399	674	888	1162	6e-069	253
MUS-Fbxl19	PTR-Kdm2A	51.62	277	131	3	399	674	888	1162	6e-069	253
MUS-Fbxl19	RNO-Kdm2A	51.62	277	131	3	399	674	887	1161	6e-069	253
MUS-Fbxl19	GGO-Kdm2A	51.62	277	131	3	399	674	1007	1281	1e-068	253
MUS-Fbxl19	MUS-Kdm2A	51.26	277	132	3	399	674	887	1161	1e-068	252
MUS-Fbxl19	PPY-Kdm2A	51.62	277	131	3	399	674	395	669	2e-068	251
MUS-Fbxl19	PPY-Kdm2B	72.13	122	33	1	28	149	622	742	7e-054	203

BLASTP 2.2.20 [Feb-08-2009]
Query: PPY-Fbxl19
Database: 559_protein.db
Query id, Subject id, % identity, alignment length, mismatches, gap openings, q. start, q. end, s. start, s. end, e-value, bit score

PPY-Fbxl19	PPY-Fbxl19	100.00	602	0	0	29	630	29	630	0.0	880
PPY-Fbxl19	HSA-Fbxl19	93.03	646	1	1	29	630	49	694	0.0	858
PPY-Fbxl19	CJA-Fbxl19	92.26	646	6	1	29	630	49	694	0.0	853
PPY-Fbxl19	RNO-Fbxl19	91.02	646	14	1	29	630	29	674	0.0	849
PPY-Fbxl19	MUS-Fbxl19	90.71	646	16	1	29	630	29	674	0.0	847
PPY-Fbxl19	GGO-Fbxl19	92.20	590	2	1	29	574	31	620	0.0	800
PPY-Fbxl19	MMU-Fbxl19	85.96	627	1	2	48	630	1	584	0.0	743
PPY-Fbxl19	PTR-Fbxl19	98.58	422	6	0	209	630	1	422	1e-178	618
PPY-Fbxl19	MUS-Kdm2B	59.18	245	100	0	334	578	1012	1256	7e-079	286
PPY-Fbxl19	GGO-Kdm2B	58.94	246	101	0	334	579	955	1200	3e-078	284
PPY-Fbxl19	HSA-Kdm2B	58.94	246	101	0	334	579	1039	1284	4e-078	284
PPY-Fbxl19	PTR-Kdm2B	58.94	246	101	0	334	579	1039	1284	4e-078	284
PPY-Fbxl19	CJA-Kdm2B	58.94	246	101	0	334	579	1037	1282	5e-078	283
PPY-Fbxl19	RNO-Kdm2B	55.37	298	132	1	334	630	1008	1305	7e-078	283
PPY-Fbxl19	MMU-Kdm2B	54.70	298	131	3	334	630	1038	1332	6e-074	270
PPY-Fbxl19	RNO-Kdm2A	51.62	277	131	3	355	630	887	1161	9e-069	253
PPY-Fbxl19	MMU-Kdm2A	51.62	277	131	3	355	630	888	1162	1e-068	253
PPY-Fbxl19	CJA-Kdm2A	51.62	277	131	3	355	630	886	1160	1e-068	253
PPY-Fbxl19	HSA-Kdm2A	51.62	277	131	3	355	630	888	1162	1e-068	253
PPY-Fbxl19	PTR-Kdm2A	51.62	277	131	3	355	630	888	1162	1e-068	253
PPY-Fbxl19	GGO-Kdm2A	51.62	277	131	3	355	630	1007	1281	1e-068	252
PPY-Fbxl19	MUS-Kdm2A	51.26	277	132	3	355	630	887	1161	2e-068	252
PPY-Fbxl19	PPY-Kdm2A	51.62	277	131	3	355	630	395	669	3e-068	251

BLASTP 2.2.20 [Feb-08-2009]
Query: PTR-Fbxl19
Database: 559_protein.db
Query id, Subject id, % identity, alignment length, mismatches, gap openings, q. start, q. end, s. start, s. end, e-value, bit score

PTR-Fbxl19	PTR-Fbxl19	100.00	422	0	0	1	422	1	422	0.0	630
PTR-Fbxl19	HSA-Fbxl19	98.82	422	5	0	1	422	273	694	1e-179	620
PTR-Fbxl19	RNO-Fbxl19	98.10	422	8	0	1	422	253	674	6e-179	618
PTR-Fbxl19	PPY-Fbxl19	98.58	422	6	0	1	422	209	630	8e-179	618

PTR-Fbxl19	MUS-Fbxl19	97.87	422	9	0	1	422	253	674	8e-179	618
PTR-Fbxl19	CJA-Fbxl19	98.34	422	7	0	1	422	273	694	1e-178	617
PTR-Fbxl19	MMU-Fbxl19	100.00	411	0	0	12	422	174	584	1e-176	610
PTR-Fbxl19	GGO-Fbxl19	98.36	366	6	0	1	366	255	620	5e-162	562
PTR-Fbxl19	MUS-Kdm2B	59.18	245	100	0	126	370	1012	1256	6e-079	286
PTR-Fbxl19	HSA-Kdm2B	55.74	296	130	1	126	420	1039	1334	1e-078	285
PTR-Fbxl19	PTR-Kdm2B	55.74	296	130	1	126	420	1039	1334	2e-078	285
PTR-Fbxl19	GGO-Kdm2B	55.74	296	130	1	126	420	955	1250	2e-078	284
PTR-Fbxl19	CJA-Kdm2B	55.74	296	130	1	126	420	1037	1332	3e-078	284
PTR-Fbxl19	RNO-Kdm2B	55.41	296	131	1	126	420	1008	1303	3e-078	284
PTR-Fbxl19	MMU-Kdm2B	54.73	296	130	3	126	420	1038	1330	3e-074	270
PTR-Fbxl19	MMU-Kdm2A	51.62	277	131	3	147	422	888	1162	5e-069	253
PTR-Fbxl19	RNO-Kdm2A	51.62	277	131	3	147	422	887	1161	5e-069	253
PTR-Fbxl19	CJA-Kdm2A	51.62	277	131	3	147	422	886	1160	5e-069	253
PTR-Fbxl19	HSA-Kdm2A	51.62	277	131	3	147	422	888	1162	6e-069	253
PTR-Fbxl19	PTR-Kdm2A	51.62	277	131	3	147	422	888	1162	6e-069	253
PTR-Fbxl19	GGO-Kdm2A	51.62	277	131	3	147	422	1007	1281	8e-069	252
PTR-Fbxl19	MUS-Kdm2A	51.26	277	132	3	147	422	887	1161	1e-068	251
PTR-Fbxl19	PPY-Kdm2A	51.62	277	131	3	147	422	395	669	1e-068	251

BLASTP 2.2.20 [Feb-08-2009]
Query: RNO-Fbxl19
Database: 559_protein.db
Query id, Subject id, % identity, alignment length, mismatches, gap openings, q. start, q. end, s. start, s. end, e-value, bit score

RNO-Fbxl19	RNO-Fbxl19	100.00	646	0	0	29	674	29	674	0.0	1015
RNO-Fbxl19	MUS-Fbxl19	99.54	646	3	0	29	674	29	674	0.0	1011
RNO-Fbxl19	HSA-Fbxl19	97.83	646	14	0	29	674	49	694	0.0	1001
RNO-Fbxl19	CJA-Fbxl19	97.37	646	17	0	29	674	49	694	0.0	997
RNO-Fbxl19	GGO-Fbxl19	97.46	590	15	0	29	618	31	620	0.0	944
RNO-Fbxl19	PPY-Fbxl19	91.02	646	14	1	29	674	29	630	0.0	890
RNO-Fbxl19	MMU-Fbxl19	91.23	627	12	1	48	674	1	584	0.0	853
RNO-Fbxl19	PTR-Fbxl19	98.10	422	8	0	253	674	1	422	7e-179	619
RNO-Fbxl19	MUS-Kdm2B	59.59	245	99	0	378	622	1012	1256	7e-079	286
RNO-Fbxl19	MUS-Kdm2B	72.95	122	32	1	28	149	595	715	3e-054	204
RNO-Fbxl19	GGO-Kdm2B	59.35	246	100	0	378	623	955	1200	3e-078	284
RNO-Fbxl19	GGO-Kdm2B	72.95	122	32	1	28	149	538	658	3e-054	204
RNO-Fbxl19	HSA-Kdm2B	59.35	246	100	0	378	623	1039	1284	4e-078	284
RNO-Fbxl19	HSA-Kdm2B	72.95	122	32	1	28	149	622	742	4e-054	204
RNO-Fbxl19	PTR-Kdm2B	59.35	246	100	0	378	623	1039	1284	4e-078	284
RNO-Fbxl19	PTR-Kdm2B	72.95	122	32	1	28	149	622	742	4e-054	204
RNO-Fbxl19	CJA-Kdm2B	59.35	246	100	0	378	623	1037	1282	5e-078	283
RNO-Fbxl19	CJA-Kdm2B	71.77	124	33	1	28	149	616	739	4e-054	204
RNO-Fbxl19	RNO-Kdm2B	58.94	246	101	0	378	623	1008	1253	8e-078	283
RNO-Fbxl19	RNO-Kdm2B	72.95	122	32	1	28	149	591	711	3e-054	204
RNO-Fbxl19	MMU-Kdm2B	58.13	246	100	2	378	623	1038	1280	7e-074	270
RNO-Fbxl19	MMU-Kdm2B	72.95	122	32	1	28	149	621	741	5e-054	204

RNO-Fbxl19	RNO-Kdm2A	51.62	277	131	3	399	674	887	1161	8e-069	253
RNO-Fbxl19	CJA-Kdm2A	51.62	277	131	3	399	674	886	1160	8e-069	253
RNO-Fbxl19	MMU-Kdm2A	51.62	277	131	3	399	674	888	1162	9e-069	253
RNO-Fbxl19	HSA-Kdm2A	51.62	277	131	3	399	674	888	1162	9e-069	253
RNO-Fbxl19	PTR-Kdm2A	51.62	277	131	3	399	674	888	1162	9e-069	253
RNO-Fbxl19	GGO-Kdm2A	51.62	277	131	3	399	674	1007	1281	1e-068	252
RNO-Fbxl19	MUS-Kdm2A	51.26	277	132	3	399	674	887	1161	1e-068	252
RNO-Fbxl19	PPY-Kdm2A	51.62	277	131	3	399	674	395	669	3e-068	251
RNO-Fbxl19	PPY-Kdm2B	72.95	122	32	1	28	149	622	742	3e-054	204

BLASTP 2.2.20 [Feb-08-2009]
Query: HSA-Fbxl19
Database: 559_protein.db
Query id, Subject id, % identity, alignment length, mismatches, gap openings, q. start, q. end, s. start, s. end, e-value, bit score

HSA-Fbxl19	HSA-Fbxl19	100.00	694	0	0	1	694	1	694	0.0	989
HSA-Fbxl19	CJA-Fbxl19	98.85	694	8	0	1	694	1	694	0.0	980
HSA-Fbxl19	RNO-Fbxl19	97.83	646	14	0	49	694	29	674	0.0	961
HSA-Fbxl19	MUS-Fbxl19	97.37	646	17	0	49	694	29	674	0.0	957
HSA-Fbxl19	GGO-Fbxl19	99.83	590	1	0	49	638	31	620	0.0	912
HSA-Fbxl19	PPY-Fbxl19	93.03	646	1	1	49	694	29	630	0.0	857
HSA-Fbxl19	MMU-Fbxl19	93.14	627	0	1	68	694	1	584	0.0	857
HSA-Fbxl19	PTR-Fbxl19	98.82	422	5	0	273	694	1	422	2e-179	620
HSA-Fbxl19	MUS-Kdm2B	59.18	245	100	0	398	642	1012	1256	8e-079	286
HSA-Fbxl19	MUS-Kdm2B	72.73	121	32	1	49	169	596	715	2e-053	202
HSA-Fbxl19	PTR-Kdm2B	58.94	246	101	0	398	643	1039	1284	3e-078	284
HSA-Fbxl19	PTR-Kdm2B	72.73	121	32	1	49	169	623	742	2e-053	202
HSA-Fbxl19	HSA-Kdm2B	58.94	246	101	0	398	643	1039	1284	3e-078	284
HSA-Fbxl19	HSA-Kdm2B	72.73	121	32	1	49	169	623	742	2e-053	202
HSA-Fbxl19	GGO-Kdm2B	58.94	246	101	0	398	643	955	1200	4e-078	284
HSA-Fbxl19	GGO-Kdm2B	72.73	121	32	1	49	169	539	658	2e-053	202
HSA-Fbxl19	CJA-Kdm2B	58.94	246	101	0	398	643	1037	1282	6e-078	283
HSA-Fbxl19	CJA-Kdm2B	71.54	123	33	1	49	169	617	739	2e-053	201
HSA-Fbxl19	RNO-Kdm2B	58.54	246	102	0	398	643	1008	1253	9e-078	283
HSA-Fbxl19	RNO-Kdm2B	72.73	121	32	1	49	169	592	711	2e-053	202
HSA-Fbxl19	MMU-Kdm2B	57.72	246	101	2	398	643	1038	1280	7e-074	270
HSA-Fbxl19	MMU-Kdm2B	72.73	121	32	1	49	169	622	741	2e-053	201
HSA-Fbxl19	HSA-Kdm2A	51.62	277	131	3	419	694	888	1162	8e-069	253
HSA-Fbxl19	PTR-Kdm2A	51.62	277	131	3	419	694	888	1162	8e-069	253
HSA-Fbxl19	MMU-Kdm2A	51.62	277	131	3	419	694	888	1162	8e-069	253
HSA-Fbxl19	RNO-Kdm2A	51.62	277	131	3	419	694	887	1161	9e-069	253
HSA-Fbxl19	CJA-Kdm2A	51.62	277	131	3	419	694	886	1160	1e-068	252
HSA-Fbxl19	GGO-Kdm2A	51.62	277	131	3	419	694	1007	1281	1e-068	252
HSA-Fbxl19	MUS-Kdm2A	51.26	277	132	3	419	694	887	1161	2e-068	252
HSA-Fbxl19	PPY-Kdm2A	51.62	277	131	3	419	694	395	669	3e-068	251
HSA-Fbxl19	PPY-Kdm2B	72.73	121	32	1	49	169	623	742	2e-053	202

BLASTP 2.2.20 [Feb-08-2009]

```
# Query: CJA-Fbxl2
# Database: 559_protein.db
# Query id, Subject id, % identity, alignment length, mismatches, gap openings, q. start, q. end, s. start, s. end, e-value, bit score
CJA-Fbxl2   CJA-Fbxl2   100.00      405         0           0           1           405         1           405         0.0         818
CJA-Fbxl2   HSA-Fbxl2   95.31       405         18          1           1           405         20          423         0.0         773
CJA-Fbxl2   PTR-Fbxl2   95.31       405         18          1           1           405         20          423         0.0         773
CJA-Fbxl2   PPY-Fbxl2   95.31       405         18          1           1           405         20          423         0.0         773
CJA-Fbxl2   GGO-Fbxl2   95.31       405         18          1           1           405         20          423         0.0         773
CJA-Fbxl2   MUS-Fbxl2   91.85       405         32          1           1           405         20          423         0.0         755
CJA-Fbxl2   RNO-Fbxl2   91.60       405         33          1           1           405         19          422         0.0         750
CJA-Fbxl2   HSA-Fbxl20  74.32       405         103         1           1           405         33          436         0.0         633
CJA-Fbxl2   PTR-Fbxl20  74.32       405         103         1           1           405         33          436         0.0         633
CJA-Fbxl2   CJA-Fbxl20  74.32       405         103         1           1           405         33          436         0.0         633
CJA-Fbxl2   MUS-Fbxl20  74.07       405         104         1           1           405         33          436         0.0         630
CJA-Fbxl2   PPY-Fbxl20  74.01       404         104         1           2           405         13          415         0.0         630
CJA-Fbxl2   RNO-Fbxl20  74.07       405         104         1           1           405         34          437         0.0         629
CJA-Fbxl2   GGO-Fbxl20  73.59       409         104         2           1           405         19          427         0.0         625
CJA-Fbxl2   MMU-Fbxl20  66.67       405         134         1           1           405         20          423         2e-162      563
# BLASTP 2.2.20 [Feb-08-2009]
# Query: GGO-Fbxl2
# Database: 559_protein.db
# Query id, Subject id, % identity, alignment length, mismatches, gap openings, q. start, q. end, s. start, s. end, e-value, bit score
GGO-Fbxl2   HSA-Fbxl2   100.00      423         0           0           1           423         1           423         0.0         849
GGO-Fbxl2   PTR-Fbxl2   100.00      423         0           0           1           423         1           423         0.0         849
GGO-Fbxl2   PPY-Fbxl2   100.00      423         0           0           1           423         1           423         0.0         849
GGO-Fbxl2   GGO-Fbxl2   100.00      423         0           0           1           423         1           423         0.0         849
GGO-Fbxl2   MUS-Fbxl2   95.98       423         17          0           1           423         1           423         0.0         828
GGO-Fbxl2   RNO-Fbxl2   95.97       422         17          0           2           423         1           422         0.0         823
GGO-Fbxl2   CJA-Fbxl2   95.31       405         18          1           20          423         1           405         0.0         773
GGO-Fbxl2   HSA-Fbxl20  78.20       422         92          0           2           423         15          436         0.0         694
GGO-Fbxl2   PTR-Fbxl20  78.20       422         92          0           2           423         15          436         0.0         694
GGO-Fbxl2   CJA-Fbxl20  78.20       422         92          0           2           423         15          436         0.0         694
GGO-Fbxl2   MUS-Fbxl20  77.96       422         93          0           2           423         15          436         0.0         692
GGO-Fbxl2   RNO-Fbxl20  77.96       422         93          0           2           423         16          437         0.0         692
GGO-Fbxl2   GGO-Fbxl20  77.28       427         92          3           2           423         1           427         0.0         679
GGO-Fbxl2   PPY-Fbxl20  77.67       403         90          0           21          423         13          415         0.0         658
GGO-Fbxl2   MMU-Fbxl20  70.85       422         123         0           2           423         2           423         0.0         624
GGO-Fbxl2   MMU-Fbxl2   50.84       419         174         10          16          423         14          411         6e-097      346
# BLASTP 2.2.20 [Feb-08-2009]
# Query: MMU-Fbxl2
# Database: 559_protein.db
# Query id, Subject id, % identity, alignment length, mismatches, gap openings, q. start, q. end, s. start, s. end, e-value, bit score
MMU-Fbxl2   MMU-Fbxl2   100.00      411         0           0           1           411         1           411         0.0         825
MMU-Fbxl2   HSA-Fbxl2   50.84       419         174         10          14          411         16          423         5e-097      346
MMU-Fbxl2   PTR-Fbxl2   50.84       419         174         10          14          411         16          423         5e-097      346
```

```
MMU-Fbxl2   PPY-Fbxl2   50.84   419   174   10   14   411   16   423   5e-097   346
MMU-Fbxl2   GGO-Fbxl2   50.84   419   174   10   14   411   16   423   5e-097   346
# BLASTP 2.2.20 [Feb-08-2009]
# Query: MUS-Fbxl2
# Database: 559_protein.db
# Query id, Subject id, % identity, alignment length, mismatches, gap openings, q. start, q. end, s. start, s. end, e-value, bit score
MUS-Fbxl2   MUS-Fbxl2   100.00   423   0   0   1   423   1   423   0.0   850
MUS-Fbxl2   RNO-Fbxl2   99.05   422   4   0   2   423   1   422   0.0   841
MUS-Fbxl2   HSA-Fbxl2   95.98   423   17   0   1   423   1   423   0.0   828
MUS-Fbxl2   PTR-Fbxl2   95.98   423   17   0   1   423   1   423   0.0   828
MUS-Fbxl2   PPY-Fbxl2   95.98   423   17   0   1   423   1   423   0.0   828
MUS-Fbxl2   GGO-Fbxl2   95.98   423   17   0   1   423   1   423   0.0   828
MUS-Fbxl2   CJA-Fbxl2   91.85   405   32   1   20   423   1   405   0.0   755
MUS-Fbxl2   HSA-Fbxl20   77.01   422   97   0   2   423   15   436   0.0   691
MUS-Fbxl2   PTR-Fbxl20   77.01   422   97   0   2   423   15   436   0.0   691
MUS-Fbxl2   CJA-Fbxl20   77.01   422   97   0   2   423   15   436   0.0   691
MUS-Fbxl2   MUS-Fbxl20   76.78   422   98   0   2   423   15   436   0.0   689
MUS-Fbxl2   RNO-Fbxl20   76.78   422   98   0   2   423   16   437   0.0   689
MUS-Fbxl2   GGO-Fbxl20   76.11   427   97   3   2   423   1   427   0.0   677
MUS-Fbxl2   PPY-Fbxl20   76.43   403   95   0   21   423   13   415   0.0   656
MUS-Fbxl2   MMU-Fbxl20   69.91   422   127   0   2   423   2   423   1e-180   624
# BLASTP 2.2.20 [Feb-08-2009]
# Query: PPY-Fbxl2
# Database: 559_protein.db
# Query id, Subject id, % identity, alignment length, mismatches, gap openings, q. start, q. end, s. start, s. end, e-value, bit score
PPY-Fbxl2   HSA-Fbxl2   100.00   423   0   0   1   423   1   423   0.0   849
PPY-Fbxl2   PTR-Fbxl2   100.00   423   0   0   1   423   1   423   0.0   849
PPY-Fbxl2   PPY-Fbxl2   100.00   423   0   0   1   423   1   423   0.0   849
PPY-Fbxl2   GGO-Fbxl2   100.00   423   0   0   1   423   1   423   0.0   849
PPY-Fbxl2   MUS-Fbxl2   95.98   423   17   0   1   423   1   423   0.0   828
PPY-Fbxl2   RNO-Fbxl2   95.97   422   17   0   2   423   1   422   0.0   823
PPY-Fbxl2   CJA-Fbxl2   95.31   405   18   1   20   423   1   405   0.0   773
PPY-Fbxl2   HSA-Fbxl20   78.20   422   92   0   2   423   15   436   0.0   694
PPY-Fbxl2   PTR-Fbxl20   78.20   422   92   0   2   423   15   436   0.0   694
PPY-Fbxl2   CJA-Fbxl20   78.20   422   92   0   2   423   15   436   0.0   694
PPY-Fbxl2   MUS-Fbxl20   77.96   422   93   0   2   423   15   436   0.0   692
PPY-Fbxl2   RNO-Fbxl20   77.96   422   93   0   2   423   16   437   0.0   692
PPY-Fbxl2   GGO-Fbxl20   77.28   427   92   3   2   423   1   427   0.0   679
PPY-Fbxl2   PPY-Fbxl20   77.67   403   90   0   21   423   13   415   0.0   658
PPY-Fbxl2   MMU-Fbxl20   70.85   422   123   0   2   423   2   423   0.0   624
PPY-Fbxl2   MMU-Fbxl2   50.84   419   174   10   16   423   14   411   6e-097   346
# BLASTP 2.2.20 [Feb-08-2009]
# Query: PTR-Fbxl2
# Database: 559_protein.db
# Query id, Subject id, % identity, alignment length, mismatches, gap openings, q. start, q. end, s. start, s. end, e-value, bit score
```

PTR-Fbxl2	HSA-Fbxl2	100.00	423	0	0	1	423	1	423	0.0	849
PTR-Fbxl2	PTR-Fbxl2	100.00	423	0	0	1	423	1	423	0.0	849
PTR-Fbxl2	PPY-Fbxl2	100.00	423	0	0	1	423	1	423	0.0	849
PTR-Fbxl2	GGO-Fbxl2	100.00	423	0	0	1	423	1	423	0.0	849
PTR-Fbxl2	MUS-Fbxl2	95.98	423	17	0	1	423	1	423	0.0	828
PTR-Fbxl2	RNO-Fbxl2	95.97	422	17	0	2	423	1	422	0.0	823
PTR-Fbxl2	CJA-Fbxl2	95.31	405	18	1	20	423	1	405	0.0	773
PTR-Fbxl2	HSA-Fbxl20	78.20	422	92	0	2	423	15	436	0.0	694
PTR-Fbxl2	PTR-Fbxl20	78.20	422	92	0	2	423	15	436	0.0	694
PTR-Fbxl2	CJA-Fbxl20	78.20	422	92	0	2	423	15	436	0.0	694
PTR-Fbxl2	MUS-Fbxl20	77.96	422	93	0	2	423	15	436	0.0	692
PTR-Fbxl2	RNO-Fbxl20	77.96	422	93	0	2	423	16	437	0.0	692
PTR-Fbxl2	GGO-Fbxl20	77.28	427	92	3	2	423	1	427	0.0	679
PTR-Fbxl2	PPY-Fbxl20	77.67	403	90	0	21	423	13	415	0.0	658
PTR-Fbxl2	MMU-Fbxl20	70.85	422	123	0	2	423	2	423	0.0	624
PTR-Fbxl2	MMU-Fbxl2	50.84	419	174	10	16	423	14	411	6e-097	346

BLASTP 2.2.20 [Feb-08-2009]
Query: RNO-Fbxl2
Database: 559_protein.db
Query id, Subject id, % identity, alignment length, mismatches, gap openings, q. start, q. end, s. start, s. end, e-value, bit score

RNO-Fbxl2	RNO-Fbxl2	100.00	422	0	0	1	422	1	422	0.0	847
RNO-Fbxl2	MUS-Fbxl2	99.05	422	4	0	1	422	2	423	0.0	841
RNO-Fbxl2	HSA-Fbxl2	95.97	422	17	0	1	422	2	423	0.0	823
RNO-Fbxl2	PTR-Fbxl2	95.97	422	17	0	1	422	2	423	0.0	823
RNO-Fbxl2	PPY-Fbxl2	95.97	422	17	0	1	422	2	423	0.0	823
RNO-Fbxl2	GGO-Fbxl2	95.97	422	17	0	1	422	2	423	0.0	823
RNO-Fbxl2	CJA-Fbxl2	91.60	405	33	1	19	422	1	405	0.0	750
RNO-Fbxl2	HSA-Fbxl20	76.78	422	98	0	1	422	15	436	0.0	688
RNO-Fbxl2	PTR-Fbxl20	76.78	422	98	0	1	422	15	436	0.0	688
RNO-Fbxl2	CJA-Fbxl20	76.78	422	98	0	1	422	15	436	0.0	688
RNO-Fbxl2	RNO-Fbxl20	76.54	422	99	0	1	422	16	437	0.0	686
RNO-Fbxl2	MUS-Fbxl20	76.54	422	99	0	1	422	15	436	0.0	686
RNO-Fbxl2	GGO-Fbxl20	75.88	427	98	3	1	422	1	427	0.0	673
RNO-Fbxl2	PPY-Fbxl20	76.43	403	95	0	20	422	13	415	0.0	654
RNO-Fbxl2	MMU-Fbxl20	69.67	422	128	0	1	422	2	423	1e-179	620

BLASTP 2.2.20 [Feb-08-2009]
Query: HSA-Fbxl2
Database: 559_protein.db
Query id, Subject id, % identity, alignment length, mismatches, gap openings, q. start, q. end, s. start, s. end, e-value, bit score

HSA-Fbxl2	HSA-Fbxl2	100.00	423	0	0	1	423	1	423	0.0	849
HSA-Fbxl2	PTR-Fbxl2	100.00	423	0	0	1	423	1	423	0.0	849
HSA-Fbxl2	PPY-Fbxl2	100.00	423	0	0	1	423	1	423	0.0	849
HSA-Fbxl2	GGO-Fbxl2	100.00	423	0	0	1	423	1	423	0.0	849
HSA-Fbxl2	MUS-Fbxl2	95.98	423	17	0	1	423	1	423	0.0	828
HSA-Fbxl2	RNO-Fbxl2	95.97	422	17	0	2	423	1	422	0.0	823

HSA-Fbxl2	CJA-Fbxl2	95.31	405	18	1	20	423	1	405	0.0	773
HSA-Fbxl2	HSA-Fbxl20	78.20	422	92	0	2	423	15	436	0.0	694
HSA-Fbxl2	PTR-Fbxl20	78.20	422	92	0	2	423	15	436	0.0	694
HSA-Fbxl2	CJA-Fbxl20	78.20	422	92	0	2	423	15	436	0.0	694
HSA-Fbxl2	MUS-Fbxl20	77.96	422	93	0	2	423	15	436	0.0	692
HSA-Fbxl2	RNO-Fbxl20	77.96	422	93	0	2	423	16	437	0.0	692
HSA-Fbxl2	GGO-Fbxl20	77.28	427	92	3	2	423	1	427	0.0	679
HSA-Fbxl2	PPY-Fbxl20	77.67	403	90	0	21	423	13	415	0.0	658
HSA-Fbxl2	MMU-Fbxl20	70.85	422	123	0	2	423	2	423	0.0	624
HSA-Fbxl2	MMU-Fbxl2	50.84	419	174	10	16	423	14	411	6e-097	346

BLASTP 2.2.20 [Feb-08-2009]
Query: CJA-Fbxl20
Database: 559_protein.db
Query id, Subject id, % identity, alignment length, mismatches, gap openings, q. start, q. end, s. start, s. end, e-value, bit score

CJA-Fbxl20	HSA-Fbxl20	100.00	436	0	0	1	436	1	436	0.0	884
CJA-Fbxl20	PTR-Fbxl20	100.00	436	0	0	1	436	1	436	0.0	884
CJA-Fbxl20	CJA-Fbxl20	100.00	436	0	0	1	436	1	436	0.0	884
CJA-Fbxl20	MUS-Fbxl20	99.77	436	1	0	1	436	1	436	0.0	882
CJA-Fbxl20	RNO-Fbxl20	99.29	424	3	0	13	436	14	437	0.0	853
CJA-Fbxl20	GGO-Fbxl20	98.83	427	0	3	15	436	1	427	0.0	839
CJA-Fbxl20	PPY-Fbxl20	95.18	436	0	1	1	436	1	415	0.0	829
CJA-Fbxl20	MMU-Fbxl20	90.54	423	40	0	14	436	1	423	0.0	774
CJA-Fbxl20	HSA-Fbxl2	78.20	422	92	0	15	436	2	423	0.0	694
CJA-Fbxl20	PTR-Fbxl2	78.20	422	92	0	15	436	2	423	0.0	694
CJA-Fbxl20	PPY-Fbxl2	78.20	422	92	0	15	436	2	423	0.0	694
CJA-Fbxl20	GGO-Fbxl2	78.20	422	92	0	15	436	2	423	0.0	694
CJA-Fbxl20	MUS-Fbxl2	77.01	422	97	0	15	436	2	423	0.0	691
CJA-Fbxl20	RNO-Fbxl2	76.78	422	98	0	15	436	1	422	0.0	688
CJA-Fbxl20	CJA-Fbxl2	74.32	405	103	1	33	436	1	405	0.0	633

BLASTP 2.2.20 [Feb-08-2009]
Query: GGO-Fbxl20
Database: 559_protein.db
Query id, Subject id, % identity, alignment length, mismatches, gap openings, q. start, q. end, s. start, s. end, e-value, bit score

GGO-Fbxl20	GGO-Fbxl20	100.00	427	0	0	1	427	1	427	0.0	863
GGO-Fbxl20	HSA-Fbxl20	98.83	427	0	3	1	427	15	436	0.0	839
GGO-Fbxl20	PTR-Fbxl20	98.83	427	0	3	1	427	15	436	0.0	839
GGO-Fbxl20	CJA-Fbxl20	98.83	427	0	3	1	427	15	436	0.0	839
GGO-Fbxl20	MUS-Fbxl20	98.59	427	1	3	1	427	15	436	0.0	838
GGO-Fbxl20	RNO-Fbxl20	98.59	427	1	3	1	427	16	437	0.0	837
GGO-Fbxl20	PPY-Fbxl20	98.28	408	2	3	20	427	13	415	0.0	798
GGO-Fbxl20	MMU-Fbxl20	89.70	427	39	3	1	427	2	423	0.0	763
GGO-Fbxl20	HSA-Fbxl2	77.28	427	92	3	1	427	2	423	0.0	679
GGO-Fbxl20	PTR-Fbxl2	77.28	427	92	3	1	427	2	423	0.0	679
GGO-Fbxl20	PPY-Fbxl2	77.28	427	92	3	1	427	2	423	0.0	679
GGO-Fbxl20	GGO-Fbxl2	77.28	427	92	3	1	427	2	423	0.0	679

```
GGO-Fbxl20  MUS-Fbxl2   76.11   427   97    3   1    427   2    423   0.0   677
GGO-Fbxl20  RNO-Fbxl2   75.88   427   98    3   1    427   1    422   0.0   673
GGO-Fbxl20  CJA-Fbxl2   73.59   409   104   2   19   427   1    405   0.0   625
# BLASTP 2.2.20 [Feb-08-2009]
# Query: MUS-Fbxl20
# Database: 559_protein.db
# Query id, Subject id, % identity, alignment length, mismatches, gap openings, q. start, q. end, s. start, s. end, e-value, bit score
MUS-Fbxl20  MUS-Fbxl20  100.00  436   0     0   1    436   1    436   0.0   883
MUS-Fbxl20  HSA-Fbxl20  99.77   436   1     0   1    436   1    436   0.0   882
MUS-Fbxl20  PTR-Fbxl20  99.77   436   1     0   1    436   1    436   0.0   882
MUS-Fbxl20  CJA-Fbxl20  99.77   436   1     0   1    436   1    436   0.0   882
MUS-Fbxl20  RNO-Fbxl20  99.53   424   2     0   13   436   14   437   0.0   854
MUS-Fbxl20  GGO-Fbxl20  98.59   427   1     3   15   436   1    427   0.0   838
MUS-Fbxl20  PPY-Fbxl20  94.95   436   1     1   1    436   1    415   0.0   827
MUS-Fbxl20  MMU-Fbxl20  90.31   423   41    0   14   436   1    423   0.0   772
MUS-Fbxl20  HSA-Fbxl2   77.96   422   93    0   15   436   2    423   0.0   692
MUS-Fbxl20  PTR-Fbxl2   77.96   422   93    0   15   436   2    423   0.0   692
MUS-Fbxl20  PPY-Fbxl2   77.96   422   93    0   15   436   2    423   0.0   692
MUS-Fbxl20  GGO-Fbxl2   77.96   422   93    0   15   436   2    423   0.0   692
MUS-Fbxl20  MUS-Fbxl2   76.78   422   98    0   15   436   2    423   0.0   689
MUS-Fbxl20  RNO-Fbxl2   76.54   422   99    0   15   436   1    422   0.0   686
MUS-Fbxl20  CJA-Fbxl2   74.07   405   104   1   33   436   1    405   0.0   630
# BLASTP 2.2.20 [Feb-08-2009]
# Query: PPY-Fbxl20
# Database: 559_protein.db
# Query id, Subject id, % identity, alignment length, mismatches, gap openings, q. start, q. end, s. start, s. end, e-value, bit score
PPY-Fbxl20  PPY-Fbxl20  100.00  415   0     0   1    415   1    415   0.0   839
PPY-Fbxl20  HSA-Fbxl20  95.18   436   0     1   1    415   1    436   0.0   829
PPY-Fbxl20  PTR-Fbxl20  95.18   436   0     1   1    415   1    436   0.0   829
PPY-Fbxl20  CJA-Fbxl20  95.18   436   0     1   1    415   1    436   0.0   829
PPY-Fbxl20  MUS-Fbxl20  94.95   436   1     1   1    415   1    436   0.0   827
PPY-Fbxl20  RNO-Fbxl20  99.26   403   3     0   13   415   35   437   0.0   810
PPY-Fbxl20  GGO-Fbxl20  98.28   408   2     3   13   415   20   427   0.0   798
PPY-Fbxl20  MMU-Fbxl20  89.83   403   41    0   13   415   21   423   0.0   732
PPY-Fbxl20  HSA-Fbxl2   77.67   403   90    0   13   415   21   423   0.0   658
PPY-Fbxl20  PTR-Fbxl2   77.67   403   90    0   13   415   21   423   0.0   658
PPY-Fbxl20  PPY-Fbxl2   77.67   403   90    0   13   415   21   423   0.0   658
PPY-Fbxl20  GGO-Fbxl2   77.67   403   90    0   13   415   21   423   0.0   658
PPY-Fbxl20  MUS-Fbxl2   76.43   403   95    0   13   415   21   423   0.0   656
PPY-Fbxl20  RNO-Fbxl2   76.43   403   95    0   13   415   20   422   0.0   654
PPY-Fbxl20  CJA-Fbxl2   74.01   404   104   1   13   415   2    405   0.0   630
# BLASTP 2.2.20 [Feb-08-2009]
# Query: PTR-Fbxl20
# Database: 559_protein.db
# Query id, Subject id, % identity, alignment length, mismatches, gap openings, q. start, q. end, s. start, s. end, e-value, bit score
```

```
PTR-Fbxl20  HSA-Fbxl20  100.00     436        0          0          1          436        1          436        0.0          884
PTR-Fbxl20  PTR-Fbxl20  100.00     436        0          0          1          436        1          436        0.0          884
PTR-Fbxl20  CJA-Fbxl20  100.00     436        0          0          1          436        1          436        0.0          884
PTR-Fbxl20  MUS-Fbxl20  99.77      436        1          0          1          436        1          436        0.0          882
PTR-Fbxl20  RNO-Fbxl20  99.29      424        3          0          13         436        14         437        0.0          853
PTR-Fbxl20  GGO-Fbxl20  98.83      427        0          3          15         436        1          427        0.0          839
PTR-Fbxl20  PPY-Fbxl20  95.18      436        0          1          1          436        1          415        0.0          829
PTR-Fbxl20  MMU-Fbxl20  90.54      423        40         0          14         436        1          423        0.0          774
PTR-Fbxl20  HSA-Fbxl2   78.20      422        92         0          15         436        2          423        0.0          694
PTR-Fbxl20  PTR-Fbxl2   78.20      422        92         0          15         436        2          423        0.0          694
PTR-Fbxl20  PPY-Fbxl2   78.20      422        92         0          15         436        2          423        0.0          694
PTR-Fbxl20  GGO-Fbxl2   78.20      422        92         0          15         436        2          423        0.0          694
PTR-Fbxl20  MUS-Fbxl2   77.01      422        97         0          15         436        2          423        0.0          691
PTR-Fbxl20  RNO-Fbxl2   76.78      422        98         0          15         436        1          422        0.0          688
PTR-Fbxl20  CJA-Fbxl2   74.32      405        103        1          33         436        1          405        0.0          633
# BLASTP 2.2.20 [Feb-08-2009]
# Query: RNO-Fbxl20
# Database: 559_protein.db
# Query id, Subject id, % identity, alignment length, mismatches, gap openings, q. start, q. end, s. start, s. end, e-value, bit score
RNO-Fbxl20  RNO-Fbxl20  100.00     437        0          0          1          437        1          437        0.0          884
RNO-Fbxl20  MUS-Fbxl20  99.53      424        2          0          14         437        13         436        0.0          854
RNO-Fbxl20  HSA-Fbxl20  99.29      424        3          0          14         437        13         436        0.0          853
RNO-Fbxl20  PTR-Fbxl20  99.29      424        3          0          14         437        13         436        0.0          853
RNO-Fbxl20  CJA-Fbxl20  99.29      424        3          0          14         437        13         436        0.0          853
RNO-Fbxl20  GGO-Fbxl20  98.59      427        1          3          16         437        1          427        0.0          837
RNO-Fbxl20  PPY-Fbxl20  99.26      403        3          0          35         437        13         415        0.0          810
RNO-Fbxl20  MMU-Fbxl20  90.54      423        40         0          15         437        1          423        0.0          773
RNO-Fbxl20  HSA-Fbxl2   77.96      422        93         0          16         437        2          423        0.0          692
RNO-Fbxl20  PTR-Fbxl2   77.96      422        93         0          16         437        2          423        0.0          692
RNO-Fbxl20  PPY-Fbxl2   77.96      422        93         0          16         437        2          423        0.0          692
RNO-Fbxl20  GGO-Fbxl2   77.96      422        93         0          16         437        2          423        0.0          692
RNO-Fbxl20  MUS-Fbxl2   76.78      422        98         0          16         437        2          423        0.0          689
RNO-Fbxl20  RNO-Fbxl2   76.54      422        99         0          16         437        1          422        0.0          686
RNO-Fbxl20  CJA-Fbxl2   74.07      405        104        1          34         437        1          405        0.0          629
# BLASTP 2.2.20 [Feb-08-2009]
# Query: HSA-Fbxl20
# Database: 559_protein.db
# Query id, Subject id, % identity, alignment length, mismatches, gap openings, q. start, q. end, s. start, s. end, e-value, bit score
HSA-Fbxl20  HSA-Fbxl20  100.00     436        0          0          1          436        1          436        0.0          884
HSA-Fbxl20  PTR-Fbxl20  100.00     436        0          0          1          436        1          436        0.0          884
HSA-Fbxl20  CJA-Fbxl20  100.00     436        0          0          1          436        1          436        0.0          884
HSA-Fbxl20  MUS-Fbxl20  99.77      436        1          0          1          436        1          436        0.0          882
HSA-Fbxl20  RNO-Fbxl20  99.29      424        3          0          13         436        14         437        0.0          853
HSA-Fbxl20  GGO-Fbxl20  98.83      427        0          3          15         436        1          427        0.0          839
HSA-Fbxl20  PPY-Fbxl20  95.18      436        0          1          1          436        1          415        0.0          829
```

```
HSA-Fbxl20  MMU-Fbxl20  90.54   423   40    0   14   436   1    423   0.0      774
HSA-Fbxl20  HSA-Fbxl2   78.20   422   92    0   15   436   2    423   0.0      694
HSA-Fbxl20  PTR-Fbxl2   78.20   422   92    0   15   436   2    423   0.0      694
HSA-Fbxl20  PPY-Fbxl2   78.20   422   92    0   15   436   2    423   0.0      694
HSA-Fbxl20  GGO-Fbxl2   78.20   422   92    0   15   436   2    423   0.0      694
HSA-Fbxl20  MUS-Fbxl2   77.01   422   97    0   15   436   2    423   0.0      691
HSA-Fbxl20  RNO-Fbxl2   76.78   422   98    0   15   436   1    422   0.0      688
HSA-Fbxl20  CJA-Fbxl2   74.32   405   103   1   33   436   1    405   0.0      633
# BLASTP 2.2.20 [Feb-08-2009]
# Query: MMU-Fbxl20
# Database: 559_protein.db
# Query id, Subject id, % identity, alignment length, mismatches, gap openings, q. start, q. end, s. start, s. end, e-value, bit score
MMU-Fbxl20  MMU-Fbxl20  100.00  423   0     0   1    423   1    423   0.0      862
MMU-Fbxl20  HSA-Fbxl20  90.54   423   40    0   1    423   14   436   0.0      774
MMU-Fbxl20  PTR-Fbxl20  90.54   423   40    0   1    423   14   436   0.0      774
MMU-Fbxl20  CJA-Fbxl20  90.54   423   40    0   1    423   14   436   0.0      774
MMU-Fbxl20  RNO-Fbxl20  90.54   423   40    0   1    423   15   437   0.0      773
MMU-Fbxl20  MUS-Fbxl20  90.31   423   41    0   1    423   14   436   0.0      772
MMU-Fbxl20  GGO-Fbxl20  89.70   427   39    3   2    423   1    427   0.0      763
MMU-Fbxl20  PPY-Fbxl20  89.83   403   41    0   21   423   13   415   0.0      732
MMU-Fbxl20  HSA-Fbxl2   70.85   422   123   0   2    423   2    423   0.0      624
MMU-Fbxl20  PTR-Fbxl2   70.85   422   123   0   2    423   2    423   0.0      624
MMU-Fbxl20  PPY-Fbxl2   70.85   422   123   0   2    423   2    423   0.0      624
MMU-Fbxl20  GGO-Fbxl2   70.85   422   123   0   2    423   2    423   0.0      624
MMU-Fbxl20  MUS-Fbxl2   69.91   422   127   0   2    423   2    423   1e-180   624
MMU-Fbxl20  RNO-Fbxl2   69.67   422   128   0   2    423   1    422   1e-179   620
MMU-Fbxl20  CJA-Fbxl2   66.67   405   134   1   20   423   1    405   2e-162   563
# BLASTP 2.2.20 [Feb-08-2009]
# Query: MMU-Fbxl21
# Database: 559_protein.db
# Query id, Subject id, % identity, alignment length, mismatches, gap openings, q. start, q. end, s. start, s. end, e-value, bit score
MMU-Fbxl21  MMU-Fbxl21  100.00  434   0     0   1    434   1    434   0.0      854
MMU-Fbxl21  HSA-Fbxl21  93.09   434   30    0   1    434   1    434   0.0      796
MMU-Fbxl21  PTR-Fbxl21  92.63   434   32    0   1    434   1    434   0.0      793
MMU-Fbxl21  MUS-Fbxl21  87.33   434   55    0   1    434   27   460   0.0      754
MMU-Fbxl21  PPY-Fbxl21  94.43   377   21    0   57   433   1    377   0.0      701
MMU-Fbxl21  RNO-Fbxl21  77.47   435   96    2   1    434   1    434   0.0      650
MMU-Fbxl21  CJA-Fbxl3   71.66   434   117   1   1    434   1    428   4e-174   602
MMU-Fbxl21  HSA-Fbxl3   71.66   434   117   1   1    434   1    428   4e-174   602
MMU-Fbxl21  PTR-Fbxl3   71.66   434   117   1   1    434   1    428   4e-174   602
MMU-Fbxl21  PPY-Fbxl3   71.66   434   117   1   1    434   1    428   4e-174   602
MMU-Fbxl21  GGO-Fbxl3   71.66   434   117   1   1    434   5    432   4e-174   602
MMU-Fbxl21  MMU-Fbxl3   75.31   405   100   0   30   434   24   428   6e-174   601
MMU-Fbxl21  RNO-Fbxl3   71.89   434   116   2   1    434   1    428   4e-173   599
MMU-Fbxl21  MUS-Fbxl3   71.66   434   117   2   1    434   1    428   4e-173   598
```

```
# BLASTP 2.2.20 [Feb-08-2009]
# Query: MUS-Fbxl21
# Database: 559_protein.db
# Query id, Subject id, % identity, alignment length, mismatches, gap openings, q. start, q. end, s. start, s. end, e-value, bit score
MUS-Fbxl21  MUS-Fbxl21  100.00      460         0           0           1           460         1           460         0.0         909
MUS-Fbxl21  MMU-Fbxl21  87.33       434         55          0           27          460         1           434         0.0         754
MUS-Fbxl21  PTR-Fbxl21  85.94       434         61          0           27          460         1           434         0.0         739
MUS-Fbxl21  HSA-Fbxl21  85.94       434         61          0           27          460         1           434         0.0         739
MUS-Fbxl21  RNO-Fbxl21  85.06       435         63          2           27          460         1           434         0.0         714
MUS-Fbxl21  PPY-Fbxl21  89.92       377         38          0           83          459         1           377         0.0         675
MUS-Fbxl21  GGO-Fbxl3   72.87       435         112         1           26          460         4           432         4e-179      619
MUS-Fbxl21  MMU-Fbxl3   76.79       405         94          0           56          460         24          428         1e-178      617
MUS-Fbxl21  HSA-Fbxl3   76.79       405         94          0           56          460         24          428         1e-178      617
MUS-Fbxl21  PTR-Fbxl3   76.79       405         94          0           56          460         24          428         1e-178      617
MUS-Fbxl21  PPY-Fbxl3   76.79       405         94          0           56          460         24          428         1e-178      617
MUS-Fbxl21  CJA-Fbxl3   76.79       405         94          0           56          460         24          428         2e-178      617
MUS-Fbxl21  RNO-Fbxl3   74.29       424         101         2           40          460         10          428         4e-178      615
MUS-Fbxl21  MUS-Fbxl3   72.58       434         113         2           27          460         1           428         1e-177      614
# BLASTP 2.2.20 [Feb-08-2009]
# Query: PPY-Fbxl21
# Database: 559_protein.db
# Query id, Subject id, % identity, alignment length, mismatches, gap openings, q. start, q. end, s. start, s. end, e-value, bit score
PPY-Fbxl21  PPY-Fbxl21  100.00      377         0           0           1           377         1           377         0.0         715
PPY-Fbxl21  MMU-Fbxl21  94.43       377         21          0           1           377         57          433         0.0         683
PPY-Fbxl21  PTR-Fbxl21  94.43       377         21          0           1           377         57          433         0.0         673
PPY-Fbxl21  HSA-Fbxl21  94.16       377         22          0           1           377         57          433         0.0         669
PPY-Fbxl21  MUS-Fbxl21  89.92       377         38          0           1           377         83          459         0.0         657
PPY-Fbxl21  RNO-Fbxl21  79.63       378         75          2           1           377         57          433         8e-162      561
PPY-Fbxl21  MUS-Fbxl3   78.72       376         80          0           1           376         51          426         4e-161      559
PPY-Fbxl21  GGO-Fbxl3   78.46       376         81          0           1           376         55          430         4e-161      558
PPY-Fbxl21  HSA-Fbxl3   78.46       376         81          0           1           376         51          426         4e-161      558
PPY-Fbxl21  PTR-Fbxl3   78.46       376         81          0           1           376         51          426         4e-161      558
PPY-Fbxl21  PPY-Fbxl3   78.46       376         81          0           1           376         51          426         4e-161      558
PPY-Fbxl21  MMU-Fbxl3   78.46       376         81          0           1           376         51          426         4e-161      558
PPY-Fbxl21  CJA-Fbxl3   78.46       376         81          0           1           376         51          426         5e-161      558
PPY-Fbxl21  RNO-Fbxl3   78.72       376         80          0           1           376         51          426         5e-161      558
# BLASTP 2.2.20 [Feb-08-2009]
# Query: RNO-Fbxl21
# Database: 559_protein.db
# Query id, Subject id, % identity, alignment length, mismatches, gap openings, q. start, q. end, s. start, s. end, e-value, bit score
RNO-Fbxl21  RNO-Fbxl21  100.00      434         0           0           1           434         1           434         0.0         843
RNO-Fbxl21  MUS-Fbxl21  85.06       435         63          2           1           434         27          460         0.0         688
RNO-Fbxl21  MMU-Fbxl21  77.47       435         96          2           1           434         1           434         0.0         624
RNO-Fbxl21  PTR-Fbxl21  77.70       435         95          2           1           434         1           434         5e-180      622
RNO-Fbxl21  HSA-Fbxl21  75.63       435         104         2           1           434         1           434         4e-174      602
```

RNO-Fbxl21	PPY-Fbxl21	79.63	378	75	2	57	433	1	377	4e-159	552
RNO-Fbxl21	HSA-Fbxl3	65.06	435	144	3	1	434	1	428	1e-152	531
RNO-Fbxl21	PTR-Fbxl3	65.06	435	144	3	1	434	1	428	1e-152	531
RNO-Fbxl21	PPY-Fbxl3	65.06	435	144	3	1	434	1	428	1e-152	531
RNO-Fbxl21	GGO-Fbxl3	65.06	435	144	3	1	434	5	432	1e-152	531
RNO-Fbxl21	CJA-Fbxl3	65.06	435	144	3	1	434	1	428	1e-152	530
RNO-Fbxl21	MUS-Fbxl3	65.52	435	142	4	1	434	1	428	1e-152	530
RNO-Fbxl21	MMU-Fbxl3	68.23	406	127	2	30	434	24	428	2e-152	530
RNO-Fbxl21	RNO-Fbxl3	68.15	405	127	2	31	434	25	428	2e-151	527

BLASTP 2.2.20 [Feb-08-2009]
Query: HSA-Fbxl21
Database: 559_protein.db
Query id, Subject id, % identity, alignment length, mismatches, gap openings, q. start, q. end, s. start, s. end, e-value, bit score

HSA-Fbxl21	HSA-Fbxl21	100.00	434	0	0	1	434	1	434	0.0	835
HSA-Fbxl21	PTR-Fbxl21	96.77	434	14	0	1	434	1	434	0.0	801
HSA-Fbxl21	MMU-Fbxl21	93.09	434	30	0	1	434	1	434	0.0	778
HSA-Fbxl21	MUS-Fbxl21	85.94	434	61	0	1	434	27	460	0.0	721
HSA-Fbxl21	PPY-Fbxl21	94.16	377	22	0	57	433	1	377	0.0	669
HSA-Fbxl21	RNO-Fbxl21	75.63	435	104	2	1	434	1	434	2e-176	610
HSA-Fbxl21	CJA-Fbxl3	70.74	434	121	1	1	434	1	428	7e-166	575
HSA-Fbxl21	HSA-Fbxl3	70.74	434	121	1	1	434	1	428	8e-166	575
HSA-Fbxl21	PTR-Fbxl3	70.74	434	121	1	1	434	1	428	8e-166	575
HSA-Fbxl21	PPY-Fbxl3	70.74	434	121	1	1	434	1	428	8e-166	575
HSA-Fbxl21	GGO-Fbxl3	70.74	434	121	1	1	434	5	432	8e-166	574
HSA-Fbxl21	MMU-Fbxl3	74.32	405	104	0	30	434	24	428	2e-165	573
HSA-Fbxl21	RNO-Fbxl3	70.97	434	120	2	1	434	1	428	2e-164	570
HSA-Fbxl21	MUS-Fbxl3	70.74	434	121	2	1	434	1	428	2e-164	570

BLASTP 2.2.20 [Feb-08-2009]
Query: PTR-Fbxl21
Database: 559_protein.db
Query id, Subject id, % identity, alignment length, mismatches, gap openings, q. start, q. end, s. start, s. end, e-value, bit score

PTR-Fbxl21	PTR-Fbxl21	100.00	434	0	0	1	434	1	434	0.0	801
PTR-Fbxl21	HSA-Fbxl21	96.77	434	14	0	1	434	1	434	0.0	770
PTR-Fbxl21	MMU-Fbxl21	92.63	434	32	0	1	434	1	434	0.0	751
PTR-Fbxl21	MUS-Fbxl21	85.94	434	61	0	1	434	27	460	0.0	692
PTR-Fbxl21	PPY-Fbxl21	94.43	377	21	0	57	433	1	377	0.0	645
PTR-Fbxl21	RNO-Fbxl21	77.70	435	95	2	1	434	1	434	1e-173	600
PTR-Fbxl21	CJA-Fbxl3	70.51	434	122	1	1	434	1	428	9e-157	544
PTR-Fbxl21	HSA-Fbxl3	70.51	434	122	1	1	434	1	428	1e-156	544
PTR-Fbxl21	PTR-Fbxl3	70.51	434	122	1	1	434	1	428	1e-156	544
PTR-Fbxl21	PPY-Fbxl3	70.51	434	122	1	1	434	1	428	1e-156	544
PTR-Fbxl21	GGO-Fbxl3	70.51	434	122	1	1	434	5	432	1e-156	544
PTR-Fbxl21	MMU-Fbxl3	74.07	405	105	0	30	434	24	428	2e-156	543
PTR-Fbxl21	MUS-Fbxl3	70.51	434	122	2	1	434	1	428	2e-155	540
PTR-Fbxl21	RNO-Fbxl3	72.73	418	109	1	17	434	16	428	2e-155	540

```
# BLASTP 2.2.20 [Feb-08-2009]
# Query: CJA-Fbxl22
# Database: 559_protein.db
# Query id, Subject id, % identity, alignment length, mismatches, gap openings, q. start, q. end, s. start, s. end, e-value, bit score
CJA-Fbxl22  CJA-Fbxl22  100.00  227  0   0  1  227  1  227  2e-102  363
CJA-Fbxl22  GGO-Fbxl22  77.27   242  39  6  1  227  1  241  3e-076  275
CJA-Fbxl22  HSA-Fbxl22  79.34   242  34  6  1  227  1  241  8e-076  274
CJA-Fbxl22  MMU-Fbxl22  79.15   235  40  6  1  227  1  234  3e-072  263
CJA-Fbxl22  PPY-Fbxl22  76.27   236  45  5  1  227  1  234  8e-069  251
CJA-Fbxl22  PTR-Fbxl22  93.69   111  4   1  1  108  7  117  8e-055  204
CJA-Fbxl22  RNO-Fbxl22  86.49   111  12  1  1  108  1  111  5e-051  192
# BLASTP 2.2.20 [Feb-08-2009]
# Query: GGO-Fbxl22
# Database: 559_protein.db
# Query id, Subject id, % identity, alignment length, mismatches, gap openings, q. start, q. end, s. start, s. end, e-value, bit score
GGO-Fbxl22  GGO-Fbxl22  100.00  241  0   0  1  241  1  241  1e-115  407
GGO-Fbxl22  HSA-Fbxl22  98.34   241  4   0  1  241  1  241  2e-113  400
GGO-Fbxl22  MMU-Fbxl22  87.97   241  22  1  1  241  1  234  1e-097  347
GGO-Fbxl22  PPY-Fbxl22  89.26   242  17  4  1  241  1  234  3e-096  342
GGO-Fbxl22  CJA-Fbxl22  78.10   242  37  6  1  241  1  227  4e-080  289
GGO-Fbxl22  PTR-Fbxl22  99.12   113  1   0  1  113  7  119  5e-062  228
GGO-Fbxl22  RNO-Fbxl22  89.29   112  12  0  1  112  1  112  2e-055  207
GGO-Fbxl22  MUS-Fbxl22  86.61   112  15  0  1  112  1  112  1e-053  201
# BLASTP 2.2.20 [Feb-08-2009]
# Query: MMU-Fbxl22
# Database: 559_protein.db
# Query id, Subject id, % identity, alignment length, mismatches, gap openings, q. start, q. end, s. start, s. end, e-value, bit score
MMU-Fbxl22  MMU-Fbxl22  100.00  234  0   0  1  234  1  234  9e-119  417
MMU-Fbxl22  GGO-Fbxl22  87.97   241  22  1  1  234  1  241  8e-097  344
MMU-Fbxl22  HSA-Fbxl22  87.14   241  24  1  1  234  1  241  5e-096  342
MMU-Fbxl22  PPY-Fbxl22  84.26   235  35  2  1  234  1  234  1e-089  320
MMU-Fbxl22  CJA-Fbxl22  79.15   235  40  6  1  234  1  227  6e-074  268
MMU-Fbxl22  PTR-Fbxl22  95.69   116  5   0  1  116  7  122  1e-061  228
MMU-Fbxl22  RNO-Fbxl22  90.18   112  11  0  1  112  1  112  4e-056  209
MMU-Fbxl22  MUS-Fbxl22  87.50   112  14  0  1  112  1  112  4e-054  202
# BLASTP 2.2.20 [Feb-08-2009]
# Query: MUS-Fbxl22
# Database: 559_protein.db
# Query id, Subject id, % identity, alignment length, mismatches, gap openings, q. start, q. end, s. start, s. end, e-value, bit score
MUS-Fbxl22  MUS-Fbxl22  100.00  236  0   0  1  236  1  236  9e-131  457
MUS-Fbxl22  RNO-Fbxl22  91.95   236  19  0  1  236  1  236  1e-120  423
MUS-Fbxl22  MMU-Fbxl22  87.50   112  14  0  1  112  1  112  2e-054  203
MUS-Fbxl22  GGO-Fbxl22  86.61   112  15  0  1  112  1  112  1e-053  201
MUS-Fbxl22  HSA-Fbxl22  86.61   112  15  0  1  112  1  112  1e-053  201
MUS-Fbxl22  PPY-Fbxl22  86.61   112  15  0  1  112  1  112  2e-053  200
```

```
MUS-Fbxl22  PTR-Fbxl22  80.49   123   24   0   1   123   7   129   3e-053   199
# BLASTP 2.2.20 [Feb-08-2009]
# Query: PPY-Fbxl22
# Database: 559_protein.db
# Query id, Subject id, % identity, alignment length, mismatches, gap openings, q. start, q. end, s. start, s. end, e-value, bit score
PPY-Fbxl22  PPY-Fbxl22  100.00  234   0    0   1   234   1   234   1e-097   347
PPY-Fbxl22  GGO-Fbxl22  89.26   242   17   4   1   234   1   241   3e-083   299
PPY-Fbxl22  HSA-Fbxl22  89.26   242   17   4   1   234   1   241   3e-083   299
PPY-Fbxl22  MMU-Fbxl22  84.26   235   35   2   1   234   1   234   3e-078   282
PPY-Fbxl22  CJA-Fbxl22  75.42   236   47   5   1   234   1   227   4e-063   232
PPY-Fbxl22  PTR-Fbxl22  99.12   113   1    0   1   113   7   119   5e-061   225
PPY-Fbxl22  RNO-Fbxl22  89.29   112   12   0   1   112   1   112   1e-054   204
PPY-Fbxl22  MUS-Fbxl22  86.61   112   15   0   1   112   1   112   1e-052   197
# BLASTP 2.2.20 [Feb-08-2009]
# Query: RNO-Fbxl22
# Database: 559_protein.db
# Query id, Subject id, % identity, alignment length, mismatches, gap openings, q. start, q. end, s. start, s. end, e-value, bit score
RNO-Fbxl22  RNO-Fbxl22  100.00  236   0    0   1   236   1   236   9e-137   477
RNO-Fbxl22  MUS-Fbxl22  91.95   236   19   0   1   236   1   236   7e-124   434
RNO-Fbxl22  MMU-Fbxl22  90.18   112   11   0   1   112   1   112   1e-056   211
RNO-Fbxl22  GGO-Fbxl22  89.29   112   12   0   1   112   1   112   7e-056   208
RNO-Fbxl22  HSA-Fbxl22  89.29   112   12   0   1   112   1   112   8e-056   208
RNO-Fbxl22  PPY-Fbxl22  89.29   112   12   0   1   112   1   112   1e-055   207
RNO-Fbxl22  PTR-Fbxl22  82.93   123   21   0   1   123   7   129   4e-055   206
RNO-Fbxl22  CJA-Fbxl22  86.49   111   12   1   1   111   1   108   1e-051   194
# BLASTP 2.2.20 [Feb-08-2009]
# Query: HSA-Fbxl22
# Database: 559_protein.db
# Query id, Subject id, % identity, alignment length, mismatches, gap openings, q. start, q. end, s. start, s. end, e-value, bit score
HSA-Fbxl22  HSA-Fbxl22  100.00  241   0    0   1   241   1   241   5e-103   365
HSA-Fbxl22  GGO-Fbxl22  98.34   241   4    0   1   241   1   241   1e-101   360
HSA-Fbxl22  MMU-Fbxl22  87.14   241   24   1   1   241   1   234   2e-085   306
HSA-Fbxl22  PPY-Fbxl22  89.26   242   17   4   1   241   1   234   6e-084   301
HSA-Fbxl22  CJA-Fbxl22  77.27   242   39   6   1   241   1   227   1e-069   254
HSA-Fbxl22  PTR-Fbxl22  99.12   113   1    0   1   113   7   119   2e-061   227
HSA-Fbxl22  RNO-Fbxl22  89.29   112   12   0   1   112   1   112   6e-055   205
HSA-Fbxl22  MUS-Fbxl22  86.61   112   15   0   1   112   1   112   5e-053   199
# BLASTP 2.2.20 [Feb-08-2009]
# Query: PTR-Fbxl22
# Database: 559_protein.db
# Query id, Subject id, % identity, alignment length, mismatches, gap openings, q. start, q. end, s. start, s. end, e-value, bit score
PTR-Fbxl22  PTR-Fbxl22  100.00  130   0    0   1   130   1   130   9e-073   263
PTR-Fbxl22  HSA-Fbxl22  99.12   113   1    0   7   119   1   113   3e-063   231
PTR-Fbxl22  GGO-Fbxl22  99.12   113   1    0   7   119   1   113   3e-063   231
PTR-Fbxl22  PPY-Fbxl22  99.12   113   1    0   7   119   1   113   7e-063   230
```

```
PTR-Fbxl22  MMU-Fbxl22  98.23   113   2     0   7    119   1    113   2e-062   229
PTR-Fbxl22  CJA-Fbxl22  93.69   111   4     1   7    117   1    108   7e-056   207
PTR-Fbxl22  RNO-Fbxl22  82.93   123   21    0   7    129   1    123   2e-055   206
PTR-Fbxl22  MUS-Fbxl22  80.49   123   24    0   7    129   1    123   9e-054   200
# BLASTP 2.2.20 [Feb-08-2009]
# Query: CJA-Fbxl3
# Database: 559_protein.db
# Query id, Subject id, % identity, alignment length, mismatches, gap openings, q. start, q. end, s. start, s. end, e-value, bit score
CJA-Fbxl3   CJA-Fbxl3   100.00  428   0     0   1    428   1    428   0.0      858
CJA-Fbxl3   GGO-Fbxl3   99.77   428   1     0   1    428   5    432   0.0      856
CJA-Fbxl3   HSA-Fbxl3   99.77   428   1     0   1    428   1    428   0.0      856
CJA-Fbxl3   PTR-Fbxl3   99.77   428   1     0   1    428   1    428   0.0      856
CJA-Fbxl3   PPY-Fbxl3   99.77   428   1     0   1    428   1    428   0.0      856
CJA-Fbxl3   MMU-Fbxl3   99.53   428   2     0   1    428   1    428   0.0      855
CJA-Fbxl3   MUS-Fbxl3   97.20   428   12    0   1    428   1    428   0.0      835
CJA-Fbxl3   RNO-Fbxl3   97.43   428   11    0   1    428   1    428   0.0      833
CJA-Fbxl3   MUS-Fbxl21  76.79   405   94    0   24   428   56   460   9e-178   614
CJA-Fbxl3   MMU-Fbxl21  71.66   434   117   1   1    428   1    434   3e-173   599
CJA-Fbxl3   HSA-Fbxl21  70.74   434   121   1   1    428   1    434   5e-170   588
CJA-Fbxl3   PTR-Fbxl21  70.51   434   122   1   1    428   1    434   3e-169   586
CJA-Fbxl3   PPY-Fbxl21  78.46   376   81    0   51   426   1    376   2e-165   573
CJA-Fbxl3   RNO-Fbxl21  65.06   435   144   3   1    428   1    434   1e-159   554
# BLASTP 2.2.20 [Feb-08-2009]
# Query: GGO-Fbxl3
# Database: 559_protein.db
# Query id, Subject id, % identity, alignment length, mismatches, gap openings, q. start, q. end, s. start, s. end, e-value, bit score
GGO-Fbxl3   GGO-Fbxl3   100.00  432   0     0   1    432   1    432   0.0      868
GGO-Fbxl3   HSA-Fbxl3   100.00  428   0     0   5    432   1    428   0.0      858
GGO-Fbxl3   PTR-Fbxl3   100.00  428   0     0   5    432   1    428   0.0      858
GGO-Fbxl3   PPY-Fbxl3   100.00  428   0     0   5    432   1    428   0.0      858
GGO-Fbxl3   MMU-Fbxl3   99.77   428   1     0   5    432   1    428   0.0      857
GGO-Fbxl3   CJA-Fbxl3   99.77   428   1     0   5    432   1    428   0.0      856
GGO-Fbxl3   MUS-Fbxl3   97.20   428   12    0   5    432   1    428   0.0      837
GGO-Fbxl3   RNO-Fbxl3   97.43   428   11    0   5    432   1    428   0.0      833
GGO-Fbxl3   MUS-Fbxl21  72.87   435   112   1   4    432   26   460   2e-178   616
GGO-Fbxl3   MMU-Fbxl21  71.66   434   117   1   5    432   1    434   2e-173   600
GGO-Fbxl3   HSA-Fbxl21  70.74   434   121   1   5    432   1    434   5e-170   588
GGO-Fbxl3   PTR-Fbxl21  72.49   418   110   1   20   432   17   434   3e-169   586
GGO-Fbxl3   PPY-Fbxl21  78.46   376   81    0   55   430   1    376   3e-165   572
GGO-Fbxl3   RNO-Fbxl21  65.06   435   144   3   5    432   1    434   2e-159   553
# BLASTP 2.2.20 [Feb-08-2009]
# Query: MMU-Fbxl3
# Database: 559_protein.db
# Query id, Subject id, % identity, alignment length, mismatches, gap openings, q. start, q. end, s. start, s. end, e-value, bit score
MMU-Fbxl3   MMU-Fbxl3   100.00  428   0     0   1    428   1    428   0.0      858
```

```
MMU-Fbxl3	HSA-Fbxl3	99.77	428	1	0	1	428	1	428	0.0	857
MMU-Fbxl3	PTR-Fbxl3	99.77	428	1	0	1	428	1	428	0.0	857
MMU-Fbxl3	PPY-Fbxl3	99.77	428	1	0	1	428	1	428	0.0	857
MMU-Fbxl3	GGO-Fbxl3	99.77	428	1	0	1	428	5	432	0.0	857
MMU-Fbxl3	CJA-Fbxl3	99.53	428	2	0	1	428	1	428	0.0	855
MMU-Fbxl3	MUS-Fbxl3	96.96	428	13	0	1	428	1	428	0.0	834
MMU-Fbxl3	RNO-Fbxl3	97.20	428	12	0	1	428	1	428	0.0	832
MMU-Fbxl3	MUS-Fbxl21	76.79	405	94	0	24	428	56	460	8e-178	614
MMU-Fbxl3	MMU-Fbxl21	75.31	405	100	0	24	428	30	434	4e-173	598
MMU-Fbxl3	HSA-Fbxl21	74.32	405	104	0	24	428	30	434	1e-169	587
MMU-Fbxl3	PTR-Fbxl21	74.07	405	105	0	24	428	30	434	5e-169	585
MMU-Fbxl3	PPY-Fbxl21	78.46	376	81	0	51	426	1	376	2e-165	573
MMU-Fbxl3	RNO-Fbxl21	68.23	406	127	2	24	428	30	434	2e-159	553
# BLASTP 2.2.20 [Feb-08-2009]
# Query: MUS-Fbxl3
# Database: 559_protein.db
# Query id, Subject id, % identity, alignment length, mismatches, gap openings, q. start, q. end, s. start, s. end, e-value, bit score
MUS-Fbxl3	MUS-Fbxl3	100.00	428	0	0	1	428	1	428	0.0	859
MUS-Fbxl3	RNO-Fbxl3	99.07	428	4	0	1	428	1	428	0.0	848
MUS-Fbxl3	GGO-Fbxl3	97.20	428	12	0	1	428	5	432	0.0	836
MUS-Fbxl3	HSA-Fbxl3	97.20	428	12	0	1	428	1	428	0.0	835
MUS-Fbxl3	PTR-Fbxl3	97.20	428	12	0	1	428	1	428	0.0	835
MUS-Fbxl3	PPY-Fbxl3	97.20	428	12	0	1	428	1	428	0.0	835
MUS-Fbxl3	CJA-Fbxl3	97.20	428	12	0	1	428	1	428	0.0	835
MUS-Fbxl3	MMU-Fbxl3	96.96	428	13	0	1	428	1	428	0.0	834
MUS-Fbxl3	MUS-Fbxl21	72.58	434	113	2	1	428	27	460	6e-177	612
MUS-Fbxl3	MMU-Fbxl21	71.66	434	117	2	1	428	1	434	2e-172	596
MUS-Fbxl3	HSA-Fbxl21	70.74	434	121	2	1	428	1	434	1e-168	584
MUS-Fbxl3	PTR-Fbxl21	72.49	418	110	1	16	428	17	434	7e-168	581
MUS-Fbxl3	PPY-Fbxl21	78.72	376	80	0	51	426	1	376	1e-165	573
MUS-Fbxl3	RNO-Fbxl21	65.52	435	142	4	1	428	1	434	2e-159	553
# BLASTP 2.2.20 [Feb-08-2009]
# Query: PPY-Fbxl3
# Database: 559_protein.db
# Query id, Subject id, % identity, alignment length, mismatches, gap openings, q. start, q. end, s. start, s. end, e-value, bit score
PPY-Fbxl3	GGO-Fbxl3	100.00	428	0	0	1	428	5	432	0.0	858
PPY-Fbxl3	HSA-Fbxl3	100.00	428	0	0	1	428	1	428	0.0	858
PPY-Fbxl3	PTR-Fbxl3	100.00	428	0	0	1	428	1	428	0.0	858
PPY-Fbxl3	PPY-Fbxl3	100.00	428	0	0	1	428	1	428	0.0	858
PPY-Fbxl3	MMU-Fbxl3	99.77	428	1	0	1	428	1	428	0.0	857
PPY-Fbxl3	CJA-Fbxl3	99.77	428	1	0	1	428	1	428	0.0	856
PPY-Fbxl3	MUS-Fbxl3	97.20	428	12	0	1	428	1	428	0.0	836
PPY-Fbxl3	RNO-Fbxl3	97.43	428	11	0	1	428	1	428	0.0	834
PPY-Fbxl3	MUS-Fbxl21	76.79	405	94	0	24	428	56	460	8e-178	614
PPY-Fbxl3	MMU-Fbxl21	71.66	434	117	1	1	428	1	434	3e-173	599
```

```
PPY-Fbxl3   HSA-Fbxl21  70.74       434         121         1           1           428         1           434         4e-170      588
PPY-Fbxl3   PTR-Fbxl21  70.51       434         122         1           1           428         1           434         3e-169      586
PPY-Fbxl3   PPY-Fbxl21  78.46       376         81          0           51          426         1           376         2e-165      573
PPY-Fbxl3   RNO-Fbxl21  65.06       435         144         3           1           428         1           434         1e-159      554
# BLASTP 2.2.20 [Feb-08-2009]
# Query: PTR-Fbxl3
# Database: 559_protein.db
# Query id, Subject id, % identity, alignment length, mismatches, gap openings, q. start, q. end, s. start, s. end, e-value, bit score
PTR-Fbxl3   GGO-Fbxl3   100.00      428         0           0           1           428         5           432         0.0         858
PTR-Fbxl3   HSA-Fbxl3   100.00      428         0           0           1           428         1           428         0.0         858
PTR-Fbxl3   PTR-Fbxl3   100.00      428         0           0           1           428         1           428         0.0         858
PTR-Fbxl3   PPY-Fbxl3   100.00      428         0           0           1           428         1           428         0.0         858
PTR-Fbxl3   MMU-Fbxl3   99.77       428         1           0           1           428         1           428         0.0         857
PTR-Fbxl3   CJA-Fbxl3   99.77       428         1           0           1           428         1           428         0.0         856
PTR-Fbxl3   MUS-Fbxl3   97.20       428         12          0           1           428         1           428         0.0         836
PTR-Fbxl3   RNO-Fbxl3   97.43       428         11          0           1           428         1           428         0.0         834
PTR-Fbxl3   MUS-Fbxl21  76.79       405         94          0           24          428         56          460         8e-178      614
PTR-Fbxl3   MMU-Fbxl21  71.66       434         117         1           1           428         1           434         3e-173      599
PTR-Fbxl3   HSA-Fbxl21  70.74       434         121         1           1           428         1           434         4e-170      588
PTR-Fbxl3   PTR-Fbxl21  70.51       434         122         1           1           428         1           434         3e-169      586
PTR-Fbxl3   PPY-Fbxl21  78.46       376         81          0           51          426         1           376         2e-165      573
PTR-Fbxl3   RNO-Fbxl21  65.06       435         144         3           1           428         1           434         1e-159      554
# BLASTP 2.2.20 [Feb-08-2009]
# Query: RNO-Fbxl3
# Database: 559_protein.db
# Query id, Subject id, % identity, alignment length, mismatches, gap openings, q. start, q. end, s. start, s. end, e-value, bit score
RNO-Fbxl3   RNO-Fbxl3   100.00      428         0           0           1           428         1           428         0.0         858
RNO-Fbxl3   MUS-Fbxl3   99.07       428         4           0           1           428         1           428         0.0         848
RNO-Fbxl3   HSA-Fbxl3   97.43       428         11          0           1           428         1           428         0.0         834
RNO-Fbxl3   PTR-Fbxl3   97.43       428         11          0           1           428         1           428         0.0         834
RNO-Fbxl3   PPY-Fbxl3   97.43       428         11          0           1           428         1           428         0.0         834
RNO-Fbxl3   CJA-Fbxl3   97.43       428         11          0           1           428         1           428         0.0         833
RNO-Fbxl3   GGO-Fbxl3   97.43       428         11          0           1           428         5           432         0.0         833
RNO-Fbxl3   MMU-Fbxl3   97.20       428         12          0           1           428         1           428         0.0         832
RNO-Fbxl3   MUS-Fbxl21  74.29       424         101         2           10          428         40          460         2e-177      613
RNO-Fbxl3   MMU-Fbxl21  71.89       434         116         2           1           428         1           434         2e-172      596
RNO-Fbxl3   HSA-Fbxl21  70.97       434         120         2           1           428         1           434         1e-168      584
RNO-Fbxl3   PTR-Fbxl21  72.73       418         109         1           16          428         17          434         4e-168      582
RNO-Fbxl3   PPY-Fbxl21  78.72       376         80          0           51          426         1           376         3e-165      572
RNO-Fbxl3   RNO-Fbxl21  68.15       405         127         2           25          428         31          434         3e-158      549
# BLASTP 2.2.20 [Feb-08-2009]
# Query: HSA-Fbxl3
# Database: 559_protein.db
# Query id, Subject id, % identity, alignment length, mismatches, gap openings, q. start, q. end, s. start, s. end, e-value, bit score
HSA-Fbxl3   GGO-Fbxl3   100.00      428         0           0           1           428         5           432         0.0         858
```

```
HSA-Fbxl3   HSA-Fbxl3   100.00   428   0     0   1    428   1    428   0.0      858
HSA-Fbxl3   PTR-Fbxl3   100.00   428   0     0   1    428   1    428   0.0      858
HSA-Fbxl3   PPY-Fbxl3   100.00   428   0     0   1    428   1    428   0.0      858
HSA-Fbxl3   MMU-Fbxl3   99.77    428   1     0   1    428   1    428   0.0      857
HSA-Fbxl3   CJA-Fbxl3   99.77    428   1     0   1    428   1    428   0.0      856
HSA-Fbxl3   MUS-Fbxl3   97.20    428   12    0   1    428   1    428   0.0      836
HSA-Fbxl3   RNO-Fbxl3   97.43    428   11    0   1    428   1    428   0.0      834
HSA-Fbxl3   MUS-Fbxl21  76.79    405   94    0   24   428   56   460   8e-178   614
HSA-Fbxl3   MMU-Fbxl21  71.66    434   117   1   1    428   1    434   3e-173   599
HSA-Fbxl3   HSA-Fbxl21  70.74    434   121   1   1    428   1    434   4e-170   588
HSA-Fbxl3   PTR-Fbxl21  70.51    434   122   1   1    428   1    434   3e-169   586
HSA-Fbxl3   PPY-Fbxl21  78.46    376   81    0   51   426   1    376   2e-165   573
HSA-Fbxl3   RNO-Fbxl21  65.06    435   144   3   1    428   1    434   1e-159   554
# BLASTP 2.2.20 [Feb-08-2009]
# Query: CJA-Fbxl4
# Database: 559_protein.db
# Query id, Subject id, % identity, alignment length, mismatches, gap openings, q. start, q. end, s. start, s. end, e-value, bit score
CJA-Fbxl4   CJA-Fbxl4   100.00   621   0     0   1    621   1    621   0.0      1285
CJA-Fbxl4   PPY-Fbxl4   98.39    621   10    0   1    621   1    621   0.0      1266
CJA-Fbxl4   MMU-Fbxl4   98.23    621   11    0   1    621   1    621   0.0      1266
CJA-Fbxl4   GGO-Fbxl4   97.91    621   13    0   1    621   1    621   0.0      1239
CJA-Fbxl4   HSA-Fbxl4   97.58    621   15    0   1    621   1    621   0.0      1239
CJA-Fbxl4   PTR-Fbxl4   97.58    621   15    0   1    621   1    621   0.0      1237
CJA-Fbxl4   MUS-Fbxl4   93.88    621   38    0   1    621   1    621   0.0      1196
CJA-Fbxl4   RNO-Fbxl4   93.40    621   41    0   1    621   1    621   0.0      1187
# BLASTP 2.2.20 [Feb-08-2009]
# Query: GGO-Fbxl4
# Database: 559_protein.db
# Query id, Subject id, % identity, alignment length, mismatches, gap openings, q. start, q. end, s. start, s. end, e-value, bit score
GGO-Fbxl4   GGO-Fbxl4   100.00   621   0     0   1    621   1    621   0.0      1286
GGO-Fbxl4   HSA-Fbxl4   98.39    621   10    0   1    621   1    621   0.0      1267
GGO-Fbxl4   PTR-Fbxl4   98.23    621   11    0   1    621   1    621   0.0      1264
GGO-Fbxl4   PPY-Fbxl4   98.23    621   11    0   1    621   1    621   0.0      1262
GGO-Fbxl4   MMU-Fbxl4   98.07    621   12    0   1    621   1    621   0.0      1260
GGO-Fbxl4   CJA-Fbxl4   97.91    621   13    0   1    621   1    621   0.0      1240
GGO-Fbxl4   MUS-Fbxl4   94.20    621   36    0   1    621   1    621   0.0      1202
GGO-Fbxl4   RNO-Fbxl4   93.72    621   39    0   1    621   1    621   0.0      1194
# BLASTP 2.2.20 [Feb-08-2009]
# Query: MMU-Fbxl4
# Database: 559_protein.db
# Query id, Subject id, % identity, alignment length, mismatches, gap openings, q. start, q. end, s. start, s. end, e-value, bit score
MMU-Fbxl4   MMU-Fbxl4   100.00   621   0     0   1    621   1    621   0.0      1286
MMU-Fbxl4   CJA-Fbxl4   98.23    621   11    0   1    621   1    621   0.0      1266
MMU-Fbxl4   PPY-Fbxl4   98.23    621   11    0   1    621   1    621   0.0      1263
MMU-Fbxl4   GGO-Fbxl4   98.07    621   12    0   1    621   1    621   0.0      1260
```

```
MMU-Fbxl4   HSA-Fbxl4   97.75   621   14   0   1   621   1   621   0.0   1238
MMU-Fbxl4   PTR-Fbxl4   97.58   621   15   0   1   621   1   621   0.0   1235
MMU-Fbxl4   MUS-Fbxl4   93.56   621   40   0   1   621   1   621   0.0   1191
MMU-Fbxl4   RNO-Fbxl4   93.08   621   43   0   1   621   1   621   0.0   1182
# BLASTP 2.2.20 [Feb-08-2009]
# Query: MUS-Fbxl4
# Database: 559_protein.db
# Query id, Subject id, % identity, alignment length, mismatches, gap openings, q. start, q. end, s. start, s. end, e-value, bit score
MUS-Fbxl4   MUS-Fbxl4   100.00   621   0   0   1   621   1   621   0.0   1288
MUS-Fbxl4   RNO-Fbxl4   97.58   621   15   0   1   621   1   621   0.0   1236
MUS-Fbxl4   GGO-Fbxl4   94.20   621   36   0   1   621   1   621   0.0   1202
MUS-Fbxl4   CJA-Fbxl4   93.88   621   38   0   1   621   1   621   0.0   1196
MUS-Fbxl4   HSA-Fbxl4   93.40   621   41   0   1   621   1   621   0.0   1194
MUS-Fbxl4   PTR-Fbxl4   93.40   621   41   0   1   621   1   621   0.0   1193
MUS-Fbxl4   PPY-Fbxl4   93.56   621   40   0   1   621   1   621   0.0   1193
MUS-Fbxl4   MMU-Fbxl4   93.56   621   40   0   1   621   1   621   0.0   1191
# BLASTP 2.2.20 [Feb-08-2009]
# Query: PPY-Fbxl4
# Database: 559_protein.db
# Query id, Subject id, % identity, alignment length, mismatches, gap openings, q. start, q. end, s. start, s. end, e-value, bit score
PPY-Fbxl4   PPY-Fbxl4   100.00   621   0   0   1   621   1   621   0.0   1285
PPY-Fbxl4   CJA-Fbxl4   98.39   621   10   0   1   621   1   621   0.0   1266
PPY-Fbxl4   MMU-Fbxl4   98.23   621   11   0   1   621   1   621   0.0   1263
PPY-Fbxl4   GGO-Fbxl4   98.23   621   11   0   1   621   1   621   0.0   1262
PPY-Fbxl4   HSA-Fbxl4   97.91   621   13   0   1   621   1   621   0.0   1240
PPY-Fbxl4   PTR-Fbxl4   97.75   621   14   0   1   621   1   621   0.0   1238
PPY-Fbxl4   MUS-Fbxl4   93.56   621   40   0   1   621   1   621   0.0   1193
PPY-Fbxl4   RNO-Fbxl4   93.08   621   43   0   1   621   1   621   0.0   1184
# BLASTP 2.2.20 [Feb-08-2009]
# Query: PTR-Fbxl4
# Database: 559_protein.db
# Query id, Subject id, % identity, alignment length, mismatches, gap openings, q. start, q. end, s. start, s. end, e-value, bit score
PTR-Fbxl4   PTR-Fbxl4   100.00   621   0   0   1   621   1   621   0.0   1285
PTR-Fbxl4   HSA-Fbxl4   98.55   621   9   0   1   621   1   621   0.0   1269
PTR-Fbxl4   GGO-Fbxl4   98.23   621   11   0   1   621   1   621   0.0   1264
PTR-Fbxl4   PPY-Fbxl4   97.75   621   14   0   1   621   1   621   0.0   1238
PTR-Fbxl4   CJA-Fbxl4   97.58   621   15   0   1   621   1   621   0.0   1238
PTR-Fbxl4   MMU-Fbxl4   97.58   621   15   0   1   621   1   621   0.0   1234
PTR-Fbxl4   MUS-Fbxl4   93.40   621   41   0   1   621   1   621   0.0   1193
PTR-Fbxl4   RNO-Fbxl4   92.91   621   44   0   1   621   1   621   0.0   1184
# BLASTP 2.2.20 [Feb-08-2009]
# Query: RNO-Fbxl4
# Database: 559_protein.db
# Query id, Subject id, % identity, alignment length, mismatches, gap openings, q. start, q. end, s. start, s. end, e-value, bit score
RNO-Fbxl4   RNO-Fbxl4   100.00   621   0   0   1   621   1   621   0.0   1286
```

```
RNO-Fbxl4   MUS-Fbxl4   97.58   621   15   0   1   621   1     621   0.0   1237
RNO-Fbxl4   GGO-Fbxl4   93.72   621   39   0   1   621   1     621   0.0   1195
RNO-Fbxl4   CJA-Fbxl4   93.40   621   41   0   1   621   1     621   0.0   1188
RNO-Fbxl4   HSA-Fbxl4   92.91   621   44   0   1   621   1     621   0.0   1186
RNO-Fbxl4   PTR-Fbxl4   92.91   621   44   0   1   621   1     621   0.0   1185
RNO-Fbxl4   PPY-Fbxl4   93.08   621   43   0   1   621   1     621   0.0   1184
RNO-Fbxl4   MMU-Fbxl4   93.08   621   43   0   1   621   1     621   0.0   1183
# BLASTP 2.2.20 [Feb-08-2009]
# Query: HSA-Fbxl4
# Database: 559_protein.db
# Query id, Subject id, % identity, alignment length, mismatches, gap openings, q. start, q. end, s. start, s. end, e-value, bit score
HSA-Fbxl4   HSA-Fbxl4   100.00  621   0    0   1   621   1     621   0.0   1286
HSA-Fbxl4   PTR-Fbxl4   98.55   621   9    0   1   621   1     621   0.0   1269
HSA-Fbxl4   GGO-Fbxl4   98.39   621   10   0   1   621   1     621   0.0   1267
HSA-Fbxl4   PPY-Fbxl4   97.91   621   13   0   1   621   1     621   0.0   1238
HSA-Fbxl4   CJA-Fbxl4   97.58   621   15   0   1   621   1     621   0.0   1237
HSA-Fbxl4   MMU-Fbxl4   97.75   621   14   0   1   621   1     621   0.0   1237
HSA-Fbxl4   MUS-Fbxl4   93.40   621   41   0   1   621   1     621   0.0   1194
HSA-Fbxl4   RNO-Fbxl4   92.91   621   44   0   1   621   1     621   0.0   1185
# BLASTP 2.2.20 [Feb-08-2009]
# Query: CJA-Fbxl5
# Database: 559_protein.db
# Query id, Subject id, % identity, alignment length, mismatches, gap openings, q. start, q. end, s. start, s. end, e-value, bit score
CJA-Fbxl5   CJA-Fbxl5   100.00  565   0    0   1   565   1     565   0.0   1095
CJA-Fbxl5   HSA-Fbxl5   96.81   565   18   0   1   565   127   691   0.0   1070
CJA-Fbxl5   PPY-Fbxl5   97.35   565   15   0   1   565   100   664   0.0   1056
CJA-Fbxl5   PTR-Fbxl5   97.17   565   16   0   1   565   127   691   0.0   1053
CJA-Fbxl5   MMU-Fbxl5   96.64   565   19   0   1   565   127   691   0.0   1050
CJA-Fbxl5   GGO-Fbxl5   94.72   568   27   1   1   565   127   694   0.0   1022
CJA-Fbxl5   RNO-Fbxl5   92.57   565   41   1   1   565   127   690   0.0   1018
CJA-Fbxl5   MUS-Fbxl5   91.86   565   45   1   1   565   127   690   0.0   1013
# BLASTP 2.2.20 [Feb-08-2009]
# Query: GGO-Fbxl5
# Database: 559_protein.db
# Query id, Subject id, % identity, alignment length, mismatches, gap openings, q. start, q. end, s. start, s. end, e-value, bit score
GGO-Fbxl5   GGO-Fbxl5   100.00  694   0    0   1     694   1   694   0.0   1363
GGO-Fbxl5   HSA-Fbxl5   97.69   694   13   1   1     694   1   691   0.0   1328
GGO-Fbxl5   PTR-Fbxl5   97.98   694   11   1   1     694   1   691   0.0   1326
GGO-Fbxl5   MMU-Fbxl5   96.83   694   19   1   1     694   1   691   0.0   1318
GGO-Fbxl5   MUS-Fbxl5   91.07   694   58   2   1     694   1   690   0.0   1266
GGO-Fbxl5   RNO-Fbxl5   91.21   694   57   2   1     694   1   690   0.0   1264
GGO-Fbxl5   PPY-Fbxl5   97.30   667   15   1   28    694   1   664   0.0   1262
GGO-Fbxl5   CJA-Fbxl5   94.72   568   27   1   127   694   1   565   0.0   1042
# BLASTP 2.2.20 [Feb-08-2009]
# Query: MMU-Fbxl5
```

```
# Database: 559_protein.db
# Query id, Subject id, % identity, alignment length, mismatches, gap openings, q. start, q. end, s. start, s. end, e-value, bit score
MMU-Fbxl5   MMU-Fbxl5   100.00   691   0    0   1     691   1    691   0.0   1359
MMU-Fbxl5   HSA-Fbxl5   98.55    691   10   0   1     691   1    691   0.0   1350
MMU-Fbxl5   PTR-Fbxl5   98.84    691   8    0   1     691   1    691   0.0   1347
MMU-Fbxl5   GGO-Fbxl5   96.83    694   19   1   1     691   1    694   0.0   1316
MMU-Fbxl5   MUS-Fbxl5   93.34    691   45   1   1     691   1    690   0.0   1294
MMU-Fbxl5   RNO-Fbxl5   93.49    691   44   1   1     691   1    690   0.0   1289
MMU-Fbxl5   PPY-Fbxl5   98.64    664   9    0   28    691   1    664   0.0   1286
MMU-Fbxl5   CJA-Fbxl5   96.64    565   19   0   127   691   1    565   0.0   1069
# BLASTP 2.2.20 [Feb-08-2009]
# Query: MUS-Fbxl5
# Database: 559_protein.db
# Query id, Subject id, % identity, alignment length, mismatches, gap openings, q. start, q. end, s. start, s. end, e-value, bit score
MUS-Fbxl5   MUS-Fbxl5   100.00   690   0    0   1     690   1    690   0.0   1364
MUS-Fbxl5   RNO-Fbxl5   96.96    690   21   0   1     690   1    690   0.0   1328
MUS-Fbxl5   HSA-Fbxl5   93.63    691   43   1   1     690   1    691   0.0   1284
MUS-Fbxl5   PTR-Fbxl5   93.63    691   43   1   1     690   1    691   0.0   1273
MUS-Fbxl5   MMU-Fbxl5   93.34    691   45   1   1     690   1    691   0.0   1272
MUS-Fbxl5   GGO-Fbxl5   91.21    694   57   2   1     690   1    694   0.0   1242
MUS-Fbxl5   PPY-Fbxl5   93.37    664   43   1   28    690   1    664   0.0   1215
MUS-Fbxl5   CJA-Fbxl5   91.86    565   45   1   127   690   1    565   0.0   1012
# BLASTP 2.2.20 [Feb-08-2009]
# Query: PPY-Fbxl5
# Database: 559_protein.db
# Query id, Subject id, % identity, alignment length, mismatches, gap openings, q. start, q. end, s. start, s. end, e-value, bit score
PPY-Fbxl5   PPY-Fbxl5   100.00   664   0    0   1     664   1    664   0.0   1300
PPY-Fbxl5   HSA-Fbxl5   99.10    664   6    0   1     664   28   691   0.0   1296
PPY-Fbxl5   PTR-Fbxl5   99.40    664   4    0   1     664   28   691   0.0   1294
PPY-Fbxl5   MMU-Fbxl5   98.64    664   9    0   1     664   28   691   0.0   1287
PPY-Fbxl5   GGO-Fbxl5   97.30    667   15   1   1     664   28   694   0.0   1262
PPY-Fbxl5   MUS-Fbxl5   93.37    664   43   1   1     664   28   690   0.0   1238
PPY-Fbxl5   RNO-Fbxl5   93.83    664   40   1   1     664   28   690   0.0   1237
PPY-Fbxl5   CJA-Fbxl5   97.35    565   15   0   100   664   1    565   0.0   1077
# BLASTP 2.2.20 [Feb-08-2009]
# Query: PTR-Fbxl5
# Database: 559_protein.db
# Query id, Subject id, % identity, alignment length, mismatches, gap openings, q. start, q. end, s. start, s. end, e-value, bit score
PTR-Fbxl5   HSA-Fbxl5   99.71    691   2    0   1     691   1    691   0.0   1361
PTR-Fbxl5   PTR-Fbxl5   100.00   691   0    0   1     691   1    691   0.0   1357
PTR-Fbxl5   MMU-Fbxl5   98.84    691   8    0   1     691   1    691   0.0   1350
PTR-Fbxl5   GGO-Fbxl5   97.98    694   11   1   1     691   1    694   0.0   1326
PTR-Fbxl5   MUS-Fbxl5   93.63    691   43   1   1     691   1    690   0.0   1298
PTR-Fbxl5   RNO-Fbxl5   93.92    691   41   1   1     691   1    690   0.0   1295
PTR-Fbxl5   PPY-Fbxl5   99.40    664   4    0   28    691   1    664   0.0   1294
```

```
PTR-Fbxl5   CJA-Fbxl5   97.17       565         16          0           127         691         1           565         0.0         1073
# BLASTP 2.2.20 [Feb-08-2009]
# Query: RNO-Fbxl5
# Database: 559_protein.db
# Query id, Subject id, % identity, alignment length, mismatches, gap openings, q. start, q. end, s. start, s. end, e-value, bit score
RNO-Fbxl5   RNO-Fbxl5   100.00      690         0           0           1           690         1           690         0.0         1362
RNO-Fbxl5   MUS-Fbxl5   96.96       690         21          0           1           690         1           690         0.0         1328
RNO-Fbxl5   HSA-Fbxl5   93.92       691         41          1           1           690         1           691         0.0         1285
RNO-Fbxl5   PTR-Fbxl5   93.92       691         41          1           1           690         1           691         0.0         1271
RNO-Fbxl5   MMU-Fbxl5   93.49       691         44          1           1           690         1           691         0.0         1267
RNO-Fbxl5   GGO-Fbxl5   91.35       694         56          2           1           690         1           694         0.0         1240
RNO-Fbxl5   PPY-Fbxl5   93.83       664         40          1           28          690         1           664         0.0         1213
RNO-Fbxl5   CJA-Fbxl5   92.57       565         41          1           127         690         1           565         0.0         1017
# BLASTP 2.2.20 [Feb-08-2009]
# Query: HSA-Fbxl5
# Database: 559_protein.db
# Query id, Subject id, % identity, alignment length, mismatches, gap openings, q. start, q. end, s. start, s. end, e-value, bit score
HSA-Fbxl5   HSA-Fbxl5   100.00      691         0           0           1           691         1           691         0.0         1391
HSA-Fbxl5   PTR-Fbxl5   99.71       691         2           0           1           691         1           691         0.0         1366
HSA-Fbxl5   MMU-Fbxl5   98.55       691         10          0           1           691         1           691         0.0         1357
HSA-Fbxl5   GGO-Fbxl5   97.69       694         13          1           1           691         1           694         0.0         1334
HSA-Fbxl5   RNO-Fbxl5   93.92       691         41          1           1           691         1           690         0.0         1312
HSA-Fbxl5   MUS-Fbxl5   93.63       691         43          1           1           691         1           690         0.0         1312
HSA-Fbxl5   PPY-Fbxl5   99.10       664         6           0           28          691         1           664         0.0         1302
HSA-Fbxl5   CJA-Fbxl5   96.81       565         18          0           127         691         1           565         0.0         1097
# BLASTP 2.2.20 [Feb-08-2009]
# Query: CJA-Fbxl6
# Database: 559_protein.db
# Query id, Subject id, % identity, alignment length, mismatches, gap openings, q. start, q. end, s. start, s. end, e-value, bit score
CJA-Fbxl6   CJA-Fbxl6   100.00      490         0           0           23          512         23          512         0.0         768
CJA-Fbxl6   HSA-Fbxl6   77.95       517         87          6           23          512         23          539         0.0         648
CJA-Fbxl6   MMU-Fbxl6   77.39       513         93          6           23          512         23          535         0.0         647
CJA-Fbxl6   GGO-Fbxl6   77.46       519         88          6           23          512         23          541         0.0         644
CJA-Fbxl6   PTR-Fbxl6   77.56       517         89          6           23          512         23          539         0.0         644
CJA-Fbxl6   PPY-Fbxl6   66.80       512         76          7           23          512         23          462         3e-149      520
CJA-Fbxl6   RNO-Fbxl6   70.00       300         82          2           23          321         27          319         1e-108      385
CJA-Fbxl6   RNO-Fbxl6   87.79       131         16          0           382         512         401         531         4e-051      194
CJA-Fbxl6   MUS-Fbxl6   69.67       300         83          2           23          321         27          319         4e-108      383
CJA-Fbxl6   MUS-Fbxl6   89.84       128         13          0           382         509         401         528         2e-053      201
# BLASTP 2.2.20 [Feb-08-2009]
# Query: GGO-Fbxl6
# Database: 559_protein.db
# Query id, Subject id, % identity, alignment length, mismatches, gap openings, q. start, q. end, s. start, s. end, e-value, bit score
GGO-Fbxl6   GGO-Fbxl6   100.00      519         0           0           23          541         23          541         0.0         892
GGO-Fbxl6   HSA-Fbxl6   98.46       519         6           1           23          541         23          539         0.0         877
```

```
GGO-Fbxl6   PTR-Fbxl6   97.88    519    9      1    23     541    23     539    0.0       863
GGO-Fbxl6   MMU-Fbxl6   92.29    519    34     1    23     541    23     535    0.0       852
GGO-Fbxl6   MUS-Fbxl6   79.65    516    91     1    23     538    27     528    0.0       736
GGO-Fbxl6   RNO-Fbxl6   79.58    519    92     1    23     541    27     531    0.0       728
GGO-Fbxl6   PPY-Fbxl6   88.30    436    7      1    106    541    71     462    0.0       711
GGO-Fbxl6   CJA-Fbxl6   81.71    432    57     5    110    541    103    512    2e-178    617
# BLASTP 2.2.20 [Feb-08-2009]
# Query: MMU-Fbxl6
# Database: 559_protein.db
# Query id, Subject id, % identity, alignment length, mismatches, gap openings, q. start, q. end, s. start, s. end, e-value, bit score
MMU-Fbxl6   MMU-Fbxl6   100.00   513    0      0    23     535    23     535    0.0       895
MMU-Fbxl6   HSA-Fbxl6   92.46    517    35     1    23     535    23     539    0.0       851
MMU-Fbxl6   GGO-Fbxl6   91.05    525    29     2    23     535    23     541    0.0       849
MMU-Fbxl6   PTR-Fbxl6   98.16    434    8      0    102    535    106    539    0.0       817
MMU-Fbxl6   MUS-Fbxl6   79.61    510    96     1    23     532    27     528    0.0       736
MMU-Fbxl6   RNO-Fbxl6   80.00    510    94     1    23     532    27     528    0.0       731
MMU-Fbxl6   PPY-Fbxl6   79.92    513    30     2    23     535    23     462    0.0       728
MMU-Fbxl6   CJA-Fbxl6   75.24    513    104    6    23     535    23     512    0.0       640
# BLASTP 2.2.20 [Feb-08-2009]
# Query: MUS-Fbxl6
# Database: 559_protein.db
# Query id, Subject id, % identity, alignment length, mismatches, gap openings, q. start, q. end, s. start, s. end, e-value, bit score
MUS-Fbxl6   MUS-Fbxl6   100.00   513    0      0    23     535    23     535    0.0       955
MUS-Fbxl6   RNO-Fbxl6   93.57    513    33     0    23     535    23     535    0.0       904
MUS-Fbxl6   GGO-Fbxl6   79.65    516    91     1    27     528    23     538    0.0       763
MUS-Fbxl6   HSA-Fbxl6   80.16    514    90     1    27     528    23     536    0.0       762
MUS-Fbxl6   PTR-Fbxl6   79.77    514    92     1    27     528    23     536    0.0       756
MUS-Fbxl6   MMU-Fbxl6   78.24    510    103    2    27     528    23     532    0.0       752
MUS-Fbxl6   PPY-Fbxl6   71.17    503    80     2    26     528    22     459    0.0       646
MUS-Fbxl6   CJA-Fbxl6   66.80    509    140    6    27     528    23     509    3e-167    580
# BLASTP 2.2.20 [Feb-08-2009]
# Query: PTR-Fbxl6
# Database: 559_protein.db
# Query id, Subject id, % identity, alignment length, mismatches, gap openings, q. start, q. end, s. start, s. end, e-value, bit score
PTR-Fbxl6   PTR-Fbxl6   100.00   517    0      0    23     539    23     539    0.0       880
PTR-Fbxl6   HSA-Fbxl6   98.84    517    6      0    23     539    23     539    0.0       873
PTR-Fbxl6   GGO-Fbxl6   97.88    519    9      1    23     539    23     541    0.0       865
PTR-Fbxl6   MMU-Fbxl6   92.65    517    34     1    23     539    23     535    0.0       851
PTR-Fbxl6   MUS-Fbxl6   79.77    514    92     1    23     536    27     528    0.0       728
PTR-Fbxl6   RNO-Fbxl6   79.69    517    93     1    23     539    27     531    0.0       722
PTR-Fbxl6   PPY-Fbxl6   87.79    434    9      1    106    539    73     462    0.0       704
PTR-Fbxl6   CJA-Fbxl6   80.88    434    61     5    106    539    101    512    7e-178    615
# BLASTP 2.2.20 [Feb-08-2009]
# Query: RNO-Fbxl6
# Database: 559_protein.db
```

```
# Query id, Subject id, % identity, alignment length, mismatches, gap openings, q. start, q. end, s. start, s. end, e-value, bit score
RNO-Fbxl6   RNO-Fbxl6   100.00      520         0           0           16          535         16          535         0.0         961
RNO-Fbxl6   MUS-Fbxl6   93.62       517         33          0           19          535         19          535         0.0         903
RNO-Fbxl6   GGO-Fbxl6   79.58       519         92          1           27          531         23          541         0.0         743
RNO-Fbxl6   HSA-Fbxl6   80.08       517         91          1           27          531         23          539         0.0         741
RNO-Fbxl6   MMU-Fbxl6   78.63       510         101         2           27          528         23          532         0.0         739
RNO-Fbxl6   PTR-Fbxl6   79.69       517         93          1           27          531         23          539         0.0         735
RNO-Fbxl6   PPY-Fbxl6   71.34       506         80          2           26          531         22          462         0.0         631
RNO-Fbxl6   CJA-Fbxl6   66.15       514         141         6           27          531         23          512         4e-162      562
# BLASTP 2.2.20 [Feb-08-2009]
# Query: HSA-Fbxl6
# Database: 559_protein.db
# Query id, Subject id, % identity, alignment length, mismatches, gap openings, q. start, q. end, s. start, s. end, e-value, bit score
HSA-Fbxl6   HSA-Fbxl6   100.00      517         0           0           23          539         23          539         0.0         911
HSA-Fbxl6   GGO-Fbxl6   98.46       519         6           1           23          539         23          541         0.0         899
HSA-Fbxl6   PTR-Fbxl6   98.84       517         6           0           23          539         23          539         0.0         898
HSA-Fbxl6   MMU-Fbxl6   92.73       523         22          2           23          539         23          535         0.0         865
HSA-Fbxl6   MUS-Fbxl6   80.16       514         90          1           23          536         27          528         0.0         754
HSA-Fbxl6   RNO-Fbxl6   80.08       517         91          1           23          539         27          531         0.0         747
HSA-Fbxl6   PPY-Fbxl6   88.53       436         6           1           104         539         71          462         0.0         714
HSA-Fbxl6   CJA-Fbxl6   81.38       435         59          5           105         539         100         512         1e-178      618
# BLASTP 2.2.20 [Feb-08-2009]
# Query: PPY-Fbxl6
# Database: 559_protein.db
# Query id, Subject id, % identity, alignment length, mismatches, gap openings, q. start, q. end, s. start, s. end, e-value, bit score
PPY-Fbxl6   PPY-Fbxl6   100.00      462         0           0           1           462         1           462         0.0         771
PPY-Fbxl6   MMU-Fbxl6   79.73       513         31          2           23          462         23          535         0.0         697
PPY-Fbxl6   HSA-Fbxl6   88.53       436         6           1           71          462         104         539         0.0         678
PPY-Fbxl6   GGO-Fbxl6   88.30       436         7           1           71          462         106         541         0.0         675
PPY-Fbxl6   PTR-Fbxl6   87.79       434         9           1           73          462         106         539         0.0         671
PPY-Fbxl6   MUS-Fbxl6   71.17       503         80          2           22          459         26          528         2e-175      607
PPY-Fbxl6   RNO-Fbxl6   71.34       506         80          2           22          462         26          531         2e-173      600
PPY-Fbxl6   CJA-Fbxl6   65.43       512         83          6           23          462         23          512         8e-152      528
# BLASTP 2.2.20 [Feb-08-2009]
# Query: CJA-Fbxl7
# Database: 559_protein.db
# Query id, Subject id, % identity, alignment length, mismatches, gap openings, q. start, q. end, s. start, s. end, e-value, bit score
CJA-Fbxl7   CJA-Fbxl7   100.00      449         0           0           1           449         1           449         0.0         902
CJA-Fbxl7   HSA-Fbxl7   98.89       449         5           0           1           449         43          491         0.0         895
CJA-Fbxl7   PTR-Fbxl7   98.89       449         5           0           1           449         43          491         0.0         895
CJA-Fbxl7   MMU-Fbxl7   98.89       449         5           0           1           449         31          479         0.0         894
CJA-Fbxl7   PPY-Fbxl7   98.66       449         6           0           1           449         1           449         0.0         891
CJA-Fbxl7   GGO-Fbxl7   98.66       449         6           0           1           449         1           449         0.0         891
CJA-Fbxl7   MUS-Fbxl7   97.55       449         11          0           1           449         43          491         0.0         883
CJA-Fbxl7   RNO-Fbxl7   97.55       449         11          0           1           449         1           449         0.0         880
```

```
# BLASTP 2.2.20 [Feb-08-2009]
# Query: GGO-Fbxl7
# Database: 559_protein.db
# Query id, Subject id, % identity, alignment length, mismatches, gap openings, q. start, q. end, s. start, s. end, e-value, bit score
GGO-Fbxl7   HSA-Fbxl7   99.78       449         1           0           1           449         43          491         0.0         909
GGO-Fbxl7   PTR-Fbxl7   99.78       449         1           0           1           449         43          491         0.0         909
GGO-Fbxl7   MMU-Fbxl7   99.78       449         1           0           1           449         31          479         0.0         906
GGO-Fbxl7   GGO-Fbxl7   100.00      449         0           0           1           449         1           449         0.0         904
GGO-Fbxl7   PPY-Fbxl7   99.55       449         2           0           1           449         1           449         0.0         899
GGO-Fbxl7   MUS-Fbxl7   98.89       449         5           0           1           449         43          491         0.0         892
GGO-Fbxl7   CJA-Fbxl7   98.66       449         6           0           1           449         1           449         0.0         891
GGO-Fbxl7   RNO-Fbxl7   98.89       449         5           0           1           449         1           449         0.0         889
# BLASTP 2.2.20 [Feb-08-2009]
# Query: MMU-Fbxl7
# Database: 559_protein.db
# Query id, Subject id, % identity, alignment length, mismatches, gap openings, q. start, q. end, s. start, s. end, e-value, bit score
MMU-Fbxl7   HSA-Fbxl7   100.00      479         0           0           1           479         13          491         0.0         939
MMU-Fbxl7   PTR-Fbxl7   100.00      479         0           0           1           479         13          491         0.0         939
MMU-Fbxl7   MMU-Fbxl7   100.00      479         0           0           1           479         1           479         0.0         938
MMU-Fbxl7   MUS-Fbxl7   98.33       479         8           0           1           479         13          491         0.0         927
MMU-Fbxl7   GGO-Fbxl7   99.78       449         1           0           31          479         1           449         0.0         906
MMU-Fbxl7   PPY-Fbxl7   99.78       449         1           0           31          479         1           449         0.0         905
MMU-Fbxl7   CJA-Fbxl7   98.89       449         5           0           31          479         1           449         0.0         894
MMU-Fbxl7   RNO-Fbxl7   98.66       449         6           0           31          479         1           449         0.0         891
# BLASTP 2.2.20 [Feb-08-2009]
# Query: MUS-Fbxl7
# Database: 559_protein.db
# Query id, Subject id, % identity, alignment length, mismatches, gap openings, q. start, q. end, s. start, s. end, e-value, bit score
MUS-Fbxl7   MUS-Fbxl7   100.00      491         0           0           1           491         1           491         0.0         965
MUS-Fbxl7   HSA-Fbxl7   98.37       491         8           0           1           491         1           491         0.0         954
MUS-Fbxl7   PTR-Fbxl7   98.37       491         8           0           1           491         1           491         0.0         954
MUS-Fbxl7   MMU-Fbxl7   98.33       479         8           0           13          491         1           479         0.0         927
MUS-Fbxl7   RNO-Fbxl7   99.78       449         1           0           43          491         1           449         0.0         904
MUS-Fbxl7   GGO-Fbxl7   98.89       449         5           0           43          491         1           449         0.0         892
MUS-Fbxl7   PPY-Fbxl7   98.44       449         7           0           43          491         1           449         0.0         891
MUS-Fbxl7   CJA-Fbxl7   97.55       449         11          0           43          491         1           449         0.0         883
# BLASTP 2.2.20 [Feb-08-2009]
# Query: PPY-Fbxl7
# Database: 559_protein.db
# Query id, Subject id, % identity, alignment length, mismatches, gap openings, q. start, q. end, s. start, s. end, e-value, bit score
PPY-Fbxl7   HSA-Fbxl7   99.78       449         1           0           1           449         43          491         0.0         908
PPY-Fbxl7   PTR-Fbxl7   99.78       449         1           0           1           449         43          491         0.0         908
PPY-Fbxl7   MMU-Fbxl7   99.78       449         1           0           1           449         31          479         0.0         906
PPY-Fbxl7   PPY-Fbxl7   100.00      449         0           0           1           449         1           449         0.0         904
PPY-Fbxl7   GGO-Fbxl7   99.55       449         2           0           1           449         1           449         0.0         899
```

```
PPY-Fbxl7   CJA-Fbxl7   98.66   449   6    0   1    449   1    449   0.0      891
PPY-Fbxl7   MUS-Fbxl7   98.44   449   7    0   1    449   43   491   0.0      891
PPY-Fbxl7   RNO-Fbxl7   98.44   449   7    0   1    449   1    449   0.0      888
# BLASTP 2.2.20 [Feb-08-2009]
# Query: PTR-Fbxl7
# Database: 559_protein.db
# Query id, Subject id, % identity, alignment length, mismatches, gap openings, q. start, q. end, s. start, s. end, e-value, bit score
PTR-Fbxl7   HSA-Fbxl7   100.00  491   0    0   1    491   1    491   0.0      951
PTR-Fbxl7   PTR-Fbxl7   100.00  491   0    0   1    491   1    491   0.0      951
PTR-Fbxl7   MMU-Fbxl7   100.00  465   0    0   27   491   15   479   0.0      941
PTR-Fbxl7   MUS-Fbxl7   98.37   491   8    0   1    491   1    491   0.0      938
PTR-Fbxl7   GGO-Fbxl7   99.78   449   1    0   43   491   1    449   0.0      906
PTR-Fbxl7   PPY-Fbxl7   99.78   449   1    0   43   491   1    449   0.0      905
PTR-Fbxl7   CJA-Fbxl7   98.89   449   5    0   43   491   1    449   0.0      894
PTR-Fbxl7   RNO-Fbxl7   98.66   449   6    0   43   491   1    449   0.0      892
# BLASTP 2.2.20 [Feb-08-2009]
# Query: RNO-Fbxl7
# Database: 559_protein.db
# Query id, Subject id, % identity, alignment length, mismatches, gap openings, q. start, q. end, s. start, s. end, e-value, bit score
RNO-Fbxl7   MUS-Fbxl7   99.78   449   1    0   1    449   43   491   0.0      907
RNO-Fbxl7   RNO-Fbxl7   100.00  449   0    0   1    449   1    449   0.0      905
RNO-Fbxl7   HSA-Fbxl7   98.66   449   6    0   1    449   43   491   0.0      892
RNO-Fbxl7   PTR-Fbxl7   98.66   449   6    0   1    449   43   491   0.0      892
RNO-Fbxl7   MMU-Fbxl7   98.66   449   6    0   1    449   31   479   0.0      891
RNO-Fbxl7   GGO-Fbxl7   98.89   449   5    0   1    449   1    449   0.0      889
RNO-Fbxl7   PPY-Fbxl7   98.44   449   7    0   1    449   1    449   0.0      888
RNO-Fbxl7   CJA-Fbxl7   97.55   449   11   0   1    449   1    449   0.0      880
# BLASTP 2.2.20 [Feb-08-2009]
# Query: HSA-Fbxl7
# Database: 559_protein.db
# Query id, Subject id, % identity, alignment length, mismatches, gap openings, q. start, q. end, s. start, s. end, e-value, bit score
HSA-Fbxl7   HSA-Fbxl7   100.00  491   0    0   1    491   1    491   0.0      951
HSA-Fbxl7   PTR-Fbxl7   100.00  491   0    0   1    491   1    491   0.0      951
HSA-Fbxl7   MMU-Fbxl7   100.00  465   0    0   27   491   15   479   0.0      941
HSA-Fbxl7   MUS-Fbxl7   98.37   491   8    0   1    491   1    491   0.0      938
HSA-Fbxl7   GGO-Fbxl7   99.78   449   1    0   43   491   1    449   0.0      906
HSA-Fbxl7   PPY-Fbxl7   99.78   449   1    0   43   491   1    449   0.0      905
HSA-Fbxl7   CJA-Fbxl7   98.89   449   5    0   43   491   1    449   0.0      894
HSA-Fbxl7   RNO-Fbxl7   98.66   449   6    0   43   491   1    449   0.0      892
# BLASTP 2.2.20 [Feb-08-2009]
# Query: CJA-Fbxl8
# Database: 559_protein.db
# Query id, Subject id, % identity, alignment length, mismatches, gap openings, q. start, q. end, s. start, s. end, e-value, bit score
CJA-Fbxl8   CJA-Fbxl8   100.00  374   0    0   1    374   1    374   4e-155   538
CJA-Fbxl8   HSA-Fbxl8   90.91   374   34   0   1    374   1    374   2e-137   480
```

```
CJA-Fbxl8   PTR-Fbxl8   91.18      374        33         0          1          374        1          374        3e-137     479
CJA-Fbxl8   PPY-Fbxl8   90.64      374        35         0          1          374        1          374        2e-136     476
CJA-Fbxl8   MMU-Fbxl8   90.64      374        35         0          1          374        1          374        8e-136     474
CJA-Fbxl8   GGO-Fbxl8   90.11      374        37         0          1          374        8          381        9e-136     474
CJA-Fbxl8   MUS-Fbxl8   76.68      373        87         0          1          373        1          373        1e-115     407
CJA-Fbxl8   RNO-Fbxl8   75.74      371        90         0          1          371        1          371        2e-113     400
# BLASTP 2.2.20 [Feb-08-2009]
# Query: GGO-Fbxl8
# Database: 559_protein.db
# Query id, Subject id, % identity, alignment length, mismatches, gap openings, q. start, q. end, s. start, s. end, e-value, bit score
GGO-Fbxl8   GGO-Fbxl8   100.00     381        0          0          1          381        1          381        6e-159     551
GGO-Fbxl8   HSA-Fbxl8   98.13      374        7          0          8          381        1          374        1e-151     527
GGO-Fbxl8   PTR-Fbxl8   98.40      374        6          0          8          381        1          374        2e-151     526
GGO-Fbxl8   PPY-Fbxl8   97.33      374        10         0          8          381        1          374        7e-149     518
GGO-Fbxl8   MMU-Fbxl8   94.39      374        21         0          8          381        1          374        1e-142     497
GGO-Fbxl8   CJA-Fbxl8   90.11      374        37         0          8          381        1          374        2e-138     483
GGO-Fbxl8   MUS-Fbxl8   78.28      373        81         0          8          380        1          373        1e-119     421
GGO-Fbxl8   RNO-Fbxl8   78.28      373        81         0          8          380        1          373        9e-119     418
# BLASTP 2.2.20 [Feb-08-2009]
# Query: MMU-Fbxl8
# Database: 559_protein.db
# Query id, Subject id, % identity, alignment length, mismatches, gap openings, q. start, q. end, s. start, s. end, e-value, bit score
MMU-Fbxl8   MMU-Fbxl8   100.00     386        0          0          1          386        1          386        6e-154     535
MMU-Fbxl8   HSA-Fbxl8   95.19      374        18         0          1          374        1          374        4e-137     479
MMU-Fbxl8   PTR-Fbxl8   95.45      374        17         0          1          374        1          374        7e-137     478
MMU-Fbxl8   PPY-Fbxl8   94.92      374        19         0          1          374        1          374        8e-136     474
MMU-Fbxl8   GGO-Fbxl8   94.39      374        21         0          1          374        8          381        9e-136     474
MMU-Fbxl8   CJA-Fbxl8   90.64      374        35         0          1          374        1          374        6e-131     458
MMU-Fbxl8   MUS-Fbxl8   78.28      373        81         0          1          373        1          373        8e-112     395
MMU-Fbxl8   RNO-Fbxl8   77.21      373        85         0          1          373        1          373        3e-109     386
# BLASTP 2.2.20 [Feb-08-2009]
# Query: MUS-Fbxl8
# Database: 559_protein.db
# Query id, Subject id, % identity, alignment length, mismatches, gap openings, q. start, q. end, s. start, s. end, e-value, bit score
MUS-Fbxl8   MUS-Fbxl8   100.00     374        0          0          1          374        1          374        0.0        645
MUS-Fbxl8   RNO-Fbxl8   90.91      374        34         0          1          374        1          374        1e-170     590
MUS-Fbxl8   CJA-Fbxl8   76.68      373        87         0          1          373        1          373        1e-135     474
MUS-Fbxl8   HSA-Fbxl8   78.82      373        79         0          1          373        1          373        5e-135     472
MUS-Fbxl8   GGO-Fbxl8   78.28      373        81         0          1          373        8          380        5e-135     472
MUS-Fbxl8   MMU-Fbxl8   78.28      373        81         0          1          373        1          373        8e-135     471
MUS-Fbxl8   PTR-Fbxl8   78.55      373        80         0          1          373        1          373        1e-134     471
MUS-Fbxl8   PPY-Fbxl8   78.82      373        79         0          1          373        1          373        5e-134     469
# BLASTP 2.2.20 [Feb-08-2009]
# Query: PTR-Fbxl8
# Database: 559_protein.db
```

```
# Query id, Subject id, % identity, alignment length, mismatches, gap openings, q. start, q. end, s. start, s. end, e-value, bit score
PTR-Fbxl8	PTR-Fbxl8	100.00	374	0	0	1	374	1	374	7e-155	538
PTR-Fbxl8	HSA-Fbxl8	99.20	374	3	0	1	374	1	374	9e-153	531
PTR-Fbxl8	GGO-Fbxl8	98.40	374	6	0	1	374	8	381	2e-151	526
PTR-Fbxl8	PPY-Fbxl8	98.40	374	6	0	1	374	1	374	2e-150	523
PTR-Fbxl8	MMU-Fbxl8	95.45	374	17	0	1	374	1	374	4e-144	502
PTR-Fbxl8	CJA-Fbxl8	91.18	374	33	0	1	374	1	374	5e-140	488
PTR-Fbxl8	MUS-Fbxl8	78.55	373	80	0	1	373	1	373	1e-119	421
PTR-Fbxl8	RNO-Fbxl8	78.28	373	81	0	1	373	1	373	4e-118	416
# BLASTP 2.2.20 [Feb-08-2009]
# Query: RNO-Fbxl8
# Database: 559_protein.db
# Query id, Subject id, % identity, alignment length, mismatches, gap openings, q. start, q. end, s. start, s. end, e-value, bit score
RNO-Fbxl8	RNO-Fbxl8	100.00	374	0	0	1	374	1	374	0.0	628
RNO-Fbxl8	MUS-Fbxl8	90.91	374	34	0	1	374	1	374	1e-164	570
RNO-Fbxl8	GGO-Fbxl8	78.28	373	81	0	1	373	8	380	6e-131	458
RNO-Fbxl8	HSA-Fbxl8	78.55	373	80	0	1	373	1	373	1e-130	457
RNO-Fbxl8	PTR-Fbxl8	78.28	373	81	0	1	373	1	373	4e-130	456
RNO-Fbxl8	PPY-Fbxl8	78.28	373	81	0	1	373	1	373	2e-129	453
RNO-Fbxl8	CJA-Fbxl8	75.74	371	90	0	1	371	1	371	3e-129	453
RNO-Fbxl8	MMU-Fbxl8	77.21	373	85	0	1	373	1	373	5e-129	452
# BLASTP 2.2.20 [Feb-08-2009]
# Query: HSA-Fbxl8
# Database: 559_protein.db
# Query id, Subject id, % identity, alignment length, mismatches, gap openings, q. start, q. end, s. start, s. end, e-value, bit score
HSA-Fbxl8	HSA-Fbxl8	100.00	374	0	0	1	374	1	374	1e-154	537
HSA-Fbxl8	PTR-Fbxl8	99.20	374	3	0	1	374	1	374	8e-153	531
HSA-Fbxl8	GGO-Fbxl8	98.13	374	7	0	1	374	8	381	2e-151	526
HSA-Fbxl8	PPY-Fbxl8	98.13	374	7	0	1	374	1	374	2e-150	523
HSA-Fbxl8	MMU-Fbxl8	95.19	374	18	0	1	374	1	374	3e-144	503
HSA-Fbxl8	CJA-Fbxl8	90.91	374	34	0	1	374	1	374	6e-140	488
HSA-Fbxl8	MUS-Fbxl8	78.82	373	79	0	1	373	1	373	8e-120	421
HSA-Fbxl8	RNO-Fbxl8	78.55	373	80	0	1	373	1	373	2e-118	417
# BLASTP 2.2.20 [Feb-08-2009]
# Query: PPY-Fbxl8
# Database: 559_protein.db
# Query id, Subject id, % identity, alignment length, mismatches, gap openings, q. start, q. end, s. start, s. end, e-value, bit score
PPY-Fbxl8	PPY-Fbxl8	100.00	374	0	0	1	374	1	374	1e-140	490
PPY-Fbxl8	HSA-Fbxl8	98.13	374	7	0	1	374	1	374	3e-137	479
PPY-Fbxl8	PTR-Fbxl8	98.40	374	6	0	1	374	1	374	4e-137	479
PPY-Fbxl8	GGO-Fbxl8	97.33	374	10	0	1	374	8	381	1e-135	474
PPY-Fbxl8	MMU-Fbxl8	94.92	374	19	0	1	374	1	374	1e-129	454
PPY-Fbxl8	CJA-Fbxl8	90.64	374	35	0	1	374	1	374	2e-127	446
PPY-Fbxl8	MUS-Fbxl8	78.82	373	79	0	1	373	1	373	3e-108	383
PPY-Fbxl8	RNO-Fbxl8	78.28	373	81	0	1	373	1	373	1e-106	378
```

```
# BLASTP 2.2.20 [Feb-08-2009]
# Query: CJA-Fbxo10
# Database: 559_protein.db
# Query id, Subject id, % identity, alignment length, mismatches, gap openings, q. start, q. end, s. start, s. end, e-value, bit score
CJA-Fbxo10  CJA-Fbxo10  100.00      643         0           0           1           643         1           643         0.0         1198
CJA-Fbxo10  MMU-Fbxo10  97.05       643         19          0           1           643         1           643         0.0         1164
CJA-Fbxo10  HSA-Fbxo10  96.73       643         21          0           1           643         1           643         0.0         1158
CJA-Fbxo10  GGO-Fbxo10  96.73       643         21          0           1           643         1           643         0.0         1158
CJA-Fbxo10  MUS-Fbxo10  92.38       643         45          1           1           643         1           639         0.0         1158
CJA-Fbxo10  PTR-Fbxo10  96.58       643         22          0           1           643         1           643         0.0         1156
CJA-Fbxo10  PPY-Fbxo10  96.73       643         19          1           1           643         1           641         0.0         1155
CJA-Fbxo10  RNO-Fbxo10  91.45       643         51          1           1           643         1           639         0.0         1149
# BLASTP 2.2.20 [Feb-08-2009]
# Query: GGO-Fbxo10
# Database: 559_protein.db
# Query id, Subject id, % identity, alignment length, mismatches, gap openings, q. start, q. end, s. start, s. end, e-value, bit score
GGO-Fbxo10  GGO-Fbxo10  100.00      956         0           0           1           956         1           956         0.0         1828
GGO-Fbxo10  HSA-Fbxo10  99.90       956         1           0           1           956         1           956         0.0         1827
GGO-Fbxo10  PTR-Fbxo10  99.79       956         2           0           1           956         1           956         0.0         1825
GGO-Fbxo10  MMU-Fbxo10  99.27       956         7           0           1           956         1           956         0.0         1816
GGO-Fbxo10  PPY-Fbxo10  99.16       956         6           1           1           956         1           954         0.0         1812
GGO-Fbxo10  MUS-Fbxo10  92.47       956         66          2           1           956         1           950         0.0         1739
GGO-Fbxo10  RNO-Fbxo10  91.53       956         75          2           1           956         1           950         0.0         1726
GGO-Fbxo10  CJA-Fbxo10  96.73       643         21          0           1           643         1           643         0.0         1162
# BLASTP 2.2.20 [Feb-08-2009]
# Query: MMU-Fbxo10
# Database: 559_protein.db
# Query id, Subject id, % identity, alignment length, mismatches, gap openings, q. start, q. end, s. start, s. end, e-value, bit score
MMU-Fbxo10  MMU-Fbxo10  100.00      956         0           0           1           956         1           956         0.0         1830
MMU-Fbxo10  GGO-Fbxo10  99.27       956         7           0           1           956         1           956         0.0         1816
MMU-Fbxo10  HSA-Fbxo10  99.16       956         8           0           1           956         1           956         0.0         1816
MMU-Fbxo10  PTR-Fbxo10  99.06       956         9           0           1           956         1           956         0.0         1814
MMU-Fbxo10  PPY-Fbxo10  98.85       956         9           1           1           956         1           954         0.0         1810
MMU-Fbxo10  MUS-Fbxo10  92.57       956         65          2           1           956         1           950         0.0         1741
MMU-Fbxo10  RNO-Fbxo10  91.63       956         74          2           1           956         1           950         0.0         1729
MMU-Fbxo10  CJA-Fbxo10  97.05       643         19          0           1           643         1           643         0.0         1167
# BLASTP 2.2.20 [Feb-08-2009]
# Query: MUS-Fbxo10
# Database: 559_protein.db
# Query id, Subject id, % identity, alignment length, mismatches, gap openings, q. start, q. end, s. start, s. end, e-value, bit score
MUS-Fbxo10  MUS-Fbxo10  100.00      950         0           0           1           950         1           950         0.0         1884
MUS-Fbxo10  RNO-Fbxo10  98.32       950         16          0           1           950         1           950         0.0         1821
MUS-Fbxo10  MMU-Fbxo10  92.57       956         65          2           1           950         1           956         0.0         1748
MUS-Fbxo10  HSA-Fbxo10  92.57       956         65          2           1           950         1           956         0.0         1746
MUS-Fbxo10  GGO-Fbxo10  92.47       956         66          2           1           950         1           956         0.0         1745
```

```
MUS-Fbxo10  PTR-Fbxo10  92.47       956         66          2           1           950         1           956         0.0         1743
MUS-Fbxo10  PPY-Fbxo10  92.26       956         66          3           1           950         1           954         0.0         1738
MUS-Fbxo10  CJA-Fbxo10  93.93       643         35          1           1           639         1           643         0.0         1172
# BLASTP 2.2.20 [Feb-08-2009]
# Query: PPY-Fbxo10
# Database: 559_protein.db
# Query id, Subject id, % identity, alignment length, mismatches, gap openings, q. start, q. end, s. start, s. end, e-value, bit score
PPY-Fbxo10  PPY-Fbxo10  100.00      954         0           0           1           954         1           954         0.0         1825
PPY-Fbxo10  GGO-Fbxo10  99.16       956         6           1           1           954         1           956         0.0         1812
PPY-Fbxo10  HSA-Fbxo10  99.27       956         5           1           1           954         1           956         0.0         1811
PPY-Fbxo10  PTR-Fbxo10  99.16       956         6           1           1           954         1           956         0.0         1810
PPY-Fbxo10  MMU-Fbxo10  98.85       956         9           1           1           954         1           956         0.0         1810
PPY-Fbxo10  MUS-Fbxo10  92.26       956         66          3           1           954         1           950         0.0         1731
PPY-Fbxo10  RNO-Fbxo10  91.53       956         73          3           1           954         1           950         0.0         1723
PPY-Fbxo10  CJA-Fbxo10  96.73       643         19          1           1           641         1           643         0.0         1160
# BLASTP 2.2.20 [Feb-08-2009]
# Query: PTR-Fbxo10
# Database: 559_protein.db
# Query id, Subject id, % identity, alignment length, mismatches, gap openings, q. start, q. end, s. start, s. end, e-value, bit score
PTR-Fbxo10  PTR-Fbxo10  100.00      956         0           0           1           956         1           956         0.0         1828
PTR-Fbxo10  GGO-Fbxo10  99.79       956         2           0           1           956         1           956         0.0         1825
PTR-Fbxo10  HSA-Fbxo10  99.90       956         1           0           1           956         1           956         0.0         1825
PTR-Fbxo10  MMU-Fbxo10  99.06       956         9           0           1           956         1           956         0.0         1813
PTR-Fbxo10  PPY-Fbxo10  99.16       956         6           1           1           956         1           954         0.0         1810
PTR-Fbxo10  MUS-Fbxo10  92.47       956         66          2           1           956         1           950         0.0         1737
PTR-Fbxo10  RNO-Fbxo10  91.53       956         75          2           1           956         1           950         0.0         1724
PTR-Fbxo10  CJA-Fbxo10  96.58       643         22          0           1           643         1           643         0.0         1160
# BLASTP 2.2.20 [Feb-08-2009]
# Query: RNO-Fbxo10
# Database: 559_protein.db
# Query id, Subject id, % identity, alignment length, mismatches, gap openings, q. start, q. end, s. start, s. end, e-value, bit score
RNO-Fbxo10  RNO-Fbxo10  100.00      950         0           0           1           950         1           950         0.0         1843
RNO-Fbxo10  MUS-Fbxo10  98.32       950         16          0           1           950         1           950         0.0         1822
RNO-Fbxo10  MMU-Fbxo10  92.89       956         62          2           1           950         1           956         0.0         1731
RNO-Fbxo10  HSA-Fbxo10  92.89       956         62          2           1           950         1           956         0.0         1729
RNO-Fbxo10  GGO-Fbxo10  92.78       956         63          2           1           950         1           956         0.0         1729
RNO-Fbxo10  PTR-Fbxo10  92.78       956         63          2           1           950         1           956         0.0         1728
RNO-Fbxo10  PPY-Fbxo10  92.78       956         61          3           1           950         1           954         0.0         1725
RNO-Fbxo10  CJA-Fbxo10  93.16       643         40          1           1           639         1           643         0.0         1156
# BLASTP 2.2.20 [Feb-08-2009]
# Query: HSA-Fbxo10
# Database: 559_protein.db
# Query id, Subject id, % identity, alignment length, mismatches, gap openings, q. start, q. end, s. start, s. end, e-value, bit score
HSA-Fbxo10  HSA-Fbxo10  100.00      956         0           0           1           956         1           956         0.0         1827
HSA-Fbxo10  GGO-Fbxo10  99.90       956         1           0           1           956         1           956         0.0         1827
```

```
HSA-Fbxo10  PTR-Fbxo10  99.90      956        1          0          1          956        1          956        0.0           1825
HSA-Fbxo10  MMU-Fbxo10  99.16      956        8          0          1          956        1          956        0.0           1816
HSA-Fbxo10  PPY-Fbxo10  99.27      956        5          1          1          956        1          954        0.0           1811
HSA-Fbxo10  MUS-Fbxo10  92.57      956        65         2          1          956        1          950        0.0           1739
HSA-Fbxo10  RNO-Fbxo10  91.63      956        74         2          1          956        1          950        0.0           1726
HSA-Fbxo10  CJA-Fbxo10  96.73      643        21         0          1          643        1          643        0.0           1162
# BLASTP 2.2.20 [Feb-08-2009]
# Query: CJA-Fbxo11
# Database: 559_protein.db
# Query id, Subject id, % identity, alignment length, mismatches, gap openings, q. start, q. end, s. start, s. end, e-value, bit score
CJA-Fbxo11  GGO-Fbxo11  100.00     858        0          0          70         927        68         925        0.0           1793
CJA-Fbxo11  HSA-Fbxo11  100.00     858        0          0          70         927        70         927        0.0           1792
CJA-Fbxo11  MMU-Fbxo11  100.00     858        0          0          70         927        69         926        0.0           1792
CJA-Fbxo11  CJA-Fbxo11  100.00     858        0          0          70         927        70         927        0.0           1792
CJA-Fbxo11  MUS-Fbxo11  99.42      858        4          1          70         927        74         930        0.0           1780
CJA-Fbxo11  RNO-Fbxo11  99.88      849        1          0          79         927        1          849        0.0           1773
CJA-Fbxo11  PTR-Fbxo11  100.00     843        0          0          85         927        1          843        0.0           1759
CJA-Fbxo11  PPY-Fbxo11  100.00     843        0          0          85         927        1          843        0.0           1759
# BLASTP 2.2.20 [Feb-08-2009]
# Query: GGO-Fbxo11
# Database: 559_protein.db
# Query id, Subject id, % identity, alignment length, mismatches, gap openings, q. start, q. end, s. start, s. end, e-value, bit score
GGO-Fbxo11  GGO-Fbxo11  100.00     858        0          0          68         925        68         925        0.0           1793
GGO-Fbxo11  HSA-Fbxo11  100.00     858        0          0          68         925        70         927        0.0           1792
GGO-Fbxo11  MMU-Fbxo11  100.00     858        0          0          68         925        69         926        0.0           1792
GGO-Fbxo11  CJA-Fbxo11  100.00     858        0          0          68         925        70         927        0.0           1792
GGO-Fbxo11  MUS-Fbxo11  99.42      858        4          1          68         925        74         930        0.0           1780
GGO-Fbxo11  RNO-Fbxo11  99.88      849        1          0          77         925        1          849        0.0           1773
GGO-Fbxo11  PTR-Fbxo11  100.00     843        0          0          83         925        1          843        0.0           1759
GGO-Fbxo11  PPY-Fbxo11  100.00     843        0          0          83         925        1          843        0.0           1759
# BLASTP 2.2.20 [Feb-08-2009]
# Query: MMU-Fbxo11
# Database: 559_protein.db
# Query id, Subject id, % identity, alignment length, mismatches, gap openings, q. start, q. end, s. start, s. end, e-value, bit score
MMU-Fbxo11  GGO-Fbxo11  100.00     858        0          0          69         926        68         925        0.0           1793
MMU-Fbxo11  HSA-Fbxo11  100.00     858        0          0          69         926        70         927        0.0           1792
MMU-Fbxo11  MMU-Fbxo11  100.00     858        0          0          69         926        69         926        0.0           1792
MMU-Fbxo11  CJA-Fbxo11  100.00     858        0          0          69         926        70         927        0.0           1792
MMU-Fbxo11  MUS-Fbxo11  99.42      858        4          1          69         926        74         930        0.0           1780
MMU-Fbxo11  RNO-Fbxo11  99.88      849        1          0          78         926        1          849        0.0           1773
MMU-Fbxo11  PTR-Fbxo11  100.00     843        0          0          84         926        1          843        0.0           1759
MMU-Fbxo11  PPY-Fbxo11  100.00     843        0          0          84         926        1          843        0.0           1759
# BLASTP 2.2.20 [Feb-08-2009]
# Query: MUS-Fbxo11
# Database: 559_protein.db
```

```
# Query id, Subject id, % identity, alignment length, mismatches, gap openings, q. start, q. end, s. start, s. end, e-value, bit score
MUS-Fbxo11  MUS-Fbxo11  100.00      857         0           0           74          930         74          930         0.0         1790
MUS-Fbxo11  HSA-Fbxo11  99.42       858         4           1           74          930         70          927         0.0         1780
MUS-Fbxo11  MMU-Fbxo11  99.42       858         4           1           74          930         69          926         0.0         1780
MUS-Fbxo11  GGO-Fbxo11  99.42       858         4           1           74          930         68          925         0.0         1780
MUS-Fbxo11  CJA-Fbxo11  99.42       858         4           1           74          930         70          927         0.0         1780
MUS-Fbxo11  RNO-Fbxo11  99.88       849         1           0           82          930         1           849         0.0         1772
MUS-Fbxo11  PTR-Fbxo11  99.76       843         2           0           88          930         1           843         0.0         1758
MUS-Fbxo11  PPY-Fbxo11  99.76       843         2           0           88          930         1           843         0.0         1758
# BLASTP 2.2.20 [Feb-08-2009]
# Query: PPY-Fbxo11
# Database: 559_protein.db
# Query id, Subject id, % identity, alignment length, mismatches, gap openings, q. start, q. end, s. start, s. end, e-value, bit score
PPY-Fbxo11  HSA-Fbxo11  100.00      843         0           0           1           843         85          927         0.0         1763
PPY-Fbxo11  CJA-Fbxo11  100.00      843         0           0           1           843         85          927         0.0         1763
PPY-Fbxo11  MMU-Fbxo11  100.00      843         0           0           1           843         84          926         0.0         1763
PPY-Fbxo11  GGO-Fbxo11  100.00      843         0           0           1           843         83          925         0.0         1763
PPY-Fbxo11  RNO-Fbxo11  99.88       843         1           0           1           843         7           849         0.0         1760
PPY-Fbxo11  PTR-Fbxo11  100.00      843         0           0           1           843         1           843         0.0         1759
PPY-Fbxo11  PPY-Fbxo11  100.00      843         0           0           1           843         1           843         0.0         1759
PPY-Fbxo11  MUS-Fbxo11  99.76       843         2           0           1           843         88          930         0.0         1759
# BLASTP 2.2.20 [Feb-08-2009]
# Query: PTR-Fbxo11
# Database: 559_protein.db
# Query id, Subject id, % identity, alignment length, mismatches, gap openings, q. start, q. end, s. start, s. end, e-value, bit score
PTR-Fbxo11  HSA-Fbxo11  100.00      843         0           0           1           843         85          927         0.0         1763
PTR-Fbxo11  CJA-Fbxo11  100.00      843         0           0           1           843         85          927         0.0         1763
PTR-Fbxo11  MMU-Fbxo11  100.00      843         0           0           1           843         84          926         0.0         1763
PTR-Fbxo11  GGO-Fbxo11  100.00      843         0           0           1           843         83          925         0.0         1763
PTR-Fbxo11  RNO-Fbxo11  99.88       843         1           0           1           843         7           849         0.0         1760
PTR-Fbxo11  PTR-Fbxo11  100.00      843         0           0           1           843         1           843         0.0         1759
PTR-Fbxo11  PPY-Fbxo11  100.00      843         0           0           1           843         1           843         0.0         1759
PTR-Fbxo11  MUS-Fbxo11  99.76       843         2           0           1           843         88          930         0.0         1759
# BLASTP 2.2.20 [Feb-08-2009]
# Query: RNO-Fbxo11
# Database: 559_protein.db
# Query id, Subject id, % identity, alignment length, mismatches, gap openings, q. start, q. end, s. start, s. end, e-value, bit score
RNO-Fbxo11  HSA-Fbxo11  99.88       849         1           0           1           849         79          927         0.0         1774
RNO-Fbxo11  CJA-Fbxo11  99.88       849         1           0           1           849         79          927         0.0         1774
RNO-Fbxo11  RNO-Fbxo11  100.00      849         0           0           1           849         1           849         0.0         1774
RNO-Fbxo11  MMU-Fbxo11  99.88       849         1           0           1           849         78          926         0.0         1774
RNO-Fbxo11  GGO-Fbxo11  99.88       849         1           0           1           849         77          925         0.0         1773
RNO-Fbxo11  MUS-Fbxo11  99.88       849         1           0           1           849         82          930         0.0         1773
RNO-Fbxo11  PTR-Fbxo11  99.88       843         1           0           7           849         1           843         0.0         1760
RNO-Fbxo11  PPY-Fbxo11  99.88       843         1           0           7           849         1           843         0.0         1760
```

```
# BLASTP 2.2.20 [Feb-08-2009]
# Query: HSA-Fbxo11
# Database: 559_protein.db
# Query id, Subject id, % identity, alignment length, mismatches, gap openings, q. start, q. end, s. start, s. end, e-value, bit score
HSA-Fbxo11  GGO-Fbxo11  100.00      858         0           0           70          927         68          925         0.0         1793
HSA-Fbxo11  HSA-Fbxo11  100.00      858         0           0           70          927         70          927         0.0         1792
HSA-Fbxo11  MMU-Fbxo11  100.00      858         0           0           70          927         69          926         0.0         1792
HSA-Fbxo11  CJA-Fbxo11  100.00      858         0           0           70          927         70          927         0.0         1792
HSA-Fbxo11  MUS-Fbxo11  99.42       858         4           1           70          927         74          930         0.0         1780
HSA-Fbxo11  RNO-Fbxo11  99.88       849         1           0           79          927         1           849         0.0         1773
HSA-Fbxo11  PTR-Fbxo11  100.00      843         0           0           85          927         1           843         0.0         1759
HSA-Fbxo11  PPY-Fbxo11  100.00      843         0           0           85          927         1           843         0.0         1759
# BLASTP 2.2.20 [Feb-08-2009]
# Query: CJA-Fbxo15
# Database: 559_protein.db
# Query id, Subject id, % identity, alignment length, mismatches, gap openings, q. start, q. end, s. start, s. end, e-value, bit score
CJA-Fbxo15  CJA-Fbxo15  100.00      434         0           0           1           434         1           434         0.0         898
CJA-Fbxo15  MMU-Fbxo15  94.70       434         23          0           1           434         1           434         0.0         856
CJA-Fbxo15  GGO-Fbxo15  94.01       434         26          0           1           434         33          466         0.0         848
CJA-Fbxo15  PPY-Fbxo15  93.09       434         30          0           1           434         1           434         0.0         844
CJA-Fbxo15  HSA-Fbxo15  93.32       434         29          0           1           434         77          510         0.0         841
CJA-Fbxo15  PTR-Fbxo15  94.78       345         18          0           1           345         77          421         0.0         674
CJA-Fbxo15  RNO-Fbxo15  71.72       435         122         1           1           434         25          459         0.0         647
CJA-Fbxo15  MUS-Fbxo15  59.73       437         168         3           1           433         46          478         3e-155      540
# BLASTP 2.2.20 [Feb-08-2009]
# Query: GGO-Fbxo15
# Database: 559_protein.db
# Query id, Subject id, % identity, alignment length, mismatches, gap openings, q. start, q. end, s. start, s. end, e-value, bit score
GGO-Fbxo15  GGO-Fbxo15  100.00      466         0           0           1           466         1           466         0.0         964
GGO-Fbxo15  HSA-Fbxo15  98.93       466         5           0           1           466         45          510         0.0         954
GGO-Fbxo15  PPY-Fbxo15  97.24       434         12          0           33          466         1           434         0.0         877
GGO-Fbxo15  MMU-Fbxo15  95.85       434         18          0           33          466         1           434         0.0         868
GGO-Fbxo15  CJA-Fbxo15  94.01       434         26          0           33          466         1           434         0.0         848
GGO-Fbxo15  PTR-Fbxo15  99.47       377         2           0           1           377         45          421         0.0         775
GGO-Fbxo15  RNO-Fbxo15  70.33       455         134         1           13          466         5           459         0.0         657
GGO-Fbxo15  MUS-Fbxo15  57.32       471         191         3           1           465         12          478         1e-155      541
# BLASTP 2.2.20 [Feb-08-2009]
# Query: MMU-Fbxo15
# Database: 559_protein.db
# Query id, Subject id, % identity, alignment length, mismatches, gap openings, q. start, q. end, s. start, s. end, e-value, bit score
MMU-Fbxo15  MMU-Fbxo15  100.00      434         0           0           1           434         1           434         0.0         897
MMU-Fbxo15  GGO-Fbxo15  95.85       434         18          0           1           434         33          466         0.0         868
MMU-Fbxo15  PPY-Fbxo15  94.93       434         22          0           1           434         1           434         0.0         863
MMU-Fbxo15  HSA-Fbxo15  94.93       434         22          0           1           434         77          510         0.0         860
MMU-Fbxo15  CJA-Fbxo15  94.70       434         23          0           1           434         1           434         0.0         856
```

```
MMU-Fbxo15  PTR-Fbxo15  96.23    345     13      0       1       345     77      421     0.0     688
MMU-Fbxo15  RNO-Fbxo15  71.95    435     121     1       1       434     25      459     0.0     644
MMU-Fbxo15  MUS-Fbxo15  59.73    437     168     2       1       433     46      478     2e-155  540
# BLASTP 2.2.20 [Feb-08-2009]
# Query: MUS-Fbxo15
# Database: 559_protein.db
# Query id, Subject id, % identity, alignment length, mismatches, gap openings, q. start, q. end, s. start, s. end, e-value, bit score
MUS-Fbxo15  MUS-Fbxo15  100.00   478     0       0       1       478     1       478     0.0     981
MUS-Fbxo15  RNO-Fbxo15  62.12    462     164     4       22      478     3       458     1e-161  561
MUS-Fbxo15  GGO-Fbxo15  57.32    471     191     3       12      478     1       465     1e-155  541
MUS-Fbxo15  MMU-Fbxo15  59.73    437     168     2       46      478     1       433     2e-155  540
MUS-Fbxo15  CJA-Fbxo15  59.73    437     168     3       46      478     1       433     3e-155  540
MUS-Fbxo15  HSA-Fbxo15  56.90    471     193     3       12      478     45      509     3e-154  536
MUS-Fbxo15  PPY-Fbxo15  58.35    437     174     2       46      478     1       433     1e-152  531
MUS-Fbxo15  PTR-Fbxo15  59.53    383     149     2       12      394     45      421     4e-130  456
# BLASTP 2.2.20 [Feb-08-2009]
# Query: PPY-Fbxo15
# Database: 559_protein.db
# Query id, Subject id, % identity, alignment length, mismatches, gap openings, q. start, q. end, s. start, s. end, e-value, bit score
PPY-Fbxo15  PPY-Fbxo15  100.00   434     0       0       1       434     1       434     0.0     898
PPY-Fbxo15  HSA-Fbxo15  97.00    434     13      0       1       434     77      510     0.0     877
PPY-Fbxo15  GGO-Fbxo15  97.24    434     12      0       1       434     33      466     0.0     877
PPY-Fbxo15  MMU-Fbxo15  94.93    434     22      0       1       434     1       434     0.0     863
PPY-Fbxo15  CJA-Fbxo15  93.09    434     30      0       1       434     1       434     0.0     844
PPY-Fbxo15  PTR-Fbxo15  97.39    345     9       0       1       345     77      421     0.0     697
PPY-Fbxo15  RNO-Fbxo15  71.26    435     124     1       1       434     25      459     0.0     642
PPY-Fbxo15  MUS-Fbxo15  58.35    437     174     2       1       433     46      478     9e-153  531
# BLASTP 2.2.20 [Feb-08-2009]
# Query: PTR-Fbxo15
# Database: 559_protein.db
# Query id, Subject id, % identity, alignment length, mismatches, gap openings, q. start, q. end, s. start, s. end, e-value, bit score
PTR-Fbxo15  PTR-Fbxo15  100.00   421     0       0       1       421     1       421     0.0     840
PTR-Fbxo15  HSA-Fbxo15  99.05    421     4       0       1       421     1       421     0.0     834
PTR-Fbxo15  GGO-Fbxo15  99.47    377     2       0       45      421     1       377     0.0     774
PTR-Fbxo15  PPY-Fbxo15  97.39    345     9       0       77      421     1       345     0.0     697
PTR-Fbxo15  MMU-Fbxo15  96.23    345     13      0       77      421     1       345     0.0     688
PTR-Fbxo15  CJA-Fbxo15  94.78    345     18      0       77      421     1       345     0.0     674
PTR-Fbxo15  RNO-Fbxo15  68.58    366     114     1       57      421     5       370     1e-146  511
PTR-Fbxo15  MUS-Fbxo15  59.53    383     149     2       45      421     12      394     4e-130  456
# BLASTP 2.2.20 [Feb-08-2009]
# Query: RNO-Fbxo15
# Database: 559_protein.db
# Query id, Subject id, % identity, alignment length, mismatches, gap openings, q. start, q. end, s. start, s. end, e-value, bit score
RNO-Fbxo15  RNO-Fbxo15  100.00   459     0       0       1       459     1       459     0.0     923
RNO-Fbxo15  GGO-Fbxo15  69.45    455     138     1       5       459     13      466     0.0     639
```

RNO-Fbxo15	HSA-Fbxo15	69.23	455	139	1	5	459	57	510	0.0	635
RNO-Fbxo15	CJA-Fbxo15	70.80	435	126	1	25	459	1	434	0.0	629
RNO-Fbxo15	MMU-Fbxo15	71.03	435	125	1	25	459	1	434	0.0	627
RNO-Fbxo15	PPY-Fbxo15	70.34	435	128	1	25	459	1	434	0.0	625
RNO-Fbxo15	MUS-Fbxo15	61.61	461	168	3	3	458	22	478	2e-158	550
RNO-Fbxo15	PTR-Fbxo15	67.49	366	118	1	5	370	57	421	4e-141	493

BLASTP 2.2.20 [Feb-08-2009]
Query: HSA-Fbxo15
Database: 559_protein.db
Query id, Subject id, % identity, alignment length, mismatches, gap openings, q. start, q. end, s. start, s. end, e-value, bit score

HSA-Fbxo15	HSA-Fbxo15	100.00	510	0	0	1	510	1	510	0.0	1026
HSA-Fbxo15	GGO-Fbxo15	98.93	466	5	0	45	510	1	466	0.0	954
HSA-Fbxo15	PPY-Fbxo15	97.00	434	13	0	77	510	1	434	0.0	877
HSA-Fbxo15	MMU-Fbxo15	94.93	434	22	0	77	510	1	434	0.0	860
HSA-Fbxo15	CJA-Fbxo15	93.32	434	29	0	77	510	1	434	0.0	841
HSA-Fbxo15	PTR-Fbxo15	99.05	421	4	0	1	421	1	421	0.0	834
HSA-Fbxo15	RNO-Fbxo15	70.11	455	135	1	57	510	5	459	0.0	652
HSA-Fbxo15	MUS-Fbxo15	56.90	471	193	3	45	509	12	478	3e-154	536

BLASTP 2.2.20 [Feb-08-2009]
Query: CJA-Fbxo16
Database: 559_protein.db
Query id, Subject id, % identity, alignment length, mismatches, gap openings, q. start, q. end, s. start, s. end, e-value, bit score

CJA-Fbxo16	CJA-Fbxo16	100.00	286	0	0	1	286	1	286	1e-156	543
CJA-Fbxo16	HSA-Fbxo16	83.33	282	41	1	1	276	1	282	4e-126	442
CJA-Fbxo16	PTR-Fbxo16	82.69	283	43	1	1	277	1	283	3e-125	439
CJA-Fbxo16	GGO-Fbxo16	82.33	283	44	1	1	277	1	283	6e-125	438
CJA-Fbxo16	PPY-Fbxo16	78.45	283	43	2	1	277	1	271	1e-114	404
CJA-Fbxo16	MUS-Fbxo16	72.95	292	72	2	1	286	1	291	3e-114	402
CJA-Fbxo16	MMU-Fbxo16	78.85	279	41	2	1	273	1	267	5e-114	402
CJA-Fbxo16	RNO-Fbxo16	72.60	292	73	2	1	286	1	291	6e-113	398

BLASTP 2.2.20 [Feb-08-2009]
Query: GGO-Fbxo16
Database: 559_protein.db
Query id, Subject id, % identity, alignment length, mismatches, gap openings, q. start, q. end, s. start, s. end, e-value, bit score

GGO-Fbxo16	GGO-Fbxo16	100.00	292	0	0	1	292	1	292	3e-163	565
GGO-Fbxo16	PTR-Fbxo16	98.97	292	3	0	1	292	1	292	2e-161	559
GGO-Fbxo16	HSA-Fbxo16	97.20	286	8	0	1	286	1	286	2e-153	532
GGO-Fbxo16	PPY-Fbxo16	94.18	292	5	1	1	292	1	280	1e-150	523
GGO-Fbxo16	MMU-Fbxo16	91.38	290	13	1	1	290	1	278	6e-142	494
GGO-Fbxo16	MUS-Fbxo16	86.06	287	39	1	1	287	1	286	3e-137	479
GGO-Fbxo16	RNO-Fbxo16	86.06	287	39	1	1	287	1	286	7e-137	478
GGO-Fbxo16	CJA-Fbxo16	82.33	283	44	1	1	283	1	277	5e-125	438

BLASTP 2.2.20 [Feb-08-2009]
Query: MUS-Fbxo16
Database: 559_protein.db

```
# Query id, Subject id, % identity, alignment length, mismatches, gap openings, q. start, q. end, s. start, s. end, e-value, bit score
MUS-Fbxo16  MUS-Fbxo16  100.00      322         0           0           1           322         1           322         0.0         632
MUS-Fbxo16  RNO-Fbxo16  94.72       322         17          0           1           322         1           322         2e-173      599
MUS-Fbxo16  GGO-Fbxo16  86.06       287         39          1           1           286         1           287         9e-140      488
MUS-Fbxo16  PTR-Fbxo16  85.71       287         40          1           1           286         1           287         4e-139      485
MUS-Fbxo16  HSA-Fbxo16  86.52       282         37          1           1           281         1           282         1e-138      484
MUS-Fbxo16  MMU-Fbxo16  77.54       325         58          3           1           322         1           313         3e-135      473
MUS-Fbxo16  PPY-Fbxo16  81.53       287         40          2           1           286         1           275         2e-128      449
MUS-Fbxo16  CJA-Fbxo16  72.95       292         72          2           1           291         1           286         1e-114      404
# BLASTP 2.2.20 [Feb-08-2009]
# Query: MMU-Fbxo16
# Database: 559_protein.db
# Query id, Subject id, % identity, alignment length, mismatches, gap openings, q. start, q. end, s. start, s. end, e-value, bit score
MMU-Fbxo16  MMU-Fbxo16  100.00      316         0           0           1           316         1           316         2e-171      592
MMU-Fbxo16  PPY-Fbxo16  95.68       278         12          0           1           278         1           278         2e-144      503
MMU-Fbxo16  PTR-Fbxo16  91.72       290         12          1           1           278         1           290         2e-142      496
MMU-Fbxo16  GGO-Fbxo16  91.38       290         13          1           1           278         1           290         4e-142      495
MMU-Fbxo16  HSA-Fbxo16  92.83       279         8           1           1           267         1           279         1e-140      490
MMU-Fbxo16  MUS-Fbxo16  77.13       328         60          3           1           316         1           325         3e-133      466
MMU-Fbxo16  RNO-Fbxo16  76.60       329         54          5           1           313         1           322         6e-131      458
MMU-Fbxo16  CJA-Fbxo16  78.85       279         41          2           1           267         1           273         4e-114      402
# BLASTP 2.2.20 [Feb-08-2009]
# Query: PTR-Fbxo16
# Database: 559_protein.db
# Query id, Subject id, % identity, alignment length, mismatches, gap openings, q. start, q. end, s. start, s. end, e-value, bit score
PTR-Fbxo16  PTR-Fbxo16  100.00      292         0           0           1           292         1           292         3e-163      565
PTR-Fbxo16  GGO-Fbxo16  98.97       292         3           0           1           292         1           292         1e-161      560
PTR-Fbxo16  HSA-Fbxo16  97.55       286         7           0           1           286         1           286         9e-154      534
PTR-Fbxo16  PPY-Fbxo16  94.52       292         4           1           1           292         1           280         4e-151      525
PTR-Fbxo16  MMU-Fbxo16  91.72       290         12          1           1           290         1           278         2e-142      496
PTR-Fbxo16  MUS-Fbxo16  85.71       287         40          1           1           287         1           286         1e-136      477
PTR-Fbxo16  RNO-Fbxo16  85.71       287         40          1           1           287         1           286         2e-136      476
PTR-Fbxo16  CJA-Fbxo16  82.69       283         43          1           1           283         1           277         2e-125      439
# BLASTP 2.2.20 [Feb-08-2009]
# Query: RNO-Fbxo16
# Database: 559_protein.db
# Query id, Subject id, % identity, alignment length, mismatches, gap openings, q. start, q. end, s. start, s. end, e-value, bit score
RNO-Fbxo16  RNO-Fbxo16  100.00      322         0           0           1           322         1           322         0.0         635
RNO-Fbxo16  MUS-Fbxo16  94.72       322         17          0           1           322         1           322         2e-173      600
RNO-Fbxo16  GGO-Fbxo16  86.06       287         39          1           1           286         1           287         2e-139      486
RNO-Fbxo16  PTR-Fbxo16  85.71       287         40          1           1           286         1           287         9e-139      484
RNO-Fbxo16  HSA-Fbxo16  86.17       282         38          1           1           281         1           282         3e-137      479
RNO-Fbxo16  MMU-Fbxo16  76.60       329         54          5           1           322         1           313         2e-133      466
RNO-Fbxo16  PPY-Fbxo16  81.53       287         40          2           1           286         1           275         6e-128      448
RNO-Fbxo16  CJA-Fbxo16  74.04       285         67          2           1           284         1           279         3e-113      399
```

```
# BLASTP 2.2.20 [Feb-08-2009]
# Query: HSA-Fbxo16
# Database: 559_protein.db
# Query id, Subject id, % identity, alignment length, mismatches, gap openings, q. start, q. end, s. start, s. end, e-value, bit score
HSA-Fbxo16  HSA-Fbxo16  100.00  349  0    0  1   349  1   349  0.0       687
HSA-Fbxo16  PTR-Fbxo16  97.55   286  7    0  1   286  1   286  1e-153    534
HSA-Fbxo16  GGO-Fbxo16  97.20   286  8    0  1   286  1   286  2e-153    533
HSA-Fbxo16  PPY-Fbxo16  92.66   286  9    1  1   286  1   274  1e-142    497
HSA-Fbxo16  MMU-Fbxo16  92.83   279  8    1  1   279  1   267  6e-141    491
HSA-Fbxo16  MUS-Fbxo16  86.52   282  37   1  1   282  1   281  5e-136    475
HSA-Fbxo16  RNO-Fbxo16  86.17   282  38   1  1   282  1   281  9e-135    471
HSA-Fbxo16  CJA-Fbxo16  83.33   282  41   1  1   282  1   276  3e-126    443
# BLASTP 2.2.20 [Feb-08-2009]
# Query: PPY-Fbxo16
# Database: 559_protein.db
# Query id, Subject id, % identity, alignment length, mismatches, gap openings, q. start, q. end, s. start, s. end, e-value, bit score
PPY-Fbxo16  PPY-Fbxo16  100.00  280  0    0  1   280  1   280  2e-156    542
PPY-Fbxo16  PTR-Fbxo16  94.52   292  4    1  1   280  1   292  6e-151    524
PPY-Fbxo16  GGO-Fbxo16  94.18   292  5    1  1   280  1   292  2e-150    523
PPY-Fbxo16  MMU-Fbxo16  95.68   278  12   0  1   278  1   278  2e-144    503
PPY-Fbxo16  HSA-Fbxo16  92.66   286  9    1  1   274  1   286  1e-142    497
PPY-Fbxo16  MUS-Fbxo16  81.53   287  40   2  1   275  1   286  5e-126    441
PPY-Fbxo16  RNO-Fbxo16  81.53   287  40   2  1   275  1   286  1e-125    440
PPY-Fbxo16  CJA-Fbxo16  78.45   283  43   2  1   271  1   277  8e-115    404
# BLASTP 2.2.20 [Feb-08-2009]
# Query: CJA-Fbxo17
# Database: 559_protein.db
# Query id, Subject id, % identity, alignment length, mismatches, gap openings, q. start, q. end, s. start, s. end, e-value, bit score
CJA-Fbxo17  CJA-Fbxo17  100.00  280  0    0  1   280  1   280  4e-152    528
CJA-Fbxo17  GGO-Fbxo17  87.86   280  32   1  1   280  1   278  3e-142    495
CJA-Fbxo17  HSA-Fbxo17  87.86   280  32   1  1   280  10  287  4e-142    495
CJA-Fbxo17  PPY-Fbxo17  87.50   280  33   1  1   280  7   284  1e-141    493
CJA-Fbxo17  MUS-Fbxo17  81.60   288  43   2  1   280  1   286  2e-135    473
CJA-Fbxo17  RNO-Fbxo17  78.72   235  48   1  1   235  1   233  2e-106    376
CJA-Fbxo17  HSA-Fbxo27  57.26   248  103  1  33  280  39  283  6e-084    301
CJA-Fbxo17  GGO-Fbxo27  57.26   248  103  1  33  280  39  283  6e-084    301
CJA-Fbxo17  PPY-Fbxo27  57.20   250  103  2  31  280  22  267  2e-083    300
CJA-Fbxo17  MUS-Fbxo27  57.79   244  100  1  34  277  37  277  4e-083    299
CJA-Fbxo17  CJA-Fbxo27  56.85   248  104  1  31  278  37  281  4e-083    299
CJA-Fbxo17  MMU-Fbxo27  56.72   238  100  1  43  280  3   237  2e-079    286
# BLASTP 2.2.20 [Feb-08-2009]
# Query: MUS-Fbxo17
# Database: 559_protein.db
# Query id, Subject id, % identity, alignment length, mismatches, gap openings, q. start, q. end, s. start, s. end, e-value, bit score
MUS-Fbxo17  MUS-Fbxo17  100.00  286  0    0  1   286  1   286  2e-162    562
```

MUS-Fbxo17	CJA-Fbxo17	84.38	288	35	2	1	286	1	280	2e-141	493
MUS-Fbxo17	GGO-Fbxo17	83.57	286	39	1	1	286	1	278	3e-140	489
MUS-Fbxo17	HSA-Fbxo17	83.57	286	39	1	1	286	10	287	3e-140	488
MUS-Fbxo17	PPY-Fbxo17	83.22	286	40	1	1	286	7	284	1e-139	487
MUS-Fbxo17	RNO-Fbxo17	87.55	241	22	1	1	241	1	233	3e-115	406
MUS-Fbxo17	CJA-Fbxo27	57.62	269	103	1	16	284	24	281	9e-090	321
MUS-Fbxo17	PPY-Fbxo27	58.71	264	97	2	23	286	16	267	4e-088	315
MUS-Fbxo17	HSA-Fbxo27	58.59	256	95	1	31	286	39	283	2e-086	310
MUS-Fbxo17	GGO-Fbxo27	58.59	256	95	1	31	286	39	283	2e-086	310
MUS-Fbxo17	MUS-Fbxo27	57.94	252	95	2	32	283	37	277	3e-083	299
MUS-Fbxo17	MMU-Fbxo27	58.13	246	92	1	41	286	3	237	7e-082	295

BLASTP 2.2.20 [Feb-08-2009]
Query: PPY-Fbxo17
Database: 559_protein.db
Query id, Subject id, % identity, alignment length, mismatches, gap openings, q. start, q. end, s. start, s. end, e-value, bit score

PPY-Fbxo17	PPY-Fbxo17	100.00	284	0	0	1	284	1	284	3e-167	578
PPY-Fbxo17	HSA-Fbxo17	98.59	284	4	0	1	284	4	287	1e-165	573
PPY-Fbxo17	GGO-Fbxo17	98.56	278	4	0	7	284	1	278	4e-162	561
PPY-Fbxo17	CJA-Fbxo17	92.86	280	18	1	7	284	1	280	2e-152	530
PPY-Fbxo17	MUS-Fbxo17	83.22	286	40	1	7	284	1	286	1e-139	487
PPY-Fbxo17	RNO-Fbxo17	81.12	233	44	0	7	239	1	233	3e-106	376
PPY-Fbxo17	CJA-Fbxo27	56.87	262	110	1	21	282	23	281	8e-088	315
PPY-Fbxo17	PPY-Fbxo27	57.81	256	104	2	29	284	16	267	2e-086	310
PPY-Fbxo17	MUS-Fbxo27	55.71	280	116	4	7	281	1	277	5e-085	305
PPY-Fbxo17	HSA-Fbxo27	58.06	248	101	1	37	284	39	283	6e-085	305
PPY-Fbxo17	GGO-Fbxo27	58.06	248	101	1	37	284	39	283	6e-085	305
PPY-Fbxo17	MMU-Fbxo27	57.56	238	98	1	47	284	3	237	2e-080	290
PPY-Fbxo17	MMU-Fbxo17	95.87	121	5	0	2	122	1	121	7e-063	232

BLASTP 2.2.20 [Feb-08-2009]
Query: RNO-Fbxo17
Database: 559_protein.db
Query id, Subject id, % identity, alignment length, mismatches, gap openings, q. start, q. end, s. start, s. end, e-value, bit score

RNO-Fbxo17	RNO-Fbxo17	100.00	250	0	0	1	250	1	250	9e-140	487
RNO-Fbxo17	MUS-Fbxo17	87.55	241	22	1	1	233	1	241	2e-115	406
RNO-Fbxo17	CJA-Fbxo17	81.70	235	41	1	1	233	1	235	5e-107	378
RNO-Fbxo17	PPY-Fbxo17	81.12	233	44	0	1	233	7	239	2e-106	376
RNO-Fbxo17	GGO-Fbxo17	81.55	233	43	0	1	233	1	233	3e-106	375
RNO-Fbxo17	HSA-Fbxo17	81.55	233	43	0	1	233	10	242	4e-106	375
RNO-Fbxo17	MUS-Fbxo27	61.27	204	72	3	32	233	37	235	3e-068	249
RNO-Fbxo17	CJA-Fbxo27	59.71	206	78	2	29	233	37	238	9e-068	248
RNO-Fbxo17	HSA-Fbxo27	58.82	204	79	2	31	233	39	238	2e-067	247
RNO-Fbxo17	GGO-Fbxo27	58.82	204	79	2	31	233	39	238	2e-067	247
RNO-Fbxo17	PPY-Fbxo27	58.74	206	79	3	29	233	22	222	6e-067	245
RNO-Fbxo17	MMU-Fbxo27	58.76	194	75	2	41	233	3	192	4e-063	233

BLASTP 2.2.20 [Feb-08-2009]

```
# Query: HSA-Fbxo17
# Database: 559_protein.db
# Query id, Subject id, % identity, alignment length, mismatches, gap openings, q. start, q. end, s. start, s. end, e-value, bit score
HSA-Fbxo17  HSA-Fbxo17  100.00      287         0           0           1           287         1           287         1e-157       546
HSA-Fbxo17  PPY-Fbxo17  98.59       284         4           0           4           287         1           284         2e-154       535
HSA-Fbxo17  GGO-Fbxo17  100.00      278         0           0           10          287         1           278         3e-152       528
HSA-Fbxo17  CJA-Fbxo17  93.57       280         16          1           10          287         1           280         2e-142       496
HSA-Fbxo17  MUS-Fbxo17  83.57       286         39          1           10          287         1           286         7e-134       468
HSA-Fbxo17  RNO-Fbxo17  81.55       233         43          0           10          242         1           233         1e-105       374
HSA-Fbxo17  HSA-Fbxo27  58.06       248         101         1           40          287         39          283         5e-085       305
HSA-Fbxo17  GGO-Fbxo27  58.06       248         101         1           40          287         39          283         5e-085       305
HSA-Fbxo17  PPY-Fbxo27  58.00       250         101         2           38          287         22          267         2e-084       304
HSA-Fbxo17  CJA-Fbxo27  57.66       248         102         1           38          285         37          281         3e-084       303
HSA-Fbxo17  MUS-Fbxo27  58.61       244         98          2           41          284         37          277         3e-083       300
HSA-Fbxo17  MMU-Fbxo27  57.56       238         98          1           50          287         3           237         2e-080       290
HSA-Fbxo17  MMU-Fbxo17  97.52       121         3           0           5           125         1           121         4e-053       199
# BLASTP 2.2.20 [Feb-08-2009]
# Query: GGO-Fbxo17
# Database: 559_protein.db
# Query id, Subject id, % identity, alignment length, mismatches, gap openings, q. start, q. end, s. start, s. end, e-value, bit score
GGO-Fbxo17  GGO-Fbxo17  100.00      278         0           0           1           278         1           278         3e-152       529
GGO-Fbxo17  HSA-Fbxo17  100.00      278         0           0           1           278         10          287         4e-152       528
GGO-Fbxo17  PPY-Fbxo17  98.56       278         4           0           1           278         7           284         8e-151       524
GGO-Fbxo17  CJA-Fbxo17  93.57       280         16          1           1           278         1           280         2e-142       496
GGO-Fbxo17  MUS-Fbxo17  83.57       286         39          1           1           278         1           286         6e-134       468
GGO-Fbxo17  RNO-Fbxo17  81.55       233         43          0           1           233         1           233         8e-106       374
GGO-Fbxo17  HSA-Fbxo27  58.06       248         101         1           31          278         39          283         5e-085       305
GGO-Fbxo17  GGO-Fbxo27  58.06       248         101         1           31          278         39          283         5e-085       305
GGO-Fbxo17  PPY-Fbxo27  58.00       250         101         2           29          278         22          267         1e-084       304
GGO-Fbxo17  CJA-Fbxo27  57.66       248         102         1           29          276         37          281         4e-084       302
GGO-Fbxo17  MUS-Fbxo27  58.61       244         98          2           32          275         37          277         2e-083       300
GGO-Fbxo17  MMU-Fbxo27  57.56       238         98          1           41          278         3           237         2e-080       290
GGO-Fbxo17  MMU-Fbxo17  98.28       116         2           0           1           116         6           121         2e-051       194
# BLASTP 2.2.20 [Feb-08-2009]
# Query: CJA-Fbxo18
# Database: 559_protein.db
# Query id, Subject id, % identity, alignment length, mismatches, gap openings, q. start, q. end, s. start, s. end, e-value, bit score
CJA-Fbxo18  CJA-Fbxo18  100.00      1045        0           0           1           1045        1           1045        0.0         2183
CJA-Fbxo18  HSA-Fbxo18  97.13       1045        28          1           1           1045        52          1094        0.0         2113
CJA-Fbxo18  PTR-Fbxo18  97.29       1034        28          0           1           1034        52          1085        0.0         2100
CJA-Fbxo18  GGO-Fbxo18  93.73       1052        59          3           1           1045        52          1103        0.0         2033
CJA-Fbxo18  PPY-Fbxo18  97.17       989         28          0           1           989         52          1040        0.0         2003
CJA-Fbxo18  MUS-Fbxo18  90.34       1046        96          2           1           1045        1           1042        0.0         1944
CJA-Fbxo18  RNO-Fbxo18  88.91       1046        110         3           1           1045        1           1041        0.0         1910
CJA-Fbxo18  MMU-Fbxo18  93.48       598         35          1           1           598         50          643         0.0         1157
```

```
# BLASTP 2.2.20 [Feb-08-2009]
# Query: GGO-Fbxo18
# Database: 559_protein.db
# Query id, Subject id, % identity, alignment length, mismatches, gap openings, q. start, q. end, s. start, s. end, e-value, bit score
GGO-Fbxo18  GGO-Fbxo18  100.00      1103        0           0           1           1103        1           1103        0.0         2226
GGO-Fbxo18  HSA-Fbxo18  94.53       1097        51          4           7           1103        7           1094        0.0         2082
GGO-Fbxo18  PTR-Fbxo18  95.67       1086        40          3           7           1092        7           1085        0.0         2064
GGO-Fbxo18  CJA-Fbxo18  93.73       1052        59          3           52          1103        1           1045        0.0         1964
GGO-Fbxo18  PPY-Fbxo18  94.24       1041        53          3           7           1047        7           1040        0.0         1947
GGO-Fbxo18  MUS-Fbxo18  86.23       1053        133         5           52          1103        1           1042        0.0         1785
GGO-Fbxo18  RNO-Fbxo18  84.81       1053        147         6           52          1103        1           1041        0.0         1754
GGO-Fbxo18  MMU-Fbxo18  94.76       649         28          3           1           649         1           643         0.0         1191
# BLASTP 2.2.20 [Feb-08-2009]
# Query: MMU-Fbxo18
# Database: 559_protein.db
# Query id, Subject id, % identity, alignment length, mismatches, gap openings, q. start, q. end, s. start, s. end, e-value, bit score
MMU-Fbxo18  MMU-Fbxo18  100.00      643         0           0           1           643         1           643         0.0         1301
MMU-Fbxo18  GGO-Fbxo18  94.76       649         28          3           1           643         1           649         0.0         1222
MMU-Fbxo18  PTR-Fbxo18  94.25       643         31          3           7           643         7           649         0.0         1203
MMU-Fbxo18  PPY-Fbxo18  94.09       643         32          3           7           643         7           649         0.0         1201
MMU-Fbxo18  HSA-Fbxo18  93.78       643         32          4           7           643         7           647         0.0         1195
MMU-Fbxo18  CJA-Fbxo18  93.48       598         35          1           50          643         1           598         0.0         1117
MMU-Fbxo18  MUS-Fbxo18  83.64       599         89          3           50          643         1           595         0.0          970
MMU-Fbxo18  RNO-Fbxo18  83.06       602         86          5           50          643         1           594         0.0          962
# BLASTP 2.2.20 [Feb-08-2009]
# Query: MUS-Fbxo18
# Database: 559_protein.db
# Query id, Subject id, % identity, alignment length, mismatches, gap openings, q. start, q. end, s. start, s. end, e-value, bit score
MUS-Fbxo18  MUS-Fbxo18  100.00      1042        0           0           1           1042        1           1042        0.0         2176
MUS-Fbxo18  RNO-Fbxo18  95.49       1042        46          1           1           1042        1           1041        0.0         2053
MUS-Fbxo18  CJA-Fbxo18  90.34       1046        96          2           1           1042        1           1045        0.0         1964
MUS-Fbxo18  HSA-Fbxo18  89.87       1046        99          3           1           1042        52          1094        0.0         1943
MUS-Fbxo18  PTR-Fbxo18  90.05       1035        98          2           1           1031        52          1085        0.0         1930
MUS-Fbxo18  GGO-Fbxo18  86.42       1053        131         5           1           1042        52          1103        0.0         1858
MUS-Fbxo18  PPY-Fbxo18  90.00       990         94          2           1           986         52          1040        0.0         1842
MUS-Fbxo18  MMU-Fbxo18  83.97       599         87          3           1           595         50          643         0.0         1020
# BLASTP 2.2.20 [Feb-08-2009]
# Query: PPY-Fbxo18
# Database: 559_protein.db
# Query id, Subject id, % identity, alignment length, mismatches, gap openings, q. start, q. end, s. start, s. end, e-value, bit score
PPY-Fbxo18  PPY-Fbxo18  100.00      1040        0           0           1           1040        1           1040        0.0         2076
PPY-Fbxo18  PTR-Fbxo18  97.79       1040        23          0           1           1040        1           1040        0.0         2025
PPY-Fbxo18  HSA-Fbxo18  97.40       1040        25          1           1           1040        1           1038        0.0         2014
PPY-Fbxo18  GGO-Fbxo18  94.24       1041        53          3           7           1040        7           1047        0.0         1928
PPY-Fbxo18  CJA-Fbxo18  97.17       989         28          0           52          1040        1           989         0.0         1912
```

```
PPY-Fbxo18  MUS-Fbxo18  89.80   990   96   2  52  1040   1   986  0.0  1743
PPY-Fbxo18  RNO-Fbxo18  88.28   990   110  3  52  1040   1   985  0.0  1706
PPY-Fbxo18  MMU-Fbxo18  94.09   643   32   3  7   649    7   643  0.0  1154
# BLASTP 2.2.20 [Feb-08-2009]
# Query: PTR-Fbxo18
# Database: 559_protein.db
# Query id, Subject id, % identity, alignment length, mismatches, gap openings, q. start, q. end, s. start, s. end, e-value, bit score
PTR-Fbxo18  PTR-Fbxo18  100.00  1085  0    0  1   1085   1   1085  0.0  2189
PTR-Fbxo18  HSA-Fbxo18  98.16   1085  18   1  1   1085   1   1083  0.0  2164
PTR-Fbxo18  GGO-Fbxo18  95.67   1086  40   3  7   1085   7   1092  0.0  2064
PTR-Fbxo18  PPY-Fbxo18  97.79   1040  23   0  1   1040   1   1040  0.0  2045
PTR-Fbxo18  CJA-Fbxo18  97.29   1034  28   0  52  1085   1   1034  0.0  2031
PTR-Fbxo18  MUS-Fbxo18  89.86   1035  100  2  52  1085   1   1031  0.0  1856
PTR-Fbxo18  RNO-Fbxo18  88.31   1035  115  3  52  1085   1   1030  0.0  1823
PTR-Fbxo18  MMU-Fbxo18  94.25   643   31   3  7   649    7   643   0.0  1172
# BLASTP 2.2.20 [Feb-08-2009]
# Query: RNO-Fbxo18
# Database: 559_protein.db
# Query id, Subject id, % identity, alignment length, mismatches, gap openings, q. start, q. end, s. start, s. end, e-value, bit score
RNO-Fbxo18  RNO-Fbxo18  100.00  1041  0    0  1   1041   1   1041  0.0  2172
RNO-Fbxo18  MUS-Fbxo18  95.49   1042  46   1  1   1041   1   1042  0.0  2053
RNO-Fbxo18  CJA-Fbxo18  88.91   1046  110  3  1   1041   1   1045  0.0  1929
RNO-Fbxo18  HSA-Fbxo18  88.72   1046  110  4  1   1041   52  1094  0.0  1915
RNO-Fbxo18  PTR-Fbxo18  88.60   1035  112  3  1   1030   52  1085  0.0  1894
RNO-Fbxo18  GGO-Fbxo18  85.09   1053  144  6  1   1041   52  1103  0.0  1823
RNO-Fbxo18  PPY-Fbxo18  88.48   990   108  3  1   985    52  1040  0.0  1806
RNO-Fbxo18  MMU-Fbxo18  83.39   602   84   5  1   594    50  643   0.0  1012
# BLASTP 2.2.20 [Feb-08-2009]
# Query: HSA-Fbxo18
# Database: 559_protein.db
# Query id, Subject id, % identity, alignment length, mismatches, gap openings, q. start, q. end, s. start, s. end, e-value, bit score
HSA-Fbxo18  HSA-Fbxo18  100.00  1094  0    0  1   1094   1   1094  0.0  2211
HSA-Fbxo18  PTR-Fbxo18  98.25   1085  17   1  1   1083   1   1085  0.0  2167
HSA-Fbxo18  GGO-Fbxo18  94.62   1097  50   4  7   1094   7   1103  0.0  2086
HSA-Fbxo18  CJA-Fbxo18  96.65   1045  33   1  52  1094   1   1045  0.0  2057
HSA-Fbxo18  PPY-Fbxo18  96.73   1040  32   1  1   1038   1   1040  0.0  2044
HSA-Fbxo18  MUS-Fbxo18  89.67   1046  101  3  52  1094   1   1042  0.0  1877
HSA-Fbxo18  RNO-Fbxo18  87.69   1048  117  4  52  1094   1   1041  0.0  1847
HSA-Fbxo18  MMU-Fbxo18  93.62   643   33   4  7   647    7   643   0.0  1177
# BLASTP 2.2.20 [Feb-08-2009]
# Query: CJA-Fbxo2
# Database: 559_protein.db
# Query id, Subject id, % identity, alignment length, mismatches, gap openings, q. start, q. end, s. start, s. end, e-value, bit score
CJA-Fbxo2   CJA-Fbxo2   100.00  300   0    0  1   300    1   300  8e-137  478
CJA-Fbxo2   GGO-Fbxo2   97.38   229   6    0  72  300    70  298  2e-131  459
```

```
CJA-Fbxo2   PPY-Fbxo2   96.90    226    7     0    72    297    70    295    7e-129    451
CJA-Fbxo2   HSA-Fbxo2   96.46    226    8     0    72    297    71    296    1e-128    450
CJA-Fbxo2   PTR-Fbxo2   96.46    226    8     0    72    297    70    295    2e-128    450
CJA-Fbxo2   RNO-Fbxo2   91.59    226    16    1    72    297    74    296    1e-121    427
CJA-Fbxo2   MUS-Fbxo2   89.82    226    20    1    72    297    75    297    2e-118    416
CJA-Fbxo2   PPY-Fbxo6   51.77    226    106   3    72    297    37    259    2e-065    240
CJA-Fbxo2   GGO-Fbxo6   51.77    226    106   3    72    297    37    259    2e-065    240
CJA-Fbxo2   PTR-Fbxo6   51.33    226    107   3    72    297    37    259    5e-065    239
CJA-Fbxo2   HSA-Fbxo6   51.33    226    107   3    72    297    37    259    8e-065    238
CJA-Fbxo2   MMU-Fbxo6   52.25    222    103   3    72    293    37    255    8e-065    238
CJA-Fbxo2   RNO-Fbxo6   52.25    222    103   3    72    293    28    246    2e-063    234
CJA-Fbxo2   MUS-Fbxo6   52.68    224    103   3    72    295    28    248    3e-063    233
CJA-Fbxo2   RNO-Fbxo44  51.09    229    109   3    72    300    46    271    2e-062    231
CJA-Fbxo2   GGO-Fbxo44  50.22    229    111   3    72    300    40    265    4e-062    229
CJA-Fbxo2   MMU-Fbxo44  50.22    229    111   3    72    300    30    255    4e-062    229
# BLASTP 2.2.20 [Feb-08-2009]
# Query: GGO-Fbxo2
# Database: 559_protein.db
# Query id, Subject id, % identity, alignment length, mismatches, gap openings, q. start, q. end, s. start, s. end, e-value, bit score
GGO-Fbxo2   GGO-Fbxo2   100.00   298    0     0    1     298    1     298    2e-127    446
GGO-Fbxo2   CJA-Fbxo2   97.38    229    6     0    70    298    72    300    7e-124    434
GGO-Fbxo2   PPY-Fbxo2   97.29    295    8     0    1     295    1     295    1e-122    430
GGO-Fbxo2   HSA-Fbxo2   98.23    226    4     0    70    295    71    296    2e-122    429
GGO-Fbxo2   PTR-Fbxo2   97.97    295    6     0    1     295    1     295    3e-122    429
GGO-Fbxo2   RNO-Fbxo2   91.59    226    16    1    70    295    74    296    2e-115    406
GGO-Fbxo2   MUS-Fbxo2   90.27    226    19    1    70    295    75    297    8e-113    398
GGO-Fbxo2   GGO-Fbxo6   51.32    228    108   3    70    297    37    261    8e-063    232
GGO-Fbxo2   PPY-Fbxo6   51.32    228    108   3    70    297    37    261    1e-062    231
GGO-Fbxo2   HSA-Fbxo6   50.88    228    109   3    70    297    37    261    2e-062    231
GGO-Fbxo2   PTR-Fbxo6   50.88    228    109   3    70    297    37    261    4e-062    229
GGO-Fbxo2   MMU-Fbxo6   52.25    222    103   3    70    291    37    255    1e-061    228
GGO-Fbxo2   RNO-Fbxo44  51.97    229    107   3    70    298    46    271    2e-061    227
GGO-Fbxo2   MMU-Fbxo44  51.09    229    109   3    70    298    30    255    5e-061    226
GGO-Fbxo2   GGO-Fbxo44  51.09    229    109   3    70    298    40    265    5e-061    226
GGO-Fbxo2   MUS-Fbxo6   52.44    225    104   3    70    294    28    249    1e-060    224
GGO-Fbxo2   RNO-Fbxo6   52.25    222    103   3    70    291    28    246    3e-060    223
GGO-Fbxo2   PTR-Fbxo44  50.66    229    110   3    70    298    30    255    5e-060    222
# BLASTP 2.2.20 [Feb-08-2009]
# Query: MUS-Fbxo2
# Database: 559_protein.db
# Query id, Subject id, % identity, alignment length, mismatches, gap openings, q. start, q. end, s. start, s. end, e-value, bit score
MUS-Fbxo2   MUS-Fbxo2   100.00   297    0     0    1     297    1     297    2e-138    483
MUS-Fbxo2   RNO-Fbxo2   96.51    229    8     0    69    297    68    296    8e-130    454
MUS-Fbxo2   PPY-Fbxo2   91.38    232    17    1    69    297    64    295    2e-124    436
MUS-Fbxo2   HSA-Fbxo2   90.95    232    18    1    69    297    65    296    4e-124    435
```

MUS-Fbxo2	PTR-Fbxo2	90.95	232	18	1	69	297	64	295	5e-124	435
MUS-Fbxo2	GGO-Fbxo2	90.09	232	20	1	69	297	64	295	3e-122	429
MUS-Fbxo2	CJA-Fbxo2	89.66	232	21	1	69	297	66	297	2e-121	426
MUS-Fbxo2	GGO-Fbxo6	50.87	230	109	3	70	297	32	259	2e-065	241
MUS-Fbxo2	PTR-Fbxo6	50.87	230	109	3	70	297	32	259	2e-065	240
MUS-Fbxo2	PPY-Fbxo6	50.87	230	109	3	70	297	32	259	5e-065	239
MUS-Fbxo2	RNO-Fbxo6	51.75	228	106	3	70	295	23	248	6e-065	239
MUS-Fbxo2	HSA-Fbxo6	50.43	230	110	3	70	297	32	259	8e-065	238
MUS-Fbxo2	MMU-Fbxo6	50.88	228	108	3	70	295	32	257	3e-064	236
MUS-Fbxo2	MUS-Fbxo6	50.88	228	108	3	70	295	23	248	2e-063	233
MUS-Fbxo2	RNO-Fbxo44	50.87	230	109	3	70	297	41	268	2e-062	231
MUS-Fbxo2	GGO-Fbxo44	50.00	230	111	3	70	297	35	262	5e-062	229
MUS-Fbxo2	MMU-Fbxo44	50.00	230	111	3	70	297	25	252	6e-062	229

BLASTP 2.2.20 [Feb-08-2009]
Query: PPY-Fbxo2
Database: 559_protein.db
Query id, Subject id, % identity, alignment length, mismatches, gap openings, q. start, q. end, s. start, s. end, e-value, bit score

PPY-Fbxo2	PPY-Fbxo2	100.00	295	0	0	1	295	1	295	4e-137	478
PPY-Fbxo2	PTR-Fbxo2	97.97	295	6	0	1	295	1	295	3e-135	473
PPY-Fbxo2	HSA-Fbxo2	96.62	296	9	1	1	295	1	296	5e-134	468
PPY-Fbxo2	GGO-Fbxo2	97.29	295	8	0	1	295	1	295	1e-131	460
PPY-Fbxo2	CJA-Fbxo2	89.23	297	30	1	1	295	1	297	1e-129	454
PPY-Fbxo2	RNO-Fbxo2	93.36	226	12	1	70	295	74	296	2e-124	436
PPY-Fbxo2	MUS-Fbxo2	92.04	226	15	1	70	295	75	297	7e-122	428
PPY-Fbxo2	GGO-Fbxo6	52.65	226	104	3	70	295	37	259	4e-066	243
PPY-Fbxo2	PPY-Fbxo6	52.65	226	104	3	70	295	37	259	5e-066	242
PPY-Fbxo2	PTR-Fbxo6	52.21	226	105	3	70	295	37	259	1e-065	241
PPY-Fbxo2	HSA-Fbxo6	52.21	226	105	3	70	295	37	259	2e-065	240
PPY-Fbxo2	MMU-Fbxo6	52.68	224	103	3	70	293	37	257	4e-065	239
PPY-Fbxo2	RNO-Fbxo6	52.68	224	103	3	70	293	28	248	2e-064	237
PPY-Fbxo2	MUS-Fbxo6	53.13	224	102	3	70	293	28	248	4e-064	236
PPY-Fbxo2	RNO-Fbxo44	50.88	226	108	3	70	295	46	268	9e-062	228
PPY-Fbxo2	GGO-Fbxo44	50.00	226	110	3	70	295	40	262	3e-061	227
PPY-Fbxo2	MMU-Fbxo44	50.00	226	110	3	70	295	30	252	3e-061	226

BLASTP 2.2.20 [Feb-08-2009]
Query: PTR-Fbxo2
Database: 559_protein.db
Query id, Subject id, % identity, alignment length, mismatches, gap openings, q. start, q. end, s. start, s. end, e-value, bit score

PTR-Fbxo2	PTR-Fbxo2	100.00	295	0	0	1	295	1	295	1e-125	441
PTR-Fbxo2	HSA-Fbxo2	100.00	226	0	0	70	295	71	296	3e-125	439
PTR-Fbxo2	PPY-Fbxo2	97.97	295	6	0	1	295	1	295	8e-125	437
PTR-Fbxo2	GGO-Fbxo2	97.97	295	6	0	1	295	1	295	2e-122	430
PTR-Fbxo2	CJA-Fbxo2	96.46	226	8	0	70	295	72	297	4e-121	425
PTR-Fbxo2	RNO-Fbxo2	92.48	226	14	1	70	295	74	296	3e-117	413
PTR-Fbxo2	MUS-Fbxo2	91.15	226	17	1	70	295	75	297	1e-114	404

PTR-Fbxo2	GGO-Fbxo6	52.21	226	105	3	70	295	37	259	6e-063	232
PTR-Fbxo2	PPY-Fbxo6	52.21	226	105	3	70	295	37	259	1e-062	231
PTR-Fbxo2	HSA-Fbxo6	51.77	226	106	3	70	295	37	259	2e-062	231
PTR-Fbxo2	PTR-Fbxo6	51.77	226	106	3	70	295	37	259	4e-062	229
PTR-Fbxo2	MMU-Fbxo6	52.23	224	104	3	70	293	37	257	1e-061	228
PTR-Fbxo2	MUS-Fbxo6	52.68	224	103	3	70	293	28	248	1e-060	225
PTR-Fbxo2	RNO-Fbxo6	52.23	224	104	3	70	293	28	248	2e-060	224
PTR-Fbxo2	RNO-Fbxo44	51.33	226	107	3	70	295	46	268	3e-059	219
PTR-Fbxo2	GGO-Fbxo44	50.44	226	109	3	70	295	40	262	1e-058	218
PTR-Fbxo2	MMU-Fbxo44	50.44	226	109	3	70	295	30	252	1e-058	218
PTR-Fbxo2	PTR-Fbxo44	50.00	226	110	3	70	295	30	252	1e-057	214

BLASTP 2.2.20 [Feb-08-2009]
Query: RNO-Fbxo2
Database: 559_protein.db
Query id, Subject id, % identity, alignment length, mismatches, gap openings, q. start, q. end, s. start, s. end, e-value, bit score

RNO-Fbxo2	RNO-Fbxo2	100.00	296	0	0	1	296	1	296	6e-138	481
RNO-Fbxo2	MUS-Fbxo2	96.51	229	8	0	68	296	69	297	5e-130	455
RNO-Fbxo2	PPY-Fbxo2	92.67	232	14	1	68	296	64	295	5e-127	445
RNO-Fbxo2	HSA-Fbxo2	92.24	232	15	1	68	296	65	296	1e-126	444
RNO-Fbxo2	PTR-Fbxo2	92.24	232	15	1	68	296	64	295	1e-126	444
RNO-Fbxo2	GGO-Fbxo2	91.38	232	17	1	68	296	64	295	7e-125	438
RNO-Fbxo2	CJA-Fbxo2	91.38	232	17	1	68	296	66	297	1e-124	437
RNO-Fbxo2	GGO-Fbxo6	51.30	230	108	3	69	296	32	259	2e-065	240
RNO-Fbxo2	PTR-Fbxo6	51.30	230	108	3	69	296	32	259	3e-065	239
RNO-Fbxo2	PPY-Fbxo6	51.30	230	108	3	69	296	32	259	6e-065	239
RNO-Fbxo2	HSA-Fbxo6	50.87	230	109	3	69	296	32	259	2e-064	237
RNO-Fbxo2	RNO-Fbxo6	51.32	228	107	3	69	294	23	248	2e-064	237
RNO-Fbxo2	MMU-Fbxo6	51.32	228	107	3	69	294	32	257	7e-064	235
RNO-Fbxo2	MUS-Fbxo6	50.88	228	108	3	69	294	23	248	1e-063	234
RNO-Fbxo2	RNO-Fbxo44	50.87	230	109	3	69	296	41	268	3e-062	230
RNO-Fbxo2	MMU-Fbxo44	50.00	230	111	3	69	296	25	252	6e-062	229
RNO-Fbxo2	GGO-Fbxo44	50.00	230	111	3	69	296	35	262	7e-062	228

BLASTP 2.2.20 [Feb-08-2009]
Query: HSA-Fbxo2
Database: 559_protein.db
Query id, Subject id, % identity, alignment length, mismatches, gap openings, q. start, q. end, s. start, s. end, e-value, bit score

HSA-Fbxo2	HSA-Fbxo2	100.00	296	0	0	1	296	1	296	5e-126	441
HSA-Fbxo2	PTR-Fbxo2	100.00	226	0	0	71	296	70	295	4e-125	439
HSA-Fbxo2	PPY-Fbxo2	98.67	226	3	0	71	296	70	295	3e-124	436
HSA-Fbxo2	GGO-Fbxo2	98.23	226	4	0	71	296	70	295	3e-122	429
HSA-Fbxo2	CJA-Fbxo2	96.46	226	8	0	71	296	72	297	4e-121	425
HSA-Fbxo2	RNO-Fbxo2	92.48	226	14	1	71	296	74	296	3e-117	413
HSA-Fbxo2	MUS-Fbxo2	91.15	226	17	1	71	296	75	297	1e-114	404
HSA-Fbxo2	GGO-Fbxo6	52.21	226	105	3	71	296	37	259	6e-063	232
HSA-Fbxo2	PPY-Fbxo6	52.21	226	105	3	71	296	37	259	1e-062	231

```
HSA-Fbxo2   HSA-Fbxo6   51.77   226   106   3   71   296   37   259   2e-062   231
HSA-Fbxo2   PTR-Fbxo6   51.77   226   106   3   71   296   37   259   5e-062   229
HSA-Fbxo2   MMU-Fbxo6   52.23   224   104   3   71   294   37   257   1e-061   228
HSA-Fbxo2   MUS-Fbxo6   52.68   224   103   3   71   294   28   248   1e-060   225
HSA-Fbxo2   RNO-Fbxo6   52.23   224   104   3   71   294   28   248   2e-060   224
HSA-Fbxo2   RNO-Fbxo44  51.33   226   107   3   71   296   46   268   3e-059   219
HSA-Fbxo2   GGO-Fbxo44  50.44   226   109   3   71   296   40   262   1e-058   218
HSA-Fbxo2   MMU-Fbxo44  50.44   226   109   3   71   296   30   252   1e-058   218
HSA-Fbxo2   PTR-Fbxo44  50.00   226   110   3   71   296   30   252   1e-057   214
# BLASTP 2.2.20 [Feb-08-2009]
# Query: GGO-Fbxo21
# Database: 559_protein.db
# Query id, Subject id, % identity, alignment length, mismatches, gap openings, q. start, q. end, s. start, s. end, e-value, bit score
GGO-Fbxo21  HSA-Fbxo21  100.00  628   0    0   1   628   1   628   0.0   1243
GGO-Fbxo21  PTR-Fbxo21  100.00  628   0    0   1   628   1   628   0.0   1243
GGO-Fbxo21  GGO-Fbxo21  100.00  628   0    0   1   628   1   628   0.0   1243
GGO-Fbxo21  MMU-Fbxo21  99.68   628   2    0   1   628   1   628   0.0   1241
GGO-Fbxo21  PPY-Fbxo21  98.89   628   0    1   1   628   1   621   0.0   1222
GGO-Fbxo21  RNO-Fbxo21  96.17   627   24   0   1   627   1   627   0.0   1201
GGO-Fbxo21  MUS-Fbxo21  95.69   627   27   0   1   627   1   627   0.0   1199
# BLASTP 2.2.20 [Feb-08-2009]
# Query: MMU-Fbxo21
# Database: 559_protein.db
# Query id, Subject id, % identity, alignment length, mismatches, gap openings, q. start, q. end, s. start, s. end, e-value, bit score
MMU-Fbxo21  HSA-Fbxo21  99.68   628   2    0   1   628   1   628   0.0   1259
MMU-Fbxo21  PTR-Fbxo21  99.68   628   2    0   1   628   1   628   0.0   1259
MMU-Fbxo21  GGO-Fbxo21  99.68   628   2    0   1   628   1   628   0.0   1259
MMU-Fbxo21  MMU-Fbxo21  100.00  628   0    0   1   628   1   628   0.0   1258
MMU-Fbxo21  PPY-Fbxo21  98.57   628   2    1   1   628   1   621   0.0   1238
MMU-Fbxo21  RNO-Fbxo21  96.01   627   25   0   1   627   1   627   0.0   1212
MMU-Fbxo21  MUS-Fbxo21  95.53   627   28   0   1   627   1   627   0.0   1210
# BLASTP 2.2.20 [Feb-08-2009]
# Query: MUS-Fbxo21
# Database: 559_protein.db
# Query id, Subject id, % identity, alignment length, mismatches, gap openings, q. start, q. end, s. start, s. end, e-value, bit score
MUS-Fbxo21  MUS-Fbxo21  100.00  627   0    0   1   627   1   627   0.0   1258
MUS-Fbxo21  RNO-Fbxo21  99.36   627   4    0   1   627   1   627   0.0   1251
MUS-Fbxo21  HSA-Fbxo21  95.69   627   27   0   1   627   1   627   0.0   1215
MUS-Fbxo21  PTR-Fbxo21  95.69   627   27   0   1   627   1   627   0.0   1215
MUS-Fbxo21  GGO-Fbxo21  95.69   627   27   0   1   627   1   627   0.0   1215
MUS-Fbxo21  MMU-Fbxo21  95.53   627   28   0   1   627   1   627   0.0   1214
MUS-Fbxo21  PPY-Fbxo21  94.58   627   27   1   1   627   1   620   0.0   1193
# BLASTP 2.2.20 [Feb-08-2009]
# Query: PPY-Fbxo21
# Database: 559_protein.db
```

```
# Query id, Subject id, % identity, alignment length, mismatches, gap openings, q. start, q. end, s. start, s. end, e-value, bit score
PPY-Fbxo21  PPY-Fbxo21  100.00      621         0           0           1           621         1           621         0.0         1231
PPY-Fbxo21  HSA-Fbxo21  98.89       628         0           1           1           621         1           628         0.0         1223
PPY-Fbxo21  PTR-Fbxo21  98.89       628         0           1           1           621         1           628         0.0         1223
PPY-Fbxo21  GGO-Fbxo21  98.89       628         0           1           1           621         1           628         0.0         1223
PPY-Fbxo21  MMU-Fbxo21  98.57       628         2           1           1           621         1           628         0.0         1221
PPY-Fbxo21  RNO-Fbxo21  95.06       627         24          1           1           620         1           627         0.0         1181
PPY-Fbxo21  MUS-Fbxo21  94.58       627         27          1           1           620         1           627         0.0         1178
# BLASTP 2.2.20 [Feb-08-2009]
# Query: PTR-Fbxo21
# Database: 559_protein.db
# Query id, Subject id, % identity, alignment length, mismatches, gap openings, q. start, q. end, s. start, s. end, e-value, bit score
PTR-Fbxo21  HSA-Fbxo21  100.00      628         0           0           1           628         1           628         0.0         1243
PTR-Fbxo21  PTR-Fbxo21  100.00      628         0           0           1           628         1           628         0.0         1243
PTR-Fbxo21  GGO-Fbxo21  100.00      628         0           0           1           628         1           628         0.0         1243
PTR-Fbxo21  MMU-Fbxo21  99.68       628         2           0           1           628         1           628         0.0         1241
PTR-Fbxo21  PPY-Fbxo21  98.89       628         0           1           1           628         1           621         0.0         1222
PTR-Fbxo21  RNO-Fbxo21  96.17       627         24          0           1           627         1           627         0.0         1201
PTR-Fbxo21  MUS-Fbxo21  95.69       627         27          0           1           627         1           627         0.0         1199
# BLASTP 2.2.20 [Feb-08-2009]
# Query: RNO-Fbxo21
# Database: 559_protein.db
# Query id, Subject id, % identity, alignment length, mismatches, gap openings, q. start, q. end, s. start, s. end, e-value, bit score
RNO-Fbxo21  RNO-Fbxo21  100.00      627         0           0           1           627         1           627         0.0         1255
RNO-Fbxo21  MUS-Fbxo21  99.36       627         4           0           1           627         1           627         0.0         1251
RNO-Fbxo21  HSA-Fbxo21  96.17       627         24          0           1           627         1           627         0.0         1219
RNO-Fbxo21  PTR-Fbxo21  96.17       627         24          0           1           627         1           627         0.0         1219
RNO-Fbxo21  GGO-Fbxo21  96.17       627         24          0           1           627         1           627         0.0         1219
RNO-Fbxo21  MMU-Fbxo21  96.01       627         25          0           1           627         1           627         0.0         1218
RNO-Fbxo21  PPY-Fbxo21  95.06       627         24          1           1           627         1           620         0.0         1197
# BLASTP 2.2.20 [Feb-08-2009]
# Query: HSA-Fbxo21
# Database: 559_protein.db
# Query id, Subject id, % identity, alignment length, mismatches, gap openings, q. start, q. end, s. start, s. end, e-value, bit score
HSA-Fbxo21  HSA-Fbxo21  100.00      628         0           0           1           628         1           628         0.0         1243
HSA-Fbxo21  PTR-Fbxo21  100.00      628         0           0           1           628         1           628         0.0         1243
HSA-Fbxo21  GGO-Fbxo21  100.00      628         0           0           1           628         1           628         0.0         1243
HSA-Fbxo21  MMU-Fbxo21  99.68       628         2           0           1           628         1           628         0.0         1241
HSA-Fbxo21  PPY-Fbxo21  98.89       628         0           1           1           628         1           621         0.0         1222
HSA-Fbxo21  RNO-Fbxo21  96.17       627         24          0           1           627         1           627         0.0         1201
HSA-Fbxo21  MUS-Fbxo21  95.69       627         27          0           1           627         1           627         0.0         1199
# BLASTP 2.2.20 [Feb-08-2009]
# Query: CJA-Fbxo22
# Database: 559_protein.db
# Query id, Subject id, % identity, alignment length, mismatches, gap openings, q. start, q. end, s. start, s. end, e-value, bit score
```

```
CJA-Fbxo22  CJA-Fbxo22  100.00      404         0           0           1           404         1           404         0.0          798
CJA-Fbxo22  GGO-Fbxo22  97.16       387         11          0           18          404         17          403         0.0          780
CJA-Fbxo22  PTR-Fbxo22  96.90       387         12          0           18          404         17          403         0.0          778
CJA-Fbxo22  MMU-Fbxo22  96.92       389         10          1           18          404         18          406         0.0          777
CJA-Fbxo22  HSA-Fbxo22  96.90       387         12          0           18          404         17          403         0.0          775
CJA-Fbxo22  PPY-Fbxo22  96.66       389         11          1           18          404         17          405         0.0          774
CJA-Fbxo22  MUS-Fbxo22  92.76       387         28          0           18          404         16          402         0.0          748
CJA-Fbxo22  RNO-Fbxo22  92.51       387         29          0           18          404         13          399         0.0          745
# BLASTP 2.2.20 [Feb-08-2009]
# Query: GGO-Fbxo22
# Database: 559_protein.db
# Query id, Subject id, % identity, alignment length, mismatches, gap openings, q. start, q. end, s. start, s. end, e-value, bit score
GGO-Fbxo22  GGO-Fbxo22  100.00      403         0           0           1           403         1           403         0.0          830
GGO-Fbxo22  PTR-Fbxo22  99.50       403         2           0           1           403         1           403         0.0          827
GGO-Fbxo22  HSA-Fbxo22  99.50       403         2           0           1           403         1           403         0.0          825
GGO-Fbxo22  PPY-Fbxo22  98.52       405         4           1           1           403         1           405         0.0          819
GGO-Fbxo22  MMU-Fbxo22  97.54       406         7           2           1           403         1           406         0.0          805
GGO-Fbxo22  CJA-Fbxo22  95.79       404         16          1           1           403         1           404         0.0          795
GGO-Fbxo22  RNO-Fbxo22  93.61       391         25          0           13          403         9           399         0.0          757
GGO-Fbxo22  MUS-Fbxo22  93.61       391         25          0           13          403         12          402         0.0          757
# BLASTP 2.2.20 [Feb-08-2009]
# Query: MMU-Fbxo22
# Database: 559_protein.db
# Query id, Subject id, % identity, alignment length, mismatches, gap openings, q. start, q. end, s. start, s. end, e-value, bit score
MMU-Fbxo22  MMU-Fbxo22  100.00      406         0           0           1           406         1           406         0.0          836
MMU-Fbxo22  PPY-Fbxo22  97.54       406         9           1           1           406         1           405         0.0          809
MMU-Fbxo22  GGO-Fbxo22  97.54       406         7           2           1           406         1           403         0.0          805
MMU-Fbxo22  PTR-Fbxo22  97.29       406         8           2           1           406         1           403         0.0          803
MMU-Fbxo22  HSA-Fbxo22  97.29       406         8           2           1           406         1           403         0.0          801
MMU-Fbxo22  CJA-Fbxo22  95.81       406         15          1           1           406         1           404         0.0          800
MMU-Fbxo22  RNO-Fbxo22  93.42       395         24          1           12          406         7           399         0.0          760
MMU-Fbxo22  MUS-Fbxo22  93.40       394         24          1           13          406         11          402         0.0          757
# BLASTP 2.2.20 [Feb-08-2009]
# Query: MUS-Fbxo22
# Database: 559_protein.db
# Query id, Subject id, % identity, alignment length, mismatches, gap openings, q. start, q. end, s. start, s. end, e-value, bit score
MUS-Fbxo22  MUS-Fbxo22  100.00      402         0           0           1           402         1           402         0.0          828
MUS-Fbxo22  RNO-Fbxo22  97.01       402         9           1           1           402         1           399         0.0          800
MUS-Fbxo22  HSA-Fbxo22  92.31       403         30          1           1           402         1           403         0.0          763
MUS-Fbxo22  CJA-Fbxo22  91.34       404         33          1           1           402         1           404         0.0          761
MUS-Fbxo22  PTR-Fbxo22  92.31       403         30          1           1           402         1           403         0.0          761
MUS-Fbxo22  MMU-Fbxo22  91.63       406         30          2           1           402         1           406         0.0          756
MUS-Fbxo22  GGO-Fbxo22  91.81       403         32          1           1           402         1           403         0.0          756
MUS-Fbxo22  PPY-Fbxo22  91.36       405         32          2           1           402         1           405         0.0          753
# BLASTP 2.2.20 [Feb-08-2009]
```

```
# Query: PPY-Fbxo22
# Database: 559_protein.db
# Query id, Subject id, % identity, alignment length, mismatches, gap openings, q. start, q. end, s. start, s. end, e-value, bit score
PPY-Fbxo22  PPY-Fbxo22  100.00      405         0           0           1           405         1           405         0.0         835
PPY-Fbxo22  GGO-Fbxo22  98.52       405         4           1           1           405         1           403         0.0         819
PPY-Fbxo22  PTR-Fbxo22  98.27       405         5           1           1           405         1           403         0.0         816
PPY-Fbxo22  HSA-Fbxo22  98.27       405         5           1           1           405         1           403         0.0         815
PPY-Fbxo22  MMU-Fbxo22  97.54       406         9           1           1           405         1           406         0.0         809
PPY-Fbxo22  CJA-Fbxo22  95.57       406         15          2           1           405         1           404         0.0         793
PPY-Fbxo22  MUS-Fbxo22  93.13       393         25          1           13          405         12          402         0.0         753
PPY-Fbxo22  RNO-Fbxo22  93.13       393         25          1           13          405         9           399         0.0         753
# BLASTP 2.2.20 [Feb-08-2009]
# Query: PTR-Fbxo22
# Database: 559_protein.db
# Query id, Subject id, % identity, alignment length, mismatches, gap openings, q. start, q. end, s. start, s. end, e-value, bit score
PTR-Fbxo22  PTR-Fbxo22  100.00      403         0           0           1           403         1           403         0.0         830
PTR-Fbxo22  GGO-Fbxo22  99.50       403         2           0           1           403         1           403         0.0         827
PTR-Fbxo22  HSA-Fbxo22  99.50       403         2           0           1           403         1           403         0.0         826
PTR-Fbxo22  PPY-Fbxo22  98.27       405         5           1           1           403         1           405         0.0         816
PTR-Fbxo22  MMU-Fbxo22  97.29       406         8           2           1           403         1           406         0.0         803
PTR-Fbxo22  CJA-Fbxo22  95.79       404         16          1           1           403         1           404         0.0         797
PTR-Fbxo22  RNO-Fbxo22  92.06       403         28          1           1           403         1           399         0.0         759
PTR-Fbxo22  MUS-Fbxo22  93.86       391         24          0           13          403         12          402         0.0         757
# BLASTP 2.2.20 [Feb-08-2009]
# Query: RNO-Fbxo22
# Database: 559_protein.db
# Query id, Subject id, % identity, alignment length, mismatches, gap openings, q. start, q. end, s. start, s. end, e-value, bit score
RNO-Fbxo22  RNO-Fbxo22  100.00      399         0           0           1           399         1           399         0.0         823
RNO-Fbxo22  MUS-Fbxo22  97.01       402         9           1           1           399         1           402         0.0         793
RNO-Fbxo22  HSA-Fbxo22  92.06       403         28          1           1           399         1           403         0.0         761
RNO-Fbxo22  MMU-Fbxo22  93.42       395         24          1           7           399         12          406         0.0         760
RNO-Fbxo22  CJA-Fbxo22  91.09       404         31          1           1           399         1           404         0.0         759
RNO-Fbxo22  PTR-Fbxo22  92.06       403         28          1           1           399         1           403         0.0         759
RNO-Fbxo22  GGO-Fbxo22  93.61       391         25          0           9           399         13          403         0.0         757
RNO-Fbxo22  PPY-Fbxo22  93.13       393         25          1           9           399         13          405         0.0         753
# BLASTP 2.2.20 [Feb-08-2009]
# Query: HSA-Fbxo22
# Database: 559_protein.db
# Query id, Subject id, % identity, alignment length, mismatches, gap openings, q. start, q. end, s. start, s. end, e-value, bit score
HSA-Fbxo22  HSA-Fbxo22  100.00      403         0           0           1           403         1           403         0.0         830
HSA-Fbxo22  PTR-Fbxo22  99.50       403         2           0           1           403         1           403         0.0         826
HSA-Fbxo22  GGO-Fbxo22  99.50       403         2           0           1           403         1           403         0.0         825
HSA-Fbxo22  PPY-Fbxo22  98.27       405         5           1           1           403         1           405         0.0         815
HSA-Fbxo22  MMU-Fbxo22  97.29       406         8           2           1           403         1           406         0.0         801
HSA-Fbxo22  CJA-Fbxo22  95.79       404         16          1           1           403         1           404         0.0         795
```

```
HSA-Fbxo22  RNO-Fbxo22  92.06       403         28          1           1           403         1           399         0.0           761
HSA-Fbxo22  MUS-Fbxo22  93.86       391         24          0           13          403         12          402         0.0           759
# BLASTP 2.2.20 [Feb-08-2009]
# Query: CJA-Fbxo24
# Database: 559_protein.db
# Query id, Subject id, % identity, alignment length, mismatches, gap openings, q. start, q. end, s. start, s. end, e-value, bit score
CJA-Fbxo24  CJA-Fbxo24  100.00      582         0           0           34          615         34          615         0.0          1146
CJA-Fbxo24  GGO-Fbxo24  91.78       584         45          2           34          615         35          617         0.0          1041
CJA-Fbxo24  HSA-Fbxo24  91.44       584         47          2           34          615         36          618         0.0          1032
CJA-Fbxo24  PTR-Fbxo24  92.25       568         41          2           50          615         14          580         0.0          1020
CJA-Fbxo24  RNO-Fbxo24  86.76       589         54          3           50          615         2           589         0.0           988
CJA-Fbxo24  MUS-Fbxo24  86.76       589         54          3           50          615         2           589         0.0           986
CJA-Fbxo24  MMU-Fbxo24  95.91       391         15          1           50          440         14          403         0.0           751
CJA-Fbxo24  PPY-Fbxo24  88.89       306         19          2           50          355         14          304         5e-150        523
# BLASTP 2.2.20 [Feb-08-2009]
# Query: GGO-Fbxo24
# Database: 559_protein.db
# Query id, Subject id, % identity, alignment length, mismatches, gap openings, q. start, q. end, s. start, s. end, e-value, bit score
GGO-Fbxo24  GGO-Fbxo24  100.00      617         0           0           1           617         1           617         0.0          1178
GGO-Fbxo24  HSA-Fbxo24  98.82       594         7           0           24          617         25          618         0.0          1159
GGO-Fbxo24  PTR-Fbxo24  99.30       568         4           0           50          617         13          580         0.0          1118
GGO-Fbxo24  CJA-Fbxo24  91.26       595         48          3           24          617         24          615         0.0          1067
GGO-Fbxo24  RNO-Fbxo24  87.33       576         49          3           51          603         2           576         0.0          1001
GGO-Fbxo24  MUS-Fbxo24  87.50       576         48          3           51          603         2           576         0.0          1000
GGO-Fbxo24  MMU-Fbxo24  98.98       391         4           0           50          440         13          403         0.0           802
GGO-Fbxo24  PPY-Fbxo24  94.44       306         3           1           50          355         13          304         2e-168        583
# BLASTP 2.2.20 [Feb-08-2009]
# Query: MMU-Fbxo24
# Database: 559_protein.db
# Query id, Subject id, % identity, alignment length, mismatches, gap openings, q. start, q. end, s. start, s. end, e-value, bit score
MMU-Fbxo24  MMU-Fbxo24  100.00      403         0           0           1           403         1           403         0.0           805
MMU-Fbxo24  PTR-Fbxo24  98.76       403         5           0           1           403         1           403         0.0           796
MMU-Fbxo24  GGO-Fbxo24  98.98       391         4           0           13          403         50          440         0.0           775
MMU-Fbxo24  HSA-Fbxo24  98.72       391         5           0           13          403         51          441         0.0           773
MMU-Fbxo24  CJA-Fbxo24  95.91       391         15          1           14          403         50          440         0.0           751
MMU-Fbxo24  RNO-Fbxo24  88.14       413         25          3           14          403         2           413         0.0           712
MMU-Fbxo24  MUS-Fbxo24  88.14       413         25          3           14          403         2           413         0.0           712
MMU-Fbxo24  PPY-Fbxo24  94.03       318         5           1           1           318         1           304         4e-166        575
# BLASTP 2.2.20 [Feb-08-2009]
# Query: MUS-Fbxo24
# Database: 559_protein.db
# Query id, Subject id, % identity, alignment length, mismatches, gap openings, q. start, q. end, s. start, s. end, e-value, bit score
MUS-Fbxo24  MUS-Fbxo24  100.00      589         0           0           1           589         1           589         0.0          1159
MUS-Fbxo24  RNO-Fbxo24  97.45       589         15          0           1           589         1           589         0.0          1127
MUS-Fbxo24  CJA-Fbxo24  86.76       589         54          3           2           589         50          615         0.0           987
```

```
MUS-Fbxo24  GGO-Fbxo24  87.50   576   48   3   2    576   51   603   0.0       974
MUS-Fbxo24  PTR-Fbxo24  87.50   576   48   3   2    576   14   566   0.0       974
MUS-Fbxo24  HSA-Fbxo24  87.33   576   49   3   2    576   52   604   0.0       969
MUS-Fbxo24  MMU-Fbxo24  88.14   413   25   3   2    413   14   403   0.0       712
MUS-Fbxo24  PPY-Fbxo24  86.41   309   26   3   2    309   14   307   3e-145    506
# BLASTP 2.2.20 [Feb-08-2009]
# Query: PPY-Fbxo24
# Database: 559_protein.db
# Query id, Subject id, % identity, alignment length, mismatches, gap openings, q. start, q. end, s. start, s. end, e-value, bit score
PPY-Fbxo24  PPY-Fbxo24  100.00  315   0    0   1    315   1    315   3e-180    622
PPY-Fbxo24  PTR-Fbxo24  94.34   318   4    1   1    304   1    318   2e-166    576
PPY-Fbxo24  MMU-Fbxo24  94.03   318   5    1   1    304   1    318   3e-166    575
PPY-Fbxo24  GGO-Fbxo24  94.44   306   3    1   13   304   50   355   1e-160    556
PPY-Fbxo24  HSA-Fbxo24  94.12   306   4    1   13   304   51   356   8e-160    554
PPY-Fbxo24  CJA-Fbxo24  88.89   306   19   2   14   304   50   355   2e-150    523
PPY-Fbxo24  MUS-Fbxo24  86.41   309   26   3   14   307   2    309   3e-145    506
PPY-Fbxo24  RNO-Fbxo24  86.56   305   25   3   14   303   2    305   2e-144    503
# BLASTP 2.2.20 [Feb-08-2009]
# Query: PTR-Fbxo24
# Database: 559_protein.db
# Query id, Subject id, % identity, alignment length, mismatches, gap openings, q. start, q. end, s. start, s. end, e-value, bit score
PTR-Fbxo24  PTR-Fbxo24  100.00  580   0    0   1    580   1    580   0.0       1149
PTR-Fbxo24  GGO-Fbxo24  99.30   568   4    0   13   580   50   617   0.0       1119
PTR-Fbxo24  HSA-Fbxo24  98.94   568   6    0   13   580   51   618   0.0       1110
PTR-Fbxo24  CJA-Fbxo24  92.25   568   41   2   14   580   50   615   0.0       1040
PTR-Fbxo24  RNO-Fbxo24  87.33   576   49   3   14   566   2    576   0.0       1000
PTR-Fbxo24  MUS-Fbxo24  87.50   576   48   3   14   566   2    576   0.0       999
PTR-Fbxo24  MMU-Fbxo24  98.76   403   5    0   1    403   1    403   0.0       823
PTR-Fbxo24  PPY-Fbxo24  94.34   318   4    1   1    318   1    304   3e-174    603
# BLASTP 2.2.20 [Feb-08-2009]
# Query: RNO-Fbxo24
# Database: 559_protein.db
# Query id, Subject id, % identity, alignment length, mismatches, gap openings, q. start, q. end, s. start, s. end, e-value, bit score
RNO-Fbxo24  RNO-Fbxo24  100.00  575   0    0   1    575   1    575   0.0       1133
RNO-Fbxo24  MUS-Fbxo24  97.91   575   12   0   1    575   1    575   0.0       1106
RNO-Fbxo24  CJA-Fbxo24  87.83   575   47   2   2    575   50   602   0.0       983
RNO-Fbxo24  GGO-Fbxo24  87.30   575   49   3   2    575   51   602   0.0       972
RNO-Fbxo24  PTR-Fbxo24  87.30   575   49   3   2    575   14   565   0.0       971
RNO-Fbxo24  HSA-Fbxo24  87.13   575   50   3   2    575   52   603   0.0       965
RNO-Fbxo24  MMU-Fbxo24  88.14   413   25   3   2    413   14   403   0.0       713
RNO-Fbxo24  PPY-Fbxo24  86.56   305   25   3   2    305   14   303   5e-144    503
# BLASTP 2.2.20 [Feb-08-2009]
# Query: HSA-Fbxo24
# Database: 559_protein.db
# Query id, Subject id, % identity, alignment length, mismatches, gap openings, q. start, q. end, s. start, s. end, e-value, bit score
```

```
HSA-Fbxo24  HSA-Fbxo24  100.00      594         0           0           25          618         25          618         0.0            1177
HSA-Fbxo24  GGO-Fbxo24  98.82       594         7           0           25          618         24          617         0.0            1159
HSA-Fbxo24  PTR-Fbxo24  98.94       568         6           0           51          618         13          580         0.0            1110
HSA-Fbxo24  CJA-Fbxo24  91.09       595         49          3           25          618         24          615         0.0            1063
HSA-Fbxo24  MUS-Fbxo24  87.33       576         49          3           52          604         2           576         0.0             994
HSA-Fbxo24  RNO-Fbxo24  87.15       576         50          3           52          604         2           576         0.0             994
HSA-Fbxo24  MMU-Fbxo24  98.72       391         5           0           51          441         13          403         0.0             800
HSA-Fbxo24  PPY-Fbxo24  94.12       306         4           1           51          356         13          304         2e-167          580
# BLASTP 2.2.20 [Feb-08-2009]
# Query: CJA-Fbxo25
# Database: 559_protein.db
# Query id, Subject id, % identity, alignment length, mismatches, gap openings, q. start, q. end, s. start, s. end, e-value, bit score
CJA-Fbxo25  CJA-Fbxo25  100.00      367         0           0           1           367         1           367         0.0             725
CJA-Fbxo25  PTR-Fbxo25  98.91       367         4           0           1           367         1           367         0.0             719
CJA-Fbxo25  MMU-Fbxo25  98.91       367         4           0           1           367         1           367         0.0             719
CJA-Fbxo25  HSA-Fbxo25  98.64       367         5           0           1           367         1           367         0.0             717
CJA-Fbxo25  PPY-Fbxo25  98.64       367         4           1           1           367         1           366         0.0             712
CJA-Fbxo25  MUS-Fbxo25  87.74       367         43          2           1           367         1           365         0.0             638
CJA-Fbxo25  GGO-Fbxo25  97.83       322         7           0           46          367         1           322         1e-178          617
CJA-Fbxo25  CJA-Fbxo32  59.89       369         132         4           1           367         1           355         8e-120          421
CJA-Fbxo25  MMU-Fbxo32  59.89       369         132         4           1           367         1           355         9e-120          421
CJA-Fbxo25  GGO-Fbxo32  59.89       369         132         4           1           367         1           355         9e-120          421
CJA-Fbxo25  HSA-Fbxo32  59.62       369         133         4           1           367         1           355         2e-119          420
CJA-Fbxo25  PTR-Fbxo32  59.35       369         134         4           1           367         1           355         5e-119          419
CJA-Fbxo25  MUS-Fbxo32  58.54       369         137         3           1           367         1           355         1e-117          414
CJA-Fbxo25  RNO-Fbxo32  57.99       369         139         3           1           367         1           355         2e-116          410
CJA-Fbxo25  RNO-Fbxo25  92.47       146         11          0           222         367         2           147         4e-081          293
# BLASTP 2.2.20 [Feb-08-2009]
# Query: GGO-Fbxo25
# Database: 559_protein.db
# Query id, Subject id, % identity, alignment length, mismatches, gap openings, q. start, q. end, s. start, s. end, e-value, bit score
GGO-Fbxo25  GGO-Fbxo25  100.00      322         0           0           1           322         1           322         0.0             654
GGO-Fbxo25  HSA-Fbxo25  99.07       322         3           0           1           322         46          367         0.0             646
GGO-Fbxo25  PTR-Fbxo25  99.07       322         3           0           1           322         46          367         0.0             645
GGO-Fbxo25  MMU-Fbxo25  99.07       322         3           0           1           322         46          367         0.0             645
GGO-Fbxo25  PPY-Fbxo25  98.76       322         3           1           1           322         46          366         0.0             639
GGO-Fbxo25  CJA-Fbxo25  97.83       322         7           0           1           322         46          367         0.0             637
GGO-Fbxo25  MUS-Fbxo25  89.75       322         31          2           1           322         46          365         4e-167          578
GGO-Fbxo25  CJA-Fbxo32  61.80       322         112         2           1           322         45          355         5e-113          399
GGO-Fbxo25  MMU-Fbxo32  61.80       322         112         2           1           322         45          355         6e-113          398
GGO-Fbxo25  GGO-Fbxo32  61.80       322         112         2           1           322         45          355         6e-113          398
GGO-Fbxo25  HSA-Fbxo32  61.80       322         112         2           1           322         45          355         6e-113          398
GGO-Fbxo25  PTR-Fbxo32  61.49       322         113         2           1           322         45          355         3e-112          396
GGO-Fbxo25  RNO-Fbxo32  61.15       314         113         1           9           322         51          355         4e-110          389
GGO-Fbxo25  MUS-Fbxo32  60.83       314         114         1           9           322         51          355         7e-110          388
```

```
GGO-Fbxo25  RNO-Fbxo25  93.20     147     10     0     176     322     1     147     2e-082     297
# BLASTP 2.2.20 [Feb-08-2009]
# Query: MMU-Fbxo25
# Database: 559_protein.db
# Query id, Subject id, % identity, alignment length, mismatches, gap openings, q. start, q. end, s. start, s. end, e-value, bit score
MMU-Fbxo25  PTR-Fbxo25  100.00    367     0      0     1       367     1     367     0.0        747
MMU-Fbxo25  MMU-Fbxo25  100.00    367     0      0     1       367     1     367     0.0        747
MMU-Fbxo25  HSA-Fbxo25  99.73     367     1      0     1       367     1     367     0.0        744
MMU-Fbxo25  PPY-Fbxo25  99.73     367     0      1     1       367     1     366     0.0        741
MMU-Fbxo25  CJA-Fbxo25  98.91     367     4      0     1       367     1     367     0.0        739
MMU-Fbxo25  MUS-Fbxo25  88.01     367     42     2     1       367     1     365     0.0        654
MMU-Fbxo25  GGO-Fbxo25  99.07     322     3      0     46      367     1     322     0.0        645
MMU-Fbxo25  MMU-Fbxo32  60.16     369     131    4     1       367     1     355     8e-125     438
MMU-Fbxo25  GGO-Fbxo32  60.16     369     131    4     1       367     1     355     8e-125     438
MMU-Fbxo25  CJA-Fbxo32  60.16     369     131    4     1       367     1     355     9e-125     438
MMU-Fbxo25  HSA-Fbxo32  59.89     369     132    4     1       367     1     355     2e-124     436
MMU-Fbxo25  PTR-Fbxo32  59.62     369     133    4     1       367     1     355     8e-124     434
MMU-Fbxo25  MUS-Fbxo32  58.27     369     138    4     1       367     1     355     5e-121     426
MMU-Fbxo25  RNO-Fbxo32  58.54     369     137    4     1       367     1     355     8e-121     424
MMU-Fbxo25  RNO-Fbxo25  93.20     147     10     0     221     367     1     147     3e-082     296
# BLASTP 2.2.20 [Feb-08-2009]
# Query: MUS-Fbxo25
# Database: 559_protein.db
# Query id, Subject id, % identity, alignment length, mismatches, gap openings, q. start, q. end, s. start, s. end, e-value, bit score
MUS-Fbxo25  MUS-Fbxo25  100.00    365     0      0     1       365     1     365     0.0        762
MUS-Fbxo25  HSA-Fbxo25  88.01     367     42     2     1       365     1     367     0.0        671
MUS-Fbxo25  PTR-Fbxo25  88.01     367     42     2     1       365     1     367     0.0        671
MUS-Fbxo25  MMU-Fbxo25  88.01     367     42     2     1       365     1     367     0.0        671
MUS-Fbxo25  CJA-Fbxo25  87.74     367     43     2     1       365     1     367     0.0        670
MUS-Fbxo25  PPY-Fbxo25  87.74     367     42     3     1       365     1     366     0.0        663
MUS-Fbxo25  GGO-Fbxo25  89.75     322     31     2     46      365     1     322     4e-172     595
MUS-Fbxo25  CJA-Fbxo32  60.05     368     131    6     1       365     1     355     1e-123     434
MUS-Fbxo25  MMU-Fbxo32  60.05     368     131    6     1       365     1     355     1e-123     434
MUS-Fbxo25  GGO-Fbxo32  60.05     368     131    6     1       365     1     355     1e-123     434
MUS-Fbxo25  HSA-Fbxo32  59.78     368     132    6     1       365     1     355     3e-123     433
MUS-Fbxo25  PTR-Fbxo32  59.51     368     133    6     1       365     1     355     1e-122     431
MUS-Fbxo25  RNO-Fbxo32  58.70     368     136    5     1       365     1     355     7e-121     425
MUS-Fbxo25  MUS-Fbxo32  58.70     368     136    6     1       365     1     355     2e-120     424
MUS-Fbxo25  RNO-Fbxo25  98.64     147     2      0     219     365     1     147     1e-086     311
# BLASTP 2.2.20 [Feb-08-2009]
# Query: PPY-Fbxo25
# Database: 559_protein.db
# Query id, Subject id, % identity, alignment length, mismatches, gap openings, q. start, q. end, s. start, s. end, e-value, bit score
PPY-Fbxo25  PPY-Fbxo25  100.00    366     0      0     1       366     1     366     0.0        745
PPY-Fbxo25  PTR-Fbxo25  99.73     367     0      1     1       366     1     367     0.0        741
```

```
PPY-Fbxo25  MMU-Fbxo25  99.73   367   0     1   1     366   1     367   0.0       741
PPY-Fbxo25  HSA-Fbxo25  99.46   367   1     1   1     366   1     367   0.0       738
PPY-Fbxo25  CJA-Fbxo25  98.64   367   4     1   1     366   1     367   0.0       733
PPY-Fbxo25  MUS-Fbxo25  87.74   367   42    3   1     366   1     365   0.0       648
PPY-Fbxo25  GGO-Fbxo25  98.76   322   3     1   46    366   1     322   0.0       639
PPY-Fbxo25  CJA-Fbxo32  60.16   369   130   5   1     366   1     355   3e-123    432
PPY-Fbxo25  MMU-Fbxo32  60.16   369   130   5   1     366   1     355   3e-123    432
PPY-Fbxo25  GGO-Fbxo32  60.16   369   130   5   1     366   1     355   3e-123    432
PPY-Fbxo25  HSA-Fbxo32  59.89   369   131   5   1     366   1     355   9e-123    431
PPY-Fbxo25  PTR-Fbxo32  59.62   369   132   5   1     366   1     355   3e-122    429
PPY-Fbxo25  MUS-Fbxo32  58.27   369   137   5   1     366   1     355   2e-119    421
PPY-Fbxo25  RNO-Fbxo32  58.54   369   136   5   1     366   1     355   3e-119    419
PPY-Fbxo25  RNO-Fbxo25  92.52   147   10    1   221   366   1     147   3e-080    290
# BLASTP 2.2.20 [Feb-08-2009]
# Query: PTR-Fbxo25
# Database: 559_protein.db
# Query id, Subject id, % identity, alignment length, mismatches, gap openings, q. start, q. end, s. start, s. end, e-value, bit score
PTR-Fbxo25  PTR-Fbxo25  100.00  367   0     0   1     367   1     367   0.0       747
PTR-Fbxo25  MMU-Fbxo25  100.00  367   0     0   1     367   1     367   0.0       747
PTR-Fbxo25  HSA-Fbxo25  99.73   367   1     0   1     367   1     367   0.0       744
PTR-Fbxo25  PPY-Fbxo25  99.73   367   0     1   1     367   1     366   0.0       741
PTR-Fbxo25  CJA-Fbxo25  98.91   367   4     0   1     367   1     367   0.0       739
PTR-Fbxo25  MUS-Fbxo25  88.01   367   42    2   1     367   1     365   0.0       654
PTR-Fbxo25  GGO-Fbxo25  99.07   322   3     0   46    367   1     322   0.0       645
PTR-Fbxo25  MMU-Fbxo32  60.16   369   131   4   1     367   1     355   8e-125    438
PTR-Fbxo25  GGO-Fbxo32  60.16   369   131   4   1     367   1     355   8e-125    438
PTR-Fbxo25  CJA-Fbxo32  60.16   369   131   4   1     367   1     355   9e-125    438
PTR-Fbxo25  HSA-Fbxo32  59.89   369   132   4   1     367   1     355   2e-124    436
PTR-Fbxo25  PTR-Fbxo32  59.62   369   133   4   1     367   1     355   8e-124    434
PTR-Fbxo25  MUS-Fbxo32  58.27   369   138   4   1     367   1     355   5e-121    426
PTR-Fbxo25  RNO-Fbxo32  58.54   369   137   4   1     367   1     355   8e-121    424
PTR-Fbxo25  RNO-Fbxo25  93.20   147   10    0   221   367   1     147   3e-082    296
# BLASTP 2.2.20 [Feb-08-2009]
# Query: RNO-Fbxo25
# Database: 559_protein.db
# Query id, Subject id, % identity, alignment length, mismatches, gap openings, q. start, q. end, s. start, s. end, e-value, bit score
RNO-Fbxo25  MUS-Fbxo25  98.64   147   2     0   1     147   219   365   3e-087    311
RNO-Fbxo25  RNO-Fbxo25  100.00  147   0     0   1     147   1     147   2e-086    308
RNO-Fbxo25  GGO-Fbxo25  93.20   147   10    0   1     147   176   322   5e-083    297
RNO-Fbxo25  HSA-Fbxo25  93.20   147   10    0   1     147   221   367   6e-083    297
RNO-Fbxo25  PTR-Fbxo25  93.20   147   10    0   1     147   221   367   7e-083    297
RNO-Fbxo25  MMU-Fbxo25  93.20   147   10    0   1     147   221   367   7e-083    297
RNO-Fbxo25  CJA-Fbxo25  92.52   147   11    0   1     147   221   367   2e-082    295
RNO-Fbxo25  PPY-Fbxo25  92.52   147   10    1   1     147   221   366   6e-081    290
# BLASTP 2.2.20 [Feb-08-2009]
```

```
# Query: HSA-Fbxo25
# Database: 559_protein.db
# Query id, Subject id, % identity, alignment length, mismatches, gap openings, q. start, q. end, s. start, s. end, e-value, bit score
HSA-Fbxo25  HSA-Fbxo25  100.00      367         0           0           1           367         1           367         0.0          747
HSA-Fbxo25  PTR-Fbxo25  99.73       367         1           0           1           367         1           367         0.0          744
HSA-Fbxo25  MMU-Fbxo25  99.73       367         1           0           1           367         1           367         0.0          744
HSA-Fbxo25  PPY-Fbxo25  99.46       367         1           1           1           367         1           366         0.0          738
HSA-Fbxo25  CJA-Fbxo25  98.64       367         5           0           1           367         1           367         0.0          737
HSA-Fbxo25  MUS-Fbxo25  88.01       367         42          2           1           367         1           365         0.0          655
HSA-Fbxo25  GGO-Fbxo25  99.07       322         3           0           46          367         1           322         0.0          646
HSA-Fbxo25  CJA-Fbxo32  60.16       369         131         4           1           367         1           355         3e-124       436
HSA-Fbxo25  MMU-Fbxo32  60.16       369         131         4           1           367         1           355         3e-124       436
HSA-Fbxo25  GGO-Fbxo32  60.16       369         131         4           1           367         1           355         3e-124       436
HSA-Fbxo25  HSA-Fbxo32  59.89       369         132         4           1           367         1           355         7e-124       435
HSA-Fbxo25  PTR-Fbxo32  59.62       369         133         4           1           367         1           355         2e-123       433
HSA-Fbxo25  MUS-Fbxo32  58.27       369         138         4           1           367         1           355         1e-120       424
HSA-Fbxo25  RNO-Fbxo32  58.54       369         137         4           1           367         1           355         3e-120       423
HSA-Fbxo25  RNO-Fbxo25  93.20       147         10          0           221         367         1           147         3e-082       296
# BLASTP 2.2.20 [Feb-08-2009]
# Query: CJA-Fbxo27
# Database: 559_protein.db
# Query id, Subject id, % identity, alignment length, mismatches, gap openings, q. start, q. end, s. start, s. end, e-value, bit score
CJA-Fbxo27  CJA-Fbxo27  100.00      262         0           0           22          283         22          283         5e-155       538
CJA-Fbxo27  PPY-Fbxo27  93.05       259         16          2           23          281         9           265         7e-139       484
CJA-Fbxo27  HSA-Fbxo27  93.80       258         16          0           24          281         24          281         2e-136       476
CJA-Fbxo27  GGO-Fbxo27  93.80       258         16          0           24          281         24          281         2e-136       476
CJA-Fbxo27  MMU-Fbxo27  94.87       234         12          0           47          280         1           234         9e-132       461
CJA-Fbxo27  MUS-Fbxo27  78.84       241         51          0           40          280         37          277         1e-113       400
CJA-Fbxo27  MUS-Fbxo17  57.62       269         103         1           24          281         16          284         1e-089       321
CJA-Fbxo27  GGO-Fbxo17  57.25       262         109         1           23          281         15          276         3e-088       316
CJA-Fbxo27  HSA-Fbxo17  57.25       262         109         1           23          281         24          285         3e-088       316
CJA-Fbxo27  PPY-Fbxo17  56.87       262         110         1           23          281         21          282         8e-088       315
CJA-Fbxo27  CJA-Fbxo17  56.70       261         110         1           24          281         18          278         8e-087       311
CJA-Fbxo27  RNO-Fbxo17  59.71       206         78          2           37          238         29          233         1e-067       248
# BLASTP 2.2.20 [Feb-08-2009]
# Query: GGO-Fbxo27
# Database: 559_protein.db
# Query id, Subject id, % identity, alignment length, mismatches, gap openings, q. start, q. end, s. start, s. end, e-value, bit score
GGO-Fbxo27  HSA-Fbxo27  100.00      283         0           0           1           283         1           283         2e-142       496
GGO-Fbxo27  GGO-Fbxo27  100.00      283         0           0           1           283         1           283         2e-142       496
GGO-Fbxo27  PPY-Fbxo27  99.58       240         0           1           44          283         29          267         2e-140       489
GGO-Fbxo27  MMU-Fbxo27  98.31       237         4           0           47          283         1           237         1e-136       477
GGO-Fbxo27  CJA-Fbxo27  93.70       238         15          0           44          281         44          281         1e-131       460
GGO-Fbxo27  MUS-Fbxo27  80.17       237         47          0           44          280         41          277         2e-111       393
GGO-Fbxo27  MUS-Fbxo17  57.77       251         95          1           44          283         36          286         1e-082       297
```

```
GGO-Fbxo27  GGO-Fbxo17  57.20   243   101   1   44    283   36    278   2e-081   293
GGO-Fbxo27  HSA-Fbxo17  57.20   243   101   1   44    283   45    287   2e-081   293
GGO-Fbxo27  PPY-Fbxo17  57.20   243   101   1   44    283   42    284   3e-081   293
GGO-Fbxo27  CJA-Fbxo17  56.38   243   103   1   44    283   38    280   4e-080   289
GGO-Fbxo27  RNO-Fbxo17  57.79   199   79    2   44    238   36    233   7e-064   235
GGO-Fbxo27  PTR-Fbxo27  100.00  92    0     0   192   283   122   213   2e-051   194
# BLASTP 2.2.20 [Feb-08-2009]
# Query: MUS-Fbxo27
# Database: 559_protein.db
# Query id, Subject id, % identity, alignment length, mismatches, gap openings, q. start, q. end, s. start, s. end, e-value, bit score
MUS-Fbxo27  MUS-Fbxo27  100.00  280   0     0   1     280   1     280   2e-141   493
MUS-Fbxo27  CJA-Fbxo27  76.43   280   63    1   1     277   1     280   5e-107   379
MUS-Fbxo27  MMU-Fbxo27  81.20   234   44    0   44    277   1     234   5e-106   375
MUS-Fbxo27  HSA-Fbxo27  80.17   237   47    0   41    277   44    280   3e-105   372
MUS-Fbxo27  GGO-Fbxo27  80.17   237   47    0   41    277   44    280   3e-105   372
MUS-Fbxo27  PPY-Fbxo27  79.75   237   47    1   41    277   29    264   2e-103   366
MUS-Fbxo27  MUS-Fbxo17  57.66   248   94    2   41    277   36    283   4e-074   269
MUS-Fbxo27  GGO-Fbxo17  58.21   280   109   4   1     277   1     275   8e-074   268
MUS-Fbxo27  CJA-Fbxo17  57.08   240   100   1   41    277   38    277   9e-074   268
MUS-Fbxo27  HSA-Fbxo17  58.21   280   109   4   1     277   10    284   1e-073   268
MUS-Fbxo27  PPY-Fbxo17  57.86   280   110   4   1     277   7     281   1e-073   267
MUS-Fbxo27  RNO-Fbxo17  60.10   198   76    2   41    235   36    233   1e-059   221
# BLASTP 2.2.20 [Feb-08-2009]
# Query: PPY-Fbxo27
# Database: 559_protein.db
# Query id, Subject id, % identity, alignment length, mismatches, gap openings, q. start, q. end, s. start, s. end, e-value, bit score
PPY-Fbxo27  PPY-Fbxo27  100.00  255   0     0   13    267   13    267   1e-150   523
PPY-Fbxo27  HSA-Fbxo27  99.59   245   0     1   24    267   39    283   1e-143   500
PPY-Fbxo27  GGO-Fbxo27  99.59   245   0     1   24    267   39    283   1e-143   500
PPY-Fbxo27  CJA-Fbxo27  93.63   251   15    1   16    265   31    281   3e-137   479
PPY-Fbxo27  MMU-Fbxo27  97.89   237   4     1   32    267   1     237   1e-134   470
PPY-Fbxo27  MUS-Fbxo27  80.08   241   47    1   25    264   37    277   2e-112   397
PPY-Fbxo27  MUS-Fbxo17  58.71   264   97    2   16    267   23    286   5e-088   315
PPY-Fbxo27  GGO-Fbxo17  58.20   256   103   2   16    267   23    278   1e-086   311
PPY-Fbxo27  HSA-Fbxo17  58.20   256   103   2   16    267   32    287   1e-086   310
PPY-Fbxo27  PPY-Fbxo17  57.81   256   104   2   16    267   29    284   4e-086   309
PPY-Fbxo27  CJA-Fbxo17  57.42   256   105   2   16    267   25    280   3e-085   306
PPY-Fbxo27  RNO-Fbxo17  58.74   206   79    3   22    222   29    233   7e-067   245
PPY-Fbxo27  PTR-Fbxo27  100.00  92    0     0   176   267   122   213   2e-051   193
# BLASTP 2.2.20 [Feb-08-2009]
# Query: PTR-Fbxo27
# Database: 559_protein.db
# Query id, Subject id, % identity, alignment length, mismatches, gap openings, q. start, q. end, s. start, s. end, e-value, bit score
PTR-Fbxo27  PTR-Fbxo27  100.00  213   0     0   1     213   1     213   3e-102   362
PTR-Fbxo27  RNO-Fbxo27  77.25   167   36    1   44    210   41    205   6e-072   261
```

```
PTR-Fbxo27  HSA-Fbxo27  100.00      92          0           0           122         213         192         283         1e-051       194
PTR-Fbxo27  GGO-Fbxo27  100.00      92          0           0           122         213         192         283         1e-051       194
PTR-Fbxo27  PPY-Fbxo27  100.00      92          0           0           122         213         176         267         2e-051       193
# BLASTP 2.2.20 [Feb-08-2009]
# Query: HSA-Fbxo27
# Database: 559_protein.db
# Query id, Subject id, % identity, alignment length, mismatches, gap openings, q. start, q. end, s. start, s. end, e-value, bit score
HSA-Fbxo27  HSA-Fbxo27  100.00      283         0           0           1           283         1           283         2e-142       496
HSA-Fbxo27  GGO-Fbxo27  100.00      283         0           0           1           283         1           283         2e-142       496
HSA-Fbxo27  PPY-Fbxo27  99.58       240         0           1           44          283         29          267         2e-140       489
HSA-Fbxo27  MMU-Fbxo27  98.31       237         4           0           47          283         1           237         1e-136       477
HSA-Fbxo27  CJA-Fbxo27  93.70       238         15          0           44          281         44          281         1e-131       460
HSA-Fbxo27  MUS-Fbxo27  80.17       237         47          0           44          280         41          277         2e-111       393
HSA-Fbxo27  MUS-Fbxo17  57.77       251         95          1           44          283         36          286         1e-082       297
HSA-Fbxo27  GGO-Fbxo17  57.20       243         101         1           44          283         36          278         2e-081       293
HSA-Fbxo27  HSA-Fbxo17  57.20       243         101         1           44          283         45          287         2e-081       293
HSA-Fbxo27  PPY-Fbxo17  57.20       243         101         1           44          283         42          284         3e-081       293
HSA-Fbxo27  CJA-Fbxo17  56.38       243         103         1           44          283         38          280         4e-080       289
HSA-Fbxo27  RNO-Fbxo17  57.79       199         79          2           44          238         36          233         7e-064       235
HSA-Fbxo27  PTR-Fbxo27  100.00      92          0           0           192         283         122         213         2e-051       194
# BLASTP 2.2.20 [Feb-08-2009]
# Query: RNO-Fbxo27
# Database: 559_protein.db
# Query id, Subject id, % identity, alignment length, mismatches, gap openings, q. start, q. end, s. start, s. end, e-value, bit score
RNO-Fbxo27  RNO-Fbxo27  100.00      208         0           0           1           208         1           208         5e-095       338
RNO-Fbxo27  PTR-Fbxo27  77.11       166         36          1           42          205         45          210         4e-063       232
# BLASTP 2.2.20 [Feb-08-2009]
# Query: MMU-Fbxo27
# Database: 559_protein.db
# Query id, Subject id, % identity, alignment length, mismatches, gap openings, q. start, q. end, s. start, s. end, e-value, bit score
MMU-Fbxo27  MMU-Fbxo27  100.00      237         0           0           1           237         1           237         5e-140       488
MMU-Fbxo27  HSA-Fbxo27  98.31       237         4           0           1           237         47          283         4e-137       478
MMU-Fbxo27  GGO-Fbxo27  98.31       237         4           0           1           237         47          283         4e-137       478
MMU-Fbxo27  PPY-Fbxo27  97.89       237         4           1           1           237         32          267         4e-135       471
MMU-Fbxo27  CJA-Fbxo27  94.87       234         12          0           1           234         47          280         3e-132       462
MMU-Fbxo27  MUS-Fbxo27  81.20       234         44          0           1           234         44          277         2e-112       396
MMU-Fbxo27  MUS-Fbxo17  58.13       246         92          1           3           237         41          286         5e-082       295
MMU-Fbxo27  PPY-Fbxo17  57.56       238         98          1           3           237         47          284         2e-080       290
MMU-Fbxo27  HSA-Fbxo17  57.56       238         98          1           3           237         50          287         2e-080       290
MMU-Fbxo27  GGO-Fbxo17  57.56       238         98          1           3           237         41          278         2e-080       290
MMU-Fbxo27  CJA-Fbxo17  56.72       238         100         1           3           237         43          280         2e-079       286
MMU-Fbxo27  RNO-Fbxo17  58.76       194         75          2           3           192         41          233         3e-063       233
# BLASTP 2.2.20 [Feb-08-2009]
# Query: CJA-Fbxo28
# Database: 559_protein.db
```

```
# Query id, Subject id, % identity, alignment length, mismatches, gap openings, q. start, q. end, s. start, s. end, e-value, bit score
CJA-Fbxo28  CJA-Fbxo28  100.00      308         0           0           63          370         63          370         8e-162       561
CJA-Fbxo28  MMU-Fbxo28  99.03       308         3           0           63          370         62          369         4e-160       555
CJA-Fbxo28  HSA-Fbxo28  99.03       308         3           0           63          370         61          368         4e-160       555
CJA-Fbxo28  PTR-Fbxo28  99.03       308         3           0           63          370         61          368         4e-160       555
CJA-Fbxo28  PPY-Fbxo28  99.03       308         3           0           63          370         61          368         4e-160       555
CJA-Fbxo28  RNO-Fbxo28  98.05       308         6           0           63          370         61          368         2e-159       553
CJA-Fbxo28  MUS-Fbxo28  97.40       308         8           0           63          370         61          368         1e-158       550
CJA-Fbxo28  GGO-Fbxo28  76.28       312         68          4           63          370         40          349         6e-110       389
# BLASTP 2.2.20 [Feb-08-2009]
# Query: GGO-Fbxo28
# Database: 559_protein.db
# Query id, Subject id, % identity, alignment length, mismatches, gap openings, q. start, q. end, s. start, s. end, e-value, bit score
GGO-Fbxo28  GGO-Fbxo28  100.00      337         0           0           1           337         1           337         4e-140       489
GGO-Fbxo28  HSA-Fbxo28  79.06       339         65          4           1           337         22          356         3e-098       350
GGO-Fbxo28  PTR-Fbxo28  79.06       339         65          4           1           337         22          356         3e-098       350
GGO-Fbxo28  PPY-Fbxo28  78.76       339         66          4           1           337         22          356         3e-098       350
GGO-Fbxo28  MMU-Fbxo28  76.33       300         65          4           40          337         62          357         6e-098       348
GGO-Fbxo28  RNO-Fbxo28  75.33       300         68          4           40          337         61          356         2e-097       347
GGO-Fbxo28  MUS-Fbxo28  75.00       300         69          4           40          337         61          356         4e-097       346
GGO-Fbxo28  CJA-Fbxo28  75.67       300         67          4           40          337         63          358         1e-096       345
# BLASTP 2.2.20 [Feb-08-2009]
# Query: MMU-Fbxo28
# Database: 559_protein.db
# Query id, Subject id, % identity, alignment length, mismatches, gap openings, q. start, q. end, s. start, s. end, e-value, bit score
MMU-Fbxo28  MMU-Fbxo28  100.00      296         0           0           62          357         62          357         3e-155       539
MMU-Fbxo28  HSA-Fbxo28  100.00      296         0           0           62          357         61          356         3e-155       539
MMU-Fbxo28  PTR-Fbxo28  100.00      296         0           0           62          357         61          356         3e-155       539
MMU-Fbxo28  PPY-Fbxo28  100.00      296         0           0           62          357         61          356         3e-155       539
MMU-Fbxo28  RNO-Fbxo28  98.99       296         3           0           62          357         61          356         1e-154       537
MMU-Fbxo28  MUS-Fbxo28  98.31       296         5           0           62          357         61          356         6e-154       535
MMU-Fbxo28  CJA-Fbxo28  99.32       296         2           0           62          357         63          358         6e-154       535
MMU-Fbxo28  GGO-Fbxo28  76.33       300         65          4           62          357         40          337         3e-105       373
# BLASTP 2.2.20 [Feb-08-2009]
# Query: MUS-Fbxo28
# Database: 559_protein.db
# Query id, Subject id, % identity, alignment length, mismatches, gap openings, q. start, q. end, s. start, s. end, e-value, bit score
MUS-Fbxo28  MUS-Fbxo28  100.00      301         0           0           56          356         56          356         6e-160       555
MUS-Fbxo28  RNO-Fbxo28  99.34       301         2           0           56          356         56          356         3e-159       552
MUS-Fbxo28  MMU-Fbxo28  98.01       301         6           0           56          356         57          357         1e-157       547
MUS-Fbxo28  HSA-Fbxo28  98.01       301         6           0           56          356         56          356         1e-157       546
MUS-Fbxo28  PTR-Fbxo28  98.01       301         6           0           56          356         56          356         1e-157       546
MUS-Fbxo28  PPY-Fbxo28  98.01       301         6           0           56          356         56          356         1e-157       546
MUS-Fbxo28  CJA-Fbxo28  97.34       301         8           0           56          356         58          358         2e-156       543
MUS-Fbxo28  GGO-Fbxo28  75.08       305         70          4           56          356         35          337         1e-108       384
```

```
# BLASTP 2.2.20 [Feb-08-2009]
# Query: PPY-Fbxo28
# Database: 559_protein.db
# Query id, Subject id, % identity, alignment length, mismatches, gap openings, q. start, q. end, s. start, s. end, e-value, bit score
PPY-Fbxo28  MMU-Fbxo28  100.00  296  0   0  61  356  62  357  3e-155  539
PPY-Fbxo28  HSA-Fbxo28  100.00  296  0   0  61  356  61  356  3e-155  539
PPY-Fbxo28  PTR-Fbxo28  100.00  296  0   0  61  356  61  356  3e-155  539
PPY-Fbxo28  PPY-Fbxo28  100.00  296  0   0  61  356  61  356  3e-155  539
PPY-Fbxo28  RNO-Fbxo28  98.99   296  3   0  61  356  61  356  1e-154  537
PPY-Fbxo28  MUS-Fbxo28  98.31   296  5   0  61  356  61  356  6e-154  535
PPY-Fbxo28  CJA-Fbxo28  99.32   296  2   0  61  356  63  358  6e-154  535
PPY-Fbxo28  GGO-Fbxo28  76.33   300  65  4  61  356  40  337  3e-105  373
# BLASTP 2.2.20 [Feb-08-2009]
# Query: PTR-Fbxo28
# Database: 559_protein.db
# Query id, Subject id, % identity, alignment length, mismatches, gap openings, q. start, q. end, s. start, s. end, e-value, bit score
PTR-Fbxo28  MMU-Fbxo28  100.00  296  0   0  61  356  62  357  3e-155  539
PTR-Fbxo28  HSA-Fbxo28  100.00  296  0   0  61  356  61  356  3e-155  539
PTR-Fbxo28  PTR-Fbxo28  100.00  296  0   0  61  356  61  356  3e-155  539
PTR-Fbxo28  PPY-Fbxo28  100.00  296  0   0  61  356  61  356  3e-155  539
PTR-Fbxo28  RNO-Fbxo28  98.99   296  3   0  61  356  61  356  1e-154  537
PTR-Fbxo28  MUS-Fbxo28  98.31   296  5   0  61  356  61  356  6e-154  535
PTR-Fbxo28  CJA-Fbxo28  99.32   296  2   0  61  356  63  358  6e-154  535
PTR-Fbxo28  GGO-Fbxo28  76.33   300  65  4  61  356  40  337  3e-105  373
# BLASTP 2.2.20 [Feb-08-2009]
# Query: RNO-Fbxo28
# Database: 559_protein.db
# Query id, Subject id, % identity, alignment length, mismatches, gap openings, q. start, q. end, s. start, s. end, e-value, bit score
RNO-Fbxo28  RNO-Fbxo28  100.00  327  0   0  30  356  30  356  3e-161  559
RNO-Fbxo28  MUS-Fbxo28  99.34   301  2   0  56  356  56  356  2e-159  553
RNO-Fbxo28  HSA-Fbxo28  98.67   301  4   0  56  356  56  356  2e-158  549
RNO-Fbxo28  PTR-Fbxo28  98.67   301  4   0  56  356  56  356  2e-158  549
RNO-Fbxo28  PPY-Fbxo28  98.67   301  4   0  56  356  56  356  2e-158  549
RNO-Fbxo28  MMU-Fbxo28  98.67   301  4   0  56  356  57  357  3e-158  549
RNO-Fbxo28  CJA-Fbxo28  98.01   301  6   0  56  356  58  358  5e-157  545
RNO-Fbxo28  GGO-Fbxo28  75.41   305  69  4  56  356  35  337  6e-109  385
# BLASTP 2.2.20 [Feb-08-2009]
# Query: HSA-Fbxo28
# Database: 559_protein.db
# Query id, Subject id, % identity, alignment length, mismatches, gap openings, q. start, q. end, s. start, s. end, e-value, bit score
HSA-Fbxo28  MMU-Fbxo28  100.00  296  0   0  61  356  62  357  3e-155  539
HSA-Fbxo28  HSA-Fbxo28  100.00  296  0   0  61  356  61  356  3e-155  539
HSA-Fbxo28  PTR-Fbxo28  100.00  296  0   0  61  356  61  356  3e-155  539
HSA-Fbxo28  PPY-Fbxo28  100.00  296  0   0  61  356  61  356  3e-155  539
HSA-Fbxo28  RNO-Fbxo28  98.99   296  3   0  61  356  61  356  1e-154  537
```

```
HSA-Fbxo28  MUS-Fbxo28  98.31   296   5    0   61   356   61   356   6e-154   535
HSA-Fbxo28  CJA-Fbxo28  99.32   296   2    0   61   356   63   358   6e-154   535
HSA-Fbxo28  GGO-Fbxo28  76.33   300   65   4   61   356   40   337   3e-105   373
# BLASTP 2.2.20 [Feb-08-2009]
# Query: CJA-Fbxo3
# Database: 559_protein.db
# Query id, Subject id, % identity, alignment length, mismatches, gap openings, q. start, q. end, s. start, s. end, e-value, bit score
CJA-Fbxo3   CJA-Fbxo3   100.00  417   0    0   1    417   1    417   0.0   818
CJA-Fbxo3   HSA-Fbxo3   99.52   417   2    0   1    417   1    417   0.0   814
CJA-Fbxo3   PTR-Fbxo3   99.52   417   2    0   1    417   1    417   0.0   814
CJA-Fbxo3   PPY-Fbxo3   99.52   417   2    0   1    417   1    417   0.0   814
CJA-Fbxo3   GGO-Fbxo3   99.52   417   2    0   1    417   1    417   0.0   814
CJA-Fbxo3   MMU-Fbxo3   99.04   417   4    0   1    417   1    417   0.0   810
CJA-Fbxo3   RNO-Fbxo3   96.16   417   16   0   1    417   1    417   0.0   795
CJA-Fbxo3   MUS-Fbxo3   95.92   417   17   0   1    417   1    417   0.0   792
# BLASTP 2.2.20 [Feb-08-2009]
# Query: GGO-Fbxo3
# Database: 559_protein.db
# Query id, Subject id, % identity, alignment length, mismatches, gap openings, q. start, q. end, s. start, s. end, e-value, bit score
GGO-Fbxo3   HSA-Fbxo3   100.00  417   0    0   1    417   1    417   0.0   817
GGO-Fbxo3   PTR-Fbxo3   100.00  417   0    0   1    417   1    417   0.0   817
GGO-Fbxo3   PPY-Fbxo3   100.00  417   0    0   1    417   1    417   0.0   817
GGO-Fbxo3   GGO-Fbxo3   100.00  417   0    0   1    417   1    417   0.0   817
GGO-Fbxo3   CJA-Fbxo3   99.52   417   2    0   1    417   1    417   0.0   814
GGO-Fbxo3   MMU-Fbxo3   99.52   417   2    0   1    417   1    417   0.0   813
GGO-Fbxo3   RNO-Fbxo3   96.16   417   16   0   1    417   1    417   0.0   793
GGO-Fbxo3   MUS-Fbxo3   95.92   417   17   0   1    417   1    417   0.0   791
# BLASTP 2.2.20 [Feb-08-2009]
# Query: MMU-Fbxo3
# Database: 559_protein.db
# Query id, Subject id, % identity, alignment length, mismatches, gap openings, q. start, q. end, s. start, s. end, e-value, bit score
MMU-Fbxo3   MMU-Fbxo3   100.00  417   0    0   1    417   1    417   0.0   817
MMU-Fbxo3   HSA-Fbxo3   99.52   417   2    0   1    417   1    417   0.0   813
MMU-Fbxo3   PTR-Fbxo3   99.52   417   2    0   1    417   1    417   0.0   813
MMU-Fbxo3   PPY-Fbxo3   99.52   417   2    0   1    417   1    417   0.0   813
MMU-Fbxo3   GGO-Fbxo3   99.52   417   2    0   1    417   1    417   0.0   813
MMU-Fbxo3   CJA-Fbxo3   99.04   417   4    0   1    417   1    417   0.0   810
MMU-Fbxo3   RNO-Fbxo3   95.92   417   17   0   1    417   1    417   0.0   793
MMU-Fbxo3   MUS-Fbxo3   95.68   417   18   0   1    417   1    417   0.0   791
# BLASTP 2.2.20 [Feb-08-2009]
# Query: MUS-Fbxo3
# Database: 559_protein.db
# Query id, Subject id, % identity, alignment length, mismatches, gap openings, q. start, q. end, s. start, s. end, e-value, bit score
MUS-Fbxo3   MUS-Fbxo3   100.00  417   0    0   1    417   1    417   0.0   817
MUS-Fbxo3   RNO-Fbxo3   98.32   417   7    0   1    417   1    417   0.0   803
```

```
MUS-Fbxo3   CJA-Fbxo3   95.92    417    17    0    1    417    1    417    0.0    790
MUS-Fbxo3   HSA-Fbxo3   95.92    417    17    0    1    417    1    417    0.0    789
MUS-Fbxo3   PTR-Fbxo3   95.92    417    17    0    1    417    1    417    0.0    789
MUS-Fbxo3   PPY-Fbxo3   95.92    417    17    0    1    417    1    417    0.0    789
MUS-Fbxo3   GGO-Fbxo3   95.92    417    17    0    1    417    1    417    0.0    789
MUS-Fbxo3   MMU-Fbxo3   95.68    417    18    0    1    417    1    417    0.0    789
# BLASTP 2.2.20 [Feb-08-2009]
# Query: PPY-Fbxo3
# Database: 559_protein.db
# Query id, Subject id, % identity, alignment length, mismatches, gap openings, q. start, q. end, s. start, s. end, e-value, bit score
PPY-Fbxo3   HSA-Fbxo3   100.00   417    0     0    1    417    1    417    0.0    817
PPY-Fbxo3   PTR-Fbxo3   100.00   417    0     0    1    417    1    417    0.0    817
PPY-Fbxo3   PPY-Fbxo3   100.00   417    0     0    1    417    1    417    0.0    817
PPY-Fbxo3   GGO-Fbxo3   100.00   417    0     0    1    417    1    417    0.0    817
PPY-Fbxo3   CJA-Fbxo3   99.52    417    2     0    1    417    1    417    0.0    814
PPY-Fbxo3   MMU-Fbxo3   99.52    417    2     0    1    417    1    417    0.0    813
PPY-Fbxo3   RNO-Fbxo3   96.16    417    16    0    1    417    1    417    0.0    793
PPY-Fbxo3   MUS-Fbxo3   95.92    417    17    0    1    417    1    417    0.0    791
# BLASTP 2.2.20 [Feb-08-2009]
# Query: PTR-Fbxo3
# Database: 559_protein.db
# Query id, Subject id, % identity, alignment length, mismatches, gap openings, q. start, q. end, s. start, s. end, e-value, bit score
PTR-Fbxo3   HSA-Fbxo3   100.00   417    0     0    1    417    1    417    0.0    817
PTR-Fbxo3   PTR-Fbxo3   100.00   417    0     0    1    417    1    417    0.0    817
PTR-Fbxo3   PPY-Fbxo3   100.00   417    0     0    1    417    1    417    0.0    817
PTR-Fbxo3   GGO-Fbxo3   100.00   417    0     0    1    417    1    417    0.0    817
PTR-Fbxo3   CJA-Fbxo3   99.52    417    2     0    1    417    1    417    0.0    814
PTR-Fbxo3   MMU-Fbxo3   99.52    417    2     0    1    417    1    417    0.0    813
PTR-Fbxo3   RNO-Fbxo3   96.16    417    16    0    1    417    1    417    0.0    793
PTR-Fbxo3   MUS-Fbxo3   95.92    417    17    0    1    417    1    417    0.0    791
# BLASTP 2.2.20 [Feb-08-2009]
# Query: RNO-Fbxo3
# Database: 559_protein.db
# Query id, Subject id, % identity, alignment length, mismatches, gap openings, q. start, q. end, s. start, s. end, e-value, bit score
RNO-Fbxo3   RNO-Fbxo3   100.00   417    0     0    1    417    1    417    0.0    817
RNO-Fbxo3   MUS-Fbxo3   98.32    417    7     0    1    417    1    417    0.0    805
RNO-Fbxo3   CJA-Fbxo3   96.16    417    16    0    1    417    1    417    0.0    794
RNO-Fbxo3   HSA-Fbxo3   96.16    417    16    0    1    417    1    417    0.0    793
RNO-Fbxo3   PTR-Fbxo3   96.16    417    16    0    1    417    1    417    0.0    793
RNO-Fbxo3   PPY-Fbxo3   96.16    417    16    0    1    417    1    417    0.0    793
RNO-Fbxo3   GGO-Fbxo3   96.16    417    16    0    1    417    1    417    0.0    793
RNO-Fbxo3   MMU-Fbxo3   95.92    417    17    0    1    417    1    417    0.0    792
# BLASTP 2.2.20 [Feb-08-2009]
# Query: HSA-Fbxo3
# Database: 559_protein.db
```

```
# Query id, Subject id, % identity, alignment length, mismatches, gap openings, q. start, q. end, s. start, s. end, e-value, bit score
HSA-Fbxo3   HSA-Fbxo3   100.00      417         0           0           1           417         1           417         0.0         817
HSA-Fbxo3   PTR-Fbxo3   100.00      417         0           0           1           417         1           417         0.0         817
HSA-Fbxo3   PPY-Fbxo3   100.00      417         0           0           1           417         1           417         0.0         817
HSA-Fbxo3   GGO-Fbxo3   100.00      417         0           0           1           417         1           417         0.0         817
HSA-Fbxo3   CJA-Fbxo3   99.52       417         2           0           1           417         1           417         0.0         814
HSA-Fbxo3   MMU-Fbxo3   99.52       417         2           0           1           417         1           417         0.0         813
HSA-Fbxo3   RNO-Fbxo3   96.16       417         16          0           1           417         1           417         0.0         793
HSA-Fbxo3   MUS-Fbxo3   95.92       417         17          0           1           417         1           417         0.0         791
# BLASTP 2.2.20 [Feb-08-2009]
# Query: CJA-Fbxo30
# Database: 559_protein.db
# Query id, Subject id, % identity, alignment length, mismatches, gap openings, q. start, q. end, s. start, s. end, e-value, bit score
CJA-Fbxo30  CJA-Fbxo30  100.00      747         0           0           1           747         1           747         0.0         1551
CJA-Fbxo30  HSA-Fbxo30  95.45       748         30          2           1           747         1           745         0.0         1483
CJA-Fbxo30  MMU-Fbxo30  95.31       747         32          1           1           747         1           744         0.0         1482
CJA-Fbxo30  PTR-Fbxo30  95.32       748         31          2           1           747         1           745         0.0         1481
CJA-Fbxo30  GGO-Fbxo30  95.32       748         31          2           1           747         1           745         0.0         1481
CJA-Fbxo30  PPY-Fbxo30  89.84       748         35          3           1           747         1           708         0.0         1373
CJA-Fbxo30  MUS-Fbxo30  87.17       748         93          3           1           747         1           746         0.0         1357
CJA-Fbxo30  RNO-Fbxo30  86.23       748         96          5           1           747         1           742         0.0         1339
# BLASTP 2.2.20 [Feb-08-2009]
# Query: GGO-Fbxo30
# Database: 559_protein.db
# Query id, Subject id, % identity, alignment length, mismatches, gap openings, q. start, q. end, s. start, s. end, e-value, bit score
GGO-Fbxo30  GGO-Fbxo30  100.00      745         0           0           1           745         1           745         0.0         1550
GGO-Fbxo30  HSA-Fbxo30  99.87       745         1           0           1           745         1           745         0.0         1548
GGO-Fbxo30  PTR-Fbxo30  99.60       745         3           0           1           745         1           745         0.0         1546
GGO-Fbxo30  MMU-Fbxo30  98.52       745         10          1           1           745         1           744         0.0         1527
GGO-Fbxo30  CJA-Fbxo30  95.32       748         31          2           1           745         1           747         0.0         1481
GGO-Fbxo30  PPY-Fbxo30  94.09       745         7           1           1           745         1           708         0.0         1440
GGO-Fbxo30  MUS-Fbxo30  87.72       749         85          5           1           745         1           746         0.0         1363
GGO-Fbxo30  RNO-Fbxo30  86.92       749         87          7           1           745         1           742         0.0         1346
# BLASTP 2.2.20 [Feb-08-2009]
# Query: MMU-Fbxo30
# Database: 559_protein.db
# Query id, Subject id, % identity, alignment length, mismatches, gap openings, q. start, q. end, s. start, s. end, e-value, bit score
MMU-Fbxo30  MMU-Fbxo30  100.00      744         0           0           1           744         1           744         0.0         1549
MMU-Fbxo30  GGO-Fbxo30  98.52       745         10          1           1           744         1           745         0.0         1527
MMU-Fbxo30  HSA-Fbxo30  98.39       745         11          1           1           744         1           745         0.0         1525
MMU-Fbxo30  PTR-Fbxo30  98.26       745         12          1           1           744         1           745         0.0         1524
MMU-Fbxo30  CJA-Fbxo30  95.31       747         32          1           1           744         1           747         0.0         1482
MMU-Fbxo30  PPY-Fbxo30  92.89       745         15          2           1           744         1           708         0.0         1419
MMU-Fbxo30  MUS-Fbxo30  87.83       748         85          4           1           744         1           746         0.0         1363
MMU-Fbxo30  RNO-Fbxo30  86.90       748         88          6           1           744         1           742         0.0         1346
```

```
# BLASTP 2.2.20 [Feb-08-2009]
# Query: MUS-Fbxo30
# Database: 559_protein.db
# Query id, Subject id, % identity, alignment length, mismatches, gap openings, q. start, q. end, s. start, s. end, e-value, bit score
MUS-Fbxo30  MUS-Fbxo30  100.00      746         0           0           1           746         1           746         0.0         1555
MUS-Fbxo30  RNO-Fbxo30  93.57       746         44          2           1           746         1           742         0.0         1453
MUS-Fbxo30  MMU-Fbxo30  87.83       748         85          4           1           746         1           744         0.0         1363
MUS-Fbxo30  GGO-Fbxo30  87.72       749         85          5           1           746         1           745         0.0         1363
MUS-Fbxo30  HSA-Fbxo30  87.58       749         86          5           1           746         1           745         0.0         1361
MUS-Fbxo30  PTR-Fbxo30  87.45       749         87          5           1           746         1           745         0.0         1358
MUS-Fbxo30  CJA-Fbxo30  87.17       748         93          3           1           746         1           747         0.0         1357
MUS-Fbxo30  PPY-Fbxo30  83.04       749         83          6           1           746         1           708         0.0         1266
# BLASTP 2.2.20 [Feb-08-2009]
# Query: PPY-Fbxo30
# Database: 559_protein.db
# Query id, Subject id, % identity, alignment length, mismatches, gap openings, q. start, q. end, s. start, s. end, e-value, bit score
PPY-Fbxo30  PPY-Fbxo30  100.00      708         0           0           1           708         1           708         0.0         1472
PPY-Fbxo30  GGO-Fbxo30  94.09       745         7           1           1           708         1           745         0.0         1440
PPY-Fbxo30  HSA-Fbxo30  93.96       745         8           1           1           708         1           745         0.0         1438
PPY-Fbxo30  PTR-Fbxo30  93.83       745         9           1           1           708         1           745         0.0         1437
PPY-Fbxo30  MMU-Fbxo30  92.89       745         15          2           1           708         1           744         0.0         1419
PPY-Fbxo30  CJA-Fbxo30  89.84       748         35          3           1           708         1           747         0.0         1373
PPY-Fbxo30  MUS-Fbxo30  83.04       749         83          6           1           708         1           746         0.0         1266
PPY-Fbxo30  RNO-Fbxo30  82.11       749         86          8           1           708         1           742         0.0         1245
# BLASTP 2.2.20 [Feb-08-2009]
# Query: PTR-Fbxo30
# Database: 559_protein.db
# Query id, Subject id, % identity, alignment length, mismatches, gap openings, q. start, q. end, s. start, s. end, e-value, bit score
PTR-Fbxo30  PTR-Fbxo30  100.00      745         0           0           1           745         1           745         0.0         1551
PTR-Fbxo30  HSA-Fbxo30  99.73       745         2           0           1           745         1           745         0.0         1547
PTR-Fbxo30  GGO-Fbxo30  99.60       745         3           0           1           745         1           745         0.0         1546
PTR-Fbxo30  MMU-Fbxo30  98.26       745         12          1           1           745         1           744         0.0         1524
PTR-Fbxo30  CJA-Fbxo30  95.32       748         31          2           1           745         1           747         0.0         1481
PTR-Fbxo30  PPY-Fbxo30  93.83       745         9           1           1           745         1           708         0.0         1437
PTR-Fbxo30  MUS-Fbxo30  87.45       749         87          5           1           745         1           746         0.0         1358
PTR-Fbxo30  RNO-Fbxo30  86.92       749         87          7           1           745         1           742         0.0         1345
# BLASTP 2.2.20 [Feb-08-2009]
# Query: RNO-Fbxo30
# Database: 559_protein.db
# Query id, Subject id, % identity, alignment length, mismatches, gap openings, q. start, q. end, s. start, s. end, e-value, bit score
RNO-Fbxo30  RNO-Fbxo30  100.00      742         0           0           1           742         1           742         0.0         1546
RNO-Fbxo30  MUS-Fbxo30  93.57       746         44          2           1           742         1           746         0.0         1453
RNO-Fbxo30  HSA-Fbxo30  87.05       749         86          7           1           742         1           745         0.0         1347
RNO-Fbxo30  MMU-Fbxo30  86.90       748         88          6           1           742         1           744         0.0         1346
RNO-Fbxo30  GGO-Fbxo30  86.92       749         87          7           1           742         1           745         0.0         1346
```

```
RNO-Fbxo30  PTR-Fbxo30  86.92       749         87          7           1           742         1           745         0.0          1345
RNO-Fbxo30  CJA-Fbxo30  86.23       748         96          5           1           742         1           747         0.0          1339
RNO-Fbxo30  PPY-Fbxo30  82.11       749         86          8           1           742         1           708         0.0          1245
# BLASTP 2.2.20 [Feb-08-2009]
# Query: HSA-Fbxo30
# Database: 559_protein.db
# Query id, Subject id, % identity, alignment length, mismatches, gap openings, q. start, q. end, s. start, s. end, e-value, bit score
HSA-Fbxo30  HSA-Fbxo30  100.00      745         0           0           1           745         1           745         0.0          1550
HSA-Fbxo30  GGO-Fbxo30  99.87       745         1           0           1           745         1           745         0.0          1548
HSA-Fbxo30  PTR-Fbxo30  99.73       745         2           0           1           745         1           745         0.0          1547
HSA-Fbxo30  MMU-Fbxo30  98.39       745         11          1           1           745         1           744         0.0          1525
HSA-Fbxo30  CJA-Fbxo30  95.45       748         30          2           1           745         1           747         0.0          1483
HSA-Fbxo30  PPY-Fbxo30  93.96       745         8           1           1           745         1           708         0.0          1438
HSA-Fbxo30  MUS-Fbxo30  87.58       749         86          5           1           745         1           746         0.0          1361
HSA-Fbxo30  RNO-Fbxo30  87.05       749         86          7           1           745         1           742         0.0          1347
# BLASTP 2.2.20 [Feb-08-2009]
# Query: CJA-Fbxo31
# Database: 559_protein.db
# Query id, Subject id, % identity, alignment length, mismatches, gap openings, q. start, q. end, s. start, s. end, e-value, bit score
CJA-Fbxo31  CJA-Fbxo31  100.00      544         0           0           1           544         1           544         0.0           915
CJA-Fbxo31  GGO-Fbxo31  95.22       544         25          1           1           544         1           543         0.0           900
CJA-Fbxo31  PPY-Fbxo31  94.85       544         27          1           1           544         1           543         0.0           898
CJA-Fbxo31  MMU-Fbxo31  94.30       544         30          1           1           544         1           543         0.0           897
CJA-Fbxo31  HSA-Fbxo31  91.18       544         43          2           1           544         1           539         0.0           895
CJA-Fbxo31  PTR-Fbxo31  94.67       544         28          1           1           544         1           543         0.0           895
CJA-Fbxo31  MUS-Fbxo31  82.35       544         59          3           1           544         1           507         0.0           830
# BLASTP 2.2.20 [Feb-08-2009]
# Query: GGO-Fbxo31
# Database: 559_protein.db
# Query id, Subject id, % identity, alignment length, mismatches, gap openings, q. start, q. end, s. start, s. end, e-value, bit score
GGO-Fbxo31  GGO-Fbxo31  100.00      543         0           0           1           543         1           543         0.0           903
GGO-Fbxo31  PPY-Fbxo31  99.63       543         2           0           1           543         1           543         0.0           901
GGO-Fbxo31  PTR-Fbxo31  99.45       543         3           0           1           543         1           543         0.0           898
GGO-Fbxo31  MMU-Fbxo31  98.34       543         9           0           1           543         1           543         0.0           898
GGO-Fbxo31  HSA-Fbxo31  95.40       543         21          1           1           543         1           539         0.0           897
GGO-Fbxo31  CJA-Fbxo31  95.22       544         25          1           1           543         1           544         0.0           889
GGO-Fbxo31  MUS-Fbxo31  82.69       543         58          3           1           543         1           507         0.0           825
# BLASTP 2.2.20 [Feb-08-2009]
# Query: MMU-Fbxo31
# Database: 559_protein.db
# Query id, Subject id, % identity, alignment length, mismatches, gap openings, q. start, q. end, s. start, s. end, e-value, bit score
MMU-Fbxo31  MMU-Fbxo31  100.00      543         0           0           1           543         1           543         0.0           938
MMU-Fbxo31  GGO-Fbxo31  98.34       543         9           0           1           543         1           543         0.0           925
MMU-Fbxo31  PPY-Fbxo31  98.16       543         10          0           1           543         1           543         0.0           923
MMU-Fbxo31  PTR-Fbxo31  97.79       543         12          0           1           543         1           543         0.0           920
```

```
MMU-Fbxo31  HSA-Fbxo31  93.74   543   30   1   1   543   1   539   0.0      920
MMU-Fbxo31  CJA-Fbxo31  94.30   544   30   1   1   543   1   544   0.0      910
MMU-Fbxo31  MUS-Fbxo31  81.40   543   65   3   1   543   1   507   0.0      830
# BLASTP 2.2.20 [Feb-08-2009]
# Query: MUS-Fbxo31
# Database: 559_protein.db
# Query id, Subject id, % identity, alignment length, mismatches, gap openings, q. start, q. end, s. start, s. end, e-value, bit score
MUS-Fbxo31  MUS-Fbxo31  100.00  507   0    0   1   507   1   507   0.0      847
MUS-Fbxo31  CJA-Fbxo31  90.93   375   16   1   1   357   1   375   4e-180   622
MUS-Fbxo31  HSA-Fbxo31  91.91   371   16   1   1   357   1   371   1e-179   621
MUS-Fbxo31  PTR-Fbxo31  90.67   375   17   1   1   357   1   375   1e-179   620
MUS-Fbxo31  GGO-Fbxo31  90.93   375   16   1   1   357   1   375   2e-179   620
MUS-Fbxo31  MMU-Fbxo31  90.67   375   17   1   1   357   1   375   3e-179   619
MUS-Fbxo31  PPY-Fbxo31  90.67   375   17   1   1   357   1   375   1e-178   617
# BLASTP 2.2.20 [Feb-08-2009]
# Query: PPY-Fbxo31
# Database: 559_protein.db
# Query id, Subject id, % identity, alignment length, mismatches, gap openings, q. start, q. end, s. start, s. end, e-value, bit score
PPY-Fbxo31  PPY-Fbxo31  100.00  543   0    0   1   543   1   543   0.0      952
PPY-Fbxo31  GGO-Fbxo31  99.63   543   2    0   1   543   1   543   0.0      946
PPY-Fbxo31  PTR-Fbxo31  99.08   543   5    0   1   543   1   543   0.0      942
PPY-Fbxo31  HSA-Fbxo31  95.03   543   23   1   1   543   1   539   0.0      941
PPY-Fbxo31  MMU-Fbxo31  98.16   543   10   0   1   543   1   543   0.0      938
PPY-Fbxo31  CJA-Fbxo31  94.85   544   27   1   1   543   1   544   0.0      927
PPY-Fbxo31  MUS-Fbxo31  82.32   543   60   3   1   543   1   507   0.0      848
# BLASTP 2.2.20 [Feb-08-2009]
# Query: PTR-Fbxo31
# Database: 559_protein.db
# Query id, Subject id, % identity, alignment length, mismatches, gap openings, q. start, q. end, s. start, s. end, e-value, bit score
PTR-Fbxo31  PTR-Fbxo31  100.00  543   0    0   1   543   1   543   0.0      905
PTR-Fbxo31  GGO-Fbxo31  99.45   543   3    0   1   543   1   543   0.0      899
PTR-Fbxo31  PPY-Fbxo31  99.08   543   5    0   1   543   1   543   0.0      897
PTR-Fbxo31  HSA-Fbxo31  94.84   543   24   1   1   543   1   539   0.0      894
PTR-Fbxo31  MMU-Fbxo31  97.79   543   12   0   1   543   1   543   0.0      894
PTR-Fbxo31  CJA-Fbxo31  94.67   544   28   1   1   543   1   544   0.0      884
PTR-Fbxo31  MUS-Fbxo31  82.50   543   59   3   1   543   1   507   0.0      825
# BLASTP 2.2.20 [Feb-08-2009]
# Query: HSA-Fbxo31
# Database: 559_protein.db
# Query id, Subject id, % identity, alignment length, mismatches, gap openings, q. start, q. end, s. start, s. end, e-value, bit score
HSA-Fbxo31  HSA-Fbxo31  100.00  539   0    0   1   539   1   539   0.0      913
HSA-Fbxo31  GGO-Fbxo31  99.26   543   0    1   1   539   1   543   0.0      908
HSA-Fbxo31  PPY-Fbxo31  98.90   543   2    1   1   539   1   543   0.0      905
HSA-Fbxo31  PTR-Fbxo31  98.71   543   3    1   1   539   1   543   0.0      904
HSA-Fbxo31  MMU-Fbxo31  97.61   543   9    1   1   539   1   543   0.0      904
```

```
HSA-Fbxo31  CJA-Fbxo31  94.49       544         25          2           1           539         1           544         0.0         892
HSA-Fbxo31  MUS-Fbxo31  85.34       539         47          3           1           539         1           507         0.0         830
# BLASTP 2.2.20 [Feb-08-2009]
# Query: CJA-Fbxo32
# Database: 559_protein.db
# Query id, Subject id, % identity, alignment length, mismatches, gap openings, q. start, q. end, s. start, s. end, e-value, bit score
CJA-Fbxo32  CJA-Fbxo32  100.00      355         0           0           1           355         1           355         0.0         737
CJA-Fbxo32  MMU-Fbxo32  99.72       355         1           0           1           355         1           355         0.0         736
CJA-Fbxo32  GGO-Fbxo32  99.72       355         1           0           1           355         1           355         0.0         736
CJA-Fbxo32  HSA-Fbxo32  99.44       355         2           0           1           355         1           355         0.0         734
CJA-Fbxo32  PTR-Fbxo32  98.59       355         5           0           1           355         1           355         0.0         728
CJA-Fbxo32  MUS-Fbxo32  96.62       355         12          0           1           355         1           355         0.0         717
CJA-Fbxo32  RNO-Fbxo32  95.77       355         15          0           1           355         1           355         0.0         707
CJA-Fbxo32  PTR-Fbxo25  60.16       369         131         4           1           355         1           367         1e-128      451
CJA-Fbxo32  MMU-Fbxo25  60.16       369         131         4           1           355         1           367         1e-128      451
CJA-Fbxo32  HSA-Fbxo25  60.16       369         131         4           1           355         1           367         3e-128      449
CJA-Fbxo32  CJA-Fbxo25  59.89       369         132         4           1           355         1           367         4e-128      449
CJA-Fbxo32  PPY-Fbxo25  60.16       369         130         5           1           355         1           366         4e-127      446
CJA-Fbxo32  MUS-Fbxo25  60.05       368         131         6           1           355         1           365         1e-123      434
CJA-Fbxo32  GGO-Fbxo25  61.80       322         112         2           45          355         1           322         9e-117      411
# BLASTP 2.2.20 [Feb-08-2009]
# Query: GGO-Fbxo32
# Database: 559_protein.db
# Query id, Subject id, % identity, alignment length, mismatches, gap openings, q. start, q. end, s. start, s. end, e-value, bit score
GGO-Fbxo32  MMU-Fbxo32  100.00      355         0           0           1           355         1           355         0.0         737
GGO-Fbxo32  GGO-Fbxo32  100.00      355         0           0           1           355         1           355         0.0         737
GGO-Fbxo32  CJA-Fbxo32  99.72       355         1           0           1           355         1           355         0.0         736
GGO-Fbxo32  HSA-Fbxo32  99.72       355         1           0           1           355         1           355         0.0         736
GGO-Fbxo32  PTR-Fbxo32  98.87       355         4           0           1           355         1           355         0.0         729
GGO-Fbxo32  MUS-Fbxo32  96.90       355         11          0           1           355         1           355         0.0         718
GGO-Fbxo32  RNO-Fbxo32  96.06       355         14          0           1           355         1           355         0.0         709
GGO-Fbxo32  PTR-Fbxo25  60.16       369         131         4           1           355         1           367         1e-128      451
GGO-Fbxo32  MMU-Fbxo25  60.16       369         131         4           1           355         1           367         1e-128      451
GGO-Fbxo32  HSA-Fbxo25  60.16       369         131         4           1           355         1           367         3e-128      449
GGO-Fbxo32  CJA-Fbxo25  59.89       369         132         4           1           355         1           367         4e-128      449
GGO-Fbxo32  PPY-Fbxo25  60.16       369         130         5           1           355         1           366         4e-127      446
GGO-Fbxo32  MUS-Fbxo25  60.05       368         131         6           1           355         1           365         1e-123      434
GGO-Fbxo32  GGO-Fbxo25  61.80       322         112         2           45          355         1           322         1e-116      411
# BLASTP 2.2.20 [Feb-08-2009]
# Query: MMU-Fbxo32
# Database: 559_protein.db
# Query id, Subject id, % identity, alignment length, mismatches, gap openings, q. start, q. end, s. start, s. end, e-value, bit score
MMU-Fbxo32  MMU-Fbxo32  100.00      355         0           0           1           355         1           355         0.0         737
MMU-Fbxo32  GGO-Fbxo32  100.00      355         0           0           1           355         1           355         0.0         737
MMU-Fbxo32  CJA-Fbxo32  99.72       355         1           0           1           355         1           355         0.0         736
```

```
MMU-Fbxo32  HSA-Fbxo32  99.72     355     1       0       1       355     1       355     0.0       736
MMU-Fbxo32  PTR-Fbxo32  98.87     355     4       0       1       355     1       355     0.0       729
MMU-Fbxo32  MUS-Fbxo32  96.90     355     11      0       1       355     1       355     0.0       718
MMU-Fbxo32  RNO-Fbxo32  96.06     355     14      0       1       355     1       355     0.0       709
MMU-Fbxo32  PTR-Fbxo25  60.16     369     131     4       1       355     1       367     1e-128    451
MMU-Fbxo32  MMU-Fbxo25  60.16     369     131     4       1       355     1       367     1e-128    451
MMU-Fbxo32  HSA-Fbxo25  60.16     369     131     4       1       355     1       367     3e-128    449
MMU-Fbxo32  CJA-Fbxo25  59.89     369     132     4       1       355     1       367     4e-128    449
MMU-Fbxo32  PPY-Fbxo25  60.16     369     130     5       1       355     1       366     4e-127    446
MMU-Fbxo32  MUS-Fbxo25  60.05     368     131     6       1       355     1       365     1e-123    434
MMU-Fbxo32  GGO-Fbxo25  61.80     322     112     2       45      355     1       322     1e-116    411
# BLASTP 2.2.20 [Feb-08-2009]
# Query: MUS-Fbxo32
# Database: 559_protein.db
# Query id, Subject id, % identity, alignment length, mismatches, gap openings, q. start, q. end, s. start, s. end, e-value, bit score
MUS-Fbxo32  MUS-Fbxo32  100.00    355     0       0       1       355     1       355     0.0       736
MUS-Fbxo32  RNO-Fbxo32  98.03     355     7       0       1       355     1       355     0.0       720
MUS-Fbxo32  MMU-Fbxo32  96.90     355     11      0       1       355     1       355     0.0       718
MUS-Fbxo32  GGO-Fbxo32  96.90     355     11      0       1       355     1       355     0.0       718
MUS-Fbxo32  CJA-Fbxo32  96.62     355     12      0       1       355     1       355     0.0       717
MUS-Fbxo32  HSA-Fbxo32  96.62     355     12      0       1       355     1       355     0.0       717
MUS-Fbxo32  PTR-Fbxo32  96.34     355     13      0       1       355     1       355     0.0       712
MUS-Fbxo32  CJA-Fbxo25  58.54     369     137     3       1       355     1       367     2e-125    440
MUS-Fbxo32  PTR-Fbxo25  58.27     369     138     4       1       355     1       367     8e-125    438
MUS-Fbxo32  MMU-Fbxo25  58.27     369     138     4       1       355     1       367     8e-125    438
MUS-Fbxo32  HSA-Fbxo25  58.27     369     138     4       1       355     1       367     3e-124    436
MUS-Fbxo32  PPY-Fbxo25  58.27     369     137     5       1       355     1       366     3e-123    432
MUS-Fbxo32  MUS-Fbxo25  58.70     368     136     6       1       355     1       365     2e-120    424
MUS-Fbxo32  GGO-Fbxo25  60.83     314     114     1       51      355     9       322     2e-113    400
# BLASTP 2.2.20 [Feb-08-2009]
# Query: PTR-Fbxo32
# Database: 559_protein.db
# Query id, Subject id, % identity, alignment length, mismatches, gap openings, q. start, q. end, s. start, s. end, e-value, bit score
PTR-Fbxo32  PTR-Fbxo32  100.00    355     0       0       1       355     1       355     0.0       736
PTR-Fbxo32  HSA-Fbxo32  99.15     355     3       0       1       355     1       355     0.0       731
PTR-Fbxo32  MMU-Fbxo32  98.87     355     4       0       1       355     1       355     0.0       729
PTR-Fbxo32  GGO-Fbxo32  98.87     355     4       0       1       355     1       355     0.0       729
PTR-Fbxo32  CJA-Fbxo32  98.59     355     5       0       1       355     1       355     0.0       728
PTR-Fbxo32  MUS-Fbxo32  96.34     355     13      0       1       355     1       355     0.0       712
PTR-Fbxo32  RNO-Fbxo32  95.49     355     16      0       1       355     1       355     0.0       703
PTR-Fbxo32  PTR-Fbxo25  59.62     369     133     4       1       355     1       367     1e-127    447
PTR-Fbxo32  MMU-Fbxo25  59.62     369     133     4       1       355     1       367     1e-127    447
PTR-Fbxo32  HSA-Fbxo25  59.62     369     133     4       1       355     1       367     4e-127    446
PTR-Fbxo32  CJA-Fbxo25  59.35     369     134     4       1       355     1       367     4e-127    446
PTR-Fbxo32  PPY-Fbxo25  59.62     369     132     5       1       355     1       366     6e-126    442
```

```
PTR-Fbxo32  MUS-Fbxo25  59.51   368     133     6       1       355     1       365     1e-122  431
PTR-Fbxo32  GGO-Fbxo25  61.49   322     113     2       45      355     1       322     4e-116  409
# BLASTP 2.2.20 [Feb-08-2009]
# Query: RNO-Fbxo32
# Database: 559_protein.db
# Query id, Subject id, % identity, alignment length, mismatches, gap openings, q. start, q. end, s. start, s. end, e-value, bit score
RNO-Fbxo32  RNO-Fbxo32  100.00  355     0       0       1       355     1       355     0.0     737
RNO-Fbxo32  MUS-Fbxo32  98.03   355     7       0       1       355     1       355     0.0     720
RNO-Fbxo32  MMU-Fbxo32  96.06   355     14      0       1       355     1       355     0.0     709
RNO-Fbxo32  GGO-Fbxo32  96.06   355     14      0       1       355     1       355     0.0     709
RNO-Fbxo32  CJA-Fbxo32  95.77   355     15      0       1       355     1       355     0.0     707
RNO-Fbxo32  HSA-Fbxo32  95.77   355     15      0       1       355     1       355     0.0     707
RNO-Fbxo32  PTR-Fbxo32  95.49   355     16      0       1       355     1       355     0.0     703
RNO-Fbxo32  PTR-Fbxo25  58.54   369     137     4       1       355     1       367     2e-124  437
RNO-Fbxo32  MMU-Fbxo25  58.54   369     137     4       1       355     1       367     2e-124  437
RNO-Fbxo32  CJA-Fbxo25  57.99   369     139     3       1       355     1       367     3e-124  436
RNO-Fbxo32  HSA-Fbxo25  58.54   369     137     4       1       355     1       367     4e-124  436
RNO-Fbxo32  PPY-Fbxo25  58.54   369     136     5       1       355     1       366     6e-123  432
RNO-Fbxo32  MUS-Fbxo25  58.70   368     136     5       1       355     1       365     7e-121  425
RNO-Fbxo32  GGO-Fbxo25  61.15   314     113     1       51      355     9       322     9e-114  401
# BLASTP 2.2.20 [Feb-08-2009]
# Query: HSA-Fbxo32
# Database: 559_protein.db
# Query id, Subject id, % identity, alignment length, mismatches, gap openings, q. start, q. end, s. start, s. end, e-value, bit score
HSA-Fbxo32  HSA-Fbxo32  100.00  355     0       0       1       355     1       355     0.0     738
HSA-Fbxo32  MMU-Fbxo32  99.72   355     1       0       1       355     1       355     0.0     736
HSA-Fbxo32  GGO-Fbxo32  99.72   355     1       0       1       355     1       355     0.0     736
HSA-Fbxo32  CJA-Fbxo32  99.44   355     2       0       1       355     1       355     0.0     734
HSA-Fbxo32  PTR-Fbxo32  99.15   355     3       0       1       355     1       355     0.0     731
HSA-Fbxo32  MUS-Fbxo32  96.62   355     12      0       1       355     1       355     0.0     717
HSA-Fbxo32  RNO-Fbxo32  95.77   355     15      0       1       355     1       355     0.0     707
HSA-Fbxo32  PTR-Fbxo25  59.89   369     132     4       1       355     1       367     3e-128  449
HSA-Fbxo32  MMU-Fbxo25  59.89   369     132     4       1       355     1       367     3e-128  449
HSA-Fbxo32  HSA-Fbxo25  59.89   369     132     4       1       355     1       367     9e-128  447
HSA-Fbxo32  CJA-Fbxo25  59.62   369     133     4       1       355     1       367     1e-127  447
HSA-Fbxo32  PPY-Fbxo25  59.89   369     131     5       1       355     1       366     1e-126  444
HSA-Fbxo32  MUS-Fbxo25  59.78   368     132     6       1       355     1       365     3e-123  433
HSA-Fbxo32  GGO-Fbxo25  61.80   322     112     2       45      355     1       322     1e-116  411
# BLASTP 2.2.20 [Feb-08-2009]
# Query: CJA-Fbxo33
# Database: 559_protein.db
# Query id, Subject id, % identity, alignment length, mismatches, gap openings, q. start, q. end, s. start, s. end, e-value, bit score
CJA-Fbxo33  CJA-Fbxo33  100.00  558     0       0       1       558     1       558     0.0     1004
CJA-Fbxo33  HSA-Fbxo33  95.34   558     23      1       1       558     1       555     0.0     987
CJA-Fbxo33  GGO-Fbxo33  95.34   558     23      1       1       558     1       555     0.0     987
```

```
CJA-Fbxo33  PPY-Fbxo33  98.01   503   10   0   56   558   53   555   0.0   984
CJA-Fbxo33  PTR-Fbxo33  95.70   558   23   1   1   558   1   557   0.0   983
CJA-Fbxo33  MMU-Fbxo33  94.09   558   30   1   1   558   1   555   0.0   979
CJA-Fbxo33  RNO-Fbxo33  90.59   563   48   1   1   558   1   563   0.0   931
CJA-Fbxo33  MUS-Fbxo33  90.04   562   52   1   1   558   1   562   0.0   930
# BLASTP 2.2.20 [Feb-08-2009]
# Query: GGO-Fbxo33
# Database: 559_protein.db
# Query id, Subject id, % identity, alignment length, mismatches, gap openings, q. start, q. end, s. start, s. end, e-value, bit score
GGO-Fbxo33  GGO-Fbxo33  100.00   497   0   0   59   555   59   555   0.0   979
GGO-Fbxo33  PPY-Fbxo33  99.20   497   4   0   59   555   59   555   0.0   978
GGO-Fbxo33  HSA-Fbxo33  99.60   497   2   0   59   555   59   555   0.0   978
GGO-Fbxo33  PTR-Fbxo33  99.60   497   2   0   59   555   61   557   0.0   977
GGO-Fbxo33  CJA-Fbxo33  98.59   497   7   0   59   555   62   558   0.0   972
GGO-Fbxo33  MMU-Fbxo33  98.79   497   6   0   59   555   59   555   0.0   971
GGO-Fbxo33  MUS-Fbxo33  92.22   501   35   1   59   555   62   562   0.0   927
GGO-Fbxo33  RNO-Fbxo33  92.43   502   33   1   59   555   62   563   0.0   921
# BLASTP 2.2.20 [Feb-08-2009]
# Query: MMU-Fbxo33
# Database: 559_protein.db
# Query id, Subject id, % identity, alignment length, mismatches, gap openings, q. start, q. end, s. start, s. end, e-value, bit score
MMU-Fbxo33  MMU-Fbxo33  100.00   506   0   0   50   555   50   555   0.0   1000
MMU-Fbxo33  PPY-Fbxo33  98.42   505   8   0   51   555   51   555   0.0   988
MMU-Fbxo33  GGO-Fbxo33  98.61   505   7   0   51   555   51   555   0.0   987
MMU-Fbxo33  HSA-Fbxo33  98.61   505   7   0   51   555   51   555   0.0   986
MMU-Fbxo33  PTR-Fbxo33  98.61   505   7   0   51   555   53   557   0.0   986
MMU-Fbxo33  CJA-Fbxo33  97.43   505   13   0   51   555   54   558   0.0   979
MMU-Fbxo33  MUS-Fbxo33  91.05   503   41   1   57   555   60   562   0.0   923
MMU-Fbxo33  RNO-Fbxo33  90.82   512   40   2   51   555   52   563   0.0   921
# BLASTP 2.2.20 [Feb-08-2009]
# Query: MUS-Fbxo33
# Database: 559_protein.db
# Query id, Subject id, % identity, alignment length, mismatches, gap openings, q. start, q. end, s. start, s. end, e-value, bit score
MUS-Fbxo33  MUS-Fbxo33  100.00   504   0   0   59   562   59   562   0.0   974
MUS-Fbxo33  RNO-Fbxo33  96.23   504   18   1   60   562   60   563   0.0   935
MUS-Fbxo33  PPY-Fbxo33  91.65   503   38   1   60   562   57   555   0.0   931
MUS-Fbxo33  HSA-Fbxo33  91.85   503   37   1   60   562   57   555   0.0   931
MUS-Fbxo33  GGO-Fbxo33  92.05   503   36   1   60   562   57   555   0.0   929
MUS-Fbxo33  PTR-Fbxo33  91.85   503   37   1   60   562   59   557   0.0   927
MUS-Fbxo33  CJA-Fbxo33  90.85   503   42   1   60   562   60   558   0.0   925
MUS-Fbxo33  MMU-Fbxo33  91.05   503   41   1   60   562   57   555   0.0   923
# BLASTP 2.2.20 [Feb-08-2009]
# Query: PPY-Fbxo33
# Database: 559_protein.db
# Query id, Subject id, % identity, alignment length, mismatches, gap openings, q. start, q. end, s. start, s. end, e-value, bit score
```

```
PPY-Fbxo33  PPY-Fbxo33  100.00   497   0    0   59   555   59   555   0.0   975
PPY-Fbxo33  HSA-Fbxo33  99.20    497   4    0   59   555   59   555   0.0   972
PPY-Fbxo33  GGO-Fbxo33  99.20    497   4    0   59   555   59   555   0.0   971
PPY-Fbxo33  PTR-Fbxo33  99.20    497   4    0   59   555   61   557   0.0   969
PPY-Fbxo33  CJA-Fbxo33  98.19    497   9    0   59   555   62   558   0.0   967
PPY-Fbxo33  MMU-Fbxo33  98.79    497   6    0   59   555   59   555   0.0   966
PPY-Fbxo33  MUS-Fbxo33  91.82    501   37   1   59   555   62   562   0.0   928
PPY-Fbxo33  RNO-Fbxo33  92.03    502   35   1   59   555   62   563   0.0   916
# BLASTP 2.2.20 [Feb-08-2009]
# Query: PTR-Fbxo33
# Database: 559_protein.db
# Query id, Subject id, % identity, alignment length, mismatches, gap openings, q. start, q. end, s. start, s. end, e-value, bit score
PTR-Fbxo33  GGO-Fbxo33  99.60    497   2    0   61   557   59   555   0.0   979
PTR-Fbxo33  PPY-Fbxo33  99.20    497   4    0   61   557   59   555   0.0   978
PTR-Fbxo33  HSA-Fbxo33  99.60    497   2    0   61   557   59   555   0.0   978
PTR-Fbxo33  PTR-Fbxo33  100.00   497   0    0   61   557   61   557   0.0   977
PTR-Fbxo33  CJA-Fbxo33  98.79    497   6    0   61   557   62   558   0.0   972
PTR-Fbxo33  MMU-Fbxo33  98.79    497   6    0   61   557   59   555   0.0   971
PTR-Fbxo33  MUS-Fbxo33  92.02    501   36   1   61   557   62   562   0.0   927
PTR-Fbxo33  RNO-Fbxo33  92.23    502   34   1   61   557   62   563   0.0   921
# BLASTP 2.2.20 [Feb-08-2009]
# Query: RNO-Fbxo33
# Database: 559_protein.db
# Query id, Subject id, % identity, alignment length, mismatches, gap openings, q. start, q. end, s. start, s. end, e-value, bit score
RNO-Fbxo33  RNO-Fbxo33  100.00   563   0    0   1    563   1    563   0.0   958
RNO-Fbxo33  MUS-Fbxo33  95.56    563   24   1   1    563   1    562   0.0   939
RNO-Fbxo33  GGO-Fbxo33  89.17    563   53   2   1    563   1    555   0.0   925
RNO-Fbxo33  HSA-Fbxo33  88.99    563   54   2   1    563   1    555   0.0   924
RNO-Fbxo33  PTR-Fbxo33  88.10    563   61   2   1    563   1    557   0.0   922
RNO-Fbxo33  PPY-Fbxo33  88.63    563   56   2   1    563   1    555   0.0   920
RNO-Fbxo33  CJA-Fbxo33  90.59    563   48   1   1    563   1    558   0.0   919
RNO-Fbxo33  MMU-Fbxo33  87.92    563   60   2   1    563   1    555   0.0   917
# BLASTP 2.2.20 [Feb-08-2009]
# Query: HSA-Fbxo33
# Database: 559_protein.db
# Query id, Subject id, % identity, alignment length, mismatches, gap openings, q. start, q. end, s. start, s. end, e-value, bit score
HSA-Fbxo33  HSA-Fbxo33  100.00   497   0    0   59   555   59   555   0.0   982
HSA-Fbxo33  PPY-Fbxo33  99.20    497   4    0   59   555   59   555   0.0   979
HSA-Fbxo33  GGO-Fbxo33  99.60    497   2    0   59   555   59   555   0.0   978
HSA-Fbxo33  PTR-Fbxo33  99.60    497   2    0   59   555   61   557   0.0   976
HSA-Fbxo33  CJA-Fbxo33  98.59    497   7    0   59   555   62   558   0.0   973
HSA-Fbxo33  MMU-Fbxo33  98.79    497   6    0   59   555   59   555   0.0   971
HSA-Fbxo33  MUS-Fbxo33  92.02    501   36   1   59   555   62   562   0.0   928
HSA-Fbxo33  RNO-Fbxo33  92.23    502   34   1   59   555   62   563   0.0   920
# BLASTP 2.2.20 [Feb-08-2009]
```

```
# Query: CJA-Fbxo34
# Database: 559_protein.db
# Query id, Subject id, % identity, alignment length, mismatches, gap openings, q. start, q. end, s. start, s. end, e-value, bit score
CJA-Fbxo34  CJA-Fbxo34  100.00      709         0           0           1           709         1           709         0.0         1404
CJA-Fbxo34  PTR-Fbxo34  93.54       712         42          3           1           709         1           711         0.0         1298
CJA-Fbxo34  HSA-Fbxo34  93.40       712         43          3           1           709         1           711         0.0         1295
CJA-Fbxo34  MMU-Fbxo34  93.40       712         41          3           1           709         1           709         0.0         1295
CJA-Fbxo34  GGO-Fbxo34  93.12       712         45          3           1           709         1           711         0.0         1294
CJA-Fbxo34  PPY-Fbxo34  94.12       578         33          1           133         709         1           578         0.0         1055
CJA-Fbxo34  MUS-Fbxo34  71.49       712         183         7           1           709         52          746         0.0          958
CJA-Fbxo34  RNO-Fbxo34  70.56       710         179         8           1           709         52          732         0.0          926
CJA-Fbxo34  CJA-Fbxo46  66.20       142         48          0           550         691         450         591         1e-060       226
CJA-Fbxo34  RNO-Fbxo46  66.20       142         48          0           550         691         450         591         1e-060       226
CJA-Fbxo34  MUS-Fbxo46  66.20       142         48          0           550         691         450         591         1e-060       226
CJA-Fbxo34  GGO-Fbxo46  66.20       142         48          0           550         691         450         591         1e-060       226
CJA-Fbxo34  HSA-Fbxo46  66.20       142         48          0           550         691         450         591         1e-060       226
CJA-Fbxo34  MMU-Fbxo46  66.20       142         48          0           550         691         450         591         1e-060       226
CJA-Fbxo34  PPY-Fbxo46  66.20       142         48          0           550         691         450         591         2e-060       226
CJA-Fbxo34  PTR-Fbxo46  66.20       142         48          0           550         691         404         545         3e-060       224
# BLASTP 2.2.20 [Feb-08-2009]
# Query: GGO-Fbxo34
# Database: 559_protein.db
# Query id, Subject id, % identity, alignment length, mismatches, gap openings, q. start, q. end, s. start, s. end, e-value, bit score
GGO-Fbxo34  GGO-Fbxo34  100.00      711         0           0           1           711         1           711         0.0         1476
GGO-Fbxo34  PTR-Fbxo34  99.30       711         5           0           1           711         1           711         0.0         1467
GGO-Fbxo34  HSA-Fbxo34  99.02       711         7           0           1           711         1           711         0.0         1463
GGO-Fbxo34  MMU-Fbxo34  96.07       712         24          2           1           711         1           709         0.0         1419
GGO-Fbxo34  CJA-Fbxo34  93.12       712         45          3           1           711         1           709         0.0         1361
GGO-Fbxo34  PPY-Fbxo34  97.92       578         11          1           135         711         1           578         0.0         1174
GGO-Fbxo34  MUS-Fbxo34  71.59       711         186         4           1           711         52          746         0.0         1026
GGO-Fbxo34  RNO-Fbxo34  71.03       711         176         6           1           711         52          732         0.0         1001
GGO-Fbxo34  RNO-Fbxo46  67.38       141         46          0           552         692         450         590         3e-061       228
GGO-Fbxo34  CJA-Fbxo46  66.90       142         47          0           552         693         450         591         3e-061       228
GGO-Fbxo34  MMU-Fbxo46  66.90       142         47          0           552         693         450         591         4e-061       227
GGO-Fbxo34  PPY-Fbxo46  66.90       142         47          0           552         693         450         591         5e-061       227
GGO-Fbxo34  MUS-Fbxo46  67.38       141         46          0           552         692         450         590         5e-061       227
GGO-Fbxo34  GGO-Fbxo46  66.90       142         47          0           552         693         450         591         5e-061       227
GGO-Fbxo34  HSA-Fbxo46  66.90       142         47          0           552         693         450         591         5e-061       227
GGO-Fbxo34  PTR-Fbxo46  66.90       142         47          0           552         693         404         545         1e-060       226
# BLASTP 2.2.20 [Feb-08-2009]
# Query: MMU-Fbxo34
# Database: 559_protein.db
# Query id, Subject id, % identity, alignment length, mismatches, gap openings, q. start, q. end, s. start, s. end, e-value, bit score
MMU-Fbxo34  MMU-Fbxo34  100.00      709         0           0           1           709         1           709         0.0         1440
MMU-Fbxo34  PTR-Fbxo34  96.63       712         20          2           1           709         1           711         0.0         1395
```

```
MMU-Fbxo34  HSA-Fbxo34  96.49   712  21   2  1    709  1    711  0.0     1390
MMU-Fbxo34  GGO-Fbxo34  96.07   712  24   2  1    709  1    711  0.0     1388
MMU-Fbxo34  CJA-Fbxo34  93.40   712  41   3  1    709  1    709  0.0     1332
MMU-Fbxo34  PPY-Fbxo34  96.71   578  16   1  135  709  1    578  0.0     1132
MMU-Fbxo34  MUS-Fbxo34  72.19   712  178  6  1    709  52   746  0.0      996
MMU-Fbxo34  RNO-Fbxo34  70.79   712  174  8  1    709  52   732  0.0      960
MMU-Fbxo34  CJA-Fbxo46  66.90   142  47   0  550  691  450  591  4e-061   228
MMU-Fbxo34  RNO-Fbxo46  66.90   142  47   0  550  691  450  591  4e-061   228
MMU-Fbxo34  MUS-Fbxo46  66.90   142  47   0  550  691  450  591  5e-061   227
MMU-Fbxo34  MMU-Fbxo46  66.90   142  47   0  550  691  450  591  5e-061   227
MMU-Fbxo34  HSA-Fbxo46  66.90   142  47   0  550  691  450  591  5e-061   227
MMU-Fbxo34  GGO-Fbxo46  66.90   142  47   0  550  691  450  591  5e-061   227
MMU-Fbxo34  PPY-Fbxo46  66.90   142  47   0  550  691  450  591  6e-061   227
MMU-Fbxo34  PTR-Fbxo46  66.90   142  47   0  550  691  404  545  1e-060   226
# BLASTP 2.2.20 [Feb-08-2009]
# Query: MUS-Fbxo34
# Database: 559_protein.db
# Query id, Subject id, % identity, alignment length, mismatches, gap openings, q. start, q. end, s. start, s. end, e-value, bit score
MUS-Fbxo34  MUS-Fbxo34  100.00  746  0    0  1    746  1    746  0.0     1460
MUS-Fbxo34  RNO-Fbxo34  86.71   700  79   3  47   746  47   732  0.0     1233
MUS-Fbxo34  PTR-Fbxo34  72.01   711  183  4  52   746  1    711  0.0     1031
MUS-Fbxo34  HSA-Fbxo34  71.87   711  184  4  52   746  1    711  0.0     1027
MUS-Fbxo34  GGO-Fbxo34  71.59   711  186  4  52   746  1    711  0.0     1026
MUS-Fbxo34  MMU-Fbxo34  72.19   712  178  6  52   746  1    709  0.0     1021
MUS-Fbxo34  CJA-Fbxo34  71.49   712  183  7  52   746  1    709  0.0     1011
MUS-Fbxo34  PPY-Fbxo34  71.28   578  150  4  185  746  1    578  0.0      828
MUS-Fbxo34  PTR-Fbxo46  50.90   222  75   5  510  728  355  545  3e-060   224
MUS-Fbxo34  MUS-Fbxo46  59.15   164  64   2  567  727  427  590  6e-060   224
MUS-Fbxo34  CJA-Fbxo46  59.15   164  64   2  568  728  428  591  6e-060   223
MUS-Fbxo34  MMU-Fbxo46  59.15   164  64   2  568  728  428  591  7e-060   223
MUS-Fbxo34  GGO-Fbxo46  59.15   164  64   2  568  728  428  591  7e-060   223
MUS-Fbxo34  PPY-Fbxo46  59.15   164  64   2  568  728  428  591  8e-060   223
MUS-Fbxo34  HSA-Fbxo46  59.15   164  64   2  568  728  428  591  8e-060   223
MUS-Fbxo34  RNO-Fbxo46  50.00   220  78   4  510  727  401  590  1e-059   223
# BLASTP 2.2.20 [Feb-08-2009]
# Query: PTR-Fbxo34
# Database: 559_protein.db
# Query id, Subject id, % identity, alignment length, mismatches, gap openings, q. start, q. end, s. start, s. end, e-value, bit score
PTR-Fbxo34  PTR-Fbxo34  100.00  711  0    0  1    711  1    711  0.0     1477
PTR-Fbxo34  GGO-Fbxo34  99.30   711  5    0  1    711  1    711  0.0     1467
PTR-Fbxo34  HSA-Fbxo34  99.30   711  5    0  1    711  1    711  0.0     1464
PTR-Fbxo34  MMU-Fbxo34  96.63   712  20   2  1    711  1    709  0.0     1427
PTR-Fbxo34  CJA-Fbxo34  93.54   712  42   3  1    711  1    709  0.0     1367
PTR-Fbxo34  PPY-Fbxo34  98.27   578  9    1  135  711  1    578  0.0     1176
PTR-Fbxo34  MUS-Fbxo34  72.01   711  183  4  1    711  52   746  0.0     1031
```

```
PTR-Fbxo34  RNO-Fbxo34  71.17    711   175   6   1     711   52    732   0.0       1005
PTR-Fbxo34  RNO-Fbxo46  67.38    141   46    0   552   692   450   590   4e-061    228
PTR-Fbxo34  CJA-Fbxo46  66.90    142   47    0   552   693   450   591   4e-061    228
PTR-Fbxo34  MMU-Fbxo46  66.90    142   47    0   552   693   450   591   4e-061    228
PTR-Fbxo34  MUS-Fbxo46  67.38    141   46    0   552   692   450   590   5e-061    227
PTR-Fbxo34  HSA-Fbxo46  66.90    142   47    0   552   693   450   591   5e-061    227
PTR-Fbxo34  GGO-Fbxo46  66.90    142   47    0   552   693   450   591   5e-061    227
PTR-Fbxo34  PPY-Fbxo46  66.90    142   47    0   552   693   450   591   5e-061    227
PTR-Fbxo34  PTR-Fbxo46  66.90    142   47    0   552   693   404   545   9e-061    226
# BLASTP 2.2.20 [Feb-08-2009]
# Query: RNO-Fbxo34
# Database: 559_protein.db
# Query id, Subject id, % identity, alignment length, mismatches, gap openings, q. start, q. end, s. start, s. end, e-value, bit score
RNO-Fbxo34  RNO-Fbxo34  100.00   732   0     0   1     732   1     732   0.0       1403
RNO-Fbxo34  MUS-Fbxo34  86.86    746   84    3   1     732   1     746   0.0       1219
RNO-Fbxo34  PTR-Fbxo34  71.17    711   175   6   52    732   1     711   0.0       981
RNO-Fbxo34  HSA-Fbxo34  71.03    711   176   6   52    732   1     711   0.0       979
RNO-Fbxo34  GGO-Fbxo34  71.03    711   176   6   52    732   1     711   0.0       977
RNO-Fbxo34  MMU-Fbxo34  70.79    712   174   8   52    732   1     709   0.0       964
RNO-Fbxo34  CJA-Fbxo34  70.56    710   179   8   52    732   1     709   0.0       959
RNO-Fbxo34  PPY-Fbxo34  72.66    578   139   5   174   732   1     578   0.0       839
RNO-Fbxo34  PTR-Fbxo46  52.02    223   77    4   492   713   351   544   3e-061    228
RNO-Fbxo34  RNO-Fbxo46  50.00    224   80    4   492   713   397   590   7e-060    223
RNO-Fbxo34  CJA-Fbxo46  50.90    222   81    4   492   713   397   590   8e-060    223
RNO-Fbxo34  MMU-Fbxo46  50.90    222   81    4   492   713   397   590   8e-060    223
RNO-Fbxo34  PPY-Fbxo46  50.90    222   81    4   492   713   397   590   9e-060    223
RNO-Fbxo34  HSA-Fbxo46  50.90    222   81    4   492   713   397   590   1e-059    223
RNO-Fbxo34  GGO-Fbxo46  50.90    222   81    4   492   713   397   590   1e-059    223
RNO-Fbxo34  MUS-Fbxo46  61.78    157   54    2   559   713   438   590   3e-059    221
# BLASTP 2.2.20 [Feb-08-2009]
# Query: HSA-Fbxo34
# Database: 559_protein.db
# Query id, Subject id, % identity, alignment length, mismatches, gap openings, q. start, q. end, s. start, s. end, e-value, bit score
HSA-Fbxo34  HSA-Fbxo34  100.00   711   0     0   1     711   1     711   0.0       1476
HSA-Fbxo34  PTR-Fbxo34  99.30    711   5     0   1     711   1     711   0.0       1464
HSA-Fbxo34  GGO-Fbxo34  99.02    711   7     0   1     711   1     711   0.0       1463
HSA-Fbxo34  MMU-Fbxo34  96.49    712   21    2   1     711   1     709   0.0       1420
HSA-Fbxo34  CJA-Fbxo34  93.40    712   43    3   1     711   1     709   0.0       1362
HSA-Fbxo34  PPY-Fbxo34  98.27    578   9     1   135   711   1     578   0.0       1177
HSA-Fbxo34  MUS-Fbxo34  71.87    711   184   4   1     711   52    746   0.0       1027
HSA-Fbxo34  RNO-Fbxo34  71.03    711   176   6   1     711   52    732   0.0       1001
HSA-Fbxo34  CJA-Fbxo46  66.90    142   47    0   552   693   450   591   4e-061    228
HSA-Fbxo34  RNO-Fbxo46  67.38    141   46    0   552   692   450   590   4e-061    228
HSA-Fbxo34  MMU-Fbxo46  66.90    142   47    0   552   693   450   591   4e-061    227
HSA-Fbxo34  MUS-Fbxo46  67.38    141   46    0   552   692   450   590   5e-061    227
```

```
HSA-Fbxo34  PPY-Fbxo46  66.90       142         47          0           552         693         450         591         5e-061       227
HSA-Fbxo34  HSA-Fbxo46  66.90       142         47          0           552         693         450         591         5e-061       227
HSA-Fbxo34  GGO-Fbxo46  66.90       142         47          0           552         693         450         591         6e-061       227
HSA-Fbxo34  PTR-Fbxo46  66.90       142         47          0           552         693         404         545         1e-060       226
# BLASTP 2.2.20 [Feb-08-2009]
# Query: PPY-Fbxo34
# Database: 559_protein.db
# Query id, Subject id, % identity, alignment length, mismatches, gap openings, q. start, q. end, s. start, s. end, e-value, bit score
PPY-Fbxo34  PPY-Fbxo34  100.00      578         0           0           1           578         1           578         0.0         1198
PPY-Fbxo34  HSA-Fbxo34  98.27       578         9           1           1           578         135         711         0.0         1177
PPY-Fbxo34  PTR-Fbxo34  98.27       578         9           1           1           578         135         711         0.0         1176
PPY-Fbxo34  GGO-Fbxo34  97.92       578         11          1           1           578         135         711         0.0         1174
PPY-Fbxo34  MMU-Fbxo34  96.71       578         16          1           1           578         135         709         0.0         1164
PPY-Fbxo34  CJA-Fbxo34  94.12       578         33          1           1           578         133         709         0.0         1122
PPY-Fbxo34  RNO-Fbxo34  72.66       578         139         5           1           578         174         732         0.0          840
PPY-Fbxo34  MUS-Fbxo34  71.28       578         150         4           1           578         185         746         0.0          828
PPY-Fbxo34  RNO-Fbxo46  67.38       141         46          0           419         559         450         590         5e-061       227
PPY-Fbxo34  MUS-Fbxo46  67.38       141         46          0           419         559         450         590         5e-061       227
# BLASTP 2.2.20 [Feb-08-2009]
# Query: CJA-Fbxo36
# Database: 559_protein.db
# Query id, Subject id, % identity, alignment length, mismatches, gap openings, q. start, q. end, s. start, s. end, e-value, bit score
CJA-Fbxo36  CJA-Fbxo36  100.00      159         0           0           1           159         1           159         2e-093       333
CJA-Fbxo36  PTR-Fbxo36  90.45       157         15          0           1           157         1           157         2e-083       299
CJA-Fbxo36  HSA-Fbxo36  89.81       157         16          0           1           157         1           157         1e-082       296
CJA-Fbxo36  PPY-Fbxo36  88.68       159         16          1           1           157         1           159         2e-081       293
CJA-Fbxo36  RNO-Fbxo36  80.25       157         31          0           1           157         1           157         3e-075       272
CJA-Fbxo36  MUS-Fbxo36  78.34       157         34          0           1           157         1           157         1e-073       266
# BLASTP 2.2.20 [Feb-08-2009]
# Query: GGO-Fbxo36
# Database: 559_protein.db
# Query id, Subject id, % identity, alignment length, mismatches, gap openings, q. start, q. end, s. start, s. end, e-value, bit score
GGO-Fbxo36  GGO-Fbxo36  100.00      119         0           0           1           119         1           119         4e-058       214
GGO-Fbxo36  PPY-Fbxo36  99.16       119         1           0           1           119         72          190         8e-058       213
GGO-Fbxo36  PTR-Fbxo36  99.16       119         1           0           1           119         70          188         1e-057       213
GGO-Fbxo36  HSA-Fbxo36  98.32       119         2           0           1           119         70          188         4e-057       211
GGO-Fbxo36  MMU-Fbxo36  97.48       119         3           0           1           119         2           120         3e-056       208
# BLASTP 2.2.20 [Feb-08-2009]
# Query: MMU-Fbxo36
# Database: 559_protein.db
# Query id, Subject id, % identity, alignment length, mismatches, gap openings, q. start, q. end, s. start, s. end, e-value, bit score
MMU-Fbxo36  MMU-Fbxo36  100.00      120         0           0           1           120         1           120         9e-059       216
MMU-Fbxo36  PPY-Fbxo36  96.67       120         4           0           1           120         71          190         1e-056       209
MMU-Fbxo36  PTR-Fbxo36  96.67       120         4           0           1           120         69          188         2e-056       209
MMU-Fbxo36  GGO-Fbxo36  97.48       119         3           0           2           120         1           119         3e-056       208
```

```
MMU-Fbxo36  HSA-Fbxo36  95.83       120         5           0           1           120         69          188         7e-056       206
# BLASTP 2.2.20 [Feb-08-2009]
# Query: MUS-Fbxo36
# Database: 559_protein.db
# Query id, Subject id, % identity, alignment length, mismatches, gap openings, q. start, q. end, s. start, s. end, e-value, bit score
MUS-Fbxo36  MUS-Fbxo36  100.00      188         0           0           1           188         1           188         6e-102       361
MUS-Fbxo36  RNO-Fbxo36  91.62       167         14          0           1           167         1           167         3e-090       322
MUS-Fbxo36  PTR-Fbxo36  78.44       167         36          0           1           167         1           167         3e-079       285
MUS-Fbxo36  HSA-Fbxo36  78.44       167         36          0           1           167         1           167         3e-079       285
MUS-Fbxo36  PPY-Fbxo36  76.92       169         37          1           1           167         1           169         2e-077       279
MUS-Fbxo36  CJA-Fbxo36  78.34       157         34          0           1           157         1           157         1e-073       267
# BLASTP 2.2.20 [Feb-08-2009]
# Query: PPY-Fbxo36
# Database: 559_protein.db
# Query id, Subject id, % identity, alignment length, mismatches, gap openings, q. start, q. end, s. start, s. end, e-value, bit score
PPY-Fbxo36  PPY-Fbxo36  100.00      190         0           0           1           190         1           190         6e-103       364
PPY-Fbxo36  PTR-Fbxo36  98.42       190         1           1           1           190         1           188         6e-100       354
PPY-Fbxo36  HSA-Fbxo36  97.89       190         2           1           1           190         1           188         3e-099       352
PPY-Fbxo36  CJA-Fbxo36  88.68       159         16          1           1           159         1           157         2e-081       292
PPY-Fbxo36  RNO-Fbxo36  80.24       167         31          1           1           167         1           165         2e-079       286
PPY-Fbxo36  MUS-Fbxo36  77.84       167         35          1           1           167         1           165         3e-077       278
PPY-Fbxo36  GGO-Fbxo36  99.16       119         1           0           72          190         1           119         1e-057       214
PPY-Fbxo36  MMU-Fbxo36  96.67       120         4           0           71          190         1           120         1e-056       210
# BLASTP 2.2.20 [Feb-08-2009]
# Query: PTR-Fbxo36
# Database: 559_protein.db
# Query id, Subject id, % identity, alignment length, mismatches, gap openings, q. start, q. end, s. start, s. end, e-value, bit score
PTR-Fbxo36  PTR-Fbxo36  100.00      188         0           0           1           188         1           188         1e-101       360
PTR-Fbxo36  HSA-Fbxo36  99.47       188         1           0           1           188         1           188         6e-101       357
PTR-Fbxo36  PPY-Fbxo36  98.42       190         1           1           1           188         1           190         5e-100       354
PTR-Fbxo36  CJA-Fbxo36  90.45       157         15          0           1           157         1           157         3e-083       298
PTR-Fbxo36  RNO-Fbxo36  81.82       165         30          0           1           165         1           165         3e-081       292
PTR-Fbxo36  MUS-Fbxo36  79.39       165         34          0           1           165         1           165         5e-079       285
PTR-Fbxo36  GGO-Fbxo36  99.16       119         1           0           70          188         1           119         1e-057       214
PTR-Fbxo36  MMU-Fbxo36  96.67       120         4           0           69          188         1           120         2e-056       210
# BLASTP 2.2.20 [Feb-08-2009]
# Query: RNO-Fbxo36
# Database: 559_protein.db
# Query id, Subject id, % identity, alignment length, mismatches, gap openings, q. start, q. end, s. start, s. end, e-value, bit score
RNO-Fbxo36  RNO-Fbxo36  100.00      188         0           0           1           188         1           188         8e-101       357
RNO-Fbxo36  MUS-Fbxo36  92.12       165         13          0           1           165         1           165         1e-089       320
RNO-Fbxo36  PTR-Fbxo36  81.82       165         30          0           1           165         1           165         4e-081       291
RNO-Fbxo36  HSA-Fbxo36  81.82       165         30          0           1           165         1           165         4e-081       291
RNO-Fbxo36  PPY-Fbxo36  80.24       167         31          1           1           165         1           167         3e-079       285
RNO-Fbxo36  CJA-Fbxo36  80.25       157         31          0           1           157         1           157         4e-075       271
```

```
# BLASTP 2.2.20 [Feb-08-2009]
# Query: HSA-Fbxo36
# Database: 559_protein.db
# Query id, Subject id, % identity, alignment length, mismatches, gap openings, q. start, q. end, s. start, s. end, e-value, bit score
HSA-Fbxo36  HSA-Fbxo36  100.00      188         0           0           1           188         1           188         2e-101        359
HSA-Fbxo36  PTR-Fbxo36  99.47       188         1           0           1           188         1           188         6e-101        357
HSA-Fbxo36  PPY-Fbxo36  97.89       190         2           1           1           188         1           190         3e-099        352
HSA-Fbxo36  CJA-Fbxo36  89.81       157         16          0           1           157         1           157         2e-082        296
HSA-Fbxo36  RNO-Fbxo36  81.82       165         30          0           1           165         1           165         4e-081        292
HSA-Fbxo36  MUS-Fbxo36  79.39       165         34          0           1           165         1           165         5e-079        285
HSA-Fbxo36  GGO-Fbxo36  98.32       119         2           0           70          188         1           119         5e-057        211
HSA-Fbxo36  MMU-Fbxo36  95.83       120         5           0           69          188         1           120         7e-056        207
# BLASTP 2.2.20 [Feb-08-2009]
# Query: CJA-Fbxo38
# Database: 559_protein.db
# Query id, Subject id, % identity, alignment length, mismatches, gap openings, q. start, q. end, s. start, s. end, e-value, bit score
CJA-Fbxo38  PTR-Fbxo38  97.82       1190        24          2           1           1190        1           1188        0.0          2342
CJA-Fbxo38  CJA-Fbxo38  100.00      1190        0           0           1           1190        1           1190        0.0          2338
CJA-Fbxo38  HSA-Fbxo38  97.65       1190        26          2           1           1190        1           1188        0.0          2338
CJA-Fbxo38  MMU-Fbxo38  97.82       1190        24          2           1           1190        1           1188        0.0          2319
CJA-Fbxo38  PPY-Fbxo38  97.56       1190        27          2           1           1190        1           1188        0.0          2315
CJA-Fbxo38  GGO-Fbxo38  94.20       1190        68          1           1           1190        1           1189        0.0          2246
CJA-Fbxo38  RNO-Fbxo38  95.30       1191        53          3           1           1190        1           1189        0.0          2229
CJA-Fbxo38  MUS-Fbxo38  93.89       1195        67          3           1           1190        1           1194        0.0          2184
# BLASTP 2.2.20 [Feb-08-2009]
# Query: GGO-Fbxo38
# Database: 559_protein.db
# Query id, Subject id, % identity, alignment length, mismatches, gap openings, q. start, q. end, s. start, s. end, e-value, bit score
GGO-Fbxo38  GGO-Fbxo38  100.00      1189        0           0           1           1189        1           1189        0.0          2358
GGO-Fbxo38  PTR-Fbxo38  95.96       1189        47          1           1           1189        1           1188        0.0          2246
GGO-Fbxo38  HSA-Fbxo38  95.88       1189        48          1           1           1189        1           1188        0.0          2246
GGO-Fbxo38  MMU-Fbxo38  96.05       1189        46          1           1           1189        1           1188        0.0          2226
GGO-Fbxo38  CJA-Fbxo38  94.71       1190        62          1           1           1189        1           1190        0.0          2224
GGO-Fbxo38  PPY-Fbxo38  95.79       1189        49          1           1           1189        1           1188        0.0          2219
GGO-Fbxo38  RNO-Fbxo38  90.43       1191        110         4           1           1189        1           1189        0.0          2120
GGO-Fbxo38  MUS-Fbxo38  89.12       1195        123         4           1           1189        1           1194        0.0          2075
# BLASTP 2.2.20 [Feb-08-2009]
# Query: MMU-Fbxo38
# Database: 559_protein.db
# Query id, Subject id, % identity, alignment length, mismatches, gap openings, q. start, q. end, s. start, s. end, e-value, bit score
MMU-Fbxo38  PTR-Fbxo38  99.75       1188        3           0           1           1188        1           1188        0.0          2354
MMU-Fbxo38  HSA-Fbxo38  99.66       1188        4           0           1           1188        1           1188        0.0          2353
MMU-Fbxo38  MMU-Fbxo38  100.00      1188        0           0           1           1188        1           1188        0.0          2337
MMU-Fbxo38  PPY-Fbxo38  99.58       1188        5           0           1           1188        1           1188        0.0          2328
MMU-Fbxo38  CJA-Fbxo38  98.32       1190        18          2           1           1188        1           1190        0.0          2319
```

```
MMU-Fbxo38  GGO-Fbxo38  96.05   1189   46    1   1   1188   1   1189   0.0   2248
MMU-Fbxo38  RNO-Fbxo38  94.29   1190   65    3   1   1188   1   1189   0.0   2229
MMU-Fbxo38  MUS-Fbxo38  92.96   1194   78    3   1   1188   1   1194   0.0   2187
# BLASTP 2.2.20 [Feb-08-2009]
# Query: MUS-Fbxo38
# Database: 559_protein.db
# Query id, Subject id, % identity, alignment length, mismatches, gap openings, q. start, q. end, s. start, s. end, e-value, bit score
MUS-Fbxo38  MUS-Fbxo38  100.00  1194   0     0   1   1194   1   1194   0.0   2270
MUS-Fbxo38  RNO-Fbxo38  96.48   1194   37    2   1   1194   1   1189   0.0   2192
MUS-Fbxo38  PTR-Fbxo38  91.88   1194   91    3   1   1194   1   1188   0.0   2154
MUS-Fbxo38  HSA-Fbxo38  91.79   1194   92    3   1   1194   1   1188   0.0   2152
MUS-Fbxo38  MMU-Fbxo38  91.88   1194   91    3   1   1194   1   1188   0.0   2147
MUS-Fbxo38  CJA-Fbxo38  93.89   1195   67    3   1   1194   1   1190   0.0   2145
MUS-Fbxo38  PPY-Fbxo38  91.62   1194   94    3   1   1194   1   1188   0.0   2141
MUS-Fbxo38  GGO-Fbxo38  88.12   1195   135   4   1   1194   1   1189   0.0   2044
# BLASTP 2.2.20 [Feb-08-2009]
# Query: PPY-Fbxo38
# Database: 559_protein.db
# Query id, Subject id, % identity, alignment length, mismatches, gap openings, q. start, q. end, s. start, s. end, e-value, bit score
PPY-Fbxo38  PTR-Fbxo38  99.49   1188   6     0   1   1188   1   1188   0.0   2350
PPY-Fbxo38  HSA-Fbxo38  99.41   1188   7     0   1   1188   1   1188   0.0   2347
PPY-Fbxo38  PPY-Fbxo38  100.00  1188   0     0   1   1188   1   1188   0.0   2337
PPY-Fbxo38  MMU-Fbxo38  99.58   1188   5     0   1   1188   1   1188   0.0   2328
PPY-Fbxo38  CJA-Fbxo38  98.07   1190   21    2   1   1188   1   1190   0.0   2315
PPY-Fbxo38  GGO-Fbxo38  95.79   1189   49    1   1   1188   1   1189   0.0   2241
PPY-Fbxo38  RNO-Fbxo38  94.03   1190   68    3   1   1188   1   1189   0.0   2223
PPY-Fbxo38  MUS-Fbxo38  92.71   1194   81    3   1   1188   1   1194   0.0   2180
# BLASTP 2.2.20 [Feb-08-2009]
# Query: PTR-Fbxo38
# Database: 559_protein.db
# Query id, Subject id, % identity, alignment length, mismatches, gap openings, q. start, q. end, s. start, s. end, e-value, bit score
PTR-Fbxo38  PTR-Fbxo38  100.00  1188   0     0   1   1188   1   1188   0.0   2359
PTR-Fbxo38  HSA-Fbxo38  99.75   1188   3     0   1   1188   1   1188   0.0   2355
PTR-Fbxo38  MMU-Fbxo38  99.75   1188   3     0   1   1188   1   1188   0.0   2335
PTR-Fbxo38  PPY-Fbxo38  99.49   1188   6     0   1   1188   1   1188   0.0   2327
PTR-Fbxo38  CJA-Fbxo38  98.32   1190   18    2   1   1188   1   1190   0.0   2320
PTR-Fbxo38  GGO-Fbxo38  95.96   1189   47    1   1   1188   1   1189   0.0   2245
PTR-Fbxo38  RNO-Fbxo38  94.20   1190   66    3   1   1188   1   1189   0.0   2227
PTR-Fbxo38  MUS-Fbxo38  92.88   1194   79    3   1   1188   1   1194   0.0   2184
# BLASTP 2.2.20 [Feb-08-2009]
# Query: RNO-Fbxo38
# Database: 559_protein.db
# Query id, Subject id, % identity, alignment length, mismatches, gap openings, q. start, q. end, s. start, s. end, e-value, bit score
RNO-Fbxo38  RNO-Fbxo38  100.00  1189   0     0   1   1189   1   1189   0.0   2307
RNO-Fbxo38  MUS-Fbxo38  96.48   1194   37    2   1   1189   1   1194   0.0   2216
```

```
RNO-Fbxo38  PTR-Fbxo38  93.78   1190    71      3       1       1189    1       1188    0.0     2206
RNO-Fbxo38  HSA-Fbxo38  93.78   1190    71      3       1       1189    1       1188    0.0     2204
RNO-Fbxo38  MMU-Fbxo38  93.87   1190    70      3       1       1189    1       1188    0.0     2202
RNO-Fbxo38  CJA-Fbxo38  95.30   1191    53      3       1       1189    1       1190    0.0     2201
RNO-Fbxo38  PPY-Fbxo38  93.61   1190    73      3       1       1189    1       1188    0.0     2197
RNO-Fbxo38  GGO-Fbxo38  90.01   1191    115     4       1       1189    1       1189    0.0     2098
# BLASTP 2.2.20 [Feb-08-2009]
# Query: HSA-Fbxo38
# Database: 559_protein.db
# Query id, Subject id, % identity, alignment length, mismatches, gap openings, q. start, q. end, s. start, s. end, e-value, bit score
HSA-Fbxo38  HSA-Fbxo38  100.00  1188    0       0       1       1188    1       1188    0.0     2360
HSA-Fbxo38  PTR-Fbxo38  99.75   1188    3       0       1       1188    1       1188    0.0     2355
HSA-Fbxo38  MMU-Fbxo38  99.66   1188    4       0       1       1188    1       1188    0.0     2332
HSA-Fbxo38  PPY-Fbxo38  99.41   1188    7       0       1       1188    1       1188    0.0     2325
HSA-Fbxo38  CJA-Fbxo38  98.15   1190    20      2       1       1188    1       1190    0.0     2318
HSA-Fbxo38  GGO-Fbxo38  95.88   1189    48      1       1       1188    1       1189    0.0     2246
HSA-Fbxo38  RNO-Fbxo38  94.20   1190    66      3       1       1188    1       1189    0.0     2228
HSA-Fbxo38  MUS-Fbxo38  92.80   1194    80      3       1       1188    1       1194    0.0     2182
# BLASTP 2.2.20 [Feb-08-2009]
# Query: CJA-Fbxo39
# Database: 559_protein.db
# Query id, Subject id, % identity, alignment length, mismatches, gap openings, q. start, q. end, s. start, s. end, e-value, bit score
CJA-Fbxo39  CJA-Fbxo39  100.00  443     0       0       1       443     1       443     0.0      916
CJA-Fbxo39  MMU-Fbxo39  92.33   443     33      1       1       443     1       442     0.0      847
CJA-Fbxo39  RNO-Fbxo39  87.58   443     55      0       1       443     1       443     0.0      822
CJA-Fbxo39  PPY-Fbxo39  89.16   443     47      1       1       443     1       442     0.0      812
CJA-Fbxo39  MUS-Fbxo39  85.55   443     64      0       1       443     1       443     0.0      804
CJA-Fbxo39  HSA-Fbxo39  88.71   443     49      1       1       443     1       442     0.0      790
CJA-Fbxo39  PTR-Fbxo39  88.04   443     52      1       1       443     1       442     0.0      783
CJA-Fbxo39  GGO-Fbxo39  88.59   403     45      1       1       403     1       402     0.0      717
# BLASTP 2.2.20 [Feb-08-2009]
# Query: GGO-Fbxo39
# Database: 559_protein.db
# Query id, Subject id, % identity, alignment length, mismatches, gap openings, q. start, q. end, s. start, s. end, e-value, bit score
GGO-Fbxo39  GGO-Fbxo39  100.00  402     0       0       1       402     1       402     0.0      799
GGO-Fbxo39  PTR-Fbxo39  98.51   402     6       0       1       402     1       402     0.0      794
GGO-Fbxo39  HSA-Fbxo39  98.76   402     5       0       1       402     1       402     0.0      793
GGO-Fbxo39  PPY-Fbxo39  96.02   402     16      0       1       402     1       402     0.0      776
GGO-Fbxo39  MMU-Fbxo39  93.78   402     25      0       1       402     1       402     0.0      759
GGO-Fbxo39  CJA-Fbxo39  88.59   403     45      1       1       402     1       403     0.0      717
GGO-Fbxo39  RNO-Fbxo39  86.10   403     55      1       1       402     1       403     0.0      704
GGO-Fbxo39  MUS-Fbxo39  84.12   403     63      1       1       402     1       403     0.0      687
# BLASTP 2.2.20 [Feb-08-2009]
# Query: MMU-Fbxo39
# Database: 559_protein.db
```

```
# Query id, Subject id, % identity, alignment length, mismatches, gap openings, q. start, q. end, s. start, s. end, e-value, bit score
MMU-Fbxo39  MMU-Fbxo39  100.00      442         0           0           1           442         1           442         0.0         846
MMU-Fbxo39  PPY-Fbxo39  95.25       442         21          0           1           442         1           442         0.0         805
MMU-Fbxo39  PTR-Fbxo39  92.99       442         31          0           1           442         1           442         0.0         791
MMU-Fbxo39  HSA-Fbxo39  93.21       442         30          0           1           442         1           442         0.0         791
MMU-Fbxo39  CJA-Fbxo39  92.33       443         33          1           1           442         1           443         0.0         790
MMU-Fbxo39  RNO-Fbxo39  88.71       443         49          1           1           442         1           443         0.0         766
MMU-Fbxo39  MUS-Fbxo39  86.68       443         58          1           1           442         1           443         0.0         748
MMU-Fbxo39  GGO-Fbxo39  93.78       402         25          0           1           402         1           402         0.0         724
# BLASTP 2.2.20 [Feb-08-2009]
# Query: MUS-Fbxo39
# Database: 559_protein.db
# Query id, Subject id, % identity, alignment length, mismatches, gap openings, q. start, q. end, s. start, s. end, e-value, bit score
MUS-Fbxo39  MUS-Fbxo39  100.00      443         0           0           1           443         1           443         0.0         920
MUS-Fbxo39  RNO-Fbxo39  97.97       443         9           0           1           443         1           443         0.0         902
MUS-Fbxo39  MMU-Fbxo39  86.68       443         58          1           1           443         1           442         0.0         807
MUS-Fbxo39  CJA-Fbxo39  85.55       443         64          0           1           443         1           443         0.0         804
MUS-Fbxo39  PPY-Fbxo39  84.88       443         66          1           1           443         1           442         0.0         786
MUS-Fbxo39  HSA-Fbxo39  83.75       443         71          1           1           443         1           442         0.0         756
MUS-Fbxo39  PTR-Fbxo39  83.30       443         73          1           1           443         1           442         0.0         754
MUS-Fbxo39  GGO-Fbxo39  84.12       403         63          1           1           403         1           402         0.0         687
# BLASTP 2.2.20 [Feb-08-2009]
# Query: PPY-Fbxo39
# Database: 559_protein.db
# Query id, Subject id, % identity, alignment length, mismatches, gap openings, q. start, q. end, s. start, s. end, e-value, bit score
PPY-Fbxo39  PPY-Fbxo39  100.00      442         0           0           1           442         1           442         0.0         808
PPY-Fbxo39  HSA-Fbxo39  94.57       442         24          0           1           442         1           442         0.0         769
PPY-Fbxo39  PTR-Fbxo39  94.12       442         26          0           1           442         1           442         0.0         768
PPY-Fbxo39  MMU-Fbxo39  95.25       442         21          0           1           442         1           442         0.0         768
PPY-Fbxo39  GGO-Fbxo39  96.02       402         16          0           1           402         1           402         0.0         742
PPY-Fbxo39  CJA-Fbxo39  89.16       443         47          1           1           442         1           443         0.0         721
PPY-Fbxo39  RNO-Fbxo39  86.68       443         58          1           1           442         1           443         0.0         710
PPY-Fbxo39  MUS-Fbxo39  84.88       443         66          1           1           442         1           443         0.0         692
# BLASTP 2.2.20 [Feb-08-2009]
# Query: PTR-Fbxo39
# Database: 559_protein.db
# Query id, Subject id, % identity, alignment length, mismatches, gap openings, q. start, q. end, s. start, s. end, e-value, bit score
PTR-Fbxo39  PTR-Fbxo39  100.00      442         0           0           1           442         1           442         0.0         846
PTR-Fbxo39  HSA-Fbxo39  99.32       442         3           0           1           442         1           442         0.0         839
PTR-Fbxo39  PPY-Fbxo39  94.12       442         26          0           1           442         1           442         0.0         803
PTR-Fbxo39  MMU-Fbxo39  92.99       442         31          0           1           442         1           442         0.0         791
PTR-Fbxo39  GGO-Fbxo39  98.51       402         6           0           1           402         1           402         0.0         760
PTR-Fbxo39  CJA-Fbxo39  88.04       443         52          1           1           442         1           443         0.0         754
PTR-Fbxo39  RNO-Fbxo39  85.33       443         64          1           1           442         1           443         0.0         738
PTR-Fbxo39  MUS-Fbxo39  83.30       443         73          1           1           442         1           443         0.0         721
```

```
# BLASTP 2.2.20 [Feb-08-2009]
# Query: RNO-Fbxo39
# Database: 559_protein.db
# Query id, Subject id, % identity, alignment length, mismatches, gap openings, q. start, q. end, s. start, s. end, e-value, bit score
RNO-Fbxo39  RNO-Fbxo39  100.00      443         0           0           1           443         1           443         0.0         920
RNO-Fbxo39  MUS-Fbxo39  97.97       443         9           0           1           443         1           443         0.0         902
RNO-Fbxo39  MMU-Fbxo39  88.71       443         49          1           1           443         1           442         0.0         825
RNO-Fbxo39  CJA-Fbxo39  87.58       443         55          0           1           443         1           443         0.0         822
RNO-Fbxo39  PPY-Fbxo39  86.68       443         58          1           1           443         1           442         0.0         804
RNO-Fbxo39  HSA-Fbxo39  85.78       443         62          1           1           443         1           442         0.0         773
RNO-Fbxo39  PTR-Fbxo39  85.33       443         64          1           1           443         1           442         0.0         771
RNO-Fbxo39  GGO-Fbxo39  86.10       403         55          1           1           403         1           402         0.0         704
# BLASTP 2.2.20 [Feb-08-2009]
# Query: HSA-Fbxo39
# Database: 559_protein.db
# Query id, Subject id, % identity, alignment length, mismatches, gap openings, q. start, q. end, s. start, s. end, e-value, bit score
HSA-Fbxo39  HSA-Fbxo39  100.00      442         0           0           1           442         1           442         0.0         845
HSA-Fbxo39  PTR-Fbxo39  99.32       442         3           0           1           442         1           442         0.0         838
HSA-Fbxo39  PPY-Fbxo39  94.57       442         24          0           1           442         1           442         0.0         805
HSA-Fbxo39  MMU-Fbxo39  93.21       442         30          0           1           442         1           442         0.0         791
HSA-Fbxo39  CJA-Fbxo39  88.71       443         49          1           1           442         1           443         0.0         761
HSA-Fbxo39  GGO-Fbxo39  98.76       402         5           0           1           402         1           402         0.0         759
HSA-Fbxo39  RNO-Fbxo39  85.78       443         62          1           1           442         1           443         0.0         739
HSA-Fbxo39  MUS-Fbxo39  83.75       443         71          1           1           442         1           443         0.0         722
# BLASTP 2.2.20 [Feb-08-2009]
# Query: CJA-Fbxo4
# Database: 559_protein.db
# Query id, Subject id, % identity, alignment length, mismatches, gap openings, q. start, q. end, s. start, s. end, e-value, bit score
CJA-Fbxo4   CJA-Fbxo4   100.00      387         0           0           1           387         1           387         0.0         781
CJA-Fbxo4   MMU-Fbxo4   96.38       387         14          0           1           387         1           387         0.0         761
CJA-Fbxo4   HSA-Fbxo4   95.87       387         16          0           1           387         1           387         0.0         757
CJA-Fbxo4   PPY-Fbxo4   95.87       387         16          0           1           387         1           387         0.0         757
CJA-Fbxo4   GGO-Fbxo4   95.09       387         19          0           1           387         1           387         0.0         752
CJA-Fbxo4   PTR-Fbxo4   90.70       387         16          1           1           387         1           367         0.0         702
CJA-Fbxo4   RNO-Fbxo4   88.37       387         43          1           1           387         47          431         0.0         690
CJA-Fbxo4   MUS-Fbxo4   87.34       387         47          1           1           387         1           385         0.0         686
# BLASTP 2.2.20 [Feb-08-2009]
# Query: GGO-Fbxo4
# Database: 559_protein.db
# Query id, Subject id, % identity, alignment length, mismatches, gap openings, q. start, q. end, s. start, s. end, e-value, bit score
GGO-Fbxo4   GGO-Fbxo4   100.00      387         0           0           1           387         1           387         0.0         763
GGO-Fbxo4   HSA-Fbxo4   99.22       387         3           0           1           387         1           387         0.0         758
GGO-Fbxo4   PPY-Fbxo4   98.19       387         7           0           1           387         1           387         0.0         750
GGO-Fbxo4   MMU-Fbxo4   97.67       387         9           0           1           387         1           387         0.0         748
GGO-Fbxo4   CJA-Fbxo4   95.09       387         19          0           1           387         1           387         0.0         736
```

```
GGO-Fbxo4   PTR-Fbxo4   93.80       387         4           2           1           387         1           367         0.0         715
GGO-Fbxo4   MUS-Fbxo4   87.86       387         45          2           1           387         1           385         0.0         671
GGO-Fbxo4   RNO-Fbxo4   87.86       387         45          2           1           387         47          431         0.0         669
# BLASTP 2.2.20 [Feb-08-2009]
# Query: MMU-Fbxo4
# Database: 559_protein.db
# Query id, Subject id, % identity, alignment length, mismatches, gap openings, q. start, q. end, s. start, s. end, e-value, bit score
MMU-Fbxo4   MMU-Fbxo4   100.00      387         0           0           1           387         1           387         0.0         763
MMU-Fbxo4   PPY-Fbxo4   98.45       387         6           0           1           387         1           387         0.0         754
MMU-Fbxo4   HSA-Fbxo4   98.45       387         6           0           1           387         1           387         0.0         753
MMU-Fbxo4   GGO-Fbxo4   97.67       387         9           0           1           387         1           387         0.0         748
MMU-Fbxo4   CJA-Fbxo4   96.38       387         14          0           1           387         1           387         0.0         746
MMU-Fbxo4   PTR-Fbxo4   92.51       387         9           2           1           387         1           367         0.0         707
MMU-Fbxo4   MUS-Fbxo4   87.60       387         46          1           1           387         1           385         0.0         675
MMU-Fbxo4   RNO-Fbxo4   87.60       387         46          1           1           387         47          431         0.0         672
# BLASTP 2.2.20 [Feb-08-2009]
# Query: MUS-Fbxo4
# Database: 559_protein.db
# Query id, Subject id, % identity, alignment length, mismatches, gap openings, q. start, q. end, s. start, s. end, e-value, bit score
MUS-Fbxo4   MUS-Fbxo4   100.00      385         0           0           1           385         1           385         0.0         804
MUS-Fbxo4   RNO-Fbxo4   95.58       385         17          0           1           385         47          431         0.0         769
MUS-Fbxo4   MMU-Fbxo4   87.60       387         46          1           1           385         1           387         0.0         716
MUS-Fbxo4   HSA-Fbxo4   88.11       387         44          2           1           385         1           387         0.0         715
MUS-Fbxo4   PPY-Fbxo4   87.60       387         46          1           1           385         1           387         0.0         715
MUS-Fbxo4   GGO-Fbxo4   87.86       387         45          2           1           385         1           387         0.0         713
MUS-Fbxo4   CJA-Fbxo4   87.34       387         47          1           1           385         1           387         0.0         710
MUS-Fbxo4   PTR-Fbxo4   83.46       387         42          3           1           385         1           367         0.0         663
# BLASTP 2.2.20 [Feb-08-2009]
# Query: PPY-Fbxo4
# Database: 559_protein.db
# Query id, Subject id, % identity, alignment length, mismatches, gap openings, q. start, q. end, s. start, s. end, e-value, bit score
PPY-Fbxo4   PPY-Fbxo4   100.00      387         0           0           1           387         1           387         0.0         763
PPY-Fbxo4   HSA-Fbxo4   98.97       387         4           0           1           387         1           387         0.0         755
PPY-Fbxo4   MMU-Fbxo4   98.45       387         6           0           1           387         1           387         0.0         753
PPY-Fbxo4   GGO-Fbxo4   98.19       387         7           0           1           387         1           387         0.0         750
PPY-Fbxo4   CJA-Fbxo4   95.87       387         16          0           1           387         1           387         0.0         742
PPY-Fbxo4   PTR-Fbxo4   93.02       387         7           2           1           387         1           367         0.0         708
PPY-Fbxo4   RNO-Fbxo4   88.11       387         44          1           1           387         47          431         0.0         676
PPY-Fbxo4   MUS-Fbxo4   87.60       387         46          1           1           387         1           385         0.0         673
# BLASTP 2.2.20 [Feb-08-2009]
# Query: PTR-Fbxo4
# Database: 559_protein.db
# Query id, Subject id, % identity, alignment length, mismatches, gap openings, q. start, q. end, s. start, s. end, e-value, bit score
PTR-Fbxo4   PTR-Fbxo4   100.00      367         0           0           1           367         1           367         0.0         740
PTR-Fbxo4   HSA-Fbxo4   94.32       387         2           1           1           367         1           387         0.0         724
```

```
PTR-Fbxo4   GGO-Fbxo4   94.06    387    3     1    1    367    1     387    0.0    723
PTR-Fbxo4   PPY-Fbxo4   93.28    387    6     1    1    367    1     387    0.0    716
PTR-Fbxo4   MMU-Fbxo4   92.76    387    8     1    1    367    1     387    0.0    714
PTR-Fbxo4   CJA-Fbxo4   90.70    387    16    1    1    367    1     387    0.0    702
PTR-Fbxo4   MUS-Fbxo4   83.46    387    42    3    1    367    1     385    0.0    640
PTR-Fbxo4   RNO-Fbxo4   83.72    387    41    3    1    367    47    431    0.0    638
# BLASTP 2.2.20 [Feb-08-2009]
# Query: RNO-Fbxo4
# Database: 559_protein.db
# Query id, Subject id, % identity, alignment length, mismatches, gap openings, q. start, q. end, s. start, s. end, e-value, bit score
RNO-Fbxo4   RNO-Fbxo4   100.00   431    0     0    1    431    1     431    0.0    861
RNO-Fbxo4   MUS-Fbxo4   95.58    385    17    0    47   431    1     385    0.0    769
RNO-Fbxo4   PPY-Fbxo4   88.11    387    44    1    47   431    1     387    0.0    715
RNO-Fbxo4   CJA-Fbxo4   88.37    387    43    1    47   431    1     387    0.0    713
RNO-Fbxo4   MMU-Fbxo4   87.60    387    46    1    47   431    1     387    0.0    712
RNO-Fbxo4   HSA-Fbxo4   88.11    387    44    2    47   431    1     387    0.0    711
RNO-Fbxo4   GGO-Fbxo4   87.86    387    45    2    47   431    1     387    0.0    709
RNO-Fbxo4   PTR-Fbxo4   83.72    387    41    3    47   431    1     367    0.0    662
# BLASTP 2.2.20 [Feb-08-2009]
# Query: HSA-Fbxo4
# Database: 559_protein.db
# Query id, Subject id, % identity, alignment length, mismatches, gap openings, q. start, q. end, s. start, s. end, e-value, bit score
HSA-Fbxo4   HSA-Fbxo4   100.00   387    0     0    1    387    1     387    0.0    763
HSA-Fbxo4   GGO-Fbxo4   99.22    387    3     0    1    387    1     387    0.0    758
HSA-Fbxo4   PPY-Fbxo4   98.97    387    4     0    1    387    1     387    0.0    755
HSA-Fbxo4   MMU-Fbxo4   98.45    387    6     0    1    387    1     387    0.0    753
HSA-Fbxo4   CJA-Fbxo4   95.87    387    16    0    1    387    1     387    0.0    742
HSA-Fbxo4   PTR-Fbxo4   94.06    387    3     2    1    387    1     367    0.0    716
HSA-Fbxo4   MUS-Fbxo4   88.11    387    44    2    1    387    1     385    0.0    673
HSA-Fbxo4   RNO-Fbxo4   87.60    387    46    1    1    387    47    431    0.0    671
# BLASTP 2.2.20 [Feb-08-2009]
# Query: CJA-Fbxo40
# Database: 559_protein.db
# Query id, Subject id, % identity, alignment length, mismatches, gap openings, q. start, q. end, s. start, s. end, e-value, bit score
CJA-Fbxo40  CJA-Fbxo40  100.00   709    0     0    1    709    1     709    0.0    1354
CJA-Fbxo40  MMU-Fbxo40  95.20    709    34    0    1    709    1     709    0.0    1297
CJA-Fbxo40  HSA-Fbxo40  95.06    709    35    0    1    709    1     709    0.0    1291
CJA-Fbxo40  PTR-Fbxo40  95.20    709    34    0    1    709    1     709    0.0    1290
CJA-Fbxo40  PPY-Fbxo40  94.78    709    37    0    1    709    1     709    0.0    1289
CJA-Fbxo40  GGO-Fbxo40  95.06    708    35    0    1    708    1     708    0.0    1284
CJA-Fbxo40  MUS-Fbxo40  83.50    709    115   2    1    707    1     709    0.0    1138
CJA-Fbxo40  RNO-Fbxo40  83.76    708    113   2    2    707    1     708    0.0    1137
# BLASTP 2.2.20 [Feb-08-2009]
# Query: GGO-Fbxo40
# Database: 559_protein.db
```

```
# Query id, Subject id, % identity, alignment length, mismatches, gap openings, q. start, q. end, s. start, s. end, e-value, bit score
GGO-Fbxo40  GGO-Fbxo40  100.00      709         0           0           1           709         1           709         0.0         1383
GGO-Fbxo40  HSA-Fbxo40  99.29       708         5           0           1           708         1           708         0.0         1373
GGO-Fbxo40  PTR-Fbxo40  99.01       708         7           0           1           708         1           708         0.0         1368
GGO-Fbxo40  PPY-Fbxo40  98.16       708         13          0           1           708         1           708         0.0         1356
GGO-Fbxo40  MMU-Fbxo40  97.46       708         18          0           1           708         1           708         0.0         1346
GGO-Fbxo40  CJA-Fbxo40  95.06       708         35          0           1           708         1           708         0.0         1312
GGO-Fbxo40  RNO-Fbxo40  85.03       708         104         2           2           707         1           708         0.0         1181
GGO-Fbxo40  MUS-Fbxo40  84.77       709         106         2           1           707         1           709         0.0         1176
# BLASTP 2.2.20 [Feb-08-2009]
# Query: MMU-Fbxo40
# Database: 559_protein.db
# Query id, Subject id, % identity, alignment length, mismatches, gap openings, q. start, q. end, s. start, s. end, e-value, bit score
MMU-Fbxo40  MMU-Fbxo40  100.00      709         0           0           1           709         1           709         0.0         1344
MMU-Fbxo40  PPY-Fbxo40  97.88       709         15          0           1           709         1           709         0.0         1323
MMU-Fbxo40  HSA-Fbxo40  97.74       709         16          0           1           709         1           709         0.0         1318
MMU-Fbxo40  PTR-Fbxo40  97.46       709         18          0           1           709         1           709         0.0         1313
MMU-Fbxo40  GGO-Fbxo40  97.46       708         18          0           1           708         1           708         0.0         1311
MMU-Fbxo40  CJA-Fbxo40  95.20       709         34          0           1           709         1           709         0.0         1290
MMU-Fbxo40  RNO-Fbxo40  85.45       708         101         2           2           707         1           708         0.0         1160
MMU-Fbxo40  MUS-Fbxo40  85.05       709         104         2           1           707         1           709         0.0         1151
# BLASTP 2.2.20 [Feb-08-2009]
# Query: MUS-Fbxo40
# Database: 559_protein.db
# Query id, Subject id, % identity, alignment length, mismatches, gap openings, q. start, q. end, s. start, s. end, e-value, bit score
MUS-Fbxo40  MUS-Fbxo40  100.00      710         0           0           1           710         1           710         0.0         1461
MUS-Fbxo40  RNO-Fbxo40  93.93       708         43          0           2           709         1           708         0.0         1361
MUS-Fbxo40  MMU-Fbxo40  85.05       709         104         2           1           709         1           707         0.0         1239
MUS-Fbxo40  HSA-Fbxo40  84.91       709         105         2           1           709         1           707         0.0         1236
MUS-Fbxo40  GGO-Fbxo40  84.77       709         106         2           1           709         1           707         0.0         1234
MUS-Fbxo40  PPY-Fbxo40  84.77       709         106         2           1           709         1           707         0.0         1234
MUS-Fbxo40  PTR-Fbxo40  84.77       709         106         2           1           709         1           707         0.0         1232
MUS-Fbxo40  CJA-Fbxo40  83.50       709         115         2           1           709         1           707         0.0         1216
# BLASTP 2.2.20 [Feb-08-2009]
# Query: PPY-Fbxo40
# Database: 559_protein.db
# Query id, Subject id, % identity, alignment length, mismatches, gap openings, q. start, q. end, s. start, s. end, e-value, bit score
PPY-Fbxo40  PPY-Fbxo40  100.00      709         0           0           1           709         1           709         0.0         1383
PPY-Fbxo40  HSA-Fbxo40  98.59       709         10          0           1           709         1           709         0.0         1364
PPY-Fbxo40  PTR-Fbxo40  98.31       709         12          0           1           709         1           709         0.0         1360
PPY-Fbxo40  GGO-Fbxo40  98.16       708         13          0           1           708         1           708         0.0         1355
PPY-Fbxo40  MMU-Fbxo40  97.88       709         15          0           1           709         1           709         0.0         1355
PPY-Fbxo40  CJA-Fbxo40  94.78       709         37          0           1           709         1           709         0.0         1313
PPY-Fbxo40  MUS-Fbxo40  84.77       709         106         2           1           707         1           709         0.0         1176
PPY-Fbxo40  RNO-Fbxo40  84.60       708         107         2           2           707         1           708         0.0         1174
```

```
# BLASTP 2.2.20 [Feb-08-2009]
# Query: PTR-Fbxo40
# Database: 559_protein.db
# Query id, Subject id, % identity, alignment length, mismatches, gap openings, q. start, q. end, s. start, s. end, e-value, bit score
PTR-Fbxo40  PTR-Fbxo40  100.00  709  0    0  1   709  1    709  0.0  1383
PTR-Fbxo40  HSA-Fbxo40  99.44   709  4    0  1   709  1    709  0.0  1377
PTR-Fbxo40  GGO-Fbxo40  99.01   708  7    0  1   708  1    708  0.0  1368
PTR-Fbxo40  PPY-Fbxo40  98.31   709  12   0  1   709  1    709  0.0  1360
PTR-Fbxo40  MMU-Fbxo40  97.46   709  18   0  1   709  1    709  0.0  1349
PTR-Fbxo40  CJA-Fbxo40  95.20   709  34   0  1   709  1    709  0.0  1318
PTR-Fbxo40  RNO-Fbxo40  85.03   708  104  2  2   707  1    708  0.0  1179
PTR-Fbxo40  MUS-Fbxo40  84.77   709  106  2  1   707  1    709  0.0  1174
# BLASTP 2.2.20 [Feb-08-2009]
# Query: RNO-Fbxo40
# Database: 559_protein.db
# Query id, Subject id, % identity, alignment length, mismatches, gap openings, q. start, q. end, s. start, s. end, e-value, bit score
RNO-Fbxo40  RNO-Fbxo40  100.00  700  0    0  10  709  10   709  0.0  1409
RNO-Fbxo40  MUS-Fbxo40  93.99   699  42   0  10  708  11   709  0.0  1319
RNO-Fbxo40  MMU-Fbxo40  85.67   698  98   2  11  708  12   707  0.0  1204
RNO-Fbxo40  HSA-Fbxo40  85.53   698  99   2  11  708  12   707  0.0  1198
RNO-Fbxo40  GGO-Fbxo40  85.39   698  100  2  11  708  12   707  0.0  1196
RNO-Fbxo40  PTR-Fbxo40  85.39   698  100  2  11  708  12   707  0.0  1195
RNO-Fbxo40  PPY-Fbxo40  84.81   698  104  2  11  708  12   707  0.0  1190
RNO-Fbxo40  CJA-Fbxo40  84.24   698  108  2  11  708  12   707  0.0  1181
# BLASTP 2.2.20 [Feb-08-2009]
# Query: HSA-Fbxo40
# Database: 559_protein.db
# Query id, Subject id, % identity, alignment length, mismatches, gap openings, q. start, q. end, s. start, s. end, e-value, bit score
HSA-Fbxo40  HSA-Fbxo40  100.00  709  0    0  1   709  1    709  0.0  1382
HSA-Fbxo40  PTR-Fbxo40  99.44   709  4    0  1   709  1    709  0.0  1377
HSA-Fbxo40  GGO-Fbxo40  99.29   708  5    0  1   708  1    708  0.0  1374
HSA-Fbxo40  PPY-Fbxo40  98.59   709  10   0  1   709  1    709  0.0  1365
HSA-Fbxo40  MMU-Fbxo40  97.74   709  16   0  1   709  1    709  0.0  1353
HSA-Fbxo40  CJA-Fbxo40  95.06   709  35   0  1   709  1    709  0.0  1318
HSA-Fbxo40  RNO-Fbxo40  85.17   708  103  2  2   707  1    708  0.0  1182
HSA-Fbxo40  MUS-Fbxo40  84.91   709  105  2  1   707  1    709  0.0  1177
# BLASTP 2.2.20 [Feb-08-2009]
# Query: CJA-Fbxo41
# Database: 559_protein.db
# Query id, Subject id, % identity, alignment length, mismatches, gap openings, q. start, q. end, s. start, s. end, e-value, bit score
CJA-Fbxo41  CJA-Fbxo41  100.00  832  0    0  2   833  2    833  0.0  1274
CJA-Fbxo41  HSA-Fbxo41  97.60   833  19   1  2   833  104  936  0.0  1261
CJA-Fbxo41  MMU-Fbxo41  97.84   833  17   1  2   833  43   875  0.0  1260
CJA-Fbxo41  GGO-Fbxo41  97.62   798  19   0  36  833  59   856  0.0  1251
CJA-Fbxo41  PTR-Fbxo41  98.94   758  8    0  76  833  28   785  0.0  1247
```

```
CJA-Fbxo41  MUS-Fbxo41  95.56       833         34          2           2           833         43          873         0.0         1221
CJA-Fbxo41  RNO-Fbxo41  89.56       814         81          3           2           813         43          854         0.0         1116
CJA-Fbxo41  PPY-Fbxo41  80.50       836         90          12          2           830         43          812         0.0          989
# BLASTP 2.2.20 [Feb-08-2009]
# Query: GGO-Fbxo41
# Database: 559_protein.db
# Query id, Subject id, % identity, alignment length, mismatches, gap openings, q. start, q. end, s. start, s. end, e-value, bit score
GGO-Fbxo41  GGO-Fbxo41  100.00      856         0           0           1           856         1           856         0.0         1422
GGO-Fbxo41  HSA-Fbxo41  99.75       809         2           0           48          856         128         936         0.0         1319
GGO-Fbxo41  MMU-Fbxo41  99.26       809         6           0           48          856         67          875         0.0         1309
GGO-Fbxo41  PTR-Fbxo41  99.75       785         2           0           72          856         1           785         0.0         1277
GGO-Fbxo41  CJA-Fbxo41  97.53       809         20          0           48          856         25          833         0.0         1277
GGO-Fbxo41  MUS-Fbxo41  96.42       809         27          1           48          856         67          873         0.0         1269
GGO-Fbxo41  RNO-Fbxo41  90.38       790         73          2           48          836         67          854         0.0         1163
GGO-Fbxo41  PPY-Fbxo41  81.65       812         77          11          48          853         67          812         0.0         1032
# BLASTP 2.2.20 [Feb-08-2009]
# Query: MMU-Fbxo41
# Database: 559_protein.db
# Query id, Subject id, % identity, alignment length, mismatches, gap openings, q. start, q. end, s. start, s. end, e-value, bit score
MMU-Fbxo41  MMU-Fbxo41  100.00      875         0           0           1           875         1           875         0.0         1394
MMU-Fbxo41  HSA-Fbxo41  99.54       875         4           0           1           875         62          936         0.0         1390
MMU-Fbxo41  MUS-Fbxo41  97.37       875         21          1           1           875         1           873         0.0         1353
MMU-Fbxo41  GGO-Fbxo41  99.37       798         5           0           78          875         59          856         0.0         1283
MMU-Fbxo41  PTR-Fbxo41  99.36       785         5           0           91          875         1           785         0.0         1260
MMU-Fbxo41  CJA-Fbxo41  97.84       833         17          1           43          875         2           833         0.0         1260
MMU-Fbxo41  RNO-Fbxo41  91.82       856         67          2           1           855         1           854         0.0         1246
MMU-Fbxo41  PPY-Fbxo41  83.14       878         76          11          1           872         1           812         0.0         1117
# BLASTP 2.2.20 [Feb-08-2009]
# Query: MUS-Fbxo41
# Database: 559_protein.db
# Query id, Subject id, % identity, alignment length, mismatches, gap openings, q. start, q. end, s. start, s. end, e-value, bit score
MUS-Fbxo41  MUS-Fbxo41  100.00      873         0           0           1           873         1           873         0.0         1326
MUS-Fbxo41  MMU-Fbxo41  97.37       875         21          1           1           873         1           875         0.0         1307
MUS-Fbxo41  HSA-Fbxo41  96.91       875         25          1           1           873         62          936         0.0         1304
MUS-Fbxo41  RNO-Fbxo41  92.86       854         60          1           1           853         1           854         0.0         1207
MUS-Fbxo41  GGO-Fbxo41  96.49       798         26          1           78          873         59          856         0.0         1195
MUS-Fbxo41  CJA-Fbxo41  95.56       833         34          2           43          873         2           833         0.0         1176
MUS-Fbxo41  PTR-Fbxo41  96.43       785         26          1           91          873         1           785         0.0         1173
MUS-Fbxo41  PPY-Fbxo41  81.21       878         91          12          1           870         1           812         0.0         1042
# BLASTP 2.2.20 [Feb-08-2009]
# Query: PTR-Fbxo41
# Database: 559_protein.db
# Query id, Subject id, % identity, alignment length, mismatches, gap openings, q. start, q. end, s. start, s. end, e-value, bit score
PTR-Fbxo41  HSA-Fbxo41  99.87       785         1           0           1           785         152         936         0.0         1283
PTR-Fbxo41  PTR-Fbxo41  100.00      785         0           0           1           785         1           785         0.0         1283
```

```
PTR-Fbxo41  GGO-Fbxo41  99.75   785   2    0    1    785   72   856   0.0   1280
PTR-Fbxo41  MMU-Fbxo41  99.36   785   5    0    1    785   91   875   0.0   1272
PTR-Fbxo41  CJA-Fbxo41  98.94   758   8    0    28   785   76   833   0.0   1262
PTR-Fbxo41  MUS-Fbxo41  96.43   785   26   1    1    785   91   873   0.0   1232
PTR-Fbxo41  RNO-Fbxo41  90.21   766   72   2    1    765   91   854   0.0   1127
PTR-Fbxo41  PPY-Fbxo41  81.22   788   76   11   1    782   91   812   0.0    996
# BLASTP 2.2.20 [Feb-08-2009]
# Query: RNO-Fbxo41
# Database: 559_protein.db
# Query id, Subject id, % identity, alignment length, mismatches, gap openings, q. start, q. end, s. start, s. end, e-value, bit score
RNO-Fbxo41  RNO-Fbxo41  100.00  854   0    0    1    854   1    854   0.0   1367
RNO-Fbxo41  MUS-Fbxo41  92.86   854   60   1    1    854   1    853   0.0   1223
RNO-Fbxo41  MMU-Fbxo41  91.82   856   67   2    1    854   1    855   0.0   1214
RNO-Fbxo41  HSA-Fbxo41  91.36   856   71   2    1    854   62   916   0.0   1212
RNO-Fbxo41  GGO-Fbxo41  90.37   779   72   2    78   854   59   836   0.0   1105
RNO-Fbxo41  CJA-Fbxo41  89.56   814   81   3    43   854   2    813   0.0   1088
RNO-Fbxo41  PTR-Fbxo41  90.08   736   70   2    121  854   31   765   0.0   1084
RNO-Fbxo41  PPY-Fbxo41  79.56   866   93   13   1    854   1    794   0.0    978
# BLASTP 2.2.20 [Feb-08-2009]
# Query: HSA-Fbxo41
# Database: 559_protein.db
# Query id, Subject id, % identity, alignment length, mismatches, gap openings, q. start, q. end, s. start, s. end, e-value, bit score
HSA-Fbxo41  HSA-Fbxo41  100.00  936   0    0    1    936   1    936   0.0   1529
HSA-Fbxo41  MMU-Fbxo41  99.54   875   4    0    62   936   1    875   0.0   1398
HSA-Fbxo41  MUS-Fbxo41  96.91   875   25   1    62   936   1    873   0.0   1358
HSA-Fbxo41  GGO-Fbxo41  99.87   798   1    0    139  936   59   856   0.0   1301
HSA-Fbxo41  PTR-Fbxo41  99.87   785   1    0    152  936   1    785   0.0   1278
HSA-Fbxo41  CJA-Fbxo41  97.60   833   19   1    104  936   2    833   0.0   1271
HSA-Fbxo41  RNO-Fbxo41  91.36   856   71   2    62   916   1    854   0.0   1252
HSA-Fbxo41  PPY-Fbxo41  83.26   878   75   11   62   933   1    812   0.0   1121
# BLASTP 2.2.20 [Feb-08-2009]
# Query: PPY-Fbxo41
# Database: 559_protein.db
# Query id, Subject id, % identity, alignment length, mismatches, gap openings, q. start, q. end, s. start, s. end, e-value, bit score
PPY-Fbxo41  PPY-Fbxo41  100.00  876   0    0    1    876   1    876   0.0   1511
PPY-Fbxo41  HSA-Fbxo41  82.69   878   80   11   1    812   62   933   0.0   1120
PPY-Fbxo41  MMU-Fbxo41  82.57   878   81   11   1    812   1    872   0.0   1117
PPY-Fbxo41  MUS-Fbxo41  81.21   878   91   12   1    812   1    870   0.0   1095
PPY-Fbxo41  RNO-Fbxo41  80.47   860   96   11   1    794   1    854   0.0   1060
PPY-Fbxo41  GGO-Fbxo41  80.90   801   81   11   78   812   59   853   0.0   1014
PPY-Fbxo41  PTR-Fbxo41  81.35   788   75   11   91   812   1    782   0.0   1003
PPY-Fbxo41  CJA-Fbxo41  80.62   836   89   12   43   812   2    830   0.0   1000
# BLASTP 2.2.20 [Feb-08-2009]
# Query: CJA-Fbxo42
# Database: 559_protein.db
```

```
# Query id, Subject id, % identity, alignment length, mismatches, gap openings, q. start, q. end, s. start, s. end, e-value, bit score
CJA-Fbxo42  CJA-Fbxo42  100.00      717         0           0           1           717         1           717         0.0         1360
CJA-Fbxo42  PPY-Fbxo42  98.61       717         10          0           1           717         1           717         0.0         1342
CJA-Fbxo42  PTR-Fbxo42  98.61       717         10          0           1           717         1           717         0.0         1342
CJA-Fbxo42  GGO-Fbxo42  98.47       717         11          0           1           717         1           717         0.0         1340
CJA-Fbxo42  HSA-Fbxo42  98.47       717         11          0           1           717         1           717         0.0         1339
CJA-Fbxo42  MMU-Fbxo42  98.47       717         10          1           1           717         1           716         0.0         1334
CJA-Fbxo42  RNO-Fbxo42  95.54       717         32          0           1           717         1           717         0.0         1301
CJA-Fbxo42  MUS-Fbxo42  94.98       717         36          0           1           717         1           717         0.0         1287
# BLASTP 2.2.20 [Feb-08-2009]
# Query: GGO-Fbxo42
# Database: 559_protein.db
# Query id, Subject id, % identity, alignment length, mismatches, gap openings, q. start, q. end, s. start, s. end, e-value, bit score
GGO-Fbxo42  GGO-Fbxo42  100.00      717         0           0           1           717         1           717         0.0         1359
GGO-Fbxo42  PTR-Fbxo42  99.72       717         2           0           1           717         1           717         0.0         1357
GGO-Fbxo42  PPY-Fbxo42  99.44       717         4           0           1           717         1           717         0.0         1355
GGO-Fbxo42  HSA-Fbxo42  99.58       717         3           0           1           717         1           717         0.0         1354
GGO-Fbxo42  MMU-Fbxo42  99.02       717         6           1           1           717         1           716         0.0         1344
GGO-Fbxo42  CJA-Fbxo42  98.47       717         11          0           1           717         1           717         0.0         1339
GGO-Fbxo42  RNO-Fbxo42  95.54       717         32          0           1           717         1           717         0.0         1303
GGO-Fbxo42  MUS-Fbxo42  95.12       717         35          0           1           717         1           717         0.0         1289
# BLASTP 2.2.20 [Feb-08-2009]
# Query: MMU-Fbxo42
# Database: 559_protein.db
# Query id, Subject id, % identity, alignment length, mismatches, gap openings, q. start, q. end, s. start, s. end, e-value, bit score
MMU-Fbxo42  MMU-Fbxo42  100.00      716         0           0           1           716         1           716         0.0         1359
MMU-Fbxo42  PPY-Fbxo42  99.30       717         4           1           1           716         1           717         0.0         1349
MMU-Fbxo42  PTR-Fbxo42  99.30       717         4           1           1           716         1           717         0.0         1348
MMU-Fbxo42  GGO-Fbxo42  99.02       717         6           1           1           716         1           717         0.0         1346
MMU-Fbxo42  HSA-Fbxo42  99.16       717         5           1           1           716         1           717         0.0         1345
MMU-Fbxo42  CJA-Fbxo42  98.47       717         10          1           1           716         1           717         0.0         1334
MMU-Fbxo42  RNO-Fbxo42  95.96       717         28          1           1           716         1           717         0.0         1308
MMU-Fbxo42  MUS-Fbxo42  95.54       717         31          1           1           716         1           717         0.0         1291
# BLASTP 2.2.20 [Feb-08-2009]
# Query: MUS-Fbxo42
# Database: 559_protein.db
# Query id, Subject id, % identity, alignment length, mismatches, gap openings, q. start, q. end, s. start, s. end, e-value, bit score
MUS-Fbxo42  MUS-Fbxo42  100.00      717         0           0           1           717         1           717         0.0         1313
MUS-Fbxo42  RNO-Fbxo42  98.19       717         13          0           1           717         1           717         0.0         1295
MUS-Fbxo42  PPY-Fbxo42  95.40       717         33          0           1           717         1           717         0.0         1273
MUS-Fbxo42  PTR-Fbxo42  95.40       717         33          0           1           717         1           717         0.0         1272
MUS-Fbxo42  GGO-Fbxo42  95.12       717         35          0           1           717         1           717         0.0         1271
MUS-Fbxo42  HSA-Fbxo42  95.40       717         33          0           1           717         1           717         0.0         1271
MUS-Fbxo42  MMU-Fbxo42  95.54       717         31          1           1           717         1           716         0.0         1269
MUS-Fbxo42  CJA-Fbxo42  94.98       717         36          0           1           717         1           717         0.0         1263
```

```
# BLASTP 2.2.20 [Feb-08-2009]
# Query: PPY-Fbxo42
# Database: 559_protein.db
# Query id, Subject id, % identity, alignment length, mismatches, gap openings, q. start, q. end, s. start, s. end, e-value, bit score
PPY-Fbxo42  PPY-Fbxo42  100.00  717  0   0  1  717  1  717  0.0  1360
PPY-Fbxo42  PTR-Fbxo42  99.72   717  2   0  1  717  1  717  0.0  1358
PPY-Fbxo42  GGO-Fbxo42  99.44   717  4   0  1  717  1  717  0.0  1357
PPY-Fbxo42  HSA-Fbxo42  99.58   717  3   0  1  717  1  717  0.0  1356
PPY-Fbxo42  MMU-Fbxo42  99.30   717  4   1  1  717  1  716  0.0  1349
PPY-Fbxo42  CJA-Fbxo42  98.61   717  10  0  1  717  1  717  0.0  1342
PPY-Fbxo42  RNO-Fbxo42  95.82   717  30  0  1  717  1  717  0.0  1311
PPY-Fbxo42  MUS-Fbxo42  95.40   717  33  0  1  717  1  717  0.0  1296
# BLASTP 2.2.20 [Feb-08-2009]
# Query: PTR-Fbxo42
# Database: 559_protein.db
# Query id, Subject id, % identity, alignment length, mismatches, gap openings, q. start, q. end, s. start, s. end, e-value, bit score
PTR-Fbxo42  PTR-Fbxo42  100.00  717  0   0  1  717  1  717  0.0  1360
PTR-Fbxo42  GGO-Fbxo42  99.72   717  2   0  1  717  1  717  0.0  1359
PTR-Fbxo42  PPY-Fbxo42  99.72   717  2   0  1  717  1  717  0.0  1358
PTR-Fbxo42  HSA-Fbxo42  99.86   717  1   0  1  717  1  717  0.0  1357
PTR-Fbxo42  MMU-Fbxo42  99.30   717  4   1  1  717  1  716  0.0  1348
PTR-Fbxo42  CJA-Fbxo42  98.61   717  10  0  1  717  1  717  0.0  1341
PTR-Fbxo42  RNO-Fbxo42  95.82   717  30  0  1  717  1  717  0.0  1311
PTR-Fbxo42  MUS-Fbxo42  95.40   717  33  0  1  717  1  717  0.0  1295
# BLASTP 2.2.20 [Feb-08-2009]
# Query: RNO-Fbxo42
# Database: 559_protein.db
# Query id, Subject id, % identity, alignment length, mismatches, gap openings, q. start, q. end, s. start, s. end, e-value, bit score
RNO-Fbxo42  RNO-Fbxo42  100.00  717  0   0  1  717  1  717  0.0  1296
RNO-Fbxo42  MUS-Fbxo42  98.19   717  13  0  1  717  1  717  0.0  1274
RNO-Fbxo42  PTR-Fbxo42  95.82   717  30  0  1  717  1  717  0.0  1269
RNO-Fbxo42  PPY-Fbxo42  95.82   717  30  0  1  717  1  717  0.0  1269
RNO-Fbxo42  GGO-Fbxo42  95.54   717  32  0  1  717  1  717  0.0  1268
RNO-Fbxo42  HSA-Fbxo42  95.82   717  30  0  1  717  1  717  0.0  1268
RNO-Fbxo42  MMU-Fbxo42  95.26   717  33  1  1  717  1  716  0.0  1266
RNO-Fbxo42  CJA-Fbxo42  95.54   717  32  0  1  717  1  717  0.0  1256
# BLASTP 2.2.20 [Feb-08-2009]
# Query: HSA-Fbxo42
# Database: 559_protein.db
# Query id, Subject id, % identity, alignment length, mismatches, gap openings, q. start, q. end, s. start, s. end, e-value, bit score
HSA-Fbxo42  HSA-Fbxo42  100.00  717  0   0  1  717  1  717  0.0  1360
HSA-Fbxo42  PTR-Fbxo42  99.86   717  1   0  1  717  1  717  0.0  1357
HSA-Fbxo42  PPY-Fbxo42  99.58   717  3   0  1  717  1  717  0.0  1356
HSA-Fbxo42  GGO-Fbxo42  99.58   717  3   0  1  717  1  717  0.0  1356
HSA-Fbxo42  MMU-Fbxo42  99.16   717  5   1  1  717  1  716  0.0  1344
```

```
HSA-Fbxo42  CJA-Fbxo42  98.47    717    11    0    1    717    1    717    0.0    1338
HSA-Fbxo42  RNO-Fbxo42  95.82    717    30    0    1    717    1    717    0.0    1308
HSA-Fbxo42  MUS-Fbxo42  95.40    717    33    0    1    717    1    717    0.0    1293
# BLASTP 2.2.20 [Feb-08-2009]
# Query: CJA-Fbxo43
# Database: 559_protein.db
# Query id, Subject id, % identity, alignment length, mismatches, gap openings, q. start, q. end, s. start, s. end, e-value, bit score
CJA-Fbxo43  CJA-Fbxo43  100.00   674    0     0    1    674    1    674    0.0    1320
CJA-Fbxo43  HSA-Fbxo43  92.43    674    51    0    1    674    35   708    0.0    1221
CJA-Fbxo43  PTR-Fbxo43  92.28    674    52    0    1    674    35   708    0.0    1217
CJA-Fbxo43  PPY-Fbxo43  92.43    674    51    0    1    674    1    674    0.0    1217
CJA-Fbxo43  GGO-Fbxo43  91.39    674    58    0    1    674    35   708    0.0    1202
CJA-Fbxo43  MMU-Fbxo43  92.14    674    53    0    1    674    1    674    0.0    1199
# BLASTP 2.2.20 [Feb-08-2009]
# Query: GGO-Fbxo43
# Database: 559_protein.db
# Query id, Subject id, % identity, alignment length, mismatches, gap openings, q. start, q. end, s. start, s. end, e-value, bit score
GGO-Fbxo43  GGO-Fbxo43  100.00   708    0     0    1    708    1    708    0.0    1308
GGO-Fbxo43  HSA-Fbxo43  98.73    708    9     0    1    708    1    708    0.0    1287
GGO-Fbxo43  PTR-Fbxo43  98.59    708    10    0    1    708    1    708    0.0    1285
GGO-Fbxo43  PPY-Fbxo43  98.07    674    13    0    35   708    1    674    0.0    1211
GGO-Fbxo43  MMU-Fbxo43  95.99    674    27    0    35   708    1    674    0.0    1170
GGO-Fbxo43  CJA-Fbxo43  91.39    674    58    0    35   708    1    674    0.0    1130
# BLASTP 2.2.20 [Feb-08-2009]
# Query: MMU-Fbxo43
# Database: 559_protein.db
# Query id, Subject id, % identity, alignment length, mismatches, gap openings, q. start, q. end, s. start, s. end, e-value, bit score
MMU-Fbxo43  MMU-Fbxo43  100.00   674    0     0    1    674    1    674    0.0    1295
MMU-Fbxo43  HSA-Fbxo43  97.03    674    20    0    1    674    35   708    0.0    1268
MMU-Fbxo43  PTR-Fbxo43  96.88    674    21    0    1    674    35   708    0.0    1266
MMU-Fbxo43  PPY-Fbxo43  96.74    674    22    0    1    674    1    674    0.0    1262
MMU-Fbxo43  GGO-Fbxo43  95.99    674    27    0    1    674    35   708    0.0    1248
MMU-Fbxo43  CJA-Fbxo43  92.14    674    53    0    1    674    1    674    0.0    1199
# BLASTP 2.2.20 [Feb-08-2009]
# Query: PPY-Fbxo43
# Database: 559_protein.db
# Query id, Subject id, % identity, alignment length, mismatches, gap openings, q. start, q. end, s. start, s. end, e-value, bit score
PPY-Fbxo43  PPY-Fbxo43  100.00   674    0     0    1    674    1    674    0.0    1274
PPY-Fbxo43  HSA-Fbxo43  99.11    674    6     0    1    674    35   708    0.0    1265
PPY-Fbxo43  PTR-Fbxo43  98.96    674    7     0    1    674    35   708    0.0    1262
PPY-Fbxo43  GGO-Fbxo43  98.07    674    13    0    1    674    35   708    0.0    1246
PPY-Fbxo43  MMU-Fbxo43  96.74    674    22    0    1    674    1    674    0.0    1217
PPY-Fbxo43  CJA-Fbxo43  92.43    674    51    0    1    674    1    674    0.0    1172
# BLASTP 2.2.20 [Feb-08-2009]
# Query: PTR-Fbxo43
```

```
# Database: 559_protein.db
# Query id, Subject id, % identity, alignment length, mismatches, gap openings, q. start, q. end, s. start, s. end, e-value, bit score
PTR-Fbxo43  PTR-Fbxo43  100.00      708         0           0           1           708         1           708         0.0         1345
PTR-Fbxo43  HSA-Fbxo43  99.58       708         3           0           1           708         1           708         0.0         1339
PTR-Fbxo43  GGO-Fbxo43  98.59       708         10          0           1           708         1           708         0.0         1318
PTR-Fbxo43  PPY-Fbxo43  98.96       674         7           0           35          708         1           674         0.0         1261
PTR-Fbxo43  MMU-Fbxo43  96.88       674         21          0           35          708         1           674         0.0         1220
PTR-Fbxo43  CJA-Fbxo43  92.28       674         52          0           35          708         1           674         0.0         1172
# BLASTP 2.2.20 [Feb-08-2009]
# Query: HSA-Fbxo43
# Database: 559_protein.db
# Query id, Subject id, % identity, alignment length, mismatches, gap openings, q. start, q. end, s. start, s. end, e-value, bit score
HSA-Fbxo43  HSA-Fbxo43  100.00      708         0           0           1           708         1           708         0.0         1343
HSA-Fbxo43  PTR-Fbxo43  99.58       708         3           0           1           708         1           708         0.0         1338
HSA-Fbxo43  GGO-Fbxo43  98.73       708         9           0           1           708         1           708         0.0         1320
HSA-Fbxo43  PPY-Fbxo43  99.11       674         6           0           35          708         1           674         0.0         1264
HSA-Fbxo43  MMU-Fbxo43  97.03       674         20          0           35          708         1           674         0.0         1224
HSA-Fbxo43  CJA-Fbxo43  92.43       674         51          0           35          708         1           674         0.0         1176
# BLASTP 2.2.20 [Feb-08-2009]
# Query: GGO-Fbxo44
# Database: 559_protein.db
# Query id, Subject id, % identity, alignment length, mismatches, gap openings, q. start, q. end, s. start, s. end, e-value, bit score
GGO-Fbxo44  GGO-Fbxo44  100.00      265         0           0           1           265         1           265         1e-150      523
GGO-Fbxo44  MMU-Fbxo44  100.00      255         0           0           11          265         1           255         1e-144      503
GGO-Fbxo44  PTR-Fbxo44  99.61       255         1           0           11          265         1           255         7e-144      501
GGO-Fbxo44  RNO-Fbxo44  94.23       260         15          0           6           265         12          271         3e-140      489
GGO-Fbxo44  MUS-Fbxo44  95.69       209         9           0           11          219         1           209         1e-109      387
GGO-Fbxo44  PPY-Fbxo6   75.11       237         59          0           28          264         25          261         3e-105      372
GGO-Fbxo44  PTR-Fbxo6   74.68       237         60          0           28          264         25          261         6e-105      371
GGO-Fbxo44  HSA-Fbxo6   74.68       237         60          0           28          264         25          261         7e-105      371
GGO-Fbxo44  MMU-Fbxo6   75.64       234         57          0           28          261         25          258         2e-104      370
GGO-Fbxo44  GGO-Fbxo6   74.26       237         61          0           28          264         25          261         1e-103      367
GGO-Fbxo44  CJA-Fbxo6   71.37       234         67          0           28          261         25          258         7e-098      348
GGO-Fbxo44  RNO-Fbxo6   69.66       234         71          0           28          261         16          249         2e-096      343
GGO-Fbxo44  MUS-Fbxo6   67.52       234         76          0           28          261         16          249         5e-092      328
GGO-Fbxo44  HSA-Fbxo44  100.00      121         0           0           11          131         1           121         7e-062      228
GGO-Fbxo44  PPY-Fbxo44  83.67       147         22          1           11          155         1           147         2e-061      227
# BLASTP 2.2.20 [Feb-08-2009]
# Query: MMU-Fbxo44
# Database: 559_protein.db
# Query id, Subject id, % identity, alignment length, mismatches, gap openings, q. start, q. end, s. start, s. end, e-value, bit score
MMU-Fbxo44  GGO-Fbxo44  100.00      255         0           0           1           255         11          265         1e-144      503
MMU-Fbxo44  MMU-Fbxo44  100.00      255         0           0           1           255         1           255         2e-144      503
MMU-Fbxo44  PTR-Fbxo44  99.61       255         1           0           1           255         1           255         1e-143      500
MMU-Fbxo44  RNO-Fbxo44  96.08       255         10          0           1           255         17          271         9e-140      487
```

MMU-Fbxo44	MUS-Fbxo44	95.69	209	9	0	1	209	1	209	2e-109	386
MMU-Fbxo44	PPY-Fbxo6	75.11	237	59	0	18	254	25	261	5e-105	372
MMU-Fbxo44	PTR-Fbxo6	74.68	237	60	0	18	254	25	261	1e-104	370
MMU-Fbxo44	HSA-Fbxo6	74.68	237	60	0	18	254	25	261	1e-104	370
MMU-Fbxo44	MMU-Fbxo6	75.64	234	57	0	18	251	25	258	4e-104	369
MMU-Fbxo44	GGO-Fbxo6	74.26	237	61	0	18	254	25	261	2e-103	366
MMU-Fbxo44	CJA-Fbxo6	71.37	234	67	0	18	251	25	258	1e-097	347
MMU-Fbxo44	RNO-Fbxo6	69.66	234	71	0	18	251	16	249	3e-096	342
MMU-Fbxo44	MUS-Fbxo6	67.52	234	76	0	18	251	16	249	6e-092	328
MMU-Fbxo44	HSA-Fbxo44	100.00	121	0	0	1	121	1	121	9e-062	228
MMU-Fbxo44	PPY-Fbxo44	83.67	147	22	1	1	145	1	147	2e-061	226

BLASTP 2.2.20 [Feb-08-2009]
Query: MUS-Fbxo44
Database: 559_protein.db
Query id, Subject id, % identity, alignment length, mismatches, gap openings, q. start, q. end, s. start, s. end, e-value, bit score

MUS-Fbxo44	MUS-Fbxo44	100.00	276	0	0	1	276	1	276	5e-155	538
MUS-Fbxo44	RNO-Fbxo44	99.52	209	1	0	1	209	17	225	3e-113	399
MUS-Fbxo44	GGO-Fbxo44	95.69	209	9	0	1	209	11	219	9e-109	384
MUS-Fbxo44	MMU-Fbxo44	95.69	209	9	0	1	209	1	209	1e-108	384
MUS-Fbxo44	PTR-Fbxo44	95.22	209	10	0	1	209	1	209	8e-108	381
MUS-Fbxo44	MMU-Fbxo6	72.11	190	53	0	20	209	27	216	9e-079	285
MUS-Fbxo44	PPY-Fbxo6	72.11	190	53	0	20	209	27	216	1e-078	284
MUS-Fbxo44	PTR-Fbxo6	71.58	190	54	0	20	209	27	216	3e-078	283
MUS-Fbxo44	HSA-Fbxo6	71.58	190	54	0	20	209	27	216	3e-078	283
MUS-Fbxo44	CJA-Fbxo6	56.86	255	89	2	20	253	27	281	8e-077	278
MUS-Fbxo44	GGO-Fbxo6	71.05	190	55	0	20	209	27	216	8e-077	278
MUS-Fbxo44	RNO-Fbxo6	65.79	190	65	0	20	209	18	207	4e-071	259
MUS-Fbxo44	MUS-Fbxo6	64.74	190	67	0	20	209	18	207	1e-067	247
MUS-Fbxo44	HSA-Fbxo44	94.21	121	7	0	1	121	1	121	5e-057	213
MUS-Fbxo44	PPY-Fbxo44	79.59	147	28	1	1	145	1	147	5e-057	212

BLASTP 2.2.20 [Feb-08-2009]
Query: PTR-Fbxo44
Database: 559_protein.db
Query id, Subject id, % identity, alignment length, mismatches, gap openings, q. start, q. end, s. start, s. end, e-value, bit score

PTR-Fbxo44	PTR-Fbxo44	100.00	255	0	0	1	255	1	255	1e-144	503
PTR-Fbxo44	GGO-Fbxo44	99.61	255	1	0	1	255	11	265	8e-144	500
PTR-Fbxo44	MMU-Fbxo44	99.61	255	1	0	1	255	1	255	1e-143	500
PTR-Fbxo44	RNO-Fbxo44	95.69	255	11	0	1	255	17	271	7e-139	484
PTR-Fbxo44	MUS-Fbxo44	95.22	209	10	0	1	209	1	209	9e-109	384
PTR-Fbxo44	PPY-Fbxo6	74.68	237	60	0	18	254	25	261	3e-104	369
PTR-Fbxo44	HSA-Fbxo6	74.26	237	61	0	18	254	25	261	6e-104	368
PTR-Fbxo44	PTR-Fbxo6	74.26	237	61	0	18	254	25	261	6e-104	368
PTR-Fbxo44	MMU-Fbxo6	75.21	234	58	0	18	251	25	258	2e-103	366
PTR-Fbxo44	GGO-Fbxo6	73.84	237	62	0	18	254	25	261	1e-102	364
PTR-Fbxo44	CJA-Fbxo6	70.94	234	68	0	18	251	25	258	7e-097	344

```
PTR-Fbxo44  RNO-Fbxo6   69.23   234   72    0   18   251   16    249   2e-095   340
PTR-Fbxo44  MUS-Fbxo6   67.09   234   77    0   18   251   16    249   3e-091   326
PTR-Fbxo44  HSA-Fbxo44  99.17   121   1     0   1    121   1     121   7e-061   225
PTR-Fbxo44  PPY-Fbxo44  82.99   147   23    1   1    145   1     147   1e-060   224
# BLASTP 2.2.20 [Feb-08-2009]
# Query: RNO-Fbxo44
# Database: 559_protein.db
# Query id, Subject id, % identity, alignment length, mismatches, gap openings, q. start, q. end, s. start, s. end, e-value, bit score
RNO-Fbxo44  RNO-Fbxo44  100.00  271   0     0   1    271   1     271   2e-154   536
RNO-Fbxo44  GGO-Fbxo44  94.23   260   15    0   12   271   6     265   4e-140   488
RNO-Fbxo44  MMU-Fbxo44  96.08   255   10    0   17   271   1     255   9e-140   487
RNO-Fbxo44  PTR-Fbxo44  95.69   255   11    0   17   271   1     255   7e-139   484
RNO-Fbxo44  MUS-Fbxo44  99.52   209   1     0   17   225   1     209   4e-114   402
RNO-Fbxo44  PPY-Fbxo6   72.57   237   65    0   34   270   25    261   2e-102   363
RNO-Fbxo44  PTR-Fbxo6   72.15   237   66    0   34   270   25    261   4e-102   362
RNO-Fbxo44  HSA-Fbxo6   72.15   237   66    0   34   270   25    261   4e-102   362
RNO-Fbxo44  MMU-Fbxo6   73.08   234   63    0   34   267   25    258   1e-101   360
RNO-Fbxo44  GGO-Fbxo6   71.73   237   67    0   34   270   25    261   9e-101   357
RNO-Fbxo44  CJA-Fbxo6   70.51   234   69    0   34   267   25    258   3e-098   349
RNO-Fbxo44  RNO-Fbxo6   68.38   234   74    0   34   267   16    249   2e-095   340
RNO-Fbxo44  MUS-Fbxo6   67.09   234   77    0   34   267   16    249   4e-091   325
RNO-Fbxo44  GGO-Fbxo2   50.62   241   116   3   34   271   58    298   2e-066   244
RNO-Fbxo44  PTR-Fbxo2   50.00   238   116   3   34   268   58    295   3e-064   236
RNO-Fbxo44  HSA-Fbxo2   50.00   238   116   3   34   268   59    296   3e-064   236
RNO-Fbxo44  MUS-Fbxo2   50.21   237   114   3   34   268   63    297   3e-064   236
RNO-Fbxo44  RNO-Fbxo2   50.21   237   114   3   34   268   62    296   5e-064   236
RNO-Fbxo44  HSA-Fbxo44  95.04   121   6     0   17   137   1     121   1e-058   217
RNO-Fbxo44  PPY-Fbxo44  80.27   147   27    1   17   161   1     147   1e-058   217
# BLASTP 2.2.20 [Feb-08-2009]
# Query: HSA-Fbxo44
# Database: 559_protein.db
# Query id, Subject id, % identity, alignment length, mismatches, gap openings, q. start, q. end, s. start, s. end, e-value, bit score
HSA-Fbxo44  HSA-Fbxo44  100.00  256   0     0   1    256   1     256   2e-110   390
HSA-Fbxo44  PPY-Fbxo44  84.38   256   8     1   1    256   1     224   2e-095   340
HSA-Fbxo44  GGO-Fbxo44  100.00  121   0     0   1    121   11    131   4e-061   226
HSA-Fbxo44  MMU-Fbxo44  100.00  121   0     0   1    121   1     121   6e-061   225
HSA-Fbxo44  PTR-Fbxo44  99.17   121   1     0   1    121   1     121   5e-060   222
HSA-Fbxo44  RNO-Fbxo44  95.04   121   6     0   1    121   17    137   9e-058   214
HSA-Fbxo44  MUS-Fbxo44  94.21   121   7     0   1    121   1     121   3e-057   213
# BLASTP 2.2.20 [Feb-08-2009]
# Query: PPY-Fbxo44
# Database: 559_protein.db
# Query id, Subject id, % identity, alignment length, mismatches, gap openings, q. start, q. end, s. start, s. end, e-value, bit score
PPY-Fbxo44  PPY-Fbxo44  100.00  224   0     0   1    224   1     224   1e-115   407
PPY-Fbxo44  HSA-Fbxo44  84.38   256   8     1   1    224   1     256   2e-105   373
```

```
PPY-Fbxo44  GGO-Fbxo44  83.67   147  22  1  1   147  11  155  3e-061   226
PPY-Fbxo44  MMU-Fbxo44  83.67   147  22  1  1   147  1   145  3e-061   226
PPY-Fbxo44  PTR-Fbxo44  82.99   147  23  1  1   147  1   145  2e-060   223
PPY-Fbxo44  RNO-Fbxo44  80.27   147  27  1  1   147  17  161  3e-058   216
PPY-Fbxo44  MUS-Fbxo44  79.59   147  28  1  1   147  1   145  9e-058   214
# BLASTP 2.2.20 [Feb-08-2009]
# Query: CJA-Fbxo45
# Database: 559_protein.db
# Query id, Subject id, % identity, alignment length, mismatches, gap openings, q. start, q. end, s. start, s. end, e-value, bit score
CJA-Fbxo45  CJA-Fbxo45  100.00  286  0   0  1   286  1   286  9e-155   537
CJA-Fbxo45  HSA-Fbxo45  99.60   250  0   1  37  286  38  286  2e-148   516
CJA-Fbxo45  MUS-Fbxo45  99.20   250  1   1  37  286  38  286  7e-148   514
CJA-Fbxo45  GGO-Fbxo45  99.57   233  0   1  54  286  23  254  1e-138   483
# BLASTP 2.2.20 [Feb-08-2009]
# Query: GGO-Fbxo45
# Database: 559_protein.db
# Query id, Subject id, % identity, alignment length, mismatches, gap openings, q. start, q. end, s. start, s. end, e-value, bit score
GGO-Fbxo45  HSA-Fbxo45  100.00  232  0   0  23  254  55  286  2e-140   489
GGO-Fbxo45  MUS-Fbxo45  99.14   232  2   0  23  254  55  286  6e-140   488
GGO-Fbxo45  GGO-Fbxo45  100.00  232  0   0  23  254  23  254  1e-139   487
GGO-Fbxo45  CJA-Fbxo45  99.57   233  0   1  23  254  54  286  1e-138   483
# BLASTP 2.2.20 [Feb-08-2009]
# Query: MUS-Fbxo45
# Database: 559_protein.db
# Query id, Subject id, % identity, alignment length, mismatches, gap openings, q. start, q. end, s. start, s. end, e-value, bit score
MUS-Fbxo45  MUS-Fbxo45  100.00  249  0   0  38  286  38  286  3e-150   522
MUS-Fbxo45  HSA-Fbxo45  99.20   249  2   0  38  286  38  286  1e-149   520
MUS-Fbxo45  CJA-Fbxo45  99.20   250  1   1  38  286  37  286  7e-148   514
MUS-Fbxo45  GGO-Fbxo45  99.14   233  2   0  54  286  22  254  1e-139   487
# BLASTP 2.2.20 [Feb-08-2009]
# Query: HSA-Fbxo45
# Database: 559_protein.db
# Query id, Subject id, % identity, alignment length, mismatches, gap openings, q. start, q. end, s. start, s. end, e-value, bit score
HSA-Fbxo45  HSA-Fbxo45  100.00  249  0   0  38  286  38  286  3e-150   522
HSA-Fbxo45  MUS-Fbxo45  99.20   249  2   0  38  286  38  286  9e-150   520
HSA-Fbxo45  CJA-Fbxo45  99.60   250  0   1  38  286  37  286  2e-148   516
HSA-Fbxo45  GGO-Fbxo45  100.00  233  0   0  54  286  22  254  2e-140   489
# BLASTP 2.2.20 [Feb-08-2009]
# Query: CJA-Fbxo46
# Database: 559_protein.db
# Query id, Subject id, % identity, alignment length, mismatches, gap openings, q. start, q. end, s. start, s. end, e-value, bit score
CJA-Fbxo46  CJA-Fbxo46  100.00  615  0   0  1   615  1   615  0.0      1018
CJA-Fbxo46  HSA-Fbxo46  98.47   590  9   0  1   590  1   590  0.0      987
CJA-Fbxo46  PPY-Fbxo46  98.47   590  9   0  1   590  1   590  0.0      984
CJA-Fbxo46  GGO-Fbxo46  98.14   590  11  0  1   590  1   590  0.0      984
```

CJA-Fbxo46	MMU-Fbxo46	98.14	590	11	0	1	590	1	590	0.0	982
CJA-Fbxo46	RNO-Fbxo46	90.85	590	54	0	1	590	1	590	0.0	938
CJA-Fbxo46	MUS-Fbxo46	91.53	590	50	0	1	590	1	590	0.0	931
CJA-Fbxo46	PTR-Fbxo46	88.14	590	24	1	1	590	1	544	0.0	824
CJA-Fbxo46	GGO-Fbxo34	67.38	141	46	0	450	590	552	692	7e-061	226
CJA-Fbxo46	PTR-Fbxo34	67.38	141	46	0	450	590	552	692	8e-061	226
CJA-Fbxo46	HSA-Fbxo34	67.38	141	46	0	450	590	552	692	9e-061	226
CJA-Fbxo46	MMU-Fbxo34	67.38	141	46	0	450	590	550	690	1e-060	226
CJA-Fbxo46	PPY-Fbxo34	67.38	141	46	0	450	590	419	559	1e-060	226
CJA-Fbxo46	CJA-Fbxo34	66.67	141	47	0	450	590	550	690	1e-060	226
CJA-Fbxo46	MUS-Fbxo34	65.25	141	49	0	450	590	587	727	6e-059	220
CJA-Fbxo46	RNO-Fbxo34	65.25	141	49	0	450	590	573	713	6e-059	220

BLASTP 2.2.20 [Feb-08-2009]
Query: GGO-Fbxo46
Database: 559_protein.db
Query id, Subject id, % identity, alignment length, mismatches, gap openings, q. start, q. end, s. start, s. end, e-value, bit score

GGO-Fbxo46	GGO-Fbxo46	100.00	590	0	0	1	590	1	590	0.0	944
GGO-Fbxo46	PPY-Fbxo46	99.15	590	5	0	1	590	1	590	0.0	944
GGO-Fbxo46	HSA-Fbxo46	99.66	590	2	0	1	590	1	590	0.0	944
GGO-Fbxo46	MMU-Fbxo46	98.47	590	9	0	1	590	1	590	0.0	940
GGO-Fbxo46	CJA-Fbxo46	98.14	590	11	0	1	590	1	590	0.0	933
GGO-Fbxo46	RNO-Fbxo46	91.19	590	52	0	1	590	1	590	0.0	903
GGO-Fbxo46	MUS-Fbxo46	91.69	590	49	0	1	590	1	590	0.0	894
GGO-Fbxo46	PTR-Fbxo46	99.75	403	1	0	188	590	142	544	0.0	659
GGO-Fbxo46	PTR-Fbxo46	93.80	129	8	0	1	129	1	129	3e-057	214
GGO-Fbxo46	HSA-Fbxo34	67.38	141	46	0	450	590	552	692	1e-060	225
GGO-Fbxo46	GGO-Fbxo34	67.38	141	46	0	450	590	552	692	1e-060	225
GGO-Fbxo46	PTR-Fbxo34	67.38	141	46	0	450	590	552	692	2e-060	225
GGO-Fbxo46	MMU-Fbxo34	67.38	141	46	0	450	590	550	690	2e-060	225
GGO-Fbxo46	PPY-Fbxo34	67.38	141	46	0	450	590	419	559	2e-060	225
GGO-Fbxo46	CJA-Fbxo34	66.67	141	47	0	450	590	550	690	4e-060	224
GGO-Fbxo46	RNO-Fbxo34	65.25	141	49	0	450	590	573	713	1e-058	219
GGO-Fbxo46	MUS-Fbxo34	65.25	141	49	0	450	590	587	727	1e-058	219

BLASTP 2.2.20 [Feb-08-2009]
Query: MMU-Fbxo46
Database: 559_protein.db
Query id, Subject id, % identity, alignment length, mismatches, gap openings, q. start, q. end, s. start, s. end, e-value, bit score

MMU-Fbxo46	MMU-Fbxo46	100.00	590	0	0	1	590	1	590	0.0	1001
MMU-Fbxo46	PPY-Fbxo46	98.81	590	7	0	1	590	1	590	0.0	992
MMU-Fbxo46	HSA-Fbxo46	98.81	590	7	0	1	590	1	590	0.0	991
MMU-Fbxo46	GGO-Fbxo46	98.47	590	9	0	1	590	1	590	0.0	987
MMU-Fbxo46	CJA-Fbxo46	98.14	590	11	0	1	590	1	590	0.0	984
MMU-Fbxo46	RNO-Fbxo46	90.68	590	55	0	1	590	1	590	0.0	939
MMU-Fbxo46	MUS-Fbxo46	91.19	590	52	0	1	590	1	590	0.0	931
MMU-Fbxo46	PTR-Fbxo46	88.81	590	20	1	1	590	1	544	0.0	834

```
MMU-Fbxo46  GGO-Fbxo34  67.38   141  46  0  450  590  552  692  6e-061  226
MMU-Fbxo46  PTR-Fbxo34  67.38   141  46  0  450  590  552  692  7e-061  226
MMU-Fbxo46  HSA-Fbxo34  67.38   141  46  0  450  590  552  692  7e-061  226
MMU-Fbxo46  MMU-Fbxo34  67.38   141  46  0  450  590  550  690  1e-060  226
MMU-Fbxo46  PPY-Fbxo34  67.38   141  46  0  450  590  419  559  1e-060  226
MMU-Fbxo46  CJA-Fbxo34  66.67   141  47  0  450  590  550  690  2e-060  225
MMU-Fbxo46  RNO-Fbxo34  65.25   141  49  0  450  590  573  713  6e-059  220
MMU-Fbxo46  MUS-Fbxo34  65.25   141  49  0  450  590  587  727  6e-059  220
# BLASTP 2.2.20 [Feb-08-2009]
# Query: MUS-Fbxo46
# Database: 559_protein.db
# Query id, Subject id, % identity, alignment length, mismatches, gap openings, q. start, q. end, s. start, s. end, e-value, bit score
MUS-Fbxo46  MUS-Fbxo46  100.00  590  0   0  1    590  1    590  0.0     1055
MUS-Fbxo46  RNO-Fbxo46  96.78   590  19  0  1    590  1    590  0.0     1030
MUS-Fbxo46  GGO-Fbxo46  91.69   590  49  0  1    590  1    590  0.0     963
MUS-Fbxo46  PPY-Fbxo46  91.69   590  49  0  1    590  1    590  0.0     962
MUS-Fbxo46  HSA-Fbxo46  91.36   590  51  0  1    590  1    590  0.0     959
MUS-Fbxo46  CJA-Fbxo46  91.53   590  50  0  1    590  1    590  0.0     958
MUS-Fbxo46  MMU-Fbxo46  91.19   590  52  0  1    590  1    590  0.0     956
MUS-Fbxo46  PTR-Fbxo46  82.37   590  58  2  1    590  1    544  0.0     807
MUS-Fbxo46  GGO-Fbxo34  67.38   141  46  0  450  590  552  692  4e-061  227
MUS-Fbxo46  HSA-Fbxo34  67.38   141  46  0  450  590  552  692  4e-061  227
MUS-Fbxo46  PTR-Fbxo34  67.38   141  46  0  450  590  552  692  5e-061  227
MUS-Fbxo46  MMU-Fbxo34  67.38   141  46  0  450  590  550  690  5e-061  227
MUS-Fbxo46  PPY-Fbxo34  67.38   141  46  0  450  590  419  559  6e-061  226
MUS-Fbxo46  CJA-Fbxo34  66.67   141  47  0  450  590  550  690  1e-060  226
MUS-Fbxo46  MUS-Fbxo34  59.15   164  64  2  427  590  567  727  5e-060  224
MUS-Fbxo46  RNO-Fbxo34  61.94   155  57  1  438  590  559  713  2e-059  221
# BLASTP 2.2.20 [Feb-08-2009]
# Query: PTR-Fbxo46
# Database: 559_protein.db
# Query id, Subject id, % identity, alignment length, mismatches, gap openings, q. start, q. end, s. start, s. end, e-value, bit score
PTR-Fbxo46  PTR-Fbxo46  100.00  544  0   0  1    544  1    544  0.0     858
PTR-Fbxo46  PPY-Fbxo46  89.49   590  16  1  1    544  1    590  0.0     820
PTR-Fbxo46  HSA-Fbxo46  89.49   590  16  1  1    544  1    590  0.0     819
PTR-Fbxo46  GGO-Fbxo46  89.32   590  17  1  1    544  1    590  0.0     818
PTR-Fbxo46  MMU-Fbxo46  88.98   590  19  1  1    544  1    590  0.0     817
PTR-Fbxo46  CJA-Fbxo46  88.31   590  23  1  1    544  1    590  0.0     808
PTR-Fbxo46  RNO-Fbxo46  93.55   403  26  0  142  544  188  590  0.0     643
PTR-Fbxo46  MUS-Fbxo46  94.54   403  22  0  142  544  188  590  0.0     639
PTR-Fbxo46  GGO-Fbxo34  67.38   141  46  0  404  544  552  692  3e-060  224
PTR-Fbxo46  PTR-Fbxo34  67.38   141  46  0  404  544  552  692  3e-060  224
PTR-Fbxo46  HSA-Fbxo34  67.38   141  46  0  404  544  552  692  3e-060  224
PTR-Fbxo46  PPY-Fbxo34  67.38   141  46  0  404  544  419  559  4e-060  224
PTR-Fbxo46  MMU-Fbxo34  67.38   141  46  0  404  544  550  690  4e-060  224
```

```
PTR-Fbxo46  CJA-Fbxo34  66.67     141       47        0         404       544       550       690       8e-060     223
PTR-Fbxo46  MUS-Fbxo34  65.25     141       49        0         404       544       587       727       2e-058     218
PTR-Fbxo46  RNO-Fbxo34  65.25     141       49        0         404       544       573       713       2e-058     218
# BLASTP 2.2.20 [Feb-08-2009]
# Query: RNO-Fbxo46
# Database: 559_protein.db
# Query id, Subject id, % identity, alignment length, mismatches, gap openings, q. start, q. end, s. start, s. end, e-value, bit score
RNO-Fbxo46  RNO-Fbxo46  100.00    590       0         0         1         590       1         590       0.0        1103
RNO-Fbxo46  MUS-Fbxo46  96.78     590       19        0         1         590       1         590       0.0        1070
RNO-Fbxo46  PPY-Fbxo46  91.19     590       52        0         1         590       1         590       0.0         998
RNO-Fbxo46  GGO-Fbxo46  91.19     590       52        0         1         590       1         590       0.0         996
RNO-Fbxo46  HSA-Fbxo46  90.85     590       54        0         1         590       1         590       0.0         993
RNO-Fbxo46  CJA-Fbxo46  90.85     590       54        0         1         590       1         590       0.0         992
RNO-Fbxo46  MMU-Fbxo46  90.68     590       55        0         1         590       1         590       0.0         991
RNO-Fbxo46  PTR-Fbxo46  74.79     599       87        4         1         590       1         544       0.0         750
RNO-Fbxo46  PTR-Fbxo34  67.38     141       46        0         450       590       552       692       4e-061      227
RNO-Fbxo46  GGO-Fbxo34  67.38     141       46        0         450       590       552       692       4e-061      227
RNO-Fbxo46  HSA-Fbxo34  67.38     141       46        0         450       590       552       692       5e-061      227
RNO-Fbxo46  MMU-Fbxo34  67.38     141       46        0         450       590       550       690       6e-061      227
RNO-Fbxo46  PPY-Fbxo34  67.38     141       46        0         450       590       419       559       6e-061      226
RNO-Fbxo46  CJA-Fbxo34  66.67     141       47        0         450       590       550       690       1e-060      226
RNO-Fbxo46  RNO-Fbxo34  50.00     222       83        3         397       590       492       713       6e-060      223
RNO-Fbxo46  MUS-Fbxo34  51.38     218       78        4         401       590       510       727       1e-059      223
# BLASTP 2.2.20 [Feb-08-2009]
# Query: HSA-Fbxo46
# Database: 559_protein.db
# Query id, Subject id, % identity, alignment length, mismatches, gap openings, q. start, q. end, s. start, s. end, e-value, bit score
HSA-Fbxo46  PPY-Fbxo46  99.49     590       3         0         1         590       1         590       0.0         948
HSA-Fbxo46  HSA-Fbxo46  100.00    590       0         0         1         590       1         590       0.0         947
HSA-Fbxo46  GGO-Fbxo46  99.66     590       2         0         1         590       1         590       0.0         946
HSA-Fbxo46  MMU-Fbxo46  98.81     590       7         0         1         590       1         590       0.0         946
HSA-Fbxo46  CJA-Fbxo46  98.47     590       9         0         1         590       1         590       0.0         938
HSA-Fbxo46  RNO-Fbxo46  90.85     590       54        0         1         590       1         590       0.0         904
HSA-Fbxo46  MUS-Fbxo46  91.36     590       51        0         1         590       1         590       0.0         895
HSA-Fbxo46  PTR-Fbxo46  99.75     403       1         0         188       590       142       544       0.0         659
HSA-Fbxo46  PTR-Fbxo46  94.57     129       7         0         1         129       1         129       8e-058      216
HSA-Fbxo46  PTR-Fbxo34  67.38     141       46        0         450       590       552       692       1e-060      225
HSA-Fbxo46  GGO-Fbxo34  67.38     141       46        0         450       590       552       692       1e-060      225
HSA-Fbxo46  HSA-Fbxo34  67.38     141       46        0         450       590       552       692       2e-060      225
HSA-Fbxo46  MMU-Fbxo34  67.38     141       46        0         450       590       550       690       2e-060      225
HSA-Fbxo46  PPY-Fbxo34  67.38     141       46        0         450       590       419       559       2e-060      224
HSA-Fbxo46  CJA-Fbxo34  66.67     141       47        0         450       590       550       690       3e-060      224
HSA-Fbxo46  RNO-Fbxo34  65.25     141       49        0         450       590       573       713       1e-058      219
HSA-Fbxo46  MUS-Fbxo34  65.25     141       49        0         450       590       587       727       1e-058      219
# BLASTP 2.2.20 [Feb-08-2009]
```

```
# Query: PPY-Fbxo46
# Database: 559_protein.db
# Query id, Subject id, % identity, alignment length, mismatches, gap openings, q. start, q. end, s. start, s. end, e-value, bit score
PPY-Fbxo46  PPY-Fbxo46  100.00      590         0           0           1           590         1           590         0.0         999
PPY-Fbxo46  HSA-Fbxo46  99.49       590         3           0           1           590         1           590         0.0         993
PPY-Fbxo46  GGO-Fbxo46  99.15       590         5           0           1           590         1           590         0.0         990
PPY-Fbxo46  MMU-Fbxo46  98.81       590         7           0           1           590         1           590         0.0         989
PPY-Fbxo46  CJA-Fbxo46  98.47       590         9           0           1           590         1           590         0.0         984
PPY-Fbxo46  RNO-Fbxo46  91.19       590         52          0           1           590         1           590         0.0         942
PPY-Fbxo46  MUS-Fbxo46  91.69       590         49          0           1           590         1           590         0.0         935
PPY-Fbxo46  PTR-Fbxo46  89.32       590         17          1           1           590         1           544         0.0         835
PPY-Fbxo46  GGO-Fbxo34  67.38       141         46          0           450         590         552         692         8e-061      226
PPY-Fbxo46  PTR-Fbxo34  67.38       141         46          0           450         590         552         692         9e-061      226
PPY-Fbxo46  HSA-Fbxo34  67.38       141         46          0           450         590         552         692         1e-060      226
PPY-Fbxo46  MMU-Fbxo34  67.38       141         46          0           450         590         550         690         1e-060      226
PPY-Fbxo46  PPY-Fbxo34  67.38       141         46          0           450         590         419         559         1e-060      226
PPY-Fbxo46  CJA-Fbxo34  66.67       141         47          0           450         590         550         690         2e-060      225
PPY-Fbxo46  RNO-Fbxo34  65.25       141         49          0           450         590         573         713         7e-059      220
PPY-Fbxo46  MUS-Fbxo34  65.25       141         49          0           450         590         587         727         7e-059      219
# BLASTP 2.2.20 [Feb-08-2009]
# Query: CJA-Fbxo47
# Database: 559_protein.db
# Query id, Subject id, % identity, alignment length, mismatches, gap openings, q. start, q. end, s. start, s. end, e-value, bit score
CJA-Fbxo47  CJA-Fbxo47  100.00      452         0           0           1           452         1           452         0.0         944
CJA-Fbxo47  GGO-Fbxo47  93.14       452         31          0           1           452         1           452         0.0         891
CJA-Fbxo47  PPY-Fbxo47  93.36       452         30          0           1           452         1           452         0.0         890
CJA-Fbxo47  PTR-Fbxo47  92.92       452         32          0           1           452         1           452         0.0         890
CJA-Fbxo47  HSA-Fbxo47  92.70       452         33          0           1           452         1           452         0.0         888
CJA-Fbxo47  MMU-Fbxo47  93.14       452         31          0           1           452         1           452         0.0         887
CJA-Fbxo47  MUS-Fbxo47  82.14       448         80          0           1           448         1           448         0.0         771
CJA-Fbxo47  RNO-Fbxo47  73.94       449         116         1           1           448         1           449         0.0         688
# BLASTP 2.2.20 [Feb-08-2009]
# Query: GGO-Fbxo47
# Database: 559_protein.db
# Query id, Subject id, % identity, alignment length, mismatches, gap openings, q. start, q. end, s. start, s. end, e-value, bit score
GGO-Fbxo47  GGO-Fbxo47  100.00      452         0           0           1           452         1           452         0.0         940
GGO-Fbxo47  PTR-Fbxo47  99.78       452         1           0           1           452         1           452         0.0         939
GGO-Fbxo47  HSA-Fbxo47  99.56       452         2           0           1           452         1           452         0.0         937
GGO-Fbxo47  PPY-Fbxo47  98.01       452         9           0           1           452         1           452         0.0         926
GGO-Fbxo47  MMU-Fbxo47  98.23       452         8           0           1           452         1           452         0.0         925
GGO-Fbxo47  CJA-Fbxo47  93.14       452         31          0           1           452         1           452         0.0         891
GGO-Fbxo47  MUS-Fbxo47  83.93       448         72          0           1           448         1           448         0.0         780
GGO-Fbxo47  RNO-Fbxo47  75.28       449         110         1           1           448         1           449         0.0         694
# BLASTP 2.2.20 [Feb-08-2009]
# Query: MMU-Fbxo47
```

```
# Database: 559_protein.db
# Query id, Subject id, % identity, alignment length, mismatches, gap openings, q. start, q. end, s. start, s. end, e-value, bit score
MMU-Fbxo47  MMU-Fbxo47  100.00      452         0           0           1           452         1           452         0.0         940
MMU-Fbxo47  GGO-Fbxo47  98.23       452         8           0           1           452         1           452         0.0         925
MMU-Fbxo47  PTR-Fbxo47  98.01       452         9           0           1           452         1           452         0.0         923
MMU-Fbxo47  HSA-Fbxo47  97.79       452         10          0           1           452         1           452         0.0         921
MMU-Fbxo47  PPY-Fbxo47  98.01       452         9           0           1           452         1           452         0.0         920
MMU-Fbxo47  CJA-Fbxo47  93.14       452         31          0           1           452         1           452         0.0         887
MMU-Fbxo47  MUS-Fbxo47  83.26       448         75          0           1           448         1           448         0.0         774
MMU-Fbxo47  RNO-Fbxo47  75.06       449         111         1           1           448         1           449         0.0         689
# BLASTP 2.2.20 [Feb-08-2009]
# Query: MUS-Fbxo47
# Database: 559_protein.db
# Query id, Subject id, % identity, alignment length, mismatches, gap openings, q. start, q. end, s. start, s. end, e-value, bit score
MUS-Fbxo47  MUS-Fbxo47  100.00      451         0           0           1           451         1           451         0.0         936
MUS-Fbxo47  RNO-Fbxo47  88.05       452         53          1           1           451         1           452         0.0         809
MUS-Fbxo47  PTR-Fbxo47  84.15       448         71          0           1           448         1           448         0.0         802
MUS-Fbxo47  GGO-Fbxo47  83.93       448         72          0           1           448         1           448         0.0         801
MUS-Fbxo47  HSA-Fbxo47  83.48       448         74          0           1           448         1           448         0.0         798
MUS-Fbxo47  PPY-Fbxo47  83.26       448         75          0           1           448         1           448         0.0         796
MUS-Fbxo47  MMU-Fbxo47  83.26       448         75          0           1           448         1           448         0.0         794
MUS-Fbxo47  CJA-Fbxo47  82.14       448         80          0           1           448         1           448         0.0         791
# BLASTP 2.2.20 [Feb-08-2009]
# Query: PPY-Fbxo47
# Database: 559_protein.db
# Query id, Subject id, % identity, alignment length, mismatches, gap openings, q. start, q. end, s. start, s. end, e-value, bit score
PPY-Fbxo47  PPY-Fbxo47  100.00      452         0           0           1           452         1           452         0.0         911
PPY-Fbxo47  GGO-Fbxo47  98.01       452         9           0           1           452         1           452         0.0         898
PPY-Fbxo47  PTR-Fbxo47  97.79       452         10          0           1           452         1           452         0.0         896
PPY-Fbxo47  HSA-Fbxo47  97.57       452         11          0           1           452         1           452         0.0         894
PPY-Fbxo47  MMU-Fbxo47  98.01       452         9           0           1           452         1           452         0.0         893
PPY-Fbxo47  CJA-Fbxo47  93.36       452         30          0           1           452         1           452         0.0         862
PPY-Fbxo47  MUS-Fbxo47  83.26       448         75          0           1           448         1           448         0.0         746
PPY-Fbxo47  RNO-Fbxo47  73.27       449         119         1           1           448         1           449         0.0         664
# BLASTP 2.2.20 [Feb-08-2009]
# Query: PTR-Fbxo47
# Database: 559_protein.db
# Query id, Subject id, % identity, alignment length, mismatches, gap openings, q. start, q. end, s. start, s. end, e-value, bit score
PTR-Fbxo47  PTR-Fbxo47  100.00      452         0           0           1           452         1           452         0.0         940
PTR-Fbxo47  GGO-Fbxo47  99.78       452         1           0           1           452         1           452         0.0         939
PTR-Fbxo47  HSA-Fbxo47  99.34       452         3           0           1           452         1           452         0.0         936
PTR-Fbxo47  PPY-Fbxo47  97.79       452         10          0           1           452         1           452         0.0         924
PTR-Fbxo47  MMU-Fbxo47  98.01       452         9           0           1           452         1           452         0.0         923
PTR-Fbxo47  CJA-Fbxo47  92.92       452         32          0           1           452         1           452         0.0         890
PTR-Fbxo47  MUS-Fbxo47  84.15       448         71          0           1           448         1           448         0.0         781
```

```
PTR-Fbxo47  RNO-Fbxo47  75.50    449    109    1    1    448    1    449    0.0    695
# BLASTP 2.2.20 [Feb-08-2009]
# Query: RNO-Fbxo47
# Database: 559_protein.db
# Query id, Subject id, % identity, alignment length, mismatches, gap openings, q. start, q. end, s. start, s. end, e-value, bit score
RNO-Fbxo47  RNO-Fbxo47  100.00   452    0      0    1    452    1    452    0.0    915
RNO-Fbxo47  MUS-Fbxo47  88.05    452    53     1    1    452    1    451    0.0    785
RNO-Fbxo47  PTR-Fbxo47  75.50    449    109    1    1    449    1    448    0.0    695
RNO-Fbxo47  GGO-Fbxo47  75.28    449    110    1    1    449    1    448    0.0    694
RNO-Fbxo47  HSA-Fbxo47  75.06    449    111    1    1    449    1    448    0.0    692
RNO-Fbxo47  PPY-Fbxo47  75.28    449    110    1    1    449    1    448    0.0    691
RNO-Fbxo47  MMU-Fbxo47  75.06    449    111    1    1    449    1    448    0.0    689
RNO-Fbxo47  CJA-Fbxo47  73.94    449    116    1    1    449    1    448    0.0    688
# BLASTP 2.2.20 [Feb-08-2009]
# Query: HSA-Fbxo47
# Database: 559_protein.db
# Query id, Subject id, % identity, alignment length, mismatches, gap openings, q. start, q. end, s. start, s. end, e-value, bit score
HSA-Fbxo47  HSA-Fbxo47  100.00   452    0      0    1    452    1    452    0.0    940
HSA-Fbxo47  GGO-Fbxo47  99.56    452    2      0    1    452    1    452    0.0    937
HSA-Fbxo47  PTR-Fbxo47  99.34    452    3      0    1    452    1    452    0.0    936
HSA-Fbxo47  PPY-Fbxo47  97.57    452    11     0    1    452    1    452    0.0    922
HSA-Fbxo47  MMU-Fbxo47  97.79    452    10     0    1    452    1    452    0.0    921
HSA-Fbxo47  CJA-Fbxo47  92.70    452    33     0    1    452    1    452    0.0    888
HSA-Fbxo47  MUS-Fbxo47  83.48    448    74     0    1    448    1    448    0.0    777
HSA-Fbxo47  RNO-Fbxo47  75.06    449    111    1    1    448    1    449    0.0    692
# BLASTP 2.2.20 [Feb-08-2009]
# Query: CJA-Fbxo48
# Database: 559_protein.db
# Query id, Subject id, % identity, alignment length, mismatches, gap openings, q. start, q. end, s. start, s. end, e-value, bit score
CJA-Fbxo48  CJA-Fbxo48  100.00   155    0      0    1    155    1    155    8e-083    297
CJA-Fbxo48  PTR-Fbxo48  89.51    143    15     0    13   155    13   155    8e-075    270
CJA-Fbxo48  GGO-Fbxo48  89.51    143    15     0    13   155    13   155    8e-075    270
CJA-Fbxo48  HSA-Fbxo48  89.51    143    15     0    13   155    13   155    9e-075    270
CJA-Fbxo48  PPY-Fbxo48a 89.51    143    15     0    13   155    13   155    2e-074    269
CJA-Fbxo48  PPY-Fbxo48b 89.51    143    15     0    13   155    13   155    2e-074    269
CJA-Fbxo48  MMU-Fbxo48  87.41    143    18     0    13   155    13   155    3e-073    265
CJA-Fbxo48  MUS-Fbxo48  73.43    143    38     0    13   155    13   155    1e-061    227
CJA-Fbxo48  RNO-Fbxo48  71.33    143    41     0    13   155    12   154    5e-059    218
# BLASTP 2.2.20 [Feb-08-2009]
# Query: GGO-Fbxo48
# Database: 559_protein.db
# Query id, Subject id, % identity, alignment length, mismatches, gap openings, q. start, q. end, s. start, s. end, e-value, bit score
GGO-Fbxo48  GGO-Fbxo48  100.00   155    0      0    1    155    1    155    8e-084    300
GGO-Fbxo48  PTR-Fbxo48  98.71    155    2      0    1    155    1    155    9e-084    300
GGO-Fbxo48  HSA-Fbxo48  98.71    155    2      0    1    155    1    155    1e-083    299
```

```
GGO-Fbxo48  PPY-Fbxo48a 96.77      155         5           0           1           155         1           155         3e-081      291
GGO-Fbxo48  PPY-Fbxo48b 96.77      155         5           0           1           155         1           155         3e-081      291
GGO-Fbxo48  MMU-Fbxo48  95.48      155         7           0           1           155         1           155         1e-079      286
GGO-Fbxo48  CJA-Fbxo48  89.51      143         15          0           13          155         13          155         7e-075      270
GGO-Fbxo48  MUS-Fbxo48  76.92      143         33          0           13          155         13          155         9e-065      237
GGO-Fbxo48  RNO-Fbxo48  73.43      143         38          0           13          155         12          154         3e-061      225
# BLASTP 2.2.20 [Feb-08-2009]
# Query: MMU-Fbxo48
# Database: 559_protein.db
# Query id, Subject id, % identity, alignment length, mismatches, gap openings, q. start, q. end, s. start, s. end, e-value, bit score
MMU-Fbxo48  MMU-Fbxo48  100.00     155         0           0           1           155         1           155         1e-083      299
MMU-Fbxo48  PPY-Fbxo48a 95.48      155         7           0           1           155         1           155         9e-080      286
MMU-Fbxo48  PPY-Fbxo48b 95.48      155         7           0           1           155         1           155         9e-080      286
MMU-Fbxo48  GGO-Fbxo48  95.48      155         7           0           1           155         1           155         1e-079      286
MMU-Fbxo48  PTR-Fbxo48  94.19      155         9           0           1           155         1           155         2e-079      286
MMU-Fbxo48  HSA-Fbxo48  94.19      155         9           0           1           155         1           155         2e-079      285
MMU-Fbxo48  CJA-Fbxo48  87.41      143         18          0           13          155         13          155         2e-073      265
MMU-Fbxo48  MUS-Fbxo48  76.92      143         33          0           13          155         13          155         4e-065      238
MMU-Fbxo48  RNO-Fbxo48  73.43      143         38          0           13          155         12          154         7e-062      227
# BLASTP 2.2.20 [Feb-08-2009]
# Query: MUS-Fbxo48
# Database: 559_protein.db
# Query id, Subject id, % identity, alignment length, mismatches, gap openings, q. start, q. end, s. start, s. end, e-value, bit score
MUS-Fbxo48  MUS-Fbxo48  100.00     161         0           0           1           161         1           161         4e-094      335
MUS-Fbxo48  RNO-Fbxo48  84.47      161         24          1           1           161         1           160         4e-078      281
MUS-Fbxo48  PPY-Fbxo48a 76.13      155         37          0           1           155         1           155         1e-069      253
MUS-Fbxo48  PPY-Fbxo48b 76.13      155         37          0           1           155         1           155         1e-069      253
MUS-Fbxo48  HSA-Fbxo48  75.48      155         38          0           1           155         1           155         3e-069      252
MUS-Fbxo48  MMU-Fbxo48  74.84      155         39          0           1           155         1           155         1e-068      250
MUS-Fbxo48  PTR-Fbxo48  75.48      155         38          0           1           155         1           155         2e-068      249
MUS-Fbxo48  GGO-Fbxo48  74.84      155         39          0           1           155         1           155         2e-068      249
MUS-Fbxo48  CJA-Fbxo48  71.61      155         44          0           1           155         1           155         1e-065      240
# BLASTP 2.2.20 [Feb-08-2009]
# Query: PPY-Fbxo48b
# Database: 559_protein.db
# Query id, Subject id, % identity, alignment length, mismatches, gap openings, q. start, q. end, s. start, s. end, e-value, bit score
PPY-Fbxo48b PPY-Fbxo48a 100.00     155         0           0           1           155         1           155         3e-083      298
PPY-Fbxo48b PPY-Fbxo48b 100.00     155         0           0           1           155         1           155         3e-083      298
PPY-Fbxo48b GGO-Fbxo48  96.77      155         5           0           1           155         1           155         3e-081      291
PPY-Fbxo48b PTR-Fbxo48  96.77      155         5           0           1           155         1           155         4e-081      291
PPY-Fbxo48b HSA-Fbxo48  96.77      155         5           0           1           155         1           155         5e-081      291
PPY-Fbxo48b MMU-Fbxo48  95.48      155         7           0           1           155         1           155         9e-080      286
PPY-Fbxo48b CJA-Fbxo48  89.51      143         15          0           13          155         13          155         2e-074      269
PPY-Fbxo48b MUS-Fbxo48  77.62      143         32          0           13          155         13          155         3e-065      238
PPY-Fbxo48b RNO-Fbxo48  74.13      143         37          0           13          155         12          154         7e-062      227
```

```
# BLASTP 2.2.20 [Feb-08-2009]
# Query: PPY-Fbxo48a
# Database: 559_protein.db
# Query id, Subject id, % identity, alignment length, mismatches, gap openings, q. start, q. end, s. start, s. end, e-value, bit score
PPY-Fbxo48a PPY-Fbxo48a 100.00      155         0           0           1           155         1           155         3e-083      298
PPY-Fbxo48a PPY-Fbxo48b 100.00      155         0           0           1           155         1           155         3e-083      298
PPY-Fbxo48a GGO-Fbxo48  96.77       155         5           0           1           155         1           155         3e-081      291
PPY-Fbxo48a PTR-Fbxo48  96.77       155         5           0           1           155         1           155         4e-081      291
PPY-Fbxo48a HSA-Fbxo48  96.77       155         5           0           1           155         1           155         5e-081      291
PPY-Fbxo48a MMU-Fbxo48  95.48       155         7           0           1           155         1           155         9e-080      286
PPY-Fbxo48a CJA-Fbxo48  89.51       143         15          0           13          155         13          155         2e-074      269
PPY-Fbxo48a MUS-Fbxo48  77.62       143         32          0           13          155         13          155         3e-065      238
PPY-Fbxo48a RNO-Fbxo48  74.13       143         37          0           13          155         12          154         7e-062      227
# BLASTP 2.2.20 [Feb-08-2009]
# Query: PTR-Fbxo48
# Database: 559_protein.db
# Query id, Subject id, % identity, alignment length, mismatches, gap openings, q. start, q. end, s. start, s. end, e-value, bit score
PTR-Fbxo48  PTR-Fbxo48  100.00      155         0           0           1           155         1           155         2e-090      322
PTR-Fbxo48  GGO-Fbxo48  98.71       155         2           0           1           155         1           155         6e-089      317
PTR-Fbxo48  HSA-Fbxo48  98.71       155         2           0           1           155         1           155         9e-089      317
PTR-Fbxo48  PPY-Fbxo48a 96.77       155         5           0           1           155         1           155         5e-087      311
PTR-Fbxo48  PPY-Fbxo48b 96.77       155         5           0           1           155         1           155         5e-087      311
PTR-Fbxo48  MMU-Fbxo48  94.19       155         9           0           1           155         1           155         7e-085      303
PTR-Fbxo48  CJA-Fbxo48  88.39       155         18          0           1           155         1           155         8e-080      287
PTR-Fbxo48  MUS-Fbxo48  75.48       155         38          0           1           155         1           155         1e-068      249
PTR-Fbxo48  RNO-Fbxo48  70.59       153         45          0           3           155         2           154         4e-063      231
# BLASTP 2.2.20 [Feb-08-2009]
# Query: RNO-Fbxo48
# Database: 559_protein.db
# Query id, Subject id, % identity, alignment length, mismatches, gap openings, q. start, q. end, s. start, s. end, e-value, bit score
RNO-Fbxo48  RNO-Fbxo48  100.00      160         0           0           1           160         1           160         1e-093      333
RNO-Fbxo48  MUS-Fbxo48  84.47       161         24          1           1           160         1           161         4e-078      281
RNO-Fbxo48  MMU-Fbxo48  71.24       153         44          0           2           154         3           155         9e-065      237
RNO-Fbxo48  PPY-Fbxo48a 71.24       153         44          0           2           154         3           155         3e-064      235
RNO-Fbxo48  PPY-Fbxo48b 71.24       153         44          0           2           154         3           155         3e-064      235
RNO-Fbxo48  GGO-Fbxo48  71.24       153         44          0           2           154         3           155         5e-064      234
RNO-Fbxo48  HSA-Fbxo48  70.59       153         45          0           2           154         3           155         2e-063      232
RNO-Fbxo48  PTR-Fbxo48  70.59       153         45          0           2           154         3           155         4e-063      231
RNO-Fbxo48  CJA-Fbxo48  69.68       155         46          1           1           154         1           155         3e-061      225
# BLASTP 2.2.20 [Feb-08-2009]
# Query: HSA-Fbxo48
# Database: 559_protein.db
# Query id, Subject id, % identity, alignment length, mismatches, gap openings, q. start, q. end, s. start, s. end, e-value, bit score
HSA-Fbxo48  HSA-Fbxo48  100.00      155         0           0           1           155         1           155         3e-090      322
HSA-Fbxo48  GGO-Fbxo48  98.71       155         2           0           1           155         1           155         6e-089      317
```

```
HSA-Fbxo48  PTR-Fbxo48  98.71   155  2    0  1   155  1   155  9e-089   317
HSA-Fbxo48  PPY-Fbxo48a 96.77   155  5    0  1   155  1   155  6e-087   310
HSA-Fbxo48  PPY-Fbxo48b 96.77   155  5    0  1   155  1   155  6e-087   310
HSA-Fbxo48  MMU-Fbxo48  94.19   155  9    0  1   155  1   155  8e-085   303
HSA-Fbxo48  CJA-Fbxo48  88.39   155  18   0  1   155  1   155  7e-080   287
HSA-Fbxo48  MUS-Fbxo48  75.48   155  38   0  1   155  1   155  3e-069   252
HSA-Fbxo48  RNO-Fbxo48  70.59   153  45   0  3   155  2   154  2e-063   232
# BLASTP 2.2.20 [Feb-08-2009]
# Query: CJA-Fbxo5
# Database: 559_protein.db
# Query id, Subject id, % identity, alignment length, mismatches, gap openings, q. start, q. end, s. start, s. end, e-value, bit score
CJA-Fbxo5   CJA-Fbxo5   100.00  440  0    0  1   440  1   440  0.0      850
CJA-Fbxo5   MMU-Fbxo5   93.26   445  15   3  10  440  5   448  0.0      789
CJA-Fbxo5   PTR-Fbxo5   93.41   440  24   4  4   440  10  447  0.0      777
CJA-Fbxo5   GGO-Fbxo5   92.95   440  26   4  4   440  10  447  0.0      775
CJA-Fbxo5   PPY-Fbxo5   93.18   440  25   4  4   440  10  447  0.0      773
CJA-Fbxo5   HSA-Fbxo5   92.73   440  27   4  4   440  10  447  0.0      772
CJA-Fbxo5   MUS-Fbxo5   62.61   436  138  6  5   440  11  421  1e-143   501
CJA-Fbxo5   RNO-Fbxo5   59.73   437  146  9  5   440  11  418  5e-131   459
# BLASTP 2.2.20 [Feb-08-2009]
# Query: GGO-Fbxo5
# Database: 559_protein.db
# Query id, Subject id, % identity, alignment length, mismatches, gap openings, q. start, q. end, s. start, s. end, e-value, bit score
GGO-Fbxo5   GGO-Fbxo5   100.00  447  0    0  1   447  1   447  0.0      850
GGO-Fbxo5   PTR-Fbxo5   99.55   447  2    0  1   447  1   447  0.0      848
GGO-Fbxo5   HSA-Fbxo5   98.88   447  5    0  1   447  1   447  0.0      843
GGO-Fbxo5   PPY-Fbxo5   97.99   447  9    0  1   447  1   447  0.0      833
GGO-Fbxo5   MMU-Fbxo5   97.54   448  10   1  1   447  1   448  0.0      828
GGO-Fbxo5   CJA-Fbxo5   91.46   445  22   4  5   447  10  440  0.0      784
GGO-Fbxo5   MUS-Fbxo5   65.22   414  126  4  34  447  26  421  5e-147   512
GGO-Fbxo5   RNO-Fbxo5   61.25   431  132  8  30  447  10  418  3e-135   473
# BLASTP 2.2.20 [Feb-08-2009]
# Query: MMU-Fbxo5
# Database: 559_protein.db
# Query id, Subject id, % identity, alignment length, mismatches, gap openings, q. start, q. end, s. start, s. end, e-value, bit score
MMU-Fbxo5   MMU-Fbxo5   100.00  425  0    0  24  448  24  448  0.0      836
MMU-Fbxo5   PTR-Fbxo5   98.35   425  6    1  24  448  24  447  0.0      819
MMU-Fbxo5   GGO-Fbxo5   97.88   425  8    1  24  448  24  447  0.0      817
MMU-Fbxo5   HSA-Fbxo5   97.65   425  9    1  24  448  24  447  0.0      815
MMU-Fbxo5   PPY-Fbxo5   97.65   425  9    1  24  448  24  447  0.0      812
MMU-Fbxo5   CJA-Fbxo5   96.01   426  15   2  24  448  16  440  0.0      801
MMU-Fbxo5   MUS-Fbxo5   65.06   415  126  5  34  448  26  421  7e-147   511
MMU-Fbxo5   RNO-Fbxo5   60.88   432  133  9  30  448  10  418  4e-134   469
# BLASTP 2.2.20 [Feb-08-2009]
# Query: MUS-Fbxo5
```

```
# Database: 559_protein.db
# Query id, Subject id, % identity, alignment length, mismatches, gap openings, q. start, q. end, s. start, s. end, e-value, bit score
MUS-Fbxo5   MUS-Fbxo5   100.00   421   0     0   1    421   1    421   0.0      868
MUS-Fbxo5   RNO-Fbxo5   86.97    422   50    3   1    421   1    418   0.0      739
MUS-Fbxo5   PPY-Fbxo5   63.76    447   136   5   1    421   1    447   4e-155   539
MUS-Fbxo5   GGO-Fbxo5   63.53    447   137   5   1    421   1    447   1e-154   537
MUS-Fbxo5   PTR-Fbxo5   63.53    447   137   5   1    421   1    447   1e-154   537
MUS-Fbxo5   MMU-Fbxo5   63.39    448   137   6   1    421   1    448   8e-154   535
MUS-Fbxo5   HSA-Fbxo5   63.31    447   138   5   1    421   1    447   9e-154   535
MUS-Fbxo5   CJA-Fbxo5   63.53    436   134   6   11   421   5    440   1e-150   524
# BLASTP 2.2.20 [Feb-08-2009]
# Query: PPY-Fbxo5
# Database: 559_protein.db
# Query id, Subject id, % identity, alignment length, mismatches, gap openings, q. start, q. end, s. start, s. end, e-value, bit score
PPY-Fbxo5   PPY-Fbxo5   100.00   447   0     0   1    447   1    447   0.0      849
PPY-Fbxo5   PTR-Fbxo5   98.43    447   7     0   1    447   1    447   0.0      835
PPY-Fbxo5   GGO-Fbxo5   97.99    447   9     0   1    447   1    447   0.0      833
PPY-Fbxo5   HSA-Fbxo5   97.76    447   10    0   1    447   1    447   0.0      830
PPY-Fbxo5   MMU-Fbxo5   97.32    448   11    1   1    447   1    448   0.0      823
PPY-Fbxo5   CJA-Fbxo5   91.69    445   21    4   5    447   10   440   0.0      781
PPY-Fbxo5   MUS-Fbxo5   65.46    414   125   4   34   447   26   421   1e-147   514
PPY-Fbxo5   RNO-Fbxo5   61.06    434   134   8   27   447   7    418   5e-136   476
# BLASTP 2.2.20 [Feb-08-2009]
# Query: PTR-Fbxo5
# Database: 559_protein.db
# Query id, Subject id, % identity, alignment length, mismatches, gap openings, q. start, q. end, s. start, s. end, e-value, bit score
PTR-Fbxo5   PTR-Fbxo5   100.00   447   0     0   1    447   1    447   0.0      851
PTR-Fbxo5   GGO-Fbxo5   99.55    447   2     0   1    447   1    447   0.0      848
PTR-Fbxo5   HSA-Fbxo5   99.33    447   3     0   1    447   1    447   0.0      845
PTR-Fbxo5   PPY-Fbxo5   98.43    447   7     0   1    447   1    447   0.0      836
PTR-Fbxo5   MMU-Fbxo5   97.99    448   8     1   1    447   1    448   0.0      832
PTR-Fbxo5   CJA-Fbxo5   91.91    445   20    4   5    447   10   440   0.0      787
PTR-Fbxo5   MUS-Fbxo5   65.22    414   126   4   34   447   26   421   4e-147   513
PTR-Fbxo5   RNO-Fbxo5   61.25    431   132   8   30   447   10   418   3e-135   473
# BLASTP 2.2.20 [Feb-08-2009]
# Query: RNO-Fbxo5
# Database: 559_protein.db
# Query id, Subject id, % identity, alignment length, mismatches, gap openings, q. start, q. end, s. start, s. end, e-value, bit score
RNO-Fbxo5   RNO-Fbxo5   100.00   418   0     0   1    418   1    418   0.0      835
RNO-Fbxo5   MUS-Fbxo5   86.49    422   52    3   1    418   1    421   0.0      721
RNO-Fbxo5   PPY-Fbxo5   60.71    448   145   8   1    418   1    447   1e-138   484
RNO-Fbxo5   PTR-Fbxo5   60.49    448   146   8   1    418   1    447   1e-137   481
RNO-Fbxo5   GGO-Fbxo5   60.49    448   146   8   1    418   1    447   1e-137   481
RNO-Fbxo5   MMU-Fbxo5   60.36    449   146   9   1    418   1    448   3e-137   479
RNO-Fbxo5   HSA-Fbxo5   60.49    448   146   9   1    418   1    447   7e-137   478
```

```
RNO-Fbxo5	CJA-Fbxo5	60.18	437	144	9	11	418	5	440	2e-132	464
# BLASTP 2.2.20 [Feb-08-2009]
# Query: HSA-Fbxo5
# Database: 559_protein.db
# Query id, Subject id, % identity, alignment length, mismatches, gap openings, q. start, q. end, s. start, s. end, e-value, bit score
HSA-Fbxo5	HSA-Fbxo5	100.00	447	0	0	1	447	1	447	0.0	850
HSA-Fbxo5	PTR-Fbxo5	99.33	447	3	0	1	447	1	447	0.0	846
HSA-Fbxo5	GGO-Fbxo5	98.88	447	5	0	1	447	1	447	0.0	844
HSA-Fbxo5	PPY-Fbxo5	97.76	447	10	0	1	447	1	447	0.0	831
HSA-Fbxo5	MMU-Fbxo5	97.32	448	11	1	1	447	1	448	0.0	828
HSA-Fbxo5	CJA-Fbxo5	91.24	445	23	4	5	447	10	440	0.0	783
HSA-Fbxo5	MUS-Fbxo5	64.98	414	127	4	34	447	26	421	3e-147	513
HSA-Fbxo5	RNO-Fbxo5	61.02	431	133	8	30	447	10	418	3e-135	473
# BLASTP 2.2.20 [Feb-08-2009]
# Query: CJA-Fbxo6
# Database: 559_protein.db
# Query id, Subject id, % identity, alignment length, mismatches, gap openings, q. start, q. end, s. start, s. end, e-value, bit score
CJA-Fbxo6	CJA-Fbxo6	100.00	285	0	0	1	285	1	285	4e-162	561
CJA-Fbxo6	HSA-Fbxo6	83.22	292	42	1	1	285	1	292	7e-134	468
CJA-Fbxo6	MMU-Fbxo6	82.53	292	44	1	1	285	1	292	2e-133	466
CJA-Fbxo6	PPY-Fbxo6	82.88	292	43	1	1	285	1	292	3e-133	466
CJA-Fbxo6	PTR-Fbxo6	82.53	292	44	1	1	285	1	292	6e-133	464
CJA-Fbxo6	GGO-Fbxo6	82.19	292	45	1	1	285	1	292	1e-131	460
CJA-Fbxo6	RNO-Fbxo6	71.98	257	72	0	27	283	18	274	4e-112	395
CJA-Fbxo6	MUS-Fbxo6	71.08	249	72	0	27	275	18	266	2e-107	380
CJA-Fbxo6	RNO-Fbxo44	70.69	232	68	0	27	258	36	267	1e-097	347
CJA-Fbxo6	GGO-Fbxo44	71.55	232	66	0	27	258	30	261	3e-097	346
CJA-Fbxo6	MMU-Fbxo44	71.55	232	66	0	27	258	20	251	5e-097	345
CJA-Fbxo6	PTR-Fbxo44	71.12	232	67	0	27	258	20	251	3e-096	343
CJA-Fbxo6	MUS-Fbxo44	56.86	255	89	2	27	281	20	253	7e-077	278
# BLASTP 2.2.20 [Feb-08-2009]
# Query: GGO-Fbxo6
# Database: 559_protein.db
# Query id, Subject id, % identity, alignment length, mismatches, gap openings, q. start, q. end, s. start, s. end, e-value, bit score
GGO-Fbxo6	GGO-Fbxo6	100.00	293	0	0	1	293	1	293	1e-169	586
GGO-Fbxo6	PTR-Fbxo6	98.98	293	3	0	1	293	1	293	2e-167	579
GGO-Fbxo6	HSA-Fbxo6	98.98	293	3	0	1	293	1	293	2e-167	579
GGO-Fbxo6	PPY-Fbxo6	97.61	293	7	0	1	293	1	293	3e-165	572
GGO-Fbxo6	MMU-Fbxo6	96.25	293	11	0	1	293	1	293	3e-162	562
GGO-Fbxo6	CJA-Fbxo6	82.19	292	45	1	1	292	1	285	2e-132	463
GGO-Fbxo6	RNO-Fbxo6	77.20	250	57	0	25	274	16	265	1e-118	417
GGO-Fbxo6	MUS-Fbxo6	74.00	250	65	0	25	274	16	265	5e-114	402
GGO-Fbxo6	GGO-Fbxo44	74.26	237	61	0	25	261	28	264	2e-103	367
GGO-Fbxo6	MMU-Fbxo44	74.26	237	61	0	25	261	18	254	2e-103	366
GGO-Fbxo6	PTR-Fbxo44	73.84	237	62	0	25	261	18	254	1e-102	364
```

GGO-Fbxo6	RNO-Fbxo44	71.73	237	67	0	25	261	34	270	8e-101	358
GGO-Fbxo6	MUS-Fbxo44	70.83	192	56	0	25	216	18	209	2e-077	280
GGO-Fbxo6	PPY-Fbxo2	51.91	235	110	3	28	259	61	295	3e-068	249
GGO-Fbxo6	PTR-Fbxo2	51.49	235	111	3	28	259	61	295	5e-068	249
GGO-Fbxo6	HSA-Fbxo2	51.49	235	111	3	28	259	62	296	5e-068	249
GGO-Fbxo6	GGO-Fbxo2	50.63	237	114	3	28	261	61	297	6e-068	249
GGO-Fbxo6	CJA-Fbxo2	51.06	235	112	3	28	259	63	297	2e-067	247
GGO-Fbxo6	MUS-Fbxo2	50.21	237	114	3	25	259	63	297	4e-067	246
GGO-Fbxo6	RNO-Fbxo2	50.63	237	113	3	25	259	62	296	8e-067	245

BLASTP 2.2.20 [Feb-08-2009]
Query: MMU-Fbxo6
Database: 559_protein.db
Query id, Subject id, % identity, alignment length, mismatches, gap openings, q. start, q. end, s. start, s. end, e-value, bit score

MMU-Fbxo6	MMU-Fbxo6	100.00	293	0	0	1	293	1	293	2e-169	586
MMU-Fbxo6	PPY-Fbxo6	97.95	293	6	0	1	293	1	293	1e-165	573
MMU-Fbxo6	PTR-Fbxo6	96.59	293	10	0	1	293	1	293	1e-163	566
MMU-Fbxo6	HSA-Fbxo6	96.59	293	10	0	1	293	1	293	2e-163	566
MMU-Fbxo6	GGO-Fbxo6	96.25	293	11	0	1	293	1	293	2e-162	562
MMU-Fbxo6	CJA-Fbxo6	82.53	292	44	1	1	292	1	285	3e-134	469
MMU-Fbxo6	RNO-Fbxo6	77.42	248	56	0	25	272	16	263	1e-117	414
MMU-Fbxo6	MUS-Fbxo6	74.19	248	64	0	25	272	16	263	2e-113	399
MMU-Fbxo6	GGO-Fbxo44	75.64	234	57	0	25	258	28	261	2e-104	370
MMU-Fbxo6	MMU-Fbxo44	75.64	234	57	0	25	258	18	251	3e-104	369
MMU-Fbxo6	PTR-Fbxo44	75.21	234	58	0	25	258	18	251	2e-103	366
MMU-Fbxo6	RNO-Fbxo44	73.08	234	63	0	25	258	34	267	1e-101	361
MMU-Fbxo6	MUS-Fbxo44	71.88	192	54	0	25	216	18	209	2e-079	286
MMU-Fbxo6	PPY-Fbxo2	51.93	233	109	3	28	257	61	293	3e-067	246
MMU-Fbxo6	HSA-Fbxo2	51.50	233	110	3	28	257	62	294	4e-067	246
MMU-Fbxo6	CJA-Fbxo2	50.63	237	114	3	28	261	63	299	7e-067	245
MMU-Fbxo6	PTR-Fbxo2	51.50	233	110	3	28	257	61	293	7e-067	245
MMU-Fbxo6	GGO-Fbxo2	51.52	231	109	3	28	255	61	291	9e-067	244
MMU-Fbxo6	MUS-Fbxo2	50.21	235	113	3	25	257	63	295	1e-065	241
MMU-Fbxo6	RNO-Fbxo2	50.64	235	112	3	25	257	62	294	2e-065	240

BLASTP 2.2.20 [Feb-08-2009]
Query: MUS-Fbxo6
Database: 559_protein.db
Query id, Subject id, % identity, alignment length, mismatches, gap openings, q. start, q. end, s. start, s. end, e-value, bit score

MUS-Fbxo6	MUS-Fbxo6	100.00	295	0	0	1	295	1	295	8e-170	587
MUS-Fbxo6	RNO-Fbxo6	85.99	257	36	0	16	272	16	272	1e-134	470
MUS-Fbxo6	GGO-Fbxo6	74.60	248	63	0	16	263	25	272	5e-114	402
MUS-Fbxo6	MMU-Fbxo6	74.19	248	64	0	16	263	25	272	3e-113	399
MUS-Fbxo6	HSA-Fbxo6	74.60	248	63	0	16	263	25	272	3e-113	399
MUS-Fbxo6	PPY-Fbxo6	74.60	248	63	0	16	263	25	272	4e-113	399
MUS-Fbxo6	PTR-Fbxo6	73.79	248	65	0	16	263	25	272	2e-112	396
MUS-Fbxo6	CJA-Fbxo6	70.92	251	73	0	16	266	25	275	3e-108	382

MUS-Fbxo6	GGO-Fbxo44	67.52	234	76	0	16	249	28	261	6e-092	328
MUS-Fbxo6	MMU-Fbxo44	67.52	234	76	0	16	249	18	251	9e-092	328
MUS-Fbxo6	RNO-Fbxo44	67.09	234	77	0	16	249	34	267	6e-091	325
MUS-Fbxo6	PTR-Fbxo44	67.09	234	77	0	16	249	18	251	6e-091	325
MUS-Fbxo6	MUS-Fbxo44	65.10	192	67	0	16	207	18	209	9e-069	251
MUS-Fbxo6	PPY-Fbxo2	52.79	233	107	3	19	248	61	293	3e-067	246
MUS-Fbxo6	PTR-Fbxo2	51.93	233	109	3	19	248	61	293	4e-066	243
MUS-Fbxo6	GGO-Fbxo2	51.71	234	110	3	19	249	61	294	4e-066	243
MUS-Fbxo6	HSA-Fbxo2	51.93	233	109	3	19	248	62	294	4e-066	243
MUS-Fbxo6	CJA-Fbxo2	51.93	233	109	3	19	248	63	295	8e-066	241
MUS-Fbxo6	RNO-Fbxo2	50.21	235	113	3	16	248	62	294	5e-065	239
MUS-Fbxo6	MUS-Fbxo2	50.21	235	113	3	16	248	63	295	8e-065	238

BLASTP 2.2.20 [Feb-08-2009]
Query: PPY-Fbxo6
Database: 559_protein.db
Query id, Subject id, % identity, alignment length, mismatches, gap openings, q. start, q. end, s. start, s. end, e-value, bit score

PPY-Fbxo6	PPY-Fbxo6	100.00	293	0	0	1	293	1	293	2e-169	585
PPY-Fbxo6	PTR-Fbxo6	97.95	293	6	0	1	293	1	293	2e-166	576
PPY-Fbxo6	HSA-Fbxo6	97.95	293	6	0	1	293	1	293	2e-166	576
PPY-Fbxo6	MMU-Fbxo6	97.95	293	6	0	1	293	1	293	2e-165	573
PPY-Fbxo6	GGO-Fbxo6	97.61	293	7	0	1	293	1	293	3e-165	572
PPY-Fbxo6	CJA-Fbxo6	82.88	292	43	1	1	292	1	285	3e-134	469
PPY-Fbxo6	RNO-Fbxo6	77.20	250	57	0	25	274	16	265	1e-117	414
PPY-Fbxo6	MUS-Fbxo6	74.00	250	65	0	25	274	16	265	3e-113	399
PPY-Fbxo6	GGO-Fbxo44	75.11	237	59	0	25	261	28	264	3e-105	373
PPY-Fbxo6	MMU-Fbxo44	75.11	237	59	0	25	261	18	254	5e-105	372
PPY-Fbxo6	PTR-Fbxo44	74.68	237	60	0	25	261	18	254	3e-104	369
PPY-Fbxo6	RNO-Fbxo44	72.57	237	65	0	25	261	34	270	2e-102	363
PPY-Fbxo6	MUS-Fbxo44	71.88	192	54	0	25	216	18	209	3e-079	286
PPY-Fbxo6	PPY-Fbxo2	51.91	235	110	3	28	259	61	295	3e-068	249
PPY-Fbxo6	HSA-Fbxo2	51.49	235	111	3	28	259	62	296	5e-068	249
PPY-Fbxo6	GGO-Fbxo2	50.63	237	114	3	28	261	61	297	6e-068	249
PPY-Fbxo6	PTR-Fbxo2	51.49	235	111	3	28	259	61	295	8e-068	248
PPY-Fbxo6	CJA-Fbxo2	51.06	235	112	3	28	259	63	297	2e-067	247
PPY-Fbxo6	MUS-Fbxo2	50.21	237	114	3	25	259	63	297	1e-066	244
PPY-Fbxo6	RNO-Fbxo2	50.63	237	113	3	25	259	62	296	2e-066	243

BLASTP 2.2.20 [Feb-08-2009]
Query: PTR-Fbxo6
Database: 559_protein.db
Query id, Subject id, % identity, alignment length, mismatches, gap openings, q. start, q. end, s. start, s. end, e-value, bit score

PTR-Fbxo6	PTR-Fbxo6	100.00	293	0	0	1	293	1	293	1e-169	587
PTR-Fbxo6	HSA-Fbxo6	99.32	293	2	0	1	293	1	293	1e-168	583
PTR-Fbxo6	GGO-Fbxo6	98.98	293	3	0	1	293	1	293	2e-167	579
PTR-Fbxo6	PPY-Fbxo6	97.95	293	6	0	1	293	1	293	2e-166	576
PTR-Fbxo6	MMU-Fbxo6	96.59	293	10	0	1	293	1	293	1e-163	566

PTR-Fbxo6	CJA-Fbxo6	82.53	292	44	1	1	292	1	285	7e-134	468
PTR-Fbxo6	RNO-Fbxo6	76.40	250	59	0	25	274	16	265	8e-117	411
PTR-Fbxo6	MUS-Fbxo6	73.20	250	67	0	25	274	16	265	2e-112	396
PTR-Fbxo6	GGO-Fbxo44	74.68	237	60	0	25	261	28	264	6e-105	372
PTR-Fbxo6	MMU-Fbxo44	74.68	237	60	0	25	261	18	254	1e-104	370
PTR-Fbxo6	PTR-Fbxo44	74.26	237	61	0	25	261	18	254	7e-104	368
PTR-Fbxo6	RNO-Fbxo44	72.15	237	66	0	25	261	34	270	4e-102	362
PTR-Fbxo6	MUS-Fbxo44	71.35	192	55	0	25	216	18	209	6e-079	285
PTR-Fbxo6	PPY-Fbxo2	51.49	235	111	3	28	259	61	295	8e-068	248
PTR-Fbxo6	HSA-Fbxo2	51.06	235	112	3	28	259	62	296	8e-068	248
PTR-Fbxo6	GGO-Fbxo2	50.21	237	115	3	28	261	61	297	9e-068	248
PTR-Fbxo6	PTR-Fbxo2	51.06	235	112	3	28	259	61	295	1e-067	248
PTR-Fbxo6	CJA-Fbxo2	50.64	235	113	3	28	259	63	297	4e-067	246
PTR-Fbxo6	MUS-Fbxo2	50.21	237	114	3	25	259	63	297	4e-067	246
PTR-Fbxo6	RNO-Fbxo2	50.63	237	113	3	25	259	62	296	1e-066	244

BLASTP 2.2.20 [Feb-08-2009]
Query: RNO-Fbxo6
Database: 559_protein.db
Query id, Subject id, % identity, alignment length, mismatches, gap openings, q. start, q. end, s. start, s. end, e-value, bit score

RNO-Fbxo6	RNO-Fbxo6	100.00	269	0	0	16	284	16	284	3e-162	562
RNO-Fbxo6	MUS-Fbxo6	85.99	257	36	0	16	272	16	272	1e-134	470
RNO-Fbxo6	GGO-Fbxo6	77.20	250	57	0	16	265	25	274	2e-118	416
RNO-Fbxo6	HSA-Fbxo6	77.20	250	57	0	16	265	25	274	1e-117	413
RNO-Fbxo6	PPY-Fbxo6	77.20	250	57	0	16	265	25	274	2e-117	413
RNO-Fbxo6	MMU-Fbxo6	77.42	248	56	0	16	263	25	272	2e-117	413
RNO-Fbxo6	PTR-Fbxo6	76.40	250	59	0	16	265	25	274	1e-116	410
RNO-Fbxo6	CJA-Fbxo6	71.81	259	73	0	16	274	25	283	1e-112	397
RNO-Fbxo6	GGO-Fbxo44	69.66	234	71	0	16	249	28	261	3e-096	343
RNO-Fbxo6	MMU-Fbxo44	69.66	234	71	0	16	249	18	251	3e-096	342
RNO-Fbxo6	PTR-Fbxo44	69.23	234	72	0	16	249	18	251	2e-095	340
RNO-Fbxo6	RNO-Fbxo44	68.38	234	74	0	16	249	34	267	4e-095	339
RNO-Fbxo6	MUS-Fbxo44	65.63	192	66	0	16	207	18	209	8e-072	261
RNO-Fbxo6	PPY-Fbxo2	51.69	236	111	3	16	248	58	293	2e-067	247
RNO-Fbxo6	HSA-Fbxo2	50.85	236	113	3	16	248	59	294	2e-066	244
RNO-Fbxo6	PTR-Fbxo2	50.85	236	113	3	16	248	58	293	2e-066	243
RNO-Fbxo6	MUS-Fbxo2	51.06	235	111	3	16	248	63	295	2e-066	243
RNO-Fbxo6	GGO-Fbxo2	50.85	234	112	3	16	246	58	291	4e-066	243
RNO-Fbxo6	CJA-Fbxo2	50.85	234	112	3	16	246	60	293	7e-066	241
RNO-Fbxo6	RNO-Fbxo2	50.64	235	112	3	16	248	62	294	1e-065	241

BLASTP 2.2.20 [Feb-08-2009]
Query: HSA-Fbxo6
Database: 559_protein.db
Query id, Subject id, % identity, alignment length, mismatches, gap openings, q. start, q. end, s. start, s. end, e-value, bit score

HSA-Fbxo6	HSA-Fbxo6	100.00	293	0	0	1	293	1	293	1e-169	586
HSA-Fbxo6	PTR-Fbxo6	99.32	293	2	0	1	293	1	293	1e-168	583

```
HSA-Fbxo6   GGO-Fbxo6   98.98      293        3          0          1          293        1          293        2e-167      579
HSA-Fbxo6   PPY-Fbxo6   97.95      293        6          0          1          293        1          293        2e-166      576
HSA-Fbxo6   MMU-Fbxo6   96.59      293        10         0          1          293        1          293        1e-163      566
HSA-Fbxo6   CJA-Fbxo6   83.22      292        42         1          1          292        1          285        1e-134      471
HSA-Fbxo6   RNO-Fbxo6   77.20      250        57         0          25         274        16         265        1e-117      414
HSA-Fbxo6   MUS-Fbxo6   74.00      250        65         0          25         274        16         265        2e-113      399
HSA-Fbxo6   GGO-Fbxo44  74.68      237        60         0          25         261        28         264        6e-105      372
HSA-Fbxo6   MMU-Fbxo44  74.68      237        60         0          25         261        18         254        1e-104      370
HSA-Fbxo6   PTR-Fbxo44  74.26      237        61         0          25         261        18         254        8e-104      368
HSA-Fbxo6   RNO-Fbxo44  72.15      237        66         0          25         261        34         270        4e-102      362
HSA-Fbxo6   MUS-Fbxo44  71.35      192        55         0          25         216        18         209        7e-079      285
HSA-Fbxo6   PPY-Fbxo2   51.49      235        111        3          28         259        61         295        1e-067      248
HSA-Fbxo6   HSA-Fbxo2   51.06      235        112        3          28         259        62         296        2e-067      247
HSA-Fbxo6   GGO-Fbxo2   50.21      237        115        3          28         261        61         297        2e-067      247
HSA-Fbxo6   PTR-Fbxo2   51.06      235        112        3          28         259        61         295        3e-067      246
HSA-Fbxo6   CJA-Fbxo2   50.64      235        113        3          28         259        63         297        7e-067      245
HSA-Fbxo6   RNO-Fbxo2   50.21      237        114        3          25         259        62         296        5e-066      242
# BLASTP 2.2.20 [Feb-08-2009]
# Query: CJA-Fbxo7
# Database: 559_protein.db
# Query id, Subject id, % identity, alignment length, mismatches, gap openings, q. start, q. end, s. start, s. end, e-value, bit score
CJA-Fbxo7   CJA-Fbxo7   100.00     522        0          0          1          522        1          522        0.0        1025
CJA-Fbxo7   GGO-Fbxo7   90.04      522        52         0          1          522        1          522        0.0         922
CJA-Fbxo7   HSA-Fbxo7   89.66      522        54         0          1          522        1          522        0.0         920
CJA-Fbxo7   PTR-Fbxo7   89.27      522        56         0          1          522        1          522        0.0         914
CJA-Fbxo7   MMU-Fbxo7   92.12      482        38         0          41         522        47         528        0.0         870
CJA-Fbxo7   MUS-Fbxo7   75.38      524        126        3          1          522        1          523        0.0         778
CJA-Fbxo7   RNO-Fbxo7   75.14      523        128        2          1          522        1          522        0.0         776
CJA-Fbxo7   PPY-Fbxo7   92.58      431        32         0          92         522        13         443        0.0         776
# BLASTP 2.2.20 [Feb-08-2009]
# Query: GGO-Fbxo7
# Database: 559_protein.db
# Query id, Subject id, % identity, alignment length, mismatches, gap openings, q. start, q. end, s. start, s. end, e-value, bit score
GGO-Fbxo7   GGO-Fbxo7   100.00     522        0          0          1          522        1          522        0.0         992
GGO-Fbxo7   HSA-Fbxo7   98.85      522        6          0          1          522        1          522        0.0         983
GGO-Fbxo7   PTR-Fbxo7   98.47      522        8          0          1          522        1          522        0.0         977
GGO-Fbxo7   CJA-Fbxo7   90.04      522        52         0          1          522        1          522        0.0         889
GGO-Fbxo7   MMU-Fbxo7   96.06      482        19         0          41         522        47         528        0.0         868
GGO-Fbxo7   PPY-Fbxo7   97.68      431        10         0          92         522        13         443        0.0         780
GGO-Fbxo7   MUS-Fbxo7   72.90      524        139        3          1          522        1          523        0.0         717
GGO-Fbxo7   RNO-Fbxo7   72.47      523        142        2          1          522        1          522        0.0         716
# BLASTP 2.2.20 [Feb-08-2009]
# Query: MMU-Fbxo7
# Database: 559_protein.db
# Query id, Subject id, % identity, alignment length, mismatches, gap openings, q. start, q. end, s. start, s. end, e-value, bit score
```

```
MMU-Fbxo7   MMU-Fbxo7   100.00   528   0     0   1    528   1    528   0.0      953
MMU-Fbxo7   HSA-Fbxo7   96.26    481   18    0   48   528   42   522   0.0      871
MMU-Fbxo7   GGO-Fbxo7   96.26    481   18    0   48   528   42   522   0.0      869
MMU-Fbxo7   PTR-Fbxo7   95.63    481   21    0   48   528   42   522   0.0      863
MMU-Fbxo7   CJA-Fbxo7   92.31    481   37    0   48   528   42   522   0.0      836
MMU-Fbxo7   PPY-Fbxo7   97.68    431   10    0   98   528   13   443   0.0      782
MMU-Fbxo7   MUS-Fbxo7   73.29    483   126   3   48   528   42   523   0.0      662
MMU-Fbxo7   RNO-Fbxo7   72.82    482   129   2   48   528   42   522   0.0      662
# BLASTP 2.2.20 [Feb-08-2009]
# Query: MUS-Fbxo7
# Database: 559_protein.db
# Query id, Subject id, % identity, alignment length, mismatches, gap openings, q. start, q. end, s. start, s. end, e-value, bit score
MUS-Fbxo7   MUS-Fbxo7   100.00   523   0     0   1    523   1    523   0.0      1066
MUS-Fbxo7   RNO-Fbxo7   90.25    523   50    1   1    523   1    522   0.0      967
MUS-Fbxo7   CJA-Fbxo7   75.38    524   126   3   1    523   1    522   0.0      803
MUS-Fbxo7   HSA-Fbxo7   72.90    524   139   3   1    523   1    522   0.0      773
MUS-Fbxo7   GGO-Fbxo7   72.90    524   139   3   1    523   1    522   0.0      772
MUS-Fbxo7   PTR-Fbxo7   72.71    524   140   3   1    523   1    522   0.0      769
MUS-Fbxo7   MMU-Fbxo7   73.29    483   126   3   42   523   48   528   0.0      717
MUS-Fbxo7   PPY-Fbxo7   71.59    433   120   3   92   523   13   443   0.0      625
# BLASTP 2.2.20 [Feb-08-2009]
# Query: PPY-Fbxo7
# Database: 559_protein.db
# Query id, Subject id, % identity, alignment length, mismatches, gap openings, q. start, q. end, s. start, s. end, e-value, bit score
PPY-Fbxo7   PPY-Fbxo7   100.00   443   0     0   1    443   1    443   0.0      830
PPY-Fbxo7   HSA-Fbxo7   97.68    431   10    0   13   443   92   522   0.0      783
PPY-Fbxo7   MMU-Fbxo7   97.68    431   10    0   13   443   98   528   0.0      782
PPY-Fbxo7   GGO-Fbxo7   97.68    431   10    0   13   443   92   522   0.0      780
PPY-Fbxo7   PTR-Fbxo7   96.98    431   13    0   13   443   92   522   0.0      775
PPY-Fbxo7   CJA-Fbxo7   92.58    431   32    0   13   443   92   522   0.0      741
PPY-Fbxo7   RNO-Fbxo7   71.30    432   122   2   13   443   92   522   6e-165   572
PPY-Fbxo7   MUS-Fbxo7   71.59    433   120   3   13   443   92   523   1e-164   570
# BLASTP 2.2.20 [Feb-08-2009]
# Query: PTR-Fbxo7
# Database: 559_protein.db
# Query id, Subject id, % identity, alignment length, mismatches, gap openings, q. start, q. end, s. start, s. end, e-value, bit score
PTR-Fbxo7   PTR-Fbxo7   100.00   522   0     0   1    522   1    522   0.0      992
PTR-Fbxo7   HSA-Fbxo7   98.85    522   6     0   1    522   1    522   0.0      978
PTR-Fbxo7   GGO-Fbxo7   98.47    522   8     0   1    522   1    522   0.0      978
PTR-Fbxo7   CJA-Fbxo7   89.27    522   56    0   1    522   1    522   0.0      879
PTR-Fbxo7   MMU-Fbxo7   95.63    481   21    0   42   522   48   528   0.0      864
PTR-Fbxo7   PPY-Fbxo7   96.98    431   13    0   92   522   13   443   0.0      775
PTR-Fbxo7   MUS-Fbxo7   72.71    524   140   3   1    522   1    523   0.0      714
PTR-Fbxo7   RNO-Fbxo7   72.28    523   143   2   1    522   1    522   0.0      712
# BLASTP 2.2.20 [Feb-08-2009]
```

```
# Query: RNO-Fbxo7
# Database: 559_protein.db
# Query id, Subject id, % identity, alignment length, mismatches, gap openings, q. start, q. end, s. start, s. end, e-value, bit score
RNO-Fbxo7   RNO-Fbxo7   100.00      522         0           0           1           522         1           522         0.0         1034
RNO-Fbxo7   MUS-Fbxo7   90.25       523         50          1           1           522         1           523         0.0          937
RNO-Fbxo7   CJA-Fbxo7   75.14       523         128         2           1           522         1           522         0.0          780
RNO-Fbxo7   GGO-Fbxo7   72.47       523         142         2           1           522         1           522         0.0          749
RNO-Fbxo7   HSA-Fbxo7   72.47       523         142         2           1           522         1           522         0.0          748
RNO-Fbxo7   PTR-Fbxo7   72.28       523         143         2           1           522         1           522         0.0          746
RNO-Fbxo7   MMU-Fbxo7   72.82       482         129         2           42          522         48          528         0.0          694
RNO-Fbxo7   PPY-Fbxo7   71.30       432         122         2           92          522         13          443         9e-175       605
# BLASTP 2.2.20 [Feb-08-2009]
# Query: HSA-Fbxo7
# Database: 559_protein.db
# Query id, Subject id, % identity, alignment length, mismatches, gap openings, q. start, q. end, s. start, s. end, e-value, bit score
HSA-Fbxo7   HSA-Fbxo7   100.00      522         0           0           1           522         1           522         0.0          992
HSA-Fbxo7   GGO-Fbxo7   98.85       522         6           0           1           522         1           522         0.0          983
HSA-Fbxo7   PTR-Fbxo7   98.85       522         6           0           1           522         1           522         0.0          978
HSA-Fbxo7   CJA-Fbxo7   89.66       522         54          0           1           522         1           522         0.0          885
HSA-Fbxo7   MMU-Fbxo7   96.06       482         19          0           41          522         47          528         0.0          871
HSA-Fbxo7   PPY-Fbxo7   97.68       431         10          0           92          522         13          443         0.0          783
HSA-Fbxo7   MUS-Fbxo7   72.90       524         139         3           1           522         1           523         0.0          717
HSA-Fbxo7   RNO-Fbxo7   72.47       523         142         2           1           522         1           522         0.0          716
# BLASTP 2.2.20 [Feb-08-2009]
# Query: CJA-Fbxo8
# Database: 559_protein.db
# Query id, Subject id, % identity, alignment length, mismatches, gap openings, q. start, q. end, s. start, s. end, e-value, bit score
CJA-Fbxo8   CJA-Fbxo8   100.00      319         0           0           1           319         1           319         0.0          636
CJA-Fbxo8   MMU-Fbxo8   99.37       319         2           0           1           319         1           319         0.0          633
CJA-Fbxo8   HSA-Fbxo8   99.37       319         2           0           1           319         1           319         0.0          633
CJA-Fbxo8   PTR-Fbxo8   99.37       319         2           0           1           319         1           319         0.0          633
CJA-Fbxo8   PPY-Fbxo8   99.37       319         2           0           1           319         1           319         0.0          633
CJA-Fbxo8   GGO-Fbxo8   98.13       320         5           1           1           319         1           320         0.0          629
CJA-Fbxo8   MUS-Fbxo8   95.92       319         13          0           1           319         1           319         1e-177       613
CJA-Fbxo8   RNO-Fbxo8   94.04       319         19          0           1           319         1           319         8e-174       600
# BLASTP 2.2.20 [Feb-08-2009]
# Query: GGO-Fbxo8
# Database: 559_protein.db
# Query id, Subject id, % identity, alignment length, mismatches, gap openings, q. start, q. end, s. start, s. end, e-value, bit score
GGO-Fbxo8   GGO-Fbxo8   100.00      320         0           0           1           320         1           320         0.0          671
GGO-Fbxo8   HSA-Fbxo8   99.69       320         0           1           1           320         1           319         0.0          664
GGO-Fbxo8   PTR-Fbxo8   99.69       320         0           1           1           320         1           319         0.0          664
GGO-Fbxo8   PPY-Fbxo8   99.69       320         0           1           1           320         1           319         0.0          664
GGO-Fbxo8   MMU-Fbxo8   99.06       320         2           1           1           320         1           319         0.0          661
GGO-Fbxo8   CJA-Fbxo8   99.06       320         2           1           1           320         1           319         0.0          661
```

```
GGO-Fbxo8   MUS-Fbxo8   95.63       320         13          1           1           320         1           319         0.0         642
GGO-Fbxo8   RNO-Fbxo8   93.75       320         19          1           1           320         1           319         0.0         629
# BLASTP 2.2.20 [Feb-08-2009]
# Query: MMU-Fbxo8
# Database: 559_protein.db
# Query id, Subject id, % identity, alignment length, mismatches, gap openings, q. start, q. end, s. start, s. end, e-value, bit score
MMU-Fbxo8   MMU-Fbxo8   100.00      319         0           0           1           319         1           319         0.0         636
MMU-Fbxo8   HSA-Fbxo8   99.37       319         2           0           1           319         1           319         0.0         633
MMU-Fbxo8   PTR-Fbxo8   99.37       319         2           0           1           319         1           319         0.0         633
MMU-Fbxo8   PPY-Fbxo8   99.37       319         2           0           1           319         1           319         0.0         633
MMU-Fbxo8   CJA-Fbxo8   99.37       319         2           0           1           319         1           319         0.0         633
MMU-Fbxo8   GGO-Fbxo8   98.13       320         5           1           1           319         1           320         0.0         629
MMU-Fbxo8   MUS-Fbxo8   95.30       319         15          0           1           319         1           319         9e-177      610
MMU-Fbxo8   RNO-Fbxo8   93.42       319         21          0           1           319         1           319         7e-173      597
# BLASTP 2.2.20 [Feb-08-2009]
# Query: MUS-Fbxo8
# Database: 559_protein.db
# Query id, Subject id, % identity, alignment length, mismatches, gap openings, q. start, q. end, s. start, s. end, e-value, bit score
MUS-Fbxo8   MUS-Fbxo8   100.00      319         0           0           1           319         1           319         0.0         635
MUS-Fbxo8   RNO-Fbxo8   97.18       319         9           0           1           319         1           319         4e-178      615
MUS-Fbxo8   HSA-Fbxo8   95.92       319         13          0           1           319         1           319         8e-178      613
MUS-Fbxo8   PTR-Fbxo8   95.92       319         13          0           1           319         1           319         8e-178      613
MUS-Fbxo8   PPY-Fbxo8   95.92       319         13          0           1           319         1           319         8e-178      613
MUS-Fbxo8   CJA-Fbxo8   95.92       319         13          0           1           319         1           319         8e-178      613
MUS-Fbxo8   MMU-Fbxo8   95.30       319         15          0           1           319         1           319         6e-177      611
MUS-Fbxo8   GGO-Fbxo8   94.69       320         16          1           1           319         1           320         1e-176      610
# BLASTP 2.2.20 [Feb-08-2009]
# Query: PPY-Fbxo8
# Database: 559_protein.db
# Query id, Subject id, % identity, alignment length, mismatches, gap openings, q. start, q. end, s. start, s. end, e-value, bit score
PPY-Fbxo8   HSA-Fbxo8   100.00      319         0           0           1           319         1           319         0.0         636
PPY-Fbxo8   PTR-Fbxo8   100.00      319         0           0           1           319         1           319         0.0         636
PPY-Fbxo8   PPY-Fbxo8   100.00      319         0           0           1           319         1           319         0.0         636
PPY-Fbxo8   MMU-Fbxo8   99.37       319         2           0           1           319         1           319         0.0         633
PPY-Fbxo8   CJA-Fbxo8   99.37       319         2           0           1           319         1           319         0.0         633
PPY-Fbxo8   GGO-Fbxo8   98.75       320         3           1           1           319         1           320         0.0         632
PPY-Fbxo8   MUS-Fbxo8   95.92       319         13          0           1           319         1           319         1e-177      613
PPY-Fbxo8   RNO-Fbxo8   94.04       319         19          0           1           319         1           319         8e-174      600
# BLASTP 2.2.20 [Feb-08-2009]
# Query: PTR-Fbxo8
# Database: 559_protein.db
# Query id, Subject id, % identity, alignment length, mismatches, gap openings, q. start, q. end, s. start, s. end, e-value, bit score
PTR-Fbxo8   HSA-Fbxo8   100.00      319         0           0           1           319         1           319         0.0         636
PTR-Fbxo8   PTR-Fbxo8   100.00      319         0           0           1           319         1           319         0.0         636
PTR-Fbxo8   PPY-Fbxo8   100.00      319         0           0           1           319         1           319         0.0         636
```

```
PTR-Fbxo8   MMU-Fbxo8   99.37   319   2    0   1     319   1    319   0.0      633
PTR-Fbxo8   CJA-Fbxo8   99.37   319   2    0   1     319   1    319   0.0      633
PTR-Fbxo8   GGO-Fbxo8   98.75   320   3    1   1     319   1    320   0.0      632
PTR-Fbxo8   MUS-Fbxo8   95.92   319   13   0   1     319   1    319   1e-177   613
PTR-Fbxo8   RNO-Fbxo8   94.04   319   19   0   1     319   1    319   8e-174   600
# BLASTP 2.2.20 [Feb-08-2009]
# Query: RNO-Fbxo8
# Database: 559_protein.db
# Query id, Subject id, % identity, alignment length, mismatches, gap openings, q. start, q. end, s. start, s. end, e-value, bit score
RNO-Fbxo8   RNO-Fbxo8   100.00  319   0    0   1     319   1    319   0.0      632
RNO-Fbxo8   MUS-Fbxo8   97.18   319   9    0   1     319   1    319   3e-178   615
RNO-Fbxo8   HSA-Fbxo8   94.04   319   19   0   1     319   1    319   5e-174   601
RNO-Fbxo8   PTR-Fbxo8   94.04   319   19   0   1     319   1    319   5e-174   601
RNO-Fbxo8   PPY-Fbxo8   94.04   319   19   0   1     319   1    319   5e-174   601
RNO-Fbxo8   CJA-Fbxo8   94.04   319   19   0   1     319   1    319   5e-174   601
RNO-Fbxo8   MMU-Fbxo8   93.42   319   21   0   1     319   1    319   4e-173   598
RNO-Fbxo8   GGO-Fbxo8   92.81   320   22   1   1     319   1    320   1e-172   597
# BLASTP 2.2.20 [Feb-08-2009]
# Query: HSA-Fbxo8
# Database: 559_protein.db
# Query id, Subject id, % identity, alignment length, mismatches, gap openings, q. start, q. end, s. start, s. end, e-value, bit score
HSA-Fbxo8   HSA-Fbxo8   100.00  319   0    0   1     319   1    319   0.0      636
HSA-Fbxo8   PTR-Fbxo8   100.00  319   0    0   1     319   1    319   0.0      636
HSA-Fbxo8   PPY-Fbxo8   100.00  319   0    0   1     319   1    319   0.0      636
HSA-Fbxo8   MMU-Fbxo8   99.37   319   2    0   1     319   1    319   0.0      633
HSA-Fbxo8   CJA-Fbxo8   99.37   319   2    0   1     319   1    319   0.0      633
HSA-Fbxo8   GGO-Fbxo8   98.75   320   3    1   1     319   1    320   0.0      632
HSA-Fbxo8   MUS-Fbxo8   95.92   319   13   0   1     319   1    319   1e-177   613
HSA-Fbxo8   RNO-Fbxo8   94.04   319   19   0   1     319   1    319   8e-174   600
# BLASTP 2.2.20 [Feb-08-2009]
# Query: GGO-Fbxo9
# Database: 559_protein.db
# Query id, Subject id, % identity, alignment length, mismatches, gap openings, q. start, q. end, s. start, s. end, e-value, bit score
GGO-Fbxo9   GGO-Fbxo9   100.00  539   0    0   17    555   17   555   0.0      1051
GGO-Fbxo9   PTR-Fbxo9   99.77   438   0    1   118   555   1    437   0.0      889
GGO-Fbxo9   PPY-Fbxo9   99.09   438   3    1   118   555   1    437   0.0      887
GGO-Fbxo9   HSA-Fbxo9   99.54   437   1    1   119   555   12   447   0.0      883
GGO-Fbxo9   RNO-Fbxo9   92.01   438   32   3   118   555   1    435   0.0      818
GGO-Fbxo9   MUS-Fbxo9   91.30   437   36   2   119   555   3    437   0.0      812
GGO-Fbxo9   CJA-Fbxo9   92.15   433   16   4   118   544   1    421   0.0      793
GGO-Fbxo9   MMU-Fbxo9   99.70   328   0    1   228   555   1    327   0.0      684
# BLASTP 2.2.20 [Feb-08-2009]
# Query: MMU-Fbxo9
# Database: 559_protein.db
# Query id, Subject id, % identity, alignment length, mismatches, gap openings, q. start, q. end, s. start, s. end, e-value, bit score
```

```
MMU-Fbxo9   PTR-Fbxo9   100.00   327   0    0   1     327   111   437   0.0      689
MMU-Fbxo9   PPY-Fbxo9   99.39    327   2    0   1     327   111   437   0.0      688
MMU-Fbxo9   GGO-Fbxo9   99.70    328   0    1   1     327   228   555   0.0      686
MMU-Fbxo9   HSA-Fbxo9   99.69    327   1    0   1     327   121   447   0.0      685
MMU-Fbxo9   MMU-Fbxo9   100.00   327   0    0   1     327   1     327   0.0      682
MMU-Fbxo9   RNO-Fbxo9   95.72    327   14   0   1     327   109   435   0.0      664
MMU-Fbxo9   MUS-Fbxo9   95.11    327   16   0   1     327   111   437   0.0      660
MMU-Fbxo9   CJA-Fbxo9   93.35    316   10   1   1     316   117   421   2e-178   616
# BLASTP 2.2.20 [Feb-08-2009]
# Query: MUS-Fbxo9
# Database: 559_protein.db
# Query id, Subject id, % identity, alignment length, mismatches, gap openings, q. start, q. end, s. start, s. end, e-value, bit score
MUS-Fbxo9   MUS-Fbxo9   100.00   437   0    0   1     437   1     437   0.0      915
MUS-Fbxo9   RNO-Fbxo9   95.63    435   18   1   3     437   2     435   0.0      872
MUS-Fbxo9   PPY-Fbxo9   91.51    436   36   1   3     437   2     437   0.0      837
MUS-Fbxo9   PTR-Fbxo9   91.51    436   36   1   3     437   2     437   0.0      837
MUS-Fbxo9   HSA-Fbxo9   91.28    436   37   1   3     437   12    447   0.0      833
MUS-Fbxo9   GGO-Fbxo9   91.30    437   36   2   3     437   119   555   0.0      830
MUS-Fbxo9   CJA-Fbxo9   85.85    431   43   3   3     426   2     421   0.0      758
MUS-Fbxo9   MMU-Fbxo9   95.11    327   16   0   111   437   1     327   0.0      660
# BLASTP 2.2.20 [Feb-08-2009]
# Query: PPY-Fbxo9
# Database: 559_protein.db
# Query id, Subject id, % identity, alignment length, mismatches, gap openings, q. start, q. end, s. start, s. end, e-value, bit score
PPY-Fbxo9   PPY-Fbxo9   100.00   437   0    0   1     437   1     437   0.0      895
PPY-Fbxo9   PTR-Fbxo9   99.31    437   3    0   1     437   1     437   0.0      891
PPY-Fbxo9   GGO-Fbxo9   99.09    438   3    1   1     437   118   555   0.0      886
PPY-Fbxo9   HSA-Fbxo9   99.08    436   4    0   2     437   12    447   0.0      884
PPY-Fbxo9   RNO-Fbxo9   92.22    437   32   2   1     437   1     435   0.0      824
PPY-Fbxo9   MUS-Fbxo9   91.51    436   36   1   2     437   3     437   0.0      818
PPY-Fbxo9   CJA-Fbxo9   92.13    432   17   3   1     426   1     421   0.0      796
PPY-Fbxo9   MMU-Fbxo9   99.39    327   2    0   111   437   1     327   0.0      686
# BLASTP 2.2.20 [Feb-08-2009]
# Query: PTR-Fbxo9
# Database: 559_protein.db
# Query id, Subject id, % identity, alignment length, mismatches, gap openings, q. start, q. end, s. start, s. end, e-value, bit score
PTR-Fbxo9   PTR-Fbxo9   100.00   437   0    0   1     437   1     437   0.0      894
PTR-Fbxo9   PPY-Fbxo9   99.31    437   3    0   1     437   1     437   0.0      891
PTR-Fbxo9   GGO-Fbxo9   99.77    438   0    1   1     437   118   555   0.0      889
PTR-Fbxo9   HSA-Fbxo9   99.77    436   1    0   2     437   12    447   0.0      887
PTR-Fbxo9   RNO-Fbxo9   92.22    437   32   2   1     437   1     435   0.0      825
PTR-Fbxo9   MUS-Fbxo9   91.51    436   36   1   2     437   3     437   0.0      819
PTR-Fbxo9   CJA-Fbxo9   92.36    432   16   3   1     426   1     421   0.0      796
PTR-Fbxo9   MMU-Fbxo9   100.00   327   0    0   111   437   1     327   0.0      687
# BLASTP 2.2.20 [Feb-08-2009]
```

```
# Query: RNO-Fbxo9
# Database: 559_protein.db
# Query id, Subject id, % identity, alignment length, mismatches, gap openings, q. start, q. end, s. start, s. end, e-value, bit score
RNO-Fbxo9   RNO-Fbxo9   100.00      435         0           0           1           435         1           435         0.0           909
RNO-Fbxo9   MUS-Fbxo9   95.63       435         18          1           2           435         3           437         0.0           872
RNO-Fbxo9   PTR-Fbxo9   92.22       437         32          2           1           435         1           437         0.0           843
RNO-Fbxo9   PPY-Fbxo9   92.22       437         32          2           1           435         1           437         0.0           843
RNO-Fbxo9   GGO-Fbxo9   92.01       438         32          3           1           435         118         555         0.0           838
RNO-Fbxo9   HSA-Fbxo9   91.97       436         33          2           2           435         12          447         0.0           837
RNO-Fbxo9   CJA-Fbxo9   85.88       432         42          4           1           424         1           421         0.0           759
RNO-Fbxo9   MMU-Fbxo9   95.72       327         14          0           109         435         1           327         0.0           664
# BLASTP 2.2.20 [Feb-08-2009]
# Query: HSA-Fbxo9
# Database: 559_protein.db
# Query id, Subject id, % identity, alignment length, mismatches, gap openings, q. start, q. end, s. start, s. end, e-value, bit score
HSA-Fbxo9   HSA-Fbxo9   100.00      447         0           0           1           447         1           447         0.0           913
HSA-Fbxo9   PTR-Fbxo9   99.77       436         1           0           12          447         2           437         0.0           887
HSA-Fbxo9   PPY-Fbxo9   99.08       436         4           0           12          447         2           437         0.0           884
HSA-Fbxo9   GGO-Fbxo9   99.54       437         1           1           12          447         119         555         0.0           883
HSA-Fbxo9   RNO-Fbxo9   91.97       436         33          2           12          447         2           435         0.0           818
HSA-Fbxo9   MUS-Fbxo9   91.28       436         37          1           12          447         3           437         0.0           814
HSA-Fbxo9   CJA-Fbxo9   92.11       431         17          3           12          436         2           421         0.0           791
HSA-Fbxo9   MMU-Fbxo9   99.69       327         1           0           121         447         1           327         0.0           683
# BLASTP 2.2.20 [Feb-08-2009]
# Query: CJA-Fbxo9
# Database: 559_protein.db
# Query id, Subject id, % identity, alignment length, mismatches, gap openings, q. start, q. end, s. start, s. end, e-value, bit score
CJA-Fbxo9   CJA-Fbxo9   100.00      428         0           0           1           428         1           428         0.0           896
CJA-Fbxo9   PTR-Fbxo9   92.36       432         16          3           1           421         1           426         0.0           818
CJA-Fbxo9   PPY-Fbxo9   92.13       432         17          3           1           421         1           426         0.0           818
CJA-Fbxo9   GGO-Fbxo9   92.15       433         16          4           1           421         118         544         0.0           815
CJA-Fbxo9   HSA-Fbxo9   92.11       431         17          3           2           421         12          436         0.0           812
CJA-Fbxo9   RNO-Fbxo9   85.88       432         42          4           1           421         1           424         0.0           759
CJA-Fbxo9   MUS-Fbxo9   85.85       431         43          3           2           421         3           426         0.0           758
CJA-Fbxo9   MMU-Fbxo9   93.35       316         10          1           117         421         1           316         2e-178        616
# BLASTP 2.2.20 [Feb-08-2009]
# Query: CJA-Fbxw10
# Database: 559_protein.db
# Query id, Subject id, % identity, alignment length, mismatches, gap openings, q. start, q. end, s. start, s. end, e-value, bit score
CJA-Fbxw10  CJA-Fbxw10  100.00      1048        0           0           1           1048        1           1048        0.0          2117
CJA-Fbxw10  HSA-Fbxw10  88.21       1052        120         2           1           1048        1           1052        0.0          1867
CJA-Fbxw10  PPY-Fbxw10  87.64       1052        126         2           1           1048        1           1052        0.0          1854
CJA-Fbxw10  MMU-Fbxw10  82.32       1052        127         4           1           1048        1           997         0.0          1689
CJA-Fbxw10  PTR-Fbxw10  76.39       1059        182         10          1           1048        1           1002        0.0          1538
CJA-Fbxw10  MUS-Fbxw10  68.08       1065        288         8           1           1048        1           1030        0.0          1416
```

```
CJA-Fbxw10  RNO-Fbxw10  67.87     775       221       6         1         758       1         764       0.0          1048
CJA-Fbxw10  GGO-Fbxw10  59.24     368       121       10        169       533       448       789       3e-114        404
CJA-Fbxw10  GGO-Fbxw10  86.81     144       19        0         473       616       648       791       1e-079        290
# BLASTP 2.2.20 [Feb-08-2009]
# Query: GGO-Fbxw10
# Database: 559_protein.db
# Query id, Subject id, % identity, alignment length, mismatches, gap openings, q. start, q. end, s. start, s. end, e-value, bit score
GGO-Fbxw10  GGO-Fbxw10  100.00    818       0         0         1         818       1         818       0.0          1570
GGO-Fbxw10  PTR-Fbxw10  66.33     398       80        9         448       791       169       566       5e-144        503
GGO-Fbxw10  HSA-Fbxw10  64.67     368       101       10        448       789       169       533       6e-128        449
GGO-Fbxw10  HSA-Fbxw10  94.44     144       8         0         648       791       473       616       2e-084        305
GGO-Fbxw10  PPY-Fbxw10  62.50     368       109       10        448       789       169       533       2e-123        435
GGO-Fbxw10  PPY-Fbxw10  93.06     144       10        0         648       791       473       616       7e-084        303
GGO-Fbxw10  MMU-Fbxw10  62.23     368       110       9         448       789       169       533       3e-115        407
GGO-Fbxw10  MMU-Fbxw10  87.10     155       20        0         648       802       473       627       5e-085        307
GGO-Fbxw10  CJA-Fbxw10  59.24     368       121       10        448       789       169       533       2e-114        405
GGO-Fbxw10  CJA-Fbxw10  86.81     144       19        0         648       791       473       616       8e-080        290
GGO-Fbxw10  MUS-Fbxw10  75.69     144       35        0         648       791       488       631       6e-069        254
GGO-Fbxw10  RNO-Fbxw10  77.78     144       32        0         648       791       488       631       6e-070        257
# BLASTP 2.2.20 [Feb-08-2009]
# Query: MMU-Fbxw10
# Database: 559_protein.db
# Query id, Subject id, % identity, alignment length, mismatches, gap openings, q. start, q. end, s. start, s. end, e-value, bit score
MMU-Fbxw10  MMU-Fbxw10  100.00    997       0         0         1         997       1         997       0.0          2009
MMU-Fbxw10  HSA-Fbxw10  89.35     1052      57        2         1         997       1         1052      0.0          1873
MMU-Fbxw10  PPY-Fbxw10  88.88     1052      62        2         1         997       1         1052      0.0          1866
MMU-Fbxw10  CJA-Fbxw10  82.51     1052      125       4         1         997       1         1048      0.0          1718
MMU-Fbxw10  PTR-Fbxw10  83.11     1030      113       5         1         997       1         1002      0.0          1677
MMU-Fbxw10  MUS-Fbxw10  65.48     1069      258       9         1         997       1         1030      0.0          1348
MMU-Fbxw10  RNO-Fbxw10  64.04     773       197       6         1         703       1         762       0.0           954
MMU-Fbxw10  GGO-Fbxw10  62.23     368       110       9         169       533       448       789       6e-115        407
MMU-Fbxw10  GGO-Fbxw10  87.10     155       20        0         473       627       648       802       8e-085        307
# BLASTP 2.2.20 [Feb-08-2009]
# Query: MUS-Fbxw10
# Database: 559_protein.db
# Query id, Subject id, % identity, alignment length, mismatches, gap openings, q. start, q. end, s. start, s. end, e-value, bit score
MUS-Fbxw10  MUS-Fbxw10  100.00    1030      0         0         1         1030      1         1030      0.0          2042
MUS-Fbxw10  PPY-Fbxw10  68.66     1069      279       7         1         1030      1         1052      0.0          1421
MUS-Fbxw10  HSA-Fbxw10  68.66     1069      279       7         1         1030      1         1052      0.0          1416
MUS-Fbxw10  CJA-Fbxw10  68.08     1065      288       8         1         1030      1         1048      0.0          1392
MUS-Fbxw10  RNO-Fbxw10  89.82     766       78        0         1         766       1         766       0.0          1392
MUS-Fbxw10  MMU-Fbxw10  65.39     1069      259       9         1         1030      1         997       0.0          1309
MUS-Fbxw10  PTR-Fbxw10  59.94     1076      311       15        1         1030      1         1002      0.0          1149
MUS-Fbxw10  GGO-Fbxw10  75.69     144       35        0         488       631       648       791       1e-068        253
# BLASTP 2.2.20 [Feb-08-2009]
```

```
# Query: RNO-Fbxw10
# Database: 559_protein.db
# Query id, Subject id, % identity, alignment length, mismatches, gap openings, q. start, q. end, s. start, s. end, e-value, bit score
RNO-Fbxw10  RNO-Fbxw10  100.00   766   0     0    1     766    1     766    0.0     1519
RNO-Fbxw10  MUS-Fbxw10  89.82    766   78    0    1     766    1     766    0.0     1390
RNO-Fbxw10  HSA-Fbxw10  69.47    773   208   5    1     762    1     756    0.0     1064
RNO-Fbxw10  PPY-Fbxw10  69.47    773   208   5    1     762    1     756    0.0     1061
RNO-Fbxw10  CJA-Fbxw10  67.87    775   221   6    1     764    1     758    0.0     1030
RNO-Fbxw10  MMU-Fbxw10  63.78    773   199   6    1     762    1     703    0.0      947
RNO-Fbxw10  PTR-Fbxw10  57.18    780   242   13   1     762    1     706    0.0      799
RNO-Fbxw10  GGO-Fbxw10  77.78    144   32    0    488   631    648   791    5e-070   257
# BLASTP 2.2.20 [Feb-08-2009]
# Query: HSA-Fbxw10
# Database: 559_protein.db
# Query id, Subject id, % identity, alignment length, mismatches, gap openings, q. start, q. end, s. start, s. end, e-value, bit score
HSA-Fbxw10  HSA-Fbxw10  100.00   1052  0     0    1     1052   1     1052   0.0     2153
HSA-Fbxw10  PPY-Fbxw10  96.10    1052  41    0    1     1052   1     1052   0.0     2075
HSA-Fbxw10  PTR-Fbxw10  91.08    1054  40    4    1     1052   1     1002   0.0     1912
HSA-Fbxw10  CJA-Fbxw10  88.21    1052  120   2    1     1052   1     1048   0.0     1890
HSA-Fbxw10  MMU-Fbxw10  89.07    1052  60    2    1     1052   1     997    0.0     1867
HSA-Fbxw10  MUS-Fbxw10  68.66    1069  279   7    1     1052   1     1030   0.0     1466
HSA-Fbxw10  RNO-Fbxw10  69.47    773   208   5    1     756    1     762    0.0     1083
HSA-Fbxw10  GGO-Fbxw10  64.67    368   101   10   169   533    448   789    1e-127   449
HSA-Fbxw10  GGO-Fbxw10  94.44    144   8     0    473   616    648   791    2e-084   305
# BLASTP 2.2.20 [Feb-08-2009]
# Query: PPY-Fbxw10
# Database: 559_protein.db
# Query id, Subject id, % identity, alignment length, mismatches, gap openings, q. start, q. end, s. start, s. end, e-value, bit score
PPY-Fbxw10  PPY-Fbxw10  100.00   1052  0     0    1     1052   1     1052   0.0     2159
PPY-Fbxw10  HSA-Fbxw10  96.10    1052  41    0    1     1052   1     1052   0.0     2075
PPY-Fbxw10  CJA-Fbxw10  87.64    1052  126   2    1     1052   1     1048   0.0     1880
PPY-Fbxw10  MMU-Fbxw10  88.59    1052  65    2    1     1052   1     997    0.0     1860
PPY-Fbxw10  PTR-Fbxw10  83.47    1059  111   8    1     1052   1     1002   0.0     1734
PPY-Fbxw10  MUS-Fbxw10  68.66    1069  279   7    1     1052   1     1030   0.0     1466
PPY-Fbxw10  RNO-Fbxw10  69.47    773   208   5    1     756    1     762    0.0     1080
PPY-Fbxw10  GGO-Fbxw10  62.50    368   109   10   169   533    448   789    5e-123   434
PPY-Fbxw10  GGO-Fbxw10  93.06    144   10    0    473   616    648   791    1e-083   303
# BLASTP 2.2.20 [Feb-08-2009]
# Query: PTR-Fbxw10
# Database: 559_protein.db
# Query id, Subject id, % identity, alignment length, mismatches, gap openings, q. start, q. end, s. start, s. end, e-value, bit score
PTR-Fbxw10  PTR-Fbxw10  100.00   1002  0     0    1     1002   1     1002   0.0     2051
PTR-Fbxw10  HSA-Fbxw10  94.11    1052  12    1    1     1002   1     1052   0.0     1997
PTR-Fbxw10  PPY-Fbxw10  91.35    1052  41    1    1     1002   1     1052   0.0     1942
PTR-Fbxw10  CJA-Fbxw10  83.37    1052  121   3    1     1002   1     1048   0.0     1755
```

```
PTR-Fbxw10  MMU-Fbxw10  84.32   1052    60      3       1       1002    1       997     0.0     1736
PTR-Fbxw10  MUS-Fbxw10  65.29   1069    265     8       1       1002    1       1030    0.0     1356
PTR-Fbxw10  RNO-Fbxw10  64.68   773     195     6       1       706     1       762     0.0      977
PTR-Fbxw10  GGO-Fbxw10  66.08   398     81      9       169     566     448     791     4e-143   501
# BLASTP 2.2.20 [Feb-08-2009]
# Query: CJA-Fbxw11
# Database: 559_protein.db
# Query id, Subject id, % identity, alignment length, mismatches, gap openings, q. start, q. end, s. start, s. end, e-value, bit score
CJA-Fbxw11  HSA-Fbxw11  100.00  542     0       0       1       542     1       542     0.0     1120
CJA-Fbxw11  GGO-Fbxw11  100.00  542     0       0       1       542     1       542     0.0     1120
CJA-Fbxw11  CJA-Fbxw11  100.00  542     0       0       1       542     1       542     0.0     1120
CJA-Fbxw11  PTR-Fbxw11  99.82   542     1       0       1       542     1       542     0.0     1118
CJA-Fbxw11  MUS-Fbxw11  95.91   563     2       1       1       542     1       563     0.0     1105
CJA-Fbxw11  MMU-Fbxw11  99.81   529     1       0       14      542     1       529     0.0     1090
CJA-Fbxw11  RNO-Fbxw11  99.62   527     2       0       16      542     1       527     0.0     1086
CJA-Fbxw11  CJA-Btrc    79.30   575     83      3       4       542     5       579     0.0      949
CJA-Fbxw11  HSA-Btrc    75.87   605     83      5       1       542     1       605     0.0      938
CJA-Fbxw11  MUS-Btrc    75.37   605     86      5       1       542     35      639     0.0      934
CJA-Fbxw11  PPY-Btrc    75.37   605     86      5       1       542     1       605     0.0      932
CJA-Fbxw11  RNO-Btrc    76.74   589     75      4       16      542     17      605     0.0      922
CJA-Fbxw11  GGO-Btrc    83.74   529     80      3       20      542     30      558     0.0      920
CJA-Fbxw11  PTR-Btrc    73.62   561     112     9       1       542     1       544     0.0      816
CJA-Fbxw11  MMU-Btrc    69.37   591     118     6       12      542     1       588     0.0      805
CJA-Fbxw11  PPY-Fbxw11  60.28   506     137     4       49      542     22      475     9e-173   598
# BLASTP 2.2.20 [Feb-08-2009]
# Query: GGO-Fbxw11
# Database: 559_protein.db
# Query id, Subject id, % identity, alignment length, mismatches, gap openings, q. start, q. end, s. start, s. end, e-value, bit score
GGO-Fbxw11  HSA-Fbxw11  100.00  542     0       0       1       542     1       542     0.0     1120
GGO-Fbxw11  GGO-Fbxw11  100.00  542     0       0       1       542     1       542     0.0     1120
GGO-Fbxw11  CJA-Fbxw11  100.00  542     0       0       1       542     1       542     0.0     1120
GGO-Fbxw11  PTR-Fbxw11  99.82   542     1       0       1       542     1       542     0.0     1118
GGO-Fbxw11  MUS-Fbxw11  95.91   563     2       1       1       542     1       563     0.0     1105
GGO-Fbxw11  MMU-Fbxw11  99.81   529     1       0       14      542     1       529     0.0     1090
GGO-Fbxw11  RNO-Fbxw11  99.62   527     2       0       16      542     1       527     0.0     1086
GGO-Fbxw11  CJA-Btrc    79.30   575     83      3       4       542     5       579     0.0      949
GGO-Fbxw11  HSA-Btrc    75.87   605     83      5       1       542     1       605     0.0      938
GGO-Fbxw11  MUS-Btrc    75.37   605     86      5       1       542     35      639     0.0      934
GGO-Fbxw11  PPY-Btrc    75.37   605     86      5       1       542     1       605     0.0      932
GGO-Fbxw11  RNO-Btrc    76.74   589     75      4       16      542     17      605     0.0      922
GGO-Fbxw11  GGO-Btrc    83.74   529     80      3       20      542     30      558     0.0      920
GGO-Fbxw11  PTR-Btrc    73.62   561     112     9       1       542     1       544     0.0      816
GGO-Fbxw11  MMU-Btrc    69.37   591     118     6       12      542     1       588     0.0      805
GGO-Fbxw11  PPY-Fbxw11  60.28   506     137     4       49      542     22      475     9e-173   598
# BLASTP 2.2.20 [Feb-08-2009]
```

```
# Query: MMU-Fbxw11
# Database: 559_protein.db
# Query id, Subject id, % identity, alignment length, mismatches, gap openings, q. start, q. end, s. start, s. end, e-value, bit score
MMU-Fbxw11  MMU-Fbxw11  100.00  529  0    0  1   529  1   529  0.0     1091
MMU-Fbxw11  HSA-Fbxw11  99.81   529  1    0  1   529  14  542  0.0     1090
MMU-Fbxw11  GGO-Fbxw11  99.81   529  1    0  1   529  14  542  0.0     1090
MMU-Fbxw11  CJA-Fbxw11  99.81   529  1    0  1   529  14  542  0.0     1090
MMU-Fbxw11  PTR-Fbxw11  99.62   529  2    0  1   529  14  542  0.0     1090
MMU-Fbxw11  RNO-Fbxw11  99.62   527  2    0  3   529  1   527  0.0     1085
MMU-Fbxw11  MUS-Fbxw11  95.64   550  3    1  1   529  14  563  0.0     1077
MMU-Fbxw11  CJA-Btrc    80.18   565  76   3  1   529  15  579  0.0      939
MMU-Fbxw11  HSA-Btrc    76.82   591  75   4  1   529  15  605  0.0      927
MMU-Fbxw11  MUS-Btrc    76.31   591  78   4  1   529  49  639  0.0      922
MMU-Fbxw11  RNO-Btrc    76.61   590  76   4  2   529  16  605  0.0      922
MMU-Fbxw11  GGO-Btrc    83.74   529  80   3  7   529  30  558  0.0      920
MMU-Fbxw11  PPY-Btrc    76.31   591  78   4  1   529  15  605  0.0      919
MMU-Fbxw11  MMU-Btrc    69.78   589  115  6  1   529  3   588  0.0      806
MMU-Fbxw11  PTR-Btrc    74.59   547  104  8  1   529  15  544  0.0      805
MMU-Fbxw11  PPY-Fbxw11  60.28   506  137  4  36  529  22  475  1e-172   597
# BLASTP 2.2.20 [Feb-08-2009]
# Query: MUS-Fbxw11
# Database: 559_protein.db
# Query id, Subject id, % identity, alignment length, mismatches, gap openings, q. start, q. end, s. start, s. end, e-value, bit score
MUS-Fbxw11  MUS-Fbxw11  100.00  563  0    0  1   563  1   563  0.0     1163
MUS-Fbxw11  PTR-Fbxw11  96.09   563  1    1  1   563  1   542  0.0     1107
MUS-Fbxw11  HSA-Fbxw11  95.91   563  2    1  1   563  1   542  0.0     1105
MUS-Fbxw11  GGO-Fbxw11  95.91   563  2    1  1   563  1   542  0.0     1105
MUS-Fbxw11  CJA-Fbxw11  95.91   563  2    1  1   563  1   542  0.0     1105
MUS-Fbxw11  MMU-Fbxw11  95.64   550  3    1  14  563  1   529  0.0     1077
MUS-Fbxw11  RNO-Fbxw11  96.17   548  0    1  16  563  1   527  0.0     1077
MUS-Fbxw11  HSA-Btrc    77.02   605  97   4  1   563  1   605  0.0      958
MUS-Fbxw11  MUS-Btrc    76.86   605  98   4  1   563  35  639  0.0      956
MUS-Fbxw11  CJA-Btrc    80.00   575  100  3  4   563  5   579  0.0      955
MUS-Fbxw11  PPY-Btrc    76.53   605  100  4  1   563  1   605  0.0      952
MUS-Fbxw11  RNO-Btrc    78.27   589  87   3  16  563  17  605  0.0      944
MUS-Fbxw11  GGO-Btrc    84.59   519  78   1  47  563  40  558  0.0      919
MUS-Fbxw11  MMU-Btrc    70.90   591  130  6  12  563  1   588  0.0      828
MUS-Fbxw11  PTR-Btrc    73.59   568  121  9  1   563  1   544  0.0      827
MUS-Fbxw11  PPY-Fbxw11  61.22   526  140  4  50  563  2   475  0.0      635
# BLASTP 2.2.20 [Feb-08-2009]
# Query: PPY-Fbxw11
# Database: 559_protein.db
# Query id, Subject id, % identity, alignment length, mismatches, gap openings, q. start, q. end, s. start, s. end, e-value, bit score
PPY-Fbxw11  PPY-Fbxw11  100.00  475  0    0  1   475  1   475  0.0      987
PPY-Fbxw11  MUS-Fbxw11  91.63   514  3    1  2   475  50  563  0.0      961
```

PPY-Fbxw11	HSA-Fbxw11	91.90	494	0	1	22	475	49	542	0.0	924
PPY-Fbxw11	GGO-Fbxw11	91.90	494	0	1	22	475	49	542	0.0	924
PPY-Fbxw11	CJA-Fbxw11	91.90	494	0	1	22	475	49	542	0.0	924
PPY-Fbxw11	PTR-Fbxw11	91.90	494	0	1	22	475	49	542	0.0	924
PPY-Fbxw11	MMU-Fbxw11	91.90	494	0	1	22	475	36	529	0.0	923
PPY-Fbxw11	RNO-Fbxw11	91.70	494	1	1	22	475	34	527	0.0	921
PPY-Fbxw11	CJA-Btrc	80.85	496	53	2	22	475	84	579	0.0	830
PPY-Fbxw11	HSA-Btrc	80.85	496	53	2	22	475	110	605	0.0	828
PPY-Fbxw11	GGO-Btrc	80.85	496	53	2	22	475	63	558	0.0	828
PPY-Fbxw11	MUS-Btrc	80.44	496	55	2	22	475	144	639	0.0	826
PPY-Fbxw11	RNO-Btrc	80.65	496	54	2	22	475	110	605	0.0	825
PPY-Fbxw11	PPY-Btrc	80.24	496	56	2	22	475	110	605	0.0	822
PPY-Fbxw11	MMU-Btrc	72.80	489	88	4	29	475	103	588	0.0	706
PPY-Fbxw11	PTR-Btrc	80.94	425	36	3	96	475	120	544	0.0	697

BLASTP 2.2.20 [Feb-08-2009]
Query: PTR-Fbxw11
Database: 559_protein.db
Query id, Subject id, % identity, alignment length, mismatches, gap openings, q. start, q. end, s. start, s. end, e-value, bit score

PTR-Fbxw11	PTR-Fbxw11	100.00	542	0	0	1	542	1	542	0.0	1119
PTR-Fbxw11	HSA-Fbxw11	99.82	542	1	0	1	542	1	542	0.0	1118
PTR-Fbxw11	GGO-Fbxw11	99.82	542	1	0	1	542	1	542	0.0	1118
PTR-Fbxw11	CJA-Fbxw11	99.82	542	1	0	1	542	1	542	0.0	1118
PTR-Fbxw11	MUS-Fbxw11	96.09	563	1	1	1	542	1	563	0.0	1107
PTR-Fbxw11	MMU-Fbxw11	99.62	529	2	0	14	542	1	529	0.0	1090
PTR-Fbxw11	RNO-Fbxw11	99.81	527	1	0	16	542	1	527	0.0	1087
PTR-Fbxw11	CJA-Btrc	79.30	575	83	3	4	542	5	579	0.0	949
PTR-Fbxw11	HSA-Btrc	75.87	605	83	5	1	542	1	605	0.0	939
PTR-Fbxw11	MUS-Btrc	75.54	605	85	5	1	542	35	639	0.0	935
PTR-Fbxw11	PPY-Btrc	75.37	605	86	5	1	542	1	605	0.0	932
PTR-Fbxw11	RNO-Btrc	76.91	589	74	4	16	542	17	605	0.0	923
PTR-Fbxw11	GGO-Btrc	83.74	529	80	3	20	542	30	558	0.0	920
PTR-Fbxw11	PTR-Btrc	73.62	561	112	9	1	542	1	544	0.0	816
PTR-Fbxw11	MMU-Btrc	69.37	591	118	6	12	542	1	588	0.0	805
PTR-Fbxw11	PPY-Fbxw11	60.28	506	137	4	49	542	22	475	1e-172	598

BLASTP 2.2.20 [Feb-08-2009]
Query: RNO-Fbxw11
Database: 559_protein.db
Query id, Subject id, % identity, alignment length, mismatches, gap openings, q. start, q. end, s. start, s. end, e-value, bit score

RNO-Fbxw11	RNO-Fbxw11	100.00	527	0	0	1	527	1	527	0.0	1087
RNO-Fbxw11	PTR-Fbxw11	99.81	527	1	0	1	527	16	542	0.0	1087
RNO-Fbxw11	HSA-Fbxw11	99.62	527	2	0	1	527	16	542	0.0	1086
RNO-Fbxw11	GGO-Fbxw11	99.62	527	2	0	1	527	16	542	0.0	1086
RNO-Fbxw11	CJA-Fbxw11	99.62	527	2	0	1	527	16	542	0.0	1086
RNO-Fbxw11	MMU-Fbxw11	99.62	527	2	0	1	527	3	529	0.0	1085
RNO-Fbxw11	MUS-Fbxw11	96.17	548	0	1	1	527	16	563	0.0	1077

```
RNO-Fbxw11  CJA-Btrc    80.46   563   74    3   1    527   17   579   0.0      937
RNO-Fbxw11  HSA-Btrc    77.08   589   73    4   1    527   17   605   0.0      925
RNO-Fbxw11  MUS-Btrc    76.74   589   75    4   1    527   51   639   0.0      922
RNO-Fbxw11  RNO-Btrc    76.91   589   74    4   1    527   17   605   0.0      922
RNO-Fbxw11  GGO-Btrc    83.74   529   80    3   5    527   30   558   0.0      918
RNO-Fbxw11  PPY-Btrc    76.57   589   76    4   1    527   17   605   0.0      918
RNO-Fbxw11  PTR-Btrc    74.86   545   102   8   1    527   17   544   0.0      803
RNO-Fbxw11  MMU-Btrc    69.68   587   115   6   1    527   5    588   0.0      800
RNO-Fbxw11  PPY-Fbxw11  60.08   506   138   4   34   527   22   475   4e-172   596
# BLASTP 2.2.20 [Feb-08-2009]
# Query: HSA-Fbxw11
# Database: 559_protein.db
# Query id, Subject id, % identity, alignment length, mismatches, gap openings, q. start, q. end, s. start, s. end, e-value, bit score
HSA-Fbxw11  HSA-Fbxw11  100.00  542   0     0   1    542   1    542   0.0      1120
HSA-Fbxw11  GGO-Fbxw11  100.00  542   0     0   1    542   1    542   0.0      1120
HSA-Fbxw11  CJA-Fbxw11  100.00  542   0     0   1    542   1    542   0.0      1120
HSA-Fbxw11  PTR-Fbxw11  99.82   542   1     0   1    542   1    542   0.0      1118
HSA-Fbxw11  MUS-Fbxw11  95.91   563   2     1   1    542   1    563   0.0      1105
HSA-Fbxw11  MMU-Fbxw11  99.81   529   1     0   14   542   1    529   0.0      1090
HSA-Fbxw11  RNO-Fbxw11  99.62   527   2     0   16   542   1    527   0.0      1086
HSA-Fbxw11  CJA-Btrc    79.30   575   83    3   4    542   5    579   0.0      949
HSA-Fbxw11  HSA-Btrc    75.87   605   83    5   1    542   1    605   0.0      938
HSA-Fbxw11  MUS-Btrc    75.37   605   86    5   1    542   35   639   0.0      934
HSA-Fbxw11  PPY-Btrc    75.37   605   86    5   1    542   1    605   0.0      932
HSA-Fbxw11  RNO-Btrc    76.74   589   75    4   16   542   17   605   0.0      922
HSA-Fbxw11  GGO-Btrc    83.74   529   80    3   20   542   30   558   0.0      920
HSA-Fbxw11  PTR-Btrc    73.62   561   112   9   1    542   1    544   0.0      816
HSA-Fbxw11  MMU-Btrc    69.37   591   118   6   12   542   1    588   0.0      805
HSA-Fbxw11  PPY-Fbxw11  60.28   506   137   4   49   542   22   475   9e-173   598
# BLASTP 2.2.20 [Feb-08-2009]
# Query: MMU-Fbxw12
# Database: 559_protein.db
# Query id, Subject id, % identity, alignment length, mismatches, gap openings, q. start, q. end, s. start, s. end, e-value, bit score
MMU-Fbxw12  MMU-Fbxw12  100.00  459   0     0   1    459   1    459   0.0      964
MMU-Fbxw12  GGO-Fbxw12  85.13   464   64    3   1    459   1    464   0.0      829
MMU-Fbxw12  HSA-Fbxw12  85.34   464   63    3   1    459   1    464   0.0      827
MMU-Fbxw12  PPY-Fbxw12  84.27   464   68    3   1    459   1    464   0.0      818
MMU-Fbxw12  PTR-Fbxw12  84.70   464   65    4   1    459   1    463   0.0      814
MMU-Fbxw12  CJA-Fbxw12  72.35   463   123   3   1    458   1    463   0.0      696
# BLASTP 2.2.20 [Feb-08-2009]
# Query: Mus-Fbxw13
# Database: 559_protein.db
# Query id, Subject id, % identity, alignment length, mismatches, gap openings, q. start, q. end, s. start, s. end, e-value, bit score
Mus-Fbxw13  Mus-Fbxw13  100.00  466   0     0   1    466   1    466   0.0      939
Mus-Fbxw13  MUS-Fbxw21  74.03   466   121   0   1    466   1    466   0.0      696
```

```
Mus-Fbxw13  MUS-Fbxw20  73.39   466   124   0   1   466   1   466   0.0      674
Mus-Fbxw13  MUS-Fbxw24  69.76   463   140   0   1   463   1   463   0.0      651
Mus-Fbxw13  MUS-Fbxw19  70.13   462   138   0   1   462   1   462   0.0      648
Mus-Fbxw13  MUS-Fbxw18  70.54   465   134   1   1   462   1   465   0.0      646
Mus-Fbxw13  MUS-Fbxw26  69.33   463   142   0   1   463   1   463   0.0      644
Mus-Fbxw13  MUS-Fbxw16  67.67   464   150   0   1   464   1   464   0.0      629
Mus-Fbxw13  MUS-Fbxw22  67.60   463   150   0   1   463   1   463   1e-180   624
Mus-Fbxw13  RNO-Fbxw12  65.88   466   157   1   1   464   1   466   3e-178   616
Mus-Fbxw13  MUS-Fbxw15  64.15   463   166   0   1   463   1   463   2e-169   587
Mus-Fbxw13  MUS-Fbxw28  64.44   464   163   2   1   463   1   463   5e-168   582
Mus-Fbxw13  MUS-Fbxw14  64.44   464   163   2   1   463   1   463   7e-168   582
# BLASTP 2.2.20 [Feb-08-2009]
# Query: MUS-Fbxw14
# Database: 559_protein.db
# Query id, Subject id, % identity, alignment length, mismatches, gap openings, q. start, q. end, s. start, s. end, e-value, bit score
MUS-Fbxw14  MUS-Fbxw14  100.00  466   0     0   1   466   1   466   0.0      974
MUS-Fbxw14  MUS-Fbxw28  97.85   466   10    0   1   466   1   466   0.0      956
MUS-Fbxw14  MUS-Fbxw22  84.37   467   71    2   1   466   1   466   0.0      817
MUS-Fbxw14  MUS-Fbxw19  77.73   467   102   2   1   466   1   466   0.0      733
MUS-Fbxw14  MUS-Fbxw26  75.80   467   111   2   1   466   1   466   0.0      717
MUS-Fbxw14  MUS-Fbxw16  74.78   464   115   2   1   463   1   463   0.0      702
MUS-Fbxw14  MUS-Fbxw20  72.35   463   126   2   1   462   1   462   0.0      676
MUS-Fbxw14  MUS-Fbxw21  67.03   464   151   2   1   463   1   463   0.0      630
MUS-Fbxw14  MUS-Fbxw18  67.23   470   149   3   1   466   1   469   5e-178   615
MUS-Fbxw14  MUS-Fbxw24  64.45   467   164   2   1   466   1   466   6e-172   595
MUS-Fbxw14  MUS-Fbxw15  63.60   467   168   2   1   466   1   466   1e-171   594
MUS-Fbxw14  Mus-Fbxw13  64.44   464   163   2   1   463   1   463   4e-170   589
MUS-Fbxw14  RNO-Fbxw12  62.45   466   171   3   1   463   1   465   1e-166   577
# BLASTP 2.2.20 [Feb-08-2009]
# Query: MUS-Fbxw15
# Database: 559_protein.db
# Query id, Subject id, % identity, alignment length, mismatches, gap openings, q. start, q. end, s. start, s. end, e-value, bit score
MUS-Fbxw15  MUS-Fbxw15  100.00  466   0     0   1   466   1   466   0.0      965
MUS-Fbxw15  MUS-Fbxw24  74.03   466   121   0   1   466   1   466   0.0      701
MUS-Fbxw15  MUS-Fbxw20  69.70   462   140   0   1   462   1   462   0.0      659
MUS-Fbxw15  MUS-Fbxw21  71.05   449   130   0   1   449   1   449   0.0      655
MUS-Fbxw15  MUS-Fbxw26  68.67   466   146   0   1   466   1   466   0.0      653
MUS-Fbxw15  MUS-Fbxw19  68.03   466   149   0   1   466   1   466   0.0      642
MUS-Fbxw15  MUS-Fbxw16  68.03   463   148   0   1   463   1   463   0.0      641
MUS-Fbxw15  MUS-Fbxw22  67.60   466   151   0   1   466   1   466   0.0      634
MUS-Fbxw15  MUS-Fbxw18  66.67   462   154   0   5   466   8   469   4e-179   619
MUS-Fbxw15  RNO-Fbxw12  64.73   465   162   1   1   463   1   465   5e-174   602
MUS-Fbxw15  Mus-Fbxw13  64.15   463   166   0   1   463   1   463   5e-173   598
MUS-Fbxw15  MUS-Fbxw14  63.60   467   168   2   1   466   1   466   1e-171   594
MUS-Fbxw15  MUS-Fbxw28  63.60   467   168   2   1   466   1   466   2e-171   593
```

```
# BLASTP 2.2.20 [Feb-08-2009]
# Query: MUS-Fbxw16
# Database: 559_protein.db
# Query id, Subject id, % identity, alignment length, mismatches, gap openings, q. start, q. end, s. start, s. end, e-value, bit score
MUS-Fbxw16  MUS-Fbxw16  100.00      464         0           0           1           464         1           464         0.0         971
MUS-Fbxw16  MUS-Fbxw26  82.72       463         80          0           1           463         1           463         0.0         807
MUS-Fbxw16  MUS-Fbxw19  80.56       463         90          0           1           463         1           463         0.0         777
MUS-Fbxw16  MUS-Fbxw20  77.59       464         104         0           1           464         1           464         0.0         760
MUS-Fbxw16  MUS-Fbxw22  77.32       463         105         0           1           463         1           463         0.0         743
MUS-Fbxw16  MUS-Fbxw24  73.43       463         123         0           1           463         1           463         0.0         703
MUS-Fbxw16  MUS-Fbxw21  72.20       464         129         0           1           464         1           464         0.0         702
MUS-Fbxw16  MUS-Fbxw28  74.78       464         115         2           1           463         1           463         0.0         702
MUS-Fbxw16  MUS-Fbxw14  74.78       464         115         2           1           463         1           463         0.0         702
MUS-Fbxw16  MUS-Fbxw18  71.03       466         132         1           1           463         1           466         0.0         669
MUS-Fbxw16  RNO-Fbxw12  66.95       466         152         1           1           464         1           466         0.0         652
MUS-Fbxw16  Mus-Fbxw13  67.67       464         150         0           1           464         1           464         0.0         643
MUS-Fbxw16  MUS-Fbxw15  68.03       463         148         0           1           463         1           463         0.0         641
# BLASTP 2.2.20 [Feb-08-2009]
# Query: MUS-Fbxw18
# Database: 559_protein.db
# Query id, Subject id, % identity, alignment length, mismatches, gap openings, q. start, q. end, s. start, s. end, e-value, bit score
MUS-Fbxw18  MUS-Fbxw18  100.00      469         0           0           1           469         1           469         0.0         976
MUS-Fbxw18  MUS-Fbxw20  74.19       465         117         1           1           465         1           462         0.0         697
MUS-Fbxw18  MUS-Fbxw26  72.92       469         124         1           1           469         1           466         0.0         696
MUS-Fbxw18  MUS-Fbxw21  71.46       466         130         1           1           466         1           463         0.0         692
MUS-Fbxw18  Mus-Fbxw13  70.54       465         134         1           1           465         1           462         0.0         671
MUS-Fbxw18  MUS-Fbxw16  71.03       466         132         1           1           466         1           463         0.0         669
MUS-Fbxw18  MUS-Fbxw22  70.56       462         136         0           8           469         5           466         0.0         661
MUS-Fbxw18  MUS-Fbxw19  69.08       469         142         1           1           469         1           466         0.0         654
MUS-Fbxw18  MUS-Fbxw24  69.30       469         141         1           1           469         1           466         0.0         651
MUS-Fbxw18  RNO-Fbxw12  67.09       468         149         2           1           466         1           465         0.0         639
MUS-Fbxw18  MUS-Fbxw15  66.67       462         154         0           8           469         5           466         4e-179      619
MUS-Fbxw18  MUS-Fbxw14  67.23       470         149         3           1           469         1           466         5e-178      615
MUS-Fbxw18  MUS-Fbxw28  66.60       470         152         3           1           469         1           466         1e-176      611
# BLASTP 2.2.20 [Feb-08-2009]
# Query: MUS-Fbxw19
# Database: 559_protein.db
# Query id, Subject id, % identity, alignment length, mismatches, gap openings, q. start, q. end, s. start, s. end, e-value, bit score
MUS-Fbxw19  MUS-Fbxw19  100.00      466         0           0           1           466         1           466         0.0         969
MUS-Fbxw19  MUS-Fbxw16  80.56       463         90          0           1           463         1           463         0.0         777
MUS-Fbxw19  MUS-Fbxw26  78.97       466         98          0           1           466         1           466         0.0         769
MUS-Fbxw19  MUS-Fbxw22  79.83       466         94          0           1           466         1           466         0.0         768
MUS-Fbxw19  MUS-Fbxw20  77.71       462         103         0           1           462         1           462         0.0         747
MUS-Fbxw19  MUS-Fbxw28  77.73       467         102         2           1           466         1           466         0.0         734
MUS-Fbxw19  MUS-Fbxw14  77.73       467         102         2           1           466         1           466         0.0         733
```

```
MUS-Fbxw19  MUS-Fbxw21  71.49      463         132         0           1           463         1           463         0.0          693
MUS-Fbxw19  MUS-Fbxw24  69.53      466         142         0           1           466         1           466         0.0          665
MUS-Fbxw19  Mus-Fbxw13  70.13      462         138         0           1           462         1           462         0.0          660
MUS-Fbxw19  MUS-Fbxw18  69.08      469         142         1           1           466         1           469         0.0          654
MUS-Fbxw19  MUS-Fbxw15  68.03      466         149         0           1           466         1           466         0.0          642
MUS-Fbxw19  RNO-Fbxw12  66.16      464         155         1           1           462         1           464         0.0          629
# BLASTP 2.2.20 [Feb-08-2009]
# Query: MUS-Fbxw20
# Database: 559_protein.db
# Query id, Subject id, % identity, alignment length, mismatches, gap openings, q. start, q. end, s. start, s. end, e-value, bit score
MUS-Fbxw20  MUS-Fbxw20  100.00     468         0           0           1           468         1           468         0.0          976
MUS-Fbxw20  MUS-Fbxw16  77.59      464         104         0           1           464         1           464         0.0          760
MUS-Fbxw20  MUS-Fbxw26  78.14      462         101         0           1           462         1           462         0.0          754
MUS-Fbxw20  MUS-Fbxw19  77.71      462         103         0           1           462         1           462         0.0          747
MUS-Fbxw20  MUS-Fbxw21  74.36      468         120         0           1           468         1           468         0.0          724
MUS-Fbxw20  MUS-Fbxw22  75.54      462         113         0           1           462         1           462         0.0          722
MUS-Fbxw20  MUS-Fbxw24  74.46      462         118         0           1           462         1           462         0.0          711
MUS-Fbxw20  MUS-Fbxw18  74.19      465         117         1           1           462         1           465         0.0          697
MUS-Fbxw20  Mus-Fbxw13  73.39      466         124         0           1           466         1           466         0.0          694
MUS-Fbxw20  MUS-Fbxw28  71.98      464         128         2           1           463         1           463         0.0          678
MUS-Fbxw20  MUS-Fbxw14  72.35      463         126         2           1           462         1           462         0.0          676
MUS-Fbxw20  RNO-Fbxw12  68.95      467         143         1           1           465         1           467         0.0          664
MUS-Fbxw20  MUS-Fbxw15  69.70      462         140         0           1           462         1           462         0.0          659
# BLASTP 2.2.20 [Feb-08-2009]
# Query: CJA-Fbxw12
# Database: 559_protein.db
# Query id, Subject id, % identity, alignment length, mismatches, gap openings, q. start, q. end, s. start, s. end, e-value, bit score
CJA-Fbxw12  CJA-Fbxw12  100.00     464         0           0           1           464         1           464         0.0          971
CJA-Fbxw12  HSA-Fbxw12  79.27      463         96          0           1           463         1           463         0.0          782
CJA-Fbxw12  PPY-Fbxw12  78.83      463         98          0           1           463         1           463         0.0          780
CJA-Fbxw12  GGO-Fbxw12  78.83      463         98          0           1           463         1           463         0.0          778
CJA-Fbxw12  PTR-Fbxw12  79.05      463         96          1           1           463         1           462         0.0          776
CJA-Fbxw12  MMU-Fbxw12  72.35      463         123         3           1           463         1           458         0.0          696
# BLASTP 2.2.20 [Feb-08-2009]
# Query: GGO-Fbxw12
# Database: 559_protein.db
# Query id, Subject id, % identity, alignment length, mismatches, gap openings, q. start, q. end, s. start, s. end, e-value, bit score
GGO-Fbxw12  GGO-Fbxw12  100.00     464         0           0           1           464         1           464         0.0          973
GGO-Fbxw12  HSA-Fbxw12  98.49      464         7           0           1           464         1           464         0.0          963
GGO-Fbxw12  PTR-Fbxw12  97.84      464         9           1           1           464         1           463         0.0          949
GGO-Fbxw12  PPY-Fbxw12  95.69      464         20          0           1           464         1           464         0.0          941
GGO-Fbxw12  MMU-Fbxw12  85.13      464         64          3           1           464         1           459         0.0          829
GGO-Fbxw12  CJA-Fbxw12  78.83      463         98          0           1           463         1           463         0.0          778
# BLASTP 2.2.20 [Feb-08-2009]
# Query: MUS-Fbxw21
```

```
# Database: 559_protein.db
# Query id, Subject id, % identity, alignment length, mismatches, gap openings, q. start, q. end, s. start, s. end, e-value, bit score
MUS-Fbxw21  MUS-Fbxw21  100.00      468         0           0           1           468         1           468         0.0         976
MUS-Fbxw21  MUS-Fbxw20  74.36       468         120         0           1           468         1           468         0.0         724
MUS-Fbxw21  MUS-Fbxw26  72.79       463         126         0           1           463         1           463         0.0         709
MUS-Fbxw21  Mus-Fbxw13  74.03       466         121         0           1           466         1           466         0.0         708
MUS-Fbxw21  MUS-Fbxw16  72.20       464         129         0           1           464         1           464         0.0         702
MUS-Fbxw21  MUS-Fbxw19  71.49       463         132         0           1           463         1           463         0.0         693
MUS-Fbxw21  MUS-Fbxw18  71.46       466         130         1           1           463         1           466         0.0         692
MUS-Fbxw21  MUS-Fbxw24  70.63       463         136         0           1           463         1           463         0.0         684
MUS-Fbxw21  MUS-Fbxw22  70.63       463         136         0           1           463         1           463         0.0         677
MUS-Fbxw21  MUS-Fbxw15  71.05       449         130         0           1           449         1           449         0.0         655
MUS-Fbxw21  RNO-Fbxw12  66.81       467         153         1           1           465         1           467         0.0         643
MUS-Fbxw21  MUS-Fbxw28  67.39       463         149         2           1           462         1           462         0.0         632
MUS-Fbxw21  MUS-Fbxw14  67.03       464         151         2           1           463         1           463         0.0         630
# BLASTP 2.2.20 [Feb-08-2009]
# Query: PPY-Fbxw12
# Database: 559_protein.db
# Query id, Subject id, % identity, alignment length, mismatches, gap openings, q. start, q. end, s. start, s. end, e-value, bit score
PPY-Fbxw12  PPY-Fbxw12  100.00      464         0           0           1           464         1           464         0.0         972
PPY-Fbxw12  HSA-Fbxw12  96.77       464         15          0           1           464         1           464         0.0         947
PPY-Fbxw12  GGO-Fbxw12  95.69       464         20          0           1           464         1           464         0.0         941
PPY-Fbxw12  PTR-Fbxw12  96.12       464         17          1           1           464         1           463         0.0         934
PPY-Fbxw12  MMU-Fbxw12  84.27       464         68          3           1           464         1           459         0.0         818
PPY-Fbxw12  CJA-Fbxw12  78.83       463         98          0           1           463         1           463         0.0         780
# BLASTP 2.2.20 [Feb-08-2009]
# Query: PTR-Fbxw12
# Database: 559_protein.db
# Query id, Subject id, % identity, alignment length, mismatches, gap openings, q. start, q. end, s. start, s. end, e-value, bit score
PTR-Fbxw12  PTR-Fbxw12  100.00      463         0           0           1           463         1           463         0.0         970
PTR-Fbxw12  HSA-Fbxw12  99.35       464         2           1           1           463         1           464         0.0         959
PTR-Fbxw12  GGO-Fbxw12  97.84       464         9           1           1           463         1           464         0.0         949
PTR-Fbxw12  PPY-Fbxw12  96.12       464         17          1           1           463         1           464         0.0         934
PTR-Fbxw12  MMU-Fbxw12  84.70       464         65          4           1           463         1           459         0.0         814
PTR-Fbxw12  CJA-Fbxw12  79.05       463         96          1           1           462         1           463         0.0         776
# BLASTP 2.2.20 [Feb-08-2009]
# Query: RNO-Fbxw12
# Database: 559_protein.db
# Query id, Subject id, % identity, alignment length, mismatches, gap openings, q. start, q. end, s. start, s. end, e-value, bit score
RNO-Fbxw12  RNO-Fbxw12  100.00      468         0           0           1           468         1           468         0.0         970
RNO-Fbxw12  MUS-Fbxw20  68.95       467         143         1           1           467         1           465         0.0         664
RNO-Fbxw12  MUS-Fbxw16  66.95       466         152         1           1           466         1           464         0.0         652
RNO-Fbxw12  MUS-Fbxw21  66.81       467         153         1           1           467         1           465         0.0         643
RNO-Fbxw12  MUS-Fbxw18  67.09       468         149         2           1           465         1           466         0.0         639
RNO-Fbxw12  MUS-Fbxw26  66.24       465         155         1           1           465         1           463         0.0         636
```

```
RNO-Fbxw12  MUS-Fbxw24  66.24       465         155         1           1           465         1           463         0.0         634
RNO-Fbxw12  MUS-Fbxw19  66.16       464         155         1           1           464         1           462         0.0         629
RNO-Fbxw12  Mus-Fbxw13  65.88       466         157         1           1           466         1           464         0.0         625
RNO-Fbxw12  MUS-Fbxw22  66.24       465         155         1           1           465         1           463         1e-180      624
RNO-Fbxw12  MUS-Fbxw15  64.73       465         162         1           1           465         1           463         5e-174      602
RNO-Fbxw12  MUS-Fbxw14  62.45       466         171         3           1           465         1           463         1e-166      577
RNO-Fbxw12  MUS-Fbxw28  61.80       466         174         3           1           465         1           463         1e-164      570
# BLASTP 2.2.20 [Feb-08-2009]
# Query: HSA-Fbxw12
# Database: 559_protein.db
# Query id, Subject id, % identity, alignment length, mismatches, gap openings, q. start, q. end, s. start, s. end, e-value, bit score
HSA-Fbxw12  HSA-Fbxw12  100.00      464         0           0           1           464         1           464         0.0         973
HSA-Fbxw12  GGO-Fbxw12  98.49       464         7           0           1           464         1           464         0.0         963
HSA-Fbxw12  PTR-Fbxw12  99.35       464         2           1           1           464         1           463         0.0         959
HSA-Fbxw12  PPY-Fbxw12  96.77       464         15          0           1           464         1           464         0.0         947
HSA-Fbxw12  MMU-Fbxw12  85.34       464         63          3           1           464         1           459         0.0         827
HSA-Fbxw12  CJA-Fbxw12  79.27       463         96          0           1           463         1           463         0.0         782
# BLASTP 2.2.20 [Feb-08-2009]
# Query: MUS-Fbxw22
# Database: 559_protein.db
# Query id, Subject id, % identity, alignment length, mismatches, gap openings, q. start, q. end, s. start, s. end, e-value, bit score
MUS-Fbxw22  MUS-Fbxw22  100.00      466         0           0           1           466         1           466         0.0         969
MUS-Fbxw22  MUS-Fbxw14  84.37       467         71          2           1           466         1           466         0.0         817
MUS-Fbxw22  MUS-Fbxw28  83.73       467         74          2           1           466         1           466         0.0         811
MUS-Fbxw22  MUS-Fbxw26  79.40       466         96          0           1           466         1           466         0.0         769
MUS-Fbxw22  MUS-Fbxw19  79.83       466         94          0           1           466         1           466         0.0         768
MUS-Fbxw22  MUS-Fbxw16  77.32       463         105         0           1           463         1           463         0.0         743
MUS-Fbxw22  MUS-Fbxw20  75.54       462         113         0           1           462         1           462         0.0         722
MUS-Fbxw22  MUS-Fbxw21  70.63       463         136         0           1           463         1           463         0.0         677
MUS-Fbxw22  MUS-Fbxw18  70.56       462         136         0           5           466         8           469         0.0         661
MUS-Fbxw22  MUS-Fbxw24  67.81       466         150         0           1           466         1           466         0.0         645
MUS-Fbxw22  Mus-Fbxw13  67.60       463         150         0           1           463         1           463         0.0         636
MUS-Fbxw22  MUS-Fbxw15  67.60       466         151         0           1           466         1           466         0.0         634
MUS-Fbxw22  RNO-Fbxw12  66.24       465         155         1           1           463         1           465         1e-180      624
# BLASTP 2.2.20 [Feb-08-2009]
# Query: MUS-Fbxw24
# Database: 559_protein.db
# Query id, Subject id, % identity, alignment length, mismatches, gap openings, q. start, q. end, s. start, s. end, e-value, bit score
MUS-Fbxw24  MUS-Fbxw24  100.00      466         0           0           1           466         1           466         0.0         967
MUS-Fbxw24  MUS-Fbxw20  74.46       462         118         0           1           462         1           462         0.0         711
MUS-Fbxw24  MUS-Fbxw26  73.61       466         123         0           1           466         1           466         0.0         706
MUS-Fbxw24  MUS-Fbxw16  73.43       463         123         0           1           463         1           463         0.0         703
MUS-Fbxw24  MUS-Fbxw15  74.03       466         121         0           1           466         1           466         0.0         701
MUS-Fbxw24  MUS-Fbxw21  70.63       463         136         0           1           463         1           463         0.0         684
MUS-Fbxw24  MUS-Fbxw19  69.53       466         142         0           1           466         1           466         0.0         665
```

```
MUS-Fbxw24  Mus-Fbxw13  69.76   463  140  0  1  463  1  463  0.0     665
MUS-Fbxw24  MUS-Fbxw18  69.30   469  141  1  1  466  1  469  0.0     651
MUS-Fbxw24  MUS-Fbxw22  67.81   466  150  0  1  466  1  466  0.0     645
MUS-Fbxw24  RNO-Fbxw12  66.24   465  155  1  1  463  1  465  0.0     634
MUS-Fbxw24  MUS-Fbxw14  64.45   467  164  2  1  466  1  466  6e-172  595
MUS-Fbxw24  MUS-Fbxw28  63.60   467  168  2  1  466  1  466  1e-169  587
# BLASTP 2.2.20 [Feb-08-2009]
# Query: MUS-Fbxw26
# Database: 559_protein.db
# Query id, Subject id, % identity, alignment length, mismatches, gap openings, q. start, q. end, s. start, s. end, e-value, bit score
MUS-Fbxw26  MUS-Fbxw26  100.00  466  0    0  1  466  1  466  0.0     976
MUS-Fbxw26  MUS-Fbxw16  82.72   463  80   0  1  463  1  463  0.0     807
MUS-Fbxw26  MUS-Fbxw22  79.40   466  96   0  1  466  1  466  0.0     769
MUS-Fbxw26  MUS-Fbxw19  78.97   466  98   0  1  466  1  466  0.0     769
MUS-Fbxw26  MUS-Fbxw20  78.14   462  101  0  1  462  1  462  0.0     754
MUS-Fbxw26  MUS-Fbxw14  75.80   467  111  2  1  466  1  466  0.0     717
MUS-Fbxw26  MUS-Fbxw28  75.16   467  114  2  1  466  1  466  0.0     711
MUS-Fbxw26  MUS-Fbxw21  72.79   463  126  0  1  463  1  463  0.0     709
MUS-Fbxw26  MUS-Fbxw24  73.61   466  123  0  1  466  1  466  0.0     706
MUS-Fbxw26  MUS-Fbxw18  72.92   469  124  1  1  466  1  469  0.0     696
MUS-Fbxw26  Mus-Fbxw13  69.33   463  142  0  1  463  1  463  0.0     664
MUS-Fbxw26  MUS-Fbxw15  68.67   466  146  0  1  466  1  466  0.0     653
MUS-Fbxw26  RNO-Fbxw12  66.24   465  155  1  1  463  1  465  0.0     636
# BLASTP 2.2.20 [Feb-08-2009]
# Query: MUS-Fbxw28
# Database: 559_protein.db
# Query id, Subject id, % identity, alignment length, mismatches, gap openings, q. start, q. end, s. start, s. end, e-value, bit score
MUS-Fbxw28  MUS-Fbxw28  100.00  466  0    0  1  466  1  466  0.0     974
MUS-Fbxw28  MUS-Fbxw14  97.85   466  10   0  1  466  1  466  0.0     956
MUS-Fbxw28  MUS-Fbxw22  83.73   467  74   2  1  466  1  466  0.0     811
MUS-Fbxw28  MUS-Fbxw19  77.73   467  102  2  1  466  1  466  0.0     734
MUS-Fbxw28  MUS-Fbxw26  75.16   467  114  2  1  466  1  466  0.0     711
MUS-Fbxw28  MUS-Fbxw16  74.78   464  115  2  1  463  1  463  0.0     702
MUS-Fbxw28  MUS-Fbxw20  71.98   464  128  2  1  463  1  463  0.0     678
MUS-Fbxw28  MUS-Fbxw21  67.39   463  149  2  1  462  1  462  0.0     632
MUS-Fbxw28  MUS-Fbxw18  66.60   470  152  3  1  466  1  469  1e-176  611
MUS-Fbxw28  MUS-Fbxw15  63.60   467  168  2  1  466  1  466  2e-171  593
MUS-Fbxw28  Mus-Fbxw13  64.44   464  163  2  1  463  1  463  3e-170  590
MUS-Fbxw28  MUS-Fbxw24  63.60   467  168  2  1  466  1  466  1e-169  587
MUS-Fbxw28  RNO-Fbxw12  61.80   466  174  3  1  463  1  465  1e-164  570
# BLASTP 2.2.20 [Feb-08-2009]
# Query: MUS-Fbxw17
# Database: 559_protein.db
# Query id, Subject id, % identity, alignment length, mismatches, gap openings, q. start, q. end, s. start, s. end, e-value, bit score
MUS-Fbxw17  MUS-Fbxw17  100.00  466  0    0  1  466  1  466  0.0     937
```

```
MUS-Fbxw17  RNO-Fbxw17  89.46       465         49          0           1           465         1           465         0.0          839
# BLASTP 2.2.20 [Feb-08-2009]
# Query: RNO-Fbxw17
# Database: 559_protein.db
# Query id, Subject id, % identity, alignment length, mismatches, gap openings, q. start, q. end, s. start, s. end, e-value, bit score
RNO-Fbxw17  RNO-Fbxw17  100.00      466         0           0           1           466         1           466         0.0          938
RNO-Fbxw17  MUS-Fbxw17  89.46       465         49          0           1           465         1           465         0.0          838
# BLASTP 2.2.20 [Feb-08-2009]
# Query: CJA-Fbxw2
# Database: 559_protein.db
# Query id, Subject id, % identity, alignment length, mismatches, gap openings, q. start, q. end, s. start, s. end, e-value, bit score
CJA-Fbxw2   HSA-Fbxw2   100.00      454         0           0           1           454         1           454         0.0          892
CJA-Fbxw2   PTR-Fbxw2   100.00      454         0           0           1           454         1           454         0.0          892
CJA-Fbxw2   CJA-Fbxw2   100.00      454         0           0           1           454         1           454         0.0          892
CJA-Fbxw2   PPY-Fbxw2   99.78       454         1           0           1           454         1           454         0.0          890
CJA-Fbxw2   GGO-Fbxw2   99.78       454         1           0           1           454         1           454         0.0          890
CJA-Fbxw2   RNO-Fbxw2   98.68       454         6           0           1           454         1           454         0.0          884
CJA-Fbxw2   MUS-Fbxw2   98.68       453         6           0           1           453         1           453         0.0          880
CJA-Fbxw2   MMU-Fbxw2   94.05       454         27          0           1           454         1           454         0.0          827
# BLASTP 2.2.20 [Feb-08-2009]
# Query: GGO-Fbxw2
# Database: 559_protein.db
# Query id, Subject id, % identity, alignment length, mismatches, gap openings, q. start, q. end, s. start, s. end, e-value, bit score
GGO-Fbxw2   GGO-Fbxw2   100.00      454         0           0           1           454         1           454         0.0          892
GGO-Fbxw2   HSA-Fbxw2   99.78       454         1           0           1           454         1           454         0.0          890
GGO-Fbxw2   PTR-Fbxw2   99.78       454         1           0           1           454         1           454         0.0          890
GGO-Fbxw2   CJA-Fbxw2   99.78       454         1           0           1           454         1           454         0.0          890
GGO-Fbxw2   PPY-Fbxw2   99.56       454         2           0           1           454         1           454         0.0          889
GGO-Fbxw2   RNO-Fbxw2   98.46       454         7           0           1           454         1           454         0.0          882
GGO-Fbxw2   MUS-Fbxw2   98.45       453         7           0           1           453         1           453         0.0          878
GGO-Fbxw2   MMU-Fbxw2   94.05       454         27          0           1           454         1           454         0.0          828
# BLASTP 2.2.20 [Feb-08-2009]
# Query: MMU-Fbxw2
# Database: 559_protein.db
# Query id, Subject id, % identity, alignment length, mismatches, gap openings, q. start, q. end, s. start, s. end, e-value, bit score
MMU-Fbxw2   MMU-Fbxw2   100.00      454         0           0           1           454         1           454         0.0          871
MMU-Fbxw2   GGO-Fbxw2   94.05       454         27          0           1           454         1           454         0.0          829
MMU-Fbxw2   HSA-Fbxw2   94.05       454         27          0           1           454         1           454         0.0          828
MMU-Fbxw2   PTR-Fbxw2   94.05       454         27          0           1           454         1           454         0.0          828
MMU-Fbxw2   CJA-Fbxw2   94.05       454         27          0           1           454         1           454         0.0          828
MMU-Fbxw2   PPY-Fbxw2   93.83       454         28          0           1           454         1           454         0.0          827
MMU-Fbxw2   RNO-Fbxw2   92.95       454         32          0           1           454         1           454         0.0          821
MMU-Fbxw2   MUS-Fbxw2   92.72       453         33          0           1           453         1           453         0.0          818
# BLASTP 2.2.20 [Feb-08-2009]
# Query: MUS-Fbxw2
```

```
# Database: 559_protein.db
# Query id, Subject id, % identity, alignment length, mismatches, gap openings, q. start, q. end, s. start, s. end, e-value, bit score
MUS-Fbxw2   MUS-Fbxw2   100.00      454         0           0           1           454         1           454         0.0         891
MUS-Fbxw2   RNO-Fbxw2   99.56       453         2           0           1           453         1           453         0.0         886
MUS-Fbxw2   HSA-Fbxw2   98.68       453         6           0           1           453         1           453         0.0         880
MUS-Fbxw2   PTR-Fbxw2   98.68       453         6           0           1           453         1           453         0.0         880
MUS-Fbxw2   CJA-Fbxw2   98.68       453         6           0           1           453         1           453         0.0         880
MUS-Fbxw2   PPY-Fbxw2   98.45       453         7           0           1           453         1           453         0.0         879
MUS-Fbxw2   GGO-Fbxw2   98.45       453         7           0           1           453         1           453         0.0         879
MUS-Fbxw2   MMU-Fbxw2   92.72       453         33          0           1           453         1           453         0.0         817
# BLASTP 2.2.20 [Feb-08-2009]
# Query: PPY-Fbxw2
# Database: 559_protein.db
# Query id, Subject id, % identity, alignment length, mismatches, gap openings, q. start, q. end, s. start, s. end, e-value, bit score
PPY-Fbxw2   PPY-Fbxw2   100.00      454         0           0           1           454         1           454         0.0         892
PPY-Fbxw2   HSA-Fbxw2   99.78       454         1           0           1           454         1           454         0.0         890
PPY-Fbxw2   PTR-Fbxw2   99.78       454         1           0           1           454         1           454         0.0         890
PPY-Fbxw2   CJA-Fbxw2   99.78       454         1           0           1           454         1           454         0.0         890
PPY-Fbxw2   GGO-Fbxw2   99.56       454         2           0           1           454         1           454         0.0         889
PPY-Fbxw2   RNO-Fbxw2   98.46       454         7           0           1           454         1           454         0.0         882
PPY-Fbxw2   MUS-Fbxw2   98.45       453         7           0           1           453         1           453         0.0         879
PPY-Fbxw2   MMU-Fbxw2   93.83       454         28          0           1           454         1           454         0.0         826
# BLASTP 2.2.20 [Feb-08-2009]
# Query: PTR-Fbxw2
# Database: 559_protein.db
# Query id, Subject id, % identity, alignment length, mismatches, gap openings, q. start, q. end, s. start, s. end, e-value, bit score
PTR-Fbxw2   HSA-Fbxw2   100.00      454         0           0           1           454         1           454         0.0         892
PTR-Fbxw2   PTR-Fbxw2   100.00      454         0           0           1           454         1           454         0.0         892
PTR-Fbxw2   CJA-Fbxw2   100.00      454         0           0           1           454         1           454         0.0         892
PTR-Fbxw2   PPY-Fbxw2   99.78       454         1           0           1           454         1           454         0.0         890
PTR-Fbxw2   GGO-Fbxw2   99.78       454         1           0           1           454         1           454         0.0         890
PTR-Fbxw2   RNO-Fbxw2   98.68       454         6           0           1           454         1           454         0.0         884
PTR-Fbxw2   MUS-Fbxw2   98.68       453         6           0           1           453         1           453         0.0         880
PTR-Fbxw2   MMU-Fbxw2   94.05       454         27          0           1           454         1           454         0.0         827
# BLASTP 2.2.20 [Feb-08-2009]
# Query: RNO-Fbxw2
# Database: 559_protein.db
# Query id, Subject id, % identity, alignment length, mismatches, gap openings, q. start, q. end, s. start, s. end, e-value, bit score
RNO-Fbxw2   RNO-Fbxw2   100.00      454         0           0           1           454         1           454         0.0         891
RNO-Fbxw2   MUS-Fbxw2   99.56       453         2           0           1           453         1           453         0.0         886
RNO-Fbxw2   HSA-Fbxw2   98.68       454         6           0           1           454         1           454         0.0         884
RNO-Fbxw2   PTR-Fbxw2   98.68       454         6           0           1           454         1           454         0.0         884
RNO-Fbxw2   CJA-Fbxw2   98.68       454         6           0           1           454         1           454         0.0         884
RNO-Fbxw2   PPY-Fbxw2   98.46       454         7           0           1           454         1           454         0.0         882
RNO-Fbxw2   GGO-Fbxw2   98.46       454         7           0           1           454         1           454         0.0         882
```

```
RNO-Fbxw2   MMU-Fbxw2   92.95   454   32   0   1   454   1   454   0.0   821
# BLASTP 2.2.20 [Feb-08-2009]
# Query: HSA-Fbxw2
# Database: 559_protein.db
# Query id, Subject id, % identity, alignment length, mismatches, gap openings, q. start, q. end, s. start, s. end, e-value, bit score
HSA-Fbxw2   HSA-Fbxw2   100.00   454   0   0   1   454   1   454   0.0   892
HSA-Fbxw2   PTR-Fbxw2   100.00   454   0   0   1   454   1   454   0.0   892
HSA-Fbxw2   CJA-Fbxw2   100.00   454   0   0   1   454   1   454   0.0   892
HSA-Fbxw2   PPY-Fbxw2   99.78   454   1   0   1   454   1   454   0.0   890
HSA-Fbxw2   GGO-Fbxw2   99.78   454   1   0   1   454   1   454   0.0   890
HSA-Fbxw2   RNO-Fbxw2   98.68   454   6   0   1   454   1   454   0.0   884
HSA-Fbxw2   MUS-Fbxw2   98.68   453   6   0   1   453   1   453   0.0   880
HSA-Fbxw2   MMU-Fbxw2   94.05   454   27   0   1   454   1   454   0.0   827
# BLASTP 2.2.20 [Feb-08-2009]
# Query: CJA-Fbxw4
# Database: 559_protein.db
# Query id, Subject id, % identity, alignment length, mismatches, gap openings, q. start, q. end, s. start, s. end, e-value, bit score
CJA-Fbxw4   CJA-Fbxw4   100.00   369   0   0   46   414   46   414   0.0   734
CJA-Fbxw4   PPY-Fbxw4   99.46   369   2   0   46   414   43   411   0.0   734
CJA-Fbxw4   GGO-Fbxw4   99.19   369   3   0   46   414   44   412   0.0   732
CJA-Fbxw4   MMU-Fbxw4   99.46   369   2   0   46   414   42   410   0.0   732
CJA-Fbxw4   HSA-Fbxw4   99.46   369   2   0   46   414   44   412   0.0   731
CJA-Fbxw4   RNO-Fbxw4   94.04   369   22   0   46   414   40   408   0.0   696
CJA-Fbxw4   MUS-Fbxw4   92.95   369   26   0   46   414   42   410   0.0   689
# BLASTP 2.2.20 [Feb-08-2009]
# Query: GGO-Fbxw4
# Database: 559_protein.db
# Query id, Subject id, % identity, alignment length, mismatches, gap openings, q. start, q. end, s. start, s. end, e-value, bit score
GGO-Fbxw4   GGO-Fbxw4   100.00   369   0   0   44   412   44   412   0.0   768
GGO-Fbxw4   PPY-Fbxw4   99.73   369   1   0   44   412   43   411   0.0   766
GGO-Fbxw4   CJA-Fbxw4   99.19   369   3   0   44   412   46   414   0.0   766
GGO-Fbxw4   MMU-Fbxw4   99.19   369   3   0   44   412   42   410   0.0   764
GGO-Fbxw4   HSA-Fbxw4   99.19   369   3   0   44   412   44   412   0.0   764
GGO-Fbxw4   RNO-Fbxw4   93.77   369   23   0   44   412   40   408   0.0   724
GGO-Fbxw4   MUS-Fbxw4   93.50   369   24   0   44   412   42   410   0.0   719
# BLASTP 2.2.20 [Feb-08-2009]
# Query: MMU-Fbxw4
# Database: 559_protein.db
# Query id, Subject id, % identity, alignment length, mismatches, gap openings, q. start, q. end, s. start, s. end, e-value, bit score
MMU-Fbxw4   MMU-Fbxw4   100.00   369   0   0   42   410   42   410   0.0   768
MMU-Fbxw4   CJA-Fbxw4   99.46   369   2   0   42   410   46   414   0.0   766
MMU-Fbxw4   PPY-Fbxw4   99.46   369   2   0   42   410   43   411   0.0   766
MMU-Fbxw4   GGO-Fbxw4   99.19   369   3   0   42   410   44   412   0.0   764
MMU-Fbxw4   HSA-Fbxw4   98.92   369   4   0   42   410   44   412   0.0   763
MMU-Fbxw4   RNO-Fbxw4   94.31   369   21   0   42   410   40   408   0.0   728
```

```
MMU-Fbxw4   MUS-Fbxw4   93.22   369   25   0   42   410   42   410   0.0   720
# BLASTP 2.2.20 [Feb-08-2009]
# Query: MUS-Fbxw4
# Database: 559_protein.db
# Query id, Subject id, % identity, alignment length, mismatches, gap openings, q. start, q. end, s. start, s. end, e-value, bit score
MUS-Fbxw4   MUS-Fbxw4   100.00   369   0   0   42   410   42   410   0.0   764
MUS-Fbxw4   RNO-Fbxw4   96.21   369   14   0   42   410   40   408   0.0   744
MUS-Fbxw4   MMU-Fbxw4   93.22   369   25   0   42   410   42   410   0.0   720
MUS-Fbxw4   GGO-Fbxw4   93.50   369   24   0   42   410   44   412   0.0   719
MUS-Fbxw4   PPY-Fbxw4   93.50   369   24   0   42   410   43   411   0.0   719
MUS-Fbxw4   CJA-Fbxw4   92.95   369   26   0   42   410   46   414   0.0   718
MUS-Fbxw4   HSA-Fbxw4   92.95   369   26   0   42   410   44   412   0.0   717
# BLASTP 2.2.20 [Feb-08-2009]
# Query: PPY-Fbxw4
# Database: 559_protein.db
# Query id, Subject id, % identity, alignment length, mismatches, gap openings, q. start, q. end, s. start, s. end, e-value, bit score
PPY-Fbxw4   PPY-Fbxw4   100.00   369   0   0   43   411   43   411   0.0   768
PPY-Fbxw4   CJA-Fbxw4   99.46   369   2   0   43   411   46   414   0.0   767
PPY-Fbxw4   GGO-Fbxw4   99.73   369   1   0   43   411   44   412   0.0   766
PPY-Fbxw4   HSA-Fbxw4   99.46   369   2   0   43   411   44   412   0.0   766
PPY-Fbxw4   MMU-Fbxw4   99.46   369   2   0   43   411   42   410   0.0   765
PPY-Fbxw4   RNO-Fbxw4   94.04   369   22   0   43   411   40   408   0.0   726
PPY-Fbxw4   MUS-Fbxw4   93.50   369   24   0   43   411   42   410   0.0   720
# BLASTP 2.2.20 [Feb-08-2009]
# Query: RNO-Fbxw4
# Database: 559_protein.db
# Query id, Subject id, % identity, alignment length, mismatches, gap openings, q. start, q. end, s. start, s. end, e-value, bit score
RNO-Fbxw4   RNO-Fbxw4   100.00   369   0   0   40   408   40   408   0.0   764
RNO-Fbxw4   MUS-Fbxw4   96.21   369   14   0   40   408   42   410   0.0   743
RNO-Fbxw4   MMU-Fbxw4   94.31   369   21   0   40   408   42   410   0.0   727
RNO-Fbxw4   PPY-Fbxw4   94.04   369   22   0   40   408   43   411   0.0   726
RNO-Fbxw4   CJA-Fbxw4   94.04   369   22   0   40   408   46   414   0.0   726
RNO-Fbxw4   GGO-Fbxw4   93.77   369   23   0   40   408   44   412   0.0   724
RNO-Fbxw4   HSA-Fbxw4   93.50   369   24   0   40   408   44   412   0.0   723
# BLASTP 2.2.20 [Feb-08-2009]
# Query: HSA-Fbxw4
# Database: 559_protein.db
# Query id, Subject id, % identity, alignment length, mismatches, gap openings, q. start, q. end, s. start, s. end, e-value, bit score
HSA-Fbxw4   HSA-Fbxw4   100.00   369   0   0   44   412   44   412   0.0   734
HSA-Fbxw4   PPY-Fbxw4   99.46   369   2   0   44   412   43   411   0.0   732
HSA-Fbxw4   CJA-Fbxw4   99.46   369   2   0   44   412   46   414   0.0   731
HSA-Fbxw4   GGO-Fbxw4   99.19   369   3   0   44   412   44   412   0.0   730
HSA-Fbxw4   MMU-Fbxw4   98.92   369   4   0   44   412   42   410   0.0   729
HSA-Fbxw4   RNO-Fbxw4   93.50   369   24   0   44   412   40   408   0.0   693
HSA-Fbxw4   MUS-Fbxw4   92.95   369   26   0   44   412   42   410   0.0   687
```

```
# BLASTP 2.2.20 [Feb-08-2009]
# Query: CJA-Fbxw5
# Database: 559_protein.db
# Query id, Subject id, % identity, alignment length, mismatches, gap openings, q. start, q. end, s. start, s. end, e-value, bit score
CJA-Fbxw5   CJA-Fbxw5   100.00   568   0    0   1   568   1    568   0.0   1155
CJA-Fbxw5   GGO-Fbxw5   95.42    568   24   1   1   568   1    566   0.0   1109
CJA-Fbxw5   HSA-Fbxw5   94.72    568   28   1   1   568   1    566   0.0   1106
CJA-Fbxw5   PPY-Fbxw5   94.04    570   27   5   1   568   1    565   0.0   1062
CJA-Fbxw5   MUS-Fbxw5   90.96    531   41   2   1   526   56   584   0.0    999
CJA-Fbxw5   RNO-Fbxw5   91.01    523   40   2   1   518   56   576   0.0    986
# BLASTP 2.2.20 [Feb-08-2009]
# Query: GGO-Fbxw5
# Database: 559_protein.db
# Query id, Subject id, % identity, alignment length, mismatches, gap openings, q. start, q. end, s. start, s. end, e-value, bit score
GGO-Fbxw5   GGO-Fbxw5   100.00   566   0    0   1   566   1    566   0.0   1143
GGO-Fbxw5   HSA-Fbxw5   99.29    566   4    0   1   566   1    566   0.0   1139
GGO-Fbxw5   CJA-Fbxw5   95.42    568   24   1   1   566   1    568   0.0   1078
GGO-Fbxw5   PPY-Fbxw5   96.30    568   16   4   1   566   1    565   0.0   1077
GGO-Fbxw5   MUS-Fbxw5   89.45    531   49   2   1   526   56   584   0.0    955
GGO-Fbxw5   RNO-Fbxw5   89.67    523   47   2   1   518   56   576   0.0    943
# BLASTP 2.2.20 [Feb-08-2009]
# Query: MUS-Fbxw5
# Database: 559_protein.db
# Query id, Subject id, % identity, alignment length, mismatches, gap openings, q. start, q. end, s. start, s. end, e-value, bit score
MUS-Fbxw5   MUS-Fbxw5   100.00   625   0    0   1    625   1   625   0.0   1269
MUS-Fbxw5   RNO-Fbxw5   97.57    576   14   0   1    576   1   576   0.0   1174
MUS-Fbxw5   CJA-Fbxw5   90.96    531   41   2   56   584   1   526   0.0    999
MUS-Fbxw5   GGO-Fbxw5   89.45    531   49   2   56   584   1   526   0.0    984
MUS-Fbxw5   HSA-Fbxw5   89.27    531   50   2   56   584   1   526   0.0    983
MUS-Fbxw5   PPY-Fbxw5   88.32    531   55   4   56   584   1   526   0.0    956
# BLASTP 2.2.20 [Feb-08-2009]
# Query: PPY-Fbxw5
# Database: 559_protein.db
# Query id, Subject id, % identity, alignment length, mismatches, gap openings, q. start, q. end, s. start, s. end, e-value, bit score
PPY-Fbxw5   PPY-Fbxw5   100.00   565   0    0   1   565   1    565   0.0   1147
PPY-Fbxw5   GGO-Fbxw5   96.30    568   16   4   1   565   1    566   0.0   1108
PPY-Fbxw5   HSA-Fbxw5   95.95    568   18   4   1   565   1    566   0.0   1105
PPY-Fbxw5   CJA-Fbxw5   94.04    570   27   5   1   565   1    568   0.0   1062
PPY-Fbxw5   MUS-Fbxw5   88.32    531   55   4   1   526   56   584   0.0    956
PPY-Fbxw5   RNO-Fbxw5   88.53    523   53   4   1   518   56   576   0.0    944
# BLASTP 2.2.20 [Feb-08-2009]
# Query: RNO-Fbxw5
# Database: 559_protein.db
# Query id, Subject id, % identity, alignment length, mismatches, gap openings, q. start, q. end, s. start, s. end, e-value, bit score
RNO-Fbxw5   RNO-Fbxw5   100.00   576   0    0   1   576   1    576   0.0   1170
```

```
RNO-Fbxw5   MUS-Fbxw5   97.57    576   14   0   1     576   1     576   0.0   1148
RNO-Fbxw5   CJA-Fbxw5   91.01    523   40   2   56    576   1     518   0.0   967
RNO-Fbxw5   GGO-Fbxw5   89.67    523   47   2   56    576   1     518   0.0   955
RNO-Fbxw5   HSA-Fbxw5   89.48    523   48   2   56    576   1     518   0.0   954
RNO-Fbxw5   PPY-Fbxw5   88.53    523   53   4   56    576   1     518   0.0   926
# BLASTP 2.2.20 [Feb-08-2009]
# Query: HSA-Fbxw5
# Database: 559_protein.db
# Query id, Subject id, % identity, alignment length, mismatches, gap openings, q. start, q. end, s. start, s. end, e-value, bit score
HSA-Fbxw5   HSA-Fbxw5   100.00   566   0    0   1     566   1     566   0.0   1145
HSA-Fbxw5   GGO-Fbxw5   99.29    566   4    0   1     566   1     566   0.0   1139
HSA-Fbxw5   CJA-Fbxw5   94.72    568   28   1   1     566   1     568   0.0   1075
HSA-Fbxw5   PPY-Fbxw5   95.95    568   18   4   1     566   1     565   0.0   1075
HSA-Fbxw5   MUS-Fbxw5   89.27    531   50   2   1     526   56    584   0.0   954
HSA-Fbxw5   RNO-Fbxw5   89.48    523   48   2   1     518   56    576   0.0   942
# BLASTP 2.2.20 [Feb-08-2009]
# Query: CJA-Fbxw7
# Database: 559_protein.db
# Query id, Subject id, % identity, alignment length, mismatches, gap openings, q. start, q. end, s. start, s. end, e-value, bit score
CJA-Fbxw7   CJA-Fbxw7   100.00   707   0    0   1     707   1     707   0.0   1248
CJA-Fbxw7   GGO-Fbxw7   99.29    707   5    0   1     707   1     707   0.0   1241
CJA-Fbxw7   HSA-Fbxw7   99.15    707   6    0   1     707   1     707   0.0   1241
CJA-Fbxw7   PTR-Fbxw7   99.15    707   6    0   1     707   1     707   0.0   1241
CJA-Fbxw7   MMU-Fbxw7   99.29    707   5    0   1     707   1     707   0.0   1241
CJA-Fbxw7   MUS-Fbxw7   99.28    557   4    0   151   707   154   710   0.0   1135
CJA-Fbxw7   PPY-Fbxw7   100.00   541   0    0   167   707   87    627   0.0   1101
CJA-Fbxw7   RNO-Fbxw7   97.84    510   7    1   157   666   83    588   0.0   1014
# BLASTP 2.2.20 [Feb-08-2009]
# Query: GGO-Fbxw7
# Database: 559_protein.db
# Query id, Subject id, % identity, alignment length, mismatches, gap openings, q. start, q. end, s. start, s. end, e-value, bit score
GGO-Fbxw7   MMU-Fbxw7   99.72    707   2    0   1     707   1     707   0.0   1223
GGO-Fbxw7   GGO-Fbxw7   100.00   707   0    0   1     707   1     707   0.0   1223
GGO-Fbxw7   HSA-Fbxw7   99.86    707   1    0   1     707   1     707   0.0   1222
GGO-Fbxw7   PTR-Fbxw7   99.86    707   1    0   1     707   1     707   0.0   1222
GGO-Fbxw7   CJA-Fbxw7   99.29    707   5    0   1     707   1     707   0.0   1218
GGO-Fbxw7   MUS-Fbxw7   98.93    559   6    0   149   707   152   710   0.0   1137
GGO-Fbxw7   PPY-Fbxw7   100.00   541   0    0   167   707   87    627   0.0   1101
GGO-Fbxw7   RNO-Fbxw7   99.20    500   4    0   167   666   89    588   0.0   1014
# BLASTP 2.2.20 [Feb-08-2009]
# Query: MMU-Fbxw7
# Database: 559_protein.db
# Query id, Subject id, % identity, alignment length, mismatches, gap openings, q. start, q. end, s. start, s. end, e-value, bit score
MMU-Fbxw7   MMU-Fbxw7   100.00   707   0    0   1     707   1     707   0.0   1219
MMU-Fbxw7   HSA-Fbxw7   99.86    707   1    0   1     707   1     707   0.0   1219
```

```
MMU-Fbxw7   PTR-Fbxw7   99.86    707    1    0    1    707    1    707    0.0    1219
MMU-Fbxw7   GGO-Fbxw7   99.72    707    2    0    1    707    1    707    0.0    1219
MMU-Fbxw7   CJA-Fbxw7   99.29    707    5    0    1    707    1    707    0.0    1214
MMU-Fbxw7   MUS-Fbxw7   99.28    557    4    0    151    707    154    710    0.0    1137
MMU-Fbxw7   PPY-Fbxw7   100.00    541    0    0    167    707    87    627    0.0    1102
MMU-Fbxw7   RNO-Fbxw7   99.20    500    4    0    167    666    89    588    0.0    1014
# BLASTP 2.2.20 [Feb-08-2009]
# Query: MUS-Fbxw7
# Database: 559_protein.db
# Query id, Subject id, % identity, alignment length, mismatches, gap openings, q. start, q. end, s. start, s. end, e-value, bit score
MUS-Fbxw7   MUS-Fbxw7   100.00    710    0    0    1    710    1    710    0.0    1350
MUS-Fbxw7   HSA-Fbxw7   93.94    710    40    1    1    710    1    707    0.0    1271
MUS-Fbxw7   PTR-Fbxw7   93.94    710    40    1    1    710    1    707    0.0    1271
MUS-Fbxw7   MMU-Fbxw7   93.94    710    40    1    1    710    1    707    0.0    1270
MUS-Fbxw7   GGO-Fbxw7   93.94    710    40    1    1    710    1    707    0.0    1270
MUS-Fbxw7   CJA-Fbxw7   94.08    710    39    1    1    710    1    707    0.0    1258
MUS-Fbxw7   PPY-Fbxw7   98.19    551    6    1    160    710    81    627    0.0    1101
MUS-Fbxw7   RNO-Fbxw7   98.43    510    4    1    160    669    83    588    0.0    1020
# BLASTP 2.2.20 [Feb-08-2009]
# Query: PPY-Fbxw7
# Database: 559_protein.db
# Query id, Subject id, % identity, alignment length, mismatches, gap openings, q. start, q. end, s. start, s. end, e-value, bit score
PPY-Fbxw7   PPY-Fbxw7   100.00    627    0    0    1    627    1    627    0.0    1244
PPY-Fbxw7   RNO-Fbxw7   97.44    586    15    0    1    586    3    588    0.0    1133
PPY-Fbxw7   HSA-Fbxw7   98.73    551    3    1    81    627    157    707    0.0    1110
PPY-Fbxw7   PTR-Fbxw7   98.73    551    3    1    81    627    157    707    0.0    1110
PPY-Fbxw7   CJA-Fbxw7   98.73    551    3    1    81    627    157    707    0.0    1110
PPY-Fbxw7   GGO-Fbxw7   98.73    551    3    1    81    627    157    707    0.0    1110
PPY-Fbxw7   MMU-Fbxw7   98.73    551    3    1    81    627    157    707    0.0    1109
PPY-Fbxw7   MUS-Fbxw7   98.19    551    6    1    81    627    160    710    0.0    1102
# BLASTP 2.2.20 [Feb-08-2009]
# Query: PTR-Fbxw7
# Database: 559_protein.db
# Query id, Subject id, % identity, alignment length, mismatches, gap openings, q. start, q. end, s. start, s. end, e-value, bit score
PTR-Fbxw7   MMU-Fbxw7   99.86    707    1    0    1    707    1    707    0.0    1224
PTR-Fbxw7   HSA-Fbxw7   100.00    707    0    0    1    707    1    707    0.0    1223
PTR-Fbxw7   PTR-Fbxw7   100.00    707    0    0    1    707    1    707    0.0    1223
PTR-Fbxw7   GGO-Fbxw7   99.86    707    1    0    1    707    1    707    0.0    1222
PTR-Fbxw7   CJA-Fbxw7   99.15    707    6    0    1    707    1    707    0.0    1218
PTR-Fbxw7   MUS-Fbxw7   98.93    559    6    0    149    707    152    710    0.0    1137
PTR-Fbxw7   PPY-Fbxw7   100.00    541    0    0    167    707    87    627    0.0    1101
PTR-Fbxw7   RNO-Fbxw7   99.20    500    4    0    167    666    89    588    0.0    1014
# BLASTP 2.2.20 [Feb-08-2009]
# Query: RNO-Fbxw7
# Database: 559_protein.db
```

```
# Query id, Subject id, % identity, alignment length, mismatches, gap openings, q. start, q. end, s. start, s. end, e-value, bit score
RNO-Fbxw7   RNO-Fbxw7   100.00      557         0           0           32          588         32          588         0.0         1133
RNO-Fbxw7   PPY-Fbxw7   99.28       554         4           0           35          588         33          586         0.0         1124
RNO-Fbxw7   MUS-Fbxw7   98.43       510         4           1           83          588         160         669         0.0         1027
RNO-Fbxw7   HSA-Fbxw7   97.84       510         7           1           83          588         157         666         0.0         1020
RNO-Fbxw7   PTR-Fbxw7   97.84       510         7           1           83          588         157         666         0.0         1020
RNO-Fbxw7   MMU-Fbxw7   97.84       510         7           1           83          588         157         666         0.0         1020
RNO-Fbxw7   GGO-Fbxw7   97.84       510         7           1           83          588         157         666         0.0         1019
RNO-Fbxw7   CJA-Fbxw7   97.84       510         7           1           83          588         157         666         0.0         1018
# BLASTP 2.2.20 [Feb-08-2009]
# Query: HSA-Fbxw7
# Database: 559_protein.db
# Query id, Subject id, % identity, alignment length, mismatches, gap openings, q. start, q. end, s. start, s. end, e-value, bit score
HSA-Fbxw7   MMU-Fbxw7   99.86       707         1           0           1           707         1           707         0.0         1224
HSA-Fbxw7   HSA-Fbxw7   100.00      707         0           0           1           707         1           707         0.0         1223
HSA-Fbxw7   PTR-Fbxw7   100.00      707         0           0           1           707         1           707         0.0         1223
HSA-Fbxw7   GGO-Fbxw7   99.86       707         1           0           1           707         1           707         0.0         1222
HSA-Fbxw7   CJA-Fbxw7   99.15       707         6           0           1           707         1           707         0.0         1218
HSA-Fbxw7   MUS-Fbxw7   98.93       559         6           0           149         707         152         710         0.0         1137
HSA-Fbxw7   PPY-Fbxw7   100.00      541         0           0           167         707         87          627         0.0         1101
HSA-Fbxw7   RNO-Fbxw7   99.20       500         4           0           167         666         89          588         0.0         1014
# BLASTP 2.2.20 [Feb-08-2009]
# Query: CJA-Fbxw8
# Database: 559_protein.db
# Query id, Subject id, % identity, alignment length, mismatches, gap openings, q. start, q. end, s. start, s. end, e-value, bit score
CJA-Fbxw8   CJA-Fbxw8   100.00      587         0           0           1           587         1           587         0.0         1135
CJA-Fbxw8   PTR-Fbxw8   90.62       597         46          2           1           587         1           597         0.0         1036
CJA-Fbxw8   MMU-Fbxw8   89.97       598         49          2           1           587         1           598         0.0         1032
CJA-Fbxw8   HSA-Fbxw8   90.13       598         48          2           1           587         1           598         0.0         1030
CJA-Fbxw8   PPY-Fbxw8   89.61       597         51          3           1           587         1           596         0.0         1024
CJA-Fbxw8   GGO-Fbxw8   92.48       492         36          1           97          587         15          506         0.0          946
CJA-Fbxw8   MUS-Fbxw8   74.58       598         141         3           1           587         1           598         0.0          843
CJA-Fbxw8   RNO-Fbxw8   73.87       597         145         3           1           587         1           596         0.0          840
# BLASTP 2.2.20 [Feb-08-2009]
# Query: GGO-Fbxw8
# Database: 559_protein.db
# Query id, Subject id, % identity, alignment length, mismatches, gap openings, q. start, q. end, s. start, s. end, e-value, bit score
GGO-Fbxw8   GGO-Fbxw8   100.00      506         0           0           1           506         1           506         0.0         1053
GGO-Fbxw8   PTR-Fbxw8   99.19       492         4           0           15          506         106         597         0.0         1019
GGO-Fbxw8   HSA-Fbxw8   98.98       492         5           0           15          506         107         598         0.0         1017
GGO-Fbxw8   PPY-Fbxw8   98.78       492         6           0           15          506         105         596         0.0         1010
GGO-Fbxw8   MMU-Fbxw8   98.37       492         8           0           15          506         107         598         0.0         1009
GGO-Fbxw8   CJA-Fbxw8   92.48       492         36          1           15          506         97          587         0.0          947
GGO-Fbxw8   MUS-Fbxw8   81.10       492         93          0           15          506         107         598         0.0          832
GGO-Fbxw8   RNO-Fbxw8   79.88       492         99          0           15          506         105         596         0.0          822
```

```
# BLASTP 2.2.20 [Feb-08-2009]
# Query: MMU-Fbxw8
# Database: 559_protein.db
# Query id, Subject id, % identity, alignment length, mismatches, gap openings, q. start, q. end, s. start, s. end, e-value, bit score
MMU-Fbxw8   MMU-Fbxw8   100.00      598         0           0           1           598         1           598         0.0          1143
MMU-Fbxw8   HSA-Fbxw8   97.66       598         14          0           1           598         1           598         0.0          1119
MMU-Fbxw8   PTR-Fbxw8   97.83       598         12          1           1           598         1           597         0.0          1115
MMU-Fbxw8   PPY-Fbxw8   96.66       598         18          2           1           598         1           596         0.0          1098
MMU-Fbxw8   CJA-Fbxw8   89.97       598         49          2           1           598         1           587         0.0          1028
MMU-Fbxw8   GGO-Fbxw8   98.37       492         8           0           107         598         15          506         0.0          1008
MMU-Fbxw8   MUS-Fbxw8   78.13       599         129         2           1           598         1           598         0.0           893
MMU-Fbxw8   RNO-Fbxw8   77.59       598         132         2           1           598         1           596         0.0           885
# BLASTP 2.2.20 [Feb-08-2009]
# Query: MUS-Fbxw8
# Database: 559_protein.db
# Query id, Subject id, % identity, alignment length, mismatches, gap openings, q. start, q. end, s. start, s. end, e-value, bit score
MUS-Fbxw8   MUS-Fbxw8   100.00      598         0           0           1           598         1           598         0.0          1051
MUS-Fbxw8   RNO-Fbxw8   92.14       598         45          2           1           598         1           596         0.0          1001
MUS-Fbxw8   HSA-Fbxw8   76.92       598         138         0           1           598         1           598         0.0           842
MUS-Fbxw8   PTR-Fbxw8   77.26       598         135         1           1           598         1           597         0.0           840
MUS-Fbxw8   MMU-Fbxw8   77.09       598         137         0           1           598         1           598         0.0           839
MUS-Fbxw8   PPY-Fbxw8   76.42       598         139         1           1           598         1           596         0.0           823
MUS-Fbxw8   GGO-Fbxw8   81.10       492         93          0           107         598         15          506         0.0           807
MUS-Fbxw8   CJA-Fbxw8   78.53       503         107         1           96          598         86          587         0.0           802
# BLASTP 2.2.20 [Feb-08-2009]
# Query: PPY-Fbxw8
# Database: 559_protein.db
# Query id, Subject id, % identity, alignment length, mismatches, gap openings, q. start, q. end, s. start, s. end, e-value, bit score
PPY-Fbxw8   PPY-Fbxw8   100.00      596         0           0           1           596         1           596         0.0          1151
PPY-Fbxw8   HSA-Fbxw8   97.99       598         10          2           1           596         1           598         0.0          1118
PPY-Fbxw8   PTR-Fbxw8   98.16       598         8           3           1           596         1           597         0.0          1115
PPY-Fbxw8   MMU-Fbxw8   96.66       598         18          2           1           596         1           598         0.0          1104
PPY-Fbxw8   CJA-Fbxw8   89.61       597         51          3           1           596         1           587         0.0          1023
PPY-Fbxw8   GGO-Fbxw8   98.78       492         6           0           105         596         15          506         0.0          1008
PPY-Fbxw8   MUS-Fbxw8   77.09       598         135         1           1           596         1           598         0.0           890
PPY-Fbxw8   RNO-Fbxw8   76.72       597         137         2           1           596         1           596         0.0           879
# BLASTP 2.2.20 [Feb-08-2009]
# Query: PTR-Fbxw8
# Database: 559_protein.db
# Query id, Subject id, % identity, alignment length, mismatches, gap openings, q. start, q. end, s. start, s. end, e-value, bit score
PTR-Fbxw8   PTR-Fbxw8   100.00      597         0           0           1           597         1           597         0.0          1152
PTR-Fbxw8   HSA-Fbxw8   99.50       598         2           1           1           597         1           598         0.0          1142
PTR-Fbxw8   MMU-Fbxw8   97.83       598         12          1           1           597         1           598         0.0          1124
PTR-Fbxw8   PPY-Fbxw8   98.16       598         8           3           1           597         1           596         0.0          1116
PTR-Fbxw8   CJA-Fbxw8   90.62       597         46          2           1           597         1           587         0.0          1037
```

```
PTR-Fbxw8   GGO-Fbxw8   99.19   492   4     0   106   597   15   506   0.0      1017
PTR-Fbxw8   MUS-Fbxw8   78.09   598   130   1   1     597   1    598   0.0       903
PTR-Fbxw8   RNO-Fbxw8   77.55   597   133   1   1     597   1    596   0.0       895
# BLASTP 2.2.20 [Feb-08-2009]
# Query: RNO-Fbxw8
# Database: 559_protein.db
# Query id, Subject id, % identity, alignment length, mismatches, gap openings, q. start, q. end, s. start, s. end, e-value, bit score
RNO-Fbxw8   RNO-Fbxw8   100.00  596   0     0   1     596   1    596   0.0      1073
RNO-Fbxw8   MUS-Fbxw8   95.46   507   23    0   90    596   92   598   0.0      1011
RNO-Fbxw8   PTR-Fbxw8   80.28   507   100   0   90    596   91   597   0.0       850
RNO-Fbxw8   MMU-Fbxw8   80.08   507   101   0   90    596   92   598   0.0       849
RNO-Fbxw8   HSA-Fbxw8   80.08   507   101   0   90    596   92   598   0.0       849
RNO-Fbxw8   PPY-Fbxw8   73.66   596   157   0   1     596   1    596   0.0       846
RNO-Fbxw8   GGO-Fbxw8   79.88   492   99    0   105   596   15   506   0.0       822
RNO-Fbxw8   CJA-Fbxw8   78.53   503   107   1   94    596   86   587   0.0       816
# BLASTP 2.2.20 [Feb-08-2009]
# Query: HSA-Fbxw8
# Database: 559_protein.db
# Query id, Subject id, % identity, alignment length, mismatches, gap openings, q. start, q. end, s. start, s. end, e-value, bit score
HSA-Fbxw8   HSA-Fbxw8   100.00  598   0     0   1     598   1    598   0.0      1155
HSA-Fbxw8   PTR-Fbxw8   99.50   598   2     1   1     598   1    597   0.0      1142
HSA-Fbxw8   MMU-Fbxw8   97.66   598   14    0   1     598   1    598   0.0      1127
HSA-Fbxw8   PPY-Fbxw8   97.99   598   10    2   1     598   1    596   0.0      1118
HSA-Fbxw8   CJA-Fbxw8   90.13   598   48    2   1     598   1    587   0.0      1031
HSA-Fbxw8   GGO-Fbxw8   98.98   492   5     0   107   598   15   506   0.0      1015
HSA-Fbxw8   MUS-Fbxw8   77.96   599   130   2   1     598   1    598   0.0       899
HSA-Fbxw8   RNO-Fbxw8   77.42   598   133   2   1     598   1    596   0.0       892
# BLASTP 2.2.20 [Feb-08-2009]
# Query: CJA-Fbxw9
# Database: 559_protein.db
# Query id, Subject id, % identity, alignment length, mismatches, gap openings, q. start, q. end, s. start, s. end, e-value, bit score
CJA-Fbxw9   CJA-Fbxw9   100.00  487   0     0   1     487   1    487   0.0       868
CJA-Fbxw9   GGO-Fbxw9   87.50   488   60    1   1     487   1    488   0.0       724
CJA-Fbxw9   HSA-Fbxw9   86.68   488   64    1   1     487   1    488   0.0       717
CJA-Fbxw9   MMU-Fbxw9   82.21   489   75    2   1     487   1    479   0.0       693
CJA-Fbxw9   PPY-Fbxw9   82.96   487   54    1   1     487   1    458   0.0       686
CJA-Fbxw9   PTR-Fbxw9   82.17   488   57    2   1     487   1    459   0.0       660
CJA-Fbxw9   RNO-Fbxw9   72.07   487   107   1   1     487   1    458   4e-180    622
CJA-Fbxw9   MUS-Fbxw9   73.20   459   94    1   29    487   29   458   9e-179    618
# BLASTP 2.2.20 [Feb-08-2009]
# Query: GGO-Fbxw9
# Database: 559_protein.db
# Query id, Subject id, % identity, alignment length, mismatches, gap openings, q. start, q. end, s. start, s. end, e-value, bit score
GGO-Fbxw9   GGO-Fbxw9   100.00  488   0     0   1     488   1    488   0.0       817
GGO-Fbxw9   HSA-Fbxw9   98.57   488   7     0   1     488   1    488   0.0       806
```

GGO-Fbxw9	PPY-Fbxw9	92.42	488	7	1	1	488	1	458	0.0	748
GGO-Fbxw9	PTR-Fbxw9	92.64	489	5	2	1	488	1	459	0.0	731
GGO-Fbxw9	MMU-Fbxw9	89.16	489	42	2	1	488	1	479	0.0	728
GGO-Fbxw9	CJA-Fbxw9	87.50	488	60	1	1	488	1	487	0.0	712
GGO-Fbxw9	RNO-Fbxw9	73.77	488	98	1	1	488	1	458	2e-178	617
GGO-Fbxw9	MUS-Fbxw9	75.22	460	84	1	29	488	29	458	8e-177	611

BLASTP 2.2.20 [Feb-08-2009]
Query: MMU-Fbxw9
Database: 559_protein.db
Query id, Subject id, % identity, alignment length, mismatches, gap openings, q. start, q. end, s. start, s. end, e-value, bit score

MMU-Fbxw9	MMU-Fbxw9	100.00	479	0	0	1	479	1	479	0.0	824
MMU-Fbxw9	GGO-Fbxw9	88.34	489	46	2	1	479	1	488	0.0	711
MMU-Fbxw9	HSA-Fbxw9	87.53	489	50	2	1	479	1	488	0.0	705
MMU-Fbxw9	PPY-Fbxw9	84.05	489	37	2	1	479	1	458	0.0	660
MMU-Fbxw9	CJA-Fbxw9	82.21	489	75	2	1	479	1	487	0.0	660
MMU-Fbxw9	PTR-Fbxw9	82.04	490	46	3	1	479	1	459	0.0	641
MMU-Fbxw9	RNO-Fbxw9	68.92	489	111	2	1	479	1	458	6e-164	568
MMU-Fbxw9	MUS-Fbxw9	69.63	461	99	2	29	479	29	458	4e-162	562

BLASTP 2.2.20 [Feb-08-2009]
Query: MUS-Fbxw9
Database: 559_protein.db
Query id, Subject id, % identity, alignment length, mismatches, gap openings, q. start, q. end, s. start, s. end, e-value, bit score

MUS-Fbxw9	MUS-Fbxw9	100.00	458	0	0	1	458	1	458	0.0	874
MUS-Fbxw9	RNO-Fbxw9	94.54	458	25	0	1	458	1	458	0.0	835
MUS-Fbxw9	PPY-Fbxw9	77.95	458	101	0	1	458	1	458	0.0	672
MUS-Fbxw9	PTR-Fbxw9	76.47	459	107	1	1	458	1	459	0.0	650
MUS-Fbxw9	GGO-Fbxw9	73.57	488	99	1	1	458	1	488	0.0	640
MUS-Fbxw9	HSA-Fbxw9	72.75	488	103	1	1	458	1	488	0.0	636
MUS-Fbxw9	CJA-Fbxw9	71.72	488	107	3	1	458	1	487	0.0	627
MUS-Fbxw9	MMU-Fbxw9	69.63	461	99	2	29	458	29	479	3e-175	606

BLASTP 2.2.20 [Feb-08-2009]
Query: PPY-Fbxw9
Database: 559_protein.db
Query id, Subject id, % identity, alignment length, mismatches, gap openings, q. start, q. end, s. start, s. end, e-value, bit score

PPY-Fbxw9	PPY-Fbxw9	100.00	458	0	0	1	458	1	458	0.0	749
PPY-Fbxw9	PTR-Fbxw9	97.60	459	10	1	1	458	1	459	0.0	734
PPY-Fbxw9	GGO-Fbxw9	92.42	488	7	1	1	458	1	488	0.0	720
PPY-Fbxw9	HSA-Fbxw9	92.01	488	9	1	1	458	1	488	0.0	719
PPY-Fbxw9	MMU-Fbxw9	83.44	489	40	2	1	458	1	479	0.0	643
PPY-Fbxw9	CJA-Fbxw9	82.96	487	54	1	1	458	1	487	0.0	632
PPY-Fbxw9	RNO-Fbxw9	78.17	458	100	0	1	458	1	458	1e-175	607
PPY-Fbxw9	MUS-Fbxw9	80.23	430	85	0	29	458	29	458	1e-174	604

BLASTP 2.2.20 [Feb-08-2009]
Query: PTR-Fbxw9
Database: 559_protein.db

```
# Query id, Subject id, % identity, alignment length, mismatches, gap openings, q. start, q. end, s. start, s. end, e-value, bit score
PTR-Fbxw9   PPY-Fbxw9   95.86    459   18    1   1    459   1    458    0.0      776
PTR-Fbxw9   PTR-Fbxw9   100.00   459   0     0   1    459   1    459    0.0      773
PTR-Fbxw9   HSA-Fbxw9   91.00    489   13    2   1    459   1    488    0.0      749
PTR-Fbxw9   GGO-Fbxw9   91.00    489   13    2   1    459   1    488    0.0      748
PTR-Fbxw9   CJA-Fbxw9   80.53    488   65    2   1    459   1    487    0.0      662
PTR-Fbxw9   MMU-Fbxw9   82.86    490   42    3   1    459   1    479    0.0      659
PTR-Fbxw9   RNO-Fbxw9   78.21    459   99    1   1    459   1    458    0.0      632
PTR-Fbxw9   MUS-Fbxw9   78.00    459   100   1   1    459   1    458    0.0      626
# BLASTP 2.2.20 [Feb-08-2009]
# Query: RNO-Fbxw9
# Database: 559_protein.db
# Query id, Subject id, % identity, alignment length, mismatches, gap openings, q. start, q. end, s. start, s. end, e-value, bit score
RNO-Fbxw9   RNO-Fbxw9   100.00   458   0     0   1    458   1    458    0.0      876
RNO-Fbxw9   MUS-Fbxw9   94.54    458   25    0   1    458   1    458    0.0      835
RNO-Fbxw9   PPY-Fbxw9   78.17    458   100   0   1    458   1    458    0.0      677
RNO-Fbxw9   PTR-Fbxw9   76.25    459   108   1   1    458   1    459    0.0      655
RNO-Fbxw9   GGO-Fbxw9   73.77    488   98    1   1    458   1    488    0.0      644
RNO-Fbxw9   HSA-Fbxw9   72.95    488   102   1   1    458   1    488    0.0      640
RNO-Fbxw9   CJA-Fbxw9   72.07    487   107   1   1    458   1    487    0.0      634
RNO-Fbxw9   MMU-Fbxw9   68.92    489   111   2   1    458   1    479    9e-177   611
# BLASTP 2.2.20 [Feb-08-2009]
# Query: HSA-Fbxw9
# Database: 559_protein.db
# Query id, Subject id, % identity, alignment length, mismatches, gap openings, q. start, q. end, s. start, s. end, e-value, bit score
HSA-Fbxw9   HSA-Fbxw9   100.00   488   0     0   1    488   1    488    0.0      822
HSA-Fbxw9   GGO-Fbxw9   98.57    488   7     0   1    488   1    488    0.0      810
HSA-Fbxw9   PPY-Fbxw9   92.01    488   9     1   1    488   1    458    0.0      752
HSA-Fbxw9   PTR-Fbxw9   92.64    489   5     2   1    488   1    459    0.0      738
HSA-Fbxw9   MMU-Fbxw9   88.34    489   46    2   1    488   1    479    0.0      726
HSA-Fbxw9   CJA-Fbxw9   86.68    488   64    1   1    488   1    487    0.0      707
HSA-Fbxw9   RNO-Fbxw9   72.95    488   102   1   1    488   1    458    1e-175   607
HSA-Fbxw9   MUS-Fbxw9   74.35    460   88    1   29   488   29   458    3e-174   602
# BLASTP 2.2.20 [Feb-08-2009]
# Query: CJA-Kdm2A
# Database: 559_protein.db
# Query id, Subject id, % identity, alignment length, mismatches, gap openings, q. start, q. end, s. start, s. end, e-value, bit score
CJA-Kdm2A   CJA-Kdm2A   100.00   1160   0    0   1    1160  1    1160   0.0      2145
CJA-Kdm2A   HSA-Kdm2A   98.80    1162   12   1   1    1160  1    1162   0.0      2141
CJA-Kdm2A   PTR-Kdm2A   98.80    1162   12   1   1    1160  1    1162   0.0      2141
CJA-Kdm2A   MMU-Kdm2A   98.71    1162   13   1   1    1160  1    1162   0.0      2136
CJA-Kdm2A   RNO-Kdm2A   97.42    1161   29   1   1    1160  1    1161   0.0      2108
CJA-Kdm2A   MUS-Kdm2A   96.99    1161   34   1   1    1160  1    1161   0.0      2107
CJA-Kdm2A   PPY-Kdm2A   98.36    669    9    1   494  1160  1    669    0.0      1235
CJA-Kdm2A   GGO-Kdm2A   94.08    439    8    1   722  1160  861  1281   0.0      794
```

CJA-Kdm2A	GGO-Kdm2A	75.93	241	40	4	488	727	519	742	2e-090	325
CJA-Kdm2A	GGO-Kdm2A	53.53	368	159	8	5	363	2	366	3e-079	288
CJA-Kdm2A	PTR-Kdm2B	58.75	686	231	13	36	687	66	733	0.0	746
CJA-Kdm2A	PTR-Kdm2B	67.27	278	89	2	885	1160	1059	1336	4e-107	381
CJA-Kdm2A	HSA-Kdm2B	58.45	686	233	12	36	687	66	733	0.0	745
CJA-Kdm2A	HSA-Kdm2B	67.27	278	89	2	885	1160	1059	1336	4e-107	381
CJA-Kdm2A	PPY-Kdm2B	58.31	686	234	12	36	687	66	733	0.0	741
CJA-Kdm2A	PPY-Kdm2B	51.44	278	77	5	885	1160	1059	1280	2e-067	249
CJA-Kdm2A	MMU-Kdm2B	58.55	661	222	13	61	687	90	732	0.0	711
CJA-Kdm2A	MMU-Kdm2B	65.47	278	91	4	885	1160	1058	1332	6e-101	360
CJA-Kdm2A	CJA-Kdm2B	56.03	680	250	13	40	688	68	729	0.0	675
CJA-Kdm2A	CJA-Kdm2B	67.27	278	89	2	885	1160	1057	1334	5e-107	381
CJA-Kdm2A	MUS-Kdm2B	71.15	364	100	2	1	362	1	361	2e-155	542
CJA-Kdm2A	MUS-Kdm2B	66.91	278	90	2	885	1160	1032	1309	4e-106	378
CJA-Kdm2A	MUS-Kdm2B	53.91	243	90	5	451	687	480	706	7e-060	224
CJA-Kdm2A	RNO-Kdm2B	67.58	364	113	2	1	362	1	361	2e-144	505
CJA-Kdm2A	RNO-Kdm2B	66.91	278	90	2	885	1160	1028	1305	2e-106	379
CJA-Kdm2A	RNO-Kdm2B	54.51	244	87	6	451	687	476	702	9e-061	227
CJA-Kdm2A	GGO-Kdm2B	67.27	278	89	2	885	1160	975	1252	3e-107	382
CJA-Kdm2A	GGO-Kdm2B	72.22	162	45	0	36	197	66	227	6e-063	234
CJA-Kdm2A	GGO-Kdm2B	54.10	244	88	6	451	687	423	649	1e-060	226
CJA-Kdm2A	CJA-Fbxl19	51.62	277	131	3	886	1160	419	694	4e-076	278
CJA-Kdm2A	MUS-Fbxl19	51.62	277	131	3	886	1160	399	674	5e-076	278
CJA-Kdm2A	HSA-Fbxl19	51.62	277	131	3	886	1160	419	694	6e-076	277
CJA-Kdm2A	PPY-Fbxl19	51.62	277	131	3	886	1160	355	630	6e-076	277
CJA-Kdm2A	RNO-Fbxl19	51.62	277	131	3	886	1160	399	674	8e-076	277
CJA-Kdm2A	MMU-Fbxl19	51.62	277	131	3	886	1160	309	584	9e-076	277
CJA-Kdm2A	PTR-Fbxl19	51.62	277	131	3	886	1160	147	422	9e-076	277
CJA-Kdm2A	GGO-Fbxl19	56.42	218	93	2	886	1101	401	618	4e-065	242

BLASTP 2.2.20 [Feb-08-2009]
Query: GGO-Kdm2A
Database: 559_protein.db
Query id, Subject id, % identity, alignment length, mismatches, gap openings, q. start, q. end, s. start, s. end, e-value, bit score

GGO-Kdm2A	GGO-Kdm2A	100.00	1281	0	0	1	1281	1	1281	0.0	2534
GGO-Kdm2A	MMU-Kdm2A	94.33	441	5	1	861	1281	722	1162	0.0	803
GGO-Kdm2A	MMU-Kdm2A	75.52	241	41	4	519	742	488	727	4e-090	325
GGO-Kdm2A	MMU-Kdm2A	53.53	368	159	8	2	366	5	363	8e-080	290
GGO-Kdm2A	HSA-Kdm2A	94.33	441	5	1	861	1281	722	1162	0.0	802
GGO-Kdm2A	HSA-Kdm2A	53.09	776	276	21	2	742	5	727	4e-169	587
GGO-Kdm2A	PTR-Kdm2A	94.33	441	5	1	861	1281	722	1162	0.0	802
GGO-Kdm2A	PTR-Kdm2A	53.09	776	276	21	2	742	5	727	4e-169	587
GGO-Kdm2A	PPY-Kdm2A	94.33	441	5	1	861	1281	229	669	0.0	801
GGO-Kdm2A	PPY-Kdm2A	73.02	252	50	4	525	759	1	251	4e-089	322
GGO-Kdm2A	CJA-Kdm2A	94.53	439	6	1	861	1281	722	1160	0.0	801
GGO-Kdm2A	CJA-Kdm2A	52.90	775	279	19	2	742	5	727	4e-169	587

GGO-Kdm2A	RNO-Kdm2A	92.95	440	12	1	861	1281	722	1161	0.0	790
GGO-Kdm2A	RNO-Kdm2A	52.26	775	284	18	2	742	5	727	8e-168	583
GGO-Kdm2A	MUS-Kdm2A	92.50	440	14	1	861	1281	722	1161	0.0	788
GGO-Kdm2A	MUS-Kdm2A	52.06	776	284	20	2	742	5	727	1e-166	579
GGO-Kdm2A	CJA-Fbxl19	51.62	277	131	3	1007	1281	419	694	4e-076	278
GGO-Kdm2A	HSA-Fbxl19	51.62	277	131	3	1007	1281	419	694	6e-076	278
GGO-Kdm2A	PPY-Fbxl19	51.62	277	131	3	1007	1281	355	630	7e-076	277
GGO-Kdm2A	MUS-Fbxl19	51.62	277	131	3	1007	1281	399	674	8e-076	277
GGO-Kdm2A	RNO-Fbxl19	51.62	277	131	3	1007	1281	399	674	8e-076	277
GGO-Kdm2A	PTR-Fbxl19	51.62	277	131	3	1007	1281	147	422	2e-075	276
GGO-Kdm2A	MMU-Fbxl19	51.62	277	131	3	1007	1281	309	584	2e-075	276
GGO-Kdm2A	GGO-Fbxl19	56.42	218	93	2	1007	1222	401	618	4e-065	242

BLASTP 2.2.20 [Feb-08-2009]
Query: MMU-Kdm2A
Database: 559_protein.db
Query id, Subject id, % identity, alignment length, mismatches, gap openings, q. start, q. end, s. start, s. end, e-value, bit score

MMU-Kdm2A	MMU-Kdm2A	100.00	1162	0	0	1	1162	1	1162	0.0	2142
MMU-Kdm2A	HSA-Kdm2A	99.57	1162	5	0	1	1162	1	1162	0.0	2139
MMU-Kdm2A	PTR-Kdm2A	99.57	1162	5	0	1	1162	1	1162	0.0	2139
MMU-Kdm2A	CJA-Kdm2A	98.80	1162	12	1	1	1162	1	1160	0.0	2131
MMU-Kdm2A	RNO-Kdm2A	97.16	1162	32	1	1	1162	1	1161	0.0	2104
MMU-Kdm2A	MUS-Kdm2A	96.82	1162	36	1	1	1162	1	1161	0.0	2103
MMU-Kdm2A	PPY-Kdm2A	99.70	669	2	0	494	1162	1	669	0.0	1236
MMU-Kdm2A	GGO-Kdm2A	93.65	441	8	1	722	1162	861	1281	0.0	791
MMU-Kdm2A	GGO-Kdm2A	75.52	241	41	4	488	727	519	742	5e-090	324
MMU-Kdm2A	GGO-Kdm2A	53.53	368	159	8	5	363	2	366	3e-079	288
MMU-Kdm2A	PTR-Kdm2B	58.75	686	231	13	36	687	66	733	0.0	747
MMU-Kdm2A	PTR-Kdm2B	67.27	278	89	2	887	1162	1059	1336	3e-107	381
MMU-Kdm2A	HSA-Kdm2B	58.45	686	233	12	36	687	66	733	0.0	746
MMU-Kdm2A	HSA-Kdm2B	67.27	278	89	2	887	1162	1059	1336	4e-107	381
MMU-Kdm2A	PPY-Kdm2B	58.31	686	234	12	36	687	66	733	0.0	742
MMU-Kdm2A	PPY-Kdm2B	51.44	278	77	5	887	1162	1059	1280	2e-067	249
MMU-Kdm2A	MMU-Kdm2B	58.55	661	222	13	61	687	90	732	0.0	712
MMU-Kdm2A	MMU-Kdm2B	65.47	278	91	4	887	1162	1058	1332	7e-101	360
MMU-Kdm2A	CJA-Kdm2B	56.18	680	249	13	40	688	68	729	0.0	677
MMU-Kdm2A	CJA-Kdm2B	67.27	278	89	2	887	1162	1057	1334	4e-107	381
MMU-Kdm2A	MUS-Kdm2B	71.15	364	100	2	1	362	1	361	1e-155	542
MMU-Kdm2A	MUS-Kdm2B	66.91	278	90	2	887	1162	1032	1309	3e-106	378
MMU-Kdm2A	MUS-Kdm2B	53.91	243	90	5	451	687	480	706	7e-060	224
MMU-Kdm2A	RNO-Kdm2B	67.58	364	113	2	1	362	1	361	2e-144	505
MMU-Kdm2A	RNO-Kdm2B	66.91	278	90	2	887	1162	1028	1305	2e-106	379
MMU-Kdm2A	RNO-Kdm2B	54.51	244	87	6	451	687	476	702	9e-061	227
MMU-Kdm2A	GGO-Kdm2B	67.27	278	89	2	887	1162	975	1252	3e-107	382
MMU-Kdm2A	GGO-Kdm2B	72.22	162	45	0	36	197	66	227	5e-063	234
MMU-Kdm2A	CJA-Fbxl19	51.62	277	131	3	888	1162	419	694	3e-076	278

MMU-Kdm2A	MUS-Fbxl19	51.62	277	131	3	888	1162	399	674	4e-076	278
MMU-Kdm2A	RNO-Fbxl19	51.62	277	131	3	888	1162	399	674	5e-076	278
MMU-Kdm2A	HSA-Fbxl19	51.62	277	131	3	888	1162	419	694	6e-076	278
MMU-Kdm2A	PPY-Fbxl19	51.62	277	131	3	888	1162	355	630	7e-076	277
MMU-Kdm2A	MMU-Fbxl19	51.62	277	131	3	888	1162	309	584	7e-076	277
MMU-Kdm2A	PTR-Fbxl19	51.62	277	131	3	888	1162	147	422	1e-075	277
MMU-Kdm2A	GGO-Fbxl19	56.42	218	93	2	888	1103	401	618	3e-065	242

BLASTP 2.2.20 [Feb-08-2009]
Query: MUS-Kdm2A
Database: 559_protein.db
Query id, Subject id, % identity, alignment length, mismatches, gap openings, q. start, q. end, s. start, s. end, e-value, bit score

MUS-Kdm2A	MUS-Kdm2A	100.00	1161	0	0	1	1161	1	1161	0.0	2185
MUS-Kdm2A	RNO-Kdm2A	99.05	1161	11	0	1	1161	1	1161	0.0	2170
MUS-Kdm2A	HSA-Kdm2A	96.90	1162	35	1	1	1161	1	1162	0.0	2131
MUS-Kdm2A	PTR-Kdm2A	96.90	1162	35	1	1	1161	1	1162	0.0	2131
MUS-Kdm2A	CJA-Kdm2A	96.90	1161	35	1	1	1161	1	1160	0.0	2127
MUS-Kdm2A	MMU-Kdm2A	96.82	1162	36	1	1	1161	1	1162	0.0	2126
MUS-Kdm2A	PPY-Kdm2A	97.16	669	18	1	494	1161	1	669	0.0	1215
MUS-Kdm2A	GGO-Kdm2A	92.05	440	16	1	722	1161	861	1281	0.0	779
MUS-Kdm2A	GGO-Kdm2A	75.10	241	42	4	488	727	519	742	2e-089	322
MUS-Kdm2A	GGO-Kdm2A	53.01	366	160	8	5	361	2	364	1e-077	283
MUS-Kdm2A	PTR-Kdm2B	58.60	686	232	12	36	687	66	733	0.0	748
MUS-Kdm2A	PTR-Kdm2B	67.99	278	87	2	886	1161	1059	1336	4e-108	384
MUS-Kdm2A	PPY-Kdm2B	58.52	687	231	12	36	687	66	733	0.0	747
MUS-Kdm2A	PPY-Kdm2B	52.16	278	75	5	886	1161	1059	1280	1e-068	253
MUS-Kdm2A	HSA-Kdm2B	58.45	686	233	12	36	687	66	733	0.0	747
MUS-Kdm2A	HSA-Kdm2B	67.99	278	87	2	886	1161	1059	1336	4e-108	384
MUS-Kdm2A	MUS-Kdm2B	55.65	726	263	11	1	687	1	706	0.0	737
MUS-Kdm2A	MUS-Kdm2B	67.27	278	89	2	886	1161	1032	1309	8e-107	380
MUS-Kdm2A	MMU-Kdm2B	58.61	662	220	12	61	687	90	732	0.0	716
MUS-Kdm2A	MMU-Kdm2B	66.19	278	89	4	886	1161	1058	1332	6e-102	364
MUS-Kdm2A	RNO-Kdm2B	54.06	727	269	14	1	687	1	702	0.0	704
MUS-Kdm2A	RNO-Kdm2B	67.63	278	88	2	886	1161	1028	1305	1e-107	382
MUS-Kdm2A	CJA-Kdm2B	55.98	686	241	14	40	688	68	729	0.0	679
MUS-Kdm2A	CJA-Kdm2B	67.99	278	87	2	886	1161	1057	1334	3e-108	385
MUS-Kdm2A	GGO-Kdm2B	67.99	278	87	2	886	1161	975	1252	2e-108	385
MUS-Kdm2A	GGO-Kdm2B	72.22	162	45	0	36	197	66	227	4e-063	235
MUS-Kdm2A	CJA-Fbxl19	51.26	277	132	3	887	1161	419	694	4e-076	278
MUS-Kdm2A	HSA-Fbxl19	51.26	277	132	3	887	1161	419	694	6e-076	278
MUS-Kdm2A	RNO-Fbxl19	51.26	277	132	3	887	1161	399	674	7e-076	277
MUS-Kdm2A	MUS-Fbxl19	51.26	277	132	3	887	1161	399	674	7e-076	277
MUS-Kdm2A	PPY-Fbxl19	51.26	277	132	3	887	1161	355	630	9e-076	277
MUS-Kdm2A	PTR-Fbxl19	51.26	277	132	3	887	1161	147	422	1e-075	276
MUS-Kdm2A	MMU-Fbxl19	51.26	277	132	3	887	1161	309	584	1e-075	276
MUS-Kdm2A	GGO-Fbxl19	55.96	218	94	2	887	1102	401	618	5e-065	241

```
# BLASTP 2.2.20 [Feb-08-2009]
# Query: PPY-Kdm2A
# Database: 559_protein.db
# Query id, Subject id, % identity, alignment length, mismatches, gap openings, q. start, q. end, s. start, s. end, e-value, bit score
PPY-Kdm2A   HSA-Kdm2A   100.00      669         0           0           1           669         494         1162        0.0          1240
PPY-Kdm2A   PTR-Kdm2A   100.00      669         0           0           1           669         494         1162        0.0          1240
PPY-Kdm2A   MMU-Kdm2A   99.70       669         2           0           1           669         494         1162        0.0          1239
PPY-Kdm2A   PPY-Kdm2A   100.00      669         0           0           1           669         1           669         0.0          1236
PPY-Kdm2A   CJA-Kdm2A   98.51       669         8           1           1           669         494         1160        0.0          1234
PPY-Kdm2A   RNO-Kdm2A   97.46       669         16          1           1           669         494         1161        0.0          1219
PPY-Kdm2A   MUS-Kdm2A   97.01       669         19          1           1           669         494         1161        0.0          1216
PPY-Kdm2A   GGO-Kdm2A   93.88       441         7           1           229         669         861         1281        0.0           795
PPY-Kdm2A   GGO-Kdm2A   73.02       252         50          4           1           251         525         759         2e-089        321
PPY-Kdm2A   GGO-Kdm2B   67.27       278         89          2           394         669         975         1252        2e-107        381
PPY-Kdm2A   CJA-Kdm2B   67.27       278         89          2           394         669         1057        1334        2e-107        381
PPY-Kdm2A   PTR-Kdm2B   67.27       278         89          2           394         669         1059        1336        3e-107        381
PPY-Kdm2A   HSA-Kdm2B   67.27       278         89          2           394         669         1059        1336        3e-107        381
PPY-Kdm2A   RNO-Kdm2B   66.91       278         90          2           394         669         1028        1305        1e-106        379
PPY-Kdm2A   MUS-Kdm2B   66.91       278         90          2           394         669         1032        1309        2e-106        378
PPY-Kdm2A   MMU-Kdm2B   65.47       278         91          4           394         669         1058        1332        5e-101        360
PPY-Kdm2A   CJA-Fbxl19  51.62       277         131         3           395         669         419         694         9e-076        276
PPY-Kdm2A   HSA-Fbxl19  51.62       277         131         3           395         669         419         694         1e-075        275
PPY-Kdm2A   PPY-Fbxl19  51.62       277         131         3           395         669         355         630         2e-075        275
PPY-Kdm2A   MUS-Fbxl19  51.62       277         131         3           395         669         399         674         2e-075        275
PPY-Kdm2A   RNO-Fbxl19  51.62       277         131         3           395         669         399         674         2e-075        275
PPY-Kdm2A   MMU-Fbxl19  51.62       277         131         3           395         669         309         584         3e-075        274
PPY-Kdm2A   PTR-Fbxl19  51.62       277         131         3           395         669         147         422         3e-075        274
PPY-Kdm2A   PPY-Kdm2B   51.44       278         77          5           394         669         1059        1280        9e-068        249
PPY-Kdm2A   GGO-Fbxl19  56.42       218         93          2           395         610         401         618         4e-065        241
# BLASTP 2.2.20 [Feb-08-2009]
# Query: PTR-Kdm2A
# Database: 559_protein.db
# Query id, Subject id, % identity, alignment length, mismatches, gap openings, q. start, q. end, s. start, s. end, e-value, bit score
PTR-Kdm2A   HSA-Kdm2A   100.00      1162        0           0           1           1162        1           1162        0.0          2189
PTR-Kdm2A   PTR-Kdm2A   100.00      1162        0           0           1           1162        1           1162        0.0          2189
PTR-Kdm2A   MMU-Kdm2A   99.57       1162        5           0           1           1162        1           1162        0.0          2157
PTR-Kdm2A   CJA-Kdm2A   98.88       1162        11          1           1           1162        1           1160        0.0          2152
PTR-Kdm2A   MUS-Kdm2A   96.82       1162        36          1           1           1162        1           1161        0.0          2141
PTR-Kdm2A   RNO-Kdm2A   97.25       1162        31          1           1           1162        1           1161        0.0          2125
PTR-Kdm2A   PPY-Kdm2A   100.00      669         0           0           494         1162        1           669         0.0          1242
PTR-Kdm2A   GGO-Kdm2A   93.88       441         7           1           722         1162        861         1281        0.0           796
PTR-Kdm2A   GGO-Kdm2A   75.93       241         40          4           488         727         519         742         2e-090        325
PTR-Kdm2A   GGO-Kdm2A   53.53       368         159         8           5           363         2           366         2e-079        289
PTR-Kdm2A   PTR-Kdm2B   59.12       685         230         13          36          687         66          733         0.0           754
PTR-Kdm2A   PTR-Kdm2B   67.27       278         89          2           887         1162        1059        1336        4e-107        381
```

PTR-Kdm2A	HSA-Kdm2B	58.83	685	232	12	36	687	66	733	0.0	754
PTR-Kdm2A	HSA-Kdm2B	67.27	278	89	2	887	1162	1059	1336	4e-107	381
PTR-Kdm2A	PPY-Kdm2B	58.83	685	232	12	36	687	66	733	0.0	754
PTR-Kdm2A	PPY-Kdm2B	51.44	278	77	5	887	1162	1059	1280	2e-067	249
PTR-Kdm2A	MUS-Kdm2B	55.86	725	263	12	1	687	1	706	0.0	741
PTR-Kdm2A	MUS-Kdm2B	66.91	278	90	2	887	1162	1032	1309	3e-106	378
PTR-Kdm2A	MMU-Kdm2B	59.09	660	220	13	61	687	90	732	0.0	724
PTR-Kdm2A	MMU-Kdm2B	65.47	278	91	4	887	1162	1058	1332	6e-101	360
PTR-Kdm2A	RNO-Kdm2B	54.57	722	273	12	1	687	1	702	0.0	707
PTR-Kdm2A	RNO-Kdm2B	66.91	278	90	2	887	1162	1028	1305	1e-106	379
PTR-Kdm2A	CJA-Kdm2B	56.74	682	242	15	40	688	68	729	0.0	686
PTR-Kdm2A	CJA-Kdm2B	67.27	278	89	2	887	1162	1057	1334	3e-107	381
PTR-Kdm2A	GGO-Kdm2B	67.27	278	89	2	887	1162	975	1252	2e-107	382
PTR-Kdm2A	GGO-Kdm2B	72.22	162	45	0	36	197	66	227	5e-063	234
PTR-Kdm2A	CJA-Fbxl19	51.62	277	131	3	888	1162	419	694	2e-076	279
PTR-Kdm2A	HSA-Fbxl19	51.62	277	131	3	888	1162	419	694	3e-076	278
PTR-Kdm2A	MUS-Fbxl19	51.62	277	131	3	888	1162	399	674	4e-076	278
PTR-Kdm2A	RNO-Fbxl19	51.62	277	131	3	888	1162	399	674	4e-076	278
PTR-Kdm2A	PPY-Fbxl19	51.62	277	131	3	888	1162	355	630	6e-076	278
PTR-Kdm2A	MMU-Fbxl19	51.62	277	131	3	888	1162	309	584	8e-076	277
PTR-Kdm2A	PTR-Fbxl19	51.62	277	131	3	888	1162	147	422	9e-076	277
PTR-Kdm2A	GGO-Fbxl19	56.42	218	93	2	888	1103	401	618	3e-065	242

BLASTP 2.2.20 [Feb-08-2009]
Query: RNO-Kdm2A
Database: 559_protein.db
Query id, Subject id, % identity, alignment length, mismatches, gap openings, q. start, q. end, s. start, s. end, e-value, bit score

RNO-Kdm2A	RNO-Kdm2A	100.00	1161	0	0	1	1161	1	1161	0.0	2195
RNO-Kdm2A	MUS-Kdm2A	99.05	1161	11	0	1	1161	1	1161	0.0	2181
RNO-Kdm2A	HSA-Kdm2A	97.33	1162	30	1	1	1161	1	1162	0.0	2152
RNO-Kdm2A	PTR-Kdm2A	97.33	1162	30	1	1	1161	1	1162	0.0	2152
RNO-Kdm2A	CJA-Kdm2A	97.33	1161	30	1	1	1161	1	1160	0.0	2148
RNO-Kdm2A	MMU-Kdm2A	97.25	1162	31	1	1	1161	1	1162	0.0	2147
RNO-Kdm2A	PPY-Kdm2A	97.61	669	15	1	494	1161	1	669	0.0	1220
RNO-Kdm2A	GGO-Kdm2A	92.50	440	14	1	722	1161	861	1281	0.0	783
RNO-Kdm2A	GGO-Kdm2A	50.26	776	298	21	5	727	2	742	6e-155	540
RNO-Kdm2A	PTR-Kdm2B	59.27	685	229	12	36	687	66	733	0.0	753
RNO-Kdm2A	PTR-Kdm2B	67.99	278	87	2	886	1161	1059	1336	3e-108	385
RNO-Kdm2A	PPY-Kdm2B	58.98	685	231	12	36	687	66	733	0.0	751
RNO-Kdm2A	PPY-Kdm2B	52.16	278	75	5	886	1161	1059	1280	2e-068	253
RNO-Kdm2A	HSA-Kdm2B	59.12	685	230	12	36	687	66	733	0.0	750
RNO-Kdm2A	HSA-Kdm2B	67.99	278	87	2	886	1161	1059	1336	4e-108	384
RNO-Kdm2A	MUS-Kdm2B	56.38	729	253	13	1	687	1	706	0.0	741
RNO-Kdm2A	MUS-Kdm2B	67.63	278	88	2	886	1161	1032	1309	3e-107	382
RNO-Kdm2A	MMU-Kdm2B	59.09	660	220	12	61	687	90	732	0.0	721
RNO-Kdm2A	MMU-Kdm2B	66.19	278	89	4	886	1161	1058	1332	5e-102	364

```
RNO-Kdm2A   RNO-Kdm2B   55.10   726    263   14   1     687    1      702    0.0       709
RNO-Kdm2A   RNO-Kdm2B   67.63   278    88    2    886   1161   1028   1305   2e-107    382
RNO-Kdm2A   CJA-Kdm2B   56.14   684    243   14   40    688    68     729    0.0       682
RNO-Kdm2A   CJA-Kdm2B   67.99   278    87    2    886   1161   1057   1334   3e-108    385
RNO-Kdm2A   GGO-Kdm2B   67.99   278    87    2    886   1161   975    1252   2e-108    385
RNO-Kdm2A   GGO-Kdm2B   72.22   162    45    0    36    197    66     227    6e-063    234
RNO-Kdm2A   CJA-Fbxl19  51.62   277    131   3    887   1161   419    694    2e-076    280
RNO-Kdm2A   MUS-Fbxl19  51.62   277    131   3    887   1161   399    674    3e-076    278
RNO-Kdm2A   HSA-Fbxl19  51.62   277    131   3    887   1161   419    694    3e-076    278
RNO-Kdm2A   PPY-Fbxl19  51.62   277    131   3    887   1161   355    630    4e-076    278
RNO-Kdm2A   RNO-Fbxl19  51.62   277    131   3    887   1161   399    674    4e-076    278
RNO-Kdm2A   MMU-Fbxl19  51.62   277    131   3    887   1161   309    584    6e-076    278
RNO-Kdm2A   PTR-Fbxl19  51.62   277    131   3    887   1161   147    422    6e-076    278
RNO-Kdm2A   GGO-Fbxl19  56.42   218    93    2    887   1102   401    618    3e-065    242
# BLASTP 2.2.20 [Feb-08-2009]
# Query: HSA-Kdm2A
# Database: 559_protein.db
# Query id, Subject id, % identity, alignment length, mismatches, gap openings, q. start, q. end, s. start, s. end, e-value, bit score
HSA-Kdm2A   HSA-Kdm2A   100.00  1162   0     0    1     1162   1      1162   0.0       2189
HSA-Kdm2A   PTR-Kdm2A   100.00  1162   0     0    1     1162   1      1162   0.0       2189
HSA-Kdm2A   MMU-Kdm2A   99.57   1162   5     0    1     1162   1      1162   0.0       2157
HSA-Kdm2A   CJA-Kdm2A   98.88   1162   11    1    1     1162   1      1160   0.0       2152
HSA-Kdm2A   MUS-Kdm2A   96.82   1162   36    1    1     1162   1      1161   0.0       2141
HSA-Kdm2A   RNO-Kdm2A   97.25   1162   31    1    1     1162   1      1161   0.0       2125
HSA-Kdm2A   PPY-Kdm2A   100.00  669    0     0    494   1162   1      669    0.0       1242
HSA-Kdm2A   GGO-Kdm2A   93.88   441    7     1    722   1162   861    1281   0.0       796
HSA-Kdm2A   GGO-Kdm2A   75.93   241    40    4    488   727    519    742    2e-090    325
HSA-Kdm2A   GGO-Kdm2A   53.53   368    159   8    5     363    2      366    2e-079    289
HSA-Kdm2A   PTR-Kdm2B   59.12   685    230   13   36    687    66     733    0.0       754
HSA-Kdm2A   PTR-Kdm2B   67.27   278    89    2    887   1162   1059   1336   4e-107    381
HSA-Kdm2A   HSA-Kdm2B   58.83   685    232   12   36    687    66     733    0.0       754
HSA-Kdm2A   HSA-Kdm2B   67.27   278    89    2    887   1162   1059   1336   4e-107    381
HSA-Kdm2A   PPY-Kdm2B   58.83   685    232   12   36    687    66     733    0.0       754
HSA-Kdm2A   PPY-Kdm2B   51.44   278    77    5    887   1162   1059   1280   2e-067    249
HSA-Kdm2A   MUS-Kdm2B   55.86   725    263   12   1     687    1      706    0.0       741
HSA-Kdm2A   MUS-Kdm2B   66.91   278    90    2    887   1162   1032   1309   3e-106    378
HSA-Kdm2A   MMU-Kdm2B   59.09   660    220   13   61    687    90     732    0.0       724
HSA-Kdm2A   MMU-Kdm2B   65.47   278    91    4    887   1162   1058   1332   6e-101    360
HSA-Kdm2A   RNO-Kdm2B   54.57   722    273   12   1     687    1      702    0.0       707
HSA-Kdm2A   RNO-Kdm2B   66.91   278    90    2    887   1162   1028   1305   1e-106    379
HSA-Kdm2A   CJA-Kdm2B   56.74   682    242   15   40    688    68     729    0.0       686
HSA-Kdm2A   CJA-Kdm2B   67.27   278    89    2    887   1162   1057   1334   3e-107    381
HSA-Kdm2A   GGO-Kdm2B   67.27   278    89    2    887   1162   975    1252   2e-107    382
HSA-Kdm2A   GGO-Kdm2B   72.22   162    45    0    36    197    66     227    5e-063    234
HSA-Kdm2A   CJA-Fbxl19  51.62   277    131   3    888   1162   419    694    2e-076    279
```

HSA-Kdm2A	HSA-Fbxl19	51.62	277	131	3	888	1162	419	694	3e-076	278
HSA-Kdm2A	MUS-Fbxl19	51.62	277	131	3	888	1162	399	674	4e-076	278
HSA-Kdm2A	RNO-Fbxl19	51.62	277	131	3	888	1162	399	674	4e-076	278
HSA-Kdm2A	PPY-Fbxl19	51.62	277	131	3	888	1162	355	630	6e-076	278
HSA-Kdm2A	MMU-Fbxl19	51.62	277	131	3	888	1162	309	584	8e-076	277
HSA-Kdm2A	PTR-Fbxl19	51.62	277	131	3	888	1162	147	422	9e-076	277
HSA-Kdm2A	GGO-Fbxl19	56.42	218	93	2	888	1103	401	618	3e-065	242

BLASTP 2.2.20 [Feb-08-2009]
Query: CJA-Kdm2B
Database: 559_protein.db
Query id, Subject id, % identity, alignment length, mismatches, gap openings, q. start, q. end, s. start, s. end, e-value, bit score

CJA-Kdm2B	CJA-Kdm2B	100.00	1334	0	0	1	1334	1	1334	0.0	2445
CJA-Kdm2B	PTR-Kdm2B	93.16	1345	72	7	1	1334	1	1336	0.0	2247
CJA-Kdm2B	HSA-Kdm2B	93.16	1345	72	7	1	1334	1	1336	0.0	2246
CJA-Kdm2B	MMU-Kdm2B	91.75	1345	87	10	1	1334	1	1332	0.0	2183
CJA-Kdm2B	RNO-Kdm2B	91.34	1282	93	5	62	1334	33	1305	0.0	2112
CJA-Kdm2B	MUS-Kdm2B	90.44	1286	101	6	62	1334	33	1309	0.0	2098
CJA-Kdm2B	PPY-Kdm2B	88.25	1345	82	9	1	1334	1	1280	0.0	2090
CJA-Kdm2B	GGO-Kdm2B	97.18	1029	16	3	315	1334	228	1252	0.0	1759
CJA-Kdm2B	GGO-Kdm2B	86.34	227	29	1	1	225	1	227	8e-108	384
CJA-Kdm2B	HSA-Kdm2A	56.54	688	234	16	68	729	40	688	0.0	712
CJA-Kdm2B	HSA-Kdm2A	67.15	277	89	2	1058	1334	888	1162	5e-113	401
CJA-Kdm2B	PTR-Kdm2A	56.54	688	234	16	68	729	40	688	0.0	712
CJA-Kdm2B	PTR-Kdm2A	67.15	277	89	2	1058	1334	888	1162	5e-113	401
CJA-Kdm2B	MUS-Kdm2A	56.10	688	237	15	68	729	40	688	0.0	711
CJA-Kdm2B	MUS-Kdm2A	67.87	277	87	2	1058	1334	887	1161	4e-114	404
CJA-Kdm2B	MMU-Kdm2A	56.27	686	239	15	68	729	40	688	0.0	707
CJA-Kdm2B	MMU-Kdm2A	67.15	277	89	2	1058	1334	888	1162	6e-113	400
CJA-Kdm2B	CJA-Kdm2A	56.41	686	238	16	68	729	40	688	0.0	706
CJA-Kdm2B	CJA-Kdm2A	67.15	277	89	2	1058	1334	886	1160	6e-113	400
CJA-Kdm2B	RNO-Kdm2A	56.04	687	239	14	68	729	40	688	0.0	702
CJA-Kdm2B	RNO-Kdm2A	67.87	277	87	2	1058	1334	887	1161	6e-114	404
CJA-Kdm2B	GGO-Kdm2A	67.15	277	89	2	1058	1334	1007	1281	4e-113	401
CJA-Kdm2B	PPY-Kdm2A	67.15	277	89	2	1058	1334	395	669	8e-113	400
CJA-Kdm2B	CJA-Fbxl19	56.00	300	131	1	1035	1334	396	694	8e-094	337
CJA-Kdm2B	CJA-Fbxl19	70.73	123	34	1	617	739	49	169	7e-053	201
CJA-Kdm2B	MUS-Fbxl19	56.00	300	131	1	1035	1334	376	674	1e-093	336
CJA-Kdm2B	MUS-Fbxl19	70.73	123	34	1	617	739	29	149	4e-053	202
CJA-Kdm2B	PPY-Fbxl19	55.67	300	132	1	1035	1334	332	630	1e-093	336
CJA-Kdm2B	HSA-Fbxl19	55.67	300	132	1	1035	1334	396	694	2e-093	336
CJA-Kdm2B	HSA-Fbxl19	71.54	123	33	1	617	739	49	169	3e-053	202
CJA-Kdm2B	RNO-Fbxl19	56.00	300	131	1	1035	1334	376	674	2e-093	336
CJA-Kdm2B	RNO-Fbxl19	71.54	123	33	1	617	739	29	149	2e-053	203
CJA-Kdm2B	PTR-Fbxl19	55.70	298	131	1	1035	1332	124	420	3e-093	335
CJA-Kdm2B	MMU-Fbxl19	55.67	300	132	1	1035	1334	286	584	4e-093	335

CJA-Kdm2B	GGO-Fbxl19	59.58	240	97	0	1035	1274	378	617	3e-080	292
CJA-Kdm2B	GGO-Fbxl19	71.54	123	33	1	617	739	31	151	2e-053	203

BLASTP 2.2.20 [Feb-08-2009]
Query: GGO-Kdm2B
Database: 559_protein.db
Query id, Subject id, % identity, alignment length, mismatches, gap openings, q. start, q. end, s. start, s. end, e-value, bit score

GGO-Kdm2B	GGO-Kdm2B	100.00	1252	0	0	1	1252	1	1252	0.0	2238
GGO-Kdm2B	PTR-Kdm2B	99.80	1025	2	0	228	1252	312	1336	0.0	1768
GGO-Kdm2B	PTR-Kdm2B	99.56	227	1	0	1	227	1	227	7e-133	467
GGO-Kdm2B	HSA-Kdm2B	99.80	1025	2	0	228	1252	312	1336	0.0	1765
GGO-Kdm2B	HSA-Kdm2B	99.56	227	1	0	1	227	1	227	7e-133	467
GGO-Kdm2B	MMU-Kdm2B	98.83	1025	9	2	228	1252	311	1332	0.0	1742
GGO-Kdm2B	MMU-Kdm2B	81.94	227	40	1	1	227	1	226	1e-103	369
GGO-Kdm2B	CJA-Kdm2B	97.28	1029	15	3	228	1252	315	1334	0.0	1738
GGO-Kdm2B	CJA-Kdm2B	86.34	227	29	1	1	227	1	225	7e-108	384
GGO-Kdm2B	RNO-Kdm2B	94.83	1025	53	0	228	1252	281	1305	0.0	1687
GGO-Kdm2B	RNO-Kdm2B	97.84	185	4	0	43	227	12	196	3e-105	375
GGO-Kdm2B	MUS-Kdm2B	93.49	1029	63	1	228	1252	281	1309	0.0	1660
GGO-Kdm2B	MUS-Kdm2B	97.84	185	4	0	43	227	12	196	4e-105	375
GGO-Kdm2B	PPY-Kdm2B	93.85	1025	7	2	228	1252	312	1280	0.0	1608
GGO-Kdm2B	PPY-Kdm2B	98.68	227	3	0	1	227	1	227	1e-131	462
GGO-Kdm2B	MUS-Kdm2A	67.87	277	87	2	976	1252	887	1161	2e-114	405
GGO-Kdm2B	MUS-Kdm2A	69.06	181	54	2	47	227	19	197	4e-072	265
GGO-Kdm2B	RNO-Kdm2A	67.87	277	87	2	976	1252	887	1161	3e-114	405
GGO-Kdm2B	RNO-Kdm2A	69.06	181	54	2	47	227	19	197	7e-072	264
GGO-Kdm2B	GGO-Kdm2A	67.15	277	89	2	976	1252	1007	1281	3e-113	402
GGO-Kdm2B	GGO-Kdm2A	60.34	174	67	2	42	215	11	182	4e-057	215
GGO-Kdm2B	CJA-Kdm2A	67.15	277	89	2	976	1252	886	1160	3e-113	401
GGO-Kdm2B	CJA-Kdm2A	69.06	181	54	2	47	227	19	197	7e-072	264
GGO-Kdm2B	MMU-Kdm2A	67.15	277	89	2	976	1252	888	1162	3e-113	401
GGO-Kdm2B	MMU-Kdm2A	69.06	181	54	2	47	227	19	197	8e-072	264
GGO-Kdm2B	HSA-Kdm2A	67.15	277	89	2	976	1252	888	1162	4e-113	401
GGO-Kdm2B	HSA-Kdm2A	69.06	181	54	2	47	227	19	197	7e-072	264
GGO-Kdm2B	PTR-Kdm2A	67.15	277	89	2	976	1252	888	1162	4e-113	401
GGO-Kdm2B	PTR-Kdm2A	69.06	181	54	2	47	227	19	197	7e-072	264
GGO-Kdm2B	PPY-Kdm2A	67.15	277	89	2	976	1252	395	669	7e-113	400
GGO-Kdm2B	CJA-Fbxl19	56.04	298	130	1	953	1250	396	692	3e-094	338
GGO-Kdm2B	CJA-Fbxl19	71.90	121	33	1	539	658	49	169	4e-053	202
GGO-Kdm2B	MUS-Fbxl19	56.00	300	131	1	953	1252	376	674	5e-094	337
GGO-Kdm2B	MUS-Fbxl19	71.90	121	33	1	539	658	29	149	3e-053	202
GGO-Kdm2B	PPY-Fbxl19	55.70	298	131	1	953	1250	332	628	6e-094	337
GGO-Kdm2B	HSA-Fbxl19	55.70	298	131	1	953	1250	396	692	1e-093	337
GGO-Kdm2B	HSA-Fbxl19	72.73	121	32	1	539	658	49	169	2e-053	203
GGO-Kdm2B	RNO-Fbxl19	56.04	298	130	1	953	1250	376	672	1e-093	337
GGO-Kdm2B	RNO-Fbxl19	72.73	121	32	1	539	658	29	149	1e-053	203

GGO-Kdm2B	PTR-Fbxl19	55.70	298	131	1	953	1250	124	420	2e-093	336
GGO-Kdm2B	MMU-Fbxl19	55.70	298	131	1	953	1250	286	582	2e-093	336
GGO-Kdm2B	GGO-Fbxl19	59.58	240	97	0	953	1192	378	617	2e-080	292
GGO-Kdm2B	GGO-Fbxl19	72.73	121	32	1	539	658	31	151	2e-053	203

BLASTP 2.2.20 [Feb-08-2009]
Query: MMU-Kdm2B
Database: 559_protein.db
Query id, Subject id, % identity, alignment length, mismatches, gap openings, q. start, q. end, s. start, s. end, e-value, bit score

MMU-Kdm2B	MMU-Kdm2B	100.00	1332	0	0	1	1332	1	1332	0.0	2404
MMU-Kdm2B	PTR-Kdm2B	95.96	1336	50	3	1	1332	1	1336	0.0	2279
MMU-Kdm2B	HSA-Kdm2B	95.96	1336	50	3	1	1332	1	1336	0.0	2278
MMU-Kdm2B	CJA-Kdm2B	91.82	1345	86	10	1	1332	1	1334	0.0	2158
MMU-Kdm2B	PPY-Kdm2B	92.35	1333	48	2	1	1332	1	1280	0.0	2158
MMU-Kdm2B	MUS-Kdm2B	91.57	1293	101	4	48	1332	17	1309	0.0	2094
MMU-Kdm2B	RNO-Kdm2B	91.23	1289	109	3	48	1332	17	1305	0.0	2078
MMU-Kdm2B	GGO-Kdm2B	98.83	1025	9	2	311	1332	228	1252	0.0	1736
MMU-Kdm2B	GGO-Kdm2B	81.94	227	40	1	1	226	1	227	2e-103	369
MMU-Kdm2B	HSA-Kdm2A	58.82	663	217	13	90	732	61	687	0.0	752
MMU-Kdm2B	HSA-Kdm2A	65.34	277	91	4	1059	1332	888	1162	1e-106	380
MMU-Kdm2B	PTR-Kdm2A	58.82	663	217	13	90	732	61	687	0.0	752
MMU-Kdm2B	PTR-Kdm2A	65.34	277	91	4	1059	1332	888	1162	1e-106	380
MMU-Kdm2B	MMU-Kdm2A	58.97	663	216	13	90	732	61	687	0.0	748
MMU-Kdm2B	MMU-Kdm2A	65.34	277	91	4	1059	1332	888	1162	1e-106	379
MMU-Kdm2B	MUS-Kdm2A	58.73	664	216	13	90	732	61	687	0.0	746
MMU-Kdm2B	MUS-Kdm2A	66.06	277	89	4	1059	1332	887	1161	9e-108	384
MMU-Kdm2B	CJA-Kdm2A	58.97	663	216	13	90	732	61	687	0.0	746
MMU-Kdm2B	CJA-Kdm2A	65.34	277	91	4	1059	1332	886	1160	1e-106	379
MMU-Kdm2B	RNO-Kdm2A	58.82	663	217	12	90	732	61	687	0.0	741
MMU-Kdm2B	RNO-Kdm2A	66.06	277	89	4	1059	1332	887	1161	1e-107	383
MMU-Kdm2B	GGO-Kdm2A	66.18	272	87	4	1064	1332	1012	1281	1e-106	380
MMU-Kdm2B	PPY-Kdm2A	66.18	272	87	4	1064	1332	400	669	2e-106	379
MMU-Kdm2B	CJA-Fbxl19	55.00	300	131	3	1036	1332	396	694	6e-090	324
MMU-Kdm2B	CJA-Fbxl19	71.90	121	33	1	622	741	49	169	7e-053	201
MMU-Kdm2B	MUS-Fbxl19	55.00	300	131	3	1036	1332	376	674	1e-089	323
MMU-Kdm2B	MUS-Fbxl19	71.90	121	33	1	622	741	29	149	5e-053	201
MMU-Kdm2B	PPY-Fbxl19	54.67	300	132	3	1036	1332	332	630	1e-089	323
MMU-Kdm2B	HSA-Fbxl19	54.67	300	132	3	1036	1332	396	694	2e-089	323
MMU-Kdm2B	HSA-Fbxl19	72.73	121	32	1	622	741	49	169	3e-053	202
MMU-Kdm2B	RNO-Fbxl19	55.00	300	131	3	1036	1332	376	674	2e-089	322
MMU-Kdm2B	RNO-Fbxl19	72.73	121	32	1	622	741	29	149	2e-053	203
MMU-Kdm2B	PTR-Fbxl19	54.70	298	131	3	1036	1330	124	420	3e-089	322
MMU-Kdm2B	MMU-Fbxl19	54.70	298	131	3	1036	1330	286	582	5e-089	321
MMU-Kdm2B	GGO-Fbxl19	58.33	240	97	2	1036	1272	378	617	4e-076	278
MMU-Kdm2B	GGO-Fbxl19	72.73	121	32	1	622	741	31	151	3e-053	202

BLASTP 2.2.20 [Feb-08-2009]

```
# Query: MUS-Kdm2B
# Database: 559_protein.db
# Query id, Subject id, % identity, alignment length, mismatches, gap openings, q. start, q. end, s. start, s. end, e-value, bit score
MUS-Kdm2B	MUS-Kdm2B	100.00	1309	0	0	1	1309	1	1309	0.0	2372
MUS-Kdm2B	RNO-Kdm2B	95.34	1309	57	1	1	1309	1	1305	0.0	2261
MUS-Kdm2B	PTR-Kdm2B	93.53	1298	80	1	12	1309	43	1336	0.0	2224
MUS-Kdm2B	HSA-Kdm2B	93.45	1298	81	1	12	1309	43	1336	0.0	2221
MUS-Kdm2B	MMU-Kdm2B	90.45	1298	116	4	12	1309	43	1332	0.0	2120
MUS-Kdm2B	CJA-Kdm2B	90.51	1286	100	6	33	1309	62	1334	0.0	2099
MUS-Kdm2B	PPY-Kdm2B	90.06	1298	69	3	12	1309	43	1280	0.0	2077
MUS-Kdm2B	GGO-Kdm2B	92.32	1029	75	1	281	1309	228	1252	0.0	1675
MUS-Kdm2B	GGO-Kdm2B	97.84	185	4	0	12	196	43	227	2e-105	375
MUS-Kdm2B	MUS-Kdm2A	56.16	730	253	13	1	706	1	687	0.0	774
MUS-Kdm2B	MUS-Kdm2A	67.15	277	89	2	1033	1309	887	1161	1e-112	400
MUS-Kdm2B	HSA-Kdm2A	55.89	730	255	14	1	706	1	687	0.0	773
MUS-Kdm2B	HSA-Kdm2A	66.79	277	90	2	1033	1309	888	1162	4e-112	398
MUS-Kdm2B	PTR-Kdm2A	55.89	730	255	14	1	706	1	687	0.0	773
MUS-Kdm2B	PTR-Kdm2A	66.79	277	90	2	1033	1309	888	1162	4e-112	398
MUS-Kdm2B	MMU-Kdm2A	55.89	730	255	14	1	706	1	687	0.0	764
MUS-Kdm2B	MMU-Kdm2A	66.79	277	90	2	1033	1309	888	1162	4e-112	398
MUS-Kdm2B	RNO-Kdm2A	56.15	732	250	14	1	706	1	687	0.0	763
MUS-Kdm2B	RNO-Kdm2A	67.51	277	88	2	1033	1309	887	1161	4e-113	401
MUS-Kdm2B	CJA-Kdm2A	55.74	732	253	15	1	706	1	687	0.0	759
MUS-Kdm2B	CJA-Kdm2A	66.79	277	90	2	1033	1309	886	1160	4e-112	398
MUS-Kdm2B	GGO-Kdm2A	67.65	272	86	2	1038	1309	1012	1281	4e-112	398
MUS-Kdm2B	PPY-Kdm2A	67.65	272	86	2	1038	1309	400	669	7e-112	397
MUS-Kdm2B	CJA-Fbxl19	56.04	298	130	1	1010	1307	396	692	2e-094	339
MUS-Kdm2B	CJA-Fbxl19	71.90	121	33	1	596	715	49	169	5e-053	202
MUS-Kdm2B	MUS-Fbxl19	56.00	300	131	1	1010	1309	376	674	4e-094	338
MUS-Kdm2B	MUS-Fbxl19	71.90	121	33	1	596	715	29	149	4e-053	202
MUS-Kdm2B	HSA-Fbxl19	55.70	298	131	1	1010	1307	396	692	4e-094	338
MUS-Kdm2B	HSA-Fbxl19	72.73	121	32	1	596	715	49	169	2e-053	203
MUS-Kdm2B	PPY-Fbxl19	55.70	298	131	1	1010	1307	332	628	4e-094	338
MUS-Kdm2B	PTR-Fbxl19	55.70	298	131	1	1010	1307	124	420	6e-094	337
MUS-Kdm2B	RNO-Fbxl19	56.04	298	130	1	1010	1307	376	672	7e-094	337
MUS-Kdm2B	RNO-Fbxl19	72.73	121	32	1	596	715	29	149	2e-053	203
MUS-Kdm2B	MMU-Fbxl19	55.70	298	131	1	1010	1307	286	582	7e-094	337
MUS-Kdm2B	GGO-Fbxl19	59.58	240	97	0	1010	1249	378	617	5e-081	295
MUS-Kdm2B	GGO-Fbxl19	72.73	121	32	1	596	715	31	151	2e-053	203
# BLASTP 2.2.20 [Feb-08-2009]
# Query: PPY-Kdm2B
# Database: 559_protein.db
# Query id, Subject id, % identity, alignment length, mismatches, gap openings, q. start, q. end, s. start, s. end, e-value, bit score
PPY-Kdm2B	PPY-Kdm2B	100.00	1280	0	0	1	1280	1	1280	0.0	2301
PPY-Kdm2B	PTR-Kdm2B	94.99	1336	11	2	1	1280	1	1336	0.0	2251
```

PPY-Kdm2B	HSA-Kdm2B	94.99	1336	11	2	1	1280	1	1336	0.0	2247
PPY-Kdm2B	MMU-Kdm2B	92.35	1333	48	2	1	1280	1	1332	0.0	2164
PPY-Kdm2B	CJA-Kdm2B	88.77	1345	75	9	1	1280	1	1334	0.0	2071
PPY-Kdm2B	MUS-Kdm2B	90.14	1298	68	3	43	1280	12	1309	0.0	2064
PPY-Kdm2B	RNO-Kdm2B	89.72	1294	77	2	43	1280	12	1305	0.0	2047
PPY-Kdm2B	GGO-Kdm2B	93.85	1025	7	2	312	1280	228	1252	0.0	1610
PPY-Kdm2B	GGO-Kdm2B	98.68	227	3	0	1	227	1	227	1e-131	463
PPY-Kdm2B	HSA-Kdm2A	58.27	707	237	14	47	733	19	687	0.0	786
PPY-Kdm2B	HSA-Kdm2A	51.26	277	77	5	1060	1280	888	1162	2e-073	269
PPY-Kdm2B	PTR-Kdm2A	58.27	707	237	14	47	733	19	687	0.0	786
PPY-Kdm2B	PTR-Kdm2A	51.26	277	77	5	1060	1280	888	1162	2e-073	269
PPY-Kdm2B	MMU-Kdm2A	58.42	707	236	14	47	733	19	687	0.0	782
PPY-Kdm2B	MMU-Kdm2A	51.26	277	77	5	1060	1280	888	1162	2e-073	269
PPY-Kdm2B	CJA-Kdm2A	58.42	707	236	14	47	733	19	687	0.0	780
PPY-Kdm2B	CJA-Kdm2A	51.26	277	77	5	1060	1280	886	1160	2e-073	269
PPY-Kdm2B	MUS-Kdm2A	58.05	708	237	14	47	733	19	687	0.0	780
PPY-Kdm2B	MUS-Kdm2A	51.99	277	75	5	1060	1280	887	1161	2e-074	272
PPY-Kdm2B	RNO-Kdm2A	58.13	707	238	13	47	733	19	687	0.0	773
PPY-Kdm2B	RNO-Kdm2A	51.99	277	75	5	1060	1280	887	1161	3e-074	272
PPY-Kdm2B	GGO-Kdm2A	51.26	277	77	5	1060	1280	1007	1281	2e-073	269
PPY-Kdm2B	PPY-Kdm2A	51.26	277	77	5	1060	1280	395	669	3e-073	269
PPY-Kdm2B	GGO-Fbxl19	72.73	121	32	1	623	742	31	151	1e-053	203
PPY-Kdm2B	RNO-Fbxl19	72.73	121	32	1	623	742	29	149	2e-053	203
PPY-Kdm2B	HSA-Fbxl19	72.73	121	32	1	623	742	49	169	2e-053	203
PPY-Kdm2B	MUS-Fbxl19	71.90	121	33	1	623	742	29	149	3e-053	202
PPY-Kdm2B	CJA-Fbxl19	71.90	121	33	1	623	742	49	169	5e-053	202

BLASTP 2.2.20 [Feb-08-2009]
Query: PTR-Kdm2B
Database: 559_protein.db
Query id, Subject id, % identity, alignment length, mismatches, gap openings, q. start, q. end, s. start, s. end, e-value, bit score

PTR-Kdm2B	PTR-Kdm2B	100.00	1336	0	0	1	1336	1	1336	0.0	2448
PTR-Kdm2B	HSA-Kdm2B	99.85	1336	2	0	1	1336	1	1336	0.0	2444
PTR-Kdm2B	MMU-Kdm2B	95.96	1336	50	3	1	1336	1	1332	0.0	2315
PTR-Kdm2B	PPY-Kdm2B	94.99	1336	11	2	1	1336	1	1280	0.0	2280
PTR-Kdm2B	CJA-Kdm2B	93.23	1345	71	7	1	1336	1	1334	0.0	2257
PTR-Kdm2B	MUS-Kdm2B	94.38	1298	69	1	43	1336	12	1309	0.0	2239
PTR-Kdm2B	RNO-Kdm2B	94.05	1294	77	0	43	1336	12	1305	0.0	2219
PTR-Kdm2B	GGO-Kdm2B	99.80	1025	2	0	312	1336	228	1252	0.0	1799
PTR-Kdm2B	GGO-Kdm2B	99.56	227	1	0	1	227	1	227	8e-133	466
PTR-Kdm2B	HSA-Kdm2A	58.56	707	235	15	47	733	19	687	0.0	790
PTR-Kdm2B	HSA-Kdm2A	67.15	277	89	2	1060	1336	888	1162	6e-113	400
PTR-Kdm2B	PTR-Kdm2A	58.56	707	235	15	47	733	19	687	0.0	790
PTR-Kdm2B	PTR-Kdm2A	67.15	277	89	2	1060	1336	888	1162	6e-113	400
PTR-Kdm2B	MMU-Kdm2A	58.70	707	234	15	47	733	19	687	0.0	787
PTR-Kdm2B	MMU-Kdm2A	67.15	277	89	2	1060	1336	888	1162	5e-113	401

PTR-Kdm2B	MUS-Kdm2A	58.47	708	234	15	47	733	19	687	0.0	786
PTR-Kdm2B	MUS-Kdm2A	67.87	277	87	2	1060	1336	887	1161	4e-114	404
PTR-Kdm2B	CJA-Kdm2A	58.70	707	234	15	47	733	19	687	0.0	785
PTR-Kdm2B	CJA-Kdm2A	67.15	277	89	2	1060	1336	886	1160	5e-113	401
PTR-Kdm2B	RNO-Kdm2A	58.56	707	235	14	47	733	19	687	0.0	779
PTR-Kdm2B	RNO-Kdm2A	67.87	277	87	2	1060	1336	887	1161	5e-114	404
PTR-Kdm2B	GGO-Kdm2A	68.01	272	85	2	1065	1336	1012	1281	5e-113	401
PTR-Kdm2B	PPY-Kdm2A	68.01	272	85	2	1065	1336	400	669	1e-112	400
PTR-Kdm2B	CJA-Fbxl19	56.04	298	130	1	1037	1334	396	692	4e-094	338
PTR-Kdm2B	CJA-Fbxl19	71.90	121	33	1	623	742	49	169	5e-053	201
PTR-Kdm2B	MUS-Fbxl19	56.00	300	131	1	1037	1336	376	674	7e-094	337
PTR-Kdm2B	MUS-Fbxl19	71.90	121	33	1	623	742	29	149	4e-053	202
PTR-Kdm2B	HSA-Fbxl19	55.70	298	131	1	1037	1334	396	692	9e-094	337
PTR-Kdm2B	HSA-Fbxl19	72.73	121	32	1	623	742	49	169	2e-053	202
PTR-Kdm2B	PPY-Fbxl19	55.70	298	131	1	1037	1334	332	628	1e-093	337
PTR-Kdm2B	RNO-Fbxl19	56.04	298	130	1	1037	1334	376	672	1e-093	337
PTR-Kdm2B	RNO-Fbxl19	72.73	121	32	1	623	742	29	149	2e-053	203
PTR-Kdm2B	PTR-Fbxl19	55.70	298	131	1	1037	1334	124	420	2e-093	336
PTR-Kdm2B	MMU-Fbxl19	55.70	298	131	1	1037	1334	286	582	2e-093	336
PTR-Kdm2B	GGO-Fbxl19	59.58	240	97	0	1037	1276	378	617	3e-080	292
PTR-Kdm2B	GGO-Fbxl19	72.73	121	32	1	623	742	31	151	2e-053	203

BLASTP 2.2.20 [Feb-08-2009]
Query: RNO-Kdm2B
Database: 559_protein.db
Query id, Subject id, % identity, alignment length, mismatches, gap openings, q. start, q. end, s. start, s. end, e-value, bit score

RNO-Kdm2B	RNO-Kdm2B	100.00	1305	0	0	1	1305	1	1305	0.0	2361
RNO-Kdm2B	MUS-Kdm2B	95.34	1309	57	1	1	1305	1	1309	0.0	2270
RNO-Kdm2B	PTR-Kdm2B	94.05	1294	77	0	12	1305	43	1336	0.0	2219
RNO-Kdm2B	HSA-Kdm2B	94.05	1294	77	0	12	1305	43	1336	0.0	2216
RNO-Kdm2B	CJA-Kdm2B	91.42	1282	92	5	33	1305	62	1334	0.0	2122
RNO-Kdm2B	MMU-Kdm2B	90.96	1294	113	3	12	1305	43	1332	0.0	2112
RNO-Kdm2B	PPY-Kdm2B	89.72	1294	77	2	12	1305	43	1280	0.0	2066
RNO-Kdm2B	GGO-Kdm2B	94.83	1025	53	0	281	1305	228	1252	0.0	1710
RNO-Kdm2B	GGO-Kdm2B	97.84	185	4	0	12	196	43	227	3e-105	375
RNO-Kdm2B	MUS-Kdm2A	54.82	726	265	14	1	702	1	687	0.0	741
RNO-Kdm2B	MUS-Kdm2A	67.51	277	88	2	1029	1305	887	1161	2e-113	402
RNO-Kdm2B	HSA-Kdm2A	54.76	725	267	13	1	702	1	687	0.0	738
RNO-Kdm2B	HSA-Kdm2A	66.79	277	90	2	1029	1305	888	1162	4e-112	398
RNO-Kdm2B	PTR-Kdm2A	54.76	725	267	13	1	702	1	687	0.0	738
RNO-Kdm2B	PTR-Kdm2A	66.79	277	90	2	1029	1305	888	1162	4e-112	398
RNO-Kdm2B	RNO-Kdm2A	55.28	729	257	15	1	702	1	687	0.0	735
RNO-Kdm2B	RNO-Kdm2A	67.51	277	88	2	1029	1305	887	1161	3e-113	401
RNO-Kdm2B	MMU-Kdm2A	54.62	725	268	13	1	702	1	687	0.0	730
RNO-Kdm2B	MMU-Kdm2A	66.79	277	90	2	1029	1305	888	1162	3e-112	398
RNO-Kdm2B	CJA-Kdm2A	54.62	725	268	13	1	702	1	687	0.0	729

RNO-Kdm2B	CJA-Kdm2A	66.79	277	90	2	1029	1305	886	1160	3e-112	398
RNO-Kdm2B	GGO-Kdm2A	67.65	272	86	2	1034	1305	1012	1281	2e-112	399
RNO-Kdm2B	PPY-Kdm2A	67.65	272	86	2	1034	1305	400	669	6e-112	397
RNO-Kdm2B	CJA-Fbxl19	55.70	298	131	1	1006	1303	396	692	1e-093	337
RNO-Kdm2B	CJA-Fbxl19	71.90	121	33	1	592	711	49	169	5e-053	202
RNO-Kdm2B	MUS-Fbxl19	55.67	300	132	1	1006	1305	376	674	2e-093	336
RNO-Kdm2B	MUS-Fbxl19	71.90	121	33	1	592	711	29	149	3e-053	202
RNO-Kdm2B	PPY-Fbxl19	55.37	298	132	1	1006	1303	332	628	2e-093	335
RNO-Kdm2B	HSA-Fbxl19	55.37	298	132	1	1006	1303	396	692	3e-093	335
RNO-Kdm2B	HSA-Fbxl19	72.73	121	32	1	592	711	49	169	2e-053	203
RNO-Kdm2B	RNO-Fbxl19	55.67	300	132	1	1006	1305	376	674	3e-093	335
RNO-Kdm2B	RNO-Fbxl19	72.73	121	32	1	592	711	29	149	2e-053	203
RNO-Kdm2B	PTR-Fbxl19	55.37	298	132	1	1006	1303	124	420	4e-093	335
RNO-Kdm2B	MMU-Fbxl19	55.37	298	132	1	1006	1303	286	582	5e-093	335
RNO-Kdm2B	GGO-Fbxl19	59.17	240	98	0	1006	1245	378	617	6e-080	291
RNO-Kdm2B	GGO-Fbxl19	72.73	121	32	1	592	711	31	151	1e-053	203

BLASTP 2.2.20 [Feb-08-2009]
Query: HSA-Kdm2B
Database: 559_protein.db
Query id, Subject id, % identity, alignment length, mismatches, gap openings, q. start, q. end, s. start, s. end, e-value, bit score

HSA-Kdm2B	HSA-Kdm2B	100.00	1336	0	0	1	1336	1	1336	0.0	2417
HSA-Kdm2B	PTR-Kdm2B	99.85	1336	2	0	1	1336	1	1336	0.0	2414
HSA-Kdm2B	MMU-Kdm2B	95.96	1336	50	3	1	1336	1	1332	0.0	2286
HSA-Kdm2B	PPY-Kdm2B	94.99	1336	11	2	1	1336	1	1280	0.0	2247
HSA-Kdm2B	CJA-Kdm2B	93.23	1345	71	7	1	1336	1	1334	0.0	2226
HSA-Kdm2B	MUS-Kdm2B	94.38	1298	69	1	43	1336	12	1309	0.0	2211
HSA-Kdm2B	RNO-Kdm2B	94.05	1294	77	0	43	1336	12	1305	0.0	2194
HSA-Kdm2B	GGO-Kdm2B	99.80	1025	2	0	312	1336	228	1252	0.0	1766
HSA-Kdm2B	GGO-Kdm2B	99.56	227	1	0	1	227	1	227	7e-133	467
HSA-Kdm2B	HSA-Kdm2A	58.13	707	238	14	47	733	19	687	0.0	786
HSA-Kdm2B	HSA-Kdm2A	67.15	277	89	2	1060	1336	888	1162	5e-113	401
HSA-Kdm2B	PTR-Kdm2A	58.13	707	238	14	47	733	19	687	0.0	786
HSA-Kdm2B	PTR-Kdm2A	67.15	277	89	2	1060	1336	888	1162	5e-113	401
HSA-Kdm2B	MMU-Kdm2A	58.27	707	237	14	47	733	19	687	0.0	782
HSA-Kdm2B	MMU-Kdm2A	67.15	277	89	2	1060	1336	888	1162	5e-113	401
HSA-Kdm2B	MUS-Kdm2A	57.91	708	238	14	47	733	19	687	0.0	781
HSA-Kdm2B	MUS-Kdm2A	67.87	277	87	2	1060	1336	887	1161	5e-114	404
HSA-Kdm2B	CJA-Kdm2A	58.27	707	237	14	47	733	19	687	0.0	780
HSA-Kdm2B	CJA-Kdm2A	67.15	277	89	2	1060	1336	886	1160	6e-113	400
HSA-Kdm2B	RNO-Kdm2A	57.99	707	239	13	47	733	19	687	0.0	774
HSA-Kdm2B	RNO-Kdm2A	67.87	277	87	2	1060	1336	887	1161	5e-114	404
HSA-Kdm2B	GGO-Kdm2A	68.01	272	85	2	1065	1336	1012	1281	5e-113	401
HSA-Kdm2B	PPY-Kdm2A	68.01	272	85	2	1065	1336	400	669	1e-112	400
HSA-Kdm2B	CJA-Fbxl19	56.04	298	130	1	1037	1334	396	692	4e-094	338
HSA-Kdm2B	CJA-Fbxl19	71.90	121	33	1	623	742	49	169	6e-053	201

```
HSA-Kdm2B	MUS-Fbxl19	56.00	300	131	1	1037	1336	376	674	5e-094	338
HSA-Kdm2B	MUS-Fbxl19	71.90	121	33	1	623	742	29	149	4e-053	202
HSA-Kdm2B	PPY-Fbxl19	55.70	298	131	1	1037	1334	332	628	7e-094	337
HSA-Kdm2B	HSA-Fbxl19	55.70	298	131	1	1037	1334	396	692	9e-094	337
HSA-Kdm2B	HSA-Fbxl19	72.73	121	32	1	623	742	49	169	2e-053	202
HSA-Kdm2B	RNO-Fbxl19	56.00	300	131	1	1037	1336	376	674	1e-093	337
HSA-Kdm2B	RNO-Fbxl19	72.73	121	32	1	623	742	29	149	2e-053	203
HSA-Kdm2B	PTR-Fbxl19	55.70	298	131	1	1037	1334	124	420	1e-093	336
HSA-Kdm2B	MMU-Fbxl19	55.70	298	131	1	1037	1334	286	582	2e-093	336
HSA-Kdm2B	GGO-Fbxl19	59.58	240	97	0	1037	1276	378	617	2e-080	292
HSA-Kdm2B	GGO-Fbxl19	72.73	121	32	1	623	742	31	151	2e-053	203
# BLASTP 2.2.20 [Feb-08-2009]
# Query: CJA-Lrrc29
# Database: 559_protein.db
# Query id, Subject id, % identity, alignment length, mismatches, gap openings, q. start, q. end, s. start, s. end, e-value, bit score
CJA-Lrrc29	CJA-Lrrc29	100.00	181	0	0	1	181	1	181	7e-103	363
CJA-Lrrc29	HSA-Lrrc29	87.78	180	22	0	1	180	1	180	1e-086	310
CJA-Lrrc29	PTR-Lrrc29	87.29	181	23	0	1	181	1	181	2e-086	309
CJA-Lrrc29	GGO-Lrrc29	86.11	180	24	1	1	180	1	179	2e-084	303
# BLASTP 2.2.20 [Feb-08-2009]
# Query: PTR-Lrrc29
# Database: 559_protein.db
# Query id, Subject id, % identity, alignment length, mismatches, gap openings, q. start, q. end, s. start, s. end, e-value, bit score
PTR-Lrrc29	PTR-Lrrc29	100.00	181	0	0	1	181	1	181	2e-100	355
PTR-Lrrc29	HSA-Lrrc29	97.78	180	4	0	1	180	1	180	1e-096	343
PTR-Lrrc29	GGO-Lrrc29	93.89	180	10	1	1	180	1	179	1e-090	323
PTR-Lrrc29	CJA-Lrrc29	87.29	181	23	0	1	181	1	181	2e-086	309
# BLASTP 2.2.20 [Feb-08-2009]
# Query: GGO-Lrrc29
# Database: 559_protein.db
# Query id, Subject id, % identity, alignment length, mismatches, gap openings, q. start, q. end, s. start, s. end, e-value, bit score
GGO-Lrrc29	GGO-Lrrc29	100.00	179	0	0	1	179	1	179	3e-100	355
GGO-Lrrc29	HSA-Lrrc29	95.00	180	8	1	1	179	1	180	6e-093	331
GGO-Lrrc29	PTR-Lrrc29	93.89	180	10	1	1	179	1	180	1e-090	323
GGO-Lrrc29	CJA-Lrrc29	86.11	180	24	1	1	179	1	180	2e-084	303
# BLASTP 2.2.20 [Feb-08-2009]
# Query: HSA-Lrrc29
# Database: 559_protein.db
# Query id, Subject id, % identity, alignment length, mismatches, gap openings, q. start, q. end, s. start, s. end, e-value, bit score
HSA-Lrrc29	HSA-Lrrc29	100.00	180	0	0	1	180	1	180	1e-099	353
HSA-Lrrc29	PTR-Lrrc29	97.78	180	4	0	1	180	1	180	1e-096	343
HSA-Lrrc29	GGO-Lrrc29	95.00	180	8	1	1	180	1	179	6e-093	331
HSA-Lrrc29	CJA-Lrrc29	87.78	180	22	0	1	180	1	180	1e-086	310
# BLASTP 2.2.20 [Feb-08-2009]
# Query: MMU-Fbxo17
```

```
# Database: 559_protein.db
# Query id, Subject id, % identity, alignment length, mismatches, gap openings, q. start, q. end, s. start, s. end, e-value, bit score
MMU-Fbxo17  MMU-Fbxo17  100.00      121         0           0           1           121         1           121         3e-054       201
MMU-Fbxo17  HSA-Fbxo17  97.52       121         3           0           1           121         5           125         8e-053       196
MMU-Fbxo17  PPY-Fbxo17  95.87       121         5           0           1           121         2           122         5e-052       194
MMU-Fbxo17  GGO-Fbxo17  98.28       116         2           0           6           121         1           116         5e-051       191
# BLASTP 2.2.20 [Feb-08-2009]
# Query: CJA-Skp2
# Database: 559_protein.db
# Query id, Subject id, % identity, alignment length, mismatches, gap openings, q. start, q. end, s. start, s. end, e-value, bit score
CJA-Skp2    CJA-Skp2    100.00      439         0           0           1           439         1           439         0.0          894
CJA-Skp2    GGO-Skp2    94.80       442         20          1           1           439         1           442         0.0          852
CJA-Skp2    PPY-Skp2    90.66       439         11          1           1           439         1           409         0.0          797
CJA-Skp2    HSA-Skp2    96.61       354         12          0           1           354         1           354         0.0          696
CJA-Skp2    PTR-Skp2    96.61       354         12          0           1           354         1           354         0.0          696
CJA-Skp2    RNO-Skp2    88.42       354         41          0           1           354         1           354         0.0          641
CJA-Skp2    MUS-Skp2    86.44       354         48          0           1           354         1           354         0.0          630
# BLASTP 2.2.20 [Feb-08-2009]
# Query: GGO-Skp2
# Database: 559_protein.db
# Query id, Subject id, % identity, alignment length, mismatches, gap openings, q. start, q. end, s. start, s. end, e-value, bit score
GGO-Skp2    GGO-Skp2    100.00      443         0           0           1           443         1           443         0.0          905
GGO-Skp2    CJA-Skp2    94.80       442         20          1           1           442         1           439         0.0          852
GGO-Skp2    PPY-Skp2    91.87       443         3           1           1           443         1           410         0.0          816
GGO-Skp2    HSA-Skp2    99.72       354         1           0           1           354         1           354         0.0          719
GGO-Skp2    PTR-Skp2    99.72       354         1           0           1           354         1           354         0.0          719
GGO-Skp2    RNO-Skp2    88.14       354         42          0           1           354         1           354         0.0          643
GGO-Skp2    MUS-Skp2    86.16       354         49          0           1           354         1           354         0.0          632
# BLASTP 2.2.20 [Feb-08-2009]
# Query: MUS-Skp2
# Database: 559_protein.db
# Query id, Subject id, % identity, alignment length, mismatches, gap openings, q. start, q. end, s. start, s. end, e-value, bit score
MUS-Skp2    MUS-Skp2    100.00      424         0           0           1           424         1           424         0.0          862
MUS-Skp2    RNO-Skp2    96.45       422         15          0           1           422         1           422         0.0          833
MUS-Skp2    HSA-Skp2    86.32       424         58          0           1           424         1           424         0.0          756
MUS-Skp2    PTR-Skp2    86.32       424         58          0           1           424         1           424         0.0          756
MUS-Skp2    GGO-Skp2    86.16       354         49          0           1           354         1           354         0.0          632
MUS-Skp2    PPY-Skp2    86.16       354         49          0           1           354         1           354         0.0          630
MUS-Skp2    CJA-Skp2    86.44       354         48          0           1           354         1           354         0.0          630
# BLASTP 2.2.20 [Feb-08-2009]
# Query: PPY-Skp2
# Database: 559_protein.db
# Query id, Subject id, % identity, alignment length, mismatches, gap openings, q. start, q. end, s. start, s. end, e-value, bit score
PPY-Skp2    PPY-Skp2    100.00      410         0           0           1           410         1           410         0.0          835
PPY-Skp2    GGO-Skp2    91.87       443         3           1           1           410         1           443         0.0          816
```

```
PPY-Skp2	CJA-Skp2	90.66	439	11	1	1	409	1	439	0.0	797
PPY-Skp2	HSA-Skp2	99.44	354	2	0	1	354	1	354	0.0	714
PPY-Skp2	PTR-Skp2	99.44	354	2	0	1	354	1	354	0.0	714
PPY-Skp2	RNO-Skp2	88.14	354	42	0	1	354	1	354	0.0	641
PPY-Skp2	MUS-Skp2	86.16	354	49	0	1	354	1	354	0.0	630
# BLASTP 2.2.20 [Feb-08-2009]
# Query: PTR-Skp2
# Database: 559_protein.db
# Query id, Subject id, % identity, alignment length, mismatches, gap openings, q. start, q. end, s. start, s. end, e-value, bit score
PTR-Skp2	HSA-Skp2	100.00	424	0	0	1	424	1	424	0.0	862
PTR-Skp2	PTR-Skp2	100.00	424	0	0	1	424	1	424	0.0	862
PTR-Skp2	RNO-Skp2	88.15	422	50	0	1	422	1	422	0.0	765
PTR-Skp2	MUS-Skp2	86.32	424	58	0	1	424	1	424	0.0	756
PTR-Skp2	GGO-Skp2	99.72	354	1	0	1	354	1	354	0.0	719
PTR-Skp2	PPY-Skp2	99.44	354	2	0	1	354	1	354	0.0	714
PTR-Skp2	CJA-Skp2	96.61	354	12	0	1	354	1	354	0.0	696
# BLASTP 2.2.20 [Feb-08-2009]
# Query: RNO-Skp2
# Database: 559_protein.db
# Query id, Subject id, % identity, alignment length, mismatches, gap openings, q. start, q. end, s. start, s. end, e-value, bit score
RNO-Skp2	RNO-Skp2	100.00	423	0	0	1	423	1	423	0.0	858
RNO-Skp2	MUS-Skp2	96.45	422	15	0	1	422	1	422	0.0	833
RNO-Skp2	HSA-Skp2	88.15	422	50	0	1	422	1	422	0.0	765
RNO-Skp2	PTR-Skp2	88.15	422	50	0	1	422	1	422	0.0	765
RNO-Skp2	GGO-Skp2	88.14	354	42	0	1	354	1	354	0.0	643
RNO-Skp2	CJA-Skp2	88.42	354	41	0	1	354	1	354	0.0	641
RNO-Skp2	PPY-Skp2	88.14	354	42	0	1	354	1	354	0.0	641
# BLASTP 2.2.20 [Feb-08-2009]
# Query: HSA-Skp2
# Database: 559_protein.db
# Query id, Subject id, % identity, alignment length, mismatches, gap openings, q. start, q. end, s. start, s. end, e-value, bit score
HSA-Skp2	HSA-Skp2	100.00	424	0	0	1	424	1	424	0.0	862
HSA-Skp2	PTR-Skp2	100.00	424	0	0	1	424	1	424	0.0	862
HSA-Skp2	RNO-Skp2	88.15	422	50	0	1	422	1	422	0.0	765
HSA-Skp2	MUS-Skp2	86.32	424	58	0	1	424	1	424	0.0	756
HSA-Skp2	GGO-Skp2	99.72	354	1	0	1	354	1	354	0.0	719
HSA-Skp2	PPY-Skp2	99.44	354	2	0	1	354	1	354	0.0	714
HSA-Skp2	CJA-Skp2	96.61	354	12	0	1	354	1	354	0.0	696
```
