# Supplementary material for: Evolution of the F-Box Gene Family in Euarchontoglires: Gene Number Variation and Selection Patterns
Source: PLoS One. 2014 Apr 11;9(4):e94899. doi: 10.1371/journal.pone.0094899 (PMC3984280; doi:10.1371/journal.pone.0094899)
Supplement: File S3 — Multiple sequence alignments of coding region sequences for each orthologous group. (PDF) [file pone.0094899.s008.pdf]

Multiple sequence alignment of Btrc

```
ENSMMUT00000009437     ------------------------------------CTCTCTCTGCAGTGCTCTATGCCC
ENSRNOT00000022426     TTTCAGGCTGCAGTTCCGGCTGGTGGATATTGGAGGAATAATAAAAAGTGCTCTATGCCC
ENSMUST00000065601     ATGGACCCGGCAGAGGCGGTGCTGCAGGAGAAAGCGCTTAAGTTTATGTGCTCTATGCCC
ENSPPYT00000003105     ATGGACCCGGCCGAGGCGGTGCTGCAAGAGAAAGCACTCAAGTTTATGTGCTCTATGCCC
ENSPTRT00000064287     ATGGACCCGGCCGAGGCGGTGCTGCAAGAGAAGGCACTCAAGTTTATGTGCTCTATGCCC
ENSCJAT00000034253     ATGGACTCAGCCGAGGCGGTGCTGCAAGAGAAAGCACTCAAGTTTATGTGCTCTATGCCC
ENSGGOT00000005049     ATGGACCCGGCCGAG---------------------------------------------
ENST00000370187        ATGGACCCGGCCGAGGCGGTGCTGCAAGAGAAGGCACTCAAGTTTATGTGCTCTATGCCC


ENSMMUT00000009437     AGGTCTCTGTGGCTGGGCTGCTCCAGCCTGGCGGACAGCATGCCTTCGCTGCGATGCCTG
ENSRNOT00000022426     AGGTCTCTGTGGCTGGGCTGCTCCAGCCTGGCGGACAGCATGCCCTCGCTGCGATGCCTG
ENSMUST00000065601     AGGTCTCTGTGGCTGGGCTGCTCCAGCCTGGCGGACAGCATGCCTTCGCTGCGATGCCTG
ENSPPYT00000003105     AGGTCTCTGTGGCTGGGCTGCTCCAGCCTGGCGGACAGCATGCCTTCGCTGCGATGCCTG
ENSPTRT00000064287     AGGTCTCTGTGGCTGGGCTGCTCCAGCCTGGCGGACAGCATGCCTTCGCTGCGATGCCTG
ENSCJAT00000034253     AGGTCTCTGTGGCTGGGCTGCTCCAGCCTGGCGGACAGCATGCCTTCGCTGCGATGCCTG
ENSGGOT00000005049     ------------------------------------------------------------
ENST00000370187        AGGTCTCTGTGGCTGGGCTGCTCCAGCCTGGCGGACAGCATGCCTTCGCTGCGATGCCTG


ENSMMUT00000009437     TATAACCCAGGGACTGGCGCACTCACAGCTTTCCAGAATTCCTCAGAGAGAGAAGACTGT
ENSRNOT00000022426     TATAACCCAGGGACTGGCGCACTCACAGCTTTCCAGAATTCCTCAGAGAGAGAAGACTGT
ENSMUST00000065601     TATAACCCAGGGACTGGCGCACTCACAGCTTTCCAGAATTCCTCAGAGAGAGAAGACTGT
ENSPPYT00000003105     TATAACCCAGGGACTGGCGCACTCACAGCTTTCCAGAATTCCTCAGAGAGAGAAGACTGT
ENSPTRT00000064287     TATAACCCAGGGACTGGCGCACTCACAGCTTTCCAGAATTCCTCAGAGAGAGAAGACTGT
ENSCJAT00000034253     TATAACCCAGGGACTGGCGCACTCACAGCTTTC---------------------------
ENSGGOT00000005049     ------------------------------------AATTCCTCAGAGAGAGAAGACTGT
ENST00000370187        TATAACCCAGGGACTGGCGCACTCACAGCTTTCCAGAATTCCTCAGAGAGAGAAGACTGT


ENSMMUT00000009437     AATAATGGCGAACCCCCTAGGAAGATAATACCAGAGAAGAATTCACTTAGACAGACGTAC
ENSRNOT00000022426     AATAATGGCGAACCCCCTAGGAAGATAATACCAGAGAAGAATTCACTTAGACAGACTTAC
ENSMUST00000065601     AATAATGGCGAACCCCCTAGGAAGATAATACCAGAGAAGAATTCACTTAGACAGACTTAC
ENSPPYT00000003105     AATAATGGCGAACCCCCTAGGAAGATAATACCAGAGAAGAATTCACTTAGACAGACGTAC
ENSPTRT00000064287     AATAATGGCGAACCCCCTAGGAAGATAATACCAGAGAAGAATTCACTTAGACAGACATAC
ENSCJAT00000034253     ---------------------------------------------------CAGACGTAC
ENSGGOT00000005049     AATAATGGCGAACCCCCTAGGAAGATAATACCAGAGAAGAATTCACTTAGACAGACATAC
ENST00000370187        AATAATGGCGAACCCCCTAGGAAGATAATACCAGAGAAGAATTCACTTAGACAGACATAC


ENSMMUT00000009437     AACAGCTGTGCCAGACTCTGCTTAAACCAAGAAACAGTATGTTTAGCAAGCACTGCTATG
ENSRNOT00000022426     AACAGCTGTGCCAGGCTCTGCATAAACCAAGAGACAGTATGTCTAACAAGCACTGCTATG
ENSMUST00000065601     AACAGCTGTGCCAGGCTTTGCATAAACCAAGAGACAGTATGTCTAACAAGCACTGCTATG
ENSPPYT00000003105     AACAGCTGTGCCAGACTCTGCTTAAACCAAGAAACAGTATGTTTAGCAAGCACTGCTATG
ENSPTRT00000064287     AACAGCTGTGCCAGACTCTGCTTAAACCAAGAAACAGTATGTTTAGGAAGCACTGCTATG
ENSCJAT00000034253     AATAGCTGTGCCAGACTCTGCTTAAACCAAGAAACAGTATGTTTAGCAAGCACTGCTATG
ENSGGOT00000005049     AACAGCTGTGCCAGACTCTGCTTAAACCAAGAAACAGTATGTTTAGCAAGCACTGCTATG
ENST00000370187        AACAGCTGTGCCAGACTCTGCTTAAACCAAGAAACAGTATGTTTAGCAAGCACTGCTATG


ENSMMUT00000009437     AAGACTGAGAATTGTGTGGCCAAAGTAATCTTCTTG------CTACGGGCACTCATTGTT
ENSRNOT00000022426     AAGACTGAAAATTGTGTGGCCAAAACAAAACTTGCCAATGGCACCTCCAGCATGATTGTG
ENSMUST00000065601     AAGACTGAAAATTGTGTGGCCAAAGCCAAACTTGCCAATGGCACTTCCAGCATGATTGTG
ENSPPYT00000003105     AAGACTGAGAATTGTGTGGCCAAAACAAAACTTGCCAATGGCACTTCCAGTATGATTGTG
ENSPTRT00000064287     AAGACTGAGAATTGTGTGGCCAAAACAAAACTTGCCAATGGCACTTCCAGTATGATTGTG
ENSCJAT00000034253     AAGACTGAGAATTGTGTGGCCAAAACAAAACTTGCCAATGGCACTTCCAGTATGATTGTG
ENSGGOT00000005049     AAGACTGAGAATTGTGTGGCCAAAACAAAACTTGCCAATGGCACTTCCAGTATGATTGTG
ENST00000370187        AAGACTGAGAATTGTGTGGCCAAAACAAAACTTGCCAATGGCACTTCCAGTATGATTGTG
```

```
ENSMMUT00000009437      GAACATACAAGAAAAAGAAACAAGAAACTGAAAGCTGTCAACAAAATGTGTTTAAAACTT
ENSRNOT00000022426      CCCAAGCAGCGGAAACTCTCAGCAAGCTATGAGAAGGAAAAGGAGCTGTGTGTCAAGTAC
ENSMUST00000065601      CCCAAGCAGCGGAAACTCTCAGCAAGCTATGAGAAGGAAAAGGAGCTGTGTGTCAAGTAT
ENSPPYT00000003105      CCCAAGCAACGGAAACTCTCAGCAAGCTATGAAAAGGAAAAGGAACTGTGTGTCAAATAC
ENSPTRT00000064287      CCCAAGCAACGG------------------------------------------------
ENSCJAT00000034253      CCCAAGCAACGGAAACTCTCGGCAAGCTATGAAAAGGAAAAGGAACTGTGTGTCAAATAC
ENSGGOT00000005049      CCAAAGCAACGGAAACTCTCAGCAAGCTATGAAAAGGAAAAGGAACTGTGTGTCAAATAC
ENST00000370187         CCCAAGCAACGGAAACTCTCAGCAAGCTATGAAAAGGAAAAGGAACTGTGTGTCAAATAC

ENSMMUT00000009437      TGCCCCGAGTGGGCATCAGACTGG---------CTGTTTTTAAGCCTGAAGTCCAGCCAC
ENSRNOT00000022426      TTTGAGCAGTGGTCAGAGTCTGATCAAGTGGAATTTGTAGAACACCTTATATCTCAAATG
ENSMUST00000065601      TTTGAGCAGTGGTCAGAGTCTGATCAAGTGGAATTTGTAGAACACCTTATATCCCAAATG
ENSPPYT00000003105      TTTGAGCAGTGGTCAGAGTCAGATCAAGTGGAATTTGTGGAACATCTTATATCCCAAATG
ENSPTRT00000064287      ------------------------------------------------------------
ENSCJAT00000034253      TTTGAGCAGTGGTCAGAGTCAGATCAAGTGGAATTTGTGGAACATCTTATATCCCAAATG
ENSGGOT00000005049      TTTGAGCAGTGGTCAGAGTCAGATCAAGTGGAATTTGTGGAACATCTTATATCCCAAATG
ENST00000370187         TTTGAGCAGTGGTCAGAGTCAGATCAAGTGGAATTTGTGGAACATCTTATATCCCAAATG

ENSMMUT00000009437      ATTGATTATGCACGACCTTTTGTAAAAAATCATTCACCACCCTCTGTGTTCTTTTTCTTT
ENSRNOT00000022426      TGTCATTACCAGCATGGGCACATCAACTCCTATCTAAAGCCTATGCTGCAGAGGGATTTC
ENSMUST00000065601      TGTCACTACCAGCATGGGCACATCAACTCCTACCTAAAACCTATGCTGCAGAGGGATTTC
ENSPPYT00000003105      TGTCATTACCAACATGGGCACATAAACTCGTATCTTAAACCTATGTTGCAGAGAGATTTC
ENSPTRT00000064287      ------------------------------------------------------------
ENSCJAT00000034253      TGTCATTACCAACATGGGCACATAAACTCATATCTTAAACCTATGTTGCAGAGAGATTTC
ENSGGOT00000005049      TGTCATTACCAACATGGGCACATAAACTCGTATCTTAAACCTATGTTGCAGAGAGATTTC
ENST00000370187         TGTCATTACCAACATGGGCACATAAACTCGTATCTTAAACCTATGTTGCAGAGAGATTTC

ENSMMUT00000009437      GCCTCCTCCCTCAAAGCTCGGGGTTTGGATCATATTGCTGAGAACATTCTGTCATACCTG
ENSRNOT00000022426      ATAACTGCACTGCCAGCACGGGGTCTGGACCATATTGCTGAGAACATTCTGTCATACTTG
ENSMUST00000065601      ATAACTGCACTGCCAGCACGGGGTCTGGACCACATCGCTGAGAACATTCTGTCATACTTG
ENSPPYT00000003105      ATAACTGCTCTGCCAGCTCGGGGTTTGGATCATATTGCTGAGAACATTCTGTCATACCTG
ENSPTRT00000064287      ---------------ACTCGGGGTTTGGATCATATTGCTGAGAACATTCTGTCATACCTG
ENSCJAT00000034253      ATAACCGCTCTGCCAGCTCGGGGTTTGGATCATATTGCTGAGAACATTCTGTCATACCTG
ENSGGOT00000005049      ATAACTGCTCTGCCAGCTCGGGGTTTGGATCATATTGCTGAGAACATTCTGTCATACCTG
ENST00000370187         ATAACTGCTCTGCCAGCTCGGGGATTGGATCATATTGCTGAGAACATTCTGTCATACCTG

ENSMMUT00000009437      GATGCCAAATCACTATGTGCTGCTGAACTTGTATGCAAGGAATGGTACCGAGTGACCTCT
ENSRNOT00000022426      GACGCCAGATCACTGTGTGCTGCTGAGCTTGTGTGCAAGGAATGGTACCGCGTGACGTCG
ENSMUST00000065601      GACGCCAAGTCACTGTGTGCTGCTGAGCTCGTGTGCAAGGAATGGTACCGCGTGACGTCG
ENSPPYT00000003105      GATGCCAAATCACTATGTGCTGCTGAACTTGTGTGCAAGGAATGGTACCGAGTGACCTCT
ENSPTRT00000064287      GATGCCAAATCACTATGTGCTGCTGAACTTGTGTGCAAGGAATGGTACCGAGTGACCTCT
ENSCJAT00000034253      GATGCCAAATCACTATGTGCTGCTGAACTTGTGTGCAAGGAATGGTACCGAGTGACCTCT
ENSGGOT00000005049      GATGCCAAATCACTATGTGCTGCTGAACTTGTGTGCAAGGAATGGTACCGAGTGACCTCT
ENST00000370187         GATGCCAAATCACTATGTGCTGCTGAACTTGTGTGCAAGGAATGGTACCGAGTGACCTCT

ENSMMUT00000009437      GATGGCATGCTATGGAAGAAGCTCATCGAGAGAATGGTCAGGACAGATTCTCTGTGGAGA
ENSRNOT00000022426      GACGGCATGCTGTGGAAGAAGCTCATCGAGAGGATGGTCAGGACAGACTCTCTGTGGCGA
ENSMUST00000065601      GACGGCATGCTGTGGAAAAAGCTCATCGAGAGGATGGTCAGGACGGACTCTCTGTGGCGA
ENSPPYT00000003105      GATGGCATGCTGTGGAGGAAGCTCATCGAGAGAATGGTCAGGACAGATTCTCTGTGGAGA
ENSPTRT00000064287      GATGGCATGCTGTGGAAGAAGCTTATCGAGAGAATGGTCAGGACAGATTCTCTGTGGAGA
ENSCJAT00000034253      GATGGCATGTTATGGAAGAAGCTCATCGAGAGAATGGTCAGGACAGATTCTTTGTGGAGA
ENSGGOT00000005049      GATGGCATGCTGTGGAAGAAGCTTATCGAGAGAATGGTCAGGACAGATTCTCTGTGGAGA
ENST00000370187         GATGGCATGCTGTGGAAGAAGCTTATCGAGAGAATGGTCAGGACAGATTCTCTGTGGAGA
```

ENSMMUT00000009437 GGCCTGGCAGAACGAAGAGGGTGGGGACAGTATTTATTCAAAAACAAACCTCCTGACGGG
ENSRNOT00000022426 GGCCTGGCAGAGCGCAGAGGGTGGGGACAATACTTATTCAAAAACAAACCTCCTGATGAG
ENSMUST00000065601 GGCCTGGCAGAGCGCAGAGGCTGGGGACAGTACTTATTCAAAAACAAACCTCCTGATGAG
ENSPPYT00000003105 GGCCTGGCAGAACGAAGAGGATGGGGACAGTATTTATTCAAAAACAAACCTCCTGACGGG
ENSPTRT00000064287 GGCCTGGCAGAACGAAGAGGATGGGGACAGTATTTATTCAAAAACAAACCTCCTGACGGG
ENSCJAT00000034253 GGCCTGGCAGAACGAAGAGGGTGGGGACAGTATTTATTCAAAAACAAACCTCCTGATGGG
ENSGGOT00000005049 GGCCTGGCAGAACGAAGAGGATGGGGACAGTATTTATTCAAAAACAAACCTCCTGACGGG
ENST00000370187 GGCCTGGCAGAACGAAGAGGATGGGGACAGTATTTATTCAAAAACAAACCTCCTGACGGG

ENSMMUT00000009437 AATGCTCCTCCCAACTCTTTTTATAGAGCACTTTATCCTAAAATTATACAAGACATTGAG
ENSRNOT00000022426 AATGCGCCTCCCAACTCCTTTTATAGAGCGCTTTATCCTAAAATTATACAAGACATTGAG
ENSMUST00000065601 AACGCTCCTCCCAACTCCTTTTATAGAGCGCTTTATCCTAAAATCATACAAGACATTGAG
ENSPPYT00000003105 AATGCTGCTCCCAACTCTTTTTATAGAGCACTTTATCCTAAAATTATACAAGACATTGAG
ENSPTRT00000064287 AATGCTCCTCCCAACTCTTTTTATAGAGCACTTTATCCTAAAATTATACAAGACATTGAG
ENSCJAT00000034253 AATACTCCTCCCAACTCTTTTTATAGAGCACTTTATCCTAAAATTATACAAGACATTGAG
ENSGGOT00000005049 AATGCTCCTCCCAACTCTTTTTATAGAGCACTTTATCCTAAAATTATACAAGACATTGAG
ENST00000370187 AATGCTCCTCCCAACTCTTTTTATAGAGCACTTTATCCTAAAATTATACAAGACATTGAG

ENSMMUT00000009437 ACAATAGAATCTAACTGGAGATGTGGAAGACATAGTTTACAGAGAATTCACTGCCGAAGT
ENSRNOT00000022426 ACAATAGAGTCCAATTGGAGATGTGGGCGACACAGTTTACAGAGAATCCACTGCCGGAGT
ENSMUST00000065601 ACAATAGAGTCCAATTGGAGATGTGGGCGACATAGTTTACAGAGAATCCACTGCCGGAGT
ENSPPYT00000003105 ACAATAGAATCTAATTGGAGATGTGGAAGACATAGTTTACAGAGAATTCACTGCCGAAGT
ENSPTRT00000064287 ACAATAGAATCTAATTGGAGATGTGGAAGACATAGTTTACAGAGAATTCACTGCCGAAGT
ENSCJAT00000034253 ACAATAGAATCTAATTGGAGATGTGGAAGACATAGTTTACAGAGAATCCACTGCCGAAGT
ENSGGOT00000005049 ACAATAGAATCTAATTGGAGATGTGGAAGACATAGTTTACAGAGAATTCACTGCCGAAGT
ENST00000370187 ACAATAGAATCTAATTGGAGATGTGGAAGACATAGTTTACAGAGAATTCACTGCCGAAGT

ENSMMUT00000009437 GAAACAAGCAAAGGAGTTTACTGTTTACAGTATGATGATCAGAAAATAGTAAGCGGCCTT
ENSRNOT00000022426 GAAACAAGTAAAGGGGTCTACTGTTTACAGTATGATGACCAGAAGATAGTCAGTGGCCTT
ENSMUST00000065601 GAAACAAGTAAAGGGGTTTACTGTTTACAGTACGACGACCAGAAGATAGTCAGCGGCCTT
ENSPPYT00000003105 GAAACAAGCAAAGGAGTTTACTGTTTACAGTATGATGATCAGAAAATAGTGAGCGGCCTT
ENSPTRT00000064287 GAAACAAGCAAAGGAGTTTACTGTTTACAGTATGATGATCAGAAAATAGTAAGCGGCCTT
ENSCJAT00000034253 GAAACAAGCAAAGGAGTTTACTGTTTACAGTATGATGATCAGAAAATAGTAAGCGGCCTT
ENSGGOT00000005049 GAAACAAGCAAAGGAGTTTACTGTTTACAGTATGATGATCAGAAAATAGTAAGCGGCCTT
ENST00000370187 GAAACAAGCAAAGGAGTTTACTGTTTACAGTATGATGATCAGAAAATAGTAAGCGGCCTT

ENSMMUT00000009437 CGAGACAACACAATCAAGATCTGGGATAAAAACACATTGGAATGCAAGCGAATTCTCACA
ENSRNOT00000022426 CGAGACAACACCATCAAGATCTGGGATAAAGCACACTGGAATGCAAGCGGATTCTCACA
ENSMUST00000065601 CGAGACAACACCATCAAGATCTGGGATAAAGCACACTGGAATGCAAGCGGATTCTCACG
ENSPPYT00000003105 CGAGACAACACAATCAAGATCTGGGATAAAAACACATTGGAATGCAAGCGAATTCTCACA
ENSPTRT00000064287 CGAGACAACACAATCAAGATCTGGGATAAAAACACATTGGAATGCAAGCGAATTCTCACA
ENSCJAT00000034253 CGAGACAACACAATCAAGATCTGGGATAAAAACACATTGGAATGCAAGCGAATTCTCACA
ENSGGOT00000005049 CGAGACAACACAATCAAGATCTGGGATAAAAACACATTGGAATGCAAGCGAATTCTCACA
ENST00000370187 CGAGACAACACAATCAAGATCTGGGATAAAAACACATTGGAATGCAAGCGAATTCTCACA

ENSMMUT00000009437 GGCCATACGGGTTCTGTCCTCTGTCTCCAGTATGATGAGAGAGTGATCATAACTGGATCA
ENSRNOT00000022426 GGCCACACAGGCTCTGTCCTGTGTCTGCAGTATGACGAGAGGGTGATCATCACGGGCTCC
ENSMUST00000065601 GGCCACACGGGCTCCGTCCTGTGTCTGCAGTACGATGAGAGGGTGATCATCACAGGCTCC
ENSPPYT00000003105 GGCCATACAGGTTCAGTCCTCTGTCTCCAGTATGATGAGAGAGTGATCATAACGGGATCA
ENSPTRT00000064287 GGCCATACGGGTTCAGTCCTCTGTCTCCAGTATGATGAGAGAGTGATCATAACGGGATCA
ENSCJAT00000034253 GGCCATACAGGTTCTGTCCTGTGTCTCCAGTATGATGAAAGAGTGATCATAACCGGATCA
ENSGGOT00000005049 GGCCATACGGGTTCAGTCCTCTGTCTCCAGTATGATGAGAGAGTGATCATAACGGGATCA
ENST00000370187 GGCCATACAGGTTCAGTCCTCTGTCTCCAGTATGATGAGAGAGTGATCATAACAGGATCA

```
ENSMMUT00000009437      TCGGATTCCACGGTCAGAGTGTGGGATGTAAATACAGGTGAAATGCTAAACACGTTGATT
ENSRNOT00000022426      TCAGACTCCACCGTCAGAGTGTGGGACGTAAATGCAGGGGAGATGCTAAACACATTGATT
ENSMUST00000065601      TCAGACTCCACCGTCAGAGTGTGGGATGTAAATGCAGGTGAGATGCTAAACACATTGATT
ENSPPYT00000003105      TCGGATTCCACAGTCAGAGTATGGGATGTAAATACAGGTGAAATGCTAAACACTTTGATT
ENSPTRT00000064287      TCGGATTCCACGGTCAGAGTGTGGGATGTAAATACAGGTGAAATGCTAAACACGTTGATT
ENSCJAT00000034253      TCGGATTCCACAGTCAGAGTGTGGGATGTAAATACAGGTGAAATGCTAAACACATTGATT
ENSGGOT00000005049      TCGGATTCCACGGTCAGAGTGTGGGATGTAAATACAGGTGAAATGCTAAACACGTTGATT
ENST00000370187         TCGGATTCCACGGTCAGAGTGTGGGATGTAAATACAGGTGAAATGCTAAACACGTTGATT

ENSMMUT00000009437      CACCATTGTGAAGCAGTTCTGCACTTGCGTTTCAATAATGGCATGATGGTGACCTGCTCC
ENSRNOT00000022426      CACCACTGTGAAGCCGTCCTGCACCTGCGCTTCAATAACGGCATGATGGTGACCTGCTCC
ENSMUST00000065601      CACCACTGTGAAGCCGTTCTGCACCTGCGCTTCAATAATGGCATGATGGTGACCTGTTCC
ENSPPYT00000003105      CACCATTGTGAAGCAGTTCTGCACTTGCGTTTCAATAATGGCATGATGGTGACCTGCTCC
ENSPTRT00000064287      CACCATTGTGAAGCAGTTCTGCACTTGCGTTTCAATAATGGCATGATGGTGACCTGCTCC
ENSCJAT00000034253      CACCATTGTGAAGCAGTTCTGCACTTGCGTTTCAATAATGGCATGATGGTGACGTGCTCC
ENSGGOT00000005049      CACCATTGTGAAGCAGTTCTGCACTTGCGTTTCAATAATGGCATGATGGTGACCTGCTCC
ENST00000370187         CACCATTGTGAAGCAGTTCTGCACTTGCGTTTCAATAATGGCATGATGGTGACCTGCTCC

ENSMMUT00000009437      AAAGATCGTTCCATTGCTGTATGGGATATGGCCTCCCCAACTGACATTACCCTCCGGAGG
ENSRNOT00000022426      AAAGACCGATCCATTGCTGTGTGGGATATGGCTTCCCCAACTGACATCACCCTCAGGAGG
ENSMUST00000065601      AAAGACCGTTCCATCGCTGTGTGGGATATGGCTTCCCCAACTGACATCACCCTCAGGAGG
ENSPPYT00000003105      AAAGATCGTTCCATTGCTGTATGGGATATGGCCTCCCCAACTGACATTACCCTCCGGAGG
ENSPTRT00000064287      AAAGATCGTTCCATTGCTGTATGGGATATGGCCTCCCCAACTGACATTACCCTCCGGAGG
ENSCJAT00000034253      AAAGATCGTTCCATTGCTGTATGGGATATGGCCTCCCCAACTGACATTACCCTCCGGAGG
ENSGGOT00000005049      AAAGATCGTTCCATTGCTGTATGGGATATGGCCTCCCCAACTGACATTACCCTCCGGAGG
ENST00000370187         AAAGATCGTTCCATTGCTGTATGGGATATGGCCTCCCCAACTGACATTACCCTCCGGAGG

ENSMMUT00000009437      GTGCTGGTCGGACACCGAGCTGCTGTCAATGTTGTAGACTTTGATGACAAGTACATCGTT
ENSRNOT00000022426      GTGCTGGTGGGCCACCGAGCTGCGGTCAATGTTGTCGACTTTGATGACAAGTACATCGTT
ENSMUST00000065601      GTGCTGGTGGGACACCGAGCTGCGGTCAATGTTGTAGACTTTGATGACAAGTACATCGTT
ENSPPYT00000003105      GTGCTGGTCGGACACCGAGCTGCTGTCAATGTTGTAGACTTTGATGACAAGTACATCGTT
ENSPTRT00000064287      GTGCTGGTCGGACACCGAGCTGCTGTCAATGTTGTAGACTTTGATGACAAGTACATTGTT
ENSCJAT00000034253      GTGCTGGTTGGACACCGAGCTGCTGTCAATGTTGTAGACTTTGATGACAAGTACATCGTT
ENSGGOT00000005049      GTGCTGGTCGGACACCGAGCTGCTGTCAACGTTGTAGACTTTGATGACAAGTACATTGTT
ENST00000370187         GTGCTGGTCGGACACCGAGCTGCTGTCAATGTTGTAGACTTTGATGACAAGTACATTGTT

ENSMMUT00000009437      TCTGCATCTGGGGATAGAACTATAAAGGTATGGAACACAAGTACTTGTGAATTTGTAAGG
ENSRNOT00000022426      TCTGCCTCTGGAGACAGAACCATAAAGGTGTGGAACACAAGTACCTGTGAATTTGTAAGG
ENSMUST00000065601      TCTGCCTCTGGAGATAGAACCATAAAGGTGTGGAACACAAGTACCTGTGAATTCGTAAGG
ENSPPYT00000003105      TCTGCATCTGGGGATAGAACTATAAAGGTGTGGAACACAAGTACTTGTGAATTTGTAAGG
ENSPTRT00000064287      TCTGCATCTGGGGATAGAACTATAAAGGTATGGAACACAAGTACTTGTGAATTTGTAAGG
ENSCJAT00000034253      TCTGCATCTGGGGATAGAACTATAAAGGTATGGAACACAAGTACTTGTGAATTTGTGAGG
ENSGGOT00000005049      TCTGCATCTGGGGATAGAACTATAAAGGTATGGAACACAAGTACTTGTGAATTTGTAAGG
ENST00000370187         TCTGCATCTGGGGATAGAACTATAAAGGTATGGAACACAAGTACTTGTGAATTTGTAAGG

ENSMMUT00000009437      ACCTTAAATGGACACAAACGAGGCATTGCCTGTTTGCAGTACAGGGACAGGCTGGTAGTG
ENSRNOT00000022426      ACCCTAAATGGGCACAAGCGTGGTATCGCCTGTTTGCAGTACAGAGACAGGCTGGTGGTG
ENSMUST00000065601      ACCCTAAATGGGCACAAGCGTGGCATCGCCTGTTTGCAGTACAGAGACAGGCTGGTGGTG
ENSPPYT00000003105      ACCTTAAATGGGCACAAACGAGGCATTGCCTGTTTGCAGTACAGGGACAGGCTGGTAGTG
ENSPTRT00000064287      ACCTTAAATGGACACAAACGAGGCATTGCCTGTTTGCAGTACAGGGACAGGCTGGTAGTG
ENSCJAT00000034253      ACCTTAAATGGACACAAACGAGGCATTGCCTGTTTGCAATACAGGGACAGGCTGGTAGTG
ENSGGOT00000005049      ACCTTAAATGGACACAAACGAGGCATTGCCTGTTTGCAGTACAGGGACAGGCTGGTAGTG
ENST00000370187         ACCTTAAATGGACACAAACGAGGCATTGCCTGTTTGCAGTACAGGGACAGGCTGGTAGTG
```

ENSMMUT00000009437	AGTGGCTCATCTGACAACACTATCAGATTATGGGACATAGAATGTGGTGCATGTTTACGA
ENSRNOT00000022426	AGCGGCTCCTCTGACAACACCATCAGGCTGTGGGACATAGAGTGCGGTGCATGTCTGCGA
ENSMUST00000065601	AGCGGCTCCTCTGACAACACCATCAGGCTGTGGGACATAGAGTGTGGAGCATGCCTGCGA
ENSPPYT00000003105	AGTGGCTCATCTGACAACACTATCAGATTATGGGACATAGAATGTGGTGCATGTTTACGA
ENSPTRT00000064287	AGTGGCTCATCTGACAACACTATCAGATTATGGGACATAGAATGTGGTGCATGTTTACGA
ENSCJAT00000034253	AGTGGCTCATCTGACAATACTATTAGATTATGGGACATAGAATGTGGTGCATGTTTACGA
ENSGGOT00000005049	AGTGGCTCATCTGACAACACTATCAGATTATGGGACATAGAATGTGGTGCATGTTTACGA
ENST00000370187	AGTGGCTCATCTGACAACACTATCAGATTATGGGACATAGAATGTGGTGCATGTTTACGA
ENSMMUT00000009437	GTGTTAGAAGGCCATGAGGAATTGGTGCGTTGTATTCGATTTGATAACAAGAGGATAGTC
ENSRNOT00000022426	GTGTTGGAAGGCCATGAGGAATTGGTACGCTGTATTCGATTTGATAACAAAAGGATAGTG
ENSMUST00000065601	GTGTTGGAGGGCCATGAGGAGTTGGTACGCTGCATTCGATTTGATAACAAAAGGATAGTG
ENSPPYT00000003105	GTGTTAGAAGGCCATGAGGAATTGGTGCGTTGTATTCGATTTGATAACAAGAGGATAGTC
ENSPTRT00000064287	GTGTTAGAAGGCCATGAGGAATTGGTGCGTTGTATTCGATTTGATAACAAGAGGATAGTC
ENSCJAT00000034253	GTGTTAGAAGGCCATGAGGAATTAGTGCGTTGTATTCGATTTGATAACAAGAGGATAGTC
ENSGGOT00000005049	GTGTTAGAAGGCCATGAGGAATTGGTGCGTTGTATTCGATTTGATAACAAGAGGATAGTC
ENST00000370187	GTGTTAGAAGGCCATGAGGAATTGGTGCGTTGTATTCGATTTGATAACAAGAGGATAGTC
ENSMMUT00000009437	AGTGGGGCCTATGATGGAAAAATTAAAGTGTGGGATCTTGTGGCTGCTTTGGACCCCCGT
ENSRNOT00000022426	AGTGGGGCCTATGATGGAAAAATTAAAGTGTGGGATCTTATGGCTGCTCTGGACCCGCGT
ENSMUST00000065601	AGCGGAGCCTATGATGGGAAAATTAAAGTGTGGGATCTTATGGCTGCTTTGGACCCGCGT
ENSPPYT00000003105	AGTGGGGCCTATGATGGAAAAATTAAAGTGTGGGATCTTGTGGCTGCTTTGGACCCCCGT
ENSPTRT00000064287	AGTGGGGCCTATGATGGAAAAATTAAAGTGTGGGATCTTGTGGCTGCTTTGGACCCCCGT
ENSCJAT00000034253	AGTGGGGCCTATGATGGAAAAATTAAAGTGTGGGATCTTGTGGCTGCTTTGGACCCCCGT
ENSGGOT00000005049	AGTGGGGCCTATGATGGAAAAATTAAAGTGTGGGATCTTGTGGCTGCTTTGGACCCTCGT
ENST00000370187	AGTGGGGCCTATGATGGAAAAATTAAAGTGTGGGATCTTGTGGCTGCTTTGGACCCCCGT
ENSMMUT00000009437	GCTCCTGCAGGGACACTCTGTCTACGGACCCTTGTGGAGCATTCCGGAAGAGTTTTTCGA
ENSRNOT00000022426	GCTCCTGCAGGGACTCTTTGTCTACGGACGCTTGTGGAGCATTCTGGAAGAGTTTTCCGA
ENSMUST00000065601	GCTCCAGCAGGGACTCTCTGTCTGCGGACACTTGTGGAGCATTCTGGAAGAGTTTTCCGC
ENSPPYT00000003105	GCTCCTGCAGGGACACTCTGTCTACGGACCCTTGTGGAGCATTCCGGAAGAGTTTTTCGA
ENSPTRT00000064287	GCTCCTGCAGGGACACTCTGTCTACGGACCCTTGTGGAGCATTCCGGAAGAGTTTTTCGA
ENSCJAT00000034253	GCTCCTGCAGGGACACTCTGTCTACGGACCCTCGTGGAGCATTCCGGAAGAGTTTTTCGA
ENSGGOT00000005049	GCTCCTGCAGGGACACTCTGTCTACGGACCCTTGTGGAGCATTCCGGAAGAGTTTTTCGA
ENST00000370187	GCTCCTGCAGGGACACTCTGTCTACGGACCCTTGTGGAGCATTCCGGAAGAGTTTTTCGA
ENSMMUT00000009437	CTCCAGTTTGATGAATTCCAGATTGTCAGTAGTTCACATGATGACACAATCCTCATCTGG
ENSRNOT00000022426	CTCCAGTTTGATGAATTCCAGATTGTCAGTAGTTCACATGATGACACAATTCTCATCTGG
ENSMUST00000065601	CTCCAGTTTGATGAATTCCAGATTGTCAGTAGTTCACATGATGACACAATTCTCATCTGG
ENSPPYT00000003105	CTCCAGTTTGATGAATTCCAGATTGTCAGTAGTTCACATGATGACACAATCCTCATCTGG
ENSPTRT00000064287	CTACAGTTTGATGAATTCCAGATTGTCAGTAGTTCACATGATGACACAATCCTCATCTGG
ENSCJAT00000034253	CTCCAGTTTGATGAATTCCAGATTGTCAGTAGTTCACATGATGACACAATCCTCATCTGG
ENSGGOT00000005049	CTACAGTTTGATGAATTCCAGATTGTCAGTAGTTCACATGATGACACAATCCTCATCTGG
ENST00000370187	CTACAGTTTGATGAATTCCAGATTGTCAGTAGTTCACATGATGACACAATCCTCATCTGG
ENSMMUT00000009437	GACTTCCTAAATGATCCGGCTGCCCAAGCTGAACCCCCCCGTTCCCCTTCTCGAACATAT
ENSRNOT00000022426	GACTTCCTAAATGATCCAGCCACTCATGCTGAACCGCCCCGCTCCCCTTCTCGGACATAC
ENSMUST00000065601	GACTTCCTGAATGATCCAGCTGCTCACGCTGAACCGCCCCGCTCCCCTTCTCGGACATAC
ENSPPYT00000003105	GACTTCTTAAATGATCCAGCTGCCCAAGCTGAACCCCCCCATTCCCCTTCTCGAACATAC
ENSPTRT00000064287	GACTTCCTAAATGATCCAGCTGCCCAAGCTGAACCCCCCCGTTCCCCTTCTCGAACATAC
ENSCJAT00000034253	GACTTCCTAAATGATCCAGCTGCCCAAGCTGAACCCCCCCGTTCCCCTTCTCGAACATAC
ENSGGOT00000005049	GACTTCCTAAATGATCCAGCTGCCCAACCTGAACCCCCCCGTTCCCCTTCTCGAACATAC
ENST00000370187	GACTTCCTAAATGATCCAGCTGCCCAAGCTGAACCCCCCCGTTCCCCTTCTCGAACATAC

```
ENSMMUT00000009437     ACCTACATCTCCAGA
ENSRNOT00000022426     ACCTACATCTCCAGA
ENSMUST00000065601     ACCTACATCTCCAGA
ENSPPYT00000003105     ACCTACATCTCCAGA
ENSPTRT00000064287     ACCTACATCTCCAGA
ENSCJAT00000034253     ACCTACATCTCCAGA
ENSGGOT00000005049     ACCTACATCTCCAGA
ENST00000370187        ACCTACATCTCCAGA
```

Multiple sequence alignment of Ccnf

```
ENSMUST00000115390     ATGGGGAGCGGCGGTGTGATCCATTGTAGGTGTGCCAAGTGTTTCTGTTATCCTACTAAA
ENSRNOT00000064392     ATGGGGAGCGGCGGTGTGATCCACTGTAGGTGTGCCAAGTGTTTCTGTTATCCTACGAAG
ENSCJAT00000062994     ATGGGGAGCGGCGGCGTGGTCCACTGTAGGTGTGCCAAGTGTTTCTGTTATCCTACAAAG
ENSMMUT00000000001     ATGGGGAGCGGTGGCGTGGTTCACTGTAGGTGTGCCAAGTGTTTCTGTTATCCTACAAAG
ENSPPYT00000008249     ATGGGGAGCGGCGGCGTGGTCCACTGTAGGTGTGCCAAGTGTTTCTGTTATCCTACAAAG
ENSGGOT00000011933     ATGGGGAGCGGCGGCGTGGTCCACTGTAGGTGTGCCAAGTGTTTCTGTTATCCTACAAAG
ENST00000397066        ATGGGGAGCGGCGGCGTGGTCCACTGTAGGTGTGCCAAGTGTTTCTGTTATCCTACAAAG
ENSPTRT00000067576     ATGGGGAGCGGCGGCGTGGTCCACTGTAGGTGTGCCAAGTGTTTCTGTTATCCTACAAAG


ENSMUST00000115390     CGAAGAATCAAAAGAAGACCCAGAAACTTAACCATCTTGAGTCTCCCAGAAGATGTACTC
ENSRNOT00000064392     CGAAGAATCAAAAGAAGACCCAGGAACTTGACCATCTTGAGTCTCCCAGAAGACGTGCTC
ENSCJAT00000062994     CGAAGAGTAAGAAGGAGGCCCCGAAACCTGACCATCTTGAGTCTCCCCGAAGATGTGCTG
ENSMMUT00000000001     CGAAGAATAAGGAGGAGGCCCCGAAACCTGACCATCTTGAGTCTCCCTGAAGATGTGCTC
ENSPPYT00000008249     CGAAGAATAAGGAGGAGGCCCCGAAACCTGACCATCTTGAGTCTCCCCGAAGATGTACTC
ENSGGOT00000011933     CGAAGAATAAGGAGGAGGCCCCGAAACCTGACCATCTTGAGTCTCCCCGAAGATGTGCTC
ENST00000397066        CGAAGAATAAGGAGGAGGCCCCGAAACCTGACCATCTTGAGTCTCCCCGAAGATGTGCTC
ENSPTRT00000067576     CGAAGAATAAGGAGGAGGCCCCGAAACCTGACCATCTTGAGTCTCCCCGAAGATGTGCTC


ENSMUST00000115390     TTTCATATCCTGAAATGGCTTTCTGTTGGGGACATCCTTGCTGTCCGAGCTGTCCACTCC
ENSRNOT00000064392     TTTCATATCCTGAAATGGCTTTCTGTTGGAGACATCCTGGCTGTCCGAGCTGTTCACTCC
ENSCJAT00000062994     TTTCACATCCTGAAGTGGCTTTCTGTGGAAGACATCCTGGCAGTCCGAGCTGTGCACTCC
ENSMMUT00000000001     TTTCACATCCTGAAATGGCTTTCTGTAGAGGACATCCTGGCTGTCCGAGCTGTACACTCC
ENSPPYT00000008249     TTTCACATCCTGAAATGGCTTTCTGTAGAGGACATCCTGGCTGTCCGAGCTGTACACTCC
ENSGGOT00000011933     TTTCACATCCTGAAATGGCTTTCTGTAGAGGACATCCTGGCTGTCCGAGCTGTACACTCC
ENST00000397066        TTTCACATCCTGAAATGGCTTTCTGTAGAGGACATCCTGGCCGTCCGAGCTGTACACTCC
ENSPTRT00000067576     TTTCACATCCTGAAGTGGCTTTCTGTAGAGGACATCCTGGCCGTCCGAGCTGTACACTCC


ENSMUST00000115390     CACCTCAAGTACCTGGTGGACAACCATGCCAGTGTGTGGGCATCTGCCAGCTTCCAGGAG
ENSRNOT00000064392     CACCTCAAGTACCTGGTGGACAACCATGCCAGTGTGTGGGCATCTGCCAGCTTCCAAGAG
ENSCJAT00000062994     CAGCTGAAGGACCTGGTAGACAACCATGCCAGCGTGTGGGCATGTGCCAGCTTCCAGGAG
ENSMMUT00000000001     CAGCTGAAGGACCTGGTGGACAACCACGCCAGTGTGTGGGCATGTGCCAGCTTCCAGGAG
ENSPPYT00000008249     CAGCTGAAGGACCTGGTGGACAACCACGCCACTGTGTGGGCATGTGCCAGCTTCCAGGAG
ENSGGOT00000011933     CAGCTGAAGGACCTGGTGGACAACCACGCCAGTGTGTGGGCATGTGCCAGCTTCCAGGAG
ENST00000397066        CAGCTGAAGGACCTGGTGGACAACCACGCCAGTGTGTGGGCATGTGCCAGCTTCCAGGAG
ENSPTRT00000067576     CAGCTGAAGGACCTGGTGGACAACCACGCCAGTGTGTGGGCATGTGCCAGCTTCCAGGAG


ENSMUST00000115390     CTGTGGCCTTCTCCACAGAACCTGAAGCTCTTTGAAAGGGCTGCTGAAAAGGGAAATTTT
ENSRNOT00000064392     CTGTGGCCTTCTCCACAGAACCTAAAGCTGTTTGAAAGGGCTGCCGAAAAGGGGAATTTT
ENSCJAT00000062994     CTGTGGCCATCTCCAGGGAACCTGAAGCTCTTTGAAAGGGCTGCTGAAAAGGGGAATTTC
ENSMMUT00000000001     CTGTGGCCGTCTCCAGGGAACCTGAAGCTCTTTGAAAGGGCTGCTGAAAAGGGGAATTTC
ENSPPYT00000008249     CTGTGGCCGTCTCCAGGAAACCTGAAGCTCTTTGAAAGGGCTGCCGAAAAGGGGAATTTC
ENSGGOT00000011933     CTGTGGCCGTCTCCAGGGAACCTGAAGCTCTTTGAAAGGGCTGCTGAAAAGGGGAATTTC
```

```
ENST00000397066        CTGTGGCCGTCTCCAGGGAACCTGAAGCTCTTTGAAAGGGCTGCTGAAAAGGGGAATTTC
ENSPTRT00000067576     CTGTGGCCGTCTCCAGGGAACCTGAAGCTCTTTGAAAGGGCTGCTGAAAAGGGGAATTTC

ENSMUST00000115390     GAAGCTGCTGTGAAGTTGGGGATTGCCTACCTCTACAATGAAGGCCTGTCTGTGTCAGAT
ENSRNOT00000064392     GAAGCTGCTGTGAAGTTGGGCATCGCCTACCTCTACAATGAAGGCTTGTCTGTGTCGGAT
ENSCJAT00000062994     GAAGCTGCTGTGAAGCTGGGCATAGCGTACCTCTACAATGAAGGCCTGTCTGTGTCTGAT
ENSMMUT00000000001     GAAGCTGCTGTGAAGCTGGGCATAGCCTACCTCTACAATGAAGGCCTGTCTGTGTCTGAT
ENSPPYT00000008249     GAAGCTGCTGTGAAGCTGGGCATAGCCTACCTCTACAATGAAGGCCTGTCTGTGTCTGAT
ENSGGOT00000011933     GAAGCTGCTGTGAAGCTGGGCATAGCCTACCTCTACAATGAAGGCCTGTCTGTGTCTGAT
ENST00000397066        GAAGCTGCTGTGAAGCTGGGCATAGCCTACCTCTACAATGAAGGCCTGTCTGTGTCTGAT
ENSPTRT00000067576     GAAGCTGCTGTGAAGCTGGGCATAGCCTACCTCTACAATGAAGGCCTGTCTGTGTCTGAT

ENSMUST00000115390     GAGGCCTGCGCAGAAGTGAACGGCTTGAAGGCCTCTCGCTTCTTCAGCATGGCTGAGAGA
ENSRNOT00000064392     GAGGCCTGCGCAGAAGTGAACGGCCTGAAGGCCTCTCGCTTCTTCAGCATGGCCGAGAGA
ENSCJAT00000062994     GAGGCCCGTGCGGAAGTGAATGGCCTGAAGGCCTCTCACTTCTTCAGTCTCACGGAGCGG
ENSMMUT00000000001     GAGGCCCGCGCAGAAGTGAATGGCCTGAAGGCCTCTCGCTTCTTTAGTCTCGCTGAGCGG
ENSPPYT00000008249     GAGGCCCGTGCAGAAGTGAATGGCCTGAAGGCCTCTCGCTTCTTCAGTCTCGCTGAGCGG
ENSGGOT00000011933     GAGGCCCGCGCAGAAGTGAATGGCCTGAAGGCCTCTCGCTTCTTCAGTCTCGCTGAGCGG
ENST00000397066        GAGGCCCGCGCAGAAGTGAATGGCCTGAAGGCCTCTCGCTTCTTCAGTCTCGCTGAGCGG
ENSPTRT00000067576     GAGGCCCGCGCAGAAGTGAATGGCCTGAAGGCCTCTCGCTTCTTCAGTCTCGCTGAGCGG

ENSMUST00000115390     CTGAATACGGGTTCTGAGCCCTTCATCTGGCTCTTCATCCGCCCACCGTGGTCAGTGTCC
ENSRNOT00000064392     TTGAATACGGGTTCGGACCCCTTCATCTGGCTCTTCATCCGCCCGCCGTGGTCAGTGTCT
ENSCJAT00000062994     CTGAACGCTGGTGCCGTGCCCTTCATCTGGCTGTTCATCCGCCCTCCGTGGTCGGTGAGT
ENSMMUT00000000001     CTGAACGTGGGTGCCGCGCCTTTCATCTGGCTCTTCATCCGCCCTCCATGGTCAGTGAGC
ENSPPYT00000008249     CTGAATGTGGGAGCCGCGCCTTTCATCTGGCTCTTCATCCGCCCTCCGTGGTCGGTGAGC
ENSGGOT00000011933     CTGAATGTGGGTGCCGCACCTTTCATCTGGCTCTTCATCCGCCCTCCGTGGTCGGTGAGC
ENST00000397066        CTGAATGTGGGTGCCGCACCTTTCATCTGGCTCTTCATCCGCCCTCCGTGGTCGGTGAGC
ENSPTRT00000067576     CTGAATGTGGGTGCCGCACCTTTCATCTGGCTCTTCATCCGCCCTCCGTGGTCGGTGAGC

ENSMUST00000115390     GGAAGCTGCTGCAAGGCTGTGGTTCATGACAGCCTCCGAGCGGAGTGTCAGTTACAGAGA
ENSRNOT00000064392     GGAAGCTGTTGCAAGGCCGTGGTCCATGACAGCCTCAGGGCTGAGTGTCAGTTACAAAGA
ENSCJAT00000062994     GGAAGCTGCTGCAAGGCCGTGGTTCACGAGAGCCTCAGGGCAGAGTCCCAACTGCAGAGG
ENSMMUT00000000001     GGAAGCTGCTGCAAGGCCGTGGTTCATGAGAGCCTCAGGGCAGAGTGCCAGCTGCAGAGG
ENSPPYT00000008249     GGAAGCTGCTGCAAGGCTGTGGTTCACGAGAGCCTCAGGGCAGAGTGCCAGCTGCAGAGG
ENSGGOT00000011933     GGAAGCTGCTGCAAGGCCGTGGTTCACGAGAGCCTCAGGGCAGAGTGCCAGCTGCAGAGG
ENST00000397066        GGAAGCTGCTGCAAGGCCGTGGTTCACGAGAGCCTCAGGGCAGAGTGCCAGCTGCAGAGG
ENSPTRT00000067576     GGAAGCTGCTGCAAGGCCGTGGTTCACGAGAGCCTCAGGGCAGAGTGCCAGCTGCAGAGG

ENSMUST00000115390     AGTCATAAAGCTTCCATACTACACTGCTTGGGAAGGGTGCTAAATCTTTTTGAGGACGAA
ENSRNOT00000064392     AGTCATAAAGCTTCCATACTGCACTGCTTGGGAAGAGTGCTGAATCTTTTTGAGGACGAA
ENSCJAT00000062994     ACTCACAAAGCATCCATACTGCACTGCTTGGGCAGAGTGCTGAGTCTGTTCGAGGATGAA
ENSMMUT00000000001     ACTCACAAAGCATCCATATTGCACTGCTTGGGCAGAGTGCTGAGTCTGTTCGAGGATGAA
ENSPPYT00000008249     ACTCACAAAGCATCCATATTGCACTGCTTGGGCAGAGTGCTGAGTCTGTTCGAGGATGAA
ENSGGOT00000011933     ACTCACAAAGCATCCATATTGCACTGCTTGGGCAGAGTGCTGAGTCTGTTCGAGGATGAA
ENST00000397066        ACTCACAAAGCATCCATATTGCACTGCTTGGGCAGAGTGCTGAGTCTGTTCGAGGATGAG
ENSPTRT00000067576     ACTCACAAAGCATCCATATTACACTGCTTGGGCAGAGTGCTGAGTCTGTTCGAGGATGAG

ENSMUST00000115390     GAGAAAAGGAAGCAGGCGCGTAGCCTTTTGGAAGAGTCTTCTCGTCAGGGATGCTTAATC
ENSRNOT00000064392     GAGAAAAGGAAGCAGGCTCATAACCTTTTTGAAGAATCTGCTCATCAGGGATGCTTGGCC
ENSCJAT00000062994     GAAAAGCAGCAGCAGGCTCGGGACCTGTTTGAAGAGGCTGCTCGTCAGGGATGTCTGAGC
ENSMMUT00000000001     GAGAAGCAGCAGCAGGCCCGTGACCTGTTTGAGGAGGCTGCTCATCAGGGATGTCTAACC
ENSPPYT00000008249     GAGAAGCAGCAGCAGGCCCATGACTTGTTTGAGGAGGCTGCTCATCAGGGATGTCTGGCC
ENSGGOT00000011933     GAGAAGCAGCAGCAGGCCCATGCCCTGTTTGAGGAGGCTGCTCAGCAGGGATGTCTGACC
```

ENST00000397066 GAGAAGCAGCAGCAGGCCCATGACCTGTTTGAGGAGGCTGCTCATCAGGGATGTCTGACC
ENSPTRT00000067576 GAGAAGCAGCAGCAGGCCCATGACCTGTTTGAGGAGGCTGCTCATCAGGGATGTCTGACC

ENSMUST00000115390 AGCTCATACCTCCTTTGGGAAAGCGACAGAAAGGTGGATATGTCCGACCCCGGACGATGC
ENSRNOT00000064392 AGCTCATACCTCCTTTGGGAAAGTGACAGAAAGGTGGATATGTCTGACCCCGGACGATGC
ENSCJAT00000062994 AGCGCATACCTCCTCTGGGAAAGCGACAGGAGGACAGATATGTCAGATCCTGGGCGAAGT
ENSMMUT00000000001 AGTTCCTACCTCCTCTGGGAAAGCGACAGGAGGACGGATGTGTCAGATCCTGGGCGATGC
ENSPPYT00000008249 AGCTCCTACCTCCTCTGGGAAAGCGACAGGAGGACAGATGTGTCAGATCCTGGGCGATGC
ENSGGOT00000011933 AGCTCCTACCTCCTCTGGGAAAGCGACAGGAGGACAGACGTGTCAGATCCTGGGCGATGC
ENST00000397066 AGCTCCTACCTCCTCTGGGAAAGCGACAGGAGGACAGATGTGTCAGATCCTGGGCGATGC
ENSPTRT00000067576 AGCTCCTACCTCCTCTGGGAAAGCGACAGGAGGACAGATGTGTCAGATCCTGGGCGATGC

ENSMUST00000115390 CTCCACAGCTTCCGGAAACTCAGGGACTACGCTGCGAAAGGCTGCTGGGAAGCACAGCTG
ENSRNOT00000064392 CTCCACAGCTTCCGGAAACTCAGGGACTACGCCGCCAAGGGCTGCTGGGAAGCACAGCTG
ENSCJAT00000062994 CTCCACAGCTTCCGAAAACTCAGGGACTACGCTGCCAAGGGCTGCTGGGAAGCGCAGCTG
ENSMMUT00000000001 CTCCACAGCTTCCGAAAAGTCAGGGACTACGCTGCCAAAGGGTGCTGGGAAGCGCAGCTG
ENSPPYT00000008249 CTCCACAGTTTCCGAAAACTCAGGGACTACGCTGCCAAAGGCTGCTGGGAAGCGCAGCTG
ENSGGOT00000011933 CTCCACAGCTTCCGAAAACTCAGGGACTACGCTGCCAAAGGCTGCTGGGAAGCGCAGCTG
ENST00000397066 CTCCACAGCTTCCGAAAACTCAGGGACTACGCTGCCAAAGGCTGCTGGGAAGCGCAGCTG
ENSPTRT00000067576 CTCCACAGCTTCCGAAAACTCAGGGACTACGCTGCCAAAGGCTGCTGGGAAGCGCAGCTG

ENSMUST00000115390 GCTTTGGCCAAAGCCTGTGCAGGTGGAAGCCAACTTGGACTGGAAGGGAAGGCCTGCAGT
ENSRNOT00000064392 GCTTTGGCCAAAGCCTGCGCAGGAGGAAGCCAGCTTGGACTAGAAGGGAAGGCCTGCAGT
ENSCJAT00000062994 TCTTTAGCCAAAGCCTGTGCAAATGGAAACCAGCTTGGACTGGAGGTGAGAGCTTCCAGT
ENSMMUT00000000001 TCTTTAGCCAAAGCCTGTGCAAATGGAAACCAGCTTGGACTAGAGGTGAGAGCTTCCAAT
ENSPPYT00000008249 TCTTTAGCCAAAGCCTGTGCAAATGCAAACCAGCTTGGACTGGAGGTGAGAGCTTCCAAT
ENSGGOT00000011933 TCTTTAGCCAAAGCCTGTGCAAATGCAAACCAGCTTGGACTGGAGGTGAGAGCTTCCAGT
ENST00000397066 TCTTTAGCCAAAGCCTGTGCAAATGCAAACCAGCTTGGACTGGAGGTGAGAGCTTCCAGT
ENSPTRT00000067576 TCTTTAGCCAAAGCCTGTGCAAATGCAAACCAGCTTGGACTGGAGGTGAGAGCTTCCAGT

ENSMUST00000115390 GAGTCGGTCTGCCAGCTCTTCCAGGCCTCCCAGGCTGTCAACAAGCAGCAGATATTCTCT
ENSRNOT00000064392 GAGTCAGTCTGCCAGCTCTTCCAAGCCTCCCAGGCCGTCAACAAGCAGCAGATATTCTCT
ENSCJAT00000062994 GAGATCGTCTGCCAGCTGTTTCAGGCCTCCCAGGCTGTCAGTAAGCAGCAAGTCTTCTCC
ENSMMUT00000000001 GAGATCGTCTGCCAGCTATTTCAGGCTTCCCAGGCTGTCAGTAAGCAGCAAGTCTTCTCC
ENSPPYT00000008249 GAGATCGTCTGCCAGCTATTTCAGGCTTCCCAGGCTGTCAGTAAACAGCAAGTCTTCTCC
ENSGGOT00000011933 GAGATCGTCTGCCAGCTATTTCAGGCTTCCCAGGCTGTCAGTAAACAACAAGTCTTCTCC
ENST00000397066 GAGATCGTCTGCCAGCTATTTCAGGCTTCCCAGGCTGTCAGTAAACAACAAGTCTTCTCC
ENSPTRT00000067576 GAGATCGTCTGCCAGCTATTTCAGGCTTCCCAGGCTGTCAGTAAACAACAAGTCTTCTCC

ENSMUST00000115390 GTGCAGAAGGGGCTCAGCGACACCATGAGGTACATTCTAATCGACTGGTTGGTGGAAGTT
ENSRNOT00000064392 GTGCAGAAGGGGCTCAGTGACACCATGAGGTACATTCTAATCGACTGGTTGGTGGAAGTT
ENSCJAT00000062994 GTGCAGAAGGGACTCAACGACACAATGAGGTACATTCTGATCGACTGGCTGGTGGAAGTC
ENSMMUT00000000001 GTGCAGAAGGGACTCAATGACACAATGAGGTACATTCTGATCGACTGGCTGGTGGAAGTT
ENSPPYT00000008249 GTGCAGAAGGGACTCAATGACACAATGAGGTACATTCTGATCGACTGGCTGGTGGAAGTT
ENSGGOT00000011933 GTGCAGAAGGGACTCAATGACACAATGAGGTACATTCTGATCGACTGGCTGGTGGAAGTT
ENST00000397066 GTGCAGAAGGGACTCAATGACACAATGAGGTACATTCTGATCGACTGGCTGGTGGAAGTT
ENSPTRT00000067576 GTGCAGAAGGGACTCAATGACACAATGAGGTACATTCTGATCGACTGGCTGGTGGAAGTT

ENSMUST00000115390 GCCACAATGAAGGACTTTACAAGCCTGTGTCTTCACCTGACTGTGGAGTGTGTAGACCGG
ENSRNOT00000064392 GCCACCATGAAGGACTTCACAAGCCTGTGTCTCCACCTGACAGTGGAGTGTGTAGACCGG
ENSCJAT00000062994 GCCACCATGAAGGACTTCACGAGCCTGTGCCTGCACCTGACCGTGGAGTGTGTGGACCGC
ENSMMUT00000000001 GCCACCATGAAGGACTTCACAAGCCTGTGTCTGCACCTGACCGTGGAGTGTGTGGACCGG
ENSPPYT00000008249 GCCACCATGAAGGATTTCACAAGCCTGTGCCTGCACCTGACCGTGGAGTGCGTGGACCGG
ENSGGOT00000011933 GCCACCATGAAGGACTTCACAAGCCTGTGCCTGCACCTGACCGTGGAGTGTGTGGACCGG

```
ENST00000397066         GCCACCATGAAGGACTTCACAAGCCTGTGCCTGCACCTGACCGTGGAGTGTGTGGACCGG
ENSPTRT00000067576      GCCACCATGAAGGACTTCACAAGCCTGTGCCTGCACCTGACCGTGGAGTGTGTGGACCGG

ENSMUST00000115390      TACCTGCGAAGGAGGCTGGTCCCCAGATACAAGCTGCAGCTCCTGGGTATTGCCTGCATG
ENSRNOT00000064392      TACTTGCGGAGAAGACTGGTCCCCAGATACAAGCTCCAGCTTCTGGGCATCGCCTGCATG
ENSCJAT00000062994      TACCTTCGGAGGAGGCTGGTGCCGCGATACACGCTGCAGCTGCTGGGCATTGCCTGTATG
ENSMMUT00000000001      TACCTGCGGAGGAGGCTGGTGCCGCGGTACAGGCTCCAGCTGCTGGGCATCGCCTGCATG
ENSPPYT00000008249      TACCTGCGGAGGAGGCTGGTGCCGCGGTACAGGCTCCAGCTGCTGGGCATCGCCTGCATG
ENSGGOT00000011933      TACCTGCGGAGGAGGCTGGTGCCACGGTACAGGCTCCAGCTGCTGGGCATCGCCTGCATG
ENST00000397066         TACCTGCGGAGGAGGCTGGTGCCGCGGTACAGGCTCCAGCTGCTGGGCATCGCCTGCATG
ENSPTRT00000067576      TACCTGCGGAGGAGGCTGGTGCCGCGGTACAGGCTCCAGCTGCTGGGCATCGCCTGCATG

ENSMUST00000115390      GTCATCTGTACCCGGTTCATCAGCAAAGAGATTCTGACGATTAGAGAGGCTGTGTGGCTC
ENSRNOT00000064392      GTCATCTGTACCCGGTTCATCAGCAAAGAGATTCTGACCATTAGAGAGGCTGTGTGGCTC
ENSCJAT00000062994      GTCATCTGCACCCGGTTCATCAGTAAAGAGATCCTGACAATCCGGGAGGCTGTGTGGCTC
ENSMMUT00000000001      GTCATCTGCACCCGGTTCATCAGTAAAGAGATCCTGACTATCCGGGAGGCCGTATGGCTC
ENSPPYT00000008249      GTCATCTGCACCCGGTTCATCAGTAAAGAGATCCTGACCATCCGGGAGGCCGTATGGCTC
ENSGGOT00000011933      GTCATCTGCACCCGGTTCATCAGTAAAGAGATCCTGACCATCCGGGAGGCCGTATGGCTC
ENST00000397066         GTCATCTGCACCCGGTTTATCAGTAAAGAGATCCTGACCATCCGGGAGGCCGTATGGCTC
ENSPTRT00000067576      GTCATCTGCACCCGGTTCATCAGTAAAGAGATCCTGACCATCCGGGAGGCCGTATGGCTC

ENSMUST00000115390      ACAGACAACACGTATAAATACGAGGACTTGGTGCGCGTGATGGGGGAGATCATTTCCGCC
ENSRNOT00000064392      ACAGACAACACGTATAAATACGAGGACTTGGTACGCGTGATGGGGGAGATCATCTCCGCC
ENSCJAT00000062994      ACGGATAACACGTACAAGTACGAGGACCTAGTGAGAATGATGGGCGAGATCGTCTCCGCC
ENSMMUT00000000001      ACAGACAACACGTACAAGTATGAGGACCTGGTGAGAATGATGGGCGAGATCGTCTCCGCC
ENSPPYT00000008249      ACGGACAACACGTACAAGTACGAGGACCTGGTGAGAATGATGGGCGAGATCGTCTCCGCC
ENSGGOT00000011933      ACGGACAACACGTACAAGTACGAGGACCTGGTGAGAATGATGGGCGAGATCGTCTCCGCC
ENST00000397066         ACGGACAACACTTACAAGTACGAGGACCTGGTGAGAATGATGGGCGAGATCGTCTCCGCC
ENSPTRT00000067576      ACGGACAACACGTACAAGTACGAGGACCTGGTGAGAATGATGGGCGAGATCGTCTCCGCC

ENSMUST00000115390      CTGGAAGGGAAGATTCGGATCCCTACCGTGGTTGACTATAAAGAGGTCCTGCTGACACTG
ENSRNOT00000064392      CTGGAAGGGAAGATTCGGATCCCTACGGTGGTTGACTATAAAGAGGTCCTGCTGACACTG
ENSCJAT00000062994      CTGGAAGGGAAGATTCGAGTCCCCACTGTGGTGGATTACAAGGAGGTCCTGCTGACGCTA
ENSMMUT00000000001      TTGGAAGGGAAGATTCGAGTCCCCACCGTGGTGGATTACAAGGAGGTCCTGCTGGCGCTA
ENSPPYT00000008249      TTGGAAGGGAAGATTCGAGTCCCCACTGTGGTGGATTACAAGGAGGTCCTGCTGACGCTA
ENSGGOT00000011933      TTGGAAGGGAAGATTCGAGTCCCCACTGTGGTGGATTACAAGGAGGTCCTGTTGACGCTA
ENST00000397066         TTGGAAGGGAAGATTCGAGTCCCCACTGTGGTGGATTACAAGGAGGTCCTGCTGACGCTA
ENSPTRT00000067576      TTGGAAGGGAAGATTCGAGTCCCCACTGTGGTGGATTACAAGGAGGTCCTGCTGACGCTA

ENSMUST00000115390      GTCCCCGTCGCACCCAGAACCCAGCACCTGTGCAGCTTCCTCTGTGAGCTCACCCTGCTG
ENSRNOT00000064392      GTCCCTGTCGCACCCAGAACCCAACACCTGTGCAGCTTCCTCTGTGAGCTCACCCTGCTG
ENSCJAT00000062994      GTCCCTGTGGAGCCGAGAACCCAGCACCTGTGCAGCTTCCTCTGTGAGCTCTCCCTGCTG
ENSMMUT00000000001      GTCCCCGTGGAGCTGAGAACCCAGCACCTGTGCAGCTTCCTCTGTGAGCTCTCCCTGCTG
ENSPPYT00000008249      GTCCCTGTGGAGCTGAGAACCCAGCACCTGTGCAGCTTCCTCTGCGAGCTCTCCCTGCTG
ENSGGOT00000011933      GTCCCTGTGGAGCTGAGAACCCAGCACCTGTGCAGCTTCCTCTGCGAGCTCTCCCTGCTG
ENST00000397066         GTCCCTGTGGAGCTGAGAACCCAGCACCTGTGCAGCTTCCTCTGCGAGCTCTCCCTGCTG
ENSPTRT00000067576      GTCCCTGTGGAGCTGAGAACCCAGCACCTGTGCAGCTTCCTCTGCGAGCTCTCCCTGCTG

ENSMUST00000115390      CACACCAGCCTGTCCATCTATGCCCCAGCCCGCCTGGCCTCTGCGGCCCTTCTCCTGGCC
ENSRNOT00000064392      CACACCAGCCTGTCCGTCTACGCCCCAGCCCGCCTGGCCTCTGCCGCCCTGCTCCTGGCC
ENSCJAT00000062994      CACACCAGCCTGTCTGCCTACGCCCCGGCCCGCCTGGCCGCCGCAGCCTTGCTCCTGGCC
ENSMMUT00000000001      CACACCAGCCTGTCCACCTATGCCCCAGCCCGCCTGGCTGCCGCAGCCCTGCTTCTGGCC
ENSPPYT00000008249      CACACCAGCCTGTCCACCTACGCCCCAGCCCGCCTGGCTGCCGCAGCCCTGCTCCTGGCC
ENSGGOT00000011933      CACACCAGCCTGTCCGCCTACGCCCCAGCCCGCCTGGCTGCCGCAGCCCTGCTCCTGGCC
```

```
ENST00000397066          CACACCAGCCTGTCCGCCTACGCCCCAGCCCGCCTGGCTGCCGCAGCCCTGCTCCTGGCC
ENSPTRT00000067576       CACACCAGCCTGTCCGCCTACGCCCCAGCCCGCCTGGCTGCCGCAGCCCTGCTCCTGGCC

ENSMUST00000115390       AGACTCATGCATGGGCAGACACAGCCCTGGACCACTCATCTGTGGGACCTCACTGGCTTC
ENSRNOT00000064392       AGACTGATGCATGGACACACACAGCCCTGGACCACTCAGCTGTGGGACCTCACTGGGTTC
ENSCJAT00000062994       AGGCTGACACACAGGCAGACACAGCCCTGGACCACTCAGCTGTGGGACCTCACCGGATTC
ENSMMUT00000000001       AGACTGACGCACAGGCAGACACAGCCCTGGACCACTCAGCTGTGGGACCTCACCGGATTC
ENSPPYT00000008249       AGACTGACGCATGGGCAGACACAGCCCTGGACCACTCAGCTGTGGGACCTCACTGGATTC
ENSGGOT00000011933       AGACTGACGCATGGGCAGACACAGCCCTGGACCACTCAGCTGTGGGACCTCACCGGATTC
ENST00000397066          AGACTGACGCACGGGCAGACACAGCCCTGGACCACTCAGCTGTGGGACCTCACCGGATTC
ENSPTRT00000067576       AGACTGACGCACGGGCAGACACAGCCCTGGACCACTCAGCTGTGGGACCTCACCGGATTC

ENSMUST00000115390       TCCTACAGTGACCTCGTACCCTGTGTCCTGAGCCTTCATAAGAAGTGTTTCCACGATGAT
ENSRNOT00000064392       TCCTACAGCGACCTCACGCCCTGCGTCTTGAGCCTCCATAAGAAGTGTTTCCATGATGAT
ENSCJAT00000062994       TCCTCTGAGGACCTCATTCCCTGCGTCTTGAGCCTTCATAAGAAGTGCTTCCACGATGAC
ENSMMUT00000000001       TCCTATGAGGACCTCATTCCCTGCGTCTTGAGCCTTCATAAGAAGTGCTTCCACGATGAC
ENSPPYT00000008249       TCCTATGAAGACCTCATTCCTTGCGTCTTGAGCCTTCATAAGAAGTGCTTCCACGATGAT
ENSGGOT00000011933       TCCTATGAAGACCTCATTCCCTGCGTCTTGAGCCTTCATAAGAAGTGCTTCCACGATGAT
ENST00000397066          TCCTATGAAGACCTCATTCCCTGCGTCTTGAGCCTCCATAAGAAGTGCTTCCATGATGAC
ENSPTRT00000067576       TCCTATGAAGACCTCATTCCCTGCGTCTTGAGCCTTCATAAGAAGTGCTTCCACGATGAC

ENSMUST00000115390       GCACCCAAAGACTACAGACAAGTCTCTCTGACGGCCGTGAAGCAGAGGTTCGAGGATAAG
ENSRNOT00000064392       GCACCCAAAGACTACAGACAAGTCTCTCTGACGGCCGTGAAGCAGCGGTTTGAGGATAAG
ENSCJAT00000062994       GCCCCCAAGGATTACAGGCAAGTCTCTCTGACCGCCGTGAAGCAGCGATTTGAGGACAAG
ENSMMUT00000000001       GCCCCCAAGGACTACAGGCAAGTCTCTCTGACCGCCGTGAAGCAGCGATTTGAGGACAAG
ENSPPYT00000008249       GCCCCCAAGGACTACAGGCAAGTCTCCCTGACTGCTGTGAAGCAGCGATTTGAGGACAAG
ENSGGOT00000011933       GCCCCCAAGGACTACAGGCAAGTCTCTCTGACCGCCGTGAAGCAGCGGTTTGAGGACAAG
ENST00000397066          GCCCCCAAGGACTACAGGCAAGTCTCTCTGACCGCCGTGAAGCAGCGGTTTGAGGACAAG
ENSPTRT00000067576       GCCCCCAAGGACTACAGGCAAGTCTCTCTGACCGCCGTGAAGCAGCGGTTTGAGGACAAG

ENSMUST00000115390       TGCTATGAGGAAATCAGCCGGGAAGAGGTGCTGAGCTATGCTGACTTGTGCAGTACAATA
ENSRNOT00000064392       TGCTATGAGGAGATCAGCCAGGAAGAGGTACTGAGCTATGCCGAACTGTGCAGTGCATTA
ENSCJAT00000062994       CGCTATGGAGAAATCAGCCAGGAAGAGGTGCTAAGCTACAGCCAGTTGTGTGCGGCATTA
ENSMMUT00000000001       CGCTATGGAGAAATCAGCCAGGAAAAGGTGCTGAGCTACAGCCAGTTGTGTGCGGCATTA
ENSPPYT00000008249       CGCTACGGAGAAATCAGCCAGGAAGAGGTGCTGAGCTACAGCCAGTTGTGTGCTGCATTA
ENSGGOT00000011933       CGCTACGGAGAAATCAGCCAGGAAGAGGTGCTGAGCTACAGCCAGTTGTGTGCTGCATTA
ENST00000397066          CGCTATGGAGAAATCAGCCAGGAAGAGGTGCTGAGCTACAGCCAGTTGTGTGCTGCATTA
ENSPTRT00000067576       CGCTACGGAGAAATCAGCCAGGAAGAGGTGCTGAGCTACAGCCAGTTGTGTGCTGCATTA

ENSMUST00000115390       GGAGTAAAACAGGAAAGCCCAGAGCCCCCATCTTTCCCTAGCTCAGGGGAGATCCACACC
ENSRNOT00000064392       GGGGTGAAACAGGAGAGCCCAGAGCCCCCGTCTTTCCCTAGCTCCGGGGAGATCCACACC
ENSCJAT00000062994       GGGGTGACACAAGACAGTCCCGACCCCCCAACCTTCCTCAGCACAGGAGAGATCCACGCC
ENSMMUT00000000001       GGAGTGACACAAGACAGCCCCGACCCCCCGACTTTCCTCAGCACAGGGGAGATCCACGCC
ENSPPYT00000008249       GGAGTGACACAAGACAGCCCTGACCCCCCGACTTTCCTCAGCACAGGGGAGATCCACGCC
ENSGGOT00000011933       GGAGTGACACAAGACAGCCCCGACCCCCCGACTTTCCTCAGCACAGGGGAGATCCACGCC
ENST00000397066          GGAGTGACACAAGACAGCCCCGACCCCCCGACTTTCCTCAGCACAGGGGAGATCCACGCC
ENSPTRT00000067576       GGAGTGACACAAGACAGCCCCGACCCCCCGACTTTCCTCAGCACAGGGGAGATCCACGCC

ENSMUST00000115390       TTCCTCAGCTCGCCCTCCGGGAGGAGGAGCAAACGGAAGCGAGAAAACAGCCTTCAGGAG
ENSRNOT00000064392       TTCCTCAGCTCACCCTCTGGGAGGAGAAGCAAACGGAAGCGAGAGAACAGCCTTCAGGAG
ENSCJAT00000062994       TTCCTCAGCTCTCCCTCGGGACGGAGAAGCAAACGGAAGCGGGAGAATAGCTTCCAGGAG
ENSMMUT00000000001       TTCCTCAGCTCTCCCTCGGGGCGGAGAACCAAACGGAAGCGGGAGAACAGCCTCCAGGAG
ENSPPYT00000008249       TTCCTCAGCTCTCCCTCAGGGCGGAGAACCAAACGGAAGCGGGAGAACAGCCTCCAGGAG
ENSGGOT00000011933       TTCCTCAGCTCTCCCTCGGGGCGGAGAACCAAACGGAAGCGGGAGAACAGCCTCCAGGAA
```

```
ENST00000397066          TTCCTCAGCTCTCCCTCGGGGCGGAGAACCAAACGGAAGCGGGAGAACAGCCTCCAGGAA
ENSPTRT00000067576       TTCCTCAGCTCTCCCTCGGGGCGAAGAACCAAACGGAAGCGGGAGAACAGCCTCCAGGAA

ENSMUST00000115390       GACAGAGGCAGCTTTGTCACCACACCCACCGCAGAGCTATCGAATCAGGAGGAGACACTG
ENSRNOT00000064392       GACAGGGGAAGCTTTGTTACCACACCCACCGCAGAACTATCGAACCAGGAGGAGACATTG
ENSCJAT00000062994       GACAGGGGCAGCTTCGTCACCACCCCTACTGCGGAGCTGTCCAGCCAGGAGGAGACGCTG
ENSMMUT00000000001       GACAGGGGCAGCTTTGTTACCACCCCCACTGCGGAGCTGTCCAGCCAGGAGGAGACGCTG
ENSPPYT00000008249       GACAGGGGCAGCTTCGTTACCACCCCCACTGCGGAGCTGTCCAGCCAGGAGGAGACGCTG
ENSGGOT00000011933       GACAGGGGCAGCTTCGTTACCACCCCCACTGCGGAGCTGTCCAGCCAGGAGGAGACGCTG
ENST00000397066          GACAGAGGCAGCTTCGTTACCACCCCCACTGCGGAGCTGTCCAGCCAGGAGGAGACGCTG
ENSPTRT00000067576       GACAGGGGCAGCTTCGTTACCACCCCCACTGCGGAGCTGTCCAGCCAGGAGGAGACGCTG

ENSMUST00000115390       CTAGGCAGCCTTCTGGACTGGAGCCTGGAGTGCTGCTCTGGCTATGAGGGAGACCAGGAG
ENSRNOT00000064392       CTAGGCAGCCTTCTGGACTGGAGCCTGGACTGCTGCTCTGGCTATGAGGGAGACCAGGAG
ENSCJAT00000062994       CTGGGCAGCTTCCTCGACTGGAGCCTGGACTGCTGCTCCGGCTATGAAGGTGACCAGGAG
ENSMMUT00000000001       CTGGGCAGCTTCCTCGACTGGAGCCTGGACTGCTGCTCTGGCTACGAAGGCGACCAGGAG
ENSPPYT00000008249       CTGGGCAGCTTCCTCGACTGGAGCCTGGACTGCTGCTCTGGCTACGAAGGCGACCAGGAG
ENSGGOT00000011933       CTGGGCAGCTTCCTCGACTGGAGCCTGGACTGCTGCTCTGGCTATGAAGGCGACCAGGAG
ENST00000397066          CTGGGCAGCTTCCTCGACTGGAGCCTGGACTGCTGCTCTGGCTATGAAGGCGACCAGGAG
ENSPTRT00000067576       CTGGGCAGCTTCCTCGACTGGAGCCTGGACTGCTGCTCTGGCTATGAAGGCGACCAGGAG

ENSMUST00000115390       AGCGAAGGCGAGAAGGAGGGTGATGTCACAGCTCCCAGCAGACTCCTTGATGTCACTGTG
ENSRNOT00000064392       AGCGAAGGAGAGAAGGAGGGGGATGTTACAGCTCCCAGTGGACTCCTTGACGTCACTGTG
ENSCJAT00000062994       AGTGAGGGCGAGAAGGAGGGCGATGTGACAGCTCCCAGTGGCGTCCTCGATGTCACCGTG
ENSMMUT00000000001       AGCGAGGGCGAGAAGGAGGGTGACGTGACAGCTCCCAGTGGCATCCTCGATGTCACCGTG
ENSPPYT00000008249       AGTGAGGGCGAGAAGGAGGGCGACGTGACAGCCCCCAGCGGCATCCTCGACGTCACCGTG
ENSGGOT00000011933       AGTGAGGGCGAGAAGGAGGGCGACGTGACAGCTCCCAGCGGCATCCTCGATGTCACCGTG
ENST00000397066          AGTGAGGGCGAGAAGGAGGGCGACGTGACAGCTCCCAGCGGCATCCTCGATGTCACCGTG
ENSPTRT00000067576       AGTGAGGGCGAGAAGGAGGGCGATGTGACAGCTCCCAGCGGCATCCTCGATGTCACCGTG

ENSMUST00000115390       GTCTACCTGAACCCAGAAGAACATTGCTGCCAGGAGTCCAGTGATGAGGAGGCCTGGCCA
ENSRNOT00000064392       GTCTACCTGAACCCAGAAGAACACTGCTGCCAGGAGTCCAGTGATGAGGAGGTGTGGCCA
ENSCJAT00000062994       GTCTACCTGAACCCAGAACAGCATTGCTGCCAGGAATCCAGTGATGAGGAGGCTTGCCCA
ENSMMUT00000000001       GTCTACCTGAATCCAGAACAGCATTGCTGCCAGGAATCCAGTGATGAGGAGGCTTGCCCA
ENSPPYT00000008249       GTCTACCTGAACCCAGAACAGCATTGCTGCCAGGAATCCAGTGATGAGGAGGCTTGCCCA
ENSGGOT00000011933       GTCTACCTGAACCCAGAACAGCATTGCTGCCAGGAATCCAGTGATGAGGAGGCTTGCCCA
ENST00000397066          GTCTACCTGAACCCAGAACAGCATTGCTGCCAGGAATCCAGTGATGAGGAGGCTTGTCCA
ENSPTRT00000067576       GTCTACCTGAACCCAGAACAGCATTGCTGCCAGGAATCCAGTGATGAGGAGGCTTGCCCA

ENSMUST00000115390       GAGGACAAGATCCACCCAGCACCA---------------GGCACCCAGGCACCTCCAGCC
ENSRNOT00000064392       GAGGACAAGAGCCACCCAACACCA---------------GGCACCCAGGCACCTCCAGCC
ENSCJAT00000062994       GAGGACGAGGGTCCCCAGGACCCACAGGTGTCGGTGCTGGACACCCAGATCCCTGCAGTC
ENSMMUT00000000001       GAGGACGAGAGACCCCAGGACCCACAGGTACCAGCGCTGGATACCCAGATCCCTGCAACC
ENSPPYT00000008249       GAGGATAGGGGACCCCAGGACCCACAGGCACTGGCACTGGACACCCAGATCCCTGCAACC
ENSGGOT00000011933       GAGGACAAGGGACCCCAGGACCCACAGGCACTGGCGCTGGACACCCAGATCCCTGCCACC
ENST00000397066          GAGGACAAGGGACCCCAGGACCCACAGGCACTGGCGCTGGACACCCAGATCCCTGCAACC
ENSPTRT00000067576       GAGGACAAGGGACCCCAGGACCCACAGGCACTGGCGCTGGACACCCAGATCCCTGCAACC

ENSMUST00000115390       TCAGCTCCCAGGCCCCTTCTCTGCAATCGGGGAGATCGGGCCAAGGACATTACGACCTCA
ENSRNOT00000064392       TCAGCTCCCTGGCCCCTTCCCTGCAACAGGGGAGATCCGGGCAAGGATGTCACGACCTCA
ENSCJAT00000062994       CCAGGACCCGAGTCCCCGCTCTGCACCAGCCAGCAGCCAGGAAAGGACATCACGACCTCA
ENSMMUT00000000001       CCAGGACCCAAGTCCCTGCTCCGCACCAGCCCGGAGCCAGGGAAGGACGTCACGACCTCA
ENSPPYT00000008249       CCTGGACCCAAACCCCTGCTCTGCACCAGCCGGGAGCCAGGGAAGGACGTCACGACCTCA
ENSGGOT00000011933       CCTGGACCCAAACCCCTGGTCCGCACCAGCCGGGAGCCAGGGAAGGACGTCACGACCTCA
```

```
ENST00000397066       CCTGGACCCAAACCCCTGGTCCGCACCAGCCGGGAGCCAGGGAAGGACGTCACGACCTCA
ENSPTRT00000067576    CCTGGACCCAAACCCCTGGTCCGCACCAGCCGGGAGCCAGGGAAGGATGTCACGACCTCA

ENSMUST00000115390     GGATACTCCTCTGTTAGCAGCTCGAGTCCCATAAGCTCCCTGGATGGTGGCATGGGGGGC
ENSRNOT00000064392     GGCTACTCCTCTGTCAGCAGCTCAAGTCCCATAAGCTCCCTGGATGGTGGCATGGTGGGC
ENSCJAT00000062994     GGGTACTCCTCTGTCAGCACCGCAAGTCCCACAGGCTCTGTGGATGGCAGCTTGGGGGTC
ENSMMUT00000000001     GGGTACTCCTCCGTCAGCACCACGAGTCCCACAAGCTCTGTGGATGGTGGCTTGGGGGTC
ENSPPYT00000008249     GGGTACTCCTCCGTCAGCACCGCGAGTCCCACAAGCTCCGTGGATGGTGGCTTGGGGGCC
ENSGGOT00000011933     GGGTACTCCTCCGTCAGCACCGCGAGTCCCACAAGCTCCGTGGACGGTGGCTTGGGGGCC
ENST00000397066        GGGTACTCCTCCGTCAGCACCGCAAGTCCCACAAGCTCCGTGGACGGTGGCTTGGGGGCC
ENSPTRT00000067576     GGGTACTCCTCCGTCAGCACCGCAAGTCCCACAAGCTCCGTGGACGGTGGCTTGGGGGCC

ENSMUST00000115390     TCTCCGCAATCTACCTCAGTGCTCTCTGTGGGCAGCCACTCAAGCACAAAGCCTTGCCAC
ENSRNOT00000064392     TCTCCCCGATCTACCTCAGTGCTCTCTGTGGGAAGCCACTCAAGCACAAAGCCTTGCTAC
ENSCJAT00000062994     CTGCCCCGATCTACCTCAGTGCTATCTCTGGACGGTGACTTGCGCACACAGCCCTACCAC
ENSMMUT00000000001     CTGCCCCGACCTACCTCAGTGCTGTCCCTGGACAGTGACTCGCACACACAGCCCTGCCAC
ENSPPYT00000008249     CTGCCCCGACCTACCTCAGTACTGTCCCTGGACAGTGACTCGCACACAAAGCCCTGCCAC
ENSGGOT00000011933     CTGCCCCGACCTACCTCAGTGCTGTCCCTGGACAGTGACTCGCACACACAGCCCTGCCAC
ENST00000397066        CTGCCCCAACCTACCTCAGTGCTGTCCCTGGACAGTGACTCGCACACACAGCCCTGCCAC
ENSPTRT00000067576     CTGCCCCGACCTACCTCAGTGCTGTCCCTGGACAGTGACTCGCACACACAGCCCTGCCAC

ENSMUST00000115390     CATCAGGCCAAGAAGTCATGTTTACAGTGTCGTCCCCCAAACTCCCCAGAGAGCGGTGTT
ENSRNOT00000064392     CATCAGGCCAAGAAGTCATGTTTACAGTGTCGTCCCCCAAATCCCCCAGAGAGCGGTGCT
ENSCJAT00000062994     CATCAGGCCAGGAAGCCATGTTTACAGTGTCGTCCCCCAAGTCCCCCGGAGGGCAGTGTT
ENSMMUT00000000001     CATCAGGCCAGGAAGTCATGTTTACAGTGTCGTCCCCCAAGTCCCCCCGAAAGCAGTGTT
ENSPPYT00000008249     CATCAGGCCAGGAAGTCATGTTTACAGTGTCGTCCCCCA---------GAGAGCAGTGTT
ENSGGOT00000011933     CATCAGGCCAGGAAGTCATGTTTACAGTGTCGTCCCCCAAGTCCCCCGGAGAGCAGTGGT
ENST00000397066        CATCAGGCCAGGAAGTCATGTTTACAGTGTCGTCCCCCAAGTCCCCCGGAGAGCAGTGTT
ENSPTRT00000067576     CATCAGGCCAGGAAGTCATGTTTACAGTGTCGTCCCCCAAGTCCCCCGGAGAGCAGTGTT

ENSMUST00000115390     CACCAGCAGCCTGTAAAGCGCCAAAACCTGTCTGTACACAGT---GACAAGGACATGCAC
ENSRNOT00000064392     CACCAGCAACCGGTAAAGCGCCAAAACCTGTCGGTGCACAGT---GACGAGGACACGAAC
ENSCJAT00000062994     CCCCAGCAACAGGTGAAGCGGAAGAATCTGTGTGTGCACAGTGAGGAGGAGGGCATGAAC
ENSMMUT00000000001     CCCCGGCAACAGGTGAAACGGATAAACCTATGCATACACAGTGAGGAGGAGGACACGAAC
ENSPPYT00000008249     CCCCGGCAACAGGTGAAGCGGATAAACCTATGCGTACACAGTGAGGAGGAGGACATGAAC
ENSGGOT00000011933     CCCCGGCAACAGGTGAAGCGGATAAACCTATGTGTACACAGTGAGGAGGAGGACATGAAC
ENST00000397066        CCCCAGCAACAGGTGAAGCGGATAAACCTATGCATACACAGTGAGGAGGAGGACATGAAC
ENSPTRT00000067576     CCCCAGCAACAGGTGAAGCGGATAAACCTATGCATACACAGTGAGGAGGAGGACATGAAC

ENSMUST00000115390     CTGGCTTCC---------
ENSRNOT00000064392     CTGGGCTTCCTGAAGCTC
ENSCJAT00000062994     TGGAGCTTG---AGGCTG
ENSMMUT00000000001     CTGGGCCTTGTGAGGCTG
ENSPPYT00000008249     CTGGGCCTTGTGAGGCTG
ENSGGOT00000011933     CTGGGCCTTGTGAGGCTG
ENST00000397066        CTGGGCCTTGTGAGGCTG
ENSPTRT00000067576     CTGGGCCTTGTGAGGCTG
```

Multiple sequence alignment of Ect2l

```
ENSMUST00000095817     GTGGAAAGTTTCCACACTCGGTTCAGCGCCTGGACACCTTTTAAGAACCAGTCCTTAAAC
ENSCJAT00000037417     ATGGAGAGCTTCCACATGAGATTTAGTGCCTGGACACCTTTTAACAACAAGTCATTAAAT
ENSPPYT00000019842     ATGGAGAGCTTCCACACAAGATTTAGTGCCTGGACACCTTTTAGCAACAAGTCATTAAAT
ENSGGOT00000012884     ATGGAGAGCTTCCACACCAGATTCAGTGCCTGGACACCTTTTAGCAACAAGCCATTAAAT
```

```
ENST00000423192          ATGGAGAGCTTCCACACCAGATTTAGTGCCTGGACACCTTTTAGCAACAAGTCATTAAAT
ENSPTRT00000056780       ATGGAGAGCTTCCACACCAGATTTAGTGCCTGGACACCTTTTAGCAACAAGTCATTAAAT

ENSMUST00000095817       AGACAGCTCTTTCAGGAAAGAGTTACTCTCATAAGTCACTGGTTTGACCTCTGGACCAAC
ENSCJAT00000037417       AGACAGCTCTTTCAGGAAAGAGTGGCTCTTATAAGTCATTGGTTTGACCTCTGGACTAAC
ENSPPYT00000019842       AGACAGCTCTTTCAGGAAAGAGTGGCTCTTATAAGTCATTGGTTTGACCTCTGGACTAAC
ENSGGOT00000012884       AGACAGCTCTTTCAGGAAAGAGTGGCTCTTATAAGTCATTGGTTTGACCTCTGGACTAAC
ENST00000423192          AGACAGCTCTTTCAGGAAAGAGTGGCTCTTATAAGTCATTGGTTTGACCTCTGGACTAAC
ENSPTRT00000056780       AGACAGCTCTTTCAGGAAAGAGTGGCTCTTATAAGTCATTGGTTTGACCTCTGGACTAAC

ENSMUST00000095817       AAGCAACGGCGGGAATTCTTATTCGTGATTCTTTCACAATGCTCAAAATCACAATTAAGG
ENSCJAT00000037417       AAGCAACGTCAAGAATTCTTATTCGCAATTTTTTTACGATGCACTAAATCACAACTAAGG
ENSPPYT00000019842       AAGCAACGTCAAGAATTCTTATTCACGATTTTTTTAAGATGCAGTAAATCACAATTAAGG
ENSGGOT00000012884       AAGCAACGTCAAGAATTCTTATTCGCAATTTTTTTAAGATGCACTAAATCACAATTAAGG
ENST00000423192          AAGCAACGTCAAGAATTCTTATTCGCAATTTTTTTAAGATGCACTAAATCACAATTAAGG
ENSPTRT00000056780       AAGCAACGTCAAGAATTCTTATTCGCAATTTTTTTAAGATGCACTAAATCACAATTAAGG

ENSMUST00000095817       TTCATCCAAGACTGGTTTTCAGAAAGGAAGCAGGTGGCCAGAGTGGATTTCTCAACAGTG
ENSCJAT00000037417       TTTGTCCAAGACTGGTTTTCAGAAAGGAAGCAAGTGGCCAAATTGGATTTCTCTACGGTG
ENSPPYT00000019842       TTTGTCCAAGACTGGTTTTCAGAAAGGATGCAAGTGGCCAGAGTGGACTTCTCTACAGTG
ENSGGOT00000012884       TTTGTCCAAGACTGGTTTTCAGAAAGGATGCAAGTGGCCAAAGTGGACTTCTCTACAGTG
ENST00000423192          TTTGTCCAAGACTGGTTTTCAGAAAGGATGCAAGTGGCCAAAGTGGACTTCTCTACAGTG
ENSPTRT00000056780       TTTGTCCAAGACTGGTTTTCAGAAAGGATGCAAGTGGCCAAAGTGGACTTCTCTACAGTG

ENSMUST00000095817       TTACCACGCTTCATTTCTCTTTATATCTTTTCCTTTCTGAATCCGAAAGACTTGTGTGCA
ENSCJAT00000037417       TTACCACGCTTCATTTCTCTATACATCTTTTCCTTTCTGAGTCCCAAAGATCTGTGTGCA
ENSPPYT00000019842       TTACCACGCTTCATTTCTCTATATATCTTTTCCTTTTTGAGTCCGAAAGATTTGTGTGCC
ENSGGOT00000012884       TTACCACGCTTCATTTCTCTATATATCTTTTCCTTTTTGAGTCCGAAAGATTTGTGTGCC
ENST00000423192          TTACCACGCTTCATTTCTCTATATATCTTTTCCTTTTTGAGTCCGAAAGATTTGTGTGCC
ENSPTRT00000056780       TTACCACGCTTCATTTCTCTATATATCTTTTCCTTTTTGAGTCCGAAAGATTTGTGTGCT

ENSMUST00000095817       GCCGCTCAAGTCAGCTGGCCCTGGAAGTTTCTAACGGAACAGGATTGCTTATGGATGCCG
ENSCJAT00000037417       GCCGCCCAAGTCAGCTGGGCCTGGAAGTTTTTAACTGAACAGGATTGCTTATGGATGCCC
ENSPPYT00000019842       GCTGCCCAAGTCAGCTGGCCCTGGAAGTTTTTAACTGAACAGGATTGCTTATGGATGCCC
ENSGGOT00000012884       GCTGCCCAAGTCAGCTGGCCCTGGAAGTTTTTAACTGAACAGGATTGCTTATGGATGCCC
ENST00000423192          GCTGCCCAAGTCAGCTGGCCCTGGAAGTTTTTAACTGAACAGGATTGCTTATGGATGCCC
ENSPTRT00000056780       GCTGCCCAAGTCAGCTGGCCCTGGAAGTTTTTAACTGAACAGGATTGCTTATGGATGCCC

ENSMUST00000095817       AAATGCACTAAGTTCGGATGGTTTCTGCCCTACACTCCGACACAGAATGAATACGGAGCT
ENSCJAT00000037417       AAATGCGTTAAGTTCGGATGGTTTCTGCCCTATACTCCAACAGATAACGAGTACGGTGCT
ENSPPYT00000019842       AAATGCATTAAGTTCGGATGGTTTCTGCCCTATACTCCAACAGATAATGAGTATGGCGCT
ENSGGOT00000012884       AAATGCGTTAAGTTCGGATGGTTTCTGCCCTATACTCCAACAGATAATGAGTATGGTGCT
ENST00000423192          AAATGCGTTAAGTTCGGATGGTTTCTGCCCTATACTCCAACAGATAATGAGTATGGTGCT
ENSPTRT00000056780       AAATGCGTTAAGTTCGGATGGTTTCTGCCCTATACTCCAACAGATAATGAGTATGGTGCT

ENSMUST00000095817       TGGAAGCATCACTACATTGCCTGTGTGTCCAGCTTGGACTGGCTAACACCCAGGGAAGCT
ENSCJAT00000037417       TGGAAGCACCATTACATTGCTTGTGTGTCCCACTTAGACTGGCTGACACCCAGGGAGGCT
ENSPPYT00000019842       TGGAAGCGCCATTACGTTGCTTGTGTGTCCCACTTAGACTGGCTGACACCTAGGGAGGCT
ENSGGOT00000012884       TGGAAGCGCCATTACGTTGCTTGTGTGTCCCACTTAGACTGGCTGACACCTAGGGAGGCT
ENST00000423192          TGGAAGCGCCATTACATTGCTTGTGTGTCCCACTTAGACTGGCTGACACCTAGGGAGGCT
ENSPTRT00000056780       TGGAAGCGCCATTACATTGCTTGTGTGTCCCACTTAGACTGGCTGACACCTAGGGAGGCT
```

```
ENSMUST00000095817     GCTGCTGTTTATGGGACACTGAATGAGCCCAAAACAGAGGACGAGGAATTCCAGGAGAGG
ENSCJAT00000037417     GCTGCTACTTATGGGACGCTGAATGAACCCAAAACAGAAGATGAGGAACTACTGGAGAGA
ENSPPYT00000019842     GCTGCTACTTATGGGACGCTGAATGAACCCAAAACAGAAGATGAGGAACTACTGGAGAGA
ENSGGOT00000012884     GCTGCTACTTATGGGACGCTGAATGAACCCAAAACAGAAGATGAGGAACTACTGGAGAGA
ENST00000423192        GCTGCTACTTATGGGACGCTGAATGAACCCAAAACAGAAGATGAGGAACTACTGGAGAGA
ENSPTRT00000056780     GCTGCTACTTATGGGACGCTGAATGAACCCAAAACAGAAGATGAGGAACTACTGGAGAGA

ENSMUST00000095817     CGACGAGAAAAGTGCCTGAGAAAAATTATTTGGGAGAACATTGCCTTCCGCAAGAAGGAG
ENSCJAT00000037417     CATAGAGAAAAGTGCCTAAGAAAAAGAATTTGGGAGAAAATTGCACTGCGTAGGAAGGAG
ENSPPYT00000019842     CAAAGAGAAAAGTGCCTAAGGAAAAGAATTTGGGAGAAAATTGTACTACGTAAGAAGGAG
ENSGGOT00000012884     CAAAGAGAAAAGTGCCTGAGGAAAAGAATTTGGGAGAAAATTGCACTACGTAAGAAGGAG
ENST00000423192        CAAAGAGAAAAGTGCCTGAGGAAAAGAATTTGGGAGAAAATTGCACTACGTAAGAAGGAG
ENSPTRT00000056780     CAAAGAGAAAAGTGCCTGAGGAAAAGAATTTGGGAGAAAATTGCACTACGTAAGAAGGAG

ENSMUST00000095817     TTATTCAAAGCTCGACCCCCTTGGCTGAGTGGAACGCGCTGCTCCAGGTTGCTAAAAAGT
ENSCJAT00000037417     TTATTCAAAGTTCGACCCCCTTGGGTCAGTGGAACGGGCTGCTCTAGCGTGCTAAAGCCC
ENSPPYT00000019842     TTATTCAAAGTTCGACCCCCTTGGGTGAGTGGAACTTGCTGCTCTAGCGTGCTAAAGCCC
ENSGGOT00000012884     TTATTCAAAGTTCGACCCCCTTGGGTGAGTGGAACTTGCTGCTCTAGCGTGCTAAAGCCC
ENST00000423192        TTATTCAAAGTTCGACCCCCTTGGGTGAGTGGAACTTGCTGCTCTAGCGTGCTAAAGCCC
ENSPTRT00000056780     TTATTCAAAGTTCGACCCCCTTGGGTGAGTGGAACTTGCTGCTCTAGTGTGCTAAAGCCC

ENSMUST00000095817     ACAAGCCTGCCCGGTGGTCCTCAGCTATGGAGAGACGGGACAGGGTTCTACGAAGCCTTG
ENSCJAT00000037417     AGATGCCAACCACGCCTCTCCCAGACTGTAAGGGAGCGAGCAGGATCACATGAAGCTTTG
ENSPPYT00000019842     AGATGCCAACCACGCCTCTCCCAGACTGTAAGGGAACAAGTGGGATTACATGAAGCGTTG
ENSGGOT00000012884     AGATGCCAACCACGCCTCTCCCAGACTGTAAGGGAGCGAGTGGGATTACATGAAGCTTTG
ENST00000423192        AGATGCCAACCACGCCTCTCCCAGACTGTAAGGGAGCGAGTGGGATTACATGAAGCTTTG
ENSPTRT00000056780     AGATGCCAACCACGCCTCTCCCAGACTGTAAGGGAGCGAGTGGGATTACATGAAGCTTTG

ENSMUST00000095817     GAGAGACAGTTTCTTCGGGCATCTTTAGATGCTTTGCCCAAGCGAAGCAATATCTCTGGA
ENSCJAT00000037417     GAGAAACAGCTTGTTTTGACATCATTAGAAACCTTGCCCAAGCAAAGCAATATTTCTGGA
ENSPPYT00000019842     GAGAAACAGCTTGTTTTGACATCGTTAGAAACCTTGCCCAAGCGAAGCAATATTTCTGGA
ENSGGOT00000012884     GAGAAACAGCTTGTTTTGACATCGTCAGAAACCCTGCCCAAGCGAAGCAATATTTCTGGA
ENST00000423192        GAGAAACAGCTTGTTTTGACATCGTTAGAAACCTTGCCCAAGCGAAGCAATATTTCTGGA
ENSPTRT00000056780     GAGAAACAGCTTGTTTTGACATCGTTAGAAACCTTGCCCAAGCGAAGCAATATTTCTGGA

ENSMUST00000095817     AGTCACTCCTACCCTTTATTATTAAAGAAACACCATCATGGGGTTGGTAGAACTGAT---
ENSCJAT00000037417     AGCCATTCCTACCCTTTATTATCAAAGAAAAATTGGCATGGACTGTATAAAAATGACAAC
ENSPPYT00000019842     AGCCATTCCTACCCTTTATTATCAAAGAAAAATTGGCATGGAGTTCATAAAAATGATGAC
ENSGGOT00000012884     AGCCATTCCTACCCTTTATTATCAAAGAAAAATTGGCATGGAGTTCATAAAAATGATGAC
ENST00000423192        AGCCATTCCTACCCTTTATTATCAAAGAAAAATTGGCATGGAGTTCATAAAAATGATGAC
ENSPTRT00000056780     AGCCATTCCTACCCTTTATTATCAAAGAAAAATTGGCATGGAGTTCATAAAAATGATGAC

ENSMUST00000095817     ------------GCTTTGCCACTACATGCCATCCTGATATCATCGCGGATTCCTGCATAT
ENSCJAT00000037417     AGCTCTTCATTTGCTCTCCGGCCACACGTCATATTAATATCCTCTCGGATTCCTGCATAC
ENSPPYT00000019842     AGCTCTTCATATGCTCTCCGGCCACACTTCATGTTAATATCATCCCGGATTCCTGCGTAT
ENSGGOT00000012884     AGCTCTTCATATGCTCTCCGGCCACACTTCATGTTAATATCATCCCGGATTCCTGCGTAT
ENST00000423192        AGATCTTCATATGCTCTCCGGCCACACTTCATGTTAATATCATCCCGGATTCCTGCGTAT
ENSPTRT00000056780     AGCTCTTCATATGCTCTCCGGCCACACTTCATGTTAATATCATCCCGGATTCCTGCGTAT

ENSMUST00000095817     GAGATGGTGATGGAGAGCATCAAGCCAGACGTCGTGGCTGTGCTGTACGAACACAGCGTG
ENSCJAT00000037417     GAGATGGTGGTGGAGAGCGTGAAGTCTGGTGTCATTTCTGTGGTATATGAACACAGCGTA
ENSPPYT00000019842     GAGATGGTGGTGGAGAGCGTGAAGGCTGGTGTCATTTCTGTGGTATATGAACACAACGTA
ENSGGOT00000012884     GAGATGGTGGTGGAGAGTGTGAAGGCTGGTGTTGTTTCTGTGGTATATGAACACAGCGTA
```

ENST00000423192 GAGATGGTGATGGAGAGTGTGAAGGCTGGTGTTGTTTCTGTGGTATATGAACACAGCGTA
ENSPTRT00000056780 GAGATGGTGGTGGAGAGTGTGAAGGCTGGTGTTGTTTCTGTGGTATATGAACACAGCGTA

ENSMUST00000095817 ACATTGGAGGGCCTGCTTCAGCTCACAGACAGAGCTCTACAAGGGCGGAAGGCACAGAGC
ENSCJAT00000037417 ACCTTGGAGAGCCTGCTTTATCTCATAGAAAAAGCTCTGGATGGGCAGAAGGCACAGAGC
ENSPPYT00000019842 ACCTTGGAGAGTCTGCTGTATCTCATAGAAAAAGCTCTGGCTGGGCAGAAGGCACAGAGC
ENSGGOT00000012884 ACCTTGGAAAGCCTTCTGTATCTTATAGAAAAAGCTCTGGATGGGCAGAAGGCACAGAGC
ENST00000423192 ACCTTGGAAAGCCTTCTGTATCTTATAGAAAAAGCTCTGGATGGGCAGAAGGCACAGAGC
ENSPTRT00000056780 ACCTTGGAAAGCCTTCTGTATCTTATAGAAAAAGCTCTGGATGGGCAGAAGGCACAGAGC

ENSMUST00000095817 CTGGGGATATTCAGCAGTGGAAACAGCAGAGAGATCGACTTGCTGCAAGGCTATAAAATT
ENSCJAT00000037417 ATGGGAATATTTAGCGATGGAGATAGCAGAGATATCAATTTACTCCAAGGGTATAAAATT
ENSPPYT00000019842 ATGGGAATATTTAGCGATGGAGATAGCAGAGAAATCAATTTACTCCAAGGCTATAAAATT
ENSGGOT00000012884 ATCGGAATATTTAGCGATGGAGACAGCAGAGAAATCAATTTACTCCAAGGCTATAAAATT
ENST00000423192 ATCGGAATATTTAGCGATGGAGACAGCAGAGAAATCAATTTACTCCAAGGCTATAAAATT
ENSPTRT00000056780 ATCGGAATATTTAGCGATGGAGACAGCAGAGAAATCAATTTACTCCAAGGCTATAAAATT

ENSMUST00000095817 TGTATTAAAAATGTACTGTGGCCTGAAGTGAGAGACTTCTGGGAGAAACTAGGAAGCCGT
ENSCJAT00000037417 GGTGTTAAAAATTTACTGAAGCCTGAAGTGAGAGATTTCTGGGAGAAATTAGGAAGCTAC
ENSPPYT00000019842 GGTGTTAAAAATTTGCTGAGGCCTGAAGTGAGAGATTTCTGGGAGAAATTAGGAAGCTGT
ENSGGOT00000012884 GGTGTTAAAAATTTACTGAGGCCTGAAGTGAGAGATTTCTGGGAGAAATTAGGAAGCTAT
ENST00000423192 GGTGTTAAAAATTTACTGAGGCCTGAAGTGAGAGATTTCTGGGAGAAATTAGGAAGCTAT
ENSPTRT00000056780 GGTGTTAAAAATTTACTGAGGCCTGAAGTGAGAGATTTCTGGGAGAAATTAGGAAGCTAT

ENSMUST00000095817 GTGGCCACTGAAGAGGAAGGAGGACATGTGGACTTATTTGTGCCACTTGGAGCATCAGAA
ENSCJAT00000037417 GTGGCCACTGAAGAAGAAGGGGGCCACGTGGACTTCTTCGCGCCCCTTGGAGCATCAGAG
ENSPPYT00000019842 GTGGCCACTGAAGAAGAAGGGGGTCACGTGGACTTCTTCGTGCCCCTTGGAGCATCAGAG
ENSGGOT00000012884 GTGGCCACTGAAGAAGAAGGGGGTCACGTGGACTTCTTCGTGCCCCTTGGAGCATCAGAG
ENST00000423192 GTGGCCACTGAAGAAGAAGGGGGTCACGTGGACTTCTTCGTGCCCCTTGGAGCATCAGAG
ENSPTRT00000056780 GTGGCCACTGAAGAAGAAGGGGGTCACGTGGACTTCTTCGTGCCCCTTGGAGCATCAGAG

ENSMUST00000095817 GCAGGTATAGAAGTTCTTTCTCAGCTGTCTCAACTAACTGGCACATGGTTCTCTGCCCCG
ENSCJAT00000037417 GCAGGAACTGAAGTTCTTTCTCAGCTGTCTCAACTAACTGGCACATTCTTCACGGCCCCC
ENSPPYT00000019842 GCAGGAATTGAAGTTCTTTCCCAGCTGTCTCAACTAACTGGCACGTTCTTTACGGCCCCC
ENSGGOT00000012884 GCAGGAATTGAAGTTCTTTCCCAGCTGTCTCAACTAACTGGCACGTTCTTTACGGCCCCC
ENST00000423192 GCAGGAATTGAAGTTCTTTCCCAGCTGTCTCAACTAACTGGCACGTTCTTTACGGCCCCC
ENSPTRT00000056780 GCAGGAATTGAAGTTCTTTCCCAGCTGTCTCAACTAACTGGCACGTTCTTTACGGCCCCC

ENSMUST00000095817 ACTGGAATCGCAACCGGTTCATACCAACACATTCTTAGTGACTGGCTGGGGCCACATCAG
ENSCJAT00000037417 ACCGGGATTGCGACTGGCTCCTACCAGCACATTCTTAGTGATTGGCTGGGAGCCCAATGG
ENSPPYT00000019842 ACTGGGATTGCAACTGGCTCTTACCAGCACATTCTTAGTGATTGGCTGGGATCCCAATGG
ENSGGOT00000012884 ACTGGGATTGCAACTGGCTCTTACCAGCACATTCTTAGTGATTGGCTGGGATCCCAATGG
ENST00000423192 ACTGGGATTGCAACTGGCTCTTACCAGCACATTCTTAGTGATTGGCTGGGATCCCAATGG
ENSPTRT00000056780 ACTGGGATTGCAACTGGCTCTTACCAGCACATTCTTAGTGATTGGCTGGGATCCCAATGG

ENSMUST00000095817 GATAGGACTCCTCTTTCTAACTACTTCAGCGAGTCTAAGCTGCAGGCATGGTCCAGTTTC
ENSCJAT00000037417 GGAAAGGCCCCCTCTTCCATCTACTTCAGTGAATCGAAGCTACAGACATGGTCCAGCTTC
ENSPPYT00000019842 GGAAAGGCCCCCTCTTCCATCTACTTCTGCGAATCGAAGCTACAGACGTGGTCCAGCTTC
ENSGGOT00000012884 GGAAAGGCCCCCTCTTCCATCTACTTCTGCGAATCGAAGCTACAGACGTGGTCCAGCTTC
ENST00000423192 GGAAAGGCCCCCTCTTCCATCTACTTCTGCGAATCGAAGCTACAGACGTGGTCCAGCTTC
ENSPTRT00000056780 GGAAAGGCCCCCTCTTCCATCTACTTCTGCGAATCGAAGCTACAGACGTGGTCCAGCTTC

ENSMUST00000095817 ACAGAATTCCTGGAAGACACCTTGAAGTCAGTGAGGAAAACGTTAAATCCACTCTTCAAG
ENSCJAT00000037417 ACAGACTTCCTAGAAGACACCTTGAAAGAGGTAAGGAAGCAGCTGTGTCCTCTCTTCAAG
ENSPPYT00000019842 ACAGACTTCCTAGAAGAAACCTTGAAAACAGTAAGGAAGCAGCTGTATCCTCTCTTCAAG
ENSGGOT00000012884 ACAGACTTCCTAGAAGAAACCTTGAAAACAGTAAGGAAGCAGCTGTATCCTTTCTTCAAG
ENST00000423192 ACAGACTTCCTAGAAGAAACCTTGAAAACAGTAAGGAAGCAGCTGTATCCTTTCTTCAAG
ENSPTRT00000056780 ACAGACTTCCTAGAAGAAACCTTGAAAACAGTAAGGAAGCAGCTGTATCCTTTCTTCAAG

ENSMUST00000095817 AACTTGCAGAAGAGCATCAGTGGCCGCATGATAGGGCAGTTTATGTTTGACACTCTGGGT
ENSCJAT00000037417 GAACTGCAGAAGAGCATCAGTGGCAGGATGATAGGGCAGTTTATGTTTGAAACCATGGGT
ENSPPYT00000019842 GAACTGCACAAGAGCATCAGTGGCAGGATGATAGGGCAGTTTATGTTTGACACCATGGGT
ENSGGOT00000012884 GAACTGCAGAAGAGCATCAGTGGCAGGATGATAGGGCAGTTTATGTTTGACACCATGGGT
ENST00000423192 GAACTGCAGAAGAGCATCAGTGGCAGGATGATAGGGCAGTTTATGTTTGACACCATGGGT
ENSPTRT00000056780 GAACTGCAGAAGAGCATCAGTGGCAGGATGATAGGGCAGTTTATGTTTGACACCATGGGT

ENSMUST00000095817 ATGGACAGCATCCTAAATAACCAAGGCCCTGCACAGGCTCTGGCGGATGGACTGATGGAG
ENSCJAT00000037417 ATGAGCGACATTCTGAACAACCAAGAGATTGCACAAGCGCTGGCAGATGGGCTGATGGAA
ENSPPYT00000019842 ATGACTAACATTCTAAACAACCAAGATACTGCGCAAGCTCTGGCAGATGGATTGATGGAG
ENSGGOT00000012884 ATGACCAACATTCTAAACAACCAAGATACTGCACAAGCTCTGGCAGATGGATTGATGGAG
ENST00000423192 ATGACCAACATTCTAAACAACCAAGATACTGCGCAAGCTCTGGCAGATGGATTGATGGAG
ENSPTRT00000056780 ATGACCAACATTCTAAACAACCAAGATACTGCGCAAGCTCTGGCAGATGGATTGATGGAG

ENSMUST00000095817 CTGTCAAAAGAAGGGTCTGAAGGAAGTGCCATCGAAGACAATTCTCAGGACTCGAAGTCA
ENSCJAT00000037417 TTGTCAAAAGAAGATTCTGAAAGAAATGGTATAGAAGACAATTTTTGGGACACAAAGTCA
ENSPPYT00000019842 TTGTCAAAAGAAGATTCTGAAAGAAATGTTGTTGAAGACAATTCTTGGGACACAAAGTCC
ENSGGOT00000012884 TTGTCAAAAGAAGATTCTGAAAGAAGTGTTGTAGAAGACAATTCTTGGGACACAAAGTCC
ENST00000423192 TTGTCAAAAGAAGATTCTGAAAGAAATGTTGTAGAAGACAATTCTTGGGACACAAAGTCC
ENSPTRT00000056780 TTGTCAAAAGAAGATTCTGAAAGAAATGTTGTAGAAGACAATTCTTGGGACACAAAGTCC

ENSMUST00000095817 AGTCCAGGCCAAAGCAACCTGAATGTGGAGGTTCTGGTTAAGCTGGAGAGGAAGCTCCAG
ENSCJAT00000037417 AGACTTAGCAAAAATGATTTAAATTTTGAAGCACTGATTAATCTGGAGAGAATGCTCCAG
ENSPPYT00000019842 AGGCTCAGCAAAAATGATTTAAATTTTGAAGCACTGATTAATCTGGAGAGAATACTCCAG
ENSGGOT00000012884 GGGCTCAGCAAAAATGATTTAAATTTTGAAGCACTGATTAATCTGGAGAGAATACTCCAG
ENST00000423192 AGGCTCAGCAAAAATGATTTAAATTTTGAAGCACTGATTAATCTGGAGAGAATACTCCAG
ENSPTRT00000056780 AGGCTCAGCAAAAATGATTTAAATTTTGAAGCACTGATTAATCTGGAGAGAATACTCCAG

ENSMUST00000095817 ATGAACTCAGTAGAGACACGAGCCCAAGTTGTGAGGGAGCTTGTGCAGAGCGAGAGACGG
ENSCJAT00000037417 AGAGACTCAGATGAAAAGCGAACTAGAGTTGTCAGGGAACTCTTACAGAGTGAGAGAAAA
ENSPPYT00000019842 AAGGACTCAGCAGAAAAGCGAGCTAGAGTTGTCAGGGAACTCTTACAGAGTGAGAGAAAA
ENSGGOT00000012884 AAGGACTCAGCAGAAAAGCGAGCTAGAGTTGTCAGGGAACTCTTACAGAGTGAGAGGAAA
ENST00000423192 AAGGACTCAGCAGAAAAGCGAGCTAGAGTTGTCAGAGAACTCTTACAGAGTGAGAGAAAA
ENSPTRT00000056780 AAGGACTCAGCAGAAAAGCGAGCTAGAGTTGTCAGAGAACTCTTACAGAGTGAGAGAAAA

ENSMUST00000095817 TACGTGCAGATGCTGCGAATTGTGCAGGACGTGTACGCCAAGCCCCTGAGGGCAGCGCTC
ENSCJAT00000037417 TATGTACAGATGCTGGAAATTGTGAGAGATGTTTATGTCACACCACTGAAAGCAGCACTG
ENSPPYT00000019842 TACGTGCAGATGCTGGAAATTGTGAGAAATGTTTATGTTGCACCACTGAAAGCAGCATTG
ENSGGOT00000012884 TACGTGCAGATACTGGAAATTGTGAGAGATGTTTATGTTGCACCACTGAAGGCAGCATTG
ENST00000423192 TACGTGCAGATACTGGAAATTGTGAGAGATGTTTATGTCGCACCACTGAAAGCAGCATTG
ENSPTRT00000056780 TACGTGCAGATACTGGAAATTGTGAGAGATGTTTATGTCGCACCACTGAAAGCAGCATTG

ENSMUST00000095817 TCCTCAAACAGAGCCATTCTGAGTGCTGCCAATATCCACATCATTTTTTCTGACATTCTG
ENSCJAT00000037417 TCATCAAACAGAGCAATTCTGAGTGCTGCCAATATCCAGATCCTCTTCTCTGATATTCTG
ENSPPYT00000019842 TCATCAAACAGAGCGATTCTGAGTGCTGCCAATATCCAGATCATTTTCTCTGATATTCTA
ENSGGOT00000012884 TCATCAAAC---GCGATTCTGAGTGCTGCCGATATCCAGATCATTTTCTCTGACATTCTA

```
ENST00000423192        TCATCAAACAGAGCGATTCTGAGTGCTGCCAATATCCAGATCATTTTCTGTGACATTCTA
ENSPTRT00000056780     TCATCAAACAGAGCGATTCTGAGTGCTGCCAATATCCAGATCATTTTCTGTGACATTCTA

ENSMUST00000095817     CACATCTTAAACCTCAACAGAGAGTTTCTAGATAACCTACGAGACAGACTACAGGAATGG
ENSCJAT00000037417     CGAATTTTAAGTCTCAACAGGCAGTTTCTAGATAACCTGAGAGACAGACTGCAGGAATGG
ENSPPYT00000019842     CAGATTTTAAGTCTCAACAGGCAGTTTCTAGATAACCTGAGAGACAGACTGCAGGAATGG
ENSGGOT00000012884     CAGATTTTAAATCTCAACAGGCAGTTTCTAGATAACCTGAGAGACAGACTGCAGGAATGG
ENST00000423192        CAGATTTTAAGTCTCAACAGGCAGTTTCTAGATAACCTGAGAGACAGACTGCAGGAATGG
ENSPTRT00000056780     CAGATTTTAAGTCTCAACAGGCAGTTTCTAGATAACCTGAGAGACAGACTGCAGGAATGG

ENSMUST00000095817     AGCCCAGCTCACTGTGTGGGAGAAATATTCATAAAGTTTGGAAGCCAGCTGAACATATAT
ENSCJAT00000037417     GGCCCAGCTCACTGTATTGGAGAAATAGTCACAAAGTTTGGAAGCCAGTTGAACACATAC
ENSPPYT00000019842     GGCCCAACTCACTGTGTGGGAGAAATAGTCATGAAGTTTGGAAGCCAGTTGAACACATAT
ENSGGOT00000012884     GGCCCAGCTCTCTGTGTGGGAGAAATAGTCATGAAGTTTGGAAGCCAGTTAAACACATAT
ENST00000423192        GGCCCAGCTCACTGTGTGGGAGAAATAGTCACGAAGTTTGGAAGCCAGTTAAACACATAT
ENSPTRT00000056780     GGCCCAGCTCACTGTGTGGGAGAAATAGTCACGAAGTTTGGAAGCCAGTTAAACACATAT

ENSMUST00000095817     ACCAATTTCTTCAACAATTATCCAGTTGTTCTGAAAACCATCGAAAAGTGCAGAGAAATG
ENSCJAT00000037417     ACCAATTTCTTCAACAATTACCCTGTCGTTCTGAAAACTATTGACAAGTGCAGAGAAATG
ENSPPYT00000019842     ACCAATTTCTTCAACAATTACCCTGTCATTCTGAAAACTATTGAGAAGTGCAGAGAAATG
ENSGGOT00000012884     ACCAATTTCTTCAACAATTACCCTGTCATTCTGAAAACTATTGAAAAGTGCAGAGAAATG
ENST00000423192        ACCAATTTCTTCAACAATTACCCTGTCATTCTGAAAACTATTGAGAAGTGCAGAGAAATG
ENSPTRT00000056780     ACCAATTTCTTCAACAATTACCCTGTCATTCTGAAAACTATTGAGAAGTGCAGAGAAATG

ENSMUST00000095817     ACACCCGCGTTTAGAGCATTCTTGAAGAGGCATGATAAAACCATTGTTACCAAAATGCTG
ENSCJAT00000037417     ATACCAGCCTTCCGAACTTTCCTGAAGAGGCATGATAAGACCATTGCTACAAAAATGCTG
ENSPPYT00000019842     ATACCAGCATTCCGAACTTTCCTGAAGAGGCATGATAAGACCATTGTTACCAAAATGCTG
ENSGGOT00000012884     ATACCAGCATTCCGAACTTTCCTGAAGAGGCATGATAAGACCATTGTTACCAAAATGCTG
ENST00000423192        ATACCAGCATTCCGAACTTTCCTGAAGAGGCATGATAAGACCATTGTTACCAAAATGCTG
ENSPTRT00000056780     ATACCAGCATTCCGAACTTTCCTGAAGAGGCATGATAAGACCATTGTTACCAAAATGCTG

ENSMUST00000095817     AGTCTGCCGGAGCTGCTGATATACCCGTCCCGGAGATTTGAAGAATATATTCACCTTCTC
ENSCJAT00000037417     AGTCTGCCAGAGCTGCTGCTGTACCCATCACGAAGATTTGAAGAATACCTTAATCTTCTC
ENSPPYT00000019842     AGCCTGCCAGAGCTGCTGCTGTACCCATCCCGAAGATTTGAAGAATACCTTAATCTTCTC
ENSGGOT00000012884     AGCCTGCCAGAGCTGCTGCTGTACCCATCCCGAAGATTTGAAGAATACCTTAATCTTCTC
ENST00000423192        AGCCTGCCAGAGCTGCTGCTGTACCCATCCCGAAGATTTGAAGAATACCTTAATCTTCTC
ENSPTRT00000056780     AGCCTGCCAGAGCTGCTGCTGTACCCATCCCAAAGATTTGAAGAATACCTTAATCTTCTC

ENSMUST00000095817     TATGCTCTGAGACTCCATACTCCGGCAGGACACGTGGACCGTGGAGACCTGACCACTGCA
ENSCJAT00000037417     TATGCTGTCAGGCTTCATACTCCTGCTGAGCATGTTGACCGTGGGGATTTGACCACTGCA
ENSPPYT00000019842     TATGCTGTCAGGCTTCATACCCCTGCAGAGCACGTTGACCGTGGGGACTTGACCACTGCA
ENSGGOT00000012884     TACGCTGTCAGGCTTCATACCCCTGCAGAGCATGTTGACCGTGGGGACTTGACCACTGCA
ENST00000423192        TACGCTGTCAGGCTTCATACCCCTGCAGAGCATGTTGACCGTGGGGACTTGACCACTGCA
ENSPTRT00000056780     TACGCTGTCAGGCTTCATACCCCTGCAGAGCATGTTGACCGTGGGGACTTGACCACTGCA

ENSMUST00000095817     ATTGATCAAGTCAAAAAATATAAAGGCTACATAGATCAGATAAAGGAAAACTTGAAGATG
ENSCJAT00000037417     ATTGACCAAATCAAAAAATATAAAGGTTATATAGATCAGATGAAGCAAAACATCAATATG
ENSPPYT00000019842     ATTGACCAAATCAAAAAATATAAAGGTTATATAGATCAGATGAAGCAAAACATCAGTATG
ENSGGOT00000012884     ATTGACCAAATAAAAAAATATAAAGGTTATATAGATCAGATGAAGCAAAACATCAATATG
ENST00000423192        ATTGACCAAATCAAAAAATATAAAGGTTATATAGATCAGATGAAGCAAAACATCACTATG
ENSPTRT00000056780     ATTGACCAAATCAAAAAATATAAAGGTTATATAGATCAGATGAAGCAAAACATCAATATG
```

```
ENSMUST00000095817     AGAGAGCAGCTGTCCGATGTACAGACACTCATTTGTGGCTGTCCGACTTTATCAGAAGCA
ENSCJAT00000037417     AAGGATCGTCTGTCAGATATACAGAGAATCATCTGGGGATGCCCTACTCTATCAGAAGTA
ENSPPYT00000019842     AAGGATCATCTGTCAGATATACAGAGAATCATCTGGGGATGCCCTACTATATCAGAAGTA
ENSGGOT00000012884     AAGGATCATCTGTCAGATATACAGAGAATCATCTGGGGATGCCCTACTCTATCAGAAGTA
ENST00000423192        AAGGATCATCTGTCAGATATACAGAGAATCATCTGGGGATGCCCTACTCTATCAGAAGTA
ENSPTRT00000056780     AAGGATCATCTGTCAGATATACAGAGAATCATCTGGGGATGCCCTACTCTATCAGAAGTA

ENSMUST00000095817     AACAGATACCTGATTCGGACCCAAGATGTGGTTCAACTTCACTGCTGTGATGAGAAAATG
ENSCJAT00000037417     AACAGATATTTGATTAGGGTCCAAGATGTAGCCCAACTTCATTGCTGTGATGAAGAAATA
ENSPPYT00000019842     AACAGATATCTGATTAGGGTACAAGATGTAGCCCAACTTCATTGCTGTGATGAAGAAATA
ENSGGOT00000012884     AACAGATATCTGATTAGGGTACAAGATGTAGCCCAACTTCATTGCTGTGATGAAGAAATA
ENST00000423192        AACAGATATCTGATTAGGGTACAAGATGTAGCCCAACTTCATTGCTGTGATGAAGAAATA
ENSPTRT00000056780     AACAGATATCTGATTAGGGTACAAGATGTAGCCCAACTTCATTGCTGTGATGAAGAAATA

ENSMUST00000095817     GACTTCTCTTTAAGGCTCTACGAACAGATCCGAGACCTCAGCCTCCTCCTCTTCAATGAT
ENSCJAT00000037417     AGTTTCTCTTTAAGGCTCTATGAACACATCTGCGACCTCAGCCTTTTCCTCTTCAATGAT
ENSPPYT00000019842     AGTTTCTCTTTAAGGCTCTATGAACACATCCATGATCTCAGCCTTTTCCTCTTCAGTGAT
ENSGGOT00000012884     AGTTTCTCTTTAAGGCTCTATGAACACATCCATGATCTCAGCCTTTTCCTCTTCAATGAT
ENST00000423192        AGTTTCTCTTTAAGGCTCTATGAACACATCCATGATCTCAGCCTTTTCCTCTTCAATGAT
ENSPTRT00000056780     AGTTTCTCTTTAAGGCTCTATGAACACATCCATGATCTCAGCCTTTTCCTCTTCAATGAT

ENSMUST00000095817     GTACTCCTTGTTAGCAGTCGGAGTACATCTCATACTCCATTTGAAAAGACTTCTAAAACA
ENSCJAT00000037417     GCACTGCTCGTTTCTAGTCGGGGCACATCTCATACTCCATTTGAGAGGACTTCAAAAACA
ENSPPYT00000019842     GCCCTGCTCGTTTCTAGTCGGGGCACATCTCATACTCCATTTGAGAGGACTTCAAAAACA
ENSGGOT00000012884     GCCCTGCTCATTTCTAGTCGGGGCACATCTCATACTCCATTTGAGAGGACTTCAAAAACA
ENST00000423192        GCCCTGCTCGTTTCTAGTCGGGGCACATCTCACACTCCATTTGAGAGGACTTCAAAAACA
ENSPTRT00000056780     GCCCTGCTCGTTTCTAGTCGGGGCACATCTCATACTCCATTTGAGAGGACTTCAAAAACA

ENSMUST00000095817     ACCTACCAGTTCACCGCATCAGTGGCCCTTCCCAGGCTGCTCATAGAAGACATTCCAGAC
ENSCJAT00000037417     ACCTACCAGTTCATTGCATCAGTGGCCCTTCATAGGTTACTCATAGAAAATATTCCAGAT
ENSPPYT00000019842     ACCTACCAGTTCATTGCATCAGTGGCCCTTCATCGGTTACTCATAGAAAATATTCCAGAT
ENSGGOT00000012884     ACCTACCAGTTCATTGCATCAGTGGCCCTTCATCAGTTACTCATAGAAAATATTCCAGAT
ENST00000423192        ACCTACCAGTTCATTGCATCAGTGGCCCTTCATCGGTTACTCATAGAAAATATTCCAGAT
ENSPTRT00000056780     ACCTACCAGTTCATTGCATCAGTGGCCCTTCATCGGTTACTCATAGAAAATATTCCAGAT

ENSMUST00000095817     TCCAAATACATCAAGAATGCATTTATTCTTCGAGGTTCAAAACGAGAATGGATCTGCGCT
ENSCJAT00000037417     TCCAAGTATGTCAAGAATGCATTTATTCTTCAAGGTCCAAAATATAAATGGATTTGTGCT
ENSPPYT00000019842     TCCAAGTATGTCAAGAATGCATTTATTCTTCAAGGTCCAAAATATAAATGGATTTGTGCT
ENSGGOT00000012884     TCCAAGTATGTCAAGAATGCATTTATTCTTCAAGGTCCAAAATATAAATGGATTTGTGCT
ENST00000423192        TCCAAGTATGTCAAGAATGCATTTATTCTTCAGGGTCCAAAATATAAATGGATTTGTGCT
ENSPTRT00000056780     TCCAAGTATGTCAAGAATGCATTTATTCTTCAAGGTCCAAAATATAAATGGATTTGTGCT

ENSMUST00000095817     ACCGAGGTGTCAGATGACAAATTCCTTTGGCTGTCAGTACTCCAGCATGCAATCAGAAGC
ENSCJAT00000037417     ACAGAAGCAGAGGATGATAAGTTCCTATGGCTATCAGTACTTCAAAATGCAATCAAAAGT
ENSPPYT00000019842     ACAGAAATAGAGGATGATAAGTTCCTATGGCTGTCAGTACTTCGAAATGCAATCAAAAGC
ENSGGOT00000012884     ACAGAAATAGAGGAGGATAAGTTCCTATGGCTGTCAGTACTTCGAAATGCAATCAAAAGC
ENST00000423192        ACAGAAATAGAGGATGATAAGTTCCTATGGCTGTCAGTACTTCGAAATGCAATCAAAAGC
ENSPTRT00000056780     ACAGAAATAGAGGATGATAAGTTCCTATGGCTGTCAGTACTTCGAAATGCAATCAAAAGC

ENSMUST00000095817     AGTATGAAG---
ENSCJAT00000037417     AGGATGGAGAAG
ENSPPYT00000019842     AGTATGGAGAAG
ENSGGOT00000012884     AGTATGGAGAAG
```

```
ENST00000423192        AGTATGGAGAAG
ENSPTRT00000056780     AGTATGGAGAAG
```

Multiple sequence alignment of Fbxl12

```
ENSMUST00000148631     ATGGCGACTCTGTTTGACCTCCCAGACTTGGTGCTCTTGGAGATCTTCTCTTACCTCCCT
ENSRNOT00000027631     ATGGCGACGCTGTTTGACCTCCCAGACTTGGTGCTCTTGGAGATCTTCTCTTACCTCCCT
ENSCJAT00000011843     ATGGCGACTTTGGTCGAACTGCCGGACTCGGTCCTACTCGAAATCTTCTCTTACCTCCCG
ENSGGOT00000006020     ATGGCGACTTTGATTGAACTGCCGGACTCGGTCCTGCTCGAGATCTTCTCTTACCTCCCG
ENSMMUT00000011032     ATGGCGACTTTGGTCGAACTGCCGGACTCAGTCCTGCTTGAGATCTTCTCTTACCTCCCG
ENSPPYT00000011113     ATGGCGACTTTGGTCGAACTGCCGGACTCGGTCCTGCTCGAGATCTTCTCTTACCTCCCG
ENST00000247977        ATGGCGACTTTGGTCGAACTGCCGGACTCGGTCCTGCTCGAGATCTTCTCTTACCTCCCG
ENSPTRT00000019206     ATGGCGACTTTGGTCGAACTGCCGGACTCGGTCCTGCTCGAGATCTTCTCTTACCTCCCG


ENSMUST00000148631     GTCCGGGACCGGATCCGCATCTCCAGGGTCTGTCACCGCTGGAAGAGGCTAGTGGATGAC
ENSRNOT00000027631     GTCCGGGACCGGATCCGCATCTCCAGGGTCTGTCACCGCTGGAAGAGGCTAGTGGATGAC
ENSCJAT00000011843     GTACGGGACCGGATCCGCATCTCCAGGGTCTGTCACCGCTGGAAGAGGCTGGTGGACGAC
ENSGGOT00000006020     GTACGGGACCGGATCCGCATCTCCAGGGTCTGTCACCGCTGGAAGAGGCTGGTGGACGAC
ENSMMUT00000011032     GTACGGGACCGGATCCGCATCTCCAGGGTCTGTCACCGCTGGAAGAGGCTGGTGGACGAC
ENSPPYT00000011113     GTACGGGACCGGATCCGCATCTCCAGGGTCTGTCACCGCTGGAAGAGGCTGGTGGACGAC
ENST00000247977        GTACGGGACCGGATCCGCATCTCCAGGGTCTGTCACCGCTGGAAGAGGCTGGTGGACGAC
ENSPTRT00000019206     GTACGGGACCGGATCCGCATCTCCAGGGTCTGTCACCGCTGGAAGAGGCTGGTGGACGAC


ENSMUST00000148631     CGGTGGCTATGGCGACACGTCGACCTGACGCTCTACACGATGCGGCCGAAAGTCATGTGG
ENSRNOT00000027631     CGGTGGCTGTGGCGACACGTCGACCTGACGCTCTACACGATGCGGCCGAAAGTCATGTGG
ENSCJAT00000011843     CGGTGGCTGTGGCGACATGTCGACTTGACGCTCTACACGATGCGACCTAAAGTCATGTGG
ENSGGOT00000006020     CGGTGGCTGTGGCGACATGTCGACCTGACGCTCTACACGATGCGACCTAAAGTCATGTGG
ENSMMUT00000011032     CGGTGGCTGTGGCGACATGTCGACCTGACGCTCTACACGATGCGACCTAAAGTCATGTGG
ENSPPYT00000011113     CGGTGGCTGTGGCGACATGTTGACCTCACGCTCTACACGATGCGACCTAAAGTCATGTGG
ENST00000247977        CGGTGGCTGTGGCGACATGTCGACCTGACGCTCTACACGATGCGACCTAAAGTCATGTGG
ENSPTRT00000019206     CGGTGGCTGTGGCGACATGTCGACCTGACGCTCTACACGATGCGACCTAAAGTCATGTGG


ENSMUST00000148631     CACCTCCTGCGCCGGTACATGGCGTCCCGGCTCTACTCGTTGCGCATGGGCGGCTACCTG
ENSRNOT00000027631     CACCTCCTGCGCCGGTACATGGCGTCCCGGCTCCACTCGTTGCGCATGGGCGGCTACCTA
ENSCJAT00000011843     CACCTCCTTCGAAGGTACATGGGATCCCGACTCCATTCCCTGCGAATGGGTGGTTACCTG
ENSGGOT00000006020     CACCTCCTTCGAAGGTACATGGCATCCCGGCTCCATTCCCTGCGGATGGGTGGCTACCTG
ENSMMUT00000011032     CACCTCCTTCGAAGGTACATGGCATCCCGGCTCCATTCCCTGCGGATGGGTGGCTACTTG
ENSPPYT00000011113     CACCTCCTTCGAAGGTACATGGCATCCCGGCTCCATTCCCTGCGGATGGGTGGCTACCTG
ENST00000247977        CACCTCCTTCGAAGGTACATGGCATCCCGGCTCCATTCCCTGCGGATGGGTGGCTACCTG
ENSPTRT00000019206     CACCTCCTTCGAAGGTACATGGCATCCCGGCTCCATTCCCTGCGGATGGGTGGCTACCTG


ENSMUST00000148631     TTTTCTGGCTCTCAGGCCCCACAGCTGTCCCCCGCCTTGATGAGGGCCCTGGGTCAGAAA
ENSRNOT00000027631     TTCTCTGGCTCTCAGGCCCCACAGCTGTCCCCTGCCTTGATGCGGGCCCTGGGCCAGAAA
ENSCJAT00000011843     TTCTCCGGCTCCCAGGCCCCCCAGTTGTCCCCTGCCCTGTTGAGAGCCCTGGGCCAGAAG
ENSGGOT00000006020     TTCTCTGGCTCTCAGGCCCCCCAGTTGTCCCCTGCTTTGTTGAGAGCCCTGGGCCAGAAG
ENSMMUT00000011032     TTCTCTGGCTCCCAGGCCCCCCAATTGTCCCCTGCTCTGTTGAGAGCCCTGGGCCAGAAA
ENSPPYT00000011113     TTCTCTGGCTCCCAGGCCCCCCAGTTGTCTCCTGCTCTGTTGAGAGCCCTGGGCCAGAAG
ENST00000247977        TTCTCTGGCTCCCAGGCCCCCCAGTTGTCCCCTGCTCTGTTGAGAGCCCTGGGCCAGAAG
ENSPTRT00000019206     TTCTCTGGCTCCCAGGCCCCCCAGTTGTCCCCTGCTCTGTTGAGAGCCCTGGGCCAGAAG


ENSMUST00000148631     TGCCCCAATCTGAAGCGCCTGTGCCTGCACGTGGCTGACCTGAGCATGGTGCCTATCACC
ENSRNOT00000027631     TGCCCCAACCTGAAGCGCCTGTGCTTGCATGTGGCTGACCTGAGTATGGTGCCTATCACC
ENSCJAT00000011843     TGCCCCAACCTGCAGCGGCTCTGCCTGCACGTGGCCGACCTGAGCATGGTGCCCATCACC
ENSGGOT00000006020     TGCCCCAACCTGAAGCGCCTCTGCCTGCACGTGGCCGACCTGAGCATGGTGCCCATCACC
```

```
ENSMMUT00000011032     TGCCCCAACCTGAAGCGCCTCTGCCTACACGTGGCCGACCTGAGCATGGTGCCCATCACC
ENSPPYT00000011113     TGCCCCAACCTGAAGCGCCTCTGCCTGCACGTGGCTGACCTGAGCATGGTGCCCATCACC
ENST00000247977        TGCCCCAACCTGAAGCGCCTCTGCCTGCACGTGGCCGACCTGAGCATGGTGCCCATCACC
ENSPTRT00000019206     TGCCCCAACCTGAAGCGCCTCTGCCTGCACGTGGCCGACCTGAGCATGGTGCCCATCACC

ENSMUST00000148631     AGCCTTCCTAGCACACTGAGGACCCTGGAGCTGCACAGCTGCGAAATCTCCATGATCTGG
ENSRNOT00000027631     AGCCTGCCTAGCACACTGAGGACCCTGGAGCTGCACAGCTGCGAAATCTCGATGATCTGG
ENSCJAT00000011843     AGCCTGCCTAGCACCCTGAGGACCCTGGAGCTGCACAGCTGTGAGATCTCCATGGCCTGG
ENSGGOT00000006020     AGCCTGCCCAGCACCTTGAGGACCCTGGAGCTGCACAGCTGCGAGATCTCCATGGCCTGG
ENSMMUT00000011032     AGCCTGCCCAGCACCCTGAGGACCCTGGAGCTGCACAGCTGCGAGATCTCTATGGCTTGG
ENSPPYT00000011113     AGCCTGCCCAGCACCCTGAGGACCCTGGAGCTGCACAGCTGTGAGATCTCCATGGCCTGG
ENST00000247977        AGCCTGCCCAGCACCTTGAGGACCCTGGAGCTGCACAGCTGCGAGATCTCCATGGCCTGG
ENSPTRT00000019206     AGCCTGCCCAGCACCTTGAGGACCCTGGAGCTGCACAGCTGCGAGATCTCCATGGCCTGG

ENSMUST00000148631     TTGCAGAAAGAGCAGGACCCCACGGTGCTGCCTCTGCTGGAATGCATCGTGCTGGACCGA
ENSRNOT00000027631     TTGCAGAAAGAGCAGGACCCCACGGTGCTGCCACTGCTGGAATGCATCGTGCTAGACCGA
ENSCJAT00000011843     CTCCTCAAGCAGCAGGACCCCACTGTGCTGCCCCTGCTCGAATGCATCGTGCTGGACCGT
ENSGGOT00000006020     CTCCACAAGCAGCAGGACCCCACCGTGCTGCCCCTGCTCGAATGCATCGTGCTGGACCGC
ENSMMUT00000011032     CTCCACAAGCAGCAGGACCCCACCGTGCTGCCCCTGCTCGAATGCATCGTGCTGGACCGC
ENSPPYT00000011113     CTCCACAAGCAGCAGGACCCCACCGTGCTGCCCCTGCTTGAATGCATCGTGCTGGACCGT
ENST00000247977        CTCCACAAGCAGCAGGACCCCACCGTGCTGCCCCTGCTTGAATGCATCGTGCTGGACCGC
ENSPTRT00000019206     CTCCACAAGCAGCAGGACCCCACCGTGCTGCCCCTGCTCGAATGCATCGTGCTGGACCGC

ENSMUST00000148631     GTGCCGGCCTTTCGCGATGAGCATCTGCAGGGCCTTACCCGATTCCGAGCCCTGCGCTCG
ENSRNOT00000027631     GTGCCGGCCTTCCGTGATGAGCACCTTCAGGGCCTTACCCGCTTCCGAGCCCTGCGCTCG
ENSCJAT00000011843     GTCCCTGCCTTCCGTGACGAGCACCTGCAGGGCCTGACGCGCTTCCGGGCCTTGCGTTCA
ENSGGOT00000006020     GTCCCTGCCTTCCGTGACGAGCACCTGCAGGGCCTGACGCGCTTCCGGGCCTTGCGCTCG
ENSMMUT00000011032     GTCCCCGCCTTCCGCGACGAGCACCTGCAGGGCCTGACGCGCTTCCGGGCCTTGCGCTCG
ENSPPYT00000011113     GTCCCCGCCTTCCGTGATGAGCACCTGCAGGGCCTGACGCGTTTCCGGGCCTTGCGCTCG
ENST00000247977        GTCCCCGCCTTCCGTGACGAGCACCTGCAGGGCCTGACGCGCTTCCGGGCCTTGCGCTCG
ENSPTRT00000019206     GTCCCCGCCTTCCGTGACGAGCATCTGCAGGGCCTGACGCGCTTCCGGGCCTTGCGCTCG

ENSMUST00000148631     CTGGTGCTGGGCGGCACCTACCGGGTCACTGAGACCGGGCTAGATGCCAGCCTGCAGGAG
ENSRNOT00000027631     CTGGTTCTGGGTGGCACCTACCGGGTCACTGAGACCGGGCTAGATTCCAGCCTGCAGGAG
ENSCJAT00000011843     CTGGTGCTGGGTGGTACCTACCGTGTAACCGAGACAGGGCTGGATGCCAGCCTGCAGGAG
ENSGGOT00000006020     CTGGTGCTGGGTGGTACCTACCGTGTGACCGAGACAGGGCTGGATGCTGGCCTGCAGGAG
ENSMMUT00000011032     CTGGTGCTGGGTGGCACCTACCGTGTGACTGAGACAGGGCTGGATGCTGGCCTGCAGGAG
ENSPPYT00000011113     CTGGTGCTGGGTGGCACCTACCGTGTGACCGAGACAGGGCTGGATGCTGGCCTGCAGGAG
ENST00000247977        CTGGTGCTGGGTGGTACCTACCGTGTGACCGAGACAGGGCTGGATGCTGGCCTGCAGGAG
ENSPTRT00000019206     CTGGTGCTGGGTGGTACCTACCGTGTGACCGAGACAGGGCTGGATGCTGGCCTGCAGGAA

ENSMUST00000148631     CTCAGCTACCTGCAAAGGCTTGAGGTGCTGGGCTGCACCCTGTCAGCTGACAGCACGCTG
ENSRNOT00000027631     CTGAGCTACCTGCAAAGGCTGGAGGTGCTGGGCTGCACCCTGTCAGCAGACAGCACCTTG
ENSCJAT00000011843     CTCAGCTACCTGCAGAGGCTCGAGGTGGTGGGCTGCACCCTGTCTGCTGACAGCACCCTA
ENSGGOT00000006020     CTCAGCTATCTGCAGAGGCTCGAGGTGCTGGGCTGCACCCTGTCTGCCGACAGCACCCTG
ENSMMUT00000011032     CTCAGCTATCTGCAGAGGCTCGAGGTGCTGGGCTGCACCCTGTCTGCTGACAGCACCCTG
ENSPPYT00000011113     CTCAGCTATCTGCAGAGGCTCGAGGTGCTGGGCTGCACCCTGTCTGCCGACAGCACCCTG
ENST00000247977        CTCAGCTATCTGCAGAGGCTTGAGGTGCTGGGCTGCACCCTGTCTGCCGACAGCACCCTG
ENSPTRT00000019206     CTCAGCTATCTGCAGAGGCTCGAGGTGCTGGGCTGCACCCTGTCTGCCGACAGCACCCTG

ENSMUST00000148631     CTGGCCATCAGCCGCCACCTTCGAGATGTGCGCAAGATTCGGCTGACCGTTGGGGGCCTC
ENSRNOT00000027631     CTGGCCATCAGCCGCCACCTCCGAGATGTGCGCAAGATTCGGCTGACCGTTGGAGGCCTC
ENSCJAT00000011843     CTGGCCATCAGCCGCCACCTCCGAGATGTGCGGAAGATCCGGCTGACCGTGAGGGGCCTC
ENSGGOT00000006020     CTGGCCATCAGCCGCCACCTCCGAGATGTGCGCAAGATCCGACTGACCGTGAGGGGCCTC
```

```
ENSMMUT00000011032     CTGGCTATCAGCCGCCACCTCCGAGATGTGCGCAAGATCCGGCTGACCGTGAGGGGCCTC
ENSPPYT00000011113     CTGGCCATCAGCCGCCACCTCCGACATGTGCGCAAGATCCGGCTGACCGTGAGGGGCCTC
ENST00000247977        CTGGCCATCAGCCGCCACCTCCGAGATGTGCGCAAGATCCGGCTGACCGTGAGGGGCCTC
ENSPTRT00000019206     CTGGCCATCAGCCGCCACCTCCGAGATGTGCGCAAGATCCGGCTGACCGTGAGGGGCCTC


ENSMUST00000148631     TCAGCCCAGGGCCTGGTCTTCCTGGAGGGAATGCCTGTCCTGGAGAGTTTGTGCTTCCAG
ENSRNOT00000027631     TCAGCTCAGGGCCTGGTGTTCCTGGAGGGAATGCCGGTCCTGGAGAGTTTGTGCTTCCAG
ENSCJAT00000011843     TCTGCCCCTGGTCTGGCTGTCTTGGAGGGGATGCCAGCCCTGGAGAGTCTGTGCCTGCAG
ENSGGOT00000006020     TCTGCCCCTGGCCTGGCTGTCCTGGAGGGAATGCCAGCCCTGGAGAGTCTGTGCCTGCAG
ENSMMUT00000011032     TCTGCCCCTGGCCTGGCTGTCCTGGAGGGAATGCCGGCCTTGGAGAGTCTGTGCCTGCAG
ENSPPYT00000011113     TCTGCCCCTGGCCTGGCTGTCCTGGAGGGAATGCCGGCCCTGGAGAGTCTGTGTCTGCAG
ENST00000247977        TCTGCCCCTGGCCTGGCTGTGCTGGAGGGAATGCCGGCCCTGGAGAGTCTGTGCCTGCAG
ENSPTRT00000019206     TCTGCCCCTGGCCTGGCTGTCCTGGAGGGAATGCCGGCCCTGGAGAGTCTGTGCCTGCAG


ENSMUST00000148631     GGTCCCCTTATCACCCCAGACATGCCCACACCCACTCAGATTGTGTCCTCCTGCCTCACC
ENSRNOT00000027631     GGTCCCCTTATCACCCCAGAAATGCCCACACCCGCTCAGATTGTGTCCTCCTGCCTCACC
ENSCJAT00000011843     GGTCCTCTTGTCACCCCAGAAATGCCAACCCCCACTGAAATCCTCTCCTCCTGCCTCACC
ENSGGOT00000006020     GGTCCCCTCATCACCCCAGAAATGCCCTCCCCCACTGAAATCCTCTCCTCCTGCCTCACT
ENSMMUT00000011032     GGTCCCCTCATCACCCCGGAAATGCCCTCCCCCACTGAAATCCTCTCCTCCTGCCTCACT
ENSPPYT00000011113     GGTCCCCTTGTCACCCCGGAAATGCCCTCCCCCACTGAAATACTCTCCTCCTGCCTCACT
ENST00000247977        GGTCCCCTCGTCACCCCAGAAATGCCCTCCCCCACTGAAATCCTCTCCTCCTGCCTCACT
ENSPTRT00000019206     GGTCCCCTCGTCACCCCAGAAATGCCCTCCCCCACTGAAATCCTCTCCTCCTGCCTCACT


ENSMUST00000148631     ATGCCGAAGCTCAGAGTGCTTGAGGTGCAAGGGCTGGGCTGGGAGGGTCAGGAAGCAGAG
ENSRNOT00000027631     ATGCCTAAGCTCAGAGTGCTTGAGGTGCAGGGGCTGGGCTGGGAGGGCCAGGAAGCAGAG
ENSCJAT00000011843     ATGCCCAAGCTCAGAGTGCTTGAGCTGCAGGGGCTGGGGTGGGAGGGTCAGGAGGCTGAG
ENSGGOT00000006020     ATGCCCAAGCTCAGAGTCCTTGAGCTGCAGGGGCTGGGGTGGGAGGGTCAGGAGGCGGAG
ENSMMUT00000011032     ATGCCCAAGCTCAGAGTCCTTGAGCTGCAGGGGCTGGGCTGGGAGGGTCAGGAGGCGGAG
ENSPPYT00000011113     ATGCCCAAGCTCAGAGTCCTTGAGCTGCAGGGGCTGGGGTGGGAGGGTCAGGAGGCGGAG
ENST00000247977        ATGCCCAAGCTCAGAGTCCTTGAGCTGCAGGGGCTGGGGTGGGAGGGTCAGGAGGCGGAG
ENSPTRT00000019206     ATGCCCAAGCTCAGAGTCCTTGAGCTGCAGGGGCTGGGGTGGGAGGGTCAGGAGGCGGAG


ENSMUST00000148631     AAAATCCTGTGCAAGGGCCTGCCCCACTGCATAGTTATTGTTAGGGCTTGTCCCAAAGAA
ENSRNOT00000027631     AAGGTCCTGTGCAAGGGCCTGCCCCACTGCCTAGTTATCGTCAGAGCTTGTCCCAAAGAA
ENSCJAT00000011843     AAGATCCTGTGTAAGGGGCTGCCCCACTGTATGGTCATCGTCAGGGCTTGCCCCAAAGAG
ENSGGOT00000006020     AAGATCCTGTGTAAGGGGCTGCCCCACTGTATGGTCATCGTCAGGGCTTGCCCCAAAGAG
ENSMMUT00000011032     AAGATCCTGTGTAAGGGGCTGCCCCACTGTATGGTCATTGTCAGG---TGCCCCAAAGAG
ENSPPYT00000011113     AAGATCCTGTGTAAGGGGCTGCCCCACTGTATGGTCATCGTCAGGGCTTGCCCCAAAGAG
ENST00000247977        AAGATCCTGTGTAAGGGGCTGCCCCACTGTATGGTCATCGTCAGGGCTTGCCCCAAAGAG
ENSPTRT00000019206     AAGATCCTGTGTAAGGGGCTGCCCCACTGTATGGTCATCGTCAGGGCTTGCCCCAAAGAG


ENSMUST00000148631     TCCATGGATTGGTGGATG
ENSRNOT00000027631     TCCATGGACTGGTGGATG
ENSCJAT00000011843     TCCATGGACTGGTGGATG
ENSGGOT00000006020     TCTATGGACTGGTGGATG
ENSMMUT00000011032     TCTATGGACTGGTGGATG
ENSPPYT00000011113     TCTATGGACTGGTGGATG
ENST00000247977        TCTATGGACTGGTGGATG
ENSPTRT00000019206     TCTATGGACTGGTGGATG
```

Multiple sequence alignment of Fbxl13

```
ENSMUST00000051358     GTGACTCCAGAGTTGATGATAAAAGCATGCACATTTTACACTGGACATTTAGTAAAGACA
ENSCJAT00000041745     ATGACTCCAGAATTGATGATAAAAGCATGTAGCTTTTATACTGGACATTTAGTAAAGACT
```

```
ENSMMUT00000017423     ATGACTCCAGAATTGATGATAAAAGCCTGTAGCTTTTATACTGGACATTTAGTAAAGACT
ENSPPYT00000020825     ATGACTCCGGAATTGATGATAAAAGCCTGTAGCTTTTATACTGGACATTTAGTAAAGACT
ENST00000313221        ATGACTCCGGAATTGATGATAAAAGCCTGTAGCTTTTATACTGGACATTTAGTAAAGACT
ENSPTRT00000036197     ATGACTCCGGAATTGATGATAAAAGCCTGTAGCTTTTATACTGGACATTTAGTAAAGACT

ENSMUST00000051358     CATTTCAGTGGTTGGAAAAAAGTTGCACGTGCAAATCAAGAGGAGATCATGGCTGAAAAA
ENSCJAT00000041745     CATTTTTGCACTTGGAGAGACATCACTCATAAAAATGAAAATGTTGCCCTGGCTGATAAA
ENSMMUT00000017423     CATTTTTGCACTTGGAGAGACATAGCTCGTACAAATGAAAATGTCGCCCTGGCTGAAAAA
ENSPPYT00000020825     CATTTTTGCACTTGGAGAGACATAGCTCGTACAGATGAAAATGTCGTCCTGGCTGAAAAA
ENST00000313221        CATTTTTGCACTTGGAGAGACATAGCTCGTACAAATGAAAATGTCGTCCTGGCTGAAAAA
ENSPTRT00000036197     CATTTTTGCACTTGGAGAGACATAGCTCATACAAATGAAAATGTCGTCCTGGCTGAAAAA

ENSMUST00000051358     ATGGACAAGGCAATAGCACATGACAATTTTAGATGCCAAAAATACATTTTTAATCGCTGG
ENSCJAT00000041745     ATGAACAGAGCAGTGACATGTTATGATTTCAGACTTCAAAAATCTGTATTTCATCACTGG
ENSMMUT00000017423     ATGAACAGAGCAGTGACATGCTACAATTTCAGACTTCAGAAATCTGTATTTCATCACTGG
ENSPPYT00000020825     ATGAACAGAGCAGTGACATGTTACAATTTCAGACTTCAAAAATCTGTATTTCATCACTGG
ENST00000313221        ATGAACAGAGCAGTGACATGCTACAATTTCAGACTTCAAAAATCTGTATTTCATCACTGG
ENSPTRT00000036197     ATGAACAGAGCAGTGACATGCTACAATTTCAGACTTCAAAAATCTGTATTTCATCACTGG

ENSMUST00000051358     TTCGCCTACACGGTTATGAGTAGAGAAAGACTTATAACTACACTGCTTCGGTTGCGACAT
ENSCJAT00000041745     CACTCTTATATGGAAGACCAGAAAGAAAAACTTAAAAATATGGTATTACGGATACAACGG
ENSMMUT00000017423     CACTCTTATATGGAAGACCAGAAAGAAAAACTTAAAAATACACTATTGCGGATACAACAG
ENSPPYT00000020825     CACTCTTATATGGAAGACCAGAAAGAAAAACTTAAAAATATGCTATTGCGGATACAACAG
ENST00000313221        CACTCTTATATGGAAGACCAGAAAGAAAAACTTAAAAATATTCTATTGCGGATACAACAG
ENSPTRT00000036197     CACTCTTATATGGAAGACCAGAAAGAAAAAATTAAAAATATGCTATTGCGGATACAACAG

ENSMUST00000051358     TTGTTCTATATGCAAAGACAAAGAATTATACTAGCAAAATGGAAGGAGAGAGCCAGACAC
ENSCJAT00000041745     ATCATCTATTGTCACAAGCTAACCATTATTCTAACAAAATGGCGTAATAAAGCAAGATGT
ENSMMUT00000017423     ATCATCTATTGTCACAAGCTGTCCATTATCCTAACAAAATGGCGGAATAGAGCAAGACTT
ENSPPYT00000020825     ATCATCTATTGTCACAAGCTAACCATTATCCTAACAAAATGGCGGAATACAGCAAGACAT
ENST00000313221        ATCATCTATTGTCACAAGCTAACCATTATCCTAACAAAATGGCGGAATACAGCAAGACAT
ENSPTRT00000036197     ATCATCTATTGTCACAAGCTAACCATTATCCTAACAAAATGGCGGAATACAGCAAGACAT

ENSMUST00000051358     AAGAGTAAAACAAGAGAAGACGACTTGATTTCAAAACATGAACTTCAGTTGAAAAAATGG
ENSCJAT00000041745     AAGAATAAAAAGAAAGAAGATGAGCTGGTATTAAAACATGAGCTTCAATTGAAAAAATGG
ENSMMUT00000017423     AAGAGTAAAAAGAAAGAAGATGAGCTGATACTAAAACATGAACTTCAATTGAAAAAATGG
ENSPPYT00000020825     AAGAGTAAAAAGAAAGAAGATGAGCTGATATTAAAACATGAACTTCAATTGAAAAAATGG
ENST00000313221        AAGAGTAAAAAGAAAGAAGATGAGCTGATATTAAAACATGAACTTCAATTGAAAAAATGG
ENSPTRT00000036197     AAGAGTAAAAAGAAAGAAGATGAGCTGATATTAAAACATGAACTTCAATTGAAAAAATGG

ENSMUST00000051358     AAGTTCAAGTTA---------------------GGGAAGCCGATTAGCCTTGAGGGGAGC
ENSCJAT00000041745     AGAAATAGGTTACTACTCAAAGGAGCTTCTGCAGAAAAATCCAATTTTCCCGAACAAAGT
ENSMMUT00000017423     AAAAATAGGTTAATACTCAAAAGAGCTACTGCAGAAGAATCCAATTTTCCTGAACAAAGT
ENSPPYT00000020825     AAAAATAGGTTAATACTCAAAAGAGCTGCTGCAGAAGAATCTAATTTTCCTGAACAAAGT
ENST00000313221        AAAAATAGGTTAATACTCAAAAGAGCTGCTGCAGAAGAATCCAATTTTCCTGAACGAAGT
ENSPTRT00000036197     AAAAATAGGTTAATACTCAAAAGAGCTGCTGCGGAAGAATCCAATTTTCCTGAACGAAGT

ENSMUST00000051358     CTCTCTGACATCGCTGTTGAGAACAGGCGCATAGCATTTGACATTTCAGTGCTACCGGAG
ENSCJAT00000041745     TCTTCTGAAGTGTGTCTTGTAGATGAGACTCTAGAATGTGACATTTCACAGTTACCTGAA
ENSMMUT00000017423     TCTTCTGAAGGCTCTCTTGTAGATGAGACTCTAAAATGTGACATTTCACTGTTACCTGAA
ENSPPYT00000020825     TCTTCTGAAGTCTCTCTTGTAGATGAGACTCTAAAATGTGACATTTCACTGTTACCTGAA
ENST00000313221        TCTTCTGAAGTCTTTCTTGTAGATGAGACTCTAAAATGTGACATTTCACTGTTACCTGAA
ENSPTRT00000036197     TCTTCTGAAGTCTCTCTTGTAGATGAGACTCTAAAATGTGACATTTCACTGTTACCTGAA
```

```
ENSMUST00000051358     CAAGCAATACTCCAGATTTTCTTGTACCTCACCTTTAAAGACATGATGGCGTGCAGTCGA
ENSCJAT00000041745     AGAGCAATATTACAGATTTTCTTCTACCTCAGTTTAAAAGATGTTTTAATATGTGGTCAA
ENSMMUT00000017423     AGAGCGATATTGCAGATTTTCTTCTACCTCAGTTTAAAAGATGTGATAATATGTGGCCAA
ENSPPYT00000020825     AGAGCAATATTACAGATTTTCTTCTACCTCAGTTTAAAAGATGTGATAATATGTGGTCAA
ENST00000313221        AGAGCAATATTACAGATTTTCTTCTACCTCAGTTTAAAAGATGTGATAATATGTGGTCAA
ENSPTRT00000036197     AGAGCAATATTACAGATTTTCTTCTACCTCAGTTTAAAAGATGTGATAATATGTGGTCAA

ENSMUST00000051358     GTCAACCGCTCCTGGATGGCGATGATTCAGAGAGGCTCCTTGTGGAACTCTATTGATTTT
ENSCJAT00000041745     GTTAATCATGCTTGGATGTTGATGACACAGCTAAGCTCACTGTGGAATGCTATTGATTTT
ENSMMUT00000017423     GTTAATCGTGCCTGGATGTTGATGACACAGCTGAACTCACTGTGGAATGCTATTGATTTT
ENSPPYT00000020825     GTTAGTCATGCCTGGATGTTGATGACACAACTAAACTCACTGTGGAATGCTATTGATTTT
ENST00000313221        GTTAATCATGCCTGGATGTTGATGACACAACTAAACTCACTGTGGAATGCTATTGATTTT
ENSPTRT00000036197     GTTAATCATGCCTGGATGTTGATGACACAACTAAACTCACTGTGGAATGCTATTGATTTT

ENSMUST00000051358     TCCACAGTTAAAAAC---ATTGCAGATAAATGTGTGGTGACCACTTTACAAAAGTGGCGG
ENSCJAT00000041745     TCCACAGTGAAACATGCGATTCCAGATAAATATATAGTGTCTACTTTGCAAAGGTGGCAT
ENSMMUT00000017423     TCCACAGTGAAAAATGTGATTCCAGATAAATATATACTGTCTACTTTGCAAAGGTGGCGT
ENSPPYT00000020825     TCCACAGTGAAAAATGTGATTCCAGATAAATATATAGTGTCTACTTTGCAAAGATGGCGT
ENST00000313221        TCCTCAGTGAAAAATGTGATTCCAGATAAATATATAGTGTCTACTTTGCAAAGGTGGCGT
ENSPTRT00000036197     TCCTCAGTGAAAAATGTGATTCCAGATAAATATATAGTGTCTACTTTGCAAAGGTGGCGT

ENSMUST00000051358     CTAAACGTGCTGCGTTTAAACTTCCGAGGCTGCGACTTCCGAACAAAAACCTTGAAAGCT
ENSCJAT00000041745     TTAAATGTGCTGCGTTTGAATTTTCGTGGTTGTCTTCTCCGACCCAAAACTTTCAGATCT
ENSMMUT00000017423     TTAAATGTGCTGCGTTTGAATTTTCATGGTTGTCTTCTCCGACCCAAAACTTTCAGATCT
ENSPPYT00000020825     TTAAATGTGCTGCGTTTGAATTTTCGTGGTTGTCTTCTCCGACCCAAAACTTTCAGATCT
ENST00000313221        TTAAATGTGCTGCGTTTGAATTTTCGTGGTTGTCTTCTCCGACCCAAAACTTTCAGATCT
ENSPTRT00000036197     TTAAATGTGCTGCGTTTGAATTTTCGTGGTTGTCTTCTCCGACCCAAAACTTTCAGATCT

ENSMUST00000051358     GTCAGCCACTGTAAAAACTTGCAAGAGTTGAACGTCTCTGACTGCCAGTCTTTCACAGAT
ENSCJAT00000041745     GCCAGCCACTGTAGGAACTTGCAAGAGTTGAATGTCTCTGACTGCCCAACATTCACAGAT
ENSMMUT00000017423     GTCAGCCACTGTAGGAACTTGCAAGAGCTGAATGTCTCTGACTGCCCAACATTCACAGAT
ENSPPYT00000020825     GTCAGCCACTGTAGGAACTTGCAAGAGCTGAATGTCTCTGACTGCCCAACATTCACAGAT
ENST00000313221        GTCAGCCACTGTAGGAACTTGCAAGAGTTGAATGTCTCTGACTGCCCAACATTCACAGAT
ENSPTRT00000036197     GTCAGCCACTGTAGGAACTTGCAAGAGTTGAATGTCTCTGACTGCCCAACATTCACAGAT

ENSMUST00000051358     GAATCCATGAGGCACATTTCTGAGGGCTGTCCTGGGGTCCTGTATCTCAATCTATCCAAC
ENSCJAT00000041745     GAATCAATGAGACACATTTCTGAGGGCTGTCCAGGGGTCCTGTATCTCAATCTGTCTAAC
ENSMMUT00000017423     GAATCAATGAGACACATTTCTGAGGGCTGCCCGGGGGTCCTGTATCTCAATCTGTCTAAC
ENSPPYT00000020825     GAATCAATGAGACACATTTCTGAGGGCTGCCCGGGGGTCCTGTATCTCAATCTGTCTAAC
ENST00000313221        GAATCAATGAGACACATTTCTGAGGGCTGCCCGGGGGTCCTGTGTCTCAATCTGTCTAAC
ENSPTRT00000036197     GAATCAATGAGACACATTTCTGAGGGCTGCCCGGGGGTCCTGTGTCTCAATTTGTCTAAC

ENSMUST00000051358     ACAACCATCACCAACAGGACCATGCGACTCCTACCCAGGTACTTCCACAACTTACAGAAC
ENSCJAT00000041745     ACAACTATCACCAACAGGACGATGCGACTCCTGCCGAGGCACTTCCACAACTTACAGAAT
ENSMMUT00000017423     ACAACTATCACCAACAGGACGATGCGACTCCTGCCGAGGCACTTCCACAACTTACAGAAT
ENSPPYT00000020825     ACAACTATCACCAACAGGACGATGCGACTCCTGCCGAGGCACTTCCACAACTTACAGAAT
ENST00000313221        ACAACTATCACCAACAGGACGATGCGACTCCTGCCGAGGCACTTCCACAACTTACAGAAT
ENSPTRT00000036197     ACAACTATCACCAACAGGACGATGCGACTCCTGCCGAGGCACTTCCACAACTTACAGAAT

ENSMUST00000051358     CTCAGTCTGGCTTACTGCCGAAAGTTCACCGACAAAGGCTTACAGTACCTGAACTTGGGG
ENSCJAT00000041745     CTTAGTTTGGCTTATTGCAGAAGGTTCACAGACAAAGGCTTACAGTACCTGAACTTGGGG
```

```
ENSMMUT00000017423     CTTAGTTTGGCTTATTGCAGAGGGTTCACAGATAAAGGCTTACAGTACCTGAACTTGGGG
ENSPPYT00000020825     CTTAGTTTGGCTTATTGCAGACGGTTCACAGACAAAGGCTTACAGTACCTGAACTTGGGG
ENST00000313221        CTTAGTTTGGCTTATTGCAGACGGTTCACAGACAAAGGCTTACAGTACCTGAACTTGGGG
ENSPTRT00000036197     CTTAGTTTGGCTTATTGCAGACGGTTCACAGACAAAGGCTTACAGTACCTGAACTTGGGG

ENSMUST00000051358     AACGGATGCCACAAGCTCATCTATCTGGACCTTTCCGGCTGCACCCAGGTT---------
ENSCJAT00000041745     AATGGATGCCACAAGCTCATCTATCTGGACCTCTCCGGCTGCACCCAGATTTCAGTCCAA
ENSMMUT00000017423     AATGGATGCCACAAGCTCATCTATCTGGACCTCTCCGGCTGCACCCAGATTTCAGTCCAA
ENSPPYT00000020825     AATGGATGCCACAAGCTCATCTATCTGGACCTCTCCGGCTGCACCCAGATTTCAGTCCAA
ENST00000313221        AATGGATGCCACAAGCTCATCTATCTGGACCTCTCTGGCTGCACCCAGATTTCAGTCCAA
ENSPTRT00000036197     AATGGATGCCACAAGCTCATCTATCTGGACCTCTCCGGCTGCACCCAGATTTCAGTCCAA

ENSMUST00000051358     ------------------------------------------------------------
ENSCJAT00000041745     GGCTTCAGGTACATTGCAAACAGCTGCACTGGAATTCTTCATCTTATCATTAATGACATG
ENSMMUT00000017423     GGCTTCAGGTACATTGCAAACAGCTGCACTGGAATTACGCATCTTACCATTAACGACATG
ENSPPYT00000020825     GGCTTCAGGTACATTGCAAACAGCTGCACTGGAGTTATGCATCTTACCATTAATGACATG
ENST00000313221        GGCTTCAGGTACATTGCAAACAGCTGCACTGGAATTATGCATCTTACCATTAATGACATG
ENSPTRT00000036197     GGCTTCAGGTACATTTCAAACAGCTGCACTGGAATTATGCATCTTACCATTAATGACATG

ENSMUST00000051358     ------------------------------TTGGTTGAGAAGTGTCCCCGTATTTCGTCG
ENSCJAT00000041745     CCAACTCTGACAGACAACTGTGTAAAAGCTTTAGTTGAAAAGTGCTCTCATATTACATCA
ENSMMUT00000017423     CCAACTCTGACGGACAACTGTGTAAAAGCTTTAGTTGAAAAGTGCTCTCGTATTACATCG
ENSPPYT00000020825     CCAACTCTGACGGACAACTGTGTAAAAGCTTTAGTTGAAAAATGCTCTCGTATTACATCG
ENST00000313221        CCAACTCTGACGGACAACTGTGTAAAAGCTTTAGTTGAAAAATGCTCTCGTATTACATCG
ENSPTRT00000036197     CCAACTCTGACGGACAACTGTGTAAAAGCTTTAGTTGAAAAATGCTCTCGTATTACATCG

ENSMUST00000051358     GTGGTTTTAATTGGTTCACCACACATCTCTGACTCTGCTTTCAAAGCTCTTTCCTCCTGC
ENSCJAT00000041745     ATGATTTTCACTGGTGCACCGCATATCTCTGATTGTACTTTCAAAGCTCTGTCTACTTGT
ENSMMUT00000017423     CTGGTTTTCACTGGTGCACCGCATATCTCCGATCGTACTTTCAAAGCTCTTTCTACTTGT
ENSPPYT00000020825     CTGGTTTTCACTGGTGCACCGCATATCACCGATTGTACTTTCAAAGCTCTTTCTACTTGT
ENST00000313221        CTGGTTTTCACTGGTGCACCGCATATCTCCGATTGTACTTTCAGAGCTCTTTCTGCTTGT
ENSPTRT00000036197     CTGGTTTTCACTGGTGCACCGCATATCTCCGATTGTACTTTCAGAGCTCTTTCTGCTTGT

ENSMUST00000051358     GACCTCAAAAAGATTCGATTTGAAGGAAACAAAAGGATTTCTGATGCGTGCTTTAAGTCG
ENSCJAT00000041745     AAACTCAGAAAGATCCGATTCGAAGGAAATAAAAGAATTACTGATGCATCCTTCAAATTT
ENSMMUT00000017423     AAACTCAGAAAGATCCGATTCGAAGGAAATAAAAGGGTTACTGATGCATCCTTCAAATAT
ENSPPYT00000020825     AAACTCAGAAAGATCCGATTTGAAGGAAATAAAAGGGTTACTGATGCATCCTTCAAATCT
ENST00000313221        AAACTCAGAAAGATCCGATTTGAAGGAAATAAAAGGGTTACTGATGCATCCTTCAAATTT
ENSPTRT00000036197     AAACTCAGAAAGATCCGATTTGAAGGAAATAAAAGGGTTACTGATGCATCCTTCAAATTT

ENSMUST00000051358     ATAGACAGGAATTATCCAGGCATCAATCACATTTACATGGTGGACTGCAAAGGGCTAACA
ENSCJAT00000041745     ATGGACAAGAATTATCCAGATCTCAGTCACATTTACATGGCTGACTGCAAGGGAATAACA
ENSMMUT00000017423     ATAGACAAGAATTATCCAAATCTCAGTCACATTTATATGGCTGACTGCAAGGGAATAACA
ENSPPYT00000020825     GTAGACAAGAATTATCCAAATCTCAGTCACATTTATATGGCTGACTGCAAGGGAATAACA
ENST00000313221        ATAGACAAGAATTATCCAAATCTCAGTCACATTTATATGGCTGACTGCAAGGGAATAACA
ENSPTRT00000036197     ATAGACAAGAATTATCCAAATCTCAGTCACATTTATATGGCTGACTGCAAGGGAATAACA

ENSMUST00000051358     GACAGCAGCCTCAAGTCACTTTCACTTTTGAAGCAATTGACAGTGTTGAACTTGACAAAT
ENSCJAT00000041745     GACAGCAGTCTCAGGTCTCTTTCACCTTTGAAGCAATTAACTGTGTTGAATTTGGCAAAT
ENSMMUT00000017423     GACAGCAGCCTCAGATCCCTTTCACCTTTGAGGCAACTGACTGTGTTGAATTTGGCAAAT
ENSPPYT00000020825     GACAGCAGCCTCAGATCCCTTTCACCTTTGAAGCAACTGACTGTGTTGAATTTGGCAAAT
ENST00000313221        GACAGCAGCCTCAGATCCCTTTCACCTTTGAAGCAACTGACTGTGTTGAATTTGGCAAAT
ENSPTRT00000036197     GACAGCAGCCTCAGATCCCTTTCACCTTTGAAGCAACTGACTGTGTTGAATTTGGCAAAT
```

```
ENSMUST00000051358     TGCATAAGGATTGGTGATATCGGCCTGAAGCATTTTTTTGATGGTCCTGCGAGTATCAGG
ENSCJAT00000041745     TGTGTAAGAATTGGTGATGTGGGACTAAGGCAATTTCTTGATGGTCCTGCAAGCATAAGG
ENSMMUT00000017423     TGTGTAAGAATTGGTGATATGGGGCTAAGGCAATTTCTTGATGGTCCTGCAAGCATAAGG
ENSPPYT00000020825     TGTGTAAGAATTGGTGATATGGGACTAAAGCAATTTCTTGATGGTCCTGCAAGCATAAAG
ENST00000313221        TGTGTAAGAATTGGTGATATGGGACTAAAGCAATTTCTTGATGGTCCTGCAAGCATGAGG
ENSPTRT00000036197     TGTGTAAGAATTGGTGATATGGGACTAAAGCAATTTCTTGATGGTCCTGCAAGCATAAAG

ENSMUST00000051358     CTAAGAGAGCTCAATTTGACTAACTGCTCACTGCTGGGCGATTCCTCTGTCATCAGACTG
ENSCJAT00000041745     ATAAGAGAGCTAAATTTAAGCAACTGTGTGCAGCTAAGTGATGTCTCTGTTCTGAAACTG
ENSMMUT00000017423     ATAAGAGAGCTAAATTTAAGCAACTGCGTGCGGCTAAGCGATGTCTCTGTTATGAAACTA
ENSPPYT00000020825     ATAAGAGAGCTAAATTTAAGCAACTGTGTGCAGCTAAGTGATGCCTCTGTTATGAAACTA
ENST00000313221        ATAAGAGAGCTAAATTTAAGCAACTGTGTGCGGCTAAGTGATGCCTCTGTTATGAAACTA
ENSPTRT00000036197     ATAAGAGAGCTAAATTTAAGCAACTGTGTGCGGCTAAGTGATGCCTCTGTTATGAAACTA

ENSMUST00000051358     TCAGAGCGTTGCCCTAATTTACACTACTTGAATTTACGAAATTGTGAACATCTGACTGAC
ENSCJAT00000041745     TCTGAGCGCTGCCCTAATTTAAACTACTTGAGTTTACGAAATTGTGAACATTTGACCGCC
ENSMMUT00000017423     TCTGAGCGCTGCCCTAATTTAAACTACTTGAGTTTACGAAATTGTGACCATTTGACAGCC
ENSPPYT00000020825     TCTGAGCGTTGCCCTAATTTAAACTACTTGAGTTTACGAAATTGTGAACATTTGACTGCC
ENST00000313221        TCTGAGCGCTGCCCTAATTTAAACTACTTGAGTTTACGAAATTGTGAACATTTGACTGCC
ENSPTRT00000036197     TCTGAGCGCTGCCCTAATTTAAACTACTTGAGTTTACGAAATTGTGAACATTTGACTGCC

ENSMUST00000051358     TTGGCAATTGAATATATTGCAAGCATGTTATCCTTGATATCAGTAGATCTTTCTGGAACA
ENSCJAT00000041745     CAAGGAATTGCATATATTGTAAACATCTTTTCATTAGTATCAATAGATCTCTCTGGAACA
ENSMMUT00000017423     CAAGGAATTGGATATATTGTGAACATCTTTTCCTTGGTATCAATAGATCTCTCTGGAACA
ENSPPYT00000020825     CAAGGAATTGGATATATTGTAAACATCTTTTCCTTGGTATCAATAGATCTCTCTGGAACA
ENST00000313221        CAAGGAATTGGATATATTGTAAACATCTTTTCCTTGGTATCAATAGATCTCTCTGGAACA
ENSPTRT00000036197     CAAGGAATTGGATATATTGTAAACATCTTTTCCTTGGTATCAATAGATCTCTCTGGAACA

ENSMUST00000051358     CTCATCTCAAATGAGGGCATGACCATACTTTCCAGACACAGAAAACTGAGGGAAGTTTCT
ENSCJAT00000041745     GATATCTCTAATGAGGATTTGAATGTACTTTCCAGACATAAAAAATTGAAGGAACTTTCT
ENSMMUT00000017423     GACATCTCTAATGAGGGTTTGAATGTACTTTCCAAACATAAAAAATTGAAGGAACTTTCT
ENSPPYT00000020825     GACATCTCTAATGAGGGTTTGAATGTGCTTTCCAGACATAAAAAATTGAAGGAACTTTCT
ENST00000313221        GACATCTCTAATGAGGGTTTGAATGTGCTTTCCAGACATAAAAAATTGAAGGAACTTTCT
ENSPTRT00000036197     GACATCTCTAATGAGGGTTTGAATGTGCTTTCCAGACATAAAAAATTGAAGGAACTTTCT

ENSMUST00000051358     GTGTCTGACTGTGTCAACATCACTGACTTTGGAATTCGGGCATACTGCAAAACCTCGCTG
ENSCJAT00000041745     GTATCTGCATGTTATAGAATCACTGATGATGGAATTCAGGCATTCTGCAAAAACTCACTG
ENSMMUT00000017423     GTATCCGAGTGTTATGGAATCACTGATGTTGGAATTCAGGCATTCTGCAAAAGCTCACTG
ENSPPYT00000020825     GTATCCGAATGTTATAGAATCACTGATGATGGAATTCAGGCATTCTGCAAAAGCTCAGTG
ENST00000313221        GTATCTGAATGTTATAGAATCACTGATGATGGAATTCAGGCATTCTGCAAAAGCTCACTG
ENSPTRT00000036197     GTATCCGAATGTTATAGAATCACTGATGATGGAATTCAGGCATTCTGCAAAAGCTCACTG

ENSMUST00000051358     TTGTTGGAACACTTGGACGTCTCCTACTGCTCCCAACTGACAGATGACATCATAAAGACT
ENSCJAT00000041745     ATCTTGGAATGTTTGGATGTCTCTTATTGCTCCCAGCTGTCAGATATGATTATCAAAGCA
ENSMMUT00000017423     ATCTTGGAACATTTGGATGTCTCTTATTGCTCCCAGCTGTCAGATATGATTATCAAAGCA
ENSPPYT00000020825     ATCTTGGAACATTTGGATGTCTCTTATTGCTCCCAGCTGTCAGATATGATTATCAAAGCA
ENST00000313221        ATCTTGGAACATTTGGATGTCTCTTATTGCTCCCAGCTGTCAGATATGATTATCAAAGCA
ENSPTRT00000036197     ATCTTGGAACATTTGGATGTCTCTTATTGCTCCCAGCTGTCAGATATGATTATCAAAGCA

ENSMUST00000051358     ATAGCCATTTTCTGTACTCGAATTACATCTCTTAACATCGCTGGATGCCCAAAGATTACC
ENSCJAT00000041745     TTGGCCATTTACTGCATTAACCTCACGTCTCTCAGCATTGCTGGCTGTCCAAAGATTACT
```

```
ENSMMUT00000017423     CTGGCCATTTACTGCATTAACCTCACGTCTCTCAGCGTTGCTGGCTGTCCAAAGATTACT
ENSPPYT00000020825     CTGGCCATTTACTGCATTAACCTCACATCTCTCAGCATTGCTGGCTGTCCAAAGATTACT
ENST00000313221        CTGGCCATTTACTGCATTAACCTCACATCTCTCAGCATTGCTGGCTGTCCAAAGATTACT
ENSPTRT00000036197     CTGGCCATTTACTGCATTAACCTCACATCTCTCAGCATTGCTGGCTGTCCAAAGATTACT

ENSMUST00000051358     GATGCAGGGATGGAGATATTATCTGCAAGGTGCCATTATCTGCACATTTTGGATATCTCT
ENSCJAT00000041745     GACTCAGTGATGGAGATGTTATCGGCAAAATGCCATTACCTGCACATTTTGGATATCTCT
ENSMMUT00000017423     GACTCAGCGATGGAGATGTTATCGGCAAAATGCCATTACCTGCACATTTTGGATATCTCT
ENSPPYT00000020825     GACTCAGCGATGGAGATGTTATCGGCAAAATGCCATTACCTGCACATTTTGGATATCTCT
ENST00000313221        GACTCAGCAATGGAGATGTTATCGGCAAAATGCCATTACCTGCACATTTTGGATATCTCT
ENSPTRT00000036197     GACTCAGCGATGGAGATGTTATCGGCAAAATGCCATTACCTGCACATTTTGGATATCTCT

ENSMUST00000051358     GGCTGTATCCAACTTACAGACCAGATCATTCAGGACCTTCAAATAGGCTGCAAACAACTC
ENSCJAT00000041745     GGTTGTGTCTTGCTTACTGACCAAATCCTTGATGACCTTCAGATAGGCTGCAAACAACTC
ENSMMUT00000017423     GGTTGTGTCCTGCTTACTGACCAAATCCTTGAGGACCTTCAGATAGGCTGCAAACAGCTC
ENSPPYT00000020825     GGTTGTGTCTTGCTTACTGACCAAATCCTTGAGGACCTTCAGATAGGCTGCAAACAACTC
ENST00000313221        GGTTGTGTCTTGCTTACTGACCAAATCCTTGAGGACCTTCAGATAGGCTGCAAACAACTC
ENSPTRT00000036197     GGTTGTGTCTTGCTTACTGACCAAATCCTTGAGGACCTTCAGATAGGCTGCAAACAACTC

ENSMUST00000051358     CGGATCCTCAAGATGCAATTCTGCAAGTCCATCTCTCCGGCAGCGGCTCAGAAAATGTCC
ENSCJAT00000041745     CGGATCCTTAGGATGCAATACTGCACAAATATTTCCAAGAATGCAGCTGAAAGAATGTCA
ENSMMUT00000017423     CGGATCCTTAAGATGCAATACTGCACAAATATTTCCAAGAAGGCAGCTCAGAGAATGTCA
ENSPPYT00000020825     CGGATCCTTAAGATGCAATACTGCACAAATATTTCCAAGAAGGCAGCTCAAAGAATGTCA
ENST00000313221        CGGATCCTTAAGATGCAATACTGCACAAATATTTCCAAGAAGGCAGCTCAAAGAATGTCA
ENSPTRT00000036197     CGGATCCTTAAGATGCAATACTGCACAAATATTTCCAAGAAGGCAGCTCAAAGAATGTCA

ENSMUST00000051358     TCTGTGGTCCAGCATCAGGAATACAACTCCGACAACCCTCCACATTGGTTTGGCTACGAC
ENSCJAT00000041745     TCGAAAGTTCAGCAGCAGGAATACAACTCTAATGACCCTCCACGTTGGTTTCGCTATGAT
ENSMMUT00000017423     TCTAAAGTTCAGCAGCAGGAATACAACTCTAATGACCCTCCACGTTGGTTTGGCTATGAT
ENSPPYT00000020825     TCTAAAGTTCAGCAGCAGGAATACAACTCTAATGACCCTCCACGTTGGTTTGGCTATGAT
ENST00000313221        TCTAAAGTTCAGCAGCAGGAATACAACACTAATGACCCTCCACGTTGGTTTGGCTATGAT
ENSPTRT00000036197     TCTAAAGTTCAGCAGCAGGAATACAACACTAATGACCCTCCACGTTGGTTTGGCTATGAT

ENSMUST00000051358     TCTGAAGGCAACCCTCTTGACAAGATTCATAGTCGAGTACAACTTAGAACTTACTCAAAA
ENSCJAT00000041745     AGGGAAGGAAACCCTCTTACACAGCCTGAAAACATAACATCATCTAAAGGACACTTAGAA
ENSMMUT00000017423     AGGGAAGGAGACCCTCTTACAGAGCTTGACAATGTAACACCATCTAAAGGAGCCTCAGAA
ENSPPYT00000020825     AGGGAAGGAAACCGTGTTACAGAGCTTGACAACATAACGTCATCTAAAGGAGCCTCAGAA
ENST00000313221        AGGGAAGGAAACCCTGTTACAGAGCTTGACAACATAACATCATCTAAAGGAGCCTTAGAA
ENSPTRT00000036197     AGGGAAGGAAACCCTGTTACAGAGCTTGACAACATAACATCATCTAAAGGAGCCTTAGAA

ENSMUST00000051358     CTGATAGTCAAAGAGCCATTCAGCATCGACGAGGAGGATCCAGAC
ENSCJAT00000041745     CTAACAGTGGAAAAGTCAACATACAGCAGTGAAGAGGAAGCAGTG
ENSMMUT00000017423     TTAACAGTGGAAAAGTCAACATACAGCAGTGAAGAGCAAGCAGCG
ENSPPYT00000020825     TTAACAGTGGAAAAGTCAACATACAGCAGCGAAGACCAAGCAGCG
ENST00000313221        TTAACAGTGAAAAAGTCAACATACAGCAGTGAAGACCAAGCAGCG
ENSPTRT00000036197     TTAACAGTGAAAAAGTCAACATACAGCAGTGAAGACCAAGCAGCG
```

Multiple sequence alignment of Fbxl14

```
ENSPPYT00000004904     ATGGAGACCCACATCTCATGCCTGTTCCCGGAGCTGCTGGCCATGATCTTCGGCTACCTG
ENSMMUT00000027308     ATGGAGACCCACATCTCATGCTTGTTCCCGGAGCTGCTGGCCATGATCTTCGGCTACCTG
ENST00000339235        ATGGAGACCCACATCTCATGCCTGTTCCCGGAGCTGCTGGCCATGATCTTCGGCTACCTG
ENSRNOT00000000022     ATGGAGACCCACATCTCGTGCCTGTTTCCGGAGCTGCTGGCCATGATCTTCGGCTACTTG
```

```
ENSMUST00000032094     ATGGAGACCCACATCTCGTGCCTGTTCCCCGAGCTGCTGGCCATGATCTTCGGCTACTTG
ENSCJAT00000001230     ATGGAGACCCATATATCGTGCCTGTTCCCCGAGCTGCTGGCCATGATCTTCGGCTACCTG

ENSPPYT00000004904     GACGTCCGGGACAAGGGGCGCGCGGCGCAGGTGTGCACGGCCTGGCGGGACGCCGCCTAC
ENSMMUT00000027308     GACGTCCGGGACAAGGGGCGCGCGGCGCAGGTGTGCACGGCCTGGCGGGACGCCGCCTAC
ENST00000339235        GACGTCCGGGACAAGGGGCGCGCGGCGCAGGTGTGCACCGCCTGGCGGGACGCCGCCTAC
ENSRNOT00000000022     GACGTCCGAGACAAGGGTCGCGCGGCGCAGGTGTGCACGGCCTGGCGGGACGCCGCCTAC
ENSMUST00000032094     GACGTCCGAGACAAGGGTCGCGCGGCGCAGGTGTGCACGGCCTGGCGGGACGCCGCCTAC
ENSCJAT00000001230     GACGTCCGGGACAAGGGGCGCGCGGCGCAGGTGTGCACGGCCTGGCGGGACGCCGCCTAC

ENSPPYT00000004904     CACAAGTCGGTGTGGCGGGGGGTGGAGGCGAAGCTGCACCTGCGCCGGGCCAACCCGTCG
ENSMMUT00000027308     CACAAGTCGGTGTGGCGGGGGGTGGAGGCCAAGCTGCACCTGCGCCGGGCCAACCCGTCG
ENST00000339235        CACAAGTCGGTGTGGCGGGGGGTGGAGGCCAAGCTGCACCTGCGCCGGGCCAACCCGTCG
ENSRNOT00000000022     CACAAGTCAGTGTGGCGGGGCGTGGAGGCCAAGCTGCACCTGCGCCGGGCGAACCCGTCG
ENSMUST00000032094     CACAAGTCGGTGTGGCGGGGCGTGGAGGCCAAGCTGCACCTGCGCCGGGCGAACCCGTCG
ENSCJAT00000001230     CACAAGTCGGTGTGGCGGGGGGTGGAGGCCAAGCTGCACCTGCGCCGGGCCAACCCGTCG

ENSPPYT00000004904     CTGTTCCCCAGCCTGCAGGCCCGGGGCATCCGCCGGGTGCAGATCCTGAGCCTCCGCCGC
ENSMMUT00000027308     CTGTTCCCCAGCCTGCAGGCCCGGGGCATCCGCCGCGTGCAGATCCTGAGCCTCCGCCGC
ENST00000339235        CTGTTCCCCAGCCTGCAGGCCCGGGGCATCCGCCGGGTGCAGATCCTGAGCCTCCGCCGC
ENSRNOT00000000022     CTGTTCCCCAGCCTTCAGGCCCGGGGCATCCGCCGCGTGCAGATCCTCAGCCTCCGCCGC
ENSMUST00000032094     CTGTTCCCCAGCCTGCAGGCCCGGGGCATCCGCCGCGTGCAGATCCTCAGCCTCCGCCGC
ENSCJAT00000001230     CTGTTCCCCAGCCTGCAGGCCCGGGGCATCCGCCGAGTGCAGATCCTGAGCCTCCGCCGC

ENSPPYT00000004904     AGCCTCAGCTACGTGATCCAGGGCATGGCCAACATCGAGAGCCTCAACCTCAGCGGCTGC
ENSMMUT00000027308     AGCCTCAGCTACGTGATCCAGGGCATGGCCAACATCGAGAGCCTCAACCTCAGCGGCTGC
ENST00000339235        AGCCTCAGCTACGTGATCCAGGGCATGGCCAACATCGAGAGCCTCAACCTCAGCGGCTGC
ENSRNOT00000000022     AGCCTCAGCTACGTGATCCAGGGCATGGCGAACATCGAGAGCCTCAACCTCAGCGGCTGC
ENSMUST00000032094     AGCCTCAGCTACGTGATCCAGGGCATGGCGAACATCGAGAGCCTCAACCTCAGCGGCTGC
ENSCJAT00000001230     AGCCTCAGCTACGTGATCCAGGGCATGGCCAACATCGAGAGCCTCAACCTCAGCGGCTGC

ENSPPYT00000004904     TACAACCTCACCGACAACGGGCTGGGCCACGCGTTTGTGCAGGAGATCGGCTCCCTGCGC
ENSMMUT00000027308     TACAACCTCACCGACAACGGGCTGGGCCACGCGTTTGTGCAGGAGATCGGCTCCCTGCGC
ENST00000339235        TACAACCTCACCGACAACGGGCTGGGCCACGCGTTTGTGCAGGAGATCGGCTCCCTGCGC
ENSRNOT00000000022     TACAACCTCACCGATAACGGCCTGGGCCACGCGTTCGTGCAGGAGATCGGCTCGCTGCGC
ENSMUST00000032094     TACAACCTCACCGACAACGGCCTGGGCCACGCGTTCGTGCAGGAGATCGGCTCGCTGCGC
ENSCJAT00000001230     TACAACCTCACCGACAACGGGCTGGGCCATGCGTTTGTGCAGGAGATCGGCTCCCTGCGT

ENSPPYT00000004904     GCTCTCAACCTGAGCCTCTGCAAGCAGATCACTGACAGCAGCCTGGGCCGCATAGCCCAG
ENSMMUT00000027308     GCTCTCAACCTGAGCCTCTGCAAGCAGATCACTGACAGCAGCCTGGGCCGCATAGCCCAG
ENST00000339235        GCTCTCAACCTGAGCCTCTGCAAGCAGATCACTGACAGCAGCCTGGGCCGCATAGCCCAG
ENSRNOT00000000022     GCTCTCAACTTGAGCCTCTGCAAGCAGATCACAGACAGCAGCCTGGGCCGCATAGCCCAG
ENSMUST00000032094     GCTCTCAACTTGAGCCTTTGCAAGCAGATCACCGACAGCAGCCTGGGCCGCATAGCCCAG
ENSCJAT00000001230     GCTCTCAACCTGAGTCTCTGCAAGCAGATCACTGACAGCAGCCTTGGCCGCATAGCCCAG

ENSPPYT00000004904     TACCTCAAGGGCCTGGAGGTGCTGGAGCTGGGGGGTTGCAGCAACATCACCAACACTGGC
ENSMMUT00000027308     TATCTGAAGGGCCTGGAGGTGCTGGAGCTGGGGGGTTGCAGCAACATCACCAACACTGGC
ENST00000339235        TACCTCAAGGGCCTGGAGGTGCTGGAGCTGGGAGGTTGCAGCAACATCACCAACACTGGC
ENSRNOT00000000022     TACCTCAAGGGCCTGGAGGTGCTAGAGCTGGGGGGCTGCAGCAACATCACCAACACCGGC
ENSMUST00000032094     TACCTCAAGGGCCTGGAGGTGCTGGAGCTGGGGGGCTGCAGCAACATCACCAACACCGGC
ENSCJAT00000001230     TACCTCAAGGGTCTGGAGGTGCTGGAGCTGGGAGGTTGCAGCAACATTACCAATACCGGC
```

```
ENSPPYT00000004904     CTTCTGCTCATCGCCTGGGGTCTGCAGCGCCTCAAGAGCCTTAACCTCCGCAGCTGCCGC
ENSMMUT00000027308     CTTTTGCTCATCGCCTGGGGTCTGCAGCGCCTCAAGAGCCTTAACCTCCGCAGCTGCCGC
ENST00000339235        CTTCTGCTCATCGCCTGGGGTCTGCAGCGCCTCAAGAGCCTTAACCTCCGCAGCTGCCGC
ENSRNOT00000000022     CTTCTGCTCATCGCCTGGGGGCTGCAGCGCCTCAAGAGCCTTAACCTCCGCAGCTGCCGC
ENSMUST00000032094     CTTCTGCTCATTGCCTGGGGGCTGCAGCGCCTCAAGAGCCTTAATCTCCGCAGCTGTCGC
ENSCJAT00000001230     CTTCTGCTTATCGCCTGGGGTCTACAGCGCCTCAAGAGCCTTAACCTCCGCAGCTGCCGC

ENSPPYT00000004904     CACCTTTCGGATGTGGGCATCGGGCACCTGGCCGGCATGACGCGCAGCGCGGCGGAGGGC
ENSMMUT00000027308     CACCTCTCGGATGTCGGCATCGGGCACCTGGCCGGCATGACGCGCAGCGCTGCGGAGGGC
ENST00000339235        CACCTTTCGGATGTGGGCATCGGGCACCTGGCCGGCATGACGCGCAGCGCGGCGGAGGGC
ENSRNOT00000000022     CATCTCTCAGACGTGGGCATCGGGCACCTGGCCGGCATGACGCGCAGCGCTGCCGAGGGC
ENSMUST00000032094     CACCTCTCGGATGTGGGCATCGGGCACCTGGCCGGCATGACGCGCAGCGCTGCCGAGGGC
ENSCJAT00000001230     CACCTTTCGGATGTGGGCATCGGGCACCTGGCTGGCATGACGCGTAGCGCGGCGGAGGGC

ENSPPYT00000004904     TGCCTGGGCCTGGAGCAGCTCACGCTACAGGACTGCCAGAAGCTCACAGATCTTTCTCTA
ENSMMUT00000027308     TGCCTGGGCCTGGAGCAGCTCACGCTACAGGACTGCCAGAAGCTCACAGATCTTTCTCTA
ENST00000339235        TGCCTGGGCCTGGAGCAGCTCACGCTACAGGACTGCCAGAAGCTCACAGATCTTTCTCTA
ENSRNOT00000000022     TGCCTGGGCCTGGAGCAGCTCACTCTACAGGACTGCCAGAAACTCACGGATCTTTCTCTG
ENSMUST00000032094     TGCCTGGGCCTGGAGCAGCTCACTCTTCAGGACTGCCAGAAACTCACGGATCTTTCCCTG
ENSCJAT00000001230     TGCCTGGGCCTGGAGCAGCTCACGCTGCAGGACTGCCAGAAGCTCACAGATTTGTCTCTA

ENSPPYT00000004904     AAGCACATCTCCCGAGGGCTGACGGGCCTGAGGCTCCTCAACCTCAGCTTCTGTGGGGGC
ENSMMUT00000027308     AAGCACATCTCCCGAGGGCTGACGGGCCTGAGGCTCCTCAACCTCAGCTTCTGTGGGGGC
ENST00000339235        AAGCACATCTCCCGAGGGCTGACGGGCCTGAGGCTCCTCAACCTCAGCTTCTGTGGGGGA
ENSRNOT00000000022     AAGCACATCTCGCGAGGGCTGACGGGCCTCAGGCTCCTCAACCTCAGCTTCTGTGGCGGG
ENSMUST00000032094     AAGCACATCTCGCGAGGGCTGACGGGCCTCCGGCTCCTCAACCTCAGCTTCTGCGGCGGC
ENSCJAT00000001230     AAGCACATCTCCCGAGGGCTGACGGGCCTGAGGCTCCTCAACCTCAGCTTCTGCGGGGGC

ENSPPYT00000004904     ATCTCGGACGCTGGCCTCCTGCACCTGTCGCACATGGGCAGCCTGCGCAGCCTCAACCTG
ENSMMUT00000027308     ATCTCGGACGCTGGCCTCCTGCACCTGTCGCACATGGGCAGCCTGCGCAGCCTCAACCTG
ENST00000339235        ATCTCGGACGCTGGCCTCCTGCACCTGTCGCACATGGGCAGCCTGCGCAGCCTCAACCTG
ENSRNOT00000000022     ATCTCGGACGCGGGCCTTCTGCACCTGTCGCACATGGGCAGCCTGCGCAGCCTTAACCTG
ENSMUST00000032094     ATCTCGGACGCGGGCCTTCTGCACCTGTCGCACATGGGCAGCCTGCGCAGCCTGAACCTG
ENSCJAT00000001230     ATCTCGGACGCTGGCCTCCTGCACCTGTCGCACATGGGCAGCCTGCGCAGCCTCAACCTG

ENSPPYT00000004904     CGCTCCTGTGACAACATCAGTGACACGGGCATCATGCATCTGGCCATGGGCAGCCTGCGC
ENSMMUT00000027308     CGCTCCTGTGACAACATCAGTGACACGGGCATCATGCATCTGGCCATGGGCAGCCTGCGC
ENST00000339235        CGCTCCTGTGACAACATCAGTGACACGGGCATCATGCATCTGGCCATGGGCAGCCTGCGC
ENSRNOT00000000022     CGCTCCTGCGACAACATCAGCGACACTGGCATCATGCATTTGGCCATGGGCAGCCTGCGC
ENSMUST00000032094     CGCTCCTGCGACAACATCAGCGACACCGGCATCATGCATCTGGCCATGGGCAGCCTGCGC
ENSCJAT00000001230     CGCTCCTGTGACAACATCAGTGACACGGGCATCATGCATCTGGCCATGGGCAGCCTGCGC

ENSPPYT00000004904     CTCTCGGGGCTGGATGTGTCGTTCTGTGACAAGGTGGGAGACCAGAGTCTGGCTTACATA
ENSMMUT00000027308     CTCTCGGGGCTGGATGTGTCGTTCTGTGACAAGGTGGGAGACCAGAGTCTGGCTTACATA
ENST00000339235        CTCTCGGGGCTGGATGTTTCGTTCTGTGACAAGGTGGGAGACCAGAGTCTGGCTTACATA
ENSRNOT00000000022     CTCTCCGGTCTGGATGTGTCATTCTGTGACAAGGTGGGAGATCAGAGCCTGGCTTACATA
ENSMUST00000032094     CTTTCCGGTCTGGATGTGTCGTTCTGTGACAAGGTGGGAGATCAGAGTCTGGCGTACATA
ENSCJAT00000001230     CTCTCCGGGCTGGATGTGTCGTTCTGTGACAAGGTGGGGGATCAGAGTCTGGCTTACATA

ENSPPYT00000004904     GCCCAGGGGCTGGATGGCCTCAAGTCTCTCTCCCTCTGCTCCTGCCACATCAGTGATGAT
ENSMMUT00000027308     GCCCAAGGGCTGGATGGCCTCAAGTCTCTCTCTCTCTGCTCCTGCCACATCAGTGATGAT
ENST00000339235        GCCCAGGGGCTGGATGGCCTCAAGTCTCTCTCCCTCTGCTCCTGCCACATCAGTGATGAT
ENSRNOT00000000022     GCCCAGGGACTGGACGGCCTCAAGTCCCTCTCCCTTTGCTCTTGCCATATCAGTGACGAT
```

```
ENSMUST00000032094     GCCCAGGGACTGGACGGCCTCAAGTCCCTCTCCCTTTGCTCTTGCCATATCAGTGACGAT
ENSCJAT00000001230     GCCCAGGGGCTGGATGGCCTCAAGTCCCTCTCCCTCTGCTCCTGCCACATCAGTGATGAT


ENSPPYT00000004904     GGTATCAACCGCATGGTGCGGCAGATGCACGGGCTGCGCACGCTCAACATTGGACAGTGT
ENSMMUT00000027308     GGCATCAACCGCATGGTGCGGCAGATGCACGGGCTGCGCACGCTCAACATTGGACAGTGT
ENST0000339235         GGCATCAACCGCATGGTGCGGCAGATGCACGGGCTGCGCACGCTCAACATTGGACAGTGT
ENSRNOT00000000022     GGCATCAACCGCATGGTGCGGCAGATGCACGGGCTGCGCACGCTCAACATTGGACAGTGT
ENSMUST00000032094     GGCATCAACCGCATGGTGCGGCAGATGCACGGGCTGCGCACGCTCAACATTGGACAGTGT
ENSCJAT00000001230     GGCATCAACCGCATGGTGCGGCAGATGCACGGGCTGCGCACACTCAACATTGGACAGTGT


ENSPPYT00000004904     GTGCGCATCACGGACAAGGGCCTGGAGCTGATCGCTGAGCACCTGAGCCAGCTCACCGGC
ENSMMUT00000027308     GTGCGCATCACGGACAAGGGCCTGGAGCTGATCGCTGAGCACCTGAGCCAGCTCACCGGC
ENST0000339235         GTGCGCATCACGGACAAGGGCCTGGAGCTGATCGCTGAGCACCTGAGCCAACTCACCGGC
ENSRNOT00000000022     GTGCGCATCACGGACAAGGGACTGGAGCTGATCGCTGAGCACCTGAGCCAGCTCACAGGC
ENSMUST00000032094     GTGCGCATCACGGACAAGGGCCTGGAGCTGATCGCTGAGCACCTGAGCCAGCTCACGGGC
ENSCJAT00000001230     GTGCGCATCACGGACAAGGGCCTGGAGCTGATCGCTGAGCACCTGAGCCAGCTCACCGGC


ENSPPYT00000004904     ATAGACCTGTACGGCTGCACCCGAATCACCAAACGCGGCCTGGAGCGCATCACGCAGCTG
ENSMMUT00000027308     ATAGACCTGTACGGCTGCACCCGAATCACCAAGCGCGGCCTGGAGCGCATCACGCAGCTG
ENST0000339235         ATAGACCTGTACGGCTGCACCCGAATCACCAAGCGCGGCCTGGAGCGCATCACGCAGCTG
ENSRNOT00000000022     ATAGACCTGTACGGCTGCACTAGGATCACCAAGCGCGGCCTGGAGCGCATCACGCAGCTG
ENSMUST00000032094     ATAGATCTGTACGGCTGCACCAGGATCACCAAGCGCGGCCTGGAGCGCATCACGCAGCTG
ENSCJAT00000001230     ATAGACCTGTACGGCTGCACCCGAATCACCAAGCGCGGTCTGGAGCGCATCACGCAGCTG


ENSPPYT00000004904     CCCTGCCTCAAGGTACTCAACCTGGGACTCTGGCAGATGACGGACAGTGAGAAGAGAAGG
ENSMMUT00000027308     CCCTGCCTCAAGGTACTCAACCTGGGACTCTGGCAGATGACGGACAGTGAGAAGAGAAGG
ENST0000339235         CCGTGCCTCAAGGTACTCAACCTGGGACTCTGGCAGATGACGGACAGTGAGAAGAGAAGG
ENSRNOT00000000022     CCCTGCCTCAAGGTACTTAACCTGGGACTTTGGCAGATGACGGACAGTGAGAAGGTCAGG
ENSMUST00000032094     CCCTGCCTCAAGGTACTTAACCTGGGACTTTGGCAGATGACGGACAGTGAGAAGGTCAGG
ENSCJAT00000001230     CCCTGCCTCAAGGTACTCAACCTGGGACTCTGGCAGATGACGGACAGTGAGAAG------
```

Multiple sequence alignment of Fbxl15

```
ENSMUST00000026256     ATGGAGCCACCAATGGAGCAGTCCGGAGGGGAGCAAGAACCAGGAGCTGTCAGGCTCCTG
ENSRNOT00000026471     ATGGAGCCACCAATGGAGCAGTCCGGAGGGGAGCAAGAACCAGGAGCCGTCAGGCTCCTG
ENSPPYT00000003129     ACGGAGCCACCGATGGAGCCGTCCGGAAGGGAGCAAGAGCCCGGAGCCATCAGGCTCCTG
ENSCJAT00000035103     AAGGAGCCACCGATGGAGCCGTCCGGAGGGGAGCAAGAGCCCGGAGCCGTCAGGCTCCTG
ENSMMUT00000004250     ATGGAGCCACCGATGGAGCCGTCCGGAGGGGAGCAAGAGCCCGGAGCCGTCAGGCTCCTG
ENSGGOT00000007017     ATGGAGCCACCGACGGAGCCGTCCGGAGGGGAGCAAGAGCCCGGGGCCGTCAGGTTCCTG
ENST0000224862         ATGGAGCCACCGATGGAGCCGTCCGGAGGGGAGCAAGAGCCCGGAGCCGTCAGGTTCCTG
ENSPTRT00000005510     ATGGAGCCACCGATGGAGCCGTCCGGAGGGGAGCAAGAGCCCGGAGCCGTCAGGTTCCTG


ENSMUST00000026256     GACCTGCCCTGGGAAGACGTGCTGCTCCCGCACGTCCTGAACTGGGTCCCGCTGCGCCAG
ENSRNOT00000026471     GACCTGCCCTGGGAAGACGTGCTGCTCCCGCACGTCCTGAACTGGGTCCCGCTGCGCCAG
ENSPPYT00000003129     GACCTGCCCTGGGAAGACGTGCTGCTCCCACACGTCCTGAACCGGGTCCCGCTGCGCCAG
ENSCJAT00000035103     GACCTGCCCTGGGAAGACGTGCTGCTCCCTCACGTCCTGAACCGGGTACCGCTGCGCCAG
ENSMMUT00000004250     GACCTGCCCTGGGAAGATGTGCTGCTCCCACACGTCCTGAACCGGGTCCCGCTGTGCCAG
ENSGGOT00000007017     GACCTGCCCTGGGAAGACGTGCTGCTCCCACACGTCCTGAACCGGGTCCCGCTGCGCCAG
ENST0000224862         GACCTGCCCTGGGAAGACGTGCTGCTCCCACACGTCCTGAACCGGGTCCCGCTGCGCCAG
ENSPTRT00000005510     GACCTGCCCTGGGAAGACGTGCTGCTCCCACACGTCCTGAACCGGGTCCCGCTGCGCCAG


ENSMUST00000026256     CTGCTCCGGCTGCAGCGCGTCAGTCGCGCCTTCCGGGCGCTCGTTCAGCTGCACTTGGCG
ENSRNOT00000026471     CTGCTCCGGCTGCAGCGCGTCAGTCGCGCCTTCCGGGCGCTCGTTCAGCTGCACCTGGCT
```

ENSPPYT00000003129 CTGCTCCGGCTGCAGCGCGTTAGCCGGGCCTTCCGGGCGCTGGTGCAGCTTCACCTGGCC
ENSCJAT00000035103 CTGCTCCGGCTGCAGCGCGTTAGCCGGGCCTTCCGGGCACTGGTACAGCTTCACCTGGCC
ENSMMUT00000004250 CTGCTCCGGCTGCAGCGCGTTAGCCGGGCCTTCCGGGCGCTGGTGCAGCTTCACCTGGCC
ENSGGOT00000007017 CTGCTCCGGCTGCAGCGCGTTAGCCGGGCCTTCCGGTCGCTGGTGCAGCTTCACCTGGCT
ENST00000224862 CTGCTCCGGCTGCAGCGCGTTAGCCGGGCCTTCCGGTCGCTGGTGCAGCTTCACCTGGCC
ENSPTRT00000005510 CTGCTCCGGCTGCAGCGCGTTAGCCGGGCCTTCCGGTCGCTGGTGCAGCTTCACTTGGCC

ENSMUST00000026256 AGGCTGCGCCGCTTCGACGCCGCTCAGGTGGGTCCACAGATTCCACGGGCGGCACTAGCC
ENSRNOT00000026471 AGGCTGCGCCGCTTTGACGCCGCTCAGGTGGGTCCACAGATTCCACGGGCCGCACTAGTC
ENSPPYT00000003129 GGGCTGCGTCGCTTCGATGCCGCGCAGGTGGGTCCGCAGATCCCGCGGGCCGCATTGGCC
ENSCJAT00000035103 GGGCTGCGTCGCTTCGACGCCGCGCAGGTGGGTCCGCAGATCCCACGGGCCGCATTGGCC
ENSMMUT00000004250 GGGCTGCGTCGCTTCGATGCAGCGCAGGTGGGTCTGCAGATCCCGCGGGCCGCATTGGCC
ENSGGOT00000007017 GGGCTGCGTCGCTTCGATGCCGCGCAGGTGGGTCCGCAGATCCCGCGGGCCGCATTGGCC
ENST00000224862 GGGCTGCGTCGCTTCGATGCCGCGCAGGTGGGTCCGCAGATCCCGCGGGCCGCATTGGCC
ENSPTRT00000005510 GGGCTGCGTCGCTTCGATGCCGCGCAGGTGGGTCCGCAGATCCCGCGGGCCGCATTGGCC

ENSMUST00000026256 CGGCTACTGCGGGATGCTGAGGGGCTGCAGGAGCTGGCGCTGGCTCCGTGTCACGAATGG
ENSRNOT00000026471 CGGCTACTGCGAGATGCTGAGGGGTTGCAGGAGCTGGCGCTGGCTCCGTGTCACGAATGG
ENSPPYT00000003129 AGGCTGCTGCGGGATGCCGAGGGGCTGCAGGAGCTGGCGCTGGCGCCGTGTCACGAATGG
ENSCJAT00000035103 CGGCTGCTGCGGGACGCCGAGGGGCTGCAGGAGCTGGCGCTAGCGCCGTGTCACGAATGG
ENSMMUT00000004250 CGGCTGCTGCGGGACGCCGAGGGGCTGCAGGAGCTGGCGCTGGCGCCGTGTCACGAATGG
ENSGGOT00000007017 CGGCTGCTGCGGGATGCCGAGGGGCTGCAGGAGCTGGCACTGGCGCCGTGTCACGAATGG
ENST00000224862 CGGCTGCTGCGGGATGCCGAGGGGCTGCAGGAGCTGGCACTGGCGCCGTGTCACGAATGG
ENSPTRT00000005510 CGGCTGCTGCGGGATGCCGAGGGGCTGCAGGAGCTGGCACTGGCGCCGTGTCACGAATGG

ENSMUST00000026256 CTGTCGGACGAGGACCTGGTGCCGGTGCTGGCACGGAATCCACAGCTCCGGAGCGTAGCG
ENSRNOT00000026471 CTGTTGGACGAGGACCTGGTGCCGGTGCTGGCACGGAATCCACAGCTACGGAGCGTAGCA
ENSPPYT00000003129 CTGTCAGACGAGGACCTGGTGCCGGTGCTGGCGCGGAATCCGCAGCTGCGGAGTGTGGCG
ENSCJAT00000035103 CTGTCAGACGAGGATCTGGTGCCGGTGCTAGCGCGGAACCCGCAGCTGCGGAGTGTGGCG
ENSMMUT00000004250 TTGTCAGACGAGGACCTGGTGCCGGTGCTGGCGCGTAATCCGCAGCTGCGGAGTGTGGCG
ENSGGOT00000007017 CTGTCAGACGAGGACCTGGTGCCGGTGCTGGCGCGGAATCCGCAGCTGCGGAGTGTAGCG
ENST00000224862 CTGTCAGACGAGGACCTGGTGCCGGTGCTGGCGCGGAATCCGCAGCTGCGGAGTGTGGCG
ENSPTRT00000005510 CTGTCAGACGAGGACCTGGTGCCGGTGCTGGCGCGGAATCCGCAGCTGCGGAGTGTGGCG

ENSMUST00000026256 CTGGCCGGCTGCGGCCAACTGAGTCGCCGAGCCCTGGGGGCGCTGGCTGAGGGCTGCCCG
ENSRNOT00000026471 CTGGCCGGCTGCGGCCAACTGAGTCGCCGAGCACTCGGAGCGCTGGCTGAGGGCTGCCCG
ENSPPYT00000003129 CTGGGCGGCTGCGGGCAACTGAGTCGCCGGGCGCTTGGGGCGCTGGCCGAGGGCTGCCCA
ENSCJAT00000035103 CTGGCCGGCTGCGGGCAACTGAGTCGCCGGGCGCTTGGGGCGCTGGCCGAGGGCTGCCCA
ENSMMUT00000004250 CTGGGCGGTTGCGGGCAACTGAGTCGTCGGGCGCTTGGGGCGCTGGCCGAGGGCTGCCCA
ENSGGOT00000007017 CTGGGCGGCTGCGGGCAACTGAGTCGCCGGGCGCTTGGGGCGCTGGCCGAGGGCTGCCCA
ENST00000224862 TTGGGCGGCTGCGGGCAACTGAGTCGCCGGGCGCTTGGGGCTTTGGCCGAGGGCTGCCCA
ENSPTRT00000005510 TTGGGCGGCTGCGGGCAACTGAGTCGCCGGGCGCTTGGGGCGCTGGCCGAGGGCTGCCCA

ENSMUST00000026256 CGTCTGCAGCGCCTTTCCCTCGCTCACTGTGACTGGGTGGACGGGCTGGCACTGCGTGGC
ENSRNOT00000026471 CGTCTGCAGCGCATTTCGCTCGCTCACTGTGACTGGGTGGACGGGCTGGCACTGCGTGGC
ENSPPYT00000003129 CGCCTGCAGCGCTTGTCGCTCGCGCACTGTGACTGGGTGGACGGCCTGGCGCTGCGCGGC
ENSCJAT00000035103 CGCCTGCAGCGCCTGTCGCTCGCGCACTGTGACTGGGTGGACGGGCTGGCGCTGCGCGGC
ENSMMUT00000004250 CGCCTGCAGCGCCTGTCGCTCGCGCACTGTGACTGGGTGGACGGGCTGGCGCTGCGCGGC
ENSGGOT00000007017 CGCCTGCAGCGCCTGTCGCTCGCGCACTGTGACTGGGTGGACGGGCTGGCGCTGCGCGGC
ENST00000224862 CGCCTGCAGCGCCTGTCGCTCGCGCACTGTGACTGGGTGGACGGGCTGGCGCTGCGCGGC
ENSPTRT00000005510 CGCCTGCAGCGCCTGTCGCTCGCGCACTGTGACTGGGTGGACGGGCTGGCGCTGCGCGGC

ENSMUST00000026256 CTCGCTGACCGCTGCCCGGCTCTGGAGGAGCTAGACCTCACCGCCTGTCGTCAACTCAAG
ENSRNOT00000026471 CTTGCTGACCGCTGCCCGGCTCTGGAGGAGCTAGACCTCACCGCCTGTCGTCAACTCAAG

```
ENSPPYT00000003129     CTCGCTGATCGCTGCCCGGCCCTGGAGGAGCTCGATCTCACCGCCTGCCGCCAGCTCAAG
ENSCJAT00000035103     CTCGCCGACCGCTGCCCGGCCCTGGAGGAGCTGGACCTCACCGCCTGCCGCCAGCTCAAG
ENSMMUT00000004250     CTCGCTGACCGCTGCCCGGCCCTGGAGGAGCTGGATCTCACCGCCTGCCGCCAGCTCAAG
ENSGGOT00000007017     CTCGCTGATCGCTGCCCGGCCCTGGAGGAGCTGGATCTCACCGCCTGCCGCCAGCTCAAG
ENST00000224862        CTCGCTGATCGCTGCCCGGCCCTGGAGGAGCTGGATCTCACCGCCTGCCGCCAGCTCAAG
ENSPTRT00000005510     CTCGCTGATCGCTGCCCGGCCCTGGAGGAGCTGGATCTCACCGCCTGCCGCCAGCTCAAG

ENSMUST00000026256     GACGAGGCCATCGTGTACCTGGCGCAGAGACGCGGCGCGGGCCTCCGCAGCCTCTCATTA
ENSRNOT00000026471     GACGAGGCCATCGTGTACCTGGCGCAGAGACGCGGCGCGGGCCTCCGCAGCCTCTCGTTG
ENSPPYT00000003129     GACGAGGCCATCGTGTACCTGGCGCAGAGGCGCGGCGCTGGTCTCCGCAGCCTCTCTCTG
ENSCJAT00000035103     GACGAGGCCATCGTGTACCTAGCGCAGAGGCGCGGCGCTGGCCTCCGCAGCCTCTCGCTG
ENSMMUT00000004250     GACGAGGCCATCGTGTACCTGGCGCAGAGGCGCGGCGCTGGCCTCCGCAGCCTCTCTCTG
ENSGGOT00000007017     GACGAGGCCATCGTGTACCTGGCGCAGAGGCGCGGCGCTGGTCTCCGCAGCCTCTCTCTG
ENST00000224862        GACGAGGCCATCGTGTACCTGGCGCAGAGGCGCGGCGCTGGTCTCCGCAGCCTCTCTCTG
ENSPTRT00000005510     GACGAGGCCATCGTGTACCTGGCGCAGAGGCGCGGCGCTGGTCTCCGCAGCCTCTCTCTG

ENSMUST00000026256     GCAGTCAACGCCAATGTTGGGGACACTGCGGTTCAAGAGTTGGCTCGAAACTGCCCGCAG
ENSRNOT00000026471     GCAGTCAACGCCAATGTGGGGGACACCGCGGTCCAAGAGTTGGCTCGAAACTGCCCGCAA
ENSPPYT00000003129     GCCGTCAACGCCAACGTGGGGGACGCCGCGGTTCAAGAGTTGGCCCGGAACTGCCCAGAA
ENSCJAT00000035103     GCCGTCAACGCCAACGTGGGGGACACCGCGGTTCAAGAGTTGGCTCGGAACTGCCCAGAA
ENSMMUT00000004250     GCCGTCAACGCCAACGTGGGGGACGCCGCGGTTCAAGAGTTGGCTCGGAACTGCCCAGAA
ENSGGOT00000007017     GCCGTCAACGCCAACGTGGGGGACGCCGCGGTTCAAGAGTTGGCTCGGAACTGCCCAGAA
ENST00000224862        GCCGTCAACGCCAACGTGGGGGACGCCGCGGTTCAAGAGTTGGCTCGGAACTGCCCAGAA
ENSPTRT00000005510     GCCGTCAACGCCAACGTGGGGGACGCCGCGGTTCAAGAGTTGGCTCGGAACTGCCCAGAA

ENSMUST00000026256     CTCGAGCACCTAGACCTCACCGGCTGCCTTCGGGTCGGAAGCGACGGTGTCAGGACACTG
ENSRNOT00000026471     CTCGAGCACCTAGACCTCACCGGCTGCCTTCGGGTCGGAAGCGACGGTGTCAGGACACTG
ENSPPYT00000003129     CTCCAGCACCTTGACCTCACCGGCTGCCTCCGCGTCGGAAGCGACGGTGTCAGGACATTG
ENSCJAT00000035103     CTCCAGCACCTTGACCTCACCGGCTGCCTCCGCGTCGGAAGCGACGGTGTCAGGACACTG
ENSMMUT00000004250     CTCCAGCACCTTGACCTCACCGGCTGCCTCCGCGTCGGAAGCGACGGTGTCAGGACATTG
ENSGGOT00000007017     CTCCAGCACCTTGACCTCACCGGCTGCCTCCGCGTCGGAAGCGACGGTGTCAGGACATTG
ENST00000224862        CTCCACCACCTTGACCTCACCGGCTGCCTCCGCGTCGGAAGCGACGGTGTCAGGACATTG
ENSPTRT00000005510     CTCCAGCACCTTGACCTCACCGGCTGCCTCCGCGTCGGAAGCGACGGTGTCAGGACATTG

ENSMUST00000026256     GCCGAGTACTGCCCGGCGCTGCGCTCTCTGCGGGTGCGGCACTGCCACCATGTGGCTGAA
ENSRNOT00000026471     GCGGAGTACTGCCCGGCGTTGCGCTCTCTGCGGGTGCGGCACTGCCACCATGTGGCCGAG
ENSPPYT00000003129     GCCGAGTACTGCCCCGCGCTGCGCTCGCTGCGGGTGCGGCACTGCCACCATGTGGCGGAG
ENSCJAT00000035103     GCCGAGTATTGCCCGGCGCTGCGCTCGCTGCGAGTGCGGCACTGCCACCATGTGGCCGAG
ENSMMUT00000004250     GCCGAGTACTGCCCCGCGCTGCGCTCGCTGCGGGTGCGGCACTGCCACCATGTGGCGGAG
ENSGGOT00000007017     GCCGAGTACTGCCCCGTGCTGCGCTCGCTGCGGGTGCGGCACTGCCACCATGTGGCGGAG
ENST00000224862        GCCGAGTACTGCCCCGTGCTGCGTTCGCTGCGGGTGCGGCACTGCCACCATGTGGCGGAG
ENSPTRT00000005510     GCCGAGTACTGCCCCGTGCTGCGCTCGCTGCGGGTGCGGCACTGCCACCATGTGGCGGAG

ENSMUST00000026256     CCGAGCCTGAGCCGCTTGCGGAAGCGTGGTGTGGACATCGACGTGGAACCACCCCTGCAC
ENSRNOT00000026471     CCCAGCCTGAGCCGCTTGCGGAAGCGTGGTGTGGACATCGACGTGGAGCCACCGCTGCAC
ENSPPYT00000003129     TCCAGCCTGAGCCGCTTGCGGAAGCGCGGCGTGGACATCGACGTGGAGCCGCCGCTGCAC
ENSCJAT00000035103     TCCAGCCTGAGCCGCTTGCGGAAACGCGGCGTGGACATCGACGTGGAGCCGCCGCTGCAC
ENSMMUT00000004250     TCCAGCCTGAGCCGCTTGCGGAAGCGCGGCGTGGACATCGACGTGGAGCCGCCGCTGCAC
ENSGGOT00000007017     TCCAGCCTGAGCCGCTTGCGGAAGCGCGGCGTGGACATCGACGTGGAGCCGCCGCTGCAC
ENST00000224862        TCCAGCCTGAGCCGCTTGCGGAAGCGCGGCGTGGACATCGACGTGGAGCCGCCGCTGCAC
ENSPTRT00000005510     TCCAGCCTGAGCCGCTTGCGGAAGCGCGGCGTGGACATCGACGTGGAGCCGCCGCTGCAC

ENSMUST00000026256     CAGGCTCTGGTGCTACTCCAGGACATGGCAGGCTTCGCACCCTTTGTCAACCTACAGGTC
ENSRNOT00000026471     CAGGCCCTGGTGCTACTCCAGGACATGGCTGGCTTCGCACCCTTTGTCAACCTACAGGTC
```

```
ENSPPYT00000003129     CAGGCCCTGGTGCTGCTGCAGGATATGGCGGGCTTCGCACCTTTTGTCAACCTGCAGGTC
ENSCJAT00000035103     CAGGCCTTGGTGCTGCTGCAGGATATGGCGGGCTTCGCACCTTTTGTCAACCTGCAGGTC
ENSMMUT00000004250     CAGGCCCTGGTGCTGCTGCAGGATATGGCTGGCTTCGCACCTTTTGTCAACCTGCAGGTC
ENSGGOT00000007017     CAGGCCCTGGTGCTGCTGCAGGATATGGCGGGCTTCGCACCTTTTGTCAACCTGCAGGTC
ENST00000224862        CAGGCCCTGGTGCTGCTGCAGGATATGGCGGGCTTCGCACCTTTTGTCAACCTGCAGGTC
ENSPTRT00000005510     CAGGCCCTGGTGCTGCTGCAGGATATGGCGGGCTTCGCACCTTTTGTCAACCTGCAGGTC
```

Multiple sequence alignment of Fbxl16

```
ENSMUST00000045692     ATGTCGAGCCCCGGTATCGATGGTGACCCCAAGCCTCCATGCTTGCCTCGAAACGGTCTG
ENSRNOT00000039777     ATGTCGAGCCCTGGTATCGATGGTGACCCCAAGCCTTCATGCTTGCCTCGAAACGGTCTG
ENSPTRT00000013928     ATGTCGAGCCCGGGCATCGACGGCGACCCCAAGCCTCCATGCTTGCCTCGAAACGGTCTG
ENSCJAT00000023547     ATGTCGAGCCCGGGTATCGACGGGGACCCCAAGCCTCCATGCTTGCCTCGAAACGGCCTG
ENSMMUT00000023745     ATGTCGAGCCCGGGCATCGACGGCGACCCCAAGCCTCCATGCTTGCCTCGAAATGGTCTG
ENSPPYT00000008168     ATGTCGAGCCCGGGCATCGACGGCGACCCCAAGCCTCCATGCTTGCCTCGAAACGGTCTG
ENSGGOT00000013950     ATGTCGAGCCCGGGCATCGACGGCGACCCCAAGCCTCCATGCTTGCCTCGAAACGGTCTG
ENST00000397621        ATGTCGAGCCCGGGCATCGACGGCGACCCCAAGCCTCCATGCTTGCCTCGAAACGGTCTG

ENSMUST00000045692     GTGAAGCTGCCTGGCCAGCCCAACGGCCTAGGTGCAGCCAGCATCACCAAGGGCACACCT
ENSRNOT00000039777     GTGAAGCTGCCTGGCCAGCCCAACGGCCTAGGCGCAGCCAGCATCACCAAGGGCACACCT
ENSPTRT00000013928     GTGAAGCTGCCGGGCCAGCCCAACGGCCTGGGTGCGGCCAGCATCACCAAGGGCACGCCA
ENSCJAT00000023547     GTGAAGCTGCCAGGCCAGCCCAACGGCCTGGGTGCGGCCAGCATCACCAAGGGCACGCCA
ENSMMUT00000023745     GTGAAGCTGCCAGGCCAGCCCAACGGCCTGGGTGCGGCCAGCATCACCAAGGGCACGCCA
ENSPPYT00000008168     GTGAAGCTGCCGGGCCAGCCCAACGGCCTGGGTGCCGCCAGCATCACCAAGGGCACGCCA
ENSGGOT00000013950     GTGAAGCTGCCGGGCCAGCCCAACGGCCTGGGTGCGGCCAGCATCACCAAGGGCACGCCA
ENST00000397621        GTGAAGCTGCCGGGCCAGCCCAACGGCCTGGGTGCGGCCAGCATCACCAAGGGCACGCCA

ENSMUST00000045692     GCTGCCAAGAACCGTCCTTGCCAGCCACCACCCCCACCGACCCTCCCACCTCCAAGCCTG
ENSRNOT00000039777     GCTGCCAAGAACCGTCCTTGCCAGCCACCACCCCCACCCACCCTTCCACCTCCAAGCCTG
ENSPTRT00000013928     GCCACCAAGAACCGCCCCTGCCAGCCACCACCCCCACCCACCCTCCCACCACCCAGCCTG
ENSCJAT00000023547     GCCACCAAGAACCGCCCCTGCCAGCCGCCACCCCCACCCACCCTCCCACCACCCAGCCTG
ENSMMUT00000023745     GCCACCAAGAACCGTCCCTGCCAGCCACCACCCCCACCCACCCTCCCACCACCCAGCCTG
ENSPPYT00000008168     GCCACCAAGAACCGCCCCTGCCAGCCACCACCCCCACCCACCCTCCCACCACCCAGCCTG
ENSGGOT00000013950     GCCACCAAGAACCGCCCCTGCCAGCCACCACCCCCACCCACCCTCCCACCACCCAGCCTG
ENST00000397621        GCCACCAAGAACCGCCCCTGCCAGCCACCACCCCCACCCACCCTCCCACCACCCAGCCTG

ENSMUST00000045692     GCTACACCACTATCCCGGGTTGCTCTGGCCGGGGGGCCATGCCCCCCAGCCAGTGGACCA
ENSRNOT00000039777     GCTACACCACTGTCCCGGGTTGCTCTGGCCGGGGGGCCATGCCCCCCAGCCAGTGGACCA
ENSPTRT00000013928     GCTGCTCCACTGTCCCGGGCTGCCCTGGCTGGGGGCCCGTGCCCCCCGGCAGGTGGACCA
ENSCJAT00000023547     GCTGCCCCACTGCCCCGGGCTGCCCTGGCCGGGGGTCCGTGCTCCCCGGCGGGTGGACCA
ENSMMUT00000023745     GCTGCTCCACTGCCCCGGGCTGCCCTGGCTGGGGGCCCGTGCCCCCTGGCAGGTGGACCA
ENSPPYT00000008168     GCTGCTCCACTGCCCCGGGCTGCCCTGGCCGGGGGCCCGTGCCCCCCAGCAGGTGGACCG
ENSGGOT00000013950     GCTGCTCCACTGTCCCGGGCTGCCCTGGCTGGGGGCCCGTGCCCCCCGGCAGGTGGACCA
ENST00000397621        GCTGCTCCACTGTCCCGGGCTGCCCTGGCTGGGGGCCCGTGCACCCCGGCAGGTGGACCA

ENSMUST00000045692     GCCTCGGGTCCAGTGTCTGGACCCCCAGTGGAGCGGCCACCACTGGCCACAGACGAGAAG
ENSRNOT00000039777     GCCTCAGGCCCAGTGCCTGGACCCCCCGTGGAGCGGCCACCACTGGCCACAGATGAGAAG
ENSPTRT00000013928     GCCTCAGCCTTGGCACCTGGGCACCCAGTGGAGCGGCCGCCGCTGGCCACGGACGAGAAG
ENSCJAT00000023547     GCCTCAGCCTTGACACCCGGGCCCCCAGCAGAGCGGCCGCCTCTGGCCACGGACGAGAAG
ENSMMUT00000023745     GCCTCAGCCTTGGCACCTGGGCCCCCAGCGGAGCGGCCGCCGCTGGCCACGGACGAGAAG
ENSPPYT00000008168     GCCTCAGCCTTGGCACCTGGGCCCCCAGCGGAGCGGCCGCCGCTGGCCACGGACGAGAAG
ENSGGOT00000013950     GCCTCAGCCTTGGCACCTGGGCACCCAGCGGAGCGGCCGCCGCTGGCCACGGACGAGAAG
ENST00000397621        GCCTCAGCCTTGGCACCTGGGCACCCAGCGGAGCGGCCGCCGCTGGCCACGGACGAGAAG
```

```
ENSMUST00000045692     ATCCTTAATGGGCTTTTCTGGTATTTCTCAGCATGTGAGAAGTGCATACTAGCTCAGGTG
ENSRNOT00000039777     ATCCTTAATGGACTCTTCTGGTATTTCTCAGCGTGTGAGAAGTGCATACTAGCCCAGGTG
ENSPTRT00000013928     ATCCTCAATGGGCTCTTCTGGTATTTCTCGGCCTGCGAGAAGTGTGTGCTGGCCCAGGTG
ENSCJAT00000023547     ATCCTCAATGGGCTCTTCTGGTACTTCTCGGCCTGCGAGAAGTGTGTGCTGGCCCAGGTG
ENSMMUT00000023745     ATCCTCAATGGGCTCTTCTGGTATTTCTCGGCCTGCGAGAAGTGTGTGCTGGCCCAGGTG
ENSPPYT00000008168     ATCCTCAATGGGCTCTTCTGGTATTTCTCGGCCTGCGAGAAGTGTGTGCTGGCCCAGGTG
ENSGGOT00000013950     ATCCTCAATGGGCTCTTCTGGTATTTCTCGGCCTGCGAGAAGTGTGTGCTGGCCCAGGTG
ENST00000397621        ATCCTCAATGGGCTCTTCTGGTATTTCTCGGCCTGCGAGAAGTGTGTGCTGGCCCAGGTG

ENSMUST00000045692     TGCAAGGCTTGGCGGCGTGTGCTCTACCAGCCCAAGTTCTGGGCGGGCCTCACGCCTGTG
ENSRNOT00000039777     TGCAAGGCTTGGCGGCGTGTGCTCTACCAGCCCAAGTTCTGGGCAGGCCTCACGCCTGTG
ENSPTRT00000013928     TGCAAGGCCTGGCGGCGCGTGCTGTACCAGCCCAAGTTCTGGGCAGGCCTCACGCCGGTG
ENSCJAT00000023547     TGCAAGGCCTGGCGGCGCGTGCTCTACCAGCCCAAGTTCTGGGCAGGCCTCACGCCGGTG
ENSMMUT00000023745     TGCAAGGCCTGGCGGCGCGTGCTTTACCAGCCCAAGTTCTGGGCAGGCCTCACGCCGGTG
ENSPPYT00000008168     TGCAAGGCCTGGCGGCGCGTGCTCTACCAGCCCAAGTTCTGGGCAGGCCTCACGCCGGTG
ENSGGOT00000013950     TGCAAGGCCTGGCGGCGCGTGCTGTACCAGCCCAAGTTCTGGGCAGGCCTCACGCCGGTG
ENST00000397621        TGCAAGGCCTGGCGGCGCGTGCTGTACCAGCCCAAGTTCTGGGCAGGCCTCACGCCGGTG

ENSMUST00000045692     CTGCATGCCAAGGAGCTGTACAACGTGCTGCCTGGAGGCGAGAAGGAGTTTGTGAACCTG
ENSRNOT00000039777     CTGCACGCCAAGGAGCTGTACAACGTGCTGCCTGGAGGCGAGAAGGAGTTTGTGAACCTG
ENSPTRT00000013928     CTGCATGCCAAGGAGCTCTACAACGTGCTGCCTGGTGGCGAGAAGGAGTTCGTGAACCTG
ENSCJAT00000023547     CTGCACGCCAAGGAGCTGTACAACGCGCTGCCTGGCGGCGAGAAGGAGTTCGTCAACCTG
ENSMMUT00000023745     CTGCACGCCAAGGAGCTCTACAATGTGCTGCCTGGTGGCGAGAAGGAGTTCGTGAACCTG
ENSPPYT00000008168     CTGCATGCCAAGGAGCTGTACAACGTGCTGCCTGGTGGCGAGAAGGAGTTCGTAAACCTG
ENSGGOT00000013950     CTGCATGCCAAGGAGCTCTACAACGTGCTGCCTGGTGGCGAGAAGGAGTTCGTGAACCTG
ENST00000397621        CTGCATGCCAAGGAGCTCTACAACGTGCTGCCTGGTGGCGAGAAGGAGTTCGTGAACCTG

ENSMUST00000045692     CAGGGCTTTGCTGCTAGAGGTTTTGAGGGCTTCTGCCTAGTTGGTGTCTCCGACTTAGAC
ENSRNOT00000039777     CAGGGCTTCGCTGCTAGAGGCTTTGAGGGCTTCTGCCTAGTTGGTGTCTCTGACTTAGAT
ENSPTRT00000013928     CAGGGTTTTGCCGCCAGAGGCTTCGAGGGCTTCTGCCTGGTTGGCGTCTCCGACCTGGAC
ENSCJAT00000023547     CAGGGCTTTGCCGCCAGAGGCTTCGAGGGCTTCTGCCTGGTTGGCGTCTCCGACCTGGAC
ENSMMUT00000023745     CAGGGCTTCGCTGCAAGAGGCTTCGAGGGCTTCTGCCTGGTTGGCGTCTCTGACCTGGAC
ENSPPYT00000008168     CAGGGCTTTGCCGCCAGAGGCTTCGAGGGCTTCTGCCTGGTTGGCGTCTCCGACCTGGAC
ENSGGOT00000013950     CAGGGTTTTGCCGCCAGAGGCTTCGAGGGCTTCTGCCTGGTTGGCGTCTCCGACCTGGAC
ENST00000397621        CAGGGTTTTGCCGCCAGAGGCTTCGAGGGCTTCTGCCTGGTTGGCGTCTCCGACCTGGAC

ENSMUST00000045692     ATCTGCGAGTTCATCGACAACTATTCTCTCTCTAAGAAGGGAGTCAAGGCCATGAGTCTC
ENSRNOT00000039777     ATCTGCGAATTCATCGACAACTACTCGCTCTCTAAGAAGGGAGTCAAGGCCATGAGTCTC
ENSPTRT00000013928     ATCTGTGAGTTCATTGACAACTATGCGCTCTCCAAGAAGGGTGTCAAAGCCATGAGCCTC
ENSCJAT00000023547     ATCTGCGAGTTCATTGACAACTACGCGCTCTCCAAGAAGGGCGTCAAAGCCATGAGCCTC
ENSMMUT00000023745     ATCTGTGAGTTCATTGACAACTATGCGCTCTCCAAGAAGGGTGTCAAAGCCATGAGCCTC
ENSPPYT00000008168     ATCTGTGAGTTCATTGACAACTATGCGCTCTCCAAGAAGGGTGTCAAAGCCATGAGCCTC
ENSGGOT00000013950     ATCTGTGAGTTCATTGACAACTATGCGCTCTCCAAGAAGGGTGTCAAAGCCATGAGCCTC
ENST00000397621        ATCTGTGAGTTCATTGACAACTATGCGCTCTCCAAGAAGGGTGTCAAAGCCATGAGCCTC

ENSMUST00000045692     AAGCGCTCCACCATCACTGACGCTGGTCTAGAGGTGATGCTGGAGCAGATGCAGGGAGTG
ENSRNOT00000039777     AAGCGCTCCACCATCACTGACGCTGGTCTAGAGGTGATGTTGGAGCAGATGCAGGGAGTG
ENSPTRT00000013928     AAGCGCTCCACCATCACGGACGCAGGCCTCGAGGTTATGCTTGAACAGATGCAGGGCGTG
ENSCJAT00000023547     AAGCGCTCCACCATCACGGACGCAGGCCTCGAGGTCATGCTGGAACAGATGCAGGGTGTG
ENSMMUT00000023745     AAGCGCTCCACCATCACGGACGCAGGCCTCGAGGTTATGCTTGAACAGATGCAGGGCGTG
ENSPPYT00000008168     AAGCGCTCCACCATCACGGACGCAGGCCTCGAGGTTATGCTTGAACAGATGCAGGGCGTG
ENSGGOT00000013950     AAGCGCTCCACCATCACGGACGCAGGCCTCGAGGTTATGCTTGAACAGATGCAGGGCGTG
ENST00000397621        AAGCGCTCCACCATCACGGACGCAGGCCTCGAGGTTATGCTTGAACAGATGCAGGGCGTG
```

```
ENSMUST00000045692     GTGCGTCTGGAGCTGTCAGGCTGCAACGATTTCACCGAGGCTGGCCTGTGGTCCAGCCTC
ENSRNOT00000039777     GTGCGCCTGGAGCTGTCAGGCTGTAACGATTTCACCGAGGCTGGCCTGTGGTCCAGCCTC
ENSPTRT00000013928     GTGCGTCTGGAGCTGTCGGGCTGCAACGACTTCACCGAGGCCGGGCTGTGGTCCAGCCTG
ENSCJAT00000023547     GTGCGTCTGGAGCTGTCCGGCTGCAATGACTTCACCGAGGCCGGGCTGTGGTCCAGCCTC
ENSMMUT00000023745     GTGCGTCTGGAGCTGTCGGGCTGCAACGACTTCACCGAGGCCGGGCTGTGGTCCAGCCTG
ENSPPYT00000008168     GTGCGTCTGGAGCTGTCGGGCTGCAACGACTTCACCGAGGCCGGGCTGTGGTCCAGCCTG
ENSGGOT00000013950     GTGCGTCTGGAGCTGTCGGGCTGCAACGACTTCACCGAGGCCGGGCTGTGGTCCAGCCTG
ENST00000397621        GTGCGTCTGGAGCTGTCGGGCTGCAACGACTTCACCGAGGCCGGGCTGTGGTCCAGCCTG

ENSMUST00000045692     AGTGCCCGTATCACCTCCCTGAGCGTCAGTGACTGCATCAATGTGGCAGATGATGCAATC
ENSRNOT00000039777     AGCGCACGTATCACCTCACTAAGCGTCAGTGACTGCATCAATGTGGCGGACGATGCCATC
ENSPTRT00000013928     AGCGCGCGCATCACCTCGCTGAGCGTGAGTGACTGCATCAACGTGGCCGACGACGCCATC
ENSCJAT00000023547     AGCGCCCGCATCACCTCACTGAGCGTGAGTGACTGCATCAACGTAGCCGACGACGCCATC
ENSMMUT00000023745     AGCGCGCGCATCACCTCGCTGAGCGTGAGTGACTGCATCAACGTGGCCGACGACGCCATC
ENSPPYT00000008168     AGCGCGCGCATCACCTCGCTGAGCGTGAGTGACTGCATCAACGTGGCCGACGACGCCATC
ENSGGOT00000013950     AGCGCGCGCATCACCTCGCTGAGCGTGAGTGACTGCATCAACGTGGCCGACGACGCCATC
ENST00000397621        AGCGCGCGCATCACCTCGCTGAGCGTGAGTGACTGCATCAACGTGGCCGACGACGCCATC

ENSMUST00000045692     GCTGCCATCTCACAACTTCTACCCAACCTGGCAGAGCTAAGCCTTCAGGCCTATCACGTG
ENSRNOT00000039777     GCTGCCATCTCACAACTTCTACCCAACCTGGCAGAGCTAAGCCTTCAGGCCTACCACGTG
ENSPTRT00000013928     GCGGCCATCTCGCAGCTGCTGCCCAACCTGGCGGAGCTGAGCCTGCAGGCCTACCACGTG
ENSCJAT00000023547     GCCGCCATCTCGCAGCTGCTGCCCAACCTGGCAGAGCTGAGCCTGCAGGCCTACCACGTG
ENSMMUT00000023745     GCGGCCATCTCGCAGCTGCTGCCCAACCTGGCGGAGCTGAGCCTGCAGGCCTACCATGTG
ENSPPYT00000008168     GCGGCCATCTCGCAGCTGCTGCCCAACCTGGCGGAGCTGAGCCTGCAGGCCTACCACGTG
ENSGGOT00000013950     GCGGCCATCTCGCAGCTGCTGCCCAACCTGGCGGAGCTGAGCCTGCAGGCCTACCACGTG
ENST00000397621        GCGGCCATCTCGCAGCTGCTGCCCAACCTGGCGGAGCTGAGCCTGCAGGCCTACCACGTG

ENSMUST00000045692     ACTGACACGGCCCTGGCCTACTTCACAGCACGCCAGGGCCACAGCACCCACACGCTGCGC
ENSRNOT00000039777     ACTGACACGGCACTGGCCTACTTCACAGCACGTCAGGGCCACAGCACCCACACGTTGCGC
ENSPTRT00000013928     ACGGACACGGCGCTGGCCTACTTCACGGCGCGCCAGGGCCACAGCACGCACACGCTGCGC
ENSCJAT00000023547     ACGGACACGGCGCTGGCCTACTTCACTGCGCGCCAGGGTCACAGCACACACACGCTGCGC
ENSMMUT00000023745     ACGGACACGGCGCTGGCCTACTTCACGGCGCGCCAGGGCCACAGCACGCACACGCTGCGC
ENSPPYT00000008168     ACGGACACGGCGCTGGCCTACTTCACGGCGCGCCAGGGCCACAGCACGCACACGCTGCGC
ENSGGOT00000013950     ACGGACACGGCGCTGGCCTACTTCACGGCGCGCCAGGGCCACAGCACGCACACGCTGCGC
ENST00000397621        ACGGACACGGCGCTGGCCTACTTCACGGCGCGCCAGGGCCACAGCACGCACACGCTGCGC

ENSMUST00000045692     CTGCTTTCCTGCTGGGAGATAACCAACCATGGGGTGGTCAATGTGGTGCACAGTCTGCCC
ENSRNOT00000039777     CTGCTCTCCTGCTGGGAGATAACCAACCACGGGGTGGTCAATGTGGTGCACAGTCTGCCC
ENSPTRT00000013928     CTGCTCTCCTGCTGGGAGATCACCAACCACGGCGTGGTCAACGTGGTGCACAGCCTGCCC
ENSCJAT00000023547     CTGCTCTCCTGCTGGGAGATCACCAACCACGGCGTGGTCAACGTGGTGCACAGCCTGCCC
ENSMMUT00000023745     CTACTCTCCTGCTGGGAGATCACCAACCACGGCGTGGTCAACGTGGTGCACAGCCTGCCC
ENSPPYT00000008168     CTGCTCTCCTGCTGGGAAATCACCAACCACGGCGTGGTCAACGTGGTGCACAGCCTGCCC
ENSGGOT00000013950     CTGCTCTCCTGCTGGGAGATCACCAACCACGGCGTGGTCAACGTGGTGCACAGCCTGCCC
ENST00000397621        CTGCTCTCCTGCTGGGAGATCACCAACCACGGCGTGGTCAACGTGGTGCACAGCCTGCCC

ENSMUST00000045692     AACCTCACCTCACTCAGCCTCTCAGGCTGCTCTAAGGTCACTGATGATGGAGTGGAGCTT
ENSRNOT00000039777     AACCTCACCTCACTCAGCCTCTCGGGCTGCTCTAAGGTCACTGATGATGGGGTGGAGCTT
ENSPTRT00000013928     AACCTCACCGCGCTCAGCCTCTCGGGCTGCTCCAAGGTCACCGACGACGGCGTGGAGCTC
ENSCJAT00000023547     AACCTCACTGCGCTCAGCCTCTCCGGCTGCTCCAAGGTCACCGACGACGGCGTAGAGCTC
ENSMMUT00000023745     AACCTCACCGCGCTCAGCCTCTCGGGCTGCTCCAAGGTCACCGACGACGGCGTGGAGCTC
ENSPPYT00000008168     AACCTCACCGCGCTCAGCCTCTCGGGCTGCTCCAAGGTCACCGACGACGGCGTGGAGCTC
ENSGGOT00000013950     AACCTCACCGCGCTCAGCCTCTCGGGCTGCTCCAAGGTCACCGACGACGGCGTGGAGCTC
ENST00000397621        AACCTCACCGCGCTCAGCCTCTCGGGCTGCTCCAAGGTCACCGACGACGGCGTGGAGCTC
```

```
ENSMUST00000045692     GTAGCTGAGAACCTGCGCAAATTGCGCAGCCTTGACCTTTCCTGGTGCCCTCGGATCACC
ENSRNOT00000039777     GTAGCCGAGAATCTGCGCAAATTGCGCAGTCTTGACCTTTCCTGGTGCCCTCGGATCACA
ENSPTRT00000013928     GTGGCCGAGAACCTGCGCAAGCTGCGCAGCCTTGACCTCTCGTGGTGCCCACGCATCACC
ENSCJAT00000023547     GTAGCCGAGAACCTGCGCAAGCTGCGCAGCCTCGACCTCTCGTGGTGCCCACGCATCACT
ENSMMUT00000023745     GTGGCCGAGAACCTGCGCAAGCTGCGCAGCCTTGACCTCTCATGGTGCCCACGCATCACC
ENSPPYT00000008168     GTGGCCGAGAACCTGCGCAAGCTGCGCAGCCTTGACCTCTCGTGGTGCCCACGCATCACT
ENSGGOT00000013950     GTGGCCGAGAACCTGCGCAAGCTGCGCAGCCTTGACCTCTCGTGGTGCCCACGCATCACC
ENST00000397621        GTGGCCGAGAACCTGCGCAAGCTGCGCAGCCTTGACCTCTCGTGGTGCCCACGCATCACC

ENSMUST00000045692     GATATGGCACTGGAGTATGTGGCCTGCGACTTGCACCGACTGGAGGAGCTCGTGCTGGAC
ENSRNOT00000039777     GATATGGCACTGGAGTATGTGGCCTGCGACTTGCACCGACTGGAGGAGCTCGTGCTGGAC
ENSPTRT00000013928     GACATGGCGCTGGAGTACGTGGCCTGCGACCTGCACCGCCTAGAGGAGCTCGTGCTCGAC
ENSCJAT00000023547     GACATGGCGCTGGAGTATGTGGCCTGCGACCTGCACCGCCTGGAGGAGCTCGTGCTGGAC
ENSMMUT00000023745     GACATGGCGCTGGAGTACGTGGCTTGCGACCTGCACCGCCTGGAGGAGCTCGTGCTCGAC
ENSPPYT00000008168     GACATGGCGCTGGAGTACGTGGCCTGCGACCTGCACCGCCTGGAGGAGCTCGTGCTCGAC
ENSGGOT00000013950     GACATGGCGCTGGAGTACGTGGCCTGCGACCTGCACCGCCTAGAGGAGCTCGTGCTCGAC
ENST00000397621        GACATGGCGCTGGAGTACGTGGCCTGCGACCTGCACCGCCTAGAGGAGCTCGTGCTCGAC

ENSMUST00000045692     AGGTGTGTACGCATCACGGACACTGGTCTCAGCTATTTGTCCACCATGTCGTCTCTCCGC
ENSRNOT00000039777     AGGTGTGTACGCATCACGGACACTGGTCTCAGCTACTTGTCCACCATGTCGTCCCTCCGC
ENSPTRT00000013928     AGGTGTGTACGCATCACGGACACTGGCCTCAGCTATCTGTCCACCATGTCGTCCCTCCGC
ENSCJAT00000023547     AGGTGTGTGCGCATCACGGACACTGGCCTCAGCTATCTGTCCACCATGTCGTCCCTCCGC
ENSMMUT00000023745     AGGTGTGTACGCATCACGGACACTGGCCTCAGCTATCTGTCCACCATGTCGTCCCTCCGC
ENSPPYT00000008168     AGGTGTGTACGCATCACGGACACTGGCCTCAGCTATCTGTCCACCATGTCGTCCCTCCGC
ENSGGOT00000013950     AGGTGTGTACGCATCACGGACACTGGCCTCAGCTATCTGTCCACCATGTCGTCCCTCCGC
ENST00000397621        AGGTGTGTACGCATCACGGACACTGGCCTCAGCTATCTGTCCACCATGTCGTCCCTCCGC

ENSMUST00000045692     AGCCTCTACCTGCGATGGTGCTGCCAGGTGCAGGACTTCGGGTTGAAGCACCTCTTAGCC
ENSRNOT00000039777     AGCCTCTACCTGCGATGGTGCTGCCAGGTGCAGGACTTCGGGTTGAAGCACCTCTTAGCC
ENSPTRT00000013928     AGCCTCTACATC------------------------------------------------
ENSCJAT00000023547     AGCCTCTACCTGCGATGGTGCTGCCAGGTGCAGGACTTCGGGCTGAAGCACCTCCTGGCC
ENSMMUT00000023745     AGCCTCTACCTGCGATGGTGCTGCCAGGTGCAGGACTTCGGGCTGAAGCACCTCCTGGCC
ENSPPYT00000008168     AGCCTCTACCTGCGATGGTGCTGCCAGGTGCAAGACTTCGGGCTGAAGCACCTCCTGGCC
ENSGGOT00000013950     AGCCTCTACCTGCGATGGTGCTGCCAGGTGCAAGACTTCGGGCTGAAGCACCTCCTGGCC
ENST00000397621        AGCCTCTACCTGCGATGGTGCTGCCAGGTGCAAGACTTCGGGCTGAAGCACCTCCTGGCC

ENSMUST00000045692     ATGAGAAATTTGCGACTCTTGTCTCTAGCAGGCTGCCCGCTCCTAACTACCACCGGGCTA
ENSRNOT00000039777     ATGAGAAGTTTGCGACTCTTGTCTCTAGCAGGCTGCCCGCTCCTAACCACCACCGGGCTA
ENSPTRT00000013928     ------------------------------------------------ACCACCGGGCTG
ENSCJAT00000023547     CTGGGGAGTTTGCGCCTCCTGTCTCTGGCAGGCTGCCCGCTGCTCACCACCACGGGGCTG
ENSMMUT00000023745     CTGGGGAGTTGGCGCCTCCTGTCTCTGGCAGGT---------------------------
ENSPPYT00000008168     CTGGGGAGTTTGCGCCTCCTGTCTCTGGCAGGCTGCCCGCTGCTCACCACCACCGGGCTG
ENSGGOT00000013950     CTGGGGAGTTTGCGCCTCCTGTCTCTGGCAGGCTGCCCGCTGCTCACCACCACCGGGCTG
ENST00000397621        CTGGGGAGTTTGCGCCTCCTGTCTCTGGCAGGCTGCCCGCTGCTCACCACCACCGGGCTG

ENSMUST00000045692     TCTGGCCTCGTGCAACTGCAAGAGCTGGAGGAGCTGGAGCTGACCAACTGCCCTGGAGCC
ENSRNOT00000039777     TCCGGCCTAGTGCAACTGCAAGAGCTGGAGGAGCTGGAGCTGACCAACTGCCCTGGAGCC
ENSPTRT00000013928     TATGGCTTGAGGCAACACCAGGAAGTGGAGGAGCAGGAGCTGACCACATGCCCCGGGTCC
ENSCJAT00000023547     TCGGGCCTGGTGCAGCTGCAGGAGCTGGAGGAGCTGGAGCTGACCAACTGCCCGGGCGCC
ENSMMUT00000023745     ------------------------------------------------------------
ENSPPYT00000008168     TCGGGCCTGGTGCAGCTGCAGGAGCTGGAGGAGCTGGAGCTGACCAACTGCCCCGGGGCC
ENSGGOT00000013950     TCGGGCCTGGTGCAGCTGCAGGAGTTGGAGGAGCTGGAGCTGACCAACTGCCCCGGGGCC
ENST00000397621        TCGGGCCTGGTGCAGCTGCAGGAGCTGGAGGAGCTGGAGCTGACCAACTGCCCCGGGGCC
```

```
ENSMUST00000045692     ACACCCGAGCTCTTCAAGTACTTCTCGCAGCACCTGCCGCGCTGCCTCGTCATTGAA
ENSRNOT00000039777     ACTCCCGAGCTCTTCAAGTACTTCTCGCAGCACCTGCCGCGCTGCCTCGTCATTGAA
ENSPTRT00000013928     ACCGAG------CTCAAGTAT---TCGCAGCAC---CCCCGCTGC------GTCGAG
ENSCJAT00000023547     ACCCCCGAGCTCTTCAAGTACTTCTCGCAGCACCTGCCCCGCTGCCTCGTCATCGAG
ENSMMUT00000023745     ---------------------------------------------------------
ENSPPYT00000008168     ACCCCCGAGCTCTTCAAGTACTTCTCGCAGCACCTGCCCCGCTGCCTCGTCATTGAG
ENSGGOT00000013950     ACCCCCGAGCTCTTCAAGTATTTCTCGCAGCACCTGCCCCGCTGCCTCGTCATTGAG
ENST00000397621        ACCCCCGAGCTCTTCAAGTATTTCTCGCAGCACCTGCCCCGCTGCCTCGTCATTGAG
```

Multiple sequence alignment of Fbxl17

```
ENSMUST00000024761     ATGGGCCACCTTCTCTCGAAGGAGCCGCGTAACCGCCCGAGCCAGAAGAGGCCTCGCTGC
ENSCJAT00000031332     ATGGGCCACCTTCTCTCGAAGGAGCCGCGTAACCGCCCGAGCCAGAAGAGGCCTCGCTGT
ENST00000542267        ATGGGCCACCTTCTCTCGAAGGAGCCGCGTAACCGCCCGAGCCAGAAGAGGCCTCGCTGT
ENSPTRT00000047746     ATGGGCCACGTTCTCTCGAAGGAGCCGCGTAACCGCCCGAGCCAGAAGAGGCCTCGCTGT


ENSMUST00000024761     TGCAGCTGGTGTCGCCGCCGGCGCCCTCTCCTCAGACTGCCCCGCCGGGCTCTGGCCAAG
ENSCJAT00000031332     TGCAGTTGGTGCCGCCGCCGGCGCCCTCTCCTCAGGCTGCCCCGCCGGACCCCAGCCAAG
ENST00000542267        TGCAGTTGGTGCCGCCGCCGGCGCCCTCTCCTCAGGCTGCCCCGCCGGACCCCAGCCAAG
ENSPTRT00000047746     TGCAGTTGGTGCCGCCGCCGGCGCCCTCTCCTCAGGCTGCCTCGCCGGACCCCAGCCAAG


ENSMUST00000024761     GCGTCCCCGCAGCCCGCGGCGCCCCGGAGCCGGGACTGCTTCTTCCGCGGGCCCTGCATG
ENSCJAT00000031332     GTGCCCCCTCAGCCGGCGGCGCCCCGGAGCCGGGACTGCTTCTTCCGCGGGCCCTGCATG
ENST00000542267        GTGCCCCCTCAGCCGGCGGCGCCCCGGAGCCGGGACTGCTTCTTCCGCGGGCCCTGCATG
ENSPTRT00000047746     GTGCCCCCTCAGCCGGCGGCGCCCCGGAGCCGGGACTGCTTCTTCCGCGGGCCCTGCATG


ENSMUST00000024761     CTCTGCTTCATCGTGCACAGCCCCGGCGCGCCCGCCTCCGCCGGCCTAGAGGAGGAGCCG
ENSCJAT00000031332     CTTTGCTTCATCGTGCACAGTCCCGGCGCGCCCGCCCCCGCCGGCCCAGAGGAGGAGCCG
ENST00000542267        CTCTGCTTCATCGTGCACAGTCCCGGCGCGCCCGCCCCCGCCGGCCCAGAGGAGGAGCCG
ENSPTRT00000047746     CTCTGCTTCATCGTGCACAGTCCCGGCGCGCCCGCCCCCGCCGGCCCAGAGGAGGAGCCG


ENSMUST00000024761     CCGCTCTCGCCGCCGCCGCGGGACGGGGCCTAC---GCCGCCGTCTCCTCGCAGCACTTG
ENSCJAT00000031332     CCGTTCTCGCCGCCGCCGCGGGACGGGGCCTACGCCGCCGCCTCCTCTTCTCAGCACCTG
ENST00000542267        CCGCTCTCGCCGCCGCCGCGGGACGGGGCCTACGCTGCCGCCTCCTCCTCTCAGCACCTG
ENSPTRT00000047746     CCGCTCTCGCCGCCGCCGCGGGACGGGGCCTACGCTGCAGCCTCCTCCTCTCAGCACCTG


ENSMUST00000024761     GCGCGGCGCTACGCGGCCCTGGCCGCCGAGGACTGCGCCGCCGCCGCACGCCGCTTCCTG
ENSCJAT00000031332     GCGCGGCGCTACGCGGCCCTGGCCGCCGAGGACTGCGCCGCAGCCGCCCGCCGCTTCCTG
ENST00000542267        GCGCGGCGCTACGCGGCCCTGGCCGCCGAGGACTGCGCCGCTGCTGCCCGCCGCTTCCTG
ENSPTRT00000047746     GCGCGGCGCTACGCGGCCCTGGCCGCCGAGGACTGCGCCGCTGCTGCCCGCCGCTTCCTG


ENSMUST00000024761     CTGTCCTCGGCCGCCGCCGCCGCCGCCGCT---------GCCTCGTCGCCCGCGTCCTGC
ENSCJAT00000031332     CTATCCTCGGCCGCCGCAGCCGCCGCCGCTGCTGCCTCGGCTTCGTCGCCCGCCTCTTGC
ENST00000542267        CTATCCTCGGCCGCCGCCGCCGCCGCCGCTGCCGCCTCGGCTTCGTCGCCCGCCTCCTGC
ENSPTRT00000047746     CTATCCTCGGCCGCCGCCGCCGCCGCCGCTGCCGCCTCGGCTTCGTCGCCCGCCTCCTGC


ENSMUST00000024761     TGCAAGGAGCTGGGGCTGGCCGCCGCCGCCGCCTGGGAGCAGCAGGGCCGGAGCCTCTTC
ENSCJAT00000031332     TGCAAAGAGCTGGGGCTGGCTGCGGCCGCCGCCTGGGAGCAGCAGGGCCGAAGCCTTTTC
ENST00000542267        TGCAAAGAGTTGGGGCTGGCTGCGGCCGCCGCCTGGGAGCAGCAGGGCCGAAGTCTCTTC
ENSPTRT00000047746     TGCAAAGAGTTGGGGCTGGCTGCGGCCGCCGCCTGGGAGCAGCAGGGCCGAAGTCTCTTC
```

```
ENSMUST00000024761     CTGGCCGGCGTGGGGCCCGTGCGCTTCCTGGGACCGCTGGCCGCCGTGCAGCTCTTCCGG
ENSCJAT00000031332     CTGGCCAGCTTGGGGCCGGTGCGTTTCCTGGGGCCGCCCGCCGCTGTGCAGCTCTTCCGG
ENST00000542267        CTGGCCAGCTTGGGGCCGGTGCGCTTCCTGGGGCCGCCCGCCGCCGTGCAGCTCTTCCGG
ENSPTRT00000047746     CTGGCCAGCTTGGGGCCGGTGCGCTTCCTGGGGCCGCCCGCCGCCGTGCAGCTCTTCCGG

ENSMUST00000024761     GCACCGCCGGCGCCGCCGGCCGAGCCCGCCACGGCCCTCGAGATGGTTTGCAAGCGGAAA
ENSCJAT00000031332     GGGCCG---CCGCCGCCGGCCGAGCCCCTTACGCCCCTCGAAATGGTGTGCAAGCGGAAG
ENST00000542267        GGGCCGACACCGTCACCGGCCGAGCTCCCTACGCCCCCCGAAATGGTGTGCAAGCGGAAG
ENSPTRT00000047746     GGGCCGACACCGTCACCGGCCGAGCCCCCTACGCCCCCCGAAATGGTGTGCAAGCGGAAG

ENSMUST00000024761     GGGGCCGGGGTCCCCGCCTGCACCCCCTGCAAGCAGCCCCGTTGCGGCTGCGGGGGCTGC
ENSCJAT00000031332     GGGGCCGGGGTCCCCGCCTGCACCCCCTGCAAGCAGCCCCGCTGTGGCGGCGGGGGTTGC
ENST00000542267        GGGGCCGGGGTCCCCGCCTGCACCCCCTGCAAGCAGCCCCGCTGCGGCGGCGGGGGCTGC
ENSPTRT00000047746     GGGGCCGGGGTCCCCGCCTGCACCCCCTGCAAGCAGCCCCGCTGCGGCGGCGGGGGCTGC

ENSMUST00000024761     ------GGCGGGGGCGGCGGCGGCGGGGGAGGGCCCGCGGGAGGAGGTGCCTCGCCGCCG
ENSCJAT00000031332     GGCGGTGGCGGCGGCGGCGGCGGTGGGGGAGGGCCTGCCGGGGGAGGCGCCTCGCCGCCC
ENST00000542267        GGCGGCGGCGGCGGCGGCGGTGGCGGGGGAGGGCCTGCGGGGGGAGGCGCTTCGCCGCCC
ENSPTRT00000047746     GGCGGCGGCGGCGGCGGCGGCGGCGGGGGAGGGCCTGCGGGGGGAGGCGCTTCGCCGCCC

ENSMUST00000024761     CGGCCGCCCGATGCCGGCTGCTGCCAGGCCCCGGAGCAGCCCCCGCCGCCGCTCTGCCCC
ENSCJAT00000031332     AGACCCCCCGACGCCGGCTGCTGCCAGGGTCCGGAGCAGCCCCAGCAGCCGCTCTGCCCC
ENST00000542267        CGGCCCCCCGACGCCGGCTGCTGCCAGGCCCCGGAGCAGCCCCCGCAGCCGCTCTGCCCT
ENSPTRT00000047746     CGGCCCCCCGACGCCGGCTGCTGCCAGGCCCCGGAGCAGCCCCCGCAGCCGCTCTGCCCT

ENSMUST00000024761     GCGCCCGCCTCTCCCGCCTCAGAGTGTGCCCCCATCGTGGCCGCCGGGGACACAGTCCGA
ENSCJAT00000031332     CCGCCCTCTTCTCCCACTTCCGAAGGTGCTCCCACCGAGGCTGGCGGGGACGTTGTCCGA
ENST00000542267        CCGCCCTCTTCTCCCACCTCCGAAGGTGCCCCCACCGAAGCTGGCGGGGACGCTGTCCGA
ENSPTRT00000047746     CCGCCCTCTTCTCCCACCTCCGAAGGTGCCCCCACCGAAGCTGGCGGGGACGCTGTCCGA

ENSMUST00000024761     GCCGGGGGCACCGCCCCCTCGTCTGCCCAGCAGCAGCCCGAGAGCGGCGACGCGGACTGT
ENSCJAT00000031332     GTCGGGGGCACCGCCCCCTCGTCCGCCCAGCAGCAGCGTGAATGTGGCGACGCGGACTGT
ENST00000542267        GCCGGGGGCACCGCCCCCTTGTCCGCCCAGCAGCAGCATGAATGTGGCGACGCGGACTGT
ENSPTRT00000047746     GCCGGGGGCACCGCCCCCTCGTCCGCCCAGCAGCAGCATGAATGTGGCGACGCGGACTGT

ENSMUST00000024761     CAGGAGCCCCCCGAAAACCCCTGCGACTGTCACAGGGAGCCGCCCCCCGAGATCCCAGAC
ENSCJAT00000031332     CTGGAGCCCCCCGAAAACCCCTGCGACTGTCACAGGGAGCCGCCCCCCGAAACCCCAGAC
ENST00000542267        CGGGAGTCCCCCGAAAACCCCTGCGACTGTCACAGGGAGCCGCCCCCCGAAACCCCAGAC
ENSPTRT00000047746     CGGGAGCCCCCCGAAAACCCCTGCGACTGTCACAGGGAGCCGCCCCCCGAAACCCCAGAC

ENSMUST00000024761     ATCAACCAACTGCCGCCGTCCATCCTGCTTAAGATATTTTCCAATTTGTCCCTGAACGAA
ENSCJAT00000031332     ATCAACCAGCTGCCGCCGTCCATCCTGCTCAAGATATTTTCCAATTTGTCACTGGATGAG
ENST00000542267        ATCAACCAGCTGCCGCCGTCCATCCTGCTCAAGATATTTTCCAATTTGTCACTGGATGAG
ENSPTRT00000047746     ATCAACCAGCTGCCGCCGTCCATCCTGCTCAAGATATTTTCCAATTTGTCACTGGATGAG

ENSMUST00000024761     CGCTGCCTTTCTGCATCGTTGGTTTGCAAGTACTGGCGTGACCTTTGCTTAGATTTCCAG
ENSCJAT00000031332     CGTTGCCTTTCTGCATCATTGGTTTGCAAGTACTGGCGTGACCTTTGTTTAGACTTCCAG
ENST00000542267        CGTTGCCTTTCCGCATCATTGGTTTGCAAGTACTGGCGTGACCTTTGTTTAGACTTCCAG
ENSPTRT00000047746     CGTTGCCTTTCCGCATCATTGGTTTGCAAGTACTGGCGTGACCTTTGTTTAGACTTCCAG
```

```
ENSMUST00000024761     TTTTGGAAGCAGCTGGACCTTAGTAGTCGTCAGCAGGTTACTGATGAATTATTGGAAAAA
ENSCJAT00000031332     TTTTGGAAGCAGCTGGATCTTAGTAGTCGTCAGCAGGTCACTGATGAATTATTGGAAAAA
ENST00000542267        TTTTGGAAGCAGCTGGATCTTAGTAGTCGTCAGCAGGTCACTGATGAATTGTTGGAAAAA
ENSPTRT00000047746     TTTTGGAAGCAGCTGGATCTTAGTAGTCGTCAGCAGGTCACTGATGAATTGTTGGAAAAA

ENSMUST00000024761     ATTGCCTCCAGAAGTCAGAATATAATTGAGATTAACATTTCTGATTGTCGAAGTCTGTCT
ENSCJAT00000031332     ATTGCATCAAGAAGTCAGAATATAATTGAAATCAACATTTCTGATTGTCGTAGTATGTCT
ENST00000542267        ATTGCATCAAGAAGTCAGAATATAATTGAAATCAACATTTCTGATTGTCGCAGTATGTCT
ENSPTRT00000047746     ATTGCATCAAGAAGTCAGAATATAATTGAAATCAACATTTCTGATTGTCGCAGTATGTCT

ENSMUST00000024761     GACAGTGGCGTGTGTGTCCTAGCATTTAAGTGTCCTGGACTTCTTAGGTATACAGCCTAC
ENSCJAT00000031332     GATACTGGCGTATGTGTTCTAGCATTTAAATGTCCTGGACTTCTTAGGTATACAGCCTAC
ENST00000542267        GATAATGGCGTATGTGTTTTAGCATTTAAATGTCCTGGACTTCTTAGGTATACAGCCTAC
ENSPTRT00000047746     GATAATGGCGTATGTGTTTTAGCATTTAAATGTCCTGGACTTCTTAGGTATACAGCCTAC

ENSMUST00000024761     AGGTGTAAGCAGCTTTCTGACACCTCAATCATTGCGGTCGCGTCTCACTGCCCTTTACTG
ENSCJAT00000031332     AGGTGTAAACAACTTTCTGACACCTCTATTATTGCGGTTGCCTCTCACTGTCCTTTACTT
ENST00000542267        AGGTGTAAACAGCTTTCTGACACCTCTATTATTGCGGTTGCCTCTCACTGTCCTTTACTT
ENSPTRT00000047746     AGGTGTAAACAGCTTTCTGACACCTCTATTATTGCGGTTGCCTCTCACTGTCCTTTACTT

ENSMUST00000024761     CAGAAAGTCCACGTAGGCAACCAGGACAAGCTCACCGATGAAGGACTTAAGCAGCTGGGC
ENSCJAT00000031332     CAGAAAGTGCATGTAGGCAACCAGGACAAACTTACTGATGAAGGACTCAAGCAGCTGGGC
ENST00000542267        CAGAAAGTGCATGTAGGCAACCAGGACAAACTCACTGATGAAGGACTCAAGCAGCTGGGC
ENSPTRT00000047746     CAGAAAGTGCATGTAGGCAACCAGGACAAACTCACTGATGAAGGACTCAAGCAGCTGGGC

ENSMUST00000024761     TCAAGATGCAGAGAACTCAAAGACATCCATTTTGGCCAGTGTTACAAGATCTCAGATGAA
ENSCJAT00000031332     TCAAAATGCAGAGAACTCAAAGATATTCATTTTGGCCAATGTTACAAGATCTCAGATGAA
ENST00000542267        TCAAAATGCAGAGAACTCAAAGATATTCATTTCGGCCAGTGTTACAAGATCTCAGATGAA
ENSPTRT00000047746     TCAAAATGCAGAGAACTCAAAGATATTCATTTCGGCCAGTGTTACAAGATCTCAGATGAA

ENSMUST00000024761     GGCATGATTGTCATAGCTAAGAGCTGCCTGAAGTTACAGAGGATATACATGCAGGAAAAC
ENSCJAT00000031332     GGCATGATCGTCATAGCTAAGGGCTGTCTGAAATTACAGAGAATATACATGCAGGAAAAC
ENST00000542267        GGCATGATCGTCATAGCTAAGGGCTGTCTGAAATTACAAAGGATATACATGCAGGAAAAC
ENSPTRT00000047746     GGCATGATCGTCATAGCTAAGGGCTGTCTGAAATTACAAAGGATATACATGCAGGAAAAC

ENSMUST00000024761     AAATTAGTGACAGATCAGTCAGTGAAGGCATTTGCTGAGCACTGTCCTGAACTCCAGTAT
ENSCJAT00000031332     AAATTAGTGACAGATCAGTCGGTGAAAGCATTTGCTGAACACTGTCCTGAGCTTCAATAT
ENST00000542267        AAATTAGTGACAGATCAGTCAGTGAAAGCATTTGCTGAACACTGTCCTGAGCTTCAATAT
ENSPTRT00000047746     AAATTAGTGACAGATCAGTCAGTGAAAGCATTTGCTGAACACTGTCCTGAGCTTCAATAT

ENSMUST00000024761     GTTGGCTTCATGGGTTGTTCAGTTACTTCCAAAGGAGTCATTCACTTAACCAAGCTAAGG
ENSCJAT00000031332     GTAGGCTTCATGGGTTGTTCAGTCACTTCTAAAGGAGTCATTCACCTAACCAAGCTAAGA
ENST00000542267        GTAGGCTTCATGGGTTGTTCAGTCACTTCTAAAGGAGTCATTCACCTAACCAAGCTAAGA
ENSPTRT00000047746     GTAGGCTTCATGGGTTGTTCAGTCACTTCTAAAGGAGTCATTCACCTAACCAAGCTAAGA

ENSMUST00000024761     AACCTGTCCAGCTTAGACCTCCGTCACATCACTGAACTGGATAATGAAACCGTGATGGAA
ENSCJAT00000031332     AACCTTTCCAGCTTGGACCTACGTCATATCACTGAACTGGATAATGAAACCGTGATGGAA
ENST00000542267        AACCTTTCCAGCTTGGACCTACGTCATATCACTGAACTGGATAATGAAACCGTGATGGAA
ENSPTRT00000047746     AACCTTTCCAGCTTGGACCTACGTCATATCACTGAACTGGATAATGAAACCGTGATGGAA
```

```
ENSMUST00000024761     ATCGTCAAGAGGTGCAAAAATCTCAGCTCTCTGAATCTCTGTCTGAACTGGATCATAAAC
ENSCJAT00000031332     ATTGTCAAGAGGTGCAAAAATCTTAGCTCTCTCAATCTCTGTCTGAACTGGATCATAAAT
ENST00000542267        ATTGTCAAGAGGTGCAAAAATCTTAGCTCTCTCAATCTCTGTCTGAACTGGATCATAAAT
ENSPTRT00000047746     ATTGTCAAGAGGTGCAAAAATCTTAGCTCTCTCAATCTCTGTCTGAACTGGATCATAAAT


ENSMUST00000024761     GACAGGTGTGTGGAGGTCATTGCAAAGGAAGGACAGAACCTGAAAGAGCTGTACCTGGTG
ENSCJAT00000031332     GACAGGTGTGTGGAGGTCATTGCAAAGGAAGGACAAAACCTGAAAGAGTTGTATTTGGTG
ENST00000542267        GACAGGTGTGTGGAGGTCATTGCAAAGGAAGGACAAAACCTGAAAGAGCTATATTTGGTG
ENSPTRT00000047746     GACAGGTGTGTGGAGGTCATTGCAAAGGAAGGACAAAACCTGAAAGAGCTGTATTTGGTG


ENSMUST00000024761     TCCTGTAAGATCACTGATTATGCACTGATAGCCATTGGGCGATACAGCGTGACCATAGAG
ENSCJAT00000031332     TCCTGTAAAATCACAGATTATGCACTGATAGCCATTGGGCGATACAGCATGACAATAGAG
ENST00000542267        TCCTGTAAAATCACAGATTATGCACTGATAGCCATTGGGCGATACAGCATGACAATAGAG
ENSPTRT00000047746     TCCTGTAAAATCACAGATTATGCACTGATAGCCATTGGGCGATACAGCATGACAATAGAG


ENSMUST00000024761     ACTGTGGACGTCGGATGGTGTAAAGAAATCACAGATCAAGGAGCCACCCTGATTGCACAG
ENSCJAT00000031332     ACTGTGGATGTTGGATGGTGTAAAGAAATTACAGATCAAGGAGCCACCCTGATTGCACAG
ENST00000542267        ACTGTGGATGTCGGATGGTGTAAAGAAATCACAGACCAAGGAGCCACCCTGATTGCACAG
ENSPTRT00000047746     ACTGTGGATGTTGGATGGTGTAAAGAAATCACAGACCAAGGAGCCACCCTGATTGCACAG


ENSMUST00000024761     AGCAGCAAGTCCCTGAGATACTTGGGCCTGATGAGATGTGACAAAGTCAATGAATTGACA
ENSCJAT00000031332     AGCAGCAAGTCTCTGAGATATTTGGGGCTGATGAGATGTGACAAAGTCAATGAAGTGACA
ENST00000542267        AGCAGCAAGTCTCTGAGATATTTGGGGCTGATGAGATGTGATAAAGTCAACGAAGTGACG
ENSPTRT00000047746     AGCAGCAAGTCTCTGAGATATTTGGGGCTGATGAGATGTGATAAAGTCAACGAAGTGACG


ENSMUST00000024761     GTGGAGCAGCTGGTGCAGCAGTACCCGCACATCACCTTCAGCACCGTTCTGCAGGACTGC
ENSCJAT00000031332     GTGGAACAGCTGGTGCAACAGTATCCCCACATCACCTTCAGCACTGTCCTGCAAGACTGC
ENST00000542267        GTGGAACAGCTGGTGCAGCAGTACCCCCACATCACCTTCAGCACCGTCCTGCAGGACTGC
ENSPTRT00000047746     GTGGAACAGCTGGTGCAGCAGTACCCCCACATCACCTTCAGCACCGTCCTGCAGGACTGC


ENSMUST00000024761     AAGAGGACCTTGGAGAGAGCCTACCAGATGGGCTGGACCCCCAATATGTCCGCTGCCACC
ENSCJAT00000031332     AAGAGGACCTTGGAGAGAGCCTATCAGATGGGCTGGACCCCCAACATGTCTGCCGCCTCC
ENST00000542267        AAGAGGACCTTGGAGAGAGCCTATCAGATGGGCTGGACCCCCAACATGTCTGCCGCCTCC
ENSPTRT00000047746     AAGAGGACCTTGGAGAGAGCCTATCAGATGGGCTGGACCCCCAACATGTCTGCCGCCTCC


ENSMUST00000024761     TCC
ENSCJAT00000031332     TCC
ENST00000542267        TCC
ENSPTRT00000047746     TCC
```

Multiple sequence alignment of Fbxl18

```
ENSPTRT00000034903     CAGGACATATCCAATGATGATGATGACATGCACCCTGCAGCAGCCGGGATGGCGGACGGG
ENSGGOT00000025986     GAGGACATTTCCAATGATGATGATGACATGCACCCTGCAGCAGCCGGGATGGCGGACGGG
ENST00000382368        GAGGACATATCCAATGATGATGATGACATGCACCCTGCAGCAGCCGGGATGGCAGACGGG
ENSMMUT00000007125     CAGGACATATCCAATGATGATGATGACATGCACCCTGCAGCAGCCGGGATGGCGGACGGG
ENSPPYT00000020192     GAGGACATATCCAATGATGATGATGACATGCACCCTGCAGCAGCCGGGATGGCGGATGGG
ENSMUST00000035985     ------ATGTCCAGTGAGGAGGAG------------GCAGCGGCAGAGGCAGCAGGTGAC
ENSRNOT00000001479     ------ATGTCCAATGATGATGAG------------GCAGCGGCAGAGGCAGCGGGTGAC


ENSPTRT00000034903     GTCCACCTCCTAGGGTTCTCTGATGAGATCCTCCTTCACATCCTGAGTCACGTCCCCAGC
```

ENSGGOT00000025986 GTCCACCTCCTAGGGTTCTCTGATGAGATCCTCCTTCACATCCTGAGTCACGTCCCCAGC
ENST00000382368 GTCCACCTCCTAGGGTTCTCTGATGAGATCCTCCTTCACATCCTGAGTCACGTCCCCAGC
ENSMMUT00000007125 GTCCACCTCCTGGGGTTCTCTGATGAGATTCTCCTTCACATCCTGAGTCACGTCCCCAGC
ENSPPYT00000020192 GTCCACCTCCTAGGGTTCTCTGATGAGATCCTCCTTCACATCCTGAGTCACGTCCCTAGC
ENSMUST00000035985 ACCCACCTGCTTGGCTTCTCCGATGAGATCCTCCTACACATCCTGAGCCATGTCCCCAGC
ENSRNOT00000001479 ACCCACCTACTTGGCTTCTCTGATGAGATCCTCCTACATATCCTGAGCCACGTCCCCAGC

ENSPTRT00000034903 ACAGATCTGATTCTGAACGTCCGGCGTACCTGTCGGAAGCTTGCAGCCCTGTGCCTTGAC
ENSGGOT00000025986 ACAGATCTGATTCTGAACGTCCGGCGTACCTGTCGGAAGCTTGCAGCCCTGTGCCTTGAC
ENST00000382368 ACAGATCTGATTCTGAACGTCCGGCGTACCTGTCGGAAGCTTGCAGCCCTGTGCCTTGAC
ENSMMUT00000007125 ACAGACCTGATTCTGAACGTCCGGCGTACCTGTCGGAAGCTCGCAGCCTTGTGCCTTGAT
ENSPPYT00000020192 ACAGATCTGATTCTGAACGTCAGGCGTACCTGTCGGAAGCTTGCAGCCCTGTGCCTTGAC
ENSMUST00000035985 ACGGACCTGGTTCTCAGTGTGCGGCGCACCTGCAGGAAACTGGCAGCCCTGTGCCTGGAC
ENSRNOT00000001479 ACAGACCTGGTTCTCAATGTGCGGCGTACCTGCCGGAAGCTCGCAGCCCTGTGTCTGGAC

ENSPTRT00000034903 AAGAGCCTCATCCACACCGTGTTGCTGCAAAAGGACTATCAGGCGAGCGAGGACAAAGTG
ENSGGOT00000025986 AAGAGCCTCATCCACACCGTGTTGCTGCAAAAGGACTATCAGGCGAGCGAGGACAAAGTG
ENST00000382368 AAGAGCCTCATCCACACCGTGTTGCTGCAAAAGGACTATCAGGCGAGCGAGGACAAAGTG
ENSMMUT00000007125 AAGAGCCTCATCCACACCGTGTTGCTGCAAAAGGACTATCAGGCGAGCGAGGACAAGGTG
ENSPPYT00000020192 AAAAGCCTCATCCACACCGTGTTGCTGCAAAAGGACTATCAGGCGAGCGAGGACAAAGTG
ENSMUST00000035985 AAGAGCCTGGTGCACACCGTGCTGCTGCAGAAGGACTACCAGGCCAGTGAGGAGAAGGTG
ENSRNOT00000001479 AAGAGCCTGGTGCACACTGTGCTACTGCAGAAGGACTACCAGGCCAGTGAGGAGAAGGTG

ENSPTRT00000034903 AGGCAGCTGGTGAAGGAGATCGGCCGGGAGATCCAGCAGCTGAGCATGGCTGGCTGCTAC
ENSGGOT00000025986 AGGCAGCTGGTGAAGGAGATCGGCCGGGAGATCCAGCAGCTGAGCATGGCTGGCTGCTAC
ENST00000382368 AGGCAGCTGGTGAAGGAGATCGGCCGGGAGATCCAGCAGCTGAGCATGGCTGGCTGCTAC
ENSMMUT00000007125 AGGCAGCTGGTGAAGGAGATCGGCCGGGAGATCCAGCAGCTGAGCATGGCTGGCTGCTAC
ENSPPYT00000020192 AGGCAGCTGGTGAAGGAGATCGGCCGGGAGATCCAGCAGCTGAGCATGGCTGGCTGCTAC
ENSMUST00000035985 AAGCAACTGGTGAAGGAGATTGGCCGGGAGATCCAACAGCTGAACATGGCAGGCTGCTAT
ENSRNOT00000001479 AAGCAGCTGGTGAAGGAGATTGGCCGGGAGATCCAGCAGCTGAACATGGCAGGCTGCTAT

ENSPTRT00000034903 TGGCTGCCTGGCTCCACCGTGGAACACGTGGCCCGCTGCCGCAGCCTGGTGAAGGTGAAC
ENSGGOT00000025986 TGGCTGCCTGGCTCCACCGTGGAACACGTGGCCCGCTGCCGCAGCCTGGTGAAGGTGAAC
ENST00000382368 TGGCTGCCTGGCTCCACCGTGGAACACGTGGCCCGCTGCCGCAGCCTGGTGAAGGTGAAC
ENSMMUT00000007125 TGGCTGCCTGGCTCCACCGTGGAACACGTGGCCCGCTGCCGCAGCCTGGTGAAGGTGAAC
ENSPPYT00000020192 TGGCTGCCTGGCTCCACCGTGGAACACGTGGCCCGCTGCCGCAGCCTGGTGAAGGTGAAC
ENSMUST00000035985 TGGTTGTCTGGCTCCACCATCGAGCACGTGGCCCGGTGTCACAGCCTGGTGAAGGTGAAC
ENSRNOT00000001479 TGGCTGCCCGGCTCCACCATCGAGCACGTGGCCCGCTGTCACAGCCTGGTGAAGGTGAAC

ENSPTRT00000034903 CTCTCGGGCTGCCACCTCACCTCCCTGCGCCTCTCCAAGATGCTCTCGGCCCTGCAGCAC
ENSGGOT00000025986 CTCTCGGGCTGCCACCTCACCTCCCTGCGCCTCTCCAAGATGCTCTCGGCCCTGCAGCAC
ENST00000382368 CTCTCGGGCTGCCACCTCACTTCCCTGCGCCTCTCCAAGATGCTCTCGGCCCTGCAGCAC
ENSMMUT00000007125 CTCTCGGGCTGCCACCTCACCTCCCTGCGCCTCTCCAAGATGCTCTCGGCCCTGCAGCAC
ENSPPYT00000020192 CTCTCGGGCTGCCACCTCACCTCCCTGCGCCTCTCCAAGATGCTCTCGGCCCTGCAGCAC
ENSMUST00000035985 TTGTCAGGTTGCCACCTCACCTCGCTGCGGCTGTCCAAGGTGCTCTCGGCCCTGCAGCAC
ENSRNOT00000001479 TTGTCAGGTTGTCACCTCACCTCGCTGCGGCTGTCCAGGGTGCTCTCCTGCCTGCAGCGC

ENSPTRT00000034903 CTGCGCTCGCTGGCCATCGACGTGAGCCCCGGCTTCGACGCCAGCCAGCTGAGCAGCGAG
ENSGGOT00000025986 CTGCGCTCGCTGGCCATTGACGTGAGCCCCGGCTTCGACGCCAGCCAGCTGAGCAGCGAG
ENST00000382368 CTGCGCTCGCTGGCCATCGACGTGAGCCCCGGCTTCGACGCCAGCCAGCTGAGCAGCGAG
ENSMMUT00000007125 CTGCGCTCGCTGGCCATCGACGTGAGCCCCGGCTTCGACGCCAGCCAGCTGAGCAGTGAG
ENSPPYT00000020192 CTGCGCTCGCTGGCCATCGACGTGAGCCCCGGCTTCGACGCCAGCCAGCTGAGCAGCGAG
ENSMUST00000035985 CTGCGCTCATTGGCCATTGACGTGAGCCCCGGCTTCGACGCCAGCCAGCTGAGCAGTGAG
ENSRNOT00000001479 CTGCGCTCACTGGCCATCGACGTGAGCCCTGGCTTCGACGCCAGCCAGCTGAGCAGTGAG

ENSPTRT00000034903 TGCAAGGCCACCCTGAGCCGCGTGCGGGAGCTCAAGCAGACGCTGTTCACTCCCTCCTAC
ENSGGOT00000025986 TGCAAGGCCACCCTGAGCCGCGTGCGGGAGCTCAAGCAGACGCTGTTCACTCCCTCCTAC
ENST00000382368 TGCAAGGCCACCCTGAGCCGCGTGCGGGAGCTCAAGCAGACGCTGTTCACTCCCTCCTAC
ENSMMUT00000007125 TGCAAGGCCACGCTGAGCCGCGTGCGGGAGCTCAAGCAGACGCTGTTCACGCCCTCGTAC
ENSPPYT00000020192 TGCAAGGCCACACTGAGCCGCGTGCGGGAGCTCAAGCAGACGCTGTTCACTCCCTCCTAC
ENSMUST00000035985 TGCAAGGCCACGCTAAGCCGCGTGCAGGAGCTCAAGCAGACACTCTTCACGCCGTCCTAC
ENSRNOT00000001479 TGCAAGGCCACGCTGAGCCGCGTGCAGGAGCTCAAGCAGACGCTCTTCACACCGTCCTAC

ENSPTRT00000034903 GGCGTGGTGCCCTGCTGCACCAGCCTAGAGAAGCTGCTGCTCTACTTCGAGATTCTGGAC
ENSGGOT00000025986 GGCGTGGTGCCCTGCTGCACCAGCCTAGAGAAGCTGCTGCTCTACTTCGAGATTCTGGAC
ENST00000382368 GGCGTGGTGCCCTGCTGCACCAGCCTAGAGAAGCTGCTGCTCTACTTCGAGATTCTGGAC
ENSMMUT00000007125 GGCGTGGTGCCCTGCTGCACCAGCCTGGAGAAGCTACTGCTCTACTTCGAGATTCTGGAC
ENSPPYT00000020192 GGCGTGGTGCCCTGCTGCACCAGCCTGGAGAAGCTGCTGCTCTACTTCGAGATTCTGGAC
ENSMUST00000035985 GGGGTGGTGCCCTGCTGTGCCAGCCTGCAGAAGCTGCTGCTCTACTTCGAGATCCTGGAC
ENSRNOT00000001479 GGCGTGGTGCCCTGCTGTGCCAGCCTGCAGAAGCTGCTGCTGTACTTCGAGATCCTGGAC

ENSPTRT00000034903 CGCACGCGCGAGGGCGCCATCCTCTCGGGCCAGCTTATGGTGGGCCAGAGCAACGTGCCG
ENSGGOT00000025986 CGCACGCGCGAGGGCGCCATCCTCTCGGGCCAGCTTATGGTGGGCCAGAGCAACGTGCCG
ENST00000382368 CGCACGCGCGAGGGCGCCATCCTCTCGGGCCAGCTTATGGTGGGCCAGAGCAACGTGCCG
ENSMMUT00000007125 CGCACACGCGAGGGCGCCATCCTCTCGGGCCAGCTCATGGTGGGCCAGAGCAACGTGCCG
ENSPPYT00000020192 CGCACGCGCGAGGGCGCCATCCTCTCGGGCCAGCTTATGGTGGGCCAGAGCAACGTGCCG
ENSMUST00000035985 CGCACCCGGGAGGGGGCCGTCCTCTCGGGCCAGCTCATGGTGGGCCAGAGCAACGTGCCC
ENSRNOT00000001479 CGCACCCGTGAGGGCGCCGTCCTCTCCGGCCAGCTCATGGTGGGCCAGAGCAACGTGCCC

ENSPTRT00000034903 CACTACCAGAACCTGCGGGTCTTCTATGCGCGCCTGGCCCCCGGCTACATCAACCAGGAG
ENSGGOT00000025986 CACTACCAGAACCTGCGGGTCTTCTATGCGCGCCTGGCCCCCGGCTACATCAACCAGGAG
ENST00000382368 CACTACCAGAACCTGCGGGTCTTCTATGCGCGCCTGGCCCCCGGCTACATCAACCAGGAG
ENSMMUT00000007125 CACTACCAGAACCTACGGGTCTTCTATGCGCGCCTGGCCCCCGGCTACATCAACCAGGAG
ENSPPYT00000020192 CACTACCAGAACCTGCGGGTCTTCTATGCGCGCCTGGCCCCCGGCTACATCAACCAGGAG
ENSMUST00000035985 CACTACCAGAACCTGCGTGTCTTCTATGCACGCCTGGCTCCTGGCTACATCAACCAGGAG
ENSRNOT00000001479 CACTACCAGAACCTGCGTGTCTTCTACGCACGCCTGGCCCCTGGCTACATCAACCAGGAG

ENSPTRT00000034903 GTGGTGCGGCTCTACCTGGCTGTGCTTAGCGACCGCACTCCTCAGAACCTCCACGCCTTC
ENSGGOT00000025986 GTGGTGCGGCTCTACCTGGCTGTGCTTAGCGACCGCACTCCTCAGAACCTCCACGCCTTC
ENST00000382368 GTGGTGCGGCTCTACCTGGCTGTGCTTAGCGACCGCACTCCTCAGAACCTCCACGCCTTC
ENSMMUT00000007125 GTGGTGCGGCTCTACCTGGCTGTGCTTAGTGACCGCACGCCTCAGAACCTCCACGCCTTC
ENSPPYT00000020192 GTGGTGCGGCTCTACCTGGCTGTGCTTAGCGACCGTACTCCTCAGAACCTCCACGCCTTC
ENSMUST00000035985 GTGGTGAGACTCTACCTGGCCGTGCTCAGCGACCGCACGCCTGAGAACCTGCATGCCTTC
ENSRNOT00000001479 GTGGTAAGGCTCTACCTCGCTGTGCTCAGCGACCGCACGCCTGAGAACCTGCATGCCTTC

ENSPTRT00000034903 CTCATCTCCGTCCCTGGCAGCTTCGCGGAGAGCGGCGCCACCAAGAACCTCCTGGACTCC
ENSGGOT00000025986 CTCATCTCCGTCCCTGGCAGCTTCGCGGAGAGCGGCGCCACCAAGAACCTCCTGGACTCC
ENST00000382368 CTCATCTCCGTCCCTGGCAGCTTCGCGGAGAGCGGCGCCACCAAGAACCTCCTGGACTCC
ENSMMUT00000007125 CTCATCTCCGTCCCCGGCAGCTTCGCGGAGAGCGGAGCCACCAAGAACCTCCTGGACTCC
ENSPPYT00000020192 CTCATCTCCGTCCCCGGCAGCTTCGCGGAGAGCGGCGCCACGAAGAACCTCCTGGACTCC
ENSMUST00000035985 CTCATCTCCGTCCCTGGCAGCTTTGCAGAGAGCGGGGCCACCAAAAACTTGCTGGACTCC
ENSRNOT00000001479 CTCATCTCCGTCCCCGGCAGCTTCGCAGAGAGCGGGGCCACCAAAAACCTGCTGGACTCC

ENSPTRT00000034903 ATGGCGCGCAATGTCGTGCTGGATGCCCTGCAGCTGCCCAAGTCCTGGCTGAACGGCTCT
ENSGGOT00000025986 ATGGCGCGCAATGTCGTGCTGGATGCCCTGCAGCTGCCCAAGTCCTGGCTGAACGGCTCT
ENST00000382368 ATGGCGCGCAATGTCGTGCTGGATGCCCTGCAGCTGCCCAAGTCCTGGCTGAACGGCTCT
ENSMMUT00000007125 ATGGCGCGCAACGTCGCGCTGGATGCCCTGCAGCTGCCCAAGTCCTGGCTGAACGGCTCT

```
ENSPPYT00000020192     ATGGCGCGCAATGTCGCGCTGGATGCCCTGCAGCTGCCCAAGTCCTGGCTGAACGGCTCT
ENSMUST00000035985     ATGGCCCGCAACGTGGCCCTGGATGCACTGCAGCTGCCCAAGTCCTGGCTCAATGGCTCC
ENSRNOT00000001479     ATGGCCCGCAACGTGGCCCTAGATGCCCTGCAGCTGCCCAAGTCCTGGCTGAATGGTTCC

ENSPTRT00000034903     TCCCTCCTGCAGCACATGAAATTCAACAACCCGTTCTACTTCAGTTTCAGCCGCTGTACC
ENSGGOT00000025986     TCCCTCCTGCAGCACATGAAATTCAACAACCCGTTCTACTTCAGTTTCAGCCGCTGTACC
ENST00000382368        TCCCTCCTGCAGCACATGAAATTCAACAACCCGTTCTACTTCAGTTTCAGCCGCTGTACC
ENSMMUT00000007125     TCCCTCCTGCAGCACATGAAATTCAACAACCCGTTCTACTTCAGCTTCAGCCGCTGCACC
ENSPPYT00000020192     TCCCTTCTGCAGCACATGAAATTCAACAACCCGTTCTACTTCAGTTTCAGCCGCTGTACC
ENSMUST00000035985     ACCCTCCTCCAGCATATGAAATTCAACAATCCCTTCTACTTCAGCTTCAGCCGGTGTACG
ENSRNOT00000001479     ACCCTCCTCCAGCACATGAAGTTCAACAATCCCTTCTACTTCAGCTTCAGCCGGTGTACG

ENSPTRT00000034903     CTGTCAGGCGGCCATCTGATCCAGCAGGTCATCAACGGCGGGAAGGACCTGCGGAGCCTG
ENSGGOT00000025986     CTGTCAGGCGGCCATCTGATCCAGCAGGTCATCAACGGCGGGAAGGACCTGCGGAGCCTG
ENST00000382368        CTGTCAGGCGGCCATCTGATCCAGCAGGTCATCAACGGCGGGAAGGACCTGCGGAGCCTG
ENSMMUT00000007125     CTGTCGGGCGGCCATCTGATCCAGCAGGTCATCAACGGCGGGAAGGACCTGCGGAGCCTG
ENSPPYT00000020192     CTGTCAGGCGGCCATCTGATCCAGCAGGTCATCAACGGTGGGAAGGACCTGCGGAGCCTG
ENSMUST00000035985     CTGTCAGGGGGTCACCTGATTCAGCGGCTCATCAATGGCGGGAAGGACCTGCGTAGCCTG
ENSRNOT00000001479     CTGTCAGGCGGTCACCTGATCCAGCGACTCATCAATGGGGGGAAGGACCTGCGTAGCCTG

ENSPTRT00000034903     GCCAGCTTGAACCTCAGCGGCTGCGTCCACTGCCTGTCCCCAGACTCGCTGCTCCGCAAG
ENSGGOT00000025986     GCCAGCTTGAACCTCAGCGGCTGCGTCCACTGCCTGTCCCCAGACTCGCTGCTCCGCAAG
ENST00000382368        GCCAGCTTGAACCTCAGCGGCTGCGTCCACTGCCTGTCCCCAGACTCGCTGCTCCGCAAG
ENSMMUT00000007125     GCCAGCCTGAACCTCAGCGGCTGCGTCCACTGCCTGTCCCCAGACTCGCTGCTCCGCAAG
ENSPPYT00000020192     GCCAGCTTGAACCTCAGCGGCTGCGTCCACTGCCTGTCCCCAGACTCGCTGCTCCGCAAG
ENSMUST00000035985     GCCAGCCTCAACCTCAGCGGCTGTGTGCATTGCCTGTCTGCGGACTCGTTGCTGCGCAAG
ENSRNOT00000001479     GCCAGCCTCAACCTCAGCGGCTGCGTGCACTGCCTGTCCGCGGACTCATTGCTGCGGAAG

ENSPTRT00000034903     GCGGAGGACGACATCGACAGCAGCATCCTGGAGACGCTGGTGGCGTCCTGCTGCAACCTG
ENSGGOT00000025986     GCGGAGGACGACATCGACAGCAGCATTCTGGAGACGCTGGTGGCGTCCTGCTGCAACCTG
ENST00000382368        GCGGAGGACGACATCGACAGCAGCATCCTGGAGACTCTGGTGGCGTCCTGCTGCAACCTG
ENSMMUT00000007125     GCGGAGGACGACATCGACAGCAGCATCCTGGAGACGCTGGTGGCGTCCTGCTGCAACCTG
ENSPPYT00000020192     GCGGAGGACGACATCGACAGCAGCATCCTGGAGACGCTGGTGGCGTCCTGCTGCAACCTG
ENSMUST00000035985     GCCGAGGACGACATTGACAGCAGCATCCTGGAGACTCTGGTGGAGTCCTGCTGCAACCTG
ENSRNOT00000001479     GCCGAGGACGACATCGACAGCAGCATCCTCGAGACTCTGGTGGAGTCCTGCTGCAACCTG

ENSPTRT00000034903     CGCCACCTGAACCTCTCGGCCGCCCACCACCACAGCTCGGAGGGCCTGGGCCGGCACCTC
ENSGGOT00000025986     CGCCACCTGAACCTCTCGGCCGCCCACCACCACAGCTCGGAGGGCCTGGGCCGCCACCTC
ENST00000382368        CGCCACCTGAACCTCTCGGCCGCCCACCACCACAGCTCGGAGGGCCTGGGCCGCCACCTC
ENSMMUT00000007125     CGCCACCTGAACCTCTCGGCTGCCCACCACCACAGCTCGGAGGGCCTGGGCCGCCACCTC
ENSPPYT00000020192     CGCCACCTGAACCTCTCGGCCGCCCACCACCACAGCTCGGAGGGCCTGGGCCGCCACCTC
ENSMUST00000035985     CACCACCTCAATCTCTCTGCCGCCCATCACCACAGCTCCGACGGCCTGGGCCGCCACCTC
ENSRNOT00000001479     CATCACCTCAATCTCTCAGCCGCCCATCACCACAGCTCTGACGGCCTGGGCCGCCACCTC

ENSPTRT00000034903     TGCCAGCTCCTGGCCCGGCTGCGTCACCTGCGCTCCCTCTCCCTGCCTGTCTGCTCTGTC
ENSGGOT00000025986     TGCCAGCTCCTGGCCCGGCTGCGTCACCTGCGCTCCCTCTCCCTGCCTGTCTGCTCTGTC
ENST00000382368        TGCCAGCTCCTGGCCCGGCTGCGTCACCTGCGCTCCCTCTCCCTGCCTGTCTGCTCTGTC
ENSMMUT00000007125     TGCCAGCTCCTGGCCCGGCTGCGTCACCTGCGCTCCCTCTCCCTGCCTGTCTGCTCCGTC
ENSPPYT00000020192     TGCCAGCTCCTGGCCCGGCTGCGTCACCTGCGCTCCCTCTCCCTGCCTGTCTGCTCTGTC
ENSMUST00000035985     TGCCAGCTCCTGGCTCGGCTCTGCCACCTGCGTTCCCTGTCCCTGCCTGTCTGCTCTGTG
ENSRNOT00000001479     TGCCAGCTCCTGGCTCGGCTCTGCCACCTGCGCTCCCTGTCCCTGCCTGTCTGCTCTGTG

ENSPTRT00000034903     GCTGACTCCGCCCCGCGCGCCGACCGCGCGCCCGCCCAGCCGGCCATGCACGCAGTGCCG
```

```
ENSGGOT00000025986     GCTGAC---------------------------------------ATGCACGCAGTGCCG
ENST00000382368        GCTGACTCCGCGCCGCGCGCCGACCGCGCGCCCGCCCAGCCGGCCATGCACGCAGTGCCG
ENSMMUT00000007125     GCCGACTCCGCGCCGCGCGCCGACCGTGCGCCCGCCCAGCCGGCCATGCACGCAGTGCCG
ENSPPYT00000020192     GCCGACTCTGCGCCGCGCGCGGACCGCGCGCCCGCCCAGCCGGCCATGCACGCAGTGCCG
ENSMUST00000035985     GCTGACTCGGCACCCAGGCCTGACCGTGCGCCCGCCCCGCCAGCCATGCACGCAGTACCC
ENSRNOT00000001479     GCCGACTCGGCACCCAGGCCTGACCGTGCGCCTGCCCCGCCAGCCATGCATGCAGTACCC

ENSPTRT00000034903     CGCGGCTTTGGCAAGAAAGTGCGTGTGGGCGTGCAGTCCTGTCCCAGCCCCTTCTCGGGC
ENSGGOT00000025986     CGCGGCTTTGGCAAGAAAGTGCGTGTGGGCGTGCAGTCCTGTCCCAGCCCCTTCTCCGGC
ENST00000382368        CGCGGCTTTGGCAAGAAAGTGCGTGTGGGCGTGCAGTCCTGTCCCAGCCCCTTCTCGGGC
ENSMMUT00000007125     CGTGGCTTTGGCAAGAAAGTGCGCGTGGGCGTGCAGTCCTGTCCTAGCCCCTTCTCCGGC
ENSPPYT00000020192     CGCGGTTTTGGCAAGAAAGTGCGTGTGGGCGTGCAGTCCTGTCCCAGCCCCTTCTCGGGC
ENSMUST00000035985     CGTGGATTTGGCAAGAAGGTGCGCATTGGGGTGCAGACCTGTCCCAACCCCTTCGTGGGC
ENSRNOT00000001479     CGTGGATTTGGCAAGAAGGTGCGCATTGGGGTGCAGACCTGTCCCAACCCCTTTGTGGGC

ENSPTRT00000034903     CAGGCGGGCCCCCAGCCCTCCTCCGTGTTCTGGTCTCTGCTGAAGAACCTGCCCTTCCTG
ENSGGOT00000025986     CAGGCGGGGCCCCAGCCCTCCTCCGTGTTCTGGTCTCTGCTGAAGAACCTGCCCTTCCTG
ENST00000382368        CAGGCGTGCCCCCAGCCCTCCTCCGTGTTCTGGTCTCTGCTGAAGAACCTGCCCTTCCTG
ENSMMUT00000007125     CAGGCGGGCCCCCAGCCTTCCTCCGTGTTCTGGTCTCTGCTGAAGAACCTGCCCTTCCTG
ENSPPYT00000020192     CAGGCGGGCCCCCAGCCCTCCTCCGTGTTCTGGTCTCTGCTGAAGAACCTGCCCTTCCTA
ENSMUST00000035985     CAGTCGGCCCCACAGCCTGCCTCTGTGTTCTGGTCTCTGCTGAAGAAGCTCCCATTTCTG
ENSRNOT00000001479     CAGTCGGCCCCACAGCCTGCCTCTGTGTTTTGGTCTCTGCTGAAGAAGCTCCCATTTCTG

ENSPTRT00000034903     GAACACCTCGAGCTGATCGGGTCCAACTTCTCCTCCGCCATGCCCCGCAACGAGCCCGCC
ENSGGOT00000025986     GAACACCTCGAGCTGATTGGGTCCAACTTCTCCTCTGCCATGCCCCGCAACGAGCCCGCC
ENST00000382368        GAACACCTCGAGCTGATTGGGTCCAACTTCTCCTCCGCCATGCCCCGCAACGAGCCCGCC
ENSMMUT00000007125     GAACACCTCGAACTGATCGGGTCCAACTTCTCCTCCGCCATGCCACGCAACGAGCCCGCC
ENSPPYT00000020192     GAACACCTTGAGCTGATCGGGTCCAATTTCTCCTCCGCCATGCCGCGCAACGAGCCCGCC
ENSMUST00000035985     GAGCACCTGGAACTGATCGGGTCCAATTTCTCCTCAGCCATGCCCCGCAATGAGCCTGCC
ENSRNOT00000001479     GAGCACCTGGAACTGATCGGGTCCAATTTCTCCTCAGCCATGCCCCGCAATGAGCCAGCC

ENSPTRT00000034903     ATCCGCAACTCGCTCCCACCCTGCAGCCGCGCACAGAGCGTCGGGGACTCGGAGGTGGCC
ENSGGOT00000025986     ATCCGCAACTCGCTCCCACCCTGCAGCCGCGCGCAGAGCGTCGGGGACTCGGAGGTGGCC
ENST00000382368        ATCCGCAACTCGCTCCCACCCTGCAGCCGCGCACAGAGTGTCGGGGACTCGGAGGTGGCC
ENSMMUT00000007125     ATCCGCAACTCGCTCCCGCCCTGCAGCCGCGCGCAGAGCGTCGGGGACTCGGAGGTGGCC
ENSPPYT00000020192     ATCCGCAACTCGCTCCCACCCTGCAGCCGCGCGCAGAGCGTCGGGGACTCGGAGGTGGCC
ENSMUST00000035985     ATCCGCAACTCCCTCCCACCCTGCAGTCGGGCACAGAACGTGGGGGACTCAGAGGTGGCC
ENSRNOT00000001479     ATCCGCAACTCCCTCCCGCCCTGCAGTCGGGCACAGAACGTGGGGGACTCAGAGGTGGCC

ENSPTRT00000034903     GCCATCGGCCAGCTGGCCTTCCTGCGGCACCTGACGCTCGCACAGCTGCCCAGCGTCCTT
ENSGGOT00000025986     GCCATCGGCCAGCTGGCCTTCCTGCGGCACCTGACGCTCGCACAGCTGCCCAGCGTCCTT
ENST00000382368        GCCATCGGCCAGCTGGCCTTCCTGCGGCACCTGACGCTCGCACAGCTGCCCAGCGTCCTT
ENSMMUT00000007125     GCCATTGGCCAGCTGGCCTTCCTGCGGCACCTGACGCTCGCACAGCTGCCCAGCGTCCTT
ENSPPYT00000020192     GCCATCGGTCAGCTGGCCTTCCTGCGGCACCTGACGCTCGCACAGCTGCCCAGCGTCCTT
ENSMUST00000035985     GCCATTGGCCAGCTGACCTTCCTGCGGCACCTGACTCTAGCCCAACTGCCAGGCATGCTC
ENSRNOT00000001479     GCCATTGGCCAGCTGACCTTCCTGCGGCACCTGACACTAGCCCAGCTGCCAGGCATGCTG

ENSPTRT00000034903     ACGGGCTCCGGGCTGGTCAATATCGGCCTGCAATGCCAGCAGTTACGGTCCCTGTCGCTG
ENSGGOT00000025986     ACGGGCTCCGGGCTGGTCAATATCGGCCTGCAGTGCCAGCAGTTGCGGTCCCTGTCGCTG
ENST00000382368        ACGGGCTCCGGGCTGGTCAATATCGGCCTGCAGTGCCAGCAGTTGCGGTCCCTGTCGCTG
ENSMMUT00000007125     ACGGGCTCCGGGCTGGTCAGTATCGGCCTGCAGTGCCAGCAGTTGCGGTCCCTGTCGCTG
ENSPPYT00000020192     ACGGGCTCCGGGCTGGTCAGTATCGGCCTGCAGTGCCAGCAGTTGCGGTCCCTGTCGCTG
ENSMUST00000035985     ACGGGCTCCGGACTCGTGAGCATCGGTCTCCAGTGCCAGCACCTGCAGTCTCTATCGCTG
ENSRNOT00000001479     ACGGGCTCTGGACTAGTGAGCATTGGCCTACAGTGCCAGCAGTTGCAGTCCCTGTCGCTG
```

ENSPTRT00000034903 GCCAACCTGGGCATGATGGGGAAGGTGGTGTACATGCCCGCGCTCTCAGACATGTTGAAG
ENSGGOT00000025986 GCCAACCTGGGCATGATGGGGAAGGTGGTGTACATGCCCGCGCTCTCAGACATGTTGAAG
ENST00000382368 GCCAACCTGGGCATGATGGGGAAGGTGGTGTACATGCCCGCGCTCTCAGACATGTTGAAG
ENSMMUT00000007125 GCCAACCTGGGCATGATGGGGAAGGTGGTGTACATGCCCGCGCTCTCAGACATGTTGAAG
ENSPPYT00000020192 GCCAACCTGGGCATGATGGGGAAGGTGGTGTACATGCCCGCGCTCTCAGATATGTTGAAG
ENSMUST00000035985 GCCAACCTGGGCATGATGGGGAAGGTGGTGTACATGCCTGCTCTTGCTGACATGCTGAAG
ENSRNOT00000001479 GCCAACCTGGGCATGATGGGAAAGGTGGTGTACATGCCTGCTCTTGCCGACATGCTGAAG

ENSPTRT00000034903 CACTGCAAGCGGCTGAGGGACCTCAGGCTGGAGCAGCCCTACTTCAGCGCCAACGCCCAG
ENSGGOT00000025986 CACTGCAAGCGGCTGAGGGACCTCAGGCTGGAGCAGCCCTACTTCAGCGCCAACGCCCAG
ENST00000382368 CACTGCAAGCGGCTGAGGGACCTCAGGCTGGAGCAGCCCTACTTCAGCGCCAACGCCCAG
ENSMMUT00000007125 CACTGCAAGCGGCTGAGGGACCTCAGGCTGGAGCAGCCCTACTTCAGCGCCAACGCCCAG
ENSPPYT00000020192 CACTGCAAGCGGCTGAGGGACCTCAGGCTGGAGCAGCCCTACTTCAGCGCCAACGCCCAG
ENSMUST00000035985 CACTGCAAGCGGCTGAAGGACCTCAGGTTGGAGCAGCCCTACTTCAACGCCAACGCTCAG
ENSRNOT00000001479 CACTGCAAGCGTCTGAAGGACCTGAGGCTGGAGCAGCCCTATTTCAACGCCAACGCACAG

ENSPTRT00000034903 TTCTTCCAGGCGCTGAGCCAGTGCCCCTCGCTGCAGCGCCTGTGCCTGGTCTCTCGCAGC
ENSGGOT00000025986 TTCTTCCAGGCGCTGAGCCAGTGCCCCTCGCTGCAGCGCCTGTGCCTGGTCTCTCGCAGC
ENST00000382368 TTCTTCCAGGCGCTGAGCCAGTGCCCCTCGCTGCAGCGCCTGTGCCTGGTCTCTCGCAGC
ENSMMUT00000007125 TTCTTCCAGGCGCTGAGCCAGTGCCCCTCGCTACAGCGCCTGTGCCTGGTCTCCCGCAGC
ENSPPYT00000020192 TTCTTCCAGGCGCTGAGCCAGTGCCCCTCGCTGCAGCGCCTGTGCCTGGTCTCTCGCAGT
ENSMUST00000035985 TTCTTCCAGGCACTGGGCCAGTGCTCGTCTCTGCAGCGCCTGTGCCTGGTATCTCGCAGC
ENSRNOT00000001479 TTCTTCCAGGCCCTGGGCCAGTGCTCCTCTCTGCAGCGCCTGTGCCTGGTGTCGCGCAGT

ENSPTRT00000034903 GGCACCCTCCAGCCCGATGCCGTGCTGGCTTTCATGGCTCGCTGCCTGCAGGTCGTCATG
ENSGGOT00000025986 GGCACCCTCCAGCCCGATGCTGTGCTGGCCTTCATGGCTCGCTGCCTGCAGGTCGTCATG
ENST00000382368 GGCACCCTCCAGCCCGATGCCGTGCTGGCCTTCATGGCTCGCTGCCTGCAGGTTGTCATG
ENSMMUT00000007125 GGCACCCTCCAGCCCGATGCCGTGCTGGCCTTCATGGCTCGCTGCCTACACGTCGTCATG
ENSPPYT00000020192 GGCACCCTCCAGCCTGATGCCGTTCTGGCCTTCATGACTCGCTGCCTGCACGTCGTCATG
ENSMUST00000035985 GGCACCCTGCAGCCAGATGCCGTGCTGGCCTTTATGGCCCGTTGCCTGCAGGTCGTCATG
ENSRNOT00000001479 GGCACTCTGCAGCCCGATGCCGTGTTGGCCTTTATGGCCCGTTGCCTGCAGGTCGTCATG

ENSPTRT00000034903 TGCCACCTGTTCACCGGGGAGTCCCTCGCCACCTGCAAGAGCCTGCAGCAGTCGCTTCTC
ENSGGOT00000025986 TGCCACCTGTTCACCGGGGAGTCCCTCGCCACCTGCAAGAGCCTGCAGCAGTCGCTTCTC
ENST00000382368 TGCCACCTGTTCACCGGGGAGTCCCTCGCCACCTGCAAGAGCCTGCAGCAGTCGCTTCTC
ENSMMUT00000007125 TGCCACCTGTTCACCGGGGAGTCCCTTGCCACCTGCAAGAGCCTGCAGCAGTCGCTTCTC
ENSPPYT00000020192 TGCCACCTGTTCACCGGGGAGTCCCTCGCCACCTGCAAGAGCCTGCAGCAGTCGCTTCTC
ENSMUST00000035985 TGTCACATGTTCACCGGAGAATCCCTTACCACCTGCAAGAGCCTGCAGCAGTCGCTCCTC
ENSRNOT00000001479 TGTCACATGTTCACCGGAGAATCCCTCACCACCTGCAAGAGCCTACAGCAGTCGCTCCTC

ENSPTRT00000034903 CGCAGCTTCCAGGCCGAGCGGCCCGCGTTAAACGTCGTCGTCTTCCCTCTGCTCCACGAG
ENSGGOT00000025986 CGCAGCTTCCAGGCCGAGCGGCCCGCGTTAAACGTCGTCATCTTCCCTCTGCTCCACGAG
ENST00000382368 CGCAGCTTCCAGGCCGAGCGGCCCGCGTTAAACGTCGTCATCTTCCCTCTGCTCCACGAG
ENSMMUT00000007125 CGCAGCTTCCAGGCCGAGCGGCCCGCATTGAACGTCGTCATCTTCCCTCTGCTCCACGAG
ENSPPYT00000020192 CGCAGCTTCCAGGCCGAGCGGCCCGCGTTAAACGTCATCATCTTCCCTCTGCTCCACGAG
ENSMUST00000035985 CGGAGTTTCCAGGCCGAGAGGCCCGCGTTGAACGTCGTCATCTTCCCCCTGCTCCACGAG
ENSRNOT00000001479 CGGAGTTTCCAGGCCGAGAGACCCGCGTTGAACGTCGTCATCTTCCCTCTGCTCCACGAG

ENSPTRT00000034903 GGCCTGACCGACGTCATCCGGGACGTCCCCCTGGTGCACCTGGACGAGATCACCTTATTT
ENSGGOT00000025986 GGCCTGACCGACGTCATCCGGGACGTCCCCCTGGTGCACCTGGATGAGATCACCTTATTT
ENST00000382368 GGCCTGACCGACGTCATCCGGGACGTCCCCCTGGTGCACCTGGATGAGATCACCTTATTT
ENSMMUT00000007125 GGCCTGACCGACGTCATCCGGGACGTCCCTCTGGTGCACCTGGATGAGATCACCTTATTT

```
ENSPPYT00000020192     GGCCTGACCGACGTCATCCGGGACGTCCCCCTGGTGCACCTGGATGAGATCACCTTATTT
ENSMUST00000035985     GGCCTGACAGATGTCATCCGAGATGTCCCCATGCTGCACCTGGATGAGATCACCTTGTTC
ENSRNOT00000001479     GGCCTGACAGATGTTATCCGAGATGTCCCCATGGTGCACCTGGACGAGATCACCTTATTC
```

```
ENSPTRT00000034903     AAGAGCAGAGTGGCCGAGGAACCGCCGAACCTGTGGTGG
ENSGGOT00000025986     AAGAGCAGAGTGGCTGAGGAACCGCCGAACCTGTGGTGG
ENST00000382368        AAGAGCAGAGTGGCCGAGGAACCGCCGAACCTGTGGTGG
ENSMMUT00000007125     AAGAGCAGAGTGGCCGAGGAACCGCCGAACCTGTGGTGG
ENSPPYT00000020192     AAGAGCAGAGTGGCCGAGGAACCGCCGAACCTGTGGTGG
ENSMUST00000035985     AAAAGCCGTGTGGCTGAGGAACCGCCCAACCTATGGTGG
ENSRNOT00000001479     AAAAGCAGAGTGGCCGAGGAACCCCCGAACCTGTGGTGG
```

Multiple sequence alignment of Fbxl19

```
ENSMUST00000033081     ATGTCGTCGAGCAGCCGGGGACCGGGGGCCGGAGCGCGCCGACGCCGAACCCGCTGCCGT
ENSRNOT00000049576     ATGTCGTCGAGCAGCCGGGGACCGGGGGCCGGAGCGCGCCGACGCCGAACCCGCTGCCGT
ENSCJAT00000005728     ATGGATGAAAAGGCGGTGGGG---GGTGGGGGAGAGAGAAGACGCCGAACCCGCTGCCGC
ENSGGOT00000003643     GCTGTGGATGAAAAGGTTGGTGGGGCGGGGGGGAGGAAAAGACGCCGAACCCGCTGCCGC
ENST00000562319        ATGTCGTCGAGCAGCCGGGGGCCGGGGGCCGGAGCGCGCCGACGCCGAACCCGCTGCCGC
ENSPPYT00000008581     ATGTCGTCGAGCAGCCGGGGGCCGGGGGCCGGAGCGCGCCGACGCCGAACCCGCTGCCGC
```

```
ENSMUST00000033081     CGTTGCCGGGCCTGTGTGCGAACTGAGTGCGGGGACTGCCATTTCTGCCGAGACATGAAG
ENSRNOT00000049576     CGTTGCCGGGCCTGTGTTCGAACTGAGTGCGGGGACTGCCATTTCTGCCGAGACATGAAG
ENSCJAT00000005728     CGCTGCCGGGCCTGTGTGCGAACTGAGTGCGGGGATTGCCACTTCTGCCGAGACATGAAG
ENSGGOT00000003643     CGCTGCCGGGCCTGTGTGCGAACTGAGTGCGGGGATTGCCACTTCTGCCGAGACATGAAG
ENST00000562319        CGCTGCCGGGCCTGTGTGCGAACTGAGTGCGGGGATTGCCACTTCTGCCGAGACATGAAG
ENSPPYT00000008581     CGCTGCCGGGCCTGTGTGCGAACTGAGTGCGGGGATTGCCACTTCTGCCGAGACATGAAG
```

```
ENSMUST00000033081     AAGTTCGGGGGGCCCGGGCGCATGAAGCAGTCGTGCCTGCTTCGGCAGTGCACCGCGCCC
ENSRNOT00000049576     AAGTTCGGGGGGCCCGGGCGCATGAAGCAGTCGTGCCTGCTTCGGCAGTGCACCGCGCCC
ENSCJAT00000005728     AAGTTCGGGGGGCCCGGGCGCATGAAGCAGTCGTGCCTGCTCCGCCAGTGCACTGCCCCT
ENSGGOT00000003643     AAGTTCGGGGGGCCCGGGCGCATGAAGCAGTCGTGCCTGCTCCGGCAGTGCACTGCCCCC
ENST00000562319        AAGTTCGGGGGGCCCGGGCGCATGAAGCAGTCGTGCCTGCTCCGGCAGTGCACTGCCCCC
ENSPPYT00000008581     AAGTTCGGGGGGCCCGGGCGCATGAAGCAGTCGTGCCTGCTCCGGCAGTGCACTGCCCCC
```

```
ENSMUST00000033081     GTGCTCCCACACACAGCTGTGTGCCTCTTGTGCGGGGAGGCTGGAAAGGAGGATACAGTG
ENSRNOT00000049576     GTGCTCCCACACACGGCTGTATGCCTCTTGTGTGGGGAAGCTGGGAAGGAGGATACAGTG
ENSCJAT00000005728     GTGCTCCCACACACAGCTGTGTGCCTCTTGTGTGGGGAGGCTGGGAAGGAGGACACGGTG
ENSGGOT00000003643     GTGCTCCCACACACAGCTGTGTGCCTCTTGTGTGGGGAGGCTGGGAAGGAGGACACGGTG
ENST00000562319        GTGCTCCCACACACAGCTGTGTGCCTCTTGTGTGGGGAGGCTGGGAAGGAGGACACGGTG
ENSPPYT00000008581     GTGCTCCCA---------------------------------------------------
```

```
ENSMUST00000033081     GAGGGAGAAGACGAGAAGTTTAGTTTGAGCCTCATGGAGTGTACAATCTGCAACGAGATC
ENSRNOT00000049576     GAGGGAGAAGAGGAGAAATTTAGTTTGAGCCTCATGGAGTGTACAATCTGCAACGAGATC
ENSCJAT00000005728     GAGGGAGAGGAAGAGAAATTTGGTTTGAGCCTCATGGAGTGTACAATCTGCAACGAGATC
ENSGGOT00000003643     GAGGGAGAGGAAGAGAAATTTGGTTTGAGCCTCATGGAGTGTACAATCTGCAACGAGATC
ENST00000562319        GAGGGAGAGGAAGAGAAATTTGGTTTGAGCCTCATGGAGTGTACAATCTGCAACGAGATC
ENSPPYT00000008581     ------------------------------------------------------------
```

```
ENSMUST00000033081     GTCCACCCTGGCTGCCTGAAGATGGGAAAGGCTGAGGGTGTTATCAATTCCGAGATACCC
ENSRNOT00000049576     GTCCACCCTGGCTGCCTGAAGATGGGAAAGGCTGAGGGTGTTATCAATTCAGAGATACCC
ENSCJAT00000005728     GTCCACCCCGGCTGCCTGAAGATGGGAAAGGCTGAGGGTGTCATCAATGCAGAGATCCCC
ENSGGOT00000003643     GTCCACCCCGGCTGCCTGAAGATGGGGAAGGCTGAGGGTGTCATCAATGCAGAGATCCCC
```

```
ENST00000562319         GTCCACCCCGGCTGCCTGAAGATGGGGAAGGCTGAGGGTGTCATCAATGCAGAGATCCCC
ENSPPYT00000008581      ---------------------ATGGGGAAGGCTGAGGGTGTCATCAATGCAGAGATCCCC

ENSMUST00000033081      AACTGCTGGGAGTGCCCCCGATGTACCCAGGAAGGACGGACAAGCAAGGATGCAGGTGAG
ENSRNOT00000049576      AACTGTTGGGAGTGCCCCCGATGTACCCAGGAAGGACGGACAAGCAAGGATGCAGGTGAG
ENSCJAT00000005728      AACTGCTGGGAGTGTCCTCGCTGCACCCAGGAAGGCCGCACCAGCAAGGATTCAGGTGAG
ENSGGOT00000003643      AACTGCTGGGAGTGCCCTCGCTGCACCCAGGAAGGCCGTACCAGCAAGGATTCAGGTGAG
ENST00000562319         AACTGCTGGGAGTGCCCTCGCTGCACCCAGGAAGGCCGCACCAGCAAGGATTCAGGTGAG
ENSPPYT00000008581      AACTGCTGGGAGTGCCCTCGCTGCACCCAGGAAGGCCGCACCAGCAAGGATTCAGGTGAG

ENSMUST00000033081      GGTCCAGGGCGCCGCAGGGCTGACAATGGTGAGGAAGGCGCTAACCTAGGGGGTGGATGG
ENSRNOT00000049576      GGTCCAGGGCGCCGCAGGGCTGACAATGGTGAGGAAGGCGCTAACCTAGGGGGTGGATGG
ENSCJAT00000005728      GGGCCTGGCCGCCGCAGGTCCGACAACGGCGAGGAAGGCGCCAGCCTGGGGAGCGGATGG
ENSGGOT00000003643      GGGCCTGGCCGCCGTAGGGCCGACAACGGCGAGGAGGGCGCCAGCTTGGGGAGCGGATGG
ENST00000562319         GGGCCTGGCCGCCGTAGGGCCGACAACGGCGAGGAGGGCGCCAGCTTGGGGAGCGGATGG
ENSPPYT00000008581      GGGCCTGGCCGCCGTAGGGCCGACAACGGTGAGGAGGGCGCCAGCTTGGGGAGCGGATGG

ENSMUST00000033081      AAGCTGACAGAGGAACCACCACCTCCACCACCCCTGCCCAGAAGAAAGGGACCCTTACCT
ENSRNOT00000049576      AAGCTGACAGAGGAGCCGCCACCTCCACCACCCCTGCCCAGAAGAAAGGGACCCTTACCT
ENSCJAT00000005728      AAGCTGACGGAGGAGCCGCCGCTTCCACCGCCCCCGCCCAGGCGCAAGGGCCCCCTGCCT
ENSGGOT00000003643      AAGCTGACAGAGGAGCCACCGCTTCCACCGCCCCCGCCCAGGCGCAAGGGCCCCCTGCCT
ENST00000562319         AAGCTGACAGAGGAGCCACCGCTTCCACCGCCCCCGCCCAGGCGCAAGGGCCCCCTGCCT
ENSPPYT00000008581      AAGCTGACAGAGGAGCCACCACTTCCACCGCCCCCGCCCAGGCGCAAGGGCCCCCTGCCT

ENSMUST00000033081      GCTGGCCCTACCCCTGACGATGTGCCTGGGCCCCCCAAACGGAAGGAGAGGGAGGGGGGG
ENSRNOT00000049576      GCTGGCCCTCCCCCTGACGATGTGCCTGGGCCCCCCAAACGGAAGGAGAGGGAGGGGGGG
ENSCJAT00000005728      GCCGGACCCCCAGCAGAGGACCTGCCTGGGCCCCCCAAACGAAAGGAAAGGGAGGCAGGG
ENSGGOT00000003643      GCCGGGCCCCCCCCGGAGGACGTGCCTGGGCCCCCCAAACGAAAGGAAAGGGAGGCAGGG
ENST00000562319         GCCGGGCCCCCCCCGGAGGACGTGCCTGGGCCCCCCAAACGAAAGGAAAGGGAGGCAGGG
ENSPPYT00000008581      GCCGGGCCCCCCCCAGAGGACGTGCCTGGGCCGCCCAAACGAAAGGAAAGGGAGGCAGGG

ENSMUST00000033081      AATGAGCCCCCCACCCCAAGGAAAAAGGTGAAAGGAGGCCGAGAGAGGCACTTGAAGAAG
ENSRNOT00000049576      AATGAGCCCCCCACCCCAAGGAAAAAGGTGAAAGGAGGCCGAGAGAGGCACTTGAAGAAG
ENSCJAT00000005728      AATGAGCCCCCCACCCCAAGGAAAAAGGTGAAAGGAGGCCGAGAGAGGCACCTGAAGAAG
ENSGGOT00000003643      AATGAGCCTCCCACCCCAAGGAAAAAGGTGAAAGGAGGCCGAGAGAGGCACCTGAAGAAG
ENST00000562319         AATGAGCCTCCCACCCCAAGGAAAAAGGTGAAAGGAGGCCGAGAGAGGCACCTGAAGAAG
ENSPPYT00000008581      AATGAGCCTCCCACCCCAAGGAAAAAGGTGAAAGGAGGCCGAGAGAGGCACCTGAAGAAG

ENSMUST00000033081      GTGGGTGGAGATGCCTGCCTCCTCCGAGGAGCGGACCCAGGCAGCCCCGGCCTTCTGCCC
ENSRNOT00000049576      GTGGGTGGAGATGCCTGCCTCCTCCGAGGAGCGGACCCAGGCAGCCCGGGCCTTCTGCCC
ENSCJAT00000005728      GTGGGTGGAGACGCCTGCCTCCTCCGAGGATCGGACCCAGGCGGCCCGGGCCTGCTGCCC
ENSGGOT00000003643      GTGGGTGGAGACGCCTGCCTCCTCCGAGGATCGGACCCAGGCGGCCCGGGCCTGCTGCCC
ENST00000562319         GTGGGTGGAGACGCCTGCCTCCTCCGAGGATCGGACCCAGGCGGCCCGGGCCTGCTGCCC
ENSPPYT00000008581      GTGGGTGGAGACGCCTGCCTCCTCCGAGGATCGGACCCAGGCGGCCCGGGCCTGCTGCCC

ENSMUST00000033081      CCCAGGGTTCTGAATCCGAGCCAGGCATTCTCATCCTGCCACCCTGGGCTCCCTCCCGAG
ENSRNOT00000049576      CCCAGGGTTCTGAATCCGAGCCAGGCATTCTCATCCTGCCACCCTGGGCTCCCTCCCGAG
ENSCJAT00000005728      CCCAGGGTCCTGAATCCAAGCCAGGCTTTCTCGTCCTGCCACCCTGGGCTCCCTCCCGAG
ENSGGOT00000003643      CCCAGGGTTCTGAATCCGAGCCAGGCTTTCTCATCCTGCCACCCTGGGCTCCCTCCCGAG
ENST00000562319         CCCAGGGTTCTGAATCCGAGCCAGGCTTTCTCATCCTGCCACCCTGGGCTCCCTCCCGAG
ENSPPYT00000008581      CCCAGGGTTCTGAATCCGAGCCAGGCTTTCTCATCCTGCCACCCTGGGCTCCCTCCCGAG
```

```
ENSMUST00000033081     AACTGGGAGAAACCAAAGCCACCTATAGCTTCTGCTGAGGGCCCGGCAGTGCCATCTCCA
ENSRNOT00000049576     AACTGGGAGAAACCAAAGCCACCTATAGCTTCTGCTGAGGGCCCAGCAGTGCCATCCCCA
ENSCJAT00000005728     AACTGGGAGAAACCAAAGCCACCTTTGGCCTCTGCAGAGGGCCCAGTGGTGCCATCCCCA
ENSGGOT00000003643     AACTGGGAGAAACCAAAGCCGCCTTTGGCCTCTGCAGAGGGCCCAGCGGTGCCGTCCCCG
ENST00000562319        AACTGGGAGAAACCAAAGCCGCCTTTGGCCTCTGCAGAGGGCCCAGCGGTGCCGTCCCCG
ENSPPYT00000008581     AACTGGGAGAAACCAAAGCCGCCTTTGGCCTCTGCAGAGGGCCCAGCGGTGCCGTCCCCA

ENSMUST00000033081     TCACCACAGAGGGAGAAACTGGAGCGTTTCAAGCGCATGTGCCAGCTGCTGGAGCGGGTG
ENSRNOT00000049576     TCGCCACAGAGGGAGAAGCTGGAGCGTTTCAAGCGTATGTGCCAGCTGCTGGAACGGGTG
ENSCJAT00000005728     TCCCCACAGAGGGAGAAACTAGAGCGTTTCAAGCGCATGTGCCAGCTGCTGGAACGGGTA
ENSGGOT00000003643     TCCCCGCAGAGGGAGAAGCTAGAGCGTTTCAAGCGGATGTGCCAGCTGCTGGAACGGGTG
ENST00000562319        TCCCCGCAGAGGGAGAAGCTAGAGCGTTTCAAGCGGATGTGCCAGCTGCTGGAACGGGTG
ENSPPYT00000008581     TCCCCGCAGAGGGAGAAGCTAGAGCGTTTCAAGCGGATGTGCCAGCTGCTGGAACGGGTG

ENSMUST00000033081     CCCGATACCTCTTCTTCCTCCTCGGACTCGGACTCGGATTCAGACTCATCCGGCACATCA
ENSRNOT00000049576     CCTGATACCTCTTCTTCGTCTTCGGACTCAGACTCTGATTCAGACTCATCTGGCACATCA
ENSCJAT00000005728     CCTGACACGTCCTCTTCCTCCTCGGACTCAGACTCCGACTCTGACTCTTCGGGCACATCG
ENSGGOT00000003643     CCTGACACCTCCTCTTCCTCCTCGGACTCAGACTCCGACTCCGACTCTTCCGGCACATCG
ENST00000562319        CCTGACACCTCCTCTTCCTCCTCGGACTCAGACTCCGACTCCGACTCTTCGGGCACATCG
ENSPPYT00000008581     CCTGACACCTCCTCTTCCTCCTCGGACTCAGACTCCGACTCCGACTCTTCGGGCACATCG

ENSMUST00000033081     CTCAGTGAGGATGAAGCTCCTGGAGAGGCCCGGAATGGGCGGCGGCCAGCACGGGGCAGC
ENSRNOT00000049576     CTTAGTGAGGATGAAGCTCCCGGGGAGGCCCGGAATGGGCGGCGGCCAGCGAGGGGCAGC
ENSCJAT00000005728     CTGAGTGAGGACGAAGCCCCTGGCGAGGCCCGAAATGGGCGACGGCCAGCCCGGGGCAGC
ENSGGOT00000003643     CTGAGTGAGGACGAAGCCCCCGGCGAGGCCCGGAATGGGCGACGGCCAGCCCGGGGCAGC
ENST00000562319        CTGAGTGAGGACGAAGCCCCCGGCGAGGCCCGGAATGGGCGACGGCCAGCCCGGGGCAGC
ENSPPYT00000008581     CTGAGTGAGGACGAAGCCCCCGGCGAGGCCCGGAATGGGCGACGGCCAACCCGGGGCAGC

ENSMUST00000033081     TCCGGCGAGAAGGAGAACCGTGGGGGACGGCGGGCTATACGCCCTGGCACTGGGGGGCCA
ENSRNOT00000049576     TCTGGCGAGAAGGAGAACCGTGGGGGACGGCGGGCTATACGCCCTGGCAGTGGAGGACCA
ENSCJAT00000005728     TCTGGCGAGAAGGAGAACCGTGGGGGGCGGCGGGCTGTGCGTCCTGGCAGTGGGGGGCCC
ENSGGOT00000003643     TCTGGCGAGAAGGAGAACCGTGGGGGGCGGCGGGCTGTGCGCCCTGGCAGTGGGGGGCCC
ENST00000562319        TCTGGCGAGAAGGAGAACCGTGGGGGGCGGCGGGCTGTGCGCCCTGGCAGTGGGGGGCCC
ENSPPYT00000008581     TCTGGCGAGAAGGAGAACCGTGGGGGGCGGCGGGCTGTGCGCCCTGGCAGCGGGGGGCCC

ENSMUST00000033081     CTGCTCAGCTGGCCCCTGGGCCCTGCCCCACCACCACGGCCTCCACAGTTGGAGCGACAT
ENSRNOT00000049576     CTGCTCAGCTGGCCTCTGGGCCCTGCCCCACCACCACGGCCTCCACAGTTGGAGCGGCAT
ENSCJAT00000005728     CTGCTCAGCTGGCCCCTGGGCCCCGCGCCACCACCCCGGCCTCCACAGCTGGAGCGGCAC
ENSGGOT00000003643     CTGCTCAGCTGGCCCCTGGGCCCAGCCCCACCACCCCGGCCTCCACAGCTGGAGCGGCAT
ENST00000562319        CTACTCAGCTGGCCCCTGGGCCCAGCCCCACCACCCCGGCCTCCACAGCTGGAGCGGCAC
ENSPPYT00000008581     CTGCTCAGCTGGCCCCTGGGCCCAGCCCCACCACCCCGGCCTCCACAGCTGGAGCGGCAT

ENSMUST00000033081     GTCGTGCGGCCCCCGCCTCGAAGCCCTGAGCCTGATACTCTGCCTTTGGCTGCTGGATCC
ENSRNOT00000049576     GTAGTAAGGCCCCCACCTCGAAGCCCCGAGCCTGACACTCTGCCTTTGGCTGCTGGATCC
ENSCJAT00000005728     GTGGTGCGGCCCCCACCTCGAAGCCCTGAGCCTGACACACTCCCCTTGGCTGCTGGATCC
ENSGGOT00000003643     GTGGTGCGGCCCCCGCCTCGAAGCCCTGAGCCCGACACACTCCCCTTGGCTGCTGGATCC
ENST00000562319        GTGGTGCGGCCCCCGCCTCGAAGCCCTGAGCCCGACACACTCCCCTTGGCTGCTGGATCC
ENSPPYT00000008581     GTGGTGCGGCCCCCGCCTCGAAGCCCTGAGCCCGACACACTCCCCTTGGCTGCTGGATCC

ENSMUST00000033081     GACCACCCTCTGCCTCGGGCTGCCTGGCTTCGTGTTTTCCAGCACCTTGGACCCCGTGAG
ENSRNOT00000049576     GACCACCCTCTTCCTCGGGCTGCCTGGCTTCGTGTCTTCCAGCACCTTGGACCCCGGGAG
ENSCJAT00000005728     GACCACCCCCTGCCCCGGGCTGCCTGGCTTCGCGTCTTCCAGCACCTCGGGCCCCGGGAG
ENSGGOT00000003643     GACCACCCCCTGCCCCGGGCCGCCTGGCTTCGCGTCTTCCAGCATCTCGGGCCGCGGGAG
```

```
ENST00000562319         GACCACCCCCTGCCCCGGGCCGCCTGGCTTCGCGTCTTCCAGCACCTCGGGCCGCGGGAG
ENSPPYT00000008581      GACCACCCCCTGCCCCGGGCCGCCTGGCTTCGCGTCTTCCAGCACCTCGGGCCGCGGGAG

ENSMUST00000033081      CTATGTGTCTGCATGAGAGTTTGCCGGACTTGGAGCCGCTGGTGCTACGACAAGCGTCTG
ENSRNOT00000049576      CTATGTGTCTGCATGAGAGTCTGCCGGACTTGGAGCCGCTGGTGCTATGACAAGCGTCTG
ENSCJAT00000005728      CTGTGTGTCTGCATGCGAGTCTGCCGGACTTGGAGCCGCTGGTGCTATGACAAGCGTCTG
ENSGGOT00000003643      CTGTGTATCTGCATGCGAGTCTGCCGAACTTGGAGCCGCTGGTGCTATGACAAGCGTCTG
ENST00000562319         CTGTGTATCTGCATGCGAGTCTGCCGAACTTGGAGCCGCTGGTGCTATGACAAGCGTCTG
ENSPPYT00000008581      CTGTGTATCTGCATGCGAGTCTGCCGAACTTGGAGCCGCTGGTGCTATGACAAGCGTCTG

ENSMUST00000033081      TGGCCTAGGATGGACCTAAGCCGGAGAAAGTCACTGACCCCACCCATGCTTAGTGGGGTG
ENSRNOT00000049576      TGGCCTAGGATGGACCTAAGCCGGAGGAAGTCACTGACCCCACCCATGCTTAGTGGGGTG
ENSCJAT00000005728      TGGCCTCGAATGGACCTGAGCCGGCGGAAGTCACTGACTCCACCCATGCTCAGCGGTGTG
ENSGGOT00000003643      TGGCCTCGAATGGACCTTAGCCGGCGGAAGTCACTGACCCCGCCCATGCTCAGTGGTGTG
ENST00000562319         TGGCCTCGAATGGACCTGAGCCGGCGGAAGTCACTGACCCCGCCCATGCTCAGTGGTGTG
ENSPPYT00000008581      TGGCCTCGAATGGACCTGAGCCGGCGGAAGTCACTGACCCCGCCCATGCTCAGTGGTGTG

ENSMUST00000033081      GTTCGCCGCCAGCCCCGTGCTCTGGACCTCAGCTGGACAGGTGTCTCCAAAAAGCAGCTC
ENSRNOT00000049576      GTTCGCCGCCAGCCACGTGCTCTGGACCTCAGCTGGACAGGTGTCTCCAAAAAGCAGCTC
ENSCJAT00000005728      GTTCGCCGCCAGCCCCGTGCCCTGGACCTCAGCTGGACAGGTGTCTCCAAGAAGCAGCTC
ENSGGOT00000003643      GTTCGCCGCCAGCCCCGTGCCCTGGACCTCAGCTGGACAGGTGTCTCCAAGAAGCAGCTC
ENST00000562319         GTTCGCCGCCAGCCCCGTGCCCTGGACCTCAGCTGGACAGGTGTCTCCAAGAAGCAGCTC
ENSPPYT00000008581      GTTCGCCGCCAGCCCCGTGCCCTGGACCTCAGCTGGACAGGTGTCTCCAAGAAGCAGCTC

ENSMUST00000033081      ATGTGGCTCCTGAACCGACTGCAAGGCCTGCAGGAGTTGGTGCTTTCTGGGTGCTCCTGG
ENSRNOT00000049576      ATGTGGCTCCTGAACCGACTGCAAGGCCTGCAGGAGTTGGTGCTTTCTGGGTGCTCCTGG
ENSCJAT00000005728      ATGTGGCTTCTGAACCGACTACAAGGCCTGCAGGAGCTGGTGCTCTCTGGCTGCTCCTGG
ENSGGOT00000003643      ATGTGGCTTCTGAACCGACTACAAGGCCTGCAGGAGCTGGTGCTCTCTGGCTGCTCCTGG
ENST00000562319         ATGTGGCTTCTGAACCGACTACAAGGCCTGCAGGAGCTGGTGCTCTCTGGCTGCTCCTGG
ENSPPYT00000008581      ATGTGGCTTCTGAACCGACTACAAGGCCTGCAGGAGCTGGTGCTCTCTGGCTGCTCCTGG

ENSMUST00000033081      CTCTCTGTCTCTGCCCTGGGCTCGGCCCCACTGCCAGCGCTGCGGCTCTTGGACCTCCGC
ENSRNOT00000049576      CTGTCCGTCTCTGCCCTGGGCTCAGCCCCACTGCCCGCTCTGCGGCTCTTGGACCTCCGC
ENSCJAT00000005728      CTCTCCGTCTCTGCCCTGGGCTCAGCCCCACTGCCAGCCTTGCGACTCCTGGACCTCCGC
ENSGGOT00000003643      CTCTCTGTCTCTGCCCTGGGCTCAGCCCCACTGCCAGCCCTGCGGCTCCTGGACCTCCGC
ENST00000562319         CTCTCTGTCTCTGCCCTGGGCTCAGCCCCACTGCCAGCCCTGCGGCTCCTGGACCTCCGC
ENSPPYT00000008581      CTCTCTGTCTCTGCCCTGGGCTCAGCCCCACTGCCAGCCCTGCGGCTCCTGGACCTCCGC

ENSMUST00000033081      TGGATTGAAGATGTTAAAGACTCCCAGCTTCGGGAGCTGCTGCTGCCTCCACCAGACACC
ENSRNOT00000049576      TGGATTGAAGACGTTAAAGACTCCCAGCTTCGGGAGCTGCTGCTGCCTCCACCAGACACC
ENSCJAT00000005728      TGGATCGAGGATGTTAAAGACTCCCAGCTCCGGGAGCTGCTGCTGCCTCCACCAGACACC
ENSGGOT00000003643      TGGATCGAGGATGTTAAAGACTCCCAGCTCCGGGAGTTGCTGCTGCCTCCACCAGACACC
ENST00000562319         TGGATCGAGGATGTTAAAGACTCCCAGCTCCGGGAGTTGCTGCTGCCTCCACCAGACACC
ENSPPYT00000008581      TGGATCGAGGATGTTAAAGACTCCCAGCTCCGGGAGCTGCTGCTGCCTCCACCAGACACC

ENSMUST00000033081      AAACCAGGGCAAACTGAGAGTCGAGGCCGACTGCAGGGGGTGGCAGAACTTCGCCTAGCT
ENSRNOT00000049576      AAACCAGGGCAAACAGAGAGTCGAGGTCGACTGCAGGGGGTGGCAGAACTTCGCCTAGCA
ENSCJAT00000005728      AAACCAGGGCAAACAGAGAGCCGTGGGCGGCTACAGGGGGTGGCAGAACTGCGCCTGGCA
ENSGGOT00000003643      AAACCAGGGCAAACAGAGAGCCGTGGTCGGCTGCAGGGGGTGGCAGAACTGCGTCTGGCA
ENST00000562319         AAACCAGGGCAAACAGAGAGCCGTGGTCGGCTGCAGGGGGTGGCAGAACTGCGTCTGGCA
ENSPPYT00000008581      AAACCAGGGCAAACAGAGAGCCGTGGTCGGCTGCAGGGGGTGGCAGAACTTCGCCTGGCA
```

```
ENSMUST00000033081     GGTCTGGAGCTCACAGATGCCTCCCTGAGGCTCCTGTTGCGCCACGCTCCCCAGCTCAGT
ENSRNOT00000049576     GGCCTGGAGCTCACTGATGCCTCCCTGAGGCTCCTATTGCGCCACGCTCCCCAACTCAGT
ENSCJAT00000005728     GGCCTGGAGCTGACAGACGCCTCCCTTCGGCTCCTGCTGCGCCATGCACCCCAGCTGAGC
ENSGGOT00000003643     GGTTTGGAGCTGACAGATGCCTCCCTGCGGCTCCTGCTGCGTCACGCACCCCAGCTGAGC
ENST00000562319        GGTTTGGAGCTGACAGATGCCTCCCTGCGTCTCCTGCTGCGTCACGCACCCCAGCTGAGC
ENSPPYT00000008581     GGTCTGGAGCTGACAGATGCCTCCCTGCGGCTCCTGTTGCGCCACGCACCCCAGCTGAGC

ENSMUST00000033081     GCCCTGGACCTGAGTCACTGCGCCCACGTTGGGGACCCCAGTGTCCACCTCCTCACGGCT
ENSRNOT00000049576     GCCCTGGATCTGAGTCACTGCGCCCACGTGGGGGACCCCAGTGTTCACCTCCTCACGGCT
ENSCJAT00000005728     GCCCTGGACCTGAGCCACTGCGCCCACGTCGGGGACCCCAGTGTTCACCTCCTCACGGCC
ENSGGOT00000003643     GCCCTGGACCTGAGCCACTGCGCCCACGTCGGGGACCCCAGTGTTCACCTCCTCACGGCC
ENST00000562319        GCCCTGGACCTGAGCCACTGCGCCCACGTCGGGGACCCCAGTGTTCACCTCCTCACGGCC
ENSPPYT00000008581     GCCCTGGACCTGAGCCACTGCGCCCACGTCGGGGACCCCAGTGTTCACCTCCTCACGGCC

ENSMUST00000033081     CCTACTTCCCCTCTTCGAGAAACACTGGTGCACCTCAATCTCGCTGGGTGCCACCGCCTC
ENSRNOT00000049576     CCTACTTCCCCTCTCCGAGAAACACTGGTGCACCTCAATCTCGCTGGGTGCCACCGCCTC
ENSCJAT00000005728     CCCACATCCCCACTCCGCGAGACCCTGGTGCACCTCAATCTTGCTGGTTGCCACCGCCTA
ENSGGOT00000003643     CCCACGTCCCCACTCCGCGAGACCCTGGTGCACCTCAATCTTGCTGGTAAGCAC------
ENST00000562319        CCCACGTCCCCACTCCGCGAGACCCTGGTGCACCTCAATCTTGCTGGTTGCCACCGCCTA
ENSPPYT00000008581     CCCACGTCCCCACTCCGCGAGACCCTGGTGCACCTCAATCTTGCTGGTTGCCATCGCCTA

ENSMUST00000033081     ACGGACCACTGCCTTCCACTGTTCCGTCGATGCCCACGTCTTCGCCGCCTGGACCTACGC
ENSRNOT00000049576     ACGGACCACTGCCTTCCACTGTTCCGTCGATGCCCACGTCTTCGCCGCCTGGACCTACGC
ENSCJAT00000005728     ACGGACCACTGCCTCCCGCTGTTCCGCCGCTGCCCACGTCTACGCCGCCTAGACTTGCGC
ENSGGOT00000003643     ------------------------------------------------------------
ENST00000562319        ACGGACCACTGCCTCCCGCTGTTCCGCCGCTGCCCTCGTCTACGCCGCCTAGACCTGCGC
ENSPPYT00000008581     ACGGACCACTGCCTCCCGCTGTTCCGCCGCTGCCCTCGTCTACGCCGCCTAGACCTGCGC

ENSMUST00000033081     TCCTGCCGCCAGCTCTCACCTGAAGCTTGTGCCCGGTTGGCGGCTGCTGGGCCCCCTGGC
ENSRNOT00000049576     TCCTGCCGCCAGCTCTCACCTGAAGCTTGTGCCCGGTTGGCGGCGGCCGGGCCCCCTGGC
ENSCJAT00000005728     TCCTGCCGCCAGCTCTCACCTGAAGCTTGTGCCCGGCTGGCGGCCGCCGGGCCCCCTGGC
ENSGGOT00000003643     ------------------------------------------------------------
ENST00000562319        TCCTGCCGCCAGCTCTCACCCGAAGCTTGTGCCCGGCTGGCAGCTGCCGGGCCCCCTGGC
ENSPPYT00000008581     TCCTGCCGCCAGCTCTCACCCGAAGCTTGTGCCCGGCTGGCAGCTGCCGGGCCCCCTGGC

ENSMUST00000033081     CCCTTTCGCTGCCCAGAAGAGAAACTGCTTCTCAAGGACAGC
ENSRNOT00000049576     CCCTTTCGCTGCCCAGAAGAGAAACTACTTCTCAAGGACAGC
ENSCJAT00000005728     CCTTTCCGCTGCCCTGAGGAGAAGCTGCTTCTCAAGGACAGC
ENSGGOT00000003643     ------------------------------------------
ENST00000562319        CCCTTCCGCTGCCCTGAGGAGAAGCTGCTTCTCAAGGACAGC
ENSPPYT00000008581     CCCTTCCGCTGCCCTGAGGAGAAGCTGCTTCTCAAGGACAGC
```

Multiple sequence alignment of Fbxl2

```
ENSCJAT00000023329     ---------------------------------------------------------CTC
ENSGGOT00000002541     ATGGTTTTCTCAAACAATGATGAAGGCCTTATTAACAAAAAGTTACCCAAAGAACTTCTG
ENST00000484457        ATGGTTTTCTCAAACAATGATGAAGGCCTTATTAACAAAAAGTTACCCAAAGAACTTCTG
ENSPTRT00000027553     ATGGTTTTCTCAAACAATGATGAAGGCCTTATTAACAAAAAGTTACCCAAAGAACTTCTG
ENSPPYT00000016320     ATGGTTTTCTCAAACAATGATGAAGGCCTTATTAACAAAAAGTTACCCAAAGAACTTCTG
ENSMUST00000035090     ATGGTTTTCTCAAACAGTGATGATGGCCTTATCAACAAGAAGCTACCCAAGGAGCTCCTC
ENSRNOT00000033126     ---GTTTTCTCAAACAATGATGACGGCCTTATCAACAAGAAGTTACCCAAGGAGCTCCTC

ENSCJAT00000023329     TATAGAATATTTTCCTTCTTGGATATAGTAACTTTGTGCCGATGTGCACAGATTTCCAAG
```

```
ENSGGOT00000002541     TTAAGAATATTTTCCTTCTTGGATATAGTAACTTTGTGCCGATGTGCACAGATTTCCAAG
ENST00000484457        TTAAGAATATTTTCCTTCTTGGATATAGTAACTTTGTGCCGATGTGCACAGATTTCCAAG
ENSPTRT00000027553     TTAAGAATATTTTCCTTCTTGGATATAGTAACTTTGTGCCGATGTGCACAGATTTCCAAG
ENSPPYT00000016320     TTAAGAATATTTTCCTTCTTGGATATAGTAACTTTGTGCCGATGTGCACAGATTTCCAAG
ENSMUST00000035090     TTGAGAATATTCTCCTTCTTGGACATCGTAACTCTATGCCGATGTGCACAGATCTCCAAG
ENSRNOT00000033126     CTAAGAATATTCTCCTTCTTGGATATCGTAACTCTATGTCGATGTGCACAGATTTCCAAG

ENSCJAT00000023329     GCTTGGAACATCTTAGCCCTGGATGGAAGCAACTGGCAAAGAATAGATCTTTTTAACTTT
ENSGGOT00000002541     GCTTGGAACATCTTAGCCCTGGATGGAAGCAACTGGCAAAGAATAGATCTTTTTAACTTT
ENST00000484457        GCTTGGAACATCTTAGCCCTGGATGGAAGCAACTGGCAAAGAATAGATCTTTTTAACTTT
ENSPTRT00000027553     GCTTGGAACATCTTAGCCCTGGATGGAAGCAACTGGCAAAGAATAGATCTTTTTAACTTT
ENSPPYT00000016320     GCTTGGAACATCTTAGCCCTGGATGGAAGCAACTGGCAAAGAATAGATCTTTTTAACTTT
ENSMUST00000035090     GCCTGGAACATCTTAGCCCTGGATGGCAGCAACTGGCAACGGGTGGATCTTTTTAACTTC
ENSRNOT00000033126     GCCTGGAACATCTTAGCCCTGGATGGTAGCAACTGGCAGCGAGTGGATCTTTTTAACTTT

ENSCJAT00000023329     CAAACAGATGTAGAGGGTCGAGTGGTGGAAAATATCTCGAAGCGATGTGGTGGATTCCTG
ENSGGOT00000002541     CAAACAGATGTAGAGGGTCGAGTGGTGGAAAATATCTCGAAGCGATGCGGTGGGTTCCTG
ENST00000484457        CAAACAGATGTAGAGGGTCGAGTGGTGGAAAATATCTCGAAGCGATGCGGTGGATTCCTG
ENSPTRT00000027553     CAAACAGATGTAGAGGGTCGAGTGGTGGAAAATATCTCGAAGCGATGCGGTGGATTCCTG
ENSPPYT00000016320     CAAACAGATGTAGAGGGTCGAGTGGTGGAAAATATCTCGAAGCGATGCGGTGGATTCCTG
ENSMUST00000035090     CAGACAGATGTAGAGGGCCGAGTGGTGGAAAACATCTCCAAGAGGTGCGGTGGCTTCCTT
ENSRNOT00000033126     CAAACAGATGTAGAGGGCCGAGTGGTGGAAAACATCTCCAAGAGGTGCGGGGGTTTCCTT

ENSCJAT00000023329     CGGAAGCTCAGCTTGCGAGGCTGCATTGGTGTCGGGGATTCCTCCTTGAAGACCTTTGCA
ENSGGOT00000002541     AGGAAGCTCAGCTTGCGAGGCTGCATTGGTGTTGGGGATTCCTCCTTGAAGACCTTTGCA
ENST00000484457        AGGAAGCTCAGCTTGCGAGGCTGCATTGGTGTTGGGGATTCCTCCTTGAAGACCTTTGCA
ENSPTRT00000027553     AGGAAGCTCAGCTTGCGAGGCTGCATTGGTGTTGGGGATTCCTCCTTGAAGACCTTTGCA
ENSPPYT00000016320     AGGAAGCTCAGCTTGCGAGGCTGCATTGGTGTTGGGGATTCCTCCTTGAAGACCTTTGCA
ENSMUST00000035090     AGAAAGCTCAGCCTGCGTGGCTGCATCGGAGTCGGGGACTCCTCTTTGAAGACCTTTGCA
ENSRNOT00000033126     AGAAAGCTCAGCTTGCGTGGCTGCATCGGCGTCGGGGACTCCTCCTTGAAGACCTTTGCA

ENSCJAT00000023329     CAGAACTGCCGAAACATTGAACATTTAAACCTCAATGGATGCACAAAAATCACTGACAGC
ENSGGOT00000002541     CAGAACTGCCGAAACATTGAACATTTGAACCTCAATGGATGCACAAAAATCACTGACAGC
ENST00000484457        CAGAACTGCCGAAACATTGAACATTTGAACCTCAATGGATGCACAAAAATCACTGACAGC
ENSPTRT00000027553     CAGAACTGCCGAAACATTGAACATTTGAACCTCAATGGATGCACAAAAATCACTGACAGC
ENSPPYT00000016320     CAGAATTGCCGAAACATTGAACATTTGAACCTCAATGGATGCACAAAAATCACTGACAGC
ENSMUST00000035090     CAGAACTGCCGGAACATTGAACATTTAAACCTCAATGGCTGCACGAAAATCACTGACAGC
ENSRNOT00000033126     CAGAACTGCCGGAACATTGAACACTTAAACCTCAATGGCTGCACAAAAATCACTGACAGC

ENSCJAT00000023329     ACGTGTTATAGCCTTAGCAGATTCTGTTCCAAGCTGAAGCATCTGGATCTGACCTCCTGT
ENSGGOT00000002541     ACGTGTTATAGCCTTAGCAGATTCTGTTCCAAGCTGAAACATCTGGATCTGACCTCCTGT
ENST00000484457        ACGTGTTATAGCCTTAGCAGATTCTGTTCCAAGCTGAAACATCTGGATCTGACCTCCTGT
ENSPTRT00000027553     ACGTGTTATAGCCTTAGCAGATTCTGTTCCAAGCTGAAACATCTGGATCTGACCTCCTGT
ENSPPYT00000016320     ACGTGTTATAGCCTTAGCAGATTCTGTTCCAAGCTGAAACATCTGGATCTGACCTCCTGT
ENSMUST00000035090     ACGTGTTACAGCCTTAGCAGATTCTGTTCCAAGCTGAAACACCTGGATCTCACGTCCTGC
ENSRNOT00000033126     ACGTGTTACAGCCTTAGCCGATTCTGTTCCAAGCTGAAACATCTGGATCTCACGTCCTGC

ENSCJAT00000023329     GTGTCTATTACAAACAGCTCCTTGAAGGGGATCAGTGAGGGCTGCCGAAACCTGGAATAC
ENSGGOT00000002541     GTGTCTATTACAAACAGCTCCTTGAAGGGGATCAGTGAGGGCTGCCGAAACCTGGAGTAC
ENST00000484457        GTGTCTATTACAAACAGCTCCTTGAAGGGGATCAGTGAGGGCTGCCGAAACCTGGAGTAC
ENSPTRT00000027553     GTGTCTATTACAAACAGCTCCTTGAAGGGGATCAGTGAGGGCTGCCGAAACCTGGAGTAC
ENSPPYT00000016320     GTGTCTATTACAAACAGCTCCTTGAAGGGGATCAGTGAGGGCTGCCGAAACCTGGAGTAC
ENSMUST00000035090     GTGTCTGTTACCAACAGCTCTTTAAAGGGCATCAGCGAGGGCTGCCGGAACCTGGAATAT
ENSRNOT00000033126     GTGTCTGTCACCAACAGCTCCTTAAAGGGCATCAGCGAGGGCTGCCGGAACCTGGAGTAT
```

```
ENSCJAT00000023329     CTGAACCTCTCTTGGTGTGACCAGATCACAAAGGATGGCATCGAGGCACTGGTGCGAGGT
ENSGGOT00000002541     CTGAACCTCTCTTGGTGTGATCAGATCACGAAGGATGGCATCGAGGCACTGGTGCGAGGT
ENST00000484457        CTGAACCTCTCTTGGTGTGATCAGATCACGAAGGATGGCATCGAGGCACTGGTGCGAGGT
ENSPTRT00000027553     CTGAACCTCTCTTGGTGTGATCAGATCACGAAGGATGGCATCGAGGCACTGGTGCGAGGT
ENSPPYT00000016320     CTGAACCTCTCTTGGTGTGATCAGATCACGAAGGATGGCATCGAGGCACTGGTGCGAGGT
ENSMUST00000035090     CTGAACCTCTCCTGGTGTGACCAGATCACAAAGGAAGGCATTGAGGCGCTGGTGCGGGGG
ENSRNOT00000033126     CTGAACCTCTCCTGGTGTGACCAGATCACAAAGGAAGGCATTGAGGCGCTGGTGCGGGGG

ENSCJAT00000023329     TGTCGAGGCCTGAAAGCCCTGCTCCTGAGGGGCTGCACACAGTTAGAAGATGAAGCTCTG
ENSGGOT00000002541     TGTCGAGGCCTGAAAGCCCTGCTCCTGAGAGGCTGCACACAGTTAGAAGATGAAGCTCTG
ENST00000484457        TGTCGAGGCCTGAAAGCCCTGCTCCTGAGGGGCTGCACACAGTTAGAAGATGAAGCTCTG
ENSPTRT00000027553     TGTCGAGGCCTGAAAGCCCTGCTCCTGAGGGGCTGCACACAGTTAGAAGATGAAGCTCTG
ENSPPYT00000016320     TGTCGAGGCCTGAAAGCCCTGCTCCTGAGGGGCTGCACACAGTTAGAAGATGAAGCTCTG
ENSMUST00000035090     TGCCGGGGTTTGAAAGCCCTGCTCCTGAGGGGTTGTACACAGTTAGAGGACGAAGCTCTG
ENSRNOT00000033126     TGCCGGGGTCTGAAAGCCCTGCTCCTGAGGGGTTGTACACAGTTAGAGGACGAAGCCCTG

ENSCJAT00000023329     AAACACATTCAGAATTACTGCCATGAGCTCGTGAGCCTCAACCTACAGTCCTGCTCACGC
ENSGGOT00000002541     AAACACATTCAGAATTACTGCCATGAGCTTGTGAGCCTCAACTTGCAGTCCTGCTCACGT
ENST00000484457        AAACACATTCAGAATTACTGCCATGAGCTTGTGAGCCTCAACTTGCAGTCCTGCTCACGT
ENSPTRT00000027553     AAACACATTCAGAATTACTGCCATGAGCTTGTGAGCCTCAACTTGCAGTCCTGCTCACGT
ENSPPYT00000016320     AAACACATTCAGAATTACTGCCATGAGCTTGTGAGCCTCAACTTGCAGTCCTGCTCACGT
ENSMUST00000035090     AAACACATTCAGAACCACTGCCACGAGCTGGTGAGCCTCAACCTGCAGTCCTGCTCACGC
ENSRNOT00000033126     AAACACATTCAGAACCACTGCCATGAGCTCGTGAGCCTCAACCTGCAGTCTTGCTCACGC

ENSCJAT00000023329     ATCACGGATGAAGGTGTGGTGCAGGTGTGCAGGGGCTGTCACCGGCTGCAGGCCCTCTGC
ENSGGOT00000002541     ATCACGGATGAAGGTGTGGTGCAGATATGCAGGGGCTGTCACCGGCTACAGGCTCTCTGC
ENST00000484457        ATCACGGATGAAGGTGTGGTGCAGATATGCAGGGGCTGTCACCGGCTACAGGCTCTCTGC
ENSPTRT00000027553     ATCACGGATGAAGGTGTGGTGCAGATATGCAGGGGCTGTCACCGGCTACAGGCTCTCTGC
ENSPPYT00000016320     ATCACGGATGAAGGTGTGGTGCAGATATGCAGGGGCTGTCACCGGCTGCAGGCTCTCTGC
ENSMUST00000035090     ATCACTGATGATGGCGTGGTGCAGATCTGCAGGGGCTGCCACCGGCTACAGGCTCTGTGC
ENSRNOT00000033126     ATCACTGATGACGGCGTGGTGCAGATCTGTAGGGGCTGCCACCGGCTACAGGCGCTGTGT

ENSCJAT00000023329     CTCTCGGGTTGCAGCAACCTCACAGATGCCTCCCTTACAGCCCTGGGTCTGAACTGCCCG
ENSGGOT00000002541     CTTTCGGGTTGCAGCAACCTCACAGATGCCTCTCTTACAGCCCTGGGTTTGAACTGTCCG
ENST00000484457        CTTTCGGGTTGCAGCAACCTCACAGATGCCTCTCTTACAGCCCTGGGTTTGAACTGTCCG
ENSPTRT00000027553     CTTTCGGGTTGCAGCAACCTCACAGATGCCTCTCTTACAGCCCTGGGTTTGAACTGTCCG
ENSPPYT00000016320     CTTTCGGGTTGCAGCAACCTCACAGATGCCTCTCTTACAGCTCTGGGTTTGAACTGTCCG
ENSMUST00000035090     CTCTCGGGTTGTAGCAACCTTACGGATGCATCTCTCACAGCCTTGGGCCTGAACTGCCCC
ENSRNOT00000033126     CTCTCGGGTTGTAGCAACCTTACGGATGCATCTCTCACGGCCTTGGGCCTGAACTGCCCC

ENSCJAT00000023329     CGACTGCAGATGTACGAGAGTGTAACGTGTACAAAAATGTCAGTCATGCATCCATCCCTA
ENSGGOT00000002541     CGACTGCAAATTTTGGAGGCTGCCCGATGCTCCCATTTGACTGACGCAGGTTTTACACTT
ENST00000484457        CGACTGCAAATTTTGGAGGCTGCCCGATGCTCCCATTTGACTGACGCAGGTTTTACACTT
ENSPTRT00000027553     CGACTGCAAATTTTGGAGGCTGCCCGATGCTCCCATTTGACTGACGCAGGTTTTACACTT
ENSPPYT00000016320     CGACTGCAAATTTTGGAGGCTGCCCGATGCTCCCATTTGACTGACGCAGGTTTTACACTT
ENSMUST00000035090     AGACTACAAGTTTTGGAGGCTGCCCGGTGCTCCCATCTGACCGACGCAGGCTTTACACTG
ENSRNOT00000033126     AGACTACAAGTTTTGGAGGCTGCCCGATGCTCCCATCTGACCGATGCAGGCTTCACACTG

ENSCJAT00000023329     TTAAGCACGAATTGCCACGAATTGGAGAAGATGGATCTTGAAGAATGCATCCTGATAACC
ENSGGOT00000002541     TTAGCTCGGAATTGCCACGAATTGGAGAAGATGGATCTTGAAGAATGCATCCTGATAACC
ENST00000484457        TTAGCTCGGAATTGCCACGAATTGGAGAAGATGGATCTTGAAGAATGCATCCTGATAACC
ENSPTRT00000027553     TTAGCTCGGAATTGCCACGAATTGGAGAAGATGGATCTTGAAGAATGCATCCTGATAACC
```

```
ENSPPYT00000016320    TTAGCTCGGAATTGCCACGAATTGGAGAAGATGGATCTTGAAGAATGCATCCTGATAACC
ENSMUST00000035090    CTAGCTCGGAATTGCCATGAGCTGGAGAAGATGGACCTTGAAGAATGTGTCCTGATTACC
ENSRNOT00000033126    CTGGCTCGGAATTGTCATGACCTGGAGAAGATGGACCTTGAAGAATGTGTCCTGATCACT

ENSCJAT00000023329    GACAGCACGCTCATCCAGCTCTCCATTCACTGTCCCAAACTGCAAGCCCTGAGCCTGTCC
ENSGGOT00000002541    GACAGCACACTCATCCAGCTCTCCATTCACTGTCCTAAACTGCAAGCCCTGAGCCTGTCC
ENST00000484457       GACAGCACACTCATCCAGCTCTCCATTCACTGTCCTAAACTGCAAGCCCTGAGCCTGTCC
ENSPTRT00000027553    GACAGCACACTCATCCAGCTCTCCATTCACTGTCCTAAACTGCAAGCCCTGAGCCTGTCC
ENSPPYT00000016320    GACAGCACACTCATCCAGCTCTCCATTCACTGTCCTAAACTGCAAGCCCTGAGCCTGTCC
ENSMUST00000035090    GACAGCACCCTCGTCCAGCTCTCCATCCACTGTCCCAAGCTGCAAGCCCTGAGCTTGTCC
ENSRNOT00000033126    GACAGCACTCTGATCCAGCTCTCCATCCACTGCCCCAAGCTGCAAGCCCTGAGCTTGTCC

ENSCJAT00000023329    CACTGTGAACTCATCACAGACGATGGGATCCTGCACCTGAGCAACAGCACCTGTGGCCAT
ENSGGOT00000002541    CACTGTGAACTCATCACAGATGATGGGATCCTGCACCTGAGCAACAGTACCTGTGGCCAT
ENST00000484457       CACTGTGAACTCATCACAGATGATGGGATCCTGCACCTGAGCAACAGTACCTGTGGCCAT
ENSPTRT00000027553    CACTGTGAACTCATCACAGATGATGGGATCCTGCACCTGAGCAACAGTACCTGTGGCCAT
ENSPPYT00000016320    CACTGTGAACTCATCACAGATGATGGGATCCTGCACCTGAGCAACAGCACCTGTGGCCAT
ENSMUST00000035090    CACTGTGAGCTCATCACAGATGAGGGGATCCTGCACCTGAGCAGCAGCACCTGTGGGCAC
ENSRNOT00000033126    CACTGTGAGCTCATCACCGATGAAGGGATCCTACACTTGAGCAGCAGCACCTGTGGGCAC

ENSCJAT00000023329    GAGAGGTTGCGGGTACTGGAGCTGGACAACTGCCTCCTCATCACTGATGTGGCCCTGGAA
ENSGGOT00000002541    GAGAGGCTGCGGGTACTGGAGTTGGACAACTGCCTCCTCATCACTGATGTGGCCCTGGAA
ENST00000484457       GAGAGGCTGCGGGTACTGGAGTTGGACAACTGCCTCCTCATCACTGATGTGGCCCTGGAA
ENSPTRT00000027553    GAGAGGCTGCGGGTACTGGAGTTGGACAACTGCCTCCTCATCACTGATGTGGCCCTGGAA
ENSPPYT00000016320    GAGAGGCTGCGGGTACTGGAGTTGGACAACTGCCTCCTCATCACTGATGTGGCCCTGGAA
ENSMUST00000035090    GAGAGACTCCGGGTGCTGGAGCTGGACAACTGCCTTCTTGTCACGGACGCCTCGCTGGAG
ENSRNOT00000033126    GAGAGACTCCGGGTACTGGAGCTGGACAACTGCCTTCTTGTCACTGACGCCTCACTGGAG

ENSCJAT00000023329    CACCTAGAGAACTGCCGAGGCCTGGAGCGCCTTGAGCTGTATGACTGCCAGCAGGTTACC
ENSGGOT00000002541    CACCTAGAGAACTGCCGAGGCCTGGAGCGCCTTGAGCTGTACGACTGCCAGCAGGTTACC
ENST00000484457       CACCTAGAGAACTGCCGAGGCCTGGAGCGCCTCGAGCTGTACGACTGCCAGCAGGTTACC
ENSPTRT00000027553    CACCTAGAGAACTGCCGAGGCCTGGAGCGCCTCGAGCTGTACGACTGCCAGCAGGTTACC
ENSPPYT00000016320    CACCTAGAGAACTGCCGAGGCCTGGAGCGCCTTGAGCTGTACGACTGCCAGCAGGTTACC
ENSMUST00000035090    CACCTGGAGAACTGCCGAGGCCTGGAGCGACTGGAGCTTTACGACTGCCAGCAGGTCACC
ENSRNOT00000033126    CACCTGGAGAACTGCCGGGGCTTGGAGAGACTGGAGCTGTACGACTGCCAGCAGGTCACC

ENSCJAT00000023329    CGTGCAGGCATCAAGCGGATGCGGGCTCAGCTCCCTCATGTCAAAGTCCACGCCTACTTT
ENSGGOT00000002541    CGTGCAGGCATCAAGCGGATGCGGGCTCAGCTCCCTCATGTCAAAGTCCACGCCTACTTT
ENST00000484457       CGTGCAGGCATCAAGCGGATGCGGGCTCAGCTCCCTCATGTCAAAGTCCACGCCTACTTT
ENSPTRT00000027553    CGTGCAGGCATCAAGCGGATGCGGGCTCAGCTCCCTCATGTCAAAGTCCACGCCTACTTT
ENSPPYT00000016320    CGTGCAGGCATCAAGCGGATGCGGGCTCAGCTCCCTCATGTCAAAGTCCACGCCTACTTT
ENSMUST00000035090    CGTGCAGGCATCAAGCGCATGCGGGCTCAGCTTCCTCATGTCAAAGTCCATGCCTACTTT
ENSRNOT00000033126    CGTGCAGGCATCAAGCGTATGCGGGCTCAGCTTCCTCGTGTCAAAGTCCATGCCTACTTT

ENSCJAT00000023329    GCTCCCGTCACCCCACCAACAGCAGTGGCAGGAAGTGGACAGCGACTGTGCAGGTGCTGT
ENSGGOT00000002541    GCTCCCGTCACCCCACCGACAGCAGTGGCAGGAAGTGGACAGCGACTGTGCAGGTGCTGT
ENST00000484457       GCTCCCGTCACCCCACCGACAGCAGTGGCAGGAAGTGGACAGCGACTGTGCAGGTGCTGT
ENSPTRT00000027553    GCTCCCGTCACCCCACCGACAGCAGTGGCAGGAAGTGGACAGCGACTGTGCAGGTGCTGT
ENSPPYT00000016320    GCTCCCGTCACCCCACCGACAGCAGTGGCAGGAAGTGGACAGCGACTGTGCAGGTGCTGT
ENSMUST00000035090    GCTCCAGTCACCCCTCCACCAGCAGTGGCAGGAAGTGGACATCGACTGTGCAGATGCTGT
ENSRNOT00000033126    GCTCCAGTCACCCCTCCACCAGCAGTGGCAGGAAGTGGACATCGACTGTGCAGATGCTGT

ENSCJAT00000023329    GTCATTCTC
```

```
ENSGGOT00000002541     GTCATTCTC
ENST00000484457        GTCATTCTC
ENSPTRT00000027553     GTCATTCTC
ENSPPYT00000016320     GTCATTCTC
ENSMUST00000035090     GTCATACTC
ENSRNOT00000033126     GTTATACTC
```

Multiple sequence alignment of Fbxl20

```
ENSRNOT00000064848     GAATCCAGAGACTGTATTTTCACTTTTGGGACAATTTTACAGATGTTCTCAAACAGTGAC
ENST00000577399        CCGAGCAGGGACCGCCTGCTGCACTTTGGGTTCAAGGCGACAATGTTCTCAAATAGTGAT
ENSMUST00000103143     ATGAGGAGGGACGTGAACGGAGTGACCAAGAGCAGGTTTGAGATGTTCTCAAACAGTGAT
ENSGGOT00000009737     ------------------------------------------ATGTTCTCAAATAGTGAT
ENSPPYT00000009921     ATGAGGAGGGACGTGAACGGAGTGACCAAGAGCAGGTTTGAGATATTTTCT---------
ENSCJAT00000059445     ATGAGGAGAGACGTGAACGGAGTGACCAAGAGCAGGTTTGAGATGTTCTCAAATAGCGAT
ENSPTRT00000016739     ATGAGGAGGGACGTGAACGGAGTGACCAAGAGCAGGTTTGAGATGTTCTCAAATAGTGAT


ENSRNOT00000064848     GAAGCCGTAATCAATAAAAAACTTCCCAAAGAACTCCTGCTAAGGATATTTTCTTTCCTG
ENST00000577399        GAAGCTGTAATCAATAAAAAACTTCCCAAAGAACTCCTGTTACGGATATTTTCTTTTCTA
ENSMUST00000103143     GAGGCTGTAATCAATAAAAAACTTCCCAAAGAACTCCTGTTGAGGATATTTTCTTTCCTG
ENSGGOT00000009737     GAAGCTGTAATCAATAAAAAACTTCCCAAAGAACTCCTGTTACGGATATTTTCTTTTCTA
ENSPPYT00000009921     ------------------------------------------------------TTTCTA
ENSCJAT00000059445     GAAGCTGTAATCAATAAAAAACTTCCCAAAGAACTCCTGTTAAGGATATTTTCTTTTCTA
ENSPTRT00000016739     GAAGCTGTAATCAATAAAAAACTTCCCAAAGAACTCCTGTTACGGATATTTTCTTTTCTA


ENSRNOT00000064848     GATGTTGTTACCCTTTGTCGCTGTGCTCAGGTCTCCAGGGCCTGGAATGTCCTAGCTCTG
ENST00000577399        GATGTTGTTACCCTGTGCCGCTGTGCTCAGGTCTCCAGGGCCTGGAATGTTCTGGCTCTG
ENSMUST00000103143     GATGTTGTTACCCTGTGTCGCTGTGCTCAGGTCTCCAGGGCCTGGAATGTCCTAGCTCTG
ENSGGOT00000009737     GATGTTGTTACCCTGTGCCGCTGTGCTCAGGTCTCCAGGGCCTGGAATGTTCTGGCTCTG
ENSPPYT00000009921     GATGTTGTTACCCTGTGCCGCTGTGCTCAGGTCTCCAGGGCCTGGAATGTTCTGGCTCTG
ENSCJAT00000059445     GATGTTGTTACCCTGTGTCGCTGTGCTCAGGTCTCCAGGGCCTGGAATGTTCTTGCTCTG
ENSPTRT00000016739     GATGTTGTCACCCTGTGCCGCTGTGCTCAGGTCTCCAGGGCCTGGAATGTTCTGGCTCTG


ENSRNOT00000064848     GATGGCAGTAACTGGCAACGGATAGACCTGTTCGATTTCCAGAGGGACATTGAGGGCCGG
ENST00000577399        GATGGCAGTAACTGGCAGCGAATTGACCTATTTGATTTCCAGAGGGATATTGAGGGCCGA
ENSMUST00000103143     GATGGCAGTAACTGGCAACGGATAGACCTGTTTGATTTCCAGAGGGACATTGAGGGCCGG
ENSGGOT00000009737     GATGGCAGTAACTGGCAGCGAATTGACCTATTTGATTTCCAGAGGGATATTGAGGGCCGA
ENSPPYT00000009921     GATGGCAGTAACTGGCAGCGAATTGACCTATTTGATTTCCAGAGGGATATTGAGGGCCGA
ENSCJAT00000059445     GATGGCAGTAACTGGCAGCGAATTGACCTATTTGATTTCCAGAGGGATATTGAGGGCCGG
ENSPTRT00000016739     GATGGCAGTAACTGGCAGCGAATTGACCTATTTGATTTCCAGAGGGATATTGAGGGCCGA


ENSRNOT00000064848     GTAGTGGAAAATATTTCTAAACGATGCGGAGGCTTTTTACGGAAGTTAAGTCTTCGTGGG
ENST00000577399        GTAGTGGAGAATATTTCAAAACGATGTGGGGGCTTTTTACGAAAGTTAAGTCTTCGTGGA
ENSMUST00000103143     GTAGTGGAAAATATTTCAAAACGATGTGGAGGCTTTTTACGAAAGTTAAGTCTTCGTGGG
ENSGGOT00000009737     GTAGTGGAGAATATTTCAAAACGATGTGGGGGCTTTTTACGAAAGTTAAGTCTTCGTGGA
ENSPPYT00000009921     GTAGTGGAGAACATTTCAAAACGATGTGGGGGCTTTTTACGAAAGTTAAGTCTTCGTGGA
ENSCJAT00000059445     GTAGTGGAGAATATTTCAAAACGATGTGGAGGCTTTTTACGAAAGTTAAGTCTTCGTGGA
ENSPTRT00000016739     GTAGTGGAGAATATTTCAAAACGATGTGGGGGCTTTTTACGAAAGTTAAGTCTTCGTGGA


ENSRNOT00000064848     TGTCTCGGAGTAGGAGACAATGCACTGAGGACCTTTGCACAAAACTGTAGGAATATTGAA
ENST00000577399        TGTCTTGGAGTGGGAGACAATGCATTAAGAACCTTTGCACAAAACTGCAGGAACATTGAA
ENSMUST00000103143     TGTCTTGGAGTAGGAGACAATGCATTAAGGACCTTTGCACAAAACTGTAGGAATATTGAA
ENSGGOT00000009737     TGTCTTGGAGTGGGAGACAATGCATTAAGAACCTTTGCACAAAACTGCAGGAACATTGAA
ENSPPYT00000009921     TGTCTTGGAGTGGGAGACAATGCATTAAGAACCTTTGCACAAAACTGCAGGAACATTGAA
```

ENSCJAT00000059445 TGTCTTGGAGTGGGAGACAATGCATTAAGAACCTTTGCACAAAACTGCAGGAACATTGAA
ENSPTRT00000016739 TGTCTTGGAGTGGGAGACAATGCATTAAGAACCTTTGCACAAAACTGCAGGAACATTGAA

ENSRNOT00000064848 GTACTAAGTCTCAACGGATGTACAAAGACGACAGACGCCACCTGCACTAGCCTTAGCAAG
ENST00000577399 GTACTGAATCTAAATGGGTGTACAAAGACAACAGACGCTACATGTACTAGCCTTAGCAAG
ENSMUST00000103143 GTACTAAGTCTCAACGGATGTACAAAGACAACAGATGCTACCTGCACTAGCCTTAGCAAG
ENSGGOT00000009737 GTACTGAATCTAAACGGGTGTACAAAGACAACAGACGCTACATGTACTAGCCTTAGCAAG
ENSPPYT00000009921 GTACTGAATCTAAATGGGTGTACAAAGACAACAGACGCTACATGTACTAGCCTTAGCAAG
ENSCJAT00000059445 GTACTGAATCTAAATGGGTGTACAAAGACAACAGATGCTACATGTACTAGCCTTAGCAAG
ENSPTRT00000016739 GTACTGAATCTAAATGGGTGTACAAAGACAACAGACGCTACATGTACTAGCCTTAGCAAG

ENSRNOT00000064848 TTCTGCTCCAAACTCAGGCACCTCGACCTGGCTTCCTGTACGTCAATAACAAACATGTCT
ENST00000577399 TTCTGTTCCAAACTCAGGCACCTTGACTTGGCTTCCTGTACATCAATAACAAACATGTCT
ENSMUST00000103143 TTCTGCTCCAAACTCAGACACCTCGACTTGGCTTCCTGTACGTCAATAACAAACATGTCT
ENSGGOT00000009737 TTCTGTTCCAAACTCAGGCACCTTGACTTGGCTTCCTGTACATCAATAACAAACATGTCT
ENSPPYT00000009921 TTCTGTTCCAAACTCAGGCACCTTGACTTGGCTTCCTGTACATCAATAACAAACATGTCT
ENSCJAT00000059445 TTCTGTTCCAAACTCAGGCACCTTGACTTGGCTTCCTGTACATCAATAACAAACATGTCT
ENSPTRT00000016739 TTCTGTTCCAAACTCAGGCACCTTGACTTGGCTTCCTGTACATCAATAACAAACATGTCT

ENSRNOT00000064848 CTCAAAGCTCTGAGTGAGGGCTGTCCACTGTTGGAGCAGTTGAACATCTCCTGGTGTGAC
ENST00000577399 CTAAAAGCTCTGAGTGAGGGATGTCCACTGTTGGAGCAGTTGAACATTTCCTGGTGTGAC
ENSMUST00000103143 CTAAAAGCTCTGAGTGAGGGCTGTCCACTGTTGGAGCAGTTGAACATTTCCTGGTGTGAC
ENSGGOT00000009737 CTAAAAGCTCTAAGTGAGGGATGTCCACTGTTGGAGCAGTTGAACATTTCCTGGTGTGAC
ENSPPYT00000009921 CTAAAAGCTCTGAGTGAGGGATGTCCACTGTTGGAGCAGTTGAACATTTCCTGGTGTGAC
ENSCJAT00000059445 CTTAAAGCTCTGAGTGAGGGATGTCCACTGTTGGAGCAGTTGAACATTTCCTGGTGTGAC
ENSPTRT00000016739 CTAAAAGCTCTGAGTGAGGGATGTCCACTGTTGGAGCAGTTGAACATTTCCTGGTGTGAC

ENSRNOT00000064848 CAAGTAACCAAGGATGGCATTCAAGCACTAGTGAGAGGCTGTGGGGGTCTCAAGGCCTTA
ENST00000577399 CAAGTAACCAAGGATGGCATTCAAGCACTAGTGAGGGGCTGTGGGGGTCTCAAGGCCTTA
ENSMUST00000103143 CAAGTAACCAAGGATGGCATTCAAGCACTAGTGAGAGGCTGTGGGGGTCTCAAGGCCTTA
ENSGGOT00000009737 CAAGTAACCAAGGATGGCATTCAAGCACTAGTGAGGGGCTGTGGGGGTCTCAAGGCCTTA
ENSPPYT00000009921 CAAGTAACCAAGGATGGCATTCAAGCACTAGTGAGGGGCTGTGGGGGTCTCAAGGCCTTA
ENSCJAT00000059445 CAAGTAACCAAGGATGGCATTCAAGCATTAGTGAGAGGCTGTGGGGGTCTCAAGGCCTTA
ENSPTRT00000016739 CAAGTAACCAAGGATGGCATTCAAGCACTAGTGAGGGGCTGTGGGGGTCTCAAGGCCTTA

ENSRNOT00000064848 TTCTTAAAAGGCTGCACTCAGCTAGAAGATGAAGCTCTCAAGTACATAGGTGCACACTGC
ENST00000577399 TTCTTAAAAGGCTGCACGCAGCTAGAAGATGAAGCTCTCAAGTACATAGGTGCACACTGC
ENSMUST00000103143 TTCTTAAAAGGCTGCACTCAGCTAGAAGATGAAGCTCTCAAGTACATAGGTGCACACTGC
ENSGGOT00000009737 TTCTTAAAAGGCTGCACGCAGCTAGAAGATGAAGCTCTCAAGTACATAGGTGCACACTGC
ENSPPYT00000009921 TTCTTAAAAGGCTGCACGCAGCTAGAAGATGAAGCTCTCAAGTACATAGGTGCACACTGC
ENSCJAT00000059445 TTCTTAAAAGGCTGCACGCAGCTAGAAGATGAAGCTCTCAAGTATATAGGTGCACACTGT
ENSPTRT00000016739 TTCTTAAAAGGCTGCACGCAGCTAGAAGATGAAGCTCTCAAGTACATAGGTGCACACTGC

ENSRNOT00000064848 CCTGAGCTGGTGACTTTGAACTTGCAAACTTGCTTACAAATCACAGATGAAGGCCTCATC
ENST00000577399 CCTGAACTGGTGACTTTGAACTTGCAGACTTGCTTGCAAATCACAGATGAAGGTCTCATT
ENSMUST00000103143 CCTGAGCTAGTGACTTTAAACCTGCAAACTTGCTTACAAATCACAGATGAAGGTCTCATT
ENSGGOT00000009737 CCTGAACTGGTGACTTTGAACTTGCAGACTTGCTTGCAAATCACAGATGAAGGTCTCATT
ENSPPYT00000009921 CCTGAACTGGTGACTTTGAACTTGCAGACTTGCTTGCAAATCACAGATGAAGGTCTCATT
ENSCJAT00000059445 CCTGAACTGGTGACTCTGAATTTGCAGACTTGCCTGCAAATCACGGATGAAGGTCTCATT
ENSPTRT00000016739 CCTGAACTGGTGACTTTGAACTTGCAGACTTGCTTGCAAATCACAGATGAAGGTCTCATT

ENSRNOT00000064848 ACCATATGCAGAGGGTGTCATAAGTTGCAGTCTCTCTGTGCCTCTGGGTGCTCCAACATC
ENST00000577399 ACTATATGCAGAGGGTGCCATAAGTTACAATCCCTTTGTGCCTCTGGCTGCTCCAACATC

```
ENSMUST00000103143     ACCATATGCAGAGGGTGTCATAAGTTGCAGTCCCTCTGTGCCTCTGGGTGCTCCAATATC
ENSGGOT00000009737     ACTATATGCAGAGGGTGCCATAAGTTACAATCCCTTTGTGCCTCTGGCTGCTCCAACATC
ENSPPYT00000009921     ACTATATGCAGAGGGTGCCATAAGTTACAATCCCTTTGTGCCTCTGGCTGCTCCAACATC
ENSCJAT00000059445     ACTATATGCAGAGGGTGCCATAAGTTACAATCCCTTTGTGCCTCTGGCTGCTCCAACATC
ENSPTRT00000016739     ACTATATGCAGAGGGTGCCATAAGTTACAATCCCTTTGTGCCTCTGGCTGCTCCAACATC

ENSRNOT00000064848     ACAGATGCCATCCTGAATGCTCTAGGTCAGAACTGCCCTCGGCTTAGAATATTAGAAGTG
ENST00000577399        ACAGATGCCATCCTGAATGCTCTAGGTCAGAACTGCCCACGGCTTAGAATATTGGAAGTG
ENSMUST00000103143     ACAGATGCCATCCTGAACGCTCTAGGTCAGAACTGCCCTCGACTTAGAATATTAGAAGTG
ENSGGOT00000009737     ACAGATGCCATCCTGAATGCTCTAGGTCAGAACTGCCCACGGCTTAGAATATTGGAAGTG
ENSPPYT00000009921     ACAGATGCCATCCTGAATGCTCTAGGTCAGAACTGCCCACGGCTTAGAATATTGGAAGTG
ENSCJAT00000059445     ACAGATGCCATCCTGAATGCTCTAGGTCAGAACTGCCCACGACTTAGAATATTGGAAGTG
ENSPTRT00000016739     ACAGATGCCATCCTGAATGCTCTAGGTCAGAACTGCCCACGGCTTAGAATATTGGAAGTG

ENSRNOT00000064848     GCCAGATGTTCCCAGTTAACAGATGTGGGCTTTACTACCCTGGCCAGGAATTGCCATGAA
ENST00000577399        GCAAGATGTTCTCAATTAACAGATGTGGGCTTTACCACTCTAGCCAGGAATTGCCATGAA
ENSMUST00000103143     GCCAGATGTTCCCAGTTAACAGATGTGGGCTTCACTACCCTAGCCAGGAATTGCCATGAG
ENSGGOT00000009737     GCAAGATGTTCTCAATTAACAGATGTGGGCTTCACCACTCTAGCCAGGAATTGCCATGAA
ENSPPYT00000009921     GCAAGATGTTCTCAATTAACAGATGTGGGCTTTACCACTCTAGCCAGGAATTGCCATGAA
ENSCJAT00000059445     GCAAGATGTTCTCAATTAACAGATGTGGGCTTTACCACTCTAGCCAGGAATTGCCATGAA
ENSPTRT00000016739     GCAAGATGTTCTCAATTAACAGATGTGGGCTTTACCACTCTAGCCAGGAATTGCCATGAA

ENSRNOT00000064848     CTTGAAAAGATGGACCTAGAAGAATGTGTGCAGATCACAGATAGCACATTAATCCAGCTT
ENST00000577399        CTTGAAAAGATGGACCTGGAAGAGTGTGTTCAGATAACAGATAGCACATTAATCCAACTT
ENSMUST00000103143     CTTGAGAAGATGGACCTAGAAGAATGTGTGCAGATCACGGATAGCACATTAATCCAGCTT
ENSGGOT00000009737     CTTGAAAAGATGGACCTGGAAGAGTGTGTTCAGATAACAGATAGCACATTAATCCAACTT
ENSPPYT00000009921     CTTGAAAAGATGGACCTGGAAGAGTGTGTTCAGATAACAGATAGCACATTAATCCAACTT
ENSCJAT00000059445     CTTGAAAAGATGGACCTGGAAGAGTGTGTTCAGATAACAGATAGCACATTAATCCAACTT
ENSPTRT00000016739     CTTGAAAAGATGGACCTGGAAGAGTGTGTTCAGATAACAGATAGCACATTAATCCAACTT

ENSRNOT00000064848     TCCATACACTGTCCTCGGCTTCAAGTGTTGAGTCTTTCTCACTGCGAGCTGATCACAGAT
ENST00000577399        TCTATACACTGTCCTCGACTTCAAGTATTGAGTCTGTCTCACTGTGAGCTGATCACAGAT
ENSMUST00000103143     TCCATTCACTGTCCTCGGCTTCAAGTGTTGAGTCTTTCTCACTGTGAGCTGATCACAGAC
ENSGGOT00000009737     TCTATACACTGTCCTCGACTTCAAGTATTGAGTCTGTCTCACTGTGAGCTGATCACAGAT
ENSPPYT00000009921     TCTATACACTGTCCTCGACTTCAAGTATTGAGTCTGTCTCACTGTGAGCTGATCACAGAT
ENSCJAT00000059445     TCTATACACTGTCCTCGACTTCAAGTATTGAGTCTGTCTCACTGTGAGCTGATCACAGAT
ENSPTRT00000016739     TCTATACACTGTCCTCGACTTCAAGTATTGAGTCTGTCTCACTGTGAGCTGATCACAGAT

ENSRNOT00000064848     GACGGGATTCGTCACCTAGGGAATGGAGCCTGTGCCCACGACCAACTGGAGGTGATTGAG
ENST00000577399        GATGGAATTCGTCACCTGGGGAATGGGGCCTGCGCCCATGACCAGCTGGAGGTGATTGAG
ENSMUST00000103143     GATGGAATCCGTCACCTAGGGAACGGGGCCTGTGCCCATGACCAGCTGGAGGTGATAGAG
ENSGGOT00000009737     GATGGAATTCGTCACCTGGGGAATGGGGCCTGCGCCCATGACCAGCTGGAGGTGATTGAG
ENSPPYT00000009921     GATGGAATTCGTCACCTGGGGAATGGGGCCTGCGCCCATGACCAGCTGGAGGTGATTGAG
ENSCJAT00000059445     GATGGAATTCGTCACCTGGGAAATGGGGCCTGTGCCCATGACCAGCTGGAGGTGATTGAG
ENSPTRT00000016739     GATGGAATTCGTCACCTGGGGAATGGGGCCTGCGCCCATGACCAGCTGGAGGTGATTGAG

ENSRNOT00000064848     CTGGACAATTGCCCACTAATCACAGATGCATCCCTGGAGCACTTGAAGAGCTGCCACAGC
ENST00000577399        CTGGACAACTGCCCACTAATCACAGATGCATCCCTGGAGCACTTGAAGAGCTGTCATAGC
ENSMUST00000103143     CTGGACAACTGCCCACTAATCACCGATGCATCCCTGGAGCACTTGAAGAGCTGTCACAGC
ENSGGOT00000009737     CTGGACAACTGCCCACTAATCACAGATGCATCCCTGGAGCACTTGAAGAGCTGTCATAGC
ENSPPYT00000009921     CTGGACAACTGCCCACTAATCACAGATGCATCCCTGGAGCACTTGAAGAGCTGTCATAGC
ENSCJAT00000059445     CTGGACAACTGCCCACTAATCACAGATGCATCCCTGGAGCACTTGAAAAGCTGTCACAGC
ENSPTRT00000016739     CTGGACAACTGCCCACTAATCACAGATGCATCCCTGGAGCACTTGAAGAGCTGTCATAGC
```

```
ENSRNOT00000064848     CTTGAGCGGATCGAACTCTATGACTGCCAGCAGATCACCCGGGCTGGCATCAAGAGACTC
ENST00000577399        CTTGAGCGGATAGAACTCTATGACTGCCAGCAAATCACACGGGCTGGAATCAAGAGACTC
ENSMUST00000103143     CTTGAGCGAATAGAACTCTATGACTGCCAGCAGATCACCCGGGCTGGCATCAAGAGACTC
ENSGGOT00000009737     CTTGAGCGGATAGAACTCTATGACTGCCAGCAAATCACACGGGCTGGAATCAAGAGACTC
ENSPPYT00000009921     CTTGAGCGGATAGAACTCTATGACTGCCAGCAAATCACACGGGCTGGAATCAAGAGACTC
ENSCJAT00000059445     CTTGAGCGGATAGAACTGTATGACTGCCAGCAAATCACACGGGCTGGAATCAAGAGACTC
ENSPTRT00000016739     CTTGAGCGGATAGAACTCTATGACTGCCAGCAAATCACACGGGCTGGAATCAAGAGACTC

ENSRNOT00000064848     AGGACCCATTTACCCAATATTAAAGTCCACGCCTACTTCGCCCCTGTCACTCCACCGCCA
ENST00000577399        AGGACCCATTTACCCAATATTAAAGTCCACGCCTACTTCGCACCTGTCACTCCACCCCCA
ENSMUST00000103143     AGGACCCATTTACCCAACATTAAAGTCCACGCCTACTTCGCCCCTGTCACTCCGCCCCCA
ENSGGOT00000009737     AGGACCCATTTACCCAATATTAAAGTCCACGCCTACTTCGCACCTGTCACTCCACCCCCA
ENSPPYT00000009921     AGGACCCATTTACCCAATATTAAAGTCCACGCCTACTTCGCACCTGTCACTCCACCCCCA
ENSCJAT00000059445     AGGACCCATTTACCCAATATTAAAGTCCACGCCTACTTCGCACCTGTCACTCCACCCCCA
ENSPTRT00000016739     AGGACCCATTTACCCAATATTAAAGTCCACGCCTACTTCGCACCTGTCACTCCACCCCCA

ENSRNOT00000064848     TCAGTAGGGGGCAGCAGACAGCGCTTCTGCAGATGCTGCATCATCCTA
ENST00000577399        TCAGTAGGGGGCAGCAGACAGCGCTTCTGCAGATGCTGCATCATCCTA
ENSMUST00000103143     TCAGTCGGGGGCAGCAGACAGCGCTTCTGCAGGTGCTGCATCATCCTA
ENSGGOT00000009737     TCAGTAGGGGGCAGCAGACAGCGCTTCTGCAGATGCTGCATCATCCTA
ENSPPYT00000009921     TCAGTAGGGGGCAGCAGACAGCGCTTCTGCAGATGCTGCATCATCCTA
ENSCJAT00000059445     TCAGTAGGGGGCAGCAGACAGCGCTTCTGCAGATGCTGCATCATCCTA
ENSPTRT00000016739     TCAGTAGGGGGCAGCAGACAGCGCTTCTGCAGATGCTGCATCATCCTA
```

Multiple sequence alignment of Fbxl21

```
ENSRNOT00000035839     ATGAAGAGGAATAATTTCTCCACCGTGAATAAAGTTGTTCACTCGTCACCAGCCGTGAAA
ENSMUST00000045428     ATGAAGAGGAATAATTTCTCTGCCGTGAATAAGGTTGTCCAGTCGTCACCAGTAGTGAAA
ENSMMUT00000002758     ATGAAGAGGAACAGTTTATCTGTTGAGAATAAAATTGTCCAGTTGTCAGGAGCAGTGAAA
ENST00000297158        ATGAAGAGGAACAGTTTATCTGTTGAGAATAAAATTGTCCAGTTGTCAGGAGCAGCGAAA
XM_527020.3            ATGAAGAGGAACAGTTTATCTGTTGGGAATAAAATTGTCCAGTTGTCAGGAGCAGCGAAA

ENSRNOT00000035839     CAGCCCAAACGTGGGCTCTGCACTTCCCTCCGCCAGACTCAGATGCTCTCTGCCCTTCTG
ENSMUST00000045428     CAGCCCAAACGTGGGCTCTGCTCTTCCCTCCGCCAGACCCATGCGCTCTCTGTCCTTCTG
ENSMMUT00000002758     CAGCCAAAACTTGGGTTCTACTCCTCTCTCAACCAGACTCATACACACACGGTTCGTCTA
ENST00000297158        CAGCCAAAAGTTGGGTTCTACTCTTCTCTCAACCAGACTCATACACACACGGTTCTTCTA
XM_527020.3            CAGCCAAAAGTTGGATTCTACTCTTCTCTCAACCAGACTCATACGCACACGGTTCTTCTA

ENSRNOT00000035839     GACTGGGGCACGTTACCTCACCACGTCATATTACGGATTTTTCAGTATCTTCCTTTAGTA
ENSMUST00000045428     GACTGGGGGACTTTACCTCACCATGTCATATTACAGATCTTTCAGTACCTTCCTTTAATA
ENSMMUT00000002758     GACTGGGGGAGTTTGCCTCACCATGTAGTATTACGAATTTTTCAGTATCTTCCTTTACTA
ENST00000297158        GACTGGGGGAGTTTGCCTCACCATGTAGTATTACAAATTTTTCAG---CTTCCTTTACTA
XM_527020.3            GACTGGGGGAGTTTGCCTCACCATGTAGTATTACAAATTTTTCAGTATCTTCCTTTACTA

ENSRNOT00000035839     GATCGAGCCCGGGCATCTTCTGTGTGTAGGAGTTGGAACGAAGTGTTCCACATTCCCGAT
ENSMUST00000045428     GATCGAGCCCGGGCTTCGTCCGTGTGCAGGAGATGGAATGAAGTATTCCACATCCCTGAT
ENSMMUT00000002758     GATCGGGCCCGTGCATCTTCTGTATGTAGGAGATGGAATGAAGTTTTTCATATTCCTGAC
ENST00000297158        GATCGGGCCTGTGCATCTTCTGTATGTAGGAGGTGGAATGAAGTTTTTCATATTTCTGAC
XM_527020.3            GATCGGGCCTGTGCATCTTCTGTATGTAGGAGGTGGAATGAAGTTTTCCATATTCCTGAC

ENSRNOT00000035839     CTTTGGAGGAAGTTCGAGTTCGAGCTGAACCAATCAGCAACCTCTTATTTTAAGTCCACT
ENSMUST00000045428     CTTTGGAGGAAGTTTGAGTTTGAGCTGAACCAATCAGCAACTTCTTATTTCAAGTCCACT
```

```
ENSMMUT00000002758     CTTTGGAGAAAGTTTGAATTTGAACTGAACCAGTCAGCTACTTCATATTTTAAGTCCACT
ENST00000297158        CTTTGGAGAAAGTTTGAATTTGAACTGAACCAGTCAGCTACTTCATCTTTTAAGTCCACT
XM_527020.3            CTTTGGAGAAAGTTTGAATTTGAACTGAACCAGTCAGCTACTTCATCTTTTAAGTCCACT

ENSRNOT00000035839     CATCCCGACCTCATTCAACAGATCATCAAAAAGCACGCGGCACATCTTCAGTACGTCAGC
ENSMUST00000045428     CATCCCGACCTCATTCAACAGATCATCAAAAAGCACGCTGCACATCTTCAGTATGTCAGC
ENSMMUT00000002758     CATCCTGATCTCATTCAGCAGATCATTAAAAAGCATTCTGCCCATCTTCAATATGTCAGC
ENST00000297158        CATCCTGATCTCATTCAGCAGATCATTAAAAAGCATTTTGCTCATCTTCAGTATGTCAGC
XM_527020.3            CATCCTGATCTCATTCAGCAGATCATTAAAAAGCATTCTGCTCATCTTCAGTATGTCAGC

ENSRNOT00000035839     TTTAAGGTTGATAGCAGTACTGAGTCAGCGGAAGCCGCCTGTCATATCCTTTCTCAGCTG
ENSMUST00000045428     TTTAAGGTTGATAGCAGTACTGAGTCGGCAGAAGCCGCCTGTGATATCCTTTCTCAGCTG
ENSMMUT00000002758     TTTAAGGTTGACAGTAGTGTCGAGTCAGCAGAAGCTGCCTGTGGTATACTCTCTCAGCTG
ENST00000297158        TTTAAGGTTGACAGTAGCGCTGAGTCAGCAGAAGCTGCCTGTGATATACTCTCTCAGCTG
XM_527020.3            TTTAAGGTTGACAGTAGCGCTGAGTCAGCAGAAGCTGCCTGTGATATACTCTCTCAGCTG

ENSRNOT00000035839     GTAAATTGCTCTATCCAGACCTTGGGATTGATTTCAACAGCCAAGCCCAGTTTCATGAAT
ENSMUST00000045428     GTAAATTGTTCTATCCAGACCTTGGGATTGATTTCAACAGCCAAACCAAGTTTCATGAAT
ENSMMUT00000002758     GTAAATTGTTCCATCCAGACCTTGGGCTTGATTTCAACAGCCAAGCCAAGTTTCATGAAT
ENST00000297158        GTAAATTGTTCCATCCAGACCTTGGGCTTGATTTCAACAGCCAAGCCAAGTTTCATGAAT
XM_527020.3            GTAAATTGTTCCATCCAGACCTTGGGCTTGATTTCAACAGCCAAGCCAAGTTTCATGAAT

ENSRNOT00000035839     ATGCCAAAGGTAAGGATATTACTAGCGCTAACTAGTTACTTCCTTGTTTATAAAGCACAG
ENSMUST00000045428     GTGCCCAAGTCTCACTTTGTGTCGGCACTTACGGTCGTGTTTGTCAACTCAAAATCGTTA
ENSMMUT00000002758     GTATCAGAGTCTCATTTTGTGTCAGCACTTACAGTTGTTTTTATCAACTCAAAATCATTA
ENST00000297158        GTGTCGGAGTCTCATTTTGTGTCAGCACTTACAGTTGTTTTTATCAACTCAAAATCATTA
XM_527020.3            GTGTCGGAGGTAAGGATATTATGCCACTTAACAGTTGTTTTTATCAACTCAAAATCATTA

ENSRNOT00000035839     CCCTCTGAACAGCCTGAAGAGACT---CTCTGGGATCCATTCTTGGTTTCCCTATCAGGG
ENSMUST00000045428     TCATCGATTAAAATTGAGGACACGCCAGTGGATGACCCTTCCCTGAAGATTCTTGTGGCT
ENSMMUT00000002758     TCATCAATCAAAATTGAAGATACACCGGTGGATGATCCTTCATTGAAGATTCTTGTGGCC
ENST00000297158        TCATCAATCAAAATTGAAGATACACCAGTGGATGATCCTTCATTGAAGATTCTTGTGGCC
XM_527020.3            TCATCAATCAAAATTGAAGATACGCCAGTGGATGATCCTTCATTGAAGATTCTTGTGGCC

ENSRNOT00000035839     AAAAAAGGACCAACCTTACAGTTCTTGAAAGGAACTTACAGGAAGCACGTGGCTGCGTGC
ENSMUST00000045428     AACAACAGTGACACCCTTCGACTCCTCAAAATGAGTAGCTGTCCTCACGTTTCATCTGAT
ENSMMUT00000002758     AATAATAGTGACACTCTAAGACTCCTAAAAATAAGTAGCTGTCCTCATGTTTCATCTGAT
ENST00000297158        AATAATAGTGACACTCTAAGACTCCCAAAGATGAGTAGCTGTCCTCATGTTTCATCTGAT
XM_527020.3            AATAATAGTGACACTCTAAGACTCCTAAAGATGAGTAGCTGTCCTCATGTTTCATCTGAT

ENSRNOT00000035839     CGAATCCTCTGTGTGGCGGACCACTGTCAGGGCCTCCGAGAGCTGGCGCTGAATTACTAC
ENSMUST00000045428     GGGATCCTCTGTGTGGCGGACCACTGTCAGGGCCTCCGAGAGCTGGCCCTGAATTACTAC
ENSMMUT00000002758     GGAATTCTTTGTGTAGCTGATCATTGTCAAGGCCTTAGAGAACTGGCGCTGAATTATTAC
ENST00000297158        GGAATTCTTTGTGTAGCTGACCGTTGTCAAGGCCTTAGAGAACTGGCGTTGAATTATTAC
XM_527020.3            GGAATTCTTTGTGTAGCTGATCATTGTCAAGGCCTTAGAGAACTGGCGTTGAATTATTAC

ENSRNOT00000035839     ATTCTGAGTGACGAACTCCTCCTGGCGCTCTCAAGCGAGACTCACGTGAACCTGGAACAT
ENSMUST00000045428     ATTCTGAGCGACGAAATTCTCCTTGCGCTCTCCAGCGAGACTCACGTGAACCTCGAACAT
ENSMMUT00000002758     ATCCTAACTGATGAACTTCTCTTTGCGCTCTCAAGCGAGACTCATGTTAACCTTGAACAT
ENST00000297158        ATCCTAACTGATGAACTTTTCCTTGCACTCTCAAGCGAGACTCATGTTAACCTTGAACAT
XM_527020.3            ATCCTAACTGATGAACTTTTCCTTGCACTCTCAAGCGAGACTCATGTTAACCTTGAACAT
```

```
ENSRNOT00000035839     CTCCGAATAGATGTTGTGAGTGAAAACCCTGGGCAGATCAAGTTCCACTCCATTAAGAAA
ENSMUST00000045428     CTCCGGATAGACGTTGTGAGTGAAAACCCGGGGCAGATCAAGTTCCATTCTATTAAGAAA
ENSMMUT00000002758     CTTCGAATTGATGTTGTGAGTGAAAATCCTGGACAGATTAAATTTCATGCTATTAAAAAA
ENST00000297158        CTTCGAATTGATGTTGTGAGTGAAAATCCTGGACAGATTAAATTTCATGCTGTTAAAAAA
XM_527020.3            CTTCGAATTGACGTTGTGAGTGAAAATCCTGGACAGATTAAATTTCATGCTATTAAGAAA

ENSRNOT00000035839     CCCAGCTGGGACGCACTGGTTAAACACTCCCCGGGGGTGAACGTCGTCATGTACTTCTTT
ENSMUST00000045428     CGCAGCTGGGACGCACTGATCAAACACTCCCCGAGAGTGAACGTCGTCATGTACTTCTTC
ENSMMUT00000002758     CACAGTTGGGATGCACTTATTAAACATTCCCCTAGAGTTAATGTTGTTATGTACTTCTTT
ENST00000297158        CACAGTTGGGATGCACTTATTAAACATTCCCCTAGAGTTAATGTTGTTATGCACTTCTTT
XM_527020.3            CACAGTTGGGATGCACTTATTAAACATTCCCCTAGAGTTAATGTTGTTATGCACTTCTTT

ENSRNOT00000035839     TTGTACGAAGAAGAGTTTGACACATTCTTCAAAGAGGAAACCCCCGTCACTCACCTTTAT
ENSMUST00000045428     TTGTATGAAGAAGAGTTTGAGGCATTCTTCAAAGAGGAAACCCCCGTCACTCACCTTTAT
ENSMMUT00000002758     CTATGTGAAGAGGAATTTGAGATGTTCTTCAAAGAAGAAACCCCTGTTACTCACCTTTAT
ENST00000297158        CTATATGAAGAGGAATTCGAGACGTTCTTCAAAGAAGAAACCCCTGTTACTCACCTTTAT
XM_527020.3            CTATATGAAGAGGAATTTGAGACGTTCTTCAAAGAAGAAACCCCTGTTACTCACCTTTAT

ENSRNOT00000035839     TTCGGCCGTTCAGTAAGCAGAACCATCCTGGGCCGGATCGGTCTCAACTGCCCTCGGCTG
ENSMUST00000045428     TTCGGACGTTCAGTAAGCAGAGCCATCCTGGGCCGGATAGGTCTTAACTGCCCGAGGCTG
ENSMMUT00000002758     TTTGGTTGTTCAGTCAGCAAAGTGGTTTTAGGACGGATAGCTCTCAACTGTCCTCGACTG
ENST00000297158        TTTGGTCGTTCAGTCAGCAAAGTGGTTTTAGGACGGGTAGGTCTCAACTGTCCTCGACTG
XM_527020.3            TTTGGTCGTTCAGTCAGCAGAGTGGTTTTAGGACGGGTAGGTCTCAACTGTCCTCGACTG

ENSRNOT00000035839     ATCGAGTTAGTGGTGTGTGCCAACGGCCTTCAGCCCCTCGACAGTGAGCTTATTCGCATC
ENSMUST00000045428     ATCGAGTTAGTGGTGTGTGCCAATGGCCTTCTGCCCCTGGACAGTGAACTCATCCGTATT
ENSMMUT00000002758     ACTGAGTTAGTGGTGTGTGCTAATGGTCTTCAGCCTCTTGATAATGAACTTATTTGTGTT
ENST00000297158        ATTGAGTTAGTGGTGTGTGCTAATGATCTTCAGCCTCTTGATAATGAACTTATTTGTATT
XM_527020.3            ATTGAGTTAGTGGTGTGTGCTAATGATCTTCAGCCTCTTGATAATGAACTTATTTGTATT

ENSRNOT00000035839     GCCGAGCACTGTAAAAACCTAACCGCCCTGGGCCTGAGCGAGTGTGAGGTCAGCTGCAGT
ENSMUST00000045428     GCCAAGCATTGTAAAAATCTAACCTCCTTGGGCCTGAGTGAGTGTGAAGTCAGCTGCAGC
ENSMMUT00000002758     GCTGAACACTGTACAAACCTAACAGCCTTGGGCCTCAGCGAATGTGAAGTCAGCTGCAGT
ENST00000297158        GCTGAACACTGTACAAACCTAACAGCCTTGGGCCTCAGCAAATGTGAAGTTAGCTGCAGT
XM_527020.3            GCTGAACACTGTACAAACCTAACAGCCTTGGGCCTCAGCAAATGTGAAGTTAGCTGCAGT

ENSRNOT00000035839     GCTTTCGTTGAGTTTGTGAGGCTCTGTGGCCGAAGGCTCACCCAGCTGTCTCTCATGGAG
ENSMUST00000045428     GCATTTGTAGAGTTTGTGAGACTCTGTGGCAGAAGGCTCACCCAGCTGTCTATCATGGAG
ENSMMUT00000002758     GCCTTCGTCGAGTTTGTAAGACTGTGTGGGAGAAGGCTAACACAGCTCTCTATAATGGAG
ENST00000297158        GCCTTCATCAGGTTTGTAAGACTGTGTGAGAGAAGGTTAACACAGCTCTCTGTAATGGAG
XM_527020.3            GCCTTCATCAGGTTTGTAAGACTGTGTGGGAGAAGGTTAACACAGCTCTCTGTAATGGAG

ENSRNOT00000035839     GAAGTTCTGGTCCCTGATGACAGATACACACCAGACGAAGTCCACACGGAAGTCTCTAAG
ENSMUST00000045428     GAAGTTCTGGTCCCTGACGACAGGTACACCCCAGATGAAGTCCACACTGAAGTCTCTAAG
ENSMMUT00000002758     GAAGTTTTGATCCCTGATGAGGATTATAGCCTAGATGAAATTCACACTGAAGTCTCCAAA
ENST00000297158        GAAGTTTTGATCCCTGATGAGGATTATAGCCTAGATGAAATTCACACTGAAGTCTCCAAA
XM_527020.3            GAAGTTTTGATCCCTGATGAGGATTATAGCCTAGATGAAATTCACACTGAAGTCTCCAAA

ENSRNOT00000035839     CACCTGGGAAGAGTGTGGTTCCCAGATGTGATGCCTGTCTGG
ENSMUST00000045428     CACCTGGGAAGAGTGTGGTTTCCGGATGTGATGCCCATCTGG
ENSMMUT00000002758     TACCTGGGAAGAGTATGGTTCCCTGATGTGATGCCTCTCTGG
```

```
ENST00000297158        TACCTGGGAAGAGTATGGTTCCCTGATGTGATGCCTCTCTGG
XM_527020.3            TACCTGGGAAGAGTATGGTTCCCTGATGTGATGCCTCTCTGG
```

Multiple sequence alignment of Fbxl22

```
ENSCJAT00000008740     ATGCACATAACCCAGCTCAACCGGGAGTGCCTGCTGCACCTCTTCTCCTTCCTAGACAAG
ENSMMUT00000016960     ATGCACATAACCCAGCTCAACCGGGAGTGCCTGCTGCACCTCTTCTCCTTCCTAGACAAG
ENSGGOT00000025062     ATGCACATAACCCAGCTCAACCGGGAGTGCCTGCTGCACCTCTTCTCCTTCCTAGACAAG
ENST00000360587        ATGCACATAACCCAGCTCAACCGGGAGTGCCTGCTGCACCTCTTCTCCTTCCTAGACAAG
ENSPPYT00000007725     ATGCACATAACCCAGCTCAACCGGGAGTGCCTGCTGCACCTCTTCTCCTTCCTAGACAAG


ENSCJAT00000008740     GACAGCAGGAAGAGCCTAGCCAGGACCTGCCCTCAGCTCCGCGAGGTGTTTGAGGACCCC
ENSMMUT00000016960     GACAGCAGGAAGAGCCTCGCCAGGACCTGCTCCCAGCTCCACGACGTGTTTGAGGACCCC
ENSGGOT00000025062     GACAGCAGGAAGAGCCTTGCCAGGACCTGCTCCCAGCTCCACGACGTGTTTGAGGACCCC
ENST00000360587        GACAGCAGGAAGAGCCTTGCCAGGACCTGCTCCCAGCTCCACGACGTGTTTGAGGACCCC
ENSPPYT00000007725     GACAGCAGGAAGAGCCTTGCCAGGACCTGCTCCCAGCTCCACGACGTGTTTGAGGACCCC


ENSCJAT00000008740     ACACTCTGGTCCCTACTGCACTTCCGTTCCCTCACTGAACTCCAGAAGGACAACTTCCTC
ENSMMUT00000016960     GCACTGTGGTCCCTGCTGCACTTCCGTTCCCTCACTGAACTCCAGAAGGACAACTTCCTC
ENSGGOT00000025062     GCACTCTGGTCCCTGCTGCACTTCCGTTCCCTCACTGAACTCCAGAAGGACAACTTCCTC
ENST00000360587        GCACTCTGGTCCCTGCTGCACTTCCGTTCCCTCACTGAACTCCAGAAGGACAACTTCCTC
ENSPPYT00000007725     GCACTCTGGTCCCTGCTGCACTTCCGTTCCCTCACTGAACTCCAGAAGGACAACTTCCTC


ENSCJAT00000008740     CTGGGCCCTGCTCTCCGCAGCCTCTCCATCTGCTGGCACTCCAGCCGTGTGCAGGTGTGC
ENSMMUT00000016960     CTGGGCCCGGCCCTCCGCAGCCTCTCCATCTGTTGGCACTCCAGCCGCGTGCAGGTGTGC
ENSGGOT00000025062     CTGGGCCCGGCACTCCGCAGCCTCTCCATCTGCTGGCACTCCAGCCGCGTGCAGGTGTGC
ENST00000360587        CTGGGCCCGGCACTCCGCAGCCTCTCCATCTGCTGGCACTCCAGCCGCGTGCAGGTGTGC
ENSPPYT00000007725     CTGGGCCCGGCACTCCGCAGCCTCTCCATCTGCTGGCACTCCAGCCGCGTGCAGGTGTGC


ENSCJAT00000008740     AGCATTGAGGACTGGCTCAAGAGT---------AGGAGTATCTGCAGCCGGCACGAGAGC
ENSMMUT00000016960     AGCATCGAGGACTGGCTCAAGAGTGCCTTCCAGAGAAGCATCTGCAGCCGGCACGAGAGC
ENSGGOT00000025062     AGCATTGAGGACTGGCTCAAGAGTGCCTTCCAGAGAAGCATCTGCAGCCGGCACGAGAGC
ENST00000360587        AGCATTGAGGACTGGCTCAAGAGTGCCTTCCAGAGAAGCATCTGCAGCCGGCACGAGAGC
ENSPPYT00000007725     AGCATTGAGGACTGGCTCAAGAGTGCCTTCCAGAGAAGCATCTGCAGCCGGCACGAGAGC


ENSCJAT00000008740     CTAGTCAATGATTTCCTCCTCCGGGTGTGCGACAGTCTCTGTGCGGTGCGCCCCCAACGG
ENSMMUT00000016960     CTGGTCAATGATTTCCTCCTCCAGGTGTGCGACAGGCTTTCTGCTGTGCGCTCCCCACGG
ENSGGOT00000025062     CTGGTCAATGATTTCCTCCTCCGGGTGTGCGACAGGCTTTCTGCTGTGCGCTCCCCACGG
ENST00000360587        CTGGTCAATGATTTCCTCCTCCGGGTGTGCGACAGGCTTTCTGCTGTGCGCTCCCCACGG
ENSPPYT00000007725     CTGGTCAATGATTTCCTCCTCCGGGTGTGCGACAGGCTTTCTCCTGCGCGCTCCCCACGG


ENSCJAT00000008740     CGGCGGGAGGCACCT---CCGTCCTCGGGGACTCTGCTCGCGGTTGGGTGGAAATCACCT
ENSMMUT00000016960     CGGCGGGAGGCGCCTGCACCTTTCTCGGGGACTCCGATCGCCGTTGAATCGAAATCACCT
ENSGGOT00000025062     AGGCGGGAGGCGCCTGCACCGTCCTCGGGGACTCCGATCGCCGTTGGATCGAAATCACCT
ENST00000360587        AGGCGGGAGGCGCCTGCACCGTCCTCGGGGACTCCGATCGCCGTTGGACCGAAATCACCT
ENSPPYT00000007725     AGACGGGAGGCGCCTGCACCGTCCTCGGGGACTCCGATCGCCGTTGGATCGAAATCACCT


ENSCJAT00000008740     CGGTGGGGAGGGCCTCACCACTCGGAGCTCGCC---TTGCGCGCGGGGGTGAGAGAGGCC
ENSMMUT00000016960     CGGTGGGGAGGACCTGACCACTCGGAGTTCGCGGGCTTGCGCGCAGGGGTGACTGGGGCC
ENSGGOT00000025062     CGGTGGGGAGGACCTGACCACTCGGAGTTTGCCGACTTGCGCGCGGGGGTGACGGGGGCC
ENST00000360587        CGGTGGGGAGGACCTGACCACTCGGAGTTCGCCGACTTGCGCTCGGGGGTGACGGGGGCC
ENSPPYT00000007725     CGGTGGGGAGGACATGACCACTCGGAGTTCGCCGCCTTGCGCGCGGGGGTGACGGGGGCC
```

```
ENSCJAT00000008740     AGGGCTGCCGTGCGCAGGGGCCTGGAGAGCCTCCGGGCAGAGCGACCCCTCGAGACCCAG
ENSMMUT00000016960     AGGGCTGCCGCGCGCAGGGGTCTGGAGAGCCTCCGGGCGGAGCGACCCCGCGAGACTCGG
ENSGGOT00000025062     AGGGCTGCCCCGCGCAGGGGTCTGGGGAGCCTCCGGGCGGAGCGACCCAGCGAGACCCCG
ENST00000360587        AGGGCTGCCGCGCGCAGGGGTCTGGGGAGCCTCCGGGCGGAGCGACCCAGCGAGACCCCG
ENSPPYT00000007725     AGGGCTGCCGCGCGCAGGGGTCTGGGGAGCTTCCGGGCGGAGCGACCCAGCGAGACCCGG

ENSCJAT00000008740     CCGGCTCCCGGAGTGTCCTGGGGTCCGTCGCCTCCAGGGCCCCCAGTAGTGATCTCAGTG
ENSMMUT00000016960     TCGGCTCCCGGAGTGCCCTGGGGACGGCCGTCTCCAGGGCCCCCGGTAGTGATCTCGGTA
ENSGGOT00000025062     CCGGCTCCCGGAGTGTCCTGGGGACCGCCACCTCCAGGGGCCCCGGTAGTGATCTCGGTG
ENST00000360587        CCGGCTCCCGGAGTGTCCTGGGGACCGCCACCTCCAGGAGCCCCGGTGGTGATCTCGGTG
ENSPPYT00000007725     CCGGCTCCCGGAGTGTCCTGGGGACCGCCACCTCCAGGGGCCCCGGCAGTGATCTCGGTG

ENSCJAT00000008740     AAGCAGGAGGAGGGAAAGTGGGGT------GCCCCTCCCTGCGGTTTGCCGCGCGCGCTT
ENSMMUT00000016960     AAGCAGGAGGAGGGGAAGCAGGGGCAGAAAGAGCCACCGAGCCGTTTTGCCCGCACGCGC
ENSGGOT00000025062     AAGCAGGAGGAGGGGAAGCAGGGGCGCACGGCTCCTCCTTGCGGTTTTGCCCGCACGCGC
ENST00000360587        AAGCAGGAGGAGGGGAAGCAGGGGCGCACGGCTCCTCCTTGCGGTTTTGCCCGCACGCGC
ENSPPYT00000007725     AAGCAGGAG------CGCAGGGGCAGAAAGGCTCCTCCTGCGGTT---TGCCGCACGCGC

ENSCJAT00000008740     GCCTGCTCGCCT---TTGCTGGGAGCAGATGAGTTTCCCGAG
ENSMMUT00000016960     GGCTGCCTGCCCACCTTTCTTGGGGCGGATGCGCTCCCGGAG
ENSGGOT00000025062     GTCTGCCCGCCCACCTTTCCTGGGGCGGATGCGTTCCCGGAG
ENST00000360587        GTCTGCCCGCCCACCTTTCCTGGGGCGGATGCGTTCCCGCAG
ENSPPYT00000007725     GTCTGCCGCCCACCTTCCTGGGGT---GATGCGTTCCCGCAG
```

Multiple sequence alignment of Fbxl3

```
ENSGGOT00000007033     ATGAAACGAGGAGGAAGAGATAGTGACCGTAATTCATCAGAAGAAGGAACTGCAGAGAAA
ENSMMUT00000030204     ATGAAACGAGGAGGAAGAGATAGTGACCGTAATTCATCAGAAGAAGGAACTACAGAGAAA
ENSCJAT00000041690     ATGAAACGAGGAGGAAGAGATAGTGACCATAATTCATCAGAAGAAGGAACTGCAGAGAAA
ENST00000355619        ATGAAACGAGGAGGAAGAGATAGTGACCGTAATTCATCAGAAGAAGGAACTGCAGAGAAA
ENSPTRT00000047025     ATGAAACGAGGAGGAAGAGATAGTGACCGTAATTCATCAGAAGAAGGAACTGCAGAGAAA
ENSPPYT00000006425     ATGAAACGAGGAGGAAGAGATAGTGACCGTAATTCATCAGAAGAAGGAACTGCAGAGAAA
ENSMUST00000145693     ATGAAACGAGGAGGAAGAGATAGTGACCAGGACTCTGCTGAGGAAGGCACTGCCGAGAAG
ENSRNOT00000057789     ATGAAACGAGGAGGAAGAGATAGTGACCAGGACTCACCTGAGGAAGGCACTGCTGAGAAG

ENSGGOT00000007033     TCCAAGAAACTGAGGACTACAAATGAGCATTCTCAGACTTGTGATTGGGGTAATCTCCTT
ENSMMUT00000030204     TCCAAGAAACTGAGGACTACAAATGAGCATTCTCAGACTTGTGATTGGGGTAATCTCCTT
ENSCJAT00000041690     TCCAAGAAACTGAGGACTACAAATGAGCATTCTCAGACTTGTGACTGGGGTAATCTCCTT
ENST00000355619        TCCAAGAAACTGAGGACTACAAATGAGCATTCTCAGACTTGTGATTGGGGTAATCTCCTT
ENSPTRT00000047025     TCCAAGAAACTGAGGACTACAAATGAGCATTCTCAGACTTGTGATTGGGGTAATCTCCTT
ENSPPYT00000006425     TCCAAGAAACTGAGGACTACAAATGAGCATTCTCAGACTTGTGATTGGGGTAATCTCCTT
ENSMUST00000145693     CCCAAGAGACCGAGGACGACACAGGAGCGCTCGCAGCCTTGTGACTGGGGTAACCTCCTG
ENSRNOT00000057789     CCTAAGAAACCAAGGACAACACATGAGTGCTCGCAGCCTTGTGACTGGGGTAACCTTCTT

ENSGGOT00000007033     CAGGACATTATTCTCCAAGTATTTAAGTATTTGCCTCTTCTTGACCGGGCTCATGCTTCA
ENSMMUT00000030204     CAGGACATTATTCTCCAAGTATTTAAGTATTTGCCTCTTCTTGACCGGGCTCATGCTTCA
ENSCJAT00000041690     CAGGACATTATTCTCCAAGTGTTTAAGTATTTGCCTCTTCTTGACCGGGCTCATGCATCA
ENST00000355619        CAGGACATTATTCTCCAAGTATTTAAATATTTGCCTCTTCTTGACCGGGCTCATGCTTCA
ENSPTRT00000047025     CAGGACATTATTCTCCAAGTATTTAAGTATTTGCCTCTTCTTGACCGGGCTCATGCTTCA
ENSPPYT00000006425     CAGGACATTATTCTCCAAGTATTTAAGTATTTGCCTCTTCTTGACCGGGCTCATGCTTCA
ENSMUST00000145693     CAGGACATCGTCCTCCATGTTTTTAAGTATTTGCCTCTTCTTGACCGGGCTCATGCTTCC
ENSRNOT00000057789     CAGGACATTGTCCTCCACGTTTTTAAGTATTTGCCTCTTCTTGACCGGGCTCATGCTTCT
```

```
ENSGGOT00000007033    CAAGTTTGCCGCAACTGGAACCAGGTATTTCACATGCCTGACTTGTGGAGATGTTTTGAA
ENSMMUT00000030204    CAAGTTTGCCGCAACTGGAACCAGGTATTTCACATGCCTGACTTGTGGAGATGTTTTGAA
ENSCJAT00000041690    CAAGTTTGCCGCAACTGGAACCAGGTATTTCACATGCCTGACTTGTGGAGATGTTTTGAA
ENST00000355619       CAAGTTTGCCGCAACTGGAACCAGGTATTTCACATGCCTGACTTGTGGAGATGTTTTGAA
ENSPTRT00000047025    CAAGTTTGCCGCAACTGGAACCAGGTATTTCACATGCCTGACTTGTGGAGATGTTTTGAA
ENSPPYT00000006425    CAAGTTTGCCGCAACTGGAACCAGGTATTTCACATGCCTGACTTGTGGAGATGTTTTGAA
ENSMUST00000145693    CAAGTGTGCCGGAATTGGAATCAGGTATTTCACATGCCTGACTTGTGGCGATGTTTTGAA
ENSRNOT00000057789    CAAGTGTGCCGGAATTGGAATCAGGTATTTCACATGCCTGACTTGTGGCGATGTTTTGAA

ENSGGOT00000007033    TTTGAACTGAATCAGCCAGCTACATCTTATTTGAAAGCTACCCATCCAGAGCTGATCAAA
ENSMMUT00000030204    TTTGAACTGAATCAGCCAGCTACATCTTACTTGAAAGCTACCCATCCAGAGCTGATCAAA
ENSCJAT00000041690    TTTGAACTGAATCAGCCAGCTACATCTTATTTGAAAGCTACCCATCCAGAGCTGATCAAA
ENST00000355619       TTTGAACTGAATCAGCCAGCTACATCTTATTTGAAAGCTACCCATCCAGAGCTGATCAAA
ENSPTRT00000047025    TTTGAACTGAATCAGCCAGCTACATCTTACTTGAAAGCTACCCATCCAGAGCTGATCAAA
ENSPPYT00000006425    TTTGAACTGAATCAGCCAGCTACATCTTATTTGAAAGCTACCCATCCAGAGCTGATCAAA
ENSMUST00000145693    TTTGAACTGAATCAGCCAGCGACATCTTACTTGAAAGCCACACACCCAGAGCTGATCAAA
ENSRNOT00000057789    TTTGAACTGAATCAGCCAGCTACATCTTACTTGAAAGCTACACATCCAGAGCTGATCAAA

ENSGGOT00000007033    CAGATTATTAAAAGACATTCAAACCATCTACAATATGTCAGCTTCAAGGTGGACAGCAGC
ENSMMUT00000030204    CAGATAATTAAAAGACATTCAAACCATCTACAATATGTCAGCTTCAAGGTGGACAGCAGC
ENSCJAT00000041690    CAGATTATTAAAAGACATTCAAACCATCTACAATATGTCAGCTTCAAGGTGGACAGCAGC
ENST00000355619       CAGATTATTAAAAGACATTCAAACCATCTACAATATGTCAGCTTCAAGGTGGACAGCAGC
ENSPTRT00000047025    CAGATTATTAAAAGACATTCAAACCATCTACAATATGTCAGCTTCAAGGTGGACAGCAGC
ENSPPYT00000006425    CAGATTATTAAAAGACATTCAAACCATCTACAATATGTCAGCTTCAAGGTGGACAGCAGC
ENSMUST00000145693    CAGATTATCAAAAGGCACTCAAACCACCTACAGTATGTCAGCTTCAAGGTGGACAGCAGC
ENSRNOT00000057789    CAGATTATTAAGAGGCATTCAAATCATCTGCAGTATGTCAGCTTCAAGGTGGACAGCAGC

ENSGGOT00000007033    AAGGAATCAGCTGAAGCAGCTTGTGATATACTATCGCAACTTGTGAATTGCTCTTTAAAA
ENSMMUT00000030204    AAGGAATCAGCTGAAGCAGCTTGTGATATACTATCGCAACTTGTGAATTGCTCTTTAAAA
ENSCJAT00000041690    AAGGAATCAGCTGAAGCAGCTTGTGATATACTGTCGCAACTTGTGAATTGCTCTTTAAAA
ENST00000355619       AAGGAATCAGCTGAAGCAGCTTGTGATATACTATCGCAACTTGTGAATTGCTCTTTAAAA
ENSPTRT00000047025    AAGGAATCAGCTGAAGCAGCTTGTGATATACTATCGCAACTTGTGAATTGCTCTTTAAAA
ENSPPYT00000006425    AAGGAATCAGCTGAAGCAGCTTGTGATATACTATCGCAACTTGTGAATTGCTCTTTAAAA
ENSMUST00000145693    AAAGAATCAGCTGAAGCTGCTTGTGACATACTGTCGCAGCTTGTGAATTGCTCTTTAAAA
ENSRNOT00000057789    AAAGAATCCGCTGAAGCAGCTTGTGATATATTATCGCAGCTTGTGAATTGCTCTTTAAAA

ENSGGOT00000007033    ACACTTGGACTTATTTCAACTGCTCGACCAAGCTTTATGGATTTACCAAAGTCTCACTTT
ENSMMUT00000030204    ACACTTGGACTTATTTCAACTGCTCGACCAAGCTTTATGGATTTACCAAAGTCTCACTTT
ENSCJAT00000041690    ACACTTGGACTTATTTCAACTGCTCGACCAAGCTTTATGGATTTACCAAAGTCTCACTTT
ENST00000355619       ACACTTGGACTTATTTCAACTGCTCGACCAAGCTTTATGGATTTACCAAAGTCTCACTTT
ENSPTRT00000047025    ACACTTGGACTTATTTCAACTGCTCGACCAAGCTTTATGGATTTACCAAAGTCTCACTTT
ENSPPYT00000006425    ACACTTGGACTTATTTCAACTGCTCGACCAAGCTTTATGGATTTACCAAAGTCTCACTTT
ENSMUST00000145693    ACTCTCGGACTTATCTCGACTGCCCGGCCAAGCTTTATGGATCTACCAAAGTCTCACTTT
ENSRNOT00000057789    ACGCTTGGACTTATTTCAACTGCTCGGCCAAGCTTTATGGATCTACCAAAGTCTCACTTT

ENSGGOT00000007033    ATCTCTGCACTGACAGTTGTGTTTGTAAACTCCAAATCCCTGTCTTCGCTTAAGATAGAT
ENSMMUT00000030204    ATCTCTGCACTGACAGTTGTGTTTGTAAACTCCAAATCCCTGTCTTCGCTTAAGATAGAT
ENSCJAT00000041690    ATCTCTGCACTGACAGTTGTGTTTGTAAACTCCAAATCCCTGTCCTCGCTTAAGATAGAT
ENST00000355619       ATCTCTGCACTGACAGTTGTGTTCGTAAACTCCAAATCCCTGTCTTCGCTTAAGATAGAT
ENSPTRT00000047025    ATCTCTGCACTGACGGTTGTGTTCGTAAACTCCAAATCCCTGTCTTCGCTTAAGATAGAT
ENSPPYT00000006425    ATCTCTGCACTGACAGTTGTGTTCGTAAACTCCAAATCCCTGTCTTCGCTTAAGATAGAT
ENSMUST00000145693    ATCTCTGCACTGACAGTTGTGTTTGTAAACTCCAAGTCCCTGTCCTCACTTAAGATAGAC
ENSRNOT00000057789    ATCTCTGCACTGACAGTTGTGTTTGTAAACTCCAAATCCCTGTCCTCACTTAAGATAGAC
```

```
ENSGGOT00000007033      GATACTCCAGTAGATGATCCATCTCTCAAAGTACTAGTGGCCAACAATAGTGATACACTC
ENSMMUT00000030204      GATACTCCAGTAGATGATCCATCTCTCAAAGTACTAGTGGCCAACAACAGTGATACACTC
ENSCJAT00000041690      GATACTCCAGTAGACGATCCATCTCTCAAAGTACTAGTGGCCAACAACAGTGATACACTG
ENST00000355619         GATACTCCAGTAGATGATCCATCTCTCAAAGTACTAGTGGCCAACAATAGTGATACACTC
ENSPTRT00000047025      GATACTCCAGTAGATGATCCATCTCTCAAAGTACTAGTGGCCAACAATAGTGATACACTC
ENSPPYT00000006425      GATACTCCAGTAGATGATCCATCTCTCAAAGTACTAGTGGCCAACAATAGTGATACACTC
ENSMUST00000145693      GACACCCCAGTCGATGACCCATCCCTTAAAGTCCTCGTGGCCAACAACAGCGACACACTC
ENSRNOT00000057789      GACACCCCAGTAGATGACCCATCCCTTAAAGTCCTAGTGGCCAACAACAGTGACACACTC

ENSGGOT00000007033      AAGCTGTTGAAAATGAGCAGCTGTCCTCATGTCTCTCCAGCAGGTATCCTTTGTGTGGCT
ENSMMUT00000030204      AAGCTGTTGAAAATGAGCAGCTGTCCTCATGTCTCTCCAGCAGGTATCCTTTGTGTGGCT
ENSCJAT00000041690      AAACTGTTGAAAATGAGCAGCTGTCCTCATGTCTCTCCAGCAGGTATCCTTTGTGTGGCT
ENST00000355619         AAGCTGTTGAAAATGAGCAGCTGTCCTCATGTCTCTCCAGCAGGTATCCTTTGTGTGGCT
ENSPTRT00000047025      AAGCTGCTGAAAATGAGCAGCTGTCCTCATGTCTCTCCAGCAGGTATCCTTTGTGTGGCT
ENSPPYT00000006425      AAGCTGTTGAAAATGAGCAGCTGTCCTCATGTCTCTCCAGCAGGTATCCTTTGTGTGGCT
ENSMUST00000145693      AAGCTGTTGAAAATGAGCAGCTGTCCTCACGTCTCTCCAGCAGGCATTCTGTGTGTGGCT
ENSRNOT00000057789      AAGCTGCTGAAAATGAGCAGCTGTCCTCATGTCTCTCCAGCAGGGATTCTGTGTGTGGCT

ENSGGOT00000007033      GATCAGTGTCACGGCTTAAGAGAACTAGCCCTGAACTACCACTTATTGAGTGATGAGTTG
ENSMMUT00000030204      GATCAGTGTCATGGCTTAAGAGAACTAGCCCTGAACTACCACTTATTGAGTGATGAGTTG
ENSCJAT00000041690      GATCAGTGCCATGGCTTAAGGGAACTAGCCCTGAACTACCACTTACTAAGTGATGAGTTG
ENST00000355619         GATCAGTGTCACGGCTTAAGAGAACTAGCCCTGAACTACCACTTATTGAGTGATGAGTTG
ENSPTRT00000047025      GATCAGTGTCACGGCTTAAGAGAACTAGCCCTGAACTACCACTTATTGAGTGATGAGTTG
ENSPPYT00000006425      GATCAGTGTCATGGCTTAAGAGAACTAGCCCTGAACTACCACTTATTGAGTGATGAGTTG
ENSMUST00000145693      GACCAGTGTCATGGCTTACGAGAACTGGCCCTGAACTACCATTTGCTAAGTGATGAGTTG
ENSRNOT00000057789      GATCAGTGTCATGGCTTACGGGAGCTGGCCCTGAACTACCATTTGCTCAGTGATGAGCTG

ENSGGOT00000007033      TTACTTGCTTTGTCTTCTGAAAAACATGTTCGATTAGAACATTTGCGCATTGATGTAGTC
ENSMMUT00000030204      TTACTTGCTTTGTCTTCTGAAAAACATGTTCGATTAGAACATTTGCGCATTGATGTAGTC
ENSCJAT00000041690      TTACTTGCTTTGTCTTCTGAAAAACATGTTCGATTAGAACATTTGCGTATTGATGTAGTC
ENST00000355619         TTACTTGCATTGTCTTCTGAAAAACATGTTCGATTAGAACATTTGCGCATTGATGTAGTC
ENSPTRT00000047025      TTACTTGCATTGTCTTCTGAAAAACATGTTCGATTAGAACATTTGCGCATTGATGTAGTC
ENSPPYT00000006425      TTACTTGCTTTGTCTTCTGAAAAACATGTTCGATTAGAACATTTGCGCATTGATGTAGTC
ENSMUST00000145693      TTGCTCGCACTGTCTTCTGAAAAGCATGTTCGCTTAGAACATTTGCGCATTGATGTTGTC
ENSRNOT00000057789      TTGCTCGCACTGTCTTCTGAAAAGCACGTTCGCTTAGAACATTTGCGCATTGATGTTGTC

ENSGGOT00000007033      AGTGAGAATCCTGGACAGACACACTTCCATACTATTCAGAAGAGCAGCTGGGATGCTTTC
ENSMMUT00000030204      AGTGAGAATCCTGGACAGACACACTTCCATACTATTCAGAAGAGCAGCTGGGATGCTTTC
ENSCJAT00000041690      AGTGAGAATCCTGGACAGACACATTTCCATACTATTCAGAAGAGCAGCTGGGATGCTTTC
ENST00000355619         AGTGAGAATCCTGGACAGACACACTTCCATACTATTCAGAAGAGTAGCTGGGATGCTTTC
ENSPTRT00000047025      AGTGAGAATCCTGGACAGACACACTTCCATACTATTCAGAAGAGTAGCTGGGATGCTTTC
ENSPPYT00000006425      AGTGAGAATCCTGGACAGACACACTTCCATACTATTCAGAAGAGCAGCTGGGATGCTTTC
ENSMUST00000145693      AGTGAGAACCCGGGACAGACACACTTCCACACTATCCAGAAGAGCAGCTGGGATGCCTTC
ENSRNOT00000057789      AGTGAGAACCCGGGACAGACACACTTCCACACGATCCAGAAGAGCAGCTGGGACGCCTTC

ENSGGOT00000007033      ATCAGACATTCACCCAAAGTGAACTTAGTGATGTATTTTTTTTTATATGAAGAAGAATTT
ENSMMUT00000030204      ATCAGACATTCACCCAAAGTGAACTTAGTGATGTATTTTTTTTTATATGAAGAAGAATTT
ENSCJAT00000041690      ATCAGACATTCACCCAAAGTGAACTTGGTGATGTATTTTTTTTTATATGAAGAAGAATTT
ENST00000355619         ATCAGACATTCACCCAAAGTGAACTTAGTGATGTATTTTTTTTTATATGAAGAAGAATTT
ENSPTRT00000047025      ATCAGACATTCACCCAAAGTGAACTTAGTGATGTATTTTTTTTTATATGAAGAAGAATTT
ENSPPYT00000006425      ATCAGACATTCACCCAAAGTGAACTTAGTGATGTATTTTTTTTTATATGAAGAAGAATTT
ENSMUST00000145693      ATCAAACACTCACCGAAAGTCAACCTAGTGATGTATTTTTTTTTATACGAAGAGGAATTT
ENSRNOT00000057789      ATCAAGCACTCACCGAAGGTCAACCTAGTGATGTATTTTTTCCTGTACGAAGAGGAGTTT
```

```
ENSGGOT00000007033     GACCCATTCTTTCGCTATGAAATACCTGCCACCCATCTGTACTTTGGGAGATCAGTAAGC
ENSMMUT00000030204     GACCCATTCTTTCGCTATGAAATACCTGCCACCCATCTGTATTTTGGGAGATCAGTAAGC
ENSCJAT00000041690     GACCCATTCTTTCGCTATGAAATACCTGCCACCCATTTGTACTTTGGGAGATCAGTAAGT
ENST00000355619        GACCCCTTCTTTCGCTATGAAATACCTGCCACCCATCTGTACTTTGGGAGATCAGTAAGC
ENSPTRT00000047025     GACCCATTCTTTCGCTATGAAATACCTGCCACCCATCTGTACTTTGGGAGATCAGTAAGC
ENSPPYT00000006425     GACCCATTCTTTCGCTATGAAATACCTGCCACCCATCTGTACTTTGGGAGATCAGTAAGC
ENSMUST00000145693     GACCCATTCTTTCGTTATGAAATACCTGCTACGCATCTTTACTTTGGGAGATCAGTCAGC
ENSRNOT00000057789     GACCCGTTCTTCCGTTATGAAATCCCTGCCACTCATCTTTACTTTGGGAGATCAGTCAGC

ENSGGOT00000007033     AAAGATGTGCTTGGCCGTGTGGGAATGACATGCCCTAGACTGGTTGAACTAGTAGTGTGT
ENSMMUT00000030204     AAAGATGTGCTTGGCCGTGTGGGAATGACATGCCCTAGACTAGTTGAACTAGTAGTGTGT
ENSCJAT00000041690     AAAGATGTGCTTGGCCGTGTGGGAATGACATGCCCTAGACTAGTTGAACTAGTAGTGTGT
ENST00000355619        AAAGATGTGCTTGGCCGTGTGGGAATGACATGCCCTAGACTGGTTGAACTAGTAGTGTGT
ENSPTRT00000047025     AAAGATGTGCTTGGCCGTGTGGGAATGACATGCCCTAGACTGGTTGAACTAGTAGTGTGT
ENSPPYT00000006425     AAAGATGTGCTTGGCCGTGTGGGAATGACATGCCCTAGACTGGTTGAACTCGTAGTGTGT
ENSMUST00000145693     AAAGATGTGCTTGGCCGTGTGGGCATGACCTGCCCGAGACTAGTGGAACTGGTAGTGTGT
ENSRNOT00000057789     AAAGATGTGCTTGGCCGCGTGGGCATGACCTGCCCCAGACTAGTAGAACTGGTAGTGTGT

ENSGGOT00000007033     GCAAATGGATTACGGCCACTTGATGAAGAGTTAATTCGCATTGCAGAACGTTGCAAAAAT
ENSMMUT00000030204     GCAAATGGATTACGGCCACTTGACGAAGAGTTAATTCGCATTGCAGAACGTTGCAAAAAT
ENSCJAT00000041690     GCAAATGGATTACGGCCACTTGATGAAGAGTTAATTCGCATTGCAGAACGTTGCAAAAAT
ENST00000355619        GCAAATGGATTACGGCCACTTGATGAAGAGTTAATTCGCATTGCAGAACGTTGCAAAAAT
ENSPTRT00000047025     GCAAATGGATTACGGCCACTTGATGAAGAGTTAATTCGCATTGCAGAACGTTGCAAAAAT
ENSPPYT00000006425     GCAAATGGATTACGGCCACTTGATGAAGAGTTAATTCGCATTGCAGAACGTTGCAAAAAT
ENSMUST00000145693     GCCAATGGGTTGCGGCCTCTTGATGAAGAGTTAATTCGCATTGCGGAACGTTGCAAAAAT
ENSRNOT00000057789     GCCAATGGCTTGCGGCCCCTTGATGAAGAGTTGATTCGCATTGCAGAACGTTGCAAAAAT

ENSGGOT00000007033     TTGTCAGCTATTGGACTAGGGGAATGTGAAGTCTCATGTAGTGCCTTTGTTGAGTTTGTG
ENSMMUT00000030204     TTGTCAGCTATTGGACTAGGGGAATGTGAAGTCTCATGTAGTGCCTTTGTTGAGTTTGTG
ENSCJAT00000041690     TTGTCAGCTATTGGACTAGGGGAATGTGAAGTCTCATGTAGTGCCTTTGTTGAGTTTGTG
ENST00000355619        TTGTCAGCTATTGGACTAGGGGAATGTGAAGTCTCATGTAGTGCCTTTGTTGAGTTTGTG
ENSPTRT00000047025     TTGTCAGCTATTGGACTAGGGGAATGTGAAGTCTCATGTAGTGCCTTTGTTGAGTTTGTG
ENSPPYT00000006425     TTGTCAGCTATTGGACTAGGGGAATGTGAAGTCTCATGTAGTGCCTTTGTTGAGTTTGTG
ENSMUST00000145693     TTGTCAGCTATTGGGCTGGGGGAATGTGAAGTCTCATGTAGTGCCTTTGTTGAGTTTGTG
ENSRNOT00000057789     CTGTCAGCTATTGGGCTGGGGGAGTGTGAGGTCTCATGTAGTGCCTTTGTCGAGTTTGTA

ENSGGOT00000007033     AAGATGTGTGGTGGCCGCCTATCTCAATTATCCATTATGGAAGAAGTACTAATTCCTGAC
ENSMMUT00000030204     AAGATGTGTGGTGGCCGCCTATCTCAATTATCCATTATGGAAGAAGTACTAATTCCTGAC
ENSCJAT00000041690     AAGATGTGTGGTGGCCGCCTATCTCAATTATCTATTATGGAAGAAGTATTAATTCCTGAC
ENST00000355619        AAGATGTGTGGTGGCCGCCTATCTCAATTATCCATTATGGAAGAAGTACTAATTCCTGAC
ENSPTRT00000047025     AAGATGTGTGGTGGCCGCCTATCTCAATTATCCATTATGGAAGAAGTACTAATTCCTGAC
ENSPPYT00000006425     AAGATGTGTGGTGGCCGCCTATCTCAGTTATCCATTATGGAAGAAGTACTAATTCCTGAC
ENSMUST00000145693     AAGATGTGTGGGGGCCGCCTGTCTCAGTTATCCATTATGGAAGAAGTGTTAATTCCTGAC
ENSRNOT00000057789     AAGATGTGTGGGGGCCGCCTGTCTCAGTTATCCATTATGGAAGAAGTGTTAATTCCTGAC

ENSGGOT00000007033     CAAAAGTATAGTTTGGAGCAGATTCACTGGGAAGTGTCCAAGCATCTTGGTAGGGTGTGG
ENSMMUT00000030204     CAAAAGTATAGTTTGGAGCAGATTCATTGGGAAGTGTCCAAGCATCTTGGTAGGGTGTGG
ENSCJAT00000041690     CAAAAGTATAGTTTGGAGCAGATTCACTGGGAAGTGTCCAAGCATCTTGGTAGGGTGTGG
ENST00000355619        CAAAAGTATAGTTTGGAGCAGATTCACTGGGAAGTGTCCAAGCATCTTGGTAGGGTGTGG
ENSPTRT00000047025     CAAAAGTATAGTTTGGAGCAGATTCACTGGGAAGTGTCCAAGCATCTTGGTAGGGTGTGG
ENSPPYT00000006425     CAAAAGTATAGTTTGGAGCAGATTCACTGGGAAGTGTCCAAGCATCTTGGTAGGGTGTGG
ENSMUST00000145693     CAAAAGTATAGTTTGGAACAAATTCATTGGGAAGTGTCTAAGCATCTTGGCAGGGTATGG
ENSRNOT00000057789     CAGAAGTATAGTTTGGAACAGATTCATTGGGAAGTGTCCAAGCATCTTGGCAGGGTGTGG
```

```
ENSGGOT00000007033     TTTCCCGACATGATGCCCACTTGG
ENSMMUT00000030204     TTTCCCGACATGATGCCCACTTGG
ENSCJAT00000041690     TTTCCTGACATGATGCCCACTTGG
ENST00000355619        TTTCCCGACATGATGCCCACTTGG
ENSPTRT00000047025     TTTCCCGACATGATGCCCACTTGG
ENSPPYT00000006425     TTTCCCGACATGATGCCCACTTGG
ENSMUST00000145693     TTCCCAGACATGATGCCTACTTGG
ENSRNOT00000057789     TTTCCAGACATGATGCCTACGTGG
```

Multiple sequence alignment of Fbxl4

```
ENSGGOT00000001195     ATGTCACCGGTCTTTCCCATGTTAACAGTTCTGACCATGTTTTATTATATATGCCTTCGG
ENST00000229971        ATGTCACCGGTCTTTCCCATGTTAACAGTTCTGACCATGTTTTATTATATATGCCTTCGG
ENSPTRT00000034076     ATGTCACCGGTCTTTCCCATGTTAACAGTTCTGACCATGTTTTATTATATATGCCTTCGG
ENSPPYT00000019619     ATGTCACCGGTCTTTCCCATGTTAACAGTTCTGACCATGTTTTATTATATATGCCTTCGG
ENSCJAT00000059721     ATGTCACCAGTCTTTCCCATGTTAACAGTTCTGACTATGTTTTATTATATATGCCTTCGG
ENSMMUT00000002355     ATGTCACCGGTCTTTCCCATGTTAACAGTTCTGACTATGTTTTATTATATATGCCTTCGG
ENSMUST00000039234     ATGTCACCAGTCTTTCCCATGTTAACAGTTCTGACCATGTTTTATTATATGTGCCTTCGG
ENSRNOT00000061128     ATGTCACCAGTGTTTCCCATGTTAACAGTTCTGACCATGTTTTATTATATGTGCCTTCGG


ENSGGOT00000001195     CGCCGAGCCAGGACAGCTACAAGAGGAGAAATGATGAACAGCCATAGAACTATAGAATCA
ENST00000229971        CGCCGAGCCAGGACAGCTACAAGAGGAGAAATGATGAACACCCATAGAGCTATAGAATCA
ENSPTRT00000034076     CGCCGAGCCAGGACAGCTACAAGAGGAGAAATGATGAACACCCATAGAACTATAGAATCA
ENSPPYT00000019619     CGCCGAGCCAGGACAGCTACAAGAGGAGAAATGATGAACAGCCATAGAACTATAGAATCA
ENSCJAT00000059721     CGCCGAGCCAGGACAGCTACAAGAGGAGAGATGATGAACAGCCATAGAACCATAGAATCA
ENSMMUT00000002355     CGCCGAGCCAGGACAGCTACAAGAGGAGAGATGATGAACAGCCATAGAACTATAGAATCA
ENSMUST00000039234     CGTCGAGCCAGGACAGCTACAAGAGGAGATATGATGAACAGCCATAGGACTATAGTGTCA
ENSRNOT00000061128     CGTCGAGCCAGGACAGCTACGAGAGGAGATATGATGAGCAGCCATAGGACTATAGTATCA


ENSGGOT00000001195     AACAGCCGGACTTCCCCTCTCAATGCAGAGGTAGTCCAGTATGCCAAAGAAGTAGTGGAT
ENST00000229971        AACAGCCAGACTTCCCCTCTCAATGCAGAGGTAGTCCAGTATGCCAAAGAAGTAGTGGAT
ENSPTRT00000034076     AACAGCCGGACTTCCCCTCTCAATGCAGAGGTAGTCCAGTATGCCAAAGAAGTAGTGGAT
ENSPPYT00000019619     AACAGCCGGCCTTCCCCTCTCAATGCAGAGGTAGTCCAGTATGCCAAAGAAGTAGTGGAT
ENSCJAT00000059721     AACAGCCGGACTTCCCCTCTCAATGCAGAGGTGGTCCAGTATGCCAAAGAAGTAGTGGAT
ENSMMUT00000002355     AACAGCCGGACTTCCCCTCTCAATGCGGAGGTAGTCCAGTATGCCAAAGAAGTAGTGGAT
ENSMUST00000039234     AACAGCCGGACTTCCCCTCTCAATGCAGAGGTGGTCCAGTATGCTAAAGAAGTGGTGGAT
ENSRNOT00000061128     AACAGCCGGACTTCCCCTCTCAATGCAGAGGTGGTCCAGTATGCTAAGGAAGTGGTGGAT


ENSGGOT00000001195     TTCAGTTCCCATTATGGAAATGAGAATAGTATGTCCTATACTATGTGGAATTTGGCTGGT
ENST00000229971        TTCAGTTCCCATTATGGAAGTGAGAATAGTATGTCCTATACTATGTGGAATTTGGCTGGT
ENSPTRT00000034076     TTCAGTTCCCATTATGGAAGTGAGAATAGTATGTCCTATACTATGTGGAATTTGGCTGGT
ENSPPYT00000019619     TTCAGTTCCCATTATGGAAGTGAGAATAGTATGTCCTATACTATGTGGAATTTGGCTGGT
ENSCJAT00000059721     TTCAGTTCCCATTATGGAAGTGAGAATAGTATGTCCTATACTATGTGGAATTTGGCTGGT
ENSMMUT00000002355     TTCAGTTCCCATTATGGAAGTGAGAATAGTATGTCCTATACTATGAGGAATTTGGCTGGC
ENSMUST00000039234     TTCAGTTCCCATTACGGCAGTGAGAACAGTATGTCCTATACCATGTGGAATTTGGCAGGT
ENSRNOT00000061128     TTCAGTTCCCACTACGGCAGCGAGAACAGCATGTCCTATACCATGTGGAATTTGGCAGGT


ENSGGOT00000001195     GTACCAAATGTATTCCCAAGTTCTGGTGACTTTACTCAGACAGCTGTGTTTCGAACTTAT
ENST00000229971        GTACCAAATGTATTCCCAAGTTCTGGTGACTTTACTCAGACAGCTGTGTTTCGAACTTAT
ENSPTRT00000034076     GTACCAAATGTATTCCCAAGTTCTGGTGACTTTACTCAGACAGCTGTGTTTCGAACTTAT
ENSPPYT00000019619     GTACCAAATGTATTCCCAAGTTCTGGTGACTTTACTCAGACAGCTGTGTTTCGAACTTAT
ENSCJAT00000059721     GTACCAAATGTATTCCCAAGTTCTGGTGACTTTACTCAGACGGCTGTGTTTCGAACTTAT
ENSMMUT00000002355     GTACCAAATGTATTCCCAAGTTCTGGTGACTTTACTCAGACAGCTGTGTTTCGAACTTAT
```

ENSMUST00000039234	GTACCAAATGTCTTCCCAAGTTCTGGTGACTTCACGCAGACGGCTGTGTTTCGAACTTAT
ENSRNOT00000061128	GTACCAAATGTCTTCCCAAGTTCTGGCGACTTCACGCAGACGGCTGTGTTTCGAACTTAC
ENSGGOT00000001195	GGGACATGGTGGGATCAGTGTCCTAGTGCTTCCTTGCCATTCAGGAGGACGCCACCTAAT
ENST0000229971	GGGACATGGTGGGATCAGTGTCCTAGTGCTTCCTTGCCATTCAAGAGGACGCCACCTAAT
ENSPTRT00000034076	GGGACATGGTGGGATCAGTGTCCTAGTGCTTCCTTGCCATTCAAGAGGACGCCACCTAAT
ENSPPYT00000019619	GGGACATGGTGGGATCAGTGTCCTAGTGCTTCCTTGCCATTCAAGAGGACGCCACCTAAT
ENSCJAT00000059721	GGGACATGGTGGGATCAGTGTCCTAGTGCTTCCTTGCCATTCAAGAGGACGCCACCTAAT
ENSMMUT00000002355	GGGACATGGTGGGATCAGTGTCCTAGTGCTTCCTTGCCATTCAAGAGGACACCACCTAAT
ENSMUST00000039234	GGGACGTGGTGGGATCAGTGTCCTAGTGCTTCCTTGCCATTCAGAAGGACACCGTCCAGT
ENSRNOT00000061128	GGAACATGGTGGGATCAGTGTCCTAGTGCATCCTTGCCATTCAGAAGAACGCCGTCCAGT
ENSGGOT00000001195	TTTCAGAGCCAGGACTATGTGGAACTTACTTTTGAACAACAGGTGTATCCTACAGCTGTT
ENST0000229971	TTTCAGAGCCAGGACTATGTGGAACTTACTTTTGAACAACAGGTGTATCCTACAGCTGTA
ENSPTRT00000034076	TTTCAGAGCCAGGACTATGTGGAACTTACTTTTGAACAACAGGTGTATCCTACAGCTCTT
ENSPPYT00000019619	TTTCAGAGCCAGGACTATGTGGAACTTGCTTTTGAACAACAGGTGTATCCTACAGCTGTT
ENSCJAT00000059721	TTTCAGAGCCAGGACTATGTGGAACTTACTTTTGAACAACAGGTGTACCCTACAGCTGTT
ENSMMUT00000002355	TTTCAGAGCCAGGACTATGTGGAACTTACTTTTGAACAACAGGTGTATCCTACAGCTGTT
ENSMUST00000039234	TTCCAGAGCCAGGACTATGTGGAACTGACTTTTGAGCAGCAAGTGTACCCCACGGCTGTC
ENSRNOT00000061128	TTTCAGAGCCAGGACTATGTGGAACTGACTTTTGAGCAACAAGTGTATCCCACAGCTGTC
ENSGGOT00000001195	CATGTTCTAGAAACCTATCATCCCGGAGCAGTCATTAGAATTCTCGCTTGTTCTGCAAAT
ENST0000229971	CATGTTCTAGAAACCTATCATCCCGGAGCAGTCATTAGAATTCTCGCTTGTTCTGCAAAT
ENSPTRT00000034076	CATGTTCTAGAAACCTATCATCCCGGAGCAGTCATTAGAATTCTCGCTTGTTCTGCAAAT
ENSPPYT00000019619	CATGTTCTAGAAACCTATCATCCCGGAGCAGTCATTAGAATTCTCGCTTGTTCTGCAAAT
ENSCJAT00000059721	TATGTTCTAGAAACCTATCATCCTGGAGCAGTCATTAGAATTCTTGCTTGTTCTGCAAAT
ENSMMUT00000002355	CATGTTCTAGAAACCTATCATCCTGGAGCAGTCATCAGAATTCTTGCTTGTTCTGCAAAT
ENSMUST00000039234	CATGTGCTGGAGACTTACCATCCTGGAGCAGTCATCAGAATTCTGGCCTGCTCAGCAAAT
ENSRNOT00000061128	CATGTGCTGGAGACCTACCATCCTGGAGCAGTCATCAGAATTCTGGCCTGCTCGGCAAAT
ENSGGOT00000001195	CCTTATTCCCCAAATCCACCAGCTGAAGTAAGATGGGAGATTCTTTGGTCAGAGAGACCT
ENST0000229971	CCTTATTCCCCAAATCCACCAGCTGAAGTAAGATGGGAGATTCTTTGGTCAGAGAGACCT
ENSPTRT00000034076	CCCTATTCCCCAAATCCACCAGCTGAAGTAAGATGGGAGATTCTTTGGTCAGAGAGACCT
ENSPPYT00000019619	CCTTATTCCCCAAATCCACCAGCTGAAGTAAGATGGGAGATTCTTTGGTCAGAGAGACCT
ENSCJAT00000059721	CCCTATTCCCCAAATCCACCAGCTGAAGTAAGATGGGAGATTCTTTGGTCAGAGAGACCT
ENSMMUT00000002355	CCTTATTCCCCAAATCCACCAACTGAAGTAAGATGGGAGATTCTTTGGTCAGAGAGACCT
ENSMUST00000039234	CCCTACTCCCCAAATCCTCCAGCTGAAGTAAGATGGGAGATTCTTTGGTCAGAGAGACCT
ENSRNOT00000061128	CCCTACTCCCCAAATCCTCCAGCAGAAGTAAGATGGGAGACTCTTTGGTCCGAGAGACCC
ENSGGOT00000001195	ACGAAGGTGAATGCTTCCCAAGCTCGCCAGTTTAAACCTTGTATTAAGCAGATAAATTTC
ENST0000229971	ACGAAGGTGAATGCTTCCCAAGCTCGCCAGTTTAAACCTTGTATTAAGCAGATAAATTTC
ENSPTRT00000034076	ACGAAGGTGAATGCTTCCCAAGCTCGCCAGTTTAAACCTTGTATTAAGCAGATAAATTTC
ENSPPYT00000019619	ACGAAGGTGAATGCTTCCCAAGCTCGCCAGTTTAAACCTTGTATTAAGCAGATAAATTTC
ENSCJAT00000059721	ACGAAGGTGAATGCTTCCCAAGCTCGCCAGTTTAAACCTTGTATTAAGCAGATAAATTTT
ENSMMUT00000002355	ACGAAGTTGAATGCTTCCCAAGCTCGCCAGTTTAAACCTTGTATTAAGCAGATAAATTTT
ENSMUST00000039234	ATGAAGGTGAATGCTTCCCAGGCTCGTCAGTTTAAACCTTGTATTAAGCAAATAAATTTC
ENSRNOT00000061128	ACGAAGGTGAATGCTTCCCAGGCTCGCCAGTTTAAACCTCGTATTAAGCAAATAAATTTC
ENSGGOT00000001195	CCCACAAATCTTATACGACTGGAAGTAAATAGTTCTCTTCTGGATTATTACACTGAATTA
ENST0000229971	CCCACAAATCTTATACGACTGGAAGTAAATAGTTCTCTTCTGGAATATTACACTGAATTA
ENSPTRT00000034076	CCCACAAATCTTATACGACTGGAAGTAAATAGTTCACTTCTGGATTATTACACTGAATTA
ENSPPYT00000019619	CCCACAAATCTTATACGACTGGAAGTAAATAGTTCTCTTCTGGATTATTACACTGAATTA
ENSCJAT00000059721	CCCACAAATCTTATACGACTGGAAGTAAATAGTTCTCTTCTGGATTATTACACTGAATTA
ENSMMUT00000002355	CCCACAAATCTTATACGACTGGAAGTAAATAGTTCTCTTCTGGATTATTACACTGAATTA

```
ENSMUST00000039234     CCTACAAACCTCATACGACTGGAAGTCAATAGTTCTCTTCTGGATTATTACACCGAACTA
ENSRNOT00000061128     CCCACAAACCTTATACGACTGGAAGTAAACAGTTCTCTTCTGGATTATTACACCGAATTA

ENSGGOT00000001195     GATGCAGTTGTGCTACATGGTGTGAAGGACAAGCCAGTGCTGTCTCATAAGACTTCACTT
ENST0000229971         GATGCAGTTGTGCTACATGGTGTGAAGGACAAGCCAGTGCTTTCTCTCAAGACTTCACTT
ENSPTRT00000034076     GATGCAGTTGTGCTACATGGTGTGAAGGACAAGCCAGTGATTTCTCTCAAGACTTCACTT
ENSPPYT00000019619     GATGCAGTTGTGCTACATGGTGTGAAGGACAAGCCAGTGCTTTCTCTCAAGACTTCACTT
ENSCJAT00000059721     GATGCAGTTGTGCTACATGGTGTGAAGGACAAGCCAGTGCTTTCTCTCAAGACTTCACTT
ENSMMUT00000002355     GATGCAGTTGTGCTACATGGTGTGAAGGACAAGCCAGTGCTTTCTCTCAAGACTTCACTT
ENSMUST00000039234     GATGCAGTTGTGCTGCATGGTACGAAGGACAAGCCACTGCTCTCTCTCAAGACTGCTCTT
ENSRNOT00000061128     GATGCAGTTGTGCTACATGGTACGAAGGACAAGCCACTGCTCTCTCTCAAGACTGCACTT

ENSGGOT00000001195     ATTGACATGAATGATATAGAAGATGATGCCTATGAAGAAAAGGATGGTTGTCGAATGGAC
ENST0000229971         ATTGACATGAATGATATAGAAGATGATGCCTATGCAGAAAAGGATGGTTGTGGAATGGAC
ENSPTRT00000034076     ATTGACATCAATGATAGAGAAGATGATGCCTATGCAGAAAAGGATGGTTGTGGAATGGAC
ENSPPYT00000019619     ATTGACATGAATGATATAGAAGATGATGCCTATGAAGAAAAGGATGGTTGTGGAATGGAC
ENSCJAT00000059721     ATTGACATGAATGATATAGAAGATGATGCCTATGAAGAAAAGAATGGCTGTGGAATGGAC
ENSMMUT00000002355     ATTGACATGAATGATATAGATGATGATGCCTATGAAGAAAAGGATGGTTGTGGAATGGAC
ENSMUST00000039234     GTAGACATGAATGATCTAGAAGATGACGACTATGAAGAGAAGGATGGCTGTGAGATGGAT
ENSRNOT00000061128     GTAGACATGAATGATCTAGAAGATGATGACTATGAAGAGAAGGACGGCTGTGAAATGGAC

ENSGGOT00000001195     AGTCTTAACAAAAAGTTTAGCAGTGCTGTCCTCGGGGAAGGGCCAAATAATGGGTATTTT
ENST0000229971         AGTCTTAACAAAAAGTTTAGCAGTGCTGTCCTCGGGGAAGGGCCAAATAATGGGTATTTT
ENSPTRT00000034076     AGTCTTAACAAAAAGTTTAGCAGTGCTGTCCTCGGGGAAGGGCCAAATAATGGGTATTTT
ENSPPYT00000019619     AGTCTTAACAAACAGTTTAGCAGTGCTGTCCTCAGGGAAGGGCCAAATAATGGGTATTTT
ENSCJAT00000059721     AATCTTAACAAAAAGTTTAGCAGTGCTGTCCTCAGGGAAGGGCCAAATAATGGGTATTTT
ENSMMUT00000002355     AATCTTAACAAAAAGTTTAGCAGTGCTGTCCTCAGGGAAGGGCCAAATAATGGATATTTT
ENSMUST00000039234     GCTCTTAACAAAAAGTTTAGCAGTGCTGCCCTTGGGGATGGGCCACATAATGGGTATTTT
ENSRNOT00000061128     GCTCTTAACAAAAAGTTTAGCAGTGCTACCCTTGGGGATGGGCCAAGTAACGGGTATTTT

ENSGGOT00000001195     GATAAACTACCTTATGAGCTTATTCAACTGATTCTGAATCATCTTACACTACCAGACCTG
ENST0000229971         GATAAACTACCTTATGAGCTTATTCAGCTGATTCTGAATCATCTTACACTACCAGACCTG
ENSPTRT00000034076     GATAAACTACCTTATGAGCTTATTCAACTGATTCTGAATCATCTTACACTACCAGACCTG
ENSPPYT00000019619     GATAAACTACCTTATGAGCTTATTCAACTGATTCTGAATCATCTTACACTACCAGACCTG
ENSCJAT00000059721     GATAAACTACCTTATGAGCTTATTCAACTGATTCTGAATCATCTTACACTACCAGACCTG
ENSMMUT00000002355     GATAAACTACCTTATGAGCTTATTCAACTGATTCTGAATCATCTTACACTACCAGACCTG
ENSMUST00000039234     GACAAACTACCATATGAGCTCATTCAACTGATTCTGAATCATCTTTCACTACCAGACCTG
ENSRNOT00000061128     GATAAACTACCATATGAGCTCATTCAACTGATTCTGAATCATCTTTCACTACCAGACCTG

ENSGGOT00000001195     TGTAGATTAGCACAGACTTGCAAACTACTGAGCCAGCATTGCTGTGATCCTCTGCAATAC
ENST0000229971         TGTAGATTAGCACAGACTTGCAAACTACTGAGCCAGCATTGCTGTGATCCTCTGCAATAC
ENSPTRT00000034076     TGTAGATTAGCACAGACTTGCAAACTACTGAGCCAGCATTGCTGTGATCCTCTGCAATAC
ENSPPYT00000019619     TGTAGATTAGCACAGACTTGCAAACTACTGAGCCAGCATTGCTGTGATCCTCTGCAATAC
ENSCJAT00000059721     TGTAGATTAGCACAGACTTGCAAACTACTGAGCCAGCATTGCTGTGATCCTCTACAATAC
ENSMMUT00000002355     TGTAGATTAGCACAGACTTGCAAACTACTGAGCCAGCATTGCTGTGATCCTCTACAATAC
ENSMUST00000039234     TGTAGATTAGCCCAGACTTGCAGGCTTCTCCACCAGCATTGCTGTGATCCTCTGCAATAT
ENSRNOT00000061128     TGTAGATTAGCACAGACTTGCAGGCTCCTCCACCAGCATTGCTGTGACCCTCTACAGTAT

ENSGGOT00000001195     ATCCACCTCAATCTGCAACCATACTGGGCAAAACTAGATGACACTTCTCTGGAATTTCTA
ENST0000229971         ATCCACCTCAATCTGCAACCATACTGGGCAAAACTAGATGACACTTCTCTGGAATTTCTA
ENSPTRT00000034076     ATCCACCTCAATCTGCAACCATACTGGGCAAAACTAGGTGACACTTCTCTGGAATTTCTA
ENSPPYT00000019619     ATCCACCTTAATCTGCAACCATACTGGGCAAAACTAGATGACACTTCTCTGGAATTTCTA
ENSCJAT00000059721     ATCCACCTCAATCTGCAACCATACTGGGCAAAACTAAATGACACCTCTCTGGAATTTCTC
ENSMMUT00000002355     ATCCATCTCAATCTGCAACCATACTGGGCAAAACTAGATGACACCTCTTTGGAATTTCTA
```

```
ENSMUST00000039234     ATCCACCTCAACCTGCAGCCATACTGGGCCCGGCTGGACGACACGTCACTGGAGTTCCTA
ENSRNOT00000061128     ATCCACCTCAACCTGCAGCCATACTGGGCCAAGCTAGATGATACTTCACTGGAGTTCCTA

ENSGGOT00000001195     CAGTCTCGCTGCACTCTTGTCCAGTGGCTTAATTTATCTTGGACTGGCAATAGAGGCTTC
ENST00000229971        CAGTCTCGCTGCACTCTTGTCCAGTGGCTTAATTTATCTTGGACTGGCAATAGAGGCTTC
ENSPTRT00000034076     CAGTCTCGCTGCACTCTTGTCCAGTGGCTTAATTTATCTTGGACTGGCAATAGAGGCTTC
ENSPPYT00000019619     CAGTCTCGATGCACTCTTGTCCAGTGGCTTAATTTATCTTGGACTGGCAATAGAGGCTTC
ENSCJAT00000059721     CAGGCTCGATGCACTCTGGTCCAGTGGCTTAATTTATCTTGGACTGGCAATAGAGGCTTC
ENSMMUT00000002355     CAGTCTCGATGCACTCTTGTCCAGTGGCTTAATTTATCTTGGACTGGCAATAGAGGCTTC
ENSMUST00000039234     CAGGCTCGCTGTGTGCTTGTCCAGTGGCTTAATCTGTCTTGGACTGGCAACAGAGGGTTC
ENSRNOT00000061128     CAGGCTCGCTGTGCTCTTGTCCAGTGGCTTAATCTGTCTTGGACTGGCAACAGAGGGTTC

ENSGGOT00000001195     ATCTCTGTTGCAGGATTTAGCAGGTTTCTGAAGGTTTGTGGATCTGAATTAGTACGCCTT
ENST00000229971        ATCTCTGTTGCAGGATTTAGCAGGTTTCTGAAGGTTTGTGGATCCGAATTAGTACGCCTT
ENSPTRT00000034076     ATCTCTGTTGCAGGATTTAGCAGGTTTCTGAAGGTTTGTGGATCTGAATTAGTACGCCTT
ENSPPYT00000019619     ATCTCTGTTGCAGGATTTAGCAGGTTTCTGAAGGTTTGTGGATCTGAATTAGTACGCCTT
ENSCJAT00000059721     ATCTCTGTTACAGGATTTAGCAGGTTTCTGAAGGTTTGTGGATCTGAATTAGTACGACTT
ENSMMUT00000002355     ATCTCTGTTGCAGGATTTAGCAGGTTTCTGAAGGTTTGTGGATCTGAATTAGTACGCCTT
ENSMUST00000039234     ATCTCTGTATCAGGATTTAGCAGGTTTCTAAAGGTGTGTGGGTCTGAGTTAGTACGCCTT
ENSRNOT00000061128     ATCTCTGTGTCAGGATTTAGCAGGTTTCTGAAGGTGTGTGGATCTGAATTAGTACGCCTT

ENSGGOT00000001195     GAATTGTCTTGCAGCCACTTTCTTAATGAAACTTGCTTAGAAGTTATTTCTGAGATGTGT
ENST00000229971        GAATTGTCTTGCAGCCACTTTCTTAATGAAACTTGCTTAGAAGTTATTTCTGAGATGTGT
ENSPTRT00000034076     GAATTGTCTTGCAGCCACTTTCTTAATGAAACTTGCTTAGAAGTTATTTCTGAGATGTGT
ENSPPYT00000019619     GAATTATCTTGCAGCCACTTTCTTAATGAAACTTCCTTAGAAGTTATTTCTGAGATGTGT
ENSCJAT00000059721     GAATTGTCTTGCAGCCACTTTCTTAATGAAACTTGTTTAGAAGTTATTTCTGAGATGTGT
ENSMMUT00000002355     GAATTGTCTTGCAGCCACTTTCTTAATGAAACTTGCTTAGAAGTTATTTCTGAGATGTGT
ENSMUST00000039234     GAGTTATCCTGCAGCCACTTTCTTAATGACACTTGCTTGGAGGTTATTTCTGAAATGTGC
ENSRNOT00000061128     GAGTTGTCTTGCAGCCACTTTCTTAATGACGCTTGCTTGGAGGTGATTTCTGAAATGTGC

ENSGGOT00000001195     CCAAATCTACAGGCCTTAAATCTCTCCTCCTGTGATAAGCTACCACCTCAAGCTTTCAAC
ENST00000229971        CCAAATCTACAGGCCTTAAATCTCTCCTCCTGTGATAAGCTACCACCTCAAGCTTTCAAC
ENSPTRT00000034076     CCAAATCTACAGGCCTTAAATCTCTCCTCCTGTGATAAGCTACCACCTCAAGCTTTCAAC
ENSPPYT00000019619     CCAAATCTACAGGCCTTAAATCTCTCCTCCTGTGATAAGCTACCACCTCAAGCTTTCAAC
ENSCJAT00000059721     CCAAATTTACAGGCCTTAAATCTCTCCTCCTGTGACAAGCTACCACCTCAAGCTTTCAAC
ENSMMUT00000002355     CCAAATCTACAAGCCTTAAATCTCTCCTCCTGTGATAAGCTACCACCTCAAGCTTTCAAC
ENSMUST00000039234     CCAAATCTACAAGACTTAAATCTCTCATCCTGCGATAAACTCCCACCTCAGGCTTTTGGC
ENSRNOT00000061128     CCAAATCTACAAGACTTAAATCTCTCATCCTGCGATAAACTGCCACCTCAGGCTTTTGGC

ENSGGOT00000001195     CACATTGCCAAGTTATGCAGCCTTAAACGACTTGTTCTCTATCGAACAAAAGTAGAGCAA
ENST00000229971        CACATTGCCAAGTTATGCAGCCTTAAACGACTTGTTCTCTATCGAACAAAAGTAGAGCAA
ENSPTRT00000034076     CACATTGCCAAGTTATGCAGCCTTAAACGACTTGTTCTCTATCGAACAAAAGTAGAGCAA
ENSPPYT00000019619     CACATTTCCAAGTTATGCAGCCTTAAACGACTTGTTCTCTATCGAACAAAAGTAGAGCAA
ENSCJAT00000059721     CACATTTCCAAGTTATGCAGCCTTAAACGACTTGTTCTCTATCGAACAAAAGTAGAGCAA
ENSMMUT00000002355     CACATTGCCAAGTTATGCAGCCTTAAACGACTTGTTCTCTATCGAACAAAAGTAGAGCAA
ENSMUST00000039234     CATATTGCCAAGTTATGCAGCCTCAAGAGGCTTGTTCTCTATCGGACAAAAGTAGAGCAA
ENSRNOT00000061128     CATATTGCCAAGTTACGCAGCCTCAAGAGGCTCATTCTCTATCGGACAAAAGTAGAGCAA

ENSGGOT00000001195     ACAGCACTGCTCAGCATTTTGAACTTCTGTTCAGAGCTTCAGCACCTCAGTTTAGGCAGT
ENST00000229971        ACAGCACTGCTCAGCATTTTGAACTTCTGTTCAGAGCTTCAGCACCTCAGTTTAGGCAGT
ENSPTRT00000034076     ACAGCACTGCTCAGCATTTTGAACTTCTGTTCAGAGCTTCAGCACCTCAGTTTAGGCAGT
ENSPPYT00000019619     ACAGCACTGCTCAGCATTTTGAACTTCTGTTCAGAGCTTCAGCACCTCAGTTTGGGCAGT
ENSCJAT00000059721     ACAGCACTGCTCAGCATTTTGAACTTCTGTTCAGAGCTTCAGCACCTCAGTTTGGGCAGT
ENSMMUT00000002355     ACAGCACTGCTCAGCATTTTGAACTTCTGTTCAGAGCTTCAGCACCTCAGTTTGGGCAGT
```

```
ENSMUST00000039234     ACAGCACTACTGAGCATTTTGAACTTCTGTGCAGAGCTTCAGCACCTCAGCCTGGGCAGC
ENSRNOT00000061128     ACAGCACTGCTGAGCATTTTGAACTTCTGTGCAGAGCTTCAGCACCTCAGCCTGGGCAGC

ENSGGOT00000001195     TGTGTCATGATTGAAGACTATGATGTGATAGCTAGCATGATAGGAGCCAAGTGTAAAAAA
ENST0000229971         TGTGTCATGATTGAAGACTATGATGTGATAGCTAGCATGATAGGAGCCAAGTGTAAAAAA
ENSPTRT00000034076     TGTGTCATGATTGAAGACTATGATGTGATAGCTAGCATGATAGGAGCCAAGTGTAAAAAA
ENSPPYT00000019619     TGTGTCATGATTGAAGACTATGATGTGATAGCTAGCATGATAGGAGCCAAGTGTAAAAAA
ENSCJAT00000059721     TGTGTCATGATTGAAGACTATGATGTGATAGCTAGCATGATAGGAGCCAAGTGTAAAAAA
ENSMMUT00000002355     TGTGTCATGATTGAAGACTATGATGTGATAGCTAGCATGATAGGAGCCAAGTGTAAAAAA
ENSMUST00000039234     TGTGTGATGATTGAAGATTATGATGTGATAGCCAGCATGATAGGAGCCAAGTGTAAAAAC
ENSRNOT00000061128     TGCGTGATGATTGAAGACTATGATGTGATAGCCAGCATGATTGGAGCCAAGTGTAAAAGC

ENSGGOT00000001195     CTCCGGACACTGGATCTGTGGAGATGTAAGAATATTACTGAGAATGGAATAGCAGAACTG
ENST0000229971         CTCCGGACCCTGGATCTGTGGAGATGTAAGAATATTACTGAGAATGGAATAGCAGAACTG
ENSPTRT00000034076     CTCCGGACCCTGGATCTGTGGAGATGTAAGAATATTACTGAGAATGGAATAGCAGAACTG
ENSPPYT00000019619     CTCCGGACCCTGGATCTGTGGAGATGTAAGAATATTACTGAGAATGGAATAGCAGAACTG
ENSCJAT00000059721     CTCCGGACCCTGGATCTATGGAGATGTAAGAATATTACTGAGAATGGAATAGCAGAACTG
ENSMMUT00000002355     CTCCGGACCCTGGATCTGTGGAGATGTAAGAATATTACTGAGAATGGAATAGCAGAACTG
ENSMUST00000039234     CTGCGGACTTTGGATCTGTGGAGATGCAAGAATATCACCGAGAATGGGATAGCAGAGTTG
ENSRNOT00000061128     CTCCGGACCTTGGATCTCTGGAGATGCAAGAATATTACGGAGAATGGGATCGCAGAGTTG

ENSGGOT00000001195     GCTTCTGGGTGTCCACTACTGGAGGAGCTTGACCTTGGCTGGTGCCCAACTCTGCAGAGC
ENST0000229971         GCTTCTGGGTGTCCACTACTGGAGGAGCTTGACCTTGGCTGGTGCCCAACTCTGCAGAGC
ENSPTRT00000034076     GCTTCTGGGTGTCCACTACTGGAGGAGCTTGACCTTGGCTGGTGCCCAACTCTGCAGAGC
ENSPPYT00000019619     GCTTCTGGGTGTCCACTTCTGGAGGAACTTGACCTTGGCTGGTGCCCAACTCTGCAGAGC
ENSCJAT00000059721     GCTTCTGGGTGTCCACTTCTGGAGGAACTTGACCTCGGCTGGTGCCCAACTCTGCAGAGC
ENSMMUT00000002355     GCTTCTGGGTGTCCACTTCTGGAGGAACTTGACCTCGGCTGGTGCCCAACTCTGCAGAGC
ENSMUST00000039234     GCTTCTGGCTGTGTTCTTCTTGAGGAACTTGACCTTGGCTGGTGCCCCACCCTTCAGAGC
ENSRNOT00000061128     GCTTCCGGGTGTGCTCTTCTTGAGGAACTTGACCTTGGCTGGTGCCCCACCCTTCAGAGC

ENSGGOT00000001195     AGCACCGGGTGCTTCACCAGACTGGCACGCCAGCTCCCAAACTTGCAAAAACTCTTTCTT
ENST0000229971         AGCACCGGGTGCTTCACCAGACTGGCACACCAGCTCCCAAACTTGCAAAAACTCTTTCTT
ENSPTRT00000034076     AGCACCGGGTGCTTCACCAGACTGGCACGCCAGCTCCCAAACTTGCAAAAACTCTTTCTT
ENSPPYT00000019619     AGTACCGGGTGCTTCACCAGACTGGCACGCCAGCTCCCAAATTTGCAAAAACTCTTTCTT
ENSCJAT00000059721     AGCACTGGGTGCTTCACCAGACTGGCACGCCAGCTCCCAAACTTGCAAAAACTCTTTCTT
ENSMMUT00000002355     AGCACCGGGTGCTTCACCAGACTGGCACGCCAGCTCCCACACTTGCAAAAACTCTTTCTT
ENSMUST00000039234     AGCACTGGGTGCTTTGTCAGACTGGCACGCCAGCTCCCAAACTTGCAGAAACTCTTTCTT
ENSRNOT00000061128     AGTACTGGGTGCTTTGCCAGACTGGCACGCCAGCTCCCAAACTTGCAGAAACTCTTTCTT

ENSGGOT00000001195     ACAGCTAACAGATCTGTATGTGACACAGACATTGATGAATTGGCATGTAATTGTACCAGG
ENST0000229971         ACAGCTAATAGATCTGTGTGTGACACAGACATTGATGAATTGGCATGTAATTGTACCAGG
ENSPTRT00000034076     ACAGCTAACAGATCTGTATGTGACACAGACATTGATGAATTGGCATGTAATTGTACCAGG
ENSPPYT00000019619     ACAGCTAACAGATCTGTATGTGACACAGACATTGAAGAATTGGCATGTAATTGTACCAGG
ENSCJAT00000059721     ACAGCTAACAGATCTGTGTGTGACACAGACATTGAAGAATTGGCATGTAATTGTACCAGG
ENSMMUT00000002355     ACAGCTAACAGATCTGTATGTGACACAGACATTGAAGAATTGGCATGTAATTGTACCAGG
ENSMUST00000039234     ACAGCTAACAGGTCTGTGTGTGACACAGACATAGAAGAACTGGCATCTAATTGTACCAGA
ENSRNOT00000061128     ACAGCTAACAGGTCTGTGTGTGACACAGACATAGAAGAACTGGCATCTAATTGTACCAGA

ENSGGOT00000001195     TTACAGCAGCTGGACATATTAGGAACAAGAATGGTAAGTCCGGCATCCTTAAGAAAACTC
ENST0000229971         TTACAGCAGCTGGACATATTAGGAACAAGAATGGTAAGTCCGGCATCCTTAAGAAAACTC
ENSPTRT00000034076     TTACAGCAGCTGGACATATTAGGAACAAGAATGGTAAGTCCGGCATCCTTAAGAAAACTC
ENSPPYT00000019619     TTACAGCAGCTGGACATATTAGGAACAAGAATGGTAAGTCCGGCATCCTTAAGAAAACTC
ENSCJAT00000059721     TTACAGCAGCTGGACATATTAGGAACAAGAATGGTAAGTCCGGCATCCTTAAGAAAACTC
ENSMMUT00000002355     TTACAGCAGCTGGACATACTAGGAACAAGAATGGTAAGTCCGGCATCCTTAAGAAAACTC
```

```
ENSMUST00000039234     TTACAGCAACTGGACATATTAGGAACAAGAATGGTAAGTCCAGCATCCTTGAGAAAACTC
ENSRNOT00000061128     TTACAGCAACTGGACATATTAGGAACAAGAATGGTAAGTCCGGCCTCCTTGAGAAAACTC


ENSGGOT00000001195     CTGGAATCTTGTAAAGATCTTTCTTTACTTGATGTGTCCTTCTGTTCGCAGATTGATAAC
ENST00000229971        CTGGAATCTTGTAAAGATCTTTCTTTACTTGATGTGTCCTTCTGTTCGCAGATTGATAAC
ENSPTRT00000034076     CTGGAATCTTGTAAAGATCTTTCTTTACTTGATGTGTCCTTCTGTTCGCAGATTGATAAC
ENSPPYT00000019619     CTGGAATCTTGTAAAGATCTTTCTTTACTTGATGTGTCCTTCTGTTCGCAGATTGATAAC
ENSCJAT00000059721     CTGGAATCTTGTAAAGATCTTTCCTTACTTGATGTGTCCTTCTGTTCACAAATTGATAAC
ENSMMUT00000002355     CTGGAATCTTGTAAAGATCTTTCTTTACTTGATGTGTCCTTCTGTTCGCAGATTGATAAC
ENSMUST00000039234     CTGGAATCTTGCAAAGATCTTTCCCTACTTGACGTGTCCTTCTGTTCCCAGATTGATAAC
ENSRNOT00000061128     CTGGAATCTTGCAAAGATCTTTCCCTACTTGACGTGTCCTTCTGTTCTCAGATTGATAAC


ENSGGOT00000001195     AGAGCTGTGCTAGAACTGAATGCAAGCTTTCCAAAAGTGTTCATAAAGAAGAGCTTTACT
ENST00000229971        AGAGCTGTGCTAGAACTGAATGCAAGCTTTCCAAAAGTGTTCATAAAAAAGAGCTTTACT
ENSPTRT00000034076     AGAGCTGTGCTAGAACTGAATGCAAGCTTTCCAAAAGTGTTCATAAAAAAGAGCTTTACT
ENSPPYT00000019619     AGAGCTGTGCTAGAACTGAATGCAAGCTTTCCAAAAGTGTTCATAAAAAAGAGCTTTACT
ENSCJAT00000059721     AGAGCTGTGCTAGAACTGAATGCAAGTTTTCCAAAAGTGTTCATAAAAAAGAGCTTTACT
ENSMMUT00000002355     AGAGCTGTGCTAGAACTGAATGCAAGCTTTCCAAAAGTGTTCATAAAAAAGAGCTTTACT
ENSMUST00000039234     AAGGCTGTGTTAGAGCTCAACGCGAGCTTTCCGAAAGTCTTCATAAAGAAGAGCTTTACT
ENSRNOT00000061128     AGGGCTGTGTTGGAACTCAACGCAAGCTTTCCAAAAGTATTCATAAAAAAGAGCTTTACT


ENSGGOT00000001195     CAG
ENST00000229971        CAG
ENSPTRT00000034076     CAG
ENSPPYT00000019619     CAG
ENSCJAT00000059721     CAG
ENSMMUT00000002355     CAG
ENSMUST00000039234     CAA
ENSRNOT00000061128     CAA
```

Multiple sequence alignment of Fbxl5

```
ENSMMUT00000042407     ATGGCGCCCTTCCCTGAAGAAGTGGACGTCTTCACCGCCCCACACTGGCGGATGAAGCAG
ENSPPYT00000016994     ------------------------------------------------------------
ENST00000341285        ATGGCGCCCTTTCCTGAAGAAGTGGACGTCTTCACCGCCCCACACTGGCGGATGAAGCAG
ENSGGOT00000011004     ATGGCGCCCTTCCCTGAAGAAGTGGACGTCTTCACCGCCCCACACTGGCGGATGAAGCAG
ENSPTRT00000029732     ATGGCGCCCTTCCCTGAAGAAGTGGACGTCTTCACCGCCCCACACTGGCGGATGAAGCAG
ENSMUST00000047857     ATGGCGCCCTTCCCCGATGAAGTGGACGTCTTCACCGCCCCGCACTGGCGGATGAAGCAG
ENSRNOT00000007016     ATGGCGCCCTTCCCCGATGAAGTGGACGTCTTCACCGCCCCACACTGGCGGATGAAGCAG


ENSMMUT00000042407     CTGGTGGGGCTCTACTGCGACAAGCTTTCTAAAACCAATTTTTCCAACAACAACGATTTC
ENSPPYT00000016994     ---------------------CAGCTTTCTAAAACCAATTTTTCCAACAACAACGATTTC
ENST00000341285        CTGGTGGGGCTCTACTGCGACAAGCTTTCTAAAACCAATTTTTCCAACAACAACGATTTC
ENSGGOT00000011004     CTGGTGGGGCTCTACTGCGACAAGCTTTCTAAAACCAATTTTTCCAACAACAACGATTTC
ENSPTRT00000029732     CTGGTGGGGCTCTACTGCGACAAGCTATCTAAAACCAATTTTTCCAACAACAACGATTTC
ENSMUST00000047857     CTGGTGGGGCGCTACTGCGACAAGCTTTCTAAAACCAACTTTTCCAACAACAATGACTTC
ENSRNOT00000007016     CTGGTGGGGCGCTACTGCGACAAGCTTTCTAAAACCAACTTTTCCAACAACAATGACTTC


ENSMMUT00000042407     CGTGCTCTTCTGCAGTCTTTGTATGCTACTTTCAAGGAGTTCAAAATGCATGAGCAGATT
ENSPPYT00000016994     CGTGCTCTTCTGCAGTCTTTGTATGCTACTTTCAAGGAGTTCAAAATGCATGAGCAGATT
ENST00000341285        CGTGCTCTTCTGCAGTCTTTGTATGCTACTTTCAAGGAGTTCAAAATGCATGAGCAGATT
ENSGGOT00000011004     CGTGCTCTTCTGCAGTCTTTGTATGCTACTTTCAAGGAGTTCAAAATGCATGAGCAGATT
ENSPTRT00000029732     CGTGCTCTTCTGCAGTCTTTGTATGCTACTTTCAAGGAGTTCAAAATGCATGAGCAGATT
ENSMUST00000047857     CGTGCTCTTCTCCAGTCTTTATATGCTACTTTCAAGGAGTTTAAAATGCATGAACAAATT
```

ENSRNOT00000007016 CGTGCTCTTCTCCAGTCTTTATATGCTACTTTCAAGGAGTTTAAAATGCATGAACAAATT

ENSMMUT00000042407 GAAAATGAATACATTATTGGTTTGCTTCAACAACGCAGCCAGACCATTTATAATGTACAT
ENSPPYT00000016994 GAAAATGAATACATTATTGGTTTGCTTCAACAACGCAGCCAGACCATTTATAATGTACAT
ENST00000341285 GAAAATGAATACATTATTGGTTTGCTTCAACAACGCAGCCAGACCATTTATAATGTACAT
ENSGGOT00000011004 GAAAATGAATACATTATTGGTTTGCTTCAACAACGCAGCCAGACCATTTATAATGTACAT
ENSPTRT00000029732 GAAAATGAATACATTATTGGTTTGCTTCAACAACGCAGCCAGACCATTTATAATGTACAT
ENSMUST00000047857 GAAAATGAATACATTATCGGTTTACTTCAGCAGCGCAGTCAGACCATATATAATGTACAC
ENSRNOT00000007016 GAAAATGAATACATTATTGGTTTACTTCAGCAACGCAGTCAGACCATTTACAATGTGCAT

ENSMMUT00000042407 TCTGACAATAAACTCTCTGAGATGCTTAGCCTCTTTGAAAAGGGACTGAAGAATGTTAAG
ENSPPYT00000016994 TCTGACAATAAACTCTCTGAGATGCTTAGCCTCTTTGAAAAGGGACTGAAGAATGTTAAG
ENST00000341285 TCTGACAATAAACTCTCCGAGATGCTTAGCCTCTTTGAAAAGGGACTGAAGAATGTTAAG
ENSGGOT00000011004 TCTGACAATAAACTCTCCGAGATGCTTAGCCTCTTTGAAAAGGGACTGAAGAATGTTAAG
ENSPTRT00000029732 TCTGACAATAAACTCTCCGAGATGCTTAGCCTCTTTGAAAAGGGACTGAAGAATGTTAAG
ENSMUST00000047857 TCTGACAATAAGCTCTCCGAGATGCTGAGTCTCTTTGAAAAGGGGCTGAAGAATGTCAAG
ENSRNOT00000007016 TCTGACAATAAGCTCTCCGAGATGCTTAGTCTCTTTGAAAAGGGGCTGAAGAATGTTAAG

ENSMMUT00000042407 AATGAATATGAACAGTTAAATTATGCAAAACAACTGAAAGAGAGATTGGAGGCTTTTACA
ENSPPYT00000016994 AATGAATATGAACAGTTAAATTATGCAAAACAACTGAAAGAGAGATTGGAGGCTTTTACA
ENST00000341285 AATGAATATGAACAGTTAAATTATGCAAAACAACTGAAAGAGAGATTGGAGGCTTTTACA
ENSGGOT00000011004 AATGAATATGAACAGTTAAATTATGCAAAACAACTGAAAGAGAGATTGGAGGCTTTTACA
ENSPTRT00000029732 AATGAATATGAACAGTTAAATTATGCAAAACAACTGAAAGAGAGATTGGAGGCTTTTACA
ENSMUST00000047857 AATGAGTACGAACAGTTAAATTATGCAAAACAGCTGAAAGAGAGATTGGAGGCTTTTACA
ENSRNOT00000007016 AATGAGTGTGAACAGTTGAATTATGCAAAGCAGCTGAAGGAGAGATTGGAGGCCTTCACG

ENSMMUT00000042407 AGAGATTTTCTTCCTCACATGAAAGAGGAAGAGGAGGTTTTTCAGCCTATGTTAATGGAA
ENSPPYT00000016994 AGAGATTTTCTTCCTCACATGAAAGAGGAAGAGGAGGTTTTTCAGCCGATGTTAATGGAA
ENST00000341285 AGAGATTTTCTTCCTCACATGAAAGAGGAAGAGGAGGTTTTTCAGCCCATGTTAATGGAA
ENSGGOT00000011004 AGAGATTTTCTTCCTCACATGAAAGAGGAAGAGGAGGTTTTTCAGCCCATGTTAATGGAA
ENSPTRT00000029732 AGAGATTTTCTTCCTCACATGAAAGAGGAAGAGGAGGTTTTTCAGCCCATGTTAATGGAA
ENSMUST00000047857 AGAGATTTTCTTCCTCACATGAAAGAGGAAGAAGAGGTTTTTCAGCCCATGTTAATGGAA
ENSRNOT00000007016 AGAGATTTCCTTCCCCACATGAAAGAGGAGGAGGAGGTTTTTCAGCCCATGTTAATGGAA

ENSMMUT00000042407 TATTTTACCTATGAAGAGCTTAAGGATATTAAAAAGAAAGTGATTGCACAACACTGCTCT
ENSPPYT00000016994 TATTTTACCTATGAAGAGCTTAAGGATATTAAAAAGAAAGTGATTGCACAACACTGCTCT
ENST00000341285 TATTTTACCTATGAAGAGCTTAAGGATATTAAAAAGAAAGTGATTGCACAACACTGCTCT
ENSGGOT00000011004 TATTTTACCTATGAAGAGCTTAAGGATATTAAAAAGAAAGTGATTGCACAACACTGCTCT
ENSPTRT00000029732 TATTTTACCTATGAAGAGCTTAAGGATATTAAAAAGAAAGTGATTGCACAACACTGCTCT
ENSMUST00000047857 TATTTTACCTACGAGGAGCTTAAAGATATCAAAAAGAAAGTGATTGCACAGCACTGCTCA
ENSRNOT00000007016 TATTTTACCTATGAAGAGCTTAAAGATATCAAAAAGAAAGTGATCGCACAACACTGCTCA

ENSMMUT00000042407 CAGAAGGACACTGCAGAACTCCTTAGAGGTCTTAGCCTATGGAATCATGCTGAAGAGCGA
ENSPPYT00000016994 CAGAAGGATACTGCAGAACTCCTTAGAGGTCTTAGCCTATGGAATCATGCTGAAGAGCGA
ENST00000341285 CAGAAGGATACTGCAGAACTCCTTAGAGGTCTTAGCCTATGGAATCATGCTGAAGAGCGA
ENSGGOT00000011004 CAGAAGGATACTGCAGAACTCCTTAGAGGTCTTAGCCTATGGAATCATGCTGAAGAGCGA
ENSPTRT00000029732 CAGAAGGATACTGCAGAACTCCTTAGAGGTCTTAGCCTATGGAATCATGCTGAAGAGCGA
ENSMUST00000047857 CAGAAGGACACGGCAGAGCTCCTCAGAGGGCTCAGTCTGTGGAACCAAGCTGAGGAGCGA
ENSRNOT00000007016 CAGAAGGACACAGCAGAACTCCTCAGAGGGCTCAGTCTGTGGAATCAAGCTGAAGAGCGA

ENSMMUT00000042407 CAGAAATATTTTAAATATTCTGTGGATGAAAAGTCAGATAAAGAAGCTGAAGTGTCAGAA
ENSPPYT00000016994 CAGAAGTTTTTTAAATATTCCGTGGATGAAAAGTCAGATAAAGAAGCAGAAGTGTCAGAA
ENST00000341285 CAGAAGTTTTTTAAATATTCCGTGGATGAAAAGTCAGATAAAGAAGCAGAAGTGTCAGAA

ENSGGOT00000011004	CAGAAGTTTTTAAATATTCCGTGGATGAAAAGTCAGATAAAGAAGCAGAAGTGTCAGAA
ENSPTRT00000029732	CAGAAGTTTTTAAATATTCCGTGGATGAAAAGTCAGATAAAGAAGCAGAAGTGTCAGAA
ENSMUST00000047857	CAGAAAGTTCTCAAGTACTCTGTGGATGAGAAGGCAGATACAGAAGCGGAAGTGTCAGAA
ENSRNOT00000007016	CAGAAAGTTTTCAAATATTCTGTGGATGAGAAGTCAGATGCAGAAGCGGAAGTGTCAGAA

ENSMMUT00000042407	CAATCCACAGGTATAACCCATCTTCCTCCTGAGGTAATGCTGTCAATTTTCAGCTATCTT
ENSPPYT00000016994	CACTCCACAGGTATAACCCATCTTCCTCCTGAGGTAATGCTGTCAATTTTCAGCTATCTT
ENST00000341285	CACTCCACAGGTATAACCCATCTTCCTCCTGAGGTAATGCTGTCAATTTTCAGCTATCTT
ENSGGOT00000011004	CACTCCACAGGTATAACCCATCTTCCTCCTGAGGTAATGCTGTCAATTTTCAGCTATCTT
ENSPTRT00000029732	CACTCCACAGGTATAACCCATCTTCCTCCTGAGGTAATGCTGTCAATTTTCAGCTATCTT
ENSMUST00000047857	CACTCCACAGGTATAACCCATCTTCCTCCTGAGGTAATGCTGTCCATTTTCAGTTACCTT
ENSRNOT00000007016	CACTCCACAGGTATAACCCATCTTCCTCCTGAGGTAATGCTGTCCATTTTCAGTTACCTT

ENSMMUT00000042407	AATCCTCAAGAGTTATGTCGATGCAGTCAAGTAAGCATGAAATGGTCTCAGCTGACAAAA
ENSPPYT00000016994	AATCCTCAAGAGTTATGTCGATGCAGTCAAGTAAGCATGAAATGGTCTCAGCTGACAAAA
ENST00000341285	AATCCTCAAGAGTTATGTCGATGCAGTCAAGTAAGCATGAAATGGTCTCAGCTGACAAAA
ENSGGOT00000011004	AATCCTCAAGAGTTATGTCGATGCAGTCAAGTAAGCATGAAATGGTCTCAGCTGACAAAA
ENSPTRT00000029732	AATCCTCAAGAGTTATGTCGATGCAGTCAAGTAAGCATGAAATGGTCTCAGCTGACAAAA
ENSMUST00000047857	AATCCTCAAGAATTGTGTCGGTGTAGTCAAGTCAGTACTAAGTGGTCTCAGCTGGCAAAA
ENSRNOT00000007016	AATCCTCAAGAATTGTGTCGGTGCAGTCAAGTCAGCACTAAGTGGTCTCAGCTGGCAAAG

ENSMMUT00000042407	GCTGGATCGCTTTGGAAACATCTGTACCCTGTTCATTGGGCCAGAGGTGACTGGTATAGT
ENSPPYT00000016994	ACTGGATCGCTTTGGAAACATCTTTACCCTGTTCATTGGGCCAGAGGTGACTGGTATAGT
ENST00000341285	ACGGGATCGCTTTGGAAACATCTTTACCCTGTTCATTGGGCCAGAGGTGACTGGTATAGT
ENSGGOT00000011004	ACGGGATCGCTTTGGAAACATCTTTACCCTGTTCATTGGGCCAGAGGTGACTGGTATAGT
ENSPTRT00000029732	ACGGGATCGCTTTGGAAACATCTTTACCCTGTTCATTGGGCCAGAGGTGACTGGTATAGT
ENSMUST00000047857	ACAGGATCTTTGTGGAAACATCTTTACCCTGTTCATTGGGCAAGAGGTGACTGGTATAGT
ENSRNOT00000007016	ACAGGATCTTTGTGGAAACATCTTTACCCTGTTCATTGGGCAAGAGGTGACTGGTATAGT

ENSMMUT00000042407	GGTCCCGCAACTGAACTTGATACTGAACCTGATGAAGAATGGGTGAAAAATAGGAAAGAT
ENSPPYT00000016994	GGTCCTGCAACTGAACTTGATACTGAACCTGATGAAGAATGGGTGAAAAATAGGAAAGAT
ENST00000341285	GGTCCCGCAACTGAACTTGATACTGAACCTGATGATGAATGGGTGAAAAATAGGAAAGAT
ENSGGOT00000011004	GGTCCCGCAACTGAACTTGATACTGAACCTGATGAAGAATGGGTGAAAAATAGGAAAGAT
ENSPTRT00000029732	GGTCCTGCAACTGAACTTGATACTGAACCTGATGAAGAATGGGTGAAAAATAGGAAAGAT
ENSMUST00000047857	GGTCCTGCCACTGAACTTGATACTGAGCCTGATGAGGAATGGGTGAGAAATAGGAAAGAT
ENSRNOT00000007016	GGCCCTGCCACTGAACTTGATACTGAACCTGATGAGGAATGGGTGAGAAGTAGGAAAGAT

ENSMMUT00000042407	GAAAGTCGTGCTTTTCATGAGTGGGATGAAGATGCTGACATAGATGAATCTGAAGAGTCT
ENSPPYT00000016994	GAAAGTCGTGCTTTTCATGAGTGGGATGAAGATGCTGACATAGATGAATCTGAAGAGTCT
ENST00000341285	GAAAGTCGTGCTTTTCATGAGTGGGATGAAGATGCTGACATTGATGAATCTGAAGAGTCT
ENSGGOT00000011004	GAAAGTCGTGCTTTTCATGAGTGGGATGAAGATGCTGACATAGATGAATCTGAAGAGTCT
ENSPTRT00000029732	GAAAGTCGTGCTTTTCATGAGTGGGATGAAGATGCTGACATAGATGAATCTGAAGAGTCT
ENSMUST00000047857	GAAAGTCGTGCTTTCCAGGAGTGGGATGAAGATGCCGATATAGATGAATCTGAAGAGTCT
ENSRNOT00000007016	GAAAGTCGTGCTTTCCAGGAATGGGACGAAGATGCCGATATAGATGAATCTGAAGAGTCT

ENSMMUT00000042407	CCGGAGGAATCGATTGCTATCAGCATTGCACAAATGGAAAAACGTTTACTCCATGGCTTA
ENSPPYT00000016994	GCGGAGGAATCAATTGCTATCAGCATTGCACAAATGGAAAAACGTTTACTCCATGGCTTA
ENST00000341285	GCGGAGGAATCAATTGCTATCAGCATTGCACAAATGGAAAAACGTTTACTCCATGGCTTA
ENSGGOT00000011004	GCGGAGGAATCAATTGCTATCAGCATTGCACAAATGGAAAAACGTTTACTCCATGGCTTA
ENSPTRT00000029732	GCGGAGGAATCAATTGCTATCAGCATTGCACAAATGGAAAAACGTTTACTCCATGGCTTA
ENSMUST00000047857	GCAGAGGAATCGGTTGCTATCAGCATTGCACAAATGGAAAAACGTGTACTCCATGGCTTA
ENSRNOT00000007016	GCAGAGGAATCGGTTGCTATCAGCATTGCACAAATGGAAAAACGTTTACTCCATGGCTTA

```
ENSMMUT00000042407      ATTCATAACGTTCTACCATATGTTGGTACTTCTGTAAAAACCTTAGTATTAGCATACAGC
ENSPPYT00000016994      ATTCATAACGTTCTACCGTATGTTGGTACTTCTGTAAAAACCTTAGTATTAGCATACAGC
ENST00000341285         ATTCATAACGTTCTACCATATGTTGGTACTTCTGTAAAAACCTTAGTATTAGCATACAGC
ENSGGOT00000011004      ATTCATAACGTTCTACCATATGTTGGTACTTCTGTAAAAACCTTAGTATTAGCATACAGC
ENSPTRT00000029732      ATTCATAACGTTCTACCATATGTTGGTACTTCTGTAAAAACCTTAGTATTAGCATACAGC
ENSMUST00000047857      ATTCATAATGTTCTGCCATATGTTGGTACCTCTGTAAAAACTTTAGTGTTAGCGTACAGC
ENSRNOT00000007016      ATTCATAATGTTCTGCCATATGTTGGTACCTCTGTAAAAACTTTAGTGTTAGCATACAGC

ENSMMUT00000042407      TCTGCAGTTTCCAGCAAAATGGTTAGGCAGATTTTAGAGCTTTGTCCTAACCTGGAGCAT
ENSPPYT00000016994      TCTGCAGTTTCCAGCAAAATGGTTAGGCAGATTTTAGAGCTTTGTCCTAACCTGGAGCAT
ENST00000341285         TCTGCAGTTTCCAGCAAAATGGTTAGGCAGATTTTAGAGCTTTGTCCTAACCTGGAGCAT
ENSGGOT00000011004      TCTGCAGTTTCCAGCAAAATGGTTAGGCAGATTTTAGAGCTTTGTCCTAACCTGGAGCAT
ENSPTRT00000029732      TCTGCAGTTTCCAGCAAAATGGTTAGGCAGATTTTAGAGCTTTGTCCTAACCTGGAGCAT
ENSMUST00000047857      TCTGCAGTCTCCAGCAAAATGGTTAGGCAGATTTTAGAGCTTTGTCCTAACCTGGAACAT
ENSRNOT00000007016      TCTGCAGTCTCCAGCAAAATGGTTAGGCAGATTTTAGAGCTTTGTCCTAATCTGGAACAT

ENSMMUT00000042407      CTGGATCTTACCCAGACTGACATTTCAGATTCTGCATTTGACAGTTGGTCTTGGCTTGGT
ENSPPYT00000016994      CTGGATCTTACCCAGACTGACATTTCAGATTCTGCATTTGACAGTTGGTCTTGGCTTGGT
ENST00000341285         CTGGATCTTACCCAGACTGACATTTCAGATTCTGCATTTGACAGTTGGTCTTGGCTTGGT
ENSGGOT00000011004      CTGGATCTTACCCAGACTGACATTTCAGATTCTGCATTTGACAGTTGGTCTTGGCTTGGT
ENSPTRT00000029732      CTGGATCTTACCCAGACTGACATTTCAGATTCTGCATTTGACAGTTGGTCTTGGCTTGGT
ENSMUST00000047857      CTGGATCTTACCCAGACCGACATTTCTGATTCTGCATTTGACAGTTGGTCTTGGCTCGGT
ENSRNOT00000007016      CTGGATCTTACCCAGACTGACATTTCCGATTCTGCATTTGACAGTTGGTCTTGGCTTGGC

ENSMMUT00000042407      TGCTGCCAGAGTCTTCGGCATCTTGATCTGTCTGGTTGTGAGAAAATCACAGATGTGGCC
ENSPPYT00000016994      TGCTGCCAGAGTCTTCGGCATCTTGATCTGTCTGGTTGTGAGAAAATCACAGATGTGGCC
ENST00000341285         TGCTGCCAGAGTCTTCGGCATCTTGATCTGTCTGGTTGTGAGAAAATCACAGATGTGGCC
ENSGGOT00000011004      TGCTGCCAGAGTCTTCGGCATCTTGATCTGTCTGGTTGTGAGAAAATCACAGATGTGGCC
ENSPTRT00000029732      TGCTGCCAGAGTCTTCGGCATCTTGACCTGTCTGGTTGTGAGAAAATCACAGATGTGGCC
ENSMUST00000047857      TGCTGCCAGAGTCTTCGGCATCTTGATCTGTCTGGATGTGAAAAAATCACAGACATGGCT
ENSRNOT00000007016      TGCTGCCAGAGTCTTCGGCATCTTGACTTGTCTGGATGTGAAAAAATCACAGACATGGCT

ENSMMUT00000042407      CTAGAGAAGATTTCCAGAGCTCTTGGAATCCTGACATCTCATCAAAGTGGCTTTTTGAAA
ENSPPYT00000016994      CTAGAGAAGATTTCCAGAGCTCTTGGAATTCTGACATCTCATCAAAGTGGCTTTTTGAAA
ENST00000341285         CTAGAGAAGATTTCCAGAGCTCTTGGAATTCTGACATCTCATCAAAGTGGCTTTTTGAAA
ENSGGOT00000011004      CTAGAGAAGATTTCCAGAGCTCTTGGAATTCTGACATCTCATCAAAGTGGCTTTTTGAAA
ENSPTRT00000029732      CTAGAGAAGATTTCCAGAGCTCTTGGAATTCTGACATCTCATCAAAGTGGCTTTTTGAAA
ENSMUST00000047857      CTAGAGAAGATTTCTAGAGCTCTTGGAGTTCTGACATCTCATCAGAGTGGCTTTCTGAAA
ENSRNOT00000007016      CTGGAGAAGATTTCTAGAGCTCTTGGAGTTCTGACATCTCATCAGAGTGGCGTTCTGAAA

ENSMMUT00000042407      ACATCTACAAGCAAAATTACTTCAACTACATGGAAAAATAAAGACATTACCATGCAGTCC
ENSPPYT00000016994      ACATCTACGAGCAAAATTACTTCAACTACATGGAAAAATAAAGACGTTACCATGCAGTCC
ENST00000341285         ACATCTACAAGCAAAATTACTTCAACTGCGTGGAAAAATAAAGACATTACCATGCAGTCC
ENSGGOT00000011004      ACATCTACAAGCAAAATTACTTCAACTACGTGGAAAAATAAAGACATTACCATGCAGTCC
ENSPTRT00000029732      ACATCTACAAGCAAAATTACTTCAACTACGTGGAAAAATAAAGACATTACCATGCAGTCC
ENSMUST00000047857      ---AGTGCAGGCAAGGCTGCTTCAACTCCATGGACAAGTAAAGACATTACCATGCCATCC
ENSRNOT00000007016      ---AGTGCAGGCAAGACTACTTCAACTCCATGGACAAATAAAGACATTACCATGCCATCC

ENSMMUT00000042407      ACCAAGCAGTATGCCTGTTTGCACGATTTAACTAACAAGGGCATTGGAGAAGAAATAGAT
ENSPPYT00000016994      ACCAAGCAGTATGCCTGTTTGCACGATTTAACTAACAAGGGCATTGGAGAAGAAATAGAT
ENST00000341285         ACCAAGCAGTATGCCTGTTTGCACGATTTAACTAACAAGGGCATTGGAGAAGAAATAGAT
ENSGGOT00000011004      ACCAAGCAGTATGCCTGTTTGCACGATTTAACTAACAAGGGCATTGGAGAAGAAATAGAT
ENSPTRT00000029732      ACCAAGCAGTACGCCTGTTTGCACGATTTAACTAACAAGGGCATTGGAGAAGAAATAGAT
ENSMUST00000047857      ACCACGCAGTATGCCTGTTTGCACAATTTAACTGACAAAGGCATTGGTGAAGAAATAGAC
```

ENSRNOT00000007016 ACCACGCAGTATGCCTGTTTGCACAATTTAACTAACAAAGGCGTTGGTGAAGAAATCGAC

ENSMMUT00000042407 AATGAACACCCCTGGACTAAGCCTGTTTCTTCTGAGAATTTCACTTCTCCTTATGTGTGG
ENSPPYT00000016994 AATGAACACCCCTGGACTAAGCCTGTTTCTTCTGAGAATTTCACTTCTCCTTATGTGTGG
ENST00000341285 AATGAACACCCCTGGACTAAGCCTGTTTCTTCTGAGAATTTCACTTCTCCTTATGTGTGG
ENSGGOT00000011004 AATGAACACCCCTGGACTAAGCCTGTTTCTTCTGAGAATTTCACTTCTCCTTATGTGTGG
ENSPTRT00000029732 AATGAACACCCCTGGACTAAGCCTGTTTCTTCTGAGAATTTCACTTCTCCTTATGTGTGG
ENSMUST00000047857 AACGAGCACTCTTGGACTGAACCTGTGTCTTCTGAAAGTTTGACTTCTCCTTATGTGTGG
ENSRNOT00000007016 AGCGAACACACTTGGACTGAACCTGTGTCTTCTGAAAGTTTCACTCCTCCCTACGTGTGG

ENSMMUT00000042407 ATGTTAGATGCTGAAGATTTGGCTGATATTGAAGATACTGTGGAATGGAAACATAGAAAT
ENSPPYT00000016994 ATGTTAGATGCTGAAGATTTGGCTGATATTGAAGATACTGTGGAATGGAGACATAGAAAT
ENST00000341285 ATGTTAGATGCTGAAGATTTGGCTGATATTGAAGATACTGTGGAATGGAGACATAGAAAT
ENSGGOT00000011004 ATGTTAGATGCTGAAGATTTGGCTGATATTGAAGATACTGTGGAATGGAGACATAGAAAT
ENSPTRT00000029732 ATGTTAGATGCTGAAGATTTGGCTGATATTGAAGATACTGTGGAATGGAGACATAGAAAT
ENSMUST00000047857 ATGCTAGATGCTGAAGATTTGGCTGATATTGAAGATGCTGTAGAGTGGAGACACAGAAAT
ENSRNOT00000007016 ATGCTAGATGCTGAAGATTTGGCCGATATCGAAGATGCTGTAGAATGGAGGCACAGAAAC

ENSMMUT00000042407 GTTGAAAGTCTTTGTGTAATGGAAACAGCATCCAACTTTAGTTGTTCCACATCTGGTTGT
ENSPPYT00000016994 GTTGAAAGTCTTTGTGTAATGGAAACAGCATCCAACTTTAGTTGTTCCACCTCTGGTTGT
ENST00000341285 GTTGAAAGTCTTTGTGTAATGGAAACAGCATCCAACTTTAGTTGTTCCACCTCTGGTTGT
ENSGGOT00000011004 GTTGAAAGTCTTTGTGTAATGGAAACAGCATCCAACTTTAGTTGTTCCACCTCTGGTTGT
ENSPTRT00000029732 GTTGAAAGTCTTTGTGTAATGGAAACAGCATCCAACTTTAGTTGTTCCACCTCTGGTTGT
ENSMUST00000047857 GTTGAGAGTCTCTGTGTGATGGAAACAGCCTCCAACTTTGGTTGTTCCTCATCTGGTTGT
ENSRNOT00000007016 GTTGAAAGTCTCTGTGTGATGGAAACAGCCTCCAACTTTGGTTGTTCCTCCTCTGGTTGT

ENSMMUT00000042407 TATAGTAAGGACATTGTTGGACTAAGGACTAGTGTCTGTTGGCAGCAGCATTGTGCTTCT
ENSPPYT00000016994 TTTAGTAAGGACATTGTTGGACTAAGGACTAGTGTCTGTTGGCAGCAGCATTGTGCTTCT
ENST00000341285 TTTAGTAAGGACATTGTTGGACTAAGGACTAGTGTCTGTTGGCAGCAGCATTGTGCTTCT
ENSGGOT00000011004 TTTAGTAAGGACATTGTTGGACTAAGGACTAGTGTCTGTTGGCAGCAGCATTGTGCTTCT
ENSPTRT00000029732 TTTAGTAAGGACATTGTTGGACTAAGGACTAGTGTCTGTTGGCAGCAGCATTGTGCTTCT
ENSMUST00000047857 TACAGTAAGGACATTGTGGGATTAAGGACTAGTGTCTGTTGGCAGCAGCATTGTGCTTCT
ENSRNOT00000007016 TACAGCAAGGACATTGTGGGATTAAGGACTAGTGTCTGTTGGCAGCAGCATTGTGCTTCT

ENSMMUT00000042407 CCAGCTTTTGCATATTGTGGTCACTCATTTTGTTGTACAGGAACAGCTTTAAGAACTATG
ENSPPYT00000016994 CCAGCCTTTGCATATTGTGGTCACTCATTTTGTTGTACAGGAACAGCTTTAAGAACTATG
ENST00000341285 CCAGCCTTTGCGTATTGTGGTCACTCATTTTGTTGTACAGGAACAGCTTTAAGAACTATG
ENSGGOT00000011004 CCAGCCTTTGCATATTGTGGTCACTCATTTTGTTGTACAGGAACAGCTTTAAGAACTATG
ENSPTRT00000029732 CCAGCCTTTGCATATTGTGGTCACTCATTTTGTTGTACAGGAACAGCTTTAAGAACTATG
ENSMUST00000047857 CCAGCCTTTGCATATTGTGGTCATTCATTCTGTTGTACAGGGACAGCTTTAAGAACTATG
ENSRNOT00000007016 CCAGCCTTTGCGTATTGTGGTCATTCATTCTGTTGCACAGGGACAGCTCTAAGAACTATG

ENSMMUT00000042407 TCAACACTCCCAGAATCTTCTGCAATGTGTAGAAAAGCATCAAGGACTAGATTGCCTAGG
ENSPPYT00000016994 TCAGCACTCCCAGAATCTTCTGCAATGTGTAGAAAAGCATCAAGGACTAGATTGCCTAGG
ENST00000341285 TCATCACTCCCAGAATCTTCTGCAATGTGTAGAAAAGCAGCAAGGACTAGATTGCCTAGG
ENSGGOT00000011004 TCATCACTCCCAGAATCTTATGCAATGTGTAGAAAAGTAGCAAGGACTAGATTGCCTAGG
ENSPTRT00000029732 TCATCACTCCCAGAATCTTCTGCAATGTGTAGAAAAGCAGCAAGGACTAGATTGCCTAGG
ENSMUST00000047857 ACTACACTCCCAGCGACTTCTGCAATGTGTAGAAAAGCATTAAGGACTACATTGCCCAGG
ENSRNOT00000007016 TCTGCACTCCCAGCGGCTTCTGCAGTGTGTAGAAAAGCATTAAGGACTACGTTGCCCAGG

ENSMMUT00000042407 GGAAAAGACTTAATTTACTTTGGGAGTGAAAAATCTGATCAAGAGACTGGACGTGTACTT
ENSPPYT00000016994 GGAAAAGACTTAATTTACTTTGGGAGTGAAAAATCTGATCAAGAGACTGGACGTGTACTT
ENST00000341285 GGAAAAGACTTAATTTACTTTGGGAGTGAAAAATCTGATCAAGAGACTGGACGTGTACTT

ENSGGOT00000011004   GGAAAAGACTTAATTTACTTTGGGAGTGAAAAATCTGATCAAGAGACTGGACGTGTACTT
ENSPTRT00000029732   GGAAAAGACTTAATTTACTTTGGGAGTGAAAAATCTGATCAAGAGACTGGACGTGTACTT
ENSMUST00000047857   GGGAAAGACTTAATTTATTTTGGGAGTGAAAAGTCTGACCAAGAGACTGGACGAGTACTT
ENSRNOT00000007016   GGGAAAGACTTAATTTATTTTGGGAGTGAAAAGTCTGACCAAGAGACTGGACGAGTACTT

ENSMMUT00000042407   CTGTTTCTCAGTTTGTCTGGATGTTATCAGATCACAGACCATGGTCTCAGGGTTTTGACT
ENSPPYT00000016994   CTGTTTCTCAGTTTATCTGGATGTTACCAGATCACAGACCATGGTCTCAGGGTTTTGACT
ENST00000341285   CTGTTTCTCAGTTTATCTGGATGTTATCAGATCACAGACCATGGTCTCAGGGTTTTGACT
ENSGGOT00000011004   CTGTTTCTCAGTTTATCTGGATGTTATCAGATCACAGACCATGGTCTCAGGGTTTTGACT
ENSPTRT00000029732   CTGTTTCTCAGTTTATCTGGATGTTATCAGATCACAGACCATGGTCTCAGGGTTTTGACT
ENSMUST00000047857   TTGTTCCTCAGTCTGTCTGGATGTTACCAGATCACAGACCATGGTCTCAGGGCTCTGACT
ENSRNOT00000007016   TTGTTCCTCAGTCTGTCTGGATGTTACCAGATCACAGACCATGGTCTCAGGGTTCTGACT

ENSMMUT00000042407   CTGGGAGGAGGGCTGCCTTATTTGGAGCACCTTAATCTCTCTGGTTGTCTTACTATAACT
ENSPPYT00000016994   CTGGGAGGAGGGCTGCCTTATTTGGAGCACCTTAATCTCTCTGGTTGTCTTACTATAACT
ENST00000341285   CTGGGAGGAGGGCTGCCTTATTTGGAGCACCTTAATCTCTCTGGTTGTCTTACTATAACT
ENSGGOT00000011004   CTGGGAGGAGGGCTGCCTTATTTGGAGCACCTTAATCTCTCTGGTTGTCTTACTATAACT
ENSPTRT00000029732   CTGGGAGGAGGGCTGCCTTATTTGGAGCACCTTAATCTCTCTGGTTGTCTTACTATAACT
ENSMUST00000047857   CTGGGAGGAGGGCTGCCTTACCTGGAGCACCTGAATCTCTCTGGCTGTCTGACTGTAACT
ENSRNOT00000007016   CTGGGAGGAGGACTGCCTTACTTGGAGCACCTGAATCTCTCTGGCTGTCTGACTGTAACT

ENSMMUT00000042407   GGTGCAGGCCTGCAGGATTTGGTTTCAGCATGTCCTTCTCTGAATGATGAATACTTTTAC
ENSPPYT00000016994   GGTGCAGGCCTGCAGGATTTGGTTTCAGCATGTCCTTCTCTGAATGATGAATACTTTTAC
ENST00000341285   GGTGCAGGCCTGCAGGATTTGGTTTCAGCATGTCCTTCTCTGAATGATGAATACTTTTAC
ENSGGOT00000011004   GGTGCAGGCCTGCAGGATTTGGTTTCAGCATGTCCTTCTCTGAATGATGAATACTTTTAC
ENSPTRT00000029732   GGTGCAGGCCTGCAGGATTTGGTTTCAGCATGTCCTTCTCTGAATGATGAATACTTTTAC
ENSMUST00000047857   GGTGCAGGGCTGCAGGATTTGGTCTCAGCGTGTCCTTCCCTAAATGACGAATACTTTTAC
ENSRNOT00000007016   GGTGCAGGACTGCAGGATTTGGTCTCAGCATGTCCTTCCCTAAATGACGAATACTTCTAC

ENSMMUT00000042407   TACTGTGACAACATTAACGGTCCTCATGCTGATACCGCCAGTGGATGCCAGAATTTGCAG
ENSPPYT00000016994   TACTGTGACAACATTAACGGTCCTCATGCTGATACCGCCAGTGGATGCCAGAATTTGCAG
ENST00000341285   TACTGTGACAACATTAACGGTCCTCATGCTGATACCGCCAGTGGATGCCAGAATTTGCAG
ENSGGOT00000011004   TACTGTGACAACATTAACGGTCCTCATGCTGATACCGCCAGTGGATGCCAGAATTTGCAG
ENSPTRT00000029732   TACTGTGACAACATTAACGGTCCTCATGCTGATACCGCCAGTGGATGCCAGAATTTGCAG
ENSMUST00000047857   TACTGTGACAACATTAACGGTCCTCATGCTGACACCGCCAGTGGATGCCAGAATTTGCAG
ENSRNOT00000007016   TACTGTGACAACATTAACGGTCCTCATGCTGACACCGCCAGTGGATGCCAGAATTTGCAG

ENSMMUT00000042407   TGTGGTTTTCGAGCCTGCTGCCGCTCTGGCGAA
ENSPPYT00000016994   TGTGGTTTTCGAGCCTGCTGCCGCTCTGGCGAA
ENST00000341285   TGTGGTTTTCGAGCCTGCTGCCGCTCTGGCGAA
ENSGGOT00000011004   TGTGGTTTTCGAGCCTGCTGCCGCTCTGGCGAA
ENSPTRT00000029732   TGTGGTTTTCGAGCCTGCTGCCGCTCTGGCGAA
ENSMUST00000047857   TGTGGTTTTCGAGCCTGCTGCCGCTCTGGCGAA
ENSRNOT00000007016   TGTGGTTTTCGAGCCTGCTGCCGCTCTGGCGAA

Multiple sequence alignment of Fbxl6

ENSMUST00000023219   ATGGCTCCCGTGGCCTCTAGGCGAGTTAGACGTCGAGTTCGAAGCTCCAAGCGGCCCCGC
ENSRNOT00000035199   ATGGCTCCTGTGGCCCCTGGGCGAGTTAGGCGTCGAGTTCGAGGCTCCAAGCGGCCCCGC
XM_002819557.1   ATGGCTGCTCCAGCCTCCGGGCAGGTCCGACGCAGAGCCCGGGCAGCGCCGCGGCCCCGC
ENSMMUT00000010304   ATGGCTTCTCCGGCCTCCCGGAAGGTCCGGCGCAGAGCTCGGGCAGCGCCGCGGGCCCGC
ENSPTRT00000038291   ATGGCTGCCCCAGCCTCCCGGCAGGTCCGACGCAGAGCTCGGGCAGCGCCGCGGCCCCGC
ENSGGOT00000016219   ATGGCTGCCCCAGCCTCCCGGCAGGTCCGACGCAGAGCCCGGGCAGCGCCGCGGCCCCGC
ENST00000331890   ATGGCTGCCCCAGCCTCCCGGCAGGTCCGACGCAGAGCCCGGGCAGCGCCGCGGCCCCGC

```
ENSMUST00000023219     TCGGCGGAGGACTGGTGGTGGGACCGCCTGGCACCTCGCGGCTCTGGGTACCACCTGCTG
ENSRNOT00000035199     TCGGCCGAGGACTGGTGGTGGGACCGGCTGGCCCCTCGCGGCTCTGGGTACCACCTGCTG
XM_002819557.1         AAGGCCGAGGACTGGTGGTGGGACCGGCTGGCGCCGAGGGGCTCGGGGTACCACCTGCTG
ENSMMUT00000010304     TCGGCCGAGGACTGGTGGTGGGACCGGCTGGCGCCGAGCGGCTCGGGGTACCACCTGCTT
ENSPTRT00000038291     TCGGCCGAGGACTGGTGGTGGGACCGGCTGGCGCCGAGGGGCTCGGGGTACCACCTGCTG
ENSGGOT00000016219     TCGGCCGAGGACTGGTGGTGGGATCGGCTGGCGCCGAGGGGCTCGGGGTACCACCTGCTG
ENST00000331890        TCGGCCGAGGACTGGTGGTGGGACCGGCTGGCGCCGAGGGGCTCGGGGTACCACCTGCTG

ENSMUST00000023219     CAAGCGGACAGCATGTTGCTCGTGCTGCCGGACCTGGAGCCTCCCCGCGCTCGTGCGCAC
ENSRNOT00000035199     CAAGCGGACAGCATGCTGCTCGTGCTGCCGGACCTGGAGCCTACCCGCGCTCGTGCTCAC
XM_002819557.1         CAGTCCGACAACATGCTGCTGGTGCTGTCCGAACCCGGCCCCGCCCGGCCCCTCTCTAAG
ENSMMUT00000010304     CCGTCCGACAGCATGCTGCTGGTGCTGTCCGAACCCGGACCCGCCCGGACCCGCGCACAG
ENSPTRT00000038291     CAGTCCGACAGTATGCTGCTGGTGCTGTCCGAACCCGGCCCCGCCCGGCCCCGCGCACAG
ENSGGOT00000016219     CAGTCCGACAGCATGCTGCTGGTGCTGTCCGAACCCGGCCCCGCCCGGCCCCGCGCACAG
ENST00000331890        CAGTCCGACAGCATGCTGCTGGTGCTGTCCGAACCCGGCCCCGCCCGGCCCCGCGCACAG

ENSMUST00000023219     CGGCGCGCCCGTCGCCGCGCTCCGAGGTCACTGGCTCGTGGCCCCACGGCTGTCGCCAAG
ENSRNOT00000035199     AGGCGCGCCCCTCGCCGCGCTCCGAGGTCACTGGCTCGTGGCCCCACAGCTGTCGCCAAG
XM_002819557.1         GGACGCGCTGCCCGCCGCACTCCCCGGCAGCCGCCCCCGGGCCTCCGCGCCGCGGCCAAG
ENSMMUT00000010304     CGACGCGCTGCCCGCCGCACTCCCCGGCAGCCGCCCCCGGGC---CGCGCCGCGGCCAAG
ENSPTRT00000038291     CGGCGCGCTTCCCGCCGCACTCCCCGGCAGGCGCCCCCGGGCCCCAGCGCCGCGGCCAAG
ENSGGOT00000016219     CGGCGCGCTTCCCGCCGCACTCCCCGGCAGCCGCCCCCGGGCCCCAGCGCCGCGGCCAAA
ENST00000331890        CGGCGCGCTTCCCGCCGCACTCCCCGGCAGCCGCCCCGGGGCCCCAGCGCCGCGGCCAAG

ENSMUST00000023219     CCCAGGACCAAACCCCGACCGGAG------CCGTCGCTCGATCAGGGCCTGGACTCTGGT
ENSRNOT00000035199     CCCAGGGCCAAACCCCGACCAGAG------CCATCACTTGACCAGGGCCTGGACTCTGGT
XM_002819557.1         CCCAAGGCCAGGCTCAGGCCCAAGCCCACGCCCACGCCCGAGGAAGGGCAAGACGCGGGC
ENSMMUT00000010304     CCCAAGGCCACGCTCAGGCCCGACCCGGCGCCCTCGCCCGAGGAAGGGCCCGACGCGGGC
ENSPTRT00000038291     CCCAAGGCCGGGCTCAGGCCCGAGCCCACGCCCACGCCCGAGGAAGGACCCGACGCGGGC
ENSGGOT00000016219     CCCAAGGCCAGGCTCAGGCCCGAGCCCACGCCCACGCCCGAGGAAAGGCCCGACGCGGGC
ENST00000331890        CCCAAGGCCGGGCTCAGGTCCGAGCCCACGCCCACGCCCGAGGAAGGGCCCGACGCGGGC

ENSMUST00000023219     TGGGGAGACCGTATTCCCTTAGAAGTCCTGGTGCATATTTTCGGGTTGTTGGTTGCGGCT
ENSRNOT00000035199     TGGGGAGATCGTATTCCCTTAGAAGTCCTGGTGCATATTTTCGGGTTGTTGGTTGCGGCT
XM_002819557.1         TGGGGAGACCGCATTCCCTTGGAAATCCTGGTGCAGATTTTCGGGTTGTTGGTGGCGGCG
ENSMMUT00000010304     TGGGGAGACCGCATTCCCTTGGAAATCCTGGTGCAGATTTTCGGGTTGTTGGTGGCGGCG
ENSPTRT00000038291     TGGGGAGACCGCATTCCCTTGGAAATCCTGGTGCAGATTTTCGGGTTGTTGGTGGCGGCG
ENSGGOT00000016219     TGGGGAGACCGCATTCCCTTGGAAATCCTGGTGCAGATTTTCGGGTTGTTGGTGGCGGCG
ENST00000331890        TGGGGAGACCGCATTCCCTTGGAAATCCTGGTGCAGATTTTCGGGTTGTTGGTGGCGGCG

ENSMUST00000023219     CATGGGCCCATGCCGTTTCTTGGCAGGGCTGCACGCGTTTGCCGTCACTGGCACGAAGCC
ENSRNOT00000035199     CATGGGCCCATGCCGTTTCTTGGCAGGGCTGCACGCGTTTGCCGTCATTGGCATGAAGCC
XM_002819557.1         GACGGCCCCATGCCCTTCCTGGGCAGGGCTGCGCGCGTGTGCCGCCGCTGGCAGGAGGCC
ENSMMUT00000010304     GACGGGCCCATGCCCTTCCTCGGCAGGGCTGCGCGCGTGTGCCGCCGCTGGCAGGAGGCC
ENSPTRT00000038291     GACGGCCCCATGCCCTTCCTGGGCAGGGCTGCGCGCGTGTGCCGCCGCTGGCAGGAGGCC
ENSGGOT00000016219     GACGGCCCCATGCCCTTCCTGGGCAGGGCTGCGCGCGTGTGCCGCCGCTGGCAGGAGGCC
ENST00000331890        GACGGCCCCATGCCCTTCCTGGGCAGGGCTGCGCGCGTGTGCCGCCGCTGGCAGGAGGCC

ENSMUST00000023219     ACCTCCCATCCTTCACTCTGGCATACTGTGACCCTGTCACCCTCGCTGGTTGGCCGGGCT
ENSRNOT00000035199     ACCTCTCATCCTTCACTCTGGCATACTGTGACCCTGTCACCGGCGCTGGTTGGCCGGGCT
XM_002819557.1         GCTTCCCAACCCGCGCTCTGGCATACCGTGACCCTGTCGTCCCCGCTGGTCGGCCGGCCT
ENSMMUT00000010304     GCTTCCCAACCCGCGCTCTGGCACACCGTGACCCTGTCGTCCCCGCTGGCCGGCCGGCCT
```

```
ENSPTRT00000038291     GCTTCCCAACCCGCGCTCTGGCACACCGTGACCCTGTCGTCCCCGCTGGTCGGCCGGCCT
ENSGGOT00000016219     GCTTCCCAACCCGCGCTCTGGCACACCGTGACCCTGTCGTCCCCGCTGGTCGGCCGGCCT
ENST00000331890        GCTTCCCAACCCGCGCTCTGGCACACCGTGACCCTGTCGTCCCCGCTGGTCGGCCGGCCT

ENSMUST00000023219     GGCAAGGGCAACCTTAAGGGAGAGAAGAAGCTCCTTGCTTGCCTGGAGTGGCTCGTACCC
ENSRNOT00000035199     GCTAAGGGCAACCTTAAGGGAGAGAAGAAGCTCCTTGCTTGCCTGGAGTGGCTCATACCC
XM_002819557.1         GCCAAGGGCGGGGTCAAGGCGGAGAAGAAGCTCCTTGCTTCCCTGGAGTGGCTTATGCCC
ENSMMUT00000010304     GCCAAGGGCGGGGTCAAGGCGGAGAAGAAGCTTCTTGCTTCCCTGGAGTGGCTTATGCCT
ENSPTRT00000038291     GCCAAGGGCGGGGTCAAGGCGGAGAAGAAGCTCCTTGCTTCCCTGGAGTGGCTTATGCCC
ENSGGOT00000016219     GCCAAGGGAGGGGTCAAGGCGGAGAAGAAGCTCCTTGCTTCCCTGGAGTGGCTTATGCCC
ENST00000331890        GCCAAGGGCGGGGTCAAGGCGGAGAAGAAGCTCCTTGCTTCCCTGGAGTGGCTTATGCCC

ENSMUST00000023219     AATCGGTTCTCTCAGCTCCAGAGCTTGACCCTCATCCACTGGAAGTCTCAAGTACACTCT
ENSRNOT00000035199     AATCGGTTCTCACAGCTCCAGAGGTTGACCCTCATCCACTGGAAGTCTCAAGTACACTCC
XM_002819557.1         AATCGGTTTTCACAGCTCCAGAGGCTGACCCTCATCCACTGGAAGTCTCAGGTACACCCC
ENSMMUT00000010304     AATCGGTTTTCACAGCTCCAGAGGCTGACCCTCATCCACTGGAAGTCTCAGGTACACCCC
ENSPTRT00000038291     AATCGGTTTTCACAGCTCCAGAGGCTGACCCTCATCCACTGGAAGTCTCAGGTACACCCC
ENSGGOT00000016219     AATCGGTTTTCACAGCTCCAGAGGCTGACCCTCATCCACTGGAAGTCTCAGGTACACCCC
ENST00000331890        AATCGGTTTTCACAGCTCCAGAGGCTGACCCTCATCCACTGGAAGTCTCAGGTACACCCC

ENSMUST00000023219     GTGTTGGAGCTGGTTAGCAAGTTCTGCCCTCGGCTCACCTTCCTCAAGCTTTCAGACTGC
ENSRNOT00000035199     GTGTTGGAGCTGGTTAGCAAGTTCTGTCCACGGCTCACCTTCCTGAAGCTTTCAGATTGC
XM_002819557.1         GTGTTGAAGCTGGTAGGTGAGTGCTGTCCTCGGCTCACTTTCCTCAAGCTTTCCGGCTGC
ENSMMUT00000010304     GTGCTGAAGCTGGTAGGCGAGTCCTGTCCTCGGCTCACTTTCCTAAAGCTCTCCGGCTGC
ENSPTRT00000038291     GTGTTGAAGCTGGTAGGTGAGTGCTGTCCTCGGCTCACTTTCCTCAAGCTCTCCGGCTGC
ENSGGOT00000016219     GTGTTGAAGCTGGTAGGTGAGTGCTGTCCTCGGCTCACTTTCCTCAAGCTCTCCGGCTGC
ENST00000331890        GTGTTGAAGCTGGTAGGTGAGTGCTGTCCTCGGCTCACTTTCCTCAAGCTCTCCGGCTGC

ENSMUST00000023219     CACACTGTGACTGCTGAAACTCTGGTCATGTTAGCGAGAGCCTGCTGCCAGCTCCACAGC
ENSRNOT00000035199     CACGGTGTGACAGCTGAAACGCTGGTCATGTTAGCAAAAGCCTGCTGTCAGCTCCACAGC
XM_002819557.1         CACNGCGTGACTGCTGACGCTCTGGTCATGCTAGCCAAAGCCTGCTGCCAGCTCCATAGC
ENSMMUT00000010304     CACGGTGTGACTGCTGATGCTCTGGTCATGCTAGCCAAAGCCTGCTGCCAGCTCCATAGC
ENSPTRT00000038291     CACGGTGTGACTGCTGACGCTCTGGTCATGCTAGCCAAAGCCTGCTGCCAGCTCCATAGC
ENSGGOT00000016219     CACGGTGTGACTGCTGACGCTCTGGTCATGCTAGCCAAAGCCTGCTGCCAGCTCCATAGC
ENST00000331890        CACGGTGTGACTGCTGACGCTCTGGTCATGCTAGCCAAAGCCTGCTGCCAGCTCCATAGC

ENSMUST00000023219     CTGGACCTACATCATTCCATGGTGGAGTCCACAGCTGTGGTGAGCTTCTTGGAGGAGGCC
ENSRNOT00000035199     CTAGATCTACACCATTCCATGGTCGAGTCCACAGCTGTGGTGAGCTTCTTGGAGGAGGCG
XM_002819557.1         CTGGACCTACAGCACTCCATGGTGGAGTCCACAGCTGTGGTGAGCTTCTTGGAGGAGGCA
ENSMMUT00000010304     CTGGACCTACAGCACTCCATGGTGGAGTCCACAGCTGTGGTAAGCTTCTTGGAGGAGGCA
ENSPTRT00000038291     CTGGACCTACAGCACTCCATGGTGGAGTCCACAGCTGTGGTGAGCTTCTTGAAGGAGGCA
ENSGGOT00000016219     CTGGACCTACAGCACTCCATGGTGGAGTCTACAGCTGTGGTGAGCTTCTTGGAGGAGGCA
ENST00000331890        CTGGACCTACAGCACTCCATGGTGGAGTCCACAGCTGTGGTGAGCTTCTTGGAGGAGGCA

ENSMUST00000023219     GGGTCCCGAATGCGTAAACTGTGGCTGACCTACAGTTCCCAGACGACGGCCATCTTGGGC
ENSRNOT00000035199     GGGTCCCGGATGCGGAGGCTGTGGCTGACCTACAGTTCCCAGACCACAGCCATCTTGGGT
XM_002819557.1         GGGTCCCGAATGCGCAGGTTGTGGCTGACCTACAGCTCCCAGACGACAGCCATCCTGGGC
ENSMMUT00000010304     GGGTCCCGAATGCGCAAGTTGTGGCTGACTTACAGCTCCCAGACGACAGCCATCCTGGGT
ENSPTRT00000038291     GGGTCCCGAATGCGCAAGTTCTGGCTGACCTACAGCTCCCAGACCACAGCCATCCTGGGC
ENSGGOT00000016219     GGGTCCCGAATGCGCAAGTTGTGGCTGACCTACAGCTCCCAGACGACAGCCATCCTGGGC
ENST00000331890        GGGTCCCGAATGCGCAAGTTGTGGCTGACCTACAGCTCCCAGACGACAGCCATCCTGGGC

ENSMUST00000023219     GCACTGCTGGACAACTGCTGCCCACAGCTCCAAGTCCTCCAGGTCAGCACTGGCATGAAC
```

```
ENSRNOT00000035199     GCACTGCTGGGCAACTGCTGCTCCCAGCTCCAAGTCCTCGAGGTCAGCGCTGGCATGAGC
XM_002819557.1         GCACTGCTGGGCAGCTGCTGCCCGCAGCTCCAGGTCCTGGAGGTGAGCACCGGTATCAAC
ENSMMUT00000010304     GCACTGCTGGGCAGCTGCTGCCCCCAGCTCCAGGTTCTGGAGGTGAGCAGCGGCATCAAC
ENSPTRT00000038291     GCACTGCTGGGCAGCTGCTGCCCCCAGCTCCAGGTCCTGGAGGTGAGCACCGGCATCAAC
ENSGGOT00000016219     GCACTGCTGGGCAGCTGCTGCCCCCAGCTCCAGGTCCTGGAGGTGAGCACCGGCATCAAC
ENST00000331890        GCACTGCTGGGCAGCTGCTGCCCCCAGCTCCAGGTCCTGGAGGTGAGCACCGGCATCAAC

ENSMUST00000023219     TGCAACAACACACCCCTGCAGCTGCCTGTGGAAGCCCTGCAGAAAGGCTGCCCCCAGCTG
ENSRNOT00000035199     TGCAACAACACACCCTTGCAGCTGCCTGTGGAAGCCCTACAGAGAGGCTGCCCCCAGCTG
XM_002819557.1         CGTAATAGCATTCCCCTTCAGCTGCCTGTCGAGGCTCTGCAGAAAGGCTGCCCTCAGCTC
ENSMMUT00000010304     CGTAACAGCATTCCCCTTCAGCTGCCTGTTGAGGCTCTGCAGAAAGGCTGCCCCCAGCTC
ENSPTRT00000038291     CGTAATAGCATTCCCCTTCAGCTGCCTGTCGAGGCTCTGCAGAAAGGCTGCCCTCAGCTC
ENSGGOT00000016219     CGTAATAGCATTCCCCTTCAGCTGCCTGTCGAGGCTCTGCAGAAAGGCTGCCCTCAGCTC
ENST00000331890        CGTAATAGCATTCCCCTTCAGCTGCCTGTCGAGGCTCTGCAGAAAGGCTGCCCTCAGCTC

ENSMUST00000023219     CAGGTGCTGCGGCTGCTGAATCTGATTTGGCTTCCCAAGCCGTGTGGCCGAGGCGTGCCC
ENSRNOT00000035199     CAGGTGCTACGGCTGCTGAATCTGATTTGGCTTCCCAAGCCTTGTGGCCGAGGAGCGCCC
XM_002819557.1         CAGGTGCTGCGGCTGCTGAACCTGATGTGGCTGCCCAAGCCTCCAGGACGAGGGGCGGCT
ENSMMUT00000010304     CAGGTGCTGCGGCTGCTGAACCTGATGTGGCTGCCCAAGCCTCTGGGACGAGGGGTGGCT
ENSPTRT00000038291     CAGGTGCTGCGGCTGCTGAACCTGATGTGGCTGCCCAAGCCTCTGGGACGAGGGGTGGCT
ENSGGOT00000016219     CAGGTGCTGCGGCTGCTGAACCTGATGTGGCTGCCCAAGCCTCCGGGACGAGGTGTGGCT
ENST00000331890        CAGGTGCTGCGGCTGTTGAACCTGATGTGGCTGCCCAAGCCTCCGGGACGAGGGGTGGCT

ENSMUST00000023219     CAGGGACCAGGCTTCCCCAGTCTTGAGGAGCTCTGTTTGGCTGGCTCCACCTGCAACTTT
ENSRNOT00000035199     CAGGGACCAGGATTCCCCAGTCTGGAGGAGCTCTGCTTGGCTGGCTCCACCTGCAGCTTT
XM_002819557.1         CCCGGACCAGGCTTCCCCAGCCTGGAGGAGCTCTGCCTGGCGAGTTCAACCTGCAACTTT
ENSMMUT00000010304     CCCGGACCAGGCTTCCCTAGCCTGGAGGAACTCTGCCTGGCGAGCTCAGCCTGCAACTTT
ENSPTRT00000038291     CCCGGACCAGGCTTCCCTAGCCTAGAGGAGCTCTGCCTGGCGAGCTCAACCTGCAACTTT
ENSGGOT00000016219     CCCGGACCAGGCTTCCCTAGCCTGGAGGAGCTCTGCCTGGCGAGCTCAACCTGCAACTTT
ENST00000331890        CCCGGACCAGGCTTCCCTAGCCTAGAGGAGCTCTGCCTGGCGAGCTCAACCTGCAACTTT

ENSMUST00000023219     GTGAGCAATGAGGTTCTGGGCCGATTGCTCCATCGCTCCCCGAAACTGCGCATGCTGGAC
ENSRNOT00000035199     GTGAGCAATGAGGTTCTGGGCCGCTTGCTCCACTGCTCCCCCAAACTGCGCCTGCTGGAT
XM_002819557.1         GTGAGCAACGAGGTCCTGGGCCGCCTACTCCACGGCTCTCCCAACCTGCGCTTACTGGAT
ENSMMUT00000010304     GTAAGCAACGAGGTCCTGGGCCGCCTACTCCACGGCTCTCCCAACTTGCGCTTACTAGAT
ENSPTRT00000038291     GTGAGCAACGAGGTCCTGGGCCGCCTGCTCCACGGCTCTCCCAACCTGCGCTTACTGGAT
ENSGGOT00000016219     GTGAGCAACGAGGTCCTGGGCCGCCTACTCCACGGCTCTCCCAACCTGCGCTTACTGGAT
ENST00000331890        GTGAGCAACGAGGTCCTGGGCCGCCTACTCCACGGCTCTCCCAACCTGCGCTTACTGGAT

ENSMUST00000023219     CTTCGAGGCTGCGCTAGGGTCACTCCTAGCGGTCTGTGTCATCTGCCATGTCAAGAGCTG
ENSRNOT00000035199     CTTCGAGGCTGTGCTAGAATCACTCCTACTGGGCTGTGTCATTTGCCGTGTCAAGAGTTG
XM_002819557.1         CTTCGTGGCTGTGCCCGCATCACGCCGGCTGGCCTTCAGGATCTGCCATGTCGGGAGCTG
ENSMMUT00000010304     CTTCGTGGCTGTGCGCGCATCACGCCGGCTGGCCTTCAGGATCTGCCGTGTCGGGAGCTG
ENSPTRT00000038291     CTTCGTGGCTGTGCGCGCATCACGCCGGCTGGCCTTCAGGATCTGCCGTGTCGGGAGCTG
ENSGGOT00000016219     CTTCGTGGCTGTGCGCGCATCACGCCGGCTGGCCTTCAGGATCTGCCATGTCGGGAGCTG
ENST00000331890        CTTCGTGGCTGTGCGCGCATCACGCCGGCTGGCCTTCAGGATCTGCCATGTCGGGAGCTG

ENSMUST00000023219     GAGCAACTCTACCTGGGCCTGTATGGCATATCTGATGGCTTGACCCTGGCTAAGGATGGC
ENSRNOT00000035199     GAGCAGCTCTACCTGGGCCTGTATGGCATGTCTGATGGGTTGGCTCTAGCCAAGGATGGC
XM_002819557.1         GAGCAGCTTCATCTGGGCCTGTATGGCACGTCAGACCGGCTGACTCTAGCCAAGGAGGGC
ENSMMUT00000010304     GAGCAGCTTCATCTGGGCCTGTATGGCACGTCAGACCGGCTGACTCTAGCCAAGGAGGGC
ENSPTRT00000038291     GAGCAGCTTCATCTGGGCCTGTATGGCACATCAGACCGGCTGACTCTAGCCAAGGAGGGC
ENSGGOT00000016219     GAGCAGCTTCATCTGGGCCTGTATGGCACGTCAGACCGGCTGACTCTAGCCAAGGAGGGC
ENST00000331890        GAGCAGCTTCATCTGGGCCTGTATGGCACGTCAGACCGGCTGACTCTAGCCAAGGAGGGC
```

```
ENSMUST00000023219     AGCCCCCTGTTGACCCGGAAGTGGTATCACACCCTGAGGGAGCTGGACTTCAGTGGCCAA
ENSRNOT00000035199     AGCCCCCTGTTGACCCAGAAGTGGTATCACACCCTGAGGGAGCTGGACTTCAGTGGCCAA
XM_002819557.1         AGCCCCTTGTTGACCCAGAAGTGGTGCCATACACTGCGAGAACTGGACTTGAGTGGCCAG
ENSMMUT00000010304     AGCCCCCTGTTGACTCAGAAGTGGTGCCATACACTGCGAGAACTGGACTTGAGTGGCCAG
ENSPTRT00000038291     AGCCCCTTTTTGACCCAGAAGTGGTGCCATACATTGCGAGAACTGGACTTGAGCGGCCAG
ENSGGOT00000016219     AGCCCCTTTTTGACCCAGAAGTGGTGCCATACACTGCGAGAACTGGACTTGAGTGGCCAG
ENST00000331890        AGCCCCTTTTTGACCCAGAAGTGGTGCCATACACTGCGAGAACTGGACTTGAGTGGCCAG

ENSMUST00000023219     GGCTTCAGCGAGAAGGACTTGGAACAGGCCCTGGCTGTTTTCTCGGGCACCCCTGGGGGC
ENSRNOT00000035199     GGCTTCAGCGAGAAAGACTTGGAACAGGCCCTGGCTGTTTTCTCAGGCACCACTGAGGGC
XM_002819557.1         TGGTTCAGTGAGAAGGACCTGGAGCAGGCCCTGGCTGCCTTCTTAAGCACCCCTGGAAGC
ENSMMUT00000010304     GGTTTCAGTGAGAAGGACCTGGAACAGGCCCTGGCTGCCTTCTTAAGCACCCCTGGGGGC
ENSPTRT00000038291     GGGTTCAGTGAGAAGGACCTGGAGCAGGCCTTGGCTGCCTTCTTAAGCACCCCTGGGGGC
ENSGGOT00000016219     GGGTTCAGTGAGAAGGACCTGGAGCAGGCCCTGGCTGCCTTCTTAAGCACCCCTGGGGGC
ENST00000331890        GGGTTCAGTGAGAAGGACCTGGAGCAGGCCCTGGCTGCCTTCTTAAGCACCCCTGGGGGC

ENSMUST00000023219     TTACACCCAGCCTTGTGCTCCCTCAACCTGAGGGGTACCCGAGTTACCCCAAGCACGGTC
ENSRNOT00000035199     TTACCCCCAGCCTTGTGTTCCCTCAACCTAAGGGGTACCCGAGTTACACCAAGCACAGTC
XM_002819557.1         TCACACCCAGCCCTGTGCTCTCTTAATCTCAGGGGCACCCGGGTCACACCAAGCACGGTC
ENSMMUT00000010304     TCACACCCAGCCCTGTGCTCTCTTAACCTCAGGGGCACCCGGGTCACACCCAGCACGGTC
ENSPTRT00000038291     TCACACCCAGCCCTGTGCTCTCTTAACCTCAGGGGCACCCGGGTCACACCAAGCACTGTC
ENSGGOT00000016219     TCACACCCAGCCCTGTGCTCTCTTAACCTCAGGGGCACCCGGGTCACACCAAGCACTGTC
ENST00000331890        TCACACCCAGCCCTGTGCTCTCTTAACCTCAGGGGCACCCGGGTCACACCAAGCACTGTC

ENSMUST00000023219     AGTTCTGTGATTAGTGGTTGCCCGGGGCTGCTGTATCTTAACCTGGAGTCCTGTCGTTGC
ENSRNOT00000035199     AGTTCTGTGATTAGCAGTTGCCCGGGGCTGCTGTATCTCAATCTGGAGTCCTGTCGTTGT
XM_002819557.1         AGCTCTGTGATCAGCAGCTGCCCAGGCCTGCTCTACCTCAACCTGGAGTCCTGCCGCTGC
ENSMMUT00000010304     AGCTCTGTGATCAGCAGCTGCCCAGGCCTGCTCTACCTCAACCTGGAGTCCTGCCGCTGC
ENSPTRT00000038291     AGCTCTGTGATCAGCAGCTGCCCGGGCCTGCTCTACCTCAACCTGGAGTCCTGCCGCTGC
ENSGGOT00000016219     AGCTCTGTGATCAGCAGCTGCCCGGGCCTGCTCTACCTCAACCTGGAGTCCTGCCGCTGC
ENST00000331890        AGCTCTGTGATCAGCAGCTGCCCGGGCCTGCTCTACCTCAACCTGGAGTCCTGCCGCTGC

ENSMUST00000023219     CTCCCCCGAGGTCTGAAGCGCGTCTACCGGGGCCTGGAGGAAGTCCAGTGGTGTCTAGAG
ENSRNOT00000035199     CTCCCACGAGGTCTGAAGCGTGCCTACAGGGGCCTGGAGGAAGTCCAGTGGTGTCTAGAG
XM_002819557.1         CTTCCCCGGGGTCTGAAGCGGGCCTACCGGGGCCTGGAGGAAGTCCAGTGGTGTCTGGAG
ENSMMUT00000010304     CTTCCCCGGGGTCTGAAGCGGGCCTACCGGGGCCTGGAGGAAGTCCAGTGGTGTCTGGAG
ENSPTRT00000038291     CTTCCCCGGGGTTTGAAGCGGGCCTACCGGGGCCTGGAGGAAGTCCAGTGGTGTCTGGAG
ENSGGOT00000016219     CTTCCCCGGGGTCTGAAGCGGGCCTACCGGGGCCTGGAGGAAGTCCAGTGGTGTCTGGAG
ENST00000331890        CTTCCCCGGGGTCTGAAGCGGGCCTACCGGGGCCTGGAGGAAGTCCAGTGGTGTCTGGAG

ENSMUST00000023219     CAGCTACTTACCAGCCCGCCCTCTGCC
ENSRNOT00000035199     CAGCTACTTACGAGCCCGCCCTCTTCC
XM_002819557.1         CAGCTGCTCACCAGCCCCTCACCCAGC
ENSMMUT00000010304     CAGCTGCTCACCAGCCCCTTGCCCAGC
ENSPTRT00000038291     CAGCTGCTCACCAGCCCCTCACCCAGC
ENSGGOT00000016219     CAGCTGCTCACCAGCCCCTCACCCAGC
ENST00000331890        CAGCTGCTCACCAGCCCCTCACCCAGC
```

Multiple sequence alignment of Fbxl7

```
ENSCJAT00000021097     GACTCCGACCTGAGCATGCGCACCCTGAGCACGCCCAGCCCAGCCCTGATATGTCCTCCG
ENSMUST00000059204     GATTCTGACTTGAGCATGCGCACACTGAGCACGCCCAGCCCAGCCTTGATATGCCCACCG
```

```
ENSRNOT00000031170     GATTCTGACTTGAGCATGCGCACACTGAGCACGCCCAGCCCAGCCTTGATATGTCCACCA
ENSPPYT00000017847     ---------------ATGCGCACACTGAGCACGCCCAGCCCAGCCCTGATATGTCCACCG
ENSGGOT00000011198     GACTCCGACCTGAGCATGCGCACACTGAGCACGCCCAGCCCAGCCCTGATATGTCCACCG
ENST00000504595        GACTCCGACCTGAGCATGCGCACACTGAGCACGCCCAGCCCAGCCCTGATATGTCCACCG
ENSMMUT00000018754     GACTCCGACCTGAGCATGCGCACACTGAGCACGCCCAGCCCAGCCCTGATATGTCCACCG
ENSPTRT00000031097     GACTCCGACCTGAGCATGCGCACACTGAGCACGCCCAGCCCAGCCCTGATATGTCCACCG

ENSCJAT00000021097     AATCTGCCAGGATTTCAGAATGGAAGGGGCTCGTCCACTTCCTCGTCCTCCATCACTGGG
ENSMUST00000059204     ACTTTGCCAGGATTTCAGAATGGAAGGGGTTCGTCCACATCTTCATCCTCCATCACCGGG
ENSRNOT00000031170     GCTTTGCCAGGATTTCAGAATGGAAGGGGCTCGTCCACATCTTCATCCTCCATCACTGGG
ENSPPYT00000017847     AATCTGCCAGGATTTCAGAATGGAAGGGGCTCGTCCACCTCCTCGTCCTCTATCACCGGG
ENSGGOT00000011198     AATCTCCCAGGATTTCAGAATGGAAGGGGCTCGTCCACCTCCTCGTCCTCCATCACCGGG
ENST00000504595        AATCTCCCAGGATTTCAGAATGGAAGGGGCTCGTCCACCTCCTCGTCCTCCATCACCGGG
ENSMMUT00000018754     AATCTGCCAGGATTTCAGAATGGAAGGGGCTCGTCCACCTCCTCGTCCTCCATCACTGGG
ENSPTRT00000031097     AATCTCCCAGGATTTCAGAATGGAAGGGGCTCGTCCACCTCCTCGTCCTCCATCACCGGG

ENSCJAT00000021097     GAGACGGTGGCCATGGTCCACTCCCCGCCCCCGACCCGCCTCACGCACCCCCTCATCCGG
ENSMUST00000059204     GAGACAGTGGCTATGGTTCACTCCCCGCCCCCGACCCGCCTCACTCACCCACTCATTCGG
ENSRNOT00000031170     GAGACAGTGGCTATGGTTCATTCCCCACCCCCGACCCGTCTCACTCACCCACTCATTCGG
ENSPPYT00000017847     GAGACGGTGGCCATGGTGCACTCCCCGCCCCCGACCCGCCTCACACACCCGCTCATCCGG
ENSGGOT00000011198     GAGACGGTGGCCATGGTGCACTCCCCGCCCCCGACCCGCCTCACACACCCGCTCATCCGG
ENST00000504595        GAGACGGTGGCCATGGTGCACTCCCCGCCCCCGACCCGCCTCACACACCCGCTCATCCGG
ENSMMUT00000018754     GAGACGGTGGCCATGGTCCACTCCCCGCCCCCGACCCGCCTCACACACCCGCTCATCCGG
ENSPTRT00000031097     GAGACGGTGGCCATGGTGCACTCCCCGCCCCCGACCCGCCTCACACACCCGCTCATCCGG

ENSCJAT00000021097     CTCGCCTCCAGACCCCAGAAGGAGCAGGCCAGCATCGACCGGCTCCCGGACCACTCCGTG
ENSMUST00000059204     CTCGCCTCCAGACCCCAGAAAGAGCAAGCCAGCATAGACAGGCTCCCGGACCACTCCATG
ENSRNOT00000031170     CTCGCTTCCAGACCCCAGAAAGAGCAAGCCAGCATAGACCGACTCCCGGACCACTCCATG
ENSPPYT00000017847     CTCGCCTCCAAACCCCAGAAGGAGCAGGCCAGCATAGACCGGCTCCCGGACCACTCCATG
ENSGGOT00000011198     CTTGCCTCCAGACCCCAGAAGGAGCAGGCCAGCATAGACCGGCTCCCGGACCACTCCATG
ENST00000504595        CTCGCCTCCAGACCCCAGAAGGAGCAGGCCAGCATAGACCGGCTCCCGGACCACTCCATG
ENSMMUT00000018754     CTCGCCTCCAGACCCCAGAAGGAGCAGGCCAGCATAGACCGGCTCCCGGACCACTCCATG
ENSPTRT00000031097     CTCGCCTCCAGACCCCAGAAGGAGCAGGCCAGCATAGACCGGCTCCCGGACCACTCCATG

ENSCJAT00000021097     GTGCACATCTTCTCCTTCCTGCCCACCAACCAGCTGTGCCGCTGCGCGCGCGTGTGCCGC
ENSMUST00000059204     GTGCAGATCTTCTCCTTCCTGCCCACCAACCAGCTATGTCGCTGTGCACGTGTGTGCCGC
ENSRNOT00000031170     GTGCAGATCTTCTCCTTCCTGCCCACCAACCAGCTATGTCGCTGTGCACGTGTGTGCCGC
ENSPPYT00000017847     GTGCAGATCTTCTCCTTCCTGCCTACCAACCAGCTGTGCCGCTGCGCGCGCGTGTGCCGC
ENSGGOT00000011198     GTGCAGATCTTCTCCTTCCTGCCCACCAACCAGCTGTGCCGCTGCGCGCGAGTGTGCCGC
ENST00000504595        GTGCAGATCTTCTCCTTCCTGCCCACCAACCAGCTGTGCCGCTGCGCGCGAGTGTGCCGC
ENSMMUT00000018754     GTGCAGATCTTCTCCTTCCTGCCCACCAACCAGCTGTGCCGCTGCGCGCGCGTGTGCCGC
ENSPTRT00000031097     GTGCAGATCTTCTCCTTCCTGCCCACCAACCAGCTGTGCCGCTGCGCGCGAGTGTGCCGC

ENSCJAT00000021097     CGCTGGTACAACCTGGCGTGGGATCCGCGCCTCTGGAGGACTATCCGCCTGACGGGCGAA
ENSMUST00000059204     CGCTGGTACAACCTGGCCTGGGACCCGCGCCTCTGGAGGACTATCCGTCTCACTGGAGAG
ENSRNOT00000031170     CGCTGGTACAACCTGGCCTGGGACCCGCGCCTCTGGAGGACTATCCGTCTCACTGGCGAG
ENSPPYT00000017847     CGCTGGTACAACCTGGCCTGGGACCCGCGGCTCTGGAGGACTATCCGCCTGACGGGCGAG
ENSGGOT00000011198     CGCTGGTACAACCTGGCCTGGGACCCGCGGCTGTGGAGGACTATCCGCCTGACGGGCGAG
ENST00000504595        CGCTGGTACAACCTGGCCTGGGACCCGCGGCTCTGGAGGACTATCCGCCTGACGGGCGAG
ENSMMUT00000018754     CGCTGGTACAACCTCGCCTGGGACCCTCGGCTCTGGAGGACTATCCGCCTGACGGGCGAG
ENSPTRT00000031097     CGCTGGTACAACCTGGCCTGGGACCCGCGGCTCTGGAGGACTATCCGCCTGACGGGCGAG

ENSCJAT00000021097     ACCATCAACGTGGACCGCGCCCTCAAAGTGCTGAGCCGCAGACTCTGCCAGGACACCCCC
ENSMUST00000059204     ACCATCAATGTGGACCGTGCCCTGAAAGTACTGACCCGCAGGCTTTGCCAGGACACCCCC
```

ENSRNOT00000031170   ACCATCAATGTGGACCGTGCCCTGAAAGTACTGACCCGCAGGCTTTGCCAGGACACTCCC
ENSPPYT00000017847   ACCATCAACGTGGACCGCGCCCTCAAGGTGCTGACCCGCAGACTCTGCCAGGACACGCCC
ENSGGOT00000011198   ACCATCAACGTGGACCGCGCCCTCAAGGTGCTGACCCGCAGACTCTGCCAGGACACCCCC
ENST00000504595   ACCATCAACGTGGACCGCGCCCTCAAGGTGCTGACCCGCAGACTCTGCCAGGACACCCCC
ENSMMUT00000018754   ACCATCAACGTGGACCGCGCCCTCAAGGTGCTGACCCGCAGACTCTGCCAGGACACCCCC
ENSPTRT00000031097   ACCATCAACGTGGACCGCGCCCTCAAGGTGCTGACCCGCAGACTCTGCCAGGACACCCCC

ENSCJAT00000021097   AACGTGTGTCTCATGCTGGAAACCGTAACTGTCAGTGGCTGCAGGCGGCTCACAGACCGA
ENSMUST00000059204   AATGTGTGTCTCATGCTGGAGACTGTCATTGTCAGTGGCTGCCGGCGGCTCACAGACCGG
ENSRNOT00000031170   AATGTGTGTCTGATGTTGGAAACTGTCATTGTCAGTGGCTGCCGGCGGCTCACAGACCGA
ENSPPYT00000017847   AACGTGTGTCTCATGCTGGAAACCGTAACTGTCAGTGGCTGCAGGCGGCTCACAGACCGA
ENSGGOT00000011198   AACGTGTGTCTCATGCTGGAAACCGTAACTGTCAGTGGCTGCAGGCGGCTCACAGACCGA
ENST00000504595   AACGTGTGTCTCATGCTGGAAACCGTAACTGTCAGTGGCTGCAGGCGGCTCACAGACCGA
ENSMMUT00000018754   AACGTGTGTCTCATGCTGGAAACCGTTACTGTCAGTGGCTGCAGGCGGCTCACAGACCGA
ENSPTRT00000031097   AACGTGTGTCTCATGCTGGAAACCGTAACTGTCAGTGGCTGCAGGCGGCTCACAGACCGA

ENSCJAT00000021097   GGGCTGTACATCATATCCCAGTGCTGTCCCGAACTGAGGCGACTGGAAGTCTCAGGCTGT
ENSMUST00000059204   GGGCTTTATACCATTGCACAGTGCTGTCCAGAACTGAGGCGCCTGGAAGTCTCAGGATGT
ENSRNOT00000031170   GGGCTTTATACCATTGCACAGTGCTGTCCGGAACTGAGGCGCCTGGAAGTCTCAGGATGT
ENSPPYT00000017847   GGGCTGTACACCATCGCCCAGTGCTGCCCCGAACTGAGGCGACTGGAAGTCTCAGGCTGT
ENSGGOT00000011198   GGGCTGTACACCATCGCCCAGTGCTGCCCCGAACTGAGGCGACTGGAAGTCTCAGGCTGT
ENST00000504595   GGGCTGTACACCATCGCCCAGTGCTGCCCCGAACTGAGGCGACTGGAAGTCTCAGGCTGT
ENSMMUT00000018754   GGGCTGTACACCATCGCCCAGTGCTGCCCCGAACTGAGGCGACTGGAAGTCTCAGGCTGT
ENSPTRT00000031097   GGGCTGTACACCATCGCCCAGTGCTGCCCCGAACTGAGGCGACTGGAAGTCTCAGGCTGT

ENSCJAT00000021097   TACAATATCTCCAACGAGGCCGTCTTTGACGTGGTGTCCCTGTGCCCCAACCTGGAGCAC
ENSMUST00000059204   TACAATATCTCGAACGAGGCTGTCTTTGATGTGGTGTCACTCTGTCCCAACCTGGAGCAT
ENSRNOT00000031170   TACAATATCTCCAATGAGGCGGTCTTTGATGTGGTGTCCCTCTGCCCCAATCTGGAGCAT
ENSPPYT00000017847   TACAATATCTCCAACGAGGCCGTCTTTGACGTGGTGTCCCTCTGCCCCAATCTGGAGCAC
ENSGGOT00000011198   TACAATATCTCCAACGAGGCCGTCTTTGACGTGGTGTCCCTCTGCCCTAATCTGGAACAC
ENST00000504595   TACAATATCTCCAACGAGGCCGTCTTTGATGTGGTGTCCCTCTGCCCTAATCTGGAGCAC
ENSMMUT00000018754   TACAATATCTCCAACGAGGCCGTCTTTGACGTGGTGTCCCTCTGCCCCAATCTGGAGCAC
ENSPTRT00000031097   TACAATATCTCCAACGAGGCCGTCTTTGACGTGGTGTCCCTCTGCCCTAATCTGGAGCAC

ENSCJAT00000021097   CTGGATGTGTCAGGGTGCTCCAAAGTGACCTGCATCAGCTTGACTCGGGAGGCCTCCATT
ENSMUST00000059204   CTGGATGTGTCAGGGTGCTCCAAAGTGACCTGCATCAGCTTGACCCGGGAGGCCTCCATT
ENSRNOT00000031170   CTGGATGTGTCAGGATGCTCCAAAGTTACGTGCATCAGCTTGACCCGGGAGGCCTCCATT
ENSPPYT00000017847   CTGGATGTGTCAGGATGCTCCAAAGTGACCTGCATCAGCTTGACCCGGGAGGCCTCCATT
ENSGGOT00000011198   CTGGATGTGTCAGGATGCTCCAAAGTGACCTGCATCAGCTTGACCCGGGAGGCCTCCATT
ENST00000504595   CTGGATGTGTCAGGATGCTCCAAAGTGACCTGCATCAGCTTGACCCGGGAGGCCTCCATT
ENSMMUT00000018754   CTGGATGTGTCAGGGTGCTCCAAAGTGACCTGCATCAGCTTGACCCGGGAGGCCTCCATT
ENSPTRT00000031097   CTGGATGTGTCAGGATGCTCCAAAGTGACCTGCATCAGCTTGACCCGGGAGGCCTCCATT

ENSCJAT00000021097   AAACTGTCCCCCTTGCATGGCAAACAGATTTCCATCCGCTACCTGGACATGACGGACTGC
ENSMUST00000059204   AAACTGTCCCCCTTACATGGCAAACAGATTTCCATCCGATACCTTGATATGACGGACTGC
ENSRNOT00000031170   AAACTGTCCCCCTTGCATGGCAAACAGATTTCCATCCGATACCTTGATATGACGGACTGC
ENSPPYT00000017847   AAACTGTCCCCCTTGCATGGCAAACAGATTTCCATCCGCTACCTAGACATGACGGACTGC
ENSGGOT00000011198   AAACTGTCCCCCTTGCATGGCAAACAGATTTCCATCCGCTACCTGGACATGACGGACTGC
ENST00000504595   AAACTGTCACCCTTGCATGGCAAACAGATTTCCATCCGCTACCTGGACATGACGGACTGC
ENSMMUT00000018754   AAACTGTCCCCCTTACATGGCAAACAGATATCCATCCGCTACCTGGACATGACGGACTGC
ENSPTRT00000031097   AAACTGTCCCCCTTGCATGGCAAACAGATTTCCATTCGCTACCTGGACATGACGGACTGC

ENSCJAT00000021097   TTCGTGCTGGAGGACGAAGGCCTGCACACCATCGCGGCGCACTGCACGCAGCTCACCCAC
ENSMUST00000059204   TTCGTGCTGGAGGACGAAGGCTTACACACCATCGCAGCTCACTGCACGCAGCTCACTCAC

ENSRNOT00000031170 TTCGTGCTGGAGGACGAAGGCTTGCACACCATCGCAGCTCACTGCACGCAGCTCACACAC
ENSPPYT00000017847 TTCGTGCTGGAGGACGAAGGCCTGCACACCATCGCGGCACACTGCACGCAGCTCACCCAC
ENSGGOT00000011198 TTCGTGCTGGAGGACGAAGGCCTGCACACCATCGCGGCGCACTGCACGCAGCTCACCCAC
ENST00000504595 TTCGTGCTGGAGGACGAAGGCCTGCACACCATCGCGGCGCACTGCACGCAGCTCACCCAC
ENSMMUT00000018754 TTCGTGCTGGAGGACGAAGGCCTGCACACCATCGCGGCACACTGCACGCAGCTCACCCAC
ENSPTRT00000031097 TTCGTGCTGGAGGACGAAGGCCTGCACACCATCGCGGCGCACTGCACGCAGCTCACCCAC

ENSCJAT00000021097 CTGTACCTGCGCCGCTGCGTCCGCCTGACCGACGAGGGCCTGCGCTACCTGGTCATCTAC
ENSMUST00000059204 CTCTATCTGCGCCGCTGCGTCCGCCTCACAGATGAGGGTCTCCGCTACCTGGTGATCTAC
ENSRNOT00000031170 CTCTATCTGCGCCGCTGTGTCCGTCTCACGGATGAGGGTCTCCGCTACCTGGTGATCTAC
ENSPPYT00000017847 CTCTACCTGCGCCGCTGCGTCCGCCTGACCGACGAGGGCCTGCGCTACCTGGTGATCTAC
ENSGGOT00000011198 CTCTACCTGCGCCGCTGCGTCCGCCTGACCGACGAAGGCCTGCGCTACCTGGTGATCTAC
ENST00000504595 CTCTACCTGCGCCGCTGCGTCCGCCTGACCGACGAAGGCCTGCGCTACCTGGTGATCTAC
ENSMMUT00000018754 CTCTACCTGCGCCGCTGCGTCCGCCTGACCGACGAGGGACTGCGCTACCTGGTGATCTAC
ENSPTRT00000031097 CTCTACCTGCGCCGCTGCGTCCGCCTGACCGACGAAGGCCTGCGCTACCTGGTGATCTAC

ENSCJAT00000021097 TGTGCCTCCATCAAGGAGCTGAGCGTCAGCGACTGCCGCTTCGTCAGCGACTTCGGCCTG
ENSMUST00000059204 TGCACTTCCATCAAGGAGCTGAGTGTCAGCGACTGCCGCTTTGTCAGTGACTTTGGCCTG
ENSRNOT00000031170 TGCACTTCCATCAAGGAGCTAAGTGTAAGCGACTGCCGCTTTGTCAGTGACTTTGGCCTG
ENSPPYT00000017847 TGCGCCTCCATCAAGGAGCTGAGCGTCAGCGACTGCCGCTTCGTCAGCGACTTCGGCCTG
ENSGGOT00000011198 TGCGCCTCCATCAAGGAGCTGAGCGTCAGCGACTGCCGCTTCGTCAGCGACTTCGGCCTG
ENST00000504595 TGCGCCTCCATCAAGGAGCTGAGCGTCAGCGACTGCCGCTTCGTCAGCGACTTCGGCCTG
ENSMMUT00000018754 TGCGCCTCTATCAAGGAGCTGAGCGTCAGCGACTGCCGCTTCGTCAGCGACTTCGGCCTG
ENSPTRT00000031097 TGCGCCTCCATCAAGGAGCTGAGCGTCAGCGACTGCCGCTTCGTCAGCGACTTCGGCCTG

ENSCJAT00000021097 CGGGAGATCGCCAAGCTGGAGTCCCGCCTGCGGTACCTGAGCATCGCGCACTGCGGCCGG
ENSMUST00000059204 CGGGAGATTGCTAAACTGGAGTCCCGCTTGAGGTACCTCAGCATCGCCCACTGCGGCCGC
ENSRNOT00000031170 CGGGAGATTGCTAAACTGGAGTCCCGCTTGAGGTACCTCAGCATCGCCCATTGCGGCCGC
ENSPPYT00000017847 CGGGAGATCGCCAAGCTGGAGTCCCGCCTGCGGTACCTGAGCATCGCGCACTGCGGTCGG
ENSGGOT00000011198 CGGGAGATCGCCAAGCTGGAGTCCCGCCTGCGGTACCTGAGCATCGCGCACTGCGGCCGG
ENST00000504595 CGGGAGATCGCCAAGCTGGAGTCCCGCCTGCGGTACCTGAGCATCGCGCACTGCGGCCGG
ENSMMUT00000018754 CGGGAGATCGCCAAACTGGAGTCCCGCCTGCGGTACCTGAGCATCGCGCACTGCGGCCGG
ENSPTRT00000031097 CGGGAGATCGCCAAGCTGGAGTCCCGCCTGCGGTACCTGAGCATCGCGCACTGCGGCCGG

ENSCJAT00000021097 GTCACCGACGTGGGCATCCGCTACGTGGCCAAGTACTGCAGCAAGCTGCGCTACCTCAAC
ENSMUST00000059204 ATCACGGATGTGGGCATTCGTTATGTGGCTAAATACTGCAGCAAATTACGCTACCTCAAT
ENSRNOT00000031170 ATCACGGATGTGGGCATTCGTTACGTGGCTAAATACTGCAGCAAATTGCGCTACCTCAAC
ENSPPYT00000017847 GTCACCGACGTGGGCATCCGCTACGTGGCCAAGTACTGCAGCAAGCTGCGCTACCTCAAC
ENSGGOT00000011198 ATCACCGACGTGGGCATCCGCTACGTGGCCAAGTACTGCAGCAAGCTGCGCTACCTCAAC
ENST00000504595 GTCACCGACGTGGGCATCCGCTACGTGGCCAAGTACTGCAGCAAGCTGCGCTACCTCAAC
ENSMMUT00000018754 GTCACAGACGTGGGCATCCGCTACGTGGCCAAGTACTGCAGCAAGCTGCGCTACCTCAAC
ENSPTRT00000031097 GTCACCGACGTGGGCATCCGCTACGTGGCCAAGTACTGCAGCAAGCTGCGCTACCTCAAC

ENSCJAT00000021097 GCGAGGGGCTGCGAGGGCATCACGGACCACGGCGTGGAGTACCTCGCCAAGAACTGCACC
ENSMUST00000059204 GCGAGGGGCTGCGAGGGCATCACGGACCATGGTGTGGAGTACCTCGCCAAGAACTGTACA
ENSRNOT00000031170 GCGAGGGGCTGCGAGGGAATCACGGACCATGGTGTGGAGTACCTTGCCAAGAACTGCACA
ENSPPYT00000017847 GCGAGGGGCTGCGAGGGCATCACGGACCACGGTGTGGAGTACCTCGCCAAGAACTGCACC
ENSGGOT00000011198 GCGAGGGGCTGCGAGGGCATCACGGACCACGGTGTGGAGTACCTCGCCAAGAACTGCACC
ENST00000504595 GCGAGGGGCTGCGAGGGCATCACGGACCACGGTGTGGAGTACCTCGCCAAGAACTGCACC
ENSMMUT00000018754 GCGAGGGGCTGCGAGGGCATCACGGACCACGGCGTGGAGTACCTCGCCAAGAACTGCACC
ENSPTRT00000031097 GCGAGGGGCTGCGAGGGCATCACGGACCACGGTGTGGAGTACCTCGCCAAGAACTGCACC

ENSCJAT00000021097 AAACTCAAGTCCCTGGATATCGGCAAATGCCCTTTGGTATCCGACACGGGCCTGGAGTGC
ENSMUST00000059204 AAACTTAAGTCGCTGGACATTGGTAAATGTCCCCTGGTCTCCGACACGGGCCTGGAATCC

ENSRNOT00000031170   AAACTTAAGTCTCTGGACATTGGCAAATGTCCCCTGGTCTCTGACACAGGCCTGGAATCC
ENSPPYT00000017847   AAACTCAAATCCCTGGATATCGGTAAATGCCCTTTGGTATCCGACACGGGCCTGGAGTGC
ENSGGOT00000011198   AAACTCAAATCCCTGGATATCGGCAAATGCCCTTTGGTATCCGACACGGGCCTGGAGTGC
ENST00000504595   AAACTCAAATCCCTGGATATCGGCAAATGCCCTTTGGTATCCGACACGGGCCTGGAGTGC
ENSMMUT00000018754   AAACTCAAGTCCCTGGATATCGGCAAATGCCCTTTGGTATCCGACACGGGCCTGGAGTGC
ENSPTRT00000031097   AAACTCAAATCCCTGGATATCGGCAAATGCCCTTTGGTATCCGACACGGGCCTGGAGTGC

ENSCJAT00000021097   CTGGCCCTGAACTGCTTCAACCTCAAGCGGCTCAGCCTCAAGTCCTGCGAGAGCATCACC
ENSMUST00000059204   CTGGCTTTGAACTGCTTCAATCTCAAGAGGCTAAGCCTCAAGTCCTGTGAGAGCATCACC
ENSRNOT00000031170   CTGGCATTGAACTGCTTCAATCTCAAGAGGCTAAGCCTCAAGTCCTGTGAGAGCATCACT
ENSPPYT00000017847   CTGGCCCTGAACTGCTTCAACCTCAAGCGGCTCAGCCTCAAGTCCTGTGAGAGCATCACC
ENSGGOT00000011198   CTGGCCCTGAACTGCTTCAACCTCAAGCGGCTCAGCCTCAAGTCCTGCGAGAGCATCACC
ENST00000504595   CTGGCCCTGAACTGCTTCAACCTCAAGCGGCTCAGCCTCAAGTCCTGCGAGAGCATCACC
ENSMMUT00000018754   CTGGCCCTGAACTGCTTCAACCTCAAGCGGCTCAGCCTCAAGTCCTGCGAGAGCATCACC
ENSPTRT00000031097   CTGGCCCTGAACTGCTTCAACCTCAAGCGGCTCAGCCTCAAGTCCTGCGAGAGCATCACC

ENSCJAT00000021097   GGCCAGGGCTTGCAGATCGTGGCCGCCAACTGCTTCGACCTCCAGACGCTGAATGTCCAG
ENSMUST00000059204   GGCCAGGGCCTGCAGATCGTGGCTGCCAACTGCTTCGACCTGCAGATGTTAAATGTCCAG
ENSRNOT00000031170   GGCCAGGGCCTGCAGATCGTGGCTGCCAACTGCTTTGACCTGCAAATGTTAAATGTCCAG
ENSPPYT00000017847   GGCCAGGGCTTGCAGATCGTGGCCGCCAACTGCTTTGACCTGCAGACGCTGAATGTCCAG
ENSGGOT00000011198   GGCCAGGGCTTGCAGATCGTGGCCGCCAACTGCTTTGACCTCCAGACGCTGAATGTCCAG
ENST00000504595   GGCCAGGGCTTGCAGATCGTGGCCGCCAACTGCTTTGACCTCCAGACGCTGAATGTCCAG
ENSMMUT00000018754   GGCCAGGGCTTGCAGATCGTGGCCGCCAACTGCTTTGACCTCCAGACGCTGAATGTCCAG
ENSPTRT00000031097   GGCCAGGGCTTGCAGATCGTGGCCGCCAACTGCTTTGACCTCCAGACGCTGAATGTCCAG

ENSCJAT00000021097   GACTGCGAGGTCTCCGTGGAGGCCCTGCGCTTCGTCAAACGCCACTGCAAGCGCTGCGTC
ENSMUST00000059204   GACTGTGAAGTTTCCGTGGAGGCCCTGCGGTTTGTGAAGCGCCATTGCAAGCGCTGTGTC
ENSRNOT00000031170   GACTGTGAAGTTTCGGTAGAGGCCCTGCGGTTTGTGAAGCGCCATTGCAAGCGCTGTGTC
ENSPPYT00000017847   GACTGCGAGGTCTCCGTGGAGGCCCTGCGCTTTGTCAAACGCCACTGCAAGCGCTGCGTC
ENSGGOT00000011198   GACTGCGAGGTCTCCGTGGAGGCCCTGCGCTTTGTCAAACGCCACTGCAAGCGCTGCGTC
ENST00000504595   GACTGCGAGGTCTCCGTGGAGGCCCTGCGCTTTGTCAAACGCCACTGCAAGCGCTGCGTC
ENSMMUT00000018754   GACTGCGAGGTCTCCGTGGAGGCCTTGCGCTTTGTCAAACGCCACTGCAAGCGCTGTGTC
ENSPTRT00000031097   GACTGCGAGGTCTCCGTGGAGGCCCTGCGCTTTGTCAAACGCCACTGCAAGCGCTGCGTC

ENSCJAT00000021097   ATCGAGCACACCAACCCGGCTTTCTTC
ENSMUST00000059204   ATTGAGCATACCAACCCCGCTTTCTTC
ENSRNOT00000031170   ATTGAGCATACCAACCCTGCTTTCTTC
ENSPPYT00000017847   ATCGAGCACACCAACCCGGCTTTCTTC
ENSGGOT00000011198   ATCGAGCACACCAACCCGGCTTTCTTC
ENST00000504595   ATCGAGCACACCAACCCGGCTTTCTTC
ENSMMUT00000018754   ATCGAGCACACCAACCCGGCTTTCTTC
ENSPTRT00000031097   ATCGAGCACACCAACCCGGCTTTCTTC

Multiple sequence alignment of Fbxl8

ENSCJAT00000032069   ATGGCTGAGCCTGGAGAGCGACTGCCAGAGGAGGTGCTGGCGCTCATCTTCCGCCACCTG
ENSMMUT00000008262   ATGGCCGAGACTGGAGAGGGACTGCCAGAGGAGGTGTTGGCGCTCATCTTCCGCCACCTG
ENSGGOT00000011930   ATGGCCGAGCCTGGAGAGGGACTGCCAGAGGAGGTGCTGGCACTCATCTTCCGCCACCTG
XM_002826519.1   ATGGCCGAGCCTGGAGAGGGACTGCCAGAGGAGGTGCTGGCACTCATCTTCCGCCACCTG
ENST00000519917   ATGGCCGAGCCTGGAGAGGGACTGCCAGAGGAGGTGCTGGCACTCATCTTCCGCCACCTG
ENSPTRT00000015146   ATGGCCGAGCCTGGAGAGGGACTGCCAGAGGAGGTGCTGGCACTCATCTTCCGCCACCTG
ENSMUST00000036221   ATGGGGGAGCTGGTCGACAATCTGCCAGAGGAAGTGTTGGCGCTCATCTTCCGTGACCTG
ENSRNOT00000020440   ATGGTGGAGCTGATTGAAAAGCTACCAGAGGAAGTTTTGGGGCTCATCTTCCGTGACCTG

```
ENSCJAT00000032069     TCCCTGAAGGACCGTGCTGCAGCCGCCAGGGTCTGCAGGGCCTGGGCTGACGCTGCTAAC
ENSMMUT00000008262     CCCCTGAGAGACCGTGCTGCCGCCGCCAGGGTCTGCAGGGCCTGGGCCGCCGCTGCCACC
ENSGGOT00000011930     TCCCTGAGAGACCGTGCTGCCGCCGCCAGGGTCTGCAGGGCCTGGGCCGCCGCTGCTACC
XM_002826519.1         TCCTTGAGAGACCGTGCTGCCGCCGCCAGGGTCTGCAGGGCCTGGGCCGCCGCTGCTACC
ENST00000519917        TCCCTGAGAGACCGTGCTGCCGCCGCCAGGGTCTGCAGGGCCTGGGCCGCCGCTGCTACC
ENSPTRT00000015146     TCCCTGAGAGACCGTGCTGCCGCCGCCAGGGTCTGCAGGGCCTGGGCCGCCGCTGCTACC
ENSMUST00000036221     CCTCTCAGGGACCTTGCTGTAGCCACCAGAGTCTGCAGGGCCTGGGCGGCGGCTGCCGCC
ENSRNOT00000020440     CCTCTCAGGGACCGTGCTGTAGCAGCCAGAGTCTGCAGGGCCTGGGCTGCCGCTGCCACT

ENSCJAT00000032069     TCCAGCGCAGTGTGGCACGACACAAAAATCAGTTGCGAATGTGAGCTGGAAGGCATGCTG
ENSMMUT00000008262     TGCAGCACTGTGTGGCACGACACAAAAATCAGTTGCGAATGTGAGCTGGAAGGCATGCTG
ENSGGOT00000011930     TGCAGCGCCGTGTGGCACGACACAAAAATCAGTTGCGAATGTGAGCTGGAAGGCATGCTG
XM_002826519.1         TGCAGCGCCGTGTGGCACGACACAAAAATCAGTTGCGAATGTGAGCTGGAAGGCATGCTG
ENST00000519917        TGCAGCGCCGTGTGGCACGACACAAAAATCAGTTGCGAATGTGAGCTGGAAGGCATGCTG
ENSPTRT00000015146     TGCAGCGCCGTGTGGCACGACACAAAAATCAGTTGCGAATGTGAGCTGGAAGGCATGCTG
ENSMUST00000036221     AACAGCACCGTGTGGTCTGATAAAAGCATCAGTTGTGACTGTGAGCTGGAAGACTTGTTG
ENSRNOT00000020440     AACAGTGCTGTGTGGTATGATACAAGCATCAGTTGTGACTGTGAGCTGGAAAACTTGTTG

ENSCJAT00000032069     CCACCTTATCTGTCCGCCTGCCTTGACCACGTTCACAACCTACGGCTGGAATTTGAGCCG
ENSMMUT00000008262     CCACTTTATCTGTCCACCTGCCTGGACCACGTTCACAACCTCCGGCTGGAATTTGAGCCA
ENSGGOT00000011930     CCACCTTATCTGTCCGCCTGCCTCGACCACGTTCACAACCTACGGCTGGAATTTGAGCCA
XM_002826519.1         CCACCTTATCTGTCCGCCTGCCTCGACCACGTTCACAAACTACGGCTGGAATTTGAGCCA
ENST00000519917        CCACCTTATCTGTCCGCCTGCCTCGACCACATTCACAACCTACGGCTGGAATTTGAGCCA
ENSPTRT00000015146     CCACCTTATCTGTCCGCCTGCCTCGACCACGTTCACAACCTACGGCTGGAATTTGAGCCA
ENSMUST00000036221     CCACCATATCTGTCCTCCTGCCTGGACCACATTCACAACCTAAGGCTGGAATATGAGCCA
ENSRNOT00000020440     CCACCGGGTTTGTCCGCCTGCCTAGACCACATTCACAACCTGAGCCTGGAATATGAACCA

ENSCJAT00000032069     TCGAGGGAGCCGAGTCGCCGGGCGGCCATCAAACTGCTGATGACTCTGGCGGACCGTGCC
ENSMMUT00000008262     TCGAGGGAGGTGAGCCGCCGGGCGGCCATCGAGCTGCTGATGGTTCTGGCGGGCCGTGCC
ENSGGOT00000011930     TCGAGGAAGCCGAGCCGCCGGGCGGCCATCGAGCTGCTGATGGTTCTGGCGGGCCGTGCC
XM_002826519.1         TCGAGGAAGCCGAGCCGCCGGGCGGCCATCGAGCTGCTGATGGTTCTGGCGGGCCGTGCC
ENST00000519917        TCGAGGAAGCCGAGCCGCCGGGCGGCCATCGAGCTGCTGATGGTTCTGGCGGGCCGTGCC
ENSPTRT00000015146     TCGAGGAAGCCGAGCCGCCGGGCGGCCATCGAGCTGCTGATGGTTCTGGCGGGCCGTGCC
ENSMUST00000036221     TCAAAGAAGCCCAGCCGCGGAACGGCCACCGAGCTGCTGACCGCTCTGGCCAGCCGAGCC
ENSRNOT00000020440     TCAAAGAAGCCCAGCCGCAGAACGGCCACCGAGCTGCTGACTGCCCTGGCCAGCCGAGCC

ENSCJAT00000032069     CTAGGGCTGCGAGGCCTCTGCCTGGAGTGCCGCGGAGAAAAGCCGCTCTTCGACTCGGGC
ENSMMUT00000008262     CTGGGGCTGCGAGGCCTGCGCCTGGAGTGCCGCGGAGAGAAGCCGCTCTTCGACGCGGGC
ENSGGOT00000011930     CCGGGGCTGCGAGGCCTGCGCCTGGAATGCCGCGGAGAAAAACCGCTCTTCGACGCGGGC
XM_002826519.1         CTGGGGCTGCGAGGCCTGCGCCTGGAGTGCCGCGGAGAAAAACCGCTCTTCGACGCGGGC
ENST00000519917        CCGGGGCTGCGAGGCCTGCGCCTGGAGTGCCGCGGAGAAAAACCGCTCTTCGACGCGGGC
ENSPTRT00000015146     CCGGGGCTGCGAGGCCTGCGCCTGGAGTGCCGCGGAGAAAAACCGCTCTTCTACGCGGGC
ENSMUST00000036221     CCAAGGCTCCGAGGCCTGCGCTTGGAGTGCCGCGGAGAGAAACCGCTTTTTGATGCGGGC
ENSRNOT00000020440     CCGAGGCTTCGAGGCCTGCGCTTGGAATGTCGGGGAGAGAAGCCGCTTTTTGATGCGGGC

ENSCJAT00000032069     CACGACGTCCTGGAGGCTGTGCACGCAGTATGCGGGGCGGCCCGAGAGCTGCGCCACCTC
ENSMMUT00000008262     CGCGACGTCCTGGAGGCTGTGCACGCTGTGTGCGGGGCGGCCCGCGAGCTACGCCACCTC
ENSGGOT00000011930     CGCGACGTCCTGGAGGCTGTGCACGCTGTATGCGGGGCGGCCAGCCAGCTACGCCACCTC
XM_002826519.1         CGCGACATCCTGGAGGCTGTGCACGCTGTATGCGGGGCGGCCAGCCAGCTACGCCACCTC
ENST00000519917        CGCGACGTCCTGGAGGCTGTGCACGCTGTATGCGGGGCGGCCAGCCAGCTACGCCACCTC
ENSPTRT00000015146     CGCGACGTCCTGGAGGCTGTGCACGCTGTATGCGGGGCGGCCAGCCAGCTACGCCACCTC
ENSMUST00000036221     CAGGACATCCTGGGAGCTGTGCACGCAGTCTGCGGAGCTGCTCACCAACTTCGCCACCTC
ENSRNOT00000020440     CGCGACATCCTGGGCGCCCTGCACACAGTCTGCGGAGCCGCTCACCAACTGCGCCACCTC
```

```
ENSCJAT00000032069     GACCTGCGACGCTTGCCCTTCACACTGGACGATGCGCTGGTGCTGCGGGCGGCGCGCAGC
ENSMMUT00000008262     GACCTGCGGCGCTTGCCCTTCACACTGGACGACGCGCTAGTGCTGCAGGCGGCGCGCGGC
ENSGGOT00000011930     GACCTGCGGCGCTTGCCCTTCACACTGGACAACGCGCTGGTGCTGCAGGCGGCACGCAGC
XM_002826519.1         GACCTGCGGTGCTTGCCCTTCACACTGGACGACGCGCTGGTGCTGCAGGCGGCGCGCAGC
ENST00000519917        GACCTGCGGCGCTTGTCCTTCACACTGGACGACGCGCTGGTGCTGCAGGCGGCGCGCAGC
ENSPTRT00000015146     GACCTGCGGCGCTTGCCCTTCACACTGGACGACGCGCTGGTGCTGCAGGCGGCGCGCAGC
ENSMUST00000036221     GACCTGCGCCACTTGCCCTACACGCTGGACGACACTCTGGTGCTTAAGGCTGCTGCTGGC
ENSRNOT00000020440     GACCTGCGCCACTTGCCCTACACAGTGGACGACACTTTGGTGCTTAAGGTTGCTGGTGGT

ENSCJAT00000032069     TGTCCCGAGCTCCACAGCCTTTTTCTGGATAACAGTACCCTAGTGGACAGCGTGGGTCCC
ENSMMUT00000008262     TGTCCCGAGCTCCACAGCCTTTTTCTGGACAACAGTACCCTAGTGGGCAGCGTGGGTCCC
ENSGGOT00000011930     TGTCCCGAGCTCCACAGCCTTTTTCTGGACAACAGTACCCTAGTGGGCAGCGTGGGTCCC
XM_002826519.1         TGTCCCGAGCTCCACAGCCTTTTTCTGGACAACAGTACCCTAGTGGGCAGCGTGGGTCCC
ENST00000519917        TGTCCCGAGCTCCACAGCCTTTTTCTGGACAACAGTACCCTAGTGGGCAGCGTGGGTCCC
ENSPTRT00000015146     TGTCCCGAGCTCCACAGCCTTTTTCTGGACAACAGTACCCTAGTGGGCAGCGTGGGTCCC
ENSMUST00000036221     TGTCCCGAGCTCCGCAGTCTTTTCCTGGACAACCATGCACTGGTGAACAGCGTGCAGCCC
ENSRNOT00000020440     TGTCCCGAGCTCCGCAGTCTTTTCCTGGACAACCATGCACTAGTGAACAGCGTACAGCCC

ENSCJAT00000032069     GGATCAGTGCTCGAACTACTGGGGGCCTGCCCGCGCCTGCGCGCTCTCGGCCTGCACCTA
ENSMMUT00000008262     GGCTCAGTGCTCGAGCTACTGGAGGCCTGCCCGCGCCTGCGCGCTCTCGGCCTGCACCTA
ENSGGOT00000011930     GGCTCAGTGCTCGAGCTACTGGAGGCCTGCCCGCGCCTGCGCGCTCTCGGCCTGCACCTA
XM_002826519.1         GGCTCAGTGCTCAAGCTACTGGAGGCCTGCCCGCGCCTGCGCGCTCTCGGCCTGCACCTA
ENST00000519917        GGCTCAGTGCTCGAGCTACTGGAGGCCTGCCCGCGCCTGCGCGCTCTCGGCCTGCACCTA
ENSPTRT00000015146     GGCTCAGTGCTCGAGCTACTGGAGGCCTGCCCGCGCCTGCGCGCTCTCGGCCTGCACCTA
ENSMUST00000036221     ACTTCTGTGCTCAAACTACTGGAGGCCTGTCCTCACTTGCGCGCCCTTGGACTGCACCTT
ENSRNOT00000020440     GCCTCTGTGCTTAGGCTATTGGAGGCTTGCCCTCACCTGCGCGCCCTTGGACTGCACCTT

ENSCJAT00000032069     GCCAGTCTGTCGCACGCCACCCTCGAAGTGCTGGCCGCGCCAGATCGCTCGCCTTTCGCG
ENSMMUT00000008262     GCCAGTTTGTCCCACGCTATCCTCGAAGCGCTGGCGGCGCCAGACCGCGCGCCTTTCGCG
ENSGGOT00000011930     GCCAGTTTGTCGCACGCCATCCTCGAAGCACTGGCGGCGCCAGACCGAGCGCCTTTCGCG
XM_002826519.1         GCCAGTTTGTCGCACGCCATCCTCGAAGCGCTGGCGGCGCCAGACCGCGCGCCTTTCGCG
ENST00000519917        GCCAGTTTGTCGCACGCCATCCTCGAAGCACTGGCGGCGCCAGACCGAGCGCCTTTCGCG
ENSPTRT00000015146     GCCAGTTTGTCGCACGCCATCCTCGAAGCACTGGCGGCGCCAGACCGAGCGCCTTTCGCG
ENSMUST00000036221     GCTAGTATGTCGCGCGCCGCGCTGGAATTGCTAGCCGCTCCGCATCGCTCCCCTTTTGCA
ENSRNOT00000020440     GCTAGTATGTCGCGTGCCGCGTTAGAATTGCTAGCCGCTCCGCATCGCGCCCCTTTTACA

ENSCJAT00000032069     CTCCTGACTCTGCGCTGCGCGTGCCCCGAAGATGCACGCGCGTCCCCGCTGCCCAACGAA
ENSMMUT00000008262     CTCCTGGCTCTGCGGTGCGCGTGCCCCGAGGATGCACGCGCGTCCCCGCTGCCCAACGAA
ENSGGOT00000011930     CTCTTGGCTCTGCGGTGCGCGTGCCCCGAAGACGCACGCGCGTCCCCGCTGCCCAACGAA
XM_002826519.1         CTCCTGGCTCTGCGGTGCGCGTGCCCCGAAGATGCACGCGCGTCCCCGCTGCCCAACGAA
ENST00000519917        CTCTTGGCTCTGCGGTGCGCGTGCCCCGAAGATGCACGCGCGTCCCCGCTGCCCAACGAA
ENSPTRT00000015146     CTCTTGGCTCTGCGGTGCGCGTGCCCCGAAGATGCACGCGCGTCCCCGCTGCCCAACGAA
ENSMUST00000036221     CTTCTGGCACTGAGGTGCGCGTGCCCCGAAGATGCTCGTGCTTCCCCTCTGCCTGATGAA
ENSRNOT00000020440     CTTCTGGCACTGAAGTGCGCGTGCCCCGAAGATGCTCGTGCTTCCCCTCTGCCTGATGAA

ENSCJAT00000032069     ACCTGGGCTGCGTTGCGCCGCCGCCACCCTGGGCTGGCCGTGGAGCTGGAGCTGGAGCCC
ENSMMUT00000008262     GCCTGGGCAGCGTTGCGCCGCCGCCACCCTGGGCTGGCAGTGGAGCTGGAGCTGGAGCCC
ENSGGOT00000011930     GCCTGGGTCTCGTTGCGCCGCCGCCACCCTGGGCTGGCAGTGGAGCTGGAGCTGGAGCCC
XM_002826519.1         GCCTGGGTCGCGTTGCGCCGCCGCCACCCTGGGCTGGCAGTGGAGCTGGAGCTGGAGCCC
ENST00000519917        GCCTGGGTCGCGTTGCGCCGCCGCCACCCTGGGCTGGCAGTGGAGCTGGAGCTGGAGCCC
ENSPTRT00000015146     GCCTGGGTCGCGTTGCGCCGCCGCCACCCTGGGCTGGCAGTGGAGCTGGAGCTGGAGCCC
ENSMUST00000036221     GCCTGGGCGACGCTGAGTTGCCGCCATCCTGGGCTGGAGGTGGAGCTGGAGCTGGAGCCT
ENSRNOT00000020440     GCCTGGGCGACACTAACTTGCTATCATCCCGGGCTGAAGGTGGAGCTGGAGCTGGAGCCT
```

```
ENSCJAT00000032069     GCGCTGCCAGCCGAGAGCGTGACACGCATCCTGCAGCCAGCGGTCCCCGTGGCTGCGCTA
ENSMMUT00000008262     GCGCTGCCCTTTGAGAGCGTGACGCGCGTCCTGCAGCCAGCGGTCCCCGTGGCTGCTCTG
ENSGGOT00000011930     GCGCTGCCCGCTGAGAGCGTGACGCGCGTCCTGCAGCCAGCCGTCCCCGTGGCTGCGCTG
XM_002826519.1         GCGCTGCCCGCTGAGAGCGTGACGCGCGTCCTGCAGCCAGCTGTCCCCGTGGCTGCGCTG
ENST00000519917        GCGCTGCCCGCTGAGAGCGTGACGCGCGTCCTGCAGCCAGCCGTCCCCGTGGCTGCGCTG
ENSPTRT00000015146     GCGCTGCCCGCTGAGAGCGTGACGCGCGTCCTGCAGCCAGCCGTCCCCGTGGCTGCGCTG
ENSMUST00000036221     GTGCTTCCAGATGAGGCCGTGACGCGCATCCTGCAACCAGCAGTACCAGTGGCTGTGCTG
ENSRNOT00000020440     GTGCTTCCAGATGAGGCCGTGTCTCGCATCCTGCAACCAGCAGTACCAGTGGCTGTGCTG

ENSCJAT00000032069     CGCCTCAACCTCTCAGGCGACACCATAGGCCCAGTGCGCTTTGCGGCGCACCACTATGCC
ENSMMUT00000008262     CGCCTCAACCTCTCAGGCGACACCGTAGGCCCAGTGCGCTTCGCAGCGCACCACTACGCC
ENSGGOT00000011930     CGCCTCAACCTCTCAGGCGACACCGTAGGCCCACTGCGCTTCGCAGCGCACCACTACGCC
XM_002826519.1         CGCCTCAACCTCTCAGGCGACACCGTAGGCCCAGTGCGCTTCGCAGCGCACCACTACGCC
ENST00000519917        CGCCTCAACCTCTCAGGCGACACCGTAGGCCCAGTGCGCTTCGCAGCACACCACTACGCC
ENSPTRT00000015146     CGCCTCAACCTCTCAGGCGACACCGTAGGCCCAGTGCGCTTCGCAGCGCACCACTACGCC
ENSMUST00000036221     CGTCTCAACCTCTCCGGTGACACTGTAGGACCCGTGCGCTTCGCAACGCGCCACTACGCT
ENSRNOT00000020440     CGTCTCAACCTCTCTGGTGACACAGTAGGACCGGTGCGCTTCGCAGCGCGCCACTACGCC

ENSCJAT00000032069     GCAACCCTGCGCGCGCTCGAGGTGCGCGCAGCCGCTTCAGCCGGGCTAAATGCCGCGCTG
ENSMMUT00000008262     GCAACCCTGCGCGCACTCGAGGTGCGCGCAGCCGCCTCAGCCAAGCTGAACGCCGCGCTG
ENSGGOT00000011930     GCAACCCTGTGCGCGCTCGAGGTGCGCGCAGCCGCTTCGGCCGAGCTGAACGCCGCGCTG
XM_002826519.1         GCAACCCTGTGCGCGCTCGAGGTGCGAGCAGCCGCTTCAGCCGAGCTGAACGCCGCGCTG
ENST00000519917        GCAACCCTGTGCGCGCTCGAGGTGCGCGCAGCCGCTTCGGCCGAGCTGAACGCCGCGCTG
ENSPTRT00000015146     GCAACCCTGTGCGCGCTCGAGGTGCGCGCAGCCGCTTCGGCCGAGCTGAACGCCGCGCTG
ENSMUST00000036221     GAAACTTTGCGCGCCCTCGAGGTGCGCGCGTCCGCATCCCCGGAGTTGCACACCGCGCTG
ENSRNOT00000020440     GAAACTTTGCGCGCCCTCGAGGTGCGCGCGTCCGCTTCCACCGAGCTGCACACCGCGCTG

ENSCJAT00000032069     GAGGAGCTGGCGGCGCGCTGCGCGGGCCTGCGCGAGGTGCATTGCTTCTGCGTGGTGAGC
ENSMMUT00000008262     GAGGAGCTGGCGGCGCGCTGCGCGGCCCTGCGCGAGGTGCATTGCTTCTGCGTGGTGAGC
ENSGGOT00000011930     GAGGAGCTGGCGGCGCGCTGCGCGGCCCTGCGCGAGGTGCATTGCTTCTGCGTGGTGAGC
XM_002826519.1         GAGGAGCTGGCGGCGCGCTGCGCGGCCCTGCGCGAGGTGCATTGCTTCTGCGTGGTGAGC
ENST00000519917        GAGGAGCTGGCGGCGCGCTGCGCGGCCCTGCGCGAGGTGCATTGTTTCTGCGTGGTGAGC
ENSPTRT00000015146     GAGGAGCTGGCGGCGCGCTGCGCGGCCCTGCGCGAGGTGCATTGCTTCTGCGTGGTGAGC
ENSMUST00000036221     GAGGAGCTGGCGGCGCGCTGCGCGGGCTTGAGAGAAATACACTGCTTCTGCGTGGTGAGA
ENSRNOT00000020440     GAGGAGCTGGCGGCGCGCTGCGCGGGCTTGAGAGAGATACACTGCTTCTGCGTGGTGAGA

ENSCJAT00000032069     CCCTCCGTGTTGGACGCCTTCCGCGCGCACTGCCCGCGCCTGCGCAACTATACCCTCAAG
ENSMMUT00000008262     CACTCGGTGCTGGAAGCCTTCCGCGCGCATTGTCCGCGCCTGCGCACCTATACCCTCAAG
ENSGGOT00000011930     CACTCGGTGCTGGACGCCTTCCGCGCGCACTGCCCGCGCCTGCGCACCTATACCCTCAAG
XM_002826519.1         CACTCGGTGCTGGACGCCTTCCGCGCGCACTGCCCGCGCCTGCGCACCTATACCCTCAAG
ENST00000519917        CACTCGGTGCTGGACGCCTTCCGCGCGCACTGCCCGCGCCTGCGCACCTATACCCTCAAG
ENSPTRT00000015146     CACTCGGTGCTGGACGCCTTCCGCGCGCACTGCCCGCGCCTGCGCACCTATACCCTCAAG
ENSMUST00000036221     CCCTCGGTACTGGACGCCTTCCGAGCGCACTGTCCGCGCCTGCGCAGCTATACGCTCAAG
ENSRNOT00000020440     CCCTCTGTACTGGATGCCTTCCGCGCGCATTGTCCGCGCCTGCGCAGCTATACGCTCAAA

ENSCJAT00000032069     CTTACTCGCGAGCCGCATCCCTGGCGGCCCACGCCAGTGGCA
ENSMMUT00000008262     CTCACGCGCGAACCGCATCCCTGGCGGCCCACGCTGGTGGCG
ENSGGOT00000011930     CTCAAGCGCGAGCCGCATCCCTGGAGGCCTACGCTCCTGGCG
XM_002826519.1         CTCACGCGCGAGCCGCATCCCTGGCGGCCTACGCTCGTGGCG
ENST00000519917        CTCACGCGCGAGCCGCATCCCTGGAGGCCTACGCTCGTGGCG
ENSPTRT00000015146     CTCACGCGCGAGCCGCATCCCTGGAGGCCTACGCTCGTGGCG
ENSMUST00000036221     CTAAAGCGTGAGCCGCATCCCTGGCGGCCCACACTGGTGCGG
ENSRNOT00000020440     CTAAAGCGTGAACCGCATCCCTGGCGGCCGACACTGATGCGG
```

Multiple sequence alignment of Fbxo10

```
ENSPPYT00000022222     ATGGAGGCTGGTGGCCTCCCTTTGGAGCTATGGCGCATGATCTTAGCCTACTTGCACCTT
ENSMMUT00000025134     ATGGAGGCTGGTGGCCTGCCCTTGGAGCTGTGGCGCATGATCTTAGCCTACTTGCACCTT
ENSGGOT00000001898     ATGGAGGCTGGTGGCCTCCCCTTGGAGCTATGGCGCATGATCTTAGCCTACTTGCACCTT
ENST00000432825        ATGGAGGCTGGTGGCCTCCCCTTGGAGCTGTGGCGCATGATCTTAGCCTACTTGCACCTT
ENSPTRT00000038754     ATGGAGGCTGGTGGCCTCCCCTTGGAGCTATGGCGCATGATCTTAGCCTACTTGCACCTT
ENSMUST00000052236     ATGGAAACGGGTGGCCTCCCCCTGGAGCTGTGGCGTATGATCTTAGCCTACCTACATCTC
ENSRNOT00000016875     ATGGAAACGGGCGGCCTCCCCCTGGAGCTGTGGCGTGTGATCTTAGCCTACCTGCATCTT


ENSPPYT00000022222     CCCGACCTGGGCCGCTGCAGCCTGGTATGCAGGGCCTGGTATGAACTGATCCTCAGTCTC
ENSMMUT00000025134     CCCGACCTGGGCCGCTGCAGCCTGGTATGCAGGGCCTGGTATGAACTGATCCTCAGTCTC
ENSGGOT00000001898     CCCGACCTGGGCCGCTGCAGCCTGGTATGCAGGGCCTGGTATGAACTGATCCTCAGTCTT
ENST00000432825        CCCGACCTGGGCCGCTGCAGCCTGGTATGCAGGGCCTGGTATGAACTGATCCTCAGTCTC
ENSPTRT00000038754     CCCGACCTGGGCCGCTGCAGCCTGGTATGCAGGGCCTGGTATGAACTGATCCTCAGTCTC
ENSMUST00000052236     CCTGACCTGGGCCGCTGCAGCCTGGTTTGCAGGGCCTGGTATGAGCTGATCCTCAGCCTG
ENSRNOT00000016875     CCTGACCTGGGCCGCTGCAGTCTGGTGTGCAGGGCCTGGTATGAGCTGATCCTCAGCCTG


ENSPPYT00000022222     GACAGCACCCGCTGGCGGCAGCTGTGTCTGGGTTGCACCGAGTGCCGCCATCCCAATTGG
ENSMMUT00000025134     GACAGCACCCGCTGGCGGCAGCTATGTCTGGGTTGCACCGAGTGCCGCCATCCCAATTGG
ENSGGOT00000001898     GACAGCACCCGCTGGCGGCAGCTGTGTCTGGGTTGCACCGAGTGCCGCCATCCCAATTGG
ENST00000432825        GACAGCACCCGCTGGCGGCAGCTGTGTCTGGGTTGCACCGAGTGCCGCCATCCCAATTGG
ENSPTRT00000038754     GACAGCACCCGCTGGCGGCAGCTGTGTCTGGGTTGCACCGAGTGCCGCCATCCCAATTGG
ENSMUST00000052236     GACAGTACCCGCTGGCGGCAGCTGTGTCTGGGCTGCACCGAGTGCCGCCACCCCAACTGG
ENSRNOT00000016875     GACAGTACCCGTTGGCGACAGCTGTGTCTGGGCTGCACTGAGTGCCGCCACCCCAACTGG


ENSPPYT00000022222     CCCAACCAGCCAGATGTGGAGCCTGAGTCTTGGAGAGAAGCCTTCAAGCAGCATTACCTT
ENSMMUT00000025134     CCCAACCAGCCAGATGTGGAGCCTGAGTCTTGGAGAGAAGCCTTCAAGCAGCATTACCTT
ENSGGOT00000001898     CCCAACCAGCCAGATGTGGAGCCTGAGTCTTGGAGAGAAGCCTTCAAGCAGCATTACCTC
ENST00000432825        CCCAACCAGCCAGATGTGGAGCCTGAGTCTTGGAGAGAAGCCTTCAAGCAGCATTACCTT
ENSPTRT00000038754     CCCAACCAGCCAGATGTGGAGCCTGAGTCTTGGAGAGAAGCCTTCAAGCAGCATTACCTT
ENSMUST00000052236     CCCAACCAGCCTGATGTGGAGCCCGAGTCCTGGAGGGAGGCCTTCAAGCAACACTACCTT
ENSRNOT00000016875     CCCAACCAGCCCGATGTGGAGCCCGAGTCCTGGAGGGAGGCCTTCAAGCAGCACTATCTT


ENSPPYT00000022222     GCATCCAAGACATGGACCAAGAATGCCTTGGACTTGGAGTCTTCCATCTGCTTTTCTCTA
ENSMMUT00000025134     GCCTCCAAGACATGGACCAAGAATGCCTTGGACTTGGAGTCTTCCGTCTGCTTTTCTCTG
ENSGGOT00000001898     GCATCCAAGACATGGACCAAGAATGCCTTGGACTTGGAGTCTTCCATCTGCTTTTCTCTA
ENST00000432825        GCATCCAAGACATGGACCAAGAATGCCTTGGACTTGGAGTCTTCCATCTGCTTTTCTCTA
ENSPTRT00000038754     GCATCCAAGACATGGACCAAGAATGCCTTGGACTTGGAGTCTTCCATCTGCTTTTCTCTA
ENSMUST00000052236     GCCTCTAAGACCTGGACCAAGAATGCACTGGACTTGGAGTCCTCCATCTGCTTCTCTCTG
ENSRNOT00000016875     GCCTCTAAGACCTGGGCTAAGAACGCACTGGACTTGGAGTCTTCTATCTGCTTCTCTCTG


ENSPPYT00000022222     TTCCGCCGGAGGAGGGAACGACGTACCCTGAGTGTCGGGCCAGGCCATGAGTTTGACAGC
ENSMMUT00000025134     TTCCGCCGGAGGAGGGAACGACGTACCCTGAGTGTTGGGCCAGGCCATGAGTTTGACAGC
ENSGGOT00000001898     TTCCGCCGGAGGAGGGAACGACGTACCCTGAGTGTTGGGCCAGGCCGTGAGTTTGACAGC
ENST00000432825        TTCCGCCGGAGGAGGGAACGACGTACCCTGAGTGTTGGGCCAGGCCGTGAGTTTGACAGC
ENSPTRT00000038754     TTCCGCCGGAGGAGGGAACGACGTACCCTGAGTGTTGGGCCAGGCCGTGAGTTTGACAGC
ENSMUST00000052236     TTCCGCAGGAAGAAGGAGAGACGCACCTTGAGTGTTGGGCCAGGCCATGAGTTTGACAGC
ENSRNOT00000016875     TTCCGCAGGAAGAAGGAGCGACGTACCTTGAGTGTTGGGCCAGGCCATGAGTTTGACAGC


ENSPPYT00000022222     CTGGGCAGTGCCTTGGCCATGGCCAGCCTGTATGACCGAATTGTACTCTTCCCAGGTGTG
ENSMMUT00000025134     CTGGGCAGTGCCTTGGCCATGGCCAGCCTGTATGACCGAATTGTGCTCTTCCCAGGTGTG
ENSGGOT00000001898     CTGGGCAGTGCCTTGGCCATGGCCAGCCTGTATGACCGAATTGTGCTCTTCCCAGGTGTG
ENST00000432825        CTGGGCAGTGCCTTGGCCATGGCCAGCCTGTATGACCGAATTGTGCTCTTCCCAGGTGTG
```

```
ENSPTRT00000038754     CTGGGCAGTGCCTTGGCCATGGCCAGCCTGTATGACCGAATTGTGCTCTTCCCAGGTGTG
ENSMUST00000052236     TTGGGCAGTGCCTTAGCCATGGCCAGCCTCTATGATCGAATCGTGCTTTTCCCAGGTGTG
ENSRNOT00000016875     TTGGGCAGTGCCTTAGCCATGGCCAGCCTCTATGATCGAATCGTGCTTTTCCCAGGTGTG

ENSPPYT00000022222     TACGAAGAGCAAGGTGAAATCATCTTGAAGGTGCCCGTGGAGATTGTAGGGCAGGGGAAG
ENSMMUT00000025134     TACGAAGAGCAAGGTGAAATCATCCTGAAGGTGCCCGTGGAGATTGTAGGGCAGGGGAAG
ENSGGOT00000001898     TACGAAGAGCAAGGTGAAATCATCTTGAAGGTGCCCGTGGAGATTGTAGGGCAGGGGAAG
ENST00000432825        TACGAAGAGCAAGGTGAAATCATCTTGAAGGTGCCTGTGGAGATTGTAGGGCAGGGGAAG
ENSPTRT00000038754     TACGAAGAGCAAGGTGAAATCATCTTGAAGGTGCCTGTGGAGATTGTAGGGCAGGGGAAG
ENSMUST00000052236     TACGAGGAGCAAGGGGAAATCATCCTGAAGGTGCCGGTGGAGATTGTGGGCCAAGGGAAG
ENSRNOT00000016875     TACGAGGAGCAAGGGGAAATCATCCTGAAGGTGCCAGTGGAGATTGTGGGCCAAGGGAAG

ENSPPYT00000022222     TTGGGTGAAGTGGCGCTGCTGGCCAGCATTGATCAGCACTGCTCAACCACACGCCTGTGC
ENSMMUT00000025134     TTGGGTGAAGTGGCCCTGCTGGCCAGCATTGATCAGCACTGCTCAACCACACGCCTGTGC
ENSGGOT00000001898     TTGGGTGAAGTGGCCCTGCTGGCCAGCATTGATCAGCACTGCTCAACCACACGCCTGTGC
ENST00000432825        TTGGGTGAAGTGGCCCTGCTGGCCAGCATTGATCAGCACTGCTCAACCACACGCCTGTGC
ENSPTRT00000038754     TTGGGTGAAGTGGCCCTGCTGGCCAGCATTGATCAGCACTGCTCAACCACACGCCTGTGC
ENSMUST00000052236     TTGGGTGAGGTGGCCCTGCTTGCCAGCATTGACCAGCACTGCTCAACCACACGTGTGTGC
ENSRNOT00000016875     TTGGGTGAGGTGGTCTTGCTTGCCAGCATTGACCAGCACTGCTCAACCACCCGTGTGTGC

ENSPPYT00000022222     AACCTCGTCTTCATGCCAGCCTGGTTCTCACCCATCATGTATAAGACAACATCAGGTCAT
ENSMMUT00000025134     AACCTCGTCTTCATGCCAGCCTGGTTCTCACCCATCATGTATAAGACAACATCAGGTCAT
ENSGGOT00000001898     AACCTCGTCTTCACGCCAGCCTGGTTCTCACCCATCATGTATAAGACAACATCAGGTCAT
ENST00000432825        AACCTCGTCTTCACGCCAGCCTGGTTCTCACCCATCATGTATAAGACAACATCAGGTCAC
ENSPTRT00000038754     AACCTCGTCTTCACGCCAGCCTGGTTCTCACCCATCATGTATAAGACAACATCAGGTCAC
ENSMUST00000052236     AATCTTGTCTTCATGCCAGCCTGGTTCTCACCAATCATGTATAAGACCACATCAGGTCAC
ENSRNOT00000016875     AATCTTGTCTTCATGCCAGCCTGGTTCTCACCAATCATGTATAAGACCACATCAGGTCAC

ENSPPYT00000022222     GTCCAGTTTGACAACTGCAACTTTGAGAATGGGCACATCCAGGTCCATGGCCCAGGTACT
ENSMMUT00000025134     GTCCAATTTGACAACTGCAACTTTGAGAATGGGCACATCCAGGTCCACGGTCCGGGTACT
ENSGGOT00000001898     GTCCAGTTTGACAACTGCAACTTTGAGAATGGGCACATCCAGGTCCATGGCCCGGGTACT
ENST00000432825        GTCCAGTTTGACAACTGCAACTTTGAGAACGGGCACATCCAGGTCCATGGCCCGGGTACT
ENSPTRT00000038754     GTCCAGTTTGACAACTGCAACTTTGAGAACGGGCACATCCAGGTCCATGGCCCGGGTACT
ENSMUST00000052236     ATCCAGTTTGACAACTGCAACTTTGAGAACGGACACATCCAGGTCCATGGTCCAGGTACC
ENSRNOT00000016875     ATCCAGTTTGACAACTGCAACTTTGAGAATGGACACATCCAGGTCCATGGCCCAGGTACC

ENSPPYT00000022222     TGCCAAGTGAAGTTCTGTACCTTCAAAAACACCCATATCTTCCTGCACAACGTGCCCCTG
ENSMMUT00000025134     TGCCAAGTGAAGTTCTGTACCTTCAAAAACACTCATATCTTCTTGCACAACGTGCCCCTG
ENSGGOT00000001898     TGCCAAGTGAAGTTCTGTACCTTCAAAAACACCCATATCTTCCTGCACAACGTGCCCCTG
ENST00000432825        TGCCAAGTGAAGTTCTGTACCTTCAAAAACACCCATATCTTCCTGCACAACGTGCCCCTG
ENSPTRT00000038754     TGCCAAGTGAAGTTCTGTACCTTCAAAAACACCCATATCTTCCTGCACACCGTGCCCCTG
ENSMUST00000052236     TGCCAAGTGAAGTTTTGTACCTTCAAAAACACTCATGTCTTTTTGCACAACGTGCCCTTA
ENSRNOT00000016875     TGCCAAGTGAAATTTTGTACCTTCAAAAATACCCATGTCTTTTTGCACAACGTGCCCTTA

ENSPPYT00000022222     TGTGTCCTGGAAAACTGTGAATTTGTGGGCAGTGAAAACAACTCTGTGACTGTTGAGGGT
ENSMMUT00000025134     TGTGTCCTGGAAAACTGTGAATTTGTGGGCAGCGAAAACAACTCTGTGACTGTTGAGGGT
ENSGGOT00000001898     TGTGTCCTGGAAAACTGTGAATTTGTGGGCAGTGAAAACAACTCTGTGACTGTTGAGGGT
ENST00000432825        TGTGTCCTGGAAAACTGTGAATTTGTGGGCAGTGAAAACAACTCTGTGACTGTTGAGGGT
ENSPTRT00000038754     TGTGTCCTGGAAAACTGTGAATTTGTGGGCAGTGAAAACAACTCTGTGACTGTTGAGGGT
ENSMUST00000052236     TGCATGTTGGAAAACTGTGAATTTGTGGGCAGCGAAAACAACTGTGTGACTGTGGAGGGT
ENSRNOT00000016875     TGTATGTTGGAAAACTGTGAATTTGTGGGCAGCGAAAACAACTCTGTGACCGTTGAGGGT

ENSPPYT00000022222     CACCCATCTGCAGATAAGAACTGGGCCTACAAGTATCTACTAGGGCTTATCAAGTCCTCG
```

```
ENSMMUT00000025134    CACCCATCTGCAGATAAGAACTGGGCCTACAAGTATCTACTAGGGCTTATCAAGTCCTCA
ENSGGOT00000001898    CACCCATCTGCAGATAAGAACTGGGCCTACAAGTATCTACTAGGGCTTATCAAGTCCTCG
ENST00000432825       CACCCATCTGCAGATAAGAACTGGGCCTACAAGTATCTACTAGGGCTTATCAAGTCCTCA
ENSPTRT00000038754    CACCCATCTGCAGATAAGAACTGGGCCTACAAGTATCTACTAGGGCTTATCAAGTCCTCA
ENSMUST00000052236    CACCCGTCGGCGGATAAGAACTGGGCCTACAAGTACCTGCTGGGCCTTATCAAGTCCTCA
ENSRNOT00000016875    CACCCGTCAGCCGACAAGAACTGGGCCTATAAGTACCTGCTGGGCCTTATCAAGGCCTCT

ENSPPYT00000022222    CCCACTTTTCTCCCCACAGAGGACTCTGACTTTTTAATGTCCCTGGACCTAGAGAGCCGG
ENSMMUT00000025134    CCCACTTTTCTCCCCACAGAGGACTCTGACTTTTTAATGTCCCTGGACCTAGAGAGCCGG
ENSGGOT00000001898    CCCACTTTTCTCCCCACAGAGGACTCTGACTTTTTAATGTCCCTGGACCTAGAGAGCCGG
ENST00000432825       CCCACTTTTCTCCCCACAGAGGACTCTGACTTTTTAATGTCCCTGGACCTAGAGAGCCGG
ENSPTRT00000038754    CCCACTTTTCTCCCCACAGAGGACTCTGACTTTTTAATGTCTCTGGACCTAGAGAGCCGG
ENSMUST00000052236    CCCATCTTTCTCCCCGCAGAGGACCATGACTTTTTAATGTCCTTGGACTTAGAGAGTCGG
ENSRNOT00000016875    CCCATCTTCCTCCCTGCAGAGGACAACGACTTTTTAATGTCCTTGGACTTAGAGAGCCAG

ENSPPYT00000022222    GACCAGGCCTGGAGCCCAAAGACCTGTGACATTGTTATCGAGGGCAGCCAGAGCCCTACC
ENSMMUT00000025134    GACCAGGCCTGGAGCCCAAAGACCTGTGACATTGTTATCGAGGGCAGCCAGAGCCCTACC
ENSGGOT00000001898    GACCAGGCCTGGAGCCCAAAGACCTGTGACATTGTTATCGAGGGCAGCCAGAGCCCTACC
ENST00000432825       GACCAGGCCTGGAGCCCAAAGACCTGTGACATTGTTATCGAGGGCAGCCAGAGCCCTACC
ENSPTRT00000038754    GACCAGGCCTGGAGCCCAAAGACCTGTGACATTGTTATCGAGGGCAGCCAGAGCCCTACC
ENSMUST00000052236    GACCAGGCCTGGAGCCCAAGGACCTGTGACATTGTGATCGAGGGCAGCCAGAGTCCTACA
ENSRNOT00000016875    GACCAGGCCTGGAGCCCAAGGACCTGTGACATTGTCATCGAGGGCAGCCAGAGTCCTACA

ENSPPYT00000022222    AGCCCAGCCTCTAGCTCCCCAAAGCCAGGCTCCAAGGCTGGCTCACAGGAGGCAGAGGTG
ENSMMUT00000025134    AGCCCAGCCTCTAGCTCCCCAAAGCCAGGCTCCAAGGCTGGCTCACAGGAGGCAGAAGTG
ENSGGOT00000001898    AGCCCAGCCTCTAGCTCCCCAAAGCCAGGCTCCAAGGCTGGCTCACAGGAGGCAGAGGTG
ENST00000432825       AGCCCAGCCTCTAGCTCCCCAAAGCCAGGCTCCAAGGCTGGCTCACAGGAGGCAGAGGTG
ENSPTRT00000038754    AGCCCAGCCTCTAGCTCCCCAAAGCCAGGCTCCAAGGCTGGCTCACAGGAGGCAGAGGTG
ENSMUST00000052236    AGCCCAGTCTGTAGCTCTCCCAAGCCAGGCTCCAAG------------GAGGCAGAGGTG
ENSRNOT00000016875    AGTCCAGTCTCTAGCTCTCCAAAGCCAGGCTCCAAG------------GAGGGAGAGGTG

ENSPPYT00000022222    GGTAGTGATGGTGAAAGGGTGGCCCAGACTCCAGACAGCAGCGATGGAGGCCTGAGTCCC
ENSMMUT00000025134    GGTAGTGATGGTGAAAGGGTGGCCCAGACCCCAGACAGCAGCGATGGAGGCCTGAGTCCC
ENSGGOT00000001898    GGTAGTGATGGTGAAAGGGTGGCCCAGACCCCGGACAGCAGCGATGGAGGCCTGAGTCCC
ENST00000432825       GGTAGTGATGGTGAAAGGGTGGCCCAGACCCCGGACAGCAGCGATGGAGGCCTGAGTCCC
ENSPTRT00000038754    GGTAGTGATGGTGAAAGGGTGGCCCAGACCCCGGACAGCAGCGATGGAGGCCTGAGTCCC
ENSMUST00000052236    GGCAGCGATGGGGAGAGAGTGGCCCAGACTCCAGACAGCAGTGACGGTGGCCTGAGCCCC
ENSRNOT00000016875    GGCAGCGATGGGGAGAGAGTGGCCCAGACTCCAGACAGCAGTGACGGCGGCCTGAGCCCC

ENSPPYT00000022222    AGCGGTGAGGATGAAGATGAGGACCAGCTGATGTACAGACTATCCTACCAAGTGCAGGGC
ENSMMUT00000025134    AGCGGTGAGGATGAAGATGAGGACCAGCTGATGTATAGACTATCCTATCAAGTGCAGGGC
ENSGGOT00000001898    AGCGGTGAGGATGAAGATGAGGACCAGCTGATGTACAGACTATCCTACCAAGTGCAGGGC
ENST00000432825       AGCGGTGAGGATGAAGATGAGGACCAGCTGATGTACAGACTATCCTACCAAGTGCAGGGC
ENSPTRT00000038754    AGCGGTGAGGATGAAGATGAGGACCAGCTGATGTACAGACTATCCTACCAAGTGCAGGGC
ENSMUST00000052236    AGCGGGGAGGATGAGGATGACGAACAGCTCACATACAGACTGTCCTACCAGGTGCAGGGC
ENSRNOT00000016875    AGCGGTGAGGATGAGGATGATGAGCAGCTCACCTACAGACTGTCCTACCAGGTGCAGGGC

ENSPPYT00000022222    CCACGCCCTGTATTGGGGGGCTCATTTCTGGGCCCACCTCTACCAGGAGCATCCATTCAG
ENSMMUT00000025134    CCACGCCCTGTATTGGGGGGCTCATTTCTGGGCCCACCTCTACCAGGAGCATCCATTCAG
ENSGGOT00000001898    CCACGCCCTGTATTGGGGGGCTCATTTCTGGGCCCACCTCTACCAGGAGCATCCATTCAG
ENST00000432825       CCACGCCCTGTATTGGGGGGCTCATTTCTGGGCCCACCTCTACCAGGAGCATCCATTCAG
ENSPTRT00000038754    CCACGCCCTGTACTGGGGGGCTCATTTCTGGGCCCACCTCTACCAGGAGCATCCATTCAG
ENSMUST00000052236    CCACGCCCTGTCCTGGGGGGCTCATTTCTGGGCCCTCCGCTACCTGGAGCATCTATTCAG
ENSRNOT00000016875    CCCCGCCCTGTTCTGGGCGGCTCCTTTCTGGGCCCTCCCCTACCTGGAGCATCCATTCAG
```

```
ENSPPYT00000022222     CTGCCCAGCTGCCTAGTGCTGAACTCACTGCAGCAGGAGCTGCAGAAGGACAAGGAGGCC
ENSMMUT00000025134     CTGCCCAGCTGCCTAGTGCTGAACTCACTGCAGCAGGAGCTGCAGAAGGACAAGGAGGCC
ENSGGOT00000001898     CTGCCCAGCTGCCTAGTGCTGAACTCACTGCAGCAGGAGCTACAGAAGGATAAGGAGGCC
ENST00000432825        CTGCCCAGCTGCCTAGTGCTGAACTCACTGCAGCAGGAGCTGCAGAAGGATAAGGAGGCC
ENSPTRT00000038754     CTGCCCAGCTGCCTAGTGCTGAACTCACTGCAGCAGGAGCTGCAGAAGGATAAGGAGGCC
ENSMUST00000052236     CTGCCCAGCTGTCTAGTGCTGAATTCACTGCATCAGGAGCTGCAGAAAGACAAGGAAGCC
ENSRNOT00000016875     CTCCCCAGCTGTCTAGTTCTGAACTCACTGCACCAGGAGCTGCAGAAAGACAAGGAAGCC

ENSPPYT00000022222     ATGGCACTGGCCAACTCTGTGCAGGGCTGCCTCATCCGCAAGTGCCTCTTCCGGGACGGG
ENSMMUT00000025134     ATGGCACTGGCCAACTCTGTGCAGGGCTGCCTCATCCGCAAGTGCCTCTTCCGCGATGGG
ENSGGOT00000001898     ATGGCACTGGCCAACTCCGTGCAGGGCTGCCTCATCCGCAAGTGCCTCTTCCGGGACGGG
ENST00000432825        ATGGCACTGGCCAACTCCGTGCAGGGCTGCCTCATCCGCAAGTGCCTCTTCCGGGACGGG
ENSPTRT00000038754     ATGGCACTGGCCAACTCTGTGCAGGGCTGCCTCATCCGCAAGTGCCTCTTCCGGGACGGG
ENSMUST00000052236     ATGGCGCTAGCCAGCTCTGTGCAGGGCTGCCTTATCCGCAAGTGCCTCTTCCGGGATGGG
ENSRNOT00000016875     ATGGCGCTAGCCAGCTCCGTGCAGGGCTGCCTTATCCGCAAGTGCCTCTTCCGGGACGGG

ENSPPYT00000022222     AAGGGAGGCGTCTTCGTCTGCTCCCATGGCAGAGCCAAGATGGAAGGAAACATCTTCCGG
ENSMMUT00000025134     AAGGGAGGCGTCTTCGTCTGCTCCCACGGCAGAGCCAAGATGGAAGGAAACATCTTCCGG
ENSGGOT00000001898     AAGGGAGGCGTCTTCGTCTGCTCCCACGGCAGAGCCAAGATGGAAGGAAACATCTTCCGG
ENST00000432825        AAGGGAGGCGTCTTCGTCTGCTCCCACGGCAGAGCCAAGATGGAAGGAAACATCTTCCGG
ENSPTRT00000038754     AAGGGAGGCGTCTTCGTCTGCTCCCACGGCAGAGCCAAGATGGAAGGAAACATCTTCCGG
ENSMUST00000052236     AAGGGCGGCGTCTTCGTCTGCTCTTATGGCCGTGCCAAGATGGAAGGAAACGTCTTTCGG
ENSRNOT00000016875     AAGGGCGGTGTCTTCGTCTGCTCTTATGGCCGCGCCAAGATGGAAGGAAACGTCTTTCGG

ENSPPYT00000022222     AACCTGACTTACGCAGTGCGGTGTATACATAATAGCAAGATCATCATGCTCAGGAACGAC
ENSMMUT00000025134     AACCTGACTTACGCAGTGCGGTGTATACATAATAGCAAGATCATCATGCTCAGGAACGAC
ENSGGOT00000001898     AACCTGACTTACGCAGTGCGGTGTATACATAATAGCAAGATCATCATGCTCAGGAACGAC
ENST00000432825        AACCTGACTTACGCAGTGCGGTGTATACATAATAGCAAGATCATCATGCTCAGGAACGAC
ENSPTRT00000038754     AACCTGACTTACGCAGTGCGGTGTATACATAATAGCAAGATCATCATGCTCAGAAACGAC
ENSMUST00000052236     AACCTGACTTACGCGGTGCGGTGTATACACAATAGCAAGATCGTCATGCTCAGGAATGAT
ENSRNOT00000016875     AACCTGACTTATGCAGTGCGATGTATACACAATAGCAAGATCGTCATGCTCAGGAATGAT

ENSPPYT00000022222     ATTTACCGCTGCCGAGCGTCAGGCATCTTTCTTCGCTTGGAGGGCGGTGGCTTGATTGCC
ENSMMUT00000025134     ATTTACCGCTGCCGAGCGTCAGGCATCTTTCTTCGCTTGGAGGGCGGTGGCTTGATTGCC
ENSGGOT00000001898     ATTTACCGCTGCCGAGCGTCAGGCATCTTTCTTCGCTTGGAGGGCGGTGGCTTGATTGCC
ENST00000432825        ATTTACCGCTGCCGAGCGTCAGGCATCTTTCTTCGCTTGGAGGGCGGTGGCTTGATTGCC
ENSPTRT00000038754     ATTTACCGCTGCCGAGCGTCAGGCATCTTTCTTCGCTTGGAGGGCGGTGGCTTGATTGCC
ENSMUST00000052236     ATTTACCGCTGCCGGGCATCAGGCATCTTTCTTCGACTGGAGGGTGGAGGCTTGATTGCT
ENSRNOT00000016875     ATTTACCGCTGCCGGGCATCAGGCATCTTTCTTCGATTGGAGGGCGGAGGCTTGATCGCT

ENSPPYT00000022222     GGCAACAACATTTACCACAATGCAGAGGCTGGTGTAGACATCCGGAAAAAGTCCAACCCA
ENSMMUT00000025134     GGCAACAACATTTACCACAATGCAGAGGCTGGTGTAGACATCCGGAAAAAGTCCAACCCA
ENSGGOT00000001898     GGCAACAACATTTACCACAATGCAGAGGCTGGTGTAGACATCCGGAAAAAGTCCAACCCA
ENST00000432825        GGCAACAACATTTACCACAATGCAGAGGCTGGTGTAGACATCCGGAAAAAGTCCAACCCA
ENSPTRT00000038754     GGCAACAACATTTACCACAATGCAGAGGCTGGTGTAGACATCCGGAAAAAGTCCAACCCA
ENSMUST00000052236     GGTAACAACATTTACCACAATGCAGAGGCTGGTGTGGACATTCGGAAGAAATCTAATCCA
ENSRNOT00000016875     GGCAACAATATTTACCACAATGCAGAGGCTGGCGTGGACATTCGGAAGAAATCCAACCCA

ENSPPYT00000022222     CTCATACTGTGTAACCAGATCCACCACGGCCTTCGCTCTGGCATTGTCGTCCTTGGCAAT
ENSMMUT00000025134     CTCATACTGTGTAACCAGATCCACCATGGCCTTCGCTCTGGCATTGTCGTCCTTGGCAAT
ENSGGOT00000001898     CTCATACTGTGTAACCAGATCCACCATGGCCTTCGCTCTGGCATTGTCGTCCTTGGCAAT
ENST00000432825        CTCATACTGTGTAACCAGATCCACCATGGCCTTCGCTCTGGCATTGTCGTCCTTGGCAAT
```

```
ENSPTRT00000038754     CTCATACTGTGTAACCAGATCCACCATGGCCTTCGCTCTGGCATTGTCGTCCTTGGCAAT
ENSMUST00000052236     CTCATCCTGTGTAACCAGATCCACCACGGTCTTCGATCTGGCATTGTTGTCCTTGGCAAT
ENSRNOT00000016875     CTCATCCTGTGTAACCAGATCCACCACGGTCTTCGATCTGGCATTGTTGTCCTTGGCAAT

ENSPPYT00000022222     GGGAAAGGCATCATCCGGAACAATCAAATCTTTTCCAATAAGGAGGCTGGCATTTACATC
ENSMMUT00000025134     GGGAAAGGCATCATCCGGAACAATCAAATCTTTTCCAATAAGGAGGCTGGCATTTACATC
ENSGGOT00000001898     GGGAAAGGCATCATCCGGAACAATCAGATCTTTTCCAATAAGGAGGCTGGTATTTACATC
ENST00000432825        GGGAAAGGCATCATCCGGAACAATCAAATCTTTTCCAATAAGGAGGCTGGCATTTACATC
ENSPTRT00000038754     GGGAAAGGCATCATCCGGAACAATCAAATCTTTTCCAATAAGGAGGCTGGCATTTACATC
ENSMUST00000052236     GGGAAAGGCGTCATCAGGAACAACCAAATCTTTTCAAATAAGGAGGCTGGCATTTATATC
ENSRNOT00000016875     GGGAAAGGTGTCATCAGGAACAACCAAATCTTTTCTAATAAGGAGGCTGGCATTTATATC

ENSPPYT00000022222     CTGTACCACGGAAACCCCGTTGTGAGCGGGAACCACATTTTCAAGGGCCGTGCTGCCGGC
ENSMMUT00000025134     CTGTACCACGGAAACCCCATCGTGAGCGGGAACCACATCTTCAAGGGCCGTGCTGCCGGC
ENSGGOT00000001898     CTGTACCACGGAAACCCCGTTGTGAGCGGGAACCACATCTTCAAGGGCCGTGCTGCCGGC
ENST00000432825        CTGTACCACGGAAACCCCGTTGTGAGCGGGAACCACATCTTCAAGGGCCGTGCTGCCGGC
ENSPTRT00000038754     CTGTACCACGGAAACCCCGTTGTGAGCGGGAACCACATCTTCAAGGGCCGTGCTGCCGGC
ENSMUST00000052236     CTGTACCATGGAAATCCAATTGTGAGTGGAAACCACATCTTCAAGGGGCGCGCAGCTGGC
ENSRNOT00000016875     CTGTACCACGGAAATCCGATTGTGAGCGGAAACCACATCTTCAAGGGGCGTGCAGCTGGC

ENSPPYT00000022222     ATAGCAGTGAATGAGAACGGCAAAGGCCTCATCACAGAAAATGTCATCCGTGAGAATCAG
ENSMMUT00000025134     ATAGCAGTGAATGAGAACGGCAAAGGCCTCATCACAGAAAATGTCATCCGTGAGAATCAG
ENSGGOT00000001898     ATAGCAGTGAATGAGAACGGCAAAGGCCTCATCACAGAAAATGTCATCCGTGAGAATCAG
ENST00000432825        ATAGCAGTGAATGAGAACGGCAAAGGCCTCATCACAGAAAATGTCATCCGTGAGAATCAG
ENSPTRT00000038754     ATAGCAGTGAATGAGAACGGCAAAGGCCTCATCACAGAAAATGTCATCCGTGAGAATCAG
ENSMUST00000052236     ATAGCAGTGAACGAGAATGGCAAAGGCCTCATCACAGAAAACGTCATCCGTGAGAATCAA
ENSRNOT00000016875     ATAGCAGTGAACGAGAATGGCAAAGGCCTCATCACAGAAAACGTCATCCGCGAGAACCAG

ENSPPYT00000022222     TGGGGAGGTGTGGACATCCGCCGTGGAGGGATCCCCGTTCTCAGGAGTAACCTCATCTGC
ENSMMUT00000025134     TGGGGAGGTGTGGACATCCGCCGTGGAGGGATCCCGGTTCTGAGGAGTAACCTCATCTGC
ENSGGOT00000001898     TGGGGAGGTGTGGACATCCGCCGTGGAGGGATCCCCGTTCTCAGGAGTAACCTCATCTGC
ENST00000432825        TGGGGAGGTGTGGACATCCGCCGTGGAGGGATCCCCGTTCTCAGGAGTAACCTCATCTGC
ENSPTRT00000038754     TGGGGAGGTGTGGACATCCGCCGTGGAGGGATCCCCGTTCTCAGGAGTAACCTCATCTGC
ENSMUST00000052236     TGGGGAGGTGTAGACATCCGCCGCGGCGGCGTCCCGATCCTCAGGAGCAACCTCATCTGC
ENSRNOT00000016875     TGGGGAGGTGTAGACATCCGCCGAGGCGGCGTCCCGATCCTCAGGAGCAACCTCATCTGC

ENSPPYT00000022222     TTTGGCTATTCAGATGGTGTGGTTGTGGGAGACGAAGGCAAAGGCCTCATAGAAGGAAAT
ENSMMUT00000025134     TTTGGCTATTCAGATGGCGTGGTTGTGGGAGACGAAGGCAAAGGCCTCATAGAAGGAAAT
ENSGGOT00000001898     TTTGGCTATTCAGATGGTGTGGTTGTGGGAGACGAAGGCAAAGGCCTCATAGAAGGAAAT
ENST00000432825        TTTGGCTATTCAGATGGTGTGGTTGTGGGAGACGAAGGCAAAGGCCTCATAGAAGGAAAT
ENSPTRT00000038754     TTTGGCTATTCAGATGGTGTGGTTGTGGGAGATGAAGGCAAAGGCCTCATAGAAGGAAAT
ENSMUST00000052236     TTTGGCTACTCCGACGGCGTGGTTGTGGGGGATGAAGGCAAAGGACTGATAGAGGGGAAC
ENSRNOT00000016875     TTTGGCTACTCAGATGGCGTGGTGGTGGGGGATGAAGGCAGAGGACTGATAGAAGGGAAT

ENSPPYT00000022222     ACCATCTACGCTAACAAGGGGTGTGGTGTGTGGATGATGTCGTCCAGCATCCCCCCATGT
ENSMMUT00000025134     ACCATCTACGCTAACAAGGGCTGTGGTGTGTGGATGATGTCGTCCAGCCTCCCCCACGTC
ENSGGOT00000001898     ACCATCTACGCTAACAAGGGCTGTGGTGTGTGGATGATGTCGTCCAGCCTCCCCCATGTC
ENST00000432825        ACCATCTACGCTAACAAGGGCTGTGGTGTGTGGATGATGTCGTCCAGCCTCCCCCATGTC
ENSPTRT00000038754     ACCATCTACGCTAACAAGGGCTGTGGTGTGTGGATGATGTCGTCCAGCCTCCCCCATGTC
ENSMUST00000052236     ACCATCTATGCTAACAAAGGATGTGGCGTGTGGATGATGTCCTCTAGCCTGCCCCACGTC
ENSRNOT00000016875     ACCATCTATGCTAACAAAGGATGTGGCGTGTGGATGATGTCCTCCAGCCTGCCCCATGTC

ENSPPYT00000022222     CTCCAGCAACCACGCTCAGCTACAATGGCCATGGAGTGGCAGTATTTAGCCAGAAGGGAT
```

```
ENSMMUT00000025134     ACCAGCAACCACGTCAGCTACAATGGCCTGTATGGAGTGGCAGTGTTTAGCCAGAAGGAT
ENSGGOT00000001898     ACCAGCAACCACGTCAGCTACAATGGCCTGTATGGAGTGGCAGTATTTAGCCAGAAGGAT
ENST00000432825        ACCAGCAACCACGTCAGCTACAATGGCCTGTATGGAGTGGCAGTATTTAGCCAGAAGGAT
ENSPTRT00000038754     ACCAGCAACCACGTCAGCTACAATGGCCTGTATGGAGTGGCAGTATTTAGCCAGAAGGAT
ENSMUST00000052236     ACCAGCAACCATGTGAGCTACAATGGCCTGTATGGAGTGGCAGTGTTCAGCCAGAAAGAT
ENSRNOT00000016875     TCCAGCAACCATGTGAGCTACAACGGCCTGTATGGGGTGGCAGTGTTCAGCCAGAAAGAC

ENSPPYT00000022222     GGCTCCAGCGAGTTACCTCGAGGCCACAGGGCTCAAGAGAACTTCAGCGAGGATGGGGAT
ENSMMUT00000025134     GGCTCCAGCGAGTTACCTCGAGGCCATAGGGCTCAAGAGAACTTCAGCGAGGATGGGGAC
ENSGGOT00000001898     GGCTCCAGCGAGTTACCTCGAGGCCACAGGGCTCAAGAGAACTTCAGCGAGGATGGGGAC
ENST00000432825        GGCTCCAGCGAGTTACCTCGAGGCCACAGGGCTCAAGAGAACTTCAGCGAGGATGGGGAC
ENSPTRT00000038754     GGCTCCAGCGAGTTACCTCGAGGCCACAGGGCTCAAGAGAACTTCAGCGAGGATGGGGAC
ENSMUST00000052236     GGC------GAGTTCCCTGGAGGCCACGGGGCCCAGGAGAACTTCAGTGAGGACGGAGAT
ENSRNOT00000016875     GGT------GAGTTCCCTGGGGGCCACGGGGCTCAGGAGAACTTCAGTGAGGACGGGGAC

ENSPPYT00000022222     GCCATCCTCTGGGAGACAGAGCTGGAGAAGGAGGACGACCCACTGCGCCGGCCCATCACC
ENSMMUT00000025134     GCCATCCTCTGGGAGACAGAGCTGGAGAAGGAGGACGACCCACTGCGCCGGCCCATCACC
ENSGGOT00000001898     GCCATCCTCTGGGAGACAGAGCTGGAGAAGGAGGACGACCCACTGCGCCGGCCCATCACC
ENST00000432825        GCCATCCTCTGGGAGACAGAGCTGGAGAAGGAGGACGACCCACTGCGCCGGCCCATCACC
ENSPTRT00000038754     GCCATCCTCTGGGAGACAGAGCTGGAGAAGGAGGACGACCCACTGCGCCGGCCCATCACC
ENSMUST00000052236     GCCATCCTCTGGGAGGCGGAGCTGGAGAAGGAGGATGACCCACTGCGCCGGCCCATCACC
ENSRNOT00000016875     GCCATCCTCTGGGAGACGGAGCTGGAGAAGGAGGATGACCCACTGCGCCGGCCCGTCACC

ENSPPYT00000022222     ATAGCTCTTGTTGAATCTAACAGTATTAATCACAATGGAGCCTCAGGACTCTATGTCCAG
ENSMMUT00000025134     ATAGCTCTTGTTGAGTCTAACAGTATTAATCACAATGGAGCCTCAGGACTCTATGTCCAG
ENSGGOT00000001898     ATAGCTCTTGTTGAGTCTAACAGTATTAATCACAATGGAGCCTCAGGACTCTATGTCCAG
ENST00000432825        ATAGCTCTTGTTGAGTCTAACAGTATTAATCACAATGGAGCCTCAGGACTCTATGTCCAG
ENSPTRT00000038754     ATAGCTCTTGTTGAGTCTAACAGTATTAATCACAATGGAGCGTCAGGACTCTATGTCCAG
ENSMUST00000052236     GTAGCGCTCGTGGAGTCCAACAGTATTAACCACAATGGAGCATCAGGAATCTTTGTCCAG
ENSRNOT00000016875     GTAGCTCTCGTGGAGTCCAACAGTATTAATCACAATGGAGCATCAGGAATCTTTGTCCAG

ENSPPYT00000022222     AGCAGCGAGGCACTGCATGTCATCACCAATGTGATCCACGCGAATGGGGACAGAGGCATT
ENSMMUT00000025134     AGCAGCGAGGCACTGCATGTCATCACCAATGTGATCCACGCGAATGGGGACAGAGGCATT
ENSGGOT00000001898     AGCAGCGAGGCACTGCACGTCATCACCAATGTGATCCACGCGAATGGGGACAGAGGCATT
ENST00000432825        AGCAGCGAGGCACTGCATGTCATCACCAATGTGATCCACGCGAATGGGGACAGAGGCATT
ENSPTRT00000038754     AGCAGCGAGGCACTGCATGTCATCACCAATGTGATCCACGCGAATGGGGACAGAGGCATT
ENSMUST00000052236     AGCAGCGAGGCACTTCAGGTCGTTGCCAATGTGATCCACGCCAACGGGGACAGAGGCATC
ENSRNOT00000016875     AGCAGCGAGGCACTTCAGGTCACCGCCAATGTGATCCATGCCAATGGGGACAGAGGCGTC

ENSPPYT00000022222     ACTGTGGCCCAGAGCAGCCAACCCACTCGAGTGGCCAACAACAGCATCTCCTGCAACCGG
ENSMMUT00000025134     ACTGTGGCCCAGAGCAGCCATCCCACCCGAGTGGCCAACAACAGCATCTCCTGCAACCGG
ENSGGOT00000001898     ACTGTGGCCCAGAGCAGCCAACCCACCCGAGTGGCCAACAACAGCATCTCCTGCAACCGG
ENST00000432825        ACTGTGGCCCAGAGCAGCCAACCCACCCGAGTGGCCAACAACAGCATCTCCTGCAACCGG
ENSPTRT00000038754     ACTGTGGCCCAGAGCAGCCAACCCACCCGAGTGGCCAACAACAGCATCTCCTGCAACCGG
ENSMUST00000052236     ACCATTGTTCAGAGCAGCCAGCTCACACGGGTGGCCAACAACAGCATCTCCTGCAATCGC
ENSRNOT00000016875     ACCATTGTTCAGAGCAGCCAGCTCACACGAGTGGCTAACAACAGCATCTCCTGCAATCGC

ENSPPYT00000022222     CAAAGTGGGGTCAAGGTTGAGGCCCAGTGCAAAGTGGAGCTCCGGGGCAATGGTATCTAT
ENSMMUT00000025134     CAAAGTGGGGTCAAGGTTGAGGCCCAGTGCAAAGTGGAGCTCCGGGGCAATGGTATCTAT
ENSGGOT00000001898     CAAAGTGGGGTCAAGGTTGAGGCCCAGTGCAAAGTGGAGCTCCGGGGCAATGGTATCTAT
ENST00000432825        CAAAGTGGGGTCAAGGTTGAGGCCCAGTGCAAAGTGGAGCTCCGGGGCAATGGTATCTAT
ENSPTRT00000038754     CAAAGTGGGGTCAAGGTTGAGGCCCAGTGCAAAGTGGAGCTCCGGGGCAATGGTATCTAT
ENSMUST00000052236     CAGAGTGGGGTCAAGGTGGAATTTCAGTGCAAGGTGGAGCTCCGGGGCAATGGTATCTAT
ENSRNOT00000016875     CAGAGTGGGGTCAAGGTGGAATTTCAATGCAAGGTGGAGCTCCGGGGCAATGGTATCTAT
```

```
ENSPPYT00000022222     GACAACAGAGGCCACGGCATTATCACCAAGGGCGACAGCACCATCGTCATTGAAAACGAT
ENSMMUT00000025134     GACAACAGAGGCCATGGCATTATCACCAAGGGCGACAGCACCATCGTCATTGAAAACGAT
ENSGGOT00000001898     GACAACAGAGGCCACGGCATTATCACCAAGGGCGACAGCACCATCGTCATTGAAAACGAT
ENST00000432825        GACAACAGAGGCCACGGCATTATCACCAAGGGCGACAGCACCATCGTCATTGAAAACGAT
ENSPTRT00000038754     GACAACAGAGGCCACGGCATTATCACCAAGGGCGACAGCACCATCGTCATTGAAAACGAT
ENSMUST00000052236     GACAACAGAGGCCATGGCATCATTACCAAGGGTGATGGTACCGCCGTTGTGGAGAATGAC
ENSRNOT00000016875     GACAACAGAGGCCATGGCATCATTACTAAGGGCGATGGTACCGCTGTTGTGGAAAATGAC

ENSPPYT00000022222     ATCATTGGCAACCGGGGCAGCGGGCTGCAGCTGCTGCCCAGGTCCGACACTAAAGTAATA
ENSMMUT00000025134     ATCATTGGCAATCGGGGCAGCGGGCTGCAGCTGCTGCCTAGGTCCGACACTAAAGTAATA
ENSGGOT00000001898     ATCATTGGCAACCGGGGCAGCGGGCTGCAGCTGCTGCCCAGGTCCGACACTAAAGTAATA
ENST00000432825        ATCATTGGCAACCGGGGCAGCGGGCTGCAGCTGCTGCCCAGGTCCGACACTAAAGTAATA
ENSPTRT00000038754     ATCATTGGCAACCGGGGCAGCGGGCTGCAGCTGCTGCCCAGGTCCGACACTAAAGTAATA
ENSMUST00000052236     ATCATCGGCAACCGCGGCAGCGGACTGCAGTTGCTGCCCAGGTCTGACACTAAGGTACTA
ENSRNOT00000016875     ATCATCGGCAACCGCGGCAGTGGACTGCAGTTGCTGCCCAGGTCTGACACTAAGGTACTA

ENSPPYT00000022222     AAGAACCGGATCCACTCGTTCCGGGCCTACGGCATCGCAGTGCGGGGCCGTGCCAAGGCC
ENSMMUT00000025134     AAGAACCGGATCCACTCATTCCGGGCCTATGGCATTGCAGTGCGGGGCCGTGCCAAGGCC
ENSGGOT00000001898     AAGAACCGGATCCACTCGTTCCGGGCCTACGGCATCGCCGTGCGGGGCCGTGCCAAGGCC
ENST00000432825        AAGAACCGGATCCACTCGTTCCGGGCCTACGGCATCGCCGTGCGGGGCCGTGCCAAGGCC
ENSPTRT00000038754     AAGAACCGGATCCACTCGTTCCGGGCCTACGGCATCGCCGTGCGGGGCCGTGCCAAGGCC
ENSMUST00000052236     AAGAACCGGATCCACTCCTTCCGGGCCTATGGCATTGCAGTGCGGGGCCGTGTTAAGGCC
ENSRNOT00000016875     AAGAACCGGATCCACTCCTTCCGGGCCTATGGCATTGCAGTGCGGGGCCGTGTTAAGGCC

ENSPPYT00000022222     CTGGTGCAGGAAAACATCATCTTCCAGGGCAAAACCAGTAAGACCATCTTTCAGCAGATC
ENSMMUT00000025134     CTGGTGCAGGAAAACATCATCTTCCAGGGCAAAACAAATAAGACCATCTTTCAGCAGATC
ENSGGOT00000001898     CTGGTGCAGGAAAACATCATCTTCCAGGGCAAAACCAGTAAGACCATCTTTCAGCAGATC
ENST00000432825        CTGGTGCAGGAAAACATCATCTTCCAGGGCAAAACCAGTAAGACCATCTTTCAGCAGATC
ENSPTRT00000038754     CTGGTGCAGGAAAACATCATCTTCCAGGGCAAAACCAGTAAGACCATCTTTCAGCAGATC
ENSMUST00000052236     CTGGTTCAGGAGAACATCATCTTCCAGGGCAAGACCAACAAGACCATCTTCCAGCAGATC
ENSRNOT00000016875     CTGGTACAGGAGAACATCATCTTCCAGGGCAAGACCAACAAGACCATCTTCCAGCAGATC

ENSPPYT00000022222     TCAAACAACCGAGAATGCATCATGCAAAACAACAAGTTCCTGGTCTTCAAGAAAAAGTCT
ENSMMUT00000025134     TCAAACAACCGAGAATGTGTCATGCAAAACAACAAGTTCCTGGTCTTCAAGAAAAAGTCT
ENSGGOT00000001898     TCAAACAACCGAGAATGCGTCATGCAAAACAACAAGTTCCTGGTCTTCAAGAAAAAGTCT
ENST00000432825        TCAAACAACCGAGAATGCATCATGCAAAACAACAAGTTCCTGGTCTTCAAGAAAAAGTCT
ENSPTRT00000038754     TCAAACAACCGAGAATGCATCATGCAAAACAACAAGTTCCTGGTCTTCAAGAAAAAGTCT
ENSMUST00000052236     ACAAACAACAGAGAGTGCATCATGCAAAACAACAAGTTCCTTGTCTTCAAGAAGAAGTCT
ENSRNOT00000016875     ACAAACAACCGAGAGTGCATCATGCAAAACAACAAGTTCCTTGTCTTCAAGAAGAAGTCT

ENSPPYT00000022222     GATACGTGGCGCCTGGTGAACCCACCAGCACGGCCCCACCTTGAAAATTCTCTCAGACGT
ENSMMUT00000025134     GATACGTGGCGCCTGGTGAACCCGCCAGCACGGCCCCACCTCGAAACCTCTCTGAGACGC
ENSGGOT00000001898     GATACGTGGCGCCTGGTGAACCCACCAGCACGGCCCCACCTTGAAAATTCTCTCAGACGT
ENST00000432825        GATACGTGGCGCCTGGTGAACCCACCAGCACGGCCCCACCTTGAAAATTCTCTCAGACGT
ENSPTRT00000038754     GATACGTGGCGCCTGGTGAACCCACCAGCACGGCCCCACCTTGAAAATTCTCTCAGACGT
ENSMUST00000052236     GATACCTGGCGCCTGGTGAACCCACCGGCACGGCCCCACCTTGAAAACTCACTCAGGGGC
ENSRNOT00000016875     GACACCTGGCGCCTGGTGAACCCACCAGCACGGCCCCACCTGGAAAACTCACTCCGGGGC

ENSPPYT00000022222     CCCTCGGCAGCCCACAATGGGCAGAAGGTGACAGCCATGGCAACGAGGATCACAGCCCGG
ENSMMUT00000025134     CCCTCGGCAGCCCACAATGGGCAGAAGGTGACAGCCATGGCAACAAGGATCACGGCCCGG
ENSGGOT00000001898     CCCTCGGCAGCCCACAATGGGCAGAAGGTGACAGCCATGGCAACGAGGATCACAGCCCGG
ENST00000432825        CCCTCGGCAGCCCACAATGGGCAGAAGGTGACAGCCATGGCAACGAGGATCACAGCCCGG
```

```
ENSPTRT00000038754     CCCTCGGCAGCCCACAATGGGCAGAAGGTGACAGCCATGGCAACGAGGATCACAGCCCGG
ENSMUST00000052236     TCCTCTGCAGCCCACAGTGGGCATAAGGTGACAGCCATGGCAACCAGGATCACAGCCCGT
ENSRNOT00000016875     TCCTCTGCAGCCCACAGTGGGCACAAGGTGGCAGCCATGGCAACCAGGATCACAGCCCGT


ENSPPYT00000022222     GTGGAAGGTGGTTTCCACAGCAACCGCAGTGTCTTCTGCACCATCCTG
ENSMMUT00000025134     GTGGAAGGTGGTTACCACAGCAACCGCAGTGTCTTCTGCACCATCCTG
ENSGGOT00000001898     GTGGAAGGTGGTTACCACAGCAACCGCAGTGTCTTCTGCACCATCCTG
ENST00000432825        GTGGAAGGTGGTTACCACAGCAACCGCAGTGTCTTCTGCACCATCCTG
ENSPTRT00000038754     GTGGAAGGTGGTTACCACAGCAACCGCAGTGTCTTCTGCACCATCCTG
ENSMUST00000052236     GTGGAAGGTGGCTACCATAGCAACCGCAGCATCTTCTGTACCATCCTG
ENSRNOT00000016875     GTGGAAGGTGGCTATCACAGCAACCGTAGCATCTTCTGTACCATCCTG
```

Multiple sequence alignment of Fbxo11

```
ENSPTRT00000022151     ------------------------------------------------------------
ENSMMUT00000032157     ATGAACTCCGTCCGAGCCGCCAACCGGAGACCCAGGCGAGTGTCGCGGCCGCGCCCGGTG
ENSCJAT00000021472     ATGAACTCCGTCCGAGCCGCCAACCGGAGACCCAGGCGAGTGTCGCGGCCGCGCCCGGTG
ENSGGOT00000011655     ATGAACTCCGTCCGAGCCGCCAACCGGAGACCCAGGCGAGTGTCGCGGCCGCGCCCGGTG
ENST00000403359        ATGAACTCCGTCCGAGCCGCCAACCGGAGACCCAGGCGAGTGTCGCGGCCGCGCCCGGTG
ENSPPYT00000014431     ATGAACTCCGTCCGAGCCGCCAACCGGAGACCCAGGCGAGTGTCGCGGCCGCGCCCGGTG
ENSMUST00000005504     ATGAACTCCGTCCGAGCCGCCAACCGGAGACCCAGGCGAGTGTCGCGGCCGCGCCCGGTG
ENSRNOT00000021998     ------------------------------------------------------------


ENSPTRT00000022151     ------------------------------------------------------------
ENSMMUT00000032157     CAGCAACAGCAGCAGCAGCCCCCGCAGCAGCCGCCGCCGCAGCCACCCCAGCAGCAGCCG
ENSCJAT00000021472     CAGCAACAGCAGCAGCAGCCCCCGCAGCAGCCGCCGCCGCAACCGCCTCAGCAGCAGCCG
ENSGGOT00000011655     CAGCAACAGCAGCAGCAGCCCCCGCAGCAGCCGCCGCCGCAGCCGCCCCAGCAGCAACCG
ENST00000403359        CAGCAACAGCAGCAGCAGCCCCCGCAGCAGCCGCCGCCGCAGCCGCCCCAGCAGCAGCCG
ENSPPYT00000014431     CAGCAACAGCAGCAGCAGCCCCCGCAGCAGCCGCCGCCGCAGCCGCCCCAGCAGCAGCCG
ENSMUST00000005504     CAACAGCAGCAGCAGCAGCCCCCGCAGCAGCCGCCGCCGCAGCCGCCTCAACAGCAGCCG
ENSRNOT00000021998     ------------------------------------------------------------


ENSPTRT00000022151     ------------------------------------------------------------
ENSMMUT00000032157     CCCCAGCAGCAGCCTCCGCCGCCGCCACAGCAGCAGCCTCCGCCGCCGCCACCGCCGCCT
ENSCJAT00000021472     CCCCAGCAGCAGCCTCCGCCGCCGCCGCAGCAGCAGCCTCCGCCGCCGCCACCGCCGCCT
ENSGGOT00000011655     CCCCAGCAGCAGCCTCCGCCGCCGCCGCAGCAGCAACCTCCGCCGCCCCCACCGCCCCCT
ENST00000403359        CCCCAGCAGCAGCCTCCGCCGCCGCCGCAGCAGCAGCCTCCGCCGCCGCCACCGCCGCCT
ENSPPYT00000014431     CCCCAGCAGCAGCCTCCGCCGCCGCCGCAACAGCAGCCTCCGCCGCCGCCACCGCCGCCT
ENSMUST00000005504     CCGCAGCAGCAGCCCCCGCCGCCGCCGCAGCAGCAGCCCCCGCCGCCGCCCCCGCCGCCT
ENSRNOT00000021998     ------------------------------------------------------------


ENSPTRT00000022151     ---------------------------------------------------------ATG
ENSMMUT00000032157     CCGCCGCTGCCTCAGGAGCGGAACAACGTCGGCGAGCGGGATGATGTGCCTGCAGATATG
ENSCJAT00000021472     CCGCCGCTGCCTCAGGAGCGGAACAACGTCGGCGAGCGGGATGATGTGCCTGCAGATATG
ENSGGOT00000011655     CCGCCGCTGCCTCAGGAGCGGAACAACGTCGGCGAGCGGGATGATGTGCCTGCAGATATG
ENST00000403359        CCGCCGCTGCCTCAGGAGCGGAACAACGTCGGCGAGCGGGATGATGTGCCTGCAGATATG
ENSPPYT00000014431     CCGCCGCTGCCTCAGGAGCGGAACAACGTCGGCGAGCGGGATGATGTGCCTGCAGATATG
ENSMUST00000005504     CCGCCGCCGCCTCAGGATCGGAACAACGCCGGCGAGAGGGATGATGTTCCTGCAGATATG
ENSRNOT00000021998     ---------------------------------------GATGATGTTCCTGCAGATATG


ENSPTRT00000022151     GTTGCAGAAGAATCAGGTCCTGGTGCACAAAATAGTCCGTACCAACTTCGTAGAAAAACT
ENSMMUT00000032157     GTTGCAGAAGAATCAGGTCCTGGTGCACAAAATAGTCCATACCAACTTCGTAGAAAAACT
ENSCJAT00000021472     GTTGCAGAAGAATCAGGTCCTGGTGCACAAAATAGTCCATACCAACTTCGTAGAAAAACT
ENSGGOT00000011655     GTTGCAGAAGAATCAGGTCCTGGTGCACAAAATAGTCCATACCAACTTCGTAGAAAAACT
```

```
ENST00000403359        GTTGCAGAAGAATCAGGTCCTGGTGCACAAAATAGTCCATACCAACTTCGTAGAAAAACT
ENSPPYT00000014431     GTTGCAGAAGAATCAGGTCCTGGTGCACAAAATAGTCCATACCAACTTCGTAGAAAAACT
ENSMUST00000005504     GTTGCAGAAGAATCAGGTCCTGGTGCACAAAATAGTCCATACCAACTTCGCAGAAAAACT
ENSRNOT00000021998     GTTGCAGAAGAATCAGGTCCTGGTGCACAAAACAGTCCATACCAGCTTCGTAGAAAAACT

ENSPTRT00000022151     CTTTTGCCGAAAAGAACAGCGTGTCCCACAAAGAACAGTATGGAGGGCGCCTCAACTTCA
ENSMMUT00000032157     CTTTTGCCGAAAAGAACAGCGTGTCCCACAAAGAACAGTATGGAGGGCGCCTCAACTTCA
ENSCJAT00000021472     CTTTTGCCAAAAAGAACAGCGTGTCCCACAAAGAACAGTATGGAGGGTGCCTCAACTTCA
ENSGGOT00000011655     CTTTTGCCGAAAAGAACAGCGTGTCCCACAAAGAACAGTATGGAGGGCGCCTCAACTTCA
ENST00000403359        CTTTTGCCGAAAAGAACAGCGTGTCCCACAAAGAACAGTATGGAGGGCGCCTCAACTTCA
ENSPPYT00000014431     CTTTTGCCGAAAAGAACAGCGTGTCCCACAAAGAACAGTATGGAGGGCGCCTCAACTTCA
ENSMUST00000005504     CTTTTGCCAAAAAGAACAGCGTGTCCTACAAAGAGCAGTATGGAGGGTGCCTCTACTTCG
ENSRNOT00000021998     CTTTTGCCAAAAAGAACAGCGTGTCCTACAAAGAACAGCATGGAGGGCGCCTCTACTTCA

ENSPTRT00000022151     ACTACAGAAAACTTTGGTCATCGTGCAAAACGTGCAAGAGTGTCTGGAAAATCACAAGAT
ENSMMUT00000032157     ACTACAGAAAACTTCGGTCATCGTGCAAAACGTGCCCGAGTGTCTGGAAAATCACAAGAT
ENSCJAT00000021472     ACTACAGAAAACTTTGGTCATCGTGCAAAACGTGCAAGAGTGTCTGGAAAATCACAAGAT
ENSGGOT00000011655     ACTACAGAAAACTTTGGTCATCGTGCAAAACGTGCAAGAGTGTCTGGAAAATCACAAGAT
ENST00000403359        ACTACAGAAAACTTTGGTCATCGTGCAAAACGTGCAAGAGTGTCTGGAAAATCACAAGAT
ENSPPYT00000014431     ACTACAGAAAACTTTGGTCATCGTGCAAAACGTGCAAGAGTGTCTGGAAAATCACAAGAT
ENSMUST00000005504     ACTACAGAAAACTTTGGTCATCGAGCAAAGCGTGCAAGAGTGTCTGGAAAGTCACAAGAT
ENSRNOT00000021998     ACTACAGAAAACTTCGGCCATCGAGCGAAGCGGGCAAGAGTGTCTGGGAAGTCACAAGAT

ENSPTRT00000022151     CTATCAGCAGCACCTGCTGAACAGTATCTTCAGGAGAAACTGCCAGATGAAGTGGTTCTA
ENSMMUT00000032157     CTATCAGCAGCACCTGCTGAACAGTATCTTCAGGAGAAACTGCCAGATGAAGTGGTTCTA
ENSCJAT00000021472     CTATCAGCAGCACCTGCTGAACAGTATCTTCAGGAAAAACTGCCAGATGAAGTGGTTCTA
ENSGGOT00000011655     CTATCAGCAGCACCTGCTGAACAGTATCTTCAGGAGAAACTGCCAGATGAAGTGGTTCTA
ENST00000403359        CTATCAGCAGCACCTGCTGAACAGTATCTTCAGGAGAAACTGCCAGATGAAGTGGTTCTA
ENSPPYT00000014431     CTATCAGCAGCACCTGCTGAACAGTATCTTCAGGAGAAACTGCCAGATGAAGTGGTTCTA
ENSMUST00000005504     CTTTCAGCAGCACCCGCTGAACAGTATCTTCAGGAGAAGCTGCCAGATGAAGTAGTTTTA
ENSRNOT00000021998     CTGTCAGCAGCACCCGCTGAACAGTATCTTCAGGAGAAGCTACCAGATGAAGTAGTTTTA

ENSPTRT00000022151     AAAATCTTCTCTTACTTGCTGGAACAGGATCTTTGTAGAGCAGCTTGTGTATGTAAACGC
ENSMMUT00000032157     AAAATCTTCTCTTACTTGCTGGAACAGGATCTTTGTAGAGCAGCTTGTGTATGTAAACGC
ENSCJAT00000021472     AAAATCTTCTCTTACTTGCTGGAACAGGATCTTTGTAGAGCAGCTTGTGTATGTAAACGC
ENSGGOT00000011655     AAAATCTTCTCTTACTTGCTGGAACAGGATCTTTGTAGAGCAGCTTGTGTATGTAAACGC
ENST00000403359        AAAATCTTCTCTTACTTGCTGGAACAGGATCTTTGTAGAGCAGCTTGTGTATGTAAACGC
ENSPPYT00000014431     AAAATCTTCTCTTACTTGCTGGAACAGGATCTTTGTAGAGCAGCTTGTGTATGTAAACGC
ENSMUST00000005504     AAAATATTTTCTTATTTGCTGGAACAAGACCTTTGTAGAGCTGCTTGCGTGTGTAAACGC
ENSRNOT00000021998     AAAATATTCTCTTATTTGCTGGAACAAGATCTTTGTAGAGCTGCTTGTGTGTGTAAACGC

ENSPTRT00000022151     TTCAGTGAACTTGCTAATGATCCAATTTTGTGGAAACGATTATATATGGAAGTATTTGAA
ENSMMUT00000032157     TTCAGTGAACTTGCTAATGATCCAATTTTGTGGAAACGATTATATATGGAAGTATTTGAA
ENSCJAT00000021472     TTCAGTGAACTTGCTAATGACCCTATTTTGTGGAAACGATTATACATGGAAGTATTTGAA
ENSGGOT00000011655     TTCAGTGAACTTGCTAATGATCCAATTTTGTGGAAACGATTATATATGGAAGTATTTGAA
ENST00000403359        TTCAGTGAACTTGCTAATGATCCAATTTTGTGGAAACGATTATATATGGAAGTATTTGAA
ENSPPYT00000014431     TTCAGTGAACTTGCTAATGATCCAATTTTGTGGAAACGATTATATATGGAAGTATTTGAA
ENSMUST00000005504     TTCAGTGAGCTTGCTAATGATCCAATTTTGTGGAAACGATTATATATGGAAGTATTTGAA
ENSRNOT00000021998     TTCAGTGAACTTGCTAATGATCCAATTTTGTGGAAACGATTATATATGGAAGTATTTGAA

ENSPTRT00000022151     TATACTCGCCCTATGATGCATCCTGAACCTGGAAAATTCTACCAGATTAATCCAGAAGAG
ENSMMUT00000032157     TATACTCGCCCTATGATGCATCCTGAACCTGGTAAATTCTACCAGATTAATCCAGAAGAG
ENSCJAT00000021472     TATACTCGTCCTATGATGCATCCTGAACCTGGTAAATTCTACCAGATTAATCCAGAAGAG
ENSGGOT00000011655     TATACTCGCCCTATGATGCATCCTGAACCTGGAAAATTCTACCAGATTAATCCAGAAGAG
```

```
ENST00000403359        TATACTCGCCCTATGATGCATCCTGAACCTGGAAAATTCTACCAGATTAATCCAGAAGAG
ENSPPYT00000014431     TATACTCGCCCTATGATGCATCCTGAACCTGGTAAATTCTACCAGATTAATCCAGAAGAG
ENSMUST00000005504     TATACCCGGCCGATGATGCATCCTGAACCTGGTAAATTCTACCAGATTAATCCAGAAGAA
ENSRNOT00000021998     TATACCCGGCCTATGATGCATCCTGAACCTGGTAAATTCTACCAGATTAATCCAGAAGAA

ENSPTRT00000022151     TATGAACATCCAAATCCCTGGAAAGAGAGTTTCCAGCAGTTGTATAAAGGTGCACATGTA
ENSMMUT00000032157     TATGAACATCCAAATCCCTGGAAAGAGAGTTTCCAGCAGTTGTATAAAGGTGCACATGTA
ENSCJAT00000021472     TATGAACACCCAAATCCCTGGAAAGAGAGTTTCCAGCAGTTGTATAAAGGTGCACATGTA
ENSGGOT00000011655     TATGAACATCCAAATCCCTGGAAAGAGAGTTTCCAGCAGTTGTATAAAGGTGCACATGTA
ENST00000403359        TATGAACATCCAAATCCCTGGAAAGAGAGTTTCCAGCAGTTGTATAAAGGTGCACATGTA
ENSPPYT00000014431     TATGAACATCCAAACCCCTGGAAAGAGAGTTTCCAGCAGTTGTATAAAGGTGCCCATGTA
ENSMUST00000005504     TATGAACATCCAAATCCATGGAAAGAAAGTTTTCAACAGTTGTATAAAGGTGCGCATGTA
ENSRNOT00000021998     TATGAACATCCAAATCCATGGAAAGAGAGTTTTCAACAGTTGTATAAAGGTGCACACGTA

ENSPTRT00000022151     AAGCCAGGATTTGCTGAACATTTCTACAGTAACCCTGCAAGATATAAAGGAAGAGAAAAT
ENSMMUT00000032157     AAGCCAGGATTTGCTGAACATTTCTACAGTAACCCTGCAAGATATAAAGGAAGAGAAAAT
ENSCJAT00000021472     AAGCCAGGATTTGCTGAACATTTCTACAGTAACCCTGCAAGATATAAAGGAAGAGAAAAT
ENSGGOT00000011655     AAGCCAGGATTTGCTGAACATTTCTACAGTAACCCTGCAAGATATAAAGGAAGAGAAAAT
ENST00000403359        AAGCCAGGATTTGCTGAACATTTCTACAGTAACCCTGCAAGATATAAAGGAAGAGAAAAT
ENSPPYT00000014431     AAGCCAGGATTTGCTGAACATTTCTACAGTAACCCTGCAAGATATAAAGGAAGAGAAAAT
ENSMUST00000005504     AAGCCAGGATTTGCGGAACATTTCTATAGTAATCCTGCAAGATACAAAGGAAGAGAAAAC
ENSRNOT00000021998     AAGCCGGGATTTGCGGAACATTTCTATAGTAATCCTGCCAGATACAAAGGGAGAGAAAAC

ENSPTRT00000022151     ATGTTGTATTATGATACTATTGAAGATGCCCTTGGTGGGGTACAAGAGGCTCATTTTGAT
ENSMMUT00000032157     ATGTTGTATTATGATACTATTGAAGATGCCCTTGGTGGGGTACAAGAGGCTCATTTTGAT
ENSCJAT00000021472     ATGTTGTATTATGATACCATTGAAGATGCCCTTGGTGGGGTACAAGAGGCTCATTTTGAT
ENSGGOT00000011655     ATGTTGTATTATGATACTATTGAAGATGCCCTTGGTGGGGTACAAGAGGCTCATTTTGAT
ENST00000403359        ATGTTGTATTATGATACTATTGAAGATGCCCTTGGTGGGGTACAAGAGGCTCATTTTGAT
ENSPPYT00000014431     ATGTTGTATTATGATACTATTGAAGATGCCCTTGGTGGGGTACAAGAGGCTCATTTTGAT
ENSMUST00000005504     ATGTTGTATTATGATACTATTGAAGATGCCCTTGGAGGAGTACAAGAAGCACATTTTGAT
ENSRNOT00000021998     ATGTTGTATTATGATACTATTGAAGATGCCCTTGGCGGAGTACAAGAAGCACATTTTGAT

ENSPTRT00000022151     GGACTTATCTTTGTTCATTCTGGAATATATACTGATGAATGGATATATATTGAATCTCCA
ENSMMUT00000032157     GGACTTATCTTTGTTCATTCTGGAATATATACTGATGAATGGATATATATTGAATCTCCA
ENSCJAT00000021472     GGACTCATTTTTGTTCATTCTGGAATATATACTGATGAATGGATATATATTGAATCTCCA
ENSGGOT00000011655     GGACTTATCTTTGTTCATTCTGGAATATATACTGATGAATGGATATATATTGAATCTCCA
ENST00000403359        GGACTTATCTTTGTTCATTCTGGAATATATACTGATGAATGGATATATATTGAATCTCCA
ENSPPYT00000014431     GGACTTATCTTTGTTCATTCTGGAATATATACTGATGAATGGATATATATTGAATCTCCA
ENSMUST00000005504     GGGCTTATCTTTGTTCATTCTGGAATATATACTGATGAATGGATATATATTGAATCTCCA
ENSRNOT00000021998     GGGCTTATCTTTGTTCATTCTGGAATATATACTGATGAATGGATATATATTGAATCTCCA

ENSPTRT00000022151     ATCACCATGATTGGTGCAGCACCTGGGAAAGTGGCAGACAAAGTTATAATTGAAAACACT
ENSMMUT00000032157     ATCACCATGATTGGTGCAGCACCTGGGAAAGTGGCAGACAAAGTTATAATTGAAAACACT
ENSCJAT00000021472     ATCACCATGATTGGTGCAGCACCTGGGAAAGTGGCAGACAAAGTTATAATTGAAAACACT
ENSGGOT00000011655     ATCACCATGATTGGTGCAGCACCTGGGAAAGTGGCAGACAAAGTTATAATTGAAAACACT
ENST00000403359        ATCACCATGATTGGTGCAGCACCTGGGAAAGTGGCAGACAAAGTTATAATTGAAAACACT
ENSPPYT00000014431     ATCACCATGATTGGTGCAGCACCTGGGAAAGTGGCAGACAAAGTTATAATTGAAAACACT
ENSMUST00000005504     ATTACTATGATTGGTGCAGCACCTGGCAAGGTTGCAGACAAGGTCATCATTGAGAACACT
ENSRNOT00000021998     ATCACTATGATTGGTGCAGCACCTGGGAAGGTTGCAGACAAGGTCATCATTGAGAACACT

ENSPTRT00000022151     AGAGATTCAACCTTCGTTTTTATGGAAGGCTCTGAAGATGCTTATGTTGGATATATGACA
ENSMMUT00000032157     AGAGATTCAACCTTCGTTTTTATGGAAGGCTCTGAAGATGCTTATGTTGGATATATGACA
ENSCJAT00000021472     AGAGATTCAACCTTTGTTTTTATGGAAGGCTCTGAAGATGCTTATGTTGGATATATGACA
ENSGGOT00000011655     AGAGATTCAACCTTCGTTTTTATGGAAGGCTCTGAAGATGCTTATGTTGGATATATGACA
```

ENST00000403359 AGAGATTCAACCTTCGTTTTTATGGAAGGCTCTGAAGATGCTTATGTTGGATATATGACA
ENSPPYT00000014431 AGAGATTCAACCTTCGTTTTTATGGAAGGCTCTGAAGATGCTTATGTTGGATATATGACA
ENSMUST00000005504 AGAGATTCGACCTTCGTGTTCATGGAAGGCTCTGAAGATGCTTATGTTGGATACATGACG
ENSRNOT00000021998 AGAGACTCCACTTTCGTTTTTATGGAAGGTTCTGAGGACGCTTACGTTGGATATATGACA

ENSPTRT00000022151 ATAAGGTTTAACCCTGATGACAAATCTGCACAACACCACAATGCACACCACTGCTTAGAG
ENSMMUT00000032157 ATAAGGTTTAACCCTGATGACAAATCTGCACAACACCACAATGCACACCACTGCTTAGAG
ENSCJAT00000021472 ATAAGGTTTAACCCTGATGACAAATCTGCACAACACCACAATGCACACCACTGCTTAGAG
ENSGGOT00000011655 ATAAGGTTTAACCCTGATGACAAATCTGCACAACACCACAATGCACACCACTGCTTAGAG
ENST00000403359 ATAAGGTTTAACCCTGATGACAAATCTGCACAACACCACAATGCACACCACTGCTTAGAG
ENSPPYT00000014431 ATAAGGTTTAACCCTGATGACAAATCTGCACAACACCACAATGCACACCACTGCTTAGAG
ENSMUST00000005504 ATAAGATTCAACCCTGATGACAAATCTGCTCAGCATCACAACGCACACCACTGCTTAGAG
ENSRNOT00000021998 ATAAGATTCAACCCTGATGACAAATCTGCCCAGCATCACAACGCGCACCACTGCCTAGAG

ENSPTRT00000022151 ATTACAGTAAATTGTAGCCCTATTATTGATCACTGTATCATCCGAAGTACATGTACAGTT
ENSMMUT00000032157 ATTACGGTAAATTGTAGCCCTATTATTGATCACTGTATCATCCGAAGTACATGTACAGTT
ENSCJAT00000021472 ATTACAGTAAATTGTAGCCCTATTATTGATCACTGTATCATTCGAAGTACATGTACAGTT
ENSGGOT00000011655 ATTACAGTAAATTGTAGCCCTATTATTGATCACTGTATCATCCGAAGTACATGTACGGTT
ENST00000403359 ATTACAGTAAATTGTAGCCCTATTATTGATCACTGTATCATCCGAAGTACATGTACAGTT
ENSPPYT00000014431 ATTACAGTAAATTGTAGCCCTATTATTGATCACTGTATCATCCGAAGTACATGTACAGTT
ENSMUST00000005504 ATCACAGTCAACTGCAGCCCTATTATTGACCACTGTATAATCCGCAGCACATGCACAGTT
ENSRNOT00000021998 ATCACAGTCAACTGCAGCCCCATTATCGACCACTGTATCATCCGAAGCACATGCACAGTT

ENSPTRT00000022151 GGTTCTGCAGTATGTGTTAGTGGTCAAGGAGCGTGTCCCACCATCAAGCACTGTAACATC
ENSMMUT00000032157 GGTTCTGCAGTATGTGTTAGTGGTCAAGGAGCATGTCCCACTATCAAGCACTGTAACATC
ENSCJAT00000021472 GGTTCTGCAGTATGTGTTAGTGGTCAAGGAGCATGTCCCACTATCAAGCACTGTAACATC
ENSGGOT00000011655 GGTTCTGCAGTATGTGTTAGTGGTCAAGGAGCATGTCCCACCATCAAGCACTGTAACATC
ENST00000403359 GGTTCTGCAGTATGTGTTAGTGGTCAAGGAGCATGTCCCACCATCAAGCACTGTAACATC
ENSPPYT00000014431 GGTTCTGCAGTATGTGTTAGTGGTCAAGGAGCATGTCCCACCATCAAGCACTGTAACATC
ENSMUST00000005504 GGTTCTGCAGTGTGTGTCAGTGGCCAGGGGGCGTGTCCCACTATCAAGCATTGTAACATC
ENSRNOT00000021998 GGCTCTGCAGTGTGTGTCAGCGGTCAGGGGGCGTGTCCCACTATCAAGCATTGTAACATC

ENSPTRT00000022151 AGTGACTGTGAAAATGTTGGACTGTATATAACAGATCATGCACAGGGAATATATGAGGAT
ENSMMUT00000032157 AGCGACTGTGAAAATGTTGGACTGTATATAACAGATCATGCACAGGGAATATATGAGGAT
ENSCJAT00000021472 AGCGACTGTGAAAATGTTGGACTGTATATAACAGATCATGCACAGGGAATATATGAGGAT
ENSGGOT00000011655 AGTGACTGTGAAAATGTTGGACTATATATAACAGATCATGCACAGGGAATATATGAGGAT
ENST00000403359 AGTGACTGTGAAAATGTTGGACTATATATAACAGATCATGCACAGGGAATATATGAGGAT
ENSPPYT00000014431 AGCGACTGTGAAAATGTTGGACTATATATAACAGATCATGCACAGGGAATATATGAGGAT
ENSMUST00000005504 AGTGACTGTGAAAACGTCGGACTTTATATAACAGATCATGCACAGGGAATATATGAAGAT
ENSRNOT00000021998 AGTGACTGTGAAAACGTCGGCCTCTATATAACAGATCATGCACAGGGAATATATGAAGAT

ENSPTRT00000022151 AATGAAATTTCCAATAATGCGTTAGCTGGGATTTGGGTTAAAAATCATGGAAACCCAATT
ENSMMUT00000032157 AATGAAATTTCCAATAATGCATTAGCTGGGATTTGGGTTAAAAATCATGGAAACCCAATT
ENSCJAT00000021472 AATGAAATTTCCAATAATGCGTTAGCTGGGATTTGGGTTAAAAATCATGGAAACCCAATT
ENSGGOT00000011655 AATGAAATTTCCAATAATGCGTTAGCTGGGATTTGGGTTAAAAATCATGGAAACCCAATT
ENST00000403359 AATGAAATTTCCAATAATGCGTTAGCTGGGATTTGGGTTAAAAATCATGGAAACCCAATT
ENSPPYT00000014431 AATGAAATTTCCAATAATGCGTTAGCTGGGATTTGGGTTAAAAATCATGGAAACCCAATA
ENSMUST00000005504 AATGAAATTTCTAATAATGCGTTAGCTGGGATTTGGGTTAAAAATCATGGAAATCCAATT
ENSRNOT00000021998 AATGAAATTTCTAATAATGCATTAGCTGGGATTTGGGTTAAAAATCATGGAAATCCAATT

ENSPTRT00000022151 ATTAGACGGAATCATATTCATCATGGACGTGATGTTGGTGTGTTCACATTTGATCATGGC
ENSMMUT00000032157 ATTAGACGGAATCATATTCATCATGGACGTGATGTTGGTGTGTTCACATTTGATCATGGC
ENSCJAT00000021472 ATTAGACGGAATCATATTCATCATGGACGCGATGTTGGTGTGTTCACATTTGATCATGGC
ENSGGOT00000011655 ATTAGACGGAATCATATTCATCATGGACGTGATGTTGGTGTGTTCACATTTGATCATGGC

```
ENST00000403359         ATTAGACGGAATCATATTCATCATGGACGTGATGTTGGTGTGTTCACATTTGATCATGGC
ENSPPYT00000014431      ATTAGACGGAATCATATTCATCATGGACGTGATGTTGGTGTGTTCACATTTGATCATGGC
ENSMUST00000005504      ATTAGACGGAATCACATTCATCATGGACGTGATGTTGGTGTGTTCACATTTGATCATGGC
ENSRNOT00000021998      ATTAGACGGAATCACATTCATCATGGACGTGATGTTGGTGTATTCACATTTGATCATGGC

ENSPTRT00000022151      ATGGGTTACTTTGAAAGTTGCAATATACACAGAAATAGGATAGCAGGCTTTGAAGTAAAA
ENSMMUT00000032157      ATGGGTTACTTTGAAAGTTGCAACATACACAGAAATAGGATAGCAGGCTTTGAAGTAAAA
ENSCJAT00000021472      ATGGGTTACTTTGAAAGTTGCAACATACACAGAAATAGGATAGCAGGCTTTGAAGTAAAA
ENSGGOT00000011655      ATGGGTTACTTTGAAAGTTGCAATATACACAGAAATAGGATAGCAGGCTTTGAAGTAAAA
ENST00000403359         ATGGGTTACTTTGAAAGTTGCAATATACACAGAAATAGGATAGCAGGCTTTGAAGTAAAA
ENSPPYT00000014431      ATGGGTTACTTTGAAAGTTGCAACATACACAGAAATAGGATAGCAGGCTTTGAAGTAAAA
ENSMUST00000005504      ATGGGCTACTTTGAAAGTTGCAACATACACAGAAACAGGATAGCAGGCTTTGAGGTAAAA
ENSRNOT00000021998      ATGGGCTACTTTGAAAGTTGCAACATACACAGAAACAGGATAGCAGGCTTTGAGGTAAAA

ENSPTRT00000022151      GCCTATGCTAACCCTACAGTGGTTCGATGTGAAATTCACCATGGGCAGACTGGAGGAATA
ENSMMUT00000032157      GCCTATGCTAACCCCACAGTGGTTCGATGTGAAATTCACCATGGACAGACTGGAGGAATA
ENSCJAT00000021472      GCCTATGCTAACCCCACAGTGGTTCGATGTGAAATTCACCATGGGCAGACTGGAGGAATA
ENSGGOT00000011655      GCCTATGCTAACCCTACAGTGGTTCGATGTGAAATTCACCATGGGCAGACTGGAGGAATA
ENST00000403359         GCCTATGCTAACCCTACAGTGGTTCGATGTGAAATTCACCATGGGCAGACTGGAGGAATA
ENSPPYT00000014431      GCCTATGCTAACCCCACAGTGGTTCGATGTGAAATTCACCATGGGCAGACTGGAGGAATA
ENSMUST00000005504      GCCTATGCCAATCCCACAGTGGTTCGATGTGAGATTCACCACGGGCAGACTGGAGGAATA
ENSRNOT00000021998      GCCTACGCTAATCCCACAGTGGTTCGATGTGAAATTCACCATGGGCAGACTGGAGGAATA

ENSPTRT00000022151      TATGTCCATGAAAAAGGAAGAGGACAATTCATAGAGAATAAAATCTATGCAAACAACTTT
ENSMMUT00000032157      TATGTCCATGAAAAAGGAAGAGGACAATTCATAGAGAATAAAATCTATGCAAACAACTTT
ENSCJAT00000021472      TATGTCCATGAAAAAGGAAGAGGACAATTCATAGAGAATAAAATCTATGCAAACAACTTT
ENSGGOT00000011655      TATGTCCATGAAAAAGGAAGAGGACAATTCATAGAGAATAAAATCTATGCAAACAACTTT
ENST00000403359         TATGTCCATGAAAAAGGAAGAGGACAATTCATAGAGAATAAAATCTATGCAAACAACTTT
ENSPPYT00000014431      TATGTCCATGAAAAAGGAAGAGGACAATTCATAGAGAATAAAATCTATGCAAACAACTTT
ENSMUST00000005504      TACGTCCACGAGAAAGGAAGAGGACAGTTCATAGAGAACAAGATCTATGCAAACAATTTC
ENSRNOT00000021998      TATGTCCATGAGAAAGGAAGAGGACAATTTATAGAGAATAAAATCTATGCAAACAACTTC

ENSPTRT00000022151      GCAGGTGTATGGATTACCTCAAATAGTGACCCAACAATAAGGGGAAATTCTATATTTAAT
ENSMMUT00000032157      GCAGGTGTATGGATTACCTCAAATAGCGACCCAACAATAAGGGGAAATTCTATATTTAAT
ENSCJAT00000021472      GCAGGTGTATGGATTACCTCAAATAGTGACCCAACAATAAGGGGAAATTCTATATTTAAT
ENSGGOT00000011655      GCAGGTGTATGGATTACCTCAAATAGTGACCCAACAATAAGGGGAAATTCTATATTTAAT
ENST00000403359         GCAGGTGTATGGATTACCTCAAATAGTGACCCAACAATAAGGGGAAATTCTATATTTAAT
ENSPPYT00000014431      GCAGGTGTATGGATTACCTCAAATAGTGACCCAACAATAAGGGGAAATTCTATATTTAAT
ENSMUST00000005504      GCAGGCGTGTGGATTACCTCAAATAGTGACCCAACAATAAGGGGGAATTCTATATTTAAT
ENSRNOT00000021998      GCAGGCGTATGGATTACCTCAAATAGTGACCCAACAATAAGGGGGAATTCTATATTTAAT

ENSPTRT00000022151      GGAAATCAAGGAGGAGTTTACATCTTTGGTGATGGACGAGGCCTTATTGAAGGAAATGAC
ENSMMUT00000032157      GGAAATCAAGGAGGAGTTTACATCTTTGGTGATGGACGAGGCCTTATTGAAGGAAATGAC
ENSCJAT00000021472      GGAAATCAAGGAGGAGTTTACATCTTTGGTGATGGGCGAGGCCTTATTGAAGGAAATGAC
ENSGGOT00000011655      GGAAATCAAGGAGGAGTTTACATCTTTGGTGATGGACGAGGCCTTATTGAAGGAAATGAC
ENST00000403359         GGAAATCAAGGAGGAGTTTACATCTTTGGTGATGGACGAGGCCTTATTGAAGGAAATGAC
ENSPPYT00000014431      GGAAATCAAGGAGGAGTTTACATCTTTGGTGATGGACGAGGCCTTATTGAAGGAAATGAC
ENSMUST00000005504      GGAAATCAAGGAGGAGTTTACATCTTTGGTGACGGACGAGGCCTTATAGAAGGAAATGAC
ENSRNOT00000021998      GGAAATCAAGGAGGAGTTTACATCTTTGGTGATGGACGAGGCCTTATAGAAGGAAATGAC

ENSPTRT00000022151      ATTTATGGCAATGCATTAGCAGGAATTCAAATTAGGACAAACAGTTGTCCAATTGTTCGG
ENSMMUT00000032157      ATTTATGGCAATGCATTAGCAGGAATACAAATTAGGACAAACAGTTGTCCAATTGTTCGG
ENSCJAT00000021472      ATTTATGGCAATGCATTAGCAGGAATTCAAATTAGGACAAACAGTTGTCCAATTGTTCGG
ENSGGOT00000011655      ATTTATGGCAATGCATTAGCAGGAATTCAAATTAGGACAAACAGTTGTCCAATTGTTCGG
```

```
ENST00000403359         ATTTATGGCAATGCATTAGCAGGAATTCAAATTAGGACAAACAGTTGTCCAATTGTTCGG
ENSPPYT00000014431      ATTTATGGCAATGCATTAGCAGGAATTCAGATTAGGACAAACAGTTGTCCAATTGTTCGG
ENSMUST00000005504      ATTTATGGTAATGCGTTAGCAGGAATTCAGATCAGGACAAACAGTTGTCCTATTGTTCGA
ENSRNOT00000021998      ATTTATGGTAATGCGTTAGCAGGAATTCAGATTAGGACAAACAGTTGTCCTATTGTTCGA

ENSPTRT00000022151      CATAACAAAATTCATGATGGCCAGCATGGTGGGATTTATGTGCATGAAAAGGGACAAGGA
ENSMMUT00000032157      CATAACAAAATTCATGATGGCCAGCATGGTGGGATTTATGTGCATGAAAAGGGACAAGGA
ENSCJAT00000021472      CATAACAAAATTCATGATGGCCAGCATGGTGGGATTTATGTGCATGAAAAGGGACAAGGA
ENSGGOT00000011655      CATAACAAAATTCATGATGGCCAGCATGGTGGGATTTATGTGCATGAAAAGGGACAAGGA
ENST00000403359         CATAACAAAATTCATGATGGCCAGCATGGTGGGATTTATGTGCATGAAAAGGGACAAGGA
ENSPPYT00000014431      CATAACAAAATTCATGATGGCCAGCATGGTGGGATTTATGTGCATGAAAAGGGACAAGGA
ENSMUST00000005504      CATAACAAAATTCATGATGGACAGCATGGTGGGATTTATGTGCATGAAAAGGGGCAAGGA
ENSRNOT00000021998      CATAACAAAATTCACGATGGACAGCATGGTGGGATTTATGTGCATGAAAAGGGACAAGGT

ENSPTRT00000022151      GTAATAGAAGAGAATGAAGTTTATAGTAACACTCTAGCTGGAGTCTGGGTGACAACTGGC
ENSMMUT00000032157      GTAATAGAAGAGAATGAAGTTTATAGTAACACTCTGGCTGGAGTCTGGGTGACAACTGGC
ENSCJAT00000021472      GTAATAGAAGAGAATGAAGTTTATAGTAATACTCTGGCTGGAGTCTGGGTGACAACTGGC
ENSGGOT00000011655      GTAATAGAAGAGAATGAAGTTTATAGTAACACTCTAGCTGGAGTCTGGGTGACAACTGGC
ENST00000403359         GTAATAGAAGAGAATGAAGTTTATAGTAACACTCTAGCTGGAGTCTGGGTGACAACTGGC
ENSPPYT00000014431      GTAATAGAAGAGAATGAAGTTTATAGTAACACTCTAGCTGGAGTCTGGGTGACAACTGGC
ENSMUST00000005504      GTAATAGAAGAGAATGAAGTTTACAGCAACACTCTGGCTGGCGTCTGGGTGACAACTGGC
ENSRNOT00000021998      GTAATAGAAGAAAATGAAGTTTACAGCAACACCCTGGCTGGCGTCTGGGTGACAACTGGC

ENSPTRT00000022151      AGCACTCCAGTACTGAGAAGAAACCGGATACACAGTGGCAAGCAGGTTGGTGTTTATTTT
ENSMMUT00000032157      AGCACTCCAGTACTGAGAAGAAACCGGATACATAGTGGCAAGCAGGTTGGTGTTTATTTT
ENSCJAT00000021472      AGCACTCCAGTACTGAGAAGAAACCGGATACACAGTGGCAAGCAGGTTGGTGTTTATTTC
ENSGGOT00000011655      AGCACTCCAGTACTGAGAAGAAACCGGATACACAGTGGCAAGCAGGTTGGTGTTTATTTT
ENST00000403359         AGCACTCCAGTACTGAGAAGAAACCGGATACACAGTGGCAAGCAGGTTGGTGTTTATTTT
ENSPPYT00000014431      AGCACTCCAGTACTGAGAAGAAACCGGATACACAGTGGCAAGCAGGTTGGTGTTTATTTT
ENSMUST00000005504      AGCACACCAGTTCTAAGAAGAAACCGGATACACAGTGGCAAGCAGGTTGGTGTTTATTTC
ENSRNOT00000021998      AGCACGCCAGTACTAAGAAGAAATCGGATACACAGTGGCAAGCAGGTCGGTGTTTATTTC

ENSPTRT00000022151      TATGACAATGGACATGGAGTGCTAGAAGACAATGATATCTATAATCATATGTATTCAGGG
ENSMMUT00000032157      TATGACAATGGACACGGAGTGCTAGAAGACAATGATATCTATAATCACATGTATTCAGGG
ENSCJAT00000021472      TATGACAATGGACATGGAGTGCTAGAAGACAATGATATCTATAATCATATGTATTCAGGG
ENSGGOT00000011655      TATGACAATGGACACGGAGTGCTAGAAGACAATGATATCTATAATCATATGTATTCAGGG
ENST00000403359         TATGACAATGGACATGGAGTGCTAGAAGACAATGATATCTATAATCATATGTATTCAGGG
ENSPPYT00000014431      TATGACAATGGACATGGAGTGCTAGAAGACAATGATATCTATAATCATATGTATTCAGGG
ENSMUST00000005504      TATGACAATGGACATGGTGTTCTAGAAGATAACGACATCTACAATCACATGTATTCAGGG
ENSRNOT00000021998      TATGACAATGGACATGGTGTTCTAGAAGATAACGATATCTACAATCATATGTATTCAGGG

ENSPTRT00000022151      GTTCAGATAAGGACTGGAAGCAACCCCAAAATCAGACGCAACAAAATCTGGGGAGGACAG
ENSMMUT00000032157      GTTCAGATAAGGACTGGAAGCAACCCCAAAATTAGACGCAACAAAATCTGGGGAGGACAG
ENSCJAT00000021472      GTTCAGATAAGGACTGGAAGCAACCCCAAAATTAGACGCAACAAAATATGGGGAGGACAG
ENSGGOT00000011655      GTTCAGATAAGGACTGGAAGCAACCCCAAAATTAGACGCAACAAAATCTGGGGAGGACAG
ENST00000403359         GTTCAGATAAGGACTGGAAGCAACCCCAAAATTAGACGCAACAAAATCTGGGGAGGACAG
ENSPPYT00000014431      GTTCAGATAAGGACTGGAAGCAACCCCAAAATTAGACGCAACAAAATCTGGGGAGGACAG
ENSMUST00000005504      GTTCAGATAAGAACTGGAAGCAACCCCAAAATTAGACGCAACAAAATTTGGGGAGGACAG
ENSRNOT00000021998      GTTCAGATAAGGACTGGAAGCAACCCCAAAATTAGAAGAAACAAAATTTGGGGAGGACAA

ENSPTRT00000022151      AATGGTGGAATTCTAGTTTATAATTCTGGTCTAGGCTGTATAGAAGACAATGAAATATTT
ENSMMUT00000032157      AATGGTGGAATTCTAGTTTATAATTCTGGTCTAGGCTGTATAGAAGACAATGAAATATTT
ENSCJAT00000021472      AATGGTGGAATTCTAGTTTATAATTCCGGTCTAGGCTGTATAGAAGACAATGAAATATTT
ENSGGOT00000011655      AATGGTGGAATTCTAGTTTATAATTCTGGTCTAGGCTGTATAGAAGACAATGAAATATTT
```

ENST00000403359	AATGGTGGAATTCTAGTTTATAATTCTGGTCTAGGCTGTATAGAAGACAATGAAATATTT
ENSPPYT00000014431	AATGGTGGAATTCTAGTTTATAATTCTGGTCTAGGCTGTATAGAAGACAATGAAATATTT
ENSMUST00000005504	AATGGTGGAATTCTAGTTTATAATTCTGGTCTAGGCTGTATAGAAGACAATGAAATATTT
ENSRNOT00000021998	AATGGTGGAATTCTAGTTTATAATTCTGGTCTAGGCTGTATAGAAGACAATGAAATCTTC
ENSPTRT00000022151	GACAATGCAATGGCTGGAGTCTGGATTAAGACAGATAGTAATCCTACACTAAGAAGAAAT
ENSMMUT00000032157	GACAATGCAATGGCTGGAGTCTGGATTAAGACAGATAGTAATCCTACACTAAGAAGAAAT
ENSCJAT00000021472	GACAATGCAATGGCTGGAGTCTGGATTAAGACAGACAGTAATCCCACACTAAGAAGAAAT
ENSGGOT00000011655	GACAATGCAATGGCTGGAGTCTGGATTAAGACAGATAGTAATCCTACACTAAGAAGAAAT
ENST00000403359	GACAATGCAATGGCTGGAGTCTGGATTAAGACAGATAGTAATCCTACACTAAGAAGAAAT
ENSPPYT00000014431	GACAATGCAATGGCTGGAGTCTGGATTAAGACAGATAGTAATCCTACACTAAGAAGAAAT
ENSMUST00000005504	GACAACGCGATGGCTGGAGTCTGGATTAAAACAGATAGTAATCCTACGCTAAGAAGAAAT
ENSRNOT00000021998	GACAACGCAATGGCTGGCGTCTGGATTAAAACAGACAGCAATCCTACGCTGAGACGGAAT
ENSPTRT00000022151	AAAATCCATGATGGAAGAGATGGTGGCATCTGTATATTTAATGGGGGTCGAGGTCTCCTT
ENSMMUT00000032157	AAAATCCATGATGGAAGAGATGGTGGCATCTGTATATTTAATGGGGGTCGAGGTCTCCTT
ENSCJAT00000021472	AAAATCCATGATGGAAGAGATGGTGGCATCTGTATATTTAATGGGGGTCGAGGTCTCCTT
ENSGGOT00000011655	AAAATCCATGATGGAAGAGATGGTGGCATCTGTATATTTAATGGGGGTCGAGGTCTCCTT
ENST00000403359	AAAATCCATGATGGAAGAGATGGTGGCATCTGTATATTTAATGGGGGTCGAGGTCTCCTT
ENSPPYT00000014431	AAAATCCATGATGGAAGAGATGGTGGCATCTGTATATTTAATGGGGGTCGAGGTCTCCTT
ENSMUST00000005504	AAAATCCATGATGGAAGAGATGGTGGCATCTGTATATTTAATGGGGGTCGAGGTCTCCTT
ENSRNOT00000021998	AAAATCCACGATGGAAGGGATGGCGGCATCTGTATATTTAATGGGGGTCGAGGTCTCCTT
ENSPTRT00000022151	GAAGAAAATGATATTTTCAGGAATGCTCAAGCAGGTGTTCTCATCAGCACTAATAGTCAT
ENSMMUT00000032157	GAAGAAAATGATATTTTCAGGAATGCTCAAGCAGGTGTTCTCATCAGCACTAATAGTCAT
ENSCJAT00000021472	GAAGAAAATGACATTTTCAGGAATGCTCAAGCAGGTGTTCTCATCAGCACTAATAGTCAC
ENSGGOT00000011655	GAAGAAAATGATATTTTCAGGAATGCTCAAGCAGGTGTTCTCATCAGCACTAATAGTCAT
ENST00000403359	GAAGAAAATGATATTTTCAGGAATGCTCAAGCAGGTGTTCTCATCAGCACTAATAGTCAT
ENSPPYT00000014431	GAAGAAAATGATATTTTCAGGAATGCTCAAGCAGGTGTTCTCATCAGCACTAACAGTCAT
ENSMUST00000005504	GAAGAAAATGATATTTTCAGGAACGCTCAAGCAGGTGTCCTCATCAGCACTAACAGTCAT
ENSRNOT00000021998	GAAGAAAATGATATTTTCAGGAACGCTCAAGCAGGTGTTCTTATCAGCACCAACAGTCAT
ENSPTRT00000022151	CCAATCTTAAGGAAAAACAGAATATTTGATGGATTTGCTGCAGGTATTGAAATTACAAAT
ENSMMUT00000032157	CCAATCTTAAGGAAAAACAGAATATTTGATGGATTTGCTGCAGGTATTGAAATTACAAAT
ENSCJAT00000021472	CCAATCTTAAGGAAAAACAGAATATTTGATGGATTTGCTGCAGGTATTGAAATTACAAAT
ENSGGOT00000011655	CCAATCTTAAGGAAAAACAGAATATTTGATGGATTTGCTGCAGGTATTGAAATTACAAAT
ENST00000403359	CCAATCTTAAGGAAAAACAGAATATTTGATGGATTTGCCGCAGGTATTGAAATTACAAAT
ENSPPYT00000014431	CCAATCTTAAGGAAAAACAGAATATTTGATGGATTTGCTGCAGGTATTGAAATTACAAAT
ENSMUST00000005504	CCAGTATTAAGGAAAAACAGAATATTTGATGGATTTGCTGCAGGTATTGAAATTACAAAT
ENSRNOT00000021998	CCAGTCTTAAGAAAAAACAGAATATTCGATGGATTTGCTGCAGGTATTGAAATTACAAAT
ENSPTRT00000022151	CACGCAACTGCAACACTAGAAGGCAATCAGATTTTTAACAACCGGTTTGGAGGCTTATTT
ENSMMUT00000032157	CACGCAACTGCAACACTAGAAGGCAATCAGATTTTTAACAACCGATTTGGAGGCTTATTT
ENSCJAT00000021472	CACGCAACTGCAACACTAGAAGGCAATCAGATTTTTAACAACCGGTTTGGAGGCTTATTT
ENSGGOT00000011655	CACGCAACTGCAACACTAGAAGGCAATCAGATTTTTAACAACCGATTTGGAGGCTTATTT
ENST00000403359	CACGCAACTGCAACACTAGAAGGCAATCAGATTTTTAACAACCGGTTTGGAGGCTTATTT
ENSPPYT00000014431	CACGCAACTGCAACACTAGAAGGCAATCAGATTTTTAATAACCGGTTTGGAGGCTTATTT
ENSMUST00000005504	CATGCAACTGCAACACTCGAAGGCAATCAGATCTTTAACAACCGATTTGGAGGCTTATTT
ENSRNOT00000021998	CATGCAACTGCAACACTAGAAGGCAATCAGATTTTTAACAACAGATTTGGAGGCTTATTT
ENSPTRT00000022151	TTAGCATCTGGTGTTAATGTGACAATGAAAGATAACAAAATAATGAACAATCAAGATGCC
ENSMMUT00000032157	TTAGCATCTGGTGTTAATGTGACAATGAAAGATAACAAAATAATGAACAATCAAGATGCC
ENSCJAT00000021472	TTAGCATCTGGTGTTAATGTGACAATGAAAGATAACAAAATAATGAACAATCAGGATGCC
ENSGGOT00000011655	TTAGCATCTGGTGTTAATGTGACAATGAAAGATAACAAAATAATGAACAATCAAGATGCC

```
ENST00000403359       TTAGCATCTGGTGTTAATGTGACAATGAAAGATAACAAAATAATGAACAATCAAGATGCC
ENSPPYT00000014431    TTAGCATCTGGGGTTAATGTGACAATGAAAGATAACAAAATAATGAACAACCAAGATGCC
ENSMUST00000005504    TTAGCATCTGGTGTTAATGTGACAATGAAAGATAACAAAATAATGAACAATCAAGATGCC
ENSRNOT00000021998    TTAGCATCTGGTGTTAACGTGACAATGAAAGATAACAAGATAATGAACAATCAAGATGCC

ENSPTRT00000022151    ATAGAAAAGGCTGTTAGTAGAGGCCAATGTTTATATAAAATATCAAGTTATACCAGCTAT
ENSMMUT00000032157    ATAGAAAAGGCTGTTAGTAGAGGCCAATGTTTATATAAAATATCAAGTTATACCAGCTAT
ENSCJAT00000021472    ATAGAAAAGGCTGTTAGTAGAGGCCAGTGTTTATATAAAATATCAAGTTATACCAGCTAT
ENSGGOT00000011655    ATAGAAAAGGCTGTTAGTAGAGGCCAATGTTTATATAAAATATCAAGTTATACCAGCTAT
ENST00000403359       ATAGAAAAGGCTGTTAGTAGAGGCCAATGTTTATATAAAATATCAAGTTATACCAGCTAT
ENSPPYT00000014431    ATAGAAAAGGCTGTTAGTAGAGGCCAATGTTTATATAAAATATCAAGTTATACCAGCTAT
ENSMUST00000005504    ATAGAAAAGGCTGTTAGTAGAGGACAATGTCTATATAAAATATCAAGTTATACCAGCTAC
ENSRNOT00000021998    ATAGAAAAGGCTGTTAGTAGAGGACAATGTTTATATAAAATATCAAGTTATACCAGCTAT

ENSPTRT00000022151    CCCATGCATGATTTCTACAGATGTCATACTTGTAACACCACAGATCGAAATGCCATATGT
ENSMMUT00000032157    CCCATGCATGATTTCTACAGATGTCATACTTGTAACACCACAGATCGAAATGCCATATGT
ENSCJAT00000021472    CCCATGCATGATTTCTACAGATGTCACACTTGTAACACCACAGATCGAAATGCCATATGT
ENSGGOT00000011655    CCCATGCATGATTTCTACAGATGTCATACTTGTAACACCACAGATCGAAATGCCATATGT
ENST00000403359       CCCATGCATGATTTCTACAGATGTCATACTTGTAACACCACAGATCGAAATGCCATATGT
ENSPPYT00000014431    CCCATGCATGATTTCTACAGATGTCATACTTGTAACACCACAGATCGAAATGCCATATGT
ENSMUST00000005504    CCCATGCATGACTTCTACAGATGTCATACTTGTAACACCACAGATCGAAATGCCATATGT
ENSRNOT00000021998    CCCATGCATGACTTCTACAGATGTCATACTTGTAACACCACAGATCGAAATGCCATATGT

ENSPTRT00000022151    GTGAACTGCATTAAGAAGTGCCATCAGGGACATGATGTAGAGTTTATTAGACATGATAGG
ENSMMUT00000032157    GTGAACTGCATTAAGAAGTGCCATCAGGGACATGATGTAGAGTTTATTAGACATGATAGG
ENSCJAT00000021472    GTGAACTGCATTAAGAAGTGCCATCAGGGACATGATGTAGAGTTTATTAGACATGATAGG
ENSGGOT00000011655    GTGAACTGCATTAAGAAGTGCCATCAGGGACATGATGTAGAGTTTATTAGACATGATAGG
ENST00000403359       GTGAACTGCATTAAGAAGTGCCATCAGGGACATGATGTAGAGTTTATTAGACATGATAGG
ENSPPYT00000014431    GTGAACTGCATTAAGAAGTGCCATCAGGGACATGACGTAGAGTTTATTAGACATGATAGG
ENSMUST00000005504    GTGAACTGCATTAAGAAGTGCCATCAGGGACATGATGTAGAGTTCATTAGACATGATAGG
ENSRNOT00000021998    GTGAACTGCATTAAGAAGTGCCATCAGGGACATGATGTAGAGTTCATTAGACATGATAGG

ENSPTRT00000022151    TTTTTCTGTGACTGTGGTGCTGGAACACTGTCTAATCCTTGTACATTAGCTGGTGAGCCT
ENSMMUT00000032157    TTTTTCTGTGACTGTGGTGCTGGAACACTGTCTAATCCTTGTACATTAGCTGGTGAGCCT
ENSCJAT00000021472    TTTTTCTGTGACTGTGGTGCTGGAACACTGTCTAATCCTTGTACATTAGCTGGTGAGCCT
ENSGGOT00000011655    TTTTTCTGTGACTGTGGTGCTGGAACACTGTCTAATCCTTGTACATTAGCTGGTGAGCCT
ENST00000403359       TTTTTCTGTGACTGTGGTGCTGGAACACTGTCTAATCCTTGTACATTAGCTGGTGAGCCT
ENSPPYT00000014431    TTTTTCTGTGACTGTGGTGCTGGAACACTGTCTAATCCTTGTACATTAGCTGGTGAGCCT
ENSMUST00000005504    TTTTTCTGTGACTGTGGTGCTGGAACGCTGTCTAATCCTTGTACCTTAGCTGGCGAGCCT
ENSRNOT00000021998    TTTTTCTGTGACTGTGGTGCTGGAACACTGTCCAATCCTTGTACATTAGCTGGTGAGCCA

ENSPTRT00000022151    ACACATGATACAGATACACTATATGACTCTGCTCCACCTATAGAATCTAATACATTGCAG
ENSMMUT00000032157    ACACATGATACAGATACACTATATGACTCTGCTCCACCTATAGAATCTAATACATTGCAG
ENSCJAT00000021472    ACACATGATACAGATACACTATATGACTCTGCTCCACCTATAGAATCAAATACATTGCAG
ENSGGOT00000011655    ACACATGATACAGATACACTATATGACTCTGCTCCACCTATAGAATCTAATACATTGCAG
ENST00000403359       ACACATGATACAGATACACTATATGACTCTGCTCCACCTATAGAATCTAATACATTGCAG
ENSPPYT00000014431    ACACATGATACAGATACACTATATGACTCTGCTCCACCTATAGAATCTAATACATTGCAG
ENSMUST00000005504    ACACACGACACAGACACACTCTATGACTCTGCCCCACCTATAGAATCTAATACGTTGCAG
ENSRNOT00000021998    ACACATGATACAGACACACTATATGACTCTGCCCCACCTATAGAGTCTAATACGTTGCAG

ENSPTRT00000022151    CACAAC
ENSMMUT00000032157    CACAAC
ENSCJAT00000021472    CACAAC
ENSGGOT00000011655    CACAAC
```

```
ENST00000403359        CACAAC
ENSPPYT00000014431     CACAAC
ENSMUST00000005504     CACAAC
ENSRNOT00000021998     CACAAC
```

Multiple sequence alignment of Fbxo15

```
ENSMUST00000037718     ATGCCATCGGAAATCTTGGTGAAGATACTTTCTTACTTGGATGCGGTGACCTTGGTGTGC
ENSRNOT00000058199     ATGCCCTCTGAAATCTTACTGAATATATTTTCTTACTTGGATGTGGTATCCTTGCTGTGC
ENSCJAT00000006055     ATGCCTTCAGAAATCTTGCTGAAGATATTTTCCTACTTGGATGCTGTGAGCCTGCTGTGT
ENSMMUT00000032776     ATGCCTTCAGAAATCTTGCTGAAGATATTTTCCTACTTGGATGCTGTGAGCCTGCTGTGT
ENSPPYT00000010778     ATGCCTTCAGAAATCTTGCTGAAGATATTTTCCTACTTGGATGCTGTGAGCCTTCTGTGT
ENST00000269500        ATGCCTTCAGAAATCTTGCTGAAGATATTTTCCTACTTGGATGCTGTGAGCCTTCTGTGT
ENSGGOT00000013533     ATGCCTTCAGAAATCTTGCTGAAGATATTTTCCTACTTGGATGCTGTGAGCCTTCTGTGT

ENSMUST00000037718     ATTGGATGTGTGAGCAGACGCTTTTATCATTTGGCTGATGACAATCTTATTTGGGTCAGG
ENSRNOT00000058199     GTTGGATGTGTGAACAGACGCTTTTATCATTTGACCAGTGACAATCTTATTTGGGTCAAA
ENSCJAT00000006055     ACTGGATGTGTGAGCAGGCGCTTTTATCATCTAGCCAATGACAATTTTATTTGGATCAGA
ENSMMUT00000032776     GCTGGATGTGTGAGCAGGCGCTTTTACCATCTAGCCAATGACAATTTTATTTGGATCAGA
ENSPPYT00000010778     ACTGGATGTGTGAGCAGGCGCTTTTATCATCTAGCCAATGACAATTTTATTTGGATTGGA
ENST00000269500        ACTGGATGTGTGAGCAGGCGCTTTTATCATCTAGCCAATGACAATTTTATTTGGATCGGA
ENSGGOT00000013533     ACTGGATGTGTGAGCAGGCGCTTTTATCATCTAGCCAATGACAATTTTATTTGGATCAGA

ENSMUST00000037718     AAGTACGCAGCTGCATTTAGATCAAAAAGATCACGTTGGAAAGCTACTTCAGTGGAGGAA
ENSRNOT00000058199     ATCTACTCAACTGCATTTTCATCAAAAAGACGATATTGGAAGCTTACTTCCGTGGAGGAA
ENSCJAT00000006055     ATCTACTCAACTGCTTTTTCATCTACAAGATCAAATCGGAAAGTTAGTTCAGTACAGAAG
ENSMMUT00000032776     ATCTACTCAACCGCTTTTTCACCTACAAGATCAAATTGGAAAGTTAATTCAGTAGAGAAG
ENSPPYT00000010778     ATCTACTCAACTGCTTTTTCACCTACAAGATCAAATTGGAAAGTTAATTCAGTAGAGAAG
ENST00000269500        ATCTACTCAACTGCTTTTTCACCTGCAAGATCAAATTGGAAATTTAATTCAGTAGAGAAG
ENSGGOT00000013533     ATCTACTCAACTGCTTTTTCACCTACAAGATCAAATTGGAAATTTAATTCAGTAGAGAAG

ENSMUST00000037718     ACAGCCACAAGTCTGAGCTTGCTGTCAGTTTGGGATAAAGAAGATGGATACTGGAAGAAA
ENSRNOT00000058199     ACAGCCACATGTGTGAGCTTACTGTCAGTTGAGGATAAAGAAGTTGGATACTGGAAGAAA
ENSCJAT00000006055     ATAGCTACGTCTATGAGCTTTCTGTCAGTTCAGAATAAAGAAGCTGGTTATTGGAAGAAA
ENSMMUT00000032776     ATAGCTGTGTCTGTGAGCTTTCTGTCAGTTCAGGATAAAGAAGCTGGTTATTGGAAGAAA
ENSPPYT00000010778     ATAGCTATGTCTATGAGCTTTCTGTCAGTTCAGGATAAAGAAGCTGGTTATTGGAAGAAA
ENST00000269500        ATAGCTATGTCTATGAGCTTTCTGTCAGTTCAGGATAAAGAAGCTGGTTATTGGAAGAAA
ENSGGOT00000013533     ATAGCTATGTCTATGAGCTTTCTGTCAGTTCAGGATAAAGAAGCTGGTTACTGGAAGAAA

ENSMUST00000037718     GAATATATTACAAAGCAGATCTCATCTGTGAAAGCAGCCCTCACCAACAGCCTCAGTCCT
ENSRNOT00000058199     GAATATATCACAAGGCAGATCTCATCTTTGAAATCAGCACTTGCCCACATTATCATGCCT
ENSCJAT00000006055     GAATATATCACAAAACAAATAGCATCTGTAAAAGCTGCACTAGCCGACATTCTCAAACCT
ENSMMUT00000032776     GAATATATCACAAAACAAATAGCATCTGTAAAAGCCGCACTAGCTGACATTCTCAAACCT
ENSPPYT00000010778     GAATATATCACAAAACAAATAGCATCTGTAAAAGCCGCACTAGCTGACATTCTCAAACCT
ENST00000269500        GAATATATCACAAAACAAATAGCATCTGTAAAAGCCGCACTAGCTGACATTCTCAAACCT
ENSGGOT00000013533     GAATATATCACAAAACAAATAGCATCTGTAAAAGCCGCACTAGCTGACATTCTCAAACCT

ENSMUST00000037718     GTCAAACGCCGCACAAGCCTTCCTTCGAAAACCAAAGAGTCCCTCAGAATATCTGGCTTA
ENSRNOT00000058199     GTCAACCCTTACACAGGTCTTCCTGTGAAAACCAAAGAGGCCCTCAGAGTATTTGGCTTA
ENSCJAT00000006055     GTTAACCCTTATACAGGCCTTCCAGTTAAGACCAAAGAGGCCCTCAGAATATTTGGTTTA
ENSMMUT00000032776     GTCAACCCTTACACAGGCCTTCCAGTTAAGACCAAAGAGGCCCTCAGAATGTTTGGTTTA
ENSPPYT00000010778     GTCAACCCTTACACAGGCCTTCCAGTTAAGACCAAAGAGGCCCTCAGAATATTTGGTTTA
ENST00000269500        GTCAACCCTTACACAGGCCTTCCAGTTAAGACCAAAGAGGCCCTCAGAATATTTGGTTTA
ENSGGOT00000013533     GTCAACCCTTACACAGGCCTTCCAGTTAAGACCAAAGAGGCCCTCAGAATATTTGGTTTA
```

```
ENSMUST00000037718     GGTTGGACAATCATCTTAAGAGAAGCCAGTGGCAAAGAACACATCATGCAGCATTCGAAT
ENSRNOT00000058199     GGTTGGGCAATTATCTTAAGAGAAAAAAATGGAAAAGAATACATCATGCGGCATGCTGAT
ENSCJAT00000006055     GGTTGGGCAATTATACTGAAAGAAAAAAGTGGAAAAGAACATATCATGGAGCATGTGGAT
ENSMMUT00000032776     GGTTGGGCAATTATACTGAAAGAAAAAAGTGGAAAAGAATATATCATGGAGCATGTTGAT
ENSPPYT00000010778     GGTTGGGCAATTATACTCAAAGAAAAAAATGGAAAAGAATATATCATGGAGCATGCTGAT
ENST00000269500        GGTTGGGCAATTATACTGAAAGAAAAAGGTGGAAAAGAATATATCATGGAGCATGTTGAT
ENSGGOT00000013533     GGTTGGGCAATTATACTGAAAGAAAAAAGTGGAAAAGAATATATCATGGAGCATGTTGAT

ENSMUST00000037718     CTTTCCGTAAATGACAACTCTGTCACTGTTTTTTGGCATGACAAAAATTGGCCACATGTA
ENSRNOT00000058199     CATTCTGTAAATGATACATCTGTCACAGTTGTGTGGTATGACAAAAATTGGCCACATTTA
ENSCJAT00000006055     CTTTCCATAAATGACACCTCAGTTACTGTTATATGGTATGGCAAAAATTGGCCGTGCCTA
ENSMMUT00000032776     CTTTCCGTAAATGACACATCAGTTACTGTTATATGGTATGGCAAAAACTGGCCGTGCCTA
ENSPPYT00000010778     CTTTCCATAAATGACACATCAGTTACTGTTATATGGTATGGCAAAAAATGGCCGTGCCTA
ENST00000269500        CTTTCCATAAATGACACATCAGTTACTGTTATATGGTATGGCAAAAAATGGCCATGCCTA
ENSGGOT00000013533     CTTTCCATAAATGACACATCAGTTACTGTTATATGGTATGGCAAAAAATGGCCGTGCCTA

ENSMUST00000037718     GACACGTTGTCCACCCTGGATTTGTATGGTGCCACACCAATTTTTATGGAGCAGTATAAA
ENSRNOT00000058199     GCCACATTGTCCACCCTGAATTTGTATGGTGTGACACCACTTTTTATGGACCAGTATTTA
ENSCJAT00000006055     GCATCATTGTCAACTTTAGATTTATGTGGTGTGACACCAGTTTTTATGGACTGGTATAAA
ENSMMUT00000032776     GCGTCATTGTCAACCTTAGATTTATGTGGTGTGACACCAGTTTTTATGGACTGGTATAAA
ENSPPYT00000010778     GCATCATTGTCAACCTTAGATTTATGTGGCATGACACCAGTTTTTACAGACTGGTATAAA
ENST00000269500        GCATCATTGTCAACCTTAGATTTATGTGGCATGACACCAGTTTTTACCGACTGGTATAAA
ENSGGOT00000013533     GCATCATTGTCAACCTTAGATTTATGTGGCATGACACCAGTTTTTACCGACTGGTATAAA

ENSMUST00000037718     GGCCCTAACACAAGTTGTCCACGATGGCTGTCTTTAATTGAAAAGTACGATCTGAGTAAT
ENSRNOT00000058199     ACCCCTGATCCAAACAGTCCACGATGGCTGTCTTTAATTGGAAAGTATGACCTGAGTAAT
ENSCJAT00000006055     ACTCCCACCAAACATAGACTCCGATGGCATTCTTTAATTGCAAAGTACAATTTGAGTCAT
ENSMMUT00000032776     ACTCCCACCAAACATAGACTCCGATGGCATTCTTTAATTGCAAAGTACAATCTGAGTCAT
ENSPPYT00000010778     ACTCCCACCAAACATAGACTCCGATGGCATTCTTTAATTGCAAAGTACGATCTGAGTCAT
ENST00000269500        ACTCCCACCAAACATAGACTCCGATGGCATTCTTTAATTGCAAAGTACAATCTGAGTCAT
ENSGGOT00000013533     ACTCCCACCAAACATAGACTCCGATGGCATTCTTTAATTGCAAAGTACAATCTGAGTCAT

ENSMUST00000037718     TTACGCAAGTCTGCTATGATTGGCTGCGACAGACATGTTCGGGTATTCTGTGTAAATCCT
ENSRNOT00000058199     TTAAGTGAGTCTACTATGATGGGCTGCGACGGACTTATTCGGATATTCTGCCTAAATCCT
ENSCJAT00000006055     TTGACTGAATCTACCATGATTGGCTGTGACAGACTTATTCGGATCTTCTGCCTGCACCCT
ENSMMUT00000032776     TTGACCGTATCTACCATGATTGGCTGTGACAGACTCATTCGGATCTTCTGCCTGCACCCT
ENSPPYT00000010778     TTGACTGTATCTACCATGATTGGCTGTGACAGACTCATTCGGATCTTCTGCCTGCACCCT
ENST00000269500        TTGACCATATCTACCATGATTGGCTGTGACAGACTCATTCGGATCTTCTGCCTGCACCCT
ENSGGOT00000013533     TTGACCGTATCTACCATGATTGGCTGTGACAGACTCATTCGGATCTTCTGCCTGCACCCT

ENSMUST00000037718     GGCCTCCTGGTGGGGCTGTGGCAGGAGAATGGTGGACTAGCTTTTGTCATGGCAAATATT
ENSRNOT00000058199     GGCCTCCTAGTGGGGCTGTGGCAGAAGGAGGACAATCTAGCTTTTGTCATGGCAAATCTT
ENSCJAT00000006055     GGCCTCCTGGTTGGACTCTGGAAGAAGGAGGAAGAACTGGCTTTTCTTATGGCAAATCTT
ENSMMUT00000032776     GGCCTCCTGGTGGGAGTGTGGAAGAAGGAGGAAGAATTGGCTTTTGTTATGGCAAATCTT
ENSPPYT00000010778     GGCCTCCTGGTGGGAGTGTGGAAGAAGGAGGAAGAACTGGCTTTTGTTATGGCAAATCTT
ENST00000269500        GGCCTCCTGGTGGGAGTGTGGAAGAAGGAGGAAGAACTGGCTTTTGTTATGGCAAATCTT
ENSGGOT00000013533     GGCCTCCTGGTGGGAGTGTGGAAGAAGGAGGAAGAACTGGCTTTTGTTATGGCAAATCTT

ENSMUST00000037718     CATTCCCATGGCCTTTTCGAGAGAAGCATAATGGGCTCAGACACTATTCCCTATACATTG
ENSRNOT00000058199     CATTTCCATCACCTTGTGGAGAGGAGCACATTGGGATCAGCCACTTTGCCTTCCATGCTC
ENSCJAT00000006055     CACTTCCATCACCTTGTGGAGAGGAGCACATTAGGCTCGGCCACTATCCCCTATGAACTG
ENSMMUT00000032776     CATTTTCATCACCTTGTGGAGAGAAGCACATTAGGCTCGGCCACTATTCCCTATGAACTA
```

```
ENSPPYT00000010778     CATTTTCATCACCTTGTGGAGAGGAGCACATTAGGCTCGGCCACTATCCCCTATGAACTG
ENST00000269500        CATTTTCATCACCTTGTGGAGAGGAGCACATTAGGCTCGGCTACTATCCCCTATGAACTG
ENSGGOT00000013533     CATTTTCATCACCTTGTGGAGAGGAGCACATTAGGCTCGGCTACTATCCCCTATGAACTG

ENSMUST00000037718     CCTCCCGACACTACATTTGTGGATAACTACCCAGACTATGGAGATAAAGGCTTTCAGCTG
ENSRNOT00000058199     CCCTCGCATTCACCTCTGTGTTATTGGTTTCCTAGGTATGGTCAAATTGTTTATCAGCTT
ENSCJAT00000006055     CCTCCACATAGCCCCTTTTTGGATGACAGTCCTGAGTATGGACTGCACGGCTACCAGCTC
ENSMMUT00000032776     CCTCCACATAGCCCGTTTTTGGATGATAGCCCCGAGTATGGACTGCACGGCTACCAACTC
ENSPPYT00000010778     CCTCCACATAGCCCCTTTCTGGATGATGGCCCCGAGTATGGATTGCACGGCTACCAACTC
ENST00000269500        CCTCCACATAGCCCCTTTTTGGATGATAGCCCCGAGTATGGACTGCACGGCTACCAACTC
ENSGGOT00000013533     CCTCCACACAGCCCCTTTTTGGATGATAGCCCCGAGTATGGACTGCACGGCTACCAACTC

ENSMUST00000037718     CATATCGACATTCATGGCAGTAAGACTTACTTCCTGTGTAGCACCTTCCACAATCTCTTC
ENSRNOT00000058199     TACATCGATATTCATAAGGTTAGCATTTTTAGCCTCTGTTCCACATTTCGAGTGTTATTC
ENSCJAT00000006055     CATGTAGATCTGCACAGCGGTGGGGTTTTCTACTTGTGTGGTACATTTCGAAATCTCTTC
ENSMMUT00000032776     CATGTTGATCTGCACAGTGGTGGAGTTTTCTACCTGTGTGGTACATTTCGCAATCTCTTC
ENSPPYT00000010778     CATGTTGATCTGCACAGCGGTGGGGTTTTCTTCCTATGTGGTACATTTCGCAATCTCTTC
ENST00000269500        CATGTTGATCTGCACAGCGGTGGGGTTTTCTACCTATGTGGTACATTTCGCAATCTCTTC
ENSGGOT00000013533     CATGTTGATCTGCACAGCGGTGGGGTTTTCTACCTATGTGGTACATTTCGCAATCTCTTC

ENSMUST00000037718     TGCAGGAGAGCGGGCATTAACAATGGATATGTGAAGTTCTTGATGATAAACTTAAAAAAT
ENSRNOT00000058199     CACCGTAGAGAGTGCATTGAGAATGGATATGTGAAGTTCGTGGTGATAAGTTTAAAAAAT
ENSCJAT00000006055     ACCAAAAAAGGAAATATTGAAAATGGACATGTGAAGCTCATTGTTATAAATTTAAAAAAT
ENSMMUT00000032776     ACCAAGAAAGGAAATATTGAAAATGGACATGTGAAGCTCATTGTTATACATTTAAAAAAT
ENSPPYT00000010778     ACCAAGAAAGGAAATATTGAAAATGGACACGTGAAGCTCATTGTTATACATTTAAGAAAT
ENST00000269500        ACCAAGAGAGGAAATATTGAAAATGGACATGTGAAGCTCATTGTTATACATTTAAAAAAT
ENSGGOT00000013533     ACCAAGAGAGGAAATATTGAAAATGGACACGTGAAGCTCATTGTTATACATTTAAAAAAT

ENSMUST00000037718     AACAGAGAACACCTACCTCTTGTTGGAAAAGTTGGCCTTGAATGGAGAACTGACTGTTTA
ENSRNOT00000058199     AACAGAGAACACCTACCTCTTATTGGAAAAGTTGGCCTTGCATGGAGAACTAATGTTTTT
ENSCJAT00000006055     AACAGAGAACACCTGCCTCTTATTGGAAAAGTTGGCCTCTCATGGAAAACTGATATTTTT
ENSMMUT00000032776     AACAGAGAACACCTACCTCTTATTGGAAAAGTTGGCCTCTCGTGGAAAACTGATATTTTG
ENSPPYT00000010778     AACAGAGAACACCTACCTCTTATTGGAAAAGTTGGCCTCTCGTGGAAAACTGATATTTTT
ENST00000269500        AACAGAGAACACCTACCTCTTATTGGAAAAGTTGGCCTCTCGTGGAAAACTGATATTTTT
ENSGGOT00000013533     AACAGAGAACACCTACCTCTTATTGGAAAAGTTGGCCTCTCGTGGAAAACTGATATTTTT

ENSMUST00000037718     AATGGCCGTATTGAGAGTTGCATTGTAGTGGATATGACCTTGCTGGATGAGGACAAGAAG
ENSRNOT00000058199     GATGGCTTTATTGAGAGTTGCTTTGTAGTGGATGTGACCCTTCTGGATGAGCACAGGAAG
ENSCJAT00000006055     GATGGCTGTATAAAGAGTTGTTCTATGATGGACGTAACTCTTTTGGATGAACATGGGAGA
ENSMMUT00000032776     GATGGCTGTATAAAGAGTTGTTCCATGATGGACGTGACTCTTTTGGATGAACATGGGAAA
ENSPPYT00000010778     GATGGCTGTATAAAGAGTTGTTCCATGATGGACATAACTCTTTTGGATGAACATGGGAAA
ENST00000269500        GATGGCTGTATAAAGAGTTGTTCCATGATGGACGTAACTCTTTTGGATGAACATGGGAAA
ENSGGOT00000013533     GATGGCTGTATAAAGAGTTGTTCCATGATGGACGTAACTCTTTTGGATGAACATGGGAAA

ENSMUST00000037718     CCCATCTGGTATGTGAGTTCTCCAGTGTGCTTGAGATCTGCCTGCCTTCCT---------
ENSRNOT00000058199     CCCTTTTGGTGTGTGAGTTCTCCAGTATGCATGAGATCTCCTGCCTACCCCTCCGATGGT
ENSCJAT00000006055     CCCTTTTGGTGTTTCAGTTCCCCAGTGTGCATGAGATTGCCTGCCACACCCTCTGACGGC
ENSMMUT00000032776     CCCTTTTGGTGTTTCAGTTCCCCGGTGTGCATGAGATCGCCTGCCACACCCGCTGATGGC
ENSPPYT00000010778     CCCTTTTGGTGTTTCAGTTCCCCGGTGTGCATGAGATCGCCTGCCACACCCTCTGACAGC
ENST00000269500        CCCTTTTGGTGTTTCAGTTCCCCGGTGTGCCTGAGATCGCCTGCCACACCCTCTGACAGC
ENSGGOT00000013533     CCCTTTTGGTGTTTCAGTTCCCCGGTGTGCCTGAGATCGCCTGCCACACCCTCTGACAGC

ENSMUST00000037718     ---GATTTCCCGCAGCCGGCTTACTCTTTCGAGTACATGGACAGCGTAGGAGGAGTGTGC
```

```
ENSRNOT00000058199     TCTAGTTTCCTGGGGAACACTTACTATGTCGACTACATGGACAAAGAAGGAGGAGTGCAC
ENSCJAT00000006055     CCTAGCCTCTTGGGACAGACGTACAGCGTGGACTATGTCGATGCAGAGGGAAGAGTGCAC
ENSMMUT00000032776     CCTAGCTTCCTGGGACAGACGTACAGCGTGGACTACGTCGATGCGGAAGGAAGAGTGCAC
ENSPPYT00000010778     CCTAGCTTCTTGGGACAGACATACAACGTGGACTACGTTGATGCGGAAGGAAGAGTGCAC
ENST00000269500        TCTAGCTTCTTGGGACAGACATACAACGTGGACTACGTTGATGCGGAAGGAAGAGTGCAC
ENSGGOT00000013533     TCTAGCTTCTTGGGACAGACATACAACGTGGACTACGTTGATGCGGAAGGAAGAGTGCAC


ENSMUST00000037718     GCAGACCTAGGGTGGTTTGAAAATACCGATGAATACTTCATTGTCAGACTGGACATTTAC
ENSRNOT00000058199     GCAGAGCTAGTGTGGATCGAAGAGACTGAAGAGTACTTCATTGTCAGCCTGGCCCTTTAC
ENSCJAT00000006055     GTGGAGCTGGTGTGGATCAGAGAGACCGAAGAATACTTTATTGTCAGCCTGGTCCTTTAC
ENSMMUT00000032776     GTGGAGCTGGTGTGGATCAGAGAGACCGAAGAATACCTTATTGTCAACCTGGTCCTTTAC
ENSPPYT00000010778     GTGGAGCTGGTGTGGATCAGAGAAACCGAAGAATACCTTATTGTCAACCTGGTCCTTTAC
ENST00000269500        GTGGAGCTGGTGTGGATCAGAGAGACCGAAGAATACCTTATTGTCAACCTGGTCCTTTAT
ENSGGOT00000013533     GTGGAGCTGGTGTGGATCAGAGAGACCGAAGAATACCTTATTGTCAACCTGGTCCTTTAC


ENSMUST00000037718     CTCAGTGTAGCAAAATTACAACAATGGTTTGGGAGGCAA---
ENSRNOT00000058199     CTCAGTGTAGCAAAAATAAACCATTGGTTTGGGACAAAATAC
ENSCJAT00000006055     CTTAGTGTTGCAAAAATCAACCACTGGTTTGGAACTGAATAT
ENSMMUT00000032776     CTTAGTGTCGCAAAAATCAACCGTTGGTTTGGGACTGAATAT
ENSPPYT00000010778     CTTAGTATCGCAAAAATCAACCATTGGTTTGGGACTGAGTAT
ENST00000269500        CTTAGTATCGCAAAAATCAACCATTGGTTTGGGACTGAATAT
ENSGGOT00000013533     CTTAGTATCGCAAAAATCAACCATTGGTTTGGGACTGAATAT
```

Multiple sequence alignment of Fbxo16

```
ENSMUST00000169656     ATGATGGCCTTTGCACCTCCAAAAAGCATCGATGGCCCCAAAATGCAGACAAAGATGAGT
ENSRNOT00000018762     ATGATGGCATTTGCACCTCCCAAAAGCATCGATGGCCCAAAAATGCAGACAAAGATGAGT
ENSCJAT00000030340     ATGATGGCATTTGCACCTCCAAAAAACACAGATGGTCCTAAAATGCAGACAAAGATGAGC
ENSMMUT00000039082     ATGATGGCATTTGCACCTCCAAAAAACCTAGATGGTCCCAAAATGCAAACAAAGATGAGC
ENST00000346498        ATGATGGCATTTGCACCTCCAAAAAACACAGATGGTCCCAAAATGCAGACAAAGATGAGC
XM_002818951.2         ATGATGGCATTTGCACCTCCGAAAAACACAGATGGTCCCAAAATGCACACAAAGATGAGC
ENSGGOT00000016399     ATGATGGCATTTGCACCTCCAAAAAGCACAGATGGTCCCAAAATGCAGACAAAGATGAGC
ENSPTRT00000067150     ATGATGGCATTTGCACCTCCAAAAAACACAGATGGTCCCAAAATGCAGACAAAGATGAGC


ENSMUST00000169656     ACCTGGACACCTCTCAACCATCAGCTTCTGAATGACCAGGTATTTGAAGAACGAAGAGCT
ENSRNOT00000018762     ACCTGGACACCTCTAAATCATCAGCTTCTGAACGACCAGGTGTTTGAAGAACGAAGAGCG
ENSCJAT00000030340     ACTTGGACACCCTTAAACCATCAGCTCTTGAATGACAGGGTATTTGAAGAAAGAAGAGCC
ENSMMUT00000039082     ACCTGGACACCCTTAAACCATCAGCTATTGAATGACC-----------------------
ENST00000346498        ACCTGGACACCCCTAAACCATCAGCTATTGAATGACC-----------------------
XM_002818951.2         ACCTGGACACCCCTAAACCATCAGCTATTGAATGACCGGGTATTTGAAGAAAGAAGAGCC
ENSGGOT00000016399     ACCTGGACACCCCTAAACCATCAGCTATTGAATGACCGGGTATTTGAAGAAAGAAGAGCC
ENSPTRT00000067150     ACCTGGACACCCCTAAACCATCAGCTATTGAATGACCGGGTATTTGAAGAAAGAAGAGCC


ENSMUST00000169656     CTGCTGGGAAAATGGTTTGACAAATGGACAGACTCTCAAAGGAGAAGAATTCTTACAGGC
ENSRNOT00000018762     CTGCTTGGAAAATGGTTTGACAAATGGACAGACTCTCAAAGGAGAAGAATTCTTACAGGC
ENSCJAT00000030340     CTGCTTGGAAAATGGTTTGACAAGTGGACAGACTCTCAAAGAAGAAGAATCCTCACGGGC
ENSMMUT00000039082     -------------GGTTTGACAAATGGACAGACTCTCAGAGAAGAAGAATCCTCACAGGC
ENST00000346498        -------------GGTTTGACAAATGGACAGACTCTCAAAGAAGAAGAATCCTCACAGGC
XM_002818951.2         CTGCTTGGCAAATGGTTTGACAAATGGACAGACTCTCAAAGAAGAAGAATCCTCACAGGC
ENSGGOT00000016399     CTGCTTGGCAAATGGTTTGACAAATGGACAGACTCTCAAAGAAGAAGAATCCTCACAGGC
ENSPTRT00000067150     CTGCTTGGCAAATGGTTTGACAAATGGACAGACTCTCAAAGAAGAAGAATCCTCACAGGC


ENSMUST00000169656     CTACTAGAGCGCTGTTCCCTGTCCCAGCAGAAGTTCTGTTGTCGAAAGCTGCAGGAAAAA
ENSRNOT00000018762     CTGCTAGAGCGCTGCTCCCTGTCCCAGCAGAAGTTCTGTTGTCGAAAACTGCAGGAAAAA
```

```
ENSCJAT00000030340     CTGTTGGAGCTCTGCTCGCTGTCCCAGCAAAAGTTCTGCTGTCGAAAGCTTCAGGAGAAA
ENSMMUT00000039082     CTGTTGGAGCGCTGCTCGCTGTCCCAGCAAAAGTTCTGCTGTCGAAAGCTTCAAGAGAAA
ENST00000346498        CTGTTGGAGCGCTGCTCGCTGTCCCAGCAAAAGTTCTGCTGTCGAAAGCTTCAAGAGAAA
XM_002818951.2         CTGTTGGAGCGCTGCTCACTGTCCCAGCAAAAGTTCTGCTGTCGAAAGCTTCAAGAGAAA
ENSGGOT00000016399     CTGTTGGAGCGCTGCTCGCTGTCCCAGCAAAAGTTCTGCTGTCGAAAGCTTCAAGAGAAA
ENSPTRT00000067150     CTGTTGGAGCGCTGCTCGCTGTCCCAGCAAATGTTCTGCTGTCGAAAGCTTCAAGAGAAA

ENSMUST00000169656     ATCCCAGCAGAGGCCCTGGATTTTACTACCAAGCTTCCAAGGGTGTTATCTGTCTACATC
ENSRNOT00000018762     ATTCCAGCAGAGGCCCTGGACTTTACTACCAAGCTTCCGAGGGTGTTATCTGTCTACATC
ENSCJAT00000030340     ATTCCAGCAGAAGCCCTGGACTTTACGACCAAGCTTCCAAGGGTGTTATCTTTATACATC
ENSMMUT00000039082     ATTCCAGCAGAAGCCCTGGACTTTACAACCAAGCTTCCAAGGGTGTTATCTTTATACATC
ENST00000346498        ATTCCAGCAGAAGCCCTGGACTTTACAACCAAGCTTCCAAGGGTGTTATCTTTATACATC
XM_002818951.2         ATTCCAGCAGAAGCCCTGGACTTTACGACCAAGCTTCCAAGGGTGTTATCTTTATACATC
ENSGGOT00000016399     ATTCCAGCAGAAGCCCTGGACTTTACGACCAAGCTTCCAAGGGTGTTATCTTTATATATC
ENSPTRT00000067150     ATTCCAGCAGAAGCCCTGGACTTTACGACCAAGCTTCCAAGGGTGTTATCTTTATACATC

ENSMUST00000169656     TTTTCCTTCCTGGATCCCCGGAGTCTTTGCCGTTGTGCACAGGTGAGCTGGTACTGGAAG
ENSRNOT00000018762     TTTTCCTTCCTGGATCCCCGGAGCCTTTGCCGTTGTGCACAGGTAACCNGGTACTGGAAG
ENSCJAT00000030340     TTTTCTTTCCTGGACCCCCGAAGCCTTTGTCGTTGTGCACAGGTGTGTTGGCACTGGAAG
ENSMMUT00000039082     TTTTCTTTCCTGGACCCCCGGAGCCTCTGTCGTTGTGCACAGGTGTGCTGGCATTGGAAG
ENST00000346498        TTTTCTTTCCTGGACCCTCGGAGCCTTTGTCGTTGTGCACAGGTGTGCTGGCATTGGAAG
XM_002818951.2         TTTTCTTTCCTGGACCCCCGGAGCCTTTGTCGTTGTGCACAGGTGTGCTGGCATTGGAAG
ENSGGOT00000016399     TTTTCTTTCCTGGACCCCCGGAGCCTTTGTCGTTGTGCACAGGTGTGCTGGCATTGGAAG
ENSPTRT00000067150     TTTTCTTTCCTGGACCCCCGGAGCCTTTGTCGTTGTGCACAGGTGTGCTGGCATTGGAAG

ENSMUST00000169656     AGCTTGGCTGAGTTGGACCAGCTCTGGATGCTCAAGTGCCTGCGCTTCAACTGGTACATC
ENSRNOT00000018762     AGCTTGGCTGAGCTGGACCAGCTCTGGATGCTCAAGTGCCTGCGTTTTAACTGGTACATC
ENSCJAT00000030340     AACCTAGCTGAGCTGGACCAGCTCTGGATGCTGAAATGTTTACGGTTCAACTGGTGCATC
ENSMMUT00000039082     AACCTAGCCGAGCTGGATCAGCTCTGGATGCTGAAATGTTTACGGTTTAACTGGTACATC
ENST00000346498        AACCTTGCTGAGCTGGACCAGCTCTGGATGCTGAAATGTTTACGGTTTAACTGGTACATC
XM_002818951.2         AACCTTGCTGAGCTGGACCAGCTCTGGATGCTGAAATGTTTACGGTTTAACTGGTACATC
ENSGGOT00000016399     AACCTTGCTGAGCTGGACCAGCTCTGGATGCTGAAATGTTTACGGTTTAACTGGTACATC
ENSPTRT00000067150     AACCTTGCTGAGCTGGACCAGCTCTGGATGCTGAAATGTTTACGGTTTAACTGGTACATC

ENSMUST00000169656     AGCTTCTCCCCAACGCCCTTTGAACAGGGCGTCTGGAAAAAGCACTACATCCAGATGGTG
ENSRNOT00000018762     AGCTTCTCCCCAACGCCCTTTGAGCAGGGTGTCTGGAAAAAGCACTACATTCAGATGGTG
ENSCJAT00000030340     AATTTCTCTCCAACTCCCTTTGAGCAGGGGATCTGGAAGAAGCACTATATTCAAATGGTG
ENSMMUT00000039082     AATTTCTCTCCAACTCCTTTTGAGCAGGGGATCTGGAAGAAGCACTATATTCAAATGGTG
ENST00000346498        AATTTCTCTCCAACTCCCTTTGAGCAGGGGATCTGGAAGAAGCACTATATTCAAATGGTG
XM_002818951.2         AATTTCTCTCCAACTCCGTTTGAGCAGGGGATCTGGAAGAAGCACTATATTCAAATGGTG
ENSGGOT00000016399     AATTTCTCTCCAACTCCCTTTGAGCAGGGGATCTGGAAGAAGCACTATATTCAAATGGTG
ENSPTRT00000067150     AATTTCTCTCCAACTCCCTTTGAGCAGGGGATCTGGAAGAAGCACTATATTCAAATGGTG

ENSMUST00000169656     AGAGAACTTCACGTCACCAAGCCCAAGACACCTCCAAAGGATGAATTCACAACTGCCGAT
ENSRNOT00000018762     AGAGAACTTCACGTCACCAAGCCCAAGACACCTCCAAAGGATGGATTCATAACTGCCGAT
ENSCJAT00000030340     AAAGAACTTCATGTTACCAAGCCTAAGACACCTCCAAAAGATGGATTTGTAA--------
ENSMMUT00000039082     AAAGAACTTCATGTTACCAAGCCTAAGACACCCCCAAAGGATGGATTTGTAATCGCTGAC
ENST00000346498        AAAGAACTTCATATTACCAAGCCTAAGACACCCCCAAAGGATGGATTTGTAATCGCTGAC
XM_002818951.2         AAAGAACTTCATGTTACCAAGCCTAAGACACCCCCAAAGGATGGATTTGTAATCGCTGAC
ENSGGOT00000016399     AAAGAACTTCATATTACCAAGCCTAAGACACCCCCAAAGAATGGATTTGTAATCGCTGAC
ENSPTRT00000067150     AAAGAACTTCATATTACCAAGCCTAAGACACCCCCAAAGGATGGATTTGTAATCGCTGAT

ENSMUST00000169656     GTTCAGCCAATTCCTGGCAATTCTCCAGATGAGAAGCAGTCCCCTTCCTTAGCTTTTCGG
ENSRNOT00000018762     GTTCAGCCCATCCCTGGCAGTTCTCCAGAGGAGAAGCAGTCCCCTTCCTTAGCTTTTCGG
```

```
ENSCJAT00000030340     ----------TTACAAGCAATTCTCCAGAGGAAAAGCAGTCCCCTTTGTCAGCTTCTCAG
ENSMMUT00000039082     GTTCAACTAGTTACAAGCAATTCTCCAGAGGAAAAACAGTCCCCTTTATCAGCTTTTCGG
ENST00000346498        GTTCAACTAGTTACAAGCAATTCTCCAGAGGAAAAACAGTCCCCTTTATCAGCTTTTCGG
XM_002818951.2         GTTCAACTAGTTACAAGCAATTCTCCAGAGGAAAAACAGTCCCCTTTATCAGCTTTTCGG
ENSGGOT00000016399     GTTCAACTAGTTACAAGCAATTCTCCAGAGGAAAAACAGTCCCCTTTATCAGCTTTTCGG
ENSPTRT00000067150     GTTCAACTAGTTACAAGCAATTCTCCAGAGGAAAAACAGTCCCCTTTATCAGCTTTTCGG

ENSMUST00000169656     TCCTCCTCCTCCTTAAGAAAGAAGAATAACCCCGGGGAGAAAGAGCTTCCACCTTGGCGA
ENSRNOT00000018762     TCCTCCTCCTCCTTAAGAAAGAAGAATAACCCTGGGGAGAAAGAGCTTCCACCATGGCGA
ENSCJAT00000030340     TCCTCTTCCTCTTTAA-AAAGAAGAATAA-----AAAGTAAAGCGCTGACCCTATGTTTA
ENSMMUT00000039082     TCCTCTTCCTCTTTAAGAAAGAAGAATAACTCAGGGCAGAAAGCACTTCCACCCTGGCGA
ENST00000346498        TCCTCTTCCTCTTTAAGAAAGAAGAATAACTCAGGGGAGAAAGCACTTCCACCCTGGCGA
XM_002818951.2         TCCTCTTCCTCTTTAAGAAAGAAGAATAACTCAGGGGAGAAAGCACTTCCACCCTGGCGA
ENSGGOT00000016399     TCCTCTTCCTCTTTAAGAAAGAAGAATAACTCAGGGGAGAAAGCACTTCCACCCTGGCGG
ENSPTRT00000067150     TCCTCTTCCTCTTTAAGAAAGAAGAATAACTCAGGGGAGAAAGCACTTCCACCCTGGCGA

ENSMUST00000169656     TCGTCAGATAAGCATCCCACTGACATCATCCGCTTTAATTATCTAGACAACTGTGACCCT
ENSRNOT00000018762     TCGTCAGACAAGCATCCCACAGACATCATCCGCTTTAATTATCTAGACAACTGTGACCCA
ENSCJAT00000030340     CCCTATCTCACACACACAACATCT-TCATTTTATTCTATGATTTAGAAAAATATGGCTCC
ENSMMUT00000039082     TCTTCTGATAAGCATCCAACAGATATCATTCGTTTTAATTACCTAGACAACTGTGACCCC
ENST00000346498        TCTTCTGATAAGCACCCAACAGATATCATTCGTTTTAATTACCTAGACAACCGTGACCCC
XM_002818951.2         TCTTCTGATAAGCATCCGACAGATATCATTCGTTTTAATTACCTAGACAACTGTGACCCC
ENSGGOT00000016399     TCTTCTGATAAGCACCCAACAGATATCATTCGTTTTAATTACCTAGACAACTGTGACCCC
ENSPTRT00000067150     TCTTCTGATAAGCACCCAACAGATATCATTCGTTTTAATTACCTAGACAACTGTGACCCC

ENSMUST00000169656     GAGCTCTTCAGGCTAGGAAGAAGAAAAAGAAGCGAAGTGACCCCAGACTTCAAGCGCCAG
ENSRNOT00000018762     GAGCTCTTCAGGCTAGGAAGAAGAAAAAGAAAGGAAGTGACCCCAGACTTCAAGCAGCAG
ENSCJAT00000030340     AAAATTAAATATACAAAAAGAAGAAAGAGAAATGAAATGACACCAGATTTCAGCCGACAG
ENSMMUT00000039082     GAGACTGTCTGGCAAGGAAGAAAAAAAAGAAACGAAATGACCCCAGATTTCAGCCGACAG
ENST00000346498        GAGACTGTCCAGCAAGGAAGAAGAAAAAGAAACCAAATGACCCCAGACTTCAGCCGACAG
XM_002818951.2         GAGACTGTCTGGCAAGGAGGAAGAAAAAGAAACCAAATGACCCCAGATTTCAGCCGACAG
ENSGGOT00000016399     GAGACTGTCTGGCAAGGAAGAAGAAAAAGAAACCAAATGACCCCAGATTTCAGCCGACAG
ENSPTRT00000067150     GAGACTGTCTGGCAAGGAAGAAGAAAAAGAAACCAAATGACCCCAGATTTCAGCCGACAG

ENSMUST00000169656     CTGCGAGATAAGAAAAATAAGCTTCAAGACAGAGCCAGGCTCAGGAAAGCACAGTCATTG
ENSRNOT00000018762     TTTCGAGATAAGAAAAATAAGCTTCAAGACAGAGCCAGGCTCAGGAAAGCACAGTCATTG
ENSCJAT00000030340     TCACCTGATAAGAAAAATAAATTGCAGGACAGAACTAGGCTAAGAAAAGCACAATCAATG
ENSMMUT00000039082     TCACATGATAAGAAAAATAAGTTGCAGGACAGAACTAGGCTAAGAAAAGCACAATCAATG
ENST00000346498        TCACATGATAAGAAAAATAAATTGCAGGACAGAACTAGGCTAAGAAAAGCACAATCAATG
XM_002818951.2         TCACATGATAAGAAAAACAAATTGCAGGACAGAACTAGGCTAAGAAAAGCACAATCAATG
ENSGGOT00000016399     TCACATGATAAGAAAAATAAATTGCAGGACAGAACTAGGCTAAGAAAAGCACAATCAATG
ENSPTRT00000067150     TCACATGATAAGAAAAATAAATTGCAGGACAGAACTAGGCTAAGAAAAGCACAATCAATG

ENSMUST00000169656     ATCTCCCTGAGAAGTTCCCACTCCTGGAGCATC
ENSRNOT00000018762     ATGTCCCTGAGAAGTTCCTTCTCACGGAGCACC
ENSCJAT00000030340     GT---------AAGTAC-TGCTTATGAAATATT
ENSMMUT00000039082     ATGTCCCTAAGAAGTTCCTTCTCATGCAGCATC
ENST00000346498        ATGTCCCTAAGAAGTTCCTTCTCATGCAGCATC
XM_002818951.2         ATGTCGAGGAGAAATCCCTTCCCACTATGTCCC
ENSGGOT00000016399     ATGTCGAGGAGAAATCCCTTCCCACTATGTCCC
ENSPTRT00000067150     ATGTCGAGGAGAAATCCCTTCCCACTATGTCCC
```

Multiple sequence alignment of Fbxo17

ENSRNOT00000027006   ATGGGAGCCCAGCCCTCGCGACGGCGGATGACCGAAGCTCAGCACATGGCTTTGGCCGAG
ENSMUST00000108278   ATGGGAGCGCGGCCCTCGCGACGGCAGATGACTGAAGCTCGGCGCCTGGCTCTAACCAAG
ENSCJAT00000027192   ATGGGCGCCCGGCCCTCGCGGCGACGGCTGCCAGCGGACCCACCCCTGGCCTTGGACGCG
ENST0000292852   ATGGGCGCCCGGCTATCGCGGCGACGGCTGCCGGCGGACCCATCCCTGGCCCTGGACGCG
ENSPPYT00000011576   ATGGGCGCCCGGCTCTCGCGGCGACGGCTGCCGGCGGACCCGTCCCTGACCCTGGACGCG

ENSRNOT00000027006   CTGCCCCCGGAGTTGCTGCTGCAGGTGCTGAGCCATGTGCCTCCTCGCGCGTTAGTGACC
ENSMUST00000108278   CTGCCCCCTGAGCTGCTGGTGCAGGTGCTGAGCCACGTGCCGCCTCGCGCGTTAGTGACC
ENSCJAT00000027192   CTGCCCCCGGAGCTGCTGGTGCAGGTGCTGAGCCACGTGCCGCCACGCGCCTTGGTCATG
ENST0000292852   CTGCCCCCGGAGCTGCTGGTGCAGGTGCTGAGCCACGTGCCGCCACGCTCCTTGGTCACG
ENSPPYT00000011576   CTGCCCCCGGAGCTGCTGGTGCAGCTGCTGAGCCACGTGCCGCCACGCGCCTTGGTCACG

ENSRNOT00000027006   CGCTGCCGGCCGGTGTGCCGCGCCTGGCGCGACCTAGTAGATGGCCCAAGCGTATGGCTG
ENSMUST00000108278   CGCTGCCGGCCAGTGTGCCGGGCCTGGCGCGACCTGGTAGATGGCCCAAGTATATGGCTG
ENSCJAT00000027192   CAATGCCGCCCAGTGTGCCGCGCCTGGCGCGACGTGGTGGACGGGCCCACCGTGTGGCTG
ENST0000292852   CGATGCCGCCCAGTGTGCCGCGCCTGGCGCGACATAGTGGACGGGCCCACTGTGTGGCTG
ENSPPYT00000011576   CGATGCCGCCCAGTGTGCCGCGCCTGGCGCGACATAGTGGACGGGCCCACTGTGTGGTTG

ENSRNOT00000027006   CTGCAACTGGCTCGCGACCGCAGCGCGGAGGGCAGAGCCCTCTACGCCTTGGCCCAGAGC
ENSMUST00000108278   CTGCAACTGGCTCGCGACCGCAGCGCGGAGGGCAGAGCCCTCTACGCCTTGGCCCAGCGC
ENSCJAT00000027192   CTGCAGCTGGCCCGCGACCGCAGCGCCGAGGGCCGCGCACTCTATGCAGTGGTCCAGCGC
ENST0000292852   CTGCAGCTGGCCCGCGACCGCAGCGCCGAGGGCCGCGCACTCTACGCAGTGGCTCAACGC
ENSPPYT00000011576   CTGCAGCTGGCCCGCGACCGCAGCGCCGAGGGCCGCGCACTCTATGCAGTGGCTCAACGC

ENSRNOT00000027006   TGCCCGGCCGACCGCAACCACGACGACTTCCCACTCTGTGCTCTGGCCCGCTTCTGCCTG
ENSMUST00000108278   TGTCCCGCCGACGCCGACCACGACGAGTTCCCATTCTGCGCTCTGGCCCGATTCTGCCTG
ENSCJAT00000027192   TGCCCCAGCAACGAGGACGAGGAGGAGTTTCCGCTGTGCGCCCTGGCGCGCTACTGTCTG
ENST0000292852   TGCCCCAGCAACGAAGACAAGGAGGAGTTCCCGCTGTGCGCCCTGGCGCGCTACTGTCTG
ENSPPYT00000011576   TGCCCCAGCAAGGAAGACAAGGAGGAGTTCCCGCTGTGCGCCCTGGCGCGCTACTGTCTG

ENSRNOT00000027006   CTAGCGCCGCTTGGCCGCAACCTCATCTACAACTCCTGCGGAGAACAGGGCTTCAGAGGT
ENSMUST00000108278   AGAGCGCCGTTTGGCCGCAACCTCATCCACAACTCCTGCGGAGAACAGGGCTTCAGAGGT
ENSCJAT00000027192   CGCGCGCCCTTCGGTCGCAACCTCATCTTCAACTCCTGCGGAGAGCAGGGCTTCAAAGGC
ENST0000292852   CGCGCGCCCTTCGGCCGCAATCTCATCTTCAACTCCTGCGGAGAGCAGGGCTTCAGAGGC
ENSPPYT00000011576   CGAGCGCCCTTCGGCCGCAATCTCATCTTCAACTCCTGCGGAGAGCAGGGCTTCAGAGGC

ENSRNOT00000027006   TGGGAAGTGGAACATGGCGGGAACGGCTGGGCTGTGGAAAAGAATCTGACGATGGTGCCA
ENSMUST00000108278   TGGGAAGTGGAGCACGGCGGAAATGGTTGGGCCGTGGAAAAGAATTTGACGCTGGTACCA
ENSCJAT00000027192   TGGGAGGTGGAGCACGGCGGGAACGGCTGGGCCATAGAAAAGAACCTCACACTGGTGCCT
ENST0000292852   TGGGAGGTGGAGCATGGCGGGAACGGCTGGGCCATAGAAAAGAACCTAACACCGGTGCCT
ENSPPYT00000011576   TGGGAGGTGGAGCATGGCGGGAACGGCTGGGCCATAGAAAAGAACCTAACACCGGTGCCT

ENSRNOT00000027006   GGGGCTCCTTCTCAAACCTGCTTCGTGACTTCTTTTGAGTGGTGCTTCAAGAGGCAGCTT
ENSMUST00000108278   GGGGCTCCTTCCCAGACCTGCTTTGTGACTTCTTTTGAGTGGTGCTCCAAGAGGCAGCTT
ENSCJAT00000027192   GGGGCTCCTTCGCAGACCTGCTTCGTGACCTCTTTCGAATGGTGCTTCAAGAGGCAGCTT
ENST0000292852   GGGGCTCCTTCGCAGACCTGCTTCGTGACCTCTTTCGAATGGTGCTCCAAGAGGCAGCTT
ENSPPYT00000011576   GGGGCTCCTTCGCAGACCTGCTTCGTGACCTCTTTCGAATGGTGCTCCAAGAGGCAGCTT

ENSRNOT00000027006   GTAGACCTGGTGAAGGAAGGAGTGTGGCAGGAGCTGCTGGACAGTGCCCAGATTGAGATC
ENSMUST00000108278   GTAGACCTGGTGAAGGAAGGAGTATGGCAGGAGCTGTTGGACAGTGGCCAGATTGAGATC
ENSCJAT00000027192   GTGGACCTGGTGATGGAGGGGGTGTGGCAGGAGCTGCTGGACAGCGCCCAGATCGAGATC
ENST0000292852   GTGGACCTGGTGATGGAAGGGGTGTGGCAGGAGCTGCTGGACAGCGCCCAGATTGAGATC

```
ENSPPYT00000011576     GTGGACCTGGTGATGGAAGGGGTGTGGCAGGAGCTGCTGGACAGCGCCCAGATTGAGATC


ENSRNOT00000027006     TACATAGCTGACTGGTGGGGCGCCCGTGAGAACTGCGGCTGCATCTACAGGCTTCGGGTC
ENSMUST00000108278     TGCATAGCTGACTGGTGGGGCGCCCGTGAGAACTGCGGCTGCATCTACCGGCTTCGAGTT
ENSCJAT00000027192     TGTGTGGCTGACTGGTGGGGCGCCCGAGAGAACTGCGGCTGCGTCTATCGGCTCCGAGTC
ENST0000292852         TGTGTGGCTGACTGGTGGGGCGCTCGAGAGAACTGCGGCTGCGTCTACCAGCTCCGGGTC
ENSPPYT00000011576     TGTGTGGCTGACTGGTGGGGTGCTCGGGAGAACTGCGGCTGCGTCTACCAGCTCCGGGTC


ENSRNOT00000027006     CGCCTACTGGACGAACATGAAAACGAAGTGGTCAAGTTCTCTGCCTCACCCAACCCAGTC
ENSMUST00000108278     CGCCTACTGGATGAATATGAAAACGAAGTGGTCAAGTTCTCTGCCTCACCCAACCCAGTC
ENSCJAT00000027192     CGCCTTCTGGACGTGTACGAAAATGAAGTGGTCAAGTTCTCAGCCTCACCCAACCCGGTC
ENST0000292852         CGCCTTCTGGATGTGTATGAAAAGGAAGTGGTCAAGTTCTCAGCCTCACCTGACCCGGTC
ENSPPYT00000011576     CGCCTTCTGGACGTGTATGAAAAGGAAGTGGTCAAGTTCTCAGCCTCACCTGACCCGGTC


ENSRNOT00000027006     CTTCAGTGGACTGAGAGGAGCTGCCGACAAGTGAGT------------------------
ENSMUST00000108278     CTTCAGTGGACTGAGAGGAGCTGCCGACAAGTCTCTCACGTCTTCACTAACTTTGGCAAG
ENSCJAT00000027192     CTTCAGTGGACTGAGAGGGGCTGCCGACAGGTCTCCCATGTCTTCACCAACTTTGGCAAG
ENST0000292852         CTTCAGTGGACTGAGAGGGGCTGCCGACAGGTCTCCCACGTCTTCACCAACTTTGGCAAG
ENSPPYT00000011576     CTTCAGTGGACTGAGAGGGGCTGCCGACAGGTCTCCCACGTCTTCACCAACTTTGGCAAG


ENSRNOT00000027006     ---------------------------------CCGGACTGCTCACTCAACCCTGGCCTG
ENSMUST00000108278     GGCATCCGGTATGTGTCTTTTGAGCAGTATGGGAGAGACACGCGTTCCTGGGTGGGACAC
ENSCJAT00000027192     GGCATCCGATACGTATCTTTTGAGCAGTACGGGAGAGACACGCGTTCCTGGGTGGGGCAC
ENST0000292852         GGCATCCGCTACGTATCTTTTGAGCAGTACGGGAGAGACGTGAGTTCCTGGGTGGGGCAC
ENSPPYT00000011576     GGCATCCGCTACGTATCTTTTGAGCAGTATGGGAGAGACGTGAGTTCCTGGGTGGGGCAC


ENSRNOT00000027006     CTCCAAGGTCTCTCA---------------------------CGTCTTCAC
ENSMUST00000108278     TACGGTGCCCTTGTAACTCACTCCAGTGTGCGGGTCAGGATCCGTCTGTCC
ENSCJAT00000027192     TATGGTGCCCTTGTGACCCACTCCAGTGTGAGGGTCAGGATCCGTCTGTCC
ENST0000292852         TATGGCGCCCTTGTGACCCACTCCAGTGTGAGGGTCAGGATCCGTCTGTCC
ENSPPYT00000011576     TACGGCGCCCTTGTGACCCACTCCAGTGTGAGGGTCAGGATCCGTCTGTCC
```

Multiple sequence alignment of Fbxo18

```
ENSPPYT00000002460     ATGAGACGGTTTAAGCGGAAGCATCTTACTGCCATCGACTGCCAGCATTTGGCTCGGAGT
ENSCJAT00000040364     ATGAGACGGTTTAAGCGGAAGCATCTTACTGCCATCGACTGCCAGCATTTGGCTCGGAGT
ENST00000379999        ATGAGACGGTTTAAGCGGAAGCATCTTACTGCCATTGACTGCCAGCATTTGGCTCGGAGT
ENSPTRT00000004144     ATGAGACGGTTTAAGCGGAAGCATCTTACTGCCATTGACTGCCAGCATTTGGCTCGGAGT
ENSMUST00000071564     ATGAGACGGTTTAAGCGGAAGCATCTTACAGTGGTCGACTGCCATCATTTGGCCCGGAGT
ENSRNOT00000025228     GTGAGACGGTTTAAGCGGAAGCATCTTACAGTGGTCGACTGCCAGCAACTGGCCCGGAGT


ENSPPYT00000002460     CACTTGGCTGTGACCCAGCCCTTCGGTCAAAGATGGACAAACAGAGATCCGAACCATGGT
ENSCJAT00000040364     CACTTGGCTGTGACCCAGCCCTTCGGTCAAAGATGGACAAACAGAGATCCGAACCATGGT
ENST00000379999        CACTTGGCTGTGACCCAGCCCTTCGGTCAAAGATGGACAAACAGAGATCCGAACCATGGT
ENSPTRT00000004144     CACTTGGCTGTGACCCAGCCCTTCGGTCAAAGATGGACAAACAGAGATGCGAACCATGGT
ENSMUST00000071564     CATTTGGCTGTGACTCAGCCCTTCAGTCAGAGATGGACAAACAGAGACCCTAACCATGGT
ENSRNOT00000025228     CATTTGGCTGCGACTCAGCCCTTCAGTCAGAGATGGACAAACAGAGACCCTAACCATGGT


ENSPPYT00000002460     CTCTATCCTAAACCCAGAACAAAAAGAGGGAGTAGGGGTCAGGGACGTCAAAGATGCATC
ENSCJAT00000040364     CTCTATCCTAAACCCAGAACAAAAAGAGGGAGTAGGGGTCAAGGATGTCAAAGATGCATC
ENST00000379999        CTCTATCCTAAACCGAGAACAAAAAGAGGGAGTAGGGGTCAGGGAAGTCAAAGATGCATC
ENSPTRT00000004144     CTCTATCCTAAACCGAGAACAAAAAGAGGGAGTAGGGGTCAGGGAAGTCAAAGATGCATC
```

```
ENSMUST00000071564     CTCTATCCTAGACCCAGAACAAAAGGACGGAATAGGGGTCGAGGATGTCAGAGATATATT
ENSRNOT00000025228     CTCTATCCTAGAGCCAGAACAAAAGGACGGAATAGGGGTCGAGGATGTCAGAGATACATT

ENSPPYT00000002460     CCTGAGTTCTTCCTAGCAGGCAAGTGGCCGTGCACCAATGACATGGCCAAAAGCAATTCT
ENSCJAT00000040364     CCTGAGTTCTTCCTAGCAGGCAAGCAGCCGTGGGCCAATGACATGGCCAGAAGCAATTCT
ENST00000379999        CCTGAGTTCTTCCTAGCAGGCAAGCAGCCGTGCACCAATGACATGGCCAAAAGCAATTCT
ENSPTRT00000004144     CCTGAGTTCTTCCTAGCAGGCAAGCGGCCGTGCACCAATGACATGGCCAAAAGCAATTCT
ENSMUST00000071564     TCTGAGTTCTTCCTAGCTGGCCACCAGCACTGTACCAATGACATGGCCAAAAGCAATTCT
ENSRNOT00000025228     TCTGAGTTCTTCCTAGCTAGCCACCAGCATTGTACCAATGACATGGCCAAAAGCAATTCT

ENSPPYT00000002460     GTTGGCCAGGACAGCTGTCAGGACTCTGAGGGTGACATGATCTCTCCTGCAGAGAGCAGC
ENSCJAT00000040364     GTTGGCCAGGACAGCTGTCAGGACTCTGAGGGTGACATGATCTTTCCTGCAGAGAACAGC
ENST00000379999        GTTGGCCAGGACAGCTGTCAGGACTCTGAGGGTGACATGATCTTTCCTGCAGAGAGCAGC
ENSPTRT00000004144     GTTGGCCAGGACAGCTGTCAGGACTCTGAGGGTGACATGATCTTTCCTGCAGAGAGCAGC
ENSMUST00000071564     GTTGGCCAGGACAGCTGTCAGGACGCTGAGGGTGACATGATTTTAACTGCAGAGAGCAGT
ENSRNOT00000025228     GTTGGCCAGGACAGCTGTCAGGACTCTGAGAATGATATGATTTTTCCTGCCGAGAGCAGT

ENSPPYT00000002460     TGTGCACTGCCTCAGGAAGGCAGTGGAGAAGCATGGCTGGGCTCATCAGGGTCTGCCCCG
ENSCJAT00000040364     TGTGCACTGCCTCAGGAAGGCAGTGGAGAAGCACGGCTGGGCGCTTCAGGGTCTGTCCCG
ENST00000379999        TGTGCACTGCCTCAGGAAGGCAGTGCAGGGCCG------GGCTCACCAGGGTCTGCCCCG
ENSPTRT00000004144     TGTGCACTGCCTCAGGAAGGCAGTGGAGAAGCAGGGCTGGGCTCATCAGGGTCTGCCCCG
ENSMUST00000071564     TGTACTCTGCCTCAGGTGGATAATGGAGAAGCGAGGCTGGGCTCATCAGGATCTGCACAG
ENSRNOT00000025228     TGTTCTCTGCCTCAGGTGGAAAATGGAGAAGTGAGGCTGGGCTCACCAGGATCTGCTCAG

ENSPPYT00000002460     CCCTCCAGGAAGCGGTCTCGGTCCTCTGAGGAAGCTACCGGGACCAGCCAGTGGGATGGA
ENSCJAT00000040364     CCCTCCAGGAAGCGGGCTCGGTCCTTTGAGGAAGCTACGGGGACCAGCCGGTGGGATGGA
ENST00000379999        CCCTCCAGGAAGCGGTCTTGGTCCTCTGAGGAAGCTACCGGGACCAGCCGGTGGGATGGA
ENSPTRT00000004144     CCCTCCAGGAAGCGGTCTTGGTCCTCTGAGGAAGCTACCGGGACCAGCTGGTGGGATGGA
ENSMUST00000071564     CCTGCCAGGAAGCGGGCTCACTGCTTTGAGGAAGCCACAGAGAGCGGCCAGTGGGATGGC
ENSRNOT00000025228     CCCGCCAGGAAGCGGCCTCGTTCCTTGGAGGAAGCCACAGAGAGCGGCCAGTGGGATGGG

ENSPPYT00000002460     GTTTCTAAGAAAGCTCTGCGGCACCATTTGTCTGTGCCATGCACAAGGCCTAGGGAGGCC
ENSCJAT00000040364     GTTTCTAAGAAAGCCCCGAGGCATCATTTGTCTGTGCCATGCACAAGGCCTAGGGAGGCC
ENST00000379999        GTTTCTAAGAAAGCTCCACGGCACCATTTGTCTGTGCCATGCACAAGGCCTAGGGAGGCC
ENSPTRT00000004144     GTTTCTAAGAAAGCTCCGCGGCACCATTTGTCTGTGCCATGCACAAGGCCTAGGGAGGCC
ENSMUST00000071564     GTTACTAAGAAGACACCGCGCCATCGTTTGTTCCCATCGTGTTCAAGGCTTAGGGAGGCC
ENSRNOT00000025228     GTTACTAAGAAGACGCCACGCCATCGTTTGTTCCCATCGTGTGCAAGGCTTAGGGAGGTC

ENSPPYT00000002460     AGGCAAGAAGCAGAGGACAGTATGTCTCGGCTCTCTGCAGAGTCTGGAGAAACCGACCAA
ENSCJAT00000040364     AGGCGAGAAGCAGAGGACAGTACGTCTCGGCTCTCTGCAGAGTCTGGAGAAACTGACCAA
ENST00000379999        AGGCAAGAAGCAGAGGACAGTACGTCTCGGCTCTCTGCGGAGTCTGGTGAAACCGACCAA
ENSPTRT00000004144     AGGCAAGAAGCAGAGGACAGTACGTCTCGGCTCTCTGCGGAGTCTGGTGAAACCGACCAA
ENSMUST00000071564     AGGCAAGGGGCAGAGGACAGTTTGTCCCAGTGCTCCCCAGTGCCGGGGGAAGCTGGCCGA
ENSRNOT00000025228     AGGCAAGGAGCAGAGGATGGTTTGTCACAGAGCTCTCCAGTACATGGAGAAGCTGCCCAA

ENSPPYT00000002460     GATGCTGGGGACATGGGTCCTGATCTCGTTCCTGACTCATACTATGGGCTTCTTGGGACC
ENSCJAT00000040364     GATGCTGGGGACCTGGGTCCTGATCCCGTTCCTGACTCATACTATGGGCTTCTTGGGACC
ENST00000379999        GATGCTGGGGACGTGGGTCCTGATCCCATTCCTGACTCATACTATGGGCTTCTTGGGACC
ENSPTRT00000004144     GATGCTGGGGACATGGGTCCTGATCCCATTCCTGACTCATACTATGGGCTTCTTGGGACC
ENSMUST00000071564     GACATTGAGGACATTGGTCCTGATCCCTTGCCTGACTCATACTATGGGCTTCTTGGAATG
ENSRNOT00000025228     GACATTGAGGACATTGGTCCTGATCCCTTGCCTGATTCATACTATGGGCTTCTTGGAACG
```

```
ENSPPYT00000002460     TTGCCCTGCCAGGAAGCACCGAGCCACATTTGCAGCCTGCCTAGTGAGGTCCTGAGGCAC
ENSCJAT00000040364     TTGCCCTGCCAGGAATCGCTGAGCCACATTTGCAGCCTGCCTAGTGAGGTCCTGAGGCAC
ENST00000379999        TTGCCCTGCCAGGAAGCACTGAGCCACATTTGCAGCCTGCCTAGTGAGGTCCTGAGGCAC
ENSPTRT00000004144     TTGCCCTGCCAGGAAGCACTGAGCCACATTTGCAGCCTGCCTAGTGAGGTCCTGAGGCAC
ENSMUST00000071564     TTGCCTTGTCAGGAAGTGCCCAGCCACATTTGCAGGCTGCCAAGTGAAGTGCTGAGACAC
ENSRNOT00000025228     TTGCCTTGCCAGGAAGTGCCCAGCCACATCTGCAGGCTGCCAAGTGAAGTCCTGAGGCAC

ENSPPYT00000002460     GTGTTTGCCTTCCTCCCGGTGGAAGACCTCTATTGGAACCTGAGCTTGGTGTGCCACCTG
ENSCJAT00000040364     GTGTTTGCCTTCCTCCCAGTAGAAGACCTCTACTGGAACCTGAGCCTGGTGTGCCACCTG
ENST00000379999        GTGTTTGCCTTCCTCCCGGTGGAAGACCTCTATTGGAACCTGAGCTTGGTGTGCCACTTG
ENSPTRT00000004144     GTGTTTGCCTTCCTCCCGGTGGAAGACCTCTATTGGAACCTGAGTTTGGTGTGCCACTTG
ENSMUST00000071564     ATCTTTGCTTTCCTGCCAGTGGAGGACCTCTACTGGAATCTCAGCCTGGTGTGCCACCTG
ENSRNOT00000025228     ATCTTTGCTTTCCTGCCAGTGGAGGACCTCTACTGGAACCTCAGCCTGGTGTGCCACCTG

ENSPPYT00000002460     TGGAGGGAGATCATCAGTGACCCACTGTTCATTCCTTGGAAGAAGCTGTACCATCGATAC
ENSCJAT00000040364     TGGAGGGAGATTATCAGCGACCCACTGTTCATTCCTTGGAAGAAGCTGTACCATCGATAC
ENST00000379999        TGGAGGGAGATCATCAGTGACCCGCTGTTCATTCCTTGGAAGAAGCTGTACCATCGATAC
ENSPTRT00000004144     TGGAGGGAGATCATCAGTGACCCGCTGTTCATTCCTTGGAAGAAGCTGTACCATCGATAC
ENSMUST00000071564     TGGAGGGAAATAATCAATGACCCGCTGTTCATTCCTTGGAAGAAGCTATATCATCGATAC
ENSRNOT00000025228     TGGAGGGAAATAGTCAATGACCCGCTGTTCATTCCTTGGAAGAAGCTGTATCATCGGTAC

ENSPPYT00000002460     CTGATGAATGAAGAGCAAGCTGTCAGCAAAGTGGACGGCATCCTGTCTAACTGTGGCATA
ENSCJAT00000040364     CTGATGAATGAAGAGCAAGCTGTCAGCAAAGTGGATGGCATCCTGTCTAACTGTGGCATA
ENST00000379999        CTGATGAATGAAGAGCAAGCTGTCAGCAAAGTGGACGGCATCCTGTCTAACTGTGGCATA
ENSPTRT00000004144     CTGATGAATGAAGAGCAAGCTGTCAGCAAAGTGGACGGCATCCTTTCTAACTGTGGCATA
ENSMUST00000071564     TTGATCAATGAAGAGCAGGCTGTCAGCAAAGTGGATGGCATTCTCTCGAGCCATGGCATA
ENSRNOT00000025228     TTGATCAACGAAGAGCAGGCTGTCAGCAAAGTGGACGGCATTCTCTCCAGCCACGGCATA

ENSPPYT00000002460     GAAAAGGAGTCAGACCTGTGTGTGCTGAACCTCATACGATACACAGCCACCACTAAGTGC
ENSCJAT00000040364     GAAAAGGAGTCAGACTTGTGTGTGCTGAACCTCATACGATACGCAGCCACCACTAAGTGC
ENST00000379999        GAAAAGGAGTCAGACCTGTGTGTGCTGAACCTCATACGATACACAGCCACCACTAAGTGC
ENSPTRT00000004144     GAAAAGGAGTCAGACCTGTGTGTGCTGAACCTCATACGATACACAGCCACCACTAAGTGC
ENSMUST00000071564     GAAAAGGATTCGGACCTATGTGTGCTGAACCTCATACGATACACGGCCACCACCAAATGC
ENSRNOT00000025228     GAAAAGGATTCGGACTTGTGTGTGCTGAACCTCATACGATACACAGCCACCACCAAGTGC

ENSPPYT00000002460     TCCCCGAGTGTGGATCCCGAGAGGGTGCTGTGGAGTCTGAGGGACCACCCCCTCCTCCCC
ENSCJAT00000040364     TCCCCGAGCGTGGATCCCGAGAGGGTGCTGTGGAGTCTGAGGGACCACCCCCTCCTCCCC
ENST00000379999        TCTCCGAGTGTGGATCCCGAGAGGGTGCTGTGGAGTCTGAGGGACCACCCCCTCCTCCCC
ENSPTRT00000004144     TCCTCGAGTGTGGATCCCGAGAGGGTGCTGTGGAGTCTGAGGGACCACCCCCTCCTCCCC
ENSMUST00000071564     TCCCCAAGTGTTGACCCTGAGAGGGTGCTGTGGAGCCTGAGGGATCATCCTCTACTTTTG
ENSRNOT00000025228     TCCCCAAGTGTGGACCCTGAGAGGGTGCTATGGAGCCTGAGGGATCACCCTCTACTTTTG

ENSPPYT00000002460     GAGGCTCAGGCGTGTGTGCGGCAACACCTCCCCGACCTCTATGCTGCTGCCGGGGGTGTC
ENSCJAT00000040364     GAGGCCAAGGCATGCATGCGGCAACACCTCCCCGACCTCTATGCCGCTGCCGGGGGTGTC
ENST00000379999        GAGGCTGAGGCGTGTGTGCGGCAACACCTCCCCGACCTCTACGCTGCTGCCGGGGGTGTC
ENSPTRT00000004144     GAGGCTGAGGCGTGTGTGCGGCAACACCTCCCCGACCTCTACGCTGCTGCCGGGGGTGTC
ENSMUST00000071564     GAGGCTGAGGCGTGCATGCGGCAACAACTACCTGACCTCTATGCAGCTGCTGGGGGCATT
ENSRNOT00000025228     GAGGCTGAGGCGTGCATGCGTCAACAACTACCTGACCTCTATGCAGCTGCTGGGGGCATT

ENSPPYT00000002460     AACGTCTGGGCCCTGGTGGCGGCTGTGGTGCTCCTCTCCAGCAGTGTGAATGACATCCAG
ENSCJAT00000040364     AACGTCTGGGCTCTGGTGGCGGCCATGGTGCTCCTCTCCAGCAGCGTGAATGACATCCAG
ENST00000379999        AACATCTGGGCCCTGGTGGCGGCTGTGGTGCTCCTCTCCAGCAGTGTGAATGACATCCAG
ENSPTRT00000004144     AATATCTGGGCCCTGGTGGCGGCTGTGGTGCTCCTCTCCAGCAGTGTGAACGACATCCAG
```

```
ENSMUST00000071564     AACGTCTGGGCCTTGGTGGCAGCCATGGTGCTCCTCTCCAGCTGTGTGAACGACATCCAG
ENSRNOT00000025228     AACGTCTGGGCCTTGGTGGCCGCCATGGTGCTCCTCTCCAGCTGTGTGAACGACATCCAG

ENSPPYT00000002460     CGACTGCTCTTCTGCCTCCGGAGACCCAGCTCCACGGTGACCATGCCAGATGTCACTGAG
ENSCJAT00000040364     CGGCTGCTCTTCTGCCTCCGGAGACCCAGCTCCACGGTGACCATGCCAGACGTCACCGAG
ENST00000379999        CGACTGCTCTTCTGCCTCCGGAGACCCAGCTCCACGGTGACCATGCCAGATGTCACCGAG
ENSPTRT00000004144     CGACTGCTCTTCTGCCTCCGGAGACCCAGCTCCACAGTGACCATGCCAGATGTCACCGAG
ENSMUST00000071564     CATCTGCTCTTCTGCCTCAGGAGACCCAGCTCCACAGTGACCATGCCAGATGTCACCGAG
ENSRNOT00000025228     CACCTGCTCTTCTGCCTCAGGAGACCCAGGTCCACAGTGACCATGCCAGACGTCACCGAG

ENSPPYT00000002460     ACCCTGTACTGCATAGCCGTGCTTCTCTACGCCATGAGGGAGAAGGGGATTAACATCAGC
ENSCJAT00000040364     ACCCTGTACTGCATAGCCGTGCTTCTCTACGCCATGAGGGAGAAGGGGATTAACATCAGC
ENST00000379999        ACCCTGTACTGCATAGCCGTGCTTCTCTACGCCATGAGGGAGAAGGGGATTAACATCAGC
ENSPTRT00000004144     ACCCTGTACTGCATAGCCGTGCTTCTCTACGCCATGAGGGAGAAGGGGATTAACATCAGC
ENSMUST00000071564     ACCTTATACTGCATAGCTGTGCTCCTTTATGCCATGAGGGAGAAGGGGATTAACATCAGC
ENSRNOT00000025228     ACCTTATACTGCATAGCTGTGCTCCTCTATGCCATGAGGGAGAAGGGGATTAACATCAGC

ENSPPYT00000002460     AATAGGATTCACTACAACATTTTCTATTGCCTATATCTTCAGGAGAATTCCTGCACTCAG
ENSCJAT00000040364     AATAGGATTCACTACAACATTTTCTATTGCCTATATCTTCAGGAGAATTCCTGCACTCAG
ENST00000379999        AATAGGATTCACTACAACATTTTCTATTGCCTATATCTTCAGGAGAATTCCTGCACTCAG
ENSPTRT00000004144     AATAGGATTCACTACAACATTTTCTATTGCCTATATCTTCAGGAGAATTCCTGCACTCAG
ENSMUST00000071564     AATAGGATTCACTACAACATTTTCTATTGCCTATATCTTCAGGAGAATTCCTGTACTCAG
ENSRNOT00000025228     AACAGGATTCACTACAACATTTTCTATTGCCTATATCTTCAGGAGAATTCCTGTACTCAG

ENSPPYT00000002460     GCCACAAAAGTTAAAGAGGAGCCATCTGTCTGGCCAGGCAAGAAAACCATCCAACTTACA
ENSCJAT00000040364     GCTACAAAAGTTAAAGAGGAGCCATCTGTCTGGCCGGGCAAGAAAACCATCCAGCTTACA
ENST00000379999        GCCACAAAAGTTAAAGAGGAGCCATCTGTCTGGCCAGGCAAGAAAACCATCCAACTTACA
ENSPTRT00000004144     GCCACAAAAGTTAAAGAGGAGCCATCTGTCTGGCCAGGCAAGAAAACCATCCAACTTACA
ENSMUST00000071564     GCCACCAAAGTTACAGAGGAGCCATCTGTCTGGCCAGGCAAGAAAACAATCCAGCTAACA
ENSRNOT00000025228     GCCACCAAAGTTACAGAGGAGCCGTCTGTCTGGCCAGGCAAGAAAACAATCCAACTAACA

ENSPPYT00000002460     CATGAACAACAGCTGATTCTGAATCACAAGATGGAACCTCTCCAGGTGGTGAAAATTATG
ENSCJAT00000040364     CATGAGCAACAGCTGATTCTGAATCATAAGATGGAACCTCTCCAGGTGGTAAAAATTATG
ENST00000379999        CATGAACAACAGCTGATTCTGAATCACAAGATGGAACCTCTCCAGGTGGTGAAAATTATG
ENSPTRT00000004144     CATGAACAACAGCTGATTCTGAATCACAAGATGGAACCTCTCCAGGTGGTGAAAATTATG
ENSMUST00000071564     CATGAACAACAGCTGATTCTGAACCATAAGATGGAGCCTCTCCAGGTGGTAAAAATTATG
ENSRNOT00000025228     CATGAACAGCAGCTGATTCTGAACCATAAGATGGAGCCTCTCCAGGTGGTAAAAATTATG

ENSPPYT00000002460     GCCTTTGCCGGCACTGGGAAGACCTCAACCCTGGTCAAGTACGCAGAGAAGTGGTCTCAG
ENSCJAT00000040364     GCCTTTGCAGGCACTGGGAAGACCTCAACCCTGGTCAAGTATGCAGAGAAGTGGTCTCAG
ENST00000379999        GCCTTTGCCGGCACTGGGAAGACCTCAACGCTGGTCAAGTATGCAGAGAAGTGGTCTCAG
ENSPTRT00000004144     GCCTTTGCCGGCACTGGGAAGACCTCAACGCTGGTCAAGTACGCAGAGAAGTGGTCTCAG
ENSMUST00000071564     GCATTTGCAGGCACCGGGAAGACTTCTACCCTGGTCAAGTATGCTGAGAAGTGGTCTCAG
ENSRNOT00000025228     GCATTTGCAGGCACCGGGAAGACGTCTACCCTGGTCAAGTATGCTGAGAAGTGGTCTCAG

ENSPPYT00000002460     AGCAGGTTTCTGTATGTGACATTCAACAAGAGCATCGCAAAGCAGGCCGAACGCGTCTTC
ENSCJAT00000040364     AGCAGGTTTCTGTATGTGACGTTCAACAAGAGCATCGCAAAGCAGGCTGAGCGTGTCTTC
ENST00000379999        AGCAGGTTTCTGTATGTGACATTCAACAAGAGCATCGCAAAGCAGGCCGAACGCGTCTTC
ENSPTRT00000004144     AGCAGGTTTCTGTATGTGACATTCAACAAGAGCATCGCAAAGCAGGCCGAACGCGTCTTC
ENSMUST00000071564     AGCAGGTTTCTGTATGTCACGTTCAACAAGAGCATCGCAAAGCAGGCAGAGCTTGTCTTC
ENSRNOT00000025228     AGCAGGTTTCTGTATGTCACCTTCAACAAGAGCATCGCCAAGCAGGCAGAG---GTCTTC
```

ENSPPYT00000002460	CCCAGCAACGTCATCTGCAAAACCTTCCATTCCATGGCCTATGGGCACGTAGGGCGGAAG
ENSCJAT00000040364	CCCAGCAACGTCATCTGCAAAACCTTCCATTCCATGGCCTACGGGCACATAGGGCGGAAG
ENST00000379999	CCCAGCAACGTCATCTGCAAAACCTTCCACTCCATGGCCTACGGGCACATAGGGCGGAAG
ENSPTRT00000004144	CCCAGCAACGTCATCTGCAAAACCTTCCATTCCATGGCCTACGGGCACATAGGGCGGAAG
ENSMUST00000071564	CCCAGCAACGTCATCTGCAAGACCTTCCATTCCATGGCCTACAGTCACGTCGGGCGGAAG
ENSRNOT00000025228	CCCAGCAACGTCATCTGCAACACAGTGTCTGCAAGAGCCATGTCATTCACTGGGGACAGG
ENSPPYT00000002460	TACCAGTCAAAGAAGAAGTTGAATCTCTTCAAGTTAACACCCTTCATGGTCAACTCCGTC
ENSCJAT00000040364	TACCAGTCGAAGAAAAAGTTGAATCTGTTCAAGTTAACACCCTTTATGGTCAACTCCGTC
ENST00000379999	TACCAGTCAAAGAAGAAGTTGAATCTCTTCAAGTTAACACCCTTCATGGTCAACTCCGTC
ENSPTRT00000004144	TACCAGTCAAAGAAGAAGTTGAATCTCTTCAAGTTAACACCCTTCATGGTCAACTCCGTC
ENSMUST00000071564	TACCAGCTGAAGAAGAAGTTGAATCTCTTCAAGTTGACACCCTTTATGGTTAACTCCGTC
ENSRNOT00000025228	TACCAGCTGAAGAAGAAGCTCAATCTCTTCAAGCTGACACCCTTCATGGTCAACTCTGTC
ENSPPYT00000002460	CTTGCTGAAGGGAAGGGTGGATTCATAAGAGCCAAGCTCGTGTGTAAGACTCTAGAAAAC
ENSCJAT00000040364	CTTGCCGAAGGGAAAGGTGGATTCATAAGAGCCAAGCTCGTGTGTAAGACTCTAGAAAAC
ENST00000379999	CTTGCTGAAGGGAAGGGTGGATTCATAAGAGCCAAGCTTGTGTGTAAGACTCTAGAAAAC
ENSPTRT00000004144	CTTGCTGAAGGGAAGGGTGGATTCATAAGAGCCAAGCTTGTGTGTAAGACTCTAGAAAAC
ENSMUST00000071564	CTTGCTGAAGGAAAAGGTGGATTTATAAGGGCCAAGTTAGTGTGTAAGACTTTAGAAAAC
ENSRNOT00000025228	CTTGCTGAAGGAAAAGGTGGATTCATAAGGGCCAAGTTAGTGTGTAAGACATTAGAAAAC
ENSPPYT00000002460	TTCTTTGCCTCGGCTGACGAAGAGCTGACCATTGATCACGTGCCTATTTGGTGTAAGAAC
ENSCJAT00000040364	TTCTTTGCCTCGGCTGATGAAGAGCTGACCATTGATCACGTGCCCATCTGGTGTAAGAAC
ENST00000379999	TTCTTTGCCTCGGCTGACGAAGAGCTGACCATTGATCACGTGCCTATTTGGTGTAAGAAC
ENSPTRT00000004144	TTCTTTGCCTCGGCTGACGAAGAGCTGACCATTGATCACGTGCCTATTTGGTGTAAGAAC
ENSMUST00000071564	TTCTTTGCATCAGCTGATGAAGAGCTAACTATCGATCATGTGCCCATTTGGTGCAAGAAT
ENSRNOT00000025228	TTCTTTGCATCAGCTGATGAAGAGCTTACTATCGATCATGTGCCCATTTGGTGCAAGAAT
ENSPPYT00000002460	AGCCAAGGACAGAGAGTCATGGTTGAGCAGAGTGAAAAACTGAATGGTGTCCTTGAAGCA
ENSCJAT00000040364	AGCCAAGGACAGAGAGTCATGGTTGAGCAGAGTGAAAAACTGAATGGTGTCCTTGAAGCA
ENST00000379999	AGCCAAGGACAGAGAGTCATGGTTGAGCAGAGTGAAAAACTGAATGGTGTCCTTGAAGCG
ENSPTRT00000004144	AGCCAAGGACAGAGAGTCATGGTTGAGCAGAGTGAAAAACTGAATGGTGTCCTTGAAGCG
ENSMUST00000071564	AGCCACGGGCAGAGAGTCATGGTGGAGCAGAGTGAGAAACTGAATGGTGTCCTTGAAGCA
ENSRNOT00000025228	AGCCAAGGACAGAGAGTCATGGTTGAACAGAGTGAGAAGCTGAATGGTGTTCTTGAAGCA
ENSPPYT00000002460	AGCCGCCTCTGGGATAACATGCGGAAGCTGGGGGAGTGCACAGAAGAGGCGTACCAGATG
ENSCJAT00000040364	AGCCGCCTCTGGGATAACATGCGAAAGCTGGGGGAGTGCACAGAAGAGGCGTACCAGATG
ENST00000379999	AGCCGCCTCTGGGATAACATGCGGAAGCTGGGGGAGTGCACAGAAGAGGCGCACCAGATG
ENSPTRT00000004144	AGCCGCCTCTGGGATAACATGCGGAAGCTGGGGGAGTGCACAGAAGAGGCGTACCAGATG
ENSMUST00000071564	AGCCGACTCTGGGACAACATGCGGAAGCTGGGGGAATGCAAAGAAGAGGCATACCAAATG
ENSRNOT00000025228	AGCCGACTCTGGGACAACATGAGGAAGCTGGGGGAATGCAAAGAAGAGGCATACCAAATG
ENSPPYT00000002460	ACTCATGACGGCTACTTGAAACTCTGGCAGCTGAGCAAACCTTTGCTGGCCTCTTTTGAT
ENSCJAT00000040364	ACTCATGATGGCTACTTGAAACTCTGGCAGCTGAGCAAGCCTTTGCTGGCCTCTTTTGAT
ENST00000379999	ACTCATGACGGCTACTTGAAACTCTGGCAGCTGAGCAAGCCTTCGCTGGCCTCTTTTGAC
ENSPTRT00000004144	ACTCATGACGGCTACTTGAAACTCTGGCAGCTGAGCAAGCCTTTGCTGGCCTCTTTTGAC
ENSMUST00000071564	ACACATGATGGCTATTTGAAACTCTGGCAGCTGAGCAAACCTTTGCTTGCCTCTTTTGAC
ENSRNOT00000025228	ACACATGACGGCTATTTGAAACTCTGGCAGCTGAGCAAACCTTTGCTTGCCTCTTTTGAC
ENSPPYT00000002460	GCCATCTTTGTGGATGAGGCCCAGGACTGCACACCAGCTATCATGAACATAGTTCTGTCT
ENSCJAT00000040364	GCCATCTTTGTGGATGAGGCCCAGGACTGCACACCAGCTATCATGAACATAGTTCTGTCT
ENST00000379999	GCCATCTTTGTGGATGAGGCCCAGGACTGCACACCAGCTATCATGAACATAGTTCTGTCT
ENSPTRT00000004144	GCCATCTTTGTGGATGAGGCCCAGGACTGCACCCCAGCTATCATGAACATAGTTCTGTCT

```
ENSMUST00000071564     GCCATCTTTGTGGATGAGGCCCAGGACTGTACCCCAGCTATCATGAACATAGTTCTGTCT
ENSRNOT00000025228     GCCATCTTTGTGGATGAGGCCCAGGACTGTACCCCAGCTATCATGAACATAGTTCTGTCT

ENSPPYT00000002460     CAGCCATGTGGGAAAATCTTTGTAGGGGACCCGCACCAGCAGATCTATACCTTCCGGGGT
ENSCJAT00000040364     CAGCCATGTGGGAAAATCTTTGTAGGGGACCCGCACCAGCAGATCTATACCTTCCGGGGT
ENST00000379999        CAGCCATGTGGGAAAATCTTTGTAGGGGACCCGCACCAGCAGATCTATACCTTCCGGGGT
ENSPTRT00000004144     CAGCCATGTGGGAAAATCTTTGTAGGGGACCCGCACCAGCAGATCTATACCTTCCGGGGT
ENSMUST00000071564     CAGCCATGTGGGAAGATCTTTGTAGGGGACCCCCACCAGCAGATCTATACCTTCCGAGGT
ENSRNOT00000025228     CAGCCATGCGGGAAGATCTTTGTAGGAGACCCCCACCAACAGATCTATACCTTCCGAGGT

ENSPPYT00000002460     GCAGTCAACGCCCTGTTCACAGTGCCCCACACCCACGTCTTCTATCTCACGCAGAGTTTT
ENSCJAT00000040364     GCGGTCAACGCCCTGTTCACAGTGCCCCACACCCACGTCTTCTATCTCACGCAGAGTTTT
ENST00000379999        GCGGTCAACGCCCTGTTCACAGTGCCCCACACCCACGTCTTCTATCTCACGCAGAGTTTT
ENSPTRT00000004144     GCGGTCAACGCCCTGTTCACAGTGCCCCACACCCACGTCTTCTATCTCACGCAGAGTTTT
ENSMUST00000071564     GCAGTGAATGCTCTGTTCACAGTTCCCCACACCCACGTCTTCTATCTCACACAGAGTTTT
ENSRNOT00000025228     GCAGTGAATGCCCTGTTCACAGTCCCCCACACTCACGTCTTCTATCTCACACAGAGCTTT

ENSPPYT00000002460     CGGTTTGGTGTGGAAATAGCTTATGTGGGAGCTACTATCTTGGATGTTTGCAAGAGAGTC
ENSCJAT00000040364     CGGTTTGGTGTGGAAATAGCTTATGTGGGAGCTACTATCTTGGATGTTTGCAAGAGAGTC
ENST00000379999        CGGTTTGGTGTGGAAATAGCTTATGTGGGAGCTACTATCTTGGATGTTTGCAAGAGAGTC
ENSPTRT00000004144     CGGTTTGGTGTGGAAATAGCTTATGTGGGAGCTACTATCTTGGATGTTTGCAAGAGAGTC
ENSMUST00000071564     AGATTTGGTGTGGAAATAGCCTATGTGGGAGCTACCATTTTGGATGTCTGCAAGAGAGTC
ENSRNOT00000025228     AGGTTTGGTGTGGAAATAGCCTATGTGGGAGCTACCATTTTGGATGTCTGCAAGAGAGTC

ENSPPYT00000002460     AGGAAAAAGACTTTGGTTGGAGGAAACCATCAGAGTGGCATTAGAGGTGACGCAAAGGGG
ENSCJAT00000040364     AGGAAAAAGACCTTGGTTGGAGGAAACCATCAGAGTGGCATTAGAGGTGATGTAAAGGGG
ENST00000379999        AGGAAAAAGACTTTGGTTGGAGGAAACCATCAGAGTGGCATTAGAGGTGACGCAAAGGGG
ENSPTRT00000004144     AGGAAAAAGACTTTGGTTGGAGGAAACCATCAGAGTGGCATTAGAGGTGACGCAAAGGGG
ENSMUST00000071564     AGGAAGAAGACGTTGGTTGGAGGGAACCATCAGAGTGGCATAAGAGGTGACATAAAGGGA
ENSRNOT00000025228     AGGAAAAAGACGCTGGTTGGAGGGAACCATCAGAGTGGCATAAGAGGTGATGTAAAGGGA

ENSPPYT00000002460     CAAGTGGCCTTGTTGTCCCGGACCAACGCCAACGTGTTTGATGAGGCTGTACGGGTGACG
ENSCJAT00000040364     CAAGTGGCCTTGTTGTCCCGGACCAATGCCAACGTGTTTGATGAGGCCGTACGGGTGACG
ENST00000379999        CAAGTGGCCTTGTTGTCCCGGACCAACGCCAACGTGTTTGATGAGGCCGTACGGGTGACG
ENSPTRT00000004144     CAAGTGGCCTTGTTGTCCCGGACCAACGCCAACGTGTTTGATGAGGCCGTACGGGTGACG
ENSMUST00000071564     CAAGTAGCTCTGCTGTCCAGGACCAATGCCAATGTGTTTGACGAGGCTGTCCGGGTAACA
ENSRNOT00000025228     CAAGTGGCTCTGCTGTCTCGGACCAATGCCAATGTGTTTGACGAGGCCGTCCGGGTGACA

ENSPPYT00000002460     GAAGGGGAAGTCCCTTCAAGGATACATTTGATTGGGGGGATTAAATCATTTGGATTGGAC
ENSCJAT00000040364     GAAGGGGAAGTCCCTTCGAGGATACATTTGATTGGGGGGATTAAATCATTTGGATTGGAC
ENST00000379999        GAAGGGGAATTCCCTTCAAGGATACATTTGATTGGGGGGATTAAATCATTTGGATTGGAC
ENSPTRT00000004144     GAAGGGGAATTCCCTTCAAGGATACATTTGATTGGGGGGATTAAATCATTTGGATTGGAC
ENSMUST00000071564     GAAGGAGAATCCCCTGCAAGGATACATTTGATTGGGGGGATTAAATCGTTTGGATTGGAC
ENSRNOT00000025228     GAAGGAGAATCGCCTGCACGGATACATTTGATCGGCGGGATTAAATCATTTGGATTGGAC

ENSPPYT00000002460     AGAATCATTGATATTTGGATCCTTCTTCAGCCAGAGGAAGAACGGAGGAAACAAAACCTC
ENSCJAT00000040364     AGAATCATTGATATTTGGATCCTTCTTCAGCCAGAGGAAGAACGGAGGAAACAAAACCTC
ENST00000379999        AGAATCATTGATATTTGGATCCTTCTTCAGCCAGAGGAAGAACGGAGGAAACAAAACCTC
ENSPTRT00000004144     AGAATCATTGATATTTGGATCCTTCTTCAGCCAGAGGAAGAACGGAGGAAACAAAACCTC
ENSMUST00000071564     AGAATCATTGACATTTGGACCCTTCTTCAGCCAGAGGAAGAACGGAGGAAACGAGACCTC
ENSRNOT00000025228     AGAATCATTGACATTTGGACTCTTCTTCAACCAGAGGAAGAACGGAGGAAACGAGACCTC
```

```
ENSPPYT00000002460     GTCATTAAAGACAAATTTATCAGAAGATGGGTGCACAAAGAAGGCTTTAGTGGCTTCAAG
ENSCJAT00000040364     GTCATTAAAGACAGATTTATCAGAAGATGGGTGCACAAAGAAGGCTTTAGTGGCTTCAAG
ENST00000379999        GTCATTAAAGACAAATTTATCAGAAGATGGGTGCACAAAGAAGGCTTTAGTGGCTTCAAG
ENSPTRT00000004144     GTCATTAAAGACAAATTTATCAGAAGATGGGTGCACAAAGAAGGCTTTAGTGGCTTCAAG
ENSMUST00000071564     ATCATTAAAGACAGGTTCATCAGAAGATGGGTGCATAAAGAAGGCTTCAGTGGCTTCAAG
ENSRNOT00000025228     ATCATTAAAGACAGGTTCATCAGAAGATGGGTGCATAAAGAAGGCTTCAGTGGCTTCAAG

ENSPPYT00000002460     AGGTATGTGACCGCTGCCGAGGACAAGGAGCTTGAAGCCAAGATCGCAGTTGTTGAAAAG
ENSCJAT00000040364     AGGTATGTGACCGCTGCCGAGGACAAGGAGCTTGAAGCCAAGATCGCAGTAGTTGAAAAG
ENST00000379999        AGGTATGTGACCGCTGCCGAGGACAAGGAGCTTGAAGCCAAGATCGCAGTTGTTGAAAAG
ENSPTRT00000004144     AGGTATGTGACTGCTGCTGAGGACAAGGAGCTTGAAGCCAAGATCGCAGTTGTTGAAAAG
ENSMUST00000071564     AGGTATGTGACTGCTGCCGAGGACAAGGAACTGGAAGCGAAGATTGCGGTAGTTGAAAAA
ENSRNOT00000025228     AGGTATGTGACTGCTGCAGAGGACAAGGAGCTGGAAGCGAAGATCGCAGTAGTTGAAAAA

ENSPPYT00000002460     TATAACATCAGGATTCCAGAGCTGGTGCAAAGGATAGAAAAATGCCATATAGAAGATTTG
ENSCJAT00000040364     TATAACATCAGGATTCCAGAGCTGGTGCAAAGGATAGAAAAATGCCATATAGAAGATTTG
ENST00000379999        TATAACATCAGGATTCCAGAGCTGGTGCAAAGGATAGAAAAATGCCATATAGAAGATTTG
ENSPTRT00000004144     TATAACATCAGGATTCCAGAGCTGGTGCAAAGGATAGAAAAATGCCATATAGAAGATTTG
ENSMUST00000071564     TACAATATCAGGATTCCAGAACTGGTAGAAAGGATAGAGAGATGCCACATAGACGATTTG
ENSRNOT00000025228     TACAATGTCAGGATCCCAGAACTGGTAGAAAGGATAGAGAGATGCCACATAGAAGATTTG

ENSPPYT00000002460     GACTTTGCAGAGTACATTCTGGGCACTGTGCACAAAGCCAAAGGCCTGGAGTTTGACACT
ENSCJAT00000040364     GACTTTGCAGAGTACATTCTGGGCACTGTGCACAAAGCCAAAGGCCTGGAGTTTGACACT
ENST00000379999        GACTTTGCAGAGTACATTCTGGGCACTGTGCACAAAGCCAAAGGCCTGGAGTTTGACACT
ENSPTRT00000004144     GACTTTGCAGAGTACATTCTGGGCACTGTGCACAAAGCCAAAGGCCTGGAGTTTGACACT
ENSMUST00000071564     GACTTTGCAGAGTACATTTTGGGCACCGTGCACAAAGCCAAAGGGTTAGAGTTTGACACT
ENSRNOT00000025228     GACTTTGCAGAGTACATTTTGGGCACCGTGCACAAAGCCAAAGGGTTAGAGTTTGACACG

ENSPPYT00000002460     GTGCATGTTTTGGATGATTTTGTGAAAGTGCCTTGTGCCCGGCATAACCTGCCCCAGCTT
ENSCJAT00000040364     GTGCATGTTTTGGATGATTTTGTGAAAGTGCCTTGTGCCCGGCATAACCTGGCCCAGCTT
ENST00000379999        GTGCATGTTTTGGATGATTTTGTGAAAGTGCCTTGTGCCCGGCATAACCTGCCCCAGCTT
ENSPTRT00000004144     GTGCATGTTTTGGATGATTTTGTGAAAGTGCCTTGTGCCCGGCATAACCTGCCCCAGCTT
ENSMUST00000071564     GTGCACGTTTTGGATGATTTCGTGAAAGTGCCTTGTGCCAGGCATAATCTTGCCCAGCTT
ENSRNOT00000025228     GTGCATGTTTTGGATGATTTCGTGAAAGTGCCTTGTGCCAGGCATAACCTTGGTCAGCTT

ENSPPYT00000002460     CCCCACTTCAGAGTTGAGTCATTTTCTGAGGATGAATGGAATTTACTGTATGTTGCAGTA
ENSCJAT00000040364     CCCCACTTCAGAGTTGAGTCATTTTCTGAGGATGAATGGAATTTGCTGTATGTTGCAGTA
ENST00000379999        CCGCACTTCAGAGTTGAGTCATTTTCTGAGGATGAATGGAATTTACTGTATGTTGCAGTA
ENSPTRT00000004144     CCGCACTTCAGAGTTGAGTCATTTTCTGAGGATGAATGGAATTTACTGTATGTTGCAGTA
ENSMUST00000071564     CCTCACTTCAGAGTTGAGTCGTTTTCTGAGGATGAATGGAATTTACTGTATGTTGCTGTT
ENSRNOT00000025228     CCTCACTTCAGAGTTGAGTCGTTTTCTGAGGATGAATGGAATTTACTCTATGTTGCTGTT

ENSPPYT00000002460     ACTCGAGCCAAGAAGCGTCTCATCATGACCAAATCATTGGAAAACATTTTGACTTTGGCT
ENSCJAT00000040364     ACTCGTGCCAAGAAGCGTCTCATCATGACCAAATCATTGGAAAACATTTTGACTTTGGCT
ENST00000379999        ACTCGAGCCAAGAAGCGTCTCATCATGACCAAATCATTGGAAAACATTTTGACTTTGGCT
ENSPTRT00000004144     ACTCGAGCCAAGAAGCGTCTCATCATGACCAAATCATTGGAAAACATTTTGACTTTGGCT
ENSMUST00000071564     ACTCGTGCCAAGAAGCGGCTCATAATGACCAAATCCCTGGAGAACATCCTGACTCTGGCT
ENSRNOT00000025228     ACTCGTGCCAAGAAGCGGCTTATAATGACCAAATCACTGGAGAACATTCTGACTCTGGCT

ENSPPYT00000002460     GGGGAGTACTTCTTGCAAGCAGAGCTGACAAGCAATGTCTTAAAAACAGGCGTGGTGCGC
ENSCJAT00000040364     GGGGAGTACTTCTTGCAAGCAGAGCTGACAAGCAATGTCTTAAAAACAGGCGTGGTGCGC
ENST00000379999        GGGGAGTACTTCTTGCAAGCAGAGCTGACAAGCAACGTCTTAAAAACAGGCGTGGTGCGC
ENSPTRT00000004144     GGGGAGTACTTCTTGCAAGCAGAGCTGACAAGCAACGTCTTAAAAACAGGCGTGGTGCGC
```

```
ENSMUST00000071564     GGGGAGTATTTCTTGCAAGCAGAGTTGACAAGTAACGTTTTGAAAACAGGAGTGGTCCAC
ENSRNOT00000025228     GGGGAGTATTTCTTGCAGGCAGAGCTGACAAGTAACGTCTTGAAAACAGGAGTGGTCCAC


ENSPPYT00000002460     TGCTGCGTGGGACAGTGCAACAATGCCATCCCTGTTGACACCGTCCTCACCATGAAGAAG
ENSCJAT00000040364     TGCTGCGTGGGACAGTGCAACAATGCCATCCCTGTTGACACCGTCCTCACCATGAAGAAG
ENST00000379999        TGCTGCGTGGGACAGTGCAACAATGCCATCCCTGTTGACACCGTCCTTACCATGAAGAAG
ENSPTRT00000004144     TGCTGCGTGGGACAGTGCAACAATGCCATCCCTGTTGACACCGTCCTCACCATGAAGAAG
ENSMUST00000071564     TGCTGTGTGGGGCAGTGCAACAACACCATCCCTGTGGACACCATCCTTACCATGAAGAAA
ENSRNOT00000025228     TGCTGTGTGGGGCAGTGCAACAACACCATCCCTGTGGACACCATCCTTACCATGAGGAAA


ENSPPYT00000002460     CTGCCCATCACCTAT---------------------------------------------
ENSCJAT00000040364     CTGCCCATAACCTACAGCAACAGGAAGGAAAACAAGGGGGGCTACCTCTGCCACTCCTGC
ENST00000379999        CTGCCCATCACCTATAGCAACAGGAAGGAAAACAAGGGGGGCTACCTCTGCCACTCCTGT
ENSPTRT00000004144     CTGCCCATCACCTATAGCAACAGGAAGGAAAACAAGGGGGGCTACCTCTGCCACTCCTGC
ENSMUST00000071564     CTGCCTATCACTTATAGCAACAGGAAGGAAAACAAGGGTGGCTACCTCTGCCACTCATGT
ENSRNOT00000025228     CTGCCTATCACCTATAGCAACAGGAAGGAAAACAAAGGTGGCTACCTGTGCCACTCCTGC


ENSPPYT00000002460     ---------------------------------------------------GTACGTCTG
ENSCJAT00000040364     GCGGAGCAGCGCATCGGGCCCCTGGCGTTCCTGACGGCCTCCCCGGAGCAGGTGCGCGCC
ENST00000379999        GCGGAGCAGCGCATCGGGCCCCTGGCGTTCCTGACAGCCTCCCCGGAGCAGGTGCGCGCC
ENSPTRT00000004144     GCGGAGCAGCGCATCGGGCCCCTGGCGTTCCTGACAGCCTCCCCGGAGCAGGTGCGCGCC
ENSMUST00000071564     GCAGAGCAGCGCATCGGGCCTTTGGCATTCCTGACTGCCTCCCCAGAGCAGGTGCGTGCC
ENSRNOT00000025228     GCAGAACAGCGGATCGGGCCTTTGGCATTCCTGACTGCCTCCCCAGAGCAGGTGCGCGCC


ENSPPYT00000002460     CTGTCTGTGGAACTTAATTCAGCCATTTGCATTTTTTGCTTG
ENSCJAT00000040364     ATGGAGCGCACCGTGGAGAACATCGTGCTGTTCCTTGTCTTC
ENST00000379999        ATGGAGCGCACTGTGGAGAACATCGTACTGTTCCTCGTCTTC
ENSPTRT00000004144     ATGGAGCGCACTGTGGAGAACATCGTACTG------------
ENSMUST00000071564     ATGGAACGCACTGTGGAAGACATAGTGCTGTTCCTTGTCTTC
ENSRNOT00000025228     ATGGAACGCACGGTGGAAGACCTCGTGCTGTTCCTCGTCTTC
```

Multiple sequence alignment of Fbxo2

```
ENSCJAT00000025715     ATGGACGGAGACGGTGACCCAGAGAGCGTGGGCCAGCCGGAGGAGGCAAGCCCAGAGGAG
ENSGGOT00000002108     ATGGACGGAGACGGTGACCCAGACAGCGTGGGCCAGCCCGAGGAGGCAAGCCCGGAGGAG
ENSPPYT00000002254     ATGGACGGAGACGGTGACCCAGAGAGCGTGGGCCAGCCCAAGGAGGCAAGCCCGGAGGAG
ENSPTRT00000000329     ATGGACGGAGACGGTGACCCAGAGAGCGTGGGCCAGCCCGAGGAGGCAAGCCCGGAGGAG
ENST00000354287        ATGGACGGAGACGGTGACCCAGAGAGCGTGGGCCAGCCCGAGGAGGCAAGCCCGGAGGAG
ENSMUST00000047951     ATGGATGGAGATGGTGATCCAGAGAGTGTGAGCCACCCCGAAGAAGCGAGCCCAGAGGAG
ENSRNOT00000012670     ATGGATGGAGATGGTGATCCAGAGAGTGTGAGCCACCCCGAAGAGGCGAGCCCGGAGGAG


ENSCJAT00000025715     CAGCCGGAGGAGGTGAGCACCGAGGAGGAGCGGCCGGAGGATCAGGAGGAGGAGGAGGAG
ENSGGOT00000002108     CAGCCAGAGGAGGCGAGTGCTGAGGAGGAGCGGCCGGAGGACCAGGAGGAGGAGGAGGCG
ENSPPYT00000002254     CAGCCAGAGGAGGCGAGCGCTGAGGAGGAGCGGCCGGAGGACCAGGAGGAGGAGGAGGCG
ENSPTRT00000000329     CAGCCAGAGGAGGCGAGTGCTGAGGAGGAGCGGCTGGAGGACCAGGAGGAGGAGGAGGCG
ENST00000354287        CAGCCAGAGGAGGCGAGTGCTGAGGAGGAGCGGCCGGAGGACCAGCAGGAGGAGGAGGCG
ENSMUST00000047951     CAGCCAGAGGAGGCGAGTGCGGAGGAGGAGCAGCTCCGGGAGGCGGAGGAGGAGGAAGCG
ENSRNOT00000012670     CAGCCTGAGGAGGCGAGTGCGGAGGAGGAGCAGCCCAGGGAGGAGGAGGAGGAAGAGACA


ENSCJAT00000025715     GAGGCGGCCGCGTACCTGGACGAGCTGCCCGAGCCGCTGCTGCTGCGCGTGCTGGCCGCG
ENSGGOT00000002108     GCGGCCGCCGCGTACCTGGACGAGCTGCCCGAGCCGCTGCTGCTGCGCGTGCTGGCCGCA
ENSPPYT00000002254     GCGGCCGCCGCGTACCTAGACGAGCTGCCCGAGCCGCTGCTGCTGCGCGTGCTGGCCGCA
ENSPTRT00000000329     GCGGCCGCCGCGTACCTGGACGAGCTGCCCGAGCCGCTGCTGCTGCGCGTGCTGGCCGCA
```

```
ENST00000354287         GCCGCCGCCGCGTACCTGGACGAGCTGCCCGAGCCGCTGCTGCTGCGCGTGCTGGCCGCA
ENSMUST00000047951      GAGGCCGTGGAGTACCTGGCCGAGCTGCCGGAGCCACTGCTGCTGCGCGTGCTGGCCGAG
ENSRNOT00000012670      GAGGCCGTGGAGTACCTTGCCGAGTTGCCTGAGCCACTGCTGCTGCGCGTGCTGGCAGAG

ENSCJAT00000025715      CTCCCGGCCGCCGAGCTGGTGCAGGCCTGCCGCCTGGTGTGCCTGCGCTGGAAGGAGCTG
ENSGGOT00000002108      CTGCCGGCCGCCGAGCTGGTGCAGGCCTGCCGCCTGGTGTGCCTGCGCTGGAAGGAGCTG
ENSPPYT00000002254      CTGCCGGCCGCCGAGCTGGTGCGGGCCTGCCGCCTGGTGTGCCTGCGCTGGAAGGAGCTG
ENSPTRT00000000329      CTGCCGGCCGCCGAGCTGGTGCAGGCCTGCCGCCTGGTGTGCCTGCGCTGGAAGGAGCTG
ENST00000354287         CTGCCGGCCGCCGAGCTGGTGCAGGCCTGCCGCCTGGTGTGCCTGCGCTGGAAGGAGCTG
ENSMUST00000047951      CTGCCAGCTACGGAGCTGGTGCAGGCCTGCCGCCTGGTGTGCCTGCGCTGGAAGGAGCTG
ENSRNOT00000012670      CTGCCCGCTACGGAGCTAGTGCAAGCCTGCCGCCTGGTGTGCCTGCGCTGGAAGGAGCTG

ENSCJAT00000025715      GTGGACGGTGCCCCGCTGTGGCTGCTCAAGTGCCAGCAGGAGGGGCTGGTGCCCGAGGGC
ENSGGOT00000002108      GTGGACGGCGCCCCGCTGTGGCTGCTCAAGTGCCAGCAGGAGGGGCTGGTGCCCGAGGGC
ENSPPYT00000002254      GTGGACGGCGCCCCGCTGTGGCTGCTCAAGTGCCAGCAGGAGGGGCTGGTGCCCGAGGGC
ENSPTRT00000000329      GTGGACGGCGCCCCGCTGTGGCTGCTCAAGTGCCAGCAGGAGGGGCTGGTGCCCGAGGGC
ENST00000354287         GTGGACGGCGCCCCGCTGTGGCTGCTCAAGTGCCAGCAGGAGGGGCTGGTGCCCGAGGGC
ENSMUST00000047951      GTGGACGGCGCCCCACTGTGGCTGCTCAAGTGCCAGCAGGAGGGGCTGGTGCCCGAGGGC
ENSRNOT00000012670      GTGGACGGCGCCCCACTGTGGCTGCTCAAGTGTCAGCAGGAGGGGCTGGTGCCTGAGGGC

ENSCJAT00000025715      GGCGCGGAGGAGGAGCGCGACCACTGGCAGCGGTTCTACTTCCTAAGCAAGCGGCGCCGC
ENSGGOT00000002108      GGCGTGGAGGAGGAGCGCGACCACTGGCAGCAGTTCTACTTCCTGAGCAAGCGGCGCCGC
ENSPPYT00000002254      GGCGCGGAGGAGGAGCGCGACCACTGGCAGCAGTTCTACTTCCTGAGCAAGCGGCGCCGC
ENSPTRT00000000329      GGCGTGGAGGAGGAGCGCGACCACTGGCAGCAGTTCTACTTCCTGAGCAAGCGGCGCCGC
ENST00000354287         GGCGTGGAGGAGGAGCGCGACCACTGGCAGCAGTTCTACTTCCTGAGCAAGCGGCGCCGC
ENSMUST00000047951      AGCGCTGATGAGGAGCGGGACCACTGGCAACAGTTCTACTTTCTGAGCAAGAGGAGGCGC
ENSRNOT00000012670      AGCGCTGATGACGAGCGGGACCACTGGCAACAGTTCTACTTTCTGAGCAAGAGGAGGCGC

ENSCJAT00000025715      AATCTGCTGCGCAACCCGTGCGGGGAAGAGGACTTGGAAGGCTGGTGTGACGTGGAGCAT
ENSGGOT00000002108      AACCTTCTGCGTAACCCGTGTGGGGAAGAGGACTTGGAAGGCTGGTGTGACGTGGAGCAT
ENSPPYT00000002254      AACCTTCTGCGTAACCCGTGTGGGGAAGAGGACTTGGAAGGCTGGTGTGACGTGGAGCAC
ENSPTRT00000000329      AACCTTCTGCGTAACCCGTGTGGGGAAGAGGACTTGGAAGGCTGGTGTGACGTGGAGCAT
ENST00000354287         AACCTTCTGCGTAACCCGTGTGGGGAAGAGGACTTGGAAGGCTGGTGTGACGTGGAGCAT
ENSMUST00000047951      AACCTGCTGCGCAACCCTTGTGGGGAAGAGGACTTGGAGGGCTGGAGCGACGTGGAGCAC
ENSRNOT00000012670      AACCTGCTGCGTAACCCGTGTGGGGAAGAGGACTTGGAGGGCTGGTGCGACGTGGAGCAC

ENSCJAT00000025715      GGTGGGGACGGCTGGAGGGTGGAGGAGCTGCCTGGAGACAGTGGGGTGGAATTTACCCAT
ENSGGOT00000002108      GGTGGGGACGGCTGGAGGGTGGAGGAGCTGCCTGGAGACAGTGGGGTGGAGTTCACCCAC
ENSPPYT00000002254      GGTGGGGACGGCTGGAGGGTGGAGGAGCTGCCTGGAGACAGTGGGGTGGAATTCACCCAC
ENSPTRT00000000329      GGTGGGGACGGCTGGAGGGTGGAGGAGCTGCCTGGAGACAGTGGGGTGGAGTTCACCCAC
ENST00000354287         GGTGGGGACGGCTGGAGGGTGGAGGAGCTGCCTGGAGACAGTGGGGTGGAGTTCACCCAC
ENSMUST00000047951      GGTGGGGACGGCTGGAGGGTGGAGGAACTGCCCGGAGACAATGGGGTGGAATTTACCCAA
ENSRNOT00000012670      GGTGGGGACGGCTGGAGAGTGGAGGAACTGCCCGGAGACAGTGGGGTGGAATTTACCCAC

ENSCJAT00000025715      GATGAGAGCGTCAAGAAGTACTTCGCCTCCTCCTTTGAGTGGTGCCGCAAAGCACAGGTC
ENSGGOT00000002108      GATGAGAGCGTCAAGAAGTACTTCGCCTCCTCCTTTGAGTGGTGTCGCAAAGCACAGGTG
ENSPPYT00000002254      GATGAGAGTGTCAAGAAGTACTTCGCCTCCTCCTTTGAGTGGTGTCGCAAAGCACAGGTC
ENSPTRT00000000329      GATGAGAGCGTCAAGAAGTACTTCGCCTCCTCCTTTGAGTGGTGTCGCAAAGCACAGGTG
ENST00000354287         GATGAGAGCGTCAAGAAGTACTTCGCCTCCTCCTTTGAGTGGTGTCGCAAAGCACAGGTC
ENSMUST00000047951      GATGACAGCGTTAAGAAATACTTCGCCTCCTCCTTCGAGTGGTGTCGCAAAGCGCAGGTC
ENSRNOT00000012670      GACGACAGCGTTAAGAAGTACTTCGCCTCCTCCTTCGAGTGGTGTCGCAAAGCGCAGGTC

ENSCJAT00000025715      ATTGACCTGCATGCCGAGGGCTACTGGGAGGAGCTACTGGATACGACTCAGCCGGCCATC
```

```
ENSGGOT00000002108    ATTGACCTGCAGGCTGAGGGCTACTGGGAGGAGCTGCTGGACACGACTCAGCCGGCCATC
ENSPPYT00000002254    ATTGACCTGCAGGCTGAGGGCTACTGGGAGGAGCTGCTGGACACAACTCAGCCGGCCATC
ENSPTRT00000000329    ATTGACCTGCAGGCTGAGGGCTACTGGGAGGAGCTGCTGGACACGACTCAGCCGGCCATC
ENST00000354287       ATTGACCTGCAGGCTGAGGGCTACTGGGAGGAGCTGCTGGACACGACTCAGCCGGCCATC
ENSMUST00000047951    ATTGATCTGCAGGCAGAGGGCTACTGGGAGGAGCTACTGGACACCACCCAGCCCGCCATC
ENSRNOT00000012670    ATTGATCTGCAGGCTGAGGGCTACTGGGAGGAGCTACTGGACACCACCCAGCCCGCCATC

ENSCJAT00000025715    GTGGTGAAGGACTGGTACTCTGGCCGCAGCGACGCTGGCTGCCTCTACGAGCTCACCGTG
ENSGGOT00000002108    GTGGTGAAGGACTGGTACTCGGGCCGCAGCGACGCTGGTTGCCTCTACGAGCTCACCGTT
ENSPPYT00000002254    GTGGTGAAGGACTGGTACTCGGGCCGCAGCGACGCTGGTTGCCTCTACGAGCTCACCGTT
ENSPTRT00000000329    GTGGTGAAGGACTGGTACTCGGGCCGCAGCGACGCTGGTTGCCTCTACGAGCTCACCGTT
ENST00000354287       GTGGTGAAGGACTGGTACTCGGGCCGCAGCGACGCTGGTTGCCTCTACGAGCTCACCGTT
ENSMUST00000047951    GTGGTGAAGGACTGGTACTCGGGTCGCACTGATGCGGGCAGCCTGTATGAGCTCACTGTG
ENSRNOT00000012670    GTGGTGAAGGACTGGTACTCGGGCCGCACGGATGCCGGCAGCCTGTATGAGCTCACTGTG

ENSCJAT00000025715    AAGCTACTGTCCGAGCACGAGGATGTGCTGGCCGAGTTCAACAGCGGGCAGGTGGCAGTG
ENSGGOT00000002108    AAGCTACTGTCCGAGCACGAGGACGTGCTGGCTGAGTTCAGCAGCGGGCAGGTGGCAGTG
ENSPPYT00000002254    AAGCTACTGTCCGAGCACGAGGACGTGCTGGCTGAGTTCAGCAGCGGGCAAGTGGCAGTG
ENSPTRT00000000329    AAGCTACTGTCCGAGCACGAGAACGTGCTGGCTGAGTTCAGCAGCGGGCAGGTGGCAGTG
ENST00000354287       AAGCTACTGTCCGAGCACGAGAACGTGCTGGCTGAGTTCAGCAGCGGGCAGGTGGCAGTG
ENSMUST00000047951    AGGCTGCTGTCGGAGAACGAAGATGTGCTGGCGGAGTTCGCTACAGGACAGGTGGCTGTG
ENSRNOT00000012670    AGGCTGCTGTCGGAGCATGAAGATGTGCTGGCGGAGTTCAATACGGGACAGGTGGCTGTG

ENSCJAT00000025715    CCCCAAGACAGTGACGGCGGAGGCTGGATGGAAATCTCCCACACCTTCACTGACTACGGG
ENSGGOT00000002108    CCCCAAGACAGTGACGGCGGGGGCTGGATGGAGATCTCCCACACCTTCACCGACTACGGG
ENSPPYT00000002254    CCCCAAGACAATGACGGCGGGGGCTGGATGGAGATCTCCCACACCTTCACCGACTACGGG
ENSPTRT00000000329    CCCCAAGACAGTGACGGCGGGGGCTGGATGGAGATCTCCCACACCTTCACCGACTACGGG
ENST00000354287       CCCCAAGACAGTGACGGCGGGGGCTGGATGGAGATCTCCCACACCTTCACCGACTACGGG
ENSMUST00000047951    CCAGAGGAC---------GGCAGTTGGATGGAGATCTCCCACACCTTCATCGACTACGGG
ENSRNOT00000012670    CCAGAGGAT---------GGCAGCTGGATGGAGATCTCCCACACTTTCACCGACTACGGG

ENSCJAT00000025715    CCGGGCGTCCGCTTCGTTCGCTTCGAGCACGGGGGGCAGGACTCCGTCTACTGGAAGGGC
ENSGGOT00000002108    CCGGGCGTCCGCTTCGTCCGCTTCGAGCACGGGGGGCAGGACTCCGTCTACTGGAAGGGC
ENSPPYT00000002254    CCGGGCGTCCGCTTCGTCCGCTTCGAGCACGGGGGGCAGGACTCCGTCTACTGGAAGGGC
ENSPTRT00000000329    CCGGGCGTCCGCTTCGTCCGCTTCGAGCACGGGGGGCAGGACTCCGTCTACTGGAAGGGC
ENST00000354287       CCGGGCGTCCGCTTCGTCCGCTTCGAGCACGGGGGGCAGGACTCCGTCTACTGGAAGGGC
ENSMUST00000047951    CCAGGTGTCCGCTTTGTCCGCTTCGAGCACGGAGGGCAGGACTCGGTCTACTGGAAGGGC
ENSRNOT00000012670    CCAGGCGTCCGCTACGTCCGCTTCGAGCACGGAGGGCAGGACTCGGTCTACTGGAAGGGC

ENSCJAT00000025715    TGGTTCGGGGCCCGGGTGACCAACAGCAGCGTGTGGGTAGAACCC
ENSGGOT00000002108    TGGTTCGGGGCCCGGGTGACCAACAGCAGCGTTCAGCTGGGCCCG
ENSPPYT00000002254    TGGTTCGGGGCCCGGGTGACCAACAGCAGCGTGTGGGTAGAACCC
ENSPTRT00000000329    TGGTTCGGGGCCCGGGTGACCAACAGCAGCGTGTGGGTAGAACCC
ENST00000354287       TGGTTCGGGGCCCGGGTGACCAACAGCAGCGTGTGGGTAGAACCC
ENSMUST00000047951    TGGTTCGGGGCCCGGGTGACCAACAGTAGCGTGTGGGTGGAACCC
ENSRNOT00000012670    TGGTTCGGGGCCCGGGTGACCAACAGTAGCGTGTGGGTGGAACCC
```

Multiple sequence alignment of Fbxo21

```
ENSMMUT00000024578    ATGGCGGCGGCAGCAGTCGACAGCGCGATGGAGGTGGTGCCGGCGCTACCGGAGGAGGCC
ENSGGOT00000023531    ATGGCGGCGGCAGCAGTCGACAGCGCAATGGAGGTGGTGCCGGCGCTGGCGGAGGAGGCC
ENST00000330622       ATGGCGGCGGCAGCAGTCGACAGCGCGATGGAGGTGGTGCCGGCGCTGGCGGAGGAGGCC
ENSPTRT00000010103    ATGGCGGCGGCAGCAGTCGACAGCGCGATGGAGGTGGTGCCGGCGCTGGCGGAGGAGGCC
ENSPPYT00000005923    ATGGCGGCGGCAGCAGTCGACAGCGCGATGGAGGTGGTGCCGGCGCTGGCGGAGGAGGCC
```

```
ENSMUST00000035579     ATGGCGTCGGTAGCGGGGGACAGCGCTATGGAGGTGGTGCCGGCGCTGGCCGAGGAGGCC
ENSRNOT00000001491     ATGGCGGCGGTAGCGGGGGACAGCGCTATGGAGGTGGTCCCGGCGCTGGCTGAAGAGGCC

ENSMMUT00000024578     GCGCCGGAGATAGCGGGCCTCAGCTGCCTGGTCAACCTGCCGGGTGAGGTGCTGGAGTAC
ENSGGOT00000023531     GCGCCGGAGGTAGCGGGCCTCAGCTGCCTCGTCAACCTGCCGGGTGAGGTGCTGGAGTAC
ENST00000330622        GCGCCGGAGGTAGCGGGCCTCAGCTGCCTCGTCAACCTGCCGGGTGAGGTGCTGGAGTAC
ENSPTRT00000010103     GCGCCGGAGGTAGCGGGCCTCAGCTGCCTCGTCAACCTGCCGGGTGAGGTGCTGGAGTAC
ENSPPYT00000005923     GCGCCGGAGGTAGCGGGCCTCAGCTGCCTCGTCAACCTGCCGGGTGAGGTGCTGGAGTAC
ENSMUST00000035579     GCGGCTGAGGCGACGGGCCCCAGCTGCCTGGTGCAGCTGCCGGGCGAGGTGCTGGAGTAC
ENSRNOT00000001491     GCGGCCGAGGCGTCGGGCCCCAGCTGCCTGGTGCAACTGCCAGGCGAGGTGCTGGAGTAC

ENSMMUT00000024578     ATCCTATGCTGCGGCTCGCTGACGGCCGCCGACATTGGCCGTGTCTCCAGCACCTGCCGG
ENSGGOT00000023531     ATCCTGTGCTGCGGCTCGCTGACGGCCGCCGACATCGGCCGTGTCTCCAGCACCTGCCGG
ENST00000330622        ATCCTGTGCTGCGGCTCGCTGACGGCCGCCGACATCGGCCGTGTCTCCAGCACCTGCCGG
ENSPTRT00000010103     ATCCTGTGCTGCGGCTCGCTGACGGCCGCCGACATCGGCCGTGTCTCCAGCACCTGCCGG
ENSPPYT00000005923     ATCCTGTGCTGCGGCTCGCTGACGGCCGCCGACATCGGCCGTGTCTCCAGCACCTGCCGG
ENSMUST00000035579     ATCCTGTGCAGCGGCTCGCTCACGGCGCTGGACATCGGCCGCGTGTCCAGCACCTGCCGC
ENSRNOT00000001491     ATCCTGTGCAGCGGCTCGCTCACGGCGCTCGACATCGGCCGCGTGTCCAGCACCTGCCGC

ENSMMUT00000024578     CGGCTGCGCGAGCTGTGCCAGAGCAGCGGGAAGGTGTGGAAGGAGCAGTTCCGGGTGAGG
ENSGGOT00000023531     CGGCTGCGCGAGCTGTGCCAGAGCAGCGGGAAGGTGTGGAAGGAGCAGTTCCGGGTGAGG
ENST00000330622        CGGCTGCGCGAGCTGTGCCAGAGCAGCGGGAAGGTGTGGAAGGAGCAGTTCCGGGTGAGG
ENSPTRT00000010103     CGGCTGCGCGAGCTGTGCCAGAGCAGCGGGAAGGTGTGGAAGGAGCAGTTCCGGGTGAGG
ENSPPYT00000005923     CGGCTGCGCGAGCTGTGCCAGAGCAGCGGGAAGGTGTGGAAGGAGCAGTTCCGGGTGAGG
ENSMUST00000035579     CGCCTGCGCGAGGTGTGCCAGAGCAGCGGGCAGGTGTGGAAGGAGCAGTTCCGGGTGAGG
ENSRNOT00000001491     CGCCTGCGCGAGGTGTGCCAGAGCAGCGGGCAGGTGTGGAAGGAGCAGTTCCGGGTGAGG

ENSMMUT00000024578     TGGCCTTCCCTTATGAAACACTACAGCCCCACCGACTACGTCAATTGGTTGGAAGAGTAT
ENSGGOT00000023531     TGGCCTTCCCTTATGAAACACTACAGCCCCACCGACTACGTCAATTGGTTGGAAGAATAT
ENST00000330622        TGGCCTTCCCTTATGAAACACTACAGCCCCACCGACTACGTCAATTGGTTGGAAGAGTAT
ENSPTRT00000010103     TGGCCTTCCCTTATGAAACACTACAGCCCCACCGACTACGTCAATTGGTTGGAAGAGTAT
ENSPPYT00000005923     TGGCCTTCCCTTATGAAACACTACAGCCCCACCGACTACGTCAATTGGTTGGAAGAGTAT
ENSMUST00000035579     TGGCCCTCCCTGATGAAGCACTACAGCCCCACCGACTACGTCAATTGGTTAGAGGAATAT
ENSRNOT00000001491     TGGCCCTCTCTGATGAAGCACTACAGTCCCACTGACTACGTCAATTGGTTAGAGGAATAT

ENSMMUT00000024578     AAAGTTCGGCAAAAAGCTGGGTTGGAAGCGCGGAAGATTGTAGCCTCGTTCTCAAAGAGG
ENSGGOT00000023531     AAAGTTCGGCAAAAAGCTGGGTTAGAAGCGCGGAAGATTGTAGCCTCGTTCTCAAAGAGG
ENST00000330622        AAAGTTCGGCAAAAAGCTGGGTTAGAAGCGCGGAAGATTGTAGCCTCGTTCTCAAAGAGG
ENSPTRT00000010103     AAAGTTCGGCAAAAAGCTGGGTTAGAAGCGCGGAAGATTGTAGCCTCGTTCTCAAAGAGG
ENSPPYT00000005923     AAAGTTCGGCAAAAAGCTGGGTTAGAAGCGCGGAAGATTGTAGCCTCGTTCTCAAAGAGG
ENSMUST00000035579     AAAGTTCGGCAGAAAGCTGGGTTAGAAGCCCGGAAGATTGTAGCTTCCTTCTCCAAGCGG
ENSRNOT00000001491     AAAGTTCGGCAGAAAGCTGGGTTAGAAGCCCGGAAGATTGTAGCCTCCTTCTCCAAACGG

ENSMMUT00000024578     TTCTTTTCAGAGCACGTTCCTTGTAATGGCTTCAGTGACATTGAGAACCTTGAAGGACCA
ENSGGOT00000023531     TTCTTTTCAGAGCACGTTCCTTGTAATGGCTTCAGTGACATTGAGAACCTTGAAGGACCA
ENST00000330622        TTCTTTTCAGAGCACGTTCCTTGTAATGGCTTCAGTGACATTGAGAACCTTGAAGGACCA
ENSPTRT00000010103     TTCTTTTCAGAGCACGTTCCTTGTAATGGCTTCAGTGACATTGAGAACCTTGAAGGACCA
ENSPPYT00000005923     TTCTTTTCAGAGCACGTTCCTTGTAATGGCTTCAGTGACATTGAGAACCTTGAAGGACCA
ENSMUST00000035579     TTCTTTTCAGAGCACGTCCCCTGTAATGGCTTCAGTGACATTGAAAACCTTGAGGGCCCA
ENSRNOT00000001491     TTCTTTTCAGAGCACGTTCCTTGTAACGGCTTCAGTGACATTGAGAACCTTGAGGGCCCA

ENSMMUT00000024578     GAGATTTTTTTTGAGGATGAACTGGTGTGTATCCTAAATATGGAAGGAAGAAAAGCTTTG
ENSGGOT00000023531     GAGATTTTTTTTGAGGATGAACTGGTGTGTATCCTAAATATGGAAGGAAGAAAAGCTTTG
```

ENST00000330622	GAGATTTTTTTTGAGGATGAACTGGTGTGTATCCTAAATATGGAAGGAAGAAAAGCTTTG
ENSPTRT00000010103	GAGATTTTTTTTGAGGATGAACTGGTGTGTATCCTAAATATGGAAGGAAGAAAAGCTTTG
ENSPPYT00000005923	GAGATTTTTTTTGAGGATGAACTGGTGTGTATCCTAAATATGGAAGGAAGAAAAGCTTTG
ENSMUST00000035579	GAGATCTTTTTTGAGGATGAGCTGGTGTGTATCCTAAACATGGAAGGAAGGAAAGCTTTG
ENSRNOT00000001491	GAGATCTTTTTTGAGGATGAACTGGTGTGCATCCTAAACATGGAAGGAAGGAAAGCTCTG

ENSMMUT00000024578	ACCTGGAAATACTACGCGAAAAAAATTCTTTACTACCTGCGGCAACAGAAAATTTTAAAT
ENSGGOT00000023531	ACCTGGAAATACTACGCAAAAAAAATTCTTTACTACCTGCGGCAACAGAAGATTTTAAAT
ENST00000330622	ACCTGGAAATACTACGCAAAAAAAATTCTTTACTACCTGCGGCAACAGAAGATCTTAAAT
ENSPTRT00000010103	ACCTGGAAATACTACGCAAAAAAAATTCTTTACTACCTGCGGCAACAGAAGATTTTAAAT
ENSPPYT00000005923	ACCTGGAAATACTACGCAAAAAAAATTCTTTACTACCTGCGGCAACAGAAAATTTTAAAT
ENSMUST00000035579	ACCTGGAAATACTACGCAAAGAAAATTCTCTACTACCTGCGGCAGCAGAAAATCCTCAAT
ENSRNOT00000001491	ACCTGGAAGTACTACGCTAAGAAGATTCTCTACTACCTGCGGCAGCAGAAAATCCTCAAC

ENSMMUT00000024578	AATCTTAAGGCCTTTCTTCAGCAGCCAGATGACTATGAGTCATATCTTGAAGGTGCTGTA
ENSGGOT00000023531	AATCTTAAGGCCTTTCTTCAGCAGCCAGATGACTATGAGTCATATCTTGAAGGTGCTGTA
ENST00000330622	AATCTTAAGGCCTTTCTTCAGCAGCCAGATGACTATGAGTCGTATCTTGAAGGTGCTGTA
ENSPTRT00000010103	AATCTTAAGGCCTTTCTTCAGCAGCCAGATGACTATGAGTCATATCTTGAAGGTGCTGTA
ENSPPYT00000005923	AATCTTAAGGCCTTTCTTCAGCAGCCAGATGACTATGAGTCATATCTTGAAGGTGCTGTA
ENSMUST00000035579	AACCTCAAGGCGTTCCTGCAGCAGCCCGACGATTACGAGTCCTATCTCGAAGGTGCCGTT
ENSRNOT00000001491	AACCTCAAGGCTTTCCTGCAGCAGCCAGATGACTACGAGTCCTATCTTGAAGGTGCCGTT

ENSMMUT00000024578	TATATTGACCAGTACTGCAATCCTCTCTCCGATATCAGCCTCAAAGACATCCAGGCCCAA
ENSGGOT00000023531	TATATTGACCAGTACTGCAATCCTCTCTCCGACATCAGCCTCAAAGACATCCAGGCCCAA
ENST00000330622	TATATTGACCAGTACTGCAATCCTCTCTCCGACATCAGCCTCAAAGACATCCAGGCCCAA
ENSPTRT00000010103	TATATTGACCAGTACTGCAATCCTCTCTCCGACATCAGCCTCAAAGACATCCAGGCCCAA
ENSPPYT00000005923	TATATTGACCAGTACTGCAATCCTCTCTCTGACATCAGCCTCAAAGACATCCAGGCCCAA
ENSMUST00000035579	TACATCGACCAGTACTGCAACCCACTCTCCGACATCAGCTTCAGAGACATCCAGGCCCAG
ENSRNOT00000001491	TACATCGACCAGTACTGCAACCCGCTCTCCGACATCAGCCTCAGAGACATCCAGGCCCAG

ENSMMUT00000024578	ATTGACAGCATCGTAGAGCTCGTTTGCAAAACTCTTCGGGGCATAAACAGTCGCCACCCC
ENSGGOT00000023531	ATTGACAGCATCGTGGAGCTTGTTTGCAAAACCCTTCGGGGCATAAACAGTCGCCACCCC
ENST00000330622	ATTGACAGCATCGTGGAGCTTGTTTGCAAAACCCTTCGGGGCATAAACAGTCGCCACCCC
ENSPTRT00000010103	ATTGACAGCATCGTGGAGCTTGTTTGCAAAACCCTTCGGGGCATAAACAGTCGCCACCCC
ENSPPYT00000005923	ATTGACAGCATCGTGGAGCTTGTTTGCAAAACCCTTCGGGGCATAAACAGTCGCCACCCC
ENSMUST00000035579	ATCCACAGCATTGTGGAGCTTGTGTGCAAAACCCTCCGTGGCATCAACAGCCGCCACCCC
ENSRNOT00000001491	ATCCACAGCATCGTGGAGCTTGTGTGCAAAACCCTCCGTGGCATCAACAGTCGTCACCCG

ENSMMUT00000024578	AGCTTGGCCTTCAAGGCAGGTGAATCATCCATGATAATGGAAATAGAACTCCAGAGCCAG
ENSGGOT00000023531	AGCTTGGCCTTCAAGGCAGGTGAATCATCCATGATAATGGAAATAGAACTCCAGAGCCAG
ENST00000330622	AGCTTGGCCTTCAAGGCAGGTGAATCATCCATGATAATGGAAATAGAACTCCAGAGCCAG
ENSPTRT00000010103	AGCTTGGCCTTCAAGGCAGGTGAATCATCCATGATAATGGAAATAGAACTCCAGAGCCAG
ENSPPYT00000005923	AGCTTGGCCTTCAAGGCAGGTGAATCATCCATGATAATGGAAATAGAACTCCAGAGCCAG
ENSMUST00000035579	AGCCTGACCTTCAGGGCAGGTGAGTCCTCTATGATAATGGAGATAGAACTTCAGAGCCAA
ENSRNOT00000001491	AGCCTGACCTTCAGGGCAGGCGAGTCCTCTATGATAATGGAGATAGAACTCCAGAGCCAA

ENSMMUT00000024578	GTGCTGGATGCCATGAACTATGTCCTTTACGACCAACTGAAGTTCAAGGGGAATCGAATG
ENSGGOT00000023531	GTGCTGGATGCCATGAACTATGTCCTTTATGACCAACTGAAGTTCAAGGGGAATCGAATG
ENST00000330622	GTGCTGGATGCCATGAACTATGTCCTTTACGACCAACTGAAGTTCAAGGGGAATCGAATG
ENSPTRT00000010103	GTGCTGGATGCCATGAACTATGTCCTTTACGACCAACTGAAGTTCAAGGGGAATCGAATG
ENSPPYT00000005923	GTACTGGATGCCATGAACTATGTCCTTTATGACCAACTGAAGTTCAAGGGGAATCGAATG
ENSMUST00000035579	GTGCTGGACGCTATCAACTATGTCCTGTATGACCAGCTGAAGTTCAAAGGGAACCGCATG
ENSRNOT00000001491	GTGCTGGACGCCATCAACTATGTCCTGTACGACCAGCTCAAGTTCAAAGGGAACCGCATG

```
ENSMMUT00000024578     GATTACTATAATGCCCTAAACTTATATATGCATCAGGTTTTGATTCGCAGAACAGGAATC
ENSGGOT00000023531     GATTACTATAACGCCCTCAACTTATATATGCATCAGGTTTTGATTCGCAGAACAGGAATC
ENST00000330622        GATTACTATAATGCCCTCAACTTATATATGCATCAGGTTTTGATTCGCAGAACAGGAATC
ENSPTRT00000010103     GATTACTATAATGCCCTCAACTTATATATGCATCAGGTTTTGATTCGCAGAACAGGAATC
ENSPPYT00000005923     GATTACTATAATGCCCTAAACTTATATATGCATCAGGTTTTGATTCGCAGAACAGGAATC
ENSMUST00000035579     GACTACTATAATGCCTTGAACCTCTACATGCACCAGGTGTTGACCCGCAGAACAGGAATC
ENSRNOT00000001491     GACTACTACAATGCCTTGAACCTCTACATGCACCAGGTATTGACTCGCAGAACGGGAATC

ENSMMUT00000024578     CCAATCAGCATGTCTCTGCTCTATTTGACGATTGCTCGGCAGTTGGGAGTCCCACTGGAG
ENSGGOT00000023531     CCAATCAGCATGTCTCTGCTCTATTTGACAATTGCTCGGCAGTTGGGAGTCCCACTGGAG
ENST00000330622        CCAATCAGCATGTCTCTGCTCTATTTGACAATTGCTCGGCAGTTGGGAGTCCCACTGGAG
ENSPTRT00000010103     CCAATCAGCATGTCTCTGCTCTATTTGACAATTGCTCGGCAGTTGGGAGTCCCACTGGAG
ENSPPYT00000005923     CCAATCAGCATGTCTCTGCTCTATTTGACAATTGCTCGGCAATTGGGAGTCCCACTGGAG
ENSMUST00000035579     CCCATCAGCATGTCTCTGCTCTACCTGACCGTCGCCCGGCAGCTGGGGGTTCCCCTGGAG
ENSRNOT00000001491     CCCATCAGCATGTCTCTGCTCTACCTGACCATCGCCCGGCAGCTGGGCGTCCCTCTGGAG

ENSMMUT00000024578     CCTGTCAACTTCCCAAGTCACTTCTTACTAAGGTGGTGCCAAGGCGCAGAAGGGGCGACC
ENSGGOT00000023531     CCTGTCAACTTCCCAAGTCACTTCTTATTAAGGTGGTGCCAAGGCGCAGAAGGGGCGACC
ENST00000330622        CCTGTCAACTTCCCAAGTCACTTCTTATTAAGGTGGTGCCAAGGCGCAGAAGGGGCGACC
ENSPTRT00000010103     CCTGTCAACTTCCCAAGTCACTTCTTATTAAGGTGGTGCCAAGGCGCAGAAGGGGCGACC
ENSPPYT00000005923     CCTGTCAACTTCCCAAGTCACTTCTTATTAAGGTGGTGCCAAGGCGCAGAAGGGGCGACC
ENSMUST00000035579     CCTGTCAACTTCCCAAGCCACTTCCTGCTGCGGTGGTGCCAAGGTGCAGAAGGGGCAACC
ENSRNOT00000001491     CCTGTCAACTTCCCAAGCCACTTCCTGCTGAGGTGGTGCCAAGGCGCAGAAGGGGCAACC

ENSMMUT00000024578     CTGGACATCTTTGACTACATCTACATAGATGCTTTTGGGAAAGGCAAGCAGCTGACAGTG
ENSGGOT00000023531     CTGGACATCTTTGACTACATCTATATAGATGCTTTTGGGAAAGGCAAGCAGCTGACAGTG
ENST00000330622        CTGGACATCTTTGACTACATCTACATAGATGCTTTTGGGAAAGGCAAGCAGCTGACAGTG
ENSPTRT00000010103     CTGGACATCTTTGACTACATCTACATAGATGCTTTTGGGAAAGGCAAGCAGCTGACAGTG
ENSPPYT00000005923     CTGGACATCTTTGACTACATCTACATAGATGCTTTTGGGAAAGGCAAGCAGCTGACAGTG
ENSMUST00000035579     CTGGACATCTTTGACTACATCTACATAGATGCTTTTGGGAAGGGCAAGCAGCTGACAGTG
ENSRNOT00000001491     CTGGACATCTTCGACTACATCTACATAGATGCTTTTGGGAAGGGCAAGCAACTGACAGTG

ENSMMUT00000024578     AAAGAATGCGAGTACTTGATCGGCCAGCACGTGACTGCGGCACTGTATGGTGTGGTCAAT
ENSGGOT00000023531     AAAGAATGCGAGTACTTGATCGGCCAGCACGTGACTGCAGCACTGTATGGGGTGGTCAAT
ENST00000330622        AAAGAATGCGAGTACTTGATCGGCCAGCACGTGACTGCAGCACTGTATGGGGTGGTCAAT
ENSPTRT00000010103     AAAGAATGCGAGTACTTGATCGGCCAGCACGTGACTGCAGCACTGTATGGGGTGGTCAAT
ENSPPYT00000005923     AAAGAATGCGAGTACTTGATCGGCCAGCATGTGACTGCAGCACTGTATGGGGTGGTCAAT
ENSMUST00000035579     AAAGAGTGTGAGTACCTGATTGGCCAGCACGTGACGGCAGCACTCTACGGCGTGGTGAAT
ENSRNOT00000001491     AAAGAGTGTGAGTACTTGATTGGCCAGCACGTGACGGCAGCTCTCTATGGCGTGGTGAAC

ENSMMUT00000024578     GTCAAGAAGGTGTTACAGAGAATGGTGGGAAACCTGTTAAGCCTGGGGAAGCGGGAAGGC
ENSGGOT00000023531     GTCAAGAAGGTGTTACAGAGAATGGTGGGAAACCTGTTAAGCCTGGGGAAGCGGGAAGGC
ENST00000330622        GTCAAGAAGGTGTTACAGAGAATGGTGGGAAACCTGTTAAGCCTGGGGAAGCGGGAAGGC
ENSPTRT00000010103     GTCAAGAAGGTGTTACAGAGAATGGTGGGAAACCTGTTAAGCCTGGGGAAGCGGGAAGGC
ENSPPYT00000005923     GTCAAGAAGGTGTTACAGAGAATGGTGGGAAACCTGTTAAGCCTGGGAAAACGGGAAGGC
ENSMUST00000035579     GTGAAGAAGGTGCTACAGCGGATGGTGGGCAACTTGCTGAGCCTGGGCAAGAGGGAAGGC
ENSRNOT00000001491     GTGAAGAAGGTGCTGCAGCGGATGGTGGGCAACCTGCTGAGCCTGGGCAAGAGGGAAGGC

ENSMMUT00000024578     ATCGACCAGTCGTACCAGCTCCTGAGAGACTCGCTGGATCTCTATCTGGCAATGTACCCG
ENSGGOT00000023531     ATCGACCAGTCATACCAGCTCCTGAGAGACTCGCTGGATCTCTATCTGGCAATGTACCCG
ENST00000330622        ATCGACCAGTCATACCAGCTCCTGAGAGACTCGCTGGATCTCTATCTGGCAATGTACCCG
ENSPTRT00000010103     ATCGACCAGTCATACCAGCTCCTGAGAGACTCGCTGGATCTCTATCTGGCAATGTACCCG
ENSPPYT00000005923     ATCGACCAGTCATACCAGCTCCTGAGAGACTCGCTGGATCTCTATCTGGCAATGTACCCG
```

```
ENSMUST00000035579     ATTGACCAGTCCTACCAGCTCCTCAGAGACTCCTTGGACCTGTACCTGGCGATGTACCCG
ENSRNOT00000001491     ATTGACCAGTCCTACCAGCTCCTCAGAGACTCCTTGGACCTGTACCTGGCGATGTACCCG

ENSMMUT00000024578     GACCAGGTGCAGCTTCTCCTCCTCCAAGCCAGGCTTTACTTCCACCTGGGCATCTGGCCA
ENSGGOT00000023531     GACCAGGTGCAGCTTCTCCTCCTCCAAGCCAGGCTTTACTTCCACCTGGGAATCTGGCCA
ENST00000330622        GACCAGGTGCAGCTTCTCCTCCTCCAAGCCAGGCTTTACTTCCACCTGGGAATCTGGCCA
ENSPTRT00000010103     GACCAGGTGCAGCTTCTCCTCCTCCAAGCCAGGCTTTACTTCCACCTGGGAATCTGGCCA
ENSPPYT00000005923     GACCAGGTGCAGCTTCTCCTCCTCCAAGCCAGGCTTTACTTCCACCTGGGAATCTGGCCA
ENSMUST00000035579     GACCAGGTGCAGCTCCTGCTCCTCCAAGCCAGACTCTACTTCCACCTGGGCATCTGGCCA
ENSRNOT00000001491     GACCAGGTGCAGCTCCTGCTCCTGCAAGCCAGGCTCTACTTCCACCTGGGCATCTGGCCA

ENSMMUT00000024578     GAGAAGTCTTTCTGTCTTGTCTTGAAGGTGCTTGACATCCTCCAGCACATCCAAACCCTA
ENSGGOT00000023531     GAGAAGTCTTTCTGTCTTGTTTTGAAGGTGCTTGACATCCTCCAGCACATCCAAACCCTA
ENST00000330622        GAGAAGTCTTTCTGTCTTGTTTTGAAGGTGCTTGACATCCTCCAGCACATCCAAACCCTA
ENSPTRT00000010103     GAGAAGTCTTTCTGTCTTGTTTTGAAGGTGCTTGACATCCTCCAGCACATCCAAACCCTA
ENSPPYT00000005923     GAG---------------------AAGGTGCTTGACATCCTCCAGCACATCCAAACCCTA
ENSMUST00000035579     GAGAAGTCTTTCTGTCTTGTCTTGAAGGTGCTTGACATCCTCCAGCACATCCAGACCCTG
ENSRNOT00000001491     GAGAAGTCTTTCTGTCTTGTCTTGAAGGTGCTTGACATCCTCCAGCACATCCAGACCCTC

ENSMMUT00000024578     GACCCGGGGCAGCACGGGGCGGTGGGCTACCTGGTGCAGCACACTCTAGAGCACATTGAG
ENSGGOT00000023531     GACCCGGGGCAGCACGGGGCGGTGGGCTACCTGGTGCAGCACACTCTAGAGCACATTGAG
ENST00000330622        GACCCGGGGCAGCACGGGGCGGTGGGCTACCTGGTGCAGCACACTCTAGAGCACATTGAG
ENSPTRT00000010103     GACCCGGGGCAGCACGGGGCGGTGGGCTACCTGGTGCAGCACACTCTAGAGCACATTGAG
ENSPPYT00000005923     GACCCGGGGCAGCACGGGGCGGTGGGCTACCTGGTGCAGCACACTCTAGAGCACATTGAG
ENSMUST00000035579     GACCCCGGGCAGCACGGGGCGGTGGGCTATCTCGTGCAGCATACCTTAGAGCACATTGAG
ENSRNOT00000001491     GACCCCGGGCAGCACGGGGCGGTGGGGTATCTAGTGCAGCACACGCTAGAGCACATTGAG

ENSMMUT00000024578     CGCAAAAAGGAGGAGGTGGGCGTGGAGGTGAAGCTGCGCTCCGATGAGAAGCACAGGGAT
ENSGGOT00000023531     CGCAAAAAGGAGGAGGTGGGCGTAGAGGTGAAGCTGCGCTCCGATGAGAAGCACAGAGAT
ENST00000330622        CGCAAAAAGGAGGAGGTGGGCGTAGAGGTGAAGCTGCGCTCCGATGAGAAGCACAGAGAT
ENSPTRT00000010103     CGCAAAAAGGAGGAGGTGGGCGTAGAGGTGAAGCTGCGCTCCGATGAGAAGCACAGAGAT
ENSPPYT00000005923     CGCAAAAAGGAGGAGGTGGGCGTAGAGGTGAAGCTGCGCTCTGATGAGAAGCACAGGGAT
ENSMUST00000035579     CGTAAGAAGGAGGAGGTGGGCGTGGAGGTGAAGCTGCGCTCCGAGGAGAAGCACAGGGAT
ENSRNOT00000001491     CGCAAGAAGGAGGAGGTGGGCGTGGAGGTGAAGCTGCGCTCCGAGGAGAAGCACAGGGAT

ENSMMUT00000024578     GTCTGCTACTCCATCGGGCTCATTATGAAGCATAAGAGGTATGGCTATAACTGTGTGATC
ENSGGOT00000023531     GTCTGCTACTCCATCGGGCTCATTATGAAGCATAAGAGGTATGGCTATAACTGTGTGATC
ENST00000330622        GTCTGCTACTCCATCGGGCTCATTATGAAGCATAAGAGGTATGGCTATAACTGTGTGATC
ENSPTRT00000010103     GTCTGCTACTCCATCGGGCTCATTATGAAGCATAAGAGGTATGGCTATAACTGTGTGATC
ENSPPYT00000005923     GTCTGCTACTCCATCGGGCTCATTATGAAGCATAAGAGGTATGGCTATAACTGTGTGATC
ENSMUST00000035579     GTCTGCTACTCCATCGGGCTTGTGATGAAGCATAAGAGGTACGGCTACAACTGTGTGATC
ENSRNOT00000001491     GTCTGCTACTCCATCGGGCTCGTTATGAAGCACAAGAGGTACGGCTACAACTGTGTGATC

ENSMMUT00000024578     TACGGCTGGGACCCCACCTGCATGATGGGACACGAGTGGATCCGGAACATGAACGTCCAC
ENSGGOT00000023531     TACGGCTGGGACCCCACCTGCATGATGGGACACGAGTGGATCCGGAACATGAACGTCCAC
ENST00000330622        TACGGCTGGGACCCCACCTGCATGATGGGACACGAGTGGATCCGGAACATGAACGTCCAC
ENSPTRT00000010103     TACGGCTGGGACCCCACCTGCATGATGGGACACGAGTGGATCCGGAACATGAACGTCCAC
ENSPPYT00000005923     TACGGCTGGGACCCCACCTGCATGATGGGACACGAGTGGATCCGGAACATGAACGTCCAC
ENSMUST00000035579     TACGGCTGGGACCCTACGTGCATGATGGGGCATGAGTGGATCCGAAACATGAATGTGCAC
ENSRNOT00000001491     TACGGCTGGGACCCCACGTGCATGATGGGGCACGAGTGGATCCGAAACATGAATGTGCAC

ENSMMUT00000024578     AGCCTACCGCACGGCCACCACCAGCCTTTCTATAACGTGCTGGTGGAGGACGGCTCCTGT
ENSGGOT00000023531     AGCCTGCCGCACGGCCACCACCAGCCTTTCTATAACGTGCTGGTGGAGGACGGCTCCTGT
```

```
ENST00000330622       AGCCTGCCGCACGGCCACCACCAGCCTTTCTATAACGTGCTGGTGGAGGACGGCTCCTGT
ENSPTRT00000010103    AGCCTGCCGCACGGCCACCACCAGCCTTTCTATAACGTGCTGGTGGAGGACGGCTCCTGT
ENSPPYT00000005923    AGCCTGCCGCATGGCCACCACCAGCCTTTCTATAACGTGCTGGTGGAGGACGGCTCCTGT
ENSMUST00000035579    AGCCTGCCTCATGGCCACCATCAGCCCTTCTACAACGTCCTGGTGGAGGATGGCTCCTGC
ENSRNOT00000001491     AGTCTGCCTCACGGCCACCATCAGCCCTTCTACAATGTGCTGGTGGAGGACGGCTCCTGC

ENSMMUT00000024578    CGATACGCAGCCCAAGAAAACTTGGAGTATAACGTGGAGCCTCAAGAAATCTCACACCCT
ENSGGOT00000023531    CGATACGCAGCCCAAGAAAACTTGGAATATAACGTGGAGCCTCAAGAAATCTCACACCCT
ENST00000330622       CGATACGCAGCCCAAGAAAACTTGGAATATAACGTGGAGCCTCAAGAAATCTCACACCCT
ENSPTRT00000010103    CGATACGCAGCCCAAGAAAACTTGGAATATAACGTGGAGCCTCAAGAAATCTCACACCCT
ENSPPYT00000005923    CGATACGCAGCCCAAGAAAACTTGGAATATAACGTGGAGCCTCAAGAAATCTCACACCCT
ENSMUST00000035579    AGATATGCAGCCCAAGAAAACCTGGAATACAACGTGGAGCCTCAGGAAATCTCGCACCCA
ENSRNOT00000001491    AGATACGCAGCTCAAGAAAACCTGGAATACAACGTGGAGCCTCAGGAAATCTCGCACCCA

ENSMMUT00000024578    GATGTGGGACGCTATTTCTCAGAGTTTACTGGCACTCACTACATCCCAAACGCAGAGCTG
ENSGGOT00000023531    GACGTGGGACGCTATTTCTCAGAGTTTACTGGCACTCACTACATCCCAAACGCAGAGCTG
ENST00000330622       GACGTGGGACGCTATTTCTCAGAGTTTACTGGCACTCACTACATCCCAAACGCAGAGCTG
ENSPTRT00000010103    GACGTGGGACGCTATTTCTCAGAGTTTACTGGCACTCACTACATCCCAAACGCAGAGCTG
ENSPPYT00000005923    GACGTGGGACGCTATTTCTCAGAGTTTACTGGCACTCACTACATCCCAAACGCAGAGCTG
ENSMUST00000035579    GATGTGGGGCGCTACTTCTCGGAGTTCACAGGCACACACTACATCCCAAATGCAGAGCTG
ENSRNOT00000001491    GATGTGGGGCGCTACTTCTCGGAGTTCACGGGCACACACTACATCCCAAATGCAGAGCTG

ENSMMUT00000024578    GAGATCCGGTATCCAGAAGATCTGGAGTTCGTCTATGAAACGGTGCAAAATATTTACAGT
ENSGGOT00000023531    GAGATCCGGTATCCAGAAGATCTGGAGTTTGTCTATGAAACGGTGCAGAATATTTACAGT
ENST00000330622       GAGATCCGGTATCCAGAAGATCTGGAGTTTGTCTATGAAACGGTGCAGAATATTTACAGT
ENSPTRT00000010103    GAGATCCGGTATCCAGAAGATCTGGAGTTTGTCTATGAAACGGTGCAGAATATTTACAGT
ENSPPYT00000005923    GAGATCCGGTATCCAGAAGATCTGGAGTTTGTCTATGAAACGGTGCAGAATATTTACAGT
ENSMUST00000035579    GAGATCCGGTACCCCGAGGACCTGGAGTTTGTGTACGAGACAGTGCAGAATATTTACAGT
ENSRNOT00000001491    GAGATCCGGTACCCCGAGGACCTGGAGTTTGTGTATGAGACAGTGCAGAACATTTACAGT

ENSMMUT00000024578    GCAAAGAAAGAGAACATAGATGAG
ENSGGOT00000023531    GCAAAGAAAGAGAACATAGATGAG
ENST00000330622       GCAAAGAAAGAGAACATAGATGAG
ENSPTRT00000010103    GCAAAGAAAGAGAACATAGATGAG
ENSPPYT00000005923    GCAAAGAAAGAGAACATAGATGAG
ENSMUST00000035579    GCAAAGGAGGACACAGCCGAG---
ENSRNOT00000001491    GCAAAGGAGGACACAGCCGAG---
```

Multiple sequence alignment of Fbxo22

```
ENSMUST00000034859    ATGGAGCCGGCAGGCGGCGGTGGCGGC---GTCTCCTCCTCCACAGATCCGCGAAGCACC
ENSRNOT00000020309    ATGGAGCCAGCAGGCGGC------------GGCTCCTCCTCCACAGATCCGCGAGGCACC
ENSCJAT00000023917    ATGGAGCCGGTGGGTGGCTGCGGCGACTGCGGCTCCTCCTCGGTAGACCCACGGAGCACC
ENSMMUT00000012815    ATGGAGCTGGTGGGCAGCAGCGGCGAGTGCGGCTCCTCCTCCGTAGACCCACGGAGCACC
ENSPPYT00000007875    ATGGAGTCGGTGGGCTGCTGCGGCGACTGCCGCGGCTCCTCCGTAGACCCACGGAGCACC
ENSGGOT00000001089    ATGGAGACGGTAGGCTGTTGCGGCGAGTGCCGCGGCTCCTCCGTAGACCCGCGGAGCACC
ENSPTRT00000013488    ATGGAGCCGGTAGGCTGCTGTGGCGAGTGCCGCGGCTCCTCCGTAGACCCGCGGAGCACC
ENST00000308275       ATGGAGCCGGTAGGCTGCTGCGGCGAGTGCCGCGGCTCCTCCGTAGACCCGCGGAGCACC

ENSMUST00000034859    TACGTGCTGAGTAACCTTGCGGAGGTGGTGGAGCGTGTGTTTACCTTCCTGCCGGCCAAA
ENSRNOT00000020309    TATGTGCTGAGTAACCTTGCGGAGGTGGTGGAGCGTGTGTTTACCTTCCTGCCGGCCAAA
ENSCJAT00000023917    TTCGTGTTGAGTAACCTGGCGGAGGTGGTGGAGCGTGTGCTCACCTTCCTACCCGCCAAG
ENSMMUT00000012815    TTCGTGTTGAGTAACCTGGCGGAGGTGGTGGAGCGTGTGCTCACCTTCCTGCCCGCCAAG
ENSPPYT00000007875    TTCGTGTTGAGTAACCTGGCGGAGGTGGTGGAGCGTGTGCTCACCTTCCTGCCCGCCAAG
```

ENSGGOT00000001089 TTCGTGTTGAGTAACCTGGCGGAGGTGGTGGAGCGTGTGCTCACCTTCCTGCCCGCCAAG
ENSPTRT00000013488 TTCGTGTTGAGTAACCTGGCGGAGGTGGTGGAGCGTGTGCTCACCTTCCTGCCCGCCAAG
ENST00000308275 TTCGTGTTGAGTAACCTGGCGGAGGTGGTGGAGCGTGTGCTCACCTTCCTGCCCGCCAAG

ENSMUST00000034859 GCGCTGCTTCGGGTAGCCGGAGTATGCCGCCTGTGGAGGGAGTGTGTACGCAGAGTGCTG
ENSRNOT00000020309 GCGTTGCTTCGGGTAGCCGGAGTATGCCGCCTGTGGAGAGAGTGTGTGCGTAGAGTGCTG
ENSCJAT00000023917 GCATTGCTGCGGGTGGCCTGCGTGTGCCGCTTATGGAGAGAGTGTGTGCGCCGAGTGTTG
ENSMMUT00000012815 GCGTTGCTGCGGGTGGCCTGCGTGTGCCGCTTATGGAGGGAGTGTGTGCGCAGAGTGTTG
ENSPPYT00000007875 GCGTTGCTGCGGGTGGCCTGCGTGTGCCGCTTATGGAGAGAGTGTGTGCGCAGAGTATTG
ENSGGOT00000001089 GCGTTGCTGCGGGTGGCCTGCGTGTGCCGCTTATGGAGGGAGTGTGTGCGCAGAGTATTG
ENSPTRT00000013488 GCGTTGCTGCGGGTGGCCTGCGTGTGCCGCTTATGGAGGGAGTGTGTGCGCAGAGTATTG
ENST00000308275 GCGTTGCTGCGGGTGGCCTGCGTGTGCCGCTTATGGAGGGAGTGTGTGCGCAGAGTATTG

ENSMUST00000034859 CGGACCCATCGCAGCGTGACCTGGATCTCCGCGGGTGTGGCTGAAGCGGGCCACCTGGAG
ENSRNOT00000020309 CGGACCCATCGCAGCGTGACCTGGATCTCCGCGGGTGTGGCGGAGGCGGGCCACCTGGAG
ENSCJAT00000023917 CGGACCCATCGGAGCTTGACCTGGATCTCCGCAGGCCTGGCGGATGCCGGCCACCTGGAG
ENSMMUT00000012815 CGGACCCATCGGAGCGTAACCTGGATCTCCGCAGGCCTGGCCGAGGCCGGCCACCTGGAG
ENSPPYT00000007875 CGGACCCATCGGAGCGTAACCTGGATCTCCGCAGGCCTGGCCGAGGCCAGCCACCTGGAG
ENSGGOT00000001089 CGGACCCATCGGAGCGTAACCTGGATCTCCGCAGGCCTGGCGGAGGCCGGCCACCTGGAG
ENSPTRT00000013488 CGGACCCATCGGAGCGTAACCTGGATCTCCGCAGGCGTGGCGGAGGCCGGCCACCTGGAG
ENST00000308275 CGGACCCATCGGAGCGTAACCTGGATCTCCGCAGGCCTGGCGGAGGCCGGCCACCTGGAG

ENSMUST00000034859 GGACATTGCTTGGTGCGCGTGGTAGCTGAGGCGCTTGAGAATGTTCGAATCTTACCACAG
ENSRNOT00000020309 GGACATTGCTTGGTCCGCGTGGTAGCTGAAGCGCTTGAGAATGTCCGCATCTTACCACGG
ENSCJAT00000023917 AGGCATTGCTTGGTTCGCGTGGTAGCCGAGGAGCTTGAGAATGTTCGCATTTTACCACAT
ENSMMUT00000012815 AGGCATTGCTTGGTTCGCGTAGTAGCAGAGGAGCTTGAGAATGTTCGCATCTTACCACAT
ENSPPYT00000007875 AGGCATTGCTTGGTTCGAGTGGTAGCAGAGGAGCTTGAGAATGTTCGCATCTTACCACAT
ENSGGOT00000001089 AGGCATTGCTTGGTTCGCGTGGTAGCAGAGGAGCTTGAGAATGTTCGCATCTTACCACAT
ENSPTRT00000013488 AGGCATTGCTTGGTTCGCGTGGTAGCAGAGGAGCTTGAGAATGTTCGCATCTTACCACAT
ENST00000308275 GGGCATTGCTTGGTTCGCGTGGTAGCAGAGGAGCTTGAGAATGTTCGCATCTTACCACAT

ENSMUST00000034859 ACAGTTCTCTACATGGCAGATTCTGAAACTTTCATCAGCCTGGAAGAGTGTCGTGGCCAT
ENSRNOT00000020309 ACAGTTCTCTACATGGCAGATTCTGAAACGTTCATCAGCCTGGAGGAGTGCCGGGGCCAT
ENSCJAT00000023917 ACAGTTCTTTACATGGCTGATTCAGAAACTTTCATTAGTCTGGAAGAGTGTCGTGGCCAT
ENSMMUT00000012815 ACAGTTCTTTACATGGCTGATTCAGAAACTTTCATTAGTCTGGAAGAGTGTCGTGGCCAT
ENSPPYT00000007875 ACGGTTCTTTACATGGCTGATTCAGAAACTTTCATTAGTCTGGAAGAGTGTCGTGGCCAT
ENSGGOT00000001089 ACAGTTCTTTACATGGCTGATTCAGAAACTTTCATTAGTCTGGAAGAGTGTCGTGGCCAT
ENSPTRT00000013488 ACAGTTCTTTACATGGCTGATTCAGAAACTTTCATTAGTCTGGAAGAGTGTCGTGGCCAT
ENST00000308275 ACAGTTCTTTACATGGCTGATTCAGAAACTTTCATTAGTCTGGAAGAGTGTCGTGGCCAT

ENSMUST00000034859 AAAAGAGCGAGGAAGAGAACTACTATGGAGACAGCATGTGCCCTGGAGAAGCTTTTCCCC
ENSRNOT00000020309 AAAAGAGCAAGGAAAAGAACTACTATGGAGACAGCATGTGCCCTGGAGAAGCTGTTCCCC
ENSCJAT00000023917 AAGAGAGCAAGGAAAAGAACTAGTATGGAAACAGCACTTGCCCTTGAGAAGCTATTCCCC
ENSMMUT00000012815 AAGAGAGCAAGGAAAAGAACTAGTATGGAAACAGCACTTGCCCTTGAGAAGCTATTCCCC
ENSPPYT00000007875 AAGAGAGCAAGGAAAAGAACTAGTATGGAAACAGCACTTGCCCTTGAGAAGCTATTCCCC
ENSGGOT00000001089 AAGAGAGCAAGGAAAAGAACTAGTATGGAAACAGCACTTGCCCTTGAGAAGCTATTCCCC
ENSPTRT00000013488 AAGAGAGCAAGGAAAAGAACTAGTATGGAAACAGCACTTGCCCTTGAGAAGCTATTCCCC
ENST00000308275 AAGAGAGCAAGGAAAAGAACTAGTATGGAAACAGCACTTGCCCTTGAGAAGCTATTCCCC

ENSMUST00000034859 AAGCAGTGCCAAGTCCTTGGGATTGTGACCCCGGGAATTGTGGTGACTCCAATGGGATCA
ENSRNOT00000020309 AAACAGTGTCAGGTCCTTGGGATTGTGACCCCAGGAATTGTAGTGACTCCAATGGGATCA
ENSCJAT00000023917 AAACGATGCCAGGTCCTTGGGATTGTGACCCCAGGAATTGTAGTGACTCCAATGGGATCA
ENSMMUT00000012815 AAACAATGCCAGGTCCTTGGGATTGTGACCCCAGGAATTGTAGTGACTCCAATGGGATCA
ENSPPYT00000007875 AAACAATGCCAAGTCCTTGGGATTGTGACTCCAGGAATTGTAGTGACTCCAATGGGATCA

ENSGGOT00000001089 AAACAATGCCAAGTCCTTGGGATTGTGACCCCAGGAATTGTAGTGACTCCAATGGGATCA
ENSPTRT00000013488 AAACAATGCCAAGTCCTTGGGATTGTGACCCCAGGAATTGTAGTGACTCCAATGGGATCA
ENST00000308275 AAACAATGCCAAGTCCTTGGGATTGTGACCCCAGGAATTGTAGTGACTCCAATGGGATCA

ENSMUST00000034859 GGTAGCAATCGACCTCAGGAAATAGAAATTGGAGAATCTGGTTTTGCTTTATTATTCCCT
ENSRNOT00000020309 GGTAGCAATCGGCCTCAGGAAATAGAAATTGGAGAATCTGGTTTTGCTTTATTATTCCCT
ENSCJAT00000023917 GGTAGCAATCGACCTCAGGAAATAGAAATTGGAGAATCTGGTTTTGCTTTATTATTCCCT
ENSMMUT00000012815 GGTAGCAATCGACCTCAGGAAATAGAAATTGGAGAATCTGGTTTTGCTTTATTATTCCCT
ENSPPYT00000007875 GGTAGCAATCGACCTCAGGAAATAGAAATTGGAGAATCTGGTTTTGCTTTATTATTCCCT
ENSGGOT00000001089 GGTAGCAATCGACCTCAGGAAATAGAAATTGGAGAATCTGGTTTTGCTTTATTATTCCCT
ENSPTRT00000013488 GGTAGCAATCGACCTCAGGAAATAGAAATTGGAGAATCTGGTTTTGCTTTATTATTCCCT
ENST00000308275 GGTAGCAATCGACCTCAGGAAATAGAAATTGGAGAATCTGGTTTTGCTTTATTATTCCCT

ENSMUST00000034859 CAAATTGAAGGAATAAAAATTCAACCCTTTCATTTTATTAAGGACTCCAAGAATTTAACA
ENSRNOT00000020309 CAAATTGAAGGAATAAAAATTCAACCCTTTCATTTTATTAAGGACCCCAAGAATTTAACT
ENSCJAT00000023917 CAAATTGAAGGAATAAAAATACAACCCTTTCATTTTATTAAGGATCCAAAGAATTTAGCA
ENSMMUT00000012815 CAAATTGAAGGAATAAAAATACAACCCTTTCATTTTATTAAGGATCCAAAGAATTTAACA
ENSPPYT00000007875 CAAATTGAAGGAATAAAAATACAACCCTTTCATTTTATTAAGGATCCAAAGAATTTAACA
ENSGGOT00000001089 CAAATTGAAGGAATAAAAATACAACCCTTTCATTTTATTAAGGATCCAAAGAATTTAACA
ENSPTRT00000013488 CAAATTGAAGGAATAAAAATACAACCCTTTCATTTTATTAAGGATCCAAAGAATTTAACA
ENST00000308275 CAAATTGAAGGAATAAAAATACAACCCTTTCATTTTATTAAGGATCCAAAGAATTTAACA

ENSMUST00000034859 CTCGAAAGACACCAGCTTACTGAAGTAGGTCTTCTGGACAACCCTGAACTTCGTGTGGTC
ENSRNOT00000020309 CTCGAAAGACATCAACTTACTGAAGTGGGTCTTCTGGATAACCCCGAACTCCGTGTGGTT
ENSCJAT00000023917 TTAGAAAGACATCAACTCACTGAAGTAGGTCTTTTAGATAACCCTGAACTTCGTGTGGTC
ENSMMUT00000012815 TTAGAAAGACATCAGCTCACTGAAGTAGGTCTTTTAGATAACCCTGAACTTCGTGTGGTC
ENSPPYT00000007875 TTAGAAAGACATCAACTCACTGAAGTAGGTCTTTTAGATAACCCTGAACTTCGTGTGGTC
ENSGGOT00000001089 TTAGAAAGACATCAACTCACTGAAGTAGGTCTTTTAGATAACCCTGAACTTCGTGTGGTC
ENSPTRT00000013488 TTAGAAAGACATCAACTCACTGAAGTAGGTCTTTTAGATAACCCTGAACTTCGTGTGGTC
ENST00000308275 TTAGAAAGACATCAACTCACTGAAGTAGGTCTTTTAGATAACCCTGAACTTCGTGTGGTC

ENSMUST00000034859 CTTGTCTTTGGCTATAACTGCTGTAAGGTGGGAGCCAGTAATTACCTGCATCGGGTAGTC
ENSRNOT00000020309 CTTGTCTTTGGCTATAACTGCTGTAAGGTGGGAGCCAGTAATTACCTGCACCGGGTGGTC
ENSCJAT00000023917 CTTGTCTTTGGTTATAATTGCTGTAAGGTGGGAGCCAATAATTTTCTGCAGCAAGTAGTC
ENSMMUT00000012815 CTTGTCTTTGGTTATAATTGCTGTAAGGTGGGAGCCAGTAATTATCTGCAGCAAGTAGTC
ENSPPYT00000007875 CTTGTCTTTGGTTATAATTGCTGTAAGGTGGGAGCCAGTAATTATCTGCAGCAAGTAGTC
ENSGGOT00000001089 CTTGTCTTTGGTTATAATTGCTGTAAGGTGGGAGCCAGTAATTATCTGCAGCAAGTAGTC
ENSPTRT00000013488 CTTGTCTTTGGTTATAATTGCTGTAAGGTGGGAGCCAGTAATTATCTGCAGCAAGTAGTC
ENST00000308275 CTTGTCTTTGGTTATAATTGCTGTAAGGTGGGAGCCAGTAATTATCTGCAGCAAGTAGTC

ENSMUST00000034859 AGCACTTTCAGCGATATGAATATCATCTTGGCTGGAGGCCAGGTGGACAATTTGTCTTCA
ENSRNOT00000020309 AGCACTTTCAGCAACATGAACATCATCTTGGCTGGAGGCCAGGTGGACAACTTGTCTTCA
ENSCJAT00000023917 AGCACTTTCAGTGATATGAATATCATCTTGGCTGGAGGCCAGGTGGACAACCTGTCATCA
ENSMMUT00000012815 AGCACTTTCAGTGATATGAATATCATCTTGGCTGGAGGCCAGGTGGACAACCTGTCATCA
ENSPPYT00000007875 AGCACTTTCAGTGATATGAATATCATCTTGGCTGGAGGCCAGGTGGACAACCTGTCATCA
ENSGGOT00000001089 AGCACTTTCAGTGATATGAATATCATCTTGGCTGGAGGCCAGGTGGACAACCTGTCATCA
ENSPTRT00000013488 AGCACTTTCAGTGATATGAATATCATCTTGGCTGGAGGCCAGGTGGACAACCTGTCATCA
ENST00000308275 AGCACTTTCAGTGATATGAATATCATCTTGGCTGGAGGCCAGGTGGACAACCTGTCATCA

ENSMUST00000034859 CTTACTTGTGAGAAGAACCCTCTGGATATTGATGCCACAGGTGTGGTTGGACTGTCATTT
ENSRNOT00000020309 CTGACTTGTGAGAAGAACCCTCTGGATATTGACGCCACAGGTGTGGTCGGACTGTCATTT
ENSCJAT00000023917 CTGACTTCTGAAAAGAACCCTCTGGATATTGATGCCACTGGTGTGGTTGGACTGTCATTT
ENSMMUT00000012815 CTGACTTCTGAAAAGAACCCTCTGGATATTGATGCCGCAGGTGTGGTTGGACTGTCATTT
ENSPPYT00000007875 CTGACTTCTGAAAAGAACCCTCTGGATATTGATGCCTCAGGTGTGGTTGGACTGTCATTT

```
ENSGGOT00000001089    CTGACTTCTGAAAAGAACCCTCTGGATATTGATGCCTCGGGTGTGGTTGGACTGTCATTT
ENSPTRT00000013488    CTGACTTCTGAAAAGAACCCTCTGGATATTGATGCCTCGGGTGTGGTTGGACTGTCATTT
ENST00000308275       CTGACTTCTGAAAAGAACCCTCTGGATATTGATGCCTCGGGTGTGGTTGGACTGTCATTT

ENSMUST00000034859    AGTGGGCACCGAATCCAGAGTGCCACAGTTCTCCTCACTGAGGATGTAAATGATGCCAAG
ENSRNOT00000020309    AGTGGGCACCGAATCCAGAGTGCCACGGTTCTCCTCAATGAGGATGTAAATGATACCAAG
ENSCJAT00000023917    AGTGGACACCGAATCCAGAGTGCTACTGTGCTCCTCAGCGAGGACGTCAATGATGAGAAG
ENSMMUT00000012815    AGTGGACACCGAATCCAGAGTGCCACTGTGCTCCTCAACGAGGACGTCAGTGATGAGAAG
ENSPPYT00000007875    AGTGGACACCGAATCCAGAGTGCCACTGTGCTCCTCAACGAGGACGTCAGTGATGAGAAG
ENSGGOT00000001089    AGTGGACACCGAATCCAGAGTGCCACTGTGCTCCTCAACGAGGACGTCAGTGATGAGAAG
ENSPTRT00000013488    AGTGGACACCGAATCCAGAGTGCCACTGTGCTCCTCAACGAGGACGTCAGTGATGAGAAG
ENST00000308275       AGTGGACACCGAATCCAGAGTGCCACTGTGCTCCTCAACGAGGACGTCAGTGATGAGAAG

ENSMUST00000034859    ACCGTCGAGGCTGCTATGCAGCGCCTCAAAGCAGCCAACATCCCTGAACAGAACACCATT
ENSRNOT00000020309    ACCGCTGAGGCCGCCATGCAGCGCCTCAAAGCAGCCAACATCCCTGAACAGAACACCATT
ENSCJAT00000023917    ACTGCTGAGGCTGCGATGCAGCGCCTCAAAGCGGCCAACATTCCAGAGCAGAACACCATT
ENSMMUT00000012815    ACTGCGGAGGCTGCAATGCAGCGCCTCAAAGCGGCCAACATTCCAGAGCAGAACACCATT
ENSPPYT00000007875    ACTGCTGAGGCTGCGATGCAGCGCCTCAAAGCGGCCAACATTCCAGAGCAGAACACCATT
ENSGGOT00000001089    ACTGCTGAGGCTGCGATGCAGCGCCTCAAAGCGGCCAACATTCCAGAGCATAACACCATT
ENSPTRT00000013488    ACTGCTGAGGCTGCGATGCAGCGCCTCAAAGCGGCCAACATTCCAGAGCATAACACCATT
ENST00000308275       ACTGCTGAGGCTGCGATGCAGCGCCTCAAAGCGGCCAACATTCCAGAGCATAACACCATT

ENSMUST00000034859    GGCTTCATGTTTGCATGCGTTGGCCGAGGCTTTCAGTACTACAGAGCCAAGGGGAATGTT
ENSRNOT00000020309    GGCTTCATGTTTGCGTGTGTTGGCCGAGGCTTTCAGTACTACAGAGCCAAGGGGAATGTT
ENSCJAT00000023917    GGCTTCATGTTTGCATGTGTTGGCAGGGGCTTTCAGTATTACAGAGCCAAGGGGAATGTT
ENSMMUT00000012815    GGCTTCATGTTTGCATGCGTTGGCAGGGGCTTTCAGTATTACAGAGCCAAGGGGAATGTT
ENSPPYT00000007875    GGCTTCATGTTTGCATGCGTTGGCAGGGGCTTTCAGTATTACAGAGCCAAAGGGAATGTT
ENSGGOT00000001089    GGCTTCATGTTTGCATGCGTTGGCAGGGGCTTTCAGTATTACAGAGCCAAGGGGAATGTT
ENSPTRT00000013488    GGCTTCATGTTTGCATGCGTTGGCAGGGGCTTTCAGTATTACAGAGCCAAGGGGAATGTT
ENST00000308275       GGCTTCATGTTTGCATGCGTTGGCAGGGGCTTTCAGTATTACAGAGCCAAGGGGAATGTT

ENSMUST00000034859    GAAGCTGATGCATTTAGAAAGTTCTTTCCCAGTGTCCCCTTATTTGGCTTCTTTGGAAAT
ENSRNOT00000020309    GAGGCTGATGCATTTAGAAAGTTTTTTCCCAATGTCCCCTTATTTGGCTTCTTTGGAAAT
ENSCJAT00000023917    GAGGCTGATGCATTTAGAAAGTATTTTCCTAGTGTGCCCTTATTCGGCTTCTTTGGAAAT
ENSMMUT00000012815    GAGGCTGATGCGTTCAGAAAGTTTTTTCCTAGTGTTCCCCTATTCGGCTTCTTTGGAAAT
ENSPPYT00000007875    GAGGCTGATGCGTTTAGAAAGTTTTTTCCTAGTGTTCCCTTATTTGGCTTCTTTGGAAAT
ENSGGOT00000001089    GAGGCTGATGCATTTAGAAAGTTTTTTCCTAGTGTTCCCTTATTCGGCTTCTTTGGAAAT
ENSPTRT00000013488    GAGGCTGATGCATTTAGAAAGTTTTTTCCTAGTGTTCCCTTATTCGGCTTCTTTGGAAAT
ENST00000308275       GAGGCTGATGCATTTAGAAAGTTTTTTCCTAGTGTTCCCTTATTCGGCTTCTTTGGAAAT

ENSMUST00000034859    GGGGAGATTGGCTGCGACCGGATAGTCACTGGGAACTTTATACTGAGGAGATGTAATGAG
ENSRNOT00000020309    GGGGAGATCGGCTGCGACCGGATAGTCACTGGGAACTTTATCCTGAGGAGGTGTAATGAG
ENSCJAT00000023917    GGAGAAATTGGATGTGATCGGATAGTCACTGGGAACTTTATATTGAGGAAATGTAATGAG
ENSMMUT00000012815    GGAGAAATTGGGTGTGATCGGATAGTCACTGGGAACTTCATATTGAGGAAATGTAATGAG
ENSPPYT00000007875    GGAGAAATTGGATGTGATCGGATAGTCACTGGGAACTTTATATTGAGGAAATGTAATGAG
ENSGGOT00000001089    GGAGAAATTGGATGTGATCGGATAGTCACTGGGAACTTTATATTGAGGAAATGTAATGAG
ENSPTRT00000013488    GGAGAAATTGGATGTGATCGGATAGTCACTGGGAACTTTATATTGAGGAAATGTAATGAG
ENST00000308275       GGAGAAATTGGATGTGATCGGATAGTCACTGGGAACTTTATATTGAGGAAATGTAATGAG

ENSMUST00000034859    GTAAAGGAAGAGGACCTGTTCCATAGCTACACAACCATCATGGCTCTCGTTCACCTGGGA
ENSRNOT00000020309    GTGAAGGAGGAGGATCTGTTCCATAGCTACACAACCATCATGGCTCTCGTTCACCTGGGG
ENSCJAT00000023917    GTAAAAGATGATGATCTGTTTCATAGCTATACAACCATAATGGCGCTCATACATCTGGGG
ENSMMUT00000012815    GTAAAAGATGATGATCTGTTTCATAGCTATACAACAATAATGGCACTCATACATCTGGGG
ENSPPYT00000007875    GTAAAAGATGATGATCTGTTTCATAGCTATACAACAATAATGGCACTCATACATCTGGGG
```

ENSGGOT00000001089 GTAAAAGATGATGATCTGTTTCATAGCTATACAACAATAATGGCACTCATACATCTGGGG
ENSPTRT00000013488 GTAAAAGATGATGATCTGTTTCATAGCTATACAACAATAATGGCACTCATACATCTGGGG
ENST00000308275 GTAAAAGATGATGATCTGTTTCATAGCTATACAACAATAATGGCACTCATACATCTGGGG

ENSMUST00000034859 ACCTCTAAA
ENSRNOT00000020309 GCCTCCAAG
ENSCJAT00000023917 TCATCTAAA
ENSMMUT00000012815 TCATCTAAA
ENSPPYT00000007875 TCGTCTAAA
ENSGGOT00000001089 TCATCTAAA
ENSPTRT00000013488 TCATCTAAA
ENST00000308275 TCATCTAAA

Multiple sequence alignment of Fbxo24

ENSMUST00000031732 ATGGTGAAGCGCAGCTGCCCTTCTTGTGGCCTGGAGGCTGGGAGTGAAAAGAAGGAAAGA
ENSRNOT00000038397 ATGGTGAAGCGCAGCTGCCCTTCTTGTGGCCTGGAGGCTGGAGGTGAAAAGAAGGAGAGA
ENSCJAT00000057702 TGGGTGAAGAGAAGCTGCCCTTCTTGTGGCCCGGAGCTTGGGGGTGAGAAGAAGGGGAGA
ENSPTRT00000036103 CGGGTGAAGAGAAGCTGCCCTTCTTGTGGCTCGGAGCTTGGGGTTGAGAAGAGGGGGAAA
ENST00000427939 CGGGTGAAGAGAAGCTGCCCTTCTTGTGGCTCGGAGCTTGGGGTTGAGAAGAGGGGGAAA
ENSGGOT00000028534 CGGGTGAAGAGAAGCTGCCCTTCTTGTGGCTCGGAGCTTGGGGTTGAGAAGAGGGGGAAA

ENSMUST00000031732 GGGAACCCTATTTCTGTTCAGTTGTTCCCCCCAGAGCTGGTGGAACACATTGTCTCCTTC
ENSRNOT00000038397 GGGAACCCTATTTCTGTTCAGTTGTTCCCCCCAGAGCTGGTGGAACACATCGTCTCCTTC
ENSCJAT00000057702 GGGAATCCGATTTCTGTCCAGTTGTTCCCCCCAGAGCTGGTGGAACATATCATCTCATTC
ENSPTRT00000036103 GGAAATCCGATTTCCATCCAGTTGTTCCCCCCAGAGCTGGTGGAGCATATCATCTCATTC
ENST00000427939 GGAAATCCGATTTCCATCCAGTTGTTCCCCCCAGAGCTGGTGGAGCATATCATCTCATTC
ENSGGOT00000028534 GGGAATCCGATTTCCATCCAGTTGTTCCCCCCAGAGCTGGTGGAGCATATCATCTCATTC

ENSMUST00000031732 CTCCCAGTCAAAGACTTGGTCGCCCTAGGCCAGACCTGCCACTACTTCCATGAAGTGTGT
ENSRNOT00000038397 CTCCCGGTCAAAGACGTGGTCGCCCTAGGCCAGACCTGCCACTACTTCCATGAAGTGTGT
ENSCJAT00000057702 CTCCCAGTCAGAGACCTTGTCGCCCTCGGCCAGACCTGCCGCTACTTCCACAAAGTGTGC
ENSPTRT00000036103 CTCCCAGTCAGAGACCTTGTTGCCCTCGGCCAGACCTGCCGCTACTTCCACGAAGTGTGC
ENST00000427939 CTCCCAGTCAGAGACCTTGTTGCCCTCGGCCAGACCTGCCGCTACTTCCACGAAGTGTGC
ENSGGOT00000028534 CTCCCAGTCAGAGACCTTGTTGCCCTTGGCCAGACCTGCCGCTACTTCCACGAAGTGTGC

ENSMUST00000031732 GATGCTGAGGGCGTGTGGAGGCGCATCTGTCGAAGGCTCAGCCCTCGAATCCGTGACCAG
ENSRNOT00000038397 GACGCCGAGGGCGTGTGGAGGCGAATATGTCGGAGGCTCAGCCCACGCATCCGTGACCAG
ENSCJAT00000057702 GATGCAGAGGGGGTGTGGAGACTCATCTGTCGCAGGCTGAGTCTGCGCCTCCGAGATCAG
ENSPTRT00000036103 GATGGGGAAGGCGTGTGGAGACGCATCTGTCGCAGACTCAGTCCGCGCCTCCAAGATCAG
ENST00000427939 GATGGGGAAGGCGTGTGGAGACGCATCTGTCGCAGACTCAGTCCGCGCCTCCAAGATCAG
ENSGGOT00000028534 GATGGGGAAGGCGTGTGGAGACGCATCTGTCGCAGACTCAGTCCGCGCCTCCAAGATCAG

ENSMUST00000031732 AGTTCTGGTGCCCGGCCCTGGAAGAGAGCTGCCATTCTTAACTATACAAAGGGCCTGTAT
ENSRNOT00000038397 GGTTCCGGCGCTCGGCCCTGGAAGAGAGCTGCCATTCTTAACTATACGAAGGGCCTGTAT
ENSCJAT00000057702 GGTTCCGGAGTCCGGCCCTGGAAGAGAGCTGCCATTCTGAACTACACGAAGGGCCTGTAT
ENSPTRT00000036103 GGTTCTGGAGTCCGGCCCTGGAAGAGAGCTGCCATTCTGAACTACACGAAGTGCCTGTAT
ENST00000427939 GGTTCTGGAGTCCGGCCCTGGAAGAGAGCTGCCATTCTGAACTACACGAAGGGCCTGTAT
ENSGGOT00000028534 GGTTCTGGAGTCCGGCCCTGGAAGAGAGCTGCCATTCTGAACTACACGAAGGGCCTGTAT

ENSMUST00000031732 TTCCAGGCATTCGGAGGACGCCGCCGATGTCTCAGCAAGAGTGTGGCCCCCCATGCTAGCC
ENSRNOT00000038397 TTCCAGGCTTTCGGAGGACGCCGCCGCTGCCTCAGCAAGAGTGTGGCCCCCCATGCTAGCC
ENSCJAT00000057702 TTCCAGGCATTTGGAGGCCGCCGCCGATGTCTCAGCAAGAGTGTAGCCCCCCCTGCTAGCC

```
ENSPTRT00000036103     TTCCAGGCATTTGGAGGCCGCCGCCGATGTCTCAGCAAGAGCGTGGCCCCCTTGCTAGCC
ENST00000427939        TTCCAGGCATTTGGAGGCCGCCGCCGATGTCTCAGCAAGAGCGTGGCCCCCTTGCTAGCC
ENSGGOT00000028534     TTCCAGGCATTTGGAGGCCGCCGCCGATGTCTCAGCAAGAGCGTGGCCCCCTTGCTAGCC

ENSMUST00000031732     CATGGCTACCGCCGCTTCTTACCCACAAAGGACCACGTGTTCATTCTTGACTACGTGGGG
ENSRNOT00000038397     CATGGCTACCGCCGCTTCCTACCTACGAAGGACCACGTGTTCATTCTCGACTATGTGGGG
ENSCJAT00000057702     CACGGCTACCGCCGCTTCTTGCCCACCAAGGACCACGTCTTCATTCTTGACTATGTGGGG
ENSPTRT00000036103     CACGGCTACCGCCGCTTCTTGCCCACCAAGGATCACGTCTTCATTCTTGACTACGTGGGG
ENST00000427939        CACGGCTACCGCCGCTTCTTGCCCACCAAGGATCACGTCTTCATTCTTGACTACGTGGGG
ENSGGOT00000028534     CACGGCTACCGCCGCTTCTTGCCCACCAAGGATCACGTCTTCATTCTTGACTACGTGGGG

ENSMUST00000031732     ACCCTCTTCTTCCTCAAAAATGCTCTGGTCTCCACCCTCGGCCAGATCCAGTGGAAGCGG
ENSRNOT00000038397     ACCCTCTTCTTCCTCAAAAATGCTCTGGTGTCCACCCTTGGCCAGATCCAGTGGAAGCGA
ENSCJAT00000057702     ACGCTCTTCTTCCTCAAAAATGCCCTGGTCTCCACCCTTGGCCAGATGCAGTGGAAGCGG
ENSPTRT00000036103     ACCCTCTTCTTCCTCAAAAATGCCCTGGTCTCCACCCTCGGCCAGATGCAGTGGAAGCGG
ENST00000427939        ACCCTCTTCTTCCTCAAAAATGCCCTGGTCTCCACCCTCGGCCAGATGCAGTGGAAGCGG
ENSGGOT00000028534     ACCCTCTTCTTCCTCAAAAATGCCCTGGTCTCCACCCTCGGCCAGATGCAGTGGAAGCGG

ENSMUST00000031732     GCCTGCCGCTACGTGGTGTTGTGTCGAGGCGCCAAGGATTTTGCGTCCGACCCAAGATGT
ENSRNOT00000038397     GCCTGCCGCTACGTGGTGCTGTGTCGTGGTGCCAAGGATTTTGCGTCCGACCCGAGATGC
ENSCJAT00000057702     GCCTGTCGCTATGTTGTGTTGTGTTGTGGAGCGAAGGATTTTGCCTCAGACCCAAGGTGT
ENSPTRT00000036103     GCCTGTCGCTATGTTGTGTTGTGTCGTGGAGCCAAGGATTTTGCCTCGGACCCAAGGTGT
ENST00000427939        GCCTGTCGCTATGTTGTGTTGTGTCGTGGAGCCAAGGATTTTGCCTCGGACCCAAGGTGT
ENSGGOT00000028534     GCCTGTCGCTATGTTGTGTTGTGTCGTGGAGCCAAGGATTTTGCCTCGGACCCAAGGTGT

ENSMUST00000031732     GACACGGTTTACCGGAAGTACCTCTATGTTTTGGCTACTCGGGAACAGCCGGCAGTGGTG
ENSRNOT00000038397     GACACGGTTTACCGGAAGTACCTCTATGTTTTGGCTACTCGGGAACAGCAGGCAGTGGTG
ENSCJAT00000057702     GACACAGCTTACCGAAAATACCTCTATGTCTTGGCCACTCGGGAGCAGCAGGGAGTGGTG
ENSPTRT00000036103     GACACAGTTTACCGTAAATACCTCTATGTCTTGGCCACTCGGGAGCAGCAGGAAGTGGTG
ENST00000427939        GACACAGTTTACCGTAAATACCTCTACGTCTTGGCCACTCGGGAGCCGCAGGAAGTGGTG
ENSGGOT00000028534     GACACAGTTTACCGTAAATACCTCTATGTCTTGGCCACTCGGGAGCAGCAGGAAGTGGTG

ENSMUST00000031732     GGCACCACCGGTAGCCGCGCCTGTGACTGTGTGGAGGTCTATCTGCAGTCCAGTGGGCAG
ENSRNOT00000038397     GGCTCCACCGGTAGCCGGGCCTGTGACTGTGTGGAGGTCTATCTGCAGTCCAGTGGGCAG
ENSCJAT00000057702     GGTACCACCAGCAGCCGGACCTGTGACTGCGTGGAGGTCTACTTGCAGTCTAGTGGGCAG
ENSPTRT00000036103     GGTACCACCAGCAGCCGGGCCTGTGACTGTGTTGAGGTCTATCTGCAGTCTAGTGGGCAG
ENST00000427939        GGTACCACCAGCAGCCGGGCCTGTGACTGTGTTGAGGTCTATCTGCAGTCTAGTGGGCAG
ENSGGOT00000028534     GGTACCACCAGCAGCCGGGCCTGTGACTGTGTTGAGGTCTATCTGCAGTCTAGTGGGCAG

ENSMUST00000031732     CGGGTCTTCAAGATGACATTCCACCACTCCATGAGCTTCAAGCAGATCGTGCTGGTTGGC
ENSRNOT00000038397     CGGGTCTTCAAGATGACATTCCACCACTCCATGAGCTTCAAGCAGATCGTGCTGGTTGGC
ENSCJAT00000057702     CGGGTCTTCAAGATGACATTCCACCACTCCATGAGCTTCAAGCAGATCGTGTTGGTTGGT
ENSPTRT00000036103     CGGGTCTTCAAGATGACATTCCACCACTCAATGACCTTCAAGCAGATCGTGCTGGTTGGT
ENST00000427939        CGGGTCTTCAAGATGACATTCCACCACTCAATGACCTTCAAGCAGATCGTGCTGGTTGGT
ENSGGOT00000028534     CGGGTCTTCAAGATGACATTCCACCACTCAATGACCTTCAAGCAGATCGTGCTGGTTGGT

ENSMUST00000031732     CAGGAGACCCAGCGGGCCCTACTGCTCCTCACAGAGGAAGGAAAGATCTACTCCTTGGTT
ENSRNOT00000038397     CAGGAGACCCAGCGGGCCCTACTGCTGCTCACAGAGGAAGGGAAGATATACTCTTTGGTT
ENSCJAT00000057702     CAGGAGACCCAGCGGGCTCTATTGCTCCTCACAGAGGAAGGAAAGATCTACTCTTTGATA
ENSPTRT00000036103     CAGGAGACCCAGCGGGCTCTACTGCTCCTCACAGAGGAAGGAAAGATCTACTCTTTGGTA
ENST00000427939        CAGGAGACCCAGCGGGCTCTACTGCTCCTCACAGAGGAAGGAAAGATCTACTCTTTGGTA
ENSGGOT00000028534     CAGGAGACCCAGCGGGCTCTACTGCTCCTCACAGAGGAAGGAAAGATCTACTCTTTGGTA
```

```
ENSMUST00000031732     GTGAATGAGACCCAGCTGGACCAGCCACGCTCCTATACAGTGCAGTTAGCCCTCAGGAAG
ENSRNOT00000038397     GTGAACGAAACCCAGCTGGACCAGCCGCGCTCCTATACAGTGCAGTTGGCCCTCAGGAAG
ENSCJAT00000057702     GTGAATGAGACCCAGCTGGACCAGCCACGCTCCTACACGGTTCAGCTGGCCCTGAGAAAG
ENSPTRT00000036103     GTGAATGAGACCCAGCTTGACCAGCCACGCTCCTACACGGTTCAGCTGGCCCTGAGGAAG
ENST00000427939        GTGAATGAGACCCAGCTTGACCAGCCACGCTCCTACACGGTTCAGCTGGCCCTGAGGAAG
ENSGGOT00000028534     GTGAATGAGACCCAGCTTGACCAGCCACGCTCCTACACGGTTCAGCTGGCCCTGAGGAAG

ENSMUST00000031732     GTGTCTCGATGCCTGCCTCACCTGCGTGTGACCTGCATGGCTTCCAACCAGAGCAGTACC
ENSRNOT00000038397     GTGTCTCGATGCCTGCCTCACCTGCGCGTGACCTGCATGGCTTCCAACCAGAGCAGCACC
ENSCJAT00000057702     GTGTCCCACTGCCTGCCTCACCTGCGCGTGGCCTGTATGACTTCCAACCAAAGCAGCACG
ENSPTRT00000036103     GTGTCCCACTACCTGCCTCACCTGCGCGTGGCCTGCATGACTTCCAACCAGAGCAGCACC
ENST00000427939        GTGTCCCACTACCTGCCTCACCTGCGCGTGGCCTGCATGACTTCCAACCAGAGCAGCACC
ENSGGOT00000028534     GTGTCCCACTACCTGCCTCACCTGCGCGTGGCCTGCATGACTTCCAACCAGAGCAGCACC

ENSMUST00000031732     CTCTATATCACGGACCAGGGGGGAGTGTATTTTGAGGTGCATACCCCCGGGGTGTATCGT
ENSRNOT00000038397     CTCTATATCACGGACCAGGGGGGAGTATATTTTGAGGTGCACACCCCAGGGGTGTATCGT
ENSCJAT00000057702     CTCTACATCACTGAACAGGGGGGAGTGTATTTCGAGGTGCATACCCCAGGGGTGTATCGC
ENSPTRT00000036103     CTCTACGTCACAGACCAGGGGGGAGTGTATTTTGAGGTGCATACCCCAGGGGTGTATCGC
ENST00000427939        CTCTACGTCACAGACCAGGGGGGAGTGTATTTTGAGGTGCATACCCCAGGGGTGTATCGC
ENSGGOT00000028534     CTCTACGTCACAGACCAGGGGGGAGTGTATTTTGAGGTGCATACCCCAGGGGTGTATCGC

ENSMUST00000031732     GATCTCTTTGGGACCCTTCAAGCCTTTGACCCCCTGGACCACCAGATGCCACTGGCTCTC
ENSRNOT00000038397     GATCTCTTTGGGACCCTTCAAGCCTTTGACCCTCTGGACCACCAGATGCCGCTTGCTCTC
ENSCJAT00000057702     GATCTCTTTGGGACTCTTCAAGCCTTTGACCCCCTGGACCAGCAGATGCCGCTTGCTCTC
ENSPTRT00000036103     GATCTCTTTGGGACCCTTCAAGCCTTTGACCCCCTGGACCAGCAGATGCCGCTTGCTCTC
ENST00000427939        GATCTCTTTGGGACCCTTCAAGCCTTTGACCCCCTGGACCAGCAGATGCCGCTTGCTCTC
ENSGGOT00000028534     GATCTCTTTGGGACCCTTCAAGCCTTTGACCCCCTGGACCAGCAGATGCCGCTTGCTCTC

ENSMUST00000031732     TCCTTGCCTGCCAAGGTCCTCTTCTGTGCTCTTGGCTATAATCATCTTGGCCTGGTAGAT
ENSRNOT00000038397     TCCCTGCCTGCCAAGGTCCTCTTCTGCGCTCTTGGCTATAACCATCTTGGCCTGGTAGAT
ENSCJAT00000057702     TCACTGCCTGCCAAGATCCTGTTCTGTGCTCTTGGCTACAATCACCTTGGCCTGGTGGAT
ENSPTRT00000036103     TCACTGCCTGCCAAGATCCTATTCTGTGCTCTTGGCTACAACCACCTTGGCCTGGTGGAT
ENST00000427939        TCACTGCCTGCCAAGATCCTATTCTGTGCTCTTGGCTACAACCACCTTGGCCTGGTGGAT
ENSGGOT00000028534     TCACTGCCTGCCAAGATCCTATTCTGTGCTCTTGGCTACAACCACCTTGGCCTGGTGGAT

ENSMUST00000031732     GAATTTGGCCGGATCTTTATGCAGGGAAATAACAGATACGGGCAGCTGGGAACTGGAGAC
ENSRNOT00000038397     GAGTTTGGCCGAATCTTTATGCAGGGAAATAACAGATACGGGCAGCTGGGAACTGGAGAC
ENSCJAT00000057702     GAATTTGGCCGAATCTTTATGCAAGGAAATAACAGATACGGGCAGCTAGGAACAGGGGAC
ENSPTRT00000036103     GAATTTGGCCGAATCTTCATGCAAGGAAATAACAGATACGGGCAGCTAGGAACAGGGGAC
ENST00000427939        GAATTTGGCCGAATCTTCATGCAAGGAAATAACAGATACGGGCAGCTAGGAACAGGGGAC
ENSGGOT00000028534     GAATTTGGCCGAATCTTCATGCAAGGAAATAACAGATACGGGCAGCTAGGAACAGGGGAC

ENSMUST00000031732     AAAATGGACCGAGGGGAGCCCACACAGGTTCACTATCTGCAACGCCCCATCGCCCTGTGG
ENSRNOT00000038397     AAAATGGACCGAGGGGAACCCACGCAGGTTCACTATCTGCACCGCCCCATTGCCCTGTGG
ENSCJAT00000057702     AAAATGGACCGAGGGGAACCCACACAGGTTCGTTACCTGCAGCGGCCCATTGCCCTGTGG
ENSPTRT00000036103     AAAATGGACCGAGGGGAACCCACACAGGTTCGTTACCTGCAGCGGCCCGTCACCCTGTGG
ENST00000427939        AAAATGGACCGAGGGGAACCCACACAGGTTTGTTACCTGCAGCGGCCCATCACCCTGTGG
ENSGGOT00000028534     AAAATGGACCGAGGGGAACCCACACAGGTTCGTTACCTGCAGCGGCCCATCACCCTGTGG

ENSMUST00000031732     TGTGGCCTCAACCATTCCTTGGTGCTGAGTCAGACCTCGGATTTCAGCAAGGAGCTGCTG
ENSRNOT00000038397     TGCGGCCTCAACCATTCCTTGGTGCTGAGTCAGACCTCGGATTTCAGCAAGGAGCTGCTG
ENSCJAT00000057702     TGTGGCCTCAACCACTCCCTAGTGCTGAGCCAGAGCTCAGACTTCAGCAAGGAGCTGCTA
```

```
ENSPTRT00000036103     TGCGGCCTCAACCACTCCCTGGTGCTGAGCCAGAGCTCAGAGTTCAGCAAGGAGCTGCTG
ENST00000427939        TGCGGCCTCAACCACTCCCTGGTGCTGAGCCAGAGCTCAGAGTTCAGCAAGGAGCTGCTG
ENSGGOT00000028534     TGCGGCCTCAACCACTCCCTGGTGCTGAGCCAGAGCTCAGAGTTCAGCAAGGAGCTGCTG

ENSMUST00000031732     GGGTGCGGCTGTGGGGCCGGAGGCCGTCTTCCTGGCTGGCCCAAGGGGAGCGCCTCCTTC
ENSRNOT00000038397     GGGTGCGGCTGTGGGGCCGGAGGCCGTCTTCCTGGCTGGCCCAAGGGGAGTGCCTCCTTT
ENSCJAT00000057702     GGCTGCGGCTGCGGAGCTGGGGGCCGCCTCCCCGGCTGGCCCAAGGGGAGTGCCTCCTTC
ENSPTRT00000036103     GGCTGCGGCTGTGGGGCTGGGGGCCGCCTCCCAGGCTGGCCCAAGGGGAGTGCCTCCTTC
ENST00000427939        GGCTGCGGCTGTGGGGCTGGGGGCCGCCTCCCAGGCTGGCCCAAGGGGAGTGCCTCCTTC
ENSGGOT00000028534     GGCTGCGGCTGTGGGGCTGGGGGCCGCCTCCCAGGCTGGCCCAAGGGGAGTGCCTCCTTC

ENSMUST00000031732     GTCAAGCTGCACATCAAGGTCCCTTTGTGTGCCTGCTCCCTCTGCTCCACCAGAGAGTGT
ENSRNOT00000038397     GTCAAACTGCACATCAAGGTCCCCTTGTGTGCCTGCTCCCTCTGCTCCACCAGAGAGTGT
ENSCJAT00000057702     GTCAAGCTCCAAGTCAAGGTCCCTTTGTGTGCCTGTGCCCTCTGCGCCACCAGGGACTGT
ENSPTRT00000036103     GTCAAGCTCCAAGTCAAGGTCCCTCTGTGTGCCTGTGCCCTCTGTGCCACCAGGGAGTGC
ENST00000427939        GTCAAGCTCCAAGTCAAGGTCCCTCTGTGTGCCTGTGCCCTCTGTGCCACCAGGGAGTGC
ENSGGOT00000028534     GTCAAGCTCCAAGTCAAGGTCCCTCTGTGTGCCTGTGCCCTCTGTGCCACCAGGGAGTGC

ENSMUST00000031732     CTCTATATGCTGTCCAGCCACGACATCGAACAGTGCCCTGTCTATCGAGACCTGCCAGCC
ENSRNOT00000038397     CTCTATATGCTGTCCAGCCACGACATCGAACAGTGCCCTGCCTATCGAGACCTGCCAGCC
ENSCJAT00000057702     CTCTACATGCTATCCAGCCATGACATTGAGGAGCACACCTCCTATCGCGACCTGCCGGCC
ENSPTRT00000036103     CTATACATCCTGTCCAGCCACGACATTGAGCAGCACGCCCCCTATCGCGACCTGCCAGCC
ENST00000427939        CTATACATCCTGTCCAGCCACGACATTGAGCAGCACGCCCCCTATCGCCACCTGCCAGCC
ENSGGOT00000028534     CTATACATCCTGTCCAGCCACGACATCGAGCAGCACGCCCCCTATCGCGACCTGCCGGCC

ENSMUST00000031732     AGCAGGGTGGGGGGGAGCCCTGAACCTAGCCAGGGGGCTGGAGCCCCCCAAGACCCTGGG
ENSRNOT00000038397     AGCAGGGTGGGAGGGAGCCCTGAACCTAGTCAGGGGGCTGGAGCCCCCCAAGACCCTGGG
ENSCJAT00000057702     AGCAGGGTGATGGGGACTCCTGAGCCCAGCCTAGGGGCTGGAGCACCCCAGGACCCTGGG
ENSPTRT00000036103     AGCAGGGTGGTGGGGACTCCCGAGCCCAGCCTGGGGGCCAGAGCACCCCAGGACCCCGGG
ENST00000427939        AGCAGGGTGGTGGGGACTCCTGAGCCCAGCCTGGGGGCCAGAGCACCCCAGGACCCCGGG
ENSGGOT00000028534     AACAGGGTGGTGGGGACTCCCGAGCCCAGCCTGGGGGCCAGAGCACCCCAGGACCCCGGG

ENSMUST00000031732     GGAACAGCCCAGGCCTGTGAGGAGTACCTCAGCCAGATCCACAGTTGCCCCACGTTGCAG
ENSRNOT00000038397     GGAACAGCCCAGGCCTGTGAGGAGTACCTCAGCCAGATCCACAGTTGCCCCACGTTGCAG
ENSCJAT00000057702     GGAACGGCCCGGGCCTGCGAGGAGTACCTCAGCCAGATCCACAGTTGCCACACGTGGCAG
ENSPTRT00000036103     GGGATGGCCCAGGCCTGCGAGGAGTACCTCAGCCAGATCCACAGTTGCCAAACGTTGCAG
ENST00000427939        GGGATGGCCCAGGCCTGCGAGGAGTACCTCAGCCAGATCCACAGTTGCCAAACGTTGCAG
ENSGGOT00000028534     GGGATGGCCCAGGCCTGCGAGGAGTACCTCAGCCAGATCTACAGTTGCCAAACGTTGCAG

ENSMUST00000031732     GACCGAATGGAGAAGATGAAGGAGATTGTGGGCTGGATGCCCTTGATGGCTGCCCAGAAA
ENSRNOT00000038397     GACCGGATGGAGAAGATGAAGGAGATTGTGGGCTGGATGCCCTTGATGGCTGCCCAGAAA
ENSCJAT00000057702     GACCGTATGGAGAAGATGAAGGAGATCATAGGGTGGATGCCCCTGATGGCCGCACAGAAG
ENSPTRT00000036103     GACCGCATGGAGAAGATGAAGGAGATCGTAGGGTGGATGCCCCTGATGGCCGCACAGAAG
ENST00000427939        GACCGCACGGAGAAGATGAAGGAGATCGTAGGGTGGATGCCCCTGATGGCCGCACAGAAG
ENSGGOT00000028534     GACCGCATGGAGAAGATGAAGGAGATCGTAGGGTGGATGCCCCTGATGGCCGCACAGAAG

ENSMUST00000031732     GATTTCTTCTGGGAGGCACTGGACATGTTGCAGAGGGCTGCTGGAGGGGCTGGCCCAGAC
ENSRNOT00000038397     GATTTCTTCTGGGAGGCACTGGACATGTTGCAGAGGGCTGCTAGCGGGGGTGGTCCAGGC
ENSCJAT00000057702     GACTTCTTCTGGGAGGCCCTGGACATGCTGCAGCAGGATGTG---GGAGGTGGGGTGGCG
ENSPTRT00000036103     GACTTCTTCTGGGAGGCCCTGGACATGCTGCAGAGGGCTGAAGGCGGGGGTGGTGTAGGG
ENST00000427939        GACTTCTTCTGGGAGGCCCTGGACATGCTGCAGAGGGCTGAAGGCGGGGGTGGTGTAGGG
ENSGGOT00000028534     GACTTCTTCTGGGAGGCCCTGGACATGCTGCAGAGGGCTGAAGGCGGGGGTGGTGTAGGG
```

```
ENSMUST00000031732      ACCTCGACTCCCGAGAGC
ENSRNOT00000038397      ACCTCCACCCCTGAGAGC
ENSCJAT00000057702      CCCCCAGTCCCTGAGAGC
ENSPTRT00000036103      CCCCCAGCCCCTGAGACC
ENST00000427939         CCCCCAGCCCCTGAGACC
ENSGGOT00000028534      CCCCCAGCCCCTGAGACC
```

Multiple sequence alignment of Fbxo25

```
ENSMUST00000043520      ATGCCGTTTCTGGGTCAGGACTGGAGGTCCCCTGGATGGAGCTGGATTAAAACCGAGGAT
ENSCJAT00000023185      ATGCCATTTTTGGGTCAGGACTGGAGATCTCCTGGATGGAGTTGGATTAAAACAGAAGAT
ENSGGOT00000014225      ------------------------------------------------------------
ENST00000276326         ATGCCATTTTTGGGTCAGGACTGGAGATCTCCTGGATGGAGTTGGATTAAGACAGAAGAT
ENSMMUT00000015522      ATGCCATTTTTGGGTCAGGACTGGAGATCTCCTGGATGGAGTTGGATTAAGACAGAAGAT
ENSPTRT00000036947      ATGCCATTTTTGGGTCAGGACTGGAGATCTCCTGGATGGAGTTGGATTAAGACAGAAGAT
ENSPPYT00000021318      ATGCCATTTTTGGGTCAGGACTGGAGATCTCCTGGATGGAGTTGGATTAAGACAGAAGAT

ENSMUST00000043520      GGGTGGAAAAGGTGTGACCCCTGTAGCCACGAGCTCAGGAGCGAGGATAGCCAGTACACC
ENSCJAT00000023185      GGCTGGAAGAGATGTGAATCCTGTAGTCAGAAACTTGAAAGAGAGAATAACCATTGTAAC
ENSGGOT00000014225      ------------------------------------------------------------
ENST00000276326         GGCTGGAAGAGATGTGAATCTTGTAGTCAGAAACTTGAAAGAGAGAATAACCGTTGTAAC
ENSMMUT00000015522      GGCTGGAAGAGATGTGAATCCTGTAGTCAGAAACTTGAAAGAGAGAATAACCATTGTAAC
ENSPTRT00000036947      GGCTGGAAGAGATGTGAATCTTGTAGTCAGAAACTTGAAAGAGAGAATAACCATTGTAAC
ENSPPYT00000021318      GGCTGGAAGAGATGTGAATCCTGTAGTCAAAAACTTGAAAGAGAGAATAACCATTGTAAC

ENSMUST00000043520      ATCAACCACAGCATTATCTTAAATAGTGGTGAAGAAGAAATATTCAACAAT---GAGTGT
ENSCJAT00000023185      ATCAGTCACAGCATTATCTTAAATAGTGAAGATGAAGAAATATTCAGTAATGAAGAACAT
ENSGGOT00000014225      ---------------ATCTTAAATAGCGAAGATGGAGAAATATTCAATAATGAAGAGCAT
ENST00000276326         ATCAGTCACAGCATTATCTTAAATAGTGAAGATGGAGAAATATTCAATAATGAAGAGCAT
ENSMMUT00000015522      ATCAGTCACAGCATTATCTTAAATAGTGAAGATGGAGAAATATTCAATAATGAAGAGCAT
ENSPTRT00000036947      ATCAGTCACAGCATTATCTTAAATAGTGAAGATGGAGAAATATTCAATAATGAAGAGCAT
ENSPPYT00000021318      ATCAGTCACAGCATTATCTTAAATAGTGAAGATGGAGAAATATTCAATAATGAAGAGCAT

ENSMUST00000043520      GAATACGCAGCCAAAAAGAGGAAGAAGGAACATTTTGGAAATGACACAGCTGCTCACAGT
ENSCJAT00000023185      GAATATGCATCCAAAAAAAGGAAAAAGGACCATTTTAGAAATGACACAAATACTCAAAGT
ENSGGOT00000014225      GAATATGCATCCAAAAAAAGGAAAAAGGACCATTTTAGAAATGACACAAATACTCAAAGT
ENST00000276326         GAATATGCATCGAAAAAAAGGAAAAAGGACCATTTTAGAAATGACACAAATACTCAAAGT
ENSMMUT00000015522      GAATATGCATCCAAAAAAAGGAAAAAGGACCATTTTAGAAATGACACAAATACTCAAAGT
ENSPTRT00000036947      GAATATGCATCGAAAAAAAGGAAAAAGGACCATTTTAGAAATGACACAAATACTCAAAGT
ENSPPYT00000021318      GAATATGCATCCAAAAAAAGGAAAAAGGACCATTTTAGAAATGACACAAATACTCAAAGT

ENSMUST00000043520      TTTTATCGTGAAAAATGGATCTACGTCCATAAGGAAAGCACAAAGGAAAGGCATGGCTAC
ENSCJAT00000023185      TTTTATCGTGAAAAATGGATCTATGTCCATAAAGAAAGCACAAAAGAAAGGCATGGCTAT
ENSGGOT00000014225      TTTTATCGTGAAAAATGGATCTATGTCCATAAAGAAAGCACAAAAGAAAGGCATGGCTAT
ENST00000276326         TTTTATCGTGAAAAATGGATCTATGTCCATAAAGAAAGCACAAAGGAAAGGCATGGCTAT
ENSMMUT00000015522      TTTTATCGTGAAAAATGGATCTATGTCCATAAAGAAAGCACAAAAGAAAGGCATGGCTAT
ENSPTRT00000036947      TTTTATCGTGAAAAATGGATCTATGTCCATAAAGAAAGCACAAAGGAAAGGCATGGCTAT
ENSPPYT00000021318      TTTTATCGTGAAAAATGGATCTATGTCCATAAAGAAAGCACAAAAGAAAGGCATGGCTAT

ENSMUST00000043520      TGCACCCTGGGAGAAGCCTTCAACCGGCTAGACTTCTCAAGTGCCATTCAGGACATCAGG
ENSCJAT00000023185      TGCACCTTGGGAGAAGCTTTTAATCGGTTAGATTTCTCAAGTGCAATTCAAGATATCCGA
ENSGGOT00000014225      TGCACCTTGGGAGAAGCCTTTAATCGGTTAGACTTCTCAAGTGCAATTCAAGATACCCGA
ENST00000276326         TGCACCTTGGGAGAAGCCTTTAATCGGTTAGACTTCTCAAGTGCAATTCAAGATATCCGA
```

```
ENSMMUT00000015522     TGCACCTTGGGAGAGGCCTTTAATCGGTTAGACTTCTCAAGTGCAATTCAAGATATCCGA
ENSPTRT00000036947     TGCACCTTGGGAGAAGCCTTTAATCGGTTAGACTTCTCAAGTGCAATTCAAGATATCCGA
ENSPPYT00000021318     TGCACCTTGGGAGAAGCCTTTAATCGGTTAGACTTCTCAAGTGCAATTCAAGATATCCGA


ENSMUST00000043520     AGGTTCACTTACGTGGTCAAACTACTGCAGCTAATTGCGAAGTCCCAGTTAACCTCACTG
ENSCJAT00000023185     AGGTTCAATTATGTGGTCAAATTATTGCAGCTAATTGCAAAATCCCAGTTAACTTCATTG
ENSGGOT00000014225     AGGTTCAATTATGTGGTCAAACTGTTGCAGCTAATTGCAAAATCCCAGTTAACTTCATTG
ENST00000276326        AGGTTCAATTATGTGGTCAAACTGTTGCAGCTAATTGCAAAATCCCAGTTAACTTCATTG
ENSMMUT00000015522     AGATTTAATTATGTGGTCAAACTATTGCAGCTAATTGCAAAATCCCAGTTAACTTCATTG
ENSPTRT00000036947     AGGTTCAATTATGTGGTCAAACTGTTGCAGCTAATTGCAAAATCCCAGTTAACTTCATTG
ENSPPYT00000021318     AGGTTCAATTATGTGGTCAAACTGTTGCAGCTAATTGCAAAATCCCAGTTAACTTCATTG


ENSMUST00000043520     AGTGGAGTGGCACAGAAGAACTACTTCAACATTTTGGATAAAATCGTCCAAAAGGTTCTT
ENSCJAT00000023185     AGCGGTGTGGCACAAAAGAATTACTTCAACATTTTGGATAAAATTGTTCAAAAGGTTCTT
ENSGGOT00000014225     AGTGGCGTGGCACAGAAGAATTACTTCAACATTTTGGATAAAATCGTTCAAAAGGTTCTT
ENST00000276326        AGTGGCGTGGCACAGAAGAATTACTTCAACATTTTGGATAAAATCGTTCAAAAGGTTCTT
ENSMMUT00000015522     AGTGGTGTGGCACAAAAGAATTACTTCAACATTTTGGATAAAATCGTTCAAAAGGTTCTT
ENSPTRT00000036947     AGTGGCGTGGCACAGAAGAATTACTTCAACATTTTGGATAAAATCGTTCAAAAGGTTCTT
ENSPPYT00000021318     AGTGGTGTGGCACAAAAGAATTACTTCAACATTTTGGATAAAATCGTTCAAAAGGTTCTT


ENSMUST00000043520     GATGACCATCAGAACCCTCGCCTGATCAAAGGCCTCCTCCAGGACCTGAGCTCCACCCTT
ENSCJAT00000023185     GATGATCACCACAATCCTCGCCTAATCAAAGATCTTCTGCAAGACCTAAGCTCCACCCTC
ENSGGOT00000014225     GATGACCACCACAATCCTCGCTTAATCAAAGATCTTCTGCAAGACCTAAGCTCTACCCTC
ENST00000276326        GATGACCACCACAATCCTCGCTTAATCAAAGATCTTCTGCAAGACCTAAGCTCTACCCTC
ENSMMUT00000015522     GATGACCACCACAATCCTCGCTTAATCAAAGATCTTCTGCAAGACCTAAGCTCTACCCTC
ENSPTRT00000036947     GATGACCATCACAATCCTCGCTTAATCAAAGATCTTCTGCAAGACCTAAGCTCTACCCTC
ENSPPYT00000021318     GATGACCACCACAATCCTCGCTTAATCAAAGATCTTCTGCAAGACCTAAGCTCTACCCTC


ENSMUST00000043520     GGCATCCTGGTGAGAGGGGTGGGGAAGTCAGTGCTAGTCGGGAACATCAACATCTGGATC
ENSCJAT00000023185     TGCATTCTTATTAGAGGAGTAGGGAAGTCTGTATTAGTGGGAAACATCAATATTTGGATT
ENSGGOT00000014225     TGCATTCTTATTAGAGGAGTAGGGAAGTCTGTATTAGTGGGAAACATCAATATTTGGATT
ENST00000276326        TGCATTCTTATTAGAGGAGTAGGGAAGTCTGTATTAGTGGGAAACATCAATATTTGGATT
ENSMMUT00000015522     TGCATTCTTATTAGAGGAGTAGGGAAGTCTGTATTAGTGGGAAACATCAATATTTGGATT
ENSPTRT00000036947     TGCATTCTTATTAGAGGAGTAGGGAAGTCTGTATTAGTGGGAAACATCAATATTTGGATT
ENSPPYT00000021318     TGCATTCTTATTAGAGGAGTAGGGAAGTCTGTATTAGTGGGAAACATCAATATTTGGATT


ENSMUST00000043520     TGCCGCCTGGAGACCGTCCTCAGCTGGCAGCAGCAGCTGCAGAACCTCCAGGTGACAAAG
ENSCJAT00000023185     TGCCGATTAGAAACTATTCTCACCTGGCAACAACAGCTACAGGATCTTCAGATGACTAAG
ENSGGOT00000014225     TGCCGATTAGAAACTATTCTCGCCTGGCAACAACAGCTACAGGATCTTCAGATGACTAAG
ENST00000276326        TGCCGATTAGAAACTATTCTCGCCTGGCAACAACAGCTACAGGATCTTCAGATGACTAAG
ENSMMUT00000015522     TGCCGATTAGAAACTATTCTCGCCTGGCAACAACAGCTACAGGATCTTCAGATGACTAAG
ENSPTRT00000036947     TGCCGATTAGAAACTATTCTCGCCTGGCAACAACAGCTACAGGATCTTCAGATGACTAAG
ENSPPYT00000021318     TGCCGATTAGAAACTATTCTCGCCTGGCAGCAACAGCTACAGGATCTTCAGATGACTAAG


ENSMUST00000043520     CAGGTAAACACTGGCCTCACACTCAGTGACCTGCCCCTGCACATGCTGAACAACATCCTG
ENSCJAT00000023185     CAAGTGAACAACGGCCTCACCCTCAGCGACCTTCCTCTGCACATGCTGAACAACATCCTA
ENSGGOT00000014225     CAAGTGAACAATGGCCTCACCCTCAGTGACCTTCCTCTGCACATGCTGAACAACATCCTA
ENST00000276326        CAAGTGAACAATGGCCTCACCCTCAGTGACCTTCCTCTGCACATGCTGAACAACATCCTA
ENSMMUT00000015522     CAAGTGAACAATGGCCTCACCCTCAGTGACCTTCCTCTGCACATGCTGAACAACATCCTG
ENSPTRT00000036947     CAAGTGAACAATGGCCTCACCCTCAGTGACCTTCCTCTGCACATGCTGAACAACATCCTA
ENSPPYT00000021318     CAAGTGAACAATGGCCTCACCCTCAGTGACCTTCCTCTGCACATGCTGAACAACATCCTA


ENSMUST00000043520     TACCGTTTCTCAGATGGCTGGGACATTGTCACCCTGGGCCAGGTAACACCAACCCTGTAC
```

```
ENSCJAT00000023185     TACCGGTTCTCAGATGGATGGGACATCATCACCTTAGGCCAGGTGACCCCCACGTTGTAT
ENSGGOT00000014225     TACCGGTTCTCAGACGGATGGGACATCGTCACCTTAGGCCAGGTGACCCCCACGTTGTAT
ENST00000276326        TACCGGTTCTCAGACGGATGGGACATCATCACCTTAGGCCAGGTGACCCCCACGTTGTAT
ENSMMUT00000015522     TACCGGTTCTCAGACGGATGGGACATCATCACCTTAGGCCAGGTGACCCCCACGCTGTAT
ENSPTRT00000036947     TACCGGTTCTCAGACGGATGGGACATCATCACCTTAGGCCAGGTGACCCCCACGTTGTAT
ENSPPYT00000021318     TACCGGTTCTCAGACGGATGGGACATCATCACCTTAGGCCAGGTGACCCCCACGTTGTAT

ENSMUST00000043520     ATGCTCAGTGAAGACAGGCGGCTGTGGAAGCGACTGTGTCAGTACCACTTTGCTGAGCAG
ENSCJAT00000023185     ATGCTCAGTGAAGACAGACAGCTATGGAAGAAGCTATGTCAGTACCATTTTGCTGAAAAG
ENSGGOT00000014225     ATGCTCAGTGAAGACAGACAGCTGTGGAAGAAGCTATGTCAGTACCATTTTGCTGAAAAG
ENST00000276326        ATGCTTAGTGAAGACAGACAGCTGTGGAAGAAGCTTTGTCAGTACCATTTTGCTGAAAAG
ENSMMUT00000015522     ATGCTCAGTGAAGACAGACAACTGTGGAAGAAGCTGTGTCAGTACCATTTTGCTGAAAAG
ENSPTRT00000036947     ATGCTTAGTGAAGACAGACAGCTGTGGAAGAAGCTTTGTCAGTACCATTTTGCTGAAAAG
ENSPPYT00000021318     ATGCTCAGTGAAGACAGACAGCTGTGGAAGAAGCTATGTCAGTACCATTTTGCTGAAAAG

ENSMUST00000043520     CAGTTTTGTAGACATTTGATCCTTTCAGAAAAAGGTCATATCGAGTGGAAGCTGATGTAT
ENSCJAT00000023185     CAGTTTTGTAGACATTTGATCCTTTCAGAAAAAGGACATATTGAGTGGAAGTTGATGTAC
ENSGGOT00000014225     CAGTTTTGTAGACATTTGATCCTTTCAGAAAAAGGTCATATTGAATGGAAGTTGATGTAC
ENST00000276326        CAGTTTTGTAGACATTTGATCCTTTCAGAAAAAGGTCATATTGAATGGAAGTTGATGTAC
ENSMMUT00000015522     CAGTTTTGTAGACATTTGATCCTTTCAGAAAAAGGTCATATTGAATGGAAGTTGATGTAC
ENSPTRT00000036947     CAGTTTTGTAGACATTTGATCCTTTCAGAAAAAGGTCATATTGAATGGAAGTTGATGTAC
ENSPPYT00000021318     CAGTTTTGTAGACATTTGATCCTTTCA---AAAGGTCATATTGAATGGAAGTTGATGTAC

ENSMUST00000043520     TTCACGCTTCAGAAGTATTACCCGACCAAAGAGCAGTACGGGGACACGCTACACTTCTGC
ENSCJAT00000023185     TTTGCACTTAAGAAACATTACCCAGCAAAGGAGCAGTATGGAGACACGCTGCATTTCTGT
ENSGGOT00000014225     TTTGCACTTCAGAAACATTACCCAGCGAAGGAGCAGTACGGAGACACACTGCATTTCTGT
ENST00000276326        TTTGCACTTCAGAAACATTACCCAGCGAAGGAGCAGTACGGAGACACACTGCATTTCTGT
ENSMMUT00000015522     TTTGCACTTCAGAAACATTACCCAGCGAAGGAGCAGTACGGAGACACGCTGCATTTCTGT
ENSPTRT00000036947     TTTGCACTTCAGAAACATTACCCAGCGAAGGAGCAGTACGGAGACACACTGCATTTCTGT
ENSPPYT00000021318     TTTGCACTTCAGAAACATTACCCAGCGAAGGAGCAGTACGGAGACACGCTGCATTTCTGT

ENSMUST00000043520     CGCCACTGCAGCATCCTCTTCTGG---------------------------AAGGACTCG
ENSCJAT00000023185     CGGCACTGCAGCATTCTCTTTTGGAAGGACTACCATCTTGCTTTACTATTCAAGGACTCA
ENSGGOT00000014225     CGGCACTGCAGCATTCTCTTTTGGAAGGACTACCATCTTGCTTTACTATTCAAGGACTCA
ENST00000276326        CGGCACTGCAGCATTCTCTTTTGGAAGGACTACCATCTTGCTTTACTATTCAAGGACTCA
ENSMMUT00000015522     CGGCACTGCAGCATTCTCTTTTGGAAGGACTACCACCTTGCTTTACTATTCAAGGACTCA
ENSPTRT00000036947     CGGCATTGCAGCATTCTCTTTTGGAAGGACTACCATCTTGCTTTACTATTCAAGGACTCA
ENSPPYT00000021318     CGGCACTGCAGCATTCTCTTTTGG---------------------------AAGGACTCA

ENSMUST00000043520     GGACACCCCTGCACCGCAGCTGACCCCGACAGCTGCTTCACTCCTGTGTCTCCGGAGCAC
ENSCJAT00000023185     GGACACCCCTGCACGGCGGCTGATCCCGACAGCTGCTTCACACCTGTGTCTCCGCAGCAC
ENSGGOT00000014225     GGACACCCCTGCACGGCAGCCGACCCTGACAGCTGCTTCATGCCTGTGTCTCCGCAGCAC
ENST00000276326        GGACACCCCTGCACGGCGGCCGACCCTGACAGCTGCTTCACGCCTGTGTCTCCGCAGCAC
ENSMMUT00000015522     GGACACCCCTGCACGGCGGCTGACCCCGACAGCTGCTTCACGCCTGTGTCTCCGCAGCAC
ENSPTRT00000036947     GGACACCCCTGCACGGCGGCCGACCCTGACAGCTGCTTCACGCCTGTGTCTCCGCAGCAC
ENSPPYT00000021318     GGACACCCCTGCACGGCGGCCGACCCTGACAGCTGCTTCACGCCTGTGTCTCCACAGCAC

ENSMUST00000043520     TTCATTGACCTCTTCAAGTTC
ENSCJAT00000023185     TTCATCGACCTCTTCAAGTTT
ENSGGOT00000014225     TTCATCGACCTCTTCAAGTTT
ENST00000276326        TTCATCGACCTCTTCAAGTTT
ENSMMUT00000015522     TTCATCGACCTCTTCAAGTTT
ENSPTRT00000036947     TTCATCGACCTCTTCAAGTTT
ENSPPYT00000021318     TTCATCGACCTCTTCAAGTTT
```

Multiple sequence alignment of Fbxo27

```
ENSMUST00000108281     ACGCCGGAGCCCGACCCCCAGGAGGTCCTGGACCTGAGCCGTCCCCCGGAGCTGCTCCTG
ENSCJAT00000038548     GCGCCGGAGCCGGAACCCGAAGGGGCACTGGACCTGAGCCAACCCCCGGAGCTGCTTCTG
ENSMMUT00000020405     ------------------------------------------------------------
ENSGGOT00000004855     GCGCCGGAGCCGGAACCCGAAGAGGCGCTGGACCTGAGCCAACCCCCGGAGCTGCTTCTG
ENST00000509137        GCGCCGGAGCCGGAACCCGAAGAGGCGCTGGACCTGAGCCAACCCCCAGAGCTGCTTCTG
ENSPPYT00000011578     GCGCTGGAGCTGGAAGACGAAGAGGCGCTGGACCTGAGCCAATCCCCGGAGTTACTTCTG


ENSMUST00000108281     CTGGTACTGAGCCACGTGCCCCCGCGCACGCTGCTGATGCACTGCCGCCGAGTGTGCCGC
ENSCJAT00000038548     GTGGTGCTGAGTCACGTCCCCCCGCGCACGCTGCTGGGGCGCTGCCGCCAAGTGTGCCGG
ENSMMUT00000020405     ---------------------------------CTCGGGCGCTGCCGCCAAGTGTGCCGG
ENSGGOT00000004855     GTGGTGCTGAGCCACGTCCCCCCGCGCACGCTGCTCGGGCGCTGCCGCCAAGTGTGCCGG
ENST00000509137        GTGGTGCTGAGCCACGTCCCCCCGCGCACGCTGCTCGGGCGCTGCCGCCAAGTGTGCCGG
ENSPPYT00000011578     GTGGTGCTGAGCCACGTCCCCCCGCGCACGCTGCTCGGGCGCTGCCGCCAAGTGTGCCGG


ENSMUST00000108281     GCCTGGCGCGCCCTGGTAGATGGCCAGGCCCTATGGTTGCTGCTGCTGGCCCGTGACCAC
ENSCJAT00000038548     GGTTGGCGCGACCTGGTGGACAGCCAGGCCCTGTGGCTGCTGATCTTGGCCCGGGACCAC
ENSMMUT00000020405     GGCTGGCGAGCCCTGGTGGACGGCCAGGCCCTGTGGCTGCTGATCCTGGCCCGCGATCAC
ENSGGOT00000004855     GGCTGGCGGGCCCTGGTGGACGGCCAGGCCCTGTGGCTGCTGATCCTGGCCCGCGACCAC
ENST00000509137        GGCTGGCGAGCCCTGGTGGACGGCCAGGCCCTGTGGCTGCTGATCCTGGCCCGCGACCAC
ENSPPYT00000011578     GGCTGGCGAGCCCTGGTGGACGGCCAGGCCCTGTGGCTGCTGATC---GCCCGCGACCAC


ENSMUST00000108281     AGCGCAGCGGGCCGCGCCCTGCTGACACTAGCCCGCCGCTGTCTGCCCCCTGCCCACGAA
ENSCJAT00000038548     AGCGCCACCGGCCGCGCGCTACTGCAGCTCGCCCGCAGCTGCCAGTCTGCCTCCCGCAAC
ENSMMUT00000020405     AGCGCCACCGGCCGCGCGCTACTGCACCTCGCCCGCAGCTGCCAGTCTCCCGCCCGTAAC
ENSGGOT00000004855     GGCGCCACCGGCCGCGCGCTGCTGCACCTCGCCCGCAGCTGCCAGTCTCCCGCCCGTAAC
ENST00000509137        GGCGCCACCGGCCGCGCGCTGCTGCACCTCGCCCGCAGCTGCCAGTCTCCCGCCCGTAAC
ENSPPYT00000011578     GGCGCCACCGGCCGCGCGCTGCTGCACCTCGCCCGCAGCTGCCAGTCTCCCGCCCGTAAC


ENSMUST00000108281     GACACGCCCTGCCCGCTGGGCCAATTCTGCGCACTGAGACCGCTAGGACGCAACCTCATC
ENSCJAT00000038548     GCCAGGCCCTGCCCGCTGGGCCGCTTCTGCGCGCGCAGACCCATTGGACGCAACCTTATT
ENSMMUT00000020405     GCCAGGCCTTGCCCCCTTGGCCGCTTCTGCGCGCGCAGACCCATCGGACGCAACCTTATT
ENSGGOT00000004855     GCCAGGCCTTGCCCCCTGGGCCGCTTCTGCGCGCGCAGACCCATCGGACGCAACCTTATT
ENST00000509137        GCCAGGCCTTGCCCCCTGGGCCGCTTCTGCGCGCGCAGACCCATCGGACGCAACCTTATT
ENSPPYT00000011578     GCCAGGCCTTGCCCCCTGGGCCGCTTCTGCGCGCGCAGACCCATCGGACGCAACCTTATT


ENSMUST00000108281     AGCAACCCCTGTGGCCAAGAAGGCCTGCGCAAATGGATGGTGCGGCACGGTGGGGATGGA
ENSCJAT00000038548     CGCAACCCCTGCGGCCAAGAAGGCCTCCGAAAGTGGATGGTGCAGCATGGTGGGGACGGC
ENSMMUT00000020405     CGCAACCCCTGCGGCCAAGAAGGCCTCCGAAAGTGGATGGTGCAGCACGGTGGGGACGGC
ENSGGOT00000004855     CGCAACCCCTGCGGCCAAGAAGGCCTCCGAAAGTGGATGGTGCAGCACGGTGGGGACGGC
ENST00000509137        CGCAACCCCTGCGGCCAAGAAGGCCTCCGAAAGTGGATGGTGCAACACGGTGGGGACGGC
ENSPPYT00000011578     CGCAACCCCTGTGGCCAAGAAGGCCTCCGAAAATGGATGGTGCAGCACGGTGGGGACGGC


ENSMUST00000108281     TGGGTGGTGGAGAAGAACAGGAAACCTGTACCTGGGGCCCCTTCACAGACCTGCTTCGTG
ENSCJAT00000038548     TGGGTGGTAGAGGAAAACAGGACAACTGTGCGTGGGGCCCCTTCTCAGACATGCTTCGTG
ENSMMUT00000020405     TGGGTGGTGGAGGAAAACAGGACAACCGTGCCTGGGGCCCCTTCTCAGACGTGCTTCGTG
ENSGGOT00000004855     TGGGTGGTGGAGGAAAACAGGACAACCGTGCCTGGGGCCCCTTCTCAGACGTGCTTCGTG
ENST00000509137        TGGGTGGTGGAGGAAAACAGGACAACCGTGCCTGGGGCCCCTTCTCAGACGTGCTTCGTG
ENSPPYT00000011578     TGGGTGGTGGAGGAAAACAGGACAACCGTGCCTGGGGCCCCTTCTCAGACGTGCTTCGTG
```

```
ENSMUST00000108281     ACTTCCTTCAGCTGGTGTCGCAAGAAGCAGGTCGTGGACCTGGTGGAGAAGGGTCTGTGG
ENSCJAT00000038548     ACTTCATTCAGCTGGTGTCGCAAGAAGCAGGTCTTGGACCTAGAGGAGGAGGGTCTGTGG
ENSMMUT00000020405     ACCTCATTCAGCTGGTGTCGCAAGAAGCAGGTCTTGGACCTAGAGGAGGAGGGTCTGTGG
ENSGGOT00000004855     ACTTCATTCAGCTGGTGTTGCAAGAAGCAGGTCTTGGACCTAGAGGAGGAGGGTCTGTGG
ENST00000509137        ACTTCATTCAGCTGGTGTTGCAAGAAGCAGGTCTTGGACCTAGAGGAGGAGGGTCTGTGG
ENSPPYT00000011578     ACTTCATTCAGCTGGTGTTGCAAGAAGCAGGTCTTGGACCTAGAGGAGGAGGGTCTGTGG

ENSMUST00000108281     CCAGAGCTGCTGGACAGTGGTGGTGTGGAGATTGCTGTCTCTGACTGGTGGGGAGCTCGA
ENSCJAT00000038548     CCAGAACTGCTGGATAGTGGCAGGATTGAGATTTGTGTCTCTGACTGGTGGGGAGCCCGA
ENSMMUT00000020405     CCAGAACTGCTGGATAGTGGCAGGATTGAGATTTGTGTCTCTGACTGGTGGGGAGCCCGA
ENSGGOT00000004855     CCAGAACTGCTGGATAGTGGCAGGATTGAGATTTGTGTCTCTGACTGGTGGGGAGCCCGA
ENST00000509137        CCAGAACTGCTGGATAGTGGCAGGATTGAGATTTGTGTCTCTGACTGGTGGGGAGCCCGA
ENSPPYT00000011578     CCTGAACTGCTGGATAGTGGCAGGATTGAGATTTGTGTCTCTGACTGGTGGGGAGCCCGA

ENSMUST00000108281     CATGACAGTGGCTGTAAGTACCGTCTCTTTGTCACGCTTCTTGATGCCCACCAGAACGTC
ENSCJAT00000038548     CACGACAGTGGCTGTAGGTATCGACTCCTTGTCCAACTTCTAGATGCCAACCAGACTGTT
ENSMMUT00000020405     CACGACAGTGGCTGTATGTATAGACTACTTGTCCAACTTCTAGACGCCAACCAGACTGTT
ENSGGOT00000004855     CACGACAGCGGCTGTATGTACAGACTCCTCGTCCAACTTCTAGACGCCAACCAGACTGTT
ENST00000509137        CACGACAGCGGCTGTATGTACAGACTCCTCGTCCAACTTCTAGACGCCAACCAGACTGTT
ENSPPYT00000011578     CACGACAGCGGCTGTATGTACAGACTCCTTGTCCAACTTCTAGACGCCAACCAGACTGTT

ENSMUST00000108281     ATAGATAAGTTCTCCGCTGTGCCAGATCCCATTGAACAGTGGAACAATGATATCTACCTG
ENSCJAT00000038548     CTAGATAAATTCTCTGCTGTGCCTGATCCCATCCCCCAGTGGAACAACAATGTCTGCCTT
ENSMMUT00000020405     CTAGATAAATTCTCTGCTGTGCCTGATCCCATCCCACAGTGGAACAACAATGCCTGCCTT
ENSGGOT00000004855     CTGGATAAATTCTCTGCTGTGCCTGATCCCATCCCGCAGTGGAACAACAATGCCTGCCTT
ENST00000509137        CTAGATAAATTCTCTGCTGTGCCTGATCCCATCCCGCAGTGGAACAACAATGCCTGCCTT
ENSPPYT00000011578     CTAGATAAATTCTCTGCTGTGCCTGATCCCATCCCGCAGTGGAACAACAATGCCTGCCTT

ENSMUST00000108281     CAGGTCACCCACGTGTTCTCCGGCATCAGGAGGGGCATACGTTTTGTCTCTTTTGAACAC
ENSCJAT00000038548     CACGTCACTCACGTGTTCTCCAACATCAAGATGGGTATCCGCTTTGTGTCTTTTGAACAC
ENSMMUT00000020405     CACGTCACTCACGTGTTCTCCAACATCAAGATGGGCGTCCGCTTTGTGTCTTTCGAACAC
ENSGGOT00000004855     CACGTCACCCACGTGTTCTCCAACATCAAGATGGGCGTCCGCTTTGTGTCTTTCGAACAC
ENST00000509137        CACGTCACCCACGTGTTCTCCAACATCAAGATGGGCGTCCGCTTTGTGTCTTTCGAACAC
ENSPPYT00000011578     CACGTCACCCACGTGTTCTCCAACATCAAGATGGGTGTCCGCTTTGTGTCTTTCGAACAC

ENSMUST00000108281     TGGGGCCAGGACACACAGTTCTGGGCTGGCCACTATGGGGCCAGAGTGACGAACTCCAGT
ENSCJAT00000038548     TGGGGCCAGGACACACAGTTCTGGGCCGGCCACTATGGAGCCCGTCTGACCAACTCCAGT
ENSMMUT00000020405     TGGGGCCAGGACACACAGTTCTGGGCTGGCCACTATGGAGCCCGTGTGACCAACTCCAGT
ENSGGOT00000004855     CGGGGCCAGGACACACAGTTCTGGGCTGGCCACTATGGAGCCCGTGTGACCAACTCCAGT
ENST00000509137        CGGGGCCAGGACACACAGTTCTGGGCTGGCCACTATGGAGCCCGTGTGACCAACTCCAGT
ENSPPYT00000011578     CGGGGCCAGGACACACAGTTCTGGGCTGGCCACTATGGAGCCCGTGTGACCAACTCCAGT

ENSMUST00000108281     GTGATCATACGAGTCTGTCAGTCC
ENSCJAT00000038548     GTGATGGTGCAAGTTCGCCCACCC
ENSMMUT00000020405     GTGATCGTGCGAGTCCATCTGTCC
ENSGGOT00000004855     GTGATCGTGCGAGTCCGTCTGTCC
ENST00000509137        GTGATCGTGCGAGTCCGTCTGTCC
ENSPPYT00000011578     GTGATCGTGCGAGTCCGTCTGTCC
```

Multiple sequence alignment of Fbxo28

```
ENSGGOT00000034250     ------------------------------------------------------------
ENSMUST00000051431     ATGGCGGCGGCGTCTGAGGAGCGGATGGCTGAGGAAGGAGGCGGCGGCCACGGCGACGGC
```

```
ENSRNOT00000000074     ATGGCGGCGGCGTCTGAGGAGCGGATGGCTGAGGAAGGAGGCGGCGGCCACGGCGACGGC
ENSCJAT00000015845     GCGGCGGCGGCGGCGGAGGAGCGGATGGCAGAGGAAGGAGGCGGTGGCCACGGCGACGGC
ENSMMUT00000000972     GCGGCGGCGGCGGCGGAGGACCGGATGGCAGAGGAAGGAGGCGGCAGCCACGGCGACGGC
ENST0000366862         ATGGCGGCAGCGGCGGAGGAGCGGATGGCAGAGGAAGGAGGCGGCGGCCAAGGCGACGGC
ENSPTRT00000003674     ATGGCGGCAGCGGCGGAGGAGCGGATGGCAGAGGAAGGAGGCGGCGGCCAAGGCGACGGC
ENSPPYT00000000200     ATGGCGGCGGCGGCGGAGGAGCGGATGGCAGAGGAAGGAGGCGGCGGCCAAGGCGACGGC

ENSGGOT00000034250     ---TCCTCTTTGGCCTCCGGCTCTACCCAGCGACAGCCCCCACCGCCCGCGCCACAGCAC
ENSMUST00000051431     GGCTCCTGTTCGGCTGCCGGCTCCGCTCAGCGACAGCCCCCGGCGCCCCCGTCGCAGGCC
ENSRNOT00000000074     GGCTCCTGTTCGGCCGCCAGCTCCGCTCAGCGACAACCCCCGACACCCCCGTCGCAGGCC
ENSCJAT00000015845     AGCTCCTCTTTGGCCGCCGGCTCTACCCAGCGGCAGCCCCCACCGCCTCCGCCCCAGCAC
ENSMMUT00000000972     GGCTCCTCTTTGGCCTCCGGCTCTACCCAGCGACAGCCCCCACCGCCCCCGCCACAACAC
ENST0000366862         GGTTCCTCTTTGGCCTCCGGCTCTACCCAGCGACAGCCTCCACCGCCCGCGCCACAGCAC
ENSPTRT00000003674     GGCTCCTCTTTGGCCTCCGGCTCTACCCAGCGACAGCCCCCACCGCCCGCGCCACAGCAC
ENSPPYT00000000200     GGCTCCTCTTTGGCCTCCGGCTCTACCCAGCGACAGCCCCCACCGCCCCCGCCACAGCAT

ENSGGOT00000034250     CCGCAGCCGGGGTCCCAGGCGCTCCCAGCCCCCGCACTGGCTCCGGACCAGCTGCCTCAA
ENSMUST00000051431     CCGCCGCCCGGGTCCCAGGCACCCGCCGCGCCCGCGCTGGCACCGGACCACCTGCCCCAA
ENSRNOT00000000074     CTGCAGCCGGGGTCTCAGGCACCCGCCGCGCCCGCGCTGGCACCGGACCACCTGCCTCAA
ENSCJAT00000015845     CCGCAGCCGGGGTCCCAGGCGCTCCCAGCGCCGGCGCTGGCTCCAGACCAGCTGCCTCAA
ENSMMUT00000000972     CCACAACCGGGGTCCCAGGCGCTGCCAGCCCCGGCGCTGGCTCCGGACCAGCTGCCTCAA
ENST0000366862         CCGCAGCCGGGGTCCCAGGCGCTCCCAGCCCCCGCGCTGGCTCCGGACCAGCTGCCTCAA
ENSPTRT00000003674     CCGCAGCCGGGGTCCCAGGCGCTCCCAGCCCCCGCGCTGGCTCCGGACCAGCTGCCTCAA
ENSPPYT00000000200     CCGCAGCCGGGGTCCCAGGCGCTCCCAGCCCCGGCGCTGGCTCCGGACCAGCTGCCTCAA

ENSGGOT00000034250     AACAACACGCTTGTGGCGCTGCCCATCGTAGCCATCGAGAACATCCTCAGCTTTATGTCC
ENSMUST00000051431     AACAACACACTGGTGGCGCTGCCCATCGTAGCCATCGAGAACATCCTCAGCTTTATGTCC
ENSRNOT00000000074     AACAACACACTGGTGGCGCTGCCCATCGTAGCCATCGAGAACATCCTCAGCTTTATGTCC
ENSCJAT00000015845     AACAACACGCTTGTGGCGCTGCCCATCGTAGCCATCGAGAACATCCTCAGCTTTATGTCC
ENSMMUT00000000972     AACAACACGCTTGTGGCGCTGCCCATCGTAGCCATCGAGAACATCCTCAGCTTTATGTCC
ENST0000366862         AACAACACGCTTGTGGCGCTGCCCATCGTAGCCATCGAGAACATCCTCAGCTTTATGTCC
ENSPTRT00000003674     AACAACACGCTTGTGGCGCTGCCCATCGTAGCCATCGAGAACATCCTCAGCTTTATGTCC
ENSPPYT00000000200     AACAACACGCTTGTGGCGCTGCCCATCGTAGCCATCGAGAACATCCTCAGCTTTATGTCC

ENSGGOT00000034250     TACGACGAAATTAGCCAGCTCCGCCTGGTCTGTAGAAGAAAGTCCAACTTCTGCAGAAGG
ENSMUST00000051431     TACGACGAAATTAGCCAGCTCCGCCTGGTTTGTAAGAGAATGGACCTGGTCTGTCAGAGA
ENSRNOT00000000074     TACGACGAAATTAGCCAGCTCCGCCTGGTTTGTAAAAGAATGGACTTGGTCTGTCAGAGA
ENSCJAT00000015845     TACGACGAAATCAGCCAGCTCCGTCTGGTTTGTAAAAGAATGGACTTGGTTTGCCAGAGA
ENSMMUT00000000972     TACGACGAAATTAGCCAGCTCCGCCTGGTTTGTAAAAGAATGGACTTGGTCTGCCAGAGA
ENST0000366862         TACGACGAAATTAGCCAGCTCCGCCTGGTTTGTAAAAGAATGGACTTGGTCTGCCAGAGA
ENSPTRT00000003674     TACGACGAAATTAGCCAGCTCCGCCTGGTTTGTAAAAGAATGGACTTGGTCTGCCAGAGA
ENSPPYT00000000200     TACGACGAAATTAGCCAGCTCCGCCTGGTTTGTAAAAGAATGGACTTGGTCTGCCAGAGA

ENSGGOT00000034250     ATACTGAATCAGAGTTACATACAGCGTTATAGATCATATAAACTTTGTAAGAGATTGTTA
ENSMUST00000051431     ATGTTGAATCAGGGGTTTCTGAAAGTGGAGAGGTTCCATAACCTGTGTCAGAAGCAAGTT
ENSRNOT00000000074     ATGCTGAATCAGGGCTTTCTGAAAGTGGAGAGGTACCATAACCTGTGTCAGAAACAAGTT
ENSCJAT00000015845     ATGTTGAATCAGGGATTTCTGAAAGTGGAGAGGTACCATAATCTATGTCAGAAACAAGTG
ENSMMUT00000000972     ATGTTGAATCAGGGATTTCTTAAAGTGGAGAGGTACCATAATCTATGTCAGAAACAAGTT
ENST0000366862         ATGTTGAATCAGGGATTTCTGAAAGTGGAGAGGTACCATAATCTATGTCAGAAACAAGTT
ENSPTRT00000003674     ATGTTGAATCAGGGATTTCTGAAAGTGGAGAGGTACCATAATCTATGTCAGAAACAAGTT
ENSPPYT00000000200     ATGTTGAATCAGGGATTTCTGAAAGTGGAGAGGTACCATAATCTATGTCAGAAACAAGTT

ENSGGOT00000034250     AAAAGTTTAAAGGAGAGGAGAGAGTCAGAAAGGAGAAACCATTCATTAGCTCGTCATGCA
ENSMUST00000051431     AAAGCACAGCTACCGAGGAGAGAGTCAGAAAGAAGAAACCATTCTTTAGCTCGTCATGCA
```

```
ENSRNOT00000000074     AAAGCACAACTACCAAGGAGGGAGTCAGAAAGGAGGAACCACTCTTTAGCTCGCCATGCA
ENSCJAT00000015845     AAAGCACAGCTCCCAAGGAGAGAGTCAGAAAGGAGAAACCATTCATTAGCTCGTCATGCA
ENSMMUT00000000972     AAAGCACAACTCCCAAGGAGAGAGTCAGAAAGGAGAAACCATTCATTAGCTCGTCATGCA
ENST00000366862        AAAGCACAACTCCCAAGGAGAGAGTCAGAAAGGAGAAACCATTCATTAGCTCGTCATGCA
ENSPTRT00000003674     AAAGCACAACTCCCAAGGAGAGAGTCAGAAAGGAGAAACCATTCATTAGCTCGTCATGCA
ENSPPYT00000000200     AAAGCACAACTCCCAAGGAGAGAGTCAGAAAGGAGAAACCATTCATTAGCTCGTCATGCA

ENSGGOT00000034250     GACATTCTTGCTGCTGTTGAAACAAGGCTGTCACTATTAAATATGACTTTCATGAAATAC
ENSMUST00000051431     GACATCCTTGCCGCTGTGGAAACGAGGTTGTCACTGTTGAATATGACCTTCATGAAGTAT
ENSRNOT00000000074     GACATCCTTGCTGCTGTGGAAACGCGGCTGTCACTGTTAAACATGACCTTCATGAAGTAT
ENSCJAT00000015845     GACATTCTTGCTGCTGTTGAAACAAGGCTGTCACTATTAAATATGACTTTCATGAAATAT
ENSMMUT00000000972     GACATTCTTGCTGCTGTTGAAACAAGGCTGTCACTATTAAATATGACTTTCATGAAATAT
ENST00000366862        GACATTCTTGCTGCTGTTGAAACAAGGCTGTCACTATTAAATATGACTTTCATGAAATAT
ENSPTRT00000003674     GACATTCTTGCTGCTGTTGAAACAAGGCTGTCACTATTAAATATGACTTTCATGAAATAT
ENSPPYT00000000200     GACATTCTTGCTGCTGTTGAAACAAGGCTGTCACTATTAAATATGACTTTCATGAAATAT

ENSGGOT00000034250     GTGGATTCCAATCTCTGTTGCTTCATCCCAGGAAAGCTTCTCTCAGAACTATATAGAATG
ENSMUST00000051431     GTGGATTCCAACCTCTGTTGCTTCATCCCAGGAAAGGTGATTGATGAGATTTACCGTGTA
ENSRNOT00000000074     GTGGACTCCAACCTCTGTTGCTTCATCCCAGGAAAGGTGATTGATGAGATTTACCGTGTA
ENSCJAT00000015845     GTGGATTCCAATCTCTGTTGCTTCATCCCAGGAAAGGTGATTGATGAGATTTATCGAGTG
ENSMMUT00000000972     GTGGATTCCAATCTCTGTTGCTTCATCCCAGGAAAGGTGATTGATGAGATTTATCGCGTG
ENST00000366862        GTGGATTCCAATCTCTGTTGCTTCATCCCAGGAAAGGTGATTGATGAGATTTATCGTGTG
ENSPTRT00000003674     GTGGATTCCAATCTCTGTTGCTTCATCCCAGGAAAGGTGATTGATGAGATTTATCGTGTG
ENSPPYT00000000200     GTGGATTCCAATCTCTGTTGCTTCATCCCAGGAAAGGTGATTGATGAGATTTATCGTGTG

ENSGGOT00000034250     TTGGCTTACTCCTTTCAAACATACATGCCATATAAAGAATATAAAATTTTAGATGCACTT
ENSMUST00000051431     CTGAGATATGTCAATTCTACCAGAGCCCCTCAGCGAGCTCACGAGGTGCTGCAGGAGTTA
ENSRNOT00000000074     CTGAGATATGTCAATTCTACCAGAGCCCCTCAGCGAGCTCATGAAGTCCTGCAGGAGTTG
ENSCJAT00000015845     TTGAGATATGTCAATTCTACCAGAGCCCCACAACGAGCTCATGAAGTACTTCAAGAATTA
ENSMMUT00000000972     TTGAGATATGTCAATTCTACCAGAGCCCCTCAGCGAGCTCATGAAGTACTTCAAGAATTA
ENST00000366862        TTGAGATATGTCAATTCTACCAGAGCCCCTCAACGAGCTCATGAAGTACTTCAAGAATTA
ENSPTRT00000003674     TTGAGATATGTCAATTCTACCAGAGCCCCTCAACGAGCTCATGAAGTACTTCAAGAATTA
ENSPPYT00000000200     TTGAGATATGTCAATTCTACCAGAGCCCCTCAACGAGCTCATGAAGTACTTCAAGAATTA

ENSGGOT00000034250     AAGTTTTTAAATTCTTACAGCCTGGGATATCTTGATATGAAAGTT------ATTCTGAAG
ENSMUST00000051431     CGGGATATCTCCTCCATGGCGATGGAGTACTTTGATGAGAAGATTGTTCCAATTTTGAAG
ENSRNOT00000000074     CGGGACATCTCCTCCATGGCAATGGAGTACTTTGATGAGAAGATTGTTCCGATCTTGAAG
ENSCJAT00000015845     AGGGATATATCCTCCATGGCAATGGAGTACTTTGATGAAAAGATTGTTCCAGTTTTAAAG
ENSMMUT00000000972     AGGGATATATCCTCTATGGCAATGGAGTACTTTGATGAAAAGATTGTTCCAATTTTAAAG
ENST00000366862        AGGGATATATCCTCTATGGCAATGGAGTACTTTGATGAAAAGATTGTTCCAATTTTAAAG
ENSPTRT00000003674     AGGGATATATCCTCTATGGCAATGGAGTACTTTGATGAAAAGATTGTTCCAATTTTAAAG
ENSPPYT00000000200     AGGGATATATCCTCCATGGCAATGGAGTACTTTGATGAAAAGATTGTTCCAATTTTAAAG

ENSGGOT00000034250     AATAAGTATCATGGAAAAAGAGTAAGTGGCACTCCTTTAAATTATTTTCCTGTTCCAGGA
ENSMUST00000051431     AGGAAATTGCCAGGATCAGATGTGTCAGGAAGACTCATGGGCTCTCCTCCAGTTCCCGGG
ENSRNOT00000000074     AGGAAATTGCCAGGATCCGATGTGTCTGGAAGACTCATGGGCTCTCCTCCAGTTCCCGGA
ENSCJAT00000015845     AGGAAATTACCAGGATCAGATGTGTCTGGAAGACTCATGGGCTCTCCTCCAGTTCCAGGA
ENSMMUT00000000972     AGGAAATTACCAGGATCAGATGTTTCTGGAAGACTCATGGGCTCTCCTCCAGTTCCAGGA
ENST00000366862        AGGAAATTACCAGGATCAGATGTTTCTGGAAGACTCATGGGCTCTCCTCCAGTTCCAGGA
ENSPTRT00000003674     AGGAAATTACCAGGATCAGATGTTTCTGGAAGACTCATGGGCTCTCCTCCAGTTCCAGGA
ENSPPYT00000000200     AGGAAATTACCAGGATCAGATGTTTCTGGAAGACTCATGGGCTCTCCTCCAGTTCCAGGA

ENSGGOT00000034250     CCGTCTGCAGCCCTAACAACAATGCAGCTCTTCTCCAAGCAAAATCCTTCAAGACAAGAG
ENSMUST00000051431     CCATCCGCAGCCCTGACAACAATGCAGCTCTTCTCCAAGCAAAACCCGTCCAGACAAGAG
```

ENSRNOT00000000074 CCATCCGCAGCCCTGACAACCATGCAGCTCTTCTCCAAGCAAAACCCGTCCAGACAAGAG
ENSCJAT00000015845 CCGTCTGCAGCCCTAACGACAATGCAGCTCTTCTCCAAGCAAAACCCTTCACGACAAGAA
ENSMMUT00000000972 CCATCTGCAGCCCTAACAACAATGCAGCTCTTCTCCAAGCAAAACCCTTCAAGACAAGAG
ENST00000366862 CCGTCTGCAGCCCTAACAACAATGCAGCTCTTCTCCAAGCAAAATCCTTCAAGACAAGAG
ENSPTRT00000003674 CCGTCTGCAGCCCTAACAACAATGCAGCTCTTCTCCAAGCAAAATCCTTCAAGACAAGAG
ENSPPYT00000000200 CCATCTGCAGCCCTAACAACAATGCAGCTCTTCTCCAAGCAAAATCCTTCAAGACAAGAG

ENSGGOT00000034250 GTTACCAAACTCCAGCAGCAGGTTAAAACAAATGGTGCTGGCGTGACTGTTCTCAGGCGT
ENSMUST00000051431 GTTACCAAACTGCAGCAGCAGGTCAGAACCAACGGCGCTGGCGTGACTGTCCTCAGGCGT
ENSRNOT00000000074 GTTACCAAACTGCAGCAGCAGGTCAAAACCAATGGTGCTGGCGTGACTGTCCTCAGGCGT
ENSCJAT00000015845 GTTACCAAACTCCAGCAGCAGGTTAAAACAAATGGTGCTGGCGTGACTGTTCTCAGGCGT
ENSMMUT00000000972 GTTACCAAACTCCAGCAGCAGGTTAAAACAAATGGTGCTGGCGTGACTGTTCTCAGGCGT
ENST00000366862 GTTACCAAACTCCAGCAGCAGGTTAAAACAAATGGTGCTGGCGTGACTGTTCTCAGGCGT
ENSPTRT00000003674 GTTACCAAACTCCAGCAGCAGGTTAAAACAAATGGTGCTGGCGTGACTGTTCTCAGGCGT
ENSPPYT00000000200 GTTACCAAACTCCAGCAGCAGGTTAAAACAAATGGTGCTGGCGTGACTGTTCTCAGGCGT

ENSGGOT00000034250 GAAATTTCTGAGCTTCGCACCAAAGTGCAAGAACAGCAAAAACAGCTTCAAGACCAGGAC
ENSMUST00000051431 GAGATTTCTGAGCTTCGCACCAAAGTGCAAGAGCAGCAGAAACAGCTTCAAGACCAAGAC
ENSRNOT00000000074 GAGATTTCTGAGCTCCGCACCAAAGTGCAAGAACAGCAGAAACAGCTGCAAGACCAAGAC
ENSCJAT00000015845 GAAATTTCTGAGCTTCGCACCAAAGTGCAAGAACAGCAAAAACAGCTTCAAGACCAGGAC
ENSMMUT00000000972 GAAATTTCTGAGCTTCGCACCAAAGTGCAAGAACAGCAAAAACAGCTTCAAGACCAGGAC
ENST00000366862 GAAATTTCTGAGCTTCGCACCAAAGTGCAAGAACAGCAAAAACAGCTTCAAGACCAGGAC
ENSPTRT00000003674 GAAATTTCTGAGCTTCGCACCAAAGTGCAAGAACAGCAAAAACAGCTTCAAGACCAGGAC
ENSPPYT00000000200 GAAATTTCTGAGCTTCGCACCAAAGTGCAAGAACAGCAAAAACAGCTTCAAGACCAGGAC

ENSGGOT00000034250 CAGAAACTGCTAGAGCAGACCCAGATCATAGGTGAACAAAATGCACGGTTGGCAGAGCTA
ENSMUST00000051431 CAGAAACTGCTCGAGCAGACCCAGATCATAGGCGAACAGAACGCACGGCTGGCAGAGCTG
ENSRNOT00000000074 CAGAAACTGCTAGAGCAGACCCAGATCATAGGCGAGCAAAACGCTCGGCTGGCGGAGCTG
ENSCJAT00000015845 CAGAAACTGCTAGAGCAGACCCAGATCATAGGTGAACAGAATGCACGGTTGGCAGAGCTA
ENSMMUT00000000972 CAGAAACTGCTAGAGCAGACCCAGATCATAGGTGAACAAAATGCACGGTTGGCAGAGCTA
ENST00000366862 CAGAAACTGCTAGAGCAGACCCAGATCATAGGTGAACAAAATGCACGGTTGGCAGAGCTA
ENSPTRT00000003674 CAGAAACTGCTAGAGCAGACCCAGATCATAGGTGAACAAAATGCACGGTTGGCAGAGCTA
ENSPPYT00000000200 CAGAAACTGCTAGAGCAGACCCAGATCATAGGTGAACAAAATGCACGGTTGGCAGAGCTA

ENSGGOT00000034250 GAACGCAAACTACGAGAAGTAATGGAAAGTGCTGTAGGAAATTCCTCAGGGTCCGGGCAG
ENSMUST00000051431 GAACGCAAGCTCCGGGAAGTCATGGAAAGTGCAGTAGGGACCTCCTCAGGGTCTGGGCAG
ENSRNOT00000000074 GAGCGGAAGCTCCGAGAAGTCATGGAAAGTGCTGTAGGAACATCCTCAGGGTCCGGGCAG
ENSCJAT00000015845 GAACGCAAATTACGAGAAGTAATGGAAAGTGCTGTAGGAAATTCCTCAGGGTCTGGGCAG
ENSMMUT00000000972 GAACGCAAACTACGAGAAGTAATGGAAAGTGCTGTAGGAAATTCCTCAGGGTCTGGGCAG
ENST00000366862 GAACGCAAACTACGAGAAGTAATGGAAAGTGCTGTAGGAAATTCCTCAGGGTCCGGGCAG
ENSPTRT00000003674 GAACGCAAACTCCGAGAAGTAATGGAAAGTGCTGTAGGAAATTCCTCAGGGTCCGGGCAG
ENSPPYT00000000200 GAACGCAAACTACGAGAAGTAATGGAAAGTGCTGTAGGAAATTCCTCAGGGTCTGGGCAG

ENSGGOT00000034250 AATGAGGAGTCTCCTCGGAAACGAAAAAAGGCCACGGAAGCCATAGACTCTCTTAGGAAA
ENSMUST00000051431 AGTGAGGAGTCCCCTCGGAAACGACGGAAGGCAACGGAAGCCATAGACTCTCTTAGGAAA
ENSRNOT00000000074 AGCGAGGAGTCTCCTCGGAAACGAAGGAAGGCAACAGAAGCCATAGACTCACTTAGGAAA
ENSCJAT00000015845 AATGAGGAGTCCCCTCGGAAACGAAAAAAGGCTGGGGAAGCCATAGACTGTCTTAGGAAA
ENSMMUT00000000972 AATGAGGAGTCTCCTCGGAAACGAAAAAAGGCCACAGAAGCCATAGACTCTCTTAGGAAA
ENST00000366862 AATGAGGAGTCTCCTCGGAAACGAAAAAAGGCCACGGAAGCCATAGACTCTCTTAGGAAA
ENSPTRT00000003674 AATGAGGAGTCTCCTCGGAAACGAAAAAAGGCCACGGAAGCCATAGACTCTCTTAGGAAA
ENSPPYT00000000200 AATGAGGAGTCTCCTCGGAAACGAAAAAAGGCCACGGAAGCCATAGACTCTCTTAGGAAA

ENSGGOT00000034250 TCTAAACGTCTTCGGAATAGAAAG
ENSMUST00000051431 TCCAAACGACTTCGGAATAGGAAG

```
ENSRNOT00000000074     TCCAAACGACTGCGGAATAGGAAG
ENSCJAT00000015845     TCTAAACGTCTTCGGAATAGAAAG
ENSMMUT00000000972     TCTAAACGTCTTCGGAATAGAAAG
ENST00000366862        TCTAAACGTCTTCGGAATAGAAAG
ENSPTRT00000003674     TCTAAACGTCTTCGGAATAGAAAG
ENSPPYT00000000200     TCTAAACGTCTTCGGAATAGAAAG
```

Multiple sequence alignment of Fbxo3

```
ENSCJAT00000020487     ATGGCGGCCATGGAGACCGAGACGGCGCCGCTGACCCTAGAGTCGCTGCCCACCGATCCC
ENSMMUT00000008476     ATGGCGGCCATGGAGACGGACACGGCGCAGCTGACCCTAGAGTCGCTGCCCACCGATCCC
ENSGGOT00000023868     ATGGCGGCCATGGAGACCGAGACGGCGCCGCTGACCCTAGAGTCGCTGCCCACCGATCCC
ENST00000265651        ATGGCGGCCATGGAGACCGAGACGGCGCCGCTGACCCTAGAGTCGCTGCCCACCGATCCC
ENSPTRT00000006572     ATGGCGGCCATGGAGACCGAGACGGCGCCGCTGACCCTAGAGTCGCTGCCCACCGATCCC
ENSPPYT00000004018     ATGGCGGCCATGGAGACCGAGACGGCGCCGCTGACCCTGGAGTCGCTGCCCACCGATCCC
ENSMUST00000028603     ATGGCTGCCGTAGAGGCCGAGACGGGGCTGCTGACCCTGGAGTCGCTGCCCACCGACCCT
ENSRNOT00000014478     ATGGCTGCCGTAGAGGCCGAGACAGGGTTGCTGACCCTGGAGTCGCTACCCACCGACCCT


ENSCJAT00000020487     CTGCTCCTCATCTTATCCTTTTTGGACTATCGGGACCTAATCAACTGTTGCTATGTCAGT
ENSMMUT00000008476     CTGCTCCTCATCTTATCCTTTTTGGACTATCGGGACCTAATCAACTGTTGCTATGTCAGT
ENSGGOT00000023868     CTGCTCCTCATCTTATCCTTTTTGGACTATCGGGACCTAATCAACTGTTGTTATGTCAGT
ENST00000265651        CTGCTCCTCATCTTATCCTTTTTGGACTATCGGGATCTAATCAACTGTTGTTATGTCAGT
ENSPTRT00000006572     CTGCTCCTCATCTTATCCTTTTTGGACTATCGGGACCTAATCAACTGTTGTTATGTCAGT
ENSPPYT00000004018     CTGCTCCTCATCTTATCCTTTTTGGACTATCGGGACCTAATCAACTGTTGTTATGTCAGT
ENSMUST00000028603     CTGCTGCTCATCTTATCCTTCGTGGACTACAGGGACCTAATCAATTGTTGCTATGTTAGT
ENSRNOT00000014478     CTGCTCCTCATCTTATCCTTCGTGGACTACAGGGACCTAATCAATTGTTGCTATGTTAGT


ENSCJAT00000020487     CGAAGACTTAGCCAGCTATCAAGTCATGATCCGCTGTGGAGAAGACATTGCAAAAAATAC
ENSMMUT00000008476     CGAAGACTTAGCCAACTATCAAGTCATGATCCGCTCTGGAGAAGACATTGCAAAAAATAC
ENSGGOT00000023868     CGAAGACTTAGCCAGCTATCAAGTCATGATCCGCTGTGGAGAAGACATTGCAAAAAATAC
ENST00000265651        CGAAGACTTAGCCAGCTATCAAGTCATGATCCGCTGTGGAGAAGACATTGCAAAAAATAC
ENSPTRT00000006572     CGAAGACTTAGCCAGCTATCAAGTCATGATCCGCTGTGGAGAAGACATTGCAAAAAATAC
ENSPPYT00000004018     CGAAGACTTAGCCAGCTATCAAGTCATGATCCGCTGTGGAGAAGACATTGCAAAAAATAC
ENSMUST00000028603     CGAAGACTTAGCCAGCTTTCAACTCATGATCCACTGTGGAGAAGACATTGCAAAAAATAC
ENSRNOT00000014478     CGAAGACTTAGCCAGCTATCAACTCATGATCCACTGTGGAGAAGACATTGCAAAAAATAC


ENSCJAT00000020487     TGGCTGATATCTGAGGACGAGAAAACACAGAAGAATCAGTGTTGGAAATCTCTCTTCATA
ENSMMUT00000008476     TGGCTGATATCTGAGGAAGAGAAAACACAGAAGAATCAGTGTTGGAAATCTCTCTTCATA
ENSGGOT00000023868     TGGCTGATATCTGAGGAAGAGAAAACACAGAAGAATCAGTGTTGGAAATCTCTCTTCATA
ENST00000265651        TGGCTGATATCTGAGGAAGAGAAAACACAGAAGAATCAGTGTTGGAAATCTCTCTTCATA
ENSPTRT00000006572     TGGCTGATATCTGAGGAAGAGAAAACACAGAAGAATCAGTGTTGGAAATCTCTCTTCATA
ENSPPYT00000004018     TGGCTGATATCTGAGGAAGAGAAAACACAGAAGAATCAGTGTTGGAAATCTCTCTTCATA
ENSMUST00000028603     TGGCTGATATCTGAGGAAGAAAAAGCCGGGAAGAGTCAGTGCTGGAGATCCCTCTTCATA
ENSRNOT00000014478     TGGCTGATAACTGAGGAAGAGAAAGCCCGGAAGAATCAGTGCTGGAAATCTCTCTTCATA


ENSCJAT00000020487     GATACTTACTCTGATGTAGGAAGATACATTGACCATTATGCTGCTATTAAAAAGGCCTGG
ENSMMUT00000008476     GATACTTACTCTGATGTAGGAAGATACATTGACCATTATGCTGCTATTAAAAAGGCCTGG
ENSGGOT00000023868     GATACTTACTCTGATGTAGGAAGATACATTGACCATTATGCTGCTATTAAAAAGGCCTGG
ENST00000265651        GATACTTACTCTGATGTAGGAAGATACATTGACCATTATGCTGCTATTAAAAAGGCCTGG
ENSPTRT00000006572     GATACTTACTCTGATGTAGGAAGATACATTGACCATTATGCTGCTATTAAAAAGGCCTGG
ENSPPYT00000004018     GATACTTACTCTGATGTAGGAAGATACATTGACCATTATGCTGCTATTAAAAAGGCCTGG
ENSMUST00000028603     GAGACCTACTCAGACGTGGGGAGGTACATCGACCATTACGCCGCCATTAAAAAGGCCTGG
ENSRNOT00000014478     GCTACTTACTCAGATGTAGGAAGATACATCAACCATTATGCTGCTATTAAAAAGGCCTGG
```

```
ENSCJAT00000020487     GATGATCTCAAGAAATATTTGGAGCCCAGGTGTCCTCGGATGGTTTTATCTCTGAAAGAG
ENSMMUT00000008476     GATGATCTCAAGAAATATTTGGAGCCCAGGTGTCCTCGGATGGTTTTATCTCTGAAAGAG
ENSGGOT00000023868     GATGATCTCAAGAAATATTTGGAGCCCAGGTGTCCTCGGATGGTTTTATCTCTGAAAGAG
ENST00000265651        GATGATCTCAAGAAATATTTGGAGCCCAGGTGTCCTCGGATGGTTTTATCTCTGAAAGAG
ENSPTRT00000006572     GATGATCTCAAGAAATATTTGGAGCCCAGGTGTCCTCGGATGGTTTTATCTCTGAAAGAG
ENSPPYT00000004018     GATGATCTCAAGAAATATTTGGAGCCCAGGTGTCCTCGGATGGTTTTATCTCTGAAAGAG
ENSMUST00000028603     CGTGACCTCAAGAAGTACTTGGAGCCCAGGTGTCCTCGGATGGTTTTATCTCTGAAAGAG
ENSRNOT00000014478     GATGACCTCAAGAAGTACTTGGAGCCTAGGTGTCCTCGGATGGTTTTATCTCTGAAAGAG

ENSCJAT00000020487     GGTGCTCGAGAGGAAGACCTTGATGCTGTGGAAGCGCAGATTGGCTGCAAGCTTCCTGAT
ENSMMUT00000008476     GGTGCTCGAGAGGAAGACCTTGACGCTGTGGAAGCGCAGATTGGCTGCAAGCTTCCTGAC
ENSGGOT00000023868     GGTGCTCGAGAGGAAGACCTCGATGCTGTAGAAGCGCAGATTGGCTGCAAGCTTCCTGAC
ENST00000265651        GGTGCTCGAGAGGAAGACCTCGATGCTGTGGAAGCGCAGATTGGCTGCAAGCTTCCTGAC
ENSPTRT00000006572     GGTGCTCGAGAGGAAGACCTCGATGCTGTGGAAGCGCAGATTGGCTGCAAGCTTCCTGAC
ENSPPYT00000004018     GGTGCTCGAGAGGAAGACCTCGATGCTGTGGAAGCGCAGATTGGCTGCAAGCTTCCTGAC
ENSMUST00000028603     GGCGCACGTGAGGAAGACCTGGATGCTGTCGAAGCTCAGATCGGTTGCAAGCTCCCGGAT
ENSRNOT00000014478     GGTGCGCGTGAGGAAGACCTGGATGCAGTAGAAGCTCAGATCGGTTGCAAGCTCCCCGAT

ENSCJAT00000020487     GATTATCGATGTTCATACAGAATCCACAATGGACAGAAATTAGTGGTTCCTGGGTTATTG
ENSMMUT00000008476     GATTATCGATGTTCATACCGGATCCACAATGGACAGAAGTTAGTGGTTCCTGGGTTATTG
ENSGGOT00000023868     GATTATCGATGTTCATACCGAATTCACAATGGACAGAAGTTAGTGGTTCCTGGGTTATTG
ENST00000265651        GATTATCGATGTTCATACCGAATTCACAATGGACAGAAGTTAGTGGTTCCTGGGTTATTG
ENSPTRT00000006572     GATTATCGATGTTCATACCGAATTCACAATGGACAGAAGTTAGTGGTTCCTGGGTTATTG
ENSPPYT00000004018     GATTATCGATGTTCATACCGAATTCACAATGGACAGAAGTTAGTGGTTCCTGGGTTATTG
ENSMUST00000028603     GATTATCGCTGTTCATACCGGATCCACAACGGGCAAAAGTTAGTGGTGCCGGGGTTGCTG
ENSRNOT00000014478     GACTATCGCTGCTCATACCGGATCCACAATGGGCAGAAGTTAGTGGTTCCAGGATTGCTG

ENSCJAT00000020487     GGAAGCATGGCGCTGTCTAATCACTATCGTTCTGAAGATTTGTTAGACGTCGATACAGCT
ENSMMUT00000008476     GGAAGCATGGCACTGTCTAATCACTATCGTTCTGAAGATTTGTTAGACGTCGATACAGCT
ENSGGOT00000023868     GGAAGCATGGCACTGTCTAATCACTATCGTTCTGAAGATTTGTTAGACGTCGATACAGCT
ENST00000265651        GGAAGCATGGCACTGTCTAATCACTATCGTTCTGAAGATTTGTTAGACGTCGATACAGCT
ENSPTRT00000006572     GGAAGCATGGCACTGTCTAATCACTATCGTTCTGAAGATTTGTTAGACGTCGATACAGCT
ENSPPYT00000004018     GGAAGCATGGCACTGTCTAATCACTATCGTTCTGAAGATTTGTTAGACGTCGATACAGCT
ENSMUST00000028603     GGAAGTATGGCACTGTCCAATCACTACCGCTCTGAAGATCTACTAGATGTTGATACTGCT
ENSRNOT00000014478     GGAAGCATGGCACTGTCAAATCACTACCGCTCTGAAGATTTATTAGACGTTGACACTGCT

ENSCJAT00000020487     GCCGGAGGATTCCAGCAGAGACAGGGACTGAAATACTGTCTCCCTTTAACTTTTTGCATA
ENSMMUT00000008476     GCCGGAGGATTCCAGCAGAGACAGGGATTGAAATACTGTCTGCCTTTAACTTTTTGCATA
ENSGGOT00000023868     GCCGGAGGATTCCAGCAGAGACAGGGACTGAAATACTGTCTCCCTTTAACTTTTTGCATA
ENST00000265651        GCCGGAGGATTCCAGCAGAGACAGGGACTGAAATACTGTCTCCCTTTAACTTTTTGCATA
ENSPTRT00000006572     GCCGGAGGATTCCAGCAGAGACAGGGACTGAAATACTGTCTCCCTTTAACTTTTTGCATA
ENSPPYT00000004018     GCCGGAGGATTCCAGCAGAGACAGGGACTGAAATACTGTCTCCCTTTAACTTTTTGCATA
ENSMUST00000028603     GCAGGAGGATTTCAGCAGAGACAGGGACTGAAATACTGTCTCCCTTTAACCTTTTGCATA
ENSRNOT00000014478     GCCGGAGGATTTCAGCAGAGACAGGGACTGAAATACTGTCTCCCTTTAACCTTTTGCATA

ENSCJAT00000020487     CATACTGGTTTGAGTCAGTACATAGCAGTGGAAGCTGCAGAGGGCCGAAACAAAAATGAA
ENSMMUT00000008476     CATACTGGTTTGAGTCAGTACATAGCAGTGGAAGCTGCAGAGGGCCGAAACAAAAATGAA
ENSGGOT00000023868     CATACTGGTTTGAGTCAGTACATAGCAGTGGAAGCTGCAGAGGGCCGAAACAAAAATGAA
ENST00000265651        CATACTGGTTTGAGTCAGTACATAGCAGTGGAAGCTGCAGAGGGCCGAAACAAAAATGAA
ENSPTRT00000006572     CATACTGGTTTGAGTCAGTACATAGCAGTGGAAGCTGCAGAGGGCCGAAACAAAAATGAA
ENSPPYT00000004018     CATACTGGTTTGAGTCAGTACATAGCAGTGGAAGCTGCAGAGGGCCGAAACAAAAATGAA
ENSMUST00000028603     CACACCGGCTTGAGTCAGTACATAGCTGTGGAGGCTGCAGAGGGGCGGAATAAGAACGAA
ENSRNOT00000014478     CATACTGGCTTGAGTCAGTACATAGCTGTGGAAGCTGCAGAGGGCCGAAATAAAAACGAA
```

```
ENSCJAT00000020487     GTTTTCTACCAATGTCCAGACCAAATGGCTCGAAATCCAGCTGCTATTGACATGTTTATC
ENSMMUT00000008476     GTTTTCTACCAATGTCCAGACCAAATGGCTCGAAATCCAGCTGCTATTGACATGTTTATT
ENSGGOT00000023868     GTTTTCTACCAATGTCCAGACCAAATGGCTCGAAATCCAGCTGCTATTGACATGTTTATT
ENST00000265651        GTTTTCTACCAATGTCCAGACCAAATGGCTCGAAATCCAGCTGCTATTGACATGTTTATT
ENSPTRT00000006572     GTTTTCTACCAATGTCCAGACCAAATGGCTCGAAATCCAGCTGCTATTGACATGTTTATT
ENSPPYT00000004018     GTTTTCTACCAATGTCCAGACCAAATGGCTCGAAATCCAGCTGCTATTGACATGTTTATT
ENSMUST00000028603     GTGTTCTACCAATGTCCAGACCAAATGGCTCGGAATCCTGCTGCTATTGACATGTTTATC
ENSRNOT00000014478     GTGTTCTACCAGTGCCCAGATCAAATGGCTCGGAATCCTGCTGCTATTGACATGTTTATC

ENSCJAT00000020487     ATAGGTGCTACTTTTACTGACTGGTTTACATCTTATGTCAACAATGTTGTATCAGGTGGC
ENSMMUT00000008476     ATAGGTGCTACTTTTACTGACTGGTTTACCTCTTATGTCAAAAATGTTGTATCAGGTGGC
ENSGGOT00000023868     ATAGGTGCTACTTTTACTGACTGGTTTACCTCTTATGTCAAAAATGTTGTATCAGGTGGC
ENST00000265651        ATAGGTGCTACTTTTACTGACTGGTTTACCTCTTATGTCAAAAATGTTGTATCAGGTGGC
ENSPTRT00000006572     ATAGGTGCTACTTTTACTGACTGGTTTACCTCTTATGTCAAAAATGTTGTATCAGGTGGC
ENSPPYT00000004018     ATAGGTGCTACTTTTACTGACTGGTTTACCTCTTATGTCAAAAATGTTGTATCAGGTGGC
ENSMUST00000028603     ATAGGTGCTACTTTTACCGACTGGTTTACATCCTATGTCAACAATGTTGTATCAGGTGGT
ENSRNOT00000014478     ATAGGTGCTACTTTTACTGACTGGTTTACATCCTATGTCAACAATGTTGTATCAGGTGGC

ENSCJAT00000020487     TTCCCCATCATCAGAGACCAAATTTTCAGATATGTTCATGATCCAGAGTGTGTAGCAACA
ENSMMUT00000008476     TTCCCTATCATCAGAGACCAAATTTTCAGATATGTTCACGATCCAGAGTGTGTAGCAACA
ENSGGOT00000023868     TTCCCCATCATCAGAGACCAAATTTTCAGATATGTTCACGATCCAGAATGTGTAGCAACA
ENST00000265651        TTCCCCATCATCAGAGACCAAATTTTCAGATATGTTCACGATCCAGAATGTGTAGCAACA
ENSPTRT00000006572     TTCCCCATCATCAGAGACCAAATTTTCAGATATGTTCACGATCCAGAATGTGTAGCAACA
ENSPPYT00000004018     TTCCCCATCATCAGAGACCAAATTTTCAGATATGTTCACGATCCAGAGTGTGTAGCAACA
ENSMUST00000028603     TTCCCCATCATCAGAGACCAGATTTTCAGATATATTCATGATCCAGAATGTGTGGCAACA
ENSRNOT00000014478     TTCCCCATCATCAGAGACCAGATTTTCAGATATATTCATGACCCAGAGTGTGTAGCAACA

ENSCJAT00000020487     ACTGGGGATATTACCGTTTCTGTTTCCACATCATTTCTGCCAGAACTGAGCTCTGTACAT
ENSMMUT00000008476     ACTGGGGATATTACTGTTTCCGTTTCCACATCGTTTCTGCCAGAACTTAGCTCTGTACAT
ENSGGOT00000023868     ACTGGGGATATTACTGTGTCAGTTTCCACATCGTTTCTGCCAGAACTTAGCTCTGTACAT
ENST00000265651        ACTGGGGATATTACTGTGTCAGTTTCCACATCGTTTCTGCCAGAACTTAGCTCTGTACAT
ENSPTRT00000006572     ACTGGGGATATTACTGTGTCAGTTTCCACATCGTTTCTGCCAGAACTTAGCTCTGTACAT
ENSPPYT00000004018     ACTGGGGATATTACTGTGTCAGTTTCCACATCGTTTCTGCCAGAACTTAGCTCTGTACAT
ENSMUST00000028603     ACTGGAGATATTACAGTTTCAGTGTCCACATCGTTTCTGCCAGAACTCAGCTCTGTGCAT
ENSRNOT00000014478     ACTGGAGATATTACAGTCTCAGTGTCCACATCGTTTCTGCCAGAACTCAGTTCTGTGCAC

ENSCJAT00000020487     CCACCCCACTATTTCTTCACATACCGAATCAGGATTGAAATGTCAAAGGATGCACTTCCT
ENSMMUT00000008476     CCACCCCACTATTTCTTCACATACCGAATCAGGATTGAAATGTCAAAGGATGCACTTCCT
ENSGGOT00000023868     CCACCCCACTATTTCTTCACATACCGAATCAGGATTGAAATGTCAAAAGATGCCCTTCCT
ENST00000265651        CCACCCCACTATTTCTTCACATACCGAATCAGGATTGAAATGTCAAAAGATGCACTTCCT
ENSPTRT00000006572     CCACCCCACTATTTCTTCACATACCGAATCAGGATTGAAATGTCAAAAGATGCACTTCCT
ENSPPYT00000004018     CCACCCCACTATTTCTTCACATACCGAATCAGGATTGAAATGTCAAAAGATGCACTTCCT
ENSMUST00000028603     CCGCCACACTATTTCTTCACATACCGAATCAGGATTGAAATGTCAAGGGATGCTCTTCCT
ENSRNOT00000014478     CCACCACACTATTTCTTCACATACCGAATCAGGATTGAAATGTCAAGGGATGCTCTTCCT

ENSCJAT00000020487     GAGAAGGCCTGTCAGTTGGACAGTCGCTATTGGAGAATAACAAATGCTAAGGGTGACGTG
ENSMMUT00000008476     GAGAAGGCCTGTCAGTTGGACAGTCGCTATTGGAGAATAACAAATGCTAAGGGTGACGTG
ENSGGOT00000023868     GAGAAGGCCTGTCAGTTGGACAGTCGCTATTGGAGAATAACAAATGCTAAGGGTGACGTG
ENST00000265651        GAGAAGGCCTGTCAGTTGGACAGTCGCTATTGGAGAATAACAAATGCTAAGGGTGACGTG
ENSPTRT00000006572     GAGAAGGCCTGTCAGTTGGACAGTCGCTATTGGAGAATAACAAATGCTAAGGGTGATGTG
ENSPPYT00000004018     GAGAAGGCCTGTCAGTTGGACAGTCGCTATTGGAGAATAACAAATGCTAAGGGTGACGTG
ENSMUST00000028603     GAGAAGGCCTGTCAGTTGGACAGTCGCTACTGGAGGATAACAAACGCTAAAGGGGATGTG
ENSRNOT00000014478     GAGAAGGCCTGTCAGTTGGACAGTCGCTACTGGAGGATAACAAATGCTAAAGGGGATGTG
```

```
ENSCJAT00000020487     GAAGAAGTTCAAGGACCTGGAGTAGTTGGTGAATTTCCAATCATCAGCCCAGGTCGGGTA
ENSMMUT00000008476     GAAGAAGTTCAAGGACCTGGAGTAGTTGGTGAATTTCCAATCATCAGCCCAGGTCGGGTA
ENSGGOT00000023868     GAAGAAGTCCAAGGACCTGGAGTAGTTGGTGAATTTCCAATCATCAGCCCAGGTCGGGTA
ENST00000265651        GAAGAAGTTCAAGGACCTGGAGTAGTTGGTGAATTTCCAATCATCAGCCCAGGTCGGGTA
ENSPTRT00000006572     GAAGAAGTTCAAGGACCTGGAGTAGTTGGTGAATTTCCAATCATCAGCCCAGGTCGGGTA
ENSPPYT00000004018     GAAGAAGTTCAAGGACCTGGAGTAGTTGGTGAATTTCCAATCATCAGCCCAGGTCGGGTA
ENSMUST00000028603     GAAGAAGTCCAGGGACCTGGAGTAGTCGGTGAATTTCCAATTATTAGCCCAGGTCGGATA
ENSRNOT00000014478     GAGGAAGTCCAGGGACCTGGAGTAGTTGGTGAATTTCCAATTATTAGCCCAGGTCGGATA

ENSCJAT00000020487     TATGAATACACAAGCTGTACCACATTCTCTACAACATCAGGATACATGGAAGGATATTAT
ENSMMUT00000008476     TATGAATACACAAGCTGTACCACATTCTCTACAACATCAGGATATATGGAAGGATATTAT
ENSGGOT00000023868     TATGAATACACAAGCTGTACCACATTCTCTACAACATCAGGATACATGGAAGGATATTAT
ENST00000265651        TATGAATACACAAGCTGTACCACATTCTCTACAACATCAGGATACATGGAAGGATATTAT
ENSPTRT00000006572     TACGAATACACAAGCTGTACCACATTCTCTACAACATCAGGATACATGGAAGGATATTAT
ENSPPYT00000004018     TATGAATACACAAGCTGTACCACATTCTCTACAACATCAGGATACATGGAAGGATATTAT
ENSMUST00000028603     TATGAATACACAAGCTGTACCACATTTTCTACAACATCAGGCTACATGGAAGGGTATTAC
ENSRNOT00000014478     TATGAATACACCAGCTGCACCACATTTTCTACAACATCAGGCTATATGGAAGGGTATTAT

ENSCJAT00000020487     ACCTTCCATTTTCTTTACTTTAAAGACAAGATCTTTAATGTTGCCATTCCCCGATTCCAT
ENSMMUT00000008476     ACCTTCCACTTTCTTTACTTTAAAGACAAGATCTTTAATGTTGCCATTCCCCGATTCCAT
ENSGGOT00000023868     ACCTTCCATTTTCTTTACTTTAAAGACAAGATCTTTAATGTTGCCATTCCCCGATTCCAT
ENST00000265651        ACCTTCCATTTTCTTTACTTTAAAGACAAGATCTTTAATGTTGCCATTCCCCGATTCCAT
ENSPTRT00000006572     ACCTTCCATTTTCTTTACTTTAAAGACAAGATCTTTAATGTTGCCATTCCCCGATTCCAT
ENSPPYT00000004018     ACCTTCCATTTTCTTTACTTTAAAGACAAGATCTTTAATGTTGCCATTCCCCGATTCCAT
ENSMUST00000028603     ACCTTCCATTTTCTGTACTTTAAGGACAAGGTCTTTAATGTTGCCATCCCCCGATTCCAC
ENSRNOT00000014478     ACCTTCCATTTTCTGTACTTTAAGGACAAGGTCTTTAATGTTGCCATCCCACGATTCCAT

ENSCJAT00000020487     ATGGCATGCCCAACATTCAGGGTGTCTATAGCCCGATTGGAAATGGGTCCTGATGAATAT
ENSMMUT00000008476     ATGGCATGTCCAACATTCAGGGTGTCTATAGCCCGATTGGAAATGGGTCCTGATGAATAT
ENSGGOT00000023868     ATGGCATGTCCAACATTCAGGGTGTCTATAGCCCGATTGGAAATGGGTCCTGATGAATAT
ENST00000265651        ATGGCATGTCCAACATTCAGGGTGTCTATAGCCCGATTGGAAATGGGTCCTGATGAATAT
ENSPTRT00000006572     ATGGCATGTCCAACATTCAGGGTGTCTATAGCCCGATTGGAAATGGGTCCTGATGAATAT
ENSPPYT00000004018     ATGGCATGTCCAACATTCAGGGTGTCTATAGCCCGATTGGAAATGGGTCCTGATGAATAT
ENSMUST00000028603     ATGGCATGTCCAACATTCAGGGTGTCTATAGCTCGATTGGAAATGGGTCCTGATGAATAT
ENSRNOT00000014478     ATGGCATGCCCAACATTCAGGGTGTCTATAGCCCGATTGGAAATGGGTCCTGATGAATAT

ENSCJAT00000020487     GAAGAGATGGAAGAAGAGGAGGAGGAGGAAGAAGAGGAAGATGAGGATGATGATTCAGCA
ENSMMUT00000008476     GAAGAGATGGAAGAAGAGGAGGAGGAGGAAGAAGAGGAAGACGAGGATGATGATTCAGCA
ENSGGOT00000023868     GAAGAGATGGAAGAAGAGGAGGAGGAGGAAGAGGAGGAAGACGAGGATGATGATTCAGCA
ENST00000265651        GAAGAGATGGAAGAAGAGGAGGAGGAGGAAGAGGAGGAAGACGAGGATGATGATTCAGCA
ENSPTRT00000006572     GAAGAGATGGAAGAAGAGGAGGAGGAGGAAGAGGAGGAAGACGAGGATGATGATTCAGCA
ENSPPYT00000004018     GAAGAGATGGAAGAAGAGGAGGAGGAGGAAGAGGAGGAAGATGAGGATGATGATTCAGCA
ENSMUST00000028603     GAAGAGATGGAGGAGGAAGCTGAGGAGGAGGAGGAGGAA---GAAAATGATGACTCTGCA
ENSRNOT00000014478     GAAGAGATGGAGGAGGAAGCTGAGGAGGAAGAGGAAGAG---GAAAACGATGACTCAGCA

ENSCJAT00000020487     GATATGGATGAATCAGATGAAGAAGATGAAGAGGAGAGACGAAGGAGAGTCTTTGATGTT
ENSMMUT00000008476     GATATGGATGAATCAGATGAAGATGATGAAGAGGAGAGACGGAGGAGAGTCTTTGATGTT
ENSGGOT00000023868     GATATGGATGAATCAGATGAAGATGATGAAGAAGAGAGACGGAGGAGAGTCTTTGATGTT
ENST00000265651        GATATGGATGAATCAGATGAAGATGATGAAGAGGAGAGACGGAGGAGAGTCTTTGATGTT
ENSPTRT00000006572     GATATGGATGAATCAGATGAAGATGATGAAGAGGAGAGACGGAGGAGAGTCTTTGATGTT
ENSPPYT00000004018     GATATGGATGAATCAGATGAAGATGATGAAGAGGAGAGACGGAGGAGAGTCTTTGATGTT
ENSMUST00000028603     GACATGGATGAATCCGATGAGTCAGATGCGGATGAGAGACGGAGGAGGGTCTTTGATGTT
ENSRNOT00000014478     GACATGGATGAATCCGATGAATCAGATGAGGATGAGAGAAGGAGGAGGGTCTTTGATGTT
```

```
ENSCJAT00000020487     CCCATTCGCCGACGCCGCTGCTCACGCCTTTTT
ENSMMUT00000008476     CCCATTCGCAGACGCCGTTGCTCACGCCTTTTT
ENSGGOT00000023868     CCCATTCGCAGACGCCGCTGCTCACGCCTTTTT
ENST00000265651        CCCATTCGCAGACGCCGCTGCTCACGCCTTTTT
ENSPTRT00000006572     CCCATTCGCAGACGCCGCTGCTCACGCCTTTTT
ENSPPYT00000004018     CCCATTCGCAGACGCCGCTGCTCACGCCTTTTT
ENSMUST00000028603     CCCATTCGCAGACGCCGCTGCTCTCGTCTGTTT
ENSRNOT00000014478     CCCATCCGCAGACGCCGCTGCTCTCGTCTTTTT
```

Multiple sequence alignment of Fbxo30

```
ENSCJAT00000038021     ATGGAGGAAGAGCTGCAGCATTCACATTGTGTGAATTGTGTCAGTAGACGGTGTATGACC
ENSGGOT00000005955     ATGGAGGAGGAGCTGCAGCATTCCCATTGTGTGAATTGTGTCAGTAGACGGTGCATGACC
ENST00000237281        ATGGAGGAGGAGCTGCAGCATTCCCATTGTGTGAATTGTGTCAGTAGACGGTGCATGACC
ENSPTRT00000034492     ATGGAGGAGGAGCTGCAGCATTCCCATTGTGTGAATTGTGTCAGTAGACGGTGCATGACC
ENSMMUT00000030864     ATGGAGGAGCAGCTGCAGCATTCCCATTGTGTGAATTGTGTCAGTAGACGGTGCATGACT
ENSMUST00000129456     ATGGAGGAAGAGGTACAGCACTCACACTGTATGAACTGTGTCAGTAGACGGTGTATGACC
ENSRNOT00000019918     ATGGAGGAAGAGGTACAGCACTCACACTGTGTGAACTGTGTCAGTAGAAGGTGTATGACC

ENSCJAT00000038021     AGACCAGAGCCTGGGATTTCCTGTGATTTGATTGGTTGTCCATTGGTTTGTGGTGCCGTT
ENSGGOT00000005955     AGGCCAGAGCCAGGGATTTCCTGTGATTTGATTGGTTGTCCATTGGTTTGTGGTGCAGTT
ENST00000237281        AGGCCAGAGCCAGGGATTTCCTGTGATTTGATTGGTTGTCCATTGGTTTGTGGTGCAGTT
ENSPTRT00000034492     AGGCCAGAGCCAGGGATTTCCTGTGATTTGATTGGTTGTCCATTGGTTTGTGGTGCAGTT
ENSMMUT00000030864     AGACCAGAGCCTGGGATTTCCTGTGATTTGATTGGTTGTCCATTGGTTTGTGGTGCAGTT
ENSMUST00000129456     AGACCAGAGCCTGGGGTTTCCTGTGACTTGATTGGCTGTCCATTGGTTTGTGGAGCAGTT
ENSRNOT00000019918     AGACCAGAGCCTGGCGTTTCCTGTGACTTGATTGGTTGTCCATTGGTTTGTGGAGCAGTC

ENSCJAT00000038021     TTCCACTCTTGTAAAGCCGATGAGCATCGACTTTTATGTCCATTTGAACGAGTGCCTTGC
ENSGGOT00000005955     TTCCATTCTTGTAAAGCTGATGAGCATCGACTTTTATGTCCATTTGAACGAGTGCCTTGC
ENST00000237281        TTCCATTCTTGTAAAGCTGATGAGCATCGACTTTTATGTCCATTTGAACGAGTGCCTTGC
ENSPTRT00000034492     TTCCATTCTTGTAAAGCTGATGAACATCGACTTTTATGTCCATTTGAACGAGTGCCTTGC
ENSMMUT00000030864     TTCCATTCTTGTAAAGCTGAAGAGCATCGACTTTTATGTCCATTTGAACGAGTGCCTTGC
ENSMUST00000129456     TTCCATTCTTGTAAAGCTGACGAACATCGGCTCTTGTGTCCCTTTGAACGAGTAGCTTGC
ENSRNOT00000019918     TTCCATTCTTGTAAAGCTGATGAGCATCGACTCTTGTGTCCTTTTGAACGAGTACCCTGC

ENSCJAT00000038021     TTAAATAGTGACTTTGGATGTCCATTTACCATGGCCCGAAATAAAGTTGCTGAACATCTA
ENSGGOT00000005955     TTAAATAGTGACTTTGGATGTCCATTTACCATGGCCCGAAATAAAGTTGCTGAACATCTA
ENST00000237281        TTAAATAGTGACTTTGGATGTCCATTTACCATGGCCCGAAATAAAGTTGCTGAACATCTA
ENSPTRT00000034492     TTAAATAGTGACTTTGGATGTCCATTTACCATGGCCCGAAATAAAGTTGCTGAACATCTA
ENSMMUT00000030864     TTAAATAGTGACTTTGGATGTCCGTTTACCATGGCCCGAAATAAAGTTGCTGAACATCTG
ENSMUST00000129456     TTAAATAGAAACTTTGGATGTCCATTTACCCTGGCACGAAATAAAGTTGCTGAACATCTA
ENSRNOT00000019918     TTAAATAGTAACTTTGGGTGTCCATTTACCCTGGCACGAAATAAAGTTGCTGAACATCTC

ENSCJAT00000038021     GAAATGTGTCCTGCAAGTGTGGTGTGCTGTACTATGGAATGGAACCGATGGCCAGTTAGT
ENSGGOT00000005955     GAAATGTGTCCTGCAAGTGTGGTGTGCTGTACTATGGAATGGAATCGATGGCCAGTTAGT
ENST00000237281        GAAATGTGTCCTGCAAGTGTGGTGTGCTGTACTATGGAATGGAATCGATGGCCAGTTAGT
ENSPTRT00000034492     GAAATGTGTCCTGCAAGTGTGGTGTGCTGTACTATGGAATGGAATCGATGGCCAGTTAGT
ENSMMUT00000030864     GAAATGTGTCCTGCAAGTGTGGTGTGCTGTACTATGGAATGGAATCGATGGCCAGTTAGT
ENSMUST00000129456     GAAATGTGTCCTGCAAGTGTGGTGTGCTGTACTATGGAATGGAATCGATGGCCCGTTAGT
ENSRNOT00000019918     GAAATGTGTCCTGCAAGTGTGGTGTGCTGCACTATGGAGTGGAACCGATGGCCCGTTAGT

ENSCJAT00000038021     TATGCAGACCGAAAATCATATGAAAATCTAAGCAGAGATGTTGATGAAGTGGCACAGTTA
ENSGGOT00000005955     TATGCAGACCGGAAATCATATGAAAATCTAAGCAGAGATGTCGATGAAGTGGCACAATTG
ENST00000237281        TATGCAGACCGGAAATCATATGAAAATCTAAGCAGAGATGTCGATGAAGTGGCACAATTG
```

```
ENSPTRT00000034492    TATGCAGACCGGAAATCATATGAAAATCTAAGCAGAGATGTCGATGAAGTGGCACAATTG
ENSMMUT00000030864    TATGCAGACCGGAAATCATATGAAAATCTAAGCAGAGATGTTGATGAAGTGGCACAATTG
ENSMUST00000129456    TATTCAGACCGGAAGTCATATGAAAGTCTAAGCAGAGATGTTGATGAAGTGGCACAATTA
ENSRNOT00000019918    TATTCAGACCGCAAGTCATATGAAAGTCTAAGCAGAGATGCTGATGAAGTGTCACAGTTA

ENSCJAT00000038021    GATATGGCCTTGGCTCTTCAAGACCAAAGAATGCTCTTAGAATCTCTCAAAGTAGCCACG
ENSGGOT00000005955    GATATGGCCTTGGCTCTTCAAGACCAAAGGATGCTCTTAGAATCCCTCAAAGTAGCCACC
ENST00000237281       GATATGGCCTTGGCTCTTCAAGACCAAAGGATGCTCTTAGAATCCCTCAAAGTAGCCACC
ENSPTRT00000034492    GATATGGCCTTGGCTCTTCAAGACCAAAGGATGCTCTTAGAATCCCTCAAAGTAGCCACC
ENSMMUT00000030864    GATATGGCCTTGGCTCTTCAAGACCAAAGGATGCTCTTAGAATCCCTCAAAGTAGCCACC
ENSMUST00000129456    GACATGGCTTTGGCTCTTCAAGATCAAAGAATGCTTTTGGAATCTCTTAAAGTAGCCACC
ENSRNOT00000019918    GACATGGCTTTGGCCCTTCAAGATCAAAGAATGCTTTTGGAATCTCTCAAAGTAGCCACC

ENSCJAT00000038021    ATGATGTCAAAAGGAACTGATAAAGTTTCCAAACCTAGAGAACAAATCTCAGTTAAATCA
ENSGGOT00000005955    ATGATGTCAAAAGCAACTGATAAAGTATCCAAACCTAGAGAACAAATCTCAGTTAAATCA
ENST00000237281       ATGATGTCAAAAGCAACTGATAAAGTATCCAAACCTAGAGAACAAATCTCAGTTAAATCA
ENSPTRT00000034492    ATGATGTCAAAAGCAACTGATAAAGTATCCAAACCTAGAGAACAAATCTCAGTTAAATCA
ENSMMUT00000030864    ATGATGTCAAAAGCAACTGATAAAGTATCCAAACCTAGAGAACAAATCTCAGTTAAATCA
ENSMUST00000129456    ATGATGTCAAAAGCAACTGATAAAATATCTGAACCTAGAGAACAAATCTCAGTGAAATCA
ENSRNOT00000019918    ATGATGTCAAAAGCAACTGGTAAAATATCTAAACCTAGAGAACAGATCTCAGTGAACTCA

ENSCJAT00000038021    AGTGTTCCAGAAATACCACATGCTAATGGTTTGGTGTCTGTTGATGAAGAATCTTATGGT
ENSGGOT00000005955    AGTGTCCCAGAAATACCGCATACTAATGGTTTGGTATCTGTTGATGAAGAATCTTATGGT
ENST00000237281       AGTGTCCCAGAAATACCACATGCTAATGGTTTAGTGTCTGTTGATGAAGAATCTTATGGT
ENSPTRT00000034492    AGTGTCCCAGAAATACCACATGCTAATGGTTTGGTGTCTGTTGATGAAGAATCTTATGGT
ENSMMUT00000030864    AGTGTCCCAGAAATACCACATACTAATGGTTTGGTGTCTGTTGATGAAGAATCTTATGGT
ENSMUST00000129456    AGTGTTCAGGAAATACCACGTACTAACGGGTTGGTATCCGTTGATGAAGAATCCTATGGT
ENSRNOT00000019918    AGTGTTCAGGAAGTACCACGTGCTAATGGGTTGGTGTCCGCTGACGAAGAGTCATATGGT

ENSCJAT00000038021    GCACTCTATCAAGCTACTGTAGAAACAACCAGAAGTTTGGCTGCTGCTTTGGATATCCTG
ENSGGOT00000005955    GCACTTTATCAAGCTACTGTAGAAACAACCAGAAGTTTGGCTGCTGCTTTGGATATCCTG
ENST00000237281       GCACTTTATCAAGCTACTGTAGAAACAACCAGAAGTTTGGCTGCTGCTTTGGATATCCTG
ENSPTRT00000034492    GCACTTTATCAAGCTACTGTAGAAACAACCAGAAGTTTGGCTGCTGCTTTGGATATCCTG
ENSMMUT00000030864    GCACTTTATCAAGCTACTGTAGAAACAACCAGAAGTTTGGCTGCTGCTTTGGATATCCTG
ENSMUST00000129456    GCACTTTATCAAGCTACTGTAGAAACAACCAGAAGTTTGGCTGCTGCTTTAGATATCCTA
ENSRNOT00000019918    GCACTTTATGAAGCTACTGTAGAAACAACCAGAAGCTTGGCTGCTGCTTTAGATATCCTA

ENSCJAT00000038021    AATACTGCTACAAGAGACATTGGCATTTTAAATACAAGTGTCCCAAATGAAATGGATGAA
ENSGGOT00000005955    AATACTGCTACAAGAGACATTGGCATGTTAAATACAAGTGTCCCAAATGACATGGATGAA
ENST00000237281       AATACTGCTACAAGAGACATTGGCATGTTAAATACAAGTGTCCCAAATGACATGGATGAA
ENSPTRT00000034492    AATACTGCTACAAGAGACATTGGCATGTTAAATACAAGTGTCCCAAATGACATGGATGAA
ENSMMUT00000030864    AATACTGCTACAAGAGACATTGGCATGTTAAATACAAGTATCCCAAATGACATGGATGAA
ENSMUST00000129456    AATTCTGCCACAAGAGACATTGGCATGTTAAATACAAGTCTCGCGAATGAAATGGATGAG
ENSRNOT00000019918    AACTCTGCCACAAGAGATATTGGCATGTTAAATACAAGTCTCACAAATGAAATGGATGAG

ENSCJAT00000038021    GAGCAAAATGCAAGAGAAAGCTTACAGGATCAAAACTTGAAAGACCAGGATCATCTTTAT
ENSGGOT00000005955    CAGCAAAATGCAAGAGAAAGCTTAGAGGATCAAAACTTGAAAGACCAAGATCATCTTTAT
ENST00000237281       CAGCAAAATGCGAGAGAAAGCTTAGAGGATCAAAACTTGAAAGACCAAGATCATCTTTAT
ENSPTRT00000034492    CAGCAAGATGCGAGAGAAAGCTTAGAGGATCAAAACTTGAAAGACCAAGATCATCTTTAT
ENSMMUT00000030864    GAGCAAAATGCGAGAGAAAGCTTAGAGGATCAAAACTTGAAAGACCAAGATCATCTTTAT
ENSMUST00000129456    GAGAATAAC---AAAGAAAGCTTCCAGGACAAAAACTTGAAAGACCAGGACCACCTTGAT
ENSRNOT00000019918    GAGAGTAAC---AGAGAAAGCTCCCAGGACAGAAACGCGAAAGACCAGGATCACCTTGAC
```

```
ENSCJAT00000038021      GAGGAGGAGATAGGAGCAGTGGGTGGAATTGACCACAATGACACAAGTCAGAATGCCCGG
ENSGGOT00000005955      GAGGAGGAAATAGGAGCAGTAGGTGGAATTGACTACAATGACACAAATCAGAATGCCCAG
ENST00000237281         GAGGAGGAAATAGGAGCAGTAGGTGGAATTGACTACAATGACACAAATCAGAATGCCCAG
ENSPTRT00000034492      GAGGAGGAAATAGGAGCAGTAGGTGGAATTGACTACAATGACACAAATCAGAATGCCCAG
ENSMMUT00000030864      GAGGAGGAGATAGGAGCAGTAGGTGGAATTGACTACAATGACACAGATCAGAATGCCCAG
ENSMUST00000129456      GAGGGTGAGATAGGGGCAGTAGGTGGAGTTGATTATAGTGGCACAAGTCAGAATGCTCAG
ENSRNOT00000019918      GAGGGTGAGATAGGGGCAGTAGGTGGAATTGATTATACTGGCACAAGTCAGAATGCTCAG

ENSCJAT00000038021      TCTGAACGAAATGGTTCAAGTGATTTATTATGTGACCTGAATACAAGTTCTTGTGACACT
ENSGGOT00000005955      TCTGAACAAAATGGTTCAAGTGATTTATTATGTGACTTGAATACAAGTTCTTATGACACT
ENST00000237281         TCTGAACAAAATGGTTCAAGTGATTTATTATGTGACTTGAATACAAGTTCTTATGACACT
ENSPTRT00000034492      TCTGAACAAAATGGTTCAAGTGATTTATTATGTGACTTGAATACAAGTTCTTATGACACT
ENSMMUT00000030864      TCTGAACAAAATGGTTCAAGTGATTTATTATGTGACTTGAATACAAGTTCTTATGACACT
ENSMUST00000129456      GCTGAACAAAATGGCTCAAGTGATTTGTTATGTGACTTAAATCCAAGTTCTAATGGTACT
ENSRNOT00000019918      GCTGAACAAAATGGCTCAAGTGATTTATTATGCAACTTAAATACAAGTTCTTACGATACT

ENSCJAT00000038021      TCTGCTCTTTGTAATGGCTTTCCTTTGAAAAATATATGTCCGCAGGTCATAGACCAGAAT
ENSGGOT00000005955      TCTGCTCTTTGTAATGGCTTTCCTTTGGAAAATATATGTACCCAGGTCATAGACCAGAAT
ENST00000237281         TCTGCTCTTTGTAATGGCTTTCCTTTGGAAAATATATGTACCCAGGTCATAGACCAGAAT
ENSPTRT00000034492      TCTGCTCTTTGTAATGGCTTTCCTTTGGAAAATATATGTACCCAGGTCATAGACCAGAAT
ENSMMUT00000030864      TCACCTCTTTGTAATGGCTTTCCTTTGGAAAATATATGTACCCAGGTCATAGACCAGAAT
ENSMUST00000129456      TCTGCTCTTTGCAATGGCTTTCCTTTGGAAAAGATGTGTATTCAGGTCAAAGGCCAGGAT
ENSRNOT00000019918      TCTGCTCTTTGTAATGGCTTTCCTTTGGAAAAGATGTGTACACAGGTCAAAGACCAGGAT

ENSCJAT00000038021      CATAATTTACATGGTGATTCAAAACAAAGTAACTTAACAAATGGAGCCTGTGTGGCATCA
ENSGGOT00000005955      CAGAATTTACATGGTGATTCAAAACAAAGTAACTTAACAAATGGAGACTGTGTGGCATCA
ENST00000237281         CAGAATTTACATGGTGATTCAAAACAAAGTAACTTAACAAATGGAGACTGTGTGGCATCA
ENSPTRT00000034492      CAGAATTTACATGGTGATTCAAAACAAAGTAACTTAACAAATGGAGACTGTGTGGCATCA
ENSMMUT00000030864      CAGAATTTACATGGTGATTCAAAACAAAGTAACTTAACAAATGGAGACCGTGTGGCATCA
ENSMUST00000129456      CAAAATTTTCATGGTGATTCCACAGAAAGTAATATAACAAATGGAGATTGTGTGGAAGCT
ENSRNOT00000019918      CAAAATTTTCATGGTGATTCCACAGAAAGTAACATAACAAATGGAGACTGTGTGGAAGCT

ENSCJAT00000038021      GATGGCGCTTCAAAACCTTCCAGCTCACTTGTGGTAGCAGCACAACTTAGGGAAGTAATA
ENSGGOT00000005955      GATGGCACTTCAAAACCTTCCAGCTCACTTGCGGTGGCAGCACAACTTAGGGAAATAATA
ENST00000237281         GATGGCACTTCAAAACCTTCCAGCTCACTTGCGGTGGCAGCACAACTTAGGGAAATAATA
ENSPTRT00000034492      GATGGCACTTCAAAACCTTCCAGCTCACTTGCGGTGGCAGCACAACTTAGGGAAATAATA
ENSMMUT00000030864      GATGGCACTTCAAAACCTTCCAGCTCACTTGCGGTAGCAGCACAACTTAGGGAAATAATA
ENSMUST00000129456      GATGGTACTTCAGAACCTTCCAGTTCACTTGTAGTACCAGAACAACTGAGGGAAATAAGT
ENSRNOT00000019918      GATGGTACTTCAGAACCTCCCAGTTCACTTTTAGTAGCAGAACAACTTAAGGAA------

ENSCJAT00000038021      CCATCCAATGCTTTGCTTAATGGCACAGTTCAGCATATCCTTATACCTGATGATGAGGAT
ENSGGOT00000005955      CCATCCAGTGCTTTGCCTAATGGCACAGTTCAGCATATCCTCATGCCAGATGATGAGGGT
ENST00000237281         CCATCCAGTGCTTTGCCTAATGGCACAGTTCAGCATATCCTCATGCCAGATGATGAAGGT
ENSPTRT00000034492      CCATCCAGTGCTTTGCCTAATGGCACAGTTCAGCATATCCTCATGCCAGATGATGAGGGT
ENSMMUT00000030864      CCATCCAGTGCTTTGCCTAATGGCACGGTTCAGCATATCCTTATGCCAGATGATGAGGAT
ENSMUST00000129456      CCTTTCAGTGCTTTGCCAGATAGTACATTTCAGCAAATCCTCATGCCAGATGAAGACGAT
ENSRNOT00000019918      ---GGAAGTGCTTTGCCAGATAGTACCTATCAACATATCCTCATGCCGGACGAAGATGAT

ENSCJAT00000038021      GAAGGTGAATTGTGTTGGAAAAAAGTAGACTTAGGGGACTTGAAGAATATGGATGTCTTA
ENSGGOT00000005955      GAAGGTGAATTGTGTTGGAAAAAAGTAGACTTAGGGGACGTGAAGAATGTGGATGTCTTA
ENST00000237281         GAAGGTGAATTGTGTTGGAAAAAAGTAGACTTAGGGGACGTGAAGAATGTGGATGTCTTA
ENSPTRT00000034492      GAAGGTGAATTGTGTTGGAAAAAAGTAGACTTAGGGGACATGAAGAATGTGGATGTCTTA
ENSMMUT00000030864      GAAGGTGAATTGTGTTGGAAAAAAGTAGACTTAGGGGACTTGAAGAATGTGGATGTCTTA
ENSMUST00000129456      GAGAAGGACTTGTGTTGGAAAAAAGTAGACTTGGGAGACTTGAAGGATGTTAATGGTTCA
```

```
ENSRNOT00000019918      GATGAGGACTTGTGTTGGAAAAAA---GACTTAGGGGACTCGAAAGATGTTAATGGTTCA

ENSCJAT00000038021      TCTTTCAGTTCTGCTCCTTCATTCAATTTTCTTTCTAATTCATGTTGGTCTAAACCAAAG
ENSGGOT00000005955      TCTTTCAGTCATGCTCCTTCATTCAATTTTCTTTCTAATTCATGTTGGTCTAAACCAAAG
ENST00000237281         TCTTTCAGTCATGCTCCTTCATTCAATTTTCTTTCTAATTCATGTTGGTCTAAACCAAAG
ENSPTRT00000034492      TCTTTCAGTCATGCTCCTTCATTCAATTTTCTTTCTAATTCATGTTGGTCTAAACCAAAG
ENSMMUT00000030864      TCTTTCAGTCATGCTCCTTCATTCAATTTTCTTTCTAATTCATGTTGGTCTAAACCAAAG
ENSMUST00000129456      CCTTTCAGTCATGCGCCTTCATTCAAGTTTCTTTCTAATTCA---TGGTATATACCAAAG
ENSRNOT00000019918      CCTTTGAGTCACGCGACTTCATTCAAGTTTCTTTCTAATTCA---TGGTATATACCAAAG

ENSCJAT00000038021      GAAGATAAAGCAGTAGATACATCAGATTTGGAAGTTGCAGAAGATCCCATGGGCCTCCAA
ENSGGOT00000005955      GAAGATAAAGCAGTAGATACATCAGATTTGGAAGTTGCAGAAGATCCTATGGGCCTCCAA
ENST00000237281         GAAGATAAAGCAGTAGATACATCAGATTTGGAAGTTGCAGAAGATCCTATGGGCCTCCAA
ENSPTRT00000034492      GAAGATAAAGCAGTAGATACATCAGATTTGGAAGTTGCAGAAGATCCTATGGGCCTCCAA
ENSMMUT00000030864      GAAGATAAAGCAGTAGATACATCAGATTTGGAAGTTGCAGAAGATCCCATGGGCCTCCAA
ENSMUST00000129456      GAAGACAAAGCAGTTGATACATCAGATTTAGAAGTTGCAGAAGATCCCATGGGCCTCCAA
ENSRNOT00000019918      GAAGACAAAGCAGTCGATACTTCAGATTTAGAGGTTGCAGAAGATCCCATGGGCCTCCAA

ENSCJAT00000038021      GGAATAGATCTGATCACAGCAGCATTGCTATTTTGTCTAGGAGATTCTCCAGGAGGGAGG
ENSGGOT00000005955      GGAATAGATCTGATCACAGCAGCATTGCTTTTTTGTCTAGGAGATTCTCCAGGAGGGAGG
ENST00000237281         GGAATAGATCTGATCACAGCAGCATTGCTTTTTTGTCTAGGAGATTCTCCAGGAGGGAGG
ENSPTRT00000034492      GGAATAGATCTGATCACAGCAGCATTGCTTTTTTGTCTAGGAGATTCTCCAGGAGGGAGG
ENSMMUT00000030864      GGAATAGATCTGATCACAGCAGCGCTGCTGTTTTGTCTAGGAGATTCTCCAGGAGGTCGG
ENSMUST00000129456      GGAATAGATCTGATTACAGCAGCATTACTGTTTTGTCTGGGAGATTCTCCAGGTGGGAGA
ENSRNOT00000019918      GGAATAGATCTGATTACAGCAGCATTACTGTTTTGTCTGGGAGATTCTCCAGGTGGGAGG

ENSCJAT00000038021      GGTATATCTGATAGCCGCATGGTTGATGTTTATCACATTGACTTTGGGACCCAGACTTTT
ENSGGOT00000005955      GGTATATCTGATAGCCGCATGGCTGATATTTATCACATTGACGTTGGGACTCAGACTTTT
ENST00000237281         GGTATATCTGATAGCCGCATGGCTGATATTTATCACATTGACGTTGGGACTCAGACTTTT
ENSPTRT00000034492      GGTATATCTGATAGCCGCATGGCTGATATTTATCACATTGACGTTGGGACTCAGACTTTT
ENSMMUT00000030864      GGTATATCTGATAGCCGCATGGCTGATATTTATCATATTGACGTTGGGACTCAGACTTTT
ENSMUST00000129456      GGTATATCTGATAGCCGCATGACTGATGTTTATCACGTTGACTTTGGGACACAGACTTTT
ENSRNOT00000019918      GGCATATCTGATAGCCGCATGGTTGATGTTTATCACGTTGACTTTGGGACACAGACTTTT

ENSCJAT00000038021      TCACTTCCATCTGCAATATTAGCTACAAATACAATGGTTGGGGAAATAGCTTCAGCCTCA
ENSGGOT00000005955      TCACTTCCATCTGCAATATTAGCTACAAGTACAATGGTTGGGGAGATAGCTTCAGCTTCA
ENST00000237281         TCACTTCCATCTGCAATATTAGCTACAAGTACAATGGTTGGGGAGATAGCTTCAGCTTCA
ENSPTRT00000034492      TCACTTCCATCTGCAATATTAGCTACAAGTACAATGGTTGGGGAGATAGCTTCAGCTTCA
ENSMMUT00000030864      TCACTTCCATCTGCAATATTAGCTACAAATACAATGGTTGGGGAGATAGCTTCAGCTTCA
ENSMUST00000129456      TCACTTCCATCTGCAATATTAGCTACAAATACAATGGTTGGAGAAATAGCTTCGGCTTCA
ENSRNOT00000019918      TCACTTCCATCTGCAATATTAGCTACAAATACAATGGTTGGAGAAATAGCTTCAGCTTCA

ENSCJAT00000038021      GCTTGTGATCATGCCAATCCACAGCTTTCAAATCCAAGTCCATTTCAGACACTTGGGCTG
ENSGGOT00000005955      GCTTGTGATCATGCCAATCCACAGCTTTCAAATCCAAGTCCGTTTCAGACACTTGGGCTG
ENST00000237281         GCTTGTGATCATGCCAATCCACAGCTTTCAAATCCAAGTCCGTTTCAGACACTTGGGCTG
ENSPTRT00000034492      GCTTGTGATCATGCCAACCCACAGCTTTCAAATCCAAGTCCGTTTCAGACACTTGGGCTG
ENSMMUT00000030864      GCTTGTGATCATGCCAATCCACAGCTTTCAAATCCAAGTCCATTTCAGACACTTGGGCTG
ENSMUST00000129456      GCTTGTGATCATGCCAATCCACAGCTTTCAAATCCAAGCCCTTTTCAGACACTTGGGCTG
ENSRNOT00000019918      GCTTGTGATCATGCCAATCCACAGCTTTCAAATCCAAGCCCTTTTCAGACACTTGGGCTG

ENSCJAT00000038021      GATTTAGTATTGGAATGTGTCGCTAGATACCAACCCAAGCAGCGTTCAATGTTTACCTTT
ENSGGOT00000005955      GATTTAGTATTGGAATGTGTCGCTAGGTACCAACCCAAGCAGCGTTCAATGTTTACCTTT
ENST00000237281         GATTTAGTATTGGAATGTGTCGCTAGGTACCAACCCAAGCAGCGTTCAATGTTTACCTTT
```

ENSPTRT00000034492 GATTTAGTATTGGAATGTGTCGCTAGGTACCAACCCAAGCAGCGTTCAATGTTTACCTTT
ENSMMUT00000030864 GATTTAGTATTGGAATGTGTCGCTAGGTACCAACCCAAGCAGCGTTCAATGTTTACCTTT
ENSMUST00000129456 GACTTAGTTTTGGAATGTGTTGCTAGGTACCAGCCTAAGCAGCGTTCAATGTTTACCTTT
ENSRNOT00000019918 GACTTAGTTTTGGAATGTGTTGCTAGGTACCAGCCTAAGCAGCGTTCAATGTTTACCTTT

ENSCJAT00000038021 GTGTGTGGACAGTTATTTAGAAGGAAAGAATTTTCTTCTCATTTTAAGAATGTGCATGGT
ENSGGOT00000005955 GTGTGTGGACAGTTATTTAGAAGGAAAGAATTTTCTTCCCACTTTAAGAATGTGCATGGT
ENST00000237281 GTGTGTGGACAGTTATTTAGAAGGAAAGAATTTTCTTCCCACTTTAAGAATGTGCATGGT
ENSPTRT00000034492 GTGTGTGGACAGTTATTTAGAAGGAAAGAATTTTCTTCCCACTTTAAGAATGTGCATGGT
ENSMMUT00000030864 GTGTGTGGACAGTTATTTAGAAGGAAAGAATTTTCTTCCCATTTTAAGAATGTGCATGGT
ENSMUST00000129456 GTATGCGGACAGCTATTTAGAAGAAAAGAATTTTCTTCCCATTTTAAGAATGTGCATGGT
ENSRNOT00000019918 GTATGCGGACAGTTATTTAGAAGAAAAGAATTTTCTTCCCATTTTAAGAACGTGCATGGT

ENSCJAT00000038021 GATATTCATGCTGGACTCAATGGCTGGATGGAACAGAGGTGCCCTTTAGCATACTATGGT
ENSGGOT00000005955 GACATTCATGCTGGACTCAATGGCTGGATGGAACAGAGGTGCCCTTTAGCTTACTATGGT
ENST00000237281 GACATTCATGCTGGACTCAATGGCTGGATGGAACAGAGGTGCCCTTTAGCTTACTATGGT
ENSPTRT00000034492 GACATTCATGCTGGACTCAATGGCTGGATGGAACAGAGGTGCCCTTTAGCTTACTATGGT
ENSMMUT00000030864 GACATTCATGCTGGACTCAATGGCTGGATGGAACAGAGGTGCCCTTTAGCTTACTATGGT
ENSMUST00000129456 GACATTCATGCTGGACTCAATGGATGGATGGAACAGAGGTGTCCTTTAGCTTATTATGGT
ENSRNOT00000019918 GACATTCACGCTGGACTCAATGGATGGATGGAGCAGAGGTGTCCTTTAGCTTATTATGGT

ENSCJAT00000038021 TGTACCTATTCTCAGCGTAGATTTTGTCCATCAATACAAGGAGCGAAGATTATCCATGAC
ENSGGOT00000005955 TGTACCTATTCTCAGCGTAGATTTTGTCCATCAATACAAGGAGCAAAGATTATACATGAC
ENST00000237281 TGTACCTATTCTCAGCGTAGATTTTGTCCATCAATACAAGGAGCAAAGATTATACATGAC
ENSPTRT00000034492 TGTACCTATTCTCAGCGTAGATTTTGTCCATCAATACAAGGAGCAAAGATTATACATGAC
ENSMMUT00000030864 TGTACCTATTCTCAGCGTAGATTTTGTCCATCAATACAAGGAGCGAAGATTATACATGAT
ENSMUST00000129456 TGTACCTATTCTCAGCGTAGATTTTGCCCATCGACACAAGGAGCAAAGATCATACATGAT
ENSRNOT00000019918 TGTACGTATTCTCAGCGTAGATTTTGCCCTTCAACACAAGGAGCAAAGATCATACATGAC

ENSCJAT00000038021 CGCCATCTGAGGTCATTTGGAGTTCAGCCATGTGTATCTACAGTATTAGTAGAGCCTGCT
ENSGGOT00000005955 CGCCATTTGAGGTCGTTTGGAGTTCAGCCATGTGTATCTACAGTATTAGTGGAGCCTGCT
ENST00000237281 CGCCATTTGAGGTCATTTGGAGTTCAGCCATGTGTATCTACAGTATTAGTGGAGCCTGCT
ENSPTRT00000034492 CGCCATTTGAGGTCATTTGGAGTTCAGCCATGTGTATCTACAGTATTAGTGGAGCCTGCT
ENSMMUT00000030864 CGCCATTTGCGGTCATTTGGAGTTCAGCCATGTGTATCTACAGTATTAGTGGAGCCTGCT
ENSMUST00000129456 CGCCATTTAAGGTCATTTGGAGTTCAGCCATGTGTATCTACAGTATTAGAAGAACCTTCT
ENSRNOT00000019918 CGCCATTTAAGGTCATTTGGAGTTCAGCCATGTGTATCTACAGCATTAGAAGAGCCTTCT

ENSCJAT00000038021 AGAAACTGTGTATTGGGATTACATAGTGACCATCTAAGTAGTCTTCCCTTTGAGGTCTTG
ENSGGOT00000005955 AGAAACTGTGTGTTAGGATTACATAATGACCATCTAAGTAGTCTTCCTTTTGAGGTCCTG
ENST00000237281 AGAAACTGTGTGTTGGGATTACATAATGACCATCTAAGTAGTCTTCCTTTTGAGGTCCTG
ENSPTRT00000034492 AGAAACTGTGTGTTGGGATTACATAATGACCATCTAAGTAGTCTTCCTTTTGAGGTCCTG
ENSMMUT00000030864 AGAAACTGTGTGTTGGGATTACATAATGACCATCTAAGTAGTCTTCCTTTTGAGGTCCTG
ENSMUST00000129456 AGAAACTGTGTGTTGGGATTGCGTAGTGACCATCTAAGTAGTCTCCCTTTTGAGGTACTC
ENSRNOT00000019918 AGAAACTGTGTGTTGGGATTACGTAGTGACCATCTAAGTAGTCTCCCTTTTGAGGTACTC

ENSCJAT00000038021 CAGCATATTGCAGGCTTTCTTGATGGCTTCAGTTTATGCCAGCTCTCATGTGTATCCAAG
ENSGGOT00000005955 CAGCATATTGCAGGCTTTCTCGATGGCTTCAGCTTATGCCAGCTCTCATGTGTATCCAAG
ENST00000237281 CAGCATATTGCAGGCTTTCTCGATGGCTTCAGCTTATGTCAGCTCTCATGTGTATCCAAG
ENSPTRT00000034492 CAGCATATTGCAGGCTTTCTCGATGGCTTCAGCTTATGCCAGCTCTCATGTGTATCCAAG
ENSMMUT00000030864 CAGCATATTGCAGGCTTTCTTGATGGCTTCAGTTTATGCCAGCTCTCATGTGTATCCAAG
ENSMUST00000129456 CAACATATTGCAGGCTTCCTTGACGGCTTCAGTTTATGCCAGCTTGCATGTGTATCCCGG
ENSRNOT00000019918 CAACATATTGCAGGCTTCCTTGATGGCTTCAGTTTATGCCAGCTTGCATGTGTATCCAGG

```
ENSCJAT00000038021     TTAATGAGGGATGTGTGTGGCAGCCTGCTTCAGTCTCGTGGCATGGTCATACTGCAGTGG
ENSGGOT00000005955     TTAATGAGGGATGTGTGTGGCAGCCTGCTTCAGTCTCGTGGCATGGTCATACTGCAGTGG
ENST00000237281        TTAATGAGGGATGTGTGTGGCAGCCTGCTTCAGTCTCGTGGCATGGTCATACTGCAGTGG
ENSPTRT00000034492     TTAATGAGGGATGTGTGTGGCAGCCTGCTTCAGTCTCGTGGCATGGTCATACTGCAGTGG
ENSMMUT00000030864     TTAATGAGGGATGTGTGTGGCAGCCTGCTTCAGTCTCGTGGCATGGTCATACTGCAGTGG
ENSMUST00000129456     CTAATGAGGGACGTTTGTGGCAGTTTACTTCAGTCTCGTGGAATGGTAATCTTGCAGTGG
ENSRNOT00000019918     CTAATGAGGGACATTTGTGGCAGTTTACTTCAGTCTCGTGGAATGGTAATATTGCAGTGG

ENSCJAT00000038021     GGGAAAAGGAAGTATCCAGAAGGAAATTCATCATGGCAGATAAAAGAAAAGGTATGGCGA
ENSGGOT00000005955     GGGAAAAGGAAGTATCCAGAAGGAAATTCATCATGGCAGATAAAAGAAAAGGTATGGCGA
ENST00000237281        GGGAAAAGGAAGTATCCAGAAGGAAATTCATCATGGCAGATAAAAGAAAAGGTATGGCGA
ENSPTRT00000034492     GGGAAAAGGAAGTATCCAGAAGGAAATTCATCATGGCAGATAAAAGAAAAGGTATGGCGA
ENSMMUT00000030864     GGGAAAAGGAAGTATCCAGAAGGAAATTCATCATGGCAGATAAAAGAAAAGGTATGGCGA
ENSMUST00000129456     GGGAAGAAGAAGTATCCGGAAGGAAATTCATCATGGCAGATAAAAGAGAAGGTATGGCGA
ENSRNOT00000019918     GGGAAAAAGAAGTATCCAGAAGGAAATTCATCCTGGCAGATAAAAGAAAAGGTGTGGCGA

ENSCJAT00000038021     TTTAGTACTGCATTTTGTTCTGTTAATGAATGGAGATTTGCTGACATCCTAAGCATGGCT
ENSGGOT00000005955     TTTAGTACTGCATTTTGTTCTGTTAATGAATGGAAATTTGCTGACATCCTAAGCATGGCA
ENST00000237281        TTTAGTACTGCATTTTGTTCTGTTAATGAATGGAAATTTGCTGACATCCTAAGCATGGCA
ENSPTRT00000034492     TTTAGTACTGCATTTTGTTCTGTTAATGAATGGAAATTTGCTGACATCCTAAGCATGGCA
ENSMMUT00000030864     TTTAGTACTGCATTTTGTTCTGTTAATGAATGGAAATTTGCTGACATCCTAAGCATGGCC
ENSMUST00000129456     TTCAGTACTGCATTTTGTTCTGTTAATGACTGGAAATTTGCTGACATCCTGAGCATGGCT
ENSRNOT00000019918     TTCAGTACTGCATTTTGTTCTGTTAATGACTGGAAGTTTGCAGACATCCTGAGCATGGCT

ENSCJAT00000038021     GACCACTTGAAGAAATGCAGTTATAATGTTGTCGAGAAACGGGAGGAAGCAATCCCATTG
ENSGGOT00000005955     GACCACTTGAAGAAATGCAGTTACAATGTTGTCGAGAAACGGGAGGAAGCAATCCCTTTG
ENST00000237281        GACCACTTGAAGAAATGCAGTTACAATGTTGTCGAGAAACGGGAGGAAGCAATCCCTTTG
ENSPTRT00000034492     GACCACTTGAAGAAATGCAGTTACAATGTTGTCGAGAAACGAGAGGAAGCAATCCCTTTG
ENSMMUT00000030864     GACCACTTGAAGAAATGCAGTTACAATGTTGTCGAGAAACGGGAGGAAGCAATCCCTTTG
ENSMUST00000129456     GACCACTTGAAGAACTGCAGTTACAACGTGATAGAGAAACGGGAGGAAGCCATCCCATTG
ENSRNOT00000019918     GACCACTTGAAGAAATGCAGTTACAATGTTATAGAGAAGCGGGAGGAAGCAATCCCATTG

ENSCJAT00000038021     CCATGTATGTGTGTGACACGAGAACTCACTAAAGAAGGACGTTCACTACGCTCAGTTTTA
ENSGGOT00000005955     CCATGTATGTGTGTGACACGAGAACTCACTAAAGAAGGACGTTCACTACGCTCAGTTTTA
ENST00000237281        CCATGTATGTGTGTGACACGAGAACTCACTAAAGAAGGACGTTCACTACGCTCAGTTTTA
ENSPTRT00000034492     CCATGTATGTGTGTGACACGAGAACTCACTAAAGAAGGACGTTCACTACGCTCAGTTTTA
ENSMMUT00000030864     CCATGTATGTGTGTGACACGAGAACTCACTAAAGAAGGACGTTCACTACGCTCAGTTTTA
ENSMUST00000129456     CCGTGTATGTGTGTGACTCGAGAGCTTACAAAAGAAGGACGTTCCCTGCGCTCAGTTTTA
ENSRNOT00000019918     CCATGTATGTGCGTGACTCGAGAACTCACAAAAGAAGGACGTTCCCTGCGCTCAGTTTTA

ENSCJAT00000038021     AAACCTGTACTT
ENSGGOT00000005955     AAACCTGTACTT
ENST00000237281        AAACCTGTACTT
ENSPTRT00000034492     AAACCTGTACTT
ENSMMUT00000030864     AAACCTGTACTT
ENSMUST00000129456     AAGCCTGTGCTT
ENSRNOT00000019918     AAGCCCGTACTG
```

Multiple sequence alignment of Fbxo31

```
ENSMUST00000034268     ATGGCGGTGTGTGCTCGGCTCTGCGGCGTGGGCCCCGCGCGTGGGTGCCGCCGCCGCCAG
ENSCJAT00000008607     ATGGCGGTGTGCGCTCGCCTCTGCGGCGTGGGCCCGTCGCGCGGATGCCGGCGCCGCCAG
ENSMMUT00000024762     ATGGCGGTGTGCGCTCGCCTCTGCGGCGTGGGCCCGTCTCGCGGATGCCGGCGCCGCCAG
ENSPTRT00000050728     ATGGCGGTGTGTGCTCGCCTTTGCGGCGTGGGTCCGTCGCGCGGATGTCGGCGCCGCCAG
```

ENSPPYT00000008952 ATGGCGGTGTGCGCTCGCCTCTGCGGCGTGGGCCCGTCGCGCGGATGCCGGCGCCGCCAG
ENSGGOT00000009426 ATGGCGGTGTGTGCTCGCCTTTGCGGCGTGGGCCCGTCGCGCGGATGCCGGCGCCGCCAG
ENST00000311635 ATGGCGGTGTGTGCTCGCCTTTGCGGCGTGGGCCCGTCGCGCGGATGTCGGCGCCGCCAG

ENSMUST00000034268 CAGCGCCGCGGCCCGGCCGAGACTGCGGCGGCGGACAGTGAGGCGGACACGGACCCCGAG
ENSCJAT00000008607 CAACGGCGGGGCCCGGCCGAGACGGCGGCGGCCGATAGCGAGCCGGACACCGATCCCGAG
ENSMMUT00000024762 CAGCGCCGGGGCCCGGCCGAGACGGCAGCGGCCGACAGCGAGCCGGACACAGACCCCGAG
ENSPTRT00000050728 CAGCGCCGGGGCCCGGCCGAGACGGCGGCAGCCGACAGCGAGCCGGACACAGACCCCGAG
ENSPPYT00000008952 CAGCGCCGGGGCCCGGCCGAGACGGCGGCGGCCGACAGCGAGCCGGACACAGACCCGGAG
ENSGGOT00000009426 CAGCGCCGGGGCCCGGCCGAGACGGCGGCGGCCGACAGCGAGCCGGACACAGACCCCGAG
ENST00000311635 CAGCGCCGGGGCCCGGCCGAGACGGCGGCGGCCGACAGCGAGCCGGACACAGACCCCGAG

ENSMUST00000034268 GAGGAGCGCATCGAGGCGGGG---------------------------------------
ENSCJAT00000008607 GAAGAGCGCATCGAGGCGGGCGCGGCGGTGCTGGCCGGGGTGGGGGGCGGCATGTGCCCC
ENSMMUT00000024762 GAGGAGCGCATCGAGGCGAGCGCGGCGGTGCTGGCCGGGGTAGGGGGCGGCTTGTGCGCG
ENSPTRT00000050728 GAGGAGCGCATCGAGGCTAGCGCCGCGGTGTTGGCCGGGGTGGGGGGCGGCTTGTGCGCG
ENSPPYT00000008952 GAGGAGCGCATCGAGGCTAGCGCTGCGGTGCTGGCCGGGGTGGGGGGCGGCTTGTGCGCG
ENSGGOT00000009426 GAGGAGCGCATCGAGGCTAGCGCCGCGGTGTTGGCCGGGGTGGGGGGCGGCTTGTGCGCG
ENST00000311635 GAGGAGCGCATCGAGGCTAGC------------GCCGGGGTCGGGGGCGGCTTGTGCGCG

ENSMUST00000034268 ---------------CCGGCGCGTTGCTCTCTGCTGGAGCTCCCGCCTGAGCTGCTCGTG
ENSCJAT00000008607 GGCCCCTCACCGTCGCCCCCGCGCTGCTCGCTGCTGGAGCTGCCGCCCGAGCTGCTGGTG
ENSMMUT00000024762 GGCCCCTCGTCGCCGCCCCCGCGCTGCTCGCTGCTGGAGCTGCCGCCGGAGCTGCTGGTG
ENSPTRT00000050728 GGCCCCTCGCCGCCGCCCCCGCGCTGCTCGCTGCTGGAGCTGCCGCCCGAGCTGCTGGTG
ENSPPYT00000008952 GGCCCCTCGCCGCCGCCCCCGCGCTGCTCGCTGCTGGAGCTGCCGCCCGAGCTGCTGGTG
ENSGGOT00000009426 GGCCCCTCGCCGCCGCCCCCGCGCTGCTCGCTGCTGGAGCTGCCGCCCGAGCTGCTGGTG
ENST00000311635 GGCCCCTCGCCGCCGCCCCCGCGCTGCTCGCTGCTGGAGCTGCCGCCCGAGCTGCTGGTG

ENSMUST00000034268 GAGATCTTCGCGTCGCTGCCCGGCACCGACCTGCCCAGCCTGGCTCAGGTCTGCAGCAGG
ENSCJAT00000008607 GAGATCTTCGCGTCGCTGCCCGGCACAGACCTGCCCAGCCTGGCCCAGGTCTGCACCAAG
ENSMMUT00000024762 GAGATCTTCGCGTCGCTGCCGGGCACCGACCTACCCAGCTTGGCCCAGGTCTGCACGAAG
ENSPTRT00000050728 GAGATCTTCGCGTCGCTGCCGGGCACGGACCTACCCAGCTTGGCCCAGGTCTGCACGAAG
ENSPPYT00000008952 GAGATCTTCGCGTCGCTGCCGGGCACCGACCTACCCAGCTTGGCCCAGGTCTGCACGAAG
ENSGGOT00000009426 GAGATCTTCGCGTCGCTGCCGGGCACGGACCTACCCAGCTTGGCCCAGGTCTGCACGAAG
ENST00000311635 GAGATCTTCGCGTCGCTGCCGGGCACGGACCTACCCAGCTTGGCCCAGGTCTGCACGAAG

ENSMUST00000034268 TTCCGCCGAATCTTGCACACGGACACCATCTGGAGACGGCGCTGCCGCGAGGAGTATGGC
ENSCJAT00000008607 TTCCGGCGCATCCTCCACACCGACACCATCTGGAGGAGGCGCTGCCGCGAAGAGTACGGT
ENSMMUT00000024762 TTCCGGCGCATCCTGCACACCGACACCATCTGGAGGAGGCGTTGCCGCGAGGAGTACGGT
ENSPTRT00000050728 TTCCGGCGCATCCTCCACACCGACACCATCTGGAGGAGGCGTTGCCGTCAGAAGTATGGT
ENSPPYT00000008952 TTCCGGCACATCCTCCACACCGACACCATCTGGAGGAGGCGTTGCCGCGAGGAGTATGGT
ENSGGOT00000009426 TTCCGGCGCATCCTCCACACCGACACCATCTGGAGGAGGCGTTGCCGTGAGGAGTATGGT
ENST00000311635 TTCCGGCGCATCCTCCACACCGACACCATCTGGAGGAGGCGTTGCCGTGAGGAGTATGGT

ENSMUST00000034268 GTTTGTGAGAACCTGCGGAAGCTGGAGATCACAGGTGTGTCTTGCCGGGACGTCTATGCA
ENSCJAT00000008607 GTTTGTGAAAACTTGCGGAAGCTGGAGATCACAGGCGTGTCCTGTCGGGACGTCTACGCG
ENSMMUT00000024762 GTTTGTGAAAACTTGCGGAAGTTGGAGATCACAGGCGTGTCTTGTCGGGACGTCTACGCG
ENSPTRT00000050728 GTTTGCGAAAACTTGCGGAAGCTGGAGATCACAGGCGTGTCTTGTCGGGACGTCTATGCG
ENSPPYT00000008952 GTTTGCGAAAACTTGCGGAAGCTGGAGATCACAGGCGTGTCTTGTCGGGACGTCTATGCG
ENSGGOT00000009426 GTTTGCGAAAACTTGCGGAAGCTGGAGATCACAGGCGTGTCTTGTCGGGACGTCTATGCG
ENST00000311635 GTTTGCGAAAACTTGCGGAAGCTGGAGATCACAGGCGTGTCTTGTCGGGACGTCTATGCG

ENSMUST00000034268 AAACTGCTTCACCGATACAGACACATTTTGGGGCTGTGGCAGCCAGATATCGGGCCGTAC

ENSCJAT00000008607 AAGTTGCTTCACCGATACAGACACATTTTGGGATTGTGGCAGCCAGATATCGGGCCATAT
ENSMMUT00000024762 AAGCTGCTTCACCGATACAGACACATTTTGGGATTGTGGCAGCCAGATATCGGGCCGTAT
ENSPTRT00000050728 AAGCTGCTTCACCGATATAGACACATTTTGGGATTGTGGCAGCCAGATATCGGGCCATAC
ENSPPYT00000008952 AAGCTGCTTCACCGATATAGACACATTTTGGGATTGTGGCAGCCAGATATCGGGCCATAC
ENSGGOT00000009426 AAGCTGCTTCACCGATATAGACACATTTTGGGATTGTGGCAGCCAGATATCGGGCCATAC
ENST00000311635 AAGCTGCTTCACCGATATAGACACATTTTGGGATTGTGGCAGCCAGATATCGGGCCATAC

ENSMUST00000034268 GGAGGATTGCTGAACGTCGTGGTGGACGGACTGTTCATCATTGGCTGGATGTACCTGCCA
ENSCJAT00000008607 GGAGGACTGCTGAACGTGGTGGTGGACGGCTTGTTCATCATTGGGTGGATGTACCTGCCT
ENSMMUT00000024762 GGAGGACTGCTGAACGTGGTGGTGGATGGTTTGTTCATCATCGGGTGGATGTACCTGCCT
ENSPTRT00000050728 GGAGGACTGCTGAACGTGGTGGTGGACGGCCTGTTCATCATCGGGTGGATGTACCTGCCT
ENSPPYT00000008952 GGAGGACTGCTGAACGTGGTGGTGGATGGCTTGTTCATCATCGGGTGGATGTACCTGCCT
ENSGGOT00000009426 GGAGGACTGCTGAACGTGGTGGTGGATGGCCTGTTCATCATCGGGTGGATGTACCTGCCT
ENST00000311635 GGAGGACTGCTGAACGTGGTGGTGGACGGCCTGTTCATCATCGGGTGGATGTACCTGCCT

ENSMUST00000034268 CCTCATGACCCCCACGTGGGAGACCCCATGCGGTTCAAGCCACTGTTTAGAATCCATCTG
ENSCJAT00000008607 CCCCACGACCCCCACGTCAATGACCCCATGAGATTCAAGCCTCTGTTCAGGATTCACCTG
ENSMMUT00000024762 CCCCATGACCCCCACGTCGATGACCCCATGAGATTCAAGCCTCTGTTCAGGATCCACCTG
ENSPTRT00000050728 CCCCATGACCCCCACGTCGATGACCCCATGAGATTCAAGCCTCTGTTCAGGATTCACCTG
ENSPPYT00000008952 CCCCATGACCCCCACGTCGATGACCCCATGAGATTCAAGCCTCTGTTCAGGATCCACCTG
ENSGGOT00000009426 CCCCATGACCCCCATGTCGATGACCCCATGAGATTCAAGCCTCTGTTCAGGATCCATCTG
ENST00000311635 CCCCATGACCCCCACGTCGATGACCCTATGAGATTCAAGCCTCTGTTCAGGATCCACCTG

ENSMUST00000034268 ATGGAGAGGAAGTCGGCTACAGTGGAGTGTATGTACGGCCACAAAGGGCCCCACAACGGC
ENSCJAT00000008607 ATGGAGAGGAAGGCTGCGACGGTGGAGTGCATGTATGGCCACAAAGGACCCCACCACGGC
ENSMMUT00000024762 ATGGAGAGGAAGGCCGCCACAGTGGAGTGCATGTACGGCCACAAAGGACCCCACCACGGC
ENSPTRT00000050728 ATGGAGAGGAAGGCTGCCACAGTGGAGTGCATGTACGGCCACAAAGGGCCCCACCACGGC
ENSPPYT00000008952 ATGGAGAGGAAGGCTGCCACAGTGGAGTGCATGTACGGCCACAAAGGGCCCCACCATGGC
ENSGGOT00000009426 ATGGAGAGGAAGGCTGCCACAGTGGAGTGCATGTACGGCCACAAAGGGCCCCACCACGGC
ENST00000311635 ATGGAGAGGAAGGCTGCCACAGTGGAGTGCATGTACGGCCACAAAGGGCCCCACCACGGC

ENSMUST00000034268 CACATCCAGATTGTGAAGAGGGACGAGTTCTCCACCAAGTGTAACCAGACAGATCACCAC
ENSCJAT00000008607 CACATCCAGATTGTGAAGAAGGATGAGTTCTCCACCAAGTGCAACCAGACGGACCACCAC
ENSMMUT00000024762 CACATCCAGATTGTGAAGAAGGATGAGTTCTCCACCAAGTGCAACCAGACGGACCACCAC
ENSPTRT00000050728 CACATCCAGATTGTGAAGAAGGATGAGTTCTCCACCAAGTGCAACCAGACGGACCACCAC
ENSPPYT00000008952 CACATCCAGATTGTGAAGAAGGATGAGTTCTCCACCAAGTGCAACCAGACGGACCACCAT
ENSGGOT00000009426 CACATCCAGATTGTGAAGAAGGATGAGTTCTCCACCAAGTGCAACCAGACGGACCACCAC
ENST00000311635 CACATCCAGATTGTGAAGAAGGATGAGTTCTCCACCAAGTGCAACCAGACGGACCACCAC

ENSMUST00000034268 AGGATGTCCGGTGGGAGGCAGGAGGAGTTTCGGACGTGGCTGAGGGAGGAGTGGGGCCGC
ENSCJAT00000008607 AGGATGTCTGGCGGGAGGCAGGAGGAATTTCGGACATGGCTGAGGGAGGAATGGGGGCGC
ENSMMUT00000024762 AGGATGTCCGGCGGGAGGCAGGAGGAGTTTCGGACGTGGCTGAGGGAGGAATGGGGGCGC
ENSPTRT00000050728 AGGATGTCCGGCGGGAGGCAGGAGGAGTTTCGGACGTGGCTGAGGGAGGAATGGGGGCGC
ENSPPYT00000008952 AGGATGTCTGGCGGGAGGCAGGAGGAGTTTCGGACGTGGCTGAGGGAGGAATGGGGGCGC
ENSGGOT00000009426 AGGATGTCCGGCGGGAGGCAGGAGGAGTTTCGGACGTGGCTGAGGGAGGAATGGGGGCGC
ENST00000311635 AGGATGTCCGGCGGGAGGCAGGAGGAGTTTCGGACGTGGCTGAGGGAGGAATGGGGGCGC

ENSMUST00000034268 ACGCTGGAAGACATCTTCCACGAGCACATGCAGGAGCTGATTCTGATGAAGTTCATCTAC
ENSCJAT00000008607 ACGCTGGAGGACATCTTCCATGAGCACATGCAGGAGCTCATCCTGATGAAGTTCATCTAC
ENSMMUT00000024762 ACGCTGGAGGACATCTTCCACGAGCACATGCAGGAGCTCATCCTGATGAAGTTCATCTAC
ENSPTRT00000050728 ACGCTGGAGGACATTTTCCACGAGCACATGCAGGAGCTAATCCTGATGAAGTTCATCTAC
ENSPPYT00000008952 ACACTGGAGGACATCTTCCACGAGCACATGCAGGAGCTCATCCTGATGAAGTTCATCTAC
ENSGGOT00000009426 ACGCTGGAGGACATCTTCCACGAGCACATGCAGGAGCTCATCCTGATGAAGTTTATCTAC
ENST00000311635 ACGCTGGAGGACATCTTCCACGAGCACATGCAGGAGCTCATCCTGATGAAGTTCATCTAC

ENSMUST00000034268 ACCAGTCAGTACGACAACTGCCTGACCTACCGACGGATCTACCTCCCGCCCAGCCACCCT
ENSCJAT00000008607 ACCAGTCAGTACGACAACTGCCTGACCTACCGCCGTATCTACCTGCCACCAAGCCGCCCT
ENSMMUT00000024762 ACCAGTCAGTACGACAACTGCCTGACCTACCGCCGCATCTACTTGCCGCCCAGCCGCCCC
ENSPTRT00000050728 ACCAGTCAGTACGACAACTGCCTGACCTACCGCCGCATCTACCTGCCGCCCAGCCACCCC
ENSPPYT00000008952 ACCAGTCAGTACGACAACTGCCTGACCTACCGCCGCATCTACCTGCCGCCGAGCCGCCCC
ENSGGOT00000009426 ACCAGTCAGTACGACAACTGCCTGACCTACCGTCGCATCTACCTGCCGCCCAGCCGCCCC
ENST00000311635 ACCAGTCAGTACGACAACTGCCTGACCTACCGCCGCATCTACCTGCCGCCCAGCCGCCCC

ENSMUST00000034268 GACGACCTCATCAAGCCCGGCCTCTTCAAGGGCACCTATGGCAGCCACGGGCTGGAGATT
ENSCJAT00000008607 GATGACCTCATCAAGCCAGGCCTCTTCAAAGGCACCTATGGCAGCCATGGCCTGGAGATT
ENSMMUT00000024762 GACGACCTCATCAAGCCTGGCCTCTTCAAAGGTACCTATGGCAGCCACGGCCTGGAGATC
ENSPTRT00000050728 GACGACCTCATCAAGCCTGGCCTCTTCAAAGGTACCTATGGCAGCCACGGCCTGGAGATC
ENSPPYT00000008952 GACGACCTCATCAAGCCTGGCCTCTTCAAAGGTACCTATGGCAGCCACGGCCTGGAGATC
ENSGGOT00000009426 GACGACCTCATCAAGCCTGGCCTCTTCAAAGGTACCTATGGCAGCCACGGCCTGGAGATT
ENST00000311635 GACGACCTCATCAAGCCTGGCCTCTTCAAAGGTACCTATGGCAGCCACGGCCTGGAGATT

ENSMUST00000034268 GTGATGCTCAGCTTCCACGGCTCACGCGCCAGGGGCACCAAGATCACGGGCGACCCCAAC
ENSCJAT00000008607 GTGATGCTCAGCTTCCACGGCCGGCGTGCCAGGGGCACCAAGATCACGGGTGACCCCAAC
ENSMMUT00000024762 GTGATGCTCAGCTTCCACGGCCGGCGTGCCAGGGGCACCAAGATCACGGGCGACCCCAAC
ENSPTRT00000050728 GTGATGCTCAGCTTCCACGGCCGGCGTGCCAGGGGCACCAAGATCACGGGCGACCCCAAC
ENSPPYT00000008952 GTGATGCTCAGCTTCCACGGCCGGCGTGCCAGGGGCACCAAGATCACGGGCGACCCCAAC
ENSGGOT00000009426 GTGATGCTCAGCTTCCACGGCCGGCGTGCCAGGGGCACCAAGATCACGGGTGACCCCAAC
ENST00000311635 GTGATGCTCAGCTTCCACGGCCGGCGTGCCAGGGGCACCAAGATCACGGGCGACCCCAAC

ENSMUST00000034268 ATCCCCGCGGGGCAGCAGACTGTAGAGATTGACCTGCAGCGCCGCATCCAGCTGCCGGAC
ENSCJAT00000008607 ATCCCCGCTGGGCAGCAGACAGTAGAGATCGACTTGAGACACCAGATCCAGCTGCCTGAC
ENSMMUT00000024762 ATCCCTGCCGGGCAGCAGACGGTGGAGATCGACCTGAGGCACCGGATTCAGCTGCCCGAC
ENSPTRT00000050728 ATCCCCGCTGGGCAGCAGACAGTGGAGATCGACCTGAGGCATCGGATCCAGCTGCCCGAC
ENSPPYT00000008952 ATCCCCGCCGGGCAGCAGACGGTGGAGATCGACCTGAGGCACCGGATCCAGCTGCCCGAC
ENSGGOT00000009426 ATCCCCGCTGGGCAGCAGACAGTGGAGATCGACCTGAGGCATCGGATCCAGCTGCCCGAC
ENST00000311635 ATCCCCGCTGGGCAGCAGACAGTGGAGATCGACCTGAGGCATCGGATCCAGCTGCCCGAC

ENSMUST00000034268 GTGGAGAACCTCCGAAACTTCAACGAGCTCTCCAGGATTGTCCTGGAGGTCCGGGAGCAG
ENSCJAT00000008607 CTCGAAAACCAGCGCAACTTCAACGAGCTGTCCCGCATCGTCCTGGAGGTGCGAGAGCGG
ENSMMUT00000024762 CTCGAGAACCAGCGCAACTTCAACGAGCTCTCCCGCCTTGTCCTGGAGGTGCGCGAGCGG
ENSPTRT00000050728 CTCGAGAACCAGCGCAACTTCAATGAGCTCTCCCGCATCGTCCTGGAGGTGCGCGAGAGG
ENSPPYT00000008952 CTCGAGAACCAGCGCAACTTCAATGAGCTCTCCCGCATTGTCCTGGAGGTGCGCGAGCGG
ENSGGOT00000009426 CTCGAGAACCAGCGCAACTTCAATGAGCTCTCCCGCATCGTCCTGGAGGTGCGCGAGAGG
ENST00000311635 CTCGAGAACCAGCGCAACTTCAATGAGCTCTCCCGCATCGTCCTGGAGGTGCGCGAGAGG

ENSMUST00000034268 GTGCGGCAGGAG---------------CAGGAGGCCGGCGAGGGC---------------
ENSCJAT00000008607 GTGCGCCAGGAGCAGCAGGAAGGCGGGTATGAGGCAGGAGAGGGACGTGGCCGACAGGGC
ENSMMUT00000024762 GTGCGCCAGGAGCAGCAGGAAGGCGGGCACGAGGCGGGCGAGGGTCGCGGCCAGCAGGGA
ENSPTRT00000050728 GTGCGCCAGGAGCAGCAGGAAGGCGGGCACGAGGCGGGCGAGGGTCGTGGCCGGCAGGGC
ENSPPYT00000008952 GTGCGCCAGGAGCAGCAGGAAGGCGGGCACGAGGCGGGCGAGGGTCGCGGCCGGCAGGGC
ENSGGOT00000009426 GTGCGCCAGGAGCAGCAGGAAGGCGGGCACGAGGCGGGCGAGGGTCGTGGCCGGCAGGGC
ENST00000311635 GTGCGCCAGGAGCAGCAGGAAGGCGGGCACGAGGCGGGCGAGGGTCGTGGCCGGCAGGGC

ENSMUST00000034268 ------------------------GCCGCGCCACCCCGGGAGCCTTCAGCCAAGGCCGCT
ENSCJAT00000008607 CTCCGGGAGTCCCAGCCAAGTCCTGCTCAGCCCAGGGCAGAGGCACCCAGCAGGGGCCCA
ENSMMUT00000024762 CCCCGGGAGTCCCAGCCAAGCCCTGCCCAGCCCAGGGTGGAGGCGCCCAGCAGGGGCCCA
ENSPTRT00000050728 CCCCGGGAGTCCCAGCCAAGCCCTGCCCAGCCCAGGGCAGAGGCGCCCAGCAAGGGCCCA

```
ENSPPYT00000008952     CCCCGGGAGTCCCAGCCAAGCCCCGCTCAGCCCAGGGCAGAGGCGCCCAGCAAGGGCCCA
ENSGGOT00000009426     CCCCGGGAGTCCCAGCCAAGCCCTGCCCAGCCCAGGGCAGAGGCGCCCAGCAAGGGCCCC
ENST00000311635        CCCCGGGAGTCCCAGCCAAGCCCTGCCCAGCCCAGGGCAGAGGCGCCCAGCAAGGGCCCA

ENSMUST00000034268     GATGGGCCACCTGCTAAGGACGGCAAAGAGCCTGGAGGTGGAGCCGAGGCAGCTGAGCAG
ENSCJAT00000008607     GACGGGACACCTGCTGAGGACGGCGGTGAACCTGGGGATGCCGCTGCTGTGGCCGAACAG
ENSMMUT00000024762     GACGGGACACCTGGTGAGGACAGTGGCAAGCCCGGGGATGCCTCGGCCGCAGCTGAGCCG
ENSPTRT00000050728     GATGGGACACCTGGTGAGGATGGTGGCGAGCCTGGGGATGCCGTAGCTGCGGCCGAGCAG
ENSPPYT00000008952     GACGGGACACCTGGTGAGGATGTTGGCGAGCCTGGGGATGCCGTGGCTGCGGCCGAGCAG
ENSGGOT00000009426     GATGGGACACCTGGTGAGGATGGTGGCGAGCCTGGGGATGCTGTAGCTGCGGCCGAGCAG
ENST00000311635        GATGGGACACCTGGTGAGGATGGTGGCGAGCCTGGGGATGCCGTAGCTGCGGCCGAGCAG

ENSMUST00000034268     TCGGCCTCGTCTGGGCAGGGGCAGCCGTTTGTGCTTCCTGTGGGTGTCAGCTCGAGGAAC
ENSCJAT00000008607     CCTGCCCAGAGTGGGCAGGGGCAGCCATTTGTGTTGCCCGTGGGTGTGAGCTCCAGGAAT
ENSMMUT00000024762     CCTGCCCAGTGTGGGCAGGGGCAGCCATTTGTGCTGCCCGTGGGCGTGAGCTCCAGGAAT
ENSPTRT00000050728     CCTGCCCAGTGTGGGCAGGGGCAGCCGTTCGTGCTGCCCGTGGGCGTGAGCTCCAGGAAT
ENSPPYT00000008952     CCTGCCCAGTGTGGGCAGGGGCAGCCGTTCGTGCTGCCTGTGGGCGTGAGCTCCAGGAAT
ENSGGOT00000009426     CCTGCCCAGTGTGGGCAGGGGCAGCCGTTCGTGCTGCCCGTGGGCGTGAGCTCCAGGAAT
ENST00000311635        CCTGCCCAGTGTGGGCAGGGGCAGCCGTTCGTGCTGCCCGTGGGCGTGAGCTCCAGGAAT

ENSMUST00000034268     GAGGATTACCCCCGCACTTGCCGCCTATGTTTCTATGGCACAGGCCTCATCGCTGGCCAC
ENSCJAT00000008607     GAAGACTACCCCCGAACCTGCAGGATGTGTTTCTATGGCACAGGCCTCATCGCGGGCCAT
ENSMMUT00000024762     GAGGACTACCCCCGAACCTGCAGGATGTGTTTTTATGGCACAGGCCTCATCGCGGGCCAC
ENSPTRT00000050728     GAGGACTACCCCCGAACCTGCAGGATGTGTTTTTATGGCACAGGCCTCATCGCGGGCCAC
ENSPPYT00000008952     GAGGACTACCCCCGAACCTGCAGGATGTGTTTTTATGGCACAGGCCTCATCGCGGGCCAC
ENSGGOT00000009426     GAGGACTACCCCCGAACCTGCAGGATGTGTTTTTATGGCACAGGCCTCATCGCGGGCCAC
ENST00000311635        GAGGACTACCCCCGAACCTGCAGGATGTGTTTTTATGGCACAGGCCTCATCGCGGGCCAC

ENSMUST00000034268     GGCTTTACCAGCCCTGAGCGCACCCCCGGAGTCTTCGTCCTGTTTGATGAGGACCGCTTT
ENSCJAT00000008607     GGCTTCACCAGCCCTGAACGCACCCCTGGGGTCTTCATCCTCTTCGACGAGGACCGCTTC
ENSMMUT00000024762     GGCTTCACCAGCCCTGAACGCACCCCCGGGGTCTTCATCCTCTTCGATGAGGACCGCTTC
ENSPTRT00000050728     GGCTTCACCAGCCCTGAACGCACCCCCGGGGTCTTCATCCTCTTCGATGAGGACCGCTTC
ENSPPYT00000008952     GGCTTCACCAGCCCTGAACGCACCCCCGGGGTCTTCATCCTCTTTGATGAGGACCGCTTC
ENSGGOT00000009426     GGCTTCACCAGCCCTGAACGCACCCCCGGGGTCTTCATCCTCTTCGATGAGGACCGCTTC
ENST00000311635        GGCTTCACCAGCCCTGAACGCACCCCCGGGGTCTTCATCCTCTTCGATGAGGACCGCTTC

ENSMUST00000034268     GGATTTCTGTGGCTGGAATTGAAGTCCTTCAGCTTGTACAGCCGAGTCCAGGCCACCTTC
ENSCJAT00000008607     GGGTTTGTCTGGCTGGAGCTGAAATCCTTCAGCCTGTACAGCCGGGTCCAGGCCACCTTC
ENSMMUT00000024762     GGGTTCGTCTGGCTGGAGCTGAAATCCTTCAGCCTGTACAGCCGGGTCCAGGCCACCTTC
ENSPTRT00000050728     GGGTTCGTCTGGCTGGAGCTGAAATCCTTCAGCCTGTACAGCCGGGTCCAGGCCACCTTC
ENSPPYT00000008952     GGGTTCGTCTGGCTGGAGCTGAAATCCTTCAGCCTGTACAGCCGGGTCCAGGCCACCTTC
ENSGGOT00000009426     GGGTTCGTCTGGCTGGAGCTGAAATCCTTCAGCCTGTACAGCCGGGTCCAGGCCACCTTC
ENST00000311635        GGGTTCGTCTGGCTGGAGCTGAAATCCTTCAGCCTGTACAGCCGGGTCCAGGCCACCTTC

ENSMUST00000034268     CAGAACGCCGCCGCGCCGTCGCCGCAGGCCTTTGACGAGATGCTCAGGAACATCCAGTCT
ENSCJAT00000008607     CGGAACGCAGATGCGCCGTCCCCACAGGCCTTTGATGAAATGCTCAAGAACATTCAGTCC
ENSMMUT00000024762     CGGAACGCAGATGCGCCGTCCCCACAGGCCTTCGATGAGATGCTCAAGAACATTCAGTCC
ENSPTRT00000050728     CGGAACGCAGATGCGCCTTCCCCACAGGCCTTCGATGAGATGCTCAAGAACATTCAGTCC
ENSPPYT00000008952     CGGAACGCAGATGCGCCGTCCCCACAGGCCTTCGATGAGATGCTCAAGAACATTCAGTCC
ENSGGOT00000009426     CGGAACGCAGATGCGCCGTCCCCACAGGCCTTCGATGAGATGCTCAAGAACATTCAGTCC
ENST00000311635        CGGAACGCAGATGCGCCGTCCCCACAGGCCTTCGATGAGATGCTCAAGAACATTCAGTCC

ENSMUST00000034268     CTCACCTCC
```

```
ENSCJAT00000008607     CTCACCTCC
ENSMMUT00000024762     CTCACTTCC
ENSPTRT00000050728     CTCACCTCC
ENSPPYT00000008952     CTCACCTCC
ENSGGOT00000009426     CTCACTTCC
ENST00000311635        CTCACCTCC
```

Multiple sequence alignment of Fbxo32

```
ENSPTRT00000038027     ATGCCATTCCTCGGGCAGGACTGGCGGTCCCCCGGGCAGAACTGGGTGAAGACGGCCGAC
ENST00000517956        ATGCCATTCCTCGGGCAGGACTGGCGGTCCCCCGGGCAGAACTGGGTGAAGACGGCCGAC
ENSCJAT00000001556     ATGCCATTCCTGGGCCAGGACTGGCGGTCCCCCGGGCAGAGCTGGGTGAAGACGGCCGAC
ENSGGOT00000002992     ATGCCATTCCTCGGGCAGGACTGGCGGTCCCCCGGGCAGAGCTGGGTGAAGACGGCCGAC
ENSMMUT00000033464     ATGCCATTCCTCGGGCAGGACTGGCGGTCCCCTGGGCAGAGCTGGGTGAAGACGGCCGAC
ENSMUST00000022986     ATGCCGTTCCTTGGGCAGGACTGGCGGTCCCCGGGCCAGAGCTGGGTGAAGACGGCGGAC
ENSRNOT00000056983     ATGCCGTTCCTTGGTCAGGACTGGCGGTCCCCGGGCCAGAGCTGGGTGAAGACGGCGGAC

ENSPTRT00000038027     GGCTGGAAGCGCTTCCTGGATGAGAAGAGCGGCAGTTTCGTGAGCGACCTCAGCAGTTAC
ENST00000517956        GGCTGGAAGCGCTTCCTGGATGAGAAGAGCGGCAGTTTCGTGAGCGACCTCAGCAGTTAC
ENSCJAT00000001556     GGCTGGAAGCGCTTCCTGGATGAGAAGAGCGGCAGCTTCGTAAGCGACCTCAGCAGTTAC
ENSGGOT00000002992     GGCTGGAAGCGCTTCCTGGATGAGAAGAGCGGCAGTTTCGTGAGCGACCTCAGCAGTTAC
ENSMMUT00000033464     GGCTGGAAGCGCTTCCTGGATGAGAAGAGCGGCAGCTTCGTGAGCGACCTCAGCAGTTAC
ENSMUST00000022986     GGCTGGAAGCGCTTCTTGGATGAGAAAAGCGGCAGCTTCGTGAGCGACCTCAGCAGTTAC
ENSRNOT00000056983     GGCTGGAAGCGCTTCTTGGATGAGAAAAGCGGCACCTTCGTGAGCGACCTCAGCAGTTAC

ENSPTRT00000038027     TGCAACAAGGAGGTATACAATAAGGAGAATCTTTTCAACAGCCTGAACTATGATGTTGCA
ENST00000517956        TGCAACAAGGAGGTATACAATAAGGAGAATCTTTTCAACAGCCTGAACTATGATGTTGCA
ENSCJAT00000001556     TGCAACAAGGAGGTATACAATAAGGAGAATCTTTTCAACAGCCTGAACTATGATGTTGCA
ENSGGOT00000002992     TGCAACAAGGAGGTATACAATAAGGAGAATCTTTTCAACAGCCTGAACTATGATGTTGCA
ENSMMUT00000033464     TGCAACAAGGAGGTATACAATAAGGAGAATCTTTTCAACAGCCTGAACTATGATGTTGCA
ENSMUST00000022986     TGCAACAAGGAGGTATACAGTAAGGAGAATCTGTTCAGCAGCCTGAACTACGACGTCGCA
ENSRNOT00000056983     TGCTTTAAGCGTTACTGCAACAAGGAGAATCTGTTCAACAGCCTGAACTACGATGTTGCA

ENSPTRT00000038027     GCCAAGAAGAGAAAGAAGGACATGCTGAATAGCAAAACCAAAACTCAGTATTTCCACCAA
ENST00000517956        GCCAAGAAGAGAAAGAAGGACATGCTGAATAGCAAAACCAAAACTCAGTATTTCCACCAA
ENSCJAT00000001556     GCCAAGAAGAGAAAGAAGGACATGCTGAATAGCAAAACCAAAACTCAGTATTTCCACCAA
ENSGGOT00000002992     GCCAAGAAGAGAAAGAAGGACATGCTGAATAGCAAAACCAAAACTCAGTATTTCCACCAA
ENSMMUT00000033464     GCCAAGAAGAGAAAGAAGGACATGCTGAATAGCAAAACCAAAACTCAGTATTTCCACCAA
ENSMUST00000022986     GCCAAGAAGAGAAAGAAAGACATTCAGAACAGCAAAACCAAAACTCAGTACTTCCATCAA
ENSRNOT00000056983     GCCAAGAAGAGAAAGAAAGACATACAGAACAGCAAAACCAAAACTCAGTATTTCCATCAG

ENSPTRT00000038027     GAAAAATGGATCTATGTTCACAAAGGAAGTACTAAAGAGCGCCATGGATATTGCACCCTG
ENST00000517956        GAAAAATGGATCTATGTTCACAAAGGAAGTACTAAAGAGCGCCATGGATATTGCACCCTG
ENSCJAT00000001556     GAAAAATGGATCTATGTTCACAAAGGAAGCACGAAAGAGCGCCATGGATATTGCACTCTG
ENSGGOT00000002992     GAAAAATGGATCTATGTTCACAAAGGAAGTACTAAAGAGCGCCATGGATATTGCACCCTG
ENSMMUT00000033464     GAAAAATGGATCTATGTTCACAAAGGAAGTACTAAAGAGCGCCATGGATATTGCACTCTG
ENSMUST00000022986     GAAAAGTGGATCTATGTTCACAAAGGAAGTACGAAGGAGCGCCATGGATACTGTACTTTG
ENSRNOT00000056983     GAGAAGTGGATCTATGTTCACAAAGGGAGTACTAAGGAGCGCCATGGATACTGCACTTTG

ENSPTRT00000038027     GGGGAAGCTTTCAACAGACTGGACTTCTCAACTGCCATTCTGGATTCCAGAAGATTTAAC
ENST00000517956        GGGGAAGCTTTCAACAGACTGGACTTCTCAACTGCCATTCTGGATTCCAGAAGATTTAAC
ENSCJAT00000001556     GGTGAAGCTTTCAACAGACTGGACTTCTCAACTGCCATTCTGGATTCCCGACGATTTAAC
ENSGGOT00000002992     GGGGAAGCTTTCAACAGACTGGACTTCTCAACTGCCATTCTGGATTCCAGAAGATTTAAC
ENSMMUT00000033464     GGGGAAGCTTTCAACAGACTGGACTTCTCAACTGCCATTTTGGATTCCAGAAGATTTAAC
```

```
ENSMUST00000022986     GGGGAAGCTTTCAACAGACTGGACTTCTCGACTGCCATCCTGGATTCCAGAAGATTCAAC
ENSRNOT00000056983     GGGGAAGCTTTCAACAGACTGGACTTCTCGACTGCCATCCTGGATTCCAGAAGATTCAAC

ENSPTRT00000038027     TACGTGGTCCGGCTGTTGGAGCTGATAGCAAAGTCACAGCTCACATCCCTGAGTGGCATC
ENST00000517956        TACGTGGTCCGGCTGTTGGAGCTGATAGCAAAGTCACAGCTCACATCCCTGAGTGGCATC
ENSCJAT00000001556     TATGTGGTCCGGCTGTTGGAGCTGATAGCAAAGTCACAGCTCACATCCCTGAGTGGCATC
ENSGGOT00000002992     TACGTGGTCCGGCTGTTGGAGCTGATAGCAAAGTCACAGCTCACATCCCTGAGTGGCATC
ENSMMUT00000033464     TACGTGGTCCGGCTGCTGGAGCTGATAGCAAAGTCACAGCTCACATCCCTGAGTGGCATC
ENSMUST00000022986     TACGTAGTAAGGCTGTTGGAGCTGATAGCAAAGTCACAGCTCACATCCCTGAGTGGCATC
ENSRNOT00000056983     TACGTAGTAAGGCTGTTGGAGCTGATAGCAAAGTCACAGCTCACATCCCTGAGTGGCATC

ENSPTRT00000038027     GCCCAAAAGAACTTCATGAATATTTTGGAAAAAGTGGTACTGAAAGTCCTTGAAGACCAG
ENST00000517956        GCCCAAAAGAACTTCATGAATATTTTGGAAAAAGTGGTACTGAAAGTCCTTGAAGACCAG
ENSCJAT00000001556     GCCCAAAAGAACTTCATGAATATTTTGGAAAAAGTGGTACTGAAAGTCCTTGAAGACCAG
ENSGGOT00000002992     GCCCAAAAGAACTTCATGAATATTTTGGAAAAAGTGGTACTGAAAGTCCTTGAAGACCAG
ENSMMUT00000033464     GCCCAGAAGAACTTCATGAATATTTTGGAAAAAGTGGTACTGAAAGTCCTTGAAGACCAG
ENSMUST00000022986     GCCCAAAAGAACTTCATGAACATTTTGGAAAAAGTGGTACTGAAAGTTCTTGAAGACCAG
ENSRNOT00000056983     GCCCAAAAGAACTTCATGAACATTTTGGAAAAAGTAGTACTGAAAGTTCTTGAAGACCAG

ENSPTRT00000038027     CAAAACATTAGACTAATAAGGGAACTACTCCAGACCCTCTACACATCCTTATGTACACTG
ENST00000517956        CAAAACATTAGACTAATAAGGGAACTACTCCAGACCCTCTACACATCCTTATGTACACTG
ENSCJAT00000001556     CAAAACATTAGACTAATAAGGGAGCTACTCCAGACCCTCTACACGTCCCTATGTACACTG
ENSGGOT00000002992     CAAAACATTAGACTAATAAGGGAACTACTCCAGACCCTCTACACATCCTTATGTACACTG
ENSMMUT00000033464     CAAAACATTAGACTAATAAGGGAACTACTCCAGACCCTCTACACGTCCTTATGTACACTG
ENSMUST00000022986     CAAAACATAAGACTTATACGGGAACTTCTCCAGACTCTCTACACATCCTTATGCACACTG
ENSRNOT00000056983     CAAAACATAAGACTCATACGGGAACTTCTCCAGACCCTCTACACATCCTTATGCACGCTG

ENSPTRT00000038027     GTCCAAAGAGTCGGCAAGTCTGTGCTGGTCGGGAACATTAACATGTGGGTGTATCGGATG
ENST00000517956        GTCCAAAGAGTCGGCAAGTCTGTGCTGGTCGGGAACATTAACATGTGGGTGTATCGGATG
ENSCJAT00000001556     GTCCAAAGAGTTGGCAAGTCTGTGCTGGTCGGGAACATTAACATGTGGGTGTATCGGATG
ENSGGOT00000002992     GTCCAAAGAGTCGGCAAGTCTGTGCTGGTCGGGAACATTAACATGTGGGTGTATCGGATG
ENSMMUT00000033464     GTCCAAAGAGTCGGCAAGTCCGTGCTGGTTGGCAACATTAACATGTGGGTGTATCGGATG
ENSMUST00000022986     GTGCAGAGAGTCGGCAAGTCTGTGCTGGTGGGCAACATTAACATGTGGGTGTATCGGATG
ENSRNOT00000056983     GTCCAGAGAGTCGGCAAGTCCGTGCTGGTGGGCAACATCAACATGTGGGTGTATCGAATG

ENSPTRT00000038027     GAGACGATTCTCCACTGGCAGCAGCAGCTGAACAACATTCAGATCAGCAGGCCTTTTTTG
ENST00000517956        GAGACGATTCTCCACTGGCAGCAGCAGCTGAACAACATTCAGATCACCAGGCCTGCCTTC
ENSCJAT00000001556     GAGACGATTCTCCACTGGCAGCAGCAGCTGAACAACATTCAGATCACCAGGCCTGCCTTC
ENSGGOT00000002992     GAGACGATTCTCCACTGGCAGCAGCAGCTGAACAACATTCAGATCACCAGGCCTGCCTTC
ENSMMUT00000033464     GAGACGATTCTCCACTGGCAGCAGCAGCTGAACAACATTCAGATCACCAGGCCTGCCTTC
ENSMUST00000022986     GAGACCATTCTACACTGGCAGCAGCAGCTGAATAGCATCCAGATCAGCAGGCCTGCCTTC
ENSRNOT00000056983     GAGACCATTCTACACTGGCAACAGCAGCTGAACAGCATCCAGATCAGCAGGCCGGCCTTC

ENSPTRT00000038027     AAAGGCCTCACCTTCACTGACCTGCCTTTGTGCCTTCAACTGAACATCATGCAGAGGCTG
ENST00000517956        AAAGGCCTCACCTTCACTGACCTGCCTTTGTGCCTACAACTGAACATCATGCAGAGGCTG
ENSCJAT00000001556     AAAGGCCTCACCTTCACTGACTTGCCTTTGTGCCTACAACTGAACATCATGCAGAGGCTG
ENSGGOT00000002992     AAAGGCCTCACCTTCACTGACCTGCCTTTGTGCCTACAACTGAACATCATGCAGAGGCTG
ENSMMUT00000033464     AAAGGCCTCACCTTCACAGACCTGCCTTTGTGCCTACAACTGAACATCATGCAGAGGCTG
ENSMUST00000022986     AAAGGCCTCACGATCACCGACCTGCCTGTGTGCTTACAACTGAACATCATGCAGAGGCTG
ENSRNOT00000056983     AAAGGCCTCACGATCACCGACCTGCCTGTGTGCTTACAACTGAACATCATGCAGAGGCTG

ENSPTRT00000038027     AGCGACGGGCGGGACCTGGTCAGCCTGGGCCAGGCTGCCCCCGACCTGCACGTGCTCAGC
ENST00000517956        AGCGACGGGCGGGACCTGGTCAGCCTGGGCCAGGCTGCCCCCGACCTGCACGTGCTCAGC
```

```
ENSCJAT00000001556     AGCGACGGCCGGGACCTGGTCAGCCTGGGCCAGGCGGCCCCCGACCTGCACGTGCTCAGC
ENSGGOT00000002992     AGCGACGGGCGGGACCTGGTCAGCCTGGGCCAGGCTGCCCCTGACCTGCACGTGCTCAGC
ENSMMUT00000033464     AGCGACGGGCGGGACCTGGTCAGCCTGGGCCAGGCGGCCCCCGACCTGCACGTGCTCAGC
ENSMUST00000022986     AGTGACGGGCGGGACCTGGTCAGCCTGGGCCAGGCAGCCCCAGACCTGCATGTGCTCAGT
ENSRNOT00000056983     AGCGATGGGCGGGACCTGGTCAGCCTGGGCCAGGCAGCCCCAGACCTGCATGTGCTCAGT


ENSPTRT00000038027     GAAGACCGGCTGCTGTGGAAGAAACTCTGCCAGTACCACTTCTCCGAGCGGCAGATCCGC
ENST00000517956        GAAGACCGGCTGCTGTGGAAGAAACTCTGCCAGTACCACTTCTCCGAGCGGCAGATCCGC
ENSCJAT00000001556     GAAGACCGGCTGCTGTGGAAGAAACTCTGCCAGTACCACTTCTCCGAGCGGCAGATCCGC
ENSGGOT00000002992     GAAGACCGGCTGCTGTGGAAGAAACTCTGCCAGTACCACTTCTCCGAGCGGCAGATCCGC
ENSMMUT00000033464     GAAGACCGGCTGCTGTGGAAGAAACTCTGCCAGTACCACTTCTCTGAGCGGCAGATCCGC
ENSMUST00000022986     GAGGACCGGCTACTGTGGAAGAGACTCTGCCAGTACCACTTCTCAGAGAGGCAGATTCGC
ENSRNOT00000056983     GAAGACCGGCTACTGTGGAAGAGACTCTGCCAGTACCACTTCTCAGAGCGGCAGATCCGC


ENSPTRT00000038027     AAACGATTAATTCTGTCAGACAAAGGGCAGCTGGATTGGAAGAAGATGTATTTCAAACTT
ENST00000517956        AAACGATTAATTCTGTCAGACAAAGGGCAGCTGGATTGGAAGAAGATGTATTTCAAACTT
ENSCJAT00000001556     AAACGATTAATTCTGTCAGACAAAGGGCAGCTGGATTGGAAGAGGATGTATTTTAAACTT
ENSGGOT00000002992     AAACGATTAATTCTGTCAGACAAAGGGCAGCTGGATTGGAAGAAGATGTATTTCAAACTT
ENSMMUT00000033464     AAACGATTAATTCTGTCAGACAAAGGGCAGCTGGATTGGAAGAAGATGTATTTCAAACTT
ENSMUST00000022986     AAGCGTTTGATCTTGTCTGACAAAGGGCAGCTGGATTGGAAGAAGATGTATTTTAAGCTT
ENSRNOT00000056983     AAGCGATTGATCTTGTCTGACAAAGGGCAGCTGGATTGGAAGAAGATGTACTTTAAGCTT


ENSPTRT00000038027     GTCCGATGTTACCCAAGAAAAGAGCAGTATGGAGATACCCTTCAGCTCTGCAAACACTGT
ENST00000517956        GTCCGATGTTACCCAAGGAAAGAGCAGTATGGAGATACCCTTCAGCTCTGCAAACACTGT
ENSCJAT00000001556     GTCCGATGTTACCCAAGGAAAGAGCAGTATGGAGATACCCTTCAGCTTTGCAAACACTGT
ENSGGOT00000002992     GTCCGATGTTACCCAAGGAAAGAGCAGTATGGAGATACCCTTCAGCTCTGCAAACACTGT
ENSMMUT00000033464     GTCCGATGTTACCCAAGGAAAGAGCAGTATGGAGATACCCTTCAGCTCTGCAAACACTGT
ENSMUST00000022986     GTACGATGTTACCCAAGAAGAGAGCAGTATGGGGTCACCCTGCAGCTTTGCAAACACTGC
ENSRNOT00000056983     GTGCGATGTTACCCAAGAAGAGAACAGTATGGGGTCACCCTGCAGCTTTGCAAACACTGC


ENSPTRT00000038027     CACATCCTTTCCTGGAAGGGCACTGACCATCCGTGCACTGCCAATAACCCAGAGAGCTGC
ENST00000517956        CACATCCTTTCCTGGAAGGGCACTGACCATCCGTGCACTGCCAATAACCCAGAGAGCTGC
ENSCJAT00000001556     CACATCCTTTCCTGGAAGGGCACTGACCATCCATGCACTGCCAATAACCCAGAGAGCTGC
ENSGGOT00000002992     CACATCCTTTCCTGGAAGGGCACTGACCATCCGTGCACTGCCAATAACCCAGAGAGCTGC
ENSMMUT00000033464     CACATCCTTTCCTGGAAGGGCACTGACCATCCGTGCACTGCCAATAACCCAGAGAGCTGC
ENSMUST00000022986     CACATTCTCTCCTGGAAGGGCACTGACCATCCGTGCACGGCCAACAACCCAGAGAGCTGC
ENSRNOT00000056983     CACATTCTCTCCTGGAAGGGCACTGACCATCCATGCACGGCCAACAACCCAGAGAGCTGC


ENSPTRT00000038027     TCCGTTTCACTTTCACCCCAGGACTTTATCAACTTGTTCAAGTTC
ENST00000517956        TCCGTTTCACTTTCACCCCAGGACTTTATCAACTTGTTCAAGTTC
ENSCJAT00000001556     TCCGTTTCACTTTCACCCCAAGACTTTATCAACTTGTTCAAGTTC
ENSGGOT00000002992     TCCGTTTCACTTTCACCCCAGGACTTTATCAACTTGTTCAAGTTC
ENSMMUT00000033464     TCCGTTTCACTTTCACCCCAGGACTTTATCAACTTGTTCAAGTTC
ENSMUST00000022986     TCCGTCTCACTTTCCCCTCAAGACTTTATCAATTTGTTCAAGTTC
ENSRNOT00000056983     TCCGTCTCACTTTCACCCCAAGACTTTATTAACTTGTTCAAGTTC
```

Multiple sequence alignment of Fbxo33

```
ENSCJAT00000031314     ATGTTGTTGTTCTTGTCAGTGCCGCAGCCCCGACACCCGGGAGCTCGAACCCGAGCCGGG
ENSMMUT00000009759     ATGTTGTTGTTCTTGTCAGTGCCGCAGCCCCGACCGCCGGGAGCTCGAACCCGAGCCGGG
ENSPPYT00000006818     ATGTTGTTGTTCTTGTCAGTGCTGCAGCCCCGACCGCCGGGAGCTCGAACCCGAGCCGGG
ENST00000298097        ATGTTGTTGTTCTTGTCAGTGCCGCAGCCCCGACCGCCGGGAGCTCGAACCCGAGCCGGG
ENSGGOT00000010826     ATGTTGTTGTTCTTGTCAGTGCCGCAGCCCCGACCGCCGGGAGCTCGAACCCGAGCCGGG
ENSPTRT00000011544     ATGTTGTTGTTCTTGTCAGTGCCGCAGCCCCGACCGCCGGGAGCTCGAACCCGAGCCGGG
```

```
ENSMUST00000043204     ATGTTGTTGTTCTTGTCAGTGCCGCAACCCCGACCGCCGGGAGCGCGAACCCGAGCCGGG
ENSRNOT00000007221     ATGTTGTTGTTCTTGTCAGTGCCGCAGCCCCGACCGCCGGGAGCGCGAACCCGAGCCGGG

ENSCJAT00000031314     GCAGCCCGGGTGGCGCGGTGGCGGCGGCTGCGGCTGCAGCAGCTGCGACGGCTGCGGGGG
ENSMMUT00000009759     GCCGCCCGGGTGGCGCGGTGGCGGTGGCTGCGGCTGCAGCAGCTGCGACGGCTGCGGGGG
ENSPPYT00000006818     GCCGCCCGGGTGGCGCGGTGGCGGCGGCTGCGGCTGCAGCAGCTGCGACGGCTGCGGGGG
ENST00000298097        GCCGCCCGGGTGGCGCGGTGGCGGCGGCTGCGGCTGCAGCAGCTGCGACGGCTGCGGGGG
ENSGGOT00000010826     GCCGCCCGGGTGGCGCGGTGGCGGCGGCTGCGGCTGCAGCAGCTGCGACGGCTGCGGGGG
ENSPTRT00000011544     GCCGCCCGGGTGGCGCGGTGGCGGCGGCTGCGGCTGCAGCAGCTGCGACGGCTGCGGGGG
ENSMUST00000043204     GCCGCCCGGTTGGTGCGGTGGCGGCGGCTGCGGCTGCTGCAGCTGCGCCGGCTGCGGGGG
ENSRNOT00000007221     GCCGCCCGGGTGGTGCGGTGGCGGCGGCTGCGGCTGCGGCAGCTGCGCCGGCTGCGGGGG

ENSCJAT00000031314     CTGCTCCGGGGACTGCGGGGGCGGCCGGGAGCCGGCAGCCAGCGGCGGGGCCGAATGGCT
ENSMMUT00000009759     CTGCTCCGGGTACTGCGGGGGCGGCCGGAAGCCGGCACCCGGCGACGGGGCCGGATGGCT
ENSPPYT00000006818     CTGCTCCGGGTACTGCGGGGGCGGCCGGGAACCGGCAGCCGGCGGCGGGGCCGGATGGCT
ENST00000298097        CTGCTCCGGGTACTGCGGGGGCGGCCGGGAGCCGGCAGCCGGCGGCGGGGCCGGATGGCT
ENSGGOT00000010826     CTGCTCCGGGTACTGCGGGGGCGGCCGGGAGCCGGCAGCCGGCGGCGGGGCCGGATGGCT
ENSPTRT00000011544     CTGCTCCGGGTACTGCGGGGGCGGCCGGGAGCCGGCAGCCGGCGGCGGGGCCGGATGGCT
ENSMUST00000043204     CTGCTGCGAGGACTGCGCAGGCGGCCGGGGACGGGCGGCCGGCGGCCGAGCCGGATGGCT
ENSRNOT00000007221     CTGCTGCGAGGACTGCGCGGGCGGTCGGGGACGAGCGGCCGGCGGCGGAGCCGGATGGCT

ENSCJAT00000031314     CTGTGCGGGCAGGCGGCTGGCGCCGCTTCGCTGCCCAGCGAGCTAATCGTGCACATCTTC
ENSMMUT00000009759     CTGTGCGGGCAGGCGGCGGGCGCTGCGTCGCTGCCCAGCGAGCTGATCGTGCACATCTTC
ENSPPYT00000006818     CTGTGCGGCCAGGCGGCGGGCGCTGCGTCGCTGCCCAGCGAGCTGATCGTGCACATCTTC
ENST00000298097        CTGTGCGGGCAGGCGGCGGGCGCTGCGTCGCTGCCCAGCGAGCTGATCGTGCACATCTTC
ENSGGOT00000010826     CTGTGCGGGCAGGCGGCGGGCGCTGCGTCGCTGCCCAGCGAGCTGATCGTGCACATCTTC
ENSPTRT00000011544     CTGTGCGGGCAGGCGGCGGGCGCTGCGTCGCTGCCCAGCGAGCTGATCGTGCACATCTTC
ENSMUST00000043204     CTGTGCGGGCAGGCGGCCGGCGCCGCGTCGCTGCCCAGCGAGCTCATCGTGCACATCTTC
ENSRNOT00000007221     CTGTGCGGGCAGGCGGCCGGCGCCGCGTCGCTGCCCAGCGAACTCATCGTGCACATCTTC

ENSCJAT00000031314     TCCTTCCTGCCGGCGCCCGACCGGCTGCGGGCCTCGGCCTCCTGTTCGCACTGGCGTGAG
ENSMMUT00000009759     TCTTTTCTGCCGGCGCCCGACCGGCTGCGGGCCTCGGCCTCCTGCTCCCACTGGCGTGAG
ENSPPYT00000006818     TCTTTTCTGCCGGCGCCCGACCGGCTGCGGGCCTCGGCCTCCTGCTCGCACTGGCGTGAG
ENST00000298097        TCTTTTCTGCCGGCGCCCGACCGGCTGCGGGCCTCGGCCTCCTGCTCGCACTGGCGTGAG
ENSGGOT00000010826     TCTTTTCTGCCGGCGCCCGACCGGCTGCGGGCCTCGGCTTCCTGCTCGCACTGGCGTGAG
ENSPTRT00000011544     TCTTTTCTGCCGGCGCCCGACCGGCTGCGGGCCTCGGCCTCCTGCTCGCACTGGCGTGAG
ENSMUST00000043204     TCCTTTCTGCCGGCGCCCGACCGGCTGCGGGCCTCGGCCTCCTGCTCGCACTGGCGGGAG
ENSRNOT00000007221     TCCTTTCTGCCGGCGCCCGACCGGCTGCGGGCCTCGGCCTCCTGCTCGCACTGGCGGGAG

ENSCJAT00000031314     TGCCTCTTCTATCCGGCCCTCTGGCCCCAGCTCCGCATCTGCCTTCGCGTCTCGCCGGCA
ENSMMUT00000009759     TGCCTCTTCTATCCGGCCCTCTGGCCCCAGCTCCGCATCTGCCTCCGCGTCTCGCCCGCG
ENSPPYT00000006818     TGCCTCTTCTATCCGGCCCTCTGGCCTCAGCTCCGCATCTGCCTCCGCGTCTCGCCCGCG
ENST00000298097        TGCCTCTTCTATCCGGCCCTGTGGCCCCAGCTCCGCATCTGCCTCCGCGTCTCGCCCGCG
ENSGGOT00000010826     TGCCTCTTCTATCCGGCCCTGTGGCCCCAGCTCCGCATCTGCCTCCGCGTCTCGCCCGCG
ENSPTRT00000011544     TGCCTCTTCTATCCGGCCCTGTGGCCCCAGCTCCGCATCTGCCTCCGCGTCTCGCCCGCG
ENSMUST00000043204     TGCCTCTTCTACCCAGCGCTGTGGCCCCAGCTCCGCATCTGCCTCCGCGTCTCGCCCGCG
ENSRNOT00000007221     TGCCTCTTCTACCCAGCCCTGTGGCCCCAGCTCCGCATCTGCCTCCGCGTCTCGCCAGCG

ENSCJAT00000031314     GAGCAGCCTCGGCTGGAATTCCTCATGCGCAAGTGCGGCTGGTTCGTGCGAGAGCTGCGT
ENSMMUT00000009759     GAGCAGCCTCGGCTGGAATTCCTCATGCGCAAGTGCGGCTGGTTCGTGCGGGAGCTGCAT
ENSPPYT00000006818     GAGCAGCCTCGGCTGGAATTCCTCATGCGCAAGTGCGGCTGGTTCGTGCGAGAGCTGCGT
ENST00000298097        GAGCAGCCTCGGCTGGAATTCCTCATGCGCAAGTGCGGCTGGTTCGTGCGAGAGCTGCGT
ENSGGOT00000010826     GAGCAGCCTCGGCTGGAATTCCTCATGCGCAAGTGCGGCTGGTTCGTGCGAGAGCTGCGT
ENSPTRT00000011544     GAGCAGCCTCGGCTGGAATTCCTCATGCGCAAGTGCGGCTGGTTCGTGCGAGAGCTGCGT
```

```
ENSMUST00000043204     GAGCAGCCGCGGCTAGAGTTCCTCATGCGCAAGTGCGGCTGGTTCGTGCGGGAGCTGCGG
ENSRNOT00000007221     GAGCAGCCGCGGCTCGAGTTTCTCATGCGCAAGTGCGGCTGGTTCGTACGGGAGCTACGG

ENSCJAT00000031314     GTTGAGTTCGCCGCCGAGAATTACCTGAGCGGCTGCGGCCCAGGCGACGGAGGTGGCGCG
ENSMMUT00000009759     GTTGAATTCGCCGCGGAGAACTACCTGAGCGGCGGCGGCCCAGGAGACGGAGGTGGCGCG
ENSPPYT00000006818     GTTGAATTCGCCGCGGAGAACTACCTGAGCGGCGGTGGCCCAGGGGACGGAGGTGGCGTG
ENST00000298097        GTTGAATTCGCCGCCGAGAACTATCTGAGCGGCGGTGGCCCAGGGGACGGAGGTGGCGCG
ENSGGOT00000010826     GTTGAATTCGCCGCCGAGAACTACCTGAGCGGCGGTGGCCCAGGGGACGGAGGTGGCGCG
ENSPTRT00000011544     GTTGAATTCGCCGCCGAGAACTATCTGAGCGGCAGTGGCCCAGGGGACGGAGGTGGCGCG
ENSMUST00000043204     GTTGAGTTCGCCGCGGAGAATTATCTGAGCGGCGGCGGCCCGGGCGACGGCGGCGGCACC
ENSRNOT00000007221     GTTGAGTTCGCCGCGGAGAATTATCTGAGCGGCGGCGGCCCGGGCGACGGCGGGGGCACC

ENSCJAT00000031314     GACACCGGGACTGGAGGGGAGGAAGTCGAGGCCCTGCAGCTCTCAGCTCGTTGGCTGGAA
ENSMMUT00000009759     GACACCGGGACTGGAGGGGAGGAAGTCGAGGCCCTGCAGCTCTCTGCTCGTTGGCTGGAA
ENSPPYT00000006818     GACACCGGGACTGGAGGGGAGGAAGTCGAGGCCCTGCAGCTCTCAGCTCGTTGGCTGGAA
ENST00000298097        GACACCGGGACTGGAGGGGAGGAAGTCGAGGCCCTGCAGCTCTCAGCTCGTTGGCTGGAA
ENSGGOT00000010826     GACAGCGGGACTGGAGGGGAGGAAGTCGAGGCCCTGCAGCTCTCAGCTCGTTGGCTGGAA
ENSPTRT00000011544     GACACCGGGACTGGAGGGGAGGAAGTCGAGGCCCTGCAGCTCTCAGCTCGTTGGCTGGAA
ENSMUST00000043204     GACACGGGCACCGGAGGGGAAGACGGGGAGGCCCTGCAGCTCTCCTCCCGCTGGCTGGAA
ENSRNOT00000007221     GACACGGGCAACGGAGGGGAAGAAAGTGAGGCCCTGCAACTCTCCTCCCGCTGGCTAGAA

ENSCJAT00000031314     GTGCTGCGCACCTACTTGGAGCTGGTGCTGTGCGTGCTGGTCAGCATCAGGAACAACAGA
ENSMMUT00000009759     GTGCTGCGCACCTACTTGGAGCTGGTGCTTTGCGTCCTGGTCAGCATCCGGAACAACAGG
ENSPPYT00000006818     GTGCTGCGCACCTACTTGGAGCTGGTGCTTTGCGTGCTGGTCAGCATCCGGAACAACAGG
ENST00000298097        GTGCTGCGCACCTACTTGGAGCTGGTGCTTTGCGTGCTGGTCAGCATCCGGAACAACAGG
ENSGGOT00000010826     GTGCTGCGCACCTACTTGGAGCTGGTGCTTTGCGTGCTGGTCAGCATCCGGAACAACAGG
ENSPTRT00000011544     GTGCTGCGCACCTACTTGGAGCTGGTGCTTTGCGTGCTGGTCAGCATCCGGAACAACAGG
ENSMUST00000043204     GTACTGCGCATCTACTTGGAGCTGGTGTTGTGCGTGCTGCTCAGCATCCGGAACAACAGG
ENSRNOT00000007221     GTGCTGCGCATCTACTTGGAGCTGGTGTTGTGCGTGCTGCTCAGCATCCGGAACAACAGG

ENSCJAT00000031314     AACCTTCAGAAGTTTAGTCTTTTTGGAGACATAAGCATTCTACAACAGCAAGGAAGTTTG
ENSMMUT00000009759     AACCTTCAGAAGTTTAGTCTTTTTGGAGACATAAGTGTTCTACAACAGCAAGGAAGTTTG
ENSPPYT00000006818     AACCTTCAGAAGTTTAGTCTTTTTGGAGACATAAGTGTTCTACAACAGCAAGGAAGTTTG
ENST00000298097        AACCTTCAGAAGTTTAGTCTTTTTGGAGACATAAGTGTTCTACAACAGCAAGGAAGTTTG
ENSGGOT00000010826     AACCTTCAGAAGTTTAGTCTTTTTGGAGACATAAGTGTTCTACAACAGCAAGGAAGTTTG
ENSPTRT00000011544     AACCTTCAGAAGTTTAGTCTTTTTGGAGACATAAGTGTTCTACAACAGCAAGGAAGTTTG
ENSMUST00000043204     AATCTCCAGAAGTTTAGTCTTTTTGGAGACATAAGTGTTGTACACCAGCAAGGAAGTTTG
ENSRNOT00000007221     AACCTCCAGAAGTTTAGTCTTTTTGGAGATATAAGTGTCGTTCAGCAGCAAGGAAGTCTG

ENSCJAT00000031314     TCAAATACATACCTCAGCAAGGTGGACCCTGATGGCAAAAAAATTAAACAAATTCAGCAA
ENSMMUT00000009759     TCAAATACATACCTCAGCAAGGTGGACCCTGATGGCAAAAAAATTAAACAAATTCAGCAA
ENSPPYT00000006818     TCAAATACATACCTCAGCAAAGTGGACCCTGATGGCAAAAAAATTAAACAAATTCAGCAA
ENST00000298097        TCAAATACATACCTCAGCAAGGTGGACCCTGATGGCAAAAAAATTAAACAAATTCAGCAA
ENSGGOT00000010826     TCAAATACATACCTCAGCAAGGTGGACCCTGATGGCAAAAAAATTAAACAAATTCAGCAA
ENSPTRT00000011544     TCAAATACATACCTCAGCAAGGTGGACCCTGATGGCAAAAAAATTAAACAAATTCAGCAA
ENSMUST00000043204     TCCAGCACGTACCTCAGCAGGGTAGACCCTGACGGCAAGAAGATTAAGCAAATTCAACAA
ENSRNOT00000007221     TCCAGCACATACCTCAGCAGAGTAGACCCTGACGGCAAGAAGATTAAGCAAATTCAGCAG

ENSCJAT00000031314     CTGTTTGAAGAAATCCTGAGTAATAGTAGGCAAATGAAATGGCTGTCCTGTGGGTTTATG
ENSMMUT00000009759     CTGTTTGAAGAAATCCTGAGTAATAGTAGGCAACTGAAATGGCTGTCCTGTGGGTTTATG
ENSPPYT00000006818     CTGTTTGAAGAAATCCTGAGTAATAGTAGGCAACTGAAATGGCTGTCCTGTGGGTTTATG
ENST00000298097        CTGTTTGAAGAAATCCTGAGTAATAGTAGGCAACTGAAATGGCTGTCCTGTGGGTTTATG
ENSGGOT00000010826     CTGTTTGAAGAAATCCTGAGTAATAGTAGGCAACTGAAATGGCTGTCCTGTGGGTTTATG
ENSPTRT00000011544     CTGTTTGAAGAAATCCTGAGTAATAGTAGGCAACTGAAATGGCTGTCCTGTGGGTTTATG
```

```
ENSMUST00000043204     CTGTTTGAAGAAATACTGAGCAATAGTAGGCAACTAAAATGGCTGTCCTGTGGGTTTATG
ENSRNOT00000007221     CTGTTTGAAGAAATACTGAGCAATAGTAGGCAACTAAAATGGCTGTCCTGTGGGTTTATG

ENSCJAT00000031314     CTGGAAATAGTAACCCCAACATCACTGTCATCTCTCTCTAATGCTGTTGCCAACACCATG
ENSMMUT00000009759     CTGGAAATAGTAACGCCAACATCACTGTCATCTCTCTCTAATGCTGTTGCCAGCACCATG
ENSPPYT00000006818     CTGGAAATAGTAACCCCAACATCACTGTCATCTCTCTCTAATGCTGTTGCCAACACCATG
ENST00000298097        CTGGAAATAGTAACCCCAACATCACTGTCATCTCTCTCTAATGCTGTTGCCAACACCATG
ENSGGOT00000010826     CTGGAAATAGTAACCCCAACATCACTGTCATCTCTCTCTAATGCTGTTGCCAACACCATG
ENSPTRT00000011544     CTGGAAATAGTAACCCCAACATCACTGTCATCTCTCTCTAATGCTGTTGCCAACACCATG
ENSMUST00000043204     CTGGAAATAGTAACCCCAACATCACTGTCGTCTCTGTCTAACCCAATTGCCAACACCATG
ENSRNOT00000007221     CTGGAAATAGTAACCCCATCATCACTGTCGTCTCTGTCTAACTCCATTGCCAACACCATG

ENSCJAT00000031314     GAGCACCTGAGTTTACTGGACAATAATATTCCTGGTAACAGCACTCTCATTACTGCAGTT
ENSMMUT00000009759     GAGCACCTGAGTTTACTTGACAATAATATTCCTGGTAACAGCACTCTTATTACTGCAGTT
ENSPPYT00000006818     GAGCATCTGAGTTTACTGGACAATAATATTCCTGGTAACAGCACTCTTATTACTGCAGTT
ENST00000298097        GAGCACCTCAGTTTACTGGACAATAATATTCCTGGTAACAGCACTCTTATTACTGCAGTT
ENSGGOT00000010826     GAGCACCTGAGTTTACTGGACAATAATATTCCTGGTAACAGCACTCTTATTACTGCAGTT
ENSPTRT00000011544     GAGCACCTCAGTTTACTGGACAATAATATTCCTGGTAACAGCACTCTTATTACTGCAGTT
ENSMUST00000043204     GAACACCTGAGTTTACTGGACAACAACATTCCTGGCAATAGCACTCTGATCACTGCAGTG
ENSRNOT00000007221     GAACACCTGAGTTTACTGGACAACAACATTCCTGGTAACAGCACGCTCATCACCGCAGTC

ENSCJAT00000031314     GAACTGGAGCGATTTGTGAATCTGCACTCACTTGCCTTGGATTTTTGTGACTTTACAGCT
ENSMMUT00000009759     GAACTGGAGCGATTCGTGAATCTGCACTCACTTGCCTTGGATTTTTGTGACTTTACAGCT
ENSPPYT00000006818     GAACTGGAGCGATTTGTGAATCTGCACTCACTTGCCTTGGATTTTTGTGACTTTACAGCT
ENST00000298097        GAACTGGAGCGATTTGTGAATCTGCACTCACTTGCCTTGGATTTTTGTGACTTTACAGCT
ENSGGOT00000010826     GAACTGGAGCGATTTGTGAATCTGCACTCACTTGCCTTGGATTTTTGTGACTTTACAGCT
ENSPTRT00000011544     GAACTGGAGCGATTTGTGAATCTGCACTCACTTGCCTTGGATTTTTGTGACTTTACAGCT
ENSMUST00000043204     GAACTAGAGCGGTTTGTAAATCTACGCTCACTTGCCCTGGATTTCTGTGACTTTACAGCT
ENSRNOT00000007221     GAACTAGAGCGCTTTGTAAATCTGCGCTCACTTGCCCTGGATTTCTGTGACTTTACAGCT

ENSCJAT00000031314     GAGATGGCAAGAGTCTTAACTGATAGCAACCATGTGCCTTTGCAGCGACTGTCTCTTCTG
ENSMMUT00000009759     GAGATGGCAAGAGTCTTAACTGATAGCAACCATGTGCCTTTGCAGCGACTGTCTCTTCTG
ENSPPYT00000006818     GAGATGGCAAGAGTCTTAACTGATAGCAACCATGTGCCTTTGCAACGACTGTCTCTTCTG
ENST00000298097        GAGATGGCAAGAGTCTTAACTGATAGCAACCATGTGCCTTTGCAACGACTGTCTCTTCTG
ENSGGOT00000010826     GAGATGGCAAGAGTCTTAACTGATAGCAACCATGTGCCTTTGCAACGACTGTCTCTTCTG
ENSPTRT00000011544     GAGATGGCAAGAGTCTTAACTGATAGCAACCATGTGCCTTTGCAACGACTGTCTCTTCTG
ENSMUST00000043204     GAAATGGCGAGAGTCTTGACCGATAGCAACCATGTGCCTTTGCAGCGACTGTCTCTTCTG
ENSRNOT00000007221     GAGATGGCGAGAGTCCTGACCGACAGCAACCATGTGCCTTTGCAGCGACTGTCTCTTCTG

ENSCJAT00000031314     GTTCACAATGTTTCCGTAATGCACAAGTCTCTAGAGAACATGCCAAATGATGAGCATTGG
ENSMMUT00000009759     GTTCACAGTGTTTCCATAATGCACAAGTCTCTGGACAACATGCCAAATGATGAGCATTGG
ENSPPYT00000006818     GTTCACAATGTTTCCATAATGCACAAGTCTCTGGACAACATGCCAAATGATGAGCACTGG
ENST00000298097        GTTCACAATGTTTCTGTAATGCACAAGTCTCTGGACAACATGCCAAATGATGAGCATTGG
ENSGGOT00000010826     GTTCACAATGTTTCTGTAATGCACAAGTCTCTGGACAACATGCCAAATGATGAGCATTGG
ENSPTRT00000011544     GTTCACAATGTTTCTGTAATGCACAAGTCTCTGGACAACATGCCAAATGATGAGCATTGG
ENSMUST00000043204     GTCCACAATGCTTCAGTGATGCTCAAGTCATTAGACAACATGCCAAATGATGAGCACTGG
ENSRNOT00000007221     GTCCACAATGCTTCAGTGATGCTCAAGTCATTAGACAACATGCCAAACGATGAGCACTGG

ENSCJAT00000031314     AAAGCCCTGTCACGAAAGAGCACCAGCCTTCGGGTCTACATAATGGCTTTTGATACCAAG
ENSMMUT00000009759     AAAGCCCTGTCACGAAAGAGCACCAACCTTCGGGTCTATATAATGGCTTTTGATATCAAG
ENSPPYT00000006818     AAAGCCCTGTCACGAAAGAGCACCAGCCTTCGGGTCTATATAATGGCTTTTGATATCAAG
ENST00000298097        AAAGCCCTGTCACGAAAGAGCACCAGCTTTCGGGTCTATATAATGGCTTTTGATATCAAG
ENSGGOT00000010826     AAAGCCCTGTCACGAAAGAGCACCAGCCTTCGGGTCTATATAATGGCTTTTGATATCAAG
ENSPTRT00000011544     AAAGCCCTGTCACGAAAGAGCACCAGCCTTCGGGTCTATATAATGGCTTTTGATATCAAG
```

```
ENSMUST00000043204     AAAGCCCTGTCACGAAAGAGCTCCAGCCTTCGGGTCTATCTAATGGTTTTTGATATTAAA
ENSRNOT00000007221     AAGGCCCTGTCACGAAAGAGCTCCAGCCTCCGGGTCTATCTAATGGCTTTTGATGTTAAA

ENSCJAT00000031314     AGTGAAGATATGTTAAAGATTTTGAAACCTAGTATGCCACTAGAGAGGATTCATTTTGAC
ENSMMUT00000009759     AGTGAAGATATGTTAAAGATTCTAAAACCCAGTATACCACTAGAGAGGATTCATTTTGAT
ENSPPYT00000006818     AGTGAAGATATGTTAAAGATTCTGAAACCCAGTATACCACTAGAGAGGATTCATTTTGAT
ENST00000298097        AGTGAAGATATGTTAAAGATTCTGAAACCCAGTATACCACTAGAGAGGATTCATTTTGAT
ENSGGOT00000010826     AGTGAAGATATGTTAAAGATTCTGAAACCCAGTATACCACTAGAGAGGATTCATTTTGAT
ENSPTRT00000011544     AGTGAAGATATGTTAAAGATTCTGAAACCCAGTATACCACTAGAGAGGATTCATTTTGAT
ENSMUST00000043204     AGTGAAGACATGCTAAAGATTCTGAAACCCAGTATACCACTTGAGAGGGTTCACTTTGAC
ENSRNOT00000007221     AGTGAAGACATGCTAAAGATTCTGAAACCCAGTATACCACTTGAGAGGGTTCACTTTGAC

ENSCJAT00000031314     AGCTATATCACTTGTGTTTCGGGGGCTATTGTTGATCTCATATCCAGGCAATATGACAAG
ENSMMUT00000009759     AGCTATATCACTTGTGTTTCAGGGGCTATTGTTGATCTTATATCCAGGCAATATGACAAG
ENSPPYT00000006818     AGCTATATCACTTGTGTTTCAGGGGCTATTGTTGATCTTATATCCAGGCAATATGACAAG
ENST00000298097        AGCTATATCACTTGTGTTTCAGGGGCTATTGTTGATCTTATATCCAGGCAATATGACAAG
ENSGGOT00000010826     AGCTATATCACTTGTGTTTCAGGGGCTATTGTTGATCTTATATCCAGGCAATATGACAAG
ENSPTRT00000011544     AGCTATATCACTTGTGTTTCAGGGGCTATTGTTGATCTTATATCCAGGCAATATGACAAA
ENSMUST00000043204     AGCTACGTCACTTGTGTCTCAGGGGCTATTGTTGATCTTATATCCAGGCAGTATGACAAG
ENSRNOT00000007221     AGCTACGTCACTTGTGTCTCAGGGGCTATTGTTGATCTTATATCCAGGCAGTATGACAAG

ENSCJAT00000031314     TTCCTTACTCATTTTATTTTAATGAATGATGTGATTGACACGTCTGGTTTTCCAGATCTT
ENSMMUT00000009759     TTCCTCACTCATTTTATTTTAATGAATGATGTGATTGACACGTCTGGTTTTCCAGATCTT
ENSPPYT00000006818     TTCCTTACTCATTTTATTTTAATGAATGATGTGATTGACACGTCTGGTTTTCCAGATCTT
ENST00000298097        TTCCTCACTCATTTTATTTTAATGAATGATGTGATTGACACATCTGGTTTTCCAGATCTT
ENSGGOT00000010826     TTCCTCACTCATTTTATTTTAATGAATGATGTGATTGACACGTCTGGTTTTCCAGATCTT
ENSPTRT00000011544     TTCCTCACTCATTTTATTTTAATGAATGATGTGATTGACACATCTGGTTTTCCAGATCTT
ENSMUST00000043204     TTCCTTACCCATTTTATATTGATGAATGATATGATTGACACATCTGGTTTTCCAGATCTT
ENSRNOT00000007221     TTCCTCACCCATTTTATATTGATGAATGATATGATTGATACGTCTGGTTTTCCGGATCTT

ENSCJAT00000031314     AGTGACAACCGAAATGAAGATCCGTTGGTTTTATTAGCATGGAGGTGCACAAAGCTCTCT
ENSMMUT00000009759     AGTGACAACCGAAATGAAGATCCGTTGGTTTTATTAGCATGGAGGTGCACAAAGCTCTCT
ENSPPYT00000006818     AGTGACAACCGAAATGAAGATCCGTTGGTTTTATTAGCATGGAGGTGCACAAAGCTCTCT
ENST00000298097        AGTGACAACCGAAATGAAGATCCGTTGGTTTTATTAGCATGGAGGTGCACAAAGCTCTCT
ENSGGOT00000010826     AGTGACAACCGAAATGAAGATCCGTTGGTTTTATTAGCATGGAGGTGCACAAAGCTCTCT
ENSPTRT00000011544     AGTGACAACCGAAATGAAGATCCGTTGGTTTTATTAGCATGGAGGTGCACAAAGCTCTCT
ENSMUST00000043204     AGTGACAACCGAAACGAAGATCCATTGGTTTTGTTAGCATGGAGGTGCACAAAGCTCACC
ENSRNOT00000007221     AGTGACAACCGAAATGAAGATCCATTGGTTTTATTGGCATGGCGGTGCACAAAGCTCACT

ENSCJAT00000031314     CTTCTGGCAATTCATGGTTACACCGTCTGGGCACACAACCTCATTGCCATTGCTCGTCTT
ENSMMUT00000009759     CTTCTGGCAATTCATGGTTACACGGTGTGGGCACATAACCTCATTGCCATTGCTCGTCTT
ENSPPYT00000006818     CTTCTGGCAGTTCATGGTTACACAGTGTGGGCACACAACCTCATTGCCATTGCTCGTCTT
ENST00000298097        CTTCTGGCAATTCATGGTTACACGGTGTGGGCACACAACCTCATTGCCATTGCTCGTCTT
ENSGGOT00000010826     CTTCTGGCAATTCATGGTTACACGGTGTGGGCACACAACCTCATTGCCATTGCTCGTCTT
ENSPTRT00000011544     CTTCTGGCAATTCATGGTTACACGGTGTGGGCACACAACCTCATTGCCATTGCTCGTCTT
ENSMUST00000043204     CTTTTGGCAATTCATGGTTACACCGTGTGGGCGCACAACCTCATTGCCATTGCTCGTCTT
ENSRNOT00000007221     CTTTTGGCAATTCATGGTTACACCGTGTGGGCACACAACCTCATTGCCATTGCTCGTCTT

ENSCJAT00000031314     CGGGGCTCTGATCTGAAAGTGCTCGAAGTCACTGAAGAAAGCATTGATTTTGACCAAGGT
ENSMMUT00000009759     CGGGGCTCTGATCTGAAAGTGCTTGAAGTCACCGAAGAAAGCATTGATTTTGACCAAGGT
ENSPPYT00000006818     CGGGGCTCTGATCTGAAAGTGCTTGAAGTCACCGAAGAAAGCATTGATTTTGACCAAGGT
ENST00000298097        CGGGGCTCTGATCTGAAAGTGCTTGAAGTCACCGAAGAAAGCATTGATTTTGACCAAGGT
ENSGGOT00000010826     CGGGGCTCTGATCTGAAAGTGCTTGAAGTCACCGAAGAAAGCATTGATTTTGACCAAGGT
ENSPTRT00000011544     CGGGGCTCTGATCTGAAAGTGCTTGAAGTCACCGAGGAAAGCATTGATTTTGACCAAGGT
```

```
ENSMUST00000043204     CGGGGCTCTGACCTAAAAGTGCTTCAAGTCACCGAAGAAAGCATTGATTTTGACCAAGGT
ENSRNOT00000007221     CGTGGCTCTGACCTAAAAGTGCTTGAAGTCACCGAAGAAAGCATTGATTTTGACCAAGGT

ENSCJAT00000031314     GAACTGGCCGACCAGGATGTAGATCCAGTGCATAACCTTATTGAGCAGGTATCCCTGGGC
ENSMMUT00000009759     GAACTGGCCGACCAGGATGTAGATCCAGTGCATAACCTTATTGAGCAGGTATCCCTGGGC
ENSPPYT00000006818     GAACTGGCCGACCAGGATGTAGATCCAGTGCATAACCTTATTGAGCAGGTATCCCTGGGC
ENST00000298097        GAACTGGCCGACCAGGATGTAGATCCAGTGCATAACCTTATTGAGCAGGTATCCCTGGGC
ENSGGOT00000010826     GAACTGGCCGACCAGGATGTAGATCCAGTGCATAACCTTATTGAGCAGGTATCCCTGGGC
ENSPTRT00000011544     GAACTGGCCGACCAGGATGTAGATCCAGTGCATAACCTTATTGAGCAGGTATCCCTGGGC
ENSMUST00000043204     GAACTAGCCGACCAGGATGTGGATCCAGTACAGAACCTTATTGAGCAGGTATCACTGGGC
ENSRNOT00000007221     GAACTAGCCGACCAGGATGTGGATCCCGTACATAACCTTCTTGAGCAGGTATCCCTGGGC

ENSCJAT00000031314     CTGGGTCAACCTTGGCATGCAGTCATGGACATCGAATCACTCAGTGTCTTCACTGAACCA
ENSMMUT00000009759     CTGGGTCAACCTTGGCACGCAGTCATGGACATCGAATCACTCAGTGTCTTCACTGAACCA
ENSPPYT00000006818     CTGGGTCAACCTTGGCATGCAGTCATGGACATCGAATCACTCAGTGTCTTCACTGAACCA
ENST00000298097        CTGGGTCAACCTTGGCATGCAGTCATGGACATCGAATCACTCAGTGTCTTCACTGAACCA
ENSGGOT00000010826     CTGGGTCAACCTTGGCATGCAGTCATGGACATCGAATCACTCAGTGTCTTCACTGAACCA
ENSPTRT00000011544     CTGGGTCAACCTTGGCATGCAGTCATGGACATCGAATCACTCAGTGTCTTCACTGAACCA
ENSMUST00000043204     CTTGGTCAGTCTTGGCACGCAGTCTTGGACATTGAATCACTCAGTGTCTTCACTGAACCA
ENSRNOT00000007221     CTTGGTCAGTCTTGGCACGCAGTCTTGGACATCGAATCACTCAGTGTCTTCACTGAACCA

ENSCJAT00000031314     AATCGTCATTTTTATAGAGAGATGCAAAGCTTCAGTGAAGACATT
ENSMMUT00000009759     AATCGTCATTTTTACAGAGAGATGCAAAGCTTCAGTGAAGACATT
ENSPPYT00000006818     AATCGTCATTTTTACAGAGAGATGCAAAGCTTCAGTGAAGACATT
ENST00000298097        AATCGTCATTTTTACAGAGAGATGCAAAGCTTCAGTGAAGACATT
ENSGGOT00000010826     AATCGTCATTTTTACAGAGAGATGCAAAGCTTCAGTGAAGACATT
ENSPTRT00000011544     AATCGTCATTTTTACAGAGAGATGCAAAGCTTCAGTGAAGACATT
ENSMUST00000043204     AATCGTCATTTTTACAGAGAGATGCAAAGCTTCAGCGAAGACATT
ENSRNOT00000007221     AATCGTCATTTTTACAGAGAGATGCAAAGCTTCAGCGAAGACATT
```

Multiple sequence alignment of Fbxo34

```
ENSCJAT00000059757     ATGCACCTAAAGCCATATTGGAAGCTCCAGAAGAAAGAGCACCCC------CTCAGCAGG
ENSMMUT00000032712     ATGCACCTAAAGCCATATTGGAAGCTCCAGAAGAAAGAGCACCCCCCGGAAGTCAGCAGG
ENSGGOT00000006879     ATGCACCTAAAGCCATATTGGAAGCTCCAGAAGAAAGAGCACCCCCCGGAAGTCAGCAGG
ENSPTRT00000011674     ATGCACCTAAAGCCATATTGGAAGCTCCAGAAGAAAGAGCACCCCCCGGAAGTCAGCAGG
ENST00000440021        ATGCACCTAAAGCCATATTGGAAGCTCCAGAAGAAAGAGCACCCCCCGGAAGTCAGCAGG
ENSMUST00000043112     ATGCACCTTAAGCTGTACTGGAAACTCCAGAAGAAAGAGCGCCCTCTTGAGGTCAGCAGG
ENSRNOT00000015778     ATGCACCTTAAGCCATACTGGAAGCTCCAGAAGAAAGAGCGGCCT---------------

ENSCJAT00000059757     GAAACGCAGAGAACTCCTATGAACCACCAAAAGGCTGTCAATGATGAAACATGCAACCCT
ENSMMUT00000032712     GAAACGCAGAGAACTCCTATGAACCACCAAAAGGCTGTAAATGATGAAACATGCAAAGCT
ENSGGOT00000006879     GAAACGCAGAGAACTCCTATGAACCACCAAAAGGCTGTAAATGATGAAACATGCAAAGCT
ENSPTRT00000011674     GAAACGCAGAGAACTCCTATGAACCACCAAAAGGCTGTAAATGATGAAACATGCAAAGCT
ENST00000440021        GAAACGCAGAGAACTCCTATGAACCACCAAAAGGCTGTAAATGATGAAACATGCAAAGCT
ENSMUST00000043112     GACACCCTGAGAACGCCTATGAGCCACGGAAAGGCCAACGGTGATGTGAAGGCCAGAGCC
ENSRNOT00000015778     ------------------CTGAGCCACGGAAACACGGACGGTGATGCGAAGGGCAGAGCT

ENSCJAT00000059757     AGCCACATAACACCAAGTGTCTTTCCTTCAGCCTCTCTTGGTAAAACATTGTCTCGAAAG
ENSMMUT00000032712     AGCCACGTAACACCAAGTGTCTTTCCTTCAGCCTCTCTTGGTAAAGCATCATCTCGAAAG
ENSGGOT00000006879     AGCCACATAACACCAAGTGTCTTTCCTTCAGCCTCTCTCAGTAAAGCATCATCTCGAAAG
ENSPTRT00000011674     AGCCACATAACACCAAGTGTCTTTCCTTCAGCCTCTCTCGGTAAAGCATCATCTCGAAAG
ENST00000440021        AGCCACATAACATCAAGTGTCTTTCCTTCAGCCTCTCTCGGTAAAGCATCATCTCGAAAG
ENSMUST00000043112     AGCTACATGAAACCCACCGTCCTTCCTTCAGCCTCCCTTGTCAAAGCATCGTCTAGAAAA
```

```
ENSRNOT00000015778     AACTGCATGAAACCCACCCCCTCCCCTTCCGCCTCTCCTGTCAAAGCATCATCTAGGAAG

ENSCJAT00000059757     CCTTTTGGGATCCTTTCTCCAAATGTTCTGTGCAGTATGAGTGGGAAGAGTCCTGTAGAG
ENSMMUT00000032712     CCGTTTGGGATCCTTTCTCCAAATGTTCTGTGCAGTATGAGTGGGAAGAGTCCTGTAGAG
ENSGGOT00000006879     CCATTTGGGATCCTTTCTCCAAATGTTCTGTGCAGTATGAGTGGGAAGAGTCCTGTAGAG
ENSPTRT00000011674     CCATTTGGGATCCTTTCTCCAAATGTTCTGTGCAGTATGAGTGGGAAGAGTCCTGTAGAG
ENST00000440021        CCATTTGGGATCCTTTCTCCAAATGTTCTGTGCAGTATGAGTGGGAAGAGTCCTGTAGAG
ENSMUST00000043112     CCTTTTGGGATCCTTTCTCCAAATGTTCTGTGCAGTATGAGTGGGAAGAGCCCTGTGGAG
ENSRNOT00000015778     CCTTTCGGGATCCTTTCTCCAAATGTCCTGTGCAGCATGAGTGGGAAGAGCCCCGTGGAG

ENSCJAT00000059757     AGCAGCTTGAATGTTAAAACCACAAAGAATGCACCATCTGCAACCATCCACCAGGGTGAA
ENSMMUT00000032712     AGCAGCTTGAATGTTAAAACCAAAAAGAATGCACCATCTGCAACGATCCACCAGGGCGAA
ENSGGOT00000006879     AGCAGCTTGAATGTTAAAACCAAAAAGAATGCACCATCTGCAACGATCCACCAGGGCGAA
ENSPTRT00000011674     AGCAGCTTGAATGTTAAAACCAAAAAGAATGCACCATCTGCAACGATCCACCAGGGCGAA
ENST00000440021        AGCAGCTTGAATGTTAAAACCAAAAAGAATGCACCATCTGCAACGATCCACCAGGGCGAA
ENSMUST00000043112     AACAGCTTGAATGTTAAAGCCAAAAAGAACGTGCTGTCTGCAGCCGTGCACCAGAGTGAG
ENSRNOT00000015778     AACAGCTTGAGTGTTAAAGCCACAAGGAATGCGCCGTCTGCAGCCGTGCAGCAGACTGAA

ENSCJAT00000059757     GAAGAAGGACCTCTTGATATCTGGGCTGTTGTGAAACCTGGGAATACAAAGGAAAAAATT
ENSMMUT00000032712     GAAGAAGGACCACTTGATATCTGGGCTGTTGTGAAACCTGGAAATACCAAGGAAAAAATT
ENSGGOT00000006879     GAAGAAGGACCACTTGATATCTGGGCTGTTGTGAAACCTGGAAATACCAAGGAAAAAATT
ENSPTRT00000011674     GAAGAAGGACCACTTGATATCTGGGCTGTTGTGAAACCTGGAAATACCAAGGAAAAAATT
ENST00000440021        GAAGAAGGACCACTTGATATCTGGGCTGTTGTGAAACCTGGAAATACCAAGGAAAAAATT
ENSMUST00000043112     GAG---GGGCTGCCTGGGACCTGGGCTATTGTCAAACCTGGGAACACGAAGGAGAAAATT
ENSRNOT00000015778     GGA---GGCCTGCCGGGGAGCTGGGCTATTGTCAAACCTGGAAACACCAAGGAGAAAATT

ENSCJAT00000059757     GCATTCTTTGCAGCCCACCAGTGTAGTAACAGGATAGGATCTATGAAAATAAAAAGTTCC
ENSMMUT00000032712     GCATTCTTTGCAGCCCACCAGTGTAGTAACAGGATAGGATCTATGAAAATAAAAAGTTCC
ENSGGOT00000006879     GCATTCTTTGCATCCCACCAGTGTAGTAACAGGATAGGATCTATGAAAATAAAAAGTTCC
ENSPTRT00000011674     GCATTCTTTGCATCCCACCAGTGTAGTAACAGGATAGGATCTATGAAAATAAAAAGTTCC
ENST00000440021        GCATTCTTTGCATCCCACCAGTGTAGTAACAGGATAGGATCTATGAAAATAAAAAGTTCC
ENSMUST00000043112     GCATTCTTTGCAGCCCACCAGTATAGCAATAGGATAGGCTCTATGAAAATTAAAAGTTCC
ENSRNOT00000015778     GCGTTCTTTGCAGCCCACCAGTGCAGCAATAGGATAGGCTCTATGAAAATAAAAAGCTCC

ENSCJAT00000059757     TGGGATATTGATGGGAGAGCTACTAAGAGAAGGAAAAAATCAGGGGATCTTAAAAAAGCA
ENSMMUT00000032712     TGGGATATTGATGGGAGAGCTACTAAGAGAAGGAAAAAATCAGGGGATCTTAGAAAAGCC
ENSGGOT00000006879     TGGGATATTGATGGGAGAGCTACTAAGAGAAGGAAAAAATCAGGGGATCTTAAAAAGGCC
ENSPTRT00000011674     TGGGATATTGATGGGAGAGCTACTAAGAGAAGGAAAAAATCAGGGGATCTTAAAAAAGCC
ENST00000440021        TGGGATATTGATGGGAGAGCTACTAAGAGAAGGAAAAAATCAGGGGATCTTAAAAAAGCC
ENSMUST00000043112     TGGGATATCGATGGGAGAGCCACTAAAAGAAGGAAAAAATCAGGAGATCTTAAGAAAGCC
ENSRNOT00000015778     TGGGATATCGATGGGAGAGCCACTAAACGAAGGAAAAAATCAGGGGATCTTAAGAAAGCC

ENSCJAT00000059757     AAGGTCCAGGTAGAAAGGATGAGGGAGGTTAACAGCAGGTGCTACCAACCTGAGCCTTTT
ENSMMUT00000032712     AAGGTACAGGTTGAAAGGATGAAGGAAGTTAACAGCAGGTGCTACCAACCTGAGCCTTTT
ENSGGOT00000006879     AAGGTACAGGTGGAAAGGATGAGGGAGGTTAACAGCAGGTGCTACCAACCTGAGCCTTTT
ENSPTRT00000011674     AAGGTACAGGTGGAAAGGATGAGGGAGGTTAACAGCAGGTGCTACCAACCTGAGCCTTTT
ENST00000440021        AAGGTACAGGTGGAAAGGATGAGGGAGGTTAACAGCAGGTGCTACCAACCTGAGCCTTTT
ENSMUST00000043112     AAGTTACAGCTAGAAATGATGAGAGAGATCAACAGCCAGTGCTACCAGTCTGAGCCGTTT
ENSRNOT00000015778     AAGTTACAGCTAGAAATGATGAGGGAGATCAACAGCCAGTGCTACCAGTCGGAGCCATTT

ENSCJAT00000059757     GCATGTGGCATTGAGCACTGTTCTGTCCATTATGTGAGTGACAGTTGTGATGGAGTCTAT
ENSMMUT00000032712     GCATGTGGCATTGAGCACTGTTCTGTGCATTATGTGAGTGACAGTGGGGATGGAGTCTAT
ENSGGOT00000006879     GCATGTGGCATTGAGCACTGTTCTGTGCACTATGTGAGTGACAGTGGGGATGGAGTCTAT
```

```
ENSPTRT00000011674     GCATGTGGCATTGAGCACTGTTCTGTGCACTATGTGAGTGACAGTGGGGATGGAGTCTAT
ENST00000440021        GCATGTGGCATTGAGCACTGTTCTGTGCACTATGTGAGTGACAGTGGGGATGGAGTCTAT
ENSMUST00000043112     GTGTGTGGCGTGGAGCACTGTTCTGTGCACTACATGAGTGACAGCGGGGACGGTGTCTGT
ENSRNOT00000015778     GCCTGTGGTGTCGAGCACTGTTCTGTGCATTACATGAGTGACAGTGGGGACGGTGTCTAT

ENSCJAT00000059757     GCCGGGAGGCCTCTGTCGGTTATACAGATGGTTGCCTTCTTGGAGCAAAGAGCCAGTGCT
ENSMMUT00000032712     GCCGGGAGGCCTCTGTCAGTTATACAGATGGTTGCCTTCTTGGAGCAAAGAGCCAGTGCT
ENSGGOT00000006879     GCTGGGAGGCCTCTGTCAGTTATACAGATGGTTGCCTTCTTGGAGCAAAGAGCCAGTGCT
ENSPTRT00000011674     GCTGGGAGGCCTCTGTCAGTTATACAGATGGTTGCCTTCTTGGAGCAAAGAGCCAGTGCT
ENST00000440021        GCTGGGAGGCCTCTGTCAGTTATACAGATGGTTGCCTTCTTGGAGCAAAGAGCCAGTGCT
ENSMUST00000043112     GCTGCGAGGCCTCTGTCGGTGATACAGATGGTTGCCTTCCTTGAGCAGAAGGCCACTGCT
ENSRNOT00000015778     GCTGCGAGGCCTCTGTCAGTGATACAGATGGTTGCCTTCCTCGAGCAGAGAGCCACTGCT

ENSCJAT00000059757     TTGCTAGCTAGTTGTACAAGAAACTGCACAAACTCACCTGCTATTGTGAGGTTTTCTGGC
ENSMMUT00000032712     CTGCTAGCTAGCTGTTCAAAAAACTGTACAAACTCACCTGCCATTGTGAGGTTTTCTGGC
ENSGGOT00000006879     CTGCTAGCTAGCTGTTCAAAAAACTGCACAAACTCACCTGCAATCGTGAGGTTTTCTGGC
ENSPTRT00000011674     CTGCTAGCTAGCTGTTCAAAAAACTGCACAAACTCACCTGCAATTGTGAGGTTTTCTGGC
ENST00000440021        CTGCTAGCTAGCTGTTCAAAAAACTGCACAAACTCACCTGCAATTGTGAGGTTTTCTGGC
ENSMUST00000043112     CTGCTAGCTAGCTGCACAAAAAACTGCACAAATTCGCCTGCCATTGTGAAGATTTCTGGC
ENSRNOT00000015778     CTGCTAGCTAGCTGCACAAAAAACTGCACAAATTCGCCTGCCATTGTGAAGATTTCTGGC

ENSCJAT00000059757     CAATCCAGAGGTGTGCCCGCAGCCTCTGAGTCCTATTCTGCCCCAGGAGCTTGTGAAGAA
ENSMMUT00000032712     CAATCCAGAGGTGTGCCCGCAGCCACCGAGTCCTGTTCTGCCCCAGGAGCTTGTGAA---
ENSGGOT00000006879     CAATCCAGAGGTGTGCCTGCAGTGTCTGAGTCCTATTCTGCCCCAGGAGCTTGTGAAGAA
ENSPTRT00000011674     CAATCCAGAGGTGTGCCTGCAGTGTCTGAGTCCTATTCTGCCCCAGGAGCTTGTGAAGAA
ENST00000440021        CAATCCAGAGGTGTGCCTGCAGTGTCTGAGTCCTATTCTGCCCCAGGAGCTTGTGAAGAA
ENSMUST00000043112     CAGTCCCGAGGTATACCGCCTGCACCTGAGCCCTTCTCTGCTCCGGAAACTTGTGAAGAA
ENSRNOT00000015778     CAGTCCCGAGGTATGCCACCTGTACCTGAGCCCTTCTCTGCTCCAGACACTTGTGAAGAA

ENSCJAT00000059757     CCCACAGAAAGGGGAAATCCTCAGGCTGTTGAACCACAAAGCGAACCAGTCCGTGTCCTC
ENSMMUT00000032712     ------GAAAGGGGAAATCTTGAGGTTGGTGAACCACAGAGCGAACCAGTCCGTGTCCTC
ENSGGOT00000006879     CCCACAGAAAGGGGAAATCTTGAGGTTGGTGAACCACAGAGCGAACCAGTCCGTGTCCTT
ENSPTRT00000011674     CCCACAGAAAGGGGAAATCTTGAGGTTGGTGAACCACAGAGCGAACCAGTCCGTGTCCTT
ENST00000440021        CCCACAGAAAGGGGAAATCTTGAGGTTGGTGAACCACAGAGCGAACCAGTCCGTGTCCTT
ENSMUST00000043112     CCCAAGGAACAAGAAAATCCCGAGACTGGCAGATCCCAGGGTGAGCCAGTCCGTGTCCTT
ENSRNOT00000015778     TCCAAGGAAAGAGAAAATCCTGAGATTGGCAGATCCCAGAGTGAGCCAGTCCGTGTCCTT

ENSCJAT00000059757     GACATGGTGGCCAAGTTGGAATCTGAGTGCCTGAAGCGGCAGAGCCACCGTGAGCCTGGG
ENSMMUT00000032712     GACATGGTAGCCAAGTTGGAGTCTGAGTGCCTGAAGCGGCAGGGCCAGCGTGAGCCTGGG
ENSGGOT00000006879     GACATGGTAGCCAAGTTGGAGTCTGAGTGCCTGAAGCGGCAGGGCCAGCGTGAGCCTGGG
ENSPTRT00000011674     GACATGGTAGCCAAGTTGGAGTCTGAGTGCCTGAAGCGGCAGGGCCAGCGTGAGCCTGGG
ENST00000440021        GACATGGTAGCCAAGTTGGAGTCTGAGTGCCTGAAGCGGCAGGGCCAGCGTGAGCCTGGG
ENSMUST00000043112     GACATGGTAGCCAGGTTGGAGTCTGAGTGCCTGAAGCACCAGGGCCAGCGGGAACCCGGG
ENSRNOT00000015778     GACATGGTAGCCAGGTTGGAGTCCGAGTGCCTGAAGCACCAGGGCCAGCGGGAACCTGGG

ENSCJAT00000059757     AGCCTCTCAAGGAATAACAGCTTCCGTCGAAATGTGGGTAGAGTATTGCTTGCAAATAGC
ENSMMUT00000032712     AGCCTCTCAAGGAATAACAGCTTTCGTCGAAATGTGGGCAGAGTTTTGCTTGCAAATAGC
ENSGGOT00000006879     AGCCTCTCAAGGAATAACAGCTTCCGTCGAAATGTGGGCAGAGTATTGCTTGCAAATAGC
ENSPTRT00000011674     AGCCTCTCAAGGAATAACAGCTTCCGTCGAAATGTGGGCAGAGTATTGCTTGCAAATAGC
ENST00000440021        AGCCTCTCAAGGAATAACAGCTTCCGTCGAAATGTGGGCAGAGTATTGCTTGCAAATAGC
ENSMUST00000043112     AGCTTGTCACGCAATAACAGTTTCCGTCGAAATGTAGGCAGGGTGTTGCTCACTAATGGT
ENSRNOT00000015778     AGCTTGTCACGGAATAACAGCTTCCGTCGGAATGTAGGCAGGGTGTTGCTCACAAACGGC
```

```
ENSCJAT00000059757    ACTCAGGCTGATGGAGGCAAAACAAAGAAAGGCGCCTTGGAGGTACCCGACACTCAGGTG
ENSMMUT00000032712    ACTCAGGCTGATGAAGGCAAAACAAAGAAAGGCGCCTTGGAGGCACCCGACACTCAGGTG
ENSGGOT00000006879    ACTCAGGCTGATGAAGGCAAAACAAAGAAAGGCGTCTTGGAGGCACCTGACACTCAGGTG
ENSPTRT00000011674    ACTCAGGCTGATGAAGGTAAAACAAAGAAAGGCGTCTTGGAGGCACCTGACACTCAGGTG
ENST00000440021       ACTCAGGCTGATGAAGGCAAAACAAAGAAAGGCGTCTTGGAGGCACCTGACACTCAGGTG
ENSMUST00000043112    TCTCAGGCCAGCGAC---AAAAGTGAAGAGGGCTCTGCAGACACAGCCGACCCTCAGGAG
ENSRNOT00000015778    TCTCAGGCCAGCGAC---AAAAACGGAGAGGGCTCTGCAGACACGCCCGGCACTCAGGTG

ENSCJAT00000059757    AATCCCATGGGGTCTGTATCTGTGGATTGTGGCCCCTCAAGAGCTGATCATTACTCTCCT
ENSMMUT00000032712    AATCCTGTGGGGTCTGTATCTGTGGATTGTGGCCCCTCAAGAGCTGATCATTGTTCTCCT
ENSGGOT00000006879    AATCCTGTGGGGTCTGTATCTGTGGATTGTGGCCCTTCAAGAGCTGATCGTTGTTCTCCT
ENSPTRT00000011674    AATCCTGTGGGGTCTGTATCTGTGGATTGTGGCCCTTCAAGAGCTGATCGTTGTTCTCCT
ENST00000440021       AATCCTGTGGGGTCTGTATCTGTGGATTGTGGCCCTTCAAGAGCTGATCGTTGTTCTCCT
ENSMUST00000043112    AATCCCTTGCAGCCGGTATCTGTGGGTGAAGAACCCTCCGTAACTGAGCATCATTCTGTA
ENSRNOT00000015778    CACCCCTTGCAGCTGGTATCTGTGGGTGAAGGACCCCCTGCAACTGACCGT---TCTGCA

ENSCJAT00000059757    AAAGAGGACCAGGCCTGGGATGGTGCTTCTCAGGACTGCCCCTCATTGCCAGCAGGTGTG
ENSMMUT00000032712    AAAGAGGACCAGGCCTGGGACAGTGCTTCTCAGGACTGCCCCCCATTGCCAGCAGGAGTG
ENSGGOT00000006879    AAGGAGGACCAGGCCTGGGACGGTGCTTCTCAGGACTGCCCCCCATTGCCAGCAGGAGTG
ENSPTRT00000011674    AAGGAGGACCAGGCCTGGGACGGTGCTTCTCAGGACTGCCCCCCATTGCCAGCAGGAGTG
ENST00000440021       AAGGAGGACCAGGCCTGGGACGGTGCTTCTCAGGACTGCCCCCCATTGCCAGCAGGAGTG
ENSMUST00000043112    GGGGAG---CAGGCCTGGGATGGCACTTCTCAAAGCTGTCCCTCATTGCCAGCCACTGTG
ENSRNOT00000015778    GGGGAG---CAGGCCTGGGATGGCACTTCTCAGGGCTGTCCCTCATTGCCGGCTGATGTG

ENSCJAT00000059757    AGTTTCCACATAGACAGTGCAGAGTTGGAGCTGGGTTCGCAAACTGCCATGAAAAACAGC
ENSMMUT00000032712    AGTTTCCACATAGACAGTGCAGAGTTAGAGCCGGGTTCACAAACTGCCATAAAAAACAGC
ENSGGOT00000006879    AGTTTCCACATAGACAGTGCAGAGTTAGAGCCGGGTTCGCAAACTGCCGTGAAAAACAGC
ENSPTRT00000011674    AGTTTCCACATAGACAGTGCAGAGTTAGAGCCGGGTTCGCAAACTGCCGTGAAAAACAGC
ENST00000440021       AGTTTCCACATAGACAGTGCAGAGTTAGAGCCGGGTTCGCAAACTGCCGTGAAAAACAGC
ENSMUST00000043112    AGCTTCCACATGGACAGCACAGATCTAGAGCCGGGTCAGCAAACAGCTATGAAGAGCTGC
ENSRNOT00000015778    AGCTTCCACACAGACAGCACCGAATCAGAGCCGGCTCTGCATACAGCTGTGAAACACTGC

ENSCJAT00000059757    AACAGGTATGATGTGGAAATGACAGATGAACTTGTTGGGTTAGCTTTTTCTTCTCATACC
ENSMMUT00000032712    AACAGGTATGATGTGGAAATGACAGATGAACTCGTTGGGTTACCTTTTTCTTCTCATACC
ENSGGOT00000006879    AACAGATATGATGTGGAAATGACAGATGAACTCGTTGGGTTACCTTTTTCCTCTCATACC
ENSPTRT00000011674    AACAGATATGATGTGGAAATGACAGATGAACTCGTTGGGTTACCTTTTTCCTCTCATACC
ENST00000440021       AACAGATATGATGTGGAAATGACAGATGAACTCGTTGGGTTACCTTTTTCCTCTCATACC
ENSMUST00000043112    AGCCGGGATGACGTAGAAATGGTGGAGGAATTT---------------------------
ENSRNOT00000015778    AGCCGCAATGATGTAGAAATGGCAGAGGAATTT---------------------------

ENSCJAT00000059757    TATTCCCAAGCCTCTGAATTGCCCACAGATGCAGTTGATTGTATGAGCAGAGAGCTCGTG
ENSMMUT00000032712    TATTCCCAAGCCTCTGAATTGCCCACAGATGCTGTTGATTGTATGAGCAGAGAGCTTGTG
ENSGGOT00000006879    TATTCCCAAGCCTCTGAATTGCCCACAGGTGCTGTTGATTGTATGAGCAGAGAGCTTGTG
ENSPTRT00000011674    TATTCCCAAGCCTCTGAATTGCCCACAAATGCTGTTGATTGTATGAGCAGAGAGCTTGTG
ENST00000440021       TATTCCCAAGCCTCTGAATTGCCCACAGATGCTGTTGATTGTATGAGCAGAGAGCTTGTG
ENSMUST00000043112    ------------GATGAATTGCCTACAGATGCTGTCCGTCGCATAAGAAGAGAGCTTGTA
ENSRNOT00000015778    ------------GATGAATTGCCTACAGATGCTGTCAGCTGT------AGAGAGCTTGTA

ENSCJAT00000059757    TCCCTTACTAGCCAAAATCCTGATCAAAGAAAAGAATCTTTGTGCATTAGTATCACTGTG
ENSMMUT00000032712    TCGCTTACTAGCCACAATCCTGATCAAAGAAAAGAATCTTTGTGCATTAGTATCACTGTG
ENSGGOT00000006879    TCGCTTACTAGCCAAAATCCTGATCAAAGAAAAGAATCTTTGTGCGTTAGTATCACTGTG
ENSPTRT00000011674    TCGCTTACTAGCCAAAATCCTGATCAAAGAAAAGAATCTTTGTGCATTAGTATCACTGTG
ENST00000440021       TCCCTTACTAGCCGAAATCCTGATCAAAGAAAAGAATCTTTGTGCATTAGTATCACTGTG
ENSMUST00000043112    ACAGTCACAAAGCACAGTCCTGAGCAGAGGCAGGACCCGCTGTGCATCAGCATCACTGTG
```

```
ENSRNOT00000015778     ACACTCACAAGCCACAGTCCTGAGCAGAGACAAGAACCTCTGTGCGTTAGCATCACTGTG

ENSCJAT00000059757     TCCAAGGTAGAGGAAGACCAGCCTTCCAGTTTAAACTCCTGTGAAGACCCACTTCCAGGG
ENSMMUT00000032712     TCCAAGGTAGAGAAAGACCAGCCTTCCAGTTTAAACTCCTGTGAAGACCCACTTCCAGGG
ENSGGOT00000006879     TCCAAGATAGACAAAGACCAGCCTTCCAATTTAAACTCCTGTGAAGACCCAGTTCCAGGG
ENSPTRT00000011674     TCCAAGGTAGACAAAGACCAGCCTTCCAATTTAAACTCCTGTGAAGACCCAGTTCCAGGG
ENST00000440021        TCCAAGGTAGACAAAGACCAGCCTTCCATTTTAAACTCCTGTGAAGACCCAGTTCCAGGG
ENSMUST00000043112     TGCACGGTAGAGAAAGACCGGCCGGCGGCTTTAGACTCCCTTGAGGAGCCTCTTCCCGGG
ENSRNOT00000015778     TGCACAGTGGAGAAAGCCCAGCCATCTGCGCTAGACCCCCTTGAAGAGCCTCTTCCTGGG

ENSCJAT00000059757     ATGTTGTTTTTTTTGCCACCTGGTCAGCAGTCGTCAGACTGTTCCCAGTTGAATGGAAGC
ENSMMUT00000032712     ATGTTGTTTTTTTTGCCACCTGGTCAGCACTTGTCAGACTGTTCCCAGTTGAATGAAAGC
ENSGGOT00000006879     ATGTTGTTTTTTTTGCCACCTGGTCAGCACTTGTCAGACTATTCCCAGTTGAATGAAAGC
ENSPTRT00000011674     ATGTTGTTTTTTTTGCCACCTGGTCAGCACTTGTCAGACTATTCCCAGTTGAATGAAAGC
ENST00000440021        ATGTTGTTTTTTTTGCCACCTGGTCAGCACTTGTCAGACTATTCCCAGTTGAATGAAAGC
ENSMUST00000043112     ATGCTCTTCTTTCTGTCATCTGGACAGGACCAGCAAGCGCACCCCCAGCTGAGGGAACAC
ENSRNOT00000015778     ATGCTGTTCTTTCTGTCACCTGGGCAGGACCAGCAGGCGCACCCCCAGTTGAGTGAACAC

ENSCJAT00000059757     ACAACAAAAGAGGCTTCAGAGGCCAGCCAGCTTGAAGATGCTGCTGAGGGTGACAGTGCA
ENSMMUT00000032712     ACAACAAGAGAGGCTTCAGAGGCCAGCCAGCTTGAAGATGCTGCTGGGGGTGACAGTGCA
ENSGGOT00000006879     ACAACAAAAGAGTCTTCAGAGGCCAGCCAGCTTGAAGATGCTGCTGGGGGTGACAGTGCA
ENSPTRT00000011674     ACAACAAAAGAGTCTTCAGAGGCCAGCCAGCTTGAAGATGCTGCTGGGGGTGACAGTGCA
ENST00000440021        ACAACAAAAGAGTCTTCAGAGGCCAGCCAGCTTGAAGATGCTGCTGGGGGTGACAGTGCA
ENSMUST00000043112     CCAGCACCAGAGGCCTCAGAGGCCAGTCAGCCTCAAGATGCTGCTGAGGGCAGCAGTGCA
ENSRNOT00000015778     CCAGCACCAGAGGCGACAGAGGCCAGTCAGCCTCCAGATGCTGCTGAGGGCAGCAGTGCC

ENSCJAT00000059757     TCTGAGGAGAAAAGTGGGTCAGCTGAGCCATTTGCATCACCAGCCTCTTCTGTGGAAAGT
ENSMMUT00000032712     TCTGAGGAAAAAAGTGGGTCTGCTGAGCCATTTGTGCCGCCAGTCTCTTCTGTGGAAAGT
ENSGGOT00000006879     TCTGAGGAAAAAAGTGGGTCTGCTGAGCCATTTGTACTGCCAGCCTCTTCTGTGGAAAGT
ENSPTRT00000011674     TCTGAGGAAAAAAGTGGGTCTGCTGAGCCATTTGTACCGCCAGCCTCTTCTGTGGAAAGT
ENST00000440021        TCTGAGGAAAAAAGTGGGTCTGCTGAGCCATTTGTACTGCCAGCCTCTTCTGTGGAAAGT
ENSMUST00000043112     GGTGAGGAGAAAGATGCCTCTGTGGAGCCGCTCCTCCCAGCAGCCTCTCCGGGAGGGAGC
ENSRNOT00000015778     CGTGAGGAGAAAGATGCCTCTCTGGAGCCGCTTGTTCCAGCAGCCTCTGGGGAAGGAAGC

ENSCJAT00000059757     ACATCACCAGTGCTTGAGGCATCCAGT---AAGAAGCAGGTGTCTCATGACTTCCTGGAG
ENSMMUT00000032712     ACATTACCAGTGCTTGAGGCATCCAGTTGGAAGAAGCAGGTGTCGCATGACTTCCTGGAG
ENSGGOT00000006879     ACATTACCAGTGCTTGAGGCATCCAGTTGGAAGAAGCAGGTGTCGCATGACTTCCTGGAG
ENSPTRT00000011674     ACATTACCAGTGCTTGAGGCATCCAGTTGGAAGAAGCAGGTGTCGCATGACTTCCTGGAG
ENST00000440021        ACATTACCAGTGCTTGAGGCATCCAGTTGGAAGAAGCAGGTGTCGCATGACTTCCTGGAG
ENSMUST00000043112     ACCTCGCAGGTGCTTGAGGCAGCCACGTGCAAGAAGCAGGTGTCACAGGACTTTCTGGAG
ENSRNOT00000015778     ACCTCACAGGGGCTTGAGGCAGCCACGTGTAAGAAGCAGGTGTCTCAGGACTTCCTGGAG

ENSCJAT00000059757     ACCAGGTTTAAAATCCAGCAGCTTTTGGAGCCTCAGCAGTACCTGGCTTTTCTACCCCAC
ENSMMUT00000032712     ACCAGGTTTAAAATCCAGCAGCTTTTGGAGCCTCAGCAGTACATGGCTTTTCTGCCCCAC
ENSGGOT00000006879     ACCAGGTTTAAAATCCAGCAGCTTTTGGAGCCTCAGCAGTACATGGCTTTTCTGCCCCAC
ENSPTRT00000011674     ACCAGGTTTAAAATCCAGCAGCTTTTGGAGCCTCAGCAGTACATGGCTTTTCTGCCCCAC
ENST00000440021        ACCAGGTTTAAAATCCAGCAGCTTTTGGAGCCTCAGCAGTACATGGCTTTTCTGCCCCAC
ENSMUST00000043112     ACCAGGTTTAAGATCCAGCAGCTTCTGGAGCCTCAGCAGTACATGGCATGCCTGCCCCAC
ENSRNOT00000015778     ACCAGGTTTAAAATCCAGCAACTTCTGGAACCTCAGCAGTACATGGCATGCCTGCCTCAC

ENSCJAT00000059757     CACATTATGGTAAAAATCTTCAGGTTACTTCCCACCAAGAGTTTAGTGGCCCTTAAATGT
ENSMMUT00000032712     CACATTATGGTAAAAATCTTCAGGTTACTTCCCACCAAGAGTTTAGTGGCCCTTAAATGT
ENSGGOT00000006879     CACATTATGGTAAAAATCTTCAGGTTACTTCCCACCAAGAGTTTAGTGGCCCTTAAATGT
```

```
ENSPTRT00000011674     CACATTATGGTAAAAATCTTCAGGTTACTTCCCACCAAGAGTTTAGTGGCCCTTAAATGT
ENST00000440021        CACATTATGGTAAAAATCTTCAGGTTACTTCCCACCAAGAGTTTAGTGGCCCTTAAATGT
ENSMUST00000043112     CACATCATAGTGAAAATCTTCAGGTTACTCCCTACCCTGAGTTTAGCCATTCTTAAATGT
ENSRNOT00000015778     CACATCATGGTGAAAATCTTCAGGTTACTCCCGACCCTGAGCTTAGCAATCCTTAAATGT

ENSCJAT00000059757     ACCTGCTGCTATTTCAAGTTTATCATTGAGTACTACAATATCAGGCCAGCAGATTCTCGC
ENSMMUT00000032712     ACCTGCTGCTATTTCAAGTTTATCATTGAGTACTACAATATCAGGCCAGCAGATTCTCGC
ENSGGOT00000006879     ACCTGCTGCTATTTCAAGTTTATCATTGAGTACTACAATATCAGGCCAGCAGATTCTCGC
ENSPTRT00000011674     ACCTGCTGCTATTTCAAGTTTATCATTGAGTACTACAATATCAGGCCAGCAGATTCTCGC
ENST00000440021        ACCTGCTGCTATTTCAAGTTTATCATTGAGTACTACAATATCAGGCCAGCAGATTCTCGC
ENSMUST00000043112     ACCTGTCGCTACTTCAAGTCCATCATTGAATACTACAACATCAGGCCAGCAGATTCTCGG
ENSRNOT00000015778     ACCTGCCGCTACTTCAAGTCCATCATTGAATACTACAACATCAGGCCAGCGGACTCTCGG

ENSCJAT00000059757     TGGGTTCGAGATCCACGCTATAGAGAGGATCCTTGCAAGCAGTGCAAGAAAAAGTATGTG
ENSMMUT00000032712     TGGGTTCGAGATCCACGCTACAGAGAAGATCCTTGCAAACAGTGCAAGAAAAAGTATGTG
ENSGGOT00000006879     TGGGTTCGAGATCCACGCTATAGAGAGGATCCTTGCAAACAGTGCAAGAAAAAGTATGTG
ENSPTRT00000011674     TGGGTTCGAGATCCACGCTATAGAGAGGATCCTTGCAAACAGTGCAAGAAAAAGTATGTG
ENST00000440021        TGGGTTCGAGATCCACGCTATAGAGAGGATCCTTGCAAACAGTGCAAGAAAAAGTATGTG
ENSMUST00000043112     TGGGTTCGAGACCCACGCTATAGGGAAGACCCTTGCAAGCAGTGCAAGAAAAAGTATGTG
ENSRNOT00000015778     TGGGTCCGAGACCCGCGCTATAGAGAAGACCCTTGCAAGCAGTGCAAGAAAAAGTATGTG

ENSCJAT00000059757     AAAGGGGATGTGTCCCTGTGCCGATGGCACCCCAAGCCCTATTGCCAGGCATTGCCCTAT
ENSMMUT00000032712     AAAGGGGATGTGTCCCTGTGCCGATGGCACCCCAAGCCCTATTGCCAGGCATTGCCCTAT
ENSGGOT00000006879     AAAGGGGATGTGTCCCTGTGCCGATGGCACCCCAAGCCCTATTGCCAGGCATTGCCCTAT
ENSPTRT00000011674     AAAGGGGATGTGTCCCTGTGCCGATGGCACCCCAAGCCGTATTGCCAGGCATTGCCCTAT
ENST00000440021        AAAGGGGATGTGTCCCTGTGCCGATGGCACCCCAAGCCCTATTGCCAGGCATTGCCCTAT
ENSMUST00000043112     AAAGGGGATGTGTCCCTGTGCCGGTGGCACCCCAAACCCTATTGCCAGGCTTTGCCATAT
ENSRNOT00000015778     AAAGGGGACGTGTCCCTGTGCCGGTGGCACCCCAAACCCTACTGCCAGGCATTGCCGTAT

ENSCJAT00000059757     GGGCCAGGGTATTGGATGTGCTGCCACCGGTCTCAGAAAGGATTTCCCGGCTGTAAGCTG
ENSMMUT00000032712     GGGCCAGGGTATTGGATGTGCTGCCACCGGTCTCAGAAAGGATTCCCTGGCTGTAAGCTG
ENSGGOT00000006879     GGGCCAGGGTATTGGATGTGCTGCCACCGGTCTCAGAAAGGATTCCCTGGCTGTAAGCTG
ENSPTRT00000011674     GGGCCAGGGTATTGGATGTGCTGCCACCGGTCTCAGAAAGGATTCCCTGGTTGTAAGCTG
ENST00000440021        GGGCCAGGGTATTGGATGTGCTGCCACCGGTCTCAGAAAGGATTCCCTGGCTGTAAGCTG
ENSMUST00000043112     GGGCCTGGCTACTGGATGTGCTGCCACCAGTCTCAGAAGGGCTTTCCTGGCTGTAAGTTA
ENSRNOT00000015778     GGGCCTGGGTACTGGATGTGCTGCCACCAGTCTCAGAAGGGCTTTCCGGGCTGTAAGTTA

ENSCJAT00000059757     GGGCTTCATGACAATCACTGGGTTCCTGCCTGCCACAGCTTTAATCGGGCAATCCATAAG
ENSMMUT00000032712     GGGCTTCATGACAATCACTGGGTTCCTGCCTGCCACAGCTTTAATCGGGCAATCCATAAG
ENSGGOT00000006879     GGGCTTCATGACAATCACTGGGTTCCTGCCTGCCACAGCTTTAATCGGGCAATCCATAAG
ENSPTRT00000011674     GGGCTTCATGACAATCACTGGGTTCCTGCCTGCCACAGCTTTAATCGGGCAATCCATAAG
ENST00000440021        GGGCTTCATGACAATCACTGGGTTCCTGCCTGCCACAGCTTTAATCGGGCAATCCATAAG
ENSMUST00000043112     GGCCTTCATGACAATCACTGGTTACCTGCCTGCCATAGCTTTAACCGGGCAATCCATAAG
ENSRNOT00000015778     GGCCTTCATGACAATCACTGGTTACCCGCCTGCCACAGCTTTAATCGGGCAATCCATAAG

ENSCJAT00000059757     AAAGCAAAAGGGACTGAAGCTGAAGAGGAATAC
ENSMMUT00000032712     AAGGCAAAAGGGACTGAAGCTGAAGAGGAATAC
ENSGGOT00000006879     AAAGCAAAAGGGACTGAAGCTGAAGAGGAATAC
ENSPTRT00000011674     AAAGCAAAAGGGACTGAAGCTGAAGAGGAATAC
ENST00000440021        AAAGCAAAAGGGACTGAAGCTGAAGAGGAATAC
ENSMUST00000043112     AAGACGAGAGGGAGTGAGACGGAAGAGGAGTAC
ENSRNOT00000015778     AAATCAAGAGGGAGTGAAACGGAAGAAGAGTAC
```

Multiple sequence alignment of Fbxo36

```
ENSCJAT00000025714      ATGGCGTCGTGGCTGCCGGAGACTCTGTTCGAAATTGTAGGACAAGGCCCGCCGCCAAGC
ENSPPYT00000015408      ATGGCGTCGTGGCTGCCGGAGACTCTCTTTGAAACTGTAGGACAAGGCCCGCCGCCGAGC
ENST00000283946         ATGGCGTCGTGGCTGCCGGAGACTCTCTTTGAAACTGTAGGACAAGGCCCGCCGCCTAGC
ENSPTRT00000056028      ATGGCGTCGTGGCTGCCGGAGACTCTCTTTGAAACTGTAGGACAAGGCCCGCCGCCTAGC
ENSMUST00000097672      ATGGCGTCGTGGCTGCCGGAGACTCTGTTTGAAATTGTAGGACAAGGCCCAGCGCCGAGC
ENSRNOT00000022901      ATGGCGTCGTGGCTACCGGAGACTCTGTTTGAAATTGTAGGACAAGGCCCGGCGCCGAGC


ENSCJAT00000025714      AAAGACTATTACCAGTTACTGGTCACCCGGTCTCAGGTAATCTTTAGATGGTGGACGATC
ENSPPYT00000015408      AAAGACTATTACCAGTTACTGGTCACCCGGTCTCAGGTAATCTATAGATGGTGGAAGATC
ENST00000283946         AAAGACTATTACCAGTTACTGGTCACCCGGTCTCAGGTAATCTTTAGATGGTGGAAGATC
ENSPTRT00000056028      AAAGACTATTACCAGTTACTGGTCACCCGGTCTCAGGTAATCTTTAGATGGTGGAAGATC
ENSMUST00000097672      AAAGACTATTACCAGTTGTTGATCACCCGGACTCAGATCATCTTTAGATGGTGGAAGATT
ENSRNOT00000022901      AAAGACTATTACCAGTTGTTGATCACCCGAACTCAGATCATCTTTAGATGGTGGAAGATT

ENSCJAT00000025714      TCTCTAAGGAGTGAGTATCGATCAACAAAACCTGGAGAAACAAAAGAAACCCATGAAGAC
ENSPPYT00000015408      TCTCTAAGGAGTGAGTATCGATCAACAAAACCTGGAGAAGCAAAAGAAACCCATGAAGAC
ENST00000283946         TCTCTAAGGAGTGAGTATCGATCAACAAAACCTGGAGAAGCAAAAGAAACCCATGAAGAC
ENSPTRT00000056028      TCTCTAAGGAGTGAGTATCGATCAACAAAACCTGGAGAAGCAAAAGAAACCCATGAAGAC
ENSMUST00000097672      TCTCTGAGAAGTGAGTATCGGTCAGCAAAACCTGGAGAAACAAAGGAAAGCCATGAAGAC
ENSRNOT00000022901      TCCCTGAGAAGTGAGTATCGGTCAGCAAAACCTGGAGAGACAAAGGAAACTCACGAAGAC


ENSCJAT00000025714      TTCCTAGAGAATGCCCATCTTCAAAGTCAAACTGCCATAATATTTGGTGTAAGAATATTA
ENSPPYT00000015408      TTCCTAGAGAATTCACATCTTCAAGGTCAAACTGCCTTAATATTTGGTGCAAGAATATTA
ENST00000283946         TTCCTAGAGAATTCACATCTTCAAGGTCAAACTGCCTTAATATTTGGTGCAAGAATATTA
ENSPTRT00000056028      TTCCTAGAGAATTCACATCTTCAAGGTCAAACTGCCTTAATATTTGGCGCAAGAATATTA
ENSMUST00000097672      TTCCTAGATAACTCTCATCTCCAAGTTCAAGTCGCCGTAGTATTTGGTACAAAAATATTA
ENSRNOT00000022901      TTCCTAGATAACTCTCATCTTCAAGTTCAAGTCGCCGTAGTATTTGGTGCAAAGATATTA


ENSCJAT00000025714      GACTATGTCATCAATCTGTGCAAAGGTCAAGTCGACTTCCTTGAACGGCTCTCTGACGAT
ENSPPYT00000015408      GACTATGTCATCAATTTGTGCAAAGGTAAATTTGACTTCCTTGAACGGCTCTCAGACGAT
ENST00000283946         GACTATGTCATCAATTTGTGCAAAGGTAAATTTGACTTCCTTGAACGGCTCTCAGACGAT
ENSPTRT00000056028      GACTATGTCATCAATTTGTGCAAAGGTAAATTTGACTTCCTTGAACGGCTCTCAGACGAT
ENSMUST00000097672      GACTATGTCTTCAACCTGTGCGAAGGTAAATTTGACTACCTGGAGCGGCTCTCCGACAGA
ENSRNOT00000022901      GACTATGTCTTCAACCTGTGTGAAGGTAAATTTGACTACCTGGAGCGGCTCTCTGACAAA

ENSCJAT00000025714      TTGCTCCTGAATATCATTTCTTATCTGGATCTGGAAGACATTGCCAGGCTCTCTCAAACA
ENSPPYT00000015408      TTGCTCCTGAATATCATTTCTTATCTGGATCTTGAAGATATTGCCAGGCTTTGTCAAACA
ENST00000283946         TTGCTCCTGACTATCATTTCTTATCTGGATCTTGAAGATATTGCCAGGCTTTGTCAAACA
ENSPTRT00000056028      TTGCTCCTGAATATCATTTCTTATCTGGATCTTGAAGATATTGCCAGGCTTTGTCAAACA
ENSMUST00000097672      CTGCTGCTGAAGATCATCTGCTACCTGGACCTGGAAGATATTGCCAGCCTCTCCCAGACA
ENSRNOT00000022901      CTGCTGCTGAAGATCATGAACTACCTGGACTTGGAAGACATCGCCAGGCTCTCTCAGACA

ENSCJAT00000025714      TCACACAGATTTGCAAAGCTGTGCATGTCTGATAAACTGTCGGAACAGATAGTCCAGTCG
ENSPPYT00000015408      TCACACAGATTTGCAAAGCTGTGCATGTCTGATAAACTGTGGGAACAGATAGTCCAGTCG
ENST00000283946         TCACACAGATTTGCAAAGCTGTGCATGTCTGATAAACTGTGGGAACAGATAGTCCAGTCG
ENSPTRT00000056028      TCACACAGATTTGCAAAGCTGTGCATGTCTGATAAACTGTGGGAACAGATAGTCCAGTCG
ENSMUST00000097672      TCAAGCAAGTTTGAGAAGTTGTGCAAGAGTGACTTGCTGTGGGAACAGATAGTCCAGTCC
ENSRNOT00000022901      TCAAGCCGGTTTGAGAAGCTGTGCAAGAGTGACTCGCTGTGGGAGCAGATAGTCCAGTCC

ENSCJAT00000025714      ACTTGTGACACCATCACTCCCGACATGAAGGCCCTGGCAGCGGACGTGGG-------AAG
ENSPPYT00000015408      ACCTGCGACACCATCACTCCTGACGTGAGGGCCCTGGCGGAGGACACGGGCTGGAGACAG
ENST00000283946         ACCTGCGACACCATCACTCCTGACGTGAGGGCCCTGGCGGAGGACACAGGCTGGAGACAG
ENSPTRT00000056028      ACCTGCGACACCATCACTCCTGACGTGAGGGCCCTGGCGGAGGACACGGGCTGGAGACAG
ENSMUST00000097672      ACCTGTGACACCATCACCCCTGACATGCGGGCCTTGGCGAAAAACATGGGCTGGAGGCAG
ENSRNOT00000022901      ACCTATGACACCATCACCCCTGACATGCGGGCCCTGGCGAATGACGTGGGCTGGAGACAG
```

```
ENSCJAT00000025714      GTGTGCTTCACCAACAAGCTCCAGCCC------CAGCTCCGCAGGAGGAAACAAAAGCAC
ENSPPYT00000015408      CTGTTCTTCACCAACAAGCTCCAGCTCCAGCGTCAGCTCCGCAAGAGGAAACAAAAATAT
ENST00000283946         CTGTTCTTCACCAACAAGCTCCAGCTCCAGCGGCAGCTCCGCAAGAGGAAACAAAAATAT
ENSPTRT00000056028      CTGTTCTTCACCAACAAGCTCCAGCTTCAGCGGCAGCTCCGCAAGAGGAAACAAAAATAT
ENSMUST00000097672      ATGTTCCTCACCAACAACATCCAGCTCCAGAGGCAGACCCGCAAGAAGAAGCAAAGACAG
ENSRNOT00000022901      ATGTTCTTCACCAACAAGATCCAGCTCCAGAGGCAGATCCGCAAGAAGAAGCAAAGACAG

ENSCJAT00000025714      GGAAGCC-----------------
ENSPPYT00000015408      GGAAACCTGAGAGAAAAGCAACCT
ENST00000283946         GGAAACCTGAGAGAAAAGCAACCT
ENSPTRT00000056028      GGAAACCTGAGAGAAAAGCAACCT
ENSMUST00000097672      GAAAACCAGGCAGAGAAGCTTGCT
ENSRNOT00000022901      GGAAGCGAGACTACGAAGCCTACT
```

Multiple sequence alignment of Fbxo38

```
ENSMUST00000048688     ATGGGACCACGAAAGAAAAGTGCTAAAGTGTGTGTGATGGACAGTGAGGTCGCTGAGGAA
ENSRNOT00000025790     ATGGGACCACGAAAGAAAAGTGTTAAAGTGTGTGTGATGGACAGTGAGGTCCCCGAGGAA
ENSGGOT00000026421     ATGGGGCCACGAAAGAAAAGTGTGAAAACATGTATCATGAATAATGAAATTCCAGAAGAA
ENSPPYT00000018532     ATGGGGCCACGAAAAAAAAGTGTGAAAACATGTATCATGAATAATGAAATTCCAGAAGAA
ENSCJAT00000041759     ATGGGGCCACGAAAGAAAAGTGTGAAAACATGTATTATGAATAATGAAATTCCAGAAGAA
ENSMMUT00000023749     ATGGGGCCACGAAAGAAAAGTGTGAAAACATGTATCATGAATAATGAAATTCCAGAAGAA
ENST00000340253        ATGGGGCCACGAAAGAAAAGTGTGAAAACATGTATCATGAATAATGAAATTCCAGAAGAA
ENSPTRT00000032169     ATGGGGCCACGAAAGAAAAGTGTGAAAACATGTATCATGAATAATGAAATTCCAGAAGAA


ENSMUST00000048688     ATGACAGCCGATGAAGAGAAGGACTACATGAACCAGCTGTCACATGAAGTGCTCTGTCAT
ENSRNOT00000025790     ATGACAGCTGATGAAGAGAAGGACTACATGAACCAGCTGTCACATGAAGTGCTATGTCAT
ENSGGOT00000026421     ATGACAGCAGATGAAACAAAGGACTATATGAATCAACTTTCACATGAAGTACTTTGCCAT
ENSPPYT00000018532     ATGACAGCAGATGAAACAAAGGACTATATGAATCAACTTTCACATGAAGTACTTTGCCAT
ENSCJAT00000041759     ATGACAGCAGATGAAACAAAGGACTATATGAATCAACTTTCACATGAAGTACTTTGCCAT
ENSMMUT00000023749     ATGACAGCAGATGAAACAAAGGACTATATGAATCAACTTTCACATGAAGTACTTTGCCAT
ENST00000340253        ATGACAGCAGATGAAACAAAGGACTATATGAATCAACTTTCACATGAAGTACTTTGCCAT
ENSPTRT00000032169     ATGACAGCAGATGAAACAAAGGACTATATGAATCAACTTTCACATGAAGTACTTTGCCAT


ENSMUST00000048688     ATCTTCAGGTACCTGCCACTACAGGACATCATGTGCATGGAATGCCTCTCTCGGAAGCTA
ENSRNOT00000025790     ATTTTCAGGTACCTGCCACTGCAGGATATCATGTGCATGGAATGTCTTTCTCGGAAGCTA
ENSGGOT00000026421     ATTTTTAGGTACCTCCCTCTGCAGGATATCATGTGTATGGAATGTCTTTCCCGGAAGCTA
ENSPPYT00000018532     ATTTTTAGGTACCTGCCTCTGCAGGATATCATGTGTATGGAATGTCTTTCCCGGAAACTA
ENSCJAT00000041759     ATTTTTAGGTACCTCCCTCTACAGGATATCATGTGTATGGAATGTCTTTCCCGGAAGCTT
ENSMMUT00000023749     ATTTTTAGGTACCTCCCTCTGCAGGATATCATGTGTATGGAATGTCTTTCCCGGAAGCTA
ENST00000340253        ATTTTTAGGTACCTCCCTCTGCAGGATATCATGTGTATGGAATGTCTTTCCCGGAAGCTA
ENSPTRT00000032169     ATTTTTAGGTACCTCCCTCTGCAGGATATCATGTGTATGGAATGTCTTTCCCGGAAGCTA


ENSMUST00000048688     AAGGAGGCAGTCACTCTGTATCTGAGAGTTGTCAGAGTGGTGGATCTCTGTGCAGGGCGC
ENSRNOT00000025790     AAGGAAGCAGTTACTCTGTATCTGAGAGTTGTGAGAGTGGTGGATCTCTGCGCAGGGCGG
ENSGGOT00000026421     AAGGAAGCAGTGACCCTATATCTGCGAGTTGTGAGAGTTGTAGATCTCTGTGCAGGGCGG
ENSPPYT00000018532     AAGGAAGCAGTGACCCTATATCTGCGAGTTGTGAGAGTTGTAGATCTCTGTGCAGGGCGG
ENSCJAT00000041759     AAGGAAGCAGTGACCCTTTATCTTCGAGTTGTGAGAGTTGTGGATCTCTGTGCAGGGCGG
ENSMMUT00000023749     AAGGAAGCAGTGACCCTGTATCTGCGAGTTGTGAGAGTTGTAGATCTCTGTGCAGGGCGG
ENST00000340253        AAGGAAGCAGTGACCCTATATCTGCGAGTTGTGAGAGTTGTAGATCTCTGTGCAGGGCGG
ENSPTRT00000032169     AAGGAAGCAGTGACCCTATATCTGCGAGTTGTGAGAGTTGTAGATCTCTGTGCAGGGCGG


ENSMUST00000048688     TGGTGGGAATATATGCCAAGTGGCTTCACAGATTCCAGTTTCCTAACCCTTTTGAAGAAG
```

```
ENSRNOT00000025790     TGGTGGGAATATATGCCAAGTGGCTTCACAGATTGCAGTTTCCTGACACTTTTGAAGAAG
ENSGGOT00000026421     TGGTGGGAATACATGCCAAGTGGCTTTACAGATTCCAGTTTTCTAACACTATTAAAGAAG
ENSPPYT00000018532     TGGTGGGAATACATGCCAAGTGGCTTTACAGATTCCAGTTTTCTAACACTATTAAAGAAG
ENSCJAT00000041759     TGGTGGGAATACATGCCAAGTGGCTTTACAGATTCCAGTTTTCTAACACTATTAAAGAAG
ENSMMUT00000023749     TGGTGGGAATACATGCCAAGTGGCTTTACAGATTCCAGTTTTCTAACACTATTAAAGAAG
ENST00000340253        TGGTGGGAATACATGCCAAGTGGCTTTACAGATGCCAGTTTTCTAACACTATTAAAGAAG
ENSPTRT00000032169     TGGTGGGAATACATGCCAAGTGGCTTTACAGATTCCAGTTTTCTAACACTATTAAAGAAG

ENSMUST00000048688     ATGCCAGATGTTGAACAGCTGTATGGCCTTCACCCTAGATACCTCGAGAGGCGAAGAGTG
ENSRNOT00000025790     ATGCCGGATGTCGAGCAGCTGTACGGCCTTCACCCTAGATACCTCGAGAGGCGGAGAGTG
ENSGGOT00000026421     ATGCCAGATGTTGAACAGCTATATGGCCTTCACCCTCGATACCTTGAGAGGCGAAGAGTA
ENSPPYT00000018532     ATGCCAGATGTTGAACAGCTATATGGCCTTCACCCTCGATACCTTGAGAGGCGAAGAGTA
ENSCJAT00000041759     ATGCCAGATGTTGAACAGCTATATGGCCTTCACCCTCGGTACCTTGAGAGGCGAAGAGTA
ENSMMUT00000023749     ATGCCAGATGTTGAACAGCTATATGGCCTTCACCCTCGATACCTTGAGAGGCGAAGAGTA
ENST00000340253        ATGCCAGATGTTGAACAGCTATATGGCCTTCACCCTCGATACCTTGAGAGGCGAAGAGTA
ENSPTRT00000032169     ATGCCAGATGTTGAACAGCTATATGGCCTTCACCCTCGATACCTTGAGAGGCGAAGAGTA

ENSMUST00000048688     AGGGGCCAGGAGGCTTTCAGCATCCCAGGAGTCTTGGAAGCTTTGCAAGCGTGCCCAAAC
ENSRNOT00000025790     AGGGGCCATGAGGCTTTCAGCATTCCAGGGGTCCTGGAGGCCTTGCAAGCCTGCCCAAAC
ENSGGOT00000026421     AGGGGCCATGAGGCTTTTAGCATTCCAGGAGTCCTAGAAGCTTTGCAGGCATGCCCAAAC
ENSPPYT00000018532     AGGGGCCATGAGGCTTTTAGCATTCCAGGAGTCCTGGAAGCTTTGCAGGCATGCCCAAAC
ENSCJAT00000041759     AGGGGCCATGAGGCTTTTAGCATTCCAGGAGTCCTAGAAGCTTTGCAGGCATGCCCAAAC
ENSMMUT00000023749     AGGGGCCATGAGGCTTTTAGCATTCCAGGAGTCCTAGAAGCTTTGCAGGCATGCCCAAAC
ENST00000340253        AGGGGCCATGAGGCTTTTAGCATTCCAGGAGTCCTAGAAGCTTTGCAGGCATGCCCAAAC
ENSPTRT00000032169     AGGGGCCATGAGGCTTTTAGCATTCCAGGAGTCCTAGAAGCTTTGCAGGCATGCCCAAAC

ENSMUST00000048688     TTGGTGGGTGTAGAAACATCTCATTTGGAGTTGGTAGAATCCATTTGGACATATATGCCG
ENSRNOT00000025790     CTGGTGGGTGTAGAAACATCTCATTTGGAGTTGGTAGAATCCATTTGGACATATATGCCG
ENSGGOT00000026421     TTAGTGGGTGTGGAAACTTCTCATTTGGAGTTGGTAGAATCCATTTGGACATATATGCCC
ENSPPYT00000018532     TTAGTGGGTGTGGAAACTTCTCATTTGGAGTTGGTAGAATCCATTTGGACATATATGCCC
ENSCJAT00000041759     TTAGTGGGTGTGGAAACTTCTCATTTGGAGTTGGTAGAATCCATTTGGACATATATGCCA
ENSMMUT00000023749     TTAGTGGGTGTGGAAACTTCTCATTTGGAATTGGTAGAATCCATTTGGACATATATGCCC
ENST00000340253        TTAGTGGGTGTGGAAACTTCTCATTTGGAGTTGGTAGAATCCATTTGGACATATATGCCC
ENSPTRT00000032169     TTAGTGGGTGTGGAAACTTCTCATTTGGAGTTGGTAGAATCCATTTGGACATATATGCCC

ENSMUST00000048688     CATGTTCATATTTTGGGGAAATTTCGTAATCGTAACGGAGCATTTCCGATTCCTCCTGAA
ENSRNOT00000025790     CATGTTCATATTTTGGGGAAATTTCGTAATCGTAACGGAGCATTTCCGATTCCTCCTGAA
ENSGGOT00000026421     CATGTTCATATTTTGGGGAAATTTCGTAATCGTAATGGAGCTTTTCCAATTCCTCCTGAA
ENSPPYT00000018532     CATGTTCATATTTTGGGGAAATTTCGTAATCGTAATGGAGCTTTTCCAATTCCTCCTGAA
ENSCJAT00000041759     CATGTTCATATTTTAGGGAAATTTCGTAATCGTAATGGAGCCTTTCCAATTCCTCCTGAA
ENSMMUT00000023749     CATGTTCATATTTTGGGGAAATTCCGTAATCGTAATGGAGCCTTTCCAATTCCTCCTGAA
ENST00000340253        CATGTTCATATTTTGGGGAAATTTCGTAATCGTAATGGAGCTTTTCCAATTCCTCCTGAA
ENSPTRT00000032169     CATGTTCATATTTTGGGGAAATTTCGTAATCGTAATGGAGCTTTTCCAATTCCTCCTGAA

ENSMUST00000048688     AATAAACTGAAAATCCCCATAGGAGCCAAAATCCAAACTTTGCATTTAGTTGGTGTCAAT
ENSRNOT00000025790     AATAAACTGAAAATCCCCATAGGAGCCAAAATCCAAACTTTGCATTTAGTTGGGGTCAAT
ENSGGOT00000026421     AATAAACTGAAAATTCCTGTAGGAGCCAAAATTCAAACTTTACATTTAGTTGGGGTGAAT
ENSPPYT00000018532     AATAAACTGAAAATTCCTGTAGGAGCCAAAATCCAAACTTTACATTTAGTTGGGGTGAAT
ENSCJAT00000041759     AATAAACTGAAAATTCCTATAGGAGCCAAAATTCAAACTTTACATTTAGTTGGGGTAAAT
ENSMMUT00000023749     AATAAACTGAAAATTCCTATAGGAGCCAAAATTCAAACTTTACATTTAGTTGGGGTGAAT
ENST00000340253        AATAAACTGAAAATTCCTATAGGAGCCAAAATTCAAACTTTACATTTAGTTGGGGTGAAT
ENSPTRT00000032169     AATAAACTGAAAATTCCTATAGGAGCCAAAATTCAAACTTTACATTTAGTTGGGGTGAAT

ENSMUST00000048688     GTTCCTGAAATCCCTTGTATCCCTATGCTAAGGCACCTGTACATGAAGTGGGTGAGACTC
```

```
ENSRNOT00000025790      GTTCCTGAAATCCCTTGTATTCCTATGCTGAGGCACCTGTACATGAAGTGGGTGAGACTC
ENSGGOT00000026421      GTTCCTGAAATTCCTTGTATCCCAATGCTAAGGCACCTTTATATGAAGTGGGTAAGACTC
ENSPPYT00000018532      GTTCCTGAAATTCCTTGTATCCCAATGCTAAGGCACCTTTATATGAAGTGGGTAAGACTT
ENSCJAT00000041759      GTTCCTGAAATTCCCTGTATCCCAATGCTAAGGCACCTTTATATGAAGTGGGTAAGACTC
ENSMMUT00000023749      GTTCCTGAAATTCCTTGTATCCCAATGCTAAGGCACCTTTATATGAAGTGGGTAAGACTT
ENST00000340253         GTTCCTGAAATTCCTTGTATCCCAATGCTAAGGCACCTTTATATGAAGTGGGTAAGACTC
ENSPTRT00000032169      GTTCCTGAAATTCCTTGTATCCCAATGCTAAGGCACCTTTATATGAAGTGGGTAAGACTC

ENSMUST00000048688      ACTAAACCACAGCCGTTTAAAGATTTCCTTTGCATCAGCTTGCGAACTTTTGTCATGAGG
ENSRNOT00000025790      ACTAAACCACAGCCGTTCAAGGATTTCCTCTGCATCAGCTTGCGGACTTTTGTCATGAGG
ENSGGOT00000026421      ACTAAACCACAGCCATTTAAAGACTTCCTTTGTATCAGCTTAAGAACTTTCGTCATGAGG
ENSPPYT00000018532      ACTAAACCACAGCCATTTAAAGACTTCCTTTGTATCAGCTTAAGAACTTTCGTCATGAGG
ENSCJAT00000041759      ACTAAACCACAGCCATTTAAAGACTTCCTTTGTATCAGCTTAAGAACTTTCGTCATGAGG
ENSMMUT00000023749      ACTAAACCACAGCCATTTAAAGACTTCCTTTGTATCAGCTTAAGAACTTTCGTCATGAGG
ENST00000340253         ACTAAACCACAGCCATTTAAAGACTTCCTTTGTATCAGCTTAAGAACTTTCGTCATGAGG
ENSPTRT00000032169      ACTAAACCACAGCCATTTAAAGACTTCCTTTGTATCAGCTTAAGAACTTTCGTCATGAGG

ENSMUST00000048688      AACTGTGCAGGACCAACAAACTCCTTGAAATACGTGCCCTTGGTAACAGGTCTGGCATCT
ENSRNOT00000025790      AACTGCGCAGGACCAACAAATTCCTTGAAATACGTGCCCTTGGTAACAGGTCTGGCCTCT
ENSGGOT00000026421      AACTGTGCAGGACCCACAAATTCCTTGAAATATGTCCCTTTAGTAACAGGCTTAGCCTCT
ENSPPYT00000018532      AACTGTGCAGGACCCACAAATTCCTTGAAATATGTCCCTTTAGTAACAGGCTTAGCCTCT
ENSCJAT00000041759      AACTGTGCAGGACCCACAAATTCTTTGAAATATGTCCCTTTAGTGACAGGCTTAGCCTCT
ENSMMUT00000023749      AACTGTGCAGGACCCACAAATTCCTTGAAATATGTCCCTTTAGTAACAGGCTTAGCCTCT
ENST00000340253         AACTGTGCAGGACCCACAAATTCCTTGAAATATGTCCCTTTAGTAACAGGCTTAGCCTCT
ENSPTRT00000032169      AACTGTGCAGGACCCACAAATTCCTTGAAATATGTCCCTTTAGTAACAGGCTTAGCCTCT

ENSMUST00000048688      GCCCGTAACCTGGAACACTTGGAAATGGTTCGAGTCCCTTTCCTGGGAGGTCTCATCCAA
ENSRNOT00000025790      GCTCGGAACTTGGAACACTTGGAGATGGTTCGAGTGCCTTTCCTGGGCGGTCTCATCCAG
ENSGGOT00000026421      GCCCGAAACTTGGAACACTTAGAAATGGTTCGAGTTCCTTTCCTTGGAGGTCTTATCCAA
ENSPPYT00000018532      GCCCGAAACTTGGAACACTTAGAAATGGTTCGAGTTCCTTTCCTTGGAGGTCTTATCCAA
ENSCJAT00000041759      GCCCGAAACTTGGAACACTTAGAAATGGTTCGAGTTCCTTTCCTTGGAGGTCTTATCCAA
ENSMMUT00000023749      GCCCGAAACTTGGAACACTTAGAAATGGTTCGAGTTCCTTTCCTTGGAGGTCTTATCCAA
ENST00000340253         GCCCGAAACTTGGAACACTTAGAAATGGTTCGAGTTCCTTTCCTTGGAGGTCTTATCCAA
ENSPTRT00000032169      GCCCGAAACTTGGAACACTTAGAAATGGTTCGAGTTCCTTTCCTTGGAGGTCTTATCCAA

ENSMUST00000048688      CACGTGGTTGAAGACAGCTGGAGATCAGGTGGTTTTAGGAACTTGCACACTATTGTCTTA
ENSRNOT00000025790      CATGTGGTTGAAGACAGCTGGAGATCAGGTGGTTTTAGGAATTTGCACACTATTGTCTTA
ENSGGOT00000026421      CATGTTGTTGAAGACAGTTGGAGATCAGGTGGTTTTAGAAATTTGCACACTATTGTTCTG
ENSPPYT00000018532      CATGTTGTTGAAGACAGTTGGAGATCAGGTGGTTTTAGAAATTTGCACACTATTGTTCTG
ENSCJAT00000041759      CATGTTGTTGAAGACAGTTGGAGATCAGGTGGTTTTAGAAATTTGCACACTATTGTTCTA
ENSMMUT00000023749      CATGTTGTTGAAGACAGTTGGAGATCAGGTGGTTTTAGAAATTTGCACACTATTGTTCTG
ENST00000340253         CATGTTGTTGAAGACAGTTGGAGATCAGGTGGTTTTAGAAATTTGCACACTATTGTTCTG
ENSPTRT00000032169      CATGTTGTTGAAGACAGTTGGAGATCAGGTGGTTTTAGAAATTTGCACACTATTGTTCTG

ENSMUST00000048688      GGAGCTTGCAAGAATGCCCTGGAAGTAGACCTTGGCTACCTCATCATCACTGCTGCGCGA
ENSRNOT00000025790      GGAGCTTGCAAGAATGCCCTTGAAGTAGATCTTGGTTACCTCATCATCACTGCTGCCAGA
ENSGGOT00000026421      GGAGCTTGCAAAAATGCTCTTGAAGTAGATCTTGGTTACCTCATCATTACTGCTGCCCGT
ENSPPYT00000018532      GGAGCTTGCAAAAATGCTCTTGAAGTAGATCTTGGTTACCTCATCATTACTGCTGCCCGT
ENSCJAT00000041759      GGAGCTTGCAAAAATGCTCTTGAAGTCGATCTTGGTTACCTCATCATTACTGCTGCCCGT
ENSMMUT00000023749      GGAGCTTGCAAAAATGCTCTTGAAGTAGATCTTGGTTACCTCATCATTACTGCTGCCCGT
ENST00000340253         GGAGCTTGCAAAAATGCTCTTGAAGTAGATCTTGGTTACCTCATCATTACTGCTGCCCGT
ENSPTRT00000032169      GGAGCTTGCAAAAATGCGCTTGAAGTAGATCTTGGTTACCTCATCATTACTGCTGCCCGT

ENSMUST00000048688      AGGTTACATGAAGTTCGCATCCAGCCTTCTTTAACCAAAGATGGTGTCTTTTCTGCCCTA
```

```
ENSRNOT00000025790     AGGTTACATGAAGTTCGCATCCAGCCTTCTTTAACCAAAGATGGTGTCTTTTCTGCCCTG
ENSGGOT00000026421     AGGTTACATGAAGTTCGGATCCAGCCTTCCCTAACCAAAGATGGTGTCTTTTCTGCCCTA
ENSPPYT00000018532     AGGTTACATGAAGTTCGGATCCAGCCTTCCCTAACCAAAGATGGTGTCTTTTCTGCCCTA
ENSCJAT00000041759     AGGTTACATGAAGTTCGGATCCAGCCTTCCCTAACCAAAGATGGTGTCTTTTCTGCCCTA
ENSMMUT00000023749     AGGTTACATGAAGTTCGGATCCAGCCTTCCCTAACCAAAGATGGTGTCTTTTCTGCCCTA
ENST00000340253        AGGTTACATGAAGTTCGGATCCAGCCTTCCCTAACCAAAGATGGTGTCTTTTCTGCCCTA
ENSPTRT00000032169     AGGTTACATGAAGTTCGGATCCAGCCTTCCCTAACCAAAGATGGTGTCTTTTCTGCCCTA

ENSMUST00000048688     AAGATGGCAGAATTGGAGTTTCCCCAGTTTGAAACCCTTCATCTGGGATATGTAGATGAG
ENSRNOT00000025790     AAGATGGCAGAATTGGAGTTTCCCCAATTTGAAACCCTTCATCTGGGATATGTAGATGAG
ENSGGOT00000026421     AAGATGGCAGAGTTGGAGTTTCCCCAGTTTGAAACCCTTCATCTAGGATATGTAGATGAG
ENSPPYT00000018532     AAGATGGCAGAGTTGGAGTTCCCCCAGTTTGAAACCCTTCATCTAGGATATGTAGATGAG
ENSCJAT00000041759     AAGATGGCAGAGTTGGAGTTCCCTCAGTTTGAAACTCTTCATCTAGGATATGTAGATGAA
ENSMMUT00000023749     AAGATGGCAGAGTTGGAGTTCCCCCAGTTTGAAACCCTTCATCTAGGATATGTAGATGAG
ENST00000340253        AAGATGGCAGAGTTGGAGTTTCCCCAGTTTGAAACCCTTCATCTAGGATATGTAGATGAG
ENSPTRT00000032169     AAGATGGCAGAGTTGGAGTTTCCCCAGTTTGAAACCCTTCATCTAGGATATGTAGATGAG

ENSMUST00000048688     TTCTTGCTACAGAGCAGAATGGCGAATGCAGATTTGGTGAAGTATGGCTTGGCTGATGTG
ENSRNOT00000025790     TTCTTGCTACAGAGCAGAATGGCGAATGCAGATTTGGTGAAGTATGGTTTGGCTGATGTG
ENSGGOT00000026421     TTTTTGCTACAGAGTAAGATTTATCCCATTTTAATTCCTAGAATTACTGTTGTAGCTAAA
ENSPPYT00000018532     TTTTTGCTACAGAGCAGAATGGCTAATGCGGATCTGGTGAAGTATGGTTTGGCTGATGTG
ENSCJAT00000041759     TTTTTGCTGCAGAGCAGAATGGCTAATGCGGATCTGGTGAAGTATGGTTTGGCTGATGTG
ENSMMUT00000023749     TTTTTGCTACAGAGCAAAATGGCTAATGCGGATCTGGTGAAGTATGGTTTGGCTGATGTG
ENST00000340253        TTTTTGCTACAGAGCAGAATGGCTAATGCGGATCTGGTGAAGTATGGTTTGGCTGATGTG
ENSPTRT00000032169     TTTTTGCTACAGAGCAGAATGGCTAATGCGGATCTGGTGAAGTATGGTTTGGCTGATGTG

ENSMUST00000048688     GTAGAAAATCCTGGTATCATTACTGATATAGGGATGAAGGCAGTCAATGAAGTTTTTTCC
ENSRNOT00000025790     GTAGAAAATCCTGGTATCATTACCGATATCGGGATGAAGGCAGTCAACGAGGTTTTCTCC
ENSGGOT00000026421     GTTGTAAATCCCTGTGATATTCAGGGCAGCTTCACCAGAGTGGCAGTGTTTTTTTTGTCC
ENSPPYT00000018532     GTAGAAAATCCTGGTATCATCACTGATATAGGGATGAAAGCAGTCAATGAAGTTTTTTCC
ENSCJAT00000041759     GTAGAAAATCCCGGTATCATCACTGATATAGGGATGAAAGCAGTCAATGAAGTTTTTTCC
ENSMMUT00000023749     GTAGAAAATCCTGGTATCATCACTGATATAGGGATGAAAGCAGTCAATGAAGTTTTTTCC
ENST00000340253        GTAGAAAATCCTGGTATCATCACTGATATAGGGATGAAAGCAGTCAATGAAGTTTTTTCC
ENSPTRT00000032169     GTAGAAAATCCTGGTATCATCACTGATATAGGGATGAAAGCAGTCAATGAAGTTTTTTCC

ENSMUST00000048688     TGTATCAAATATCTGGCAATTTATAACTGTCCTCATCTACACAACCCATACAATTGGATC
ENSRNOT00000025790     TGTATCAAGTATCTGGCGATTTATAACTGTCCTCATCTACACAACCCATATAATTGGATA
ENSGGOT00000026421     TGCGTATTGTTTACCTTTCTCTACACATGTCCACACTGGGCTCCAAGAACATCATTTTAT
ENSPPYT00000018532     TGTATCAAATATCTGGCAATTTACAATTGCCCTCATCTACACAACCCATACAATTGGATC
ENSCJAT00000041759     TGTATCAAATACCTGGCAATTTACAATTGCCCTCATCTACACAACTCATACAATTGGCTC
ENSMMUT00000023749     TGTATCAAATATCTGGCAATTTACAATTGCCCTCATCTACACAACCCATACAATTGGATC
ENST00000340253        TGTATCAAATATCTGGCAATTTACAATTGCCCTCATCTACACAACCCATACAATTGGATC
ENSPTRT00000032169     TGTATCAAATATCTGGCAATTTACAATTGCCCTCATCTACACAACCCATACAATTGGATC

ENSMUST00000048688     TCAGACCACTCGAGGTGGATGCGACTGGTTGATATCAACCTCGTGCGGTGCCATGCTTTG
ENSRNOT00000025790     TCAGATCACTCTAGATGGACCCGACTGGTTGATATCAACCTCGTGCGCTGCCATGCTTTG
ENSGGOT00000026421     TCAGACCACTCAAGATGGACTCGATTGGTTGATATCAACCTAGTACGGTGCCATGCTTTG
ENSPPYT00000018532     TCAGACCACTCAAGATGGACTCGATTGGTTGATATCAACCTAGTACGGTGCCATGCTTTG
ENSCJAT00000041759     TCAGACCACTCAAGATGGACCCGATTGGTTGATATCAACCTAGTGCGGTGCCATGCTTTG
ENSMMUT00000023749     TCAGACCACTCAAGATGGACTCGATTGGTTGATATCAACCTAGTACGGTGCCATGCTTTG
ENST00000340253        TCAGACCACTCAAGATGGACTCGATTGGTTGATATCAACCTAGTACGGTGCCATGCTTTG
ENSPTRT00000032169     TCAGACCATTCAAGATGGACTCGATTGGTTGATATCAACCTAGTACGGTGCCATGCTTTG

ENSMUST00000048688     AAGCTGGATTCTTTTGGCCAGTTTGTTGAATTATTGCCCAGCCTGGAATTTATTTCTCTG
```

```
ENSRNOT00000025790     AAGCTGGATTCTTTTGGCCAGTTTGTTGAATTATTGCCCAGCCTGGAGTTTATTTCGCTG
ENSGGOT00000026421     AAGCTGGACTCTTTTGGCCAGTTCATTGAATTATTACCCAGCCTAGAGTTTATTTCACTG
ENSPPYT00000018532     AAGCTGGACTCTTTTGGCCAGTTTATTGAATTATTACCCAGCCTAGAGTTTATTTCACTG
ENSCJAT00000041759     AAGCTGGACTCTTTTGGCCAGTTTATTGAATTGTTACCCAGCCTAGAGTTTATTTCACTG
ENSMMUT00000023749     AAGCTGGACTCTTTTGGCCAGTTTATTGAATTGTTACCCAGCCTAGAGTTTATTTCACTG
ENST00000340253        AAGCTGGACTCTTTTGGCCAGTTTATTGAATTATTACCCAGCCTAGAGTTTATTTCACTG
ENSPTRT00000032169     AAGCTGGACTCTTTTGGCCAGTTTATTGAATTATTACCCAGCCTAGAGTTTATTTCACTG

ENSMUST00000048688     GACCAGATGTTCCGTGAGCCACCCAAGGGCTGTGCTCGAGTTGGTCTGAGTGCAGGAACT
ENSRNOT00000025790     GACCAGATGTTTCGTGAGCCACCCAAGGGCTGTGCTCGAGTAGGTCTGAGTGCAGGAACT
ENSGGOT00000026421     GATCAGATGTTTCGTGAACCACCCAAGGGTTGTGCTCGAGTTGGTCTGAGTGCAGGCACA
ENSPPYT00000018532     GATCAGATGTTTCGTGAACCACCCAAGGGTTGTGCTCGAGTTGGTCTGAGTGCAGGCACA
ENSCJAT00000041759     GATCAGATGTTTCGTGAACCACCCAAGGGTTGTGCTCGAGTTGGTTTGAGTGCAGGCACA
ENSMMUT00000023749     GATCAGATGTTTCGTGAACCACCCAAGGGTTGTGCTCGAGTTGGTCTGAGTGCAGGCACA
ENST00000340253        GATCAGATGTTTCGTGAACCACCCAAGGGTTGTGCTCGAGTTGGTCTGAGTGCAGGCACA
ENSPTRT00000032169     GATCAGATGTTTCGTGAACCACCCAAGGGTTGTGCTCGAGTTGGTCTGAGTGCAGGCACA

ENSMUST00000048688     GGAATTGGGGTTTCCTCAGCGCTTGTCAGCAACCAGAATTCCAACAATGACGACAATAAT
ENSRNOT00000025790     GGAATTGGGGTTTCCTCAGCGCTTGTCAGCAATCAGAATTCCAACAATGACGATAATAAT
ENSGGOT00000026421     GGAATTGGTGTTTCATCAGCTCTTGTTAGCAACCAGAACTCCAACAATGACGATAATAAT
ENSPPYT00000018532     GGAATTGGTGTTTCATCAGCTCTTGTTAGCAACCAGAACTCCAACAATGATGATAATAAT
ENSCJAT00000041759     GGAATTGGTGTTTCATCAGCTCTTGTTAGTAACCAGAACTCCAACAACGACGATAATAAT
ENSMMUT00000023749     GGAATTGGGGTTTCATCAGCTCTTGTTAGCAACCAGAACTCCAACAATGACGATAATAAT
ENST00000340253        GGAATTGGTGTTTCATCAGCTCTTGTTAGCAACCAGAACTCCAACAATGACGATAATAAT
ENSPTRT00000032169     GGAATTGGTGTTTCATCAGCTCTTGTTAGCAACCAGAACTCCAACAATGACGATAATAAT

ENSMUST00000048688     GCTCCAAATAACAACGCCAACCTCCATGATAACAATCACCATCACCCAGATGATTCGGAT
ENSRNOT00000025790     GCTCAGAATAACAACGCCAACATCCATGACAACAATCACCATCACCCAGACGACTCGGAT
ENSGGOT00000026421     GCCCAGAATAACAATGCCAACATCCACGACAACAATCACCATCACCCAGATGACTCAGAC
ENSPPYT00000018532     GCCCAGAATAACAATGCCAACATCCACGACAACAATCACCATCACCCAGATGACTCAGAC
ENSCJAT00000041759     GCCCAGAATAACAATGCCAACATCCATGACAACAATCATCATCACCCAGATGACTCAGAT
ENSMMUT00000023749     GCCCAGAATAACAATGCCAACATCCACGACAACAATCACCATCACCCAGATGACTCAGAC
ENST00000340253        GCCCAGAATAACAATGCCAACATCCACGACAACAATCACCATCATCCAGATGACTCAGAC
ENSPTRT00000032169     GCCCAGAATAACAATGCCAACATCCACGACAACAATCACCATCACCCAGATGACTCAGAT

ENSMUST00000048688     GACGACAATGACTTTCGTCCAGACCTACAAGCAGGAGAGGCACAATTTGCAGCCGATGCG
ENSRNOT00000025790     GACGACAATGACTTCCGTCAAGACCTACAACAGGGAGAGGCACAGTTTGCAGCCGATGCA
ENSGGOT00000026421     GAGGAGAATGACTTTCGGCAAGATCTGCAGCCAGGAGAGCAGCAGTTTGCAGCTGACGCA
ENSPPYT00000018532     GAGGAGAATGACTTTCGGCAAGATCTACAGCCAGGAGAGCAGCAGTTTGCAGCTGACGCG
ENSCJAT00000041759     GAGGAGAATGACTTTCGGCAAGACCTGCAGCCAGGAGAGCAGCAGTTTGCAGCTGACGCA
ENSMMUT00000023749     GAGGAGAATGACTTTCGGCAAGATCTACAGCCAGGAGAGCAGCAGTTTGCAGCTGACGCA
ENST00000340253        GAGGAGAATGACTTTCGGCAAGATCTGCAGCCAGGAGAGCAGCAGTTTGCAGCTGACGCA
ENSPTRT00000032169     GAGGAGAATGACTTTCGGCAAGATCTGCAGCCAGGAGAGCAGCAGTTTGCAGCTGACGCA

ENSMUST00000048688     TTGAATGAGATGGAAGACATGGTCCAGGAAGATGGAGAACTGGTAGCCGAGAGTGGAAAT
ENSRNOT00000025790     CTGAATGAGATGGAGGACATGGTCCAGGACGATGGAGAGGTGGTAGCCGAGAGTGGAAGC
ENSGGOT00000026421     TTAAATGAGATGGAAGACATCGTCCAAGAAGATGGAGAGGTGGTGGCCGAGAGTGGAAAT
ENSPPYT00000018532     TTAAATGAGATGGAAGACATCGTCCAAGAAGATGGAGAGGTGGTGGCCGAGAGTGGAAAT
ENSCJAT00000041759     TTAAATGAGATGGAAGACATCGTGCAAGAAGATGGAGAGGTGGTGGCCGAGAGTGGAAAT
ENSMMUT00000023749     TTAAATGAGATGGAAGACATCGTCCAAGAAGATGGAGAGGTGGTGGCCGAGAGTGGAAAT
ENST00000340253        TTAAATGAGATGGAAGACATCGTCCAAGAAGATGGAGAGGTGGTGGCCGAGAGTGGAAAT
ENSPTRT00000032169     TTAAATGAGATGGAAGACATCGTCCAAGAAGATGGAGAGGTGGTGGCCGAGAGTGGAAAT

ENSMUST00000048688     GGTATGCCAGCTCACAATCGGGAAGTTCTTCCTGTGGATGCTGATGAGGAACAAGCAGGA
```

ENSRNOT00000025790 GGTGTGCCCGCTCAC---CAGGGAGTTCTGCCTGTGGATGCCGATGAGGAGCAAGCAGGA
ENSGGOT00000026421 AATACTCCAGCTCACAGCCAGGCAATTATTCCTGTGGATGTTGATGAGGAACAAGCAGGA
ENSPPYT00000018532 AATACTCCAGCTCACAGCCAGGCAATTATTCCTGTGGATGTTGATGAGGAACAAGCAGGA
ENSCJAT00000041759 AATACTCCAGCTCACAGCCAGGCAATTATTCCTGTGGATGTTGATGAGGAACAAGCAGGA
ENSMMUT00000023749 AATACTCCAGCTCACAGCCAGGCAATTATTCCTGTGGATGTTGATGAGGAACAAGCAGGA
ENST00000340253 AATACTCCAGCTCACAGCCAGGCAATTATTCCTGTGGATGTTGATGAGGAACAAGCAGGA
ENSPTRT00000032169 AATACTCCAGCTCACAGCCAGGCAATTATTCCTGTGGATGTTGATGAGGAACAAGCAGGA

ENSMUST00000048688 CCTAGTGGTCTACAACGCGTGGTAAAACCTACACCAATTGCTGATCACGATTCAGAGAGC
ENSRNOT00000025790 CCTAGTGGTCTACAGCGTGTAGTAAAACCAACACCAATTGCTGTTCATGATTCAGAGAGC
ENSGGOT00000026421 CCCAGTGGTCTTCAGCGTGTAGTAAAACCAACCCCAATTACTGTTCATGATTCAGAGAGT
ENSPPYT00000018532 CCCAGTGGTCTTCAGCGTGTAGTAAAACCAACCCCAATTACTGTTCATGATTCAGAGAGT
ENSCJAT00000041759 CCCAGTGGTCTTCAGCGTGTAGTAAAACCAACCCCAATTACTGTTCATGATTCAGAGAGT
ENSMMUT00000023749 CCCAGTGGTCTTCAGCGTGTAGTAAAACCAACCCCAATTACTGTTCATGATTCAGAGAGC
ENST00000340253 CCCAGTGGTCTTCAGCGTGTAGTAAAACCAACCTCAATTACTGTTCATGATTCAGAGAGT
ENSPTRT00000032169 CCCAGTGGTCTTCAGCGTGTAGTAAAACCAACCCCAATTACTGTTCATGATTCAGAGAGT

ENSMUST00000048688 GATGATGAAGAAGATAGTTTAGAACTCCAAGAAGTCTGGGCTCCTAAGAATGGCACCCGG
ENSRNOT00000025790 GATGATGAAGAAGACAGTCTAGAACTCCAAGAAGTCTGGATTCCTAAGAATGGTGCTCGG
ENSGGOT00000026421 GATGATGAAGAAGATAGTCTAGAACTCCAAGAAGTCTGGATTCCTAAGAACGGTACTCGG
ENSPPYT00000018532 GATGATGAAGAAGATAGTCTAGAACTCCAAGAAGTCTGGATTCCTAAGAATGGTACTCGG
ENSCJAT00000041759 GACGATGAAGAAGATAGTCTAGAACTCCAAGAAGTCTGGATTCCTAAGAACGGTACTCGG
ENSMMUT00000023749 GATGATGAAGAAGATAGTCTAGAACTCCAAGAAGTCTGGATTCCTAAGAACGGTACTCGG
ENST00000340253 GATGATGAAGAAGATAGTCTAGAACTCCAAGAAGTCTGGATTCCTAAGAACGGTACTCGG
ENSPTRT00000032169 GATGATGAAGAAGATAGTCTAGAACTCCAAGAAGTCTGGATTCCTAAGAACGGTACTCGG

ENSMUST00000048688 CGTTACTCAGAGCGGGAAGAAAAGACTGGAGACTCAGGACAGTCCAGGGAAACGGCAGTA
ENSRNOT00000025790 CGTTACTCAGAACGGGAAGAAAAAACCGGAGACTCAGGACAGCCCAGGGAAACCGCAGTA
ENSGGOT00000026421 CGTTACTCTGAACGTGAAGAAAAAACTGGAGAGTCAGTGCAGTCCAGAGAATTGTCAGTA
ENSPPYT00000018532 CGTTACCCTGAACGTGAAGAAAAAACTGGAGAGTCAGTGCAGTCCAGAGAATTAAGCAGT
ENSCJAT00000041759 CGTTACTCTGAACGTGAAGAAAAAACTGGAGAGTCAGTACAGTCCAGGGAATCGTCAGTA
ENSMMUT00000023749 CGTTACTCTGAACGTGAAGAGAAAACTGGAGAGTCAGTGCAGTCCAGGGAATTGTCAGTA
ENST00000340253 CGTTACTCTGAACGTGAAGAAAAAACTGGAGAGTCAGTGCAGTCCAGAGAATTGTCAGTA
ENSPTRT00000032169 CGTTACTCTGAACGTGAAGAAAAAACTGGAGAGTCAGTGCAGTCCAGAGAATTGTCAGTA

ENSMUST00000048688 AGTGGGAAAGGCAAGACTCCACTTCGAAAGAGGTGCAACTCCCACCAGACGGGCCAGGCG
ENSRNOT00000025790 AGTGGAAAAGGCAAGACTCCACTTCGAAAGAGGTACAACTCTCATCAGATGGGCCAGGCG
ENSGGOT00000026421 AGTGGAAAAGGCAAGACTCCACTTCGAAAGAGGTACAACTCCCATCAGATGGGCCAGTCG
ENSPPYT00000018532 AGGGGAAAAGGCAAGACTCCACTTCGAAAGAGGTACAACTCCCATCAGATGGGCCAGTCG
ENSCJAT00000041759 AGTGGAAAAGGCAAGACTCCACTCCGAAAGAGGTACAACTCCCATCAGATGGGCCAGTCG
ENSMMUT00000023749 AGTGGAAAAGGCAAGACTCCACTTCGAAAGAGGTACAACTCCCATCAGATGGGCCAGTCG
ENST00000340253 AGTGGAAAAGGCAAGACTCCACTTCGAAAGAGGTACAACTCCCATCAGATGGGCCAGTCG
ENSPTRT00000032169 AGTGGAAAAGGCAAGACTCCACTTCGAAAGAGGTACAACTCCCATCAGATGGGCCAGTCG

ENSMUST00000048688 AAGCCGTTTCCCCTGGAGGAGAGCAGCTGTGAGAAAGGCTGTCAGGTGACCAGTGAGCAG
ENSRNOT00000025790 AAGCAGTTTCCCCTGGAGGAGAGCAGCTGTGAGAAAGGCTGTCAGGTCACCAGTGAACAG
ENSGGOT00000026421 AAGCAGTTTCCCCTCGAGGAAAGCAGCTGTGAGAAAGGCTGTCAGGTCACCAGTGAGCAG
ENSPPYT00000018532 AAGCAGTTTCCCCTCGAGGAAAGCAGCTGTGAGAAAGGCTGTCAGGTCACCAGTGAGCAG
ENSCJAT00000041759 AAGCAGTTTCCCCTCGAGGAAAGCAGCTGTGAGAAAGGCTGTCAGGTCACCAGTGAGCAG
ENSMMUT00000023749 AAGCAGTTTCCCCTCGAGGAAAGCAGCTGTGAGAAAGGCTGTCAGGTCACCAGTGAGCAG
ENST00000340253 AAGCAGTTTCCCCTCGAGGAAAGCAGCTGTGAGAAAGGCTGTCAGGTCACCAGTGAGCAG
ENSPTRT00000032169 AAGCAGTTTCCCCTCGAGGAAAGCAGCTGTGAGAAAGGCTGTCAGGTCACCAGTGAGCAG

ENSMUST00000048688 ATCAAAGCCGATATGAAAGCAGCTAGGGATGTTTCTGAAAAGAAAAAATCCAAGGATGTC

ENSRNOT00000025790 ATCAAAGCCGATATGAAAGCAGCTAGGGATATTTCTGAAAAGAAAAAAACCAAGGATGTT
ENSGGOT00000026421 ATCAAAGCCGATATGAAAGCAGCTAGGGATATTCCTGAAAAGAAAAAAAACAAGGATGTT
ENSPPYT00000018532 ATCAAAGCCGATATGAAAGCAGCTAGGGATATTCCTGAAAAGAAAAAAAACAAGGATGTT
ENSCJAT00000041759 ATCAAAGCCGATATGAAAGCAGCTAGGGATATTCCTGAAAAGAAAAAAAACAAGGATGTT
ENSMMUT00000023749 ATCAAAGCCGATATGAAAGCAGCTAGGGATATTCCTGAAAAGAAAAAAAACAAGGATGTT
ENST00000340253 ATCAAAGCCGATATGAAAGCAGCTAGGGATATTCCTGAAAAGAAAAAAAACAAGGATGTT
ENSPTRT00000032169 ATCAAAGCCGATATGAAAGCAGCTAGGGATATTCCTGAAAAGAAAAAAAACAAGGATGTT

ENSMUST00000048688 TATCCCAGCTGCAGCAGCACAGCCGCCAGCACAGCGGGAAACGCCAGCTCACCCAGCACT
ENSRNOT00000025790 TATCCCAGCTGCAGCACCACGGCCGCCAGCACCGTGGGAAACTCCAGCTCGCCCAGCACT
ENSGGOT00000026421 TATCCCAGCTGCAGCAGCACCACCGCCAGCACAGTGGGAAACTCCAGCTCACACAACACT
ENSPPYT00000018532 TATCCCAGCTGCAGCAGCACCACCGCCAGCACAGTGGGAAACTCCAGCTCACACAGCACT
ENSCJAT00000041759 TATCCCAGCTGCAGCAGCACCACCGCCAGCACAGTGGGAAACTCCAGCTCACACAGCACT
ENSMMUT00000023749 TATCCCAGCTGCAGCAGCACCACCGCCAGCACAGTGGGAAACTCCAGCTCACACAGCACT
ENST00000340253 TATCCCAGCTGCAGCAGCACCACCGCCAGCACAGTGGGAAACTCCAGCTCACACAACACT
ENSPTRT00000032169 TATCCCAGCTGCAGCAGCACCACCGCCAGCACAGTGGGAAACTCCAGCTCACACAACACT

ENSMUST00000048688 GCTTCCCAGAGCCCGGACTTTGCAAGGACGGTGACCAGCAGCGGCTCTTCCGAGCCGAGC
ENSRNOT00000025790 GCTTCTCAGAGCCCGGACTTTGTAAGGACGGTGACCAGCGGCGGCTCTTCTGAGCCTAGC
ENSGGOT00000026421 GCTTCTCAAAGCCCCGACTTTGTAAGGACGGTGAACAGCGGCGGCTCTTCCGAGCCTAGC
ENSPPYT00000018532 GCTTCTCAAAGCCCCGACTTTGTAAGGACGGTGAACAGCGGCGGCTCTTCCGAGCCTAGC
ENSCJAT00000041759 GCTTCTCAAAGCCCTGACTTTGTAAGGACGGTGAACAGCGGCGGCTCTTCCGAGCCTAGC
ENSMMUT00000023749 GCTTCTCAAAGCCCCGACTTTGTAAGGACGGTGAACAGCGGCGGCTCTTCCGAGCCTAGC
ENST00000340253 GCTTCTCAAAGCCCCGACTTTGTAAGGACGGTGAACAGCGGCGGCTCTTCCGAGCCTAGC
ENSPTRT00000032169 GCTTCTCAAAGCCCCGACTTTGTAAGGACGGTGAACAGCGGCGGCTCTTCCGAGCCTAGC

ENSMUST00000048688 CCTCCAGAAGTAGATGTATCCAGGCAGTGTGTCTGCTCCCCTGGTGGGTCAGAGGACTCT
ENSRNOT00000025790 CCTCCAGAGGTGGATGTGTCCCGGCAGTGTGTCTGCTCCCCTGGTGGGTCAGAGGACTCG
ENSGGOT00000026421 CCTACAGAAGTGGATGTGTCCAGGCAGTGTGCCTGCTCCCCCGGTGGGTCAGAGGACTCT
ENSPPYT00000018532 CCTACAGAAGTGGATGTGTCCAGGCAGTGTGCCTGCTCCCCCGGTGGGTCAGAGGACTCT
ENSCJAT00000041759 CCTACAGAAGTGGATGTGTCCAGGCAGTGTGCCTGCTCCCCCGGTGGGTCAGAGGACTCT
ENSMMUT00000023749 CCTACAGAAGTGGATGTGTCCAGGCAGTGTGCCTGCTCCCCCGGTGGGTCAGAGGACTCT
ENST00000340253 CCTACAGAAGTGGATGTGTCCAGGCAGTGTGCCTGCTCCCCCGGTGGGTCAGAGGACTCT
ENSPTRT00000032169 CCTACAGAAGTGGATGTGTCCAGGCAGTGTGCCTGCTCCCCCGGTGGGTCAGAGGACTCT

ENSMUST00000048688 GAGGCCATGGAGGAGGGAGATGCAGAGAGCTCTGTCTGCCCCAGATGCTGCTGTCTCAGG
ENSRNOT00000025790 GAGGCCATGGAGGAGGGAGATGCAGAGAGCTCCGTCTGCCCCAGATGCTGCTGTCTCAGG
ENSGGOT00000026421 GAGGCCATGGAGGAGGGAGATGCAGAGAGTTCTGTCTGCCCCAGATGCTGCTGTCACAGG
ENSPPYT00000018532 GAGGCCATGGAGGAGGGAGATGCAGAGAGTTCTGTCTGCCCCAGATGCTGCTGTCACAGG
ENSCJAT00000041759 GAGGCCTTGGAGGAGGGAGATGCAGAGAGTTCTGTCTGCCCCAGATGCTGCTGTCACAGG
ENSMMUT00000023749 GAGGCCATGGAGGAGGGAGATGCAGAGAGTTCTGTCTGCCCCAGATGCTGCTGTCACAGG
ENST00000340253 GAGGCCATGGAGGAGGGAGATGCAGAGAGTTCTGTCTGCCCCAGATGCTGCTGTCACAGG
ENSPTRT00000032169 GAGGCCGTGGAGGAGGGAGATGCAGAGAGTTCTGTCTGCCCCAGATGCTGCTGTCACAGG

ENSMUST00000048688 CCCCAGGAGTCTCAAAGGAGAACTGGCAGGTGTTCTGATGAGGAGCGTCCTTCAACCAGC
ENSRNOT00000025790 CCCCAGGAGTCTCAAAGGAGAACTAGCAGGCGTTCTGATGAGGAGCGGCCTTCGACCAGC
ENSGGOT00000026421 CCCCAGGAATCCCAAAGGAGAACTAGCAGATGTTCTGATGAGGAACGTCCTTCAACCAGC
ENSPPYT00000018532 CCTCAGGAATCCCAAAGGAGAACTAGCAGGTGTTCTGATGAGGAACGTCCTTCAACCAGC
ENSCJAT00000041759 CCCCAGGAATCCCAAAGGAGAACTAGCAGGTGTTCTGATGAGGAACGTCCTTCAACCAGC
ENSMMUT00000023749 CCCCAGGAATCCCAAAGGAGAACTAGCAGGTGTTCTGATGAGGAACGTCCTTCAACCAGC
ENST00000340253 CCCCAGGAATCCCAAAGGAGAACTAGCAGGTGTTCTGATGAGGAACGTCCTTCAACCAGC
ENSPTRT00000032169 CCCCAGGAATCCCAAAGGAGAACTAGCAGGTGTTCTGATGAGGAACGTCCTTCAACCAGC

ENSMUST00000048688 CGAGCCTGTGTTGTGAATGGCGCGGATGGTACGAGATCCGCCTTTTCCTTTAGGACTCTG

```
ENSRNOT00000025790     AGAGCCTGTGTTGTGAATGGCGCGGATGGTACGAGATCCGCCTTTTCCTTTAGGACTCTG
ENSGGOT00000026421     CGAGCCTGTGTTGTGAATGGCCCGGATGGTACGAGATCCGCCTTTTCCTTTAGGACTCTG
ENSPPYT00000018532     CGAGCCTGTGTTGTGAATGGCCCGGATGGTACGAGATCCGCCTTTTCCTTTAGGACTCTG
ENSCJAT00000041759     CGAGCCTGTGTTGTGAATGGCCCGGATGGTACGAGATCCGCCTTTTCCTTTAGGACTCTG
ENSMMUT00000023749     CGAGCCTGTGTTGTGAATGGCCCGGATGGTACGAGATCCGCCTTTTCCTTTAGGACTCTG
ENST00000340253        CGAGCCTGTGTTGTGAATGGCCCGGATGGTACGAGATCCGCCTTTTCCTTTAGGACTCTG
ENSPTRT00000032169     CGAGCCTGTGTTGTGAATGGCCCGGATGGTACGAGATCCGCCTTTTCCTTTAGGACTCTG

ENSMUST00000048688     CCACAAGGGGGGTCTTCAGGCCCAGCACATGATGAGAGGACTAATGGGAGTGGCTGTGGG
ENSRNOT00000025790     CCACAAGGGGGGTCTTCAGGCCCAGCACATGATGAGAGGACTAATGGGAGTGGCTGTGGG
ENSGGOT00000026421     CCACAAGGGGGGTCTTCAGGCCCAGCACATGATGAGAGGACTAATGGGAGTGGCTCTGGG
ENSPPYT00000018532     CCACAAGGGGGGTCTTCAGGCCCAGCACATGATGAGAGGACTAATGGGAGTGGCTCTGGG
ENSCJAT00000041759     CCACAAGGGGGGTCTTCAGGCCCAGCACATGATGAGAGGACTAATGGGAGTGGCTCTGGG
ENSMMUT00000023749     CCACAAGGGGGGTCTTCAGGCCCAGCACATGATGAGAGGACTAATGGGAGTGGCTCTGGG
ENST00000340253        CCACAAGGGGGGTCTTCAGGCCCAGCACATGATGAGAGGACTAATGGGAGTGGCTCTGGG
ENSPTRT00000032169     CCACAAGGGGGGTCTTCAGGCCCAGCACATGATGAGAGGACTAATGGGAGTGGCTCTGGG

ENSMUST00000048688     GCTACAGGTGAGGACAGGAGGGGGAGCTCCCAGCCTGAGAGTTGTGACGTGCAGTCTAAT
ENSRNOT00000025790     GCTACAGGTGAGGACAGGAGGGGGAGCTCCCAGCCTGAGAGTTGTGACGTGCAGTCTAAT
ENSGGOT00000026421     GCTACAGGTGAGGACAGGAGGGGGAGCTCCCAGCCTGAGAGTTGTGACGTGCAGTCTAAT
ENSPPYT00000018532     GCTACAGGTGAGGACAGGAGGGGGAGCTCCCAGCCTGAGAGTTGTGACGTGCAGTCTAAT
ENSCJAT00000041759     GCTACAGGTGAGGACAGGAGGGGGAGCTCCCAGCCTGAGAGTTGTGACGTGCAGTCTAAT
ENSMMUT00000023749     GCTACAGGTGAGGACAGGAGGGGGAGCTCCCAGCCTGAGAGTTGTGACGTGCAGTCTAAT
ENST00000340253        GCTACAGGTGAGGACAGGAGGGGGAGCTCCCAGCCTGAGAGTTGTGACGTGCAGTCTAAT
ENSPTRT00000032169     GCTACAGGTGAGGACAGGAGGGGGAGCTCCCAGCCTGAGAGTTGTGACGTGCAGTCTAAT

ENSMUST00000048688     GAAGACTATCCTCGGAGGCCCCTAACCAGGGCCAGGAGCAGACTGTCCCATGTACCGCTG
ENSRNOT00000025790     GAAGACTACCCTCGGAGGCCCCTAACCAGGGCCAGGAGCAGACTGTCCCATGTCCCGCTG
ENSGGOT00000026421     GAAGACTACCCTCGGAGGCCCCTAACCAGGGCCAGGAGCAGACTGTCCCATGTACTGCTG
ENSPPYT00000018532     GAAGACTACCCTCGGAGGCCCCTAACCAGGGCCAGGAGCAGACTGTCCCATGTACTGCTG
ENSCJAT00000041759     GAAGACTACCCTCGGAGGCCCCTAACCAGGGCCAGGAGCAGACTGTCCCATGTACTGCTG
ENSMMUT00000023749     GAAGACTACCCTCGGAGGCCCCTAACCAGGGCCAGGAGCAGACTGTCCCATGTACTGCTG
ENST00000340253        GAAGACTACCCTCGGAGGCCCCTAACCAGGGCCAGGAGCAGACTGTCCCATGTACTGCTG
ENSPTRT00000032169     GAAGACTACCCTCGGAGGCCCCTAACCAGGGCCAGGAGCAGACTGTCCCATGTACTGCTG

ENSMUST00000048688     ATATCTGAGTCAGAAGTTGCCAAAACAAAGCCATGCCATGCCATGAAACGAAAGCGAACT
ENSRNOT00000025790     ATACCTGAGTCAGAAATTGCCAAAACCAAGCCATGTCATGCCATGAAACGAAAGCGAACG
ENSGGOT00000026421     GTATCTGAGTCAGAAGTAGCCAAAACAAAGCCACGTCACGCCATGAAACGGAAGCGGACA
ENSPPYT00000018532     GTATCTGAGTCAGAAGTAGCCAAAACAAAGCCACGTCACGCCATGAAACGGAAGCGGACA
ENSCJAT00000041759     GTATCTGAGTCAGAAGTTGCCAAAACAAAGCCACGTCATGCCATGAAACGGAAGCGGACA
ENSMMUT00000023749     GTATCTGAGTCAGAAGTAGCCAAAACAAAGCCACGTCACGCCATGAAACGGAAGCGGACA
ENST00000340253        GTATCTGAGTCAGAAGTAGCCAAAACAAAGCCACGTCACGCCATGAAACGGAAGCGGACA
ENSPTRT00000032169     GTATCTGAGTCAGAAGTAGCCAAAACAAAGCCACGTCACGCCATGAAACGGAAGCGGACA

ENSMUST00000048688     GCAGATAAGTCTACCAGCACCAGTGACCCTGTGATTGAAGATGACCACGTACAGGTTCTT
ENSRNOT00000025790     GCAGATAAGTCGACCAGCACCAGTGACCCTGTGATCGAGGATGACCATGTGCAGGTTCTT
ENSGGOT00000026421     GCAGATAAATCCACTAGTACAAGTGATCCTGTGATCGAGGATGACCATGTGCAGGTTCTT
ENSPPYT00000018532     GCAGATAAATCCACCAGTACAAGTGATCCTGTGATCGAGGATGACCATGTGCAGGTTCTT
ENSCJAT00000041759     GCAGATAAATCCACCAGTACCAGTGATCCTGTGATTGAGGATGACCATGTGCAGGTTCTT
ENSMMUT00000023749     GCAGATAAATCCACCAGTACAAGTGATCCTGTGATCGAGGATGACCATGTGCAGGTTCTT
ENST00000340253        GCAGATAAATCCACTAGTACAAGTGATCCTGTGATCGAGGATGACCATGTGCAGGTTCTT
ENSPTRT00000032169     GCAGATAAATCCACTAGTACAAGTGATCCTGTGATCGAGGATGACCATGTGCAGGTTCTT

ENSMUST00000048688     GTATTAAAATCCAAAAATCTTGTTGGAGTCACAATGACCAATTGTGGAATCACAGATCTA
```

```
ENSRNOT00000025790     GTATTGAAATCCAAAAATCTCGTTGGAGTCACAATGACGAATTGTGGAATCACAGATCTA
ENSGGOT00000026421     GTATTAAAATCCAAGAATCTTGTTGGAGTCACTATGACCAATTGTGGAATCACAGATCTA
ENSPPYT00000018532     GTATTAAAATCCAAGAATCTTGTTGGAGTCACTATGACCAATTGTGGAATCACAGATCTA
ENSCJAT00000041759     GTATTAAAATCCAAGAATCTTGTTGGAGTCACTATGACCAATTGTGGAATCACAGATCTA
ENSMMUT00000023749     GTATTAAAATCCAAGAATCTCGTTGGAGTCACTATGACCAATTGTGGAATCACAGATCTA
ENST00000340253        GTATTAAAATCCAAGAATCTTGTTGGAGTCACTATGACCAATTGTGGAATCACAGATCTA
ENSPTRT00000032169     GTATTAAAATCCAAGAATCTTGTTGGAGTCACTATGACCAATTGTGGAATCACAGATCTA

ENSMUST00000048688     GTACTGAAAGACTGCCCCAAGATGATGTTCATCCATGCTACCAGGTGCAGGGTACTGAAG
ENSRNOT00000025790     GTGCTGAAAGACTGCCCCAAGATGATGTTCATCCATGCTACCAGGTGCAGGGTGCTGAAG
ENSGGOT00000026421     GTGCTAAAAGACTGTCCGAAGATGATGTTCATCCATGCTACCAGGTGCAGGGTACTAAAA
ENSPPYT00000018532     GTGCTAAAAGACTGTCCGAAGATGATGTTCATCCATGCTACCAGGTGCAGGGTACTAAAA
ENSCJAT00000041759     GTGCTAAAAGACTGTCCCAAGATGATGTTCATTCATGCTACCAGGTGCAGGGTATTAAAA
ENSMMUT00000023749     GTGCTAAAAGACTGTCCCAAGATGATGTTCATCCATGCTACCAGGTGCAGGGTACTAAAA
ENST00000340253        GTGCTAAAAGACTGTCCAAAGATGATGTTCATCCATGCTACCAGGTGCAGGGTACTAAAA
ENSPTRT00000032169     GTGCTAAAAGACTGTCCGAAGATGATGTTCATCCATGCTACCAGGTGCAGGGTACTAAAA

ENSMUST00000048688     CATTTAAAGGTAGAAAATGCACCAATTGTAAACCGATTTGACTATGCACAGTGCAAGAAA
ENSRNOT00000025790     CACTTAAAGGTGGAAAATGCACCGATTGTAAACCGCTTTGACTACGCACAGTGCAAGAAA
ENSGGOT00000026421     CATTTAAAGGTAGAAAATGCACCAATTGTAAACCGATTTGACTATGCACAGTGCAAGAAA
ENSPPYT00000018532     CATTTAAAAGTAGAAAATGCACCAATTGTAAACCGATTTGACTATGCACAGTGCAAGAAA
ENSCJAT00000041759     CATTTAAAAGTAGAAAATGCACCAATTGTAAATCGATTTGACTATGCACAGTGCAAGAAA
ENSMMUT00000023749     CATTTAAAAGTAGAAAATGCACCAATTGTAAACCGATTTGACTACGCACAGTGCAAGAAA
ENST00000340253        CATTTAAAGGTAGAAAATGCACCAATTGTAAACCGATTTGACTATGCACAGTGCAAGAAA
ENSPTRT00000032169     CATTTAAAGGTAGAAAATGCACCAATTGTAAACCGATTTGACTATGCACAGTGCAAGAAA

ENSMUST00000048688     CTAAATATGGACCAGGTACTAGACCAAATACTGAGGATGCCTCCGGAGAGGAACCGGATC
ENSRNOT00000025790     CTAAATATGGACCAGGTACTCGACCAAATACTGAGGATGCCTCCGGAGAGGAACCGCATC
ENSGGOT00000026421     CTGAACATGGATCAGGTACTAGACCAGATACTAAGAATGCCACCCGAGAGAAACCGCATC
ENSPPYT00000018532     CTGAACATGGATCAGGTACTAGACCAGATACTAAGAATGCCACCCGAGAGAAACCGCATC
ENSCJAT00000041759     CTGAACATGGATCAGGTACTAGACCAGATACTAAGAATGCCACCCGAGAGAAACCGCATC
ENSMMUT00000023749     CTGAACATGGATCAGGTACTAGACCAGATACTAAGAATGCCACCCGAGAGAAACCGCATC
ENST00000340253        CTGAACATGGATCAGGTACTAGACCAGATACTAAGAATGCCACCCGAGAGAAACCGCATC
ENSPTRT00000032169     CTGAACATGGATCAGGTACTAGACCAGATACTAAGAATGCCACCCGAGAGAAACCGCATC

ENSMUST00000048688     ATCTACCTGCGCCCAATGCAGCAGGTAGACACACTAACTCTGGAGCAGAAGCTCTTCAGC
ENSRNOT00000025790     ATCTACCTGCGCCCGATGCAGCAGGTAGACACACTAACTCTGGAGCAGAAGCTCTTCAGC
ENSGGOT00000026421     ATATACCTACGCCCAATGCAGCAGGTGGACACTCTAACTTTGGAGCAGAAGCTATTTAGT
ENSPPYT00000018532     ATATACCTACGCCCAATGCAGCAGGTGGACACTCTAACTTTGGAGCAGAAGCTATTTAGT
ENSCJAT00000041759     ATATACCTACGCCCAATGCAGCAGGTGGACACTCTAACTTTGGAGCAGAAGCTGTTTAGT
ENSMMUT00000023749     ATATACCTACGCCCAATGCAGCAGGTGGACACGCTAACTTTGGAGCAGAAGCTGTTTAGT
ENST00000340253        ATATACCTACGCCCAATGCAGCAGGTGGACACTCTAACTTTGGAGCAGAAGCTATTTAGT
ENSPTRT00000032169     ATATACCTACGCCCAATGCAGCAGGTGGACACTCTAACTTTGGAGCAGAAGCTATTTAGT

ENSMUST00000048688     GGGCCCTACCCCTATCACATCTGCATCATCCACGAGTTCAGCAATCCTCCCAACGTGCGG
ENSRNOT00000025790     GGCCCCTACCCCTACCACATCTGCGTCATCCATGAGTTCAGCAATCCTCCCAACGTGCGC
ENSGGOT00000026421     GGTCCCTACCCCTATCACATCTGTATTATCCATGAATTCAGTAACCCTCCCAATGTCCGG
ENSPPYT00000018532     GGTCCCTACCCCTATCACATCTGTATTATCCATGAATTCAGTAACCCTCCCAATGTCCGG
ENSCJAT00000041759     GGTCCCTACCCCTATCACATCTGTATTATTCATGAATTCAGTAACCCTCCCAATGTCCGA
ENSMMUT00000023749     GGTCCCTACCCCTATCACATCTGTATTATCCATGAATTCAGCAACCCTCCCAATGTCCGG
ENST00000340253        GGTCCCTACCCCTATCACATCTGTATTATCCATGAATTCAGTAACCCTCCCAATGTCCGG
ENSPTRT00000032169     GGTCCCTACCCCTATCACATCTGTATTATCCATGAATTCAGTAACCCTCCCAATGTCCGG

ENSMUST00000048688     AACAAGGTGCGCATCCGCAATTGGATGGACACGATAGCCAACATCAATCAAGAGCTCATT
```

```
ENSRNOT00000025790     AACAAGGTGCGCATTCGCAATTGGATGGACACGATCGCCAACATCAATCAAGAGCTCATT
ENSGGOT00000026421     AATAAGGTGCGCATTCGCAGCTGGATGGACACTATAGCAAACATCAATCAAGAGCTCATT
ENSPPYT00000018532     AATAAGGTGCGCATTCGCAGCTGGATGGACACTATAGCAAACATCAATCAAGAGCTCATT
ENSCJAT00000041759     AATAAGGTGCGAATTCGCAGTTGGATGGACACTATAGCAAACATCAATCAAGAGCTCATT
ENSMMUT00000023749     AATAAGGTGCGCATTCGCAGCTGGATGGACACTATAGCAAACATCAATCAAGAGCTCATT
ENST00000340253        AATAAGGTGCGCATTCGCAGCTGGATGGACACTATAGCAAACATCAATCAAGAGCTCATT
ENSPTRT00000032169     AATAAGGTGCGCATTCGCAGCTGGATGGACACTATAGCAAACATCAATCAAGAGCTCATT

ENSMUST00000048688     AAATACGAATTCTTCCTGGAAGCCACTCGGACTGAAGAGGATTTAAAGAAGTACCCTAAG
ENSRNOT00000025790     AAATATGAATTCTTCCTGGAAGCCACTCGAACTGAAGAAGATTTAAAGAAGTACCCTAAG
ENSGGOT00000026421     AAATATGAATTCTTCCCTGAAGCCACTCGAAGTGAAGAAGACTTAAAGAAATACCCCAAG
ENSPPYT00000018532     AAATATGAATTCTTCCCTGAAGCCACTCGAAGTGAAGAAGACTTAAAGAAATACCCCAAG
ENSCJAT00000041759     AAATATGAATTCTTCCCTGAAGCCACTCGAAGTGAAGAAGACTTAAAGAAGTACCCCAAG
ENSMMUT00000023749     AAATATGAATTCTTCCCTGAAGCCACTCGAAGTGAAGAAGACTTAAAGAAATACCCCAAG
ENST00000340253        AAATATGAATTCTTCCCTGAAGCCACTCGAAGTGAAGAAGACTTAAAGAAATACCCCAAG
ENSPTRT00000032169     AAATATGAATTCTTCCCTGAAGCCACTCGAAGTGAAGAAGACTTAAAGAAATACCCCAAG

ENSMUST00000048688     TATCCCTGGGGGAGAGAAATCTACACTTTAGAGGGTGTTGTAGATGGAGCGCCGTATTCC
ENSRNOT00000025790     TATCCCTGGGGGAGAGAAATCTACACTTTAGAGGGTGTTGTTGATGGAGCTCCATACTCC
ENSGGOT00000026421     TACCCCTGGGGGAGAGAAATCTATACTTTAGAAGGTGTTGTGGATGGAGCTCCATATTCC
ENSPPYT00000018532     TACCCCTGGGGGAGAGAAATCTATACTTTAGAAGGTGTTGTGGATGGAGCTCCATATTCC
ENSCJAT00000041759     TACCCCTGGGGGAGAGAAATCTATACTTTAGAAGGTGTTGTGGATGGAGCTCCGTATTCC
ENSMMUT00000023749     TACCCCTGGGGGAGAGAAATCTATACTTTAGAAGGTGTTGTGGATGGAGCTCCATATTCC
ENST00000340253        TACCCCTGGGGGAGAGAAATCTATACTTTAGAAGGTGTTGTGGATGGAGCTCCATATTCC
ENSPTRT00000032169     TACCCCTGGGGGAGAGAAATCTATACTTTAGAAGGTGTTGTGGATGGAGCTCCATATTCC

ENSMUST00000048688     ATGATTTCCGACTTCCCTTGGCTGAGGTCACTGCGGACCGCAGAGCCCAACAGCTTTGCC
ENSRNOT00000025790     ATGATTTCCGACTTCCCTTGGCTGAGGTCACTGCGGGCAGCAGAGCCCAACAGCTTTGCC
ENSGGOT00000026421     ATGATTTCTGACTTCCCTTGGCTGAGGTCATTACGAGCTGCAGAGCCCAACAGCTTCGCT
ENSPPYT00000018532     ATGATTTCTGACTTCCCTTGGCTGAGGTCATTACGAGCTGCAGAGCCCAACAGCTTCGCT
ENSCJAT00000041759     ATGATTTCTGATTTCCCTTGGCTGAGGTCGTTACGAGCAGCAGAGCCCAACAGCTTCGCT
ENSMMUT00000023749     ATGATTTCTGACTTCCCTTGGCTGAGGTCATTACGAGCAGCAGAGCCCAACAGCTTCGCT
ENST00000340253        ATGATTTCTGACTTCCCTTGGCTGAGGTCATTACGAGCTGCAGAGCCCAACAGCTTCGCT
ENSPTRT00000032169     ATGATTTCTGACTTCCCTTGGCTGAGGTCATTACGAGCTGCAGAGCCCAACAGCTTCGCT

ENSMUST00000048688     AGATATGATTTTGAAGATGATGAAGAGAGCACCATCTATGCTCCTCGAAGAAAAGGGCAG
ENSRNOT00000025790     AGATACGACTTTGAAGATGATGAAGAAAGTACCATCTATGCTCCTCGAAGGAAAGGACAG
ENSGGOT00000026421     CGATACGACTTTGAAGACGATGAAGAAAGCACTATCTATGCTCCTAGAAGGAAAGGACAG
ENSPPYT00000018532     CGATACGACTTTGAAGACGATGAAGAAAGCACTATCTATGCTCCTAGAAGGAAAGGACAG
ENSCJAT00000041759     CGATACGACTTTGAAGATGATGAAGAAAGCACTATCTATGCTCCTAGAAGGAAAGGACAG
ENSMMUT00000023749     CGATACGACTTTGAAGATGATGAAGAAAGCACTATCTATGCTCCTAGAAGGAAAGGACAG
ENST00000340253        CGATACGACTTTGAAGACGATGAAGAAAGCACTATCTATGCTCCTAGAAGGAAAGGACAG
ENSPTRT00000032169     CGATACGACTTTGAAGACGATGAAGAAAGCACTATCTATGCTCCTAGAAGGAAAGGACAG

ENSMUST00000048688     TTGTCTGCAGACATTTGTATGGAGACAATAGGAGAGGAGATCTCAGAGATGCGCCAGATG
ENSRNOT00000025790     TTGTCAGCAGACATTTGCATGGAGACAATCGGGGAGGAGATCTCAGAGATGCGTCAGATG
ENSGGOT00000026421     CTGTCTGCAGACATCTGTATGGAAACAATAGGAGAGGAAATTTCAGAGATGCGTCAGATG
ENSPPYT00000018532     CTGTCTGCAGACATCTGTATGGAAACAATAGGAGAGGAAATTTCAGAGATGCGTCAGATG
ENSCJAT00000041759     CTGTCTGCAGACATCTGTATGGAAACAATAGGAGAGGAAATTTCAGAGATGCGTCAGATG
ENSMMUT00000023749     CTGTCTGCAGACATCTGTATGGAAACAATAGGAGAGGAAATTTCAGAGATGCGTCAGATG
ENST00000340253        CTGTCTGCAGACATCTGTATGGAAACAATAGGAGAGGAAATTTCAGAGATGCGTCAGATG
ENSPTRT00000032169     CTGTCTGCAGACATCTGTATGGAAACAATAGGAGAGGAAATTTCAGAGATGCGTCAGATG

ENSMUST00000048688     AAGAGGGGCATATTTCAGCGAGTAGTAGCGATTTTTATCCACTACTGTGATGTCAATGGA
```

```
ENSRNOT00000025790     AAGAGGGGTGTATTTCAGCGAGTAGTGGCCATTTTTATCCACTACTGTGATGTCAACGGA
ENSGGOT00000026421     AAGAAGGGTGTATTTCAGCGAGTAGTGGCAATTTTTATCCACTATTGTGATGTCAATGGA
ENSPPYT00000018532     AAGAAGGGTGTATTTCAGCGAGTAGTGGCAATTTTTATCCACTATTGTGATGTCAATGGA
ENSCJAT00000041759     AAGAAAGGTGTATTTCAGCGAGTAGTGGCCATTTTTATCCACTATTGTGATGTCAATGGA
ENSMMUT00000023749     AAGAAGGGTGTATTTCAGCGAGTAGTGGCAATTTTTATCCACTATTGTGATGTCAATGGA
ENST00000340253        AAGAAGGGTGTATTTCAGCGAGTAGTGGCAATTTTTATCCACTATTGTGATGTCAATGGA
ENSPTRT00000032169     AAGAAGGGTGTATTTCAGCGAGTAGTGGCAATTTTTATCCACTATTGTGATGTCAATGGA


ENSMUST00000048688     GAGCCAGTTGAAGACGACTACATT
ENSRNOT00000025790     GAGCCAGTTGAAGATGACTACATT
ENSGGOT00000026421     GAGCCAGTTGAAGATGACTACATT
ENSPPYT00000018532     GAGCCAGTTGAAGATGACTACATT
ENSCJAT00000041759     GAGCCAGTTGAAGATGACTACATT
ENSMMUT00000023749     GAGCCAGTTGAAGATGACTACATT
ENST00000340253        GAGCCAGTTGAAGATGACTACATT
ENSPTRT00000032169     GAGCCAGTTGAAGATGACTACATT
```

Multiple sequence alignment of Fbxo39

```
ENSMUST00000108504     ATGGATGAAGACTGTGAAGTGACCCAGCTCCAAGAGCAGAGCTGCTGGGCCACTCTGCCA
ENSRNOT00000020071     ATGGATGAAGACAGTGAAGTGACGCAGCCCCAAGATCAGAGCTGCTGGGCCACTCTGCCG
ENSCJAT00000033404     ATGGACGAAGAGAGTGAACTGATCCAGCCCGAAGACGAGAGCTGCTGGGCCAGTCTGCCT
ENSMMUT00000013403     ATGGACGAAGAAAGTGAACTGATCCAGCCCCAAGACCAGAGCTGCTGGGCCGCTCTGCCC
ENSPPYT00000009237     ATGGACGAAGAAAGTGAACTGATCCAGCCCCAAGACCAGAGCTGCTGGGCCACTCTGCCC
ENSGGOT00000031003     ATGGACGAAGAAAGTGAACTGATCCAGCCCCAAGACCAGAGCTGCTGGGCCTTTCTGCCT
ENST00000321535        ATGGACGAAGAAAGTGAACTGATCCAGCCCCAAGACCAGAGCTGCTGGGCCTTTCTGCCC
ENSPTRT00000015954     ATGGACGAAGAAAGTGAACTGATCCAGCCCCAAGACCAGAGCTGCTGGGCCTTTCTGCCC


ENSMUST00000108504     GATGTGTGCCTGCGACGTGTTTTCTGGTGGCTGGGAGACAGGGACAGGTCCAGAGCTGCC
ENSRNOT00000020071     GATGTGTGCCTACGACGTGTTTTCTGGTGGCTGGGAGACAGGGACAGGTCCAGAGCTGCC
ENSCJAT00000033404     GATCTGTGCCTGTGCCGTGTTTTCTGGTGGCTAGGAGACAGGGACAGGTCCAGGGCTGCT
ENSMMUT00000013403     GATGTGTGTCTGTGCCGTGTTTTCTGGTGGCTAGGAGACAGGGACAGGTCCAGGGCTGCT
ENSPPYT00000009237     GATGTGTGTCTGTGCCGTGTTTTCTGGTGGCTAGGAGACAGGGACAGGTCCAGGGCTGCT
ENSGGOT00000031003     GATTTGTGTCTGTGCCGTGTTTTCTGGTGGCTAGGAGACAGGGACAGGTCCAGGGCTGCT
ENST00000321535        GATTTGTGTCTGTGCCGTGTTTTCTGGTGGCTAGGAGACAGGGACAGGTCCAGGGCTGCT
ENSPTRT00000015954     GATTTGTGTCTGTGCCGTGTTTTCTGGTGGCTAGGAGACAGGGACAGGTCCAGGGCTGCT


ENSMUST00000108504     CTGGTCTGCAGAAAGTGGAACCAGATTATGTACTCAGCTGACCTCTGGCGATACAGGACC
ENSRNOT00000020071     CTGGTCTGCAGAAAATGGAACCAGATCATGTACTCAGCTGACCTCTGGCGATACAGGACC
ENSCJAT00000033404     CTTGTCTGCAGAAAATGGAACCAGATAATGTATTCTGCTGACCTCTGGCGGTATAGGACC
ENSMMUT00000013403     CTTGTCTGCAGAAAGTGGAACCAGATGATGTATTCTGCTGACCTCTGGCGGTACAGGACC
ENSPPYT00000009237     CTTGTCTGCAGAAAGTGGAACCAGATGATGTATTCTGCTGACCTCTGGCGGTACAGGACC
ENSGGOT00000031003     CTTGTCTGCAGAAAGTGGAACCAGATGATGTATTCTGCTGAGCTCTGGCGGTACAGAACC
ENST00000321535        CTTGTCTGCAGAAAGTGGAACCAGATGATGTATTCTGCTGAGCTCTGGCGGTACAGAACC
ENSPTRT00000015954     CTTGTCTGCAGAAAGTGGAACCAGATGATGTATTCTGCTGAGCTCTGGCGGTACAGAACC


ENSMUST00000108504     ATCACGTTCAGCGGAAGGCCGTCCAGGGTACACGCATCTGAATTCGAGTCGGCACTGTGG
ENSRNOT00000020071     ATCACGTTCAGCGGAAGGCCGTCCAGGGTACACGCATCTGAATTTGAGTCAGCACTCTGG
ENSCJAT00000033404     ATCACCTTCAGCGGGAGACCTTCGAGGGTACATGCATCTGAGTTTGAGTCAGCTCTTTGG
ENSMMUT00000013403     ATCACCTTCAGCGGGAGACCTTCCAGGGTACATGCATCTGAAGTTGAGTCAGCTCTTTGG
ENSPPYT00000009237     ATCACCTTCAGCGGGAGACCTTCCAGGGTACACGCATCTGAAGTTGAGTCAGCTCTTTGG
ENSGGOT00000031003     ATCACCTTCAGCGGGAGACCTTCCAGGGTACATGCATCTGAAGTTGAGTCAGCTGTTTGG
ENST00000321535        ATCACCTTCAGCGGGAGACCTTCCAGGGTACATGCATCTGAAGTTGAGTCAGCTGTTTGG
ENSPTRT00000015954     ATCACCTTCAGCGGGAGACCTTCCAGGGTACATGCATCTGAAGTTGAGTCAGCTGTTTGG
```

```
ENSMUST00000108504     TACATTAAGAAATTTGGCCGATACCTGGAACACTTGGAAATCAAGTTCCTGAACCCTTAC
ENSRNOT00000020071     TACGTTAAGAAATTTGGCCGATACCTGGAACACTTGGAAATCAAGTTCCTGAACCCTTAC
ENSCJAT00000033404     TATGTTAAGAAGTTTGGTCGTTATCTGGAGCACCTGGAAGTCAAATTCCTGAATCCTTAC
ENSMMUT00000013403     TATGTTAAGAAGTTTGGTCGTTATCTGGAGCGCCTGGAGGTCAAATTCCTGAATCCTTAC
ENSPPYT00000009237     TACGTTAAGAAGTTTGGTCGTTATCTGGAGCGCCTGGAGGTCAAATTCCTGAATCCTTAC
ENSGGOT00000031003     TATGTTAAGAAGTTTGGTCGTTATCTGGAGCACCTGGAGGTCAAATTCATGAATCCTTAC
ENST00000321535        TATGTTAAGAAGTTTGGTCGTTATCTGGAGCACCTGGAGGTCAAATTCATGAATCCTTAC
ENSPTRT00000015954     TATGTTAAGAAGTTTGGTCGTTATCTGGAGCACCTGGAGGTCAAATTCATGAATCCTTAC

ENSMUST00000108504     AATGCCGTCTTGACCAAAAAGTTCCAGGTCACCATGCGAGGCCTCCTGTCATGCCTGGGC
ENSRNOT00000020071     AATGCGGTCTTGACCAAGAAGTTCCAGGTCACCATGCGAGGCCTCCTGTCGTGCCTGGGC
ENSCJAT00000033404     AATGCTGTCTTGACCAAGATGTTCCAGGTCACTATGCAGGGCCTTCTGTCTCGTCTGGGC
ENSMMUT00000013403     AATGCTGTCTTGACCAAGAAGTTCCAGGTCACCATGCGGGGCCTCCTGTCTTGTCTGAGT
ENSPPYT00000009237     AATGCTGTCTTGACCAAGAAGTTCCAGGTCACAATGCGGGGCCTCCTGTCTTGTCTGAGT
ENSGGOT00000031003     AATGCTGTCTTGACCAAGAAGTTCCAGGTCACCATGCGGGGCCTCCTGTCTTGTCTGAGT
ENST00000321535        AATGCTGTCTTGACCAAGAAGTTCCAGGTCACCATGCGGGGCCTCCTGTCTTGTCTGAGT
ENSPTRT00000015954     AATGCTGTCTTGACCAAGAAGTTCCAGGTCACCATGCGGGGCCTCCTGTCTTGTCTGAGT

ENSMUST00000108504     AAGAGTAACAACCGCCTGCGGTCACTCTCTATCCAACACCTGGAGCTGGATCGGCTGGTC
ENSRNOT00000020071     AAGAGTAACAACCGCCTGCGGTCACTGTCCATCCAGCATTTAGAACTGGATCGTCTGGTC
ENSCJAT00000033404     AAAAGCAACAACCGTCTGAAATCTCTTTCCATCCAACACCTGGAGCTGGACCGCCTGGTA
ENSMMUT00000013403     AAGAGCAACAACCGTCTGAAATCTCTTTCCATCCAACACCTGGAGCTGGACCGCCTGGTG
ENSPPYT00000009237     AAGAGCAACAACCGTCTGAAATCTCTTTCCATCCAACACCTGGAGCTGGACCGCCTGGTG
ENSGGOT00000031003     AAGAGCAACAACCGTCTGAAAGCTCTTTCCATCCAATACCTGGAGCTGGACCGCCTGGTA
ENST00000321535        AAGAGCAACAACCGTCTGAAATCTCTTTCCATCCAATACCTGGAGCTGGACCGCCTGGTA
ENSPTRT00000015954     AAGAGCAACAACCGTCTGAAATCTCTTTCCATCCAATACCTGGAGCTGGACCGCCTGGTA

ENSMUST00000108504     TGGAGAAATAGCATTAGGGGTTCACTCATCAAGAGCCTGAGTTTTTTCTTAAAGAAAATG
ENSRNOT00000020071     TGGAGAAACAGCATTAGGGGTTCGCTCATCAAGAGCCTGAGTTTTTTCTTAAAGAAAATG
ENSCJAT00000033404     TGGAGGAACAGTATCAGGAGATCGTTTGTCAGGAGCTTGAGCTTCTTCTTGAAGAAGATA
ENSMMUT00000013403     TGGAGGAACAGCATCAGGAGCTCATTCATCAGGAGCTTGAGCTTCTTCTTAAAGAAGATG
ENSPPYT00000009237     TGGAGGAACAGCATCAGGAGCTCATTCATCAGGAGCTTGAGCTTCTTCTTAAAGAAGATG
ENSGGOT00000031003     TGGAGGAACAGCATCAGGAGCTCATTAATCAGCAGCTTGAGCTTCTTCTTAAAGAAGATG
ENST00000321535        TGGAGGAACAGCATCAGGAGCTCATTCATCAGCAGCTTGAGCTTCTTCTTAAAGAAGATG
ENSPTRT00000015954     TGGAGGAACAGCATCAGGAGCTCATTCATCAGCAGCTTGAGTTTCTTCTTAAAGAAGATG

ENSMUST00000108504     GGCAAACACCTGGACCATCTCAGCCTGAAAGGAGCCCGGCTGACAGTGGAGCAAGGCTGC
ENSRNOT00000020071     GGCAAACACCTGGACCATCTCAGCCTAAAAGGAGCCCGGCTGACAGTGGAGCAAGGCTGC
ENSCJAT00000033404     GGCAAACACCTGGATTATCTCAACCTAAAAGGGGCCAGGCTGACGGTGGAGCAAGGCTGC
ENSMMUT00000013403     GGCAAACACCTGGATTACCTCAACCTAAAAGGGGCCAGGCTGACCGTGGAGCAAGGCTGC
ENSPPYT00000009237     GGCAAACGCCTGGATTATCTCAACCTAAAAGGGGCCAGGCTGACCGTGGAGCAAGGCTGC
ENSGGOT00000031003     GGCAAACGCCTGGATTATCTCAACCTAAAAGGGGCCAGGCTGACCGTGGAGCAAGGCTGC
ENST00000321535        GGCAAACGCCTGGATTATCTCAACCTAAAAGGGGCCAGGCTGACCGTGGAGCAAGGCTGC
ENSPTRT00000015954     GGCAAACGCCTGGATTATCTCAACCTAAAAGGGGCCAGGCTGACCGTGGAGCAAGGCTGC

ENSMUST00000108504     CACATCCTCAACTCCCTGAGCTACATGCAGAATGAGAACATGGCTTCAGAGCTCAACATT
ENSRNOT00000020071     CACATCCTCAACTCCCTGAGCTACATGCGGAATGAGAACGTGGCCTCAGAGCTCAACATT
ENSCJAT00000033404     CACGTTCTCAACTCCCTCAGCTACATGAGGAATAAGAATGTGATCTCGGAGCTCAACATC
ENSMMUT00000013403     CACATTCTCGACTCCCTCAGCCACCTGAGGAATGAGAATGTGATATCGGAACTCAACATC
ENSPPYT00000009237     CAAATTCTCGACTCCCTCAGCTGCATGAGGAATGAGAATGTGATCTCAGAGCTCAACATC
ENSGGOT00000031003     CAAATTCTCGACTCCCTCAGCTACATGAGGAATGAGAATGTGATCTCAGAGCTCAACATC
ENST00000321535        CAAATTCTCGACTCCCTCAGCTACATGAGGAATGAGAATGTGATCTCAGAGCTCAACATC
ENSPTRT00000015954     CAAATTCTCGACTCCCTCAGCTACGTGAGGAATGAGAATGTGATCTCAGAGCTCAACATC
```

```
ENSMUST00000108504     GAAGACTTCTTCAGCCATCACCTGGCTGTCTATGGCAGCTCCCAGTTCAACAAGGCCATG
ENSRNOT00000020071     GAAGACTTCTTCAGCCATCATCTGGCTGTCTATGGCAGCTCCCAGTTCAACAAGGCCATG
ENSCJAT00000033404     GAGGACTACTTCAGCCATCACCTTGCTGTCTACAGCAGCGCCCAATTCAAAAAGACCATG
ENSMMUT00000013403     GAGGACTACTTCAGTCATCATCTTGCTGTCTACAGCAGCCCCCAGTTCAAAAAGACCATG
ENSPPYT00000009237     GAGGACTATTTCAGCCATCACCTTGCTGTCTACAGCAGCCCCCAGTTCAAAAAGACCATG
ENSGGOT00000031003     GAGGACTATTTCAGCCATCACCTTGCTGTCTACAACAGCCCCCAGTTCAAAAAGACCATG
ENST00000321535        GAGGACTATTTCAGCCATCACCTTGCTGTCTACAACAGCCCCCAGTTCAAAAAGACCATG
ENSPTRT00000015954     GAGGACTATTTCAGCCATCACCTTGCTGTCTACAACAGCCCCCAGTTCAAAAAGACCATG

ENSMUST00000108504     GCCACCTTCCGCAACTTGACATTCCTAACCCTCAACTACAACTGTATCTCCGACGAGCTG
ENSRNOT00000020071     GCCACATTCCACAACTTGACATTCCTAACACTCAACTACAACTGTATCTCTGATGAGTTG
ENSCJAT00000033404     TCCACGTTCCACAATCTTGTGTCCCTGACCCTCAACTACAACTGTATCTCCGACGAGCTG
ENSMMUT00000013403     TCCACATTCCACAATCTTGTGTCCCTGAACCTCAATTACAACTGTATCTCCGACGAGCTG
ENSPPYT00000009237     TCCACATTCCACAATCTTGTGTCCCTGAACCTCAACTACAACTGTATCTCCGACGAGCTG
ENSGGOT00000031003     TCCACATTCCACAATCTTGTGTCCCTGAACCTCAACTACAACTGTATCTCCGACGAGCTG
ENST00000321535        TCCACATTCCACAATCTTGTGTCCCTGAACCTCAACTACAACTGTATCTCCGACGAGCTG
ENSPTRT00000015954     TCCACATTCCACAATCTTGTGTCCCTGAACCTCAACTACAACTGTATCTCCGACGAGCTG

ENSMUST00000108504     CTCGAGACCTTGAGCGAGAACGCCGGGACCCTCCGGACCATGAACATCAAATGCCACGTT
ENSRNOT00000020071     CTCGAGACCTTGAGCGAGAACGCCGGTACCCTCCGGACCATGAACATCAAGTGCCATGTT
ENSCJAT00000033404     CTTGAGAACTTGTGTGAGAATGCCAGTACCCTCTGGACCATAAACATCAAATGCCACATT
ENSMMUT00000013403     CTTGAGAATTTGTGTGAGAACGCCAGCACCCTCTGGACCATAAACATCAAATGCCACATT
ENSPPYT00000009237     CTTGAGAACTTGTGTGAGAACGCCAGCACCCTCCAGACCATCAACATCAAATGCCACATT
ENSGGOT00000031003     CTTGAGAACTTGTGTGAGAATGCCAGCACCCTCCAGACCATCAACATCAAATGCCACGTT
ENST00000321535        CTTGAGAACTTGTGTGAGAATGCCAGCACCCTCCGGACCATCAACATCAAATGCCACGTT
ENSPTRT00000015954     CTTGAGAACTTGTGTGAGAATGCCAGCACCCTCCGGACCATCAACATCAAATGCCACGTT

ENSMUST00000108504     CATGACCCTCACGGTCAGGTAGTCTGGGGTATGTCTTGGGCCAAGTTGGCCAGGCAGGCC
ENSRNOT00000020071     CATGACCCTCACGGTCAGGTAGTCTGGGGTATGTCCTGGGCCAAGCTGGCCAGGCAGGCC
ENSCJAT00000033404     CATGACCCCCATGGACAGGTCATCTGGGGTATGTCCTGGGCCAAGTTAGGCAGGGAGGCC
ENSMMUT00000013403     CATGACCCCCACGGACAGGTCATCTGGGGTATGTCCTGGGCCAAGCTGGCCAGGCAGGCC
ENSPPYT00000009237     CATGACCCCCACGGACAGGTCATCTGGGGTATGTCCTGGGCCAAGCTGGCCAGGCAGGCC
ENSGGOT00000031003     CATGACCCCCACGGACAGGTCATCTGGGGTATGTCCTGGGCTAAGCTGGCCAGGCAGGCC
ENST00000321535        CATGACCCCCACGGACAGGTCATCTGGGGTATGTCCTGGGCCAAGCTGGCCAGGCAGGCC
ENSPTRT00000015954     CATGACCCCCACGGACAGGTCATCTGGGGTATGTCCTGGGCCAAGCTGGCCAGGCAGGCC

ENSMUST00000108504     AGCAACCTGAAGGTGAACTTCTTCTTTGAGCGGGTGATGAAATATGAACGCCTGGCCCGG
ENSRNOT00000020071     AGCAACCTGAAGGTGAACTTCTTCTTTGAGAGGGTGATGAAATATGAACGCCTGGCCCGG
ENSCJAT00000033404     ACCAATCTGAAGGTGAACTTCTTCTTTGAACGGATCATGAAGTACGAGTGCTTGGCCCGG
ENSMMUT00000013403     ACCAATCTGAAGGTGAACTTCTACTTTGAACGGATCATGAAGTACGAACGCTTGGCCCGA
ENSPPYT00000009237     ACCAATCTGAAGGTGAACTTCTTCTTTGAACGGATCATGAAGTACGAACGCTTGGCCCGA
ENSGGOT00000031003     ACCAATCTGAAGGTGAACTTCTTCTTTGAACGGATCATGAAGTACGAACGCTTGGCCCGA
ENST00000321535        ACCAATCTGAAGGTGAACTTCTTCTTTGAACGGATCATGAAGTACGAACGCTTGGCCCGA
ENSPTRT00000015954     ACCAATCTGAAGGTGAACTTCTTCTTTGAACGGATCATGAAGTACGAACGCTTGGCCCGA

ENSMUST00000108504     ATCTTGCTGCAGGAGATTCCAGTCAGGAGCATCAGCCTGAGGAGTTGCTATTTCAGTGAC
ENSRNOT00000020071     ATCTTGCTTCAGGAGATTCCAGTCAGGAGCATCAGCCTGAGAAGCTGCTATTTCAGTGAC
ENSCJAT00000033404     ATCCTCTTGCAGGAGATCCCGGTCAGGAGCCTCAGTCTGAGAAGCTGTTATTTCAGTGAC
ENSMMUT00000013403     ATCCTCTTGCAGGAGATCCCAGTCAGGAGCATCAGTCTGAGAAGCTGCTATTTCAGTGAC
ENSPPYT00000009237     ATCCTCTTGCAGGAGATCCCGATCAGGAGCATCAGTCTGAGAAGCTGCTATTTCAGTGAC
ENSGGOT00000031003     ATCCTCTTGCAGGAGATCCCGATCAGGAGCATCAGTCTGAGAAGCTGCTATTTCAGTGAC
ENST00000321535        ATCCTCTTGCAGGAGATCCCGATCAGGAGCATCAGTCTGAGAAGCTGCTATTTCAGTGAC
ENSPTRT00000015954     ATCCTCTTGCAGGAGATCCCGATCAGGAGCATCAGTCTGAGAAGCTGCTATTTCAGTGAT
```

```
ENSMUST00000108504     CCGGACTGGTCCATGCGGCCCACTCTGACAGACCTCCTGCCCACCTTCCGGAACACCCTG
ENSRNOT00000020071     CCAGACTGGTCCATGAGACCCACTCTGACAGACCTCCTGCCCACCTTCCGGAACACTCTG
ENSCJAT00000033404     CCAGACTGTTCCATGAGACCCACTCTGACGGATCTCCTGCCCACCTTCCGGCATACTCTG
ENSMMUT00000013403     CCAGACTGGTCCATGAGACCCACTCTGATAGATCTCCTGCCCACCTTCCGGCACACTCTG
ENSPPYT00000009237     CCAGACTGGTCAATGAGACCCACTCTGATAGATCTCCTGCCCAGCTTCCGGCACACTCTG
ENSGGOT00000031003     CCAGACTATTCAATGAGACCCACTCTGATAGATCTCCTGCCCACCTTCCGGCACACTCTG
ENST00000321535        CCAGACTGTTCAATGAGACCCACTCTGATAGATCTCCTGCCCACCTTCCGGCACACTCTG
ENSPTRT00000015954     CCAGACTATTCAATGAGACCCACTCTGATAGATCTCCTGCCCACCTTCCGGCACACTCTG

ENSMUST00000108504     CAGAAGTTAACGTTCGAGTTCAACAATAATCACGAGTCTCTAGATGAGCAGCTGCATCTC
ENSRNOT00000020071     CAGAAGTTAACCTTCGAGTTCAACAATAACCATGAGTCTCTAGATGAGCAGCTGCACCTC
ENSCJAT00000033404     CAGAAATTAACTTTTGAATTCAACAACAACCATGAGTCACTCGACGAGGAGCTGCACGTC
ENSMMUT00000013403     CAGAAATTAACTTTTGAATTCAACAACAACCACGAGTCACTCGACGAGGAGCTGCACCTC
ENSPPYT00000009237     CAGAAATTAACTTGTGAATTCAACAACAACCATGAGTCACTCGACGAGGAGCTGCACCTC
ENSGGOT00000031003     CAGAAATTAACTTGTGAATTCAACAACAACCATGAGTCACTCGACGAGGAGCTGCACCTC
ENST00000321535        CAGAAATTAACTTGTGAATTCAACAACAACCATGAGTCACTCGACGAGGAGCTGCACCTC
ENSPTRT00000015954     CAGAAATTAACTTGTGAATTCAACAACAACCATGAGTCACTCGACGACGAGCTGCACCTC

ENSMUST00000108504     CTCATCTTGGCCTGTCGGAAGCTGTTTTACTTCAAAATCTGGGCTTTCCTGGACGTTAAG
ENSRNOT00000020071     CTCATCCTGGCCTGTCGGAAGCTGTTTTACTTCAAAATCTGGGCTTTCCTGGACGTTAAG
ENSCJAT00000033404     CTCATCTTATCCTGCAGGAAGTTGTTTTACTTCAAAATCTGGGCTTTCCTTGATGTTAGG
ENSMMUT00000013403     CTCATCGTATCCTGCAGGAAGTTGTTTTACTTCAAAATCTGGGCTTTCCTTGATGTTAGG
ENSPPYT00000009237     CTCATCATATCCTGCAGGAAGCTGTTTTACTTCAAAATCTGGGCTTTCCTTGATGTTAGT
ENSGGOT00000031003     CTCATCATATCCTGCAGGAAGTTGTTTTACTTCAAAATCTGGGCTTTCCTTGATGTTAGT
ENST00000321535        CTCATCATATCCTGCAGGAAGTTGTTTTACTTCAAAATCTGGGCTTTCCTTGATGTTAGT
ENSPTRT00000015954     CTCATCATATCCTGCAGGAAGTTGTTTTACTTCAAAATCTGGGCTTTCCTTGATGTTAGT

ENSMUST00000108504     TTTGTGGAGCGGATCCTGAAGAGCCAGGAGGAAGGGCAGTGCTCCCTGCATACACTGAAG
ENSRNOT00000020071     TTTGTGGAGCGGATCCTGAAGAGCCAGGAGGAGGGGCAGTGCTCCCTGCGCACACTGAAG
ENSCJAT00000033404     TTTGTGGAGCAGATCCTGAAGAGTCAGAAAGAAGGGAAGTGTGCCCTGCGCACACTCAAG
ENSMMUT00000013403     TTTGTGGAGCGGATCCTGAAGAGTCAGAAAGAACGGCAGTGTGCCCTGCGCACGCTCAAG
ENSPPYT00000009237     TTTGTGGAGCGGATCCTGAAGAGTCAGAAAGAACGGCAGTGTGCCCTGCGTATATTCAAG
ENSGGOT00000031003     TTTGTGGAGCGGATCCTGAAGAGTCAGAAAGAACGGCAGTGTGCCCTGCGTGTATTCAAG
ENST00000321535        TTTGTGGAGCGGATCCTGAAGAGTCAGAAAGAACGGCAGTGTGCCCTGCGTGTATTCAAG
ENSPTRT00000015954     TTTGTGGAGCGGATCCTGAAGAGTCAGAAAGAACGGCAGTGTGCCCTGCGTGTATTCAAG

ENSMUST00000108504     GTGAGAATTTATACAAACCGATATGAGACAAATGAAGAGGACAGGACCCTACGGGAAATT
ENSRNOT00000020071     GTGAGAATTTATACAAACCGCTATGAAACAAATGAAGAGGACAGGACCCTACGGGAAATT
ENSCJAT00000033404     GTGAGAATTTATACAAACAGATATGAGACGAATGAAGAGGACAGGACCCTGCGGGAAATT
ENSMMUT00000013403     GTGAGAATTTATACAAACAGATATGAGACGAATGAAGAGGACAGGACCCTGCGGGAAATT
ENSPPYT00000009237     GTGAGAATTTATACAAACAGATATGAGACGAATGAAGAGGAAAACACCCTGCGGGAAATT
ENSGGOT00000031003     GTAAGA------------------------------------------------------
ENST00000321535        GCGAGAATTTATACAAACAGATATGAGACGAATGAAGAGGACAAGACCCTGCAGGAAATT
ENSPTRT00000015954     GCAAGAATTTATACAAACAGATATGAGACGAATGAAGAGGACAAGACCCTGCAGGAAATT

ENSMUST00000108504     TACAGGAAATACAGAAAGCTGATCGATTCAGAACTTAACTATTTTGTCGTCGCCTACCCC
ENSRNOT00000020071     TACAGGAAATACAGAAAGCTGATCGATTCAGAACTTAACTATTTTGTCATCGCCTACCCC
ENSCJAT00000033404     TACAGGAAGTACAGAAAGCTGATCGATTCAGAGCTTAGCTATTTTGTCATCGCTTACCCT
ENSMMUT00000013403     TACAGGAAGTATAGAAAGCTGATCGACTCAGAGCTTAGCTATTTCGTCATCGCTTACCCT
ENSPPYT00000009237     TACAGGAAGTACAGAAAGCTGATCCAATCAGAGCTTAACTATTTTGTCATGGCTTACTCT
ENSGGOT00000031003     ------------------------------------------------------------
ENST00000321535        TACAGGAAGTACAGAAAGCTGATCGAATCAGAGCTTAGCTATTTTGTCATCGTTTACTCT
ENSPTRT00000015954     TACAGGAAGTACAGAAAGCTGATCGAATCAGAGCTTAGCTATTTTGTCATCGTTTACTCT
```

```
ENSMUST00000108504      ATGATG
ENSRNOT00000020071      ATGATG
ENSCJAT00000033404      ATGATG
ENSMMUT00000013403      ATGATG
ENSPPYT00000009237      AAAATG
ENSGGOT00000031003      ------
ENST00000321535         GTGATG
ENSPTRT00000015954      GTGATG
```

Multiple sequence alignment of Fbxo4

```
ENSCJAT00000002268      ATGGCAGGAAGCGAGCCGCGCAGCGGAACTAGCTCCCCGCCGCCGCCCTTCAGCGACTGG
ENSMMUT00000005623      ATGGCGGGAAGCGAGCCGCGCAGCGGAACTAGCTCGCCGCCGCCGCCCTTCAGCGACTGG
ENSPPYT00000017940      ATGGCGGGAAGCGAGCCGCGCAGCGGAACTAGCTCGCCGCCGCCGCCCTTCAGCGACTGG
ENSGGOT00000005532      ATGGCGGGAAGCGAGCCGCGCAGGGGCAGTAACTCGCCGCCGCCGCCGTTCAGCGACTGG
ENSPTRT00000031241      ATGGCGGGAAGCGAGCCGCGCCGCGGAACTAACTCGCCGCCGCCGCCCTTCAGCGACTGG
ENST00000281623         ATGGCGGGAAGCGAGCCGCGCAGCGGAACAAACTCGCCGCCGCCGCCCTTCAGCGACTGG
ENSMUST00000022791      ATGGCTGGAAGCGAGCCCCGC---GGAGCCGGCTCCCCGCCGCCCGCC---AGCGACTGG
ENSRNOT00000020925      ATGGCTGGAAGCGAGCCCCGC---GGAGCCGGCTCCCCGCCGCCCGCC---AGCGACTGG


ENSCJAT00000002268      GGCCGCCTGGAGGCGGCCATCCTCAGCGGCTGGAGGACCTTCTGGCAGTCAGTGAGCAAG
ENSMMUT00000005623      GGCCGCTTGGAGGCGGCCATACTCAGCGGCTGGAAGACTTTCTGGCAGTCAATGAGCAAG
ENSPPYT00000017940      GGCCGCCTGGAGGCGGCCATCCTCAGCGGCTGGAAGACCTTCTGGCAGTCAGTGAGCAAG
ENSGGOT00000005532      GGCCGCCTGGAGGCGGCCATCCTCAGCGGCTGGAAGACCTTCTGGCAGTCAGTGAGCAAG
ENSPTRT00000031241      GGCCGCCTGGAGGCGGCCATCCTCAGCGGCTGGAAGACCTTCTGGCAGTCAGTGAGCAAG
ENST00000281623         GGCCGCCTGGAGGCGGCCATCCTCAGCGGCTGGAAGACCTTCTGGCAGTCAGTGAGCAAG
ENSMUST00000022791      GGCCGCCTGGAGGCAGCCATCCTGAGCGGCTGGAGGACCTTCTGGTATTCGGTGGCCAAG
ENSRNOT00000020925      GGCCGCCTGGAGGCTGCTATCCTGAGCGGCTGGAGGACCTTCTGGAATTCGGTGGGCAAG


ENSCJAT00000002268      GAGAGGGTGGCGAGAACAGCCTCGCGGGAGGAGGTGGATGAGGCGGCCAGCACCCTGACG
ENSMMUT00000005623      GAGAGGGTGGCGCGTACGGCCTCGCGGGAGGAGGTGGATGAGGCGGCCAGCACCCTGACG
ENSPPYT00000017940      GAGAGGGTGGCGCGTACGACCTCGCGGGAGGAGGTGGATGAGGCGGCCAGCACCCTGACG
ENSGGOT00000005532      GAGAGGGTGGCGCGTACGACCTCGCGGGAGGAGGTGGATGAGGCGGCCAGCACCCTGATG
ENSPTRT00000031241      GAGAGAGTGGCGCGTACGACCTCGCGGGAGGAGGTGGATGAGGCGGCCAGCACCCTGACG
ENST00000281623         GAGAGGGTGGCGCGTACGACCTCACGGGAGGAGGTGGATGAGGCGGCCAGCACCCTGACG
ENSMUST00000022791      GAGCGGGCGACGCCGACGGCCTCCCGGAAGGAGGCGGCGGAGGAGACGAGCGCGCTGACG
ENSRNOT00000020925      GAGCGGGCGACGCGGACGGCCTCCCGGAAGGAGGAGGCGGAGGAGACGAGCGCGCTGACG


ENSCJAT00000002268      CGGTTGCCGATTGATGTACAGCTATATATTTTGTCCTTTCTTTCACCTCATGATCTGTGT
ENSMMUT00000005623      CGGCTGCCGATTGATGTGCAGCTATATATTTTGTCCTTTCTTTCACCTCATGATCTGTGT
ENSPPYT00000017940      CGGCTGCCGATTGATGTACAGCTATATATTTTGTCCTTTCTTTCACCTCATGATCTGTGT
ENSGGOT00000005532      CGGCTGCCGATTGATGTGCAGCTATATATTTTGTCCTTTCTTTCACCTCATGATCTGTGT
ENSPTRT00000031241      CGGCTGCCGATTGATGTGCAGCTATATATTTTGTCCTTTCTTTCACCTCATGATCTGTGT
ENST00000281623         CGGCTGCCGATTGATGTACAGCTATATATTTTGTCCTTTCTTTCACCTCATGATCTGTGT
ENSMUST00000022791      CGGCTGCCGGTTGATGTGCAGTTGTATATCTTGTCGTTTCTTTCACCCCACGATCTGTGC
ENSRNOT00000020925      CGGCTGCCGATTGATGTGCAGCTGTATATCTTGTCATTTCTTTCACCCCATGACCTGTGC


ENSCJAT00000002268      CAGTTGGGAAGTACAAATCATTATTGGAATGAAACTGTAAGAGATCCAATTCTGTGGAGA
ENSMMUT00000005623      CAGTTGGGAAGTACAAATCATTATTGGAATGAAACTGTAAGAGATCCAATTCTGTGGAGA
ENSPPYT00000017940      CAGTTGGGAAGTACAAATCATTATTGGAATGAAACTGTAAGAGATCCAATTCTGTGGAGA
ENSGGOT00000005532      CAGTTGGGAAGTACAAATCATTATTGGAATGAAACTGTAAGAGATCCAATTCTGTGGAGA
ENSPTRT00000031241      CAGTTGGGAAGTACAAATCATTATTGGAATGAAACTGTAAGAGATCCAATTCTGTGGAGA
ENST00000281623         CAGTTGGGAAGTACAAATCATTATTGGAATGAAACTGTAAGAGATCCAATTCTGTGGAGA
ENSMUST00000022791      CAGCTGGGAAGTACAGATCATTACTGGAACAAAACTATAAGAGACCCAATTCTCTGGAGA
```

```
ENSRNOT00000020925      CAGTTGGGAAGTACAGATCACTATTGGAATGAAACTGTAAGAGACCCAATTCTCTGGAGA

ENSCJAT00000002268      TACTTTCTGTTGAGGGATCTTCCTTCTTGGTCTTCTGTTGACTGGAAGTCTCTTCCAGAT
ENSMMUT00000005623      TACTTTTTGTTAAGGGATCTTCCTTCTTGGTCTTCTGTTGACTGGAAGTCTCTTCCAGAT
ENSPPYT00000017940      TACTTTCTGTTGAGGGATCTTCCTTCTTGGTCTTCTGTTGACTGGAAGTCTCTTCCAGAT
ENSGGOT00000005532      TACTTCCTGTTGCGGGATCTTCCTTCTTGGTCTTCTGTTGACTGGAAGTCTCTTCCAGAT
ENSPTRT00000031241      TACTTTCTGTTGAGGGATCTTCCTTCTTGGTCTTCTGTTGACTGGAAGTCTCTTCCAGAT
ENST00000281623         TACTTTTTGTTGAGGGATCTTCCTTCTTGGTCTTCTGTTGACTGGAAGTCTCTTCCAGAT
ENSMUST00000022791      TACTTTCTGTTGCGGGATCTCCCTTCTTGGTCTTCGGTTGATTGGAAGTCACTTCCAGAT
ENSRNOT00000020925      TACTTTCTGTTTCGAGATCTCCCTTCTTGGTCCTCTGTTGATTGGAAGTCACTTCCGGAT

ENSCJAT00000002268      CTAGAAATCTTAAAAAAACCTATATCTGAGGTCACTGATGGTGCATTTTTTGACTACATG
ENSMMUT00000005623      CTAGAAATCTTAAAAAAGCCTGTATCTGAAGTCACTGATGGTGCATTTTTTGACTACATG
ENSPPYT00000017940      CTAGAAATCTTAAAAAAGCCTATATCTGAGGTCACTGATGGTGCATTTTTTGACTACATG
ENSGGOT00000005532      CTAGAAATCTTAAAAAAGCCTATATCTGAGGTCACTGATGGTGCATTTTTTGACTACATG
ENSPTRT00000031241      CTAGAAATCTTAAAAAAGCCTATATCTGAGGTCACTGATGGTGCATTTTTTGATTACATG
ENST00000281623         CTAGAAATCTTAAAAAAGCCTATATCTGAGGTCACTGATGGTGCATTTTTTGACTACATG
ENSMUST00000022791      CTAGAGATCTTAAAAAAGCCAATATCTGAGGTCACCGACAGCACTTGTCTTGATTACATG
ENSRNOT00000020925      CTAGAGATATTAAAAAAGCCCATATCTGAGGTCACCGACAGCACATGTTTTGATTACATG

ENSCJAT00000002268      GCAGTCTATAAAATGTGCTGTCCATATACAAGAAAAGCTTCAAAAACCAGCCGTCCTATG
ENSMMUT00000005623      GCAGTCTATAGAATGTGCTGTCCATATACAAGAAGAGCTTCAAAATCCAGCCGTCCTATG
ENSPPYT00000017940      GCAGTCTATAGAATGTGCTGTCCATATACAAGAAGAGCTTCAAAATCCAGCCGTCCTATG
ENSGGOT00000005532      GCAGTCTATAGAATGTGCTGTCCATATACAAGAAGAGCTTCAAAATCCAGCCGTCCTATG
ENSPTRT00000031241      GCAGTCTATAGAATGTGCTGTCCATATACAAGAAGAGCTTCAAAATCCAGCCGTCCTATG
ENST00000281623         GCAGTCTATAGAATGTGCTGTCCATACACAAGAAGAGCTTCAAAATCCAGCCGTCCTATG
ENSMUST00000022791      GAGGTTTATAAAATGTGCTGTCCATATACGCGAAGAGCCTTGAAAGCCAGCCGTCCTATG
ENSRNOT00000020925      GAGGTTTATAAAATGTGCTGTCCATATACGAGAAGAGCCTTGAAAGCCAGCCGTCCTATG

ENSCJAT00000002268      TATGGAGCTGTCACTTCATTTTTACACTCCCTGATCATTCAGAATGAACCACGATTTGCT
ENSMMUT00000005623      TATGGAGCTGTCACTTCTTTTTTACACTCCCTGATCATTCAGAATGAACCACGATTTGCT
ENSPPYT00000017940      TATGGAGCTGTCACTTCTTTTTTACACTCCCTGATCATTCAGAATGAACCACGATTTGCT
ENSGGOT00000005532      TATGGAGCTGTCACTTCTTTTTTACACTCCCTGATCATTCAGAATGAACCACGATTTGCT
ENSPTRT00000031241      TATGGAGCTGTCACTTCTTTTTTACACTCGCTGATCATTCAGAATGAACCACGATTTGCT
ENST00000281623         TATGGAGCTGTCACTTCTTTTTTACACTCCCTGATCATTCAGAATGAACCACGATTTGCT
ENSMUST00000022791      TATGGAGTGGTTACCTCTTTCTTACACTCACTGATCATTCAGAATGAACCCCGGTTTGCT
ENSRNOT00000020925      TATGGAGCGGTTACCTCTTTCTTACACTCACTGATCATTCAGAATGAACCCCGGTTTGCT

ENSCJAT00000002268      ATGTTTGGACCAGGTTTGGAAGAACTGAATACCTCTTTGGTGTTGAGCTTGATGTCTTCA
ENSMMUT00000005623      ATGTTTGGACCAGGTTTGGAAGAATTGAATACCTCTTTGGTGTTGAGCTTAATGTCTTCA
ENSPPYT00000017940      ATGTTTGGACCAGGTTTGGAAGAATTGAATACCTCTTTGGTGTTGAGCTTGATGTCTTCA
ENSGGOT00000005532      ATGTTTGGACCAGGTTTGGAAGAATTGAATACCTCTTTGGTGTTGAGCTTGATGTCTTCA
ENSPTRT00000031241      ATGTTTGGACCAGGTTTGGAAGAATTGAATACCTCTTTGGTGTTGAGCTTGATGTCTTCA
ENST00000281623         ATGTTTGGACCAGGTTTGGAAGAATTGAATACCTCTTTGGTGTTGAGCTTGATGTCTTCA
ENSMUST00000022791      ATGTTTGGACCAGGTTTGGAAGAACTGAACACATCCTTGGTGTTGAGTTTGATGTCTTCT
ENSRNOT00000020925      ATGTTTGGACCAGGTTTGGAAGAACTGAACACATCTTTGGTGTTGAGTTTGATGTCTTCT

ENSCJAT00000002268      GAGGAACTTTGCCCAACAGCTGGTTTGCCTCAGAGGCAAATTGATGGTATTGGATCAGGA
ENSMMUT00000005623      GAGGAACTTTGCCCAACAGCTGGTTTGCCTCAGAGGCAGATTGATGGTATTGGATCAGGA
ENSPPYT00000017940      GAGGAACTTTGCCCAACAGCTGGTTTGCCTCAGAGGCAGATTGATGGTATTGGATCAGGA
ENSGGOT00000005532      GAGGAACTTTGCCCAACAGCTGGTTTGCCTCAGAGGCAGATTGATGGTATTGGATCAGGA
ENSPTRT00000031241      GAGGAACTTTGCCCAACAGCTGGTTTGCCTCAGAGGCAGATTGATGGTATTGGATCAGGA
ENST00000281623         GAGGAACTTTGCCCAACAGCTGGTTTGCCTCAGAGGCAGATTGATGGTATTGGATCAGGA
ENSMUST00000022791      GAGGACCTTTGCCCAACTGCTGGTTTACCTCACAGACAGATTGATGGTATTGGATCTGGA
```

ENSRNOT00000020925 GAGGATCTTTGCCCAACTGCTGGTTTACCTCATACTCAGATAGAAGGTATTGGATCTGGA

ENSCJAT00000002268 GTCAATTTTCAGTTGAGCAGCCAACATAAATTCAACATTTTGATCTTATATTCAACTACC
ENSMMUT00000005623 GTCAATTTTCAGTTGAACAACCAACATAAATTCAACATTCTAATCTTATATTCAACTACC
ENSPPYT00000017940 GTCAATTTTCAGTTGAACAACCAACATAAATTCAACATTCTAATCTTATATTCAACTACC
ENSGGOT00000005532 GTCAATTTTCAGTTGAACAACCAACATAAATTCAACATTCTAATCTTATATTCAACTACC
ENSPTRT00000031241 GTCAATTTTCAGTTGAACAACCAACATAAATTCAACATTCTAATCTTATATTCAACTACC
ENST00000281623 GTCAATTTTCAGTTGAACAACCAACATAAATTCAACATTCTAATCTTATATTCAACTACC
ENSMUST00000022791 GTCAACTTCCAGTTGAATAACCAGCAAAAATTCAACATCCTGATATTATACTCGACTACC
ENSRNOT00000020925 GTCAGTTTCCAGTTGAGCAATCAACACAAATTCAACATCCTGATACTGTACTCAACTACC

ENSCJAT00000002268 AGAAAGGAAAGAGATAGAGCAAGGGAAGAGCATACAAGTGCAGTTAACAAGATATTCAGT
ENSMMUT00000005623 AGAAAGGAAAGAGATAGAGCAAGGGAAGAGCATACAAGTGCAGTTAACAAGATATTCAGT
ENSPPYT00000017940 AGAAAGGAAAGAGATAGAGCAAGGGAAGAGCATACAAGTGCAGTTAACAAGATGTTCAGT
ENSGGOT00000005532 AGAAAGGAAAGAGATAGAGCAAGGGAAGAGCATACAAGTGCAGTTAACAAGATGTTCAGT
ENSPTRT00000031241 AGAAAGGAAAGAGATAGAGCAAGGGAAGAGCATACAAGTGCAGTTAACAAGATGTTCAGT
ENST00000281623 AGAAAGGAAAGAGATAGAGCAAGGGAAGAGCATACAAGTGCAGTTAACAAGATGTTCAGT
ENSMUST00000022791 AGAAAAGAAAGAGACAGAGCAAGGGAGGAGCACACCAGCACCGTTAACAAGATGTTCAGC
ENSRNOT00000020925 AGAAAGGAAAGAGACAGAGCGAGGGAGGAGCACACAAGCACGGTTAACAAGATGTTCAGC

ENSCJAT00000002268 CGACAGAATGAAGGTGATGATCAACAAGGAAGCCGGTATAGTGTGATTCCGCAAATTCAG
ENSMMUT00000005623 CGACAGAATGAAGGTGATGATCAACAAGGAAGCCGGTACAGTGTGATTCCACAAATTCAA
ENSPPYT00000017940 CGACAGAATGAAGGTGATGATCAACAAGGAAGCCGGTACAGTGTGATTCCACAGATTCAA
ENSGGOT00000005532 CGACACAATGAAGGCGATGATCAACAAGGAAGCCGGTACAGTGTGATTCCACAGATTCAA
ENSPTRT00000031241 CGACACAAGGAAGGTGATGATCAACAAGGAAGCCGGTACAGTGTGATTCCACAGATTCAA
ENST00000281623 CGACACAATGAAGGTGATGATCAACAAGGAAGCCGGTACAGTGTGATTCCACAGATTCAA
ENSMUST00000022791 CTACAGAGTGAGGGGGACGAGCAGCAGGGCAGCCGCTACAGTGTGATCCCGCAGATTCAG
ENSRNOT00000020925 CTACAGAGCGAGGGAGACGAGCAACAGGGGAGCCGCTACAGCGTTATCCCACAGATTCAG

ENSCJAT00000002268 AAAGTGTGTGAAGTTGTGGATGGTTTCATCTATGTTGCAAATGGTGAAGCTCATAAAAGA
ENSMMUT00000005623 AAAGTGTGTGAAGTTGTAGATGGGTTCATCTATGTTGCAAATGCTGAAGCTCATAAAAGA
ENSPPYT00000017940 AAAGTGTGTGAAGTTGTAGATGGGTTCATCTATGTTGCAAATGCTGAAGCTCATAAAAGA
ENSGGOT00000005532 AAAGTGTGTGAAGTTGTAGATGGGTTCATCTATGTTGCAAATGCTGAAGCTCATAAAAGA
ENSPTRT00000031241 AAAGTGTGTGAAGTTGTAGATGGGTTCATCTATGTTGCAAATGCTGAAGCTCATAAAAGA
ENST00000281623 AAAGTGTGTGAAGTTGTAGATGGGTTCATCTATGTTGCAAATGCTGAAGCTCATAAAAGA
ENSMUST00000022791 AAAGTGTGTGAAGTCGTAGACGGGTTCATCTACGTGGCAAACGCTGAAGCTCACCGACGT
ENSRNOT00000020925 AAAGTGTGTGAGGTTGTAGATGGGTTTATCTACGTGGCAAATGCTGAAGCTCATAGACGT

ENSCJAT00000002268 CATAAATGGCAAGATGAATTTTCTCATATTATGGCAATGACAGATCCAGCTTTTGGGTCT
ENSMMUT00000005623 CATGAATGGCAAGATGAATTTTCTCATATTATGGCAATGACAGATCCAGCCTTTGGGTCT
ENSPPYT00000017940 CATGAATGGCAAGATGAATTTTCTCATATTATGGCAATGACAGATCCAGCCTTTGGGTCT
ENSGGOT00000005532 CATGAATGGCAAGATGAATTTTCTCATATTATGGCAATGACAGATCCAGCCTTTGGGTCT
ENSPTRT00000031241 CATGAATGGCAAGATGAATTTTCTCATATTATGGCAATGACAGATCCAGCCTTTGGGTCT
ENST00000281623 CATGAATGGCAAGATGAATTTTCTCATATTATGGCAATGACAGATCCAGCCTTTGGGTCT
ENSMUST00000022791 CATGAATGGCAAGATGAATTTTCTCGGATTATGGCCATGACAGACCCAGCTTTTGGATCT
ENSRNOT00000020925 CATGAATGGCAAGATGAATTTTCTCGTATTATGGCCATGACAGATCCAGCTTTTGGATCT

ENSCJAT00000002268 TCGGGAAGACCATTGTTGGTTTTATCTTGTATTTCTCAAGGGGATGTTAAAAGAATGCCC
ENSMMUT00000005623 TCGGGAAGACCATTGTTGGTTTTATCTTGTATTTCTCAAGGGGATGTAAAAAGAATGCCC
ENSPPYT00000017940 TCGGGAAGACCATTGTTGGTTTTATCTTGTATTTCTCAAGGGGATGTAAAAAGAATGCCC
ENSGGOT00000005532 TCGGGAAGACCATTGTTGGTTTTATCTTGTATTTCTCAAGGGGATGTAAAAAGAATGCCC
ENSPTRT00000031241 TCGGGAAGACCATTGTTGGTTTTATCTTGTATTTCT------------------------
ENST00000281623 TCGGGAAGACCATTGTTGGTTTTATCTTGTATTTCTCAAGGGGATGTAAAAAGAATGCCC
ENSMUST00000022791 TCAGGAAGACCCATGCTGGTTTTATCTTGTATTTCTCAAGCAGATGTAAAGAGAATGCCT

```
ENSRNOT00000020925     TCAGGAAGACCAATGCTGGTTTTATCTTGTATTTCTCAAGCAGATGTAAAAAGAATGCCT

ENSCJAT00000002268     TGTTTTTATTTGGCCCATGAGCTGCGTCTGAGTCTTCTAAATCACCCGTGGATGGTCCAG
ENSMMUT00000005623     TGTTTTTATTTGGCTCATGAGCTGCATCTGAATCTTCTAAATCACCCATGGCTGGTCCAG
ENSPPYT00000017940     TGTTTTTATTTGGCTCATGAGCTGCATCTGAATCTTCTAAATCACCCATGGCTGGTCCAG
ENSGGOT00000005532     TGTTTTTATTTGGCTCATGAGCTGCATCTGAATCTTCTAAATCACCCATGGCTGGTCCAG
ENSPTRT00000031241     ------------------------------------CTAAATCACCCATGGCTGGTCCAG
ENST00000281623        TGTTTTTATTTGGCTCATGAGCTGCATCTGAATCTTCTAAATCACCCATGGCTGGTCCAG
ENSMUST00000022791     TGTTTTTATTTAGCTCATGAGCTGCACCTCAGTCTTCTAAACCACCCATGGATGGTCCAG
ENSRNOT00000020925     TGTTTTTATTTAGCTCATGAACTTCGTCTCAGTCTTCTGAACCACCCATGGATGGTCCAG

ENSCJAT00000002268     GATATAGAGGCTGAAACGCTAACTGGTTTTTTGAATGGCATTGAATGGATTCTTGAAGAA
ENSMMUT00000005623     GACACAGAGGCTGAAACTCTAACTGGTTTTTTGAATGGCATTGAGTGGATTCTTGAAGAA
ENSPPYT00000017940     GATACAGAGGCTGAAACTCTGACTGGTTTTTTGAATGGCATTGAGTGGATTCTTGAAGAA
ENSGGOT00000005532     GATACAGAGGCTGAAACTCTGACTGGTTTTTTGAATGGCATTGAGTGGATTCTTGAAGAA
ENSPTRT00000031241     GATACAGAGGCTGAAACTCTGACTGGTTTTTTGAATGGCATTGAGTGGATTCTTGAAGAA
ENST00000281623        GATACAGAGGCTGAAACTCTGACTGGTTTTTTGAATGGCATTGAGTGGATTCTTGAAGAA
ENSMUST00000022791     GATACAGAGGCTGAAACTCTGACTGGTTTTTTGAATGGCATTGAGTGGATTCTTGAAGAA
ENSRNOT00000020925     GATACAGAGGCTGAGACTCTGACTGGTTTCTTGAATGGCATTGAGTGGATTCTTGAAGAA

ENSCJAT00000002268     GTAGAATCTAAGCGTGCAAGA
ENSMMUT00000005623     GTAGAATCTAAGCGTGCAAGA
ENSPPYT00000017940     GTGGAATCTAAGCATTCAAGA
ENSGGOT00000005532     GTGGAATCTAAGCGTGCAAGA
ENSPTRT00000031241     GTGGAATCTAAGCGTGCAAGA
ENST00000281623        GTGGAATCTAAGCGTGCAAGA
ENSMUST00000022791     GTAGAATCTAAGCGTGCAAAA
ENSRNOT00000020925     GTAGAATCTAAGCATGCGAAA
```

Multiple sequence alignment of Fbxo40

```
ENSCJAT00000062273     ATGGGGAAAGCACGCAGAACCTCACCAGGGCAGCACAGGCATTGTGAGGGGTGCTTCAAC
ENSMMUT00000019013     ATGGGGAAAGCCCGCAGGCCTCCGCCAGGGCACCACAGGCATTGTGAGGGATGCTTCAAC
ENSPPYT00000015697     ATGGGGAAAGCCCGCAGGCCCCCGCCAGGGCACCACAGGCATTGCGAGGGATGCTTCAAC
ENSGGOT00000016842     ATGGGGAAAGCCCGCAGATCCCCGCCAGGGCACCACAGGCATTGTGAGGGATGCTTCAAC
ENST00000338040        ATGGGGAAAGCCCGCAGATCCCCGCCAGGGCACCACAGGCATTGTGAGGGATGCTTCAAC
ENSPTRT00000028534     ATGGGGAAAGCCCGCAGATCCCCGCCAGGGCACCACAGGCATTGTGAGGGATGCTTCAAC
ENSMUST00000114806     ATGGGTCGGGCACGCAAGCCTCCACCTGCGCTGCACAGGCACTGTGAGGGATGCTTCAAC
ENSRNOT00000003340     ---GGCCGAGCACGCAGGCCTCCACCTGCACTGCACAGGCACTGCGAGGGATGCATCGAC

ENSCJAT00000062273     CGCCACTGCCACATTCCTGTGGAACCCAACATCTCCTGCCTGGTAATAAGCTGCCACCTG
ENSMMUT00000019013     CGCCACTGCCACATTCCTGCGGAACCCAACGTCTCCTGCCTGGTAATAAGCTGCCACCTG
ENSPPYT00000015697     CGCCACTGCCACATTCCTGTGGAACCCAACACCTCCTGCCTGGTAATAAGCTGCCACCTG
ENSGGOT00000016842     CGCCACTGCCACATTCCTGTGGAACCCAACACCTCCTGCCTGGTAATAAGCTGCCACCTG
ENST00000338040        CGCCACTGCCACATTCCTGTGGAACCCAACACCTCCTGCCTGGTAATAAGCTGCCACCTG
ENSPTRT00000028534     CGCCACTGCCACATTCCTGTGGAACCCAACACCTCCTGCCTGGTAATAAGCTGCCACCTG
ENSMUST00000114806     CGCCACTGCCACGTTCCGGTGGAGCCCAGCGTCTCCTGCCTGGTGATAAGCTGCCACCTG
ENSRNOT00000003340     CGCCACTGCCGTGTTCCAGCGGAGCCCAGCGTCTCCTGCCTGGTGATAAGCTGTCACCTG

ENSCJAT00000062273     CTCTGTGGTGCCACCTTCCACATGTGCAAAGAGGCAGAGCACCAGCTTCTCTGCCCATTA
ENSMMUT00000019013     CTCTGTGGTGCCACCTTCCACATGTGCAAAGAGGCAGAGCACCAGCTCCTCTGCCCTTTA
ENSPPYT00000015697     CTCTGTGGTGCCACCTTCCATATGTGCAAAGAGGCAGAGCACCAGCTCCTCTGCCCTTTA
ENSGGOT00000016842     CTCTGTGGTGCCACCTTCCACATGTGCAAAGAGGCAGAGCACCAGCTCCTCTGCCCTTTA
ENST00000338040        CTCTGTGGTGCCACCTTCCACATGTGCAAAGAGGCAGAGCACCAGCTCCTCTGCCCTTTA
```

ENSPTRT00000028534 CTCTGTGGTGCCACCTTCCACATGTGCAAAGAGGCAGAGCACCAGCTCCTCTGCCCTTTA
ENSMUST00000114806 CTCTGTGGCGCCACCTTCCACATGTGCAAAGAGTCAGAGCATACACTCCTCTGCCCTCTG
ENSRNOT00000003340 CTCTGTGGCGCTACCTTCCACATGTGCAAAGAGTCAGAGCATACGCTCCTCTGCCCTCTG

ENSCJAT00000062273 GAGCAGGTTCCCTGCCTCAACTCCAAATATGGCTGCCCCCTGTCCATGTCCCGCCACAAG
ENSMMUT00000019013 GAGCAGGTTCCATGCCTCAACTCCGAATATGGCTGCCCTCTGTCCATGTCCCGCCACAAA
ENSPPYT00000015697 GAGCAGGTTCCATGCCTCAACTCTGAATATGGCTGCCCTCTGTCCATGTCCCGCCACAAA
ENSGGOT00000016842 GAGCAGGTTCCGTGCCTCAACTCCGAATATGGCTGCCCTCTGTCCATGTCCCGCCACAAA
ENST00000338040 GAGCAGGTTCCGTGCCTCAACTCCGAATATGGCTGCCCTCTGTCCATGTCCCGCCACAAA
ENSPTRT00000028534 GAGCAGGTTCCGTGCCTCAACTCCGAATATGGCTGCCCTCTGTCCATGTCCCGCCACAAA
ENSMUST00000114806 GAGCAGGTTCCCTGCCTCAACTCTGAGTATGGCTGCCCGCTCTCCATGGCACGCCACAAG
ENSRNOT00000003340 GAACAGGTTCCGTGCCTCAACTCTGAGTATGGCTGCCCGCTCTCCATGGCACGCCACAAA

ENSCJAT00000062273 CTGGCCAAGCACCTGCAGGTGTGCCCCGCCAGCGTGGTCTGCTGCTCCATGGAGTGGAAC
ENSMMUT00000019013 CTGGCCAAGCACCTGCAGGTGTGCCCCGCCAGCGTGGTCTGCTGCTCCATGGAGTGGAAC
ENSPPYT00000015697 CTGGCCAAGCACCTGCAGGTGTGCCCCGCCAGCGTGGTCTGCTGCTCCATGGAGTGGAAC
ENSGGOT00000016842 CTGGCCAAGCACCTGCAGGTGTGCCCCGCCAGCGTGGTCTGCTGCTCCATGGAGTGGAAC
ENST00000338040 CTGGCCAAGCACCTGCAGGTGTGCCCCGCCAGCGTGGTCTGCTGCTCCATGGAGTGGAAC
ENSPTRT00000028534 CTGGCCAAGCACCTGCAGGTGTGCCCCGCCAGCGTGGTCTGCTGCTCCATGGAGTGGAAC
ENSMUST00000114806 CTGGCTAAGCACCTGCAGGTATGCCCCGCCAGCGTGGTCTGCTGCTCCATGGAATGGATC
ENSRNOT00000003340 CTGGCTAAGCACCTGCAGGTGTGCCCCGCCAGCGTGGTCTGCTGCTCCATGGAATGGAAC

ENSCJAT00000062273 CGCTGGCCAAACGTGGACTCTGAAACCACCCTTCATGAGAACATCATGAAAGAAAACCCC
ENSMMUT00000019013 CGCTGGCCAAATGTGGACTCTGAAACCACCCTTCACGAGAACATCATGAAAGAGACCCCC
ENSPPYT00000015697 CGCTGGCCAAATGTGGACTCTGAAACCACCCTTCATGAGAATATCATGAAAGAGACCCCC
ENSGGOT00000016842 CGCTGGCCAAATGTGGACTCTGAAACCACCCTTCATGAAAACATAATGAAAGAGACCCCC
ENST00000338040 CGCTGGCCAAATGTGGACTCTGAAACCACCCTTCATGAAAACATCATGAAAGAGACCCCC
ENSPTRT00000028534 CGCTGGCCAAATGTGGACTCTGAAACCACCCTTCATGAAAACATCATGAAAGAGACCCCC
ENSMUST00000114806 CGCTGGCCAAACGTGGACTCAGAAACATTCCTTCACGAGAACATCATGAAAGAGACCCCC
ENSRNOT00000003340 CGCTGGCCAAACGTGGACTCGGAAACAGTCCTTCATGAGAACATCATGAAAGAGAGCCCC

ENSCJAT00000062273 AATGAGGAGTGTTTGGACACAGCCCTGGCCCTGCAGGACCAGAAGGTCCTCTTCAGATCC
ENSMMUT00000019013 AGTGAGGAGTGTTTGGACACAGCCCTGGCCCTGCAGGACCAGAAGGTCCTCTTCAGATCC
ENSPPYT00000015697 AGTGAGGAGTGTTTGGACACAGCCCTGGCCCTGCAGGACCAGAAGGTCCTCTTCAGATCC
ENSGGOT00000016842 AGTGAGGAGTGTTTGGACACAGCCCTGGCCCTGCAGGATCAGAAGGTCCTCTTCAGATCC
ENST00000338040 AGTGAGGAGTGTTTGGACACAGCCCTGGCCCTGCAGGATCAGAAGGTCCTCTTCAGATCC
ENSPTRT00000028534 AGTGAGGAGTGTTTGGACACAGCCCTGGCCCTGCGGGATCAGAAGGTCCTCTTCAGATCC
ENSMUST00000114806 AGTGAGGAGTGTTTGGACACAGCCCTGGCCCTCCAGGACCAGAAGGTACTCTTCAGATCC
ENSRNOT00000003340 AGCGAGGAGTGTTTGGATACAGCCCTGGCCCTCCAAGATCAGAAGGTTCTCTTCAGATCC

ENSCJAT00000062273 TTGAAAATGGTGGAACTTTTCCCAGAAACTAGAGAGGCCACTGAGGAGGAGCCAACTATG
ENSMMUT00000019013 TTGAAAATGGTGGAACTTTTCCCAGAAACTAGAGAGGCTACTGAGGAGGAACCAACTATG
ENSPPYT00000015697 TTGAAAATGGTGGAACTTTTCCCAGAAACTAGAGAGGCTACTGAGGAGGAACCAACTGTG
ENSGGOT00000016842 TTGAAAATGGTGGAACTTTTCCCAGAAACTAGAGAGGCTACTGAGGAGGAACCAACTATG
ENST00000338040 TTGAAAATGGTGGAACTTTTCCCAGAAACTAGAGAGGCTACTGAGGAGGAACCAACTATG
ENSPTRT00000028534 TTGAAAATGGTGGAACTTTTCCCAGAAACTAGAGAGGCTACTGAGGAGGAACCAACTATG
ENSMUST00000114806 TTGAAAATGGTAGAGCTTTTCCCAGAAACCAGAGACGCCACGGAGGAGGAACCAGACATG
ENSRNOT00000003340 TTGAAAATGGTAGAGCTTTTTCCAGAAACCAGAGACGCCACCGAGGAGGAGCCAGCCATG

ENSCJAT00000062273 AATGGTGAAACCAGTGTGGAGGACATGGGAGGAGCAGTGGGTGGATTGGATGTCGGTTTG
ENSMMUT00000019013 AATGGTGAAACCGGTGTGGAGGAAATGGGAGGAGCAGTGGGTGGAGTGGATATCAGTTTG
ENSPPYT00000015697 AATGGTGAAAACAGTGTGGAGGAAATGGGAGGAGCAGTGGGTGGAGTGGATATCAGTTTG
ENSGGOT00000016842 AATGGTGAAACCAGTGTGGAGGAAATGGGAGGAGCAGTGGGTGGAGTGGATATCGGTTTG
ENST00000338040 AATGGTGAAACCAGTGTGGAGGAAATGGGAGGAGCAGTGGGTGGAGTGGATATCGGTTTG

ENSPTRT00000028534   AATGGTGAAACCAGTGTGGAGGAAATGGGAGGAGCAGTGGGTGGAGTGGATATCGGTTTG
ENSMUST00000114806   AATGGTGACACCAGTTGGGAGGAGACTGGGGGCGCAGTGGGGGGAGTGGATGCCAGGCTA
ENSRNOT00000003340   AATGGCGATACCAGTTGGGAGGAGACTGCGGGCGCAGTCGGGGGCGTGGATGCAGGGCTA

ENSCJAT00000062273   GTACCACATGATTTGTCAGCAACTAATGAGGAGATGGCAGAGCTAAGTCAAGAAGAACGG
ENSMMUT00000019013   GTACCACATGGTTTGTCAGCAACTAATGGGGAGATGGCAGAGCTAAGTCAAGAAGAACGG
ENSPPYT00000015697   GTACCACATGGTCTGTCAGCAACTAATGGGGAGATGGCAGAGCTAAGTCAAGAAGAACGG
ENSGGOT00000016842   GTACCACATGGTCTGTCAGCAACTAATGGGGAGATGGCAGAGCTAAGTCAAGAAGAACGG
ENST00000338040   GTACCACATGGTCTGTCAGCAACTAATGGGGAGATGGCAGAGCTAAGTCAAGAAGAACGG
ENSPTRT00000028534   GTACCACATGGTCTGTCAGCAACTAATGGGGAGATGGCAGAGCTAAGTCAAGAAGAACGG
ENSMUST00000114806   GCACCAAATTCTTTGCCGGCAACCAGTAGGCAAATGATGGAGCTCAGTCAGGAGGAACGA
ENSRNOT00000003340   GTACCAAACTCTTTGCCGGCAACCAATGGGCAAACGATGGAGCTCAGTCAGGAGGAACGA

ENSCJAT00000062273   GAGATGCTAGCCAAAACCAAAGAAGGGATGGACCTGGACAAGTTTGGCCAGTGGGAAAAT
ENSMMUT00000019013   GAGGTGCTAGCCAAAACCAAAGAAGGGATGGACCTGGCTAAGTTTGGCCAGTGGGAAAAT
ENSPPYT00000015697   GAGGTGCTAGCCAAAACCAAAGAAGGGATGGACCTGGTCAAGTTTGGCCAGTGGGAAAAT
ENSGGOT00000016842   GAGGTGCTAGCCAAAACCAAAGAAGGGATGGACCTGGTCAAGTTTGGCCAGTGGGAAAAT
ENST00000338040   GAGGTGCTAGCCAAAACCAAAGAAGGGATGGACCTGGTCAAGTTTGGCCAGTGGGAAAAT
ENSPTRT00000028534   GAGGTGCTAGCCAAAACCAAAGAAGGGATGGACCTGGTCAAGTTTGGCCAGTGGGAAAAT
ENSMUST00000114806   GATGCACTGGCAAAAACCAAAGAAGGGATGGACCTGGACAAGTTTGGCAAGTGGGAAAGT
ENSRNOT00000003340   GATGCATTGGCCAAAACCAAAGAAGGGATGGACCTGGACAAGTTTGGCAAGTGGGAAAGT

ENSCJAT00000062273   ATTTTCAGTAAAGAGCATGCAGCCTCTGCTTTAACAAATTCATCAGTGAGCTGTGAGAGC
ENSMMUT00000019013   ATTTTCAGCAAAGAGCACGCAGCCTCTGCTTTAACAAATTCGTCAGCGAGCTGTGAGAGC
ENSPPYT00000015697   ATTTTCAGCAAAGAGCACGCAGCCTCTGCTTTAACAAATTCATCAGCGAGCTGTGAGAGC
ENSGGOT00000016842   ATTTTCAGCAAAGAGCACGCAGCCTCTGCTTTAACAAATTCATCAGCGAGCTGTGAGAGC
ENST00000338040   ATTTTCAGCAAAGAGCACGCAGCCTCTGCTTTAACAAATTCATCAGCGAGCTGTGAGAGC
ENSPTRT00000028534   ATTTTCAGCAAAGAGCACGCAGCCTCTGCTTTAACAAATTCATCAGTGAGCTGTGAGAGC
ENSMUST00000114806   ATGTTCAGCAAGGAGCACGCAGCCTCTGTTTTAACAGGTTCCTTGGGGAAGAGTGAAGAC
ENSRNOT00000003340   ATGTTCAGCAAGGAGCACGCAGCCTCTGTTTTAACAGGTTCCTCGGGGAAAAGTGAAGAC

ENSCJAT00000062273   AAGAACAAGATTGGCCCAGAGAAAGAACAGATTTCCAGTGGCAATAACACGGTAGAAGGA
ENSMMUT00000019013   AAGAACAAGAATGGCCCAGAGAAGGAACAGATTTCCAGTGGCAATAACATGGTAGAAGGA
ENSPPYT00000015697   AAGAACAAGAATGACCCAGAGAAAGAACAGATTTCCAGTGGCAACAACATGGTAGAAGGA
ENSGGOT00000016842   AAGAACAAGAATGACTCCAAGAAAGAACAGATTTCCAGTGGCCATAACATGGTAGAAGGA
ENST00000338040   AAGAACAAGAATGACTCCGAGAAAGAACAGATTTCCAGTGGCCATAACATGGTAGAAGGA
ENSPTRT00000028534   AAGAACAAGAATGACTCCGAGAAAGAACAGATTTCCAGTGGCCATAACATGGTAGAAGGA
ENSMUST00000114806   AAGAATGGGGATGTGGCTGGGAAAGAGCAGTGCTCCAGTAATGTTCGCATAGGGGATGCA
ENSRNOT00000003340   AAGAATGAGAATGTGTCTGGGAAAGAGCAGCGCTCTGGTGATGGTAGCACAGGGGATGCA

ENSCJAT00000062273   GAGGGCGTTCCCAAAAAGAAAGAACCACAGGAGAATGAGAAGCAACAGGACATTCATAAA
ENSMMUT00000019013   GAGGGCGCTCCCGAAAAGAAAGAACCACAGGAAAATCAGAAGCAGCAGAACGTTCACACA
ENSPPYT00000015697   GAGGGCGCTCCCCAAAAGAAAGAACCACAGGAAAATCAGAAGCAGCAGGACGTTCGTACA
ENSGGOT00000016842   GAGGGCGCTCCCAAAAAGAAAGAACCACAGGAAAATCAGAAGCAGCAGGACATTTGTACA
ENST00000338040   GAGGGCGCTCCCAAAAAGAAAGAACCACAGGAAAATCAGAAGCAGCAGGACGTTCGTACA
ENSPTRT00000028534   GAGGGCACTCCCAAAAAGAAAGAACCACAGGAAAATCAGAAGCAGCAGGACGTTCGTACA
ENSMUST00000114806   GAGGGTTCTGCCGAAAGGAGAGGACCACAGGAAAGCCAGAAGTCCCAGGAACTTCCCGCT
ENSRNOT00000003340   GAGTGTTCTACCGAAAGGAGAGGACCACAGGAAAGCCCGAAGCCCCAGGAACCTCCCACC

ENSCJAT00000062273   GCCGTGGAAACCGCAGGGCTTGCCCCTTGGCAGGATGGTGTTCTGGAAAGACTGAAAACA
ENSMMUT00000019013   GCTGTGGAAACCACAGGGCTTGCCCCTTGGCAGGATGGTGTTCTGGAAAGACTGAAAACA
ENSPPYT00000015697   GCCATGGAAACCACAGGGCTTGCCCCTTGGCAGGATGGTGTTCTGGAAAGACTGAAAACA
ENSGGOT00000016842   GCCATGGAAACCACAGGGCTTGCCCCTTGGCAGGATGGTGTTCTGGAAAGACTGAAAACA
ENST00000338040   GCCATGGAAACCACAGGGCTTGCCCCTTGGCAGGATGGTGTTCTGGAAAGACTGAAAACA

```
ENSPTRT00000028534     GCCATGGAAACCACAGGGCTTGCCCCTTGGCAGGATGGTGTTCTGGAAAGACTGAAAACA
ENSMUST00000114806     ACCATGGAGATGACAGGGCTTGCTCCCTGGCAAGATGGTGTTCTGGAAAGACTAAAAACA
ENSRNOT00000003340     ACCATTGAGATGACAGGGCTTGCTCCCTGGCAAGATGGCGTTCTGGAAAGACTAAAAACA

ENSCJAT00000062273     GCTGTGGATGCAAAGGACTATAACATGTATTTAGTGCACAATGGGCGGATGCTGATTCAC
ENSMMUT00000019013     GCTGTGGATGCAAAGGACTATAACATGTATCTAGTGCACAATGGGCGGATGCTGATCCAC
ENSPPYT00000015697     GCTGTGGATGCAAAGGACTATAACATGTATCTAGTGCACAATGGGCGGATGCTGATTCAC
ENSGGOT00000016842     GCTGTGGATGCAAAGGACTATAACATGTATCTAGTGCACAATGGGCGGATGCTGATTCAC
ENST00000338040        GCTGTGGATGCAAAGGACTATAACATGTATCTAGTGCACAATGGGCGGATGCTGATACAC
ENSPTRT00000028534     GCTGTGGATGCAAAGGACTATAACATGTATCTAGTGCACAATGGGCGGATGCTGATTCAC
ENSMUST00000114806     GCCGTGGATGCCAAAGACTATAACATGTATCTGGTGCACAACGGGCGGATGCTCATCCAC
ENSRNOT00000003340     GCAGTGGACGCCAAAGACTATAACATGTATCTGGTGCACAATGGACGGATGCTCATCCAC

ENSCJAT00000062273     TTCGGTCAGATGCCTGCTTGTACACCTAAGGAGAGAGACTTTGTTTATGGCAAGCTTGAA
ENSMMUT00000019013     TTTGGTCAGATGCCTGCTTGTACACCCAAGGAGAGAGACTTTGTTTATGGCAAGCTTGAG
ENSPPYT00000015697     TTTGGTCAGATGCCTGCTTGTACACCCAAGGAGAGAGACTTTGTTTATGGCAAGCTTGAG
ENSGGOT00000016842     TTTGGTCAGATGCCTGCTTGTACACCCAAGGAGAGAGACTTTGTTTATGGCAAGCTTGAG
ENST00000338040        TTTGGTCAGATGCCTGCTTGTACACCCAAGGAGAGAGACTTTGTTTATGGCAAGCTGGAG
ENSPTRT00000028534     TTTGGTCAGATGCCTGCTTGTACACCCAAGGAGAGAGACTTTGTTTATGGCAAGCTTGAG
ENSMUST00000114806     TTTGGGCAGATGCCTGCTTGTACGCCCAAGGAGAGGGACTTTGTTTATGGCAACCTAGAG
ENSRNOT00000003340     TTCGGGCAGATGCCTGCTTGTACGCCCAAGGAGAGAGACTTCGTTTACGGCAACCTCGAA

ENSCJAT00000062273     GCTCAGGAAGTTAAGACTGTGTACACCTTCAAAGTTCCTGTGAGCTACTGTGGAAAGCGA
ENSMMUT00000019013     GCTCAGGAAGTTAAGACTGTTTATACCTTCAAAGTTCCTGTGAGCTACTGTGGAAAGCGA
ENSPPYT00000015697     GCTCAGGAAGTTAAGACTGTTTACACCTTCAAAGTTCCTGTGAGCTACTGTGGAAAGCGA
ENSGGOT00000016842     GCTCAGGAAGTTAAGACTGTTTACACCTTCAAAGTTCCTGTGAGCTACTGTGGAAAGCGA
ENST00000338040        GCTCAGGAAGTTAAGACTGTTTACACCTTCAAAGTTCCTGTGAGCTACTGTGGAAAGCGA
ENSPTRT00000028534     GCTCAGGAAGTTAAGACTGTTTACACCTTCAAAGTTCCTGTGAGCTACTGTGGAAAGCGA
ENSMUST00000114806     GCTCAAGAAGTGAAAACGGTTTATACCTTCAAGATTCCCGTGAGCTACTGCGGGAAGCGA
ENSRNOT00000003340     GCTCAAGAAGTGAAGACGGTTTATACCTTCAAAATTCCCGTGAGCTACTGCGGGAAGCGA

ENSCJAT00000062273     GCTCGACTTGGAGACGCCATGTTGAGTTGTAAGCCCAGTGAACACAAGGCAGTGGATACT
ENSMMUT00000019013     GCTCGACTTGGAGATGCCATGTTGAGTTGTAAGCCGAGTGAACACAAGGCAGTGGATACT
ENSPPYT00000015697     GCTCGACTTGGAGATGCCATGTTGAGTTGTAAGCCAAGTGAACACAAGGCAGTGGATACT
ENSGGOT00000016842     GCTCGACTTGGAGATGCCATGTTGAGTTGTAAGCCAAGTGAACACAAGGCAGTGGATACT
ENST00000338040        GCTCGACTTGGAGATGCCATGTTGAGTTGTAAGCCAAGTGAACACAAGGCAGTGGATACT
ENSPTRT00000028534     GCTCGACTTGGAGATGCCATGTTGAGTTGTAAGCCAAGTGAACACAAGGCAGTGGATACT
ENSMUST00000114806     GCTCGCTTGGGAGATGCCATGTTGAAATGCAGACCAAGTGAACACAAGGCTGTGGACACT
ENSRNOT00000003340     GCTCGCTTGGGAGATGCCATGTTGAGGTGCAGACCAAGTGAGCACAAGGCCGTGGATACT

ENSCJAT00000062273     TCAGATCTGGGGATCACTGTGGAGGATCTGCCCAAATCAGATCTCATCAAGACCACCCTC
ENSMMUT00000019013     TCAGATCTGGGGATCACTGTGGAGGACCTGCCCAAATCAGATCTCATCAAGACCACCCTC
ENSPPYT00000015697     TCAGATTTGGGGATCACTGTGGAGGACCTGCCCAAATCAGATCTCATCAAGACCACCCTC
ENSGGOT00000016842     TCAGATTTGGGGATCACTGTGGAGGACCTGCCCAAATCAGATCTCATCAAGACCACCCTC
ENST00000338040        TCAGATTTGGGGATCACTGTGGAGGACCTGCCCAAATCAGATCTCATCAAGACCACCCTC
ENSPTRT00000028534     TCAGATTTGGGGATCACTGTGGAGGACCTGCCCAAATCAGATCTCATCAAGACCACCCTC
ENSMUST00000114806     TCTGATCTGGGCATCTCTGTGGAGGACCTGCCCAAATCTGATCTCATCAAGACCACCCTC
ENSRNOT00000003340     TCCGACTTGGGCATCTCTGTGGAGGACCTGCCCAAATCCGATCTCATCAAGACCACCCTC

ENSCJAT00000062273     CAGTGTGCTTTGGAAAGAGAACTCAAAGGCCACGTCATCTCTGAATCCAGAAGCATTGAT
ENSMMUT00000019013     CAGTGTGCTTTGGAAAGAGAACTCAAAGGCCACGTCATCTCTGAATCCAGAAGCATTGAT
ENSPPYT00000015697     CAGTGTGCTTTGGAAAGAGAACTCAAAGGCCACGTCATCTCTGAATCCAGAAGCATTGAT
ENSGGOT00000016842     CAGTGTGCTTTGGAAAGAGAACTCAAAGGCCACGTCATCTCTGAATCCAGAAGCATTGAT
ENST00000338040        CAGTGTGCTTTGGAAAGAGAACTCAAAGGCCACGTCATCTCTGAATCCAGAAGCATTGAT
```

ENSPTRT00000028534 CAGTGTGCTTTGGAAAGAGAACTCAAAGGCCACGTCATCTCTGAATCCAGAAGCATTGAT
ENSMUST00000114806 CAGTGTGCTTTGGAAAGAGAACTCAAAGGCCATGTCATCTCTGAATCCAGGAGCATCGAT
ENSRNOT00000003340 CAGTGTGCTTTGGAAAGAGAACTCAAAGGCCACGTCATCTCTGAGTCCAGGAGCATCGAT

ENSCJAT00000062273 GGACTGTTCATGGATTTTGCCACACAAACATACAACTTTGAGCCGGAACAGTTTTCCTCT
ENSMMUT00000019013 GGACTGTTCATGGATTTTGCCACACAAACATACAACTTTGAGCCAGAACAGTTTTCCTCT
ENSPPYT00000015697 GGACTGTTCATGGATTTTGCCACACAAACATACAACTTTGAGCCAGAACAGTTTTCCTCT
ENSGGOT00000016842 GGACTGTTCATGGATTTTGCCACACAAACATACAACTTTGAGCCAGAACAGTTTTCCTCT
ENST00000338040 GGACTGTTCATGGATTTTGCCACACAAACATACAACTTTGAGCCAGAACAGTTTTCCTCT
ENSPTRT00000028534 GGACTGTTCATGGATTTTGCCACACAAACATACAACTTTGAGCCAGAACAGTTTTCCTCT
ENSMUST00000114806 GGACTCTTTATGGACCTTGCCACCCAAACATACAACTTTGAGCCAGAACAGTTTTCCTCA
ENSRNOT00000003340 GGACTCTTTATGGATCTCGCCACCCAAACATACAACTTTGAGTCAGAACAGTTTTCCTCA

ENSCJAT00000062273 GGGACAGTGCTGGCTGACCTAGCCCCTGCCACCCCAGGGGGACTCCACGTGGAGCTCCAC
ENSMMUT00000019013 GGGACAGTGCTGGCTGACCTAACCGCTGCCACCCCAGGGGGACTCCATGTGGAGCTGCAC
ENSPPYT00000015697 GGGACAGTGCTGGCTGATCTAACCACTGCCACCCCAGGGGGACTCCACGTGGAGCTCCAC
ENSGGOT00000016842 GGGACAGTGCTGGCTGACCTAACCGCTGCCACCCCAGGGGGACTCCACGTGGAGCTCCAC
ENST00000338040 GGGACAGTGCTGGCTGACCTAACCGCTGCCACCCCAGGGGGACTCCACGTGGAGCTCCAC
ENSPTRT00000028534 GGGACAGTGCTGGCTGACCTAACTGCTGCCACCCCAGGGGGACTCCACGTGGAGCTCCAC
ENSMUST00000114806 GAGACAGTGTTGGCTGATCTAGGCACTGCCCAACCCGGGGGCCTGCACGTGGAGCTCCAC
ENSRNOT00000003340 GAGACAGTGTTGGCTGACCTAGGCACTACCCAATCCGGGGGCCTGCATGTGGAGCTCCAC

ENSCJAT00000062273 AGCGAGTGTGTGACCAGGAGACACAACAAAAGCAGCTCTGCCTTCACTTTCACTTGCAAC
ENSMMUT00000019013 AGTGAGTGTGTCACCAGGAGACACAACAAAAGCAGTTCTGCCTTCACTTTCACTTGCAAC
ENSPPYT00000015697 AGCGAGTGTGTGACCAGGAGACACAACAAAAGCAGCTCTGCCTTCACTTTCACTTGCAAC
ENSGGOT00000016842 AGTGAGTGTGTGACCAGGAGACACAACAAAAGCAGCTCTGCCTTCACTTTCACTTGCAAC
ENST00000338040 AGCGAGTGTGTGACCAGGAGACACAACAAAAGCAGCTCTGCCTTCACTTTCACTTGCAAC
ENSPTRT00000028534 AGCGAGTGTGTGACCAGGAGACACAACAAAAGCAGCTCTGCCTTCACTTTCACTTGCAAC
ENSMUST00000114806 AGCGAGTGTGTGACTAGAAGACACAACAAAAGCAGCTCAGCTTTTACGTTCACCTGCAAC
ENSRNOT00000003340 AGTGAGTGTGTGACCAGAAGACACAACAAAAGCAGCTCAGCTTTTACTTTTACCTGCAAC

ENSCJAT00000062273 AAATTCTTCAGGAGGGATGAGTTCCCCCTGCACTTCAAGAATGTTCACACAGACATTCAG
ENSMMUT00000019013 AAATTCTTCAGGAGGGATGAGTTCCCCCTGCACTTCAAGAATGTCCACACAGACATTCAG
ENSPPYT00000015697 AAATTCTTCAGGAGGGATGAGTTTCCCCTGCACTTCAAGAATGTCCACACAGACATTCAG
ENSGGOT00000016842 AAATTCTTCAGGAGGGATGAGTTCCCCCTGCACTTCAAGAATGTCCACACAGACATTCAG
ENST00000338040 AAATTCTTCAGGAGGGATGAGTTCCCCCTGCACTTCAAGAATGTCCACACAGACATTCAG
ENSPTRT00000028534 AAATTCTTCAGGAGGGATGAGTTCCCCCTGCACTTCAAGAATGTCCACACAGACATTCAG
ENSMUST00000114806 AAATTCTTCCGAAGGGATGAATTTCCCCTGCACTTCAAGAATGTCCACACTGACATCCAG
ENSRNOT00000003340 AAATTCTTCCGGAGGGATGAATTTCCCCTGCACTTCAAGAATGTCCACACCGACATTCAG

ENSCJAT00000062273 TCGTGTCTCAATGGCTGGTTCCAGCATCGATGCCCCCTTGCCTACATGGGTTGTACATTT
ENSMMUT00000019013 TCATGTCTCAACGGCTGGTTCCAGCATCGATGCCCCCTCGCCTACTTGGGATGTACATTT
ENSPPYT00000015697 TCATGTCTCAATGGCTGGTTCCAGCATCGATGCCCCCTCGCCTACTTGGGATGTACATTT
ENSGGOT00000016842 TCATGTCTCAATGGCTGGTTCCAGCATCGATGCCCCCTCGCCTACTTGGGATGTACATTT
ENST00000338040 TCATGTCTCAATGGCTGGTTCCAGCATCGATGCCCCCTCGCCTACTTGGGATGTACATTT
ENSPTRT00000028534 TCATGTCTCAATGGCTGGTTCCAGCATCGATGCCCCCTCGCCTACTTGGGATGTACATTT
ENSMUST00000114806 TCGAGTCTCGATGGCTGGTTCCAGCATCGATGCCCCCTAGCCTACTTGGGATGTACATTT
ENSRNOT00000003340 TCGTGTCTCAATGGCTGGTTCCAGCATCGATGCCCCCTCGCCTACTTGGGATGTACATTT

ENSCJAT00000062273 GTTCAAAACCATTTCCGTCCCCCAGGGCAAAAGGCAAAAGTAATCTATAGTCAGGAGCTC
ENSMMUT00000019013 GTTCAAAACCATTTCCGTCCCCCGGGGCAAAAGGCAAAAGTAATCTACAGCCAGGAGCTC
ENSPPYT00000015697 GTTCAAAACCATTTCCGTCCCCCAGGGCAAAAGGCAAAAGTAATCTATAGCCAGGAGCTC
ENSGGOT00000016842 GTTCAAAACCATTTCCGTCCCCCAGGGCAAAAGGCAAAAGTAATCTATAGCCAGGAGCTC
ENST00000338040 GTTCAAAACCATTTCCGTCCCCCAGGGCAAAAGGCAAAAGTAATCTATAGCCAGGAGCTC

ENSPTRT00000028534 GTTCAAAACCATTTCCGTCCCCCAGGGCAAAAGGCAAAAGTAATCTATAGCCAGGAGCTC
ENSMUST00000114806 GTTCAGAACCACTTCCGCCCCCCTGGACAAAAAGCAAAAGTGATCTATAGCCAGGAGCTG
ENSRNOT00000003340 GTTCAAAACCACTTCCGCCCCCCTGGACAAAAAGCAAAAGTGATCTATAGTCAGGAGCTG

ENSCJAT00000062273 AAGACCTTTGCCATTAAGCCAGAGGTTGCTCCAGAGCTGAGTGAGGGAAGGAAGAACAAC
ENSMMUT00000019013 AAGACCTTTGCCATTAAGCCGGAGGTTGCTCCAGAGCTGAGCGAACGAAGGAAGAACAAC
ENSPPYT00000015697 AAGACCTTTGCCATTAAGCCGGAGGTTGCTTCAGAGCTGAGCGAGGGAAGGAAGAACAAC
ENSGGOT00000016842 AAGACCTTTGCCATTAAGCCGGAGGTTGCTCCAGAGCTGAGTGAGGGAAGAAAGAACAAC
ENST00000338040 AAGACCTTTGCCATTAAGCCGGAGGTTGCTCCAGAGCTGAGCGAGGGAAGGAAGAACAAC
ENSPTRT00000028534 AAGACCTTTGCCATTAAGCCGGAGGTTGCTCCAGAGCTGAGCGAGGGAAGGAAGAACAAC
ENSMUST00000114806 AAGACCTTTGCCATCAAACCAGAGGTTGCTCCGGAGCTGAGTGAGAAATGGAAGAGCGAC
ENSRNOT00000003340 AAGACCTTTGCCATCAAGCCAGAGGTTGCTCCGGAACTGAGCGAGAAATGGAAAAGCGAC

ENSCJAT00000062273 CATCTTTTGGGCCATGGAGGAAAAAGTGAGAATTCTCTAACCAGCCTGCCCCTGGAGATT
ENSMMUT00000019013 CATCTTTTGGGTCATGGAGGAAAAAGCCAGAATTCTTTAACCAGCCTGCCCCTGGAGATT
ENSPPYT00000015697 CATCTTTTGGGTCATGGAGGAAAAAGCCAGAATTCTTTAACCAGCCTGCCCCTGGAGATT
ENSGGOT00000016842 CATCTTTTGGGTCATGGAGGAAAAAGCCAGAATTCTTTAACCAGCCTGCCCCTGGAGATT
ENST00000338040 CATCTTTTGGGTCATGGAGGAAAAAGCCAGAATTCTTTAACCAGCCTGCCCCTGGAGATT
ENSPTRT00000028534 CATCTTTTGGGTCATGGAGGAAAAAGCCAGAATTCTTTAACCAGCCTGCCCCTGGAGATT
ENSMUST00000114806 CATCTCTCTGGCCGTGATGGGAAAAGCCTGAATTCTCTAACCAGCCTGCCCCTGGAGGTG
ENSRNOT00000003340 CATCTCTCAGGCCGCGATGGGAAAAGCCTGAATTCTCTAACCAGCCTGCCCTTGGAGGTT

ENSCJAT00000062273 TTGCAGTACATTGCTGGGTTCTTGGACAGCGTCAGCCTGGCCCAGCTCTCCCAGGTGTCT
ENSMMUT00000019013 TTGCAGTACATTGCTGGGTTCTTGGACAGTGTCAGCCTGGCCCAGCTCTCCCAGGTGTCT
ENSPPYT00000015697 TTGAAGTACATTGCTGGGTTCTTGGACAGCGTCAGCCTGGCCCAGCTTTCCCAGGTGTCT
ENSGGOT00000016842 TTGAAGTACATTGCTGGGTTCTTGGACAGCGTCAGCCTGGCCCAGCTCTCCCAGGTGTCT
ENST00000338040 TTGAAGTACATTGCTGGGTTCTTGGACAGCGTCAGCCTGGCCCAGCTCTCCCAGGTGTCT
ENSPTRT00000028534 TTGAAGTACATTGCTGGGTTCTTGGACAGCGTCAGCCTGGCCCAGCTCTCCCAGGTGTCT
ENSMUST00000114806 TTGCAATACATTGCTGGGTTTCTGGACAGCATCAGCCTGTCCCAGCTGTCCCAGGTGTCA
ENSRNOT00000003340 TTGCAATACATTGCTGGGTTTCTGGACAGCATCAGCCTGTCCCAGTTATCCCAGGTATCA

ENSCJAT00000062273 GTGCTGATGAGGAATATCTGTGCCACTTTATTACAAGAGAGAGGGATGGTCCTCTTGCAA
ENSMMUT00000019013 GTGCTGATGAGGAATATCTGTGCCACTTTGTTACAAGAGAGAGGGATGGTCCTTTTGCAA
ENSPPYT00000015697 GTGTTGATGAGGAATATCTGTGCCACTTTGTTACAAGAGAGAGGAATGGTCCTTTTGCAA
ENSGGOT00000016842 GTGCTGATGAGGAATATCTGTGCCACTTTGTTACAAGAGAGAGGAATGGTCCTTTTGCAA
ENST00000338040 GTGCTGATGAGGAATATCTGTGCCACTTTGTTACAAGAGAGAGGAATGGTCCTTTTGCAA
ENSPTRT00000028534 GTGCTGATGAGGAATATCTGTGCCACTTTGTTACAAGAGAGAGGAATGGTCCTTTTGCAA
ENSMUST00000114806 GTACTGATGAGGAACATCTGTGCCACTTTGTTGCAAGAGAGAGGGATGGTCCTCTCGCAA
ENSRNOT00000003340 GTACTGATGAGGAACATCTGTGCAACGTTGTTGCAAGAGAGAGGGATGGTCCTCTTGCAA

ENSCJAT00000062273 TGGAAGAAGAAGAGGTATTCCCATGGAGGCTTCTCCTGGAGAGTCCACAGAGAAATCTGG
ENSMMUT00000019013 TGGAAGAAGAAGAGGTATTCCCATGGAGGCACCTCCTGGAGAGTCCACAGAGAGATCTGG
ENSPPYT00000015697 TGGAAGAAAAAGAGATATTCCCATGGAGGCACCTCCTGGAGAGTCCACAGAGAGATCTGG
ENSGGOT00000016842 TGGAAGAAAAAGAGGTATTCCCATGGAGGCACCTCCTGGAGAGTCCACAGAGAGATCTGG
ENST00000338040 TGGAAGAAAAAGAGGTATTCCCATGGAGGCACCTCCTGGAGAGTCCACAGAGAGATCTGG
ENSPTRT00000028534 TGGAAGAAAAAGAGGTATTCCCATGGAGGCACCTCCTGGAGAGTCCACAGAGAGATCTGG
ENSMUST00000114806 TGGAAGAAGAAGAGGTATTCTCACGGAGGCACCTCCTGGAAAGTCCACAATCAGATCTGG
ENSRNOT00000003340 TGGAAGAAGAAGAGGTATTCTCATGGAGGCACCTCCTGGAAAGTCCACAATCAGATCTGG

ENSCJAT00000062273 CAGTTCAGCAGCCTTTTCTCCAAAATCAAGAGCTGGGAGTTTAATGAAGTCACCTCCATG
ENSMMUT00000019013 CAGTTCAGCAGCCTCTTCTCCAAAATCAAGAGCTGGGAGTTTAATGAAGTCACCTCCATG
ENSPPYT00000015697 CAGTTCAGCAGCCTTTTCTCCAAAATCAAGAGCTGGGAGTTTAATGAAGTCACCTCCATG
ENSGGOT00000016842 CAGTTCAGCAGCCTCTTCTCCAAAATCAAGAGCTGGGAGTTTAATGAAGTCACCTCCATG
ENST00000338040 CAGTTCAGCAGCCTCTTCTCCAAAATCAAGAGCTGGGAGTTTAATGAAGTCACCTCCATG

```
ENSPTRT00000028534     CAGTTCAGCAGCCTCTTCTCCAAAATCAAGAGCTGGGAGTTTAATGAAGTCACCTCCATG
ENSMUST00000114806     CAGTTCAGCAGCCTCTTCTCAAAAATCAACAGCTGGGAGTTTAATGACGTCACCTCCATG
ENSRNOT00000003340     CAGTTCAGCAGCCTCTTCTCAAAAATCAAGAGCTGGGAGTTTAATGACGTCACCTCCATG


ENSCJAT00000062273     TCTGAGCACCTGAAGTCTTGTCCTTTCAACATTGTAGAGCACAAAACTGACCCGATTCTT
ENSMMUT00000019013     TCCGAGCACCTGAAGTCCTGTCCTTTCAACATCGTAGAGCACAAAACTGACCCGATTCTT
ENSPPYT00000015697     TCTGAGCACCTGAAGTCCTGTCCTTTCAACATTGTAGAGCACAAAACTGACCCGATTCTT
ENSGGOT00000016842     TCTGAGCACCTGAAGTCCTGTCCTTTCAACATTGTAGAGCACAAAACTGACCCGATTCTT
ENST00000338040        TCTGAGCACCTGAAGTCCTGTCCTTTCAACATTGTAGAGCACAAAACTGACCCGATTCTT
ENSPTRT00000028534     TCTGAGCACCTGAAGTCCTGTCCTTTCAACATTGTAGAGCACAAAACTGACCCGATTCTT
ENSMUST00000114806     TCTGAGCACCTGAAAACCTGTCCTTTCAACATCGTGGAGCGGAAGACTGACCCGATTCGT
ENSRNOT00000003340     TCCGAACACCTGAAAACCTGTCCTTTCAATGTCGTGGAGCATAAGACTGACCCCATTCGT


ENSCJAT00000062273     TTGACCAGCATGTGTGAGCCACGTGAGCAGGCCCGAGAGAGCTTAGTCTCCACCTTCAGA
ENSMMUT00000019013     TTGACTAGCATGTGTCAGCCCCGTGAGCAGGCCCGAGAGAGCTTAGTCTCCACCTTTAGA
ENSPPYT00000015697     TTGACCAGCATGTGTCAGCCCCGTGAGCAGGCCCGAGAGAGCTTAGTCTCCACCTTTAGA
ENSGGOT00000016842     TTGACCAGCATGTGTCAGCCCCGTGAGCAGGCCCGAGAGAGCTTAGTCTCCACCTTTAGA
ENST00000338040        TTGACTAGCATGTGTCAGCCCCGTGAGCAGGCCCGAGAGAGCTTAGTCTCCACCTTTAGA
ENSPTRT00000028534     TTGACCAGCATGTGTCAGCCCCGTGAGCAGGCCCGAGAGAGCTTAGTCTCCACCTTTAGA
ENSMUST00000114806     TTGACCAGCATGTGCCAGCCCCAAGAGAAGGCCCGGGAGAGCTTAGTGTCCACCTTTAGG
ENSRNOT00000003340     CTGACCAGCATGTGCCAGCCCCAAGAGCAGGCCAGGGAGAGCTTAGTGTCCACGTTTAGG


ENSCJAT00000062273     GTCAGACCACGAGGAAGATACGTCTCC
ENSMMUT00000019013     GTCAGACCACGAGGAAGATACGTCTCC
ENSPPYT00000015697     GTCAGACCACGAGGAAGATACGTCTCC
ENSGGOT00000016842     GTCAGACCACAAGGAAGATACGTCTGC
ENST00000338040        ATCAGACCACGAGGAAGATACGTCTCC
ENSPTRT00000028534     GTCAGACCACGAGGAAGATACGTCTCC
ENSMUST00000114806     GCCCGACCACGGGGCAGACACTTT---
ENSRNOT00000003340     GCCCGACCGCGGGGAAGGCACTCT---
```

Multiple sequence alignment of Fbxo41

```
XM_003775898.1         CGCTTCCGGAGCCTGTCGTCGCTGCGCGCGCACCTGGAATACAGCCACACCTACGAGACG
ENSRNOT00000043201     CGCTTCCGGAGCCTGTCGTCGTTGCGCGCGCACCTGGAATACAGCCACACCTACGAGACG
ENSMUST00000161078     CGCTTCCGGAGCCTATCGTCGCTGCGCGCGCACCTGGAGTACAGCCACACCTACGAGACG
ENSCJAT00000007928     ------------------------------------------------------------
ENSMMUT00000020918     CGCTTCCGCAGCCTGTCGTCGCTGCGCGCGCACCTGGAGTACAGCCACACCTACGAGACG
ENSGGOT00000007817     ATGACAACTGGCTTGTCTGATCAGCAGGTGGTCTGTGACCTTGATCACAGGGCAGTGGAA
ENST00000521871        CGCTTCCGGAGCCTGTCGTCGCTGCGCGCGCACCTGGAGTACAGCCACACCTACGAGACG
ENSPTRT00000022434     ------------------------------------------------------------


XM_003775898.1         CTCTACATCCTCTCCAAGACCAACAGCATCTGCGACGGCGCCGCCGCCGCCGCGGCCGCC
ENSRNOT00000043201     CTCTACATCCTCTCCAAGACCAACAGCATCTGCGACGGCGCGGCCGCCGCTGCAGCTGCA
ENSMUST00000161078     CTCTACATCCTTTCCAAGACCAACAGCATCTGCGACGGCGCGGCCGCCGCTGCAGCCGCT
ENSCJAT00000007928     ------------NNCAAGACCAACAGCATCTGCGACGGCGCTGCAGCAGCCGCGGCCGCT
ENSMMUT00000020918     CTCTACATCCTCTCCAAGACCAACAGCATTTGCGACGGCGCCGCCGCTGCAGCGGCCGCC
ENSGGOT00000007817     GGCCTATTGCAGGCAGTTAGGAAGAGGATGGCAGGGGCCTCGCCCGCGGTCCCCCATGAG
ENST00000521871        CTCTACATCCTCTCCAAGACCAACAGCATCTGCGACGGCGCCGCCGCCGCCGCGGCCGCC
ENSPTRT00000022434     ------------------------------------------------------------


XM_003775898.1         GCAGCGGCCGCCTCGGGCTTCCCGCTGGCTCCCGAGCCCGCCGCCCTGCTGGCCGTGCCC
ENSRNOT00000043201     GCTGCCGCAGCCTCTGGCTTCCCTCTGGCGCCTGAGCCCGCGGCCCTGCTGGCTGTCCCC
ENSMUST00000161078     GCCGCCGCAGCCTCTGGATTCCCGCTGGCGCCTGAGCCCGCCGCCTTGCTGGCTGTCCCC
```

```
ENSCJAT00000007928      GCCGCCGCC---TCCGACTTCCCGCTGGCGCCCGAGCCCGCCGCCCTGCTGGCGGTGCCT
ENSMMUT00000020918      GCGGCGGCCGCCTCGGGCTTCCCGCTGGCGCCCGAGCCCGCCGCCCTGCTGGCAGTGCCC
ENSGGOT00000007817      CGCGCCCGGCTGACCGGC------GGGGCGGCCGAGCCAGCCGCCCTGCTGGCCGTGCCC
ENST00000521871         GCCGCCGCTGCCTCGGGGTTCCCGCTGGCTCCCGAGCCCGCCGCCCTGCTGGCCGTGCCC
ENSPTRT00000022434      ------------------------------------------------------------

XM_003775898.1          GGCGCCCGGCGAGAGGTCTTCGAGAGCACTTCCTTCCAGGGCAAGGAGCAGGCGGCCGGG
ENSRNOT00000043201      GGGGCCCGGCGCGAGGTCTTCGAGAGCACGTCCTTCCAGGGCAAGGAGCAGGCAGCGGGG
ENSMUST00000161078      GGGGCCCGGCGTGAGGTCTTTGAGAGCACGTCCTTCCAGGGCAAGGAACAGGCGACCGGG
ENSCJAT00000007928      GGCGCCCGGCGTGAGGTCTTCGAGAGCACGTCTTCCAGGGCAAGGAGCAGGCGGTCGGTC
ENSMMUT00000020918      GGCGCCCGGCGCGAGGTCTTCGAGAGCACGTCCTTCCAGGGCAAGGAGCAGGCGGCCGGG
ENSGGOT00000007817      GGCGCCCGGCGAGAGGTCTTCGAGAGCACTTCCTTCCAGGGCAAGGAGCAGGCGGCCGGG
ENST00000521871         GGCGCCCGGCGAGAGGTCTTCGAGAGCACTTCCTTCCAGGGCAAGGAGCAGGCGGCCGGG
ENSPTRT00000022434      ---------------------------------------GGCAAGGAGCAGGCGGCCGGG

XM_003775898.1          CCGTCGCCCGCGGCGCCGCACCTGCTGCACCACCACCATCACCACGCGCCCCTCGCCCAC
ENSRNOT00000043201      CCAGCGCCCGCGGGGCCGCACCTGCTGCACCATCATCACCACCACGCGCCACTGGCGCAT
ENSMUST00000161078      CCCTCGCCCGCGGGGCCGCACCTGCTGCACCATCATCACCACCACGCGCCACTGGCGCAC
ENSCJAT00000007928      CTCTCGCCCGCGGCGCCGCACCTGCACCACCACCACCACCACCACGCGCCCCTCGCCCAC
ENSMMUT00000020918      CCGTCGCCCGCGGCGCCGCACCTGCTGCACCACCACCACCACCACGCGCCCCTCGCACAC
ENSGGOT00000007817      CCGTCGCCCGCGGCGCCGCACCTGCTGCACCACCACCATCACCACGCTCCCCTCGCCCAC
ENST00000521871         CCGTCGCCCGCGGCGCCGCACCTGCTGCACCACCACCATCACCACGCTCCCCTCGCCCAC
ENSPTRT00000022434      CCGTCGCCCGCGGCGCCGCACCTGCTGCACCACCACCATCACCACGCTCCCCTCGCCCAC

XM_003775898.1          TTCCCCGGCGACCTGGTGCCCGCTAGCCTGCCCTGCGAGGAGTTGGCAGAGCCGGGCCTC
ENSRNOT00000043201      TTCCCCGGTGACCTGGTGCCCGCCAGCCTGCCCTGCGAGGAGCTGGCCGAGCCGGGGCTC
ENSMUST00000161078      TTTCCCGCTGACCTGGTGCCCGCTAGCCTGCCCTGCGAGGAGCTGGCTGAGCCGGGGCTC
ENSCJAT00000007928      TTCCCCGGAGACCTGGTTCCCGCCAGCCTGCCCTGCGAGGAGTTGGCCGAGCCGGGCCTC
ENSMMUT00000020918      TTCCCCGGCGACCTGGTGCCCGCCAGCCTGCCCTGCGAGGAGTTGGCCGAGCCGGGCCTC
ENSGGOT00000007817      TTCCCCGGCGACCTGGTGCCCGCTAGCCTGCCCTGTGAGGAGTTGGCCGAGCCGGGCCTT
ENST00000521871         TTCCCCGGCGACCTGGTGCCCGCTAGCCTGCCCTGTGAGGAGTTGGCCGAGCCGGGCCTT
ENSPTRT00000022434      TTCCCCGGCGACCTGGTGCCCGCTAGCCTGCCCTGTGAGGAGTTGGCCGAGCCGGGCCTT

XM_003775898.1          GTGCCCGCCGCCGCAGCGCGCTATGCGCTGCGCGAGATCGAAATCCCGCTAGGGGAGCTG
ENSRNOT00000043201      GTGCCC------GCTGCGCGCTATGCGCTACGCGAAATCGAAATCCCACTCGGCGAGCTC
ENSMUST00000161078      GTGCCC------GCTGCGCGCTATGCGCTACGCGAAATCGAAATCCCACTCGGCGAGCTG
ENSCJAT00000007928      GTGCCCGCCGCCGCCGCACGCTACGCGCTGCGCGAGATCGAGATCCCGCTGGGGGAGCTG
ENSMMUT00000020918      GTGCCCGCCGCTGCCGCTCGCTATGCGCTGCGCGAGATCGAGATCCCGCTGGGGGAGCTG
ENSGGOT00000007817      GTGCCCGCCGCCGCAGCGCGCTATGCGCTGCGCGAGATCGAGATCCCGCTGGGGGAGCTG
ENST00000521871         GTGCCCGCCGCAGCAGCGCGCTATGCGCTGCGCGAGATCGAGATCCCGCTGGGGGAGCTG
ENSPTRT00000022434      GTGCCCGCCGCCGCAGCGCGCTATGCGCTGCGCGAGATCGAGATCCCGCTGGGGGAGCTG

XM_003775898.1          TTCGCCCGCAAGTCAGTGGCGTCCTCGGCGTGCTCGACGCCGCCGCCTGGCCCCGGCCCC
ENSRNOT00000043201      TTCGCGCGCAAGTCCGTGGCATCCTCGGCCTGCTCGACGCCGCCACCCGGACCTGGCCCT
ENSMUST00000161078      TTTGCGCGCAAGTCCGTGGCATCCTCTGCCTGCTCGACGCCGCCACCCGGACCTGGCCCT
ENSCJAT00000007928      TTTGCCCGCAAGTCCGTGGCGTCCTCGGCGTGCTCGACGCCGCCACCTGGGCCCGGCCCC
ENSMMUT00000020918      TTCGCCCGCAAGTCCGTGGCGTCCTCAGCGTGCTCGACGCCGCCGCCTGGGCCTGGCCCC
ENSGGOT00000007817      TTCGCCCGCAAGTCCGTGGCGTCCTCGGCGTGCTCGACGCCGCCGCCTGGCCCCGGCCCC
ENST00000521871         TTCGCCCGCAAGTCCGTGGCGTCCTCGGCGTGCTCGACGCCGCCGCCTGGCCCCGGCCCC
ENSPTRT00000022434      TTCGCCCGCAAGTCCGTGGCGTCCTCGGCGTGCTCGACGCCGCCGCCTGGCCCCGTCCCC

XM_003775898.1          GGCCCTTGCCCCGGGCCTGCCTCCGCTTCGCCCGCTTCCCCCTCACCCGCTGATGTGGCC
ENSRNOT00000043201      GGTCCTTGTTCCGGGCCTACCTCTGCATCGCCGGCGTCGCCATCACCTGCGGACGTCGCC
ENSMUST00000161078      GGTCCTTGTTCCGGGCCCTCCTCTGCATCGCCGGCGTCGCCATCACCTGCGGACGTCGCC
```

```
ENSCJAT00000007928     GGCCCCTGCTCCGGGCCGGCCTCCGCCTCCCCCGCGTCCCCCTCACCCGCGGATGTGGCC
ENSMMUT00000020918     GGCCCTTGCCCCGGGCCTGCCTCCGCTTCGCCCGCGTCCCCCTCACCTGCCGATGTGGCC
ENSGGOT00000007817     GGCCCTTGCCCCGGGCCTGCCTCCGCTTCGCCCGCGTCCCCCTCACCCGCTGATGTGGCC
ENST00000521871        GGCCCTTGCCCCGGGCCTGCCTCCGCTTCGCCCGCGTCCCCCTCACCCGCTGATGTGGCC
ENSPTRT00000022434     GGCCCTTGCCCCGGGCCTGCCTCCGCTTCGCCCGCGTCCCCCTCACCCGCTGATGTGGCC

XM_003775898.1         TACGAAGAGGGCCTGGCGCGCCTCAAGATCCGCGCGCTGGAGAAGCTGGAGGTGGACCGG
ENSRNOT00000043201     TACGAAGAGGGCTTGGCACGCCTCAAGATCCGCGCCCTGGAGAAGCTGGAGGTGGACCGG
ENSMUST00000161078     TACGAAGAGGGCCTGGCACGCCTTAAGATCCGCGCCCTGGAGAAGCTGGAGGTGGACCGG
ENSCJAT00000007928     TATGAAGAGGGCCTGGCGCGCCTCAAGATCCGCGCGCTGGAGAAGCTGGAGGTGGACAGG
ENSMMUT00000020918     TACGAAGAGGGCCTAGCGCGCCTCAAGATCCGCGCGCTGGAGAAGCTGGAGGTGGACCGG
ENSGGOT00000007817     TACGAAGAGGGCCTGGCGCGCCTCAAGATCCGCGCGCTGGAGAAGCTGGAGGTGGACCGG
ENST00000521871        TACGAAGAGGGCCTGGCGCGCCTCAAGATCCGCGCGCTGGAGAAGCTGGAGGTGGACCGG
ENSPTRT00000022434     TACGAAGAGGGCCTGGCGCGCCTCAAGATCCGCGCGCTGGAGAAGCTGGAGGTGGACCGG

XM_003775898.1         CGGCTGGAGCGGCTGAGCGAGGAGGTGGAGCAGAAGATCGCGGGCCAGGTGGGCCGGCTG
ENSRNOT00000043201     AGACTAGAGCGGCTGAGCGAGGAGGTGGAGCAGAAGATCGCGGGCCAGGTGGGTCGGCTA
ENSMUST00000161078     AGACTAGAGCGGCTGAGCGAGGAGGTGGAGCAGAAGATCGCGGGCCAGGTGGGTCGGCTA
ENSCJAT00000007928     CGGCTGGAGCGGCTGAGCGAGGAAGTGGAGCAGAAGATCGCTGGCCAGGTGGGCCGGCTG
ENSMMUT00000020918     CGGCTGGAGCGCCTGAGCGAGGAGGTGGAGCAGAAGATCGCGGGCCAGGTGGGCCGGCTG
ENSGGOT00000007817     CGGCTGGAGCGGCTGAGCGAGGAGGTGGAGCAGAAGATCGCGGGCCAGGTGGGCCGGCTG
ENST00000521871        CGGCTGGAGCGGCTGAGCGAGGAGGTGGAGCAGAAGATCGCGGGCCAGGTGGGCCGGCTG
ENSPTRT00000022434     CGGCTGGAGCGGCTGAGCGAGGAGGTGGAGCAGAAGATCGCGGGCCAGGTGGGCCGGCTG

XM_003775898.1         CAGGCCGAGCTGGAGCGCAAGGCGGCCGAACTGGAGACTGCGCGGCAGGAGAGTGCGAGG
ENSRNOT00000043201     CAGGCTGAACTGGAGCGCAAGGCAGCAGAGTTGGAGACTGCACGACAGGAGAGCGCGCGC
ENSMUST00000161078     CAGGCGGAGCTGGAGCGCAAGGCAGCAGAGCTAGAAACTGCAAGACAGGAGAGCGCGCGC
ENSCJAT00000007928     CAGGCCGAGCTGGAGCGCAAGGCGGCTGAACTGGAGACGGCGCGGCAGGAGAGTGCGAGG
ENSMMUT00000020918     CAGGCCGAGCTGGAGCGCAAGGCGGCGGAGCTGGAGACTGCGCGGCAGGAGAGTGCGAGG
ENSGGOT00000007817     CAGGCCGAGCTGGAGCGCAAGGCGGCCGAACTGGAGACTGCGCGGCAGGAGAGTGCGAGG
ENST00000521871        CAGGCCGAGCTGGAGCGCAAGGCGGCCGAACTGGAGACTGCGCGGCAGGAGAGTGCGAGG
ENSPTRT00000022434     CAGGCCGAGCTGGAGCGCAAGGCGGCCGAACTGGAGACTGCGCGGCAGGAGAGTGCGAGG

XM_003775898.1         CTCGGGCGCGAGAAGGAGGAGCTGGAGGAGCGCGCGTCTGAGCTCTCCCGCCAGGTGGAC
ENSRNOT00000043201     CTGGGGCGTGAGAAGGAGGAACTGGAGGAGCGCGCGTCTGAGCTCTCGCGCCAAGTGGAC
ENSMUST00000161078     CTGGGACGCGAGAAGGAGGAGCTGGAGGAGCGCGCCTCTGAGCTCTCACGCCAAGTGGAC
ENSCJAT00000007928     CTCGGGCGCGAGAAGGAGGAGCTGGAGGAGCGAGCGTCTGAGCTCTCCCGCCAGGTGGAC
ENSMMUT00000020918     CTTGGGCGCGAGAAGGAGGAGCTGGAGGAGCGCGCGTCTGAGCTCTCCCGCCAGGTGGAC
ENSGGOT00000007817     CTCGGGCGCGAGAAGGAGGAGCTGGAGGAGCGCGCGTCTGAGCTCTCCCGCCAGGTGGAC
ENST00000521871        CTCGGGCGCGAGAAGGAGGAGCTGGAGGAGCGCGCGTCTGAGCTCTCCCGCCAGGTGGAC
ENSPTRT00000022434     CTCGGGCGCGAGAAGGAGGAGCTGGAGGAGCGCGCGTCTGAGCTCTCCCGCCAGGTGGAC

XM_003775898.1         GTGAGCGTAGAGCTGCTGGCCTCACTCAAGCAGGACCTGGTGCACAAGGAACAGGAGCTG
ENSRNOT00000043201     GTGAGCGTGGAACTGCTGGCTTCGCTCAAGCAGGACCTGGTGCATAAGGAACAGGAGCTG
ENSMUST00000161078     GTGAGCGTGGAACTGCTGGCTTCACTCAAGCAGGACCTGGTGCATAAGGAACAGGAGCTG
ENSCJAT00000007928     GTGAGCGTGGAGCTGCTGGCCTCGCTCAAGCAGGACCTAGTGCACAAGGAACAGGAGCTG
ENSMMUT00000020918     GTGAGCGTAGAGCTTCTGGCCTCACTCAAGCAGGACCTGGTGCACAAGGAACAGGAGCTG
ENSGGOT00000007817     GTGAGCGTAGAGCTGCTGGCCTCACTCAAGCAGGACCTGGTGCACAAGGAACAGGAGCTG
ENST00000521871        GTGAGCGTAGAGCTGCTGGCCTCACTCAAGCAGGACCTGGTGCACAAGGAACAGGAGCTG
ENSPTRT00000022434     GTGAGCGTAGAGCTGCTGGCCTCACTCAAGCAGGACCTGGTGCACAAGGAACAGGAGCTG

XM_003775898.1         AGCCGCAAGCAGCAG---------------------TTCCTGCAAGTATGT---------
ENSRNOT00000043201     AGCCGCAAGCAGCAGAGAATTTATACTCCTCCATCCATCATAAAAAGAAGTCAGAGTAGG
ENSMUST00000161078     AGCCGCAAGCAGCAGGAGGTGGTGCAGATCGACCAGTTCCTGAAGGAGACGGCAGCTCGG
```

```
ENSCJAT00000007928     AGCCGCAAACAGCAGGAGGTGGTGCAGATTGACCAGTTCCTGAAGGAGACAGCGGCGCGG
ENSMMUT00000020918     AGCCGCAAGCAGCAGGAGGTGGTGCAGATCGACCAGTTCCTGAAGGAGACAGCGGCGCGG
ENSGGOT00000007817     AGCCGCAAGCAGCAGGAGGTGGTGCAGATCGACCAGTTCCTGAAGGAGACTGCGGCGCGG
ENST00000521871        AGCCGCAAGCAGCAGGAGGTGGTGCAGATCGACCAGTTCCTGAAGGAGACGGCGGCGCGG
ENSPTRT00000022434     AGCCGCAAGCAGCAGGAGGTGGTGCAGATCGACCAGTTCCTGAAGGAGACGGCGGCGCGG

XM_003775898.1         ------------------------------------------------------------
ENSRNOT00000043201     AACTCAAGGCAAGAACTGGAACAGAGACCAGTGGTAGAGCGCTTGCCTAGCAAACGAAAC
ENSMUST00000161078     GAGGCCAGTGCCAAGCTGCGGCTGCAACAGTTCATCGAGGAGCTCCTGGAGCGTGCAGAC
ENSCJAT00000007928     GAGGCCAGCGCTAAGCTGCGGCTACAGCAGTTCATTGAGGAGCTCCTTGAGCGGGCTGAC
ENSMMUT00000020918     GAGGCCAGCGCCAAGCTGCGGCTGCAGCAGTTCATTGAGGAACTCCTTGAGCGGGCTGAC
ENSGGOT00000007817     GAGGCCAGCGCCAAGCTGCGGCTGCAGCAGTTCATTGAGGAGCTCCTTGAGCGGGCTGAC
ENST00000521871        GAGGCCAGCGCCAAGCTGCGGCTGCAGCAGTTCATTGAGGAGCTCCTTGAGCGGGCTGAC
ENSPTRT00000022434     GAGGCCAGCGCCAAGCTGCGGCTGCAGCAGTTCATTGAGGAGCTCCTTGAGCGGGCTGAC

XM_003775898.1         ---------------TTGACTATGTTGGTGGTACTGTGC---------------------
ENSRNOT00000043201     AGAGGGGAGGCGGCTGCTCAGCCTGCTTCAACTTTTTGTGCCTGGGACCCCAGCCCATAT
ENSMUST00000161078     AGGGCCGAGCGCCAGTTGCAGGTCATCAGCAGCAGCTGTGGCAGCACACCCAGTGCCAGC
ENSCJAT00000007928     CGTGCTGAGCGGCAGCTGCAGGTCATCAGCAGCAGCTGTGGCAGCACGCCCAGCGCCAGC
ENSMMUT00000020918     CGTGCCGAGCGACAGCTGCAGGTCATCAGCAGCAGCTGTGGCAGCACGCCCAGTGCCAGC
ENSGGOT00000007817     CGTGCCGAGCGGCAGCTGCAGGTCATCAGCAGCAGCTGTGGCAGCACGCCCAGCGCCAGC
ENST00000521871        CGTGCCGAGCGGCAGCTGCAGGTCATCAGCAGCAGCTGTGGCAGCACGCCCAGCGCCAGC
ENSPTRT00000022434     CGTGCCGAGCGGCAGCTGCAGGTCATCAGCAGCAGCTGTGGCAGCACGCCCAGCGCCAGC

XM_003775898.1         ---------------------------------------CTCCATGGTTCTTCGCGTGTC
ENSRNOT00000043201     TTGTCCAGAGGTTCATCTTATTCTGTCAGAGGTCCTGAACTGNGAGGCCCAGGCAGAATG
ENSMUST00000161078     CTGGGCAGAGGAGGTGGGGGCAGTGCCTCGGGGCCTGGGGTGAGAGGCCCCGGCAGAATG
ENSCJAT00000007928     CTGGGCCGTGGAGGTGGGGGAGGTGGCGCTGGCCCCAATGCCCGGGGCGCAGGCAGAATG
ENSMMUT00000020918     CTGGGCCGTGGAGGTGGGGGCGGTGGTGCTGGCCCCAATGCCCGGGGCCCAGGCAGAATG
ENSGGOT00000007817     CTGGGCCGTGGAGGTGGGGGCGGTGGTGCTGGCCCCAATGCCCGGGGCCCAGGCAGAATG
ENST00000521871        CTGGGCCGTGGAGGTGGGGGCGGTGGTGCTGGACCCAATGCCCGGGGCCCAGGCAGAATG
ENSPTRT00000022434     CTGGGCCGTGGAGGTGGGGGCGGTGGTGCTGGCCCCAATGCCCGGGGCCCAGGCAGAATG

XM_003775898.1         CGAGAACACCACGCGGGCCCGGCCGTGCCTAACACATATGCAGTGTCACGGCATGGCTCC
ENSRNOT00000043201     CGAGAACACCATGCAGGCCCAGCTGTGCCAAGCACATATGCTGTATCACGGCATGGCTCC
ENSMUST00000161078     CGAGAACATCATGCAGGCTCAGCTGTGCCAAGCACATACGCCGTATCACGGCATGGCTCC
ENSCJAT00000007928     CGAGAACACCACGCAGGCCCGGCCATGCCTAGCACATATGCAGTGTCACGGCATGGCTCC
ENSMMUT00000020918     CGAGAACACCACGCGGGCCCGGCCGTGCCTAGCACATATGCAGTGTCACGGCATGGCTCC
ENSGGOT00000007817     CGAGAACACCACGTGGGCCCGGCCGTGCCTAACACATATGCAGTGTCACGGCATGGCTCC
ENST00000521871        CGAGAACACCACGTGGGCCCGGCCGTGCCTAACACATATGCAGTGTCACGGCATGGCTCC
ENSPTRT00000022434     CGAGAACACCACGTGGGCCCGGCCGTGCCTAACACATATGCAGTGTCACGGCATGGCTCC

XM_003775898.1         TCTCCCAGCACAGGGGCCTCCAGCCGTGTGCCAGCCGCATCCCAGAGCTCAGGCTGCTAT
ENSRNOT00000043201     TCTCCCAGCACAGGGGCCTCCAGCCGTGTGCCAGCTGCATCCCAGAGCTCAGGCTGCTAT
ENSMUST00000161078     TCTCCCAGCACAGGGGCCTCCAGTCGTGTGCCAGCTGCATCCCAGAGCTCAGGCTGCTAT
ENSCJAT00000007928     TCTCCCAGCACAGGGGCCTCCAGCCGTGTGCCAGCTGCATCCCAGAGTTCAGGCTGCTAT
ENSMMUT00000020918     TCTCCCAGCACAGGGGCCTCCAGCCGTGTGCCAGCCGCATCCCAGAGCTCAGGCTGCTAT
ENSGGOT00000007817     TCTCCCAGCACAGGGGCCTCCAGCCGTGTGCCAGCCGCATCCCAGAGCTCAGGCTGCTAT
ENST00000521871        TCTCCGAGCACAGGGGCCTCCAGCCGTGTGCCAGCCGCATCCCAGAGCTCAGGCTGCTAT
ENSPTRT00000022434     TCTCCCAGCACAGGGGCCTCCAGCCGTGTGCCAGCCGCATCCCAGAGCTCAGGCTGCTAT

XM_003775898.1         GACAGTGACAGTCTGGAGTTGCCCAGGCCAGAGGAGGGGGCCCCTGAGGACAGTGGCCCT
ENSRNOT00000043201     GACAGTGACAGTCTGGAGCTACCCCGACAAGAGGAAGGGCCCTCGGAGGACAGTGGCCCT
ENSMUST00000161078     GACAGTGACAGTCTGGAGCTGCCCCGGCCAGAGGAAGGGCCCTCGGAGGACAGTGGCCCT
```

```
ENSCJAT00000007928     GACAGTGACAGTCTGGAGCTGCCCAGGCCAGAGGAGGGGGTCCCTGAGGACAGTGGCCCT
ENSMMUT00000020918     GACAGTGACAGTCTGGAGCTGCCCAGGCCGGAGGAGGGGGCCCCTGAGGACAGTGGCCCT
ENSGGOT00000007817     GACAGTGACAGTCTGGAGCTGCCCAGGCCAGAGGAGGGGGCCCCTGAGGACAGTGGTCCT
ENST00000521871        GACAGTGACAGTCTGGAGCTGCCCAGGCCAGAGGAGGGGGCCCCTGAGGACAGTGGCCCT
ENSPTRT00000022434     GACAGTGACAGTCTGGAGCTGCCCAGGCCAGAGGAGGGGGCCCCTGAGGACAGTGGCCCT


XM_003775898.1         GGGGGCTTGGGCACACGGGCCCAGGCTGCCAACGGGGGCTCAGAGCGGTCCCAGCCCCCT
ENSRNOT00000043201     GGGGGCTTGGGTTCACGGGCCCAGGCAACCAACGGTGGTTCAGAGCGGTCCCAGCCCCCT
ENSMUST00000161078     GGGGGCTTGGGTTCACGGGCCCAGGCTACCAACGGTGGTTCAGAGCGGTCCCAGGCCCCT
ENSCJAT00000007928     GGGGGCTTGGGCACACGGGCCCAGGCTGCCAACGGGGGCTCAGAACGGTCCCAGCCCCCT
ENSMMUT00000020918     GGGGGCTTGGGCACACGGGCCCAGGCTACCAACGGGGGCTCAGAGCGGTCCCAGCCCCCT
ENSGGOT00000007817     GGGGGCTTGGGCACACGGGCCCAGGCTGCCAACGGGGGCTCAGAGTGGTCCCAGCCCCCT
ENST00000521871        GGGGGCTTGGGCACACGGGCCCAGGCTGCCAACGGGGGCTCAGAGCGGTCCCAGCCCCCT
ENSPTRT00000022434     GGGGGCTTGGGCACACGGGCCCAGGCTGCCAACGGGGGCTCAGAGCGGTCCCAGCCCCCT


XM_003775898.1         CGCAGCTCAGGCCTGCGGCGCCAGGCCATCCAGAACTGGCAGCGCAGACCCCGCCGACAC
ENSRNOT00000043201     CGTAGTTCAGGTCTACGAAGACAGGCCATCCAGAACTGGCAGCGCCGACCGCGCCGTCAT
ENSMUST00000161078     CGTAGTTCAGGCCTAAGACGACAGGCCATCCAGAACTGGCAACGCCGACCTCGTCGTCAC
ENSCJAT00000007928     CGCAGCTCAGGCCTGCGGCGCCAGGCCATCCAAAACTGGCAGCGCAGACCCCGCCGACAC
ENSMMUT00000020918     CGCAGCTCAGGCCTGCGGCGCCAGGCCATCCAGAACTGGCAGCGCAGACCCCGCCGACAC
ENSGGOT00000007817     CGCAGCTCAGGCCTGCGGCGCCAGGCCATCCAGAACTGGCAGCGCAGACCCCGCCGACAC
ENST00000521871        CGCAGCTCAGGCCTGCGGCGCCAGGCCATCCAGAACTGGCAGCGCAGACCCCGCCGACAC
ENSPTRT00000022434     CGCAGCTCAGGCCTGCGGCGCCAGGCCATCCAGAACTGGCAGCGCAGACCCCGCCGACAC


XM_003775898.1         AGCACTGAGGGGGAAGAGGGTGATGTCTCCGACGTTGGCTCCCGAACCACTGAGTCGGAG
ENSRNOT00000043201     AGCACGGAAGGGGAGGAAGGTGACGTCTCAGATGTGGGCTCCCGAACTACTGAGTCAGAG
ENSMUST00000161078     AGCACCGAGGGGGAGGAGGGTGACGTTTCGGATGTGGGCTCCCGAACTACTGAGTCAGAG
ENSCJAT00000007928     AGCACCGAGGGAGAAGAGGGTGATGTCTCTGACGTGGGCTCCCGAACCACTGAGTCAGAG
ENSMMUT00000020918     AGCACTGAGGGGGAAGAGGGTGACGTCTCTGACGTTGGCTCCCGAACCACTGAGTCAGAG
ENSGGOT00000007817     AGCACTGAGGGGGAAGAGGGTGATGTCTCCGACGTTGGCTCCCGAACCACTGAGTCAGAG
ENST00000521871        AGCACTGAGGGGGAAGAGGGTGATGTCTCCGACGTTGGCTCCCGAACCACTGAGTCAGAG
ENSPTRT00000022434     AGCACTGAGGGGGAAGAGGGTGATGTCTCCGACGTTGGCTCCCGAACCACTGAGTCAGAG


XM_003775898.1         GCTGAGGGCCCGTTGGATGCACCCCGCCCCGGGCCTGCTATGGCTGGGCCATTGAGCAGC
ENSRNOT00000043201     GCTGAAGGACCCTCTGATGTCCCACGCCCTGGACCTGCTGTGGCTGGACCCTTGAACAGC
ENSMUST00000161078     GCTGAAGGACCCTCAGATGTCCCACGCCCTGGACCTGCTGTGGCTGGCCCCTTGAACAGC
ENSCJAT00000007928     GCTGAGGGCCCGTTGGATGCGCCCCGCCCCGGGCCTGCTATGGCTGGGCCCTTGAGCAGC
ENSMMUT00000020918     GCTGAGGGCCCGTTGGATGCGCCCCGCCCCGGGCCTGCTATGGCTGGGCCATTGAGCAGC
ENSGGOT00000007817     GCTGAGGGCCCGTTGGATGCGCCCCGCCCCGGGCCTGCTATGGCTGGGCCATTGAGCAGC
ENST00000521871        GCTGAGGGCCCGTTGGATGCGCCCCGCCCCGGGCCTGCTATGGCTGGGCCATTGAGCAGC
ENSPTRT00000022434     GCTGAGGGCCCGTTGGATGCGCCCCGCCCCGGGCCTGCTATGGCTGGGCCATTGAGCAGC


XM_003775898.1         TGCCGGCTTTCAGCGCCCTTCAAGCCTGGGGCAGGA------------------------
ENSRNOT00000043201     TGCCGGCTCTCGGCCCGCCCTGAAGGAGGCAGCGGGCGAGGCCGACGGATGGAGAGAGGC
ENSMUST00000161078     TGCCGGCTCTCTGCACGTCCTGAAGGAGGCAGCGGGCGAGGCCGTCGGGTGGAGAGAGGT
ENSCJAT00000007928     TGCCGGCTCTCAGCCCGTCCCGAGGGAGGCAGTGGGCGGGGTCGGCGAGCAGAGAGGGGC
ENSMMUT00000020918     TGCCGGCTCTCAGCCCGCCCTGAGGGAGGCAGTGGACGGGGTCGGCGAGCAGAGAGGGGC
ENSGGOT00000007817     TGCCGGCTTTCAGCCCGCCCCGAGGGAGGCAGTGGGCGGGGTCGGCGAGCAGAGAGGGTC
ENST00000521871        TGCCGGCTCTCAGCCCGCCCCGAGGGAGGCAGTGGGCGGGGTCGGCGAGCAGAGAGGGTC
ENSPTRT00000022434     TGCCGGCTCTCAGCCCGCCCCGAGGGAGGCAGTGGGCGGGGTCGGCGAGCAGAGAGGGTC


XM_003775898.1         ACTCTTGTGACAGGGCAGGGCTGCAGTGGCACAAGTGTACTCAGATTGTCTGTTGCACAA
ENSRNOT00000043201     AGCCCCTCACGCTCCAATGAGGTCATCAGCCCAGAAATCCTCAAGATGCGTGCTGCCCTC
ENSMUST00000161078     AGCCCCTCACGCTCCAATGAGGTCATCAGCCCAGAAATCCTCAAGATGCGAGCTGCCCTC
```

```
ENSCJAT00000007928     AGCCCCTCACGCTCCAACGAGGTCATCAGCCCAGAGATCCTGAAGATGCGAGCCGCCCTC
ENSMMUT00000020918     AGCCCCTCACGCTCCAATGAGGTCATCAGCCCAGAGATCCTGAAGATGCGAGCCGCCCTC
ENSGGOT00000007817     AGCCCCTCACGCTCCAATGAGGTCATCAGCCCGGAGATCCTGAAGATGCGAGCTGCCCTC
ENST00000521871        AGCCCCTCACGCTCCAATGAGGTCATCAGCCCAGAGATCCTGAAGATGCGAGCTGCCCTC
ENSPTRT00000022434     AGCCCCTCACGCTCCAATGAGGTCATCAGCCCAGAGATCCTGAAGATGCGAGCTGCCCTC

XM_003775898.1         GGATGCCACATCCGGGGGGCAGGGGGGTCCATTCACCTG------------GTCTGCCGG
ENSRNOT00000043201     TTCTGTATCTTCACCTACCTGGATACCCGGACACTGCTACATGCTGCAGAGGTCTGCCGG
ENSMUST00000161078     TTCTGTATCTTCACCTACCTGGATACCCGGACACTGCTCCATGCAGCAGAGGTCTGTCGG
ENSCJAT00000007928     TTCTGCATCTTCACCTACTTGGACACCCGCACACTGCTGCATGCTGCCGAGGTCTGCCGG
ENSMMUT00000020918     TTCTGCATCTTCACCTACCTGGACACGCGCACACTGCTGCACGCCGCTGAGGTTTGCCGG
ENSGGOT00000007817     TTCTGCATCTTCACCTACCTGGACACGCGCACACTGCTGCATGCTGCCGAGGTCTGCCGA
ENST00000521871        TTCTGCATCTTCACCTACCTGGACACGCGCACACTGCTGCATGCTGCCGAGGTCTGCCGG
ENSPTRT00000022434     TTCTGCATCTTCACCTACCTGGACACGCGCACACTGCTGCATGCTGCCGAGGTCTGCCGG

XM_003775898.1         GACTGGCGCTTCGTGGCCCGCCACCCCGCAGTCTGGACAAGGGTGCTGCTTGAGAATGCC
ENSRNOT00000043201     GACTGGCGCTTTGTGGCGCGCCATCCCGCTGTCTGGACGAGAGTGCTGCTCGAGAACGCT
ENSMUST00000161078     GACTGGCGCTTTGTGGCCCGCCATCCCGCTGTCTGGACGAGAGTGCTACTTGAGAATGCC
ENSCJAT00000007928     GACTGGCGCTTCGTGGCCCGCCACCCCGCAGTCTGGACAAGGGTGCTGCTTGAGAATGCC
ENSMMUT00000020918     GACTGGCGCTTCGTGGCCCGCCACCCTGCAGTCTGGACAAGGGTGCTGCTTGAGAATGCC
ENSGGOT00000007817     GACTGGCGCTTCGTGGCCCGCCACCCCGCAGTCTGGACAAGGGTGCTGCTTGAGAATGCC
ENST00000521871        GACTGGCGCTTCGTGGCCCGCCACCCCGCAGTCTGGACAAGGGTGCTGCTTGAGAATGCC
ENSPTRT00000022434     GACTGGCGCTTCGTGGCCCGCCACCCCGCAGTCTGGACAAGGGTGCTGCTTGAGAATGCC

XM_003775898.1         CGTGTCTGCTCCAAGTTCCTGGCAATGCTGGCTCAGTGGTGCACCCAGGCCCACTCTCTG
ENSRNOT00000043201     CGAGTCTGTTCCAAGTTCCTGGCGATGTTGGCTCAGTGGTGCACCCAGGCTCACTCATTA
ENSMUST00000161078     CGAGTCTGTTCCAAGTTCCTGGCGATGTTGGCTCAGTGGTGCACCCAGGCTCACTCGTTA
ENSCJAT00000007928     CGTGTCTGCTCCAAGTTCCTGGCAATGCTGGCTCAGTGGTGCACCCAGGCTCACTCTCTG
ENSMMUT00000020918     CGTGTCTGCTCCAAGTTCCTGGCAATGCTGGCTCAGTGGTGCACCCAGGCCCACTCTCTG
ENSGGOT00000007817     CGTGTCTGCTCCAAGTTCCTGGCAATGCTGGCTCAGTGGTGCACCCAGGCCCACTCTCTG
ENST00000521871        CGTGTCTGCTCCAAGTTCCTGGCAATGCTGGCTCAGTGGTGCACCCAGGCCCACTCTCTG
ENSPTRT00000022434     CGTGTCTGCTCCAAGTTCCTGGCAATGCTGGCTCAGTGGTGCACCCAGGCCCACTCTCTG

XM_003775898.1         ACGCTGCAGAACTTGAAGCCCCGGCAGCGGGGAAAGAAGGAGAGCAAGGAGGAGTATGCC
ENSRNOT00000043201     ACATTACAGAACCTGAAGCCCCGGCAGCGGGGAAAGAAGGAAAGCAAGGAGGAGTATGCC
ENSMUST00000161078     ACATTACAGAACCTGAAGCCCCGGCAGCGGGGGAAGAAGGAAAGCAAGGAGGAGTATGCC
ENSCJAT00000007928     ACACTGCAGAACTTGAAGCCCCGGCAGCGGGGAAAGAAGGAGAGCAAGGAGGAGTATGCC
ENSMMUT00000020918     ACGCTGCAGAACTTGAAGCCCCGGCAGCGGGGAAAGAAGGAGAGCAAGGAGGAGTATGCC
ENSGGOT00000007817     ACGCTGCAGAACTTGAAGCCCCGGCAGCGGGGAAAGAAGGAGAGCAAGGAGGAGTATGCC
ENST00000521871        ACGCTGCAGAACTTGAAGCCCCGGCAGCGGGGAAAGAAGGAGAGCAAGGAGGAGTATGCC
ENSPTRT00000022434     ACGCTGCAGAACTTGAAGCCCCGGCAGCGGGGAAAGAAGGAGAGCAAGGAGGAGTATGCC

XM_003775898.1         CGGAGCACCCGGGGCTGCCTGGAAGCTGGGCTGGAGTCCCCTGCTGAAGGGCAGGGGGGT
ENSRNOT00000043201     CGCAGCACCCGGGGCTGCCTTGAAGCAGGGCTGGAGTCCCTGCTGAAGGCCGCTGGTGGG
ENSMUST00000161078     CGCAGCACCCGGGGCTGCCTTGAAGCAGGGCTGGAGTCCCTGCTGAAGGCAGCTGGTGGG
ENSCJAT00000007928     CGGAGTACCCGGGGCTGCCTGGAAGCTGGGCTGGAGTCCCTGCTGAAGGCAGCTGGGGGG
ENSMMUT00000020918     CGTAGCACCCGGGGCTGCTTGGAAGCTGGGCTGGAGTCCCTGCTGAAGGCAGCTGGGGGG
ENSGGOT00000007817     CGGAGCACCCGGGGCTGCCTGGAAGCTGGGCTGGAGTCCCTGCTGAAGGCAGCTGGGGGG
ENST00000521871        CGGAGCACCCGGGGCTGCCTGGAAGCTGGGCTGGAGTCCCTGCTGAAGGCAGCTGGGGGG
ENSPTRT00000022434     CGGAGCACCCGGGGCTGCCTGGAAGCTGGGCTGGAGTCCCTGCTGAAGGCAGCTGGGGGG

XM_003775898.1         AACCTGCTGATCCTGCGCATCTCCCACTGTCCAAACATCCTCACCGACCGCTCGCTCTGG
ENSRNOT00000043201     AACCTGCTGATCCTGCGGATCTCCCACTGCCCCAACATCCTCACTGACCGGTCACTCTGG
ENSMUST00000161078     AACCTGCTGATCCTGCGAATCTCCCACTGCCCCAACATCCTCACTGACCGGTCCCTCTGG
```

```
ENSCJAT00000007928      AACCTGCTGATCCTGCGCATCTCCCACTGTCCAAACATCCTCACCGACCGCTCGCTCTGG
ENSMMUT00000020918      AACCTGCTGATCCTGCGCATCTCCCACTGTCCAAACATCCTCACCGACCGCTCGCTCTGG
ENSGGOT00000007817      AACCTGCTGATCCTGCGCATCTCCCACTGTCCAAACATCCTCACCGACCGCTCACTCTGG
ENST00000521871         AACCTGCTGATCCTGCGCATCTCCCACTGTCCAAACATCCTCACCGACCGCTCGCTCTGG
ENSPTRT00000022434      AACCTGCTGATCCTGCGCATCTCCCACTGTCCAAACATCCTCACCGATCGCTCGCTCTGG

XM_003775898.1          CTGGCCAGCTGCTACTGCCGTGCCCTGCAGGCTGTCACGTACAGGAGTGCCACAGACCCC
ENSRNOT00000043201      CTGGCCAGCTGCTACTGCCGTGCCCTGCAGGCTGTCACCTACAGGAGTGCCACGGACCCT
ENSMUST00000161078      CTGGCCAGCTGCTACTGCCGTGCCCTGCAGGCTGTCACCTACAGGAGTGCCACGGACCCC
ENSCJAT00000007928      CTGGCCAGCTGCTACTGCCGCGCGCTGCAGGCTGTCACCTACAGGAGTGCCACAGACCCT
ENSMMUT00000020918      CTGGCCAGTTGCTACTGCCGTGCCCTGCAGGCTGTCACCTACAGGAGTGCCACAGACCCT
ENSGGOT00000007817      CTGGCCAGCTGCTACTGCCGTGCCCTGCAGGCTGTCACGTACAGGAGTGCCACAGACCCC
ENST00000521871         CTGGCCAGCTGCTACTGCCGTGCCCTGCAGGCTGTCACGTACAGGAGTGCCACAGACCCC
ENSPTRT00000022434      CTGGCCAGCTGCTACTGCCGTGCCCTGCAGGCTGTCACATACAGGAGTGCCACAGACCCC

XM_003775898.1          GTGGGCCACGAGGTCATTTGGGGCGTGGCGCAGGACTGCAGAGAAGATTCGTCTTTCAAG
ENSRNOT00000043201      GTCGGCCATGAGGTGATCTGGGCCCTGGGCGCAGGCTGCAGAGACATTGTCTCCCTCCAG
ENSMUST00000161078      GTCGGCCATGAGGTGATCTGGGCCCTGGGTGCAGGCTGCAGAGACATTGTCTCCCTCCAG
ENSCJAT00000007928      GTGGGCCACGAGGTCATTTGGGCCCTGGGAGCAGGCTGCAGAGAGATCGTCTCCCTCCAA
ENSMMUT00000020918      GTGGGCCATGAGGTCATTTGGGCCCTGGGCGCAGGCTGCAGAGAGATCGTCTCCCTCCAA
ENSGGOT00000007817      GTGGGCCACGAGGTCATTTGGGCCCTGGGCGCAGGCTGCAGAGAGATCGTCTCCCTCCAA
ENST00000521871         GTGGGCCATGAGGTCATTTGGGCCCTGGGCGCAGGCTGCAGAGAGATCGTCTCCCTCCAA
ENSPTRT00000022434      GTGGGCCACGAGGTCATTTGGGCCCTGGGCGCAGGCTGCAGAGAGATCGTCTCCCTCCAA

XM_003775898.1          TGGGCGCCACTTCACCCCTGCCAGCAGCCCACGCGCTTCAGTAACCGCTGCCTGCAGATG
ENSRNOT00000043201      GTGGCTCCACTTCACCCCTGCCAGCAGCCCACCCGGTTCAGCAACCGTTGCCTGCAGATG
ENSMUST00000161078      GTGGCGCCACTTCACCCCTGCCAGCAGCCCACCCGGTTCAGCAACCGTTGCCTGCAGATG
ENSCJAT00000007928      GTGGCGCCACTTCACCCCTGCCAGCAGCCCACGCGCTTCAGTAACCGCTGCCTGCAGATG
ENSMMUT00000020918      GTGGCACCACTTCACCCCTGCCAGCAGCCCACGCGCTTCAGTAACCGCTGCCTGCAGATG
ENSGGOT00000007817      GTGGCACCACTTCACCCCTGCCAGCAGCCCACACGCTTCAGTAACCGCTGCCTGCAGATG
ENST00000521871         GTGGCACCACTTCACCCCTGCCAGCAGCCCACACGCTTCAGTAACCGCTGCCTGCAGATG
ENSPTRT00000022434      GTGGCACCACTTCACCCCTGCCAGCAGCCCACACGCTTCAGTAACCGCTGCCTGCAGATG

XM_003775898.1          ATTGGTCGCTGTTGGCCCCACCTGCGGGCCCTGGGGGTCGGGGGTGCCGGCTGTGGGGTG
ENSRNOT00000043201      ATTGGACGCTGTTGGCCCCACCTCCGGGCCCTGGGCGTAGGTGGTGCCGGCTGTGGGGTA
ENSMUST00000161078      ATTGGACGCTGTTGGCCCCACCTCCGGGCCCTGGGCGTAGGTGGTGCCGGCTGTGGGGTA
ENSCJAT00000007928      ATTGGTCGCTGTTGGCCCCACCTGCGGGCCCTGGGGGTCGGGGGTGCCGGCTGTGGGGTA
ENSMMUT00000020918      ATTGGTCGCTGTTGGCCCCACCTGCGGGCCCTGGGGGTCGGGGGTGCCGGCTGTGGGGTG
ENSGGOT00000007817      ATTGGTCGCTGTTGGCCCCACCTGCGGGCCCTGGGGGTCGGGGGTGCCGGCTGTGGGGTG
ENST00000521871         ATTGGTCGCTGTTGGCCCCACCTGCGGGCCCTGGGGGTCGGGGGTGCCGGCTGTGGGGTG
ENSPTRT00000022434      ATTGGTCGCTGTTGGCCCCACCTGCGGGCCCTGGGGGTCGGGGGTGCCGGCTGTGGGGTG

XM_003775898.1          CAGGGCCTGGCATCACTCGCGAGAAACTGCATGCGGCTGCAGGTCCTGGAGCTTGACCAT
ENSRNOT00000043201      CAGGGCCTGGCATCACTTGCAAGAAACTGTATGCGGCTGCAGGTCCTGGAACTGGACCAC
ENSMUST00000161078      CAGGGCCTGGCATCACTTGCAAGAAACTGTATGCGGCTGCAGGTCCTGGAGCTGGACCAC
ENSCJAT00000007928      CAGGGCCTGGCATCACTCGCGAGAAACTGCATGCGGCTGCAGGTCCTGGAGCTTGACCAT
ENSMMUT00000020918      CAGGGCCTGGCATCACTTGCGAGAAACTGCATGCGGCTGCAGGTCCTGGAGCTTGACCAT
ENSGGOT00000007817      CAGGGCCTGGCATCACTCGCGAGAAACTGCATGCGGCTGCAGGTCCTGGAGCTTGACCAC
ENST00000521871         CAGGGCCTGGCATCACTCGCGAGAAACTGCATGCGGCTGCAGGTCCTGGAGCTTGACCAC
ENSPTRT00000022434      CAGGGCCTGGCATCACTCGCAAGAAACTGCATGCGGCTGCAGGTCCTGGAGCTTGACCAC

XM_003775898.1          GTGTCAGAGATTACCCAGGAGGTGGCGGCAGAGGTCTGCCGGGAAGGCCTGAAGGGACTG
ENSRNOT00000043201      GTGTCAGAGATTACCCAGGAGGTGGCGGCAGAGGTCTGCCGGGAAGGCCTGAAAGGACTG
ENSMUST00000161078      GTGTCAGAGATTACCCAGGAGGTGGCGGCTGAGGTCTGCCGGGAAGGCCTGAAGGGACTG
```

```
ENSCJAT00000007928     GTGTCAGAGATCACCCAGGAGGTGGCGGCAGAGGTCTGCCGTGAAGGCCTGAAGGGACTG
ENSMMUT00000020918     GTGTCAGAGATCACCCAGGAGGTGGCGGCAGAGGTCTGCCGGGAAGGCCTGAAGGGATTG
ENSGGOT00000007817     GTGTCAGAGATCACCCAGGAGGTGGCAGCAGAGGTCTGCCGGGAAGGCCTGAAGGGACTG
ENST00000521871        GTGTCAGAGATCACCCAGGAGGTGGCAGCAGAGGTCTGCCGGGAAGGCCTGAAGGGACTG
ENSPTRT00000022434     GTGTCAGAGATCACCCAGGAGGTGGCAGCAGAGGTCTGCCGGGAAGGCCTGAAGGGACTG


XM_003775898.1         GAGATGCTGGTGCTCACGGCGACTCCCGTCACCCCTAAGGCCCTACTGCACTTCAACAGC
ENSRNOT00000043201     GAGATGCTGGTGCTCACAGCCACCCCTGTCACCCCTAAGGCCCTGCTACATTTTAACAGC
ENSMUST00000161078     GAGATGCTGGTGCTCACAGCCACCCCTGTCACCCCTAAGGCCCTGCTTCATTTTAACAGC
ENSCJAT00000007928     GAGATGCTGGTGCTCACAGCCACCCCTGTCACCCCTAAGGCCCTGCTGCACTTCAACAGC
ENSMMUT00000020918     GAGATGCTGGTGCTCACAGCGACTCCCGTCACCCCTAAGGCCCTGCTGCATTTCAACAGC
ENSGGOT00000007817     GAGATGCTGGTGCTCACAGCGACTCCCGTCACCCCTAAGGCCCTACTGCACTTCAACAGC
ENST00000521871        GAGATGCTGGTGCTCACGGCGACTCCCGTCACCCCTAAGGCCCTACTGCACTTCAACAGC
ENSPTRT00000022434     GAGATGCTGGTGCTCACGGCGACTCCCGTCACCCCTAAGGCCCTACTGCACTTCAACAGC


XM_003775898.1         ATCTGCCGGAACCTCAAGTCCATTGGTTCCAGATTGGGATTGGCGGATTATTTCAAAGAG
ENSRNOT00000043201     ATCTGCCGGAACCTCAAATCCATTGTGGTGCAGATTGGGATTGCAGATTACTTCAAAGAG
ENSMUST00000161078     ATCTGCCGGAACCTTAAATCCATTGTGGTGCAGATTGGGATTGCAGATTACTTCAAAGAG
ENSCJAT00000007928     ATCTGTCGGAACCTCAAGTCCATTGTGGTCCAGATTGGGATTGCAGATTATTTCAAAGAA
ENSMMUT00000020918     ATCTGCCGGAACCTCAAGTCGATTGTGGTCCAGATTGGGATTGCGGATTATTTCAAAGAG
ENSGGOT00000007817     ATCTGCCGGAACCTCAAGTCCATTGTGGTCCAGATTGGGATTGCAGATTATTTCAAAGAG
ENST00000521871        ATCTGCCGGAACCTCAAGTCCATTGTGGTCCAGATTGGGATTGCGGATTATTTCAAAGAG
ENSPTRT00000022434     ATCTGCCGGAACCTCAAGTCCATTGTGGTCCAGATTGGGATTGCGGATTATTTCAAAGAG


XM_003775898.1         CCCAGCAGCCCTGAGGCCCAGAAGCTGTTTGAGGACATGGTGACAAAACTCCAGGCT---
ENSRNOT00000043201     CCCAGCAGTCCCGAGGCCCAAAAGCTGTTTGAGGATATGGTGACAAAACTCCAG------
ENSMUST00000161078     CCCAGCAGTCCCGAGGCCCAGAAGCTGTTTGAGGACATGGTGACAAAACTCCAGGCCCTG
ENSCJAT00000007928     CCCAGCAGCCCTGAGGCCCAGAAGCTGTTTGAAGACATGGTGACAAAACTCCAGGCCCTA
ENSMMUT00000020918     CCCAGCAGCCCTGAGGCCCAAAAGCTGTTTGAGGACATGGTGACAAAACTCCAGGCCCTG
ENSGGOT00000007817     CCCAGCAGCCCTGAGGCCCAGAAGCTGTTTGAGGACATGGTGACAAAACTCCAGGCTCTG
ENST00000521871        CCCAGCAGCCCTGAGGCCCAGAAGCTGTTTGAGGACATGGTGACAAAACTCCAGGCTCTG
ENSPTRT00000022434     CCCAGCAGCCCTGAGGCCCAGAAGCTGTTTGAGGACATGGTGACAAAACTCCAGGCTCTG


XM_003775898.1         CGTGCGACGGAGGGCTTCTCTAAGATTCTGCACATCAAGGTGGAAGGCCCCTGC
ENSRNOT00000043201     ------------------------------------------------------
ENSMUST00000161078     CGTCGGAGGCCTGGCTTCTCGAAGATTCTGCACATCAAGGTAGAAGGTGGTTGC
ENSCJAT00000007928     CGACGAAGGCCCGGCTTCTCTAAAATTCTGCACATCAAGGTGGAAGGCGGCTGC
ENSMMUT00000020918     CGACGGAGGCCCGGCTTCTCTAAGATTCTGCACATCAAGGTGGAAGGCGGCTGC
ENSGGOT00000007817     CGACGGAGGCCCGGCTTCTCTAAGATTCTGCACATCAAGGTGGAAGGCGGCTGC
ENST00000521871        CGACGGAGGCCCGGCTTCTCTAAGATTCTGCACATCAAGGTGGAAGGCGGCTGC
ENSPTRT00000022434     CGACGGAGGCCCGGCTTCTCTAAGATTCTGCACATCAAGGTGGAAGGCGGCTGC
```

Multiple sequence alignment of Fbxo42

```
ENSCJAT00000016175     ATGGCCAGCTCCTCGGACAGTGAAGATGACAGTTTCATGGCTGTGGACCAAGAAGAAACT
ENSMMUT00000017856     ATGGCCAGCTCCTCAGACAGTGAAGATGACAGTTTCATGGCTGTGGACCAGGAAGAAACT
ENSPPYT00000002179     ATGGCCAGCTCCTCGGACAGTGAAGATGACAGTTTCATGGCTGTGGACCAGGAAGAAACT
ENSGGOT00000014643     ATGGCCAGCTCCTCGGACAGTGAAGATGACAGTTTCATGGCTGTGGACCAGGAAGAAACT
ENST00000375592        ATGGCCAGCTCCTCGGACAGTGAAGATGACAGTTTCATGGCTGTGGACCAGGAAGAAACT
ENSPTRT00000000465     ATGGCCAGCTCCTCGGACAGTGAAGATGACAGTTTCATGGCTGTGGACCAAGAAGAAACT
ENSMUST00000030757     ATGGCCAGCTCCTCGGACAGTGAAGATGACAGTGTCATGGCCGTGGACCAAGAAGAAACT
ENSRNOT00000019195     ATGGCCAGCTCCTCAGACAGTGAAGATGACAGTTTCATGGCCGTGGACCAAGAAGAAACT


ENSCJAT00000016175     GTGTTGGAAGGGACAATGGAGCAAGATGAGGAGCCCCACCTAGCATTGGAGGCTGAGGAG
```

```
ENSMMUT00000017856     GTGCTGGAAGGAACAATGGAGCAAGATGAGGAGCCCCACCCAGTATTGGAGGCTGACGAG
ENSPPYT00000002179     GTGCTGGAAGGGACAATGGAGCAAGATGAGGAGCCCCACCCAGTATTGGAGGCTGAGGAG
ENSGGOT00000014643     GTGCTGGAAGGGACAATGGATCAAGATGAGGAGCCCCACCCACTATTGGAGGCTGAGGAG
ENST00000375592        GTGCTGGAAGGGACAATGGATCAAGATGAGGAGCCCCACCCAGTATTGGAGGCTGAGGAG
ENSPTRT00000000465     GTGCTGGAAGGGACAATGGATCAAGATGAGGAGCCCCACCCAGTATTGGAGGCTGAGGAG
ENSMUST00000030757     GCACTGGAAGGGACAATGGAGCAAGATGAGGACCCCCACCCAGTGTTGGAGGTTGAGGAA
ENSRNOT00000019195     GCACTGGAAGGGACAATGGAGCAAGATGAGGACCCACACCCAGTATTGGAGGTTGAGGAA

ENSCJAT00000016175     ACTCGACATAATAGGTCCATGTTGGAGCTGCCAGAAGAGGTTTTGGAGTATATCCTGTCC
ENSMMUT00000017856     ACTAGACATAATAGGTCCATGTCGGAGCTGCCAGAAGAGGTTTTGGAGTATATCCTGTCC
ENSPPYT00000002179     ACTAGACATAATAGGTCCATGTCGGAGCTGCCAGAAGAGGTTTTGGAGTATATCCTGTCC
ENSGGOT00000014643     ACTAGACATAATAGGTCCATGTCGGAGCTGCCAGAAGAGGTTTTGGAGTATATCCTGTCC
ENST00000375592        ACTAGACATAATAGGTCCATGTCGGAGCTGCCAGAAGAGGTTTTGGAGTATATCCTGTCC
ENSPTRT00000000465     ACTAGACATAATAGGTCCATGTCGGAGCTGCCAGAAGAGGTTTTGGAGTATATCCTGTCC
ENSMUST00000030757     ACAAGACATAATAGGTCCATGTCTGAGCTGCCGGAAGAGGTTTTGGAGTATATTCTGTCC
ENSRNOT00000019195     ACAAGACATAATAGGTCCATGTCTGAGCTGCCAGAAGAGGTTTTGGAGTATATCCTGTCC

ENSCJAT00000016175     TTTCTCTCACCGTATCAGGAACACAAAACTGCAGCCCTTGTCTGCAAACAGTGGTATCGA
ENSMMUT00000017856     TTTCTGTCACCATATCAGGAACACAAAACTGCGGCCCTTGTCTGCAAACAGTGGTATCGA
ENSPPYT00000002179     TTTCTCTCACCGTATCAGGAACACAAAACTGCGGCCCTTGTCTGCAAACAGTGGTATCGA
ENSGGOT00000014643     TTTCTCTCACCCTATCAGGAACACAAAACTGCGGCCCTTGTCTGCAAACAGTGGTATCGA
ENST00000375592        TTTCTCTCACCGTATCAGGAACACAAAACTGCGGCCCTTGTCTGCAAACAGTGGTATCGA
ENSPTRT00000000465     TTTCTCTCACCGTATCAGGAACACAAAACTGCGGCCCTTGTCTGCAAACAGTGGTATCGA
ENSMUST00000030757     TTCCTTTCACCATACCAGGAACACAAGACTGCAGCTCTGGTCTGCAAACAGTGGTATCGA
ENSRNOT00000019195     TTCCTCTCACCATATCAGGAACACAAAACCGCAGCTCTGGTCTGCAAGCAGTGGTATCGG

ENSCJAT00000016175     CTCATCAAAGGTGTAGCTCATCAGTGTTATCACGGTTTCATGAAGGCTGTCCAGGAAGGA
ENSMMUT00000017856     CTTATCAAAGGTGTAGCCCATCAATGTTATCATGGTTTCATGAAGGCTGTCCAGGAAGGA
ENSPPYT00000002179     CTTATCAAAGGTGTAGCCCATCAGTGTTATCATGGTTTCATGAAGGCTGTCCAGGAAGGA
ENSGGOT00000014643     CTTATCAAAGGTGTAGCCCATCAGTGTTATCATGGTTTCATGAAGGCTGTCCAGGAAGGA
ENST00000375592        CTTATCAAAGGTGTAGCCCATCAGTGTTATCATGGTTTCATGAAGGCTGTCCAGGAAGGA
ENSPTRT00000000465     CTTATCAAAGGTGTAGCCCATCAGTGTTATCATGGTTTCATGAAGGCTGTCCAGGAAGGA
ENSMUST00000030757     CTTATCAAAGGTGTGGCCCACCAGTGTTACCATGGCTTCATGAAGGCTGTCCAGGAAGGA
ENSRNOT00000019195     CTTATCAAAGGTGTAGCCCACCAGTGTTACCATGGTTTCATGAAGGCTGTCCAGGAAGGA

ENSCJAT00000016175     AACATTCAGTGGGAGAGCCGTACTTACCCTTATCCTGGAACTCCAATCACTCAGCGGTTC
ENSMMUT00000017856     AACATTCAGTGGGAGAGCCGTACCTATCCTTATCCTGGAACCCCAATCACTCAGCGCTTC
ENSPPYT00000002179     AACATTCAGTGGGAGAGCCGTACCTATCCTTATCCTGGAACCCCAATCACTCAGCGCTTC
ENSGGOT00000014643     AACATTCAGTGGGAGAGCCGTACCTATCCTTATCCTGGAACCCCAATCACTCAGCGCTTC
ENST00000375592        AACATTCAGTGGGAGAGCCGTACCTATCCTTATCCTGGAACCCCAATCACTCAGCGCTTC
ENSPTRT00000000465     AACATTCAGTGGGAGAGCCGTACCTATCCTTATCCTGGAACCCCAATCACTCAGCGCTTC
ENSMUST00000030757     AACATTCAGTGGGAGAGCCGGACTTACCCTTATCCGGGAACCCCTATCACTCAGCGCTTC
ENSRNOT00000019195     AACATTCAGTGGGAGAGCCGAACTTACCCTTATCCCGGAACCCCCATCACTCAGCGCTTC

ENSCJAT00000016175     TCACACAGCGCATGCTATTATGATGCTAATCAGTCTATGTACGTGTTTGGAGGCTGTACC
ENSMMUT00000017856     TCGCACAGCGCATGCTATTATGATGCTAATCAGTCTATGTATGTGTTTGGAGGCTGTACC
ENSPPYT00000002179     TCGCACAGTGCATGCTATTATGATGCTAATCAGTCTATGTATGTGTTTGGAGGCTGTACC
ENSGGOT00000014643     TCGCACAGTGCATGCTATTACGATGCTAATCAATCTATGTATGTGTTTGGAGGCTGTACC
ENST00000375592        TCGCACAGTGCATGCTATTATGATGCTAATCAGTCTATGTATGTGTTTGGAGGCTGTACC
ENSPTRT00000000465     TCGCACAGTGCATGCTATTATGATGCTAATCAGTCTATGTATGTGTTTGGAGGCTGTACC
ENSMUST00000030757     TCCCACAGTGCGTGCTATTATGATGCCAATCAGTCTATGTATGTCTTTGGTGGCTGTACG
ENSRNOT00000019195     TCCCACAGTGCATGCTATTACGATGCCAATCAGTCTATGTATGTCTTTGGAGGCTGTACC

ENSCJAT00000016175     CAGAGCAGCTGCAATGCTGCTTTCAATGACCTCTGGAGACTTGACCTAAACAGCAAAGAG
```

```
ENSMMUT00000017856     CAGAGCAGCTGCAATGCTGCTTTCAATGACCTCTGGAGACTTGACCTAAACAGCAAAGAG
ENSPPYT00000002179     CAGAGCAGCTGCAATGCTGCTTTCAATGACCTCTGGAGACTTGACCTAAACAGCAAAGAG
ENSGGOT00000014643     CAGAGCAGCTGCAATGCTGCTTTCAATGACCTCTGGAGACTTGACCTAAACAGCAAAGAG
ENST00000375592        CAGAGCAGCTGCAATGCTGCTTTCAATGACCTCTGGAGACTTGACCTAAACAGCAAAGAG
ENSPTRT00000000465     CAGAGCAGCTGCAATGCTGCTTTCAATGACCTCTGGAGACTTGACCTAAACAGCAAAGAG
ENSMUST00000030757     CAGAGCAGTTGCAATGCTGCCTTCAATGACCTCTGGAGGCTAGACCTAAATAGCAAGGAG
ENSRNOT00000019195     CAGAGCAGCTGCAATGCTGCCTTCAATGACCTCTGGAGGCTCGACCTAAATAGCAAAGAG

ENSCJAT00000016175     TGGATCCGACCTTTGGCTTCAGGCTCCTATCCTTCCCCCAAAGCTGGTGCAACTCTGGTC
ENSMMUT00000017856     TGGATCCGACCTTTGGCTTCAGGGTCCTATCCTTCCCCCAAAGCTGGAGCAACTCTGGTC
ENSPPYT00000002179     TGGATCCGACCTTTGGCTTCAGGGTCCTATCCTTCCCCCAAAGCTGGAGCAACTCTCGTC
ENSGGOT00000014643     TGGATCCGACCTTTGGCTTCAGGGTCCTATCCTTCCCCCAAAGCTGGAGCAACTCTGGTC
ENST00000375592        TGGATCCGACCTTTGGCTTCAGGGTCCTATCCTTCCCCCAAAGCTGGAGCAACTCTGGTC
ENSPTRT00000000465     TGGATCCGACCTTTGGCTTCAGGGTCCTATCCTTCCCCCAAAGCTGGAGCAACTCTGGTC
ENSMUST00000030757     TGGATTCGACCATTGGCCTCAGGGTCCTATCCTTCCCCCAAAGCTGGGGCCACTCTGGTC
ENSRNOT00000019195     TGGATTCGACCGTTGGCCTCAGGGTCCTATCCTTCCCCCAAAGCTGGGGCCACTCTGGTC

ENSCJAT00000016175     GTGTACAAGGACTTGCTAGTGCTGTTTGGTGGCTGGACGCGGCCAAGCCCTTATCCCCTA
ENSMMUT00000017856     GTGTATAAGGACTTGCTAGTGCTGTTTGGTGGCTGGACGCGGCCAAGCCCTTATCCGCTA
ENSPPYT00000002179     GTGTACAAGGACTTGCTAGTGCTGTTTGGTGGCTGGACGCGGCCAAGCCCTTATCCCCTA
ENSGGOT00000014643     GTGTACAAGGACTTGCTAGTGCTGTTTGGTGGCTGGACGCGACCAAGCCCTTATCCCCTA
ENST00000375592        GTGTACAAGGACTTGCTAGTGCTGTTTGGTGGCTGGACGCGGCCAAGCCCTTATCCCCTA
ENSPTRT00000000465     GTGTACAAGGACTTGCTAGTGCTGTTTGGTGGCTGGACGCGGCCAAGCCCTTATCCCCTA
ENSMUST00000030757     GTGTACAAGGACTTGCTAGTGCTATTTGGTGGCTGGACAAGGCCAAGTCCCTATCCCTTA
ENSRNOT00000019195     GTGTACAAGGACTTGCTAGTGCTATTTGGTGGCTGGACAAGGCCAAGTCCCTATCCCTTA

ENSCJAT00000016175     CATCAGCCAGAGAGATTCTTTGATGAAATACACACTTACTCCCCCTCTAAAAACTGGTGG
ENSMMUT00000017856     CACCAGCCAGAGAGATTCTTTGATGAAATACACACTTACTCACCCTCTAAAAATTGGTGG
ENSPPYT00000002179     CACCAGCCAGAGAGATTCTTTGATGAAATACACACTTACTCACCCTCTAAAAATTGGTGG
ENSGGOT00000014643     CACCAGCCAGAGAGATTCTTTGATGAAATACACACTTACTCACCCTCTAAAAATTGGTGG
ENST00000375592        CACCAGCCAGAGAGATTCTTTGATGAAATACACACTTACTCACCCTCTAAAAATTGGTGG
ENSPTRT00000000465     CACCAGCCAGAGAGATTCTTTGATGAAATACACACTTACTCACCCTCTAAAAATTGGTGG
ENSMUST00000030757     CACCAGCCAGAGAGATTCTTTGATGAAATTCACACGTACTCGCCGTCTAAAAACTGGTGG
ENSRNOT00000019195     CACCAACCAGAGAGATTCTTTGATGAAATTCACACATACTCACCATCTAAAAACTGGTGG

ENSCJAT00000016175     AACTGCATTGTGACAACCCATGGGCCACCTCCCATGGCTGGCCACTCCTCCTGTGTGATA
ENSMMUT00000017856     AACTGCATTGTGACAACCCATGGGCCACCTCCCATGGCTGGCCACTCCTCCTGTGTAATA
ENSPPYT00000002179     AACTGCATTGTGACAACCCATGGGCCACCTCCCATGGCTGGCCACTCCTCCTGTGTGATA
ENSGGOT00000014643     AACTGCATTGTGACAACCCATGGGCCACCTCCCATGGCTGGCCACTCCTCCTGTGTGATA
ENST00000375592        AACTGCATTGTGACAACCCATGGGCCACCTCCCATGGCTGGCCACTCCTCCTGTGTGATA
ENSPTRT00000000465     AACTGCATTGTGACAACCCATGGGCCACCTCCCATGGCTGGCCACTCCTCCTGTGTGATA
ENSMUST00000030757     AACTGCATTGTCACCACGCATGGGCCTCCTCCCATGGCTGGCCATTCCTCCTGTGTGATA
ENSRNOT00000019195     AACTGCATTGTCACCACACACGGGCCTCCTCCTATGGCTGGCCATTCCTCCTGTGTGATA

ENSCJAT00000016175     GATGATAAAATGATTGTCTTTGGTGGCTCTTTAGGATCCCGGCAAATGAGCAATGATGTC
ENSMMUT00000017856     GATGATAAAATGATTGTCTTTGGTGGCTCTTTAGGATCCCGGCAAATGAGCAATGATGTC
ENSPPYT00000002179     GATGATAAAATGATTGTCTTTGGTGGCTCTTTAGGATCCCGGCAAATGAGCAATGATGTC
ENSGGOT00000014643     GATGATAAAATGATTGTCTTTGGTGGCTCTTTAGGATCCCGGCAAATGAGCAATGATGTC
ENST00000375592        GATGATAAAATGATTGTCTTTGGTGGCTCTTTAGGATCCCGGCAAATGAGCAATGATGTC
ENSPTRT00000000465     GATGATAAAATGATTGTCTTTGGTGGCTCTTTAGGATCCCGGCAAATGAGCAATGATGTC
ENSMUST00000030757     GGTGATAAAATGATTGTCTTCGGTGGCTCCTTAGGATCCCGGCAGATGAGCAATGAAGTC
ENSRNOT00000019195     GGTGACAAAATGATTGTCTTCGGTGGCTCCTTAGGATCCCGGCAGATGAGCAATGAAGTC

ENSCJAT00000016175     TGGGTCCTTGACCTTGAGCAGTGGGCGTGGTCCAAGCCGAACATCTCCGGCCCCAGTCCT
```

ENSMMUT00000017856 TGGGTCCTTGACCTCGAGCAGTGGGCGTGGTCCAAGCCGAACATCTCCGGCCCCAGTCCT
ENSPPYT00000002179 TGGGTCCTTGACCTTGAGCAGTGGGCGTGGTCCAAGCCGAACATCTCCGGCCCCAGTCCT
ENSGGOT00000014643 TGGGTCCTTGACCTTGAGCAGTGGGCGTGGTCCAAGCCGAACATCTCCGGCCCCAGTCCT
ENST00000375592 TGGGTCCTTGACCTTGAGCAGTGGGCGTGGTCCAAGCCGAACATCTCTGGCCCCAGTCCT
ENSPTRT00000000465 TGGGTCCTTGACCTTGAGCAGTGGGCGTGGTCCAAGCCGAACATCTCCGGCCCCAGTCCT
ENSMUST00000030757 TGGGTCCTGGACCTGGAGCAGTGGGCGTGGTCCAAGCCGAACATCTCTGGCCCCAGTCCA
ENSRNOT00000019195 TGGGTCCTTGACCTGGAGCAGTGGGCGTGGTCCAAGCCGAACATCTCTGGCCCCAGCCCT

ENSCJAT00000016175 CATCCTCGAGGTGGCCAATCTCAGATTGTCATAGATGATGCAACTATCTTAATCCTCGGA
ENSMMUT00000017856 CATCCTCGAGGTGGCCAATCTCAGATTGTCATAGATGATGCAACTATCTTAATCCTCGGA
ENSPPYT00000002179 CATCCTCGAGGTGGCCAATCTCAGATTGTCATAGATGATGCAACTATCTTAATCCTCGGA
ENSGGOT00000014643 CATCCTCGAGGTGGCCAATCTCAGATTGTCATAGATGATGCAACTATCTTAATCCTCGGA
ENST00000375592 CATCCTCGAGGTGGCCAATCTCAGATTGTCATAGATGATGCAACTATCTTAATCCTCGGA
ENSPTRT00000000465 CATCCTCGAGGTGGCCAATCTCAGATTGTCATAGATGATGCAACTATCTTAATCCTCGGA
ENSMUST00000030757 CACCCTCGAGGTGGCCAGTCTCAGATTGTTATAGATGATACAACTCTTCTGATCCTTGGC
ENSRNOT00000019195 CACCCCCGAGGTGGCCAGTCTCAGATTGTTATAGATGATGCAACCATCCTGATCCTTGGT

ENSCJAT00000016175 GGGTGTGGTGGTCCCAATGCTTTGTTCAAGGATGCTTGGTTGTTGCACATGCATTCTGGT
ENSMMUT00000017856 GGGTGTGGCGGTCCCAATGCTCTGTTCAAGGATGCTTGGTTGTTGCACATGCATTCTGGT
ENSPPYT00000002179 GGGTGTGGCGGTCCCAATGCTCTATTCAAGGATGCTTGGTTGTTGCACATGCATTCTGGT
ENSGGOT00000014643 GGGTGTGGCGGTCCCAATGCTCTATTCAAGGATGCTTGGTTGTTGCACATGCATTCTGGT
ENST00000375592 GGGTGTGGCGGTCCCAATGCTCTATTCAAGGATGCTTGGTTGTTGCACATGCATTCTGGT
ENSPTRT00000000465 GGGTGTGGCGGTCCCAATGCTCTATTCAAGGATGCTTGGTTGTTGCACATGCATTCTGGT
ENSMUST00000030757 GGGTGTGGCGGTCCCAATGCGCTGTTCAAAGATGCCTGGCTGCTGCACATGCACCCAGGT
ENSRNOT00000019195 GGCTGTGGCGGTCCCAACGCTCTGTTCAAAGATGCCTGGCTGCTGCACATGCACCCGGGT

ENSCJAT00000016175 CCTTGGGCCTGGCAGCCACTCAAGGTAGAAAATGAAGAGCATGGGGCCCCAGAACTGTGG
ENSMMUT00000017856 CCTTGGGCCTGGCAGCCACTCAAGGTAGAAAATGAAGAGCATGGGGCCCCAGAACTGTGG
ENSPPYT00000002179 CCTTGGGCCTGGCAGCCACTCAAGGTAGAAAATGAAGAGCATGGGGCCCCAGAACTGTGG
ENSGGOT00000014643 CCTTGGGCCTGGCAGCCACTCAAGGTAGAAAATGAAGAGCATGGGGCCCCAGAACTGTGG
ENST00000375592 CCTTGGGCCTGGCAGCCACTCAAGGTAGAAAATGAAGAGCATGGGGCCCCAGAACTGTGG
ENSPTRT00000000465 CCTTGGGCCTGGCAGCCACTCAAGGTAGAAAATGAAGAGCATGGGGCCCCAGAACTGTGG
ENSMUST00000030757 CCCTGGGCCTGGCAGCCACTCAAGGTAGAAAATGAAGACCATGGTGCCCCGGAGCTATGG
ENSRNOT00000019195 CCCTGGGCCTGGCAGCCACTTAAGGTAGAAAACGAAGACCATGGTGCCCCGGAACTATGG

ENSCJAT00000016175 TGTCATCCAGCTTGCCGGGTGGGACAGTGTGTGGTGGTCTTCAGCCAGGCTCCTAGTGGG
ENSMMUT00000017856 TGTCATCCAGCATGCCGGGTGGGACAGTGTGTGGTGGTCTTCAGCCAGGCTCCTAGTGGG
ENSPPYT00000002179 TGCCATCCAGCTTGCCGGGTGGGACAGTGTGTGGTGGTCTTCAGCCAGGCTCCTAGTGGG
ENSGGOT00000014643 TGCCATCCAGCTTGCCGGGTGGGACAGTGTGTGGTGGTCTTCAGCCAGGCTCCTAGTGGG
ENST00000375592 TGCCATCCAGCTTGCCGGGTGGGACAGTGTGTGGTGGTCTTCAGCCAGGCTCCTAGTGGG
ENSPTRT00000000465 TGCCATCCAGCTTGCCGGGTGGGACAGTGTGTGGTGGTCTTCAGCCAGGCTCCTAGTGGG
ENSMUST00000030757 TGTCATCCAGCCTGCCGGGTGGGACAGTGTGTGGTGGTCTTCAGCCAGGCGCCAAGCGGG
ENSRNOT00000019195 TGTCATCCAGCCTGCCGGGTGGGACAGTGTGTGGTGGTCTTCAGCCAGGCGCCCAGCGGG

ENSCJAT00000016175 AGAGCCCCACTCAGCCCCAGTTTGAACTCTCGCCCATCACCTATCAGTGCCACTCCTCCA
ENSMMUT00000017856 AGAGCCCCACTCAGCCCCAGTTTGAACTCTCGCCCATCACCTATCAGTGCCACTCCTCCA
ENSPPYT00000002179 AGAGCCCCACTCAGCCCCAGTTTGAACTCCCGCCCATCACCTATCAGTGCCACTCCTCCA
ENSGGOT00000014643 AGAGCCCCACTCAGCCCCAGTTTGAACTCTCGCCCATCACCTATCAGTGCCACTCCTCCA
ENST00000375592 AGAGCCCCACTCAGCCCCAGTTTGAACTCTCGCCCATCACCTATCAGTGCCACTCCTCCA
ENSPTRT00000000465 AGAGCCCCACTCAGCCCCAGTTTGAACTCTCGCCCATCACCTATCAGTGCCACTCCTCCA
ENSMUST00000030757 AGAGCGCCCCTCAGCCCCAGTTTGAACTCTCGCCCGTCACCTATCAGTGCCACTCCTCCA
ENSRNOT00000019195 AGAGCGCCCCTCAGCCCCAGTTTGAACTCTCGCCCATCACCTATCAGTGCCACTCCTCCA

ENSCJAT00000016175 GCTCTAGTTCCCGAAACCCGCGAGTACCGCTCTCAGTCTCCAGTAAGAAGCATGGATGAA

ENSMMUT00000017856 GCTCTCGTTCCTGAAACCCGAGAGTACCGCTCTCAGTCTCCAGTAAGAAGCATGGATGAA
ENSPPYT00000002179 GCTCTCGTTCCTGAAACCCGAGAGTACCGCTCTCAGTCTCCAGTAAGAAGCATGGATGAA
ENSGGOT00000014643 GCTCTCGTTCCTGAAACCCGAGAGTACCGCTCTCAGTCTCCAGTAAGAAGCATGGATGAA
ENST00000375592 GCTCTCGTTCCTGAAACCCGAGAGTACCGCTCTCAGTCTCCAGTAAGAAGCATGGATGAA
ENSPTRT00000000465 GCTCTCGTTCCTGAAACCCGAGAGTACCGCTCTCAGTCTCCAGTAAGAAGCATGGATGAA
ENSMUST00000030757 GCCCTGGTTCCTGAGACCCGAGAGTACCGCTCCCAGTCCCCGGTGAGGAGTATGGACGAA
ENSRNOT00000019195 GCCCTGGTTCCTGAGACCCGAGAGTACCGCTCCCAGTCCCCAGTGAGGAGTATGGATGAA

ENSCJAT00000016175 GCTCCTTGTGTTAATGGCCGCTGGGGAACACTGAGACCCAGGGCTCAAAGGCAGACTCCT
ENSMMUT00000017856 GCTCCTTGTGTTAACGGCCGCTGGGGAACACTGAGACCCAGGGCTCAAAGGCAGACTCCT
ENSPPYT00000002179 GCTCCTTGTGTTAACGGCCGCTGGGGAACACTGAGACCCAGGGCTCAACGGCAGACTCCT
ENSGGOT00000014643 GCTCCTTGTGTTAACGGCCGCTGGGGAACACTGAGACCCAGGGCTCAAAGGCAGACTCCT
ENST00000375592 GCTCCTTGTGTTAACGGCCGCTGGGGAACACTGAGACCCAGGGCTCAAAGGCAGACTCCT
ENSPTRT00000000465 GCTCCTTGTGTTAACGGCCGCTGGGGAACACTGAGACCCAGGGCTCAAAGGCAGACTCCT
ENSMUST00000030757 GCGCCCTGTGTTAATGGCCGCTGGGGAACACTGAGGCCCAGGGCTCAAAGGCAGACTCCC
ENSRNOT00000019195 GCTCCTTGTGTTAATGGCCGCTGGGGAACACTGAGGCCCAGGGCTCAAAGGCAGACTCCC

ENSCJAT00000016175 TCAGGTTCCCGGGAAGGGAGCCTTTCCCCAGCCAGAGGAGATGGCTCTCCTATCCTCAAT
ENSMMUT00000017856 TCGGGTTCCCGGGAAGGGAGCCTTTCCCCAGCCAGAGGAGATGGCTCTCCTATCCTCAAT
ENSPPYT00000002179 TCAGGTTCCCGGGAAGGGAGCCTTTCCCCAGCCAGAGGAGACGGCTCTCCTATCCTCAAT
ENSGGOT00000014643 TCAGGTTCCCGGGAAGGGAGCCTTTCCCCAGCCAGAGGAGACGGCTCTCCTATCCTCAAT
ENST00000375592 TCAGGTTCCCGGGAAGGGAGCCTTTCCCCAGCCAGAGGAGACGGCTCTCCTATCCTCAAT
ENSPTRT00000000465 TCAGGTTCCCGGGAAGGGAGCCTTTCCCCAGCCAGAGGAGACGGCTCTCCTATCCTCAAT
ENSMUST00000030757 TCGGGTTCCCGGGAAGGGAGCCTCTCCCCAGCCAGAGGAGATGGCTCTCCCATTCTCAAT
ENSRNOT00000019195 TCAGGTTCCCGGGAAGGGAGCCTCTCCCCAGCCAGAGGAGATGGCTCTCCCATCCTCAAT

ENSCJAT00000016175 GGTGGGAGTTTGTCTCCAGGAACAGCAGCTGTGGGTGGCTCTTCTTTGGACAGTCCTGTA
ENSMMUT00000017856 GGTGGGAGTTTGTCTCCAGGAACGGCAGCTGTGGGTGGCTCTTCTTTGGACAGTCCTGTA
ENSPPYT00000002179 GGTGGGAGTTTGTCTCCAGGAACGGCAGCTGTGGGTGGCTCTTCTTTGGACAGTCCTGTA
ENSGGOT00000014643 GGTGGGAGTTTGTCTCCAGGAACGGCAGCTGTGGGTGGCTCTTCTTTGGACAGTCCTGTA
ENST00000375592 GGTGGGAGTTTGTCTCCAGGAACGGCAGCTGTGGGTGGCTCTTCTTTGGACAGTCCTGTA
ENSPTRT00000000465 GGTGGGAGTTTGTCTCCAGGAACGGCAGCTGTGGGTGGCTCTTCTTTGGACAGTCCTGTA
ENSMUST00000030757 GGTGGAAATTTGTCTCCAGGGACAGTAGCTGTCGGGGGTGCTTCCTTAGACAGCCCCGTA
ENSRNOT00000019195 GGTGGAAATTTGTCTCCAGGGACAGCAGCTGTCGGGGGTGCTTCCTTAGACAGCCCTGTG

ENSCJAT00000016175 CAGGCAGTATCTCCAAGTACTCCGTCTGCTGCTGAAGGATGTGACCTAAAAATGGGACTT
ENSMMUT00000017856 CAGGCCATATCTCCAAGTACTCCATCTGCTGCTGAAGGATATGACCTAAAAATGGGACTT
ENSPPYT00000002179 CAGGCCATATCTCCAAGTACTCCATCTGCTGCTGAAGGATACGACCTAAAAATAGGACTT
ENSGGOT00000014643 CAGGCCATATCTCCAAGTACTCCATCTGCTGCTGAAGGATACGACCTGAAAATAGGACTT
ENST00000375592 CAGGCCATATCTCCAAGTACTCCATCTGCTCCTGAAGGATACGACCTGAAAATAGGACTT
ENSPTRT00000000465 CAGGCCATATCTCCAAGTACTCCATCTGCTGCTGAAGGATACGACCTGAAAATAGGACTT
ENSMUST00000030757 CAGGTCGTGTCTCCAAGCACTCCATCTGCCTCTGACGGCTACGACCTAAAAGTAGGACTT
ENSRNOT00000019195 CAGGTGGTATCTCCAAGCACTCCATCTGCCTCTGACGGGTATGACCTAAAAGTAGGGCTT

ENSCJAT00000016175 TCTTTGGCCCCCCGACGAGGATCGCTACCAGATCAGAAAGATCTGCGATTAGGATCCATA
ENSMMUT00000017856 TCTTTGGCCCCCCGACGAGGATCACTACCAGATCAGAAAGATCTGAGATTAGGATCCATA
ENSPPYT00000002179 TCTTTGGCCCCCCGACGAGGATCACTACCAGATCAGAAAGATCTGAGATTAGGATCCGTA
ENSGGOT00000014643 TCTTTGGCCCCCCGACGAGGATCATTACCAGATCAGAAAGATCTGAGATTAGGATCCATA
ENST00000375592 TCTTTGGCCCCCCGACGAGGATCACTACCAGATCAGAAAGATCTGAGATTAGGATCCATA
ENSPTRT00000000465 TCTTTGGCCCCCCGACGAGGATCACTACCAGATCAGAAAGATCTGAGATTAGGATCCATA
ENSMUST00000030757 TCTCTAGCTCCTCGACGAGGGTCTTTACCAGATCAGAAGGACCTGAGGTTAAGTTCCATT
ENSRNOT00000019195 TCTCTAGTCCCCCGGCGAGGGTCTTTACCAGATCAGAAGGACCTGAGGTTAAGCTCTATT

ENSCJAT00000016175 GATCTGAATTGGGATCTGAAACCTGCTTCCGGTAGCAATCCTATGGATAGCATGGACAAC

```
ENSMMUT00000017856     GATCTGAATTGGGATCTGAAATCTGCTTCCAGTAGCAATCCCATGGATGGCATGGACAAT
ENSPPYT00000002179     GATCTGAATTGGGATCTGAAACCCGCTTCCAGTAGCAATCCCATGGATGGCATGGACAAT
ENSGGOT00000014643     GATCTGAATTGGGATCTGAAACCCGCTTCCAGTAGCAATCCCATGGATGGCATGGACAAT
ENST00000375592        GATCTGAATTGGGATCTGAAACCCGCTTCCAGTAGTAATCCCATGGATGGCATGGACAAT
ENSPTRT00000000465     GATCTGAATTGGGATCTGAAACCCGCTTCCAGTAGTAATCCCATGGATGGCATGGACAAT
ENSMUST00000030757     GATCTGAATTGGGACCTGAAGTCTGCTTCCAGTAGTAGTCACGTGGATAGTATAGACAAC
ENSRNOT00000019195     GATGTGAATTGGGACCTGAAGTCTGCATCCACTAGCAGTCGTGTGGACAGTACAGACAGC

ENSCJAT00000016175     AGGACAGTTGGGGGAAGTATGAGACACCCTCCTGAACAGACAAATGGTGTGCATACCCCA
ENSMMUT00000017856     AGGACAGTT---GGAAGTATGAGACACCCTCCTGAACAGACAAATGGTGTGCATACCCCA
ENSPPYT00000002179     AGGACAGTTGGGGGAAGTATGAGACACCCTCCTGAACAGACAAATGGTGTGCATACCCCA
ENSGGOT00000014643     AGGACAGTTGGGGGAAGTATGAGACACCCTCCTGAACAGACAAATGGTGTGCATACCCCA
ENST00000375592        AGGACAGTTGGGGGAAGTATGAGACACCCTCCTGAACAGACAAATGGTGTGCATACCCCA
ENSPTRT00000000465     AGGACAGTTGGGGGAAGTATGAGACACCCTCCTGAACAGACAAATGGTGTGCATACCCCA
ENSMUST00000030757     AGGACAGTTGCAGGGAGTGTGAGACACCCTCCAGAACAGACCAATGGTGTTCACACCCCA
ENSRNOT00000019195     AGGACAGTTGCGGGGAGTGTAAGACACCCTCCTGAACAGACCAATGGTGTTCACACCCCA

ENSCJAT00000016175     CCTCACGTGGCCAGTGCCCTTGCAGGGGCCGTCTCCCCAGGTGCCCTGCGTCGGAGTCTG
ENSMMUT00000017856     CCTCACGTGGCCAGTGCCCTTGCAGGGGCCGTCTCCCCAGGTGCCCTGCGTCGGAGTCTG
ENSPPYT00000002179     CCTCACGTAGCCAGTGCCCTTGCAGGGGCCGTCTCCCCAGGTGCCCTGCGTCGGAGTCTG
ENSGGOT00000014643     CCTCACGTGGCCAGTGCCCTTGCAGGGGCCGTCTCCCCAGGTGCCCTGCGTCGGAGTCTG
ENST00000375592        CCTCACGTGGCCAGTGCCCTTGCAGGGGCCGTCTCCCCAGGTGCCCTGCGTCGGAGTCTG
ENSPTRT00000000465     CCTCACGTGGCCAGTGCCCTTGCAGGGGCCGTCTCCCCAGGTGCCCTGCGTCGGAGTCTG
ENSMUST00000030757     CCACATGTGGCCAGTGCCCTTGCAGGGGCTGTATCTCCCGGTGCCCTGCGTCGGAGTTTG
ENSRNOT00000019195     CCGCATGTGGCCAGTGCCCTTGCCGGGGCTGTATCTCCTGGTGCCCTGCGTCGGAGTTTG

ENSCJAT00000016175     GAAGCCATCAAAGCGATGTCCTCCAAAGGCCCCTCAGCCTCTGCAGCACTAAGCCCTCCT
ENSMMUT00000017856     GAAGCCATCAAAGCGATGTCCTCCAAAGGCCCCTCGGCCTCTGCAGCACTAAGTCCTCCT
ENSPPYT00000002179     GAAGCCATCAAAGCAATGTCCTCCAAAGGCCCCTCGGCCTCTGCAGCACTAAGTCCTCCT
ENSGGOT00000014643     GAAGCCATCAAAGCGATGTCCTCCAAAGGCCTCTCGGCCTCTGCAGCACTAAGTCCTCCT
ENST00000375592        GAAGCCATCAAAGCGATGTCCTCCAAAGGCCCCTCGGCCTCTGCAGCACTAAGTCCTCCT
ENSPTRT00000000465     GAAGCCATCAAAGCGATGTCCTCCAAAGGCCCCTCGGCCTCTGCAGCACTAAGTCCTCCT
ENSMUST00000030757     GAAGCCATCAAAGCGATGTCATCCAAAGGCCCCTCAGCCTCGGCAGCACTAAGTCCTCCT
ENSRNOT00000019195     GAAGCCATCAAAGCAATGTCATCCAAAGGCCCCTCAGCCTCAGCAGCACTAAGTCCTCCT

ENSCJAT00000016175     CGTGGGTCTTCTCCAGGCTCTCCTGGGAGCCAGAGCTTGAGCAGTGGAGAAACAGTGCCC
ENSMMUT00000017856     CTTGGGTCTTCTCCAGGCTCTCCTGGGAGCCAGAGTTTGAGCAGTGGAGAAACAGTGCCC
ENSPPYT00000002179     CTTGGGTCTTCTCCAGGCTCTCCTGGGAGCCAGAGTTTGAGCAGTGGAGAAACAGTGCCC
ENSGGOT00000014643     CTTGGGTCTTCTCCAGGCTCTCCTGGGAGCCAGAGTTTGAGCAGTGGAGAAACAGTGCCC
ENST00000375592        CTTGGGTCTTCTCCAGGCTCTCCTGGGAGCCAGAGTTTGAGCAGTGGAGAAACAGTGCCC
ENSPTRT00000000465     CTTGGGTCTTCTCCAGGCTCTCCTGGGAGCCAGAGTTTGAGCAGTGGAGAAACAGTGCCC
ENSMUST00000030757     CTTGGGTCTTCTCCAAGCTCCCCTGGGAGTCAGAGCCTGAGCAGTGGAGAAACAGTGCCC
ENSRNOT00000019195     CTTGGGTCTTCTCCAAGCTCCCCTGGGAGTCAGAGTCTGAGCAGTGGAGAAACAGTACCC

ENSCJAT00000016175     ATCCCTCGCCCAGGGCCGGCCCAAGGAGATGGACATTCCTTACCTCCCATTGCCCGCCGC
ENSMMUT00000017856     ATCCCTCGCCCAGGGCCTGCCCAAGGAGATGGACATTCCTTACCTCCCATTGCCCGCCGC
ENSPPYT00000002179     ATCCCTCGCCCAGGGCCTGCCCAAGGAGATGGACATTCCTTACCTCCCATTGCCCGCCGC
ENSGGOT00000014643     ATCCCTCGCCCAGGGCCTGCCCAAGGAGATGGACATTCCTTACCTCCCATTGCTCGCCGC
ENST00000375592        ATCCCTCGCCCAGGGCCTGCCCAAGGAGATGGACATTCCTTACCTCCCATTGCTCGCCGC
ENSPTRT00000000465     ATCCCTCGCCCAGGGCCTGCCCAAGGAGATGGACATTCCTTACCTCCCATTGCTCGCCGC
ENSMUST00000030757     AACTCCCGCCCAGGACCAGCCCAAGGAGATGGTCATTCCTTACCCCCGATTGCTCGGCGC
ENSRNOT00000019195     ATCCCCCGCCCAGGACCAGCCCAAGGAGATGGTCATTCCTTACCCCCCATTGCTCGCCGC

ENSCJAT00000016175     CTGGGCCACCACCCTCCACAGTCCCTAAATGTTGGCAAACCCCTATACCAGAGTATGAAC
```

```
ENSMMUT00000017856     CTGGGCCACCACCCTCCACAGTCCCTAAATGTTGGCAAACCCCTATACCAGAGTATGAAC
ENSPPYT00000002179     CTGGGCCACCACCCTCCACAGTCCCTAAATGTTGGCAAACCCCTATACCAGAGTATGAAC
ENSGGOT00000014643     CTGGGCCACCACCCTCCACAGTCCCTAAATGTTGGCAAACCCCTATACCAGAGTATGAAC
ENST00000375592        CTGGGCCACCACCCTCCACAGTCCCTAAATGTTGGCAAACCCCTATACCAGAGTATGAAC
ENSPTRT00000000465     CTGGGCCACCACCCTCCACAGTCCCTAAATGTTGGCAAACCCCTATACCAGAGTATGAAC
ENSMUST00000030757     CTGGGCCACCACCCTCCACAGTCCCTTAATGTGGGTAAACCCTTGTACCAGAGTATGAAC
ENSRNOT00000019195     CTGGGCCACCACCCTCCACAGTCCCTCAATGTTGGCAAGCCCTTGTACCAGAGTATGAAC

ENSCJAT00000016175     TGCAAGCCCATGCAAATGTATGTGCTGGACATTAAAGACACCAAGGAGAAGGGGCGGGTC
ENSMMUT00000017856     TGCAAGCCCATGCAGATGTACGTGCTAGACATTAAAGACACTAAGGAGAAGGGGCGGGTC
ENSPPYT00000002179     TGCAAGCCCATGCAGATGTACGTGCTGGACATTAAAGACACCAAGGAGAAGGGGCGGGTC
ENSGGOT00000014643     TGCAAGCCCATGCAGATGTACGTGCTGGACATTAAAGACACCAAGGAGAAGGGGCGGGTC
ENST00000375592        TGCAAGCCCATGCAGATGTACGTGCTGGACATTAAAGACACCAAGGAGAAGGGGCGGGTC
ENSPTRT00000000465     TGCAAGCCCATGCAGATGTACGTGCTGGACATTAAAGACACCAAGGAGAAGGGGCGGGTC
ENSMUST00000030757     TGCAAACCTATGCAGATGTATGTGCTAGACATTAAAGACACCAAGGAGAAGGGCCGAGTC
ENSRNOT00000019195     TGCAAACCCATGCAGATGTATGTGCTGGACATTAAAGACACCAAGGAGAAGGGCCGGGTC

ENSCJAT00000016175     AAATGGAAAGTATTTAATAGCAGTTCTGTCGTCGGACCTCCTGAAACCAGCCTACATACC
ENSMMUT00000017856     AAATGGAAAGTATTTAATAGCAGTTCTGTGGTCGGACCTCCTGAAACCAGCCTGCATACC
ENSPPYT00000002179     AAATGGAAAGTATTTAATAGCAGTTCTGTGGTTGGACCTCCTGAAACCAGCCTGCATACC
ENSGGOT00000014643     AAATGGAAAGTATTTAATAGCAGTTCTGTGGTTGGACCTCCTGAAACCAGCCTGCATACC
ENST00000375592        AAATGGAAAGTATTTAATAGCAGTTCTGTGGTTGGACCTCCTGAAACCAGCCTGCATACC
ENSPTRT00000000465     AAATGGAAAGTATTTAATAGCAGTTCTGTGGTTGGACCTCCTGAAACCAGCCTGCATACC
ENSMUST00000030757     AAGTGGAAAGTGTTCACAAGCAGCTCGGTGGTCGGGCCTCCTGAGACCAGCCTGCACACG
ENSRNOT00000019195     AAGTGGAAAGTATTTACTAGCAGTTCTGTGGTTGGACCTCCTGAAACCAGCCTGCACACA

ENSCJAT00000016175     GTGGTACAAGGCAGGGGTGAACTCATCATATTTGGAGGACTCATGGACAAGAAACAGAAT
ENSMMUT00000017856     GTGGTACAAGGCAGGGGTGAACTCATCATATTTGGAGGACTCATGGACAAGAAACAGAAT
ENSPPYT00000002179     GTGGTACAAGGCAGGGGTGAACTCATCATATTTGGAGGACTCATGGACAAGAAACAGAAT
ENSGGOT00000014643     GTGGTACAAGGCAGGGGTGAACTCATCATATTTGGAGGACTCATGGACAAGAAACAGAAT
ENST00000375592        GTGGTACAAGGCAGGGGTGAACTCATCATATTTGGAGGACTCATGGACAAGAAACAGAAT
ENSPTRT00000000465     GTGGTACAAGGCAGGGGTGAACTCATCATATTTGGAGGACTCATGGACAAGAAACAGAAT
ENSMUST00000030757     GTGGTGCAGGGCCGAGGCGAGCTCATCGTATTTGGAGGGCTCATGGACAAGAAGCAGAAT
ENSRNOT00000019195     GTGGTACAGGGCCGAGGAGAGCTCATCATATTTGGAGGGCTCATGGACAAGAAGCAGAAT

ENSCJAT00000016175     GTGAAGTACTATCCAAAAACAAACGCTTTGTACTTTGTACGAGCAAAGAGA
ENSMMUT00000017856     GTGAAGTACTATCCAAAAACAAACGCCTTGTACTTTGTACGAGCAAAGAGA
ENSPPYT00000002179     GTGAAGTACTATCCAAAAACAAACGCCTTGTACTTTGTACGAGCAAAGAGA
ENSGGOT00000014643     GTGAAGTACTATCCAAAAACAAACGCCTTGTACTTTGTACGAGCAAAGAGA
ENST00000375592        GTGAAGTACTATCCAAAAACAAACGCCTTGTACTTTGTACGAGCAAAGAGA
ENSPTRT00000000465     GTGAAGTACTATCCAAAAACAAACGCCTTGTACTTTGTACGAGCAAAGAGA
ENSMUST00000030757     GTGAAGTACTACCCAAAGACAAACGCCTTGTACTTTGTGCGCGCAAAGAGA
ENSRNOT00000019195     GTGAAGTACTATCCAAAAACCAACGCCTTGTACTTTGTGCGAGCAAAGAGA
```

Multiple sequence alignment of Fbxo43

```
ENSCJAT00000030661     ATGTCACAAAGGCACTCAGGTCAAGCTAGCAGTGAAGCAGGAAATGGGGTGGATTGTCCT
ENSMMUT00000006227     ATGTCTCAAAAACACTCAGGTCAAGCTGGCACTGAAGCAGGAAATGGGGTGGACTCTCCT
ENSGGOT00000000299     ATGTCACAAAGGCACTCAGGTCAAGCTGGCACTGAAGCGGGAAATGGGGCGGACTCTCCT
ENST00000428847        ATGTCGCAAAGGCACTCAGGTCAAGCTGGCACTGAAGCAGGAAATGGGGCGGACTCTCCT
ENSPTRT00000059848     ATGTCACAAAGGCACTCAGGTCAAGCTGGCACTGAAGCAGGAAATGGGGCGGACTCTCCT
ENSPPYT00000021914     ATGTCACAAAGGCACTCAGGTCAAGCTGGCACTGAAGCAGGAAATGGGGCGGACTCTCCT

ENSCJAT00000030661     CCAGTTGTCAACTCCAAGTACTCTACCTTCAGAGATTGTTGTTCCACATCTTCACTTCAA
```

```
ENSMMUT00000006227     CCAATTGTCAACTCCAAGTACTCCACCTTCAGAGATTTTTGTTCCACATCTTCATTTCAA
ENSGGOT00000000299     CTAATTGTCAACTCCAAGTACTCCACCTTCAGAGATTTTCGTTCCACGTCTTCATTTCAA
ENST00000428847        CCAATTGTCAACTCCAAGTACTCCACCTTCAGAGATTTTTGTTCCACATCTTCATTTCAA
ENSPTRT00000059848     CCAATTGTCAACTCCAAGTACTCCACCTTCAGAGATTTTTGTTCCACGTCTTCATTTCAA
ENSPPYT00000021914     CCAATTGTCAACTCCAAGTACTCCACCTTCAGAGATTTTTGTTCCACATCTTCATTTCAA

ENSCJAT00000030661     GATAATGGCTACAGTGAGTTAAAATCTTGTAGCTTTGATAATACAGACAAAGACTATCTT
ENSMMUT00000006227     GATAGTGGCTACAATGAGTTAAAATCTTGTAGCTTTGATAATACAGATAAAGAATATCTT
ENSGGOT00000000299     GATAGTGGCTACAATGAGTTAAAATCTTGTAGCTTTGATAATATAGATAAAGAATATCTT
ENST00000428847        GATAGTGGCTACAATGAGTTAAAATCTTGTAGCTTTGATAATATAGATAAAGAATATCTT
ENSPTRT00000059848     GATAGTGGCTACAATGAGTTAAAATCTTGTAGCTTTGATAATATAGATAAAGAATATCTT
ENSPPYT00000021914     GATAGTGGCTACAATGAGTTAAAATCTTGTAGCTTTGATAATATAGATAAAGAATATCTT

ENSCJAT00000030661     GGAAAAAAAGAAAAAGGCCCAGCATTACTACATGAGCACCCTGAAACTTCAAGCCTGGAC
ENSMMUT00000006227     GGAAAGAAAGTAAAAGGCCCAACATTACTCCATGAGCACCCTGAAACTTCAGGCCTGGGC
ENSGGOT00000000299     GGAAAGAAAGAAAAAGGCCCAACATTACTCTATGAGCACCCTGAAACTTCAGGCCTGGGC
ENST00000428847        GGAAAGAAAGAAAAAGGCCCAACATTACTCTATGAGCACCCTGAAACTTCAGGCCTGGGC
ENSPTRT00000059848     GGAAAGAAAGAAAAAGGCCCAACATTACTCTATGAGCACCCTGAAACTTCAGGCCTGGGC
ENSPPYT00000021914     GGAAAGAAAGAAAAAGGCCCACCATTACTCTATGAGCACCCTGAAACTTCAGGCCTGGGC

ENSCJAT00000030661     TTAACACATCCTTTAGAGTCTCCCACCCAAAAAAAGAAATTTATCTTGCTTAGAAAGGAA
ENSMMUT00000006227     TTAACACATTCTTTAGAGTCTCCCACTCAAAAAAAGAAATTTATCTTGCCTAGGAAGGAA
ENSGGOT00000000299     TTAACACATCCTTTAGAGTCTCCCACTCAAAAAAAGAAATGTATCTTGCCTAGAAAGGAA
ENST00000428847        TTAACACATCCTTTAGAATCTCCCACTCAAAAAAAGAAATGTATCTTGCCTAGAAAGGAA
ENSPTRT00000059848     TTAACACATCCTTTAGAGTCTCCCACTCAAAAAAAGAAATGTATCTTGCCTAGAAAGGAA
ENSPPYT00000021914     TTAAAACATCCTTTAGAGTCTCCCACTCAAAAAAAGAAATGTATCTTGCCTAGGAAGGAA

ENSCJAT00000030661     AAGGATAAAACCCCAGAACTTTGTGAAACACCTAAAATCAGTGACAAAAAATGTTTACCT
ENSMMUT00000006227     AATGATAAAACCTCAGAACTTTGTGAAACACCTAAAATTAGTGGGAAAAAATGTTTACCT
ENSGGOT00000000299     AAGGATAAAACCCCAGAACTTTGTGAAACACCTAAAATCAGTGGGAAAAAATGTTTACCT
ENST00000428847        AAGGATAAAACCCCAGAACTTTGTGAAACACCTAAAATCAGTGGGAAAAAATGTTTACCT
ENSPTRT00000059848     AAGGATAAAACCCCAGAACTTTGTGAAACACCTAAAATCAGTGGGAAAAAATGTTTACCT
ENSPPYT00000021914     AAGGATAAAACCCCAGAACTTTGTGAAACACCTAAAATCAGTGGGAAAAAATGTTTACCT

ENSCJAT00000030661     CGCAGAAGGTTGAATCTATCTTTCTCTCTTCTAAAGGGGGACTTTGAATCACAAAATAGT
ENSMMUT00000006227     CGCAGAAGGTTGCATGTATCTTTCTCTCTTCTAAAGGGGGACTTTGAATCACAAAATAGT
ENSGGOT00000000299     CGCAGAAGGTTGAATGTATCTTTCGCTCTTCTAAAAGGGGACTTTGAATCACAAAATAGT
ENST00000428847        CGCAGAAGGTTGAATGTATCTTTCGCTCTTCTAAAAGGGGACTTTGAATCACAAAATAGT
ENSPTRT00000059848     CGCAGAAGGTTGAATGTATCTTTCGCTCTTCTAAAAGGGGACTTTGAATCACAAAATAGT
ENSPPYT00000021914     CGCAGAAGGTTGAATGTATCTTTCGCTCTTCTAAAGGGGGACTTTGAATCACAAAATAGT

ENSCJAT00000030661     TCTTTAGAAAGTAGTATAAGCCAAGTTCTCAACTTAGAAAAAAATATTCCAAGCAGTGCT
ENSMMUT00000006227     GCTCTAGAAAGTAGTATAAGCCAAGTTCTCAACTTAGAAAAAAATATTCCAAGCAGTGCT
ENSGGOT00000000299     TCTTTAGAAAGTAGTATAAGCCAAGTTATCAACTTAGAAAAAAATATTCCAAGCAGTGCT
ENST00000428847        TCTTTAGAAAGTAGTATAAGCCAAGTTATCAACTTAGAAAAAAATATTCCAAGCAGTGCT
ENSPTRT00000059848     TCTTTAGAAAGTAGTATAAGCCAAGTTATCAACTTAGAAAAAAATATTCCAAGCAGTGCT
ENSPPYT00000021914     TCTTTAGAAAGTAGTATAAGCCAAGTTATCAACTTAGAAAAAAATATTCCAAGCAGTGCC

ENSCJAT00000030661     TCAGGTTTGTCCAGGCCAAATAATTTTAGCCCTTTAGTTACTAGCACTTTAAAAACAGAA
ENSMMUT00000006227     TCAGGTTTTTCCAGGGCAAATAATTTTAGCCCTTTAGTTACTAGCACTTTAAAAACAGAA
ENSGGOT00000000299     TCAGGTTTTTCCAGGGCAAATAATTTTAGCCCTTTAGTTACTAGCACTTTAAAAACAGAA
ENST00000428847        TCAGGTTTTTCCAGGGCAAATAATTTTAGCCCTTTAGTTACTAGCACTTTAAAAACAGAA
ENSPTRT00000059848     TCAGGTTTTCCCAGGGCAAATAATTTTAGCCCTTTAGTTACTAGCACTTTAAAAACAGAA
```

```
ENSPPYT00000021914      TCAGGTTTTTCCAGGGCAAATAATTTTAGCCCTTTAGTTACTAGCACTTTAAAAACAGAA

ENSCJAT00000030661      GAAGTGACTTTGAGCAGTCAAAAATTGAGGCTTAATTTTTCTCAGCAAAAGACTTCCACA
ENSMMUT00000006227      GAAGTGACTTCATGCAGTCAAAAATTGAGGCTTAATTTTTCTCAGCAAAAGACTTCCACA
ENSGGOT00000000299      GAAGTGACTTCATGCAGTCAAAAATTGAGGCTTAATTTTTCTCAGCAAAAGACTTCCACA
ENST0000428847          GAAGTGACTTCATGCAGTCAAAAATTGAGGCTTAATTTTTCTCAGCAAAAGACTTCCACA
ENSPTRT00000059848      GAAGTGACTTCATGCAGTCAAAAATTGAGGCTTAATTTTTCTCAGCAAAAGACTTCCACA
ENSPPYT00000021914      GAAGTGACTTCATGCAGTCAAAAATTGAGGCTTAATTTTTCTCAGCAAAAGACTTCCACA

ENSCJAT00000030661      ATTGATGATTCCAAAGATGATTGTAACCTATTTGAAGTTGACTGTATATCTCCAATTCAA
ENSMMUT00000006227      ATTGATGATTCCAAAGATGATTGTAGCCTATTTGAAGTTGAATGTATATCTCCAATTCAG
ENSGGOT00000000299      ATTGATGATTCCAAACATGATTGTAGCCTATTTGAAGTTGAATGTATATCTCCAATTCAG
ENST0000428847          ATTGATGATTCCAAAGATGATTGTAGCCTATTTGAAGTTGAATGTATATCTCCAATTCAG
ENSPTRT00000059848      ATTGATGATTCCAAAGATGATTGTAGCCTATTTGAAGTTGAATGTATATCTCCAATTCAG
ENSPPYT00000021914      ATTGATGATTCCAAAGATGACTGTAGCCTATTTGAAGTTGAATGTATATCTCCAATTCAG

ENSCJAT00000030661      GGCAATAATTTTAAAGACTCTATCACACATGACTTTAGTGATAGCAGTTTATGCATTAAT
ENSMMUT00000006227      GGCAATGATTTTAAAGACTCTATCACGCATGACTTTAGTGATAGCAGTTTATCCATTAAT
ENSGGOT00000000299      GGCAATAATTTTAAAGACTCTATCACACATGACTTTAGTGATAGCAGTTTATGCATTAAT
ENST0000428847          GGCAATAATTTTAAAGACTCTATCACACATGACTTTAGTGATAGCAGTTTATGCATTAAT
ENSPTRT00000059848      GGCAATAATTTTAAAGACTCTATCACACATGACTTTAGTGATAGCAGTTTATGCATTAAT
ENSPPYT00000021914      GGCAATAATTTTAAAGACTCTATCACACATGACTTTAGTGATAGCAGTTTATGCATTAAT

ENSCJAT00000030661      GATGAGAATACATGTCCAGAGCCCCTGGGCTCCTCTGTTAGTGGAACAACTTGTGGAACA
ENSMMUT00000006227      GATGAGAATGCATGTCCAGAGCTCCTGGGCTCCTCTGTTAGTGGAACAACTTGTGGAACA
ENSGGOT00000000299      GATGAGAATGCGTGTCCAGAGCTCCTGGGCTCCTCTGTTAGTGGAACAACTTGTGGAACG
ENST0000428847          GATGAGAATGCATGTCCAGAGCTCCTGGGCTCCTCTGTTAGTGGAACAACTTGTGGAACA
ENSPTRT00000059848      GATGAGAATGCATGTCCAGAGCTCCTGGGCTCCTCTGTTAGTGGAACAACTTGTGGAACA
ENSPPYT00000021914      GATGAGAATGCATGTCCAGAGCTCCTGGGCTCCTCTGTTAGTGGGACAACTTGTGGAACA

ENSCJAT00000030661      GATGAGGACATATTTGTGACTCCAATAAGTAATCTTGTATCAAACATTAAATTTAATGCA
ENSMMUT00000006227      GATGAGGACATATTTGTGACTCCGATAAGTAATCTTGTGGCAAACATTAGATTTAATGCA
ENSGGOT00000000299      GATGAGGACATATTTGTGACTCCGATAAGTAATCTTGTGGCAAACATTAGATTTAATACA
ENST0000428847          GATGAGGACATATTTGTGACTCCGATAAGTAATCTTGTGGCAAACATTAGATTTAACGCA
ENSPTRT00000059848      GATGAGGACATATTTGTGACTCCGATAAGTAATCTTGTGGCAAACATTAGATTTAACGCA
ENSPPYT00000021914      GATGAGGACATATTTGTGACTCCGATAAGTAATCTTGTGGCAAACATTAGATTTAACGCA

ENSCJAT00000030661      ATTCAAATACTTTCTCCTTCACCTGAAGTGAAGGGCAATATTTCAACGCCTGAAGACAGT
ENSMMUT00000006227      AGTCAAATACTTTCTCCTTCACCTGAAGTGAGAGGCAATATTTCAACTCCTGAAGACAGT
ENSGGOT00000000299      AGTCAAATACTTTCTCCTTCACCTGAAGTGAGAGGCAGTATTTCAACGCCTGAAGACAGT
ENST0000428847          AGTCAAATACTTTCTCCTTCACCTGAAGTGAGAGGCAGTATTTCAACGCCTGAAGACAGT
ENSPTRT00000059848      AGTCAAATACTTTCTCCTTCACCTGAAGTGAGAGGCAGTATTTCAACGCCTGAAGACAGT
ENSPPYT00000021914      AGTCAAATACTTTCTCCTTCACCTGAAGTGAGAGGCAATATTTCAACGCCTGAAGACAGT

ENSCJAT00000030661      GGTTTTAACTCACTTAGCTTGGAGAAATCAGAATATTCCCTCTCTGACCAGGAGGGTTCT
ENSMMUT00000006227      GGTTTTAACTCACTTAGCTTGGAGAAATCAGAAGATTCCCTCTCTGACCAGGAGGGTTCT
ENSGGOT00000000299      GGTTTTAACTCACTTAGCTTGGAGAAATCAGAAGATTCCCTCTCTGACCAGGAGGGTTCT
ENST0000428847          GGTTTTAACTCACTTAGCTTGGAGAAATCAGAAGATTCCCTGTCTGACCAGGAGGGTTCT
ENSPTRT00000059848      GGTTTTAACTCACTTAGCTTGGAGAAATCAGAAGATTCCCTCTCTGACCAGGAGGGTTCT
ENSPPYT00000021914      GGTTTTAACTCACTTAGCTTGGAGAAATCAGAAGATTCCCTCTCTGACCAGGAGGGTTCT

ENSCJAT00000030661      TTTCAAGAACTATTCCAGAAACATAAGGGGACTCCCAAAGTGGGGGACACCATAAGAAAG
```

```
ENSMMUT00000006227     TTTCAAGAACTACTTCAGAAACATAAGGGGACTCCCAAAGTGGGGGACACCATAAGAAAG
ENSGGOT00000000299     TTTCAAGAACTACTGCAGAAACATAAGGGGACTTCCAAAGTTGGGGACACCATAAGAAAG
ENST00000428847        TTTCAAGAACTACTGCAGAAACATAAGGGGACTCCCAAAGTTGGGGACACCATAAGAAAG
ENSPTRT00000059848     TTTCAAGAACTACTGCAGAAACATAAGGGGACTCCCAAAGTTGGGGACACCATAAGAAAG
ENSPPYT00000021914     TTTCAAGAACTACTGCAGAAACATAAGGGGACTCCCAAAGTTGGGGACACCATAAGAAAG

ENSCJAT00000030661     ACAAGGCATCTTAAAAGGTCGAGAAGACTGTCCACCCTTCGGGAGCAAGGCTCACAGTCA
ENSMMUT00000006227     ACAAGACATCTTGGAAGGTCGAGAAGACTGTCCACCCTTCGGGAGCAAAGTTCGCAGTCA
ENSGGOT00000000299     ACAAGACATCTTGGAAGGTCGAGAAGACTATCCACCCTTCGGGAACAAAGCTCGCAGTCA
ENST00000428847        ACAAGACATCTTGGAAGGTCGAGAAGACTGTCCACCCTTCGGGAACAAAGCTCGCAGTCA
ENSPTRT00000059848     ACAAGACATCTTGGAAGGTCGAGAAGACTGTCCACCCTTCGGGAACAAAGCTCGCAGTCA
ENSPPYT00000021914     ACAAGACATCTTGGAAGGTCGAGAAGACTGTCCACCCTTCGGGAGCAAAGCTCGCAGTCA

ENSCJAT00000030661     GAGACAGAAGAGGAAAAGCAGATTGTCCACCCTGGCTCTGAAAAAAGAGCAGCAGCTGCT
ENSMMUT00000006227     GAGACAGAAGAGGAAAAGCAGATTGTCCACCCTGACTCTGAAAAAAGAGCAGCAGCTGCT
ENSGGOT00000000299     GAGACAGAAGAGGAAAAGCAGATTGTCCACCCTGACTCTGAAAAAAGAGCAGCAGCTGCT
ENST00000428847        GAGACAGAAGAGGAAAAGCAGATTGTCCACCCTGACTCTGAAAAAAGAGCAGCAGCTGCT
ENSPTRT00000059848     GAGACAGAAGAGGAAAAGCAGATTGTCCACCCTGACTCTGAAAAAAGAGCAGCAGCTGCT
ENSPPYT00000021914     GAGACAGAAGAGGAAAAGCAGATTGTCCACCCTGACTCTGAAAAAAGAGCAGCAGCTGCT

ENSCJAT00000030661     TCTGCTATCTCAGAGGGTCAACTGAACAGTGATGAGAGTGGGGATTCAACCTTTAGTTTA
ENSMMUT00000006227     TCTGCCATCTCAGGGGGTCAGCTGAGCAGTGATGAGAGTGGGGATTTGACCTTTAGCTTA
ENSGGOT00000000299     TCTGCCATCTCAGAGGGTCAGCTGAGTAGTGATGAGAGTGGGGATTTGACCTTTAGCTTA
ENST00000428847        TCTGCCATCTCAGAGGGTCAGCTGAGTAGTGATGAGAGTGGGGATTTGACCTTTAGCTTA
ENSPTRT00000059848     TCTGCCATCTCAGAGGGTCAGCTGAGTAGTGATGAGAGTGGGGATTTGACCTTTAGCTTA
ENSPPYT00000021914     TCTGCCATCTCAGAGGGTCAGCTGAGTAGTGATGAGAGTGGGGATTTGACCTTTAGCTTA

ENSCJAT00000030661     AAGAATTTATCAAAGACCCCAGCCTTGCAATTGGTGCATGAGCTCTTCATGAAAAGCAAA
ENSMMUT00000006227     AAGAATTTATCAAAGACCCCAGCCTTGCAATTGGTGCATGAGCTGTTCATGAAAAGCAAA
ENSGGOT00000000299     AAGAATTTATCAAAGACCCCAGCCTTGCAACTGGTACATGAGCTGTTAATGAAAAGCAAG
ENST00000428847        AAGAATTTATCAAAGACCCCAGCCTTGCAATTGGTACATGAGCTGTTCATGAAAAGCAAG
ENSPTRT00000059848     AAGAATTTATCAAAGACCCCAGCCTTGCAATTGGTACATGAGCTGTTCATGAAAAGCAAG
ENSPPYT00000021914     AAGAATTTATCAAAGACCCCAGCCTTGCAATTGGTACATGAGCTGTTCATGAAAAGCAAG

ENSCJAT00000030661     AGAAAAAGAAGCCAGCAAAATAGTGGACATGAATTCTTAGAGGAAGGGGATGGGGGGAAA
ENSMMUT00000006227     AGAAAAAGATTACAGGAAAATAGTGGACATGAATTCTTAGAGCAAGGGAATGGGGAGAAA
ENSGGOT00000000299     AGGAAAAGATTACAGGAAAATAGTGGACATGAATTCTTAGAGCAAGGGGATGGGGAGAAA
ENST00000428847        AGGAAAAGATTACAGGAAAATAGTGGACATGAATTCTTAGAGCAAGGGGATGGGGAGAAA
ENSPTRT00000059848     AGGAAAAGATTACAGGAAAATAGTGGACATGAATTCTTAGAGCAAGGGGATGGGGAGAAA
ENSPPYT00000021914     AGGAAAAGATTCCAGGAAAATAGTGGACATGAATTCTTAGAGCAAGGGGATGGGGAGAAA

ENSCJAT00000030661     ATAGCTGTATTACAGTGTATACTTGCAGGACTGATTGGCAAGAAGATGGGTATAGAAAAA
ENSMMUT00000006227     ATAGCTGTGCTACAGTGTATACTTGCAGGACTGATTGGCAAGAAAATGGGTATAGAAAAA
ENSGGOT00000000299     ATAGCTGTACTGCAGTGTATACTTGCAGGACTGATTGGCAAGAAAATGGGTATAGAAAAA
ENST00000428847        ATAGCTGTACTGCAGTGTATACTTGCAGGACTGATCGGCAAGAAAATGGGTATAGAAAAA
ENSPTRT00000059848     ATAGCTGTACTGCAGTGTATACTTGCGGGACTGATTGGCAAGAAAATGGGTATAGAAAAA
ENSPPYT00000021914     ATAGCTGTACTGCAGTGTATACTTGCAGGACTGATTGGCAAGAAAATGGGTATAGAAAAA

ENSCJAT00000030661     CTGGATATCTTAACAGAATTGAAATATAGAAATTTAAAGCATATTCTTGCTATGGTTTTA
ENSMMUT00000006227     CTGGACATCTTAACAGAATTAAAATACAGAAATTTAAAGCATATTCTTGCTATGGTTTTA
ENSGGOT00000000299     CTGGACATCTTAACAGAATTAAAATATAGAAACTTAAAGCATATTCTTGCTATGGTTTTA
ENST00000428847        CTGGACATCTTAACAGAATTAAAATATAGAAATTTAAAGCATATTCTTGCTATGGTTTTA
ENSPTRT00000059848     CTGGACATCTTAACAGAATTAAAATATAGAAATTTAAAGCATATTCTTGCTATGGTTTTA
```

ENSPPYT00000021914 CTGGACATCTTAACAGAATTAAAATATAGAAATTTAAAGCATATTCTTGCTATGGTTTTA

ENSCJAT00000030661 GATTCCTTGACTGCAGAGAGCCTATGCAGTGTTTGGAAGGTAAGCAGAAATTGGCGTGAA
ENSMMUT00000006227 GATTCCTTGACTGCAGAGAGCCTATGCAGTGTTTGGAAAGTAAGCAGAAATTGGCGTGAA
ENSGGOT00000000299 GAGTCCTTGACCGCAGAGAGCCTATGCAGTGTATGGAAAGTAAGCAGAAATTGGCGTGAA
ENST0000428847 GAGTCCTTGACCGCAGAGAGCCTATGCAGTGTTTGGAAAGTAAGCAGAAATTGGCGTGAA
ENSPTRT00000059848 GAGTCCTTGACCGCAGAGAGCCTATGCAGTGTTTGGAAAGTAAGCAGAAATTGGCGTGAA
ENSPPYT00000021914 GATTCCTTGACTGCAGAGAGCCTATGCAGTGTTTGGAAAGTAAGCAGAAATTGGCGTGAA

ENSCJAT00000030661 ATTGTTGTTCAAGATAAAAATGCAAATCGGAGGAGGAAATTTTATATTACGCAACTGAAA
ENSMMUT00000006227 ATTGTTGTTCAAGATAAAAATGCAAATCGGAGGAGAAAATTTTATATCACACAACTGAAA
ENSGGOT00000000299 ATTGTTGTTCAAGATAAAAATGCAAATCGGAGGAGGAAATTTTATATCACACAACTGAAA
ENST0000428847 ATTGTTGTTCAAGATAAAAATGCAAATCGGAGGAGGAAATTTTATATCACACAACTGAAA
ENSPTRT00000059848 ATTGTTGTTCAAGATAAAAATGCAAATCGGAGGAGGAAATTTTATATCACACAACTGAAA
ENSPPYT00000021914 ATTGTTGTTCAAGATAAAAATGCAAATCGGAGGAGGAAGTTTTATATCACACAACTGAAA

ENSCJAT00000030661 ACAGATTCTGAGGGGGCTGCATTAAATGTTGAGGATGCTGCCACTCGGCTCCAGCTTTTA
ENSMMUT00000006227 ACAGATTCCGAGGGGGCTGTATTAAATGTTGAGGATGCTGCCACTCGGCTCCAGCTTTTA
ENSGGOT00000000299 ACAGATTCTGAGGGTGCTGTATTAAATGTCGAGGATGCTGCCACTCGGCTCCAGCTTTTA
ENST0000428847 ACAGATTCTGAGGGGGCTGTATTAAATGTCGAGGATGCTGCCACTCGGCTCCAGCTTTTA
ENSPTRT00000059848 ACAGATTCTGAGGGGGCTGTATTAAATGTCGAGGATGCTGCCACTCGGCTCCAGCTTTTA
ENSPPYT00000021914 ACAGATTCTGAGGGGGCTGTATTAAATGTCGAGGATGCTGCCACTCGGCTCCAGCTTTTA

ENSCJAT00000030661 AATCGCTCAGCTTTAAGATCTGTGCAGGCACAGGCTAGGATACCTGGTTCTCAGAGAGAG
ENSMMUT00000006227 AATCGCTCAGCTTTAAGATCTGTGCAGGCACAGGCTAGGATACCTGGTTCTCAGAGAGAG
ENSGGOT00000000299 AATCGCTCAGCTTTAAGATCTGTGCAGGCACAGGCTAGGATACCTGGTTCTCAGAGAGAG
ENST0000428847 AATCGCTCAGCTTTAAGATCTGTGCAGGCACAGGCTAGGATACCTGGTTCTCAGAGAGAG
ENSPTRT00000059848 AATCGCTCAGCTTTAAGATCTGTGCAGGCACAGGCTAGGATACCTGGTTCTCAGAGAGAG
ENSPPYT00000021914 AATCGCTCAGCTTTAAGATCTGTGCAGGCACAGGCTAGGATACCTGGTTCTCAGAGAGAG

ENSCJAT00000030661 CAAGGGTCAACATTTTCTCCCTGGGGAGAAGTTTTGACACCTCTAGCAAGCTCTTCTGTT
ENSMMUT00000006227 CAAGGGTCAACATTATCTCCCTGGGGAGAAGTTTTGACACCTCTAGCAAGCTCTTCTGTT
ENSGGOT00000000299 CAAGGGTCAACATTATCTCCCTGGGGAGAAGTTTTGACACCTCTAGCAAGCTCTTCTGTT
ENST0000428847 CAAGGGTCAACATTATCTCCCTGGGGAGAAGTTTTGACACCTCTAGCAAGCTCTTCTGTT
ENSPTRT00000059848 CAAGGGTCAACATTATCTCCCTGGGGAGAAGTTTTGACACCTCTAGCAAGCTCTTCTGTT
ENSPPYT00000021914 CAAGGGTCAACATTATCTCCCTGGGGAGAAGTTTTGACACCTCTAGCAAGCTCTTCTGTT

ENSCJAT00000030661 ACTCACTTAAGTAGTAAGCAGGAAGAATATGTTAAGGTTGCCAAAACACTTTTTATTGAT
ENSMMUT00000006227 ACTCACTTAAGTAGTAAACAGGAAGAATATGTTAAGGTTGCCAAAACACTTTTTATTGAT
ENSGGOT00000000299 ACTCACTTAAGTAGTAAACAGGAAGAATATGTTAAGGTTGCCAAAACACTTTTTACTGAT
ENST0000428847 ACTCACTTAAGTAGTAAACAGGAAGAATATGTTAAGGTTGCCAAAACACTTTTTACTGAT
ENSPTRT00000059848 ACTCACTTAAGTAGTAAACAGGAAGAATATGTTAAGGTTGCCAAAACACTTTTTACTGAT
ENSPPYT00000021914 ACTCACTTAAGTAGTAAACAGGAAGAATATGTTAAGGTTGCCAAAACACTTTTTACTGAT

ENSCJAT00000030661 GAAGCAATAAAAACCTTGCCCAAGGTGCCAATCCCCTGCTAAGTACCAGCCATATAAGAAA
ENSMMUT00000006227 GAAGCATTAAAACCTTGCCCAAGGTGCCAGTCCCCTGCTAAATACCAGCCATATAAGAAA
ENSGGOT00000000299 GAAGCATTAAAACCTTGCCCAAGGTGCCAGTCCCCTGCTAAGTACCAGCCATATAAGAAA
ENST0000428847 GAAGCATTAAAACCTTGCCCAAGGTGCCAGTCCCCTGCTAAGTACCAGCCATATAAGAAA
ENSPTRT00000059848 GAAGCATTAAAACCTTGCCCAAGGTGCCAGTCCCCTGCTAAGTACCAGCCATATAAGAAA
ENSPPYT00000021914 GAAGCATTAAAACCTTGCCCAAGGTGCCAGTCCCCTGCTAAGTACCAGCCATATAAGAAA

ENSCJAT00000030661 AGGGGACTGTGTAGCCGCACAGCCTGTGGTTTTGACTTTTGTGTGTTATGTTTGTGTGCT

```
ENSMMUT00000006227     AGGGGACTGTGTAGCCGCACAGCCTGTGGTTTTGACTTTTGTGTGTTATGTTTGTGTGCT
ENSGGOT00000000299     AGGGGACTGTGTAGCCGAACAGCCTGTGGTTTTGACTTTTGTGTGTTATGTCTGTGTGCT
ENST00000428847        AGGGGACTGTGTAGCCGAACAGCCTGTGGTTTTGACTTTTGTGTGTTATGTCTGTGTGCT
ENSPTRT00000059848     AGGGGACTGTGTAGCCGAACAGCCTGTGGTTTTGACTTTTGTGTGTTATGTCTGTGTGCT
ENSPPYT00000021914     AGGGGACTGTGTAGCCAAACAGCCTGTGGTTTTGACTTTTGTGTGTTATGTCTGTGTGCT


ENSCJAT00000030661     TATCATGGGTCTGAAGAATGTAGTAGAGGAGCAGCAAAGCCAAGAAATAGAAAAGATGCT
ENSMMUT00000006227     TATCATGGGTCTGAAGAATGTAGTAGAGGAGCAGCAAAGCCAAGAAATAGAAAAGATGCT
ENSGGOT00000000299     TATCATGGGTCTGAAGAATGTAGTAGAGGAGCAGCAAAGCCAAGAAATAGAAAAGATACT
ENST00000428847        TATCATGGGTCTGAAGAATGTAGTAGAGGAGCAGCAAAGCCAAGAAATAGAAAAGATGCT
ENSPTRT00000059848     TATCATGGGTCTGAAGAATGTAGTAGAGGAGCAGCAAAGCCAAGAAATAGAAAAGATGCT
ENSPPYT00000021914     TATCATGGGTCTGAAGAATGTAGTAGAGGAGCAGCAAAGCCAAGAAATAGAAAAGATGCT


ENSCJAT00000030661     GTCCCAGGAAGTGCCCAGAGTAAGCGGAATTTAAAACGCCTC
ENSMMUT00000006227     CTCCCAGGAAGTGCCCAGAGTAAGCGGAATTTAAAACGCCTC
ENSGGOT00000000299     CTCCCAGGAAGTGCCCAGAGTAAGCGGAATTTAAAACGCCTC
ENST00000428847        CTCCCAGGAAGTGCCCAGAGTAAGCGGAATTTAAAACGCCTC
ENSPTRT00000059848     CTCCCAGGAAGTGCCCAGAGTAAGCGGAATTTAAAACGCCTC
ENSPPYT00000021914     CTCCCAGGAAGTGCCCAGAGTAAGCGGAATTTAAAACGCCTC
```

Multiple sequence alignment of Fbxo44

```
ENSGGOT00000002110     ATGGCTGTGGGGAACATCAACGAGCTGCCCGAGAACATCCTGCTGGAGCTGTTTACGCAC
ENSPTRT00000000334     ATGGCTGTGGGGAACATCAACGAGCTGCCCGAGAACATCCTGCTGGAGCTGTTCACGCAC
ENST00000251547        ATGGCTGTGGGGAACATCAACGAGCTGCCCGAGAACATCCTGCTGGAGCTGTTCACGCAC
ENSMMUT00000003197     ATGGCTGTGGGGAACATCAACGAGCTGCCCGAGAACATCCTGCTGGAGCTGTTCACGCAT
ENSMUST00000167160     ATGGCAGTAGGCAACATCAACGAGCTGCCAGAGAACATCCTGCTGGAACTGTTCATCCAC
ENSRNOT00000012438     ATGGCTGTAGGCAACATCAACGAGCTGCCGGAGAACATTCTGCTGGAACTGTTCACCCAC


ENSGGOT00000002110     GTGCCCGCCCGCCAGCTGCTGCTGAACTGCCGCCTGGTCTGCAGCCTCTGGCGGGACCTC
ENSPTRT00000000334     GTGCCCGCCCGCCAGCTGCTGCTGAACTGCCGCCTGGTCTGCAGCCTCTGGCGGGACCTC
ENST00000251547        GTGCCCGCCCGCCAGCTGCTGCTGAACTGCCGCCTGGTCTGCAGCCTCTGGCGGGACCTC
ENSMMUT00000003197     GTGCCCGCCCGCCAGCTGCTGCTGAACTGCCGCCTGGTCTGCAGCCTCTGGCGGGACCTC
ENSMUST00000167160     ATCCCAGCTCGCCAACTGCTGCTGCGCTGCCGACCGGTCTGCAGCCTCTGGCGAGACCTC
ENSRNOT00000012438     ATCCCGGCTCGCCAGCTACTGCTGCGCTGCCGACCAGTCTGCAGCCTCTGGAGAGACCTC


ENSGGOT00000002110     ATCGACCTCGTGACCCTCTGGAAACGCAAGTGCCTGCGAGAGGGCTTCATCACCGAGGAC
ENSPTRT00000000334     ATCGACCTCGTGACCCTCTGGAAACGCAAGTGCCTGCGAGAGGGCTTCATCACCGAGGAC
ENST00000251547        ATCGACCTCGTGACCCTCTGGAAACGCAAGTGCCTGCGAGAGGGCTTCATCACTGAGGAC
ENSMMUT00000003197     ATCGACCTGGTGACCCTCTGGAAGCGCAAGTGCCTGCGAGAGGGCTTCATCACCGAGGAC
ENSMUST00000167160     ATTGACCTGGTTACACTCTGGAAGCGCAAGTGCCTTCAAGAGGGCTTCATCACCGAGGAC
ENSRNOT00000012438     ATTGACCTGGTCACACTCTGGAAGCGCAAGTGCCTTCAGGAGGGCTTCATCACCGAGGAC


ENSGGOT00000002110     TGGGACCAGCCCGTGGCCGACTGGAAGATCTTCTACTTCTTACGGAGCCTGCACAGGAAC
ENSPTRT00000000334     TGGGACCAGCCCGTGGCCGACTGGAAGATCTTCTACTTCTTACGGAGCCTGCACAGGAAC
ENST00000251547        TGGGACCAGCCCGTGGCCGACTGGAAGATCTTCTACTTCTTACGGAGCCTGCACAGGAAC
ENSMMUT00000003197     TGGGACCAGCCCGTGGCCGACTGGAAGATCTTCTACTTCCTACGGAGCCTGCACAGGAAC
ENSMUST00000167160     TGGGACCAGCCTGTGGCTGACTGGAAGATCTTCTACTTCCTGCGCAGCCTCCAGAGGAAC
ENSRNOT00000012438     TGGGACCAGCCCGTGGCTGACTGGAAGATCTTCTATTTCCTGCGTAGCCTCCAGAGGAAC


ENSGGOT00000002110     CTCCTGCACAACCCGTGCGCTGAAGAGGGGTTCGAGTTCTGGAGCCTGGATGTGAATGGA
ENSPTRT00000000334     CTCCTGCACAACCCGTGCGCTGAAGAGGGGTTCGAGTTCTGGAGCCTGGATGTGAATGGA
ENST00000251547        CTCCTGCACAACCCGTGCGCTGAAGAGGGGTTCGAGTTCTGGAGCCTGGATGTGAATGGA
```

```
ENSMMUT00000003197     CTCCTGCACAACCCATGCGCTGAAGAGGGGTTCGAGTTCTGGAGCCTGGATGTGAATGGA
ENSMUST00000167160     CTCCTTCACAACCCTTGTGCTGAAGAGGGATTTGAGTTCTGGAGCCTGGACGTGAACGGA
ENSRNOT00000012438     CTCCTTCACAACCCTTGTGCCGAAGAGGGCTTTGAGTTCTGGAGCCTGGATGTGAATGGA

ENSGGOT00000002110     GGCGATGAGTGGAAGGTGGAGGATCTCTCTCGAGACCAGAGGAAGGAATTCCCCAATGAC
ENSPTRT00000000334     GGCGATGAGTGGAAGGGGGAGGATCTCTCTCGAGACCAGAGGAAGGAATTCCCCAATGAC
ENST00000251547        GGCGATGAGTGGAAGGTGGAGGATCTCTCTCGAGACCAGAGGAAGGAATTCCCCAATGAC
ENSMMUT00000003197     GGCGATGAGTGGAAGGTGGAGGATCTCTCTCGAGACCAGAGGAAGGAATTCCCCAATGAC
ENSMUST00000167160     GGCGATGAATGGAAGGTAGAGGATCTCTCTAAAGACCAGCGAAAGGAATTCCCCAATGAC
ENSRNOT00000012438     GGAGATGAATGGAAGGTGGAGGATCTCTCCAAGGACCAGCGGAAGGAATTCCCCAATGAC

ENSGGOT00000002110     CAGGTCAAGAAATACTTCGTTACTTCATATTACACCTGCCTCAAGTCCCAGGTGGTGGAC
ENSPTRT00000000334     CAGGTCAAGAAATACTTCGTTACTTCATATTACACCTGCCTCAAGTCCCAGGTGGTAGAC
ENST00000251547        CAGGTCAAGAAATACTTCGTTACTTCATATTACACCTGCCTCAAGTCCCAGGTGGTGGAC
ENSMMUT00000003197     CAGGTCAAGAAATACTTCGTGACTTCATATTACACCTGCCTCAAGTCCCAGGTGGTGGAC
ENSMUST00000167160     CAGGTCAAGAAATACTTCGTGACTTCCTATTACACCTGCCTCAAGTCCCAGGTGGTGGAC
ENSRNOT00000012438     CAGGTCAAGAAATACTTCGTGACTTCCTATTACACCTGCCTCAAGTCCCAGGTGGTGGAC

ENSGGOT00000002110     CTCAAGGCCGAAGGGTATTGGGAGGAGCTGATGGATACCACACGGCCGGACATCGAGGTC
ENSPTRT00000000334     CTCAAGGCCGAAGGGTATTGGGAGGAGCTGATGGATACCACACGGCCGGACATCGAGGTC
ENST00000251547        CTCAAGGCCGAAGGGTATTGGGAGGAGCTGATGGATACCACACGGCCGGACATCGAGGTC
ENSMMUT00000003197     CTCAAGGCCGAAGGGTATTGGGAGGAGCTGATGGATACCACACGGCCGGACATCGAGGTC
ENSMUST00000167160     CTCAAGGCTGAAGGGTATTGGGAGGAGCTGATGGACACCACCCGGCCGGACATCGAGGTC
ENSRNOT00000012438     CTCAAGGCTGAAGGGTATTGGGAGGAACTGATGGACACCACCCGACCGGACATCGAGGTC

ENSGGOT00000002110     AAGGACTGGTTCGCAGCCAGGCCAGATTGCGGGTCCAAGTACCAGCTGTGCGTTCAGCTC
ENSPTRT00000000334     AAGGACTGGTTCGCAGCCAGGCCAGATTGCGGGTCCAAGTACCAGCTGTGCGTTCAGCTC
ENST00000251547        AAGGACTGGTTCGCAGCCAGGCCAGATTGCGGGTCCAAGTACCAGCTGTGCGTTCAGCTC
ENSMMUT00000003197     AAGGACTGGTTCGCAGCCAGGCCAGATTGCGGGTCCAAGTACCAGCTGTGCGTTCAGCTC
ENSMUST00000167160     AAGGACTGGTTTGCAGCCAGGCCAGACTGCGGGTCCAAGTACCAGCTGTGCGTCCAACTC
ENSRNOT00000012438     AAGGACTGGTTTGCAGCCAGGCCGGACTGCGGGTCCAAGTACCAGCTGTGCGTCCAACTC

ENSGGOT00000002110     CTGTCGTCCGCACACGCGCCTCTGGGGACCTTCCAGCCAGACCCCGCGACCATCCAGCAG
ENSPTRT00000000334     CTGTCGTCCGCACACGCGCCTCTGGGGACCTTCCAGCCAGACCCGGCGACCATCCAGCAG
ENST00000251547        CTGTCGTCCGCGCACGCGCCTCTGGGGACCTTCCAGCCAGACCCGGCGACCATCCAGCAG
ENSMMUT00000003197     CTGTCGTCCGCGCACGCGCCTCTGGGGACCTTCCAGCCAGACCCGGCAACCATCCAGCAG
ENSMUST00000167160     CTGTCGTCAGCCCACGCACCACTGGGAACCTTCCAGCCGGACCCGGTGATGATTCAGCAG
ENSRNOT00000012438     CTGTCGTCAGCTCACGCACCACTGGGAACCTTCCAGCCGGACCCAGTGATGATCCAGCAG

ENSGGOT00000002110     AAGAGCGATGCCAAGTGGAGGGAGGTCTCCCACACATTCTCCAACTACCCGCCCGGCGTC
ENSPTRT00000000334     AAGAGCGATGCCAAGTGGAGGGAGGTCTCCCACACATTCTCCAACTACCCGCCCGGCGTC
ENST00000251547        AAGAGCGATGCCAAGTGGAGGGAGGTCTCCCACACATTCTCCAACTACCCGCCCGGCGTC
ENSMMUT00000003197     AAGAGCGATGCCAAGTGGAGGGAGGTCTCCCACACATTCTCCAACTATCCGCCCGGCGTC
ENSMUST00000167160     AAAAGCGATGCCAAGTGGAGGGAGGTTTCACACACATTCTCCAACTATCCTCCCGGCGTC
ENSRNOT00000012438     AAAAGCGATGCCAAGTGGAGGGAGGTCTCACACACATTCTCCAACTATCCGCCCGGCGTC

ENSGGOT00000002110     CGCTACATCTGGTTTCAGCACGGCGGCGTGGACACTCATTACTGGGCCGGCTGGTACGGC
ENSPTRT00000000334     CGCTACATCTGGTTTCAGCACGGCGGCGTGGACACTCATTACTGGGCCGGCTGGTACGGC
ENST00000251547        CGCTACATCTGGTTTCAGCACGGCGGCGTGGACACTCATTACTGGGCCGGCTGGTACGGC
ENSMMUT00000003197     CGCTACATCTGGTTTCAGCACGGCGGCGTGGACACTCATTACTGGGCCGGCTGGTACGGC
ENSMUST00000167160     CGCTACATCTGGTTTCAGCACGGAGGCGTGGACACTCACTACTGGGCCGGCTGGTACGGC
ENSRNOT00000012438     CGCTACATCTGGTTTCAGCACGGAGGCGTGGACACCCACTACTGGGCCGGCTGGTACGGC
```

```
ENSGGOT00000002110     CCGAGGGTCACCAACAGCAGCATCACCATCGGGCCCCCGCTGCCC
ENSPTRT00000000334     CCGAGGGTCACCAACAGCAGCATCACCATCGGGCCCCCGCTGCCC
ENST00000251547        CCGAGGGTCACCAACAGCAGCATCACCATCGGGCCCCCGCTGCCC
ENSMMUT00000003197     CCGAGGGTCACCAACAGCAGCATCACCATCGGGCCCCCGCTGCCC
ENSMUST00000167160     CCGAGAGTCACAAACAGCAGCATCACCATCGGGCCCCCGCTGCCC
ENSRNOT00000012438     CCGAGAGTCACCAACAGCAGCGTCATCATCGGGCCTCCGCTGCCC
```

Multiple sequence alignment of Fbxo45

```
ENSCJAT00000003176     CGGGCTGGTCGGGCGGTTTCGGCGCCCGCGCTGAGCCGCGGGGGAGGGGCGGAGGACGCC
ENSMUST00000042732     ATGGCGGCGCCGGGCCCGGGGGCTGGGGCAGCCTCGGGCGGCGCTAGTGGTGGCGGCGCC
ENSGGOT00000014245     ATGGCGGCGCCGGCCCCGGGGGCTGGGGCAGCCTCGGGCGGCGCTGGCTGTAGCGGCGGC
ENST00000311630        ATGGCGGCGCCGGCCCCGGGGGCTGGGGCAGCCTCGGGCGGCGCTGGCTGTAGCGGCGGC

ENSCJAT00000003176     GGTGCAGCCGGTGCGTCTGCCCTCAGTGCGGGCTCCGGGGCCGGGGGCCGGTTGCCCAGC
ENSMUST00000042732     GGTGCCGGCGGCGCCAGCGCGGGCTCGGGGTCCTCCGGGGTCGGCGGCCGGTTGCCCAGC
ENSGGOT00000014245     GGC---------------------------------------------------------
ENST00000311630        GGCGCGGCGGGCGCGGGCTCGGGCTCTGGGGCCGCGGGGGCCGGGGGCCGGCTGCCCAGC

ENSCJAT00000003176     CGGGTGCTGGAGTTGGTGTTCTCTTACCTGGAGCTGTCCGAGCTGCGGAGCTGCGCCCTG
ENSMUST00000042732     CGGGTGCTGGAGTTGGTGTTCTCCTACCTGGAGCTGTCCGAGCTGCGGAGCTGCGCCCTG
ENSGGOT00000014245     ------------------------------------TCCGAGCTGCGGAGCTGCGCCCTG
ENST00000311630        CGGGTGCTGGAGTTGGTGTTCTCTTACCTGGAGCTGTCCGAGCTGCGGAGCTGCGCCCTG

ENSCJAT00000003176     GTGTGCAAGCACTGGTACCGCTGCCTGCACGGCGATGAGAACAGCGAGGTGTGGCGGAGC
ENSMUST00000042732     GTTTGCAAGCACTGGTACCGCTGCCTCCACGGCGACGAGAACAGCGAGGTGTGGCGGAGC
ENSGGOT00000014245     GTGTGCAAGCACTGGTACCGCTGCCTGCACGGCGATGAGAACAGCGAGGTGTGGCGGAGC
ENST00000311630        GTGTGCAAGCACTGGTACCGCTGCCTGCACGGCGATGAGAACAGCGAGGTGTGGCGGAGC

ENSCJAT00000003176     CTGTGCGCCCGCAGCCTGGCAGAAGAGGCTCTGCGCACGGACATTCTCTGCAACCTGCCC
ENSMUST00000042732     CTGTGCGCCCGCAGCCTAGCAGAAGAGGCTCTGCGCACGGACATCCTCTGCAACCTGCCC
ENSGGOT00000014245     CTGTGCGCCCGCAGCCTGGCAGAAGAGGCTCTGCGCACGGACATCCTCTGTAACCTGCCC
ENST00000311630        CTGTGCGCCCGCAGCCTGGCAGAAGAGGCTCTGCGCACGGACATCCTGTGCAACCTGCCC

ENSCJAT00000003176     AGCTACAAGGCCAAAATACGTGCTTTCCAGCATGCCTTCAGCACTAATGACTGCTCCAGG
ENSMUST00000042732     AGCTACAAGGCCAAGGTGCGTGCTTTCCAACATGCCTTCAGCACGAATGACTGCTCCAGG
ENSGGOT00000014245     AGCTACAAGGCCAAGATACGTGCTTTTCAACATGCCTTCAGCACTAATGACTGCTCCAGG
ENST00000311630        AGCTACAAGGCCAAGATACGTGCTTTTCAACATGCCTTCAGCACTAATGACTGCTCCAGG

ENSCJAT00000003176     AATGTCTACATTAAGAAGAATGGCTTTACTTTACATCGAAACCCCATCGCTCAGAGCACT
ENSMUST00000042732     AATGTCTATATCAAGAAGAATGGTTTTACATTACATCGGAACCCCATCGCTCAGAGCACT
ENSGGOT00000014245     AATGTCTACATTAAGAAGAATGGCTTTACTTTACATCGAAACCCCATTGCTCAGAGCACT
ENST00000311630        AATGTCTACATTAAGAAGAATGGCTTTACTTTACATCGAAACCCCATTGCTCAGAGCACT

ENSCJAT00000003176     GATGGTGCAAGGACCAAGATTGGTTTCAGTGAGGGCCGCCATGCATGGGAAGTGTGGTGG
ENSMUST00000042732     GATGGTGCAAGGACCAAGATTGGTTTCAGTGAGGGCCGCCACGCCTGGGAAGTGTGGTGG
ENSGGOT00000014245     GATGGTGCAAGGACCAAGATTGGTTTCAGTGAGGGCCGCCATGCATGGGAAGTGTGGTGG
ENST00000311630        GATGGTGCAAGGACCAAGATTGGTTTCAGTGAGGGCCGCCATGCATGGGAAGTGTGGTGG

ENSCJAT00000003176     GAGGGCCCTCTGGGCACCGTGGCAGTGATTGGAATCGCCACGAAACGGGCCCCCATGCAG
```

```
ENSMUST00000042732     GAGGGCCCTTTGGGCACGGTGGCAGTGATTGGGATTGCTACGAAACGGGCCCCCATGCAG
ENSGGOT00000014245     GAGGGCCCTCTGGGCACTGTGGCAGTGATTGGAATTGCCACGAAACGGGCCCCCATGCAG
ENST00000311630        GAGGGCCCTCTGGGCACTGTGGCAGTGATTGGAATTGCCACAAAACGGGCCCCCATGCAG


ENSCJAT00000003176     TGCCAAGGTTATGTGGCATTGCTAGGCAGTGATGACCAGAGCTGGGGCTGGAATCTAGTG
ENSMUST00000042732     TGCCAAGGTTATGTGGCCTTGCTGGGAAGTGACGACCAGAGCTGGGGCTGGAATCTGGTG
ENSGGOT00000014245     TGCCAAGGTTATGTGGCATTGCTGGGCAGTGATGACCAGAGCTGGGGCTGGAATCTGGTG
ENST00000311630        TGCCAAGGTTATGTGGCATTGCTGGGCAGTGATGACCAGAGCTGGGGCTGGAATCTGGTG


ENSCJAT00000003176     GACAATAATCTACTGCATAATGGTGAAGTCAATGGCAGTTTTCCACAGTGCAACAATGCA
ENSMUST00000042732     GACAATAATCTACTACATAACGGGGAAGTCAACGGCAGCTTCCCACAGTGCAACAACGCA
ENSGGOT00000014245     GACAATAATCTACTACATAATGGAGAAGTCAATGGCAGTTTTCCACAGTGCAACAACGCA
ENST00000311630        GACAATAATCTACTACATAATGGAGAAGTCAATGGCAGTTTTCCACAGTGCAACAACGCA


ENSCJAT00000003176     CCAAAATACCAGATAGGAGAAAGAATTCGAGTCATCTTGGACATGGAAGATAAGACTTTA
ENSMUST00000042732     CCAAAGTATCAGATAGGAGAGAGAATCCGAGTCATCCTGGACATGGAGGATAAGACTTTA
ENSGGOT00000014245     CCAAAATATCAGATAGGAGAAAGAATTCGAGTCATCTTGGACATGGAAGATAAGACTTTA
ENST00000311630        CCAAAATATCAGATAGGAGAAAGAATTCGAGTCATCTTGGACATGGAAGATAAGACTTTA


ENSCJAT00000003176     GCTTTTGAACGTGGATATGAGTTCCTGGGGGTTGCATTTAGAGGACTTCCAAAGGTCTGC
ENSMUST00000042732     GCTTTTGAGCGTGGGTATGAGTTCCTGGGGGTTGCCTTTCGAGGACTTCCAAAAGCCTGC
ENSGGOT00000014245     GCTTTTGAACGTGGATATGAGTTCCTGGGGGTTGCTTTCAGAGGACTTCCAAAGGTCTGC
ENST00000311630        GCTTTTGAACGTGGATATGAGTTCCTGGGGGTTGCTTTTAGAGGACTTCCAAAGGTCTGC


ENSCJAT00000003176     TTATATCCAGCAGTTTCTGCTGTATATGGCAACACAGAAGTGACTTTGGTTTACCTTGGA
ENSMUST00000042732     TTATACCCAGCTGTCTCTGCAGTATATGGCAACACAGAAGTGACTTTGGTTTATCTTGGA
ENSGGOT00000014245     TTATACCCAGCAGTTTCTGCTGTATATGGCAACACAGAAGTGACTTTGGTTTACCTTGGA
ENST00000311630        TTATACCCAGCAGTTTCTGCTGTATATGGCAACACAGAAGTGACTTTGGTTTACCTTGGA


ENSCJAT00000003176     AAACCTTTGGATGGA
ENSMUST00000042732     AAACCTCTGGATGGA
ENSGGOT00000014245     AAACCTTTGGACGGA
ENST00000311630        AAACCTTTGGACGGA
```

Multiple sequence alignment of Fbxo46

```
ENSCJAT00000023978     ATGGACCGCGGAAGCCTCCTGCCCTTCCAGCTGTGGTGCCCCCGGCCCTTTGGCACCTAC
ENSPTRT00000072368     ATGGACCGCGGGAGCCTCCTGCCCTTCCAGCTATGGTGCCCCCGGCCCTTTGGCACCTAC
ENSMMUT00000030067     ATGGACCGCGGAAGCCTCCTGCCCTTCCAGCTATGGTGCCCCCGGCCGTTTGGCACCTAC
XM_002829425.1         ATGGACCGCGGAAGCCTCCTGCCCTTCCAACTATGGTGCCCCCGGCCGTTTGGCACCTAC
ENSGGOT00000030407     ATGGACCGCGGGAGCCTCCTGCCCTTCCAGCTATGGTGCCCCCGGCCCTTTGGCACCTAC
ENST00000317683        ATGGACCGTGGGAGCCTCCTGCCCTTCCAGCTATGGTGCCCCCGGCCCTTTGGCACCTAC
ENSMUST00000165913     ATGGACAGGGGCAGCCTCCTGCCCTTCCAACTCTGGTGCCCCCGGCCCTTCAGCAAGTAT
ENSRNOT00000012082     ATGGACAGGGGCAGCCTCCTGCCCTTCCAACTCTGGTGCCCCCGGCCCTTCAGCAAGTAT


ENSCJAT00000023978     TCACAGAACCAGCCTCGCCCAGCTTCTGCGGCCCTCAAGCCATCAGCCTGCGCTGAGCCT
ENSPTRT00000072368     TCACAGAACCAGCCACGCCCGCCTTCTGCGGCCCTCAAGCCATCAGCCTGCCCTGAGCCT
ENSMMUT00000030067     TCACAGAACCAGCCGCGCCCGCCTTCTGCGGCCCTCAAGCCTTCTGCCTGCCCTGAGCCT
XM_002829425.1         TCACAGAACCAGCCGCGCCCGCCTTCTGCGGCCCTAAAGCCATCAGCCTGCCCTGAGCCT
ENSGGOT00000030407     TCACAGAACCAGCCGCGCCCGCCTTCTGCGGCCCTCAAGCCATCAGCCTGCCCTGAGCCT
ENST00000317683        TCACAGAACCAGCCACGCCCGCCTTCTGCGGCCCTCAAGCCATCAGCCTGCCCTGAGCCT
ENSMUST00000165913     TCCCAGAACCAGCCGCGCCCACCTTCCACAGCCCTCAAGCCGCCAGTCTGTCCTGACACA
```

```
ENSRNOT00000012082     TCCCAGAACCAGCCGCGTCCACCTTCCGCAACCCTCAAGCCACCAGTCTGTCCTGACACA

ENSCJAT00000023978     GGTGGCGGAGTTGAGCCAGACCATGGGCCTGCCCACTCAGAGAACACACCACCTGCCTTG
ENSPTRT00000072368     GGTGGCGGGGCCGAGCCAGACCATGGGCCTGCCCACTCAGAGAACACACCACCTGCCTTG
ENSMMUT00000030067     GGCGGCGGGGCCGAGCCAGACCATGGGCCTGCCCACTCAGAGAACACACCACCTGCCTTG
XM_002829425.1         GGTGGCGGGGCGGAGCCAGACCATGGGCCTGCCCACTCAGAGAACACACCACCCGCCTTG
ENSGGOT00000030407     GGTGGCGGGGCCGAGCCAGACCATGGGCCTGCCCACTCAGAGAACACACCACCCGCCTTG
ENST00000317683        GGTGGCGGGGCCGAGCCAGACCATGGGCCTGCCCACTCAGAGAACACACCACCCGCCTTG
ENSMUST00000165913     AGCAGCGGGACTGAGCCCGACCACAGGCCTGCGCACTTGGAGAGCACACCACCTGCCGTG
ENSRNOT00000012082     AGCAGCGGGACTGAGCCAGACCACAGGCCTGCACACTTGGAGAGCACACCACCTGCCTTG

ENSCJAT00000023978     GCCACCGAGGCCCCTGCCTCCCAGCCGGCTCCACTCCTCTCAGCAGCAGCTGCTGGCGAT
ENSPTRT00000072368     GCCACTGAGGTCCCTGCCTCCCAGCCGGCTCCGCTCCTCTCAGCAGCAGCTGCTGGTGAT
ENSMMUT00000030067     GCCACCGAGGTCCCTGCCTCCCAGCCGGCTCCGCTCCTCTCAGCAGCAGCTGCTGGTGAT
XM_002829425.1         GCCACCGAGGTCCCTGCCTCCCAGCCGGCTCCGCTCCTCTCAGCAGCAGCTGCTGGTGAT
ENSGGOT00000030407     GCCGCTGAGGTCCCTGCCTCCCAGCCGGCTCCGCTCCTCTCAGCAGCAGCTGCTGGTGAT
ENST00000317683        GCCACTGAGGTCCCTGCCTCCCAGCCGGCTCCGCTCCTCTCAGCAGCAGCTGCTGGTGAT
ENSMUST00000165913     GCTGCAGAAGCCCCCACCTCCCAGCCTGCTCCACTCCTCTCTACAGCAGCTTCCGGTGAT
ENSRNOT00000012082     GCTGCAGAAGCCCCCACCTCCCAGCATGCTCCACTCCTCTCTACAGCAGCTTCAGGTGAT

ENSCJAT00000023978     GAGGGTCGAGTCCTCCTAGACACGTGGTATGTCATCAAGCCTGGGAATACAAAGGAGAAG
ENSPTRT00000072368     GAGGGTCGCGTCCTCCTGGACACGTGGTATGTCATCAAGCCCGGGAATACAAAGGAGAAG
ENSMMUT00000030067     GAGGGTCGAGTCCTCCTGGACACCTGGTATGTCATCAAGCCCGGGAATACAAAGGAGAAG
XM_002829425.1         GAGGGTCGAGTCCTCCTGGACACGTGGTATGTCATCAAGCCCGGGAATACAAAGGAGAAG
ENSGGOT00000030407     GAGGGTCGAGTCCTCCTGGACACGTGGTATGTCATCAAGCCCGGGAATACAAAGGAGAAG
ENST00000317683        GAGGGTCGAGTCCTCCTGGACACGTGGTATGTCATCAAGCCCGGGAATACAAAGGAGAAG
ENSMUST00000165913     GAAGGTCGGGTCCTGCTGGACACGTGGTATGTTATCAAGCCTGGAAATACAAAGGAGAAG
ENSRNOT00000012082     GAGGGTCGAGTCCTGCTGGACACGTGGTATGTTATCAAACCTGGAAATACAAAGGAGAAG

ENSCJAT00000023978     GTGGCCTTCTTTGTGGCCCACCAGTGTGGTGGGGGCAGCCGGGCCAGCTCCATGAAGGTC
ENSPTRT00000072368     GTGGCCTTCTTTGTGGCCCACCAGAGGGGTGGGGGCAGCCGGGCCAGCTCCATAAGGTCC
ENSMMUT00000030067     GTGGCCTTCTTTGTGGCCCACCAGTGTGGTGGGGGCAGCCGGGCCAGCTCCATGAAGGTC
XM_002829425.1         GTGGCCTTCTTTGTGGCTCACCAGTGTGGTGGGGGCAGCCGGGCCAGCTCCATGAAGGTT
ENSGGOT00000030407     GTGGCCTTCTTTGTGGCCCACCAGTGTGGTGGGGGCAGCCGGGCCAGCTCCATGAAGGTC
ENST00000317683        GTGGCCTTCTTTGTGGCCCACCAGTGTGGTGGGGGCAGCCGGGCCAGCTCCATGAAGGTC
ENSMUST00000165913     GTGGCCTTCTTTGTGGCCCACCAGTGTGGTGGAAGTAGCCGGGCCAGCTCTATGAAGGTC
ENSRNOT00000012082     GTGGCTTTCTTTGTGGCCCACCAGTGTGGTGGAGGTAGCCGGGCTAGCTCTATGAAGGTC

ENSCJAT00000023978     AAGGGGCACTGGGGCAGCGACAGCTCCAAGGCCAAGCGGAGACGGCGCTGTCTTGACCCC
ENSPTRT00000072368     CTAGTAAGGTGGGGCAGCGATAGCTCCAAGGCCAAGCGGCGGAGG---------------
ENSMMUT00000030067     AAGGGGCACTGGGGCAGTGATAGCTCCAAGGCCAAGCGGAGGAGGCGCTGTCTTGACCCC
XM_002829425.1         AAGGGGCACTGGGGCAGCGATAGCTCCAAGGCCAAGCGGCGGAGGCGCTGTCTTGACCCT
ENSGGOT00000030407     AAGGGGCACTGGGGCAGCGATAGCTCCAAGGCCAAGCGGCGGAGGCGCTGTCTTGACCCC
ENST00000317683        AAGGGGCACTGGGGCAGCGATAGCTCCAAGGCCAAGCGGCGGAGGCGCTGTCTTGACCCC
ENSMUST00000165913     AAAGGGCACTGGGGCAGTGACAGCTCCAAAGCTAAGAGGAGGAGGCGCTGCCTTGAGCCC
ENSRNOT00000012082     AAAGGGCACTGGGGCAGTGACAGCTCCAAAGCTAAGAGGAGGAGGCGCTGCCTTGAGCCC

ENSCJAT00000023978     ACCAAGGCTCCTCCTGATCCAGGGGGTCGGGAGGGGCCCCCTGCTACTGAGGAGGGCCCC
ENSPTRT00000072368     ------------------------------------------------------------
ENSMMUT00000030067     ACCAAAGCTCCTCCTGACCCAGGGGGCCGGGAGGGCCTGGCTACCTCTGAGGAGGGCCCT
XM_002829425.1         ACCAAGGCTCCTCCCGACCCGGGGGGCCGGGAGGGCCTCCCTGCTACTGAGGAGGGCCCC
ENSGGOT00000030407     ACCAAGGCTCCTCCTGACCCAGGGGGCCGGGAGGGCCCCCCTGCTGCTGAGGAGGGCCCC
ENST00000317683        ACCAAGGCTCCTCCTGACCCAGGGGGCCGGGAGGGCCCCCCTGCTGCTGAGGAGGGCCCC
ENSMUST00000165913     ACCAAGGCTCCTCCAGACCAAGGGGGCCGAGAAGGAACCCCTGCCACTGAGGTGACCCCC
```

```
ENSRNOT00000012082     ACCAAGGCTCCTCCAGACCAAGGGGGCCAAGAAGGGACCCCTGCCACTGAGGTGGCCCCC


ENSCJAT00000023978     TCCTCAGCTGGTGAGGATGTGGACCTGCTCTCTGTGGCCGAGATGGTGGCCCTGGTGGAA
ENSPTRT00000072368     ------------------------------------------------------------
ENSMMUT00000030067     GCCTCAGCCGGTGAGGACGTGGACCTGCTCTCTGTGGCCGAGATGGTGGCCCTCGTGGAA
XM_002829425.1         ACCTCAGCCGGTGAGGACGTCGACCTGCTCTCTGTGGCCGAGATGGTGGCCCTGGTGGAA
ENSGGOT00000030407     GCCTCAGCCGGTGACGACGTGGACCTGCTCTCTGTGGCCGAGATGGTGGCCCTGGTGGAA
ENST00000317683        GCCTCAGCCGGTGAGGACGTGGACCTGCTCTCTGTGGCCGAGATGGTGGCCCTGGTGGAA
ENSMUST00000165913     ACATCATCTGGGGATGATGTAGACCTGGTCTCTGTAGCAGAAATGGTGGCCCTGGTTGAA
ENSRNOT00000012082     ACTTCATCCGGGGATGATGTAGACCTGCTCTCTGTAGCAGAAATGGTGGCCCTGGTTGAA


ENSCJAT00000023978     CAGCGGGCAGCCCTGGCCCTGCAGAGCTACCCACGACCAGCCACCCCAGCACCTGTAGTC
ENSPTRT00000072368     ---CGCGCAGCCCTGGCCCTGCAGAGCTACCCACGACCGACCACCCCAGCGCCTGTAGTC
ENSMMUT00000030067     CAGCGGGCGGCCCTGGCCCTGCAGAGCTACCCACGACCGGCCACCCCAGCGCCTGTAGTT
XM_002829425.1         CAGCGGGCAGCCCTGGCCCTGCAGAGCTACCCACGACCGACCACCCCAGCGCCTGTAGTC
ENSGGOT00000030407     CAGCGGGCAGCCCTGGCCCTGCAGAGCTACCCACGACCGACCACCCCAGCGCCTGTAGTC
ENST00000317683        CAGCGGGCAGCCCTGGCCCTGCAGAGCTACCCACGACCGACCACCCCAGCGCCTGTAGTC
ENSMUST00000165913     CAGAGAGCTGCCCTGGCCCTGCAGAGTTATCCGCGCCCCAGTACCCCAGCTCCCGTGGTC
ENSRNOT00000012082     CAGAGAGCTGCCCTGGCCCTGCAGAGTTACCCACGCCCGAGTACCCCAGCTCCTGTGGTC


ENSCJAT00000023978     TTTGTGTCAGCTGAGCAGGGTGGACCGGCCAAGGGGGTGGGGTCCGAACGGCGGTCTGGC
ENSPTRT00000072368     TTTGTGTCCGCCGAGCAAGGTGGACCGGCCAAGGGGGTGGGATCTGAACGGCGGTCCGGT
ENSMMUT00000030067     TTTGTGTCCGCGGAGCAGGGTGGACCGGCCAAGGGGGTGGGGTCTGAACGGCGGTCCGGT
XM_002829425.1         TTTGTGTCCGCCGAGCAAGGTGGACCGGCCAAGGGGGTGGGATCTGAACGGCGGTCTGGT
ENSGGOT00000030407     TTTGTGTCCGCGGAGCAAGGTGGACCGGCCAAGGGGGTGGGATCTGAACGGCGGTCCGGT
ENST00000317683        TTTGTGTCCGCCGAGCAAGGTGGACCGGCCAAGGGGGTGGGATCTGAACGGCGGTCCGGT
ENSMUST00000165913     TTTGTGTCAGCTGATCAGGGGGGACCAGCCAAGGGGCTAGGGTCGGAAAGGCGGTCTGGT
ENSRNOT00000012082     TTTGTGTCAGCTGAGCAGGGGGGACCAGCCAAGGGGCTAGGGTCAGAAAGGCGGTCTGGT


ENSCJAT00000023978     GGTGGGGACTGCAGCCGTGTAGCCGAGGCTGTGGCCCACTTTGAGGCGCAGAGGGACAGC
ENSPTRT00000072368     GGTGGGGACTGCAGCCGTGTAGCCGAGGCCGTGGCCCACTTTGAAGCGCAGAGGGACAGC
ENSMMUT00000030067     GGAGGGGACTGCAGCCGTGTAGCGGAGGCCGTGGCCCACTTTGAGGCGCAGAGAGACAGC
XM_002829425.1         GGTGGGGACTGCAGCCGTGTAGCCGAGGCCGTGGCCCACTTTGAAGCGCAGAGGGACAGC
ENSGGOT00000030407     GGTGGGGACTGCAGCCGTGTAGCCGAGGCCGTGGCCCACTTTGAAGCGCAGAGGGACAGC
ENST00000317683        GGTGGGGACTGCAGCCGTGTAGCCGAGGCCGTGGCCCACTTTGAAGCGCAGAGGGACAGC
ENSMUST00000165913     GGTGGTGACTGCAGCCGAGTTGCTGAGGCAGTGGCCCACTTTGAGGCCCAGCGGGACAGC
ENSRNOT00000012082     GGTGGTGACTGCAGCCGAGTTGCTGAGGCAGTGGCCCACTTTGAGGCCCAGCGGGACAGC


ENSCJAT00000023978     CCTCCCACCAAGGGCCTCCGCAAGGAGGAGCGGCCCGGACCAGGCCCTGGGGAGGTGCGC
ENSPTRT00000072368     CCTCCCACCAAGGGCCTCCGCAAGGAAGAGCGGCCCGGGCCAGGCCCTGGGGAGGTGCGC
ENSMMUT00000030067     CCTCCCACCAAGGGCCTCCGCAAGGAAGAGCGACCCGGGCCAGGCCCTGGGGAGGTGCGC
XM_002829425.1         CCTCCCACCAAGGGCCTCCGCAAGGAAGAGCGGCCTGGGCCAGGCCCTGGGGAGGTGCGC
ENSGGOT00000030407     CCTCCCACCAAGGGCCTCCGCAAGGAAGAGCGGCCCGGGCCAGGCCCTGGGGAGGTGCGC
ENST00000317683        CCTCCCACCAAGGGCCTCCGCAAGGAAGAGCGGCCCGGGCCAGGCCCTGGGGAGGTGCGC
ENSMUST00000165913     CCTCCTACCAAGGGTCTCCGCAAGGAGGAACGGCCAGGCCCTGGGCCCGGCGAGGTGCGT
ENSRNOT00000012082     CCTCCTACCAAGGGTCTCCGCAAGGAGGAGCGGCCAGGCCCTGGGCCAGGAGAGGTGCGA


ENSCJAT00000023978     ATCGCCTTCCGCATCTCCAACGGCCGGGAGCCCCGTGCACCAGACAGCGGCCTGCCCAGT
ENSPTRT00000072368     ATCGCCTTCCGCATCTCCAACGGCCGGGAGCCCCGTGCACCAGACAGCGGCCTGCCCAGT
ENSMMUT00000030067     ATCGCCTTCCGCATCTCCAATGGCCGGGAGCCCCGTGCACCAGACAGCGGCCTGCCCACT
XM_002829425.1         ATCGCCTTCCGCATCTCCAACGGCCGGGAGCCCCGTGCACCAGACAGTGGCCTGCCCAGT
ENSGGOT00000030407     ATCGCCTTCCGCATCTCCAACGGCCGGGAGCCCCGTGCACCAGACAGCGGCCTGCCCAGT
ENST00000317683        ATCGCCTTCCGCATCTCCAACGGCCGGGAGCCCCGTGCACCAGACAGCGGCCTGCCCAGT
ENSMUST00000165913     ATAGCTTTCCGAATCTCCAATGTCCGAGAGCCCCATTCACCAGATGGCAACTTACCCAAT
```

```
ENSRNOT00000012082     ATAGCCTTCCGTATCTCCAATGTCCGGGAGCCCCAGTCACCAGATGGCAGCTTAGCCAAT

ENSCJAT00000023978     GGGGGCGGGGGCAGGCCTGGTTGTGCCTACCCTGGCAGCCCAGGTCCTGGGGCCCGAGCC
ENSPTRT00000072368     GGGGGCGGGGGCAGGCCTGGTTGTGCCTACCCTGGCAGCCCAGGTCCTGGGGCCCGAGCC
ENSMMUT00000030067     GGGGGCGGGGGCAGGCCTGGTTGTGCCTACCCTGGCAGCCCAGGTCCTGGGGCCCGAGCC
XM_002829425.1         GGGGGCGGGGGCAGGCCTGGTTGTGCCTACCCTGGCAGCCCAGGTCCTGGGGCCCGAGCT
ENSGGOT00000030407     GGGGGCGGGGGCAGGCCTGGTTGTGCCTACCCTGGCAGCCCAGGTCCTGGGGCCCGAGCC
ENST00000317683        GGGGGCGGGGGCAGGCCTGGTTGTGCCTACCCTGGCAGCCCAGGTCCTGGGGCCCGAGCC
ENSMUST00000165913     GGGGGTGGAGGCCGGCCTGGTTGTGCCTACCCTGGTAGTCCTGGACCTGGGACTCGAGCC
ENSRNOT00000012082     GGGGGTGGAGGTCGGCCTGCTTGTCCCTACCCTGGTAGTCCTGGGCCTGGGACTCGAGCC

ENSCJAT00000023978     AAGGACAAGATCACGTGTGACTTATACCAGCTCATCAGCCCCTCGCGGGATGCCCTCCCC
ENSPTRT00000072368     AAGGACAAGATCACATGTGACTTATACCAGCTCATCAGCCCCTCGCGGGATGCCCTCCCC
ENSMMUT00000030067     AAGGACAAGATCACATGTGACTTATACCAGCTCATCAGCCCCTCGCGGGATGCCCTCCCC
XM_002829425.1         AAGGACAAGATCACATGTGACTTATACCAGCTCATCAGCCCCTCGCGGGATGCCCTCCCC
ENSGGOT00000030407     AAGGACAAGATCACATGTGACTTATACCAGCTCATCAGCCCCTCGCGGGATGCCCTCCCC
ENST00000317683        AAGGACAAGATCACATGTGACTTATACCAGCTCATCAGCCCCTCGCGGGATGCCCTCCCC
ENSMUST00000165913     AAAGACAAAATCACCTGCGACTTGTACCAGCTTATCAGCCCCTCCAGGGATGCCCTTCCC
ENSRNOT00000012082     AAAGACAAAATCACCTGCGACTTGTACCAGCTGATCAGCCCCTCTAGGGATGCCCTTCCC

ENSCJAT00000023978     AGCAACGTGGAGTTCCTGCTGGCCAGGGCGGATGAGGCCAGCGAGGGTGAGAGCCCAGCG
ENSPTRT00000072368     AGCAACGTGGAGTTCCTGCTGGCCAGGGCGGATGAGGCCAGTGAGGGTGACAGCCCGGCA
ENSMMUT00000030067     AGCAACGTGGAGTTCCTGCTGGCCAGGGCGGATGAGGCCAGCGAGGGTGAGAGCCCGGCG
XM_002829425.1         AGCAACGTGGAGTTCCTGCTGGCCAGGGCGGACGAGGCCAGCGAGGGTGACAGCCCGGCG
ENSGGOT00000030407     AGCAACGTGGAGTTCTTGCTGGCCAGGGCGGATGAGGCCAGTGAGGGTGACAGCCCGGCA
ENST00000317683        AGCAACGTGGAGTTCCTGCTGGCCAGGGCGGATGAGGCCAGTGAGGGTGACAGCCCGGCA
ENSMUST00000165913     AGCAATGTGGAGTTCCTATTGGCCAGGGCTGATGAAGCCAGTGAGGGTGAGACACCAGCC
ENSRNOT00000012082     AGCAACGTGGAGTTCCTACTGGCCAGGGCCGATGAAGCAAGTGAGGGTGAGACACCAGCC

ENSCJAT00000023978     CCCGTGAGGCCCGAGGACACTCCTCCGGCGCCGCCTCCACCCCCTGCCCGGGACTGTGGA
ENSPTRT00000072368     CCCGCCAGGCCTGAGGACACTCCCCCGGCGCCCCCTCCGCCCCCTGCCCGAGACTGCGGA
ENSMMUT00000030067     CCCGCCAGGCCTGAGGACACTCCCCCGGCGCCCCCTCCGCCCCCTGCCCGGGACTGCGGA
XM_002829425.1         CCCGCCAGGCCCGAGGACACTCCCCCGGCGCCCCCTCCGCCGCCTGCCCGGGACTGCGGA
ENSGGOT00000030407     CCCGCCAGGCCTGAGGACACTCCCCCGGCGCCCCCTCCGCCCCCTGCCCGGGACTGCGGA
ENST00000317683        CCCGCCAGGCCTGAGGACACTCCCCCGGCGCCCCCTCCGCCCCCTGCCCGGGACTGCGGA
ENSMUST00000165913     CCCACCAGGCCTGAGGACACTCCACCAGCACCGCCTCCACCCCCTGCCCGGGACTGTGGT
ENSRNOT00000012082     CCGGCCAGGCCTGAGGACACTCCACCAGCACCCCCTCCGCCCCCTGCCCGGGACTGTGGT

ENSCJAT00000023978     GCGTCCGGCTTCCACGTGGACGTGGTGGTGACGGGCGTGGTGGATGAGTGCATCTTCTTT
ENSPTRT00000072368     GCGTCAGGCTTCCACGTGGACGTGGTGGTGACGGGCGTGGTAGATGAGTGCATCTTCTTT
ENSMMUT00000030067     GCGTCAGGCTTCCACGTGGACGTGGTGGTGACGGGCGTGGTAGATGAGTGCATCTTCTTT
XM_002829425.1         GCATCAGGCTTCCACGTGGACGTGGTGGTGACGGGCGTGGTAGATGAGTGCATCTTCTTT
ENSGGOT00000030407     GCGTCAGGCTTCCACGTGGACGTGGTGGTGACGGGCGTGGTAGATGAGTGCATCTTCTTT
ENST00000317683        GCGTCAGGCTTCCACGTGGACGTGGTGGTGACGGGCGTGGTAGATGAGTGCATCTTCTTT
ENSMUST00000165913     GCATCAGGATTCCATGTAGATGTGGTGGTAACAGGTGTAGTGGATGCGTGCATCTTCTTT
ENSRNOT00000012082     GCATCAGGATTCCACGTGGACGTGGTGGTGACAGGAGTAGTGGATGCCTGCATCTTCTTT

ENSCJAT00000023978     GGCAAGGACGGCACCAAGAACGTGAAGGAGGAGACTGTGTGCCTGACAGTCAGCCCCGAG
ENSPTRT00000072368     GGCAAGGACGGCACCAAGAACGTGAAGGAGGAGACTGTGTGCCTGACGGTCAGCCCGGAG
ENSMMUT00000030067     GGCAAGGATGGCACCAAGAACGTGAAGGAGGAGACTGTGTGCCTGACGGTCAGCCCAGAG
XM_002829425.1         GGCAAGGACGGCACCAAGAACGTGAAGGAGGAGACTGTGTGCCTGACGGTCAGCCCGGAG
ENSGGOT00000030407     GGCAAGGACGGCACCAAGAACGTGAAGGAGGAGACTGTGTGCCTGACAGTCAGCCCGGAG
ENST00000317683        GGCAAGGACGGCACCAAGAACGTGAAGGAGGAGACTGTGTGCCTGACGGTCAGCCCGGAG
ENSMUST00000165913     GGCAAAGATGGCACCAAGAACGTGAAGGAAGAGACTGTGTGCCTGACAGTGAGCCCTGAG
```

```
ENSRNOT00000012082     GGCAAAGATGGCACCAAGAATGTGAAGGAAGAGACTGTGTGCCTGACCGTGAGCCCCGAG

ENSCJAT00000023978     GAGCCGCCGCCTCCGGGCCAGCTCTTCTTCCTCCAGAACCGCGGGCCGGATGGGCCCCCA
ENSPTRT00000072368     GAACCGCCGCCTCCGGGCCAGCTCTTCTTTCTCCAGAACCGCGGGCAGGACGGGCCCCCA
ENSMMUT00000030067     GAGCCGCCGCCTCCGGGCCAGCTCTTCTTTCTCCAGAACCGCGGGCCGGACGGGCCCCCG
XM_002829425.1         GAGCCGCCGCCTCCGGGCCAGCTCTTCTTTCTCCAGAACCGCGGGCCGGACGGGCCCCCG
ENSGGOT00000030407     GAACCGCCGCCTCCGGGCCAGCTCTTCTTTCTCCAGAACCGCGGGCCGGACGGGCCCCCG
ENST00000317683        GAACCGCCGCCTCCGGGCCAGCTCTTCTTTCTCCAGAACCGCGGGCCGGACGGGCCCCCG
ENSMUST00000165913     GAGCCACCCCCACCTGGCCAGCTCTTCTTCTTGCAGTCCCGTGGTCCAGAAGGGCCTCCT
ENSRNOT00000012082     GAGCCACCCCCTCCTGGACAGCTCTTCTTCCTCCAGTCCCGGGGGCCGGAAGGGCCTCCT

ENSCJAT00000023978     GAGCCTCCCCCGGCAGACTCGCCGGCCACCGCGCCTGGCCCGGACGATGCCGAGGGCACG
ENSPTRT00000072368     GAGCCACCCCCGGCCGACTCCCCGGCCACTGCGCCCGGCCCAGACGATGCCGAGGGCACG
ENSMMUT00000030067     GAGCCACCCCCAGCAGACTCCCCGGCCACCGCGCCCGGCCCAGACGATGCCGAGGGCACG
XM_002829425.1         GAGCCACCCCCGGCCGACTCCCCGGCCACTGCGCCCGGCCCAGACGATGCCGAGGGCACG
ENSGGOT00000030407     GAGCCACCCCCGGCCGACTCCCCGGCCACTGCGCCCGGCCCAGACGATGCCGAGGGCACG
ENST00000317683        GAGCCACCCCCGGCCGACTCCCCGGCCACTGCGCCCGGCCCAGACGATGCCGAGGGCACG
ENSMUST00000165913     GAGCCACCCCCAGCTGATATACCAAGCACAGTGCCAGGCCCCGATGATTCTGAGGGCACA
ENSRNOT00000012082     GAGCCACCCCCAGCTGATACAGCAAGCAAGGTGCCAGGCCCCGAGGATTCTGAGGGCACA

ENSCJAT00000023978     GCGGATACCTCCCTGTGCCGCCTATACCGGCACGTGTCACACGACTTCCTAGAGATCCGC
ENSPTRT00000072368     GCGGACACCTCCCTGTGCCGCTTGTACCGGCACGTGTCGCACGACTTCCTAGAGATCCGC
ENSMMUT00000030067     GCGGACACCTCCCTGTGTCGCCTGTACCGGCACGTGTCGCACGACTTCCTAGAGATCCGC
XM_002829425.1         GCGGACACTTCCTTGTGCCGCCTGTACCGGCACGTGTCGCACGACTTCCTAGAGATCCGC
ENSGGOT00000030407     GCGGACACCTCCCTGTGCCGCTTGTACCGGCACGTGTCGCACGACTTCCTAGAGATCCGC
ENST00000317683        GCGGACACCTCCCTGTGCCGCTTGTACCGGCACGTGTCGCACGACTTCCTAGAGATCCGC
ENSMUST00000165913     ACGGACACCTCCCTCTGCCGCCTGTACCGGCATGTGTCACACGACTTCCTGGAGATTCGC
ENSRNOT00000012082     ACAGACACCTCCCTGTGCCGCCTGTACCGGCACGTGTCGCATGACTTCCTGGAGATTCGC

ENSCJAT00000023978     TTCAAGATTCAGCGGCTGCTGGAGCCGCGGCAGTACATGCTGCTGCTGCCCGAGCACGTG
ENSPTRT00000072368     TTCAAGATTCAGCGGCTGCTGGAGCCGCGACAGTACATGCTGCTGCTGCCCGAGCACGTG
ENSMMUT00000030067     TTCAAGATTCAGCGGCTGCTGGAGCCGCGACAGTACATGCTGCTGCTGCCAGAGCACGTG
XM_002829425.1         TTCAAGATTCAGCGGCTGCTGGAGCCGCGACAGTACATGCTGCTGCTGCCCGAGCACGTG
ENSGGOT00000030407     TTCAAGATTCAGCGGCTGCTGGAGCCGCGACAGTACATGCTGCTGCTGCCCGAGCACGTG
ENST00000317683        TTCAAGATTCAGCGGCTGCTGGAGCCGCGACAGTACATGCTGCTGCTGCCCGAGCACGTG
ENSMUST00000165913     TTTAAAATCCAGCGTCTTCTGGAGCCACGGCAGTACATGCTGCTGCTGCCTGAGCACGTG
ENSRNOT00000012082     TTCAAAATCCAGCGTCTCCTGGAGCCGAGGCAGTACATGCTGCTGCTGCCGGAGCATGTG

ENSCJAT00000023978     CTGGTCAAGATCTTCAGCTTCCTGCCCACCCGCGCGCTGGCCGCCCTCAAGTGCACCTGC
ENSPTRT00000072368     CTGGTCAAGATCTTCAGCTTCCTGCCCACGCGCGCGCTGGCCGCCCTCAAGTGCACCTGC
ENSMMUT00000030067     CTGGTCAAGATCTTCAGCTTCCTGCCCACGCGCGCGCTGGCCGCCCTCAAGTGCACCTGC
XM_002829425.1         CTGGTCAAGATCTTCAGCTTCCTGCCCACGCGCGCGCTGGCCGCCCTGAAGTGCACCTGC
ENSGGOT00000030407     CTGGTCAAGATCTTCAGCTTCCTGCCCACGCGCGCGCTGGCCGCCCTCAAGTGCACCTGC
ENST00000317683        CTGGTCAAGATCTTCAGCTTCCTGCCCACGCGCGCGCTGGCCGCCCTCAAGTGCACCTGC
ENSMUST00000165913     CTCGTCAAGATATTCAGCTTCCTGCCCACGCGGGCCTTGGCAGCCCTCAAGTGCACCTGC
ENSRNOT00000012082     CTCGTCAAGATATTCAGCTTCCTGCCCACACGGGCCCTGGCTGCCCTCAAGTGCACCTGC

ENSCJAT00000023978     CACCACTTCAAGGGCATCATCGAGGCGTTCGGCGTGCGGGCCACAGACTCGCGCTGGAGC
ENSPTRT00000072368     CACCACTTCAAGGGCATCATCGAGGCGTTTGGCGTGCGGGCCACAGACTCGCGCTGGAGC
ENSMMUT00000030067     CACCACTTCAAGGGCATCATCGAGGCATTCGGCGTACGGGCCACAGACTCGCGCTGGAGC
XM_002829425.1         CACCACTTCAAGGGCATCATCGAGGCGTTTGGCGTGCGGGCCACAGACTCGCGCTGGAGC
ENSGGOT00000030407     CACCACTTCAAGGGCATCATCGAGGCGTTTGGCGTGCGGGCCACAGACTCGCGCTGGAGC
ENST00000317683        CACCACTTCAAGGGCATCATCGAGGCGTTTGGCGTGCGGGCCACAGACTCGCGCTGGAGC
ENSMUST00000165913     CACCACTTCAAGGGGATCATCGAGGCTTTCGGTGTGCGTGCCACGGACTCTCGCTGGAGC
```

```
ENSRNOT00000012082     CACCACTTCAAGGGTATCATTGAGGCCTTTGGTGTACGAGCCACGGACTCTCGCTGGAGC


ENSCJAT00000023978     CGAGACCCGCTCTACCGCGATGACCCGTGCAAGCAGTGCCGCAAGAGATACGAGAAGGGC
ENSPTRT00000072368     CGAGACCCGCTCTACCGCGATGATCCGTGCAAACAGTGCCGCAAGAGATACGAGAAGGGA
ENSMMUT00000030067     CGAGACCCGCTCTACCGCGATGACCCGTGCAAGCAGTGCCGCAAGAGATACGAGAAGGGC
XM_002829425.1         CGAGACCCGCTCTACCGCGATGACCCGTGCAAACAGTGCCGCAAGAGATACGAGAAGGGC
ENSGGOT00000030407     CGAGACCCGCTCTACCGCGATGATCCGTGCAAACAGTGCCGCAAGAGATACGAGAAGGGC
ENST00000317683        CGAGACCCGCTCTACCGCGATGATCCGTGCAAACAGTGCCGCAAGAGATACGAGAAGGGC
ENSMUST00000165913     CGGGACCCGCTCTACCGAGACGACCCTTGTAAGCAGTGCCGCAAGAGATACGAGAAGGGT
ENSRNOT00000012082     CGGGACCCCCTCTACCGAGATGACCCTTGTAAGCAGTGCCGCAAGAGATATGAGAAGGGT


ENSCJAT00000023978     GACGTGTCGCTCTGCCGCTGGCACCCCAAGCCCTACCACCACGACCTGCCTTACGGACGT
ENSPTRT00000072368     GACGTGTCGCTCTGCCGCTGGCACCCCAAGCCCTACCACCATGACCTGCCTTACGGACGT
ENSMMUT00000030067     GACGTGTCGCTCTGCCGCTGGCACCCCAAGCCCTACCACCATGACCTGCCTTACGGACGT
XM_002829425.1         GACGTGTCGCTCTGCCGCTGGCACCCCAAGCCCTACCACCATGACCTGCCTTACGGACGT
ENSGGOT00000030407     GACGTGTCGCTCTGCCGCTGGCACCCCAAGCCCTACCACCATGACCTGCCTTACGGACGT
ENST00000317683        GACGTGTCGCTCTGCCGCTGGCACCCCAAGCCCTACCACCATGACCTGCCTTACGGACGT
ENSMUST00000165913     GATGTGTCACTCTGCCGCTGGCACCCCAAACCCTACCACCATGACCTGCCTTACGGACGT
ENSRNOT00000012082     GATGTGTCACTCTGCCGCTGGCACCCCAAACCCTACCACCACGACCTGCCTTATGGACGT


ENSCJAT00000023978     TCCTACTGGATGTGCTGCCGTCGAGCCGACCGCGAGACGCCAGGTTGCCGCCTGGGCCTC
ENSPTRT00000072368     TCCTACTGGATGTGCTGCCGCCGAGCCGACCGCGAGACTCCCGGCTGCCGCCTGGGCCTC
ENSMMUT00000030067     TCCTACTGGATGTGCTGCCGCCGAGCCGACCGCGAGACGCCCGGCTGCCGCCTGGGCCTC
XM_002829425.1         TCCTACTGGATGTGCTGCCGCCGAGCCGACCGCGAGACGCCCGGCTGCCGCCTGGGCCTC
ENSGGOT00000030407     TCCTACTGGATGTGCTGCCGCCGAGCCGACCGCGAGACTCCCGGCTGCCGCCTGGGCCTC
ENST00000317683        TCCTACTGGATGTGCTGCCGTCGAGCCGACCGCGAGACTCCCGGCTGCCGCCTGGGCCTC
ENSMUST00000165913     TCCTACTGGATGTGCTGCCGCAGAGCCGATCGTGAGACACCAGGCTGTCGCCTGGGCTTG
ENSRNOT00000012082     TCCTACTGGATGTGCTGCCGCAGAGCCGACCGCGAGACCCCAGGCTGTCGCCTGGGCTTG


ENSCJAT00000023978     CACGATAACAACTGGGTGCTGCCCTGCAATGGGCCGGGTGGGGGCCGGGCAGGCCGGGAG
ENSPTRT00000072368     CACGATAACAACTGGGTGCTGCCCTGCAATGGGCCAGGCGGGGGCCGGGCCGGCCGGGAG
ENSMMUT00000030067     CACGATAACAACTGGGTGCTGCCCTGCAATGGGCCGGGCGGAGGCCGGGCAGGCCGGGAG
XM_002829425.1         CACGATAACAACTGGGTGCTGCCCTGCAATGGGCCGGGCGGGGGCCGGGCCGGCCGGGAG
ENSGGOT00000030407     CACGATAACAACTGGGTGCTGCCCTGCAATGGGCCAGGCGGGGGCCGGGCCGGCCGGGAG
ENST00000317683        CACGATAACAACTGGGTGCTGCCCTGCAATGGGCCAGGCGGGGGCCGGGCCGGCCGGGAG
ENSMUST00000165913     CATGACAACAACTGGGTACTGCCGTGCAATGGAGTGGGTGGAGGCCGTGCTGGCCGGGAA
ENSRNOT00000012082     CATGACAACAACTGGGTACTGCCGTGTAACGGAGTAGGTGGAGGCCGTGCTGGCCGGGAA


ENSCJAT00000023978     GAGGGGAGG
ENSPTRT00000072368     GAGGGGAGG
ENSMMUT00000030067     GAGGGGAGG
XM_002829425.1         GAGGGGAGG
ENSGGOT00000030407     GAGGGGAGG
ENST00000317683        GAGGGGAGG
ENSMUST00000165913     GAGGGGAGG
ENSRNOT00000012082     GAGGGGAGG
```

Multiple sequence alignment of Fbxo47

```
ENSCJAT00000016284     ATGGCATCCAGAATAAATACCAGTTTCACTTTGATTCCCAACCAGAAACATAGACGTAGT
ENSPPYT00000009931     ATGGCATCCAGAATAAATACCAATTTCACTTTGATTCCCAACCAGAAACTTAGACGTAGT
ENSMMUT00000025551     ATGGCATCCAGAATAAATACCAATTTCACTTTGATTCCCAACCAGAAACTTAGACGTAGT
ENSGGOT00000013620     ATGGCATCCAGAATAAATACAAATTTCACTTTGATTCCCAACCAGAAACTTAGACGTAGT
ENSPTRT00000059793     ATGGCATCCAGAATAAATACAAATTTCACTTTGATTCCCAACCAGAAACTTAGACGTAGT
```

ENST00000378079 ATGGCATCCAGAATAAATACAAATTTCACTTTGATTCCCAACCAGAAACTTAGACGTAGT
ENSMUST00000093939 ATGGCGTCTAGAGTAAATACCAGTTTCACTTTGATACCCAAGCAAAAATGTAGACGTTCT
ENSRNOT00000055379 ATGGCGTCTAGAGTAAATACCACTTTCACTCTGATACCCAGCTCAAAATGTAGACGTTTG

ENSCJAT00000016284 AATCATCAAACCAGCTGTTACTCCAAGACTCTTGGCTCAGGCTTTCAACCCATATCAACA
ENSPPYT00000009931 AATCGTCAAACCAGCTGTTATTCCAAGACCCTTGGCTCAGGCTTTCAACCCACATCAACA
ENSMMUT00000025551 AATCGTCAAACCAGCTGTTGTTCCAAGACCCTTGGCTCAGGCTTTCAACCCATATCAACA
ENSGGOT00000013620 AATCGTCAAACCAGCTGTTATTCCAAGACCCTTGGCTCAGGCTTTCAACCCATATCAACA
ENSPTRT00000059793 AATCGTCAAAGCAGCTGTTATTCCAAGACCCTTGGCTCAGGCTTTCAACCCATATCAACA
ENST00000378079 AATCGTCAAACCAGCTGTTATTCCAAGACCCTTGGCTCAGGCTTTCAACCCATATCAACA
ENSMUST00000093939 AATCATCACTCCAGCTATTTTTGCAACACCCTTGATTCAGATCCTCAACTCTTATCTACA
ENSRNOT00000055379 AATCATCAATCCAGCGATTTTTGCAACACTCTTGATTCAGATCCTCAGCTCTTATCTACA

ENSCJAT00000016284 TTTGGAAATTTTAAAGCCTTACCATTGGAAATATTCCAAATAATTTTAAAATATTTGTCA
ENSPPYT00000009931 TTTGGAAATTTTAAAGCCTTACCATTGGAAATATTCCAGATAATTCTAAAATATTTGTCA
ENSMMUT00000025551 TTTGGAAATTTTAAAGCCTTACCATTGGAAATATTCCAGATAATTTTAAAATATTTGTCA
ENSGGOT00000013620 TTTGGAAATTTTAAAGCCTTACCATTGGAAATATTCCAGATAATTTTAAAATATTTGTCA
ENSPTRT00000059793 TTTGGAAATTTTAAAGCCTTACCATTGGAAATATTCCAGATAATTTTAAAATATTTGTCA
ENST00000378079 TTTGGAAATTTTAAAGCCTTACCATTGGAAATATTCCAGATAATTTTAAAATATTTGTCA
ENSMUST00000093939 CTTGGAAATTTTAAAGTCTTACCATTGGAAATATTACACATAATTTTAAGATACTTGTCA
ENSRNOT00000055379 CTTGGAAATTTTAAAGTCTTACCATTGGAAATATTACACATAATTTTAAGATACTTGTCA

ENSCJAT00000016284 GTGAAGGATATCAGCATGCTAAGCATGGTGTCTAAAACAGTCAGCCACCATATTGTTAAT
ENSPPYT00000009931 GTGAAGGATATCAGCATGCTAAGCATGGTGTCCAAAACAGTCAGCCAACACATTATTAAT
ENSMMUT00000025551 GTGAAGGATATCAGCATGCTAAGCATGGTGTCCAAAACAGTCAGCCAACACATTATTAAT
ENSGGOT00000013620 GTGAAGGATATCAGCATGCTAAGCATGGTGTCCAAAACAGTCAGCCAACACATTATTAAT
ENSPTRT00000059793 GTGAAGGATATCAGCATGCTAAGCATGGTGTCCAAAACAGTCAGCCAACACATTATTAAT
ENST00000378079 GTGAAGGATATCAGCATGCTAAGCATGGTGTCCAAAACAGTCAGCCAACACATTATTAAT
ENSMUST00000093939 GTGAAAGATATTGGCATGTTAAGTATGGTGTCTAAAACAGTCAGCCAGCACATAATTAAT
ENSRNOT00000055379 GTGAAGGATATTGGCATGTTAAGTATGGTGTCCAAAACAGTCAGCCAGCACATAATTAAT

ENSCJAT00000016284 TATATATCAACCTCATCAGGACGCAAAAGACTTCTACTACAGGACTTTCATAACCTTGAG
ENSPPYT00000009931 TATATCTCAACCTCATCAGGGAGCAAAAGACTTTTACTACAGGACTTTCATAACCTTGAG
ENSMMUT00000025551 TATATCTCAACCTCATCAGGAAGCAAAAGACTTTTACTACAGGACTTTCATAACCTTGAG
ENSGGOT00000013620 TATATCTCAACCTCATCAGGAAGCAAAAGACTTTTACTACAGGACTTTCATAACCTTGAG
ENSPTRT00000059793 TATATCTCAACCTCATCAGGAAGCAAAAGACTTTTACTACAGGACTTTCATAACCTTGAG
ENST00000378079 TATATCTCAACCTCATCAGGAAGCAAAAGACTTTTACTACAGGACTTTCATAACCTTGAG
ENSMUST00000093939 TATATCTCAACATCATCTGGAAGTAGGAGACTTTTACTACAGAACTTTCATGACCTTGAT
ENSRNOT00000055379 TACATCTCAACCTCATCTGGAAGTAGGAGACTTTTACTACAGAACTTTCATGACCTTGAT

ENSCJAT00000016284 CTGCCTGGCAGGAGACAAGACTCTGCTATACTGGAGCACTACAGATCTCTAGGTCTACTG
ENSPPYT00000009931 CTGCCTGACGGGAGACAAGACTCTGCTATACTGGAGCACTACAGATCTCTAGGTCTACTG
ENSMMUT00000025551 CTGCCTGACAGGAGACAAGACTCTGCTATACTGGAGCACTACAGATCTCTAGGTCTACTG
ENSGGOT00000013620 CTGCCTGACAGGAGACAAGACTCTGCTATACTGGAGCACTACAGATCTCTAGGTCTACTG
ENSPTRT00000059793 CTGCCTGACAGGAGACAAGACTCTGCTATACTGGAGCACTACAGATCTCTAGGTCTACTG
ENST00000378079 CTGCCTGACAGGAGACAAGACTCTGCTATACTGGAGCACTACAGATCTCTAGGTCTACTG
ENSMUST00000093939 CTGCCTGGCACAAAAGAAGAGACTGCTCTACTGGAGCACTACAGAGCTCTAGGTCTACTA
ENSRNOT00000055379 CTGCCTGGCACAAAAGAAGAGACTGCTCTATTGGAGCACTACAGAGCTCTAGGTCTACTA

ENSCJAT00000016284 TTTAAAAGATGCACATTGCTGTTACCCACCAAGGAAAGGCTAAAGTACATTCACAAGATA
ENSPPYT00000009931 TTTAAAAGATGCACATTGCTGCTACCCACCAAGGAAAGGCTAAAATACATTCACAAGATA
ENSMMUT00000025551 TTTAAAAGATGTACATTGCTGCTACCCACCAAGGAAAGGCTAAAGTACATTCACAAGATA
ENSGGOT00000013620 TTTAAAAGATGCACATTGCTGCTACCCACCAAGGAAAGGCTAAAATACATTCACAAGATA
ENSPTRT00000059793 TTTAAAAGATGCACATTGCTGCTACCCACCAAGGAAAGGCTAAAATACATTCACAAGATA

```
ENST00000378079         TTTAAAAGATGCACATTGCTGCTACCCACCAAGGAAAGGCTAAAATACATTCACAAGATA
ENSMUST00000093939      TTTAAAAGATGCACCTTGCTGCTTCCCACCAAGGAAAGACTGAAGTACATTCACAAGATA
ENSRNOT00000055379      TTTAAAAGATGCACCTTGCTGCTTCCCACCAAGGAAAGACTGAAATACATTCAGAAGATA

ENSCJAT00000016284      CTCACAGAAGTTTCCTGCTTTAAATTCAGTGGCTGTGCAGCTCCTATGCAGTGTTTAGGA
ENSPPYT00000009931      CTCACAGAAGTTTCCTGCTTTAAATTCAATGGCTGCGCAGCTCCTATGCAGTGTTTAGGA
ENSMMUT00000025551      CTCACAGAAGTTTCCTGCTTTAAATTCAATGGCTGTGCAGCTCCTATGCAGTGTTTAGGA
ENSGGOT00000013620      CTCACAGAAGTTTCCTGCTTTAAATTCAATGGCTGTGCAGCTCCTATGCAGTGTTTAGGA
ENSPTRT00000059793      CTCACAGAAGTTTCCTGCTTTAAATTCAATGGCTGTGCAGCTCCTATGCAGTGTTTAGGA
ENST00000378079         CTCACAGAAGTTTCCTGCTTTAAATTCAATGGCTGTGCAGCTCCTATGCAGTGTTTAGGA
ENSMUST00000093939      CTCTCAGAAGTATCCTGCTTTAAATTCAGTGGCTGTTCAGTTCCTTTGCAGTGTTTAGGA
ENSRNOT00000055379      CTCTCAGAAGTTCCCTGCTTCAAGTTCAGTGGCTGCTCAGCTCCTCTGCAGTGTTTAGGA

ENSCJAT00000016284      TTCACATGTTATGGCATGTTTTTACAGACCTTAACAGCAGGTTGGGATGAACTTGAGTGC
ENSPPYT00000009931      TTAACATGTTATGGCATGTTTTTACAGACCTTAACAGCAGGTTGGGATGAACTTGAGTGC
ENSMMUT00000025551      TTAACATGTTATGGCATGTTTTTACAGACCTTAACAGCAGGTTGGGATGAACTTGAGTGC
ENSGGOT00000013620      TTAACATGTTATGGCATGTTTTTACAGACCTTAACAGCAGGTTGGGATGAACTTGAGTGC
ENSPTRT00000059793      TTAACATGTTATGGCATGTTTTTACAGACCTTAACAGCAGGTTGGGATGAACTTGAGTGC
ENST00000378079         TTAACATGTTATGGCATGTTTTTACAGACCTTAACAGCAGGTTGGGATGAACTTGAGTGC
ENSMUST00000093939      TTATCATGTTACGGCATGTTTTTACAGACCTTAACAGCAGGTTGGGATGAACTTGAATGC
ENSRNOT00000055379      TTATCATGTTATGGCATGTTTTTACAGACTTTAACCGCAGGCTGGGATGAACTTGAATGT

ENSCJAT00000016284      CATCGCGTTTATAATTTCTTATGCGAACTGACCAATCTCTGCCGCAAGATGCAAATGGTT
ENSPPYT00000009931      CATCGTGTTTATAATTTCTTATGCGAACTGACTAATCTCTGCCGCAAGATGCAAATGGCT
ENSMMUT00000025551      CATCGCGTTTATAATTTCTTATGCGAACTGACTAATCTCTGCCGCAAGATGCAAATGGCT
ENSGGOT00000013620      CATCGCGTTTATAATTTCTTATGCGAACTGACTAATCTCTGCCGCAAGATGCAAATGGCT
ENSPTRT00000059793      CATCGCGTTTATAATTTCTTATGCGAACTGACTAATCTCTGCCGCAAGATGCAAATGGCT
ENST00000378079         CATCGCGTTTATAATTTCTTATGCGAACTGACTAATCTCTGCCGCAAGATACAAATGGCT
ENSMUST00000093939      CATCGTGTGTATAACTTCTTATGTGAGCTGACTAATCTCTCCCGAAAGATGCAGACAGTT
ENSRNOT00000055379      CATCGTGTGTATAACTTCTTATGTGAGCAGACTAATCTCTCCCGAAAGATGCAGACTGTT

ENSCJAT00000016284      GTCTGCAGCAAACCAGGAAGTGCCCGAAAACTGGAATTAAGGATCAGACTCTTCTGTAGG
ENSPPYT00000009931      GTCTGCAGCAAACCAGGAAGTGCCCGAAAACTGGAGTTAAGAATCAGACTCTTCTGTAGG
ENSMMUT00000025551      GTCTGCAGCAAACCAGGAAGTGCCCGAAAACTTGAGTTAAGGATCAGACTCTTCTGTAGG
ENSGGOT00000013620      GTCTGCAGCAAACCAGGAAGTGCCCGAAAACTGGAGTTAAGAATCAGACTCTTCTGTAGG
ENSPTRT00000059793      GTCTGCAGCAAACCAGGAAGTGCCCGAAAACTGGAGTTAAGAATCAGGCTCTTCTGTAGG
ENST00000378079         GTCTGCAGCAAACCAGGAAGTGCCCAAAAACTGGAGTTAAGAATCAGACTCTTCTGTAGG
ENSMUST00000093939      GTCTGCAACAAACCAGGAAGTGCTCGAAAACTAGAGTTAAGGGTTAGACTATTCTGTCGG
ENSRNOT00000055379      GTCTGTAACAAACCAGGTAAAGACTTTCAGGTAGAGTCTCGTGTTTCTTTCCTTTTGGGC

ENSCJAT00000016284      AATGTCCTGCTTGATCATTGGACACATCAAAATGATTCTGCTTTTTGGTTGACACGAATA
ENSPPYT00000009931      AATGTCCTCCTTGATCATTGGACACATCAAAGTGATTCTGCTTTTTGGTTGACACGAATA
ENSMMUT00000025551      AATGTCCTCCTTGATCATTGGACACATCGAAGTGATTCTGCTTTTTGGTTGACACGTATA
ENSGGOT00000013620      AATGTCCTCCTTGATCATTGGACACATCGAAGTGATTCTGCTTTTTGGTTGACGCGAATA
ENSPTRT00000059793      AATGTCCTCCTTGATCATTGGACACATCGAAGTGATTCTGCTTTTTGGTTGACACGAATA
ENST00000378079         AATGTCCTCCTTGATCATTGGACACATCGAAGTGATTCTGCTTTTTGGTTGACGCGAATA
ENSMUST00000093939      AATGTCCTACTTGATCACTGGACACATCGAAGTGATTCTGCATTTTGGTTAACTCGAATT
ENSRNOT00000055379      AAGGGGCTGCTGAAGGGCCTAGCTCATACCTCTGATCCCAGAATATGGAAAAAAAGGATT

ENSCJAT00000016284      TTAAAACCATGGCCAATGGTGAACCAGGCAAGATTACTGTACATCATCTTTGGGCCAATA
ENSPPYT00000009931      TTAAAACCATGGCCAATGGTGAATCAGGCAAGATTACTGTATATCATCTTTGGGCCAATA
ENSMMUT00000025551      TTAAAACCATGGCCAATGGTGAATCAGGCAAGATTACTGTATATCATCTTTGGGCCAATA
ENSGGOT00000013620      TTAAAACCATGGCCAATGGTGAATCAGGCAAGATTGCTGTATATCATCTTTGGGCCAATA
ENSPTRT00000059793      TTAAAACCATGGCCAATGGTGAATCAGGCAAGATTACTGTATATCATCTTTGGGCCAATA
```

```
ENST00000378079       TTAAAACCATGGCCAATGGTGAATCAGGCAAGATTACTGTATATCATCTTTGGGCCAATA
ENSMUST00000093939    TTAAAGCCATGGCCAATGGTGAATCAGGCAAGATTGCTATATATTATCTTTGGACCAACA
ENSRNOT00000055379    ATTAAACCTTGGAGAAATGTTAAACAACTATTTATTTTATATATAAGTTTTCATCCATCT

ENSCJAT00000016284    TCTCCTCAGGATGGACAGGTGATTTGGCAGAAAATGATAGAAGAACCTGCAGATGAATTC
ENSPPYT00000009931    TCTCCTCAAGATGGACAGGTGGTTTGGCAGAAAATGATAGAAGAACCTACAGATGAATTC
ENSMMUT00000025551    TCTCCTCAAGATGGACAGGTGGTTTGGCAGAAAATGATAGAAGAACCTACAGATGAATTC
ENSGGOT00000013620    TCTCCTCAAGATGGACAGGTGGTTTGGCAGGAAATGATAGAAGAACCTACAGATGAATTC
ENSPTRT00000059793    TCTCCTCAAGATGGACAGGTGGTTTGGCAGGAAATGATAGAAGAACCTACAGATGAATTC
ENST00000378079       TCTCCTCAAGATGGACAGGTGGTTTGGCAGGAAATGATAGAAGAACCTACAGATGAATTC
ENSMUST00000093939    TCTCCTCATGATGGGCAGGTGATTTGGCAGGAAATGATAGAAGGACCTACAGATGAATCT
ENSRNOT00000055379    TTCCCTATTCCAGGGCAGGTGATTTGGCAGGAAATGATAGAAGGGCCTACAGATGAGTCC

ENSCJAT00000016284    AATCTGAAAGGTTTGGCTGATGCCATTAAGTTATTATATGACTCTGGCACTAAAGAGTGG
ENSPPYT00000009931    AGTCTGAAAGGTTTGGCTGATGCCATTAAGTTACTATATGATGCTAGCACTAAAGAGTGG
ENSMMUT00000025551    AGTCTGAAAGGTTTGGCTGATGCCATTAAGCTACTATATGACGCTAGCACTGAAGAGTGG
ENSGGOT00000013620    AGTCTGAAAGGTTTGGCTGATGCCATTAAGTTACTATATGACGCTAGCACTAAAGAGTGG
ENSPTRT00000059793    AGTCTGAAAGGTTTGGCTGATGCCATTAAGTTACTATATGACGCTAGCACTAAAGAGTGG
ENST00000378079       AGTCTGAAAGGTTTGGCTGATGCCATTAAGTTACTATATGACGCTAGCACTAAAGAGTGG
ENSMUST00000093939    AGTCTGAAAGGTTTGGCTAATGCCATTAAGTTACTGTATGACACAGGTGCCAAAGGCTGG
ENSRNOT00000055379    AGTCTGAAAGGTTTGGCTAATGCCATTAAGTTACTGTATGACACAGGTGCCAAAGGCTGG

ENSCJAT00000016284    ACAGCAGATGATGTCATCAGTCTTGTAGATGAACTATCAGTGGTTCCCCATGAGTGGCTT
ENSPPYT00000009931    ACAGCAGATGATGTTATCAGTCTTGTAGATGATCTATCAGTGGTTCCCCGTGAATGGCTT
ENSMMUT00000025551    ACAGCAGATGATGTTATCAGTCTTGTAGATGAACTATCAGTGGTTCCCCGTGAGTGGCTT
ENSGGOT00000013620    ACAGCAGATGATGTTATCAGTCTTGTAGATGAACTATCAGTGGTTCCCCGTGAGTGGCTT
ENSPTRT00000059793    ACAGCAGATGATGTTATCAGTCTTGTAGATGAACTATCAGTGGTTCCCCGTGAGTGGCTT
ENST00000378079       ACAGCAGATGATGTTATCAGTCTTGTAGATGAACTATCAGTGGTTCCCCGTGAGTGGCTT
ENSMUST00000093939    ACAGCAGATGATGTCATCAGTCTTGTAGATGAACTATCAGTGGTTCCCCGGGAGTGGCTT
ENSRNOT00000055379    ACAGCAGATGATGTCATCAGTCTTGTAGATGAGCTGTCAGTGGTTCCCCGGGAGTGGCTT

ENSCJAT00000016284    CTAGAGAATAATGCACGTCTCCTAATGCTAACTGGAAACAACATCTGTCTCACTTTCATG
ENSPPYT00000009931    CTAGAGAATAATGCACGTCTCCTAATGCTAAGTGGAAACAACATCTGTTTCACTTTCATG
ENSMMUT00000025551    CTAGAGAATAATGCACGTCTCCTAATGCTAAGTGGAAACAACATCTGTTTCACTTTCATG
ENSGGOT00000013620    CTAGAGAATAATGCACGTCTCCTAATGCTAAGTGGAAACAACATCTGTTTCAGTTTCATG
ENSPTRT00000059793    CTAGAGAATAATGCACGTCTCCTAATGCTAAGTGGAAACAACATCTGTTTCAGTTTCATG
ENST00000378079       CTAGAGAATAATGCACGTCTCCTAATGCTAAGTGGAAACAACATCTGTTTCAGTTTCATG
ENSMUST00000093939    CTGGAGAATAATGCACGCCTCCTAATCCTGAGCGGGAACAACATCTGCTTCACTTTCATG
ENSRNOT00000055379    CTGGAGAACAATGCACGCCTCCTGATCCTGAGCGGGAACAACATCTGCTTCACTTTCATG

ENSCJAT00000016284    GCTAGTAAAGCTGTGAATGGACGGGCCATTGAACTGGCAAGGTTCATAGTCTTTTTGGCT
ENSPPYT00000009931    GCTAGTAAAGCTGTGAATGGACGGACCATTGAACTGGCAAGGCTCATAGTCTTTTTGGCT
ENSMMUT00000025551    GCTAGTAAAGCTGTGAATGGACGGACCATTGAACTGGCAAGGCTCATAGTCTTTTTAGCT
ENSGGOT00000013620    GCTAGTAAAGCTGTGAATGGACGGACCATTGAACTAGCAAGGCTCGTAGTCTTTTTGGCT
ENSPTRT00000059793    GCTAGTAAAGCTGTGAATGGAAGGACCATTGAACTGGCAAGGCTCGTAGTCTTTTTGGCT
ENST00000378079       GCTAGTAAAGCTGTGAATGGACGCACCATTGAACTGGCAAGGCTCGTAGTCTTTTTGGCT
ENSMUST00000093939    GCCAGTAAAGCTGTAAATGGACGAGCCGTTGAACTGGCAAGACTTGTAGTCTTTTTGGCT
ENSRNOT00000055379    GCTAGTAAAGCTGTAAACGGGCGAGCCATCGAGCTGGCAAGGCTCATAGTCTTTTTGGCT

ENSCJAT00000016284    TTGGTGTGTGAGAAAGAACTATACTGCATGGATTGGACAGTTAAAATGATGCAAAGAGTC
ENSPPYT00000009931    TTGGTGTGTGAGAAAGAACTATACTGCATGGATTGGACTGTTAAAATGATGCAAAAAGTC
ENSMMUT00000025551    TTGGTGTGTGAGAAAGAACTATACTGCATGGATTGGACAGTTAAAATGATGCAAAAAGTC
ENSGGOT00000013620    TTGGTGTGTGAGAAAGAACTGTACTGCATGGATTGGACAGTTAAAATGATGCAAAAAGTC
ENSPTRT00000059793    TTGGTGTGTGAGAAAGAACTGTACTGCATGGATTGGACAGTTAAAATGATGCAAAAAGTC
```

```
ENST00000378079        TTGGTGTGTGAGAAAGAACTGTACTGCATGGATTGGACAGTTAAAATGATGCAAAAAGTC
ENSMUST00000093939     TTGGTGTGTGAGAAAGAGTTGTACTGCATGGACTGGACAGTCAGAATGATGCAGAAAGTC
ENSRNOT00000055379     TTGGTGTGTGAGAAAGAGTTGTACTGCATGGACTGGACAGTTAGAATGATGCAAAAAGTC

ENSCJAT00000016284     TGTAAAATCTTTAGCACTCCAGTGGAAAGAAATAACTTCCTACAGAATGTGGCAAATGCA
ENSPPYT00000009931     TGCAAAGTCTTTAGCACTCCAGTGGAAAGAAATAACTTCCTGCAGAGTGTGGCAAATGCA
ENSMMUT00000025551     TGCAAAGTCTTTAGCACTCCAGTGGAAAGAAATAACTTCCTGCAGAATGTGGCAAATGCA
ENSGGOT00000013620     TGCAAAGTCTTTAGCACTCCAGTGGAAAGAAAGAACTTCCTGCAGAATGTGGCAAATGCA
ENSPTRT00000059793     TGCAAAGTCTTTAGCACTCCAGTAGAAAGAAAGAACTTCCTGCAGAATGTGGCAAATGCA
ENST00000378079        TGCAAAGTCTTTAGCACTCCAGTGGAAAGAAAGAACTTCCTGCAGAATGTGGCAAATGCA
ENSMUST00000093939     TGCAAAGTCTTCAGCACTGCAGCAGAGAGGAAGAGCTTCCTGCAGAGCATAGCAAATGCT
ENSRNOT00000055379     TGCAAAGTCTTCAACACTGCAACAGAGAGAAAGAGCTTCCTGCAGAGCGTAGCAAATGCC

ENSCJAT00000016284     TTTGCATGTATTACAATGGAAATGCTGCAATCAATCATGTCTGGAGATCGTGATCAAGAT
ENSPPYT00000009931     TTTGCATGTGTTATAATGGAAATGCTGCAATCAATTATGTCTGGAGACCGTGATGAAGAT
ENSMMUT00000025551     TTTGCATGTGTTATAATGGAAATGCTGCAATCAATTATGTCTGGAGACCGTGATGAAGAT
ENSGGOT00000013620     TTTGCATGTGTTATAATGGAAATGCTGCAATCAATTATGTCTGGAGACCGTGATGAAGAT
ENSPTRT00000059793     TTTGCATGTGTTATAATGGAAATGCTGCAATCAATTATGTCTGGAGACCGTGATGAAGAT
ENST00000378079        TTTGCATGTGTTATAATGGAAATGCTGCAATCAATTATGTCTGGAGACCGTGATGAAGAT
ENSMUST00000093939     TTTGCCTGTGTCACGATGGAAATGCTGCAGCCTGTTATGTCGGGAGATCGCGATGACGAT
ENSRNOT00000055379     TTTGCCTGTGTCACGATGGAAATGCTGCAGCCCGTTATGTCTGGAGAGCGTGATGATGAT

ENSCJAT00000016284     GACAGAAGCTTCTTGAATTTGTTCCATCTTGTACATGCTCAGGCTAACTTCCATAAGGAG
ENSPPYT00000009931     GACAGAAGCTTTTTGAATTTGTTCCATCTTGTACATGCTCAGGCTAACTTCCATAAGGAG
ENSMMUT00000025551     GACAGAAGCTTTCTGAATTTGTTCCATCTTGTACATGCTCAGGCTAACTTCCATAAAGAG
ENSGGOT00000013620     GACAGAAGCTTTTTGAATTTGTTCCATCTTGTACATGCTCAGGCTAACTTCCATAAGGAG
ENSPTRT00000059793     GACAGAAGCTTTTTGAATTTGTTCCATCTTGTACATGCTCAGGCTAACTTCCATAAGGAG
ENST00000378079        GACAGAAGCTTTTTGAATTTGTTCCATCTTGTACATGCTCAGGCTAACTTCCATAAGGAG
ENSMUST00000093939     GACAGAGGCTTCCTGAATCTGTTCCATCTTCTCCATGCTCAGGCGAACTTCCATAAGGAG
ENSRNOT00000055379     GACAGAGGCTTCCTGAATTTGTTCCATCTTCTCCATGCTCAGGCAAACTTCCATAAGGAG

ENSCJAT00000016284     GTTCTATATTTGACCATGAATACTCCTCTGTCTACC
ENSPPYT00000009931     GTCCTGTATTTGACCATGAATACTCCTCTGTCTACC
ENSMMUT00000025551     GTCCTGTATTTGACCATGAATACTTCTCTGTCTGCC
ENSGGOT00000013620     GTCCTGTATTTGACCATGAATACTCCTCTGTCTACC
ENSPTRT00000059793     GTCCTGTATTTGACCATGAATACTCCTTTGTCTACC
ENST00000378079        GTCCTGTATTTGACCATGAATACTCCTCTGTCTACC
ENSMUST00000093939     GTGCTGTATCTGACCATGAACGCT---ATCTCTTCC
ENSRNOT00000055379     GTGCTGTATCTGACCATGAATGCT---GTCTCTTCC
```

Multiple sequence alignment of Fbxo48

```
ENSCJAT00000012586     ATGCAGAAAAACTCCAAGAGAAGCAATAATTCAAGAGTTTCTCTCACAGACACAAACTCT
ENSMMUT00000010559     ATGCATAAAAACTCCAAGAGAAACAGTAATTCAAGAGTTTCTCACACAGAAGTGAACTCT
ENSGGOT00000007379     ATGCATAAAAACTCCAAGAGAAACAGTAATTCAAGAGTTTCTCACACAGAAGCGAACTCT
ENST00000377957        ATGCATAAAAACTCCAAGAGAAACAATAATTTAAGAGTTTCTCACACAGAAGCGAACTCT
ENSPTRT00000058862     ATGCATAAAAACTCCAAGAGAAACAATAATTCAGGAGTTTCTCACACAGAAGCGAACTCT
ENSPPYT00000014792     ATGCATAAAAACTCCAAGAGAAACAATAATTCAAGAGTTTCTCACACAGAAGTGAACTCT
ENSMUST00000061327     ATGAAGAAAACCTCCAAGAAGAACAATAATTTCAAAATTCCTGGTACAGAATTGAATTCC
ENSRNOT00000032965     ---ATGAAGAAAACTTCCAAGAACAGTAATGCCAAGGTTCCCGGTATGGAATTGAATTCC

ENSCJAT00000012586     GTGGATGCTGAGAAGGAAAATCATGAGAGTCAAAACAACTTTGTTGACCTGCTGCCTGAA
ENSMMUT00000010559     GTGGATGCTGAGAAGGAAAAAAATGAGAGTGAAAACAACTTTGTTGAACTGCTGCCTCAA
ENSGGOT00000007379     GTGGATGCTGAGAAGGAAAAAAATGAGAGTCAAAACAACTTTTTTGAACTGCTGCCTGCA
```

```
ENST00000377957        GTGGATGCTGAGAAGGAAAAAAATGAGAGTCAAAACAACTTTTTTGAACTGCTGCCTGCA
ENSPTRT00000058862     GTGGATGCTGAGAAGGAAAAAAATGAGAGTCAAAACAACTTTTTTGAACTGCTGCCTGCA
ENSPPYT00000014792     GTGGATGCTGAGAAGGAAAAAAATGAGAGTCAAAACAACTTTGTTGAACTGCTGCCTACA
ENSMUST00000061327     GCGGATGCTGAAAGGGGAAAAGAAGAGAGTCAACGTAACTTTGTTGAACTACTGCCCTTG
ENSRNOT00000032965     GAGGATGCTGAAGGGGGGAGAAAAGAGAATCAAAGTAACTTTGTTGACCTGCTGCCCCTG

ENSCJAT00000012586     GAAATCAGTTTAAAAATTTTCAGTCAGCTGGACATGCGGAGTTTGTGCAGGGCTTCCTTG
ENSMMUT00000010559     GAACTCACTTTTAAAATTTTCAGTCAGCTGGACATTCGGAGTTTATGCAGGGCTTCATTG
ENSGGOT00000007379     GAAATCACTTTTAAAATTTTCAGTCAGCTGGACATTCGGAGTCTGTGCAGGGCTTCATTG
ENST00000377957        GAAATCACTTTTAAAATTTTCAGTCAGCTGGACATTCGGAGTCTGTGCAGGGCTTCATTG
ENSPTRT00000058862     GAAATCACTTTTAAAATTTTCAGTCAACTGGACATTCGGAGTCTGTGCAGGGCTTCATTG
ENSPPYT00000014792     GAAATCACTTTTAAAATTTTCAGTCAGCTGGACATTCGGAGTCTGTGCAGGGCTTCATTG
ENSMUST00000061327     GAAGTCACTTATAAAATTTTCAGCCAGCTAGACATTCAGAGTTTATGCAGAGCTTCCAGG
ENSRNOT00000032965     GAAGTCACTTTTAACATTTTCAGCCAGCTAGACATTCCGAGTTTATGTAGCGCGTCCAGG

ENSCJAT00000012586     ACATGCAGGAGCTGGAATGACACAGTAAGAAACAATGACTCTTTATGGAAACCTCACTGC
ENSMMUT00000010559     ACATGCAGGAGCTGGAATTACACCATAAGAAACAGTGACTCTTTATGGAAACCTCACTGC
ENSGGOT00000007379     ACATGCAGGAGCTGGAATGACACAATAAGAAACAGTGACTCTTTATGGAAACCTCACTGC
ENST00000377957        ACATGCAGGAGCTGGAATGACACAATAAGAAACAGTGACTCTTTATGGAAACCTCACTGC
ENSPTRT00000058862     ACATGCAGGAGCTGGAATGACACAATAAGAAACAGTGACTCTTTATGGAAACCTCACTGC
ENSPPYT00000014792     ACATGCAGGAGCTGGAATGACAGAATAAGAAACAGTGACTCTTTATGGAAACCTCACTGC
ENSMUST00000061327     ACATGCACAGGTTGGAATTGTGCAATAAGAAACAATGACTCCTTATGGAAACCCCACTGC
ENSRNOT00000032965     ACGTGCACAAGTTGGAACCATGCAATAAGAAACAATGACTCCTTATGGAAACCTCACTGC

ENSCJAT00000012586     ATGACTCTAAGAGCTGTGTGCCGGAGAGAAATAGATGATGATCTAGAAAGTGGTTATTCT
ENSMMUT00000010559     ATGACTGTAAGAGCTGTGTGCCGAAGAGAAATAGATGATGATCTAGAAAGTGGTTATTCC
ENSGGOT00000007379     ATGACTGTAAGAGCTGTGTGCCGAAGAGAAATAGATGATGATCTAGAAAGTGGTTATTCC
ENST00000377957        ATGACTGTAAGAGCTGTGTGCCGAAGAGAAATAGATGATGATCTAGAAAGTGGTTATTCC
ENSPTRT00000058862     ATGACTGTAAGAGCTGTGTGCCGAAGAGAAATAGATGATGATCTAGAAAGTGGTTATTCC
ENSPPYT00000014792     ATGACTGTAAGAGCTGTGTGCCGAAGAGAAATAGATGACGATCTAGAAAGTGGTTATTCC
ENSMUST00000061327     TTGACCATAAGAGCTGTGTGCCAAAGAGAAATAGACGATGATATAAAAAGTGGTTATACC
ENSRNOT00000032965     TTGAGCATAAGAGCTGTGTGCCAAAGAGAAGTAGATGATGATATAGAAAATGGTTATACC

ENSCJAT00000012586     TGGAGGGTAATACTCCTGAGGAATTACCAGAAGAGTAAAGTGAAACACGAATGGCTAAGT
ENSMMUT00000010559     TGGAGGGTAATACTGCTGAGGAATTACCAGAAGAGTAAAGTGAAACACGAATGGCTAAGT
ENSGGOT00000007379     TGGAGGGTGATACTGCTGAGGAATTACCAGAAGAGTAAAGTGAAACACGAATGGCTAAGT
ENST00000377957        TGGAGGGTGATACTGCTGAGGAATTACCAGAAGAGTAAAGTGAAACACGAATGGCTAAGT
ENSPTRT00000058862     TGGAGGGTGATACTGCTGAGGAATTACCAGAAGAGTAAAGTGAAACACGAATGGCTAAGT
ENSPPYT00000014792     TGGAGGGTAATACTGCTGAGGAATTACCAGAAGAGTAAAGTGAAACACGAATGGCTAAGT
ENSMUST00000061327     TGGAGGGTAATACTCCTAAGAAATTACCAGAAGAGCAAAGTGAAATATGAGTGGCTAAGT
ENSRNOT00000032965     TGGAGGGTAATACTCCTGAGAAATTACCAGAAGAGCAAAGTGAAAAACGAGTGGCTAAGC

ENSCJAT00000012586     GGCAGATACAGCAACATTTGTTCTTCCATTAGCCTACCAGAAAAAATCATGTACCCAATG
ENSMMUT00000010559     GGCAGATACAGCAACATTTGTTCTCCCATTAGCCTACCAGAAAAAACCATGTACCCAATG
ENSGGOT00000007379     GGCAGATACAGCAACATTTGTTCTCCCATTAGCCTACCAGAAAAAATCATGTACCCAATG
ENST00000377957        GGCAGATACAGCAACATTTGTTCTCCCATTAGCCTACCAGAAAAAATCATGTACCCAATG
ENSPTRT00000058862     GGCAGATACAGCAACATTTGTTCTCCCATTAGCCTACCAGAAAAAATCATGTACCCAATG
ENSPPYT00000014792     GGCAGATACAGCAACATTTGTTCTCCCATTAGCCTACCAGAAAAAATCATGTACCCAATG
ENSMUST00000061327     GGCAGATACAGCAACATCCGTTCTCCTGTTAACTTGCCAGAAAAGGCTATGTGCCCCATG
ENSRNOT00000032965     GGCCGATACAGCAACATCCGTTCTCCCGTTAGCCTGCCAGAGAAGGCCATGTGCCCGATG

ENSCJAT00000012586     GATGCAGATACATGGGGGGAAATTCTAGAAGCAGAACTGGAAAGA
ENSMMUT00000010559     GATGCAGATACATGGGGGGAAATTCTAGAAGCAGAACTGGAAAGA
ENSGGOT00000007379     GATGCAGATACATGGGGGGAAATTCTAGAAGCAGAACTGGAAAGA
```

```
ENST00000377957        GATGCAGATACATGGGGGAAATTCTAGAAGCAGAACTGGAAAGA
ENSPTRT00000058862     GATGCAGATACATGGGGGAAATTCTAGAAGCAGAACTGGAAAGA
ENSPPYT00000014792     GATGCAGATACATGGGGGAAATTCTAGAAGCAGAACTGGAAAGA
ENSMUST00000061327     GATGCAGATACGTGGGGGAAATTCTAGATGCAGAACTGGAAAGA
ENSRNOT00000032965     GACGCAGATACGTGGGGGAAATTCTAGATGCAGAGCTGGGAAGA
```

Multiple sequence alignment of Fbxo5

```
ENSCJAT00000024816     ------------------CCCCTGCAGCTGCGCCCCACCGCCACCCCGTGCTCCTGCAGC
ENSMMUT00000026804     ATGAGCCGGCGCCCCTGCAGCTGCGCCCCACGGCCACCCCGCTGCTGCTGCAGCGCCAGC
ENSPPYT00000019921     ATGAGCCGGCGCCCCTGCAGCTGCGCCCTACGGCCACCCCGCTGCTCCTGCAGCGCCAGC
ENST00000229758        ATGAGCCGGCGCCCCTGCAGCTGCGCCCTACGGCCACCCCGCTGCTCCTGCAGCGCCAGC
ENSGGOT00000001616     ATGAGCCGGCGCCCCTGCAGCTGCGCCCTACGGCCACCCCGCTGCTCCTGCAGCGCCAGC
ENSPTRT00000034561     ATGAGCCGGCGCCCCTGCAGCTGCGCCCTACGGCCACCCCGCTGCTCCTGCAGCGCCAGC
ENSMUST00000019907     ATGAGCCGGCGCACCTGCAGTGACCTGCGCCGGCCGTCCTCCTGTCCCTGCCGTCTT---
ENSRNOT00000029416     ATGAGCCGGCGCGCCTGCGGTGATCTCCCCCGGCCGTCCTCCTGTCTCTGCCCTCTT---


ENSCJAT00000024816     GCCGTGACAGCCGCCGGACGCCCTCGTCCCTCGGACAGTTGTAAAGAAGAAAGTTCTACC
ENSMMUT00000026804     GCCGTGACAGCCGCCGGGCGTCCTCGACCCTCGGATAGTTGTAAAGAAGAAAGTTCTACC
ENSPPYT00000019921     GCCGTGACAGCCGCCGAGCGCCCTCGACCGTCGGATAGTTGTAAAGAAGAAAGTTCTACC
ENST00000229758        GCAGTGACAGCCGCCGGGCGCCCTCGACCCTCGGATAGTTGTAAAGAAGAAAGTTCTACC
ENSGGOT00000001616     GCAGTGACAGCCGCCGGGCGCCCTCGACCCTCGGATAGTTGTAAAGAAGAAAGTTCTACC
ENSPTRT00000034561     GCAGTGACAGCCGCCGGGCGCCCTCGACCCTCGGATAGTTGTAAAGAAGAAAGTTCTACC
ENSMUST00000019907     ---------------GGCGCGCGGACTACCGTGGACGGTTGTAAAGAAGAAAGCCCTGTT
ENSRNOT00000029416     ---------------GACGCGCGGACGACCGTGGACGGTTGTAAAGAAGAAAGCCCTGTT


ENSCJAT00000024816     CTTTCTGTCAAAATGAAGTGTGATTTTAATTGTAACCATGTTCATTCCGGACTGAAACTG
ENSMMUT00000026804     CTTTCTGTCAAAATGAAGTGTGATTTTAATTGTAACCATGTTCATTCCGGACTTAAACTG
ENSPPYT00000019921     CTTTCTGTCAAAATGAAGTGTGATTTTAATTGTAACAATGTTCATTCCGGACTTAAACTG
ENST00000229758        CTTTCTGTCAAAATGAAGTGTGATTTTAATTGTAACCATGTTCATTCCGGACTTAAACTG
ENSGGOT00000001616     CTTTCTGTCAAAATGAAGTGTGATTTTAATTGTAACCATGTTCATTCCGGACTTAAACTG
ENSPTRT00000034561     CTTTCTGTCAAAATGAAGTGTGATTTTAATTGTAACCATGTTCATTCCGGACTTAAACTG
ENSMUST00000019907     CTTTCTGTTACAATGAAGTGT---TTTAATTGCAACCCTGATCTTTCCGAGCTTGAAGTG
ENSRNOT00000029416     CTTTCGGTTACCATGAAGTGT---TTTAATTGCAACCCTGATCTTTTGGAGCTTGAGGTG


ENSCJAT00000024816     GTAAAACCTGATGACATTGGAAGACGAGTTTCCTACACTCCTGGATATTTGGAAGGTTCC
ENSMMUT00000026804     GTAAAACCTGATGACATTGGAAGACTAGTTTCCTACACCCCTGCATATTTGGAAGGTTCC
ENSPPYT00000019921     GTAAAACCTGATGACATTGGAAGACTAGTTTCCTACACCCCTGCATATTTGGAAGGTTCC
ENST00000229758        GTAAAACCTGATGACATTGGAAGACTAGTTTCCTACACCCCTGCATATTTGGAAGGTTCC
ENSGGOT00000001616     GTAAAACCTGATGACATTGGAAGACTAGTTTCCTACACCCCTGCATATTTGGAAGGTTCC
ENSPTRT00000034561     GTAAAACCTGATGACATTGGAAGACTAGTTTCCTACACCCCTGCATATTTGGAAGGTTCC
ENSMUST00000019907     GTGAAGCCTGAGGACAGTGGGATAGAAGCTTCCTACAGTCCCGTGTGTTTGGAACCTTCC
ENSRNOT00000029416     GTGAAGACTGAGGACAGTGGGAGGGAAGGTTCCTACAGTCCTGTGTGTTTGGAACCTTCC


ENSCJAT00000024816     TGTAAAGACTGCATTAAAGACTATGAAAGGCTGTCATGCATTGGGTCACCAGTTGTGAGC
ENSMMUT00000026804     TGTAAAGACTGCATTAAAGACTATGAAAGGCTGTCATGTATTGGGTCACCGATTGTGAGC
ENSPPYT00000019921     TGTAAAGACTGCATTAAAGACTATGAAAGGCTGTCATGTATTGGGTCACCGATTGTGAGC
ENST00000229758        TGTAAAGACTGCATTAAAGACTATGAAAGGCTGTCATGTATTGGGTCACCGATTGTGAGC
ENSGGOT00000001616     TGTAAAGACTGCATTAAAGACTATGAAAGGCTGTCATGTATTGGGTCACCGATTGTGAGC
ENSPTRT00000034561     TGTAAAGACTGCATTAAAGACTATGAAAGGCTGTCATGTATTGGGTCACCGATTGTGAGC
ENSMUST00000019907     TGTAATGACTGTGTTAGAAACCATGAGAGGTTGTCTTTCATCGACTCACCAATTGTGGGA
ENSRNOT00000029416     TGTGACAATTGTGTTGGAAACCATGAAAGATTGTCTTTTGTCGAATCACCAATTGTGGGA


ENSCJAT00000024816     CCTAGGATTGTGGAACTTGAAACTGAAAGCAAGCCTTTGCATAACAAGGAAAATCAACAT
```

```
ENSMMUT00000026804     CCTAGGATTGTAGAACTTGAAACTGAAAGCAAACCCTTGCATAACAAGGAAAATCAACAT
ENSPPYT00000019921     CCTAGGACTGTAGAACTTGAAACTGAAAGCAAGCCCTTGCATAACAAGGAAAATCAACAT
ENST00000229758        CCTAGGATTGTACAACTTGAAACTGAAAGCAAGCGCTTGCATAACAAGGAAAATCAACAT
ENSGGOT00000001616     CCTAGGATTGTAGAACTTGAAACTGAAAGCAAGCCCTTGCATAACAAGGAAAATCAACAT
ENSPTRT00000034561     CCTAGGATTGTAGAACTTGAAACTGAAAGCAAGCCCTTGCATAACAAGGAAAATCAACAT
ENSMUST00000019907     CAT------------------------------------GATAACAAGGAAAATCAACGC
ENSRNOT00000029416     CAT------------------------------------GATAACAAGGAAAATCAACGT

ENSCJAT00000024816     GTACAACAAACAAACAATAATACAAATGAAATAGAAGTACTAGAGACCAGTAGACTTTAT
ENSMMUT00000026804     GTGCAACAAACACTTAATAGTACAAATGAAATAGAAGCACTAGAGACCAGTAGACTTTAT
ENSPPYT00000019921     GTGCAACAGACACTTAATAGTACAAATGAAATAGAAGCACTAGAGACCAGTAGACTTTAT
ENST00000229758        GTGCAACAGACACTTAATAGTACAAATGAAATAGAAGCACTAGAGACCAGTAGACTTTAT
ENSGGOT00000001616     GTGCAACAGACACTTAATAGTACAAATGAATTAGAAGCACTAGAGACCAGTAGACTTTAT
ENSPTRT00000034561     GTGCAACAGACACTTAATAGTACAAATGAAATAGAAGCACTAGAGACCAGTAGACTTTAT
ENSMUST00000019907     GTACAAAACACACTAGATAGTTCAAACGAAACAGAAGAGCTAGAGGCCAGTAGACTGTAT
ENSRNOT00000029416     GTACAAAATATACTAGATAGTTCAAAGGAAGTGGAAGAGCTGGAGGCCAGCAGACTGTAT

ENSCJAT00000024816     GAAGACAGTGGCTATTCCTCATTTTCTCAATTAAGTGGCCTCAGTGAACATGAAGAAGGT
ENSMMUT00000026804     GAAGACAGTGGCTATTCCTCATTTTCTCAACAAAGTGGCCTCAGTGAACATGAAGAAGGT
ENSPPYT00000019921     GAAGACAGTGGCTATTCCTCATTTTCTCAACAAAGTGGCCTCAGTGAACATGAAGAAGGT
ENST00000229758        GAAGACAGTGGCTATTCCTCATTTTCTCTACAAAGTGGCCTCAGTGAACATGAAGAAGGT
ENSGGOT00000001616     GAAGACAGTGGCTATTCCTCATTTTCTCAACAAAGTGGCCTCAGTGAACATGAAGAAGGT
ENSPTRT00000034561     GAAGACAGTGGCTATTCCTCATTTTCTCAACAAAGTGGCCTCAGTGAACATGAAGAAGGT
ENSMUST00000019907     GAGGACAGTGGCTACTCATCTTTCACACAA------------AGTGACCGTGACGATGGC
ENSRNOT00000029416     GAGGACAGCGGCTACTCCTCATTCATACAG------------AGTGACAGTGACGATGGC

ENSCJAT00000024816     AGCCTCCTGGAGGAAAATTTCAGTGACAGTCCACAATCCTGCCTGCTACACATACAAAGC
ENSMMUT00000026804     AGCCTCCTGGAGGAAAATTTCAGTGACAGTCCACAGTCCTGCCTGCTACAAATACAAAGC
ENSPPYT00000019921     AGCCTCCTGGAGGAGAATTTCAGTGACGGTCTACAATCCTGCCTGCTACAAATACAAAGC
ENST00000229758        AGCCTCCTGGAGGAGAATTTCGGTGACAGTCTACAATCCTGCCTGCTACAAATACAAAGC
ENSGGOT00000001616     AGCCTCCTGGAGGAGAATTTCGGTGACAGTCTACAATCCTGCCTGCTACAAATACAAAGC
ENSPTRT00000034561     AGCCTCCTGGAGGAGAATTTCGGTGACAGTCTACAATCCTGCCTGCTACAAATACAAAGC
ENSMUST00000019907     ATCCTTATCCTGGAGAATTTCAGAAACAGTCCCCAGGCCCGTCTGCTGCCATCACAGAGC
ENSRNOT00000029416     ATCCTTATCCTGGAGAATTTCAGAAACAGTTCCCAGGCCCATCTGCTGTCATCGCAGAGC

ENSCJAT00000024816     CCAGACCAATATCCCAACAAAAACTTGCTGCCAGTTCTTCATTTTGAAAAAGTGGTTTGT
ENSMMUT00000026804     CCAGACCAATATCCCAACAAAAACTTGCTGCCAGTTCTTCATTTTGAAAAAGTGGTTTGT
ENSPPYT00000019921     CCAGACCAATATCCCAACAAAAACTTGCTGCCAGTTCTTCATTTTGAAAAAGTGGTCTGT
ENST00000229758        CCAGACCAATATCCCAACAAAAACTTGCTGCCAGTTCTTCATTTTGAAAAAGTGGTTTGT
ENSGGOT00000001616     CCAGACCAATATCCCAACAAAAACTTGCTGCCAGTTCTTCATTTTGAAAAAGTGGTTTGT
ENSPTRT00000034561     CCAGACCAATATCCCAACAAAAACTTGCTGCCAGTTCTTCATTTTGAAAAAGTGGTTTGT
ENSMUST00000019907     CCGGACCAGCATCCCAACAAAACCTTGCTGCCTGTCCTGCATTTTGAAAGAGTGGTTTGC
ENSRNOT00000029416     CCAGACCAGCATCCCAACAAAAACCTGCTGCCTGCCCTGCATTTTGAAAGAGTGGTTTGC

ENSCJAT00000024816     TCAACATTAAAAAAGAATGCCAAACGAAATCCTAAATTAGATCGGGAGATGCTGAAGGAA
ENSMMUT00000026804     TCAACATTAAAAAAGAATGCAAAGCGAAATCCTAAAGTAGATCGGGAGATGCTGAAGGAA
ENSPPYT00000019921     TCAACATTAAAAAAGAATGCAAAACGAAATCCTAAAGTAGATCGGGAGATGCTGAAGGAA
ENST00000229758        TCAACATTAAAAAAGAATGCAAAACGAAATCCTAAAGTAGATCGGGAGATGCTGAAGGAA
ENSGGOT00000001616     TCAACATTAAAAAAGAATGCAAAACGAAATCCTAAAGTAGATCGGGAGATGCTGAAGGAA
ENSPTRT00000034561     TCAACATTAAAAAAGAATGCAAAACGAAATCCTAAAGTAGATCGGGAGATGCTGAAGGAA
ENSMUST00000019907     TCAACACTAAAAAAGAATGGCAAGCGAAACCCTAAAGTGGATCGAGAAATGCTGAAGGAA
ENSRNOT00000029416     TCAACATTAAAAAAGAATGGCAAGCGAAACTCTAAAGTGGATCAAGAAATGCTGAAGGAA

ENSCJAT00000024816     ATTATAGCCAAAGGGAATTTTAGACTGCAGAATATAATTGGCAGAAAAATGGGCCTAGAA
```

```
ENSMMUT00000026804     ATTATAGCCAGAGGAAATTTTAGACTGCAGAATATAATTGGCAGAAAAATGGGCCTAGAA
ENSPPYT00000019921     ATTATAGCCAGAGGAAATTTTAGACTGCAGAATATAATTGGCAGAAAAATGGGCCTAGAA
ENST00000229758        ATTATAGCCAGAGGAAATTTTAGACTGCAGAATATAATTGGCAGAAAAATGGGCCTAGAA
ENSGGOT00000001616     ATTATAGCCAGAGGAAATTTTAGACTGCAGAATATAATTGGCAGAAAAATGGGCCTAGAA
ENSPTRT00000034561     ATTATAGCCAGAGGAAATTTTAGACTGCAGAATATAATTGGCAGAAAAATGGGCCTAGAA
ENSMUST00000019907     GTTATTGCCAGCGGGAACTTTAGACTGCAAAATATAATTGGCAAGAAAATGGGCCTGGAG
ENSRNOT00000029416     GTTATCGCCAGCGGAAACATTACACTGCAAAATATAATTGGCAAGAAAATGGGCCTGGAA

ENSCJAT00000024816     TGTGTAGATATTCTCAGCGAACTCTTTCTAAGAGGACTCAGACATCTCTTAGCAACTATT
ENSMMUT00000026804     TGTGTAGATATTCTCAGCGAACTCTTTCGAAGGGGACTCAGACATCTCTTAGCAACTATT
ENSPPYT00000019921     TGTGTAGATATTCTCAGCGAACTCTTTCGAAGGGGACTCAGACATCTCTTAGCAACTATT
ENST00000229758        TGTGTAGATATTCTCAGCGAACTCTTTCGAAGGGGACTCAGACATGTCTTAGCAACTATT
ENSGGOT00000001616     TGTGTAGATATTCTCAGCGAACTCTTTCGAAGGGGACTCAGACATGTCTTAGCAACTATT
ENSPTRT00000034561     TGTGTAGATATTCTCAGCGAACTCTTTCGAAGGGGACTCAGACATGTCTTAGCAACTATT
ENSMUST00000019907     CACCTAGACATCCTGGCTGAGCTCTCCCGCAGGGGATTCGTGCACCTGTTGGCTAACATT
ENSRNOT00000029416     CACCTAGATATCCTGGCTGAGCTCTCCCGGAGGGGATGTATGCACCTGTTAGCTAATATT

ENSCJAT00000024816     TTGGCACAACTCAGTGATATGGACTTAATCAATGTGTCTAAAGTGAGCACAACTTGGAAG
ENSMMUT00000026804     TTAGCACAACTCAGTGACATGGACTTAATCAATGTGTCTAAAGTGAGCACAACTTGGAAG
ENSPPYT00000019921     TTAGCACAACTCAGTGACATGGACTTAATCAATGTGTCTAAAGTGAGCACAACTTGGAAG
ENST00000229758        TTAGCACAACTCAGTGACATGGACTTAATCAATGTGTCTAAAGTGAGCACAACTTGGAAG
ENSGGOT00000001616     TTAGCACAACTCAGTGACATGGACTTAATCAATGTGTCTAAAGTGAGCACAACTTGGAAG
ENSPTRT00000034561     TTAGCACAACTCAGTGACATGGACTTAATCAATGTGTCTAAAGTGAGCACAACTTGGAAG
ENSMUST00000019907     TTGACTAAGCTCAGCGGCATGGACTTAGTAAATCTGTCTAAAGTGAGCAGAATTTGGAAG
ENSRNOT00000029416     TTCATGAAGCTCAGCGGCATGGACTTAATAAATTTGTCTAAAGTGAGCAGAATTTGGAAG

ENSCJAT00000024816     AAGATCCTAGAAGATGATAAGGGGGCATTCCAGTTGTACAGTAAAGCAATACAGAGAGTT
ENSMMUT00000026804     AAGATCCTAGAAGATGATAAGGGGGCATTCCAGTTGTACAGTAAAGCAATACAAAGAGTT
ENSPPYT00000019921     AAGATCCTAGAAGATGATAAGGGGGCATTCCAGTTGTACAGTAAAGCAATACAAAGAGTT
ENST00000229758        AAGATCCTAGAAGATGATAAGGGGGCATTCCAGTTGTACAGTAAAGCAATACAAAGAGTT
ENSGGOT00000001616     AAGATCCTAGAAGATGATAAGGGGGCATTCCAGTTGTACAGTAAAGCAATACAAAGAGTT
ENSPTRT00000034561     AAGATCCTAGAAGATGATAAGGGGGCATTCCAGTTGTACAGTAAAGCAATACAAAGAGTT
ENSMUST00000019907     AAGATACTGGAAAACAATAAGGGGGCGTTCCAGCTCTACAGCAAAACCATGCAGCGAGTC
ENSRNOT00000029416     AAGATACTAGAAAGCGATAAAGGAGCATTCCAGCAATACAGCAAAGCCATGCAGAGAGTC

ENSCJAT00000024816     ACCGAAAACAATAAATTTTCACCACATGCTTCAACCAGAGAATATGTTATGTTCAGAACC
ENSMMUT00000026804     ACCGAAAACAATAAATTTTCACCACATGCGTCAACCAGAGAATTTGTTATGTTCAGAACC
ENSPPYT00000019921     ACCGAAAACAATAAGTTTTCACCTCATGCTTCAACCAGAGAATATGTTATGTTCAGAACC
ENST00000229758        ACCGAAAACAATAAATTTTCACCTCATGCTTCAACCAGAGAATATGTTATGTTCAGAACC
ENSGGOT00000001616     ACCGAAAACAATAAATTTTCACCTCATGCTTCAACCAGAGAATATGTTATGTTCAGAACC
ENSPTRT00000034561     ACCGAAAACAATAAATTTTCACCTCATGCTTCAACCAGAGAATATGTTATGTTCAGAACC
ENSMUST00000019907     ATTGAAAGCAGTAAGTTGTCACTACATGCTACAACGAGAGGATATGTTGTGGGCAGAGCT
ENSRNOT00000029416     ATTGAAAGCAATAAGTTGTCACTACATGCTTCAACCAGAGGATATGTCGTGGGCAGAACT

ENSCJAT00000024816     CCACTGGCTTCTGTTCAGAAATCAGCAGCCCAGACTTCTCTCAAAAAAGATGCTCAAGCC
ENSMMUT00000026804     CCACTGGCTTCTGTTCAGAAATCAGCAGCCCAGACTTCTCTCAAAAAAGATGCTCAAACC
ENSPPYT00000019921     CCACTGGCTTCTGTTCAGAAATCAGCAGCCCAGACTTCTCTCAAAAAAGATGCTCAAACC
ENST00000229758        CCACTGGCTTCTGTTCAGAAATCAGCAGCCCAGACTTCTCTCAAAAAAGATGCTCAAACC
ENSGGOT00000001616     CCACTTTCTTCTGTTCAGAAATCAGCAGCCCAGACTTCTCTCAAAAAAGATGCTCAAACC
ENSPTRT00000034561     CCACTGGCTTCTGTTCAGAAATCAGCAGCCCAGACTTCTCTCAAAAAAGATGCTCAAACC
ENSMUST00000019907     GCACTAACTTGTGTTCAAAAGTCATCGACCTGGGCACCTCCCAAAAAAGATGTTCAAATC
ENSRNOT00000029416     GCACTAACCTGTGTTCAAAAGTCATCGACCTGGGCACCTCCC---AAAAATGTTCAA---

ENSCJAT00000024816     AAGGTATCCAGTCAGGGTGATCAGAAAGGTTCTACTTACAGTCGACACAATGAATTCTCT
```

```
ENSMMUT00000026804     AAGGTATCCAATCAAGGTGATCAGAAGGGTTCTACTTATAGTCGACACAATGAATTCTCT
ENSPPYT00000019921     AAGTTATCCAGTCAAGGTGATCAGAAAGGTTCTACTTATAGTCGACACAATGAATTCTCT
ENST00000229758        AAGTTATCCAATCAAGGTGATCAGAAAGGTTCTACTTATAGTCGACACAATGAATTCTCT
ENSGGOT00000001616     AAGTTATCCAATCAAGGTGATCAGAAAGGTTCTACTTATAGTCGACACAATGAATTCTCT
ENSPTRT00000034561     AAGTTATCCAATCAAGGTGATCAGAAAGGTTCTACTTATAGTCGACACAATGAATTCTCT
ENSMUST00000019907     AAGTCCTCCAGTCAGCGTGGTCAGAGAGTTTCTACCTACAGCCGGCACAATGAGTTCGTG
ENSRNOT00000029416     ------CCCAGTCAACGTGGTCAGAGGGGTTCTGTCTACAGCCGGCACAATGAGTTCCTG

ENSCJAT00000024816     GAGGTTGCCAAGACTTTAAAAAAGAACGAAAGCCTCAAAGCTTGTATTCGCTGTAATTCA
ENSMMUT00000026804     GAGGTTGCCAAGACTTTGAAAAAGAATGAAAGCCTCAAAGCCTGTATTCGCTGTAATTCA
ENSPPYT00000019921     GAGGTTGCCAAGACATTGAAAAAGAACGAAAGCCTCAAAGCCTGTATTCGCTGTAATTCA
ENST00000229758        GAGGTTGCCAAGACATTGAAAAAGAACGAAAGCCTCAAAGCCTGTATTCGCTGTAATTCA
ENSGGOT00000001616     GAGGTTGCCAAGACATTGAAAAAGAACGAAAGCCTCAAAGCCTGTATTCGCTGTAATTCA
ENSPTRT00000034561     GAGGTTGCCAAGACATTGAAAAAGAACGAAAGCCTCAAAGCCTGTATTCGCTGTAATTCA
ENSMUST00000019907     GAGGTGGCAAAGACATTGAAGAACAACGAAAGCCTCAAAGCCTGTGTTCGCTGTAATTTC
ENSRNOT00000029416     GAGGTGGCAAAGACATTGAAAAACAACGAAAGCCTCAAAGCCTGTGTTCGCTGTAATTTC

ENSCJAT00000024816     CCTGCAAAATATGATTGCTATTTACAAAGGGCAACCTGCAAACGAGAAGGCTGTGGATTT
ENSMMUT00000026804     CCTGCAAAATATGATTGCTATTTACAACGGGCAACCTGCAAACGAGAAGGCTGTGGATTT
ENSPPYT00000019921     CCTGCAAAATATGATTGCTATTTACAACGGGCAACCTGCAAACGAGAAGGCTGTGGATTT
ENST00000229758        CCTGCAAAATATGATTGCTATTTACAACGGGCAACCTGCAAACGAGAAGGCTGTGGATTT
ENSGGOT00000001616     CCTGCAAAATATGATTGCTATTTACAACGGGCAACCTGCAAACGAGAAGGCTGTGGATTT
ENSPTRT00000034561     CCTGCAAAATATGATTGCTATTTACAACGGGCAACCTGCAAACGAGAAGGCTGTGGATTT
ENSMUST00000019907     CCTGCAAAATATGACCACTATTTAGAGCGAGCAGTCTGCAAACGGGAAAGCTGTCAATTT
ENSRNOT00000029416     CCTGCAAAATATGACCATTATTTAGAGCGAGCAGTCTGCAAACGGGAAAGTTGTAAATTC

ENSCJAT00000024816     GATTATTGTACAAGGTGTCTCTGTAATTATCATACTACTAAAGACTGTTCTGATGGCAAG
ENSMMUT00000026804     GATTATTGTACGAGGTGTCTATGTAATTACCATACTACCAAAGACTGTTCAGATGGCAAG
ENSPPYT00000019921     GATTATTGTACGAAGTGTCTCTGTAATTACCATACTACTAAAGACTGTTCAGATGGCAAG
ENST00000229758        GATTATTGTACGAAGTGTCTCTGTAATTATCATACTACTAAAGACTGTTCAGATGGCAAG
ENSGGOT00000001616     GATTATTGTACGAAGTGTCTCTGTAATTATCATACTACTAAAGACTGTTCAGATGGCAAG
ENSPTRT00000034561     GATTATTGTACGAAGTGTCTCTGTAATTATCATACTACTAAAGACTGTTCAGATGGCAAG
ENSMUST00000019907     GAATATTGTACAAAGTGTCTGTGTGCTTACCATAACAACAAAGACTGTTTGAATGGCAAG
ENSRNOT00000029416     GAATATTGTACAAAGTGTCTGTGTGCTTACCATAACAACAAAGACTGTTTGAATAGCAAG

ENSCJAT00000024816     TTCCTCAAAGCCAGTTGTAAAATAGGTCCCCTGCCTGGTACAAAGAAAAGCAAAAAGAAT
ENSMMUT00000026804     CTCCTCAAAGCCAGTTGTAAAATAGGTCCCCTGCCTGGTACAAAGAAAAGCAAAAAGAAT
ENSPPYT00000019921     CTCCTCAAAGCCAGTTGTAAAATAGGTCCCCTGCCTGGTACGAAGAAAAGCAAAAAGAAT
ENST00000229758        CTCCTCAAAGCCAGTTGTAAAATAGGTCCCCTGCCTGGTACAAAGAAAAGCAAAAAGAAT
ENSGGOT00000001616     CTCCTCAAAGCCAGTTGTAAAATAGGTCCCCTGCCTGGTACAAAGAAGAGCAAAAAGAAT
ENSPTRT00000034561     CTCCTCAAAGCCAGTTGTAAAATAGGTCCCCTGCCTGGTACAAAGAAAAGCAAAAAGAAT
ENSMUST00000019907     ATCCTAAAAGCCAGCTGTAAAGTGGGTCCTTTGCCTGGAACTAAAAAGAGTAAAAAGAAC
ENSRNOT00000029416     AACCTAAAAGCCAGCTGCAAAATGGGTCCTTTGCCTGGAACTAAAAAGAGTAAAAAGAAC

ENSCJAT00000024816     TTACGAAGACTG
ENSMMUT00000026804     TTACGAAGATTG
ENSPPYT00000019921     TTACGAAGATTA
ENST00000229758        TTACGAAGATTG
ENSGGOT00000001616     TTACGAAGATTG
ENSPTRT00000034561     TTACGAAGATTG
ENSMUST00000019907     TTACAAAGATTG
ENSRNOT00000029416     TTACAAAGATTG
```

Multiple sequence alignment of Fbxo6

```
ENSCJAT00000025757     ATGGATGCTCCCCACCCCACCACAGCCCTGGCCAGCATTAACGAGCTGCCGGAGAACATC
ENSGGOT00000009440     ATGGATGCTCCCCACTCCAAGGCAGCCCTGGACAGCATTAACGAGTTGCCCGAGAACATC
ENST00000376753        ATGGATGCTCCCCACTCCAAAGCAGCCCTGGACAGCATTAACGAGCTGCCCGAGAACATC
ENSPTRT00000000335     ATGGATGCTCCCCACTCCAAAGCAGCCCTGGACAGCATTAACGAGCTGCCCGAGAACATC
ENSMMUT00000047575     ATGGATGCTCCCCACCCCAAGGCAGCCCTGGACAGCATTAACGAGCTACCCGAGAACATC
ENSPPYT00000002252     ATGGATGCTCCCCACCCCAAGGCAGCCCTGGACAGCATTAACGAGCTGCCCGAGAACATC
ENSMUST00000168503     ---------------------------ATGGTCCACATCAACGAGCTGCCAGAGAACATT
ENSRNOT00000012301     ---------------------------ATGGTCAACATCAACGAGCTGCCGGAGAACATT

ENSCJAT00000025757     CTGCTGGAGCTGTTCTTGCACGTGCCTGCCCGCCAGCTGCTGCTGAACTGCCGCCCCGTC
ENSGGOT00000009440     CTGCTGGAGCTGTTTACGCACGTGCCCGCCCGCCAGCTGCTGCTGAACTGCCGCCTGGTC
ENST00000376753        CTGCTGGAGCTGTTCACGCACGTGCCCGCCCGCCAGCTGCTGCTGAACTGCCGCCTGGTC
ENSPTRT00000000335     CTGCTGGAGCTGTTCACGCACGTGCCCGCCCGCCAGCTGCTGCTGAACTGCCGCCTGGTC
ENSMMUT00000047575     CTGCTGGAGCTGTTCACGCACGTGCCCGCCCGCCAGCTGCTGCTGAACTGCCGCCTGGTC
ENSPPYT00000002252     CTGCTGGAGCTGTTCACGCATGTGCCCGCCCGCCAGCTGCTGCTGAACTGCCGCCTGGTC
ENSMUST00000168503     CTCCTGGAGCTGTTCATCCATATCCCGGCCCCACAGCTGCTGCGCAACTGCCGCCTGGTC
ENSRNOT00000012301     CTACTGGAACTGTTCACCCACGTCCCGGCCCCACAGCTGCTGCGCAACTGCCGCCTGGTC

ENSCJAT00000025757     TGCAGCCTCTGGCGGGACCTCATTGACCTCATGATCCTCTGGAAGCGCAAGTGTCTGCGA
ENSGGOT00000009440     TGCAGCCTCTGGCGGGACCTCATCGACCTCATGACCCTCTGGAAACGCAAGTGCCTGCGA
ENST00000376753        TGCAGCCTCTGGCGGGACCTCATCGACCTCATGACCCTCTGGAAACGCAAGTGCCTGCGA
ENSPTRT00000000335     TGCAGCCTCTGGCGGGACCTCATCGACCTCATGACCCTCTGGAAACGCAAGTGCCTGCGA
ENSMMUT00000047575     TGCAGCCTCTGGCGGGATCTCATCGACCTCATGACCCTCTGGAAGCGCAAGTGCCTGCGA
ENSPPYT00000002252     TGCAGCCTCTGGCGGGACCTCATCGACCTCATGACCCTCTGGAAGCGCAAGTGCCTGCGA
ENSMUST00000168503     TGCCGCCTCTGGCGAGACCTCATCGATGTGGTGTCCCTATGGAAGCGCAAGAGTCTTCGA
ENSRNOT00000012301     TGCAGCCTCTGGCGAGACCTCATCGATGTGATGACCCTCTGGAAGCGCAAGAGCCTTCGA

ENSCJAT00000025757     GACGGCTTCATCACCAAGGACTGGGACCAGCCTGTGGAGGACTGGAAGATCTGCTACTTC
ENSGGOT00000009440     GAGGGCTTCATCACCGAGGACCGGGACCAGCCCGTGGCCGATTGGAAAATCTTCTACTTC
ENST00000376753        GAGGGCTTCATCACCAAGGACTGGGACCAGCCCGTGGCCGACTGGAAAATCTTCTACTTC
ENSPTRT00000000335     GAGGGCTTCATCACCGAGGACTGGGACCAGCCCGTGGCCAACTGGAAAATCTTCTACTTC
ENSMMUT00000047575     GAGGGCTTCATCACCGAGGACTGGGACCAGCCCGTGGCCGACTGGAAGATCTTCTACTTC
ENSPPYT00000002252     GAGGGCTTCATCACCGAGGACTGGGACCAGCCCGTGGCCGACTGGAAAATCTTCTACTTC
ENSMUST00000168503     GAGGGCTTCTTCACCAAAGACCGGTGCGAGCCCGTGGAAGACTGGAAGGTCTTCTATATC
ENSRNOT00000012301     GAGGGCTTTGTCACCAAAGACCGGGACGAGCCTGTGGATGACTGGAAGATCTTCTATATC

ENSCJAT00000025757     CTAAGGAGCCTGCAGAGGAACCTCCTGCGCAACCCGTGCGCCGAAGAGGGTATGTTAGCA
ENSGGOT00000009440     CTACAGAGCCTGCATAGGAACCTCCTGCGCAACCCGTGTGCTGAAGAGGATATGTTTGCA
ENST00000376753        CTACGGAGCCTGCATAGGAACCTCCTGCGCAACCCGTGTGCTGAAGAGGATATGTTTGCA
ENSPTRT00000000335     CTACGGAGCCTGCATAGGAACCTCCTGCGCAACCCGTGTGCTGAAGAGGATATGTTTGCA
ENSMMUT00000047575     CTACGGAGCCTGCACAGGAACCTCCTGCGCAACCCGTGCGCTGAAGAGGATATGTTTGCA
ENSPPYT00000002252     CTACGGAGCCTGCATAGGAACCTCTTGCGCAACCCGTGTGCTGAAGAGGATATGTTTGCA
ENSMUST00000168503     CTGTGCAGCCTGCAGAGGAACCTCCTTCGGAACCCGTGTGCTGAAGAGAACCTGAGCTCA
ENSRNOT00000012301     CTGTGCAGTCTGCAAAGGAACCTCCTTCGGAACCCGTGTGCCGAAGAGAACCTGAGATCA

ENSCJAT00000025757     TGGCAAATCGATACCGAAGGTGGCGACAAATGGAAGGTGGAGAGCCTCCCTGGAGCCCAC
ENSGGOT00000009440     TGGCAAATCGATTTCAATGGTGGAGACCGCTGGAAGGTGGAGAGCCTCCCTGGAGCCCAT
ENST00000376753        TGGCAAATTGATTTCAATGGTGGGGACCGCTGGAAGGTGGAGAGCCTCCCTGGAGCCCAC
ENSPTRT00000000335     TGGCAAATCGATTTCAATGGTGGGGACCGCTGGAAGGTGGAGAGCCTCCCTGGAGCCCAC
ENSMMUT00000047575     TGGCAAATCGATTTCAATGGTGGGGACCGCTGGAAGGTGGAGAGCCTCCCTGGAGCCCAC
ENSPPYT00000002252     TGGCAAATCGATTTCAATGGTGGGGACCGCTGGAAGGTGGAGAGCCTCCCTGGAGCCCAC
ENSMUST00000168503     TGGCGGATAGACTCCAACGGAGGGGATCGCTGGAAGGTGGAGACGCTCCCTGGGAGCTGT
ENSRNOT00000012301     TGGCGGATAGACTCCAATGGGGGAGATGAGTGGAAGGTGGAGAGCCTCCCTGGGGACCAC
```

```
ENSCJAT00000025757     AGGACAGCTTTTCCTGACCCCAAAGTCAAGAATTATTTTGTCACATCTTACGGGATGTGC
ENSGGOT00000009440     GGGACAGATTTTCCTGACCCCAAAGTCAAGAAGTATTTTGTCACATCCTACGAAATGTGC
ENST00000376753        GGGACAGATTTTCCTGACCCCAAAGTCAAGAAGTATTTTGTCACATCCTACGAAATGTGC
ENSPTRT00000000335     GGGACAGATTTTCCTGACCCCAAAGTCAAGAAGTATTTTGTCACATCCTACGAAATGTGC
ENSMMUT00000047575     GGGACAGATTTTCCTGACCCCAAAGTCAAGAAGTATTTTGTCACATCCTACGAAATGTGC
ENSPPYT00000002252     GGGACAGATTTTCCTGACCCCAAAGTCAAGAAGTATTTTGTCACATCCTACGAAATGTGC
ENSMUST00000168503     GGCACAAGCTTTCCTGACAACAAGGTCAAGAAGTATTTTGTCACCTCTTTTGAGATGTGC
ENSRNOT00000012301     GGCACAAGCTTTCCTGACACCAAGGTCAAGAAGTATTTTGTCACCTCTTATGGGATGTGC

ENSCJAT00000025757     CTCAAGTCCCAGCTGGTGGACCTCGTAGCCAAGGGCTACTGGGAGGAGCTACTGGACACG
ENSGGOT00000009440     CTCAAGTCCCAGCTGGTAGACCTTGTAGCCGAGGGCTACTGGGAGGAGCTACTAGACACA
ENST00000376753        CTCAAGTCCCAGCTGGTGGACCTTGTAGCCGAGGGCTACTGGGAGGAGCTACTAGACACA
ENSPTRT00000000335     CTCAAGTCCCAGCTGGTGGACCTTGTAGCCGAGGGCTACTGGGAGGAGCTACTAGACACA
ENSMMUT00000047575     CTCAAGTCCCAGCTGGTGGACCTTGTAGCTGAGGGCTACTGGGAGGAACTACTGGACACA
ENSPPYT00000002252     CTCAAGTCCCAGCTGGTGGACCTTGTAGCCGAGGGCTACTGGGAGGAGCTACTAGACACA
ENSMUST00000168503     CTCAAATCCCAGATGGTGGACCTCAAAGCTGAGGGCTACTGCGAGGAGCTGATGGACACC
ENSRNOT00000012301     CTCAAGTCCCAGATGGTGGACCTCAAAGCCGAGGGCTACTCTGAAAAGCTGCTGGACACC

ENSCJAT00000025757     TTCAGGCCAGACATCGTGGTTAAGGACTGGTTTGCTGCCAGACACGACTGTGGCTGCACC
ENSGGOT00000009440     TTCCGGCCGGACATCGTGGTTAAGGACTGGTTTGCTGCCAGAGCTGACTGTGGCTGCACC
ENST00000376753        TTCCGGCCGGACATCGTGGTTAAGGACTGGTTTGCTGCCAGAGCCGACTGTGGCTGCACC
ENSPTRT00000000335     TTCCGGCCGGACATCGTGGTTAAGGACTGGTTTGCTGCCAGAGCCGACTGTGGCTGCACC
ENSMMUT00000047575     TTCCGGCCGGACATCGTGGTTAAGGACTGGTTTGCTGCCAGAGCCGACTGTGGCTGCACC
ENSPPYT00000002252     TTCCGGCCGGACATCGTGGTTAAGGACTGGTTTGCTGCCAGAGCCGACTGTGGCTGCACC
ENSMUST00000168503     TTTCGGCCTGACATTGTGGTTAAGGACTGGGTTGCCCCCAGAGCAGACTGTGGCTGCACC
ENSRNOT00000012301     GTTCGGCCGGACATTGTGGTTAAAGACTGGTTTGCCCCACGAGCAGACTGCGGCTGTACC

ENSCJAT00000025757     TACCAACTCAAGGTGTACCTGGCCTCGGCTGACTACTTCGTGTTGGATTCCTTCGAGCCC
ENSGGOT00000009440     TACCAACTCAAAGTGCAGCTGGCCTCGGCTGACTACTTCGTGTTGGCCTCCTTCGAGCCC
ENST00000376753        TACCAACTCAAAGTGCAGCTGGCCTCGGCTGACTACTTCGTGTTGGCCTCCTTCGAGCCC
ENSPTRT00000000335     TACCAACTCAAAGTGCAGCTGGCCTCGGCTGACTACTTCGTGTTGGCCTCCTTCGAGCCC
ENSMMUT00000047575     TACCAACTCAAGGTGCAGCTGACCTCAGCTGACTACTTCGTGTTGGCCTCCTTCGAGCCC
ENSPPYT00000002252     TACCAACTCAAGGTGCAGCTGGCCTCGGCTGACTACTTCGTGTTGGCCTCCTTCGAGCCC
ENSMUST00000168503     TATCAACTCCGGGTACAGCTGGCCTCTGCGGACTACATTGTCTTGGCCTCTTTTGAGCCT
ENSRNOT00000012301     TATCACCTCCGGGTACAGCTGGCCTCTGCCGACTACATTGTCCTAGCCTCCTTTGAGCCT

ENSCJAT00000025757     CCACCCGTGACCATCAATCAGTGGAGCGATGCCACATGGACAGAGGTCTCCCACACCTTC
ENSGGOT00000009440     CCACCTGTGACCATCCAACAGTGGAACAATGCCACATGGACAGAGGTCTCCTACACCTTC
ENST00000376753        CCACCTGTGACCATCCAACAGTGGAACAATGCCACATGGACAGAGGTCTCCTACACCTTC
ENSPTRT00000000335     CCACCTGTGACCATCCAACAGTGGAACAATGCCACATGGACAGAGGTCTCCTACACCTTC
ENSMMUT00000047575     CCACCTGTGACCATCCAACAGTGGAACAATGCTGCATGGACAGAGGTCTCCTACACCTTC
ENSPPYT00000002252     CCACCTGTGACCATCCAACAGTGGAACAATGCCGCGTGGACAGAGGTCTCCTACACCTTC
ENSMUST00000168503     CCACCTGTGACATTCCAACAGTGGAATGATGCCAAATGGCAAGAGATTTCCCACACCTTC
ENSRNOT00000012301     CCGCCTGTGACCATCGAACAGTGGAATGACGCCAGCTGGCAAGAGATCTCCCACACCTTC

ENSCJAT00000025757     TCGGACTACCCTCCAGGTGTCCGCTACATCCTCTTCCGGCACGGGGGCCAGGACACCCAG
ENSGGOT00000009440     TCAGACTACCCCCGGGGTGTCCGCTACATCCTTTTCCAGCATGGGGGCAGGGACACCCAG
ENST00000376753        TCAGACTACCCCCGGGGTGTCCGCTACATCCTCTTCCAGCATGGGGGCAGGGACACCCAG
ENSPTRT00000000335     TCAGACTACCCCCGGGGTGTCCGCTACATCCTCTTCCAGCATGGGGGCAGGGACACCCAG
ENSMMUT00000047575     TCAGACTACCCCCGGGGTGTCCGCTACATCCTCTTTCAGCACGGGGGCAGTGACACCCAG
ENSPPYT00000002252     TCAGACTACCCCCGGGGTGTCCGCTACATCCTCTTCCAGCACGGGGGCAGGGACACCCAG
ENSMUST00000168503     TCTGATTACCCTCCAGGTGTCCGTCACATCCTTTTTCAACACGGGGGCCAGGACACTCAG
ENSRNOT00000012301     TCCAATTACCCTCCTGGCGTCCGTCACATCCTTTTTCAACATGGGGGCAAGGACACACAG
```

```
ENSCJAT00000025757     TTCTGGGCAGGCTGGTACGGGCCTGGAGTCACCAACAGCAGCATCGTCGTCAGCCACAAG
ENSGGOT00000009440     TACTGGGCAGGCTGGTATGGGCCCCGAGTCACCAACAGCAGCATCGTCGTCAGCCCCAAG
ENST00000376753        TACTGGGCAGGCTGGTATGGGCCCCGAGTCACCAACAGCAGCATTGTCGTCAGCCCCAAG
ENSPTRT00000000335     TACTGGGCAGGCTGGTATGGGCCCCGAGTCACCAACAGCAGCATCGTCGTCAGCCCCAAG
ENSMMUT00000047575     TACTGGGCAGGCTGGTATGGGCCCCGAGTCACCAACAGCAGCATCGTCGTCAGCCACAAG
ENSPPYT00000002252     TACTGGGCAGGCTGGTATGGGCCCCGAGTCACCAACAGCAGCATCGTCGTCAGCCCCAAG
ENSMUST00000168503     TTCTGGAAAGGCTGGTACGGCCCCCGTGTCACCAACAGCAGCATCATTATCAGCCACAGG
ENSRNOT00000012301     TTCTGGAAAGGCTGGTATGGTCCCCGTGTCACCAACAGCAGCATCATCGTCAGCCACAGG


ENSCJAT00000025757     ATGAGCAGGAACCTGGCCTCCTCTGAGGCTCAGCCTGGGCAG------------------
ENSGGOT00000009440     ATGACCAGGAACCAGGCCTCCTCCGAGGCTCAGCCTGGGCAGAAGCATGGACAGGAGGAG
ENST00000376753        ATGACCAGGAACCAGGCCTCCTCCGAGGCTCAGCCTGGGCAGAAGCATGGACAGGAGGAG
ENSPTRT00000000335     ATGACCAGGAACCAGGCCTCCTCCGAGGCTCAGCCTGGGCAGAAGCATGGACAGGAGGAG
ENSMMUT00000047575     AGGACCAGGAACCAGGCCTCCTCCGAGGCTCAGCCTGGGAAGAAGCATGGACAGGAGGAG
ENSPPYT00000002252     ATGACCAGGAACCAGGCCTCCTCCGAGACTCAGCCTGGGCAGAAGCATGGACAGGAAGAG
ENSMUST00000168503     ACAGCCAAGAACCCTCCCCCTGCCAGAACTCTACCGGAAGAAGTAATCGGAAGGAGACGG
ENSRNOT00000012301     ACAGCCAAGAACCCCGCCCCTGCCAGAACTCTACCGGAGGAAAGTAATAGGAGGAAGATA


ENSCJAT00000025757     ---ACCCAGTTGCCCTACAAAGTTGTTCTCCCAATC
ENSGGOT00000009440     GCTGCCCAATCACCCTACCGAGCTGTTGTCCAGATT
ENST00000376753        GCTGCCCAATCGCCCTACCGAGCTGTTGTCCAGATT
ENSPTRT00000000335     GCTGCCCAATCGCCCTACCGAGCTGTTGTCCAGATT
ENSMMUT00000047575     GCTGCCCAATCGCCCTACCAAGCTGTTCTCCAGATT
ENSPPYT00000002252     GCTGCCCAATCGCCCTACCAAGCTGTTCTCCAGATT
ENSMUST00000168503     CGAGCTTCGGACCTATGGCAAAGGCTAAGGCGT---
ENSRNOT00000012301     CTCAGCTTTGGCTCCTGGGAAGACCTAAGCCCT---
```

Multiple sequence alignment of Fbxo7

```
ENSCJAT00000012155     ATGAAGCTGCGGGTACGGCTTGAGAAACGGACCCAGCAGGTGGAGGTGCCAGAGGCGGAG
ENSMMUT00000009420     ------------------------------------------------------------
ENSPPYT00000013647     ATGAGGCTGCGGGTGCGGCTTCTGAAGCGGACCTGGCCGCTGGAGGTGCCCGAAACGGAG
ENSGGOT00000017216     ATGAGGCTGCGGGTGCGGCTTCTGAAGCGGACCTGGCCGCTGGAGGTGCCCGAGACGGAG
ENSPTRT00000026695     ATGAGGCTGCGGGTGCGGCTTCTGAAGCGGACCTGGCCGCTGGAGGTGCCCGAGACGGAG
ENST00000266087        ATGAGGCTGCGGGTGCGGCTTCTGAAGCGGACCTGGCCGCTGGAGGTGCCCGAGACGGAG
ENSMUST00000130320     ATGAAGCTGCGCGTGCGGCTTCAGAAGCGGACCCAGCCGCTCGAGGTGCCGGAGTCGGAG
ENSRNOT00000006366     ATGAAGCTGCGCGTGCGGCTTCAAAAGCGGACCCAGCCGCTCGAGGTGCCGGAGTCGGAG


ENSCJAT00000012155     CCGACGCTGGGGCAGCTGCGCACGCACCTGAGCCTGGCCCTGCTTCCCACCTGGGGCTAC
ENSMMUT00000009420     ---------GGGACCTTCACTGGAGGCCTCGGCTCTTCC---------------------
ENSPPYT00000013647     CCGACGCTGGGGCAGCTGCGCTGGCACCTGAGGCAGTCCCTGCTGTGCACCTGGGGGTAC
ENSGGOT00000017216     CCGACGCTGGGGCACCTGCGCTCGCACCTGAGGCAGTCCCTGCTGTGCACCTGGGGGTAC
ENSPTRT00000026695     CCGACGCTGGGGCATTTGCGCTCGCGCCTGAGGCAGTCCCTGCTGTGCACCTGGGGGTAC
ENST00000266087        CCGACGCTGGGGCATTTGCGCTCGCACCTGAGGCAGTCCCTGCTGTGCACCTGGGGGTAC
ENSMUST00000130320     CCAACGCTCGGGCAGCTGCGCGCGCACCTCAGCCAGGTCCTGCTGCCCACGTTGGGGTTC
ENSRNOT00000006366     CCGACGCTCGGGCAGCTGCGCGCGCACCTCATCCAGGACCTGCTGCCCACGTTGGGGTTC


ENSCJAT00000012155     AGTTCTGATACCCGATTTGCAATTACATTGAACAACAAGGATGCCCTCACTGGAGATGAA
ENSMMUT00000009420     GGTTCTGATACCCGATTTACAATTACATTGAACTACAAGGATGCCCTCACTGGAGATGAA
ENSPPYT00000013647     CGTTCTGATACCCGATTTACAATTACATTGAACTACAAGGATCCCCTCACTGGAGATGAA
ENSGGOT00000017216     AGTTCTAATACCCGATTTACAATTACATTGAACTACAAGGATCCCCTCACTGGAGATGAA
ENSPTRT00000026695     AGTTCTAATACCCGATTTACAATTACATTGAACTACAAGGATCCCCTCACTGGAGATGAA
ENST00000266087        AGTTCTAATACCCGATTTACAATTACATTGAACTACAAGGATCCCCTCACTGGAGATGAA
ENSMUST00000130320     AGTTCTGATACCCGATTTGCAATTACATTGAACAACAAGGATGCCCTCACTGGAGATGAA
```

```
ENSRNOT00000006366      AGTTCTGATACCCGATTTGCAATTACATTGAACAACAAGGATGCCCTCACTGGAGATGAA

ENSCJAT00000012155      GAGACCTTGGCTTCATATGGGATTGTTTCTGGGGACTTGATATGTTTGGTTCTTGAAGAT
ENSMMUT00000009420      GAGACCTTGGCTTCCTATGGGATTGTTTCTGGGGACTTGATATGTTTGATTCTTCAAGAT
ENSPPYT00000013647      GAGACCTTGGCTTCATATGGGATTGTTTCTGGGGACTTGATATGTTTGATTCTTCAAGAT
ENSGGOT00000017216      GAGACCTTGGCTTCATATGGGATTGTTTCTGGGGACTTGATATGTTTGATTCTTCAAGAT
ENSPTRT00000026695      GAGACCTTGGCTTCATATGGGATTGTTTCTGGGGACTTGATATGTTTGATTCTTCAAGAT
ENST00000266087         GAGACCTTGGCTTCATATGGGATTGTTTCTGGGGACTTGATATGTTTGATTCTTCAAGAT
ENSMUST00000130320      GAGACCTTGGCTTCATATGGGATTGTTTCTGGGGACTTGATATGTTTGGTTCTTGAAGAT
ENSRNOT00000006366      GAGACCTTGGCTTCATATGGGATTGTTTCTGGGGACTTGATATGTTTGGTTCTTGAAGAT

ENSCJAT00000012155      GCCATTCCAGCACCTAATTTACCTTCATCCACAGATTCAGAGCATTCTTCACTCCAGAAT
ENSMMUT00000009420      GACATTCCAGCGCCTAATTTACCTTCATCCACAGATTCAGAGCATTCTTCACTCCAGAAT
ENSPPYT00000013647      GACATTCCAGCGCCTAATATACCTTCATCCACAGATTCAGAGCATTCTTCACTCCAGAAT
ENSGGOT00000017216      GACATTCCAGCGCCTAATATACCTTCATCCACAGATTCAGAGCATTCTCCACTCCAGAAT
ENSPTRT00000026695      GACATTCCAGCGCCTAATATACCTTCATCCACAGATTCAGAGCATTCTTCACTCCAGAAT
ENST00000266087         GACATTCCAGCGCCTAATATACCTTCATCCACAGATTCAGAGCATTCTTCACTCCAGAAT
ENSMUST00000130320      GACATGCCGGCACCTAACTTACCTTCATCTACAGATACAGAGCATTCCTCACTCCAGGAT
ENSRNOT00000006366      GAAATGCCGGCACCTAACTTACCTTCATCTACAGATTCAGAGCATTCCTCTCTCCAGAAT

ENSCJAT00000012155      AATGACCAACCCTCTTTGGCCACTAGCTCCAATCAGACCAGCATACAGGGTGAACAACTG
ENSMMUT00000009420      AATGACCAACCCTCTTTGGCCACCAGCTCCAATGAGACTAGCATACAGGATGAACAACCA
ENSPPYT00000013647      AATGAGCAACCCTCTTTGGCCACCAGCTCCAATCAGACTAGCATACAGGATGAACAACCA
ENSGGOT00000017216      AATGAGCAACCCTCTTTGGCCACCAGCTCCAATCAGACTAGCATACAGGATGAACAACCA
ENSPTRT00000026695      AATGAGCAACCCTCTTTGGCCACCATCTCCAATCAGACTAGCATGCAGGATGAACAACCA
ENST00000266087         AATGAGCAACCCTCTTTGGCCACCAGCTCCAATCAGACTAGCATGCAGGATGAACAACCA
ENSMUST00000130320      AATGACCAACCTTCTTTGGCTGCCACCCCAAGTCAGACTAACATCCCAGATGAACAAGGG
ENSRNOT00000006366      AATGACCAACCTCCTTTGGCTGCCACCTCAAGCCAGGCTAACATCCCAGATGAACAAGGG

ENSCJAT00000012155      AGTGATTCATTCCAAGGACAGGCAGCCCAGTCTGATGTCTGGAATGACGACAATATGTCA
ENSMMUT00000009420      AGTGATTCATTCCAAGGACAGGCAGCCCAGTCTGATGTTTGGAATGATGACAGTACGTTA
ENSPPYT00000013647      AGTGATTCATTCCAAGGACAGGCAGCCCAGTCTGATGTTTGGAATGACGACAGTACGTTA
ENSGGOT00000017216      AGTGATTCATTCCAAGGACAGGCAGCCCAGTCTGGTGTTTGGAATGACGACAGTATGTTA
ENSPTRT00000026695      AGTGATTCATTCCAAGGACAGGCAGCCCAGTCTGGTGTTTGGAATGACGACAGTATGTTA
ENST00000266087         AGTGATTCATTCCAAGGACAGGCAGCCCAGTCTGGTGTTTGGAATGACGACAGTATGTTA
ENSMUST00000130320      ACTGATTCATCCCAAGGACAGGCCACCCCATTTGATGCCTGGACTGATGACAGTATGGAA
ENSRNOT00000006366      AGTGATTCATCCCACGGACAGGTCACCCAATATGATGCCTGGACCGATGACAGTATGGAA

ENSCJAT00000012155      GGGCCCAGTCAAAATTTTGAAGCTGAGTCAATTCAAGATATTGCGGTTATGGAAGAGAGC
ENSMMUT00000009420      GGGCCTAGTCAAAATTTTGAAGCTGAGTCAATTCAAGATATTGAGGATATGGCAGAGGGC
ENSPPYT00000013647      GGGCCTAGTCAAAATTTTGAAGCTGAGTCAATTCAAGATAATGCAGATATGGCAGAGGGC
ENSGGOT00000017216      GGGCCTAGTCAAAATTTTGAAGCTGAGTCAATTCAAGATAATGCGCATATGGCAGAGGGC
ENSPTRT00000026695      GGGCCTAGTCAAAATTTTGAAGCTGAGTCAATTCAAGATAATGCGCATATGGCAGAGGGC
ENST00000266087         GGGCCTAGTCAAAATTTTGAAGCTGAGTCAATTCAAGATAATGCGCATATGGCAGAGGGC
ENSMUST00000130320      GGGCCTAGTCAAAATGTTGAAGCTGAGTCAATTCAGGATGCCATGAGTATGGAAGAGGTT
ENSRNOT00000006366      GGACCTAGTCACAGTGCTGAAGCTGTGTCAATTCAGGACGCCATGAGTGTGGAAGAGGCT

ENSCJAT00000012155      ACAGGTTTCTATCCCTCAGAACCCATGCTCTGCAGTGAATCAGTGGAAGGGCAAGTGCCA
ENSMMUT00000009420      ACAGGTTTCTATCCCTCAGAACCCATGCTCTGTAGTGAATCGGTGGAAGGGCAAGTGCCA
ENSPPYT00000013647      ACAGGTTTCTGTCTCTCAGAACCCATGCTCTGTAGTGAATCGGTGGAAGGGCAAGTGCCA
ENSGGOT00000017216      ACAGGTTTCTATCCCTCAGAACCCATGCTCTGTAGTGAATCGGTGGAAGGGCAAGTGCCA
ENSPTRT00000026695      ACAGGTTTCTATCCCTCAGAACCCATGCTCTGTAGTGAATCGGTGGAAGGGCAAGTGCCA
ENST00000266087         ACAGGTTTCTATCCCTCAGAACCCATGCTCTGTAGTGAATCGGTGGAAGGGCAAGTGCCA
ENSMUST00000130320      TCTGGTTTCCATCCATTGGAACCAATGCTCTGTAATGAAACAGAGGATGGGCAGGTACCA
```

```
ENSRNOT00000006366      TCTGGTTTCCATCCACTGGAGCCAATGCTCTGCAGTGAAACAGAGGACGGGCAGGTGCCA

ENSCJAT00000012155      CATTCATTAGAGACCTTGTATCAATCAGCTAACTGTTCTGATGCCAGTGATGCCTTGATA
ENSMMUT00000009420      CATTCATTAGAGACCTTGTATCAATCAGCTGACTGTTCTGATGCCAATGATGCCTTGATA
ENSPPYT00000013647      CATTCATTAGAGACCTTGTATCAATCAGCTGACTGTTCTGATGCCAATGATGCCTTGATA
ENSGGOT00000017216      CATTCATTAGAGACCTTGTATCAATCAGCTGACTGTTCTGATGCCAATGATGCCTTGATA
ENSPTRT00000026695      CATTCATTAGAGACCTTGTATCAATCAGCTGACTGTTCTGATGCCAATGATGCCTTGATA
ENST00000266087         CATTCATTAGAGACCTTGTATCAATCAGCTGACTGTTCTGATGCCAATGATGCCTTGATA
ENSMUST00000130320      CATTCTCTAGAGACCCTGTACCAGTCAGCTGGCTGCTCCAACATCAGTGATGCCTTGATA
ENSRNOT00000006366      CATTCCCTAGAGGCCCTGTACCAGTCAGCCGGCTGCTCCACCGTCAGTGATGCCTTGATA

ENSCJAT00000012155      GTGTTGATACATCTTCTCATGTTGGAGTCAGGTTACACACCTCAGGGCACTGAAGCCAAA
ENSMMUT00000009420      GTGTTGATACATCTTCTCATGTTGGAGTCAGGTTACATGCCTCAGGGCACCGAAGCCAAA
ENSPPYT00000013647      GTGTTGATACATCTTCTCATGTTGGAGTCAGGTTACATACCTCAGGGCACCGAAGCCAAA
ENSGGOT00000017216      GTGTTGATACATCTTCTCATGTTGGAGTCAGGTTACATACCTCAGGGCACCGAAGCCAAA
ENSPTRT00000026695      GTGTTGATACATCTTCTCATGTTGGAGTCAGGTTACATACCTCAGGGCACTGAAGCCAAA
ENST00000266087         GTGTTGATACATCTTCTCATGTTGGAGTCAGGTTACATACCTCAGGGCACCGAAGCCAAA
ENSMUST00000130320      GTGCTGGTGCATCTCCTGATGCTCGAGTCAGGCTACATTCCTCAGGGAACTGAAACCAAA
ENSRNOT00000006366      GTGCTGGTGCATCTCCTTATGCTTGAGTCGGGCTACATACCTCAGGGGACTGAAGCCAAA

ENSCJAT00000012155      GCAATGTCCATGCCGGAGAAGTGGAAGTCGAGTGGGGTGTATAAGCTGCAGTACATGCAT
ENSMMUT00000009420      GCACTGTCCATGCCGGAGAAGTGGAAGTTGAGCGGGGTGTATAAGCTGCAGTACATGCAT
ENSPPYT00000013647      GCACTGTCCATGCCGGAGAAGTGGAAGTTGAGCGGGGTGTATAAGCTGCAGTACATGCAT
ENSGGOT00000017216      GCACTGTCCATGCCGGAGAAGTGGAAGTCGAGCGGGGTGTATAAGCTGCAGTACATGCAT
ENSPTRT00000026695      GCACTGTCCATGCCGGAGAAGTGGAAGTCGAGCGGGGTGTATAAGCTGCAGTACATGCAT
ENST00000266087         GCACTGTCCATGCCGGAGAAGTGGAAGTTGAGCGGGGTGTATAAGCTGCAGTACATGCAT
ENSMUST00000130320      GCAGTGACAATGCCTGAGAAGTGGAAGTCAAGTGGTGTATACAAACTGCAGTACACACAT
ENSRNOT00000006366      GCAGCATCCATGCCTGAGAAATGGAAGTCAAGTGGCGTATACAAGCTCCAGTACACACAT

ENSCJAT00000012155      CCTCTCTGCGAGGGCGGCTCTGCTGCTCTCACCTGTGTGCCTTTGGGAAACCTGATTGTT
ENSMMUT00000009420      CCTCTCTGCGAGGGCAGCTCCGCTACTCTCACCTGTGTGCCTCTGGGAAACCTGATTGTT
ENSPPYT00000013647      CCTCTCTGCGAGGGCAGCTCCGCTACTCTCACCTGTGTGCCTTTGGGAAACCTGATTGTT
ENSGGOT00000017216      CCTCTCTGCGAGGGCAGCTCCGCTACTCTCACCTGTGTGCCTTTGGGAAACCTGATTGTT
ENSPTRT00000026695      CCTCTCTGCGAGGGCAGCTCCGCTACTCTCACCTGTGTGCCTTTGGGAAACCTGATTGTT
ENST00000266087         CCTCTCTGCGAGGGCAGCTCCGCTACTCTCACCTGTGTGCCTTTGGGAAACCTGATTGTT
ENSMUST00000130320      CCTCTCTGCGAGGGCGGCTTTGCCGTGCTTACCTGTGTGCCTTTGGGAAACTTAATCATT
ENSRNOT00000006366      CCTCTCTGTGAGGGCGGCTCTGCCGTGCTTACCTGTGTACCTTTGGGAAAGCTCATCATG

ENSCJAT00000012155      GTAAATGCTACGCTAAAAATCAACAGTGAGATTAGAAGTGTGAAAAGATTGCAGCTGCTA
ENSMMUT00000009420      GTAAATGCTACACTGAAAATCAACAATGAGATTAGAAGTGTGAAAAGATTGCAGCTGCTA
ENSPPYT00000013647      GTAAATGCTACACTAAAAATCAACAATGAGATTAGAAGCGTGAAAAGATTGCAGCTGCTA
ENSGGOT00000017216      GTAAATGCTACACTAAAAATCAACAATGAGATTAGAAGTGTGAAAAGATTGCAGCTGCTA
ENSPTRT00000026695      GTAAATGCTACACTAAAAATCAACAATGAGATTAGAAGTGTGAAAAGATTGCAGCTGCTA
ENST00000266087         GTAAATGCTACACTAAAAATCAACAATGAGATTAGAAGTGTGAAAAGATTGCAGCTGCTA
ENSMUST00000130320      ATAAATGCTACAATAAAAGTCAACGGTGGGATTAAAAACGTGAAGTCAGTGCAGCTGCAG
ENSRNOT00000006366      ATAAACGCTACAATAAAAGTCAACGGTGGGATTAAAAATGTGAAGTCAGTGCAGCTGAAG

ENSCJAT00000012155      CCAGAATCTTTTATTTGCAAAGAGAAACTAGGGGAAAATGTAGCCAAGATATACAAAGAT
ENSMMUT00000009420      CCAGAATCTTTTATTTGCAAAGAGAAACTAGGGGAACATGTAGCCAAGATATACAAAGAT
ENSPPYT00000013647      CCAGAATCTTTTATTTGCAAAGAGAAACTAGGGGAAAATGTAGCCAAGATATACAAAGAT
ENSGGOT00000017216      CCAGAATCGTTTATTTGCAAAGAGAAACTAGGGGAAAATGTAGCCAACATATACAAAGAT
ENSPTRT00000026695      CCAGAATCTTTTATTTGCAAAGAGAAACTAGGGGAAAATGTAGCCAACATATACAAAGAT
ENST00000266087         CCAGAATCTTTTATTTGCAAAGAGAAACTAGGGGAAAATGTAGCCAACATATACAAAGAT
ENSMUST00000130320      CCAGGATCCTACGTTGCTGCTGGAGAACCAGGGGAAAGCGCAGCCAAAGTGTACAAAGAT
```

```
ENSRNOT00000006366      CCAGGAGCCTACGTTCGCAGAGCAGAACCAGGGGAAAGTGCAGCTAAAGTGTACAAAGAT


ENSCJAT00000012155      CTTCAGAAGCTCTCCCGCCTCTTTAAAGACCAGCTGGTGTATCCTCTTCTGGCTTTTACC
ENSMMUT00000009420      CTTCAGAAACTCTCTCGCCTCTTTAAAGACCAGCTGGTGTACCCTCTTCTGGCTTTTACC
ENSPPYT00000013647      CTTCAGAAACTCTCTCGCCTCTTTAAAGACCAGCTGGTGTATCCTCTTCTGGCTTTTACC
ENSGGOT00000017216      CTTCAGAAACTCTCTCGCCTCTTTAAAGACCAGCTGGTGTATCCTCTTCTGGCTTTTACC
ENSPTRT00000026695      CTTCAGAAACTCTCTCGCCTCTTTAAAGACCAGCTGGTGTATCCTCTTCTGGCTTTTACC
ENST0000266087          CTTCAGAAACTCTCTCGCCTCTTTAAAGACCAGCTGGTGTATCCTCTTCTGGCTTTTACC
ENSMUST00000130320      CTTAAGAAGCTCTCTCGTCTCTTCAAAGACCAGCTGGTGTACCCTCTTCTGGCTTTCACA
ENSRNOT00000006366      CTTAAGAAGCTCTCTCGTCTCTTCAAAGACCAGCTGGTGTACCCTCTTCTGGCTTTCACA


ENSCJAT00000012155      CGACAAGCACTGAACCTACCAGATGTATTTGGTTTGGTCGTCCTCCCATTGGAACTGAAA
ENSMMUT00000009420      CGACAAGCACTGAGCCTACCAGATGTATTTGGGTTGGTCGTCCTCCCGTTGGAACTGAAA
ENSPPYT00000013647      CGACAAGCACTGAACCTACCAGATGTATTTGGGTTGGTCGTCCTCCCATTGGAACTAAAA
ENSGGOT00000017216      CGACAAGCACTGAACCTACCAGATGTATTTGGGTTGGTCGTCCTCCCATTGGAACTGAAA
ENSPTRT00000026695      CGACAAGCACTGAACCTACCAGATGTATTTGGGTTGGTCGTCCTCCCATTGGAACTGAAA
ENST0000266087          CGACAAGCACTGAACCTACCAGATGTATTTGGGTTGGTCGTCCTCCCATTGGAACTGAAA
ENSMUST00000130320      CGGCAAGTACTGAACCTTCCAGATGTGTTTGGGCTGGTTGTCCTTCCACTGGAGCTGAAA
ENSRNOT00000006366      CGACAAGTGCTGAACCTCCCAGATGTGTTTGGGCTGGTTGTCCTTCCACTGGAGCTGAAA


ENSCJAT00000012155      CTACGGATCTTCCGACTCTTGGATGTTCGTTCCATCCTGTCTTTGTCTGCAGTTTGTCGT
ENSMMUT00000009420      CTACGGATCTTCCGACTTCTGGATGTTCGTTCCGTCTTGTCTTTGTCTGCAGTTTGTCGT
ENSPPYT00000013647      CTACGGATCTTCCGACTTCTGGATGTTCGTTCCGTCTTGTCTTTGTCTGCAGTTTGTCGT
ENSGGOT00000017216      CTACGGATCTTCCGACTTCTGGATGTTCGTTCCGTCTTGTCTTTGTCTGCGGTTTGTCGT
ENSPTRT00000026695      CTACGGATCTTCCGACTTCTGGATGTTCGTTCCGTCTTGTCTTTGTCTGCGGTTTGTCGT
ENST0000266087          CTACGGATCTTCCGACTTCTGGATGTTCGTTCCGTCTTGTCTTTGTCTGCGGTTTGTCGT
ENSMUST00000130320      CTACGCATCTTCCGACTTTTGGATGTTCATTCTGTCCTGGCCCTGTCTGCAGTCTGTCAT
ENSRNOT00000006366      CTACGCATCTTCCGACTTCTGGATGTTCATTCCGTCCTGGCTCTGTCTGCAGTCTGCCAT


ENSCJAT00000012155      GACCTCTTTACTGCTTCAAATGACCCACTCCTGTGGAGGTTTTTATATCTTCGTGATTTT
ENSMMUT00000009420      GACCTCTTTACTGCTTCAAATGACCCACTTTTGTGGAGGTTTTTGTATCTGCGTGATTTT
ENSPPYT00000013647      GACCTCTTTACTGCTTCAAATGACCCACTCCTGTGGAGGTTTTTATATCTGCGTGATTTT
ENSGGOT00000017216      GACCTCTTTACTGCTTCAAATGACCCACTCCTGTGGAGGTTTTTATATCTGCGTGATTTT
ENSPTRT00000026695      GACCTCTTTACTGCTTCAAATGACCCACTCCTGTGGAGGTTTTTATATCTGCGTGATTTT
ENST0000266087          GACCTCTTTACTGCTTCAAATGACCCACTCCTGTGGAGGTTTTTATATCTGCGTGATTTT
ENSMUST00000130320      GACCTCCTCATTGCGTCAAATGACCCACTGCTGTGGAGGTGTTTGTATCTGCGGGATTTT
ENSRNOT00000006366      GACCTCCTCATTGCATCAAATGACCCACTGCTGTGGAGGTGCTTGTATCTGCGGGATTTT


ENSCJAT00000012155      CGAGACAGTACTGTCAGAGTTCAAGACACAGACTGGAAAGAACTGTACAGGAAGAGGCAC
ENSMMUT00000009420      CGAGACAATACTGTCAGAGTTCAAGACACAGATTGGAAAGAACTGTACAGGAAGAGGCAC
ENSPPYT00000013647      CGAGACAATACTGTCAGAGTTCAAGACACAGATTGGAAAGAACTGTACAGGAAGAGGCAC
ENSGGOT00000017216      CGAGACAATACTGTCAGAATTCAAGACACAGATTGGAAAGAACTGTACAGGAAGAGGCAC
ENSPTRT00000026695      CGAGACAATACTGTCAGAGTTCAAGACACAGATTGGAAAGAACTGTACAGGAAGAGGCAC
ENST0000266087          CGAGACAATACTGTCAGAGTTCAAGACACAGATTGGAAAGAACTGTACAGGAAGAGGCAC
ENSMUST00000130320      CGAGATGGCACTGTCAGAGGTCCTGACACAGACTGGAAAGAATTGTATAGAAAGAAGCAC
ENSRNOT00000006366      CGAGACAGTACTATCAGAGGTCCTGACACAGATTGGAAAGAATTGTACAGAAAGAAGCAC


ENSCJAT00000012155      ATACAAAGAAAAGAATCCCCAAGAGGGCGGTTTGTGTTGCTCCTGCCATCGTCACCTCAC
ENSMMUT00000009420      ATACAAAGAAAAGAATCCCCCAAAGGGCGGTTTGTGATGCTCCTGCCATCGTCAACTCAC
ENSPPYT00000013647      ATACAAAGAAAAGAATCCCCGAAAGGGCGGTTTGTGATGCTCCTGCCATCGTCAACTCAC
ENSGGOT00000017216      ATACAAAGAAAAGAATCCCCGAAAGGGCGGTTTGTGATGCTCCTGCCATCGTCAACTCAT
ENSPTRT00000026695      ATACAAAGAAAAGAATCCCCGAAAGGGCGGTTTGTGATGCTCCGGCCATCGTCAACTCAC
ENST0000266087          ATACAAAGAAAAGAATCCCCGAAAGGGCGGTTTGTGATGCTCCTGCCATCGTCAACTCAC
ENSMUST00000130320      ATACAAAGAAAAGAGGCTCAGAGGATGCGGCATGCAATGTTCCTACCA---TCAGCCCAC
```

```
ENSRNOT00000006366     ATCCAAAGAAAAGAAGCCCAGAGGATGCGGCATGTGATGTACCTACCG---TCAGTCCAC

ENSCJAT00000012155     CCCATTCCATTCTATCCCAACCCTTTGCACCCTAGGCCATTTTCTAGCTCCCTCCTTCCT
ENSMMUT00000009420     GGCATTCCATTCTATCCCAACCCCTTGCACCCTAGGCCATTTCCTAGCTCCCGCCTTCCT
ENSPPYT00000013647     GCCATTCCATTCTATCCCAACCCCTTGCACCCTAGGCCATTTCCTAGCTCCCGCCTTCCT
ENSGGOT00000017216     ACCATTCCATTCTATCCCAACCCCTTGCACCCTAGGCCATTTCCTAGCTCCCGCCTTCCT
ENSPTRT00000026695     ACCATTCCATTCTATCCCAACCCCTTGCACCCTAGGCCATTTCCTAGCTCCCGCCTTCCT
ENST00000266087        ACCATTCCATTCTATCCCAACCCCTTGCACCCTAGGCCATTTCCTAGCTCCCGCCTTCCT
ENSMUST00000130320     CCTATCCCATTTTGTCCCATTCCCGTCTACCCCAGGGCCTACCCCACCTCGCTGCTTCCT
ENSRNOT00000006366     CCTATCCCATTCTGCCCCATTCCCGTCTACCCCAGGCCCTACCCCACCACGCTGCTTCCT

ENSCJAT00000012155     CCGGGAATTATCGGGGGTGAATATGACCAAAGACCAATACTTCCCTATGTTGGAGACCCA
ENSMMUT00000009420     CCAGGAATTATCGGGGGTGAATATGACCAAAGACCAACACTTCCCTATGTTGGAGACCCA
ENSPPYT00000013647     CCAGGAATTATCGGGGGTGAATATGACCAAAGACCAACACTTCCTTATGTTGGAGACCCA
ENSGGOT00000017216     CCAGGAATTATCGGGGGTGAATATGACCAAAGACCAACGCTTCCCTATGTTGGAGACCCA
ENSPTRT00000026695     CCAGGAATTATCGGGGGTGAATATGACCAAAGACCAACACTTCCCTATGTTGGAGACCCA
ENST00000266087        CCAGGAATTATCGGGGGTGAATATGACCAAAGACCAACACTTCCCTATGTTGGAGACCCA
ENSMUST00000130320     CCAGGAATCATTGGTGGTGAATATGATGAGAGGCCGATACTGCCTAGTGTTGGGGACCCA
ENSRNOT00000006366     CCAGGAATCATTGGTGGTGAATATGATGAGAGGCCAATATTGCCTAGTGTTGGGGACCCA

ENSCJAT00000012155     ATCAATTCACTCATCCCTGGGCCTGGGGAAACACCCAGCCAGTTTCCTCCACTCAGACCA
ENSMMUT00000009420     ATCAATTCACTCATTCCTGGTCCTGGGGAGACGCCCAGCCAGTTCCCTCCACTCAGACCA
ENSPPYT00000013647     ATCAATTCACTCATTCCTGGTCCTGGGGAGACGCCCAGCCAGTTTCCTCCACTCAGACCA
ENSGGOT00000017216     ATCAATTCACTCATTCCTGGTCCTGGGGAGACGCCCAGCCAGTTTCCTCCACTCAGACCA
ENSPTRT00000026695     ATCAATTCACTCATTCCTGGTCCTGGGGAGACGCCCAGCCAGTTTGCTCCACTCAGACCA
ENST00000266087        ATCAGTTCACTCATTCCTGGTCCTGGGGAGACGCCCAGCCAGTTTCCTCCACTGAGACCA
ENSMUST00000130320     GTCACCTCACTTATCCCAAGGCCCGGGGAGCTTCCTGGTCAGTTCCGTCCACTCAGACCA
ENSRNOT00000006366     GTCACCTCACTCATCCCAAGGCCTGGGGAGCCTCCTAGCCAGTTCCGTCCAGTCAGACCA

ENSCJAT00000012155     CGATTCGATCCAATTGGCCCCCTTCCAGGACCTAACCCCATCTTGCCGGGGCGAGGCAGC
ENSMMUT00000009420     CGCTTTGATCCAATTGGCCCACTCCCAGGACCTAACCCCATCTTGCCAGGGCGAGGCGGC
ENSPPYT00000013647     CGCTTTGATCCAATTGGCCCACTTCCAGGACCTAACCCCATCTTGCCAGGGCGAGGCGGC
ENSGGOT00000017216     CGTTTTGATCCAATTGGCCCACTTCCAGGACCTAACCCCATCTTGCCAGGGCGAGGCGGC
ENSPTRT00000026695     CGCTTTGATCCAGTTGGCCCACTTCCAGGACCTAACCCCATCTTGCCAGGGCGAGGCGGC
ENST00000266087        CGCTTTGATCCAGTTGGCCCACTTCCAGGACCTAACCCCATCTTGCCAGGGCGAGGCGGC
ENSMUST00000130320     CGTTTTGACCCTGTTGACCCACTTCCAGGACCTCACTCCCTCTTGCCAGGAAGAGCTATC
ENSRNOT00000006366     CGTTTTGATCCTGTTGGCCCACTTCCAGGATCTAACTCCCTCTTACCAGGAAGAGCTAGC

ENSCJAT00000012155     CCCAGTGACAGATTTCCCTTTAGACCCAGCAGGGGTCGGCCAACTGACAGCCGGCTTTCG
ENSMMUT00000009420     CCCAATGACAGATTTCCCTTTAGACCCAGTAGGGGTCGGCCAACTGACGGCCGCCTGTCA
ENSPPYT00000013647     CCCAATGACAGATTTCCCTTTAGACCCAGCAGGGGTCGGCCTACTGATGGCCGGCTGTCA
ENSGGOT00000017216     CCCAATGACAGATTTCCCTTTAGACCCAGCAGGGGTCGGCCAACTGATGGCCGGCTGTCA
ENSPTRT00000026695     CCCAATGACAGATTTCCCTTTAGACCCAGCAGGGGTCGGCCAACTGATGGCCGGCTGTCA
ENST00000266087        CCCAATGACAGATTTCCCTTTAGACCCAGCAGGGGTCGGCCAACTGATGGCCGGCTGTCA
ENSMUST00000130320     CCCAACAACAGATTTCCCTTCAGACCCGGCAGGGGTAGGTCAGCTGACAGCCGGCTGCCA
ENSRNOT00000006366     CCCAACAACAGATTTCCCTTCAGACCCGGCAGGGGTCGGTCAGCTGACAACCGGCTACCA

ENSCJAT00000012155     TTCATG
ENSMMUT00000009420     TTCATG
ENSPPYT00000013647     TTCATG
ENSGGOT00000017216     TTCATG
ENSPTRT00000026695     TTCATG
ENST00000266087        TTCATG
ENSMUST00000130320     TTCCTG
```

```
ENSRNOT00000006366     TACCTG
```

Multiple sequence alignment of Fbxo8

```
ENSGGOT00000005368     ATGGGTCAAGGGTTGTGGAGAGTGGTCAGAAACCAGCAGCTACAACAAGAAGGCTACAGT
ENST00000393674        ATGGGTCAAGGGTTGTGGAGAGTGGTCAGAAACCAGCAGCTGCAACAAGAAGGCTACAGT
ENSPTRT00000067771     ATGGGTCAAGGGTTGTGGAGAGTGGTCAGAAACCAGCAGCTACAACAAGAAGGCTACAGT
ENSPPYT00000017680     ATGGGTCAAGGGTTGTGGAGAGTGGTCAGAAACCAGCAGCTACAACAAGAAGGCTACAGT
ENSCJAT00000009986     ATGGGTCAAGGGCTGTGGAGAGTGGTCAGAAACCAGCAGCTACAACAAGAAGGCTACAGT
ENSMMUT00000041493     ATGGGTCAGGGGTTGTGGAGAGTTGTCAGAAACCAGCAGCTACAACAAGAAGGCTACAGT
ENSMUST00000040218     ATGGGTCAAGGACTCTGGAGAGTGGCCAGAAACCATCATCTCCAACAGGAAGCCTACAGT
ENSRNOT00000014015     GTTTCCCAGGGACTTTGGACAAATCTACAGGNTCAGCATCTCCAACAGGAAGCCTACGGT


ENSGGOT00000005368     GAGCAAGGCTACCTCACCAGAGAGCAGAGCAGGAGAATGGCTGCAAGCAACATTTCTAAC
ENST00000393674        GAGCAAGGCTACCTCACCAGAGAGCAGAGCAGGAGAATGGCTGCGAGCAACATTTCTAAC
ENSPTRT00000067771     GAGCAAGGCTACCTCACCAGAGAGCAGAGCAGGAGAATGGCTGCGAGCAACATTTCTAAC
ENSPPYT00000017680     GAGCAGGGCTACCTCACCAGAGAGCAGAGCAGGAGAATGGCTGCGAGCAACATTTCTAAC
ENSCJAT00000009986     GAGCAAGGCTACCTCACCAGAGAGCAGAGCAGGAGGATGGCTGCAAACAACATTTCTAAC
ENSMMUT00000041493     GAGCAAGGCTACCTCACTAGAGAGCAGAGCAGGAGAATGGCTGCGAACAACATTTCTAAC
ENSMUST00000040218     GAGACAGGCTACCTCAGCAGAGAACAGAGCAGGAGAGTGGCTTCAAGCAACATTTCTCAC
ENSRNOT00000014015     GAGACAGGCTACCTCAGCAGAGAACAGAGCAGGAGAGTGGCTTCAAGCAACATTTCTCAC


ENSGGOT00000005368     ACCAATCATCGTAAACAAGTCCAAGGAGGCATTGACATATATCATCTTTTGAAGGCAAGG
ENST00000393674        ACCAATCATCGTAAACAAGTCCAAGGAGGCATTGACATATATCATCTTTTGAAGGCAAGG
ENSPTRT00000067771     ACCAATCATCGTAAACAAGTCCAAGGAGGCATTGACATATATCATCTTTTGAAGGCAAGG
ENSPPYT00000017680     ACCAATCATCGTAAACAAGTCCAAGGAGGCATTGACATATATCATCTTTTGAAGGCAAGG
ENSCJAT00000009986     ACCAGTCATCGTAAACAAGTCCAAGGAGGCATTGACATATATCATCTTTTGAAGGCAAGG
ENSMMUT00000041493     AGCAATCATCGTAAACAAGTCCAAGGAGGCATTGACATATATCATCTTTTGAAGGCAAGG
ENSMUST00000040218     ACCAGCCACCGGAAACAAGCCCAAGGAGGCATTGATATCTATCATCTTCTGAAGGCAAGG
ENSRNOT00000014015     ACCAGTCACCGGAAACAAGCCCAAGGAGGCATCGATATATATCATCTTCTGAAGGCAAGG


ENSGGOT00000005368     AAATCGAAAGAACAGGAAGGATTCATTAATTTGGAAATGTTGCCTCCTGAGCTAAGCTTT
ENST00000393674        AAATCGAAAGAACAGGAAGGATTCATTAATTTGGAAATGTTGCCTCCTGAGCTAAGCTTT
ENSPTRT00000067771     AAATCGAAAGAACAGGAAGGATTCATTAATTTGGAAATGTTGCCTCCTGAGCTAAGCTTT
ENSPPYT00000017680     AAATCAAAAGAACAGGAAGGATTCATTAATTTGGAAATGTTGCCTCCTGAGCTAAGTTTT
ENSCJAT00000009986     AAATCTAAAGAACAGGAAGGATTCATTAATTTGGAAATGTTGCCTCCTGAGCTAAGCTTT
ENSMMUT00000041493     AAATCAAAAGAACAGGAAGGATTCATTAATTTGGAAATGCTGCCTCCTGAGCTAAGCTTC
ENSMUST00000040218     AAGTCTAAAGAACAGGAGGGGTTCATAAACTTGGAAATGCTGCCTCCTGAGCTGAGCTTC
ENSRNOT00000014015     AAGTCTAAAGAACAGGAAGGGTTCATTAACTTGGAAATGCTGCCTCCTGAGCTGAGCTTC


ENSGGOT00000005368     ACCATCTTGTCCTACCTGAATGCAACTGACCTTTGCTTGGCTTCATGTGTTTGGCAGGAC
ENST00000393674        ACCATCTTGTCCTACCTGAATGCAACTGACCTTTGCTTGGCTTCATGTGTTTGGCAGGAC
ENSPTRT00000067771     ACCATCTTGTCCTACCTGAATGCAACTGACCTTTGCTTGGCTTCATGTGTTTGGCAGGAC
ENSPPYT00000017680     ACCATCTTGTCCTACCTGAATGCAACTGACCTTTGCTTGGCTTCATGTGTTTGGCAGGAC
ENSCJAT00000009986     ACCATCTTGTCCTACCTGAATGCAACTGACCTTTGCTTGGCTTCATGTGTTTGGCAGGAC
ENSMMUT00000041493     ACCATCTTGTCCTACCTGAATGCAACTGACCTTTGCTTGGCTTCGTGTGTTTGGCAGGAC
ENSMUST00000040218     ACCATCTTATCCTACCTGAATGCAACTGACCTTTGCTTAGCTTCCTGTGTTTGGCAAGAC
ENSRNOT00000014015     ACCATCTTATCCTACCTGAATGCAACTGACCTCTGCTTAGCTTCCTGTGTTTGGCAAGAC


ENSGGOT00000005368     CTTGCGAATGATGAACTTCTCTGGCAAGGGTTGTGCAAATCCACTTGGGGTCACTGTTCC
ENST00000393674        CTTGCGAATGATGAACTTCTCTGGCAAGGGTTGTGCAAATCCACTTGGGGTCACTGTTCC
ENSPTRT00000067771     CTTGCGAATGATGAACTTCTCTGGCAAGGGTTGTGCAAATCCACTTGGGGTCACTGTTCC
ENSPPYT00000017680     CTTGCGAATGATGAACTTCTCTGGCAAGGGTTGTGCAAATCCACTTGGGGTCACTGTTCC
ENSCJAT00000009986     CTCGCGAATGATGAACTTCTCTGGCAAGGGTTGTGCAAATCCACTTGGGGTCACTGTTCT
```

```
ENSMMUT00000041493    CTCGCAAATGATGAACTTCTCTGGCAAGGGTTGTGCAAATCCACTTGGGGTCACTGTTCC
ENSMUST00000040218    CTCGCTAATGATGAACTTCTCTGGCAAGGGTTGTGTAAATCTACATGGGGTCACTGCTCT
ENSRNOT00000014015    CTTGCTAATGATGAACTTCTCTGGCAAGGGTTGTGTAAATCCACATGGGGTCACTGTTCT

ENSGGOT00000005368    ATATACAATAAGAACCCACCTTTAGGATTTTCTTTTAGAAAATTGTATATGCAGCTGGAT
ENST00000393674       ATATACAATAAGAACCCACCTTTAGGATTTTCTTTTAGAAAATTGTATATGCAGCTGGAT
ENSPTRT00000067771    ATATACAATAAGAACCCACCTTTAGGATTTTCTTTTAGAAAATTGTATATGCAGCTGGAT
ENSPPYT00000017680    ATATACAATAAGAACCCACCTTTAGGATTTTCTTTTAGAAAATTGTATATGCAGCTGGAT
ENSCJAT00000009986    ATATACAATAAGAACCCACCTTTAGGATTTTCTTTTAGAAAATTGTATATGCAGCTGGAT
ENSMMUT00000041493    ATATACAATAAGAACCCACCTTTAGGATTTTCTTTTAGAAAATTGTATATGCAGCTGGAT
ENSMUST00000040218    ATATACAATAAGAACCCACCTTTAGGATTTTCTTTTAGAAAATTGTATATGCAGCTAGAT
ENSRNOT00000014015    ATATACAATAAGAATCCACCTTTAGGATTTTCTTTTAGAAAATTGTATATGCAGCTAGAT

ENSGGOT00000005368    GAAGGCAGCCTCACCTTTAATGCCAACCCAGATGAGGGAGTGAACTACTTTATGTCCAAG
ENST00000393674       GAAGGCAGCCTCACCTTTAATGCCAACCCAGATGAGGGAGTGAACTACTTTATGTCCAAG
ENSPTRT00000067771    GAAGGCAGCCTCACCTTTAATGCCAACCCAGATGAGGGAGTGAACTACTTTATGTCCAAG
ENSPPYT00000017680    GAAGGCAGCCTCACCTTTAATGCCAACCCAGATGAGGGAGTGAACTACTTTATGTCCAAG
ENSCJAT00000009986    GAAGGCAGCCTCACCTTTAATGCCAACCCAGATGAGGGAGTGAACTACTTTATGTCCAAG
ENSMMUT00000041493    GAAGGCAGCCTCACCTTTAATGCCAACCCAGATGAGGGAGTGAACTACTTTATGTCCAAG
ENSMUST00000040218    GAGGGAAGCCTCACCTTTAATGCCAACCCAGAGGAGGGAGTGAGCTACTTTATGTCCAAG
ENSRNOT00000014015    GAGGGAAGCCTCACCTTTAATGCCAACCCAGAGGAGGGAGTGAGCTACTTTATGTCCAAG

ENSGGOT00000005368    GGTATCCTGGATGATTCGCCAAAGGAAATAGCAAAGTTTATCTTCTGTACAAGAACACTA
ENST00000393674       GGTATCCTGGATGATTCGCCAAAGGAAATAGCAAAGTTTATCTTCTGTACAAGAACACTA
ENSPTRT00000067771    GGTATCCTGGATGATTCGCCAAAGGAAATAGCAAAGTTTATCTTCTGTACAAGAACACTA
ENSPPYT00000017680    GGTATCCTAGATGATTCACCAAAGGAAATAGCAAAGTTTATCTTCTGTACAAGAACACTA
ENSCJAT00000009986    GGTATCCTAGATGATTCGCCAAAGGAAATAGCAAAGTTTATCTTCTGTACAAGAACTCTA
ENSMMUT00000041493    GGTATCCTAGATGATTCGCCAAAGGAAATAGCAAAGTTTATCTTCTGTACAAGAACACTA
ENSMUST00000040218    GGTATCCTAGATGATTCGCCAAAGGAAATAGCAAAATTTATCTTCTGTACCAGAACACTA
ENSRNOT00000014015    GGTATCCTAGATGATTCGCCAAAGGAAATAGCAAAATTTATCTTCTGTACCAGAACACTA

ENSGGOT00000005368    AATTGGAAAAAAACTGAGAATCTATCTTGATGAAAGGAGAGATGTCTTGGATGACCTTGTA
ENST00000393674       AATTGGAAAAAAACTGAGAATCTATCTTGATGAAAGGAGAGATGTCTTGGATGACCTTGTA
ENSPTRT00000067771    AATTGGAAAAAAACTGAGAATCTATCTTGATGAAAGGAGAGATGTCTTGGATGACCTTGTA
ENSPPYT00000017680    AATTGGAAAAAAACTGAGAATCTATCTTGATGAAAGGAGAGATGTCTTGGATGACCTTGTA
ENSCJAT00000009986    AATTGGAAAAAAACTGAGAATCTATCTTGATGAAAGGAGAGATGTCTTGGATGACCTTGTA
ENSMMUT00000041493    AATTGGAAAAAAACTGAGAATCTATCTTGATGAAAGGAGAGATGTCTTGGATGACCTTGTA
ENSMUST00000040218    AATTGGAAAAAAACTGAGAATCTATCTTGATGAAAGGAGAGATGTCTTGGATGACCTTGTA
ENSRNOT00000014015    AATTGGAAAAAAACTGAGAATCTATCTTGATGAAAGGAGAGATGTCTTGGATGACCTTGTA

ENSGGOT00000005368    ACATTGCATAATTTTAGAAATCAGTTCTTGCCAAATGCACTGAGAGAATTTTTTCGTCAT
ENST00000393674       ACATTGCATAATTTTAGAAATCAGTTCTTGCCAAATGCACTGAGAGAATTTTTTCGTCAT
ENSPTRT00000067771    ACATTGCATAATTTTAGAAATCAGTTCTTGCCAAATGCACTGAGAGAATTTTTTCGTCAT
ENSPPYT00000017680    ACATTGCATAATTTTAGAAATCAGTTCTTGCCAAATGCACTGAGAGAATTTTTTCGTCAT
ENSCJAT00000009986    ACGTTGCATAATTTTAGAAATCAGTTCTTGCCAAATGCACTGAGAGAATTTTTTCGTCAT
ENSMMUT00000041493    ACATTGCATAATTTTAGAAATCAGTTCTTGCCAAATGCACTGAGAGAATTTTTTCGTCAT
ENSMUST00000040218    ACCTTGCATAATTTTAGAAATCAGTTCTTGCCGAATGCACTGAGAGAATTTTTTCGTCAT
ENSRNOT00000014015    ACCTTGCATAATTTTAGGAATCAGTTTTTGCCAAATGCATTGCGAGAATTTTTTCGTCAC

ENSGGOT00000005368    ATCCATGCCCCTGAAGAGCGTGGAGAGTATCTTGAAACTCTTATAACAAAGTTCTCACAT
ENST00000393674       ATCCATGCCCCTGAAGAGCGTGGAGAGTATCTTGAAACTCTTATAACAAAGTTCTCACAT
ENSPTRT00000067771    ATCCATGCCCCTGAAGAGCGTGGAGAGTATCTTGAAACTCTTATAACAAAGTTCTCACAT
ENSPPYT00000017680    ATCCATGCCCCTGAAGAGCGTGGAGAGTATCTTGAAACTCTTATAACAAAGTTCTCACAT
ENSCJAT00000009986    ATCCATGCCCCTGAAGAGCGTGGAGAGTATCTTGAAACTCTTATAACAAAGTTCTCACAT
```

ENSMMUT00000041493 ATCCATGCCCCTGAAGAGCGTGGAGAGTATCTTGAAACTCTTATAACAAAGTTCTCACAT
ENSMUST00000040218 ATTCATGCCCCTGAAGAGCGTGGGGAGTACCTTGAAACTCTTATAACCAAGTTCTCACAT
ENSRNOT00000014015 ATTCATGCTCCTGAGGAACGTGGGGAGTACCTTGAAACTCTTATAACAAAGTTCTCACAT

ENSGGOT00000005368 AGATTCTGTGCTTGCAACCCTGATTTAATGCGAGAACTTGGCCTTAGTCCTGATGCTGTC
ENST00000393674 AGATTCTGTGCTTGCAACCCTGATTTAATGCGAGAACTTGGCCTTAGTCCTGATGCTGTC
ENSPTRT00000067771 AGATTCTGTGCTTGCAACCCTGATTTAATGCGAGAACTTGGCCTTAGTCCTGATGCTGTC
ENSPPYT00000017680 AGATTCTGTGCTTGCAACCCTGATTTAATGCGAGAACTTGGCCTTAGTCCTGATGCTGTC
ENSCJAT00000009986 AGGTTCTGTGCTTGTAACCCTGATTTAATGAGAGAACTTGGCCTTAGTCCCGATGCTGTC
ENSMMUT00000041493 AGATTCTGTGCTTGTAACCCTGATTTAATGCGAGAACTTGGCCTTAGTCCTGATGCTGTC
ENSMUST00000040218 AGGTTCTGTGCTTGCAATCCTGACCTCATGCGAGAACTTGGCCTTAGTCCTGATGCTGTC
ENSRNOT00000014015 AGGTTCTGTGCTTGCAATCCTGACCTAATGCGAGAACTTGGCCTTAGTCCAGATGCTGTC

ENSGGOT00000005368 TATGTACTGTGCTACTCTTTGATTCTACTTTCCATTGACCTCACTAGCCCTCATGTGAAG
ENST00000393674 TATGTACTGTGCTACTCTTTGATTCTACTTTCCATTGACCTCACTAGCCCTCATGTGAAG
ENSPTRT00000067771 TATGTACTGTGCTACTCTTTGATTCTACTTTCCATTGACCTCACTAGCCCTCATGTGAAG
ENSPPYT00000017680 TATGTACTGTGCTACTCTTTGATTCTACTTTCCATTGACCTCACTAGCCCTCATGTGAAG
ENSCJAT00000009986 TATGTACTGTGCTACTCTTTGATTCTACTTTCCATTGACCTCACTAGCCCTCATGTGAAG
ENSMMUT00000041493 TATGTACTGTGCTACTCTTTGATTCTACTTTCCATTGACCTCACTAGCCCTCATGTGAAG
ENSMUST00000040218 TATGTACTGTGCTACTCTTTGATTCTACTTTCCATTGACCTCACTAGTCCTCATGTGAAG
ENSRNOT00000014015 TATGTACTGTGCTACTCTTTGATTCTACTTTCCATTGACCTCACTAGTCCTCATGTGAAG

ENSGGOT00000005368 AATAAAATGTCAAAAAGAGAATTTATTCGAAATACCCGTCGCGCTGCTCAAAATATTAGT
ENST00000393674 AATAAAATGTCAAAAAGGGAATTTATTCGAAATACCCGTCGCGCTGCTCAAAATATTAGT
ENSPTRT00000067771 AATAAAATGTCAAAAAGAGAGTTTATTCGAAATACCCGTCGTGCTGCTCAAAATATTAGT
ENSPPYT00000017680 AATAAAATGTCAAAAAGAGAATTTATTCGAAATACCCGTCGCGCTGCTCAAAATATTAGT
ENSCJAT00000009986 AATAAAATGTCAAAAAGAGAATTTATTCGAAATACCCGTCGCGCTGCTCAGAATATTAGT
ENSMMUT00000041493 AATAAAATGTCAAAAAGAGAATTTATTCGAAATACCCGTCGCGCTGCTCAAAATATTAGT
ENSMUST00000040218 AATAAAATGTCAAAAAGAGAGTTTATTCGAAATACGCGCCGTGCTGCTCAGAACATTAGT
ENSRNOT00000014015 AATAAAATGTCAAAGAGAGAGTTTATTCGAAATACCCGCCGAGCTGCTCAGAACATTAGT

ENSGGOT00000005368 GAAGATTTTGTAGGGCATCTTTATGACAATATCTACCTTATTGGCCATGTGGCTGCA
ENST00000393674 GAAGATTTTGTAGGGCATCTTTATGACAATATCTACCTTATTGGCCATGTGGCTGCA
ENSPTRT00000067771 GAAGATTTTGTAGGGCATCTTTATGACAATATCTACCTTATTGGCCATGTGGCTGCA
ENSPPYT00000017680 GAAGATTTTGTAGGGCATCTTTATGACAATATCTACCTTATTGGCCATGTGGCTGCA
ENSCJAT00000009986 GAAGATTTTGTAGGGCACCTTTATGACAATATCTACCTTATTGGCCATGTGGCTGCA
ENSMMUT00000041493 GAAGATTTTGTAGGGCATCTTTATGACAATATCTACCTTATTGGCCATGTGGCTGCG
ENSMUST00000040218 GAAGATTTTGTAGGACACCTTTATGACAACATCTACCTTATTGGCCATGTGGCTGCA
ENSRNOT00000014015 GAAGATTTTGTAGGACACCTTTATGACAACATCTACCTTATTGGCCATGTGGCTGCA

Multiple sequence alignment of Fbxo9

ENSPPYT00000019443 ATGGCAGAAGCTGAGGAAGATTGTCATTCTGATACTGTCAGAGCAGATGATGAAGAAAAT
ENST00000244426 CAGGCAGAAGCTGAGGAAGATTGTCATTCTGATACTGTCAGAGCAGATGATGAAGAAAAT
ENSGGOT00000006553 ATGGCAGAAGCTGAGGAAGATTGTCATTCTGATACTGTCAGAGCAGATGATGAAGAAAAT
ENSPTRT00000033806 ATGGCAGAAGCTGAGGAAGATTGTCATTCTGATACTGTCAGAGCAGATGATGAAGAAAAT
ENSMUST00000001402 AGCGCAGAAGCGGAGGAAGATTGTCATTCTGATGCTGACAGAGTAGGCGATGAAGGAAAT
ENSRNOT00000010961 ATGGCAGAAGCGGAGGAAGATTGTCATTCTGATGCTGTCAGAGTAGGCGATGAAGGACAT

ENSPPYT00000019443 GAAAGTCCTGCTGAAACAGATCTGCAGGCACAACTGCAGATGTTCCGAGCTCAGTGGATG
ENST00000244426 GAAAGTCCTGCTGAAACAGATCTGCAGGCACAACTCCAGATGTTCCGAGCTCAGTGGATG
ENSGGOT00000006553 GAAAGTCCTGCTGAAACAGATCTGCAGGCGCAACTCCAGATGTTCCGAGCTCAGTGGATG
ENSPTRT00000033806 GAAAGTCCTGCTGAAACAGATCTGCAGGCACAACTCCAGATGTTCCGAGCTCAGTGGATG
ENSMUST00000001402 GAGAGTCCGGCTGAGAGAGACCTGCAGGCGCAGCTCCAGATGTTCAGAGCTCAGTGGATG

```
ENSRNOT00000010961      GAGAGTCCGGCTGAGAGAGACCTGCAGGCGCAGCTCCAGATGTTCAGAGCTCAGTGGATG

ENSPPYT00000019443      TTTGAACTTGCTCCAGGTGTAAGCTCTAGCAATTTAGAAAATCGACCTTGCAGAGCAGCA
ENST00000244426         TTTGAACTTGCTCCAGGTGTAAGCTCTAGCAATTTAGAAAATCGACCTTGCAGAGCAGCA
ENSGGOT00000006553      TTTGAACTTGCTCCAGGTGTAAGCTCTAGCAATTTAGAAAATCGACCTTGCAGAGCAGCA
ENSPTRT00000033806      TTTGAACTTGCTCCAGGTGTAAGCTCTAGCAATTTAGAAAATCGACCTTGCAGAGCAGCA
ENSMUST00000001402      TTTGAACTGACCCCAGGTGTAGGCTCCAGTCATGGAGAAACTCGGCCTTGCAGAGCAGGA
ENSRNOT00000010961      TTTGAACTTACCCCAGGCGTAGGTTCCAGTAATGTGGAAAGCCGGCCTTGCAGAGCAGGG

ENSPPYT00000019443      AGAGGCTCTCTCCAGAAAACAGCGGCAGATACCAAAGGAAAACAAGAACAGGCAAAAGAA
ENST00000244426         AGAGGCTCTCTCCAGAAAACATCGGCAGATACCAAAGGAAAACAAGAACAGGCAAAAGAA
ENSGGOT00000006553      AGAGGCTCTCTCCAGAAAACATCGGCAGATACCAAAGGAAAACAAGAACAGGCAAAAGAA
ENSPTRT00000033806      AGAGGCTCTCTCCAGAAAACATCGGCAGATACCAAAGGAAAACAAGAACAGGCAAAAGAA
ENSMUST00000001402      AGAAGCTCTATGCTGAAAGCAGCTGCAGACACCAAAGGACGACAGGAACTGGCAAAGGAA
ENSRNOT00000010961      AGAAGCTCTATACTGAAAGCAGCTGCAGAC---AAAGGAAGACAAGAATTGGCAAAGGAG

ENSPPYT00000019443      GAAAAGGCTCGAGAACTCTTCCTAAAAGCAGTAGAAGAAGAACAAAATGGAGCTCTCTAT
ENST00000244426         GAAAAGGCTCGAGAACTCTTCCTAAAAGCAGTAGAAGAAGAACAAAATGGAGCTCTCTAT
ENSGGOT00000006553      GAAAAGGCTCGAGAACTCTTCCTAAAAGCAGTAGAAGAAGAACAAAATGGAGCTCTCTAT
ENSPTRT00000033806      GAAAAGGCTCGAGAACTCTTCCTAAAAGCAGTAGAAGAAGAACAAAATGGAGCTCTCTAT
ENSMUST00000001402      GAAAAGGCTCGAGAACTCTTCCTGCAGGCAGTGGAGGAAGAACAAAATGGAGCTCTCTAT
ENSRNOT00000010961      GAAAAGGCTCGAGAACTCTTCCTGAAGGCAGTAGAGGAAGAACAAAATGGAGCTCTCTAT

ENSPPYT00000019443      GAAGCCATCAAGTTTTATCGTAGGGCTATGCAACTTGTACCTGATATAGAGTTCAAGATT
ENST00000244426         GAAGCCATCAAGTTTTATCGTAGGGCTATGCAACTTGTACCTGATATAGAGTTCAAGATT
ENSGGOT00000006553      GAAGCCATCAAGTTTTATCGTAGGGCTATGCAACTTGTACCTGATATAGAGTTCAAGATT
ENSPTRT00000033806      GAAGCCATCAAGTTTTATCGTAGGGCTATGCAACTTGTACCTGATATAGAGTTCAAGATT
ENSMUST00000001402      GAAGCCATCAAGTTCTACCGTAGGGCGATGCAGCTGGTGCCAGACATTGAGTTCAAGATC
ENSRNOT00000010961      GAAGCCATCAAGTTCTACCGTAGGGCTATGCAGCTAGTACCTGATATTGAGTTCAAGATC

ENSPPYT00000019443      ACTTATACCCGGTCTCCAGATGGTGATGGCGTTGGAAACAGCTACATTGAAGATAATGAT
ENST00000244426         ACTTATACCCGGTCTCCAGATGGTGATGGCGTTGGAAACAGCTACATTGAAGATAATGAT
ENSGGOT00000006553      ACTTATACCCGGTCTCCAGATGGTGATGGCGTTGGAAACAGCTACATTGAAGATAATGAT
ENSPTRT00000033806      ACTTATACCCGGTCTCCAGATGGTGATGGCGTTGGAAACAGCTACATTGAAGATAATGAT
ENSMUST00000001402      ACTTACACCCGGTCTCCAGACGGCGACGGCGTTGGGAGCGGCTACATCGAAGAGAACGAG
ENSRNOT00000010961      ACTTATACCCGGTCTCCAGATGGCGATGGCGTTGGAAGCAGCTACATCGAAGATAATGAG

ENSPPYT00000019443      GATGACAGCAAAATGGCAGATCTCTTGTCCTACTTCCAGCAGCAACTCACATTTCAGGAG
ENST00000244426         GATGACAGCAAAATGGCAGATCTCTTGTCCTACTTCCAGCAGCAACTCACATTTCAGGAG
ENSGGOT00000006553      GATGACAGCAAAATGGCAGATCTCTTGTCCTACTTCCAGCAGCAACTCACATTTCAGGAG
ENSPTRT00000033806      GATGACAGCAAAATGGCAGATCTCTTGTCCTACTTCCAGCAGCAACTCACATTTCAGGAG
ENSMUST00000001402      GACGCCAGCAAGATGGCCGATCTCCTGTCGTACTTCCAGCAGCAGCTCACGTTGCAGGAG
ENSRNOT00000010961      GACGCCAGCAAGATGGCCGATCTCCTGTCGTACTTCCAGCAGCAGCTCACATTTCAGGAG

ENSPPYT00000019443      TCTGTGCTCAAACTGTGTCAGCCTGAGCTTGAGAGCAGTCAGACTCACATATCAGTGCTG
ENST00000244426         TCTGTGCTTAAACTGTGTCAGCCTGAGCTTGAGAGCAGTCAGATTCACATATCAGTGCTG
ENSGGOT00000006553      TCTGTGCTTAAACTGTGTCAGCCTGAGCTTGAGAGCAGTCAGACTCACATATCAGTGCTG
ENSPTRT00000033806      TCTGTGCTTAAACTGTGTCAGCCTGAGCTTGAGAGCAGTCAGACTCACATATCAGTGTTG
ENSMUST00000001402      TCTGTGCTCAAACTCTGTCAGCCTGAGCTTGAGACCAGTCAGACTCACATATCAGTCCTG
ENSRNOT00000010961      TCTGTGCTCAAACTCTGTCAGCCTGAGCTTGAGACCAGTCAGACTCACATATCAGTCCTG

ENSPPYT00000019443      CCAATGGAGGTCCTGATGTACATCTTCCGATGGGTGGTGTCTAATGACTTGGACCTCAGA
```

```
ENST00000244426       CCAATGGAGGTCCTGATGTACATCTTCCGATGGGTGGTGTCTAGTGACTTGGACCTCAGA
ENSGGOT00000006553    CCAATGGAGGTCCTGATGTACATCTTCCGATGGGTGGTGTCTAGTGACTTGGACCTCAGA
ENSPTRT00000033806    CCAATGGAGGTCCTGATGTACATCTTCCGATGGGTGGTGTCTAGTGACTTGGACCTCAGA
ENSMUST00000001402    CCTATGGAGGTGCTGATGTACATCTTCCGATGGGTGGTATCAAGTGACTTGGACCTCAGA
ENSRNOT00000010961    CCTATGGAGGTGCTGATGTACATCTTCCGATGGGTGGTGTCAAGTGACTTGGACCTCAGA

ENSPPYT00000019443    TCATTGGAGCAATTGTCGCTGGTGTGCAGAGGATTCTACATCTGTGCCAGAGACCCTGAA
ENST00000244426       TCATTGGAGCAGTTGTCGCTGGTGTGCAGAGGATTCTACATCTGTGCCAGAGACCCTGAA
ENSGGOT00000006553    TCATTGGAGCAGTTGTCGCTGGTGTGCAGGGGATTCTACATCTGTGCCAGAGACCCTGAA
ENSPTRT00000033806    TCATTGGAGCAGTTGTCGCTGGTGTGCAGAGGATTCTACATCTGTGCCAGAGACCCTGAA
ENSMUST00000001402    TCGTTAGAGCAGTTGTCACTGGTGTGCAGAGGATTCTATATCTGTGCCAGAGACCCTGAA
ENSRNOT00000010961    TCGTTAGAGCAGTTGTCACTGGTGTGCAGAGGATTCTATATCTGTGCCAGAGACCCTGAA

ENSPPYT00000019443    ATATGGCGTCTGGCCTGCTTGAAAGTTTGGGGCAGAAGCTGTGTTAAACTTGTTCCGTAC
ENST00000244426       ATATGGCGTCTGGCCTGCTTGAAAGTTTGGGGCAGAAGCTGTATTAAACTTGTTCCGTAC
ENSGGOT00000006553    ATATGGCGTCTGGCCTGCTTGAAAGTTTGGGGCAGAAGCTGTATTAAACTTGTTCCGTAC
ENSPTRT00000033806    ATATGGCGTCTGGCCTGCTTGAAAGTTTGGGGCAGAAGCTGTATTAAACTTGTTCCGTAC
ENSMUST00000001402    ATCTGGCGTCTGGCTTGCTTGAAAGTGTGGGGCAGAAGCTGCATGAAGCTCGTTCCATAC
ENSRNOT00000010961    ATATGGCGTCTGGCTTGCTTGAAAGTGTGGGGCAGAAGCTGTATGAAACTTGTCCCATTC

ENSPPYT00000019443    ACATCCTGGAGAGAGATGTTTTTAGAACGGCCTCGTGTTCGGTTTGATGGAGTGTATATC
ENST00000244426       ACGTCCTGGAGAGAGATGTTTTTAGAACGGCCTCGTGTTCGGTTTGATGGCGTGTATATC
ENSGGOT00000006553    ACGTCCTGGAGAGAGATGTTTTTAGAACGGCCTCGTGTTCGGTTTGATGGAGTATATATC
ENSPTRT00000033806    ACGTCCTGGAGAGAGATGTTTTTAGAACGGCCTCGTGTTCGGTTTGATGGAGTGTATATC
ENSMUST00000001402    GCGTCCTGGAGAGAGATGTTTCTAGAACGGCCCCGAGTTCGGTTTGATGGAGTGTACATC
ENSRNOT00000010961    TCATCCTGGAGAGAGATGTTTCTAGAACGGCCTCGTGTTCGGTTTGACGGCGTGTACATC

ENSPPYT00000019443    AGTAAAACCACATATATTCGTCAAGGGGAACAGTCTCTTGATGGTTTCTATAGAGCCTGG
ENST00000244426       AGTAAAACCACATATATTCGTCAAGGGGAACAGTCTCTTGATGGTTTCTATAGAGCCTGG
ENSGGOT00000006553    AGTAAAACCACATATATTCGTCAAGGGGAACAGTCTCTTGATGGTTTCTATAGAGCCTGG
ENSPTRT00000033806    AGTAAAACCACATATATTCGTCAAGGGGAACAGTCTCTTGATGGTTTCTATAGAGCCTGG
ENSMUST00000001402    AGTAAAACCACGTATATTCGCCAGGGAGAGCAGTCACTTGATGGTTTCTACAGGGCGTGG
ENSRNOT00000010961    AGTAAAACCACATATATTCGCCAGGGAGAGCAGTCTCTTGATGGTTTCTATAGAGCCTGG

ENSPPYT00000019443    CACCAAGTGGAATATTACAGGTACATAAGATTCTTTCCTGATGGCCATGTGATGATGTTG
ENST00000244426       CACCAAGTGGAATATTACAGGTACATAAGATTCTTTCCTGATGGCCATGTGATGATGTTG
ENSGGOT00000006553    CACCAAGTGGAATATTACAGGTACATAAGATTCTTTCCTGATGGCCATGTGATGATGTTG
ENSPTRT00000033806    CACCAAGTGGAATATTACAGGTACATAAGATTCTTTCCTGATGGCCATGTGATGATGTTG
ENSMUST00000001402    CACCAAGTGGAATATTACAGATACATGAGATTCTTTCCTGATGGCCATGTGATGATGTTA
ENSRNOT00000010961    CACCAAGTGGAATATTACAGATACATAAGATTCTTTCCTGATGGCCATGTGATGATGTTG

ENSPPYT00000019443    ACAACCCCTGAAGAGCCTCAGTCCATTGTTCCACGTTTAAGAACTAGGAATACCAGGACT
ENST00000244426       ACAACCCCTGAAGAGCCTCAGTCCATTGTTCCACGTTTAAGAACTAGGAATACCAGGACT
ENSGGOT00000006553    ACAACCCCTGAAGAGCCTCAGTCCATTGTTCCACGTTTAAGAACTAGGAATACCAGGACT
ENSPTRT00000033806    ACAACCCCTGAAGAGCCTCAGTCCATTGTTCCACGTTTAAGAACTAGGAATACCAGGACT
ENSMUST00000001402    ACCACCCCCGAGGAGCCTCCGTCCATCGTTCCCCGGTTAAGAACCAGGAACACCAGAACG
ENSRNOT00000010961    ACAACCCCTGAGGAGCCTCCATCCATTGTTCCCCGTTTAAGAACCAGGAACACCAGAACG

ENSPPYT00000019443    GATGCAATTTTACTGGGTCACTATCGCTTGTCACAAGACACAGACAATCAGACCAAAGTA
ENST00000244426       GATGCAATTCTACTGGGTCACTATCGCTTGTCACAAGACACAGACAATCAGACCAAAGTA
ENSGGOT00000006553    GATGCAATTCTACTGGGTCACTATCGCTTGTCACAAGACACAGACAATCAGACCAAAGTA
ENSPTRT00000033806    GATGCAATTCTACTGGGTCACTATCGCTTGTCACAAGACACAGACAATCAAACCAAAGTA
ENSMUST00000001402    GATGCAATTCTGCTGGGTCATTACCGCTTGTCACAAGATGCAGACAATCAGACCAAAGTC
```

```
ENSRNOT00000010961     GATGCAATTCTGCTGGGTCATTATCGCTTGTCACAAGATGCAGACAATCAGACCAAAGTA


ENSPPYT00000019443     TTTGCTGTAATAACTAAGAAAAAAGAAGAAAAACCACTTGACTATAAATACAGATATTTT
ENST00000244426        TTTGCTGTAATAACTAAGAAAAAAGAAGAAAAACCACTTGACTATAAATACAGATATTTT
ENSGGOT00000006553     TTTGCTGTAATAACTAAGAAAAAAGAAGAAAAACCACTTGACTATAAATACAGATATTTT
ENSPTRT00000033806     TTTGCTGTAATAACTAAGAAAAAAGAAGAAAAACCACTTGACTATAAATACAGATATTTT
ENSMUST00000001402     TTTGCTGTAATAACTAAGAAAAAAGAAGAAAAGCCACTTGACCATAAATACAGGTATTTT
ENSRNOT00000010961     TTTGCTGTGATAACTAAGAAAAAAGAAGAAAAGCCACTTGACCATAAGTACAGGTATTTT


ENSPPYT00000019443     CGTCGTGTCCCTGTACAAGAAGCAGATCAGAGTTTTCATGTGGGGCTACAACTATGTTCC
ENST00000244426        CGTCGTGTCCCTGTACAAGAAGCAGATCAGAGTTTTCATGTGGGGCTACAGCTATGTTCC
ENSGGOT00000006553     CGTCGTGTTCCTGTACAAGAAGCAGATCAGAGTTTTCATGTGGGGCTACAGCTATGTTCC
ENSPTRT00000033806     CGTCGTGTCCCTGTACAAGAAGCAGATCAGAGTTTTCATGTGGGGCTACAGCTATGTTCC
ENSMUST00000001402     CGTCGTGTTCCTGTCCAAGAGGCAGATCACAGCTTCCATGTGGGACTGCAGCTGTGCTCC
ENSRNOT00000010961     CGCCGTGTTCCTGTCCAAGAGGCAGATCACAACTTTCATGTGGGGCTGCAGCTGTGCTCC


ENSPPYT00000019443     AGTGGCCACCAGAGGTTCAACAAACTCATCTGGATACATCATTCTTGTCACATTACTTAC
ENST00000244426        AGTGGTCACCAGAGGTTCAACAAACTCATCTGGATACATCATTCTTGTCACATTACTTAC
ENSGGOT00000006553     AGTGGCCACCAGAGGTTCAACAAACTCATCTGGATACATCATTCTTGTCACATTACTTAC
ENSPTRT00000033806     AGTGGTCACCAGAGGTTCAACAAACTCATCTGGATACATCATTCTTGTCACATTACTTAC
ENSMUST00000001402     AGTGGCCACCAGAGGTTCAACAAACTCATCTGGATCCACCACTCTTGTCACATCACTTAC
ENSRNOT00000010961     AGTGGCCACCAGAGGTTCAACAAACTCATCTGGATCCACCACTCTTGTCACATCACTTAC


ENSPPYT00000019443     AAATCAACTGGTGAGACTGCAGTCAGTGCTTTTGAGATTGACAAGATGTACACCCCCTTG
ENST00000244426        AAATCAACTGGTGAGACTGCAGTCAGTGCTTTTGAGATTGACAAGATGTACACCCCCTTG
ENSGGOT00000006553     AAATCAACTGGTGAGACTGCAGTCAGTGCTTTTGAGATTGACAAGATGTACACCCCCTTG
ENSPTRT00000033806     AAATCAACTGGTGAGACTGCAGTCAGTGCTTTTGAGATTGACAAGATGTACACCCCTTTG
ENSMUST00000001402     AAAGCAACTGGTGAGACTGCAGTGAGTGCTTTTGAGATTGACAAGATGTACACGCCCCTG
ENSRNOT00000010961     AGATCGACTGGTGAGACTGCAGTGAGTGCTTTTGATATTGACAAGATGTACACCCCCTTG


ENSPPYT00000019443     TTCTTCGCCAGAGTAAGGAGCTACACAGCTTTCTCAGAAAGGCCTCTG
ENST00000244426        TTCTTCGCCAGAGTAAGGAGCTACACAGCTTTCTCAGAAAGGCCTCTG
ENSGGOT00000006553     TTCTTCGCCAGAGTAAGGAGCTACACAGCTTTCTCAGAAAGGCCTCTG
ENSPTRT00000033806     TTCTTCGCCAGAGTAAGGAGCTACACAGCTTTCTCAGAAAGGCCTCTG
ENSMUST00000001402     TTGTTCGCCAGAGTGAGGAGCTACACTGCCTTCTCGGAAAGGCCTCTG
ENSRNOT00000010961     TTCTTCGCCAGAGTGAGAAGCTACACGGCGTTCTCCGAGAGGCCTCTG
```

Multiple sequence alignment of Fbxw10

```
ENSMUST00000036085     ATGGAAAACAGGGAACCAAAACTCAAGCAGGCCCCCTATTTTCGTTGTGAAAAGGGACCC
ENSCJAT00000055574     ATGGAAAACCTGGAATCAAGGCTCAAGAATGCCCCCGATTTTCGTTGTGAGAAGGGAAAC
ENSMMUT00000026976     ATGGAAAACCTGGAATCAACGCTCAAGAATGCCCCCTATTTTCATTGTGAGAAGGGAACC
ENSPPYT00000009368     ATGGAAAACCTGGAATCAAGGCTCAAGAATGCCCCCTATTTTCATTGTGAGAAGGGAACT
ENST00000308799        ATGGAAAACCTGGAATCAAGGCTCAAGAATGCCCCCTATTTTCGTTGTGAGAAGGGAACC
ENSPTRT00000016330     ATGGAAAACCTGGAATCAAGGCTCAAGAATGCCCCCTATTTTTGTTGTGAGAAGGGAACC


ENSMUST00000036085     AACTGGGTCCCCGTGTGCCAGAAGTGTGAAGCCTGTGTCTTAGCATGGAAGATCTTTGCC
ENSCJAT00000055574     GATTTCTACTCTATATACCAGAAGTGTGAGACGTGTGTCTTGGCCTGGAAGATCTTCTCT
ENSMMUT00000026976     GATTCCATCCCTCTATGCCAGAAGTGTGAGACGTGTGTCTTAGCCTGGAAGATCTTCTCT
ENSPPYT00000009368     GATTCCATCCCTCTATGCCAGAAGTGTGAGACGTGTGTCTTAGCCTGGAAGATCTTCTCT
ENST00000308799        GATTCCATCCCTCTATGCCGGAAGTGTGAGACGTGTGTCTTAGCCTGGAAGATCTTCTCT
ENSPTRT00000016330     GATTCCATCCCTCTATGCCGGAAGTGTGAGACGTGTGTCTTAGCCTGGAAGATCTTCTCT
```

```
ENSMUST00000036085     ACCAAAGAGTGGTTCCGGAGGGTCAACGACATATCCCAGAGGAGATTTCTAGTCAGCATC
ENSCJAT00000055574     ACCAAAGAGTGGTTCTGCAGGATCAATGACGTGTCTCAGAGGAGGTTTCTAGTTAGCATT
ENSMMUT00000026976     ACCAAAGAGTGGTTCTGCAGGATCAGTGACATATCACAGAGGAGGTTTCTAGTTAGCATT
ENSPPYT00000009368     ACCAAAGAGTGGTTCTGCAGGATCAATGACATATCACAGAGGAGGTTTCTAGTTGGCATT
ENST00000308799        ACCAAAGAGTGGTTCTGCAGGATCAATGACATATCACAGAGGAGGTTTCTAGTTGGCATT
ENSPTRT00000016330     ACCAAAGAGTGGTTCTGCAGGATCAATGACATATCACAGAGGAGGTTTCTAGTTGGCATT

ENSMUST00000036085     TTGGGGCAGTTAAATAGCCTGTATTTGTTACAGTATTTCCAGAATATCCTAGAGACCACC
ENSCJAT00000055574     CTGAAGCAGTTAAATAGCTTATATTTGTTACACTATTTCCAAAATATCCTTCAGACCACA
ENSMMUT00000026976     CTGAAGCAGTTAAATAGCTTGTATTTGTTACACTATTTCCAGAATATCCTTCAGACCACA
ENSPPYT00000009368     CTGAAGCAGTTAAATAGCTTGTATTTGTTACACTATTTCCAAAATATCCTTCAGACCACA
ENST00000308799        CTGAAGCAGTTAAATAGCTTATATTTGTTACACTATTTCCAAAATATCCTTCAGACCACA
ENSPTRT00000016330     CTGAAGCAGTTAAATAGCTTATATTTGTTACACTATTTCCAAAATATCCTTCAGACCACA

ENSMUST00000036085     CAGGGGAAGGACTTCATCTACAACAGGTCTCGGATCAAACTCAGTAGGAAGGGGGGGAAG
ENSCJAT00000055574     CAGGGAAAGGATTTCATCTATAACAGGTCCCTGGTCGATCTCAGCAAGAAGGAGGGGAAA
ENSMMUT00000026976     CAGGGAAAGGATTTCATCTATAACAGGTCCCGGATCGACCTGAGCAAGAAAGAGCGGAAA
ENSPPYT00000009368     CAGGGAAAGGATTTTATCTATAGCAGGTCCCGGATCGACCTCAGCAAGAAAGAGGGGAAA
ENST00000308799        CAGGGAAAGGATTTCATCTATAACAGGTCCCGGATCAACCTCAGCAAGAAAGAGGGGAAA
ENSPTRT00000016330     CAGGGAAAGGATTTCATCTATAACAGGTCCCGGATCAACCTCAGCAAGAAAGAGGGGAAA

ENSMUST00000036085     GTTGTAAAGTCCTCCTTGAACCAGATGTTGGATAAAACGGTGGAACGGAAGATGAAGGAG
ENSCJAT00000055574     GTTGCTAAGTCCTCCTTGAACCAAATGTCAGATAAAACAGTAGAACAGAAGATGAAGGAG
ENSMMUT00000026976     GTTGTGAAGTCCTTCTTGAACCAAATGTTGGATAAAACAGTAGAACAGAAGATGAAGGAG
ENSPPYT00000009368     GTTGTGAAGTCCTCCTTGAACCAAATGTTGGATGAAACAGTAGAACAGAAGATGAAAGAG
ENST00000308799        GTTGTGAAGTCCTCCTTGAACCAAATGTTGGATAAAACAGTAGAACAGAAGATGAAAGAG
ENSPTRT00000016330     GTTGTGAAGTCCTCCTTGAACCAAATGTTGGATAAAACAGTAGAACGGAAGATGAAAGAG

ENSMUST00000036085     ATCCTCTACTGGTTTGGCAACAGCACCCACCGAACTAAGGCAAATTATACTCTCTTGCTG
ENSCJAT00000055574     CTCTTGTACTGGTTTGTGAACAGCACCCACTGGACCAAGGCGAATTATACTCTCTTACTG
ENSMMUT00000026976     ATCTTGTACTGGTTTGGGAACAGCACCCACTGGACCAAGGCGAATTATACTCTCTTACTG
ENSPPYT00000009368     ATCTTGTACTGGTTTGCGAACAGCACCCAGTGGACCAAGGCAAATTATACTCTGTTACTG
ENST00000308799        ATCTTGTACTGGTTTGCGAACAGCACCCAGTGGACCAAGGCGAATTATACTCTCTTACTG
ENSPTRT00000016330     ATCTTGTACTGGTTTGCGAACAGCACCCAGTGGACCAAGGCGAATTATACTCTCTTACTG

ENSMUST00000036085     CTGCAGATGTGCGACTCAAATTTACTGCTCACTGCTGCCAATGTGATCAGAGTCCTGTTC
ENSCJAT00000055574     CTGCAGATGTGCAACCCCAAATTACTGCTCACTGCTGCCAACGTGATCAGAGTCCTGTTT
ENSMMUT00000026976     CTGCAGATGTGCAACCCCAAATTACTGCTCACTGCTGCCAATGTGATCAGAGTCCTGTTT
ENSPPYT00000009368     CTGCAGATGTGCAACCCCAAATTACTGCTCACTGCTGCCAATGTGATCAGAGTCCTGTTT
ENST00000308799        CTGCAGATGTGCAACCCCAAATTACTGCTCACTGCTGCCAATGTGATCAGAGTCCTGTTT
ENSPTRT00000016330     CTGCAGATGTGCAACCCCAAATTACTGCTCACTGCTGCCAATGTGATCAGAGTCCTGTTT

ENSMUST00000036085     ATGAAAGAGTGGAACAGTATTTCAGGCCTTCATGATGACACTCCTGATGTGATGTTCTTC
ENSCJAT00000055574     CTGAGAGAACAGAACAATATCTCAGGTCTCAATCAGGACATCACAGATGTGTGTTTTTCC
ENSMMUT00000026976     CTGAGAGAGCAGAACAATATCTCAGGGCTCAATCAAGACATCACAGATGTGTGTTTTTCC
ENSPPYT00000009368     CTGAGAGAGGAGAACAATATCTCAGGGCTCAATCAAGACATCAGAGATGTGTGTTTTTCC
ENST00000308799        CTGAGAGAGGAGAACAATATCTCAGGGCTCAATCAAGACATCACAGATGTGTGTTTTTCC
ENSPTRT00000016330     CTGAGAGAGGAGAACAATATCTCAGGGCTCAATCAAGACATCACAGATGTGTGTTTTTCC

ENSMUST00000036085     CCTGAGAAAAAGTACAGCGGTACACAGGACACCTCATATGTCTCTTGGGCAGCTAGACCC
ENSCJAT00000055574     CCGGAAAAAGACCACAGCTCCAAGTCTGCGACCTCACAAGTCTATTGGAGAACCAAAACT
ENSMMUT00000026976     CCCGAGAAAGATGACAGCTCCAAGTCTGTGACCTCACAAGTCTATTGGACAGCCAAAACT
```

ENSPPYT00000009368 CCTGAGAAAGACCACAGCTCCAAGTCTGCGACTTCACAAGTCTATTGGACAGCCAAAACT
ENST00000308799 CCTGAGAAAGACCACAGCTCCAAGTCTGCGACCTCACAAGTCTATTGGACAGCCAAAACT
ENSPTRT00000016330 CCTGAGAAAGACCACAGCTCCAAGTCTGCGACCTCACAAGTCTATTGGACAGCCAAAACT

ENSMUST00000036085 AAACCTGTGTCATTCCCAATGTCCAAACATTTAGGTAATAAA------CTCGGGACTGAA
ENSCJAT00000055574 CAGTACACGTCCTTTCCTTTGTCCAAAGCCCTAGAAAATGAACACTTGCTTGGGGCAGCA
ENSMMUT00000026976 CAGCACACATCCCTTCCTTTGTCCAAAGCCCCAGAAAATGAACACTTACTTGGGGCAGCT
ENSPPYT00000009368 CAGTACACATCCCTTCCTTTGTCCAAAGCCCCAGAAAATGCACACTTGCTTGGGGCAGCA
ENST00000308799 CAGCACACATCCCTTCCTTTGTCCAAAGCCCCAGAAAATGAACACTTGCTTGGGGCAGCA
ENSPTRT00000016330 CCGCACACATCCCTTCCTTTGTCCAAAGCCCCAGAAAATGAACACTTGCTTGGGGCAGCA

ENSMUST00000036085 AATACAACCGAGGGAAAAGGGGAGAGTTCGCTCCAGTGTATCCATGAAATGAATAGACAG
ENSCJAT00000055574 TCTAACCCGGAGGGACCATGGAGGCGTTCACTCCAGTGTATTTCCGAGATGAATAGGCTG
ENSMMUT00000026976 TCTAACCCTGAGGAACCATGGAGGAATTCACTCCAGTGTATTTCTGAAATGAATAGGCTG
ENSPPYT00000009368 TCTAACCCTGAGGAACCATGGAGGAATTCACTCCGGTGTATTTCCGAAATGAATACGCTG
ENST00000308799 TCTAACCCTGAGGAACCATGGAGGAATTCACTCCGGTGTATATCCGAAATGAATAGGCTG
ENSPTRT00000016330 TCTAACCCTGAGGAACCATGGAGGAATTCACTCCGGTGTATTTCCGAAATGAATAGGCTG

ENSMUST00000036085 ATTTTTGGAAAAGGGGGCATGTCCAGGCTGGGAGATGATCCGTGCAACCTGCTGCTGAGC
ENSCJAT00000055574 TTTTCTGAAAAAGGAGACTTAACCAAGCCAGGGTTCGATCCCTGCAATCTGTTGGTTGAC
ENSMMUT00000026976 TTTTCTGAAAAAGGAGGCATAACCAAGCCAGGGTACGATCCCTGCAATCTATTGGTTGAC
ENSPPYT00000009368 TTTTCTGGAAAAGGAGACATAACCAAGCCAGGGTACGATCCCTGCAATCTATTGGTTGAC
ENST00000308799 TTTTCTGGAAAAGGAGACATAACCAAGCCAGGGTACGATCCCTGCAATCTATTGGTTGAC
ENSPTRT00000016330 TTTTCTGGAAAAGGAGACATAACCAAGCCAGGGTACGATCCCTGCAATCTATTGGTTGAC

ENSMUST00000036085 CTGGATCATGTCCAACTCCTGTCTTCTGGGTACAGCAAATATCGGGACTTTATACGCGAC
ENSCJAT00000055574 CTGGATGACATCAGAGACCTGTCTTCTGGGTTCAGCAAATACCGAGACTTTATCCGTTAC
ENSMMUT00000026976 CTGGATGACCTCAGAGACCTGTCTTCTGGGTTCAGCAAATACCGAGACTTCATCCGTTAC
ENSPPYT00000009368 CTGGATGACATCAGAGACCTGTCTTCTGGGTTCAGCAAATACCGAGACTTCATCCGTTAC
ENST00000308799 CTGGATGACATCAGAGACCTGTCTTCTGGGTTCAGCAAATACCGAGACTTCATCCGTTAC
ENSPTRT00000016330 CTGGATGACATCAGAGACCTGTCTTCTGGGTTTAGCAAATACCGAGACTTCATCCGTTAC

ENSMUST00000036085 CTGCCTCTCCACCTCTCCAAGTACATTCTAAGAATGCTGGATAAGCACAGTCTGAACAGG
ENSCJAT00000055574 CTGCCCATCCACCTCTCCAAGTACATTCTAAGTATGCTGGATAGACACACCCTGAACAAA
ENSMMUT00000026976 CTGCCCATCCACCTCTCCAAGTACATTCTAAGTATGCTGGATAGACACACCCTGAACAAG
ENSPPYT00000009368 CTGCCCATCCACCTCTCCAAGTACATTCTAAGAATGCTGGATAAACACACCCTGAACAAG
ENST00000308799 CTGCCCATCCACCTCTCCAAGTACATTCTAAGTATGCTGGATAGACACACCCTGAACAAG
ENSPTRT00000016330 CTGCCCATCCACCTCGCCAAGTACATTCTAAGAATGCTGGATAGACACACCCTGAACAAG

ENSMUST00000036085 TGTATCTTTGTAAGCCAGCACTGGGCCACTCTGGCCCAGCAGGTCAAGGTGGACCAGTCC
ENSCJAT00000055574 TGTGCCTCTGTGAGCCAGCACTGGGCCGCACTGGCTCAACAGGTTAAGGTGGAATTGTCG
ENSMMUT00000026976 TGTGCCTCTGTGAGCCAGCACTGGGCCGCCATGGCTCAACAGGTCAAGATGGACTTGTCA
ENSPPYT00000009368 TGCGCCTCTGTGAGCCAGCACTGGGCCGCCATGGCTCAACAGGTCAAGATGGACTTGTCA
ENST00000308799 TGCGCCTCTGTGAGCCAGCACTGGGCCGCCATGGCTCAACAGGTCAAGATGGACTTGTCA
ENSPTRT00000016330 TGCGCCTCTGTGAGCCAGCACTGGGCCGCCATGGCTCAACAGGTCAAGATGGACTTGTCA

ENSMUST00000036085 ATGCACTCCTTCATTCAGAACCAGATTTCTCTCCTGCAGGGGTCCTACACAAGGGGAATA
ENSCJAT00000055574 GCGCACGGCTTCATTCAGAACCAGATTGCCTTCTTGCAGGCGTCCTACACAAGAGAAATT
ENSMMUT00000026976 GCGCACAGCTTCATTCAGAACCAGATTGTCTTCATGCAGGGGTCCTACACAAGAGGAATT
ENSPPYT00000009368 GTGCACGGCTTCATTCAGAACCAGATTACCTTCTTGCAGGGGTCCTACACAAGAGGAATT
ENST00000308799 GCGCACGGCTTCATTCAGAACCAGATTACCTTCTTGCAGGGGTCCTACACAAGAGGAATT
ENSPTRT00000016330 GCGCACGGCTTCACTCAGAACCAGATTACCTTCTTGCAGGGGTCCTACACAAGAGGAATT

```
ENSMUST00000036085    GATCCTAATTATGCCAACAAAGTCTCAATCCCAGTGCCCAAGATAGTAGATGATGGGAAG
ENSCJAT00000055574    GATCCTAATTACGCCAGTAAAGTTTCTATCCCAGTTCCTAAAATGGTAGATGACGGGAAG
ENSMMUT00000026976    GATCCTAATTATGCCAATAAGGTTTCTATCCCAGTTCCTAAAATGGTAGATGACGGGAAG
ENSPPYT00000009368    GATCCTAATTATGCCAATAAGGTTTCTATCCCAGTTCCTAAAATGGTAGATGACGGGAAG
ENST00000308799       GATCCTAATTATGCCAATAAGGTTTCTATCCCAGTTCCTAAAATGGTAGATGACGGGAAG
ENSPTRT00000016330    GATCCTAATTATGCCAATAAGGTTTCTATCCCAGTTCCTAAAATGGTAGATGACGGGAAG

ENSMUST00000036085    CGTTCACGGTCAAAAAATCAGAAGTGGAAACTGAGAACAAAGACTGATTACAACTTGTGG
ENSCJAT00000055574    CACGTGCATGTGAAACATCAGAAATGGAAGCTGAGAACGAAGACTGACTACAACCTGTGG
ENSMMUT00000026976    CGCATGCGTGTGAAACATCCGAAGTGGAAGCTGAGGACGAAGAATGAGTACAACCTATGG
ENSPPYT00000009368    CGCATGCGTGTGAAACATCCAAAGTGGGAGCTGAGAACGAAGAATGAGTACACCCTGTGG
ENST00000308799       AGCATGCGTGTGAAACATCCGAAGTGGAAGCTGAGAACGAAGAATGAGTACAACCTGTGG
ENSPTRT00000016330    AGCATGCGTGTGAAACATCCGAAGTGGAAGCTGAGAACGAAGAATGAGTACAACCTGTGG

ENSMUST00000036085    AATGCCTACCAGAACCAGGAAACCCAGCTGGTCCAGATGGAGGAGAGGAACGTTTTCTGC
ENSCJAT00000055574    ACTGCATACCAGAACCAGGAAACACAGCAGGTCCTGATGGAGGAGAGAAATGTTTTCTGT
ENSMMUT00000026976    ACTGCATACCAGAACCAGGAAACGCAGCAGGTCCTAATGGAGGAGAGAAATATTTTCTGT
ENSPPYT00000009368    ACTGCATACCAGAACCAGGAAACGCAGCAGGTCCTGATGGAGGAGAGAAATGTTTTCTGT
ENST00000308799       ACTGCATACCAGAACGAGGAAACGCAGCAGGTCCTGATAGAGGAGAGAAATGTTTTCTGT
ENSPTRT00000016330    ACTGCATACCAGAACGAGGAAACGCAGCAGGTCCTGATGGAGGAGAGAAATGTTTTCTGT

ENSMUST00000036085    GGCACCTACAACATCCGCGTCCTCTCTGACACGTTTGACCAAAACAGAATCATTCACTAT
ENSCJAT00000055574    GGGACCTACAATGTCCGCATTCTCTCTGACAGGTGGGATCAAAACAGAGTCATCCACTAC
ENSMMUT00000026976    GGAACCTACAATGTTCGCATTCTCTCTGACACGTGGGATCAAAAAAGAGTCATCCACTAT
ENSPPYT00000009368    GGGACCTACAATGTTCGCATTCTCTCTGACACGTGGGATCAAAACAGAGTCATCCACTAT
ENST00000308799       GGGACCTACAATGTTCGCATTCTCTCTGACACGTGGGATCAAAACCGAGTCATCCACTAT
ENSPTRT00000016330    GGGACCTACAATGTTCGCATTCTCTCTGACACGTGGGATCAAAACCGAGTCATCCACTAT

ENSMUST00000036085    AACGGGGGAGACCTAATGGCCATATCATCCAATCGGAAGATCCACCTTCTAGATATCATG
ENSCJAT00000055574    TCCGGGGGAGATCTGATAGCTGAGTCATCTAATCGAAAGATCCATCTTCTGGATATCATA
ENSMMUT00000026976    TCCGGGGGAGATCTGATAGCTGTGTCATCTAATCGAAAGATCCATCTTCTGGACATCATA
ENSPPYT00000009368    TCCGGGGGAGATCTGATAGCTGTGTCATCTAATCGAAAGATCCATCTTCTGGACATCATA
ENST00000308799       TCCGGGGGAGATCTGATAGCTGTGTCATCTAATCGAAAGATCCATCTTCTGGACATCATA
ENSPTRT00000016330    TCCGGGGGAGATCTGATAGCTGTGTCATCTAATCGAAAGATCCATCTTCTGGACATCATA

ENSMUST00000036085    CAGACCAAGGAGCTGCCCATCGAGTTCCGAGGCCATGCCGGCAGCGTCAGGGCTCTCTTC
ENSCJAT00000055574    CAAGTAAAAGCGATACCCATTGAGTTCCGAGGCCATGCTGGGAGTGTCCGCGCCCTCTTC
ENSMMUT00000026976    CAAGGAAAAGTGATACCCGTTGAATTCCGAGGCCATGCTGGGAGTGTCCGGGCCCTCTTC
ENSPPYT00000009368    CAAGTAAAAGCGATACCCGTTGAATTCCGAGGCCATGCTGGGAGTGTCCGGGCCCTCTTC
ENST00000308799       CAAGTGAAAGCGATACCCGTTGAATTCCGAGGCCATGCTGGGAGTGTCCGGGCCCTCTTC
ENSPTRT00000016330    CAAGTGAAAGCGATACCCGTTGAATTCCGAGGCCATGCTGGGAGTGTCCGGGCCCTCTTC

ENSMUST00000036085    CTGTCTGAGGAGGACAATATTCTCCTGAGTGGGAGTTACGATCTGAGCATAAGATACTGG
ENSCJAT00000055574    CTGTGTGAGGAGGAAAACTTTCTCCTAAGCGGGAGCTATGACCTAAGTATCAGATACTGG
ENSMMUT00000026976    CTATGTGAGGAGGAAAATTTTCTCCTAAGCGGGAGCTATGACCTAAGTATCAGATACTGG
ENSPPYT00000009368    CTGTGTGAGGAGGAAAACTTTCTCCTGAGCGGGAGCTATGACCTAAGTATCAGATACTGG
ENST00000308799       CTGTGTGAGGAGGAAAACTTTCTCCTAAGCGGGAGCTATGACCTAAGTATCAGATACTGG
ENSPTRT00000016330    CTGTGTGAGGAGGAAAACTTTCTCCTAAGCGGGAGCTATGACCTAAGTATCAGATACTGG

ENSMUST00000036085    GATGTGAAAACCGGGGCTTGTGTACGAATCTTTTATGGCCACCAGGGGACAATCACTTGC
ENSCJAT00000055574    GATCTGAGAAGTGGGGCTTGCACACGAATCTTCAATGGTCACCAGGGGACTGTCACTTGC
ENSMMUT00000026976    GATCTGAAAAGTGGGGCTTGCACACGAATCTTCGGTGGTCACCAGGGGACTATCACTTGC
```

```
ENSPPYT00000009368     GATCTGAAAAGTGGGGCTTGCACACGAATCTTCGGTGGTCACCAGGGGACTATCACTTGC
ENST00000308799        GATCTGAAAAGTGGGGTTTGCACACGAATCTTCGGTGGTCACCAGGGGACTATCACTTGC
ENSPTRT00000016330     GATCTGAAAAGTGGGGTTTGCGCACGAATCTTCGGTGGTCACCAGGGGACTATCACTTGC

ENSMUST00000036085     CTGGATGTCTATAAGAACAGGCTTGTATCTGGAGCAAAAGATGGACAGGTGAAAGAATGG
ENSCJAT00000055574     ATGGATTTATGTAAGAACAGGCTTGTATCTGGAGCAAGAGATTGCCAGGTAAAAGTGTGG
ENSMMUT00000026976     ATAGATTTGTGTAAGAACAGGCTCGTTTCGGGAGGAAAAGATTGCCAGGTAAAAGTATGG
ENSPPYT00000009368     ATGGACTTGTGTAAGAACAGGCTCGTATCTGGAGGAAGAGATTGCCAGGTAAAAGTATGG
ENST00000308799        ATGGACTTGTGTAAGAACAGGCTCGTATCTGGAGGAAGAGATTGCCAGGTAAAAGTATGG
ENSPTRT00000016330     ATGGACTTGTGTAAGAACAGGCTCGTATCTGGAGGAAGAGATTGCCAGGTA---------

ENSMUST00000036085     GACATAGAGACAGGGAAGTGCCTGAAGACCTTTAAACACAAAGATCCCATCTTGGCTGCC
ENSCJAT00000055574     GATGTAGACACAGGGAAGTGCCTGAAGACGTTTAGACACAAAGACCCCATCCTGGCCACC
ENSMMUT00000026976     GATGTAGACACAGGGAAGTGCCTGAAGACGTTTAGACACAAAGACCCCATCTTGGCCACC
ENSPPYT00000009368     GATGTAGACACAGGGAAGTGCCTGAAGACGTTTAGACACAAAGACCCCATCTTGGCCACC
ENST00000308799        GATGTAGACACAGGGAAGTGCCTGAAGACGTTTAGACACAAAGACCCCATCTTGGCCACC
ENSPTRT00000016330     ------------------------------------------------------------

ENSMUST00000036085     AAGATCAGTGAGACCTACATTGTGAGCAGCTGTGAGCGAGGCATAGTCAAGGTGTGGCAC
ENSCJAT00000055574     AGGATCAACGACACCTACATTGTGAGCGCGTGTGAGCGAGGGGTGGTAAAAGTGTGGCAC
ENSMMUT00000026976     AGGATCAATGATACCTACATTGTGAGCAGCTGTGAGCGAGGGGTGGTGAAAGTGTGGCAC
ENSPPYT00000009368     AGGATTAATGATACCTACATTGTGAGCAGCTGTGAGCGAGGGGTGGTGAAAGTGTGGCAC
ENST00000308799        AGGATCAATGATACCTACATTGTGAGCAGCTGTGAGCGAGGGCTGGTGAAAGTGTGGCAC
ENSPTRT00000016330     ------------------------------------------------------------

ENSMUST00000036085     GTTGTCACAGCTCAGCTGCAAAAGACTCTCACTGGGCACGAGGGAGCTGTGAAGTGCCTG
ENSCJAT00000055574     ATTGCTATGGGCCAGTTGGTAAAGACTCTCAGTGGCCATGAGGGAGCTGTGAAGTGTCTG
ENSMMUT00000026976     ATTGTCATGGCCCAGTTGGTAAAGACTCTCAATGGCCACGAGGGAGCCGTGAAGTGCCTG
ENSPPYT00000009368     ATTGCCATGGCCCAGTTGGTAAAGACTCTCAATGGCCACGAGGGAGCCGTGAAATGCCTG
ENST00000308799        ATTGCCATGGCCCAGTTGGTAAAGACTCTCAGTGGCCATGAGGGAGCCGTGAAATGCCTG
ENSPTRT00000016330     ---------------------AAAACTCTCAATGGCCACGAGGGAGCCGTGAAATGCCTG

ENSMUST00000036085     TTCTTTAACGAATGGCATCTCGTCTCTGGAGGTGCTGACGGCCTGGTCATGGCCTGGAGC
ENSCJAT00000055574     TTCTTTGACCAGTGGCATCTCCTCTCGGGAAGTGCTGATGGCCTGGTCATGGCCTGGAGC
ENSMMUT00000026976     TTCTTCGACCAGTGGCATCTCCTCTCGGGAAGCACTGATGGCCTGGTCATGGCCTGGAGC
ENSPPYT00000009368     TTCTTTGACCAGTGGCATCTCCTCTCAGGAAGCACTGATGGCCTGGTCATGGCCTGGAGC
ENST00000308799        TTCTTTGACCAGTGGCATCTCCTCTCAGGAAGTACTGATGGCCTGGTCATGGCCTGGAGC
ENSPTRT00000016330     TTCTTTGACCAGTGGCATCTCCTGTCAGGAAGCACTGATGGCCTGGTCATGGCCTGGAGC

ENSMUST00000036085     ATGGTGGGAAAGTACGAACGGTGCCTGATGGCCTTCAAGCATCCTAAGGAGGTGCTGCAG
ENSCJAT00000055574     ATGGCGGGGAAGTATGAGCGCTGCCTGATGGCCTTCAAGCATCCCAGGGAGGTGCTCCAC
ENSMMUT00000026976     ATGGTGGGGAAGTATGAGCGCTGCCTGATGGCCTTCAAGCATCCCAAGGAGGTGCTCCAC
ENSPPYT00000009368     ATGGTGGGGAAGTACGAGCGCTGCCTGATAGCCTTCAAGCATCCCAAGGAGGTGCTCGAC
ENST00000308799        ATGGTGGGGAAGTACGAGCGCTGCCTGATGGCCTTCAAGCATCCCAAGGAGGTGCTCGAC
ENSPTRT00000016330     ATGGTGGGGAAGTACGAGCGCTGCCTGATGGCCTTCAAGCATCCCAAGGAGGTGCTCGAC

ENSMUST00000036085     GTGTCTCTCCTCTACCTCCGGGTCATCAGTGCCTGTGGAGACGGCAAGATCCGAATTTAT
ENSCJAT00000055574     GTGTTCCTTCTCTTCCTCCGGGTCATCAGCGCCTGTGCAGATGGCAAGATCCGAATTTAC
ENSMMUT00000026976     GTGTCCCTTCTCTTCCTCCGGGTCATCAGCGCCTGCGCAGATGGCAAGATCAGAATTTAC
ENSPPYT00000009368     GTGTCGCTTCTCTTCCTCCGGGTCATCAGCGCCTGTGCAGATGGCAAGATCCGAATTTAC
ENST00000308799        GTGTCCCTTCTCTTCCTCCGGGTCATCAGCGCCTGTGCAGATGGCAAGATCCGAATTTAC
ENSPTRT00000016330     GTGTCCCTTCTCTTCCTCCGGGTCATCAGCGCCTGTGCAGATGGCAAGATCCGAATTTAC
```

```
ENSMUST00000036085     AATTTCCTCAATGGGAACTGTCTGAAGGTGATAAAAGTCGACGCCAGAGGTGACCCCGTG
ENSCJAT00000055574     AATTTCCTCAATGGGAACTGTATGAAGGTGATAAAAGCCAATGGCAGAGGCGATCCTGTG
ENSMMUT00000026976     AATTTCCTCAACGGGAACTGTATGAAGGTGATAAAAGCCAATGGCAGAGGCGATCCTGTG
ENSPPYT00000009368     AATTTCCTCAACGGGAACTGTATGAAGGTGATAAAAGCCAATGGCAGAGGCGATCCTGTG
ENST00000308799        AATTTCCTCAACGGGAACTGTATGAAGGTGTTAAAAGCCAATGGCAGAGGTGATCCTGTG
ENSPTRT00000016330     AATTTCCTCAACGGGAACTGTATGAAGGTGATAAAAGCCAATGGCAGAGGCGATCCTGTG

ENSMUST00000036085     CTATCCTTCTTTTATCAGGGCAACAGGATGGTGGCCCACACGGACAGCAACATTCTCGTG
ENSCJAT00000055574     CTGTCCTTCTTTATTCGGGGCAACAGGATGGTGGTCAACACAGAGAGCAATGTTCTCATG
ENSMMUT00000026976     CTGTCCTTCTTTATTCAGGGCAACAGGATGGTGGTCAACACAGAGAGCAATGTTCTCGTG
ENSPPYT00000009368     CTGTCCTTCTTTATTCAGGGCAACAGGATGGTGGTCAACACAGAGAGCAATGTTCTCATG
ENST00000308799        CTGTCCTTCTTTATTCAGGGCAACAGGATGGTGGTCAACACAGAGAGCAATGTTCTCATG
ENSPTRT00000016330     CTGTCCTTCTTTATTCAGGGCAACAGGATGGTGGTCAACACAGAGAGCAATGTTCTCATG

ENSMUST00000036085     TTCCAGTTTGAAAATGTCAAGTGGCAGTACAGCTCAGACAAGAACAAAGTAAAGAAGAGC
ENSCJAT00000055574     TTCCAGTTTGAGCACATAAAGTGGCAGTATGCCATGGAAAAAATAAAACAAGAGAAGAAT
ENSMMUT00000026976     TTCCAGTTCGAACACATAAAGTGGCAGTATGCCATGGAAAAAACGAAACAAAAGAATAAG
ENSPPYT00000009368     TTCCAGTTTGAGCACATAAAGTGGCAGTATGCTGTGGAAAAAACGAAACAAAAGAAGAAT
ENST00000308799        TTCCAGTTTGAGCACATAAAGTGGCAGTATGCCGTGGAAAAAACGAAACAAAAGAAGAAT
ENSPTRT00000016330     TTCCAGTTTGAGCACATAAAGTGGCAGTATGCCGTGGAAAAAACGAAACAAAAGAAGAAT

ENSMUST00000036085     AAGGACAAAGAGGAGGAGAGGGAAGAGACCAGCCTCGGGGATGAGCATTCCAGGTCTACC
ENSCJAT00000055574     AAGGAGAAAGAGGAGGAAAAAGAAGAAAATATCCTCATGGAAATTCTCTTCAAGTCTAAT
ENSMMUT00000026976     AAGGAGAAAGAGGAGGAAAAAGAAGAAAACAGCCTCATGGAAATTCTCTCTAAGTCTAAT
ENSPPYT00000009368     AAGGAGAAAGAGGAGGAAAAAGAAGAAAATGGTCTCATGGAAATTCTCTCTAAGTGTAAT
ENST00000308799        AAGGAGAAAGAGGAGGAAAAAGAAGAAAATAGTCTCATGGAAATTCTCTCTAAGTGTAAT
ENSPTRT00000016330     AAGGAGAAAGAGGAGGAAAAAGAAGAAAATAGTCTCATGGAAATTCTCTCTAAGTGTAAT

ENSMUST00000036085     ATTCAGGGGCACAGCCTGAAGGACTCTGTGTCTAGTAAACAGGAGTTC------------
ENSCJAT00000055574     ACTCAGGTTCACAGCCCGAGAGAGTCTGTATCCAGTAAACAAACTGTGATCCAGGAGTTC
ENSMMUT00000026976     ATGCAGGTTCACAGCCCGAGAGAATCTGTATCCAGTAAACAAACTGTGATCCAAGAGCTT
ENSPPYT00000009368     GTTCAGGTTCACAGCCCAAGAGAGTCTGTATCCAGTAAACAAACTGTGATCCAAGAGCTC
ENST00000308799        ATTCAGGTTCACAGCCCAAGAGAGTCTGTATCCAGTAAACAAACTGTGATCCAAGAGCTC
ENSPTRT00000016330     ATTCAGGTTCACAGCCCAAGAGAGTCTGTATCCAGTAAACAAACTGTGATCCAAGAGCTC

ENSMUST00000036085     ---------------TCAAAGTCCCGGGTTCACCTGAAACAAACCAAGAACCTATCT---
ENSCJAT00000055574     CTACCAAAGAAACCCCCCAAGTCCCGAGTACTCCTGAAGCCAGCCAAGTTATCTTCAGAT
ENSMMUT00000026976     CTACCGAGCAAACCTCCCAAGTCCCGAGTACTCCTGAAGCCAGCCAAGTTCTCTTCAGAT
ENSPPYT00000009368     CTACCAGGCAAACCTCCCAAGTCCCGAGTACTCCTGAAGCCGGCCAAGTTCTCTTCAGAT
ENST00000308799        CTACCAGGCAAACCTCCCAAGTCCCGAGTACTCCTGAAGCCGGCCAAGTTCTCTTCAGAT
ENSPTRT00000016330     CTACCAGGCAAACCTCCCAAGTCCCGAGTACTCCTGAAGCCGGCCAAGTTCTCTTCAGAT

ENSMUST00000036085     ---------------------TCAGATGATATGGAAACTCCTGTAGGCGAAGTCAGTCAT
ENSCJAT00000055574     GATGCAGAAAAAGGACAAAAACAAGAACAATTGGAAACTCCAGAGGAATTGATCAATCAC
ENSMMUT00000026976     GATGTGGAGAAAGCACCAAAACAAGGACAATTGGAAACTCCTGAAAAACTGCTCAATCAC
ENSPPYT00000009368     GATGTGGAGAAGGCACAAAAACAAGGACAATTGGAAACTCCTGGAAAACCGCTCAGTCAC
ENST00000308799        GATGTGGAGAAAGCACAAAAACAAGGACAATTGGAAACTCCTGGAAAACTGCCCAGTCAC
ENSPTRT00000016330     GATGTGGAGAAAGCACAAAAACAAGGACAATTGGAAACTCCTGGAAAACTGCCCAGTCAC

ENSMUST00000036085     CCCTTGCAAAAGCTCTGGAAAGTCCCTATGACACCTGACCGATTCCTTCTGACTATCAGT
ENSCJAT00000055574     CCAAAGAGAAAGTCTTGGAAAATCCCTATGTCACCTGATCGATTCCTCCTGACTGTCAGC
ENSMMUT00000026976     CCAAAGAAAAAGTCTTGGAAAATTCCTATGTCACCTGACCAATTCCTCCTGACTGTCAGC
```

```
ENSPPYT00000009368     CCAAAGAAAAAGTCTTGGAAAATCCCTATGTCACCTGACCAATTCCTCCTGACTGTTAGC
ENST00000308799        CCAAAGAAAAAGTCTTGGAAAATCCCTATGTCACCTGACCAATTCCTCCTGACTGTTAGC
ENSPTRT00000016330     CCAAAGAAAAAGTCTTGGAAAATCCCTATGTCACCTGACCAATTCCTCCTGACTGTTTGC

ENSMUST00000036085     GCCTTGCAACAAGCCCACAATTCTGAAGAGTTTGCCTATCCCCATAGGCCCAGACCTCAA
ENSCJAT00000055574     ACCCTGCAGCAAGCCCATAATTCTGGGGAATTTACCTATCCCTGTAGGCCCAAAACAGAA
ENSMMUT00000026976     GCCCTGCAGCAAGCCCATAATTCCGGGAAATTCGCCTATCCCTGTAGGCCCCGAACAGAA
ENSPPYT00000009368     GCCCTGCAGCAAGCCCATAATTCCGGGGAATTTGCCTATCCCTGTAGGCCCCAAACAGAA
ENST00000308799        GCCCTGCAGCACGCCCATAATTCCGGGGAATTTGCCTATCCCTGTAGGCCCCAAACAGAA
ENSPTRT00000016330     GCCCTGCAGCAAGCCCATAATTCCGGGGAATTTGCCTATCCCTGTAGGCCCCAAACAGAA

ENSMUST00000036085     GTCATTGATGCTTGGGGGCCTTCCATTCCATATCCGAGGAAAGTCCTATCACTCAAAGGA
ENSCJAT00000055574     ATCACCGATGCCTGGGAAGCTTCAATTACATACCCAAGGAAGGTCTTGAATTTCAAAGGA
ENSMMUT00000026976     ATTATTGATGTCTGGGGACCTTCAATTTCATACCCAAGGAAGGTCTTGAATTTCAAAGGA
ENSPPYT00000009368     ATTATTGATGTCTGGGGACCTTCAATTTCATACCCAAGGAAGGTCTTGAATTTCAAAGGA
ENST00000308799        ATTACTGATGTCTGGGGACCTTCAATTTCATACCCAAGGAAGGTCTTGAATTTCAAAGGA
ENSPTRT00000016330     ATTACTGATGTCTGGGGACCTTCAATTTCATACCCAAGGAAGGTCTTGAATTTCAAAGGA

ENSMUST00000036085     AAATCAGTTCAACACGCAGTTGATCAGCTGCGATCCAGCAATCTTCCTACAGGTGTGAGA
ENSCJAT00000055574     AAATCAATCCAATATGCAGTTGATCGGTTGAGATTCAGCAATCCTCCCATAGACGTGAAA
ENSMMUT00000026976     AAATCCATCCAACATGCAGTTGATCAGTTGAGATTGAGCAATCCTCCTATAGATGTGAAA
ENSPPYT00000009368     AAATCAATCCAACGTGCAGTTGATCAGTTGAGATTCAGCAATCCTTCTATAGATGTGAAA
ENST00000308799        AAATCAATCCAACGTGCAGTTGATCGGTTGAGATTGAGCAATCCTCCTATAGATGTGAAA
ENSPTRT00000016330     AAATCAATCCAACGTGCAGTTGATCGGTTGAGATTGAGCAATCCTCCTATAGATGTGAAA

ENSMUST00000036085     CAAACCAACATTCCCCTTGAAATCCAGAAACTTCAGCCCAACTTGAAAAAGTCCTTGCAC
ENSCJAT00000055574     CAAACCAGTATTCCCCTTGAAATCCGGAAACTGCAGCCCAACTTGAAGAACTCTTTGCAC
ENSMMUT00000026976     CAAACCAGTATTCCCCTTGAAATCCAGAAACTGCAGCCCAACTTGAAGATCTCTTTGCAC
ENSPPYT00000009368     CAAACCAGTATGCCCCTTGAAATCCAGAAACTGCAGCCCAACTTGAAGATCTCTTTGCAC
ENST00000308799        CGAACCAGTATTCCCCTTGAAATCCAGAAACTGCAGCCCAACTTGAAGATCTCTTTGCAC
ENSPTRT00000016330     CGAACCAGTATTCCCCTTGAAATCCAGAAACTGCAGCCCAACTTGAAGATCTCTTTGCAC

ENSMUST00000036085     AGTCCCAGAGTCCAGGCCACTGTACCCCAGCCCAGTCTTATCCGTCCCAAGGTCTCTGAC
ENSCJAT00000055574     AGTCCCAGAGTCCAGTCCACCATACCCCAGCCCATGATTATCTGCTCCAGGTTCTCTGGC
ENSMMUT00000026976     AGTCCCAGAGTCCAGTCCACTTTGCCGCAGCCCATGATTATCCGCTCCAGGTTCTCTGGC
ENSPPYT00000009368     AGTCCTAGAGTCCAGTCCACCATACCCCAGCCCATGATTATCCGCTCCAGGTTCTCTGGC
ENST00000308799        AGTCCTAGAGTCCAGTCCACCATACCCCAGCCCATGATTATCCGCTCCAGGTTCTCTGGC
ENSPTRT00000016330     AGTCCTAGAGTCCAGTCCACCATACCCCAGCCCATGATTATCCGCTCCAGGTTCTCTGGC

ENSMUST00000036085     AGCTTAAGGGGTGACGAGCACTTGACCAGTTCAATTGATGGGACAATGCGCCGTGCAGGC
ENSCJAT00000055574     AGCTTAAAGGGTGGAGACCAAGTGACCAGTTCAATTGAAAGGGCAGTGGGCAGTACGGGT
ENSMMUT00000026976     AGCTTAAAGGGTGGAGACCAAGTGACCAGTTCAATTGAAAGGGCAGTGTGCAGT------
ENSPPYT00000009368     AGCTTAAAGGGTGGAGACCAAATGACCAGTTCAATTGAAAGGGCTGTGTGCAGTACGGGT
ENST00000308799        AGCTTAAAGGGTGGAGACCAAGTGACCAGTTCAATTGAAAGGGCTGTGTGCAGTACGGGT
ENSPTRT00000016330     AGCTTAAAGGGTGGAGACCAAGTGACCAGTTCAATTGAAAGGGCTGTGTGCAGTACGGGT

ENSMUST00000036085     CCCCTGACCAGCATGCAGGTCATTAAACCAAACCGAATGCTCGCTCCTCGAGGCGGCACA
ENSCJAT00000055574     CCCCTGACCAGTATGCAGGTCATTAAACCAAACCGCATGCTAGCTCCACAAATGGGCACA
ENSMMUT00000026976     CCCCTGACCAGTATGCAGGTCATTAAACCAAACCGCATGCTAGCTCCAAAAGTGGGCACA
ENSPPYT00000009368     CCCCTGACCAGTATGCAGGTCATTAAACCAAACCGCATGCTGGCGCCACAAGTGGGCACA
ENST00000308799        CCCCTGACCAGTATGCAGGTCATTAAACCAAACCGCATGCTAGCTCCACAAGTGGGCACA
ENSPTRT00000016330     CCCCTGACTAGTATGCAGGTCATTAAACCAAACCGCATGCTAGCTCCACAAGTGGGCACA
```

```
ENSMUST00000036085     GCCACCCTGTCTCCCAAGAAAGAACGGCCTCGTTTCTACACAACCCTTGATCCTCTGAGA
ENSCJAT00000055574     GCCACCCTGTCTCTTAAGAAAGAACGGCCTTGCATGTACACAGCCCTTGATCCCCTTAGA
ENSMMUT00000026976     GCCACCCTGTCTCTTAACAAAGAACGGCCTCGCATCTACACAGCCCTTGATCCCCTTAGA
ENSPPYT00000009368     GCTACCCTGTCTCTTAAGAAAGAACGGCCTCGCATCTACACAGCCCTTGATCCTCTTAGA
ENST00000308799        GCCACCCTGTCTCTTAAGAAAGAACGGCCTCGCATCTATACAGCCCTTGATCCTTTTAGA
ENSPTRT00000016330     GCCACCCTGTCTCTTAAGAAAGAACGGCCTCGCATCTATACAGCCCTTGATCCTTTTAGA


ENSMUST00000036085     ATGAACACTGGGTTCATGCTGATGACTGTGAAGGAGGAAAAAGAATTTGCTGAAGCCAAG
ENSCJAT00000055574     GTGAACACTGAGTTCATGCTGTTGACTGTGAAGGAGGAGAAAGAGTACCAGGAAGCCAAG
ENSMMUT00000026976     GTGAACACTGAGTTCGTGCTGTTGACCGTGAAGGAGGAGAAAGAGCACCAGGAAGCCAAG
ENSPPYT00000009368     GTGAACACTGAGTTCGTGCTGTTGACCGTGAAGAGGGAGAAAGAGCACCAGGAAGCCAAG
ENST00000308799        GTGAACACTGAGTTCGTGCTGTTGACCGTGAAGGAGGAGAAGGAGCACCAGGAAGCCAAG
ENSPTRT00000016330     GTGAACACTGAGTTCGTGCTGTTGACCGTGAAGGAGGAGAAGGAGCACCAGGAAGCCAAG


ENSMUST00000036085     ATGAAGGAATATGAAGCCAGTGTATCCACTAAAGAGGTCGACCCAGGAAAAGCCAGCAAA
ENSCJAT00000055574     ATGAAGGAATATCAGGCCAGGGAGTCCACTGGAGTGGTTGATCCAGAAAAAGCTCGCAAA
ENSMMUT00000026976     ATGAAGGAATATCAGGCCAGGGAGTCCACTGGAGTGGTTGATCCAGGAAAAGCCAGCAAA
ENSPPYT00000009368     ATGAAGGAATATCAGACCAGGGAGTCCACTGGAGTGGTTGATCCAGGAAAAGCCAGCAAA
ENST00000308799        ATGAAGGAATATCAGGCCAGGGAGTCCACTGGAGTGGTTGATCCAGGAAAAGTCAGCAAA
ENSPTRT00000016330     ATGAAGGAATATCAGGCCAGGGAGTCCACTGGAGTGGTTGATCCAGGAAAAGCCAGCAAA


ENSMUST00000036085     GCTGCGTGGATCCGTAAGATCAAAGGCCTCCCTATTGATAATTTCATGAAGGAAGGGAAA
ENSCJAT00000055574     GCTGCGTGGATCAGAAAAATCAAAGGCCTGCCTATTGATAATTTCATGAAACGGGGGAAA
ENSMMUT00000026976     GCTGCATGGATCAGGAAAATCAAAGGCCTGCCTATTGATAATTTCATGAAGCAAGGGAAA
ENSPPYT00000009368     GCTGCATGGATCAGGAAGATCAAAGGCCTGCCTATTGATAATTTCATGAAACAAGGGAAA
ENST00000308799        GCTGCATGGATCAGGAAGATCAAAGGCCTGCCTATTGATAATTTCACGAAGCAAGGGAAA
ENSPTRT00000016330     GCTGCATGGATCAGGAAGATCAAAGGCCTGCCTATTGATAATTTCACGAAGCAAGGGAAA


ENSMUST00000036085     ACAGCAGCCCCCGAACTTGGACAAAATGTGTTTATC
ENSCJAT00000055574     ACAGCGGCGCCTGAACTTGGACAAAATGTGTTTATC
ENSMMUT00000026976     ACAGCGGCCCCTGAACTTGGACAAAATGTATTTATC
ENSPPYT00000009368     ACAGCCGCCCCTGAACTTGGAGAAAATGTATTTATC
ENST00000308799        ACAGCGGCCCCTGAACTTGGACAAAATGTATTTATC
ENSPTRT00000016330     ACAGCGGCCCCTGAACTTGGACAAAATGTATTTATC
```

Multiple sequence alignment of Fbxw11

```
ENSPPYT00000018671     ATGGAGCCCGACTCGGTGATTGAGGACAATACCATCGAGCTCATGAACACT---------
ENSMMUT00000022503     ----------------------------------------CTGCAGTGTTCTGTGCCAAGG
ENSMUST00000093205     ATGGAGCCCGACTCGGTGATTGAAGACAAGACCATCGAGCTCATGTGTTCTGTGCCAAGG
ENSRNOT00000006078     ---------------------------------------------TGTTCTGTGCCAAGG
ENSCJAT00000037877     ATGGAGCCCGACTCGGTGATTGAGGACAAGACCATCGAGCTCATGTGTTCTGTGCCAAGG
ENSGGOT00000007919     ATGGAGCCCGACTCGGTGATTGAGGACAAGACCATCGAGCTCATGTGTTCTGTGCCAAGG
ENST00000265094        ATGGAGCCCGACTCGGTGATTGAGGACAAGACCATCGAGCTCATGTGTTCTGTGCCAAGG
ENSPTRT00000032395     ATGGAGCCCGACTCGGTGATTGAGGACAAGACCATCGAGCTCATGTGTTCTGTGCCAAGG


ENSPPYT00000018671     ---------------------------------TCAGTTATGGAAGATCAAAATGAAGAT
ENSMMUT00000022503     TCTTTGTGGCTAGGCTGCGCCAACCTGGTAGAGAGCATGTGCGCACTGAGTTGCCTGCAG
ENSMUST00000093205     TCTTTGTGGCTAGGCTGCGCCAACCTGGTAGAGAGCATGTGCGCACTGAGTTGCCTGCAG
ENSRNOT00000006078     TCTTTGTGGCTAGGCTGCGCCAACCTGGTAGAGAGCATGTGCGCACTGAGTTGCCTGCAG
ENSCJAT00000037877     TCTTTGTGGCTAGGCTGCGCCAACCTGGTAGAGAGCATGTGCGCACTGAGTTGCCTGCAG
ENSGGOT00000007919     TCTTTGTGGCTAGGCTGCGCCAACCTGGTAGAGAGCATGTGCGCACTGAGTTGCCTGCAG
ENST00000265094        TCTTTGTGGCTAGGCTGCGCCAACCTGGTAGAGAGCATGTGCGCACTGAGTTGCCTGCAG
```

ENSPTRT00000032395   TCTTTGTGGCTAGGCTGCGCCAACCTGGTAGAGAGCATGTGCGCACTGAGTTGCCTGCAG

ENSPPYT00000018671   GAGTCCCCAAAGAAAAATACTCTTCAGATAAGTAATGGAACATCATCTGTGATCGTCTCC
ENSMMUT00000022503   AGCATGCCCAGTGTCAGATGTCTCCAGATAAGTAATGGAACATCATCTGTGATCGTCTCC
ENSMUST00000093205   AGCATGCCCAGTGTCAGATGTCTCCAGATCAGTAATGGAACGTCATCTGTGATTGTCTCC
ENSRNOT00000006078   AGCATGCCCAGTGTCAGATGTCTCCAGATAAGTAATGGAACGTCATCTGTGATTGTCTCC
ENSCJAT00000037877   AGCATGCCCAGTGTCAGATGTCTCCAGATAAGTAATGGAACATCATCTGTGATCGTCTCC
ENSGGOT00000007919   AGCATGCCCAGTGTCAGATGTCTCCAGATAAGTAATGGAACATCATCTGTGATCGTCTCC
ENST00000265094   AGCATGCCCAGTGTCAGATGTCTCCAGATAAGTAATGGAACATCATCTGTGATCGTCTCC
ENSPTRT00000032395   AGCATGCCCAGTGTCAGATGTCTCCAGATAAGTAATGGAACATCATCTGTGATCGTCTCC

ENSPPYT00000018671   AGAAAGAGGCCATCAGAAGGAAACTATCAAAAAGAAAAAGACTTGTGTATTAAATATTTT
ENSMMUT00000022503   AGAAAGAGGCCATCAGAAGGAAACTATCAAAAAGAAAAAGACTTGTGTATTAAATATTTT
ENSMUST00000093205   AGAAAGAGGCCGTCAGAGGGGAACTACCAGAAAGAAAAGGACTTGTGCATTAAGTACTTT
ENSRNOT00000006078   AGAAAGAGGCCATCGGAGGGGAACTACCAGAAAGAAAAGGACTTGTGCATCAAGTACTTT
ENSCJAT00000037877   AGAAAGAGGCCATCAGAAGGAAACTATCAAAAAGAAAAAGACTTGTGTATTAAATATTTT
ENSGGOT00000007919   AGAAAGAGGCCATCAGAAGGAAACTATCAAAAAGAAAAAGACTTGTGTATTAAGTATTTT
ENST00000265094   AGAAAGAGGCCATCAGAAGGAAACTATCAAAAAGAAAAAGACTTGTGTATTAAATATTTT
ENSPTRT00000032395   AGAAAGAGGCCATCAGAAGGAAACTATCAAAAAGAAAAAGACTTGTGTATTAAATATTTT

ENSPPYT00000018671   GACCAGTGGTCTGAATCAGATCAAGTGGAATTTGTGGAACATCTTATTTCACGAATGTGT
ENSMMUT00000022503   GACCAGTGGTCTGAATCAGATCAAGTGGAATTTGTGGAACATCTTATTTCACGAATGTGT
ENSMUST00000093205   GACCAGTGGTCTGAATCAGATCAGGTGGAATTTGTGGAGCATCTTATCTCACGGATGTGT
ENSRNOT00000006078   GACCAGTGGTCTGAATCAGATCAGGTAGAATTTGTGGAGCATCTTATCTCACGAATGTGT
ENSCJAT00000037877   GACCAGTGGTCTGAATCAGATCAAGTGGAATTTGTGGAACATCTTATTTCACGAATGTGT
ENSGGOT00000007919   GACCAGTGGTCTGAATCAGATCAAGTGGAATTTGTGGAACATCTTATTTCACGAATGTGT
ENST00000265094   GACCAGTGGTCTGAATCAGATCAAGTGGAATTTGTGGAACATCTTATTTCACGAATGTGT
ENSPTRT00000032395   GACCAGTGGTCTGAATCAGATCAAGTGGAATTTGTGGAACATCTTATTTCACGAATGTGT

ENSPPYT00000018671   CATTATCAGCATGGACATATTAACTCTTACCTGAAGCCCATGTTGCAGCGGGACTTTATT
ENSMMUT00000022503   CATTATCAGCATGGACATATTAACTCTTACCTGAAGCCCATGTTGCAGCGGGACTTTATT
ENSMUST00000093205   CATTATCAGCATGGACATATTAACTCTTACCTGAAGCCCATGTTGCAGCGGGACTTTATC
ENSRNOT00000006078   CATTATCAGCATGGACATATTAACTCTTACCTGAAGCCCATGTTGCAGCGGGACTTCATC
ENSCJAT00000037877   CATTATCAGCATGGACATATTAACTCTTACCTGAAGCCCATGTTGCAGCGAGATTTTATT
ENSGGOT00000007919   CATTATCAGCATGGACATATTAACTCTTACCTGAAGCCCATGTTGCAGCGGGACTTTATT
ENST00000265094   CATTATCAGCATGGACATATTAACTCTTACCTGAAGCCCATGTTGCAGCGGGACTTTATT
ENSPTRT00000032395   CATTATCAGCATGGACATATTAACTCTTACCTGAAGCCCATGTTGCAGCGGGACTTTATT

ENSPPYT00000018671   ACCGCTTTACCAGAGCAAGGCTTAGATCACATAGCAGAAAACATTCTTTCGTACCTGGAT
ENSMMUT00000022503   ACCGCTTTACCAGAGCAAGGCTTAGATCACATAGCAGAAAACATCCTTTCGTACCTGGAC
ENSMUST00000093205   ACTGCTTTACCAGAGCAAGGCTTAGATCACATAGCAGAAAACATTCTCTCCTACCTGGAT
ENSRNOT00000006078   ACCGCTTTACCAGAGCAAGGCTTAGATCACATAGCAGAGAACATTCTTTCCTACCTGGAT
ENSCJAT00000037877   ACCGCTTTACCAGAGCAAGGCTTAGATCACATAGCAGAAAACATTCTTTCATACCTGGAT
ENSGGOT00000007919   ACCGCTTTACCAGAGCAAGGCTTAGATCACATAGCAGAAAACATTCTTTCGTACCTGGAT
ENST00000265094   ACCGCTTTACCAGAGCAAGGCTTAGATCACATAGCAGAAAACATTCTTTCGTACCTGGAT
ENSPTRT00000032395   ACCGCTTTACCAGAGCAAGGCTTAGATCACATAGCAGAAAACATTCTTTCGTACCTGGAT

ENSPPYT00000018671   GCCAGGTCTCTGTGTGCAGCAGAGCTGGTATGTAAAGAATGGCAGCGAGTGATCTCAGAA
ENSMMUT00000022503   GCCAGGTCCCTGTGTGCAGCGGAGCTGGTGTGTAAAGAATGGCAGCGGGTGATCTCAGAA
ENSMUST00000093205   GCCAGGTCTCTGTGTGCAGCAGAGCTGGTGTGTAAAGAATGGCAGCGAGTGATCTCAGAA
ENSRNOT00000006078   GCCAGGTCTCTGTGTGCAGCAGAGCTGGTGTGTAAAGAGTGGCAGCGGGTGATCTCCGAG
ENSCJAT00000037877   GCCAGGTCTCTGTGTGCAGCAGAGCTGGTATGTAAAGAATGGCAGCGAGTGATCTCAGAA
ENSGGOT00000007919   GCCAGGTCTCTGTGTGCGGCAGAGCTGGTATGTAAAGAATGGCAGCGAGTGATCTCAGAA
ENST00000265094   GCCAGGTCTCTGTGTGCAGCAGAGCTGGTATGTAAAGAATGGCAGCGAGTGATCTCAGAA

```
ENSPTRT00000032395      GCCAGGTCTCTGTGTGCAGCAGAGCTGGTATGTAAAGAATGGCAGCGAGTGATCTCAGAA

ENSPPYT00000018671      GGAATGCTTTGGAAGAAGCTGATTGAACGAATGGTACGCACTGATCCCCTATGGAAAGGA
ENSMMUT00000022503      GGAATGCTGTGGAAGAAGCTGATTGAACGAATGGTGCGCACTGATCCCCTGTGGAAAGGA
ENSMUST00000093205      GGGATGCTTTGGAAGAAGCTGATTGAGAGGATGGTGCGCACCGACCCTCTCTGGAAGGGA
ENSRNOT00000006078      GGGATGCTTTGGAAGAAGCTGATCGAGAGGATGGTGCGCACCGACCCTCTCTGGAAGGGA
ENSCJAT00000037877      GGAATGCTTTGGAAGAAGCTGATTGAACGAATGGTACGCACTGATCCCCTATGGAAAGGA
ENSGGOT00000007919      GGAATGCTTTGGAAGAAGCTGATTGAACGAATGGTACGCACTGATCCCCTATGGAAAGGA
ENST00000265094         GGAATGCTTTGGAAGAAGCTGATTGAACGAATGGTACGCACTGATCCCCTATGGAAAGGA
ENSPTRT00000032395      GGAATGCTTTGGAAGAAGCTGATTGAACGAATGGTGCGCACTGATCCCCTATGGAAAGGA

ENSPPYT00000018671      CTTTCAGAAAGAAGAGGGTGGGATCAGTACCTGTTTAAAAACAGACCCACAGATGGCCCT
ENSMMUT00000022503      CTTTCAGAAAGAAGAGGGTGGGATCAGTACCTGTTTAAAAACAGACCCACAGATGGCCCT
ENSMUST00000093205      CTCTCAGAAAGAAGAGGCTGGGATCAGTACCTGTTTAAAAACAGACCTACAGATGGCCCT
ENSRNOT00000006078      CTCTCAGAAAGAAGAGGCTGGGATCAGTACCTGTTTAAAAACAGACCTACAGATGGCCCT
ENSCJAT00000037877      CTTTCAGAAAGAAGAGGGTGGGATCAGTACCTGTTTAAAAACAGACCCACAGATGGCCCT
ENSGGOT00000007919      CTTTCAGAAAGAAGAGGGTGGGATCAGTACCTGTTTAAAAACAGACCCACAGATGGCCCT
ENST00000265094         CTTTCAGAAAGAAGAGGGTGGGATCAGTACCTGTTTAAAAACAGACCCACAGATGGCCCT
ENSPTRT00000032395      CTTTCAGAAAGAAGAGGGTGGGATCAGTACCTGTTTAAAAACAGACCCACAGATGGCCCT

ENSPPYT00000018671      CCAAATTCATTTTATAGGTCATTATACCCAAAGATTATCCAGGATATAGAGACTATAGAA
ENSMMUT00000022503      CCAAATTCATTTTATAGGTCATTATACCCAAAGATTATCCAGGATATAGAGACTATAGAA
ENSMUST00000093205      CCCAACTCATTTTATAGATCATTATACCCAAAGATTATCCAGGACATAGAGACCATAGAA
ENSRNOT00000006078      CCCAACTCATTTTATAGATCATTATACCCAAAGATTATCCAGGACATAGAGACCATAGAA
ENSCJAT00000037877      CCAAATTCATTTTATAGGTCATTATACCCAAAGATTATCCAGGATATAGAGACTATAGAA
ENSGGOT00000007919      CCAAATTCATTTTATAGGTCATTATACCCAAAGATTATCCAGGATATAGAGACTATAGAA
ENST00000265094         CCAAATTCATTTTATAGGTCATTATACCCAAAGATTATCCAGGATATAGAGACTATAGAA
ENSPTRT00000032395      CCAAATTCATTTTATAGGTCATTATACCCAAAGATTATCCAGGATATAGAGACTATAGAA

ENSPPYT00000018671      TCTAACTGGCGGTGTGGACGACACAACTTGCAGAGGATTCAGTGCCGCTCTGAAAATAGT
ENSMMUT00000022503      TCTAACTGGCGGTGCGGACGACACAACTTGCAGAGGATTCAGTGCCGCTCTGAAAATAGT
ENSMUST00000093205      TCCAACTGGCGGTGTGGACGACACAACTTGCAGAGGATCCAGTGCCGCTCTGAAAATAGT
ENSRNOT00000006078      TCCAACTGGCGGTGTGGACGGCACAACTTGCAGAGGATCCAGTGCCGCTCTGAAAATAGT
ENSCJAT00000037877      TCTAATTGGCGGTGTGGACGACACAACTTGCAGAGGATTCAGTGCCGCTCTGAAAATAGT
ENSGGOT00000007919      TCTAACTGGCGGTGTGGACGACACAACTTGCAGAGGATTCAGTGCCGCTCTGAAAATAGT
ENST00000265094         TCTAACTGGCGGTGTGGACGACACAACTTGCAGAGGATTCAGTGCCGCTCTGAAAATAGT
ENSPTRT00000032395      TCTAACTGGCGGTGTGGACGACACAACTTGCAGAGGATTCAGTGCCGCTCTGAAAATAGT

ENSPPYT00000018671      AAAGGTGTCTACTGTTTACAGTACGACGATGAAAAAATTATCAGTGGCCTACGAGATAAT
ENSMMUT00000022503      AAAGGTGTCTACTGTTTACAGTACGATGATGAAAAAATTATCAGTGGCCTACGAGATAAT
ENSMUST00000093205      AAGGGTGTCTACTGTTTGCAATATGATGATGACAAAATTATCAGTGGCCTCCGGGACAAC
ENSRNOT00000006078      AAGGGCGTCTACTGTTTGCAATATGATGATGACAAAATTATCAGTGGCCTCCGAGACAAC
ENSCJAT00000037877      AAAGGTGTCTACTGTTTACAGTACGATGATGAAAAAATTATCAGTGGCCTACGAGATAAT
ENSGGOT00000007919      AAAGGTGTCTACTGTTTACAGTATGATGATGAAAAAATTATCAGTGGCCTACGAGATAAT
ENST00000265094         AAAGGTGTCTACTGTTTACAGTACGATGATGAAAAAATTATCAGTGGCCTACGAGATAAT
ENSPTRT00000032395      AAAGGTGTCTACTGTTTACAGTACGATGATGAAAAAATTATCAGTGGCCTACGAGATAAT

ENSPPYT00000018671      TCTATTAAG---------------------------------------------------
ENSMMUT00000022503      TCTATTAAGATTTGGGATAAAACCAGCCTGGAATGTTTGAAAGTGTTAACGGGACACACA
ENSMUST00000093205      TCTATCAAGATCTGGGATAAAAGCAGCTTGGAATGTTTGAAAGTGCTAACGGGCCACACA
ENSRNOT00000006078      TCTATCAAGATCTGGGATAAAAGCAGCTTGGAATGTTTGAAAGTGCTAACGGGACACACG
ENSCJAT00000037877      TCTATTAAGATTTGGGATAAAACCAGCCTGGAATGTTTGAAAGTGTTAACAGGACACACA
ENSGGOT00000007919      TCTATTAAGATATGGGATAAAACCAGCCTGGAATGTTTGAAAGTGTTAACAGGACACACA
ENST00000265094         TCTATTAAGATATGGGATAAAACCAGCCTGGAATGTTTGAAAGTGTTAACAGGACACACA
```

```
ENSPTRT00000032395      TCTATTAAGATATGGGATAAAAGCAGCCTGGAATGTTTGAAAGTGTTAACAGGACACACA


ENSPPYT00000018671      ------------------------------------------------------------
ENSMMUT00000022503      GGCTCTGTCCTCTGTCTGCAGTATGATGAGCGTGTCATTGTAACCGGCTCTTCAGATTCT
ENSMUST00000093205      GGCTCTGTCCTCTGCCTCCAGTATGATGAGCGAGTCATTGTAACTGGTTCTTCAGACTCC
ENSRNOT00000006078      GGCTCTGTCCTCTGCCTCCAGTATGATGAACGAGTCATTGTAACTGGTTCTTCAGACTCT
ENSCJAT00000037877      GGCTCTGTCCTCTGCCTGCAATATGATGAGCGTGTCATTGTAACTGGCTCTTCAGATTCT
ENSGGOT00000007919      GGCTCTGTCCTCTGTCTGCAGTATGATGAGCGTGTTATTGTAACCGGCTCTTCAGATTCT
ENST00000265094         GGCTCTGTCCTCTGTCTGCAGTATGATGAGCGTGTCATTGTAACTGGCTCTTCAGATTCT
ENSPTRT00000032395      GGCTCTGTCCTCTGTCTGCAGTATGATGAGCGTGTCATTGTAACCGGCTCTTCAGATTCT


ENSPPYT00000018671      ---------GTGTGGGATGTGAACACGGGTGAAGTTCTTAACACATTGATCCACCACAAT
ENSMMUT00000022503      ACGGTGAGGGTGTGGGATGTGAACACGGGTGAAGTTCTTAACACATTGATCCACCACAAT
ENSMUST00000093205      ACGGTGAGAGTCTGGGATGTGAACACTGGTGAGGTGCTCAACACACTCATCCACCACAAT
ENSRNOT00000006078      ACAGTGAGAGTCTGGGATGTGAACACGGGGGAAGTTCTCAACACACTCATCCACCACAAC
ENSCJAT00000037877      ACAGTGAGAGTGTGGGATGTGAACACGGGTGAAGTTCTTAACACATTAATCCATCACAAT
ENSGGOT00000007919      ACGGTGAGAGTGTGGGATGTGAACACGGGTGAAGTTCTTAACACATTGATCCACCACAAT
ENST00000265094         ACGGTGAGAGTGTGGGATGTGAACACGGGTGAAGTTCTTAACACATTGATCCACCACAAT
ENSPTRT00000032395      ACGGTGAGAGTGTGGGATGTGAACACGGGTGAAGTTCTTAACACATTGATCCACCACAAT


ENSPPYT00000018671      GAGGCCGTACTGCACTTACGCTTCAGCAATGGACTGATGGTGACCTGTTCCAAGGACCGC
ENSMMUT00000022503      GAGGCCGTACTGCACTTACGCTTCAGCAATGGACTGATGGTGACCTGTTCCAAGGACCGC
ENSMUST00000093205      GAAGCCGTACTGCACTTACGCTTCAGCAATGGACTGATGGTGACTTGTTCCAAGGACCGT
ENSRNOT00000006078      GAAGCCGTGCTGCACTTACGCTTCAGCAACGGACTGATGGTGACTTGTTCCAAGGACCGC
ENSCJAT00000037877      GAGGCTGTACTGCACTTACGCTTCAGCAATGGACTGATGGTGACCTGTTCCAAAGACCGC
ENSGGOT00000007919      GAGGCTGTACTGCACTTACGCTTCAGCAATGGACTGATGGTGACCTGTTCCAAGGACCGC
ENST00000265094         GAGGCTGTATTGCACTTACGCTTCAGCAATGGACTGATGGTGACCTGTTCCAAGGACCGC
ENSPTRT00000032395      GAGGCTGTACTGCACTTACGCTTCAGTAATGGACTGATGGTGACCTGTTCCAAGGACCGC


ENSPPYT00000018671      TCCATTGCTGTGTGGGACATGGCTTCTGCGACCGATATCACTTTACGCCGTGTCCTGGTT
ENSMMUT00000022503      TCCATTGCTGTGTGGGACATGGCTTCTGCGACCGATATCACTTTACGCCGTGTCCTGGTT
ENSMUST00000093205      TCCATTGCCGTGTGGGACATGGCTTCTGCCACCGATATCACTTTACGCCGTGTTCTGGTT
ENSRNOT00000006078      TCCATCGCTGTGTGGGACATGGCTTCTGCCACTGATATCACTTTACGCCGTGTCCTGGTT
ENSCJAT00000037877      TCCATTGCTGTGTGGGACATGGCTTCTGCAACTGATATCACTTTACGCCGTGTCCTGGTT
ENSGGOT00000007919      TCCATTGCTGTGTGGGACATGGCTTCTGCGACTGACATCACTTTACGCCGTGTCCTGGTT
ENST00000265094         TCCATTGCTGTGTGGGACATGGCTTCTGCGACCGACATCACTTTACGCCGTGTCCTGGTT
ENSPTRT00000032395      TCCATTGCTGTGTGGGACATGGCTTCTGCGACCGACATCACTTTACGCCGTGTCCTGGTT


ENSPPYT00000018671      GGCCACCGGGCTGCTGTCAATGTAGTAGACTTTGATGACAAGTACATCGTGTCCGCCTCT
ENSMMUT00000022503      GGCCACCGGGCTGCTGTCAATGTAGTAGACTTTGACGACAAGTACATTGTGTCCGCCTCT
ENSMUST00000093205      GGCCACCGTGCTGCTGTCAATGTAGTAGACTTTGATGATAAATACATCGTGTCTGCTTCA
ENSRNOT00000006078      GGCCACCGTGCTGCTGTCAATGTAGTAGACTTTGATGATAAGTACATCGTGTCTGCCTCT
ENSCJAT00000037877      GGCCACCGGGCTGCTGTCAACGTAGTAGACTTTGATGACAAGTACATCGTGTCTGCCTCT
ENSGGOT00000007919      GGCCACCGGGCTGCCGTCAATGTAGTAGACTTTGACGACAAGTACATCGTGTCTGCCTCT
ENST00000265094         GGCCACCGGGCTGCCGTCAATGTAGTAGACTTTGACGACAAGTACATCGTGTCTGCCTCT
ENSPTRT00000032395      GGCCACCGGGCTGCCGTCAATGTAGTAGACTTTGACGACAAGTACATCGTGTCTGCCTCT


ENSPPYT00000018671      GGTGACAGGACCATCAAAGTCTGGAGCACAAGCACCTGTGAATTTGTTCGTACTCTCAAT
ENSMMUT00000022503      GGTGACAGGACCATCAAAGTCTGGAGCACGAGCACCTGTGAATTTGTCCGTACTCTCAAT
ENSMUST00000093205      GGAGACAGGACCATTAAAGTGTGGAGCACGAGCACATGTGAGTTTGTCCGCACTCTGAAT
ENSRNOT00000006078      GGAGACAGGACTATTAAAGTGTGGAGCACGAGCACCTGTGAGTTCGTCCGCACTCTGAAT
ENSCJAT00000037877      GGCGACAGGACTATCAAAGTCTGGAGCACGAGCACCTGTGAATTTGTTCGTACTCTCAAT
ENSGGOT00000007919      GGTGACAGGACCATCAAAGTCTGGAGCACGAGCACCTGTGAATTTGTTCGTACTCTCAAT
ENST00000265094         GGTGACAGGACCATCAAAGTCTGGAGCACGAGCACCTGTGAATTTGTTCGTACTCTCAAT
```

```
ENSPTRT00000032395      GGTGACAGGACCATCAAAGTCTGGAGCACGAGCACCTGTGAATTTGTTCGTACTCTCAAT

ENSPPYT00000018671      GGGCACAAGCGAGGCATTGCCTGTCTGCAGTACAGGGATCGGCTGGTTGTTAGTGGATCA
ENSMMUT00000022503      GGGCACAAGCGAGGCATTGCCTGTCTCCAGTACAGGGATCGGCTGGTTGTTAGTGGATCA
ENSMUST00000093205      GGGCACAAGCGAGGCATCGCCTGTCTGCAGTACCGCGACCGGCTTGTTGTTAGTGGATCA
ENSRNOT00000006078      GGGCACAAGCGAGGCATTGCCTGTCTGCAGTACCGGGACCGGCTTGTTGTTAGTGGATCA
ENSCJAT00000037877      GGGCACAAGCGAGGCATAGCCTGTCTCCAGTACAGGGATCGGCTGGTTGTTAGTGGATCA
ENSGGOT00000007919      GGGCACAAGCGAGGCATTGCCTGTCTCCAGTACAGGGATCGCCTGGTTGTTAGTGGATCA
ENST00000265094         GGGCACAAGCGGGGCATTGCCTGTCTCCAGTACAGGGATCGCCTGGTTGTTAGTGGATCA
ENSPTRT00000032395      GGGCACAAGCGAGGCATTGCCTGTCTCCAGTACAGGGATCGCCTGGTTGTTAGTGGATCA

ENSPPYT00000018671      TCAGATAATACCATTAGGCTCTGGGATATTGAATGTGGTGCCTGTTTAAGAGTCCTAGAG
ENSMMUT00000022503      TCAGATAATACCATTAGGCTCTGGGATATTGAATGTGGTGCCTGTTTAAGAGTCCTAGAG
ENSMUST00000093205      TCAGATAATACCATCCGGTTATGGGATATTGAATGTGGTGCCTGTTTAAGAGTCCTAGAG
ENSRNOT00000006078      TCAGATAATACCATCCGGTTATGGGATATTGAATGTGGTGCCTGTTTAAGAGTCCTAGAG
ENSCJAT00000037877      TCAGATAATACCATCAGGCTTTGGGATATTGAATGTGGTGCCTGTTTAAGAGTCCTAGAG
ENSGGOT00000007919      TCAGATAATACCATTAGGCTCTGGGATATTGAATGTGGTGCCTGTTTAAGAGTCCTAGAG
ENST00000265094         TCAGATAATACCATTAGGCTCTGGGATATTGAATGTGGTGCCTGTTTAAGAGTCCTAGAG
ENSPTRT00000032395      TCAGATAATACCATTAGGCTCTGGGATATTGAATGTGGTGCCTGTTTAAGAGTCCTAGAG

ENSPPYT00000018671      GGACATGAAGAATTGGTCCGATGCATCCGGTTTGATAACAAGAGGATTGTCAGTGGGGCC
ENSMMUT00000022503      GGACATGAAGAATTGGTCCGATGCATCCGGTTTGATAACAAGAGGATTGTCAGTGGGGCC
ENSMUST00000093205      GGGCACGAAGAATTGGTCCGGTGCATCCGTTTTGATAACAAGAGGATTGTCAGTGGCGCC
ENSRNOT00000006078      GGGCACGAAGAGTTGGTCCGGTGCATCCGTTTTGATAACAAGAGGATTGTCAGTGGCGCC
ENSCJAT00000037877      GGACATGAAGAACTGGTTCGATGCATCCGGTTTGATAACAAGAGGATTGTCAGTGGGGCC
ENSGGOT00000007919      GGACATGAAGAATTGGTCCGATGCATCCGGTTTGATAACAAGAGGATTGTCAGTGGGGCC
ENST00000265094         GGACATGAAGAATTGGTCCGATGCATCCGGTTTGATAACAAGAGGATTGTCAGTGGGGCC
ENSPTRT00000032395      GGACATGAAGAATTGGTCCGATGCATCCGGTTTGATAACAAGAGGATTGTCAGTGGGGCC

ENSPPYT00000018671      TATGATGGGAAAATTAAAGTTTGGGACTTGCAAGCTGCTCTTGACCCTCGAGCCCCAGCA
ENSMMUT00000022503      TATGATGGGAAAATTAAAGTTTGGGACTTGCAAGCTGCTCTTGACCCTCGAGCCCCAGCG
ENSMUST00000093205      TATGATGGGAAGATTAAAGTCTGGGACTTGCAGGCTGCTCTTGACCCTCGGGCCCCAGCA
ENSRNOT00000006078      TATGACGGGAAGATTAAAGTCTGGGATTTGCAGGCTGCTCTTGACCCTCGGGCCCCAGCA
ENSCJAT00000037877      TATGATGGGAAAATTAAAGTTTGGGACTTGCAAGCTGCTCTTGACCCTCGAGCCCCAGCA
ENSGGOT00000007919      TATGATGGGAAAATTAAAGTTTGGGACTTGCAAGCTGCTCTTGACCCTCGAGCCCCAGCA
ENST00000265094         TATGATGGGAAAATTAAAGTTTGGGACTTGCAAGCTGCTCTTGACCCTCGAGCCCCAGCA
ENSPTRT00000032395      TATGATGGGAAAATTAAAGTTTGGGACTTGCAAGCTGCTCTTGACCCTCGAGCCCCAGCA

ENSPPYT00000018671      AGCACGTTGTGTTTGCGCACATTGGTGGAACATTCTGGACGTGTGTTTCGGCTCCAGTTT
ENSMMUT00000022503      AGCACATTGTGTTTGCGCACATTGGTGGAACATTCTGGACGTGTGTTTCGGCTACAGTTT
ENSMUST00000093205      AGCACATTGTGTCTGCGCACCTTGGTGGAACACTCTGGACGTGTGTTTCGGCTGCAGTTT
ENSRNOT00000006078      AGCACATTGTGTCTGCGCACCTTGGTGGAACACTCTGGACGTGTGTTTCGGCTGCAGTTT
ENSCJAT00000037877      AGCACGTTGTGTTTGCGCACATTGGTGGAACATTCTGGACGTGTGTTTCGGCTACAGTTT
ENSGGOT00000007919      AGCACATTGTGTTTGCGCACATTGGTGGAACATTCTGGACGTGTGTTTCGGCTCCAGTTT
ENST00000265094         AGCACATTGTGTTTGCGCACATTGGTGGAACATTCTGGACGTGTGTTTCGGCTCCAGTTT
ENSPTRT00000032395      AGCACATTGTGTTTGCGCACATTGGTGGAACATTCTGGACGTGTGTTTCGGCTCCAGTTT

ENSPPYT00000018671      GATGAGTTTCAGATCATCAGCAGCTCCCATGATGACACTATTTTGATTTGGGATTTCTTA
ENSMMUT00000022503      GATGAGTTTCAGATCATCAGCAGCTCCCATGATGACACTATTTTGATTTGGGATTTCTTA
ENSMUST00000093205      GATGAGTTTCAGATCATCAGCAGCTCCCATGATGACACTATTTTGATTTGGGATTTCTTA
ENSRNOT00000006078      GATGAGTTTCAGATCATCAGCAGCTCCCATGATGACACTATTTTGATTTGGGATTTTTTA
ENSCJAT00000037877      GATGAGTTTCAAATCATCAGCAGCTCCCATGATGACACTATTTTGATTTGGGATTTCTTA
ENSGGOT00000007919      GATGAGTTTCAGATCATCAGCAGCTCCCATGATGACACTATTTTGATTTGGGATTTCTTA
ENST00000265094         GATGAGTTTCAGATCATCAGCAGCTCCCATGATGACACTATTTTGATTTGGGATTTCTTA
```

```
ENSPTRT00000032395     GATGAGTTTCAGATCATCAGCAGCTCCCATGATGACACTATTTTGATTTGGGATTTCTTA


ENSPPYT00000018671     AATGTGCCTCCCAGTGCCCAGAATGAGACCCGTTCTCCCTCCAGAACATACACTTACATC
ENSMMUT00000022503     AATGTGCCTCCCAGTGCCCAGAATGAGACCCGTTCTCCCTCCAGAACATACACTTACATC
ENSMUST00000093205     AATGTGCCTCCCAGTGCCCAGAATGAGACCCGCTCTCCCTCCAGAACCTACACCTATATC
ENSRNOT00000006078     AATGTGCCTCCCAGTGCCCAGAATGAGACACGTTCTCCCTCCAGAACCTACACCTATATC
ENSCJAT00000037877     AATGTGCCTCCCAGTGCCCAGAATGAGACCCGTTCTCCCTCCAGAACATACACTTACATT
ENSGGOT00000007919     AATGTGCCTCCCAGTGCCCAGAATGAGACCCGTTCTCCCTCCAGAACATACACTTACATC
ENST00000265094        AATGTGCCTCCCAGTGCCCAGAATGAGACCCGTTCTCCCTCCAGAACATACACTTACATC
ENSPTRT00000032395     AATGTGCCTCCCAGTGCCCAGAATGAGACCCGTTCTCCCTCCAGAACATACACTTACATC


ENSPPYT00000018671     TCTAGA
ENSMMUT00000022503     TCTAGA
ENSMUST00000093205     TCCAGA
ENSRNOT00000006078     TCCAGA
ENSCJAT00000037877     TCTAGA
ENSGGOT00000007919     TCTAGA
ENST00000265094        TCTAGA
ENSPTRT00000032395     TCTAGA
```

Multiple sequence alignment of Fbxw12

```
ENSCJAT00000005278     ATGGAGATCCGATTGCCCGACTTAGCTTTGAAGCGGATCTTCTCTTTCCTGGACGTATTC
ENSMMUT00000004359     ATGGAGATCCAATTGCCTGACTTAGCTTTGAAGCAAATCTTCTCTTTCCTGGACCTGTTT
ENSPPYT00000016175     ATGGAGATCCGATTGCCTGACTTAGCTTTGAAGCGAATCTTCTCTTTCTTGGACCTGTTC
ENSGGOT00000025916     ATGGAGATCCGATTGCCTGACTTAGCTTTGAAGCGAATCTTCTCTTTCCTGGACCTGTTC
ENST00000296438        ATGGAGATCCGATTGCCTGACTTAGCTTTGAAGCGAATCTTCTCTTTCCTGGACCTGTTC
ENSPTRT00000027837     ATGGAGATCCGATTGCCTGACTTAGCTTTGAAGCGAATCTTCTCTTTCCTGGACCTGTTC
ENSMUST00000061456     ATGGAGATCCATTTGCCTAGTGTGCCGTTGATGAAAATCTTCTCCTATCTGGATGCCTAC
ENSRNOT00000028113     ATGGAGTTCCATTTGCCCAGTGTGCCGATGAGGAAAATCCTTTCCTATCTGGATGCCTTC


ENSCJAT00000005278     AGCTTGCTGCAGGTTTCCCAGGTGAACAAGGATTGGAATAGGGTTGCAGAGAGCAATTAC
ENSMMUT00000004359     GGCTTGCTGCAGGCTTCCAGAACAAATAATCATTGGAATAGGATTGCAGAGAGCGATTTC
ENSPPYT00000016175     GGCTTGCTGCAGGTTTCCCAGGTGAACAAGCATTGGAATAGGATTGCAGACAGCGATTAC
ENSGGOT00000025916     GGCTTGCTGCAGGTTTCCCAGGTGAACAAGCATTGGAATAGGATTGCAGACAGCGATTAC
ENST00000296438        GGCTTGCTGCAGGTTTCCCAGGTGAACAAGCATTGGAATAGGATTGCAGACAGTGATTAC
ENSPTRT00000027837     GGCTTGCTG---GTTTCCCAGGTGAACAAGCATTGGAATAGGATTGCAGACAGCGATTAC
ENSMUST00000061456     AGTTTGCTACAGGCTGCCCAAGTGAACAAGGACTGGAATGAACTTGCAAGCAGTGATGTC
ENSRNOT00000028113     AGTTTGCTGCAGATTGCCCAAGTGAACAAGAGATGGAATACATTTGCCAGCACTGATGTC


ENSCJAT00000005278     CTGTGGAGGGCACACTCTCTGCAGAGATGGGACTGCAACAACGTCACCGATCAACACCTG
ENSMMUT00000004359     CTG------TCGCTATCTCTGCAGAGATGGGACTGCAGCAACTTCACCAATCAACACCTG
ENSPPYT00000016175     CTGTGGAGGTCACTATCTCTGCAGAGATGGGACTGCAGCAACTTCACCTATCAACACCTG
ENSGGOT00000025916     CTGTGGAGGTCACTATCTCTGCAGAGATGGGACTGCAGCAACTTCACCAATCAACACCTG
ENST00000296438        CTGTGGAGGTCACTATCTCTGCAGAGATGGGACTGCAGCAACTTCACCAATCAACACCTG
ENSPTRT00000027837     CTGTGGAGGTCACTATCTCTGCAGAGATGGGACTGCAGCAACTTCACCAATCAACACCTG
ENSMUST00000061456     CTATGGAGGAGGTTGTGTCAGAAGAGATGGCTCTGTGACATGGTTACCCTACAACTCCTG
ENSRNOT00000028113     CTGTGGAGGAAGTTCTGTCTGAAGAGGTGGTTCTATGAAGTCGTCACCCTAGAGCTCCTC


ENSCJAT00000005278     GGCGCACACACATGGAAGCAATTTTTCCTGCACCAGAGAAGGAAGGAGCTTCGGTTGTCA
ENSMMUT00000004359     GGCACACACACATGGAAGAAATTTTTCCTGCATCAAAGAAGGAAGGAGCTTCGGTTGGCA
ENSPPYT00000016175     GGCACACACACATGGAAGCAATTTTTCCTGCATCAAAGAAGGAAGGAGCTTCGATTGGCA
ENSGGOT00000025916     GGCACACACACATGGAAGCAATTTTTCCTGCATCAAAGAAGGAAGGAGCTTCAGTTGGCA
ENST00000296438        GGCACACACACATGGAAGCAATTTTTCCTGCATCAAAGAAGGAAGGAGCTTCGGTTGGCA
```

ENSPTRT00000027837	GGCACACACACATGGAAGCAATTTTTCCTGCATCAAAGAAGGAAGGAGCTTCGGTTGGCA
ENSMUST00000061456	GGCACAGAGACATGGAAGGAGTTCTTCGTTTCCCGAACATGGCAAGAACATGCCAAGTCC
ENSRNOT00000028113	GGCACAGAGTCATGGAAGCAGTTCTTCGTTTGCCGAACACGGCAAGAACGAGCCAAGTCC

ENSCJAT00000005278	TTGGCACAGCCTGATAACTTTATCTGCAAAATAACTGAGAACACTGCCTTTGAAGCGGGT
ENSMMUT00000004359	TTGGCACAGCCACATAACTTTATCTACAAAGTCACGAAGAACATTGCATATGACATGGAG
ENSPPYT00000016175	TTGGCACAGCCGCGTAACTTTATCTACAAAGTAACTAAGAACATCGCATTTGAGACACAG
ENSGGOT00000025916	TTGGCACAGCCGCATAACTTTGTCTACAAAGTAACTAAGAACATCGCATTTGAGACGGAG
ENST00000296438	TTGGCACAGCCGCATAACTTTATCTACAAAGTAACTAAGAACATCGCATTTGAGACGGAG
ENSPTRT00000027837	TTGGCACAGCCGCATAACTTTATCTGCAAAGTAACTAAGAACATCGCATTTGAGACGGAG
ENSMUST00000061456	CGGGCAATTCGAGAAGATTTCATTTACAAGATTCCTGCGGAGTATGGAGTTCGGGCACAT
ENSRNOT00000028113	CGGGCAAAGCCAGAAGATTTCACTTACAAAATTCCCGTGGAAATTGGAGCTCCGAGAAAT

ENSCJAT00000005278	TTGGCTTATCTCTCAGGAAATAGCTTTACAATGGATGAACAGGAGAAGTCAATCATTTGC
ENSMMUT00000004359	TTGGCTTATCTCTCAGGAAATAGCCTGACAATGGATGAACAGGAGAAATCAGTCATTTGT
ENSPPYT00000016175	TTGGCTTATCTCTCAGGAAATAGCCTTACAATGGATGAACAGGAGAAATCAATCATTTGT
ENSGGOT00000025916	TTGGCTTATCTCTCAGGAAATAGACTTACAGTGGATGAACAGGAGAAATCAATCATTTGT
ENST00000296438	TTGGCTTATCTCTCAGGAAATAGACTTACAGTGGATGAACAGGAGAAATCAATCATTTGT
ENSPTRT00000027837	TTGGCTTATCTCTCAGGAAATAGACTTACAGTGGATGAACAGGAGAAATCAATCATTTGT
ENSMUST00000061456	CCATGTTATATCTCAGGGCGTGGCTTAACAAGAAACGGACAAGGCAGGCCAGTCGTCTGT
ENSRNOT00000028113	GCATATTATATCTCAGGGTGTGGCTTAACAAGAGATGGGCAAGGCAAGTCGGTTATCTGC

ENSCJAT00000005278	AGTGTCTCTCCAAAGAAAGAGCTTTGTGCCTGGGATGTGCAAGAGGGCACCATGATCTGG
ENSMMUT00000004359	GGTGTATCTCCAAAGCAAGAGCTTTGCGCCTGGGATGTGCAAGAGGGCACCATGATCTGG
ENSPPYT00000016175	AGTGTATCTCCAAAGCAAGAGCTTTGTGCCTGGGATGTGCAAGAGGGCACCATCATCTGG
ENSGGOT00000025916	AGTGTATCTCCAAAGCAAGAGCTTTGTGCCTGGGATGTGCAAGAGGGCACCATGATCTGG
ENST00000296438	AGTGTATCTCCAAAGCAAGAGCTTTGTGCCTGGGATGTGCAAGAGGGTACCATGATCTGG
ENSPTRT00000027837	AGTGTATCTCCAAAGCAAGAGCTTTGTGCCTGGGATGTGCAAGAGGATACCATGATCTGG
ENSMUST00000061456	ATGGTGACTTCTGTGAACAGGATTTCTACCTGGGATATTCAGGAGGGTGTTTTGACCTGG
ENSRNOT00000028113	GTGGTGACTTCCATGAACACTATTTCCACCTGGGATCTTCATAAGGGTGCTGTGACCTGG

ENSCJAT00000005278	TCAAGCCCAGTCCAGAAGTTCCAAATCTCAAATCTGGTAACCCTCCCTCAGATGCATCTT
ENSMMUT00000004359	TCAAGCCCAGTCCATGAGTTCTATTTCTTAAATCTGGTAACCCTTCCTCAGATGCATCTT
ENSPPYT00000016175	TCAAGCCCAGTCCAGGAGTTCCATTTATCAAATCTGGTAACCCTCCCTCAGATGCATCTC
ENSGGOT00000025916	TCAAGCCCAGTCCAGGAGTTCCATTTCTCAAATCTGGTAACCCTCCCTCAGATGCATCTC
ENST00000296438	TCAAGCCCAGTCCAGGAGTTCCATTTCTCAAATCTGGTAACCCTCCCTCAGATGCATCTC
ENSPTRT00000027837	TCAAGCCCAGTCCAGGAGTTCCATTTCTCAAATCTGGTAACCCTCCCTCAGATGCATCTC
ENSMUST00000061456	GTAAGCCCAGTACAGCAAGTCGGTATCAAGCTGTTGACTACCCTCCCTGAGATGCACATT
ENSRNOT00000028113	GTAAGCCCAGTACAGCCCAGCTACATCAAGATCTTGGCTACCCTCCCTGAGATGCACATT

ENSCJAT00000005278	GTCATCACTGAGGATAAGAGCAAAACGATCAAAGTGTGGAATTGTCAGGACAGGGATTCT
ENSMMUT00000004359	GCCATCACTATGGATCAGAAAAAAACTATCAAAGTGTGGAACTGTCAGGACAGGGATGCT
ENSPPYT00000016175	GCCGTCACTATGGATCGGAAAAAAACTATCAAAGTGTGGAACTGTCAGGACAGGGACGCT
ENSGGOT00000025916	GCCATCACTATGGATCGGAAAAAAACTATCAAAGTGTGGAACTGTCAGGACAGGGACGCT
ENST00000296438	GCCATCACTATGGATCGGAAAAAAACTATCAAAGTGTGGAACTGTCAGGACAGGGACGCT
ENSPTRT00000027837	GCCATCACTATGGATCGGAAAAAAACTATCAAAGTGTGGAACTGTCAGGACAGGGACGCT
ENSMUST00000061456	GCAGTCACTGTAGATATTCAATCAACTATCAAACTGTGGGACTGTCAGAACAGTGAAGCT
ENSRNOT00000028113	GCGGTCACTGTAGACATAGAATCAACTATCAAACTGTGGGACTGTCACAACAGGGAAGCC

ENSCJAT00000005278	CTGGCCACTCTCCCCATGCCAGAGACCTGTTATTGCATGGAAGCCCATCTCACAAAGGAT
ENSMMUT00000004359	CTGGCCATTCTCCGCATGCCAGAGTCCTGTTATGGCATGGAAGCCTATCTCACAAAGGAT
ENSPPYT00000016175	CTGGCCGTTCTCCCCATGCCAGAGCCCTGTTATTGCATGGAAGCCTATCTTACAAAGGAT
ENSGGOT00000025916	CTGGCTGTTCTCCCCATGCCACAGCCCTGTTATTGCATGGAAGCCTATCTTACAAAGGAT
ENST00000296438	CTGGCTGTTCTCCCCATGCCACAGCCCTGTTATTGCATGGAAGCCTATCTTACAAAGGAT

ENSPTRT00000027837 CTGGCTGTTCTCCCCATGCCACAGCCCTGTTATTGCATGGAAGCCTATCTTACAAAGGAT
ENSMUST00000061456 CTGGCAACTAGCAACCTGGAATCTCCATGTAAATTGCTGAAAGCTGTCTTTACCAAGGAT
ENSRNOT00000028113 CTGGCCACTAACAACATGCTCTCTTCCTGTCAATCACTGAAAGCCGTCTTCACAAAGGAT

ENSCJAT00000005278 GGCCCATTCCTGATGGTTGGCGATGCTGCAGGTGACATTTACACATTTACACTGCCGGAG
ENSMMUT00000004359 GGCCCATTCCTGACGGTTGGTGATGCTGCAGGTAACATCTACACATTTACACTGCCTGGG
ENSPPYT00000016175 GGCCCATTCCTGATGGTGGGCGATGCTGCAGGTGACATCTACACATTTACACTGCCTGGG
ENSGGOT00000025916 GGCCCATTCCTGATGGTTGGTGATGCTGCGGGTGACATCTACACATTTACACTGCCTGGG
ENST00000296438 GGCCCATTCCTGATGGTTGGCGATGCTGCAGGTGACATCTACACATTTACACTGCCTGGG
ENSPTRT00000027837 GGCCCATTCCTGATGGTTGGCGATGCTGCAGGTGACATCTACACATTTACACTGCCTGGG
ENSMUST00000061456 GGCCCGATGGTCTTGATAGGTGATATCTCCGGAAACCTCTACATCTTTAGGATTCCCGAC
ENSRNOT00000028113 GGCCCTGTTGTCTTGGCAGGTGATACCTCGGGTAACCTCTTAATCTTTAGGATTCCCGAC

ENSCJAT00000005278 TTAAGGAATATTTCTAAAGTTACTGCATTTCAATGTGCTATTGTACTTCTACGCTGCTCT
ENSMMUT00000004359 TTAAGGGATGTTTCTAAAGTTACTGCATCTCAATATGGTATTGTATTTCTACACTGCTCT
ENSPPYT00000016175 TTAAGGGATGTTTCTAAAGTTACTGCATTTCAATATGGTATTGTACTTCTACACTGCTCT
ENSGGOT00000025916 TTAAGGGATATTTCTAAAGTTACTGCATTTCAATATGGTATTGTACTTCTACACTGCTCT
ENST00000296438 TTAAGAGATGTTTCTAAAGTTACTGCATTTCAATATGGTATTGTACTTCTACACTGCTCT
ENSPTRT00000027837 TTAAGGGATGTTTCTAAAGTTACTGCATTTCAATATGGTATTGTACTTCTACACTGCTCT
ENSMUST00000061456 TTACACCTCATTTCCAAACTCAAAGTATTCCAATGCAGTATTAATCAACTCAACTGCTCT
ENSRNOT00000028113 TTGCACCTCATTTCCAGAGTCCATGTATTCCAATACGGTATTAACGAACTCTACTGCTCT

ENSCJAT00000005278 CCTGACAAGAAATGGGTGTTTGCATGTGGGACATACAGTCGTACCTTTCCACAGGTGTTC
ENSMMUT00000004359 CCTGACAAGAAATGGATATTTGCATGTGGGACATATCGTCGTACCTTGCCACAGGTATTC
ENSPPYT00000016175 CCTGACAAGAAATGGGTATTTGCATGTGGGACATACAGTCGTACCTTGCCACAGGTATTC
ENSGGOT00000025916 CCTGACAAGAAATGGGTATTTGCATGTGGGACATACAGTCGTACCTTGCCACAGGTATTC
ENST00000296438 CCTGACAAGAAATGGGTATTTGCATGTGGGACATACAGTCGTACCTTGCCACAGGTATTC
ENSPTRT00000027837 CCTGACAAGAAATGGGTATTTGCATGTGGGACATACAGTCGTACCTTGCCACAGGTATTC
ENSMUST00000061456 CCTCAGAAGAAATGGATCTTTCTGAATAGGAAACACCCACATATCTTGCCAAAGGTGTTT
ENSRNOT00000028113 CCTCAGAAGAAATGGGTCTTTCTGAGTAAGAAACACTCACAAATCTTGCCAAAGGTGTTT

ENSCJAT00000005278 CTCGCAGAGGACTTACTGAGACCATCAGAAGGCAGCGCTCCTCTGTCTACCTTTCTCCCA
ENSMMUT00000004359 CTCACAGAGTCCTTACTGAGACCATCAGAAGGCAGTGCTCCTCTATCTACCTCTCTCCCA
ENSPPYT00000016175 CTCACAGAGTCCTTACTGAGGCCATCAGAAGGCAGTGCTCCTCTGTCTACCTTTCTCCCA
ENSGGOT00000025916 CTCACAGAGTCCTTACTGAGACCATCAGAAGGCAGTGCTCCTCTGTCTACATTTCTCCCA
ENST00000296438 CTCACAGAGTCCTTACTGAGACCATCAGAAGGCAGTGTTCCTCTGTCTACCTTTCTCCCA
ENSPTRT00000027837 CTCACAGAGTCCTTACTGAGACCATCAGAAGGCAGTGTTCCTCTGTCTACCTTTCTCCCA
ENSMUST00000061456 TACATGAGCAGCTTACTGAGGACATCAGAATTCTCTGCCCCTGTGTCTACCATTCTTAAA
ENSRNOT00000028113 TTCATGAGCAGCCTACTGAGGACATCAGAATTCTCCGCCCCCGCATCTACCATTCTCTCG

ENSCJAT00000005278 CATAAATTGTGCACCACCGCCTGCTGGAACCCAAAGAAGAAAAACAGGATTACACTGATG
ENSMMUT00000004359 CATAAATTATGTGTCAGTGCCTGCTGGACCCCAAAGGTGAAAAACAGGCTGACACTGATG
ENSPPYT00000016175 CATAAATTATGTGCCAGCGCCTGCTGGGCCCCAAAGGTGAAAAACAGGATAACACTGATG
ENSGGOT00000025916 CATAAATTATGTGCCAGCGCCTGCTGGACCCCAAAGGTGAAAAACAGGATAACACTGATG
ENST00000296438 CATAAATTATGTGCCAGCGCCTGCTGGACCCCAAAGGTGAAAAACAGGATAACACTGATG
ENSPTRT00000027837 CATAAATTATGTGCCAGCGCCTGCTGGACCCCAAAGGTGAAAAACAGGATAACACTGATG
ENSMUST00000061456 TTTTCATTATGCCAAAGAGCCTTCTGGACCCCAAGAATGGAAGACAGGATAACCCTGATG
ENSRNOT00000028113 TTTCCATTATGCCAAAGAGCCTTCTGGACCCCACGAAGGGAAGACCGGATAACCCTGATG

ENSCJAT00000005278 TCCCATAATCGCCGTGGAAGAAAGACAGAAATTATCACCTTTGATCTCACAACTGAGAGG
ENSMMUT00000004359 TCCCAAAGTAACCCTAAAAAA------GAATTTGTCACCTTTGATCTAACAACCAAGAGG
ENSPPYT00000016175 TCCCAAAGTAGCACTGGAAAAAAGACAGAATTTATCACCTTTGATCTAACAACCAAGAAG
ENSGGOT00000025916 TCCCAAAGTAGCACTGGAAAAAAGACAGAATTTATCACCTTTGATATAACAACCAAGAAG
ENST00000296438 TCCCAAAGTAGCACTGGAAAAAAGACAGAATTTATCACCTTTGATCTAACAACCAAGAAG

```
ENSPTRT00000027837     TCCCAAAGTAGCACTGGAAAAAAGACAGAATTTATCACCTTTGATCTAACAACCAAGAAG
ENSMUST00000061456     TCCATACGTCGCACCCAAAAGATCACAAAGTTTATCACATTTGATATGAAGCTGAAAGAG
ENSRNOT00000028113     TCCATAAAAGGCTCCAAAAAAATCACAACATTTGTCACGTTTGATATCAAGCTGGAAAAG

ENSCJAT00000005278     ACTGGAGGCCAAACAGTCGTCCAAGCATATGAGATCTCAAGTTTCCAGTTGCCACCTCGT
ENSMMUT00000004359     ACTGGAGGCCAAACAGTCATCCAAGTACATAAGATTGCAAGTTTCCAGTTGGCAGCTCAT
ENSPPYT00000016175     ACTGGAGGCCAAACAGTCATCCAAGCACATGAGGTTGCAAGTTTCCAGGTGGCAGCTCAT
ENSGGOT00000025916     ACTGGAGGCCAAACAGTCATCCAAGCATATGAGATCGCAAGTTTCCAGGTGGCAGCTCAT
ENST00000296438        ACTGGAGGCCAAACAGTCATCCAAGCATATGAGATCGCAAGTTTCCAGGTGGCAGCTCAT
ENSPTRT00000027837     ACTGGAGGCCAAACAGTCATCCAAGCATATGAGATCGCAAGTTTCCAGGTGGCAGCTCAT
ENSMUST00000061456     ATCCAGAACAAAATGACTGTTAAAGAACAATTCGTTGCAAGCTTCTCAGTGCCAGATGAT
ENSRNOT00000028113     ATCGGGAACAAAATAAGGGTTAAAGAATATGCCATAGCGAACTTCTCGTTGAAAAAGTTC

ENSCJAT00000005278     CTGGAGCACCCTACCTGGATAGGAGCCAGTGATGGATATTTGATTGTCTTTCCCAGTGGG
ENSMMUT00000004359     CTGGAGTCCCCTGTTTGGATGGGAGCCAATGATGGATATACGATTGTCTTTACCAATGGG
ENSPPYT00000016175     CTGAAGTGCCCTATCTGGATGGGAGCCAGTGATGGATATATGATTGTCTTTACCAGTGGG
ENSGGOT00000025916     CTGAAGTGCCCTATCTGGATGGGAGCCAGTGATGGATATATGATTGTCTTTACCAGTGGG
ENST00000296438        CTGAAGTGCCCTATCTGGATGGGAGCCAGTGATGGATATATGATTGTCTTTACCAGTGGA
ENSPTRT00000027837     CTGAAGTGCCCTATCTGGATGGGAGCCAGTGATGGATATATGATTGTCTTTACCAGTGGG
ENSMUST00000061456     ATGGGGAGACCAAAATGGTTTGGCGTTAGTGGTAAGGATGTGATTGTCTGTTCAACTGGA
ENSRNOT00000028113     GAGGACACCCTGGAGTGGATGGGAGTCAGTGATAAGAACGTGATTGTTTGTTCAACTGGG

ENSCJAT00000005278     CCAGACTTGCTCCTATTCAGCATCACTGGCTTCCTGCTGCAACAATTTCAGGACCACCAG
ENSMMUT00000004359     CCATACTTGTTACTCTTCAGCATCACTGGCTTCCTGCTGCAACGATTTGAGGACCACCAG
ENSPPYT00000016175     CCATACTTGTTACTCTTCAGCATCACTGGCTTCCTGCTGCAACGATTTGAGGACCACCAG
ENSGGOT00000025916     CCATACTTGTTACTCTTCAGCATCACTGGCTTCCTGCTGCAACGATTTGAGGACCATCAG
ENST00000296438        CCATACTTGTTACTCTTCAGCATCACTGGCTTCCTGCTGCAACGATTTGAGGACCATCAG
ENSPTRT00000027837     CCATACTTGTTACTCTTCAGCATCACTGGCTTCCTGCTGCAACGATTTGAGGACCATCAG
ENSMUST00000061456     TCCTCTCTCTTGCTCTTCAACATAAAAGGTGTCTATCTGCAGACAATTATGTACTACACA
ENSRNOT00000028113     TCCTCACTCTTGATCTACAGCATCACGGGTCTCCACCTGCAGACATTTCCTTACGGCCCA

ENSCJAT00000005278     ACAGCCATCAGCAACTTCTGGGTGGATCCTCTCTATGTGCTCACCACGTCTGCTGACTCT
ENSMMUT00000004359     GCAGCCATCGACAACTTCTGGGTGGATCCTCGCTATGTGCTCACCACGTCCAAGAACTCT
ENSPPYT00000016175     GCAGCCATCAACAACTTCTGGGTGGATCCTTGCTATGTGCTCACCACATCCGAGAACTCT
ENSGGOT00000025916     GCAGCCATCAACAACTTCTGGGTGGATCCTTGCTATGTGCTCACCACATCCGAGAACTCT
ENST00000296438        GCAGCCATCAACAACTTCTGGGTGGATCCTTGCTATGTGCTCACCACATCCGAGAACTCT
ENSPTRT00000027837     GCAGCCATCAACAACTTCTGGGTGGATCCTTGCTATGTGCTCACCACATCCGAGAACTCT
ENSMUST00000061456     GACTGGATCTTGAGACTCTGGATGGACCCCATTTATGTCATTGTCACCTTTAACAATTCT
ENSRNOT00000028113     GGAGAGATCCTGAGACTGTCGGTGGACCCCCTTCATGTCATCGTCACATTTATCGATTCT

ENSCJAT00000005278     GTGCACCTCTACATGTGGGAAGAGGGAGGCTGCCCTCCATACCTCAGGAGCTGCTGTCAC
ENSMMUT00000004359     GTGCACGTGTACATGTGGGAAGAAGGAGGCCGCCATCCATACCTCAGGAGCTGCTATCGC
ENSPPYT00000016175     GTGCACGTGTACGTGTGGGAAGAAGGAGGCCGCCATCCATACCTCAGGAGCTGCTGTCAC
ENSGGOT00000025916     GTGCACGTGTACATGTGGGAAGAAGGAGGCCGCCATCCATACCTCAGGAGCTGCTGTCAC
ENST00000296438        GTGCACGTGTACATGTGGGAAGAAGGAGGCCGCCATCCATACCTCAGGAGCTGCTGTCAC
ENSPTRT00000027837     GTGCACGTGTACATGTGGGAAGAAGGAGGCCGCCATCCATACCTCAGGAGCTGCTGTCAC
ENSMUST00000061456     TTGGTTGTGTACGCGTGGGAAGAG------AGATGCCAGCAGCTCAATGGGTGTTATCAG
ENSRNOT00000028113     TTGGAGTTGTTTGCGTGGGAAGAA------AGAAGCCCACTGCTCAGGAGGTGTTACCGG

ENSCJAT00000005278     TTGGACAGTACATGGCGTGATCACATATCACACAGGTTTATCTCTTGTGTGATGTGTGAC
ENSMMUT00000004359     CTGGAAAACATATGGCATGATCCCACAACAGACAGCTGCATCTCCAGTGTGATGTGTGAT
ENSPPYT00000016175     CTGGAAAACACGTGGCATGATCACACAACAGACAGCTGCATCTCCAGTGTGATGTGTGAT
ENSGGOT00000025916     CTGGAAAACACGTGGCATGATCACACAACAGACAGCTGCATCTCCAGTGTGATGTGTGAT
ENST00000296438        CTGGAAAACACGTGGCATGATCACACAACAGACAGCTGCATCTCCAGTGTGATGTGTGAT
```

```
ENSPTRT00000027837     CTGGAAAACACGTGGCATGATCACACAACAGACAGCTGCATCTCCAGTGTGATGTGTGAT
ENSMUST00000061456     CTGCAAAGCAGGAGAAGGCTACCA---CAGCAAAGCTTCATTAACAAAACACTATGTGAT
ENSRNOT00000028113     CTGTATAACAAGAGACATATGCTG---CATCGACGCTTTGTTAACAAAACTCTATGTGAC


ENSCJAT00000005278     AATGCAAGCATAGTACTTAAGGTGAGAAATATAACTGAATCCAGCATCGTGGTGATGTAT
ENSMMUT00000004359     AATGCAAGCATAGTACTTACGGTGACAGCA---AGTGAATACAGCATGCTGGTGATGTAT
ENSPPYT00000016175     AATGCAAGCATAGTACTTAGGCTGAGGAAAGTAAGTGACTCCAGCATTCTGGTGATGTAT
ENSGGOT00000025916     AATGCAAGCATAGTACTTACGGTGACGAAAGTAAGTGACTCCAGCATTCTGGTGATGTAT
ENST00000296438        AATGCAAGCATAGTACTTAGGGTGAGGAAAGTAAGTGACTCCAGCATTCTGGTGATGTAT
ENSPTRT00000027837     AATGCAAGCATAGTACTTAGGGTGAGGAAAGTAAGTGACTCCAGCATTCTGGTGATGTAT
ENSMUST00000061456     GATATGAGCATAATTCGAGTGATGACAACTCATTCCATCCCCAGCTTTCTGATGGCATAT
ENSRNOT00000028113     GATGTGAGCATAGTTCGGGTGATGACAAATGTTCCCTCCTCCAGCTTTCTGATGGTGTAC


ENSCJAT00000005278     TCTCTGAATATG
ENSMMUT00000004359     TCTTTGAACACG
ENSPPYT00000016175     TCTTTGAATACG
ENSGGOT00000025916     TCTTTGAATACG
ENST00000296438        TCTTTGAATACG
ENSPTRT00000027837     TCTTTGAATACG
ENSMUST00000061456     ATCTTGACATCT
ENSRNOT00000028113     ACCTTGAACAAC
```

Multiple sequence alignment of Fbxw2

```
ENSMMUT00000002327     ATGGAGAGAAAGGACTTTGAGACATGGCTTGATAACATTTCTGTTACATTTCTTTCTCTG
ENSPPYT00000022812     ATGGAGAGAAAGGACTTTGAGACATGGCTTGATAACATTTCTGTTACATTTCTTTCTCTG
ENSCJAT00000039931     ATGGAGAGAAAGGACTTTGAGACATGGCTTGATAACATTTCTGTTACATTTCTTTCTCTG
ENST00000373926        ATGGAGAGAAAGGACTTTGAGACATGGCTTGATAACATTTCTGTTACATTTCTTTCTCTG
ENSPTRT00000039407     ATGGAGAGAAAGGACTTTGAGACATGGCTTGATAACATTTCTGTTACGTTTCTTTCTCTG
ENSGGOT00000007476     ATGGAGAGAAAGGACTTTGAGACATGGCTTGATAACATTTCTGTTACATTTCTTTCTCTG
ENSRNOT00000025399     ATGGAGAGAAAGGACTTTGAGACATGGCTTGATAACATTTCTGTTACATTTCTTTCTCTG
ENSMUST00000028220     ATGGAGAGAAAGGACTTTGAGACATGGCTTGATAACATTTCTGTTACATTTCTTTCTCTG


ENSMMUT00000002327     ACGGACTTGCAGAAAAAATGAAACTCTGGATCACCTGATTAGTCTGAGTGGGGCAGTCCAG
ENSPPYT00000022812     ACGGACTTGCAGAAAAAATGAAACTCTGGATCACCTGATTAGTCTGAGTGGGGCAGTCCAG
ENSCJAT00000039931     ACGGACTTGCAGAAAAAATGAAACTCTGGATCACCTGATTAGTCTGAGTGGGGCAGTCCAG
ENST00000373926        ACGGACTTGCAGAAAAAATGAAACTCTGGATCACCTGATTAGTCTGAGTGGGGCAGTCCAG
ENSPTRT00000039407     ACGGACTTGCAGAAAAAATGAAACTCTGGATCACCTGATTAGTCTGAGTGGGGCAGTCCAG
ENSGGOT00000007476     ACGGACTTGCAGAAAAAATGAAACTCTGGATCACCTGATTAGTCTGAGTGGGGCAGTCCAG
ENSRNOT00000025399     ACGGACTTGCAGAAAAAATGAAACTCTGGACCACCTGATTAGTCTGAGTGGGGCAGTCCAG
ENSMUST00000028220     ACGGACTTGCAGAAAAAATGAAACTCTGGACCACCTGATTAGTCTGAGTGGGGCAGTCCAG


ENSMMUT00000002327     CTCAGGCATCTCTCCAATAACCTAGAGACTCTCCTCAAGCGGGACTTCCTCAAACTCCTT
ENSPPYT00000022812     CTCAGGCATCTCTCCAATAACCTAGAGACTCTCCTCAAGCGGGACTTCCTCAAACTCCTT
ENSCJAT00000039931     CTCAGGCATCTCTCCAATAACCTAGAGACTCTCCTCAAGCGGGACTTCCTCAAACTCCTT
ENST00000373926        CTCAGGCATCTCTCCAATAACCTAGAGACTCTCCTCAAGCGGGACTTCCTCAAACTCCTT
ENSPTRT00000039407     CTCAGGCATCTCTCCAATAACCTAGAGACTCTCCTCAAGCGGGACTTCCTCAAACTCCTT
ENSGGOT00000007476     CTCAGGCATCTCTCCAATAACCTAGAGACTCTCCTCAAGCGGGACTTCCTCAAACTCCTT
ENSRNOT00000025399     CTCAGGCATCTCTCCAATAACCTGGAGACTCTCCTCAAGCGGGACTTCCTCAAACTCCTT
ENSMUST00000028220     CTCAGGCATCTCTCCAATAACCTGGAGACTCTCCTCAAGCGGGACTTCCTCAAACTCCTT


ENSMMUT00000002327     CCCCTGGAGCTCAGTTTTTATTTGTTAAAATGGCTCGATCCTCAGACTTTACTCACATGC
ENSPPYT00000022812     CCCCTGGAGCTCAGTTTTTATTTGTTAAAATGGCTCGATCCTCAGACTTTACTCACATGC
ENSCJAT00000039931     CCCCTGGAGCTCAGTTTTTATTTGTTAAAATGGCTCGATCCTCAGACTTTACTCACATGC
```

```
ENST00000373926        CCCCTGGAGCTCAGTTTTTATTTGTTAAAATGGCTCGATCCTCAGACTTTACTCACATGC
ENSPTRT00000039407     CCCCTGGAGCTCAGTTTTTATTTGTTAAAATGGCTCGATCCTCAGACTTTACTCACATGC
ENSGGOT00000007476     CCCCTGGAGCTCAGTTTTTATTTGTTAAAATGGCTCGATCCTCAGACTTTACTCACATGC
ENSRNOT00000025399     CCCCTGGAGCTCAGTTTTTATTTGTTAAAATGGCTCGATCCTCAGACTTTGCTCACATGC
ENSMUST00000028220     CCCCTGGAGCTCAGTTTTTATTTGTTAAAATGGCTCGATCCTCAGACTTTACTCACATGC

ENSMMUT00000002327     TGCCTCGTCTCTAAACAATGGAATAAGGTGATAAGTGCCTGTACAGAGGTGTGGCAGACT
ENSPPYT00000022812     TGCCTCGTCTCTAAACAGTGGAATAAGGTGATAAGTGCCTGTACAGAGGTGTGGCAGACT
ENSCJAT00000039931     TGCCTCGTCTCTAAACAGTGGAATAAGGTGATAAGTGCCTGCACAGAGGTGTGGCAGACT
ENST00000373926        TGCCTCGTCTCTAAACAGTGGAATAAGGTGATAAGTGCCTGTACAGAGGTGTGGCAGACT
ENSPTRT00000039407     TGCCTCGTCTCTAAACAGTGGAATAAGGTGATAAGTGCCTGTACAGAGGTGTGGCAGACT
ENSGGOT00000007476     TGCCTCGTCTCTAAACAGTGGAATAAGGTGATAAGTGCCTGTACAGAGGTGTGGCAGACT
ENSRNOT00000025399     TGCCTGGTCTCTAAGCAGTGGAATAAGGTGATAAGTGCCTGCACAGAGGTGTGGCAGACT
ENSMUST00000028220     TGCCTGGTCTCTAAGCAGTGGAATAAGGTGATAAGTGCCTGTACAGAGGTGTGGCAGACT

ENSMMUT00000002327     GCATGTAAAAATTTGGGCTGGCAGATAGATGATTCTGTTCAGGACGCTTTGCACTGGAAG
ENSPPYT00000022812     GCATGTAAAAATTTGGGCTGGCAGATAGATGATTCTGTTCAGGACGCTTTGCACTGGAAG
ENSCJAT00000039931     GCATGTAAAAATTTGGGCTGGCAGATAGATGATTCTGTTCAAGACGCTTTGCACTGGAAG
ENST00000373926        GCATGTAAAAATTTGGGCTGGCAGATAGATGATTCTGTTCAGGACGCTTTGCACTGGAAG
ENSPTRT00000039407     GCATGTAAAAATTTGGGCTGGCAGATAGATGATTCTGTTCAGGACGCTTTGCACTGGAAG
ENSGGOT00000007476     GCATGTAAAAATTTGGGCTGGCAGATAGATGATTCTGTTCAGGACGCTTTGCACTGGAAG
ENSRNOT00000025399     GCATGTAAAAATTTGGGCTGGCAAATAGATGATTCTGTTCAGGACGCATTGCACTGGAAG
ENSMUST00000028220     GCATGTAAAAATTTGGGCTGGCAGATAGATGATTCTGTTCAGGACTCATTGCACTGGAAG

ENSMMUT00000002327     AAGGTTTATTTGAAGGCTATTTTGAGAATGAAGCAACTGGAGGACCATGAAGCCTTTGAG
ENSPPYT00000022812     AAGGTTTATTTGAAGGCTATTTTGAGAATGAAGCAACTGGAGGACCATGAAGCCTTTGAA
ENSCJAT00000039931     AAGGTTTATTTGAAGGCTATTTTGAGAATGAAGCAACTGGAGGACCATGAAGCCTTTGAG
ENST00000373926        AAGGTTTATTTGAAGGCTATTTTGAGAATGAAGCAACTGGAGGACCATGAAGCCTTTGAA
ENSPTRT00000039407     AAGGTTTATTTGAAGGCTATTTTGAGAATGAAGCAACTGGAGGACCATGAAGCCTTTGAA
ENSGGOT00000007476     AAGGTTTATTTGAAGGCTATTTTGAGAATGAAGCAACTGGAGGACCATGAAGCCTTTGAA
ENSRNOT00000025399     AAGGTTTACTTGAAGGCTATTTTGAGGATGAAGCAACTGGAGGACCATGAAGCCTTTGAG
ENSMUST00000028220     AAGGTTTATTTGAAGGCTATTTTGAGGATGAAGCAACTGGAGGACCATGAAGCCTTTGAG

ENSMMUT00000002327     ACCTCATCATTAATTGGACACAGTGCCAGAGTGTATGCACTTTACTACAAAGATGGACTT
ENSPPYT00000022812     ACCTCATCATTAATTGGACACAGTGCCAGAGTGTATGCACTTTACTACAAAGATGGACTT
ENSCJAT00000039931     ACCTCATCATTAATTGGACACAGTGCCAGAGTGTATGCACTTTACTACAAAGATGGACTT
ENST00000373926        ACCTCGTCATTAATTGGACACAGTGCCAGAGTGTATGCACTTTACTACAAAGATGGACTT
ENSPTRT00000039407     ACCTCATCATTAATTGGACACAGTGCCAGAGTGTATGCACTTTACTACAAAGACGGACTT
ENSGGOT00000007476     ACCTCATCATTAATTGGACACAGTGCCAGAGTGTATGCACTTTACTACAAAGATGGACTT
ENSRNOT00000025399     ACCTCATCATTAATTGGACATAGTGCCAGAGTGTATGCACTTTACTACAAGGATGGACTT
ENSMUST00000028220     ACCTCTTCGTTAATTGGACATAGTGCCAGAGTGTATGCACTTTACTACAAAGATGGACTT

ENSMMUT00000002327     CTCTGTACAGGGTCAGATGACTTGTCTGCAAAGCTGTGGGATGTGAGCACAGGGCAGTGC
ENSPPYT00000022812     CTCTGTACAGGGTCAGATGACTTGTCTGCAAAGCTGTGGGATGTGAGCACAGGGCAGTGC
ENSCJAT00000039931     CTCTGTACAGGGTCAGATGACTTGTCTGCAAAGCTGTGGGATGTGAGCACAGGGCAGTGC
ENST00000373926        CTCTGTACAGGGTCAGATGACTTGTCTGCAAAGCTGTGGGATGTGAGCACAGGGCAGTGC
ENSPTRT00000039407     CTCTGTACAGGGTCAGATGACTTGTCTGCAAAGCTGTGGGATGTGAGCACAGGGCAGTGC
ENSGGOT00000007476     CTCTGTACAGGGTCAGATGACTTGTCTGCAAAGCTGTGGGATGTGAGCACAGGGCAGTGC
ENSRNOT00000025399     CTCTGTACAGGGTCAGATGATTTGTCTGCAAAGCTGTGGGATGTAAGCACAGGGCAGTGT
ENSMUST00000028220     CTCTGTACAGGGTCAGATGACTTGTCTGCAAAGCTGTGGGATGTAAGCACAGGGCAGTGT

ENSMMUT00000002327     GTTTATGGCATCCAGACCCACACTTGTGCAGCGGTGAAGTTTGATGAACAGAAGCTTGTG
ENSPPYT00000022812     GTTTATGGCATCCAGACCCACACTTGTGCAGCGGTGAAGTTTGATGAACAGAAGCTTGTG
ENSCJAT00000039931     GTTTATGGCATCCAGACCCACACTTGTGCAGCGGTGAAGTTTGATGAACAGAAGCTTGTG
```

ENST00000373926 GTTTATGGCATCCAGACCCACACTTGTGCAGCGGTGAAGTTTGATGAACAGAAGCTTGTG
ENSPTRT00000039407 GTTTATGGCATCCAGACCCACACTTGTGCAGCGGTGAAGTTTGATGAACAGAAGCTTGTG
ENSGGOT00000007476 GTTTATGGCATCCAGACCCACACTTGTGCAGCGGTGAAGTTTGATGAACAGAAGCTTGTG
ENSRNOT00000025399 GTTTACGGCATCCAGACCCACACTTGTGCAGCCGTGAAGTTCGATGAACAGAAGCTTGTG
ENSMUST00000028220 GTTTACGGCATCCAGACCCACACTTGTGCAGCTGTGAAGTTCGATGAACAGAAGCTTGTG

ENSMMUT00000002327 ACAGGCTCCTTTGACAACACTGTGGCTTGCTGGGAATGGAGTTCCGGAGCCAGGACCCAG
ENSPPYT00000022812 ACAGGCTCCTTTGACAACACTGTGGCTTGCTGGGAATGGAGTTCCGGAGCCAGGACCCAG
ENSCJAT00000039931 ACAGGCTCCTTTGACAACACTGTGGCTTGCTGGGAATGGAGTTCCGGAGCCAGGACCCAG
ENST00000373926 ACAGGCTCCTTTGACAACACTGTGGCTTGCTGGGAATGGAGTTCCGGAGCCAGGACCCAG
ENSPTRT00000039407 ACAGGCTCCTTTGACAACACTGTGGCTTGCTGGGAATGGAGTTCCGGAGCCAGGACCCAG
ENSGGOT00000007476 ACAGGCTCCTTTGACAACACTGTGGCTTGCTGGGAATGGAGTTCCGGAGCCAGGACCCAG
ENSRNOT00000025399 ACAGGCTCCTTTGACAACACTGTGGCCTGCTGGGAATGGAGTTCCGGAGCCAGGACCCAG
ENSMUST00000028220 ACAGGCTCCTTTGACAACACTGTGGCCTGCTGGGAGTGGAGTTCCGGAGCCAGGACCCAG

ENSMMUT00000002327 CACTTTCGGGGGCACACGGGGGCGGTATTTAGCGTGGACTACAATGATGAGCTGGATGTC
ENSPPYT00000022812 CACTTTCGGGGGCACACGGGGGCGGTATTTAGCGTGGACTACAATGATGAACTGGATATC
ENSCJAT00000039931 CACTTTCGGGGGCACACGGGGGCGGTGTTTAGTGTGGACTACAATGATGAACTGGATATC
ENST00000373926 CACTTTCGGGGGCACACGGGGGCGGTATTTAGCGTGGACTACAATGATGAACTGGATATC
ENSPTRT00000039407 CACTTTCGGGGGCACACGGGGGCGGTATTTAGCGTGGACTACAATGATGAACTGGATATC
ENSGGOT00000007476 CACTTTCGGGGGCACACGGGGGCGGTATTTAGCGTGGACTACAATGATGAACTGGATATC
ENSRNOT00000025399 CACTTCCGGGGGCACACAGGGGCGGTGTTCAGTGTGGACTACAGTGATGAACTGGATATT
ENSMUST00000028220 CACTTCCGGGGGCACACGGGGGCGGTGTTCAGTGTGGACTACAGTGATGAACTGGATATT

ENSMMUT00000002327 TTGGTGAGCGGCTCTGCAGACTTCACTGTGAAAGTATGGGCTTTATCTGCTGGGACATGC
ENSPPYT00000022812 TTGGTGAGCGGCTCTGCAGACTTCACTGTGAAAGTATGGGCTTTATCTGCTGGGACATGC
ENSCJAT00000039931 TTGGTGAGTGGCTCTGCAGACTTCACTGTGAAAGTATGGGCTTTATCTGCTGGGACATGC
ENST00000373926 TTGGTGAGCGGCTCTGCAGACTTCACTGTGAAAGTATGGGCTTTATCTGCTGGGACATGC
ENSPTRT00000039407 TTGGTGAGCGGCTCTGCAGACTTCACTGTGAAAGTATGGGCTTTATCTGCTGGGACATGC
ENSGGOT00000007476 TTGGTGAGCGGCTCTGCAGACTTCACTGTGAAAGTATGGGCTTTATCTGCTGGGACATGC
ENSRNOT00000025399 TTGGTGAGTGGCTCTGCGGACTTCGCTGTGAAAGTATGGGCTTTATCTGCTGGGACATGC
ENSMUST00000028220 TTGGTGAGTGGCTCTGCGGACTTCGCTGTGAAAGTATGGGCTTTATCTGCTGGGACATGC

ENSMMUT00000002327 CTGAACACACTCACCGGGCACACGGAATGGGTCACCAAGGTAGGAAGACTTAGAAGAAAT
ENSPPYT00000022812 CTGAACACACTCACCGGGCACACGGAATGGGTCACCAAGGTAGTTTTGCAGAAGTGCAAA
ENSCJAT00000039931 CTGAACACACTCACCGGGCACACGGAATGGGTCACCAAGGTAGTTTTGCAGAAATGCAAA
ENST00000373926 CTGAACACACTCACCGGGCACACGGAATGGGTCACCAAGGTAGTTTTGCAGAAGTGCAAA
ENSPTRT00000039407 CTGAACACACTCACCGGGCACACGGAATGGGTCACCAAGGTAGTTTTGCAGAAGTGCAAG
ENSGGOT00000007476 CTGAACACACTCACCGGGCACACGGAATGGGTCACCAAGGTAGTTTTGCAGAAGTGCAAA
ENSRNOT00000025399 CTGAACACACTCACTGGGCACACAGAATGGGTCACCAAGGTGGTTCTGCAGCAGTGCAAA
ENSMUST00000028220 CTGAATACACTCACTGGGCATACTGAATGGGTCACCAAGGTGGTTTTGCAGAAGTGCAAA

ENSMMUT00000002327 CTTCTTGTCAGGCTTTTTGAGGGAGGGAGTGGAGGATGCATGATGGTATACAACAGTGAC
ENSPPYT00000022812 GTCAAGTCTCTCTTGCACAGTCCTGGAGACTACATCCTCTTAAGTGCAGACAAATATGAG
ENSCJAT00000039931 GTCAAATCTCTCTTGCACAGTCCCGGAGACTACATCCTCTTAAGTGCAGACAAATATGAG
ENST00000373926 GTCAAGTCTCTCTTGCACAGTCCTGGAGACTACATCCTCTTAAGTGCAGACAAATATGAG
ENSPTRT00000039407 GTCAAGTCTCTCTTGCACAGTCCTGGAGACTACATCCTCTTAAGTGCAGACAAATATGAG
ENSGGOT00000007476 GTCAAGTCTCTCTTGCACAGTCCTGGAGACCACATCCTCTTAAGTGCAGACAAATATGAG
ENSRNOT00000025399 GTCAAGTCTCTCTTGCACAGCCCTGGGGACTACATCCTTTTAAGTGCAGACAAGTACGAG
ENSMUST00000028220 GTCAAGTCTCTCTTGCACAGCCCTGGAGACTACATCCTCTTAAGTGCAGACAAATATGAG

ENSMMUT00000002327 CAGCAAATTTGGCCAATTGGGAGAGAAATCAACTGCAAGTGCTTAAAGACATTGTCTGTC
ENSPPYT00000022812 ATTAAGATTTGGCCAATTGGGAGAGAAATCAACTGCAAGTGCTTAAAGACATTGTCTGTC
ENSCJAT00000039931 ATTAAGATTTGGCCAATTGGGAGAGAAATCAACTGCAAGTGCTTAAAGACATTGTCTGTC

```
ENST00000373926        ATTAAGATTTGGCCAATTGGGAGAGAAATCAACTGTAAGTGCTTAAAGACATTGTCTGTC
ENSPTRT00000039407     ATTAAGATTTGGCCAATTGGGAGAGAAATCAACTGTAAGTGCTTAAAGACATTGTCTGTC
ENSGGOT00000007476     ATTAAGATTTGGCCAATTGGGAGAGAAATCAACTGCAAGTGCTTAAAGACATTGTCTGTC
ENSRNOT00000025399     ATCAAGATTTGGCCAATTGGGAGAGAAATCAACTGTAAGTGCTTAAAGACATTGTCTGTC
ENSMUST00000028220     ATCAAGATTTGGCCAATTGGGAGAGAAATCAACTGTAAGTGCTTGAAGACACTGTCTGTC

ENSMMUT00000002327     TCTGAGGATAGAAGCATCTGCCTGCAGCCAAGACTGCATTTTGATGGGAAATACATTGTC
ENSPPYT00000022812     TCTGAGGATAGAAGTATCTGCCTGCAGCCAAGACTTCATTTTGATGGCCAATACATTGTC
ENSCJAT00000039931     TCTGAGGATAGAAGTATTTGCCTGCAGCCAAGACTTCATTTTGATGGCAAATACATTGTC
ENST00000373926        TCTGAGGATAGAAGTATCTGCCTGCAGCCAAGACTTCATTTTGATGGCAAATACATTGTC
ENSPTRT00000039407     TCTGAGGATAGAAGTATCTGCCTGCAGCCAAGACTTCATTTTGATGGCAAATACATTGTC
ENSGGOT00000007476     TCTGAGGATAGAAGTATCTGCCTGCAGCCAAGACTTCATTTTGATGGCAAATACATTGTC
ENSRNOT00000025399     TCTGAGGATAGAAGTATCTGCCTACAGCCAAGACTTCATTTTGATGGAAAATACATTGTC
ENSMUST00000028220     TCTGAGGATAGAAGTATCTGCCTGCAGCCAAGACTTCATTTTGATGGAAAATACATTGTC

ENSMMUT00000002327     TGCAGTTCAGCACTTGGTCTCTACCAGTGGGACTTTGCTAGTTATGATATTCTCAGGGTC
ENSPPYT00000022812     TGTAGTTCAGCACTTGGTCTCTACCAGTGGGACTTTGCCAGTTATGATATTCTCAGGGTC
ENSCJAT00000039931     TGTAGTTCAGCACTTGGTCTGTACCAGTGGGACTTTGCCAGTTACGATATTCTAAGGGTC
ENST00000373926        TGTAGTTCAGCACTTGGTCTCTACCAGTGGGACTTTGCCAGTTATGATATTCTCAGGGTC
ENSPTRT00000039407     TGTAGTTCAGCACTTGGTCTCTACCAGTGGGACTTTGCCAGTTATGATATTCTCAGGGTC
ENSGGOT00000007476     TGTAGTTCAGCACTTGGTCTCTATCAGTGGGACTTTGCCAGTTATGATATTCTCAGGGTC
ENSRNOT00000025399     TGTAGTTCAGCCCTGGGTCTGTACCAGTGGGACTTTGCCAGTTATGATATCCTCAGGGTC
ENSMUST00000028220     TGTAGTTCAGCCCTGGGTCTGTACCAGTGGGACTTTGCCAGTTATGATATTCTCAGGGTC

ENSMMUT00000002327     ATCAAGACACCTGAGATAGCAAACTTGGCCTTGCTTGGCTTTGGAGATATCTTTGCCCTG
ENSPPYT00000022812     ATCAAGACTCCTGAGATAGCAAACTTGGCCTTGCTTGGCTTTGGAGATATCTTTGCCCTG
ENSCJAT00000039931     ATCAAGACTCCTGAGATAGCAAACTTGGCCTTGCTTGGCTTTGGAGATATCTTTGCTCTG
ENST00000373926        ATCAAGACTCCTGAGATAGCAAACTTGGCCTTGCTTGGCTTTGGAGATATCTTTGCCCTG
ENSPTRT00000039407     ATCAAGACTCCTGAGATAGCAAACTTGGCCTTGCTTGGCTTTGGAGATATCTTTGCCCTG
ENSGGOT00000007476     ATCAAGACTCCTGAGATAGCAAACTTGGCCTTGCTTGGCTTTGGAGATATCTTTGCCCTG
ENSRNOT00000025399     ATCAAGACACCTGAGGTAGCAAACTTGGCCTTGCTTGGCTTTGGAGATGTCTTCGCTCTG
ENSMUST00000028220     ATCAAGACACCTGAGGTAGCAAACTTGGCCTTGCTTGGCTTTGGAGATGTCTTCGCCCTG

ENSMMUT00000002327     CTGTTTGACAACCGCTACCTGTACATCATGGACTTGCGGACAGAGAGCCTGATTAGTCGC
ENSPPYT00000022812     CTGTTTGACAACCGCTACCTGTACATCATGGACTTGCGGACAGAGAGCCTGATTAGTCGC
ENSCJAT00000039931     CTGTTTGACAACCGATACCTGTACATCATGGACTTGCGGACAGAGAGCCTGATTAGTCGC
ENST00000373926        CTGTTTGACAACCGCTACCTGTACATCATGGACTTGCGGACAGAGAGCCTGATTAGTCGC
ENSPTRT00000039407     CTGTTTGACAACCGCTACCTGTACATCATGGACTTGCGGACAGAGAGCCTGATTAGTCGC
ENSGGOT00000007476     CTGTTTGACAACCGCTACCTGTACATCATGGACTTGCGGACAGAGAGCCTGATTAGTCGC
ENSRNOT00000025399     CTGTTTGACAACCACTACCTATACATCATGGACTTGAGGACAGAGAGCCTAATTAGCCGC
ENSMUST00000028220     CTGTTTGACAACCACTACCTATATATCATGGACTTGAGGACAGAGAGCCTAATTAGCCGC

ENSMMUT00000002327     TGGCCTCTGCCAGAGTACAGGAAGTCAAAGAGAGGCTCAAGCTTCCTGGCAGGCGAAGCA
ENSPPYT00000022812     TGGCCTCTGCCAGAGTACAGGAAATCAAAGAGAGGCTCAAGCTTCCTGGCAGGCGAAGCA
ENSCJAT00000039931     TGGCCTCTGCCAGAGTACAGGAAATCAAAGAGAGGCTCAAGCTTCCTGGCAGGCGAAGCA
ENST00000373926        TGGCCTCTGCCAGAGTACAGGAAATCAAAGAGAGGCTCAAGCTTCCTGGCAGGCGAAGCA
ENSPTRT00000039407     TGGCCTCTGCCAGAGTACAGGAAATCAAAGAGAGGCTCAAGCTTCCTGGCAGGCGAAGCA
ENSGGOT00000007476     TGGCCTCTGCCAGAGTACAGGAAATCAAAGAGAGGCTCAAGCTTCCTGGCAGGCGAAGCA
ENSRNOT00000025399     TGGCCTCTGCCAGAGTACAGGAAATCAAAGAGAGGCTCCAGCTTCCTGGCAGGCGAAGCG
ENSMUST00000028220     TGGCCTCTGCCAGAGTACAGGAAATCAAAGAGAGGCTCCAGCTTCCTGGCAGGCGAAGCG

ENSMMUT00000002327     TCCTGGCTGAATGGATTGGATGGGCACAATGACACGGGCTTGGTCTTTGCCACCAGCATG
ENSPPYT00000022812     TCCTGGCTGAATGGACTGGATGGGCACAATGACACGGGCTTGGTCTTTGCCACCAGCATG
ENSCJAT00000039931     TCCTGGCTGAATGGACTGGATGGGCACAATGACACGGGCTTGGTCTTTGCCACCAGCATG
```

```
ENST00000373926       TCCTGGCTGAATGGACTGGATGGGCACAATGACACGGGCTTGGTCTTTGCCACCAGCATG
ENSPTRT00000039407    TCCTGGCTGAATGGACTGGATGGGCACAATGACACGGGCTTGGTCTTTGCCACCAGCATG
ENSGGOT00000007476    TCCTGGCTGAATGGACTGGATGGGCACAATGACACGGGCTTGGTCTTTGCTACCAGCATG
ENSRNOT00000025399    TCCTGGCTGAATGGACTGGATGGGCACAATGACACGGGCTTAGTCTTTGCTACCAGCATG
ENSMUST00000028220    TCCTGGTTGAATGGATTGGATGGGCACAATGACACGGGCTTAGTCTTTGCCACCAGCATG


ENSMMUT00000002327    CCTGACCACAGTATTCACCTGGTGTTGTGGAAGGAGCACGGC
ENSPPYT00000022812    CCTGACCACAGTATTCACCTGGTGTTGTGGAAGGAGCACGGC
ENSCJAT00000039931    CCTGACCACAGTATTCACTTGGTGTTGTGGAAGGAGCACGGC
ENST00000373926       CCTGACCACAGTATTCACCTGGTGTTGTGGAAGGAGCACGGC
ENSPTRT00000039407    CCTGACCACAGTATTCACCTGGTGTTGTGGAAGGAGCACGGC
ENSGGOT00000007476    CCTGACCACAGTATTCACCTGGTGTTGTGGAAGGAGCACGGC
ENSRNOT00000025399    CCTGACCACAGTATTCACCTGGTTTTATGGAAGGAGCATGGC
ENSMUST00000028220    CCTGACCACAGTATTCACCTGGTGTTATGGAAGGAGCATTGC
```

Multiple sequence alignment of Fbxw4

```
ENSCJAT00000059963    GCCAGGGCCGCTCGGGGCCGGGAAGAGCACAGAGCTGCTCGGGAGTCCGCTGCCCGCCCG
ENSMMUT00000009449    ATGGCGGCGGAGGCCGGGGAGGAGGAGGAGGAGGCGGCTCGGGAGTCGGCCGCCCGCCCG
ENSPPYT00000003110    GCCATGGCGGCGGCGGCCGAGGAGAAGGAGGAGGCGGCTCGGGAGTCGGCTGCCCGCCTG
ENSGGOT00000022150    ATGGCGGCGGCGGCCGGGGAGGAGGAGGAGGAGGCGGCTCGGGAGGCGGCTGCCCGCCCG
ENST00000389046       ATGGCGGCGGCGGCCGGGGAGGAGGAGGAGGAGGCGGCTCGGGAGTCGGCTGCCCGCCCG
ENSMUST00000046869    ATGGCGGAGGACGCGGCGGAGGATGCCGCAGCGGCGGCCGTGGAGCCGGCCACCCGTCCG
ENSRNOT00000023299    ATGGCGGCGGCAGCC------GAGGACGGGGCAGCGGCTGTGGAGCCGGCCACCCGTCCG


ENSCJAT00000059963    GCCGCGGGGCCTGCGCTCTGGCGCCTGCCGGAGGAGCTGCTGCTGCTCATCTGCTCCTAC
ENSMMUT00000009449    GCCGCGGGGCCCGCGCTCTGGCGCCTGCCGGAGGAGCTGCTGCTGCTCATCTGCTCCTAC
ENSPPYT00000003110    GCCGCGGGGCCTGCGCTCTGGCGTCTGCCGGAGGAGCTGCTGCTGCTCATCTGCTCCTAC
ENSGGOT00000022150    GCCGCGGGGCCTGCGCTCTGGCGCCTGCCGGAGGAGCTGCTGCTGCTCATCTGCTCCTAC
ENST00000389046       GCCGCGGGGCCTGCGCTCTGGCGCCTGCCGGAGGAGCTGCTGCTGCTCATCTGCTCCTAC
ENSMUST00000046869    GCCGCGGGGCCCGCGCTCTGGCGCCTGCCGGAGGAGCTGCTGCTGCTCATCTGCTCCTAC
ENSRNOT00000023299    GCCGCGGGGCCCGCGCTCTGGAGACTGCCGGAGGAGCTGCTGCTGCTCATCTGCTCCTAC


ENSCJAT00000059963    CTGGACATGCGGGCCCTCGGCCGCCTGGCCCAGGTGTGCCGCTGGCTGCGGCGCTTCACC
ENSMMUT00000009449    CTGGACATGCGGGCCCTCGGCCGCCTGGCCCAGGTGTGCCGCTGGCTGCGGCGCTTCACC
ENSPPYT00000003110    CTGGACATGCGGGCCCTCGGCCGCCTGGCCCAGGTGTGCCGCTGGCTGCGGCGCTTCACC
ENSGGOT00000022150    CTGGACATACGGGCCCTCGGCCGCCTGGCCCAGGTGTGCCGCTGGCTGCGGCGCTTCACC
ENST00000389046       CTGGACATGCGGGCCCTCGGCCGCCTGGCCCAGGTGTGCCGCTGGCTGCGGCGCTTCACC
ENSMUST00000046869    CTCGACACGCGGGCCCTCGGCCGCCTGGCCCAAGTCTGCCGCTGGCTGCGGCGCTTCACC
ENSRNOT00000023299    CTCGACATGCGGGCCCTCGGCCGCCTGGCCCAGGTGTGCCGCTGGCTGCGGCGTTTCACC


ENSCJAT00000059963    AGCTGCGACCTGCTCTGGCGCCGGATAGCTCGGGCCTCGCTCAACTCCGGCTTCACGCGG
ENSMMUT00000009449    AGCTGCGACCTGCTTTGGCGCCGGATAGCCCGGGCCTCGCTCAACTCCGGCTTCACGCGG
ENSPPYT00000003110    AGCTGCGATCTGCTCTGGCGCCGGATAGCCCGGGCCTCGCTCAACTCCGGCTTCACGCGG
ENSGGOT00000022150    AGCTGCGATCTGCTCTGGCGCCGGATAGCCCGGGCCTCGCTCAACTCCGGCTTCACGCGG
ENST00000389046       AGCTGCGATCTGCTCTGGCGCCGGATAGCCCGGGCCTCGCTCAACTCCGGCTTCACGCGG
ENSMUST00000046869    AGCTGCGACCTGCTCTGGCGCCCGATAGCCCGGGCCTCGCTCAACACCGGCTTCACGCGG
ENSRNOT00000023299    AGCTGCGACCTGCTCTGGCGCCCGATAGCCCGGGCCTCGCTCAACTCCGGCTTCACGCGG


ENSCJAT00000059963    CTCGGCACCGACCTGATGACCAGTGTCCCAGTGAAGGAACGGGTGAAGGTGTCTCAGAAC
ENSMMUT00000009449    CTCGGCACCGACCTGATGACCAGTGTCCCAGTGAAGGAACGGGTGAAGGTGTCTCAGAAC
ENSPPYT00000003110    CTCGGCACCGACCTGATGACCAGTGTCCCAGTGAAGGAACGGGTGAAGGTGTCTCAGAAC
ENSGGOT00000022150    CTCGGCACCGACCTGATGACCAGTGTCCCAGTGAAGGAACGAGTGAAGGTGTCTCAGAAC
ENST00000389046       CTCGGCACCGACCTGATGACCAGTGTCCCAGTGAAGGAACGAGTGAAGGTGTCTCAGAAC
```

```
ENSMUST00000046869      CTCGGCACCGACCTCATGGCAGGCATTCCAGTAAAGGAGCGCGTGAAACTGTCTCAGAAC
ENSRNOT00000023299      CTCGGCACCGACCTGATGACCAGCATTCCAGTAAAGGAACGAGTGAAGCTGTCTCAGAAC

ENSCJAT00000059963      TGGAGACTGGGGCGCTGCCGAGAGGGGATTCTGCTGAAGTGGAGATGCAGTCAGATGCCT
ENSMMUT00000009449      TGGAGACTGGGGCGCTGCCGAGAGGCGATTCTGCTGAAGTGGAGATGCAGTCAGATGCCC
ENSPPYT00000003110      TGGAGACTGGGGCGCTGCCGAGAGGGGATTCTGCTGAAGTGGAGATGCAGTCAGATGCCC
ENSGGOT00000022150      TGGAGACTGGGGCGCTGCCGAGAGGGGATTCTGCTGAAGTGGAGATGCAGTCAGATGCCC
ENST0000389046          TGGAGACTGGGGCGCTGCCGAGAGGGGATTCTGCTGAAGTGGAGATGCAGTCAGATGCCC
ENSMUST00000046869      TGGCGGCTGGGGCGCTGCCGAGATCGGATTCTGCTGAAGTGGAGATATAGTCAGATGCCT
ENSRNOT00000023299      TGGCGGCTGGGACGATGCCGAGATCGGATTCTACTGAAGTGGAGATACAGTCAGATGCCT

ENSCJAT00000059963      TGGATGCAGCTAGAGGATGATTCTCTGTACATATCTCAGGCTAATTTCATCCTGGCCTAC
ENSMMUT00000009449      TGGATGCAGCTAGAGGATGATTCTCTGTACATATCCCAGGCTAATTTCATCCTGGCCTAC
ENSPPYT00000003110      TGGATGCAGCTAGAGGATGATTCTCTGTACATATCCCAAGCTAATTTCATCCTGGCCTAC
ENSGGOT00000022150      TGGATGCAGCTAGAGGATGATTCTCTGTACATATCCCAGGCTAATTTCATCCTGGCCTAC
ENST0000389046          TGGATGCAGCTAGAGGATGATTCTCTGTACATATCCCAGGCTAATTTCATCCTGGCCTAC
ENSMUST00000046869      TGGATGCAGCTACAGGATGCTTCTCTGTACCTATCACAGGCTAATTTCATCCTGGCCTAC
ENSRNOT00000023299      TGGATGCAGCTAGAAGACTCTTCTCTGTACCTATCTCAGGCTAATTTCATCCTGGCCTAC

ENSCJAT00000059963      CAGTTCCGTCCAGATGGTGCCAGCTTGAACCGTCGGCCTCTGGGAGTCTTTGCTGGGCAT
ENSMMUT00000009449      CAGTTCCGTCCAGATGGTGCCAGCTTGAACCGTCGGCCTCTAGGAGTCTTTGCTGGGCAT
ENSPPYT00000003110      CAGTTCCGTCCAGATGGTGCCAGCTTGAACCGTCGGCCTCTGGGAGTCTTTGCTGGGCAT
ENSGGOT00000022150      CAGTTCCGTCCAGATGGTGCCAGCTTGAATCGTCGGCCTCTGGGAGTCTTTGCTGGGCAC
ENST0000389046          CAGTTCCGTCCAGATGGTGCCAGCTTGAATCGTCGGCCTCTGGGAGTCTTTGCTGGGCAT
ENSMUST00000046869      CAGTTCCGCCCGGATGGTGCAAGCTTGAACCGACGGCCCTTCAGAGTCTTCTCTGGGCAT
ENSRNOT00000023299      CAGTTCCGCCCGGATGGTGCAAGCTTGAACCGTCGGCCCTTCAGAGTCTTCGCTGGGCAC

ENSCJAT00000059963      GATGAGGATGTTTGCCACTTTGTGCTGGCCAACTCGCATATTATCAGTGCAGGAGGAGAT
ENSMMUT00000009449      GATGAGGACGTTTGCCACTTTGTGCTGGCCAACTCGCATATTATCAGTGCAGGAGGAGAT
ENSPPYT00000003110      GATGAGGACGTTTGCCACTTTGTGCTGGCCAACTCGCATATTGTTAGTGCAGGAGGAGAT
ENSGGOT00000022150      GATGAGGACGTTTGCCACTTTGTGCTGGCCAACTCACATATTGTTAGTGCAGGAGGAGAT
ENST0000389046          GATGAGGACGTTTGCCACTTTGTGCTGGCCAACTCGCATATTGTTAGTGCAGGAGGGGAT
ENSMUST00000046869      GATGAGGACGTCTGTCACTTTGTGCTGGCCAACTCCCACATCGTCAGTGCAGGAGGAGAC
ENSRNOT00000023299      GATGAGGACGTTTGTCACTTTGTGCTGGCCAACTCCCATATCATCAGTGCGGGAGGAGAT

ENSCJAT00000059963      GGGAAGATTGGCATTCACAAGATTCACAGCACCTTCACTGTCAAGTACTCGGCTCATGAA
ENSMMUT00000009449      GGGAAGATTGGCGTTCATAAGATTCACAGCACCTTCACTGTCAAGTACTCGGCTCATGAA
ENSPPYT00000003110      GGGAAGATTGGCGTTCATAAGATTCACAGCACCTTCACTGTCAAGTACTCGGCTCATGAA
ENSGGOT00000022150      GGGAAGATTGGCGTTCATAAGATTCACAGCACCTTCACTGTCAAGTACTCGGCTCATGAA
ENST0000389046          GGGAAGATTGGCATTCATAAGATTCACAGCACCTTCACTGTCAAGTACTCGGCTCATGAA
ENSMUST00000046869      GGGAAGATCGGTGTTCACAAGATCCACAGCACCTTCACTGTCAAGTACTCGGCTCACGAG
ENSRNOT00000023299      GGGAAGATCGGTGTTCATAAGATTCACAGCACCTTCACTGTCAAGTACTCAGCTCATGAA

ENSCJAT00000059963      CAGGAGGTGAACTGTGTGGATTGCAAAGGGGGCATCATTGTGAGTGGCTCCAGGGACAGG
ENSMMUT00000009449      CAGGAGGTGAACTGTGTGGATTGCAAAGGAGGCATCATTGTGAGTGGCTCCAGGGACAGG
ENSPPYT00000003110      CAGGAGGTGAACTGTGTGGATTGCAAAGGGGGCATCATTGTGAGTGGCTCCAGGGACAGG
ENSGGOT00000022150      CAGGAGGTGAACTGTGTGGATTGCAAAGGGGGCATCATTGTGAGTGGCTCCAGGGACAGG
ENST0000389046          CAGGAGGTGAACTGTGTGGATTGCAAAGGGGGCATCATTGTGAGTGGCTCCAGGGACAGG
ENSMUST00000046869      CAGGAGGTGAACTGTGTGGACTGTAAAGGTGGCATCATTGTGAGTGGCTCCCGGGACAGG
ENSRNOT00000023299      CAGGAGGTGAACTGTGTGGACTGCAAAGGCGGCATCATTGTGAGTGGCTCCAGGGACAGG

ENSCJAT00000059963      ACGGCCAAGGTGTGGCCTTTGGCCTCAGGCCGACTGGGGCAGTGCCTACACACCATCCAG
ENSMMUT00000009449      ACGGCCAAGGTGTGGCCTTTGGCCTCAGGCCGGCTGGGGCAGTGCTTACACACCATCCAG
```

```
ENSPPYT00000003110     ACGGCCAAGGTGTGGCCTTTGGCCTCAGGCCGGCTGGGGCAGTGCTTACACACCATCCAG
ENSGGOT00000022150     ACGGCCAAGGTGTGGCCTTTGGCCTCAGGCCGGCTGGGGCAGTGCTTACACACCATCCAG
ENST0000389046         ACGGCCAAGGTGTGGCCTTTGGCCTCAGGCCGGCTGGGGCAGTGCTTACACACCATCCAG
ENSMUST00000046869     ACAGCCAAGGTATGGCCTCTGGCCTCGGGCCGGCTGGGGCAGTGCTTACACACCATCCAG
ENSRNOT00000023299     ACGGCCAAGGTCTGGCCTTTGGCCTCGGGCCAGCTGGGGCAGTGCTTACACACCATCCAG

ENSCJAT00000059963     ACTGAAGACCGAGTCTGGTCCATTGCTATCAGCCCATTACTCAGCTCTTTTGTGACAGGG
ENSMMUT00000009449     ACTGAAGACCGAGTCTGGTCCATTGCTATCAGCCCATTACTCAGCTCTTTTGTGACAGGG
ENSPPYT00000003110     ACTGAAGACCGAGTCTGGTCCATTGCTATCAGCCCATTACTCAGCTCTTTTGTGACAGGG
ENSGGOT00000022150     ACTGAAGACCGAGTCTGGTCCATTGCTATCAGCCCATTACTCAGCTCTTTTGTGACAGGG
ENST0000389046         ACTGAAGACCGAGTCTGGTCCATTGCTATCAGCCCATTACTCAGCTCTTTTGTGACAGGG
ENSMUST00000046869     ACCGAAGACCGAGTCTGGTCCATTGCTATCAGCCCATTACTCAGCTCTTTTGTGACTGGA
ENSRNOT00000023299     ACCGAAGACAGAGTCTGGTCCATTGCTATCAGCCCAGTACTCAGCTCTTTTGTGACTGGA

ENSCJAT00000059963     ACGGCTTGTTGCGGTCACTTCTCACCCCTGAGAATCTGGGACCTCAACAGTGGGCAGCTG
ENSMMUT00000009449     ACGGCTTGTTGCGGGCACTTCTCACCCCTGAGAATCTGGGACCTCAACAGTGGGCAGCTG
ENSPPYT00000003110     ACGGCTTGTTGTGGGCACTTCTCACCCCTGAGAATCTGGGACCTCAACAGTGGGCAGCTC
ENSGGOT00000022150     ACGGCTTGTTGTGGGCACTTCTCACCCCTGAGAATCTGGGACCTCAACAGTGGGCAGCTG
ENST0000389046         ACGGCTTGTTGCGGGCACTTCTCACCCCTGAGAATCTGGGACCTCAACAGTGGGCAGCTG
ENSMUST00000046869     ACAGCTTGTTGTGGGCACTTCTCACCTCTGAGAATCTGGGACCTCAACAGTGGGCAGCTG
ENSRNOT00000023299     ACAGCTTGTTGTGGGCACTTCTCACCTCTGAGAATCTGGGACCTCACCAGTGGGCAGCTG

ENSCJAT00000059963     ATGACACACTTGGGTAGTGACTTTCCCCCAGGGGCTGGGGTGCTGGATGTCATGTATGAG
ENSMMUT00000009449     ATGACACACTTGGGCAGTGACTTTCCCCCAGGGGCTGGGGTGCTGGATGTCATGTATGAG
ENSPPYT00000003110     ATGACACACTTGGGCAGTGACTTTCCCCCAGGGGCTGGGGTGCTAGATGTCATGTATGAG
ENSGGOT00000022150     ATGACACACTTGGGCAGTGACTTTCCCCCAGGGGCTGGGGTGCTGGATGTCATGTATGAG
ENST0000389046         ATGACACACTTGGGCAGTGACTTTCCCCCAGGGGCTGGGGTGCTGGATGTCATGTATGAG
ENSMUST00000046869     ATCACACATCTGGGCAGTGACTTTCCCCCAGGGGCTGGGGTGCTAGATGTCATGTATGAG
ENSRNOT00000023299     ATCACACATCTGGGCAGTGACTTCCCCCCAGGGGCTGGGGTGCTGGATGTCATGTATGAG

ENSCJAT00000059963     TCCCCTTTCACACTGCTGTCCTGTGGCTATGACACCTACGTTCGCTACTGGGACCTCCGC
ENSMMUT00000009449     TCCCCTTTCACACTGCTGTCCTGTGGCTATGACACCTATGTTCGCTACTGGGACCTCCGC
ENSPPYT00000003110     TCCCCTTTCACACTGCTGTCCTGTGGCTATGACACCTATGTTCGCTACTGGGACCTCCGC
ENSGGOT00000022150     TCCCCTTTCACACTGCTGTCCTGTGGCTATGACACCTATGTTCGCTACTGGGACCTCCGC
ENST0000389046         TCCCCTTTCACACTGCTGTCCTGTGGCTATGACACCTATGTTCGCTACTGGGACCTCCGC
ENSMUST00000046869     TCCCCCTCTACACTGCTGTCCTGTGGTTATGACACCTATGTTCGCTATTGGGACCTCCGC
ENSRNOT00000023299     TCCCCCTCTACACTACTGTCCTGTGGTTATGATACCTATGTCCGCTACTGGGACCTCCGC

ENSCJAT00000059963     ACCAGTGTCCGGAAATGTGTCATGGAGTGGGAGGAGCCCCACGACAGCACCCTGTACTGC
ENSMMUT00000009449     ACCAGTGTCCGGAAATGTGTCATGGAGTGGGAGGAGCCCCATGACAGCACCCTGTACTGC
ENSPPYT00000003110     ACCAGTGTCCGGAAATGTGTTATGGAGTGGGAGGAGCCCCACGATAGCACCCTGTACTGC
ENSGGOT00000022150     ACCAGTGTCCGGAAATGTGTCATGGAGTGGGAGGAGCCCCACGACAGCACCCTGTACTGC
ENST0000389046         ACCAGCGTCCGGAAATGTGTCATGGAGTGGGAGGAGCCCCACGACAGCACCCTGTACTGC
ENSMUST00000046869     ACAAGTACACGGAAATGTGTCATGGAGTGGGAGGAGCCTCATGACAGTACCTTCTACTGC
ENSRNOT00000023299     ACAAGTGCCCGGAAATGTGTCATGGAGTGGGAGGAGCCTCATGACAGTACCTTCTACTGC

ENSCJAT00000059963     CTGCAGACAGATGGCAACCACCTGCTGGCCACAGGTTCCTCCTACTATGGTGTTGTACGA
ENSMMUT00000009449     CTGCAGACAGATGGCAACCACCTGCTGGCCACAGGTTCCTCCTACTACGGTGTTGTACGG
ENSPPYT00000003110     CTGCAGACAGATGGCAACCACCTGCTGGCCACAGGTTCCTCCTACTACGGTGTTGTACGG
ENSGGOT00000022150     CTGCAGACAGATGGCAACCACCTGCTGGCCACAGGTTCCTCCTACTACGGTGTTGTACGG
ENST0000389046         CTGCAGACAGATGGCAACCACCTGCTGGCCACAGGTTCCTCCTACTACGGTGTTGTACGG
ENSMUST00000046869     CTACAGACAGACGGCAACCACCTGCTGGCCACAGGCTCCTCCTACTACGGTCTCGTGCGG
ENSRNOT00000023299     CTACAGACAGACGGCAACCACCTGCTGGCCACAGGGTCCTCCTACTACGGTCTCGTGCGG
```

```
ENSCJAT00000059963     CTGTGGGACCGGCGCCAAAGGGCCTGCCTGCACGCCTTCCCGCTGACGTCAACCCCCCTC
ENSMMUT00000009449     CTGTGGGACCGGCGCCAAAGGGCTTGCCTGCATGCCTTCCCGCTGACGTCGACCCCCCTC
ENSPPYT00000003110     CTGTGGGACCGGCGCCAAAGGGCCTGCCTGCACGCCTTCCCGCTGACGTCGACTCCCCTC
ENSGGOT00000022150     CTGTGGGACCGGCGTCAAAGGGCCTGCCTGCACGCCTTCCCGCTGACGTCGACTCCCCTC
ENST00000389046        CTGTGGGACCGGCGTCAAAGGGCCTGCCTGCACGCCTTCCCGCTGACGTCGACTCCCCTC
ENSMUST00000046869     CTGTGGGACCGACGCCAGCGGGCTTGCCTGCATGCCTTCTCATTGACGTCGACCCCACTC
ENSRNOT00000023299     CTCTGGGACCGACGCCAGCGGGCTTGCCTGCATGCCTTCCCGTTGACATCGACCCCACTC

ENSCJAT00000059963     AGCAGCCCTGTGTACTGCCTGCGTCTCACCACCAAGCATCTCTATGCTGCACTGTCTTAC
ENSMMUT00000009449     AGCAGCCCTGTGTACTGCCTGCGTCTCACCACCAAGCATCTCTATGCTGCGCTGTCTTAC
ENSPPYT00000003110     AGCAGCCCTGTGTACTGCCTGCGTCTCACCACCAAGCATCTCTATGCTGCACTGTCTTAC
ENSGGOT00000022150     AGCAGCCCTGTGTACTGCCTGCGTCTCACCACCAAGCATCTCTATGCTGCACTGTCTTAC
ENST00000389046        AGCAGCCCTGTGTACTGCCTGCGTCTCACCACCAAGCATCTCTATGCTGCCCTGTCTTAC
ENSMUST00000046869     AGCAGCCCTGTGTACTGCCTGCGGTTCACCACTAGGCATCTCTATGCTGCGCTGTCTTAC
ENSRNOT00000023299     AGCAGTCCTGTGTACTGCCTGCGGTTCACCACCAGGCATCTCTATGCCGCGCTGTCTTAC

ENSCJAT00000059963     AACCTCCATGTCCTGGATTTTCAAAACCCA
ENSMMUT00000009449     AACCTCCATGTCCTGGATTTTCAAAACCCA
ENSPPYT00000003110     AACCTCCACGTCCTGGATTTTCAAAACCCA
ENSGGOT00000022150     AACCTCCACGTCCTGGATTTTCAAAACCCA
ENST00000389046        AACCTCCACGTCCTGGATTTTCAAAACCCA
ENSMUST00000046869     AACCTCCACGTCTTAGACTTTCAGAACCCA
ENSRNOT00000023299     AACCTCCACGTCTTAGACTTTGAAAACCCA
```

Multiple sequence alignment of Fbxw5

```
ENSCJAT00000025543     ATGGACGAGGGCGGTACGCCCCTGCTCCCCGACAGCCTGGTCTACCAGATCTTCCTGAGC
ENSGGOT00000004374     ATGGACGAGGGCGGCACGCCCCTGCTCCCCGACAGCCTGGTCTACCAGATCTTCCTGAGC
ENST00000325285        ATGGACGAGGGCGGCACGCCCCTGCTCCCCGACAGCCTGGTCTACCAGATCTTCCTGAGC
ENSPPYT00000023105     ATGGACGAGGGAAACACGCCCCTGCTCCCCGACAGCCTGGTCTACCAGATCTTCCTGAGC
ENSMUST00000015239     ATGGATGAGGGGGGCCTGCCCCTGCTCCCCGACAGCCTTGTCTACCAGATCTTCCTGAGT
ENSRNOT00000048494     ATGGATGAGGGGGGCATGCCCCTGCTCCCCGACAGCCTGGTCTACCAGATCTTCCTGAGC

ENSCJAT00000025543     CTGGGCCCGGCCGACGTGCTGGCCGCGGGGCTGGTGTGCCGCCAGTGGCTCGCCGTGTCG
ENSGGOT00000004374     CTGGGCCCGGCCGACGTGCTGGCCGCCGGGCTGGTGTGCCGCCAATGGCAGGCCGTGTCG
ENST00000325285        CTGGGCCCGGCCGACGTGCTGGCCGCCGGGCTGGTGTGCCGCCAATGGCAGGCCGTGTCG
ENSPPYT00000023105     CTGGGGCCGACCGACGTGCTGGCCGCCGGGCTGGTGTGCCGCCAATGGCAGGCCGTGTCG
ENSMUST00000015239     TTGGGCCCTGCAGATGTGCTGGCTGCTGGGCTGGTATGCCGCCAATGGCAGGCTGTGTCC
ENSRNOT00000048494     TTGGGCCCTGCAGATGTGCTGGCTGCTGGGCTGGTGTGCCGCCAGTGGCAGGCTGTCTCC

ENSCJAT00000025543     CGGGATGAGTTCCTGTGGAGGGAGCAGTTCTACCGCTACTACCAGGTGGCCCGAGACGTG
ENSGGOT00000004374     CGGGACGAGTTCCTGTGGAGGGAGCAGTTCTACCGCTACTACCAGGTGGCCCGCGACGTG
ENST00000325285        CGGGACGAGTTCCTGTGGAGGGAGCAGTTCTACCGCTACTACCAGGTGGCCCGCGACGTG
ENSPPYT00000023105     CGGGACGAGTTCCTGTGGAGGGAGCAGTTCTACCGCTACTACCGGGTGGCCCGCGACTTG
ENSMUST00000015239     CGGGATGAGTTCTTATGGAAGGAGCAATTCTACCGCTACTACCAGGTGGCTCGAGATGTG
ENSRNOT00000048494     CGGGACGAGTTCCTATGGAGGGAGCAGTTCTACCGCTACTACCAGGTGGCTCGCGATGTG

ENSCJAT00000025543     CCCCGACACCCAGCGGCCACGTCCTGGTTAGAGGAGTTCCAGCGGCTGTATGACATGGTG
ENSGGOT00000004374     CCCCGACACCCAGCGGCCATGTCCTGGTACGAGGAGTTCCAGCGGCTGTATGACACGGTG
ENST00000325285        CCCCGACACCCAGCGGCCATGTCCTGGTACGAGGAGTTCCAGCGGCTGTATGACACGGTG
ENSPPYT00000023105     CCCCGACACCCAGCGGCCACGTCCTGGTACGAGGAGTTCCAGCGGCTGTATGACACGGTG
ENSMUST00000015239     CCCCGCCACCCAGCGGCCACATCCTGGTATGAGGAGTTCCGGCGGCTCTATGACATGGTA
ENSRNOT00000048494     CCCCGCCACCCAGCGGCCACATCCTGGTATGAGGAGTTCCGGCGGCTCTATGACATGGTG
```

```
ENSCJAT00000025543     CCCTGTGTGGAGGTGCAGACGCTACGGGAGCACACGGATCAGGTCTTGCACCTCAGTTTC
ENSGGOT00000004374     CCCTGCGTGGAGGTGCAGACGCTGCGGGAACACACAGACCAGGTCCTGCACCTCAGCTTC
ENST00000325285        CCCTGCGTGGAGGTGCAGACGCTGCGGGAACACACAGACCAGGTCCTGCACCTCAGCTTC
ENSPPYT00000023105     CCCTGCGTGGAGGTGCAGACACTGCGGGAACACACAGACCAGGTCCTGCACCTCAGCTTC
ENSMUST00000015239     CCCTGTGTGGAGGTGCAGACACTGAAGGAGCACACTGACCAGGTCCTGCACCTCAGCTTC
ENSRNOT00000048494     CCCTGTGTGGAGGTGCAGACACTGAAGGAGCACACCGACCAGGTCCTGCACCTCAGCTTC

ENSCJAT00000025543     TCCCACTCGGGCTACCAGTTCGCGTCCTGCTCGAAGGACTGCACTGTGAAGATTTGGAGC
ENSGGOT00000004374     TCCCATTCCGGGTACCAGTTCGCGTCCTGCTCCAAGGACTGCACTGTGAAGATCTGGAGC
ENST00000325285        TCCCATTCCGGGTACCAGTTCGCGTCCTGCTCCAAGGACTGCACTGTGAAGATCTGGAGC
ENSPPYT00000023105     TCCCATTCCGGGTACCAGTTCGCGTCCTGCTCCAAGGACTGCACTGTGAAGATCTGGAGC
ENSMUST00000015239     TCCCACTCGGGGTACCAGTTTGCCTCCTGCTCCAAGGACTGCACGGTGAAGATCTGGAAC
ENSRNOT00000048494     TCCCACTCGGGGTACCAGTTTGCCTCCTGCTCCAAGGACTGCACAGTGAAGATCTGGAAC

ENSCJAT00000025543     AACGACCTGACCGTCTCGCTGCTGCACAGCGCAGACATGCGGCCATACAACTGGAGCTAC
ENSGGOT00000004374     AACGACCTGACCATCTCGCTGCTGCACAGCGCGGACATGCGGCCCTACAACTGGAGCTAC
ENST00000325285        AACGACCTGACCATCTCGCTGCTGCACAGCGCGGACATGCGGCCCTACAACTGGAGCTAC
ENSPPYT00000023105     AACGACCTGACCATCTCACTGCTGCACAGCGCAGACATGCGGCCCTACAACTGGAGCTAC
ENSMUST00000015239     AACGACCTGACCATCTCACTGCTGCACAGTGCAGATATGAGGCCATACAACTGGAGTTAC
ENSRNOT00000048494     AATGACCTGACCATCTCTCTGCTGCACAGTGCAGATATGAGGCCATACAACTGGAGTTAC

ENSCJAT00000025543     ACGCAGTTCTCGCAGTTCAACCAGGACGACTCACTGCTGCTGGCCTCAGGGGTGTTCCTG
ENSGGOT00000004374     ACCCAGTTCTCCCAGTTCAACAAGGACGACTCGCTACTGCTGGCCTCGGGGGTGTTCCTG
ENST00000325285        ACCCAGTTCTCCCAGTTCAACAAGGACGACTCGCTACTGCTGGCCTCGGGGGTGTTCCTG
ENSPPYT00000023105     ACCCAGTTCTCCCAGTTCAACAAGGACGACTCGCTGCTGCTGGCCTCGGGGGTGTTCCTG
ENSMUST00000015239     ACCCAGTTTTCCCAGTTCAACCAGGATGACTCACTGCTGCTGGCCTCAGGGGTGTTCCTG
ENSRNOT00000048494     ACCCAGTTCTCCCAGTTCAACCAGGATGACTCACTGTTGCTGGCCTCGGGGGTGTTCCTA

ENSCJAT00000025543     GGGCCACACAACTCCTCCTCTGGCGAGATCGCGGTCATCAGCCTAGACTCCTTTGCACTG
ENSGGOT00000004374     GGGCCGCACAACTCCTCATCCGGTGAGATCGCTGTCATCAGCCTAGACTCCTTCGCGCTG
ENST00000325285        GGGCCGCACAACTCCTCATCCGGCGAGATTGCTGTCATCAGCCTAGACTCCTTCGCGCTG
ENSPPYT00000023105     GGGCCGCACAACTCCTCATCCGGCGAGATTGCTGTCATCAGCCTAGACTCCTTCGCGCTG
ENSMUST00000015239     GGGCCACATAACTCCTCCTCAGGCGAGATAGCTGTCATCAGCTTAGACTCCTTCGCCCTG
ENSRNOT00000048494     GGGCCACATAACTCCTCCTCAGGCGAGATAGCTGTCATCAGCTTAGACTCCTTCGCCCTG

ENSCJAT00000025543     CTGTCCCGCGTGCGGAATAAGCCCTACGACGTGTTTGGCTGCTGGCTGACCGAGACCAGC
ENSGGOT00000004374     CTGTCCCGCGTGCGGAACAAGCCCTACGACGTGTTCGGCTGTTGGCTCACCGAGACCAGC
ENST00000325285        CTGTCCCGCGTGCGGAACAAGCCCTATGACGTGTTTGGCTGTTGGCTCACCGAGACCAGC
ENSPPYT00000023105     CTGTCCCGCGTGCGGAACAAGCCCTACGACGTGTTTGGCTGTTGGCTCACTGAGACCAGC
ENSMUST00000015239     CTGTCCCGTGTGCGAAACAAGCCCTATGATGTGTTTGGCTGCTGGCTCACAGAAACCAGC
ENSRNOT00000048494     CTGTCGCGTGTGCGAAACAAGCCCTATGATGTGTTTGGCTGCTGGCTCACAGAAACCAGC

ENSCJAT00000025543     CTCATCTCAGGGAACCTGCACCGCATCGGAGACATCACCTCCTGCTCTGTCCTGTGGCTC
ENSGGOT00000004374     CTCATCTCGGGGAACCTGCACCGCATCGGAGATATCACCTCCTGCTCGGTGCTGTGGCTC
ENST00000325285        CTCATCTCGGGGAACCTGCACCGCATCGGAGATATCACCTCCTGCTCGGTGCTGTGGCTC
ENSPPYT00000023105     CTCATCTCGGGGAACCTGCACCGCATCGGAGATATCACCTCCTGCTCGGTGCTGTGGCTC
ENSMUST00000015239     CTCATTTCGGGGAACCTGCACCGCATTGGAGATATCACCTCCTGCTCAGTGCTGTGGCTC
ENSRNOT00000048494     CTCATCTCGGGGAACCTGCACCGCATTGGAGATGTCACCTCCTGCTCAGTGCTGTGGCTC

ENSCJAT00000025543     AACAACGCCTTCCAGGACGTGGAGTCAGAGAACGTCAACGTGGTGAAGCGGCTGTTCAAG
ENSGGOT00000004374     AACAATGCCTTCCAGGACGTGGAGTCAGAGAACGTCAACGTGGTGAAGCGGCTGTTCAAG
```

```
ENST00000325285        AACAATGCCTTCCAGGATGTGGAGTCAGAGAACGTCAACGTGGTGAAGCGGCTGTTCAAG
ENSPPYT00000023105     AACAACGCCTTCCAGGACGTGGAGTCAGAAAACGTCAATGTGGTGAAGCGGCTGTTCAAG
ENSMUST00000015239     AACAATGCCTTCCAGGATGTGGAGTCAGAGAATGTCAACGTGGTAAAGCGGCTCTTTAAG
ENSRNOT00000048494     AACAATGCCTTCCAGGATGTGGAGTCAGAGAACGTCAACGTGGTAAAGCGGCTCTTTAAG

ENSCJAT00000025543     ATCCAGAACCTCAACGCCAGCACCATCCGCACTGTGATGGTGGCCGACTGCAGCCGCTTC
ENSGGOT00000004374     ATCCAGAACCTCAACGCCAGCACCGTCCGCACGGTGATGGTGGCCGACTGCAGCCGCTTC
ENST00000325285        ATCCAGAACCTCAATGCCAGCACCGTCCGCACGGTGATGGTGGCCGACTGCAGCCGCTTC
ENSPPYT00000023105     ATCCAGAACCTCAACGCCAGCACCATCCGCACGGTGATGGTGGCCGACTGCAGCCGTTTT
ENSMUST00000015239     ATCCAGAACCTCAATGCCAGCACCATCCGCACAGTGATGGTGGCTGACTGTAGCCGCTTT
ENSRNOT00000048494     ATCCAGAACCTCAATGCCAGCACCATCCGCACCGTGATGGTGGCTGACTGCAGCCGCTTT

ENSCJAT00000025543     GACAGCCCTGACCTCCTGCTGGACGCTGGCGACCCAGGCACATCCCCCTGCCGTGTCTTT
ENSGGOT00000004374     GACAGCCCTGACCTGCTGCTGGAAGCCGGCGACCCGGCCACGTCCCCCTGCCGCATCTTT
ENST00000325285        GACAGCCCTGACCTGCTGCTGGAAGCCGGTGACCCGGCCACGTCCCCCTGCCGCATCTTT
ENSPPYT00000023105     GACAGCCCTGACCTCCTGCTGGAAGCCGGCGACCCGGCCACGTCCCCCTGCCGCATCTTT
ENSMUST00000015239     GATAGCCCGGACCTCCTGCTGGATGCTAGCGACCAGGCTGGGCTCCCCTGCCGAGTCTTT
ENSRNOT00000048494     GATAGCCCGGACCTCCTGCTGGATGCTGGTGACCAGGCTGGGCTCCCCTGCCGAGTCTTT

ENSCJAT00000025543     GACCTGGGCAGCAACAACGAGGAGGAGGTGGCCAGCCCAGCCCCTGCCCACGCCAAGGAG
ENSGGOT00000004374     GACCTGGGCAGCGACAACGAGGAGGTGGTGGCTGGCCCGGCCCCCGCCCACGCCAAGGAG
ENST00000325285        GACCTGGGCAGCGACAACGAGGAGGTGGTGGCTGGCCCGGCCCCCGCCCACGCCAAGGAG
ENSPPYT00000023105     GACCTGGGCAGCGACAACGAGGAGGTGGTGGCTGGCCCGGCCCCCGCCCATGCCAGTGAG
ENSMUST00000015239     GACCTAGGTGGGGACACTGAAGAAGAGGCCACTGACCCAGGTTCTGACCACGTCAAGAAA
ENSRNOT00000048494     GACCTAGGTGGGGACACTGAAGAAGAGGCCACTGACCCAGGTTCTGGCCATGTCAAGGAA

ENSCJAT00000025543     GGCTTGCGGCACTTTCTGGACAGCGTGTTGGAGGGGCGGGCGCAGCTGTCAGACTGCGTG
ENSGGOT00000004374     GGCTTGCGGCACTTTCTGGACCGCGTGCTGGAGGGGCGGGCGCAGCTGTCGGAGCGCGTG
ENST00000325285        GGCTTGCGGCACTTTCTGGACCGCGTGCTGGAGGGGCGGGCGCAGCTGTCGGAGCGCATG
ENSPPYT00000023105     GGCTTCCGGCACTTTCTGGACCGTGTGCTGGAGGGGCGGGCACAGCTGTCGGAGCGCGTG
ENSMUST00000015239     GGTTTACGGCGTGTGTTTGATAGCGTCTTGGATGGACACGGACAGCTATCGGATTGTGCA
ENSRNOT00000048494     GGTTTACGGCGTGTGTTTGATAGCGTCCTGGATGGACATGGACAGCTGTCAGATTGTGCA

ENSCJAT00000025543     CTGGAGACCAAGGTGGCCGAGCTGCTGGCCCAGGGCCACACCAAGCCCCCTGAGCACAGC
ENSGGOT00000004374     CTAGAGACCAAGGTGGCCGAGCTGCTGGCCCAGGGCCACACCAAGCCACCTGAGCGCAGT
ENST00000325285        CTAGAGACCAAGGTGGCCGAGCTGCTGGCCCAGGGCCACACCAAGCCACCCGAGCGCAGT
ENSPPYT00000023105     CTAGAGACCAAGGTGGCCGAGCTGCTGGCCCAGGGCCACACCAAGCCCCCCGAGCGCAGT
ENSMUST00000015239     CTGGAGACCAAGGTGGCTGAGCTGCTGGCCCAGGGCCACACCAAGCCTCCAGAGTGCAAT
ENSRNOT00000048494     CTGGAGACCAAGGTGGCTGAGCTGCTGGCCCAGGGCCACACCAAGCCCCCAGAGTGTCCT

ENSCJAT00000025543     GACACAGGCACCAGGAGCAAGTACCTCATCTTCACCACTGGCTGCCTCACCTACTCCCCA
ENSGGOT00000004374     GCCACAGGCGCCAGGAGCAAGTACCTCATCTTCACCACTGGCTGCCTCACCTACTCCCCA
ENST00000325285        GCCACAGGCGCCAAGAGCAAGTACCTCATCTTCACCACTGGCTGCCTCACCTACTCCCCA
ENSPPYT00000023105     GCCACAGGCGCCAGGAGCAAGTACCTCATCTTCACCACTGGCTGCCTCACCTACTCCCCA
ENSMUST00000015239     GACGCTGACACCAGGAATAAGTACCTCATCTTCACCACTGGCTGCCTCACGTACTCACCG
ENSRNOT00000048494     GACGCTGACAGCAGGAACAAGTACCTCATCTTCACCACTGGCTGCCTCACGTACTCACCT

ENSCJAT00000025543     CACCAGATTGGCATTAAGCAGATCCTGCCACACCAGATGACCACGGCGGGGCCCGTGCTG
ENSGGOT00000004374     CACCAGATCGGCATCAAGCAGATCCTGCCACACCAGATGACCACGGCAGGGCCCGTGCTG
ENST00000325285        CACCAGATCGGCATCAAGCAGATCCTGCCACACCAGATGACCACGGCAGGGCCCGTGCTG
ENSPPYT00000023105     CACCAGATCGGCATCAAGCAGATCCTGCCACACCAGATGACCACGGCGGGGCCCGTGCTG
ENSMUST00000015239     CATCAGATCGGCATCAAGCAGATCCTGCCACACCAGATGACAACTGCAGGGCCCGTGCTG
ENSRNOT00000048494     CATCAGATCGGCATCAAGCAGATCCTGCCGCACCAGATGACAACTGCAGGGCCCGTGCTG
```

```
ENSCJAT00000025543     GGCGAGGGCCGGGGGTCGGACGCCTTCTTCGACGCGCTGGACCACGTCATAGACGTGCAC
ENSGGOT00000004374     GGTGAGGGCCGGGGCTCCGACGCCTTCTTCGACGCGCTGGACCACGTCATAGACATACAC
ENST00000325285        GGTGAGGGCCGGGGCTCCGATGCCTTCTTCGACGCGCTGGACCACGTCATAGACATACAC
ENSPPYT00000023105     GGTGAGGGCCGGGGCTCCGACGCCTTCTTCGATGCGCTGGACCACGTCATAGACGTGCAC
ENSMUST00000015239     GGCGAGGGCCGGGGCTCTGATGCCTTCTTTGATGCCCTGGACCATGTCATTGATGTGCAT
ENSRNOT00000048494     GGCGAGGGCCGGGGCTCTGATGCCTTCTTTGATGCCCTGGACCATGTCATTGATGTGCAT

ENSCJAT00000025543     GGCCACATCATTGGCATGGGCCTGTCACCCGACAACAGGTACCTGTATGTGAACAGCCGC
ENSGGOT00000004374     GGACACATCATCGGCATGGGCCTGTCGCCCGACAACAGGTACCTGTACGTGAACAGCCGT
ENST00000325285        GGACACATCATCGGCATGGGCCTGTCGCCCGACAACAGGTACCTGTACGTGAACAGCCGC
ENSPPYT00000023105     GGGCACATCATTGGCATGGGCCTGTCGCCCGACAACAGGTACCTGTACGTGAACAGCCGC
ENSMUST00000015239     GGGCACATCATTGGCATGGGCCTGTCTCCTGATAACAGGTACCTGTATGTGAATAGCCGT
ENSRNOT00000048494     GGGCACATCATTGGCATGGGCCTGTCTCCCGATAACAGGTACCTGTATGTGAATAGCCGT

ENSCJAT00000025543     GCCTGGCCCAGTGGTGCGGTGGTGGCTGACCCCATGCAGCCGCCGCCCATCGCGGAGGAG
ENSGGOT00000004374     GCGTGGCCCAACGGTGCGGTGGTGGCCGACCCCATGCAGCCGCCACCAATCGCGGAGGAG
ENST00000325285        GCCTGGCCCAACGGTGCGGTGGTGGCCGACCCCATGCAGCCGCCACCAATCGCGGAGGAG
ENSPPYT00000023105     GCCTGGCCCAGTGGTGCGGTGGTGGCCGACCCCATGCAGCCGCCACCCATTGCAGAGGAG
ENSMUST00000015239     GCCTGGCCCCCTGGCTCAGTGGTAGCCGACCCCATGCAGCCACCACCCATTGCGGAGGAG
ENSRNOT00000048494     GCCTGGCCCCCTGGCTCAGTGGTCGCCGACCCCATGCAGCCACCACCCATTGCAGAGGAG

ENSCJAT00000025543     ATTGACCTGCTGGTGTTCGACCTGAAGACCATGCGGGAGGTGAGGCGGGCGCTACGTGCG
ENSGGOT00000004374     ATTGACCTGTTGGTGTTCGACCTCAAGACCATGCGGGAGGTGAGGCGGGCTCTGCGTGCA
ENST00000325285        ATTGACCTGCTGGTGTTCGACCTCAAGACCATGCGGGAGGTGAGGCGGGCTCTGCGTGCG
ENSPPYT00000023105     ---GGCCTGCTGGTGTTCGACCTCAAG---ATGCGGGAGGTGAGGCGGGCTCTGCGTGCG
ENSMUST00000015239     ATTGACTTGCTGGTGTTTGATCTCAAGACCATGCGAGAGGTGAAGCGAGCTCTTCGAGCG
ENSRNOT00000048494     ATTGACTTGCTGGTGTTTGATCTGAAGACCATGCGGGAGGTGAAGCGAGCTCTGCGAGCA

ENSCJAT00000025543     CACCGCGCCTACACACCCAACGACGAGTGCTTCTTCATCTTCCTGGATGTCAGCAGGGAC
ENSGGOT00000004374     CACCGCGCCTACACGCCCAACGACGAGTGCTTCTTCATCTTCCTGGACGTCAGCAGGGAC
ENST00000325285        CACCGCGCCTACACGCCCAACGACGAGTGCTTCTTCATCTTCCTGGACGTCAGCAGGGAC
ENSPPYT00000023105     CACCGCGCCTACACGCCCAACGACGAGTGCTTCTTCATCTTCCTGGACGTCAGCAGGGAC
ENSMUST00000015239     CACCGTGCCTACACACCCAATGACGAGTGCTTCTTCATCTTCTTGGATGTCAGCAGGGAT
ENSRNOT00000048494     CACCGTGCCTACACACCCAATGATGAGTGCTTCTTCATCTTTCTGGATGTCAGCAGGGAT

ENSCJAT00000025543     TTTGTGGCCAGCGGGGCGGAGGACCGGCACGGCTACATCTGGGACCGCCACTACAACATC
ENSGGOT00000004374     TTCGTGGCCAGCGGGGCGGAGGACCGGCATGGCTACATCTGGGACCGCCACTACAACATC
ENST00000325285        TTCGTGGCCAGCGGGGCGGAGGACCGGCACGGCTACATCTGGGACCGCCACTACAACATC
ENSPPYT00000023105     TTCGTGGCCAGCGGGGCCGAGGACCGGCATGGCTACATCTGGGACCGCCACTACAACATC
ENSMUST00000015239     TTTGTGGCCAGTGGGGCTGAAGATCGGCATGGCTATATCTGGGACCGCCACTACAACATC
ENSRNOT00000048494     TTTGTGGCCAGTGGGGCTGAAGATCGGCATGGCTATATCTGGGACCGCCACTACAACATC

ENSCJAT00000025543     TGCCTGGCCAAGCTGCGGCACGATGATGTGGTCAACTCAGTGGTTTTCAGTCCCCAGGAG
ENSGGOT00000004374     TGCCTGGCCAGGCTGCGGCACGAGGATGTGGTCAACTCAGTGGTCTTCAGTCCCCAGGAG
ENST00000325285        TGTCTGGCCAGGCTGCGGCACGAGGATGTGGTCAACTCAGTGGTCTTCAGTCCCCAGGAG
ENSPPYT00000023105     TGCCTGGCCAAGCTGCGGCACGAGGACGTGGTCAACTCAGTGGTCTTCAGTCCCCAGGAG
ENSMUST00000015239     TGCCTAGCCAAGCTGCGGCATGAGGATGTGGTCAACTCCGTGGCTTTTAGCCCCCAGGAG
ENSRNOT00000048494     TGCCTAGCCAAGCTGCGGCATGAGGATGTGGTCAACTCAGTGGCCTTT------------

ENSCJAT00000025543     CAGGAGCTGCTGCTTACGGCCAGCGACGACGCCACCATCAAAGCCTGGCGCTCACCACGC
ENSGGOT00000004374     CAGGAGCTGCTGCTCACGGCCAGCGACGACGCCACCATCAAAGCCTGGCGCTCCCCACGC
```

```
ENST00000325285        CAGGAGCTGCTGCTCACGGCCAGCGACGACGCCACCATCAAAGCCTGGCGCTCCCCACGC
ENSPPYT00000023105     CAGGAGCTGCTGCTCACAGCCAGCGACGACGCCACCATCAAAGCCTGGCGCTCCCCACGC
ENSMUST00000015239     CAGGAACTCTTGCTGACAGCCAGCGACGATGCCACTATCAAAGCCTGGCGTTCACCACGC
ENSRNOT00000048494     ------------------------------------------------------------

ENSCJAT00000025543     ACTGTGCGCATCCTCCAGGCGCCTCGCCCTCGGCCTCGCGCCTTCTTCTGGCTTGCCAGC
ENSGGOT00000004374     ACCGTGCGCATCCTTCAGGCACCTCGCCCACGGCCTCGCACCTTCTTCTGGCTTGCCAGC
ENST00000325285        ACCATGCGCGTCCTCCAGGCACCTCGCCCACGGCCTCGCACCTTCTTCTGGCTTGCCAGC
ENSPPYT00000023105     ACCGTGCGCGTCCTCCAGGCACCTCGCCCACGGCCTCGCGCCTTCTTCTGGCTTGCCAGC
ENSMUST00000015239     ATTGTTCGTGTTCTGCAGGCTCCCCGCCCTCGCCCCCGCCCCTTCTTCTGGTTTGCCAGC
ENSRNOT00000048494     ------------------------------------------------------------

ENSCJAT00000025543     CAGAGACGC
ENSGGOT00000004374     CAGAGGCGC
ENST00000325285        CAGAGGCGC
ENSPPYT00000023105     CAGAGGCGC
ENSMUST00000015239     CATAGGCGC
ENSRNOT00000048494     ---------
```

Multiple sequence alignment of Fbxw7

```
ENSGGOT00000022707     ATGTGTGTCCCGAGAAGCGGTTTGATACTGAGCTGCATTTGCCTTTACTGTGGAGTTTTG
ENSCJAT00000004056     ATGTGTGTCCCGAGAAGCGGTTTGATACTGAGCTGCATTTGCCTTTACTGTGGAGTTTTG
ENST00000263981        ATGTGTGTCCCGAGAAGCGGTTTGATACTGAGCTGCATTTGCCTTTACTGTGGAGTTTTG
ENSPPYT00000017582     ATGTGTGTCCCGAGAAGCGGTTTGATACTGAGCTGCATTTGCCTTTACTGTGGAGTTTTG
ENSMMUT00000028596     ATGTGTGTCCCGAGAAGCAGTTTGATACTGAGCTGCATTTGCCTTTACTGTGGAGTTTTG
ENSMUST00000029727     GTGTGCGTCCCGAGCAGCGTTCTGGTTCTGAGCTGCGTCTGCTGGTGCTGGGGAGTTTTG
ENSRNOT00000015279     GTGTGCGTCCTGAGCAGCGTTCTGGTTCTGAGCTGCGTCTGCTGGTGCTGGGGAGTTTTG

ENSGGOT00000022707     TTGCCGGTTCTGCTCCCTAATCTTCCTTTTCTGACGTGCCTGAGCATGTCCACATTAGAA
ENSCJAT00000004056     TTGCCGGTTCTGCTCCCTAATCTTCCTTTTCTGACGTGCCTGAGCATGTCCACATTAGAA
ENST00000263981        TTGCCGGTTCTGCTCCCTAATCTTCCTTTTCTGACGTGCCTGAGCATGTCCACATTAGAA
ENSPPYT00000017582     TTGCCGGTTCTGCTCCCTAATCTTCCTTTTCTGACGTGCCTGAGCATGTCCACATTAGAA
ENSMMUT00000028596     TTGCCGGTTCTGCTCCCTAATCTTCCTTTTCTGACGTGCCTGAGCATGTCCACATTAGAA
ENSMUST00000029727     CTGCCGGTTCCGCTGCCTAATCTTCCTTTTCTGGCGTGCCTGAGCATGTCCACGTTAGAA
ENSRNOT00000015279     CTGCCGGTTCCGCTGCCTAATCTTCCTTTTCTGGCGTGCCTGAGCATGTCCACGTTAGAA

ENSGGOT00000022707     TCTGTGACATACCTACCTGAAAAAGGTTTATATTGTCAGAGACTGCCAAGCAGCCGGACA
ENSCJAT00000004056     TCTGTGACATACCTACCTGAAAAAGGTTTATATTGTCAGAGACTGCCAAGCAGCCGGACA
ENST00000263981        TCTGTGACATACCTACCTGAAAAAGGTTTATATTGTCAGAGACTGCCAAGCAGCCGGACA
ENSPPYT00000017582     TCTGTGACATACCTACCTGAAAAAGGTTTATATTGTCAGAGACTGCCAAGCAGCCGGACA
ENSMMUT00000028596     TCTGTGACATACCTACCTGAAAAAGGTTTATATTGTCAGAGACTGCCAAGCAGCCGGACA
ENSMUST00000029727     TCTGTGACATACCTACCTGAAAAGGGGTTATATTGTCAGAGACTGCCAAGCAGCCGGACA
ENSRNOT00000015279     TCTGTGACATACCTACCTGAAAAGGGGTTATATTGTCAGAGACTGCCAAGCAGCCGGACA

ENSGGOT00000022707     CACGGGGGCACAGAATCACTGAAGGGGAAAAATACAGAAAATATGGGTTTCTACGGCACA
ENSCJAT00000004056     CACGGGGGCACAGAATCACTGAAGGGGAAAAATACAGAAAATATGGGTTTCTACGGCACA
ENST00000263981        CACGGGGGCACAGAATCACTGAAGGGGAAAAATACAGAAAATATGGGTTTCTACGGCACA
ENSPPYT00000017582     CACGGGGGCACAGAATCACTGAAGGGGAAAAATACAGAAAATATGGGTTTCTACGGCACA
ENSMMUT00000028596     CACGGGGGCACAGAATCACTGAAGGGGAAAAATACAGAAAATATGGGTTTCTACGGCACA
ENSMUST00000029727     CACGGGGGCACAGAATCCCTGAAGGGGAAAAATACAGAAAATATGGGTTTCTACGGCACA
ENSRNOT00000015279     CACGGGGGCACAGAATCCCTGAAGGGGAAAAATACAGAAAATATGGGTTTCTACGGCACA
```

```
ENSGGOT00000022707    TTAAAAATGATTTTTTACAAAATGAAAAGAAAGTTGGACCATGGTTCTGAGGTCCGCTCT
ENSCJAT00000004056    TTAAAAATGATTTTTTACAAAATGAAAAGAAAGTTGGACCATGGTTCTGAGGTCCGCTCT
ENST00000263981       TTAAAAATGATTTTTTACAAAATGAAAAGAAAGTTGGACCATGGTTCTGAGGTCCGCTCT
ENSPPYT00000017582    TTAAAAATGATTTTTTACAAAATGAAAAGAAAGTTGGACCATGGTTCTGAGGTCCGCTCT
ENSMMUT00000028596    TTAAAAATGATTTTTTACAAAATGAAAAGAAAGTTGGACCATGGTTCTGAGGTCCGCTCT
ENSMUST00000029727    TTAAAAATGATTTTTTACAAAATGAAAAGAAAGTTGGACCATGGTTCTGAAGTTCGTTCC
ENSRNOT00000015279    TTAAAAATGATTTTTTACAAAATGAAAAGAAAGTTGGACCATGGCTCTGAGGTCCGTTCC

ENSGGOT00000022707    TTTTCTTTGGGAAAGAAACCATGCAAAGTCTCAGAATATACAAGTACCACTGGGCTTGTA
ENSCJAT00000004056    TTTTCTTTGGGAAAGAAACCATGCAAAGTCTCAGAATATACAAGTACCACTGGGCTTGTA
ENST00000263981       TTTTCTTTGGGAAAGAAACCATGCAAAGTCTCAGAATATACAAGTACCACTGGGCTTGTA
ENSPPYT00000017582    TTTTCTTTGGGAAAGAAACCATGCAAAGTCTCAGAATATACAAGTACCACTGGGCTTGTA
ENSMMUT00000028596    TTTTCTTTGGGAAAGAAACCATGCAAAGTCTCAGAATATACAAGTACCACTGGGCTTGTA
ENSMUST00000029727    TTTTCTTTGGGAAAGAAACCATGCAAAGTCTCAGATTATACCAGTACCACTGGCCTTGTA
ENSRNOT00000015279    TTTTCTTTGGGAAAGAAACCATGCAAAGTCTCAGATTATACCAGTACCACTGGGCTTGTA

ENSGGOT00000022707    CCATGTTCAGCAACACCAACAACTTTTGGGGACCTCAGAGCAGCCAATGGCCAAGGTCAA
ENSCJAT00000004056    CCATGTTCAGCAACACCAACAACTTTTGGGGACCTCAGAGCAGCCAATGGCCAAGGGCAA
ENST00000263981       CCATGTTCAGCAACACCAACAACTTTTGGGGACCTCAGAGCAGCCAATGGCCAAGGGCAA
ENSPPYT00000017582    CCATGTTCAGCAACACCAACAACTTTTGGGGACCTCAGAGCAGCCAATGGCCAAGGGCAA
ENSMMUT00000028596    CCATGTTCAGCAACACCAACAACTTTTGGGGACCTCAGAGCAGCCAATGGCCAAGGGCAA
ENSMUST00000029727    CCATGTTCAGCAACACCAACAACTTTTGGGGACCTGAGAGCAGCCAATGGGCAAGGGCAG
ENSRNOT00000015279    CCATGTTCAGCAACACCAACAACTTTTGGGGACCTGAGAGCAGCCAATGGGCAAGGGCAG

ENSGGOT00000022707    CAACGACGCCGAATTACATCTGTCCAGCCACCTACAGGCCTCCAGGAATGGCTAAAAATG
ENSCJAT00000004056    CAACGACGCCGAATTACATCTGTCCAGCCGCCTACAGGCCTCCAGGAATGGCTGAAAATG
ENST00000263981       CAACGACGCCGAATTACATCTGTCCAGCCACCTACAGGCCTCCAGGAATGGCTAAAAATG
ENSPPYT00000017582    CAACGACGCCGAATTACATCTGTCCAGCCACCTACAGGCCTCCAGGAATGGCTAAAAATG
ENSMMUT00000028596    CAACGACGCCGAATTACATCTGTCCAGCCACCTACAGGCCTCCAGGAATGGCTGAAAATG
ENSMUST00000029727    CAGCGGCGGAGGATTACATCTGTCCAACCACCCACAGGCCTTCAAGAGTGGCTGAAAATG
ENSRNOT00000015279    CAACGGCGGAGGATTACATCTGTGCAGCCGCCCACAGGCCTCCAAGAGTGGCTGAAAATG

ENSGGOT00000022707    TTTCAGAGCTGGAGTGGACCAGAGAAATTGCTTGCTTTAGATGAACTGATTGATAGTTGT
ENSCJAT00000004056    TTTCAGAGCTGGAGTGGACCAGAGAAATTGCTTGCTTTAGATGAACTCATTGATAGTTGT
ENST00000263981       TTTCAGAGCTGGAGTGGACCAGAGAAATTGCTTGCTTTAGATGAACTCATTGATAGTTGT
ENSPPYT00000017582    TTTCAGAGCTGGAGTGGACCAGAGAAATTGCTTGCTTTAGATGAACTCATTGATAGTTGT
ENSMMUT00000028596    TTTCAGAGCTGGAGCGGACCAGAGAAATTGCTTGCTTTAGATGAACTCATTGATAGTTGT
ENSMUST00000029727    TTTCAGAGCTGGAGCGGACCAGAGAAGTTGCTGGCTTTAGATGAGCTCATTGACAGCTGT
ENSRNOT00000015279    TTTCAGAGCTGGAGTGGACCAGAGAAGTTGCTGGCCTTAGATGAGCTCATCGATAGCTGT

ENSGGOT00000022707    GAACCAACACAAGTAAAACATATGATGCAAGTGATAGAACCCCAGTTTCAACGAGACTTC
ENSCJAT00000004056    GAACCAACACAAGTAAAACATATGATGCAAGTGATAGAACCCCAGTTTCAACGAGACTTC
ENST00000263981       GAACCAACACAAGTAAAACATATGATGCAAGTGATAGAACCCCAGTTTCAACGAGACTTC
ENSPPYT00000017582    GAACCAACACAAGTAAAACATATGATGCAAGTGATAGAACCCCAGTTTCAACGAGACTTC
ENSMMUT00000028596    GAACCAACACAAGTAAAACATATGATGCAAGTGATAGAACCCCAGTTTCAACGAGACTTC
ENSMUST00000029727    GAACCAACACAAGTGAAGCATATGATGCAAGTGATAGAGCCCCAGTTCCAGCGAGACTTC
ENSRNOT00000015279    GAACCCACACAAGTGAAGCACATGATGCAAGTGATCGAGCCCCAGTTCCAGCGAGACTTC

ENSGGOT00000022707    ATTTCATTGCTCCCTAAAGAGTTGGCACTCTATGTGCTTTCATTCCTGGAACCCAAAGAC
ENSCJAT00000004056    ATTTCATTGCTCCCTAAAGAGTTGGCACTTTATGTGCTTTCATTCCTGGAACCCAAAGAC
ENST00000263981       ATTTCATTGCTCCCTAAAGAGTTGGCACTCTATGTGCTTTCATTCCTGGAACCCAAAGAC
ENSPPYT00000017582    ATTTCATTGCTCCCTAAAGAGTTGGCACTCTATGTGCTTTCATTCCTGGAACCCAAAGAC
ENSMMUT00000028596    ATTTCATTGCTCCCTAAAGAGTTGGCACTCTATGTGCTTTCATTCCTGGAACCCAAAGAC
ENSMUST00000029727    ATCTCCTTGCTTCCTAAAGAGTTGGCACTCTATGTGCTTTCATTCCTGGAACCCAAAGAC
```

```
ENSRNOT00000015279     ATCTCCTTGCTCCCCAAAGAGCTGGCACTCTATGTACTTTCATTCCTGGAACCCAAAGAC


ENSGGOT00000022707     CTGCTACAAGCGGCTCAGACATGTCGCTACTGGAGAATTTTGGCTGAAGACAACCTTCTC
ENSCJAT00000004056     CTGCTGCAAGCGGCTCAGACGTGTCGCTACTGGAGAATTTTGGCTGAAGACAACCTTCTC
ENST00000263981        CTGCTACAAGCAGCTCAGACATGTCGCTACTGGAGAATTTTGGCTGAAGACAACCTTCTC
ENSPPYT00000017582     CTGCTACAAGCGGCTCAGACATGCCGCTACTGGAGAATTTTGGCTGAAGACAACCTTCTC
ENSMMUT00000028596     CTGCTACAAGCGGCTCAGACATGTCGCTATTGGAGAATTTTGGCTGAAGACAACCTTCTC
ENSMUST00000029727     CTGCTGCAAGCGGCTCAGACTTGTCGATACTGGAGAATTTTGGCTGAGGATAACCTTCTC
ENSRNOT00000015279     CTGCTGCAAGCGGCTCAGACGTGTCGATACTGGAGGATTTTGGCTGAGGATAACCTTCTC


ENSGGOT00000022707     TGGAGAGAGAAATGCAAAGAAGAGGGGATTGATGAACCATTGCACATCAAGAGAAGAAAA
ENSCJAT00000004056     TGGAGAGAAAAATGCAAAGAAGAGGGGATTGATGAACCATTGCACATCAAGAGAAGAAAA
ENST00000263981        TGGAGAGAGAAATGCAAAGAAGAGGGGATTGATGAACCATTGCACATCAAGAGAAGAAAA
ENSPPYT00000017582     TGGAGAGAGAAATGCAAAGAAGAGGGGATTGATGAACCATTGCACATCAAGAGAAGAAAA
ENSMMUT00000028596     TGGAGAGAGAAATGCAAAGAAGAGGGGATTGATGAACCGTTGCACATCAAGAGAAGAAAA
ENSMUST00000029727     TGGAGAGAGAAATGTAAAGAAGAGGGGATTGATGAACCGTTGCACATCAAGAGAAGAAAA
ENSRNOT00000015279     TGGAGAGAGAAATGTAAAGAAGAGGGGATTGATGAACCGTTGCACATCAAGAGAAGAAAA


ENSGGOT00000022707     GTAATAAAACCAGGTTTCATACACAGTCCATGGAAAAGTGCATACATCAGACAGCACAGA
ENSCJAT00000004056     GTAATAAAACCAGGTTTCATACACAGTCCGTGGAAAAGTGCATACATCAGACAGCACAGA
ENST00000263981        GTAATAAAACCAGGTTTCATACACAGTCCATGGAAAAGTGCATACATCAGACAGCACAGA
ENSPPYT00000017582     GTAATAAAACCAGGTTTCATACACAGTCCATGGAAAAGTGCATACATCAGACAGCACAGA
ENSMMUT00000028596     GTAATAAAACCAGGTTTCATACACAGTCCATGGAAAAGTGCATACATCAGACAGCACAGA
ENSMUST00000029727     ATAATAAAACCAGGTTTCATACACAGCCCATGGAAGAGTGCGTATATCAGACAGCACAGA
ENSRNOT00000015279     ATAATAAAACCAGGTTTCATACACAGCCCATGGAAGAGTGCGTACATCAGACAGCACAGA


ENSGGOT00000022707     ATTGATACTAACTGGAGGCGAGGAGAACTCAAATCTCCTAAGGTGCTGAAAGGACATGAT
ENSCJAT00000004056     ATTGATACTAACTGGAGGCGAGGAGAACTCAAATCTCCTAAGGTGCTGAAAGGACATGAT
ENST00000263981        ATTGATACTAACTGGAGGCGAGGAGAACTCAAATCTCCTAAGGTGCTGAAAGGACATGAT
ENSPPYT00000017582     ATTGATACTAACTGGAGGCGAGGAGAACTCAAATCTCCTAAGGTGCTGAAAGGACATGAT
ENSMMUT00000028596     ATTGATACTAACTGGAGGCGAGGAGAACTCAAATCTCCTAAGGTGCTGAAAGGACATGAT
ENSMUST00000029727     ATTGATACAAACTGGAGACGAGGAGAACTCAAATCTCCTAAGGTGCTGAAAGGGCATGAT
ENSRNOT00000015279     ATTGATACAAACTGGAGGCGAGGAGAACTCAGATCTCCTAAGGTGCTGAAAGGGCATGAT


ENSGGOT00000022707     GATCATGTGATCACATGCTTACAGTTTTGTGGTAACCGAATAGTTAGTGGTTCTGATGAC
ENSCJAT00000004056     GATCATGTGATCACATGCTTACAGTTTTGTGGTAACCGAATAGTTAGTGGTTCTGATGAC
ENST00000263981        GATCATGTGATCACATGCTTACAGTTTTGTGGTAACCGAATAGTTAGTGGTTCTGATGAC
ENSPPYT00000017582     GATCATGTGATCACATGCTTACAGTTTTGTGGTAACCGAATAGTTAGTGGTTCTGATGAC
ENSMMUT00000028596     GATCATGTGATCACATGCTTACAGTTTTGTGGTAACCGAATAGTTAGTGGTTCTGATGAC
ENSMUST00000029727     GACCATGTGATCACATGCCTACAGTTTTGTGGCAACCGCATAGTTAGTGGTTCTGATGAC
ENSRNOT00000015279     GATCATGTGATTACGTGCCTACAGTTTTGTGGTAACCGCATAGTTAGTGGTTCTGATGAC


ENSGGOT00000022707     AACACTTTAAAAGTTTGGTCAGCCGTCACAGGCAAATGTCTGAGAACATTAGTGGGACAT
ENSCJAT00000004056     AACACTTTAAAAGTTTGGTCAGCAGTCACGGGCAAATGTCTGAGAACATTAGTGGGACAT
ENST00000263981        AACACTTTAAAAGTTTGGTCAGCAGTCACAGGCAAATGTCTGAGAACATTAGTGGGACAT
ENSPPYT00000017582     AACACTTTAAAAGTTTGGTCAGCAGTCACGGGCAAATGTCTGAGAACATTAGTGGGACAT
ENSMMUT00000028596     AACACTCTAAAAGTTTGGTCAGCAGTCACGGGCAAATGTCTGAGAACATTAGTGGGACAT
ENSMUST00000029727     AACACTTTAAAAGTTTGGTCAGCGGTCACGGGCAAGTGTCTGAGAACGTTAGTGGGACAT
ENSRNOT00000015279     AACACTTTAAAAGTTTGGTCAGCGGTCACGGGTAAGTGTCTGAGGACGTTAGTGGGACAC


ENSGGOT00000022707     ACAGGTGGAGTATGGTCATCACAAATGAGAGACAACATCATCATTAGTGGATCTACAGAT
ENSCJAT00000004056     ACAGGTGGAGTATGGTCATCACAGATGAGAGACAACATCATCATTAGTGGATCTACAGAT
ENST00000263981        ACAGGTGGAGTATGGTCATCACAAATGAGAGACAACATCATCATTAGTGGATCTACAGAT
```

```
ENSPPYT00000017582     ACAGGTGGAGTATGGTCATCACAAATGAGAGACAACATCATCATTAGTGGATCTACAGAT
ENSMMUT00000028596     ACAGGTGGAGTATGGTCATCACAAATGAGAGACAACATCATCATTAGTGGATCTACCGAT
ENSMUST00000029727     ACAGGTGGAGTGTGGTCATCACAGATGAGAGACAATATCATCATCAGTGGATCGACTGAC
ENSRNOT00000015279     ACCGGTGGAGTGTGGTCTTCACAGATGAGAGACAATATCATCATTAGTGGATCTACTGAC

ENSGGOT00000022707     CGGACACTCAAAGTGTGGAATGCAGAGACTGGAGAATGTATACACACCTTATATGGGCAT
ENSCJAT00000004056     CGGACTCTCAAAGTGTGGAATGCAGAGACTGGAGAATGTATACACACCTTATATGGACAT
ENST00000263981        CGGACACTCAAAGTGTGGAATGCAGAGACTGGAGAATGTATACACACCTTATATGGGCAT
ENSPPYT00000017582     CGGACTCTCAAAGTGTGGAATGCAGAGACTGGAGAATGTATACACACCTTATATGGGCAT
ENSMMUT00000028596     CGGACTCTCAAAGTGTGGAATGCAGAGACTGGAGAATGTATACACACCTTATATGGGCAT
ENSMUST00000029727     CGGACTCTCAAAGTGTGGAATGCTGAAACTGGAGAGTGTATACATACTTTATATGGGCAC
ENSRNOT00000015279     CGGACTCTCAAAGTGTGGAATGCAGAGACTGGAGAGTGTATACATACGTTATATGGGCAC

ENSGGOT00000022707     ACTTCCACTGTGCGTTGTATGCATCTACATGAAAAAAGAGTTGTTAGCGGTTCTCGAGAT
ENSCJAT00000004056     ACTTCCACTGTGCGTTGTATGCATCTTCATGAAAAAAGAGTTGTTAGCGGTTCTCGAGAT
ENST00000263981        ACTTCCACTGTGCGTTGTATGCATCTTCATGAAAAAAGAGTTGTTAGCGGTTCTCGAGAT
ENSPPYT00000017582     ACTTCCACTGTGCGTTGTATGCATCTTCATGAAAAAAGAGTTGTTAGCGGTTCTCGAGAT
ENSMMUT00000028596     ACTTCCACAGTGCGTTGTATGCATCTTCATGAAAAAAGAGTTGTTAGCGGTTCCCGAGAT
ENSMUST00000029727     ACTTCTACTGTACGGTGTATGCATCTCCATGAAAAAAGGGTTGTAAGCGGTTCTCGAGAT
ENSRNOT00000015279     ACCTCCACTGTACGGTGTATGCATCTCCATGAAAAAAGGGTTGTGAGCGGTTCTCGAGAT

ENSGGOT00000022707     GCCACTCTTAGGGTTTGGGATATTGAGACAGGCCAGTGTTTACATGTTTTGATGGGTCAT
ENSCJAT00000004056     GCCACTCTTAGGGTTTGGGATATTGAGACAGGCCAGTGTTTACATGTTTTGATGGGTCAT
ENST00000263981        GCCACTCTTAGGGTTTGGGATATTGAGACAGGCCAGTGTTTACATGTTTTGATGGGTCAT
ENSPPYT00000017582     GCCACTCTTAGGGTTTGGGATATTGAGACAGGCCAGTGTTTACATGTTTTGATGGGTCAT
ENSMMUT00000028596     GCCACTCTTAGGGTTTGGGATATTGAGACAGGCCAGTGTTTACATGTTTTGATGGGTCAT
ENSMUST00000029727     GCCACTCTCAGGGTTTGGGATATTGAGACCGGCCAGTGTTTACACGTCCTGATGGGTCAC
ENSRNOT00000015279     GCCACTCTCAGGGTTTGGGATATTGAGACCGGCCAGTGTTTACACGTCTTGATGGGCCAT

ENSGGOT00000022707     GTTGCAGCAGTCCGCTGTGTTCAATATGATGGCAGGAGGGTTGTTAGTGGAGCATATGAT
ENSCJAT00000004056     GTTGCAGCAGTCCGCTGTGTTCAATATGATGGCAGGAGGGTTGTTAGTGGAGCATATGAT
ENST00000263981        GTTGCAGCAGTCCGCTGTGTTCAATATGATGGCAGGAGGGTTGTTAGTGGAGCATATGAT
ENSPPYT00000017582     GTTGCAGCAGTCCGCTGTGTTCAATATGATGGCAGGAGGGTTGTTAGTGGAGCATATGAT
ENSMMUT00000028596     GTTGCAGCAGTCCGCTGTGTTCAATATGATGGCAGGAGGGTTGTTAGTGGAGCATATGAT
ENSMUST00000029727     GTAGCAGCGGTCCGCTGCGTTCAGTATGATGGCAGGAGGGTTGTTAGTGGAGCTTATGAT
ENSRNOT00000015279     GTGGCAGCAGTCCGCTGTGTTCAGTATGATGGCAGGAGGGTTGTTAGTGGAGCATATGAT

ENSGGOT00000022707     TTTATGGTAAAGGTGTGGGATCCAGAGACTGAAACCTGTCTACACACGTTGCAGGGGCAT
ENSCJAT00000004056     TTTATGGTAAAGGTGTGGGATCCAGAGACTGAAACCTGTCTACACACGTTGCAGGGGCAT
ENST00000263981        TTTATGGTAAAGGTGTGGGATCCAGAGACTGAAACCTGTCTACACACGTTGCAGGGGCAT
ENSPPYT00000017582     TTTATGGTAAAGGTGTGGGATCCAGAGACTGAAACCTGTCTACACACGTTGCAGGGGCAT
ENSMMUT00000028596     TTTATGGTAAAGGTGTGGGATCCAGAGACTGAAACCTGTCTACACACGTTGCAGGGGCAT
ENSMUST00000029727     TTTATGGTGAAGGTGTGGGATCCAGAGACTGAGACCTGTCTACACACGTTACAGGGACAC
ENSRNOT00000015279     TTTATGGTGAAGGTGTGGGATCCAGAGACCGAGACCTGTCTACACACGTTACAGGGACAC

ENSGGOT00000022707     ACTAATAGAGTCTATTCATTACAGTTTGATGGTATCCATGTGGTGAGTGGATCTCTTGAT
ENSCJAT00000004056     ACTAATAGAGTCTATTCATTACAGTTTGATGGTATCCATGTGGTCAGTGGATCTCTTGAT
ENST00000263981        ACTAATAGAGTCTATTCATTACAGTTTGATGGTATCCATGTGGTGAGTGGATCTCTTGAT
ENSPPYT00000017582     ACTAATAGAGTCTATTCATTACAGTTTGATGGTATCCATGTGGTGAGTGGATCTCTTGAT
ENSMMUT00000028596     ACTAATAGAGTCTATTCATTACAGTTTGATGGTATCCATGTGGTGAGTGGATCTCTTGAT
ENSMUST00000029727     ACTAATAGAGTCTATTCATTACAGTTTGATGGCATCCATGTGGTGAGTGGATCTCTTGAT
ENSRNOT00000015279     ACTAATAGAGTCTATTCGTTACAGTTTGACGGCATCCATGTGGTGAGTGGATCTCTTGAT
```

```
ENSGGOT00000022707     ACATCAATCCGTGTTTGGGATGTGGAGACAGGGAATTGCATTCACACGTTAACAGGGCAC
ENSCJAT00000004056     ACATCAATCCGAGTTTGGGATGTGGAGACAGGGAATTGCATTCACACGTTAACAGGGCAT
ENST00000263981        ACATCAATCCGTGTTTGGGATGTGGAGACAGGGAATTGCATTCACACGTTAACAGGGCAC
ENSPPYT00000017582     ACATCAATCCGTGTTTGGGATGTGGAGACAGGGAATTGCATTCACACGTTAACAGGGCAC
ENSMMUT00000028596     ACATCAATTCGCGTTTGGGATGTGGAGACAGGGAATTGCATTCACACGTTAACAGGGCAC
ENSMUST00000029727     ACATCAATCCGAGTCTGGGATGTGGAGACAGGGAATTGTATTCACACGCTAACAGGACAC
ENSRNOT00000015279     ACATCAATCCGAGTCTGGGATGTGGAGACAGGGAATTGCATTCACACGCTAACAGGACAC

ENSGGOT00000022707     CAGTCGTTAACAAGTGGAATGGAACTCAAAGACAATATTCTTGTCTCTGGGAATGCAGAT
ENSCJAT00000004056     CAGTCGTTAACAAGTGGAATGGAACTCAAAGACAATATTCTTGTCTCTGGGAATGCAGAT
ENST00000263981        CAGTCGTTAACAAGTGGAATGGAACTCAAAGACAATATTCTTGTCTCTGGGAATGCAGAT
ENSPPYT00000017582     CAGTCGTTAACAAGTGGAATGGAACTCAAAGACAATATTCTTGTCTCTGGGAATGCAGAT
ENSMMUT00000028596     CAGTCGTTAACAAGTGGAATGGAACTCAAAGACAATATTCTTGTCTCTGGGAATGCAGAT
ENSMUST00000029727     CAGTCATTAACGAGTGGAATGGAACTCAAAGACAATATTCTTGTCTCTGGGAATGCAGAT
ENSRNOT00000015279     CAGTCGTTAACAAGTGGAATGGAACTCAAAGACAATATTCTGGTCTCTGGGAATGCAGAT

ENSGGOT00000022707     TCTACAGTTAAAATCTGGGATATCAAAACAGGACAGTGTTTACAAACATTGCAAGGTCCC
ENSCJAT00000004056     TCTACAGTTAAAATCTGGGATATCAAAACAGGACAGTGTTTACAAACATTGCAAGGTCCC
ENST00000263981        TCTACAGTTAAAATCTGGGATATCAAAACAGGACAGTGTTTACAAACATTGCAAGGTCCC
ENSPPYT00000017582     TCTACAGTTAAAATCTGGGATATCAAAACAGGACAGTGTTTACAAACATTGCAAGGTCCC
ENSMMUT00000028596     TCTACAGTTAAAATCTGGGATATCAAAACAGGACAGTGTTTACAAACATTGCAAGGTCCC
ENSMUST00000029727     TCTACAGTTAAGATCTGGGATATCAAAACAGGACAGTGTTTACAAACTTTGCAAGGTCCC
ENSRNOT00000015279     TCTACAGTTAAAATCTGGGATATCAAAACAGGACAGTGTTTACAGACATTGCAAGGTCCC

ENSGGOT00000022707     AACAAGCATCAGAGTGCTGTGACCTGTTTACAGTTCAACAAGAACTTTGTAATTACCAGC
ENSCJAT00000004056     AACAAGCATCAGAGTGCTGTGACCTGTTTACAGTTCAACAAGAACTTTGTAATTACCAGC
ENST00000263981        AACAAGCATCAGAGTGCTGTGACCTGTTTACAGTTCAACAAGAACTTTGTAATTACCAGC
ENSPPYT00000017582     AACAAGCATCAGAGTGCTGTGACCTGTTTACAGTTCAACAAGAACTTTGTAATTACCAGC
ENSMMUT00000028596     AACAAGCATCAGAGTGCTGTGACCTGTTTACAGTTCAACAAGAACTTTGTAATTACCAGC
ENSMUST00000029727     AGCAAGCATCAGAGCGCTGTGACCTGCTTACAGTTCAACAAGAACTTCGTAATTACCAGC
ENSRNOT00000015279     AGCAAGCATCAGAGCGCTGTGACCTGCTTACAGTTCAACAAGAACTTTGTAATTACCAGC

ENSGGOT00000022707     TCAGATGATGGAACTGTAAAACTATGGGACTTGAAAACGGGTGAATTTATTCGAAACCTA
ENSCJAT00000004056     TCAGATGATGGAACTGTAAAACTATGGGACTTGAAAACGGGTGAATTTATTCGAAACCTA
ENST00000263981        TCAGATGATGGAACTGTAAAACTATGGGACTTGAAAACGGGTGAATTTATTCGAAACCTA
ENSPPYT00000017582     TCAGATGATGGAACTGTAAAACTATGGGACTTGAAAACGGGTGAATTTATTCGAAACCTA
ENSMMUT00000028596     TCAGATGATGGAACTGTAAAACTATGGGACTTGAAAACGGGTGAATTTATTCGAAACCTA
ENSMUST00000029727     TCAGACGACGGAACGGTCAAACTCTGGGACTTGAAAACGGGTGAATTTATCCGAAACCTC
ENSRNOT00000015279     TCAGACGATGGAACGGTAAAACTCTGGGACTTGAAAACGGGTGAATTTATCCGAAACCTA

ENSGGOT00000022707     GTCACATTGGAGAGTGGGGGGAGTGGGGGAGTTGTGTGGCGGATCAGAGCCTCAAACACA
ENSCJAT00000004056     GTCACATTGGAGAGTGGGGGGAGTGGGGGAGTTGTGTGGCGGATCAGAGCCTCAAACACA
ENST00000263981        GTCACATTGGAGAGTGGGGGGAGTGGGGGAGTTGTGTGGCGGATCAGAGCCTCAAACACA
ENSPPYT00000017582     GTCACATTGGAGAGTGGGGGGAGTGGGGGAGTTGTGTGGCGGATCAGAGCCTCAAACACA
ENSMMUT00000028596     GTCACATTGGAGAGTGGGGGGAGTGGGGGAGTCGTGTGGCGGATCAGAGCCTCAAACACA
ENSMUST00000029727     GTCACATTGGAGAGTGGGGGGAGCGGGGGAGTTGTGTGGCGGATCAGGGCCTCAAACACA
ENSRNOT00000015279     GTCACATTGGAGAGTGGG------------------------------------------

ENSGGOT00000022707     AAGCTGGTGTGTGCAGTTGGGAGTCGGAATGGGACTGAAGAAACCAAGCTGCTGGTGCTG
ENSCJAT00000004056     AAGCTGGTGTGTGCAGTTGGGAGTCGGAATGGGACTGAAGAAACCAAGCTGCTGGTGCTG
ENST00000263981        AAGCTGGTGTGTGCAGTTGGGAGTCGGAATGGGACTGAAGAAACCAAGCTGCTGGTGCTG
ENSPPYT00000017582     AAGCTGGTGTGTGCAGTTGGGAGTCGGAATGGGACTGAAGAAACCAAGCTGCTGGTGCTG
ENSMMUT00000028596     AAGCTGGTGTGTGCAGTTGGGAGTCGGAATGGGACTGAAGAAACCAAGCTGCTGGTGCTG
ENSMUST00000029727     AAGCTGGTGTGTGCAGTCGGGAGTCGGAATGGAACTGAGGAAACCAAGCTCCTGGTGCTG
```

```
ENSRNOT00000015279     ------------------------------------------------------------

ENSGGOT00000022707     GACTTTGATGTGGACATGAAG
ENSCJAT00000004056     GACTTTGATGTGGACATGAAG
ENST00000263981        GACTTTGATGTGGACATGAAG
ENSPPYT00000017582     GACTTTGATGTGGACATGAAG
ENSMMUT00000028596     GACTTTGATGTGGACATGAAG
ENSMUST00000029727     GACTTTGATGTGGACATGAAA
ENSRNOT00000015279     ---------------------
```

Multiple sequence alignment of Fbxw8

```
ENSMUST00000049474     ATGGACGACCACAACCTGGAGGAGTTCCGCCGGCACTGGCAGGAGGAGTTGGCGCAGTCA
ENSRNOT00000001488     ATGGAGGACCATAACCTGGAGGAGTTCCGCCAGCGCTGGCAGGAGGAATTGGCGCACTCG
ENSCJAT00000018458     ATGGACGAATGCAGCTTAGAGGAGTTCCGTCGGCGCTGGCAGGAGGAGCTGGCGCAGGCC
ENSMMUT00000024573     ATGGACGACTGCAGCCTGGATGAGTTCCGTCGGCGCTGGCAGGAGGAGCTGGCGCAGGCC
ENSPPYT00000005920     ATGGACGACTACAGCCTGGATGAGTTCCGTCGGCGCTGGCAGGAGGAGCTGGCGCAGGCC
ENST00000309909        ATGGACGACTACAGCCTGGATGAGTTCCGTCGGCGCTGGCAGGAGGAGCTGGCGCAGGCC
ENSPTRT00000010100     ATGGACGACTACAGCCTGGATGAGTTCCGTCGGCGCTGGCAGGAGGAGCTGGCGCAGGCC

ENSMUST00000049474     CAGGCGCTGAGGCGACGGCGGCGGCTCGAGGCTGGGGAGCGGCGGCCGCGAAGGCCCGAG
ENSRNOT00000001488     CAGGTGCTGAGGCGACGGCGGCGGCTCGAGGCTGGGGAGCGGCGGCCCCGGAGACCGGAG
ENSCJAT00000018458     CAGGCGCCTAGGAAGTGGCGGCGGTCTGATGCTGCCGAGAGGCGGGCGCGACGGCCGGAG
ENSMMUT00000024573     CAGGCGCCGAGGAAGCGGCGACGGCCCGAGGCTGCCGAGAGGTGGGCTCGGCGGCCGGAG
ENSPPYT00000005920     CAGGCGCCGAAGAAGCGGCGACGGCCCGAGGTTGCTGAGAGGCGGGCTCGGCGGCCGGAG
ENST00000309909        CAGGCGCCGAAGAAGCGGCGACGGCCCGAGGCTGCCGAGAGGCGGGCTCGGCGGCCGGAG
ENSPTRT00000010100     CAGGCGCCGAAGAAGCGGCGACGGCCCGAGGCTGCCGAGAGGCGGGCTCGGCGGCCGGAG

ENSMUST00000049474     GCGGGAGCG---CGAGGCGAGCCGGCCTCGGGCTACCTGGGGCTGGCGCAGGGGCTGCTG
ENSRNOT00000001488     GCGGGAGCG---CGCGGCGAGCCGGCCTCGGGGTACCTGGGGCTGGCGCAGGGTCTGCTG
ENSCJAT00000018458     GTGGCCCCCGGGCGCAGCGAACAGGCCTTGGGAGACCCGGCGCTGGCCCAGGGTCTCCTG
ENSMMUT00000024573     GTGGTCCCCGGGCGCGGCGAACAGGCCTCGGGGGACCCGGCGCTGGCCCAGGGTCTCCTG
ENSPPYT00000005920     GTGGGCTCCGGGCACGGCGAACAGGCCTCTGGGGACCCGACGCTAGCCCAGGGTCTCCTG
ENST00000309909        GTGGGCTCCGGGCGCGGCGAACAGGCCTCGGGGGACCCGGCGCTGGCCCAGCGTCTCCTG
ENSPTRT00000010100     GTGGGCTCCGGGCGCGGCGAACAGGCCTCGGGGGACCCGGCGCTGGCCCAGGGTCTCCTG

ENSMUST00000049474     GAGGGCGCGGGCCGACCCCCGGCGCCCAGGCCTGGCCGGGGCGACAGGAAGGATACGTCC
ENSRNOT00000001488     GAGGGCGCGGGCCGACCCCCGGCGCCCAGGCCCGGCCGGACCGACAGGAAGGATGTGTCC
ENSCJAT00000018458     GAGGGCGCAGGGAGGCCTCCGGCGGCGCGGGCGACTCGGGCCGAGGCGCAGGACGTGGCG
ENSMMUT00000024573     GAGGGCGCGGGGAGGCCCCCGGCAGCGCGGGCGACTCGGACCGAGGGGCAGGACGTGGCG
ENSPPYT00000005920     GAGGGCGCGGGGAGGCCCCCGGCGGCGCGGGCGACTCGGGCCGAGGGGCAG---GTGACG
ENST00000309909        GAGGGCGCGGGGAGGCCCCCGGCGGCGCGGGCGACTCGGGCCGAGGGGCAGGACGTAGCG
ENSPTRT00000010100     GAGGGCGCGGGGAGGCCCCCGGCGGCGCGGGCGACTCGGGCCGAGGGGCAGGACGTAGCG

ENSMUST00000049474     AGCCGATCGCGCTCACCCCCAGACCGCGACGCCACGGAGCCCGAGCCGCTGGTGGATCAG
ENSRNOT00000001488     AGCCGTTCGCGCTCACCCCCGGACCGCGACGCCGCGGAGCCCGAGCCGCTGGTGGACCAG
ENSCJAT00000018458     AGCCGGTCC---------------------------GGTTTTCAGCAGCTGGTGGACCAG
ENSMMUT00000024573     AGCCGCTCCCGTTCTCCTCTGGCCCGCGAGGGTGCCGGCGGGGAGCAGCTGGTGGACCAG
ENSPPYT00000005920     AGCCGCTCACGTTCTCCTCTGGCCCGCGAGGGCGCCGGGGGGGAGCAGCTGGTGGACCAG
ENST00000309909        AGCCGCTCACGTTCTCCTCTGGCCCGCGAGGGCGCCGGCGGGGAGCAGCTGGTGGACCAG
ENSPTRT00000010100     AGCCGCTCACGTTCT---CTGGCCCGCGAGGGCGCCGGCGGGGAGCAGCTGGTGGACCAG

ENSMUST00000049474     CTCATCCGCGACTTGAATGAGCTGGATGACGTGCCCTTCTTTGATGTCCGTCTGCCTTAC
```

```
ENSRNOT00000001488      CTCATCCGCGACTTGAATGAAATGGATGATGTGCCCTTCTTTGATGTCCATCTGCCTTAT
ENSCJAT00000018458      CTAATCCGCGACCTGAATGAAATGAATGATGTGCCCTTCTTTGATATCCAACTGCCTTAC
ENSMMUT00000024573      CTCATCCGCGACCTGAATGAAATGAATGATGTGCCTTTCTTTGATATCCAACTGCCTTAT
ENSPPYT00000005920      CTCATCCGCGACCTGAATGAAATGAATGATGTGCCTTTCTTTGATATCCAACTGCCTTAC
ENST00000309909         CTCATCCGCGACCTGAATGAAATGAATGATGTGCCTTTCTTTGATATCCAACTGCCTTAC
ENSPTRT00000010100      CTCATCCGCGACCTGAATGAAATGAATGATGTGCCTTTCTTTGATATCCAACTGCCTTAC

ENSMUST00000049474      GAACTGGCCATCAATATATTCCAGTATCTGAACAGAAGAGAGCTGGGACTGTGTGCTCAG
ENSRNOT00000001488      GAACTGGCGATCAATATATTTCAGTATCTGAACAGAAGAGAGCTGGGGCTGTGTGCTCAG
ENSCJAT00000018458      GAATTGGCAATCAATATATTTCAGTATTTGGACAGGAAAGAACTAGGAAGATGTGCACAG
ENSMMUT00000024573      GAATTGGCAATCAATATATTTCAGTATCTGGACAGGAAAGAACTAGGAAGATGTGCACAG
ENSPPYT00000005920      GAATTGGCAATCAATATATTTCAGTATCTGGACAGGAAAGAACTAGGAAGATGTGCACAG
ENST00000309909         GAATTGGCAATCAATATATTTCAGTATCTGGACAGGAAAGAACTAGGAAGATGTGCACAG
ENSPTRT00000010100      GAATTGGCAATCAATATATTTCAGTATCTGGACAGGAAAGAACTAGGAAGATGTGCACAG

ENSMUST00000049474      GTGAGCAAGACATGGAAGGTGATTGCTGAAGACGAGGTGCTGTGGTACCGGCTGTGCCGA
ENSRNOT00000001488      GTGAGCAAGACATGGAAGGTGATTGCCGAAGACGAGGTGCTGTGGTACCGGCTGTGCCGA
ENSCJAT00000018458      GTGAGCAAGACGTGGAAGGTGATTGCGGAGGATGAGGTGCTGTGGTACAGGCTGTGCCAG
ENSMMUT00000024573      GTGAGCAAGACGTGGAAGGTGATTGCGGAGGATGAGGTGCTGTGGTACAGGCTGTGCCAG
ENSPPYT00000005920      GTGAGCAAGACGTGGAAGGTGATTGCAGAGGATGAAGTGCTGTGGTACAGGCTGTGCCAG
ENST00000309909         GTGAGCAAGACGTGGAAGGTGATTGCAGAGGATGAGGTGCTGTGGTACAGGCTGTGCCAG
ENSPTRT00000010100      GTGAGCAAGACGTGGAAGGTGATTGCAGAGGATGAGGTGCTGTGGTACAGGCTGTGCCAG

ENSMUST00000049474      CAGGAGGGGCACCTTCCCCACAGCAGATTCTCCGATTACACCTGCTGGAAGCTCATCTTG
ENSRNOT00000001488      CAGGAGGGGCACCTTCCCCACAGCAGATTCTCCGATTACACCTGCTGGAAGCTCATCTTG
ENSCJAT00000018458      CAGGAAGGGCACCTTCCGGGTAGCAGCATCTCCGACTGTTCTTGCTGGAAGCTCATCTTC
ENSMMUT00000024573      CAGGAAGGGCACCTTCCGGATAGCAGCATCTCTGACTATTCTTGCTGGAAGCTCATCTTC
ENSPPYT00000005920      CAGGAAGGGCACCTTCCAGATAGCAGCATCTCTGACTATTCTTGCTGGAAGCTCATCTTC
ENST00000309909         CAGGAAGGGCACCTTCCGGATAGCAGCATCTCTGACTATTCTTGCTGGAAGCTCATCTTC
ENSPTRT00000010100      CAGGAAGGGCACCTTCCGGATAGCAGCATCTCTGACTATTCTTGCTGGAAGCTCATCTTC

ENSMUST00000049474      CAAGAGTGCCTCGCCAAGGAGCACACATTAAGAGCCAACTGGAAGAATCGCAAAGGTGCC
ENSRNOT00000001488      CAAGAGTGCCTCGCCCCTGTGCACCTGATTAGACCCTCATGGATGAATCGCAAAGGTGCC
ENSCJAT00000018458      CAAGAATGCCAAGCCAAGGAACACATGTTACGAACCAACTGGAAGAACCGCAAAGGTGCC
ENSMMUT00000024573      CAAGAGTGCCGAGCCAAGGAACACATGTTACGAACCAACTGGAAGAATCGCAAAGGTGCC
ENSPPYT00000005920      CAAGAGTGCCGAGCCAAGGAACACATGTTACGAACCAACTGGAAGAATCGCAAAGGTGCT
ENST00000309909         CAAGAGTGCCGAGCCAAGGAACACATGTTACGAACCAACTGGAAGAATCGCAAAGGTGCC
ENSPTRT00000010100      CAAGAGTGCCGAGCCAAGGAACACATGTTACGAACCAACTGGAAGAATCGCAAAGGTGCC

ENSMUST00000049474      GTGAGCGAGCTGGAGCATGTTCCCGACGCTGTTTTGTGTGACGTGCGTTCTCACGATGGT
ENSRNOT00000001488      GTGAGCGAGCTGGAACACGTTCCTGACGCTGTTTTGTGTGACGTGCGTTCTCACGATGGA
ENSCJAT00000018458      GTGAGTGAGCTGGAGCACGTTCCTGACGCGGTTTTGTGTGATGTGCATTCTCATGATGGC
ENSMMUT00000024573      GTGAGTGAGCTGGAGCACATTCCTGACGCGGTTTTGTGTGATGTGCATTCTCACGATGGC
ENSPPYT00000005920      GTGAGCGAGCTGGAGCATGTTCCTGACGCAGTTTTGCGTGATGTGCATTCTCACGATGGC
ENST00000309909         GTGAGCGAGCTGGAGCATGTTCCTGACACAGTTTTGTGTGATGTGCATTCTCACGATGGT
ENSPTRT00000010100      GTGAGCGAGCTGGAGCATGTTCCTGACGCAGTTTTGTGTGATGTGCATTCTCACGATGGT

ENSMUST00000049474      GTGGTCATTGCTGGATACACGTCAGGGGATGTGCGGGTGTGGGACACCCGCACCTGGGAC
ENSRNOT00000001488      GTTGTCATCGCAGGATACACGTCAGGGGAGGTGAGGGTGTGGGACACCCGCACCTGGGAC
ENSCJAT00000018458      GTGGTCATTGCTGGATATACATCAGGGGATGTGAGAGTGTGGGACACCCGCACCTGGGAC
ENSMMUT00000024573      GTGGTCATTGCTGGATATACATCAGGGGACGTGAGAGTGTGGGACACCCGCACCTGGGAC
ENSPPYT00000005920      GTGGTCATTGCTGGATATACATCAGGGGATGTGAGAGTGTGGGACACCCGCACCTGGGAC
ENST00000309909         GTGGTCATTGCGGGATATACATCAGGGGATGTGAGAGTGTGGGACACCCGCACCTGGGAC
ENSPTRT00000010100      GTGGTCATTGCGGGATATACATCAGGGGATGTGAGAGTGTGGGACACCCGCACCTGGGAC
```

```
ENSMUST00000049474      TACGTGGCCCCCTTCCTGGAGTCTGAGTCTGAGGAGGAGGATCCTGGAATGCAGCCATAT
ENSRNOT00000001488      TATGTGGCCCCCTTCCTGGAGTCTGAGTCTGAGGAGGAGGATCCTGGAATGCAGCCATAT
ENSCJAT00000018458      TACGTAGCCCCCTTCTTGGAATCAGAGGACGAGGAGGACGAGCCTGGACTGCAGCCAAAT
ENSMMUT00000024573      TACGTAGCCCCCTTCCTGGAATCCGAGGACGAGGAGGACGAGCCTGGAATGCAGCCAAAT
ENSPPYT00000005920      TACGTAGCCCCCTTCCTGGAATCAGAGGACGAGGAGGATGAGCCTGGAATGCAGCCAAAT
ENST00000309909         TACGTAGCCCCCTTCCTGGAATCAGAGGACGAGGAGGATGAGCCTGGAATGCAGCCAAAT
ENSPTRT00000010100      TATGTAGCCCCCTTCCTGGAATCAGAGGACGAGGAGGACGAGCCTGGAATGCAGCCAAAT

ENSMUST00000049474      GTCTCCTTTGTGAGGATCAACAGCTCGCTGGCAGTGGCGGCTTACGAGGATGGGATCCTT
ENSRNOT00000001488      GTCTCCTTTGTGAGGATCAACAGCTCGCTGGCAGTGGCGGCTTACGAGGATGGAATTCTT
ENSCJAT00000018458      GTCTCCTTTGTGAGGATAAACAGCTCACTGGCAGTAGCAGCTTATGAGGATGGGTTTCTC
ENSMMUT00000024573      GTCTCCTTTGTGAGGATAAACAGCTCGCTGGCAGTAGCAGCTTATGAGGATGGGTTTCTT
ENSPPYT00000005920      GTCTCCTTTGTGAGGATAAACAGCTCGCTGGCAGTAGCAGCTTATGAGGATGGGTTTCTT
ENST00000309909         GTCTCCTTTGTGAGGATAAACAGCTCGTTGGCAGTAGCAGCTTATGAGGATGGGTTTCTT
ENSPTRT00000010100      GTCTCCTTTGTGAGGATAAACAGCTCGTTGGCAGTAGCAGCTTATGAGGATGGGTTTCTT

ENSMUST00000049474      AACATTTGGGACCTGAGAACCGGAAGGTTCCCTATCTTTCGTTTTGAGCATGACGCAAGA
ENSRNOT00000001488      AACGTTTGGGACCTGAGAACCGGGAGGTTCCCTATCTTTCGTTTTGAACACGATGCAAGA
ENSCJAT00000018458      AATATTTGGGATTTAAGAACCGGAAGGTACCCTGTTCATCGTTTTGAGCACGATGCAAGA
ENSMMUT00000024573      AATATTTGGGATTTAAGGACCGGAAAGTACCCTGTTCATCGTTTTGAACATGATGCAAGA
ENSPPYT00000005920      AATATTTGGGATTTAAGGACCGGAAAGTACCCTGTTCATCGTTTTGAGCACGATGCAAGA
ENST00000309909         AATATTTGGGATTTAAGGACCGGAAAGTACCCTGTTCATCGTTTTGAGCACGATGCAAGA
ENSPTRT00000010100      AATATTTGGGATTTAAGGACCGGAAAGTACCCTGTTCATCGTTTTGAGCACGATGCAAGA

ENSMUST00000049474      ATACAAGCCCTTGCGCTGAGCCAAGAAAAGCCCATTGTTGCCACGGCTTCTGCTTTTGAC
ENSRNOT00000001488      ATACAAGCCCTTGCGCTCAGCCAGGAAAAGCCCGTGGTTGCCACAGCTTCTGCTTTTGAC
ENSCJAT00000018458      ATACAGGCACTAGCCCTCAGCCAGGACGATGCAGCTGTGGCCACGGCTTCTGCTTTTGAC
ENSMMUT00000024573      ATACAGGCACTAGCCCTCAGCCAGGACGATGCAACTGTGGCCACAGCTTCTGCTTTTGAT
ENSPPYT00000005920      ATACAGGCACTAGCCCTCAGCCAGGACGATGCAACCGTGGCCACAGCTTCTGCTTTTGAT
ENST00000309909         ATACAGGCACTAGCCCTCAGCCAGGACGATGCAACCGTGGCCACAGCTTCTGCTTTTGAT
ENSPTRT00000010100      ATACAGGCACTAGCCCTCAGCCAGGACGATGCAACCGTGGCCACAGCTTCTGCTTTTGAT

ENSMUST00000049474      GTTGTGATGTTGTACCCCAACGAGGAGGGGCATTGGCATGTGGCCTCGGAGTTTGAAGTT
ENSRNOT00000001488      GTTGTGATGTTGTACCCCAACGAGGAGGGGAACTGGCATGTAGCCTCGGAATTTGAAGTT
ENSCJAT00000018458      ATCGTGATGTTATCCCCCAATGAGGAGGGGTACTGGCAGATAGCTGCGGAATTTGAAGTT
ENSMMUT00000024573      GTCGTGATGCTGTCCCCCAGTGAGGAGGGGTACTGGCAGATAGCTGCGGAATTTGAAGTT
ENSPPYT00000005920      GTCGTGATGTTATCCCCCAATGAGGAGGGGTACTGGCAGATAGCTGCGGAATTTGAAGTT
ENST00000309909         GTCGTGATGTTATCCCCCAATGAGGAGGGGTACTGGCAGATAGCTGCGGAATTTGAAGTT
ENSPTRT00000010100      GTCGTGATGTTATCCCCCAATGAGGAGGGGTACTGGCAGATAGCTGCGGAATTTGAAGTT

ENSMUST00000049474      CAGAAGCTGGTTGACTACCTTGAAATAGTTCCGAATACTGGGAGGTACCCTGTGGCAATA
ENSRNOT00000001488      CAGAAACTGGTTGACTACCTTGAAATAGTTCCGAACACTGGGAGGTACCCTGTAGCAATA
ENSCJAT00000018458      CCGAAACTGGTTCAGCGCCTTGAAATAGTTCCAGAAACCAGAAGGTACCCCGTGGCAATA
ENSMMUT00000024573      CCGAAACTGGTTCAGTACCTTGAAATAGTTCCAGAAACTGGAAGGTACCCTGTGGCAGTA
ENSPPYT00000005920      CCGAAACTGGTTCAGTACCTTGAAATAGTTCCAGAAACCAGAAGGTACCCTGTGGCAGTA
ENST00000309909         CCGAAACTGGTTCAGTACCTTGAAATAGTTCCAGAAACCAGAAGGTACCCTGTGGCAGTA
ENSPTRT00000010100      CCGAAACTGGTTCAGTACCTTGAAATAGTTCCAGAAACCAGAAGGTACCCTGTGGCAGTA

ENSMUST00000049474      GCCACAGCCGGGGATCTGGTGTACCTGCTGAAGGCCGACGACTCAGCCAGAACCCTTCAT
ENSRNOT00000001488      GCCACTGCCGGGGACCTGGTGTACCTGCTGAAGGCTGAGGACTCGGCCAGAACCCTTCAC
ENSCJAT00000018458      GCTGCTGCTGGAGACCTGATGTACCTGCTTAAAGCTGAAGACTCCGCGAGAACCCTCCTT
ENSMMUT00000024573      GCCGCTGCTGGAGATCTGATGTACCTGCTCAAAGCCGAAGACTCTGCCAGAACCCTCCTT
```

```
ENSPPYT00000005920     GCCGCTGCTGGAGATCTGATGTACCTGCTCAAAGCCGAAGACTCCGCCAGAACCCTCCTT
ENST00000309909        GCCGCTGCTGGAGATCTGATGTACCTGCTCAAAGCCGAAGACTCCGCCAGAACCCTCCTT
ENSPTRT00000010100     GCCGCTGCTGGAGATCTGATGTACCTGCTCAAAGCCGAAGACTCCGCCAGAACCCTCCTT

ENSMUST00000049474     TATGTCTATGGCCAGCCTGCCACATGTCTGGATGTCTCAGCCAGCCAGGTTGCCTTTGGA
ENSRNOT00000001488     TATGTCTACGGCCAGCCTGCCACGTGTCTGGATGTCTCCGCCAGCCAGGTCGCCTTTGGA
ENSCJAT00000018458     TACGCCCACGGCCCGCCTGTCACATGTCTAGATGTCTCGGCCAACCAAGTTGCTTTTGGT
ENSMMUT00000024573     TATGCCCACGGCCCGCCTGTCACATGTCTAGACGTCTCTGCCAACCAAGTTGCTTTTGGT
ENSPPYT00000005920     TACGCCCACGGCCCGCCTGTCACATGTCTAGACGTCTCGGCCAACCAAGTTGCTTTTGGT
ENST00000309909        TACGCCCACGGCCCGCCTGTCACATGTCTAGACGTCTCGGCCAACCAAGTTGCTTTTGGT
ENSPTRT00000010100     TACGCCCACGGCCCGCCTGTCACATGTCTAGACGTCTCGGCCAACCAAGTTGCTTTTGGT

ENSMUST00000049474     GTGAAGAGTCTAGGATGGGTGTATGAAGGAAACAAGATCCTGGTGTACAGCCTGGAAGCA
ENSRNOT00000001488     GTGAAGAGCCTGGGATGGGTGTATGAGGGAAACAAGATCCTGGTGTACAGCCTGGAAGCA
ENSCJAT00000018458     GTACAGGGTCTGGGATGGGTGTACGAAGGGAGCAAGATTCTGGTGTATAGCCTGGAAGCA
ENSMMUT00000024573     GTACAGGGCCTGGGATGGGTGTACGAAGGAAGCAAGATCCTGGTATATAGCCTGGAAGCA
ENSPPYT00000005920     GTACAGGGTCTGGGATGGGTGTACGAAGGAAGCAAGATCTTGGTGTATAGCCTGGAAGCA
ENST00000309909        GTACAGGGTCTGGGATGGGTGTACGAAGGAAGCAAGATCCTGGTGTATAGCCTGGAAGCA
ENSPTRT00000010100     GTACAGGGTCTGGGATGGGTGTACGAAGGAAGCAAGATCCTGGTGTATAGCCTGGAAGCA

ENSMUST00000049474     GAGCGCTGCCTCTCGAAGCTGGGCAATGCACTTGGAGACTTTACCTGTGTCAACATCCGG
ENSRNOT00000001488     GAGCGCTGCCTCTCGAAGCTGGGCAATGCACTTGGAGACTTCACCTGTGTCAACATCCGG
ENSCJAT00000018458     GGATGCCACCTCTTGAAGCTGGGTAACGTTCTCCGTGACTTCACGTGTGTCAACCTCGAC
ENSMMUT00000024573     GGACGCCGCCTCTTGAAGCTGGGTAACGTTCTCCGTGACTTCACATGTGTCAACCTCAGC
ENSPPYT00000005920     GGATGCCGCCTCTTGAAGCTGGGTAACGTTCTCCGTGACTTCACGTGTGTCAACCTCAGC
ENST00000309909        GGACGCCGCCTCTTGAAGCTGGGTAACGTTCTCCGTGACTTCACGTGTGTCAACCTCAGC
ENSPTRT00000010100     GGACGCCGCCTCTTGAAGCTGGGTAACGTTCTCCGTGACTTCACGTGTGTCAACCTCAGC

ENSMUST00000049474     GATAGCCCTCCCAACCTCATGGTCAGCGGCAACATGGACAGGAGAGTGAGGATCCATGAC
ENSRNOT00000001488     GACAGCCCTCCCAACCTCATGGTCAGCGGCAACATGGACAGGAGAGTGAGGCTTCATGAC
ENSCJAT00000018458     GACAGTCCTCCCAACCTCATGGTCAGTGGCAACAGGGATGGGAGGGTGAGGATCCACGAC
ENSMMUT00000024573     GACAGCCCTCCCAACCTCATGGTCAGTGGCAACATGGACGGGAGGGTGAGGATCCATGAC
ENSPPYT00000005920     GACAGCCCTCCCAACCTCATGGTCAGTGGCAACACGGACGGGAGGGTGAGGATCCATGAC
ENST00000309909        GACAGCCCTCCCAACCTCATGGTCAGTGGCAACATGGACGGGAGGGTGAGGATCCACGAC
ENSPTRT00000010100     GACAGCCCTCCCAACCTCATGGTCAGTGGCAACATGGACGGGAGGGTGAGGATCCATGAC

ENSMUST00000049474     CTCCGCAGCGATAAGATCGCCCTGTCGCTGTCTGCCCATCAGCTGGGGGTGTCCGCAGTC
ENSRNOT00000001488     CTCCGCACCGATAAGATTGCCCTGTCGCTGTCCGCCCATCAGCTGGGGGTGTCTGCAGTC
ENSCJAT00000018458     CTCCGCACCAGTAACGTCACGCTGTCACTCTCCGCCCACCAGCTTGGGGTCTCTGCTGTG
ENSMMUT00000024573     CTCCGCAGCGGTAACATCGCCCTGTCGCTCTCTGCCCACCAGCTTGGGGTCTCTGCCGTG
ENSPPYT00000005920     CTCCGCAGCGGTAACATCGCCCTGTCACTCTCCGCCCATCAGCTCAGGGTCTCTGCTGTG
ENST00000309909        CTCCGCAGTGGTAACATCGCCCTGTCGCTCTCCGCCCATCAGCTCAGGGTCTCTGCTGTG
ENSPTRT00000010100     CTCCGCAGTGGTAACATCGCCCTGTCGCTCTCCGCCCATCAGCTCAGGGTCTCTGCTGTG

ENSMUST00000049474     CAGATGGATGACTGGAAGGTTGTCAGTGGAGGCGAGGAGGGGCTGGTGTCTGTGTGGGAT
ENSRNOT00000001488     CAGATGGATGACTGGAAGATTGTTAGTGGAGGCGAGGAGGGGCTGGTGTCTGTGTGGGAT
ENSCJAT00000018458     AGGATGAACGACTGGAAGATTGTCAGCGGAAAGGAAGAC---CTGGTGTCTGTGTGGGAT
ENSMMUT00000024573     CAGATGGACGACTGGAAGATCGTCAGCGGAGGCGAGGAAGGCCTGGTGTCCGTGTGGGAT
ENSPPYT00000005920     CAGATGGATGACTGGAAGATCGTCAGTGGAGGCGAGGAAGGCCTGGTGTCCGTGTGGGAT
ENST00000309909        CAGATGGATGACTGGAAGATCGTCAGTGGAGGCGAGGAAGGCCTGGTGTCCGTGTGGGAT
ENSPTRT00000010100     CAGATGGATGACTGGAAGATCGTCAGTGGAGGCGAGGAAGGCCTGGTGTCCGTGTGGGAT

ENSMUST00000049474     TACCGCATGAACCAGAAGCTGTGGGAAGTGCACTCCAGGCACCCTGTGCGCTATCTCTCC
```

ENSRNOT00000001488   TACCGCATGAACCAGAAGCTGTGGGAGGTGCACTCCAGGCACCCCGTGCGCTATATCTCC
ENSCJAT00000018458   TATCGGATGAACCAGAAGCTGTGGGAGGTGTATTCTGGACACCCCGTAAAGCACATCTCA
ENSMMUT00000024573   TATCGGACGAACCAAAAGCTGTGGGAGGTGTATTCTGGACACCCCGTGCAGCACATCTCA
ENSPPYT00000005920   TATCGGACAAACCAGAAGCTGTGGGAGGTGTATTCCGGACACCCTGTGCAGCACATCTCA
ENST00000309909   TATCGGATGAACCAGAAGCTGTGGGAGGTGTATTCCGGGCACCCGGTGCAGCACATCTCA
ENSPTRT00000010100   TATCGGATGAACCAGAAGCTGTGGGAGGTGTATTCCGGGCACCCGGTGCAGCACATCTCA

ENSMUST00000049474   TTCAATAGCCACAGCCTCATCACTGCCAACGTGCCCTACGAGAAGGTGCTGCGAAACTCC
ENSRNOT00000001488   TTCAACAGCCACAGCCTGATCACCGCCAATGTGCCCTACGAGAAGGTGCTGCGAAACTCC
ENSCJAT00000018458   TTCAGCAGTCGAAGCCTCATCACAGCCAACGTGCCCTACCAGCCGGTGGTGCGGAACGCC
ENSMMUT00000024573   TTCAGCAGCCACAGCCTCATCACGGCCAACGTGCCCTACCAGACGGTGATGCGAAACGTC
ENSPPYT00000005920   TTCAGCAGCCACAGCCTCATCACGGCCAATGTGCCTTACCAGACGGTGATGCGAAACGCC
ENST00000309909   TTCAGCAGCCACAGCCTCATCACGGCCAACGTGCCTTACCAGACGGTAATGCGAAACGCC
ENSPTRT00000010100   TTCAGCAGCCACAGCCTCATCACGGCCAACGTGCCTTACCAGACGGTGATGCGAAACGCC

ENSMUST00000049474   GACCTCGACAACTTTGCCTGTCACAGGAGACATCGTGGCCTGATCCATGCCTATGAATTT
ENSRNOT00000001488   GACCTGGACAACTTTGCCTGTCACAGGAGACACCGCGGCCTGATCCATGCCTATGAATTT
ENSCJAT00000018458   GACCGGGACAACTTCACTCCTCACAGGAGACACCAGGGGCTGATCCGCGCCTATGAGTTT
ENSMMUT00000024573   GACCTGGACAGCTTCACTACTCACAGGAGACACCGGGGGCTGATCCGTGCCTACGAGTTT
ENSPPYT00000005920   GACCTGGACAGCTTCACTACTCACAGGAGACACCGGGGGCTGATCCGCGCCTACGAGTTT
ENST00000309909   GACCTGGACAGCTTCACTACTCACAGGAGACACCGGGGGCTGATCCGCGCCTATGAGTTT
ENSPTRT00000010100   GACCTGGACAGCTTCACCACTCACAGGAGACACCGGGGGCTGATCCGCGCCTACGAGTTT

ENSMUST00000049474   GCTGTGGACCAGCTGGCCTTTCAGAGCCCCCTTCCTGTCTGCCGCTTACCCCGTGACATC
ENSRNOT00000001488   GCTGTGGACCAGCTGGCCTTTCAGAGCCCTCTTCCTATCTGCCGCTTACCCCGTGACACC
ENSCJAT00000018458   GCGGTAGACCAGCTGGCCTTCCAGAGCCCACTCCCTGCCTGCCGTTCCTCCTGCGACACC
ENSMMUT00000024573   GCAGTGGACCAGCTGGCCTTCCAGAGCCCTCTCCCTGTCTGCCGTTCATCCTGTGACGCC
ENSPPYT00000005920   GCGGTGGACCAGCTGGCCTTCCAGAGCCCTCTCCCTGTCTGTCGTTCATCCTGTGACGCC
ENST00000309909   GCGGTGGACCAGCTGGCCTTCCAGAGCCCTCTCCCTGTCTGCCGTTCATCCTGTGACGCC
ENSPTRT00000010100   GCGGTGGACCAGCTGGCCTTCCAGAGCCCTCTCCCTGTCTGCCGTTCATCCTGTGACGCC

ENSMUST00000049474   ATGGCTGGATACAGCTATGACCTCGCACTGTCTTTCCCCCATGACAGTATT
ENSRNOT00000001488   GTGGCTGGATACAGCTATGACCTAGCCCTGTCTTTCCCCTATGACAGTATT
ENSCJAT00000018458   ATGGCCACTCACTACTACGACCTTGCACTGGCTTTTCCCTATAACCATGTT
ENSMMUT00000024573   ATGGCCACTCACTACTATGACCTCGCACTGGCCTTTCCCTATAACCACGTT
ENSPPYT00000005920   ATGGCCACTCACTACTACGACCTCGCGCTGGCCTTTCCCTATAACCACGTT
ENST00000309909   ATGGCCACTCACTACTACGACCTCGCACTGGCCTTTCCCTATAACCATGTT
ENSPTRT00000010100   ATGGCCACTCACTACTACGACCTCGCACTGGCCTTTCCCTATAACCATGTT

Multiple sequence alignment of Fbxw9

ENSMUST00000095220   ATGGATCTTTCCTCAGGGCGGAGTGGGGATCCTCGCTCTTGTGAGGAGGAATCTGACCCC
ENSRNOT00000005581   ATGGAGCTTCCCTCAGGGCAGTGTGGGAATCCTCGCTCTTGTGAGGACGAGTCGGACCCC
ENSCJAT00000010100   ATGGAGCTTCCCTTAGGGCCGTGCGACGATTCCCGTGGCTGGGACGATGACTCGGATCCC
ENSMMUT00000038719   ATGGAGCTTCCCCTAGGGCAGTGCCATGATTCCCGCTCCTGGGACGATGACTCGGACCCA
ENSPPYT00000011187   ATGGAGCTTCCCCTAGGGCCGTGCGATGATTCCCGCGCCTGGGACGATGACTCGGACCCA
ENST00000393261   ATGGAGCTTCCCCTAGGGCGGTGCGATGATTCCCGCACCTGGGACGATGACTCGGACCCA
ENSGGOT00000029731   ATGGAGCTTCCCCTAGGGCGGTGCGATGATTCCCGCACTTGGGACGATGACTCGGACCCA
ENSPTRT00000019434   ATGGAGCTTCCCCTAGGGCGGTGCGATGATTCCCGCACCTGGGACGATGAGTCGGACCCA

ENSMUST00000095220   GAGCCAGATCCCGACCCTGACACTCAAGCTGAGGCCTACGTAGCCCGTGTGCTTACCCCT
ENSRNOT00000005581   GAGCCAGAGCCCGACCCTGATGCTCAAGCTGAGGCCTATGTAGCACGCGTGCTCACCCCG
ENSCJAT00000010100   GAGTCAGAGACAGACCCCGACGCGCAGGCCGAGGCCTACGTGGCCCGCGTGCTCAGTCCG
ENSMMUT00000038719   GAGTCAGAGACAGACCCAGACGCGCAGGCCGAGGCCTACGTGGCCCGCGTTCTCAGTCCG

```
ENSPPYT00000011187     GAGTCAGAGACAGACCCAGACGCGCAGGCCGAGGCCTATGTGGCCCGCGTTCTCAGTCCT
ENST00000393261        GAGTCAGAGACAGACCCAGACGCGCAGGCCAAGGCCTACGTGGCCCGCGTTCTCAGTCCG
ENSGGOT00000029731     GAGTCAGAGACAGACCCAGACGCACAGGCGAAGGCCTACGTGGCCCGCGTTCTCAGTCCG
ENSPTRT00000019434     GAGTCAGAGACAGACCCAGACGCGCAGGCCAAGGCCTACGTGGCCCGCGTTCTCAGTCCG

ENSMUST00000095220     CCCAAGCTTGGCCTGACCCCGCGGCGCTCGTCACTGCAGTCCATGTTCTCCGCGTCCCTA
ENSRNOT00000005581     CCCAAACCTGGCATGACCCCGCGGCGCTCGTCACTGCAGTCCATGTTCTCCGCGTCCCTG
ENSCJAT00000010100     CCAAAATCCGAGCTGGCGACCCCGCGCCCCTCGCAGCTGTCCACGCCCGCCTCATCCCCG
ENSMMUT00000038719     CCAAAATCCGGGCTGGCGTTCCCGCGTCCCTCGCAGCTATCCACGCCCGCCGCGTCCCCG
ENSPPYT00000011187     CCAAAATCCGGGCTGGCGTTCTCGCGCCCCTCGCAGCTATCCACGCCCGCCGCGTCCCCG
ENST00000393261        CCAAAATCCGGGCTGGCGTTCTCGCGCCCCTCGCAGCTATCCACACCCGCCGCGTCCCCG
ENSGGOT00000029731     CCAAAATCCGGGCTGGCGTTCTCGCGCCCCTCGCAGCTATCCACACCCGCCGCGTCCCCG
ENSPTRT00000019434     CCAAAATCCGGGCTGGCGTTCTCGCGCCCCTCGCAGCTATCCACACCCGCCGCGTCCCCG

ENSMUST00000095220     GGCGTGCCGGAGCGAAAGGCCGCCTCCAAAGTCCCGGCCGTGCGCCTACCGGGCCTCTTG
ENSRNOT00000005581     GGCATGCCAGAGCGAAAGGATGCCTCCAAAGTACCGGCAGTGAGCCTGCCGGGCCTCCTC
ENSCJAT00000010100     AGCGCTTCGGAGCCTCGAGCCGCGTCCAAGGTTTCGACCGTAAGTGAGCCGGGCCTTCTG
ENSMMUT00000038719     AGCGCTTCGGAGCCTCGGGCCGCGTCCAAGGTTTCGGCCGTAAGTGAGCCAGGCCTTCTG
ENSPPYT00000011187     AGCGCTTCGGAGCCTCGGGCCGCGTCCAAGGTTTCGGCCGTAAGTGAGCCAGGCCTTCTG
ENST00000393261        AGCGCTTCGGAGCCTCGGGCCGCGTCCAGGGTTTCGGCCGTAAGTGAGCCGGGCCTTCTG
ENSGGOT00000029731     AGCGCTTCGGAACCTCGGGCCGCGTCCAAGGTTTCGGCCGTAAGTGAGCCGGGCCTTCTG
ENSPTRT00000019434     AGCGCTTCGGAGCCTCGGGCCGCGTCCAAGGTTTCGGCCGTAAGTGAGCCGGGCCTTCTG

ENSMUST00000095220     AGCCTTCCCCCGGAGCTGCTGCTGGAGATCTGTGCCTACCTGGATGCGCGAGTCGTGCTT
ENSRNOT00000005581     AGCCTTCCCCCGGAGCTGCTGCTGGAGATCTGTGCCTACCTGGATGCGCGGGTCGTGCTT
ENSCJAT00000010100     AGCCTTCCCCCGGAGCTGCTGCTTGAGATCTGCTCCTACCTGGACGCCCGCCTCGTGCTC
ENSMMUT00000038719     AGCCTTCCCCCGGAGCTGCTGCTCGAGATCTGCTCCTACCTGGACGCCCGCCTCGTGCTC
ENSPPYT00000011187     AGCCTTCCCCCGGAGCTGCTGCTCGAGATCTGCTCCTACCTGGACGCCCGTCTCGTGCTC
ENST00000393261        AGCCTTCCCCCGGAGCTGCTGCTCGAGATCTGCTCCTACCTGGACGCCCGCCTCGTGCTC
ENSGGOT00000029731     AGCCTTCCCCCGGAGCTGCTGCTCGAGATCTGCTCCTACCTGGACGCCCGCCTCGTGCTC
ENSPTRT00000019434     AGCCTTCCCCCGGAGCTGCTGCTCGAGATCTGCTCCTACCTGGACGCCCGCCTCGTGCTC

ENSMUST00000095220     CAAGTCCTGCCGTGCGTGTGCCAAGCGTTGCACGACCTCGTGCGTGATCGTGTCACCTGG
ENSRNOT00000005581     CATGTCCTGCCGTGCGTGTGCCAAGCGCTGCACAACCTCGTGCGTGATCGAGTCACCTGG
ENSCJAT00000010100     CACGTCCTGTCACGCGTGTGCCACGCTCTGCGTAACCTCGTGCTTGACCATGTCACCTGG
ENSMMUT00000038719     CACGTCCTGTCGCGCGTGTGCCACGCGCTGCGCGATCTCGTGTATGACCGTGTCACCTGG
ENSPPYT00000011187     CACGTCCTGTCGCGGGTGTGCCACGCGCTGCGCGACCTCGTGTCTGACCATGTCACCTGG
ENST00000393261        CACGTCCTGTCGCGGGTGTGCCACGCGCTCCGCGACCTCGTGTCTGACCATGTCACCTGG
ENSGGOT00000029731     CACGTCCTGTCGCGGGTGTGCCACGCGCTGCGCGACCTCGTGTCTGACCATGTCACCTGG
ENSPTRT00000019434     CACGTCCTGTCGCGGGTGTGCCACGCGCTGCGCGACCTCGTGTCTGACCATGTCACCTGG

ENSMUST00000095220     AGGCTACGCGCTCAGCGCCGCGTACGCGCACCCTACCCAGTGGTGGAAGAGGAGAACTTT
ENSRNOT00000005581     AGGCTACGCGCTCAGCGCCGCGTACGCTCACCCTACCCAGTGGTAGAAGAGGAGAACTTT
ENSCJAT00000010100     AGGCTACGCGCGCAACGCCGTGTACGCGCGCCCTACCCAGTGGTGGAAGGGAAGAACTTT
ENSMMUT00000038719     AGGCTACGCGCGCTACGCCGCGTACGCGCGCCCTACCCAGTGGTGGAAGAGAAGAACTTT
ENSPPYT00000011187     AGGCTACGCACGCTACGCCGCGTACGCGCGCCCTACCCAGTGGTGGAAGAGAAGAACTTT
ENST00000393261        AGGCTACGCGCGCTACGCCGCGTACGCGCGCCCTACCCAGTGGTGGAAGAGAAGAACTTT
ENSGGOT00000029731     AGGCTACGCGCGCTACGCCGCGTACGCGCGCCCTACCCAGTGGTGGAAGAGAAGAACTTT
ENSPTRT00000019434     AGGCTACGCGCGCTACGCCGCGTACGCGCGCCCTACCCAGTGGTGGAAGAGAAGAACTTT

ENSMUST00000095220     GACTGGCCAGCCGCCTGCATTGAGCTGGAGCAGCACCTGGCCCGCTGGGCAGAGGATGGA
ENSRNOT00000005581     GACTGGCCGGCCGCCTGCATTGAGCTGGAGCAGCACCTGGCCCGCTGGGCAGAGGACGGA
ENSCJAT00000010100     GACTGGCCAGCAGCCTGCACTGAGCTGGAGCAGCACCTGTCCCGCTGGGCGGAGGATGGG
ENSMMUT00000038719     GACTGGCCGGCAGCCTGCATTGCGCTGGAGGAGCACCTGTCCCGCTGGGCAGAGGATGGG
```

ENSPPYT00000011187   GACTGGCCGGCAGCCTGCATTGCGCTGGAGCAGCACCTGTCCCGCTGGGCAGAGGATGGG
ENST00000393261   GACTGGCCGGCAGCCTGCATTGCGCTGGAGCAGCACCTGTCCCGCTGGGCAGAGGATGGG
ENSGGOT00000029731   GACTGGCCGGCAGCCTGCATTGCGCTGGAGCAGCACCTGTCCCGCTGGGCAGAGGATGGG
ENSPTRT00000019434   GACTGGCCGGCAGCCTGCATTGCGCTGGAGCAGCACCTGTCCCGCTGGGCAGAGGATGGG

ENSMUST00000095220   CAGCGAACCGAGTACTTCTGCCTGGCTGATGGCCACTTTGCTTCCATTGATGCGGTGTTG
ENSRNOT00000005581   CAGCGGACCGAATACTTCTGCCTGGCTGATGGCCACTTTGCTTCCATTGATGCAGTGTTG
ENSCJAT00000010100   CGCTGGGCTGAGCACTTCTGCCTGGCCAACGGGCACATTTCATCCATTGACTCAGTGCTG
ENSMMUT00000038719   CGCCGGGCCGAATACTTCTGCTTGGCCGATGGGCACCTGGCTGCCGTTGACTCAGTGCTG
ENSPPYT00000011187   CGCTGGGTTGAATACTTCTGCCTGGCCGACGGGCACGTGGCTTCTGTTGACTCAGTGCTG
ENST00000393261   CGCTGGGTCGAATACTTCTGCCTGGCCGAAGGCCACGTGGCTTCCGTTGACTCAGTGCTG
ENSGGOT00000029731   CGCTGGGTCGAATACTTCTGCCTGGCCGACGGCCACGTGGCTTCCGTTGACTCAGTGCTG
ENSPTRT00000019434   CGCTGGGTCGAATACTTCTGCCTGGCTGAAGGCCACGTGGCTTCCGTTGACTCAGTGCTG

ENSMUST00000095220   CTGCTCCAGGGTGGGGCACTGTGTCTGTCAGGCTCCCGGGATCGGAATGTCAACCTGTGG
ENSRNOT00000005581   CTACTCCAGGGTGGGACACTGTGTTTGTCAGGCTCCCGGGATCGAAATGTCAACCTGTGG
ENSCJAT00000010100   CTGCTCCAGGGTGGGGCACTCTGTCTGTCGGGCTCCCGAGATCGCAACGTCAACTTGTGG
ENSMMUT00000038719   CTGCTCCAGGGTGGGTCACTCTGTCTGTCGGGCTCCCGAGATCGCACTGTCAACTTGTGG
ENSPPYT00000011187   CTGCTCCAGGGTGGGTCACTCTGTCTGTCGGGCTCCCGAGATCGCAACGTCAACTTGTGG
ENST00000393261   CTGCTCCAGGGTGGGTCACTCTGTCTGTCGGGCTCCCGAGATCGCAACGTCAACTTGTGG
ENSGGOT00000029731   CTGCTCCAGGGTGGGTCACTCTGTCTGTCGGGCTCCCGAGATCGCAACGTCAACTTGTGG
ENSPTRT00000019434   TTGCTCCAGGGTGGGTCACTCTGTCTGTCGGGCTCCCGAGATCGCAACGTCAACTTGTGG

ENSMUST00000095220   GACCTGCGCCATCTAGGGAAGGATCCCAGCCGAGTTCTGGTGAAGGCCTTAGGCACCCAG
ENSRNOT00000005581   GACTTGCGACATCTAGGGAAGGAGCCCAGCCGAGTTCTGGTGAAGGCCTTGGGCACCCAG
ENSCJAT00000010100   GACCTGCGGCAGCTGGGGATGGAGCCCAGCCAGGTTCTGGTCAAGACCTTGGGCACCGAT
ENSMMUT00000038719   GACCTGCGGCAGCTGGGGACGGAGCCCAGCCAGGTTCTGGTCAAGACCTTGGGTACTAAG
ENSPPYT00000011187   GACCTGCGGCAGCTGGGGACGGAGTCCAACCAGGTTCTGGTCAAGACCTTAGGCACTAAG
ENST00000393261   GACCTGCGGCAGCTGGGGACGGAGTCCAACCAGGTTCTGATCAAGACCTTAGGCACTAAG
ENSGGOT00000029731   GACCTGCGGCAGCTGGGGACGGAGTCCAGCCAGGTTCTGGTCAAGACCTTAGGCACTAAG
ENSPTRT00000019434   GACCTGCGGCAGCTGGGGACGGAGTCCAACCAGGTTCTGGTCAAGACCTTAGGCACTAAG

ENSMUST00000095220   GGCAATAGCACACACAAGGGCTGGGTATGGTCGCTAGCAGCGCAGGACCACCGTGTGTGC
ENSRNOT00000005581   GGCAATAGCACACACAAGGGCTGGGTATGGTCGCTAGCAGCACAGGACCACCGTGTGTGC
ENSCJAT00000010100   CGATTTAGTACGCACGAGGGCTGGGTGTGGTCGCTGGCGGCACAGGACCATTGTGTGTGC
ENSMMUT00000038719   CGGAATAGTACCCACGAGGGCTGGGTGTGGTCACTGGCAGCGCAGGACCACCGCGTGTGC
ENSPPYT00000011187   CGAAATAGTACCCATGAGGGCTGGGTGTGGTCACTGGCAGCACAGGACCACCGCGTGTGC
ENST00000393261   CGAAATAGTACCCATGAGGGCTGGGTGTGGTCACTGGCAGCGCAGGACCACCGCGTGTGC
ENSGGOT00000029731   CGAAATAGTACCCATGAGGGCTGGGTGTGGTCACTGGCAGCGCAGGACCACCGCGTGTGC
ENSPTRT00000019434   CGAAATAGTACCCATGAGGGCTGGGTGTGGTCACTGGCAGCGCAGGACCACCGCGTGTGC

ENSMUST00000095220   TCCGGCTCTTGGGACAGCACTGTGAAGCTGTGGGACATGGCGGCTGATGGGCAGCAGTTT
ENSRNOT00000005581   TCCGGCTCTTGGGACAGCACCGTGAAGCTGTGGGACATGGCGGCCGATGGGCAGCAGTTT
ENSCJAT00000010100   TCCGGCTCCTGGGACAGCACAGTGAAGCTCTGGGACATGGCAGCAGATGGGCAGCAGTTT
ENSMMUT00000038719   TCTGGCTCCTGGGACAGCACAGTGAAGCTCTGGGACCTGGCAGCAGATGGGCAGCAGTTC
ENSPPYT00000011187   TCCGGCTCCTGGGACAGCACAGTGAAGCTCTGGGACATGGCAGCGGATGGGCAGCAGTTC
ENST00000393261   TCCGGCTCCTGGGACAGCACAGTGAAGCTCTGGGACATGGCAGCGGATGGGCAGCAGTTC
ENSGGOT00000029731   TCCGGCTCCTGGGACAGCACAGTGAAGCTCTGGGACATGGCAGCGGATGGGCAGCAGTTC
ENSPTRT00000019434   TCCGGCTCCTGGGACAGCACAGTGAAGCTCTGGGACATGGCAGCGGATGGGCAGCAGTTC

ENSMUST00000095220   GGAGAGATCAAGGGCAAGGCGGCAGTGCTGTGCCTTTCCTACCAACCTGATATCCTGGTG
ENSRNOT00000005581   GGAGAGATCAAGGGCAAGGCGGCAGTGCTGTGCCTTTCCTACCAACCTGATATCCTGGTG
ENSCJAT00000010100   GGCGAGATAAAGGCTAACTCAGCCGTGCTGTGCCTCTCCTACCTGCCTGACATCCTGGTG
ENSMMUT00000038719   GGCGAGATAAAGACCAGCTCAGCCGTGCTGTGCCTCTCCTACCTGTCTGACGTCCTGGTG

```
ENSPPYT00000011187     GGCGAGATAAAGGCCAGCTCAGCCGTGCTGTGCCTCTCCTACCTGCCTGATATCCTGGTG
ENST0000393261         GGCGAGATAAAGGCCAGCTCAGCCGTGCTGTGCCTCTCCTACCTGCCTGACATCCTGGTG
ENSGGOT00000029731     GGCGAGATAAAGGCCAGCTCAGCCGTGCTGTGCCTCTCCTACCTGCCTGACATCCTGGTG
ENSPTRT00000019434     GGCGAGATAAAGGCCAGCTCAGCCGTGCTGTGCCTCTCCTACCTGCCTGACATCCTGGTG

ENSMUST00000095220     ACTGGTACCTATGACAAGAAGGTGACCATCTATGATCCCAGAGCTGGTTTGGCCCTGGTG
ENSRNOT00000005581     ACTGGTACCTATGACAAGAAGGTGACCATCTATGATCCCAGAGCTGGCCTGACCCTGGTG
ENSCJAT00000010100     ACCGGCACCTATGACAAGAAGGTGACCATCTACGACCCCAGAGCCGGCCCAGCCCTGTTG
ENSMMUT00000038719     ACTGGCACCTATGACAAGAAGGTGGCCATCTATGACCCCAGAGCCAGCCCAGCCCTGTTG
ENSPPYT00000011187     ACTGGCACCTATGACAAGAAGGTGACCATCTACGACCCCAGAGCCGGCCCAGCCCTGTTG
ENST0000393261         ACTGGCACCTATGACAAGAAGGTGACCATCTACGACCCCAGAGCCGGCCCAGCCCTGTTG
ENSGGOT00000029731     ACTGGCACCTATGACAAGAAGGTGACCATCTACGACCCCAGAGCCGGCCCAGCCCTGTTG
ENSPTRT00000019434     ACTGGCACCTATGACAAGAAGGTGACCATCTACGACCCCAGAGCCGGCCCAGCCCTGTTG

ENSMUST00000095220     AAGAGCCGGAGGCTGCACTCAAGCGCGGTGCTAGCAGTGCTGGCAGATGACAGGCATGTC
ENSRNOT00000005581     AAGAGCCGGAGGCTGCACTCAAGTGCTGTGCTAGCAGTGCTGGCGGATGACAGACATATC
ENSCJAT00000010100     AAGAGCCAGCGACTACACTCTAGCCCTGTGTTGGCCCTGCTGGCAGATGACCAGCACATC
ENSMMUT00000038719     AAGTGCCAGCAACTACACTCCAGACCCGTGCTGGCCCTGATGGCGGATGACCAGCACATC
ENSPPYT00000011187     AAGCGCCAGCAACTACACTCCAGACCCGTGCTGACCCTGCTGGCGGATGACCGGCACATC
ENST0000393261         AAGCACCAGCAACTACACTCCAGACCCGTGCTGACCCTGCTGGCGGATGACCGGCACATC
ENSGGOT00000029731     AAGCACCAGCAACTACACTCCAGACCCGTGCTGACCCTGCTGGCGGATGACCGGCACATC
ENSPTRT00000019434     AAGCACCAGCAACTACACTCCAGACCCGTGCTGACCCTGCTGGCGGATGACCGGCACATC

ENSMUST00000095220     ATCTCAGGCAGCGAGGACCACAGCCTTGTGGTATTTGATCGTCGAGCCAATAGCGTCCTA
ENSRNOT00000005581     ATCTCAGGTAGTGAGGACCACAGTCTTGTGGTATTTGATCGTCGAGCCAATAGCGTCCTG
ENSCJAT00000010100     ATCTCAGGCAGCGAGGACCGCACCCTGGTGGTGGTTGACCGCCGAGCCAACAGAGTCCTG
ENSMMUT00000038719     ATCTCAGGCAGCGAGGACCGCACCCTGGTAGTGGTTGACCGCCGAGCCAACAGAGTCCTG
ENSPPYT00000011187     ATCTCAGGCAGCGAGGACCACACCCTGGTGGTGGTGGACCGCCGAGCCAACAGCGTCCTG
ENST0000393261         ATCTCAGGCAGCGAGGACCACACCCTGGTGGTGGTGGACCGCCGAGCCAACAGCGTCCTG
ENSGGOT00000029731     ATCTCAGGCAGCGAGGACCACACCCTGGTGGTGGTGGACCGCCGAGCCAACAGCGTCCTG
ENSPTRT00000019434     ATCTCAGGCAGCGAGGACCACACCCTGGTGGTGGTGGACCGCCGAGCCAACAGCGTCCTG

ENSMUST00000095220     CAGCGGCTGCAGCTGGACTCCTATCTCCTGTGCATGTCTTACCAGGAGCCCCAGCTCTGG
ENSRNOT00000005581     CAGCGGCTCCAGCTGGACTCCTATCTGCTCTGCATGTCTTACCAGGAGCCCCAGCTCTGG
ENSCJAT00000010100     CAGCGGCTGCAGCTGGACTCCTACCTGCTCTGCATGTCCCACCAGGATATGCAGCTCTGG
ENSMMUT00000038719     CAGCGTCTGCAGCTGGACTCTTACCTGCTCTGCATGTCCTACCAGGAACCCCAGCTCTGG
ENSPPYT00000011187     CAGCGTCTGCAGCTGGACTCCTACCTGCTCTGCATGTCCTACCAGGAACCCCAGCTCTGG
ENST0000393261         CAGCGTCTGCAGCTGGACTCCTACCTGCTCTGCATGTCCTACCAGGAACCCCAGCTCTGG
ENSGGOT00000029731     CAGCGTCTGCAGCTGGACTCCTACCTGCTCTGCATGTCCTACCAGGAACCCCAGCTCTGG
ENSPTRT00000019434     CAGCGTCTGCAGCTGGACTCCTACCTGCTCTGCATGTCCTACCAGGAACCCCAGCTCTGG

ENSMUST00000095220     GCAGGTGACAACCAGGGTCTGCTGCACGTCTTCGCCAATCGAGATGGTTGCTTCCAGCTT
ENSRNOT00000005581     GCAGGTGACAACCAGGGCCTGCTGCACGTCTTCGCCAACCAAGATGGCTGCTTCCAGCTT
ENSCJAT00000010100     ACTGGTGACAACGAGGGCCTGCTGCACGTCTTCGCCAACCGCAACGGCTGCTTCCAGCAT
ENSMMUT00000038719     GCTGGTGACAACCACGGCCTGCTGCACGTCTTCGTCAACCGCAACGGCTGCTTACAGCTT
ENSPPYT00000011187     GCTGGTGACAACCAGGGCCTGCTGCACGTCTTCGCCAACCGCAACGGCTGCTTCCAGCTT
ENST0000393261         GCTGGTGACAACCAGGGCCTGCTGCACGTCTTCGCCAACCGCAACGGCTGCTTCCAGCTT
ENSGGOT00000029731     GCTGGTGACAACCAGGGCCTGCTGCACGTCTTCGCCAACCGCAACGGCTGCTTCCAGCTT
ENSPTRT00000019434     GCTGGTGACAACCAGGGCCTGCTGCACGTCTTTGCCAACCGCAACGGCTGCTTCCAGCTT

ENSMUST00000095220     GTTCGGTCCTTTGATGTGGGTCACCAGTCTCAGATCACAGGGATCAAACACTCGCTGGGG
ENSRNOT00000005581     GTTCGGACCTTTGATGTGGGTCACCAGTCTCAGATCACAGGGATCAAACACTCCCTGGGG
ENSCJAT00000010100     GTCCGGTCCTTCAACGTGGGCCACAGGTCTCACATCACTGGGATCCAGTACTCACTGGGA
ENSMMUT00000038719     ATCCGGTCCTTTGACGTGGGCCACAGCTTTCCCATCACTGGGATCCAGTACTCAGTGGGA
```

```
ENSPPYT00000011187     ATCCGGTCCTTTGATGTGGGCCACAGCTTTCCTATCACTGGGATCCAGTACTCAGTGGGA
ENST00000393261        ATCCGGTCCTTTGATGTGGGCCACAGCTTTCCCATCACTGGGATCCAGTACTCCGTGGGA
ENSGGOT00000029731     ATCCGGTCCTTTGATGTGGGCCACAGCTTTCCCATCACTGGGATCCAGTACTCAGTGGGA
ENSPTRT00000019434     ATCCGGTCCTTTGATGTGGGCCACAGCTTTCCCATCACTGGGATCCAGTACTCCGTGGGA


ENSMUST00000095220     ACTTTGTACACAACATCTACTGACAAGACCATTCGGGTTCACGTGCCCACAGATCCACCT
ENSRNOT00000005581     ACTCTGTACACAACATCTACTGACAAGACTATTCGGGTTCATGTGCCCACAGATCCACCT
ENSCJAT00000010100     GCCTTGTATACTGCATCCACTGACAAGACCCTCCGGGTGCACGTGCCCACAGACCCACCA
ENSMMUT00000038719     GCCTTGTACACCACATCCACTGACAGGTCCATCCGGGTGCACGTGCCCACAGACCCACCA
ENSPPYT00000011187     GCCTTGTACACCACATCCACTGACAAGACCATCCGGGTGCACGTGCCCACAGACCCACCA
ENST00000393261        GCCTTGTACACCACATCCACTGACAAGACCATCCGGGTGCACGTGCCCACAGACCCACCA
ENSGGOT00000029731     GCCTTGTACACCACATCCACTGACAAGACCATCCGGGTGCACGTGCCCACAGACCCACCA
ENSPTRT00000019434     GCCTTGTACACCACATCCACTGACAAGACCATCCGGGTGCACGTGCCCACAGACCCACCA


ENSMUST00000095220     AGGACCATCTGTACCCGAAGCCACCACAACGTGTTAAATGGGATCTGTGCTGAGGGCAAC
ENSRNOT00000005581     AAGACCATCTGTACCCGAAGGCACCACAACGTGTTAAATGGGATCTGTGCTGAGGGCAAC
ENSCJAT00000010100     AGGACCATCTGCACCCGAAGGCACGACAATGGGTTCACCAAGGTCTGTGCTGAGGGCAAC
ENSMMUT00000038719     AGGACCATCTGCACCCGAAAGTACGACAGTACGCTCAATAGGGTCTGTGCTGAGGGCAAC
ENSPPYT00000011187     AGGACCATTTGCACCCGAAAGCATGACAATGGGCTCAATAGGGTCTGTGCTGAGGGCAAC
ENST00000393261        AGGACCATTTGCACCCGAAGGCATGACAATGGGCTCAATAGGGTCTGTGCTGAGGGCAAC
ENSGGOT00000029731     AGGACCATTTGCACCCGAAGGCATGACAATGGGCTCAATAGGGTCTGTGCTGAGGGCAAC
ENSPTRT00000019434     AGGACCATTTGCACCCGAAGGCATGACAATGGGCTCAATAGGGTCTGTGCTGAGGGCAAC


ENSMUST00000095220     GTAGTGGTGGCTGCCTCTGGTGGCCTGTCATTGGAGGTCTGGAGGCTGCTGGCC
ENSRNOT00000005581     ATAGTGGTGGCTGCCTCTGGTGGCCTGTCATTGGAGGTCTGGAGGCTGTTGGCC
ENSCJAT00000010100     CTGGTGGTGGCTGCCTCTGGGGGCCTGTCGCTAGAGGTCTGGAGGCTGCAGGCC
ENSMMUT00000038719     CTCGTGGTGGCCGGCTCTGGGGACGTGTCGCTAGAGGTCTGGAGGCTGCCGGCC
ENSPPYT00000011187     CTAGTGGTGGCCGGCTCTGGAGACCTGTCGCTAGAGGTCTGGAGGCTGCAGGCC
ENST00000393261        CTGGTGGTGGCCGGCTCTGGAGACCTGTCGCTAGAGGTCTGGAGGCTGCAGGCC
ENSGGOT00000029731     CTGGTGGTGGCCGGCTCTGGAGACCTGTCGCTAGAGGTCTGGAGGCTGCAGGCC
ENSPTRT00000019434     CTGGTGGTGGCCGGCTCTGGAGACCTGTCGCTAGAGGTCTGGAGGCTGCAGGCC
```

Multiple sequence alignment of Kdm2A

```
ENSMMUT00000012253     ATGGAACCCGAAGAAGAAAGGATTCGTTACAGCCAGAGATTGCGTGGTACCATGCGACGA
ENSCJAT00000002028     ATGGAACCCGAAGAAGAAAGGATTCGTTACAGCCAGAGATTGCGTGGTACCATGCGACGG
ENST00000529006        ATGGAACCCGAAGAAGAAAGGATTCGTTACAGCCAGAGATTGCGTGGTACCATGCGACGA
ENSPTRT00000007365     ATGGAACCCGAAGAAGAAAGGATTCGTTACAGCCAGAGATTGCGTGGTACCATGCGACGA
ENSMUST00000047898     ATGGAACCTGAAGAAGAAAGGATTCGGTACAGCCAGAGATTGCGTGGTACCATGCGTCGT
ENSRNOT00000054850     ATGGAACCTGAAGAAGAAAGGATTCGGTACAGCCAGAGATTGCGTGGTACCATGCGACGT


ENSMMUT00000012253     CGCTATGAAGATGATGGCATTTCAGATGATGAAATTGAAGGGAAAAGAACTTTTGACTTG
ENSCJAT00000002028     CGCTATGAAGATGACGGCATTTCAGATGATGAAATTGAAGGGAAAAGAACTTTTGACTTG
ENST00000529006        CGCTATGAAGATGATGGCATTTCAGATGATGAAATTGAAGGAAAAAGAACTTTTGACTTG
ENSPTRT00000007365     CGCTATGAAGATGATGGCATTTCAGATGATGAAATTGAAGGAAAAAGAACTTTTGACTTG
ENSMUST00000047898     CGCTATGAAGATGATGGCATTTCAGATGATGAAATTGAAGGGAAAAGAACTTTTGACTTG
ENSRNOT00000054850     CGCTATGAAGATGATGGCATTTCAGATGATGAAATTGAAGGGAAAAGAACTTTTGACTTG


ENSMMUT00000012253     GAAGAGAAACTGCACACCAACAAATATAATGCCAATTTTGTTACTTTTATGGAAGGAAAA
ENSCJAT00000002028     GAAGAAAAACTGCATACCAACAAATATAATGCCAATTTTGTTACGTTTATGGAAGGAAAA
ENST00000529006        GAAGAGAAACTGCACACCAACAAATATAATGCCAATTTTGTTACTTTTATGGAAGGAAAA
ENSPTRT00000007365     GAAGAGAAACTGCACACCAACAAATATAATGCCAATTTTGTTACTTTTATGGAAGGAAAA
ENSMUST00000047898     GAAGAGAAGCTCCAAACCAACAAATATAATGCCAATTTTGTTACTTTTATGGAGGGAAAA
ENSRNOT00000054850     GAAGAGAAGCTCCATACCAACAAATATAATGCCAATTTTGTTACTTTTATGGAGGGAAAA
```

```
ENSMMUT00000012253     GATTTTAATGTAGAGTATATTCAGCGGGGTGGCTTGAGAGATCCTCTGATTTTCAAGAAT
ENSCJAT00000002028     GATTTTAATGTAGAGTATATCCAGCGGGGTGGTTTGAGAGATCCTCTGATTTTCAAGAAT
ENST00000529006        GATTTTAATGTAGAGTATATTCAGCGGGGTGGCTTGAGAGATCCTCTGATTTTCAAGAAT
ENSPTRT00000007365     GATTTTAATGTAGAGTATATTCAGCGGGGTGGCTTGAGAGATCCTCTGATTTTCAAGAAT
ENSMUST00000047898     GATTTTAATGTAGAGTATATCCAGCGGGGTGGCTTGAGAGACCCTCTCATTTTCAAGAAT
ENSRNOT00000054850     GATTTTAATGTAGAGTATATCCAGCGGGGTGGCTTGAGAGACCCTCTCATTTTCAAGAAT

ENSMMUT00000012253     TCTGATGGACTTGGAATAAAAATGCCGGATCCAGACTTCACTGTGAATGATGTCAAAATG
ENSCJAT00000002028     TCTGATGGACTTGGAATAAAAATGCCGGATCCAGACTTCACTGTGAATGATGTCAAAATG
ENST00000529006        TCTGATGGACTCGGAATAAAAATGCCGGATCCAGACTTCACTGTGAATGATGTCAAAATG
ENSPTRT00000007365     TCTGATGGACTCGGAATAAAAATGCCGGATCCAGACTTCACTGTGAATGATGTCAAAATG
ENSMUST00000047898     TCTGATGGACTTGGAATAAAGATGCCGGATCCAGACTTCACAGTGAATGATGTCAAAATG
ENSRNOT00000054850     TCTGATGGACTTGGAATAAAGATGCCGGATCCAGACTTCACAGTGAATGATGTCAAAATG

ENSMMUT00000012253     TGTGTAGGGAGTCGTCGCATGGTGGATGTCATGGATGTGAACACACAGAAAGGCATTGAA
ENSCJAT00000002028     TGTGTGGGGAGTCGTCGCATGGTGGATGTCATGGACGTGAACACGCAAAAAGGCATTGAA
ENST00000529006        TGTGTGGGGAGTCGTCGCATGGTGGATGTCATGGACGTGAACACACAGAAAGGCATTGAA
ENSPTRT00000007365     TGTGTGGGGAGTCGTCGCATGGTGGATGTCATGGACGTGAACACACAGAAAGGCATTGAA
ENSMUST00000047898     TGTGTGGGGAGTCGTCGGATGGTGGATGTCATGGATGTGAACACACAGAAGGGGATTGAA
ENSRNOT00000054850     TGTGTGGGGAGTCGTCGGATGGTGGATGTCATGGACGTGAACACACAGAAGGGGATTGAA

ENSMMUT00000012253     ATGACCATGGCTCAGTGGACACGCTACTATGAGACCCCAGAGGAGGAGCGAGAAAAACTC
ENSCJAT00000002028     ATGACCATGGCTCAGTGGACACGCTACTATGAGACCCCAGAGGAGGAGCGAGAGAAACTC
ENST00000529006        ATGACCATGGCTCAGTGGACACGCTACTATGAGACCCCAGAGGAGGAGCGAGAGAAACTC
ENSPTRT00000007365     ATGACCATGGCTCAGTGGACACGCTACTATGAGACCCCAGAGGAGGAGCGAGAGAAACTC
ENSMUST00000047898     ATGACCATGGCACAATGGACACGATACTATGAGACTCCAGAGGAAGAGCGAGAAAAACTC
ENSRNOT00000054850     ATGACCATGGCGCAATGGACACGATACTATGAGACTCCAGAGGAAGAGCGAGAGAAACTC

ENSMMUT00000012253     TATAATGTCATCAGCCTAGAGTTTAGCCACACCAGGCTGGAGAACATGGTGCAGAGGCCC
ENSCJAT00000002028     TATAATGTCATCAGCCTTGAGTTTAGCCACACCAGACTGGAGAACATGGTGCAGAGGCCC
ENST00000529006        TATAATGTCATCAGCCTCGAGTTTAGCCACACCAGGCTGGAGAATATGGTGCAGAGGCCC
ENSPTRT00000007365     TATAATGTCATCAGCCTCGAGTTTAGCCACACCAGGCTGGAGAATATGGTGCAGAGGCCC
ENSMUST00000047898     TATAATGTTATCAGCCTAGAGTTTAGCCACACCAGGCTTGAGAATATGGTGCAGCGGCCT
ENSRNOT00000054850     TATAATGTTATCAGCCTAGAGTTTAGCCACACCAGGCTTGAGAATATGGTACAGCGGCCT

ENSMMUT00000012253     TCCACGGTGGATTTCATTGACTGGGTAGACAACATGTGGCCAAGGCACTTGAAGGAAAGC
ENSCJAT00000002028     TCCACGGTGGATTTCATTGACTGGGTAGACAACATGTGGCCAAGGCACTTAAAGGAAAGC
ENST00000529006        TCCACGGTGGATTTCATTGACTGGGTAGACAACATGTGGCCAAGGCACTTGAAGGAAAGC
ENSPTRT00000007365     TCCACGGTGGATTTCATTGACTGGGTAGACAACATGTGGCCAAGGCACTTGAAGGAAAGC
ENSMUST00000047898     TCCACGGTGGATTTCATTGACTGGGTAGATAACATGTGGCCAAGGCACTTGAAAGAAAGT
ENSRNOT00000054850     TCCACGGTGGATTTCATTGACTGGGTAGATAACATGTGGCCAAGGCACTTGAAAGAAAGT

ENSMMUT00000012253     CAGACTGAATCAACAAATGCCATCTTGGAGATGCAGTACCCTAAAGTGCAGAAGTACTGT
ENSCJAT00000002028     CAGACTGAATCAACAAATGCCATCTTGGAGATGCAGTACCCTAAAGTGCAGAAGTACTGT
ENST00000529006        CAGACTGAATCAACAAATGCCATCTTGGAGATGCAGTACCCTAAAGTGCAGAAGTACTGT
ENSPTRT00000007365     CAGACTGAATCAACAAATGCCATCTTGGAGATGCAGTACCCTAAAGTGCAGAAGTACTGT
ENSMUST00000047898     CAGACAGAATCAACAAATGCCATCTTAGAGATGCAGTACCCTAAAGTGCAAAAGTACTGT
ENSRNOT00000054850     CAGACTGAATCAACAAATGCCATCTTAGAGATGCAGTACCCTAAAGTGCAAAAGTACTGT

ENSMMUT00000012253     CTAATGAGTGTTCGAGGCTGCTATACTGACTTCCATGTGGACTTTGGTGGTACCTCTGTT
ENSCJAT00000002028     CTAATGAGTGTTCGAGGCTGCTATACTGACTTCCATGTGGACTTTGGTGGTACCTCTGTT
```

ENST00000529006 CTAATGAGTGTTCGAGGCTGCTATACTGACTTCCATGTGGACTTTGGTGGTACCTCTGTT
ENSPTRT00000007365 CTAATGAGTGTTCGAGGCTGCTATACTGACTTCCATGTGGACTTTGGTGGTACCTCTGTT
ENSMUST00000047898 CTAATGAGTGTTCGAGGCTGCTATACTGACTTCCATGTGGATTTTGGAGGTACTTCTGTT
ENSRNOT00000054850 CTAATGAGTGTTCGAGGCTGCTATACTGACTTCCATGTGGATTTTGGAGGTACTTCTGTT

ENSMMUT00000012253 TGGTATCACATCCATCAAGGGGGAAAGGTCTTCTGGCTCATCCCCCCTACAGCCCACAAC
ENSCJAT00000002028 TGGTATCACATCCATCAAGGGGGAAAGGTCTTCTGGCTCATCCCCCCTACAGCCCACAAC
ENST00000529006 TGGTATCACATCCATCAAGGGGGAAAGGTCTTCTGGCTCATCCCCCCTACAGCCCACAAC
ENSPTRT00000007365 TGGTATCACATCCATCAAGGGGGAAAGGTCTTCTGGCTCATCCCCCCTACAGCCCACAAC
ENSMUST00000047898 TGGTATCACATCCACCAAGGTGGAAAGGTCTTCTGGCTCATCCCCCCTACAGCCCACAAC
ENSRNOT00000054850 TGGTATCACATCCACCAAGGTGGAAAGGTCTTCTGGCTCATCCCCCCTACAGCCCACAAC

ENSMMUT00000012253 CTGGAGCTGTACGAGAATTGGCTGCTGTCAGGGAAACAGGGAGACATCTTTCTGGGTGAC
ENSCJAT00000002028 CTGGAGCTGTACGAGAATTGGCTGCTCTCAGGGAAACAGGGAGACATCTTTCTGGGTGAC
ENST00000529006 CTGGAGCTGTACGAGAATTGGCTGCTGTCAGGGAAACAGGGAGACATCTTTCTGGGTGAC
ENSPTRT00000007365 CTGGAGCTGTACGAGAATTGGCTGCTGTCAGGGAAACAGGGAGACATCTTTCTGGGTGAC
ENSMUST00000047898 CTGGAGCTGTACGAGAATTGGCTGCTATCAGGGAAACAGGGAGACATCTTTCTGGGTGAC
ENSRNOT00000054850 CTGGAGCTGTACGAGAATTGGCTGCTATCAGGGAAACAGGGAGACATCTTTCTGGGTGAC

ENSMMUT00000012253 CGGGTATCAGATTGTCAGCGCATTGAGCTCAAGCAGGGCTATACCTTCGTCATTCCCTCA
ENSCJAT00000002028 CGGGTATCAGATTGCCAGCGCATTGAGCTCAAGCAGGGCTATACCTTCGTCATTCCCTCA
ENST00000529006 CGGGTATCAGATTGTCAGCGCATTGAGCTCAAGCAGGGCTATACCTTCGTCATTCCCTCA
ENSPTRT00000007365 CGGGTATCAGATTGTCAGCGCATTGAGCTCAAGCAGGGCTATACCTTCGTCATTCCCTCA
ENSMUST00000047898 CGGGTGTCAGATTGCCAACGAATTGAGCTCAAGCAGGGCTATACCTTCGTTATTCCCTCA
ENSRNOT00000054850 CGGGTGTCAGATTGCCAACGAATTGAGCTCAAGCAGGGCTATACCTTCGTTATTCCCTCA

ENSMMUT00000012253 GGCTGGATTCATGCTGTGTATACTCCTACAGACACGTTAGTGTTTGGGGGCAATTTTTTG
ENSCJAT00000002028 GGCTGGATTCATGCCGTGTATACTCCTACAGACACGTTAGTGTTTGGAGGCAATTTTTTG
ENST00000529006 GGCTGGATTCATGCTGTGTATACTCCTACAGACACATTAGTGTTTGGGGGCAATTTTTTG
ENSPTRT00000007365 GGCTGGATTCATGCTGTGTATACTCCTACAGACACATTAGTGTTTGGGGGCAATTTTTTG
ENSMUST00000047898 GGTTGGATTCATGCTGTGTATACTCCTACAGACACATTAGTGTTTGGAGGCAATTTTTTG
ENSRNOT00000054850 GGTTGGATTCATGCTGTGTATACTCCTACGGACACATTAGTGTTTGGAGGCAATTTTTTG

ENSMMUT00000012253 CATAGCTTCAACATCCCTATGCAGTTAAAAATATACAACATTGAAGATCGGACACGGGTT
ENSCJAT00000002028 CATAGCTTCAACATCCCTATGCAGTTAAAAATATACAACATTGAAGATCGGACACGGGTT
ENST00000529006 CATAGCTTCAACATCCCTATGCAGTTAAAAATATACAACATTGAAGATCGGACACGGGTT
ENSPTRT00000007365 CATAGCTTCAACATCCCTATGCAGTTAAAAATATACAACATTGAAGATCGGACACGGGTT
ENSMUST00000047898 CATAGCTTCAACATCCCCATGCAATTAAAGATATACAGCATTGAAGATCGAACACGGGTT
ENSRNOT00000054850 CATAGCTTTAACATCCCCATGCAATTAAAGATATATAGTATTGAAGATCGAACACGGGTT

ENSMMUT00000012253 CCAAATAAGTTTCGCTATCCATTCTACTATGAGATGTGTTGGTATGTGTTGGAGCGCTAT
ENSCJAT00000002028 CCAAATAAGTTTCGCTATCCATTCTACTATGAGATGTGTTGGTATGTGTTGGAACGCTAT
ENST00000529006 CCAAATAAGTTTCGCTATCCATTCTACTATGAGATGTGTTGGTATGTGTTGGAGCGCTAT
ENSPTRT00000007365 CCAAATAAGTTTCGCTATCCATTCTACTATGAGATGTGTTGGTATGTGTTGGAGCGCTAT
ENSMUST00000047898 CCAAATAAATTCCGTTACCCATTTTACTATGAAATGTGTTGGTATGTGTTGGAGCGCTAT
ENSRNOT00000054850 CCAAATAAATTCCGTTACCCATTTTACTATGAAATGTGTTGGTATGTGTTGGAGCGCTAT

ENSMMUT00000012253 GTGTACTGCATAACCAACCGTTCCCACCTAACCAAGGAATTTCAGAAAGAGTCCCTCAGC
ENSCJAT00000002028 GTGTACTGCATAACCAACCGTTCCCACCTGACCAAGGAATTTCAGAAAGAGTCCCTCAGC
ENST00000529006 GTGTACTGCATAACCAACCGTTCCCACCTAACTAAGGAATTTCAGAAAGAGTCCCTCAGC
ENSPTRT00000007365 GTGTACTGCATAACCAACCGTTCCCACCTAACCAAGGAATTTCAGAAAGAGTCCCTCAGC
ENSMUST00000047898 GTATACTGCATAACCAACCGATCCCACCTAACTAAGGATTTTCAGAAAGAATCCCTTAGC
ENSRNOT00000054850 GTATACTGCATAACCAACCGATCTCACCTAACTAAGGAATTTCAGAAAGAATCCCTTAGC

ENSMMUT00000012253	ATGGATTTGGAGTTAAATGGGTTGGAGTCTGGGAATGCGGATGAGGAAGCAGTGGATCGA
ENSCJAT00000002028	ATGGATTTGGAGTTAAATGGGTTGGAGTCTGGAAATGGGGATGAGGAAGCAGTGGATCGA
ENST00000529006	ATGGATTTGGAGTTAAATGGGTTGGAGTCTGGGAATGGGGATGAGGAAGCAGTGGATCGA
ENSPTRT00000007365	ATGGATTTGGAGTTAAATGGGTTGGAGTCTGGGAATGGGGATGAGGAAGCAGTGGATCGA
ENSMUST00000047898	ATGGATATGGAGTTAAATGAGTTGGAGTCTGGAAATGGTGATGAGGAAGGGGTGGACAGA
ENSRNOT00000054850	ATGGATATGGAATTAAATGAGTTGGAGTCTGGAAATGGTGATGAGGAAGGGGCGGACAAA
ENSMMUT00000012253	GAACCCCGACGCTTGAGCAGCAGGCGTTCTGTCCTCACTAGTCCTGTCGCGAATGGGGTC
ENSCJAT00000002028	GAACCCCGACGCTTGAGCAGCAGGCGTTCTGTCCTCACTAGCCCTGTAGCCAATGGTGTC
ENST00000529006	GAACCCCGACGCTTGAGCAGCAGGCGTTCTGTCCTCACTAGCCCTGTAGCGAATGGAGTC
ENSPTRT00000007365	GAACCCCGACGCTTGAGCAGCAGGCGTTCTGTCCTCACTAGCCCTGTAGCGAATGGAGTC
ENSMUST00000047898	GAAGCCCGACGCATGAACAATAAGCGATCTGTGCTTACCAGCCCTGTTGCTAATGGAGTG
ENSRNOT00000054850	GAAGCCCGACGCATGAACAGTAAGCGATCTGTGCTTACCAGCCCTGTGGCTAATGGAGTC
ENSMMUT00000012253	AACCTGGATTATGATGGACTGGGCAAAACCTGCCGAAGTCTTCCAAGTCTGAAGAAAACT
ENSCJAT00000002028	AACTTGGATTATGATGGACTGGGCAAAACCTGCCGAAGTCTTCCAAGTCTGAAGAAAACT
ENST00000529006	AACCTGGATTATGATGGACTGGGCAAAACCTGCCGAAGTCTTCCAAGTCTGAAGAAAACT
ENSPTRT00000007365	AACCTGGATTATGATGGACTGGGCAAAACCTGCCGAAGTCTTCCAAGTCTGAAGAAAACT
ENSMUST00000047898	AACCTGGATTACGATGGACTTGGCAAAGCCTGCCGAAGTCTTCCAAGTCTGAAGAAAACT
ENSRNOT00000054850	AACCTGGATTATGATGGACTTGGCAAAGCCTGCCGAAGTCTTCCAAATCTGAAGAAAACT
ENSMMUT00000012253	TTGTCTGGGGACTCATCTTCTGACTCTAGCCGGGGATCCCACAATGGACAAGTGTGGGAT
ENSCJAT00000002028	TTGTCTGGGGACTCATCTTCTGACTCTAGCCGGGGTTCCCACAATGGCCAAGTGTGGGAT
ENST00000529006	TTGGCTGGGGACTCATCTTCTGACTGTAGCCGGGGCTCCCACAATGGACAAGTGTGGGAT
ENSPTRT00000007365	TTGGCTGGGGACTCATCTTCTGACTGTAGCCGGGGCTCCCACAATGGACAAGTGTGGGAT
ENSMUST00000047898	TTGTCTGGAGACTCATCCTCAGACTCTACCCGGGGATCCCACAATGGCCAAGTTTGGGAT
ENSRNOT00000054850	CTGTCTGGAGACTCATCCTCAGACTCTAGTCGGGGATCCCACAATGGCCAAGTTTGGGAT
ENSMMUT00000012253	CCCCAGTGTGCTCCCCGAAAGGACAGGCAAGTGCATCTGACCCATTTTGAGCTTGAAGGC
ENSCJAT00000002028	CCCCAGTGTGGTCCCCGAAAGGACAGGCAAGTGCATCTGACCCATTTTGAGCTTGAAGGC
ENST00000529006	CCCCAGTGTGCTCCCCGAAAGGACAGGCAAGTGCATCTGACCCATTTTGAGCTTGAAGGC
ENSPTRT00000007365	CCCCAGTGTGCTCCCCGAAAGGACAGGCAAGTGCATCTGACCCATTTTGAGCTTGAAGGC
ENSMUST00000047898	CCCCAATGTAGCCCTAAAAAGGATAGGCAAGTGCATCTCACCCATTTTGAACTTGAAGGT
ENSRNOT00000054850	CCCCAATGTAGCCCTAGAAAGGATAGGCAAGTGCATCTGACCCACTTTGAACTTGAAGGT
ENSMMUT00000012253	CTTCGCTGCCTTGTAGATAAGTTGGAGTCTCTGCCACTGCACAAGAAATGTGTCCCCACA
ENSCJAT00000002028	CTTCGCTGCCTTGTAGATAAGTTGGAGTCTCTGCCACTGCACAAGAAATGTGTCCCCACA
ENST00000529006	CTTCGCTGCCTTGTAGATAAGTTGGAGTCTCTGCCACTGCACAAGAAATGTGTCCCCACA
ENSPTRT00000007365	CTTCGCTGCCTTGTAGATAAGTTGGAGTCTCTGCCACTGCACAAGAAATGTGTCCCCACA
ENSMUST00000047898	CTTCGATGTCTTGTAGATAAGTTAGAGTCACTGCCACTGCACAAGAAGTGTGTCCCCACA
ENSRNOT00000054850	CTTCGATGTCTTGTAGATAAGTTAGAGTCACTGCCACTGCACAAGAAGTGTGTCCCCACA
ENSMMUT00000012253	GGGATAGAAGATGAAGATGCTCTCATTGCTGATGTAAAGATTTTGCTGGAAGAGCTTGCC
ENSCJAT00000002028	GGGATAGAAGATGAAGATGCTCTCATTGCTGATGTCAAGATTTTGCTGGAGGAGCTTGCC
ENST00000529006	GGGATAGAAGATGAAGATGCTCTCATTGCTGATGTAAAGATTTTGCTGGAGGAGCTTGCC
ENSPTRT00000007365	GGGATAGAAGATGAAGATGCTCTCATTGCTGATGTAAAGATTTTGCTGGAGGAGCTTGCC
ENSMUST00000047898	GGAATAGAAGACGAAGATGCTCTGATTGCTGATGTAAAGATTTTGCTGGAAGAACTTGCC
ENSRNOT00000054850	GGGATAGAAGACGAAGATGCTCTGATTGCTGATGTAAAGATTTTGCTGGAAGAACTTGCC
ENSMMUT00000012253	AACAGCGATCCCAAGTTAGCCCTCACTGGAGTTCCTATAGTACAGTGGCCAAAAAGGGAT
ENSCJAT00000002028	AACAGCGATCCCAAGTTAGCCCTCACTGGAGTTCCTATAGTACAGTGGCCAAAAAGGGAT

```
ENST00000529006        AACAGCGATCCCAAGTTAGCCCTCACTGGAGTTCCTATAGTACAGTGGCCAAAAAGGGAT
ENSPTRT00000007365     AACAGCGATCCCAAGTTAGCCCTCACTGGAGTTCCTATAGTACAGTGGCCAAAAAGGGAT
ENSMUST00000047898     AGTAGCGATCCCAAGTTAGCCCTCACTGGAGTCCCTATAGTACAGTGGCCAAAAAGGGAT
ENSRNOT00000054850     AGTAGTGATCCCAAGTTAGCCCTCACTGGAGTTCCCATAGTACAGTGGCCAAAAAGGGAT

ENSMMUT00000012253     AAGCTTAAATTCCCCACTCGGCCAAAGGTGCGGGTTCCTACCATCCCCATTACAAAGCCT
ENSCJAT00000002028     AAGCTTAAATTCCCCACTCGGCCAAAGGTGCGGGTTCCTACCATCCCCATTACAAAGCCT
ENST00000529006        AAGCTTAAATTCCCCACTCGGCCAAAGGTGCGGGTTCCTACCATCCCCATTACGAAGCCT
ENSPTRT00000007365     AAGCTTAAATTCCCCACTCGGCCAAAGGTGCGGGTTCCTACCATCCCCATTACGAAGCCT
ENSMUST00000047898     AAGCTTAAATTCCCTACCAGGCCAAAGGTGAGGGTTCCTACAATTCCCATCACAAAGCCT
ENSRNOT00000054850     AAGCTTAAATTTCCGACCAGGCCAAAGGTGAGGGTTCCTACAATTCCCATCACAAAGCCT

ENSMMUT00000012253     CACACTATGAAACCAGCTCCACGGTTAACACCTGTGAGGCCAGCTGCTGCCTCCCCGATT
ENSCJAT00000002028     CACACTATGAAACCAGCTCCACGGTTAACACCTGTGAGGCCAGCTGCTGCCTCTCCGATT
ENST00000529006        CACACTATGAAACCAGCTCCACGGTTAACACCTGTGAGGCCAGCTGCTGCCTCCCCGATT
ENSPTRT00000007365     CACACTATGAAACCAGCTCCACGGTTAACACCTGTGAGGCCAGCTGCTGCCTCCCCGATT
ENSMUST00000047898     CACACCATGAAGCCAGCTCCACGCTTAACACCTGTAAGGCCTGCTGCAGCCTCCCCCATT
ENSRNOT00000054850     CACACCATGAAGCCGGCTCCGCGCCTAACACCTGTAAGGCCTGCTGCAGCCTCCCCCATT

ENSMMUT00000012253     GTGTCAGGAGCCAGACGGAGACGAGTGCGATGTCGAAAATGCAAAGCCTGTGTGCAAGGA
ENSCJAT00000002028     GTGTCAGGGGCCAGACGGAGACGAGTGCGGTGTCGAAAATGCAAAGCCTGTGTACAAGGA
ENST00000529006        GTGTCAGGAGCCAGACGGAGACGAGTGCGATGTCGAAAATGCAAAGCCTGTGTGCAAGGA
ENSPTRT00000007365     GTGTCAGGAGCCAGACGGAGACGAGTGCGATGTCGAAAATGCAAAGCCTGTGTGCAAGGA
ENSMUST00000047898     GTGTCAGGAGCCAGGCGGAGAAGAGTGCGGTGCAGGAAATGCAAAGCTTGTGTGCAAGGA
ENSRNOT00000054850     GTGTCAGGAGCCAGGCGGAGAAGAGTGCGCTGCAGGAAATGCAAAGCCTGTGTGCAAGGA

ENSMMUT00000012253     GAGTGTGGTGTTTGCCACTACTGCAGAGACATGAAGAAGTTTGGGGGGCCTGGACGCATG
ENSCJAT00000002028     GAGTGTGGTGTTTGCCACTACTGCAGAGACATGAAGAAGTTTGGGGGGCCTGGACGCATG
ENST00000529006        GAGTGTGGTGTTTGCCACTACTGCAGAGACATGAAGAAGTTTGGGGGGCCTGGACGCATG
ENSPTRT00000007365     GAGTGTGGTGTTTGCCACTACTGCAGAGACATGAAGAAGTTTGGGGGGCCTGGACGCATG
ENSMUST00000047898     GAATGTGGAGTCTGCCACTACTGCAGGGACATGAAGAAATTTGGTGGACCTGGACGCATG
ENSRNOT00000054850     GAATGTGGAGTCTGCCACTACTGCAGGGACATGAAGAAATTTGGTGGGCCTGGACGCATG

ENSMMUT00000012253     AAGCAGTCCTGTGTCCTCCGACAGTGCTTGGCACCCAGACTGCCTCACTCAGTCACATGT
ENSCJAT00000002028     AAGCAGTCCTGTGTCCTCCGACAGTGCTTGGCACCCAGACTGCCTCATTCAGTCACATGT
ENST00000529006        AAGCAGTCCTGTGTCCTCCGACAGTGCTTGGCACCCAGACTGCCTCACTCAGTCACATGT
ENSPTRT00000007365     AAGCAGTCCTGTGTCCTCCGACAGTGCTTGGCACCCAGACTGCCTCACTCAGTCACATGT
ENSMUST00000047898     AAGCAATCCTGTGTCCTCCGACAGTGCTTAGCACCCAGACTGCCTCATTCAGTTACGTGT
ENSRNOT00000054850     AAGCAGTCCTGTGTCCTCAGACAGTGCTTAGCACCTAGACTGCCTCATTCAGTTACATGT

ENSMMUT00000012253     TCCCTCTGTGGAGAGGTGGATCAGAATGAAGAGACACAAGACTTTGAGAAGAAACTCATG
ENSCJAT00000002028     TCCCTTTGTGGAGAGGTGGATCAGAATGAAGAGACACAAGACTTTGAGAAGAAACTCATG
ENST00000529006        TCCCTCTGTGGAGAGGTGGATCAGAATGAAGAGACACAAGACTTTGAGAAGAAACTCATG
ENSPTRT00000007365     TCCCTCTGTGGAGAGGTGGATCAGAATGAAGAGACACAAGACTTTGAGAAGAAACTCATG
ENSMUST00000047898     TCTCTCTGTGGAGAAGTAGATCAGAATGAAGAGACCCAGGACTTTGAAAAGAAACTCATG
ENSRNOT00000054850     TCTCTCTGTGGAGAAGTAGATCAGAATGAAGAGACACAGGACTTTGAAAAGAAACTCATG

ENSMMUT00000012253     GAATGCTGTATCTGCAATGAGATTGTTCATCCTGGCTGCCTCCAGATGGATGGAGAGGGG
ENSCJAT00000002028     GAATGCTGTATCTGCAATGAGATTGTTCATCCTGGCTGCCTCCAGATGGATGGAGAGGGG
ENST00000529006        GAATGCTGTATCTGCAATGAGATTGTTCATCCTGGCTGCCTCCAGATGGACGGAGAGGGG
ENSPTRT00000007365     GAATGCTGTATCTGCAATGAGATTGTTCATCCTGGCTGCCTCCAGATGGACGGAGAGGGG
ENSMUST00000047898     GAATGCTGCATCTGCAACGAGATAGTTCATCCTGGCTGCCTCCAGATGGATGGAGAGGGG
ENSRNOT00000054850     GAATGCTGCATCTGCAACGAGATAGTTCATCCTGGCTGCCTCCAGATGGATGGAGAGGGG
```

```
ENSMMUT00000012253     TTGCTTAATGAAGAATTGCCAAATTGCTGGGAATGTCCAAAGTGCTACCAGGAGGACAAC
ENSCJAT00000002028     TTGCTTAATGAGGAATTGCCAAATTGCTGGGAATGTCCAAAGTGCTACCAGGAGGACAGC
ENST00000529006        TTGCTTAACGAAGAATTGCCAAATTGCTGGGAATGTCCAAAGTGCTACCAGGAGGACAGC
ENSPTRT00000007365     TTGCTTAATGAAGAATTGCCAAATTGCTGGGAATGTCCAAAGTGCTACCAGGAGGACAGC
ENSMUST00000047898     TTGCTGAACGAGGAATTGCCAAATTGCTGGGAGTGTCCAAAGTGTTACCAGGAAGACAGC
ENSRNOT00000054850     TTGCTTAACGAGGAATTGCCAAATTGCTGGGAGTGTCCAAAGTGTTATCAGGAAGACAGC

ENSMMUT00000012253     TCGGAGAAAGCCCAGAAGCGGAAAATGGAAGAGAGTGACGAAGAAGCCGTGCAAGCCAAA
ENSCJAT00000002028     TCCGAAAAAGCCCAGAAGCGGAAAATGGAAGAGAGTGATGAAGAAGCCGTGCAAGCCAAA
ENST00000529006        TCGGAGAAAGCCCAGAAGCGGAAAATGGAAGAGAGTGACGAAGAAGCTGTGCAAGCCAAA
ENSPTRT00000007365     TCGGAGAAAGCCCAGAAGCGGAAAATGGAAGAGAGTGATGAAGAAGCTGTGCAAGCCAAA
ENSMUST00000047898     TCGGACAAAGCCCAGAAGCGGAAAATAGAAGAGAGTGATGAAGAAGCTGTACAAGCCAAA
ENSRNOT00000054850     TCGGAGAAAGCCCAGAAGCGGAAAATAGAAGAGAGTGATGAAGAAGCTGTACAAGCCAAA

ENSMMUT00000012253     GTCCTGCGGCCCCTGCGGAGCTGCGATGAGCCTCTCACGCCCCCGCCTCATTCACCTACT
ENSCJAT00000002028     GTCCTGCGGCCCCTGCGGAGCTGCGATGAGCCTCTCACGCCCCCGCCTCATTCACCCACT
ENST00000529006        GTCCTGCGGCCCCTGCGGAGCTGCGATGAGCCTCTCACGCCCCCGCCTCATTCACCCACT
ENSPTRT00000007365     GTCCTGCGGCCCCTGCGGAGCTGCGATGAGCCTCTCACGCCCCCGCCTCATTCACCCACT
ENSMUST00000047898     GTCTTACGGCCCCTGAGGAGCTGCGAGGAGCCTCTCACACCCCCGCCTCACTCACCTACT
ENSRNOT00000054850     GTCCTACGGCCCCTGAGGAGCTGCGAGGAGCCTCTCACACCCCCGCCTCACTCACCTACT

ENSMMUT00000012253     TCCATGCTGCAGCTCATCCATGACCCGGTTTCCCCCCGGGGTATGGTGACTCGGTCATCC
ENSCJAT00000002028     TCCATGCTGCAGCTCATCCATGACCCGGTTTCCCCCCGGGGTGTGGTGACTCGGTCATCC
ENST00000529006        TCCATGCTGCAGCTCATCCATGACCCGGTTTCCCCCCGGGGTATGGTGACTCGGTCATCC
ENSPTRT00000007365     TCCATGCTGCAGCTCATCCATGACCCGGTTTCCCCCCGGGGTATGGTGACTCGGTCATCC
ENSMUST00000047898     TCCATGCTGCAGCTCATCCACGACCCGGTTTCTCCCCGGGGTATGGTGACTCGGTCATCC
ENSRNOT00000054850     TCCATGCTGCAGCTCATCCACGACCCGGTTTCTCCCCGGGGTATGGTGACTCGGTCATCC

ENSMMUT00000012253     CCTGGGGCTGGCCCCAGCGACCACCACAGTGCCAGCCGCGATGAGCGCTTCAAACGGCGG
ENSCJAT00000002028     CCTGGGGCTGGCCCCAGCGACCACCACAGTGCCAGCCGTGATGAGCGCTTCAAACGGCGG
ENST00000529006        CCTGGGGCTGGCCCCAGCGACCACCACAGTGCCAGCCGCGATGAGCGCTTCAAACGGCGG
ENSPTRT00000007365     CCTGGGGCTGGCCCCAGCGACCACCACAGTGCCAGCCGCGATGAGCGCTTCAAACGGCGG
ENSMUST00000047898     CCTGGGGCTGGCCCCAGCGACCACCACAGTGCCAGCCGTGATGAACGCTTCAAACGGCGG
ENSRNOT00000054850     CCTGGGGCTGGCCCCAGCGACCACCACAGTGCCAGCCGTGATGAACGCTTCAAACGGCGG

ENSMMUT00000012253     CAGTTGCTGCGGCTGCAGGCCACAGAGCGCACCATGGTACGGGAAAAGGAGAACAATCCC
ENSCJAT00000002028     CAGTTGCTGCGGCTGCAGGCCACAGAGCGCACCATGGTACGGGAAAAGGAGAACAATCCC
ENST00000529006        CAGTTGCTGCGGCTGCAGGCCACAGAGCGCACCATGGTACGGGAAAAGGAGAACAATCCC
ENSPTRT00000007365     CAGTTGCTGCGGCTGCAGGCCACAGAGCGCACCATGGTACGGGAAAAGGAGAACAATCCC
ENSMUST00000047898     CAGTTGCTGCGGCTACAAGCCACCGAGCGCACCATGGTACGGGAAAAGGAGAACAATCCC
ENSRNOT00000054850     CAGTTGCTGCGGCTACAAGCCACAGAGCGCACCATGGTACGGGAAAAGGAGAACAATCCC

ENSMMUT00000012253     AGCGGCAAAAAGGAGCTGTCTGAAGTTGAGAAAGCCAAGATCCGGGGATCGTACCTCACT
ENSCJAT00000002028     AGCGGCAAAAAGGAGCTGTCTGAAGTTGAGAAAGCCAAGATCCGGGGATCGTACCTCACT
ENST00000529006        AGCGGCAAAAAGGAGCTGTCTGAAGTTGAGAAAGCCAAGATCCGGGGATCGTACCTCACT
ENSPTRT00000007365     AGCGGCAAAAAGGAGCTGTCTGAAGTTGAGAAAGCCAAGATCCGGGGATCGTACCTCACT
ENSMUST00000047898     AGCGGCAAAAAGGAGCTGTCTGAAGTTGAGAAAGCCAAGATCCGGGGATCGTACCTCACT
ENSRNOT00000054850     AGCGGCAAAAAGGAGCTGTCTGAAGTTGAGAAAGCCAAGATCCGGGGATCGTACCTCACT

ENSMMUT00000012253     GTCACGCTACAGAGGCCCACCAAAGAGCTCCACGGGACATCCATTGTGCCCAAGCTGCAG
ENSCJAT00000002028     GTCACGCTACAGAGGCCCACCAAAGAGCTCCACGGGACATCCATTGTGCCCAAGCTGCAG
```

```
ENST00000529006        GTCACGCTACAGAGGCCCACCAAAGAGCTCCACGGGACATCCATTGTGCCCAAGCTGCAG
ENSPTRT00000007365     GTCACGCTACAGAGGCCCACCAAAGAGCTCCACGGGACATCCATTGTGCCCAAGCTGCAG
ENSMUST00000047898     GTCACTCTACAGAGGCCCACCAAAGAGCTCCACGGGACATCCATTGTGCCCAAGCTGCAG
ENSRNOT00000054850     GTCACGCTACAGAGGCCCACCAAAGAGCTCCACGGGACATCCATTGTGCCCAAGCTGCAG

ENSMMUT00000012253     GCCATCACGGCCTCCTCTGCCAACCTTCGCCATTCCCCCCGTGTGCTAGTGCAGCACTGC
ENSCJAT00000002028     GCCATCACGGCCTCCTCTGCCAACCTTCGCCATTCCCCCCGTGTGCTAGTGCAGCACTGC
ENST00000529006        GCCATCACGGCCTCCTCTGCCAACCTTCGCCATTCCCCCCGTGTGCTAGTGCAGCACTGC
ENSPTRT00000007365     GCCATCACGGCCTCCTCTGCCAACCTTCGCCATTCCCCCCGTGTGCTAGTGCAGCACTGC
ENSMUST00000047898     GCCATCACGGCCTCCTCTGCCAACCTTCGCCCTAACCCCCGCGTGCTAATGCAGCACTGC
ENSRNOT00000054850     GCCATCACGGCCTCCTCTGCCAACCTTCGCCCTAACCCCCGTGTGCTAATGCAGCACTGC

ENSMMUT00000012253     CCAGCCCGAACCCCCCAGCGTGGGGATGAGGAGAGGCTGGGGGGAGAGGAGGAAGAGGAG
ENSCJAT00000002028     CCAGCCCGAACCCCCCAGCGTGGGGATGAGGAGGGGCTGGGGGGA---GAGGAGGAGGAA
ENST00000529006        CCAGCCCGAACCCCCCAGCGTGGGGATGAGGAGGGGCTGGGGGGAGAGGAGGAAGAGGAG
ENSPTRT00000007365     CCAGCCCGAACCCCCCAGCGTGGGGATGAGGAGGGGCTGGGGGGAGAGGAGGAAGAGGAG
ENSMUST00000047898     CCAGCCCGAAACCCCCAGCATGGGGATGAGGAGGGGCTTGGGGGAGAGGAGGAGGAAGAG
ENSRNOT00000054850     CCAGCCCGGAACCCCCAGCGTGGGGATGAGGAGGGGCTTGGGGGAGAGGAGGAGGAAGAG

ENSMMUT00000012253     GAGGAGGAGGAGGAAGATGACAGTGCAGAGGAGGGGGGTGCAGCCAGGCTGAATGGCCGG
ENSCJAT00000002028     GAGGAGGAGGAGGAAGATGACAGTGCAGAGGAGGGGGGTGCAGCCAGGCTGAATGGCCGG
ENST00000529006        GAGGAGGAGGAGGAAGATGACAGTGCAGAGGAGGGGGGTGCAGCCAGGCTGAATGGCCGG
ENSPTRT00000007365     GAGGAGGAGGAGGAAGATGACAGTGCAGAGGAGGGGGGTGCAGCCAGGCTGAATGGCCGG
ENSMUST00000047898     GAGGAGGAGGAGGAAGATGACAGTGCAGAGGAGGGGGGTGCAGCCAGGCTGAATGGCCGG
ENSRNOT00000054850     GAGGAGGAGGAGGAAGATGACAGTGCAGAGGAGGGGGGTGCAGCAAGGCTGAATGGCCGG

ENSMMUT00000012253     GGCAGTTGGGCTCAGGATGGAGACGAAAGCTGGATGCAGCGGGAGGTCTGGATGTCTGTC
ENSCJAT00000002028     GGCAGTTGGGCTCAGGATGGAGACGAAAGCTGGATGCAGCGGGAGGTCTGGATGTCTGTC
ENST00000529006        GGCAGTTGGGCTCAGGATGGAGACGAAAGCTGGATGCAGCGGGAGGTCTGGATGTCTGTC
ENSPTRT00000007365     GGCAGTTGGGCTCAGGATGGAGACGAAAGCTGGATGCAGCGGGAGGTCTGGATGTCTGTC
ENSMUST00000047898     GGCAGTTGGGCTCAGGATGGAGACGAAAGCTGGATGCAGCGGGAGGTCTGGATGTCTGTC
ENSRNOT00000054850     GGCAGTTGGGCTCAGGATGGAGACGAAAGCTGGATGCAGCGGGAGGTCTGGATGTCTGTC

ENSMMUT00000012253     TTCCGCTACCTCAGCCGCAGAGAACTTTGTGAATGTATGCGAGTGTGCAAGACGTGGTAT
ENSCJAT00000002028     TTCCGCTACCTCAGCCGCAGAGAACTTTGTGAATGTATGCGAGTGTGCAAGACATGGTAT
ENST00000529006        TTCCGCTACCTCAGCCGCAGAGAACTTTGTGAATGTATGCGAGTGTGCAAGACGTGGTAT
ENSPTRT00000007365     TTCCGCTACCTCAGCCGCAGAGAACTTTGTGAATGTATGCGAGTGTGCAAGACGTGGTAT
ENSMUST00000047898     TTCCGCTACCTCAGCCGCAAAGAACTTTGTGAATGTATGCGAGTGTGCAAGACATGGTAT
ENSRNOT00000054850     TTCCGCTACCTCAGCCGCAGAGAACTTTGTGAATGTATGCGAGTGTGCAAGACGTGGTAT

ENSMMUT00000012253     AAATGGTGCTGCGACAAGAGACTCTGGACAAAAATTGACTTGAGTAGGTGTAAGGCCATT
ENSCJAT00000002028     AAATGGTGCTGCGACAAGAGACTTTGGACAAAAATTGACTTGAGTAGGTGTAAGGCCATA
ENST00000529006        AAATGGTGCTGCGACAAGAGACTTTGGACAAAAATTGACTTGAGTAGGTGTAAGGCCATT
ENSPTRT00000007365     AAATGGTGCTGCGACAAGAGACTTTGGACAAAAATTGACTTGAGTAGGTGTAAGGCCATT
ENSMUST00000047898     AAATGGTGCTGTGATAAACGACTTTGGACAAAAATTGACTTGAGTAGGTGTAAGGCCATC
ENSRNOT00000054850     AAATGGTGCTGTGACAAACGACTTTGGACAAAAATTGACTTGAGTAGGTGTAAGGCCATC

ENSMMUT00000012253     GTGCCCCAGGCCCTCAGTGGCATCATCAAGAGGCAGCCAGTCAGCCTTGACCTCAGTTGG
ENSCJAT00000002028     GTGCCCCAGGCCCTCAGTGGCATCATCAAGAGGCAGCCAGTCAGCCTTGACCTCAGTTGG
ENST00000529006        GTGCCCCAGGCCCTCAGTGGCATCATCAAGAGGCAGCCAGTCAGCCTTGACCTCAGTTGG
ENSPTRT00000007365     GTGCCCCAGGCCCTCAGTGGCATCATCAAGAGGCAGCCAGTCAGCCTTGACCTCAGTTGG
ENSMUST00000047898     GTACCACAAGCTCTCAGTGGTATCATCAAGCGGCAGCCAGTAAGCCTCGACCTCAGCTGG
ENSRNOT00000054850     GTGCCCCAGGCTCTCAGTGGCATCATCAAGCGGCAGCCAGTAAGCCTGGACCTTAGTTGG
```

```
ENSMMUT00000012253     ACCAACATCTCTAAAAAGCAATTGACATGGCTTGTCAATAGGCTGCCAGGACTGAAAGAC
ENSCJAT00000002028     ACCAACATCTCTAAAAAGCAACTGACATGGCTCGTCAACAGGCTGCCAGGACTGAAAGAC
ENST00000529006        ACCAACATCTCTAAAAAGCAACTGACATGGCTCGTCAATAGGCTGCCAGGACTGAAAGAC
ENSPTRT00000007365     ACCAACATCTCTAAAAAGCAACTGACATGGCTCGTCAATAGGCTGCCAGGACTGAAAGAC
ENSMUST00000047898     ACTAACATCTCCAAAAAGCAGCTGACATGGCTGGTCAATAGGCTGCCAGGATTAAAAGAC
ENSRNOT00000054850     ACTAACATCTCCAAAAAGCAGCTGACATGGCTGGTCAATAGGCTGCCAGGATTAAAAGAC

ENSMMUT00000012253     CTCCTCCTAGCAGGCTGCTCCTGGTCTGCAGTCTCTGCCCTCAGCACCTCCAGCTGCCCC
ENSCJAT00000002028     CTCCTCCTAGCAGGCTGCTCCTGGTCTGCAGTCTCTGCCCTCAGCACCTCCAGCTGCCCC
ENST00000529006        CTCCTCCTAGCAGGCTGCTCCTGGTCTGCAGTCTCTGCCCTCAGCACCTCCAGCTGCCCC
ENSPTRT00000007365     CTCCTCCTAGCAGGCTGCTCCTGGTCTGCAGTCTCTGCCCTCAGCACCTCCAGCTGCCCC
ENSMUST00000047898     CTCCTCCTAGCAGGCTGTTCCTGGTCTGCAGTATCTGCCCTCAGCACTTCCAGCTGCCCG
ENSRNOT00000054850     CTCCTCCTAGCAGGCTGTTCCTGGTCTGCAGTATCTGCCCTCAGCACCTCCAGCTGCCCC

ENSMMUT00000012253     CTTCTCAGGACCCTTGATCTTCGGTGGGCAGTAGGAATCAAGGACCCTCAAATTCGGGAC
ENSCJAT00000002028     CTTCTCAGGACCCTTGATCTTCGGTGGGCAGTGGGAATCAAGGACCCTCAAATTCGGGAC
ENST00000529006        CTTCTCAGGACCCTTGATCTTCGGTGGGCAGTAGGAATCAAGGACCCTCAAATTCGGGAC
ENSPTRT00000007365     CTTCTCAGGACCCTTGATCTTCGGTGGGCAGTAGGAATCAAGGACCCTCAAATTCGGGAC
ENSMUST00000047898     CTTCTCAGGACCCTTGATCTTCGGTGGGCAGTAGGAATTAAAGACCCTCAAATTCGGGAC
ENSRNOT00000054850     CTTCTCAGGACCCTTGATCTTCGGTGGGCAGTAGGAATTAAAGACCCTCAAATTCGGGAC

ENSMMUT00000012253     TTGCTTACTCCACCGGCTGATAAACCAGGTCAGGACAATCGCAGCAAGCTCCGGAACATG
ENSCJAT00000002028     TTGCTCACTCCACCGGCTGATAAACCAGGTCAGGACAATCGCAGCAAGCTCCGGAACATG
ENST00000529006        TTGCTTACTCCACCGGCTGATAAACCAGGTCAGGACAATCGCAGCAAGCTCCGGAACATG
ENSPTRT00000007365     TTGCTTACTCCACCGGCTGATAAACCAGGTCAGGACAATCGCAGCAAGCTCCGGAACATG
ENSMUST00000047898     TTGCTGACTCCACCCACAGATAAGCCAGGTCAAGACAATCGAAGCAAACTCCGGAACATG
ENSRNOT00000054850     TTGCTGACTCCACCCACAGATAAGCCAGGTCAAGACAATCGAAGCAAACTCCGGAACATG

ENSMMUT00000012253     ACTGACTTCCGGCTGGCAGGCCTTGACATCACAGATGCCACGCTTCGCCTCATCATTCGC
ENSCJAT00000002028     ACCGACTTCCGGCTGGCAGGCCTTGACATCACCGATGCCACACTTCGCCTCATCATTCGC
ENST00000529006        ACCGACTTCCGGCTGGCAGGCCTTGACATCACAGATGCCACGCTTCGCCTCATAATTCGC
ENSPTRT00000007365     ACCGACTTCCGGCTGGCAGGCCTTGACATCACAGATGCCACGCTTCGCCTCATCATTCGC
ENSMUST00000047898     ACTGACTTCCGGCTGGCAGGCCTTGACATCACAGATGCTACTCTCCGACTCATCATTCGC
ENSRNOT00000054850     ACTGACTTCCGGCTGGCAGGCCTTGACATCACCGATGCCACTCTCCGACTCATTATTCGC

ENSMMUT00000012253     CACATGCCCCTCCTGTCTCGACTCGACCTCAGTCACTGCAGCCACCTTACAGATCAGTCC
ENSCJAT00000002028     CACATGCCCCTCCTGTCTCGACTCGACCTCAGTCACTGCAGCCACCTTACAGATCAGTCC
ENST00000529006        CACATGCCCCTCCTGTCTCGACTCGACCTCAGTCACTGCAGCCACCTTACAGATCAGTCC
ENSPTRT00000007365     CACATGCCCCTCCTGTCTCGACTCGACCTCAGTCACTGCAGCCACCTTACAGATCAGTCC
ENSMUST00000047898     CACATGCCCCTTTTGTCTCGACTTGACCTCAGTCACTGCAGTCACCTTACAGATCAGTCC
ENSRNOT00000054850     CACATGCCCCTTTTGTCTCGACTTGACCTCAGTCACTGCAGCCACCTTACAGATCAGTCC

ENSMMUT00000012253     TCCAATCTACTCACCGCTGTTGGGTCTTCCACTCGCTACTCTCTCACAGAGCTCAATATG
ENSCJAT00000002028     TCCAATCTACTCACTGCTGTCGGGTCTTCCACTCGCTACTCCCTCACAGAGCTCAATATG
ENST00000529006        TCCAATCTACTCACTGCTGTCGGGTCTTCCACTCGCTACTCTCTCACAGAGCTCAATATG
ENSPTRT00000007365     TCCAATCTACTCACTGCTGTCGGGTCTTCCACTCGCTACTCTCTCACAGAGCTCAATATG
ENSMUST00000047898     TCCAACCTACTAACTGCTGTCGGGTCTTCCACTCGATACTCCCTTACAGAGCTCAATATG
ENSRNOT00000054850     TCCAACCTGCTCACTGCTGTCGGGTCTTCCACTCGATACTCCCTTACAGAGCTCAACATG

ENSMMUT00000012253     GCAGGTTGCAATAAATTGACAGACCAGACCCTGATCTACCTACGGCGCATTGCCAACGTC
ENSCJAT00000002028     GCAGGTTGCAATAAATTGACAGACCAGACCCTGATCTACCTACGGCGCATTGCCAACGTC
```

```
ENST00000529006        GCAGGTTGCAATAAATTGACAGACCAGACCCTGATCTACCTACGGCGCATTGCCAACGTC
ENSPTRT00000007365     GCAGGTTGCAATAAATTGACAGACCAGACCCTGATCTACCTACGGCGCATTGCCAACGTC
ENSMUST00000047898     GCAGGTTGCAATAAATTGACAGACCAGACCCTGTTCTTCCTAAGGCGAATTGCTAATGTC
ENSRNOT00000054850     GCGGGTTGCAATAAACTGACAGACCAGACCCTGTTCTTCCTAAGGAGAATTGCTAATGTC

ENSMMUT00000012253     ACCTTGATCGACCTTCGAGGATGCAAGCAGATCACTCGAAAAGCCTGCGAGCACTTCATC
ENSCJAT00000002028     ACCTTGATCGACCTTCGAGGATGCAAGCAGATCACTCGAAAAGCCTGCGAGCACTTCATC
ENST00000529006        ACCTTGATCGACCTTCGAGGATGCAAGCAGATCACTCGAAAAGCCTGCGAGCACTTCATC
ENSPTRT00000007365     ACCTTGATCGACCTTCGAGGATGCAAGCAGATCACTCGAAAAGCCTGCGAGCACTTCATC
ENSMUST00000047898     ACCTTGATTGACCTTCGAGGATGCAAACAGATCACGAGAAAAGCCTGTGAGCACTTCATC
ENSRNOT00000054850     ACCTTGATTGACCTTCGAGGATGCAAACAGATCACAAGAAAAGCCTGTGAGCACTTCATC

ENSMMUT00000012253     TCAGACTTGTCCATCAACAGCCTCTACTGCCTGTCTGACGAGAAGCTGATACAGAAGATC
ENSCJAT00000002028     TCAGACTTGTCCATCAACAGCCTCTACTGCCTGTCTGACGAGAAGTTGATACAGAAGATC
ENST00000529006        TCAGACTTGTCCATCAACAGCCTCTACTGCCTGTCTGACGAGAAGCTGATACAGAAGATC
ENSPTRT00000007365     TCAGACTTGTCCATCAACAGCCTCTACTGCCTGTCTGACGAGAAGCTGATACAGAAGATC
ENSMUST00000047898     TCAGACTTGTCCATCAACAGCCTCTACTGCCTGTCTGATGAGAAACTGATACAGAAGATT
ENSRNOT00000054850     TCAGACTTGTCCATCAACAGCCTCTACTGCCTGTCTGATGAGAAACTGATACAGAAGATT

ENSMMUT00000012253     AGC
ENSCJAT00000002028     AGC
ENST00000529006        AGC
ENSPTRT00000007365     AGC
ENSMUST00000047898     AGC
ENSRNOT00000054850     AGC
```

Multiple sequence alignment of Kdm2B

```
ENSMMUT00000030348     ATGGCAGGTCCGCAAATGGGGGGATCTGCAGAGGATCACCCCTCACGAAAAAGACATGCA
ENSCJAT00000002986     ATGGCAGGTCCGCAAATGGGGGGATCTGCAGAGGATCACCCCCCACGAAAAAGACATGCA
ENSPPYT00000005974     ATGGCAGGTCCGCAAATGGGGGGATATGCAGAGGATCACCCCCCACGAAAAAGACATGCA
ENSGGOT00000014084     ATGGCGGGTCCGCAAATGGGGGGATCTGCAGACGATCACCCCCCACGAAAAAGACATGCA
ENST00000377071        ATGGCGGGTCCGCAAATGGGGGGATCTGCAGAGGATCACCCCCCACGAAAAAGACATGCA
ENSPTRT00000010204     ATGGCGGGTCCGCAAATGGGGGGATCTGCAGAGGATCACCCCCCACGAAAAAGACATGCA
ENSMUST00000046073     ------------------------------------------------------ATGGAG
ENSRNOT00000044071     ------------------------------------------------------ATGGAG

ENSMMUT00000030348     GCAGAAAAGCAAAAAAAGGAAACAATTATATATACAAAATGCTATGAATTTGAGTCGGCC
ENSCJAT00000002986     GCAGCAAAACAAAAAAAGAAAAAAATTATATATACAAAATGCTTTGAATTTGAGTCGGCC
ENSPPYT00000005974     GCAGAAAAGCAAAAAAAGAAAACAGTTATATATACAAAATGCTTTGAATTTGAGTCGGCC
ENSGGOT00000014084     GCAGAAAAGCAAAAAAAGAAAACAGTTATATATACAAAATGCTTTGAATTTGAGTCGGCC
ENST00000377071        GCAGAAAAGCAAAAAAAGAAAACAGTTATATATACAAAATGCTTTGAATTTGAGTCGGCC
ENSPTRT00000010204     GCAGAAAAGCAAAAAAAGAAAACAGTTATATATACAAAATGCTTTGAATTTGAGTCGGCC
ENSMUST00000046073     GCAGAGAAAGACTCTGGAAGAAGATTG---------------------------------
ENSRNOT00000044071     GCAGAGAAAGACTCTGGAAGAAGATTG---------------------------------

ENSMMUT00000030348     ACACAGAGGGGTAGCCCTGGGCAACAGGGGCAGGAACGGAGCGGGCTCGGGGTTCGCCCG
ENSCJAT00000002986     ACACAGCTGCCC------CGCCATCTCCGGGATGCGCCCGCGGCCTACAACTCGGCCCTC
ENSPPYT00000005974     ATACAGCGCCCGATTGACCGCCAGCGATACGACGAGAACGAGGACTTGTCGGACGTGGAG
ENSGGOT00000014084     ACACAGCGCCCGATTGACCGCCAGCGATACGACGAGAACGAGGACTTGTCGGACGTGGAG
ENST00000377071        ACACAGCGCCCGATTGACCGCCAGCGATACGACGAGAACGAGGACTTGTCGGACGTGGAG
ENSPTRT00000010204     ACACAGCGCCCGATTGACCGCCAGCGATACGACGAGAACGAGGACTTGTCGGACGTGGAG
ENSMUST00000046073     ------CGTGCGATTGACCGCCAGAGATACGACGAGAACGAAGACTTGTCGGACGTGGAG
ENSRNOT00000044071     ------CGTGCGATTGACCGCCAGAGATACGACGAGAACGAAGACTTGTCGGACGTGGAG
```

```
ENSMMUT00000030348     GAATCTGGGGGGCTGCGGGGCCGGGCGCTGGGCGTGAGTGTGCGCGCGCGCTTGTGG---
ENSCJAT00000002986     CAAAACAAATCTGTATCTGGCATGTGCTTAGAGTCTCAGACACTTAACCAGCTGTACCAA
ENSPPYT00000005974     GAGATCGTCAGCGTCCGCGGCTTCAGCCTGGAGGAGAAGCTTCGCAGCCAGCTGTACCAG
ENSGGOT00000014084     GAGATCGTCAGCGTCCGCGGCTTCAGCCTGGAGGAGAAGCTTCGCAGCCAGCTGTACCAG
ENST00000377071        GAGATCGTCAGCGTCCGCGGCTTCAGCCTGGAGGAGAAGCTTCGCAGCCAGCTGTACCAG
ENSPTRT00000010204     GAGATCGTCAGCGTCCGCGGCTTCAGCCTGGAGGAGAAGCTTCGCAGCCAGCTGTACCAG
ENSMUST00000046073     GAAATTGTCAGCGTCCGTGGCTTCAGCCTGGAGGAGAAGCTACGTAGCCAGTTATACCAG
ENSRNOT00000044071     GAAATTGTCAGCGTCCGTGGCTTCAGCCTGGAGGAGAAGCTACGTAGCCAGCTATACCAG

ENSMMUT00000030348     GGAGATGAGGCAGCTGCTGGTGCGTTTTGGGATTTCAACTATGAGTACGTACAGAGAGAA
ENSCJAT00000002986     GGGGACTTTGTGCACGCCGTGGAGGGCAAAGATTTCAACTATGAGTACGTACAGAGAGAA
ENSPPYT00000005974     GGGGACTTTGTGCACGCCATGGAGGGCAAAGATTTCAACTATGAGTACGTACAGAGAGAA
ENSGGOT00000014084     GGGGACTTCGTGCACGCCATGGAGGGCAAAGATTTCAACTATGAGTACGTACAGAGAGAA
ENST00000377071        GGGGACTTCGTGCACGCCATGGAGGGCAAAGATTTCAACTATGAGTACGTACAGAGAGAA
ENSPTRT00000010204     GGGGACTTCGTGCACGCCATGGAGGGCAAAGATTTCAACTATGAGTACGTACAGAGAGAA
ENSMUST00000046073     GGGGACTTCGTGCATGCTATGGAAGGCAAAGATTTTAACTATGAGTACGTACAGAGAGAA
ENSRNOT00000044071     GGGGACTTCGTGCATGCCATGGAAGGCAAAGATTTTAACTATGAGTACGTACAGAGAGAA

ENSMMUT00000030348     GCTCTCAGGGTTCCCCTGATATTTCGAGAAAAGGATGGACTGGGAATTAAGATGCCTGAC
ENSCJAT00000002986     GCTCTCAGGGTTCCCCTGATATTTCGAGAAAAGGATGGACTGGGAATTAAGATGCCTGAC
ENSPPYT00000005974     GCTCTCAGGGTTCCCCTGATATTTCGAGAAAAGGATGGACTGGGAATTAAGATGCCTGAC
ENSGGOT00000014084     GCTCTCAGGGTTCCCCTGATATTTCGAGAAAAGGATGGACTGGGAATTAAGATGCCTGAC
ENST00000377071        GCTCTCAGGGTTCCCCTGATATTTCGAGAAAAGGATGGACTGGGAATTAAGATGCCTGAC
ENSPTRT00000010204     GCTCTCAGGGTTCCCCTGATATTTCGAGAAAAGGATGGACTGGGAATTAAGATGCCTGAC
ENSMUST00000046073     GCTCTCAGGGTCCCCCTGGTTTTTCGGGACAAGGATGGACTAGGGATCAAGATGCCAGAC
ENSRNOT00000044071     GCTCTCAGGGTTCCCCTGGTTTTTCGAGACAAGGATGGACTAGGAATCAAGATGCCAGAC

ENSMMUT00000030348     CCTGATTTCACAGTCCGAGACGTCAAACTCCTAGTGGGTAATCGGCGGCTTGTGGACGTG
ENSCJAT00000002986     CCTGATTTCACAGTCCGAGACGTCAAACTCCTAGTGGGGAGCCGGCGGCTTGTGGACGTG
ENSPPYT00000005974     CCTGATTTCACAGTCCGAGACGTCAAACTCCTAGTGGGGAGCCGGCGACTTGTGGACGTG
ENSGGOT00000014084     CCTGATTTCACAGTCCGAGACGTCAAACTCCTAGTGGGGAGCCGGCGGCTTGTGGACGTG
ENST00000377071        CCTGATTTCACAGTCCGAGACGTCAAACTCCTAGTGGGGAGCCGGCGGCTTGTGGACGTG
ENSPTRT00000010204     CCTGATTTCACAGTCCGAGACGTCAAACTCCTAGTGGGGAGCCGGCGGCTTGTGGACGTG
ENSMUST00000046073     CCTGATTTCACAGTCCGAGACGTCAAACTCCTGGTGGGGAGCCGCCGTTTGGTGGATGTC
ENSRNOT00000044071     CCTGACTTCACAGTCCGAGACGTCAAACTCCTGGTGGGGAGTCGCCGGCTGGTGGACGTG

ENSMMUT00000030348     ATGGATGTGAACACCCAGAAGGGCACAGAGATGAGCATGTCCCAGTTTGTGCGTTACTAC
ENSCJAT00000002986     ATGGATGTGAACACCCAGAAGGGCACGGAGATGAGCATGTCCCAGTTTGTGAGATACTAC
ENSPPYT00000005974     ATGGATGTGAACACCCAGAAGGGCACGGAGATGAGCATGTCCCAGTTTGTGCGTTACTAC
ENSGGOT00000014084     ATGGATGTGAACACCCAGAAGGGCACGGAGATGAGCATGTCCCAGTTTGTGCGTTACTAC
ENST00000377071        ATGGATGTGAACACCCAGAAGGGCACGGAGATGAGCATGTCCCAGTTTGTGCGTTACTAC
ENSPTRT00000010204     ATGGATGTGAACACCCAGAAGGGCACGGAGATGAGCATGTCCCAGTTTGTGCGTTACTAC
ENSMUST00000046073     ATGGACGTCAACACCCAGAAGGGTACCGAGATGAGCATGTCCCAGTTTGTGCGCTACTAC
ENSRNOT00000044071     ATGGACGTGAACACCCAGAAGGGCACGGAGATGAGCATGTCCCAGTTCGTGCGTTACTAC

ENSMMUT00000030348     GAGACGCCTGAGGCCCAGCGGGACAAGCTGTACAACGTCATCAGCCTAGAGTTCAGCCAC
ENSCJAT00000002986     GAGACACCCGAGGCCCAGCGGGACAAGCTGTACAACGTCATCAGTCTGGAGTTCAGCCAC
ENSPPYT00000005974     GAGACGCCCGAGGCCCAGCGGGACAAGCTGTACAACGTCATCAGCCTAGAGTTCAGCCAC
ENSGGOT00000014084     GAGACGCCCGAGGCCCAGCGGGACAAGCTGTACAACGTCATCAGCCTAGAGTTCAGCCAC
ENST00000377071        GAGACGCCCGAGGCCCAGCGGGACAAGCTGTACAACGTCATCAGCCTAGAGTTCAGCCAC
ENSPTRT00000010204     GAGACGCCCGAGGCCCAGCGGGACAAGCTGTACAACGTCATCAGCCTAGAGTTCAGCCAC
ENSMUST00000046073     GAGACACCAGAGGCACAGCGGGATAAACTGTACAACGTCATCAGCCTCGAGTTCAGCCAT
ENSRNOT00000044071     GAGACACCAGAGGCACAGAGGGACAAACTGTACAATGTCATCAGCCTCGAGTTCAGCCAT
```

```
ENSMMUT00000030348     ACCAAGCTGGAGCACTTGGTCAAGCGTCCGACTGTGGTAGACCTGGTGGACTGGGTGGAC
ENSCJAT00000002986     ACCAAGCTGGAGCACTTGGTCAAGCGTCCGACCGTGGTAGACCTGGTGGACTGGGTGGAC
ENSPPYT00000005974     ACCAAGCTGGAGCACTTGGTCAAGCGTCCGACTGTGGTAGACCTGGTGGACTGGGTGGAC
ENSGGOT00000014084     ACCAAGCTGGAGCACTTGGTCAAGCGTCCGACTGTGGTAGACCTGGTGGACTGGGTGGAC
ENST00000377071        ACCAAGCTGGAGCACTTGGTCAAGCGTCCGACTGTGGTAGACCTGGTGGACTGGGTGGAC
ENSPTRT00000010204     ACCAAGCTGGAGCACTTGGTCAAGCGTCCGACTGTGGTAGACCTGGTGGACTGGGTGGAC
ENSMUST00000046073     ACGAAGCTGGAGCATCTGGTCAAGCGTCCCACTGTGGTGGACCTGGTCGACTGGGTGGAC
ENSRNOT00000044071     ACGAAGCTGGAGCATCTAGTCAAGCGTCCCACTGTGGTGGACCTGGTCGACTGGGTGGAC

ENSMMUT00000030348     AACATGTGGCCCCAGCATCTGAAGGAGAAGCAGACAGAAGCCACGAATGCCATTGCAGAG
ENSCJAT00000002986     AACATGTGGCCCCAGCATCTGAAGGAAAAGCAGACAGAAGCCACGAACGCCATTGCAGAG
ENSPPYT00000005974     AACATGTGGCCCCAGCATCTGAAGGAGAAGCAGACAGAAGCCACGAACGCCATTGCAGAG
ENSGGOT00000014084     AACATGTGGCCCCAGCATCTGAAGGAGAAGCAGACAGAAGCCACGAACGCCATTGCAGAG
ENST00000377071        AACATGTGGCCCCAGCATCTGAAGGAGAAGCAGACAGAAGCCACGAACGCCATTGCAGAG
ENSPTRT00000010204     AACATGTGGCCCCAGCATCTGAAGGAGAAGCAGACAGAAGCCACGAACGCCATTGCAGAG
ENSMUST00000046073     AACATGTGGCCTCAGCATCTAAAGGAAAAGCAGACAGAAGCCACCAATGCCCTTGCAGAG
ENSRNOT00000044071     AACATGTGGCCCCAACATCTAAAGGAAAAGCAGACAGAAGCCACAAACGCCCTTGCCGAG

ENSMMUT00000030348     ATGAAGTATCCGAAAGTGAAGAAGTACTGTCTGATGAGCGTGAAAGGTTGTTTCACCGAC
ENSCJAT00000002986     ATGAAGTACCCGAAAGTAAAGAAAAAATGCCAGTTGCAGTTAATAAGCAGGCTCAATGAT
ENSPPYT00000005974     ATGAAGTACCCGAAAGTGAAGAAGTACTGTCTGATGAGCGTGAAAGGTTGTTTCACCGAC
ENSGGOT00000014084     ATGAAGTACCCGAAAGTGAAG---------------------------------------
ENST00000377071        ATGAAGTACCCGAAAGTGAAAAAGTACTGTCTGATGAGCGTGAAAGGTTGTTTCACCGAC
ENSPTRT00000010204     ATGAAGTACCCGAAAGTGAAGAAGTACTGTCTGATGAGCGTGAAAGGTTGTTTCACTGAC
ENSMUST00000046073     ATGAAGTACCCCAAAGTGAAAAAGTACTGTCTGATGAGCGTGAAGGGCTGTTTCACTGAC
ENSRNOT00000044071     ATGAAATACCCCAAAGTGAAGAAGTTTACGTTCACGTCAGTGAAGAATTGTTTCTGCATA

ENSMMUT00000030348     TTCCACATCGACTTTGGAGGCACTTCCGTTTGGTACCATGTTTTCCGGGGTGGGAAGATT
ENSCJAT00000002986     TTTCATGTAGAGACAGGGTTTCATCACATATATATACATATAATTCGTCACCAGAAGGTT
ENSPPYT00000005974     TTCCACATTGACTTTGGAGGCACTTCCGTTTGGTACCATGTTTTCCGGGGTGGGAAGATT
ENSGGOT00000014084     ------------------------------------------------------------
ENST00000377071        TTCCACATCGACTTTGGAGGCACTTCCGTTTGGTACCATGTTTTCCGGGGTGGGAAGATT
ENSPTRT00000010204     TTCCACATCGACTTTGGAGGCACTTCCGTTTGGTACCATGTTTTCCGGGGTGGGAAGATT
ENSMUST00000046073     TTCCACATTGACTTTGGAGGCACCTCCGTGTGGTACCATGTGTTCCGTGGTGGCAAGATC
ENSRNOT00000044071     GTTAAAATAGACTTTGGCGGGTTGGAAGTGTGGGAAAAAATTCTGCAGGCTCCCAAGATC

ENSMMUT00000030348     TTTTGGCTGATTCCTCCAACGCTGCACAATTTGGCGCTGTACGAGGAGTGGGTGCTGTCA
ENSCJAT00000002986     TTTTGGCTGATCCCTCCAACGCGGCACAATTTGGCGCTGTATGAACAGTGGGTGCTGTCA
ENSPPYT00000005974     TTTTGGCTGATTCCTCCAACGCTGCACAATTTGGCGCTGTACGAGGAGTGGGTGCTGTCA
ENSGGOT00000014084     ------------------------------------------------------------
ENST00000377071        TTTTGGCTGATTCCTCCAACGCTGCACAATTTGGCGCTGTACGAGGAGTGGGTGCTGTCA
ENSPTRT00000010204     TTTTGGCTGATTCCTCCAACGCTGCACAATTTGGCGCTGTACGAGGAGTGGGTGCTGTCA
ENSMUST00000046073     TTTTGGCTGATCCCTCCAACCCTGCACAACTTGGCTTTGTACGAGGAGTGGGTGCTGTCT
ENSRNOT00000044071     TTTTGGCTGATCCCCCCAACCCTGCACAACTTGGCTCTGTATGAGGAGTGGGTGCTGTCT

ENSMMUT00000030348     GGCAAACAGAGTGACATCTTTCTGGGAGACCGTGTGGAACGATGCCAAAGAATTGAGCTG
ENSCJAT00000002986     GGCAAACAGAGTGACATCTTTCTGGGAGACCGTGTGGAACGATGCCAAAGAATTGAGCTG
ENSPPYT00000005974     GGCAAACAGAGTGACATCTTTCTGGGAGACCGTGTGGAACGATGCCAAAGAATTGAGCTG
ENSGGOT00000014084     ------------------------------------------------------------
ENST00000377071        GGCAAACAGAGTGACATCTTTCTGGGAGACCGTGTGGAACGATGCCAAAGAATTGAGCTG
ENSPTRT00000010204     GGCAAACAGAGTGACATCTTTCTGGGAGACCGTGTGGAACGATGCCAAAGAATTGAGCTG
ENSMUST00000046073     GGCAAACAGAGCGACATCTTTCTGGGAGACCGCGTGGAACGCTGCCAAAGAATTGAGCTG
ENSRNOT00000044071     GGCAAACAGAGTGACATCTTTCTGGGAGACCGAGTGGAACGCTGCCAAAGAATTGAGCTG
```

```
ENSMMUT00000030348     AAGCAGGGCTACACATTTTTCATCCCTTCCGGTTGGATCCATGCCGTCTACACCCCTGTA
ENSCJAT00000002986     AAACAGGGCTACACATTTTTCATCCCTTCCGGTTGGATCCATGCTGTCTACACCCCTGTA
ENSPPYT00000005974     AAGCAGGGCTACACATTTTTCATCCCTTCTGGTTGGATCCATGCTGTCTACACCCCTGTA
ENSGGOT00000014084     ---------------------------------TGGATCCATGCCGTCTACACCCCTGTA
ENST00000377071        AAGCAGGGCTACACATTTTTCATCCCTTCCGGTTGGATCCATGCCGTCTACACCCCTGTA
ENSPTRT00000010204     AAGCAGGGCTACACATTTTTCATCCCTTCCGGTTGGATCCATGCCGTCTACACCCCTGTA
ENSMUST00000046073     AAGCAAGGCTACACCTTTTTCATCCCTTCCGGTTGGATCCATGCGGTTTATACGCCTGTG
ENSRNOT00000044071     AAGCAAGGCTACACATTTTTCATCCCTTCTGGTTGGATCCATGCAGTGTACACGCCCGTG

ENSMMUT00000030348     GACTCTTTGGTATTCGGCGGAAACATCCTGCACAGCTTTAACGTGCCCATGCAGCTGCGG
ENSCJAT00000002986     GACTCGTTGGTCTTCGGCGGGAACATCCTGCATAGCTTTAACGTGCCCATGCAGCTGCGG
ENSPPYT00000005974     GACTCTTTGGTGTTCGGCGGAAACATCCTGCACAGCTTTAACGTGCCCATGCAGCTGCGG
ENSGGOT00000014084     GACTCTTTGGTGTTCGGCGGAAACATCCTGCACAGCTTTAACGTGCCCATGCAGCTGCGG
ENST00000377071        GACTCTTTGGTGTTCGGCGGAAACATCCTGCACAGCTTTAACGTGCCCATGCAGCTGCGG
ENSPTRT00000010204     GACTCTTTGGTGTTCGGCGGAAACATCCTGCACAGCTTTAACGTGCCCATGCAGCTGCGG
ENSMUST00000046073     GACTCTCTGGTGTTCGGCGGGAACATCCTGCATAGCTTCAACGTGCCCATGCAGCTGCGG
ENSRNOT00000044071     GACTCTCTGGTGTTCGGCGGGAACATCCTGCACAGCTTCAACGTGCCCATGCAGCTGCGC

ENSMMUT00000030348     ATCTACGAGATCGAGGACAGGACGCGGGTGCAGCCCAAATTCCGTTACCCCTTCTACTAT
ENSCJAT00000002986     ATCTACGAGATCGAGGACAGAACGCGGGTGCAGCCAAAATTCCGTTACCCCTTCTACTAT
ENSPPYT00000005974     ATCTACGAGATCGAGGACAGGACACGGGTGCAGCCCAAATTCCGTTACCCCTTCTACTAT
ENSGGOT00000014084     ATCTACGAGATCGAGGACAGGACGCGGGTGCAGCCCAAATTCCGTTACCCCTTCTACTAT
ENST00000377071        ATCTACGAGATCGAGGACAGGACGCGGGTGCAGCCCAAATTCCGTTACCCCTTCTACTAT
ENSPTRT00000010204     ATCTACGAGATCGAGGACAGGACGCGGGTGCAGCCCAAATTCCGTTACCCCTTCTACTAT
ENSMUST00000046073     ATCTACGAGATCGAGGACAGGACCCGGGTTCAGCCCAAGTTCCGTTACCCCTTCTACTAT
ENSRNOT00000044071     ATCTACGAGATCGAGGACAGGACCCGGGTTCAGCCCAAGTTCCGTTACCCCTTCTACTAT

ENSMMUT00000030348     GAGATGTGCTGGTATGTCCTGGAGAGATACGTGTACTGTGTGACCCAGCGCTCCCACCTC
ENSCJAT00000002986     GAGATGTGCTGGTATGTCCTGGAGAGATACGTGTACTGTGTGACCCAGCGCTCCCACCTC
ENSPPYT00000005974     GAGATGTGCTGGTATGTCCTGGAGAGATACGTGTACTGTGTGACCCAGCGCTCCCACCTC
ENSGGOT00000014084     GAGATGTGCTGGTATGTCCTGGAGAGATACGTGTACTGTGTGACCCAGCGCTCCCACCTC
ENST00000377071        GAGATGTGCTGGTATGTCCTGGAGAGATACGTGTACTGTGTGACCCAGCGCTCCCACCTC
ENSPTRT00000010204     GAGATGTGCTGGTATGTCCTGGAGAGATACGTGTACTGTGTGACCCAGCGCTCCCACCTC
ENSMUST00000046073     GAGATGTGCTGGTATGTCTTGGAGAGATATGTGTACTGTGTGACCCAGCGCTCCTACCTC
ENSRNOT00000044071     GAGATGTGCTGGTATGTCTTGGAGAGATACGTGTACTGTGTGACCCAGCGCTCCTACCTC

ENSMMUT00000030348     ACTCAGGAATACCAGAGGGAGTCGATGCTTATTGATGCCCCGAGGAAGCCCAGCATAGAC
ENSCJAT00000002986     ACTCAGGAATACCAGAGGGAGTCAATGCTTATTGATGCCCCGAGGAAGCCCAGCATAGAC
ENSPPYT00000005974     ACTCAGGAATACCAGAGGGAGTCGATGCTTATTGATGCCCCGAGGAAGCCCAGCATAGAC
ENSGGOT00000014084     ACTCAGGAATACCAGAGGGAGTCGATGCTTATTGATGCCCCGAGGAAGCCCAGCATAGAC
ENST00000377071        ACTCAGGAATACCAGAGGGAGTCGATGCTTATTGATGCCCCGAGGAAGCCCAGCATAGAC
ENSPTRT00000010204     ACTCAGGAATACCAGAGGGAGTCGATGCTTATTGATGCCCCGAGGAAGCCCAGCATAGAC
ENSMUST00000046073     ACTCAGGAATACCAGAGAGAATTAATGCTTATTGATGCCCCAAGAAAAACCAGTGTAGAC
ENSRNOT00000044071     ACTCAGGAATACCAGCGAGAATTAATGCTCATTGATGCCCCAAGAAAAAACAGTGTAGAC

ENSMMUT00000030348     GGCTTCTCTTCGGATTCCTGGCTGGAGATGGAGGAGGAGGCCTGTGATCAGCAGCCTCAG
ENSCJAT00000002986     GGTTTCTCTTCGGATTCCTGGCTGGAGATGGAGGAGGAGGCCTGTGATCAGCAGCCT---
ENSPPYT00000005974     GGCTTCTCTTCGGATTCCTGGCTGGAGATGGAGGAGGAGGCCTGTGATCAGCAGCCTCAG
ENSGGOT00000014084     GGCTTCTCTTCCGATTCCTGGCTGGAGATGGAGGAGGAGGCCTGTGATCAGCAGCCTCAG
ENST00000377071        GGCTTCTCTTCGGATTCCTGGCTGGAGATGGAGGAGGAGGCCTGTGATCAGCAGCCTCAG
ENSPTRT00000010204     GGCTTCTCTTCGGATTCCTGGCTGGAGATGGAGGAGGAGGCCTGTGATCAGCAGCCTCAG
ENSMUST00000046073     GGCTTTTCATCCGACTCCTGGCTGGACATGGAGGAGGAGTCCTGCGAGCAGCAGCCACAG
ENSRNOT00000044071     GGTTTCTCATCTGATTCCTGGCTGGAGATGGAGGAGGAGTCCCGTGAGCAGCAGCTCCAG
```

```
ENSMMUT00000030348     GAGGAGGAGGAGAAGGACGAGGAGGGCGAGGGCAGGGACAGGGCACCCAAACCGCCCACC
ENSCJAT00000002986     ------------------------GGCGAGGGCAGGGACAGGACGCCCAAGCCACCTACC
ENSPPYT00000005974     GAGGAGGAGGAGAAGGACGAGGAGGGCGAGGGCAGGGACAGGGTACCCAAACCGCCCACC
ENSGGOT00000014084     GAGGAGGAGGAGAAGGACGAGGAGGGCGAGGGCAGGGACAGGGCACCCAAACCGCCCACC
ENST00000377071        GAGGAGGAGGAGAAGGACGAGGAGGGCGAGGGCAGGGACAGGGCACCCAAACCGCCCACC
ENSPTRT00000010204     GAGGAGGAGGAGAAGGACGAGGAGGGCGAGGGCAGGGACAGGGCACCCAAACCGCCCGCC
ENSMUST00000046073     GAGGAAGAGGAGAAGGAGGAGGAAGGAGATGGTGCAGATAAAACACCCAAGCCACCCACC
ENSRNOT00000044071     GAGGAGGAGGACAAGGAAGAGGAAGGGGATGGTGCAGACAAAACACCCAAGCCACCCACC

ENSMMUT00000030348     GATGGCTCCGCTTCACCCACCAGCATGCCCTCCGAGGACCAGGAGGCCCTTGGGAAGAAG
ENSCJAT00000002986     GAGGGCTCTGCCTCACCCACCAGCACGCCCTCCGAGGACCAGGAGGCCCCCGGGAAGAAG
ENSPPYT00000005974     GATGGCTCCACTTCACCCACCAGCACACCCTCCGAGGACCAGGAGTCCCTCGGGAAGAAG
ENSGGOT00000014084     GATGGCTCCACTTCACCCACCAGCACGCCCTCTGAGGACCAGGAGGCCCTTGGGAAGAAG
ENST00000377071        GATGGCTCCACTTCACCCACCAGCACGCCCTCTGAGGACCAGGAGGCCCTCGGGAAGAAG
ENSPTRT00000010204     GATGGCTCCACTTCACCCACCAGCACGCCCTCCGAGGACCAGGAGGCCCTCGGGAAGAAG
ENSMUST00000046073     GATGACCCCACCTCACCCACCAGCACCCCGCCCGAAGACCAGGACAGCACAGGGAAGAAG
ENSRNOT00000044071     GAGGGCCCCACCTCACCCACCAGCACCCAGTCGGAAGACCAGGACAGCACAGGGAAGAAG

ENSMMUT00000030348     CCCAAAGCACCTGCCCTGCGGTTCCTCAAAAGGACTTTGTCTAACGAGTCAGAGGAGAGC
ENSCJAT00000002986     CCCAAAGCGCCTGCCCTGCGGTTCCTCAAAAGGACTTTGTCCAACGAGTCGGAGGAGAGT
ENSPPYT00000005974     CCCAAAGCACCTGCCCTGCGATTCCTCAAAAGGACTTTGTCTAACGAGTCGGAGGAGAGT
ENSGGOT00000014084     CCCAAAGCACCTGCCCTGCGATTCCTCAAAAGGACTTTGTCTAACGAGTCGGAGGAAAGT
ENST00000377071        CCCAAAGCACCTGCCCTGCGATTCCTCAAAAGGACTTTGTCTAATGAGTCGGAGGAAAGT
ENSPTRT00000010204     CCCAAAGCACCTGCCCTGCGATTCCTCAAAAGGACTTTGTCTAACGAGTCGGAGGAGAGT
ENSMUST00000046073     CCTAAAGCCCCTGCCATACGGTTCCTCAAGAGGACGTTGTCCAATGAGTCAGAGGAAAGT
ENSRNOT00000044071     CCTAAAGCCCCTGCTATGCGATTCCTCAAGAGGACTTTGTCCAATGAGTCCGAGGAAAGT

ENSMMUT00000030348     GTGAAGTCCACCACGTTGCCCGTAGACTACCCCAAGACCCCCACTGGCTCTCCCGCCACG
ENSCJAT00000002986     GTCAAGTCCACCACACTGCCCGTGGACTACCCCAAGACGCCCACTGGCTCTCCCGCCGCG
ENSPPYT00000005974     GTGAAGTCTACTACGTTGCCCGTAGACTACCCCAAGACCCCCACCGGCTCTCCCGCCACG
ENSGGOT00000014084     GTGAAGTCCACCACATTGCCTGTAGACTACCCCAAGACCCCCACCGGCTCTCCCGCCACG
ENST00000377071        GTGAAGTCCACCACATTGGCCGTAGACTACCCCAAGACCCCCACCGGCTCTCCCGCCACG
ENSPTRT00000010204     GTGAAGTCCACCACATTGGCCGTAGACTACCCCAAGACCCCCACCGGCTCTCCCGCCACG
ENSMUST00000046073     GTCAAGTCGACCTCGATGCCCACGGACGATCCCAAGACGCCCACGGGCTCCCCGGCCACC
ENSRNOT00000044071     GTCAAGTCCACAGCGATGCCCGTAGACTACCCCAAGACGCCCACAGGCTCCCCGGCCACT

ENSMMUT00000030348     GAGGTCTCTGCTAAATGGACCCATCTCACCGAGTTTGAACTGAAGGGCCTGAAAGCTCTG
ENSCJAT00000002986     GAGGCCGCTGCCAAGTGGACCCATCTCACCGAGTTTGAACTGAAGGGCCTGAAAGCTCTG
ENSPPYT00000005974     GAGGTCTCTGCCAAATGGACCCATCTCACCGAGTTTGAACTGAAGGGCCTGAAAGCTCTG
ENSGGOT00000014084     GAGGTCTCTGCCAAATGGACCCATCTCACCGAGTTTGAACTGAAGGGCCTGAAAGCTCTG
ENST00000377071        GAGGTCTCTGCCAAATGGACCCATCTCACTGAGTTTGAACTGAAGGGCCTGAAAGCTCTG
ENSPTRT00000010204     GAGGTCTCTGCCAAATGGACCCATCTCACCGAGTTTGAACTGAAGGGCCTGAAAGCTCTG
ENSMUST00000046073     GAGGTTTCTACCAAGTGGACTCACCTTACCGAATTTGAACTGAAGGGCTTGAAAGCCCTG
ENSRNOT00000044071     GAGGTTTCTACCAAGTGGACTCACCTTACCGAATTTGAACTGAAGGGCTTGAAAGCCCTG

ENSMMUT00000030348     GTGGAGAAACTGGAATCCCTCCCGGAGAACAAGAAGTGTGTCCCCGAGGGCATCGAGGAC
ENSCJAT00000002986     GTGGAGAAACTGGAATCCCTCCCAGAGAACAAGAAGTGCGTCCCCGAGGGCATCGAGGAC
ENSPPYT00000005974     GTGGAGAAACTGGAATCCCTCCCGGAGAACAAGAAGTGTGTCCCCGAGGGCATCGAGGAC
ENSGGOT00000014084     GTGGAGAAACTGGAATCCCTCCCGGAGAACAAGAAGTGTGTCCCCGAAGGCATCGAGGAC
ENST00000377071        GTGGAGAAACTGGAATCCCTCCCGGAGAACAAGAAGTGTGTCCCCGAGGGCATCGAGGAC
ENSPTRT00000010204     GTGGAGAAACTGGAATCCCTCCCGGAGAACAAGAAGTGTGTCCCCGAGGGCATCGAGGAC
ENSMUST00000046073     GTTGAAAAGCTAGAGTCCCTTCCGGAGAATAAGAAGTGTGTCCCTGAGGGAATCGAGGAC
ENSRNOT00000044071     GTTGAAAAACTAGAGTCTCTTCCGGAGAATAAGAAGTGTGTCCCTGAGGGAATTGAGGAC
```

```
ENSMMUT00000030348     CCCCAGGCACTCCTGGAGGGTGTGAAGAATGTCCTGAAGGAGCACGCGGATGATGACCCT
ENSCJAT00000002986     CCCCAGGCACTCCTGGAGGGTGTGAAGAATGTTCTGAAGGAGCACGCGGATGATGACCCT
ENSPPYT00000005974     CCCCAGGCACTCCTGGAGGGTGTGAAGAACGTCCTGAAGGAACACGCGGATGATGACCCT
ENSGGOT00000014084     CCCCAGGCACTCCTGGAGGGTGTGAAGAACGTCCTGAAGGAGCACGCGGATGATGACCCT
ENST00000377071        CCCCAGGCACTCCTGGAGGGTGTGAAGAACGTCCTGAAGGAGCACGCAGATGATGACCCT
ENSPTRT00000010204     CCCCAGGCACTCCTGGAGGGTGTGAAGAACGTCCTGAAGGAGCACGCGGATGATGATCCT
ENSMUST00000046073     CCCCAGGCCCTCCTGGAAGGTGTAAAGAATGTACTGAAAGAGCACGTGGATGACGACCCC
ENSRNOT00000044071     CCCCAGGCCCTCCTGGAAGGTGTTAAGAATGTACTGAAGGAACACGTGGATGATGACCCC

ENSMMUT00000030348     AGTCTGGCCATCACTGGGGTCCCTGTGGTGACTTGGCCAAAGAAGACTCCAAAGAACCGG
ENSCJAT00000002986     AGTCTGGCCATCACTGGGGTCCCTGTGGTGACTTGGCCAAAGAAGACTCCAAAGAACCGG
ENSPPYT00000005974     AGTCTGGCCATCACTGGGGTCCCTGTGGTGACTTGGCCAAAGAAGACTCCAAAGAACCGG
ENSGGOT00000014084     AGTCTGGCCATCACTGGGGTCCCTGTGGTGACTTGGCCAAAGAAGACTCCAAAGAACCGG
ENST00000377071        AGTCTGGCCATCACTGGGGTCCCTGTGGTGACTTGGCCAAAGAAGACTCCAAAGAACCGG
ENSPTRT00000010204     AGTCTGGCCATCACTGGGGTCCCTGTGGTGACTTGGCCAAAGAAGACTCCAAAGAACCGG
ENSMUST00000046073     ACCCTGGCCATCACCGGGGTCCCTGTGGTCAGCTGGCCAAAGAAAACTGCAAAGAACCGG
ENSRNOT00000044071     AACCTGGCCATCACCGGGGTCCCCGTGGTCAGTTGGCCAAAGAAAACTCCAAAGAACCGG

ENSMMUT00000030348     GCTGTGGGTCGGCCCAAGGGGAAGCTGGGCCCGGCCTCCGCGGTGAAGTTGGCCGCCAAC
ENSCJAT00000002986     GCTGTGGGTCGGCCCAAGGGGAAGCTGGGCCCGGCCTCCGCGGTGAAGTTGGCCGCCAAC
ENSPPYT00000005974     GCTGTGGGTCGGCCCAAGGGGAAGCTCGGCCCGGCCTCCGCGGTGAAGTTGGCCGCCAAT
ENSGGOT00000014084     GCTGTGGGTCGGCCCAAGGGGAAGCTGGGCCCGGCCTCCGCGGTGAAGTTGGCCGCCAAC
ENST00000377071        GCTGTGGGTCGGCCCAAGGGGAAGCTGGGCCCGGCCTCCGCGGTGAAGTTGGCCGCCAAC
ENSPTRT00000010204     GCTGTGGGTCGGCCCAAGGGGAAGCTGGGCCCGGCCTCCGCGGTGAAGTTGGCCGCCAAC
ENSMUST00000046073     GTGGTGGGTCGGCCTAAGGGCAAGTTGGGCCCGGCCTCAGCGGTGAAGTTGGCTGCCAAC
ENSRNOT00000044071     GTGGTGGGTCGGCCTAAGGGCAAGTTGGGCCCGGCCTCAGCGGTGAAGTTGGCTGCCAAC

ENSMMUT00000030348     CGGACAACGGCAGGAGCTCGGCGGCGCCGGACGCGATGCCGCAAGTGCGAGGCCTGCCTG
ENSCJAT00000002986     CGGACAACTGCAGGAGCTCGGCGACGCCGGACGCGATGCCGCAAGTGCGAGGCCTGCCTG
ENSPPYT00000005974     CGGACAACCGCAGGAGCTCGGCGGCGCCGGACGCGATGCCGCAAGTGCGAGGCCTGCCTG
ENSGGOT00000014084     CGGACAACGGCAGGAGCTCGGCGGCGCCGGACGCGATGCCGCAAGTGCGAGGCCTGCCTG
ENST00000377071        CGGACAACGGCAGGAGCTCGGCGGCGCCGGACGCGATGCCGCAAGTGCGAGGCCTGCCTG
ENSPTRT00000010204     CGGACAACGGCAGGAGCTCGGCGGCGCCGGACGCGATGCCGCAAGTGCGAGGCCTGCCTG
ENSMUST00000046073     CGAACAACAGCAGGAGCTCGCAGGCGCCGGACGCGATGCCGCAAGTGCGAGGCCTGCCTG
ENSRNOT00000044071     CGAACAACAGCAGGAGCTCGCAGGCGCCGGACGCGATGCCGCAAGTGCGAGGCCTGCCTG

ENSMMUT00000030348     CGGACCGAGTGCGGAGAGTGCCACTTCTGCAAGGACATGAAGAAGTTCGGGGGCCCCGGG
ENSCJAT00000002986     CGGACCGAGTGCGGAGAGTGCCACTTCTGCAAGGACATGAAGAAGTTCGGGGGCCCCGGG
ENSPPYT00000005974     CGGACCGAGTGCGGAGAGTGCCACTTCTGCAAGGACATGAAGAAGTTCGGGGGCCCCGGG
ENSGGOT00000014084     CGGACCGAGTGCGGAGAGTGCCACTTCTGCAAGGACATGAAGAAGTTCGGGGGCCCCGGG
ENST00000377071        CGGACCGAGTGCGGAGAGTGCCACTTCTGCAAGGACATGAAGAAGTTCGGGGGCCCCGGG
ENSPTRT00000010204     CGGACCGAGTGCGGAGAGTGCCACTTCTGCAAGGACATGAAGAAGTTCGGGGGCCCCGGG
ENSMUST00000046073     CGGACGGAGTGTGGAGAGTGCCACTTTTGCAAGGACATGAAGAAGTTTGGAGGTCCTGGG
ENSRNOT00000044071     CGGACGGAGTGTGGAGAGTGCCACTTTTGCAAGGACATGAAGAAGTTCGGAGGTCCTGGG

ENSMMUT00000030348     CGCATGAAGCAGAGCTGCATCATGCGGCAGTGCATCGCGCCAGTGCTCCCCCACACCGCC
ENSCJAT00000002986     CGGATGAAGCAGAGCTGCATCATGCGGCAGTGCATCGCGCCAGTGCTGCCCCACACCGCC
ENSPPYT00000005974     CGCATGAAGCAGAGCTGCATCATGCGGCAGTGCATCGCGCCAGTGCTGCCCCACACTGCC
ENSGGOT00000014084     CGCATGAAGCAGAGCTGCATCATGCGGCAGTGCATCGCGCCAGTGCTGCCCCACACCGCC
ENST00000377071        CGCATGAAGCAGAGCTGCATCATGCGGCAGTGCATCGCGCCAGTGCTGCCCCACACCGCC
ENSPTRT00000010204     CGCATGAAGCAGAGCTGCATCATGCGGCAGTGCATCGCGCCAGTGCTGCCCCACACCGCT
ENSMUST00000046073     CGCATGAAGCAGAGCTGCATCATGCGGCAGTGCATCGCGCCAGTGCTGCCCCACACCGCC
ENSRNOT00000044071     CGCATGAAGCAGAGCTGCATCATGCGGCAGTGCATCGCGCCAGTGCTGCCCCACACCGCC
```

```
ENSMMUT00000030348     GTGTGCCTTGTGTGTGGCGAGGCGGGGAAGGAAGACACAGTGGAAGAGGAGGAAGGCAAG
ENSCJAT00000002986     GTGTGCCTCGTGTGTGGCGAGGCGGGGAAGGAAGACACGGTGGAGGAAGAGGAAGGCAAG
ENSPPYT00000005974     GTGTGCCTTGTGTGTGGCGAGGCGGGGAAGGAAGACACGGTGGAAGAGGAGGAAGGCAAG
ENSGGOT00000014084     GTGTGCCTTGTGTGTGGCGAGGCGGGGAAGGAAGACACGGTGGAAGAGGAGGAAGGCAAG
ENST0000377071         GTGTGCCTTGTGTGTGGCGAGGCGGGGAAGGAAGACACGGTGGAAGAGGAGGAAGGCAAG
ENSPTRT00000010204     GTGTGCCTTGTGTGTGGCGAGGCGGGGAAGGAAGACACGGTGGAAGAGGAGGAAGGCAAG
ENSMUST00000046073     GTGTGCCTTGTGTGTGGCGAGGCAGGGAAGGAGGACACAGTGGAAGAGGAAGAAGGCAAG
ENSRNOT00000044071     GTGTGCCTTGTGTGTGGCGAGGCAGGGAAGGAGGACACAGTGGAAGAGGAAGAAGGCAAG

ENSMMUT00000030348     TTTAACCTCATGCTCATGGAGTGCTCCATCTGCAACGAAATCATCCACCCCGGATGCCTT
ENSCJAT00000002986     TTTAACCTCATGCTCATGGAGTGCTCCATCTGCAATGAAATCATCCACCCCGGATGCCTT
ENSPPYT00000005974     TTTAACCTCATGCTCATGGAGTGCTCCATCTGCAATGAAATCATCCACCCTGGATGCCTT
ENSGGOT00000014084     TTTAACCTCATGCTCATGGAGTGCTCCATCTGCAATGAAATCATCCACCCTGGATGCCTT
ENST0000377071         TTTAACCTCATGCTCATGGAGTGCTCCATCTGCAATGAAATCATCCACCCTGGATGCCTT
ENSPTRT00000010204     TTTAACCTCATGCTCATGGAGTGCTCCATCTGCAATGAAATCATCCACCCTGGATGCCTT
ENSMUST00000046073     TTTAACCTCATGCTCATGGAATGCTCCATCTGCAACGAGATCATCCACCCTGGATGCCTT
ENSRNOT00000044071     TTTAACCTCATGCTCATGGAGTGCTCCATCTGCAACGAGATCATCCACCCTGGATGCCTT

ENSMMUT00000030348     AAGATTAAGGAGTCAGAGGGTGTGGTCAACGACGAGCTTCCAAACTGCTGGGAGTGTCCG
ENSCJAT00000002986     AAGATTAAGGAATCAGAGGGTGTGGTCAACGATGAGCTTCCAAACTGCTGGGAGTGTCCG
ENSPPYT00000005974     AAGATTAAGGAGTCAGAGGGTGTGGTCAACGACGAGCTTCCAAACTGCTGGGAGTGTCCG
ENSGGOT00000014084     AAGATTAAGGAGTCAGAGGGTGTGGTCAACGACGAGCTTCCAAACTGCTGGGAGTGTCCG
ENST0000377071         AAGATTAAGGAGTCAGAGGGTGTGGTCAACGACGAGCTTCCAAACTGCTGGGAGTGTCCG
ENSPTRT00000010204     AAGATTAAGGAGTCAGAGGGTGTGGTCAACGACGAGCTTCCAAACTGCTGGGAGTGTCCG
ENSMUST00000046073     AAGATTAAGGAATCGGAGGGTGTGGTCAACGATGAGCTTCCCAACTGCTGGGAGTGTCCG
ENSRNOT00000044071     AAGATTAAGGAATCAGAGGGTGTGGTCAACGATGAGCTTCCCAACTGCTGGGAGTGTCCG

ENSMMUT00000030348     AAGTGTAACCACGCCGGCAAGACCGGGAAACAAAAGCGTGGCCCTGGCTTTAAGTACGCC
ENSCJAT00000002986     AAGTGTAACCATGCCGGCAAGACCGGGAAGCAAAAGCGTGGCCCTGGCTTTAAGTATGCC
ENSPPYT00000005974     AAGTGTAACCACGCCGGCAAGACCGGGAAACAAAAGCGTGGCCCTGGCTTTAAGTACGCC
ENSGGOT00000014084     AAGTGTAACCACGCCGGCAAGACCGGGAAACAAAAGCGTGGCCCTGGCTTTAAGTACGCC
ENST0000377071         AAGTGTAACCACGCCGGCAAGACCGGGAAACAAAAGCGTGGCCCTGGCTTTAAGTACGCC
ENSPTRT00000010204     AAGTGTAACCACGCCGGCAAGACCGGGAAACAAAAGCGTGGCCCTGGCTTTAAGTATGCC
ENSMUST00000046073     AAGTGTAACCATGCCGGCAAGACCGGGAAACAAAAGCGTGGCCCTGGCTTTAAGTATGCC
ENSRNOT00000044071     AAGTGTAACCATGCCGGCAAGACCGGGAAACAAAAGCGTGGCCCTGGCTTTAAGTATGCC

ENSMMUT00000030348     TCCAACCTGCCCGGCTCCCTGCTCAAGGAGCAGAAGATGAACCGGGACAACAAGGAAGGG
ENSCJAT00000002986     TCCAACCTGCCCGGCTCCCTGCTCAAGGAGCAGAAGATGAACCGGGACAACAAGGAAGGG
ENSPPYT00000005974     TCCAACCTGCCCGGCTCCCTGCTCAAGGAGCAGAAGATGAACCGGGACAACAAGGAAGGG
ENSGGOT00000014084     TCCAACCTGCCCGGCTCCCTGCTCAAGGAGCAGAAGATGAACCGGGACAACAAGGAAGGG
ENST0000377071         TCCAACCTGCCCGGCTCCCTGCTCAAGGAGCAGAAGATGAACCGGGACAACAAGGAAGGG
ENSPTRT00000010204     TCCAACCTGCCCGGCTCCCTGCTCAAGGAGCAGAAGATGAACCGGGACAACAAGGAAGGG
ENSMUST00000046073     TCCAACCTGCCTGGCTCCTTGCTCAAGGAGCAGAAGATGAACCGGGACAACAAGGAAGGG
ENSRNOT00000044071     TCCAACTTGCCTGGCTCCTTGCTCAAGGAGCAGAAGATGAACCGGGACAACAAGGAAGGG

ENSMMUT00000030348     CAGGAACCTGCCAAGCGGAGGAGCGAATGTGAGGAGGCACCCCGGCGCAGGTCGGACGAG
ENSCJAT00000002986     CAGGAGCCTGCCAAGCGGAGGAGTGAATGTGAGGAGGCACCCCGGCGCAGGTCGGACGAG
ENSPPYT00000005974     CAGGAACCTGCCAAGCGGAGGAGTGAGTGTGAGGAGGCGCCCCGGCGCAGGTCGGATGAG
ENSGGOT00000014084     CAGGAACCTGCCAAGCGGAGGAGTGAGTGTGAGGAGGCGCCCCGGCGCAGGTCGGATGAG
ENST0000377071         CAGGAACCTGCCAAGCGGAGGAGTGAGTGTGAGGAGGCGCCCCGGCGCAGGTCGGATGAG
ENSPTRT00000010204     CAGGAACCTGCCAAGCGGAGGAGTGAGTGTGAGGAGGCGCCCCGGCGCAGGTCGGATGAG
ENSMUST00000046073     CAAGAGCCTGCCAAGCGGAGAAGTGAGTGTGAAGAGGCTCCCCGTCGGAGGTCAGACGAG
ENSRNOT00000044071     CAGGAGCCTGCCAAGCGGAGGAGTGAGTGTGAAGAGGCCCCCCGTCGCAGGTCAGACGAG
```

```
ENSMMUT00000030348     CACCCCAAGAAGGTGCCGCCGGACGGCCTCCTGCGCAGAAAGTCTGATGACGTGCACCTG
ENSCJAT00000002986     CACCCCAAGAAGGCACCCCCAGACGGCATCCTGCGAAGGAAGTCTGACGACGTGCACCTG
ENSPPYT00000005974     CACCCTAAGAAGGTGCCGCCGGACGGCCTCCTGCGCAGAAAGTCTGACGACGTGCACCTG
ENSGGOT00000014084     CACCCGAAGAAGGTGCCGCCGGACGGCCTCCTGCGCAGAAAGTCTGACGACGTGCACCTG
ENST00000377071        CACTCGAAGAAGGTGCCGCCGGACGGCCTTCTGCGCAGAAAGTCTGACGACGTGCACCTG
ENSPTRT00000010204     CACCCGAAGAAGGTGCCGCCGGACGGCCTCCTGCGCAGAAAGTCTGATGACGTGCACCTG
ENSMUST00000046073     CACCCCAAAAAGGTGCCTGCAGATGGCATCCTCCGCCGAAAGTCTGATGATGTGCACCTG
ENSRNOT00000044071     CACCCCAAGAAGGTGCCTACAGATGGCATCCTCCGCCGAAAGTCTGACGATGTGCACCTG

ENSMMUT00000030348     AGGAAGAAGCGGAAATACGAGAAGCCCCAGGAGCTGAGTGGACGCAAGCGGGCCTCATCG
ENSCJAT00000002986     AGGAAGAAGCGGAAATATGAGAAGCCCCAGGAACTGAGTGGACGCAAGCGGGCCTCATCG
ENSPPYT00000005974     AGGAAGAAGCGGAAATACGAGAAGCCCCAGGAGCTGAGTGGGCGCAAGCGGGCCTCATCG
ENSGGOT00000014084     AGGAAGAAGCGGAAATACGAGAAGCCCCAGGAGCTGAGTGGACGCAAGCGGGCCTCATCG
ENST00000377071        AGGAAGAAGCGGAAATACGAGAAGCCCCAGGAGCTGAGTGGACGCAAGCGGGCCTCATCG
ENSPTRT00000010204     AGGAAGAAGCGGAAATACGAGAAGCCCCAGGAGCTGAGTGGACGCAAGCGGGCCTCATCG
ENSMUST00000046073     AGGAGGAAGCGGAAATACGAGAAGCCCCAAGAGCTGAGTGGACGCAAGCGAGCCTCGTCG
ENSRNOT00000044071     AGGAGGAAGCGGAAATACGAGAAGCCCCAAGAGCTGAGTGCACGCAAGCGAGCCTCGACG

ENSMMUT00000030348     CTTCAAACGTCCCCCGGTTCCTCCTCTCACCTCTCGCCGAGGCCCCCTCTAGGCAGCAGC
ENSCJAT00000002986     CTTCAAACGTCCCCCGGTTCCTCCTCTCACCTCTCGCCGAGGCCCCCTCTAGGCAGCAGC
ENSPPYT00000005974     CTTCAAACGTCCCCCGGTTCCTCCTCTCACCTCTCGCCGAGGCCCCCTCTAGGCAGCAGC
ENSGGOT00000014084     CTTCAAACGTCCCCCGGTTCCTCCTCTCACCTCTCGCCGAGGCCCCCTCTAGGCAGCAGC
ENST00000377071        CTTCAAACGTCCCCCGGTTCCTCCTCTCACCTCTCGCCGAGGCCCCCTCTAGGCAGCAGC
ENSPTRT00000010204     CTTCAAACGTCCCCCGGTTCCTCCTCTCACCTCTCGCCGAGGCCCCCTCTAGGCAGCAGC
ENSMUST00000046073     CTTCAAACGTCCCCCGGTTCCTCCTCTCACCTCTCGCCGAGGCCCCCTCTAGGCAGCAGT
ENSRNOT00000044071     CTTCAAACGTCCCCCGGTTCCTCCTCTCACCTCTCGCCGAGGCCCCCTCTAGGCAGCAGT

ENSMMUT00000030348     CTCAGCCCCTGGTGGAGATCCAGTCTCACTTACTTCCAGCAGCAGCTCAAACCTGGCAAA
ENSCJAT00000002986     CTCAGCCCCTGGTGGAGATCCAGTCTCACTTACTTCCAGCAGCAGCTTAAACCTGGCAAA
ENSPPYT00000005974     CTCAGCCCCTGGTGGAGATCCAGTCTCACTTACTTCCAGCAGCAGCTCAAACCTGGCAAA
ENSGGOT00000014084     CTCAGCCCCTGGTGGAGATCCAGTCTCACTTACTTCCAGCAGCAGCTCAAACCTGGCAAA
ENST00000377071        CTCAGCCCCTGGTGGAGATCCAGTCTCACTTACTTCCAGCAGCAGCTCAAACCTGGCAAA
ENSPTRT00000010204     CTCAGCCCCTGGTGGAGATCCAGTCTCACTTACTTCCAGCAGCAGCTCAAACCTGGCAAA
ENSMUST00000046073     CTCAGCCCTTGGTGGAGATCCAGTCTCACTTACTTCCAGCAGCAGCTAAAACCTGGCAAA
ENSRNOT00000044071     CTCAGCCCTTGGTGGAGATCCAGTCTCACTTACTTCCAGCAGCAGCTAAAACCTGGCAAA

ENSMMUT00000030348     GAAGATAAGCTTTTCAGGAAAAAGCGTCGGTCCTGGAAGAACGCCGAGGACCGCATGGCG
ENSCJAT00000002986     GAAGATAAGCTTTTCAGGAAAAAGCGGCGGTCCTGGAAGAACGCGGAGGACCGCATGGCA
ENSPPYT00000005974     GAAGATAAGCTTTTCAGGAAAAAGCGGCGGTCCTGGAAGAACGCCGAGGACCGCATGGCG
ENSGGOT00000014084     GAAGATAAGCTTTTCAGGAAAAAGCGGCGGTCCTGGAAGAACGCCGAGGACCGCATGGCG
ENST00000377071        GAAGATAAGCTTTTCAGGAAAAAGCGGCGGTCCTGGAAGAACGCCGAGGACCGCATGGCG
ENSPTRT00000010204     GAAGATAAGCTTTTCAGGAAAAAGCGGCGGTCCTGGAAGAACGCCGAGGACCGCATGGCG
ENSMUST00000046073     GAAGATAAGCTTTTCAGGAAAAAGCGGCGGTCCTGGAAGAACGCTGAGGATCGTCTGTCA
ENSRNOT00000044071     GAAGATAAGCTTCTCAGGAAAAAGCGGCGGTCCTGGAAGAACGCTGAGGATCGGCTGTCA

ENSMMUT00000030348     CTGGCCAACAAGCCCCTCCGGCGCTTCAAGCAGGAACCCGAGGACGATCTGCCCGAGGCA
ENSCJAT00000002986     CTGGCCAACAAGCCCCTCCGGCGCTTCAAGCAGGAGCCCGAGGACGATCTGCCCGAGGCA
ENSPPYT00000005974     CTGGCCAACAAGCCCCTCCGGCGCTTCAAGCAGGAGCCCGAGGACGATCTGCCCGAGGCG
ENSGGOT00000014084     CTGGCCAACAAGCCCCTCCGGCGCTTCAAGCAGGAACCCGAGGACGAACTGCCCGAGGCG
ENST00000377071        CTGGCCAACAAGCCCCTCCGGCGCTTCAAGCAGGAACCCGAGGACGAACTGCCCGAGGCG
ENSPTRT00000010204     CTGGCCAACAAGCCCCTCCGGCGCTTCAAGCAGGAACCCGAGGACGAACTGCCCGAGGCG
ENSMUST00000046073     CTGGCCAACAAGCCCCTTCGGCGCTTTAAGCAGGAACCGGAGGACGATCTGCCTGAGGCA
ENSRNOT00000044071     CTGGCCAACAAGCCCCTTCGGCGCTTCAAGCAGGAGCCAGAGGACGACCTGCCTGAGGCA
```

```
ENSMMUT00000030348     CCCCCAAAGACCAGGGAGAGCGACCACTCCCGCTCCAGCTCCCCCACCGCAGGACCCAGC
ENSCJAT00000002986     CCCCCCAAGACCAGGGAGAGCGACCACTCCCGCTCCAGCTCCCCCACCGCGGGACCCAGC
ENSPPYT00000005974     CCCCCCAAGACTAGGGAGAGCGACCACTCCCGCTCCAGCTCCCCCACCGCGGGACCCAGC
ENSGGOT00000014084     CCCCCCAAGACCAGGGAGAGCGACCACTCCCGCTCCAGCTCCCCCACCGCGGGACCCAGC
ENST00000377071        CCCCCCAAGACCAGGGAGAGCGACCACTCCCGCTCCAGCTCCCCCACCGCGGGACCCAGC
ENSPTRT00000010204     CCCCCCAAGACCAGGGAGAGCGACCACTCCCGCTCCAGCTCCCCCACCGCGGGACCCAGC
ENSMUST00000046073     CCTCCTAAGACCCGGGAGAGTGATCAGTCACGTTCCAGCTCACCCACTGCTGGTCCCAGC
ENSRNOT00000044071     CCTCCTAAGACCCGGGAGAGTGATCAGTCACGGTCCAGCTCGCCCACAGCTGGGCCCAGC

ENSMMUT00000030348     ACCGAGGGGACCGAGGGCCCGGAGGAGAAGAAGAAGGTGAAGATGCGCCGGAAGCGGCGG
ENSCJAT00000002986     ACCGAGGGGGCTGAGGGCCCAGAGGAGAAGAAGAAGGTGAAGATACGCCGGAAGCGGCGG
ENSPPYT00000005974     ACCGAAGGGGCCGAGGGCCCGGAGGAGAAGAAGAAGGTGAAGATGCGCCGGAAGCGGCGG
ENSGGOT00000014084     ACCGAAGGGGCCGAGGGCCCGGAGGAGAAGAAGAAGGTGAAGATGCGCCGGAAGCGGCGG
ENST00000377071        ACCGAAGGGGCCGAGGGCCCGGAGGAGAAGAAGAAGGTGAAGATGCGCCGGAAGCGGCGG
ENSPTRT00000010204     ACCGAAGGGGCCGAGGGCCCGGAGGAGAAGAAGAAGGTGAAGATGCGCCGGAAGCGGCGG
ENSMUST00000046073     ACTGAGGGAGCTGAAGGCCCAGAAGAGAAGAAAAAGGTGAAGATGCGCCGGAAGCGGCGA
ENSRNOT00000044071     ACTGAGGGGGCCGAGGGCCCAGAAGAGAAGAGGAAGGTGAAGATGCGCCGGAAACGGCGG

ENSMMUT00000030348     CTTCCCAACAAGGAGCTGAGCAGGGAGCTGAGCAAGGAGCTCAACCACGAGATCCAGAGG
ENSCJAT00000002986     CTCCCCAACAAGGAGCTGAGCAGGGAGCTGAGCAAGGAGCTCAACCACGAGATCCAGAGG
ENSPPYT00000005974     CTTCCCAACAAGGAGCTGAGCAGGGAGCTAAGCAAGGAGCTCAACCACGAGATCCAGAGG
ENSGGOT00000014084     CTTCCCAACAAGGAGCTGAGCAGGGAGCTGAGCAAGGAGCTCAACCACGAGATCCAGAGG
ENST00000377071        CTTCCCAACAAGGAGCTGAGCAGGGAGCTGAGCAAGGAGCTCAACCACGAGATCCAGAGG
ENSPTRT00000010204     CTTCCCAACAAGGAGCTGAGCAGGGAGCTGAGCAAGGAGCTCAACCACGAGATCCAGAGG
ENSMUST00000046073     CTTGTTAACAAGGAGCTGAGCAAAGAGCTAAGCAAGGAGCTCAACCATGAGATCCAAAAG
ENSRNOT00000044071     CTTCCCAACAAGGAGTTGAGCAAAGAGCTAAGCAAGGAGCTCAACCACGAGATCCAAAAG

ENSMMUT00000030348     ACGGAGAGCAGCCTGGCCAACGAGAACCAGCAGCCCATCAAGTCGGAGCCTGAGAGTGAG
ENSCJAT00000002986     ACGGAGAGCAGCCTGGCCAACGAGAACCAGCAGCCCATCAAGTCGGAGCCGGAGAGTGAA
ENSPPYT00000005974     ACGGAGAACAGCCTGGCCAACGAGAACCAGCAGCCCATCAAGTCGGAGCCTGAGAGCGAG
ENSGGOT00000014084     ACGGAGAACAGCCTGGCCAACGAGAACCAGCAGCCCATCAAGTCGGAGCCTGAGAGCGAG
ENST00000377071        ACGGAGAACAGCCTGGCCAACGAGAACCAGCAGCCCATCAAGTCGGAGCCTGAGAGCGAG
ENSPTRT00000010204     ACGGAGAACAGCCTGGCCAACGAGAACCAGCAGCCCATCAAGTCGGAGCCCGAGAGCGAG
ENSMUST00000046073     ACGGAGAGCACCCTGGCTCACGAGAGCCAGCAGCCCATCAAGTCAGAGCCTGAGAGCGAG
ENSRNOT00000044071     ACAGAGAGCACCTTAGCCCACGAGAACCACCAGCCTATCAAGTCAGAGCCCGAGAGTGAA

ENSMMUT00000030348     GGCGAGGAGCCCAAGCGGCCCCCAGGCGTCTGCGAGCGTCCCCACCGCTTCAGCAAGGGG
ENSCJAT00000002986     GGCGAGGAGCCTAGGCGGCCCCCGGGTGTCTGCGAGCGCCCCCACCGTTTCAGCAAGGGG
ENSPPYT00000005974     GGCGAGGAGCCCAAGCGGCCCCCGGGCATCTGCGAGCGCCCCCACCGCTTCAGCAAGGGG
ENSGGOT00000014084     GGCGAGGAGCCCAAGCGGCCCCCGGGCATCTGCGAGCGTCCCCACCGCTTCAGCAAGGGG
ENST00000377071        GGCGAGGAGCCCAAGCGGCCCCCGGGCATCTGCGAGCGTCCCCACCGCTTCAGCAAGGGG
ENSPTRT00000010204     GGCGAGGAGCCCAAGCGGCCCCCGGGCATCTGCGAGCGTCCCCACCGCTTCAGCAAGGGG
ENSMUST00000046073     AACGACGAGCCCAAGAGGCCCTTAAGCCACTGCGAGCGCCCCCACCGCTTCAGCAAAGGG
ENSRNOT00000044071     AACGAGGAGCCAAAGAGGCCCTTAAGCCACTGCGAGCGCCCGCACCGCTTCAGCAAAGGG

ENSMMUT00000030348     CTCAACGGCACCCCTCGGGAGCTGCGGCACCAGCTGGGACCCAGCCTGCGCAGCCCGCCC
ENSCJAT00000002986     CTCAACGGCACCCCCCGGGAGCTGCGGCACCAGCTGGGGCCCGGCCTGCGCAGCCCGCCC
ENSPPYT00000005974     CTCAACGGCACCCCCCGAGAGCTGCGGCACCAGCTGGGGCCCAGCCTGCGCAGCCCGCCC
ENSGGOT00000014084     CTCAACGGCACCCCCCGGGAGCTGCGGCACCAGCTGGGGCCCAGCCTGCGCAGCCCGCCC
ENST00000377071        CTCAACGGCACCCCCCGGGAGCTGCGGCACCAGCTGGGGCCCAGCCTGCGCAGCCCGCCC
ENSPTRT00000010204     CTCAACGGCACCCCCCGGGAGCTGCGGCACCAGCTGGGGCCCAGCCTGCGCAGCCCGCCC
ENSMUST00000046073     CTCAACGGCACACCTCGGGAGCTGCGGCACTCGCTGGGACCTGGCCTGCGTAGTCCACCT
ENSRNOT00000044071     CTCAACGGCACTCCTCGGGAGCTGCGGCACTCACTGGGACCTGGGCTTCGCAGCCCACCT
```

```
ENSMMUT00000030348     CGTGTCATCTCCCGGCCCCCACCCTCCGTGTCCCCGCCCAAGTGTATCCAGATGGAGCGC
ENSCJAT00000002986     CGAGTCATCTCCCGGCCCCCGCCCTCCGTGTCCCCACCCAAGTGTATCCAGATGGAGCGC
ENSPPYT00000005974     CGCGTCATCTCCCGGCCCCCACCCTCCGTGTCCCCGCCCAAGTGTATCCAGATGGAGCGC
ENSGGOT00000014084     CGTGTCATCTCCCGGCCCCCACCCTCCGTGTCCCCGCCCAAGTGTATCCAGATGGAGCGC
ENST00000377071        CGTGTCATCTCCCGGCCCCCACCCTCCGTGTCCCCGCCCAAGTGTATCCAGATGGAGCGC
ENSPTRT00000010204     CGTGTCATCTCCCGGCCCCCACCCTCCGTGTCTCCGCCCAAGTGTATCCAGATGGAGCGC
ENSMUST00000046073     CGTGTTATGTCCCGGCCCCCGCCCTCTGCATCCCCACCCAAGTGCATCCAGATGGAGCGT
ENSRNOT00000044071     CGTGTCATCTCCCGGCCCCCGCCCTCTACATCCCCACCCAAGTGCATCCAGATGGAGCGT

ENSMMUT00000030348     CATGTGATCCGGCCACCCCCCATCAGCCCCCCGCCTGACTCGCTACCCCTGGACGATGGG
ENSCJAT00000002986     CATGTGATCCGGCCACCCCCCATCAGCCCCCCACCTGACTCACTGCCCCTGGATGACGGG
ENSPPYT00000005974     CATGTGATCCGGCCACCCCCCATCAGCCCCCCGCCTGACTCGCTACCCCTGGACGATGGG
ENSGGOT00000014084     CATGTGATCCGGCCACCCCCCATCAGCCCCCCGCCTGACTCGCTACCCCTGGACGATGGG
ENST00000377071        CATGTGATCCGGCCACCCCCCATCAGCCCCCCGCCTGACTCGCTACCCCTGGACGATGGG
ENSPTRT00000010204     CATGTGATCCGGCCACCCCCCATCAGCCCCCCGCCTGACTCGCTACCCCTGGACGATGGG
ENSMUST00000046073     CACGTGATCCGGCCACCGCCCATCAGCCCCCCACCTGACTCGCTGCCCCTGGATGATGGA
ENSRNOT00000044071     CACGTGATCCGGCCACCACCCATCAGCCCCCCACCTGACTCGCTGCCCCTGGATGATGGA

ENSMMUT00000030348     GCAGCCCACGTCATGCACAGGGAGGTGTGGATGGCCGTCTTCAGCTACCTCAGCCACCAA
ENSCJAT00000002986     GCAGCCCACGTCATGCACAGGGAGGTGTGGATGGCCGTCTTCAGCTACCTCAGCCACCAA
ENSPPYT00000005974     GCAGCCCACGTCATGCACAGGGAGGTGTGGATGGCCGTCTTCAGCTACCTCAGCCACCAA
ENSGGOT00000014084     GCAGCCCACGTCATGCACAGGGAGGTGTGGATGGCCGTCTTCAGCTACCTCAGCCACCAA
ENST00000377071        GCAGCCCACGTCATGCACAGGGAGGTGTGGATGGCCGTCTTCAGCTACCTCAGCCACCAA
ENSPTRT00000010204     GCAGCCCACGTCATGCACAGGGAGGTGTGGATGGCCGTCTTCAGCTACCTCAGCCACCAA
ENSMUST00000046073     GCAGCCCACGTCATGCATAGGGAGGTGTGGATGGCAGTCTTCAGCTACCTCAGCCACCGA
ENSRNOT00000044071     GCAGCCCATGTCATGCATAGGGAGGTGTGGATGGCAGTCTTCAGCTACCTCAGCCACCAA

ENSMMUT00000030348     GACCTGTGTGTCTGCATGCGGGTCTGCAGGACCTGGAACCGCTGGTGCTGCGATAAGCGG
ENSCJAT00000002986     GACCTGTGTGTCTGCATGCGGGTCTGCAGGACCTGGAACCGCTGGTGCTGTGATAAGCGG
ENSPPYT00000005974     GACCTGTGTGTGTGCATGCGGGTCTGCAGGACCTGGAACCGC------------------
ENSGGOT00000014084     GACCTGTGTGTGTGCATGCGGGTCTGCAGGACCTGGAACCGCTGGTGCTGCGATAAGCGG
ENST00000377071        GACCTGTGTGTGTGCATGCGGGTCTGCAGGACCTGGAACCGCTGGTGCTGCGATAAGCGG
ENSPTRT00000010204     GACCTGTGTGTGTGCATGCGGGTCTGCAGGACCTGGAACCGCTGGTGCTGCGATAAGCGG
ENSMUST00000046073     GACCTGTGTGTCTGCATGCGGGTCTGCAGGACCTGGAACCGCTGGTGCTGCGATAAGCGG
ENSRNOT00000044071     GACCTGTGTGTCTGCATGCGGGTCTGCAGGACCTGGAACCGCTGGTGCTGCGATAAGCGG

ENSMMUT00000030348     TTGTGGACCCGCATTGACCTGAACCACTGCAAGTCTATCACACCCGTGATGCTGAGTGGC
ENSCJAT00000002986     TTGTGGACCCGCATTGACCTGAACCACTGCAAGTCTATCACACCCCTGATGCTGAGCGGC
ENSPPYT00000005974     ------------------------------------------------------------
ENSGGOT00000014084     TTGTGGACCCGCATTGACCTGAACCACTGCAAGTCTATCACACCCCTGATGCTGAGTGGC
ENST00000377071        TTGTGGACCCGCATTGACCTGAACCACTGCAAGTCTATCACACCCCTGATGCTGAGTGGC
ENSPTRT00000010204     TTGTGGACCCGCATTGACCTGAACCACTGCAAGTCTATCACACCCCTGATGCTGAGTGGC
ENSMUST00000046073     TTGTGGACCCGCATCGACCTGAACCGCTGCAAGTCCATCACACCCCTGATGCTGAGCGGT
ENSRNOT00000044071     TTGTGGACCCGCATCGACCTGAACCACTGCAAGTCCATCACTCCCCTGATGCTGAGCGGC

ENSMMUT00000030348     ATCATCCGGCGACAGCCTGTCTCCCTCGACGTCAGCTGGACC---ATCTGCAAGAAGCAG
ENSCJAT00000002986     ATCATCCGGCGACAGCCCGTCTCCCTCGACCTCAGCTGGACCAATATCTCCAAGAAGCAG
ENSPPYT00000005974     ------------------------------------------------------------
ENSGGOT00000014084     ATCATCCGGCGACAGCCCGTCTCCCTCGACCTCAGCTGGACCAATATCTCCAAGAAGCAG
ENST00000377071        ATCATCCGGCGACAGCCCGTCTCCCTCGACCTCAGCTGGACCAATATCTCCAAGAAGCAG
ENSPTRT00000010204     ATCATCCGGCGACAGCCCGTCTCCCTCGACCTCAGCTGGACCAATATCTCCAAGAAGCAG
ENSMUST00000046073     ATCATCCGGCGACAGCCTGTCTCCCTTGACCTCAGTTGGACCAACATCTCCAAGAAGCAG
ENSRNOT00000044071     ATCATCCGGCGGCAGCCGGTCTCCCTGGATCTCAGCTGGACCAACATCTCCAAGAAGCAG
```

```
ENSMMUT00000030348     CTGAGCTGGCTCATCAACCGGCTG------CTCCGGGACTTGGTGCTGTCAGGCTGCTCA
ENSCJAT00000002986     CTGAGCTGGCTCATCAACCGGCTGCCTGGGCTCCGGGACTTGGTGCTGTCAGGCTGCTCG
ENSPPYT00000005974     ------------------------------CTTCGGGACTTGGTGCTGTCAGGCTGCTCA
ENSGGOT00000014084     CTGAGCTGGCTCATCAACCGGCTGCCTGGGCTCCGGGACTTGGTGCTGTCAGGCTGCTCA
ENST00000377071        CTGAGCTGGCTCATCAACCGGCTGCCTGGGCTCCGGGACTTGGTGCTGTCAGGCTGCTCA
ENSPTRT00000010204     CTGAGCTGGCTCATCAACCGGCTGCCTGGGCTCCGGGACTTGGTGCTGTCAGGCTGCTCA
ENSMUST00000046073     CTGAGTTGGCTCATCAACCGGTTGCCTGGGCTCCGAGACTTGGTGCTGTCAGGCTGCTCA
ENSRNOT00000044071     CTGAGCTGGCTCATCAACCGGTTGCCTGGGCTCCGAGACTTGGTGCTGTCAGGCTGCTCA

ENSMMUT00000030348     TGGATCGCGGTCTCAGCCCTTTGCAGCTCCAGTTGTCCGCTGCTCCGGACTCTGGATGTC
ENSCJAT00000002986     TGGATCGCAGTCTCTGCCCTTTGCAGCTCCAGTTGTCCGCTGCTCCGGACCCTGGATGTT
ENSPPYT00000005974     TGGATCGCGGTCTCGGCCCTTTGCAGCTCCAGTTGTCCGCTGCTCCGGACCCTGGATGTC
ENSGGOT00000014084     TGGATCGCGGTCTCGGCCCTTTGCAGCTCCAGTTGTCCGCTGCTCCGGACCCTGGATGTC
ENST00000377071        TGGATCGCGGTCTCGGCCCTTTGCAGCTCCAGTTGTCCGCTGCTCCGGACCCTGGATGTC
ENSPTRT00000010204     TGGATCGCGGTCTCGGCCCTTTGCAGCTCCAGTTGTCCGCTGCTCCGGACCCTGGATGTC
ENSMUST00000046073     TGGATCGCTGTCTCAGCCCTCTGTAGCTCCAGTTGTCCATTGCTCCGGACCCTGGATGTC
ENSRNOT00000044071     TGGATCGCTGTCTCAGCCCTCTGTAGCTCCAGTTGTCCACTGCTCCGGACCCTGGATGTC

ENSMMUT00000030348     CAGTGGGTGGAGGGACTAAAGGATGCCCAGATGCGGGATCTCCTGTCCCCGCCCACGGAC
ENSCJAT00000002986     CAGTGGGTAGAGGGACTAAAGGATGCCCAGATGCGGGATCTCCTCTCTCCGCCCACAGAT
ENSPPYT00000005974     CAGTGGGTGGAGGGACTAAAGGATGCCCAGATGCGGGATCTCCTGTCCCCGCCCACAGAC
ENSGGOT00000014084     CAGTGGGTGGAGGGACTAAAGGATGCCCAGATGCGGGATCTCCTGTCCCCGCCCACGGAC
ENST00000377071        CAGTGGGTGGAGGGACTAAAGGATGCCCAGATGCGGGATCTCCTGTCCCCGCCCACAGAC
ENSPTRT00000010204     CAGTGGGTGGAGGGACTAAAGGATGCCCAGATGCGGGATCTCCTGTCCCCGCCCACAGAC
ENSMUST00000046073     CAGTGGGTAGAAGGACTAAAGGATGCCCAGATGAGGGATCTCCTGTCTCCACCCACAGAC
ENSRNOT00000044071     CAGTGGGTAGAAGGACTAAAGGATGCCCAGATGCGGGATCTCCTGTCTCCACCCACAGAC

ENSMMUT00000030348     AACAGGCCAGGTCAGATGGACAATCGGAGCAAGCTCCGGAACATCGTGGAGCTGCGCCTG
ENSCJAT00000002986     AACAGGCCAGGTCAGATGGACAATCGGAGCAAGCTCCGGAACATCGTGGAGCTGCGCCTG
ENSPPYT00000005974     AACAGGCCAGGTCAGATGGACAATCGGAGCAAGCTCCGGAACATCGTGGAGCTGCGACTG
ENSGGOT00000014084     AACAGGCCAGGTCAAATGGACAATCGGAGCAAGCTCCGGAACATCGTGGAGCTGCGCCTG
ENST00000377071        AACAGGCCAGGTCAGATGGACAATCGGAGCAAGCTCCGGAACATCGTGGAGCTGCGCCTG
ENSPTRT00000010204     AACAGGCCAGGTCAGATGGACAATCGGAGCAAGCTCCGGAACATCGTGGAGCTGCGCCTG
ENSMUST00000046073     AACAGGCCAGGTCAGATGGACAATCGGAGCAAGCTCCGGAACATTGTAGAGCTGCGCCTA
ENSRNOT00000044071     AACAGGCCAGGTCAAATGGACAATCGGAGCAAGCTCCGGAACATTGTGGAACTGCGCCTA

ENSMMUT00000030348     GCAGGCCTGGACATCACAGATGCCTCCCTGCGGCTCATCATCCGCCACATGCCCCTGCTC
ENSCJAT00000002986     GCAGGCCTGGACATCACGGACGCCTCCCTGCGGCTCATCATCCGCCACATGCCCCTGCTC
ENSPPYT00000005974     GCAGGCCTGGACATCACAGATGCCTCCCTGCGGCTCATCATCCGCCACATGCCCCTGCTC
ENSGGOT00000014084     GCAGGCCTGGACATCACAGATGCCTCCCTGCGGCTCATCATCCGCCACATGCCCCTGCTC
ENST00000377071        GCAGGCCTGGACATCACAGATGCCTCCCTGCGGCTCATCATCCGCCACATGCCCCTGCTC
ENSPTRT00000010204     GCAGGCCTGGACATCACAGATGCCTCCCTGCGGCTCATCATCCGCCACATGCCCCTGCTC
ENSMUST00000046073     GCTGGCCTGGACATCACAGATGTCTCCCTGCGGCTCATTATTCGCCATATGCCCCTGCTC
ENSRNOT00000044071     GCTGGCCTGGACATCACAGATGTCTCCCTGCGGCTCATTATTCGCCATATGCCCCTGCTC

ENSMMUT00000030348     TCCAAGCTCCACCTCAGTTACTGTAACCACGTCACCGACCAGTCCATCAACCTGCTCACC
ENSCJAT00000002986     TCCAAGCTCCACCTCAGTTACTGTAACCATGTCACCGACCAGTCCATCAACCTGCTCACC
ENSPPYT00000005974     TCCAAGCTCCACCTCAGTTACTGTAACCATGTCACCGACCAGTCCATCAACCTGCTCACC
ENSGGOT00000014084     TCCAAGCTCCACCTCAGTTACTGTAACCACGTCACCGACCAGTCCATCAACCTGCTCACC
ENST00000377071        TCCAAGCTCCACCTCAGTTACTGTAACCACGTCACCGACCAGTCTATCAACCTGCTCACT
ENSPTRT00000010204     TCCAAGCTCCACCTCAGTTACTGTAACCACGTCACCGACCAGTCCATCAACCTGCTCACC
ENSMUST00000046073     TCGAAGCTCCAACTCAGTTACTGTAACCACATCAATGACCAGTCCATCAACCTGCTCACT
ENSRNOT00000044071     TCCAAGCTCCACCTCAGTTACTGTAACCACGTCACTGATCAGTCCATCAACCTGCTCACT
```

```
ENSMMUT00000030348     GCTGTTGGCACCACCACCCGAGACTCCTTAACCGAGATCAACCTGTCTGACTGCAATAAG
ENSCJAT00000002986     GCTGTGGGCACCACCACCCGAGACTCCTTAACCGAGATCAACCTGTCCGACTGCAATAAG
ENSPPYT00000005974     GCTGTTGGCACCACCACCCGAGACTCCTTAACCGAGATCAACCTGTCTGACTGCAATAAG
ENSGGOT00000014084     GCTGTTGGCACCACCACCCGAGACTCCTTAACCGAGATCAACCTGTCTGACTGCAATAAG
ENST00000377071        GCTGTTGGCACCACCACCCGAGACTCCTTAACCGAGATCAACCTGTCTGACTGCAATAAG
ENSPTRT00000010204     GCTGTTGGCACCACCACCCGAGACTCCTTAACCGAGATCAACCTGTCTGACTGCAATAAG
ENSMUST00000046073     GCCGTCGGCACCACCACCCGAGACTCGCTGACAGAGGTCAACCTATCAGACTGTAATAAG
ENSRNOT00000044071     GCGGTCGGCACCACCACCCGGGACTCTCTGACAGAGATCAACCTATCGGACTGCAATAAG

ENSMMUT00000030348     GTCACTGATCAGTGCCTGTCCTTCTTCAAACGCTGTGGAAACATCTGTCACATTGACCTG
ENSCJAT00000002986     GTCACTGATCAGTGCCTGTCCTTCTTCAAACGCTGTGGAAACATCTGTCATATTGACCTG
ENSPPYT00000005974     GTCACTGATCAGTGCCTGTCCTTCTTCAAACGCTGTGGAAACATCTGTCATATTGACCTG
ENSGGOT00000014084     GTCACTGATCAGTGCCTGTCCTTCTTCAAACGCTGTGGAAACATCTGTCATATTGACCTG
ENST00000377071        GTCACTGATCAGTGCCTGTCCTTCTTCAAACGCTGTGGAAACATCTGTCATATTGACCTG
ENSPTRT00000010204     GTCACTGATCAGTGCCTGTCCTTCTTCAAACGCTGTGGAAACATCTGTCATATTGACCTG
ENSMUST00000046073     GTAACTGACCTGTGCCTGTCCTTCTTCAAACGCTGTGGAAATATCTGTCATATTGACCTG
ENSRNOT00000044071     GTCACTGACCAGTGCCTGTCCTTCTTCAAACGCTGTGGAAATATCTGTCATATTGACCTG

ENSMMUT00000030348     AGGTACTGCAAGCAAGTCACCAAGGAAGGCTGTGAGCAGTTCATAGCCGAGATGTCTGTG
ENSCJAT00000002986     AGGTACTGCAAGCAAGTTACCAAGGAAGGCTGTGAGCAGTTCATAGCCGAGATGTCTGTG
ENSPPYT00000005974     AGGTACTGCAAGCAAGTCACCAAGGAAGGCTGTGAGCAGTTCATAGCCGAGATGTCTGTG
ENSGGOT00000014084     AGGTACTGCAAGCAAGTCACCAAGGAAGGCTGTGAGCAGTTCATAGCCGAGATGTCTGTG
ENST00000377071        AGGTACTGCAAGCAAGTCACCAAGGAAGGCTGTGAGCAGTTCATAGCCGAGATGTCTGTG
ENSPTRT00000010204     AGGTACTGCAAGCAAGTCACCAAGGAAGGCTGTGAGCAGTTCATAGCCGAGATGTCTGTG
ENSMUST00000046073     AGGTACTGCAAGCAAGTCACCAAGGAAGGCTGTGAGCAATTCATAGCTGAAATGTCTGTG
ENSRNOT00000044071     AGGTACTGCAAGCAAGTCACCAAGGAAGGCTGTGAGCAGTTCATAGCTGAAATGTCTGTG

ENSMMUT00000030348     AGTGTCCAGTTTGGGCAAGTAGAAGAAAAACTCCTGCAAAAACTGAGT
ENSCJAT00000002986     AGTGTCCAGTTTGGGCAAGTGGAAGAAAAACTCCTGCAAAAACTGAGT
ENSPPYT00000005974     AGTGTCCAGTTTGGGCAAGTAGAAGAAAAACTCCTGCAAAAACTGAGT
ENSGGOT00000014084     AGTGTCCAGTTTGGGCAAGTAGAAGAAAAACTCCTGCAAAAACTGAGT
ENST00000377071        AGTGTCCAGTTTGGGCAAGTAGAAGAAAAACTCCTGCAAAAACTGAGT
ENSPTRT00000010204     AGTGTCCAGTTTGGGCAAGTAGAAGAGAAACTCCTGCAAAAACTGAGT
ENSMUST00000046073     AGTGTCCAATTTGGGCAAGTGGAAGAGAAACTCCTGCAAAAACTAAGT
ENSRNOT00000044071     AGTGTCCAGTTTGGGCAAGTGGAAGAGAAACTCCTGCAAAAACTAAGT
```

Multiple sequence alignment of Lrrc29

```
ENSCJAT00000031791     ATGGCGGAGTCGCTGCCCCAGGAGATGCTCACATATATTCTGAGCTTCCTGCCTCTGTCA
ENSGGOT00000030127     ATGGCGAAGTCGCTGCCCCTGGAGATGCTCACATATATTCTGAGCTTCCTGCCTCTGTCA
ENST00000454102        ATGGCGGAGTCGCTGCCCCTGGAGATGCTCACATATATTCTGAGCTTCCTGCCTCTGTCA
ENSPTRT00000076260     ATGGCGGAGTCGCTGCCCCTGGAGATGCTCACATATATTCTGAGCTTCCTGCCTCTGTCA

ENSCJAT00000031791     GATCAGAAAGAGGCCTCCCTCGTGAGTTGGGCTTGGTACCGTGCAGCCCAGAATGCCCTT
ENSGGOT00000030127     GATCAGAAAGAGGCCTCCCTCGTGAGTTGGGCTTGGTACCGTGCTGCCCAGAATGCCCTT
ENST00000454102        GATCAGAAAGAGGCCTCCCTCGTGAGTTGGGCTTGGTACCGTGCTGCCCAGAATGCCCTT
ENSPTRT00000076260     GATCAGAAAGAAGCCTCCCTCGTGAGTTGGGCTTGGTACCGTGCTGCCCAGAATGCCCTT

ENSCJAT00000031791     CGGGAGAGCCTGGGCCTCAAGAGCGTCTGCTGCATCAGCCTGACCAACCTGGATGACTCT
ENSGGOT00000030127     CGGGAGAGCCTGGGCCTCAGGGGCATCTGCTGCATCAGCCTGACCAACCTGGATGGCTCT
ENST00000454102        CGGGAGAGCCTGGGCCTCAGGGGCATCTGCTGCATCAGCCTGACCAACCTGGATGGCTCT
ENSPTRT00000076260     CGGGAGAGCCTGGGCCTCAGAGGCATCTGCTGCATCAGCCTGACCAACCTGGATGGCTCT
```

```
ENSCJAT00000031791     CCAGCTTCACACCAGGTGCTACAATCTGTTGCCTACCACCTGGGCCCGCACCTGCAGAGC
ENSGGOT00000030127     CCGGCTTCACACCAGGTGCTACAATCTGTTGCTTACCACCTGGGCCCATACCTGCAGAGC
ENST00000454102        CTGGCTTCACACCAGGTGCTACAATCTGTTGCTTACCACCTGGGCCCACACCTGCAGAGC
ENSPTRT00000076260     CTGGCTTCAAACCAGGTGCTACAATCTGTTGCTTACCACCTGGGCCCACACCTGCAGAGC


ENSCJAT00000031791     TTGTCCCTGGGTGGCGGCAGCCCCACAGAGGCCTCCTTTGTAGACCTGATCCTGGGCTGC
ENSGGOT00000030127     TTGTCCCTGGGTGGAGGCAGCCCCACAGAGGCCTCCTTTGTGGCCCTGATCCTGGGCTGC
ENST00000454102        TTGTCCCTGGGTGGAGGCAGCCCCACAGAGGCCTCCTCTGTGGCCCTGATCCTGGGCTGC
ENSPTRT00000076260     TTGTCCCTGGGTGGAGGCAGCCCCACAGAGGCCTCCTCTGTGGCCCTGATCCTGGGCTGC


ENSCJAT00000031791     CCAACCCTGCGTGTCCTTGACCTCAGTGGCTGCAATAGCCTCTTCACCTCGGGCACATGG
ENSGGOT00000030127     CCAGCCCTGTGTGTCTTTGACCTCAGTGGCTGCAATAGCCTCTTCACCTCGGGCACACTG
ENST00000454102        CCAGCCCTGTGTGTCCTTGACCTCAGTGGCTGCAATAGCCTCTTCACCTCGGGCACACTG
ENSPTRT00000076260     CCAGCCCTGTGTGTCCTTGACCTCAGTGGCTGCAATAGCCTCTTCACCTCGGGCATACTG


ENSCJAT00000031791     CTGGCTCAGCCCGAGACAACACACAGCATCCGGCAGACCTTGAGTGGCCTCCATGAGCTC
ENSGGOT00000030127     CTGGCTCAGCCCGAGATGGCACAGAGCGTCCAGCAGGCTTTGAGCAGCCTCCGTGAGCTC
ENST00000454102        CTGGCTCAGCCAGAGATGGCACAGAGCGTCCAGCAGGCTTTGAGCGGCCTCCGTGAGCTC
ENSPTRT00000076260     CTGGCTCAGCCCGAGATGGCACAGAGCGTCCAGCAGGCTTTGAGCGGCCTCTGTGAGCTC


ENSCJAT00000031791     AACCTGACTGGCCTGCGGGACCTGGCTGACCTTAGCTTCAACCGGCTCAGCAGCTGTGCC
ENSGGOT00000030127     AACCTGGCTGGCCTG---GACCTGGCTGACCTCAGCTTCAACCGGCGCAGCAGCTGTGCC
ENST00000454102        AACCTGGCTGGCCTGCGAGACCTGGCTGACCTCAGCTTCAACCAGCTCAGCAGCTGTGCC
ENSPTRT00000076260     AACCTGGCTGGCCTGCGAGACCTGGCTGACCTCAGCTTCAACCGGCTCAGCAGCTGTGCC


ENSCJAT00000031791     CCCAGCCTGGAGCGCCTCTCCTTGGCCTACTGCCACCTCACCTTCGAGCCGGACCCAGCC
ENSGGOT00000030127     CCCAGCCTGGAGCGCCTCTCCTTGGCATACTGCCACCTCACCTTCGAGCTGGGCCCAGCC
ENST00000454102        CCCAGCCTGGAGCGCCTCTCCTTGGCATACTGCCACCTCACCTTCGAGCTAGGCCCAGCC
ENSPTRT00000076260     CCCAGCCTGGAGCGCCTCTCCTTGGCATACTGCCACCTCACCTTCGAGCTAGGCCCAGCC
```

Multiple sequence alignment of Skp2

```
ENSMUST00000096482     ATGCATAGGAAGCACCTTCAGGAGATTCCGGACCAGAGTGGCAACGTCACCACCAGCTTC
ENSRNOT00000023099     ATGCATAGGAAGCACCTTCAGGAGATTCCGGACCAGAGTAGCAACGTCACCACCAGCTTC
ENSPPYT00000017902     ATGCACAGGAAGCACCTCCAGGAGATTCCAGACCTGAGTAGCAACGTTGCCACCAGCTTC
ENSCJAT00000035834     ATGCACAGGAAGCACCTCCAGGAGATTCCAGACCTGAGTAGCAACGTTGCCACTAGCTTC
ENSGGOT00000032293     ATGCACAGGAAGCACCTCCAGGAGATTCCAGACCTGAGTAGCAACGTTGCCACCAGCTTC
ENST00000274255        ATGCACAGGAAGCACCTCCAGGAGATTCCAGACCTGAGTAGCAACGTTGCCACCAGCTTC
ENSPTRT00000031184     ATGCACAGGAAGCACCTCCAGGAGATTCCAGACCTGAGTAGCAACGTTGCCACCAGCTTC


ENSMUST00000096482     ACGTGGGGATGGGATTCCAGCAAGACTTCTGAACTGCTATCAGGCATGGGTGTCTCGGCC
ENSRNOT00000023099     ACGTGGGGATGGGATTCCAGCAAGACTTCTGAACTGCTGTCAGGCATGGGTGTCTCTGCC
ENSPPYT00000017902     ACGTGGGGATGGGATTCCAGCAAGACTTCTGAACTGCTGTCAGGCATGGGGGTCTCCGCC
ENSCJAT00000035834     ACGTGGGGATGGGATTCCAGCAAGACTTCTGAACTGCTCTCAGGCATGGGGGTCTCCGCC
ENSGGOT00000032293     ACGTGGGGATGGGATTCCAGCAAGACTTCTGAACTGCTGTCAGGCATGGGGGTCTCCGCC
ENST00000274255        ACGTGGGGATGGGATTCCAGCAAGACTTCTGAACTGCTGTCAGGCATGGGGGTCTCCGCC
ENSPTRT00000031184     ACGTGGGGATGGGATTCCAGCAAGACTTCTGAACTGCTGTCAGGCATGGGGGTCTCCGCC


ENSMUST00000096482     TTGGAGAAGGAGGAGGTGGACAGTGAGAACATCCCACATGGACTGCTCTCAAACCTCGGC
ENSRNOT00000023099     TTGGAGAAGGAGGAGGTGGACAGTGAGAACATCCCACATGGACTGCTCTCAAACCTCGGC
```

```
ENSPPYT00000017902     CTGGAGAAAGAGGAGCCCGACAGTGAGAACATCCCCCAGGAACTGCTCTCAAACCTGGGC
ENSCJAT00000035834     CTGGAGAAAGAGGAGCCCGACAGTGAGAACATCCCCCAGGAACTGCTCTCAAACCTGGGC
ENSGGOT00000032293     CTGGAGAAAGAGGAGCCCGACAGTGAGAACATCCCCCGGGAACTGCTCTCAAACCTGGGC
ENST00000274255        CTGGAGAAAGAGGAGCCCGACAGTGAGAACATCCCCCAGGAACTGCTCTCAAACCTGGGC
ENSPTRT00000031184     CTGGAGAAAGAGGAGCCCGACAGTGAGAACATCCCCCAGGAACTGCTCTCAAACCTGGGC

ENSMUST00000096482     CACCCCCAGAGCCCTCCAAGGAAACGAGTCAAGGGCAAAGGGAGTGACAAAGACTTTGTG
ENSRNOT00000023099     CACCCCCAGAGCCCTCCAAGGAAAAGGCTAAAGAGCAAAGGGAGTGACAAAGACTTTGTG
ENSPPYT00000017902     CACCCGGAGAGCCCCCCACGGAAACGGCTGAAGAGCAAAGGGAGTGACAAAGACTTTGTG
ENSCJAT00000035834     ATCCCGCAGAGCCCCCCAAGGAAACGGCTGAAGAGCAAAGGGAGTGACAAAGACTTTGTG
ENSGGOT00000032293     CACCCGGAGAGCCCCCCACGGAAACGGCTGAAGAGCAAAGGGAGTGACAAAGACTTTGTG
ENST00000274255        CACCCGGAGAGCCCCCCACGGAAACGGCTGAAGAGCAAAGGGAGTGACAAAGACTTTGTG
ENSPTRT00000031184     CACCCGGAGAGCCCCCCACGGAAACGGCTGAAGAGCAAAGGGAGTGACAAAGACTTTGTG

ENSMUST00000096482     ATCATCCGTCGGCCGAAGCTTAGTCGGGAGAACTTTCCAGGTGTCTCCTGGGACTCCCTT
ENSRNOT00000023099     ATCATTCGTCGGCCTAAGCTCAATCGAGAGAACTTTCCAGGTGTTTCCTGGGACTCCCTT
ENSPPYT00000017902     ATTGTCCGCAGGCCTAAGCTAAATCGAGAGAACTTTCCAGGTGTTTCATGGGACTCCCTT
ENSCJAT00000035834     ATTATCCGCAGGCCTAAGCTAAATCGAGAGAACTTTCCAGGTGTTTCATGGGACTCCCTT
ENSGGOT00000032293     ATTGTCCGCAGGCCTAAGCTAAATCGAGAGAACTTTCCAGGTGTTTCATGGGACTCCCTT
ENST00000274255        ATTGTCCGCAGGCCTAAGCTAAATCGAGAGAACTTTCCAGGTGTTTCATGGGACTCCCTT
ENSPTRT00000031184     ATTGTCCGCAGGCCTAAGCTAAATCGAGAGAACTTTCCAGGTGTTTCATGGGACTCCCTT

ENSMUST00000096482     CCAGATGAGCTGCTCCTTGGGATCTTTTCCTGTCTGTGCCTCCCTGAGCTGCTGAGAGTC
ENSRNOT00000023099     CCAGATGAGCTGCTCCTGGGAATCTTTTCCTGTCTGTGCCTCCCTGAGCTTTTGAGAGTC
ENSPPYT00000017902     CCGGATGAGCTGCTCTTGGGAATCTTTTCCTGTCTGTGCCTCCCTGAGCTGCTAAAGGTC
ENSCJAT00000035834     CCGGATGAGCTGCTTTTGGGGATCTTTTCCTGTCTGTGCCTCCCTGAGCTGCTAAAGGTC
ENSGGOT00000032293     CCGGATGAGCTGCTCTTGGGAATCTTTTCCTGTCTGTGCCTCCCTGAGCTGCTAAAGGTC
ENST00000274255        CCGGATGAGCTGCTCTTGGGAATCTTTTCCTGTCTGTGCCTCCCTGAGCTGCTAAAGGTC
ENSPTRT00000031184     CCGGATGAGCTGCTCTTGGGAATCTTTTCCTGTCTGTGCCTCCCTGAGCTGCTAAAGGTC

ENSMUST00000096482     TCGGGCGTTTGCAAGAGGTGGTACCGCCTCTCGCTCGATGAGTCTCTCTGGCAGTCCCTC
ENSRNOT00000023099     TCTGGAGTTTGCAAGAGGTGGTACCGCCTCTCGCTCGATGAGTCTCTCTGGCAGTCCCTA
ENSPPYT00000017902     TCTGGTGTTTGTAAGAGGTGGTATCGCCTAGCGTCTGATGAGTCTCTATGGCAGACCTTA
ENSCJAT00000035834     TCTGGTGTTTGTAGGAGATGGTATCGCCTAGCGTTTGACGAGTCTCTGTGGCAAACCTTA
ENSGGOT00000032293     TCTGGTGTTTGTAAGAGGTGGTATCGCCTAGCGTCTGATGAGTCTCTATGGCAGACCTTA
ENST00000274255        TCTGGTGTTTGTAAGAGGTGGTATCGCCTAGCGTCTGATGAGTCTCTATGGCAGACCTTA
ENSPTRT00000031184     TCTGGTGTTTGTAAGAGGTGGTATCGCCTAGCGTCTGATGAGTCTCTATGGCAGACCTTA

ENSMUST00000096482     GACCTCGCGGGTAAAAATCTGCACCCAGACGTGACTGTGCGCTTGCTCTCCCGCGGGGTG
ENSRNOT00000023099     GACCTCGCAGGCAAAAATCTGCACCCAGACGTGACAGTGCGCCTGCTCTCTCGAGGAGTC
ENSPPYT00000017902     GACCTCACAGGTAAAAATCTGCACCCGGATGTGACTGGTCGGTTGCTGTCTCAAGGGGTG
ENSCJAT00000035834     GACCTCACAGGTAAAAATCTGCACCCGGATGTTACTGGTCGGTTGCTGTCTCAAGGGGTG
ENSGGOT00000032293     GACCTCACAGGTAAAAATCTGCACCCGGATGTGACTGGTCGGTTGCTGTCTCAAGGGGTG
ENST00000274255        GACCTCACAGGTAAAAATCTGCACCCGGATGTGACTGGTCGGTTGCTGTCTCAAGGGGTG
ENSPTRT00000031184     GACCTCACAGGTAAAAATCTGCACCCGGATGTGACTGGTCGGTTGCTGTCTCAAGGGGTG

ENSMUST00000096482     GTCGCCTTCCGCTGCCCTCGGTCCTTTATGGAGCAGCCGCTGGGTGAAAGCTTCAGCTCT
ENSRNOT00000023099     GTGGCCTTCCGCTGCCCACGGTCGTTTATGGAGCAGCCACTGGGCGAAAGCTTCAGCTCT
ENSPPYT00000017902     ATCGCCTTCCGCTGCCCACGATCATTTATGGACCAACCATTGGCTGAACATTTCAGCCCT
ENSCJAT00000035834     ATTGCCTTCCGCTGCCCACGATCATTTATGGACCAACCATTAGCTGAACATTTCAGCCCT
ENSGGOT00000032293     ATTGCCTTCCGCTGCCCACGATCATTTATGGACCAACCATTGGCTGAACATTTCAGCCCT
ENST00000274255        ATTGCCTTCCGCTGCCCACGATCATTTATGGACCAACCATTGGCTGAACATTTCAGCCCT
ENSPTRT00000031184     ATTGCCTTCCGCTGCCCACGATCATTTATGGACCAACCATTGGCTGAACATTTCAGCCCT
```

ENSMUST00000096482 TTCCGGGTACAGCACATGGACCTGTCGAACTCAGTGATAAATGTGTCGAACCTCCATAAG
ENSRNOT00000023099 TTCCGTGTACAGCACATGGACCTGTCGAACTCAGTTATAAATGTGTCAAACCTCCACGGG
ENSPPYT00000017902 TTTCGCGTACAGCACATGGACCTGTCGAACTCAGTTATAGAAGTGTCCACCCTCCACGGC
ENSCJAT00000035834 TTTCGTGTGCAGCACATGGACCTGTCAAACTCGGTTATTGAAGTGTCCACCCTCCAGGGC
ENSGGOT00000032293 TTTCGTGTACAGCACATGGACCTGTCGAACTCAGTTATAGAAGTGTCCACCCTCCACGGC
ENST00000274255 TTTCGTGTACAGCACATGGACCTATCGAACTCAGTTATAGAAGTGTCCACCCTCCACGGC
ENSPTRT00000031184 TTTCGTGTACAGCACATGGACCTGTCGAATTCAGTTATAGAAGTGTCCACCCTCCACGGC

ENSMUST00000096482 ATTCTGTCCGAGTGCTCCAAGCTGCAGAATCTAAGCCTGGAAGGCCTGCAGCTCTCAGAC
ENSRNOT00000023099 ATTCTGTCCGAGTGCTCCAAGCTGCAGAATCTGAGTCTGGAAGGCCTGCAGCTTTCAGAT
ENSPPYT00000017902 ATACTGTCTCAGTGTTCCAAGTTGCAGAATCTAAGCCTGGAAGGCCTGCGGCTTTCGGAT
ENSCJAT00000035834 ATTCTGTCTCAGTGTTCCAAGTTGCAGAATCTAAGCCTGGAAGGCCTGCGGCTGTCGGAT
ENSGGOT00000032293 ATACTGTCTCAGTGTTCCAAGTTGCAGAATCTAAGCCTGGAAGGCCTGCGGCTTTCGGAT
ENST00000274255 ATACTGTCTCAGTGTTCCAAGTTGCAGAATCTAAGCCTGGAAGGCCTGCGGCTTTCGGAT
ENSPTRT00000031184 ATACTGTCTCAGTGTTCCAAGTTGCAGAATCTAAGCCTGGAAGGCCTGCGGCTTTCGGAT

ENSMUST00000096482 CCCATTGTCAAGACTCTTGCACAGAATGAAAACTTGGTGCGACTAAACCTTTGTGGGTGC
ENSRNOT00000023099 CCCATTGTCACTACTCTTGCGCAGAATGAAAACTTGGTGCGACTGAACCTTTGTGGGTGC
ENSPPYT00000017902 CCCATTGTCAATAATCTCGCACAAAACTCAAATTTGGTGCGACTTAACCTTTCTGGGTGT
ENSCJAT00000035834 CCCATTGTCAATAATCTTGCGCAAAACTCAAATTTAGTTCGACTTAACCTTTGTGGGTGT
ENSGGOT00000032293 CCCATTGTCAATACTCTCGCAAAAAACTCAAATTTAGTGCGACTTAACCTTTCTGGGTGT
ENST00000274255 CCCATTGTCAATACTCTCGCAAAAAACTCAAATTTAGTGCGACTTAACCTTTCTGGGTGT
ENSPTRT00000031184 CCCATTGTCAATACTCTCGCAAAAAACTCAAATTTAGTGCGACTTAACCTTTCTGGGTGT

ENSMUST00000096482 TCTGGGTTTTCTGAATCTGCCGTGGCGACTCTGCTAAGCAGCTGCTCCAGACTGGATGAG
ENSRNOT00000023099 TCTGGATTTTCTGAGTCTGCCGTGGCCACCCTGCTGAGCAGCTGCTCCAGACTGGATGAA
ENSPPYT00000017902 TCTGGATTCTCTGAATTTGCCCTGCAGACTTTGCTAAGCAGCTGTTCCAGACTGGATGAG
ENSCJAT00000035834 TCTGGATTCTCTGAATTTGCCCTGCAGACTTTGCTAAGTGGCTGTTCCAGACTGGATGAG
ENSGGOT00000032293 TCTGGATTCTCTGAATTTGCCCTGCAGACTCTGCTAAGCAGCTGTTCCAGACTGGATGAG
ENST00000274255 TCTGGATTCTCTGAATTTGCCCTGCAGACTTTGCTAAGCAGCTGTTCCAGACTGGATGAG
ENSPTRT00000031184 TCTGGATTCTCTGAATTTGCCCTGCAGACTTTGCTAAGCAGCTGTTCCAGACTGGATGAG

ENSMUST00000096482 CTAAATCTCTCCTGGTGCTTTGACTTCACTGAAAAGCACGTGCAAGCGGCTGTGGCACAT
ENSRNOT00000023099 CTGAACCTCTCCTGGTGCTTTGACTTCACTGAGAAGCATGTGCAAGCGGCCGTGGCACAT
ENSPPYT00000017902 CTGAACCTCTCCTGGTGTTTTGATTTCACTGAAAAGCATGTACAGGTGGCTGTTGCGCAT
ENSCJAT00000035834 CTGAACCTCTCCTGGTGCTTTGATTTTACTGAGAAGCATGTGCAGGTGGCTGTTGCGCAT
ENSGGOT00000032293 CTGAACCTCTCCTGGTGTTTTGATTTCACTGAAAAGCATGTACAGGTGGCTGTTGCGCAT
ENST00000274255 CTGAACCTCTCCTGGTGTTTTGATTTCACTGAAAAGCATGTACAGGTGGCTGTTGCGCAT
ENSPTRT00000031184 CTGAACCTCTCCTGGTGTTTTGATTTCACTGAAAAGCATGTACAGGTGGCTGTTGCGCAT

ENSMUST00000096482 TTACCAAACACCATCACCCAGCTGAACCTCAGCGGCTACCGAAAGAACCTCCAGAAAACA
ENSRNOT00000023099 TTACCAGATACGCTCACCCAGCTGAACCTCAGCGGCTACCGAAAGAACCTCCAGAAAACA
ENSPPYT00000017902 GTGTCAGAGACCATCACCCAGCTGAATCTTAGCGGCTACAGAAAGAATCTCCAGAAATCA
ENSCJAT00000035834 GTGTCAGAGACCATCACCCAGCTGAATCTTAGTGGCTACAGAAAGAATCTCCAGAAATCA
ENSGGOT00000032293 GTGTCAGAGACCATCACCCAGCTGAATCTTAGCGGCTACAGAAAGAATCTCCAGAAATCA
ENST00000274255 GTGTCAGAGACCATCACCCAGCTGAATCTTAGCGGCTACAGAAAGAATCTCCAGAAATCA
ENSPTRT00000031184 GTGTCAGAGACCATCACCCAGCTGAATCTTAGCGGCTACAGAAAGAATCTCCAGAAATCA

ENSMUST00000096482 GATCTTTGTACCATAATTAAACGATGCCCCAACCTCATCCGCCTCGACTTAAGTGACAGT
ENSRNOT00000023099 GATCTTTGTACCCTAATTAAACGATGCCCCAACCTCGTCCGCCTCGACTTAAGTGACAGT
ENSPPYT00000017902 GATCTCTCTACTTTAGTTAGAAGATGCCCCAATCTTGTCCATCTAGACTTAAGTGATAGT
ENSCJAT00000035834 GATCTCTCTACTTTAGTTAGAAGATGCCCCAATCTTGTCCACCTAGACTTAAGTGACAGT
ENSGGOT00000032293 GATCTCTCTACTTTAGTTAGAAGATGCCCCAATCTTGTCCATCTAGACTTAAGTGATAGT

```
ENST00000274255        GATCTCTCTACTTTAGTTAGAAGATGCCCCAATCTTGTCCATCTAGACTTAAGTGATAGT
ENSPTRT00000031184     GATCTCTCTACTTTAGTTAGAAGATGCCCCAATCTTGTCCATCTAGACTTAAGTGATAGT

ENSMUST00000096482     ATCATGCTAAAGAATGACTGCTTTCCAGAATTTTTTCAACTCAACTACCTCCAACACCTC
ENSRNOT00000023099     ATCATGCTGAAGAATGACTGCTTTCCAGAATTTTTTCAACTCAACTACCTCCAACACCTC
ENSPPYT00000017902     GTCATGCTAAAGAATGACTGCTTTCAGGAATTTTTCCAGCTCAACTACCTCCAACACCTA
ENSCJAT00000035834     ATCATGCTGAAGAATGATTGCTTCCCGGAATTTTTCCAGCTCAACTACCTCCAACACCTG
ENSGGOT00000032293     GTCATGCTAAAGAATGACTGCTTTCAGGAATTTTTCCAGCTCAACTACCTCCAACACCTA
ENST00000274255        GTCATGCTAAAGAATGACTGCTTTCAGGAATTTTTCCAGCTCAACTACCTCCAACACCTA
ENSPTRT00000031184     GTCATGCTAAAGAATGACTGCTTTCAGGAATTTTTCCAGCTCAACTACCTCCAACACCTA

ENSMUST00000096482     TCGCTCAGCCGGTGCTATGATATAATACCTGATACTCTACTTGAACTTGGAGAAATTCCT
ENSRNOT00000023099     TCGCTCAGCCGGTGCTACGATATAATACCTGAGACGCTACTTGAACTCGGAGAAATCCCC
ENSPPYT00000017902     TCACTCAGTCGGTGCTATGATATAATACCTGAAACTTTACTATTAGTGACAAGAGCTGGG
ENSCJAT00000035834     TCTCTCAGTCGGTGCTATGATATAATACCTGAAACTTTACTTGAACTTGGAGAAATTCCC
ENSGGOT00000032293     TCACTCAGTCGGTGCTATGATATAATACCTGAAACTTTACTTGAACTTGGAGAAATTCCC
ENST00000274255        TCACTCAGTCGGTGCTATGATATAATACCTGAAACTTTACTTGAACTTGGAGAAATTCCC
ENSPTRT00000031184     TCACTCAGTCGGTGCTATGATATAATACCTGAAACTTTACTTGAACTTGGAGAAATTCCC

ENSMUST00000096482     ACACTAAAAACGCTACAAGTTTTTGGAATCGTGCCAGAGGGAACCCTTCAGCTACTGAGG
ENSRNOT00000023099     ACACTAAAAACACTACAAGTTTTTGGGATCGTGCCAGACGGGACCCTTCAGCTACTGAGA
ENSPPYT00000017902     GTTAGGATCCGGTTGGACTCTATCGGATGCCCTCAAACATACAGAACTTCCAAACTCAAG
ENSCJAT00000035834     ACACTAAAAACACTACAAGTTTTTGGAATTGTGCCAGATGGTACCCTTCAACTGTTAAAG
ENSGGOT00000032293     ACACTAAAAACACTACAAGTTTTTGGAATCGTGCCAGATGGTACCCTTCAACTGTTAAAG
ENST00000274255        ACACTAAAAACACTACAAGTTTTTGGAATCGTGCCAGATGGTACCCTTCAACTGTTAAAG
ENSPTRT00000031184     ACACTAAAAACACTACAAGTTTTTGGAATCGTGCCAGATGGTACCCTTCAACTGTTAAAG

ENSMUST00000096482     GAAGCTCTTCCTCGGCTGCAGATTAACTGCGCCTATTTCACCACCATTGCAAGGCCAACT
ENSRNOT00000023099     GAAGCTCTTCCTCGCCTGCAGATTAATTGTGCCTATTTCACCTCCATTGCAAGGCCAACT
ENSPPYT00000017902     TCCAGCCATAAGCTATTT---------TGCCAACATGTCAGAGTAATCTGTATTTTTGTA
ENSCJAT00000035834     GAAGCCCTTCCTCATCTACAGATTAATTGCTCCCATTTCACCACCATTGCTAGGCCAACT
ENSGGOT00000032293     GAAGCCCTTCCTCATCTACAGATTAATTGCTCCCATTTCACCACCATTGCCAGGCCAACT
ENST00000274255        GAAGCCCTTCCTCATCTACAGATTAATTGCTCCCATTTCACCACCATTGCCAGGCCAACT
ENSPTRT00000031184     GAAGCCCTTCCTCATCTACAGATTAATTGCTCCCATTTCACCACCATTGCCAGGCCAACT

ENSMUST00000096482     ATGGACAGCAAGAAGAACCTGGAGATTTGGGGTATCAAGTGCCGACTGACTCTGCAAAAG
ENSRNOT00000023099     ATGGACAACAAGAAGAACCCGGAGATTTGGGGTATCAAATGCCGACTGACCCTGCAAAAG
ENSPPYT00000017902     TGTGATTTC------------------------TACTTTTATAGACTTGTTTTAAAACAA
ENSCJAT00000035834     ATTGACAACCAAAAGAACCAGGAGATATGGGGCATCAAATGCCGACTGACCCTGCAAAAG
ENSGGOT00000032293     ATTGGCAACAAAAAGAACCAGGAGATATGGGGCATCAAATGCCGACTGACACTGCAAAAG
ENST00000274255        ATTGGCAACAAAAAGAACCAGGAGATATGGGGCATCAAATGCCGACTGACACTGCAAAAG
ENSPTRT00000031184     ATTGGCAACAAAAAGAACCAGGAGATATGGGGCATCAAATGCCGACTGACACTGCAAAAG

ENSMUST00000096482     CCCAGTTGTCTA
ENSRNOT00000023099     CCCAGT---TTA
ENSPPYT00000017902     ------------
ENSCJAT00000035834     CCCAGTTGTCTC
ENSGGOT00000032293     CCCAGTTGTCTA
ENST00000274255        CCCAGTTGTCTA
ENSPTRT00000031184     CCCAGTTGTCTA
```
