# Supplementary material for: Evolution of the F-Box Gene Family in Euarchontoglires: Gene Number Variation and Selection Patterns
Source: PLoS One. 2014 Apr 11;9(4):e94899. doi: 10.1371/journal.pone.0094899 (PMC3984280; doi:10.1371/journal.pone.0094899)
Supplement: Table S1 — F-box gene numbers and their accession numbers in the eight genomes. (DOC) [file pone.0094899.s010.doc]

Table S1. F-box gene numbers and accession numbers in eight genomes

A. F-box gene accession numbers in the 8 genomes

| Orthogroup | Ensembl or NCBI accession numbers | |  |  |  |  | | |  | |  |
| --- | --- | --- | --- | --- | --- | --- | --- | --- | --- | --- | --- |
|  | Rat | Mouse | Marmoset | Macaque | | Orangutan | Gorilla | Chimpanzee | | Human | |
| Btrc | ENSRNOG00000016280 | ENSMUSG00000025217 | ENSCJAG00000017573 | ENSMMUG00000006741 | | ENSPPYG00000002583 | ENSGGOG00000005023 | ENSPTRG00000002869 | | ENSG00000166167 | |
| Ccnf | ENSRNOG00000007483 | ENSMUSG00000072082 | ENSCJAG00000014784 | ENSMMUG00000000001 | | ENSPPYG00000006998 | ENSGGOG00000011877 | ENSPTRG00000007649 | | ENSG00000162063 | |
| Ect2l | ENSRNOG00000040139 | ENSMUSG00000071392 | ENSCJAG00000019079 | ENSMMUG00000012210 | | ENSPPYG00000017048 | ENSGGOG00000012824 | ENSPTRG00000029383 | | ENSG00000203734 | |
| Fbxl12 | ENSRNOG00000020368 | ENSMUSG00000066892 | ENSCJAG00000006105 | ENSMMUG00000007875 | | ENSPPYG00000009535 | ENSGGOG00000005995 | ENSPTRG00000010450 | | ENSG00000127452 | |
| Fbxl13 | ENSRNOG00000043035 | ENSMUSG00000048520 | ENSCJAG00000021213 | ENSMMUG00000012427 | | ENSPPYG00000017876 | ENSGGOG00000034848 | ENSPTRG00000019544 | | ENSG00000161040 | |
| Fbxl15 | ENSRNOG00000019509 | ENSMUSG00000025226 | ENSCJAG00000017990 | ENSMMUG00000002996 | | ENSPPYG00000002601 | ENSGGOG00000006993 | ENSPTRG00000002890 | | ENSG00000107872 | |
| Fbxl16 | ENSRNOG00000022248 | ENSMUSG00000025738 | ENSCJAG00000012128 | ENSMMUG00000016906 | | ENSPPYG00000006925 | ENSGGOG00000013902 | ENSPTRG00000007555 | | ENSG00000127585 | |
| Fbxl19 | ENSRNOG00000018986 | ENSMUSG00000030811 | ENSCJAG00000002985 | ENSMMUG00000003230 | | ENSPPYG00000007294 | ENSGGOG00000003625 | ENSPTRG00000008021 | | ENSG00000099364 | |
| Fbxl2 | ENSRNOG00000027099 | ENSMUSG00000032507 | ENSCJAG00000012006 | ENSMMUG00000031439 | | ENSPPYG00000014031 | ENSGGOG00000002522 | ENSPTRG00000014732 | | ENSG00000153558 | |
| Fbxl20 | ENSRNOG00000005081 | ENSMUSG00000020883 | ENSCJAG00000008532 | ENSMMUG00000030060 | | ENSPPYG00000008489 | ENSGGOG00000009694 | ENSPTRG00000009090 | | ENSG00000108306 | |
| Fbxl22 | ENSRNOG00000017684 | ENSMUSG00000050503 | ENSCJAG00000004563 | ENSMMUG00000012116 | | ENSPPYG00000006542 | ENSGGOG00000024384 | 747699 | | ENSG00000197361 | |
| Fbxl3 | ENSRNOG00000010102 | ENSMUSG00000022124 | ENSCJAG00000021193 | ENSMMUG00000021458 | | ENSPPYG00000005425 | ENSGGOG00000007005 | ENSPTRG00000022568 | | ENSG00000005812 | |
| Fbxl4 | ENSRNOG00000005641 | ENSMUSG00000040410 | ENSCJAG00000001288 | ENSMMUG00000001659 | | ENSPPYG00000016861 | ENSGGOG00000001187 | ENSPTRG00000018438 | | ENSG00000112234 | |
| Fbxl5 | ENSRNOG00000005261 | ENSMUSG00000039753 | ENSCJAG00000012393 | ENSMMUG00000009388 | | ENSPPYG00000014616 | ENSGGOG00000010962 | ENSPTRG00000015927 | | ENSG00000118564 | |
| Fbxl6 | ENSRNOG00000025497 | ENSMUSG00000022559 | ENSCJAG00000021427 | ENSMMUG00000007370 | | 100453956 | ENSGGOG00000016161 | ENSPTRG00000020696 | | ENSG00000182325 | |
| Fbxl7 | ENSRNOG00000024433 | ENSMUSG00000043556 | ENSCJAG00000010823 | ENSMMUG00000013364 | | ENSPPYG00000015354 | ENSGGOG00000011159 | ENSPTRG00000016741 | | ENSG00000183580 | |
| Fbxl8 | ENSRNOG00000015242 | ENSMUSG00000033313 | ENSCJAG00000016495 | ENSMMUG00000005907 | | 100435215 | ENSGGOG00000011893 | ENSPTRG00000008216 | | ENSG00000135722 | |
| Fbxo10 | ENSRNOG00000012634 | ENSMUSG00000048232 | ENSCJAG00000010535 | ENSMMUG00000017886 | | ENSPPYG00000019060 | ENSGGOG00000001885 | ENSPTRG00000020945 | | ENSG00000147912 | |
| Fbxo11 | ENSRNOG00000016396 | ENSMUSG00000005371 | ENSCJAG00000010907 | ENSMMUG00000022843 | | ENSPPYG00000012424 | ENSGGOG00000011606 | ENSPTRG00000011907 | | ENSG00000138081 | |
| Fbxo15 | ENSRNOG00000038225 | ENSMUSG00000034391 | ENSCJAG00000003151 | ENSMMUG00000023298 | | ENSPPYG00000009238 | ENSGGOG00000013486 | ENSPTRG00000010109 | | ENSG00000141665 | |
| Fbxo16 | ENSRNOG00000014003 | ENSMUSG00000034532 | ENSCJAG00000015578 | ENSMMUG00000007480 | | 100457353 | ENSGGOG00000016343 | ENSPTRG00000034416 | | ENSG00000214050 | |
| Fbxo18 | ENSRNOG00000018549 | ENSMUSG00000058594 | ENSCJAG00000020531 | ENSMMUG00000015143 | | ENSPPYG00000002064 | ENSGGOG00000013326 | ENSPTRG00000002261 | | ENSG00000134452 | |
| Fbxo22 | ENSRNOG00000022702 | ENSMUSG00000032309 | ENSCJAG00000012331 | ENSMMUG00000009161 | | ENSPPYG00000006671 | ENSGGOG00000001081 | ENSPTRG00000007311 | | ENSG00000167196 | |
| Fbxo24 | ENSRNOG00000024922 | ENSMUSG00000089984 | ENSCJAG00000015213 | ENSMMUG00000015309 | | ENSPPYG00000017419 | ENSGGOG00000023939 | ENSPTRG00000019496 | | ENSG00000106336 | |
| Fbxo25 | ENSRNOG00000042464 | ENSMUSG00000038365 | ENSCJAG00000011914 | ENSMMUG00000011105 | | ENSPPYG00000018301 | ENSGGOG00000014174 | ENSPTRG00000019933 | | ENSG00000147364 | |
| Fbxo27 | Annotation^a^ | ENSMUSG00000037463 | ENSCJAG00000014025 | ENSMMUG00000014544 | | ENSPPYG00000009953 | ENSGGOG00000004829 | ENSPTRG00000010950 | | ENSG00000161243 | |
| Fbxo28 | ENSRNOG00000000066 | ENSMUSG00000047539 | ENSCJAG00000008131 | ENSMMUG00000000676 | | ENSPPYG00000000185 | ENSGGOG00000010974 | ENSPTRG00000002021 | | ENSG00000143756 | |
| Fbxo3 | ENSRNOG00000009549 | ENSMUSG00000027180 | ENSCJAG00000010459 | ENSMMUG00000006063 | | ENSPPYG00000003369 | ENSGGOG00000001949 | ENSPTRG00000003490 | | ENSG00000110429 | |
| Fbxo30 | ENSRNOG00000014852 | ENSMUSG00000047648 | ENSCJAG00000019390 | ENSMMUG00000021936 | | ENSPPYG00000017073 | ENSGGOG00000005928 | ENSPTRG00000018682 | | ENSG00000118496 | |
| Fbxo31 | ENSRNOG00000042274 | ENSMUSG00000031811 | ENSCJAG00000004482 | ENSMMUG00000017610 | | ENSPPYG00000007627 | ENSGGOG00000009386 | ENSPTRG00000008444 | | ENSG00000103264 | |
| Fbxo33 | ENSRNOG00000005285 | ENSMUSG00000035329 | ENSCJAG00000016096 | ENSMMUG00000006984 | | ENSPPYG00000005770 | ENSGGOG00000010786 | ENSPTRG00000006299 | | ENSG00000165355 | |
| Fbxo34 | ENSRNOG00000011704 | ENSMUSG00000037536 | ENSCJAG00000021876 | ENSMMUG00000023258 | | ENSPPYG00000029688 | ENSGGOG00000006852 | ENSPTRG00000006373 | | ENSG00000178974 | |
| Fbxo36 | ENSRNOG00000017053 | ENSMUSG00000073633 | ENSCJAG00000013238 | ENSMMUG00000004254 | | ENSPPYG00000013250 | ENSGGOG00000015685 | ENSPTRG00000013006 | | ENSG00000153832 | |
| Fbxo38 | ENSRNOG00000019063 | ENSMUSG00000042211 | ENSCJAG00000021201 | ENSMMUG00000016908 | | ENSPPYG00000015927 | ENSGGOG00000004687 | ENSPTRG00000017388 | | ENSG00000145868 | |
| Fbxo39 | ENSRNOG00000014953 | ENSMUSG00000070388 | ENSCJAG00000017188 | ENSMMUG00000009604 | | ENSPPYG00000007885 | ENSGGOG00000023510 | ENSPTRG00000008650 | | ENSG00000177294 | |
| Fbxo4 | ENSRNOG00000015622 | ENSMUSG00000022184 | ENSCJAG00000001220 | ENSMMUG00000003966 | | ENSPPYG00000015430 | ENSGGOG00000005501 | ENSPTRG00000016833 | | ENSG00000151876 | |
| Fbxo40 | ENSRNOG00000002459 | ENSMUSG00000047746 | ENSCJAG00000008502 | ENSMMUG00000013543 | | ENSPPYG00000013496 | ENSGGOG00000016785 | ENSPTRG00000015278 | | ENSG00000163833 | |
| Fbxo41 | ENSRNOG00000033202 | ENSMUSG00000047013 | ENSCJAG00000004115 | ENSMMUG00000014911 | | 100441381 | ENSGGOG00000007779 | ENSPTRG00000012061 | | ENSG00000163013 | |
| Fbxo42 | ENSRNOG00000014305 | ENSMUSG00000028920 | ENSCJAG00000008276 | ENSMMUG00000012733 | | ENSPPYG00000001822 | ENSGGOG00000014592 | ENSPTRG00000000225 | | ENSG00000037637 | |
| Fbxo46 | ENSRNOG00000008815 | ENSMUSG00000050428 | ENSCJAG00000012379 | ENSMMUG00000021360 | | 100433727 | ENSGGOG00000027287 | ENSPTRG00000038848 | | ENSG00000177051 | |
| Fbxo47 | ENSRNOG00000036882 | ENSMUSG00000070336 | ENSCJAG00000008361 | ENSMMUG00000018180 | | ENSPPYG00000008499 | ENSGGOG00000013571 | ENSPTRG00000030923 | | ENSG00000204952 | |
| Fbxo5 | ENSRNOG00000024077 | ENSMUSG00000019773 | ENSCJAG00000012799 | ENSMMUG00000019076 | | ENSPPYG00000017116 | ENSGGOG00000001606 | ENSPTRG00000018721 | | ENSG00000112029 | |
| Fbxo6 | ENSRNOG00000009217 | ENSMUSG00000055401 | ENSCJAG00000013250 | ENSMMUG00000023668 | | ENSPPYG00000001886 | ENSGGOG00000009401 | ENSPTRG00000000148 | | ENSG00000116663 | |
| Fbxo7 | ENSRNOG00000004637 | ENSMUSG00000001786 | ENSCJAG00000006238 | ENSMMUG00000006728 | | ENSPPYG00000011755 | ENSGGOG00000017158 | ENSPTRG00000014290 | | ENSG00000100225 | |
| Fbxo8 | ENSRNOG00000010502 | ENSMUSG00000038206 | ENSCJAG00000005158 | ENSMMUG00000002331 | | ENSPPYG00000015210 | ENSGGOG00000005344 | ENSPTRG00000016604 | | ENSG00000164117 | |
| Fbxo9 | ENSRNOG00000008214 | ENSMUSG00000001366 | 100388071 | ENSMMUG00000005340 | | ENSPPYG00000016718 | ENSGGOG00000006520 | ENSPTRG00000018287 | | ENSG00000112146 | |
| Fbxw10 | ENSRNOG00000003287 | ENSMUSG00000090173 | ENSCJAG00000004384 | ENSMMUG00000019200 | | ENSPPYG00000008006 | ENSGGOG00000009625 | ENSPTRG00000008859 | | ENSG00000171931 | |
| Fbxw11 | ENSRNOG00000004395 | ENSMUSG00000020271 | ENSCJAG00000019297 | ENSMMUG00000016008 | | ENSPPYG00000016040 | ENSGGOG00000007880 | ENSPTRG00000017526 | | ENSG00000072803 | |
| Fbxw2 | ENSRNOG00000018687 | ENSMUSG00000035949 | ENSCJAG00000020331 | ENSMMUG00000001646 | | ENSPPYG00000019552 | ENSGGOG00000007441 | ENSPTRG00000021304 | | ENSG00000119402 | |
| Fbxw7 | ENSRNOG00000010889 | ENSMUSG00000028086 | ENSCJAG00000002101 | ENSMMUG00000020330 | | ENSPPYG00000015122 | ENSGGOG00000024434 | ENSPTRG00000016514 | | ENSG00000109670 | |
| Fbxw8 | ENSRNOG00000001126 | ENSMUSG00000032867 | ENSCJAG00000009513 | ENSMMUG00000017475 | | ENSPPYG00000005001 | ENSGGOG00000002728 | ENSPTRG00000005503 | | ENSG00000174989 | |
| Fbxw9 | ENSRNOG00000004212 | ENSMUSG00000008167 | ENSCJAG00000005225 | ENSMMUG00000016870 | | ENSPPYG00000009601 | ENSGGOG00000013425 | ENSPTRG00000010534 | | ENSG00000132004 | |
| Kdm2A | ENSRNOG00000019145 | ENSMUSG00000054611 | ENSCJAG00000001071 | ENSMMUG00000008771 | | ENSPPYG00000003001 | ENSGGOG00000001636 | ENSPTRG00000003945 | | ENSG00000173120 | |
| Kdm2B | ENSRNOG00000025702 | ENSMUSG00000029475 | ENSCJAG00000001555 | ENSMMUG00000021564 | | ENSPPYG00000005050 | ENSGGOG00000014021 | ENSPTRG00000005560 | | ENSG00000089094 | |
| Fbxl14 | ENSRNOG00000022886 | ENSMUSG00000030019 | ENSCJAG00000000675 | ENSMMUG00000019440 | | ENSPPYG00000004140 |  |  | | ENSG00000171823 | |
|  | ENSRNOG00000000021 |  |  |  | |  |  |  | |  | |
| Fbxl17 | ENSRNOG00000013875 | ENSMUSG00000023965 | ENSCJAG00000016107 | ENSMMUG00000013183 | | ENSPPYG00000015671 |  | ENSPTRG00000017116 | | ENSG00000145743 | |
| Fbxl18 | ENSRNOG00000001117 | ENSMUSG00000066640 |  | ENSMMUG00000005052 | | ENSPPYG00000017335 | ENSGGOG00000014037 | ENSPTRG00000018889 | | ENSG00000155034 | |
|  | ENSRNOG00000033326 |  |  |  | |  |  |  | |  | |
| Fbxl21 | ENSRNOG00000012168 | ENSMUSG00000035509 |  | ENSMMUG00000001948 | | ENSPPYG00000015805 |  | ENSPTRG00000000146 | | ENSG00000164616 | |
| Fbxo17 | ENSRNOG00000019942 | ENSMUSG00000030598 | ENSCJAG00000032831 | 721961 | | ENSPPYG00000009951 | Annotation^a^ | ENSPTRG00000005505 | | ENSG00000104835 | |
| Fbxo2 | ENSRNOG00000009409 | ENSMUSG00000041556 | ENSCJAG00000013230 |  | | ENSPPYG00000001888 | ENSGGOG00000002095 | ENSPTRG00000020555 | | ENSG00000116661 | |
| Fbxo21 | ENSRNOG00000001129 | ENSMUSG00000032898 |  | ENSMMUG00000017479 | | ENSPPYG00000005004 | ENSGGOG00000010676 | ENSPTRG00000020469 | | ENSG00000135108 | |
| Fbxo32 | ENSRNOG00000006738 | ENSMUSG00000022358 | ENSCJAG00000000844 | ENSMMUG00000023778 | |  | ENSGGOG00000002976 | ENSPTRG00000000147 | | ENSG00000156804 | |
| Fbxo43 |  |  | ENSCJAG00000015760 | ENSMMUG00000004384 | | ENSPPYG00000018785 | ENSGGOG00000000296 |  | | ENSG00000156509 | |
| Fbxo44 | ENSRNOG00000009298 | ENSMUSG00000029001 |  | ENSMMUG00000032251 | | ENSPPYG00000001887 | ENSGGOG00000002097 | ENSPTRG00000030457 | | ENSG00000132879 | |
| Fbxo45 |  | ENSMUSG00000035764 | ENSCJAG00000001685 |  | |  | ENSGGOG00000014199 |  | | ENSG00000174013 | |
| Fbxo48 | ENSRNOG00000023026 | ENSMUSG00000044966 | ENSCJAG00000006473 | ENSMMUG00000007550 | | ENSPPYG00000012736 | ENSGGOG00000007346 |  | | ENSG00000204923 | |
|  |  |  |  |  | | ENSPPYG00000012349 |  |  | |  | |
| Fbxw4 | ENSRNOG00000017343 | ENSMUSG00000040913 | ENSCJAG00000017627 | ENSMMUG00000006753 | | ENSPPYG00000002586 | ENSGGOG00000005039 | ENSPTRG00000041241 | | ENSG00000107829 | |
| Fbxw5 | ENSRNOG00000028674 | ENSMUSG00000015095 | ENSCJAG00000013148 |  | | ENSPPYG00000019791 | ENSGGOG00000004350 | ENSPTRG00000016797 | | ENSG00000159069 | |
| Lrrc29 |  |  | ENSCJAG00000016350 |  | |  | ENSGGOG00000027648 | ENSPTRG00000014883 | | ENSG00000237102 | |
| Skp2 |  | ENSMUSG00000054115 | ENSCJAG00000018275 |  | | ENSPPYG00000015400 | ENSGGOG00000015620 |  | | ENSG00000145604 | |
| Fbxw12 | ENSRNOG00000020720 |  | ENSCJAG00000002742 | ENSMMUG00000003079 | | ENSPPYG00000013907 | ENSGGOG00000022457 |  | | ENSG00000164049 | |
| Gene |  |  |  |  | |  |  |  | |  | |
| Fbxw13 |  | ENSMUSG00000049314 |  |  | |  |  |  | |  | |
| Fbxw14 |  | ENSMUSG00000054087 |  |  | |  |  |  | |  | |
| Fbxw15 |  | ENSMUSG00000074060 |  |  | |  |  |  | |  | |
| Fbxw16 |  | ENSMUSG00000074062 |  |  | |  |  |  | |  | |
| Fbxw17 | ENSRNOG00000030027 | ENSMUSG00000037816 |  |  | |  |  |  | |  | |
| Fbxw18 |  | ENSMUSG00000074059 |  |  | |  |  |  | |  | |
| Fbxw19 |  | ENSMUSG00000074061 |  |  | |  |  |  | |  | |
| Fbxw20 |  | ENSMUSG00000061701 |  |  | |  |  |  | |  | |
| Fbxw21 |  | ENSMUSG00000047237 |  |  | |  |  |  | |  | |
| Fbxw22 |  | ENSMUSG00000070324 |  |  | |  |  |  | |  | |
| Fbxw24 |  | ENSMUSG00000062275 |  |  | |  |  |  | |  | |
| Fbxw26 |  | ENSMUSG00000059547 |  |  | |  |  |  | |  | |
| Fbxw28 |  | ENSMUSG00000079284 |  |  | |  |  |  | |  | |
|  |  |  |  |  | |  |  |  | |  | |

Note ^a^ We manually annotated the gene using FGENESH+. Gene *Fbxw13*, *Fbxw14*, *Fbxw15*, *Fbxw16*, *Fbxw17*, *Fbxw18*, *Fbxw19*, *Fbxw20*, *Fbxw21*, *Fbxw22*, *Fbxw24*, *Fbxw26* and *Fbxw28* were assigned to the same orthogroup Fbxw12.

|  |  |  |
| --- | --- | --- |

B. F-box gene numbers in eight genomes

| species | Total Genes^a^ | F-box Gene Number^b^ | Percentage of F-box genes |
| --- | --- | --- | --- |
| Human | 22104 | 71 | 0.32% |
| Chimpanzee | 18746 | 66 | 0.35% |
| Gorilla | 20962 | 68 | 0.32% |
| Orangutan | 20424 | 69 | 0.34% |
| Macaque | 21905 | 66 | 0.30% |
| Marmoset | 20993 | 67 | 0.32% |
| Mouse | 22662 | 81 | 0.36% |
| Rat | 22938 | 71 | 0.31% |

^a^ Numbers of protein-coding genes that were annotated in Ensembl.

^b^ Numbers only indicated the protein-coding F-box genes (excluding F-box gene related pseudogenes).
