# Supplementary material for: Evolution of the F-Box Gene Family in Euarchontoglires: Gene Number Variation and Selection Patterns
Source: PLoS One. 2014 Apr 11;9(4):e94899. doi: 10.1371/journal.pone.0094899 (PMC3984280; doi:10.1371/journal.pone.0094899)
Supplement: Table S2 — F-box gene-related pseudogenes in the eight genomes. (DOC) [file pone.0094899.s011.doc]

Table S2. F-box gene related pseudogenes in eight genomes

| species | Pseudogene id | species presenting homologous DNA regions^a^ |
| --- | --- | --- |
| Mouse | ENSMUSG00000090407 | Rat |
|  | ENSMUSG00000090234 | Rat |
|  | ENSMUSG00000083250 | Rat |
|  | ENSMUSG00000083623 |  |
| Rat | ENSRNOG00000042145 | Chimpanzee; Macaque; Marmoset; Orangutan; Gorilla |
| Human | ENSG00000230870 |  |
|  | ENSG00000229635 | Chimpanzee ;Macaque; Marmoset; Orangutan; Gorilla |
|  | ENSG00000229693 | Macaque; Marmoset; Orangutan; Gorilla |
|  | ENSG00000230701 | Chimpanzee; Macaque; Orangutan; Gorilla |

^a^ The orthologous psedogenes from these species were not annotated in ENSEMBL, presumably owing to incomplete annotation of psedudogenes by ENSEMBL. However, homologous DNA regions were found using blastn method at NCBI database. Given the coverage of queried sequence was over 90% and sequences similarity was over 90%, the aligned DNA region was assigned as the corresponding homologous region.
