# Supplementary material for: Evolution of the F-Box Gene Family in Euarchontoglires: Gene Number Variation and Selection Patterns
Source: PLoS One. 2014 Apr 11;9(4):e94899. doi: 10.1371/journal.pone.0094899 (PMC3984280; doi:10.1371/journal.pone.0094899)
Supplement: Table S3 — Chromosomal distributions of F-box genes in the eight genomes. (DOC) [file pone.0094899.s012.doc]

Table S3. Chromosomal distributions of F-box genes in eight genomes

1. Distribution of F-box genes in human genome

| Chromosome | Physical Length | Observed No. of  F-box genes (m_i_) | Expected No. of  F-box genes (λ_i_)^a^ | Distribution  Test^b^ |
| --- | --- | --- | --- | --- |
| 1 | 249,250,621 | 5 | 6.217 | 0.411 |
| 2 | 243,199,373 | 4 | 6.066 | 0.276 |
| 3 | 198,022,430 | 4 | 4.939 | 0.451 |
| 4 | 191,154,276 | 3 | 4.768 | 0.299 |
| 5 | 180,915,260 | 7 | 4.513 | 0.088 |
| 6 | 171,115,067 | 5 | 4.268 | 0.258 |
| 7 | 159,138,663 | 3 | 3.969 | 0.439 |
| 8 | 146,364,022 | 5 | 3.651 | 0.163 |
| 9 | 141,213,431 | 3 | 3.522 | 0.532 |
| 10 | 135,534,747 | 4 | 3.381 | 0.252 |
| 11 | 135,006,516 | 2 | 3.368 | 0.346 |
| 12 | 133,851,895 | 4 | 3.339 | 0.244 |
| 13 | 115,169,878 | 1 | 2.873 | 0.219 |
| 14 | 107,349,540 | 2 | 2.678 | 0.499 |
| 15 | 102,531,392 | 2 | 2.557 | 0.529 |
| 16 | 90,354,753 | 6 | 2.254 | 0.008 |
| 17 | 81,195,210 | 4 | 2.025 | 0.055 |
| 18 | 78,077,248 | 1 | 1.948 | 0.420 |
| 19 | 59,128,983 | 5 | 1.475 | 0.004 |
| 20 | 63,025,520 | 0 | 1.572 | 0.208 |
| 21 | 48,129,895 | 0 | 1.201 | 0.301 |
| 22 | 51,304,566 | 1 | 1.280 | 0.634 |
| X | 155,270,560 | 0 | 3.873 | 0.021 |
| Y | 59,373,566 | 0 | 1.481 | 0.227 |

1. Distribution of F-box genes in Chimpanzee genome

| Chromosome | Physical Length | Observed No. of  F-box genes (m_i_) | Expected No. of  F-box genes (λ_i_)^a^ | Distribution  Test^b^ |
| --- | --- | --- | --- | --- |
| 1 | 228,333,871 | 5 | 4.789 | 0.347 |
| 2a | 113,622,374 | 3 | 2.383 | 0.218 |
| 2b | 247,518,478 | 1 | 5.192 | 0.034 |
| 3 | 202,329,955 | 3 | 4.244 | 0.387 |
| 4 | 193,495,092 | 3 | 4.058 | 0.422 |
| 5 | 182,651,097 | 7 | 3.831 | 0.042 |
| 6 | 172,623,881 | 5 | 3.621 | 0.159 |
| 7 | 161,824,586 | 3 | 3.394 | 0.560 |
| 8 | 143,986,469 | 5 | 3.020 | 0.086 |
| 9 | 137,840,987 | 2 | 2.891 | 0.448 |
| 10 | 133,524,379 | 3 | 2.801 | 0.308 |
| 11 | 133,121,534 | 2 | 2.792 | 0.471 |
| 12 | 134,246,214 | 3 | 2.816 | 0.312 |
| 13 | 115,123,233 | 1 | 2.415 | 0.305 |
| 14 | 106,544,938 | 2 | 2.235 | 0.613 |
| 15 | 99,548,318 | 2 | 2.088 | 0.653 |
| 16 | 89,983,829 | 6 | 1.887 | 0.003 |
| 17 | 82,630,442 | 4 | 1.733 | 0.032 |
| 18 | 76,611,499 | 1 | 1.607 | 0.523 |
| 19 | 63,644,993 | 4 | 1.335 | 0.012 |
| 20 | 61,729,293 | 0 | 1.295 | 0.274 |
| 21 | 32,799,110 | 0 | 0.688 | 0.503 |
| 22 | 49,737,984 | 1 | 1.043 | 0.720 |
| X | 156,848,144 | 0 | 3.290 | 0.037 |
| Y | 26,342,871 | 0 | 0.553 | 0.575 |

1. Distribution of F-box genes in Gorilla genome

| Chromosome | Physical Length | Observed No. of  F-box genes (m_i_) | Expected No. of  F-box genes (λ_i_)^a^ | Distribution  Test^b^ |
| --- | --- | --- | --- | --- |
| 1 | 229,507,203 | 5 | 5.349 | 0.555 |
| 2a | 111,351,968 | 3 | 2.595 | 0.263 |
| 2b | 131,632,457 | 1 | 3.068 | 0.189 |
| 3 | 199,944,510 | 4 | 4.660 | 0.502 |
| 4 | 201,139,530 | 3 | 4.688 | 0.312 |
| 5 | 165,930,986 | 4 | 3.867 | 0.345 |
| 6 | 171,703,152 | 5 | 4.002 | 0.215 |
| 7 | 158,137,892 | 3 | 3.686 | 0.497 |
| 8 | 145,327,772 | 5 | 3.387 | 0.128 |
| 9 | 121,947,112 | 3 | 2.842 | 0.317 |
| 10 | 147,764,049 | 4 | 3.444 | 0.264 |
| 11 | 133,470,886 | 2 | 3.111 | 0.399 |
| 12 | 133,360,231 | 3 | 3.108 | 0.623 |
| 13 | 97,499,607 | 1 | 2.272 | 0.337 |
| 14 | 88,974,843 | 2 | 2.074 | 0.657 |
| 15 | 82,026,568 | 2 | 1.912 | 0.299 |
| 16 | 80,971,650 | 6 | 1.887 | 0.003 |
| 17 | 94,257,108 | 5 | 2.197 | 0.025 |
| 18 | 78,787,515 | 1 | 1.836 | 0.452 |
| 19 | 56,181,278 | 5 | 1.309 | 0.002 |
| 20 | 62,603,092 | 0 | 1.459 | 0.232 |
| 21 | 35,451,371 | 0 | 0.826 | 0.438 |
| 22 | 35,671,106 | 1 | 0.831 | 0.203 |
| X | 154,045,127 | 0 | 3.590 | 0.028 |

1. Distribution of F-box genes in Orangutan genome

| Chromosome | Physical Length | Observed No. of  F-box genes (m_i_) | Expected No. of  F-box genes (λ_i_)^a^ | Distribution  Test^b^ |
| --- | --- | --- | --- | --- |
| 1 | 229,942,017 | 5 | 5.237 | 0.574 |
| 2a | 113,028,656 | 4 | 2.574 | 0.119 |
| 2b | 135,000,294 | 1 | 3.075 | 0.188 |
| 3 | 202,140,232 | 3 | 4.604 | 0.325 |
| 4 | 198,332,218 | 3 | 4.517 | 0.339 |
| 5 | 183,952,662 | 7 | 4.190 | 0.063 |
| 6 | 174,210,431 | 5 | 3.968 | 0.210 |
| 7 | 157,549,271 | 3 | 3.588 | 0.518 |
| 8 | 153,482,349 | 4 | 3.496 | 0.274 |
| 9 | 135,191,526 | 3 | 3.079 | 0.630 |
| 10 | 133,410,057 | 4 | 3.039 | 0.191 |
| 11 | 132,107,971 | 2 | 3.009 | 0.421 |
| 12 | 136,387,465 | 4 | 3.106 | 0.203 |
| 13 | 117,095,149 | 1 | 2.667 | 0.255 |
| 14 | 108,868,599 | 2 | 2.480 | 0.549 |
| 15 | 99,152,023 | 2 | 2.258 | 0.607 |
| 16 | 77,800,216 | 5 | 1.772 | 0.010 |
| 17 | 73,212,453 | 4 | 1.667 | 0.028 |
| 18 | 94,050,890 | 1 | 2.142 | 0.369 |
| 19 | 60,714,840 | 5 | 1.383 | 0.003 |
| 20 | 62,736,349 | 0 | 1.429 | 0.240 |
| 21 | 48,394,510 | 0 | 1.102 | 0.332 |
| 22 | 46,535,552 | 1 | 1.060 | 0.714 |
| X | 156,195,299 | 0 | 3.558 | 0.029 |

1. Distribution of F-box genes in Macaque genome

| Chromosome | Physical Length | Observed No. of  F-box genes (m_i_) | Expected No. of  F-box genes (λ_i_)^a^ | Distribution  Test^b^ |
| --- | --- | --- | --- | --- |
| 1 | 228,252,215 | 4 | 5.181 | 0.409 |
| 2 | 189,746,636 | 3 | 4.307 | 0.376 |
| 3 | 196,418,989 | 2 | 4.458 | 0.178 |
| 4 | 167,655,696 | 5 | 3.805 | 0.185 |
| 5 | 182,086,969 | 3 | 4.133 | 0.408 |
| 6 | 178,205,221 | 6 | 4.045 | 0.115 |
| 7 | 169,801,366 | 4 | 3.854 | 0.343 |
| 8 | 147,794,981 | 5 | 3.355 | 0.124 |
| 9 | 133,323,859 | 4 | 3.026 | 0.189 |
| 10 | 94,855,758 | 1 | 2.153 | 0.366 |
| 11 | 134,511,895 | 4 | 3.053 | 0.194 |
| 12 | 106,505,843 | 1 | 2.417 | 0.305 |
| 13 | 138,028,943 | 3 | 3.133 | 0.617 |
| 14 | 133,002,572 | 2 | 3.019 | 0.419 |
| 15 | 110,119,387 | 2 | 2.500 | 0.544 |
| 16 | 78,773,432 | 4 | 1.788 | 0.036 |
| 17 | 94,452,569 | 1 | 2.144 | 0.368 |
| 18 | 73,567,989 | 1 | 1.670 | 0.503 |
| 19 | 64,391,591 | 5 | 1.462 | 0.004 |
| 20 | 88,221,753 | 5 | 2.002 | 0.017 |
| X | 153,947,521 | 0 | 3.494 | 0.030 |

1. Distribution of F-box genes in Marmoset genome

| Chromosome | Physical Length | Observed No. of  F-box genes (m_i_) | Expected No. of  F-box genes (λ_i_)^a^ | Distribution  Test^b^ |
| --- | --- | --- | --- | --- |
| 1 | 210,400,635 | 4 | 4.928 | 0.453 |
| 2 | 204,313,951 | 7 | 4.785 | 0.112 |
| 3 | 190,850,796 | 3 | 4.470 | 0.347 |
| 4 | 171,630,274 | 5 | 4.020 | 0.218 |
| 5 | 159,171,411 | 3 | 3.728 | 0.488 |
| 6 | 158,406,734 | 1 | 3.710 | 0.115 |
| 7 | 155,834,243 | 4 | 3.650 | 0.303 |
| 8 | 128,169,293 | 1 | 3.002 | 0.199 |
| 9 | 124,281,992 | 4 | 2.911 | 0.170 |
| 10 | 132,174,527 | 4 | 3.096 | 0.201 |
| 11 | 130,397,257 | 2 | 3.054 | 0.411 |
| 12 | 121,768,101 | 6 | 2.852 | 0.027 |
| 13 | 117,903,854 | 3 | 2.762 | 0.300 |
| 14 | 108,792,865 | 3 | 2.548 | 0.253 |
| 15 | 98,464,013 | 3 | 2.306 | 0.202 |
| 16 | 96,796,970 | 3 | 2.267 | 0.194 |
| 17 | 74,750,902 | 1 | 1.751 | 0.478 |
| 18 | 47,448,759 | 0 | 1.111 | 0.329 |
| 19 | 49,578,535 | 1 | 1.161 | 0.677 |
| 20 | 44,557,958 | 3 | 1.044 | 0.022 |
| 21 | 50,472,720 | 0 | 1.182 | 0.307 |
| 22 | 49,145,316 | 5 | 1.151 | 0.001 |
| X | 142,054,208 | 0 | 3.327 | 0.036 |
| Y | 50,472,720 | 0 | 1.182 | 0.307 |

1. Distribution of F-box genes in Mouse genome

| Chromosome | Physical Length | Observed No. of  F-box genes (m_i_) | Expected No. of  F-box genes (λ_i_)^a^ | Distribution  Test^b^ |
| --- | --- | --- | --- | --- |
| 1 | 195,471,971 | 2 | 5.809 | 0.071 |
| 2 | 182,113,224 | 4 | 5.412 | 0.371 |
| 3 | 160,039,680 | 1 | 4.756 | 0.049 |
| 4 | 156,508,116 | 6 | 4.651 | 0.189 |
| 5 | 151,834,684 | 7 | 4.512 | 0.088 |
| 6 | 149,736,546 | 2 | 4.450 | 0.179 |
| 7 | 145,441,459 | 4 | 4.322 | 0.566 |
| 8 | 129,401,213 | 5 | 3.846 | 0.191 |
| 9 | 124,595,110 | 17 | 3.703 | 0.000 |
| 10 | 130,694,993 | 4 | 3.884 | 0.349 |
| 11 | 122,082,543 | 6 | 3.628 | 0.076 |
| 12 | 120,129,022 | 1 | 3.570 | 0.129 |
| 13 | 120,421,639 | 2 | 3.579 | 0.307 |
| 14 | 124,902,244 | 3 | 3.712 | 0.492 |
| 15 | 104,043,685 | 5 | 3.092 | 0.093 |
| 16 | 98,207,768 | 2 | 2.919 | 0.442 |
| 17 | 94,987,271 | 4 | 2.823 | 0.156 |
| 18 | 90,702,639 | 2 | 2.696 | 0.495 |
| 19 | 61,431,566 | 4 | 1.826 | 0.038 |
| X | 171,031,299 | 0 | 5.083 | 0.006 |
| Y | 91,744,698 | 0 | 2.727 | 0.065 |

1. Distribution of F-box genes in Rat genome

| Chromosome | Physical Length | Observed No. of  F-box genes (m_i_) | Expected No. of  F-box genes (λ_i_)^a^ | Distribution  Test^b^ |
| --- | --- | --- | --- | --- |
| 1 | 290,094,216 | 11 | 7.096 | 0.058 |
| 2 | 285,068,071 | 4 | 6.973 | 0.175 |
| 3 | 183,740,530 | 3 | 4.494 | 0.343 |
| 4 | 248,343,840 | 3 | 6.075 | 0.145 |
| 5 | 177,180,328 | 7 | 4.334 | 0.074 |
| 6 | 156,897,508 | 2 | 3.838 | 0.263 |
| 7 | 143,501,887 | 3 | 3.510 | 0.534 |
| 8 | 132,457,389 | 7 | 3.240 | 0.018 |
| 9 | 121,549,591 | 2 | 2.973 | 0.429 |
| 10 | 112,200,500 | 7 | 2.745 | 0.007 |
| 11 | 93,518,069 | 1 | 2.288 | 0.334 |
| 12 | 54,450,796 | 5 | 1.332 | 0.003 |
| 13 | 118,718,031 | 1 | 2.904 | 0.214 |
| 14 | 115,151,701 | 2 | 2.817 | 0.465 |
| 15 | 114,627,140 | 3 | 2.804 | 0.309 |
| 16 | 90,051,983 | 2 | 2.203 | 0.622 |
| 17 | 92,503,511 | 3 | 2.263 | 0.193 |
| 18 | 87,229,863 | 2 | 2.134 | 0.641 |
| 19 | 72,914,587 | 3 | 1.784 | 0.106 |
| 20 | 57,791,882 | 0 | 1.414 | 0.243 |
| X | 154,597,545 | 0 | 3.782 | 0.023 |

I. Adjusted p-values for F-box gene distribution in Chromosome X in 8 species^c^

| Genome | Chrome | P-value | Corrected P-value |
| --- | --- | --- | --- |
| Mouse | X | 0.006 | 0.048 |
| Macaque | X | 0.017 | 0.068 |
| Human | X | 0.021 | 0.056 |
| Rat | X | 0.023 | 0.046 |
| Gorilla | X | 0.028 | 0.046 |
| Orangutan | X | 0.029 | 0.038 |
| Marmoset | X | 0.036 | 0.041 |
| Chimpanzee | X | 0.037 | 0.037 |

^a^ The expected gene number λ_i_ on chromosome i would is a sample from a Poisson distribution, $\lambda_{i}=mL_{i}/\sum L_{j}$ , where m is the total number of F-box genes in the whole genome and$L_{i}$ is the length of chromosome i.

^b^ The probabilities p (m_i_ < λ_i_) and p (m_i_ > λ_i_) were evaluated under the cumulative Poisson distribution at α ≤ 0.05 significance levels.

^c^ The Benjamini and Hochberg False Discovery Rate correction was applied.
