# Supplementary material for: Evolution of the F-Box Gene Family in Euarchontoglires: Gene Number Variation and Selection Patterns
Source: PLoS One. 2014 Apr 11;9(4):e94899. doi: 10.1371/journal.pone.0094899 (PMC3984280; doi:10.1371/journal.pone.0094899)
Supplement: Table S4 — Events causing F-box gene number variation during the evolution of Euarchontoglires. (DOC) [file pone.0094899.s013.doc]

Table S4. Events causing F-box gene number variation during the evolution of *Euarchontoglires*

| species | Tandem duplication (No.) | Retroposition | F-box domain lost | Gene lost |
| --- | --- | --- | --- | --- |
| Murinae |  |  | *Fbxo43*; *Lrrc29* |  |
| Mouse | *Fbxw12*(11) |  |  | *Fbxl14* |
| Rat |  | *Fbxl18* | *Fbxo45* |  |
| Primate |  |  |  | *Fbxw17Fbxl14* |
| Chimpanzee |  |  | *Fbxo45 Fbxw5* | *Fbxo17 Fbxw4* |
| Gorilla |  |  | *Fbxl21 Fbxl14 Fbxl17* |  |
| Human |  |  |  |  |
| Macaque |  |  | *Skp2 Lrrc29 Fbxo45*  *Fbxo2 Fbxw5* |  |
| Marmoset |  |  | *Fbxl21 Fbxo21 Fbxl18* | *Fbxo44* |
| Orangutan | *Fbxo48*(1) |  | *Lrrc29* | *Fbxo45 Fbxo32* |
