# Supplementary material for: Evolution of the F-Box Gene Family in Euarchontoglires: Gene Number Variation and Selection Patterns
Source: PLoS One. 2014 Apr 11;9(4):e94899. doi: 10.1371/journal.pone.0094899 (PMC3984280; doi:10.1371/journal.pone.0094899)
Supplement: Table S5 — Tests of variable ω among sites for 71 orthogroups using models M0-M3 comparison. (DOC) [file pone.0094899.s014.doc]

Table S5. Tests of variable ω among sites for 71 orthogroups using models M0-M3 comparison

| Gene | | M0 (*lnL*) | M3 (*lnL*) | *2△L* | *p-*value |
| --- | --- | --- | --- | --- | --- |
| Btrc | -3014.07 | | -2995.38 | 37.38 | 1.51E-07 |
| Ccnf | -6108.67 | | -6065.66 | 86.01 | 0.00E+00 |
| Ect2l | -6359.78 | | -6328.13 | 63.30 | 5.87E-13 |
| Fbxl12 | -2250.58 | | -2243.39 | 14.37 | 6.20E-03 |
| Fbxl13 | -5353.24 | | -5323.25 | 59.97 | 2.94E-12 |
| Fbxl14 | -2037.82 | | -2037.74 | 0.16 | 9.97E-01 |
| Fbxl15 | -1876.03 | | -1875.04 | 1.99 | 7.38E-01 |
| Fbxl16 | -2552.75 | | -2543.64 | 18.22 | 1.12E-03 |
| Fbxl17 | -3742.13 | | -3735.83 | 12.60 | 1.34E-02 |
| Fbxl18 | -4376.72 | | -4374.18 | 5.08 | 2.80E-01 |
| Fbxl19 | -3747.60 | | -3679.31 | 136.58 | 0.00E+00 |
| Fbxl2 | -2646.97 | | -2635.19 | 23.57 | 9.73E-05 |
| Fbxl20 | -2209.04 | | -2209.04 | 0.00 | 1.00E+00 |
| Fbxl21 | -3554.76 | | -3524.77 | 59.98 | 2.93E-12 |
| Fbxl22 | -1493.41 | | -1476.66 | 33.50 | 9.45E-07 |
| Fbxl3 | -2624.85 | | -2616.07 | 17.56 | 1.51E-03 |
| Fbxl4 | -4144.30 | | -4128.87 | 30.87 | 3.25E-06 |
| Fbxl5 | -3895.81 | | -3886.61 | 18.40 | 1.03E-03 |
| Fbxl6 | -4105.95 | | -4087.35 | 37.19 | 1.65E-07 |
| Fbxl7 | -2703.57 | | -2699.59 | 7.97 | 9.25E-02 |
| Fbxl8 | -3318.34 | | -3293.37 | 49.94 | 3.71E-10 |
| Fbxo10 | -6150.11 | | -6126.51 | 47.20 | 1.39E-09 |
| Fbxo11 | -4545.97 | | -4545.97 | 0.00 | 1.00E+00 |
| Fbxo15 | -3349.85 | | -3325.94 | 47.82 | 1.03E-09 |
| Fbxo16 | -2248.44 | | -2227.41 | 42.08 | 1.61E-08 |
| Fbxo17 | -1984.88 | | -1960.32 | 49.11 | 5.53E-10 |
| Fbxo18 | -6918.65 | | -6860.25 | 116.79 | 0.00E+00 |
| Fbxo2 | -1946.53 | | -1942.70 | 7.65 | 1.05E-01 |
| Fbxo21 | -3642.87 | | -3635.38 | 14.98 | 4.75E-03 |
| Fbxo22 | -2712.61 | | -2695.62 | 33.99 | 7.50E-07 |
| Fbxo24 | -3792.59 | | -3781.19 | 22.79 | 1.39E-04 |
| Fbxo25 | -1932.21 | | -1929.33 | 5.76 | 2.18E-01 |
| Fbxo27 | -1666.56 | | -1661.75 | 9.63 | 4.72E-02 |
| Fbxo28 | -2877.24 | | -2858.59 | 37.28 | 1.58E-07 |
| Fbxo3 | -2915.52 | | -2906.74 | 17.56 | 1.50E-03 |
| Fbxo30 | -4935.93 | | -4922.97 | 25.94 | 3.26E-05 |
| Fbxo31 | -3364.46 | | -3344.57 | 39.77 | 4.82E-08 |
| Fbxo32 | -2153.08 | | -2146.74 | 12.66 | 1.30E-02 |
| Fbxo33 | -3608.28 | | -3603.93 | 8.70 | 6.90E-02 |
| Fbxo34 | -5503.98 | | -5476.16 | 55.64 | 2.39E-11 |
| Fbxo36 | -1572.03 | | -1540.82 | 62.43 | 8.94E-13 |
| Fbxo38 | -8162.63 | | -8065.98 | 193.30 | 0.00E+00 |
| Fbxo39 | -3070.22 | | -3056.64 | 27.15 | 1.85E-05 |
| Fbxo4 | -2626.21 | | -2622.73 | 6.95 | 1.38E-01 |
| Fbxo40 | -5335.09 | | -5271.64 | 126.89 | 0.00E+00 |
| Fbxo41 | -5449.35 | | -5379.30 | 140.09 | 0.00E+00 |
| Fbxo42 | -4562.25 | | -4555.72 | 13.07 | 1.09E-02 |
| Fbxo43 | -3593.45 | | -3587.57 | 11.76 | 1.93E-02 |
| Fbxo44 | -1437.67 | | -1436.30 | 2.75 | 6.01E-01 |
| Fbxo45 | -1477.29 | | -1459.92 | 34.74 | 5.26E-07 |
| Fbxo46 | -4120.75 | | -4105.53 | 30.43 | 4.00E-06 |
| Fbxo47 | -3617.54 | | -3602.51 | 30.06 | 4.76E-06 |
| Fbxo48 | -1350.67 | | -1327.35 | 46.65 | 1.81E-09 |
| Fbxo5 | -3541.95 | | -3513.67 | 56.57 | 1.52E-11 |
| Fbxo6 | -2593.25 | | -2544.89 | 96.72 | 0.00E+00 |
| Fbxo7 | -4209.68 | | -4189.33 | 40.69 | 3.11E-08 |
| Fbxo8 | -1945.47 | | -1937.00 | 16.93 | 1.99E-03 |
| Fbxo9 | -2683.45 | | -2676.52 | 13.86 | 7.77E-03 |
| Fbxw10 | -7475.54 | | -7427.45 | 96.18 | 0.00E+00 |
| Fbxw11 | -2989.94 | | -2944.67 | 90.54 | 0.00E+00 |
| Fbxw12 | -5496.43 | | -5446.77 | 99.32 | 0.00E+00 |
| Fbxw2 | -2737.06 | | -2704.24 | 65.64 | 1.88E-13 |
| Fbxw4 | -2764.41 | | -2731.88 | 65.07 | 2.49E-13 |
| Fbxw5 | -3525.51 | | -3512.54 | 25.95 | 3.24E-05 |
| Fbxw7 | -3254.85 | | -3253.15 | 3.40 | 4.93E-01 |
| Fbxw8 | -4782.25 | | -4766.04 | 32.41 | 1.58E-06 |
| Fbxw9 | -3950.77 | | -3932.65 | 36.24 | 2.58E-07 |
| Kdm2A | -6398.14 | | -6396.90 | 2.49 | 6.47E-01 |
| Kdm2B | -8250.74 | | -8115.32 | 270.83 | 0.00E+00 |
| Lrrc29 | -947.97 | | -947.40 | 1.14 | 8.87E-01 |
| Skp2 | -3379.06 | | -3348.72 | 60.68 | 2.09E-12 |
