# Supplementary material for: Evolution of the F-Box Gene Family in Euarchontoglires: Gene Number Variation and Selection Patterns
Source: PLoS One. 2014 Apr 11;9(4):e94899. doi: 10.1371/journal.pone.0094899 (PMC3984280; doi:10.1371/journal.pone.0094899)
Supplement: Table S6 — Lineage-specific positive selection was identified using branch-site selection models. (DOC) [file pone.0094899.s015.doc]

Table S6. Lineage-specific positive selection was identified using branch-site selection models

| Gene | Foreground branch | Brach-site models | Parameter estimates | | | | | *lnL* | *p*-value |
| --- | --- | --- | --- | --- | --- | --- | --- | --- | --- |
| Btrc | Mouse | Model A H0 | proportion | 0.769 | 0.169 | 0.051 | 0.011 | -4385.963 |  |
|  |  | (ω2=1) | background | 0.000 | 1.000 | 0.000 | 1.000 |  |  |
|  |  |  | foreground | 0.000 | 1.000 | 1.000 | 1.000 |  |  |
|  |  | Model A H1 | proportion | 0.809 | 0.169 | 0.019 | 0.004 | -4359.171 | 2.48E-13 |
|  |  |  | background | 0.000 | 1.000 | 0.000 | 1.000 |  |  |
|  |  |  | foreground | 0.000 | 1.000 | 317.074 | 317.074 |  |  |
| Btrc | Macaque | Model A H0 | proportion | 0.709 | 0.044 | 0.233 | 0.014 | -4337.759 |  |
|  |  | (ω2=1) | background | 0.007 | 1.000 | 0.007 | 1.000 |  |  |
|  |  |  | foreground | 0.007 | 1.000 | 1.000 | 1.000 |  |  |
|  |  | Model A H1 | proportion | 0.836 | 0.054 | 0.104 | 0.007 | -4192.528 | 0.00E+00 |
|  |  |  | background | 0.010 | 1.000 | 0.010 | 1.000 |  |  |
|  |  |  | foreground | 0.010 | 1.000 | 999.000 | 999.000 |  |  |
| Fbxl16 | Chimpanzee | Model A H0 | proportion | 0.731 | 0.018 | 0.245 | 0.006 | -3114.285 |  |
|  |  | (ω2=1) | background | 0.009 | 1.000 | 0.009 | 1.000 |  |  |
|  |  |  | foreground | 0.009 | 1.000 | 1.000 | 1.000 |  |  |
|  |  | Model A H1 | proportion | 0.946 | 0.028 | 0.025 | 0.001 | -3086.359 | 7.82E-14 |
|  |  |  | background | 0.008 | 1.000 | 0.008 | 1.000 |  |  |
|  |  |  | foreground | 0.008 | 1.000 | 999.000 | 999.000 |  |  |
| Fbxl8 | Mouse | Model A H0 | proportion | 0.722 | 0.226 | 0.039 | 0.012 | -3369.329 |  |
|  |  | (ω2=1) | background | 0.061 | 1.000 | 0.061 | 1.000 |  |  |
|  |  |  | foreground | 0.061 | 1.000 | 1.000 | 1.000 |  |  |
|  |  | Model A H1 | proportion | 0.757 | 0.232 | 0.009 | 0.003 | -3366.025 | 1.02E-02 |
|  |  |  | background | 0.062 | 1.000 | 0.062 | 1.000 |  |  |
|  |  |  | foreground | 0.062 | 1.000 | 150.123 | 150.123 |  |  |
| Fbxl8 | Macaque | Model A H0 | proportion | 0.744 | 0.236 | 0.016 | 0.005 | -3369.547 |  |
|  |  | (ω2=1) | background | 0.064 | 1.000 | 0.064 | 1.000 |  |  |
|  |  |  | foreground | 0.064 | 1.000 | 1.000 | 1.000 |  |  |
|  |  | Model A H1 | proportion | 0.760 | 0.232 | 0.006 | 0.002 | -3366.612 | 1.54E-02 |
|  |  |  | background | 0.066 | 1.000 | 0.066 | 1.000 |  |  |
|  |  |  | foreground | 0.066 | 1.000 | 65.689 | 65.689 |  |  |
| Fbxo15 | Mouse | Model A H0 | proportion | 0.493 | 0.471 | 0.019 | 0.018 | -4792.008 |  |
|  |  | (ω2=1) | background | 0.113 | 1.000 | 0.113 | 1.000 |  |  |
|  |  |  | foreground | 0.113 | 1.000 | 1.000 | 1.000 |  |  |
|  |  | Model A H1 | proportion | 0.505 | 0.444 | 0.027 | 0.024 | -4780.888 | 2.41E-06 |
|  |  |  | background | 0.129 | 1.000 | 0.129 | 1.000 |  |  |
|  |  |  | foreground | 0.129 | 1.000 | 103.602 | 103.602 |  |  |
| Fbxo15 | Marmoset | Model A H0 | proportion | 0.202 | 0.116 | 0.433 | 0.249 | -4771.422 |  |
|  |  | (ω2=1) | background | 0.071 | 1.000 | 0.071 | 1.000 |  |  |
|  |  |  | foreground | 0.071 | 1.000 | 1.000 | 1.000 |  |  |
|  |  | Model A H1 | proportion | 0.404 | 0.204 | 0.260 | 0.132 | -4757.627 | 1.50E-07 |
|  |  |  | background | 0.082 | 1.000 | 0.082 | 1.000 |  |  |
|  |  |  | foreground | 0.082 | 1.000 | 6.507 | 6.507 |  |  |
| Fbxo16 | Rat | Model A H0 | proportion | 0.669 | 0.136 | 0.163 | 0.033 | -3007.316 |  |
|  |  | (ω2=1) | background | 0.058 | 1.000 | 0.058 | 1.000 |  |  |
|  |  |  | foreground | 0.058 | 1.000 | 1.000 | 1.000 |  |  |
|  |  | Model A H1 | proportion | 0.740 | 0.124 | 0.116 | 0.019 | -2969.988 | 0.00E+00 |
|  |  |  | background | 0.082 | 1.000 | 0.082 | 1.000 |  |  |
|  |  |  | foreground | 0.082 | 1.000 | 998.905 | 998.905 |  |  |
| Fbxo22 | 11 | Model A H0 | proportion | 0.868 | 0.132 | 0.000 | 0.000 | -2777.171 |  |
|  |  | (ω2=1) | background | 0.016 | 1.000 | 0.016 | 1.000 |  |  |
|  |  |  | foreground | 0.016 | 1.000 | 1.000 | 1.000 |  |  |
|  |  | Model A H1 | proportion | 0.878 | 0.116 | 0.006 | 0.001 | -2770.558 | 2.76E-04 |
|  |  |  | background | 0.021 | 1.000 | 0.021 | 1.000 |  |  |
|  |  |  | foreground | 0.021 | 1.000 | 462.111 | 462.111 |  |  |
| Fbxo28 | Rat | Model A H0 | proportion | 0.777 | 0.201 | 0.018 | 0.005 | -3077.584 |  |
|  |  | (ω2=1) | background | 0.061 | 1.000 | 0.061 | 1.000 |  |  |
|  |  |  | foreground | 0.061 | 1.000 | 1.000 | 1.000 |  |  |
|  |  | Model A H1 | proportion | 0.795 | 0.197 | 0.006 | 0.001 | -3075.681 | 5.10E-02 |
|  |  |  | background | 0.062 | 1.000 | 0.062 | 1.000 |  |  |
|  |  |  | foreground | 0.062 | 1.000 | 21.111 | 21.111 |  |  |
| Fbxo28 | Gorilla | Model A H0 | proportion | 0.607 | 0.021 | 0.359 | 0.012 | -3024.355 |  |
|  |  | (ω2=1) | background | 0.035 | 1.000 | 0.035 | 1.000 |  |  |
|  |  |  | foreground | 0.035 | 1.000 | 1.000 | 1.000 |  |  |
|  |  | Model A H1 | proportion | 0.770 | 0.030 | 0.192 | 0.008 | -2957.261 | 0.00E+00 |
|  |  |  | background | 0.047 | 1.000 | 0.047 | 1.000 |  |  |
|  |  |  | foreground | 0.047 | 1.000 | 125.978 | 125.978 |  |  |
| Fbxo38 | 11 | Model A H0 | proportion | 0.080 | 0.009 | 0.822 | 0.089 | -8115.155 |  |
|  |  | (ω2=1) | background | 0.028 | 1.000 | 0.028 | 1.000 |  |  |
|  |  |  | foreground | 0.028 | 1.000 | 1.000 | 1.000 |  |  |
|  |  | Model A H1 | proportion | 0.903 | 0.097 | 0.000 | 0.000 | -8107.605 | 1.02E-04 |
|  |  |  | background | 0.027 | 1.000 | 0.027 | 1.000 |  |  |
|  |  |  | foreground | 0.027 | 1.000 | 1.000 | 1.000 |  |  |
| Fbxo38 | Orangutan | Model A H0 | proportion | 0.903 | 0.097 | 0.000 | 0.000 | -8107.605 |  |
|  |  | (ω2=1) | background | 0.027 | 1.000 | 0.027 | 1.000 |  |  |
|  |  |  | foreground | 0.027 | 1.000 | 1.000 | 1.000 |  |  |
|  |  | Model A H1 | proportion | 0.905 | 0.093 | 0.002 | 0.000 | -8091.744 | 1.78E-08 |
|  |  |  | background | 0.029 | 1.000 | 0.029 | 1.000 |  |  |
|  |  |  | foreground | 0.029 | 1.000 | 999.000 | 999.000 |  |  |
| Fbxo38 | Gorilla | Model A H0 | proportion | 0.717 | 0.028 | 0.245 | 0.010 | -8058.747 |  |
|  |  | (ω2=1) | background | 0.031 | 1.000 | 0.031 | 1.000 |  |  |
|  |  |  | foreground | 0.031 | 1.000 | 1.000 | 1.000 |  |  |
|  |  | Model A H1 | proportion | 0.923 | 0.039 | 0.037 | 0.002 | -7897.268 | 0.00E+00 |
|  |  |  | background | 0.033 | 1.000 | 0.033 | 1.000 |  |  |
|  |  |  | foreground | 0.033 | 1.000 | 999.000 | 999.000 |  |  |
| Fbxo39 | 12 | Model A H0 | proportion | 0.000 | 0.000 | 0.965 | 0.035 | -3374.019 |  |
|  |  | (ω2=1) | background | 0.105 | 1.000 | 0.105 | 1.000 |  |  |
|  |  |  | foreground | 0.105 | 1.000 | 1.000 | 1.000 |  |  |
|  |  | Model A H1 | proportion | 0.854 | 0.031 | 0.110 | 0.004 | -3371.042 | 1.47E-02 |
|  |  |  | background | 0.104 | 1.000 | 0.104 | 1.000 |  |  |
|  |  |  | foreground | 0.104 | 1.000 | 36.097 | 36.097 |  |  |
| Fbxo41 | Rat | Model A H0 | proportion | 0.829 | 0.171 | 0.000 | 0.000 | -7914.671 |  |
|  |  | (ω2=1) | background | 0.034 | 1.000 | 0.034 | 1.000 |  |  |
|  |  |  | foreground | 0.034 | 1.000 | 1.000 | 1.000 |  |  |
|  |  | Model A H1 | proportion | 0.821 | 0.169 | 0.009 | 0.002 | -7901.949 | 4.55E-07 |
|  |  |  | background | 0.030 | 1.000 | 0.030 | 1.000 |  |  |
|  |  |  | foreground | 0.030 | 1.000 | 224.361 | 224.361 |  |  |
| Fbxo41 | Mouse | Model A H0 | proportion | 0.770 | 0.107 | 0.108 | 0.015 | -7893.845 |  |
|  |  | (ω2=1) | background | 0.034 | 1.000 | 0.034 | 1.000 |  |  |
|  |  |  | foreground | 0.034 | 1.000 | 1.000 | 1.000 |  |  |
|  |  | Model A H1 | proportion | 0.828 | 0.110 | 0.054 | 0.007 | -7781.147 | 0.00E+00 |
|  |  |  | background | 0.036 | 1.000 | 0.036 | 1.000 |  |  |
|  |  |  | foreground | 0.036 | 1.000 | 999.000 | 999.000 |  |  |
| Fbxo41 | Orangutan | Model A H0 | proportion | 0.708 | 0.094 | 0.175 | 0.023 | -7862.848 |  |
|  |  | (ω2=1) | background | 0.024 | 1.000 | 0.024 | 1.000 |  |  |
|  |  |  | foreground | 0.024 | 1.000 | 1.000 | 1.000 |  |  |
|  |  | Model A H1 | proportion | 0.797 | 0.110 | 0.081 | 0.011 | -7718.643 | 0.00E+00 |
|  |  |  | background | 0.024 | 1.000 | 0.024 | 1.000 |  |  |
|  |  |  | foreground | 0.024 | 1.000 | 999.000 | 999.000 |  |  |
| Fbxo41 | Gorilla | Model A H0 | proportion | 0.541 | 0.089 | 0.318 | 0.052 | -7852.199 |  |
|  |  | (ω2=1) | background | 0.021 | 1.000 | 0.021 | 1.000 |  |  |
|  |  |  | foreground | 0.021 | 1.000 | 1.000 | 1.000 |  |  |
|  |  | Model A H1 | proportion | 0.811 | 0.138 | 0.044 | 0.007 | -7732.381 | 0.00E+00 |
|  |  |  | background | 0.020 | 1.000 | 0.020 | 1.000 |  |  |
|  |  |  | foreground | 0.020 | 1.000 | 999.000 | 999.000 |  |  |
| Fbxo46 | Chimpanzee | Model A H0 | proportion | 0.743 | 0.040 | 0.206 | 0.011 | -4662.376 |  |
|  |  | (ω2=1) | background | 0.025 | 1.000 | 0.025 | 1.000 |  |  |
|  |  |  | foreground | 0.025 | 1.000 | 1.000 | 1.000 |  |  |
|  |  | Model A H1 | proportion | 0.937 | 0.050 | 0.012 | 0.001 | -4640.412 | 3.41E-11 |
|  |  |  | background | 0.026 | 1.000 | 0.026 | 1.000 |  |  |
|  |  |  | foreground | 0.026 | 1.000 | 999.000 | 999.000 |  |  |
| Fbxo47 | Mouse | Model A H0 | proportion | 0.739 | 0.108 | 0.134 | 0.020 | -3614.670 |  |
|  |  | (ω2=1) | background | 0.129 | 1.000 | 0.129 | 1.000 |  |  |
|  |  |  | foreground | 0.129 | 1.000 | 1.000 | 1.000 |  |  |
|  |  | Model A H1 | proportion | 0.813 | 0.098 | 0.079 | 0.010 | -3568.683 | 0.00E+00 |
|  |  |  | background | 0.160 | 1.000 | 0.160 | 1.000 |  |  |
|  |  |  | foreground | 0.160 | 1.000 | 999.000 | 999.000 |  |  |
| Fbxo48 | Mouse | Model A H0 | proportion | 0.527 | 0.321 | 0.095 | 0.058 | -1364.299 |  |
|  |  | (ω2=1) | background | 0.058 | 1.000 | 0.058 | 1.000 |  |  |
|  |  |  | foreground | 0.058 | 1.000 | 1.000 | 1.000 |  |  |
|  |  | Model A H1 | proportion | 0.615 | 0.371 | 0.008 | 0.005 | -1361.382 | 1.57E-02 |
|  |  |  | background | 0.080 | 1.000 | 0.080 | 1.000 |  |  |
|  |  |  | foreground | 0.080 | 1.000 | 998.998 | 998.998 |  |  |
| Fbxo5 | Rat | Model A H0 | proportion | 0.743 | 0.257 | 0.000 | 0.000 | -3765.311 |  |
|  |  | (ω2=1) | background | 0.119 | 1.000 | 0.119 | 1.000 |  |  |
|  |  |  | foreground | 0.119 | 1.000 | 1.000 | 1.000 |  |  |
|  |  | Model A H1 | proportion | 0.725 | 0.250 | 0.019 | 0.006 | -3747.153 | 1.68E-09 |
|  |  |  | background | 0.124 | 1.000 | 0.124 | 1.000 |  |  |
|  |  |  | foreground | 0.124 | 1.000 | 100.632 | 100.632 |  |  |
| Fbxo6 | Rat | Model A H0 | proportion | 0.658 | 0.173 | 0.134 | 0.035 | -2802.521 |  |
|  |  | (ω2=1) | background | 0.058 | 1.000 | 0.058 | 1.000 |  |  |
|  |  |  | foreground | 0.058 | 1.000 | 1.000 | 1.000 |  |  |
|  |  | Model A H1 | proportion | 0.735 | 0.185 | 0.064 | 0.016 | -2797.505 | 1.54E-03 |
|  |  |  | background | 0.060 | 1.000 | 0.060 | 1.000 |  |  |
|  |  |  | foreground | 0.060 | 1.000 | 11.963 | 11.963 |  |  |
| Fbxo6 | Marmoset | Model A H0 | proportion | 0.680 | 0.195 | 0.097 | 0.028 | -2803.714 |  |
|  |  | (ω2=1) | background | 0.056 | 1.000 | 0.056 | 1.000 |  |  |
|  |  |  | foreground | 0.056 | 1.000 | 1.000 | 1.000 |  |  |
|  |  | Model A H1 | proportion | 0.746 | 0.211 | 0.034 | 0.010 | -2800.453 | 1.07E-02 |
|  |  |  | background | 0.063 | 1.000 | 0.063 | 1.000 |  |  |
|  |  |  | foreground | 0.063 | 1.000 | 998.974 | 998.974 |  |  |
| Fbxo7 | 10 | Model A H0 | proportion | 0.535 | 0.231 | 0.163 | 0.071 | -4891.128 |  |
|  |  | (ω2=1) | background | 0.129 | 1.000 | 0.129 | 1.000 |  |  |
|  |  |  | foreground | 0.129 | 1.000 | 1.000 | 1.000 |  |  |
|  |  | Model A H1 | proportion | 0.680 | 0.282 | 0.027 | 0.011 | -4889.034 | 4.07E-02 |
|  |  |  | background | 0.132 | 1.000 | 0.132 | 1.000 |  |  |
|  |  |  | foreground | 0.132 | 1.000 | 12.510 | 12.510 |  |  |
| Fbxo7 | 11 | Model A H0 | proportion | 0.386 | 0.175 | 0.302 | 0.137 | -4891.437 |  |
|  |  | (ω2=1) | background | 0.130 | 1.000 | 0.130 | 1.000 |  |  |
|  |  |  | foreground | 0.130 | 1.000 | 1.000 | 1.000 |  |  |
|  |  | Model A H1 | proportion | 0.691 | 0.288 | 0.015 | 0.006 | -4887.291 | 3.98E-03 |
|  |  |  | background | 0.136 | 1.000 | 0.136 | 1.000 |  |  |
|  |  |  | foreground | 0.136 | 1.000 | 396.096 | 396.096 |  |  |
| Fbxo7 | Macaque | Model A H0 | proportion | 0.686 | 0.314 | 0.000 | 0.000 | -4891.921 |  |
|  |  | (ω2=1) | background | 0.133 | 1.000 | 0.133 | 1.000 |  |  |
|  |  |  | foreground | 0.133 | 1.000 | 1.000 | 1.000 |  |  |
|  |  | Model A H1 | proportion | 0.697 | 0.288 | 0.011 | 0.004 | -4872.477 | 4.49E-10 |
|  |  |  | background | 0.151 | 1.000 | 0.151 | 1.000 |  |  |
|  |  |  | foreground | 0.151 | 1.000 | 999.000 | 999.000 |  |  |
| Fbxo8 | Mouse | Model A H0 | proportion | 0.884 | 0.041 | 0.072 | 0.003 | -1950.436 |  |
|  |  | (ω2=1) | background | 0.021 | 1.000 | 0.021 | 1.000 |  |  |
|  |  |  | foreground | 0.021 | 1.000 | 1.000 | 1.000 |  |  |
|  |  | Model A H1 | proportion | 0.951 | 0.028 | 0.020 | 0.001 | -1934.505 | 1.66E-08 |
|  |  |  | background | 0.033 | 1.000 | 0.033 | 1.000 |  |  |
|  |  |  | foreground | 0.033 | 1.000 | 999.000 | 999.000 |  |  |
| Fbxw11 | Macaque | Model A H0 | proportion | 0.934 | 0.054 | 0.011 | 0.001 | -3380.587 |  |
|  |  | (ω2=1) | background | 0.000 | 1.000 | 0.000 | 1.000 |  |  |
|  |  |  | foreground | 0.000 | 1.000 | 1.000 | 1.000 |  |  |
|  |  | Model A H1 | proportion | 0.945 | 0.053 | 0.002 | 0.000 | -3377.897 | 2.04E-02 |
|  |  |  | background | 0.000 | 1.000 | 0.000 | 1.000 |  |  |
|  |  |  | foreground | 0.000 | 1.000 | 75.061 | 75.061 |  |  |
| Fbxw11 | Orangutan | Model A H0 | proportion | 0.787 | 0.007 | 0.205 | 0.002 | -3345.013 |  |
|  |  | (ω2=1) | background | 0.000 | 1.000 | 0.000 | 1.000 |  |  |
|  |  |  | foreground | 0.000 | 1.000 | 1.000 | 1.000 |  |  |
|  |  | Model A H1 | proportion | 0.953 | 0.008 | 0.039 | 0.000 | -3276.693 | 0.00E+00 |
|  |  |  | background | 0.000 | 1.000 | 0.000 | 1.000 |  |  |
|  |  |  | foreground | 0.000 | 1.000 | 999.000 | 999.000 |  |  |
| Fbxw12 | Marmoset | Model A H0 | proportion | 0.297 | 0.571 | 0.045 | 0.087 | -5563.932 |  |
|  |  | (ω2=1) | background | 0.113 | 1.000 | 0.113 | 1.000 |  |  |
|  |  |  | foreground | 0.113 | 1.000 | 1.000 | 1.000 |  |  |
|  |  | Model A H1 | proportion | 0.322 | 0.592 | 0.030 | 0.056 | -5560.089 | 5.56E-03 |
|  |  |  | background | 0.119 | 1.000 | 0.119 | 1.000 |  |  |
|  |  |  | foreground | 0.119 | 1.000 | 11.327 | 11.327 |  |  |
| Fbxw12 | Macaque | Model A H0 | proportion | 0.292 | 0.560 | 0.051 | 0.097 | -5563.860 |  |
|  |  | (ω2=1) | background | 0.118 | 1.000 | 0.118 | 1.000 |  |  |
|  |  |  | foreground | 0.118 | 1.000 | 1.000 | 1.000 |  |  |
|  |  | Model A H1 | proportion | 0.336 | 0.647 | 0.006 | 0.012 | -5560.377 | 8.31E-03 |
|  |  |  | background | 0.116 | 1.000 | 0.116 | 1.000 |  |  |
|  |  |  | foreground | 0.116 | 1.000 | 21.034 | 21.034 |  |  |
| Fbxw12 | 9 | Model A H0 | proportion | 0.317 | 0.598 | 0.029 | 0.056 | -5564.086 |  |
|  |  | (ω2=1) | background | 0.110 | 1.000 | 0.110 | 1.000 |  |  |
|  |  |  | foreground | 0.110 | 1.000 | 1.000 | 1.000 |  |  |
|  |  | Model A H1 | proportion | 0.295 | 0.569 | 0.046 | 0.089 | -5560.137 | 4.95E-03 |
|  |  |  | background | 0.116 | 1.000 | 0.116 | 1.000 |  |  |
|  |  |  | foreground | 0.116 | 1.000 | 11.803 | 11.803 |  |  |
| Fbxw2 | Macaque | Model A H0 | proportion | 0.774 | 0.021 | 0.199 | 0.005 | -2894.765 |  |
|  |  | (ω2=1) | background | 0.013 | 1.000 | 0.013 | 1.000 |  |  |
|  |  |  | foreground | 0.013 | 1.000 | 1.000 | 1.000 |  |  |
|  |  | Model A H1 | proportion | 0.939 | 0.000 | 0.061 | 0.000 | -2828.098 | 0.00E+00 |
|  |  |  | background | 0.043 | 1.000 | 0.043 | 1.000 |  |  |
|  |  |  | foreground | 0.043 | 1.000 | 999.000 | 999.000 |  |  |
| Fbxw9 | Rat | Model A H0 | proportion | 0.751 | 0.093 | 0.138 | 0.017 | -3936.931 |  |
|  |  | (ω2=1) | background | 0.117 | 1.000 | 0.117 | 1.000 |  |  |
|  |  |  | foreground | 0.117 | 1.000 | 1.000 | 1.000 |  |  |
|  |  | Model A H1 | proportion | 0.872 | 0.112 | 0.014 | 0.002 | -3929.494 | 1.15E-04 |
|  |  |  | background | 0.124 | 1.000 | 0.124 | 1.000 |  |  |
|  |  |  | foreground | 0.124 | 1.000 | 201.140 | 201.140 |  |  |
| Kdm2B | Rat | Model A H0 | proportion | 0.880 | 0.098 | 0.019 | 0.002 | -9723.711 |  |
|  |  | (ω2=1) | background | 0.019 | 1.000 | 0.019 | 1.000 |  |  |
|  |  |  | foreground | 0.019 | 1.000 | 1.000 | 1.000 |  |  |
|  |  | Model A H1 | proportion | 0.886 | 0.090 | 0.022 | 0.002 | -9686.040 | 0.00E+00 |
|  |  |  | background | 0.022 | 1.000 | 0.022 | 1.000 |  |  |
|  |  |  | foreground | 0.022 | 1.000 | 839.149 | 839.149 |  |  |
| Kdm2B | Mouse | Model A H0 | proportion | 0.883 | 0.099 | 0.016 | 0.002 | -9724.251 |  |
|  |  | (ω2=1) | background | 0.020 | 1.000 | 0.020 | 1.000 |  |  |
|  |  |  | foreground | 0.020 | 1.000 | 1.000 | 1.000 |  |  |
|  |  | Model A H1 | proportion | 0.891 | 0.097 | 0.010 | 0.001 | -9701.461 | 1.47E-11 |
|  |  |  | background | 0.021 | 1.000 | 0.021 | 1.000 |  |  |
|  |  |  | foreground | 0.021 | 1.000 | 999.000 | 999.000 |  |  |
| Kdm2B | Macaque | Model A H0 | proportion | 0.856 | 0.088 | 0.051 | 0.005 | -9716.373 |  |
|  |  | (ω2=1) | background | 0.019 | 1.000 | 0.019 | 1.000 |  |  |
|  |  |  | foreground | 0.019 | 1.000 | 1.000 | 1.000 |  |  |
|  |  | Model A H1 | proportion | 0.888 | 0.083 | 0.026 | 0.002 | -9638.012 | 0.00E+00 |
|  |  |  | background | 0.023 | 1.000 | 0.023 | 1.000 |  |  |
|  |  |  | foreground | 0.023 | 1.000 | 999.000 | 999.000 |  |  |
| Kdm2B | 9 | Model A H0 | proportion | 0.894 | 0.103 | 0.003 | 0.000 | -9725.064 |  |
|  |  | (ω2=1) | background | 0.020 | 1.000 | 0.020 | 1.000 |  |  |
|  |  |  | foreground | 0.020 | 1.000 | 1.000 | 1.000 |  |  |
|  |  | Model A H1 | proportion | 0.897 | 0.097 | 0.005 | 0.001 | -9719.710 | 1.07E-03 |
|  |  |  | background | 0.021 | 1.000 | 0.021 | 1.000 |  |  |
|  |  |  | foreground | 0.021 | 1.000 | 257.293 | 257.293 |  |  |

Note: only genes showing statistically significant positive selection were shown here.
