# Supplementary material for: Evolution of the F-Box Gene Family in Euarchontoglires: Gene Number Variation and Selection Patterns
Source: PLoS One. 2014 Apr 11;9(4):e94899. doi: 10.1371/journal.pone.0094899 (PMC3984280; doi:10.1371/journal.pone.0094899)
Supplement: Table S7 — Statistical tests for differences in SNP distributions across different regions of F-box genes. (DOCX) [file pone.0094899.s016.docx]

Table S7. Statistical tests for differences in SNP distributions across different regions of F-box genes

1. Kruskal-Wallis test for differences of SNP density distribution among F-box domain, other domain and non-domain regions.

|  | df | chi-squared | *p*-value |
| --- | --- | --- | --- |
| Synonymous | 2 | 0.696 | 0.706 |
| Nonsynonymous | 2 | 18.798 | 8.28e-05 |
| All substitutions | 2 | 7.527 | 0.023 |

1. Mann-Whitney U Test for differences in SNP density between domain and non-domain regions.

|  | W | *p*-value |
| --- | --- | --- |
| Synonymous | 2845 | 0.048 |
| Non-synonymous | 1894 | 0.038 |
| All substitutions | 2315 | 0.784 |

1. Fisher’s exact test for differences in ratios of non-synonymous to synonymous SNPs between F-box and other domain regions, and domain and non-domain regions.

| Region | Non-synonymous | Synonymous | *p*-value |
| --- | --- | --- | --- |
| Fbox domains | 47 | 49 |  |
| Other domains | 123 | 172 | 0.130 (Fbox vs other domain) |
| Domains | 170 | 221 |  |
| Non-domains | 502 | 361 | 1.403e-06 (Domains vs Non-domains) |
